# Supplementary material for: Predictive performance of international COVID-19 mortality forecasting models
Source: medRxiv. 2020 Nov 19:2020.07.13.20151233. Preprint. [Version 5] doi: 10.1101/2020.07.13.20151233 (PMC7685335; doi:10.1101/2020.07.13.20151233)
Supplement: Supplement 2020 [file 91596-2020.07.13.20151233-3.pdf]

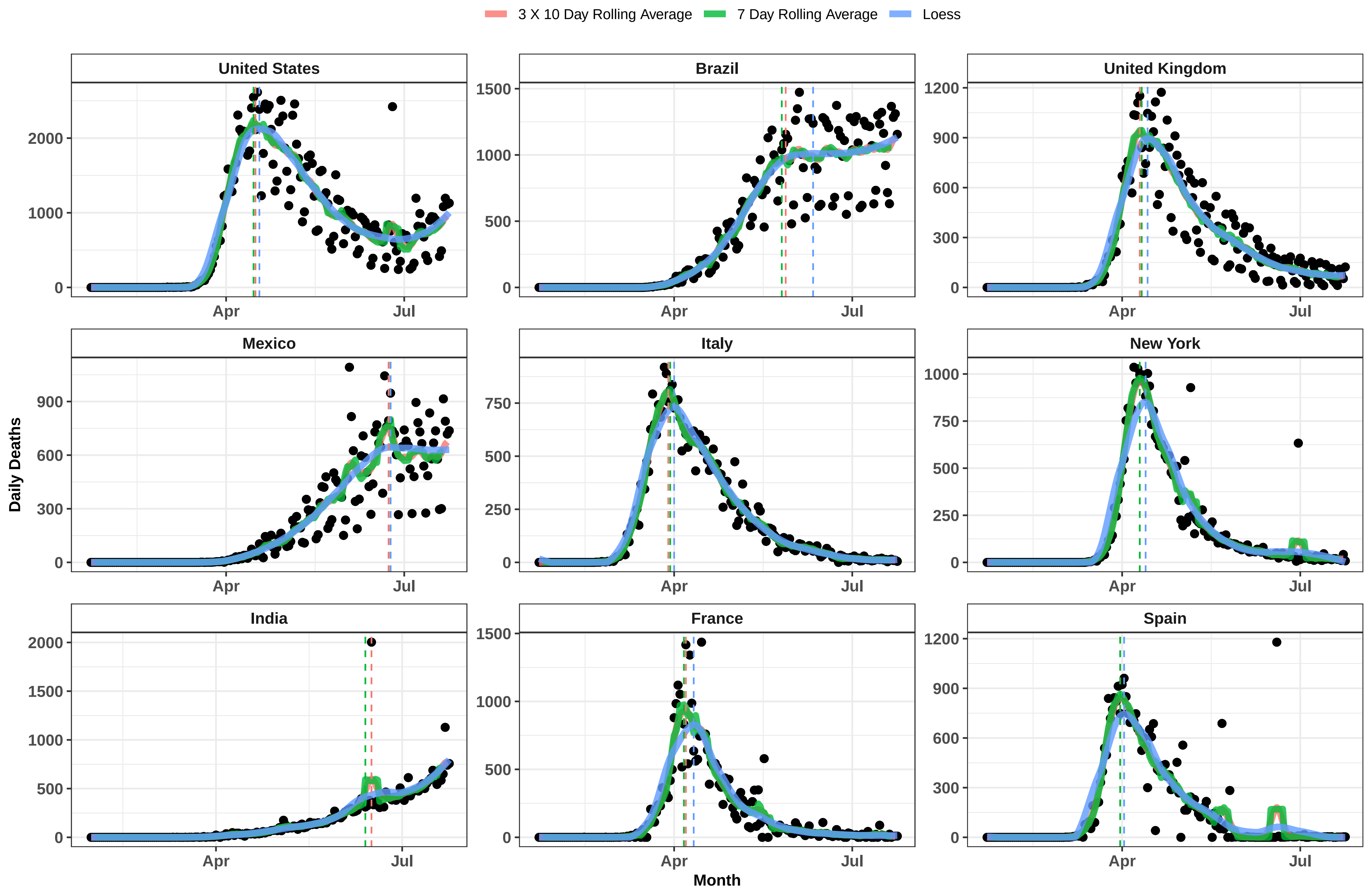

3 X 10 Day Rolling Average
7 Day Rolling Average
Loess
United States
Brazil
United Kingdom
Mexico
Italy
New York
India
France
Spain
Daily Deaths
Month
Apr
Jul

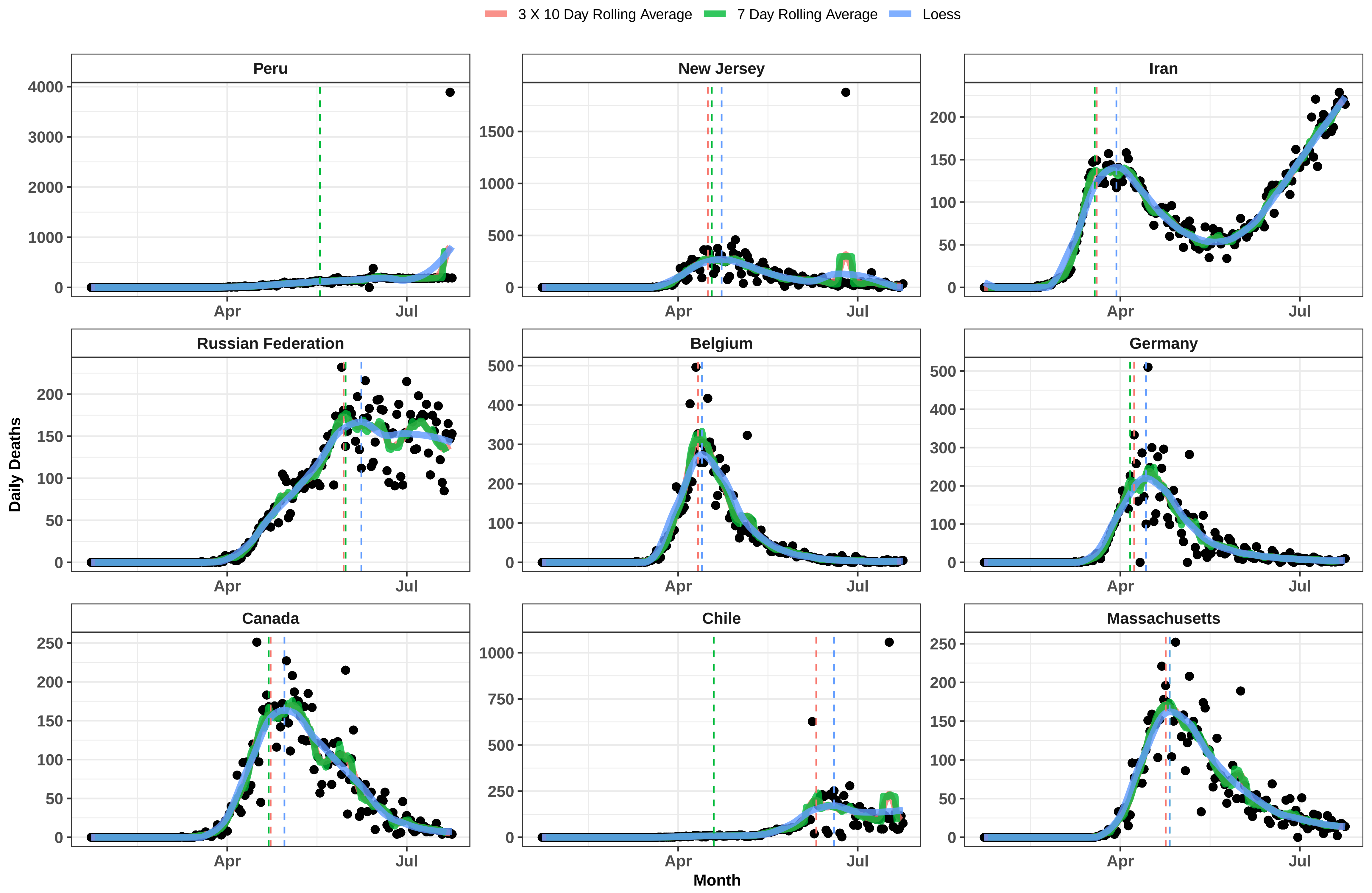

3 X 10 Day Rolling Average
7 Day Rolling Average
Loess
Peru
New Jersey
Iran
Russian Federation
Belgium
Germany
Canada
Chile
Massachusetts
Daily Deaths
Month
Apr
Jul

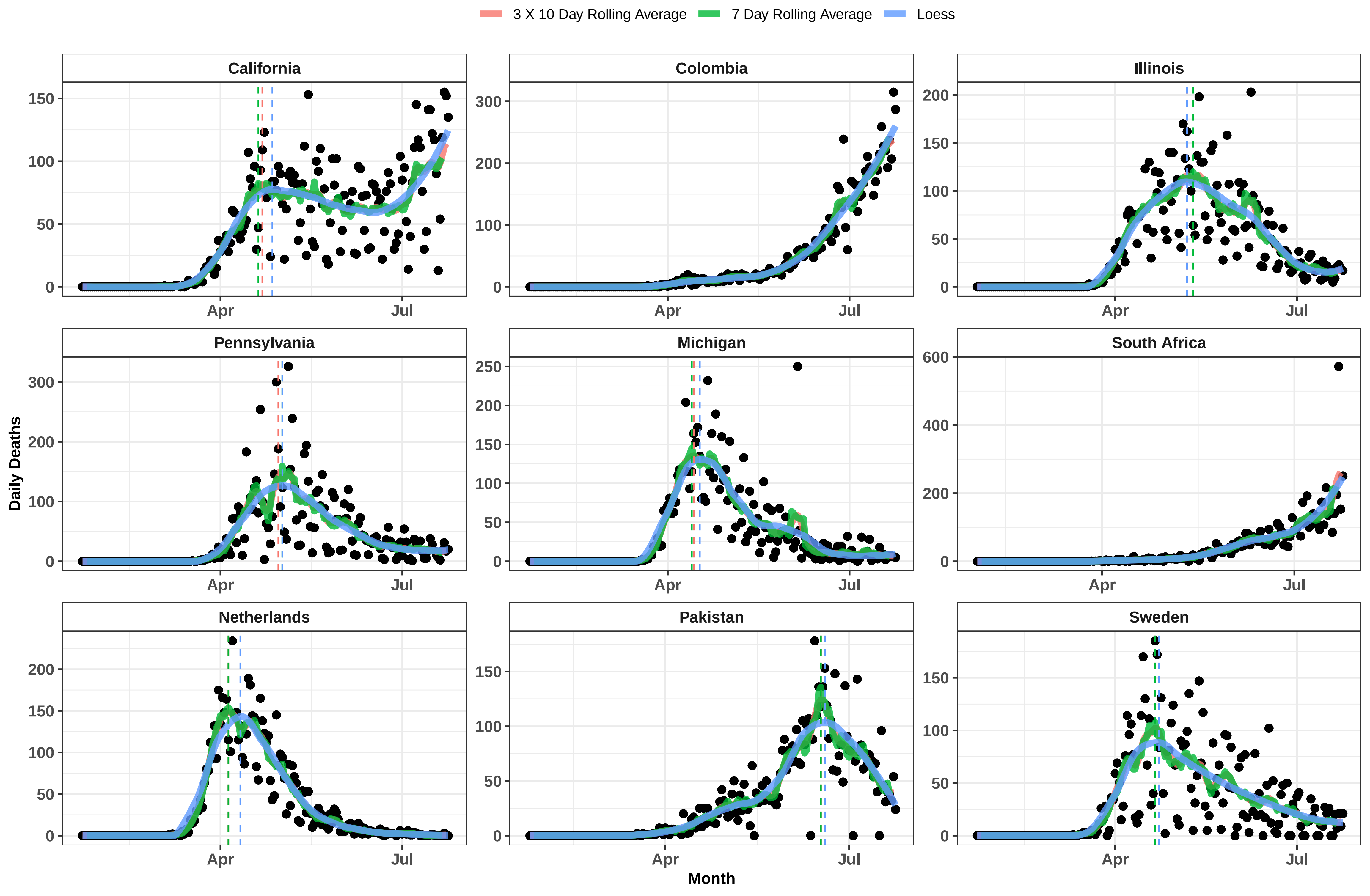
3 X 10 Day Rolling Average
7 Day Rolling Average
Loess
California
Colombia
Illinois
Pennsylvania
Michigan
South Africa
Netherlands
Pakistan
Sweden
Daily Deaths
Month
Apr
Jul
0
50
100
150
200
250
300
400
600

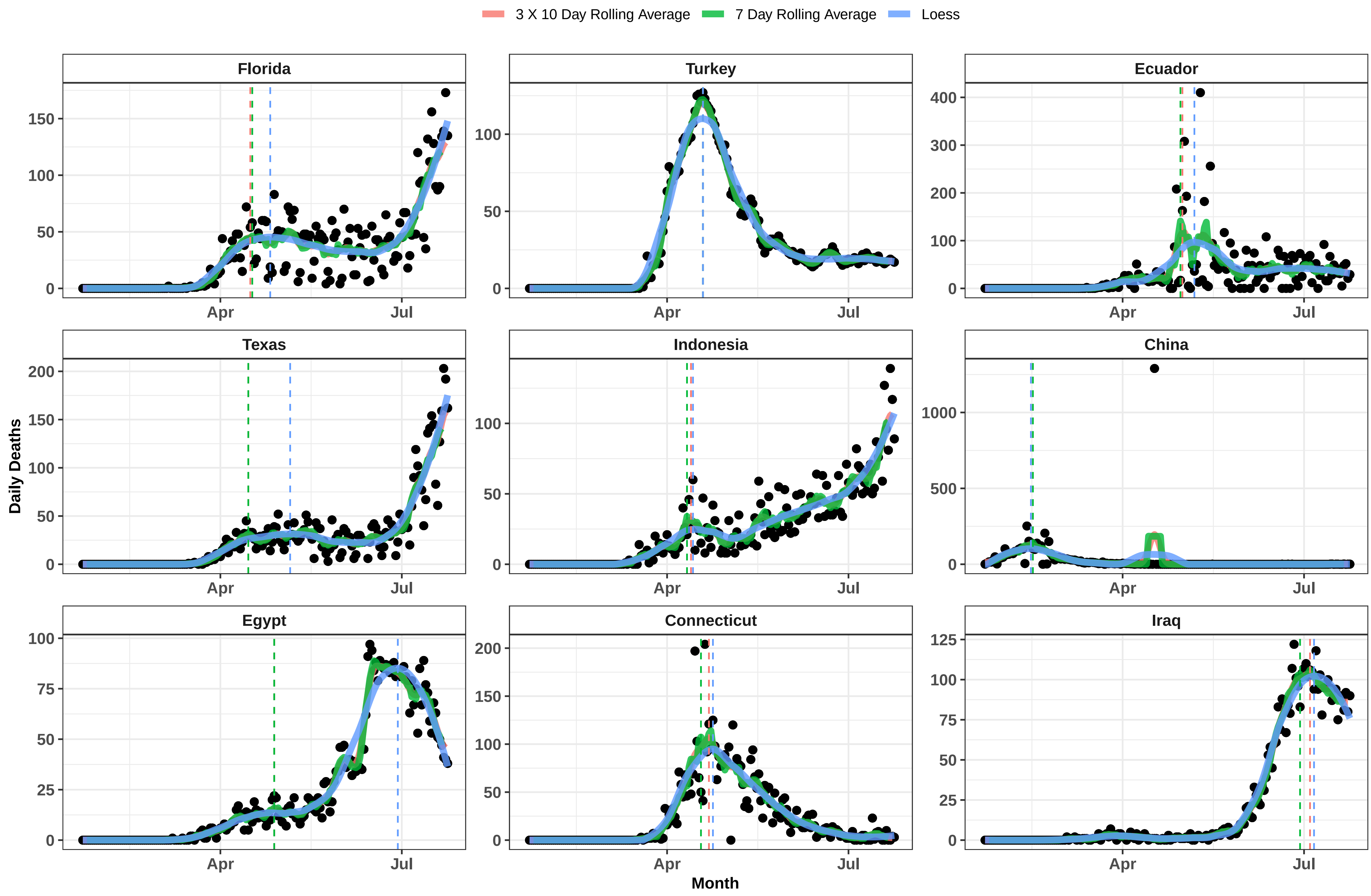

3 X 10 Day Rolling Average
7 Day Rolling Average
Loess
Florida
Turkey
Ecuador
Texas
Indonesia
China
Egypt
Connecticut
Iraq
Daily Deaths
Month
Apr
Jul

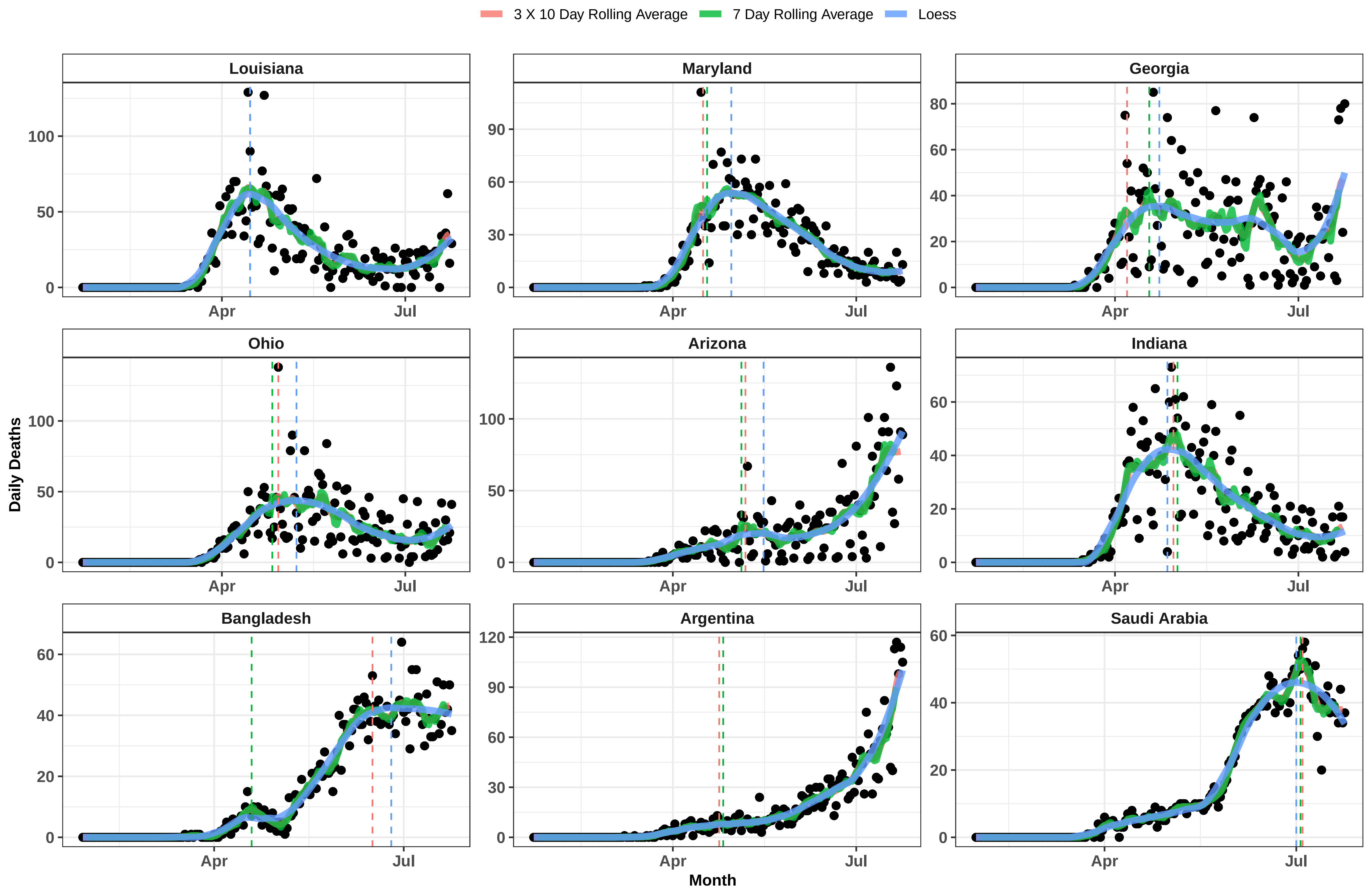

3 X 10 Day Rolling Average
7 Day Rolling Average
Loess
Louisiana
Maryland
Georgia
Ohio
Arizona
Indiana
Bangladesh
Argentina
Saudi Arabia
Daily Deaths
Month
Apr
Jul

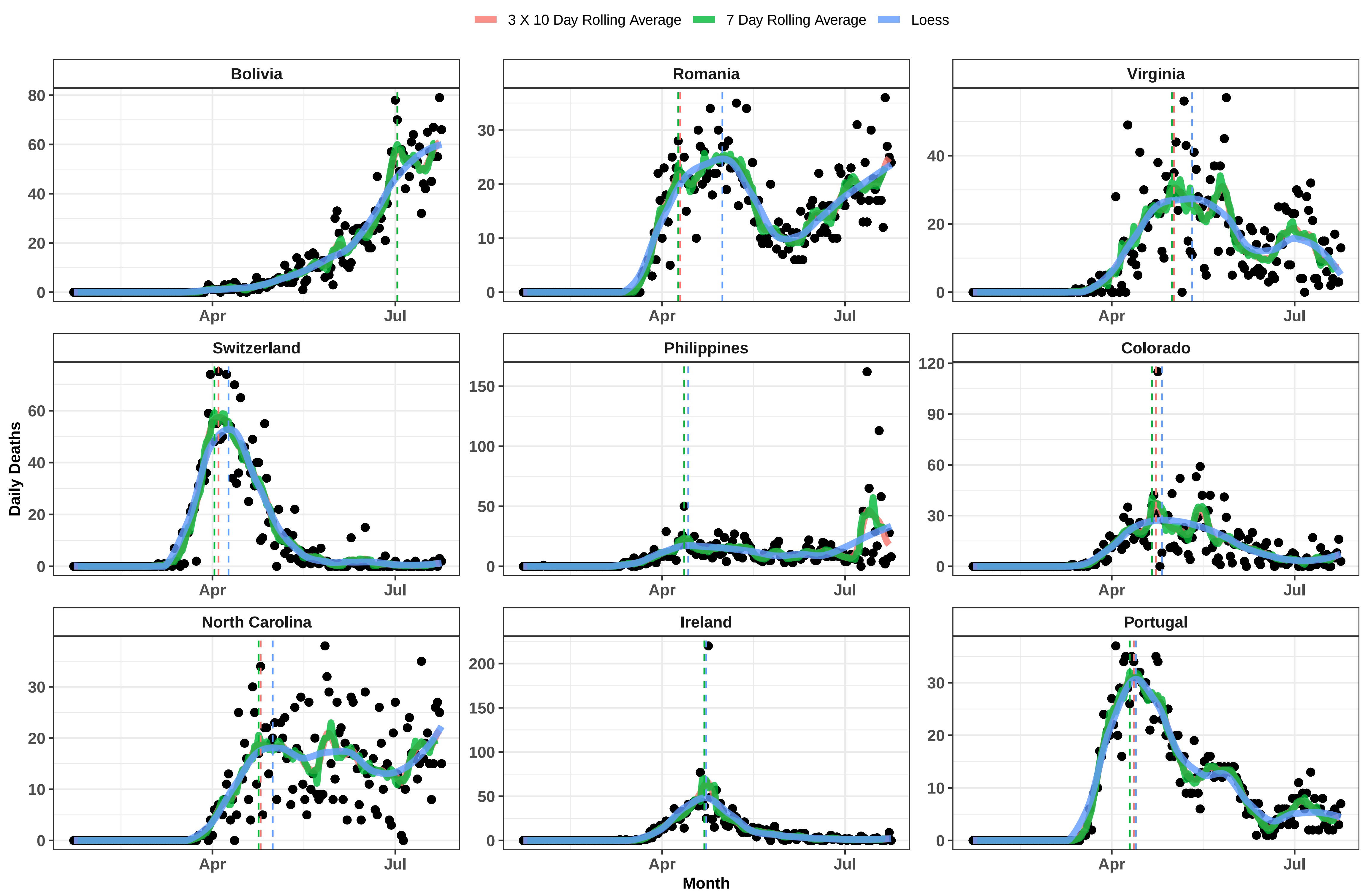

3 X 10 Day Rolling Average
7 Day Rolling Average
Loess
Bolivia
Romania
Virginia
Switzerland
Philippines
Colorado
North Carolina
Ireland
Portugal
Daily Deaths
Month
Apr
Jul

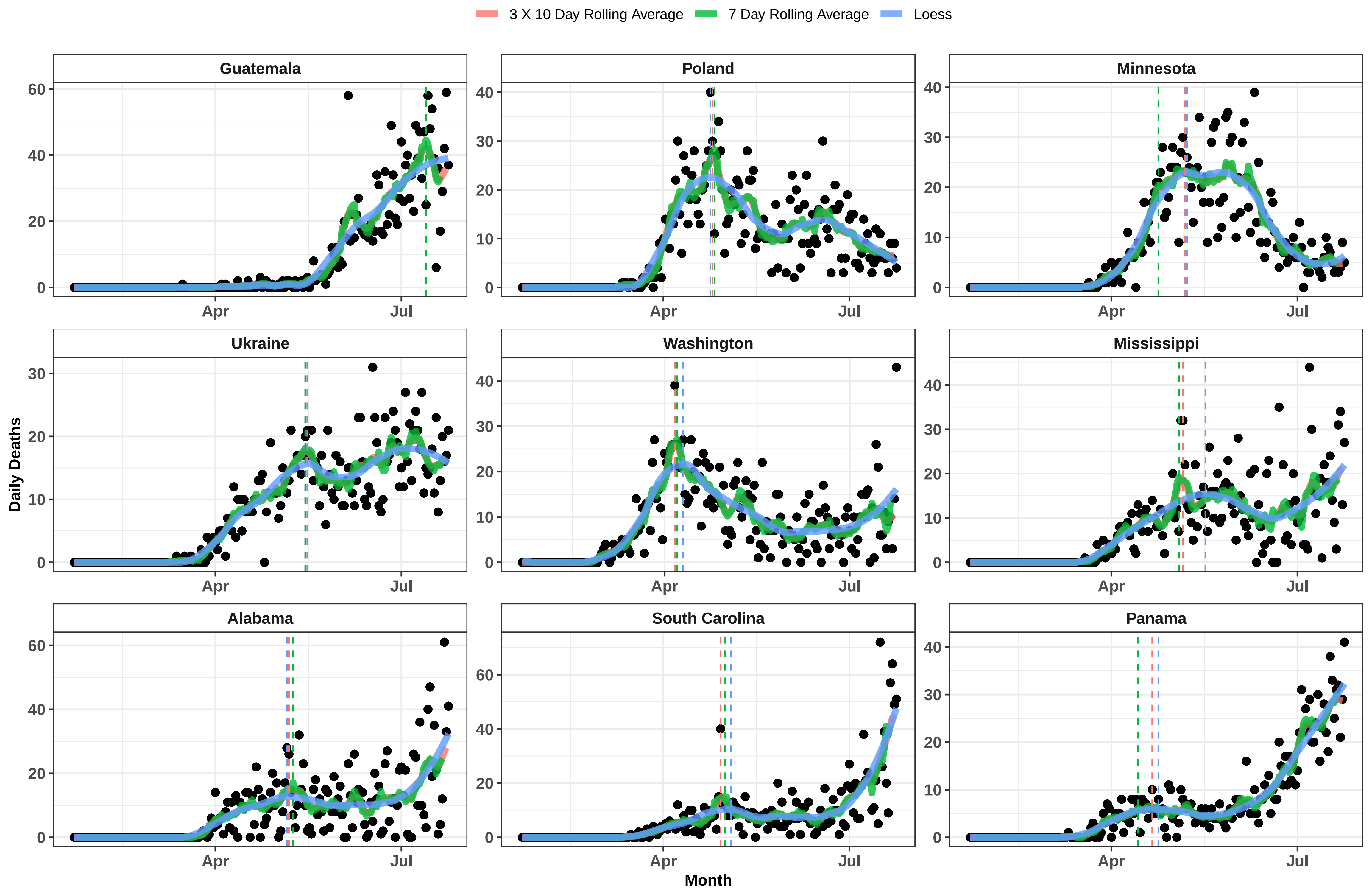
3 X 10 Day Rolling Average
7 Day Rolling Average
Loess
Guatemala
Poland
Minnesota
Ukraine
Washington
Mississippi
Alabama
South Carolina
Panama
Daily Deaths
Month
Apr
Jul

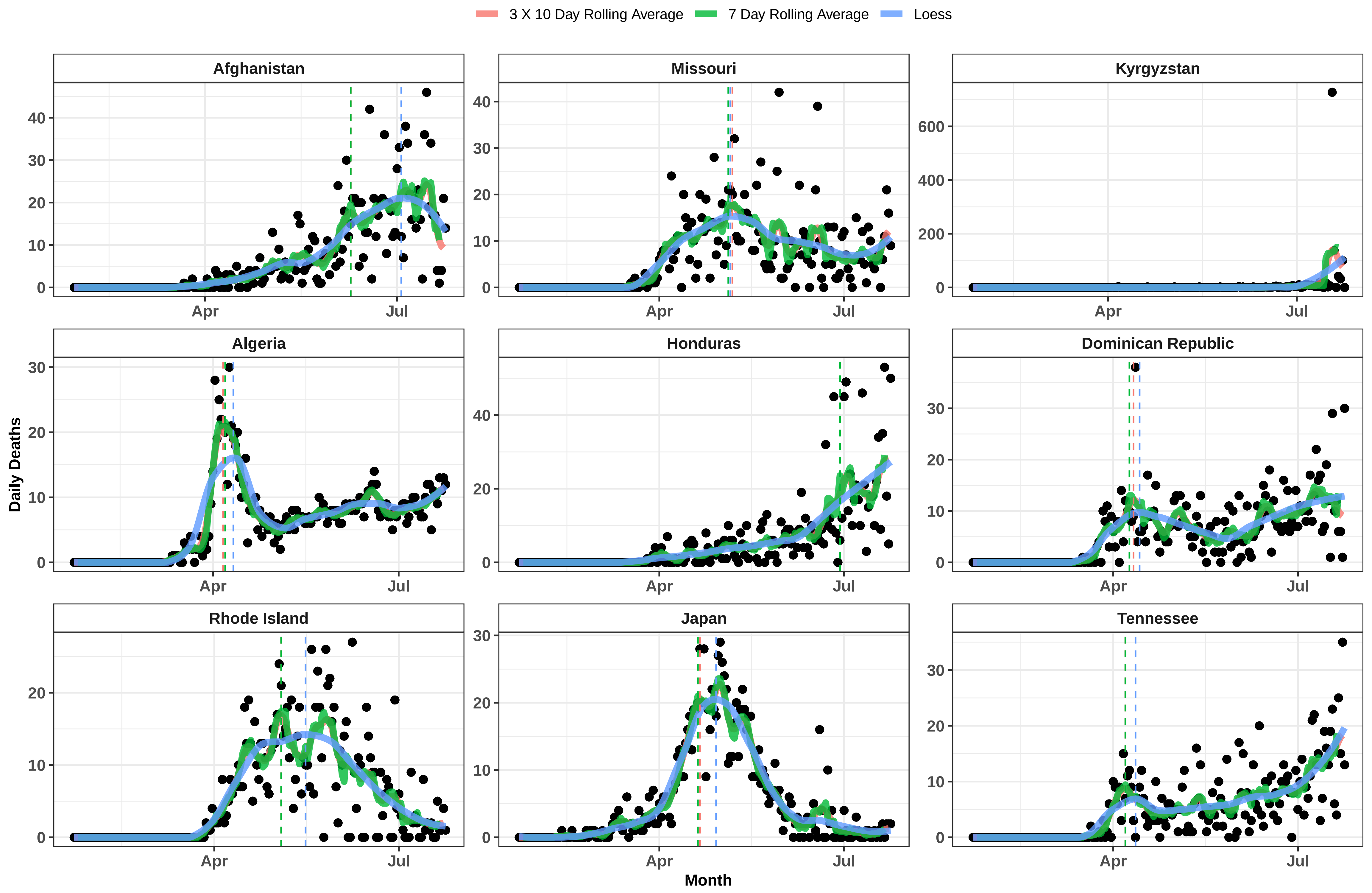

3 X 10 Day Rolling Average
7 Day Rolling Average
Loess
Afghanistan
Missouri
Kyrgyzstan
Algeria
Honduras
Dominican Republic
Rhode Island
Japan
Tennessee
Daily Deaths
Month
Apr
Jul

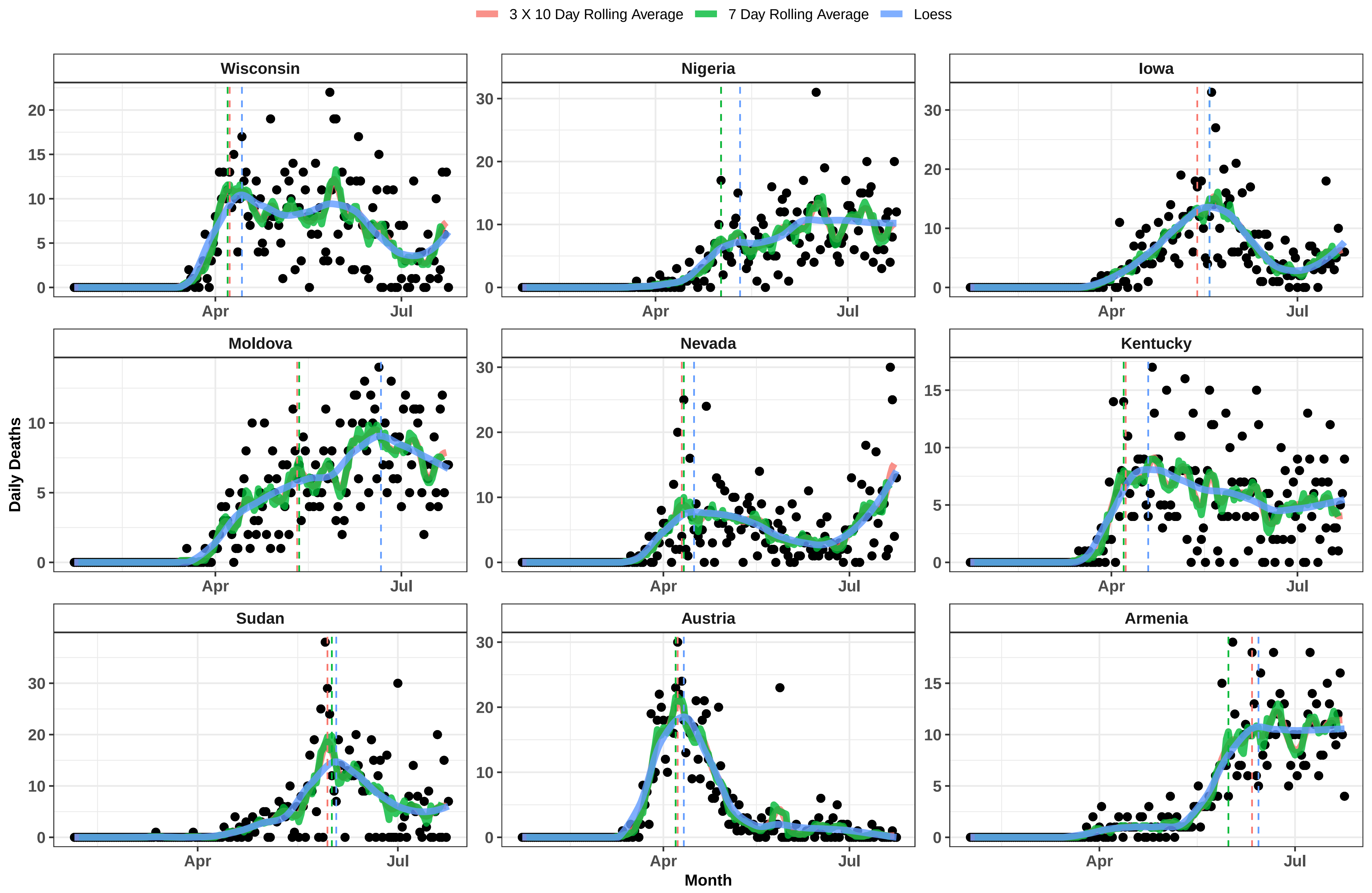

3 X 10 Day Rolling Average
7 Day Rolling Average
Loess
Wisconsin
Nigeria
Iowa
Moldova
Nevada
Kentucky
Sudan
Austria
Armenia
Daily Deaths
Month
Apr
Jul

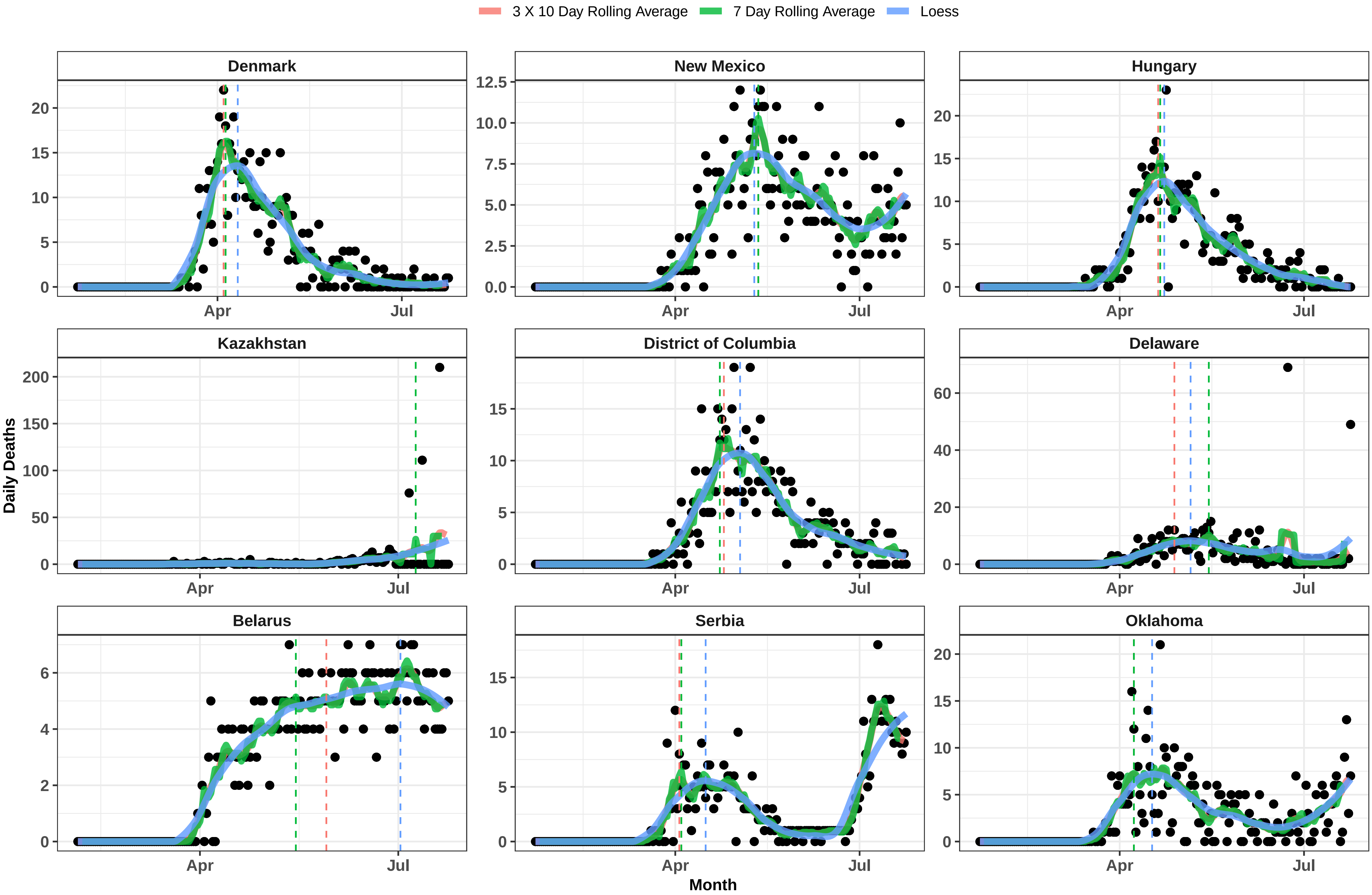

3 X 10 Day Rolling Average
7 Day Rolling Average
Loess
Denmark
New Mexico
Hungary
Kazakhstan
District of Columbia
Delaware
Belarus
Serbia
Oklahoma
Daily Deaths
Month
Apr
Jul

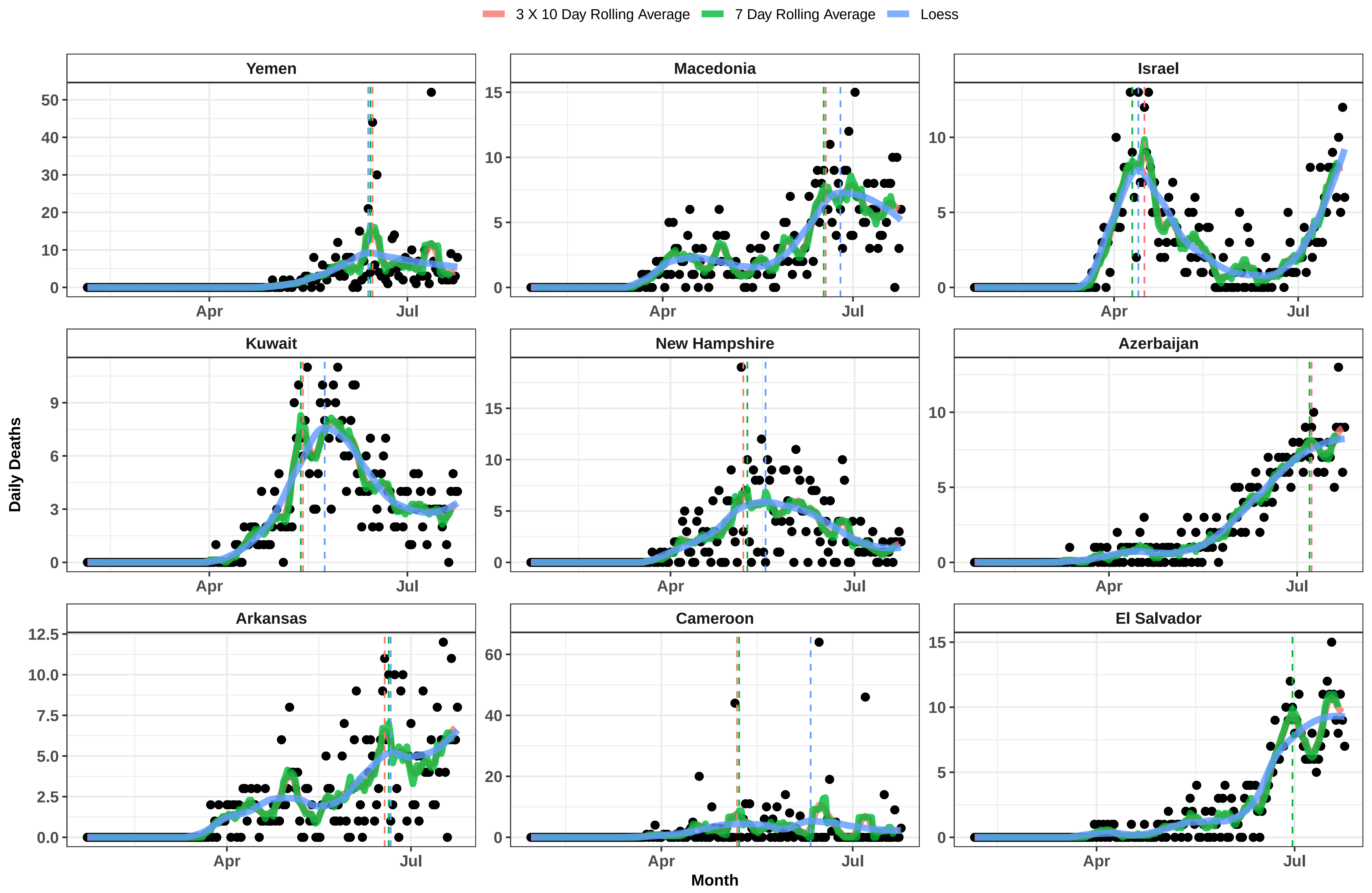

3 X 10 Day Rolling Average
7 Day Rolling Average
Loess
Yemen
Macedonia
Israel
Kuwait
New Hampshire
Azerbaijan
Arkansas
Cameroon
El Salvador
Apr
Jul
Daily Deaths
Month

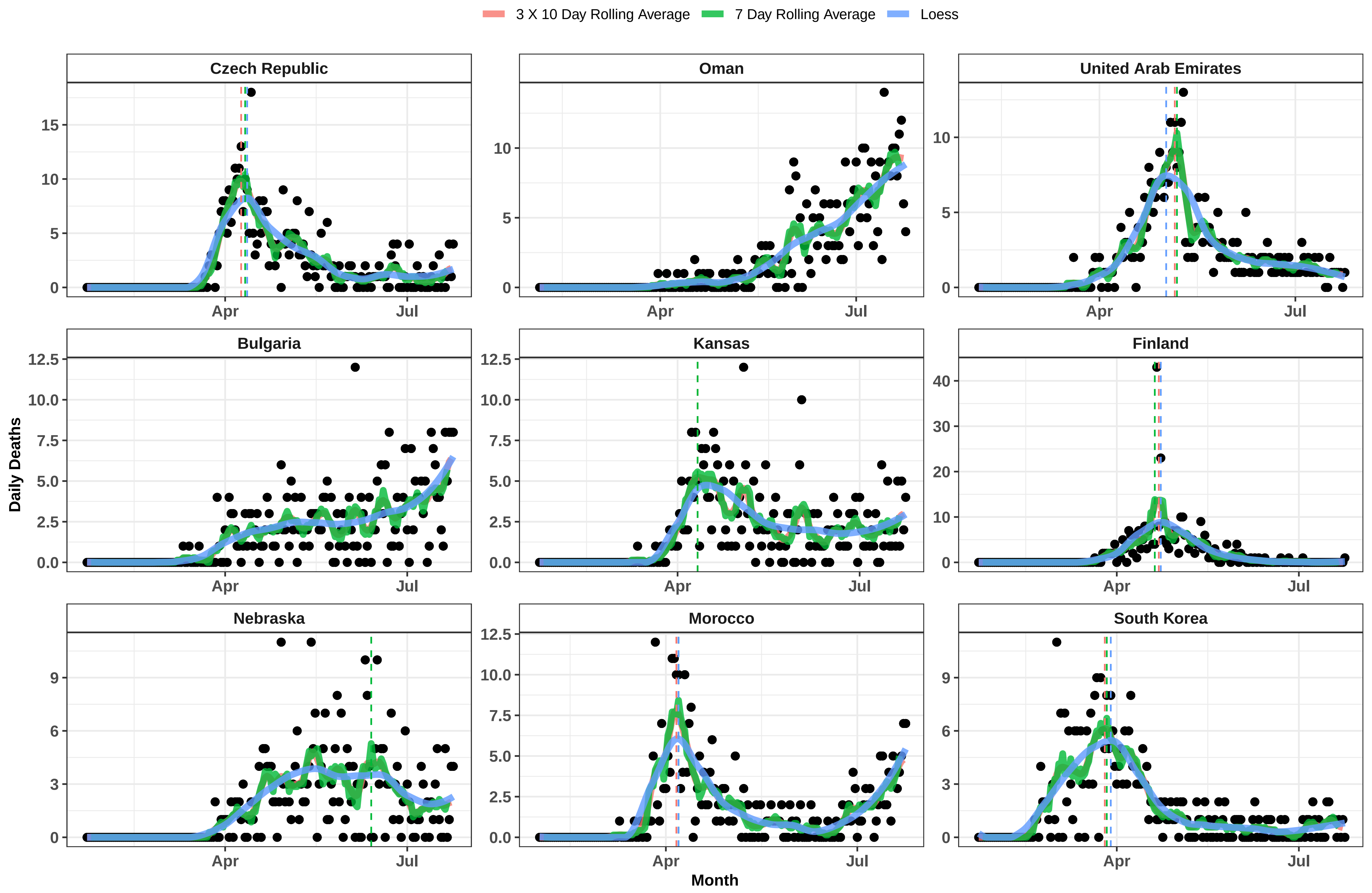

3 X 10 Day Rolling Average
7 Day Rolling Average
Loess
Czech Republic
Oman
United Arab Emirates
Bulgaria
Kansas
Finland
Nebraska
Morocco
South Korea
Daily Deaths
Month
Apr
Jul

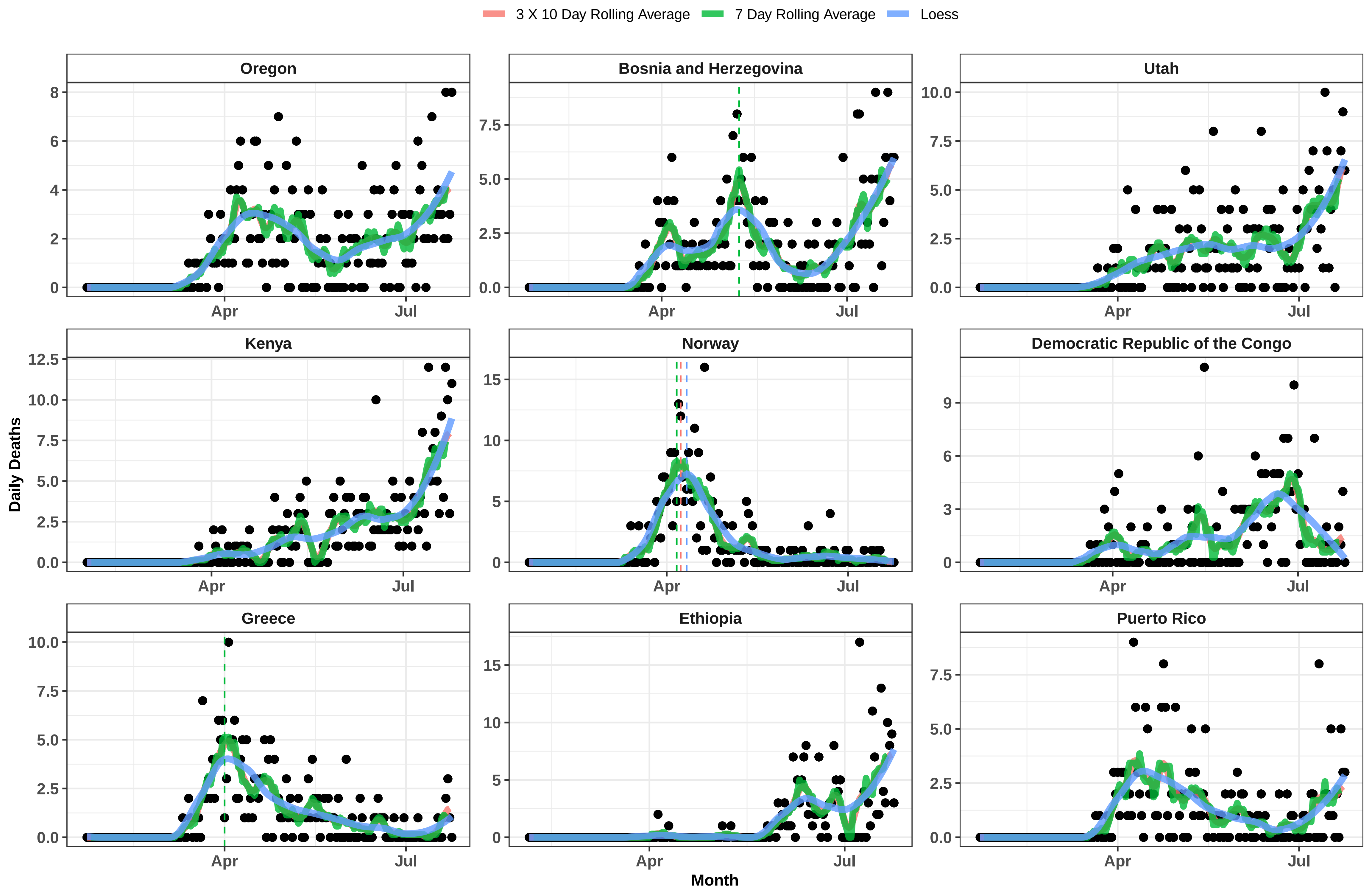

3 X 10 Day Rolling Average
7 Day Rolling Average
Loess
Oregon
Bosnia and Herzegovina
Utah
Kenya
Norway
Democratic Republic of the Congo
Greece
Ethiopia
Puerto Rico
Daily Deaths
Month
Apr
Jul

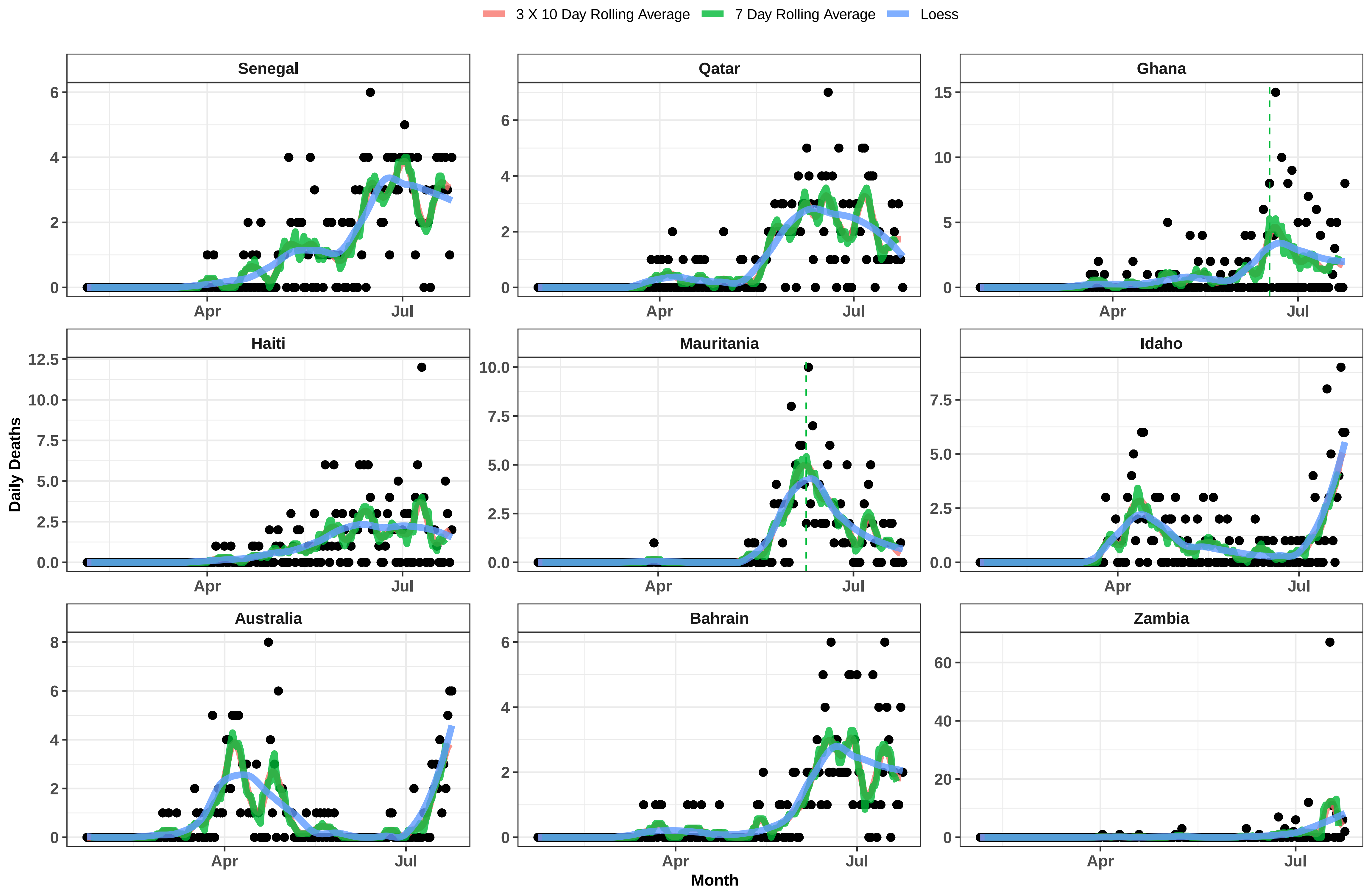

3 X 10 Day Rolling Average
7 Day Rolling Average
Loess
Senegal
Qatar
Ghana
Haiti
Mauritania
Idaho
Australia
Bahrain
Zambia
Daily Deaths
Month
Apr
Jul

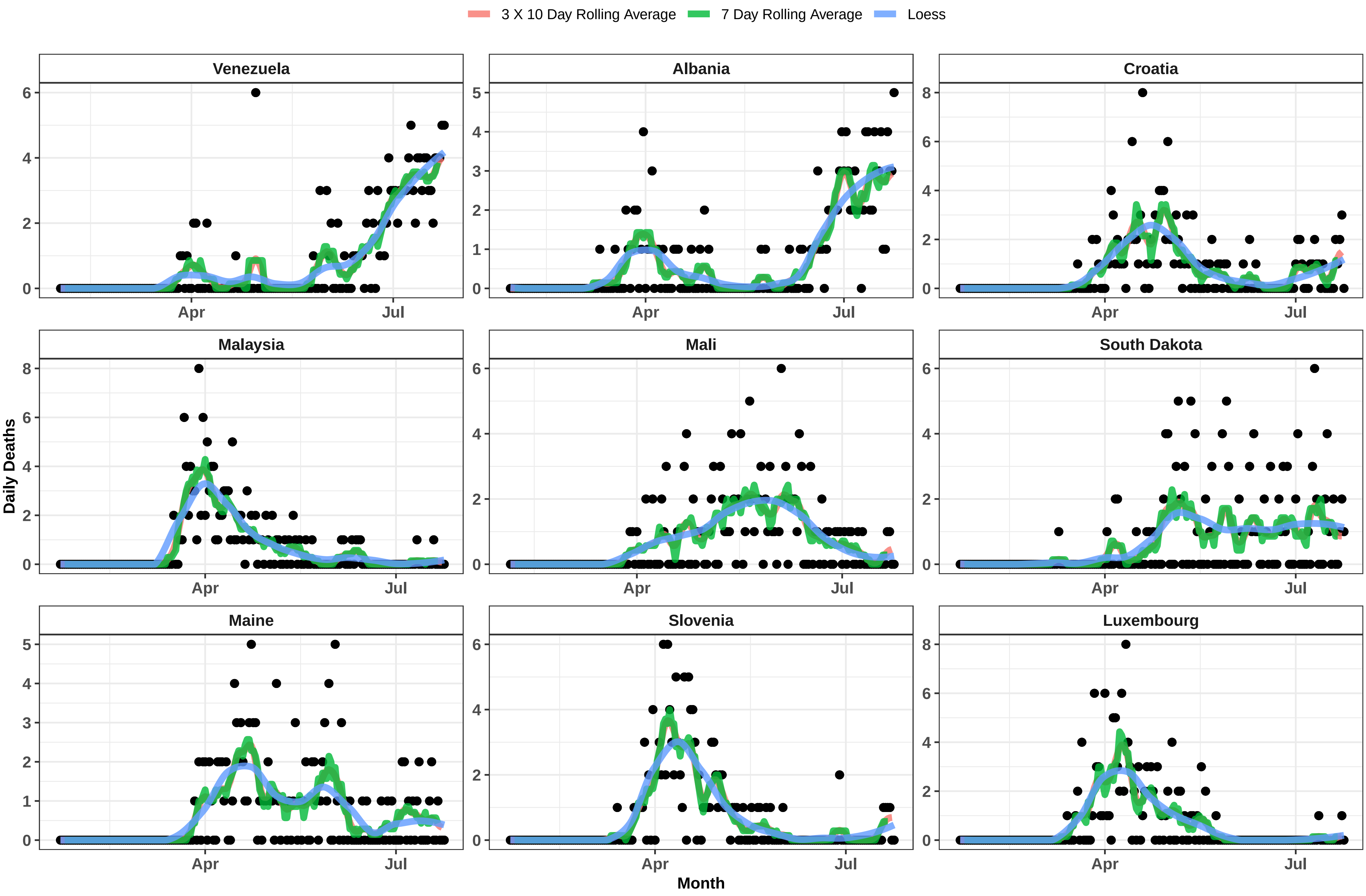

3 X 10 Day Rolling Average
7 Day Rolling Average
Loess
Venezuela
Albania
Croatia
Malaysia
Mali
South Dakota
Maine
Slovenia
Luxembourg
Daily Deaths
Month
Apr
Jul

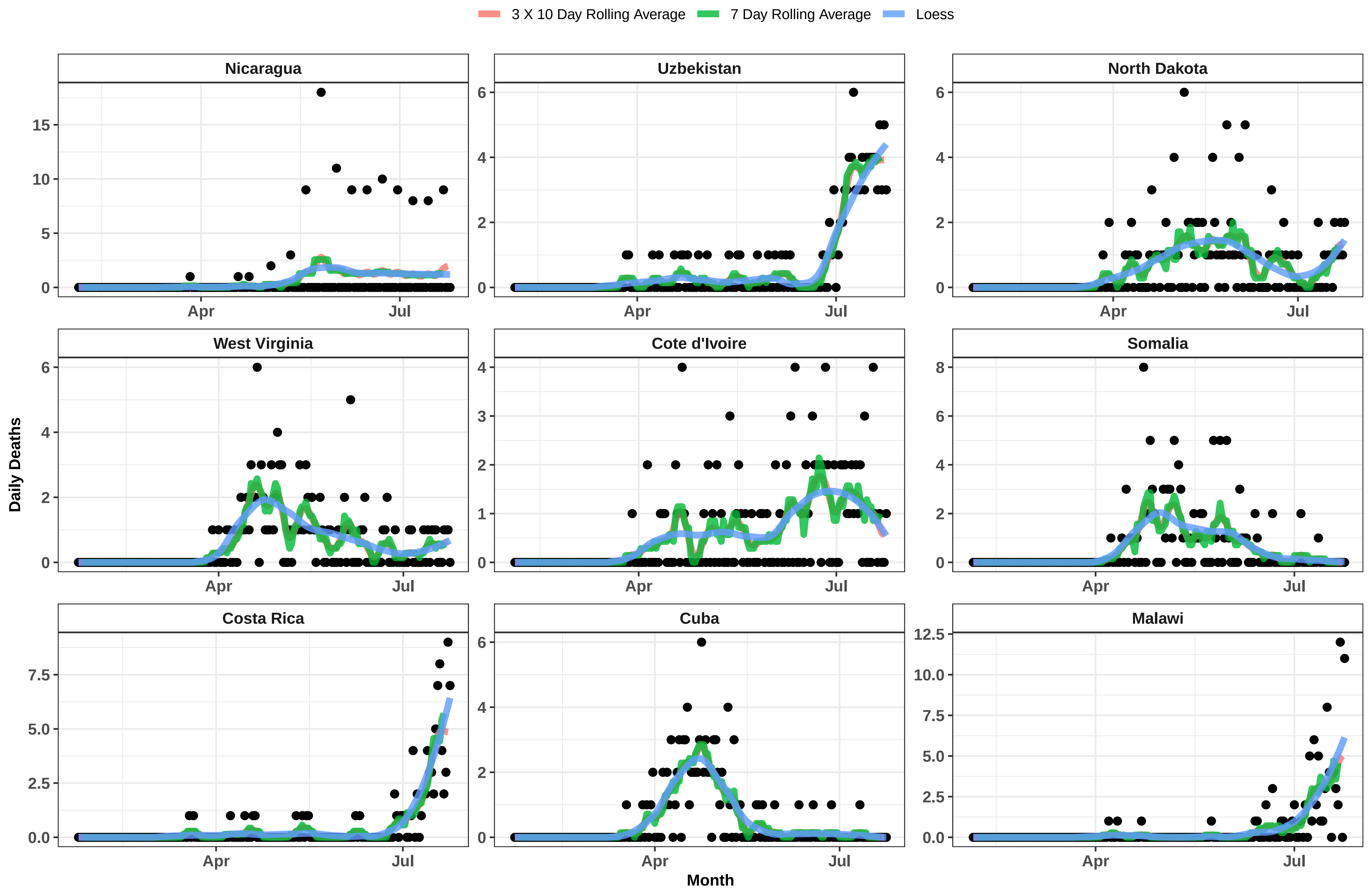

3 X 10 Day Rolling Average
7 Day Rolling Average
Loess
Nicaragua
Uzbekistan
North Dakota
West Virginia
Cote d'Ivoire
Somalia
Costa Rica
Cuba
Malawi
Daily Deaths
Month
Apr
Jul

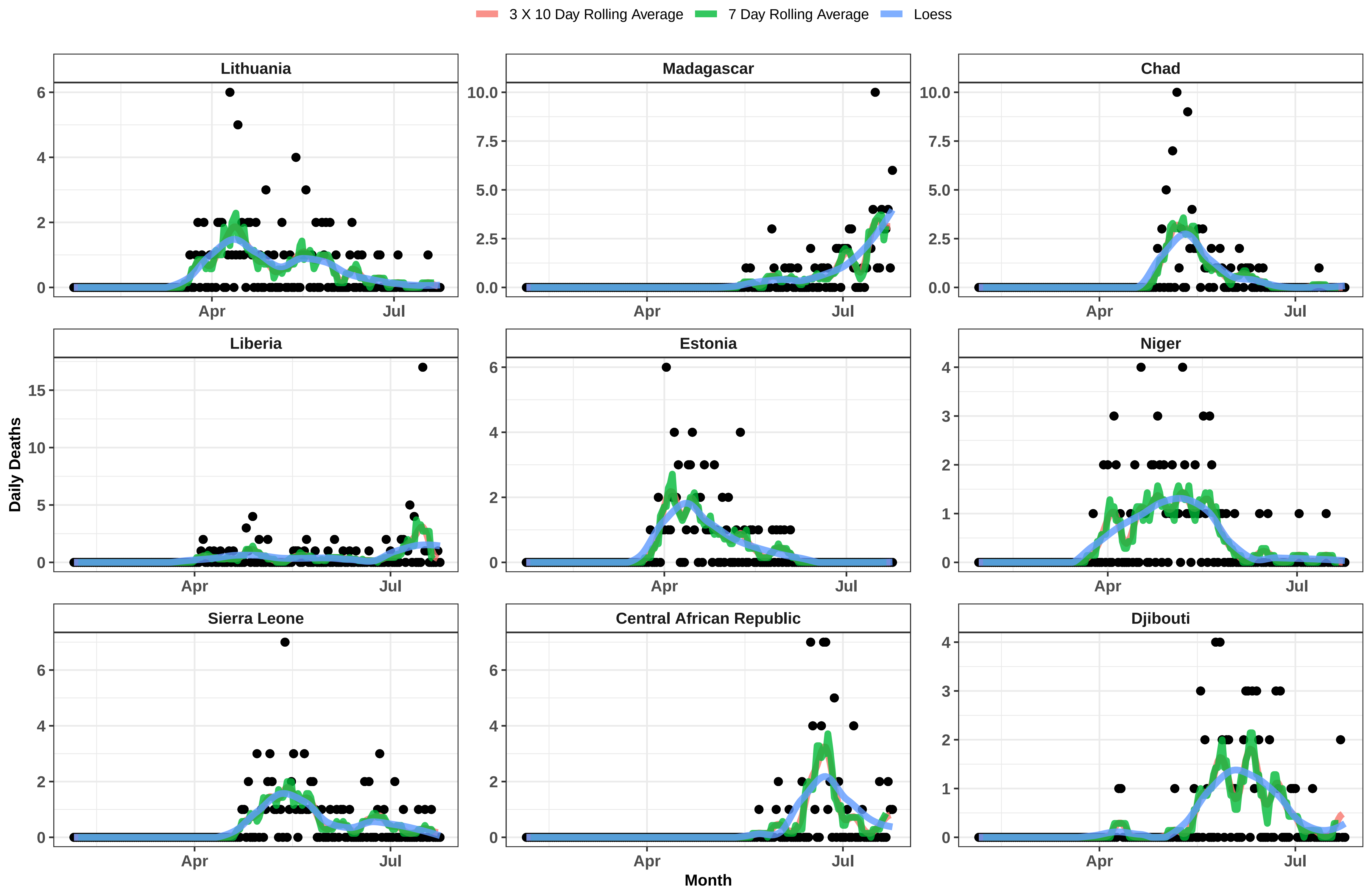

3 X 10 Day Rolling Average
7 Day Rolling Average
Loess
Lithuania
Madagascar
Chad
Liberia
Estonia
Niger
Sierra Leone
Central African Republic
Djibouti
Daily Deaths
Month
Apr
Jul

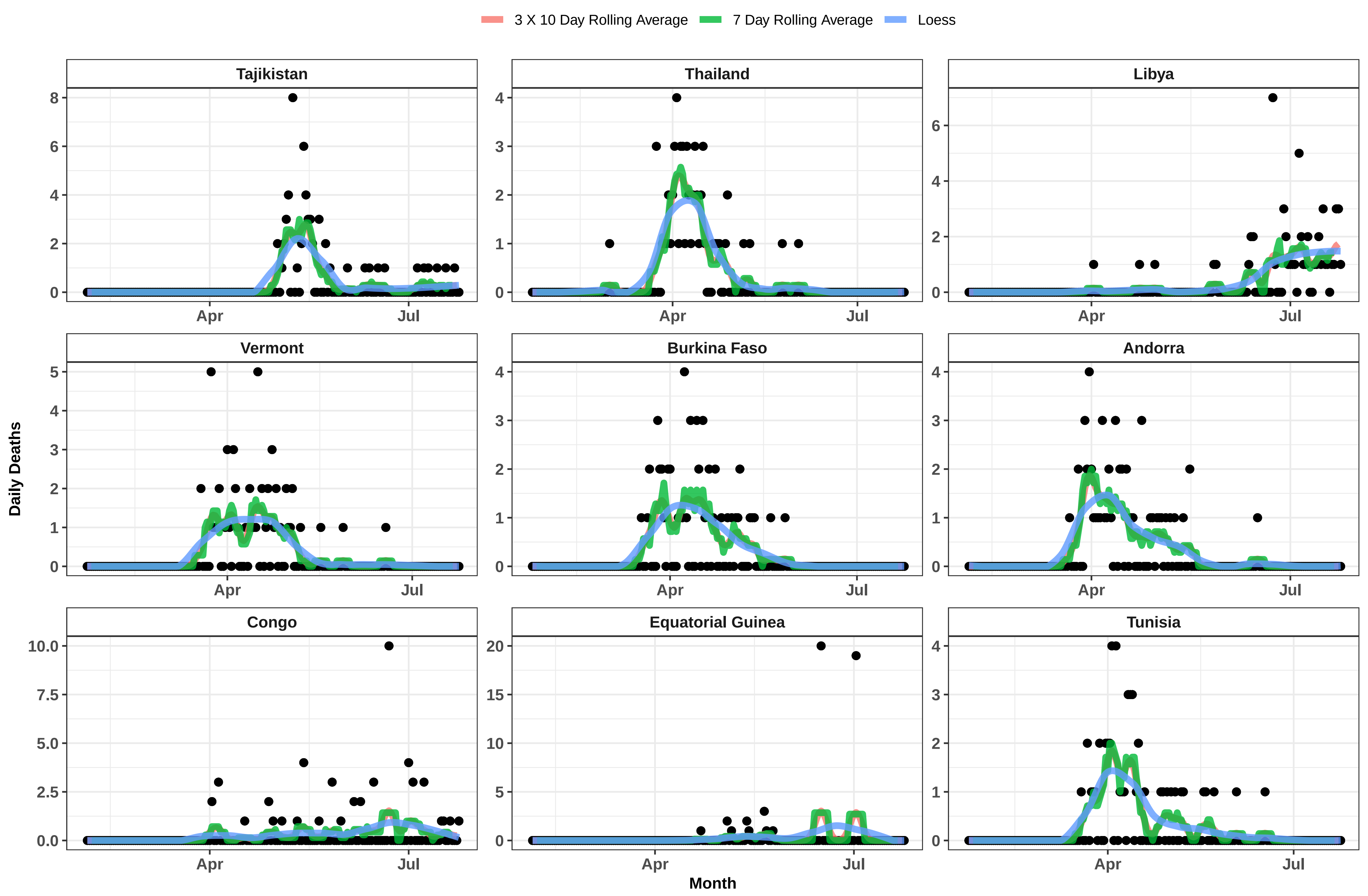

3 X 10 Day Rolling Average
7 Day Rolling Average
Loess
Tajikistan
Thailand
Libya
Vermont
Burkina Faso
Andorra
Congo
Equatorial Guinea
Tunisia
Daily Deaths
Month
Apr
Jul

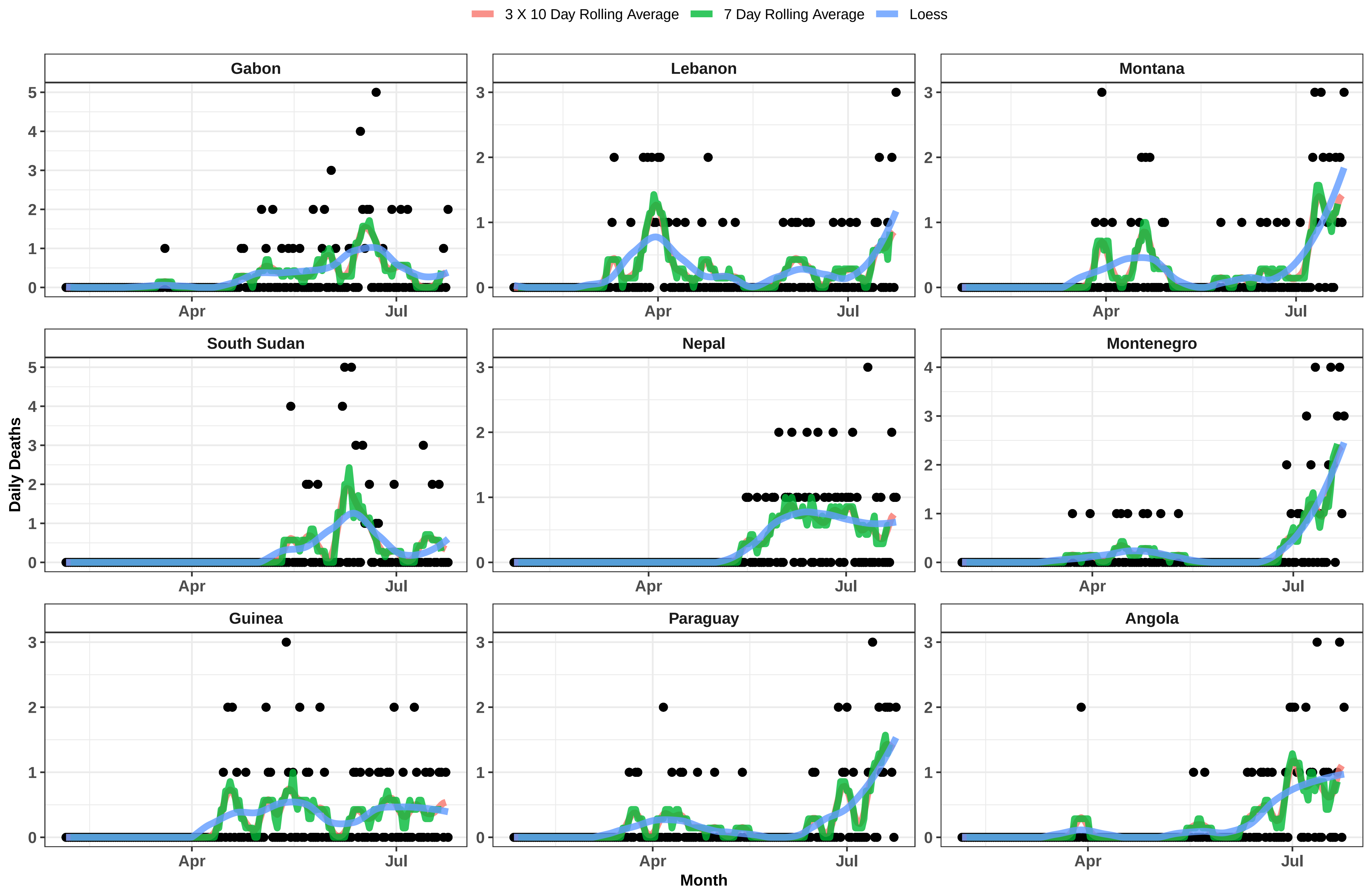

3 X 10 Day Rolling Average
7 Day Rolling Average
Loess
Gabon
Lebanon
Montana
South Sudan
Nepal
Montenegro
Guinea
Paraguay
Angola
Daily Deaths
Month
Apr
Jul

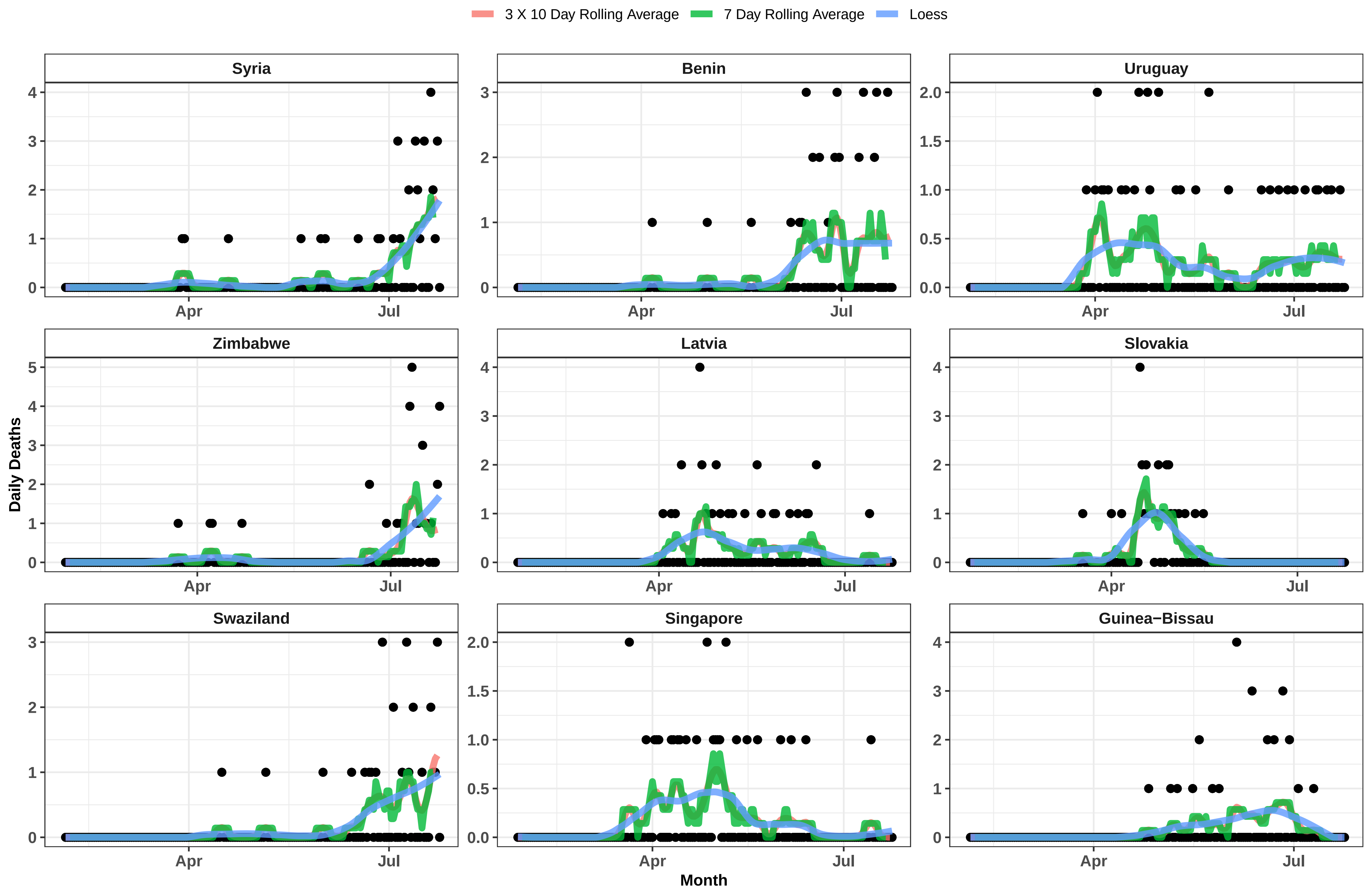

3 X 10 Day Rolling Average
7 Day Rolling Average
Loess
Syria
Benin
Uruguay
Zimbabwe
Latvia
Slovakia
Swaziland
Singapore
Guinea−Bissau
Daily Deaths
Month
Apr
Jul

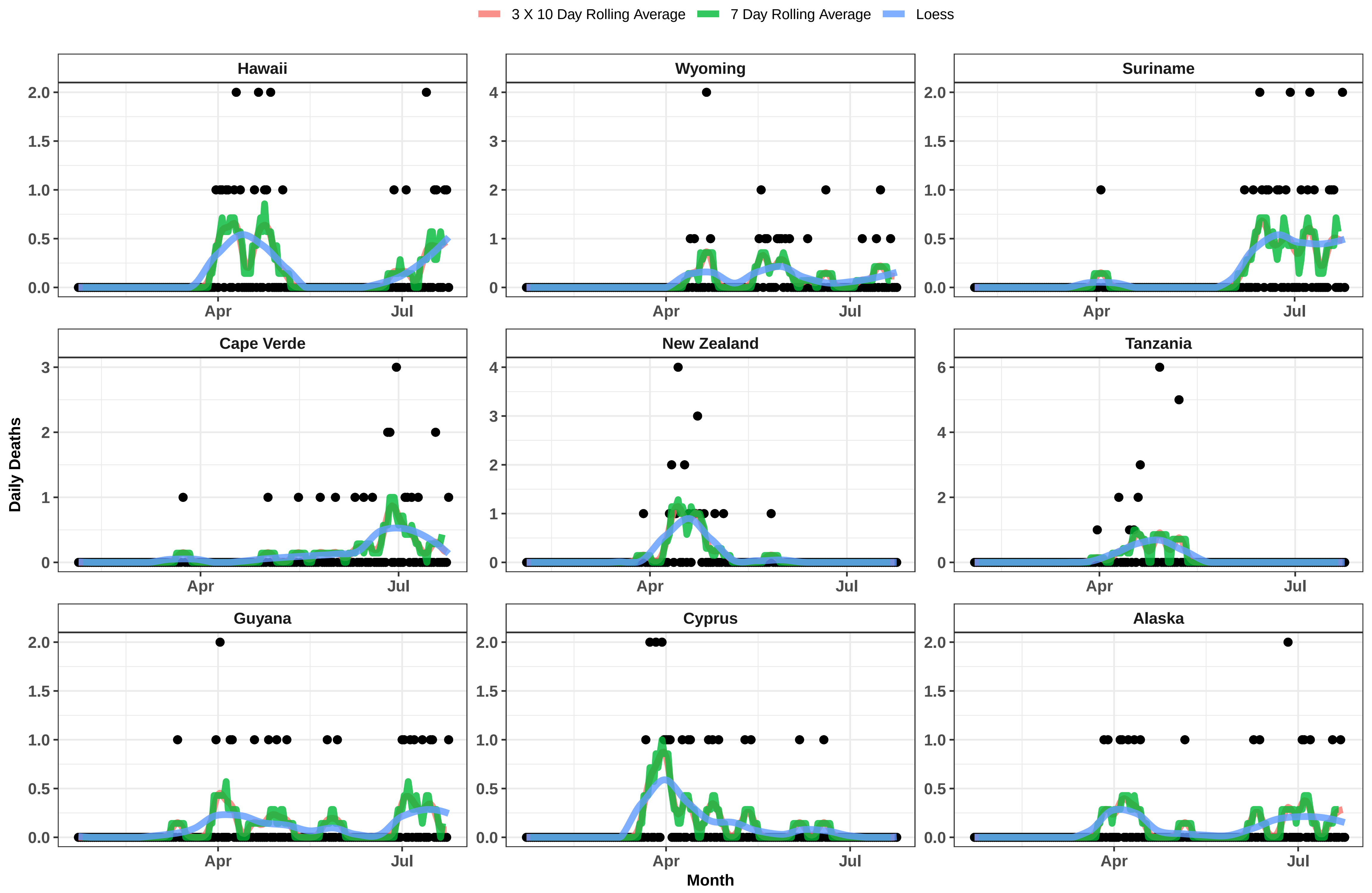

3 X 10 Day Rolling Average
7 Day Rolling Average
Loess
Hawaii
Wyoming
Suriname
Cape Verde
New Zealand
Tanzania
Guyana
Cyprus
Alaska
Daily Deaths
Month
Apr
Jul

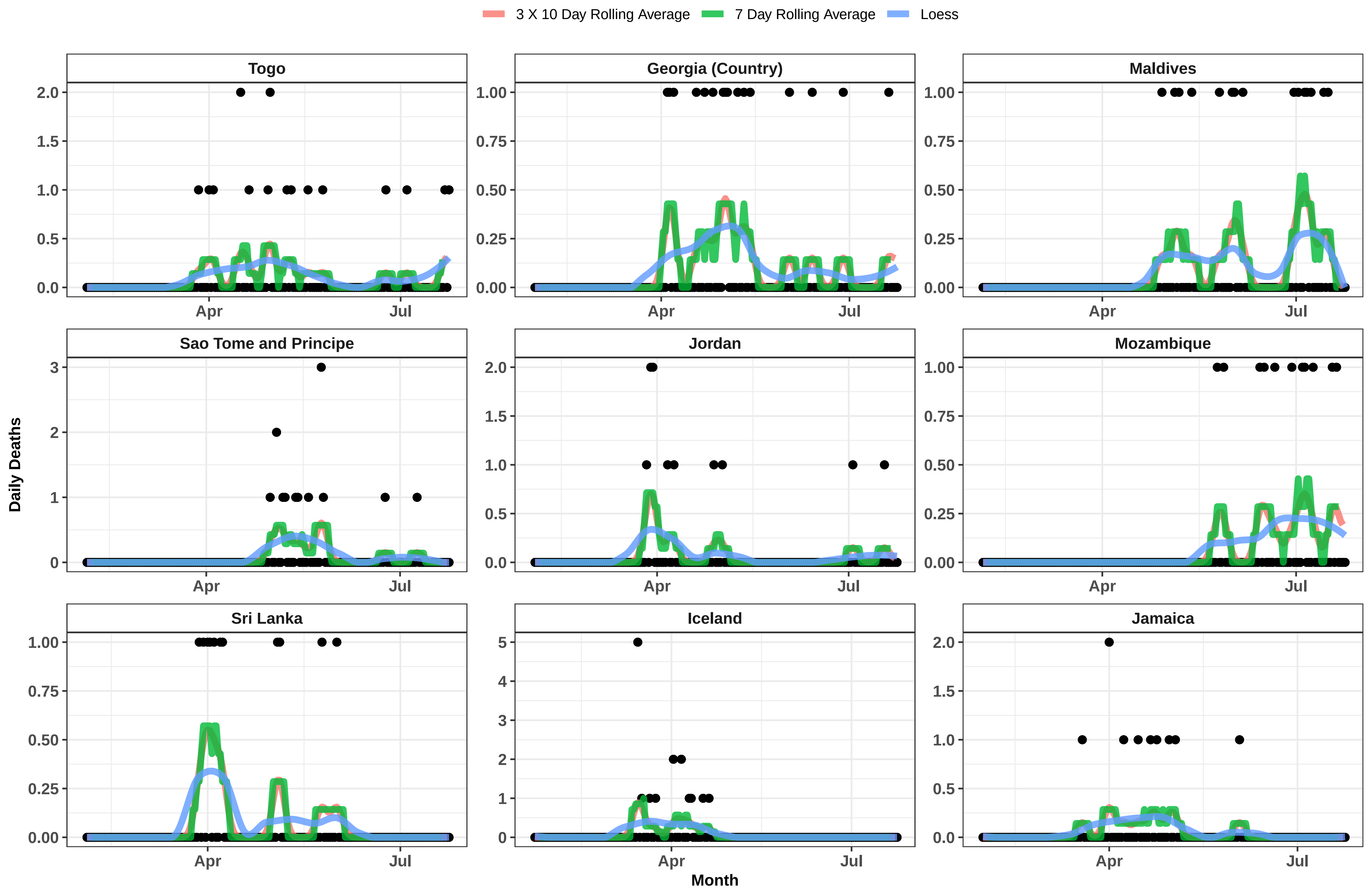

3 X 10 Day Rolling Average
7 Day Rolling Average
Loess
Togo
Georgia (Country)
Maldives
Sao Tome and Principe
Jordan
Mozambique
Sri Lanka
Iceland
Jamaica
Daily Deaths
Month
Apr
Jul

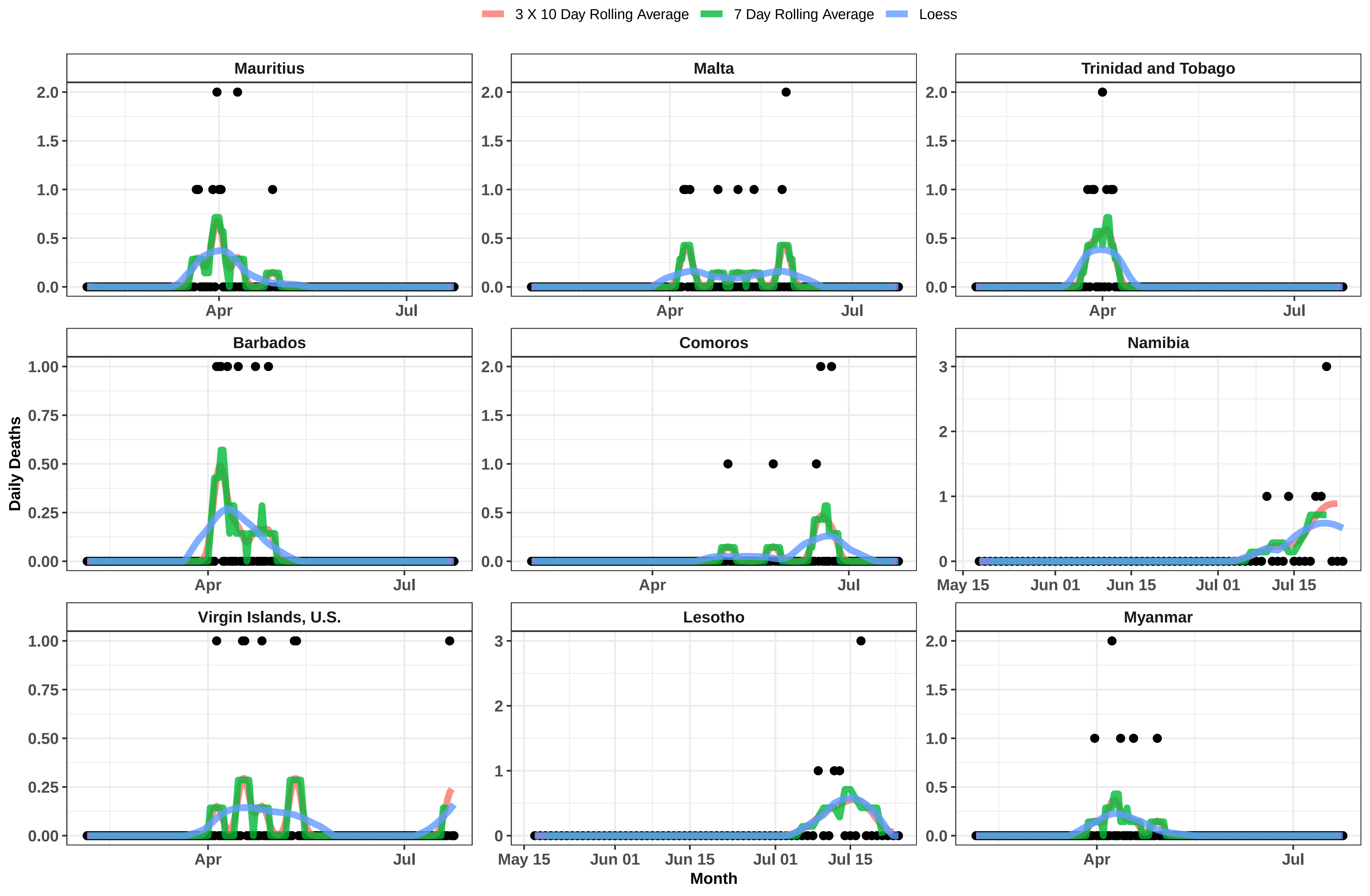
3 X 10 Day Rolling Average
7 Day Rolling Average
Loess
Mauritius
Malta
Trinidad and Tobago
Barbados
Comoros
Namibia
Virgin Islands, U.S.
Lesotho
Myanmar
Daily Deaths
Month
Apr
Jul
May 15
Jun 01
Jun 15
Jul 01
Jul 15

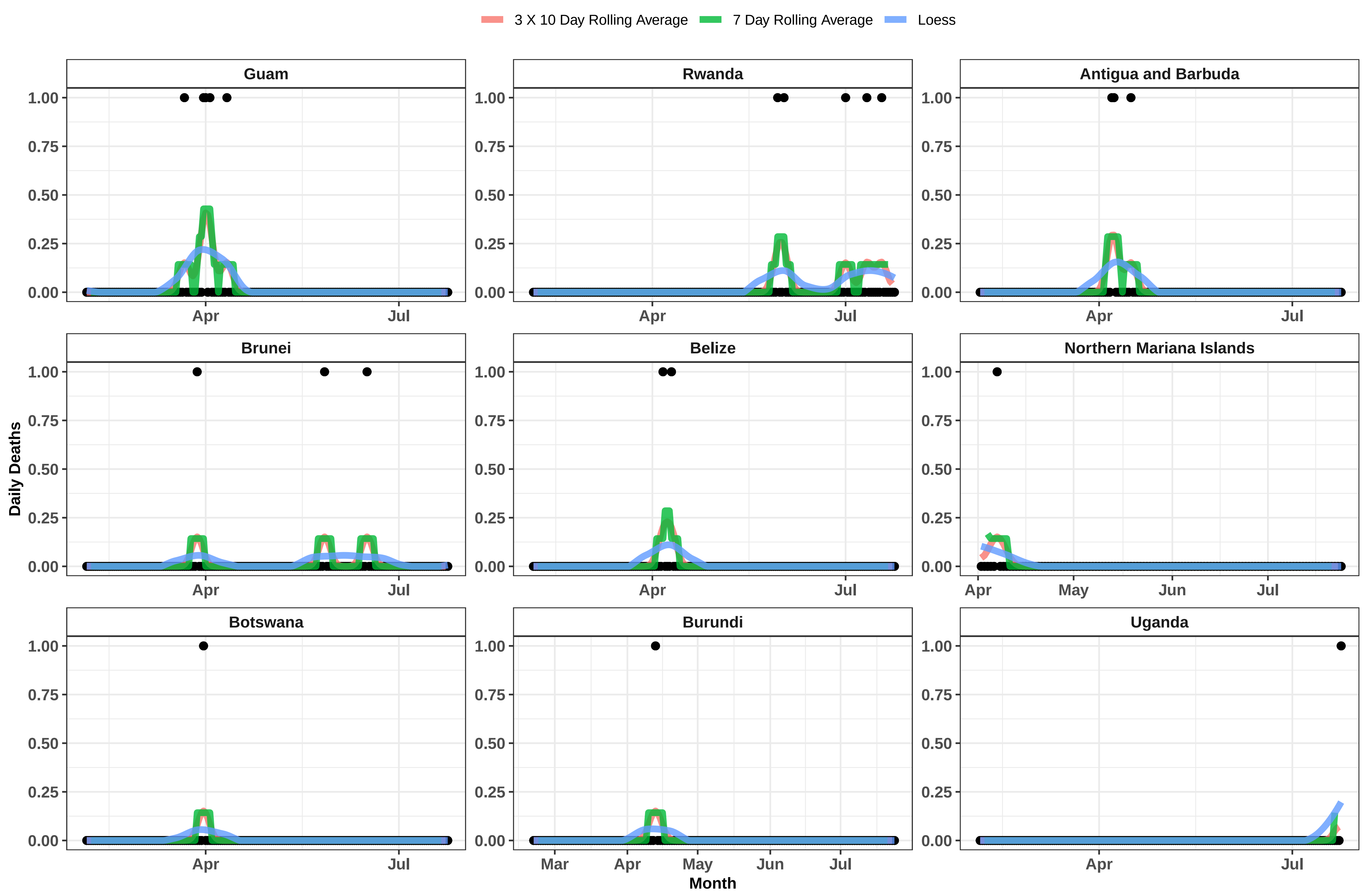

3 X 10 Day Rolling Average
7 Day Rolling Average
Loess
Guam
Rwanda
Antigua and Barbuda
Brunei
Belize
Northern Mariana Islands
Botswana
Burundi
Uganda
Daily Deaths
1.00
0.75
0.50
0.25
0.00
Mar
Apr
May
Jun
Jul
Month

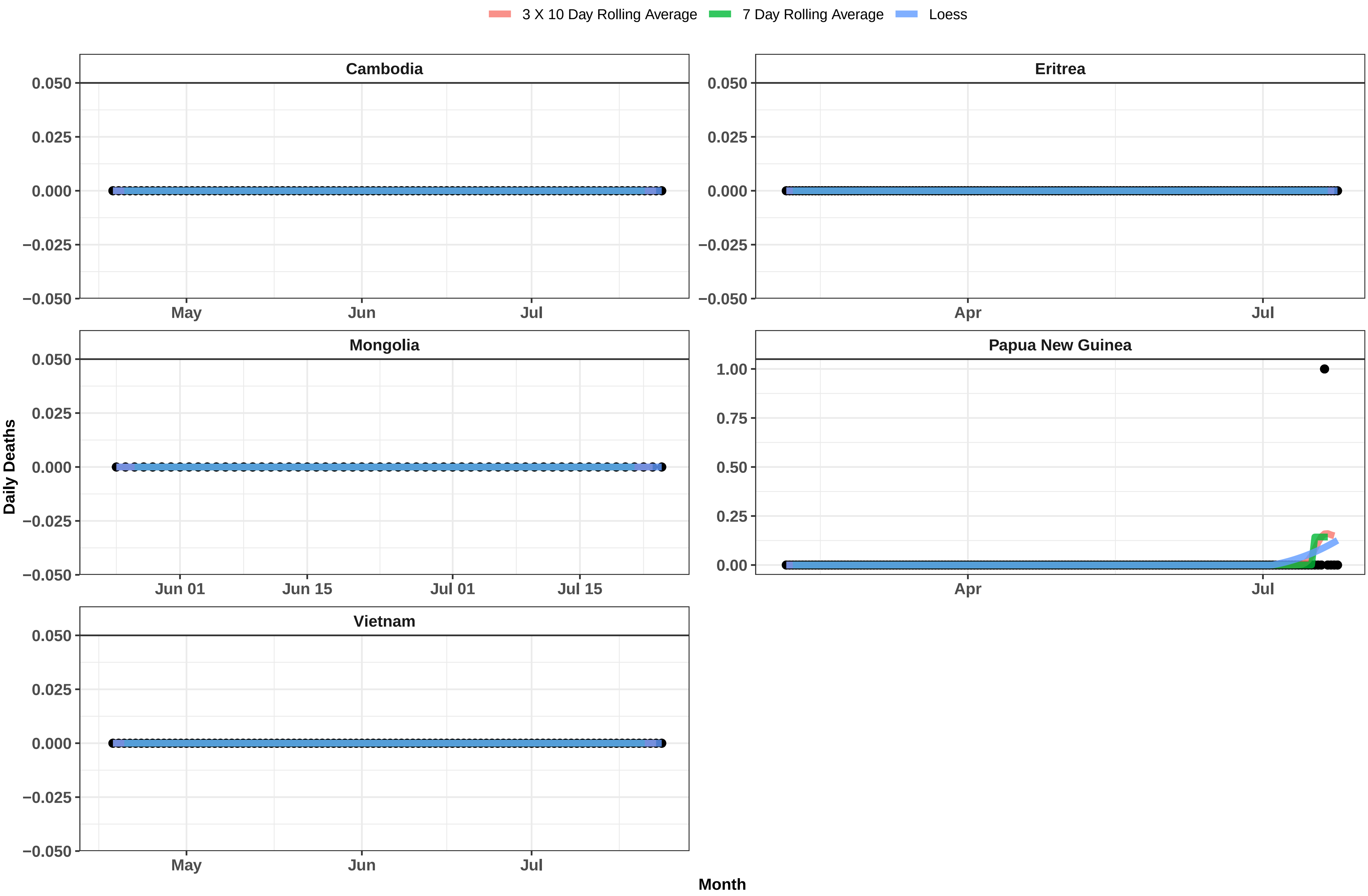
3 X 10 Day Rolling Average
7 Day Rolling Average
Loess
Cambodia
Eritrea
Mongolia
Papua New Guinea
Vietnam
0.050
0.025
0.000
−0.025
−0.050
1.00
0.75
0.50
0.25
0.00
May
Jun
Jul
Apr
Jun 01
Jun 15
Jul 01
Jul 15
Daily Deaths
Month
