# Supplementary material for: Predictive performance of international COVID-19 mortality forecasting models
Source: medRxiv. 2020 Nov 19:2020.07.13.20151233. Preprint. [Version 5] doi: 10.1101/2020.07.13.20151233 (PMC7685335; doi:10.1101/2020.07.13.20151233)
Supplement: Supplement 2020 [file 91596-2020.07.13.20151233-4.pdf]

United States

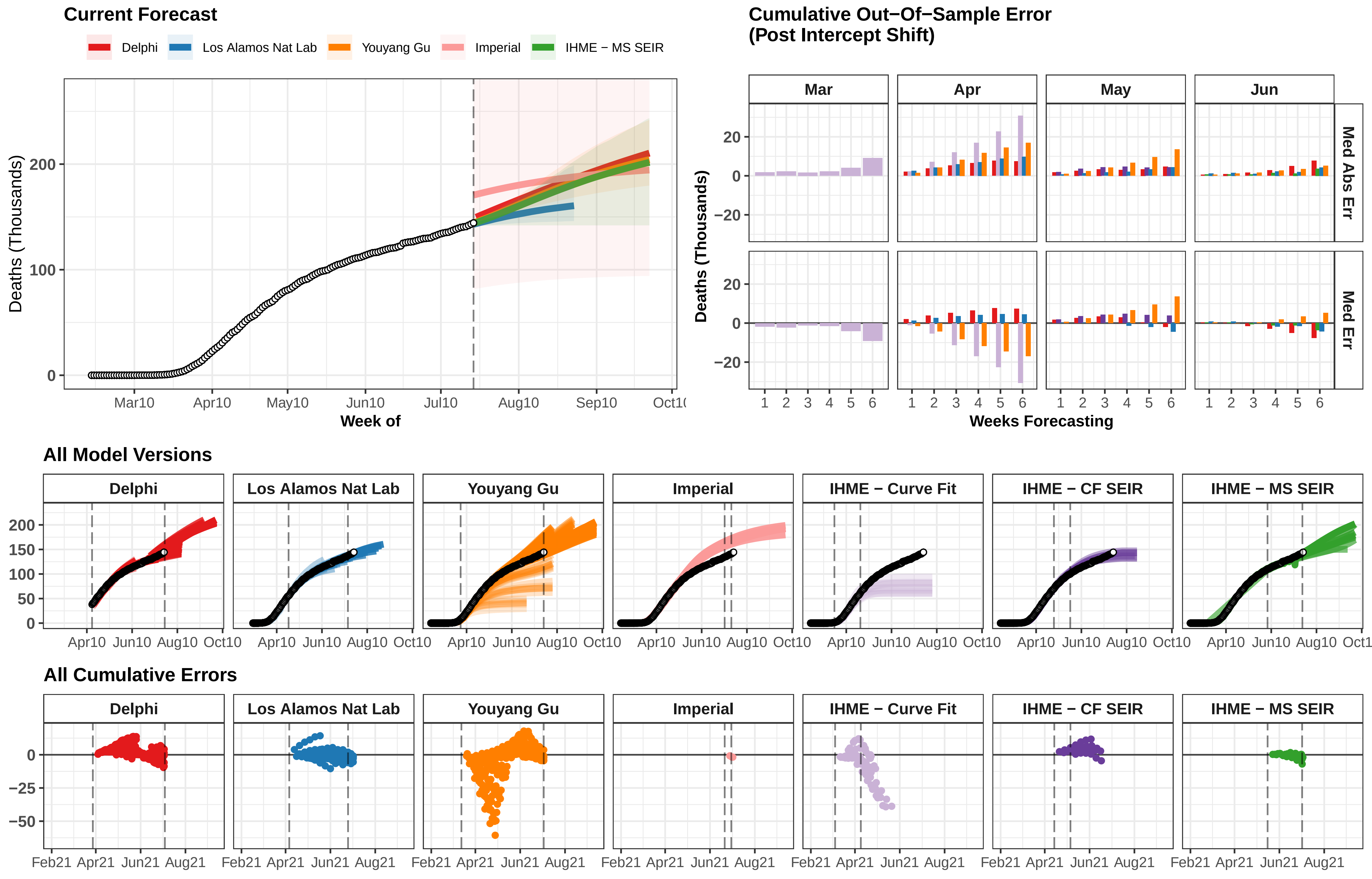

Brazil

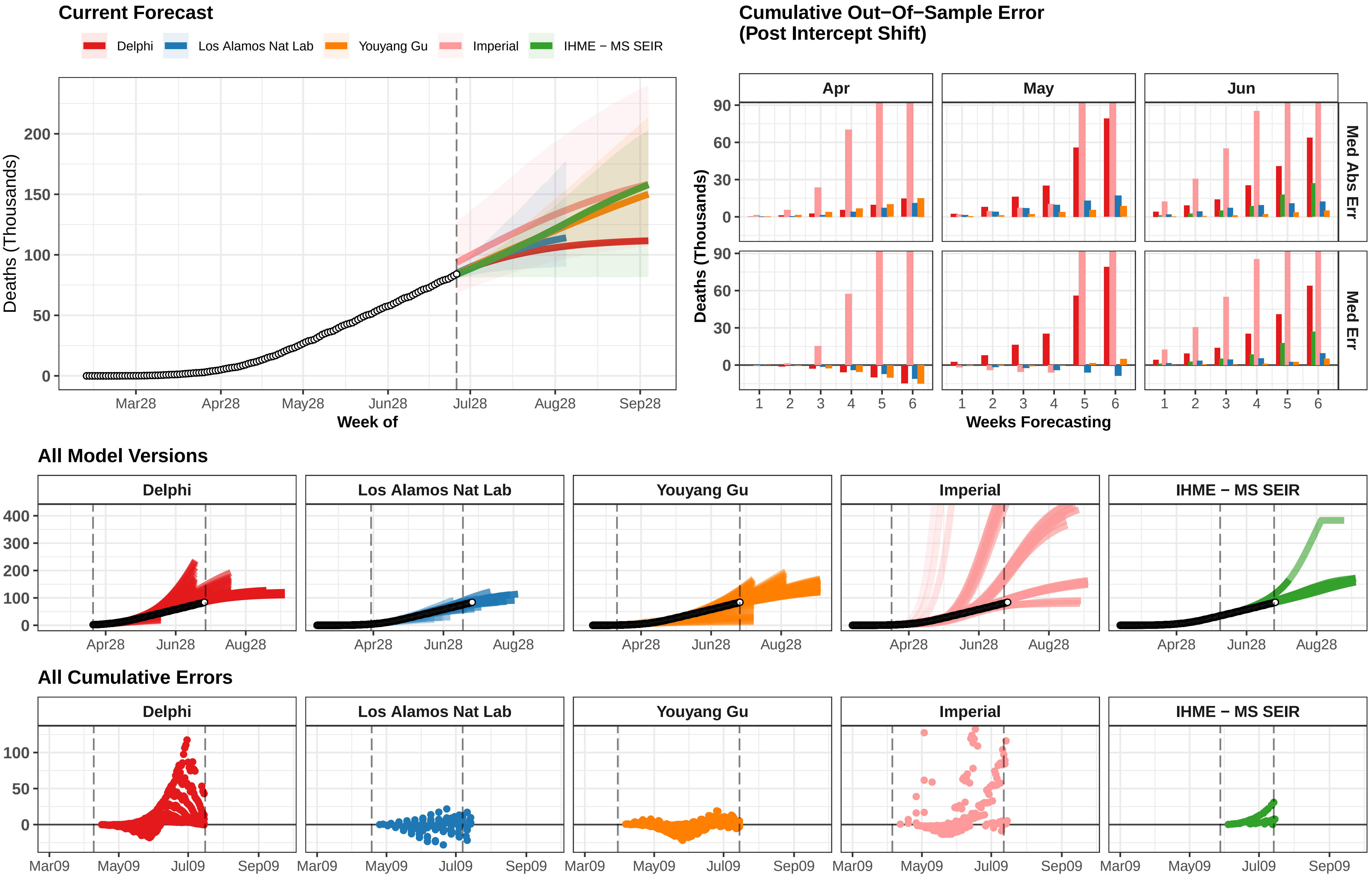
Current Forecast
Delphi
Los Alamos Nat Lab
Youyang Gu
Imperial
IHME − MS SEIR
Deaths (Thousands)
Week of
Mar28
Apr28
May28
Jun28
Jul28
Aug28
Sep28
Cumulative Out−Of−Sample Error
(Post Intercept Shift)
Apr
May
Jun
Med Abs Err
Med Err
Weeks Forecasting
All Model Versions
All Cumulative Errors
Mar09
May09
Jul09
Sep09

United Kingdom

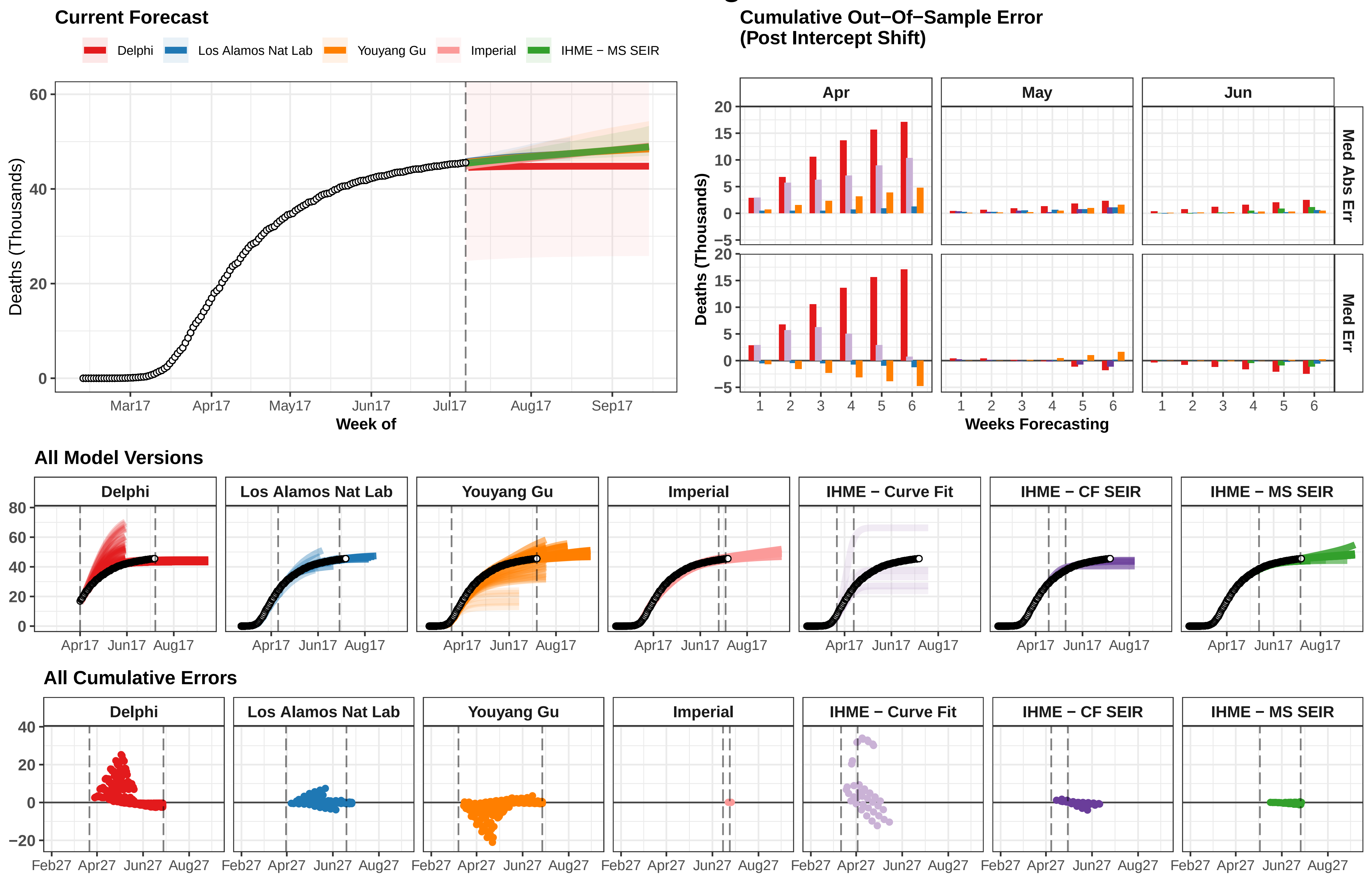

Mexico

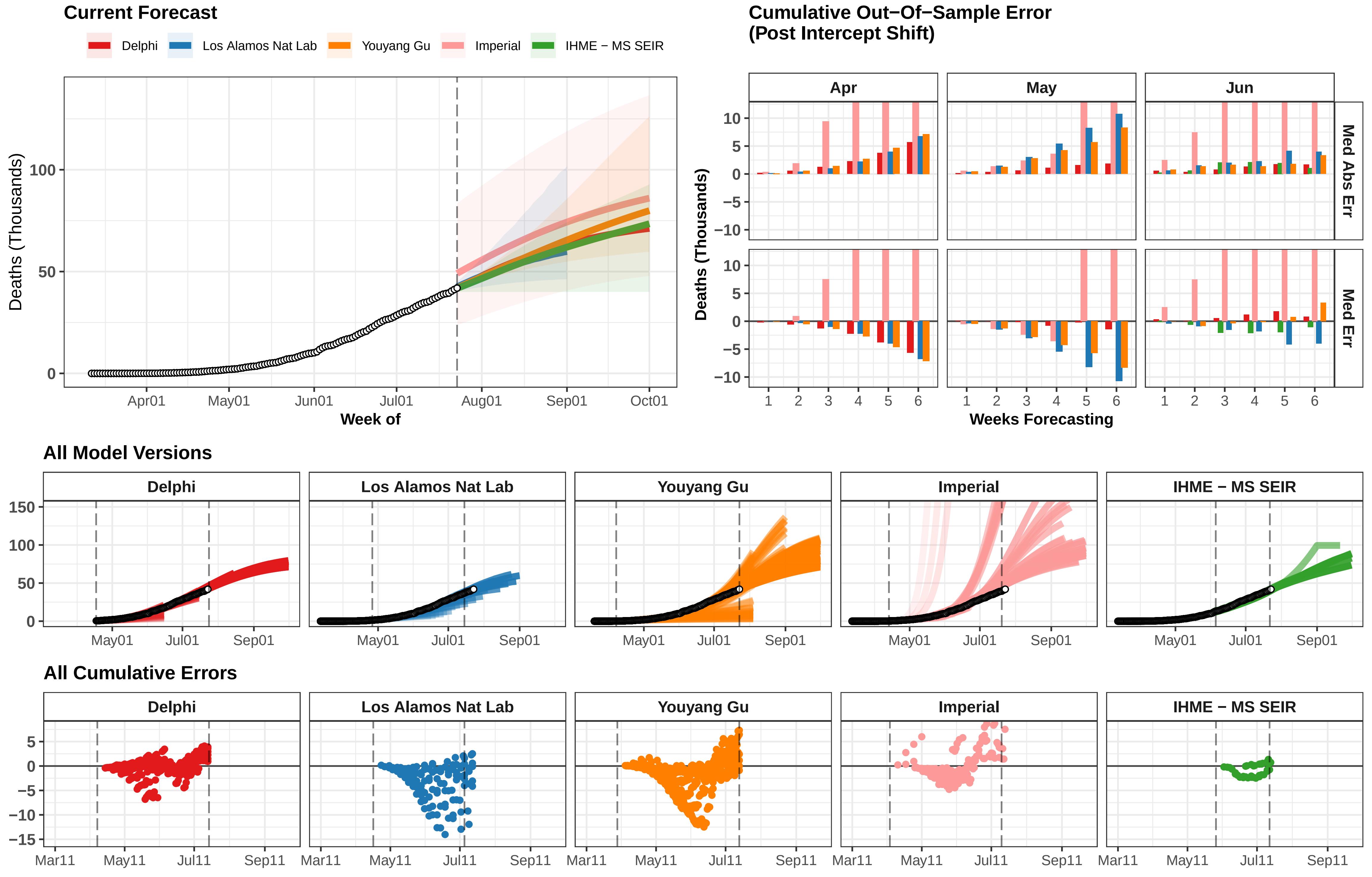

Current Forecast
Delphi
Los Alamos Nat Lab
Youyang Gu
Imperial
IHME − MS SEIR
Deaths (Thousands)
100
50
0
Apr01
May01
Jun01
Jul01
Aug01
Sep01
Oct01
Week of
Cumulative Out−Of−Sample Error
(Post Intercept Shift)
Apr
May
Jun
Med Abs Err
Med Err
10
5
0
−5
−10
1
2
3
4
5
6
Weeks Forecasting
All Model Versions
Delphi
Los Alamos Nat Lab
Youyang Gu
Imperial
IHME − MS SEIR
150
100
50
0
May01
Jul01
Sep01
All Cumulative Errors
5
0
−5
−10
−15
Mar11
May11
Jul11
Sep11

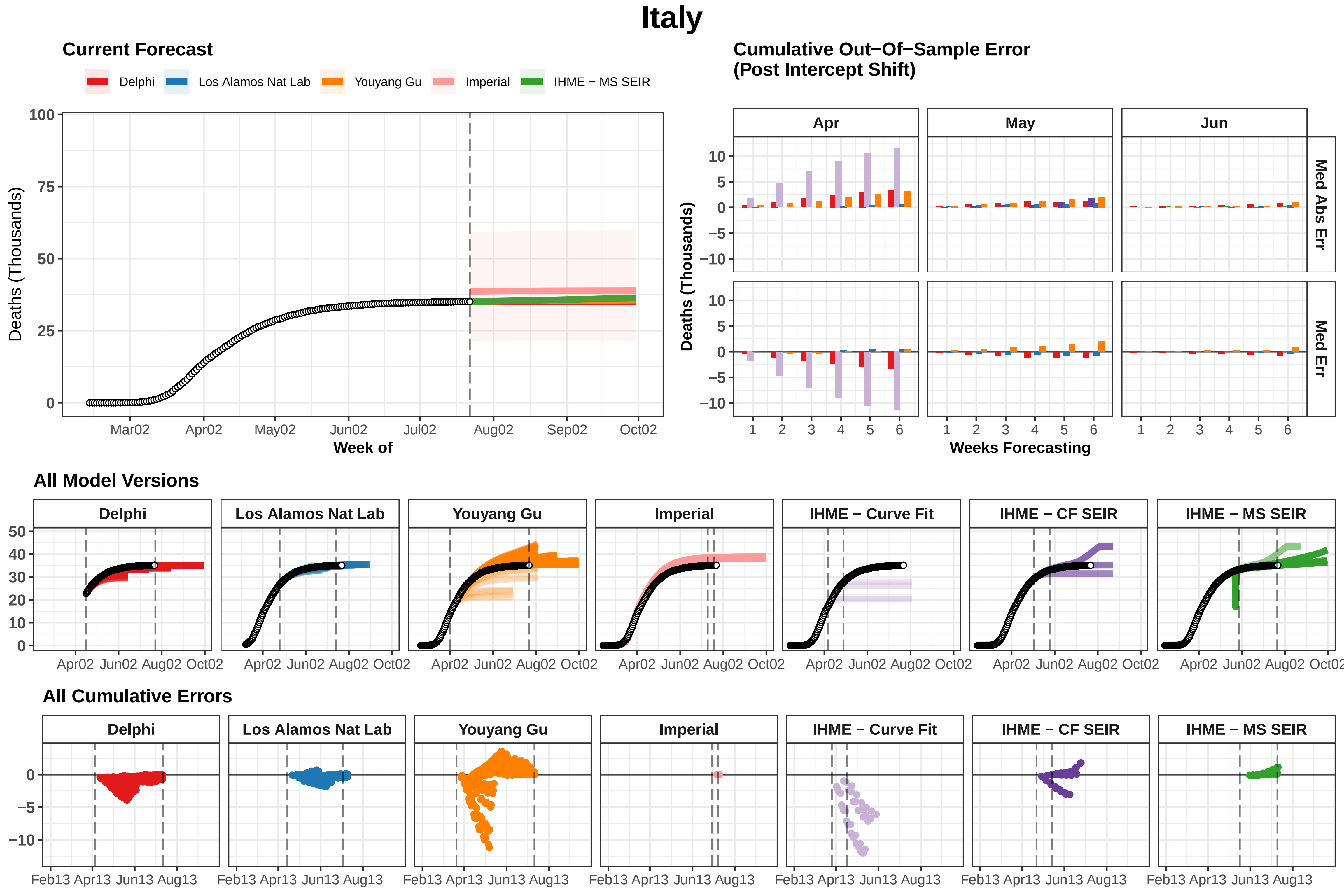

Italy
Current Forecast
Delphi
Los Alamos Nat Lab
Youyang Gu
Imperial
IHME − MS SEIR
Deaths (Thousands)
Week of
Mar02
Apr02
May02
Jun02
Jul02
Aug02
Sep02
Oct02
Cumulative Out−Of−Sample Error
(Post Intercept Shift)
Apr
May
Jun
Med Abs Err
Med Err
Weeks Forecasting
All Model Versions
IHME − Curve Fit
IHME − CF SEIR
All Cumulative Errors
Feb13
Apr13
Jun13
Aug13

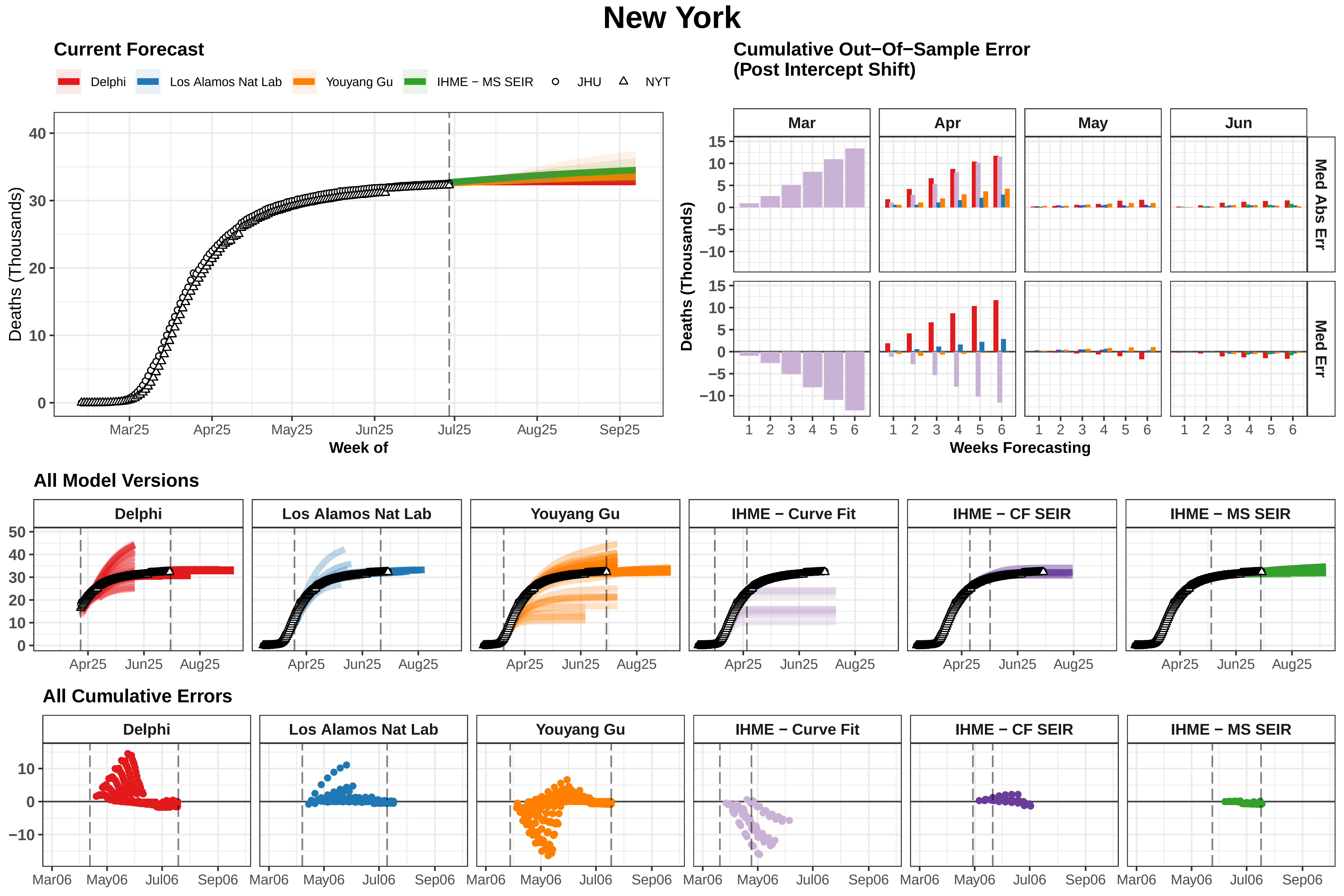
New York
Current Forecast
Delphi
Los Alamos Nat Lab
Youyang Gu
IHME − MS SEIR
JHU
NYT
Deaths (Thousands)
Week of
Mar25
Apr25
May25
Jun25
Jul25
Aug25
Sep25
Cumulative Out−Of−Sample Error
(Post Intercept Shift)
Mar
Apr
May
Jun
Med Abs Err
Med Err
Weeks Forecasting
All Model Versions
Delphi
Los Alamos Nat Lab
Youyang Gu
IHME − Curve Fit
IHME − CF SEIR
IHME − MS SEIR
All Cumulative Errors
Mar06
May06
Jul06
Sep06

India

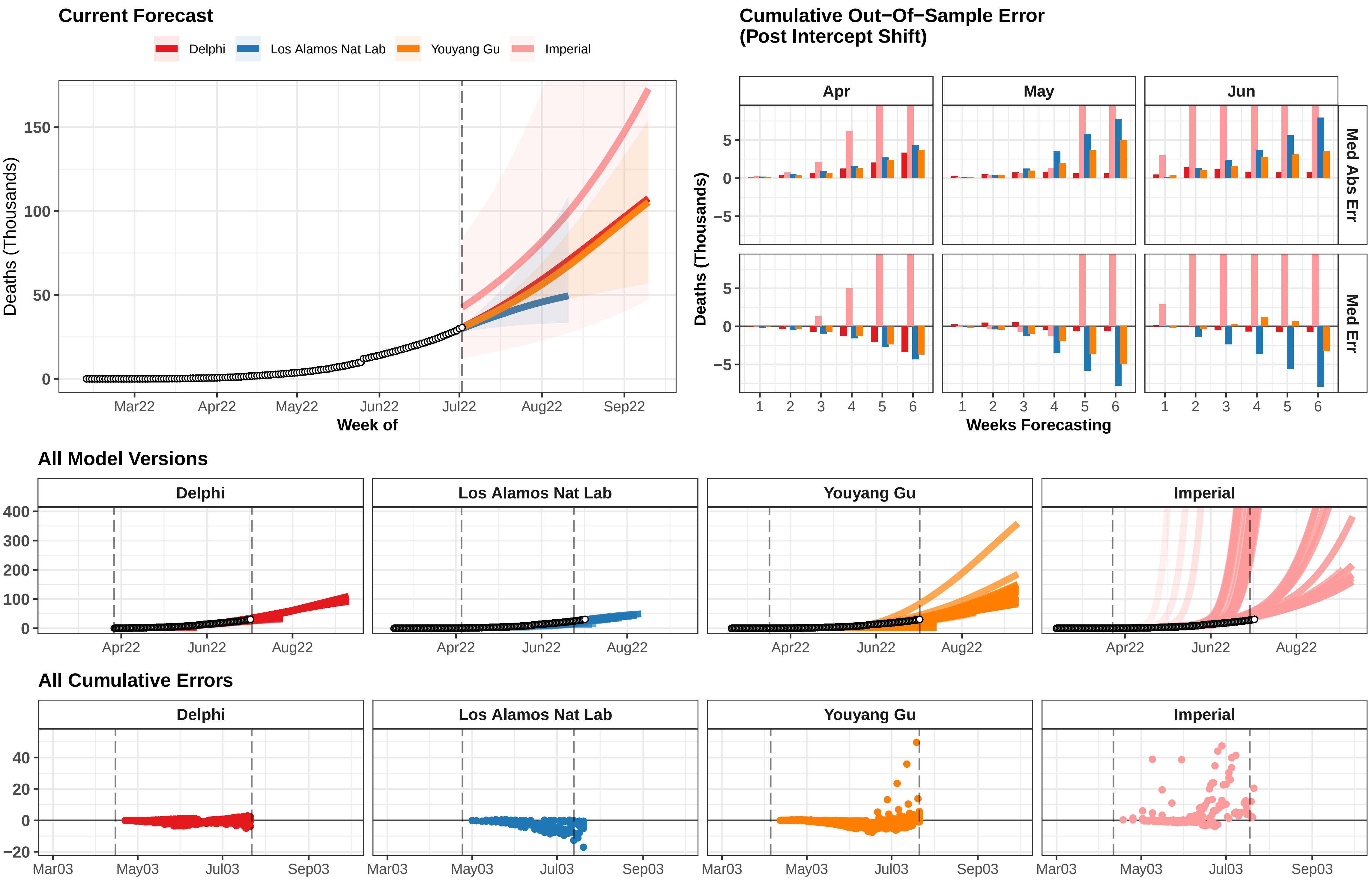
Current Forecast
Delphi
Los Alamos Nat Lab
Youyang Gu
Imperial
Deaths (Thousands)
Week of
Mar22
Apr22
May22
Jun22
Jul22
Aug22
Sep22
Cumulative Out−Of−Sample Error
(Post Intercept Shift)
Apr
May
Jun
Med Abs Err
Med Err
Weeks Forecasting
All Model Versions
Delphi
Los Alamos Nat Lab
Youyang Gu
Imperial
All Cumulative Errors
Mar03
May03
Jul03
Sep03

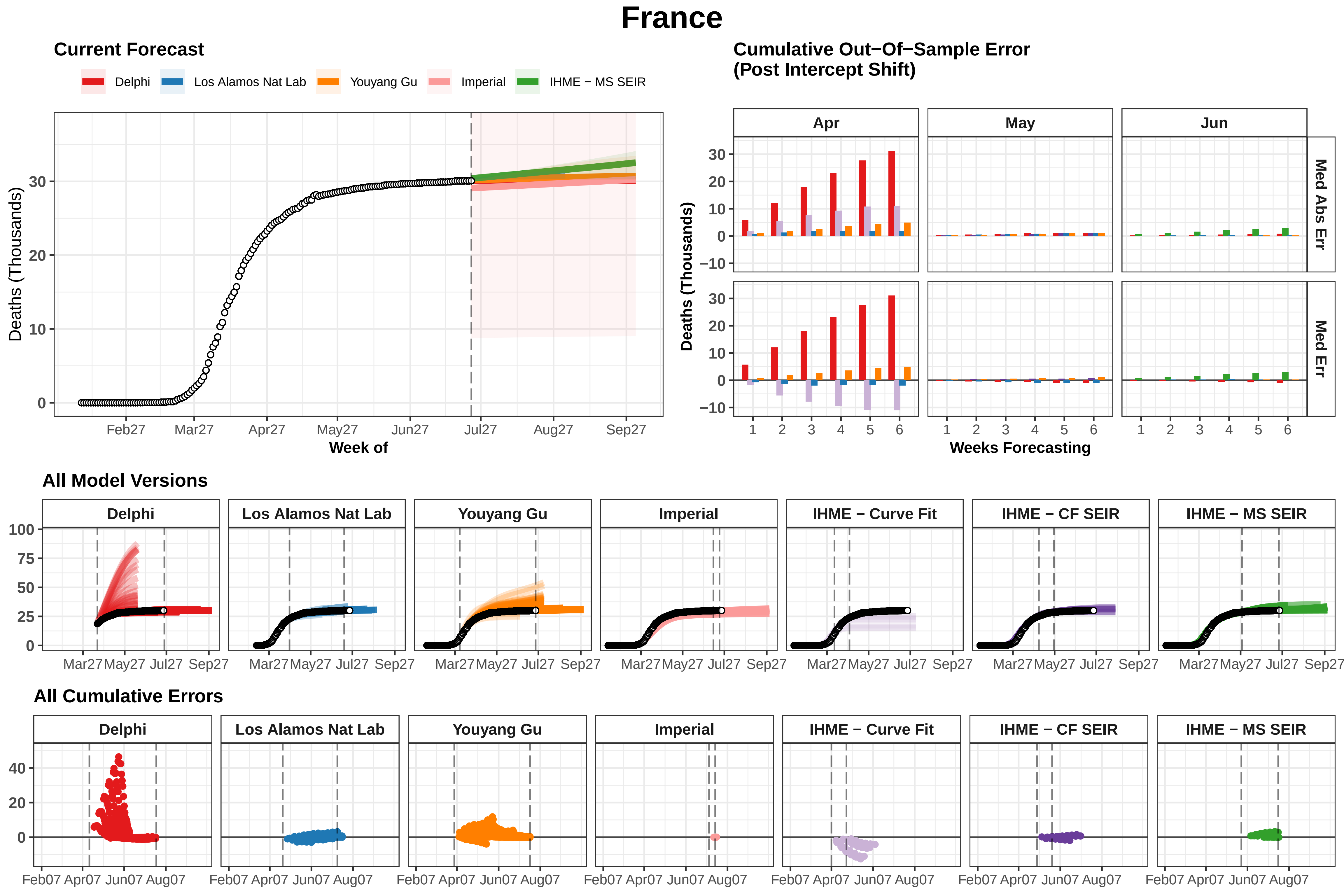
France
Current Forecast
Delphi
Los Alamos Nat Lab
Youyang Gu
Imperial
IHME − MS SEIR
Deaths (Thousands)
Week of
Feb27
Mar27
Apr27
May27
Jun27
Jul27
Aug27
Sep27
Cumulative Out−Of−Sample Error
(Post Intercept Shift)
Apr
May
Jun
Med Abs Err
Med Err
Weeks Forecasting
All Model Versions
IHME − Curve Fit
IHME − CF SEIR
All Cumulative Errors
Feb07
Apr07
Jun07
Aug07

Spain

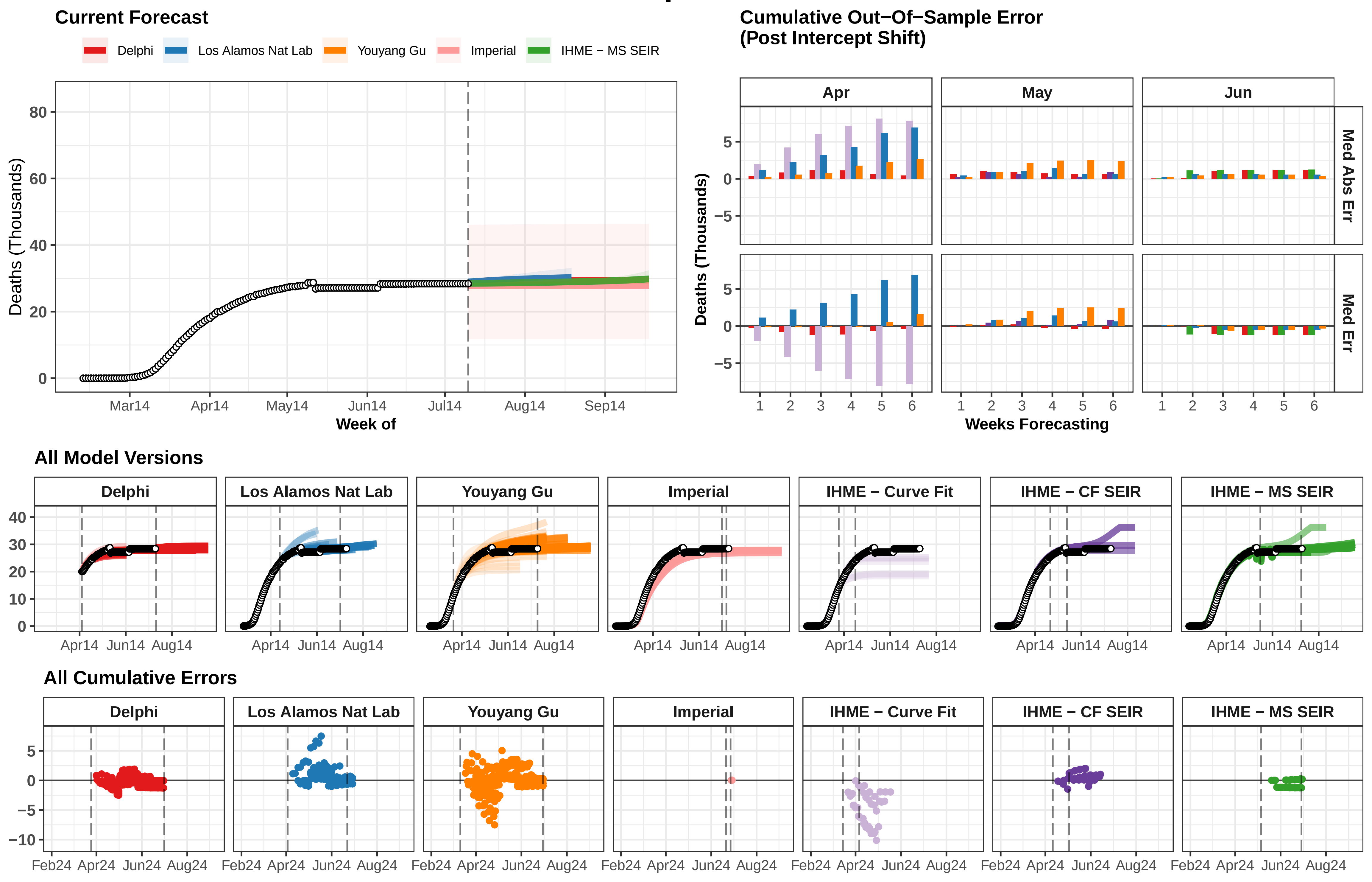
Current Forecast
Delphi
Los Alamos Nat Lab
Youyang Gu
Imperial
IHME − MS SEIR
Deaths (Thousands)
0
20
40
60
80
Mar14
Apr14
May14
Jun14
Jul14
Aug14
Sep14
Week of
Cumulative Out−Of−Sample Error
(Post Intercept Shift)
Apr
May
Jun
Med Abs Err
Med Err
Deaths (Thousands)
−5
0
5
1 2 3 4 5 6
Weeks Forecasting
All Model Versions
Delphi
Los Alamos Nat Lab
Youyang Gu
Imperial
IHME − Curve Fit
IHME − CF SEIR
IHME − MS SEIR
0
10
20
30
40
Apr14 Jun14 Aug14
All Cumulative Errors
Delphi
Los Alamos Nat Lab
Youyang Gu
Imperial
IHME − Curve Fit
IHME − CF SEIR
IHME − MS SEIR
−10
−5
0
5
Feb24 Apr24 Jun24 Aug24

New Jersey

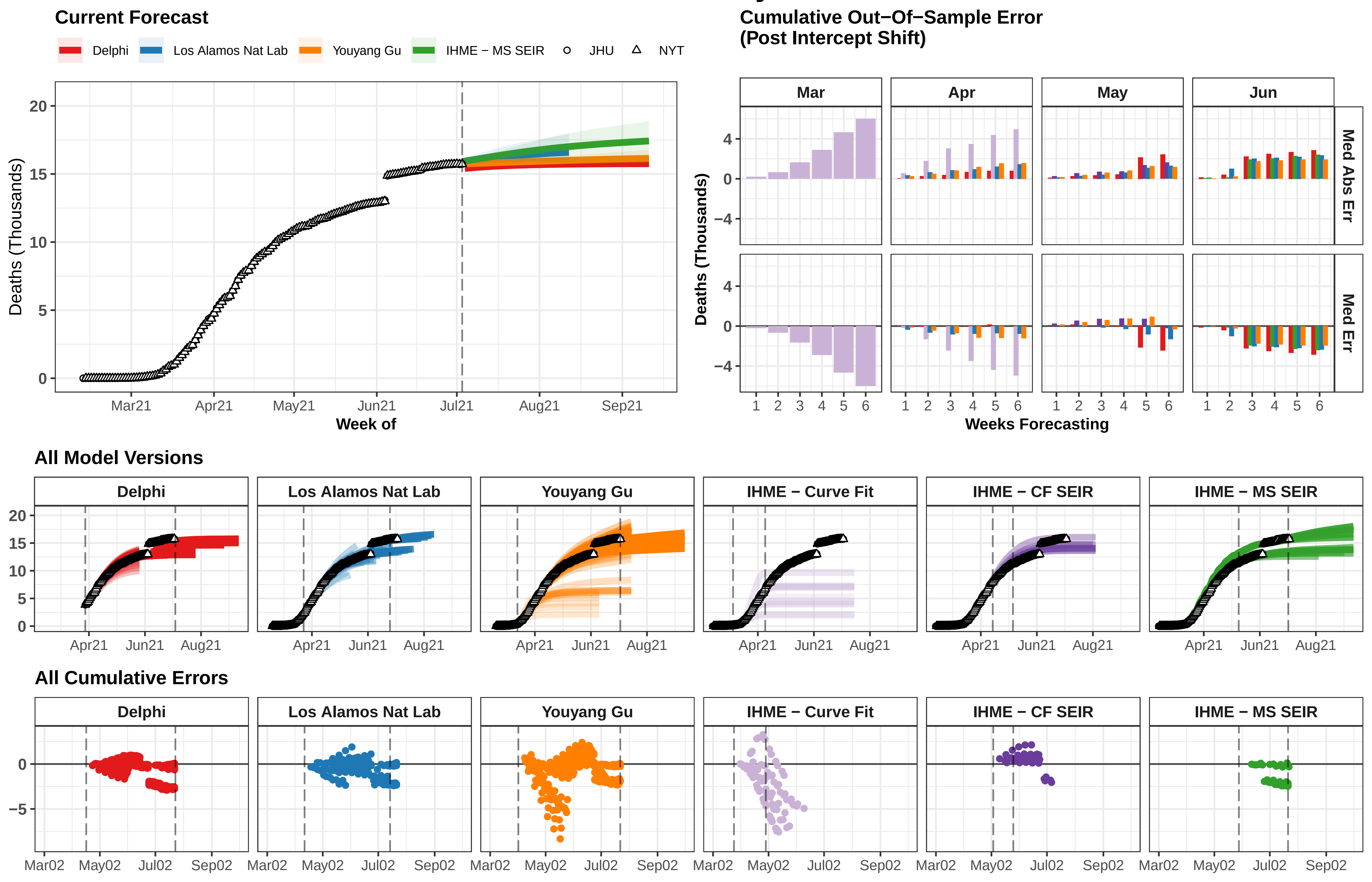

Current Forecast
Delphi
Los Alamos Nat Lab
Youyang Gu
IHME − MS SEIR
JHU
NYT
Deaths (Thousands)
0
5
10
15
20
Mar21
Apr21
May21
Jun21
Jul21
Aug21
Sep21
Week of
Cumulative Out−Of−Sample Error
(Post Intercept Shift)
Mar
Apr
May
Jun
Med Abs Err
Med Err
Deaths (Thousands)
4
0
−4
1
2
3
4
5
6
Weeks Forecasting
All Model Versions
Delphi
Los Alamos Nat Lab
Youyang Gu
IHME − Curve Fit
IHME − CF SEIR
IHME − MS SEIR
Apr21
Jun21
Aug21
All Cumulative Errors
0
−5
Mar02
May02
Jul02
Sep02

Iran

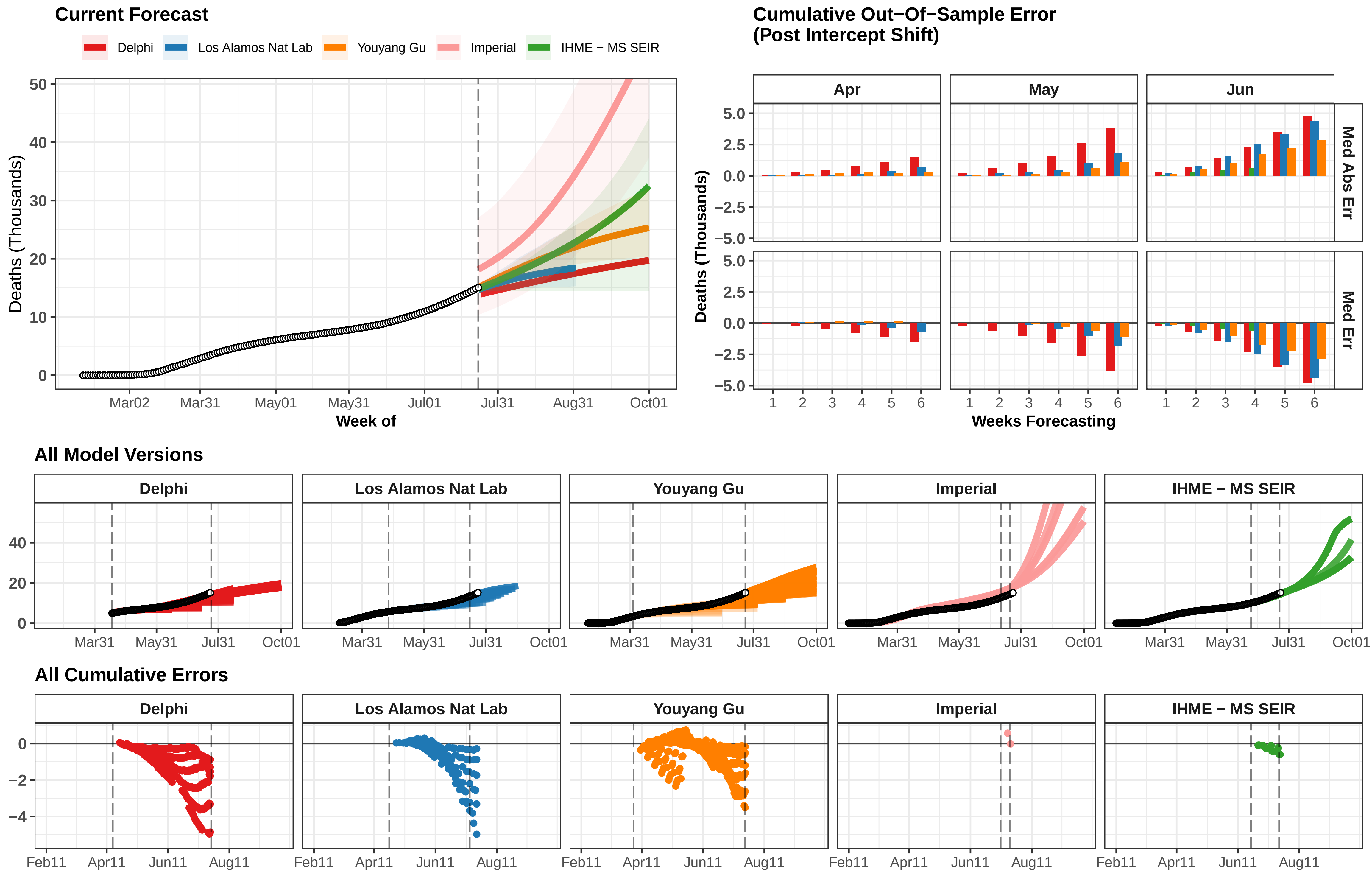
Current Forecast
Delphi
Los Alamos Nat Lab
Youyang Gu
Imperial
IHME − MS SEIR
Deaths (Thousands)
0
10
20
30
40
50
Mar02
Mar31
May01
May31
Jul01
Jul31
Aug31
Oct01
Week of
Cumulative Out−Of−Sample Error
(Post Intercept Shift)
Apr
May
Jun
Med Abs Err
Med Err
5.0
2.5
0.0
−2.5
−5.0
1
2
3
4
5
6
Weeks Forecasting
All Model Versions
Delphi
Los Alamos Nat Lab
Youyang Gu
Imperial
IHME − MS SEIR
0
20
40
Mar31
May31
Jul31
Oct01
All Cumulative Errors
0
−2
−4
Feb11
Apr11
Jun11
Aug11

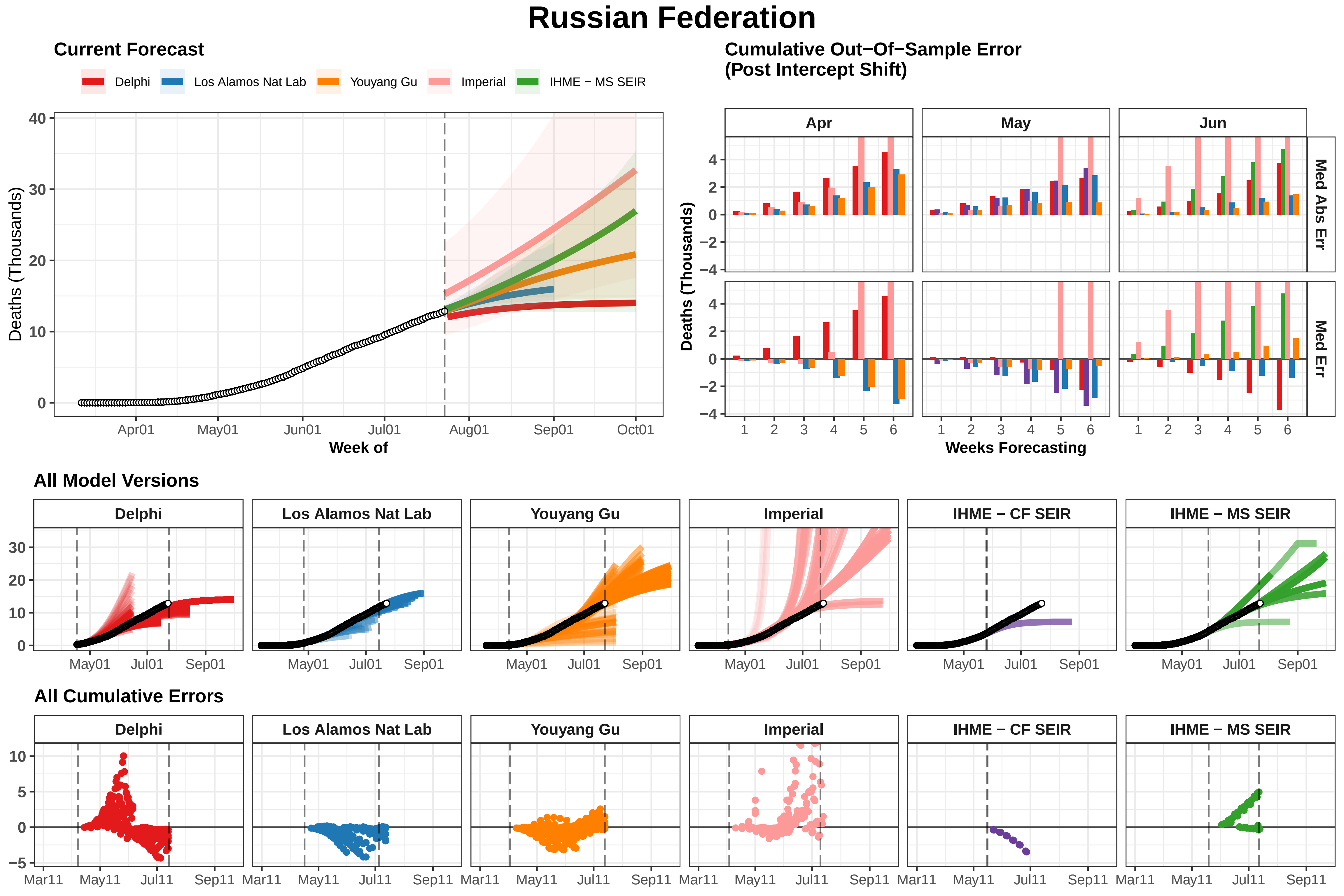

Russian Federation
Current Forecast
Delphi
Los Alamos Nat Lab
Youyang Gu
Imperial
IHME − MS SEIR
Deaths (Thousands)
Week of
Apr01
May01
Jun01
Jul01
Aug01
Sep01
Oct01
Cumulative Out−Of−Sample Error
(Post Intercept Shift)
Apr
May
Jun
Med Abs Err
Med Err
Weeks Forecasting
All Model Versions
IHME − CF SEIR
All Cumulative Errors
Mar11
May11
Jul11
Sep11

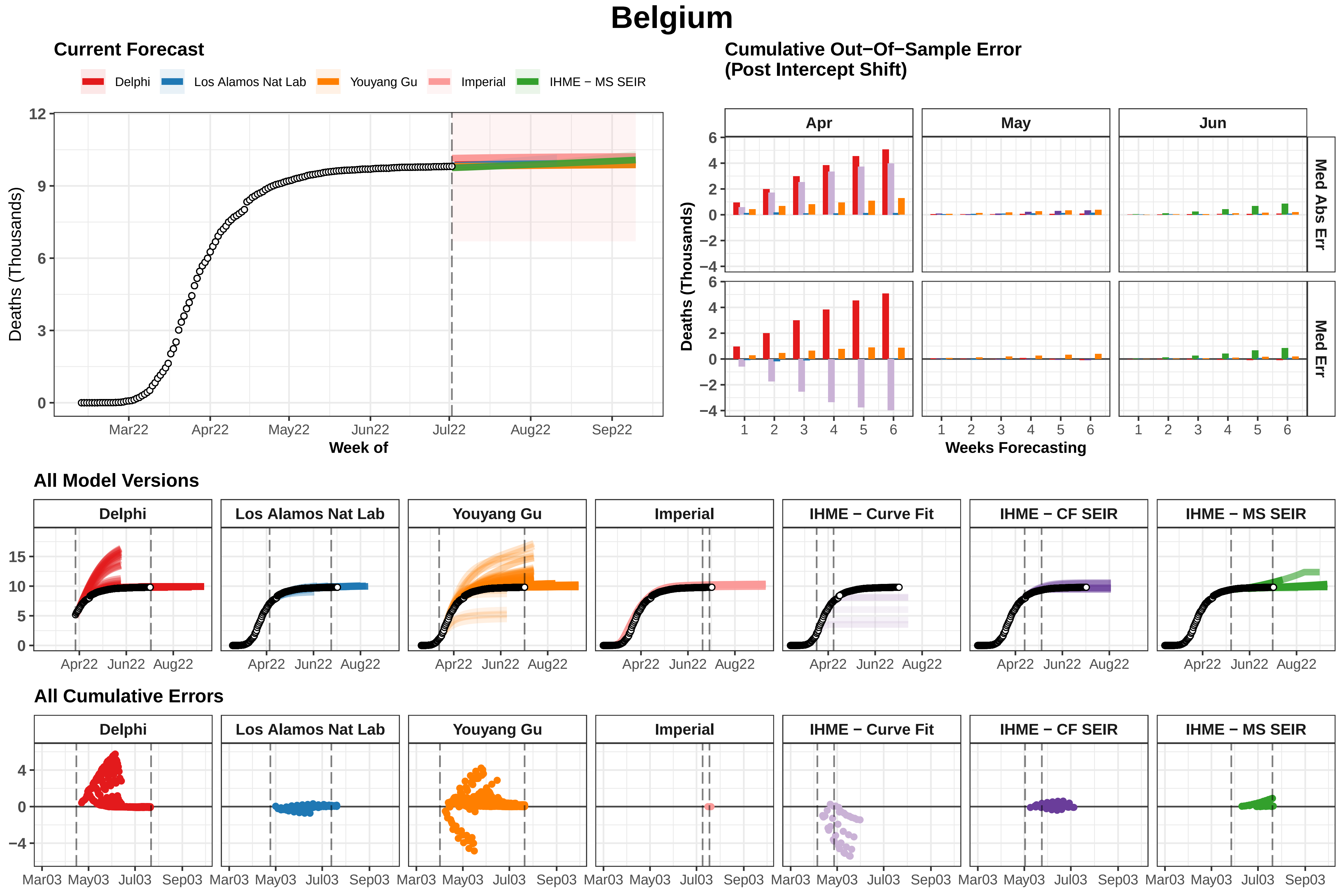

Belgium
Current Forecast
Delphi
Los Alamos Nat Lab
Youyang Gu
Imperial
IHME − MS SEIR
Deaths (Thousands)
Week of
Mar22 Apr22 May22 Jun22 Jul22 Aug22 Sep22
Cumulative Out−Of−Sample Error (Post Intercept Shift)
Apr
May
Jun
Med Abs Err
Med Err
Weeks Forecasting
All Model Versions
Delphi
Los Alamos Nat Lab
Youyang Gu
Imperial
IHME − Curve Fit
IHME − CF SEIR
IHME − MS SEIR
Apr22 Jun22 Aug22
All Cumulative Errors
Mar03 May03 Jul03 Sep03

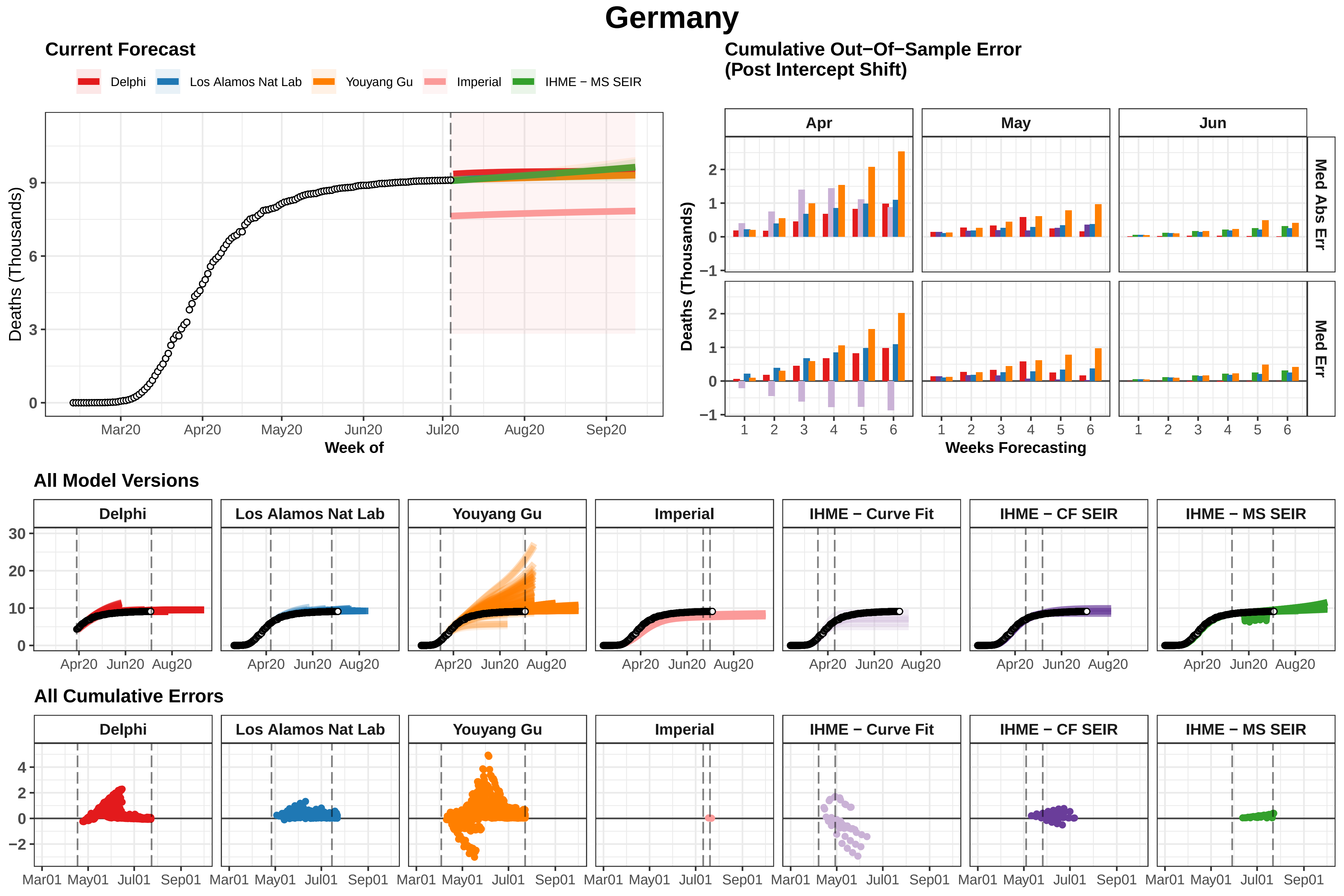

Germany
Current Forecast
Delphi
Los Alamos Nat Lab
Youyang Gu
Imperial
IHME − MS SEIR
Deaths (Thousands)
Week of
Mar20
Apr20
May20
Jun20
Jul20
Aug20
Sep20
Cumulative Out−Of−Sample Error
(Post Intercept Shift)
Apr
May
Jun
Med Abs Err
Med Err
Weeks Forecasting
All Model Versions
Delphi
Los Alamos Nat Lab
Youyang Gu
Imperial
IHME − Curve Fit
IHME − CF SEIR
IHME − MS SEIR
All Cumulative Errors
Mar01
May01
Jul01
Sep01

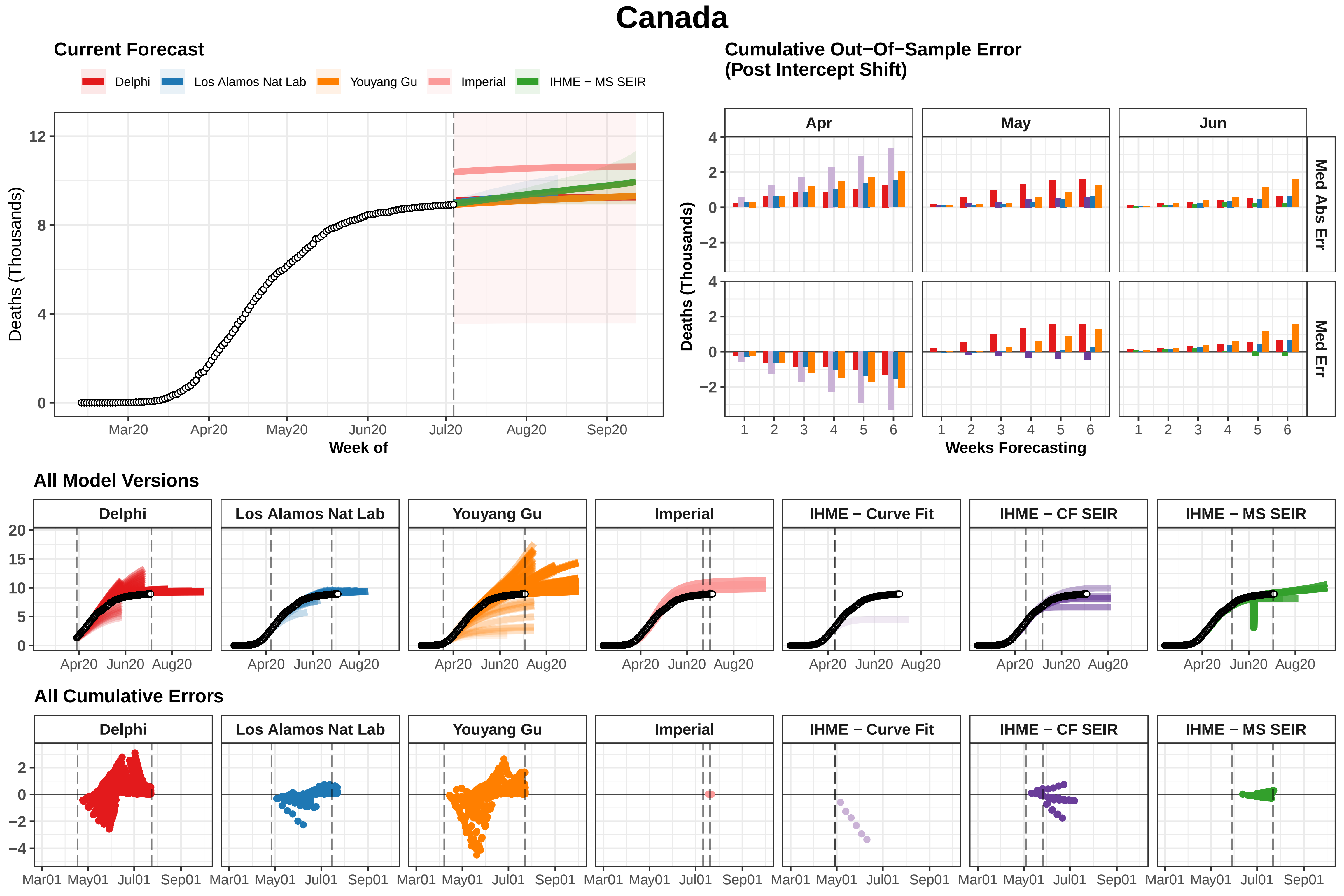
Canada
Current Forecast
Delphi
Los Alamos Nat Lab
Youyang Gu
Imperial
IHME − MS SEIR
Deaths (Thousands)
Week of
Mar20
Apr20
May20
Jun20
Jul20
Aug20
Sep20
Cumulative Out−Of−Sample Error
(Post Intercept Shift)
Apr
May
Jun
Med Abs Err
Med Err
Weeks Forecasting
All Model Versions
IHME − Curve Fit
IHME − CF SEIR
All Cumulative Errors
Mar01
May01
Jul01
Sep01

Chile

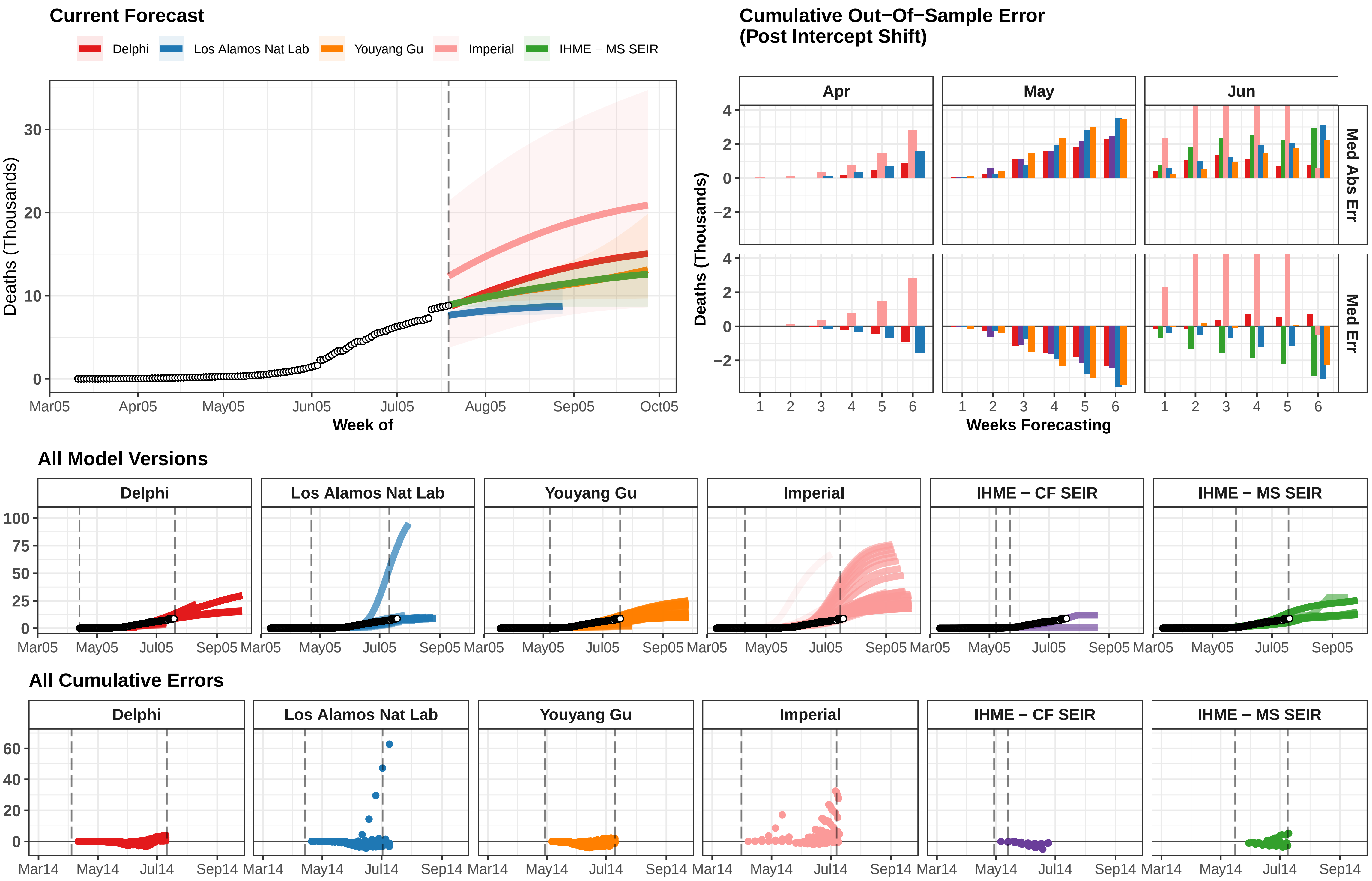
Current Forecast
Delphi
Los Alamos Nat Lab
Youyang Gu
Imperial
IHME − MS SEIR
Deaths (Thousands)
Week of
Mar05
Apr05
May05
Jun05
Jul05
Aug05
Sep05
Oct05
Cumulative Out−Of−Sample Error (Post Intercept Shift)
Apr
May
Jun
Med Abs Err
Med Err
Weeks Forecasting
All Model Versions
IHME − CF SEIR
All Cumulative Errors
Mar14
May14
Jul14
Sep14

Massachusetts

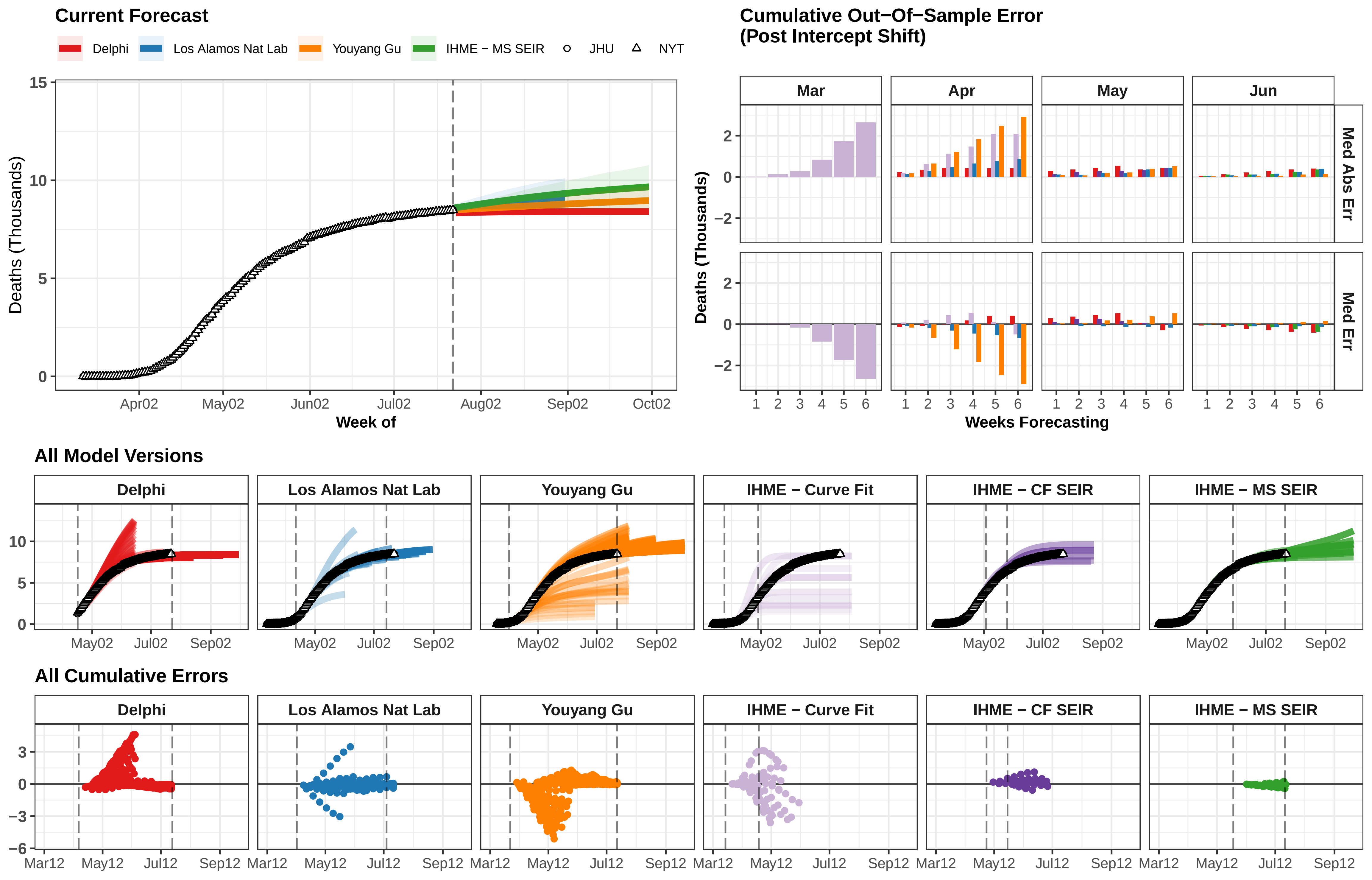

California

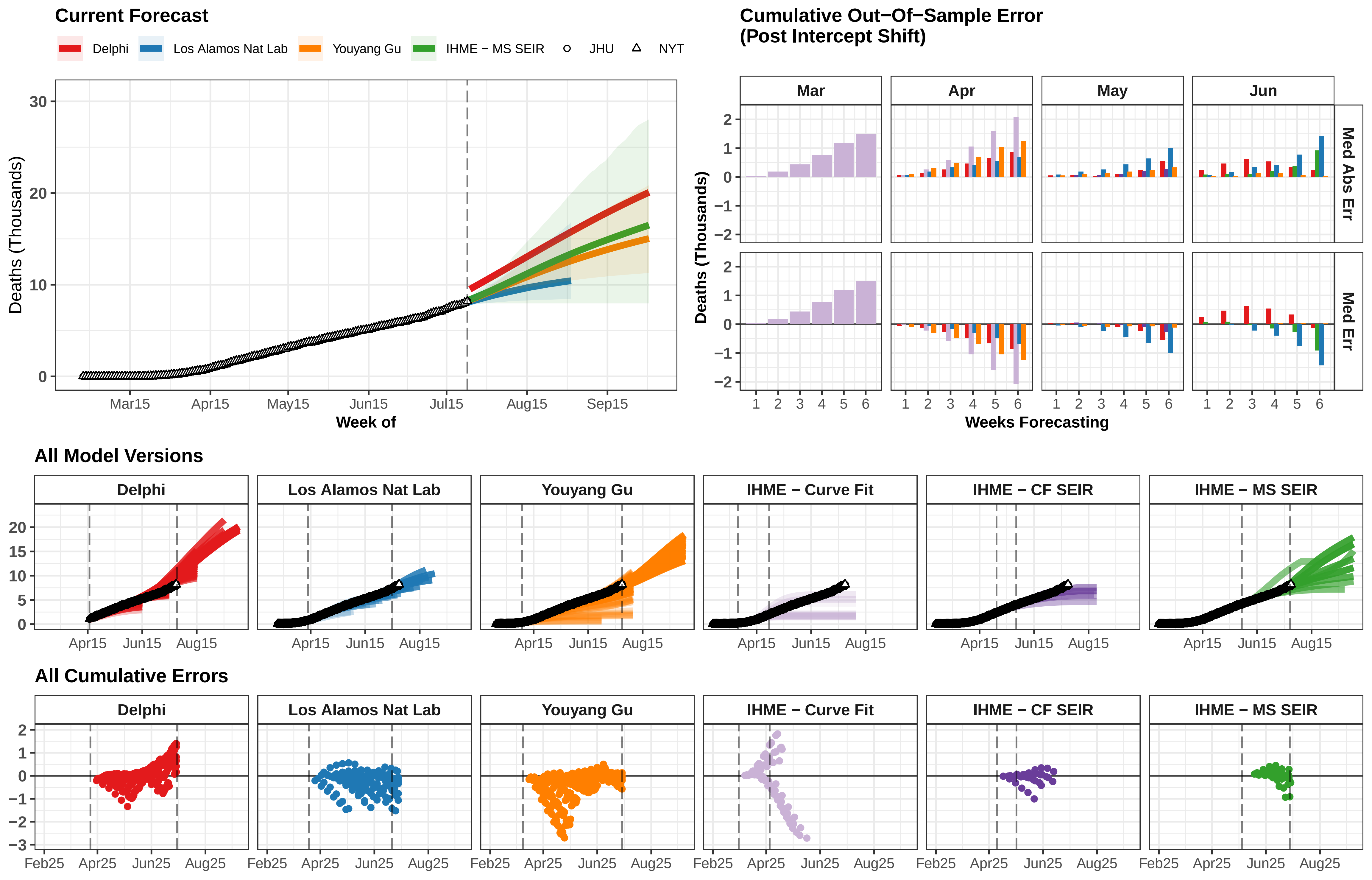
Current Forecast
Delphi
Los Alamos Nat Lab
Youyang Gu
IHME − MS SEIR
JHU
NYT
Deaths (Thousands)
30
20
10
0
Mar15
Apr15
May15
Jun15
Jul15
Aug15
Sep15
Week of
Cumulative Out−Of−Sample Error
(Post Intercept Shift)
Mar
Apr
May
Jun
Med Abs Err
Med Err
Deaths (Thousands)
2
1
0
−1
−2
1 2 3 4 5 6
Weeks Forecasting
All Model Versions
Delphi
Los Alamos Nat Lab
Youyang Gu
IHME − Curve Fit
IHME − CF SEIR
IHME − MS SEIR
20
15
10
5
0
Apr15
Jun15
Aug15
All Cumulative Errors
Delphi
Los Alamos Nat Lab
Youyang Gu
IHME − Curve Fit
IHME − CF SEIR
IHME − MS SEIR
2
1
0
−1
−2
−3
Feb25
Apr25
Jun25
Aug25

Colombia

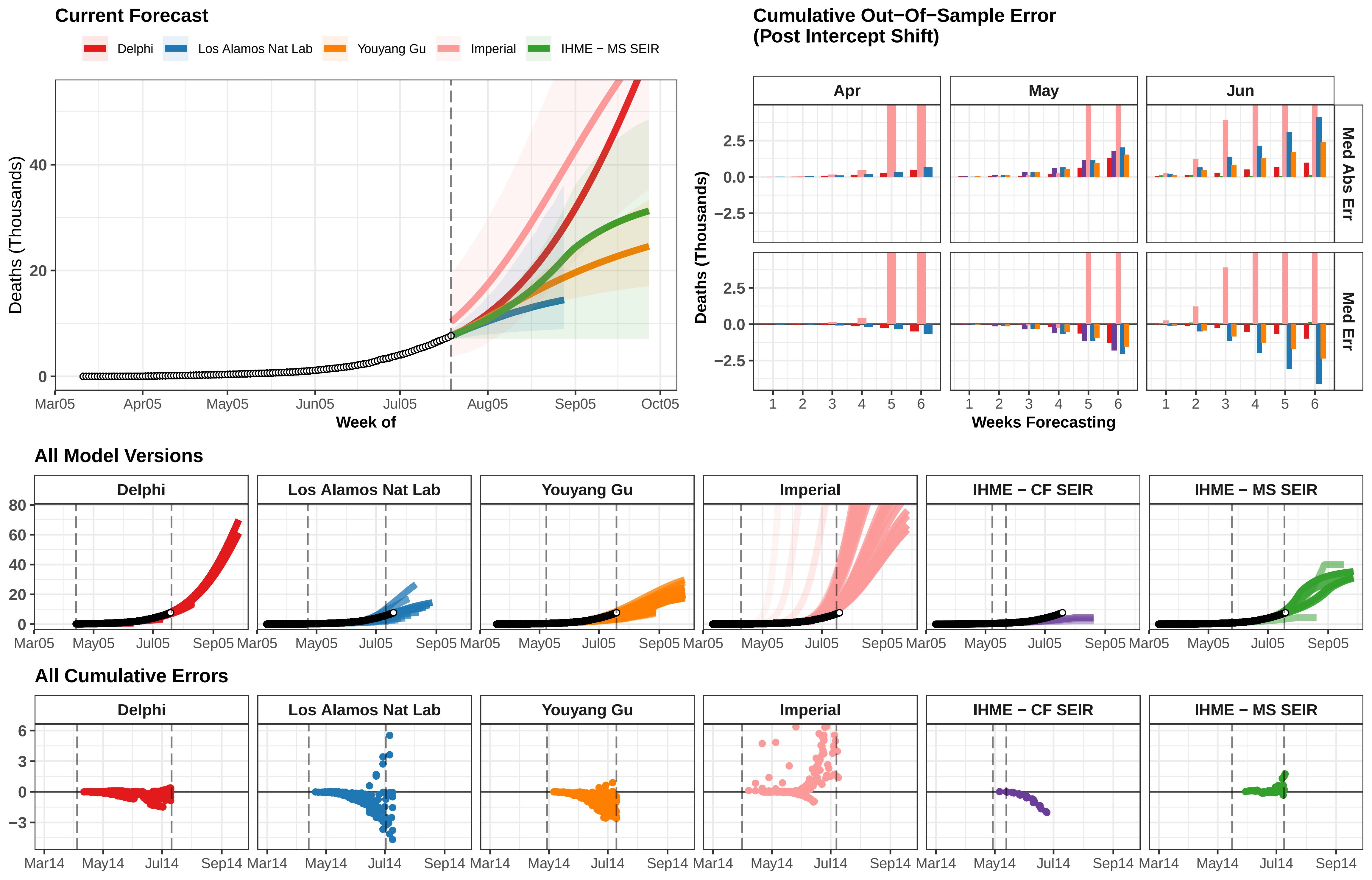

Current Forecast
Delphi
Los Alamos Nat Lab
Youyang Gu
Imperial
IHME − MS SEIR
Deaths (Thousands)
Week of
Mar05
Apr05
May05
Jun05
Jul05
Aug05
Sep05
Oct05
Cumulative Out−Of−Sample Error
(Post Intercept Shift)
Apr
May
Jun
Med Abs Err
Med Err
Weeks Forecasting
All Model Versions
IHME − CF SEIR
All Cumulative Errors
Mar14
May14
Jul14
Sep14

Illinois

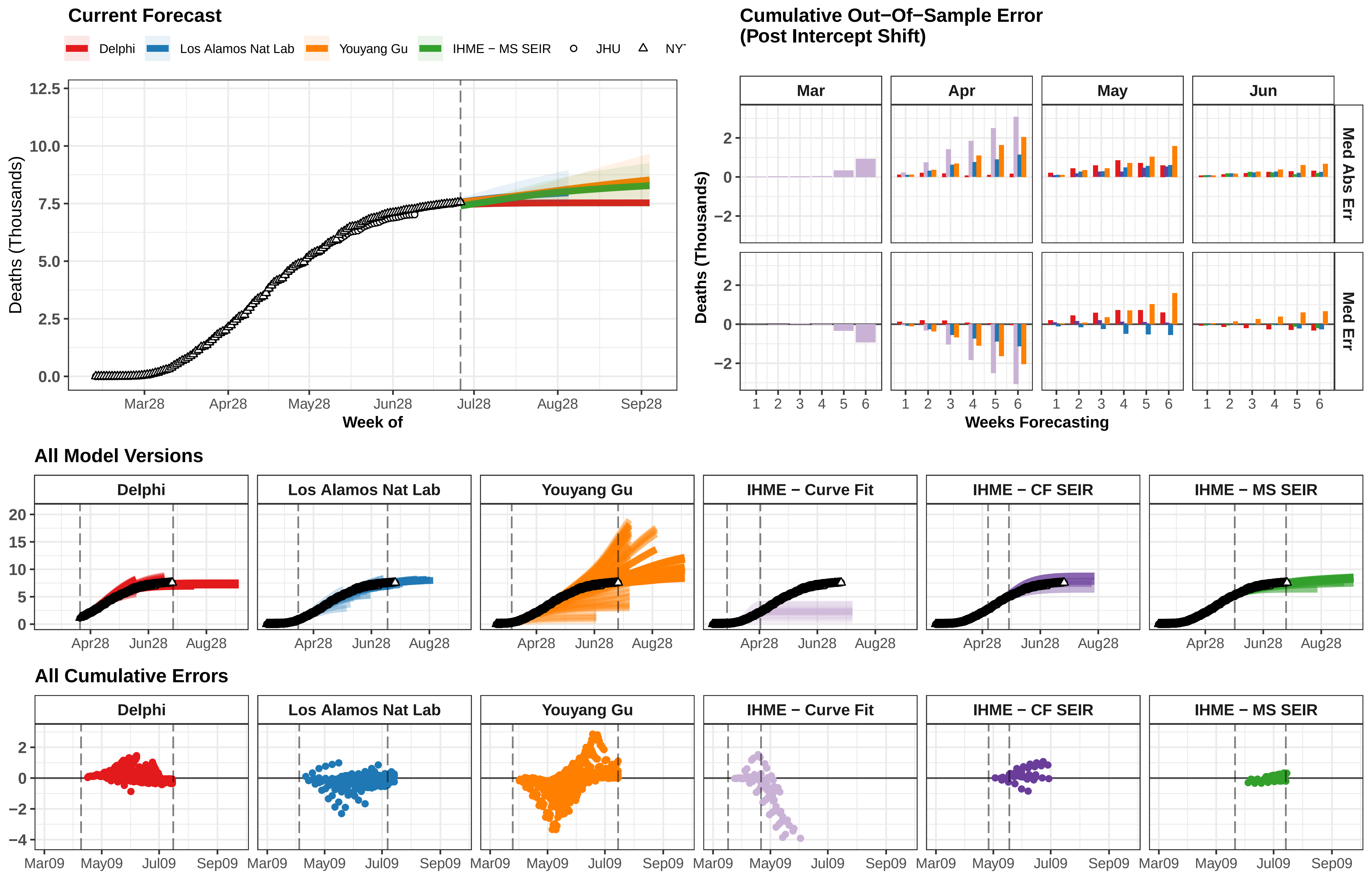
Current Forecast
Delphi
Los Alamos Nat Lab
Youyang Gu
IHME − MS SEIR
JHU
NY
Deaths (Thousands)
12.5
10.0
7.5
5.0
2.5
0.0
Mar28
Apr28
May28
Jun28
Jul28
Aug28
Sep28
Week of
Cumulative Out−Of−Sample Error
(Post Intercept Shift)
Mar
Apr
May
Jun
Med Abs Err
Med Err
Deaths (Thousands)
Weeks Forecasting
All Model Versions
Delphi
Los Alamos Nat Lab
Youyang Gu
IHME − Curve Fit
IHME − CF SEIR
IHME − MS SEIR
All Cumulative Errors
Mar09
May09
Jul09
Sep09

Pennsylvania

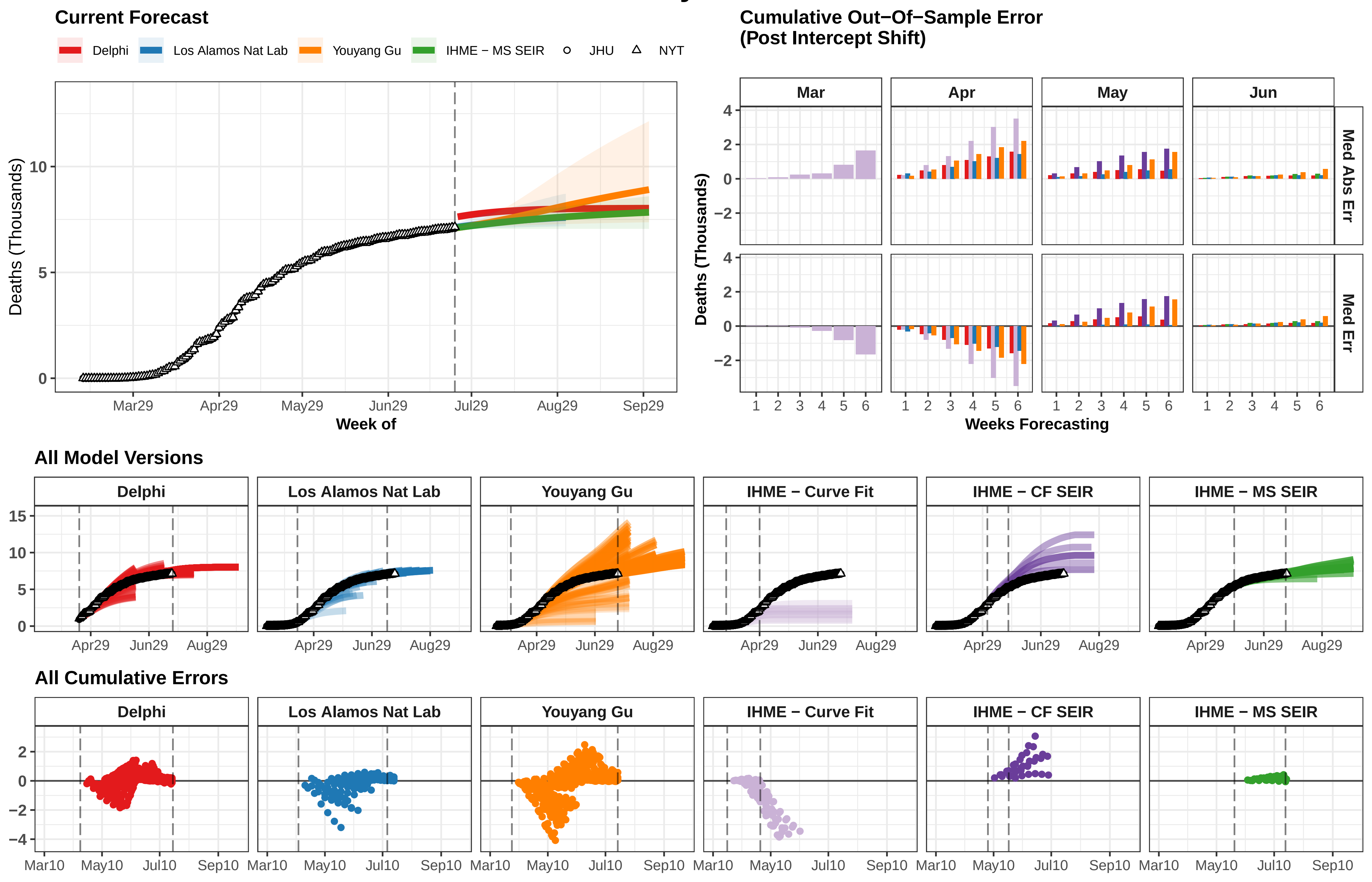

Michigan

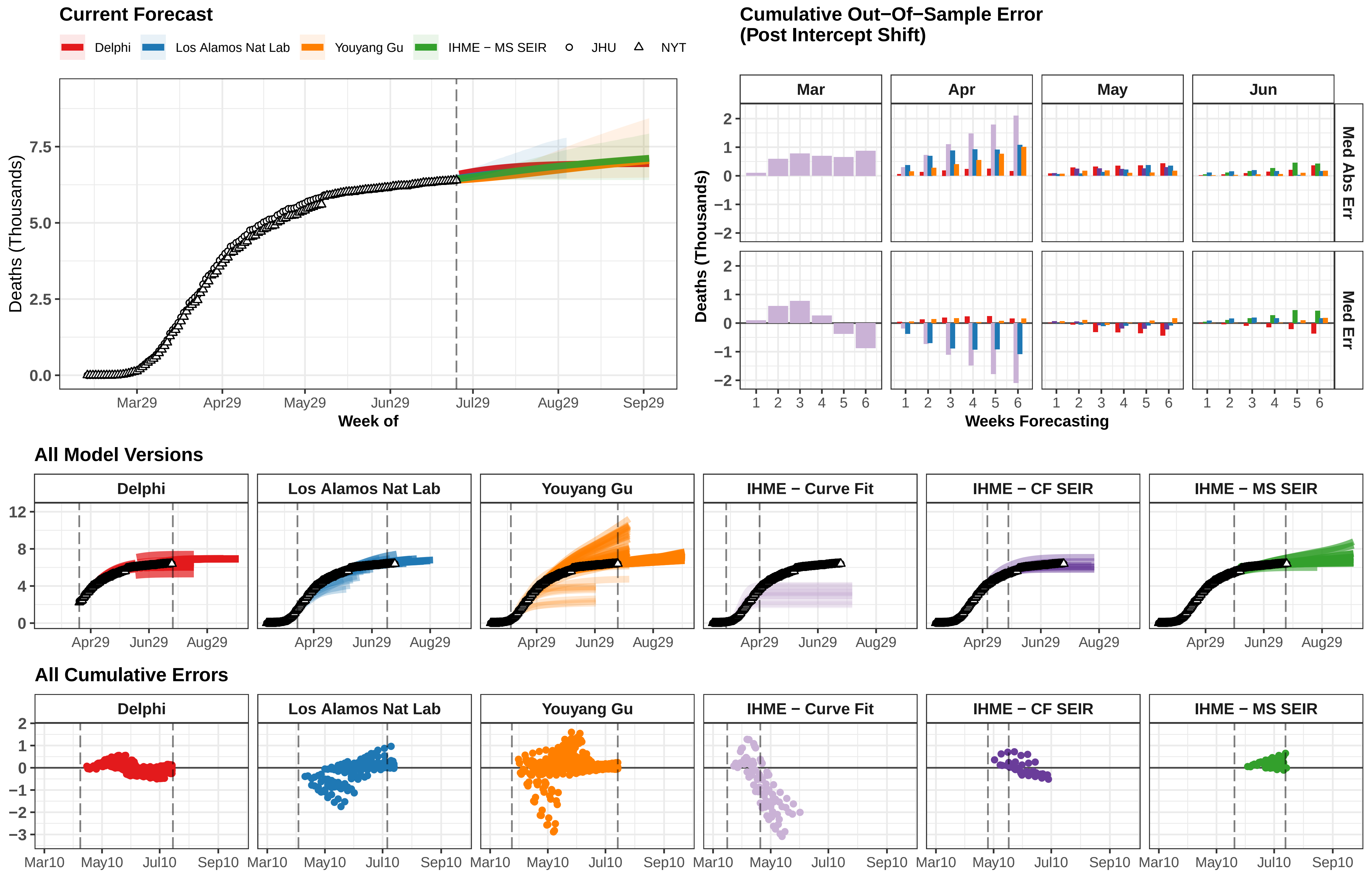

Current Forecast
Delphi
Los Alamos Nat Lab
Youyang Gu
IHME − MS SEIR
JHU
NYT
Deaths (Thousands)
7.5
5.0
2.5
0.0
Mar29
Apr29
May29
Jun29
Jul29
Aug29
Sep29
Week of
Cumulative Out−Of−Sample Error
(Post Intercept Shift)
Mar
Apr
May
Jun
Med Abs Err
Med Err
Deaths (Thousands)
2
1
0
−1
−2
1 2 3 4 5 6
Weeks Forecasting
All Model Versions
Delphi
Los Alamos Nat Lab
Youyang Gu
IHME − Curve Fit
IHME − CF SEIR
IHME − MS SEIR
12
8
4
0
Apr29
Jun29
Aug29
All Cumulative Errors
Delphi
Los Alamos Nat Lab
Youyang Gu
IHME − Curve Fit
IHME − CF SEIR
IHME − MS SEIR
2
1
0
−1
−2
−3
Mar10
May10
Jul10
Sep10

Netherlands

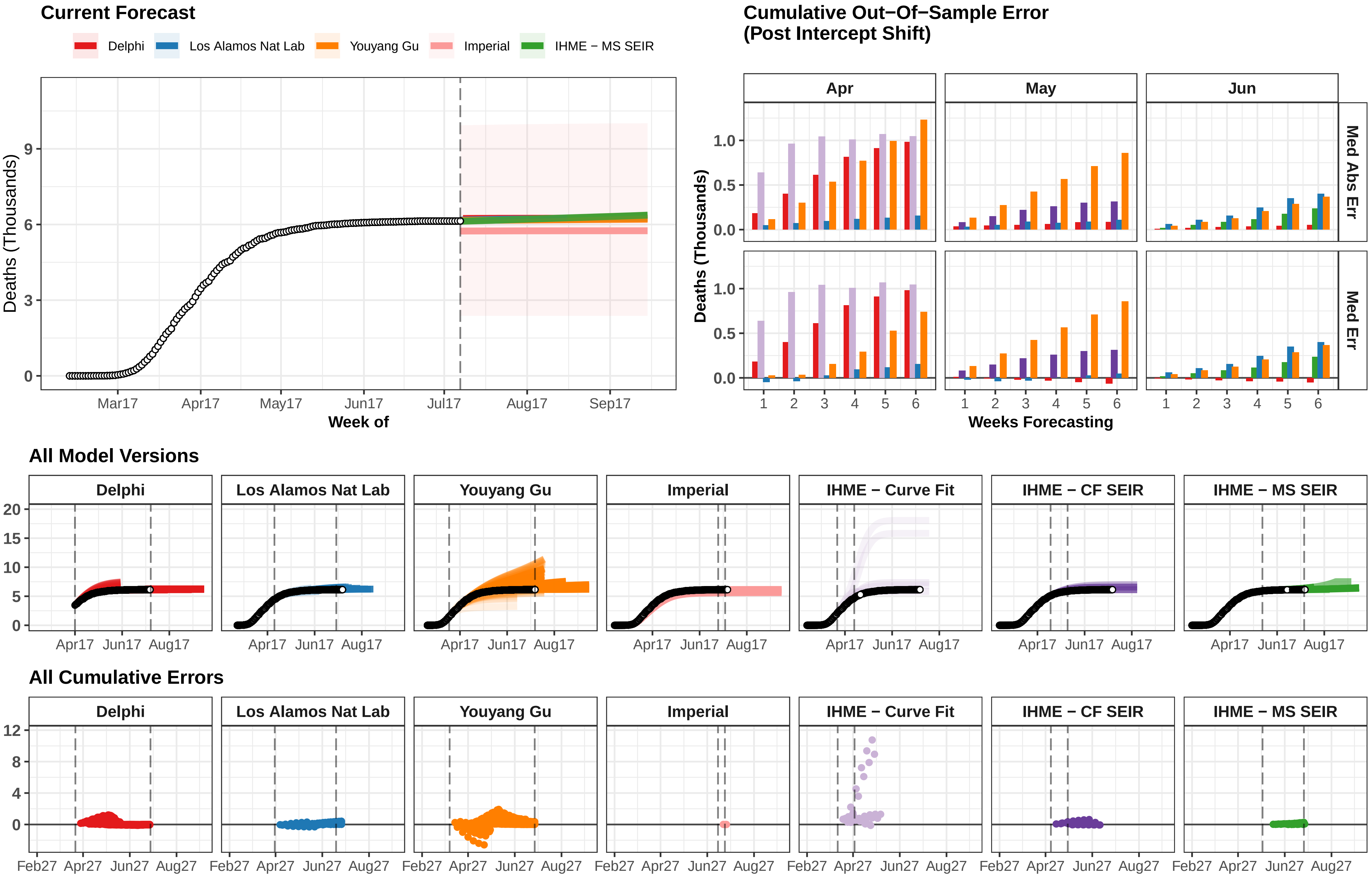

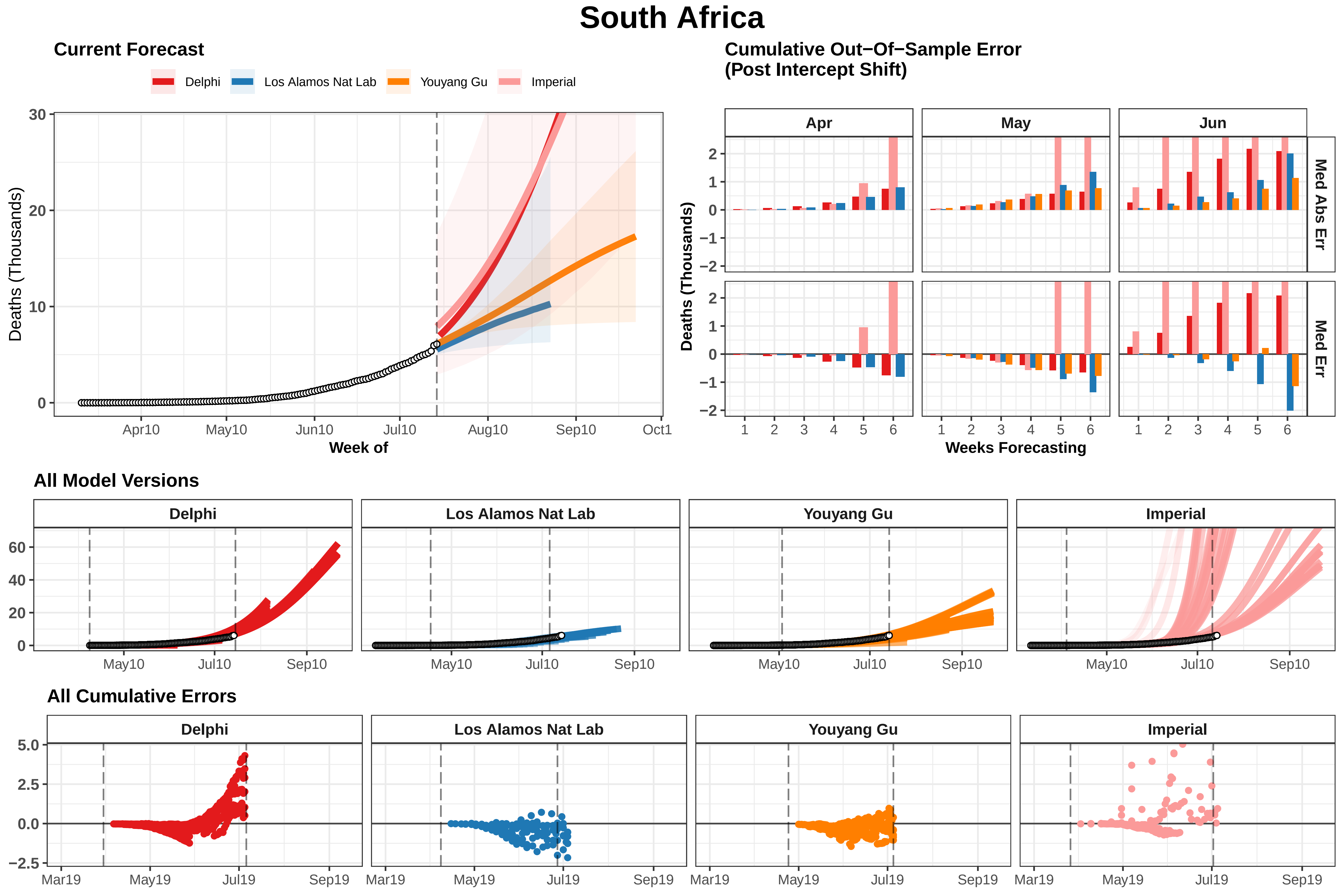
South Africa
Current Forecast
Delphi
Los Alamos Nat Lab
Youyang Gu
Imperial
Deaths (Thousands)
Week of
Apr10
May10
Jun10
Jul10
Aug10
Sep10
Oct1
Cumulative Out−Of−Sample Error
(Post Intercept Shift)
Apr
May
Jun
Med Abs Err
Med Err
Weeks Forecasting
All Model Versions
All Cumulative Errors
Mar19
May19
Jul19
Sep19

Pakistan

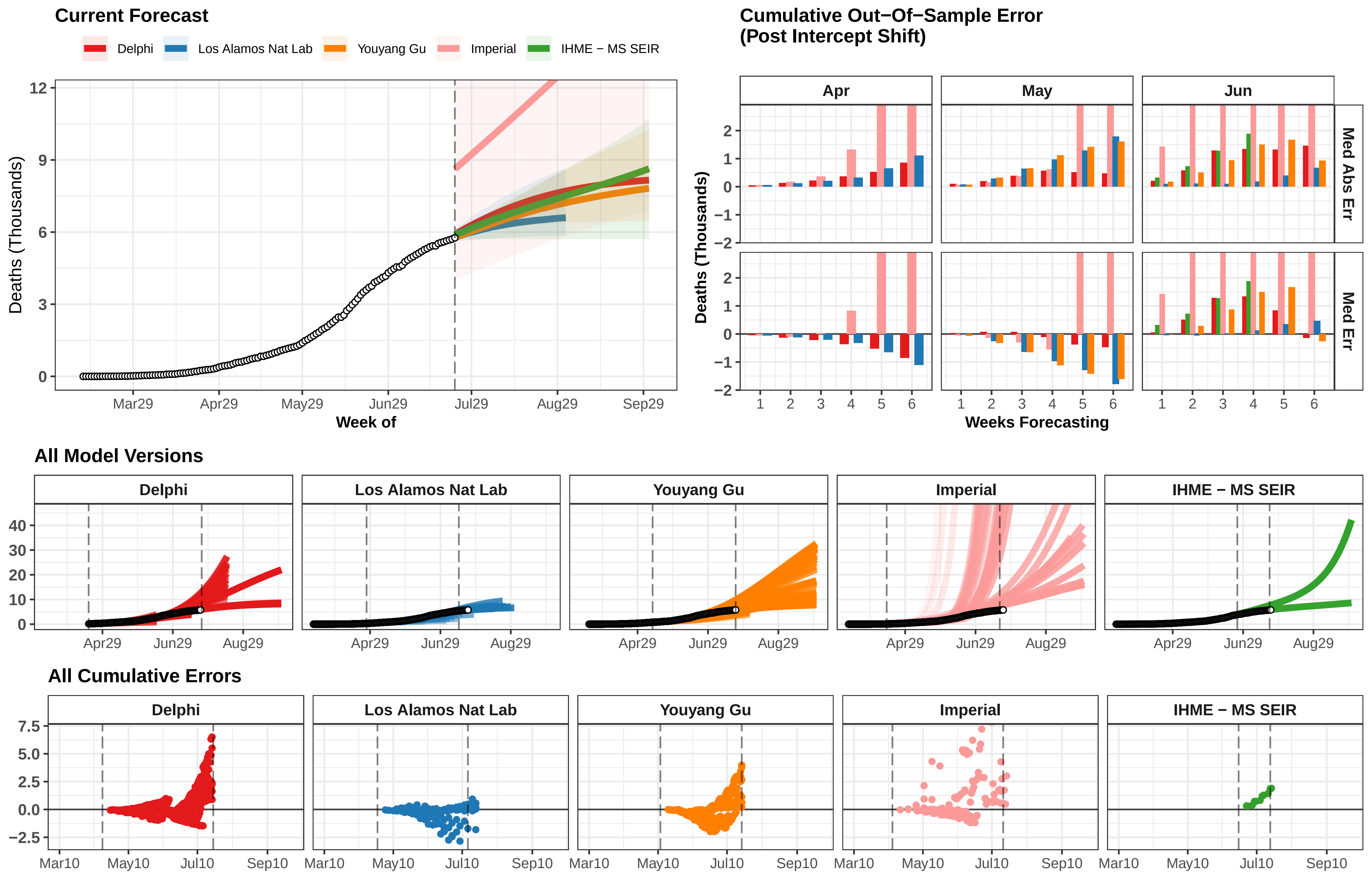
Current Forecast
Delphi
Los Alamos Nat Lab
Youyang Gu
Imperial
IHME − MS SEIR
Deaths (Thousands)
Week of
Mar29
Apr29
May29
Jun29
Jul29
Aug29
Sep29
Cumulative Out−Of−Sample Error
(Post Intercept Shift)
Apr
May
Jun
Med Abs Err
Med Err
Weeks Forecasting
All Model Versions
All Cumulative Errors
Mar10
May10
Jul10
Sep10

Sweden

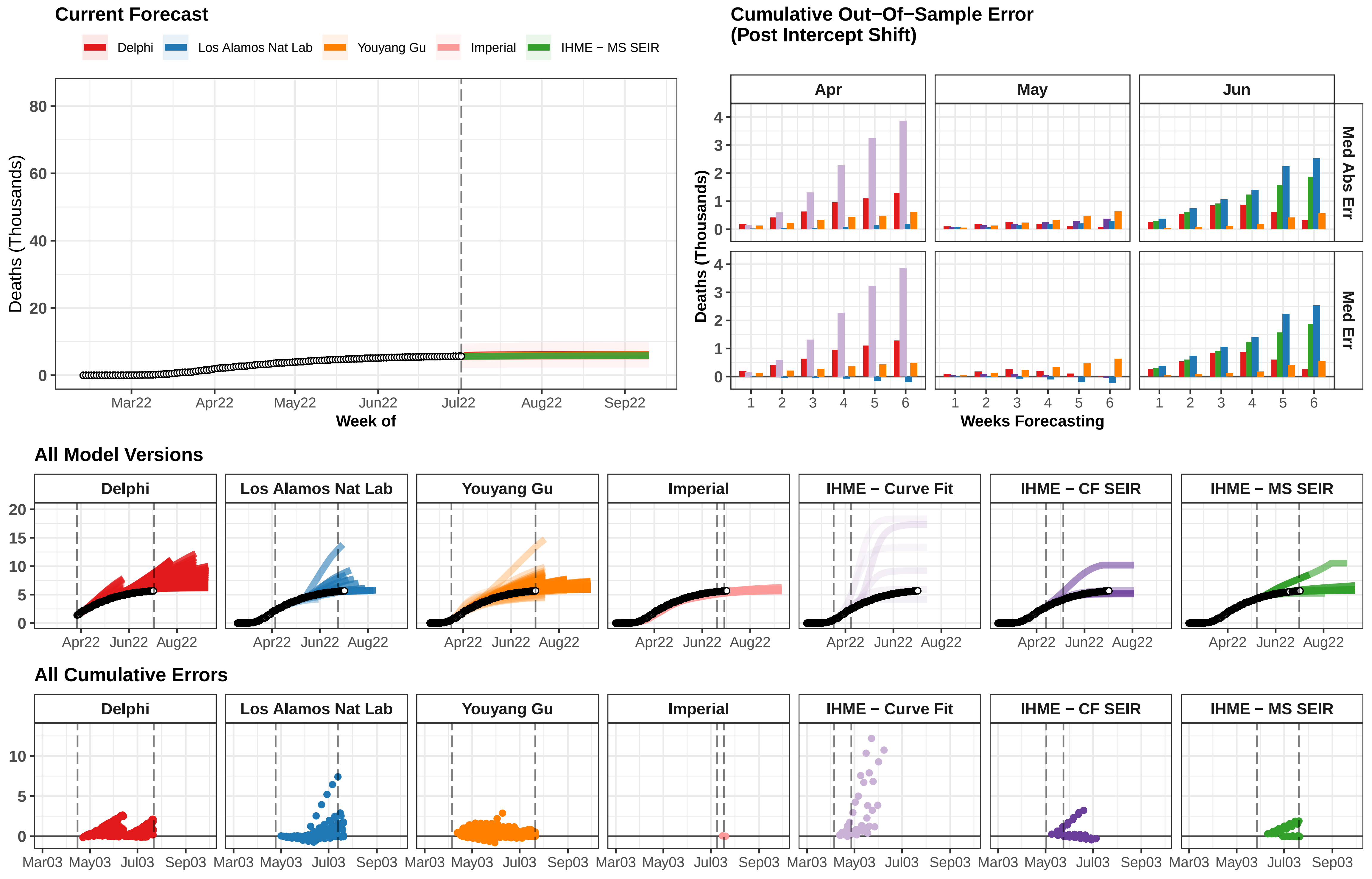

Current Forecast
Delphi
Los Alamos Nat Lab
Youyang Gu
Imperial
IHME − MS SEIR
Deaths (Thousands)
Week of
Mar22
Apr22
May22
Jun22
Jul22
Aug22
Sep22
Cumulative Out−Of−Sample Error
(Post Intercept Shift)
Apr
May
Jun
Med Abs Err
Med Err
Weeks Forecasting
All Model Versions
Delphi
Los Alamos Nat Lab
Youyang Gu
Imperial
IHME − Curve Fit
IHME − CF SEIR
IHME − MS SEIR
All Cumulative Errors
Mar03
May03
Jul03
Sep03

Turkey

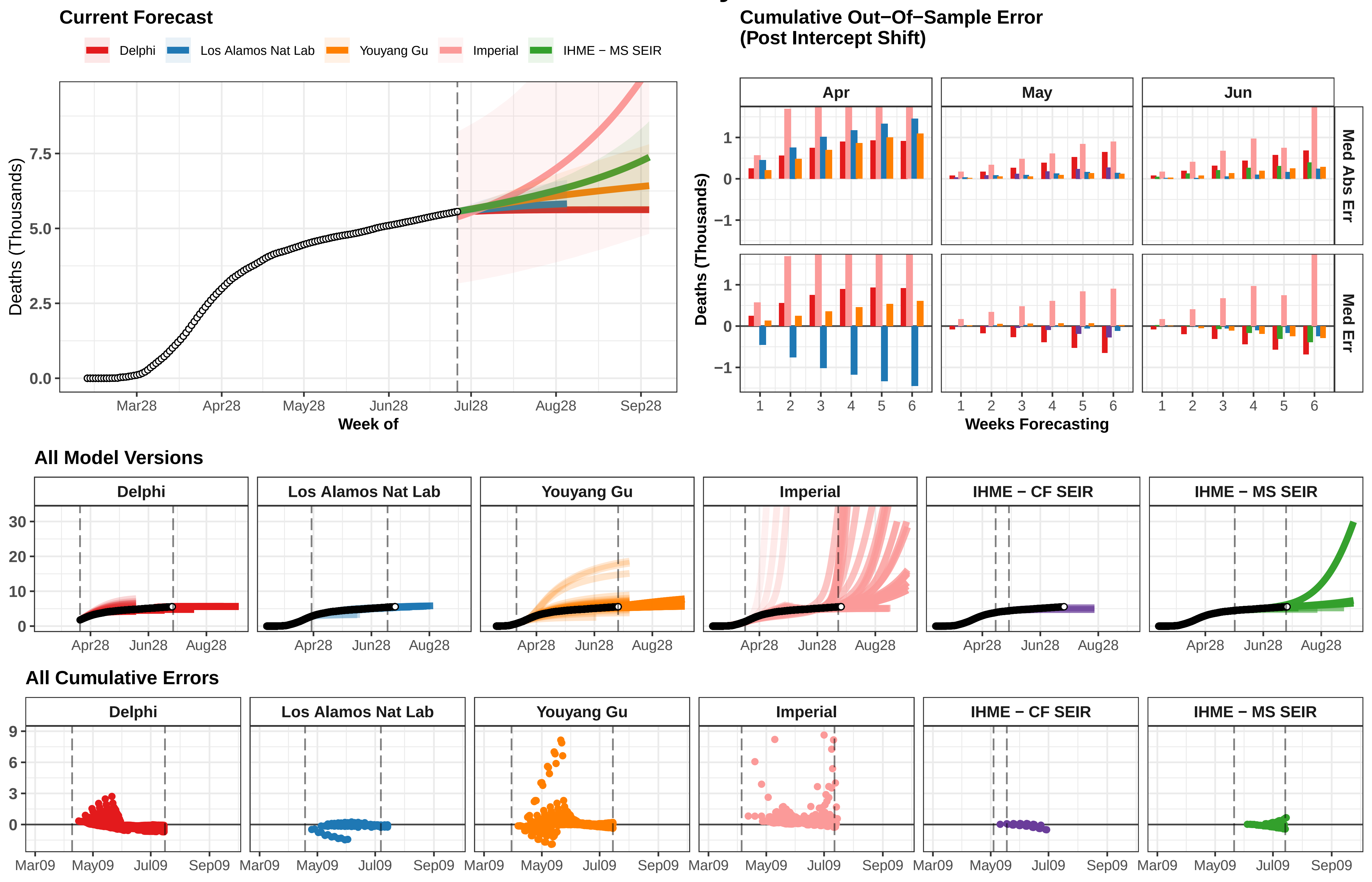
Current Forecast
Delphi
Los Alamos Nat Lab
Youyang Gu
Imperial
IHME − MS SEIR
Deaths (Thousands)
Week of
Mar28
Apr28
May28
Jun28
Jul28
Aug28
Sep28
Cumulative Out−Of−Sample Error
(Post Intercept Shift)
Apr
May
Jun
Med Abs Err
Med Err
Weeks Forecasting
All Model Versions
Delphi
Los Alamos Nat Lab
Youyang Gu
Imperial
IHME − CF SEIR
IHME − MS SEIR
All Cumulative Errors
Mar09
May09
Jul09
Sep09

Florida

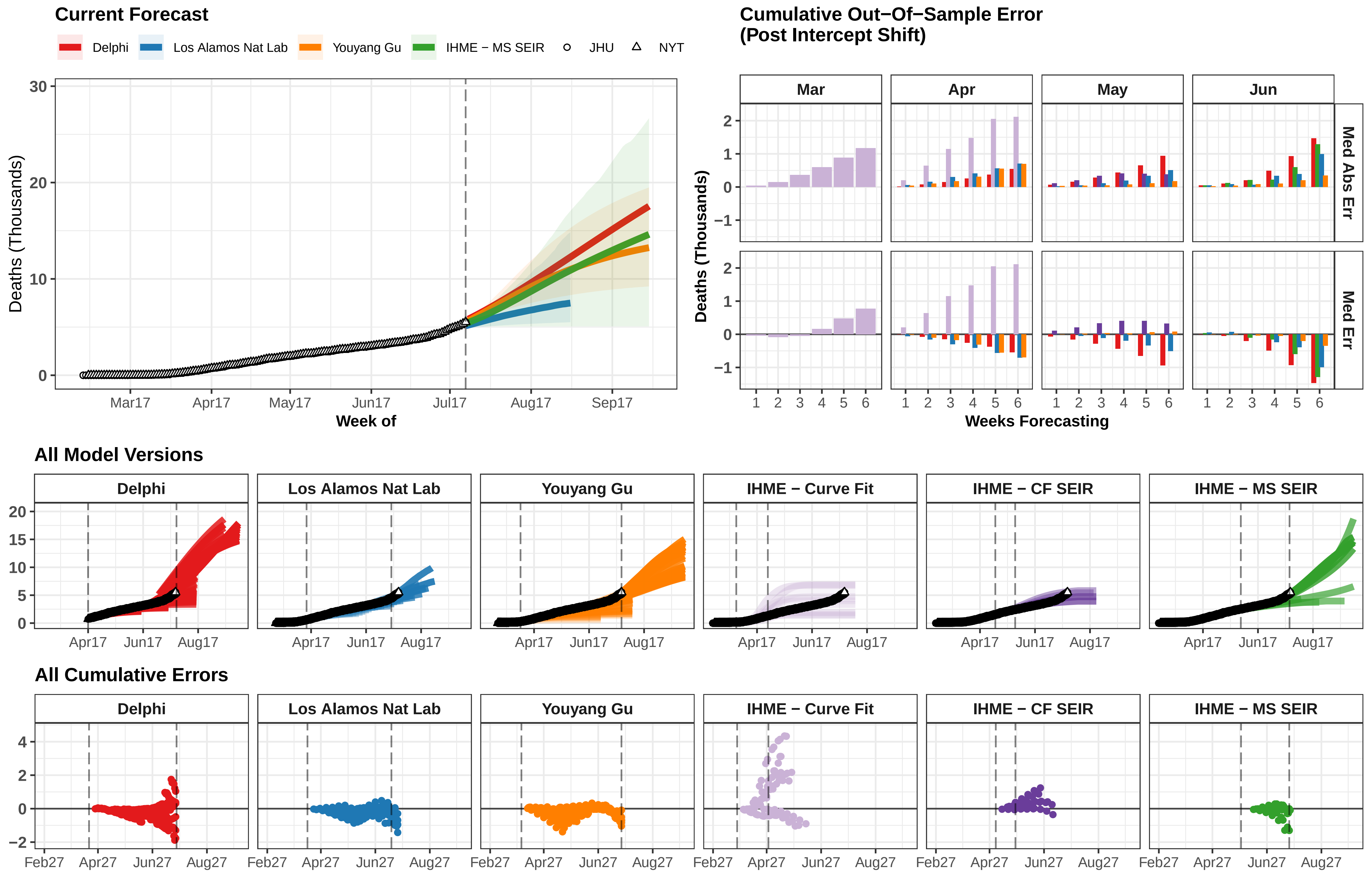
Current Forecast
Delphi
Los Alamos Nat Lab
Youyang Gu
IHME − MS SEIR
JHU
NYT
Deaths (Thousands)
30
20
10
0
Mar17
Apr17
May17
Jun17
Jul17
Aug17
Sep17
Week of
Cumulative Out−Of−Sample Error
(Post Intercept Shift)
Mar
Apr
May
Jun
Med Abs Err
Med Err
Deaths (Thousands)
2
1
0
−1
1
2
3
4
5
6
Weeks Forecasting
All Model Versions
Delphi
Los Alamos Nat Lab
Youyang Gu
IHME − Curve Fit
IHME − CF SEIR
IHME − MS SEIR
20
15
10
5
0
Apr17
Jun17
Aug17
All Cumulative Errors
Delphi
Los Alamos Nat Lab
Youyang Gu
IHME − Curve Fit
IHME − CF SEIR
IHME − MS SEIR
4
2
0
−2
Feb27
Apr27
Jun27
Aug27

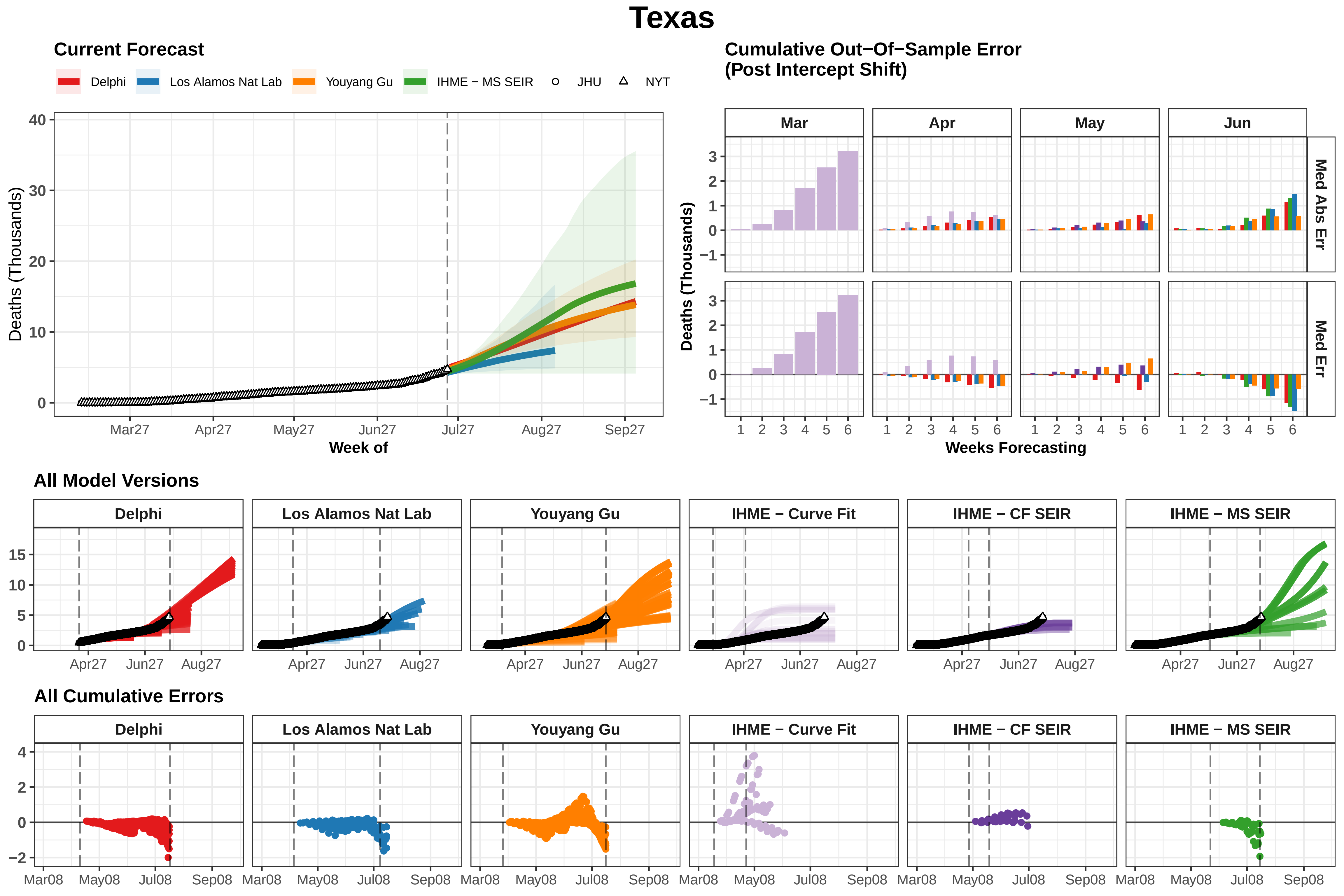
Texas
Current Forecast
Delphi
Los Alamos Nat Lab
Youyang Gu
IHME − MS SEIR
JHU
NYT
Deaths (Thousands)
Week of
Mar27
Apr27
May27
Jun27
Jul27
Aug27
Sep27
Cumulative Out−Of−Sample Error
(Post Intercept Shift)
Mar
Apr
May
Jun
Med Abs Err
Med Err
Weeks Forecasting
All Model Versions
IHME − Curve Fit
IHME − CF SEIR
All Cumulative Errors
Mar08
May08
Jul08
Sep08

China

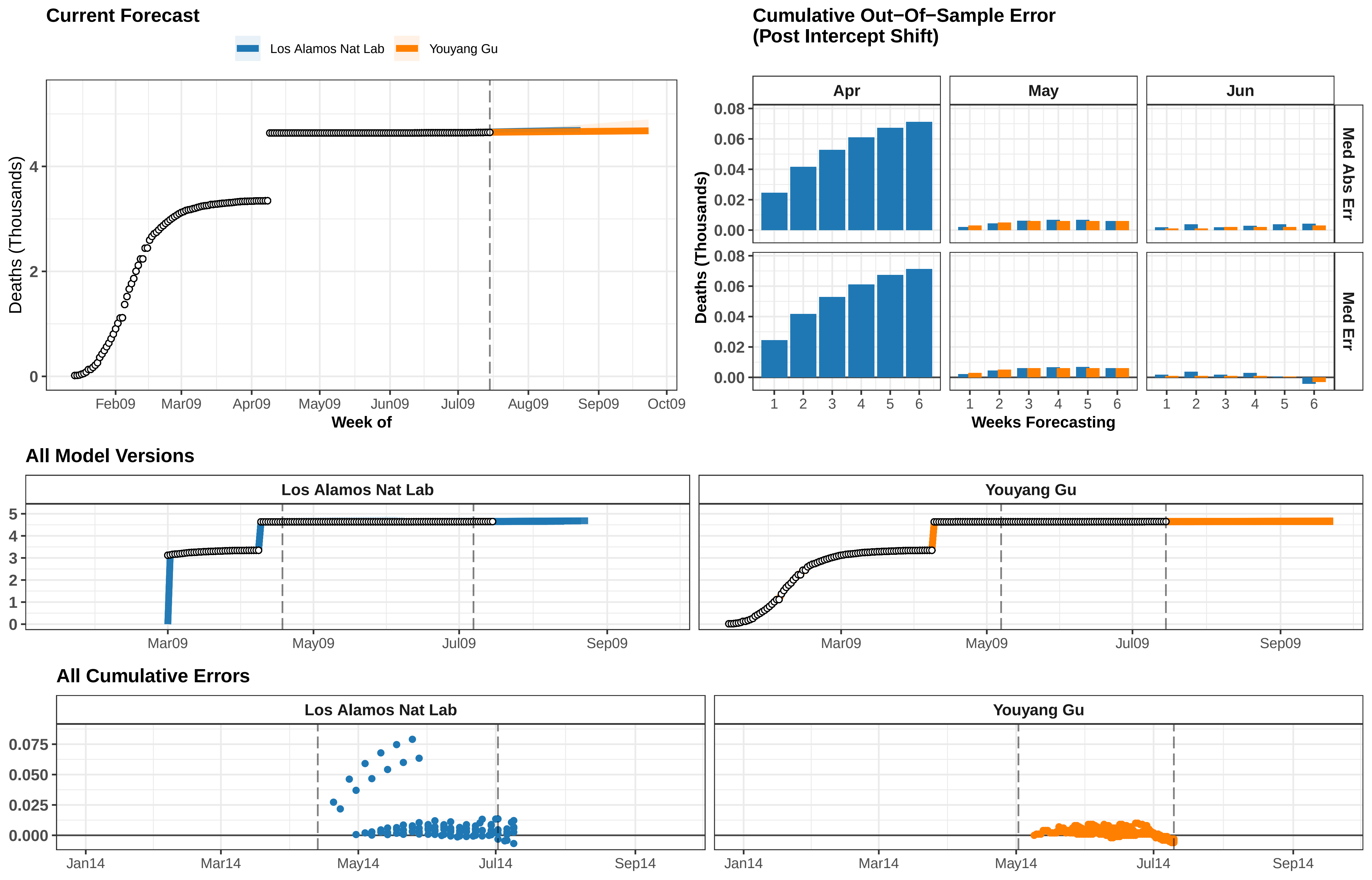
Current Forecast
Los Alamos Nat Lab
Youyang Gu
Deaths (Thousands)
0
2
4
Feb09
Mar09
Apr09
May09
Jun09
Jul09
Aug09
Sep09
Oct09
Week of
Cumulative Out−Of−Sample Error
(Post Intercept Shift)
Apr
May
Jun
Med Abs Err
Med Err
0.00
0.02
0.04
0.06
0.08
1
2
3
4
5
6
Weeks Forecasting
All Model Versions
Los Alamos Nat Lab
Youyang Gu
0
1
2
3
4
5
Mar09
May09
Jul09
Sep09
All Cumulative Errors
0.000
0.025
0.050
0.075
Jan14
Mar14
May14
Jul14
Sep14

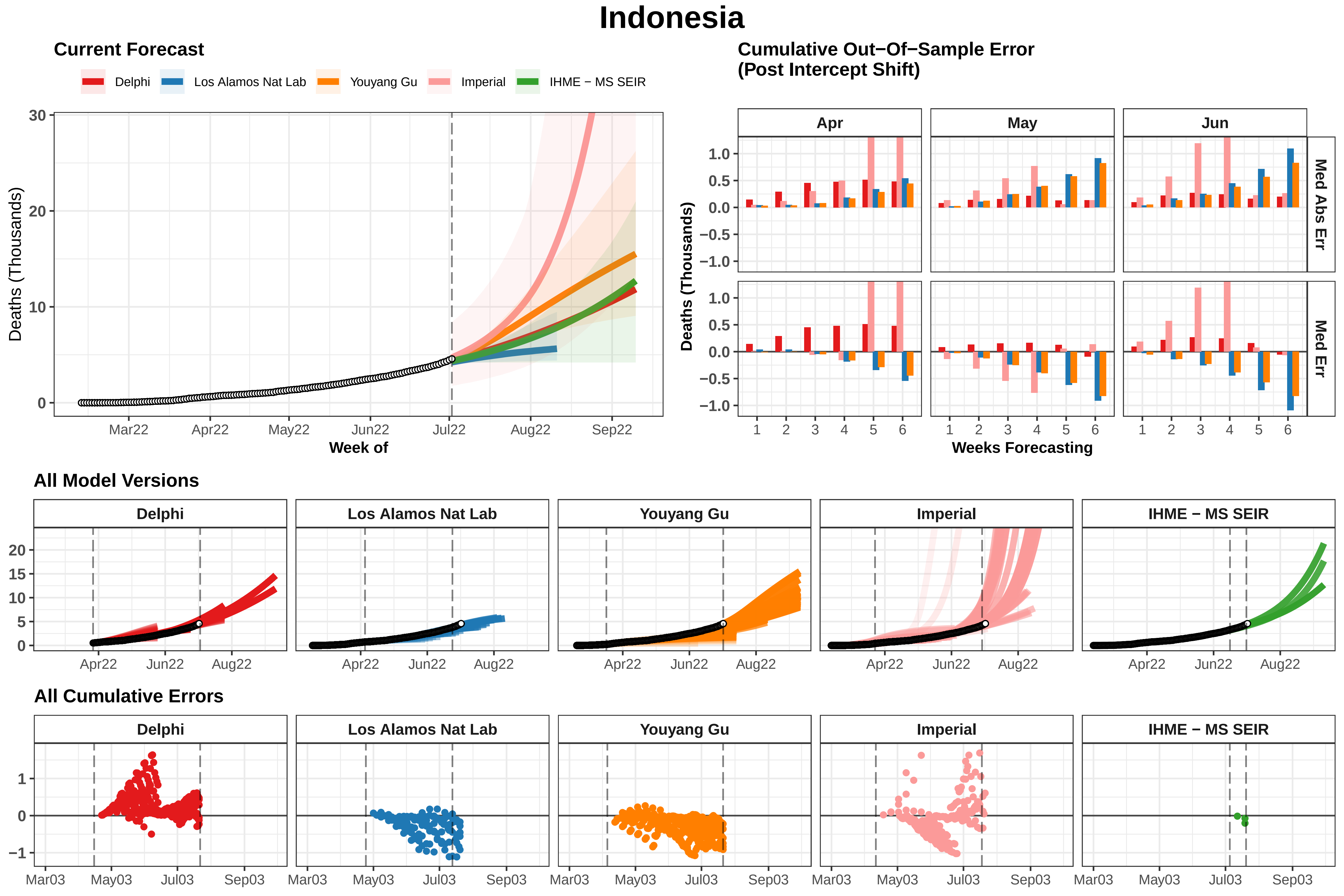
Indonesia
Current Forecast
Delphi
Los Alamos Nat Lab
Youyang Gu
Imperial
IHME − MS SEIR
Deaths (Thousands)
Week of
Mar22
Apr22
May22
Jun22
Jul22
Aug22
Sep22
Cumulative Out−Of−Sample Error
(Post Intercept Shift)
Apr
May
Jun
Med Abs Err
Med Err
Weeks Forecasting
All Model Versions
All Cumulative Errors
Mar03
May03
Jul03
Sep03

Egypt

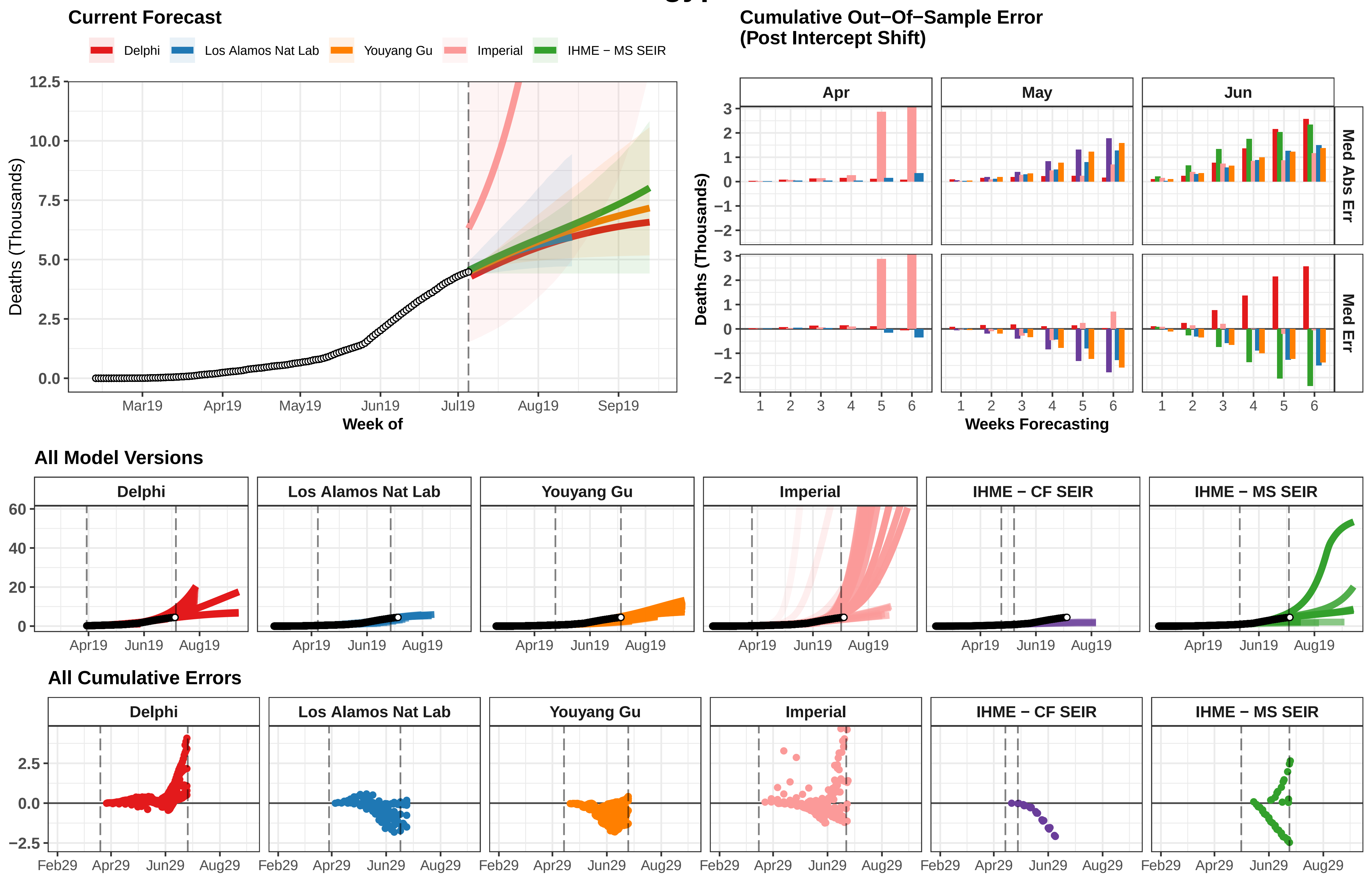

Current Forecast
Delphi
Los Alamos Nat Lab
Youyang Gu
Imperial
IHME − MS SEIR
Deaths (Thousands)
Week of
Mar19
Apr19
May19
Jun19
Jul19
Aug19
Sep19
Cumulative Out−Of−Sample Error
(Post Intercept Shift)
Apr
May
Jun
Med Abs Err
Med Err
Weeks Forecasting
All Model Versions
Delphi
Los Alamos Nat Lab
Youyang Gu
Imperial
IHME − CF SEIR
IHME − MS SEIR
All Cumulative Errors
Feb29
Apr29
Jun29
Aug29

Connecticut

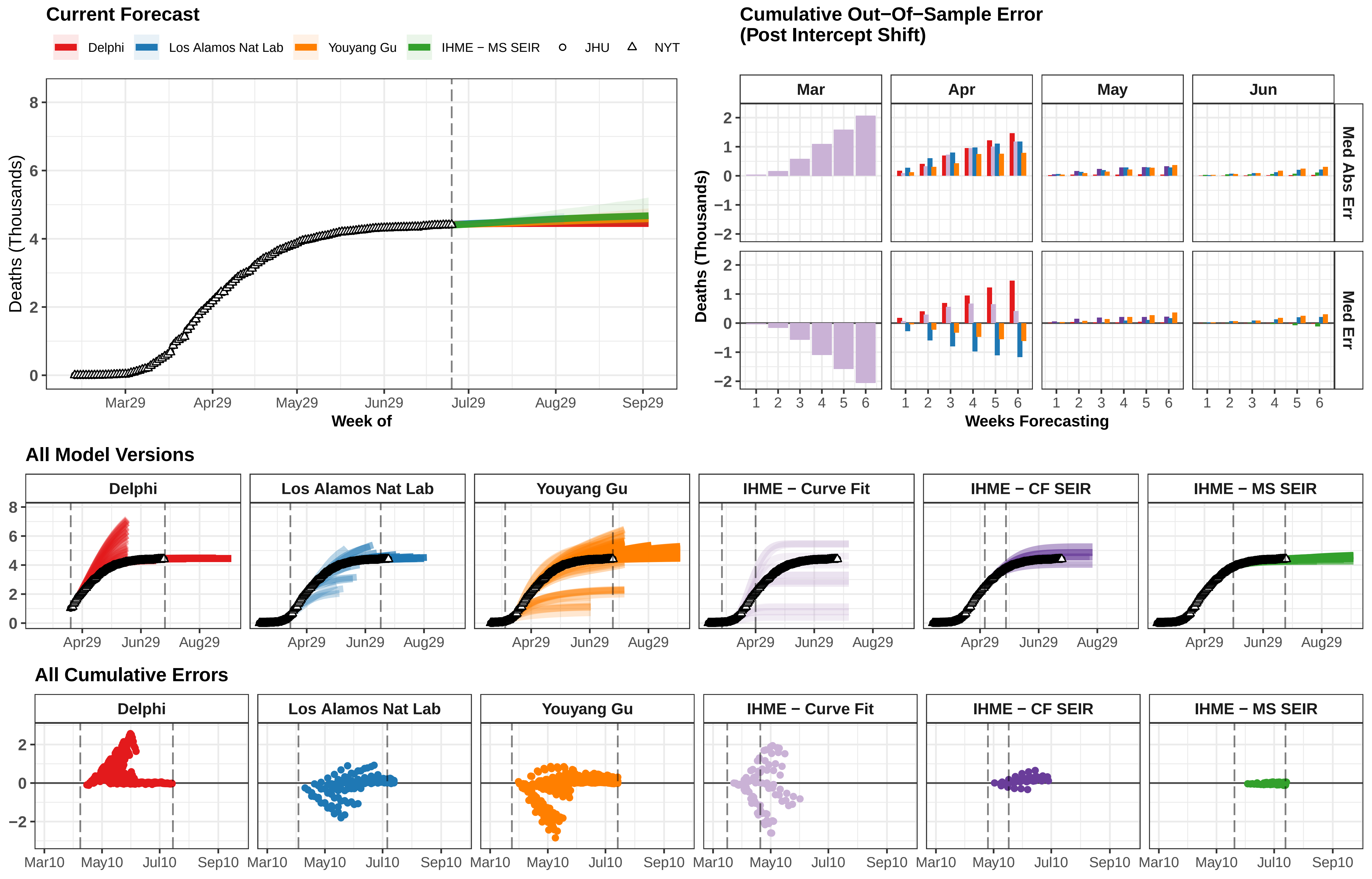

Iraq

Current Forecast

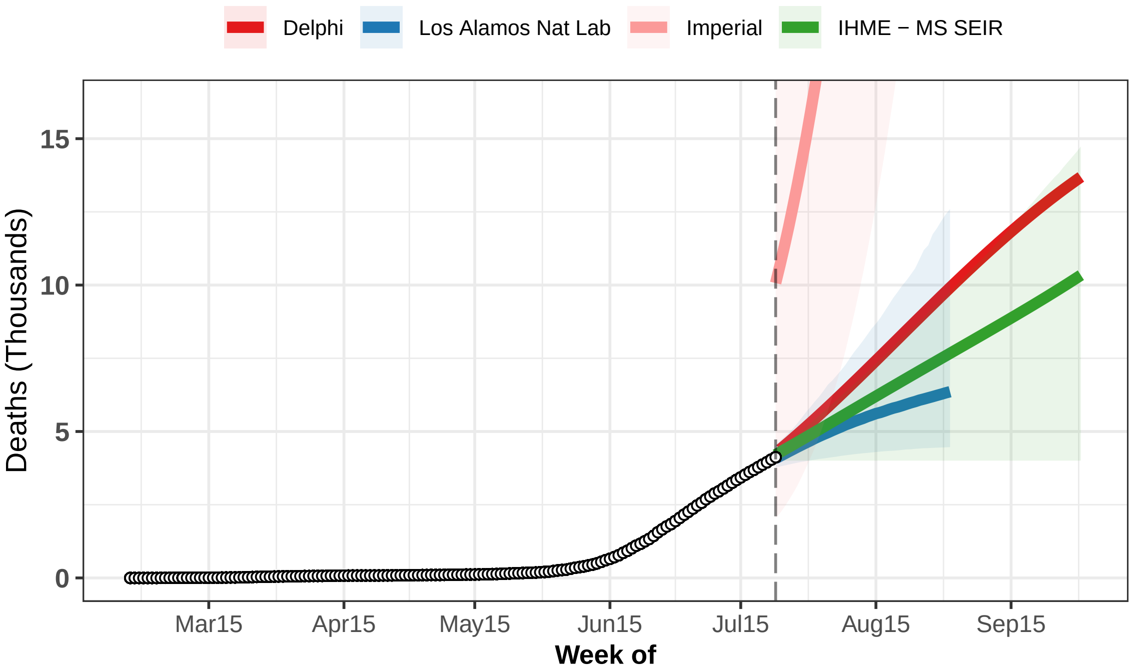

Cumulative Out−Of−Sample Error
(Post Intercept Shift)

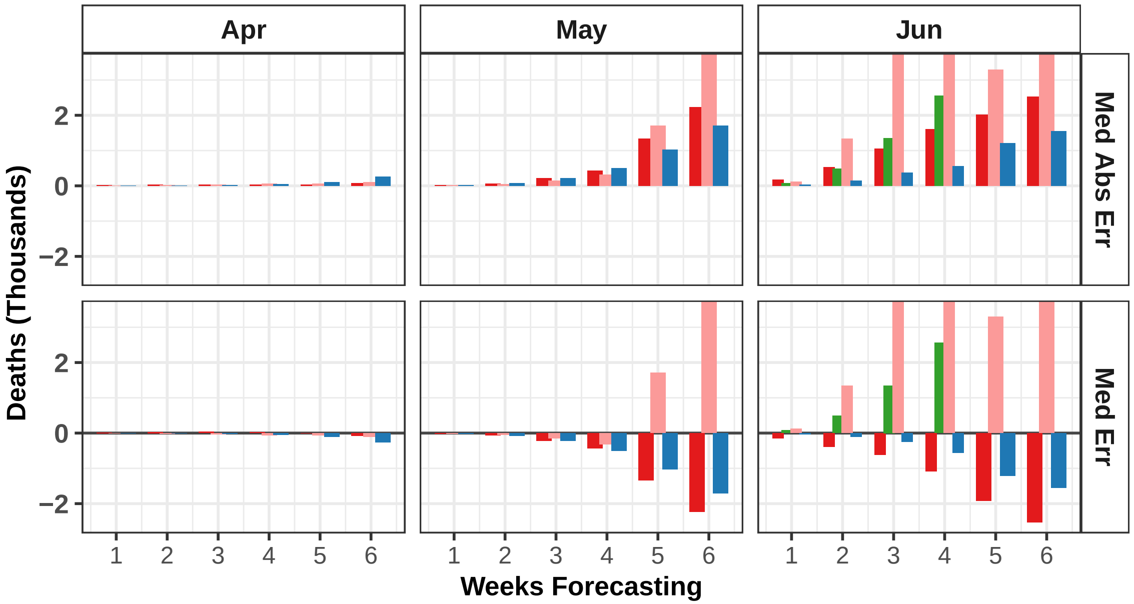

All Model Versions

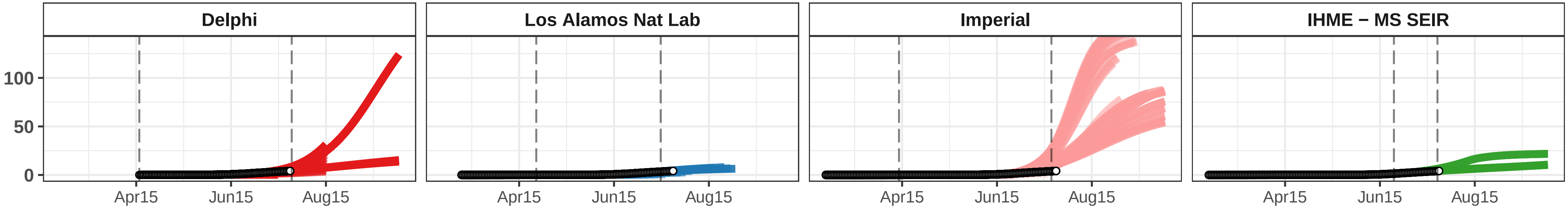

All Cumulative Errors

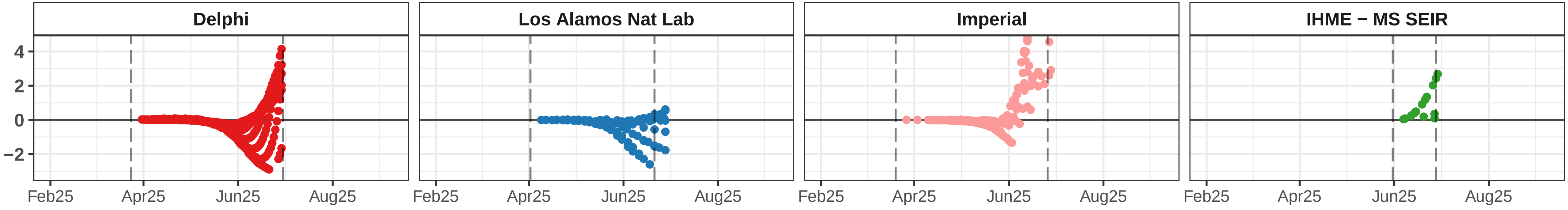

Louisiana

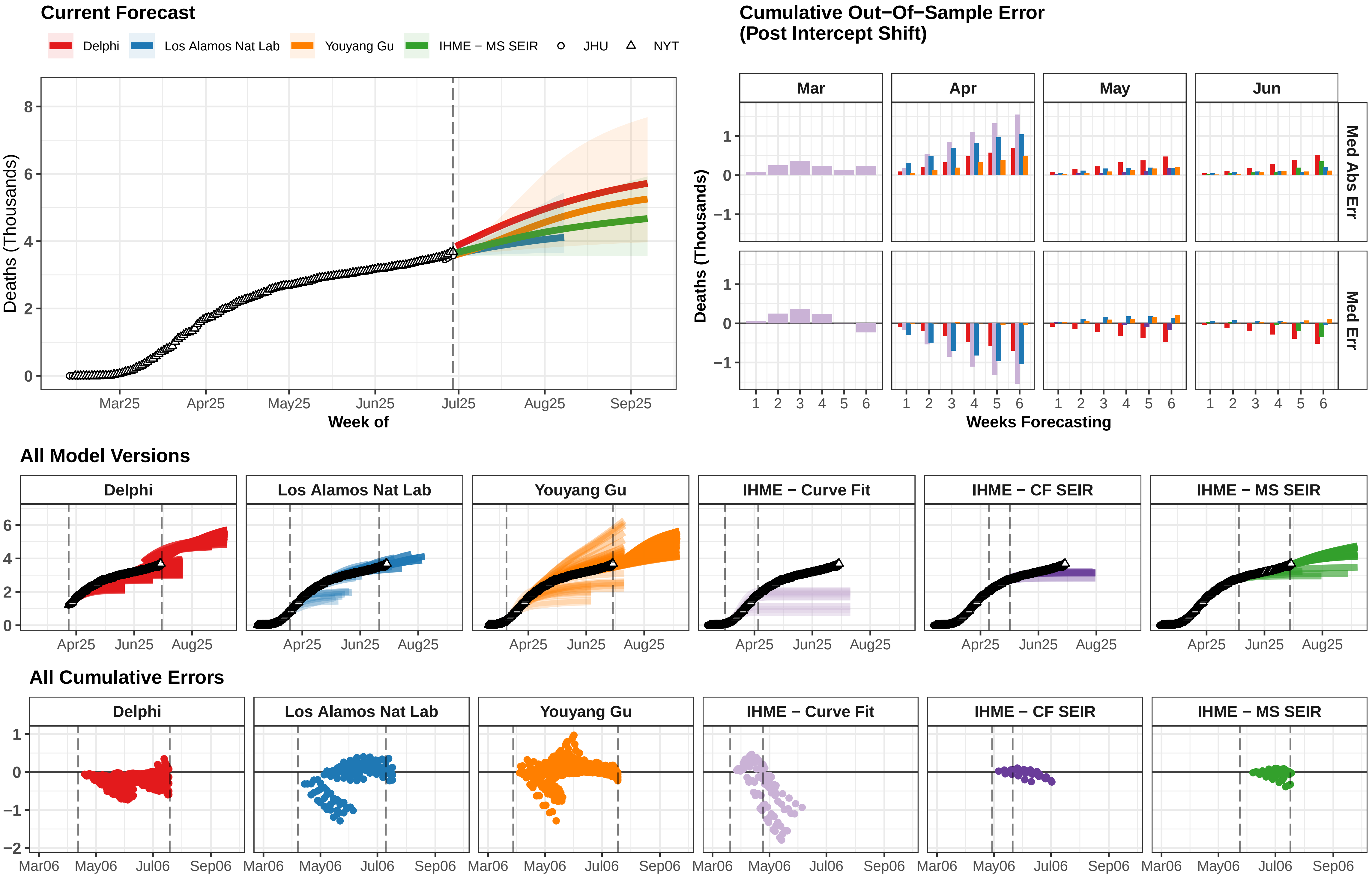

Current Forecast
Delphi
Los Alamos Nat Lab
Youyang Gu
IHME − MS SEIR
JHU
NYT
Deaths (Thousands)
Week of
Mar25
Apr25
May25
Jun25
Jul25
Aug25
Sep25
Cumulative Out−Of−Sample Error
(Post Intercept Shift)
Mar
Apr
May
Jun
Med Abs Err
Med Err
Weeks Forecasting
All Model Versions
Delphi
Los Alamos Nat Lab
Youyang Gu
IHME − Curve Fit
IHME − CF SEIR
IHME − MS SEIR
All Cumulative Errors
Mar06
May06
Jul06
Sep06

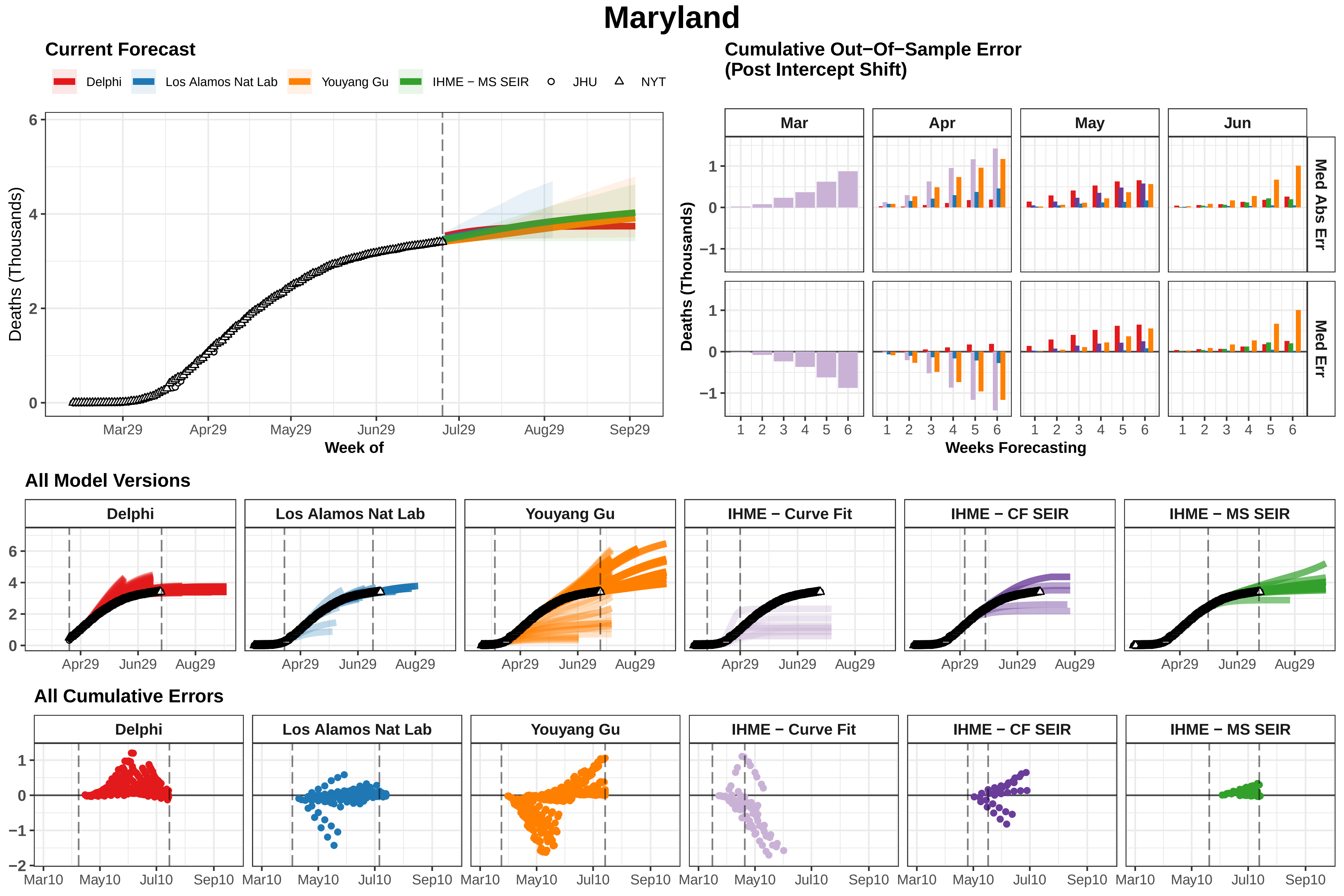

Maryland
Current Forecast
Delphi
Los Alamos Nat Lab
Youyang Gu
IHME − MS SEIR
JHU
NYT
Deaths (Thousands)
Week of
Mar29
Apr29
May29
Jun29
Jul29
Aug29
Sep29
Cumulative Out−Of−Sample Error
(Post Intercept Shift)
Mar
Apr
May
Jun
Med Abs Err
Med Err
Weeks Forecasting
All Model Versions
Delphi
Los Alamos Nat Lab
Youyang Gu
IHME − Curve Fit
IHME − CF SEIR
IHME − MS SEIR
All Cumulative Errors
Mar10
May10
Jul10
Sep10

Georgia

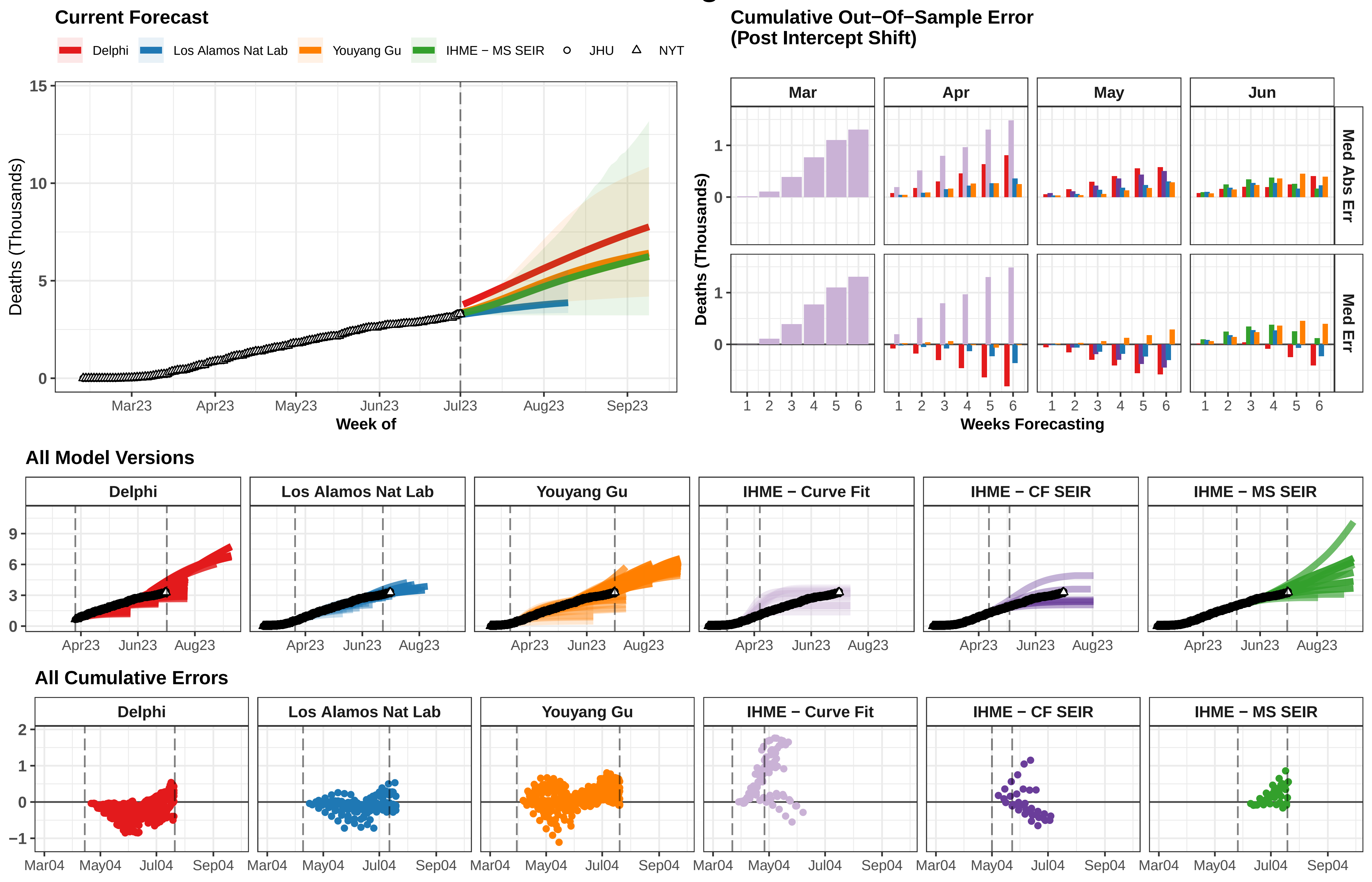

Current Forecast
Delphi
Los Alamos Nat Lab
Youyang Gu
IHME − MS SEIR
JHU
NYT
Deaths (Thousands)
Week of
Mar23
Apr23
May23
Jun23
Jul23
Aug23
Sep23
Cumulative Out−Of−Sample Error (Post Intercept Shift)
Mar
Apr
May
Jun
Med Abs Err
Med Err
Weeks Forecasting
All Model Versions
Delphi
Los Alamos Nat Lab
Youyang Gu
IHME − Curve Fit
IHME − CF SEIR
IHME − MS SEIR
All Cumulative Errors
Mar04
May04
Jul04
Sep04

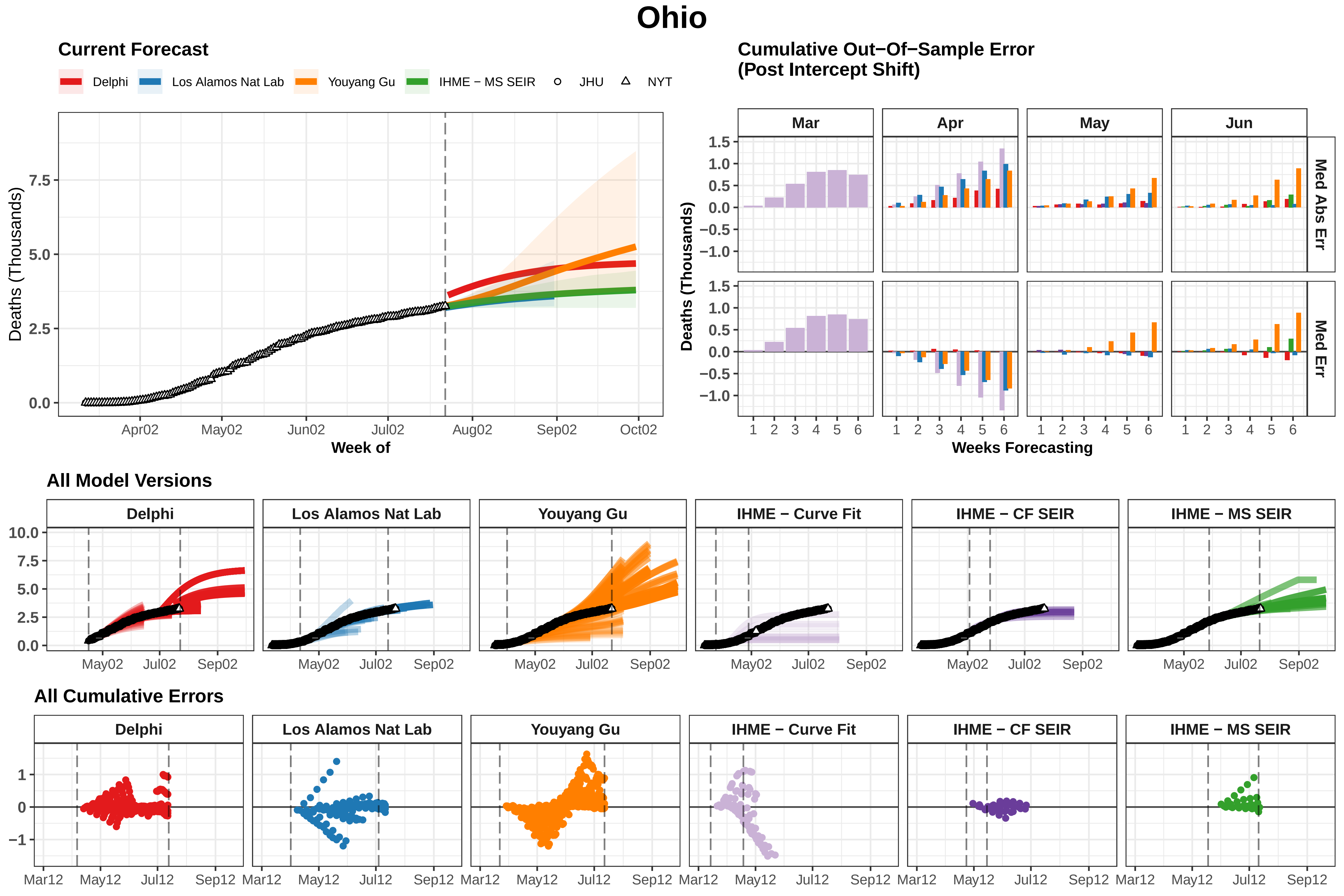

Ohio
Current Forecast
Delphi
Los Alamos Nat Lab
Youyang Gu
IHME − MS SEIR
JHU
NYT
Deaths (Thousands)
0.0
2.5
5.0
7.5
Apr02
May02
Jun02
Jul02
Aug02
Sep02
Oct02
Week of
Cumulative Out−Of−Sample Error
(Post Intercept Shift)
Mar
Apr
May
Jun
Med Abs Err
Med Err
Deaths (Thousands)
1.5
1.0
0.5
0.0
−0.5
−1.0
1 2 3 4 5 6
Weeks Forecasting
All Model Versions
Delphi
Los Alamos Nat Lab
Youyang Gu
IHME − Curve Fit
IHME − CF SEIR
IHME − MS SEIR
10.0
7.5
5.0
2.5
0.0
May02
Jul02
Sep02
All Cumulative Errors
Delphi
Los Alamos Nat Lab
Youyang Gu
IHME − Curve Fit
IHME − CF SEIR
IHME − MS SEIR
1
0
−1
Mar12
May12
Jul12
Sep12

Arizona

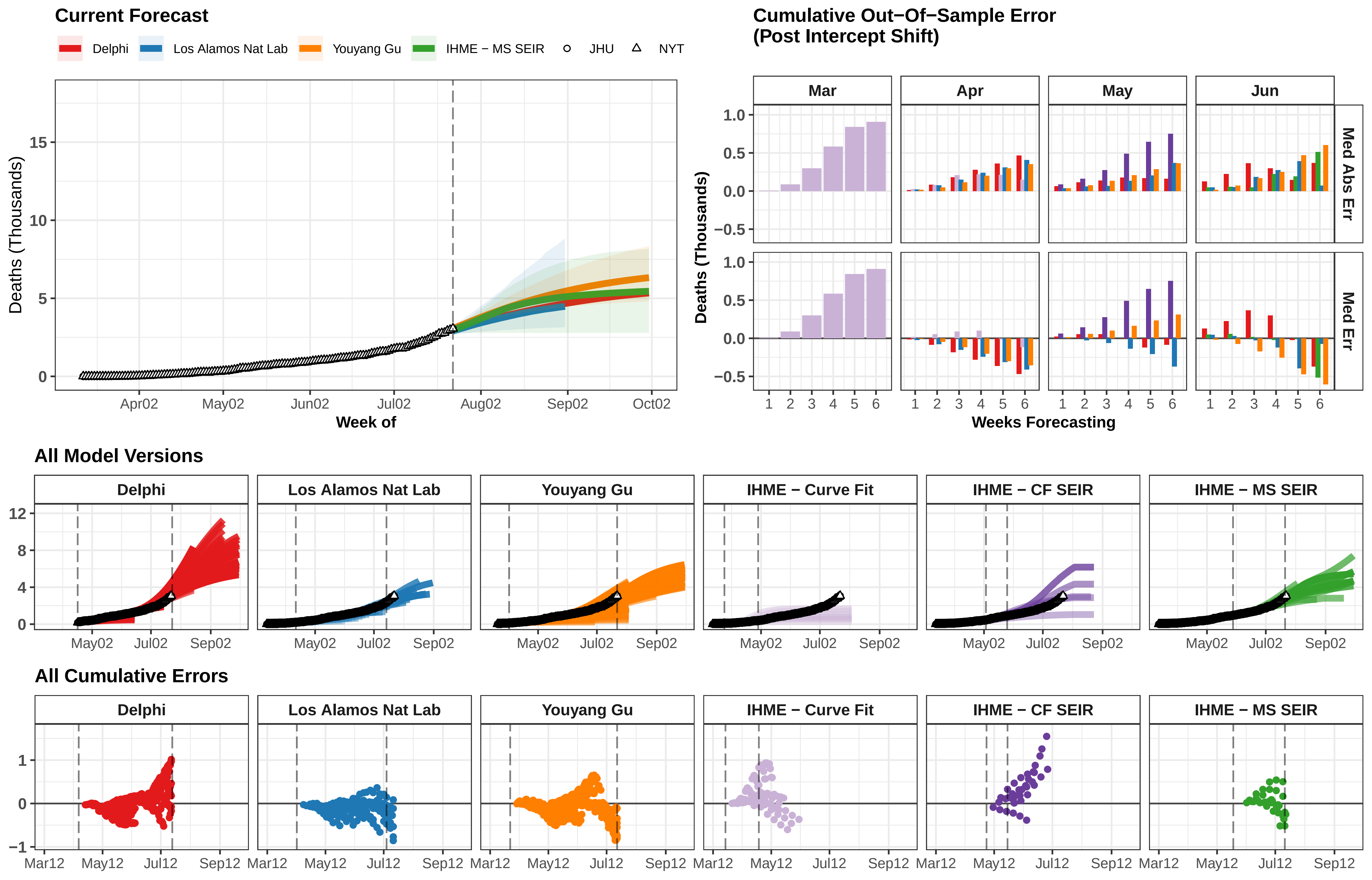
Current Forecast
Delphi
Los Alamos Nat Lab
Youyang Gu
IHME − MS SEIR
JHU
NYT
Deaths (Thousands)
Week of
Apr02
May02
Jun02
Jul02
Aug02
Sep02
Oct02
Cumulative Out−Of−Sample Error
(Post Intercept Shift)
Mar
Apr
May
Jun
Med Abs Err
Med Err
Weeks Forecasting
All Model Versions
Delphi
Los Alamos Nat Lab
Youyang Gu
IHME − Curve Fit
IHME − CF SEIR
IHME − MS SEIR
All Cumulative Errors
Mar12
May12
Jul12
Sep12

Indiana

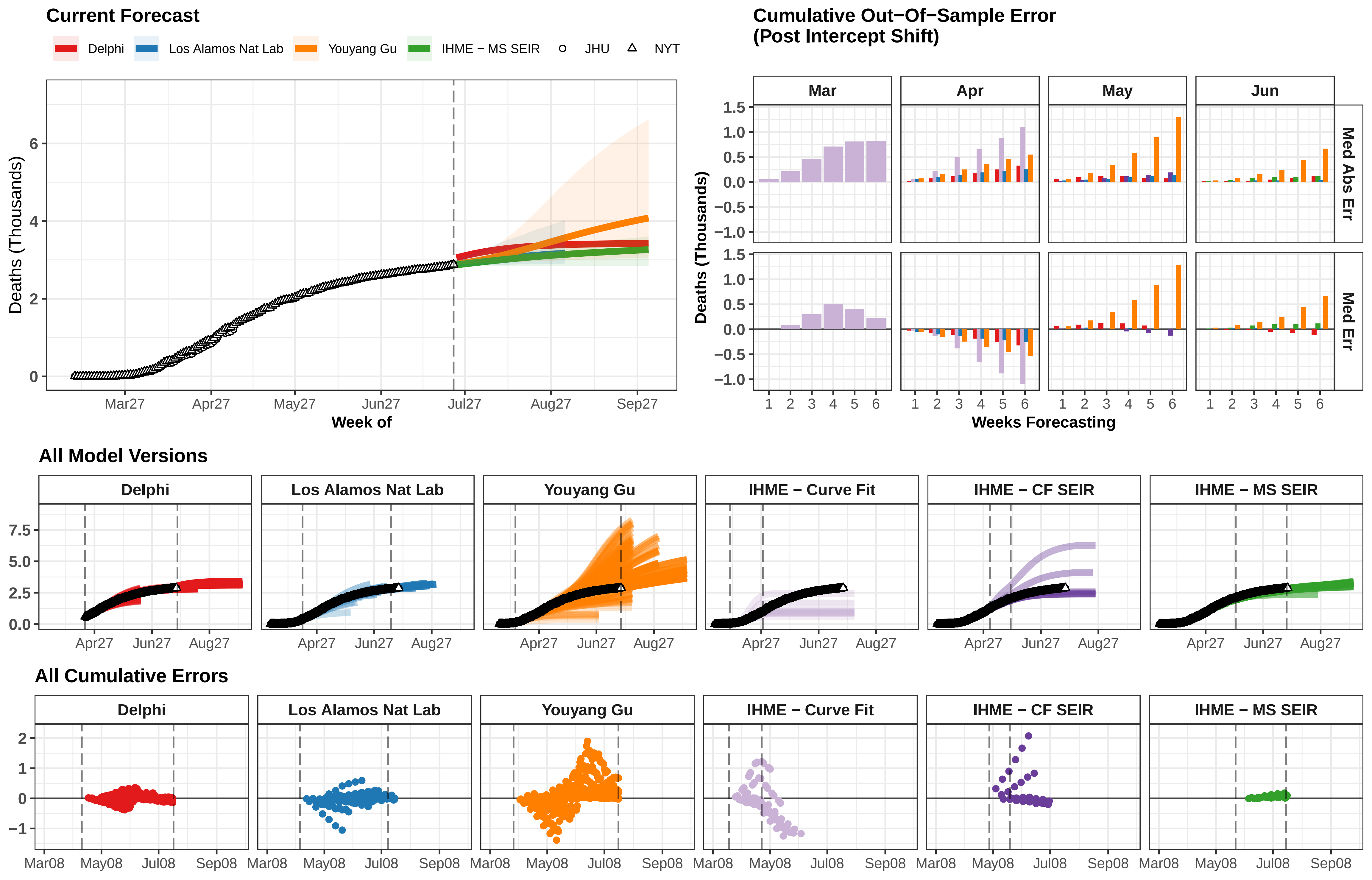

Current Forecast
Delphi
Los Alamos Nat Lab
Youyang Gu
IHME − MS SEIR
JHU
NYT
Deaths (Thousands)
Week of
Mar27
Apr27
May27
Jun27
Jul27
Aug27
Sep27
Cumulative Out−Of−Sample Error
(Post Intercept Shift)
Mar
Apr
May
Jun
Med Abs Err
Med Err
Weeks Forecasting
All Model Versions
Delphi
Los Alamos Nat Lab
Youyang Gu
IHME − Curve Fit
IHME − CF SEIR
IHME − MS SEIR
All Cumulative Errors
Mar08
May08
Jul08
Sep08

Bangladesh

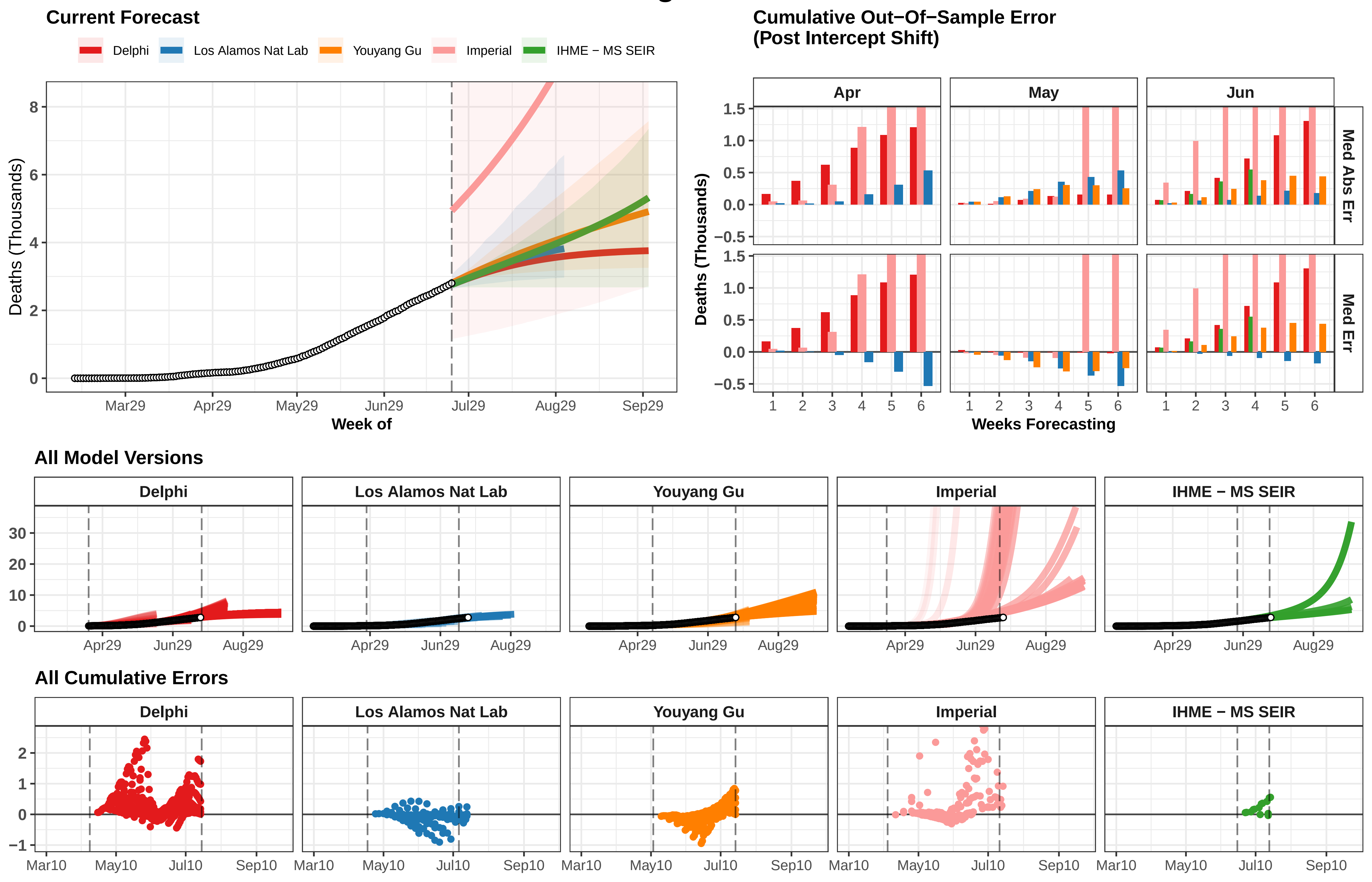

Current Forecast
Delphi
Los Alamos Nat Lab
Youyang Gu
Imperial
IHME − MS SEIR
Deaths (Thousands)
0
2
4
6
8
Mar29
Apr29
May29
Jun29
Jul29
Aug29
Sep29
Week of
Cumulative Out−Of−Sample Error
(Post Intercept Shift)
Apr
May
Jun
Med Abs Err
Med Err
Deaths (Thousands)
1.5
1.0
0.5
0.0
−0.5
1
2
3
4
5
6
Weeks Forecasting
All Model Versions
Delphi
Los Alamos Nat Lab
Youyang Gu
Imperial
IHME − MS SEIR
30
20
10
0
Apr29
Jun29
Aug29
All Cumulative Errors
Delphi
Los Alamos Nat Lab
Youyang Gu
Imperial
IHME − MS SEIR
2
1
0
−1
Mar10
May10
Jul10
Sep10

Argentina

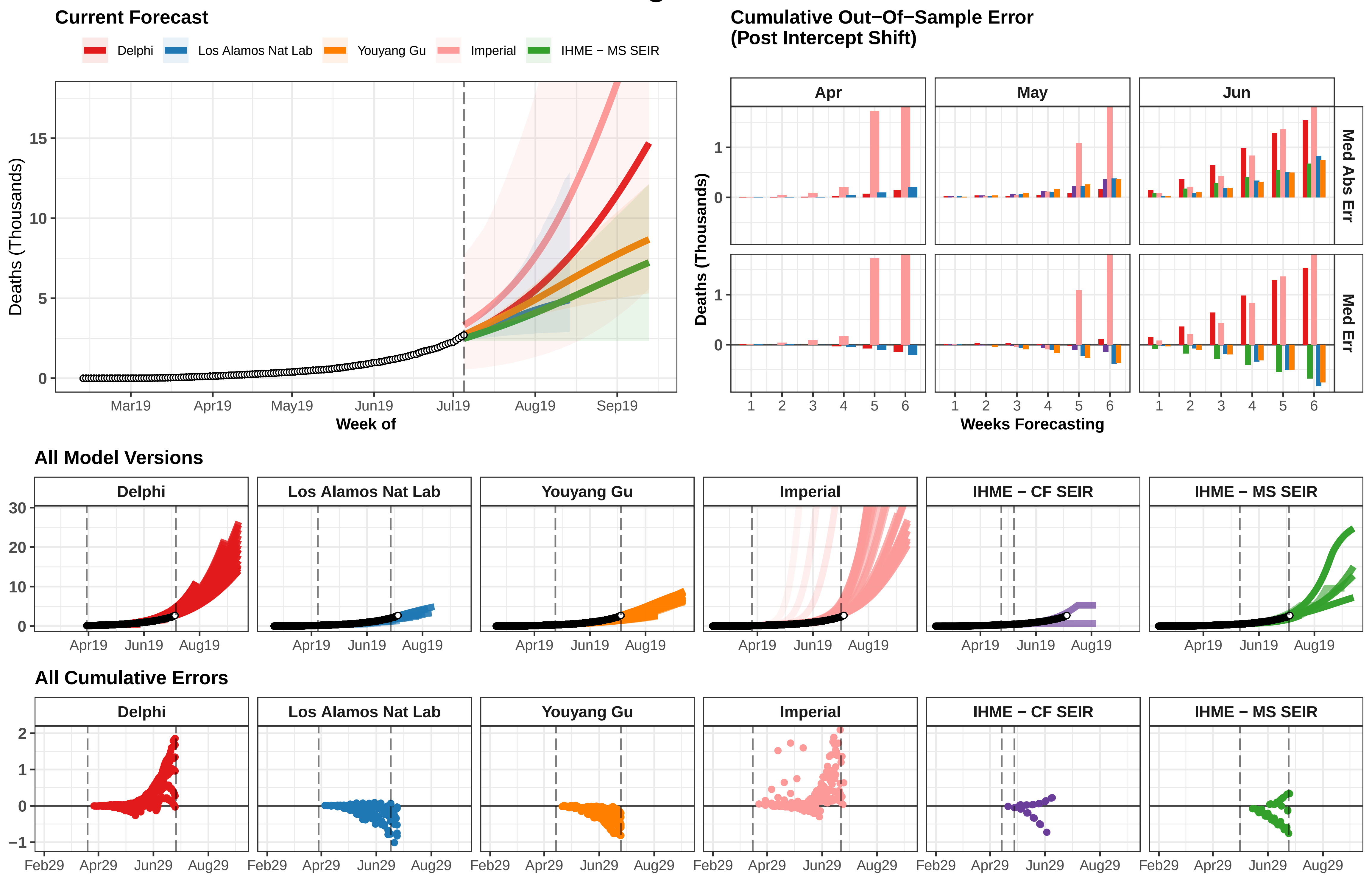

Current Forecast
Delphi
Los Alamos Nat Lab
Youyang Gu
Imperial
IHME − MS SEIR
Deaths (Thousands)
Week of
Mar19
Apr19
May19
Jun19
Jul19
Aug19
Sep19
Cumulative Out−Of−Sample Error
(Post Intercept Shift)
Apr
May
Jun
Med Abs Err
Med Err
Weeks Forecasting
All Model Versions
IHME − CF SEIR
All Cumulative Errors
Feb29
Apr29
Jun29
Aug29

Saudi Arabia

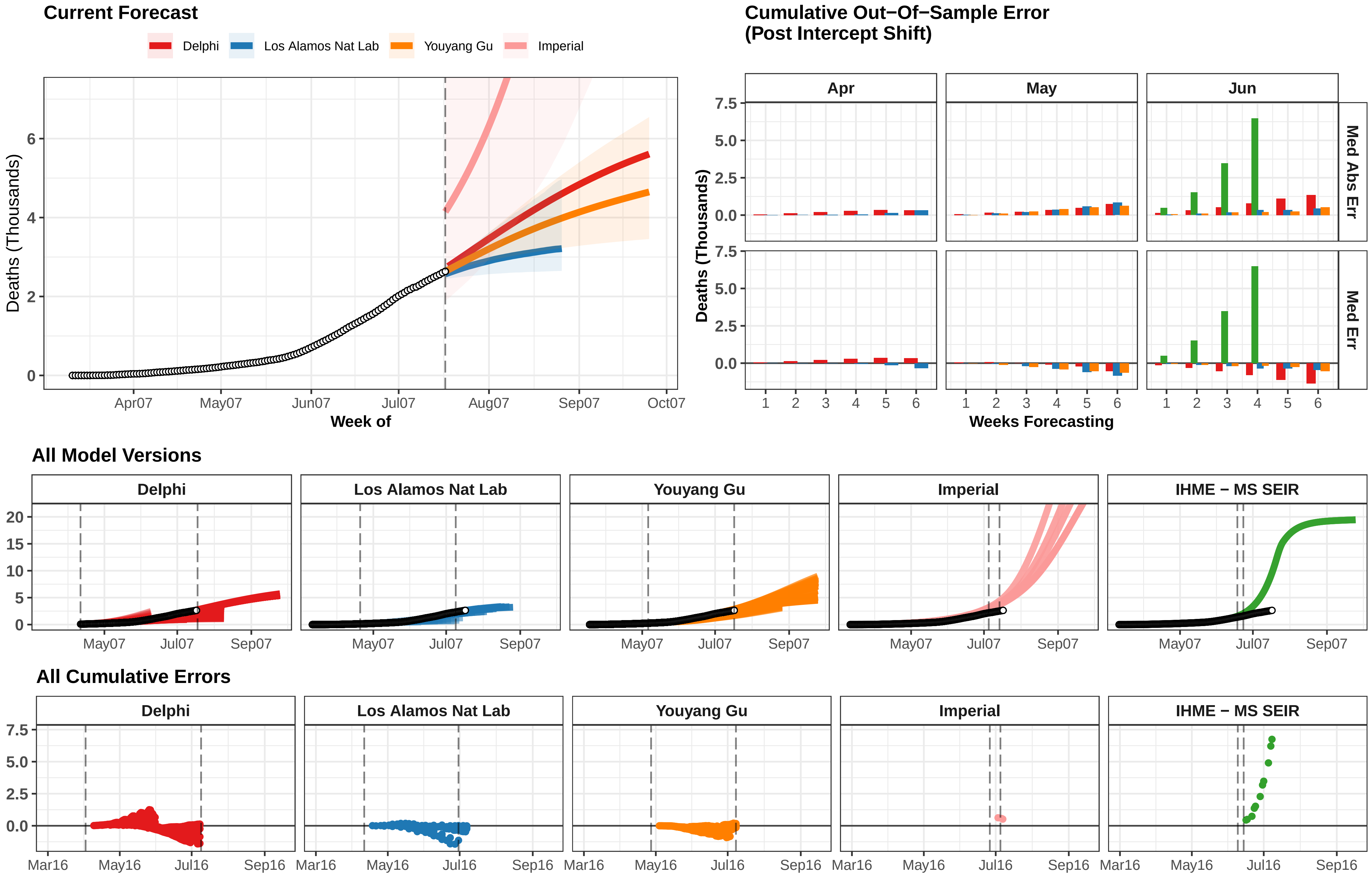

Bolivia

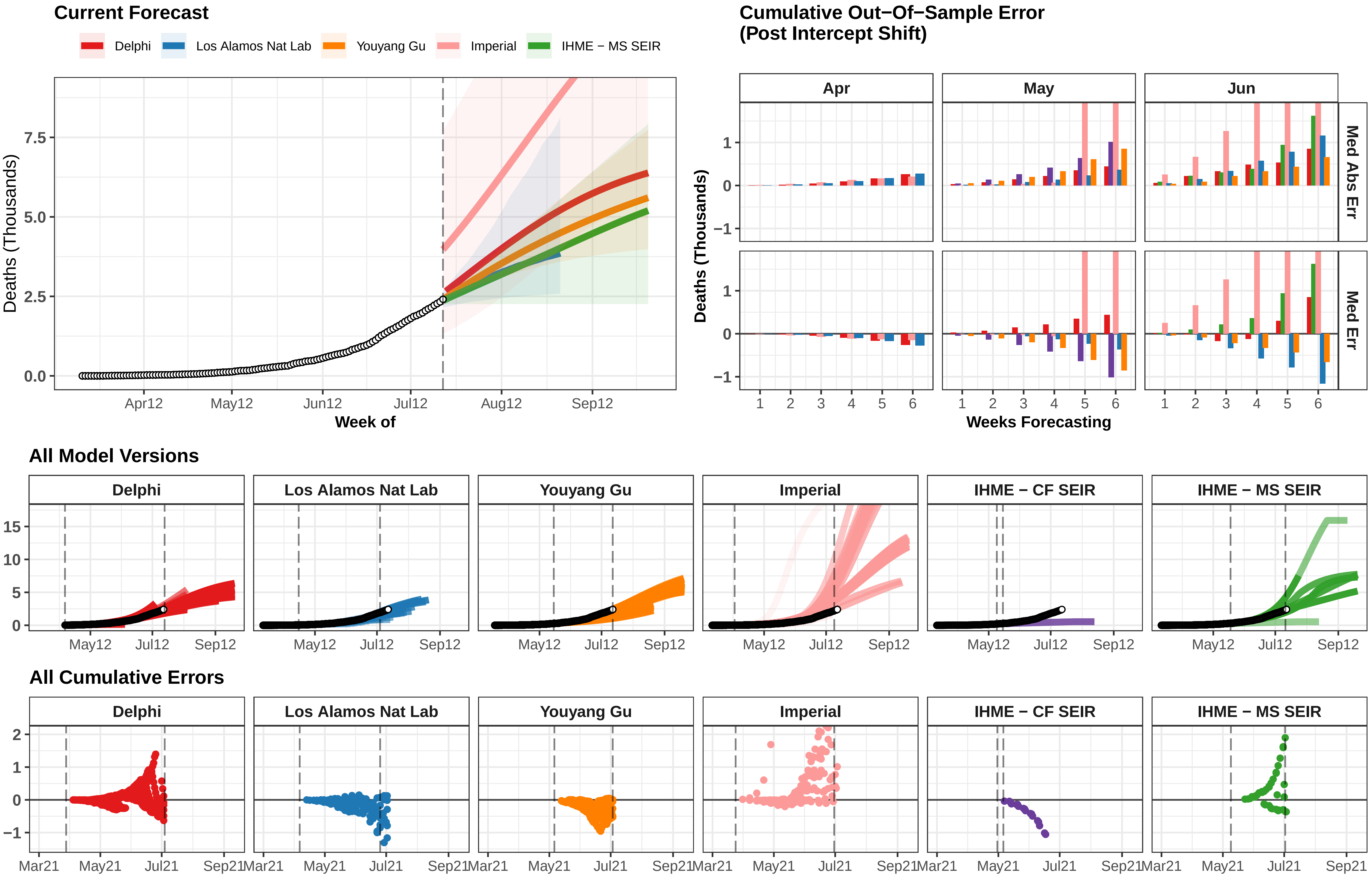
Current Forecast
Delphi
Los Alamos Nat Lab
Youyang Gu
Imperial
IHME − MS SEIR
Deaths (Thousands)
Week of
Apr12
May12
Jun12
Jul12
Aug12
Sep12
Cumulative Out−Of−Sample Error
(Post Intercept Shift)
Apr
May
Jun
Med Abs Err
Med Err
Weeks Forecasting
All Model Versions
Delphi
Los Alamos Nat Lab
Youyang Gu
Imperial
IHME − CF SEIR
IHME − MS SEIR
All Cumulative Errors
Mar21
May21
Jul21
Sep21

Romania

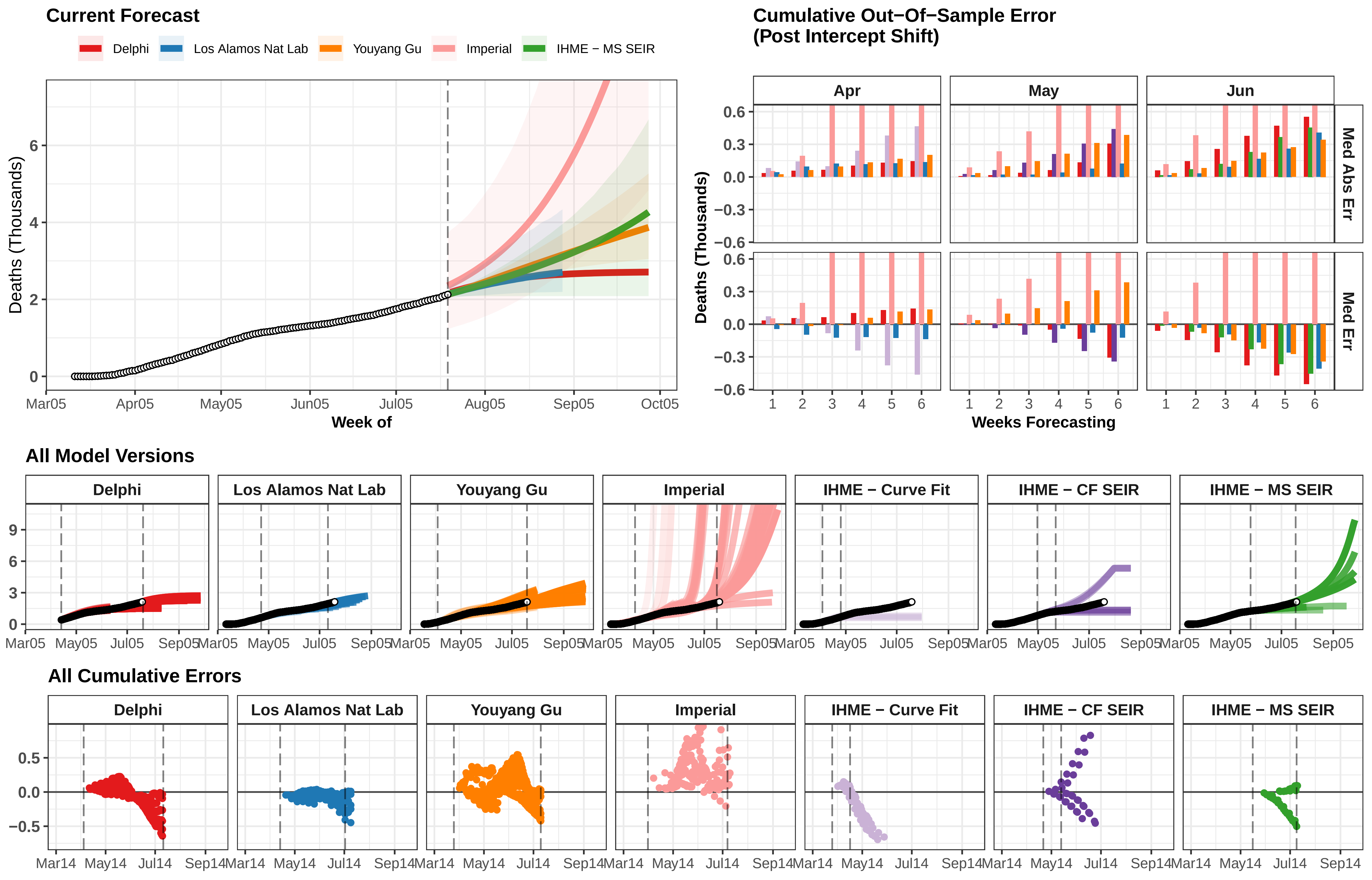
Current Forecast
Delphi
Los Alamos Nat Lab
Youyang Gu
Imperial
IHME − MS SEIR
Deaths (Thousands)
Week of
Mar05
Apr05
May05
Jun05
Jul05
Aug05
Sep05
Oct05
Cumulative Out−Of−Sample Error
(Post Intercept Shift)
Apr
May
Jun
Med Abs Err
Med Err
Weeks Forecasting
All Model Versions
Delphi
Los Alamos Nat Lab
Youyang Gu
Imperial
IHME − Curve Fit
IHME − CF SEIR
IHME − MS SEIR
All Cumulative Errors
Mar14
May14
Jul14
Sep14

Virginia

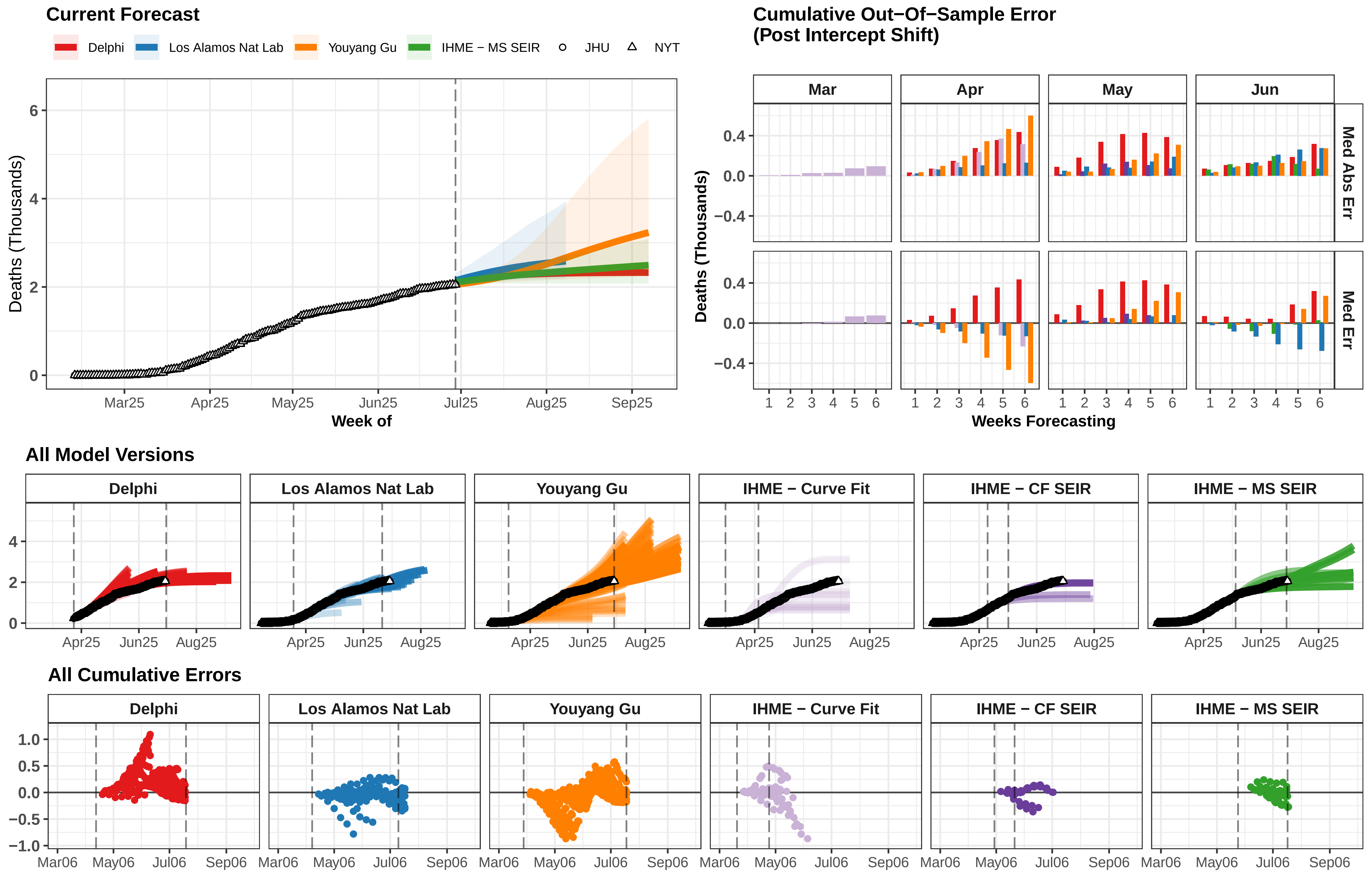

Current Forecast
Delphi
Los Alamos Nat Lab
Youyang Gu
IHME − MS SEIR
JHU
NYT
Deaths (Thousands)
Week of
Mar25
Apr25
May25
Jun25
Jul25
Aug25
Sep25
Cumulative Out−Of−Sample Error (Post Intercept Shift)
Mar
Apr
May
Jun
Med Abs Err
Med Err
Weeks Forecasting
All Model Versions
Delphi
Los Alamos Nat Lab
Youyang Gu
IHME − Curve Fit
IHME − CF SEIR
IHME − MS SEIR
All Cumulative Errors
Mar06
May06
Jul06
Sep06

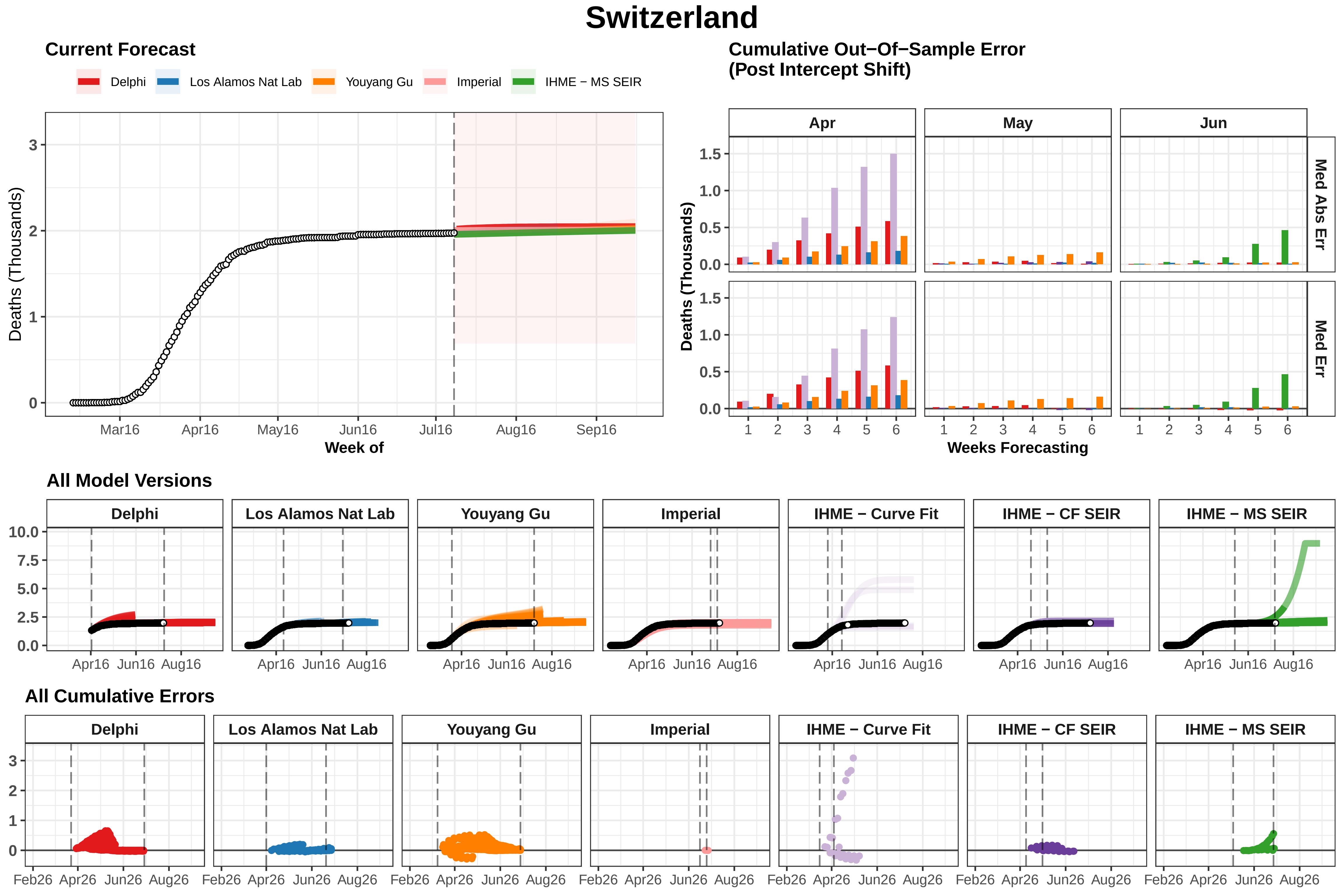
Switzerland
Current Forecast
Delphi
Los Alamos Nat Lab
Youyang Gu
Imperial
IHME − MS SEIR
Deaths (Thousands)
Week of
Mar16
Apr16
May16
Jun16
Jul16
Aug16
Sep16
Cumulative Out−Of−Sample Error (Post Intercept Shift)
Apr
May
Jun
Med Abs Err
Med Err
Weeks Forecasting
All Model Versions
Delphi
Los Alamos Nat Lab
Youyang Gu
Imperial
IHME − Curve Fit
IHME − CF SEIR
IHME − MS SEIR
All Cumulative Errors
Feb26
Apr26
Jun26
Aug26

Philippines

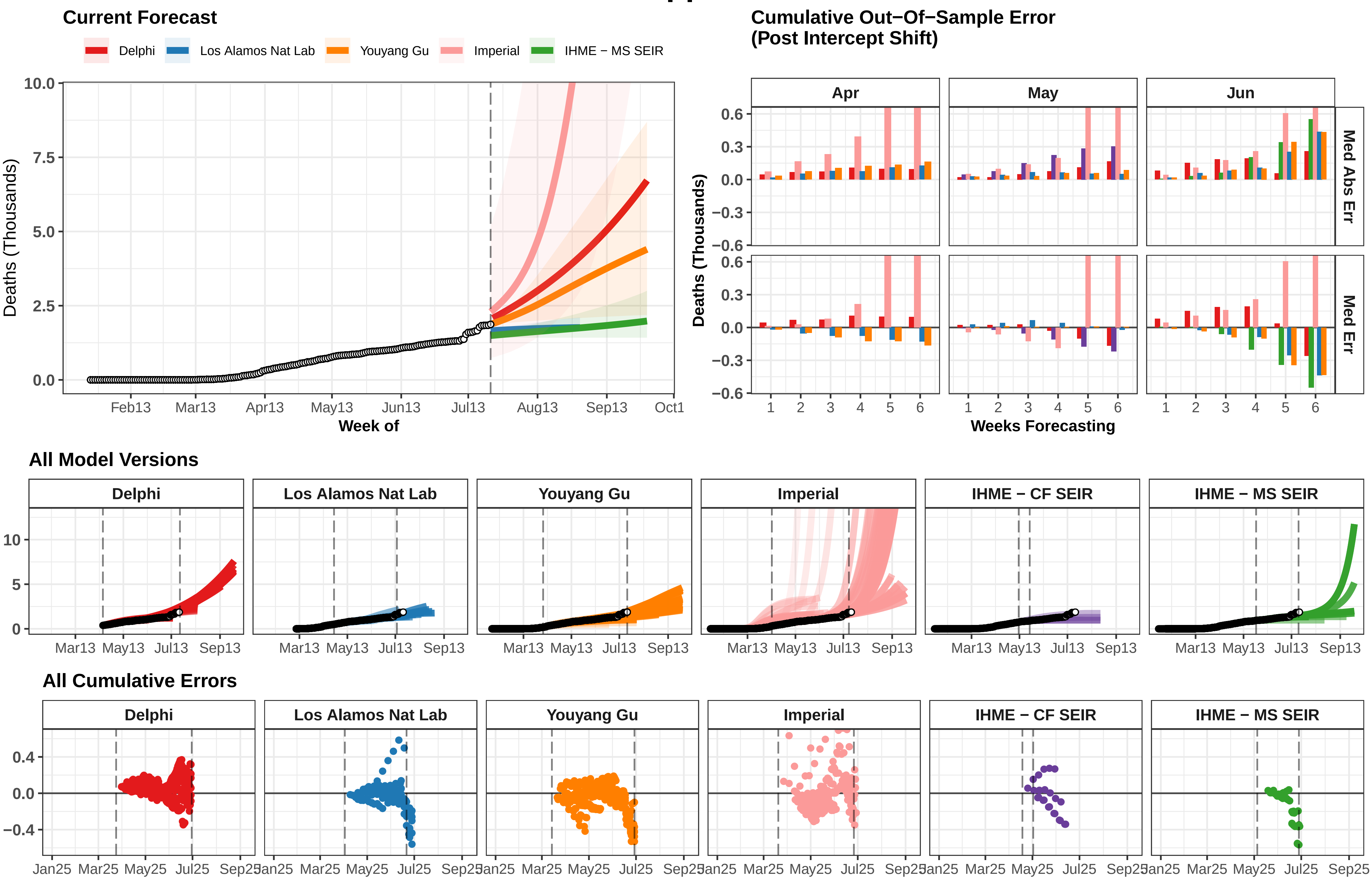

Colorado

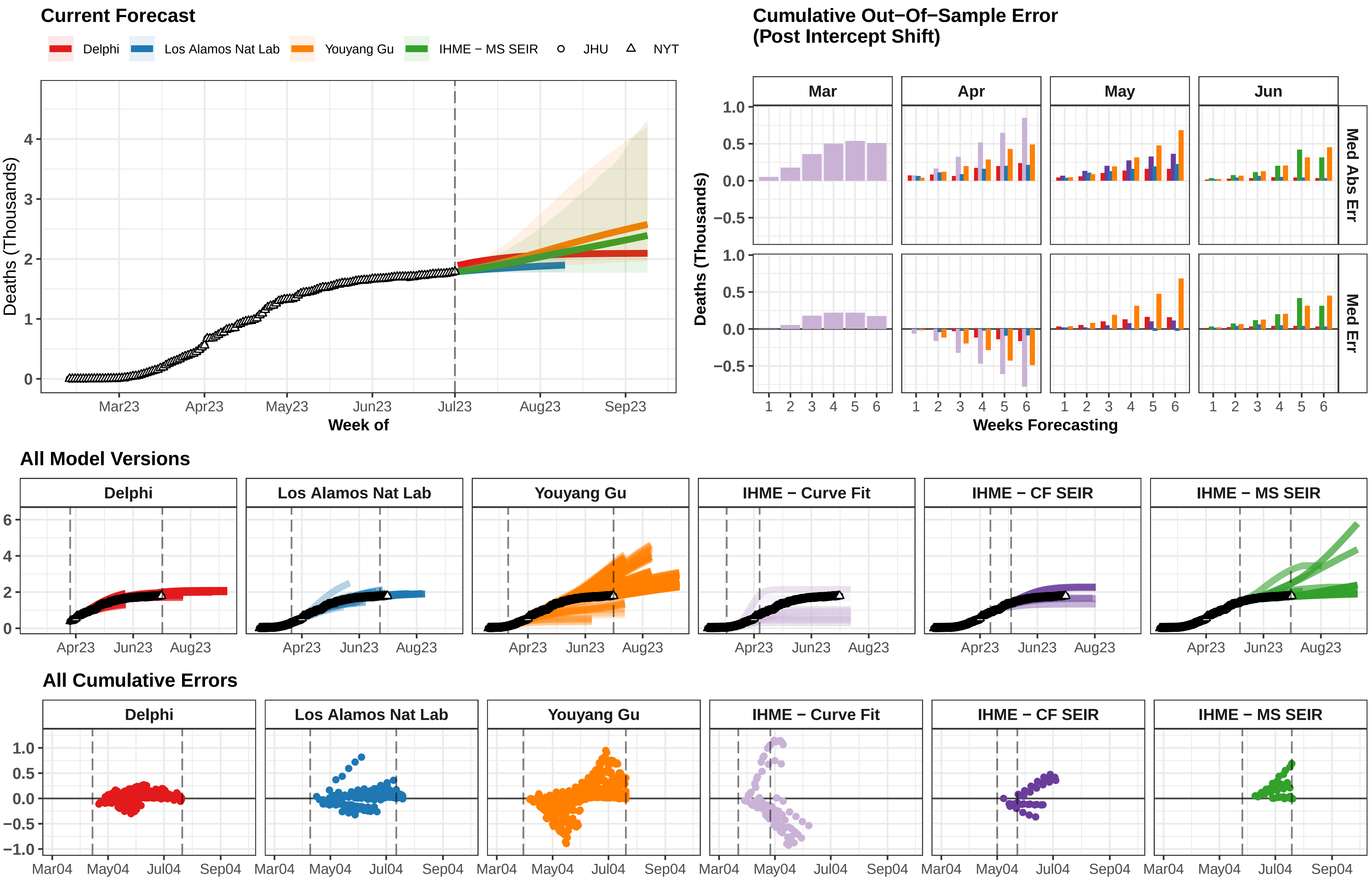
Current Forecast
Delphi
Los Alamos Nat Lab
Youyang Gu
IHME − MS SEIR
JHU
NYT
Deaths (Thousands)
Week of
Mar23
Apr23
May23
Jun23
Jul23
Aug23
Sep23
Cumulative Out−Of−Sample Error
(Post Intercept Shift)
Mar
Apr
May
Jun
Med Abs Err
Med Err
Weeks Forecasting
All Model Versions
Delphi
Los Alamos Nat Lab
Youyang Gu
IHME − Curve Fit
IHME − CF SEIR
IHME − MS SEIR
All Cumulative Errors
Mar04
May04
Jul04
Sep04

Ireland

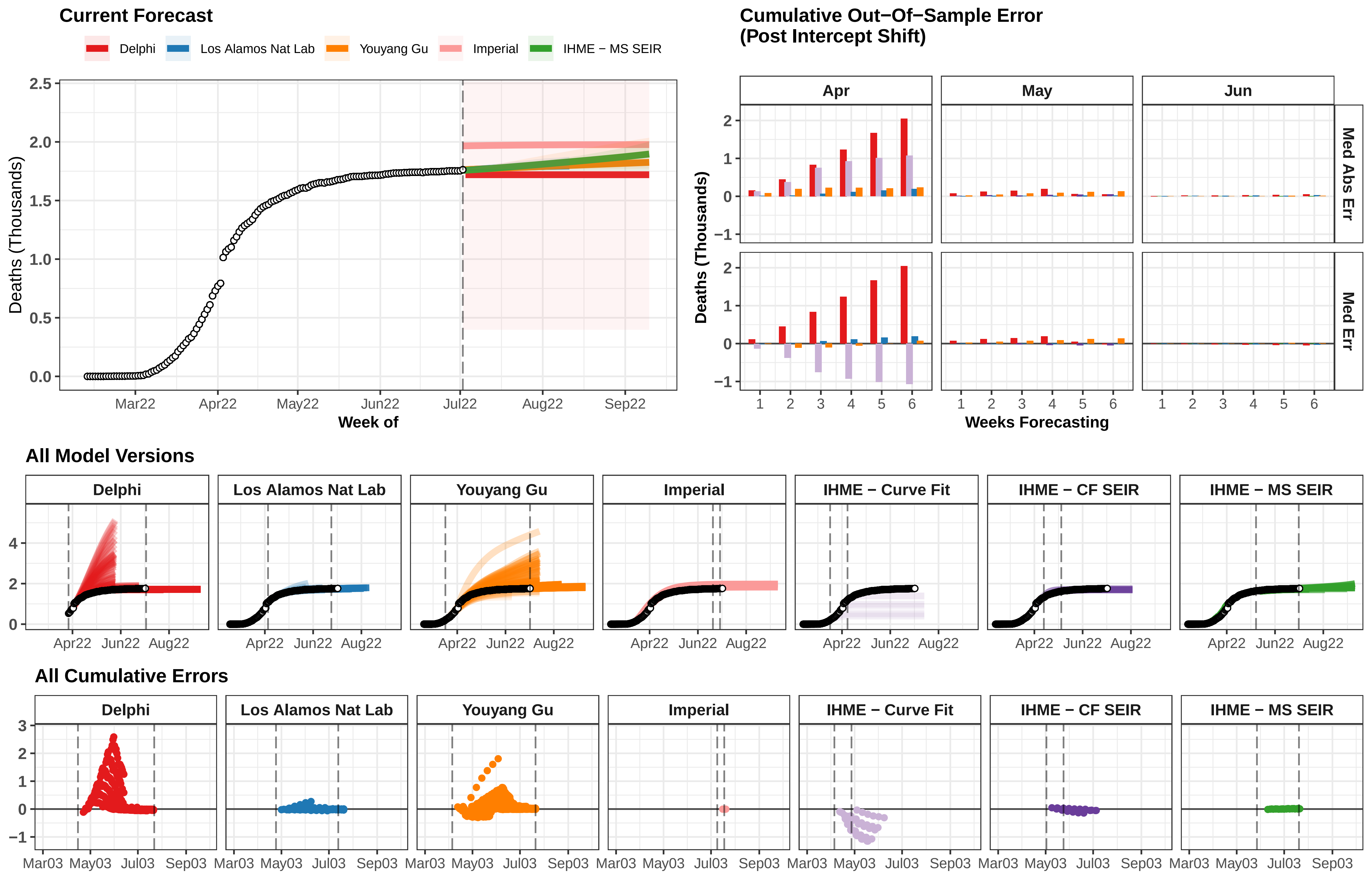

Current Forecast
Delphi
Los Alamos Nat Lab
Youyang Gu
Imperial
IHME − MS SEIR
Deaths (Thousands)
Week of
Mar22
Apr22
May22
Jun22
Jul22
Aug22
Sep22
Cumulative Out−Of−Sample Error
(Post Intercept Shift)
Apr
May
Jun
Med Abs Err
Med Err
Weeks Forecasting
All Model Versions
IHME − Curve Fit
IHME − CF SEIR
All Cumulative Errors
Mar03
May03
Jul03
Sep03

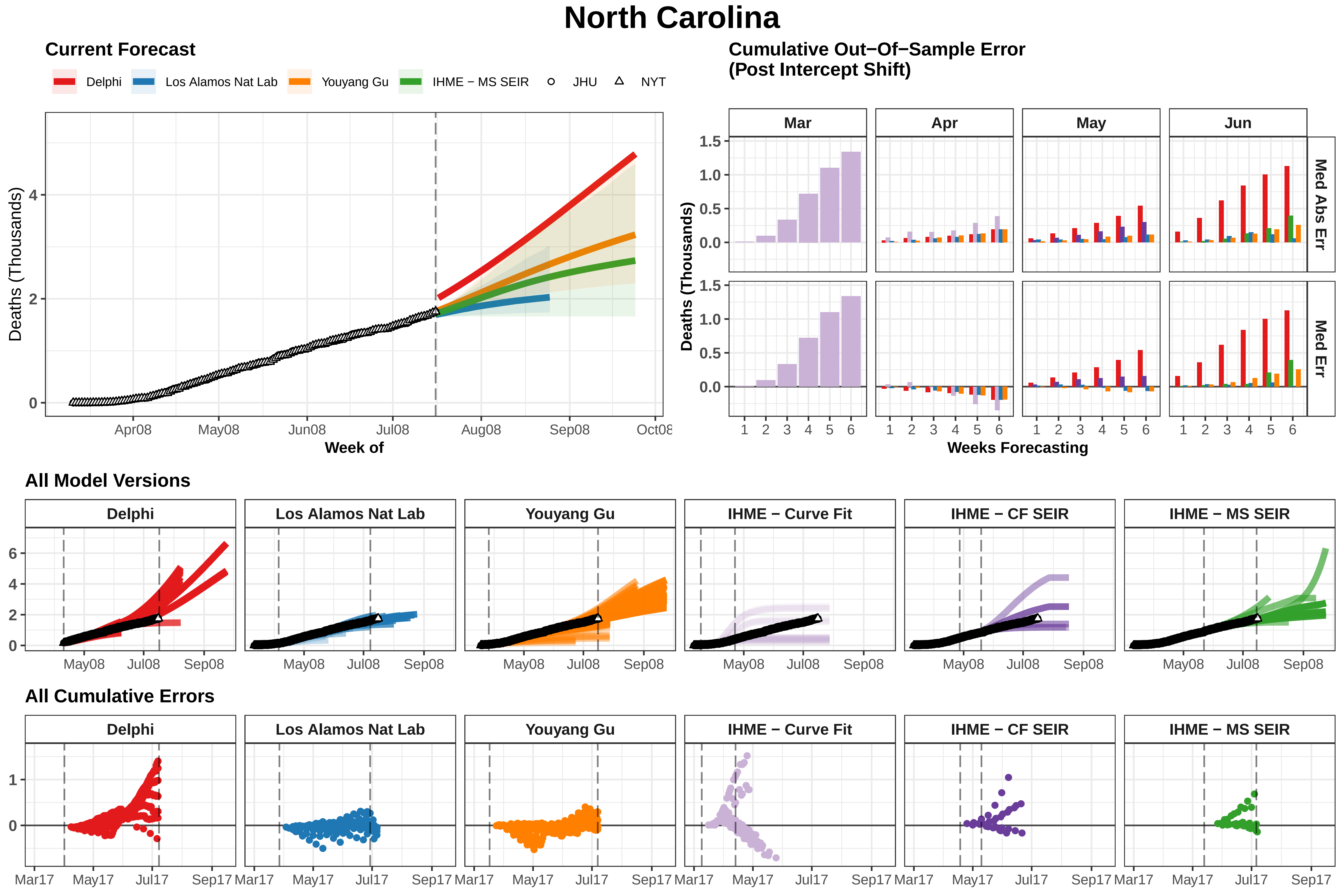
North Carolina
Current Forecast
Delphi
Los Alamos Nat Lab
Youyang Gu
IHME − MS SEIR
JHU
NYT
Deaths (Thousands)
Week of
Apr08
May08
Jun08
Jul08
Aug08
Sep08
Oct08
Cumulative Out−Of−Sample Error
(Post Intercept Shift)
Mar
Apr
May
Jun
Med Abs Err
Med Err
Weeks Forecasting
All Model Versions
Delphi
Los Alamos Nat Lab
Youyang Gu
IHME − Curve Fit
IHME − CF SEIR
IHME − MS SEIR
All Cumulative Errors
Mar17
May17
Jul17
Sep17

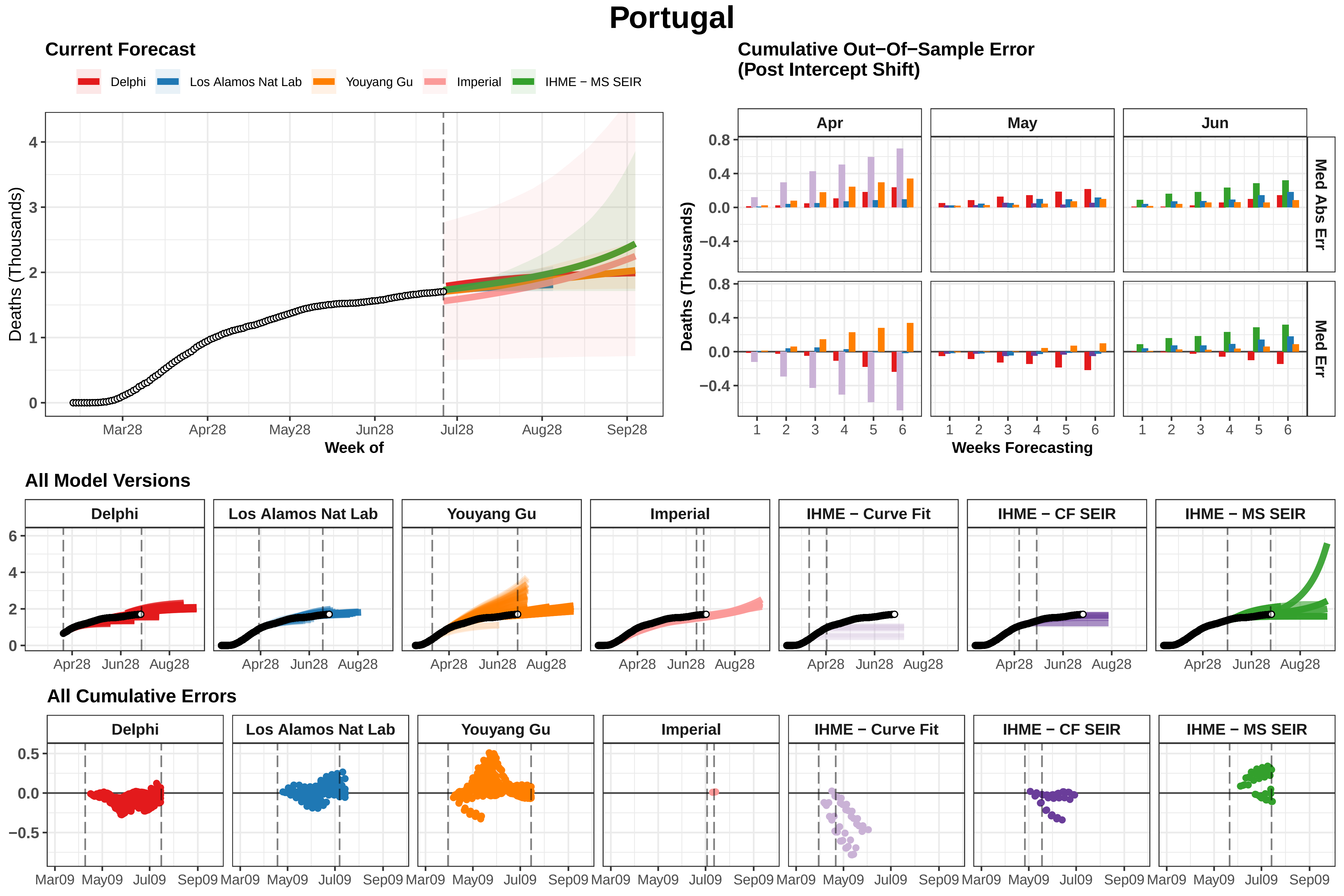

Portugal
Current Forecast
Delphi
Los Alamos Nat Lab
Youyang Gu
Imperial
IHME − MS SEIR
Deaths (Thousands)
Week of
Mar28
Apr28
May28
Jun28
Jul28
Aug28
Sep28
Cumulative Out−Of−Sample Error
(Post Intercept Shift)
Apr
May
Jun
Med Abs Err
Med Err
Weeks Forecasting
All Model Versions
Delphi
Los Alamos Nat Lab
Youyang Gu
Imperial
IHME − Curve Fit
IHME − CF SEIR
IHME − MS SEIR
All Cumulative Errors
Mar09
May09
Jul09
Sep09

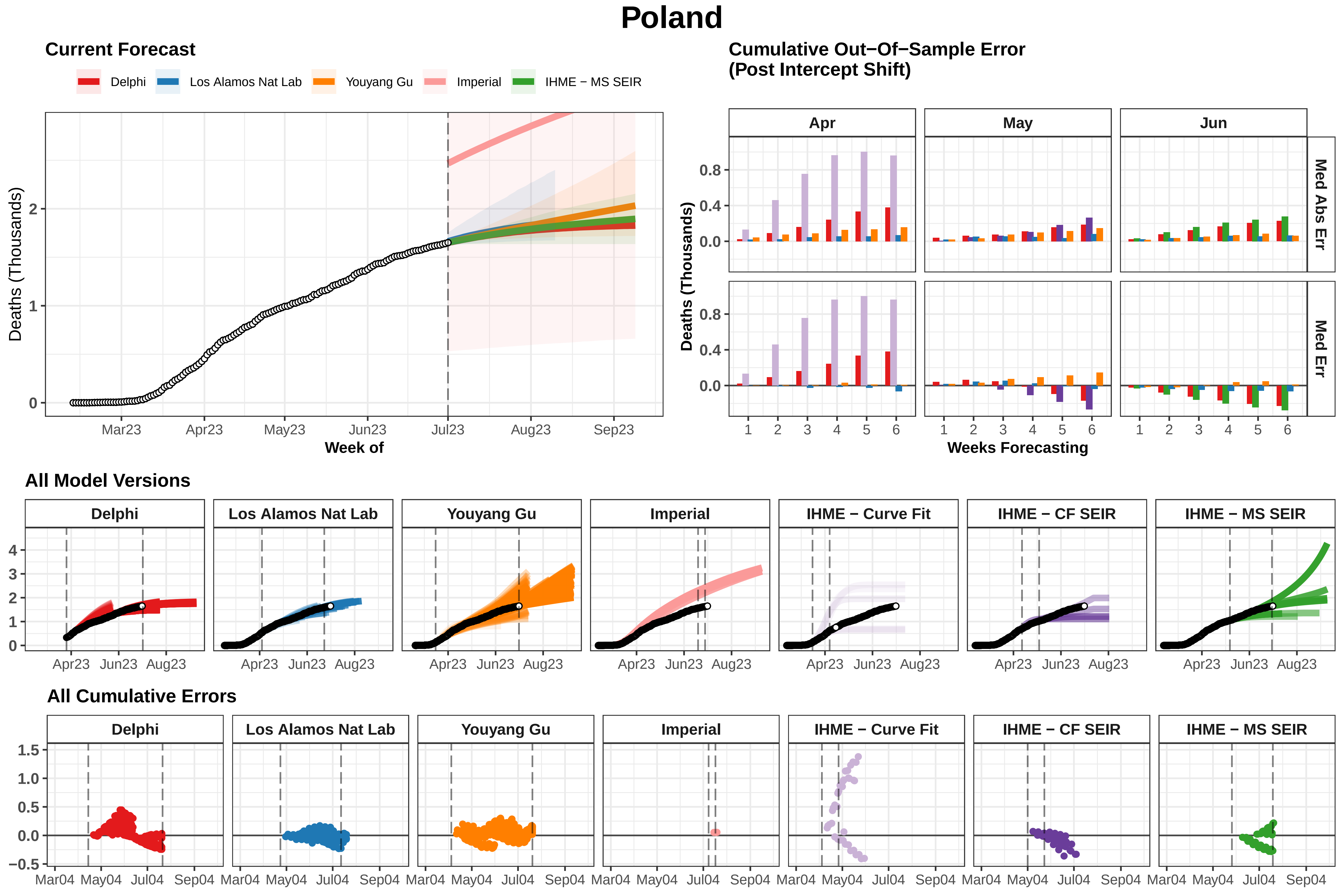

Poland
Current Forecast
Delphi
Los Alamos Nat Lab
Youyang Gu
Imperial
IHME − MS SEIR
Deaths (Thousands)
Week of
Mar23
Apr23
May23
Jun23
Jul23
Aug23
Sep23
Cumulative Out−Of−Sample Error
(Post Intercept Shift)
Apr
May
Jun
Med Abs Err
Med Err
Weeks Forecasting
All Model Versions
IHME − Curve Fit
IHME − CF SEIR
All Cumulative Errors
Mar04
May04
Jul04
Sep04

Guatemala

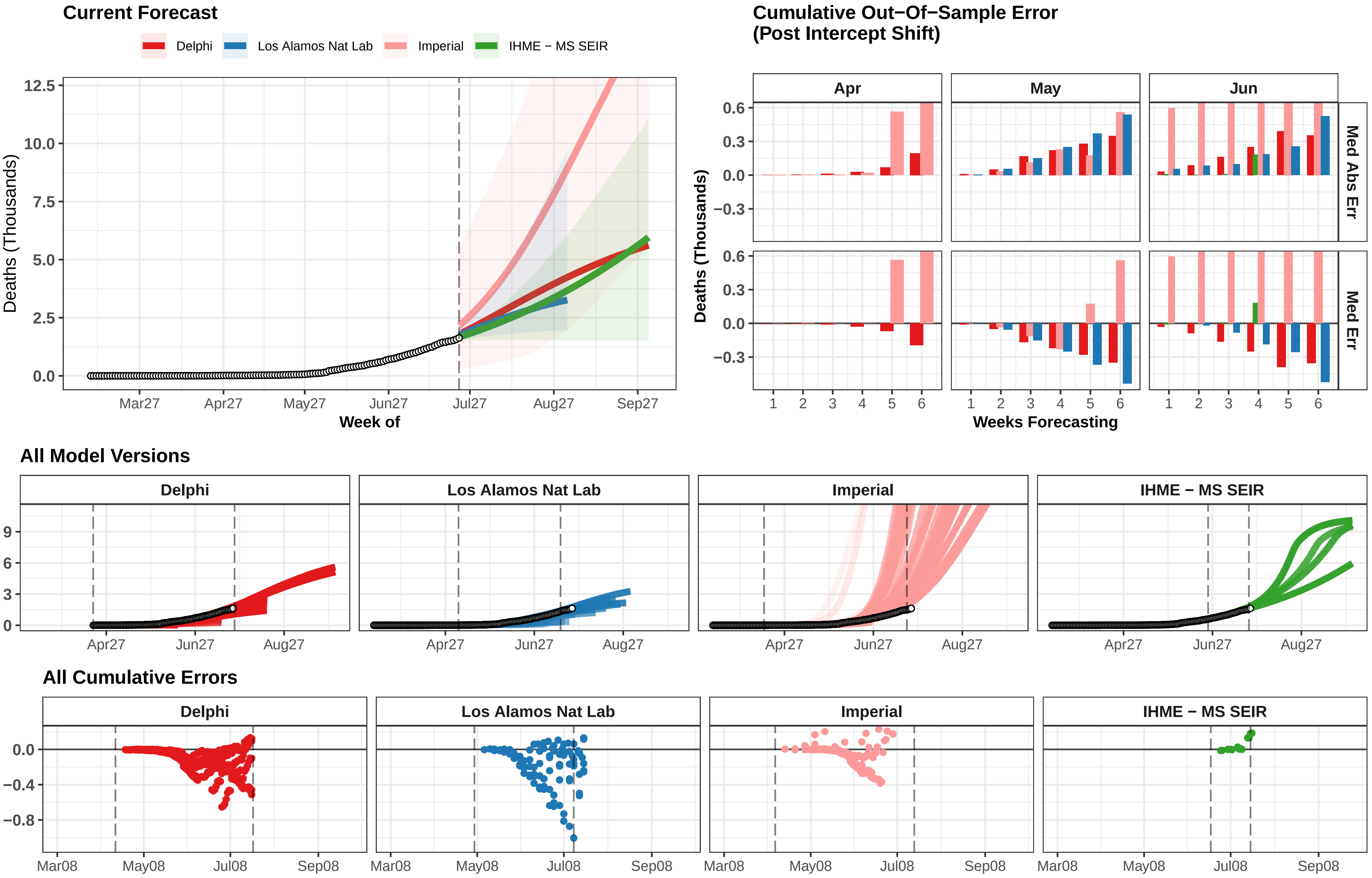

Current Forecast
Delphi
Los Alamos Nat Lab
Imperial
IHME − MS SEIR
Deaths (Thousands)
Week of
Mar27
Apr27
May27
Jun27
Jul27
Aug27
Sep27
Cumulative Out−Of−Sample Error
(Post Intercept Shift)
Apr
May
Jun
Med Abs Err
Med Err
Weeks Forecasting
All Model Versions
All Cumulative Errors
Mar08
May08
Jul08
Sep08

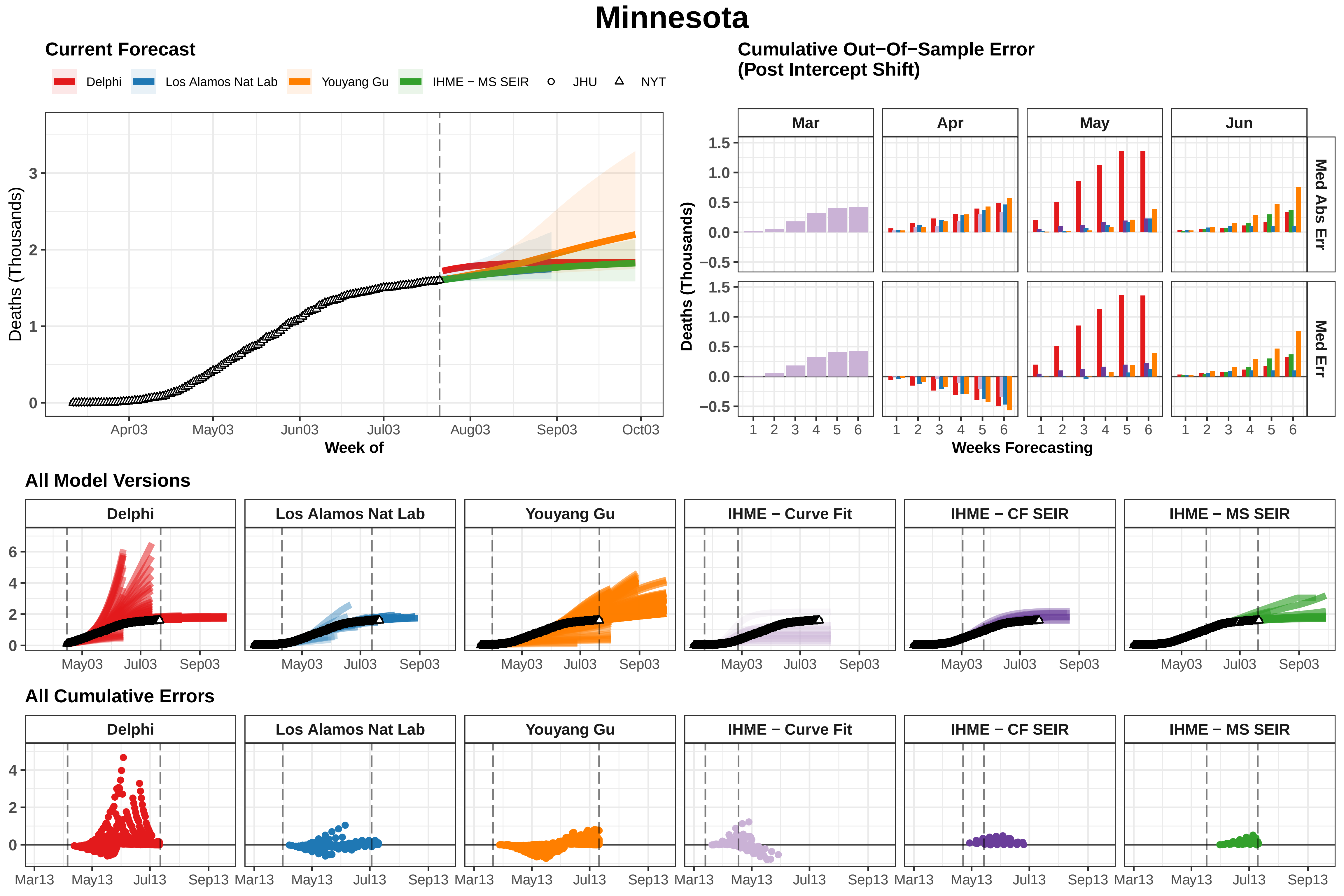

Minnesota
Current Forecast
Delphi
Los Alamos Nat Lab
Youyang Gu
IHME − MS SEIR
JHU
NYT
Deaths (Thousands)
Week of
Apr03
May03
Jun03
Jul03
Aug03
Sep03
Oct03
Cumulative Out−Of−Sample Error
(Post Intercept Shift)
Mar
Apr
May
Jun
Med Abs Err
Med Err
Weeks Forecasting
All Model Versions
Delphi
Los Alamos Nat Lab
Youyang Gu
IHME − Curve Fit
IHME − CF SEIR
IHME − MS SEIR
All Cumulative Errors
Mar13
May13
Jul13
Sep13

Ukraine

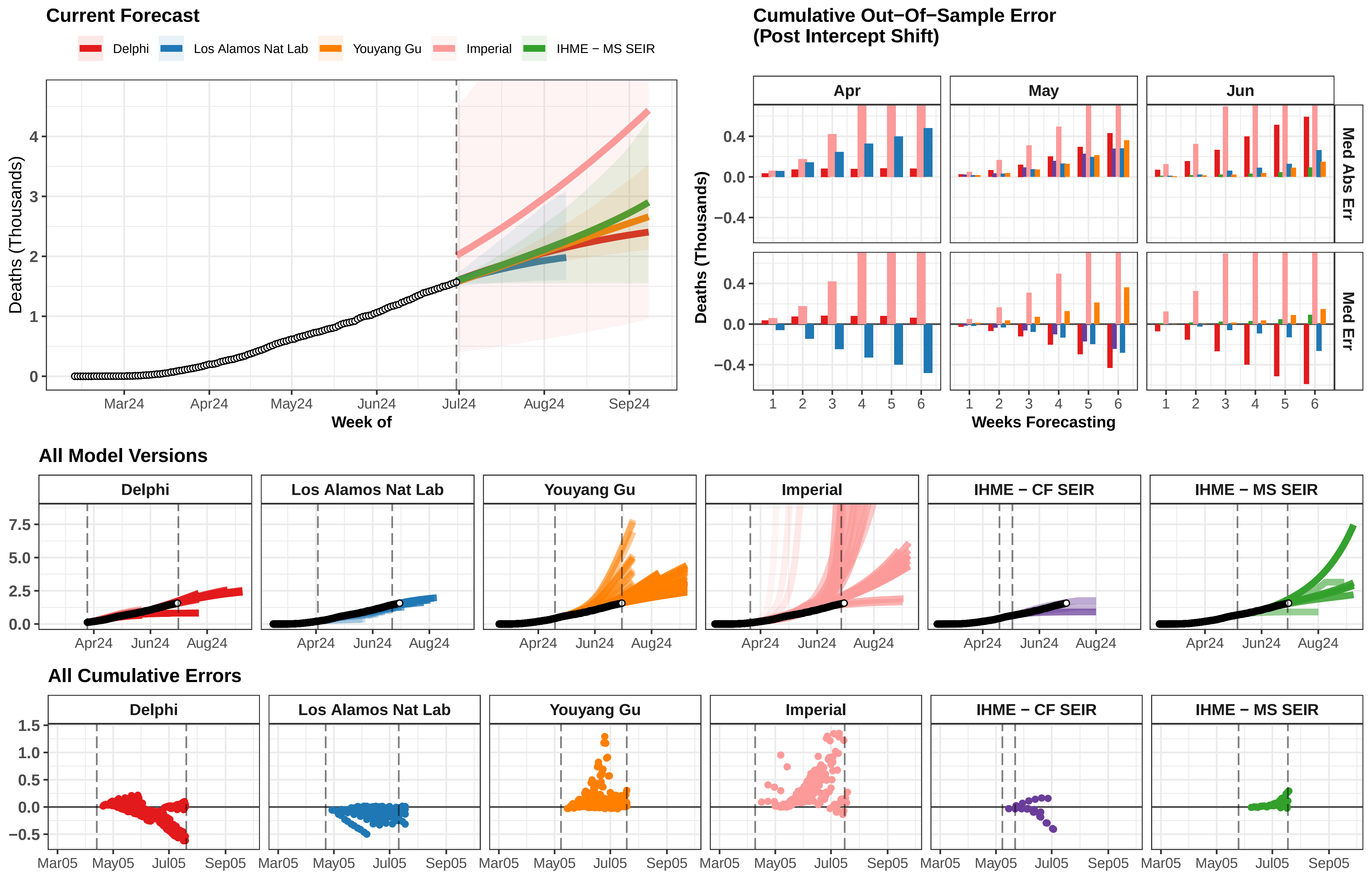

Current Forecast
Delphi
Los Alamos Nat Lab
Youyang Gu
Imperial
IHME − MS SEIR
Deaths (Thousands)
0
1
2
3
4
Mar24
Apr24
May24
Jun24
Jul24
Aug24
Sep24
Week of
Cumulative Out−Of−Sample Error
(Post Intercept Shift)
Apr
May
Jun
Med Abs Err
Med Err
0.4
0.0
−0.4
1
2
3
4
5
6
Weeks Forecasting
All Model Versions
Delphi
Los Alamos Nat Lab
Youyang Gu
Imperial
IHME − CF SEIR
IHME − MS SEIR
7.5
5.0
2.5
0.0
Apr24
Jun24
Aug24
All Cumulative Errors
1.5
1.0
0.5
0.0
−0.5
Mar05
May05
Jul05
Sep05

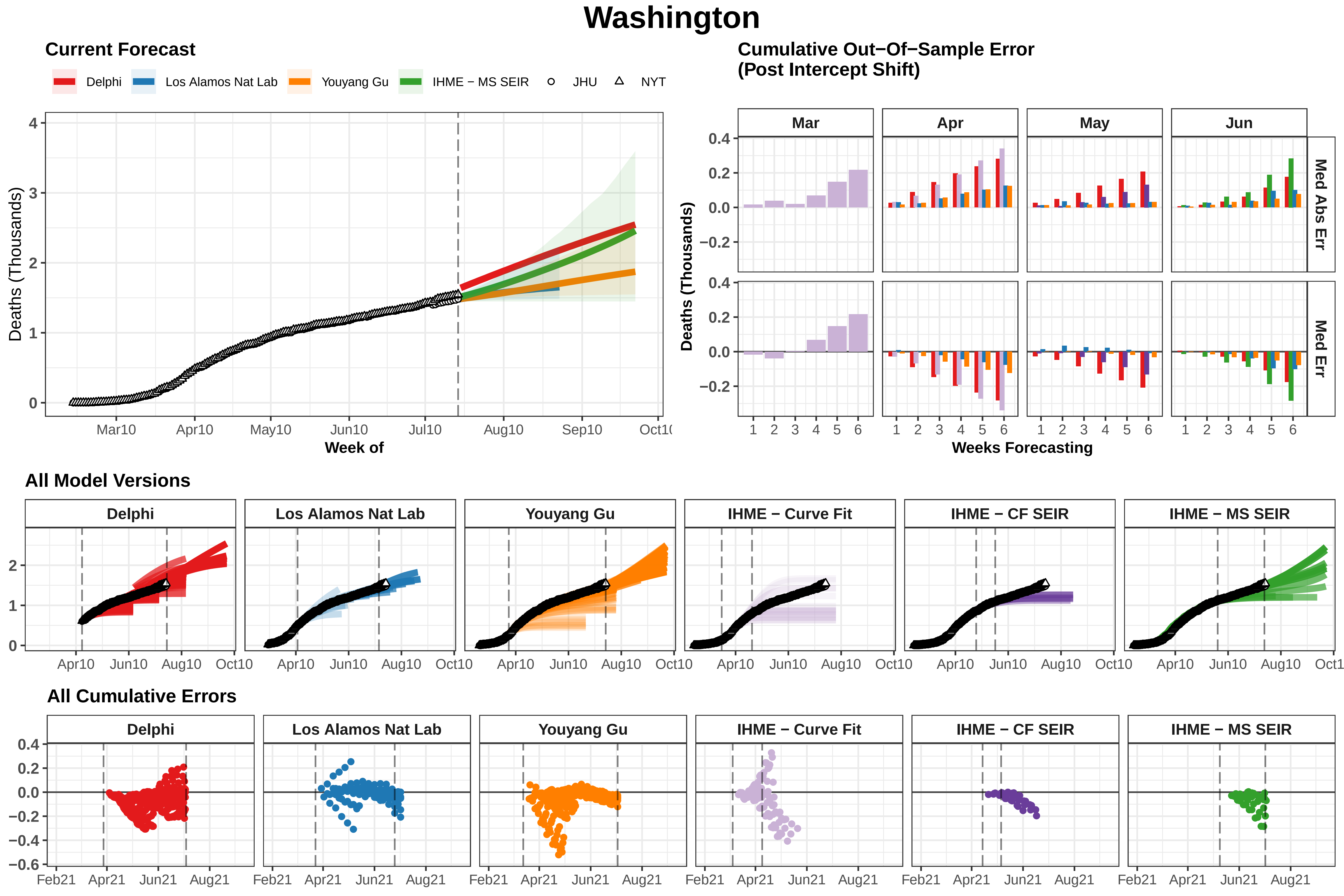
Washington
Current Forecast
Delphi
Los Alamos Nat Lab
Youyang Gu
IHME − MS SEIR
JHU
NYT
Deaths (Thousands)
Week of
Mar10
Apr10
May10
Jun10
Jul10
Aug10
Sep10
Cumulative Out−Of−Sample Error
(Post Intercept Shift)
Mar
Apr
May
Jun
Med Abs Err
Med Err
Weeks Forecasting
All Model Versions
Delphi
Los Alamos Nat Lab
Youyang Gu
IHME − Curve Fit
IHME − CF SEIR
IHME − MS SEIR
Apr10
Jun10
Aug10
Oct10
All Cumulative Errors
Feb21
Apr21
Jun21
Aug21

Mississippi

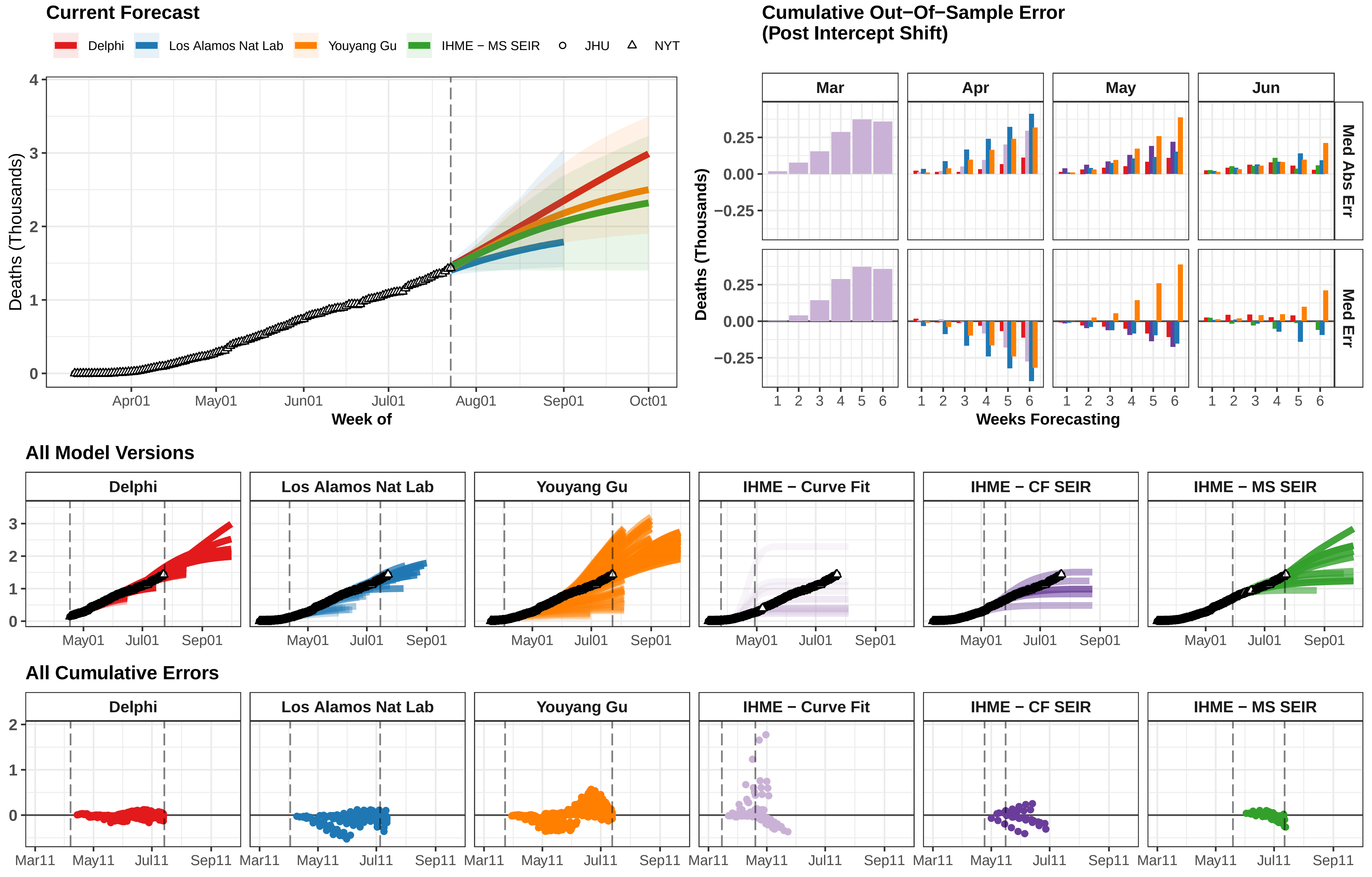

Alabama

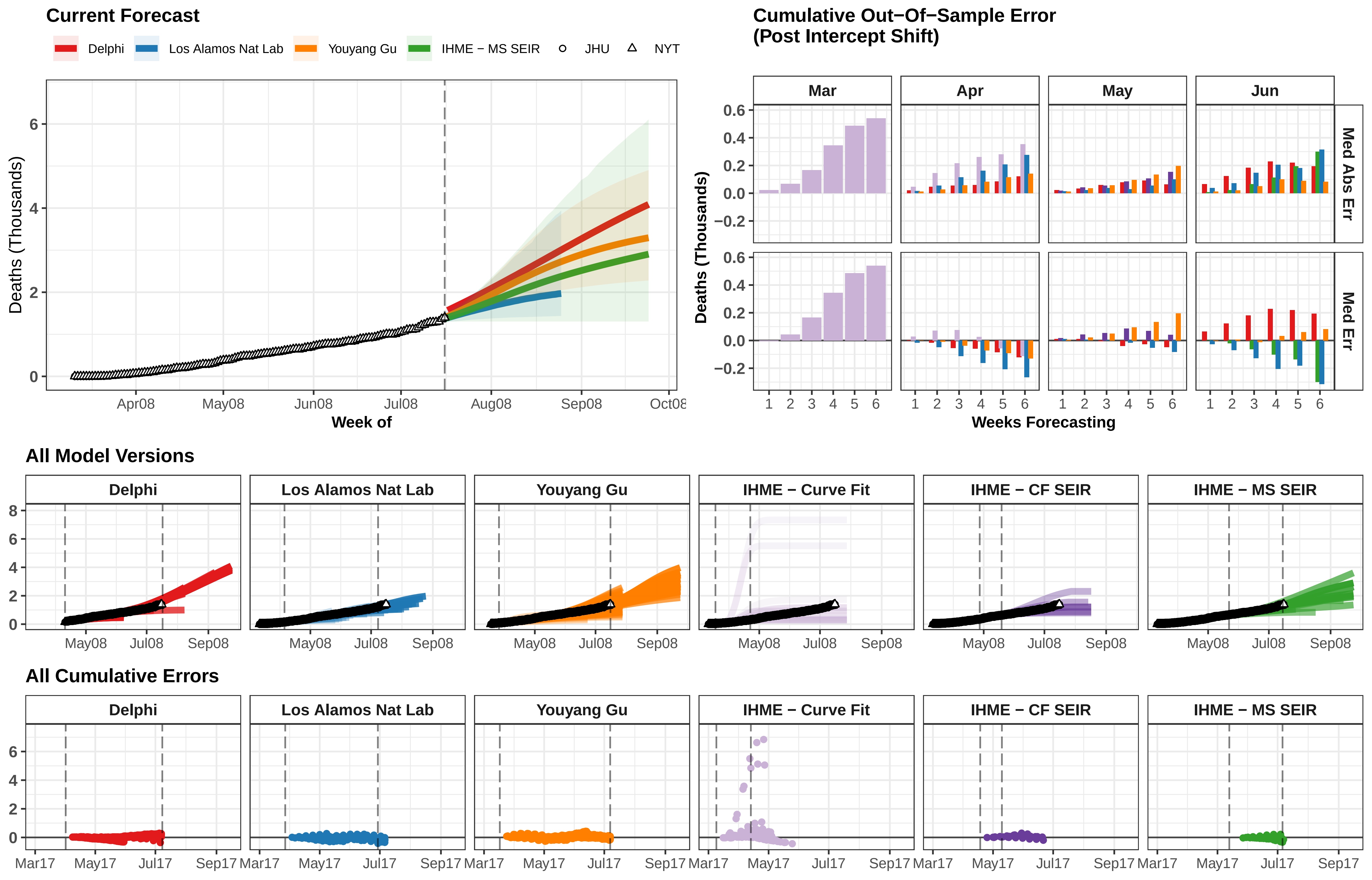
Current Forecast
Delphi
Los Alamos Nat Lab
Youyang Gu
IHME − MS SEIR
JHU
NYT
Deaths (Thousands)
Week of
Apr08
May08
Jun08
Jul08
Aug08
Sep08
Oct08
Cumulative Out−Of−Sample Error
(Post Intercept Shift)
Mar
Apr
May
Jun
Med Abs Err
Med Err
Weeks Forecasting
All Model Versions
Delphi
Los Alamos Nat Lab
Youyang Gu
IHME − Curve Fit
IHME − CF SEIR
IHME − MS SEIR
All Cumulative Errors
Mar17
May17
Jul17
Sep17

South Carolina

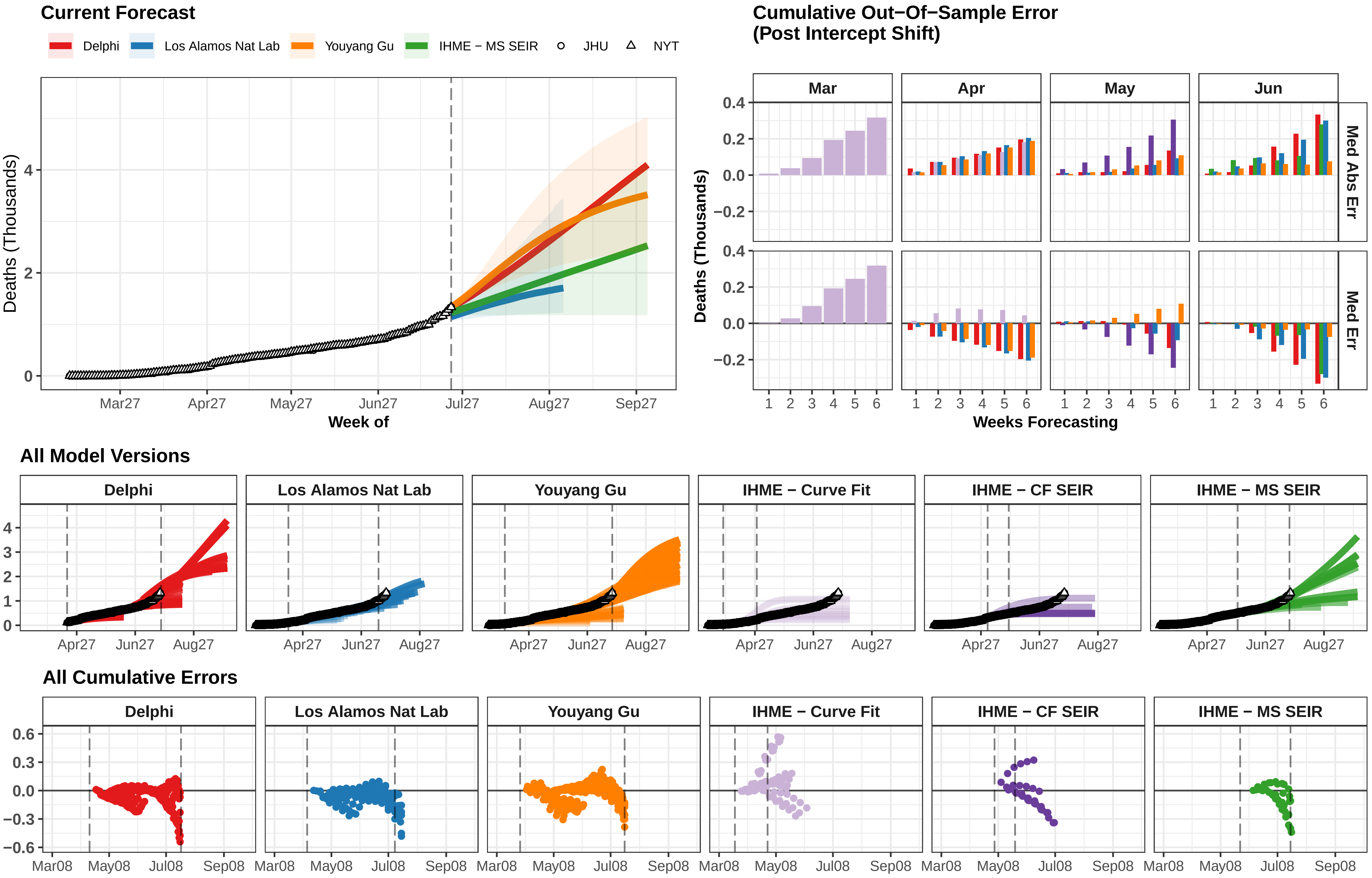

Missouri

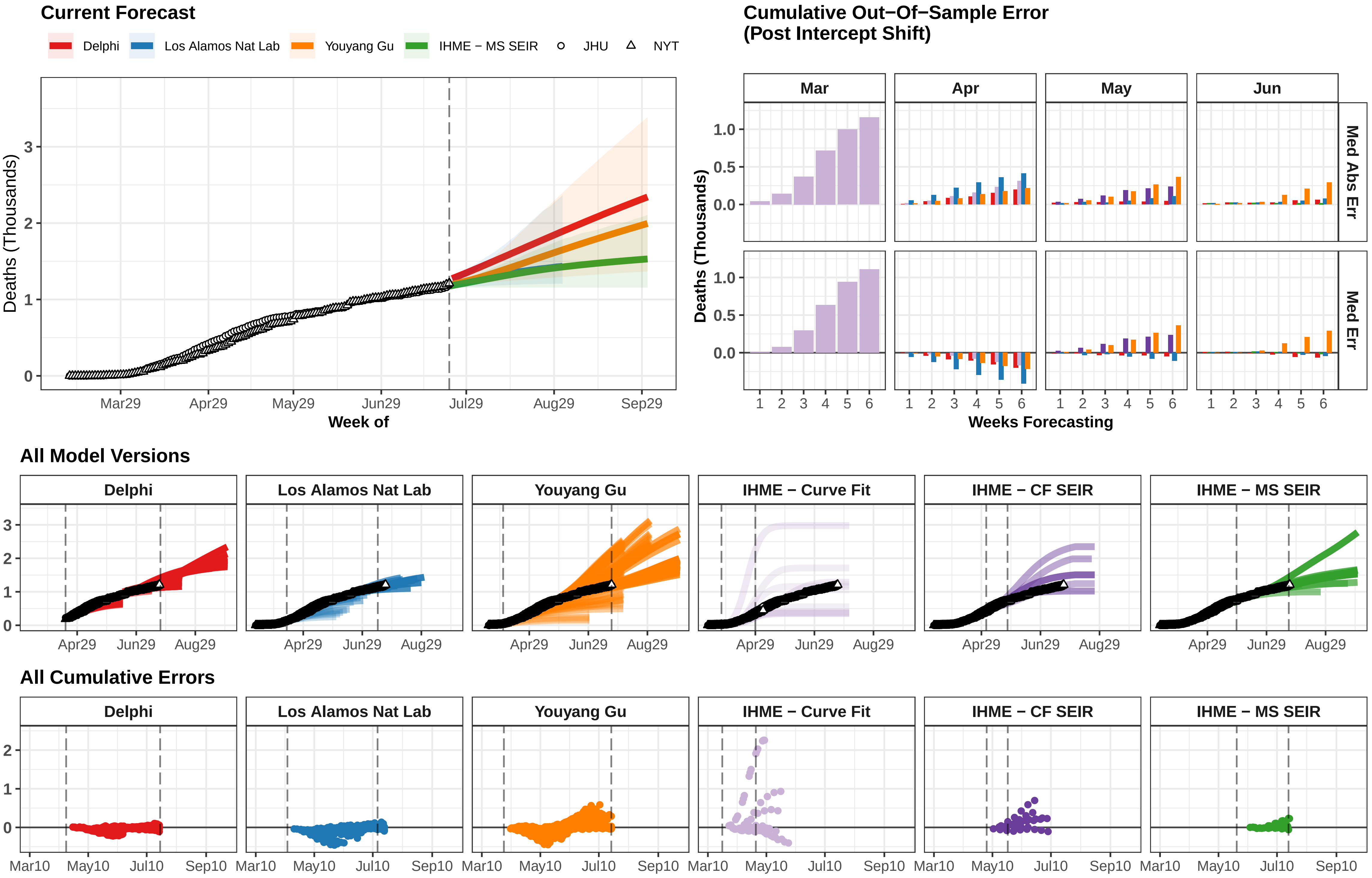

Current Forecast
Delphi
Los Alamos Nat Lab
Youyang Gu
IHME − MS SEIR
JHU
NYT
Deaths (Thousands)
Week of
Mar29
Apr29
May29
Jun29
Jul29
Aug29
Sep29
Cumulative Out−Of−Sample Error
(Post Intercept Shift)
Mar
Apr
May
Jun
Med Abs Err
Med Err
Weeks Forecasting
All Model Versions
Delphi
Los Alamos Nat Lab
Youyang Gu
IHME − Curve Fit
IHME − CF SEIR
IHME − MS SEIR
All Cumulative Errors
Mar10
May10
Jul10
Sep10

Afghanistan

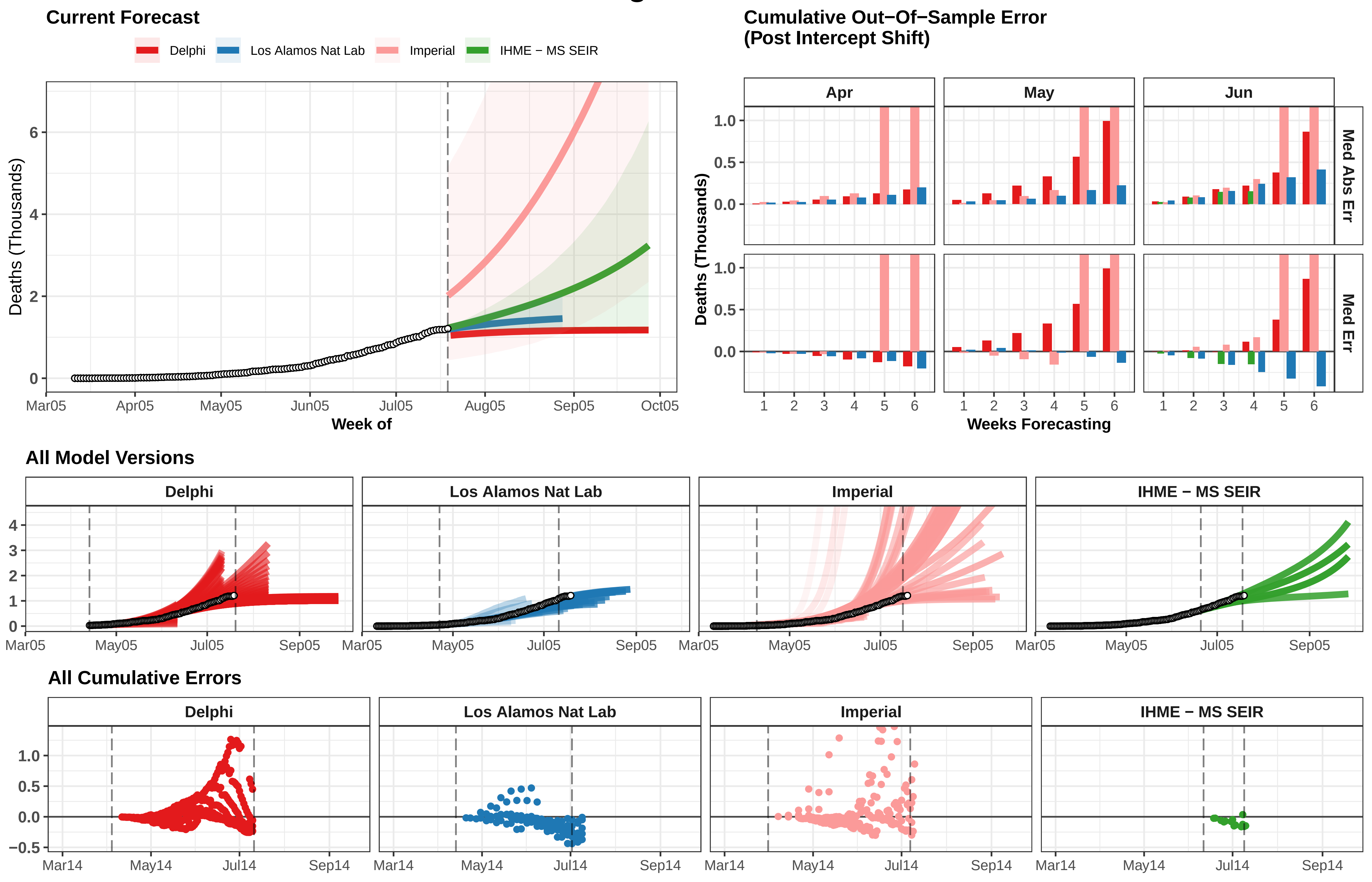

Kyrgyzstan

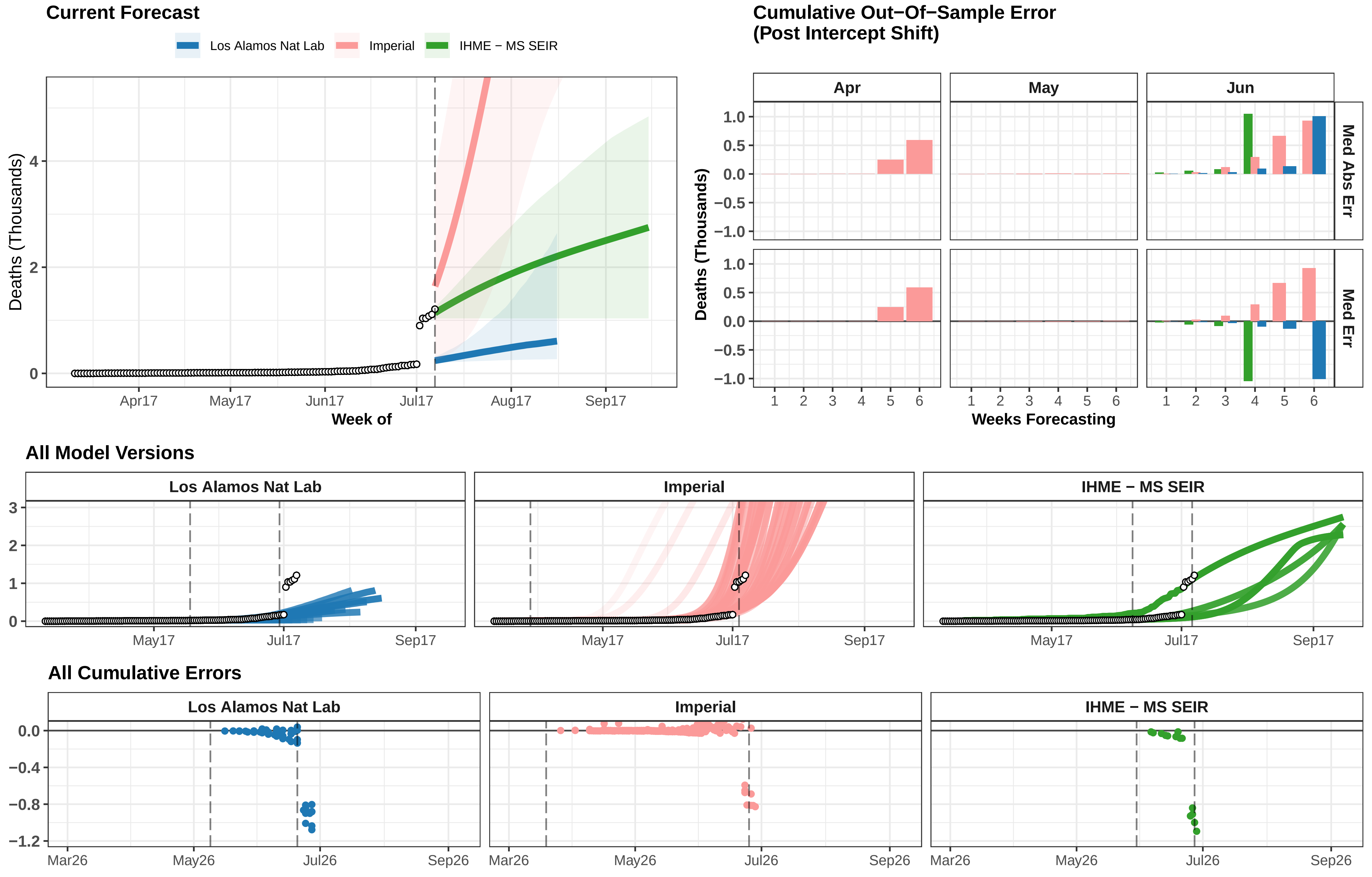
Current Forecast
Los Alamos Nat Lab
Imperial
IHME − MS SEIR
Deaths (Thousands)
Week of
Apr17
May17
Jun17
Jul17
Aug17
Sep17
Cumulative Out−Of−Sample Error
(Post Intercept Shift)
Apr
May
Jun
Med Abs Err
Med Err
Weeks Forecasting
All Model Versions
All Cumulative Errors
Mar26
May26
Jul26
Sep26

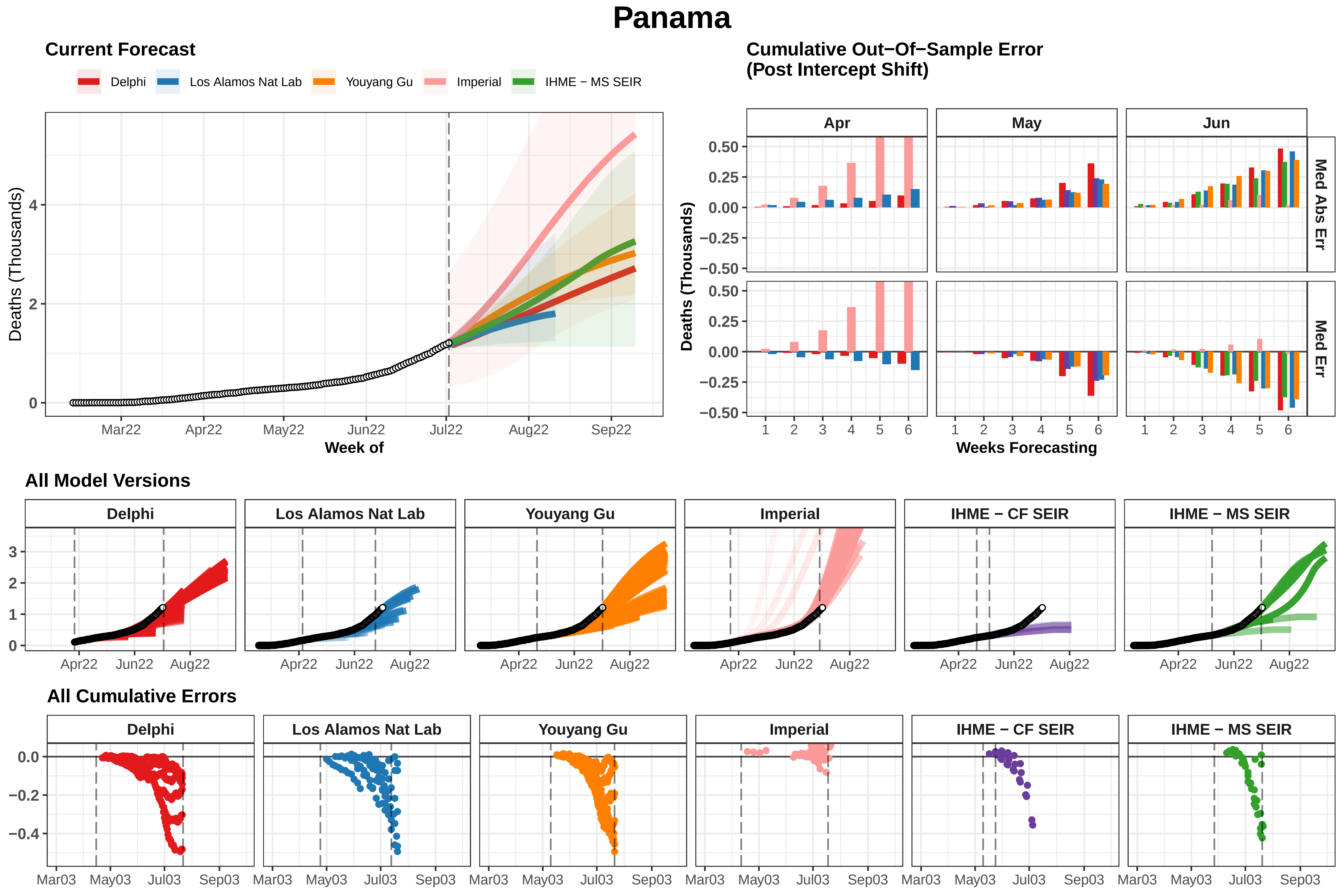

Panama
Current Forecast
Delphi
Los Alamos Nat Lab
Youyang Gu
Imperial
IHME − MS SEIR
Deaths (Thousands)
Week of
Mar22
Apr22
May22
Jun22
Jul22
Aug22
Sep22
Cumulative Out−Of−Sample Error
(Post Intercept Shift)
Apr
May
Jun
Med Abs Err
Med Err
Weeks Forecasting
All Model Versions
Delphi
Los Alamos Nat Lab
Youyang Gu
Imperial
IHME − CF SEIR
IHME − MS SEIR
All Cumulative Errors
Mar03
May03
Jul03
Sep03

Algeria

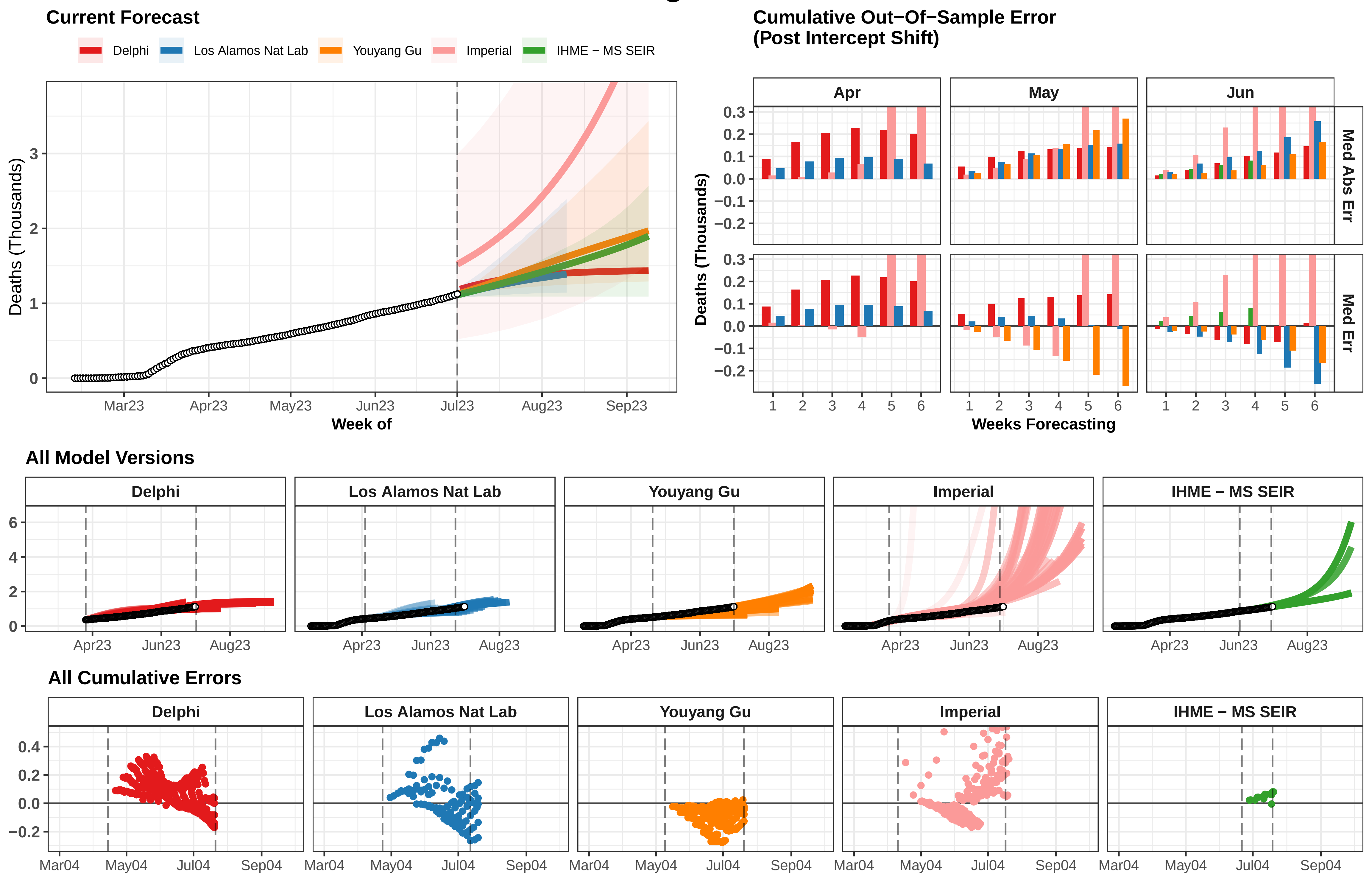

Current Forecast
Delphi
Los Alamos Nat Lab
Youyang Gu
Imperial
IHME − MS SEIR
Deaths (Thousands)
Week of
Mar23
Apr23
May23
Jun23
Jul23
Aug23
Sep23
Cumulative Out−Of−Sample Error
(Post Intercept Shift)
Apr
May
Jun
Med Abs Err
Med Err
Weeks Forecasting
All Model Versions
All Cumulative Errors
Mar04
May04
Jul04
Sep04

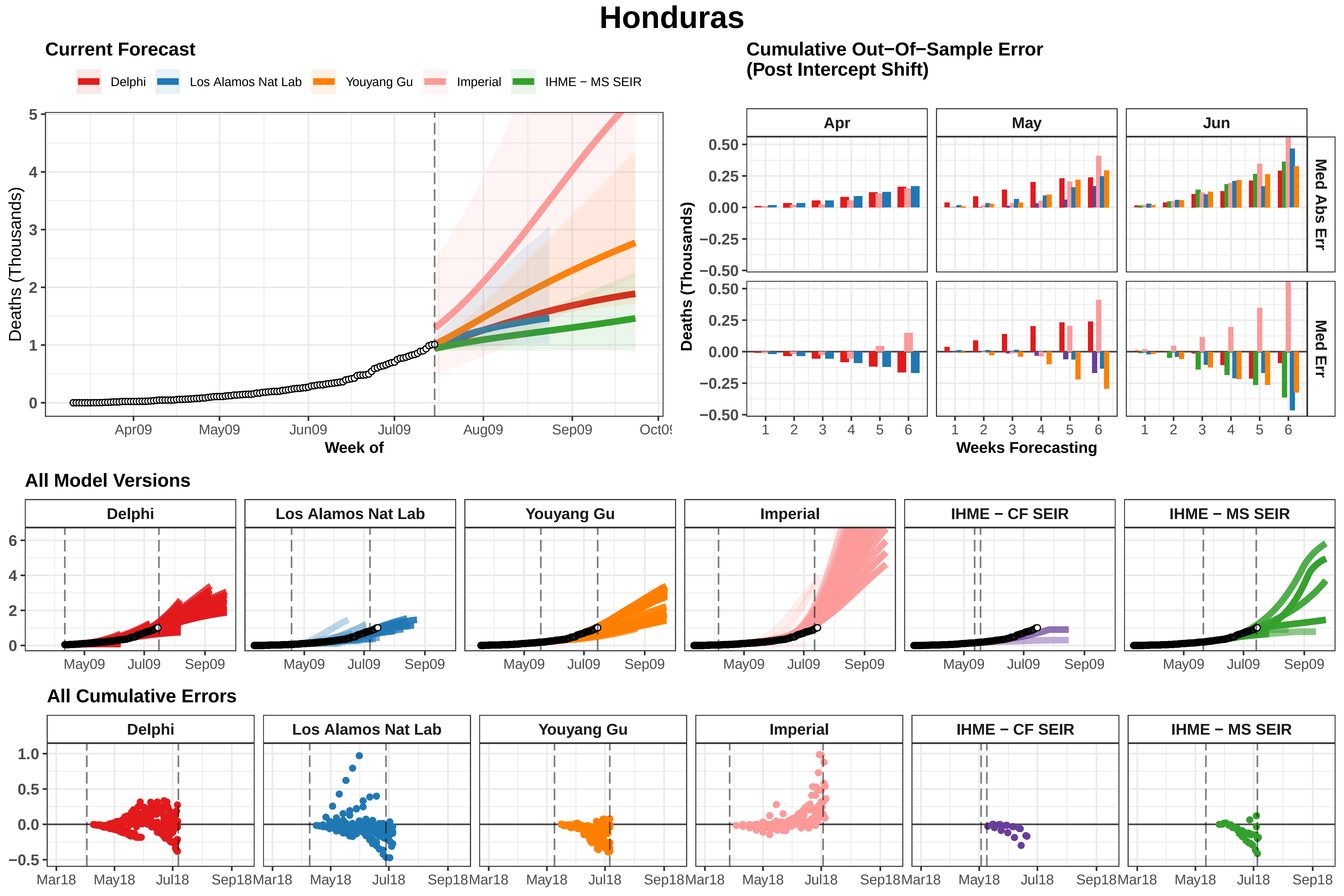
Honduras
Current Forecast
Delphi
Los Alamos Nat Lab
Youyang Gu
Imperial
IHME − MS SEIR
Deaths (Thousands)
Week of
Apr09
May09
Jun09
Jul09
Aug09
Sep09
Cumulative Out−Of−Sample Error
(Post Intercept Shift)
Apr
May
Jun
Med Abs Err
Med Err
Deaths (Thousands)
Weeks Forecasting
All Model Versions
Delphi
Los Alamos Nat Lab
Youyang Gu
Imperial
IHME − CF SEIR
IHME − MS SEIR
All Cumulative Errors
Delphi
Los Alamos Nat Lab
Youyang Gu
Imperial
IHME − CF SEIR
IHME − MS SEIR
Mar18
May18
Jul18
Sep18

Dominican Republic

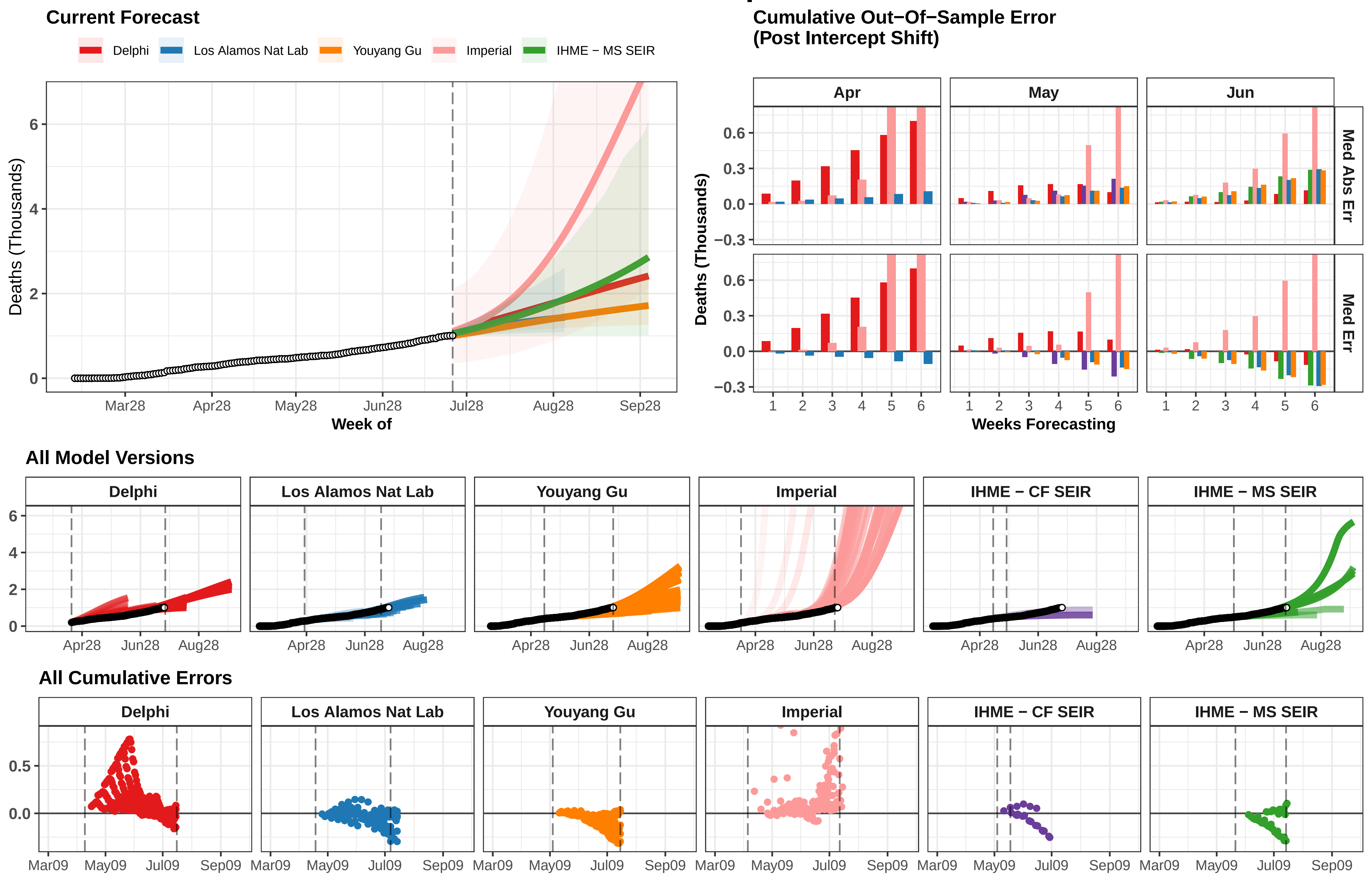

Rhode Island

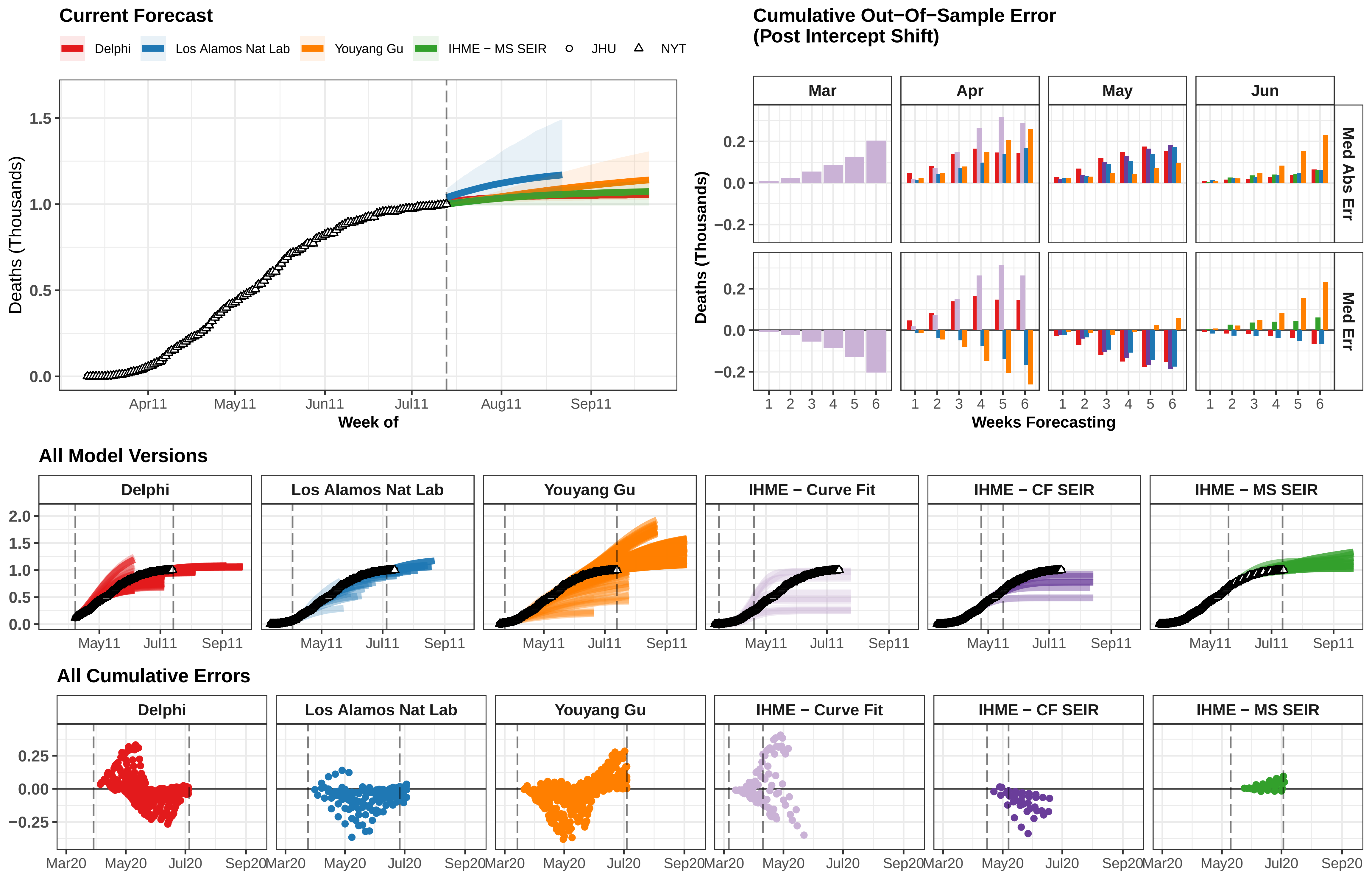

Japan

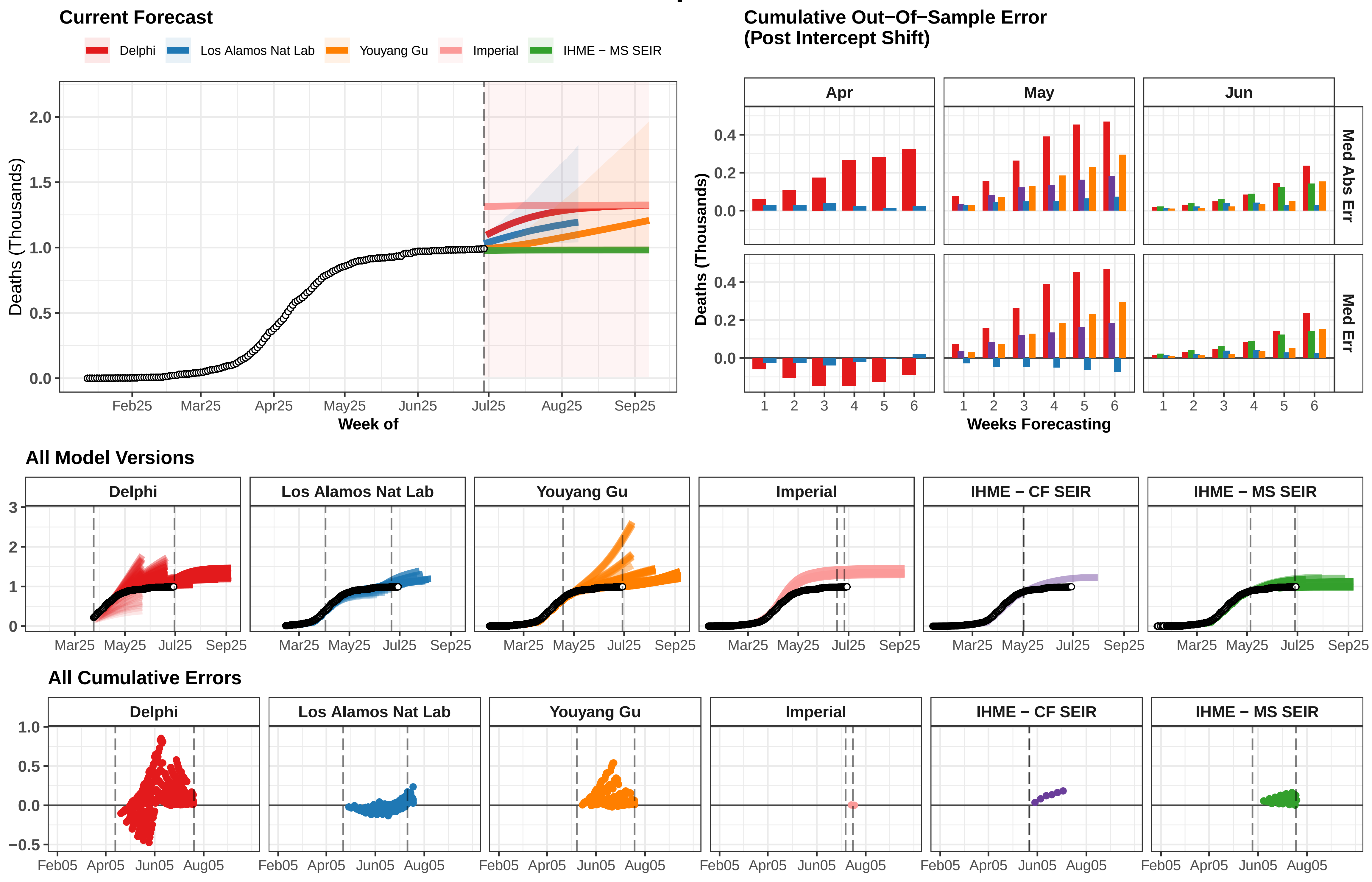
Current Forecast
Delphi
Los Alamos Nat Lab
Youyang Gu
Imperial
IHME − MS SEIR
Deaths (Thousands)
2.0
1.5
1.0
0.5
0.0
Feb25
Mar25
Apr25
May25
Jun25
Jul25
Aug25
Sep25
Week of
Cumulative Out−Of−Sample Error
(Post Intercept Shift)
Apr
May
Jun
Med Abs Err
Med Err
0.4
0.2
0.0
1
2
3
4
5
6
Weeks Forecasting
All Model Versions
Delphi
Los Alamos Nat Lab
Youyang Gu
Imperial
IHME − CF SEIR
IHME − MS SEIR
3
2
1
0
Mar25
May25
Jul25
Sep25
All Cumulative Errors
1.0
0.5
0.0
−0.5
Feb05
Apr05
Jun05
Aug05

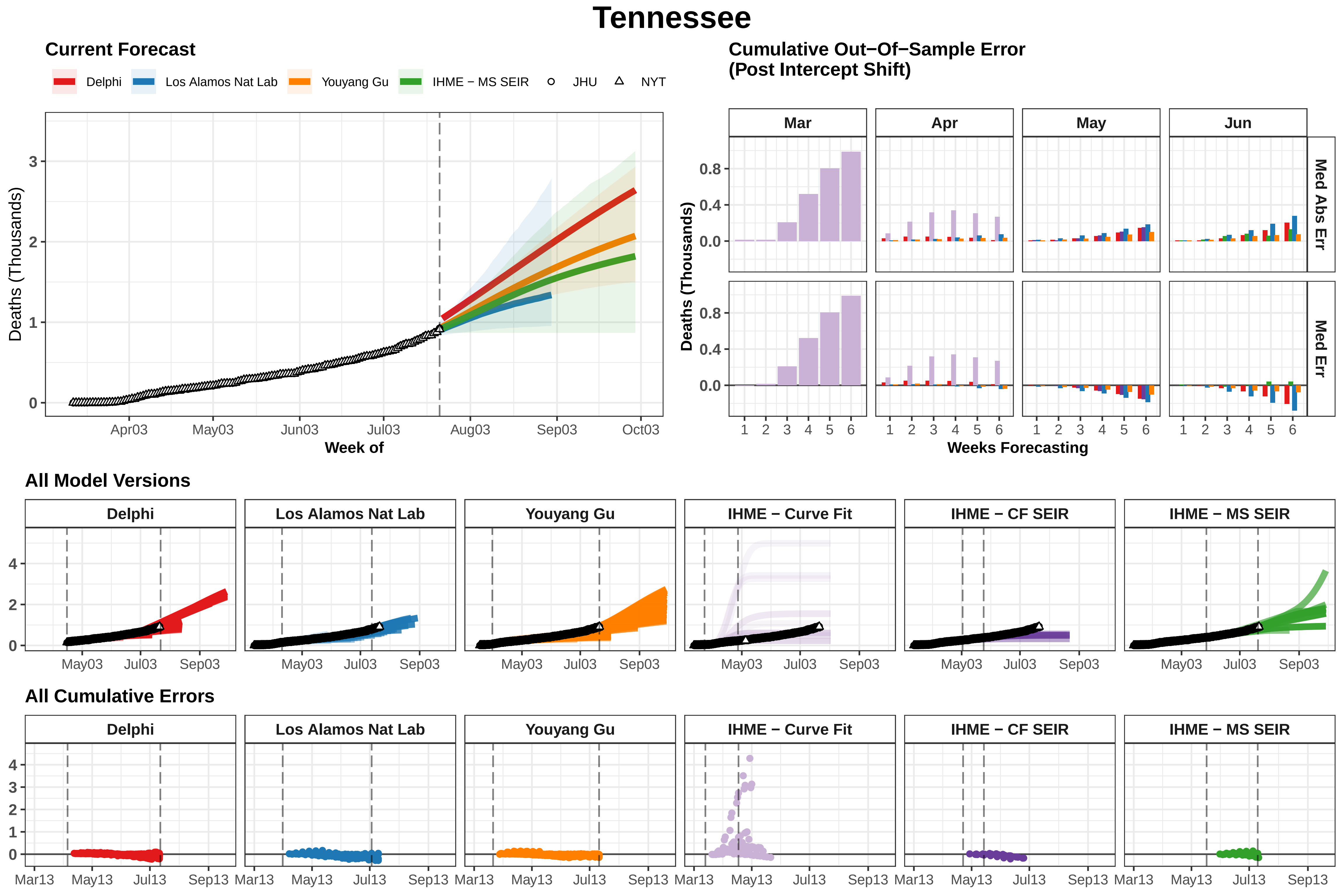
Tennessee
Current Forecast
Delphi
Los Alamos Nat Lab
Youyang Gu
IHME − MS SEIR
JHU
NYT
Deaths (Thousands)
Week of
Apr03
May03
Jun03
Jul03
Aug03
Sep03
Oct03
Cumulative Out−Of−Sample Error
(Post Intercept Shift)
Mar
Apr
May
Jun
Med Abs Err
Med Err
Weeks Forecasting
All Model Versions
Delphi
Los Alamos Nat Lab
Youyang Gu
IHME − Curve Fit
IHME − CF SEIR
IHME − MS SEIR
All Cumulative Errors
Mar13
May13
Jul13
Sep13

Wisconsin

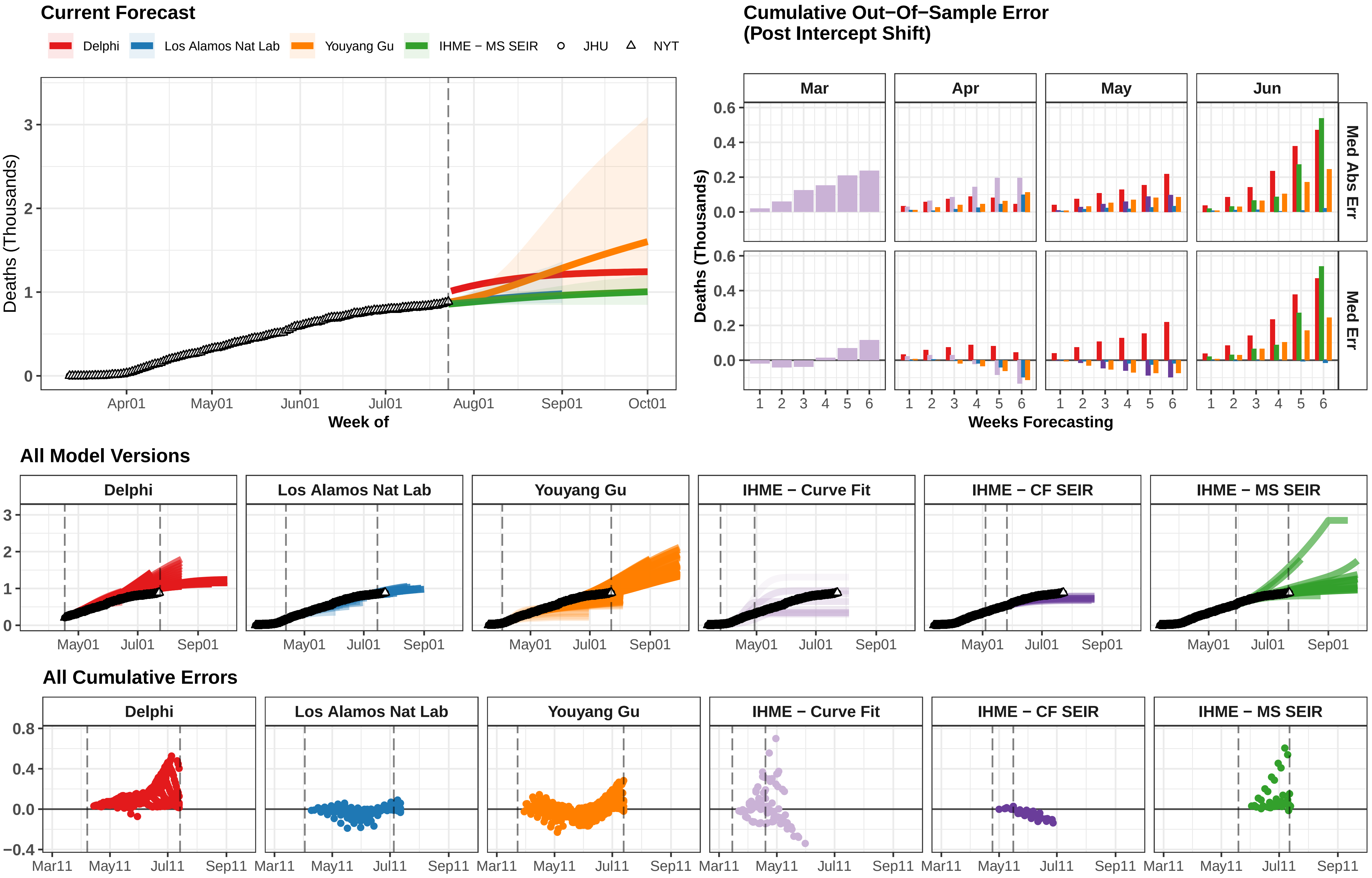
Current Forecast
Delphi
Los Alamos Nat Lab
Youyang Gu
IHME − MS SEIR
JHU
NYT
Deaths (Thousands)
Week of
Apr01
May01
Jun01
Jul01
Aug01
Sep01
Oct01
Cumulative Out−Of−Sample Error
(Post Intercept Shift)
Mar
Apr
May
Jun
Med Abs Err
Med Err
Weeks Forecasting
All Model Versions
IHME − Curve Fit
IHME − CF SEIR
All Cumulative Errors
Mar11
May11
Jul11
Sep11

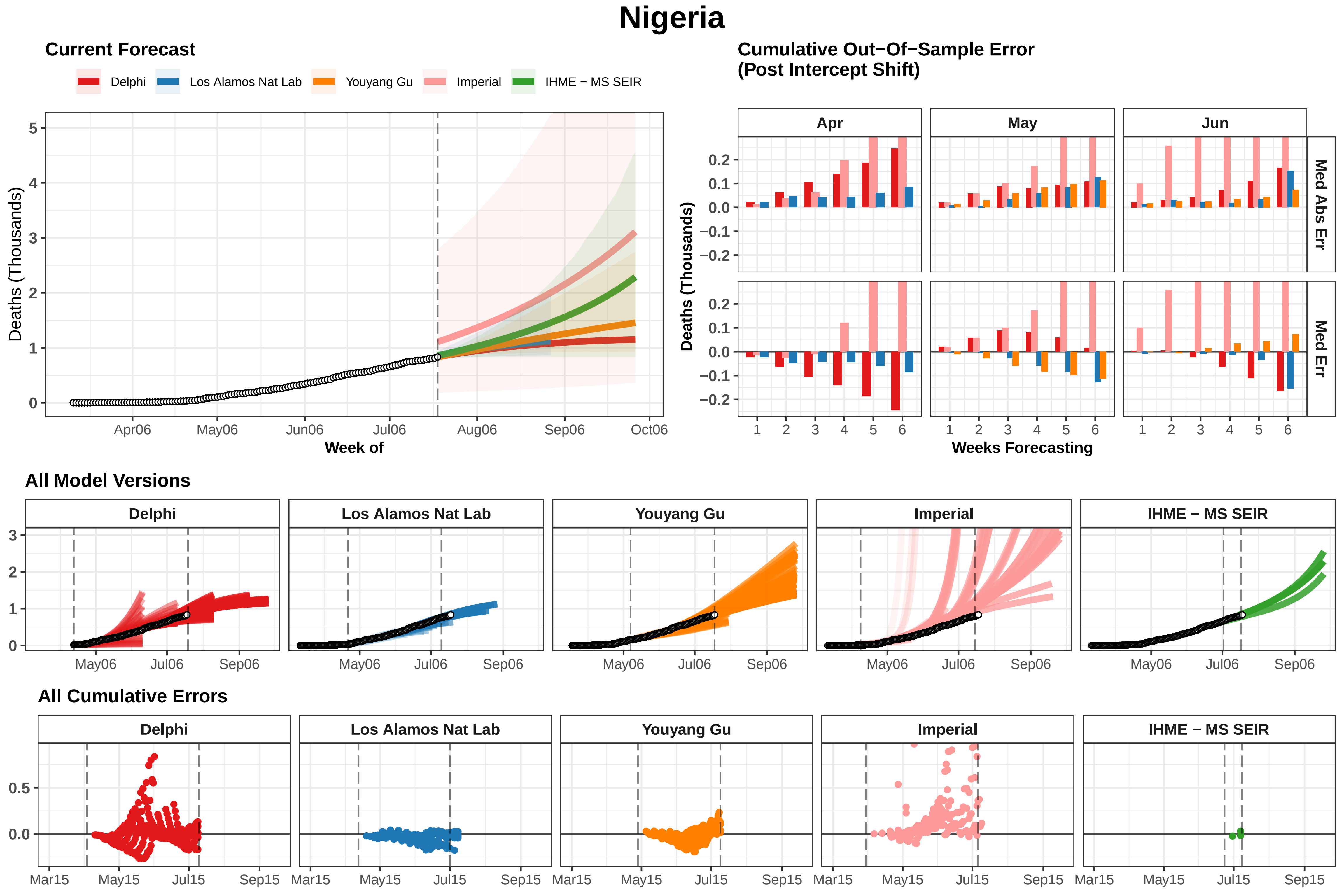

Nigeria
Current Forecast
Delphi
Los Alamos Nat Lab
Youyang Gu
Imperial
IHME − MS SEIR
Deaths (Thousands)
Week of
Apr06
May06
Jun06
Jul06
Aug06
Sep06
Oct06
Cumulative Out−Of−Sample Error
(Post Intercept Shift)
Apr
May
Jun
Med Abs Err
Med Err
Weeks Forecasting
All Model Versions
All Cumulative Errors
Mar15
May15
Jul15
Sep15

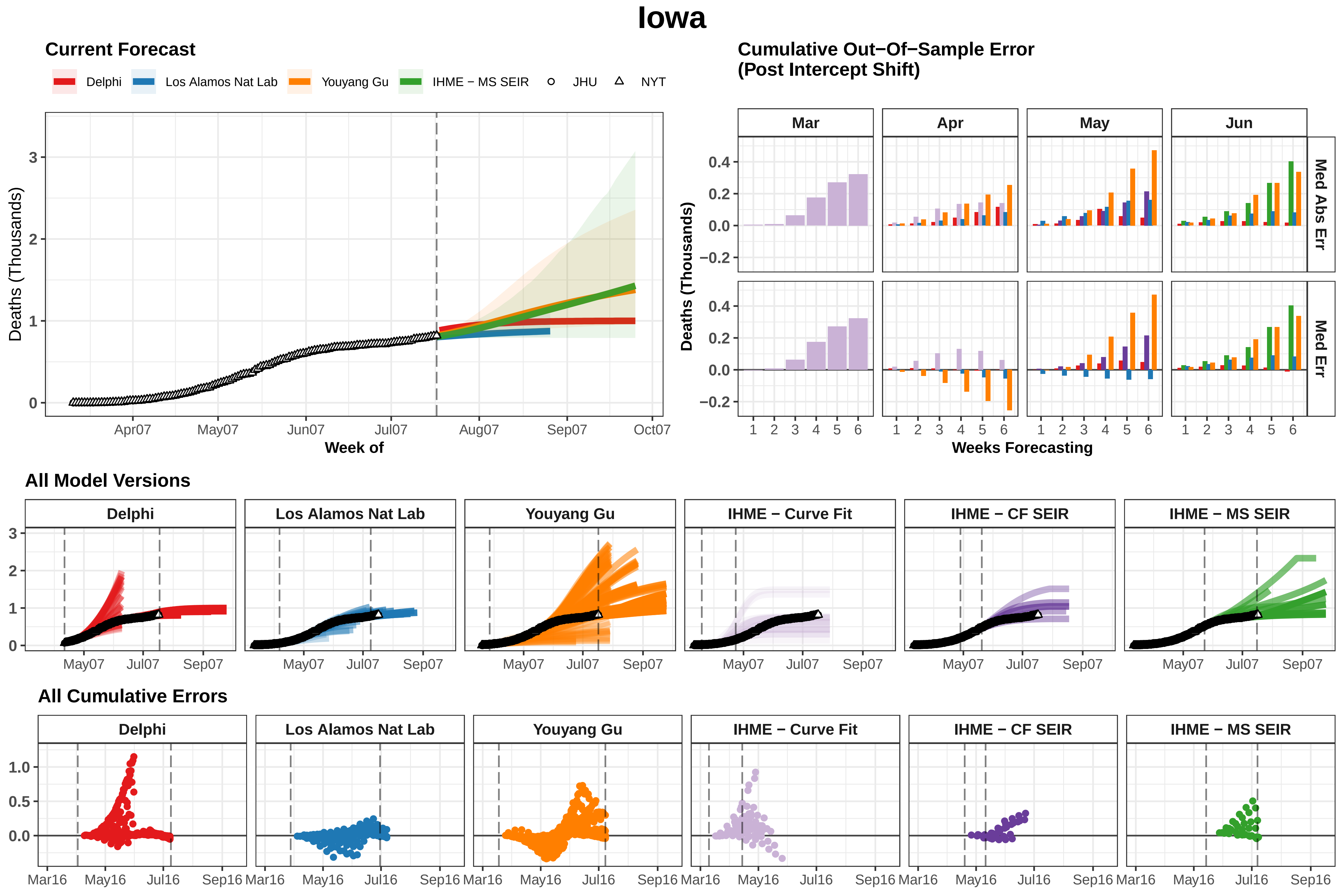
Iowa
Current Forecast
Delphi
Los Alamos Nat Lab
Youyang Gu
IHME − MS SEIR
JHU
NYT
Deaths (Thousands)
Week of
Apr07
May07
Jun07
Jul07
Aug07
Sep07
Oct07
Cumulative Out−Of−Sample Error
(Post Intercept Shift)
Mar
Apr
May
Jun
Med Abs Err
Med Err
Weeks Forecasting
All Model Versions
IHME − Curve Fit
IHME − CF SEIR
All Cumulative Errors
Mar16
May16
Jul16
Sep16

Moldova

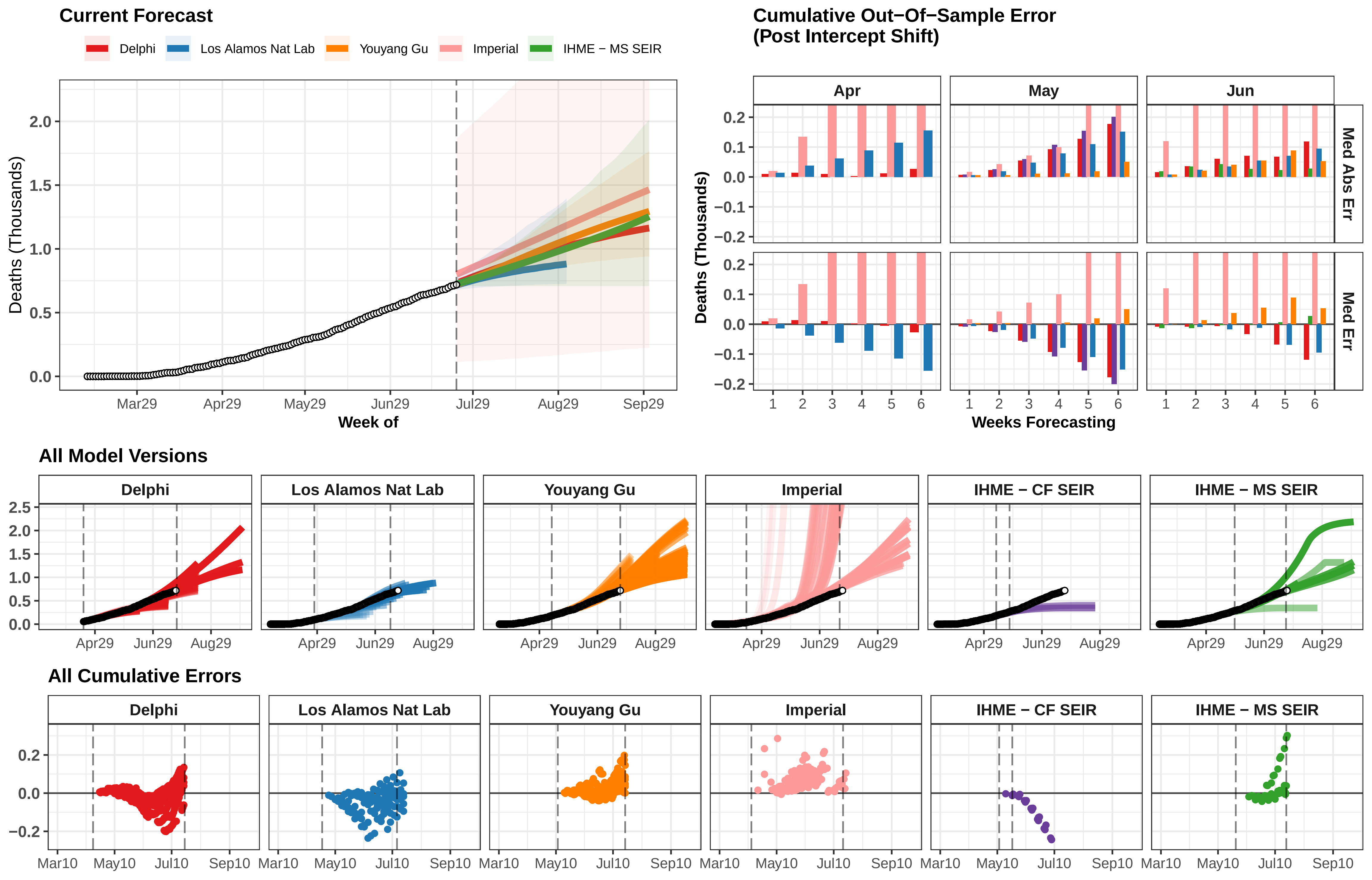

Current Forecast
Delphi
Los Alamos Nat Lab
Youyang Gu
Imperial
IHME − MS SEIR
Deaths (Thousands)
2.0
1.5
1.0
0.5
0.0
Mar29
Apr29
May29
Jun29
Jul29
Aug29
Sep29
Week of
Cumulative Out−Of−Sample Error
(Post Intercept Shift)
Apr
May
Jun
Med Abs Err
Med Err
Deaths (Thousands)
0.2
0.1
0.0
−0.1
−0.2
1
2
3
4
5
6
Weeks Forecasting
All Model Versions
Delphi
Los Alamos Nat Lab
Youyang Gu
Imperial
IHME − CF SEIR
IHME − MS SEIR
2.5
2.0
1.5
1.0
0.5
0.0
Apr29
Jun29
Aug29
All Cumulative Errors
Delphi
Los Alamos Nat Lab
Youyang Gu
Imperial
IHME − CF SEIR
IHME − MS SEIR
0.2
0.0
−0.2
Mar10
May10
Jul10
Sep10

Austria

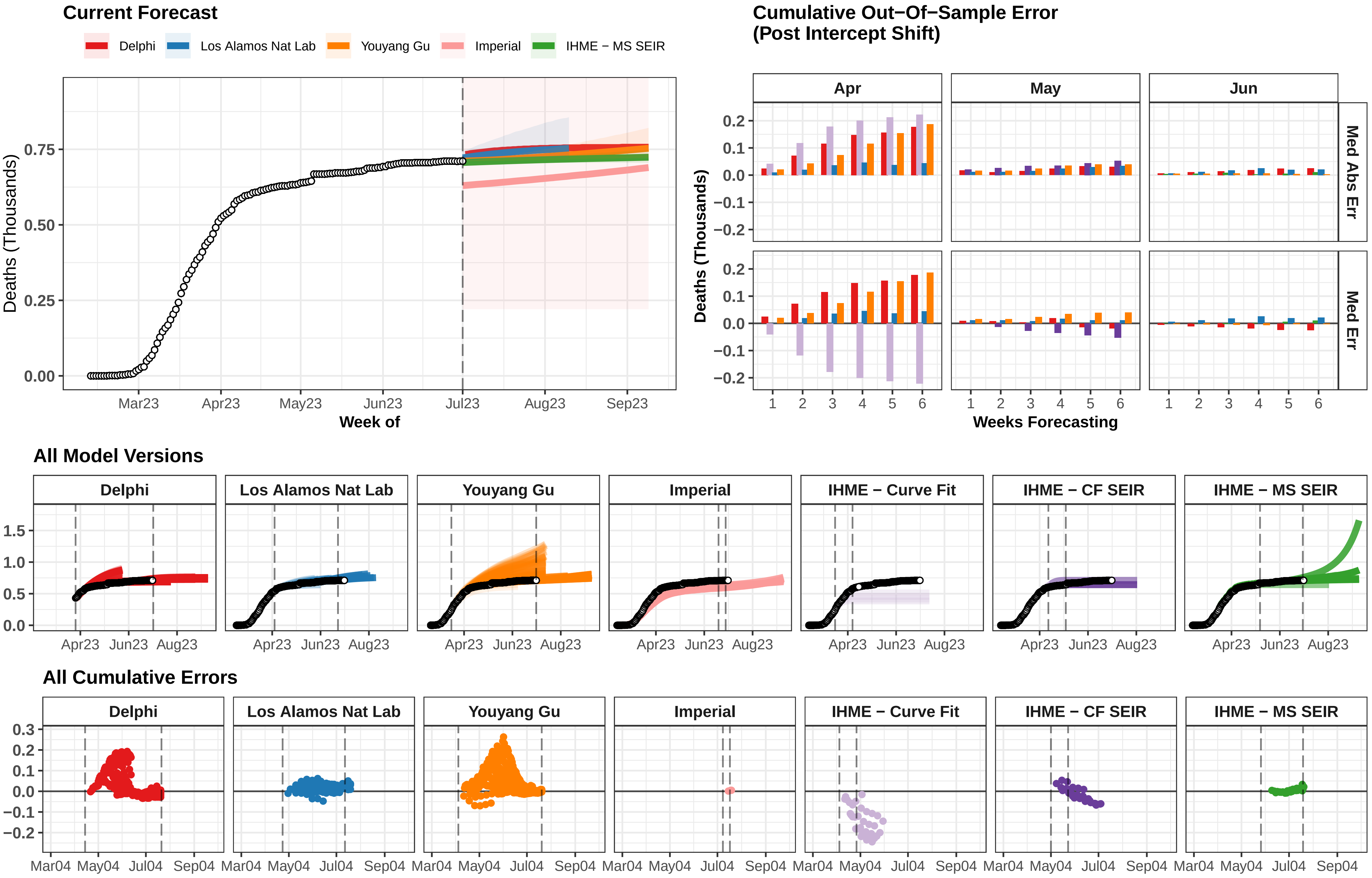

Current Forecast
Delphi
Los Alamos Nat Lab
Youyang Gu
Imperial
IHME − MS SEIR
Deaths (Thousands)
Week of
Mar23
Apr23
May23
Jun23
Jul23
Aug23
Sep23
Cumulative Out−Of−Sample Error
(Post Intercept Shift)
Apr
May
Jun
Med Abs Err
Med Err
Weeks Forecasting
All Model Versions
Delphi
Los Alamos Nat Lab
Youyang Gu
Imperial
IHME − Curve Fit
IHME − CF SEIR
IHME − MS SEIR
All Cumulative Errors
Mar04
May04
Jul04
Sep04

Nevada

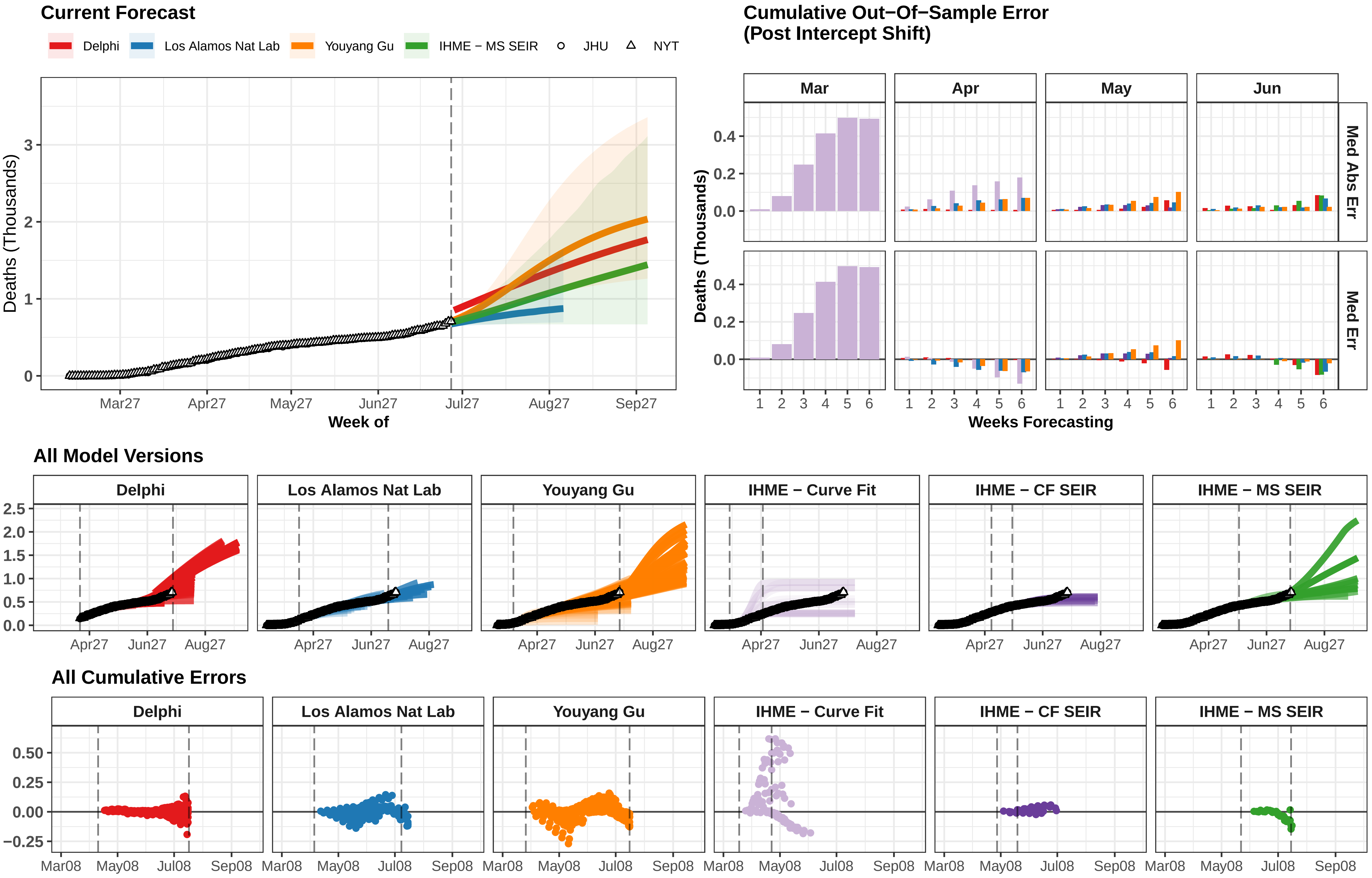
Current Forecast
Delphi
Los Alamos Nat Lab
Youyang Gu
IHME − MS SEIR
JHU
NYT
Deaths (Thousands)
Week of
Mar27
Apr27
May27
Jun27
Jul27
Aug27
Sep27
Cumulative Out−Of−Sample Error (Post Intercept Shift)
Mar
Apr
May
Jun
Med Abs Err
Med Err
Weeks Forecasting
All Model Versions
Delphi
Los Alamos Nat Lab
Youyang Gu
IHME − Curve Fit
IHME − CF SEIR
IHME − MS SEIR
All Cumulative Errors
Mar08
May08
Jul08
Sep08

Sudan

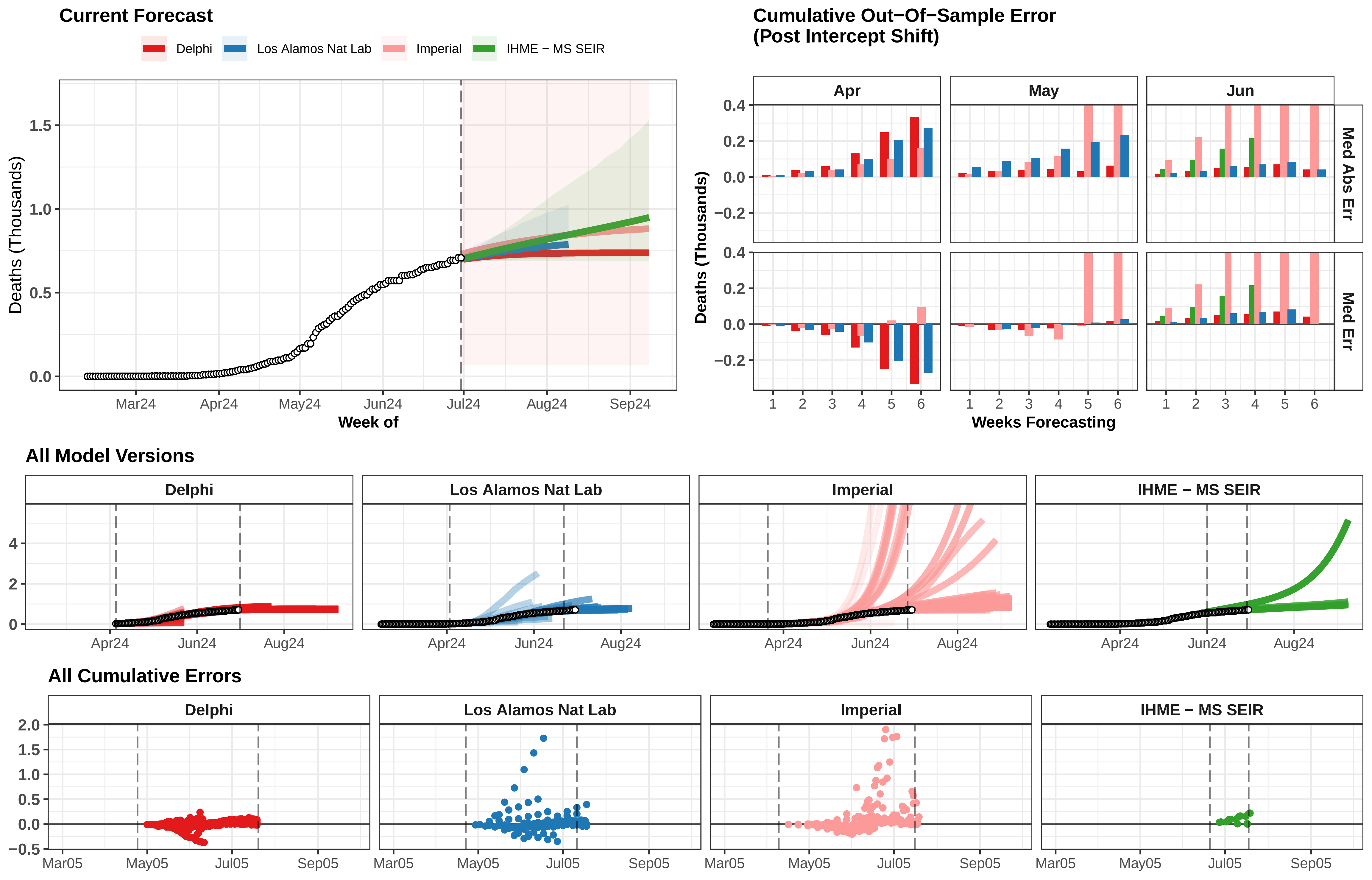
Current Forecast
Delphi
Los Alamos Nat Lab
Imperial
IHME − MS SEIR
Deaths (Thousands)
0.0
0.5
1.0
1.5
Mar24
Apr24
May24
Jun24
Jul24
Aug24
Sep24
Week of
Cumulative Out−Of−Sample Error
(Post Intercept Shift)
Apr
May
Jun
Med Abs Err
Med Err
Deaths (Thousands)
0.4
0.2
0.0
−0.2
1
2
3
4
5
6
Weeks Forecasting
All Model Versions
Delphi
Los Alamos Nat Lab
Imperial
IHME − MS SEIR
0
2
4
Apr24
Jun24
Aug24
All Cumulative Errors
Delphi
Los Alamos Nat Lab
Imperial
IHME − MS SEIR
2.0
1.5
1.0
0.5
0.0
−0.5
Mar05
May05
Jul05
Sep05

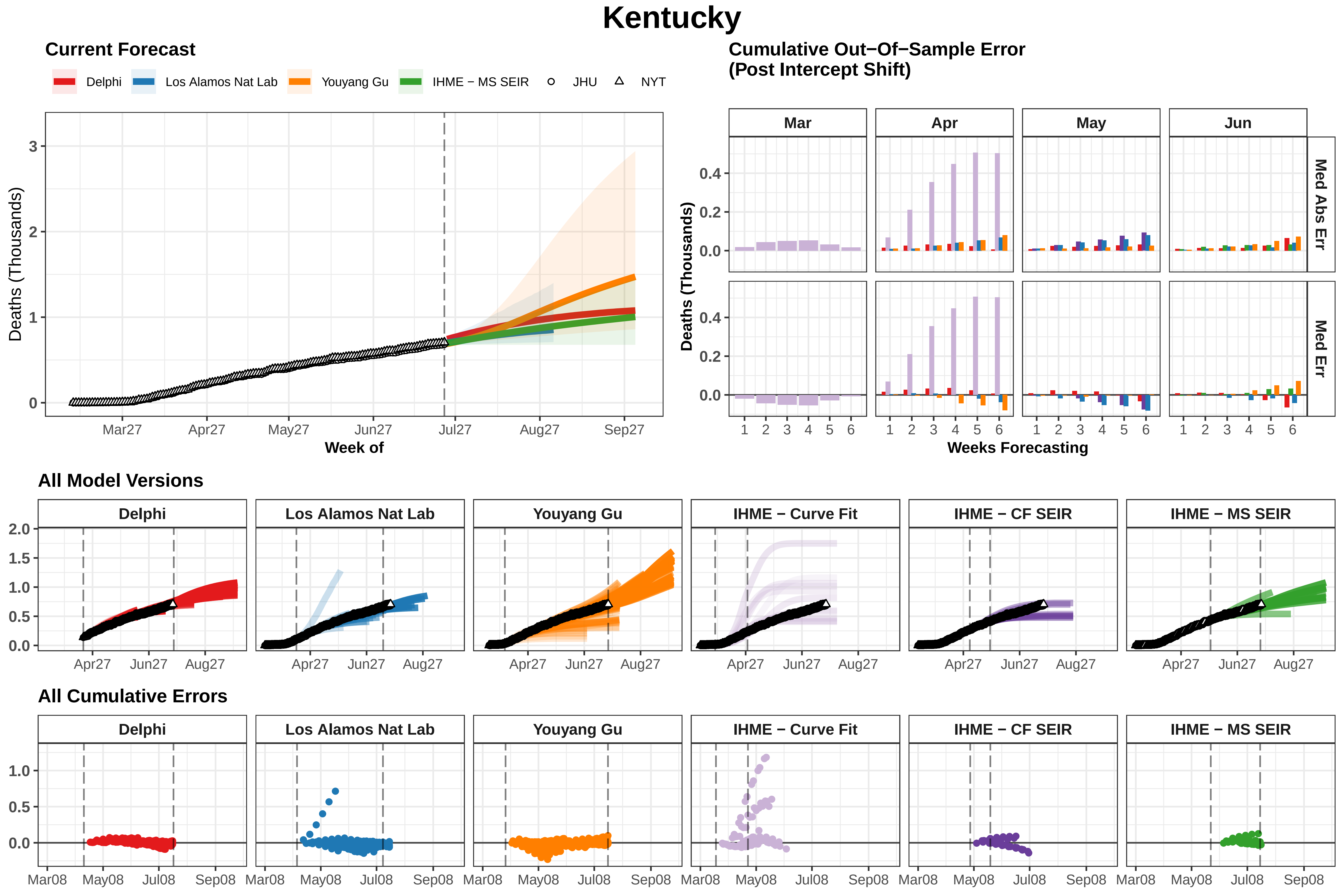

Kentucky
Current Forecast
Delphi
Los Alamos Nat Lab
Youyang Gu
IHME − MS SEIR
JHU
NYT
Deaths (Thousands)
Week of
Mar27
Apr27
May27
Jun27
Jul27
Aug27
Sep27
Cumulative Out−Of−Sample Error
(Post Intercept Shift)
Mar
Apr
May
Jun
Med Abs Err
Med Err
Weeks Forecasting
All Model Versions
Delphi
Los Alamos Nat Lab
Youyang Gu
IHME − Curve Fit
IHME − CF SEIR
IHME − MS SEIR
All Cumulative Errors
Mar08
May08
Jul08
Sep08

Armenia

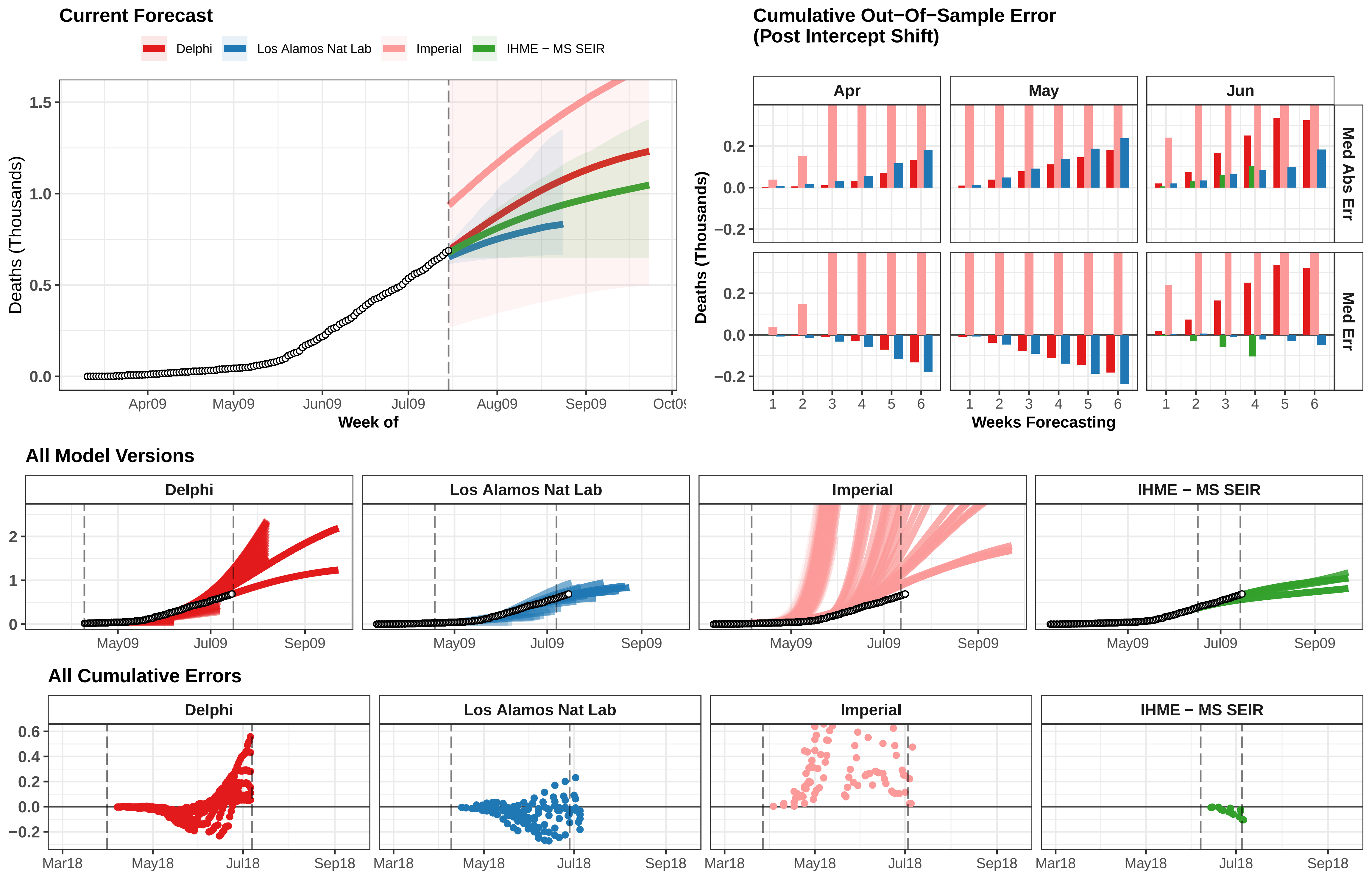

Current Forecast
Delphi
Los Alamos Nat Lab
Imperial
IHME − MS SEIR
Deaths (Thousands)
1.5
1.0
0.5
0.0
Apr09
May09
Jun09
Jul09
Aug09
Sep09
Oct09
Week of
Cumulative Out−Of−Sample Error
(Post Intercept Shift)
Apr
May
Jun
Med Abs Err
Med Err
0.2
0.0
−0.2
1
2
3
4
5
6
Weeks Forecasting
All Model Versions
2
1
0
May09
Jul09
Sep09
All Cumulative Errors
0.6
0.4
0.2
0.0
−0.2
Mar18
May18
Jul18
Sep18

Denmark

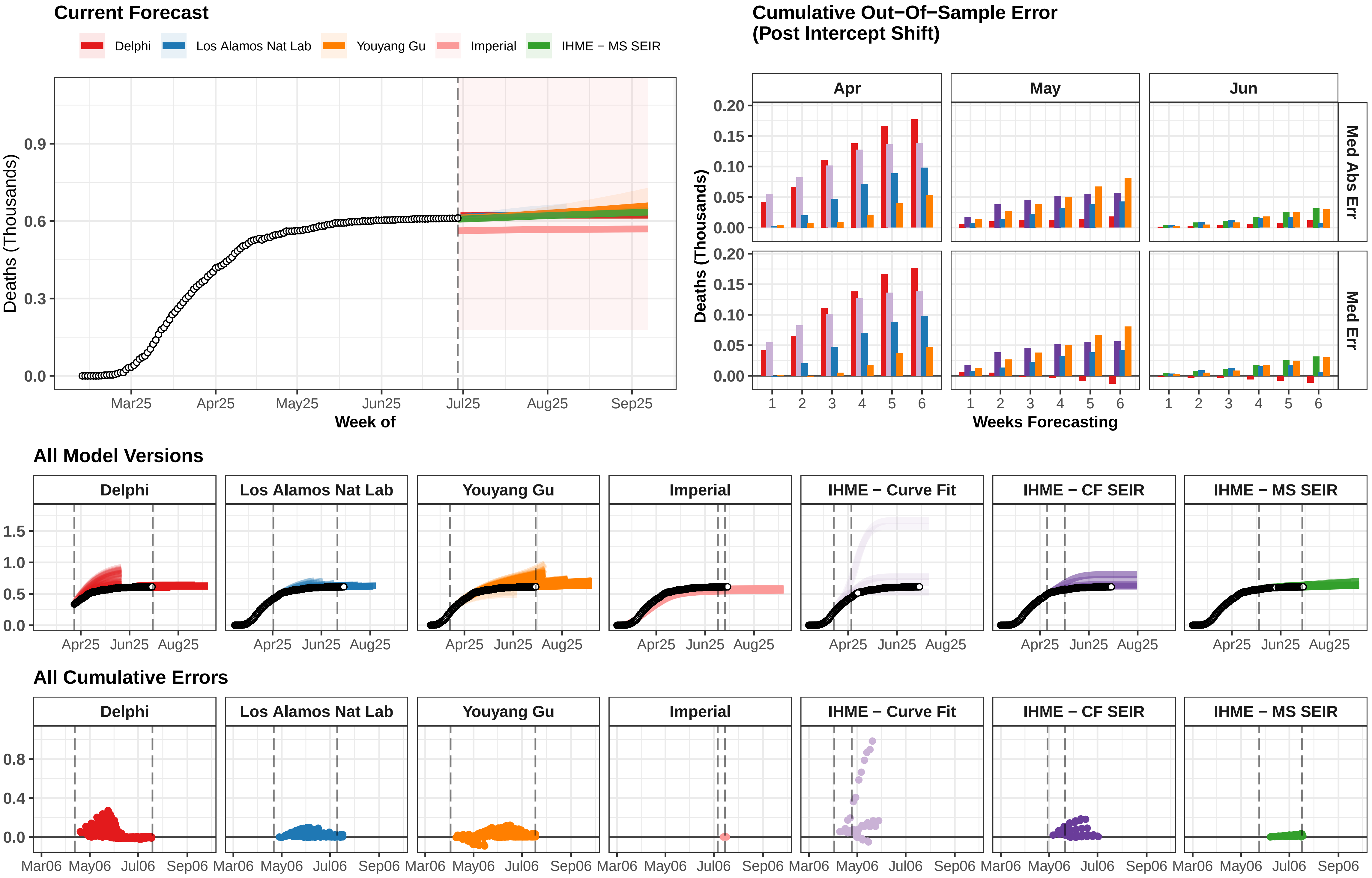
Current Forecast
Delphi
Los Alamos Nat Lab
Youyang Gu
Imperial
IHME − MS SEIR
Deaths (Thousands)
0.0
0.3
0.6
0.9
Mar25
Apr25
May25
Jun25
Jul25
Aug25
Sep25
Week of
Cumulative Out−Of−Sample Error
(Post Intercept Shift)
Apr
May
Jun
Med Abs Err
Med Err
0.00
0.05
0.10
0.15
0.20
1
2
3
4
5
6
Weeks Forecasting
All Model Versions
IHME − Curve Fit
IHME − CF SEIR
0.0
0.5
1.0
1.5
Apr25
Jun25
Aug25
All Cumulative Errors
0.0
0.4
0.8
Mar06
May06
Jul06
Sep06

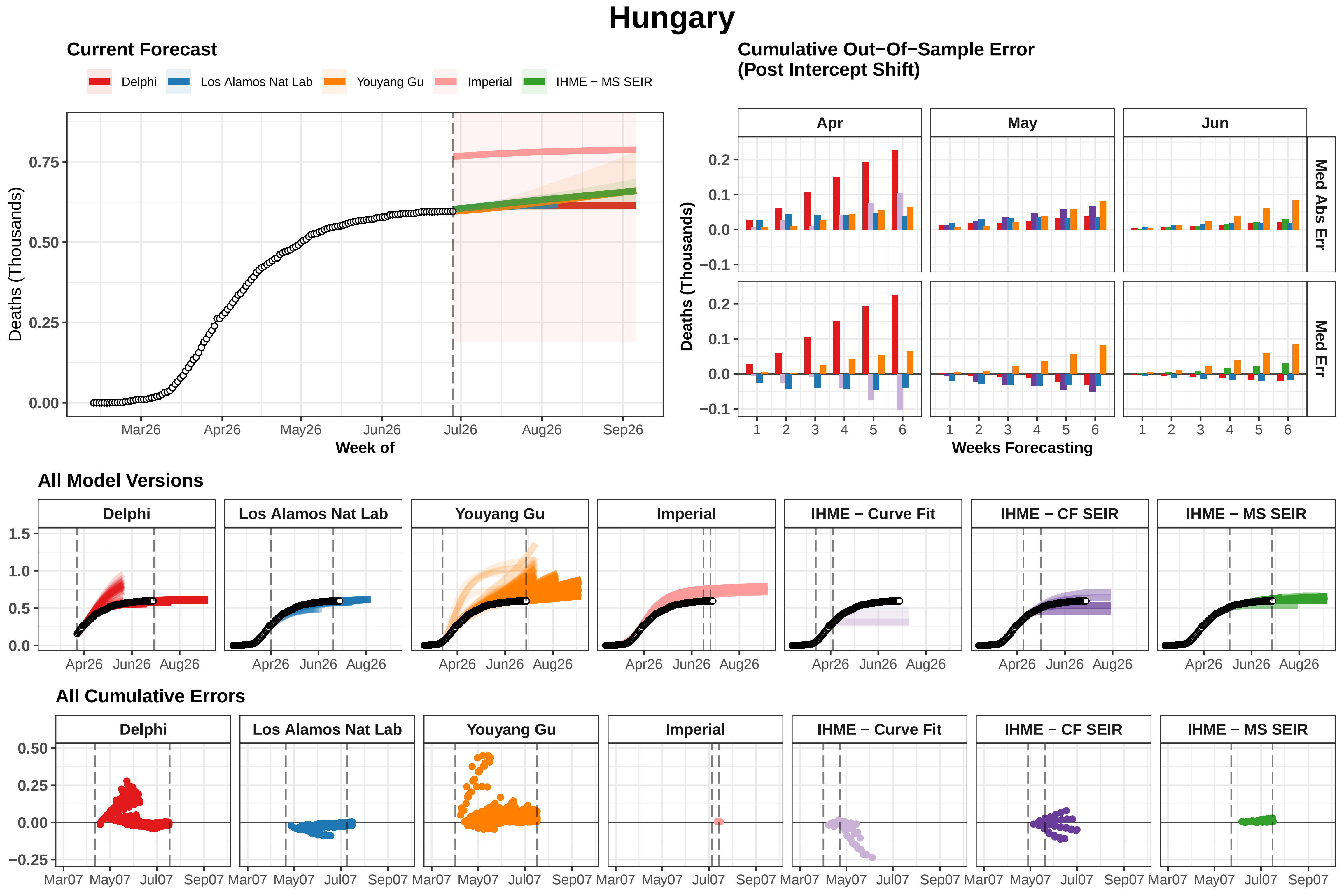
Hungary
Current Forecast
Delphi
Los Alamos Nat Lab
Youyang Gu
Imperial
IHME − MS SEIR
Deaths (Thousands)
0.00
0.25
0.50
0.75
Mar26
Apr26
May26
Jun26
Jul26
Aug26
Sep26
Week of
Cumulative Out−Of−Sample Error
(Post Intercept Shift)
Apr
May
Jun
Med Abs Err
Med Err
Deaths (Thousands)
−0.1
0.0
0.1
0.2
1
2
3
4
5
6
Weeks Forecasting
All Model Versions
Delphi
Los Alamos Nat Lab
Youyang Gu
Imperial
IHME − Curve Fit
IHME − CF SEIR
IHME − MS SEIR
0.0
0.5
1.0
1.5
Apr26
Jun26
Aug26
All Cumulative Errors
Delphi
Los Alamos Nat Lab
Youyang Gu
Imperial
IHME − Curve Fit
IHME − CF SEIR
IHME − MS SEIR
−0.25
0.00
0.25
0.50
Mar07
May07
Jul07
Sep07

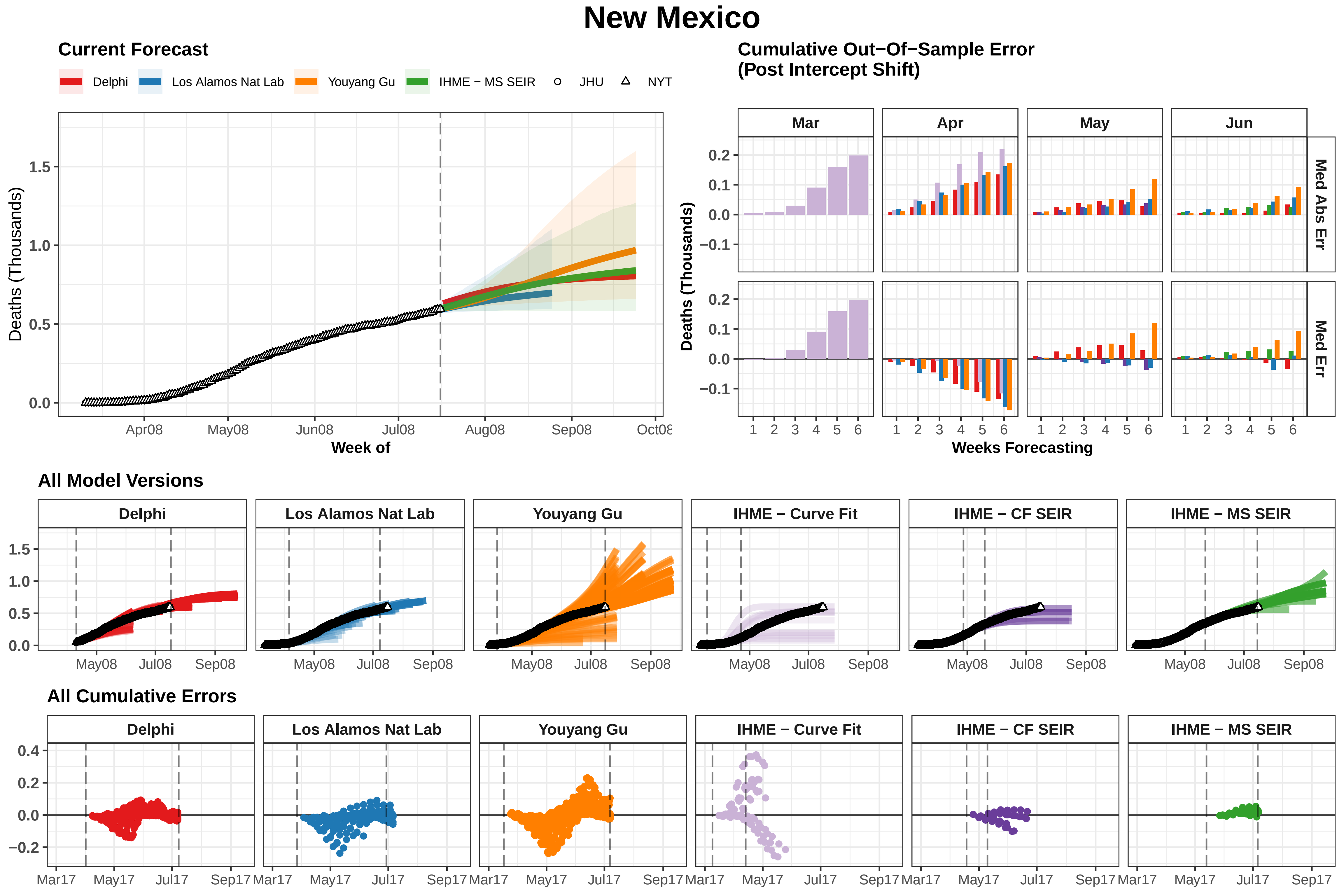
New Mexico
Current Forecast
Delphi
Los Alamos Nat Lab
Youyang Gu
IHME − MS SEIR
JHU
NYT
Deaths (Thousands)
Week of
Apr08
May08
Jun08
Jul08
Aug08
Sep08
Oct08
Cumulative Out−Of−Sample Error
(Post Intercept Shift)
Mar
Apr
May
Jun
Med Abs Err
Med Err
Weeks Forecasting
All Model Versions
IHME − Curve Fit
IHME − CF SEIR
All Cumulative Errors
Mar17
May17
Jul17
Sep17

Kazakhstan

Current Forecast

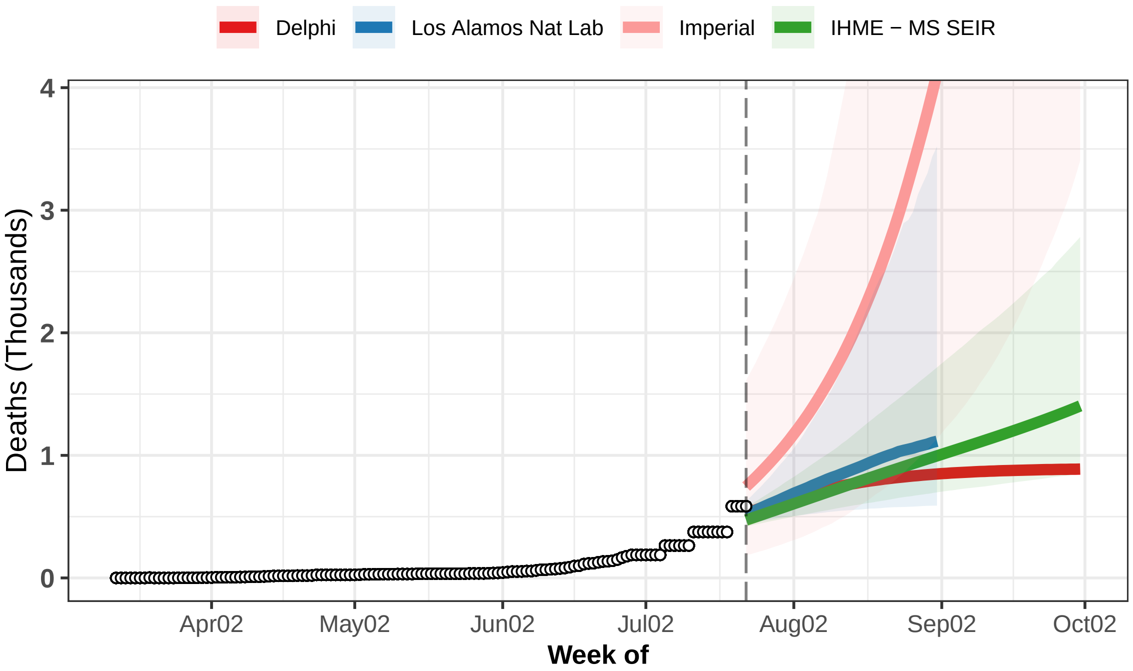

Cumulative Out−Of−Sample Error (Post Intercept Shift)

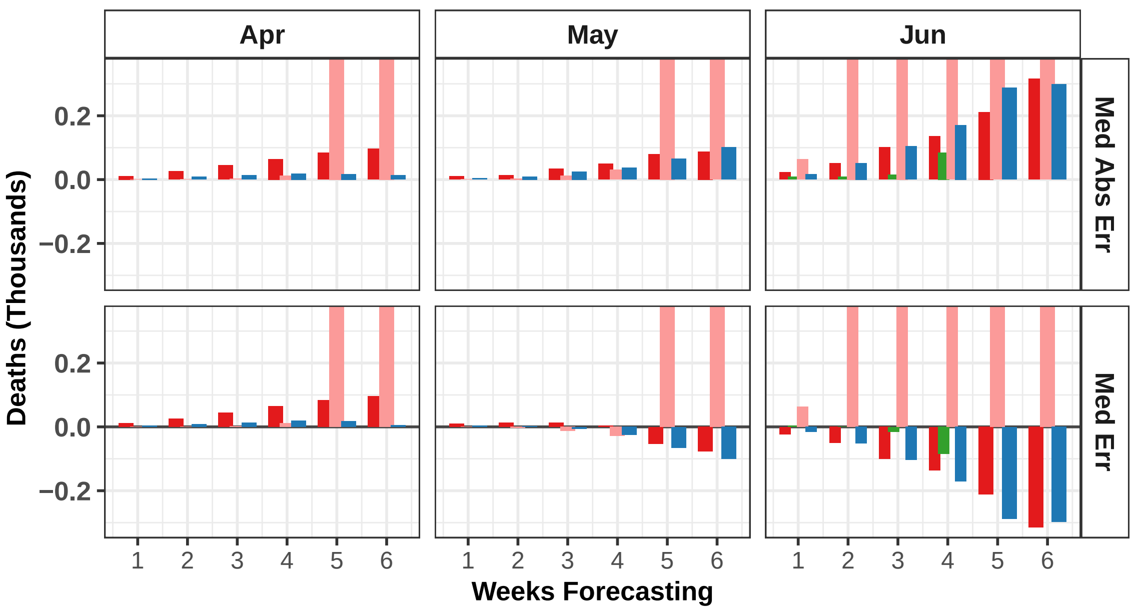

All Model Versions

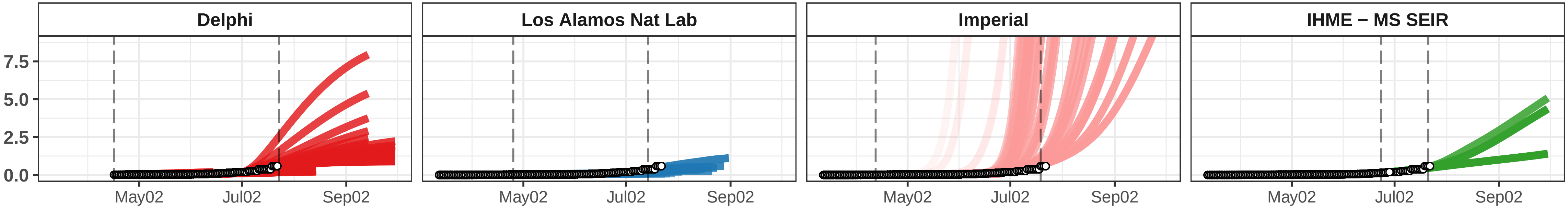

All Cumulative Errors

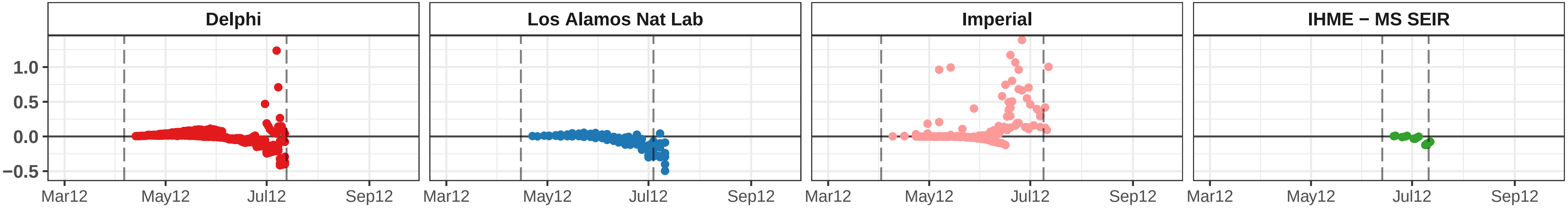

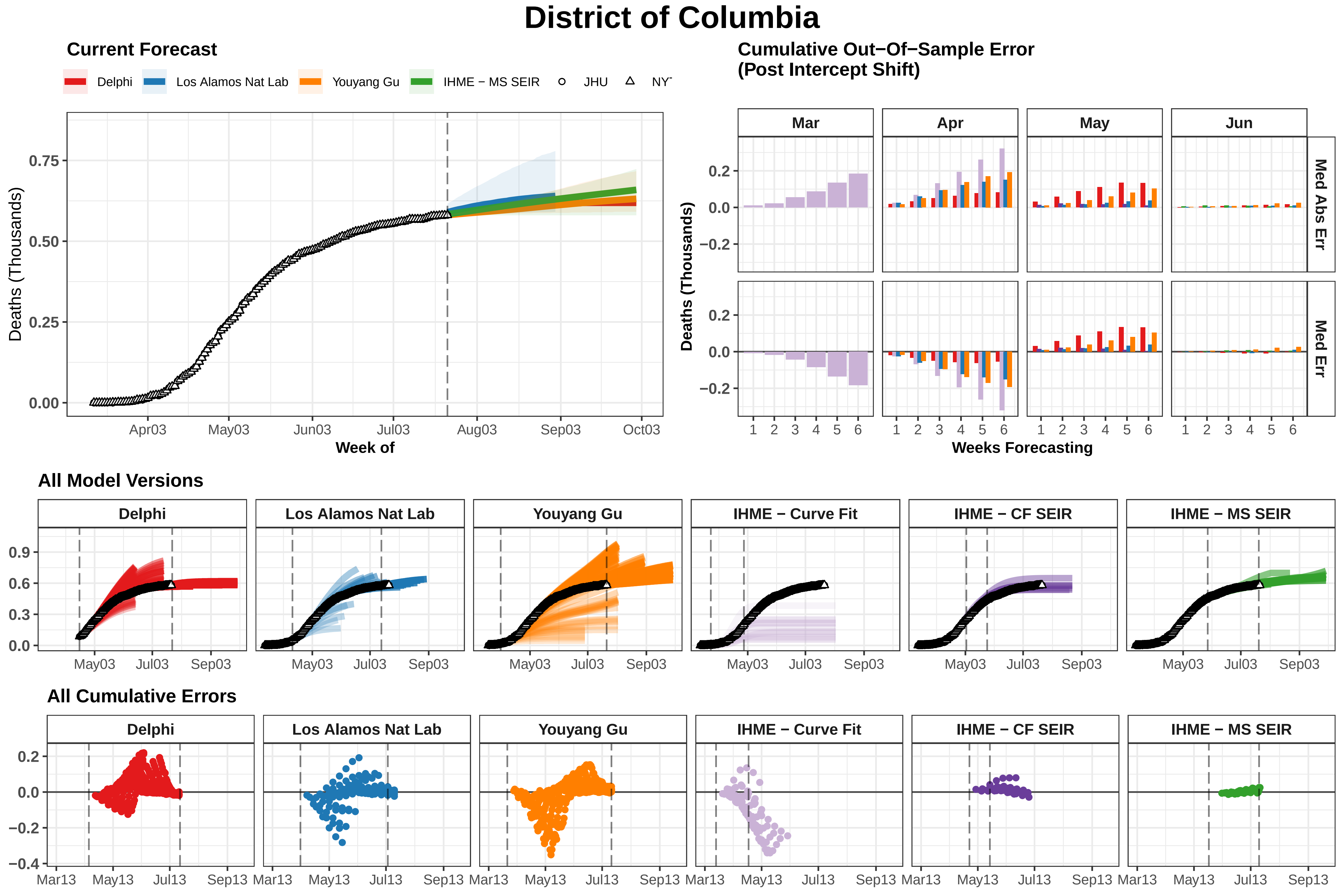

District of Columbia
Current Forecast
Delphi
Los Alamos Nat Lab
Youyang Gu
IHME − MS SEIR
JHU
NY
Deaths (Thousands)
0.75
0.50
0.25
0.00
Apr03
May03
Jun03
Jul03
Aug03
Sep03
Oct03
Week of
Cumulative Out−Of−Sample Error
(Post Intercept Shift)
Mar
Apr
May
Jun
Med Abs Err
Med Err
0.2
0.0
−0.2
1 2 3 4 5 6
Weeks Forecasting
All Model Versions
Delphi
Los Alamos Nat Lab
Youyang Gu
IHME − Curve Fit
IHME − CF SEIR
IHME − MS SEIR
0.9
0.6
0.3
0.0
May03
Jul03
Sep03
All Cumulative Errors
0.2
0.0
−0.2
−0.4
Mar13
May13
Jul13
Sep13

Delaware

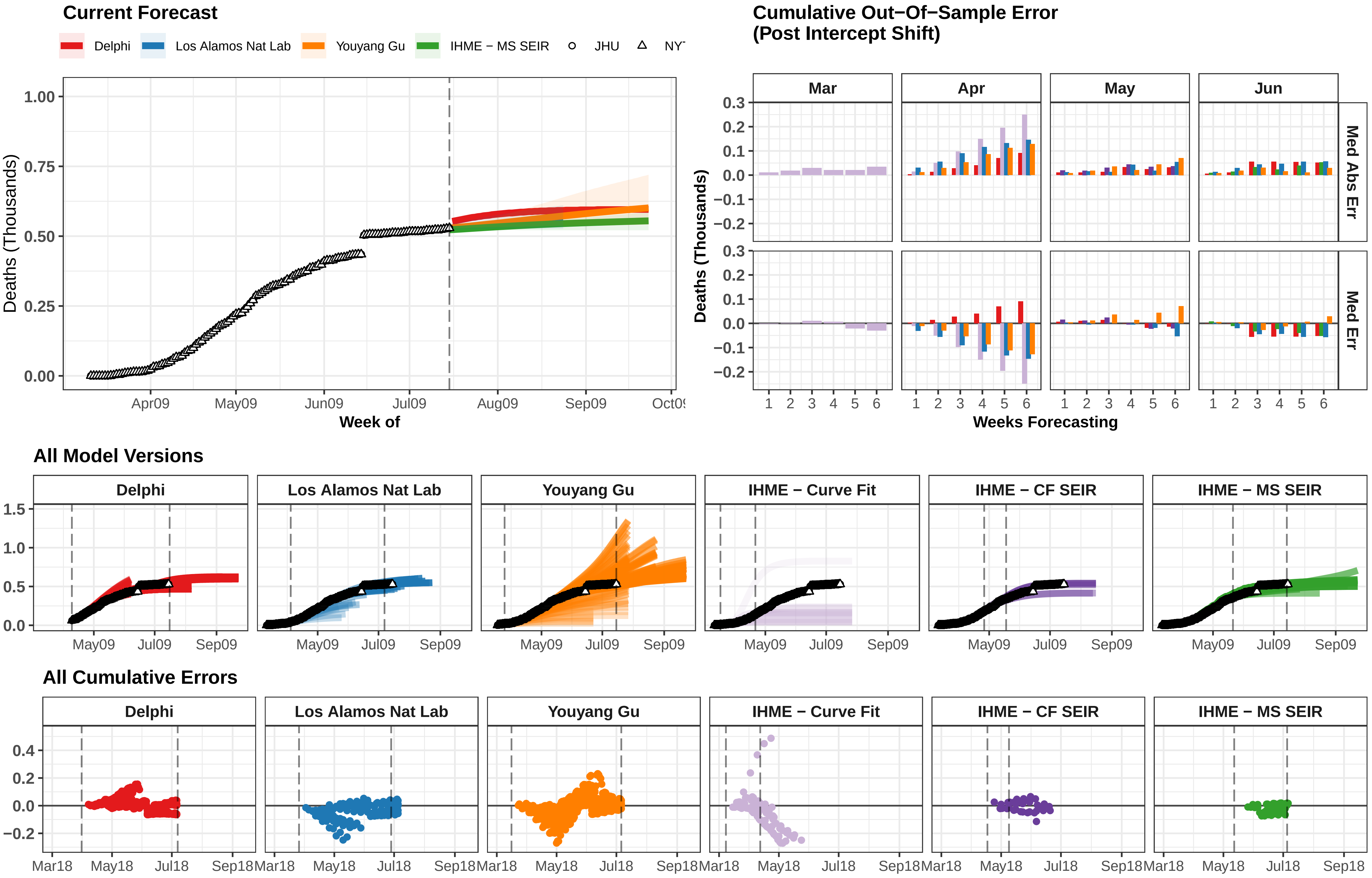

Current Forecast
Delphi
Los Alamos Nat Lab
Youyang Gu
IHME − MS SEIR
JHU
NY
Deaths (Thousands)
Week of
Apr09
May09
Jun09
Jul09
Aug09
Sep09
Cumulative Out−Of−Sample Error
(Post Intercept Shift)
Mar
Apr
May
Jun
Med Abs Err
Med Err
Weeks Forecasting
All Model Versions
Delphi
Los Alamos Nat Lab
Youyang Gu
IHME − Curve Fit
IHME − CF SEIR
IHME − MS SEIR
All Cumulative Errors
Mar18
May18
Jul18
Sep18

Belarus

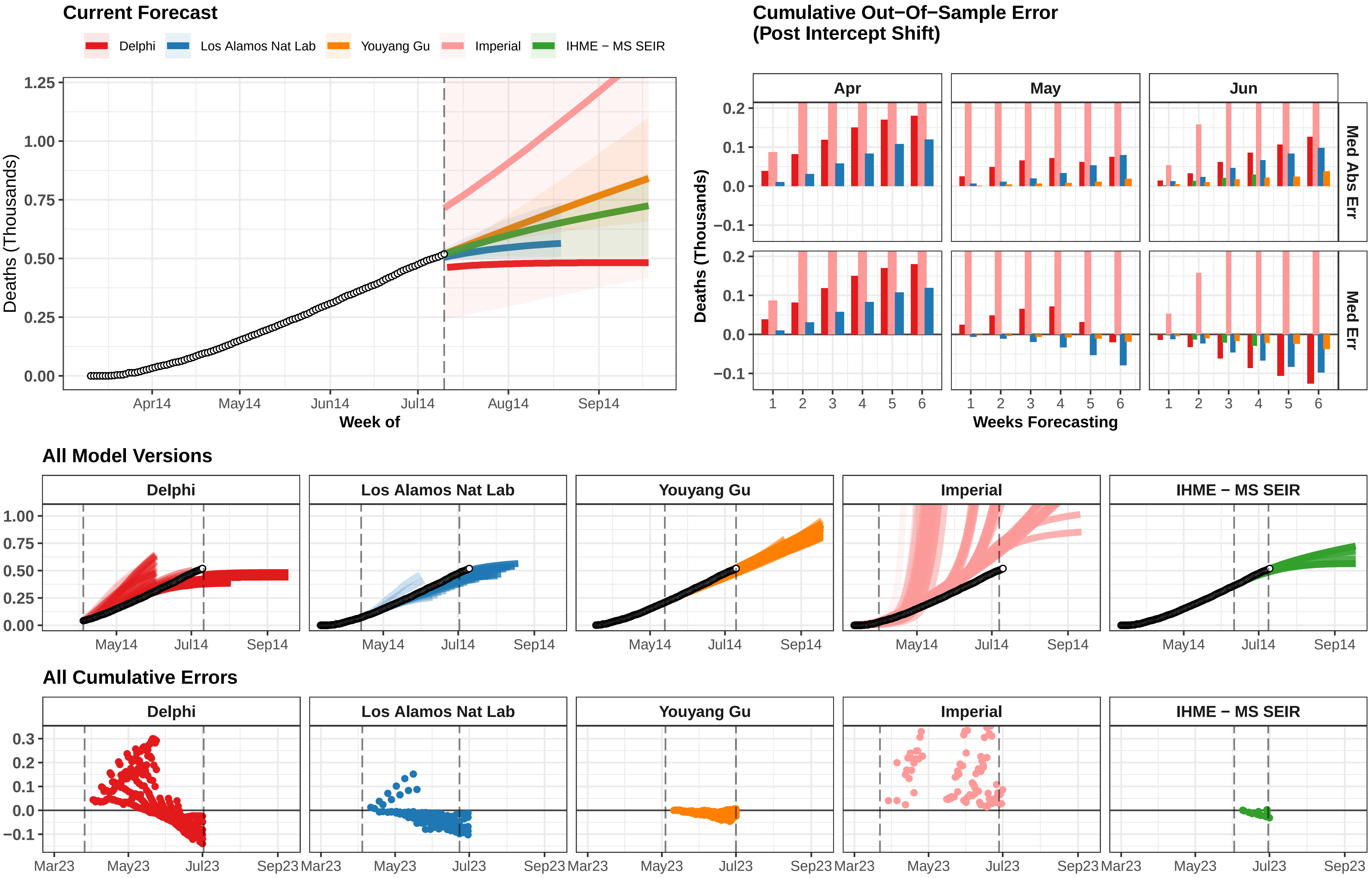

Current Forecast
Delphi
Los Alamos Nat Lab
Youyang Gu
Imperial
IHME − MS SEIR
Deaths (Thousands)
1.25
1.00
0.75
0.50
0.25
0.00
Apr14
May14
Jun14
Jul14
Aug14
Sep14
Week of
Cumulative Out−Of−Sample Error
(Post Intercept Shift)
Apr
May
Jun
Med Abs Err
Med Err
0.2
0.1
0.0
−0.1
1
2
3
4
5
6
Weeks Forecasting
All Model Versions
Delphi
Los Alamos Nat Lab
Youyang Gu
Imperial
IHME − MS SEIR
1.00
0.75
0.50
0.25
0.00
May14
Jul14
Sep14
All Cumulative Errors
0.3
0.2
0.1
0.0
−0.1
Mar23
May23
Jul23
Sep23

Serbia

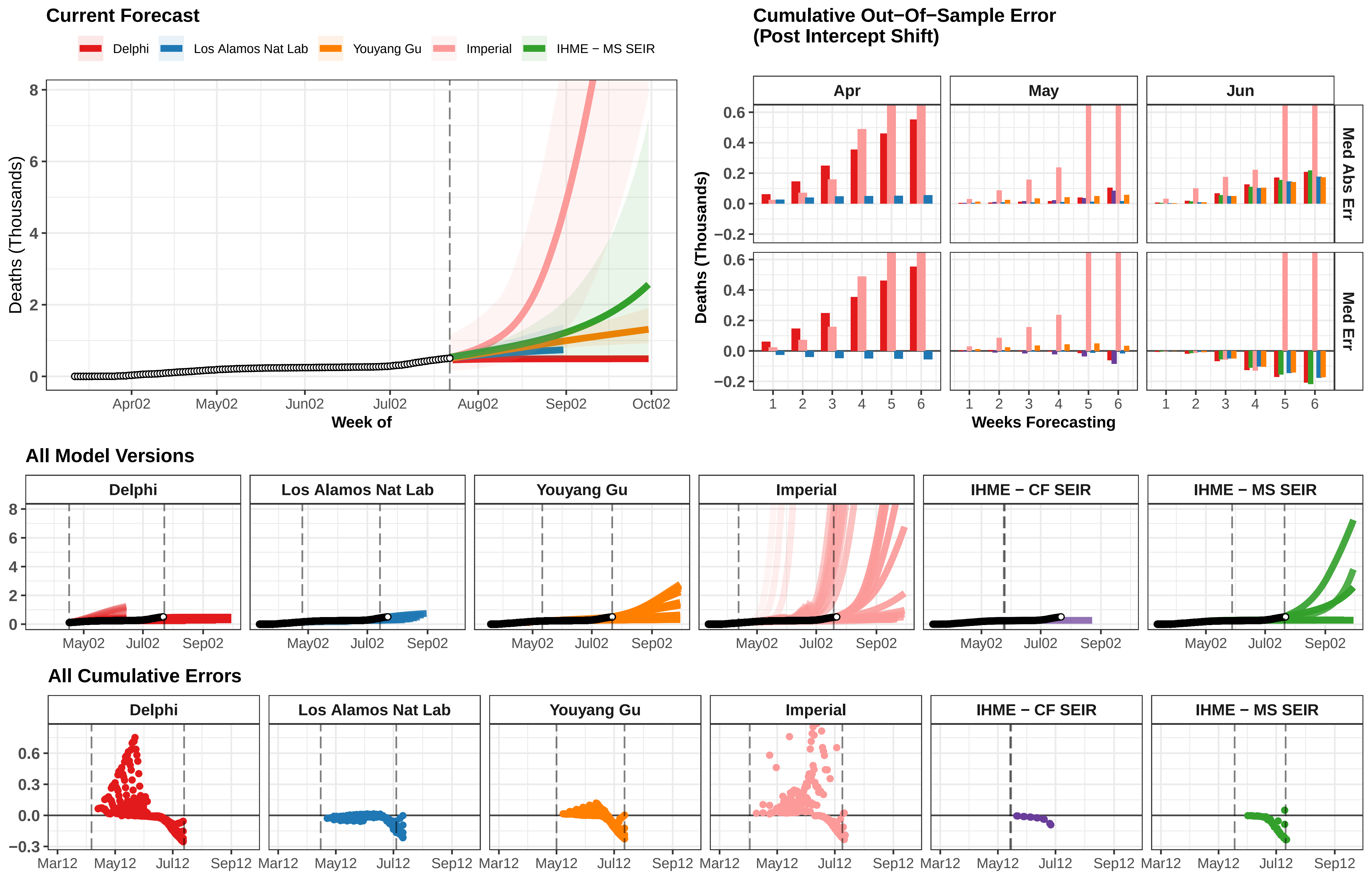
Current Forecast
Delphi
Los Alamos Nat Lab
Youyang Gu
Imperial
IHME − MS SEIR
Deaths (Thousands)
Week of
Apr02
May02
Jun02
Jul02
Aug02
Sep02
Oct02
Cumulative Out−Of−Sample Error
(Post Intercept Shift)
Apr
May
Jun
Med Abs Err
Med Err
Weeks Forecasting
All Model Versions
IHME − CF SEIR
All Cumulative Errors
Mar12
May12
Jul12
Sep12

Oklahoma

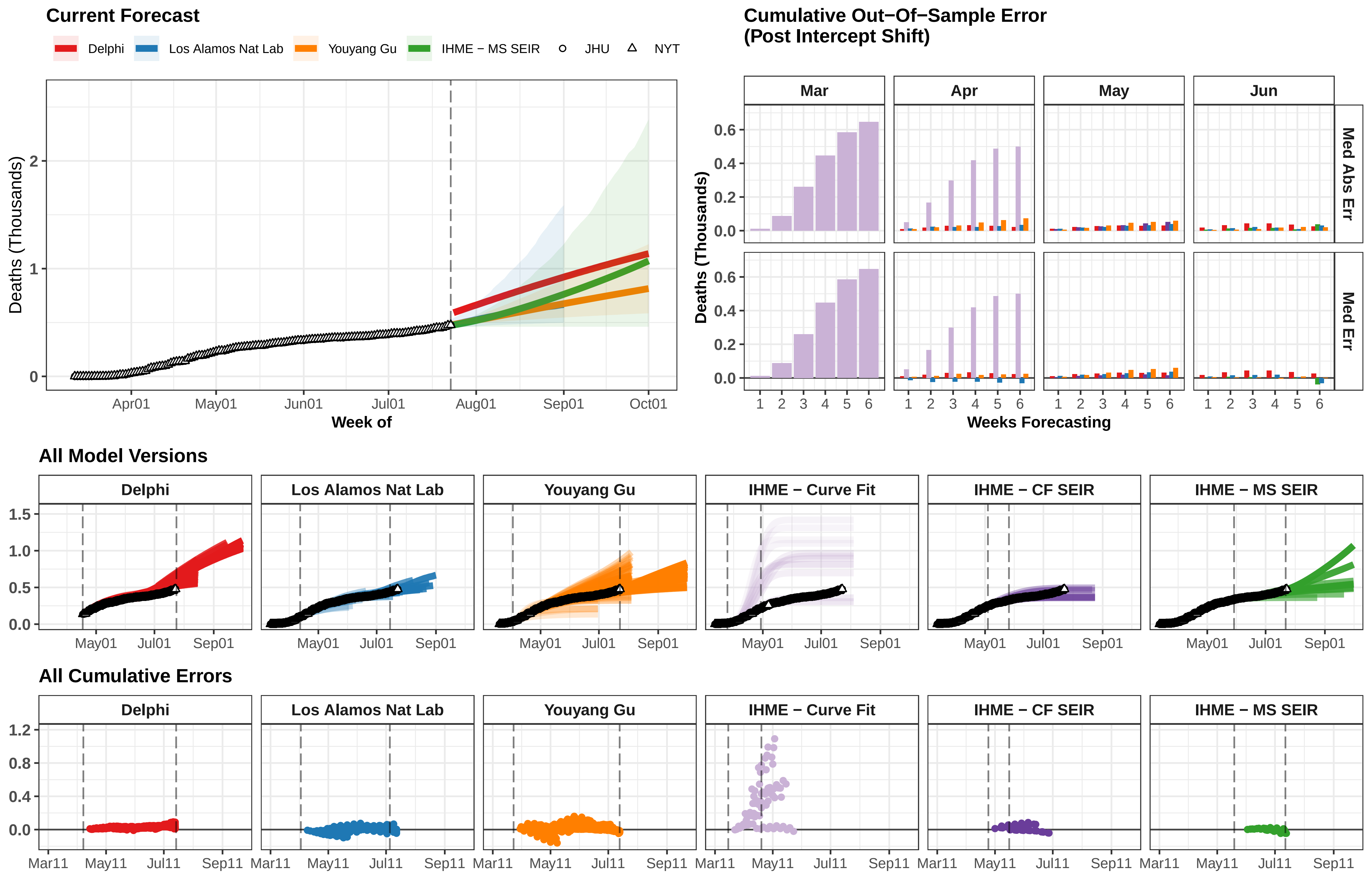

Current Forecast
Delphi
Los Alamos Nat Lab
Youyang Gu
IHME − MS SEIR
JHU
NYT
Deaths (Thousands)
0
1
2
Apr01
May01
Jun01
Jul01
Aug01
Sep01
Oct01
Week of
Cumulative Out−Of−Sample Error
(Post Intercept Shift)
Mar
Apr
May
Jun
Med Abs Err
Med Err
Deaths (Thousands)
0.0
0.2
0.4
0.6
1 2 3 4 5 6
Weeks Forecasting
All Model Versions
Delphi
Los Alamos Nat Lab
Youyang Gu
IHME − Curve Fit
IHME − CF SEIR
IHME − MS SEIR
0.0
0.5
1.0
1.5
May01
Jul01
Sep01
All Cumulative Errors
0.0
0.4
0.8
1.2
Mar11
May11
Jul11
Sep11

Yemen

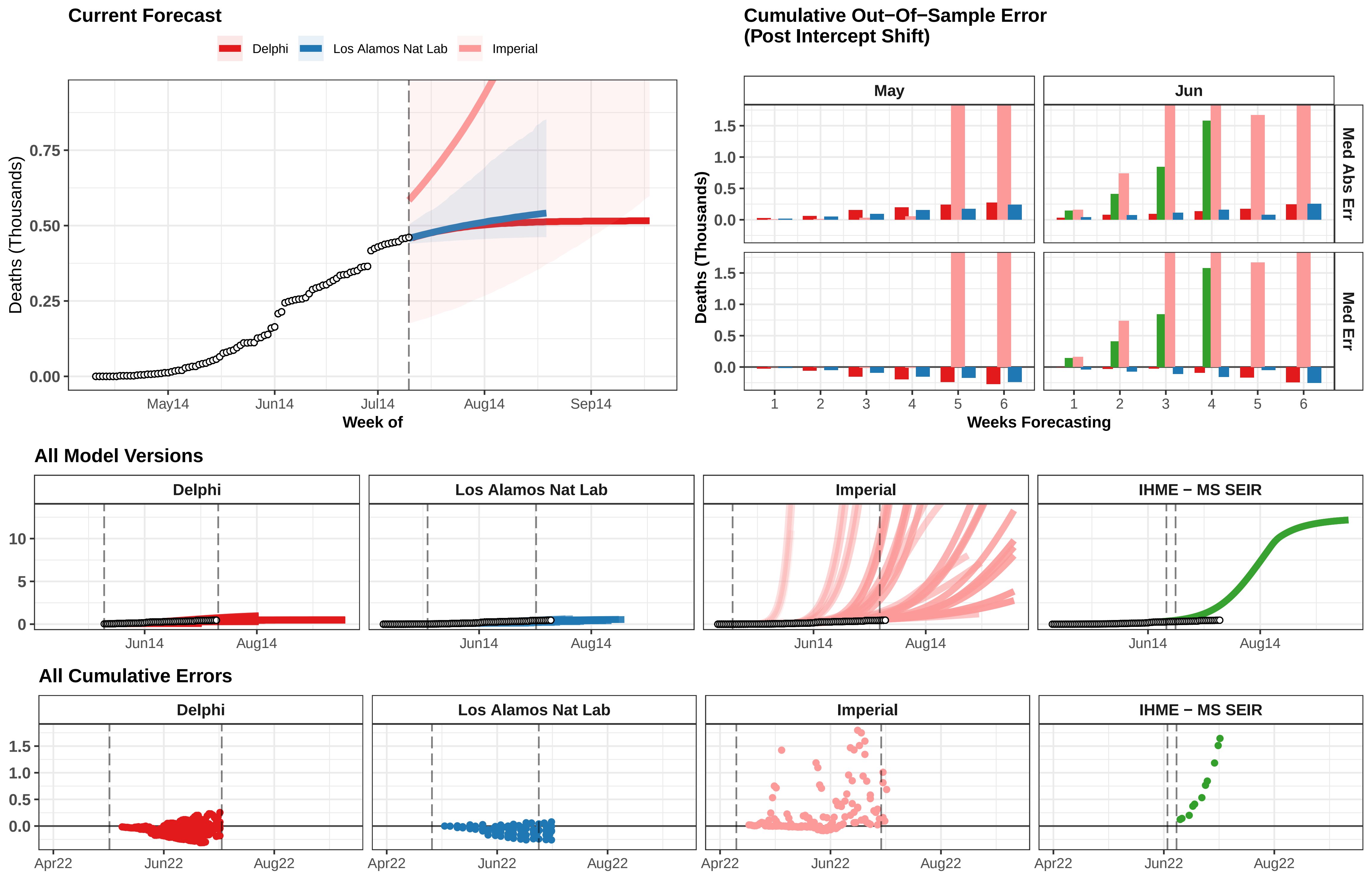
Current Forecast
Delphi
Los Alamos Nat Lab
Imperial
Deaths (Thousands)
Week of
May14
Jun14
Jul14
Aug14
Sep14
Cumulative Out−Of−Sample Error
(Post Intercept Shift)
May
Jun
Med Abs Err
Med Err
Weeks Forecasting
All Model Versions
IHME − MS SEIR
All Cumulative Errors
Apr22
Jun22
Aug22

Macedonia

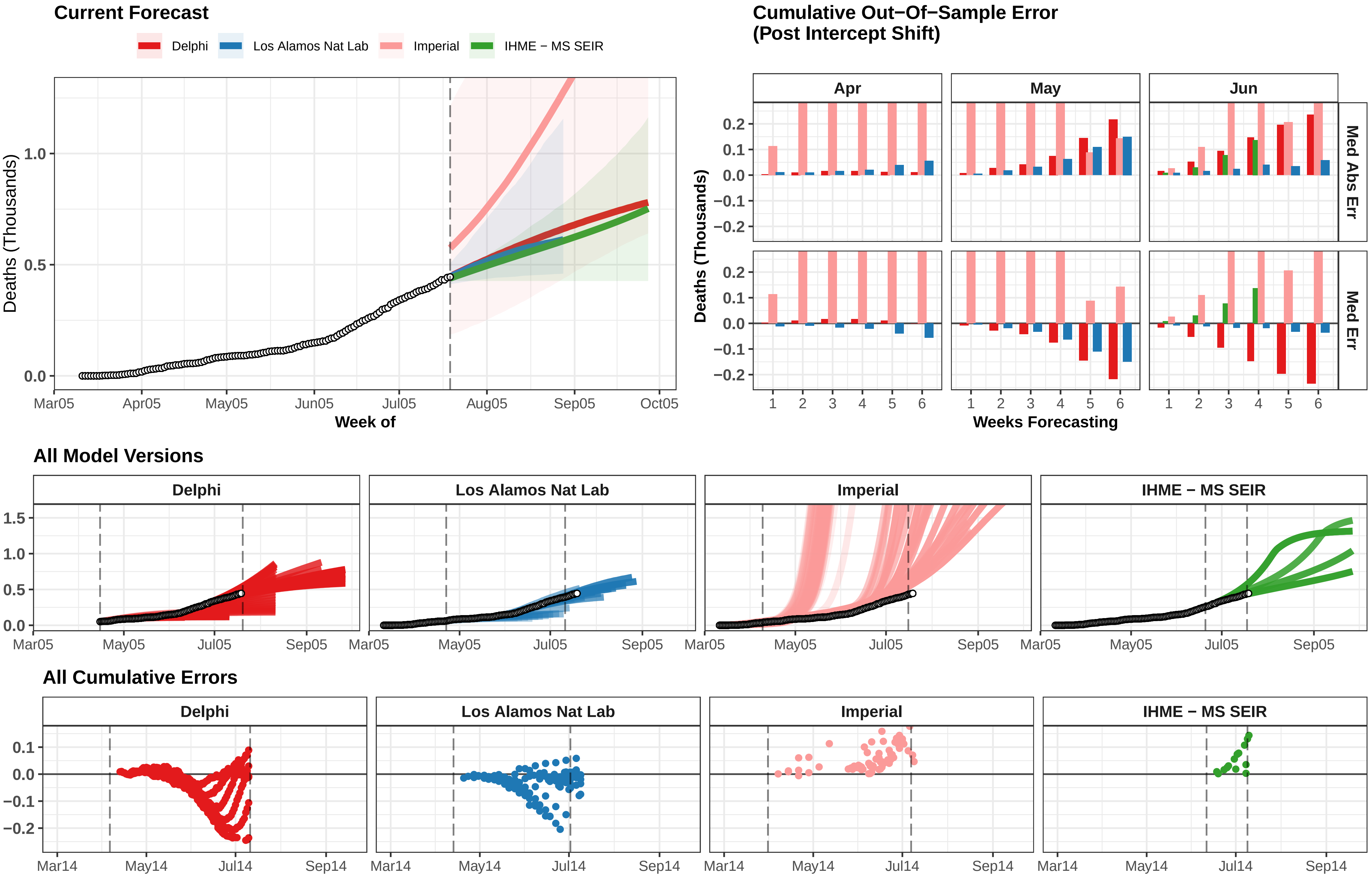

Current Forecast
Delphi
Los Alamos Nat Lab
Imperial
IHME − MS SEIR
Deaths (Thousands)
Week of
Cumulative Out−Of−Sample Error
(Post Intercept Shift)
Apr
May
Jun
Med Abs Err
Med Err
Weeks Forecasting
All Model Versions
Delphi
Los Alamos Nat Lab
Imperial
IHME − MS SEIR
All Cumulative Errors
Delphi
Los Alamos Nat Lab
Imperial
IHME − MS SEIR

Israel

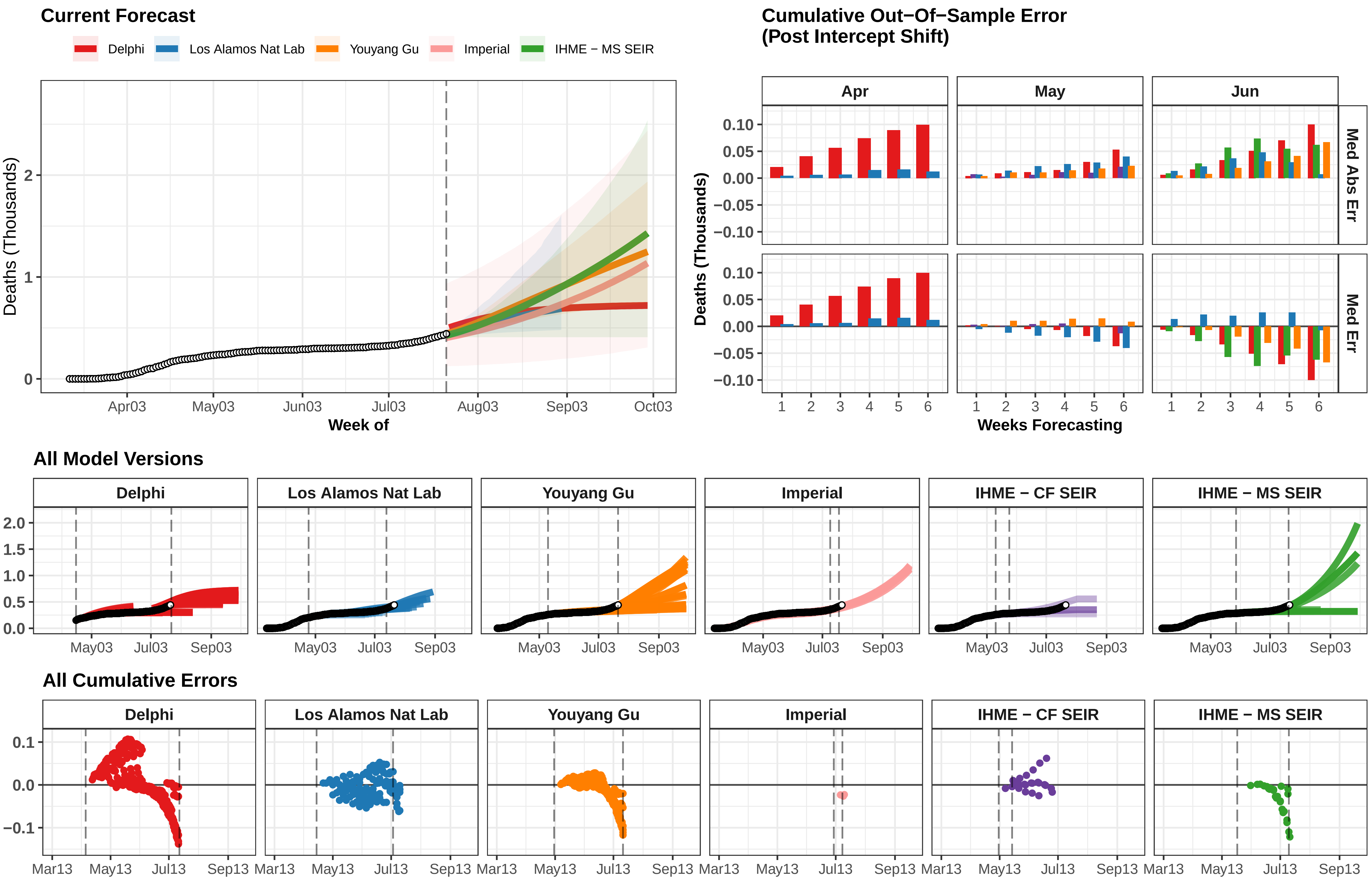
Current Forecast
Delphi
Los Alamos Nat Lab
Youyang Gu
Imperial
IHME − MS SEIR
Deaths (Thousands)
0
1
2
Apr03
May03
Jun03
Jul03
Aug03
Sep03
Oct03
Week of
Cumulative Out−Of−Sample Error
(Post Intercept Shift)
Apr
May
Jun
Med Abs Err
Med Err
0.10
0.05
0.00
−0.05
−0.10
1
2
3
4
5
6
Weeks Forecasting
All Model Versions
Delphi
Los Alamos Nat Lab
Youyang Gu
Imperial
IHME − CF SEIR
IHME − MS SEIR
2.0
1.5
1.0
0.5
0.0
May03
Jul03
Sep03
All Cumulative Errors
0.1
0.0
−0.1
Mar13
May13
Jul13
Sep13

Kuwait

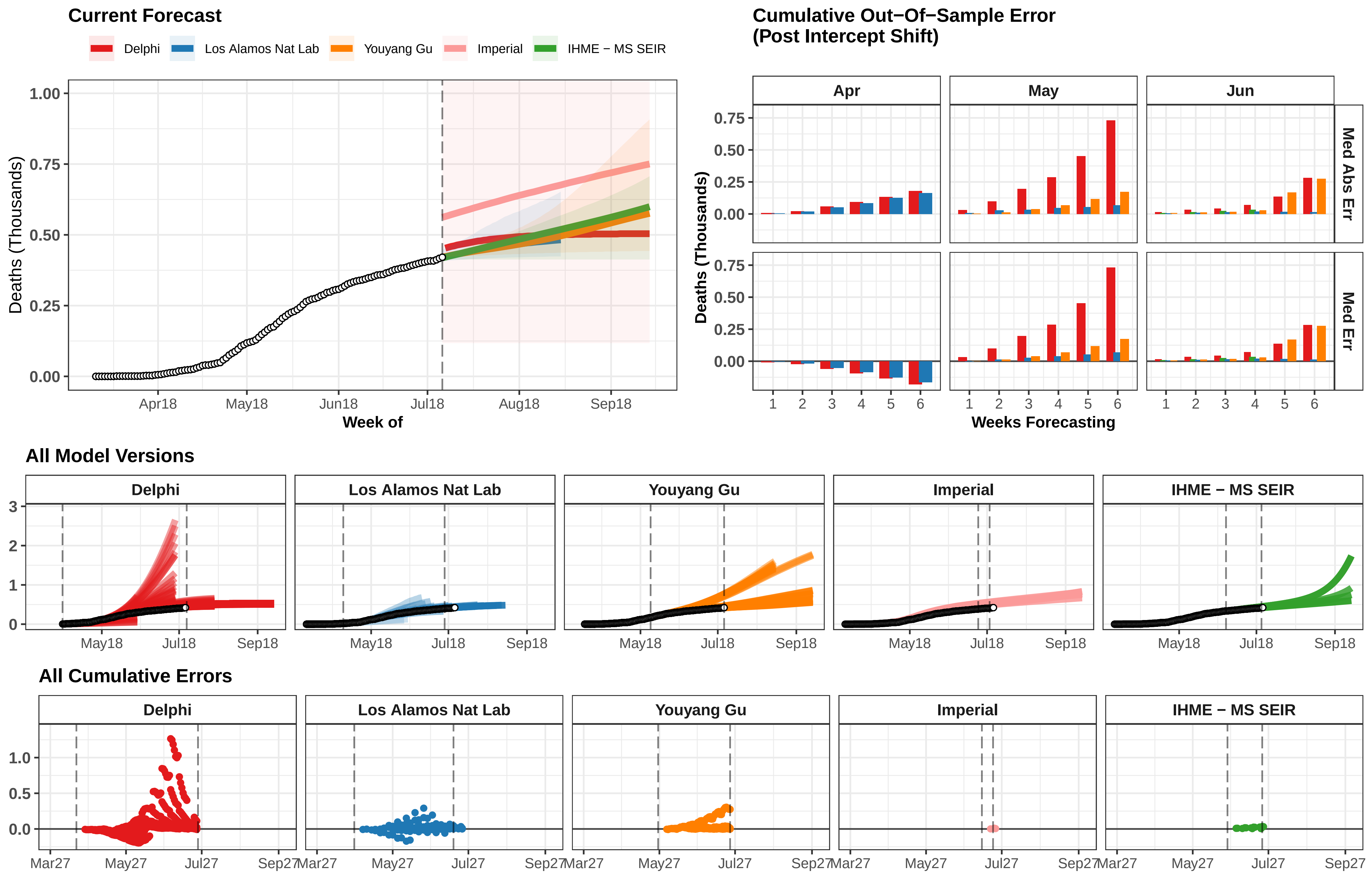

Current Forecast
Delphi
Los Alamos Nat Lab
Youyang Gu
Imperial
IHME − MS SEIR
Deaths (Thousands)
1.00
0.75
0.50
0.25
0.00
Apr18
May18
Jun18
Jul18
Aug18
Sep18
Week of
Cumulative Out−Of−Sample Error
(Post Intercept Shift)
Apr
May
Jun
Med Abs Err
Med Err
Weeks Forecasting
All Model Versions
May18
Jul18
Sep18
All Cumulative Errors
Mar27
May27
Jul27
Sep27

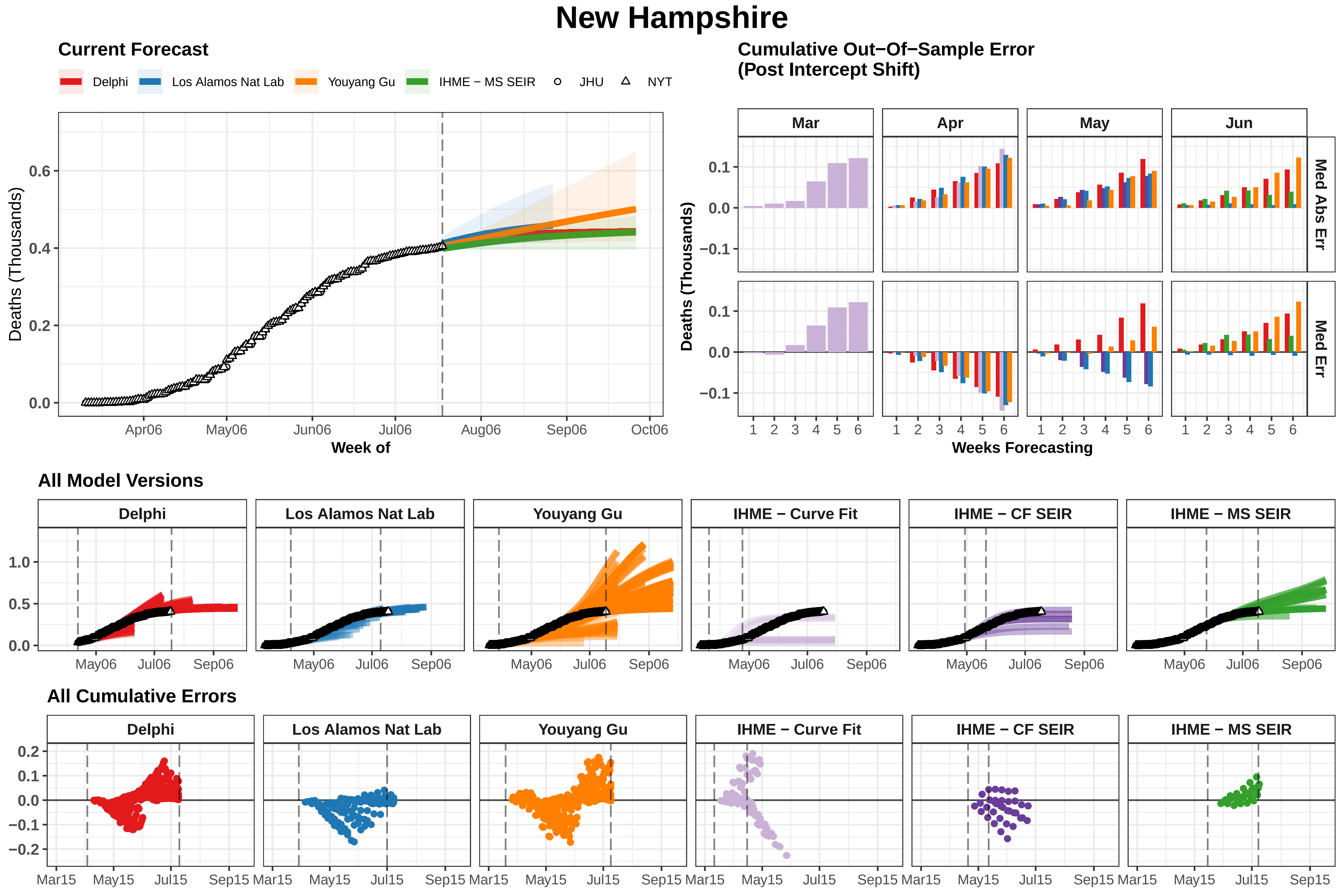
New Hampshire
Current Forecast
Delphi
Los Alamos Nat Lab
Youyang Gu
IHME − MS SEIR
JHU
NYT
Deaths (Thousands)
0.0
0.2
0.4
0.6
Apr06
May06
Jun06
Jul06
Aug06
Sep06
Oct06
Week of
Cumulative Out−Of−Sample Error
(Post Intercept Shift)
Mar
Apr
May
Jun
Med Abs Err
Med Err
−0.1
0.0
0.1
1
2
3
4
5
6
Weeks Forecasting
All Model Versions
Delphi
Los Alamos Nat Lab
Youyang Gu
IHME − Curve Fit
IHME − CF SEIR
IHME − MS SEIR
0.0
0.5
1.0
May06
Jul06
Sep06
All Cumulative Errors
−0.2
−0.1
0.0
0.1
0.2
Mar15
May15
Jul15
Sep15

Azerbaijan

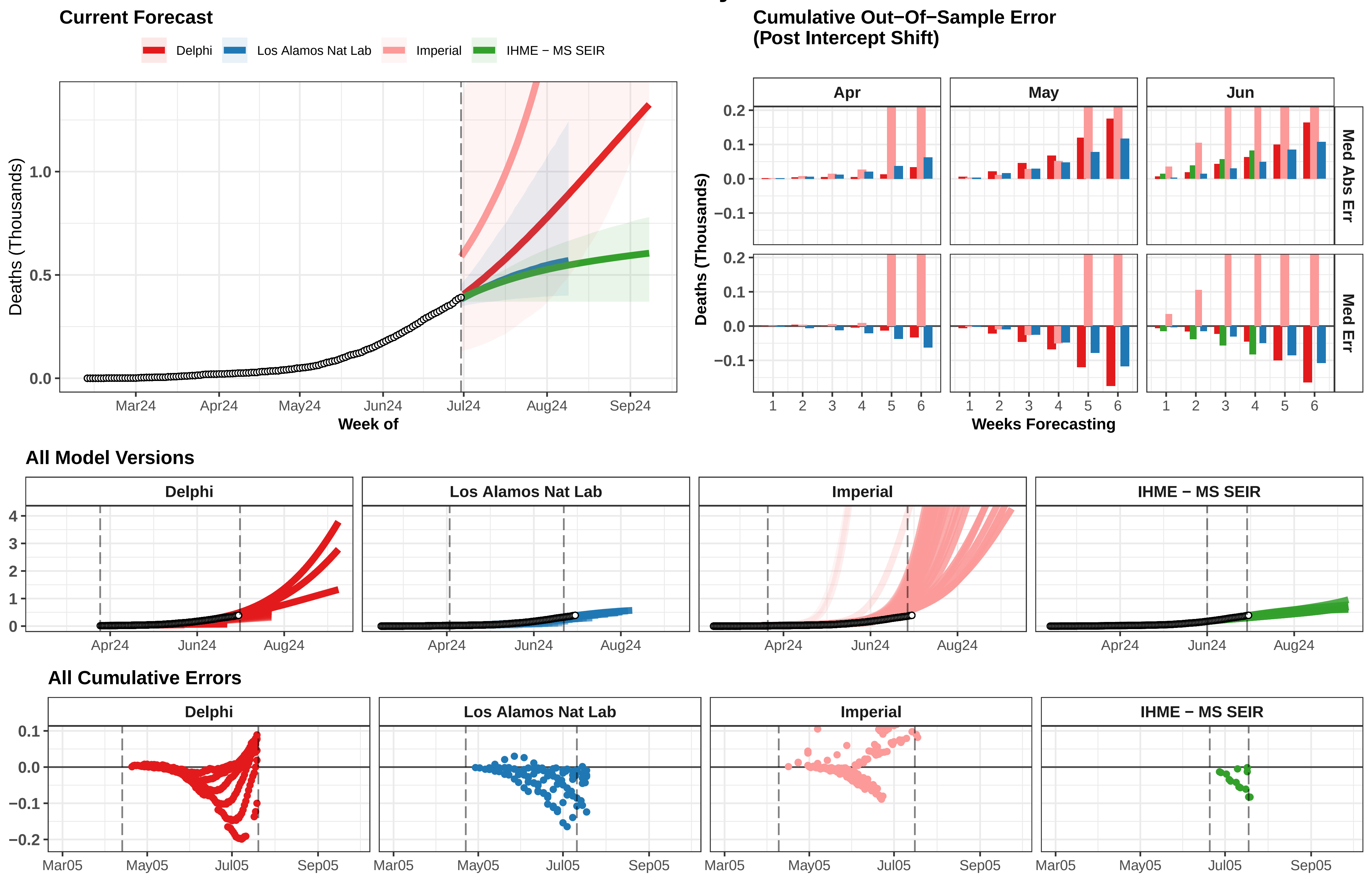

Current Forecast
Delphi
Los Alamos Nat Lab
Imperial
IHME − MS SEIR
Deaths (Thousands)
Week of
Mar24
Apr24
May24
Jun24
Jul24
Aug24
Sep24
Cumulative Out−Of−Sample Error
(Post Intercept Shift)
Apr
May
Jun
Med Abs Err
Med Err
Weeks Forecasting
All Model Versions
All Cumulative Errors
Mar05
May05
Jul05
Sep05

Arkansas

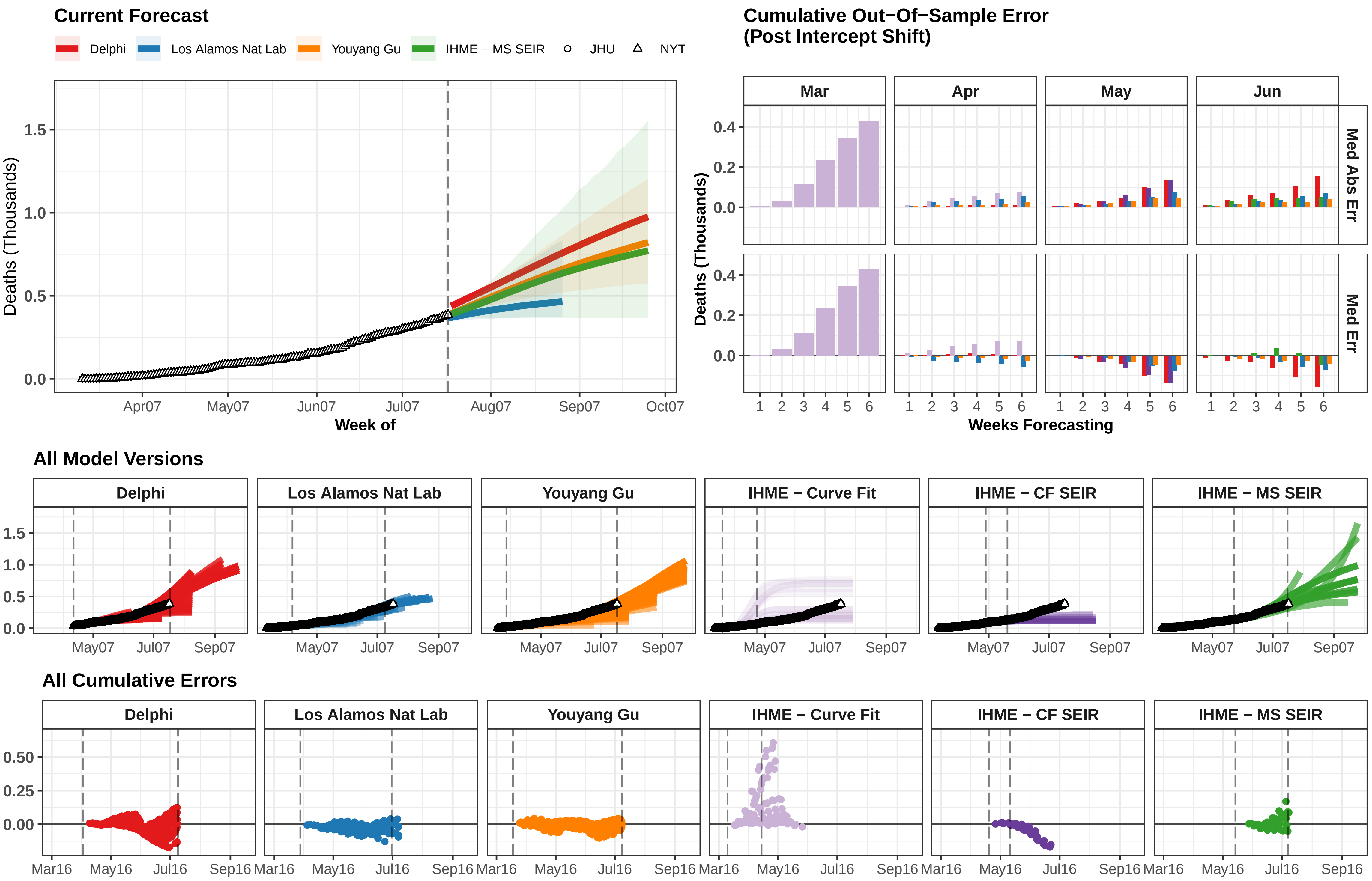
Current Forecast
Delphi
Los Alamos Nat Lab
Youyang Gu
IHME − MS SEIR
JHU
NYT
Deaths (Thousands)
Week of
Apr07
May07
Jun07
Jul07
Aug07
Sep07
Oct07
Cumulative Out−Of−Sample Error
(Post Intercept Shift)
Mar
Apr
May
Jun
Med Abs Err
Med Err
Weeks Forecasting
All Model Versions
Delphi
Los Alamos Nat Lab
Youyang Gu
IHME − Curve Fit
IHME − CF SEIR
IHME − MS SEIR
All Cumulative Errors
Mar16
May16
Jul16
Sep16

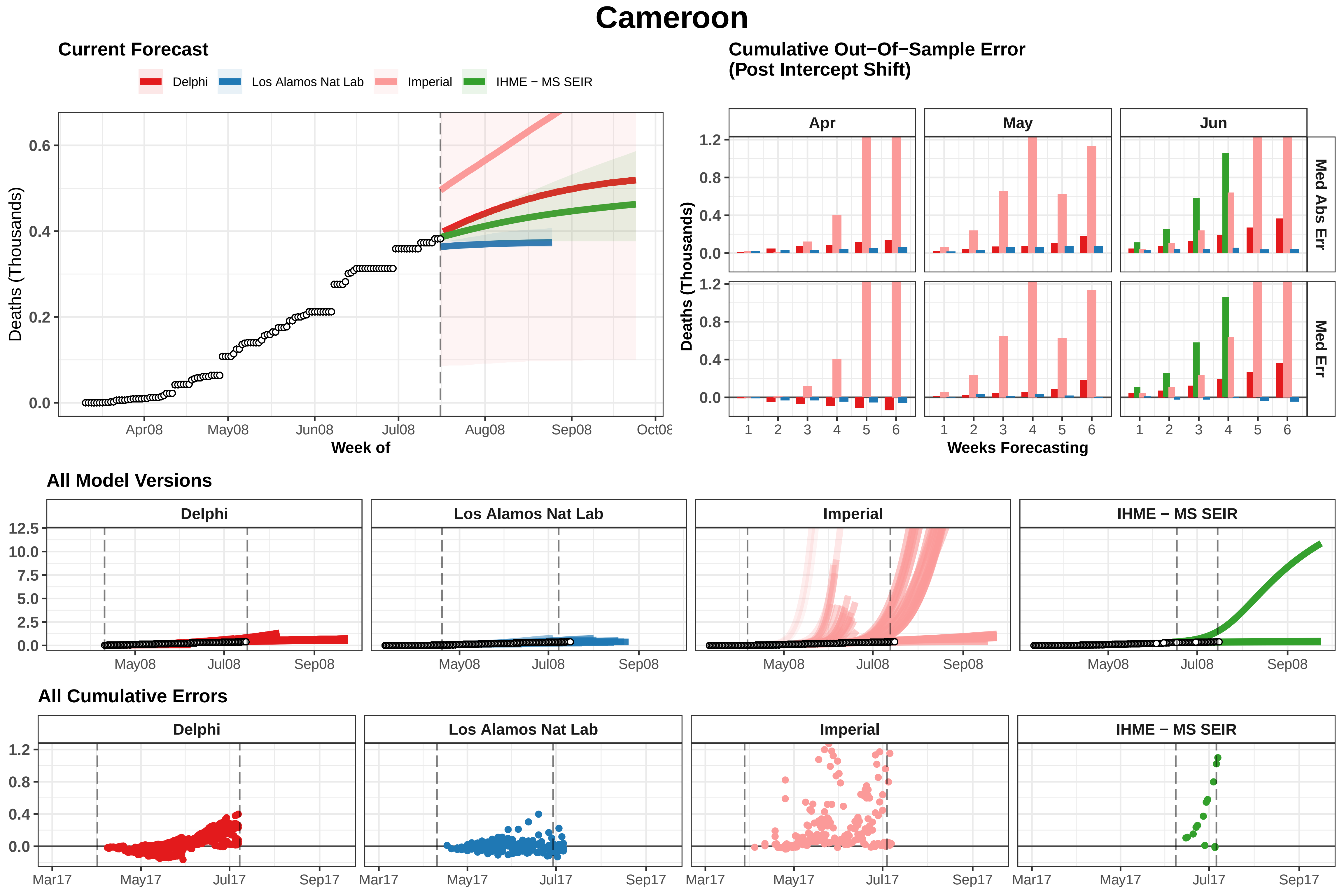
Cameroon
Current Forecast
Delphi
Los Alamos Nat Lab
Imperial
IHME − MS SEIR
Deaths (Thousands)
Week of
Apr08
May08
Jun08
Jul08
Aug08
Sep08
Oct08
Cumulative Out−Of−Sample Error
(Post Intercept Shift)
Apr
May
Jun
Med Abs Err
Med Err
Weeks Forecasting
All Model Versions
All Cumulative Errors
Mar17
May17
Jul17
Sep17

El Salvador

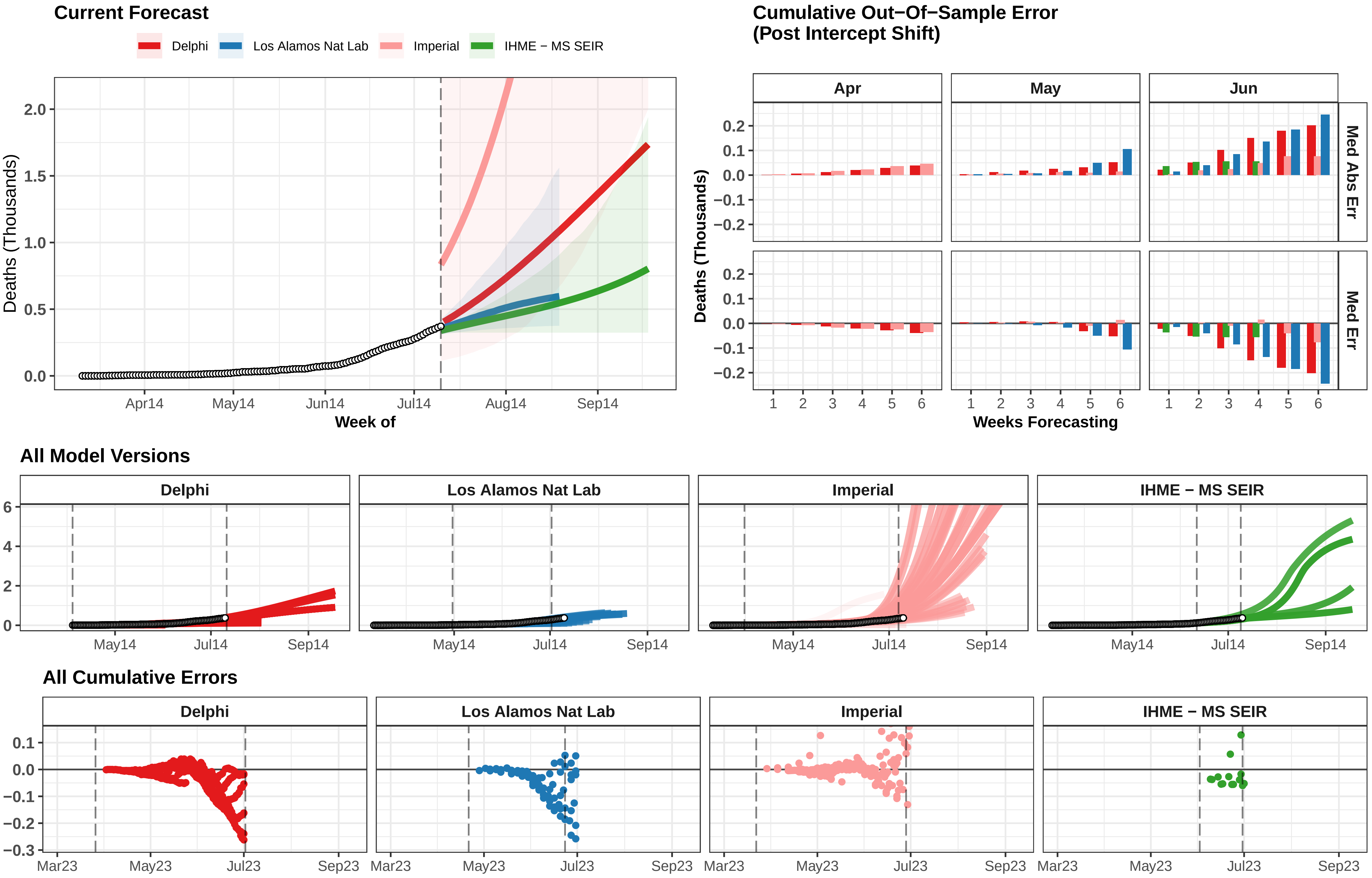
Current Forecast
Delphi
Los Alamos Nat Lab
Imperial
IHME − MS SEIR
Deaths (Thousands)
2.0
1.5
1.0
0.5
0.0
Apr14
May14
Jun14
Jul14
Aug14
Sep14
Week of
Cumulative Out−Of−Sample Error
(Post Intercept Shift)
Apr
May
Jun
Med Abs Err
Med Err
0.2
0.1
0.0
−0.1
−0.2
1
2
3
4
5
6
Weeks Forecasting
All Model Versions
Delphi
Los Alamos Nat Lab
Imperial
IHME − MS SEIR
6
4
2
0
May14
Jul14
Sep14
All Cumulative Errors
0.1
0.0
−0.1
−0.2
−0.3
Mar23
May23
Jul23
Sep23

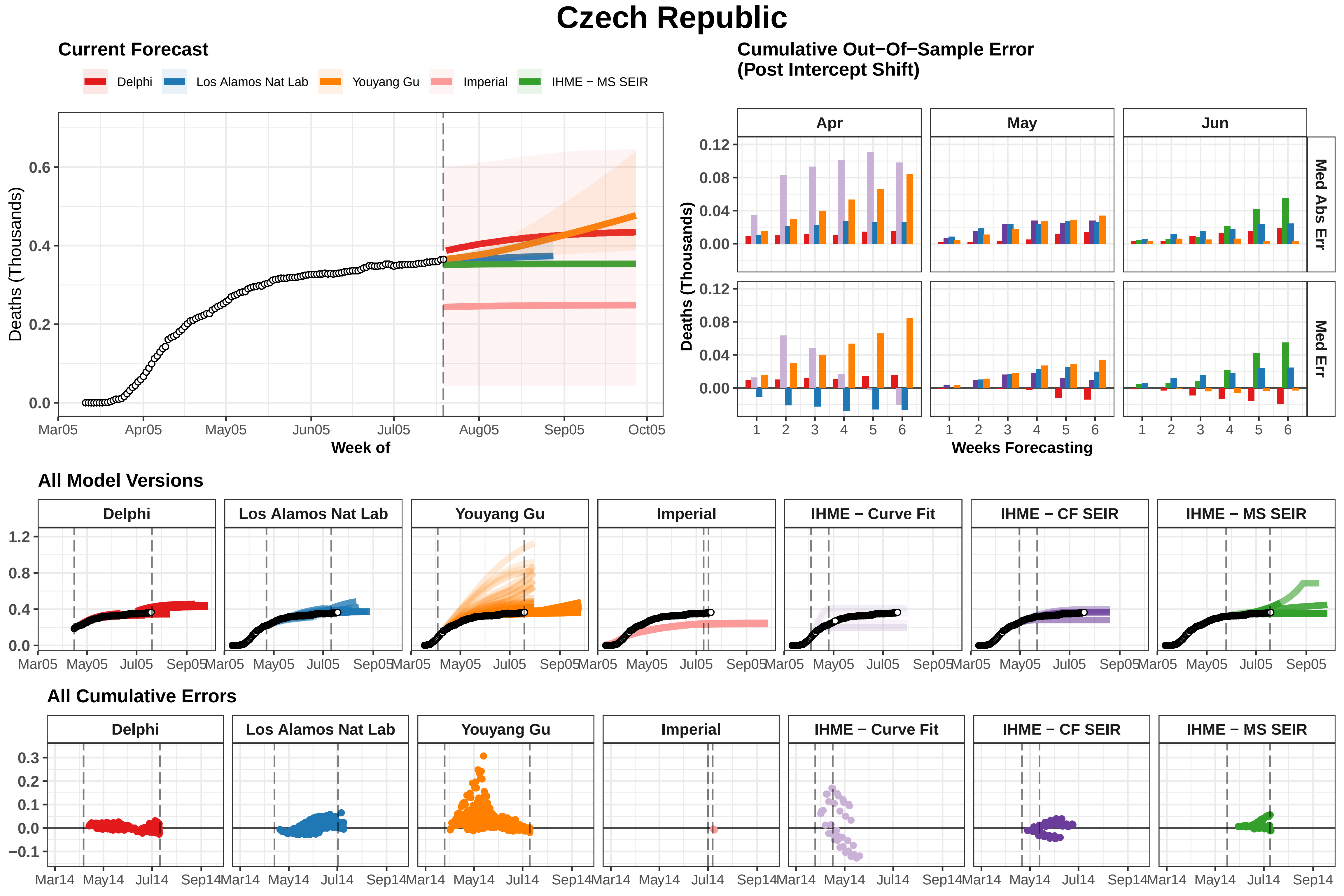

Czech Republic
Current Forecast
Delphi
Los Alamos Nat Lab
Youyang Gu
Imperial
IHME − MS SEIR
Deaths (Thousands)
Week of
Cumulative Out−Of−Sample Error
(Post Intercept Shift)
Apr
May
Jun
Med Abs Err
Med Err
Weeks Forecasting
All Model Versions
Delphi
Los Alamos Nat Lab
Youyang Gu
Imperial
IHME − Curve Fit
IHME − CF SEIR
IHME − MS SEIR
All Cumulative Errors

Oman

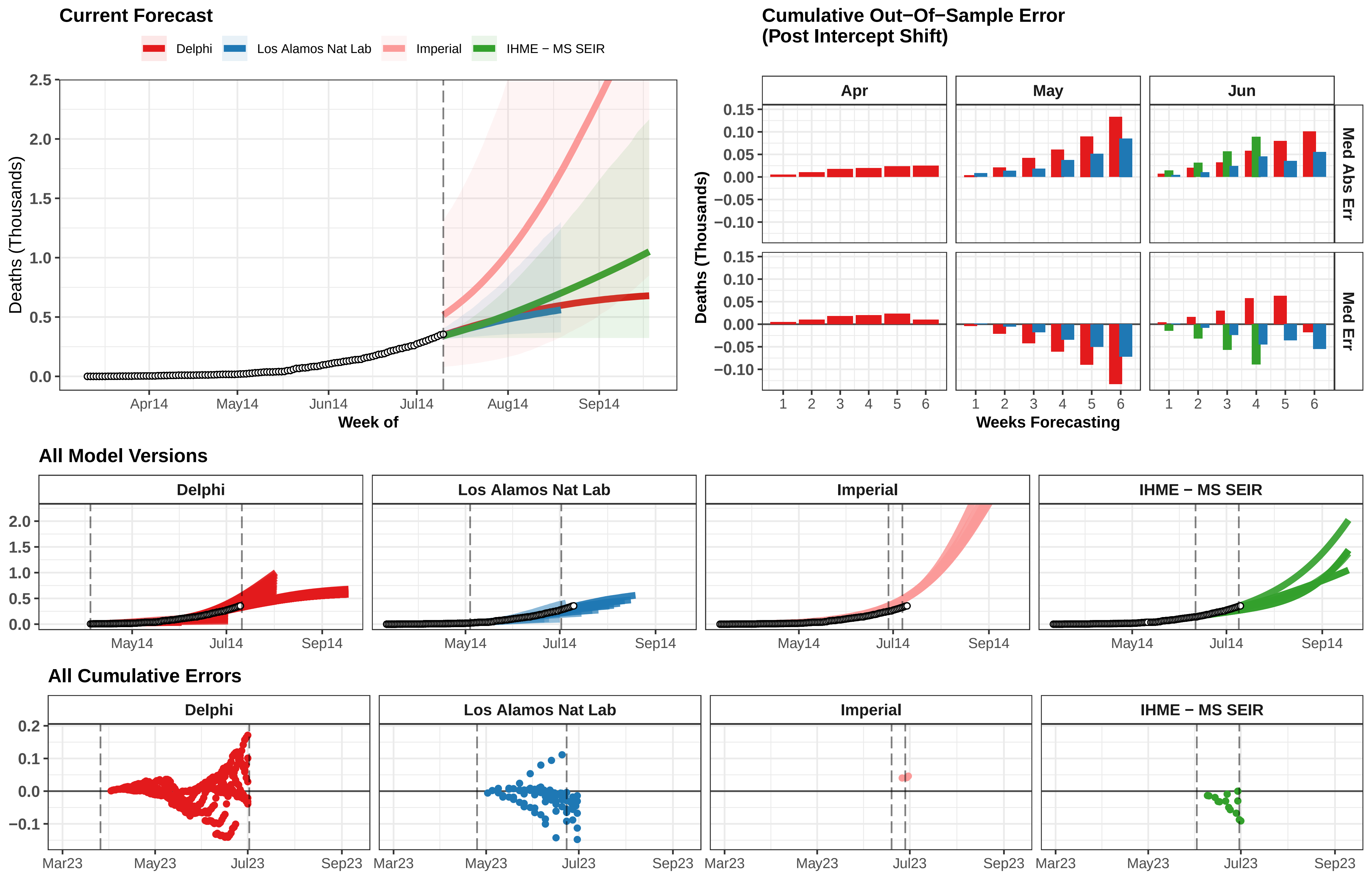
Current Forecast
Delphi
Los Alamos Nat Lab
Imperial
IHME − MS SEIR
Deaths (Thousands)
Week of
Apr14
May14
Jun14
Jul14
Aug14
Sep14
Cumulative Out−Of−Sample Error
(Post Intercept Shift)
Apr
May
Jun
Med Abs Err
Med Err
Weeks Forecasting
All Model Versions
All Cumulative Errors
Mar23
May23
Jul23
Sep23

United Arab Emirates

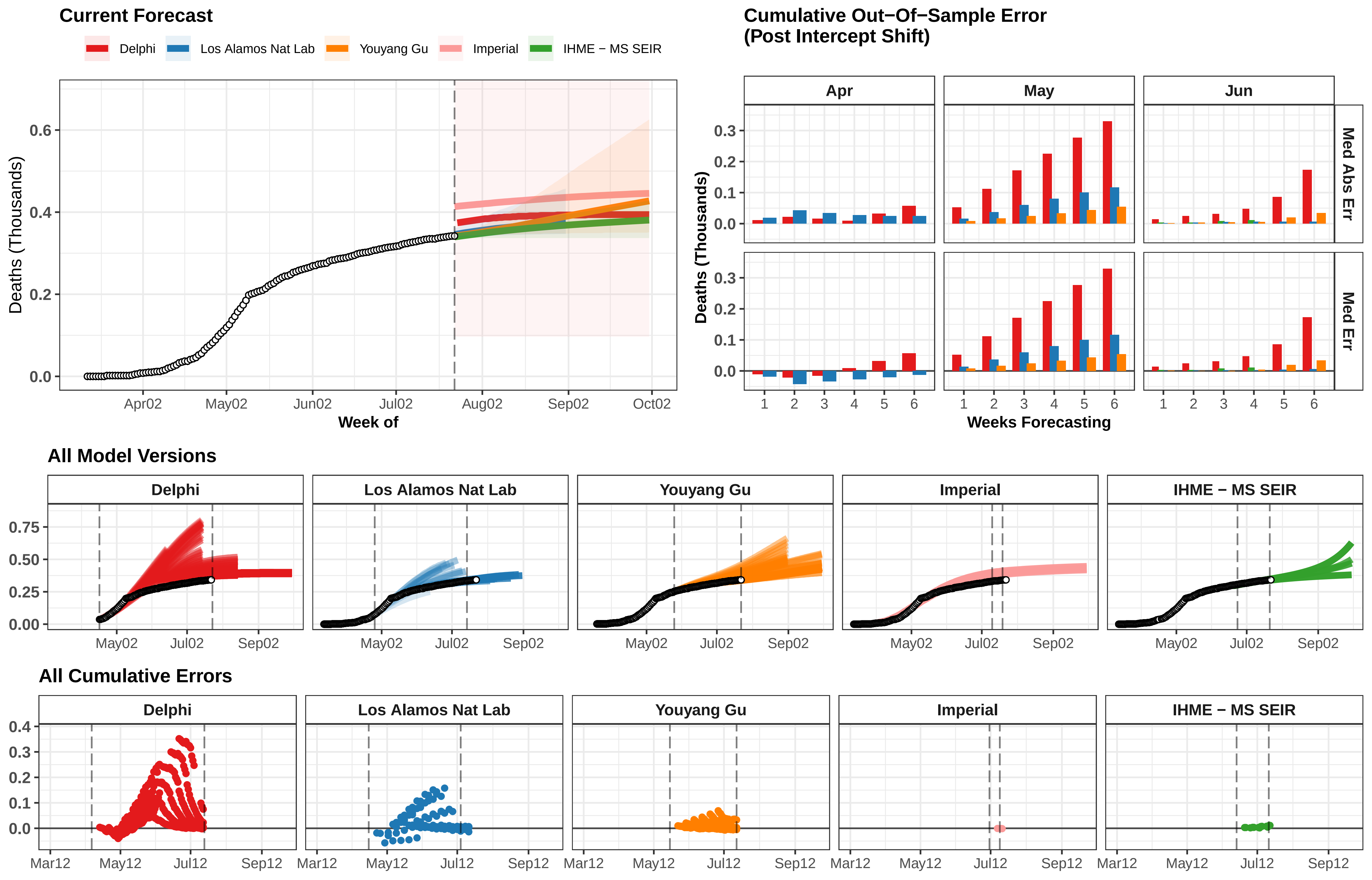

Bulgaria

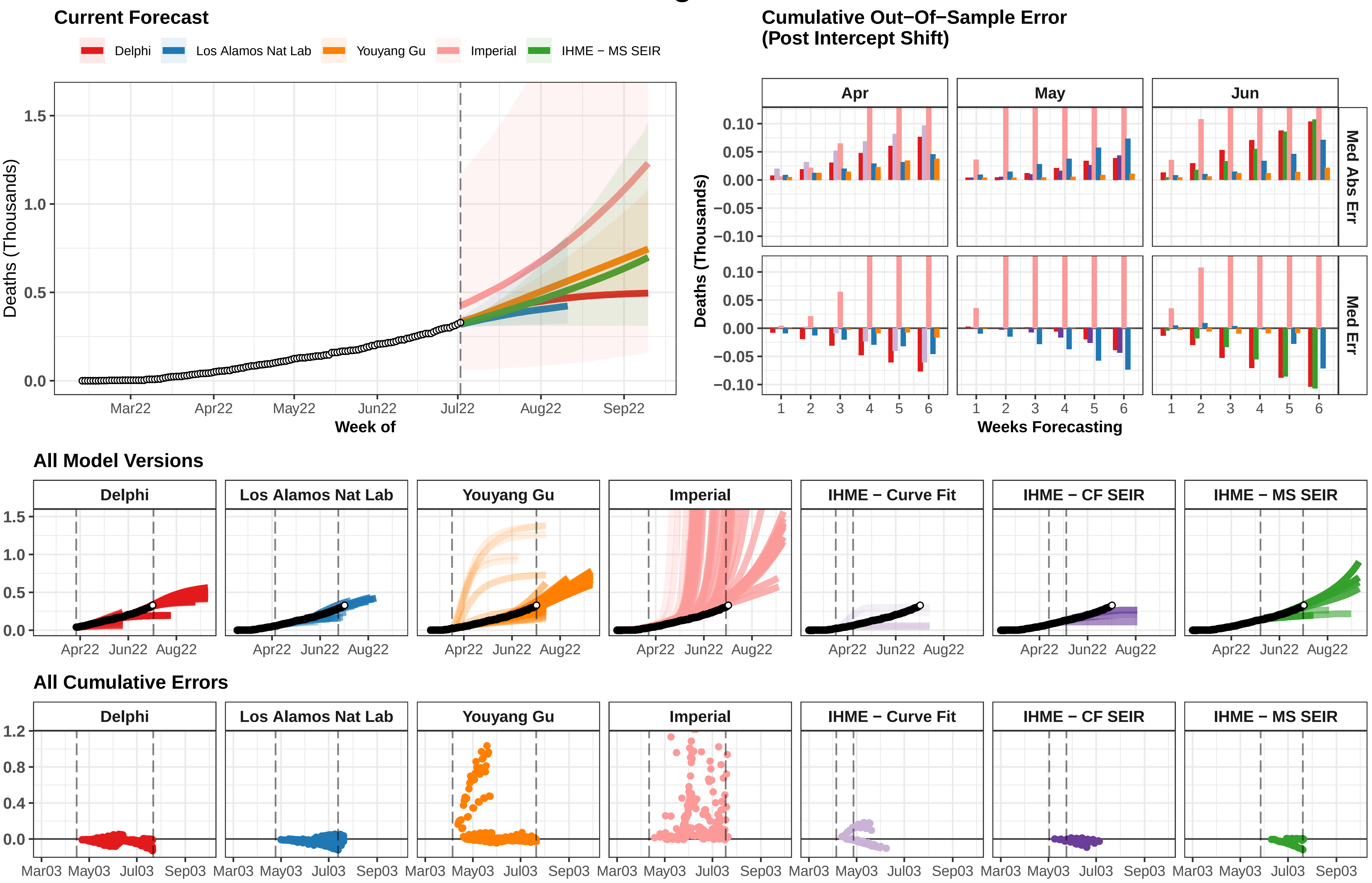

Current Forecast
Delphi
Los Alamos Nat Lab
Youyang Gu
Imperial
IHME − MS SEIR
Deaths (Thousands)
Week of
Mar22
Apr22
May22
Jun22
Jul22
Aug22
Sep22
Cumulative Out−Of−Sample Error
(Post Intercept Shift)
Apr
May
Jun
Med Abs Err
Med Err
Weeks Forecasting
All Model Versions
Delphi
Los Alamos Nat Lab
Youyang Gu
Imperial
IHME − Curve Fit
IHME − CF SEIR
IHME − MS SEIR
All Cumulative Errors
Mar03
May03
Jul03
Sep03

Finland

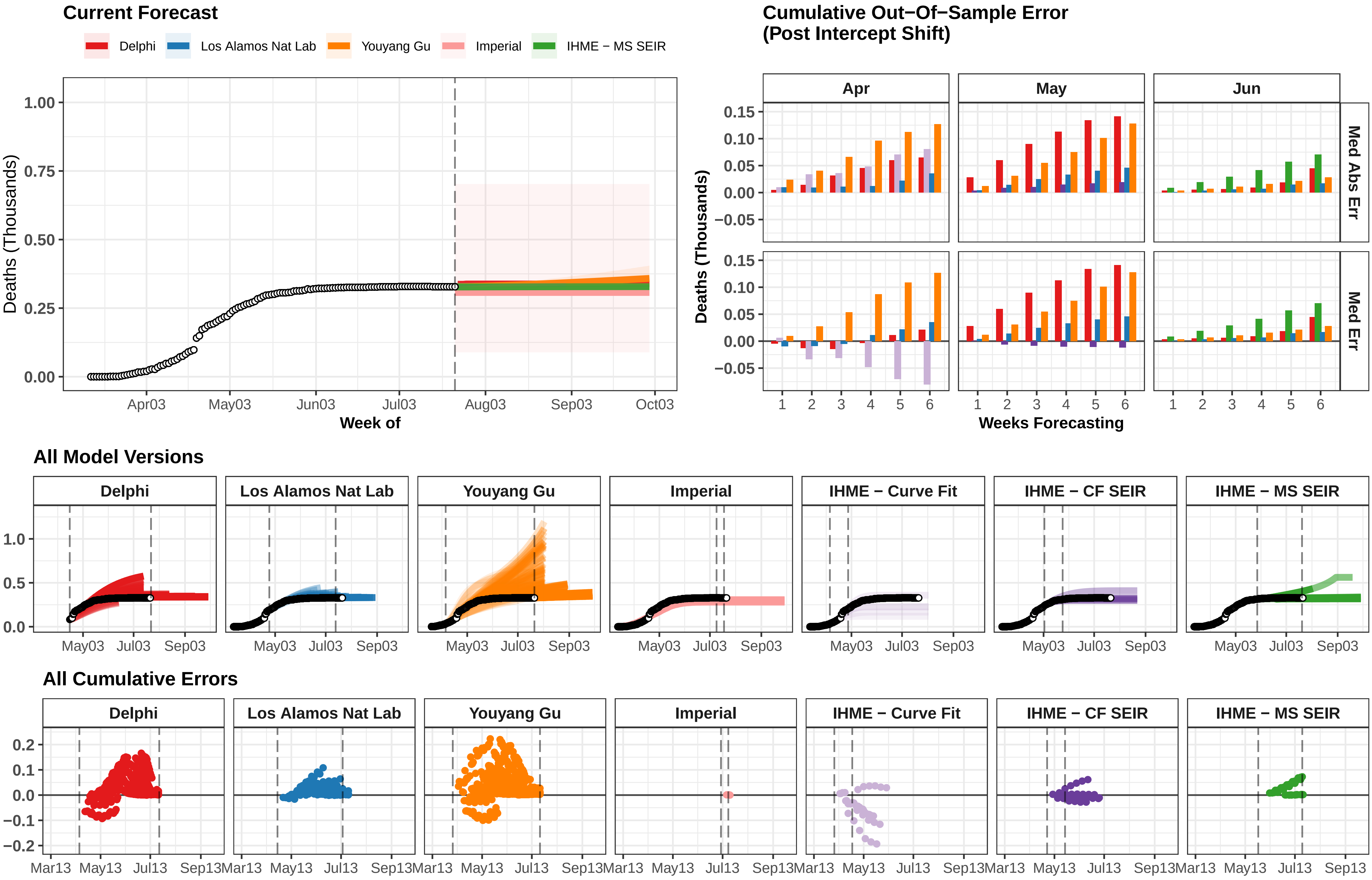

Current Forecast
Delphi
Los Alamos Nat Lab
Youyang Gu
Imperial
IHME − MS SEIR
Deaths (Thousands)
1.00
0.75
0.50
0.25
0.00
Apr03
May03
Jun03
Jul03
Aug03
Sep03
Oct03
Week of
Cumulative Out−Of−Sample Error
(Post Intercept Shift)
Apr
May
Jun
Med Abs Err
Med Err
0.15
0.10
0.05
0.00
−0.05
Weeks Forecasting
1 2 3 4 5 6
All Model Versions
Delphi
Los Alamos Nat Lab
Youyang Gu
Imperial
IHME − Curve Fit
IHME − CF SEIR
IHME − MS SEIR
1.0
0.5
0.0
May03 Jul03 Sep03
All Cumulative Errors
0.2
0.1
0.0
−0.1
−0.2
Mar13 May13 Jul13 Sep13

Kansas

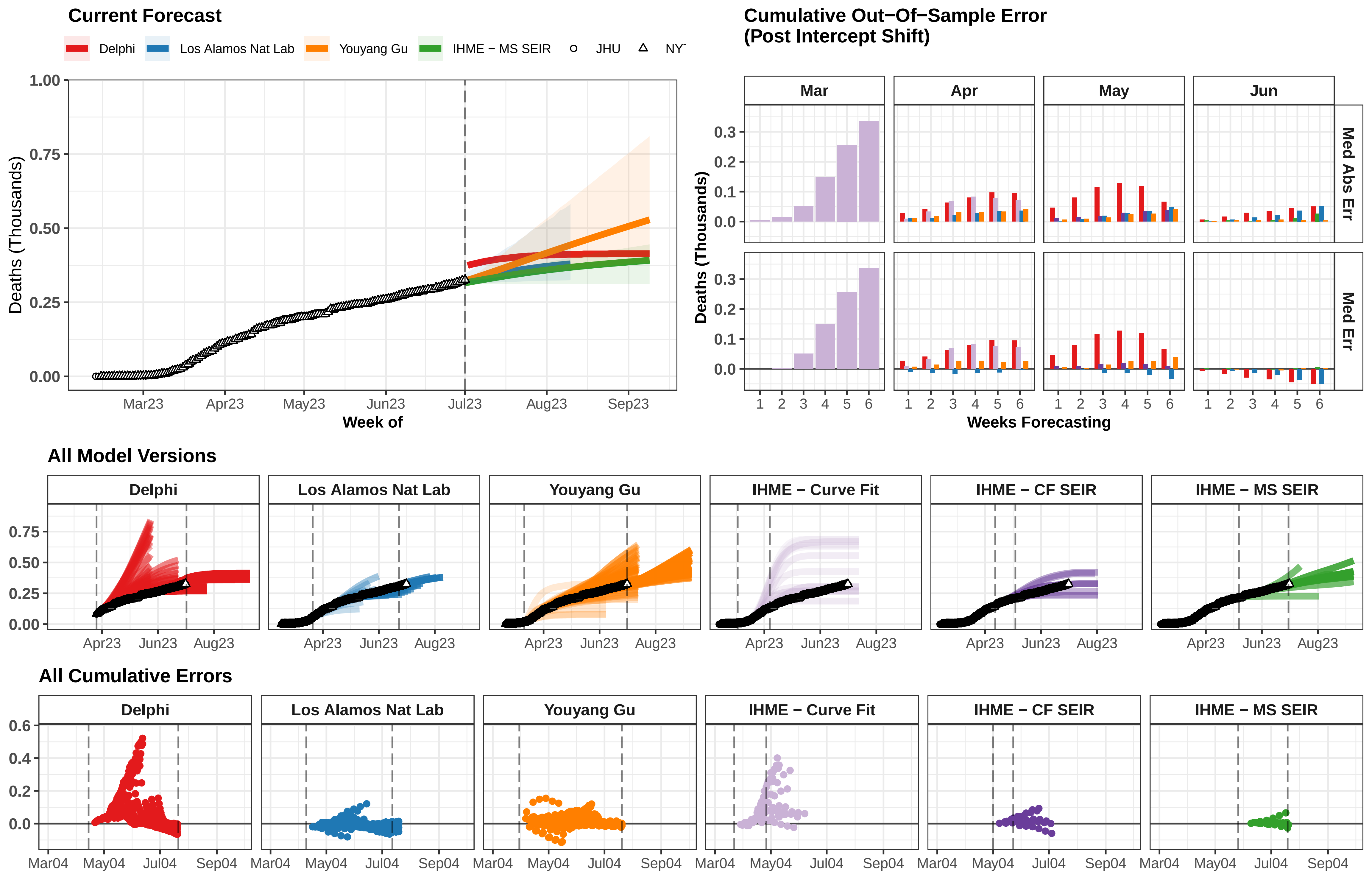
Current Forecast
Delphi
Los Alamos Nat Lab
Youyang Gu
IHME − MS SEIR
JHU
NY
Deaths (Thousands)
Week of
Mar23
Apr23
May23
Jun23
Jul23
Aug23
Sep23
Cumulative Out−Of−Sample Error
(Post Intercept Shift)
Mar
Apr
May
Jun
Med Abs Err
Med Err
Weeks Forecasting
All Model Versions
Delphi
Los Alamos Nat Lab
Youyang Gu
IHME − Curve Fit
IHME − CF SEIR
IHME − MS SEIR
All Cumulative Errors
Mar04
May04
Jul04
Sep04

Nebraska

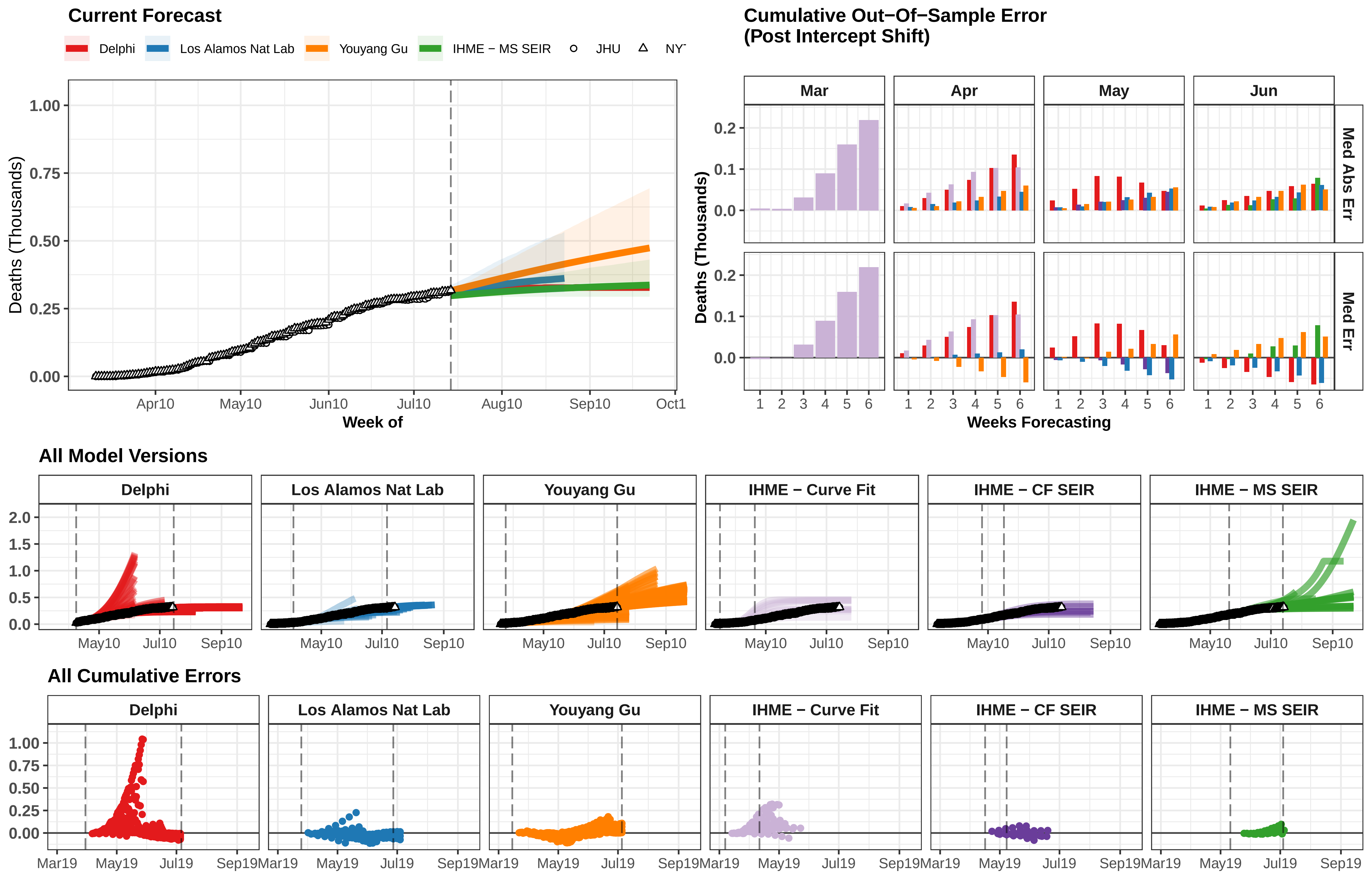

Current Forecast
Delphi
Los Alamos Nat Lab
Youyang Gu
IHME − MS SEIR
JHU
NY
Deaths (Thousands)
1.00
0.75
0.50
0.25
0.00
Apr10
May10
Jun10
Jul10
Aug10
Sep10
Oct1
Week of
Cumulative Out−Of−Sample Error
(Post Intercept Shift)
Mar
Apr
May
Jun
Med Abs Err
Med Err
Weeks Forecasting
All Model Versions
Delphi
Los Alamos Nat Lab
Youyang Gu
IHME − Curve Fit
IHME − CF SEIR
IHME − MS SEIR
All Cumulative Errors
Mar19
May19
Jul19
Sep19

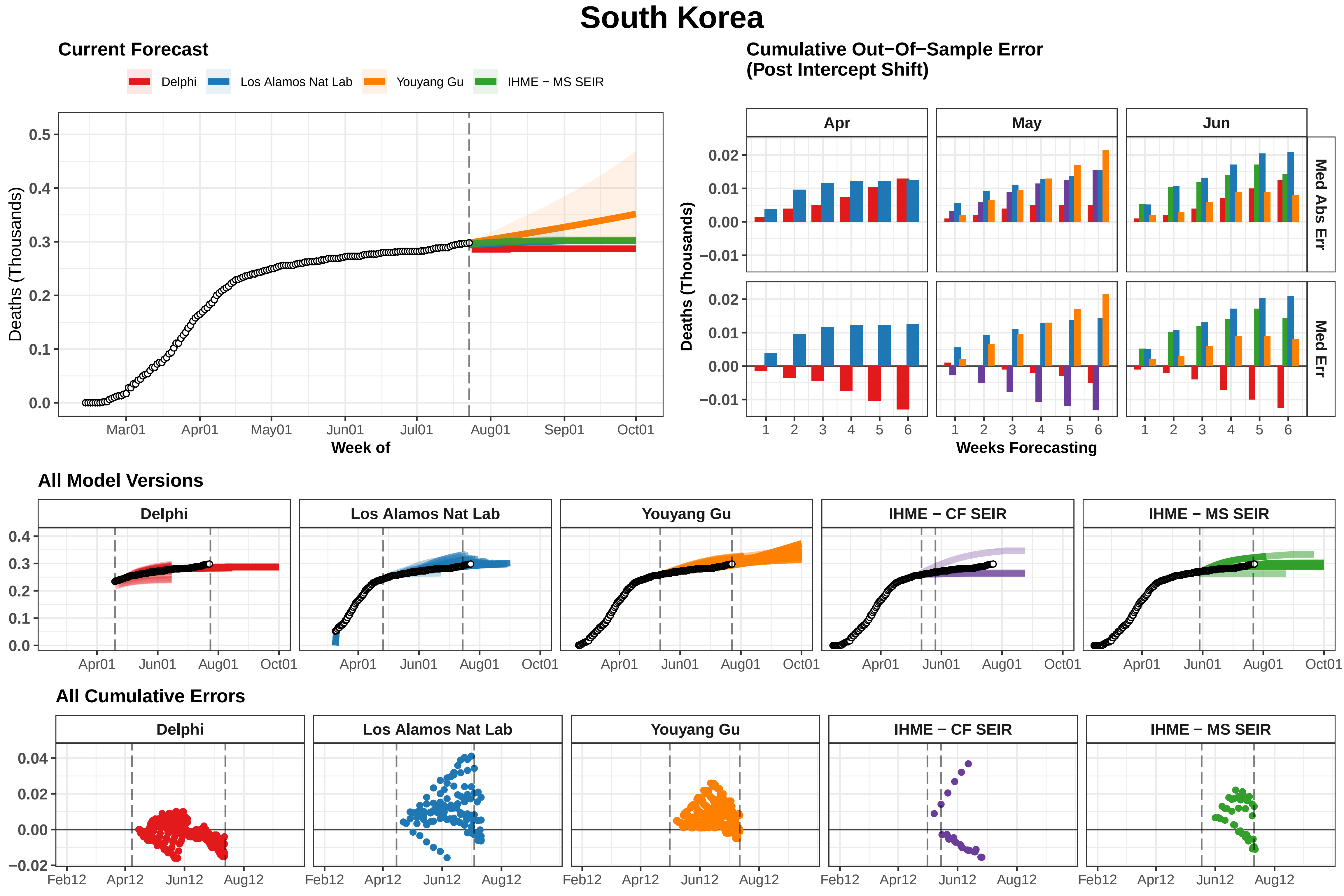
South Korea
Current Forecast
Delphi
Los Alamos Nat Lab
Youyang Gu
IHME − MS SEIR
Deaths (Thousands)
0.0
0.1
0.2
0.3
0.4
0.5
Mar01
Apr01
May01
Jun01
Jul01
Aug01
Sep01
Oct01
Week of
Cumulative Out−Of−Sample Error
(Post Intercept Shift)
Apr
May
Jun
Med Abs Err
Med Err
−0.01
0.00
0.01
0.02
1
2
3
4
5
6
Weeks Forecasting
All Model Versions
Delphi
Los Alamos Nat Lab
Youyang Gu
IHME − CF SEIR
IHME − MS SEIR
All Cumulative Errors
−0.02
0.00
0.02
0.04
Feb12
Apr12
Jun12
Aug12

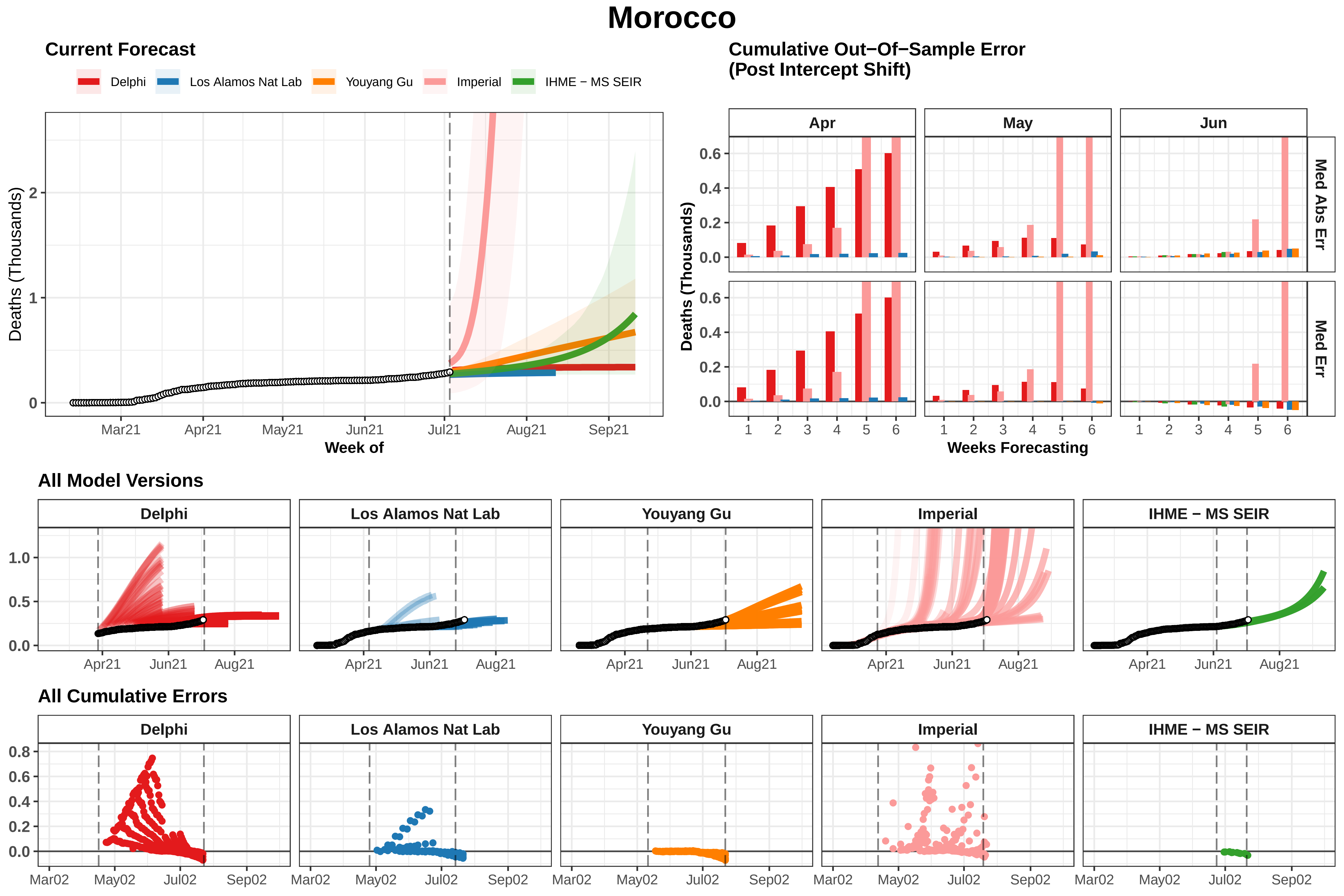

Morocco
Current Forecast
Delphi
Los Alamos Nat Lab
Youyang Gu
Imperial
IHME − MS SEIR
Deaths (Thousands)
Week of
Mar21
Apr21
May21
Jun21
Jul21
Aug21
Sep21
Cumulative Out−Of−Sample Error
(Post Intercept Shift)
Apr
May
Jun
Med Abs Err
Med Err
Weeks Forecasting
All Model Versions
All Cumulative Errors
Mar02
May02
Jul02
Sep02

Oregon

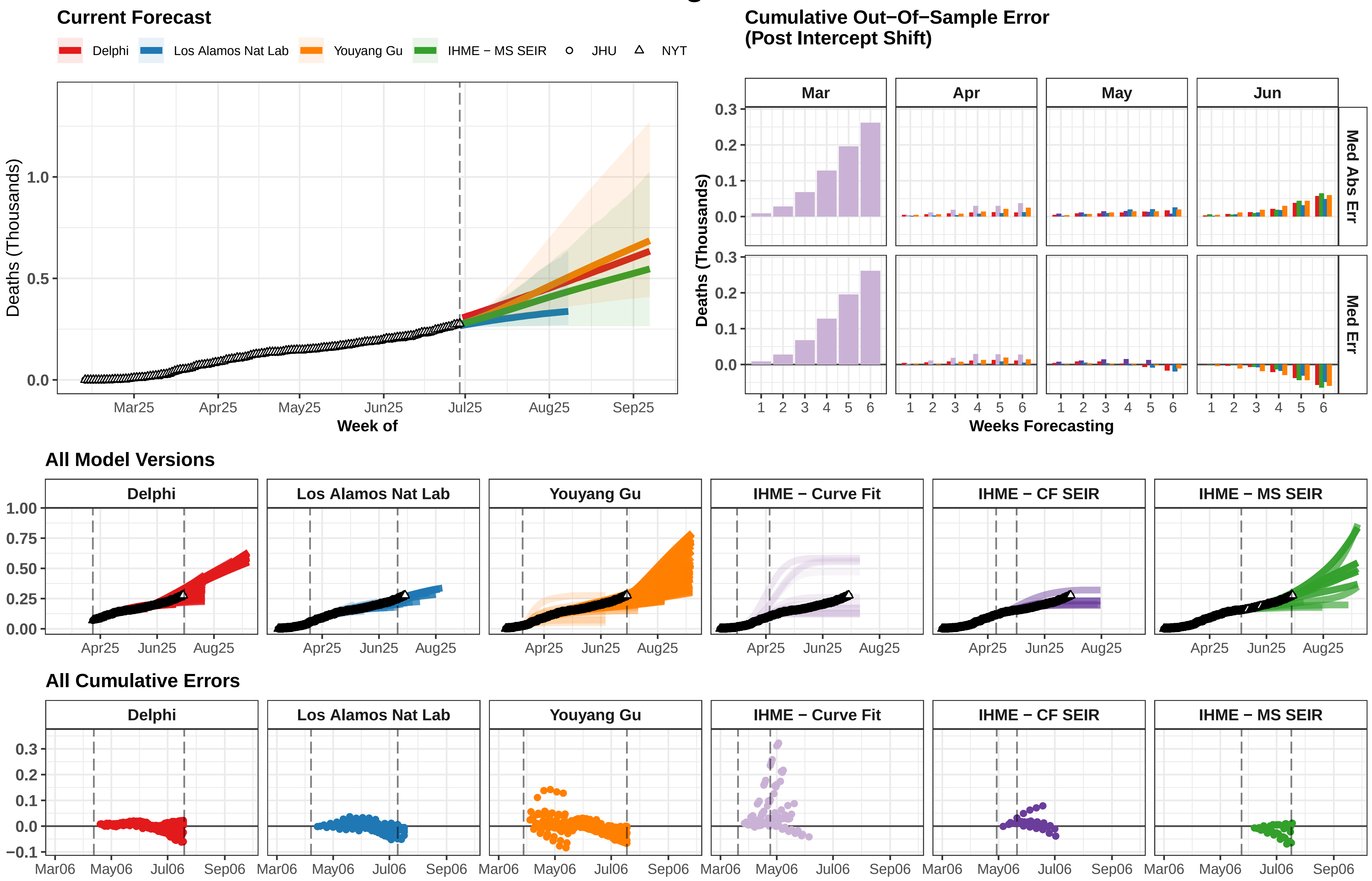

Current Forecast
Delphi
Los Alamos Nat Lab
Youyang Gu
IHME − MS SEIR
JHU
NYT
Deaths (Thousands)
1.0
0.5
0.0
Mar25
Apr25
May25
Jun25
Jul25
Aug25
Sep25
Week of
Cumulative Out−Of−Sample Error
(Post Intercept Shift)
Mar
Apr
May
Jun
Med Abs Err
Med Err
0.3
0.2
0.1
0.0
1
2
3
4
5
6
Weeks Forecasting
All Model Versions
Delphi
Los Alamos Nat Lab
Youyang Gu
IHME − Curve Fit
IHME − CF SEIR
IHME − MS SEIR
1.00
0.75
0.50
0.25
0.00
Apr25
Jun25
Aug25
All Cumulative Errors
0.3
0.2
0.1
0.0
−0.1
Mar06
May06
Jul06
Sep06

Bosnia and Herzegovina

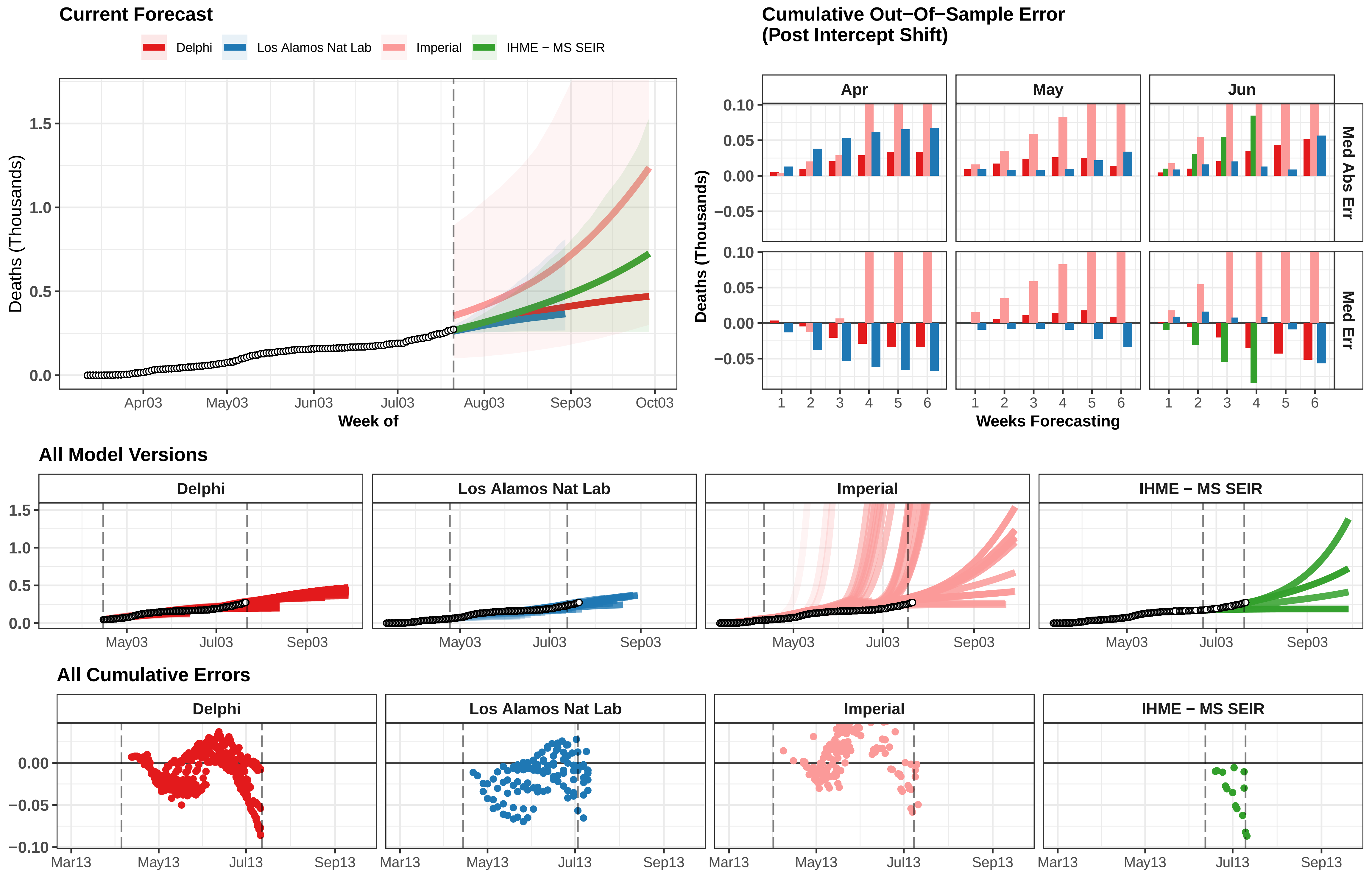

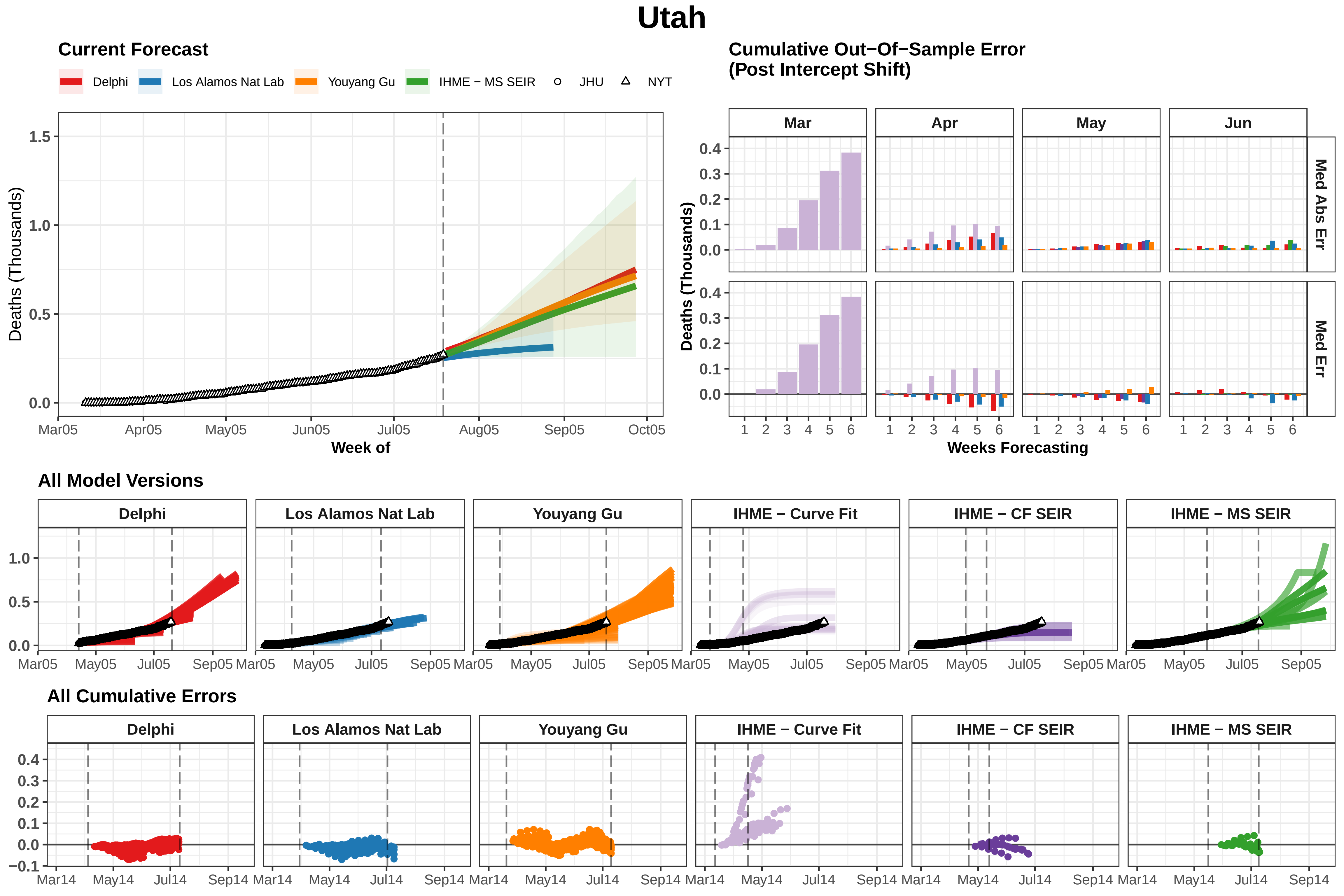
Utah
Current Forecast
Delphi
Los Alamos Nat Lab
Youyang Gu
IHME − MS SEIR
JHU
NYT
Deaths (Thousands)
Week of
Mar05
Apr05
May05
Jun05
Jul05
Aug05
Sep05
Oct05
Cumulative Out−Of−Sample Error (Post Intercept Shift)
Mar
Apr
May
Jun
Med Abs Err
Med Err
Weeks Forecasting
All Model Versions
IHME − Curve Fit
IHME − CF SEIR
All Cumulative Errors
Mar14
May14
Jul14
Sep14

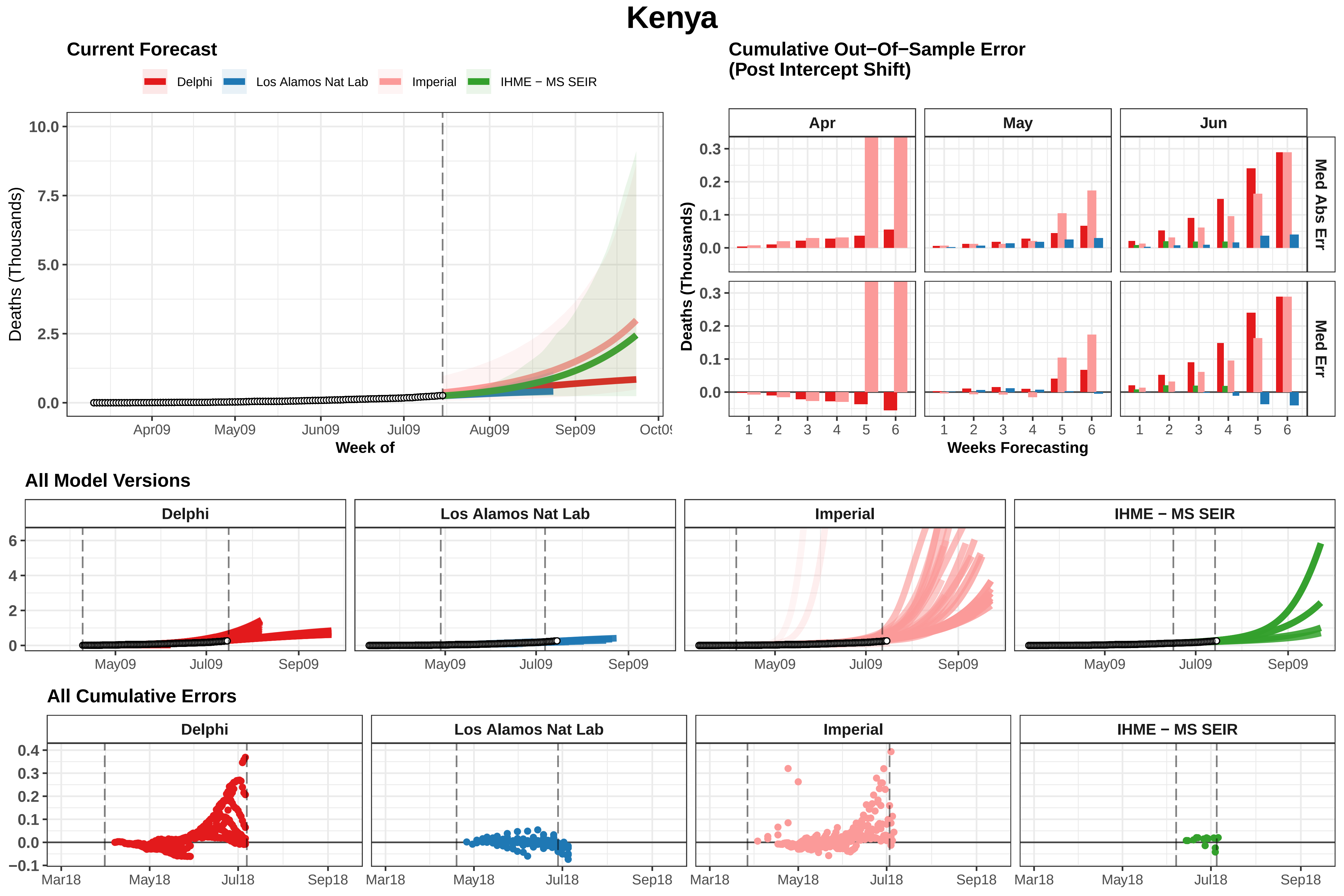
Kenya
Current Forecast
Delphi
Los Alamos Nat Lab
Imperial
IHME − MS SEIR
Deaths (Thousands)
10.0
7.5
5.0
2.5
0.0
Apr09
May09
Jun09
Jul09
Aug09
Sep09
Oct09
Week of
Cumulative Out−Of−Sample Error
(Post Intercept Shift)
Apr
May
Jun
Med Abs Err
Med Err
0.3
0.2
0.1
0.0
1
2
3
4
5
6
Weeks Forecasting
All Model Versions
Delphi
Los Alamos Nat Lab
Imperial
IHME − MS SEIR
6
4
2
0
May09
Jul09
Sep09
All Cumulative Errors
0.4
0.3
0.2
0.1
0.0
−0.1
Mar18
May18
Jul18
Sep18

Norway

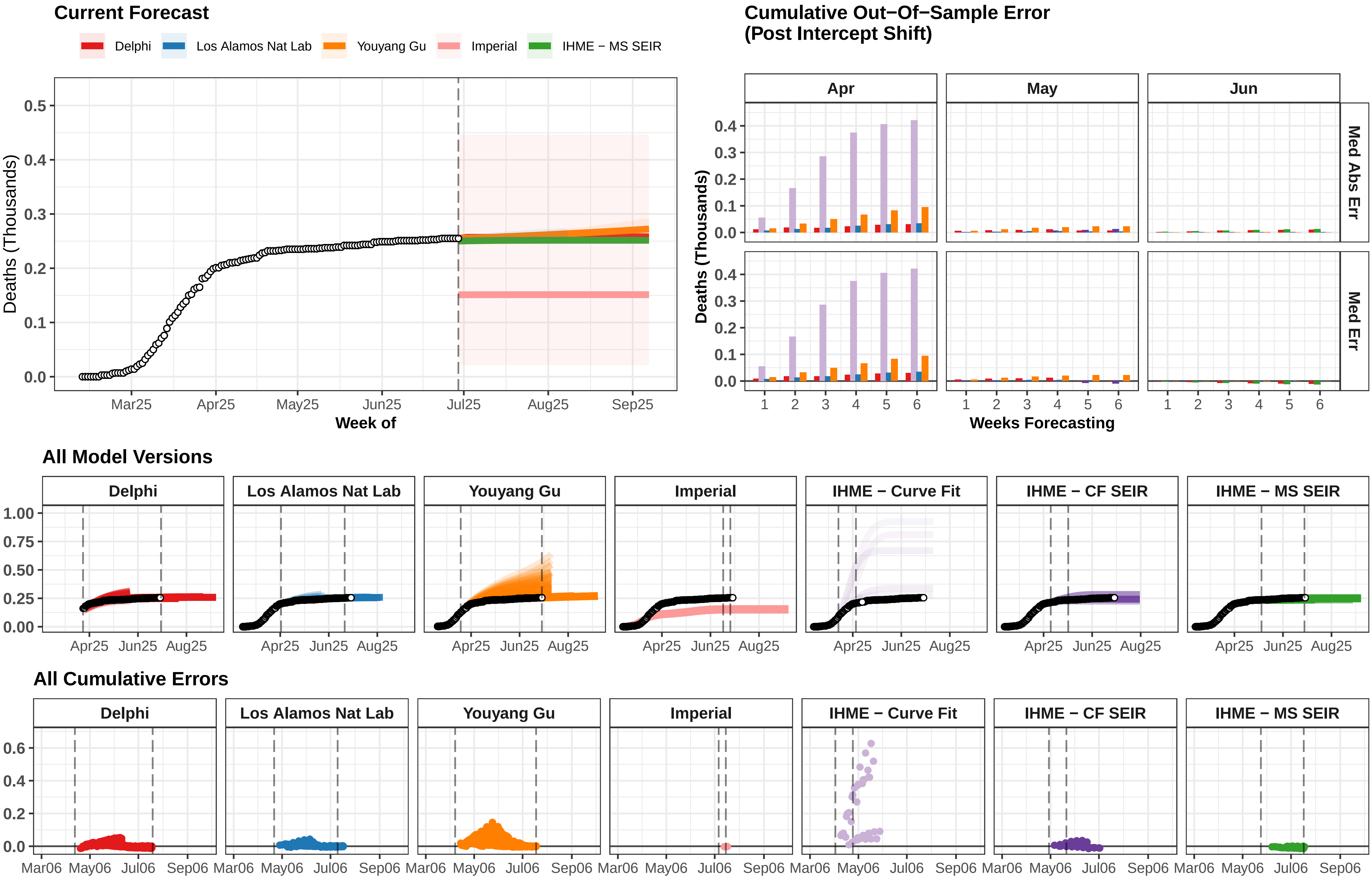

Current Forecast
Delphi
Los Alamos Nat Lab
Youyang Gu
Imperial
IHME − MS SEIR
Deaths (Thousands)
Week of
Mar25
Apr25
May25
Jun25
Jul25
Aug25
Sep25
Cumulative Out−Of−Sample Error
(Post Intercept Shift)
Apr
May
Jun
Med Abs Err
Med Err
Weeks Forecasting
All Model Versions
Delphi
Los Alamos Nat Lab
Youyang Gu
Imperial
IHME − Curve Fit
IHME − CF SEIR
IHME − MS SEIR
All Cumulative Errors
Mar06
May06
Jul06
Sep06

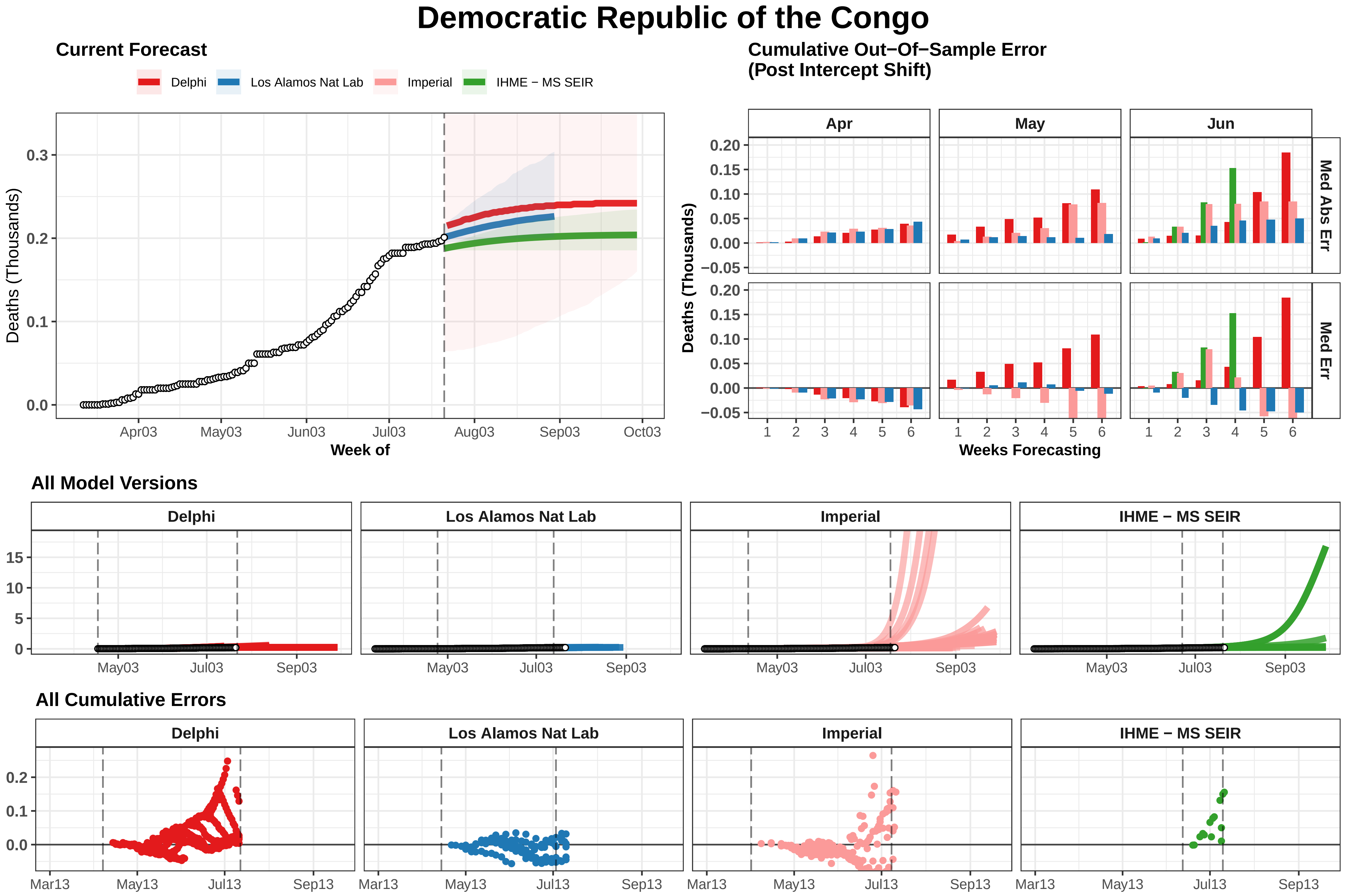

Democratic Republic of the Congo
Current Forecast
Delphi
Los Alamos Nat Lab
Imperial
IHME − MS SEIR
Deaths (Thousands)
0.0
0.1
0.2
0.3
Apr03
May03
Jun03
Jul03
Aug03
Sep03
Oct03
Week of
Cumulative Out−Of−Sample Error
(Post Intercept Shift)
Apr
May
Jun
Med Abs Err
Med Err
Deaths (Thousands)
−0.05
0.00
0.05
0.10
0.15
0.20
1
2
3
4
5
6
Weeks Forecasting
All Model Versions
Delphi
Los Alamos Nat Lab
Imperial
IHME − MS SEIR
0
5
10
15
May03
Jul03
Sep03
All Cumulative Errors
Delphi
Los Alamos Nat Lab
Imperial
IHME − MS SEIR
0.0
0.1
0.2
Mar13
May13
Jul13
Sep13

Greece

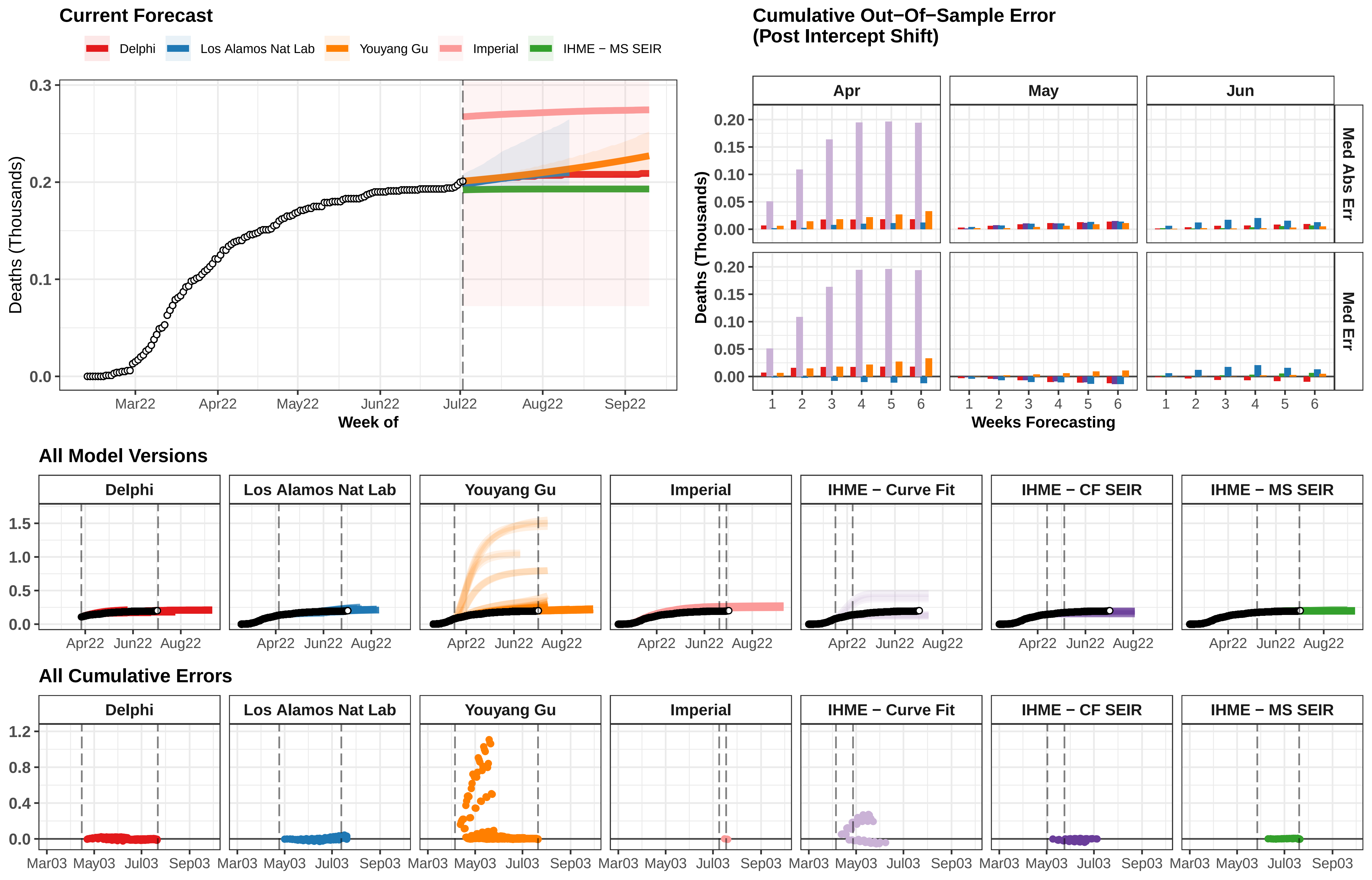

Current Forecast
Delphi
Los Alamos Nat Lab
Youyang Gu
Imperial
IHME − MS SEIR
Deaths (Thousands)
Week of
Mar22 Apr22 May22 Jun22 Jul22 Aug22 Sep22
Cumulative Out−Of−Sample Error
(Post Intercept Shift)
Apr
May
Jun
Med Abs Err
Med Err
Weeks Forecasting
All Model Versions
All Cumulative Errors
Delphi
Los Alamos Nat Lab
Youyang Gu
Imperial
IHME − Curve Fit
IHME − CF SEIR
IHME − MS SEIR
Apr22 Jun22 Aug22
Mar03 May03 Jul03 Sep03

Ethiopia

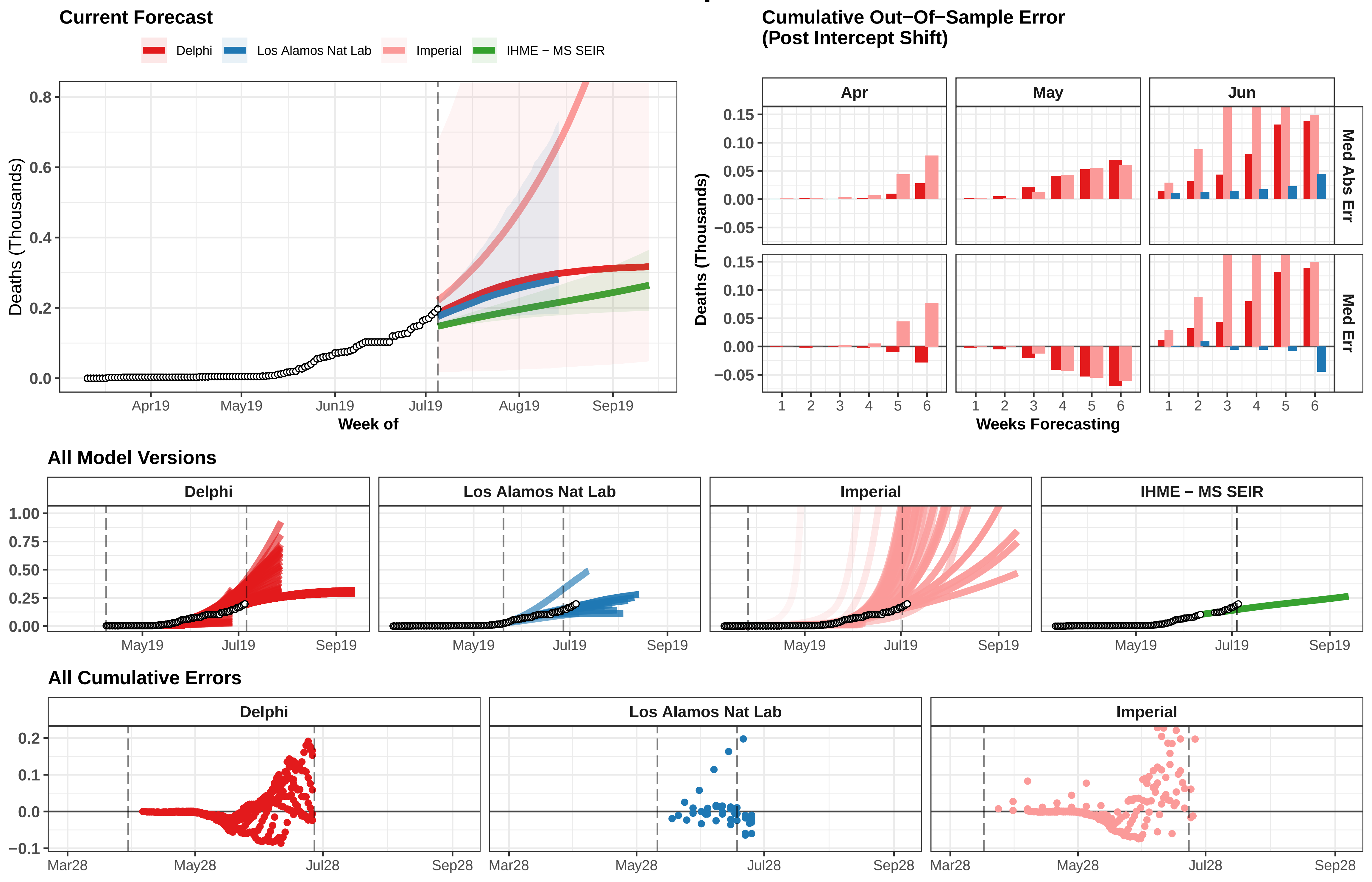

Current Forecast
Delphi
Los Alamos Nat Lab
Imperial
IHME − MS SEIR
Deaths (Thousands)
0.0
0.2
0.4
0.6
0.8
Apr19
May19
Jun19
Jul19
Aug19
Sep19
Week of
Cumulative Out−Of−Sample Error
(Post Intercept Shift)
Apr
May
Jun
Med Abs Err
Med Err
0.15
0.10
0.05
0.00
−0.05
1
2
3
4
5
6
Weeks Forecasting
All Model Versions
Delphi
Los Alamos Nat Lab
Imperial
IHME − MS SEIR
1.00
0.75
0.50
0.25
0.00
May19
Jul19
Sep19
All Cumulative Errors
Delphi
Los Alamos Nat Lab
Imperial
0.2
0.1
0.0
−0.1
Mar28
May28
Jul28
Sep28

Puerto Rico

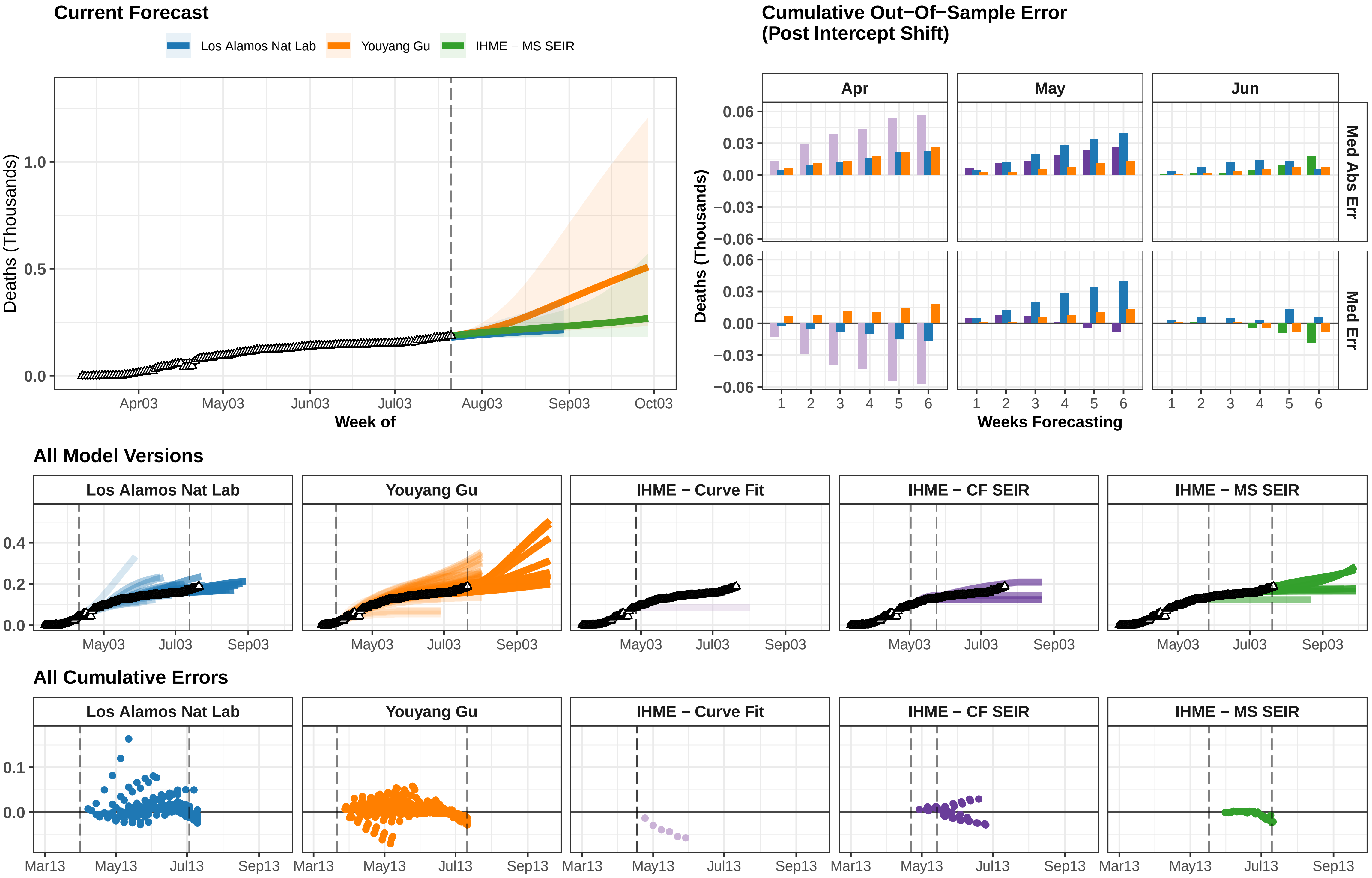
Current Forecast
Los Alamos Nat Lab
Youyang Gu
IHME − MS SEIR
Deaths (Thousands)
1.0
0.5
0.0
Apr03
May03
Jun03
Jul03
Aug03
Sep03
Oct03
Week of
Cumulative Out−Of−Sample Error
(Post Intercept Shift)
Apr
May
Jun
Med Abs Err
Med Err
0.06
0.03
0.00
−0.03
−0.06
1
2
3
4
5
6
Weeks Forecasting
All Model Versions
Los Alamos Nat Lab
Youyang Gu
IHME − Curve Fit
IHME − CF SEIR
IHME − MS SEIR
0.4
0.2
0.0
May03
Jul03
Sep03
All Cumulative Errors
0.1
0.0
Mar13
May13
Jul13
Sep13

Senegal

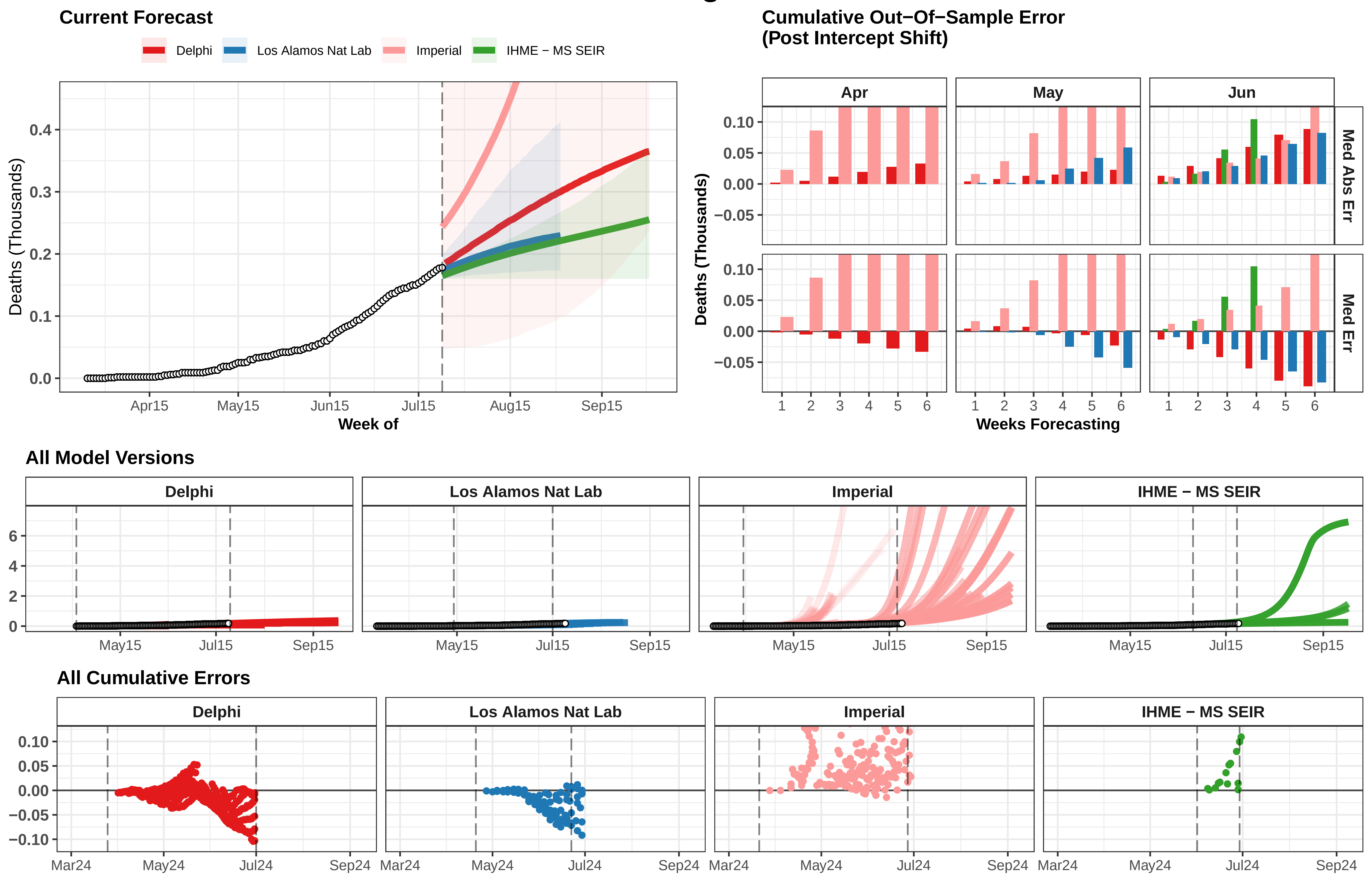

Current Forecast
Delphi
Los Alamos Nat Lab
Imperial
IHME − MS SEIR
Deaths (Thousands)
0.0
0.1
0.2
0.3
0.4
Apr15
May15
Jun15
Jul15
Aug15
Sep15
Week of
Cumulative Out−Of−Sample Error
(Post Intercept Shift)
Apr
May
Jun
Med Abs Err
Med Err
0.10
0.05
0.00
−0.05
1
2
3
4
5
6
Weeks Forecasting
All Model Versions
0
2
4
6
All Cumulative Errors
−0.10
Mar24
May24
Jul24
Sep24

Qatar

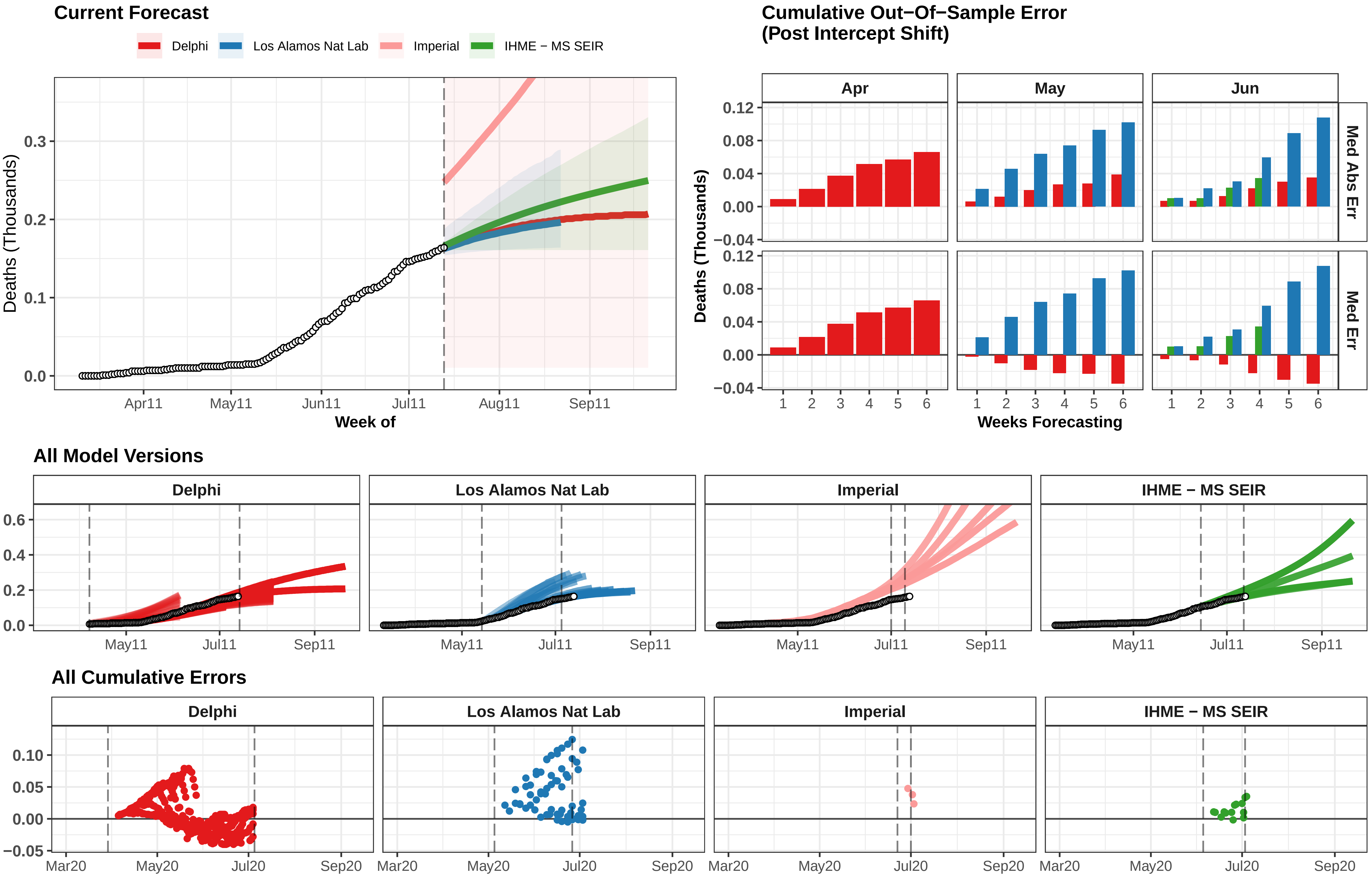

Current Forecast
Delphi
Los Alamos Nat Lab
Imperial
IHME − MS SEIR
Deaths (Thousands)
0.0
0.1
0.2
0.3
Apr11
May11
Jun11
Jul11
Aug11
Sep11
Week of
Cumulative Out−Of−Sample Error
(Post Intercept Shift)
Apr
May
Jun
Med Abs Err
Med Err
Deaths (Thousands)
0.12
0.08
0.04
0.00
−0.04
1
2
3
4
5
6
Weeks Forecasting
All Model Versions
Delphi
Los Alamos Nat Lab
Imperial
IHME − MS SEIR
0.0
0.2
0.4
0.6
May11
Jul11
Sep11
All Cumulative Errors
Delphi
Los Alamos Nat Lab
Imperial
IHME − MS SEIR
0.10
0.05
0.00
−0.05
Mar20
May20
Jul20
Sep20

Mauritania

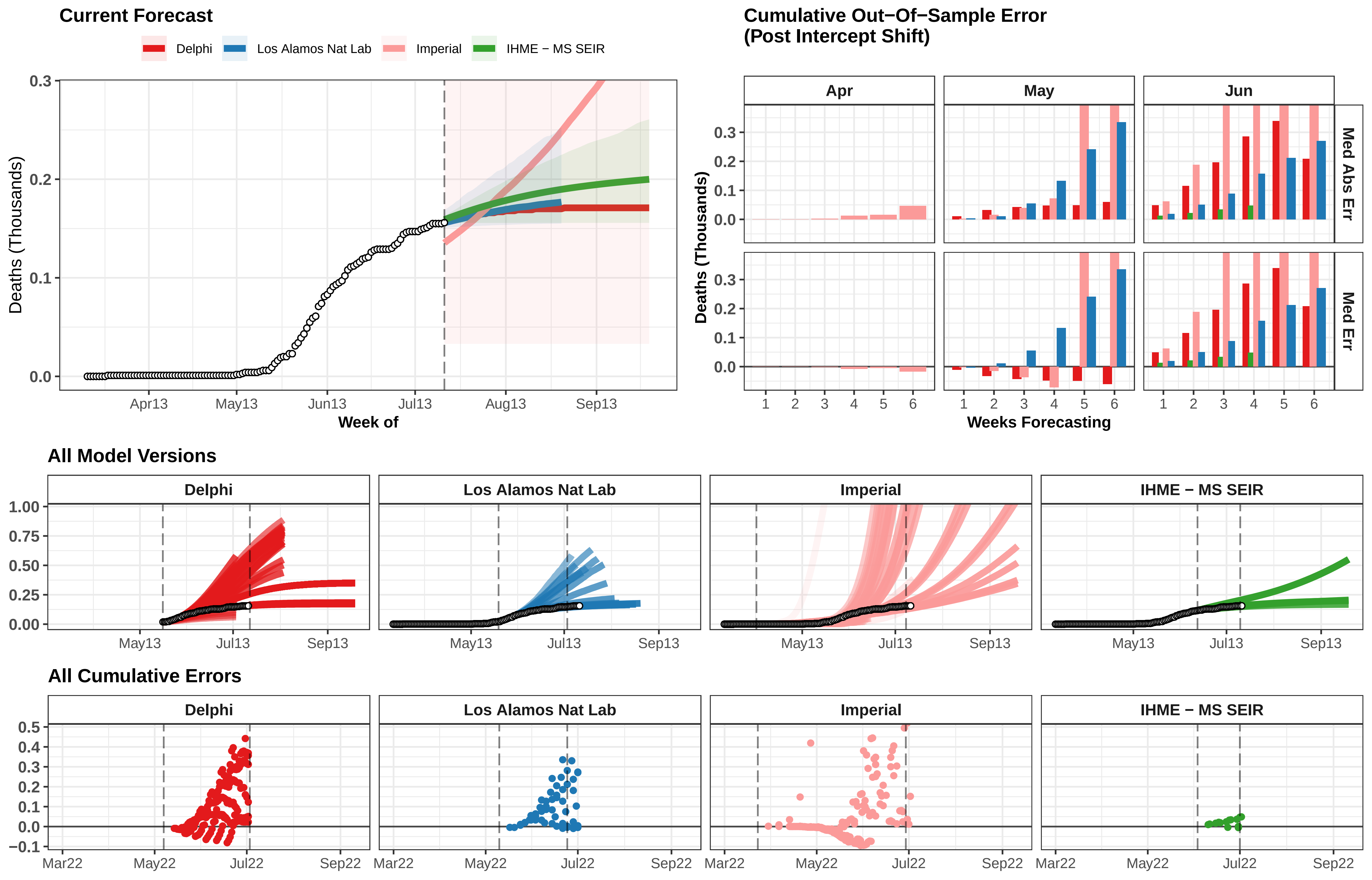
Current Forecast
Delphi
Los Alamos Nat Lab
Imperial
IHME − MS SEIR
Deaths (Thousands)
Week of
Apr13
May13
Jun13
Jul13
Aug13
Sep13
Cumulative Out−Of−Sample Error
(Post Intercept Shift)
Apr
May
Jun
Med Abs Err
Med Err
Weeks Forecasting
All Model Versions
All Cumulative Errors
Mar22
May22
Jul22
Sep22

Haiti

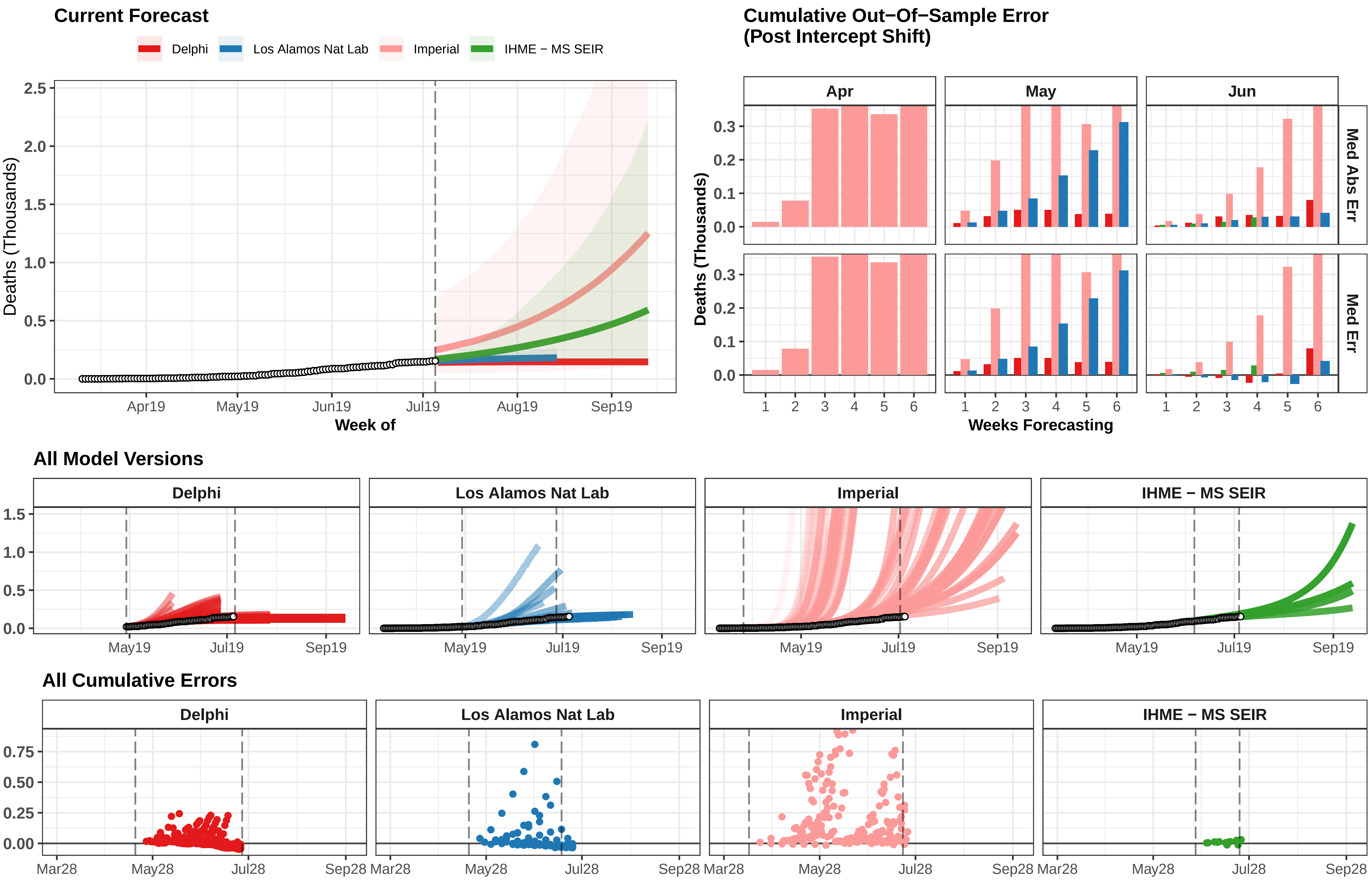

Current Forecast
Delphi
Los Alamos Nat Lab
Imperial
IHME − MS SEIR
Deaths (Thousands)
2.5
2.0
1.5
1.0
0.5
0.0
Apr19
May19
Jun19
Jul19
Aug19
Sep19
Week of
Cumulative Out−Of−Sample Error
(Post Intercept Shift)
Apr
May
Jun
Med Abs Err
Med Err
Deaths (Thousands)
0.3
0.2
0.1
0.0
1
2
3
4
5
6
Weeks Forecasting
All Model Versions
Delphi
Los Alamos Nat Lab
Imperial
IHME − MS SEIR
1.5
1.0
0.5
0.0
May19
Jul19
Sep19
All Cumulative Errors
Delphi
Los Alamos Nat Lab
Imperial
IHME − MS SEIR
0.75
0.50
0.25
0.00
Mar28
May28
Jul28
Sep28

Ghana

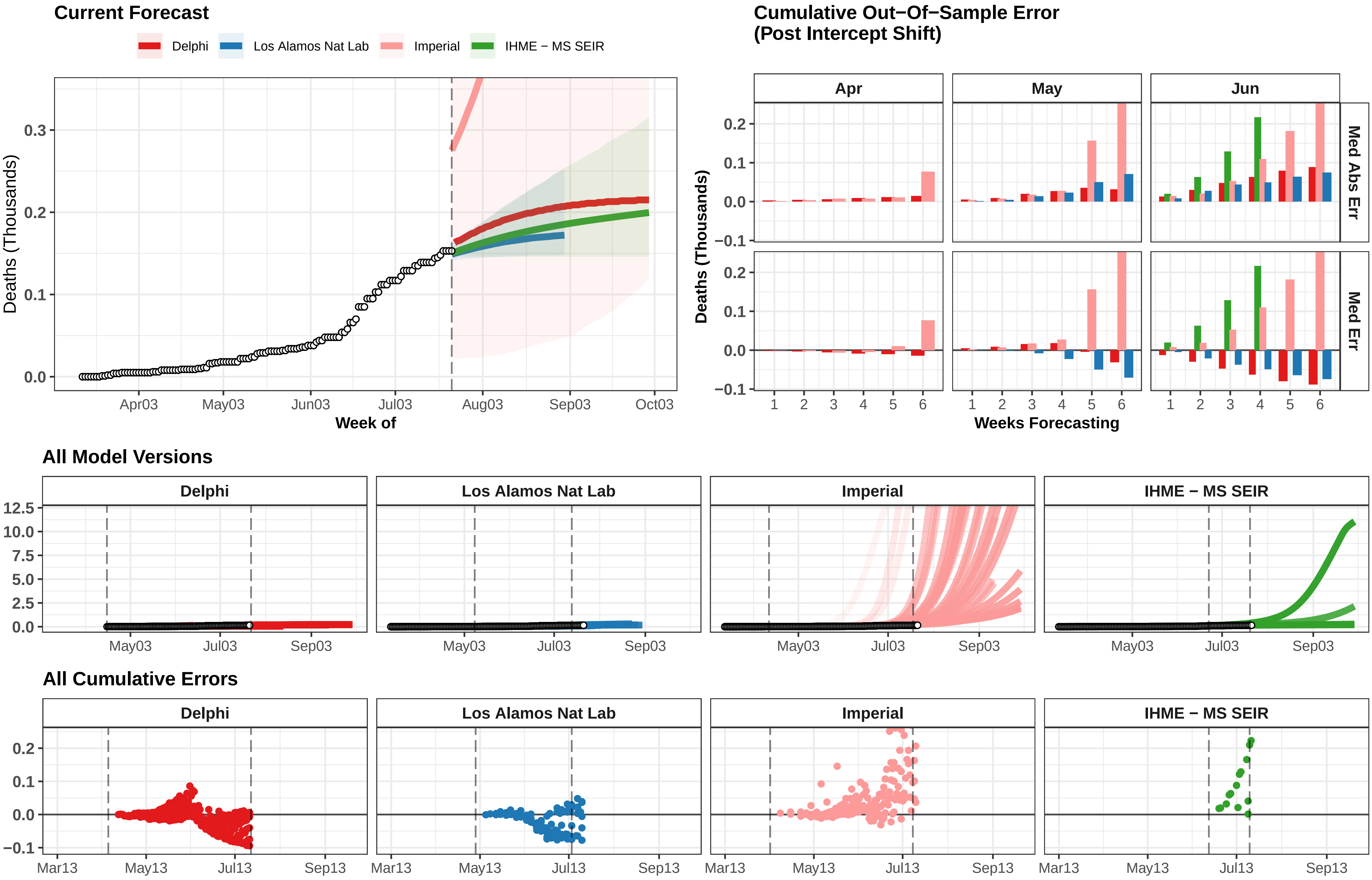
Current Forecast
Delphi
Los Alamos Nat Lab
Imperial
IHME − MS SEIR
Deaths (Thousands)
Week of
Apr03
May03
Jun03
Jul03
Aug03
Sep03
Oct03
Cumulative Out−Of−Sample Error
(Post Intercept Shift)
Apr
May
Jun
Med Abs Err
Med Err
Weeks Forecasting
All Model Versions
All Cumulative Errors
Mar13
May13
Jul13
Sep13

Idaho

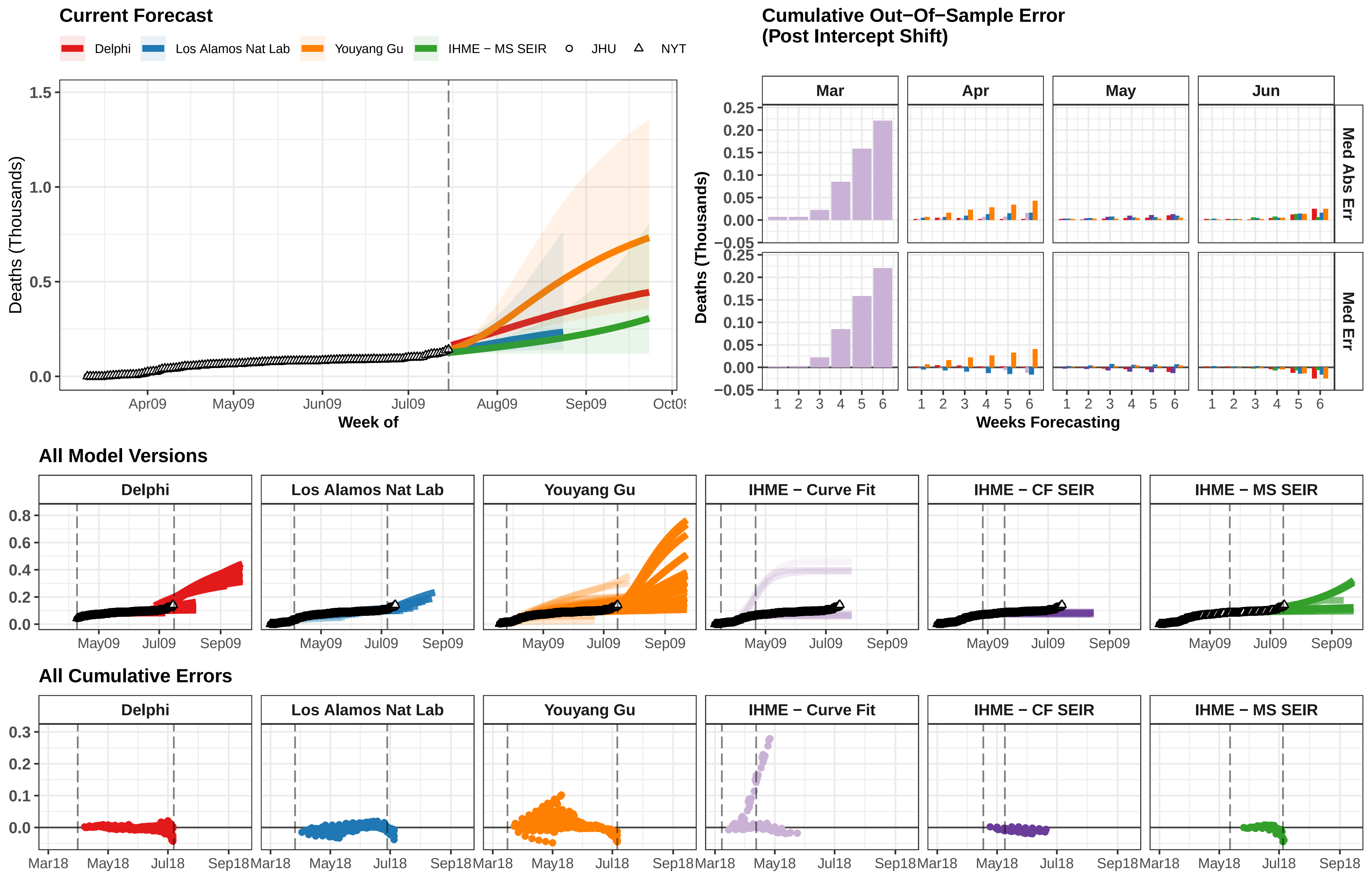
Current Forecast
Delphi
Los Alamos Nat Lab
Youyang Gu
IHME − MS SEIR
JHU
NYT
Deaths (Thousands)
Week of
Apr09
May09
Jun09
Jul09
Aug09
Sep09
Cumulative Out−Of−Sample Error
(Post Intercept Shift)
Mar
Apr
May
Jun
Med Abs Err
Med Err
Weeks Forecasting
All Model Versions
Delphi
Los Alamos Nat Lab
Youyang Gu
IHME − Curve Fit
IHME − CF SEIR
IHME − MS SEIR
All Cumulative Errors
Mar18
May18
Jul18
Sep18

Australia

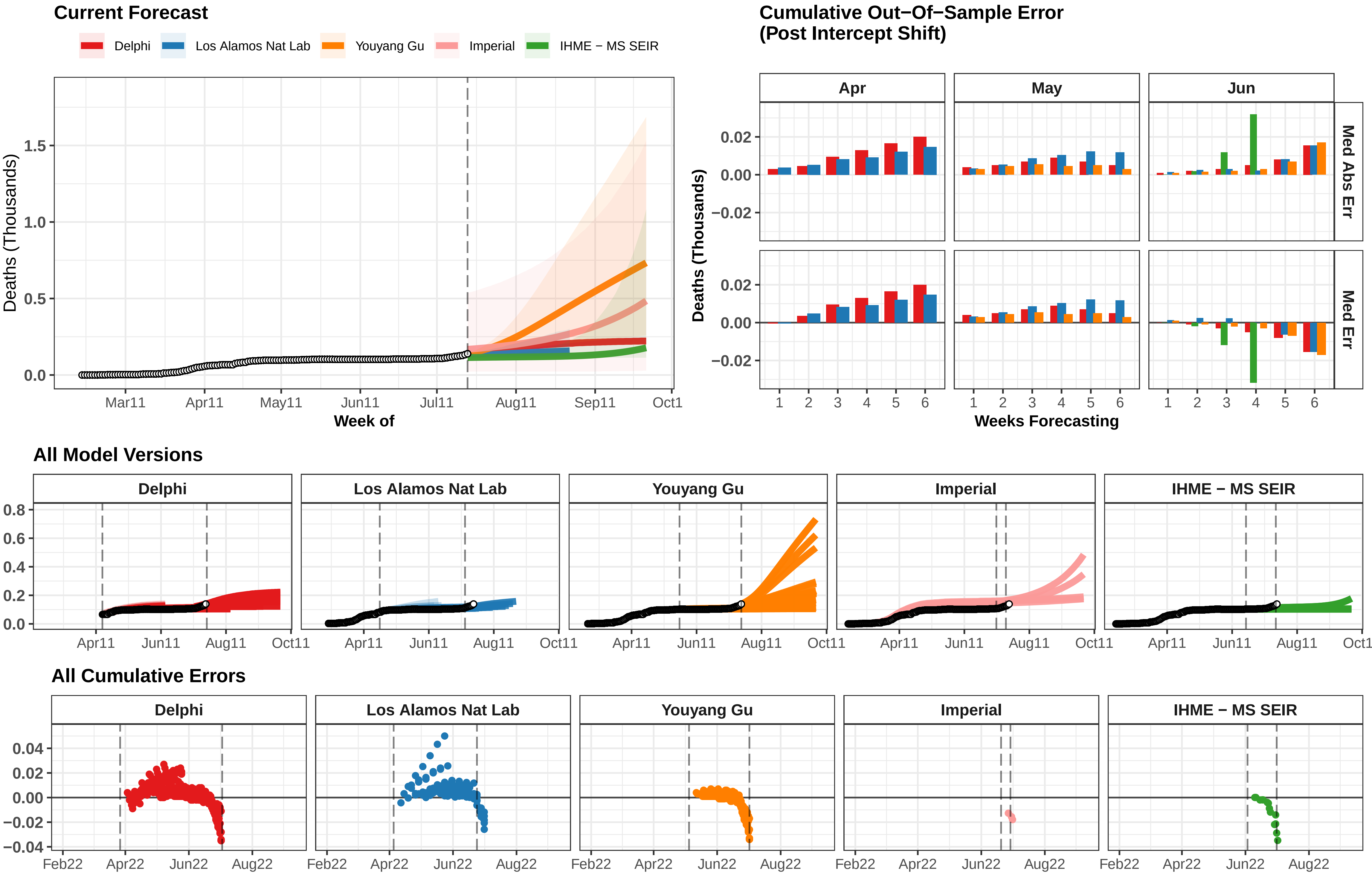
Current Forecast
Delphi
Los Alamos Nat Lab
Youyang Gu
Imperial
IHME − MS SEIR
Deaths (Thousands)
1.5
1.0
0.5
0.0
Mar11
Apr11
May11
Jun11
Jul11
Aug11
Sep11
Oct1
Week of
Cumulative Out−Of−Sample Error
(Post Intercept Shift)
Apr
May
Jun
Med Abs Err
Med Err
0.02
0.00
−0.02
Weeks Forecasting
All Model Versions
0.8
0.6
0.4
0.2
0.0
Oct11
All Cumulative Errors
0.04
0.02
0.00
−0.02
−0.04
Feb22
Apr22
Jun22
Aug22

Bahrain

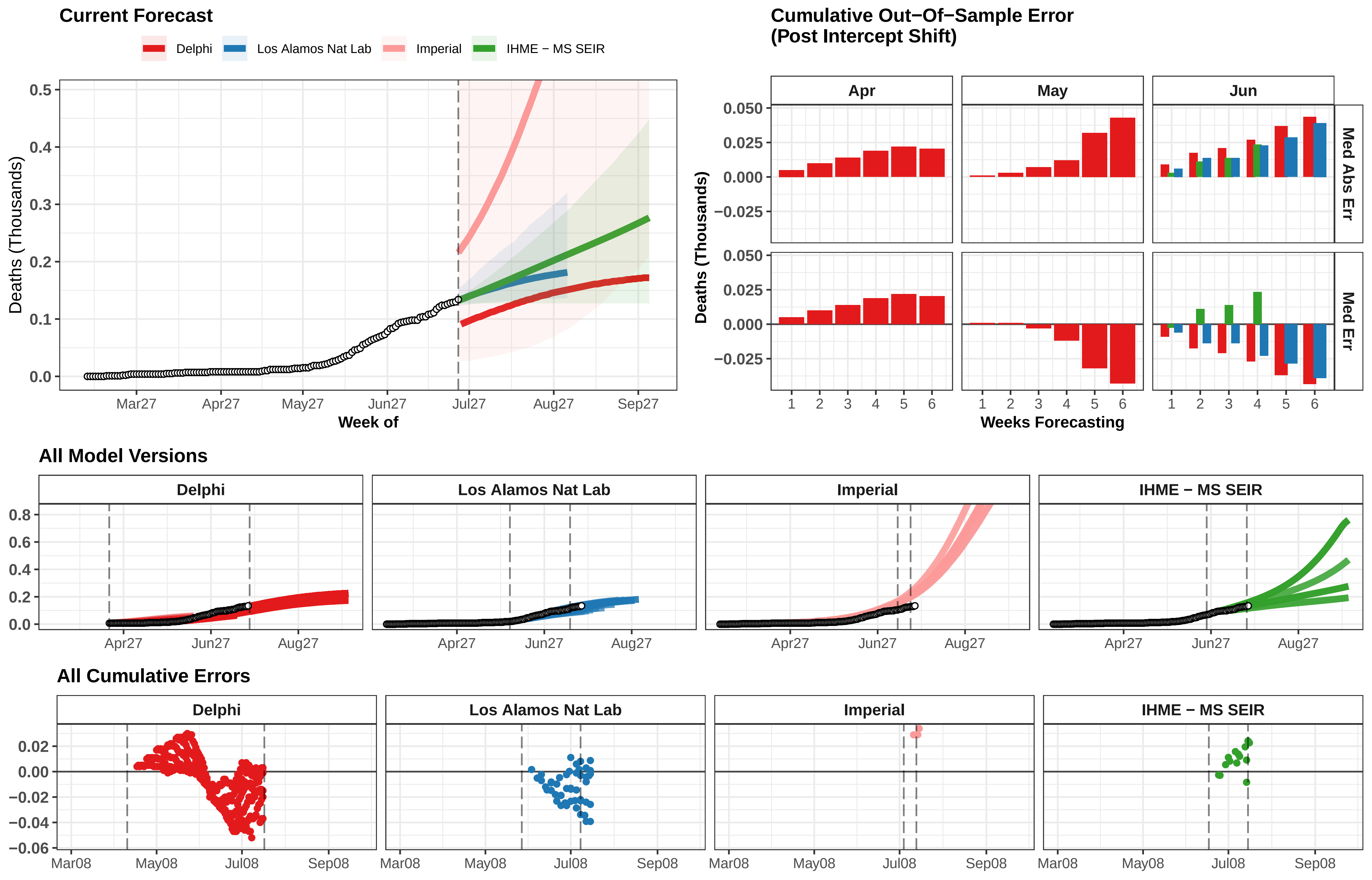
Current Forecast
Delphi
Los Alamos Nat Lab
Imperial
IHME − MS SEIR
Deaths (Thousands)
0.0
0.1
0.2
0.3
0.4
0.5
Mar27
Apr27
May27
Jun27
Jul27
Aug27
Sep27
Week of
Cumulative Out−Of−Sample Error
(Post Intercept Shift)
Apr
May
Jun
Med Abs Err
Med Err
0.050
0.025
0.000
−0.025
1
2
3
4
5
6
Weeks Forecasting
All Model Versions
Delphi
Los Alamos Nat Lab
Imperial
IHME − MS SEIR
0.8
0.6
0.4
0.2
0.0
Apr27
Jun27
Aug27
All Cumulative Errors
Delphi
Los Alamos Nat Lab
Imperial
IHME − MS SEIR
0.02
0.00
−0.02
−0.04
−0.06
Mar08
May08
Jul08
Sep08

Zambia

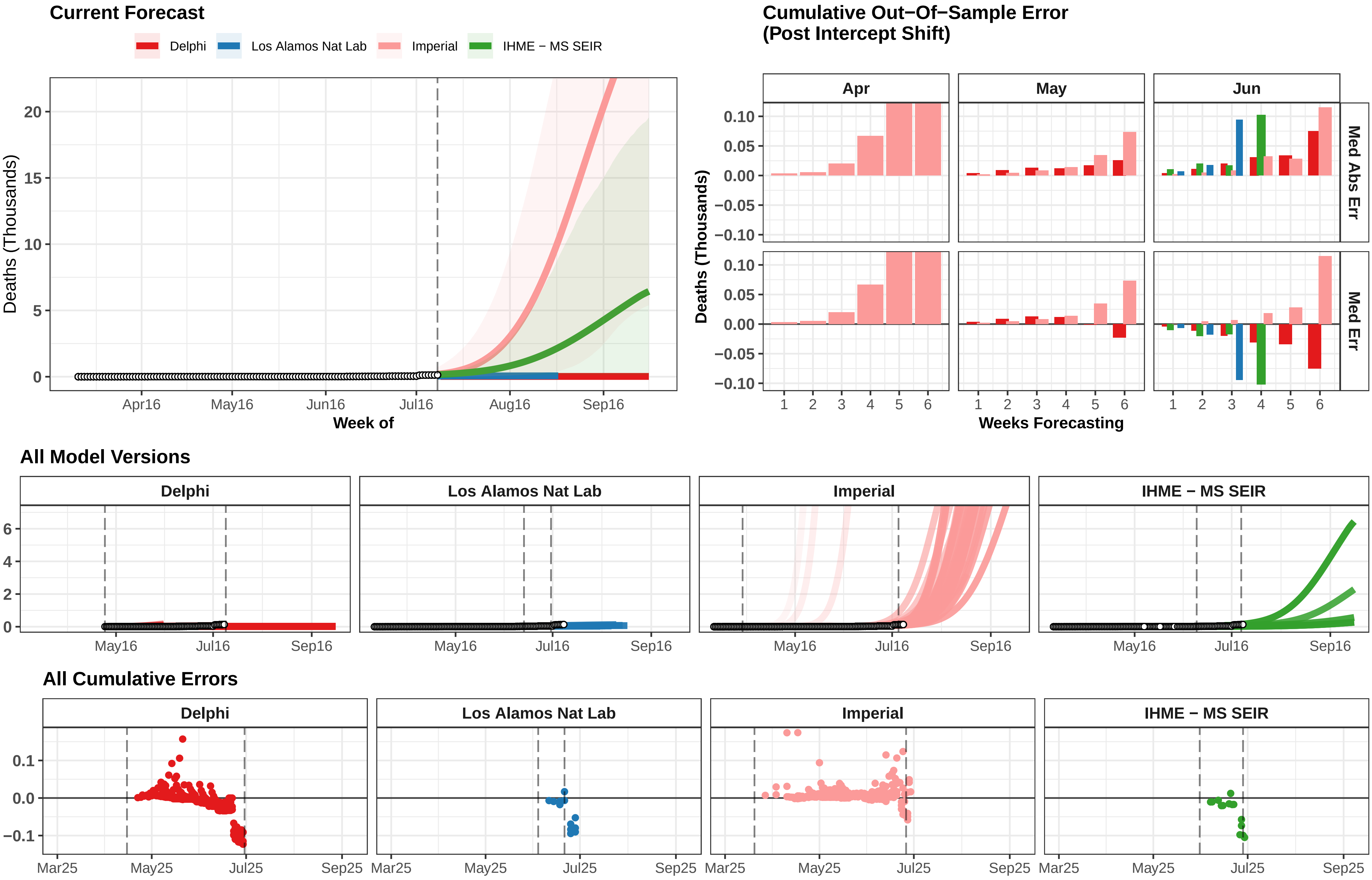
Current Forecast
Delphi
Los Alamos Nat Lab
Imperial
IHME − MS SEIR
Deaths (Thousands)
0
5
10
15
20
Apr16
May16
Jun16
Jul16
Aug16
Sep16
Week of
Cumulative Out−Of−Sample Error
(Post Intercept Shift)
Apr
May
Jun
Med Abs Err
Med Err
0.10
0.05
0.00
−0.05
−0.10
1
2
3
4
5
6
Weeks Forecasting
All Model Versions
Delphi
Los Alamos Nat Lab
Imperial
IHME − MS SEIR
0
2
4
6
May16
Jul16
Sep16
All Cumulative Errors
Delphi
Los Alamos Nat Lab
Imperial
IHME − MS SEIR
0.1
0.0
−0.1
Mar25
May25
Jul25
Sep25

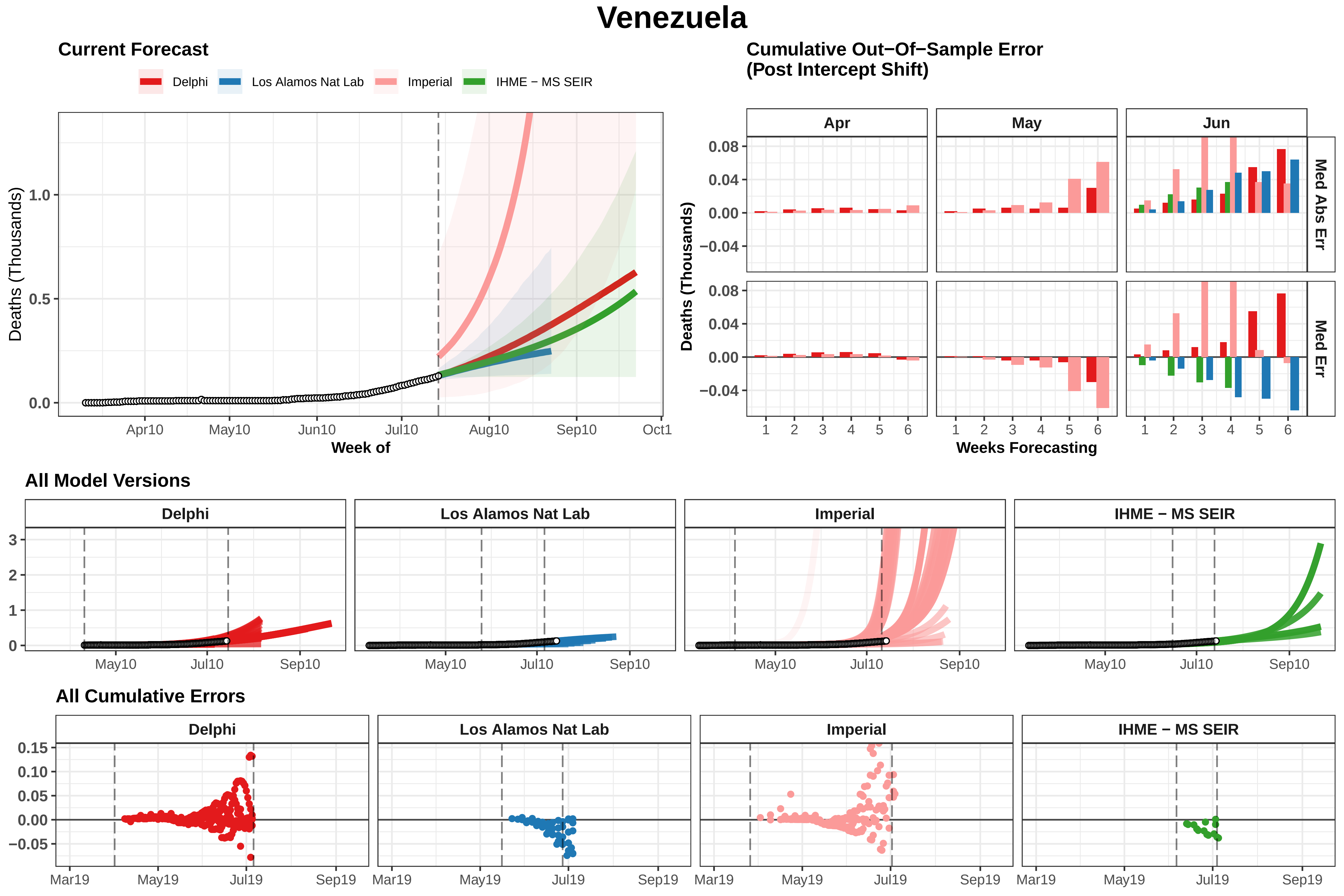
Venezuela
Current Forecast
Delphi
Los Alamos Nat Lab
Imperial
IHME − MS SEIR
Deaths (Thousands)
0.0
0.5
1.0
Apr10
May10
Jun10
Jul10
Aug10
Sep10
Oct1
Week of
Cumulative Out−Of−Sample Error
(Post Intercept Shift)
Apr
May
Jun
Med Abs Err
Med Err
0.08
0.04
0.00
−0.04
1 2 3 4 5 6
Weeks Forecasting
All Model Versions
Delphi
Los Alamos Nat Lab
Imperial
IHME − MS SEIR
0
1
2
3
May10
Jul10
Sep10
All Cumulative Errors
0.15
0.10
0.05
0.00
−0.05
Mar19
May19
Jul19
Sep19

Croatia

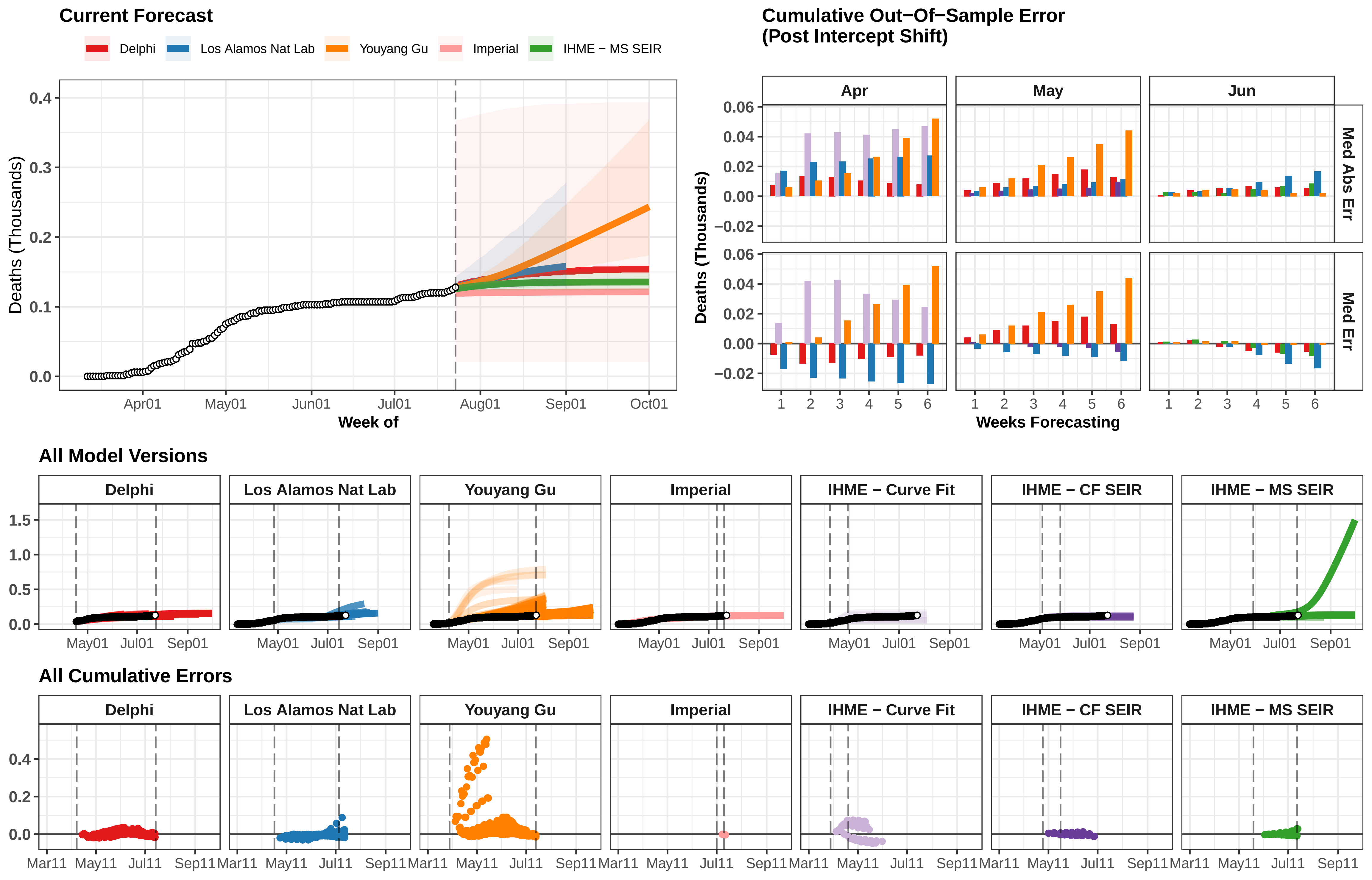

Current Forecast
Delphi
Los Alamos Nat Lab
Youyang Gu
Imperial
IHME − MS SEIR
Deaths (Thousands)
Week of
Apr01
May01
Jun01
Jul01
Aug01
Sep01
Oct01
Cumulative Out−Of−Sample Error
(Post Intercept Shift)
Apr
May
Jun
Med Abs Err
Med Err
Weeks Forecasting
All Model Versions
IHME − Curve Fit
IHME − CF SEIR
All Cumulative Errors
Mar11
May11
Jul11
Sep11

Albania

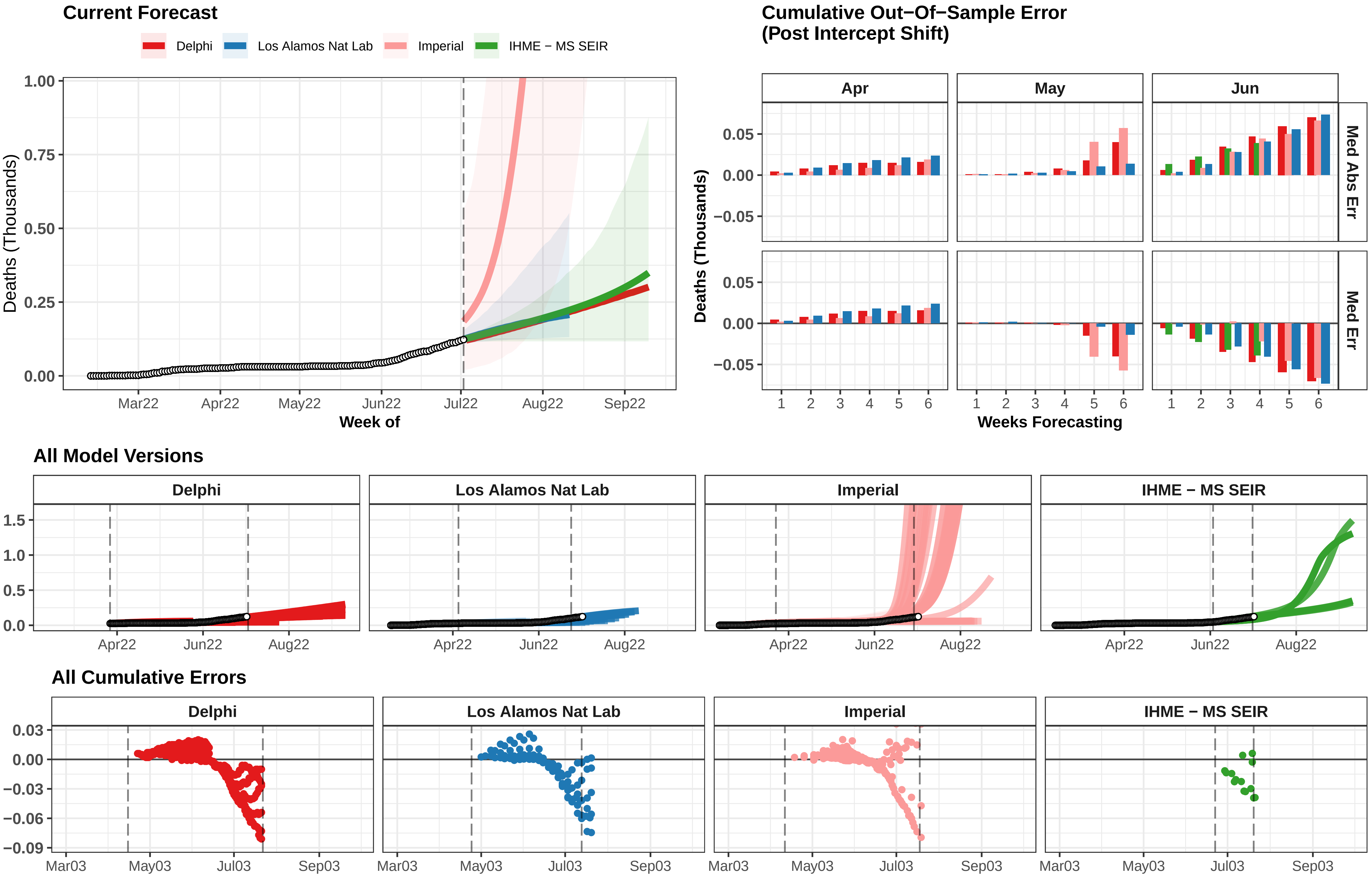
Current Forecast
Delphi
Los Alamos Nat Lab
Imperial
IHME − MS SEIR
Deaths (Thousands)
Week of
Cumulative Out−Of−Sample Error (Post Intercept Shift)
Apr
May
Jun
Med Abs Err
Med Err
Weeks Forecasting
All Model Versions
All Cumulative Errors

Malaysia

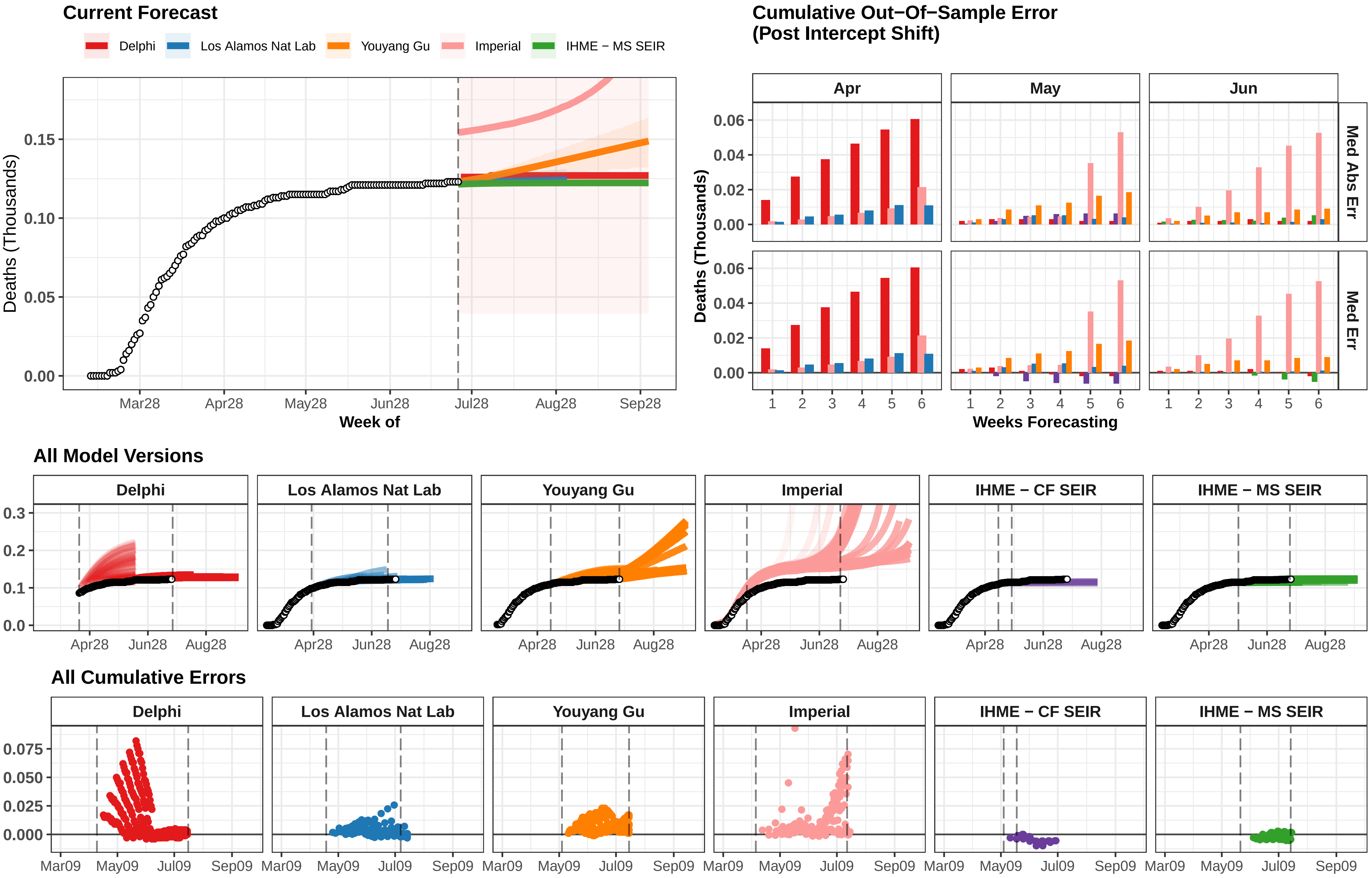

Current Forecast
Delphi
Los Alamos Nat Lab
Youyang Gu
Imperial
IHME − MS SEIR
Deaths (Thousands)
0.00
0.05
0.10
0.15
Mar28
Apr28
May28
Jun28
Jul28
Aug28
Sep28
Week of
Cumulative Out−Of−Sample Error
(Post Intercept Shift)
Apr
May
Jun
Med Abs Err
Med Err
0.00
0.02
0.04
0.06
1
2
3
4
5
6
Weeks Forecasting
All Model Versions
Delphi
Los Alamos Nat Lab
Youyang Gu
Imperial
IHME − CF SEIR
IHME − MS SEIR
0.0
0.1
0.2
0.3
Apr28
Jun28
Aug28
All Cumulative Errors
0.000
0.025
0.050
0.075
Mar09
May09
Jul09
Sep09

Mali

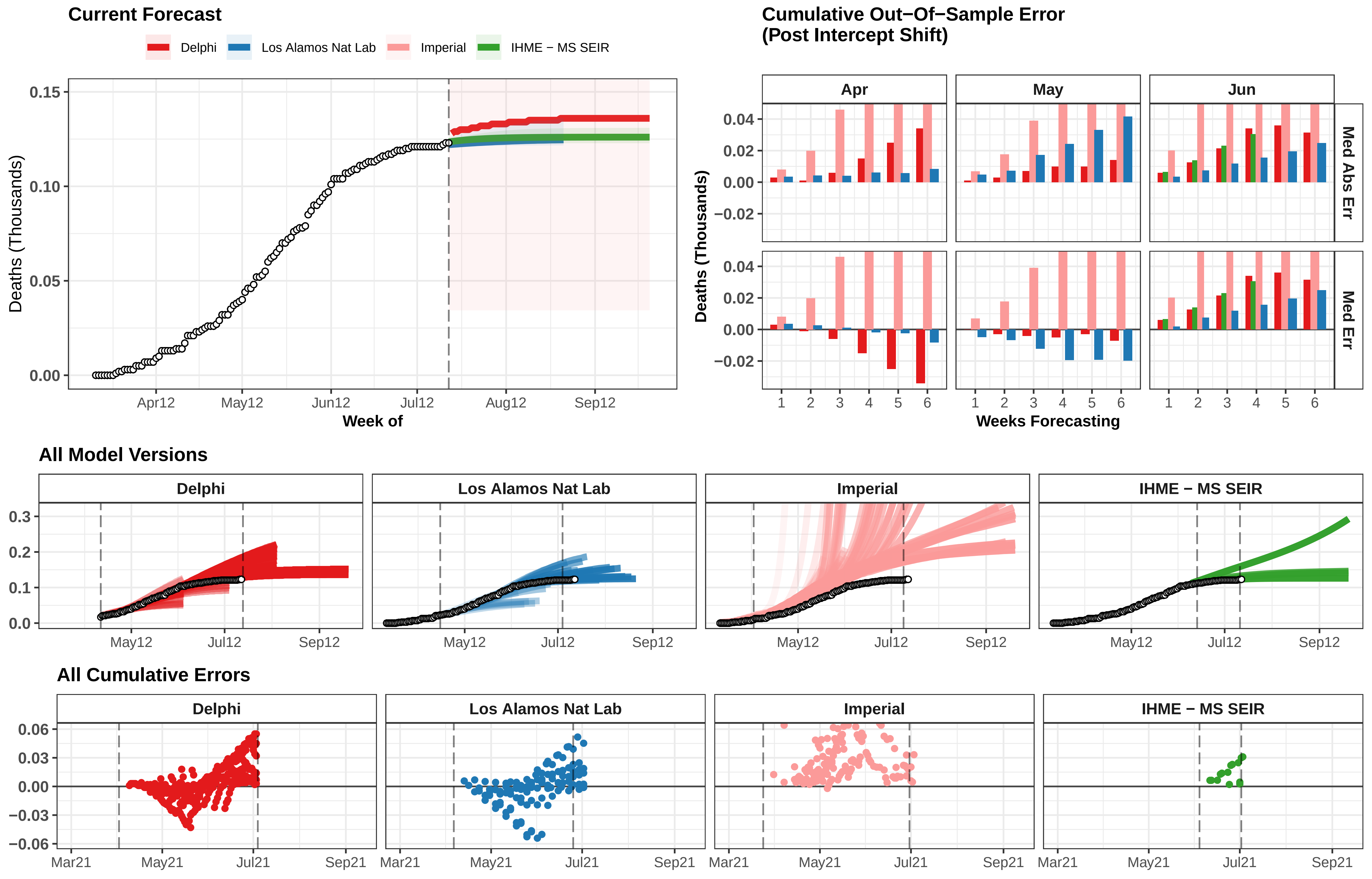
Current Forecast
Delphi
Los Alamos Nat Lab
Imperial
IHME − MS SEIR
Deaths (Thousands)
0.15
0.10
0.05
0.00
Apr12
May12
Jun12
Jul12
Aug12
Sep12
Week of
Cumulative Out−Of−Sample Error
(Post Intercept Shift)
Apr
May
Jun
Med Abs Err
Med Err
0.04
0.02
0.00
−0.02
1
2
3
4
5
6
Weeks Forecasting
All Model Versions
0.3
0.2
0.1
0.0
May12
Jul12
Sep12
All Cumulative Errors
0.06
0.03
0.00
−0.03
−0.06
Mar21
May21
Jul21
Sep21

South Dakota

Current Forecast

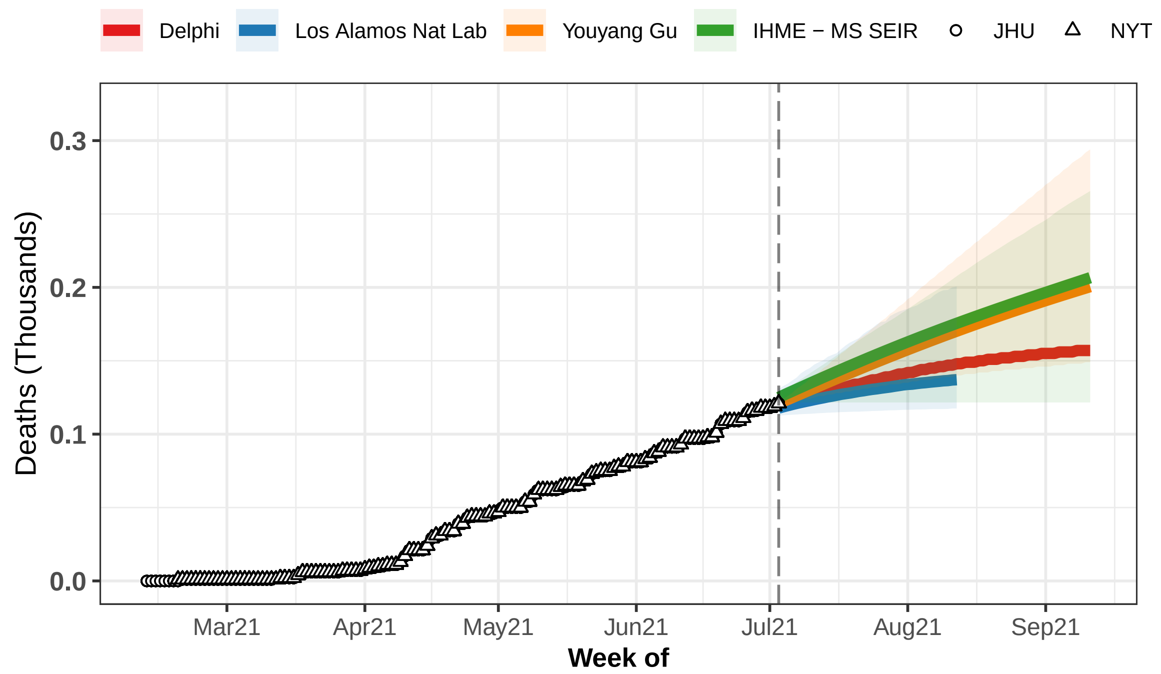

Cumulative Out−Of−Sample Error (Post Intercept Shift)

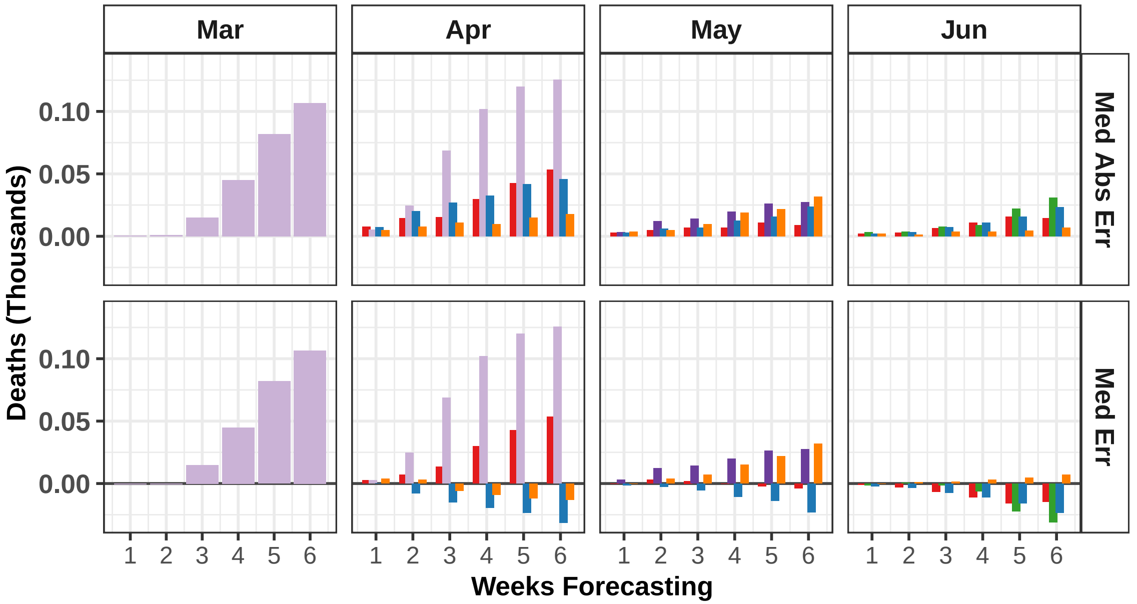

All Model Versions

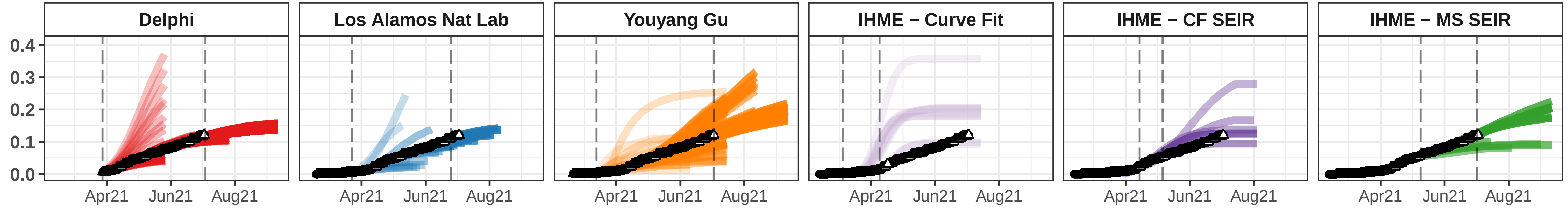

All Cumulative Errors

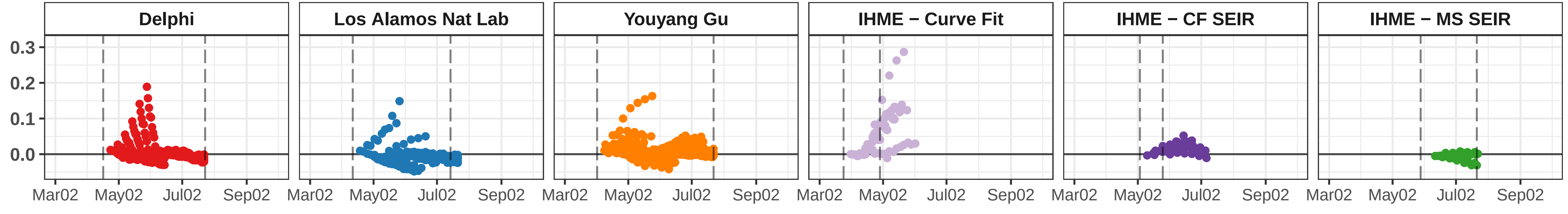

Maine

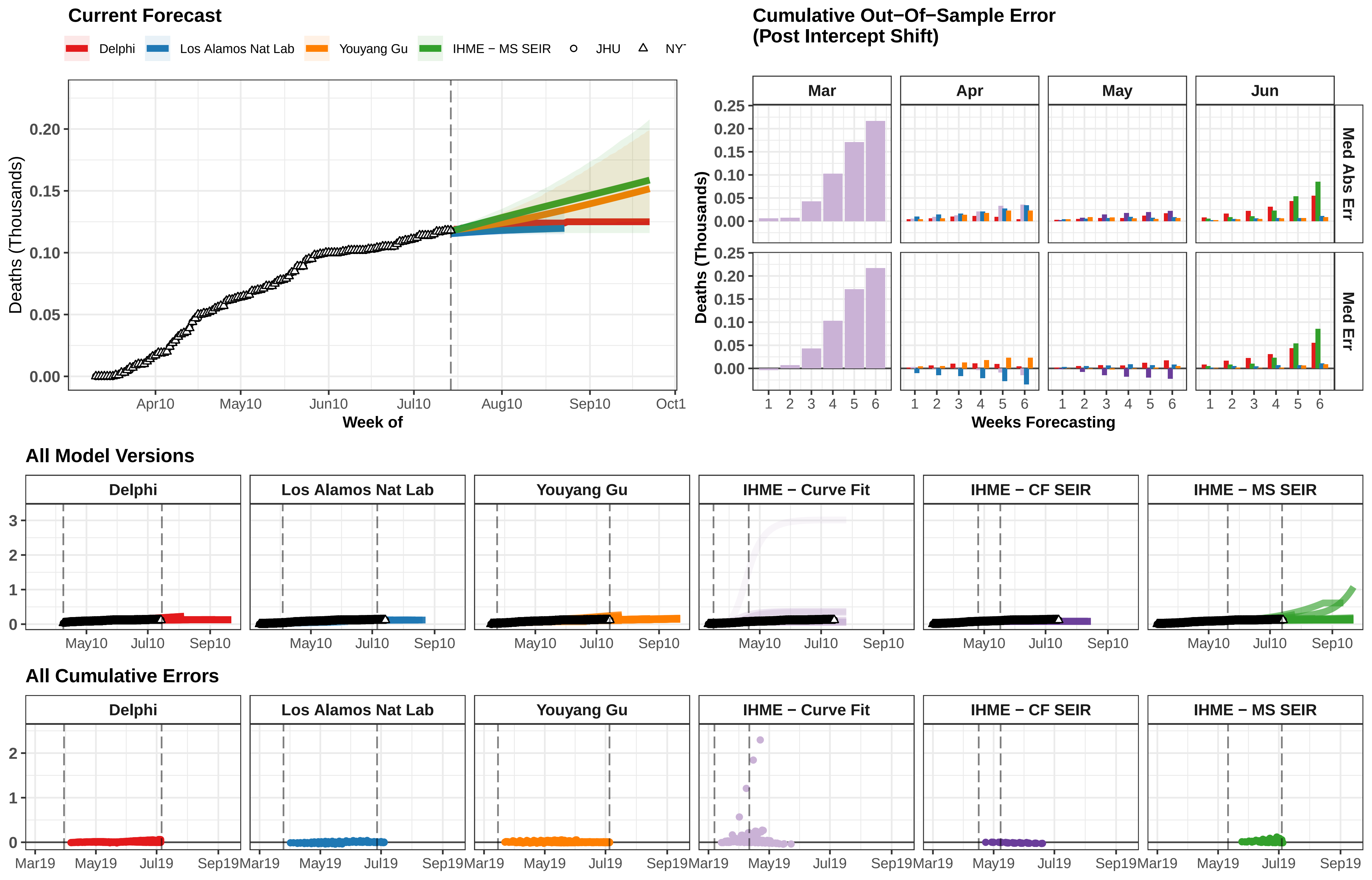

Current Forecast
Delphi
Los Alamos Nat Lab
Youyang Gu
IHME − MS SEIR
JHU
NY
Deaths (Thousands)
0.00
0.05
0.10
0.15
0.20
Apr10
May10
Jun10
Jul10
Aug10
Sep10
Oct1
Week of
Cumulative Out−Of−Sample Error
(Post Intercept Shift)
Mar
Apr
May
Jun
Med Abs Err
Med Err
0.25
Weeks Forecasting
All Model Versions
Delphi
Los Alamos Nat Lab
Youyang Gu
IHME − Curve Fit
IHME − CF SEIR
IHME − MS SEIR
0
1
2
3
All Cumulative Errors
Mar19
May19
Jul19
Sep19

Slovenia

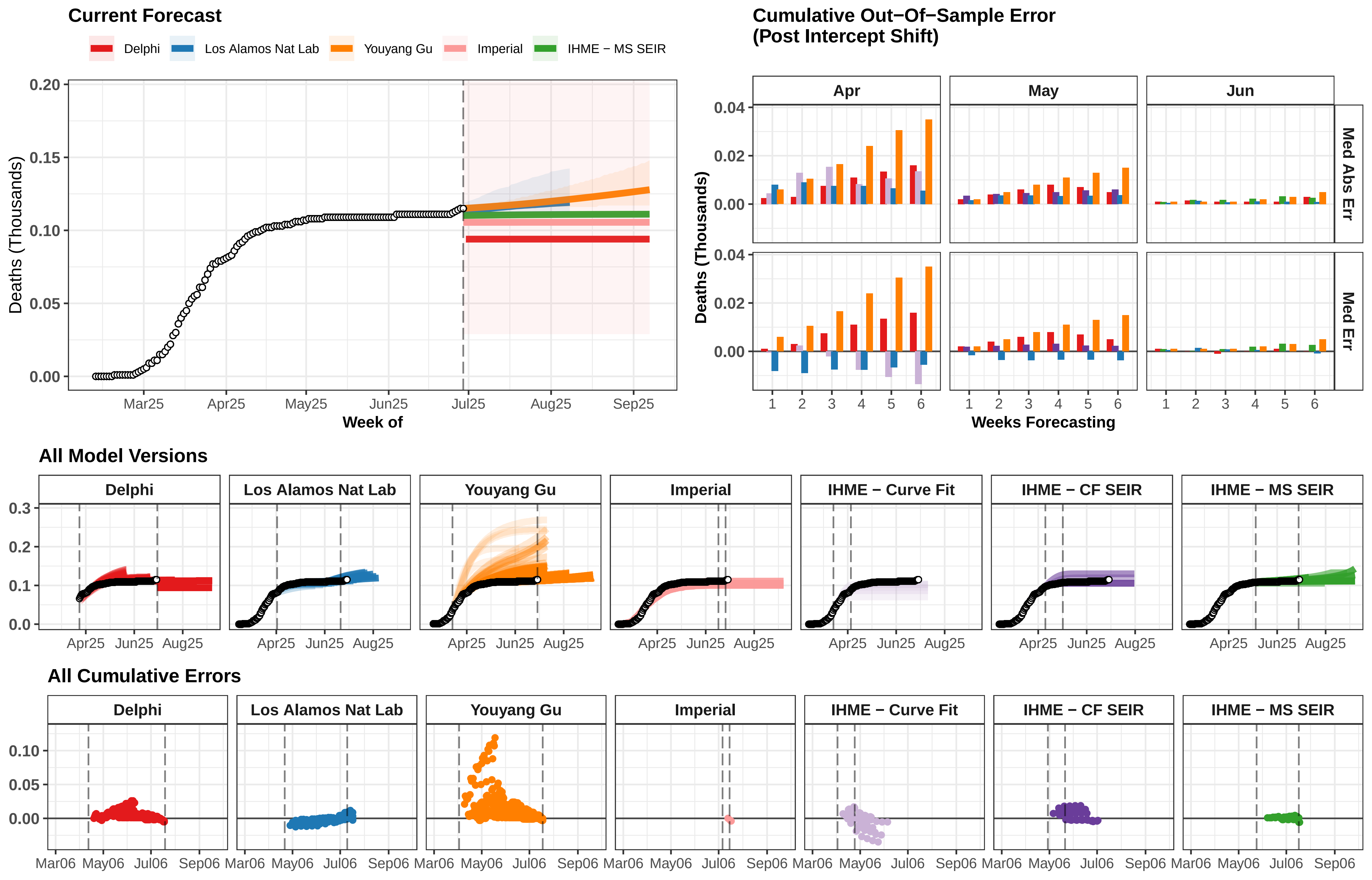
Current Forecast
Delphi
Los Alamos Nat Lab
Youyang Gu
Imperial
IHME − MS SEIR
Deaths (Thousands)
0.20
0.15
0.10
0.05
0.00
Mar25
Apr25
May25
Jun25
Jul25
Aug25
Sep25
Week of
Cumulative Out−Of−Sample Error
(Post Intercept Shift)
Apr
May
Jun
Med Abs Err
Med Err
0.04
0.02
0.00
1
2
3
4
5
6
Weeks Forecasting
All Model Versions
Delphi
Los Alamos Nat Lab
Youyang Gu
Imperial
IHME − Curve Fit
IHME − CF SEIR
IHME − MS SEIR
0.3
0.2
0.1
0.0
Apr25
Jun25
Aug25
All Cumulative Errors
0.10
0.05
0.00
Mar06
May06
Jul06
Sep06

Luxembourg

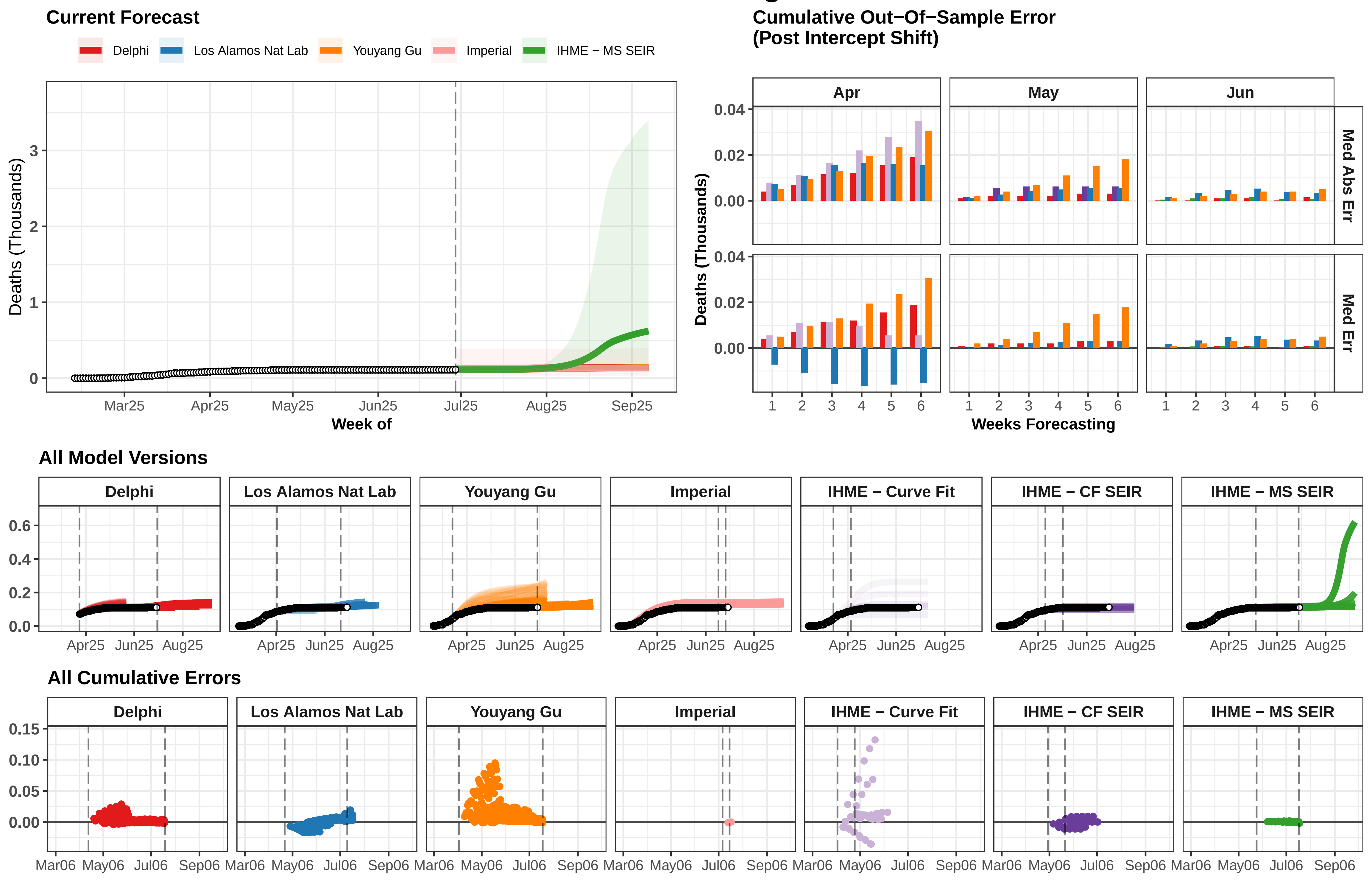
Current Forecast
Delphi
Los Alamos Nat Lab
Youyang Gu
Imperial
IHME − MS SEIR
Deaths (Thousands)
Week of
Mar25
Apr25
May25
Jun25
Jul25
Aug25
Sep25
Cumulative Out−Of−Sample Error
(Post Intercept Shift)
Apr
May
Jun
Med Abs Err
Med Err
Weeks Forecasting
All Model Versions
Delphi
Los Alamos Nat Lab
Youyang Gu
Imperial
IHME − Curve Fit
IHME − CF SEIR
IHME − MS SEIR
Apr25
Jun25
Aug25
All Cumulative Errors
Mar06
May06
Jul06
Sep06

Nicaragua

Current Forecast

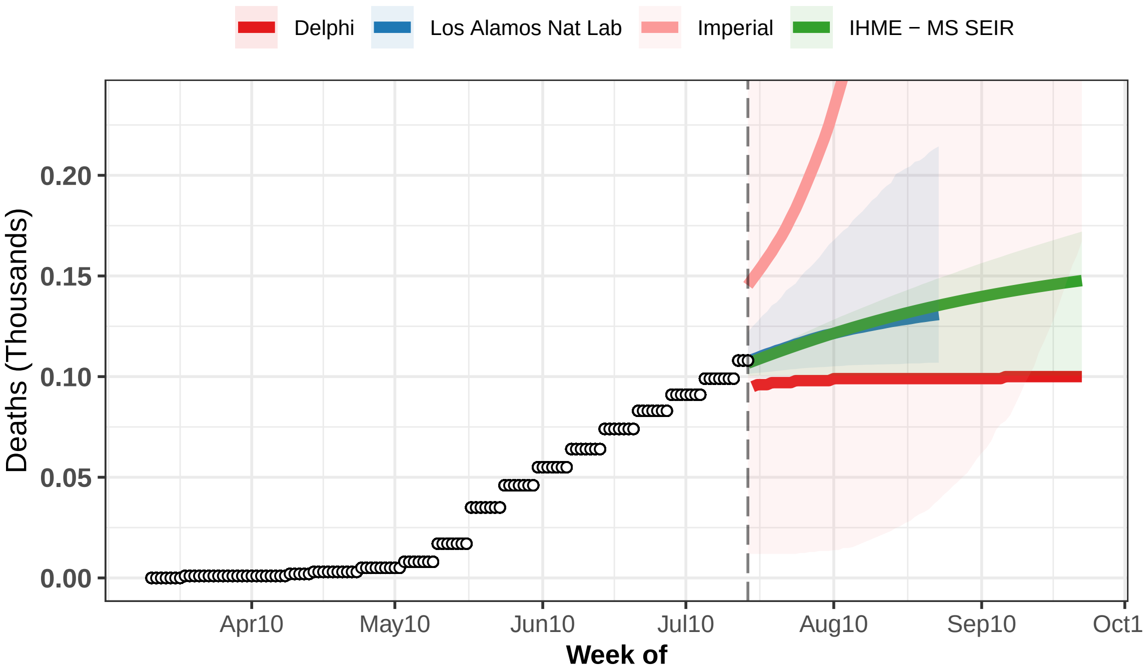

Cumulative Out−Of−Sample Error (Post Intercept Shift)

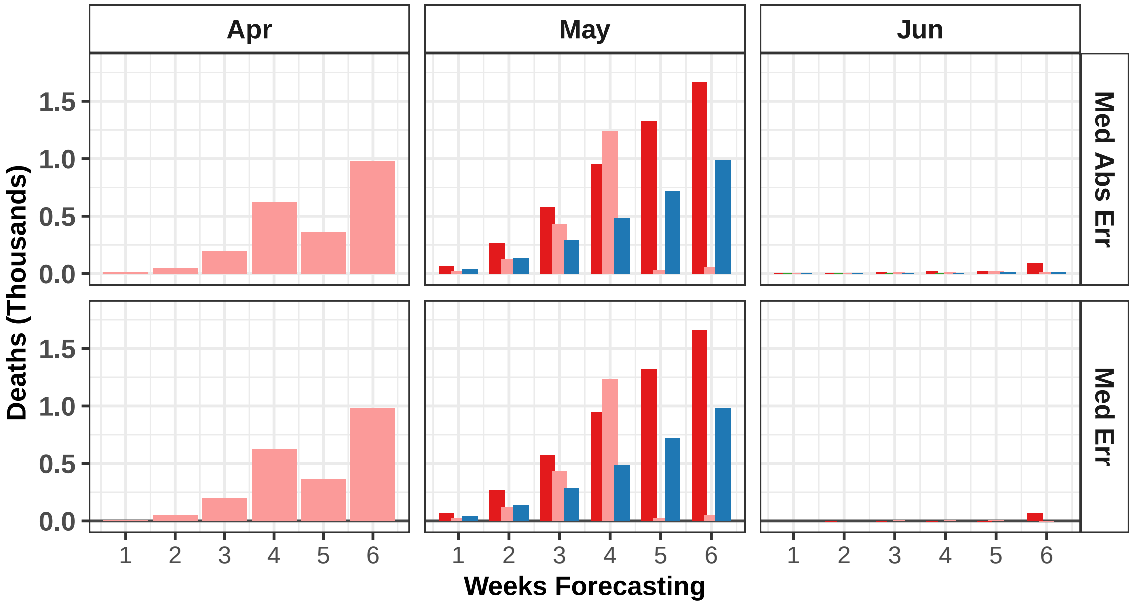

All Model Versions

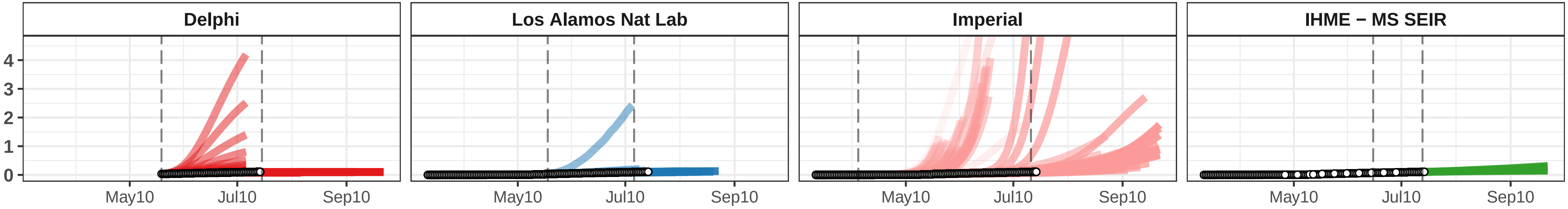

All Cumulative Errors

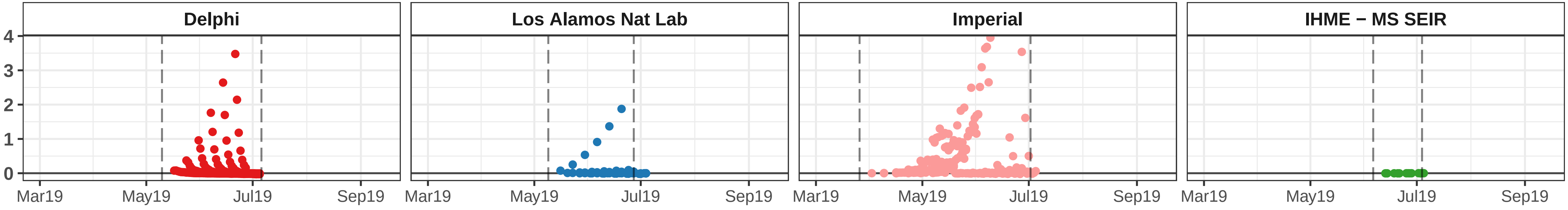

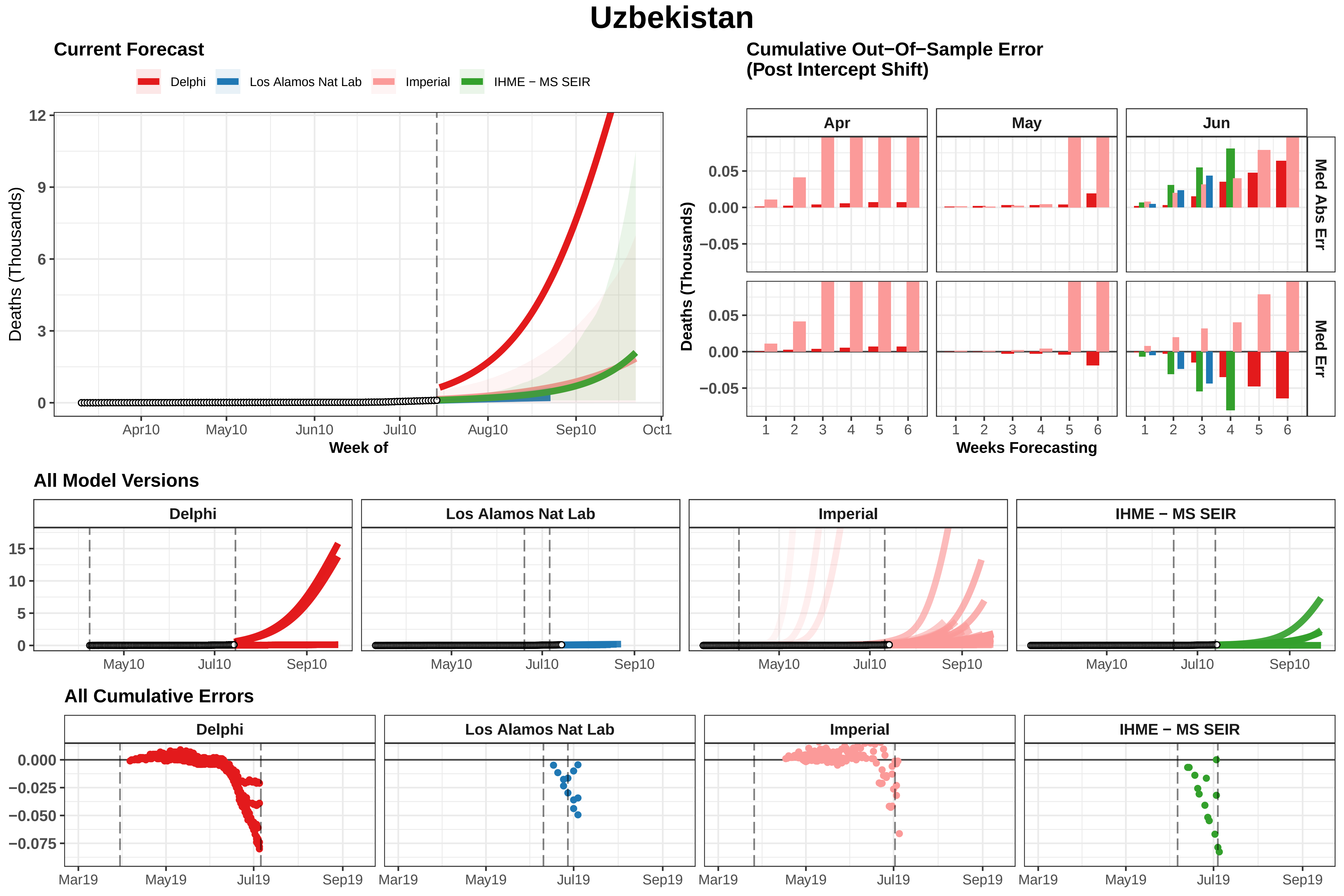
Uzbekistan
Current Forecast
Delphi
Los Alamos Nat Lab
Imperial
IHME − MS SEIR
Deaths (Thousands)
Week of
Apr10
May10
Jun10
Jul10
Aug10
Sep10
Oct1
Cumulative Out−Of−Sample Error
(Post Intercept Shift)
Apr
May
Jun
Med Abs Err
Med Err
Weeks Forecasting
All Model Versions
All Cumulative Errors
Mar19
May19
Jul19
Sep19

West Virginia

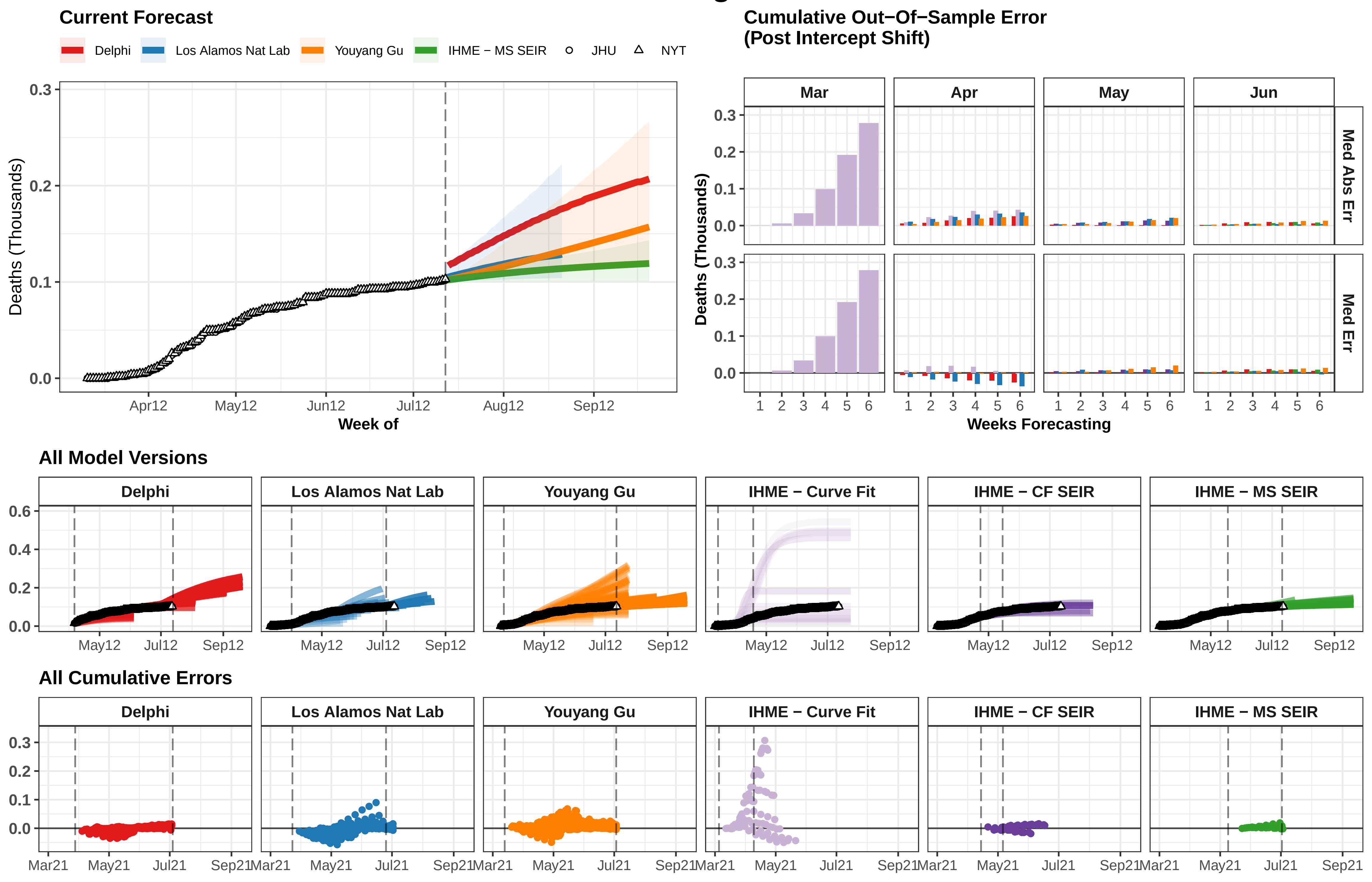

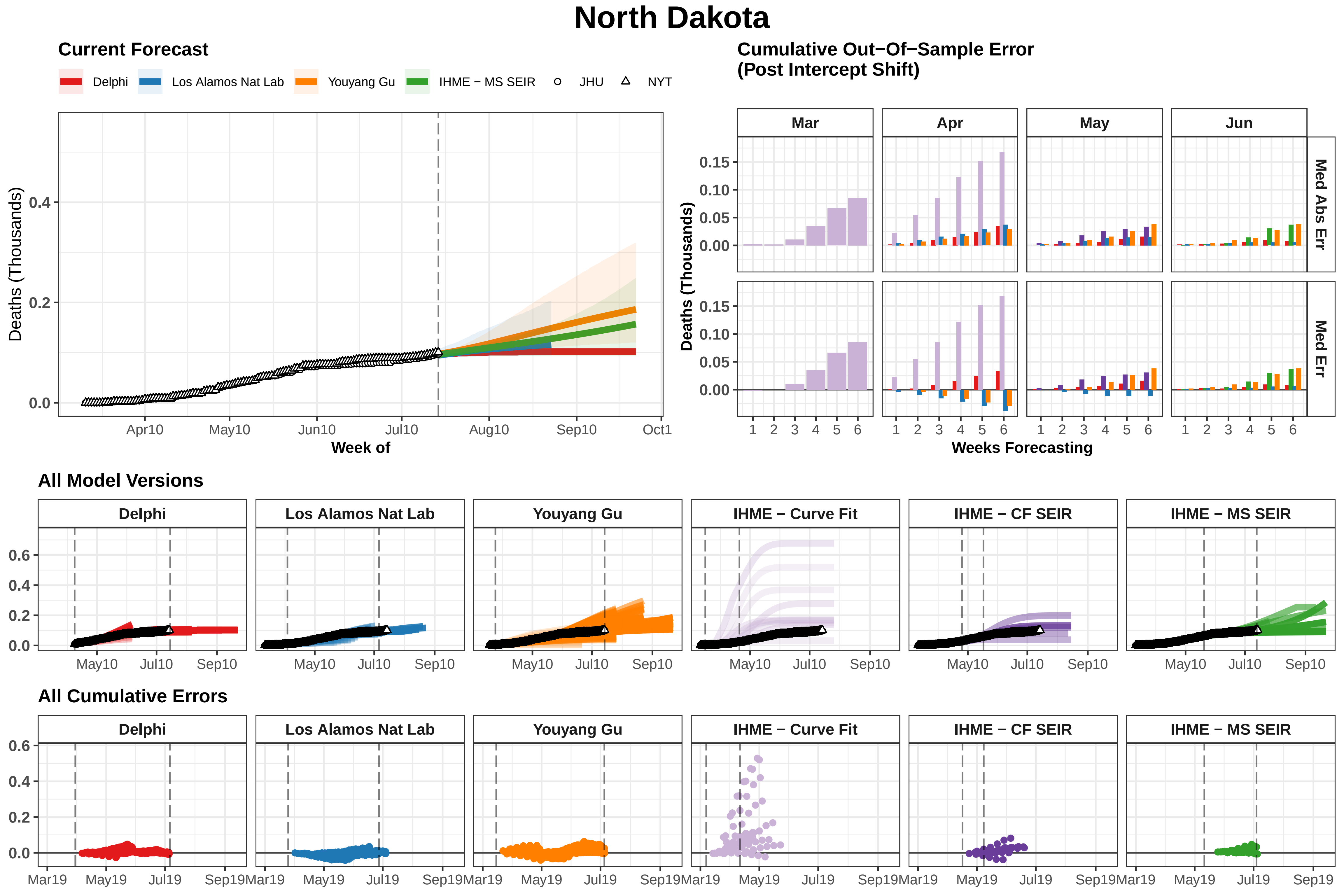
North Dakota
Current Forecast
Delphi
Los Alamos Nat Lab
Youyang Gu
IHME − MS SEIR
JHU
NYT
Deaths (Thousands)
0.0
0.2
0.4
Apr10
May10
Jun10
Jul10
Aug10
Sep10
Oct1
Week of
Cumulative Out−Of−Sample Error
(Post Intercept Shift)
Mar
Apr
May
Jun
Med Abs Err
Med Err
0.15
0.10
0.05
0.00
1
2
3
4
5
6
Weeks Forecasting
All Model Versions
Delphi
Los Alamos Nat Lab
Youyang Gu
IHME − Curve Fit
IHME − CF SEIR
IHME − MS SEIR
0.6
0.4
0.2
0.0
May10
Jul10
Sep10
All Cumulative Errors
Mar19
May19
Jul19
Sep19

Cote d'Ivoire

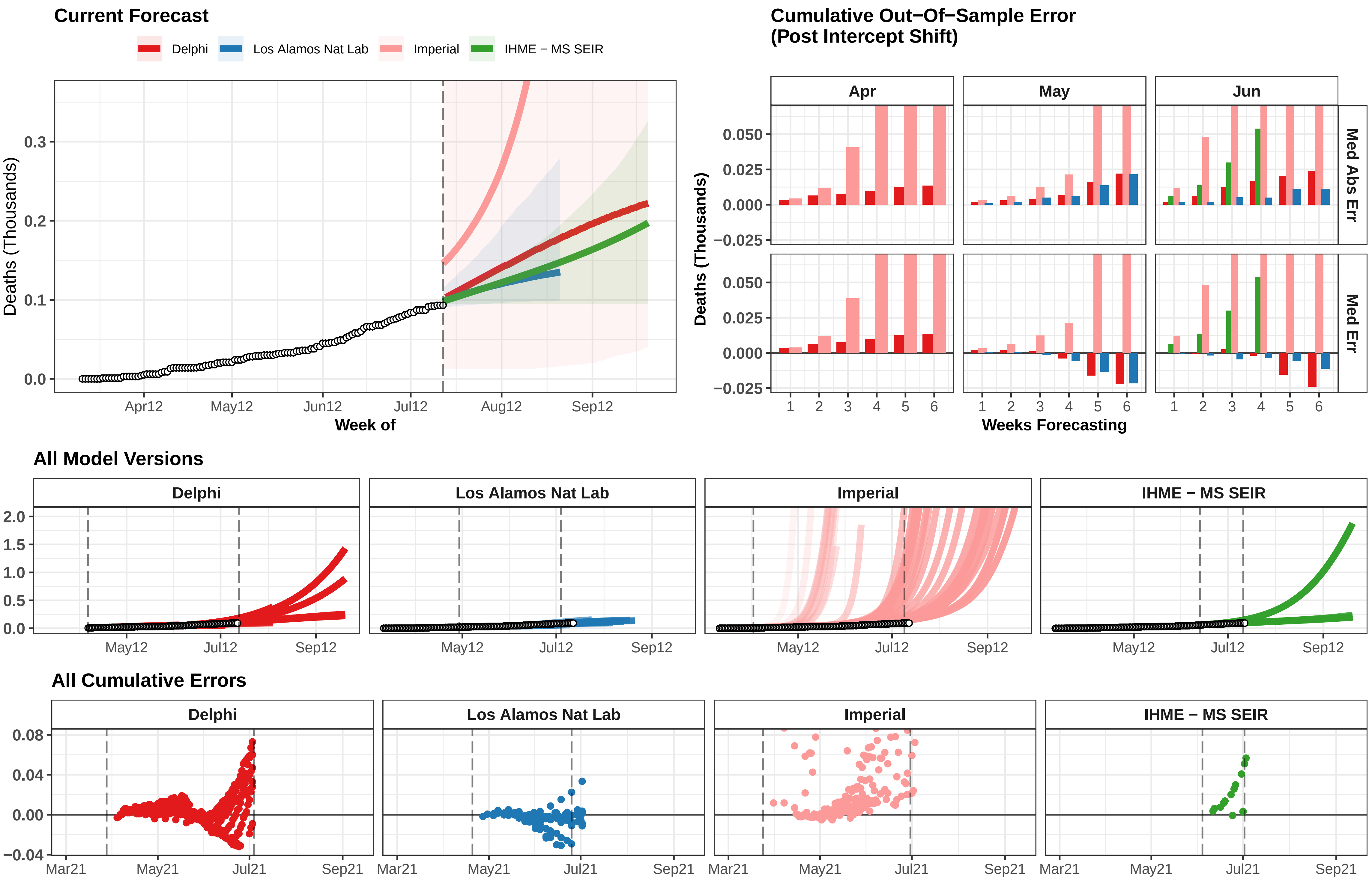

Somalia

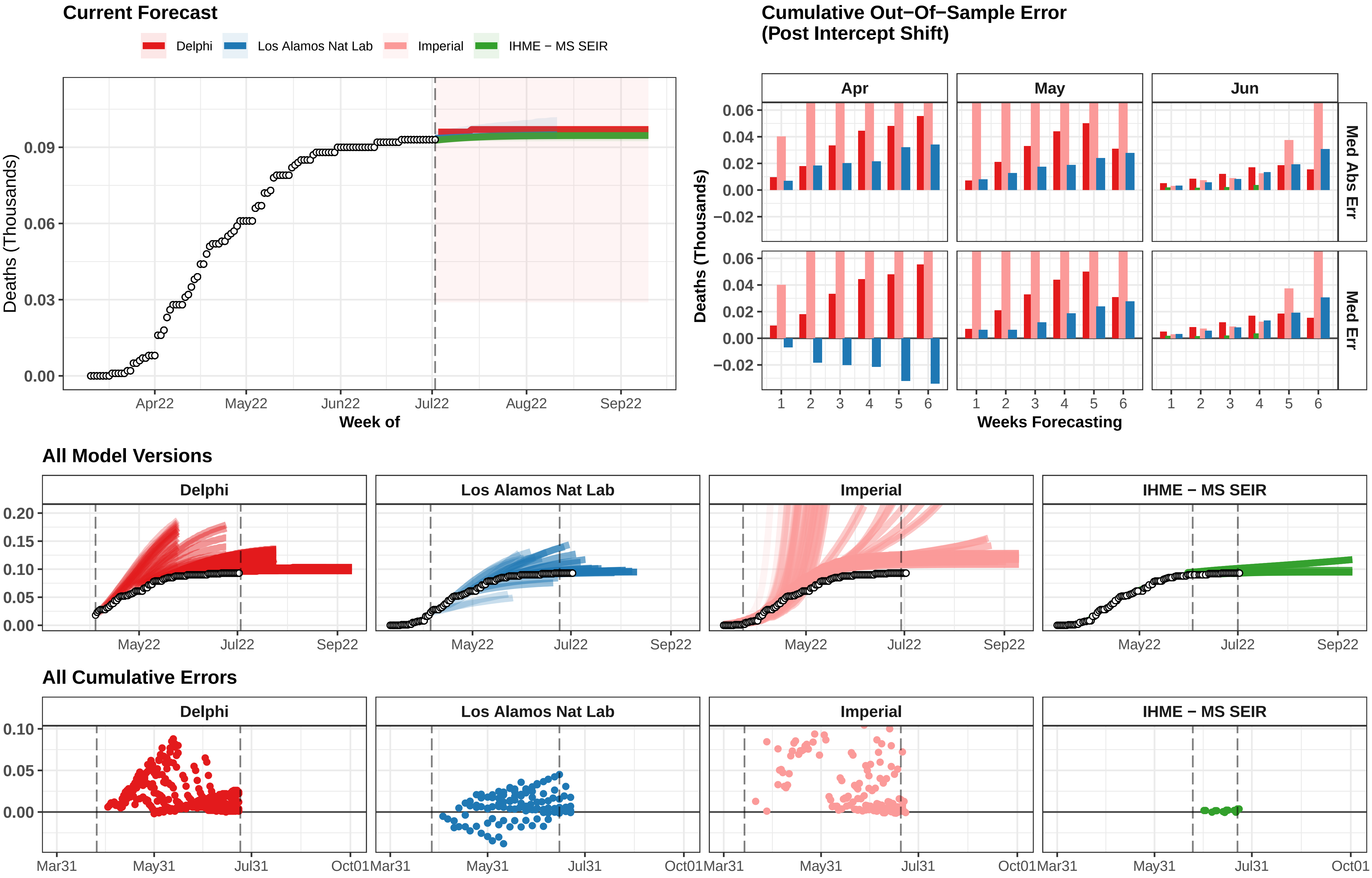
Current Forecast
Delphi
Los Alamos Nat Lab
Imperial
IHME − MS SEIR
Deaths (Thousands)
0.00
0.03
0.06
0.09
Apr22
May22
Jun22
Jul22
Aug22
Sep22
Week of
Cumulative Out−Of−Sample Error
(Post Intercept Shift)
Apr
May
Jun
Med Abs Err
Med Err
0.06
0.04
0.02
0.00
−0.02
1
2
3
4
5
6
Weeks Forecasting
All Model Versions
0.20
0.15
0.10
0.05
0.00
All Cumulative Errors
Mar31
May31
Jul31
Oct01

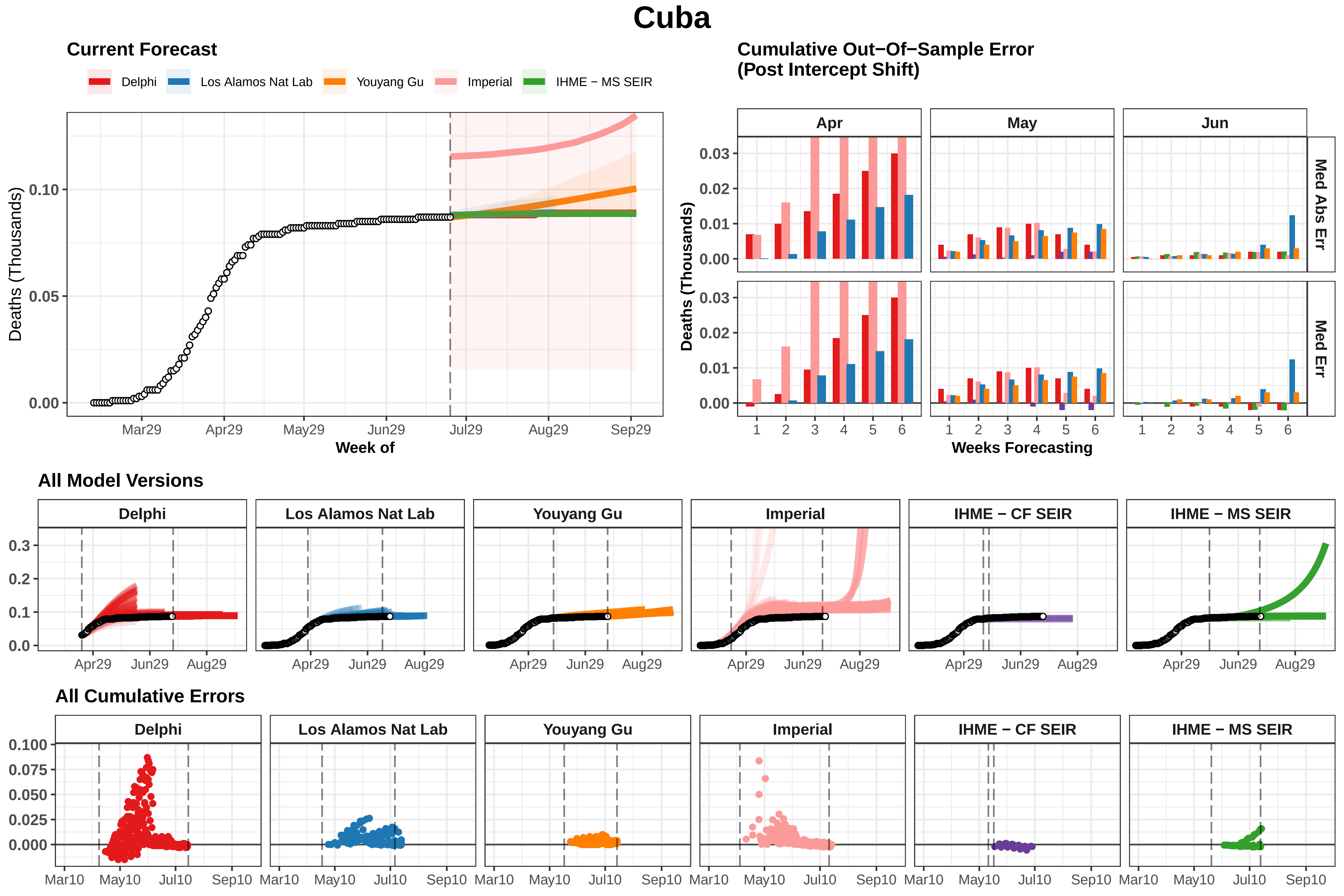
Cuba
Current Forecast
Delphi
Los Alamos Nat Lab
Youyang Gu
Imperial
IHME − MS SEIR
Deaths (Thousands)
0.10
0.05
0.00
Mar29
Apr29
May29
Jun29
Jul29
Aug29
Sep29
Week of
Cumulative Out−Of−Sample Error
(Post Intercept Shift)
Apr
May
Jun
Med Abs Err
Med Err
0.03
0.02
0.01
0.00
1
2
3
4
5
6
Weeks Forecasting
All Model Versions
IHME − CF SEIR
0.3
0.2
0.1
0.0
Jun29
All Cumulative Errors
0.100
0.075
0.050
0.025
0.000
Mar10
May10
Jul10
Sep10

Costa Rica

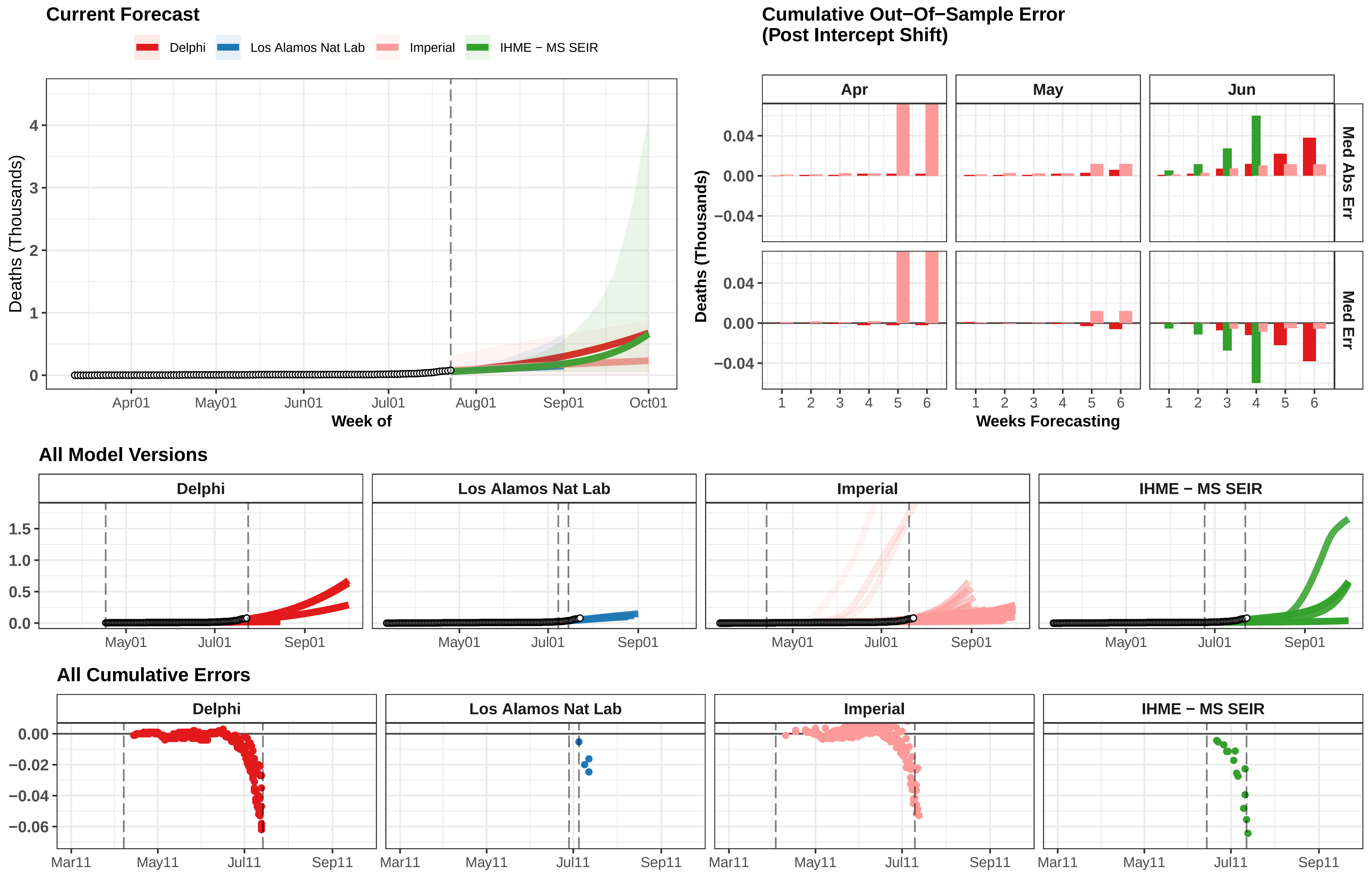

Current Forecast
Delphi
Los Alamos Nat Lab
Imperial
IHME − MS SEIR
Deaths (Thousands)
4
3
2
1
0
Apr01
May01
Jun01
Jul01
Aug01
Sep01
Oct01
Week of
Cumulative Out−Of−Sample Error
(Post Intercept Shift)
Apr
May
Jun
Med Abs Err
Med Err
0.04
0.00
−0.04
1 2 3 4 5 6
Weeks Forecasting
All Model Versions
Delphi
Los Alamos Nat Lab
Imperial
IHME − MS SEIR
1.5
1.0
0.5
0.0
May01
Jul01
Sep01
All Cumulative Errors
Delphi
Los Alamos Nat Lab
Imperial
IHME − MS SEIR
0.00
−0.02
−0.04
−0.06
Mar11
May11
Jul11
Sep11

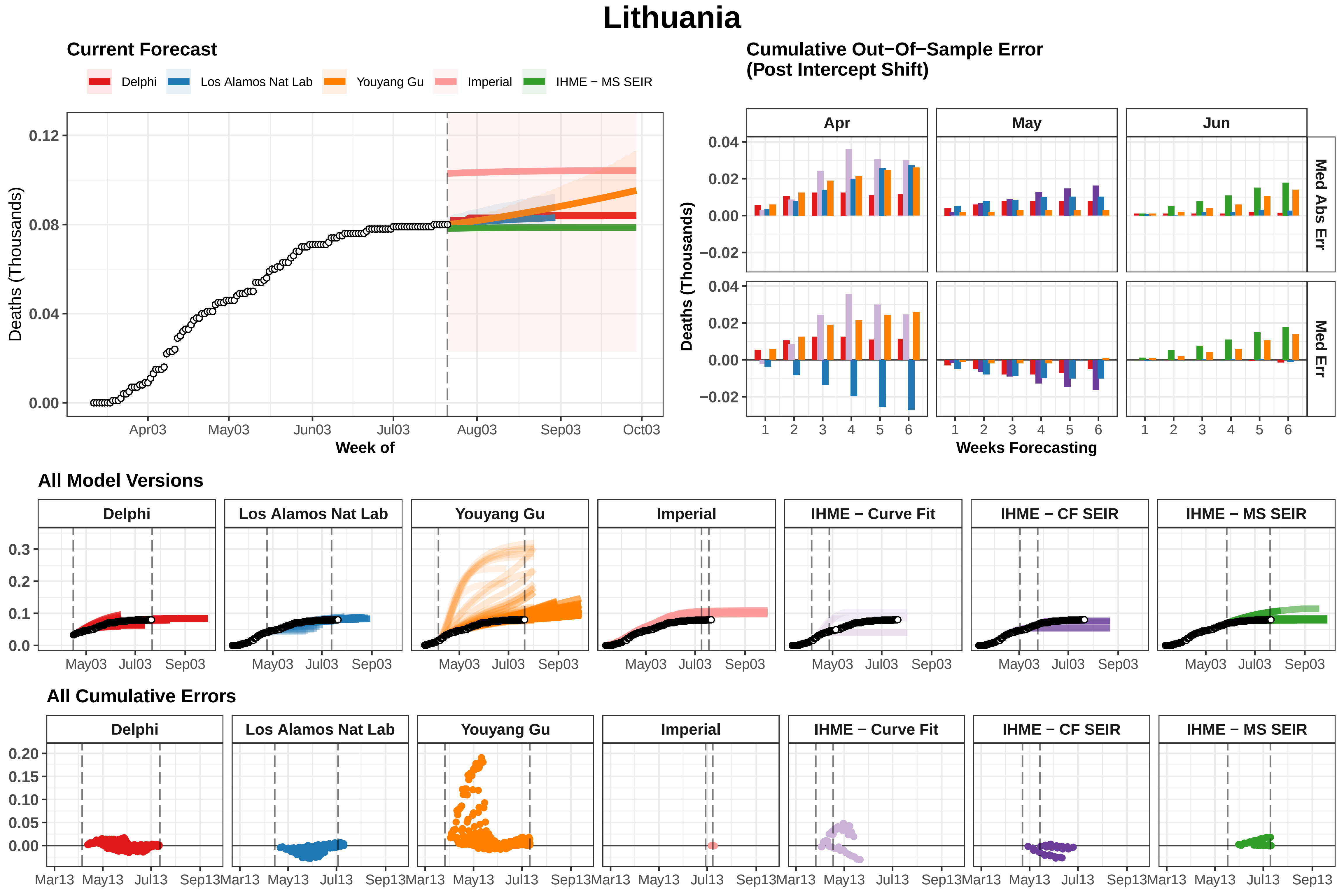

Lithuania
Current Forecast
Delphi
Los Alamos Nat Lab
Youyang Gu
Imperial
IHME − MS SEIR
Deaths (Thousands)
Week of
Apr03
May03
Jun03
Jul03
Aug03
Sep03
Oct03
Cumulative Out−Of−Sample Error
(Post Intercept Shift)
Apr
May
Jun
Med Abs Err
Med Err
Weeks Forecasting
All Model Versions
IHME − Curve Fit
IHME − CF SEIR
All Cumulative Errors
Mar13
May13
Jul13
Sep13

Malawi

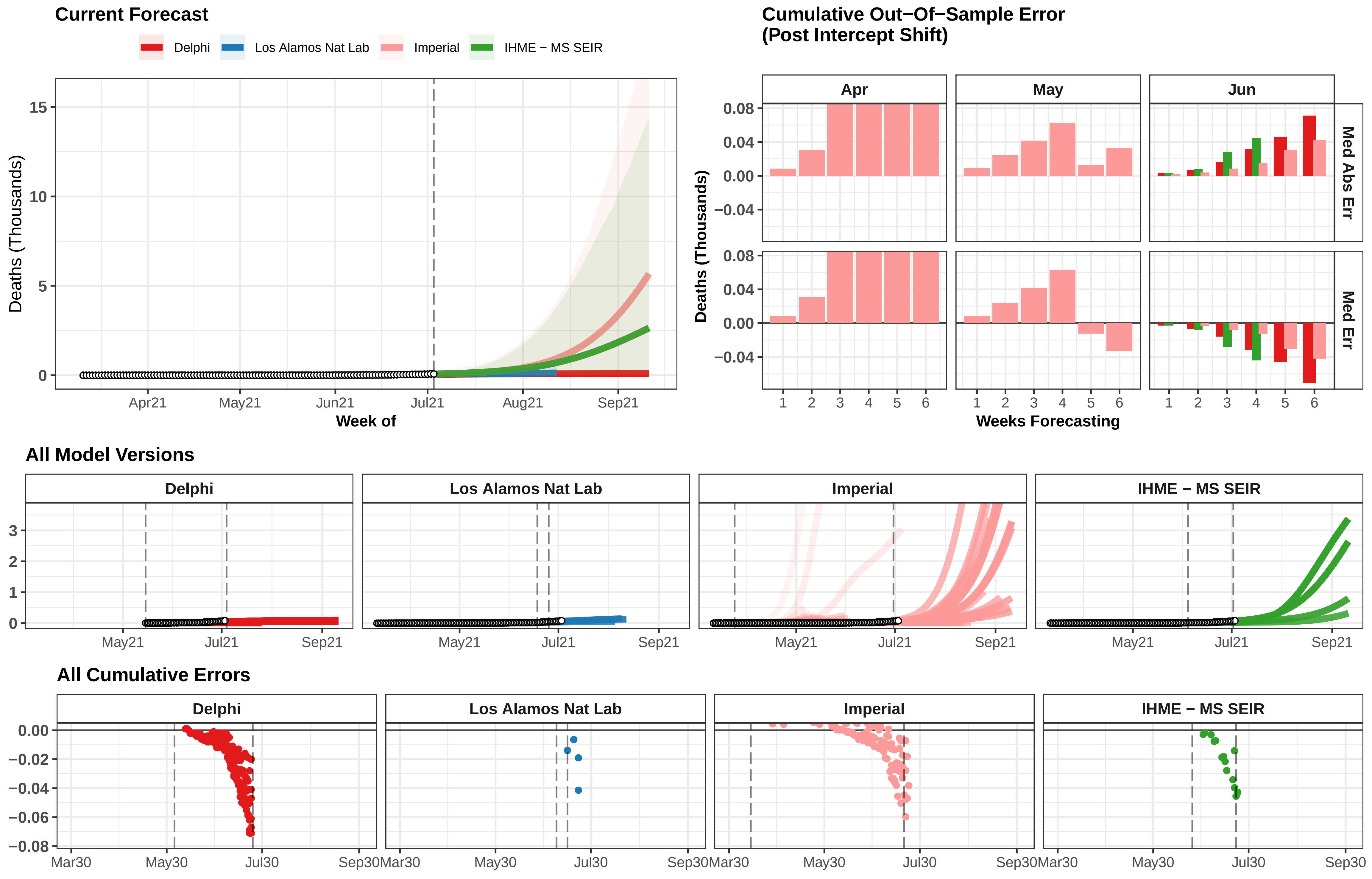

Current Forecast
Delphi
Los Alamos Nat Lab
Imperial
IHME − MS SEIR
Deaths (Thousands)
15
10
5
0
Apr21
May21
Jun21
Jul21
Aug21
Sep21
Week of
Cumulative Out−Of−Sample Error
(Post Intercept Shift)
Apr
May
Jun
Med Abs Err
Med Err
0.08
0.04
0.00
−0.04
1
2
3
4
5
6
Weeks Forecasting
All Model Versions
Delphi
Los Alamos Nat Lab
Imperial
IHME − MS SEIR
3
2
1
0
May21
Jul21
Sep21
All Cumulative Errors
0.00
−0.02
−0.04
−0.06
−0.08
Mar30
May30
Jul30
Sep30

Chad

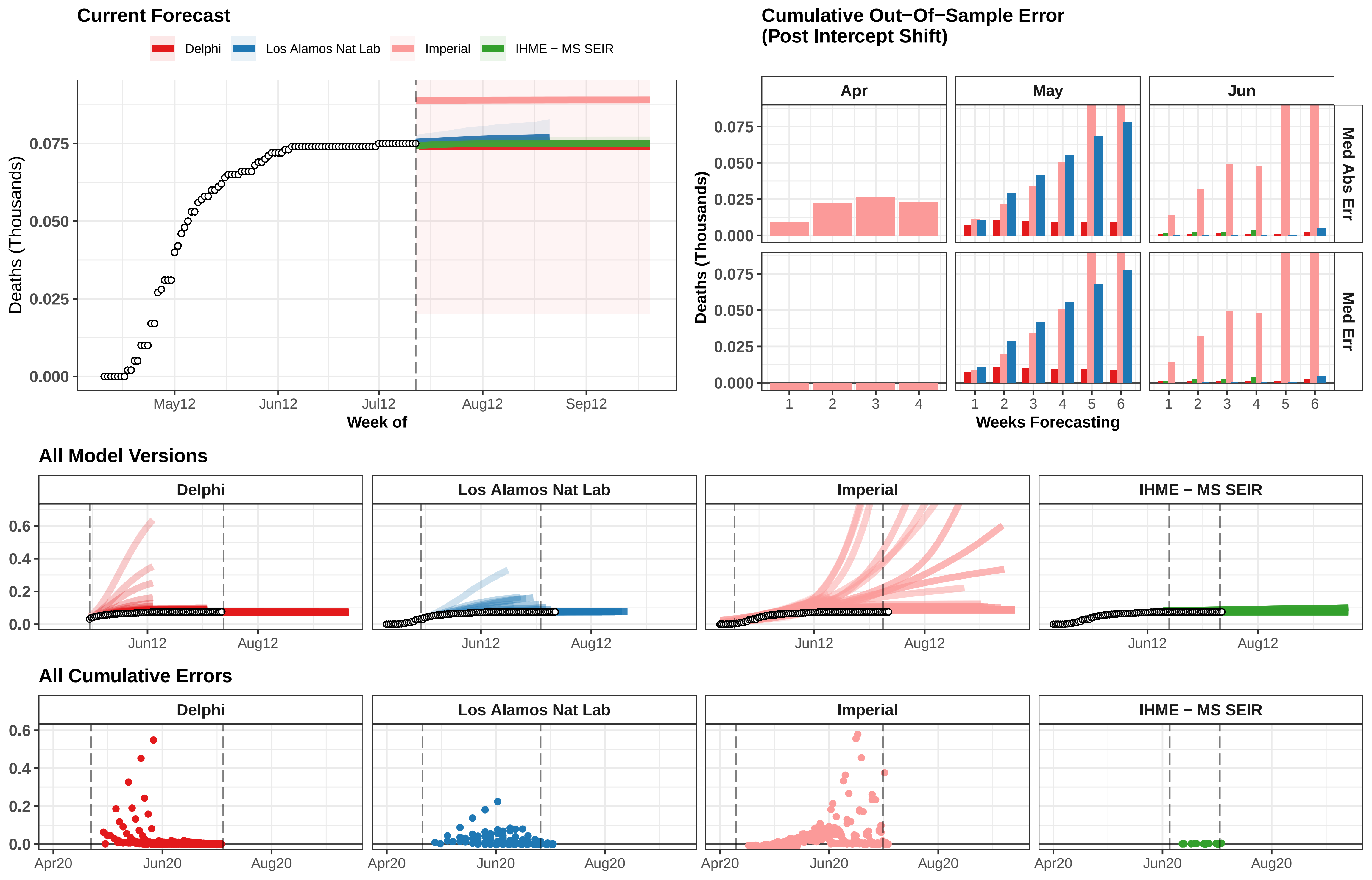

Current Forecast
Delphi
Los Alamos Nat Lab
Imperial
IHME − MS SEIR
Deaths (Thousands)
0.075
0.050
0.025
0.000
May12
Jun12
Jul12
Aug12
Sep12
Week of
Cumulative Out−Of−Sample Error
(Post Intercept Shift)
Apr
May
Jun
Med Abs Err
Med Err
Weeks Forecasting
All Model Versions
Delphi
Los Alamos Nat Lab
Imperial
IHME − MS SEIR
0.6
0.4
0.2
0.0
Jun12
Aug12
All Cumulative Errors
Apr20
Jun20
Aug20

Liberia

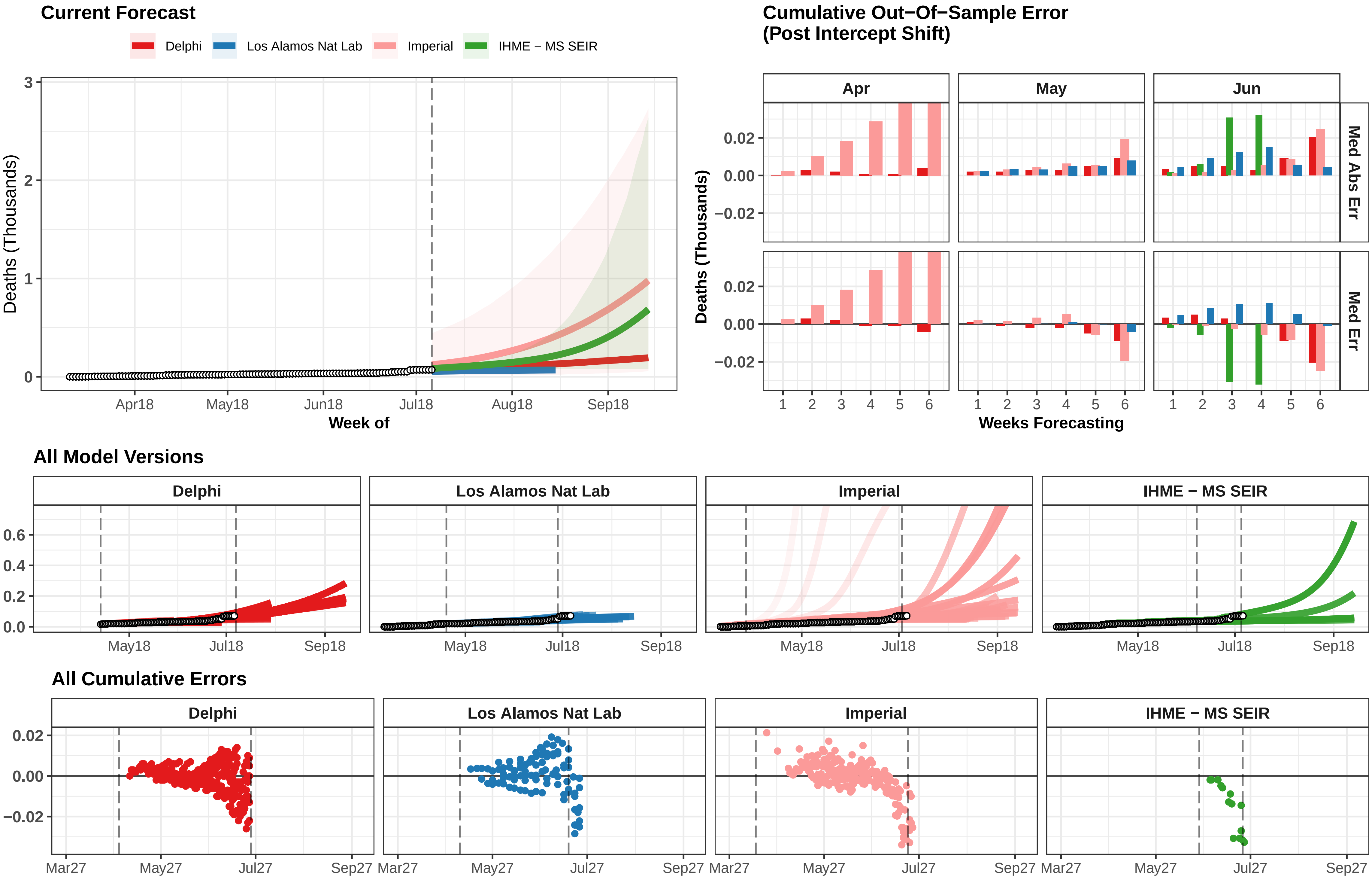

Current Forecast
Delphi
Los Alamos Nat Lab
Imperial
IHME − MS SEIR
Deaths (Thousands)
Week of
Apr18
May18
Jun18
Jul18
Aug18
Sep18
Cumulative Out−Of−Sample Error
(Post Intercept Shift)
Apr
May
Jun
Med Abs Err
Med Err
Weeks Forecasting
All Model Versions
All Cumulative Errors
Mar27
May27
Jul27
Sep27

Madagascar

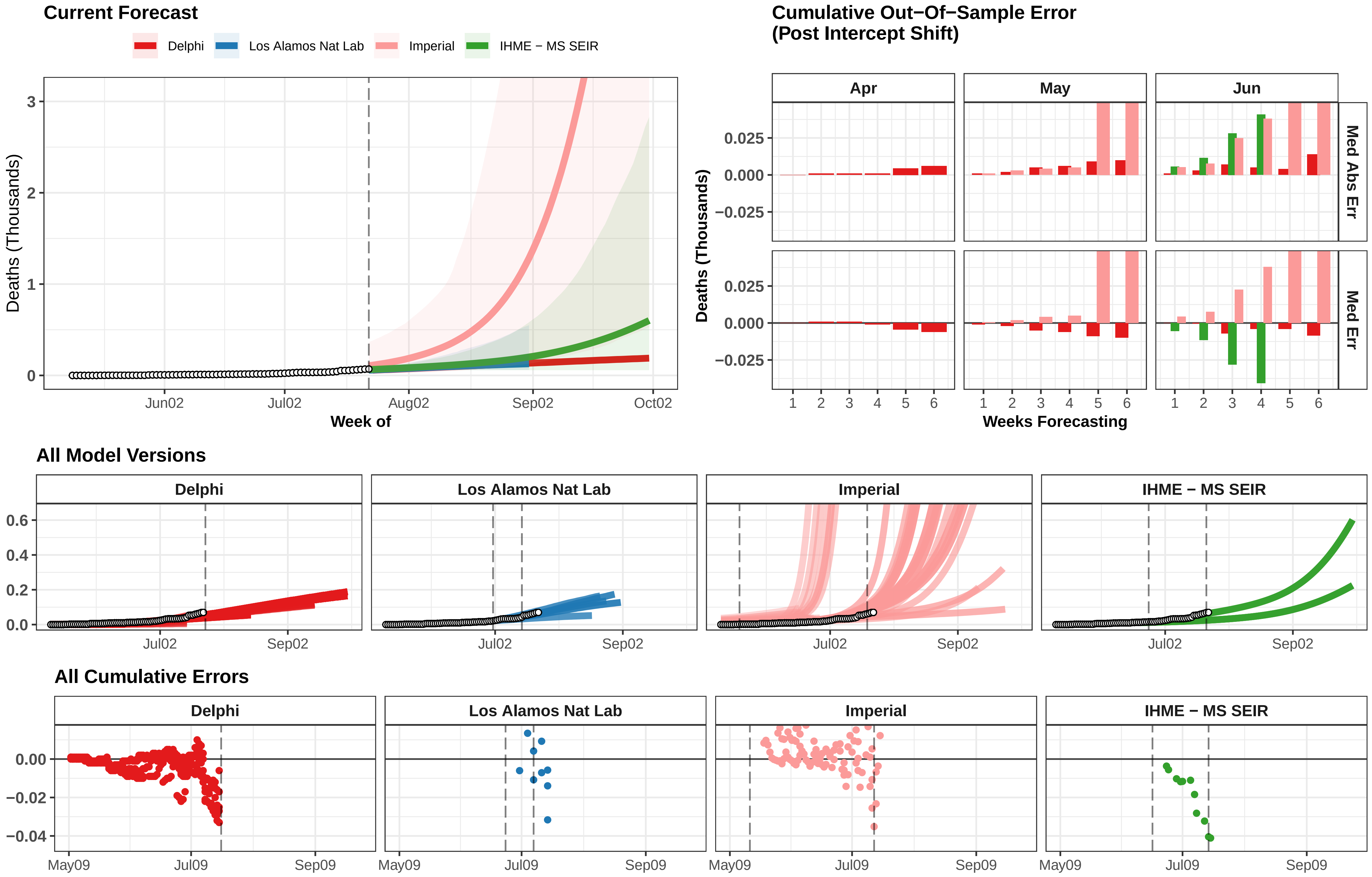
Current Forecast
Delphi
Los Alamos Nat Lab
Imperial
IHME − MS SEIR
Deaths (Thousands)
3
2
1
0
Jun02
Jul02
Aug02
Sep02
Oct02
Week of
Cumulative Out−Of−Sample Error
(Post Intercept Shift)
Apr
May
Jun
Med Abs Err
Med Err
0.025
0.000
−0.025
1 2 3 4 5 6
Weeks Forecasting
All Model Versions
Delphi
Los Alamos Nat Lab
Imperial
IHME − MS SEIR
0.6
0.4
0.2
0.0
Jul02
Sep02
All Cumulative Errors
Delphi
Los Alamos Nat Lab
Imperial
IHME − MS SEIR
0.00
−0.02
−0.04
May09
Jul09
Sep09

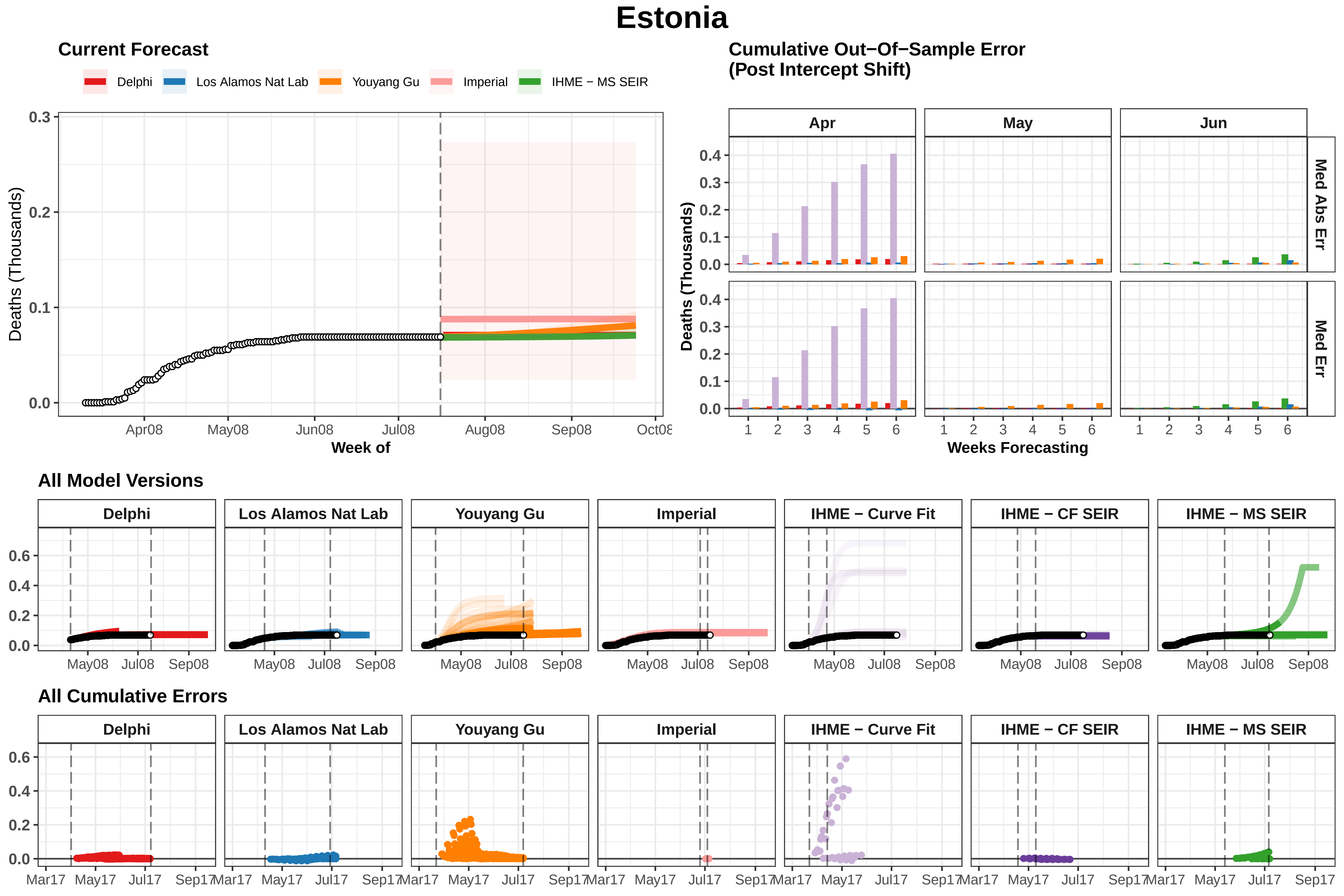
Estonia
Current Forecast
Delphi
Los Alamos Nat Lab
Youyang Gu
Imperial
IHME − MS SEIR
Deaths (Thousands)
0.0
0.1
0.2
0.3
Apr08
May08
Jun08
Jul08
Aug08
Sep08
Oct08
Week of
Cumulative Out−Of−Sample Error
(Post Intercept Shift)
Apr
May
Jun
Med Abs Err
Med Err
Deaths (Thousands)
0.0
0.1
0.2
0.3
0.4
1
2
3
4
5
6
Weeks Forecasting
All Model Versions
Delphi
Los Alamos Nat Lab
Youyang Gu
Imperial
IHME − Curve Fit
IHME − CF SEIR
IHME − MS SEIR
0.0
0.2
0.4
0.6
May08
Jul08
Sep08
All Cumulative Errors
Delphi
Los Alamos Nat Lab
Youyang Gu
Imperial
IHME − Curve Fit
IHME − CF SEIR
IHME − MS SEIR
0.0
0.2
0.4
0.6
Mar17
May17
Jul17
Sep17

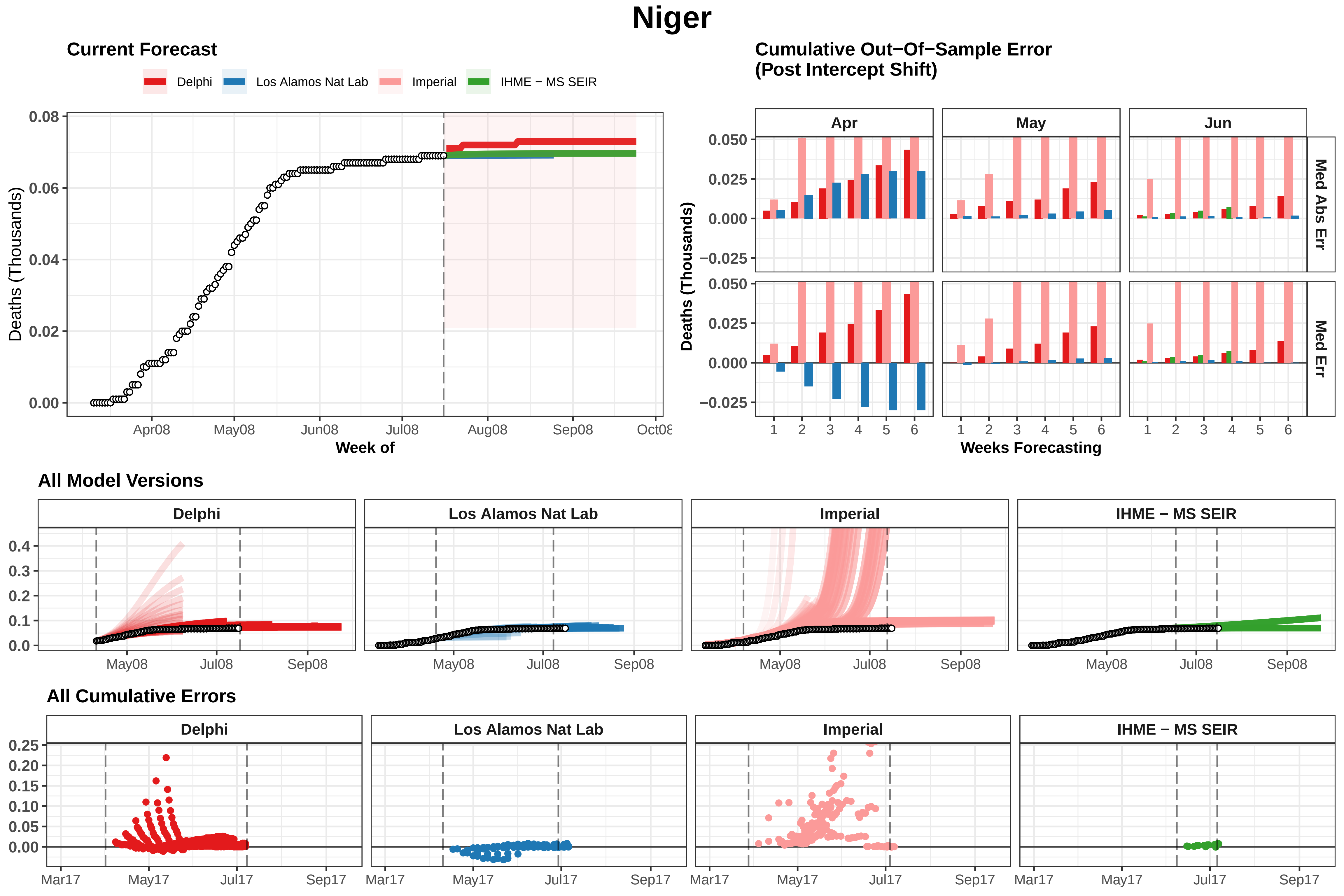

Niger
Current Forecast
Delphi
Los Alamos Nat Lab
Imperial
IHME − MS SEIR
Deaths (Thousands)
Week of
Cumulative Out−Of−Sample Error
(Post Intercept Shift)
Apr
May
Jun
Med Abs Err
Med Err
Weeks Forecasting
All Model Versions
All Cumulative Errors

Sierra Leone

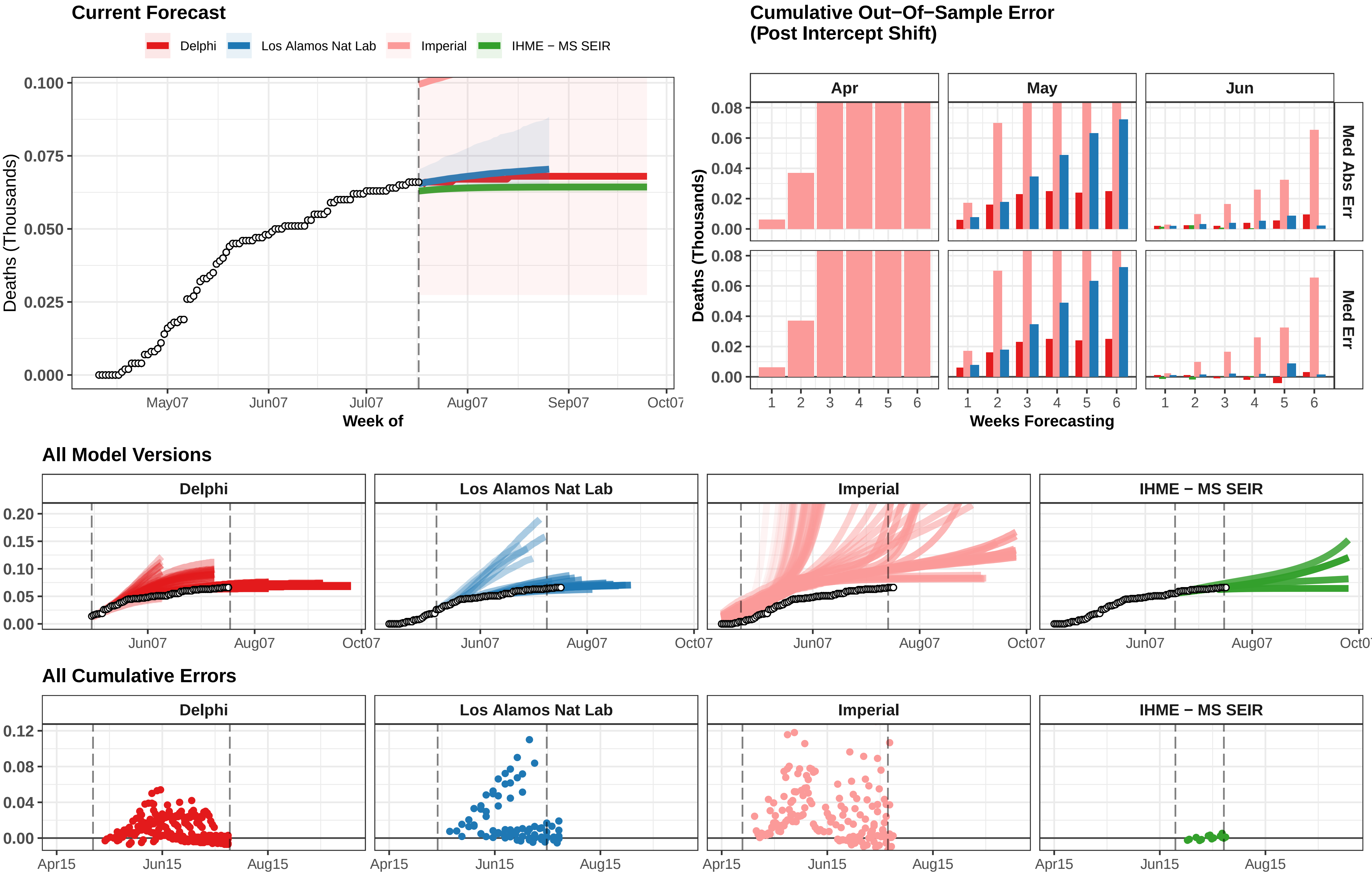

Central African Republic

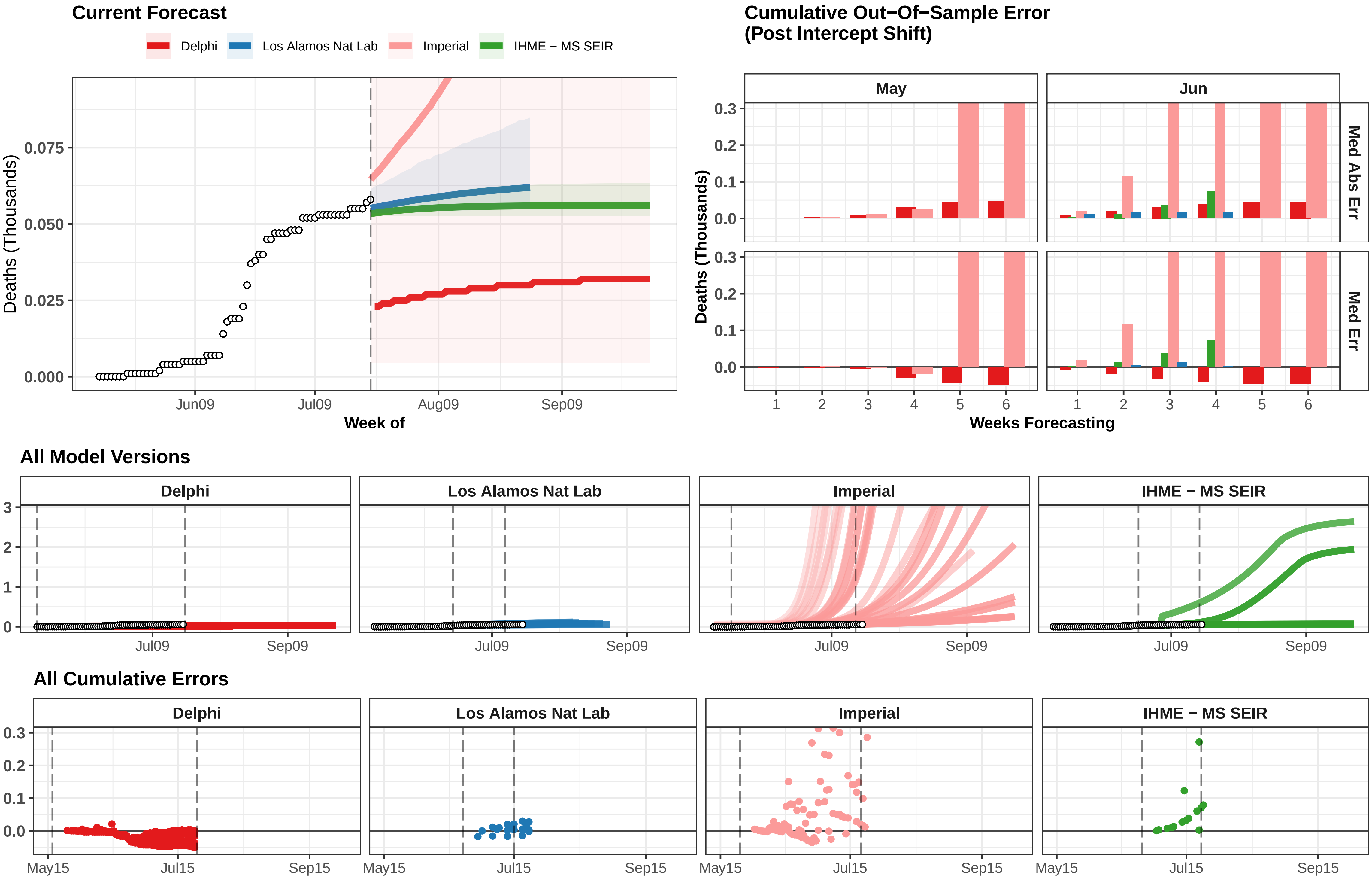

Djibouti

Current Forecast

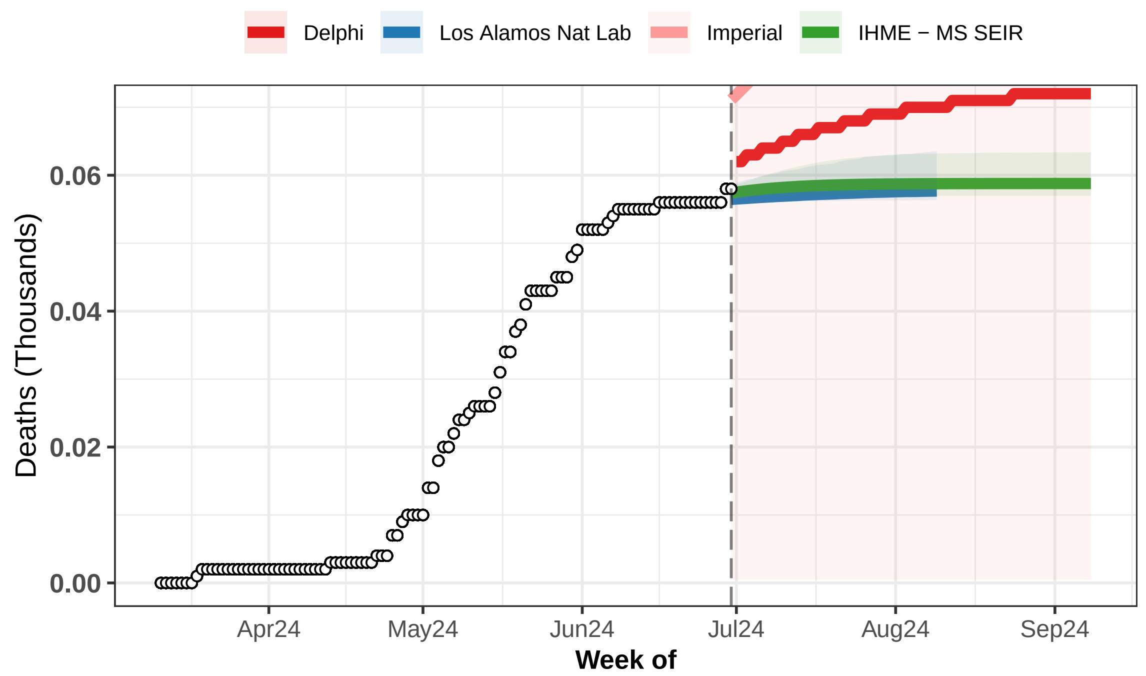

Cumulative Out−Of−Sample Error (Post Intercept Shift)

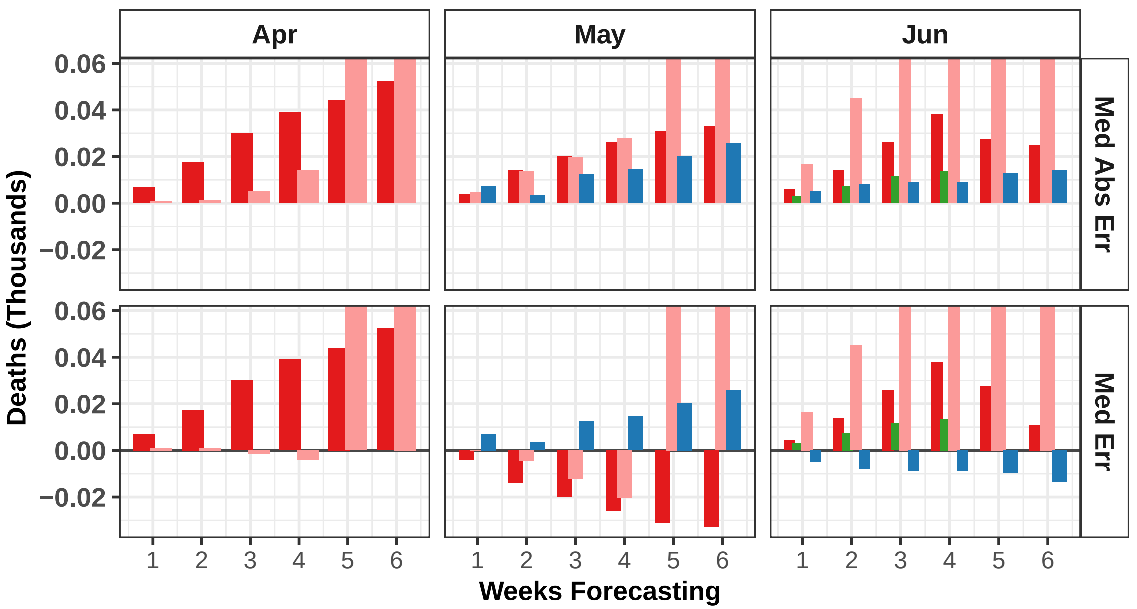

All Model Versions

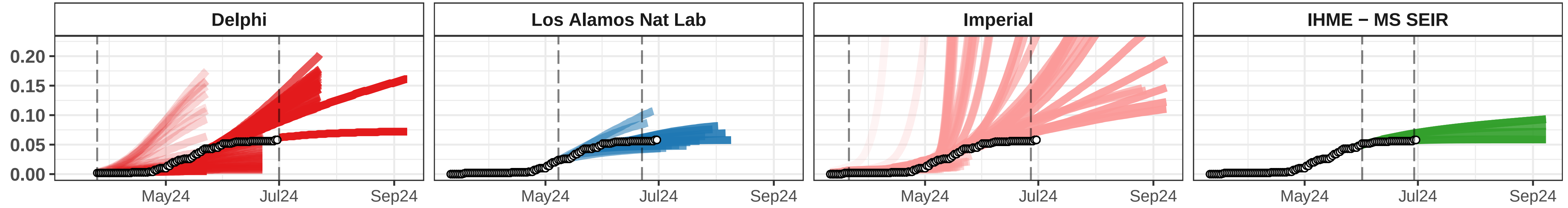

All Cumulative Errors

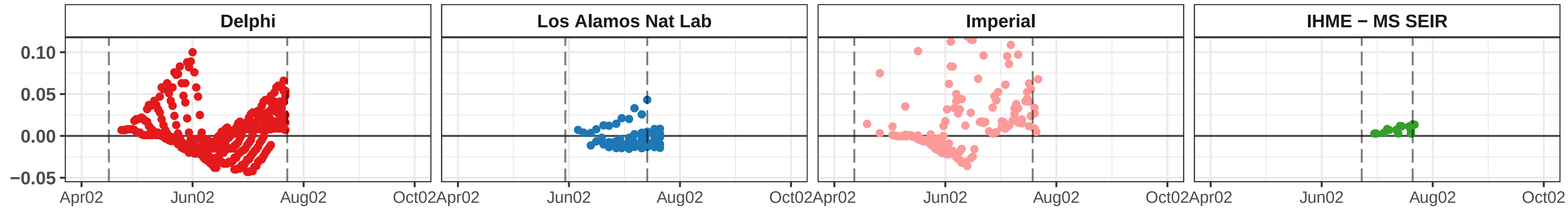

Tajikistan

Current Forecast

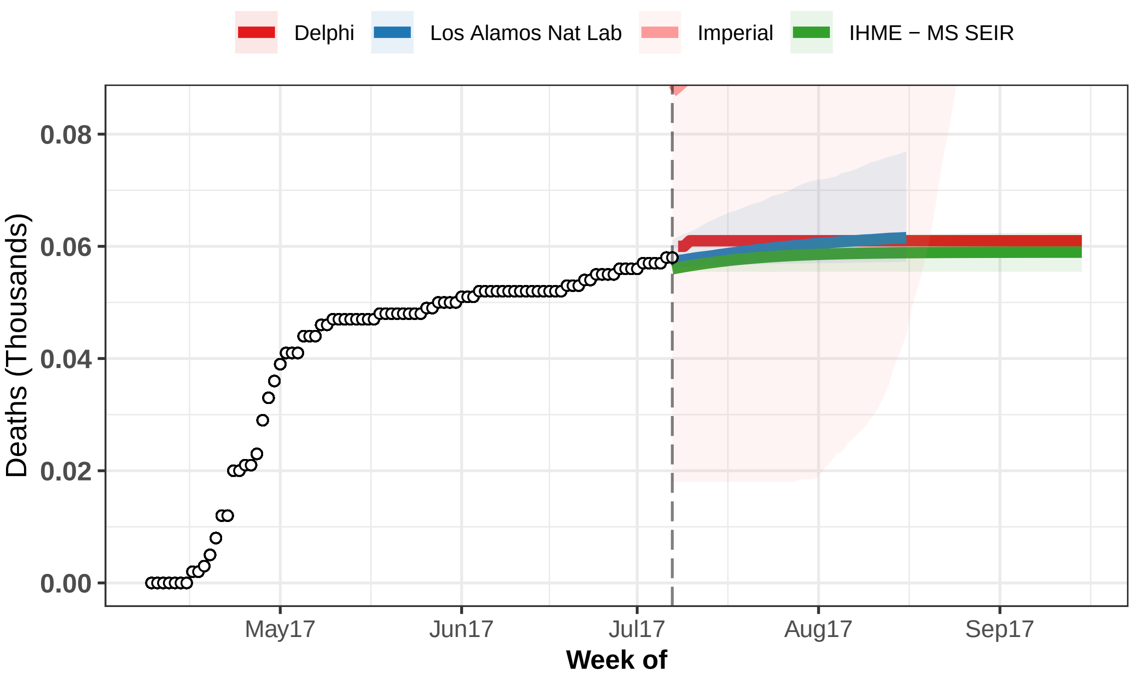

Cumulative Out−Of−Sample Error (Post Intercept Shift)

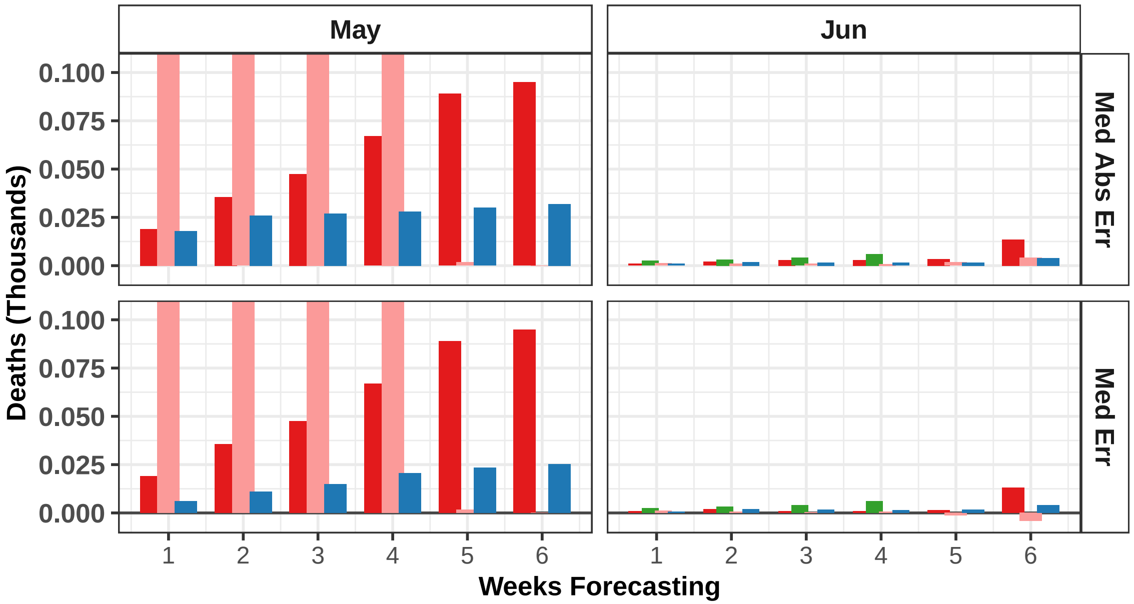

All Model Versions

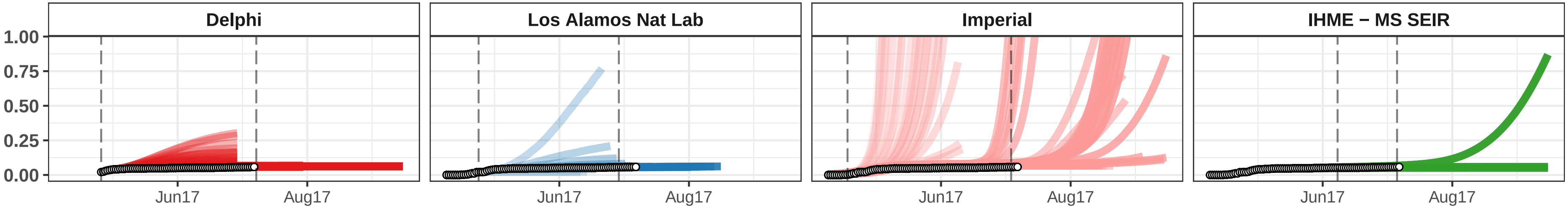

All Cumulative Errors

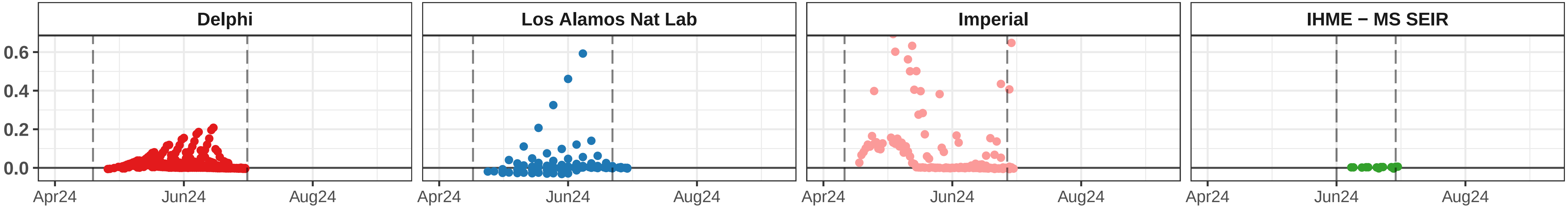

Thailand

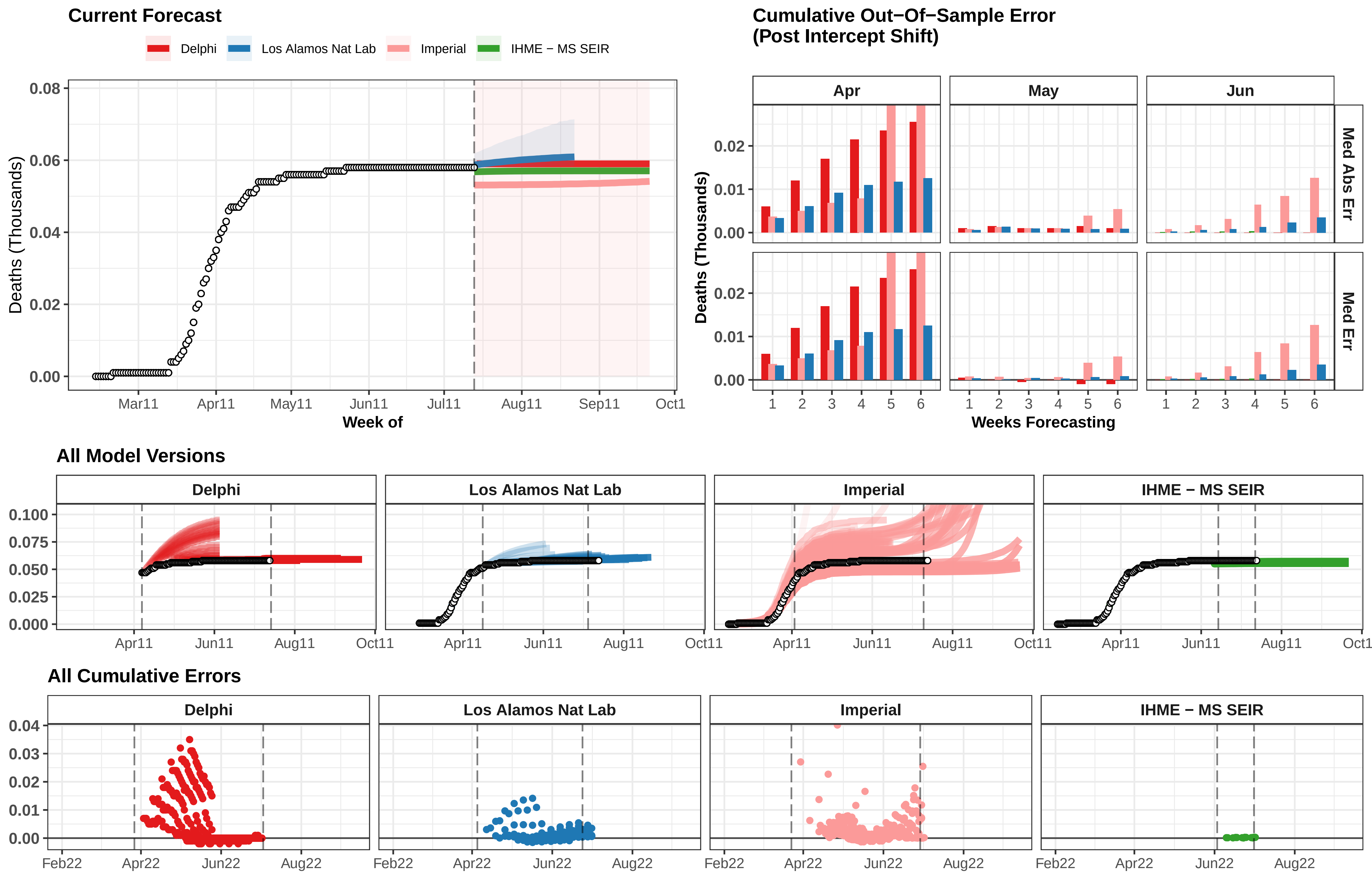
Current Forecast
Delphi
Los Alamos Nat Lab
Imperial
IHME − MS SEIR
Deaths (Thousands)
0.08
0.06
0.04
0.02
0.00
Mar11
Apr11
May11
Jun11
Jul11
Aug11
Sep11
Oct1
Week of
Cumulative Out−Of−Sample Error
(Post Intercept Shift)
Apr
May
Jun
Med Abs Err
Med Err
Deaths (Thousands)
0.02
0.01
0.00
1
2
3
4
5
6
Weeks Forecasting
All Model Versions
Delphi
Los Alamos Nat Lab
Imperial
IHME − MS SEIR
0.100
0.075
0.050
0.025
0.000
Apr11
Jun11
Aug11
Oct11
All Cumulative Errors
Delphi
Los Alamos Nat Lab
Imperial
IHME − MS SEIR
0.04
0.03
0.02
0.01
0.00
Feb22
Apr22
Jun22
Aug22

Libya

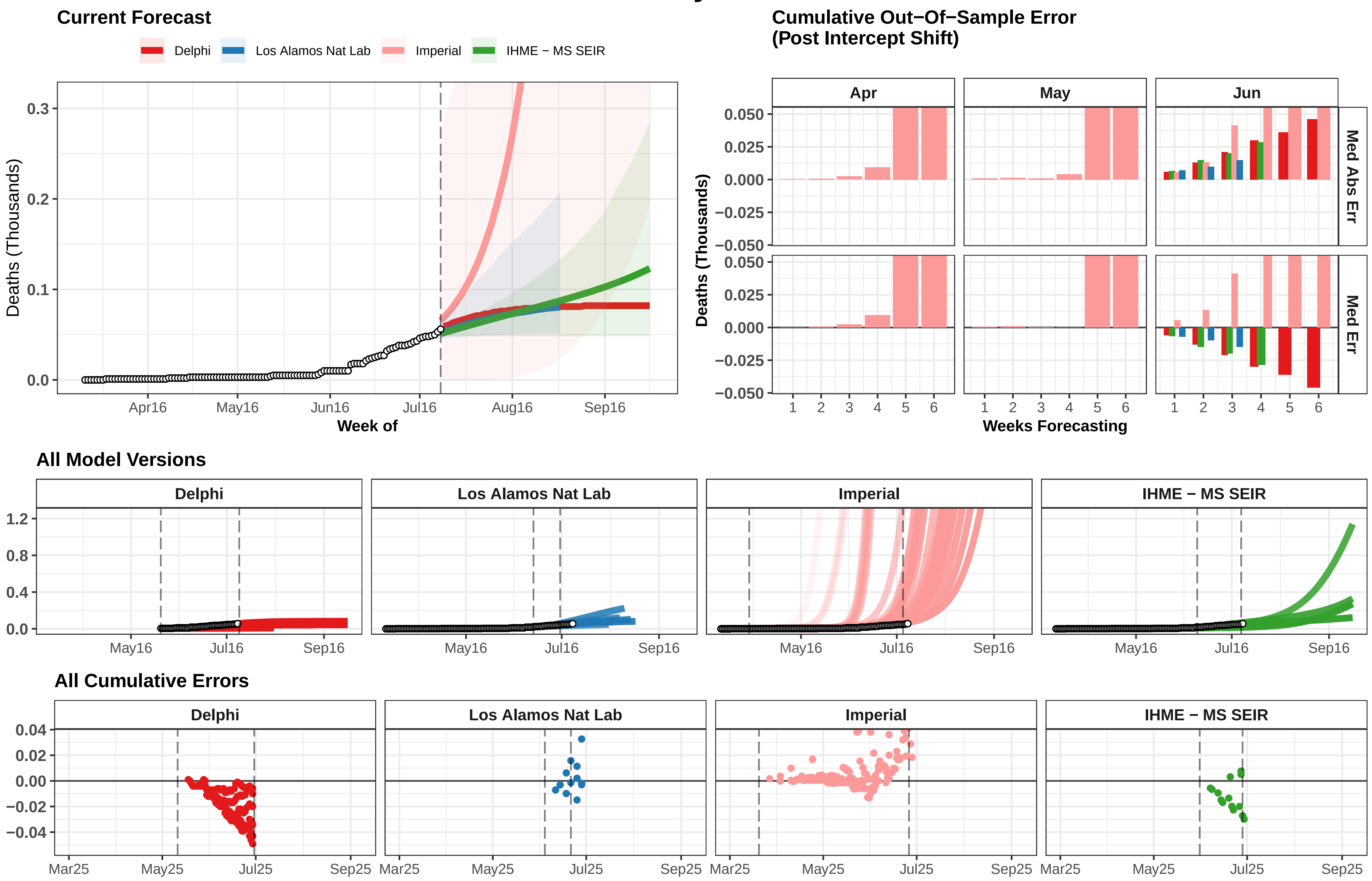

Current Forecast
Delphi
Los Alamos Nat Lab
Imperial
IHME − MS SEIR
Deaths (Thousands)
0.0
0.1
0.2
0.3
Apr16
May16
Jun16
Jul16
Aug16
Sep16
Week of
Cumulative Out−Of−Sample Error
(Post Intercept Shift)
Apr
May
Jun
Med Abs Err
Med Err
0.050
0.025
0.000
−0.025
−0.050
1
2
3
4
5
6
Weeks Forecasting
All Model Versions
1.2
0.8
0.4
0.0
All Cumulative Errors
0.04
0.02
0.00
−0.02
−0.04
Mar25
May25
Jul25
Sep25

Vermont

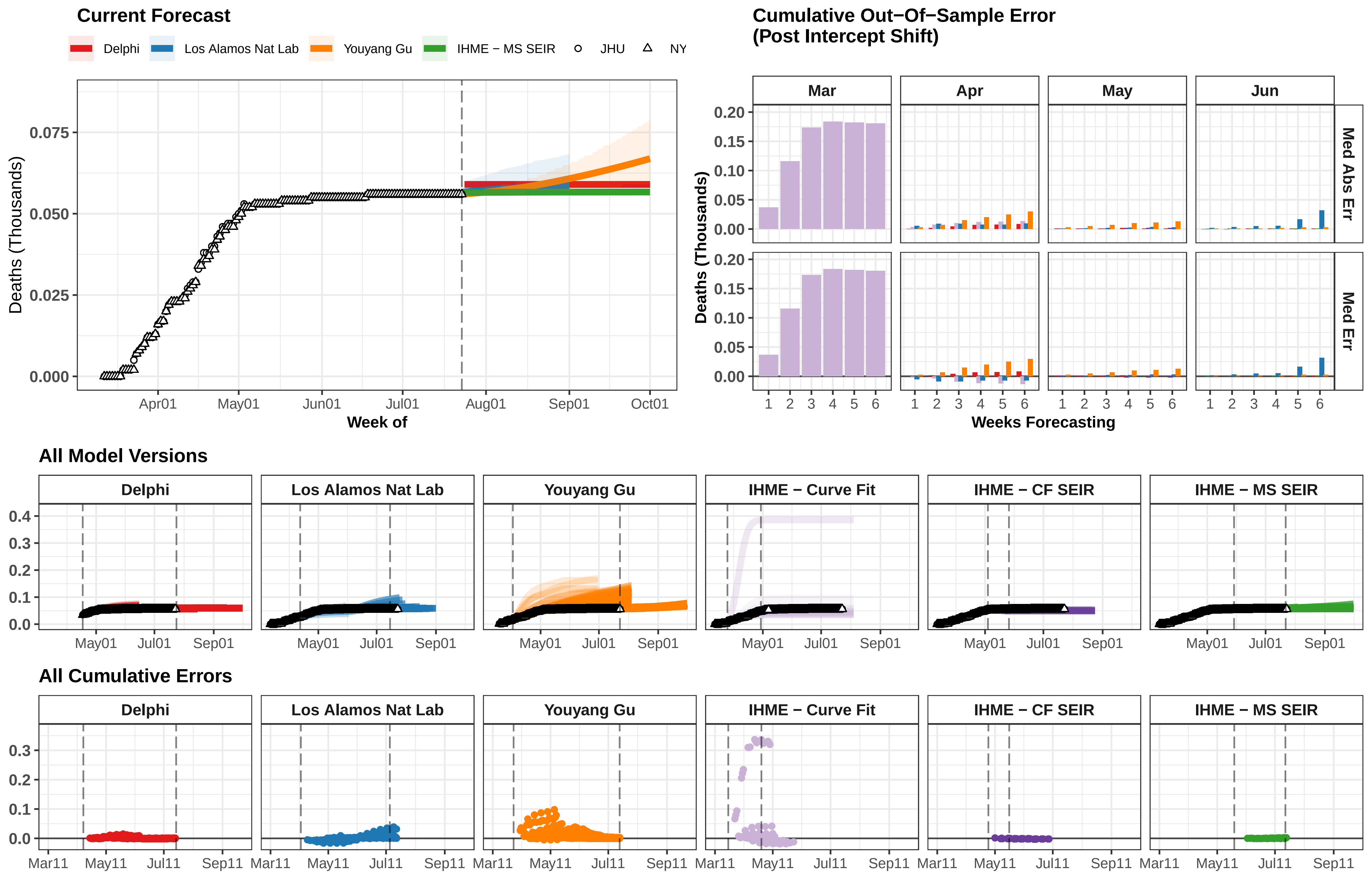
Current Forecast
Delphi
Los Alamos Nat Lab
Youyang Gu
IHME − MS SEIR
JHU
NY
Deaths (Thousands)
0.075
0.050
0.025
0.000
Apr01
May01
Jun01
Jul01
Aug01
Sep01
Oct01
Week of
Cumulative Out−Of−Sample Error
(Post Intercept Shift)
Mar
Apr
May
Jun
Med Abs Err
Med Err
Deaths (Thousands)
0.20
0.15
0.10
0.05
0.00
1
2
3
4
5
6
Weeks Forecasting
All Model Versions
Delphi
Los Alamos Nat Lab
Youyang Gu
IHME − Curve Fit
IHME − CF SEIR
IHME − MS SEIR
0.4
0.3
0.2
0.1
0.0
May01
Jul01
Sep01
All Cumulative Errors
Delphi
Los Alamos Nat Lab
Youyang Gu
IHME − Curve Fit
IHME − CF SEIR
IHME − MS SEIR
0.3
0.2
0.1
0.0
Mar11
May11
Jul11
Sep11

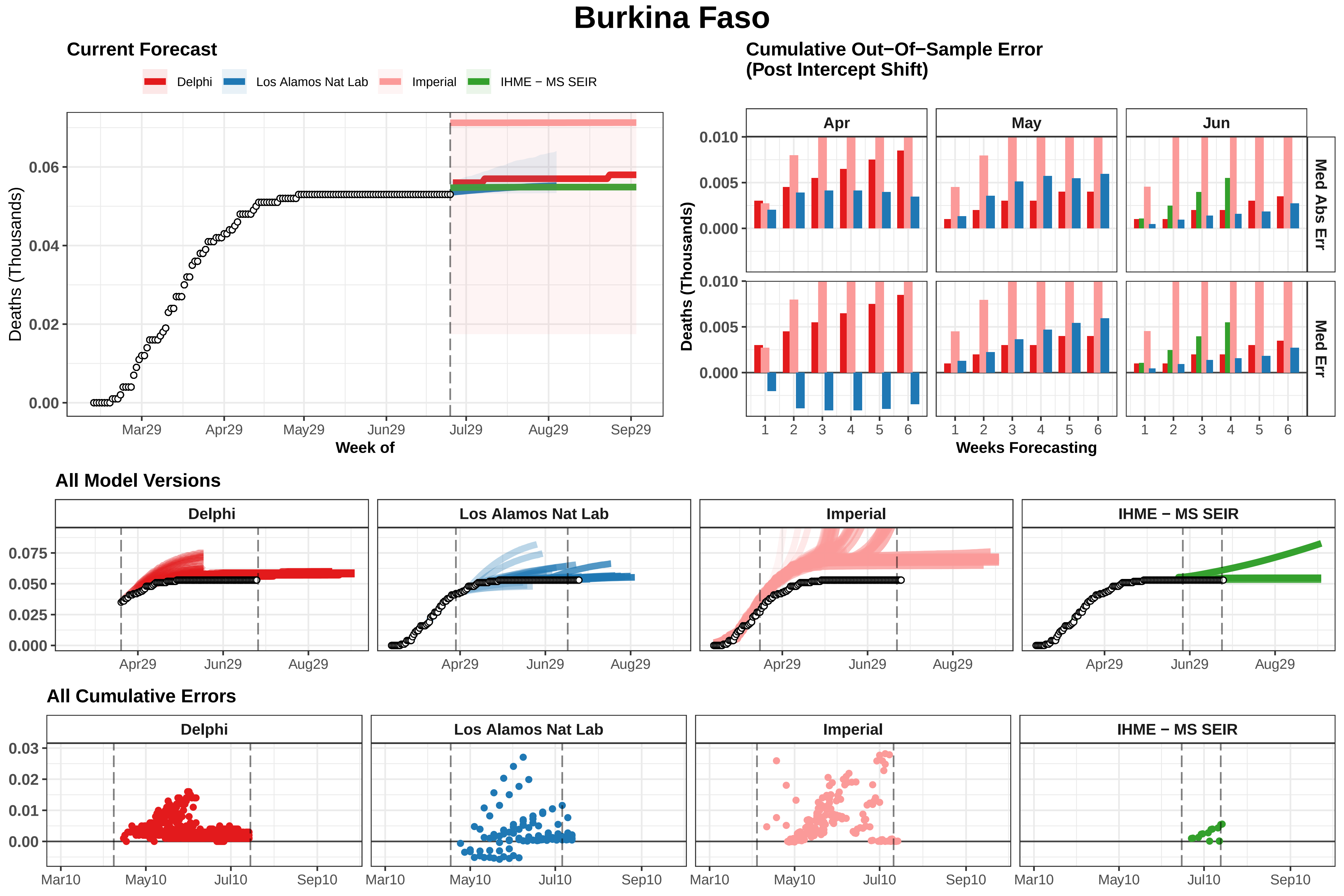

Burkina Faso
Current Forecast
Delphi
Los Alamos Nat Lab
Imperial
IHME − MS SEIR
Deaths (Thousands)
Week of
Mar29
Apr29
May29
Jun29
Jul29
Aug29
Sep29
Cumulative Out−Of−Sample Error
(Post Intercept Shift)
Apr
May
Jun
Med Abs Err
Med Err
Weeks Forecasting
All Model Versions
All Cumulative Errors
Mar10
May10
Jul10
Sep10

Andorra

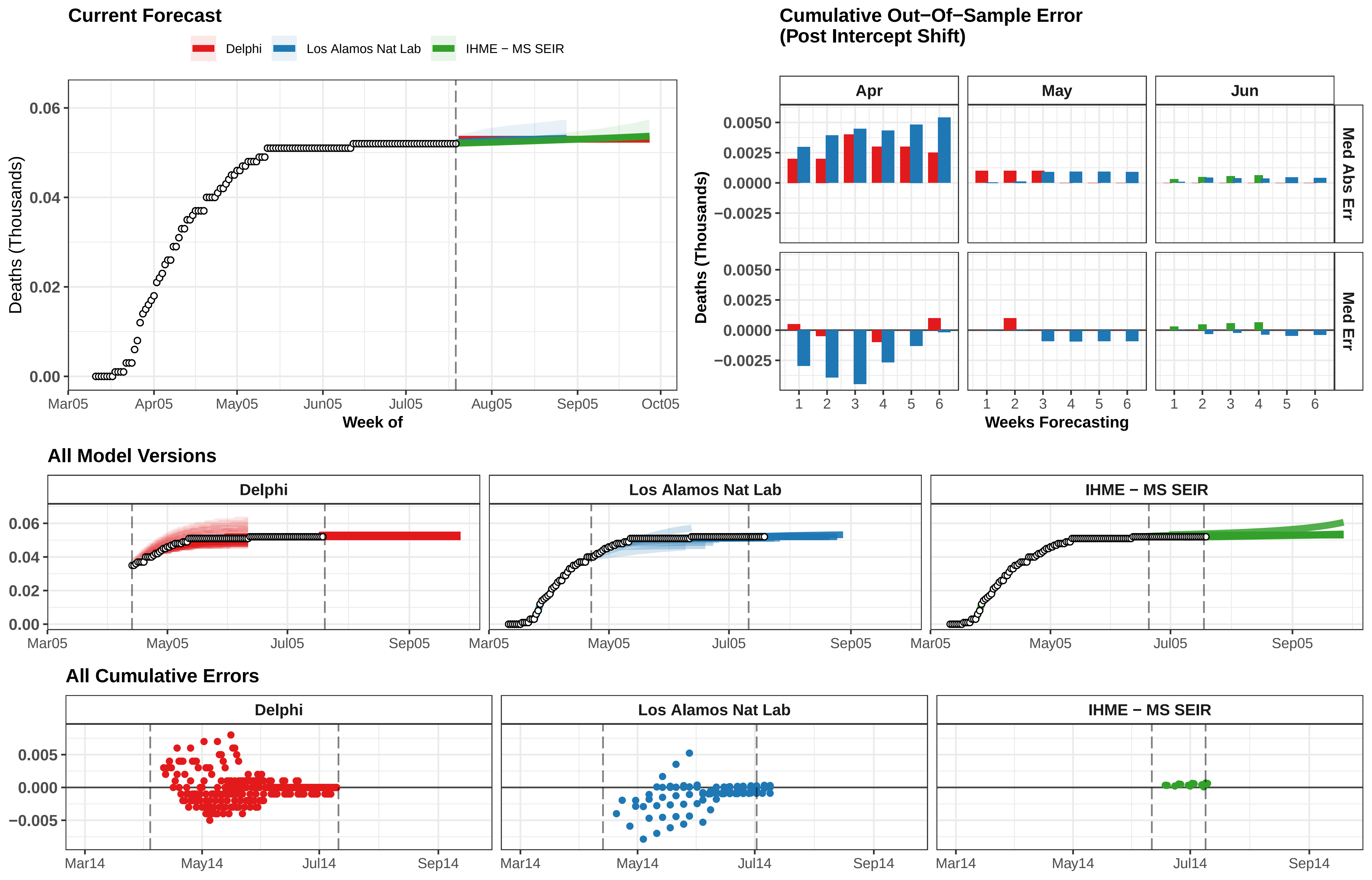
Current Forecast
Delphi
Los Alamos Nat Lab
IHME − MS SEIR
Deaths (Thousands)
0.06
0.04
0.02
0.00
Mar05
Apr05
May05
Jun05
Jul05
Aug05
Sep05
Oct05
Week of
Cumulative Out−Of−Sample Error
(Post Intercept Shift)
Apr
May
Jun
Med Abs Err
Med Err
0.0050
0.0025
0.0000
−0.0025
1
2
3
4
5
6
Weeks Forecasting
All Model Versions
All Cumulative Errors
0.005
0.000
−0.005
Mar14
May14
Jul14
Sep14

Equatorial Guinea

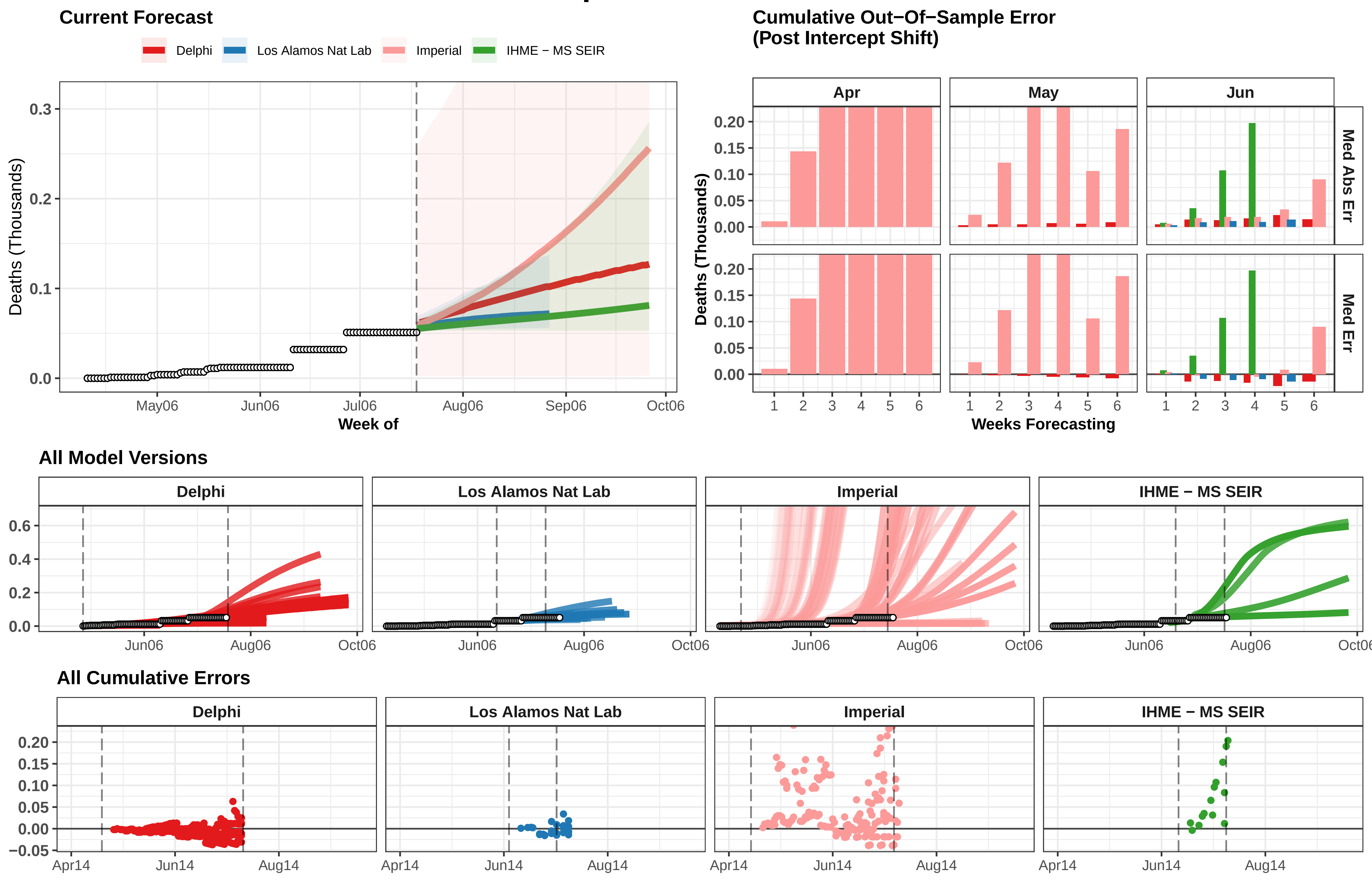

Congo

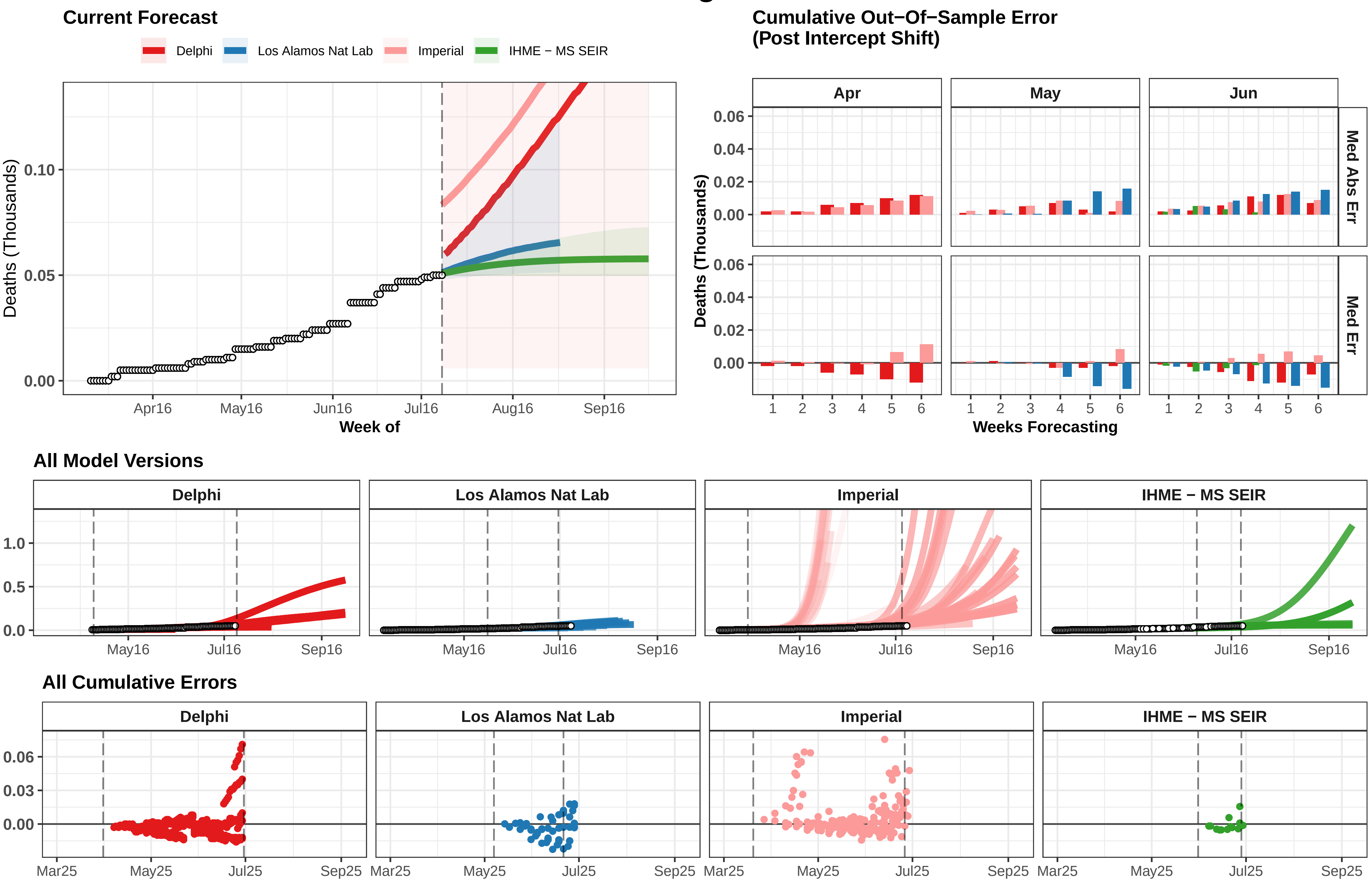

Current Forecast
Delphi
Los Alamos Nat Lab
Imperial
IHME − MS SEIR
Deaths (Thousands)
0.10
0.05
0.00
Apr16
May16
Jun16
Jul16
Aug16
Sep16
Week of
Cumulative Out−Of−Sample Error
(Post Intercept Shift)
Apr
May
Jun
Med Abs Err
Med Err
0.06
0.04
0.02
0.00
1
2
3
4
5
6
Weeks Forecasting
All Model Versions
1.0
0.5
0.0
All Cumulative Errors
0.03
Mar25
May25
Jul25
Sep25

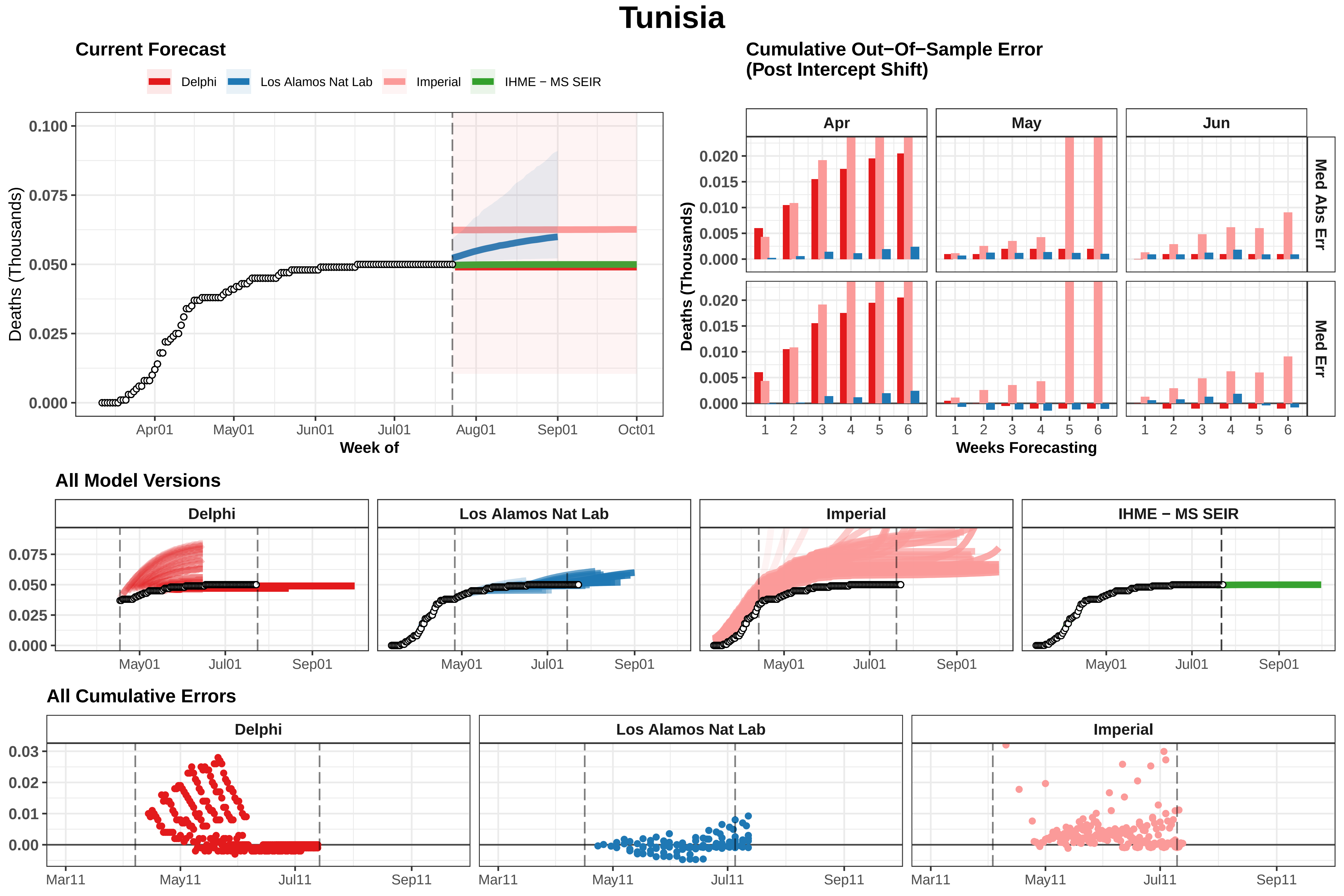

Tunisia
Current Forecast
Delphi
Los Alamos Nat Lab
Imperial
IHME − MS SEIR
Deaths (Thousands)
Week of
Apr01
May01
Jun01
Jul01
Aug01
Sep01
Oct01
Cumulative Out−Of−Sample Error
(Post Intercept Shift)
Apr
May
Jun
Med Abs Err
Med Err
Weeks Forecasting
All Model Versions
All Cumulative Errors
Mar11
May11
Jul11
Sep11

Gabon

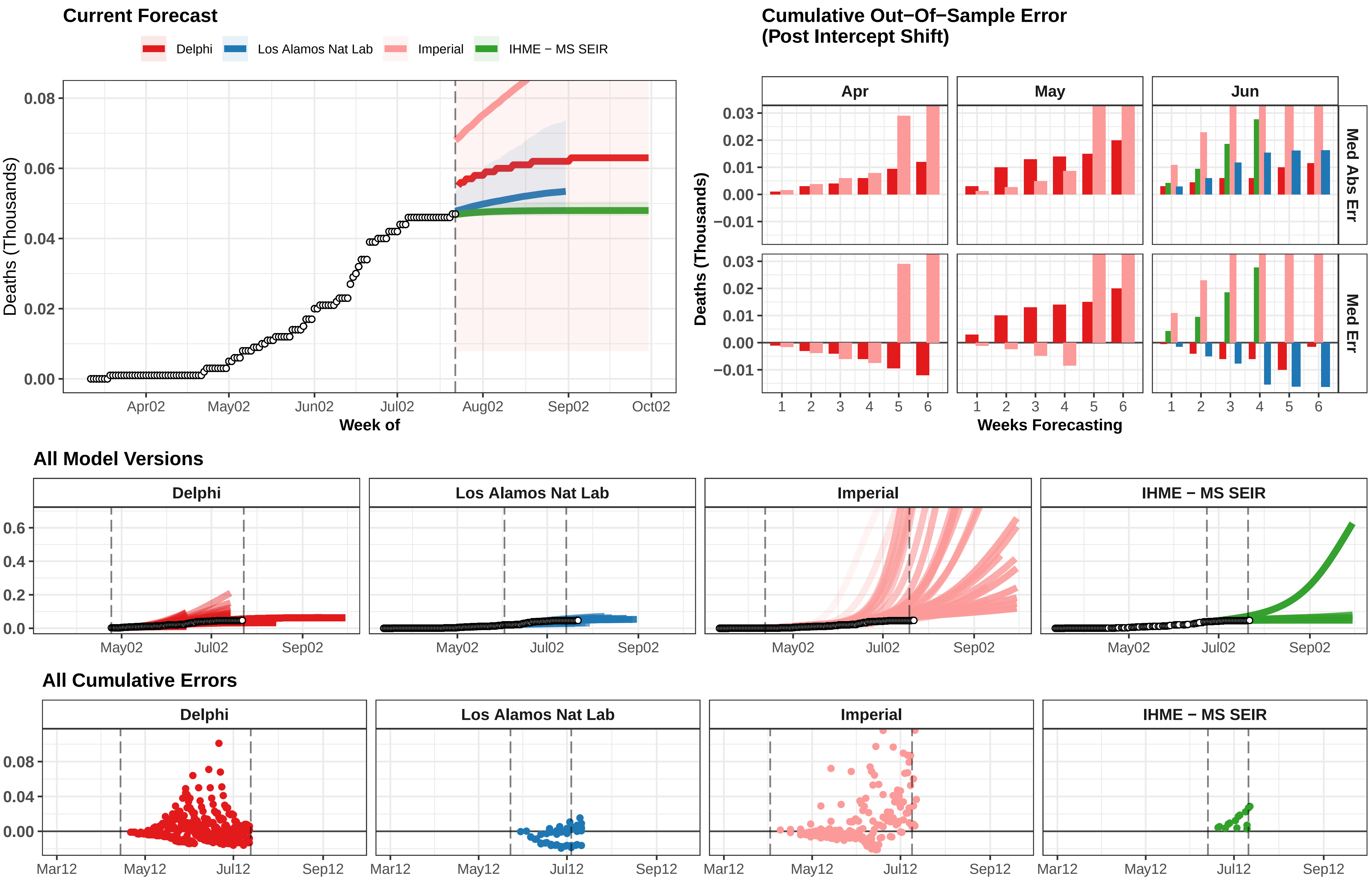

Current Forecast
Delphi
Los Alamos Nat Lab
Imperial
IHME − MS SEIR
Deaths (Thousands)
0.00
0.02
0.04
0.06
0.08
Apr02
May02
Jun02
Jul02
Aug02
Sep02
Oct02
Week of
Cumulative Out−Of−Sample Error
(Post Intercept Shift)
Apr
May
Jun
Med Abs Err
Med Err
0.03
0.02
0.01
0.00
−0.01
1
2
3
4
5
6
Weeks Forecasting
All Model Versions
0.6
0.4
0.2
0.0
All Cumulative Errors
0.08
0.04
0.00
Mar12
May12
Jul12
Sep12

South Sudan

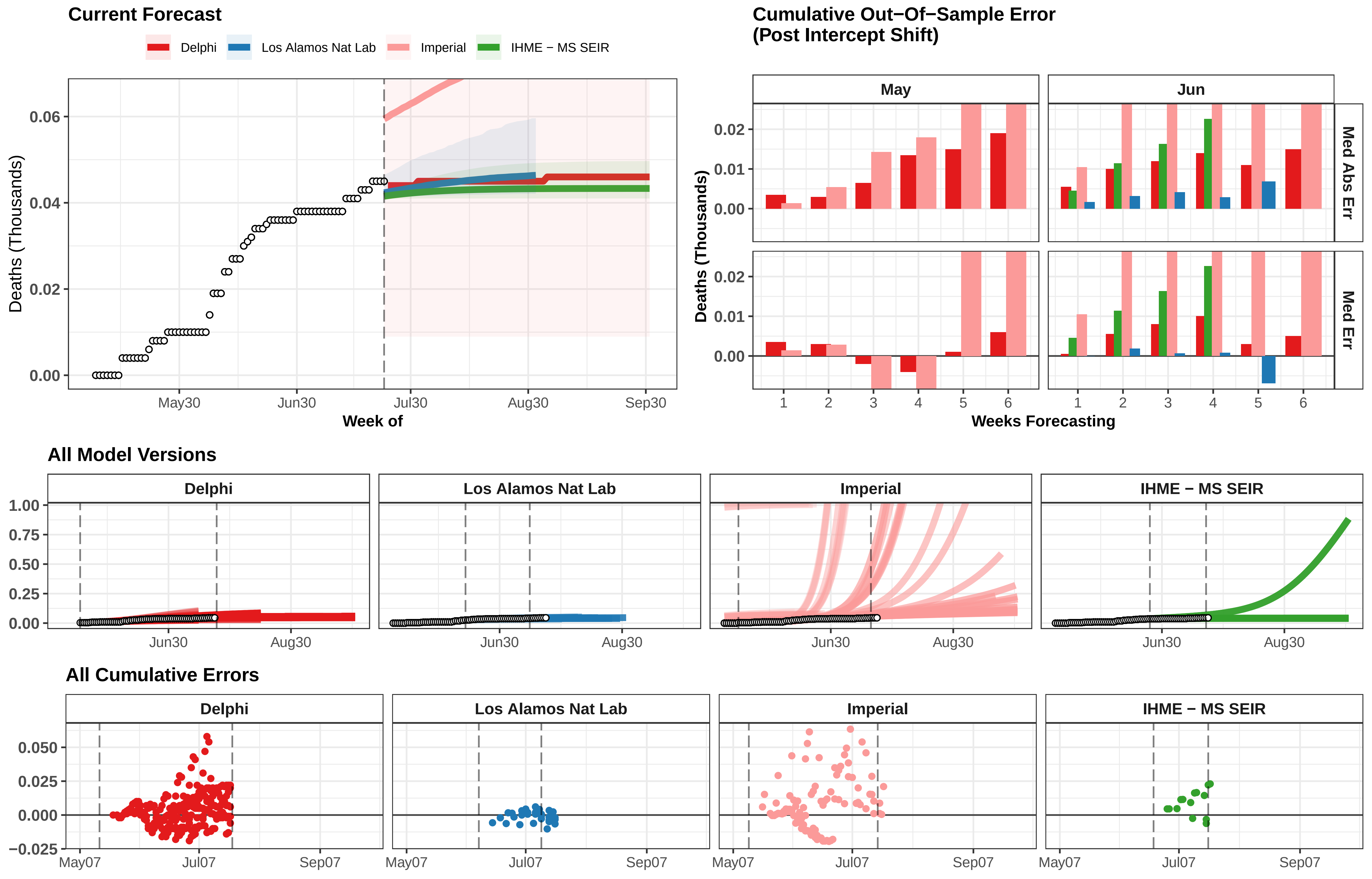

Current Forecast
Delphi
Los Alamos Nat Lab
Imperial
IHME − MS SEIR
0.06
0.04
0.02
0.00
Deaths (Thousands)
May30
Jun30
Jul30
Aug30
Sep30
Week of
Cumulative Out−Of−Sample Error
(Post Intercept Shift)
May
Jun
Med Abs Err
Med Err
0.02
0.01
0.00
Deaths (Thousands)
1
2
3
4
5
6
Weeks Forecasting
All Model Versions
Delphi
Los Alamos Nat Lab
Imperial
IHME − MS SEIR
1.00
0.75
0.50
0.25
0.00
Jun30
Aug30
All Cumulative Errors
Delphi
Los Alamos Nat Lab
Imperial
IHME − MS SEIR
0.050
0.025
0.000
−0.025
May07
Jul07
Sep07

Lebanon

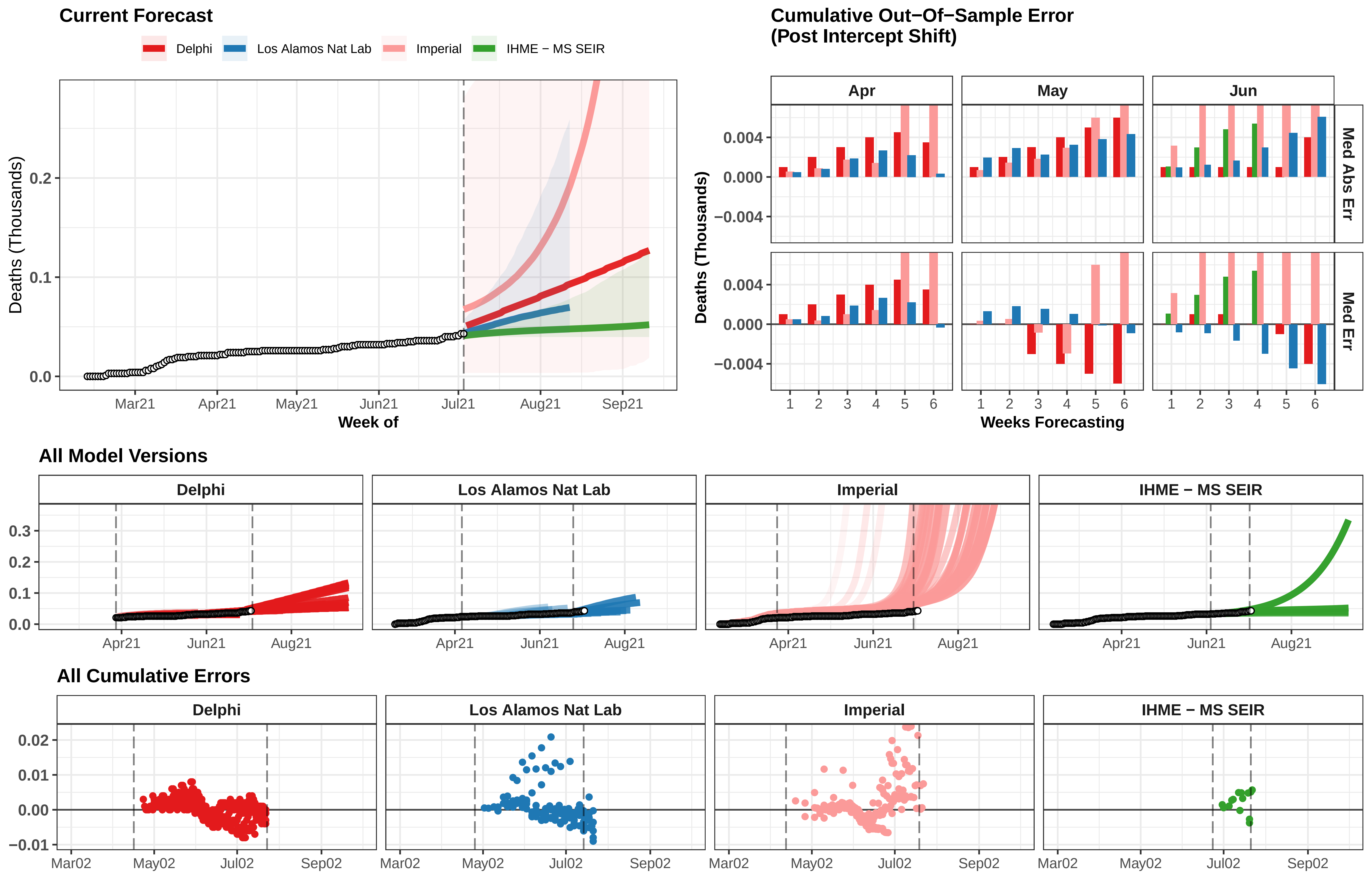

Current Forecast
Delphi
Los Alamos Nat Lab
Imperial
IHME − MS SEIR
Deaths (Thousands)
0.0
0.1
0.2
Mar21
Apr21
May21
Jun21
Jul21
Aug21
Sep21
Week of
Cumulative Out−Of−Sample Error
(Post Intercept Shift)
Apr
May
Jun
Med Abs Err
Med Err
0.004
0.000
−0.004
1
2
3
4
5
6
Weeks Forecasting
All Model Versions
Delphi
Los Alamos Nat Lab
Imperial
IHME − MS SEIR
0.0
0.1
0.2
0.3
Apr21
Jun21
Aug21
All Cumulative Errors
Delphi
Los Alamos Nat Lab
Imperial
IHME − MS SEIR
0.02
0.01
0.00
−0.01
Mar02
May02
Jul02
Sep02

Montana

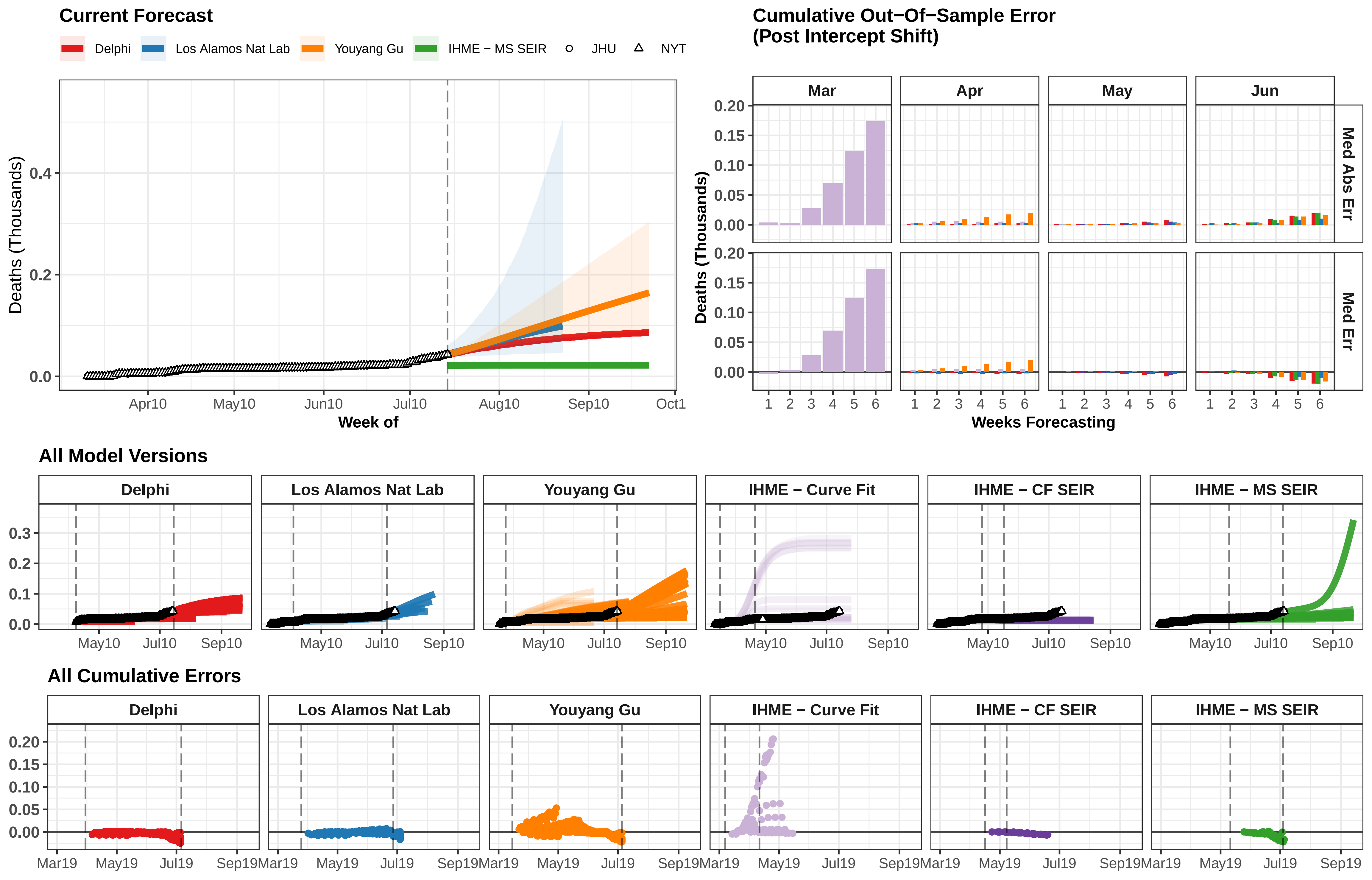

Current Forecast
Delphi
Los Alamos Nat Lab
Youyang Gu
IHME − MS SEIR
JHU
NYT
Deaths (Thousands)
0.4
0.2
0.0
Apr10
May10
Jun10
Jul10
Aug10
Sep10
Oct1
Week of
Cumulative Out−Of−Sample Error
(Post Intercept Shift)
Mar
Apr
May
Jun
Med Abs Err
Med Err
0.20
0.15
0.10
0.05
0.00
1
2
3
4
5
6
Weeks Forecasting
All Model Versions
Delphi
Los Alamos Nat Lab
Youyang Gu
IHME − Curve Fit
IHME − CF SEIR
IHME − MS SEIR
0.3
0.2
0.1
0.0
May10
Jul10
Sep10
All Cumulative Errors
Mar19
May19
Jul19
Sep19

Nepal

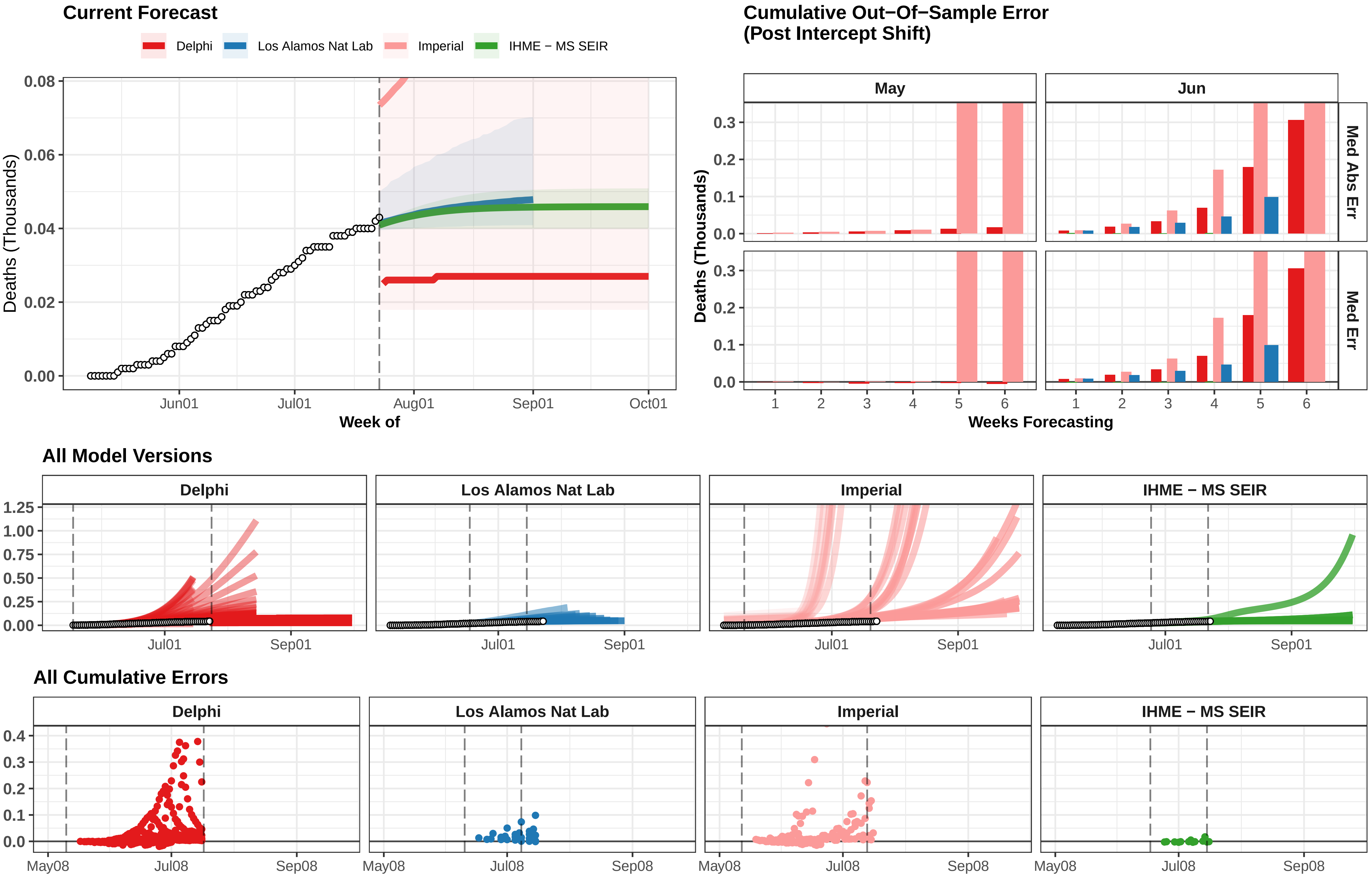
Current Forecast
Delphi
Los Alamos Nat Lab
Imperial
IHME − MS SEIR
Deaths (Thousands)
0.00
0.02
0.04
0.06
0.08
Jun01
Jul01
Aug01
Sep01
Oct01
Week of
Cumulative Out−Of−Sample Error
(Post Intercept Shift)
May
Jun
Med Abs Err
Med Err
0.0
0.1
0.2
0.3
1
2
3
4
5
6
Weeks Forecasting
All Model Versions
1.25
1.00
0.75
0.50
0.25
0.00
Jul01
Sep01
All Cumulative Errors
0.4
May08
Jul08
Sep08

Guinea

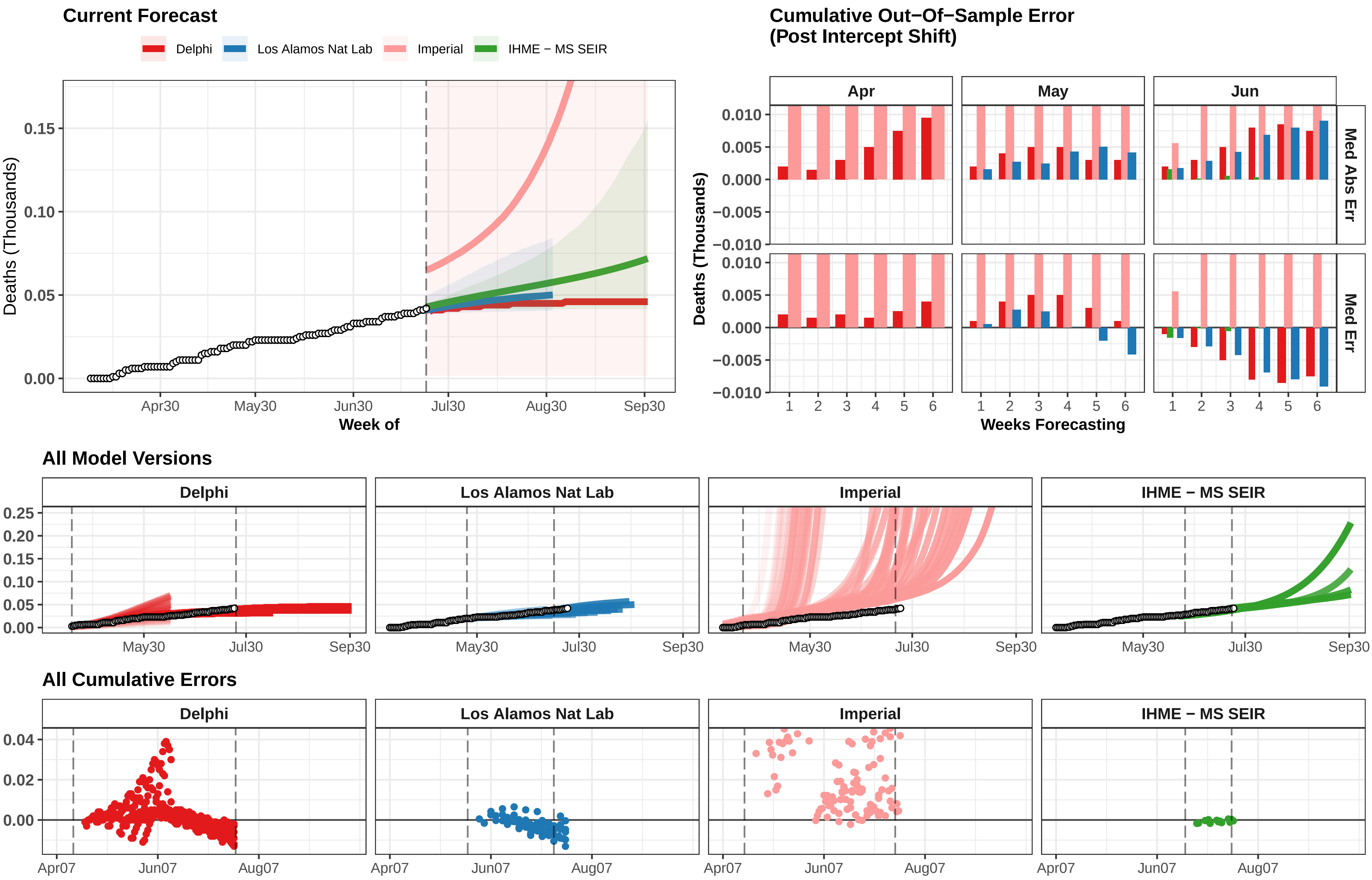
Current Forecast
Delphi
Los Alamos Nat Lab
Imperial
IHME − MS SEIR
Deaths (Thousands)
0.00
0.05
0.10
0.15
Apr30
May30
Jun30
Jul30
Aug30
Sep30
Week of
Cumulative Out−Of−Sample Error
(Post Intercept Shift)
Apr
May
Jun
Med Abs Err
Med Err
Deaths (Thousands)
0.010
0.005
0.000
−0.005
−0.010
1
2
3
4
5
6
Weeks Forecasting
All Model Versions
Delphi
Los Alamos Nat Lab
Imperial
IHME − MS SEIR
0.25
0.20
0.15
0.10
0.05
0.00
May30
Jul30
Sep30
All Cumulative Errors
Delphi
Los Alamos Nat Lab
Imperial
IHME − MS SEIR
0.04
0.02
0.00
Apr07
Jun07
Aug07

Montenegro

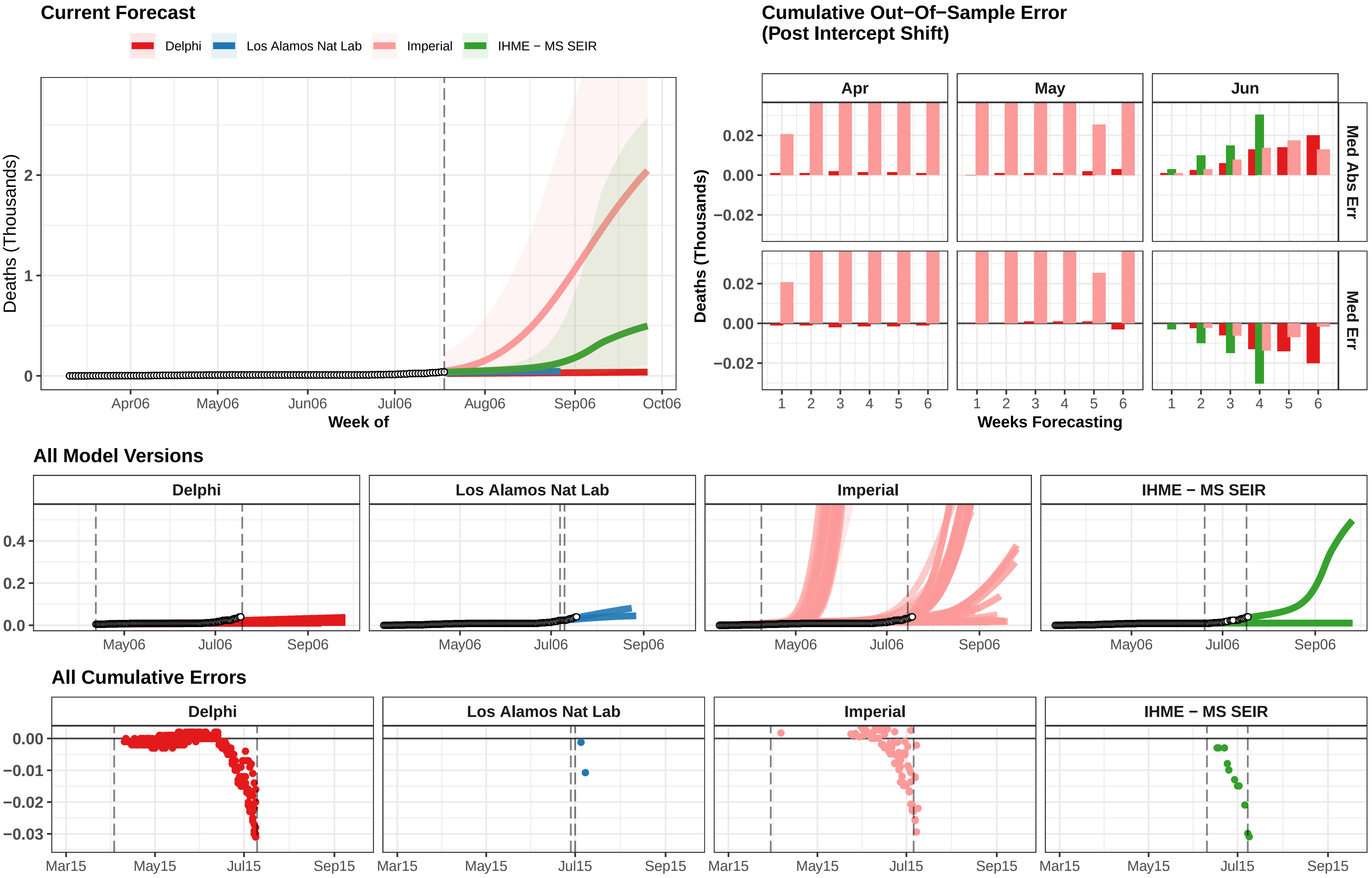
Current Forecast
Delphi
Los Alamos Nat Lab
Imperial
IHME − MS SEIR
Deaths (Thousands)
Week of
Apr06
May06
Jun06
Jul06
Aug06
Sep06
Oct06
Cumulative Out−Of−Sample Error
(Post Intercept Shift)
Apr
May
Jun
Med Abs Err
Med Err
Weeks Forecasting
All Model Versions
All Cumulative Errors
Mar15
May15
Jul15
Sep15

Paraguay

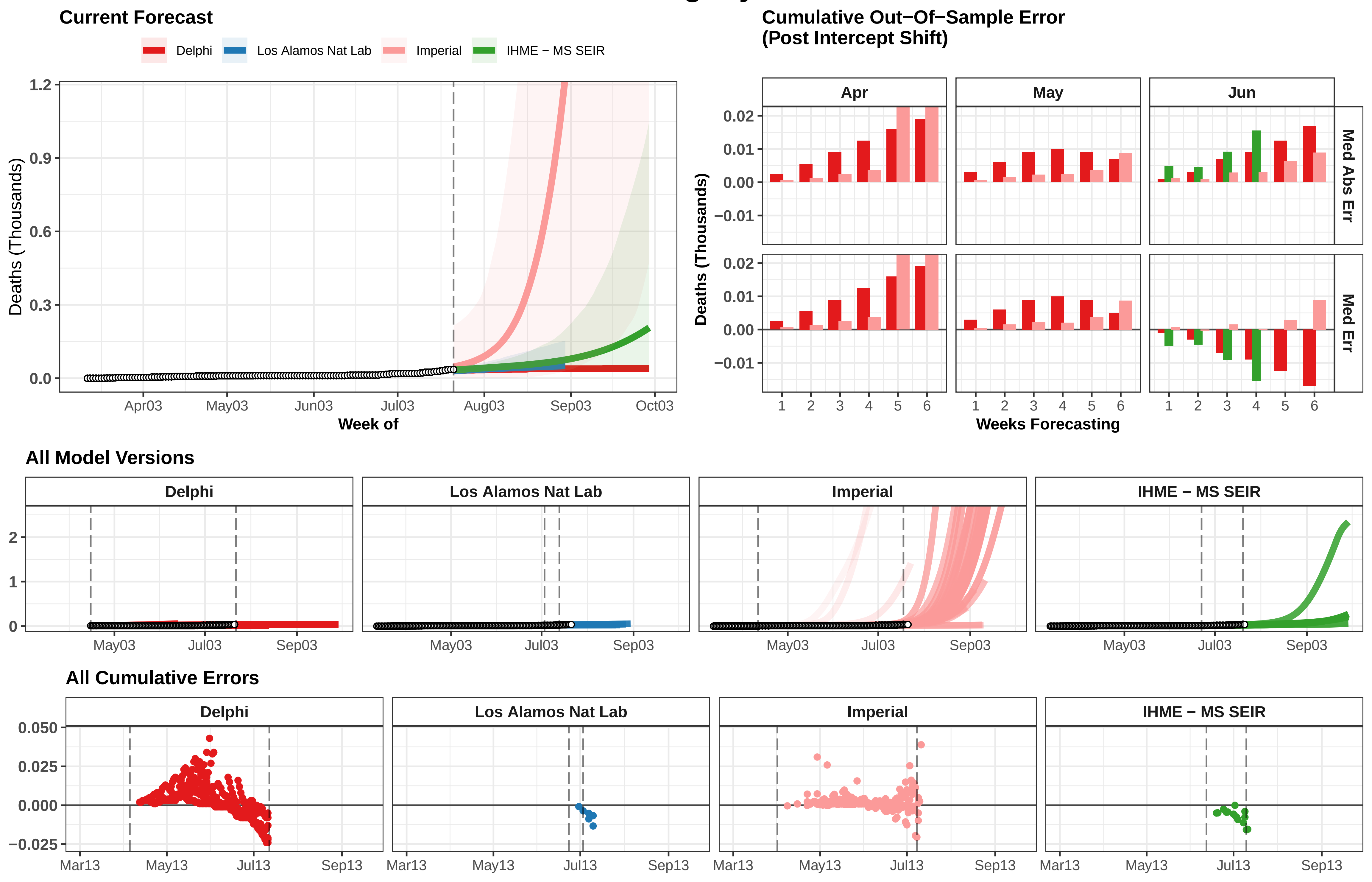

Current Forecast
Delphi
Los Alamos Nat Lab
Imperial
IHME − MS SEIR
Deaths (Thousands)
1.2
0.9
0.6
0.3
0.0
Apr03
May03
Jun03
Jul03
Aug03
Sep03
Oct03
Week of
Cumulative Out−Of−Sample Error
(Post Intercept Shift)
Apr
May
Jun
Med Abs Err
Med Err
0.02
0.01
0.00
−0.01
Weeks Forecasting
All Model Versions
Delphi
Los Alamos Nat Lab
Imperial
IHME − MS SEIR
2
1
0
May03
Jul03
Sep03
All Cumulative Errors
0.050
0.025
0.000
−0.025
Mar13
May13
Jul13
Sep13

Syria

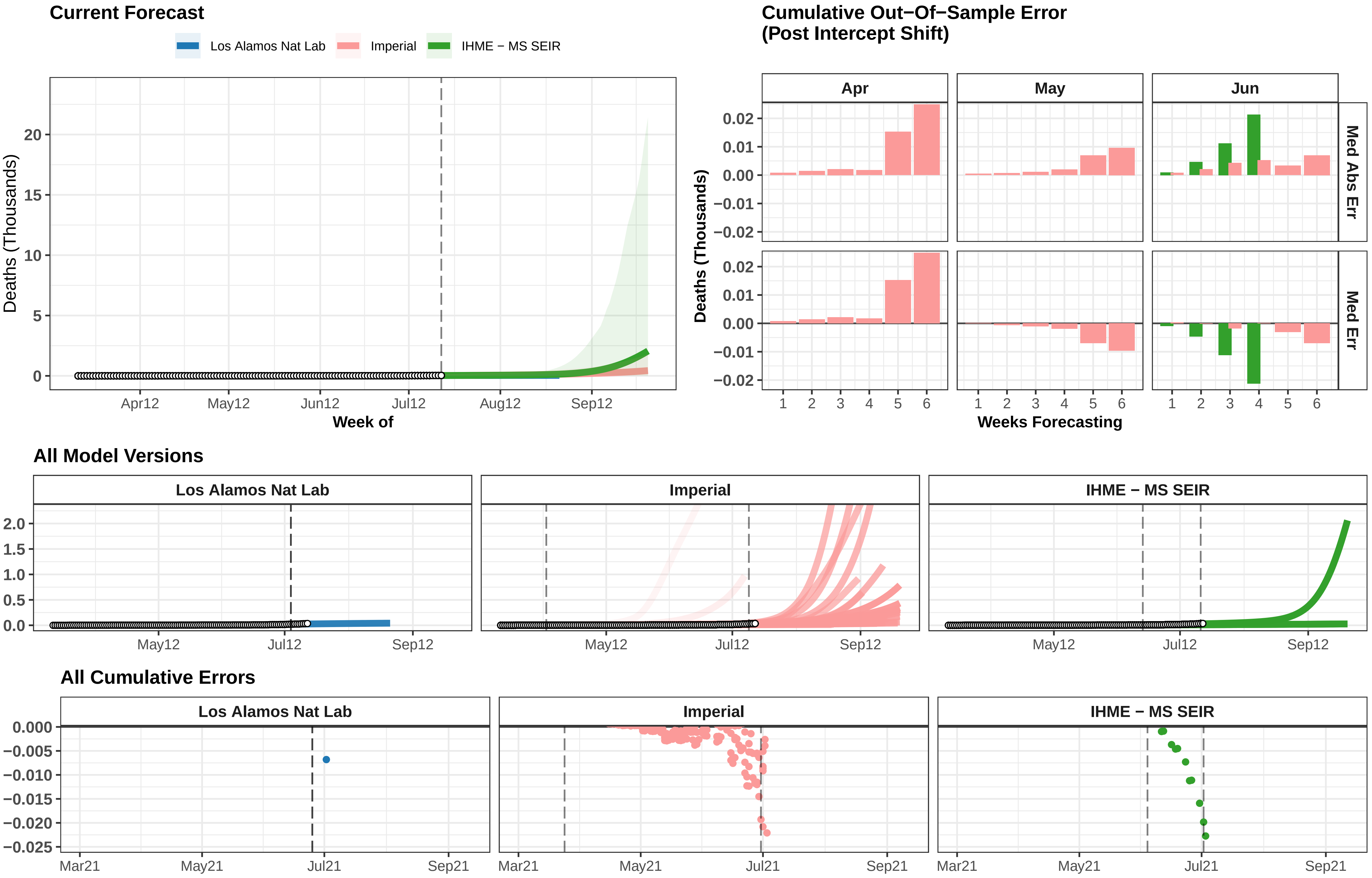
Current Forecast
Los Alamos Nat Lab
Imperial
IHME − MS SEIR
Deaths (Thousands)
0
5
10
15
20
Apr12
May12
Jun12
Jul12
Aug12
Sep12
Week of
Cumulative Out−Of−Sample Error
(Post Intercept Shift)
Apr
May
Jun
Med Abs Err
Med Err
0.02
0.01
0.00
−0.01
−0.02
1
2
3
4
5
6
Weeks Forecasting
All Model Versions
Los Alamos Nat Lab
Imperial
IHME − MS SEIR
2.0
1.5
1.0
0.5
0.0
May12
Jul12
Sep12
All Cumulative Errors
Los Alamos Nat Lab
Imperial
IHME − MS SEIR
0.000
−0.005
−0.010
−0.015
−0.020
−0.025
Mar21
May21
Jul21
Sep21

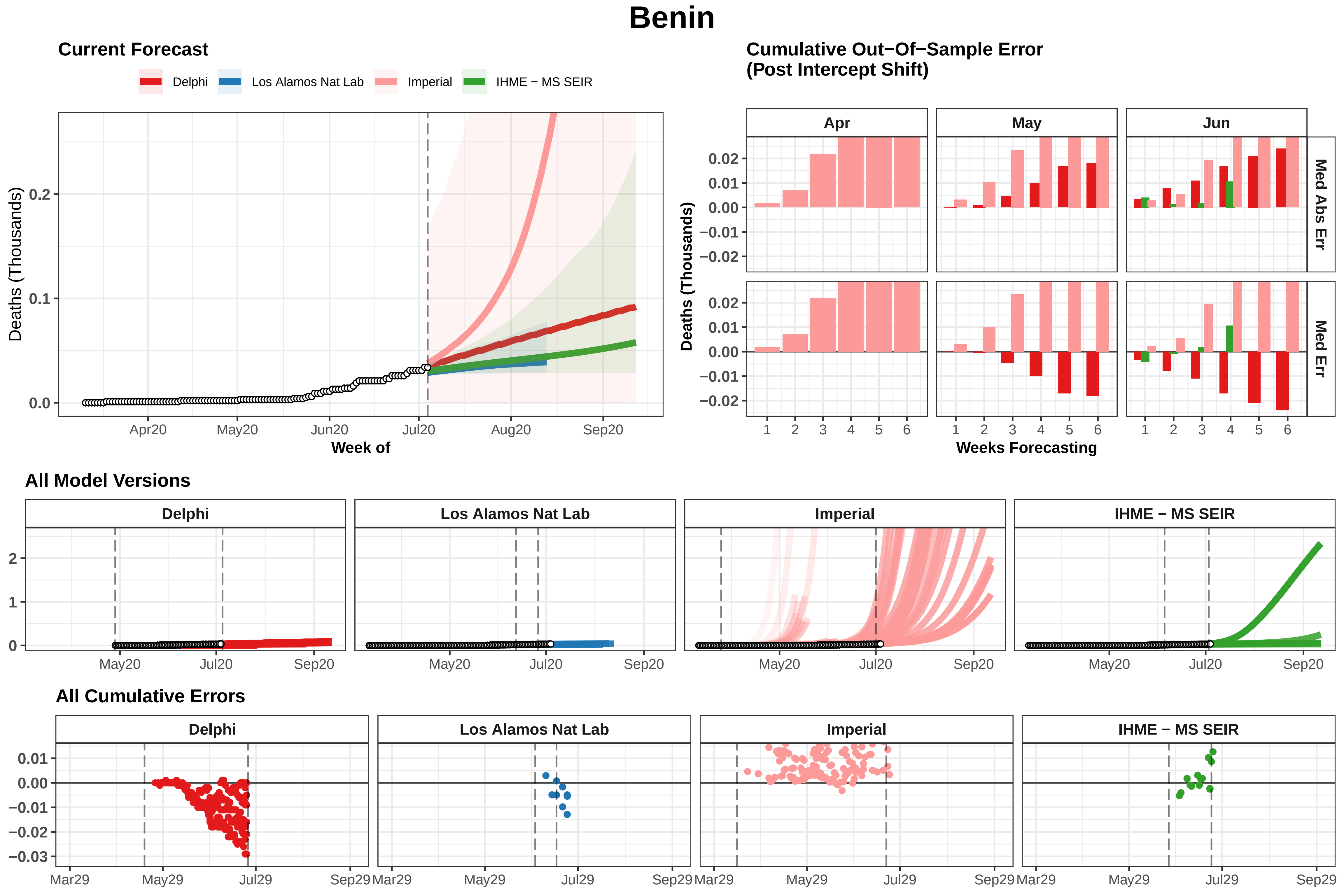
Benin
Current Forecast
Delphi
Los Alamos Nat Lab
Imperial
IHME − MS SEIR
Deaths (Thousands)
0.0
0.1
0.2
Apr20
May20
Jun20
Jul20
Aug20
Sep20
Week of
Cumulative Out−Of−Sample Error
(Post Intercept Shift)
Apr
May
Jun
Med Abs Err
Med Err
0.02
0.01
0.00
−0.01
−0.02
1
2
3
4
5
6
Weeks Forecasting
All Model Versions
Delphi
Los Alamos Nat Lab
Imperial
IHME − MS SEIR
0
1
2
May20
Jul20
Sep20
All Cumulative Errors
Delphi
Los Alamos Nat Lab
Imperial
IHME − MS SEIR
0.01
0.00
−0.01
−0.02
−0.03
Mar29
May29
Jul29
Sep29

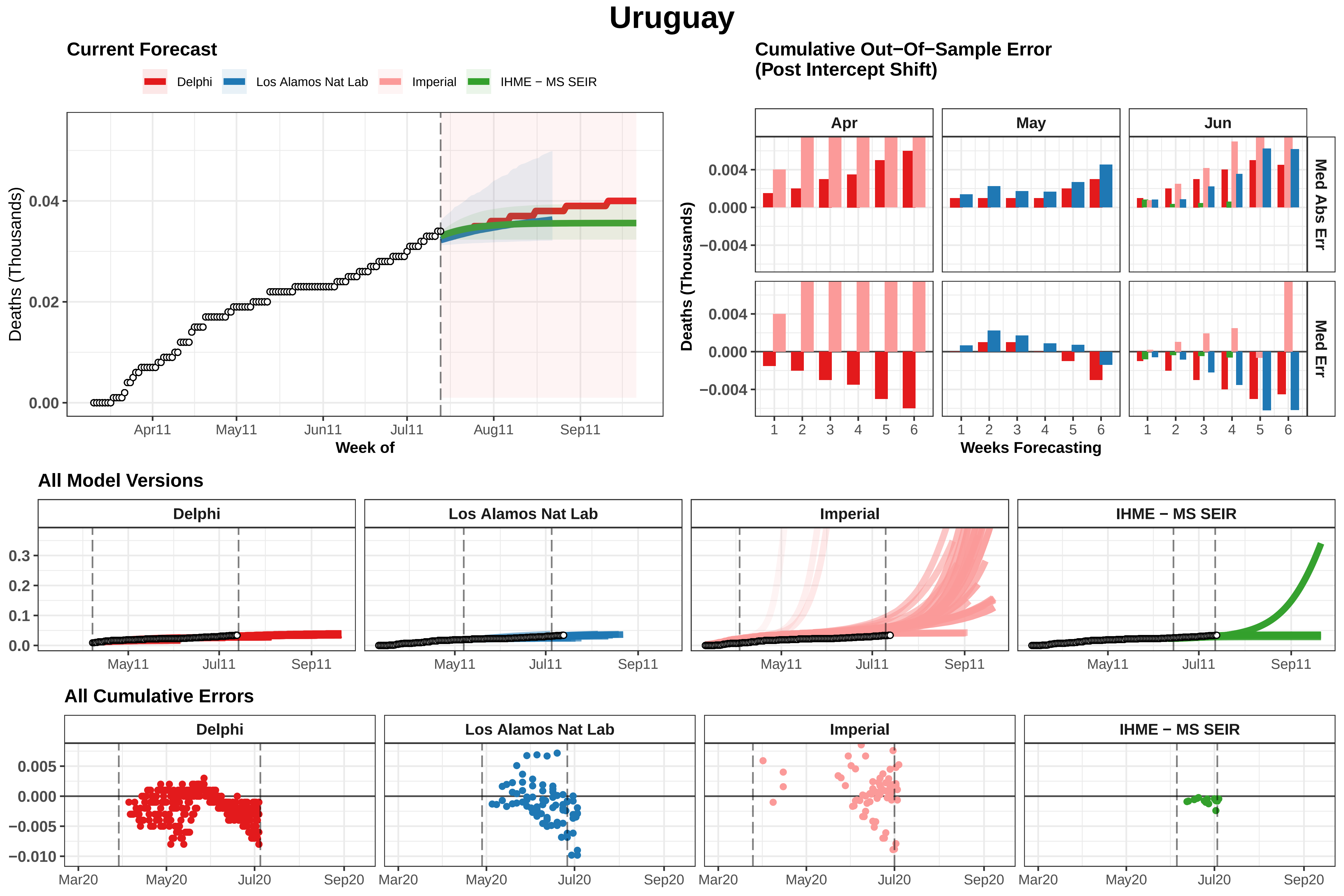

Uruguay
Current Forecast
Delphi
Los Alamos Nat Lab
Imperial
IHME − MS SEIR
Deaths (Thousands)
0.00
0.02
0.04
Apr11
May11
Jun11
Jul11
Aug11
Sep11
Week of
Cumulative Out−Of−Sample Error
(Post Intercept Shift)
Apr
May
Jun
Med Abs Err
Med Err
0.004
0.000
−0.004
1
2
3
4
5
6
Weeks Forecasting
All Model Versions
Delphi
Los Alamos Nat Lab
Imperial
IHME − MS SEIR
0.0
0.1
0.2
0.3
May11
Jul11
Sep11
All Cumulative Errors
0.005
0.000
−0.005
−0.010
Mar20
May20
Jul20
Sep20

Angola

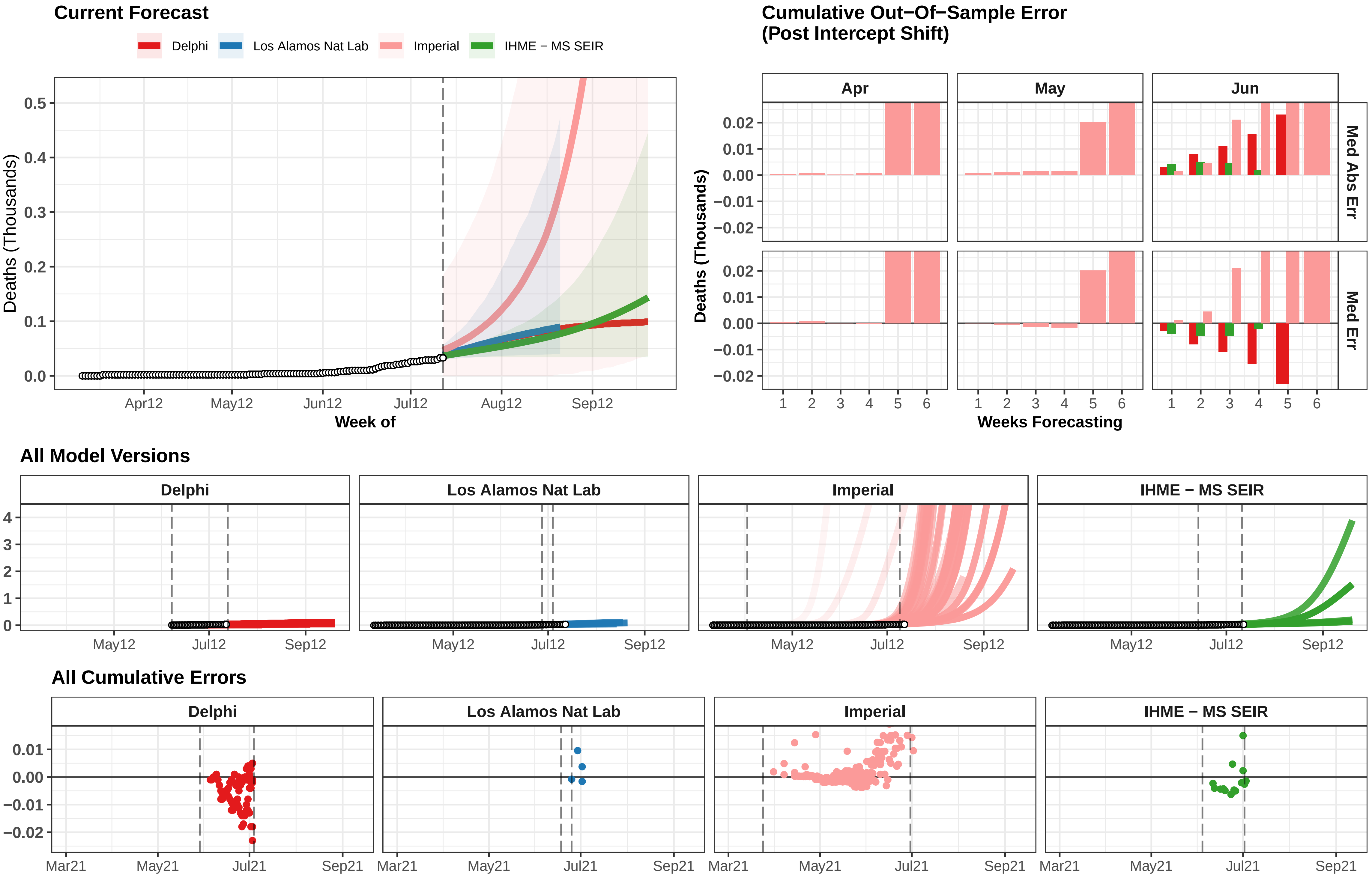

Current Forecast
Delphi
Los Alamos Nat Lab
Imperial
IHME − MS SEIR
Deaths (Thousands)
0.5
0.4
0.3
0.2
0.1
0.0
Apr12
May12
Jun12
Jul12
Aug12
Sep12
Week of
Cumulative Out−Of−Sample Error
(Post Intercept Shift)
Apr
May
Jun
Med Abs Err
Med Err
0.02
0.01
0.00
−0.01
−0.02
1
2
3
4
5
6
Weeks Forecasting
All Model Versions
Delphi
Los Alamos Nat Lab
Imperial
IHME − MS SEIR
4
3
2
1
0
May12
Jul12
Sep12
All Cumulative Errors
0.01
0.00
−0.01
−0.02
Mar21
May21
Jul21
Sep21

Latvia

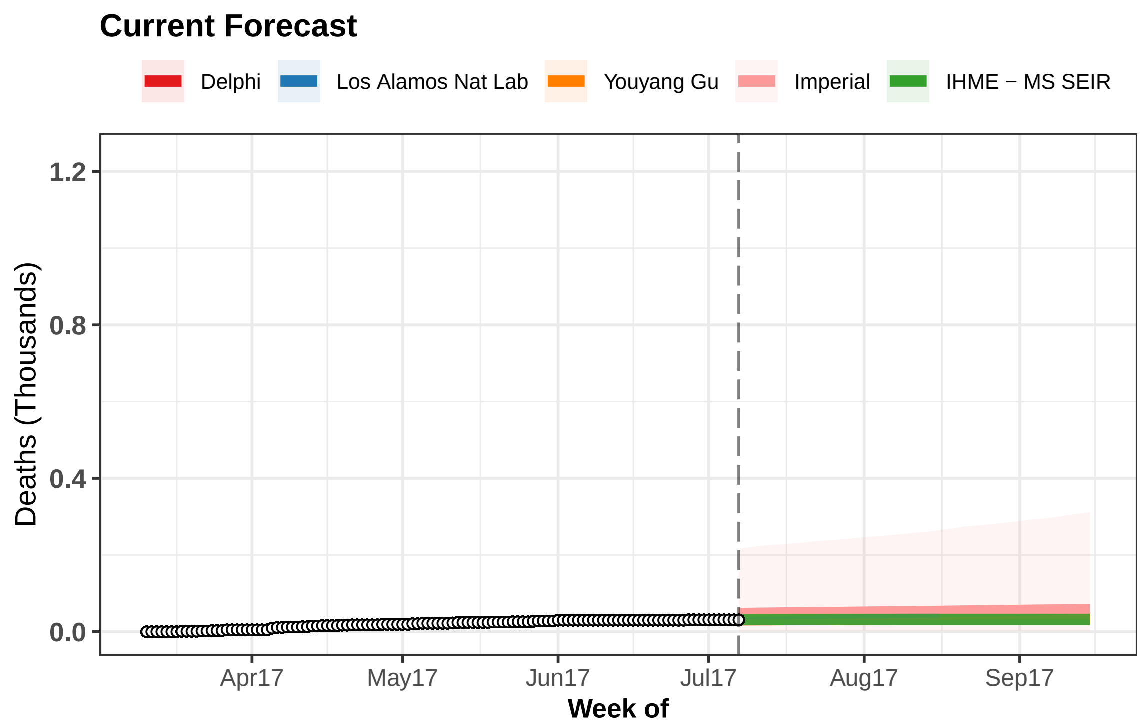

Current Forecast
Delphi
Los Alamos Nat Lab
Youyang Gu
Imperial
IHME − MS SEIR
Deaths (Thousands)
1.2
0.8
0.4
0.0
Apr17
May17
Jun17
Jul17
Aug17
Sep17
Week of

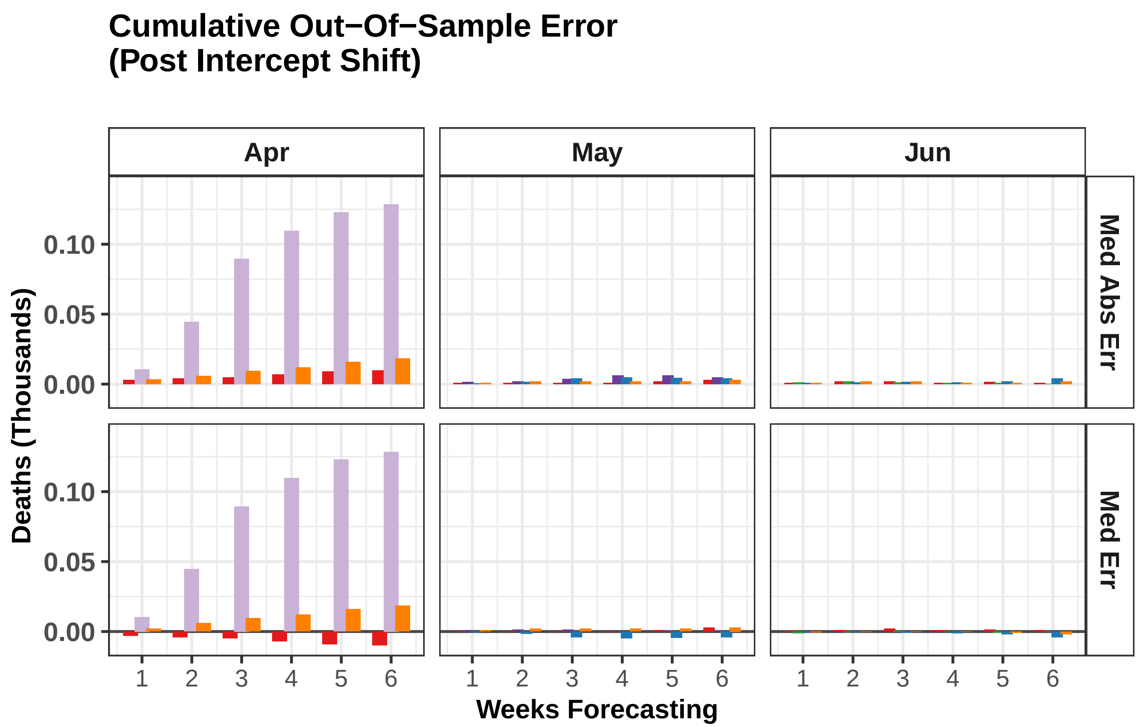

Cumulative Out−Of−Sample Error
(Post Intercept Shift)
Apr
May
Jun
Med Abs Err
Med Err
Deaths (Thousands)
0.10
0.05
0.00
1
2
3
4
5
6
Weeks Forecasting

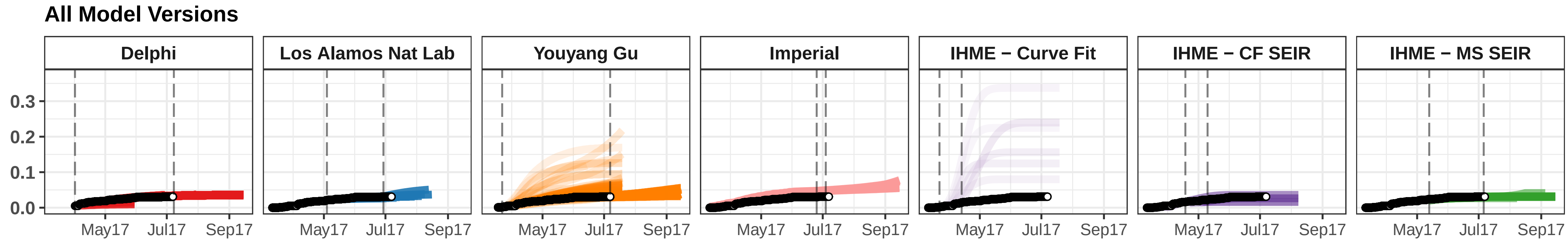

All Model Versions
Delphi
Los Alamos Nat Lab
Youyang Gu
Imperial
IHME − Curve Fit
IHME − CF SEIR
IHME − MS SEIR
0.3
0.2
0.1
0.0
May17
Jul17
Sep17

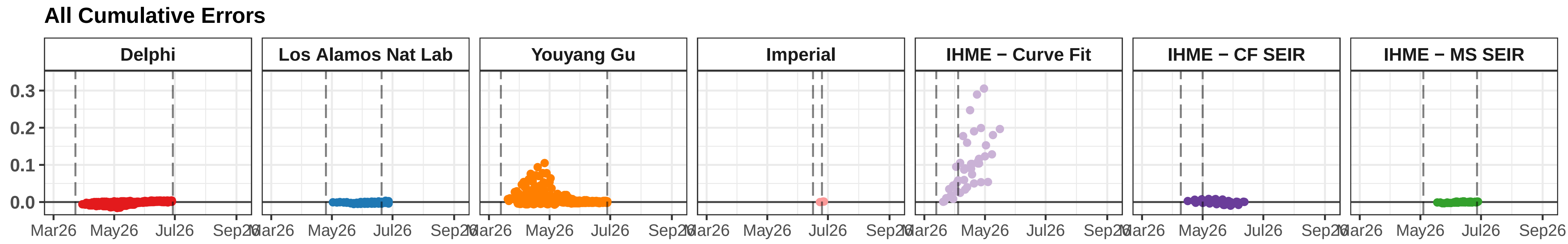

All Cumulative Errors
Delphi
Los Alamos Nat Lab
Youyang Gu
Imperial
IHME − Curve Fit
IHME − CF SEIR
IHME − MS SEIR
0.3
0.2
0.1
0.0
Mar26
May26
Jul26
Sep26

Slovakia

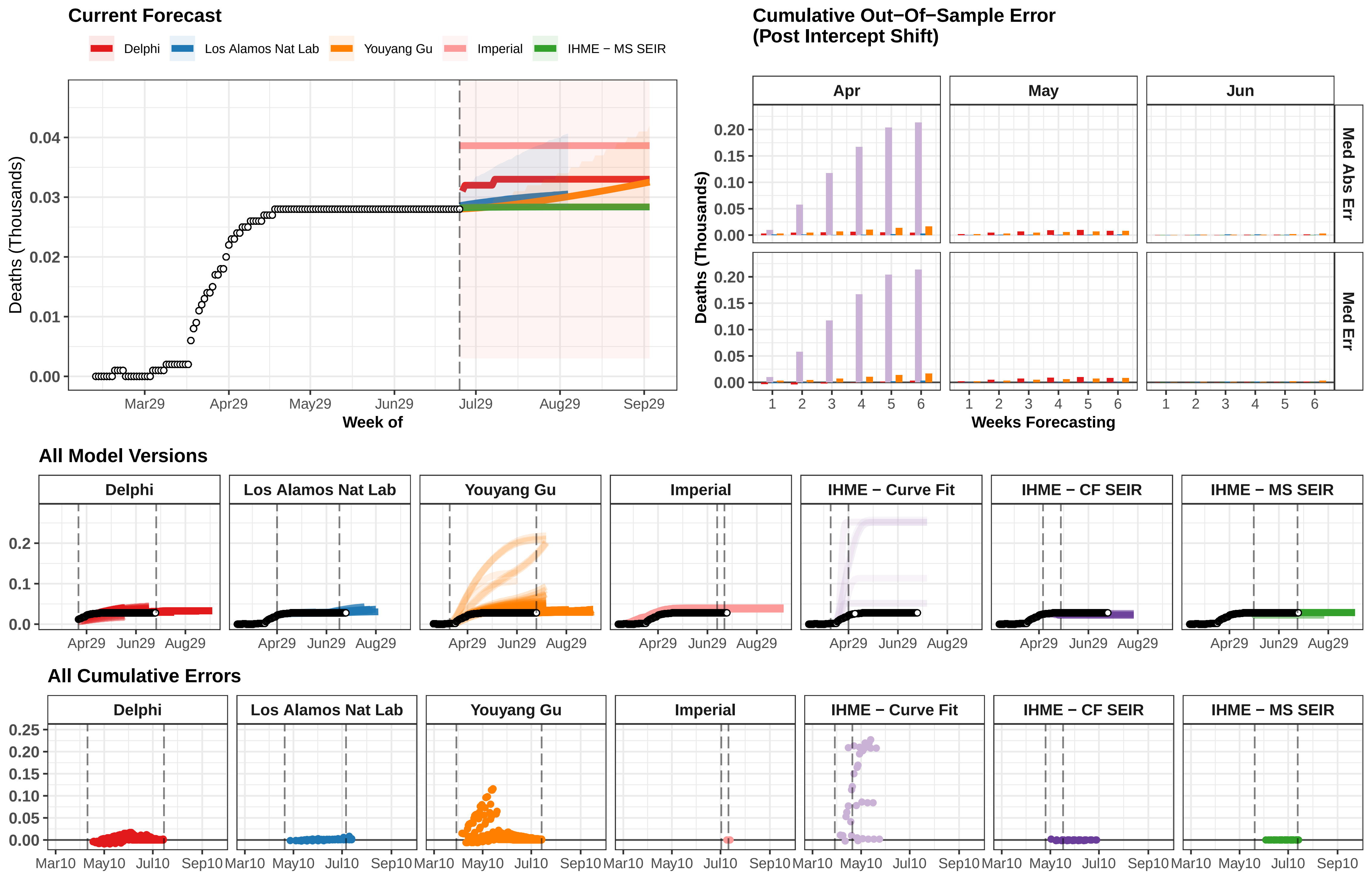

Current Forecast
Delphi
Los Alamos Nat Lab
Youyang Gu
Imperial
IHME − MS SEIR
Deaths (Thousands)
0.04
0.03
0.02
0.01
0.00
Mar29
Apr29
May29
Jun29
Jul29
Aug29
Sep29
Week of
Cumulative Out−Of−Sample Error
(Post Intercept Shift)
Apr
May
Jun
Med Abs Err
Med Err
0.20
0.15
0.10
0.05
0.00
1
2
3
4
5
6
Weeks Forecasting
All Model Versions
Delphi
Los Alamos Nat Lab
Youyang Gu
Imperial
IHME − Curve Fit
IHME − CF SEIR
IHME − MS SEIR
0.2
0.1
0.0
Apr29
Jun29
Aug29
All Cumulative Errors
0.25
0.20
0.15
0.10
0.05
0.00
Mar10
May10
Jul10
Sep10

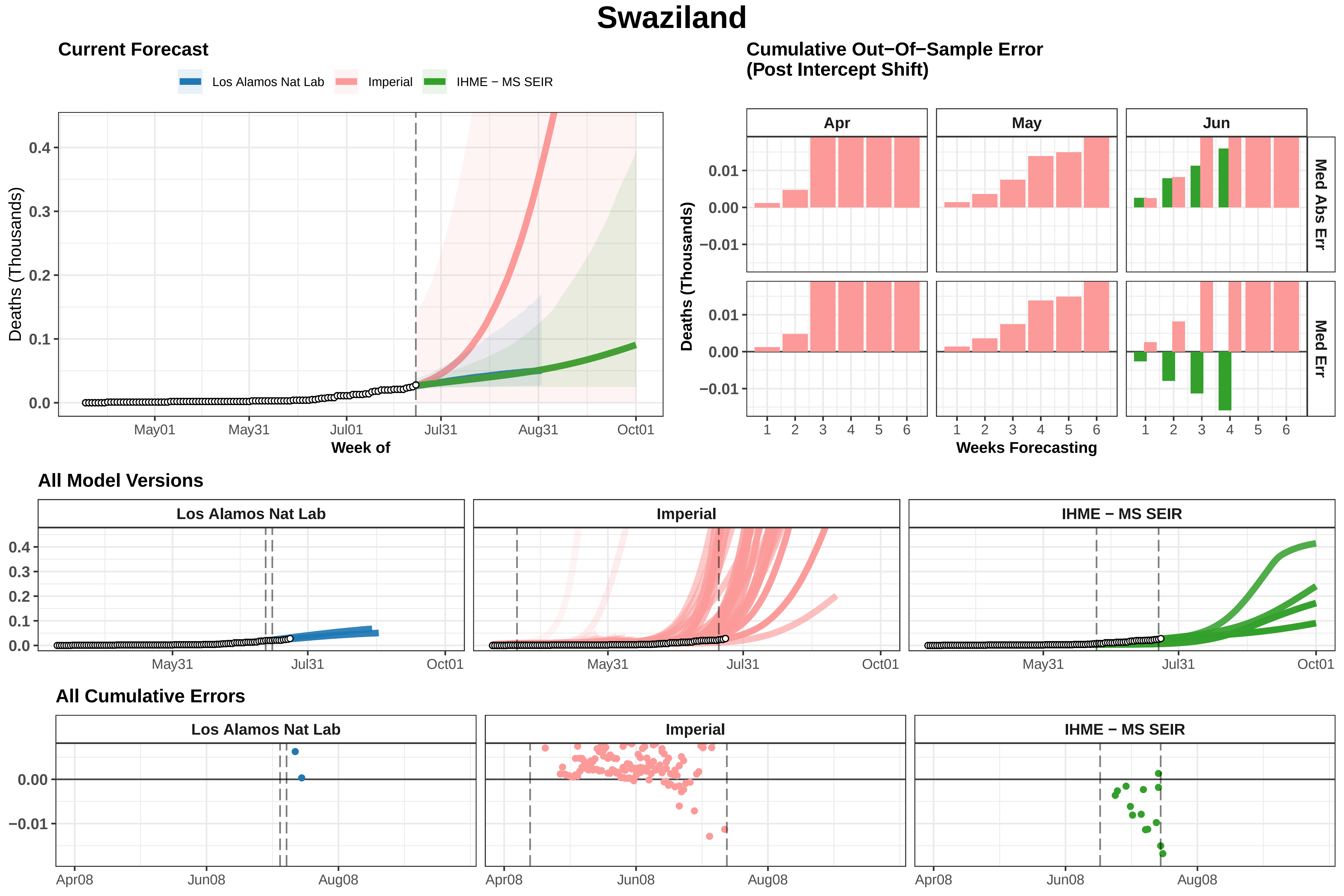
Swaziland
Current Forecast
Los Alamos Nat Lab
Imperial
IHME − MS SEIR
Deaths (Thousands)
0.0
0.1
0.2
0.3
0.4
May01
May31
Jul01
Jul31
Aug31
Oct01
Week of
Cumulative Out−Of−Sample Error
(Post Intercept Shift)
Apr
May
Jun
Med Abs Err
Med Err
−0.01
0.00
0.01
1
2
3
4
5
6
Weeks Forecasting
All Model Versions
Los Alamos Nat Lab
Imperial
IHME − MS SEIR
All Cumulative Errors
Apr08
Jun08
Aug08

Zimbabwe

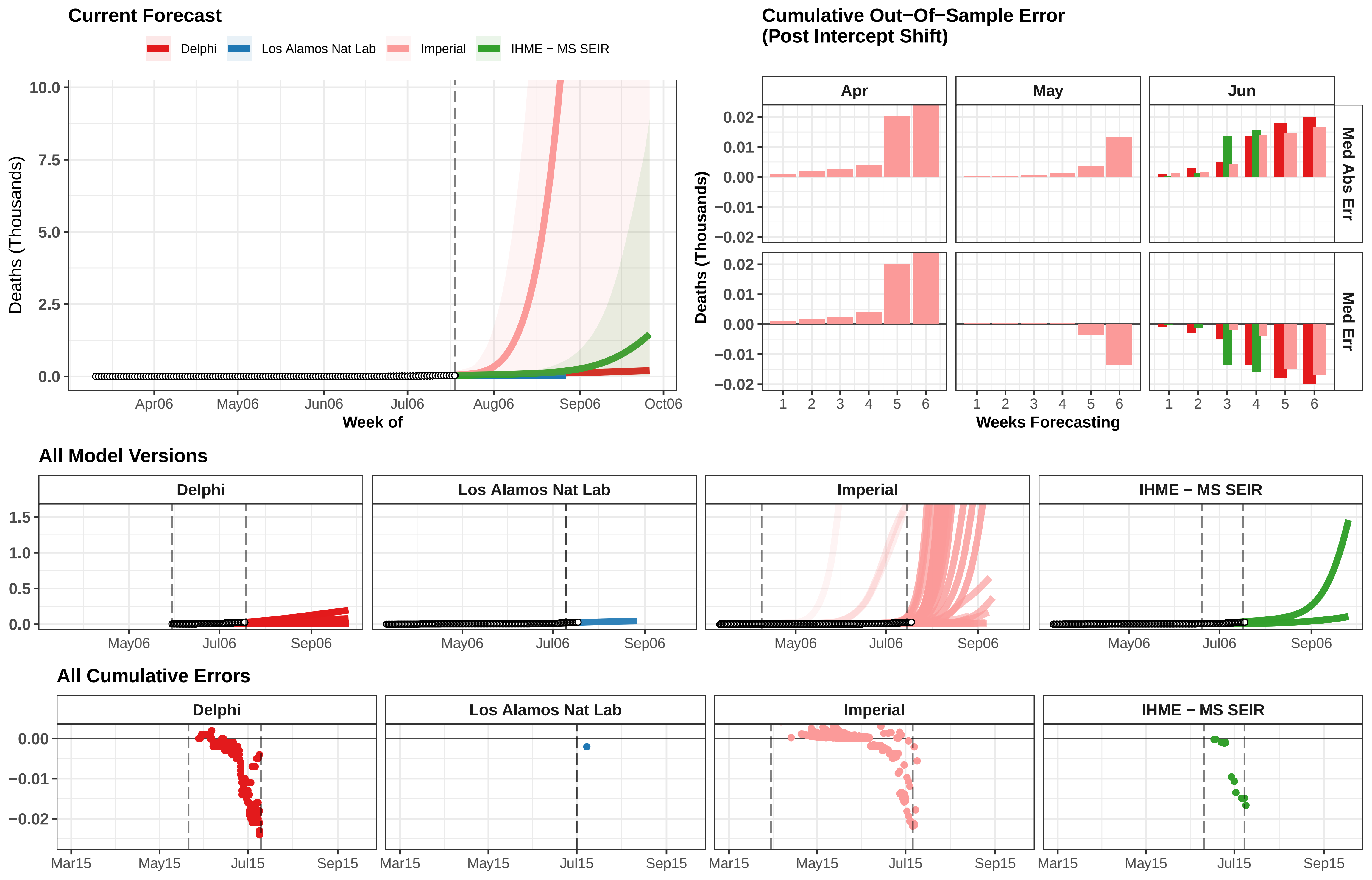
Current Forecast
Delphi
Los Alamos Nat Lab
Imperial
IHME − MS SEIR
Deaths (Thousands)
Week of
Cumulative Out−Of−Sample Error
(Post Intercept Shift)
Apr
May
Jun
Med Abs Err
Med Err
Weeks Forecasting
All Model Versions
All Cumulative Errors

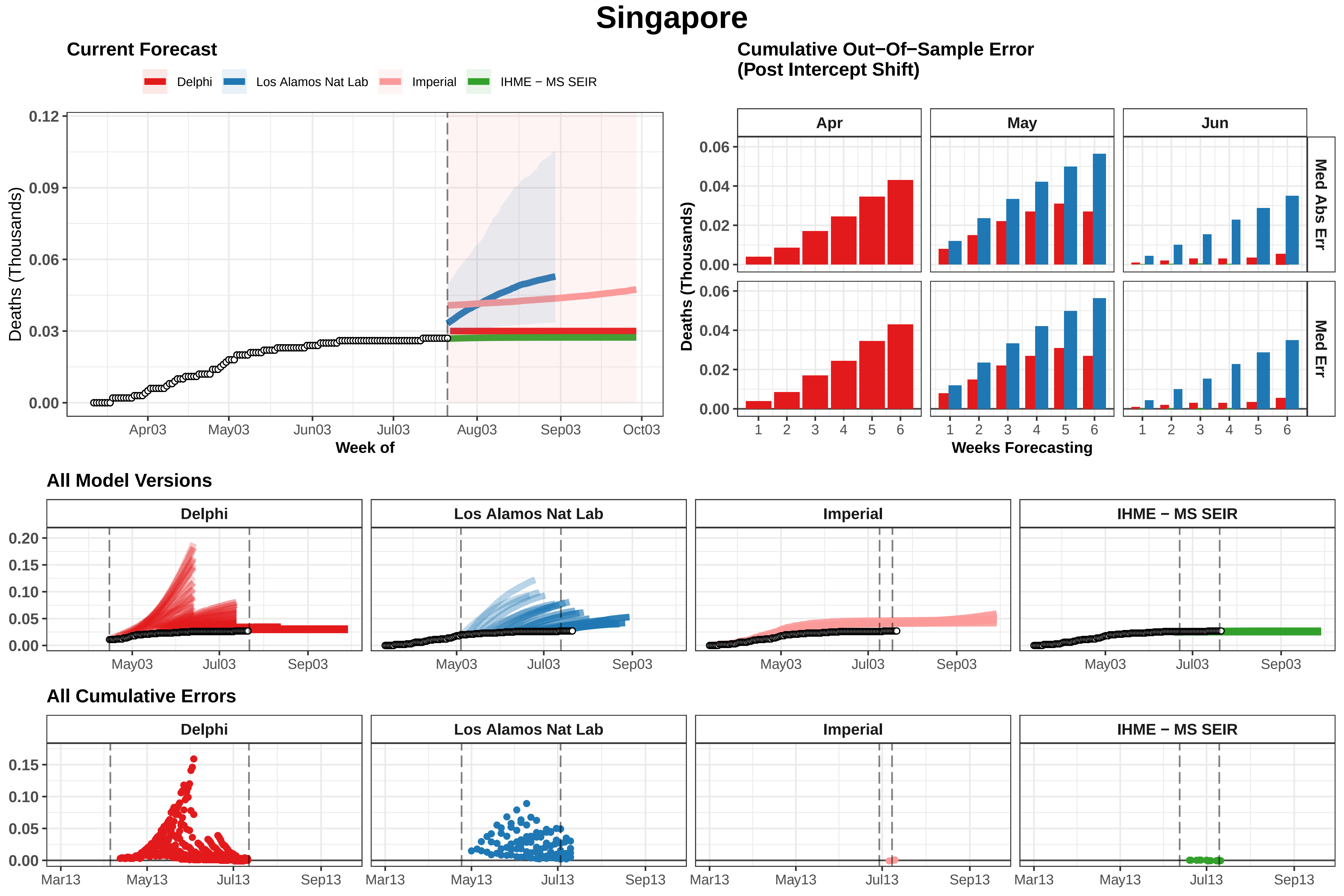

Singapore
Current Forecast
Delphi
Los Alamos Nat Lab
Imperial
IHME − MS SEIR
Deaths (Thousands)
Week of
Apr03
May03
Jun03
Jul03
Aug03
Sep03
Oct03
Cumulative Out−Of−Sample Error
(Post Intercept Shift)
Apr
May
Jun
Med Abs Err
Med Err
Weeks Forecasting
All Model Versions
All Cumulative Errors
Mar13
May13
Jul13
Sep13

Guinea−Bissau

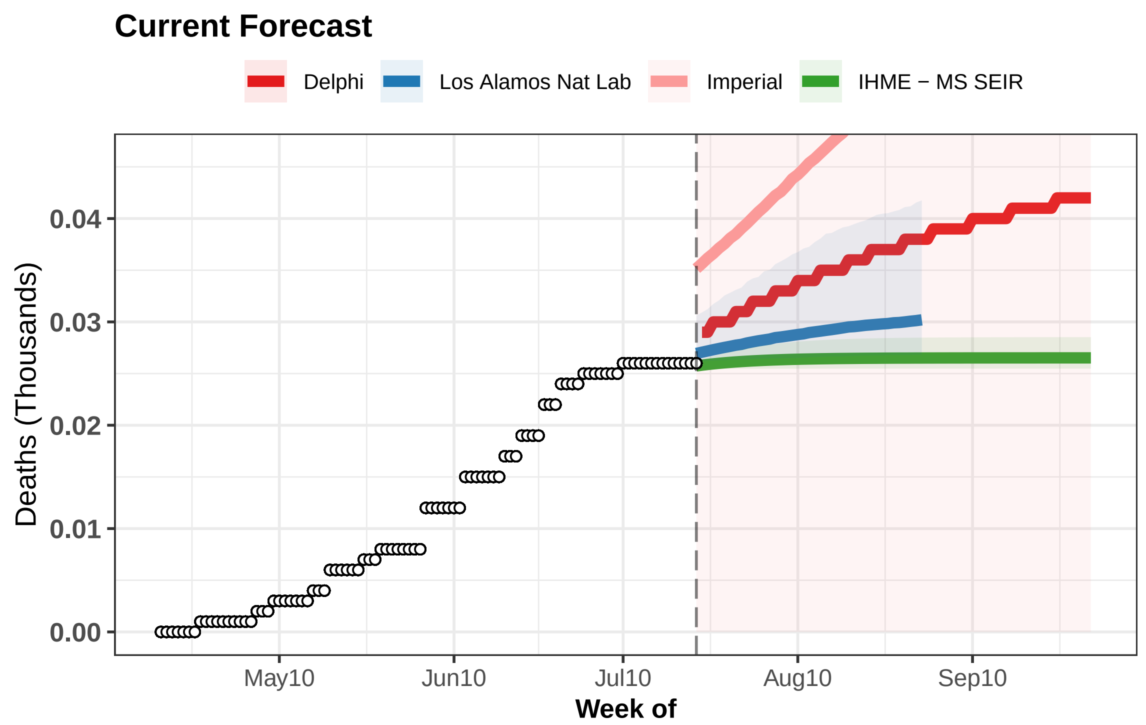

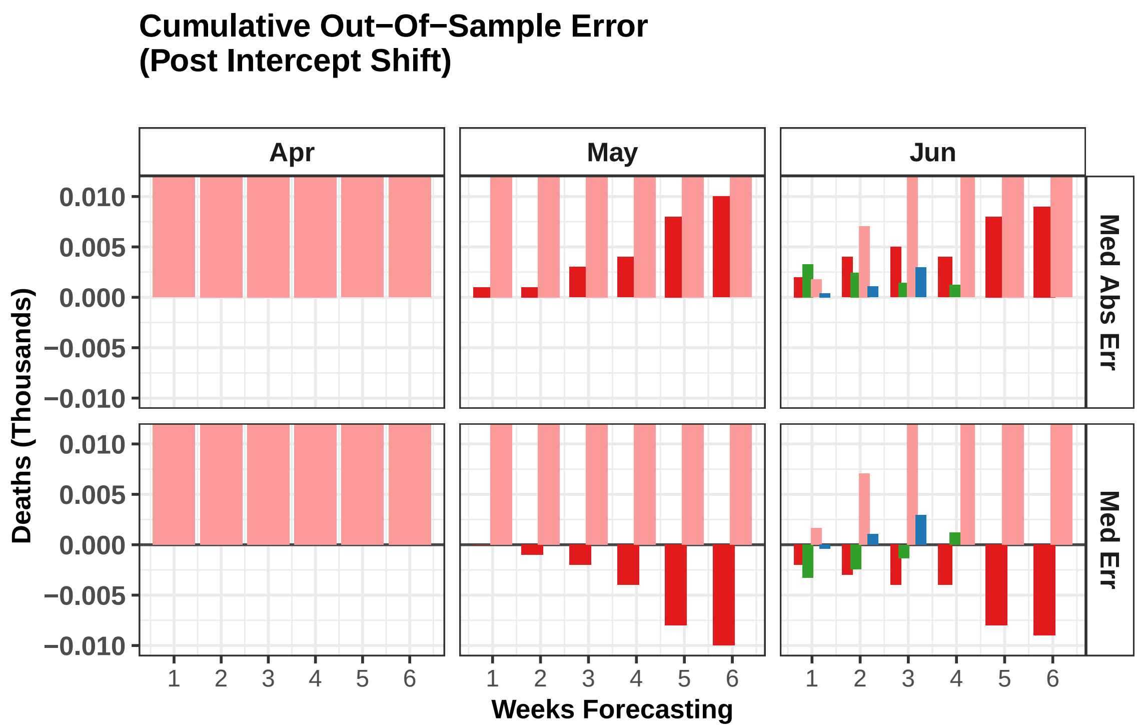

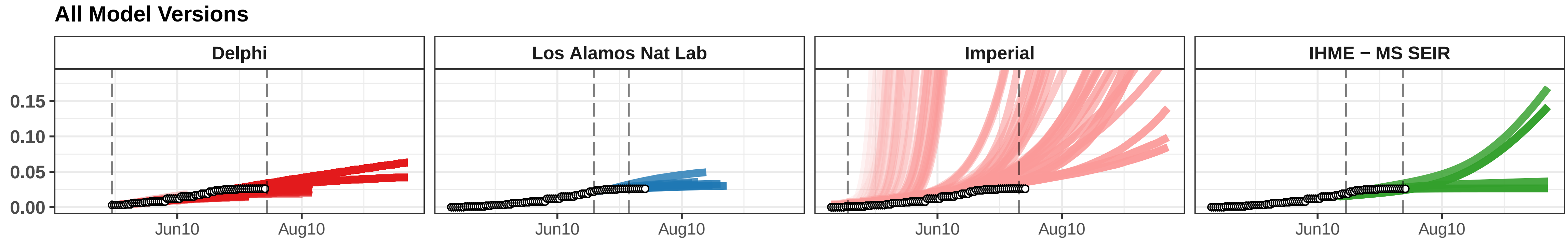

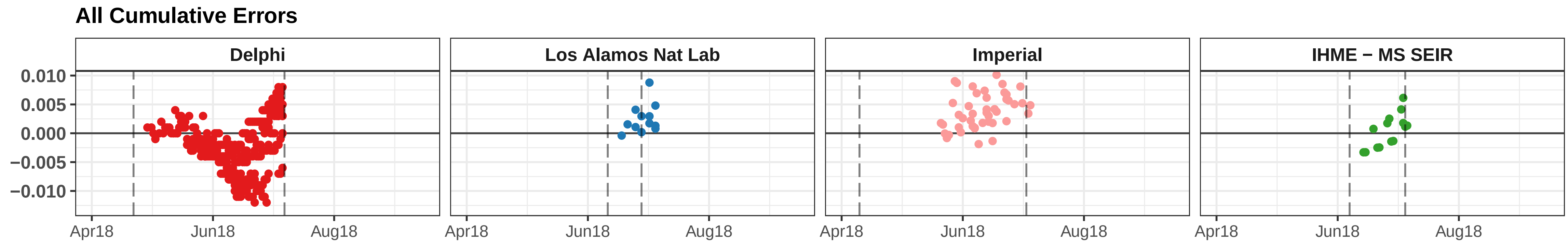

Hawaii

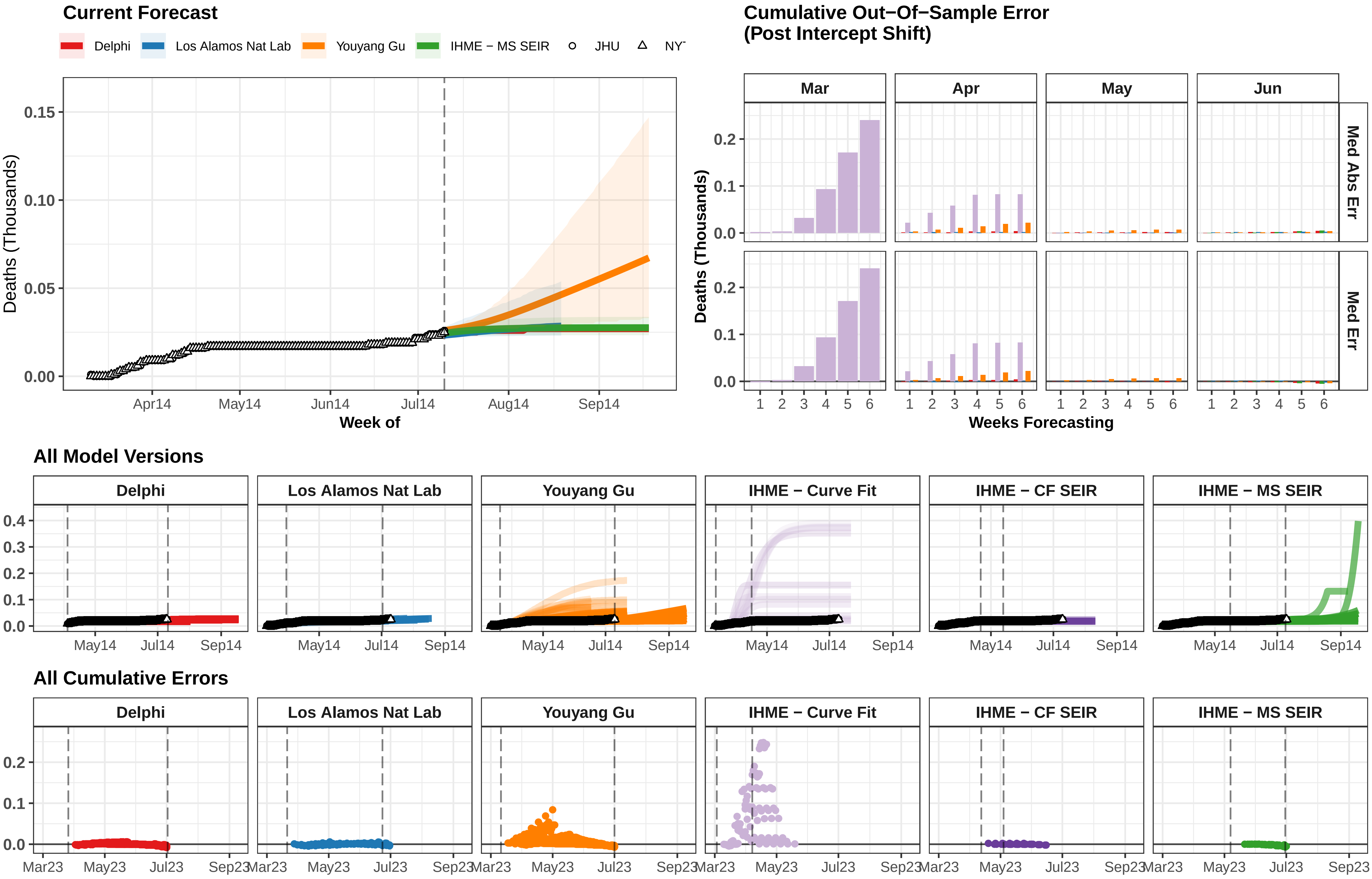

Current Forecast
Delphi
Los Alamos Nat Lab
Youyang Gu
IHME − MS SEIR
JHU
NY
0.15
0.10
0.05
0.00
Deaths (Thousands)
Apr14
May14
Jun14
Jul14
Aug14
Sep14
Week of
Cumulative Out−Of−Sample Error
(Post Intercept Shift)
Mar
Apr
May
Jun
Med Abs Err
Med Err
0.2
0.1
0.0
Deaths (Thousands)
1 2 3 4 5 6
Weeks Forecasting
All Model Versions
Delphi
Los Alamos Nat Lab
Youyang Gu
IHME − Curve Fit
IHME − CF SEIR
IHME − MS SEIR
0.4
0.3
0.2
0.1
0.0
May14
Jul14
Sep14
All Cumulative Errors
Delphi
Los Alamos Nat Lab
Youyang Gu
IHME − Curve Fit
IHME − CF SEIR
IHME − MS SEIR
0.2
0.1
0.0
Mar23
May23
Jul23
Sep23

Wyoming

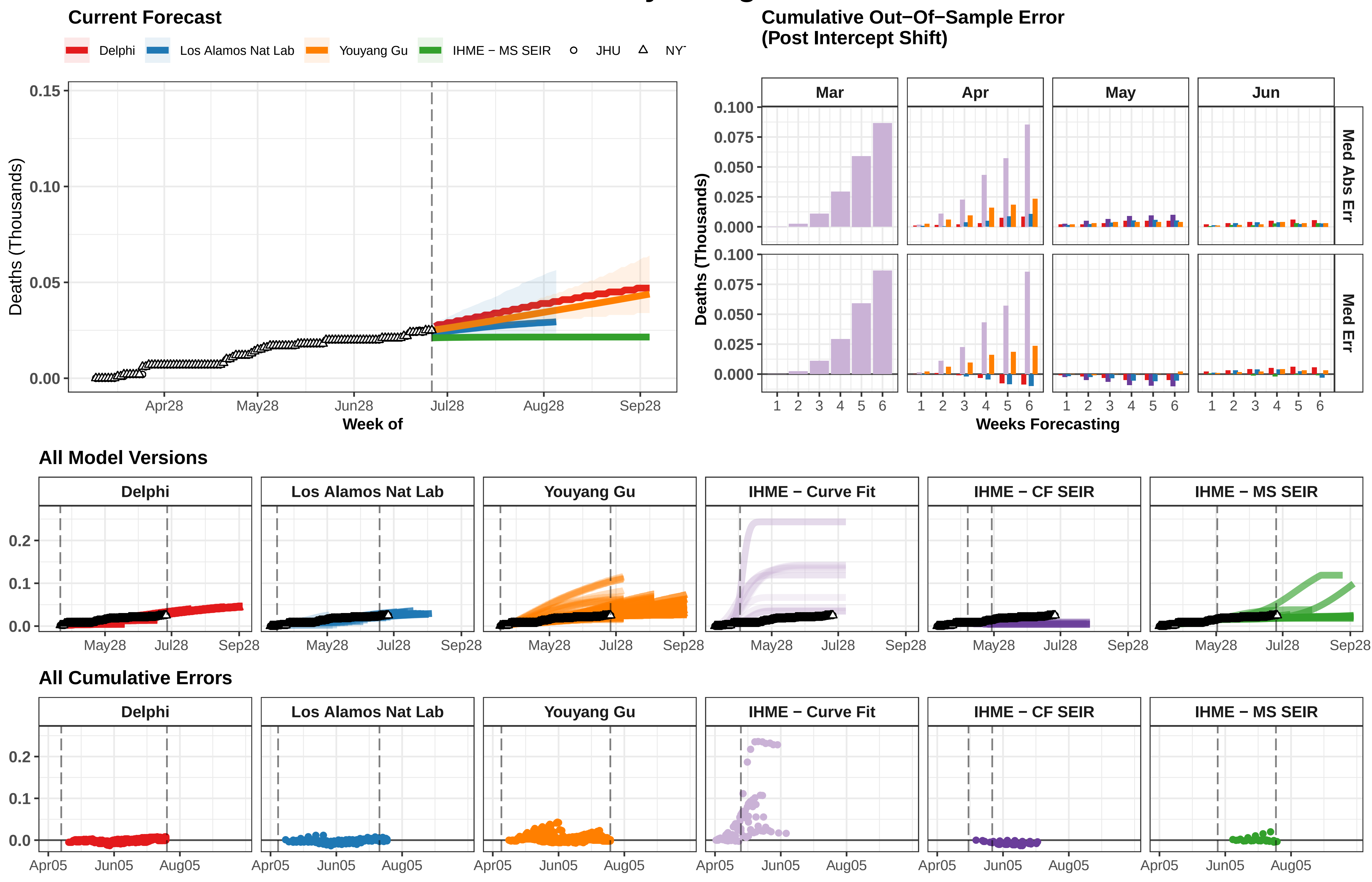
Current Forecast
Delphi
Los Alamos Nat Lab
Youyang Gu
IHME − MS SEIR
JHU
NY
Deaths (Thousands)
Week of
Apr28
May28
Jun28
Jul28
Aug28
Sep28
Cumulative Out−Of−Sample Error (Post Intercept Shift)
Mar
Apr
May
Jun
Med Abs Err
Med Err
Weeks Forecasting
All Model Versions
Delphi
Los Alamos Nat Lab
Youyang Gu
IHME − Curve Fit
IHME − CF SEIR
IHME − MS SEIR
All Cumulative Errors
Apr05
Jun05
Aug05

Suriname

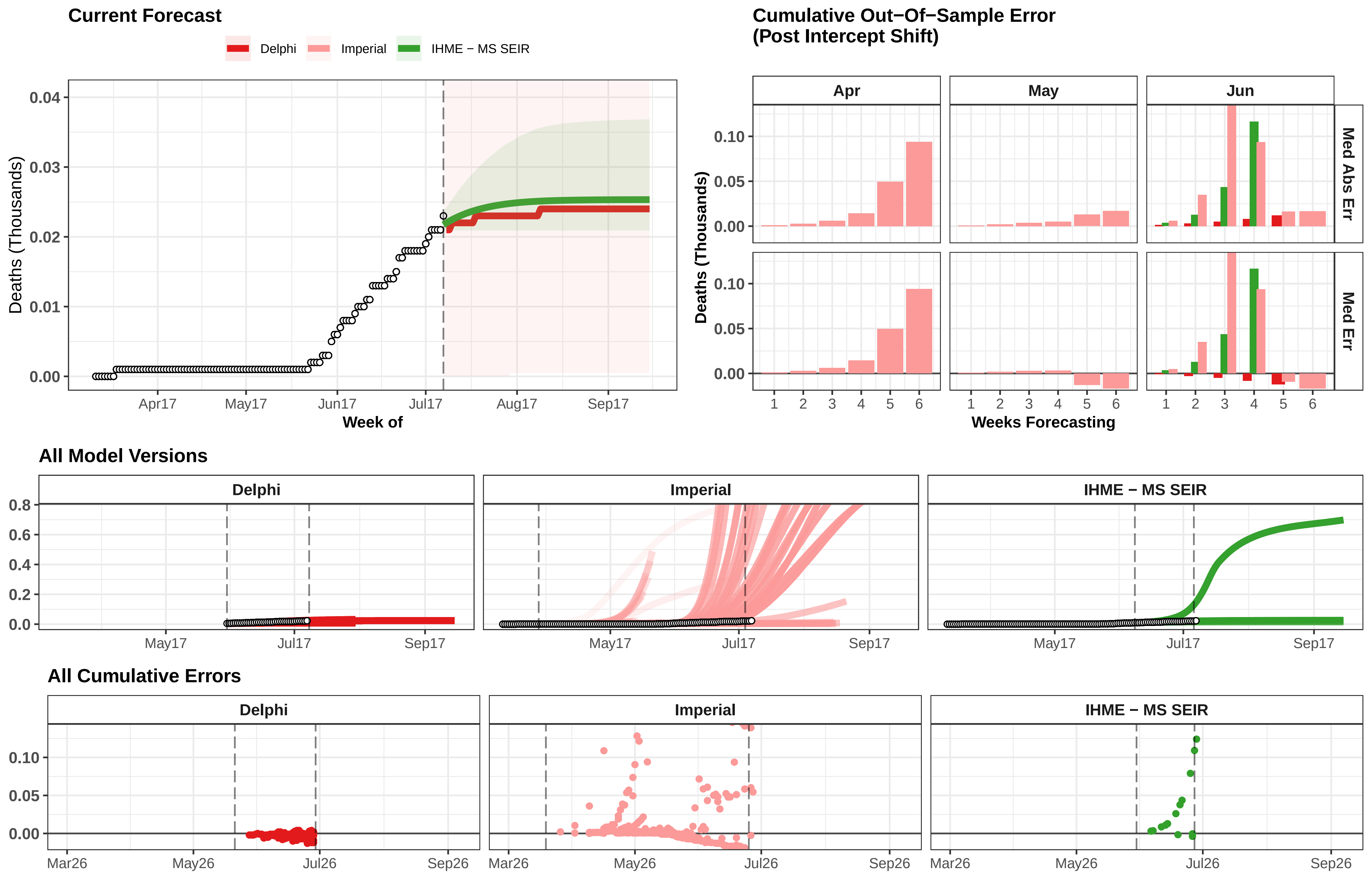

Current Forecast
Delphi
Imperial
IHME − MS SEIR
Deaths (Thousands)
0.00
0.01
0.02
0.03
0.04
Apr17
May17
Jun17
Jul17
Aug17
Sep17
Week of
Cumulative Out−Of−Sample Error
(Post Intercept Shift)
Apr
May
Jun
Med Abs Err
Med Err
Deaths (Thousands)
0.00
0.05
0.10
1
2
3
4
5
6
Weeks Forecasting
All Model Versions
Delphi
Imperial
IHME − MS SEIR
0.0
0.2
0.4
0.6
0.8
May17
Jul17
Sep17
All Cumulative Errors
Delphi
Imperial
IHME − MS SEIR
0.00
0.05
0.10
Mar26
May26
Jul26
Sep26

New Zealand

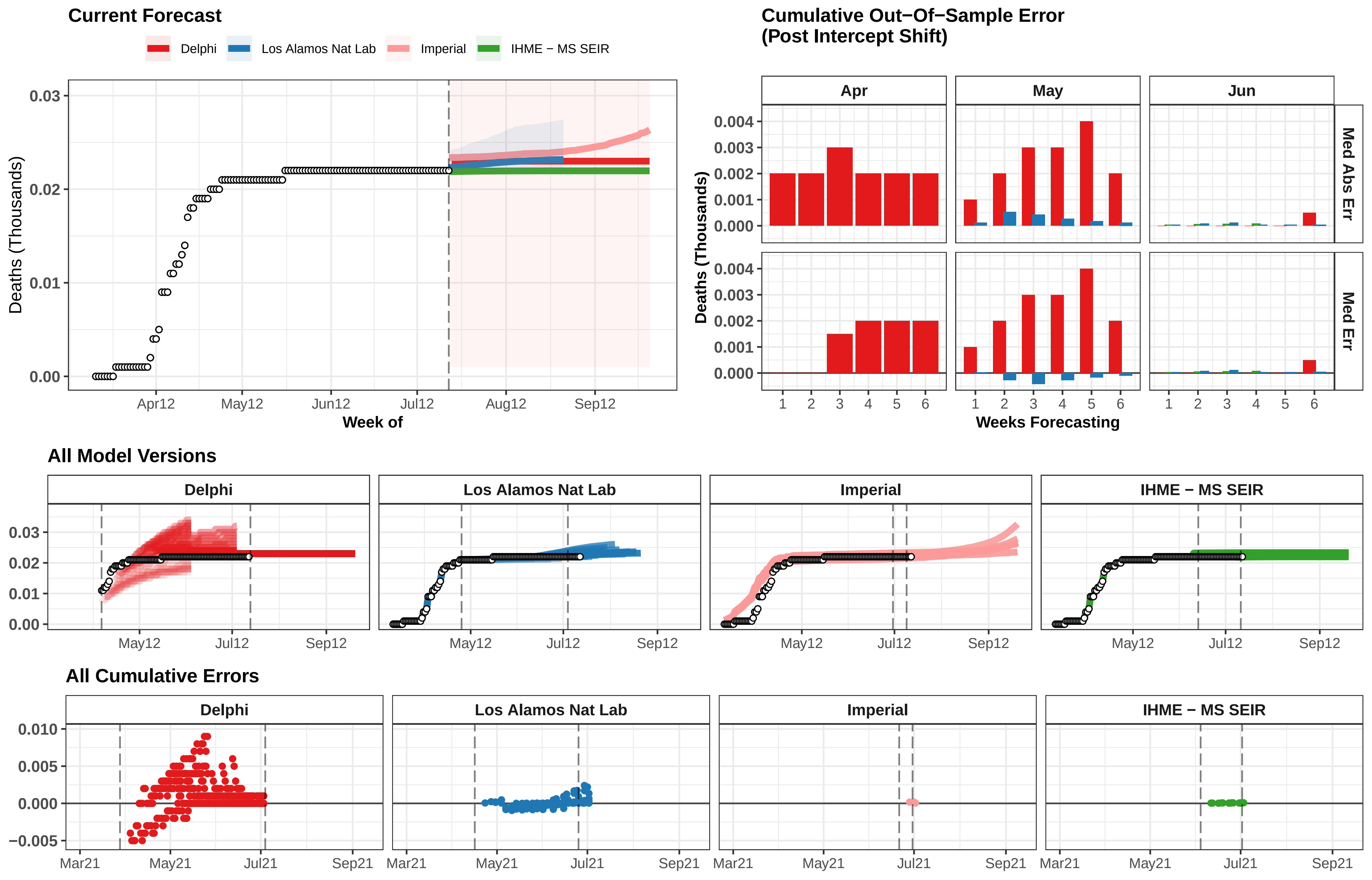

Current Forecast
Delphi
Los Alamos Nat Lab
Imperial
IHME − MS SEIR
Deaths (Thousands)
0.00
0.01
0.02
0.03
Apr12
May12
Jun12
Jul12
Aug12
Sep12
Week of
Cumulative Out−Of−Sample Error
(Post Intercept Shift)
Apr
May
Jun
Med Abs Err
Med Err
0.000
0.001
0.002
0.003
0.004
1
2
3
4
5
6
Weeks Forecasting
All Model Versions
Delphi
Los Alamos Nat Lab
Imperial
IHME − MS SEIR
May12
Jul12
Sep12
All Cumulative Errors
0.010
0.005
0.000
−0.005
Mar21
May21
Jul21
Sep21

Cape Verde

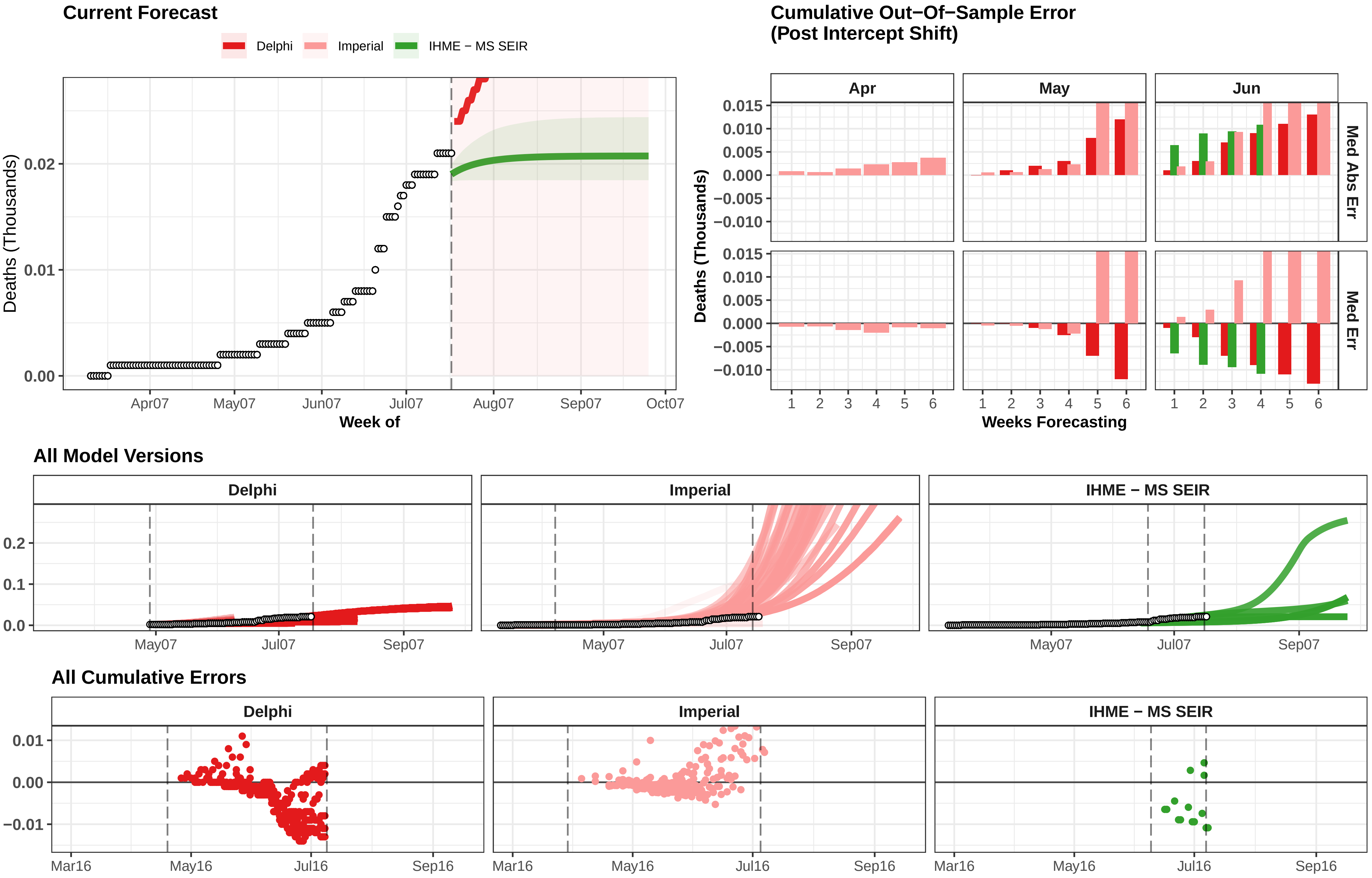
Current Forecast
Delphi
Imperial
IHME − MS SEIR
Deaths (Thousands)
0.00
0.01
0.02
Apr07
May07
Jun07
Jul07
Aug07
Sep07
Oct07
Week of
Cumulative Out−Of−Sample Error
(Post Intercept Shift)
Apr
May
Jun
Med Abs Err
Med Err
Deaths (Thousands)
0.015
0.010
0.005
0.000
−0.005
−0.010
1
2
3
4
5
6
Weeks Forecasting
All Model Versions
Delphi
Imperial
IHME − MS SEIR
0.0
0.1
0.2
May07
Jul07
Sep07
All Cumulative Errors
Delphi
Imperial
IHME − MS SEIR
0.01
0.00
−0.01
Mar16
May16
Jul16
Sep16

Tanzania

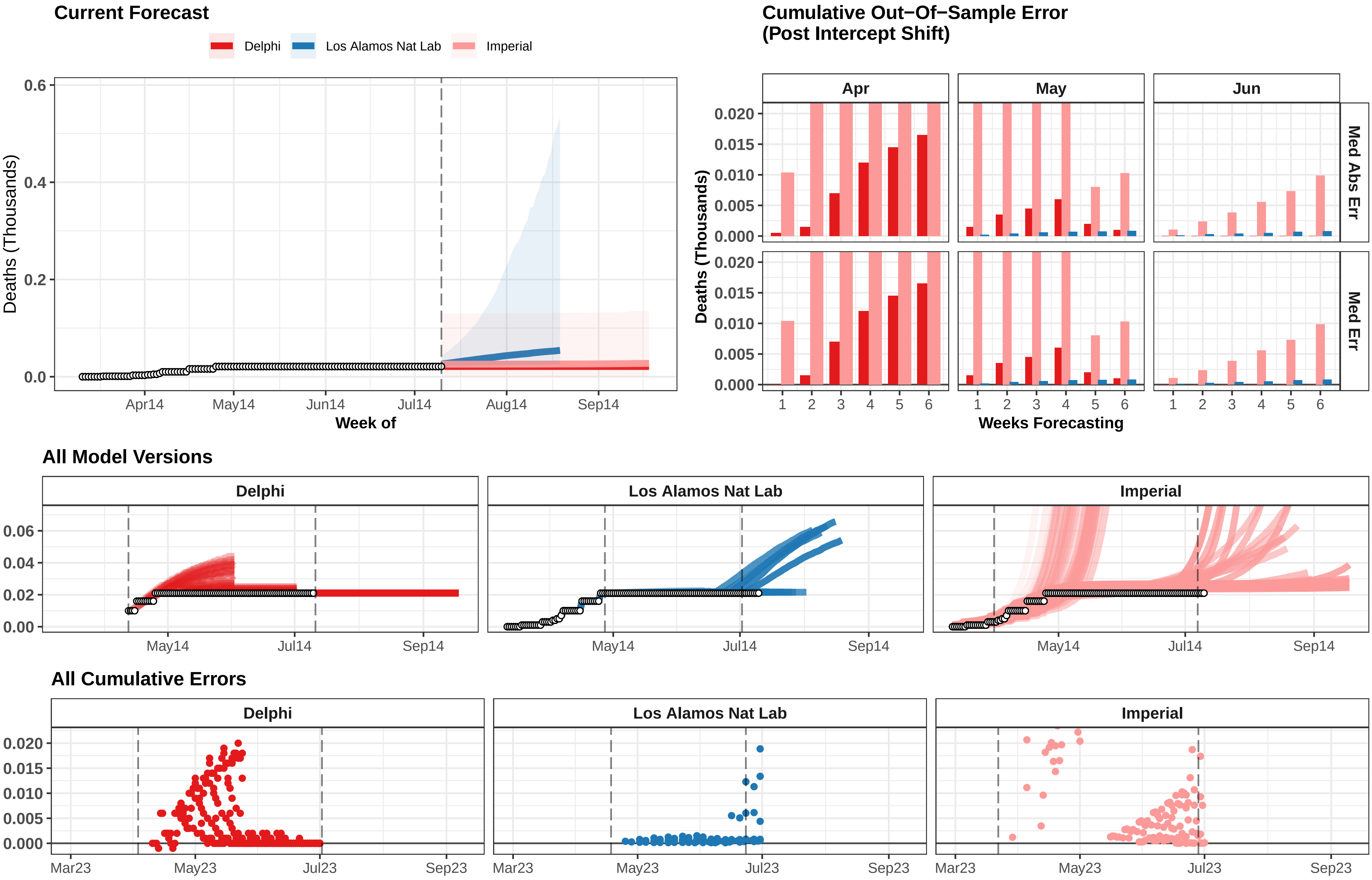
Current Forecast
Delphi
Los Alamos Nat Lab
Imperial
Deaths (Thousands)
0.6
0.4
0.2
0.0
Apr14
May14
Jun14
Jul14
Aug14
Sep14
Week of
Cumulative Out−Of−Sample Error
(Post Intercept Shift)
Apr
May
Jun
Med Abs Err
Med Err
0.020
0.015
0.010
0.005
0.000
1
2
3
4
5
6
Weeks Forecasting
All Model Versions
Delphi
Los Alamos Nat Lab
Imperial
0.06
0.04
0.02
0.00
May14
Jul14
Sep14
All Cumulative Errors
Mar23
May23
Jul23
Sep23

Cyprus

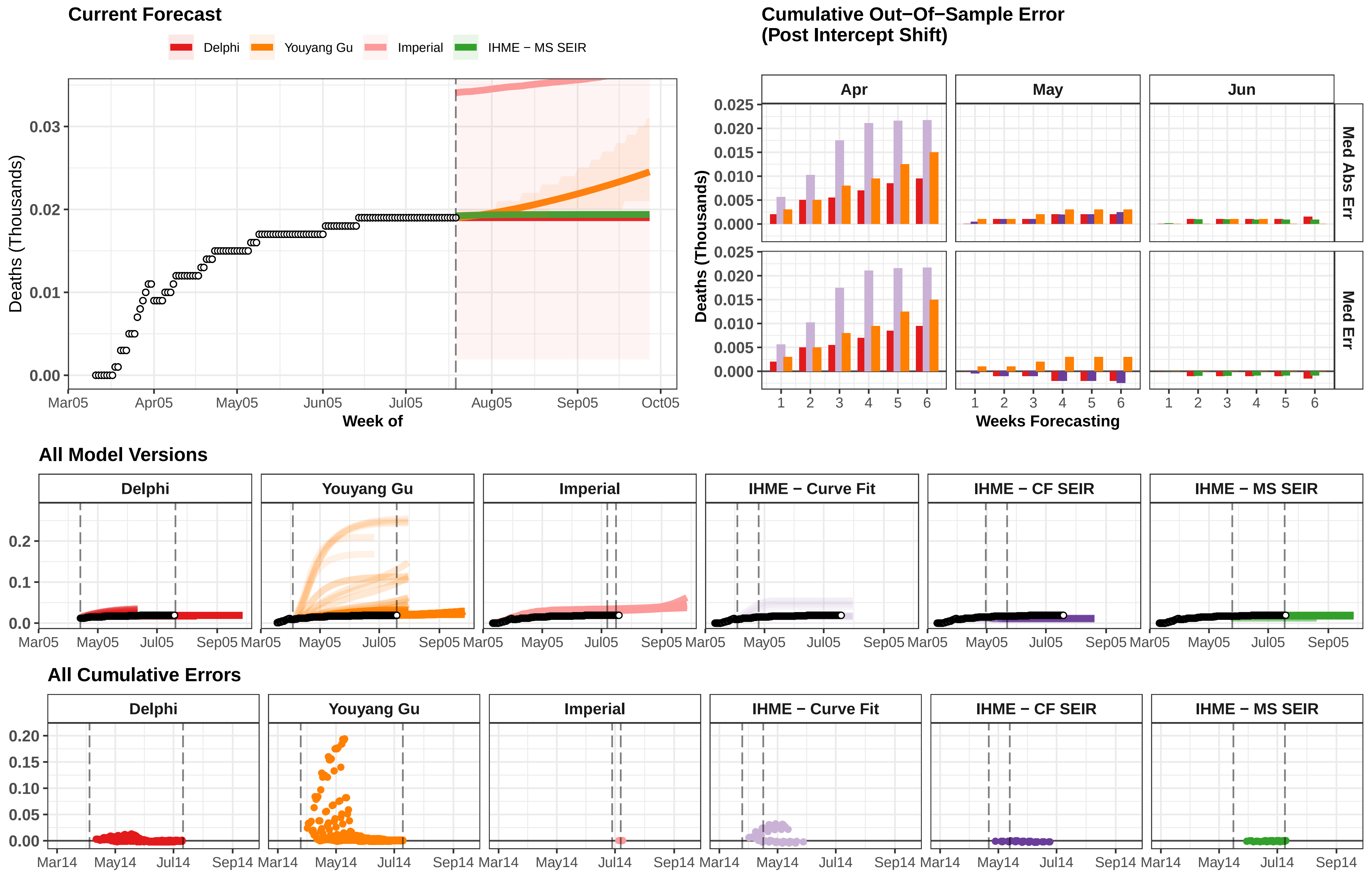

Current Forecast
Delphi
Youyang Gu
Imperial
IHME − MS SEIR
Deaths (Thousands)
0.00
0.01
0.02
0.03
Mar05
Apr05
May05
Jun05
Jul05
Aug05
Sep05
Oct05
Week of
Cumulative Out−Of−Sample Error
(Post Intercept Shift)
Apr
May
Jun
Med Abs Err
Med Err
0.000
0.005
0.010
0.015
0.020
0.025
Weeks Forecasting
1
2
3
4
5
6
All Model Versions
Delphi
Youyang Gu
Imperial
IHME − Curve Fit
IHME − CF SEIR
IHME − MS SEIR
0.0
0.1
0.2
Mar05
May05
Jul05
Sep05
All Cumulative Errors
0.00
0.05
0.10
0.15
0.20
Mar14
May14
Jul14
Sep14

Guyana

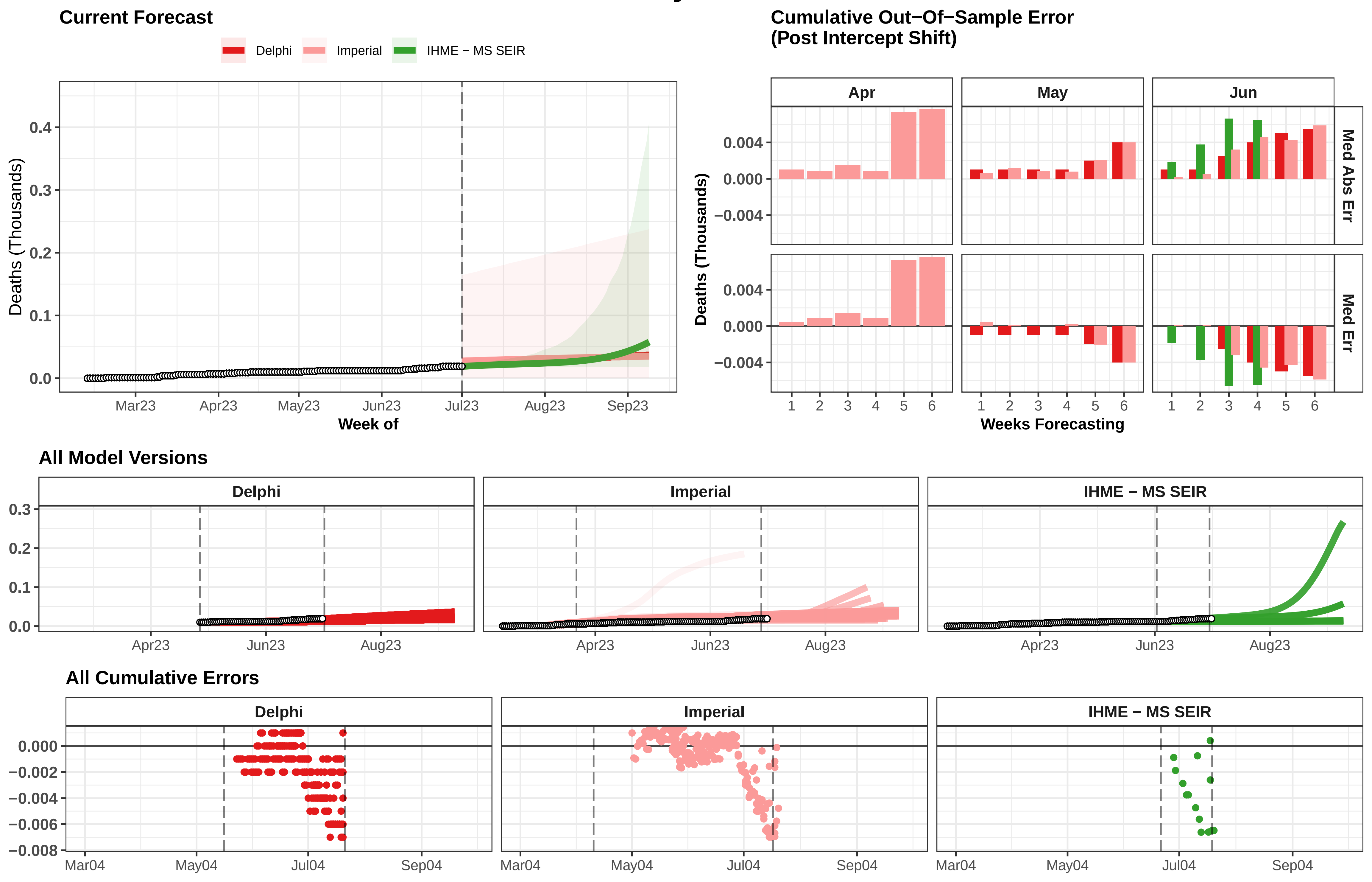

Current Forecast
Delphi
Imperial
IHME − MS SEIR
Deaths (Thousands)
0.4
0.3
0.2
0.1
0.0
Mar23
Apr23
May23
Jun23
Jul23
Aug23
Sep23
Week of
Cumulative Out−Of−Sample Error
(Post Intercept Shift)
Apr
May
Jun
Med Abs Err
Med Err
0.004
0.000
−0.004
1 2 3 4 5 6
Weeks Forecasting
All Model Versions
Delphi
Imperial
IHME − MS SEIR
0.3
0.2
0.1
0.0
Apr23
Jun23
Aug23
All Cumulative Errors
0.000
−0.002
−0.004
−0.006
−0.008
Mar04
May04
Jul04
Sep04

Alaska

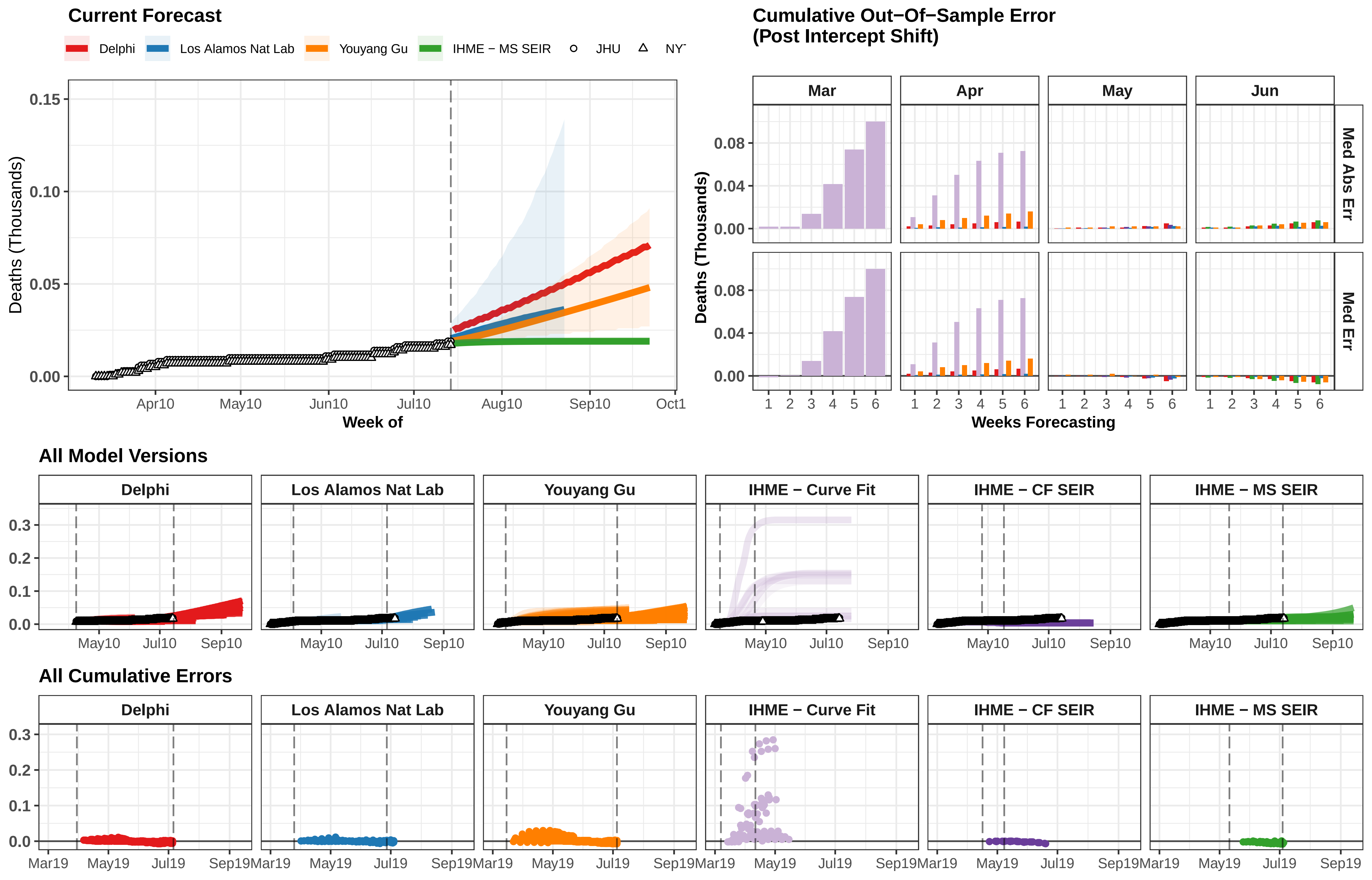

Current Forecast
Delphi
Los Alamos Nat Lab
Youyang Gu
IHME − MS SEIR
JHU
NY
Deaths (Thousands)
Week of
Apr10
May10
Jun10
Jul10
Aug10
Sep10
Oct1
Cumulative Out−Of−Sample Error
(Post Intercept Shift)
Mar
Apr
May
Jun
Med Abs Err
Med Err
Weeks Forecasting
All Model Versions
IHME − Curve Fit
IHME − CF SEIR
All Cumulative Errors
Mar19
May19
Jul19
Sep19

Georgia (Country)

Current Forecast

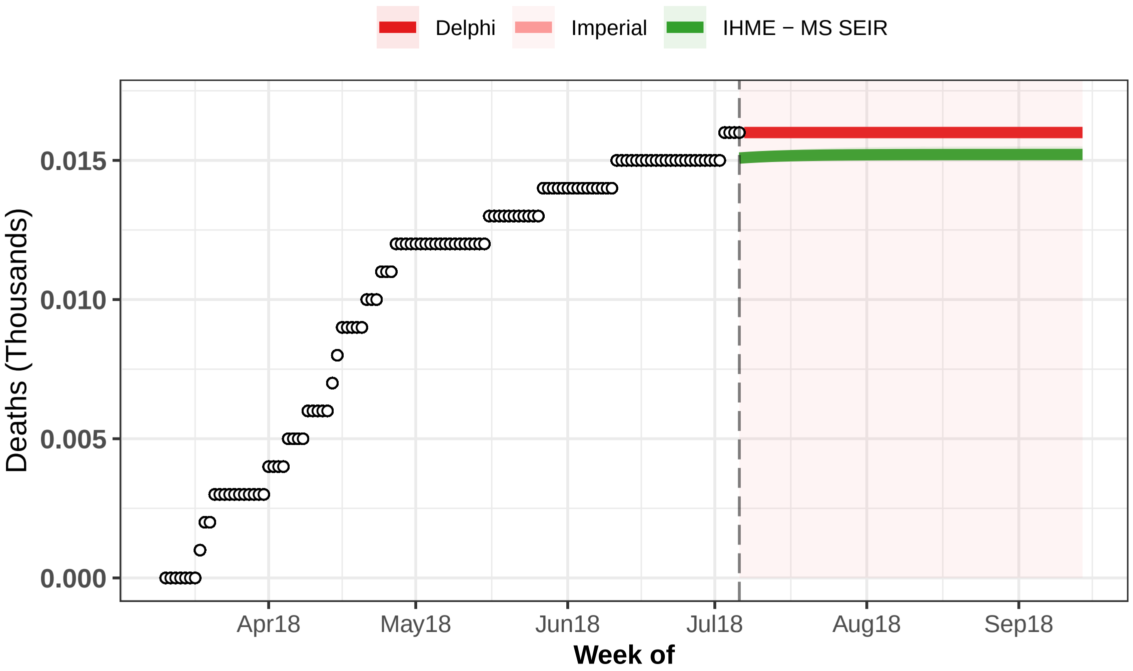

Cumulative Out−Of−Sample Error (Post Intercept Shift)

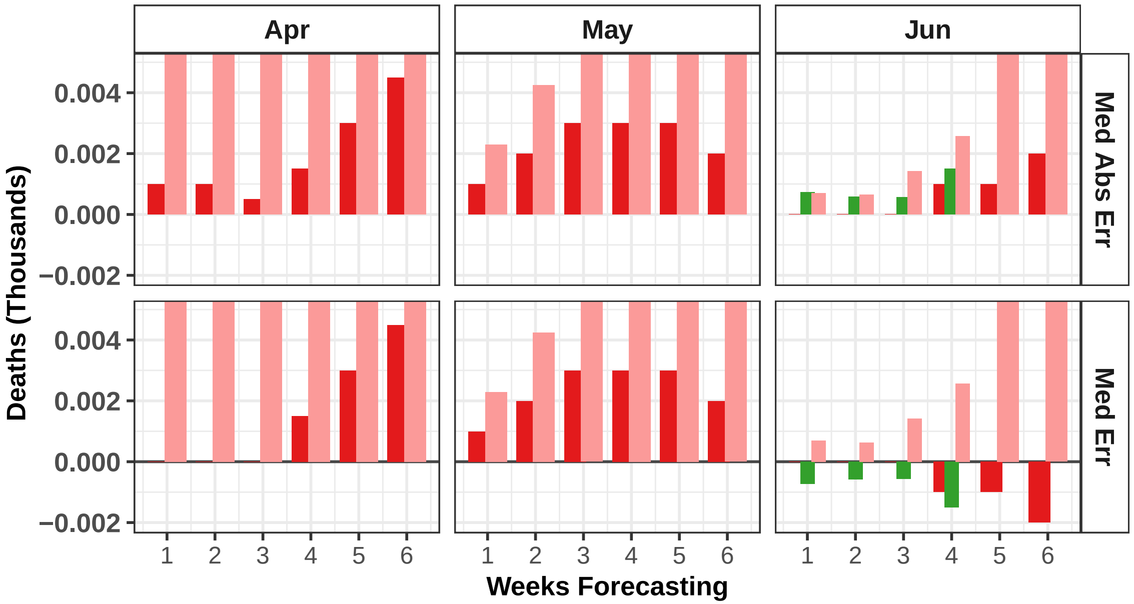

All Model Versions

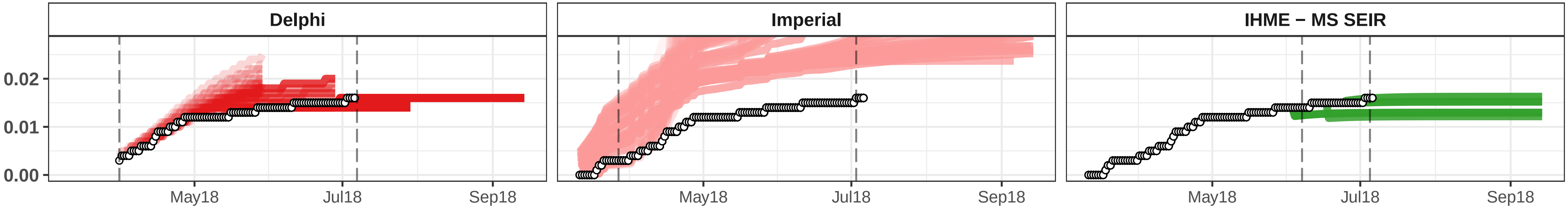

All Cumulative Errors

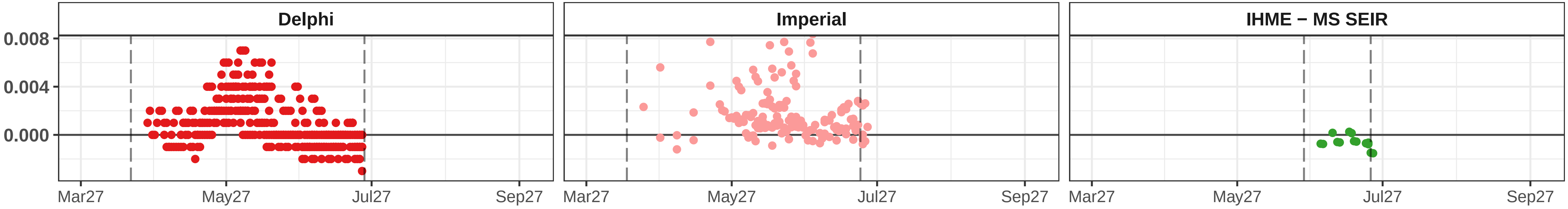

Togo

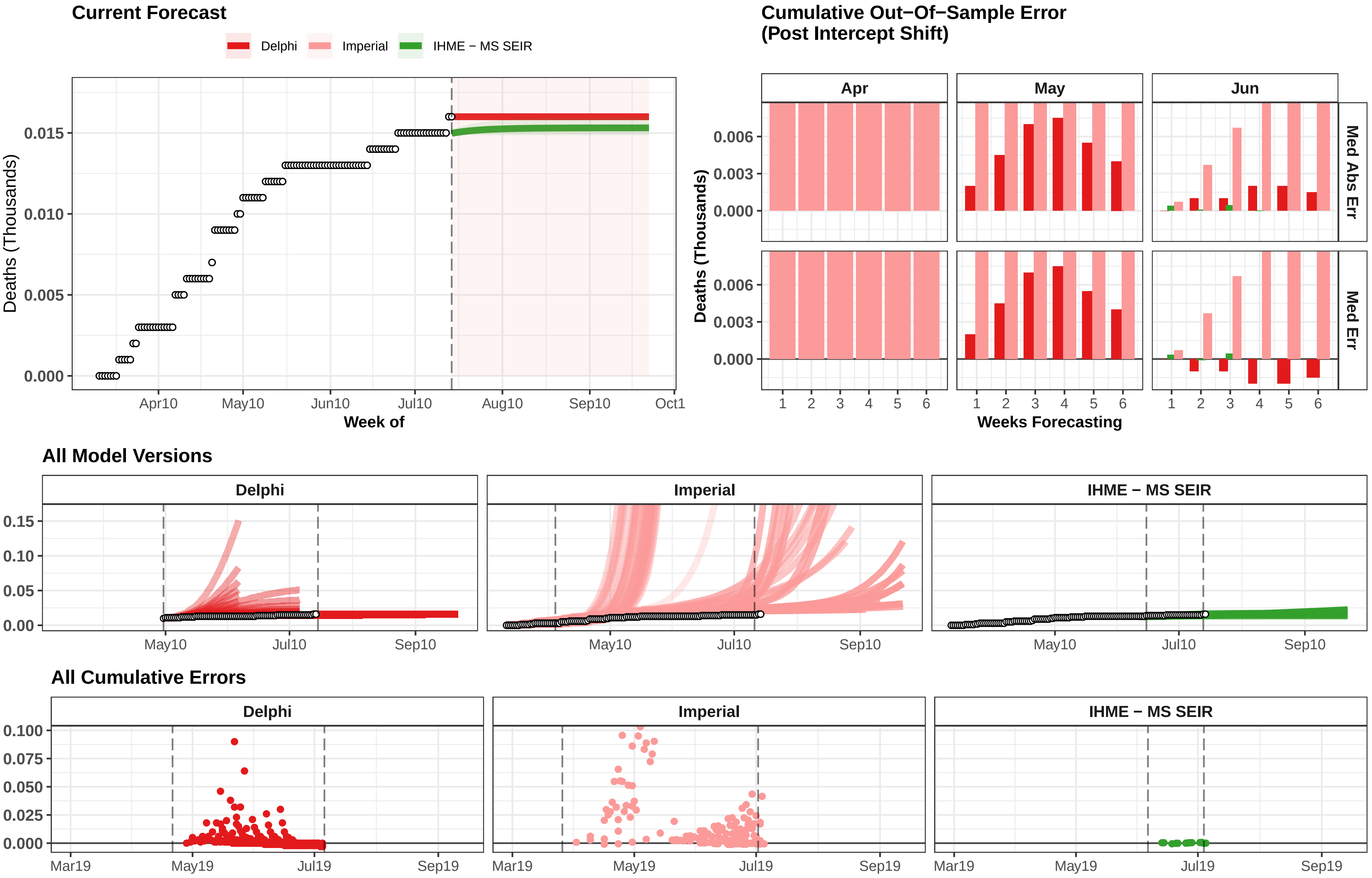

Current Forecast
Delphi
Imperial
IHME − MS SEIR
Deaths (Thousands)
0.000
0.005
0.010
0.015
Apr10
May10
Jun10
Jul10
Aug10
Sep10
Oct1
Week of
Cumulative Out−Of−Sample Error
(Post Intercept Shift)
Apr
May
Jun
Med Abs Err
Med Err
0.000
0.003
0.006
1
2
3
4
5
6
Weeks Forecasting
All Model Versions
0.00
0.05
0.10
0.15
May10
Jul10
Sep10
All Cumulative Errors
0.000
0.025
0.050
0.075
0.100
Mar19
May19
Jul19
Sep19

Maldives

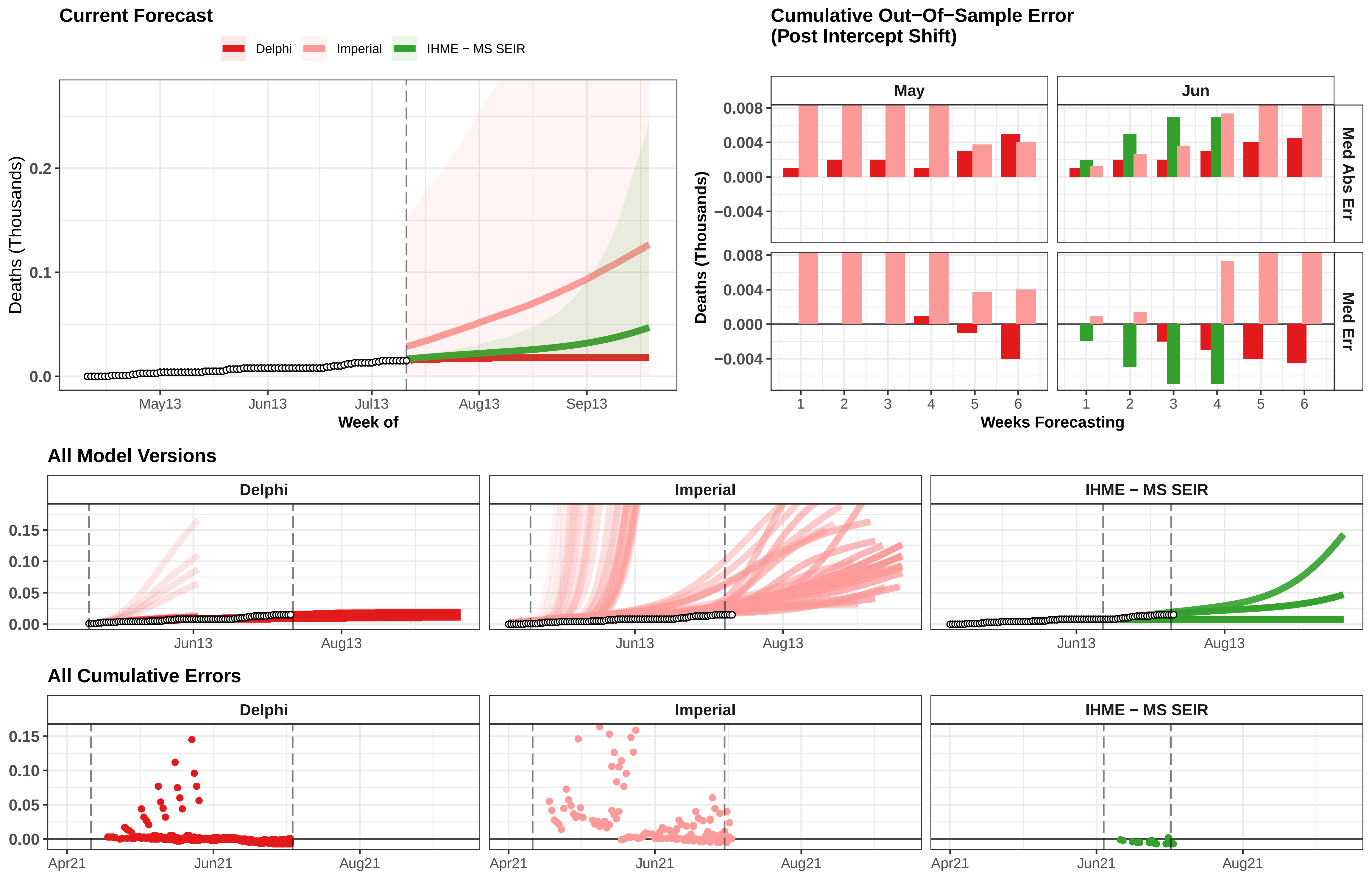

Current Forecast
Delphi
Imperial
IHME − MS SEIR
Deaths (Thousands)
0.0
0.1
0.2
May13
Jun13
Jul13
Aug13
Sep13
Week of
Cumulative Out−Of−Sample Error
(Post Intercept Shift)
May
Jun
Med Abs Err
Med Err
Deaths (Thousands)
0.008
0.004
0.000
−0.004
1
2
3
4
5
6
Weeks Forecasting
All Model Versions
Delphi
Imperial
IHME − MS SEIR
0.15
0.10
0.05
0.00
Jun13
Aug13
All Cumulative Errors
Delphi
Imperial
IHME − MS SEIR
0.15
0.10
0.05
0.00
Apr21
Jun21
Aug21

Sao Tome and Principe

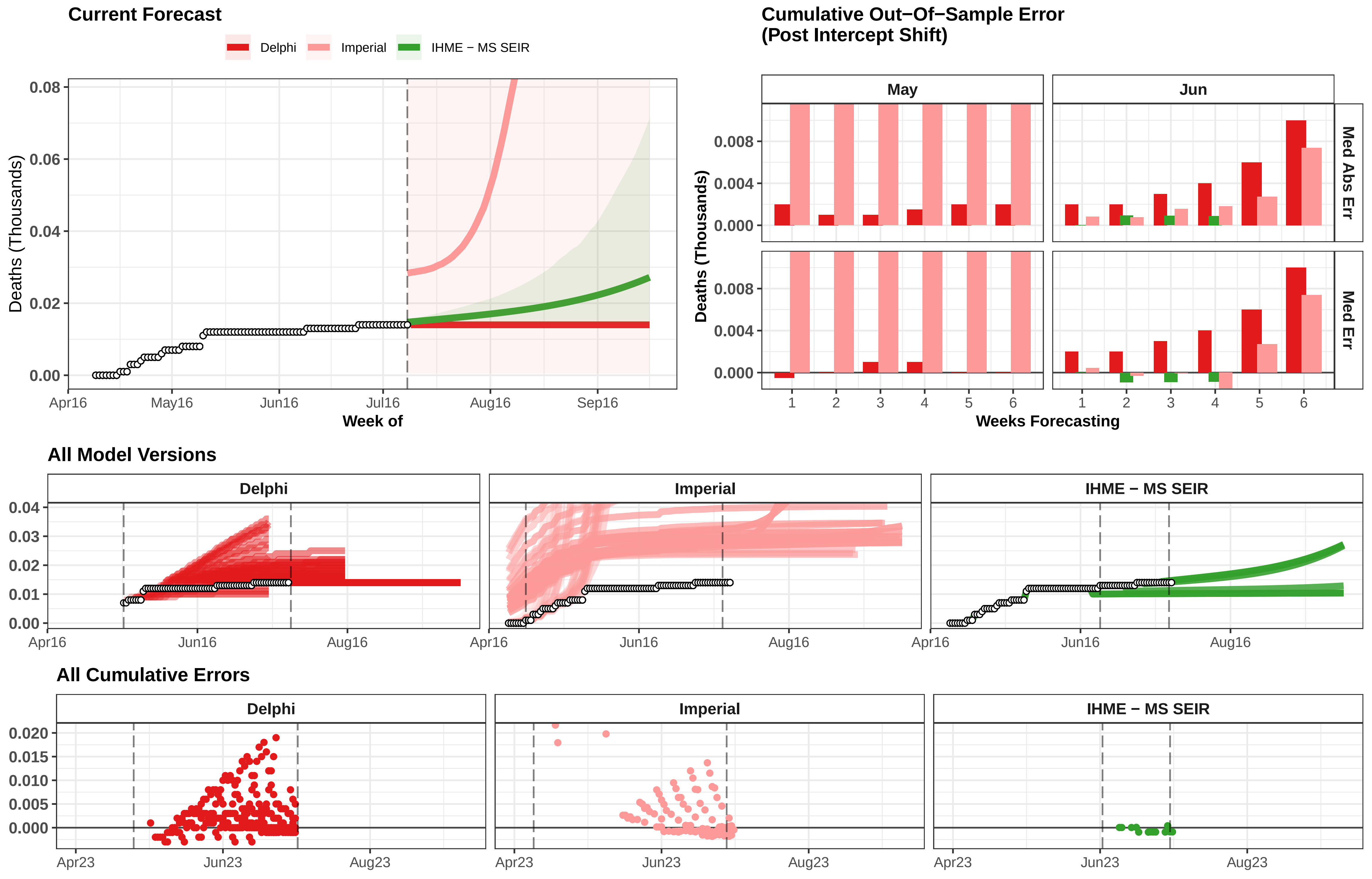

Jordan

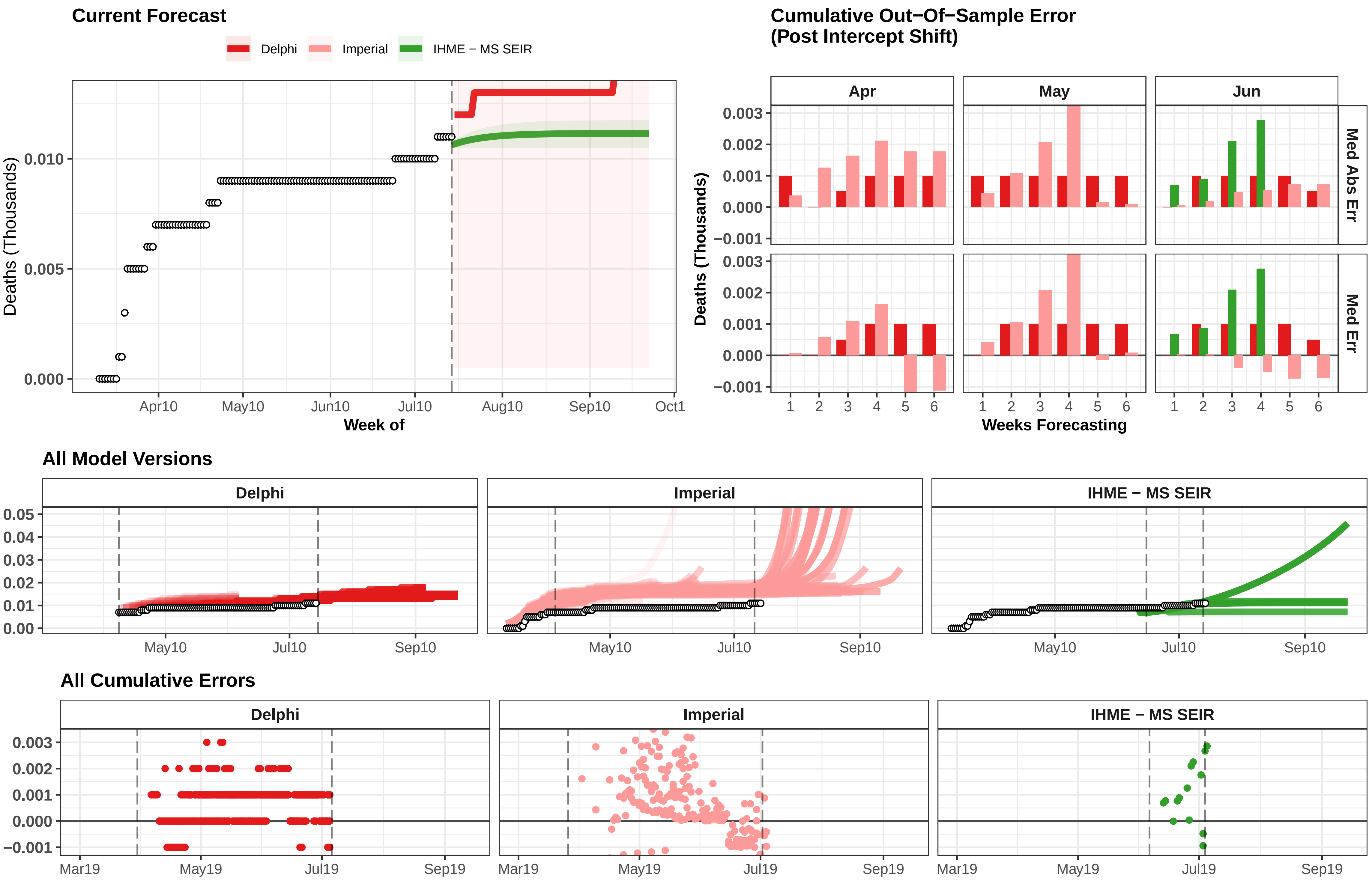
Current Forecast
Delphi
Imperial
IHME − MS SEIR
Deaths (Thousands)
0.000
0.005
0.010
Apr10
May10
Jun10
Jul10
Aug10
Sep10
Oct1
Week of
Cumulative Out−Of−Sample Error
(Post Intercept Shift)
Apr
May
Jun
Med Abs Err
Med Err
0.003
0.002
0.001
0.000
−0.001
1
2
3
4
5
6
Weeks Forecasting
All Model Versions
Delphi
Imperial
IHME − MS SEIR
0.05
0.04
0.03
0.02
0.01
0.00
May10
Jul10
Sep10
All Cumulative Errors
Mar19
May19
Jul19
Sep19

Mozambique

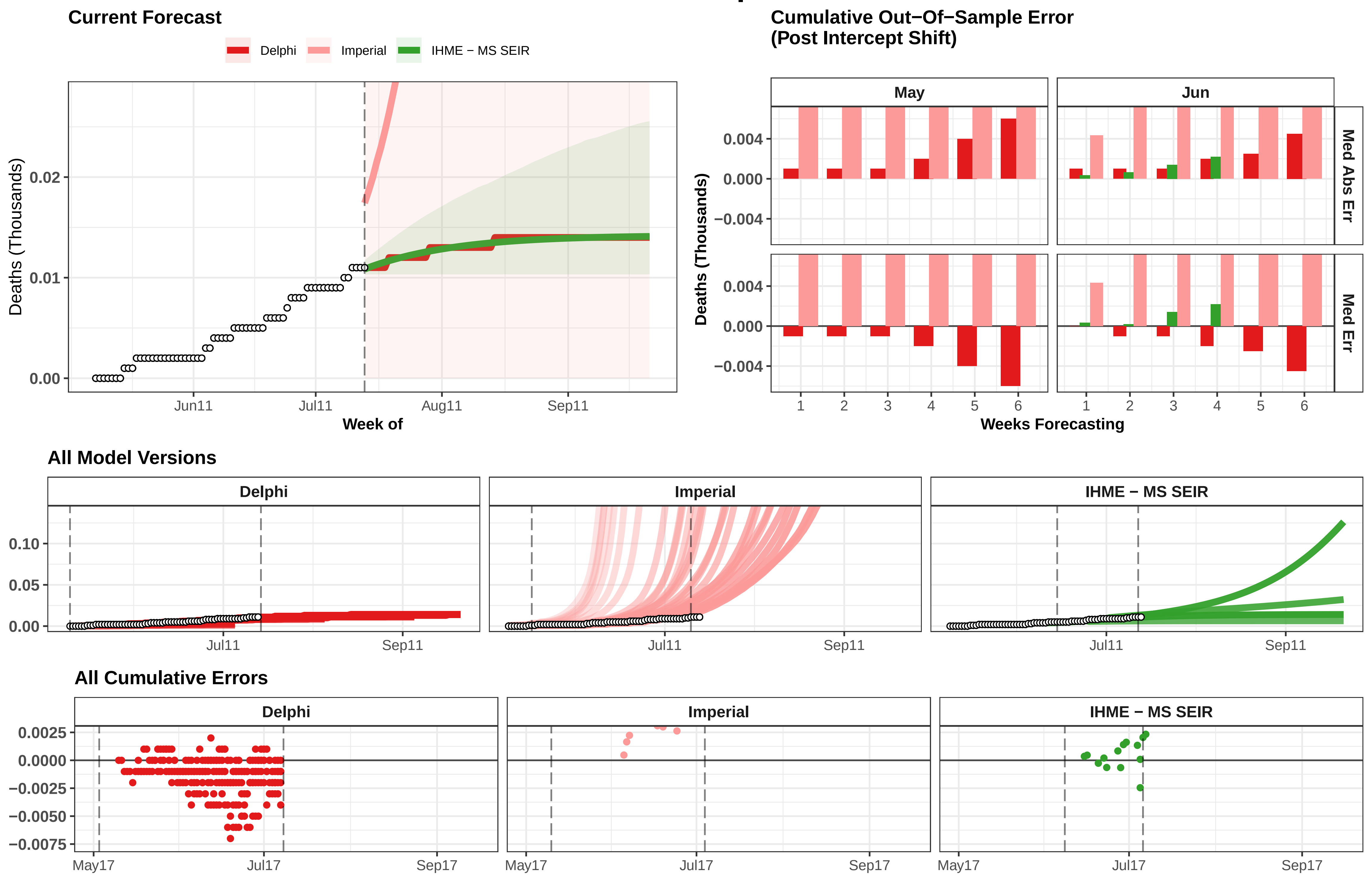
Current Forecast
Delphi
Imperial
IHME − MS SEIR
Deaths (Thousands)
0.00
0.01
0.02
Jun11
Jul11
Aug11
Sep11
Week of
Cumulative Out−Of−Sample Error
(Post Intercept Shift)
May
Jun
Med Abs Err
Med Err
0.004
0.000
−0.004
1
2
3
4
5
6
Weeks Forecasting
All Model Versions
Delphi
Imperial
IHME − MS SEIR
0.10
0.05
0.00
Jul11
Sep11
All Cumulative Errors
0.0025
0.0000
−0.0025
−0.0050
−0.0075
May17
Jul17
Sep17

Sri Lanka

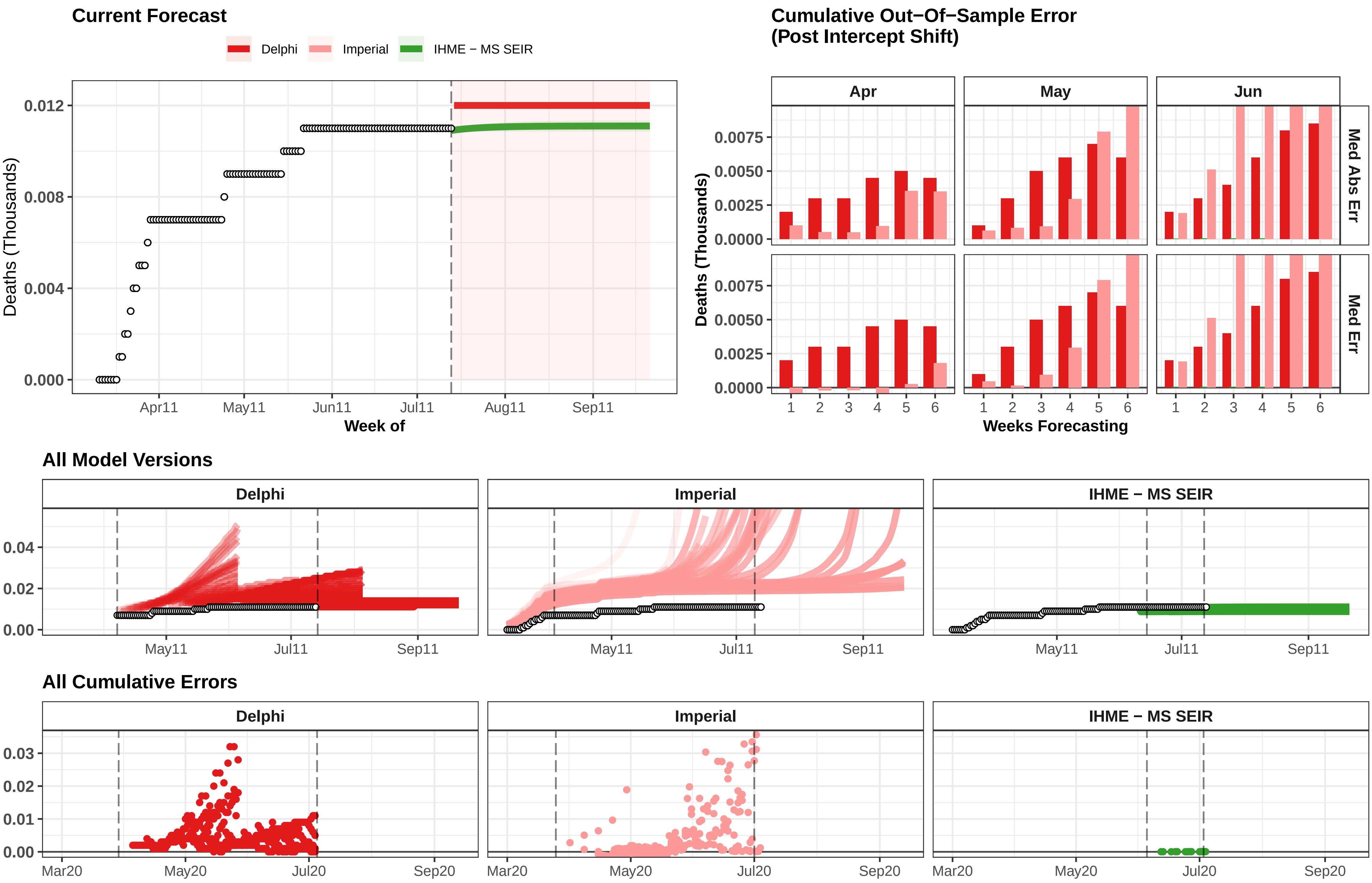

Current Forecast
Delphi
Imperial
IHME − MS SEIR
Deaths (Thousands)
0.000
0.004
0.008
0.012
Apr11
May11
Jun11
Jul11
Aug11
Sep11
Week of
Cumulative Out−Of−Sample Error
(Post Intercept Shift)
Apr
May
Jun
Med Abs Err
Med Err
0.0000
0.0025
0.0050
0.0075
1
2
3
4
5
6
Weeks Forecasting
All Model Versions
Delphi
Imperial
IHME − MS SEIR
0.00
0.02
0.04
May11
Jul11
Sep11
All Cumulative Errors
0.00
0.01
0.02
0.03
Mar20
May20
Jul20
Sep20
