# Supplementary material for: Predictive performance of international COVID-19 mortality forecasting models
Source: medRxiv. 2020 Nov 19:2020.07.13.20151233. Preprint. [Version 5] doi: 10.1101/2020.07.13.20151233 (PMC7685335; doi:10.1101/2020.07.13.20151233)
Supplement: Supplement 2020 [file 89490-2020.07.13.20151233-2.pdf]

United States

Current Forecast

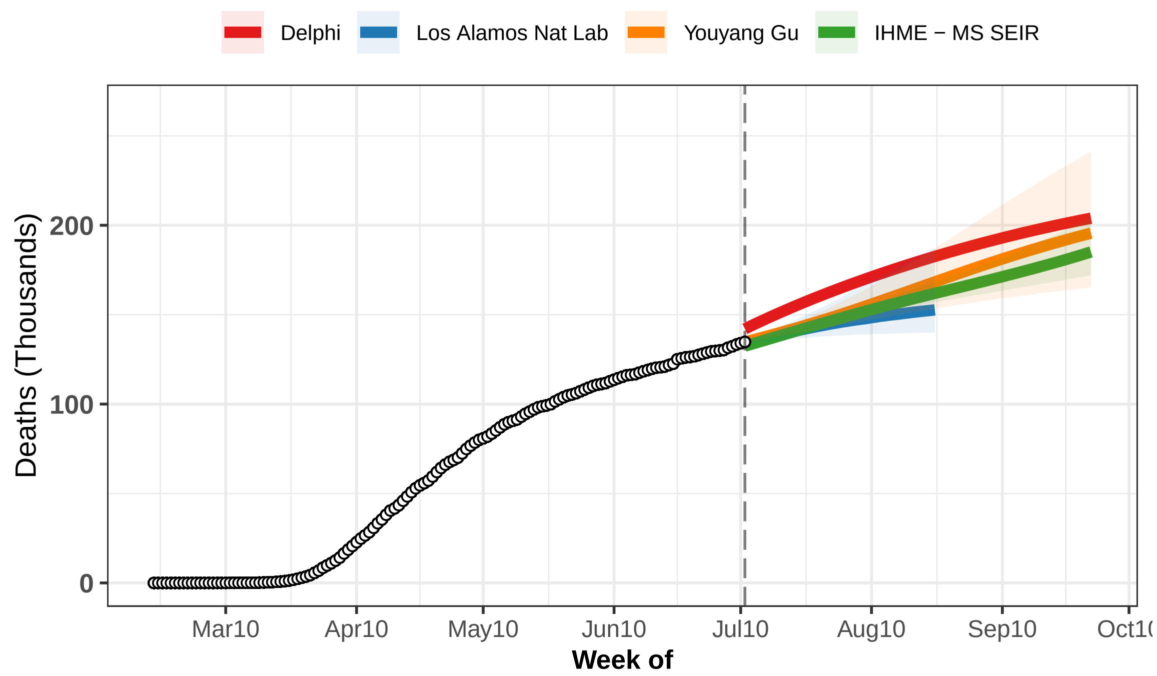

Cumulative Out−Of−Sample Error (Post Intercept Shift)

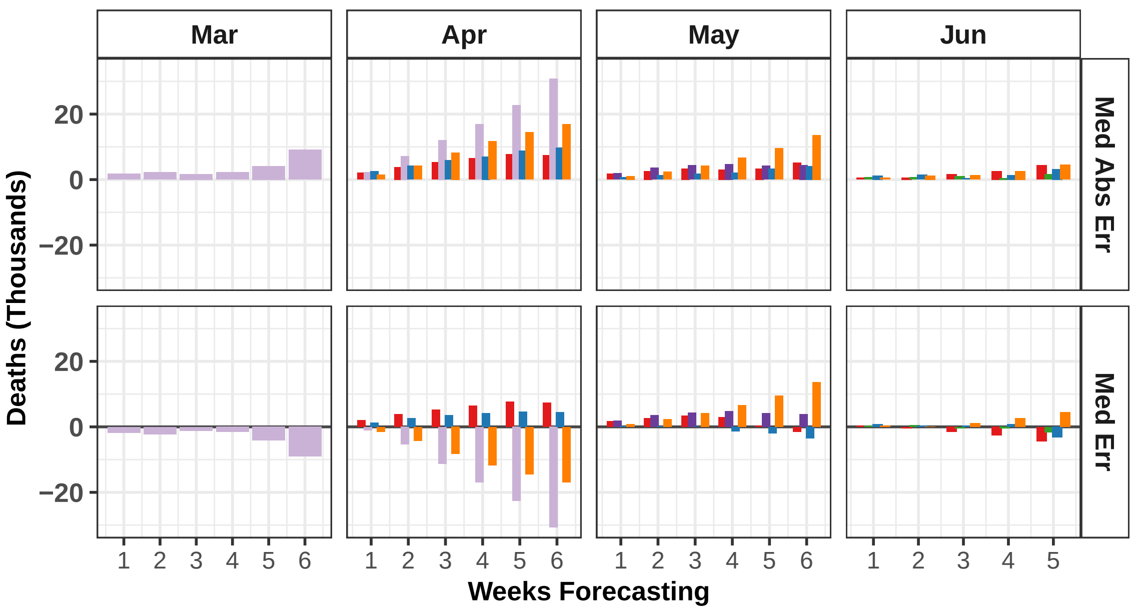

All Model Versions

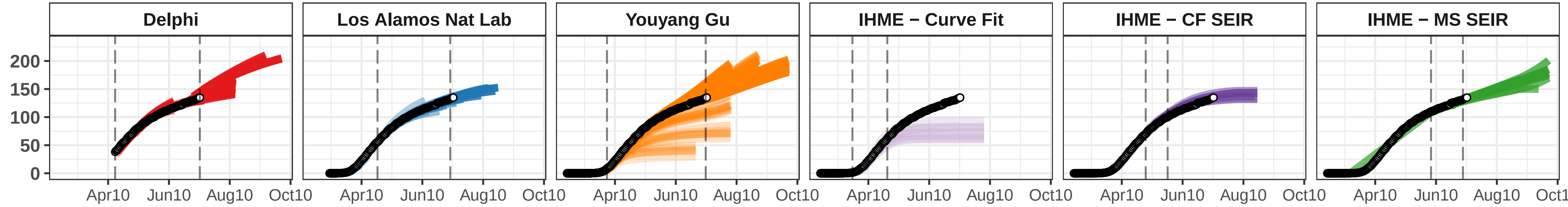

All Cumulative Errors

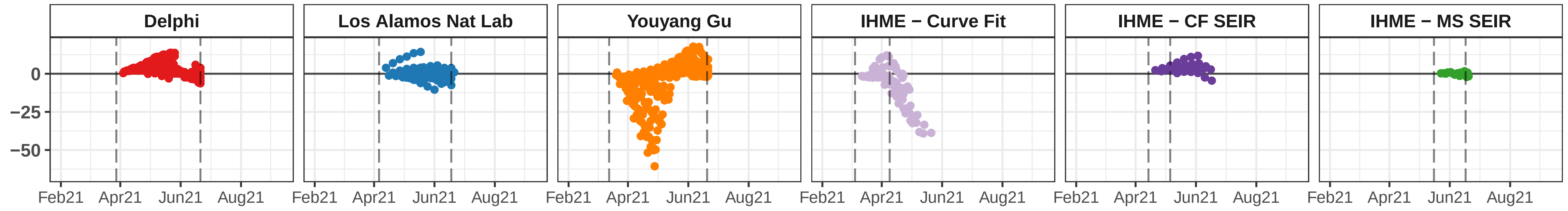

Brazil

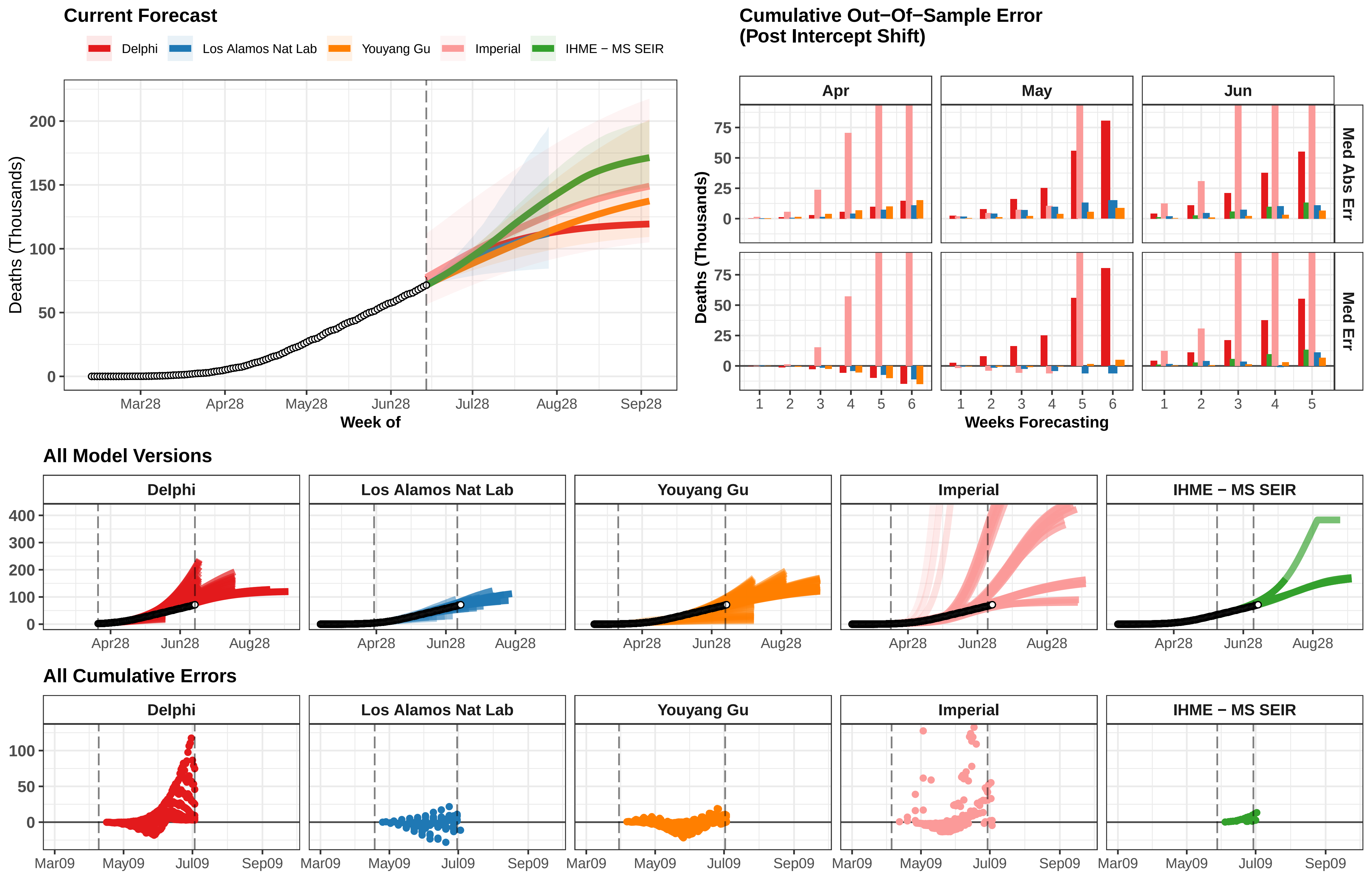

Current Forecast
Delphi
Los Alamos Nat Lab
Youyang Gu
Imperial
IHME − MS SEIR
Deaths (Thousands)
200
150
100
50
0
Mar28
Apr28
May28
Jun28
Jul28
Aug28
Sep28
Week of
Cumulative Out−Of−Sample Error
(Post Intercept Shift)
Apr
May
Jun
Med Abs Err
Med Err
75
50
25
0
1
2
3
4
5
6
Weeks Forecasting
All Model Versions
400
300
200
100
All Cumulative Errors
Mar09
May09
Jul09
Sep09

United Kingdom

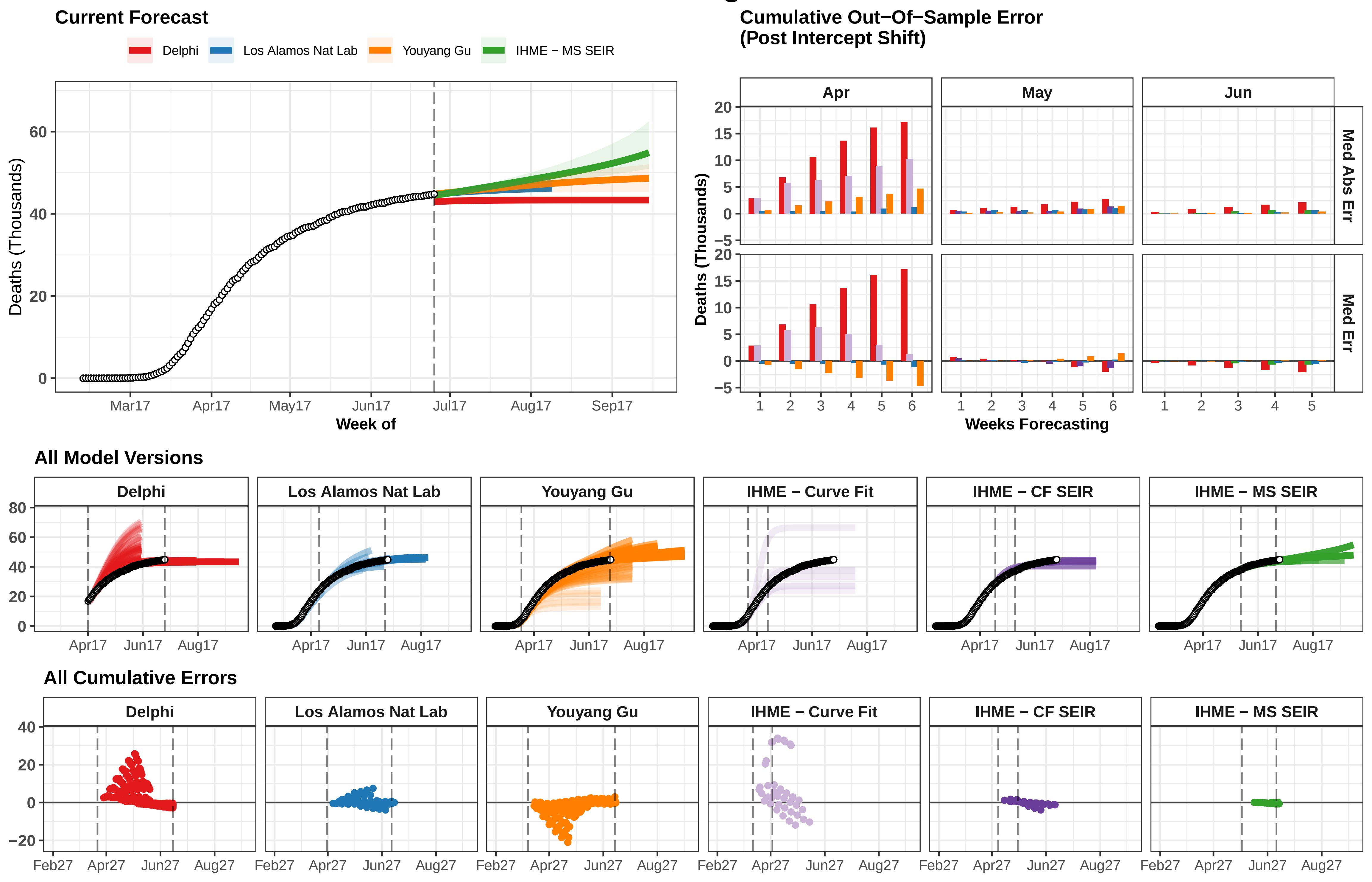

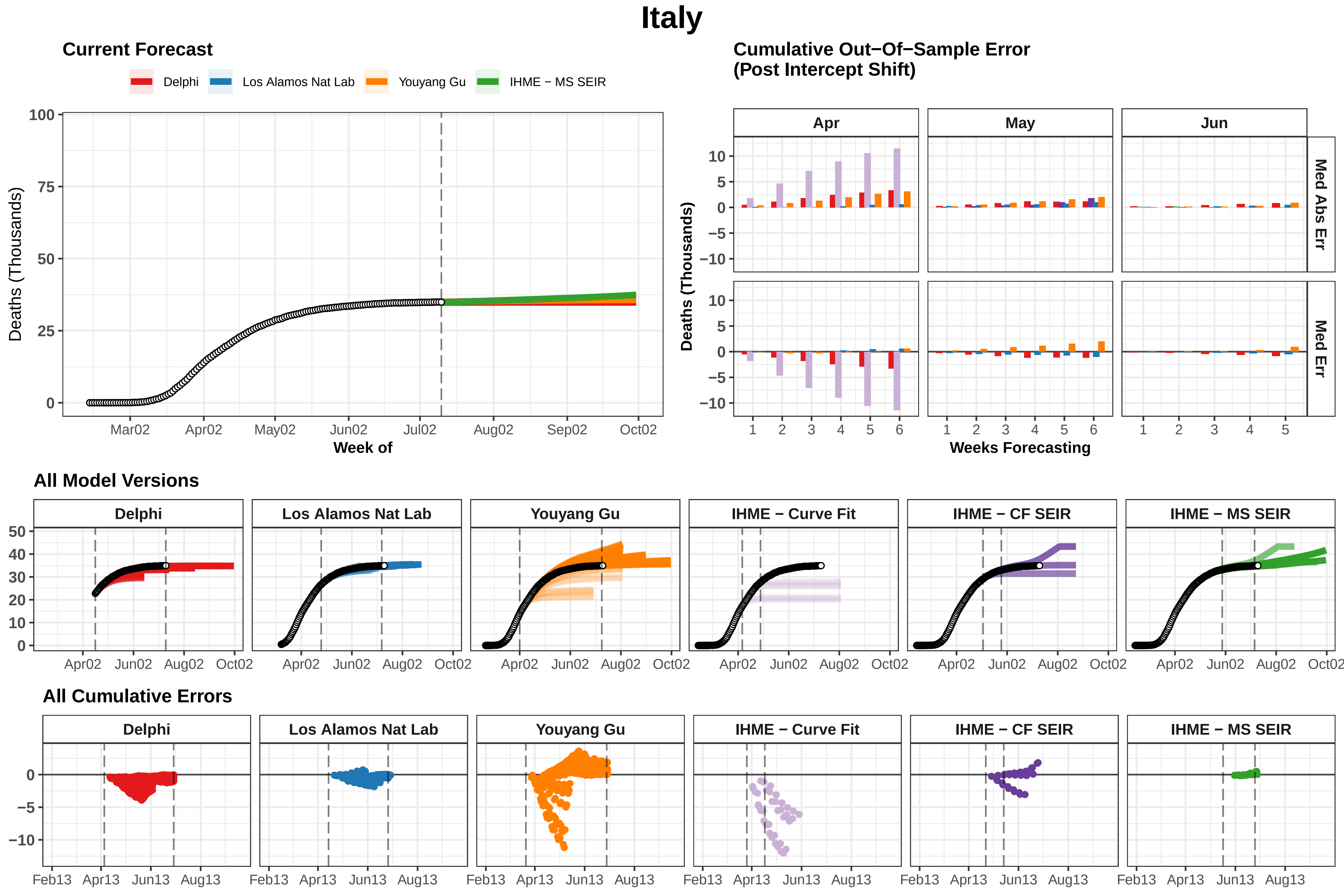
Italy
Current Forecast
Delphi
Los Alamos Nat Lab
Youyang Gu
IHME − MS SEIR
Deaths (Thousands)
100
75
50
25
0
Mar02
Apr02
May02
Jun02
Jul02
Aug02
Sep02
Oct02
Week of
Cumulative Out−Of−Sample Error
(Post Intercept Shift)
Apr
May
Jun
Med Abs Err
Med Err
Deaths (Thousands)
10
5
0
−5
−10
1
2
3
4
5
6
Weeks Forecasting
All Model Versions
Delphi
Los Alamos Nat Lab
Youyang Gu
IHME − Curve Fit
IHME − CF SEIR
IHME − MS SEIR
50
40
30
20
10
0
Apr02
Jun02
Aug02
Oct02
All Cumulative Errors
Delphi
Los Alamos Nat Lab
Youyang Gu
IHME − Curve Fit
IHME − CF SEIR
IHME − MS SEIR
0
−5
−10
Feb13
Apr13
Jun13
Aug13

Mexico

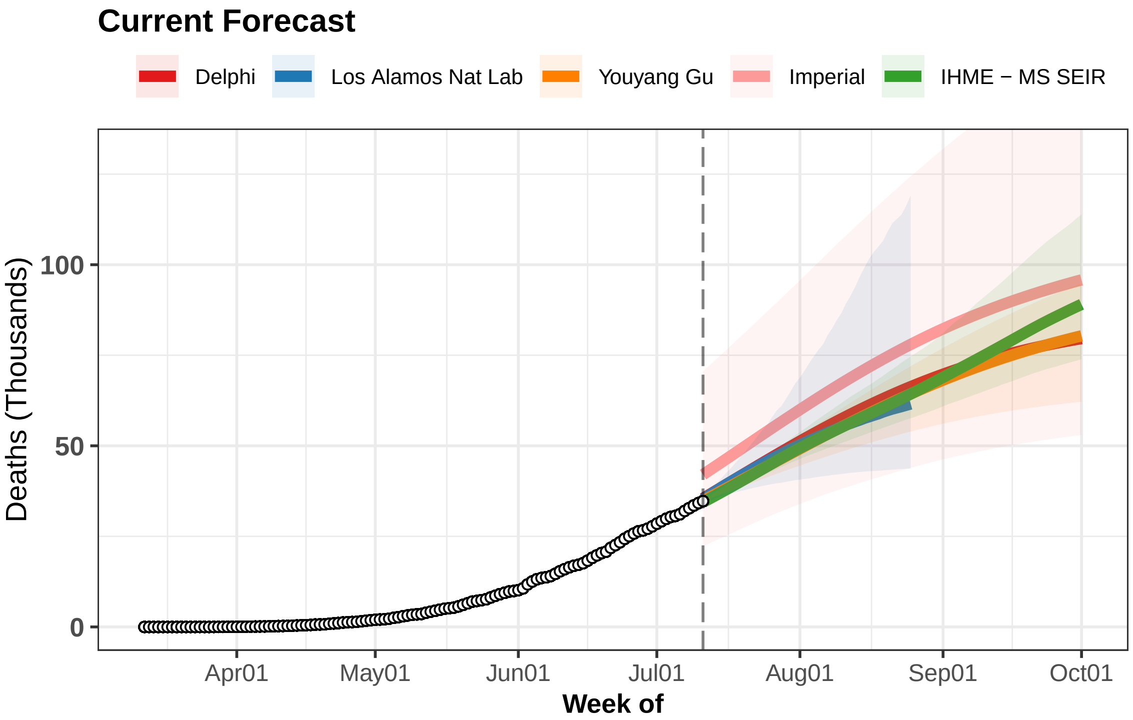
Current Forecast
Delphi
Los Alamos Nat Lab
Youyang Gu
Imperial
IHME − MS SEIR
Deaths (Thousands)
100
50
0
Apr01
May01
Jun01
Jul01
Aug01
Sep01
Oct01
Week of

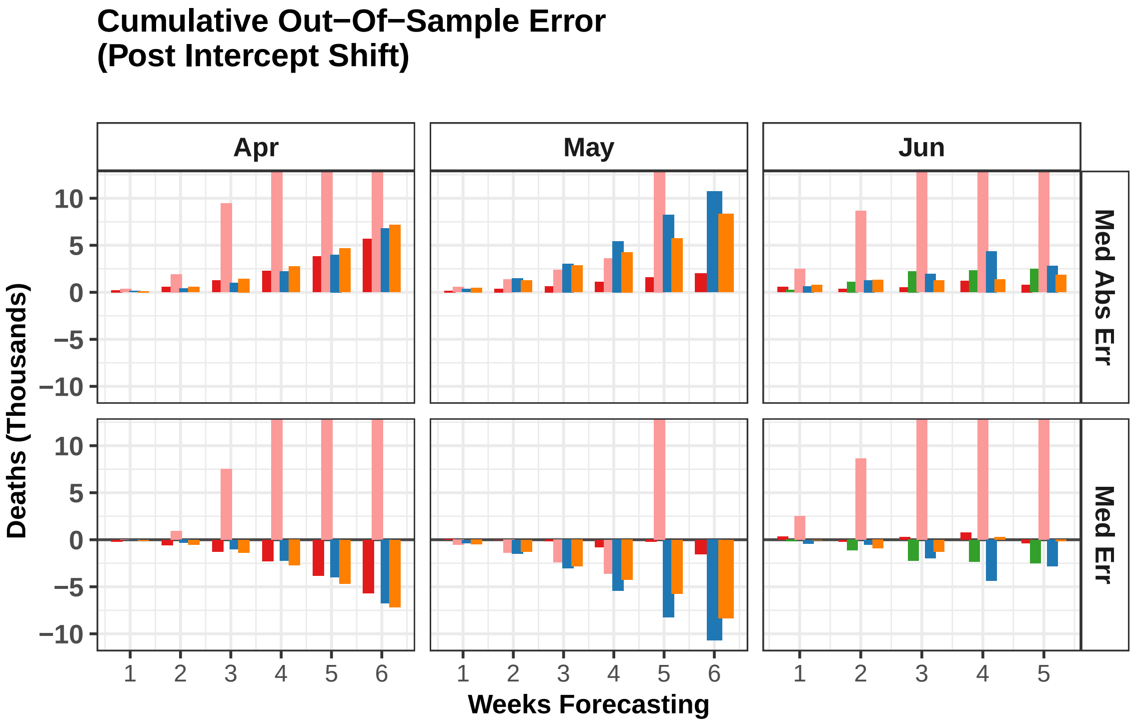
Cumulative Out−Of−Sample Error
(Post Intercept Shift)
Apr
May
Jun
Med Abs Err
Med Err
Deaths (Thousands)
10
5
0
−5
−10
1
2
3
4
5
6
Weeks Forecasting

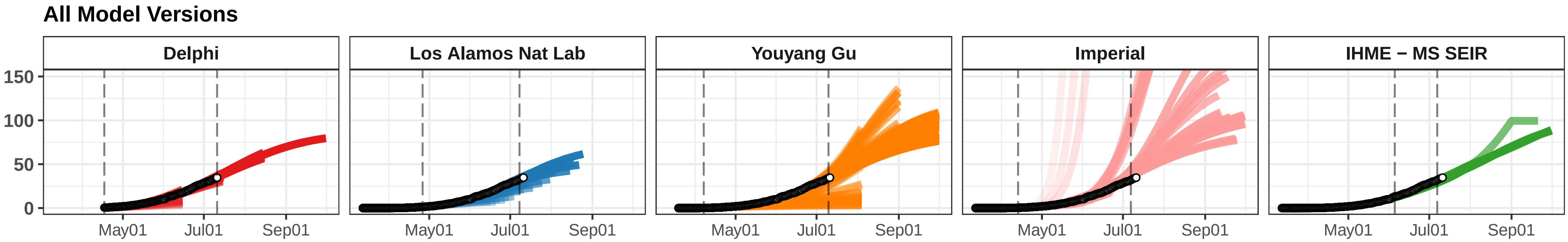
All Model Versions
Delphi
Los Alamos Nat Lab
Youyang Gu
Imperial
IHME − MS SEIR
150
100
50
0
May01
Jul01
Sep01

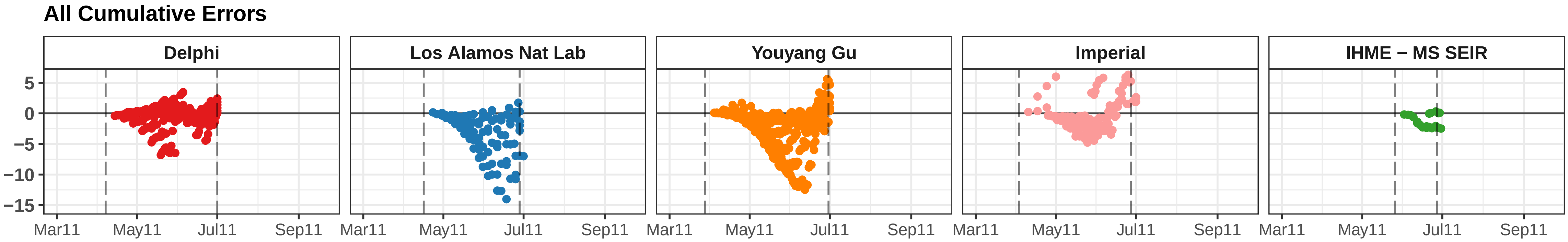
All Cumulative Errors
Delphi
Los Alamos Nat Lab
Youyang Gu
Imperial
IHME − MS SEIR
5
0
−5
−10
−15
Mar11
May11
Jul11
Sep11

New York

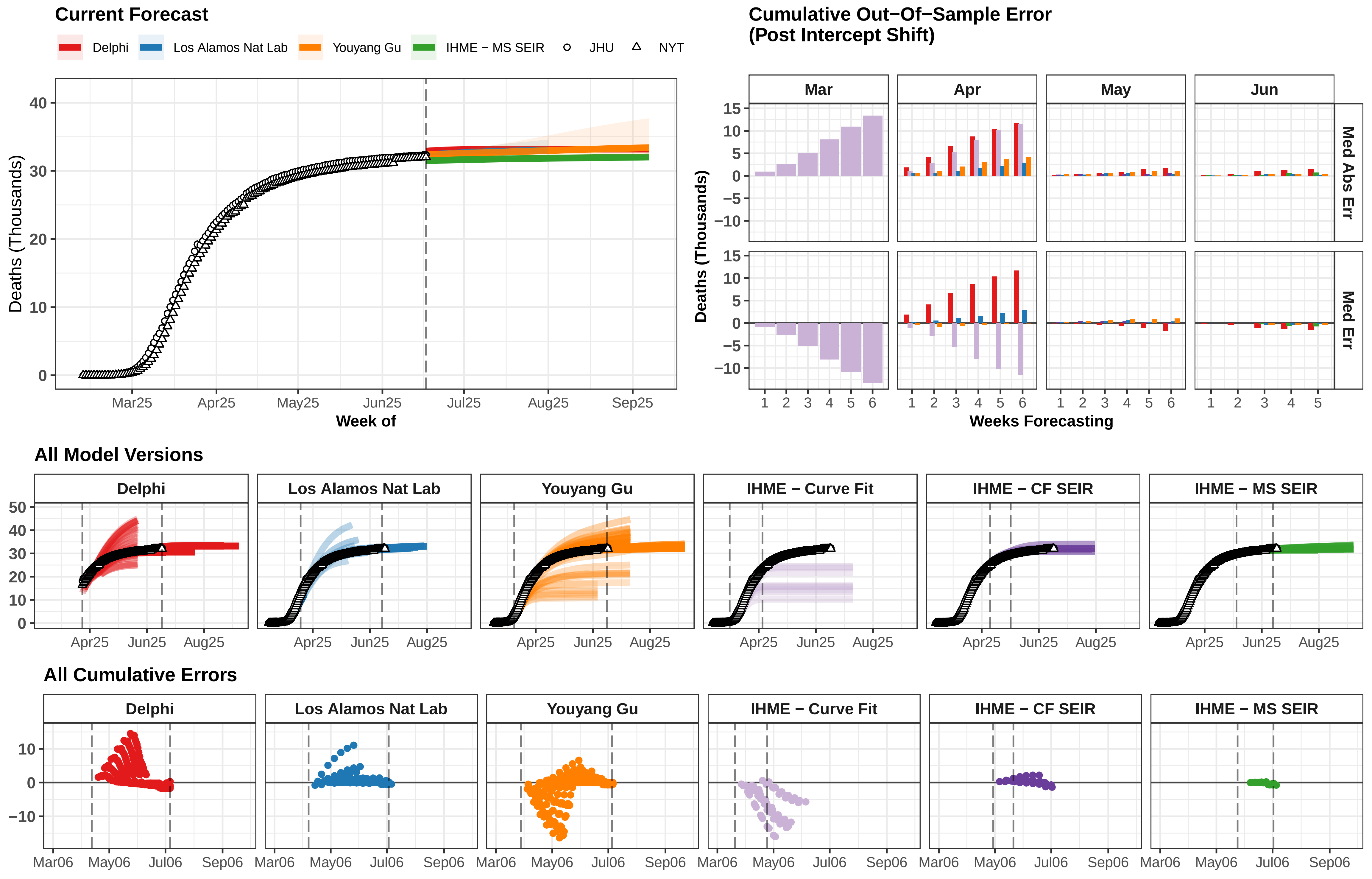

Current Forecast
Delphi
Los Alamos Nat Lab
Youyang Gu
IHME − MS SEIR
JHU
NYT
Deaths (Thousands)
Week of
Mar25
Apr25
May25
Jun25
Jul25
Aug25
Sep25
Cumulative Out−Of−Sample Error
(Post Intercept Shift)
Mar
Apr
May
Jun
Med Abs Err
Med Err
Weeks Forecasting
All Model Versions
Delphi
Los Alamos Nat Lab
Youyang Gu
IHME − Curve Fit
IHME − CF SEIR
IHME − MS SEIR
All Cumulative Errors
Mar06
May06
Jul06
Sep06

France

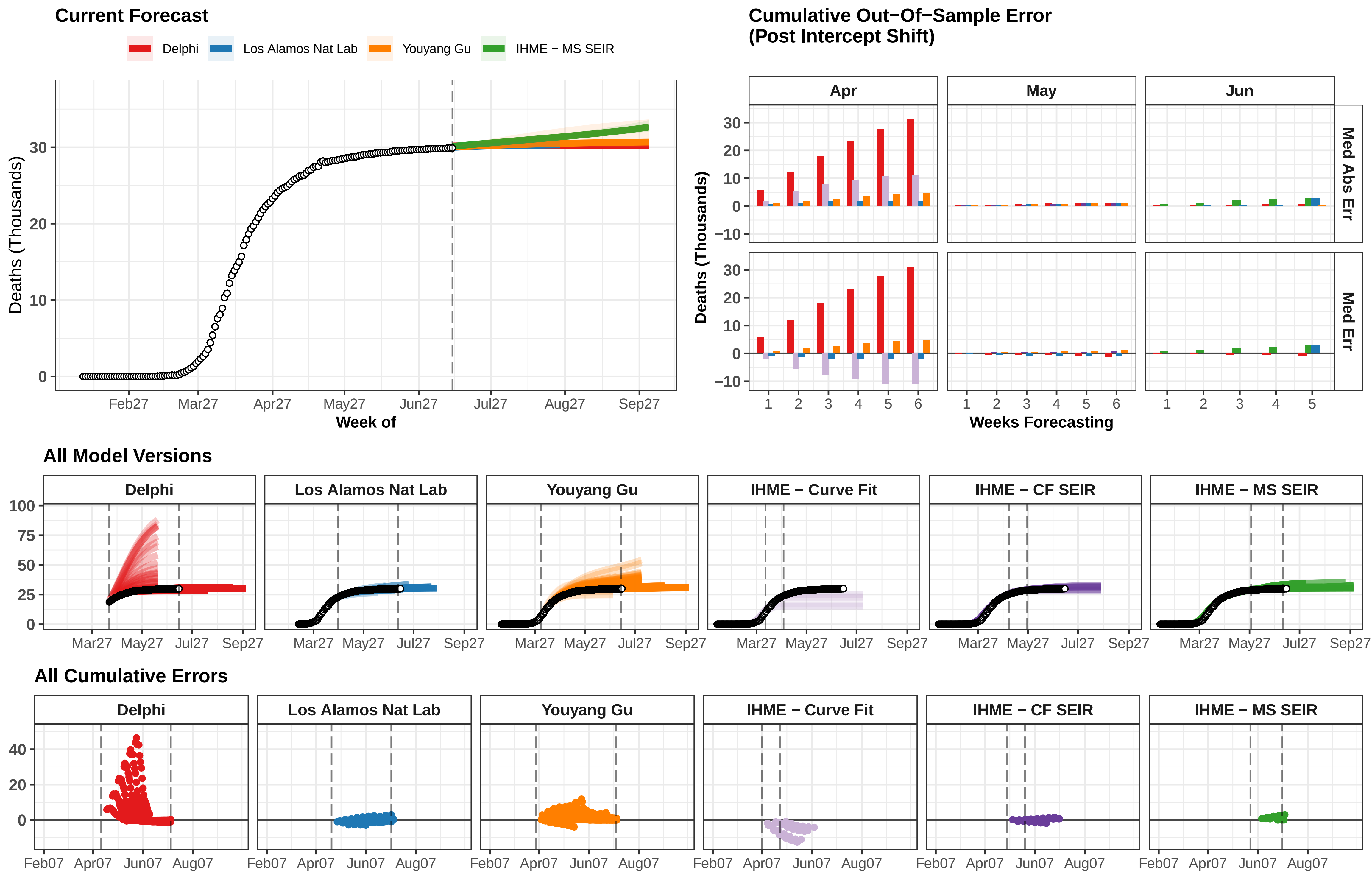

Current Forecast
Delphi
Los Alamos Nat Lab
Youyang Gu
IHME − MS SEIR
Deaths (Thousands)
Week of
Feb27
Mar27
Apr27
May27
Jun27
Jul27
Aug27
Sep27
Cumulative Out−Of−Sample Error
(Post Intercept Shift)
Apr
May
Jun
Med Abs Err
Med Err
Weeks Forecasting
All Model Versions
Delphi
Los Alamos Nat Lab
Youyang Gu
IHME − Curve Fit
IHME − CF SEIR
IHME − MS SEIR
All Cumulative Errors
Feb07
Apr07
Jun07
Aug07

Spain

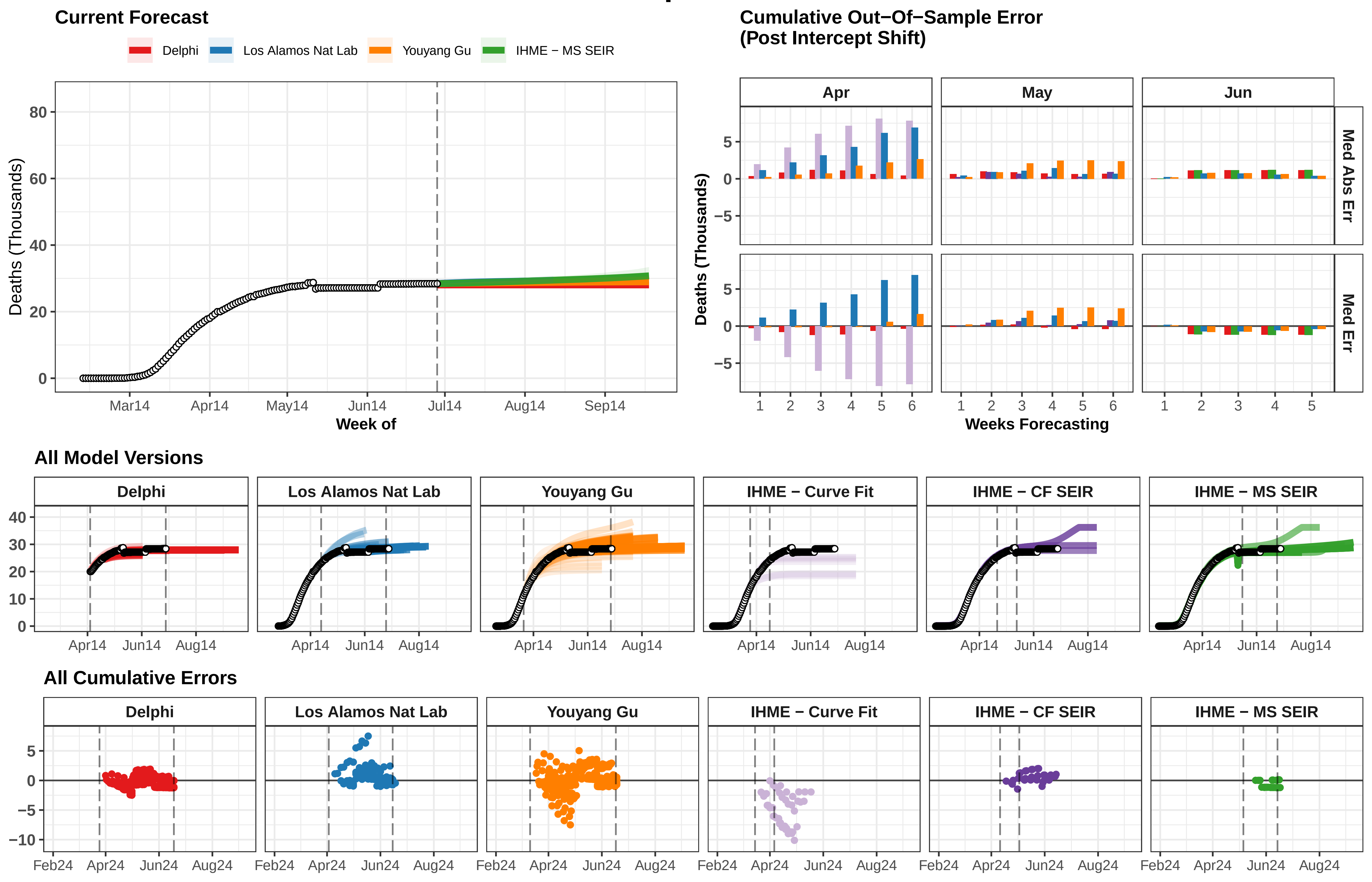

Current Forecast
Delphi
Los Alamos Nat Lab
Youyang Gu
IHME − MS SEIR
Deaths (Thousands)
80
60
40
20
0
Mar14
Apr14
May14
Jun14
Jul14
Aug14
Sep14
Week of
Cumulative Out−Of−Sample Error
(Post Intercept Shift)
Apr
May
Jun
Med Abs Err
Med Err
5
0
−5
1
2
3
4
5
6
Weeks Forecasting
All Model Versions
Delphi
Los Alamos Nat Lab
Youyang Gu
IHME − Curve Fit
IHME − CF SEIR
IHME − MS SEIR
40
30
20
10
0
Apr14
Jun14
Aug14
All Cumulative Errors
−10
Feb24
Apr24
Jun24
Aug24

India

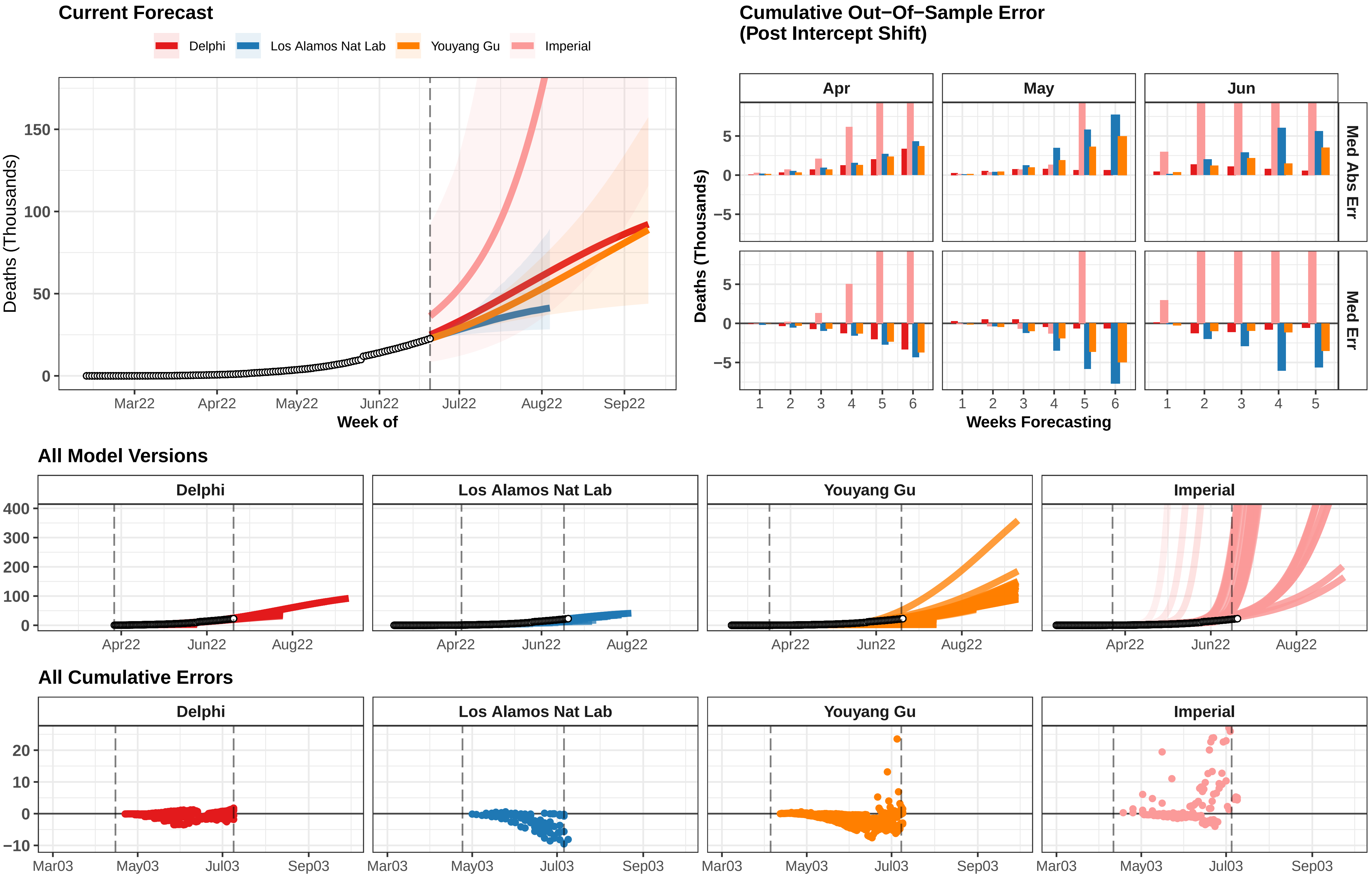
Current Forecast
Delphi
Los Alamos Nat Lab
Youyang Gu
Imperial
Deaths (Thousands)
0
50
100
150
Mar22
Apr22
May22
Jun22
Jul22
Aug22
Sep22
Week of
Cumulative Out−Of−Sample Error
(Post Intercept Shift)
Apr
May
Jun
Med Abs Err
Med Err
Deaths (Thousands)
−5
0
5
1
2
3
4
5
6
Weeks Forecasting
All Model Versions
Delphi
Los Alamos Nat Lab
Youyang Gu
Imperial
0
100
200
300
400
Apr22
Jun22
Aug22
All Cumulative Errors
Delphi
Los Alamos Nat Lab
Youyang Gu
Imperial
−10
0
10
20
Mar03
May03
Jul03
Sep03

New Jersey

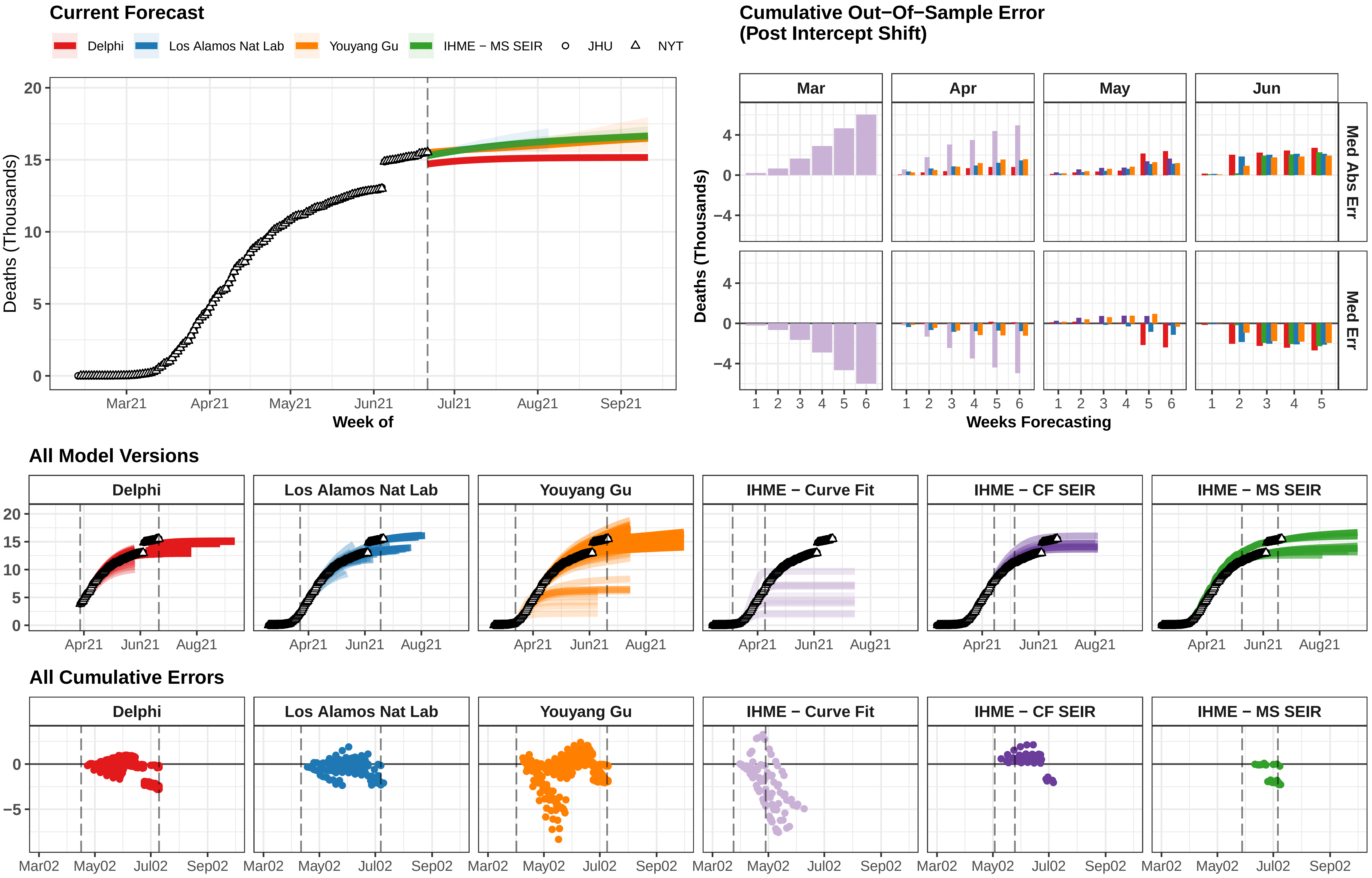

Current Forecast
Delphi
Los Alamos Nat Lab
Youyang Gu
IHME − MS SEIR
JHU
NYT
Deaths (Thousands)
Week of
Mar21
Apr21
May21
Jun21
Jul21
Aug21
Sep21
Cumulative Out−Of−Sample Error
(Post Intercept Shift)
Mar
Apr
May
Jun
Med Abs Err
Med Err
Weeks Forecasting
All Model Versions
Delphi
Los Alamos Nat Lab
Youyang Gu
IHME − Curve Fit
IHME − CF SEIR
IHME − MS SEIR
All Cumulative Errors
Mar02
May02
Jul02
Sep02

Iran

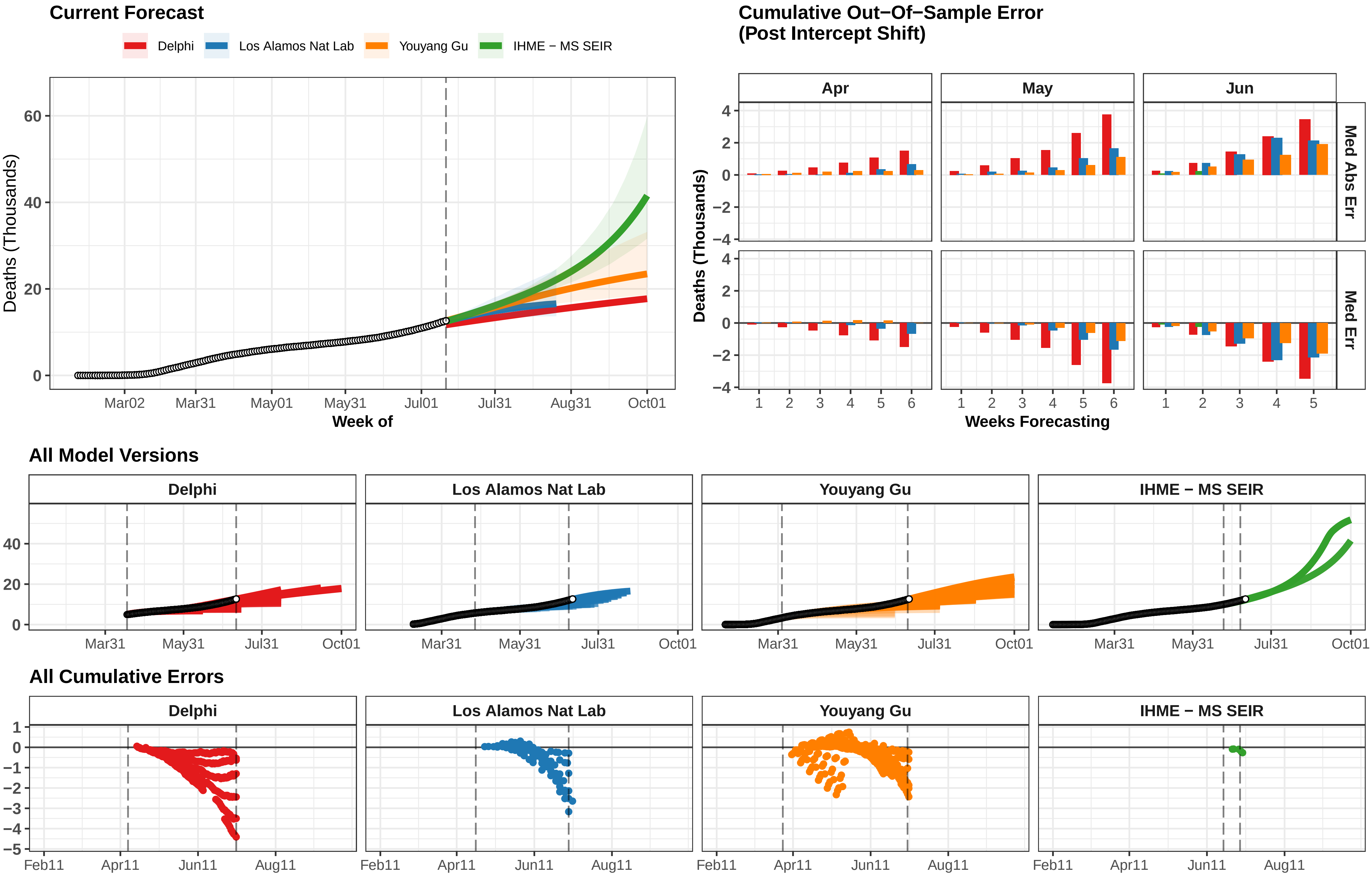
Current Forecast
Delphi
Los Alamos Nat Lab
Youyang Gu
IHME − MS SEIR
Deaths (Thousands)
60
40
20
0
Mar02
Mar31
May01
May31
Jul01
Jul31
Aug31
Oct01
Week of
Cumulative Out−Of−Sample Error
(Post Intercept Shift)
Apr
May
Jun
Med Abs Err
Med Err
Deaths (Thousands)
4
2
0
−2
−4
1
2
3
4
5
6
Weeks Forecasting
All Model Versions
Delphi
Los Alamos Nat Lab
Youyang Gu
IHME − MS SEIR
40
20
0
Mar31
May31
Jul31
Oct01
All Cumulative Errors
Delphi
Los Alamos Nat Lab
Youyang Gu
IHME − MS SEIR
1
0
−1
−2
−3
−4
−5
Feb11
Apr11
Jun11
Aug11

Peru

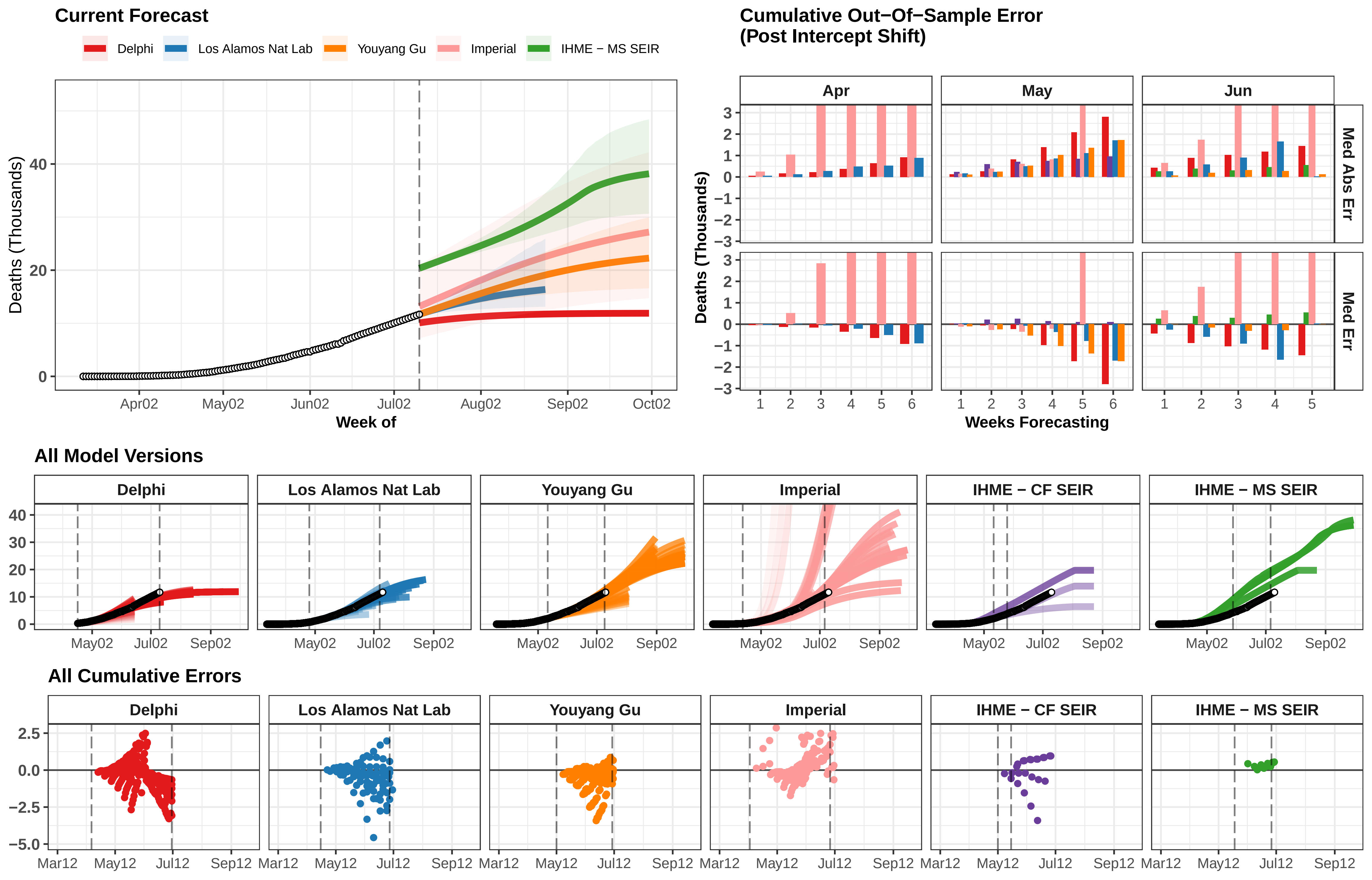
Current Forecast
Delphi
Los Alamos Nat Lab
Youyang Gu
Imperial
IHME − MS SEIR
Deaths (Thousands)
Week of
Apr02
May02
Jun02
Jul02
Aug02
Sep02
Oct02
Cumulative Out−Of−Sample Error
(Post Intercept Shift)
Apr
May
Jun
Med Abs Err
Med Err
Weeks Forecasting
All Model Versions
IHME − CF SEIR
All Cumulative Errors
Mar12
May12
Jul12
Sep12

Russian Federation

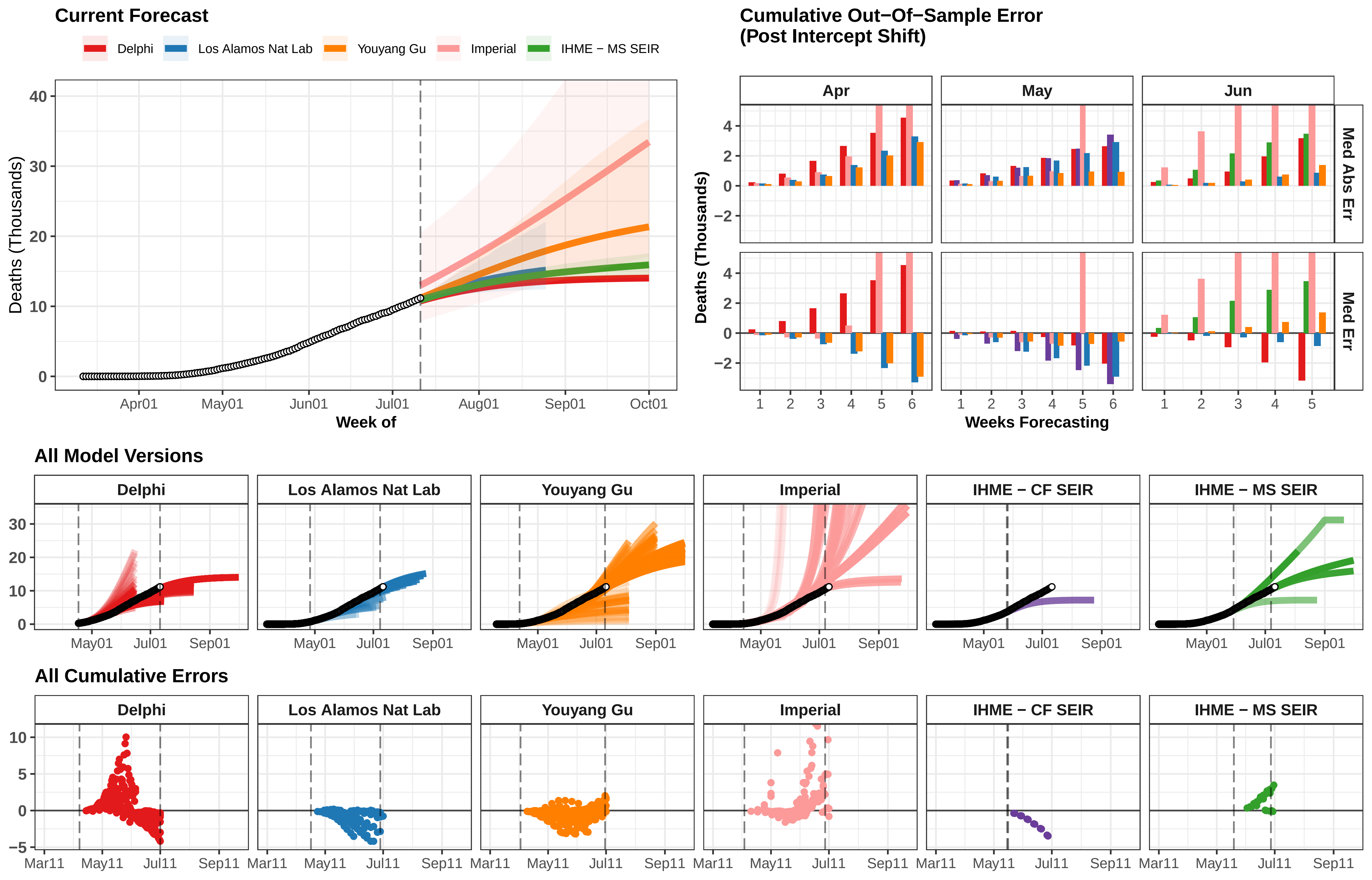

Belgium

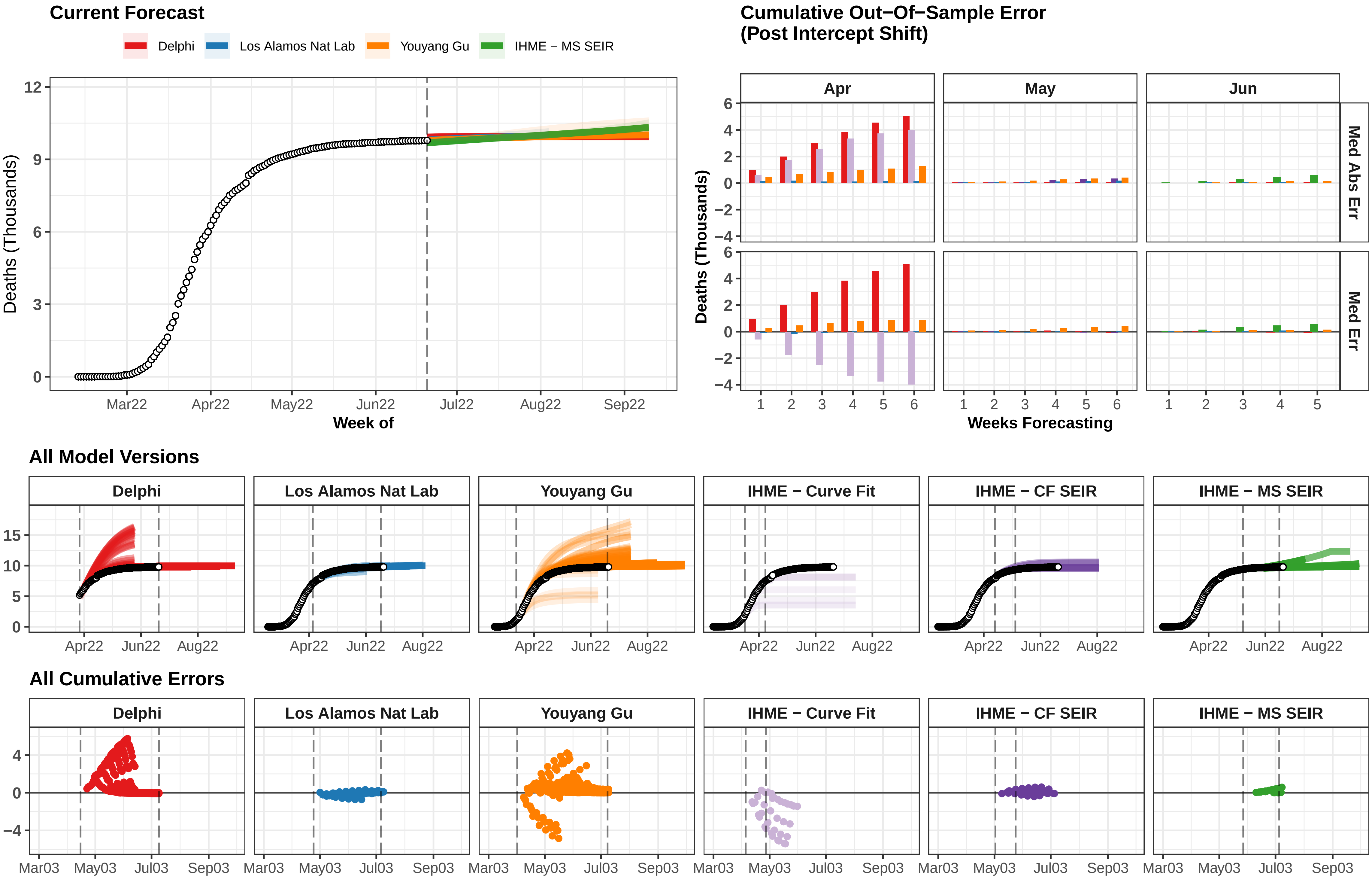

Current Forecast
Delphi
Los Alamos Nat Lab
Youyang Gu
IHME − MS SEIR
Deaths (Thousands)
Week of
Mar22 Apr22 May22 Jun22 Jul22 Aug22 Sep22
Cumulative Out−Of−Sample Error (Post Intercept Shift)
Apr
May
Jun
Med Abs Err
Med Err
Weeks Forecasting
All Model Versions
Delphi
Los Alamos Nat Lab
Youyang Gu
IHME − Curve Fit
IHME − CF SEIR
IHME − MS SEIR
All Cumulative Errors
Mar03 May03 Jul03 Sep03

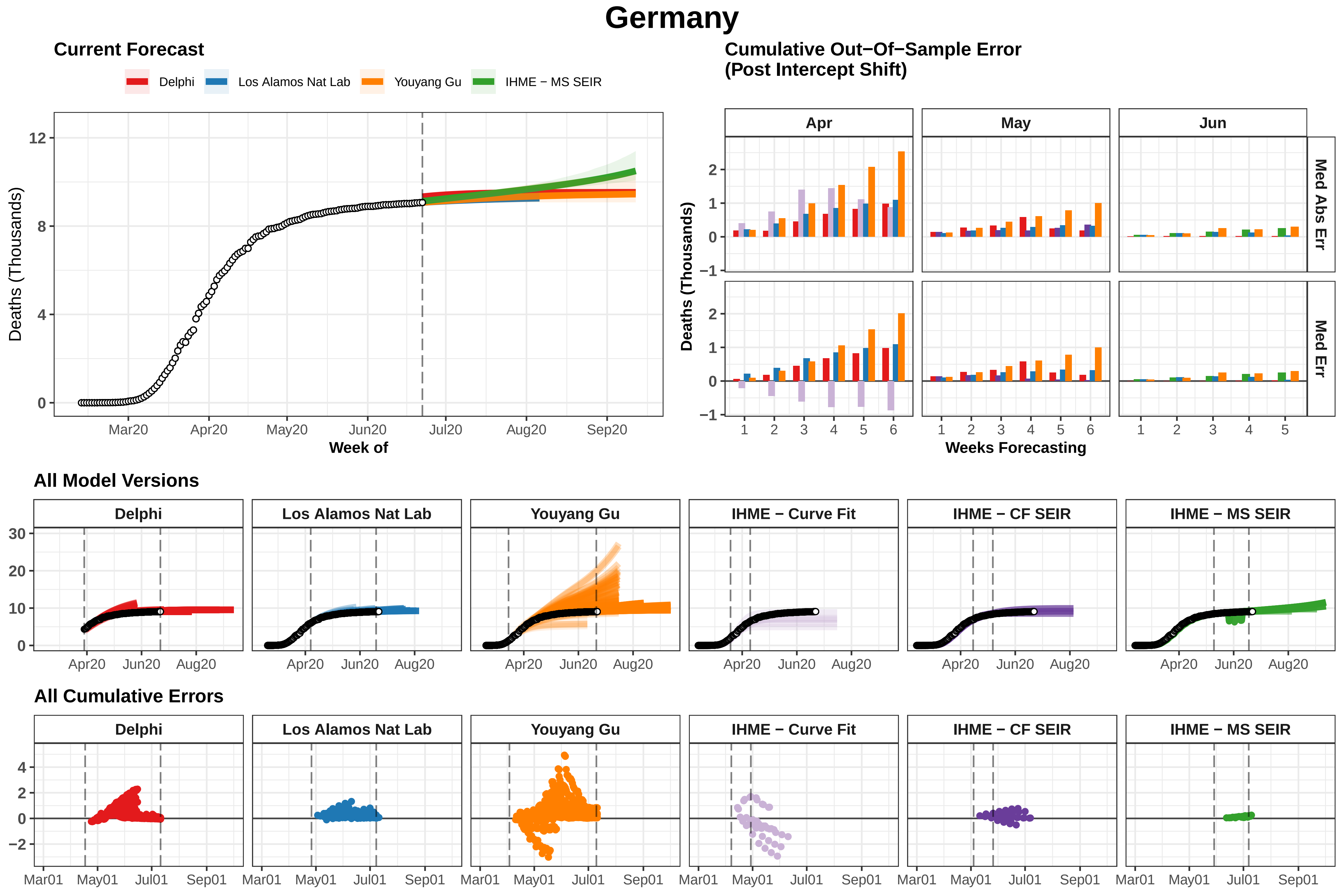
Germany
Current Forecast
Delphi
Los Alamos Nat Lab
Youyang Gu
IHME − MS SEIR
Deaths (Thousands)
0
4
8
12
Mar20
Apr20
May20
Jun20
Jul20
Aug20
Sep20
Week of
Cumulative Out−Of−Sample Error
(Post Intercept Shift)
Apr
May
Jun
Med Abs Err
Med Err
Deaths (Thousands)
−1
0
1
2
1
2
3
4
5
6
Weeks Forecasting
All Model Versions
Delphi
Los Alamos Nat Lab
Youyang Gu
IHME − Curve Fit
IHME − CF SEIR
IHME − MS SEIR
0
10
20
30
Apr20
Jun20
Aug20
All Cumulative Errors
Delphi
Los Alamos Nat Lab
Youyang Gu
IHME − Curve Fit
IHME − CF SEIR
IHME − MS SEIR
−2
0
2
4
Mar01
May01
Jul01
Sep01

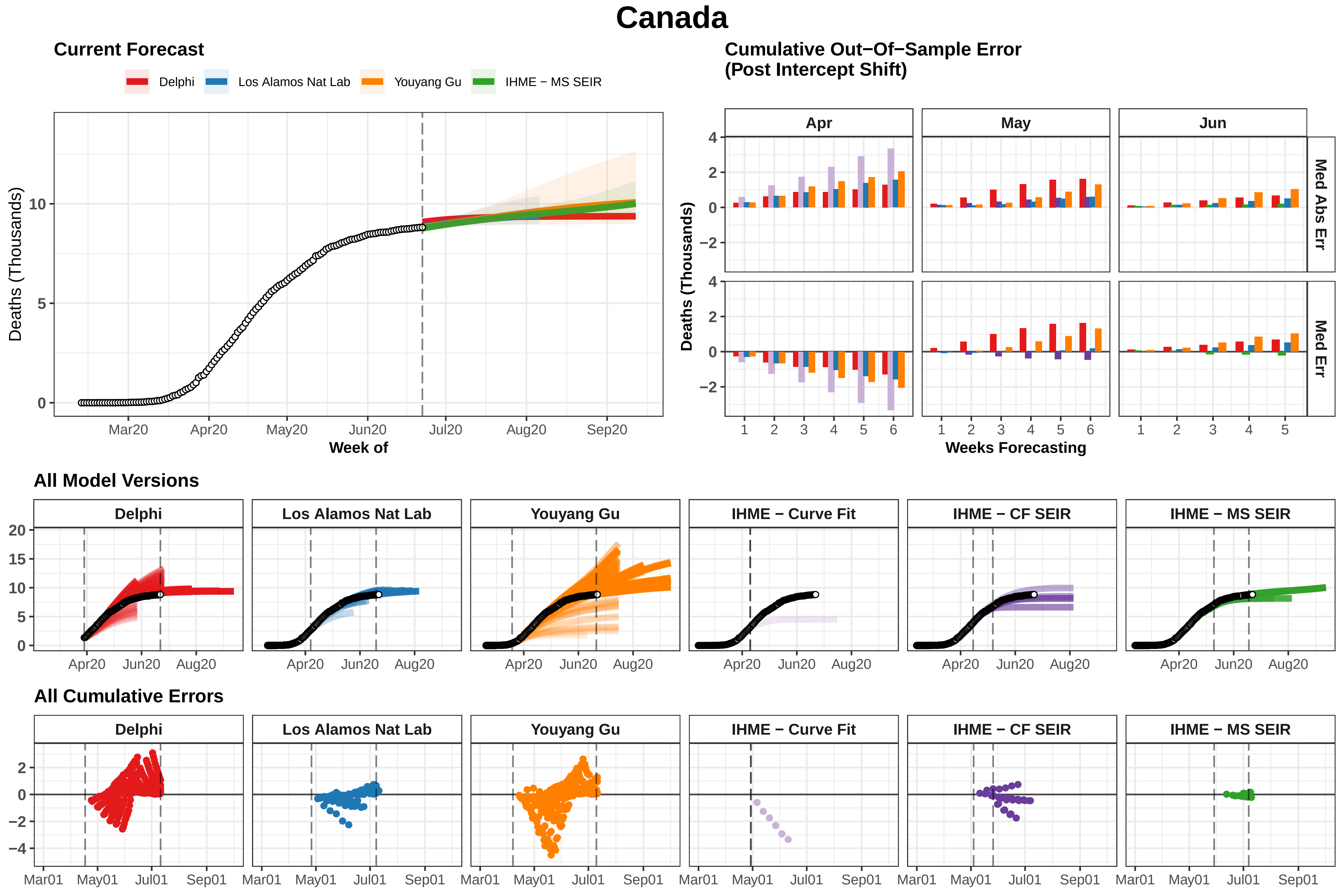

Canada
Current Forecast
Delphi
Los Alamos Nat Lab
Youyang Gu
IHME − MS SEIR
Deaths (Thousands)
Week of
Mar20
Apr20
May20
Jun20
Jul20
Aug20
Sep20
Cumulative Out−Of−Sample Error
(Post Intercept Shift)
Apr
May
Jun
Med Abs Err
Med Err
Weeks Forecasting
All Model Versions
IHME − Curve Fit
IHME − CF SEIR
All Cumulative Errors
Mar01
May01
Jul01
Sep01

Massachusetts

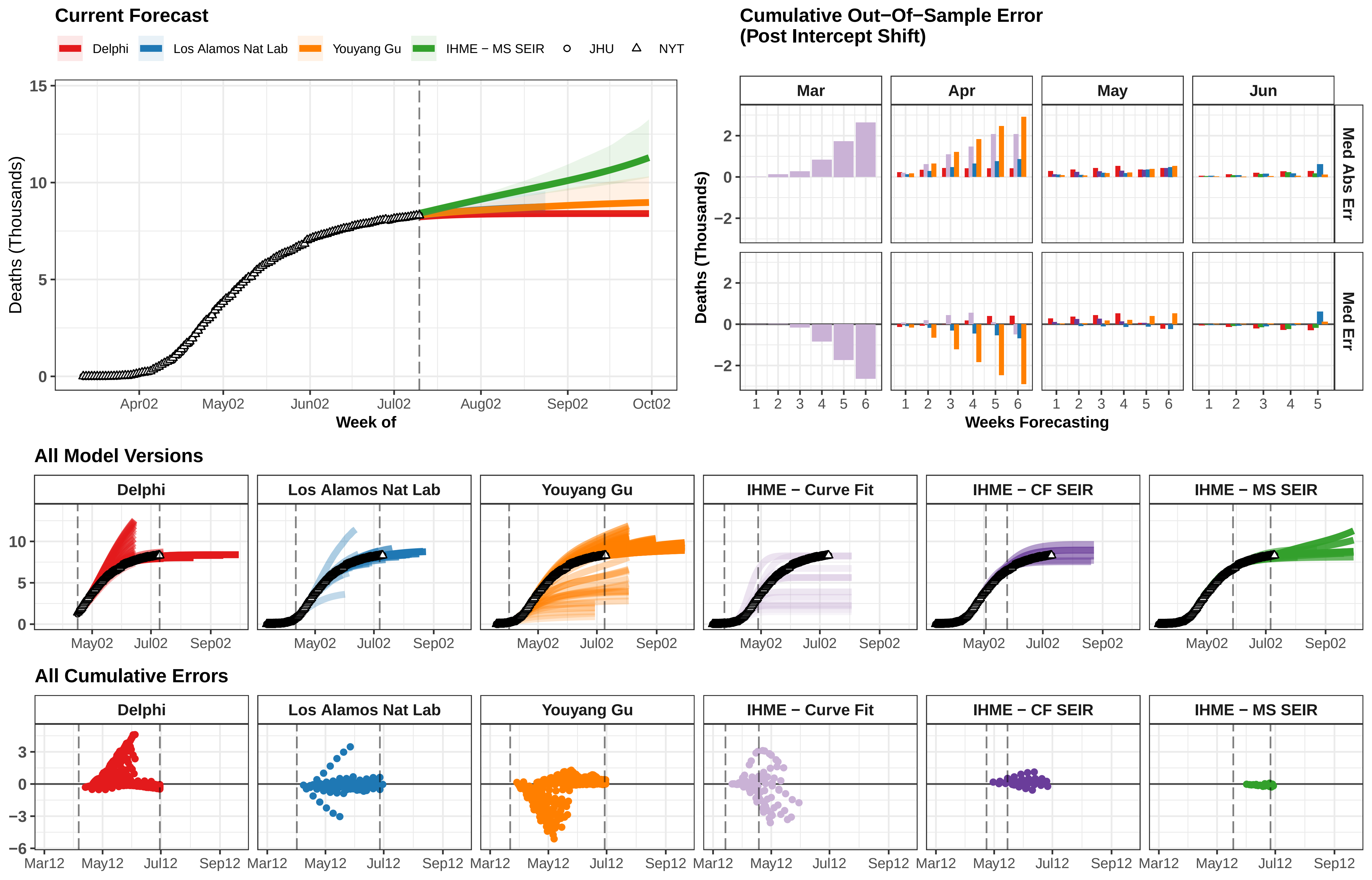

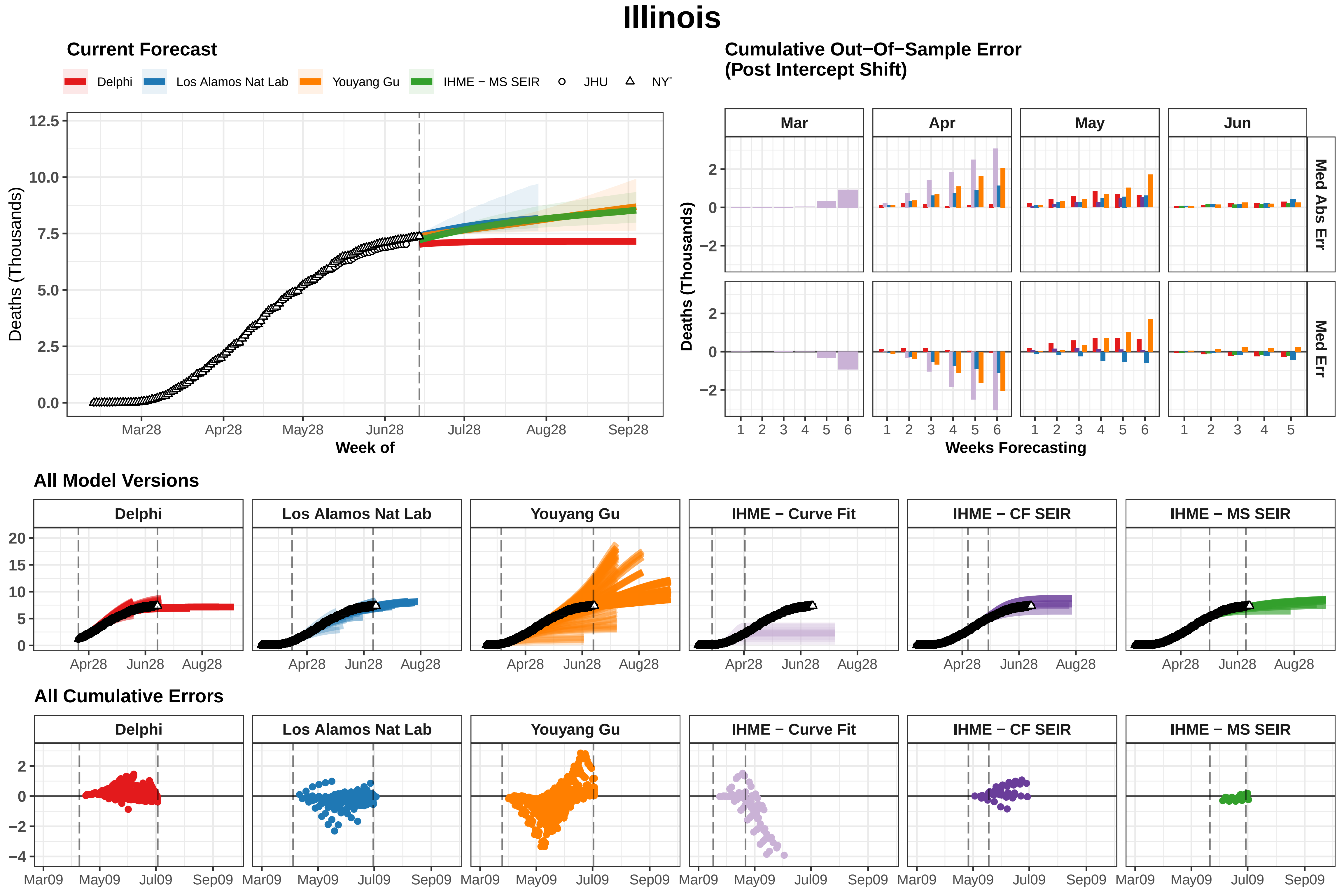

Illinois
Current Forecast
Delphi
Los Alamos Nat Lab
Youyang Gu
IHME − MS SEIR
JHU
NY
Deaths (Thousands)
Week of
Mar28
Apr28
May28
Jun28
Jul28
Aug28
Sep28
Cumulative Out−Of−Sample Error
(Post Intercept Shift)
Mar
Apr
May
Jun
Med Abs Err
Med Err
Weeks Forecasting
All Model Versions
Delphi
Los Alamos Nat Lab
Youyang Gu
IHME − Curve Fit
IHME − CF SEIR
IHME − MS SEIR
All Cumulative Errors
Mar09
May09
Jul09
Sep09

California

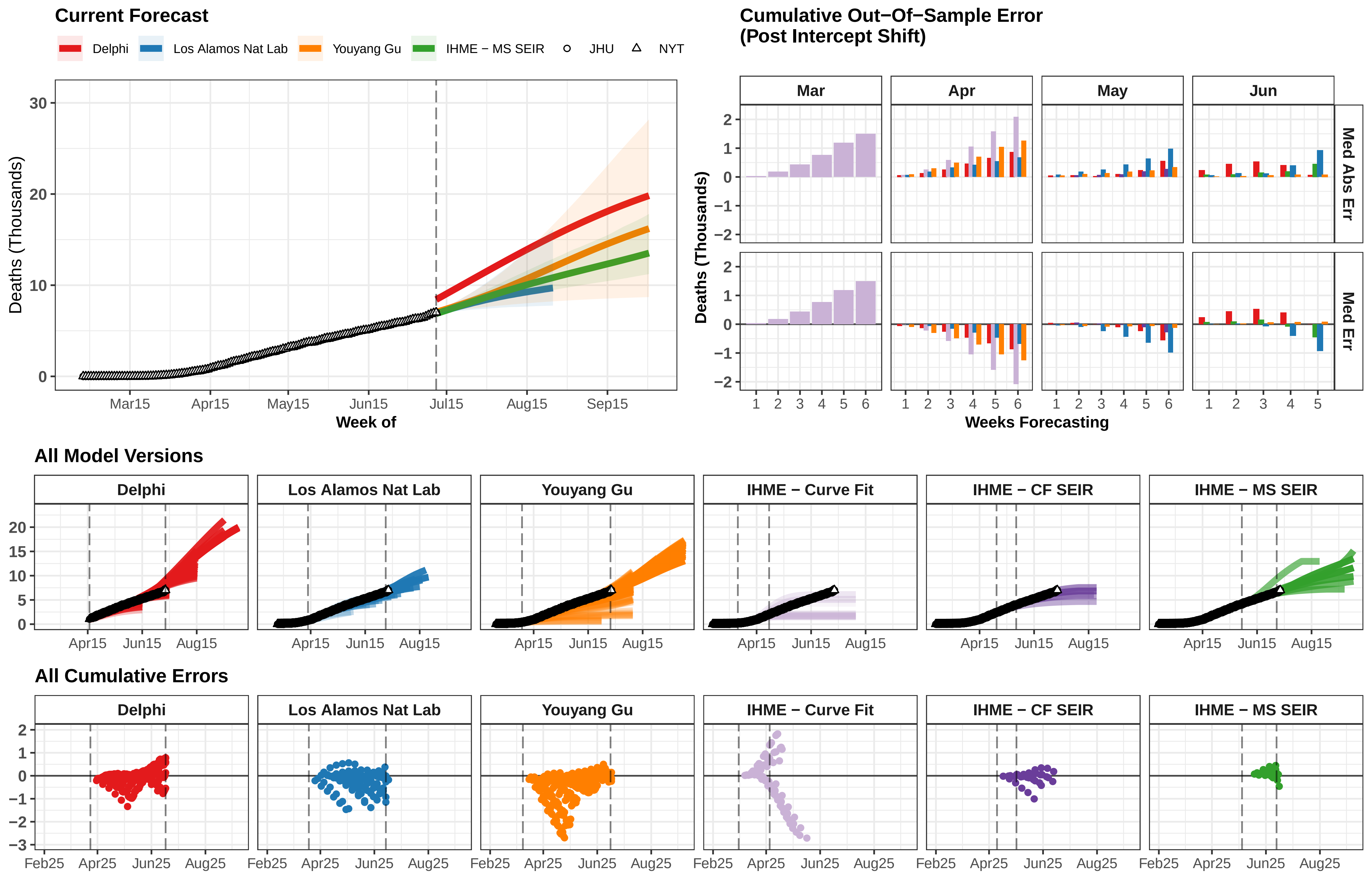
Current Forecast
Delphi
Los Alamos Nat Lab
Youyang Gu
IHME − MS SEIR
JHU
NYT
Deaths (Thousands)
Week of
Mar15
Apr15
May15
Jun15
Jul15
Aug15
Sep15
Cumulative Out−Of−Sample Error
(Post Intercept Shift)
Mar
Apr
May
Jun
Med Abs Err
Med Err
Weeks Forecasting
All Model Versions
Delphi
Los Alamos Nat Lab
Youyang Gu
IHME − Curve Fit
IHME − CF SEIR
IHME − MS SEIR
All Cumulative Errors
Feb25
Apr25
Jun25
Aug25

Pennsylvania

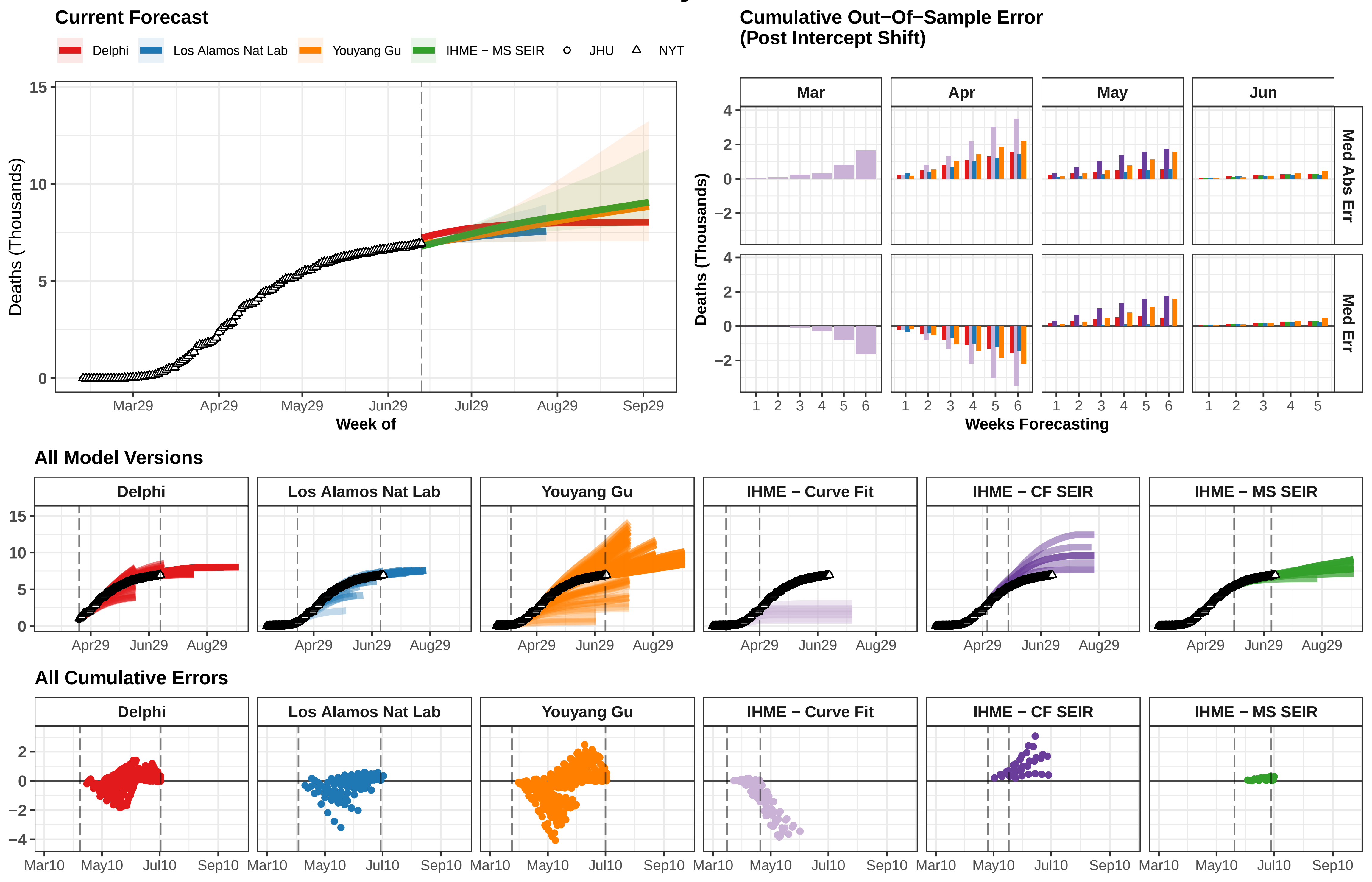

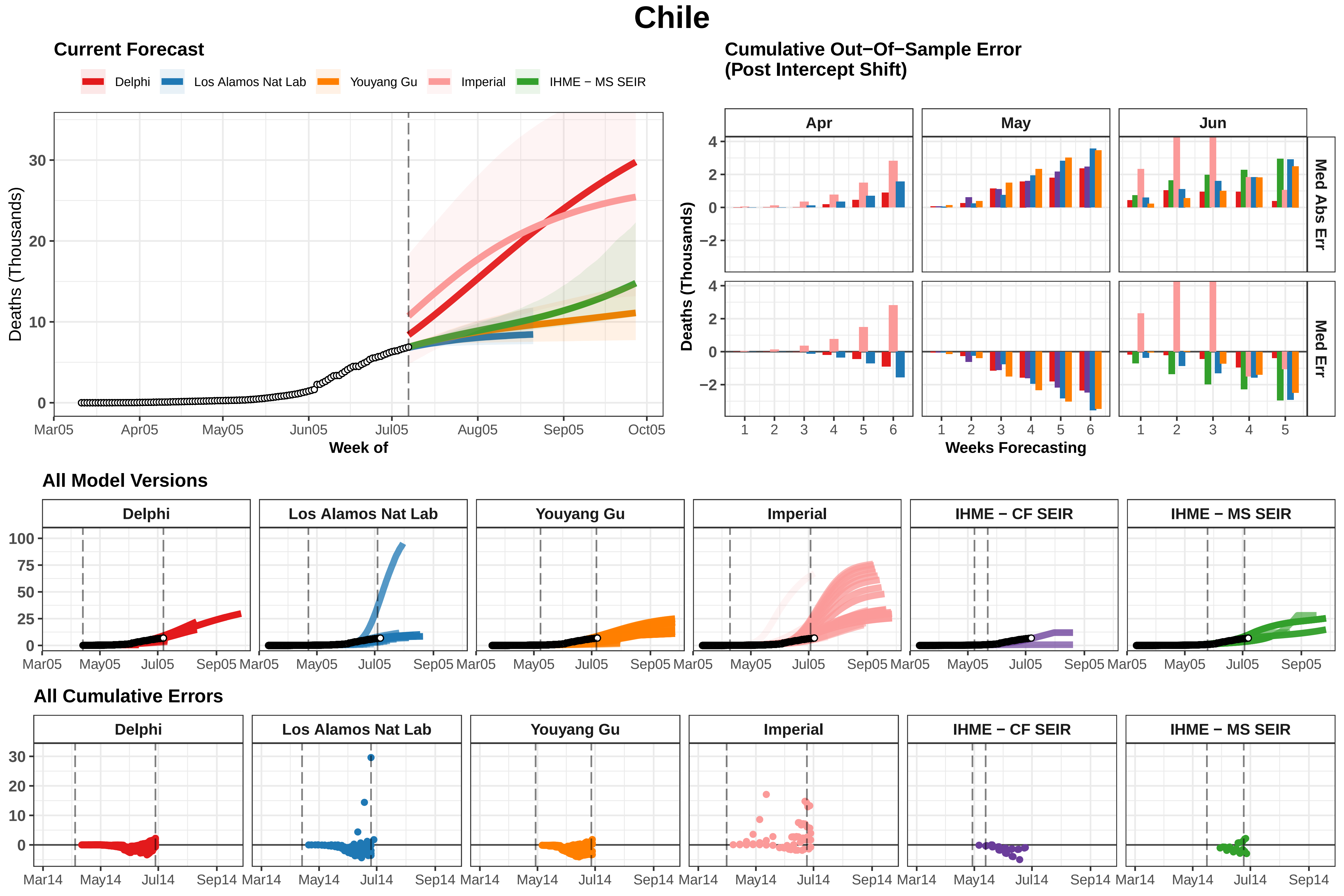
Chile
Current Forecast
Delphi
Los Alamos Nat Lab
Youyang Gu
Imperial
IHME − MS SEIR
Deaths (Thousands)
Week of
Mar05
Apr05
May05
Jun05
Jul05
Aug05
Sep05
Oct05
Cumulative Out−Of−Sample Error
(Post Intercept Shift)
Apr
May
Jun
Med Abs Err
Med Err
Weeks Forecasting
All Model Versions
IHME − CF SEIR
All Cumulative Errors
Mar14
May14
Jul14
Sep14

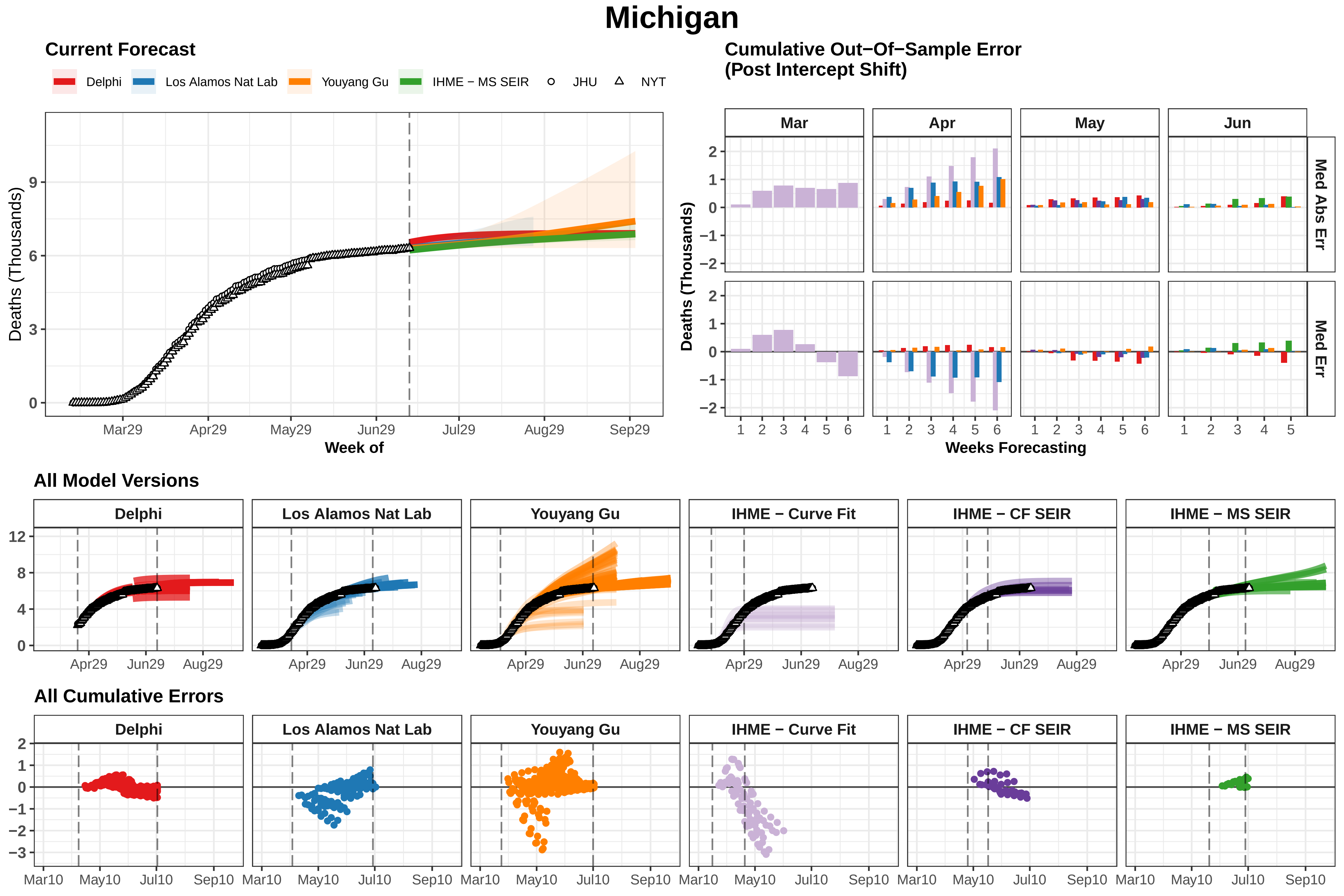

Michigan
Current Forecast
Delphi
Los Alamos Nat Lab
Youyang Gu
IHME − MS SEIR
JHU
NYT
Deaths (Thousands)
Week of
Mar29
Apr29
May29
Jun29
Jul29
Aug29
Sep29
Cumulative Out−Of−Sample Error
(Post Intercept Shift)
Mar
Apr
May
Jun
Med Abs Err
Med Err
Weeks Forecasting
All Model Versions
Delphi
Los Alamos Nat Lab
Youyang Gu
IHME − Curve Fit
IHME − CF SEIR
IHME − MS SEIR
All Cumulative Errors
Mar10
May10
Jul10
Sep10

Netherlands

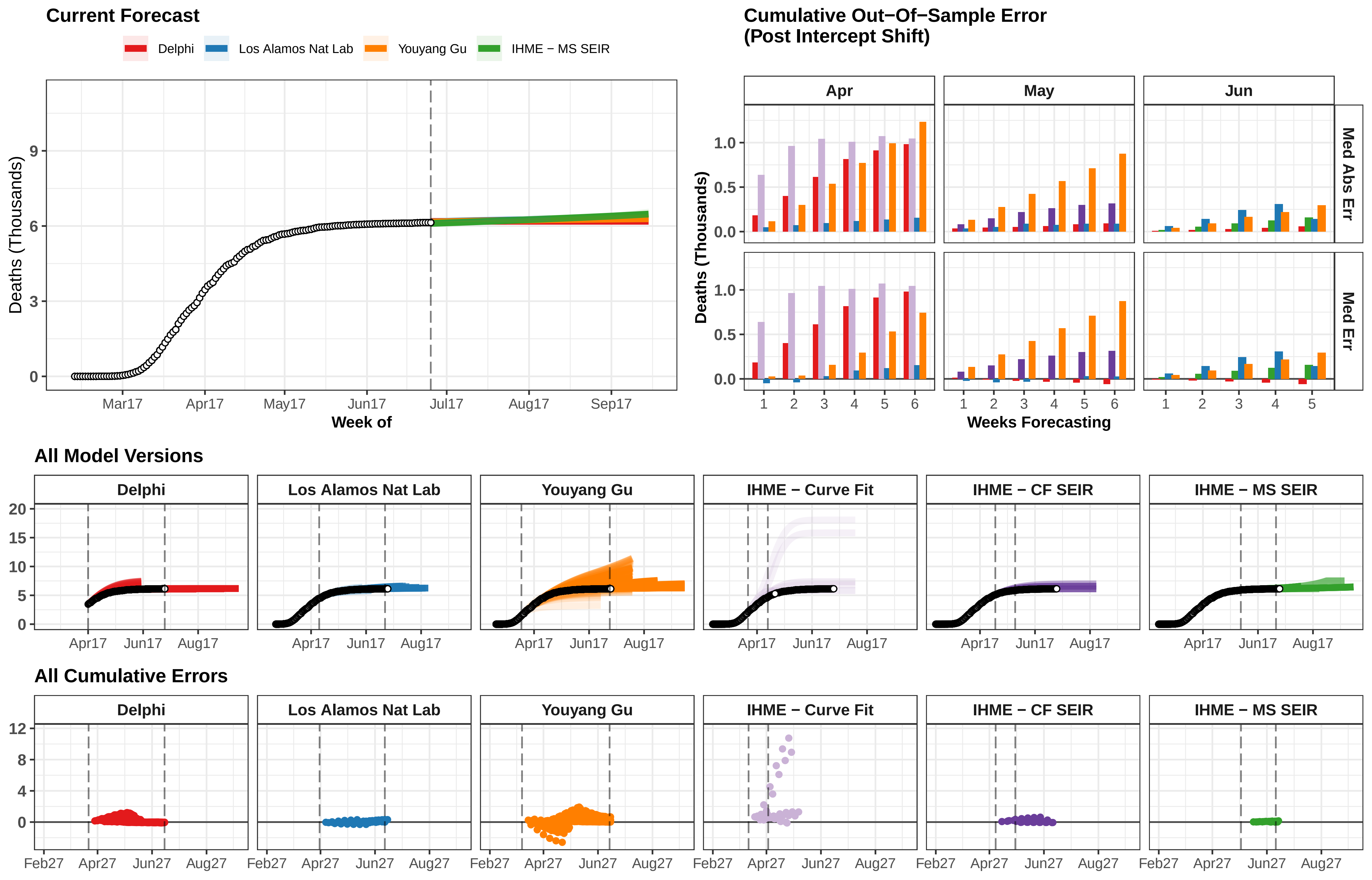

Sweden

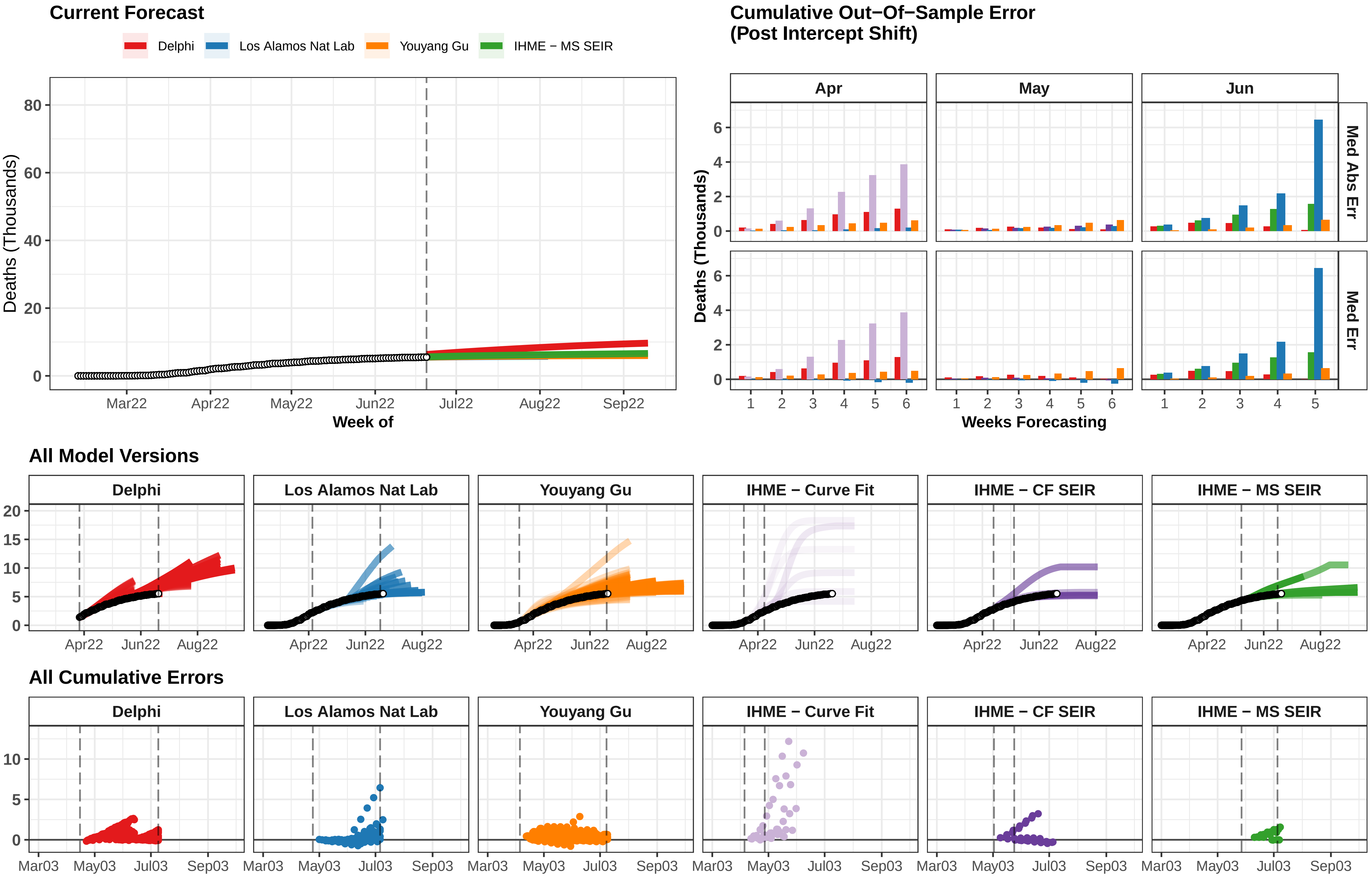
Current Forecast
Delphi
Los Alamos Nat Lab
Youyang Gu
IHME − MS SEIR
Deaths (Thousands)
Week of
Mar22
Apr22
May22
Jun22
Jul22
Aug22
Sep22
Cumulative Out−Of−Sample Error
(Post Intercept Shift)
Apr
May
Jun
Med Abs Err
Med Err
Weeks Forecasting
All Model Versions
Delphi
Los Alamos Nat Lab
Youyang Gu
IHME − Curve Fit
IHME − CF SEIR
IHME − MS SEIR
All Cumulative Errors
Mar03
May03
Jul03
Sep03

Turkey

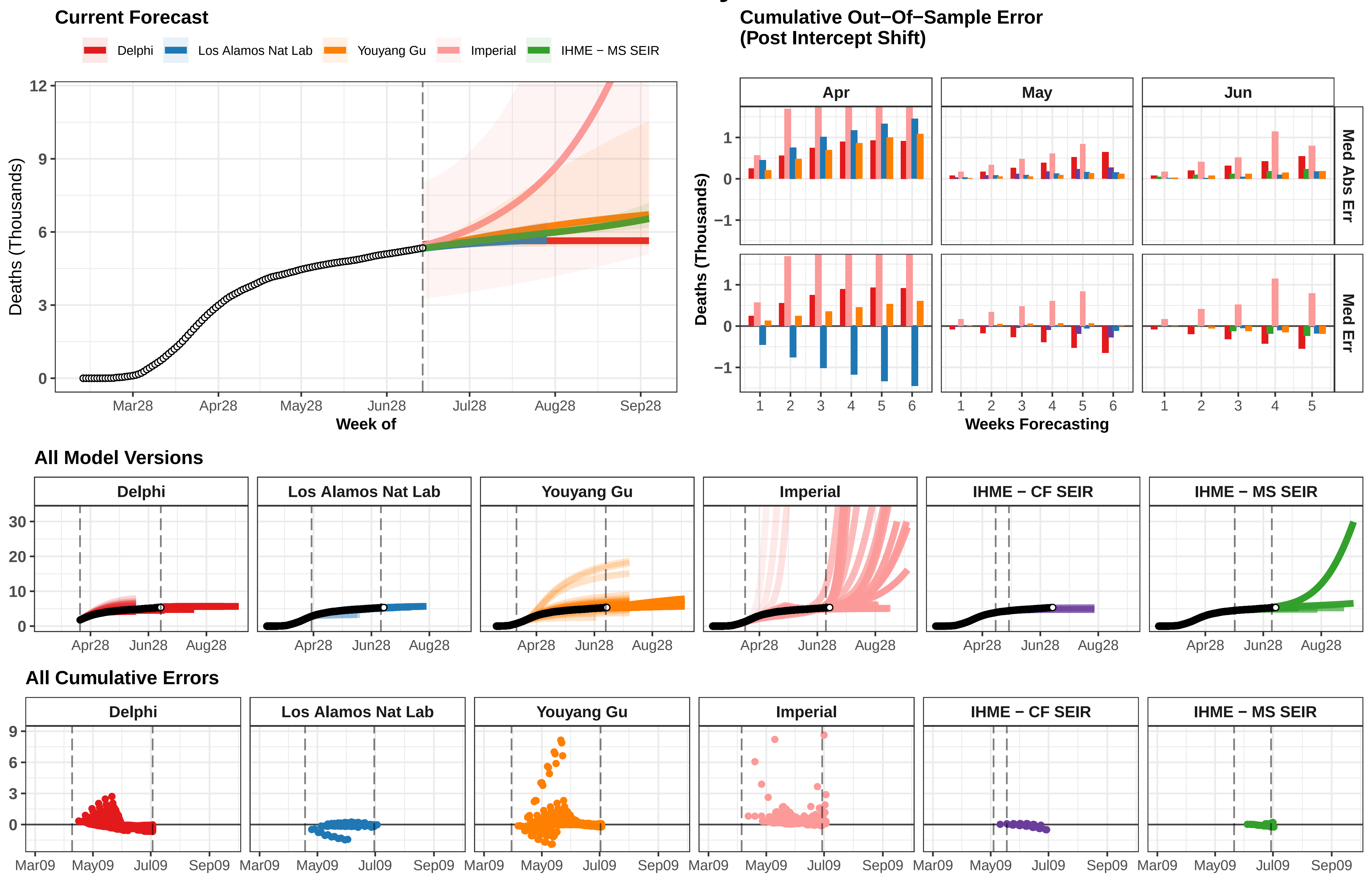
Current Forecast
Delphi
Los Alamos Nat Lab
Youyang Gu
Imperial
IHME − MS SEIR
Deaths (Thousands)
Week of
Mar28
Apr28
May28
Jun28
Jul28
Aug28
Sep28
Cumulative Out−Of−Sample Error
(Post Intercept Shift)
Apr
May
Jun
Med Abs Err
Med Err
Weeks Forecasting
All Model Versions
IHME − CF SEIR
All Cumulative Errors
Mar09
May09
Jul09
Sep09

Colombia

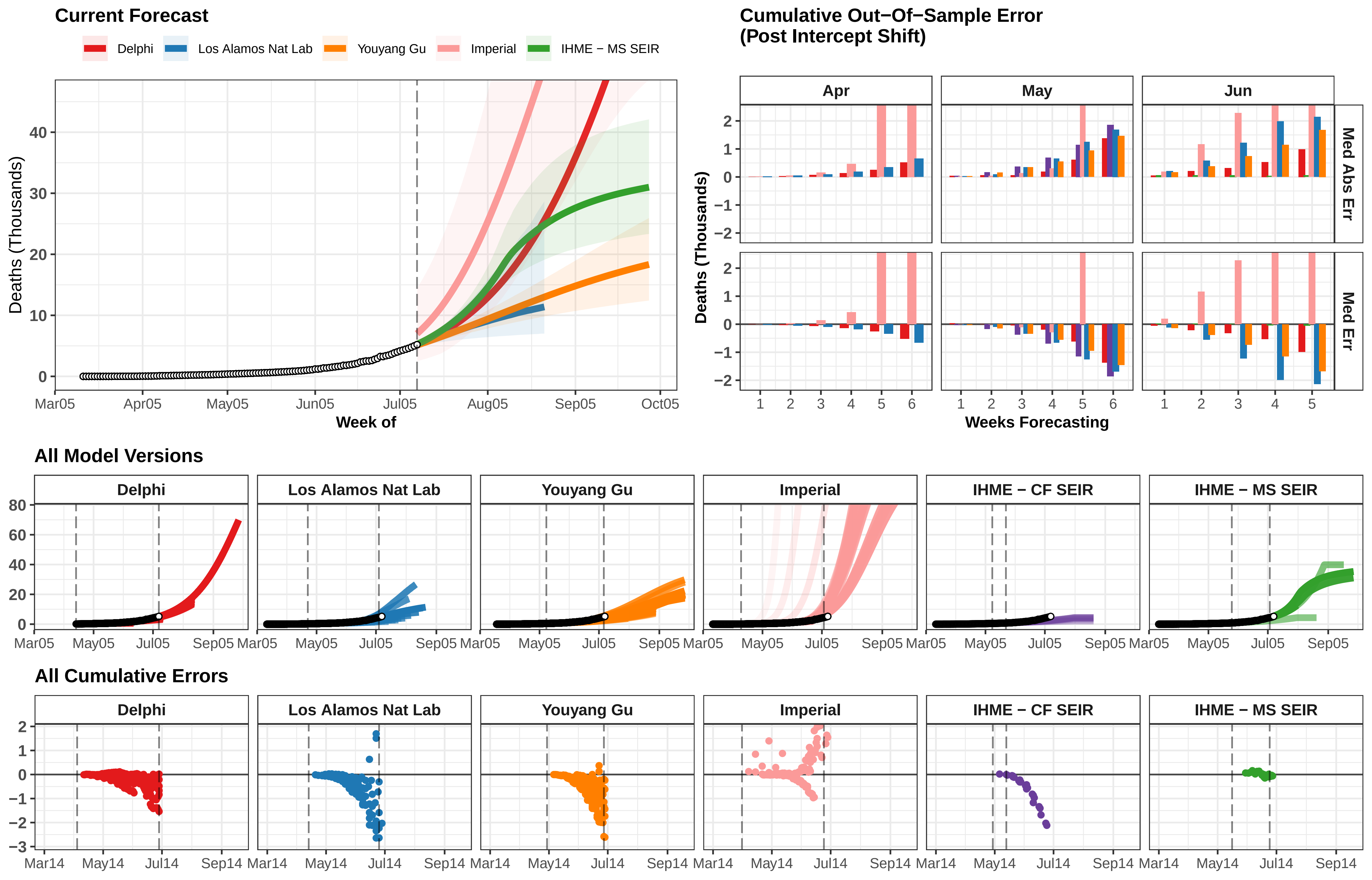

Current Forecast
Delphi
Los Alamos Nat Lab
Youyang Gu
Imperial
IHME − MS SEIR
Deaths (Thousands)
Week of
Mar05
Apr05
May05
Jun05
Jul05
Aug05
Sep05
Oct05
Cumulative Out−Of−Sample Error
(Post Intercept Shift)
Apr
May
Jun
Med Abs Err
Med Err
Weeks Forecasting
All Model Versions
IHME − CF SEIR
All Cumulative Errors
Mar14
May14
Jul14
Sep14

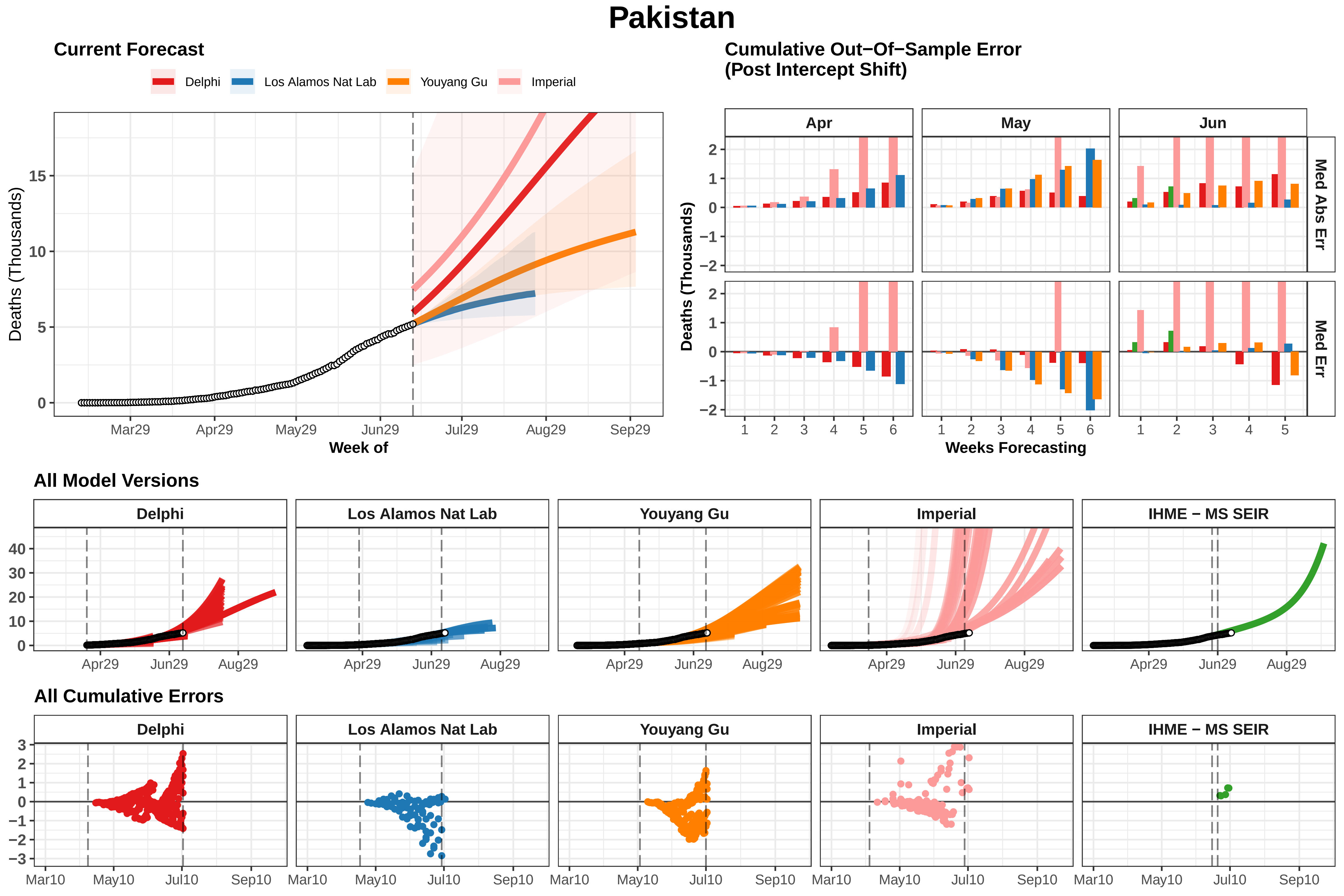

Pakistan
Current Forecast
Delphi
Los Alamos Nat Lab
Youyang Gu
Imperial
Deaths (Thousands)
0
5
10
15
Mar29
Apr29
May29
Jun29
Jul29
Aug29
Sep29
Week of
Cumulative Out−Of−Sample Error
(Post Intercept Shift)
Apr
May
Jun
Med Abs Err
Med Err
Deaths (Thousands)
2
1
0
−1
−2
1
2
3
4
5
6
Weeks Forecasting
All Model Versions
Delphi
Los Alamos Nat Lab
Youyang Gu
Imperial
IHME − MS SEIR
40
30
20
10
0
Apr29
Jun29
Aug29
All Cumulative Errors
Delphi
Los Alamos Nat Lab
Youyang Gu
Imperial
IHME − MS SEIR
3
2
1
0
−1
−2
−3
Mar10
May10
Jul10
Sep10

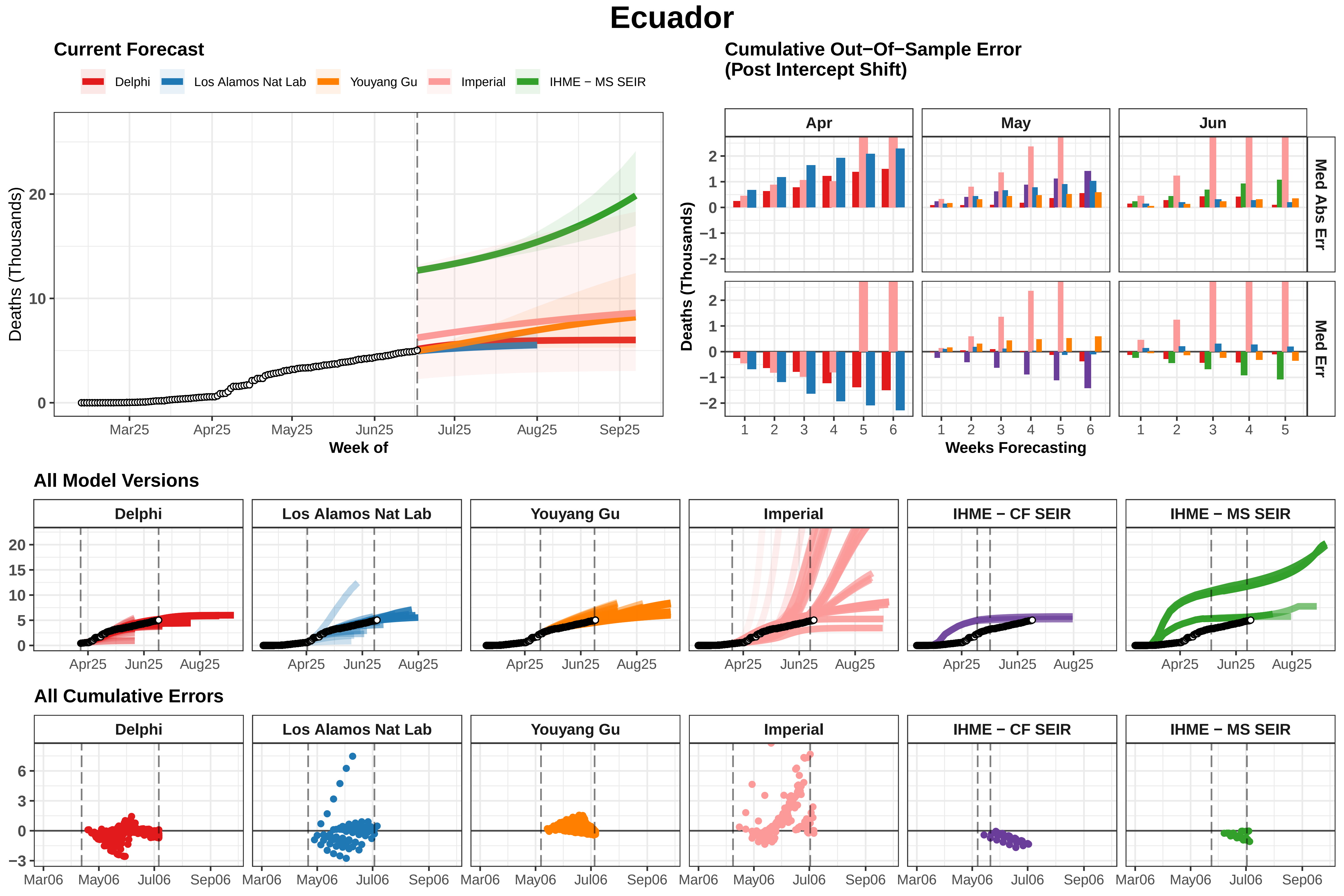

Ecuador
Current Forecast
Delphi
Los Alamos Nat Lab
Youyang Gu
Imperial
IHME − MS SEIR
Deaths (Thousands)
Week of
Mar25
Apr25
May25
Jun25
Jul25
Aug25
Sep25
Cumulative Out−Of−Sample Error
(Post Intercept Shift)
Apr
May
Jun
Med Abs Err
Med Err
Weeks Forecasting
All Model Versions
IHME − CF SEIR
All Cumulative Errors
Mar06
May06
Jul06
Sep06

China

Current Forecast

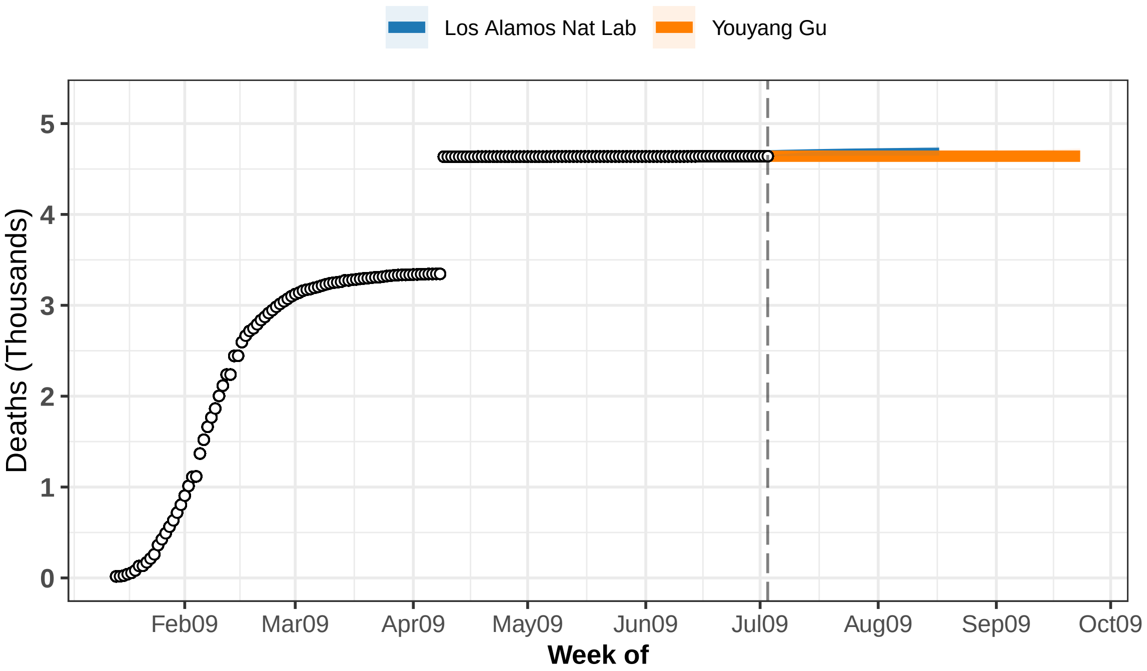

Cumulative Out−Of−Sample Error (Post Intercept Shift)

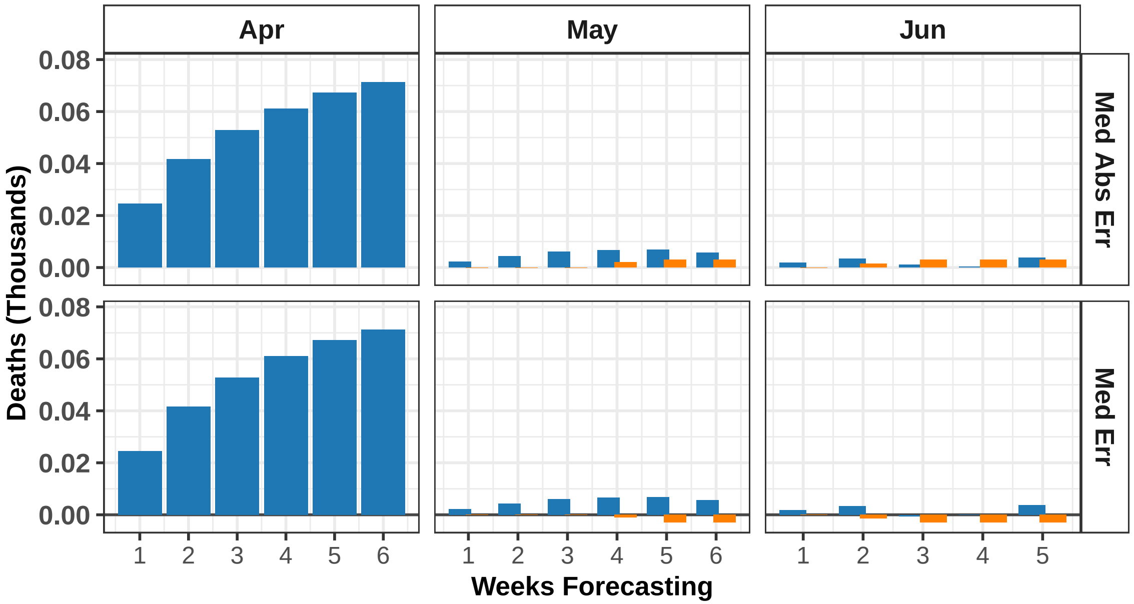

All Model Versions

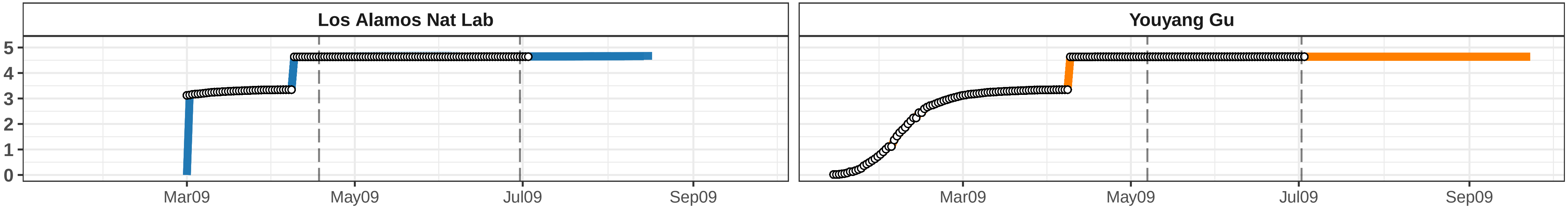

All Cumulative Errors

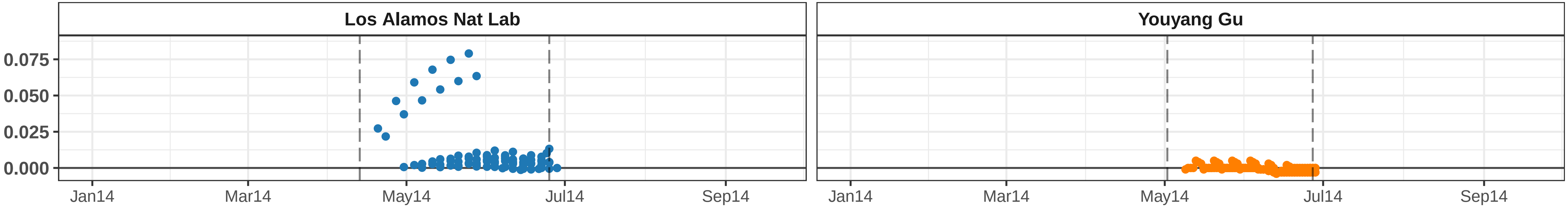

Connecticut

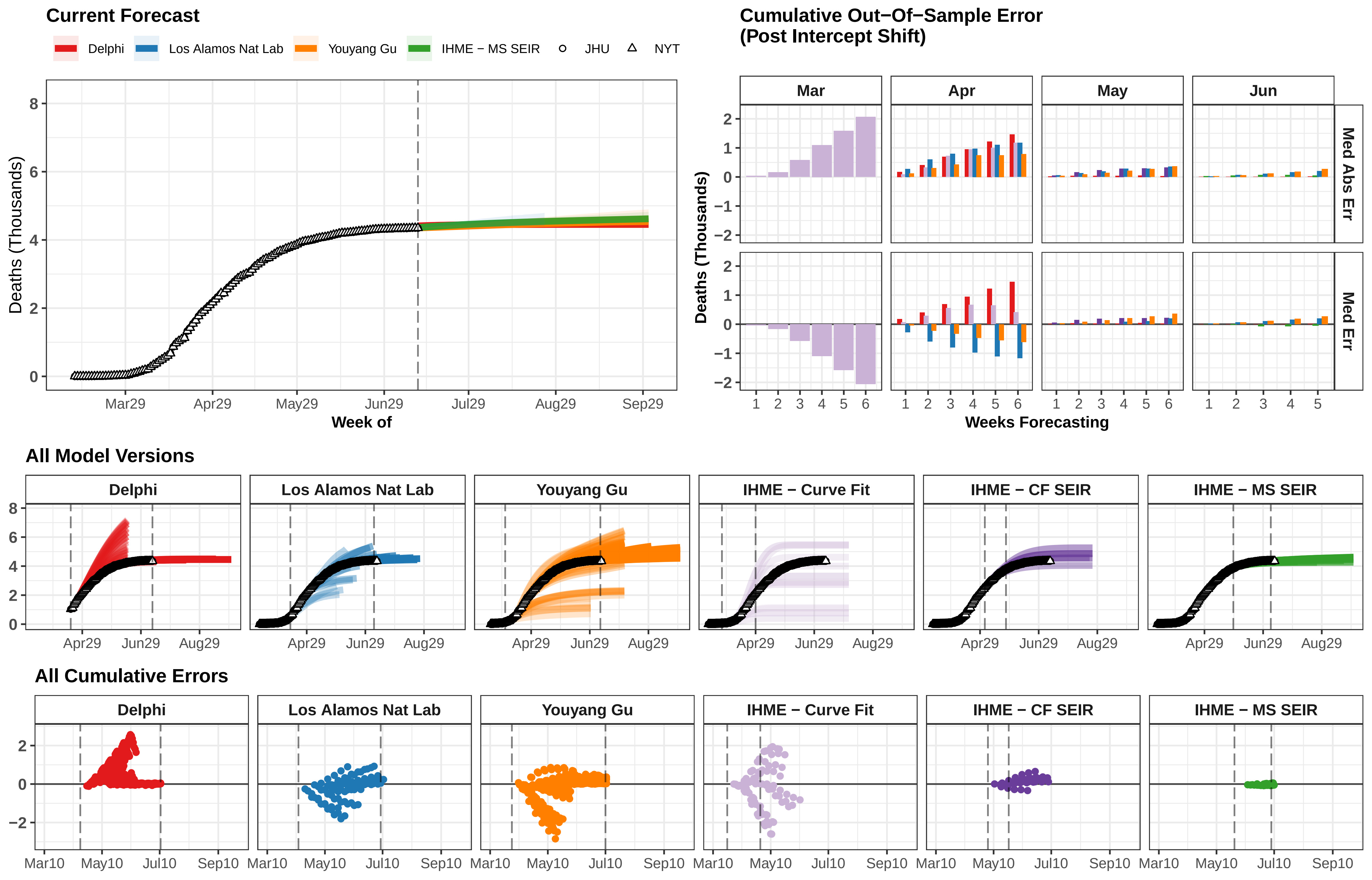

Florida

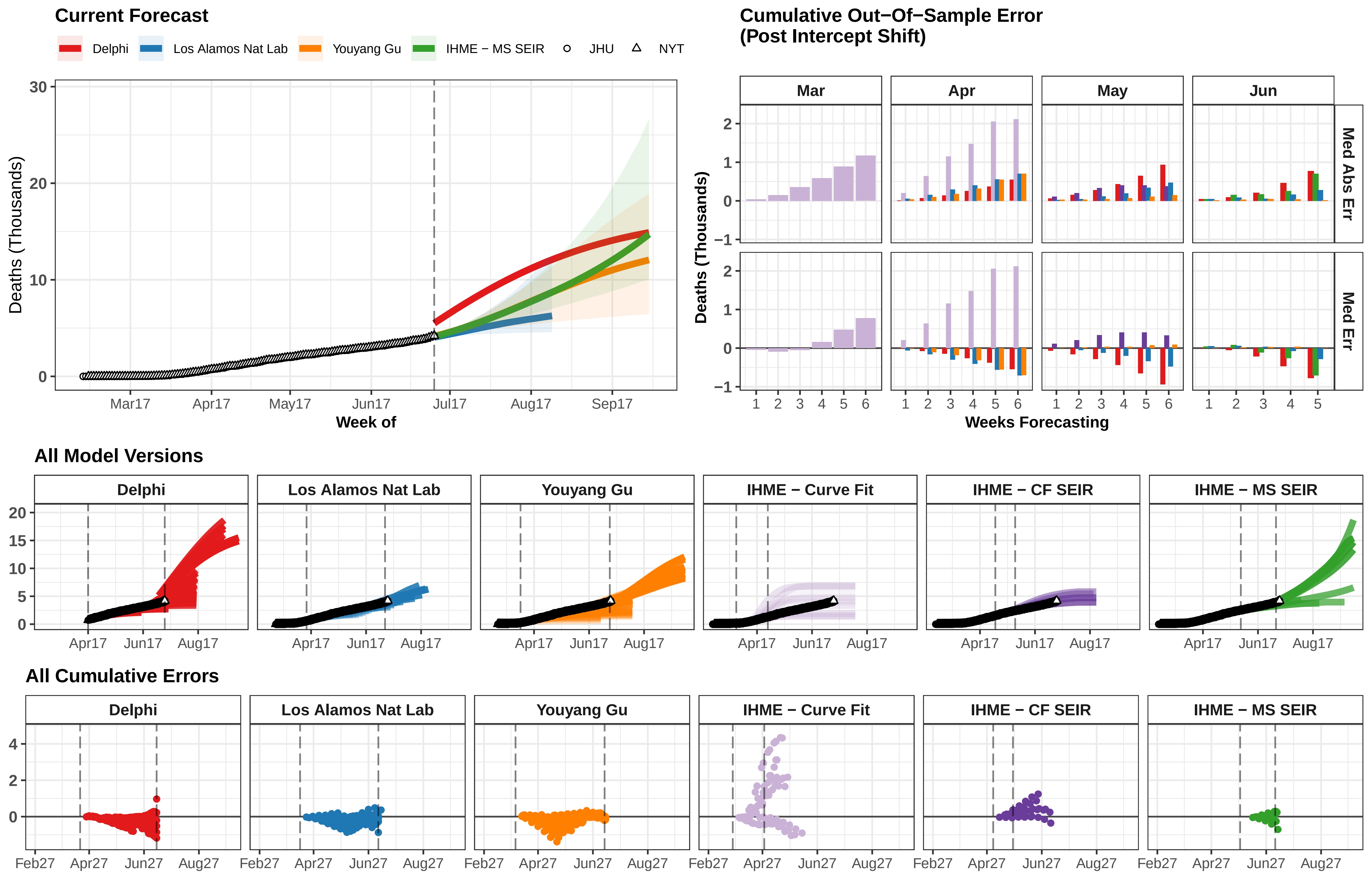
Current Forecast
Delphi
Los Alamos Nat Lab
Youyang Gu
IHME − MS SEIR
JHU
NYT
Deaths (Thousands)
Week of
Mar17
Apr17
May17
Jun17
Jul17
Aug17
Sep17
Cumulative Out−Of−Sample Error
(Post Intercept Shift)
Mar
Apr
May
Jun
Med Abs Err
Med Err
Weeks Forecasting
All Model Versions
Delphi
Los Alamos Nat Lab
Youyang Gu
IHME − Curve Fit
IHME − CF SEIR
IHME − MS SEIR
All Cumulative Errors
Feb27
Apr27
Jun27
Aug27

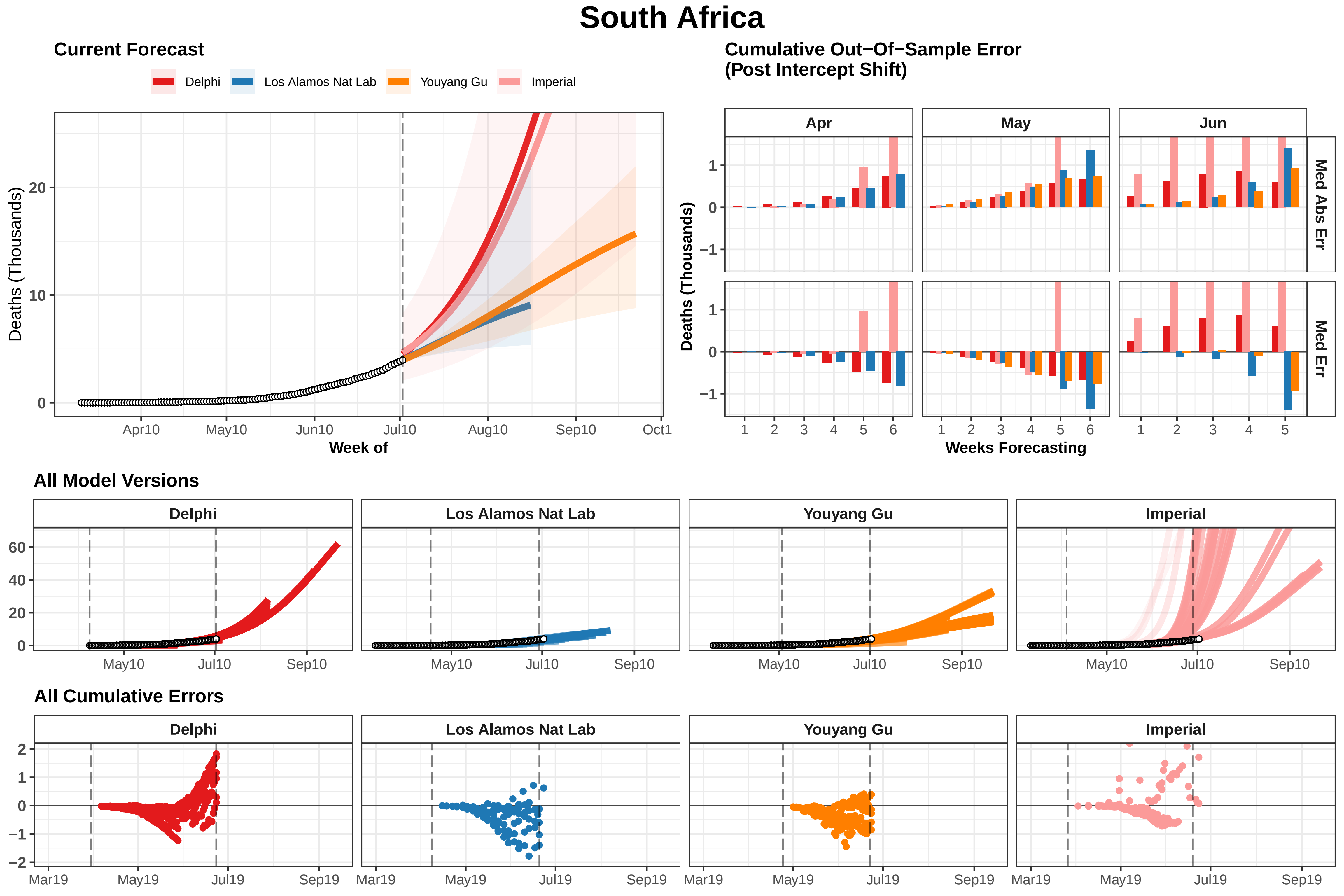
South Africa
Current Forecast
Delphi
Los Alamos Nat Lab
Youyang Gu
Imperial
Deaths (Thousands)
Week of
Apr10
May10
Jun10
Jul10
Aug10
Sep10
Oct1
Cumulative Out−Of−Sample Error
(Post Intercept Shift)
Apr
May
Jun
Med Abs Err
Med Err
Weeks Forecasting
All Model Versions
All Cumulative Errors
Mar19
May19
Jul19
Sep19

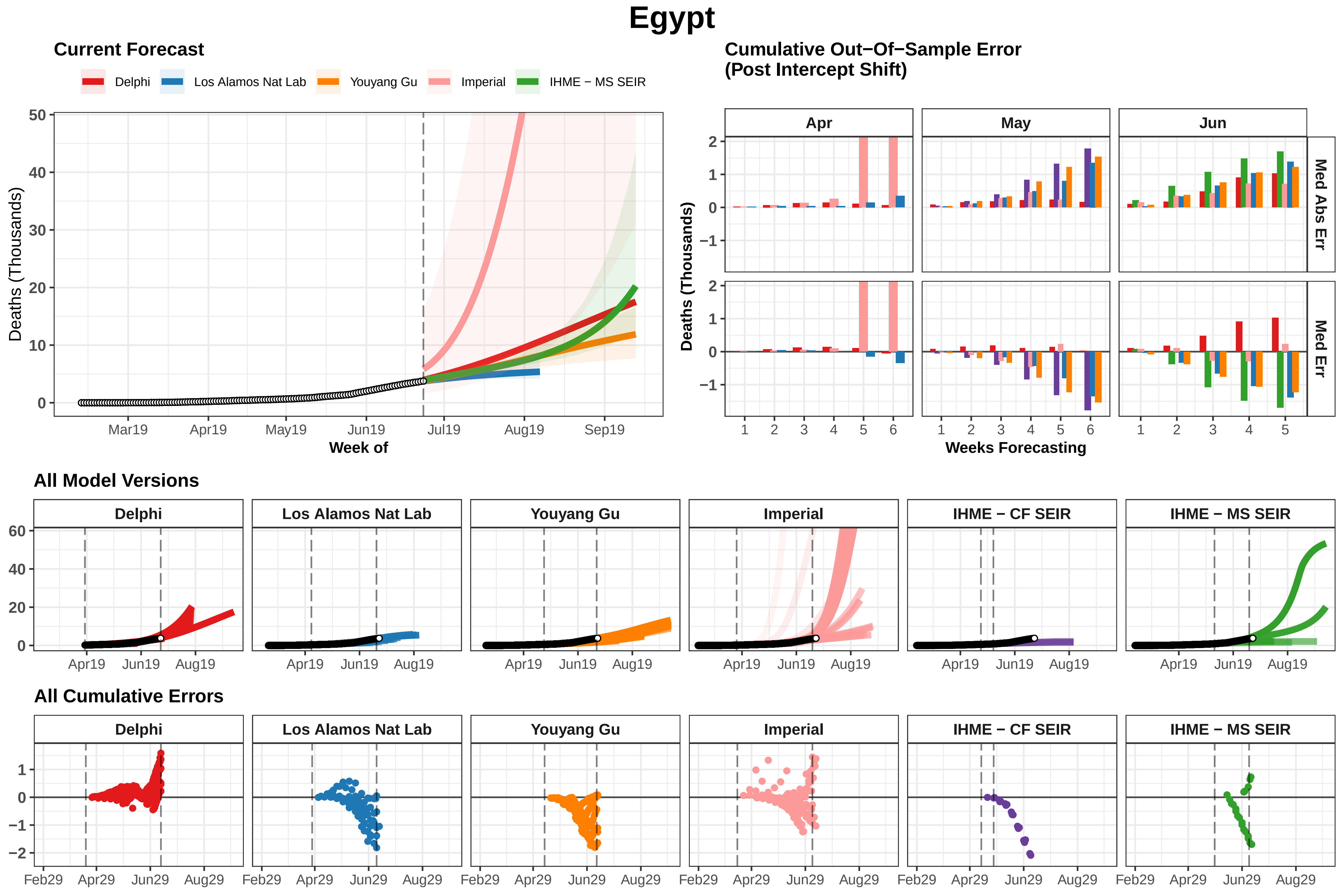
Egypt
Current Forecast
Delphi
Los Alamos Nat Lab
Youyang Gu
Imperial
IHME − MS SEIR
Deaths (Thousands)
Week of
Mar19
Apr19
May19
Jun19
Jul19
Aug19
Sep19
Cumulative Out−Of−Sample Error
(Post Intercept Shift)
Apr
May
Jun
Med Abs Err
Med Err
Weeks Forecasting
All Model Versions
Delphi
Los Alamos Nat Lab
Youyang Gu
Imperial
IHME − CF SEIR
IHME − MS SEIR
All Cumulative Errors
Feb29
Apr29
Jun29
Aug29

Indonesia

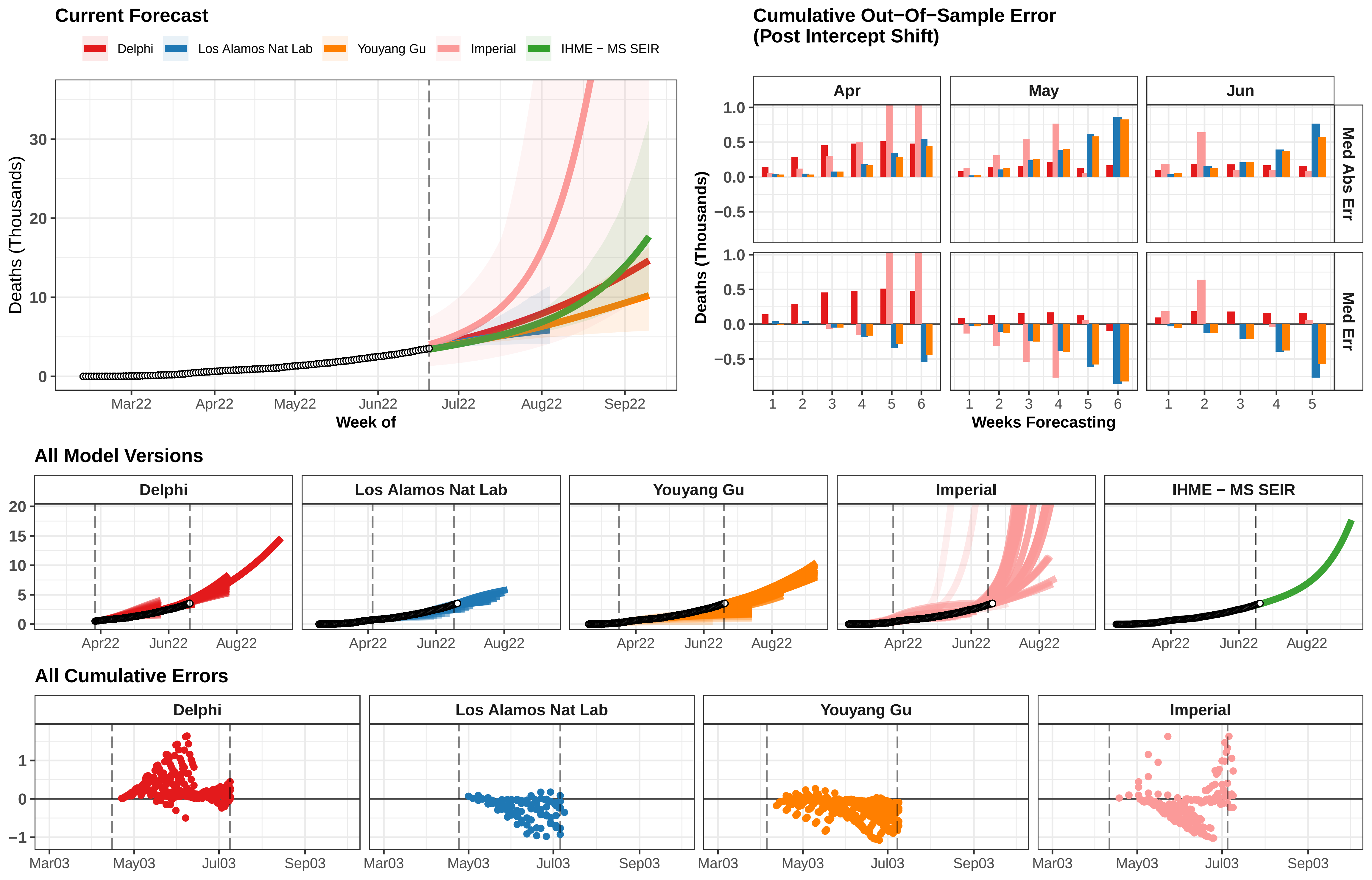

Current Forecast
Delphi
Los Alamos Nat Lab
Youyang Gu
Imperial
IHME − MS SEIR
Deaths (Thousands)
0
10
20
30
Mar22
Apr22
May22
Jun22
Jul22
Aug22
Sep22
Week of
Cumulative Out−Of−Sample Error
(Post Intercept Shift)
Apr
May
Jun
Med Abs Err
Med Err
1.0
0.5
0.0
−0.5
Deaths (Thousands)
1
2
3
4
5
6
Weeks Forecasting
All Model Versions
Delphi
Los Alamos Nat Lab
Youyang Gu
Imperial
IHME − MS SEIR
20
15
10
5
0
Apr22
Jun22
Aug22
All Cumulative Errors
Delphi
Los Alamos Nat Lab
Youyang Gu
Imperial
1
0
−1
Mar03
May03
Jul03
Sep03

Louisiana

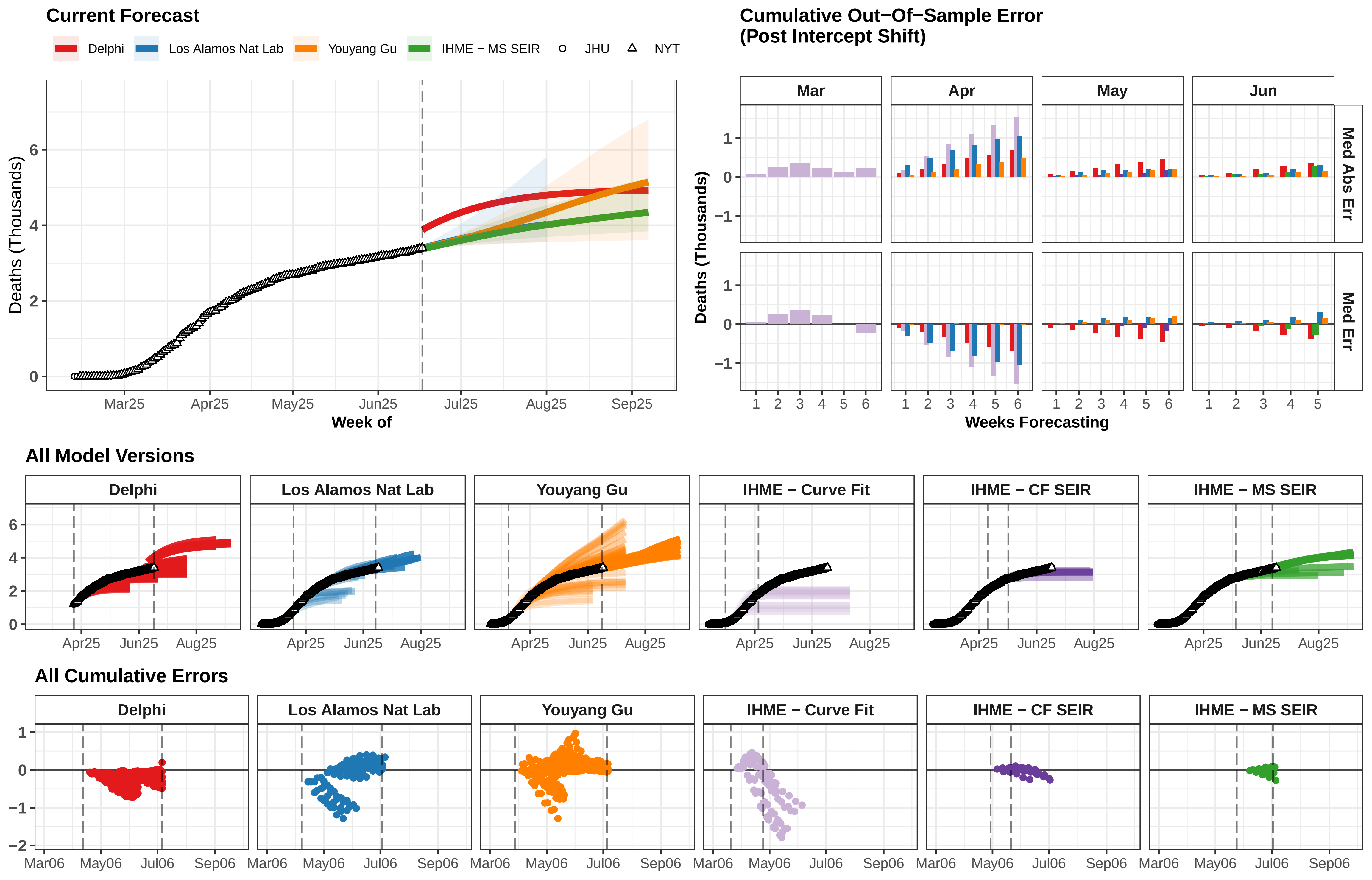
Current Forecast
Delphi
Los Alamos Nat Lab
Youyang Gu
IHME − MS SEIR
JHU
NYT
Deaths (Thousands)
Week of
Mar25
Apr25
May25
Jun25
Jul25
Aug25
Sep25
Cumulative Out−Of−Sample Error
(Post Intercept Shift)
Mar
Apr
May
Jun
Med Abs Err
Med Err
Weeks Forecasting
All Model Versions
Delphi
Los Alamos Nat Lab
Youyang Gu
IHME − Curve Fit
IHME − CF SEIR
IHME − MS SEIR
All Cumulative Errors
Mar06
May06
Jul06
Sep06

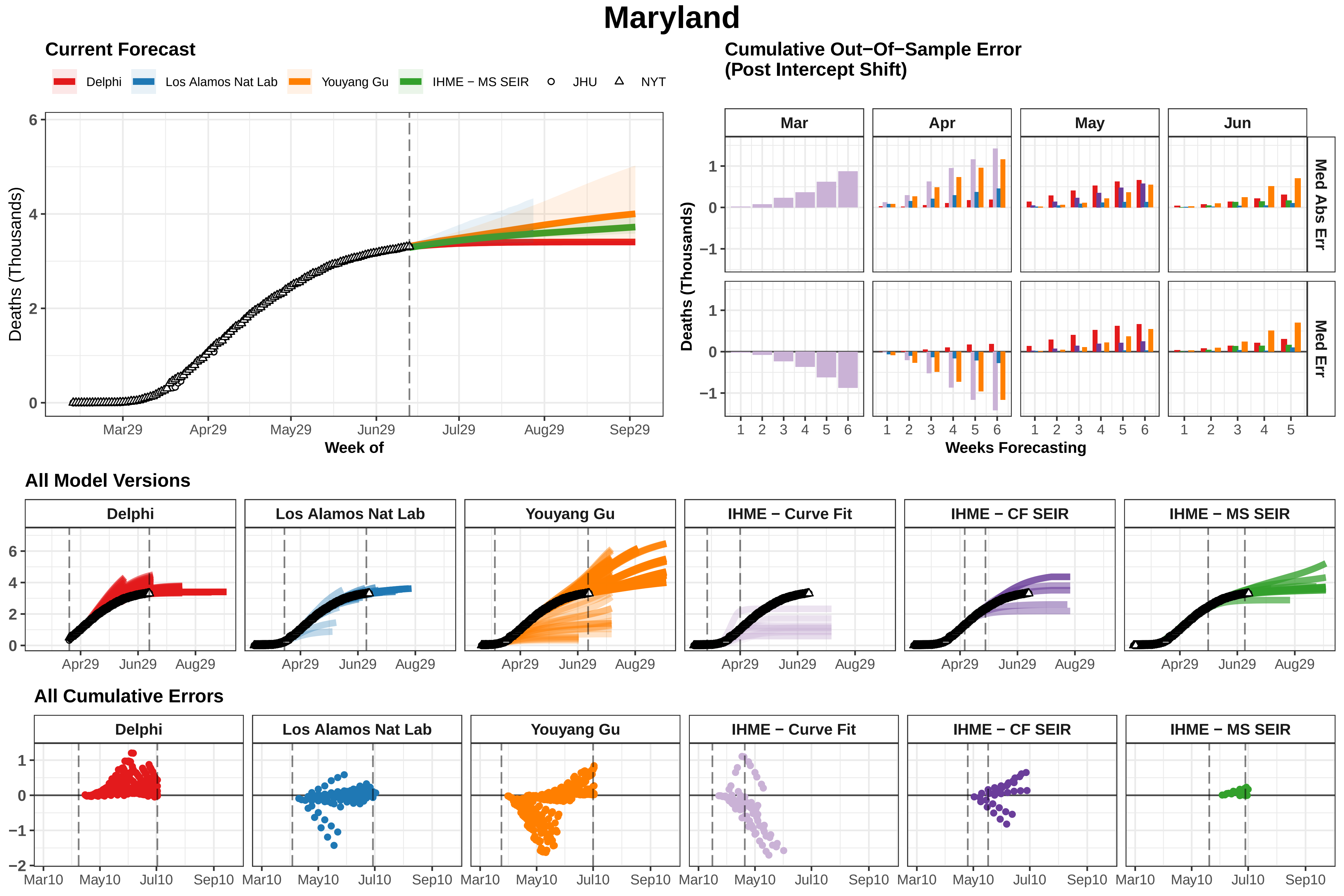

Maryland
Current Forecast
Delphi
Los Alamos Nat Lab
Youyang Gu
IHME − MS SEIR
JHU
NYT
Deaths (Thousands)
0
2
4
6
Mar29
Apr29
May29
Jun29
Jul29
Aug29
Sep29
Week of
Cumulative Out−Of−Sample Error (Post Intercept Shift)
Mar
Apr
May
Jun
Med Abs Err
Med Err
Deaths (Thousands)
1
0
−1
1 2 3 4 5 6
1 2 3 4 5
Weeks Forecasting
All Model Versions
Delphi
Los Alamos Nat Lab
Youyang Gu
IHME − Curve Fit
IHME − CF SEIR
IHME − MS SEIR
0
2
4
6
Apr29
Jun29
Aug29
All Cumulative Errors
Delphi
Los Alamos Nat Lab
Youyang Gu
IHME − Curve Fit
IHME − CF SEIR
IHME − MS SEIR
1
0
−1
−2
Mar10
May10
Jul10
Sep10

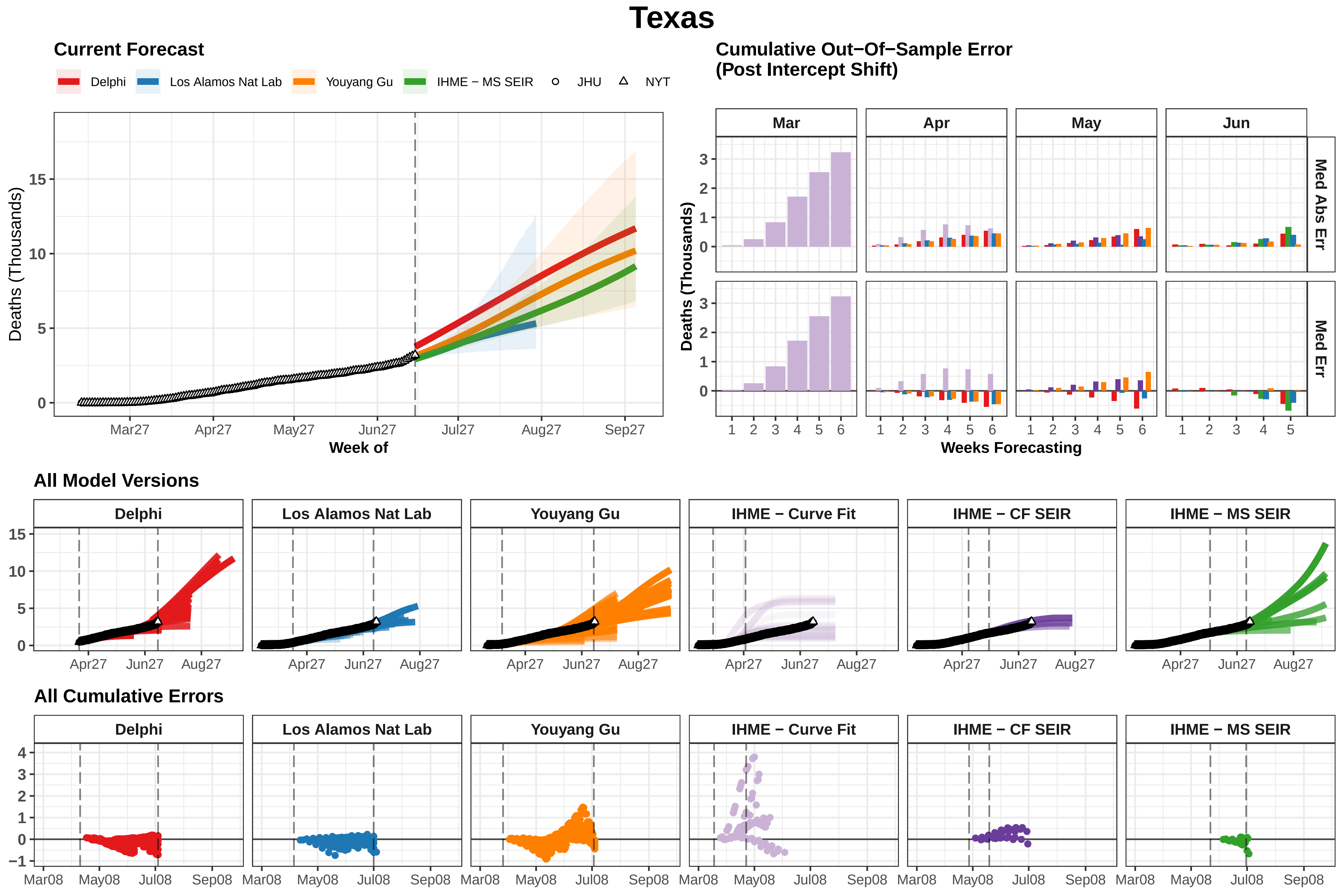

Texas
Current Forecast
Delphi
Los Alamos Nat Lab
Youyang Gu
IHME − MS SEIR
JHU
NYT
Deaths (Thousands)
Week of
Mar27
Apr27
May27
Jun27
Jul27
Aug27
Sep27
Cumulative Out−Of−Sample Error
(Post Intercept Shift)
Mar
Apr
May
Jun
Med Abs Err
Med Err
Weeks Forecasting
All Model Versions
Delphi
Los Alamos Nat Lab
Youyang Gu
IHME − Curve Fit
IHME − CF SEIR
IHME − MS SEIR
All Cumulative Errors
Mar08
May08
Jul08
Sep08

Iraq

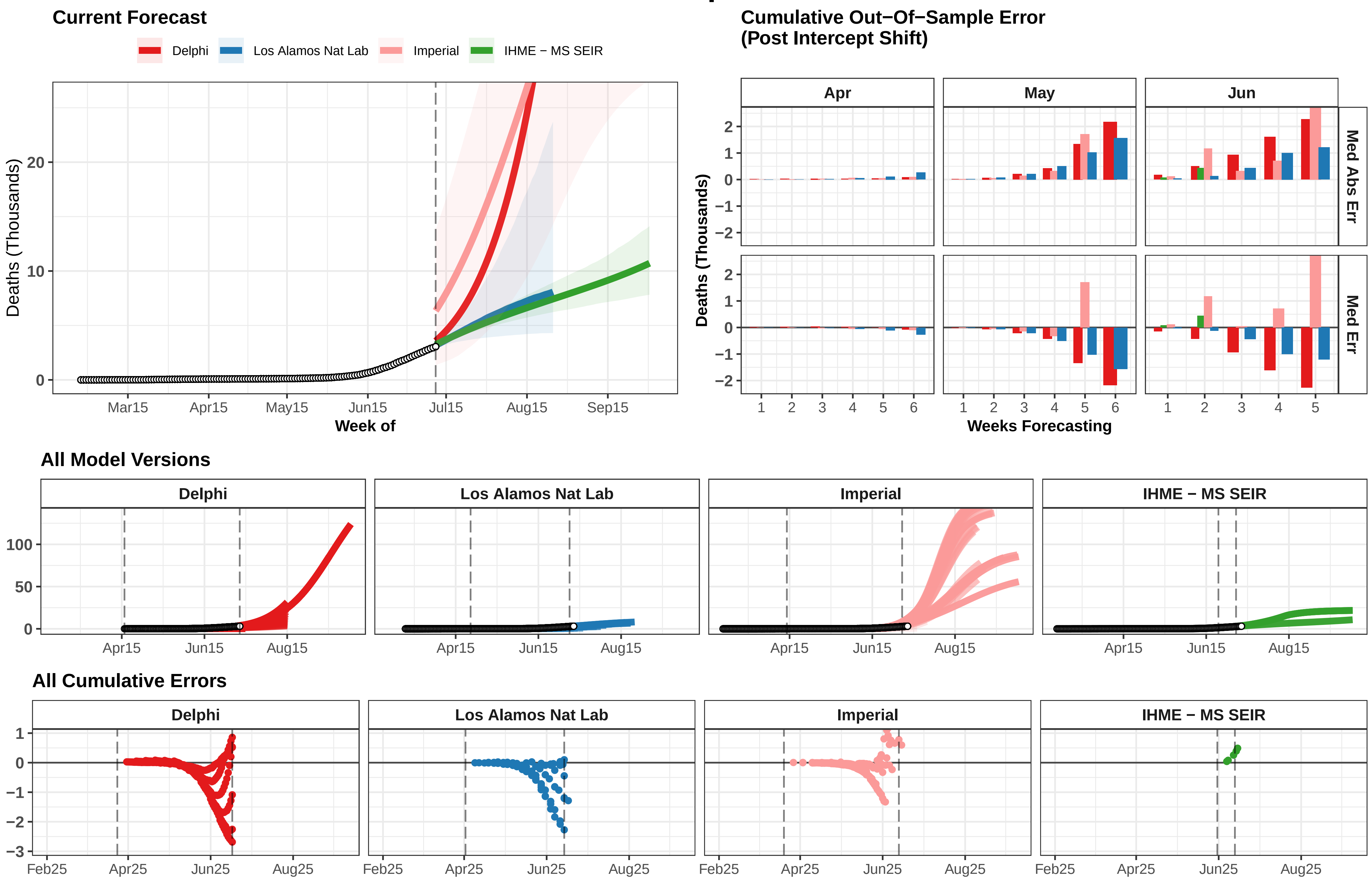

Current Forecast
Delphi
Los Alamos Nat Lab
Imperial
IHME − MS SEIR
Deaths (Thousands)
0
10
20
Mar15
Apr15
May15
Jun15
Jul15
Aug15
Sep15
Week of
Cumulative Out−Of−Sample Error
(Post Intercept Shift)
Apr
May
Jun
Med Abs Err
Med Err
Deaths (Thousands)
2
1
0
−1
−2
1
2
3
4
5
6
Weeks Forecasting
All Model Versions
Delphi
Los Alamos Nat Lab
Imperial
IHME − MS SEIR
100
50
0
Apr15
Jun15
Aug15
All Cumulative Errors
Delphi
Los Alamos Nat Lab
Imperial
IHME − MS SEIR
1
0
−1
−2
−3
Feb25
Apr25
Jun25
Aug25

Ohio

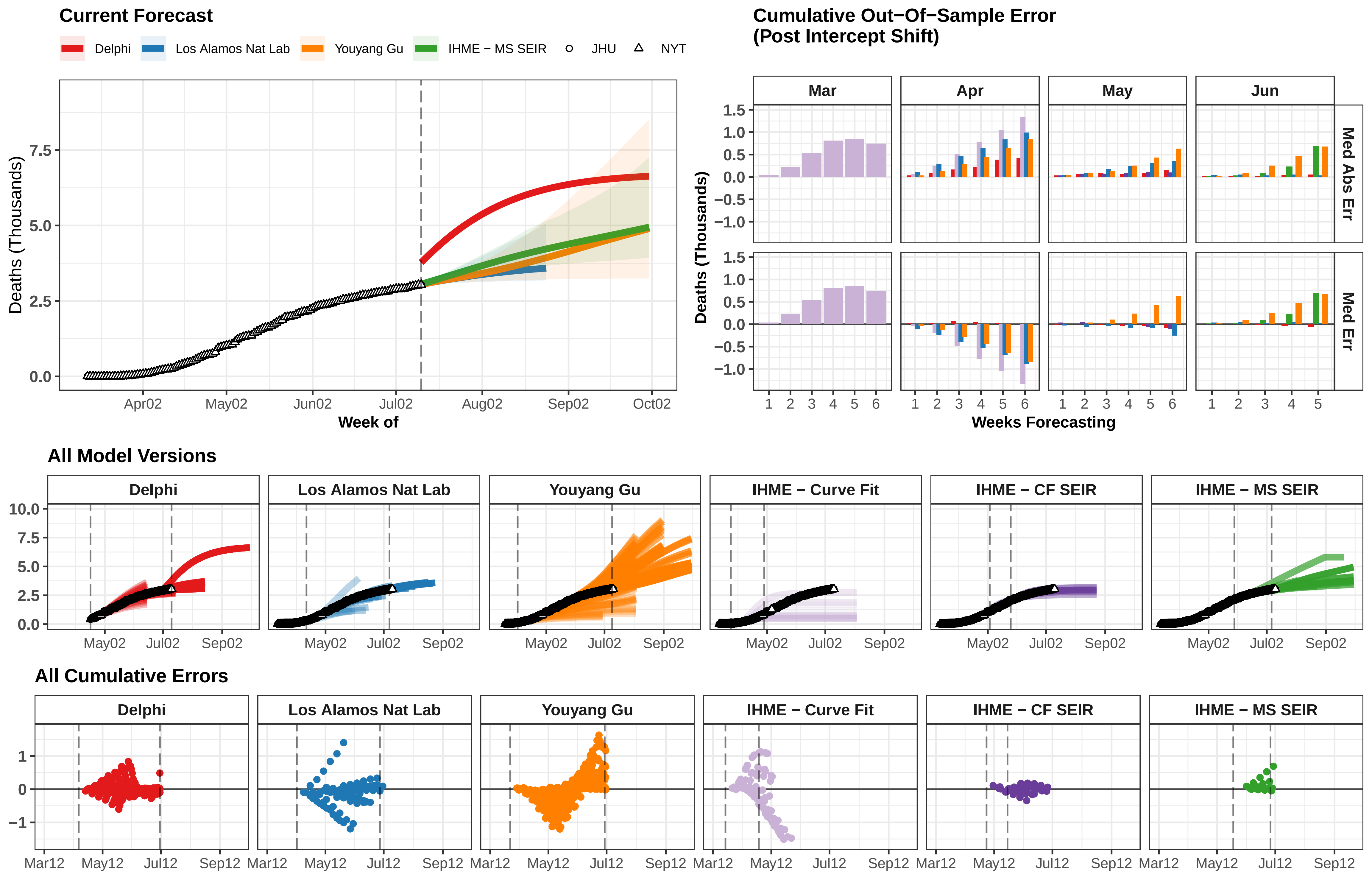

Current Forecast
Delphi
Los Alamos Nat Lab
Youyang Gu
IHME − MS SEIR
JHU
NYT
Deaths (Thousands)
7.5
5.0
2.5
0.0
Apr02
May02
Jun02
Jul02
Aug02
Sep02
Oct02
Week of
Cumulative Out−Of−Sample Error
(Post Intercept Shift)
Mar
Apr
May
Jun
Med Abs Err
Med Err
Deaths (Thousands)
1.5
1.0
0.5
0.0
−0.5
−1.0
1 2 3 4 5 6
Weeks Forecasting
All Model Versions
Delphi
Los Alamos Nat Lab
Youyang Gu
IHME − Curve Fit
IHME − CF SEIR
IHME − MS SEIR
10.0
7.5
5.0
2.5
0.0
May02
Jul02
Sep02
All Cumulative Errors
1
0
−1
Mar12
May12
Jul12
Sep12

Georgia

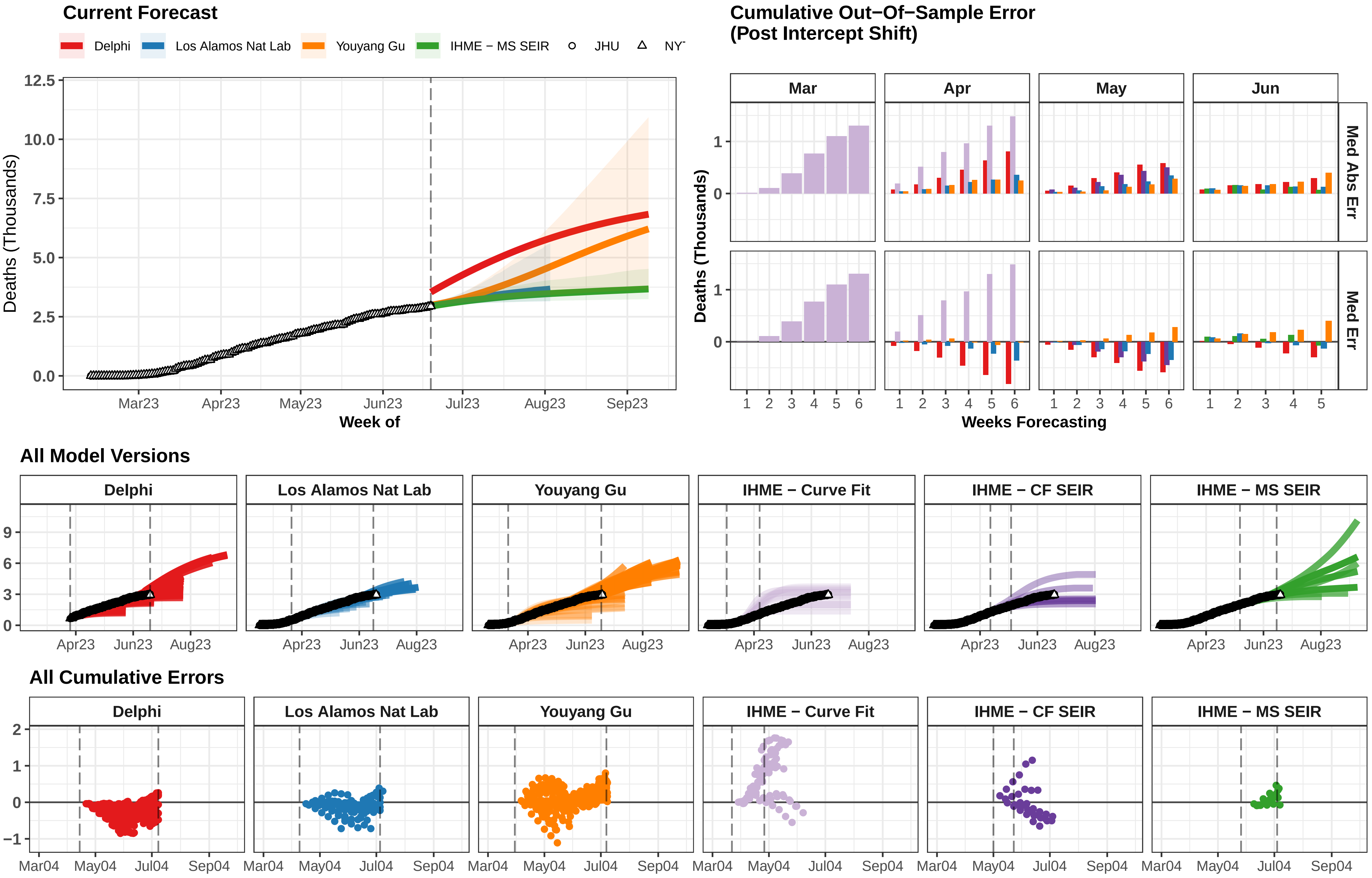
Current Forecast
Delphi
Los Alamos Nat Lab
Youyang Gu
IHME − MS SEIR
JHU
NY
12.5
10.0
7.5
5.0
2.5
0.0
Deaths (Thousands)
Mar23
Apr23
May23
Jun23
Jul23
Aug23
Sep23
Week of
Cumulative Out−Of−Sample Error
(Post Intercept Shift)
Mar
Apr
May
Jun
Med Abs Err
Med Err
Deaths (Thousands)
1
0
1 2 3 4 5 6
1 2 3 4 5
Weeks Forecasting
All Model Versions
Delphi
Los Alamos Nat Lab
Youyang Gu
IHME − Curve Fit
IHME − CF SEIR
IHME − MS SEIR
9
6
3
0
Apr23
Jun23
Aug23
All Cumulative Errors
Delphi
Los Alamos Nat Lab
Youyang Gu
IHME − Curve Fit
IHME − CF SEIR
IHME − MS SEIR
2
1
0
−1
Mar04
May04
Jul04
Sep04

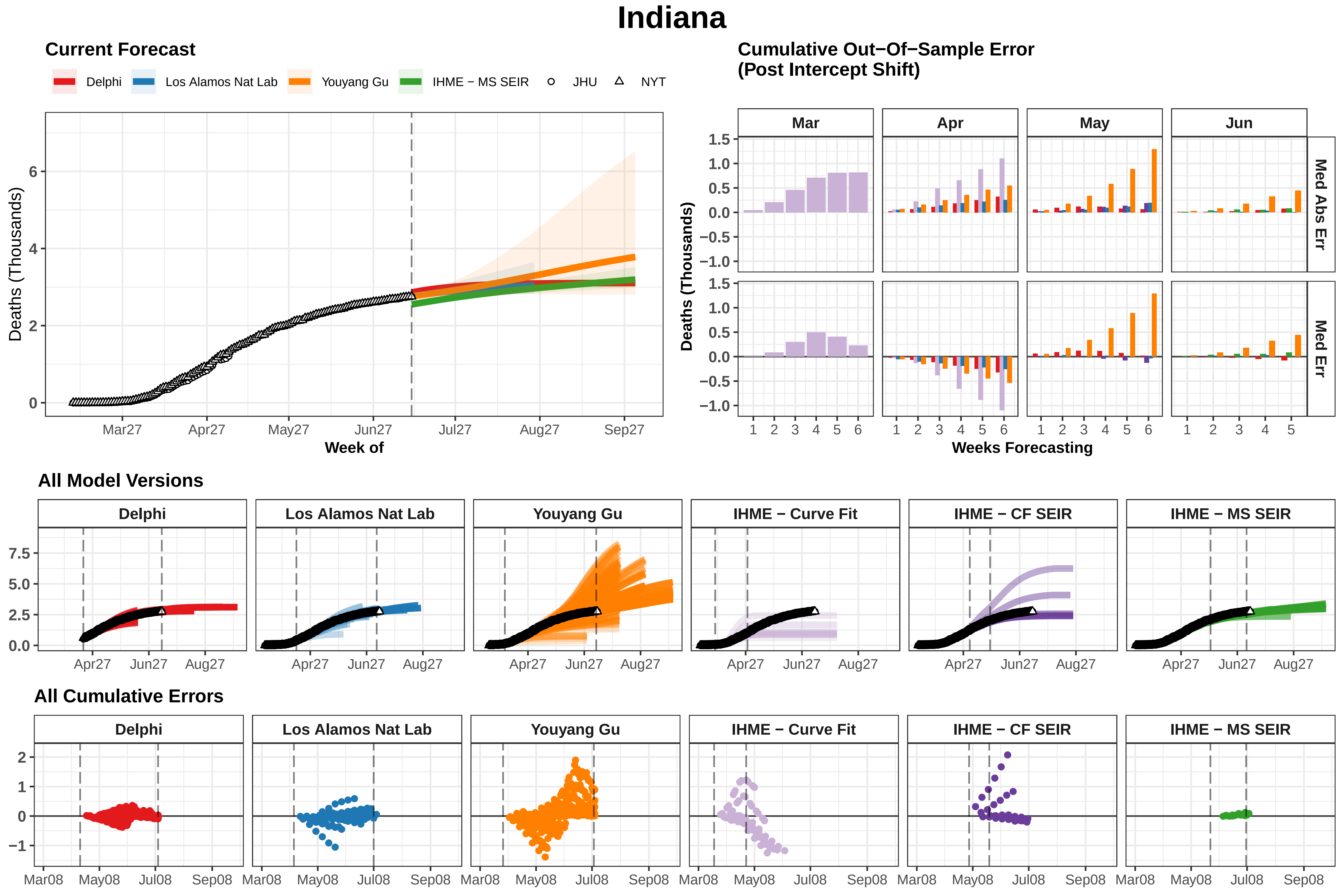

Indiana
Current Forecast
Delphi
Los Alamos Nat Lab
Youyang Gu
IHME − MS SEIR
JHU
NYT
Deaths (Thousands)
Week of
Mar27
Apr27
May27
Jun27
Jul27
Aug27
Sep27
Cumulative Out−Of−Sample Error
(Post Intercept Shift)
Mar
Apr
May
Jun
Med Abs Err
Med Err
Weeks Forecasting
All Model Versions
IHME − Curve Fit
IHME − CF SEIR
All Cumulative Errors
Mar08
May08
Jul08
Sep08

Bangladesh

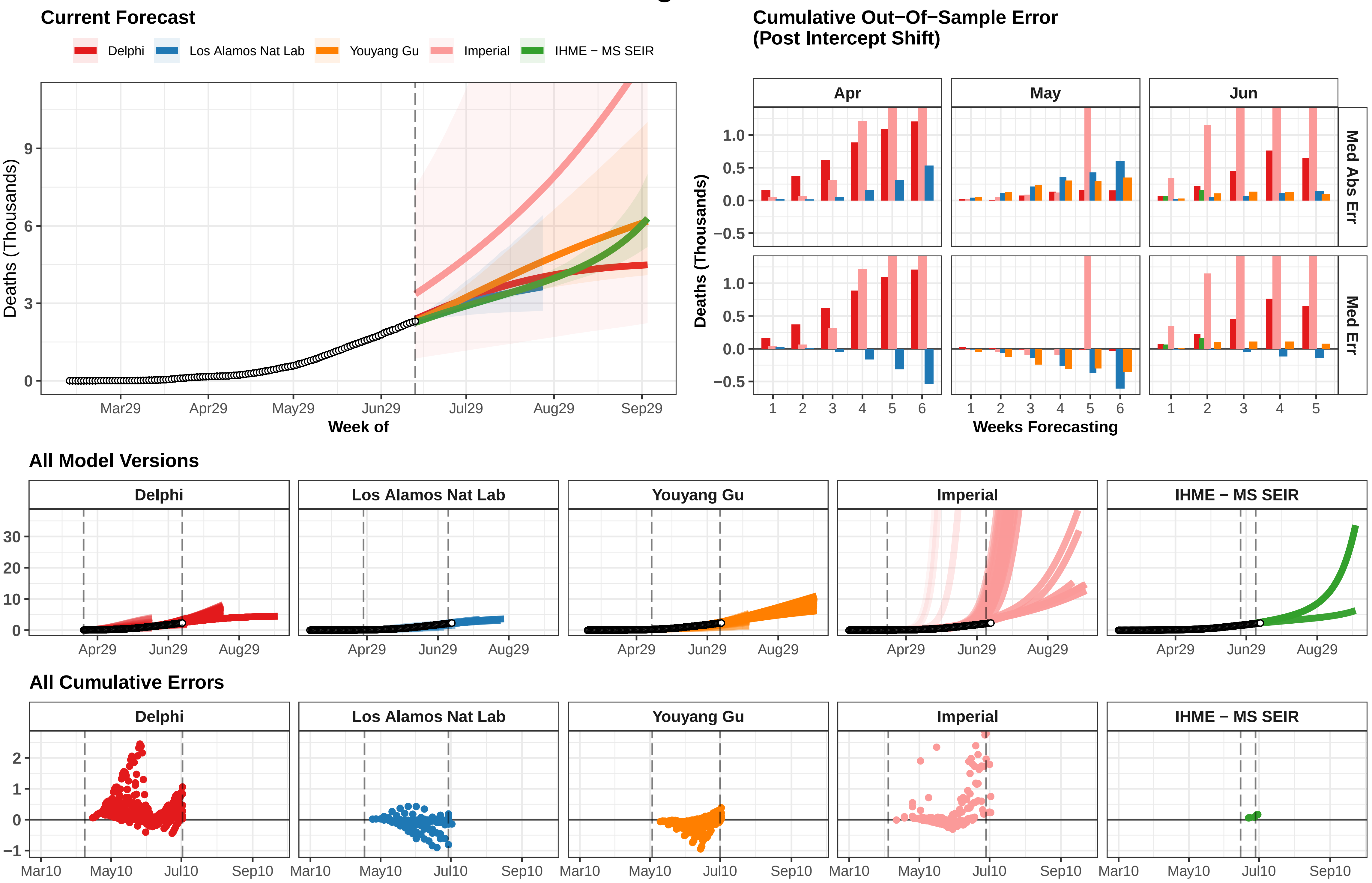
Current Forecast
Delphi
Los Alamos Nat Lab
Youyang Gu
Imperial
IHME − MS SEIR
Deaths (Thousands)
Week of
Mar29
Apr29
May29
Jun29
Jul29
Aug29
Sep29
Cumulative Out−Of−Sample Error
(Post Intercept Shift)
Apr
May
Jun
Med Abs Err
Med Err
Weeks Forecasting
All Model Versions
All Cumulative Errors
Mar10
May10
Jul10
Sep10

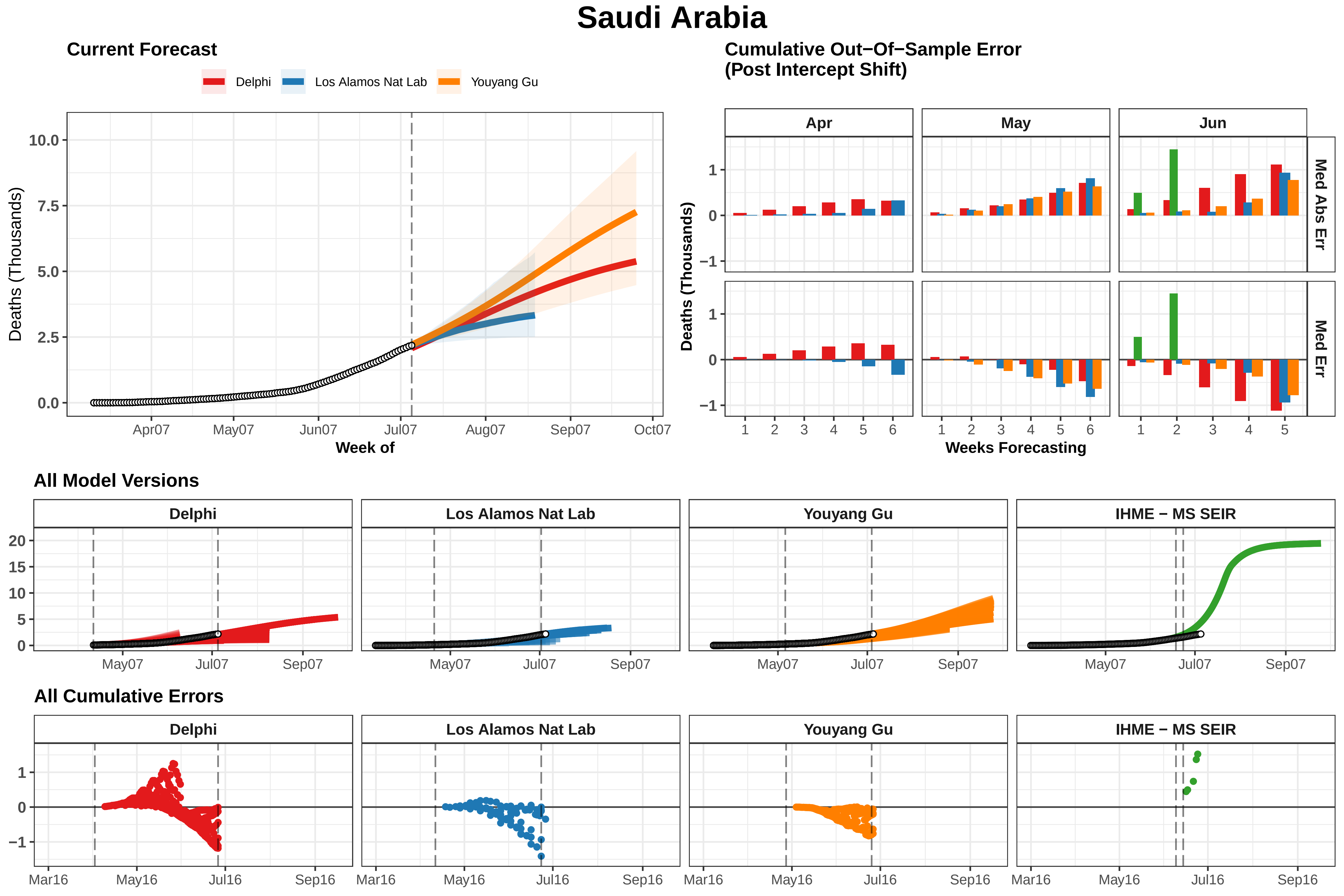

Saudi Arabia
Current Forecast
Delphi
Los Alamos Nat Lab
Youyang Gu
Deaths (Thousands)
Week of
Cumulative Out−Of−Sample Error
(Post Intercept Shift)
Apr
May
Jun
Med Abs Err
Med Err
Weeks Forecasting
All Model Versions
IHME − MS SEIR
All Cumulative Errors

Arizona

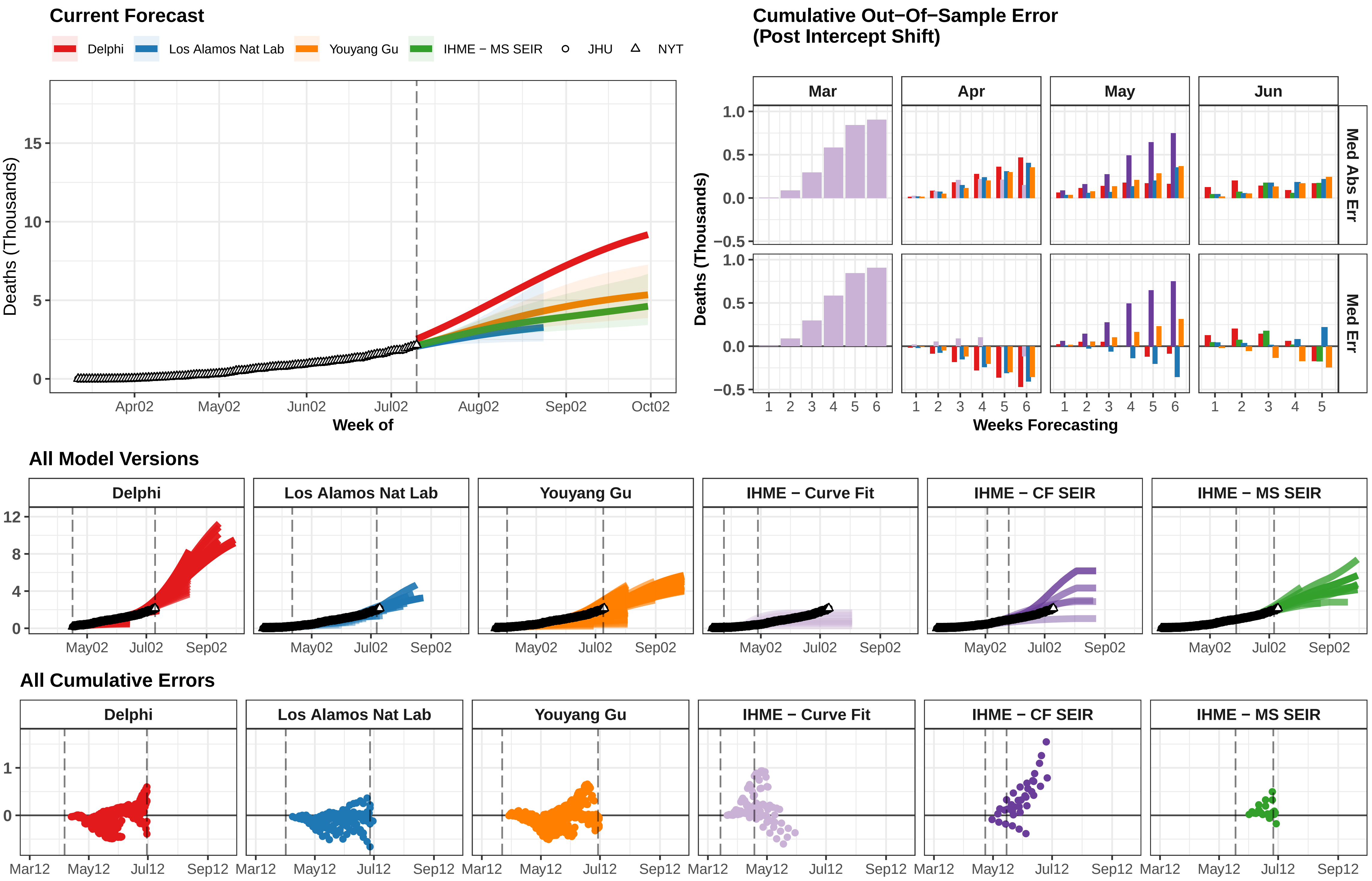

Current Forecast
Delphi
Los Alamos Nat Lab
Youyang Gu
IHME − MS SEIR
JHU
NYT
Deaths (Thousands)
0
5
10
15
Apr02
May02
Jun02
Jul02
Aug02
Sep02
Oct02
Week of
Cumulative Out−Of−Sample Error
(Post Intercept Shift)
Mar
Apr
May
Jun
Med Abs Err
Med Err
Deaths (Thousands)
−0.5
0.0
0.5
1.0
1
2
3
4
5
6
Weeks Forecasting
All Model Versions
Delphi
Los Alamos Nat Lab
Youyang Gu
IHME − Curve Fit
IHME − CF SEIR
IHME − MS SEIR
0
4
8
12
May02
Jul02
Sep02
All Cumulative Errors
0
1
Mar12
May12
Jul12
Sep12

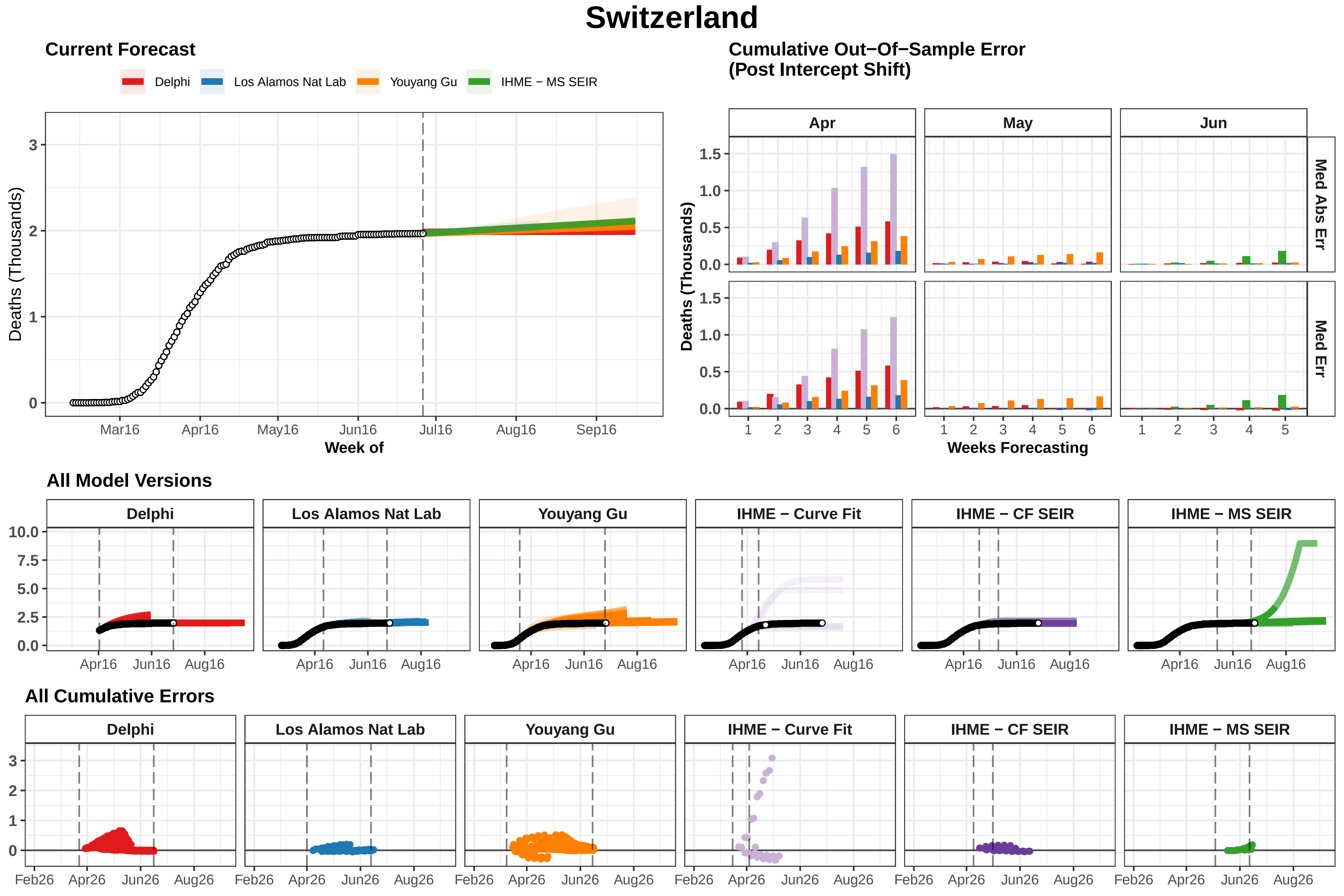

Switzerland
Current Forecast
Delphi
Los Alamos Nat Lab
Youyang Gu
IHME − MS SEIR
Deaths (Thousands)
Week of
Mar16
Apr16
May16
Jun16
Jul16
Aug16
Sep16
Cumulative Out−Of−Sample Error
(Post Intercept Shift)
Apr
May
Jun
Med Abs Err
Med Err
Weeks Forecasting
All Model Versions
Delphi
Los Alamos Nat Lab
Youyang Gu
IHME − Curve Fit
IHME − CF SEIR
IHME − MS SEIR
All Cumulative Errors
Feb26
Apr26
Jun26
Aug26

Virginia

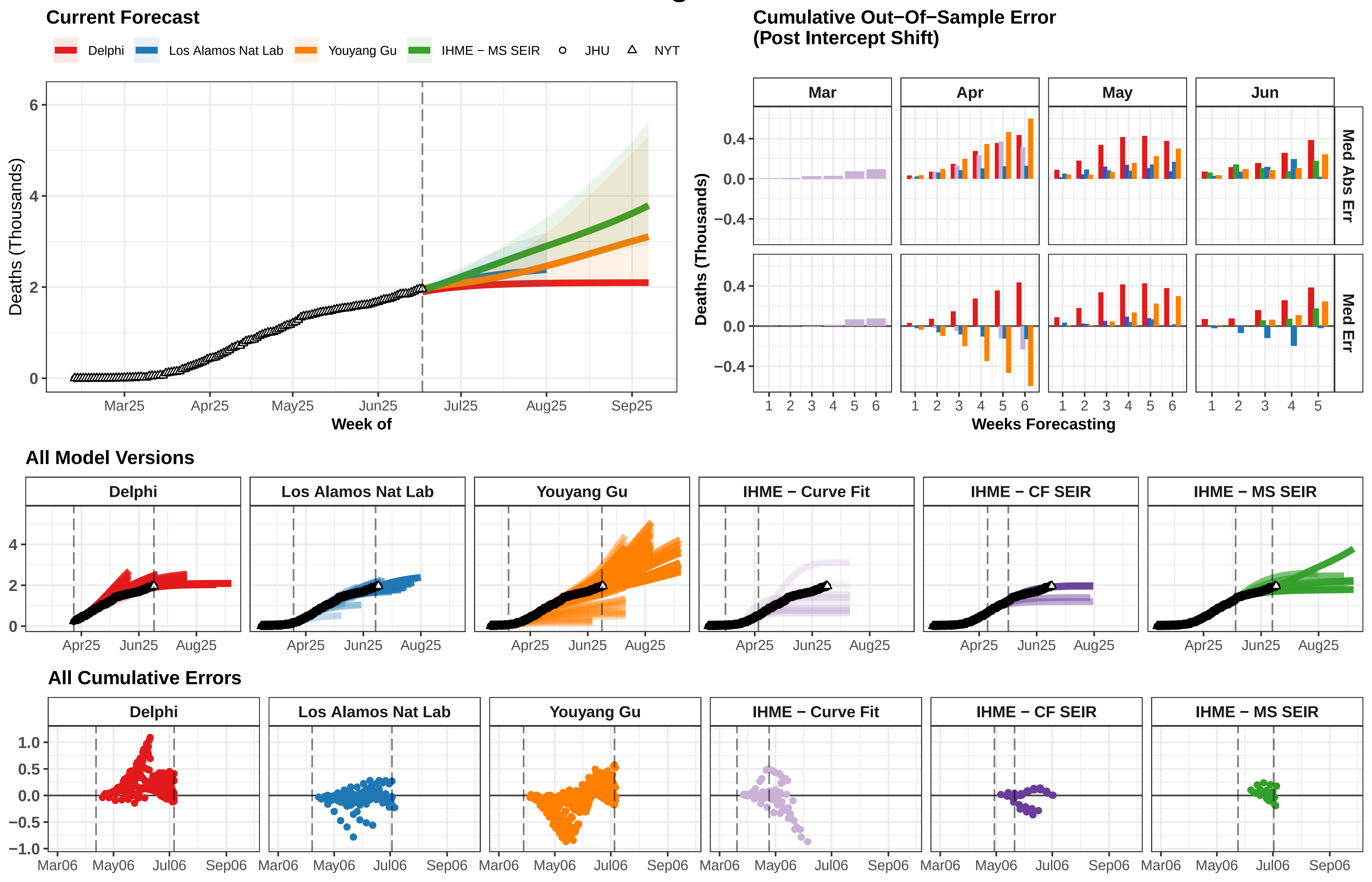

Current Forecast
Delphi
Los Alamos Nat Lab
Youyang Gu
IHME − MS SEIR
JHU
NYT
Deaths (Thousands)
6
4
2
0
Mar25
Apr25
May25
Jun25
Jul25
Aug25
Sep25
Week of
Cumulative Out−Of−Sample Error
(Post Intercept Shift)
Mar
Apr
May
Jun
Med Abs Err
Med Err
0.4
0.0
−0.4
Weeks Forecasting
All Model Versions
Delphi
Los Alamos Nat Lab
Youyang Gu
IHME − Curve Fit
IHME − CF SEIR
IHME − MS SEIR
Apr25
Jun25
Aug25
All Cumulative Errors
1.0
0.5
0.0
−0.5
−1.0
Mar06
May06
Jul06
Sep06

Romania

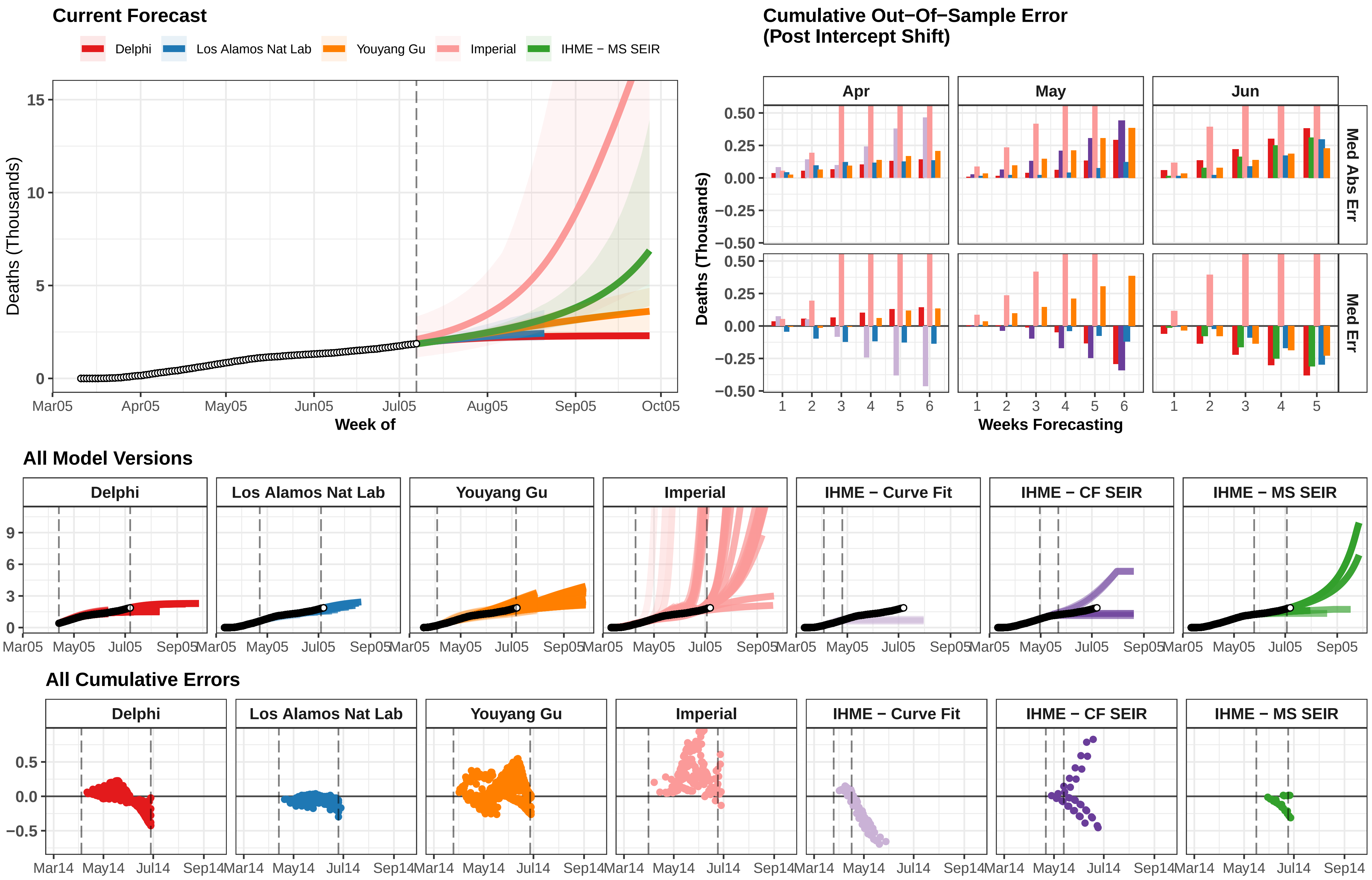
Current Forecast
Delphi
Los Alamos Nat Lab
Youyang Gu
Imperial
IHME − MS SEIR
Deaths (Thousands)
Week of
Mar05
Apr05
May05
Jun05
Jul05
Aug05
Sep05
Oct05
Cumulative Out−Of−Sample Error
(Post Intercept Shift)
Apr
May
Jun
Med Abs Err
Med Err
Weeks Forecasting
All Model Versions
IHME − Curve Fit
IHME − CF SEIR
All Cumulative Errors
Mar14
May14
Jul14
Sep14

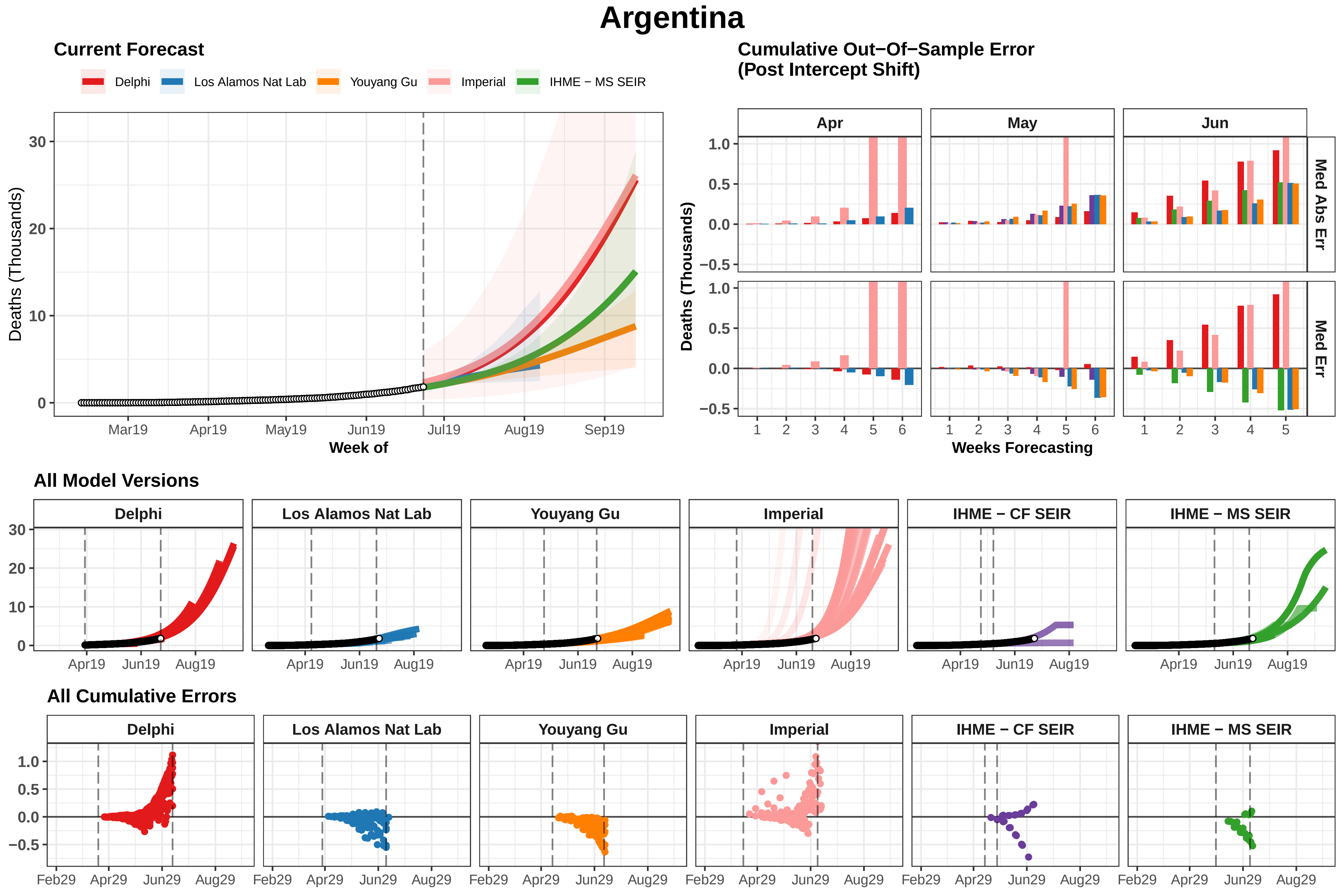
Argentina
Current Forecast
Delphi
Los Alamos Nat Lab
Youyang Gu
Imperial
IHME − MS SEIR
Deaths (Thousands)
Week of
Mar19
Apr19
May19
Jun19
Jul19
Aug19
Sep19
Cumulative Out−Of−Sample Error
(Post Intercept Shift)
Apr
May
Jun
Med Abs Err
Med Err
Weeks Forecasting
All Model Versions
IHME − CF SEIR
All Cumulative Errors
Feb29
Apr29
Jun29
Aug29

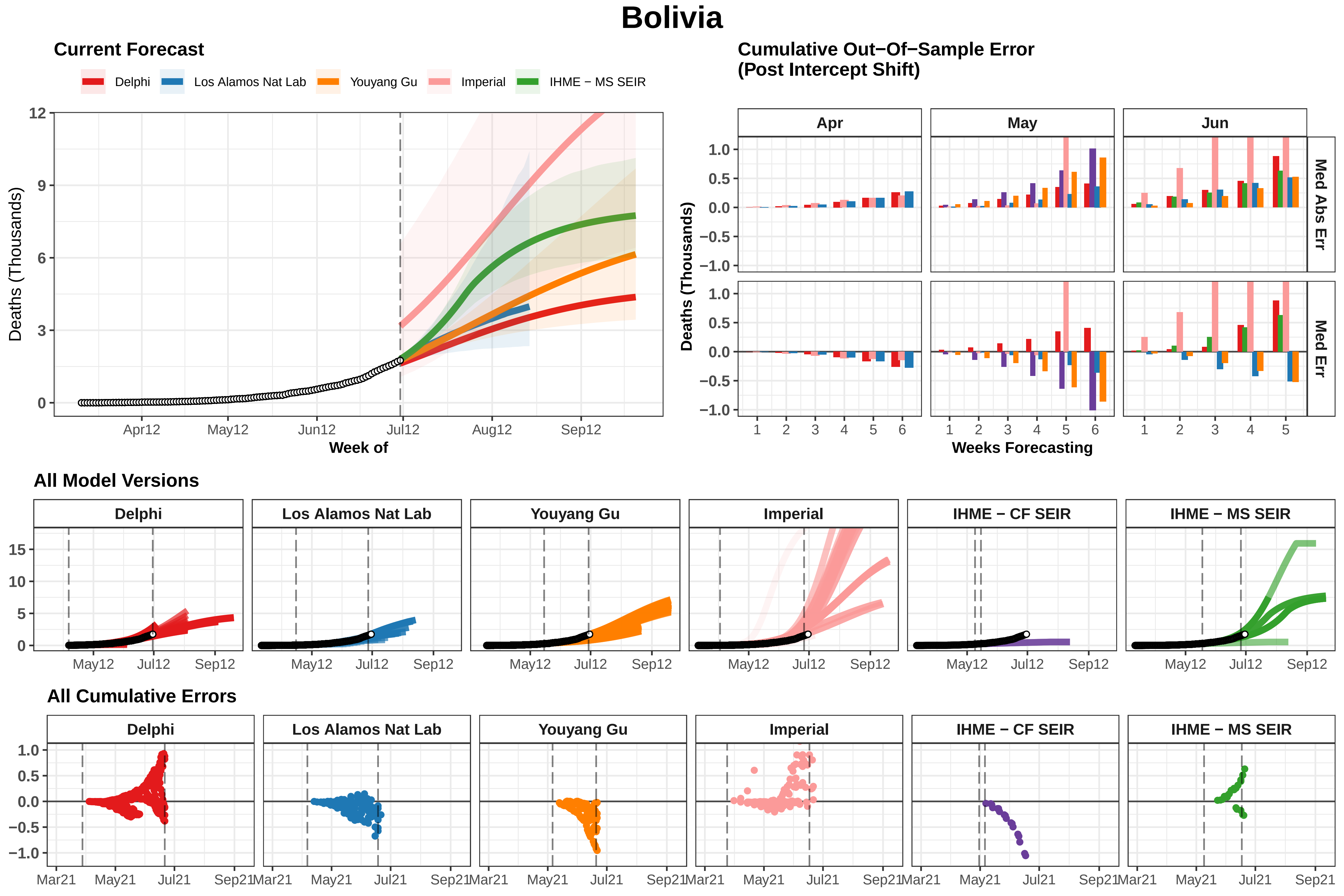
Bolivia
Current Forecast
Delphi
Los Alamos Nat Lab
Youyang Gu
Imperial
IHME − MS SEIR
Deaths (Thousands)
Week of
Apr12
May12
Jun12
Jul12
Aug12
Sep12
Cumulative Out−Of−Sample Error (Post Intercept Shift)
Apr
May
Jun
Med Abs Err
Med Err
Weeks Forecasting
All Model Versions
IHME − CF SEIR
All Cumulative Errors
Mar21
May21
Jul21
Sep21

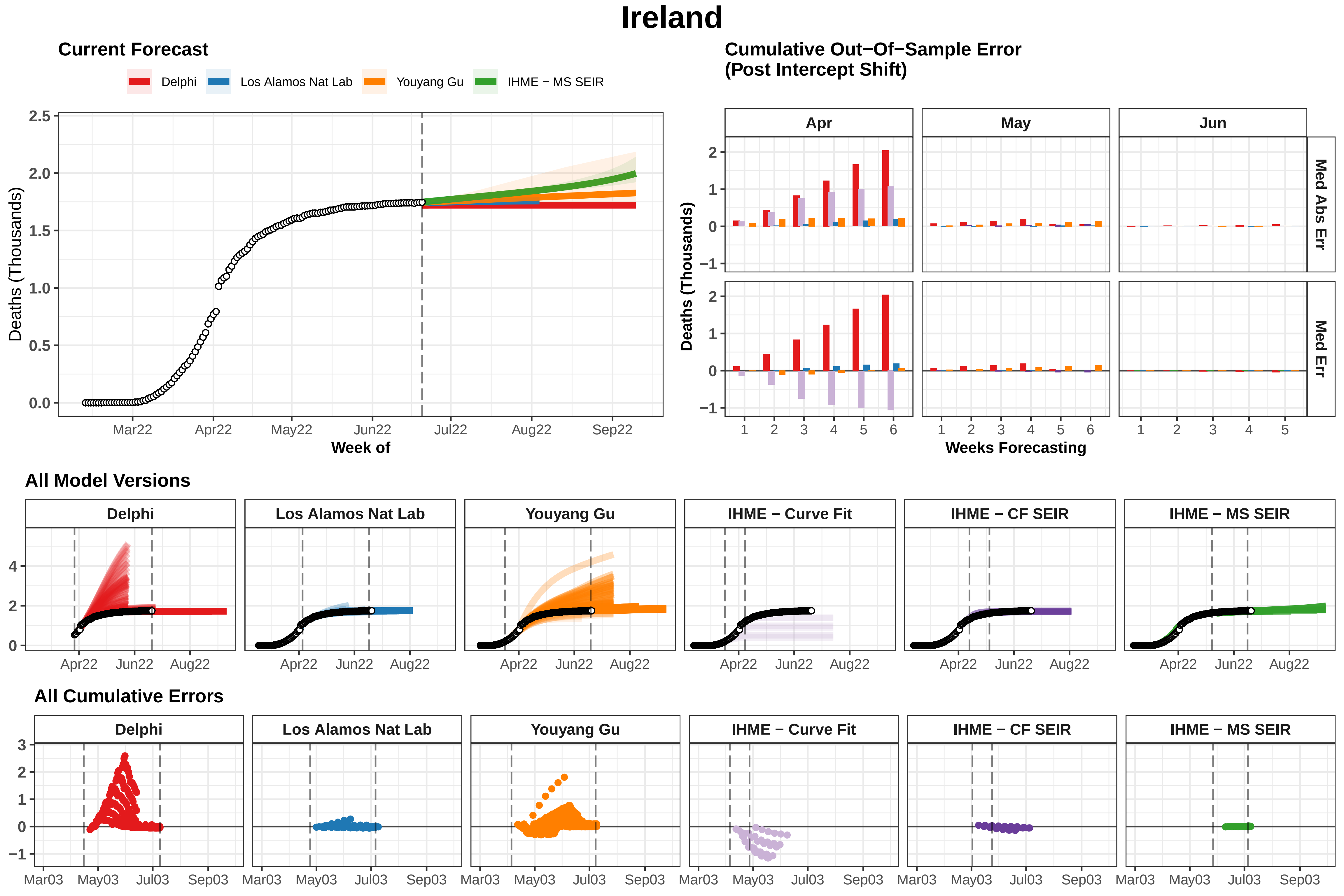
Ireland
Current Forecast
Delphi
Los Alamos Nat Lab
Youyang Gu
IHME − MS SEIR
Deaths (Thousands)
Week of
Mar22
Apr22
May22
Jun22
Jul22
Aug22
Sep22
Cumulative Out−Of−Sample Error
(Post Intercept Shift)
Apr
May
Jun
Med Abs Err
Med Err
Weeks Forecasting
All Model Versions
IHME − Curve Fit
IHME − CF SEIR
All Cumulative Errors
Mar03
May03
Jul03
Sep03

Colorado

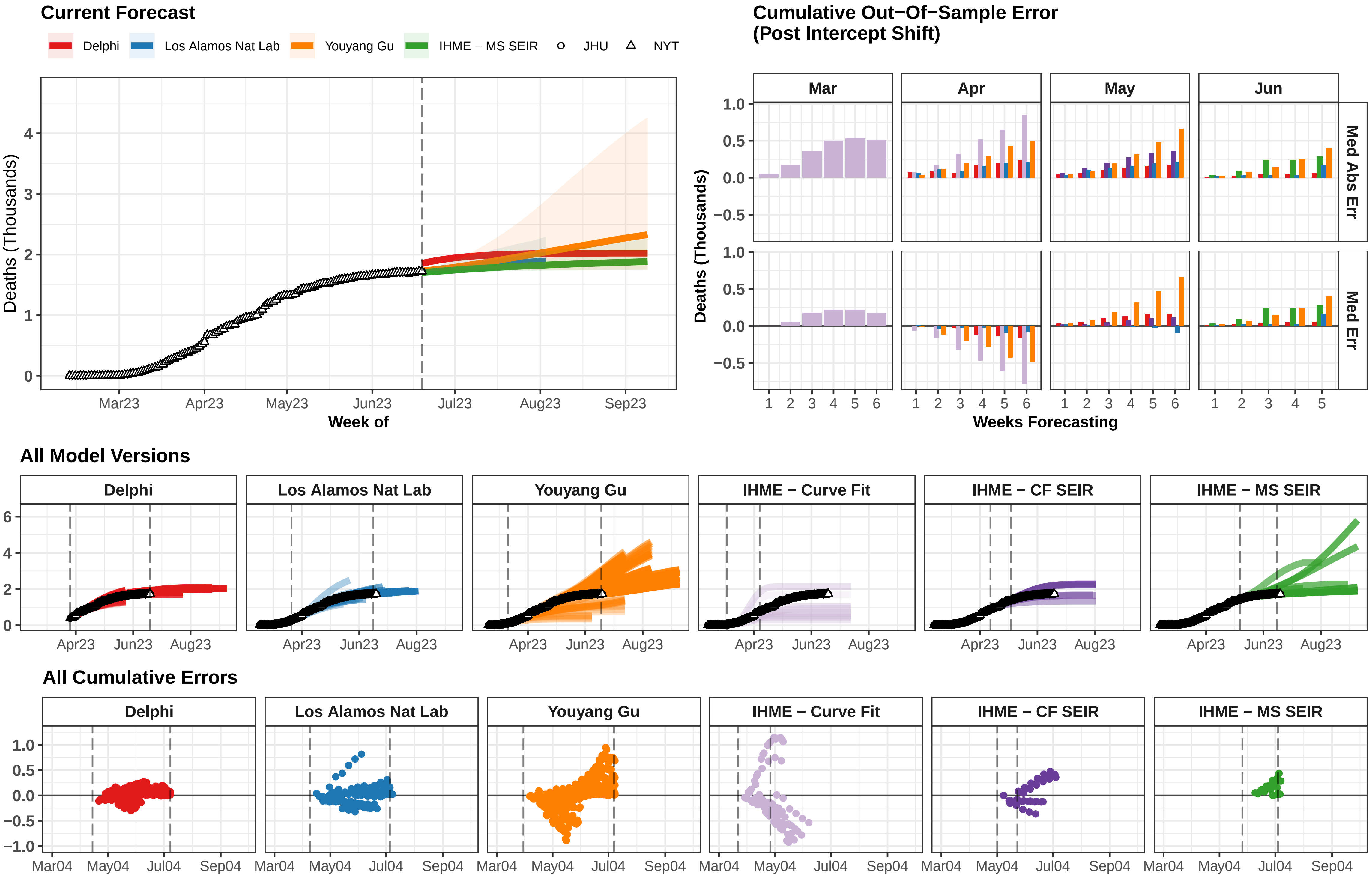

Current Forecast
Delphi
Los Alamos Nat Lab
Youyang Gu
IHME − MS SEIR
JHU
NYT
Deaths (Thousands)
Week of
Mar23
Apr23
May23
Jun23
Jul23
Aug23
Sep23
Cumulative Out−Of−Sample Error
(Post Intercept Shift)
Mar
Apr
May
Jun
Med Abs Err
Med Err
Weeks Forecasting
All Model Versions
Delphi
Los Alamos Nat Lab
Youyang Gu
IHME − Curve Fit
IHME − CF SEIR
IHME − MS SEIR
All Cumulative Errors
Mar04
May04
Jul04
Sep04

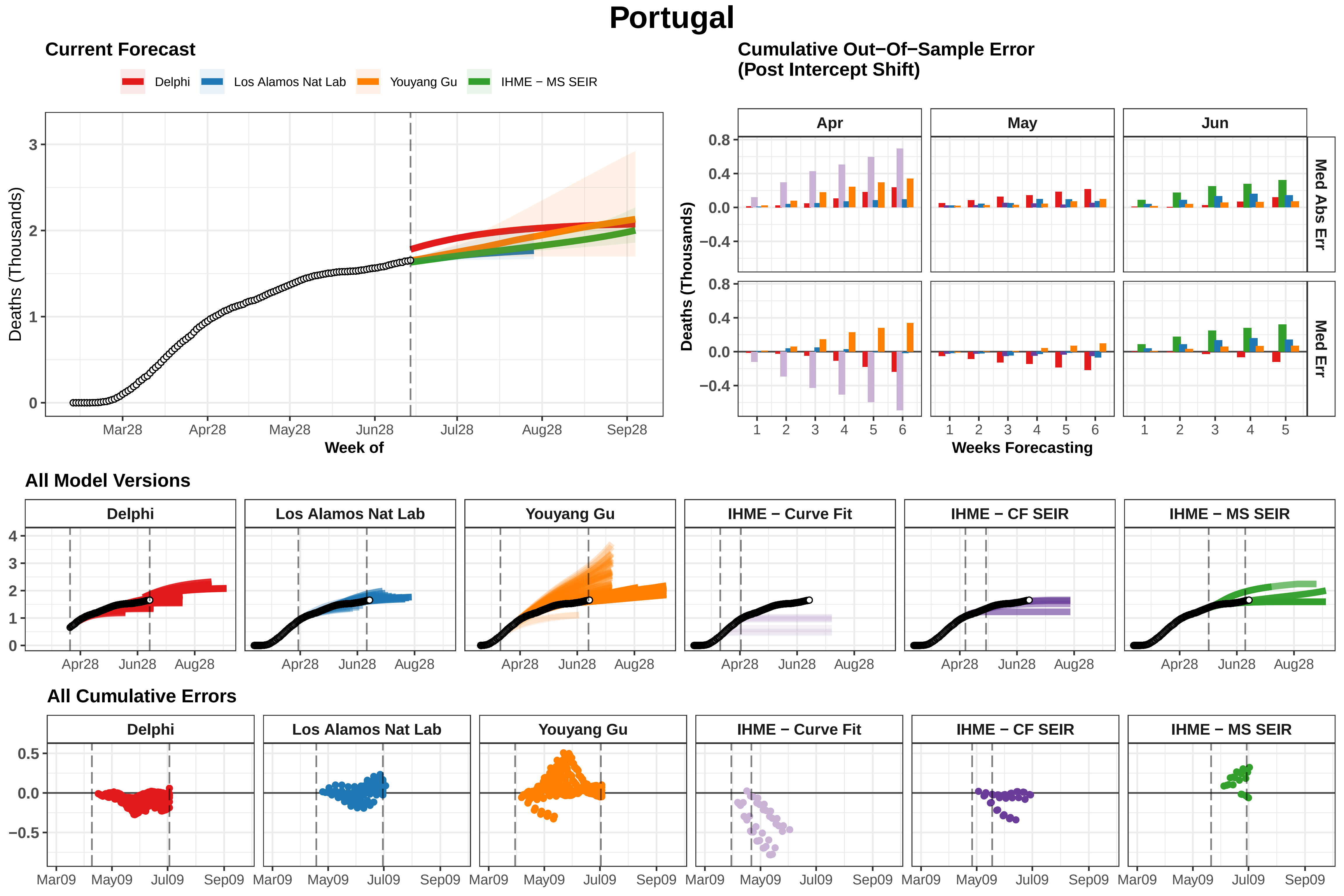
Portugal
Current Forecast
Delphi
Los Alamos Nat Lab
Youyang Gu
IHME − MS SEIR
Deaths (Thousands)
0
1
2
3
Mar28
Apr28
May28
Jun28
Jul28
Aug28
Sep28
Week of
Cumulative Out−Of−Sample Error
(Post Intercept Shift)
Apr
May
Jun
Med Abs Err
Med Err
0.8
0.4
0.0
−0.4
Weeks Forecasting
All Model Versions
Delphi
Los Alamos Nat Lab
Youyang Gu
IHME − Curve Fit
IHME − CF SEIR
IHME − MS SEIR
4
Apr28
Jun28
Aug28
All Cumulative Errors
0.5
0.0
−0.5
Mar09
May09
Jul09
Sep09

Poland

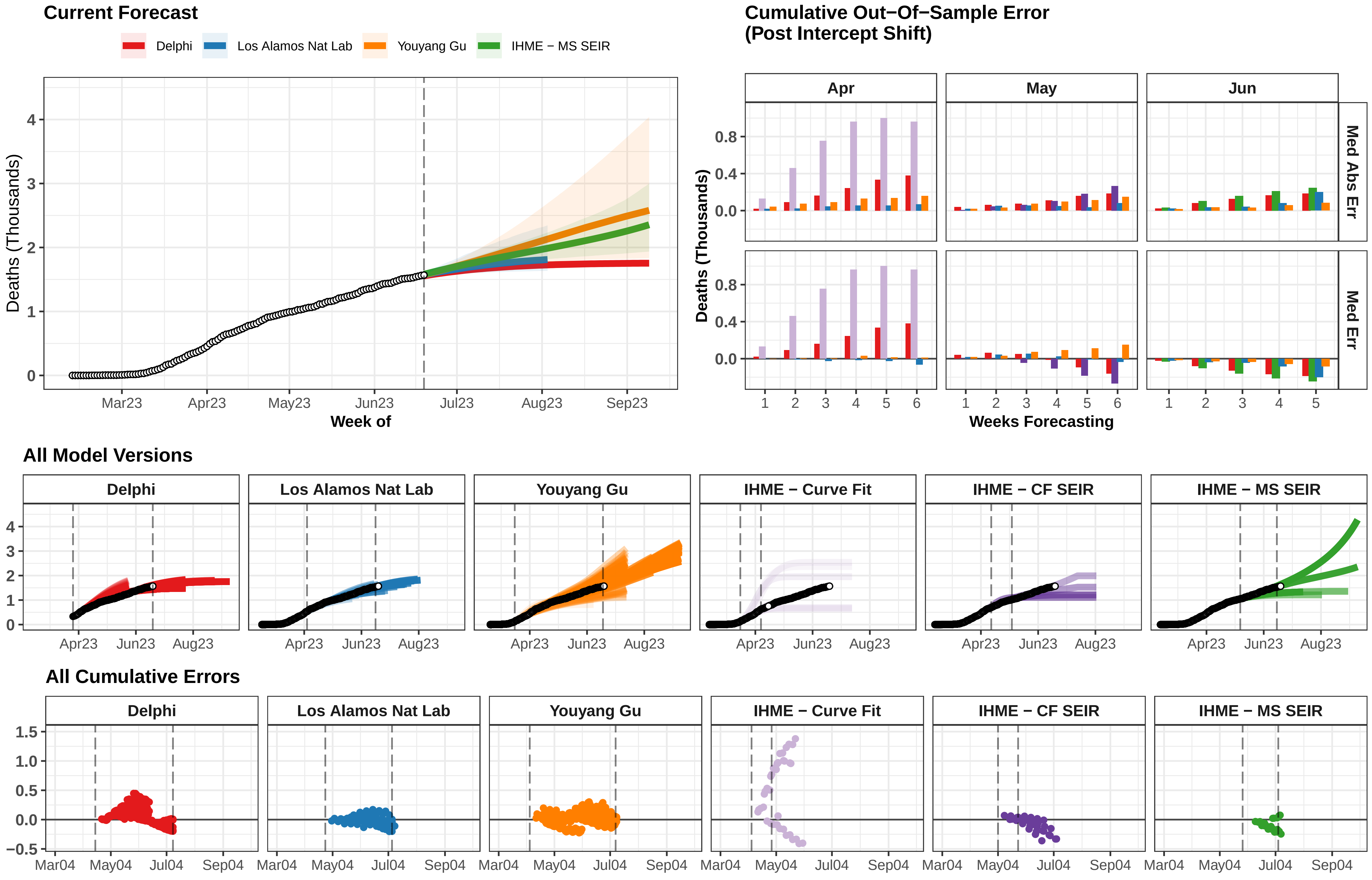
Current Forecast
Delphi
Los Alamos Nat Lab
Youyang Gu
IHME − MS SEIR
Deaths (Thousands)
Week of
Mar23
Apr23
May23
Jun23
Jul23
Aug23
Sep23
Cumulative Out−Of−Sample Error
(Post Intercept Shift)
Apr
May
Jun
Med Abs Err
Med Err
Weeks Forecasting
All Model Versions
Delphi
Los Alamos Nat Lab
Youyang Gu
IHME − Curve Fit
IHME − CF SEIR
IHME − MS SEIR
All Cumulative Errors
Mar04
May04
Jul04
Sep04

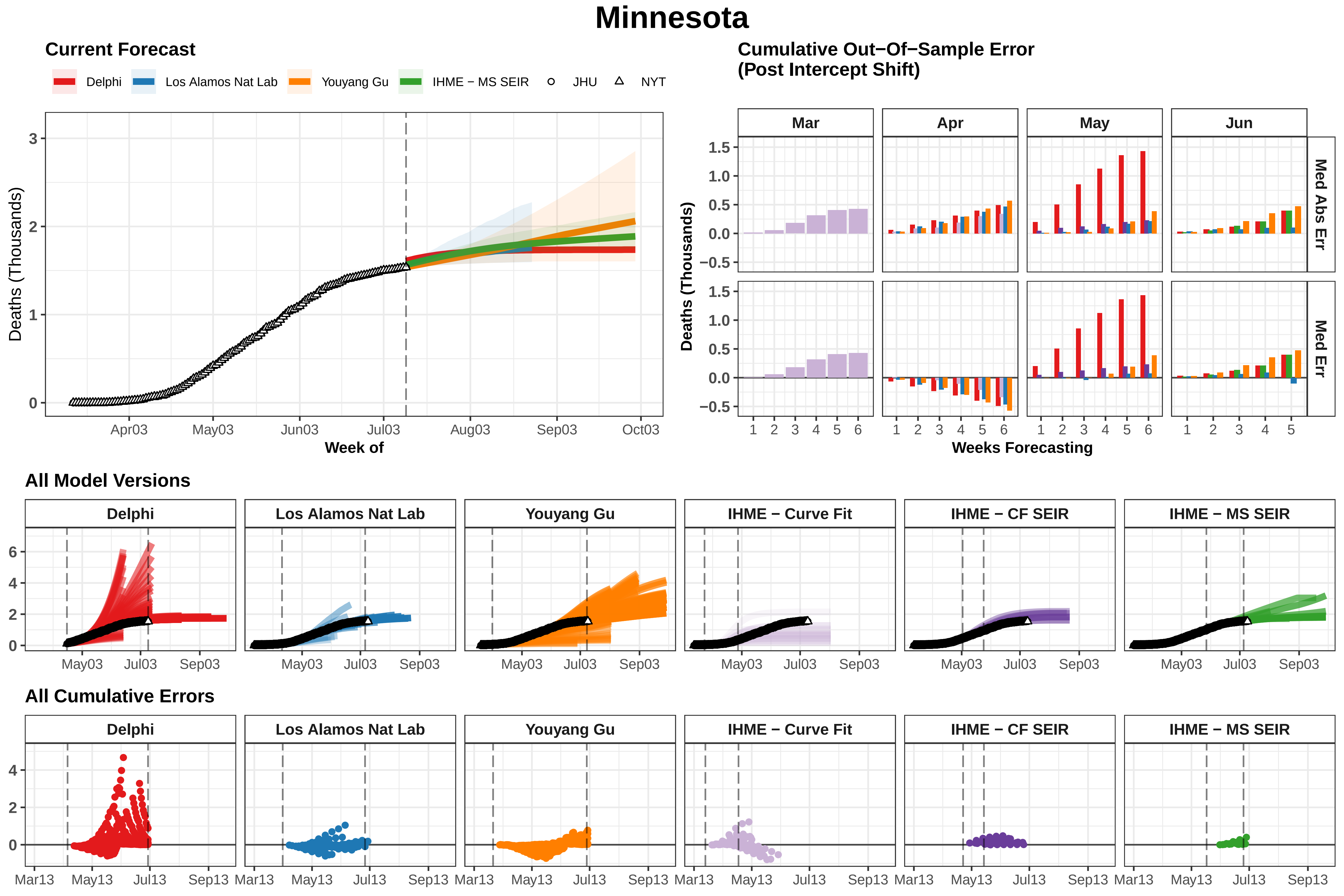
Minnesota
Current Forecast
Delphi
Los Alamos Nat Lab
Youyang Gu
IHME − MS SEIR
JHU
NYT
Deaths (Thousands)
Week of
Apr03
May03
Jun03
Jul03
Aug03
Sep03
Oct03
Cumulative Out−Of−Sample Error
(Post Intercept Shift)
Mar
Apr
May
Jun
Med Abs Err
Med Err
Weeks Forecasting
All Model Versions
Delphi
Los Alamos Nat Lab
Youyang Gu
IHME − Curve Fit
IHME − CF SEIR
IHME − MS SEIR
All Cumulative Errors
Mar13
May13
Jul13
Sep13

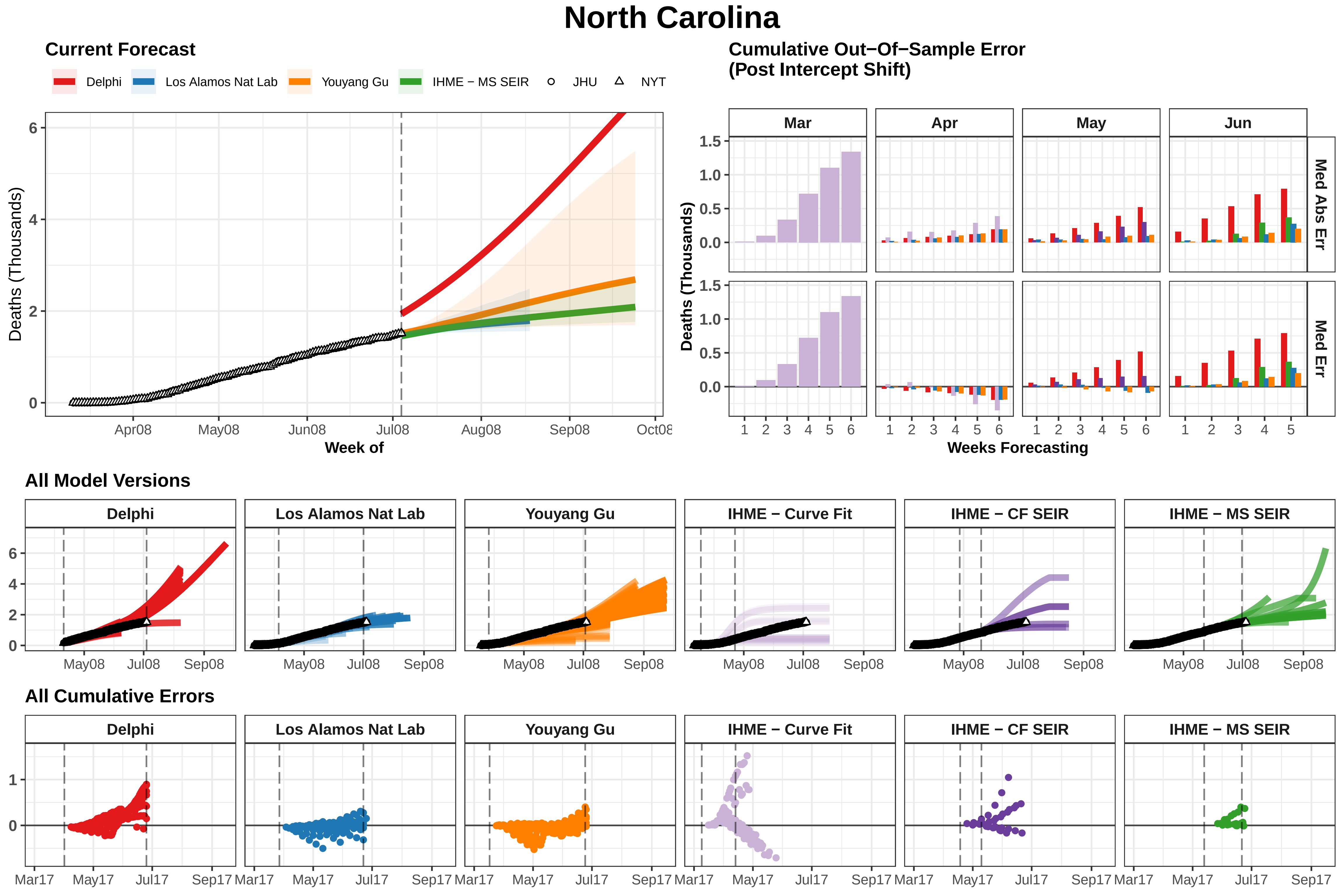
North Carolina
Current Forecast
Delphi
Los Alamos Nat Lab
Youyang Gu
IHME − MS SEIR
JHU
NYT
Deaths (Thousands)
Week of
Apr08
May08
Jun08
Jul08
Aug08
Sep08
Oct08
Cumulative Out−Of−Sample Error
(Post Intercept Shift)
Mar
Apr
May
Jun
Med Abs Err
Med Err
Weeks Forecasting
All Model Versions
Delphi
Los Alamos Nat Lab
Youyang Gu
IHME − Curve Fit
IHME − CF SEIR
IHME − MS SEIR
All Cumulative Errors
Mar17
May17
Jul17
Sep17

Washington

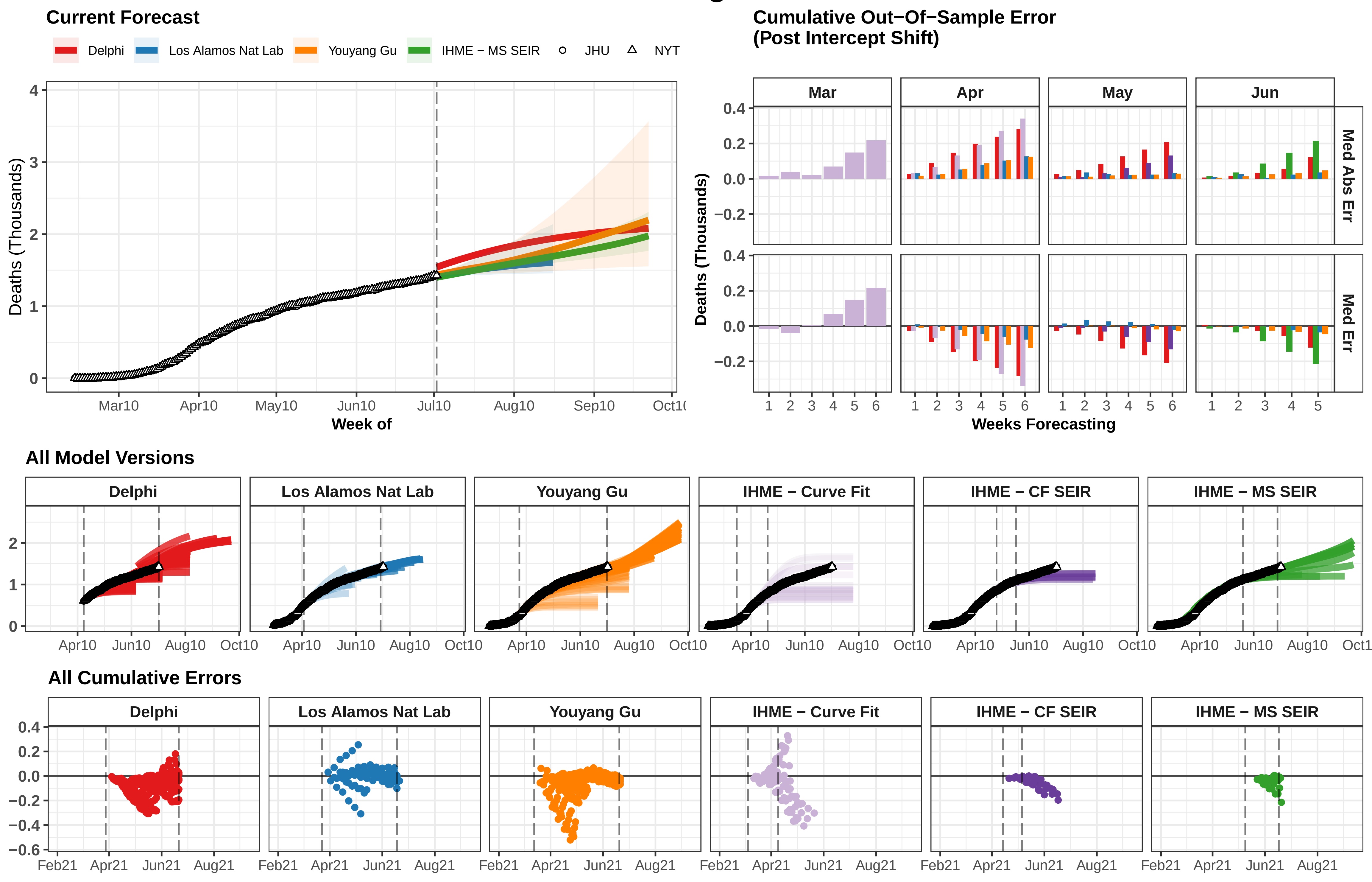
Current Forecast
Delphi
Los Alamos Nat Lab
Youyang Gu
IHME − MS SEIR
JHU
NYT
Deaths (Thousands)
Week of
Mar10
Apr10
May10
Jun10
Jul10
Aug10
Sep10
Cumulative Out−Of−Sample Error
(Post Intercept Shift)
Mar
Apr
May
Jun
Med Abs Err
Med Err
Weeks Forecasting
All Model Versions
Delphi
Los Alamos Nat Lab
Youyang Gu
IHME − Curve Fit
IHME − CF SEIR
IHME − MS SEIR
Apr10
Jun10
Aug10
Oct10
All Cumulative Errors
Feb21
Apr21
Jun21
Aug21

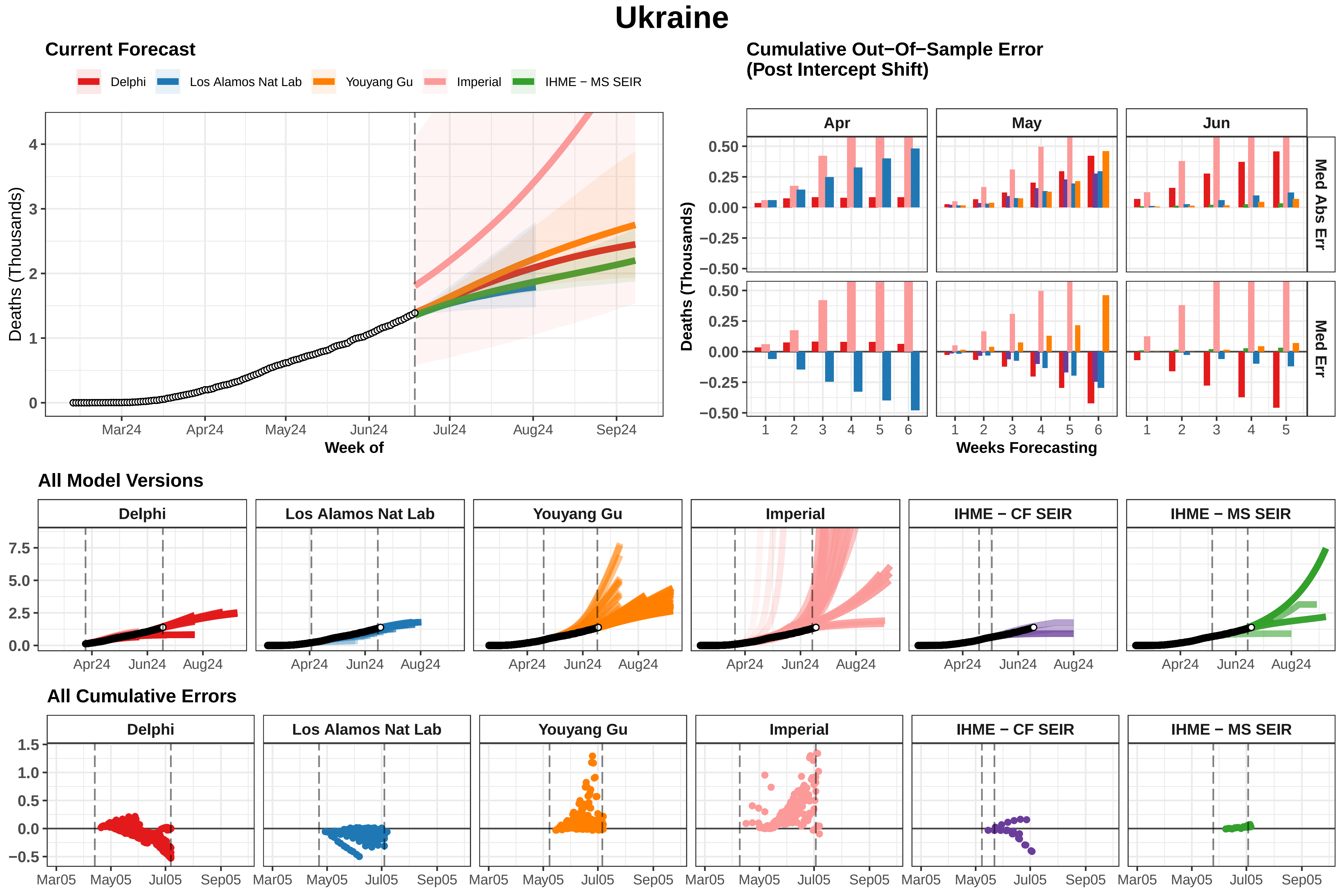
Ukraine
Current Forecast
Delphi
Los Alamos Nat Lab
Youyang Gu
Imperial
IHME − MS SEIR
Deaths (Thousands)
Week of
Mar24
Apr24
May24
Jun24
Jul24
Aug24
Sep24
Cumulative Out−Of−Sample Error
(Post Intercept Shift)
Apr
May
Jun
Med Abs Err
Med Err
Weeks Forecasting
All Model Versions
Delphi
Los Alamos Nat Lab
Youyang Gu
Imperial
IHME − CF SEIR
IHME − MS SEIR
All Cumulative Errors
Mar05
May05
Jul05
Sep05

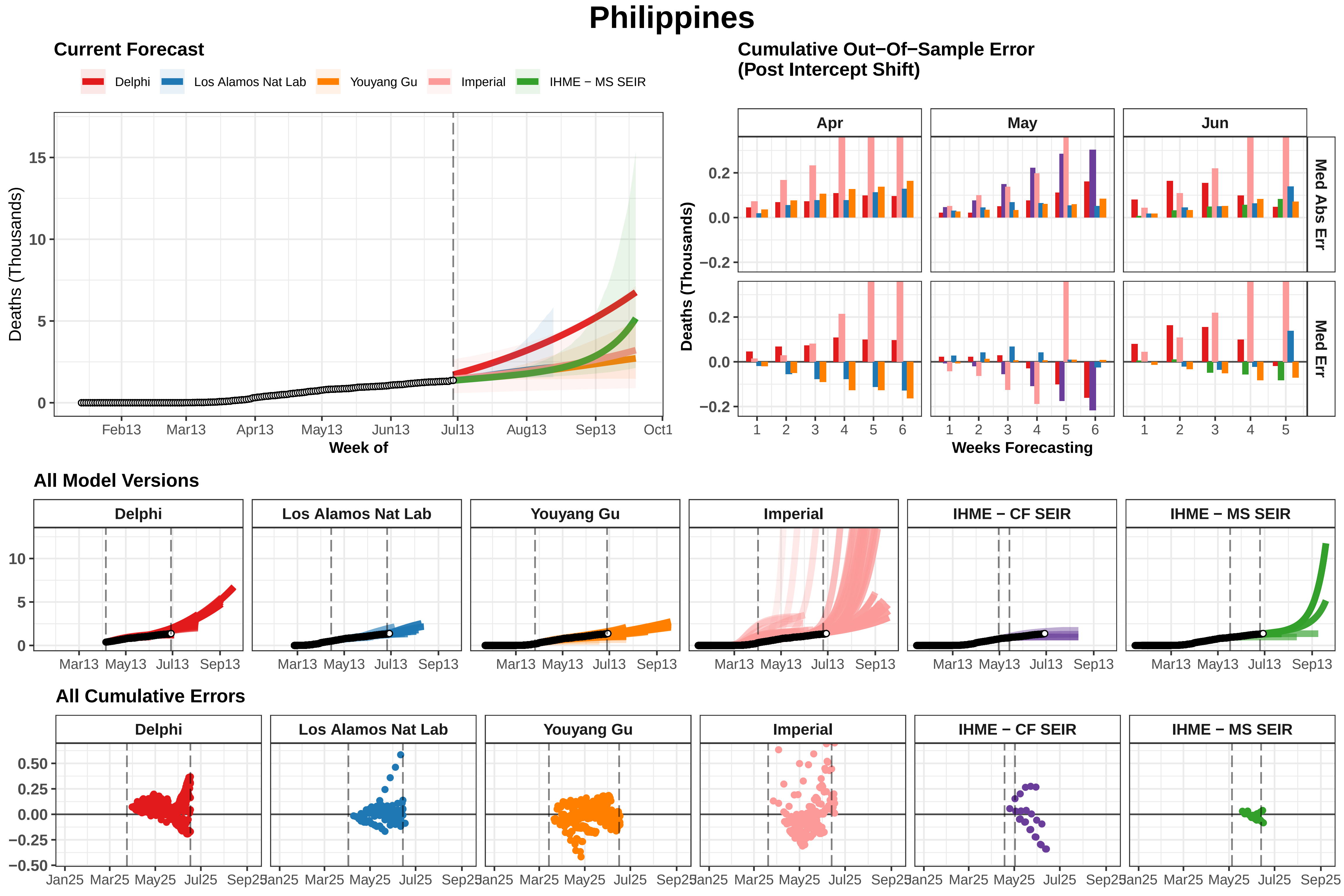

Philippines
Current Forecast
Delphi
Los Alamos Nat Lab
Youyang Gu
Imperial
IHME − MS SEIR
Deaths (Thousands)
Week of
Feb13
Mar13
Apr13
May13
Jun13
Jul13
Aug13
Sep13
Oct1
Cumulative Out−Of−Sample Error
(Post Intercept Shift)
Apr
May
Jun
Med Abs Err
Med Err
Weeks Forecasting
All Model Versions
IHME − CF SEIR
All Cumulative Errors
Jan25
Mar25
May25
Jul25
Sep25

Mississippi

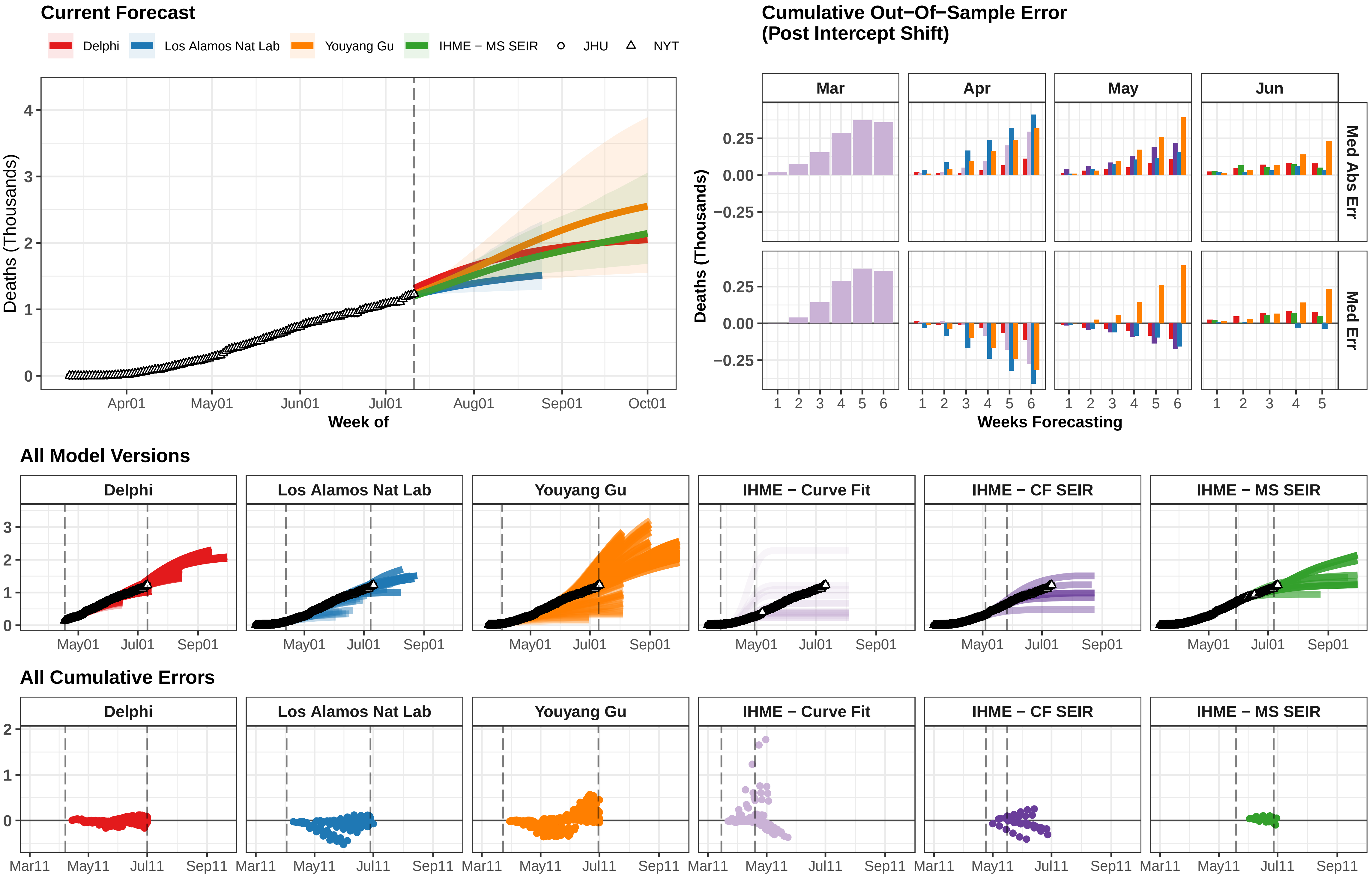

Guatemala

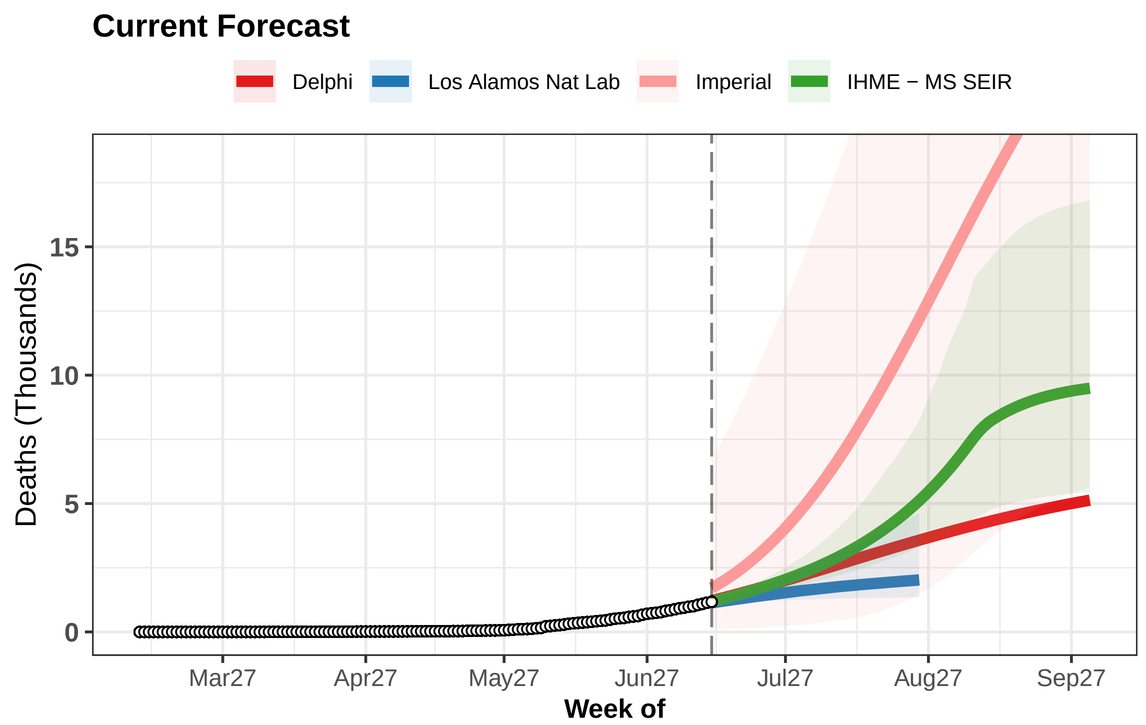
Current Forecast
Delphi
Los Alamos Nat Lab
Imperial
IHME − MS SEIR
Deaths (Thousands)
0
5
10
15
Mar27
Apr27
May27
Jun27
Jul27
Aug27
Sep27
Week of

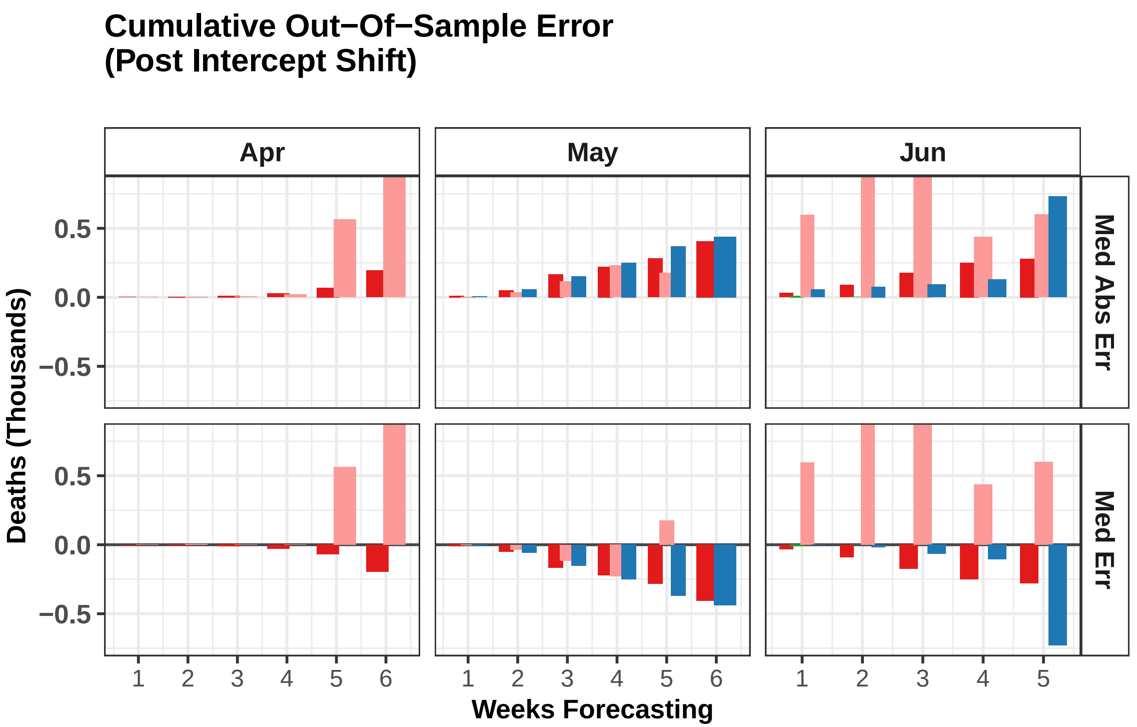
Cumulative Out−Of−Sample Error
(Post Intercept Shift)
Apr
May
Jun
Med Abs Err
Med Err
Deaths (Thousands)
0.5
0.0
−0.5
1
2
3
4
5
6
Weeks Forecasting

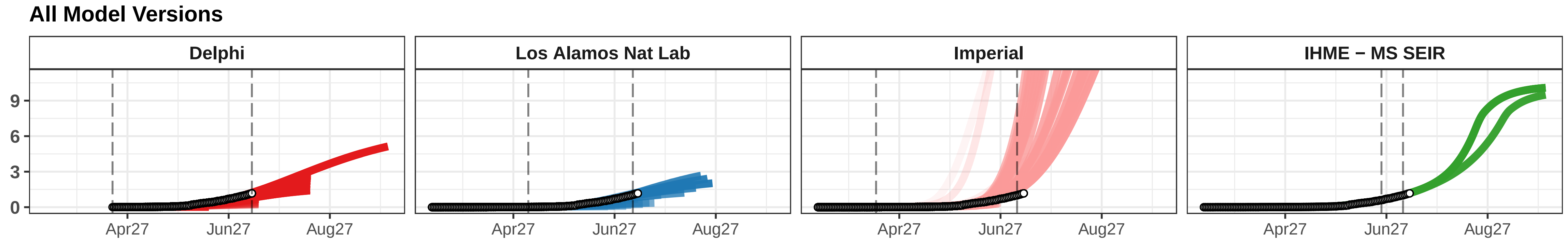
All Model Versions
Delphi
Los Alamos Nat Lab
Imperial
IHME − MS SEIR
0
3
6
9
Apr27
Jun27
Aug27

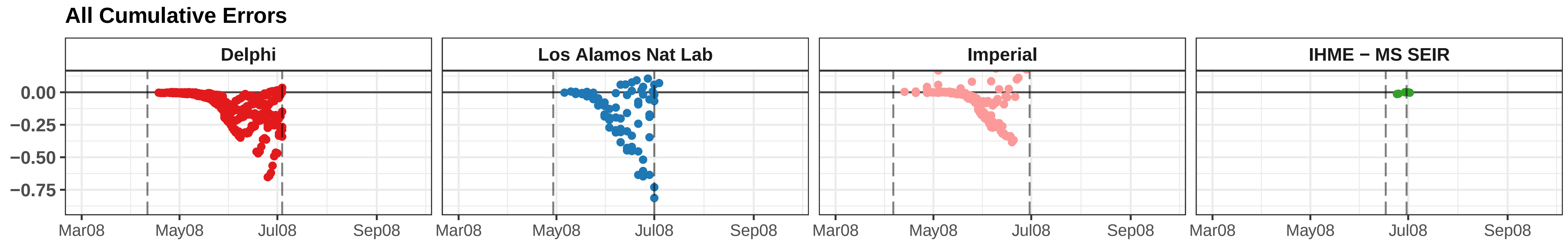
All Cumulative Errors
Delphi
Los Alamos Nat Lab
Imperial
IHME − MS SEIR
0.00
−0.25
−0.50
−0.75
Mar08
May08
Jul08
Sep08

Alabama

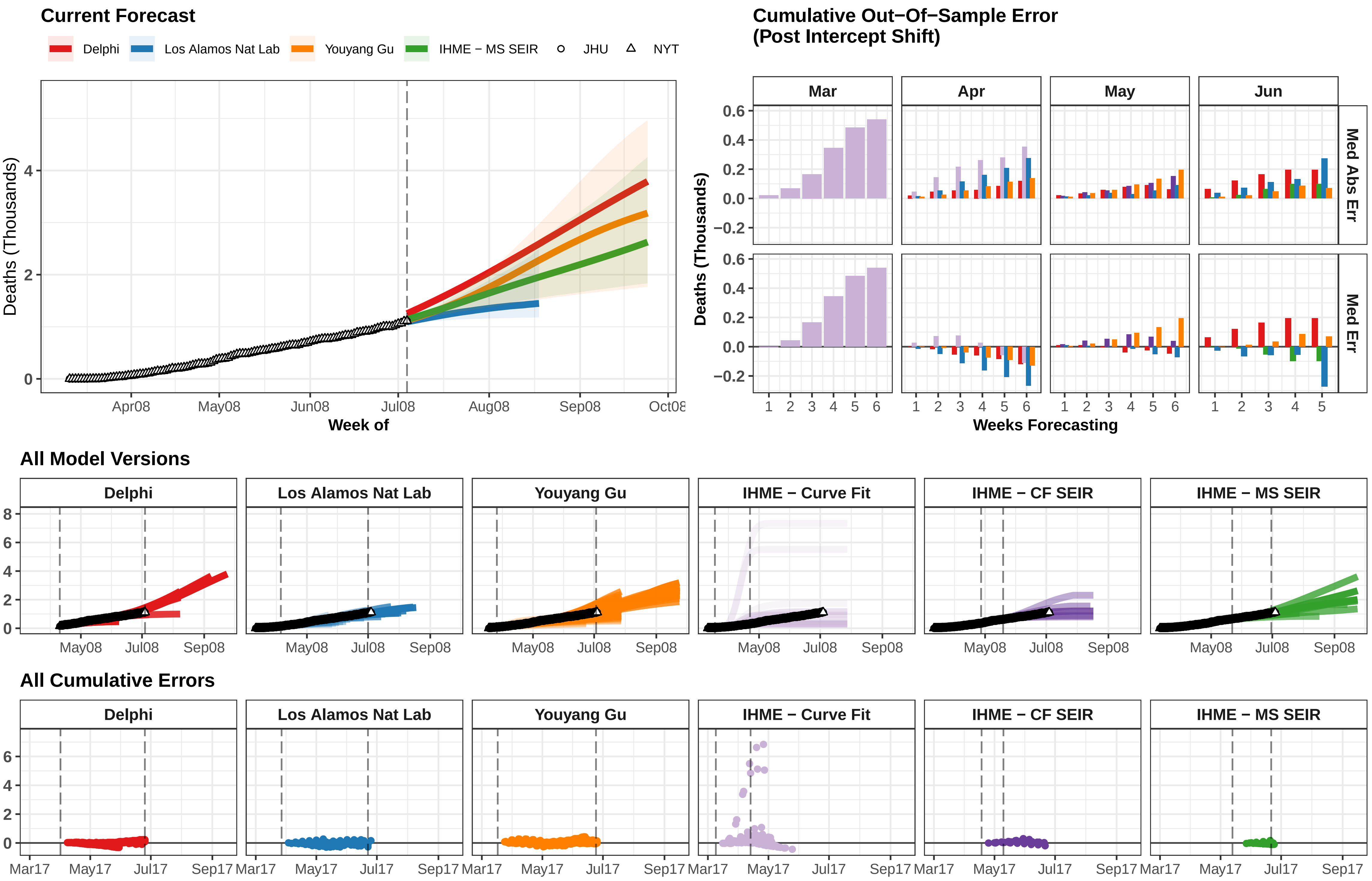
Current Forecast
Delphi
Los Alamos Nat Lab
Youyang Gu
IHME − MS SEIR
JHU
NYT
Deaths (Thousands)
Week of
Apr08
May08
Jun08
Jul08
Aug08
Sep08
Oct08
Cumulative Out−Of−Sample Error
(Post Intercept Shift)
Mar
Apr
May
Jun
Med Abs Err
Med Err
Weeks Forecasting
All Model Versions
Delphi
Los Alamos Nat Lab
Youyang Gu
IHME − Curve Fit
IHME − CF SEIR
IHME − MS SEIR
All Cumulative Errors
Mar17
May17
Jul17
Sep17

Missouri

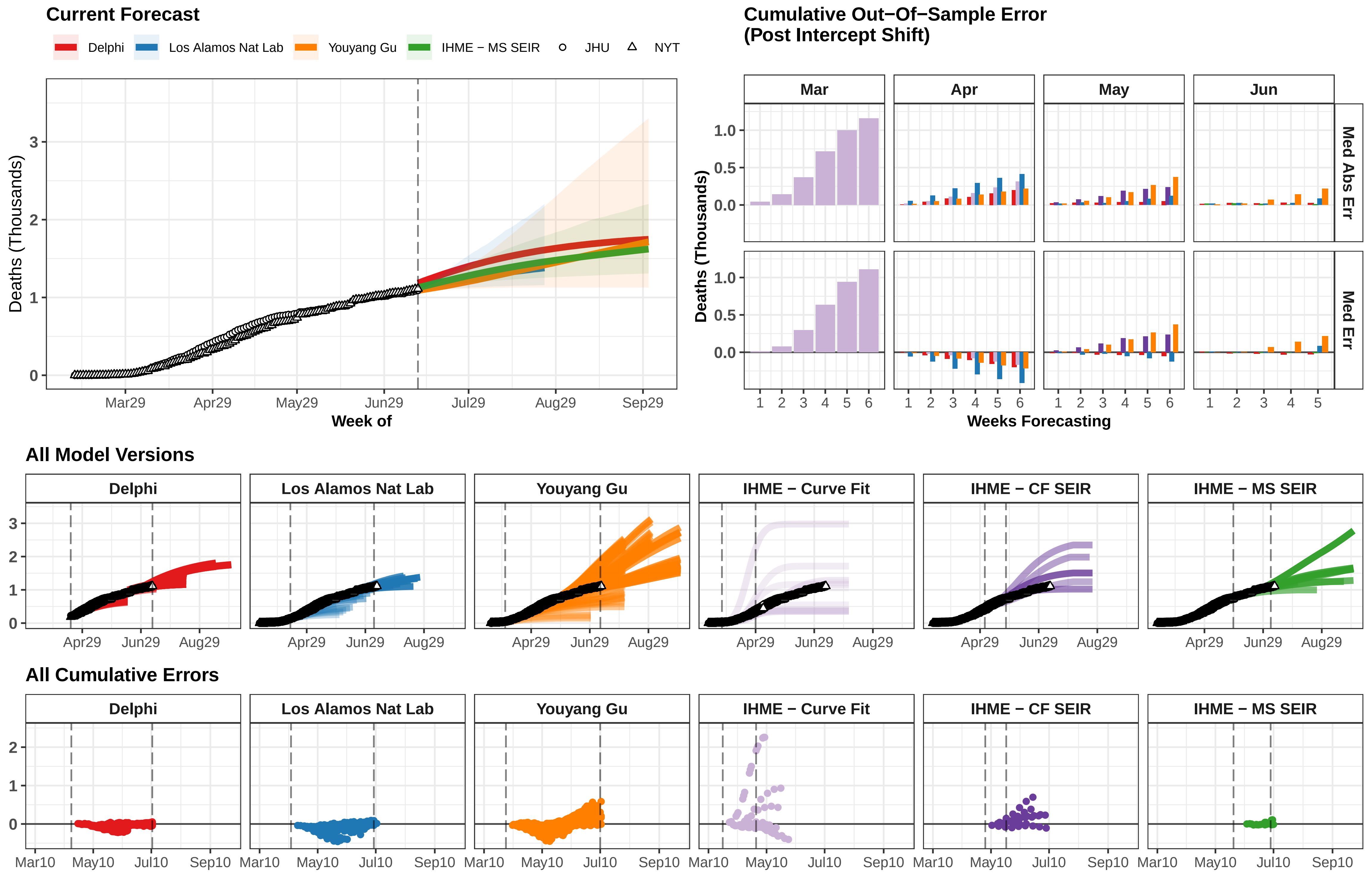

Current Forecast
Delphi
Los Alamos Nat Lab
Youyang Gu
IHME − MS SEIR
JHU
NYT
Deaths (Thousands)
Week of
Mar29
Apr29
May29
Jun29
Jul29
Aug29
Sep29
Cumulative Out−Of−Sample Error
(Post Intercept Shift)
Mar
Apr
May
Jun
Med Abs Err
Med Err
Weeks Forecasting
All Model Versions
Delphi
Los Alamos Nat Lab
Youyang Gu
IHME − Curve Fit
IHME − CF SEIR
IHME − MS SEIR
All Cumulative Errors
Mar10
May10
Jul10
Sep10

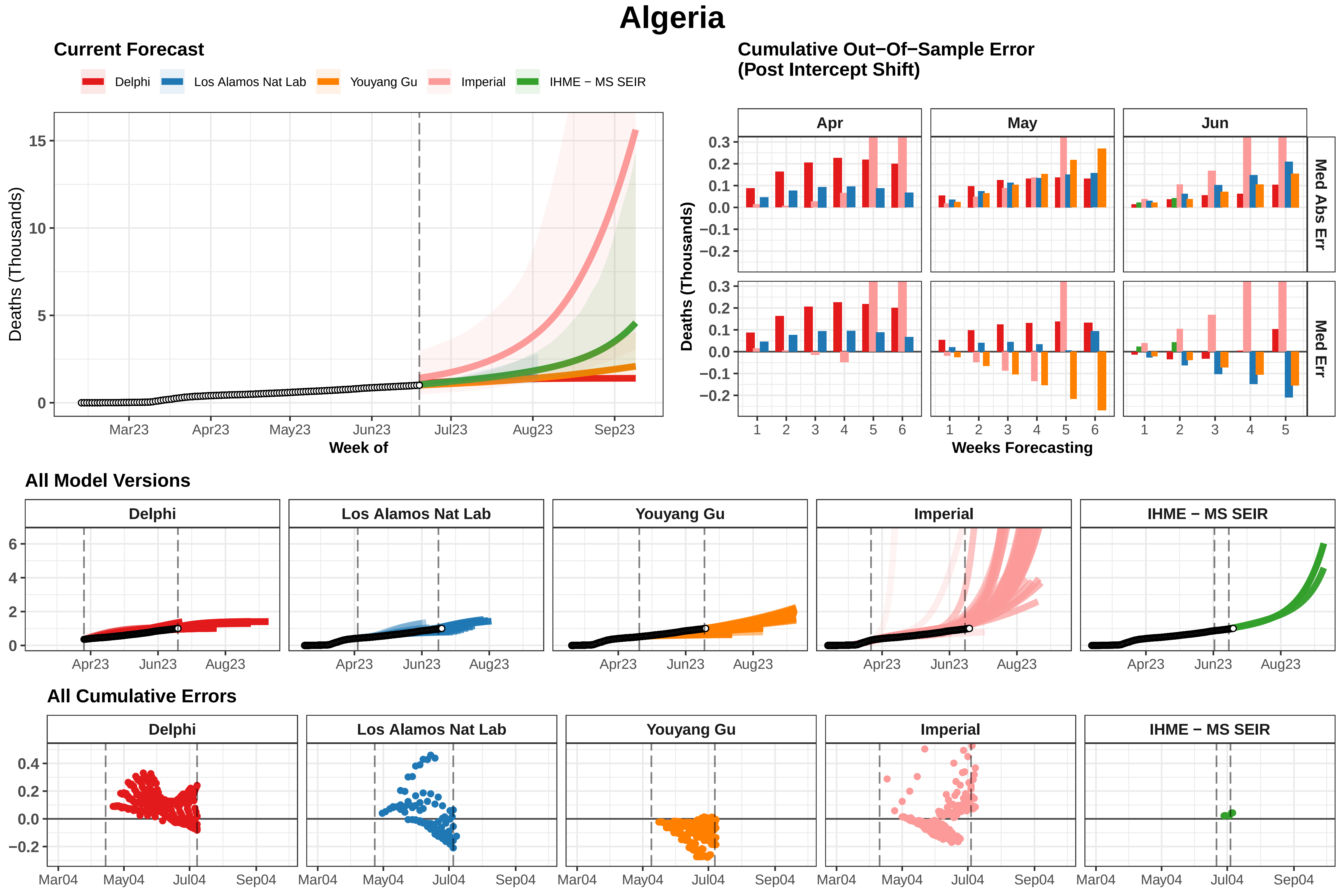
Algeria
Current Forecast
Delphi
Los Alamos Nat Lab
Youyang Gu
Imperial
IHME − MS SEIR
Deaths (Thousands)
Week of
Mar23
Apr23
May23
Jun23
Jul23
Aug23
Sep23
Cumulative Out−Of−Sample Error
(Post Intercept Shift)
Apr
May
Jun
Med Abs Err
Med Err
Weeks Forecasting
All Model Versions
All Cumulative Errors
Mar04
May04
Jul04
Sep04

Afghanistan

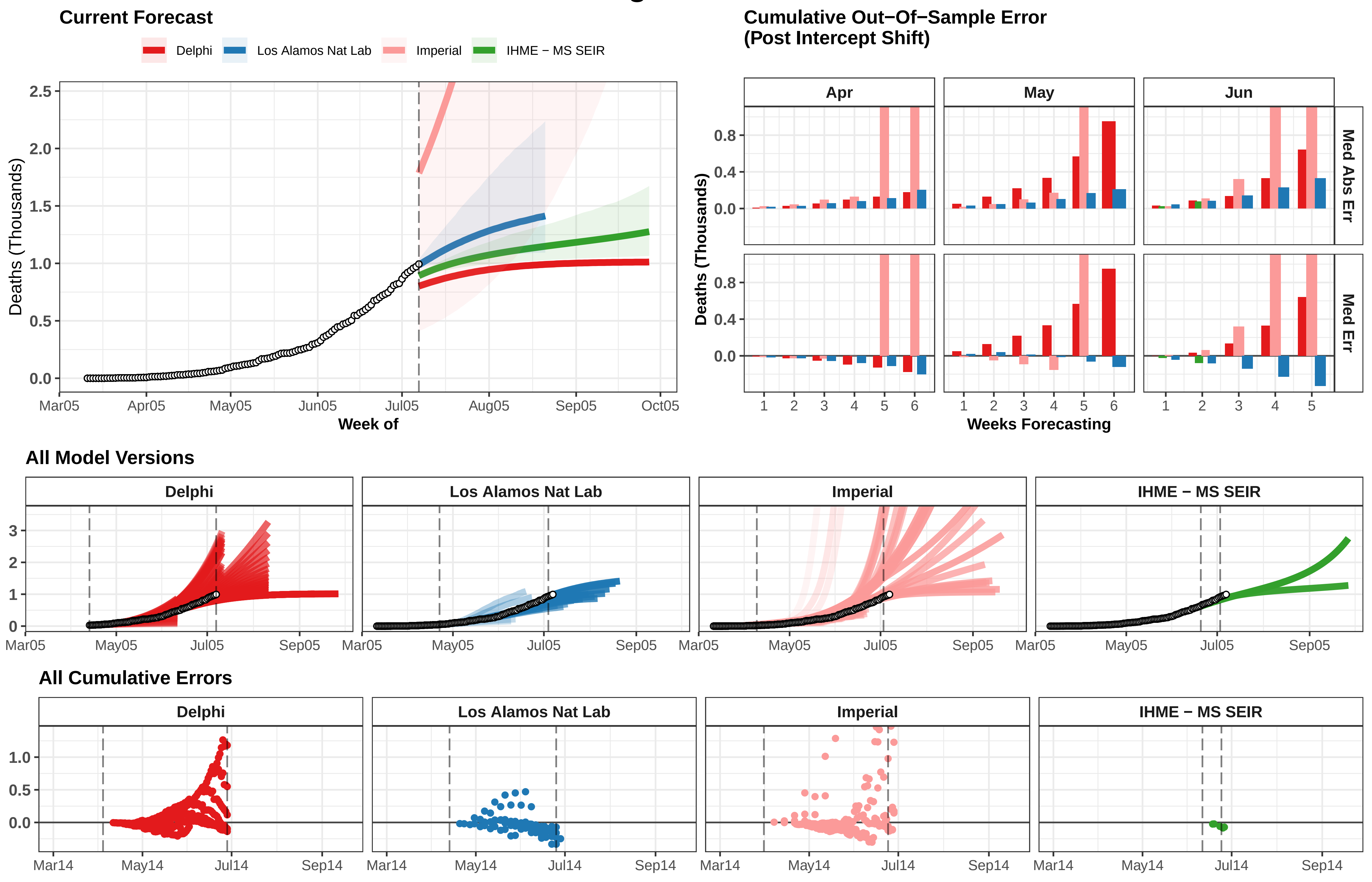

Japan

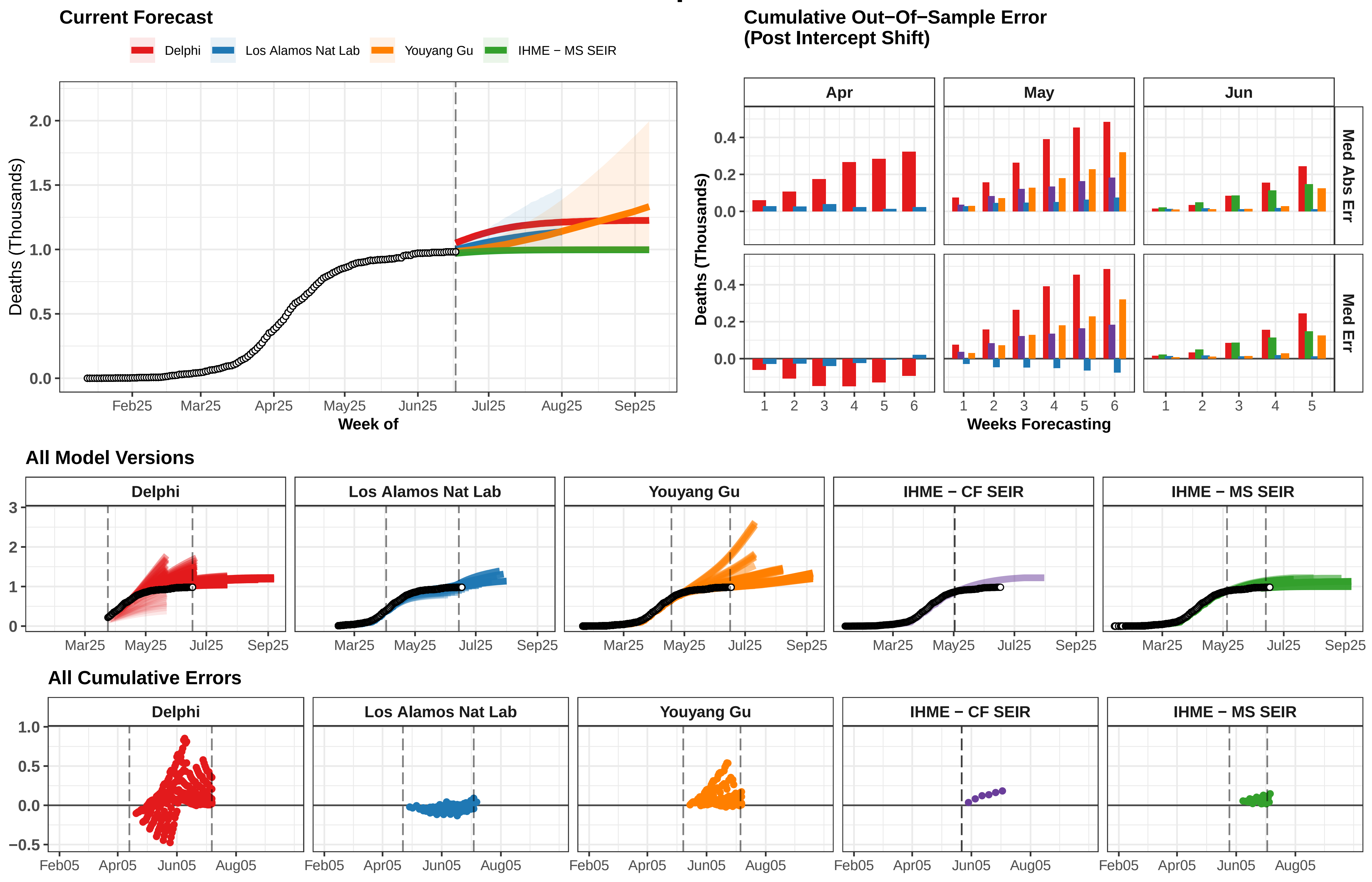

Current Forecast
Delphi
Los Alamos Nat Lab
Youyang Gu
IHME − MS SEIR
Deaths (Thousands)
2.0
1.5
1.0
0.5
0.0
Feb25
Mar25
Apr25
May25
Jun25
Jul25
Aug25
Sep25
Week of
Cumulative Out−Of−Sample Error
(Post Intercept Shift)
Apr
May
Jun
Med Abs Err
Med Err
0.4
0.2
0.0
1
2
3
4
5
6
Weeks Forecasting
All Model Versions
Delphi
Los Alamos Nat Lab
Youyang Gu
IHME − CF SEIR
IHME − MS SEIR
3
2
1
0
All Cumulative Errors
1.0
0.5
0.0
−0.5
Feb05
Apr05
Jun05
Aug05

Rhode Island

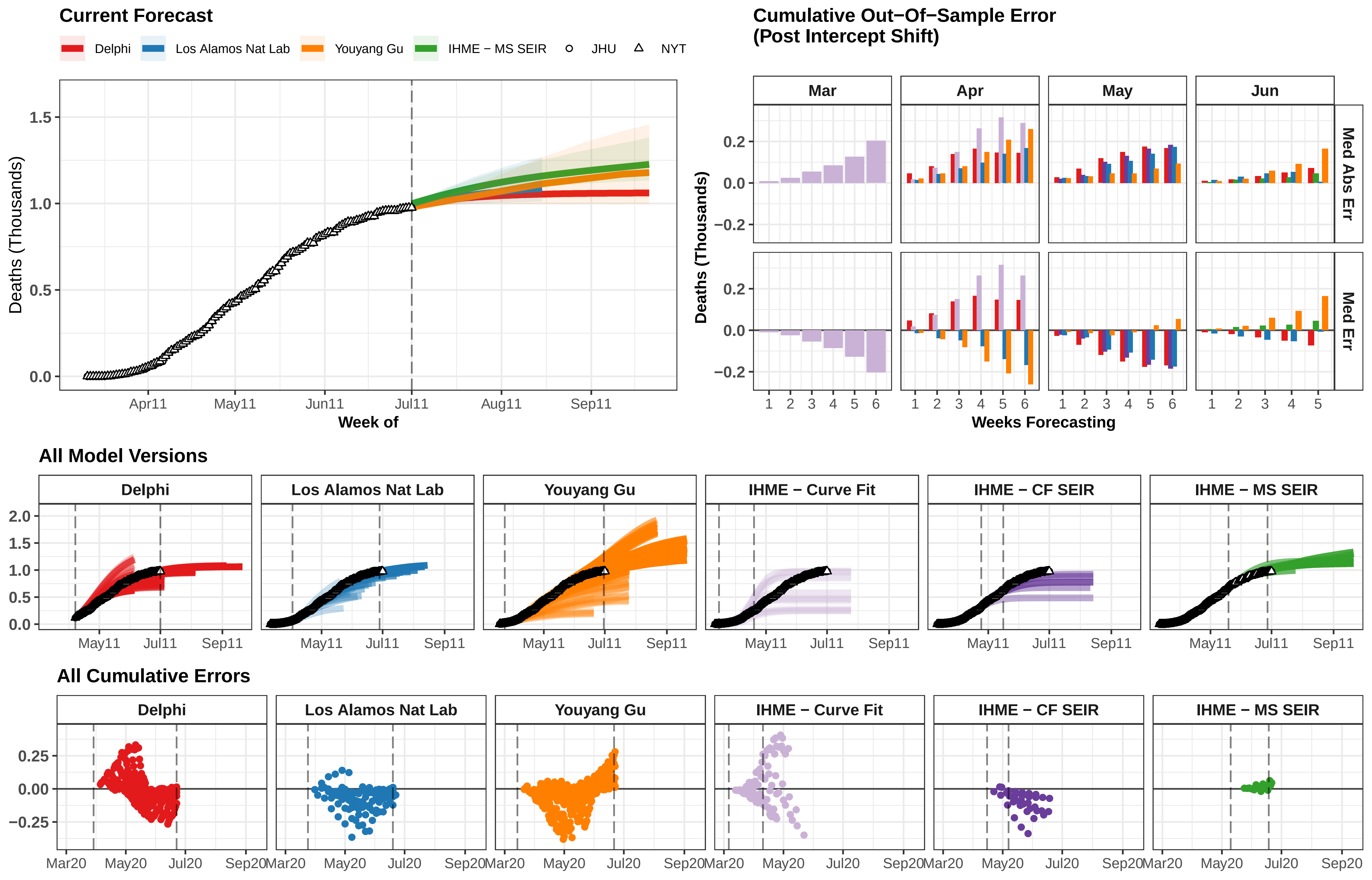

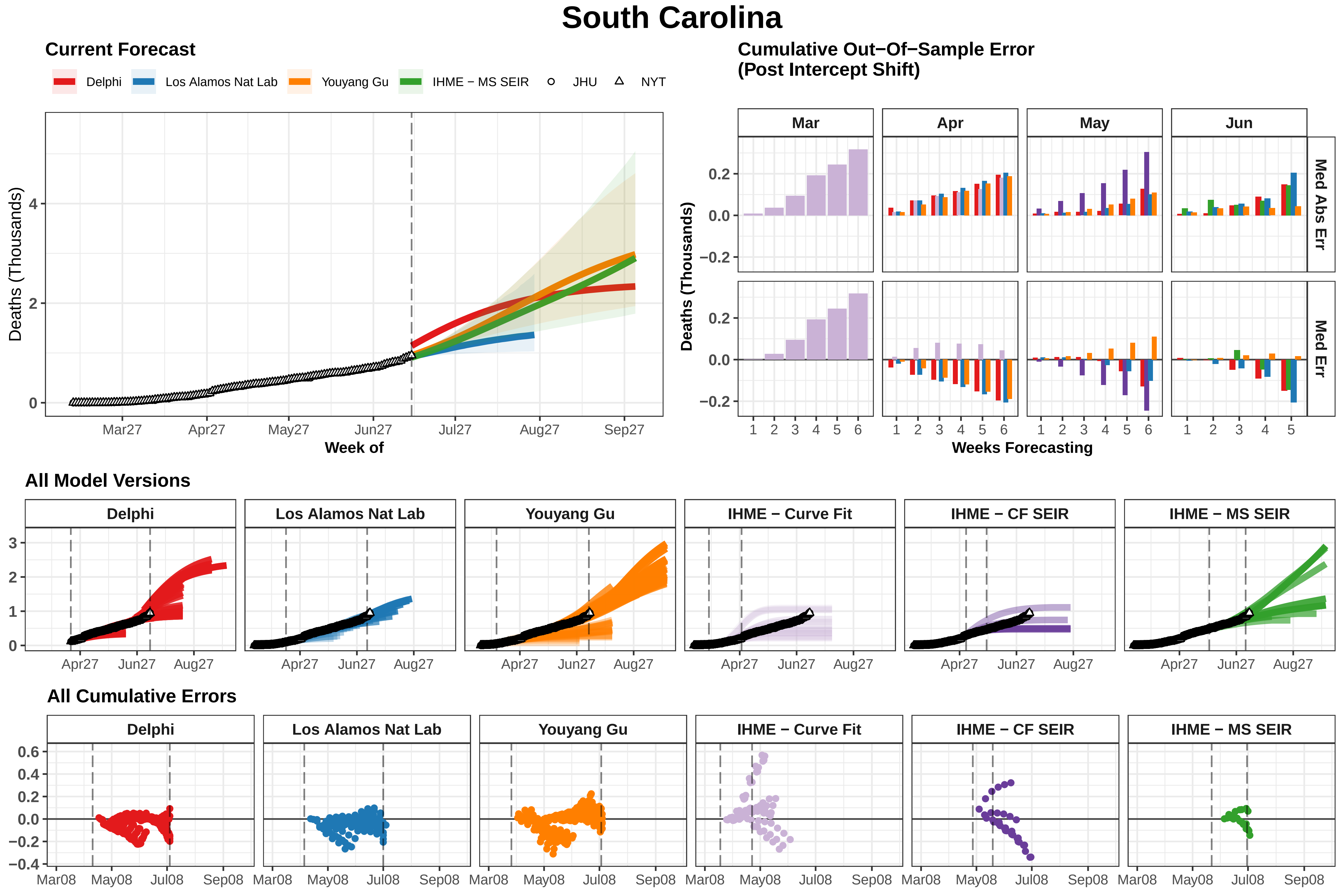
South Carolina
Current Forecast
Delphi
Los Alamos Nat Lab
Youyang Gu
IHME − MS SEIR
JHU
NYT
Deaths (Thousands)
Week of
Mar27
Apr27
May27
Jun27
Jul27
Aug27
Sep27
Cumulative Out−Of−Sample Error
(Post Intercept Shift)
Mar
Apr
May
Jun
Med Abs Err
Med Err
Weeks Forecasting
All Model Versions
IHME − Curve Fit
IHME − CF SEIR
All Cumulative Errors
Mar08
May08
Jul08
Sep08

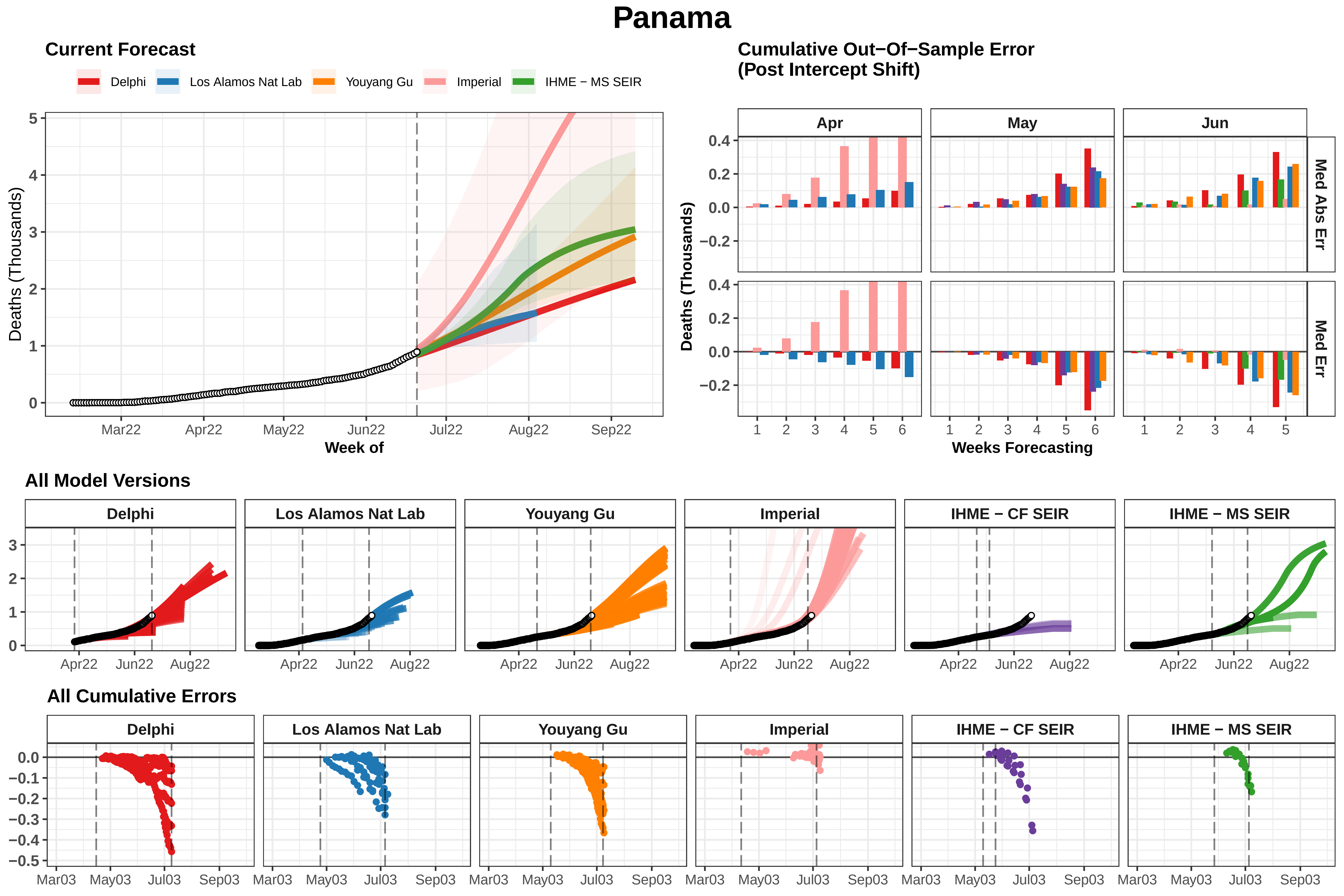

Panama
Current Forecast
Delphi
Los Alamos Nat Lab
Youyang Gu
Imperial
IHME − MS SEIR
Deaths (Thousands)
Week of
Mar22
Apr22
May22
Jun22
Jul22
Aug22
Sep22
Cumulative Out−Of−Sample Error
(Post Intercept Shift)
Apr
May
Jun
Med Abs Err
Med Err
Weeks Forecasting
All Model Versions
Delphi
Los Alamos Nat Lab
Youyang Gu
Imperial
IHME − CF SEIR
IHME − MS SEIR
All Cumulative Errors
Mar03
May03
Jul03
Sep03

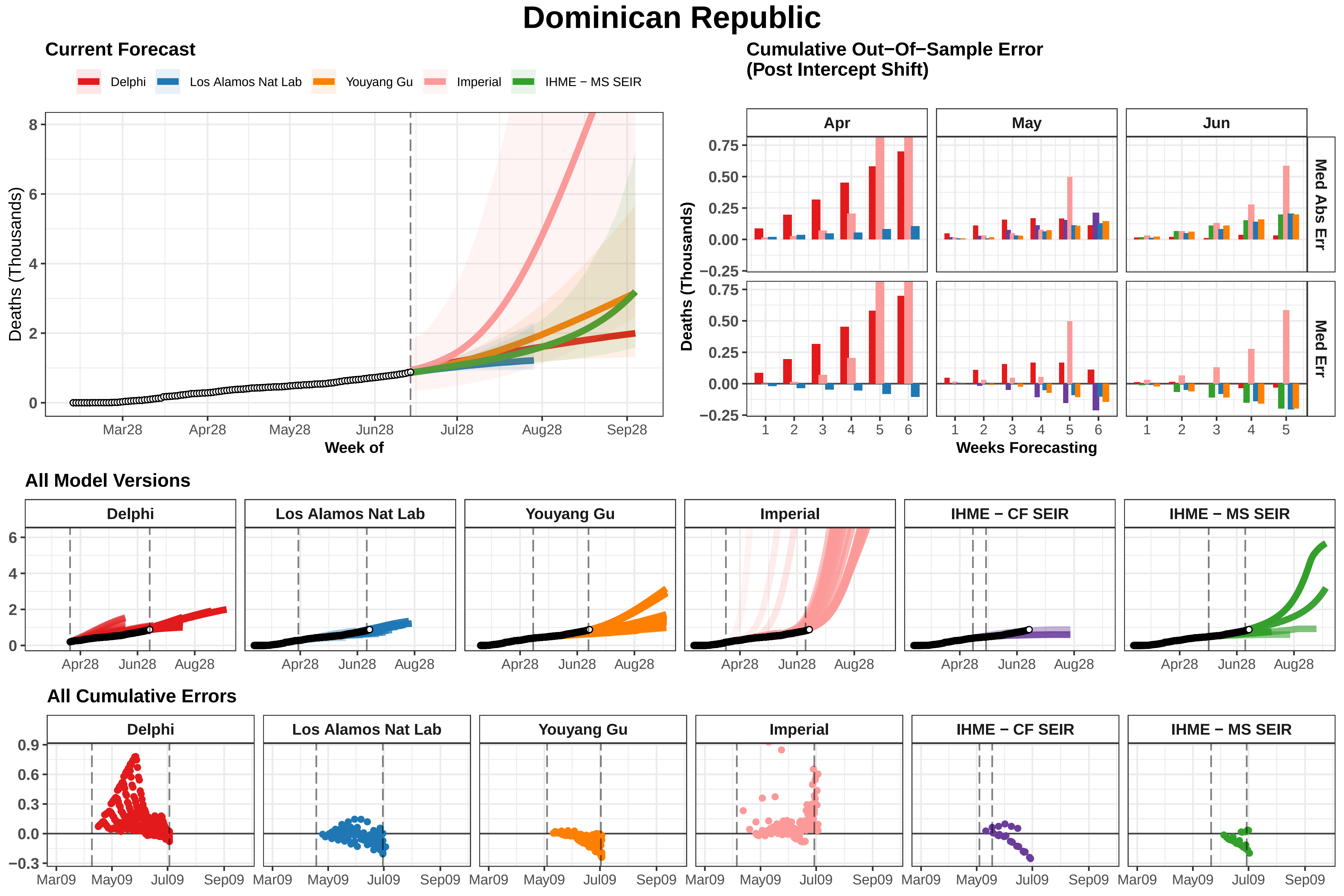

Dominican Republic
Current Forecast
Delphi
Los Alamos Nat Lab
Youyang Gu
Imperial
IHME − MS SEIR
Deaths (Thousands)
Week of
Mar28
Apr28
May28
Jun28
Jul28
Aug28
Sep28
Cumulative Out−Of−Sample Error
(Post Intercept Shift)
Apr
May
Jun
Med Abs Err
Med Err
Weeks Forecasting
All Model Versions
Delphi
Los Alamos Nat Lab
Youyang Gu
Imperial
IHME − CF SEIR
IHME − MS SEIR
All Cumulative Errors
Mar09
May09
Jul09
Sep09

Wisconsin

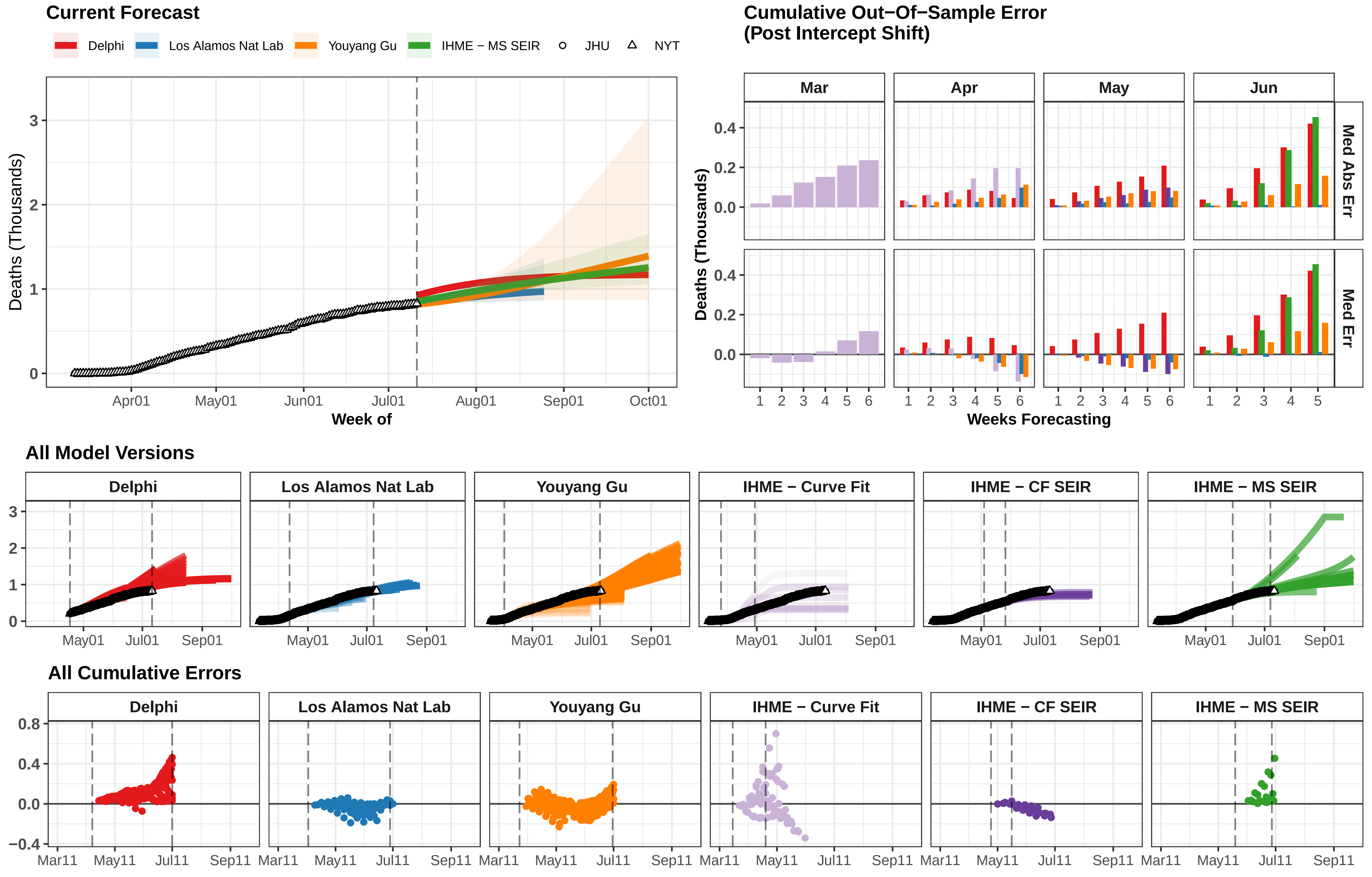

Current Forecast
Delphi
Los Alamos Nat Lab
Youyang Gu
IHME − MS SEIR
JHU
NYT
Deaths (Thousands)
0
1
2
3
Apr01
May01
Jun01
Jul01
Aug01
Sep01
Oct01
Week of
Cumulative Out−Of−Sample Error
(Post Intercept Shift)
Mar
Apr
May
Jun
Med Abs Err
Med Err
Deaths (Thousands)
0.0
0.2
0.4
1
2
3
4
5
6
Weeks Forecasting
All Model Versions
Delphi
Los Alamos Nat Lab
Youyang Gu
IHME − Curve Fit
IHME − CF SEIR
IHME − MS SEIR
0
1
2
3
May01
Jul01
Sep01
All Cumulative Errors
Delphi
Los Alamos Nat Lab
Youyang Gu
IHME − Curve Fit
IHME − CF SEIR
IHME − MS SEIR
−0.4
0.0
0.4
0.8
Mar11
May11
Jul11
Sep11

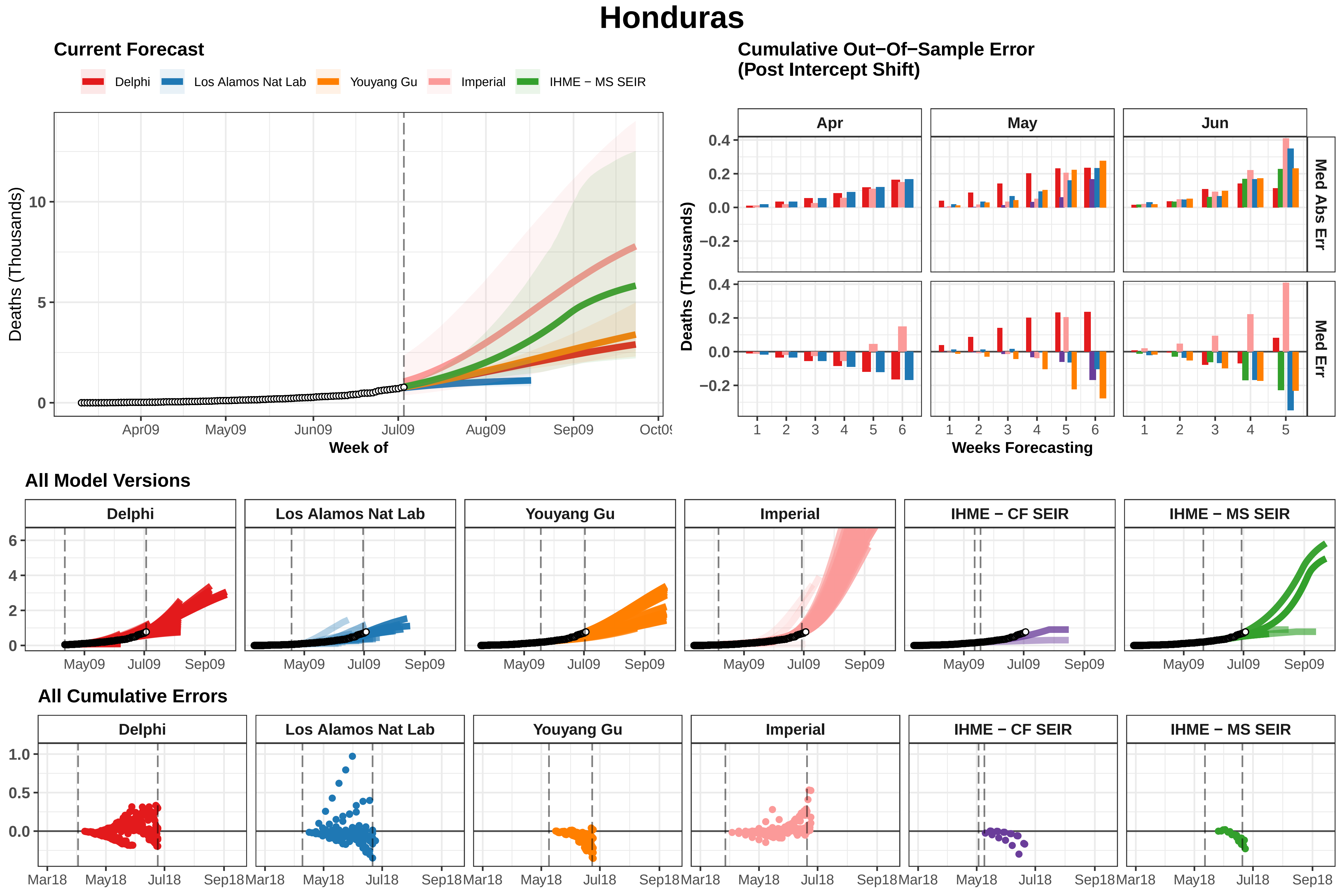

Honduras
Current Forecast
Delphi
Los Alamos Nat Lab
Youyang Gu
Imperial
IHME − MS SEIR
Deaths (Thousands)
Week of
Apr09
May09
Jun09
Jul09
Aug09
Sep09
Cumulative Out−Of−Sample Error
(Post Intercept Shift)
Apr
May
Jun
Med Abs Err
Med Err
Weeks Forecasting
All Model Versions
Delphi
Los Alamos Nat Lab
Youyang Gu
Imperial
IHME − CF SEIR
IHME − MS SEIR
All Cumulative Errors
Mar18
May18
Jul18
Sep18

Iowa

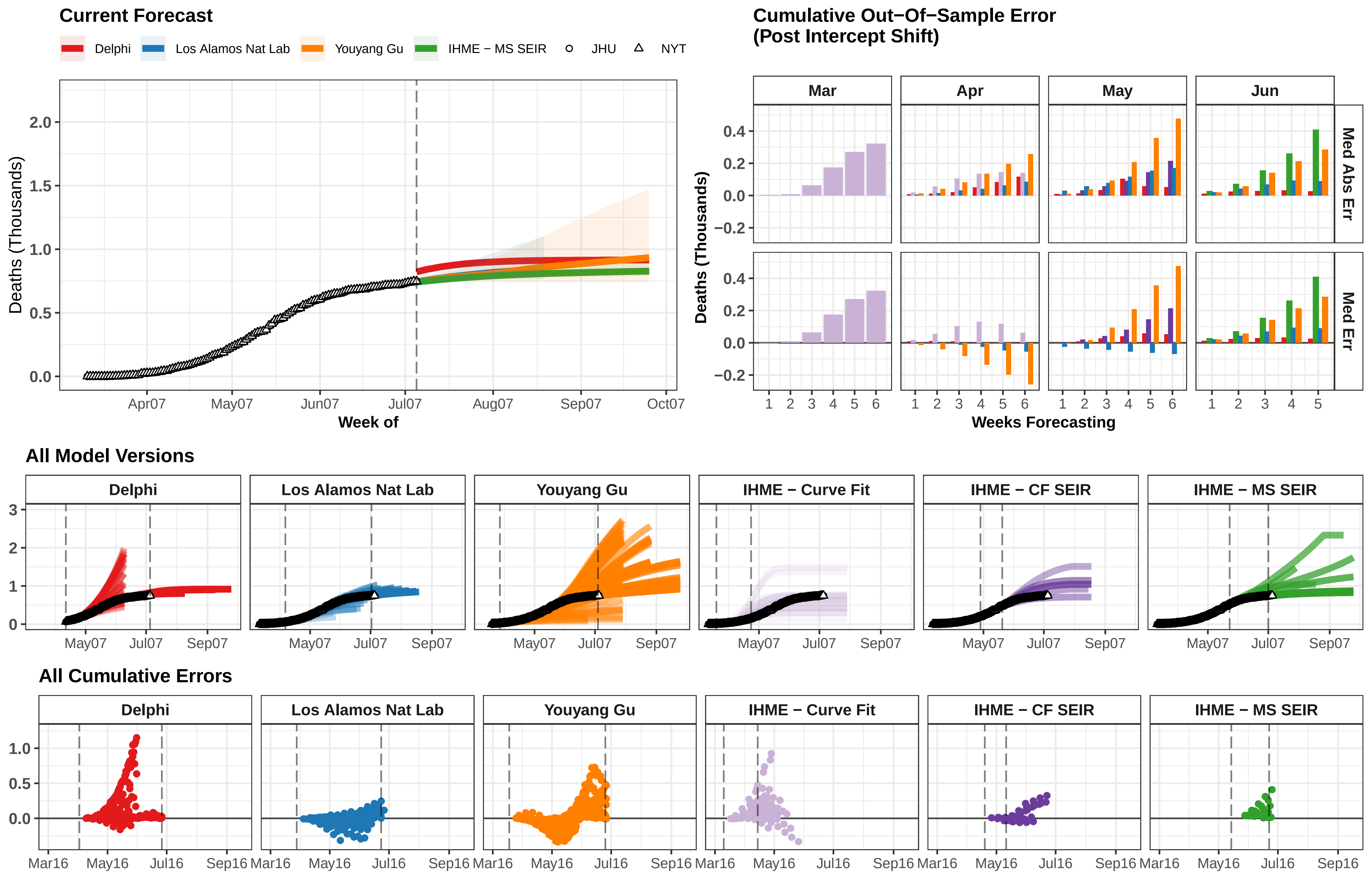

Current Forecast
Delphi
Los Alamos Nat Lab
Youyang Gu
IHME − MS SEIR
JHU
NYT
Deaths (Thousands)
Week of
Apr07
May07
Jun07
Jul07
Aug07
Sep07
Oct07
Cumulative Out−Of−Sample Error
(Post Intercept Shift)
Mar
Apr
May
Jun
Med Abs Err
Med Err
Weeks Forecasting
All Model Versions
Delphi
Los Alamos Nat Lab
Youyang Gu
IHME − Curve Fit
IHME − CF SEIR
IHME − MS SEIR
All Cumulative Errors
Mar16
May16
Jul16
Sep16

Tennessee

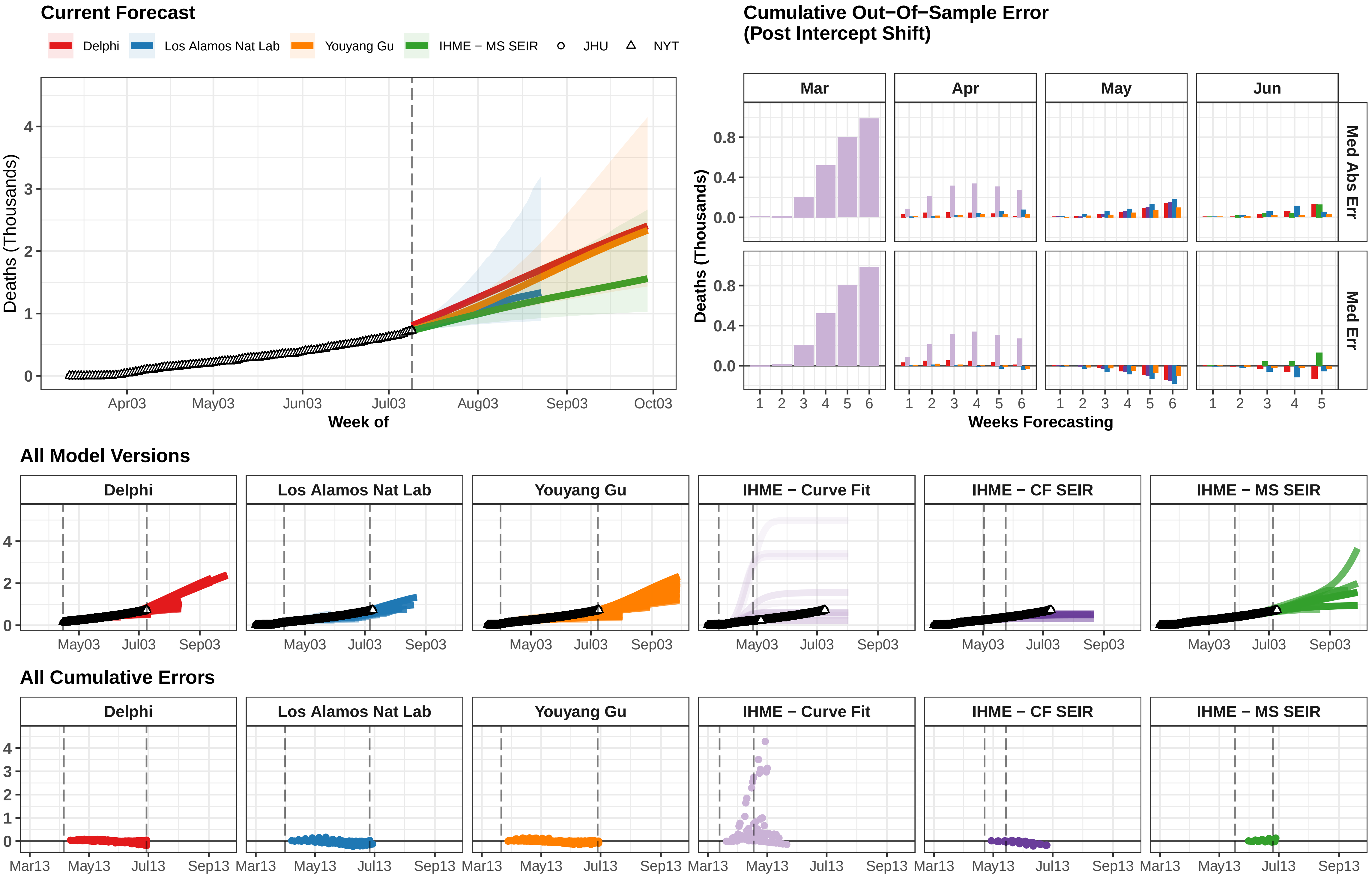
Current Forecast
Delphi
Los Alamos Nat Lab
Youyang Gu
IHME − MS SEIR
JHU
NYT
Deaths (Thousands)
Week of
Apr03
May03
Jun03
Jul03
Aug03
Sep03
Oct03
Cumulative Out−Of−Sample Error
(Post Intercept Shift)
Mar
Apr
May
Jun
Med Abs Err
Med Err
Weeks Forecasting
All Model Versions
Delphi
Los Alamos Nat Lab
Youyang Gu
IHME − Curve Fit
IHME − CF SEIR
IHME − MS SEIR
All Cumulative Errors
Mar13
May13
Jul13
Sep13

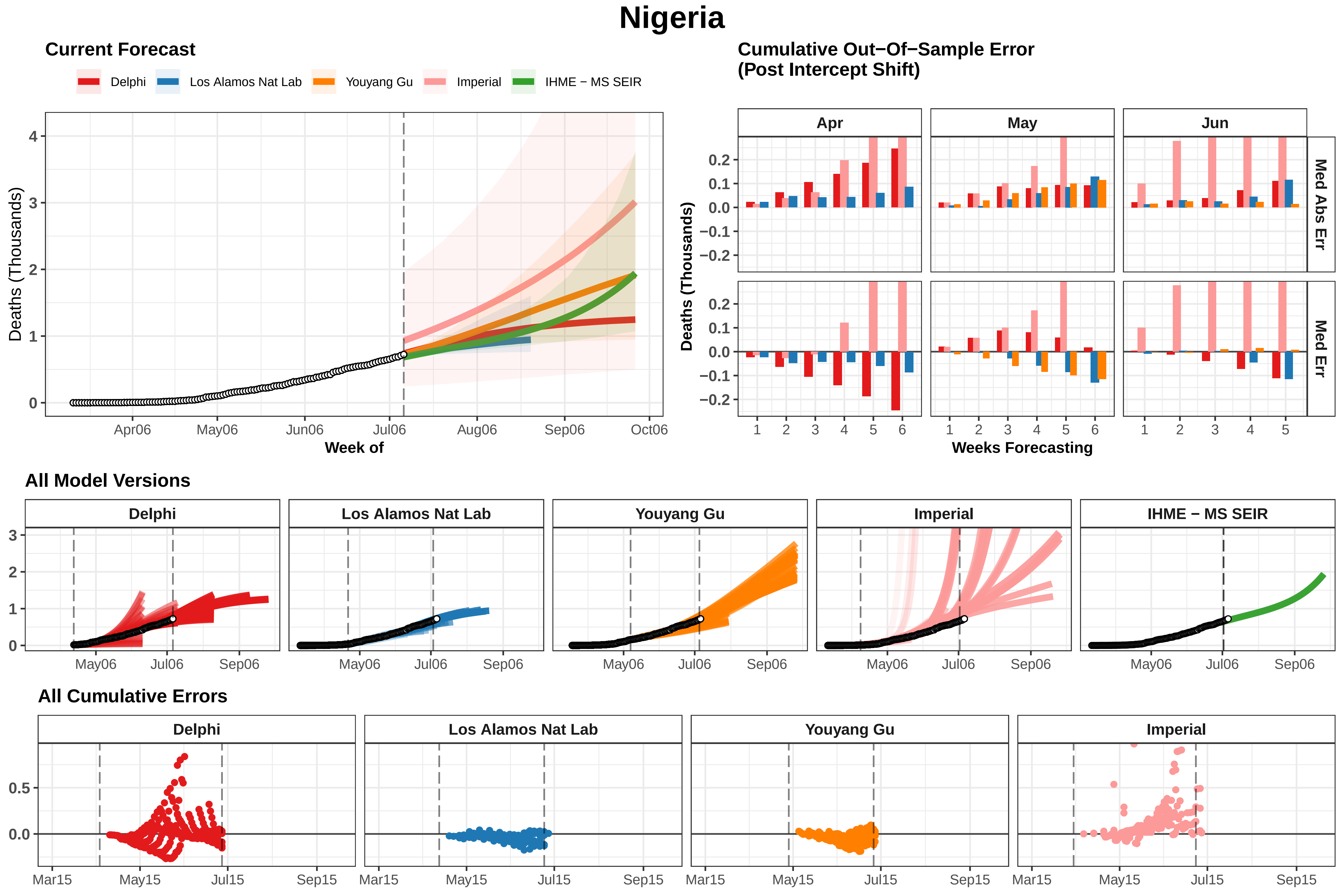

Nigeria
Current Forecast
Delphi
Los Alamos Nat Lab
Youyang Gu
Imperial
IHME − MS SEIR
Deaths (Thousands)
Week of
Apr06
May06
Jun06
Jul06
Aug06
Sep06
Oct06
Cumulative Out−Of−Sample Error
(Post Intercept Shift)
Apr
May
Jun
Med Abs Err
Med Err
Weeks Forecasting
All Model Versions
All Cumulative Errors
Mar15
May15
Jul15
Sep15

Austria

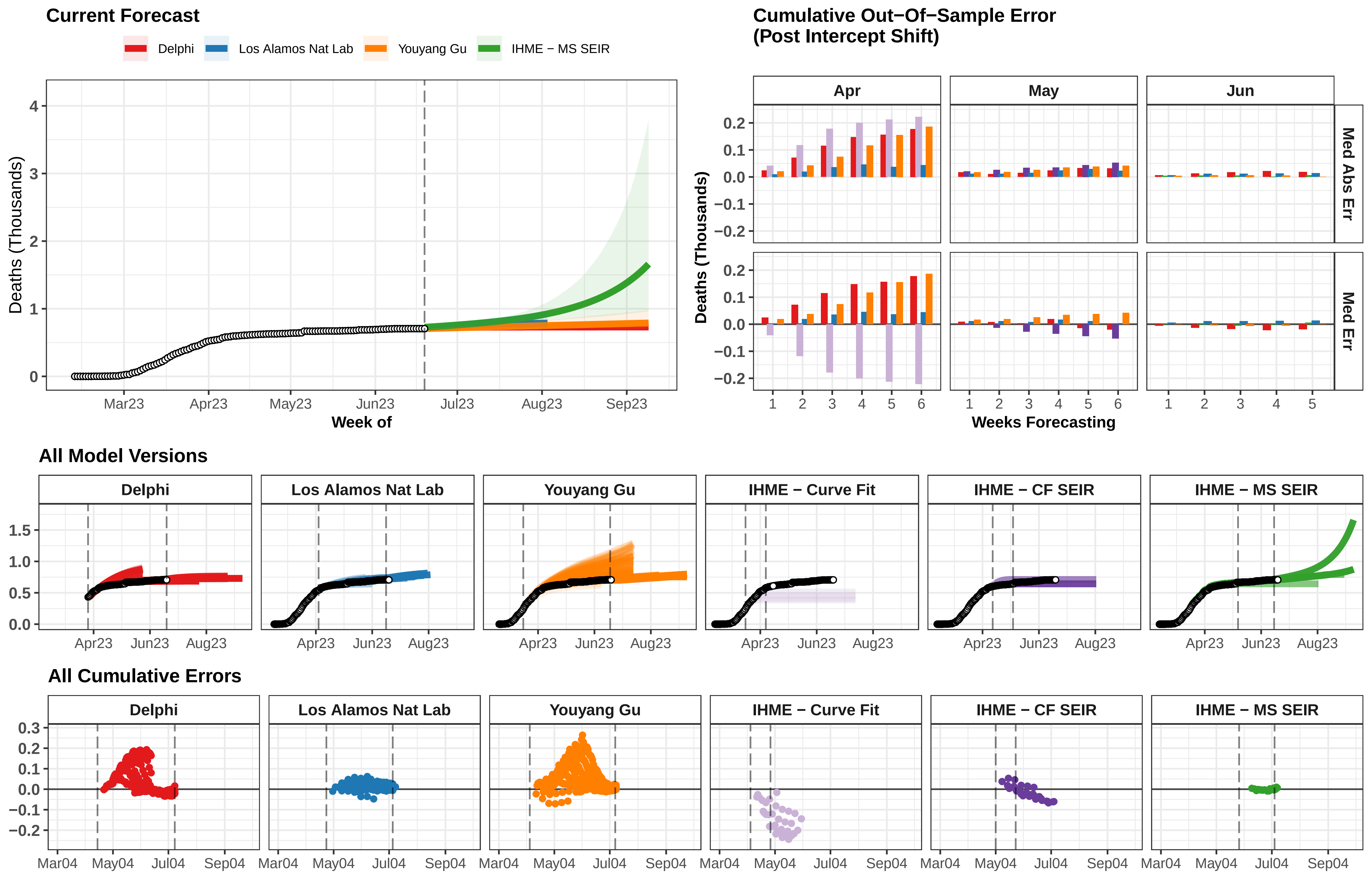

Current Forecast
Delphi
Los Alamos Nat Lab
Youyang Gu
IHME − MS SEIR
Deaths (Thousands)
0
1
2
3
4
Mar23
Apr23
May23
Jun23
Jul23
Aug23
Sep23
Week of
Cumulative Out−Of−Sample Error
(Post Intercept Shift)
Apr
May
Jun
Med Abs Err
Med Err
Deaths (Thousands)
0.2
0.1
0.0
−0.1
−0.2
1
2
3
4
5
6
Weeks Forecasting
All Model Versions
Delphi
Los Alamos Nat Lab
Youyang Gu
IHME − Curve Fit
IHME − CF SEIR
IHME − MS SEIR
1.5
1.0
0.5
0.0
Apr23
Jun23
Aug23
All Cumulative Errors
Delphi
Los Alamos Nat Lab
Youyang Gu
IHME − Curve Fit
IHME − CF SEIR
IHME − MS SEIR
0.3
0.2
0.1
0.0
−0.1
−0.2
Mar04
May04
Jul04
Sep04

Sudan

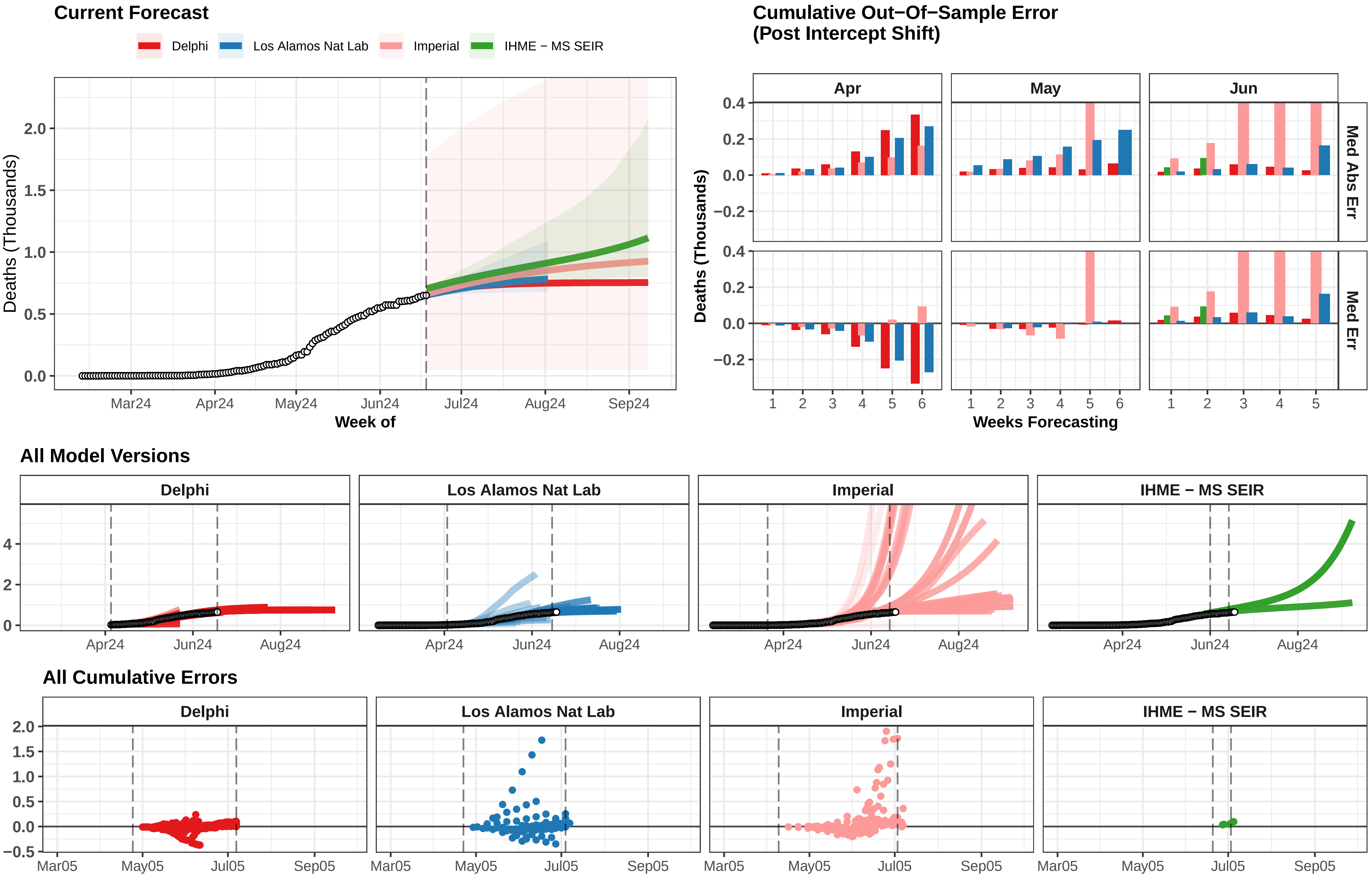
Current Forecast
Delphi
Los Alamos Nat Lab
Imperial
IHME − MS SEIR
Deaths (Thousands)
Week of
Mar24
Apr24
May24
Jun24
Jul24
Aug24
Sep24
Cumulative Out−Of−Sample Error
(Post Intercept Shift)
Apr
May
Jun
Med Abs Err
Med Err
Weeks Forecasting
All Model Versions
All Cumulative Errors
Mar05
May05
Jul05
Sep05

Kentucky

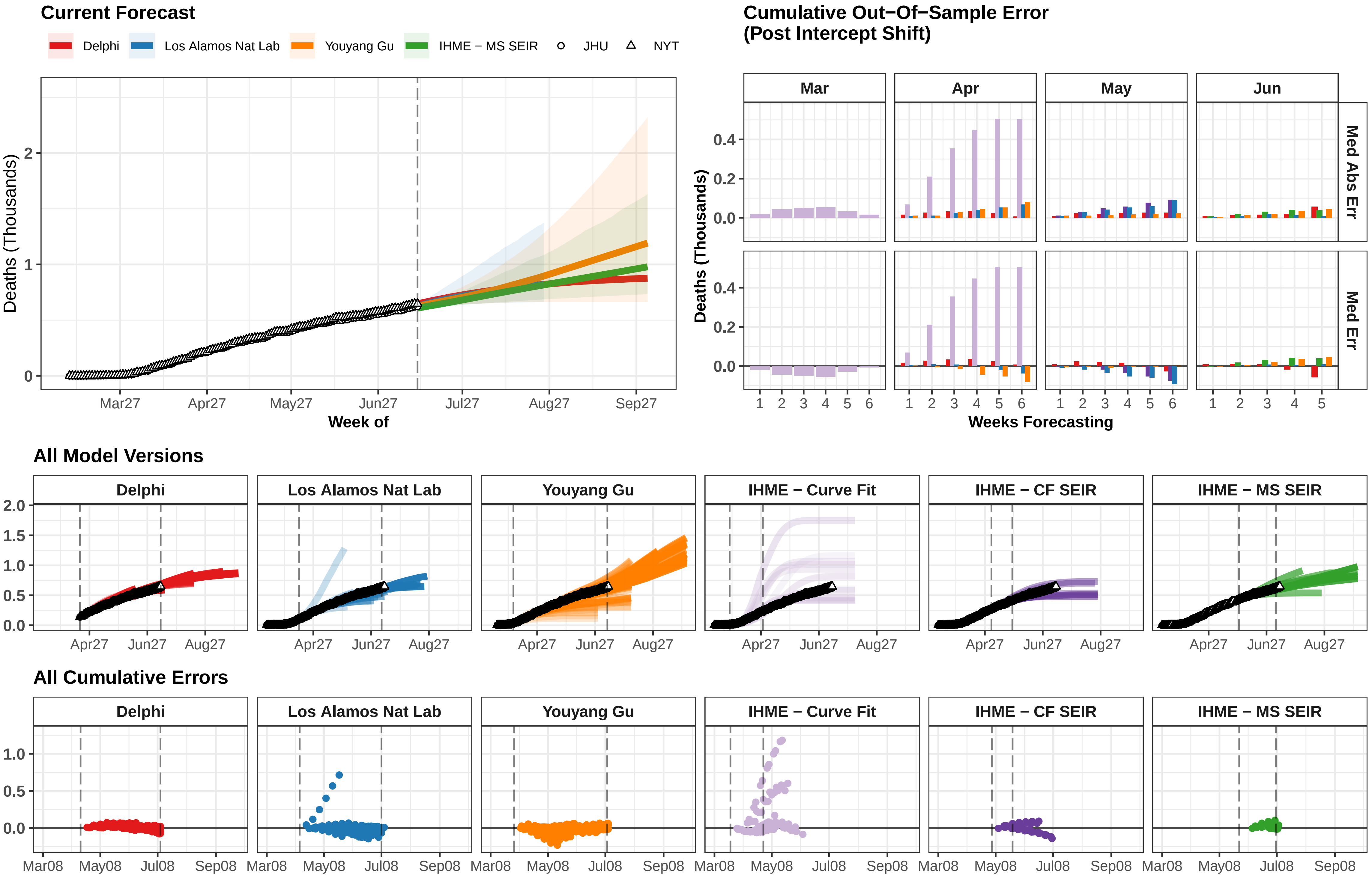
Current Forecast
Delphi
Los Alamos Nat Lab
Youyang Gu
IHME − MS SEIR
JHU
NYT
Deaths (Thousands)
Week of
Mar27
Apr27
May27
Jun27
Jul27
Aug27
Sep27
Cumulative Out−Of−Sample Error
(Post Intercept Shift)
Mar
Apr
May
Jun
Med Abs Err
Med Err
Weeks Forecasting
All Model Versions
Delphi
Los Alamos Nat Lab
Youyang Gu
IHME − Curve Fit
IHME − CF SEIR
IHME − MS SEIR
All Cumulative Errors
Mar08
May08
Jul08
Sep08

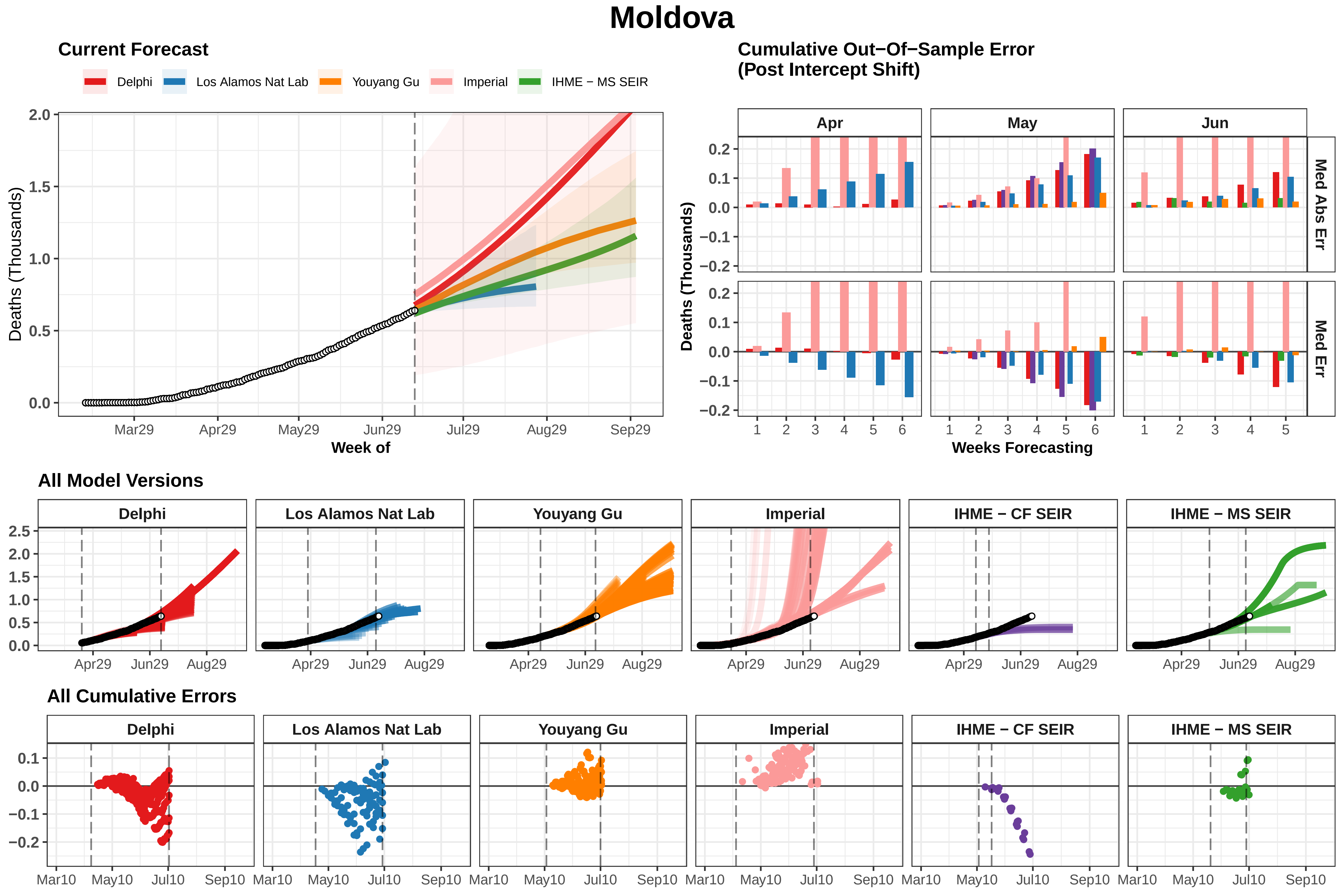
Moldova
Current Forecast
Delphi
Los Alamos Nat Lab
Youyang Gu
Imperial
IHME − MS SEIR
Deaths (Thousands)
Week of
Mar29
Apr29
May29
Jun29
Jul29
Aug29
Sep29
Cumulative Out−Of−Sample Error
(Post Intercept Shift)
Apr
May
Jun
Med Abs Err
Med Err
Weeks Forecasting
All Model Versions
Delphi
Los Alamos Nat Lab
Youyang Gu
Imperial
IHME − CF SEIR
IHME − MS SEIR
All Cumulative Errors
Mar10
May10
Jul10
Sep10

Denmark

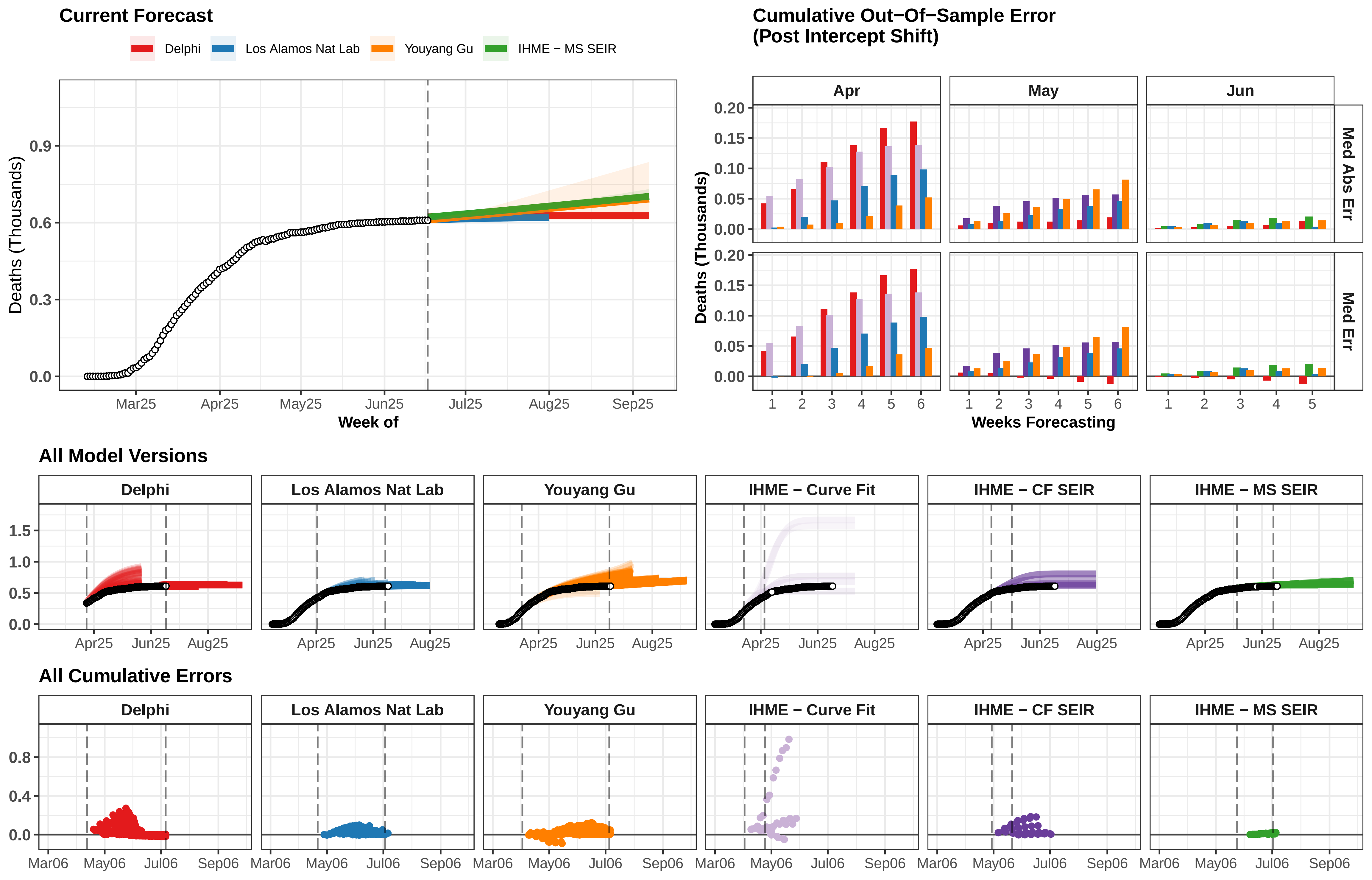

Current Forecast
Delphi
Los Alamos Nat Lab
Youyang Gu
IHME − MS SEIR
Deaths (Thousands)
0.0
0.3
0.6
0.9
Mar25
Apr25
May25
Jun25
Jul25
Aug25
Sep25
Week of
Cumulative Out−Of−Sample Error
(Post Intercept Shift)
Apr
May
Jun
Med Abs Err
Med Err
0.00
0.05
0.10
0.15
0.20
Weeks Forecasting
All Model Versions
IHME − Curve Fit
IHME − CF SEIR
0.0
0.5
1.0
1.5
Apr25
Jun25
Aug25
All Cumulative Errors
0.0
0.4
0.8
Mar06
May06
Jul06
Sep06

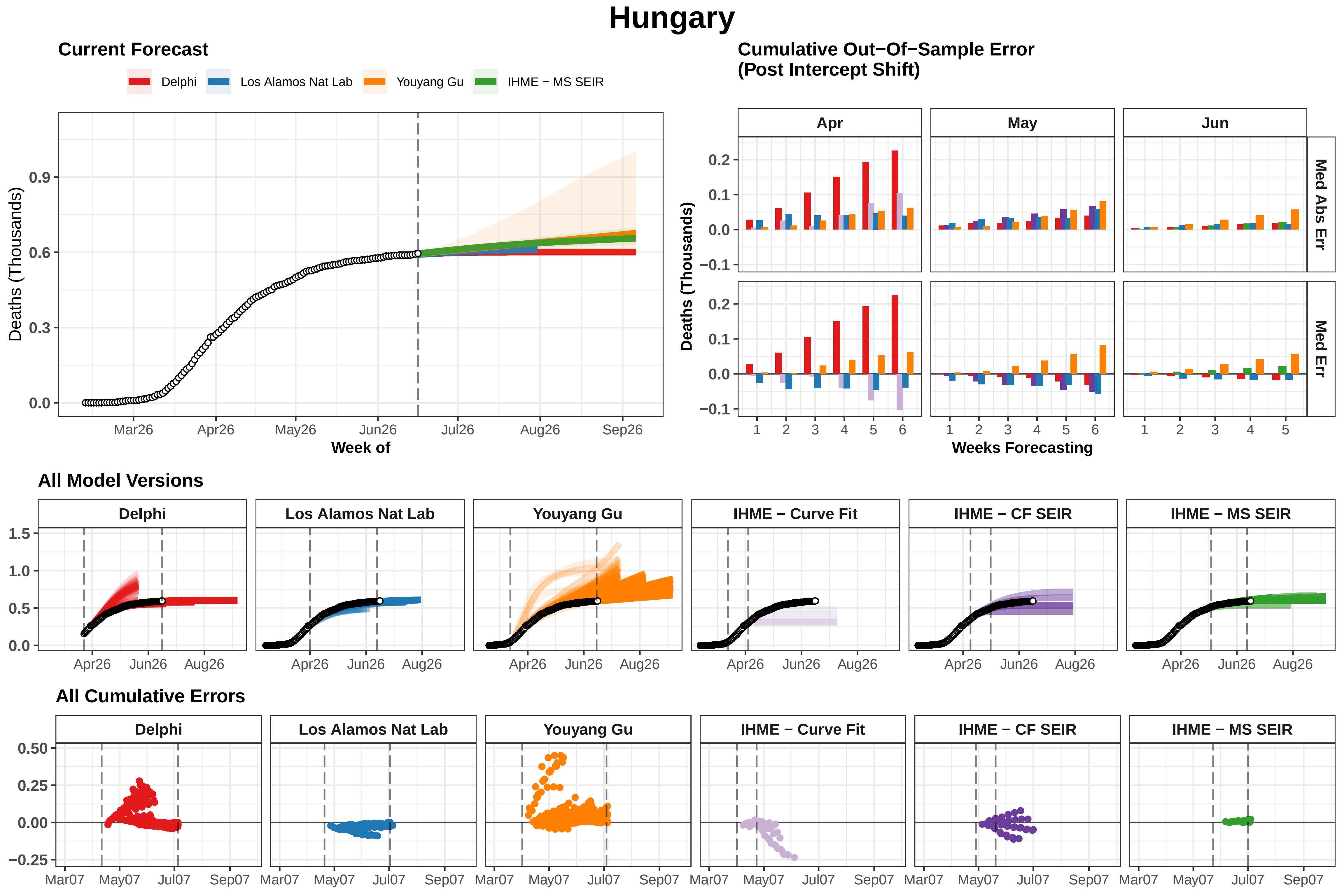

Hungary
Current Forecast
Delphi
Los Alamos Nat Lab
Youyang Gu
IHME − MS SEIR
Deaths (Thousands)
Week of
Mar26
Apr26
May26
Jun26
Jul26
Aug26
Sep26
Cumulative Out−Of−Sample Error
(Post Intercept Shift)
Apr
May
Jun
Med Abs Err
Med Err
Weeks Forecasting
All Model Versions
IHME − Curve Fit
IHME − CF SEIR
All Cumulative Errors
Mar07
May07
Jul07
Sep07

Nevada

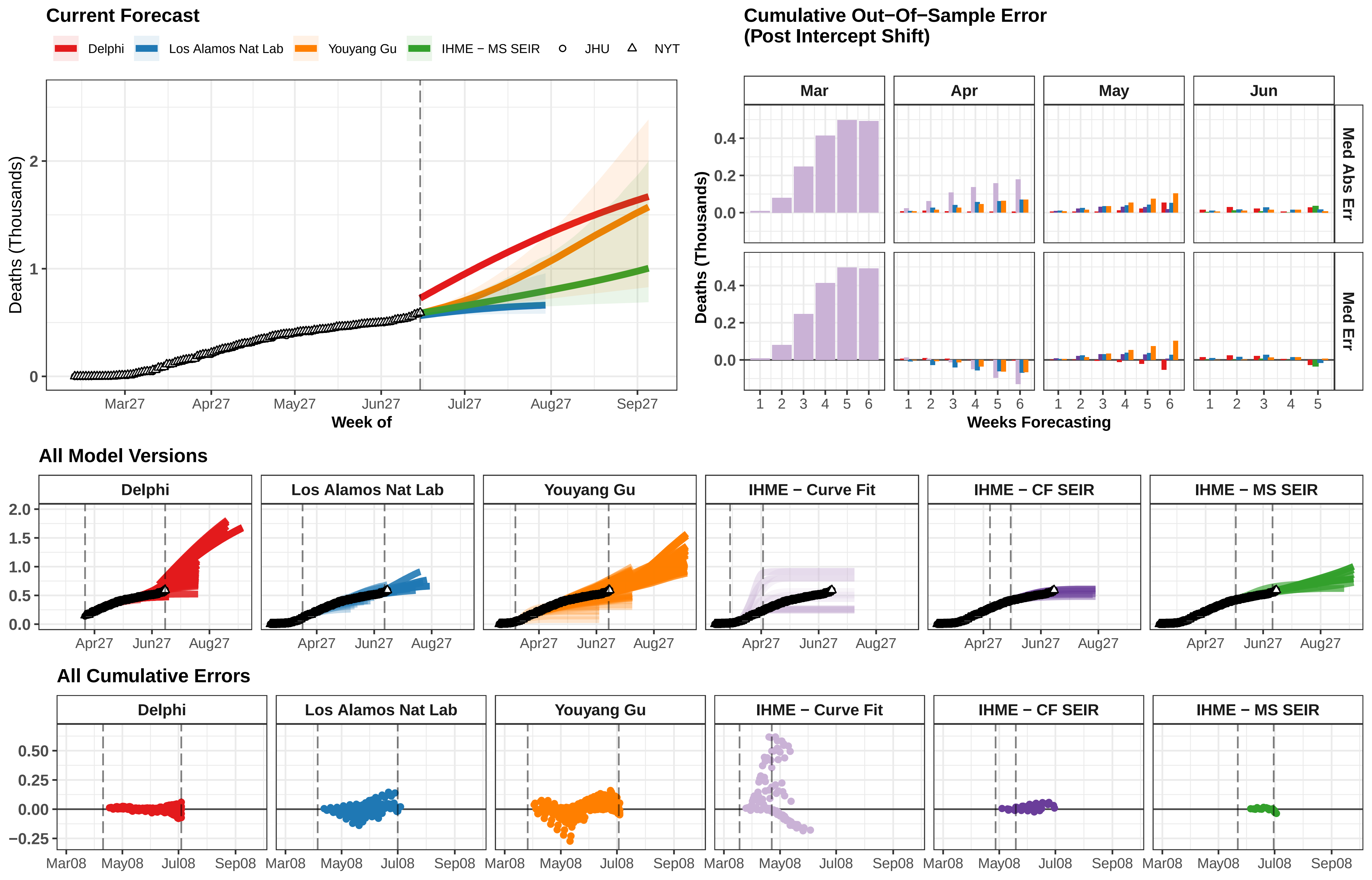

Current Forecast
Delphi
Los Alamos Nat Lab
Youyang Gu
IHME − MS SEIR
JHU
NYT
Deaths (Thousands)
Week of
Mar27
Apr27
May27
Jun27
Jul27
Aug27
Sep27
Cumulative Out−Of−Sample Error (Post Intercept Shift)
Mar
Apr
May
Jun
Med Abs Err
Med Err
Weeks Forecasting
All Model Versions
Delphi
Los Alamos Nat Lab
Youyang Gu
IHME − Curve Fit
IHME − CF SEIR
IHME − MS SEIR
All Cumulative Errors
Mar08
May08
Jul08
Sep08

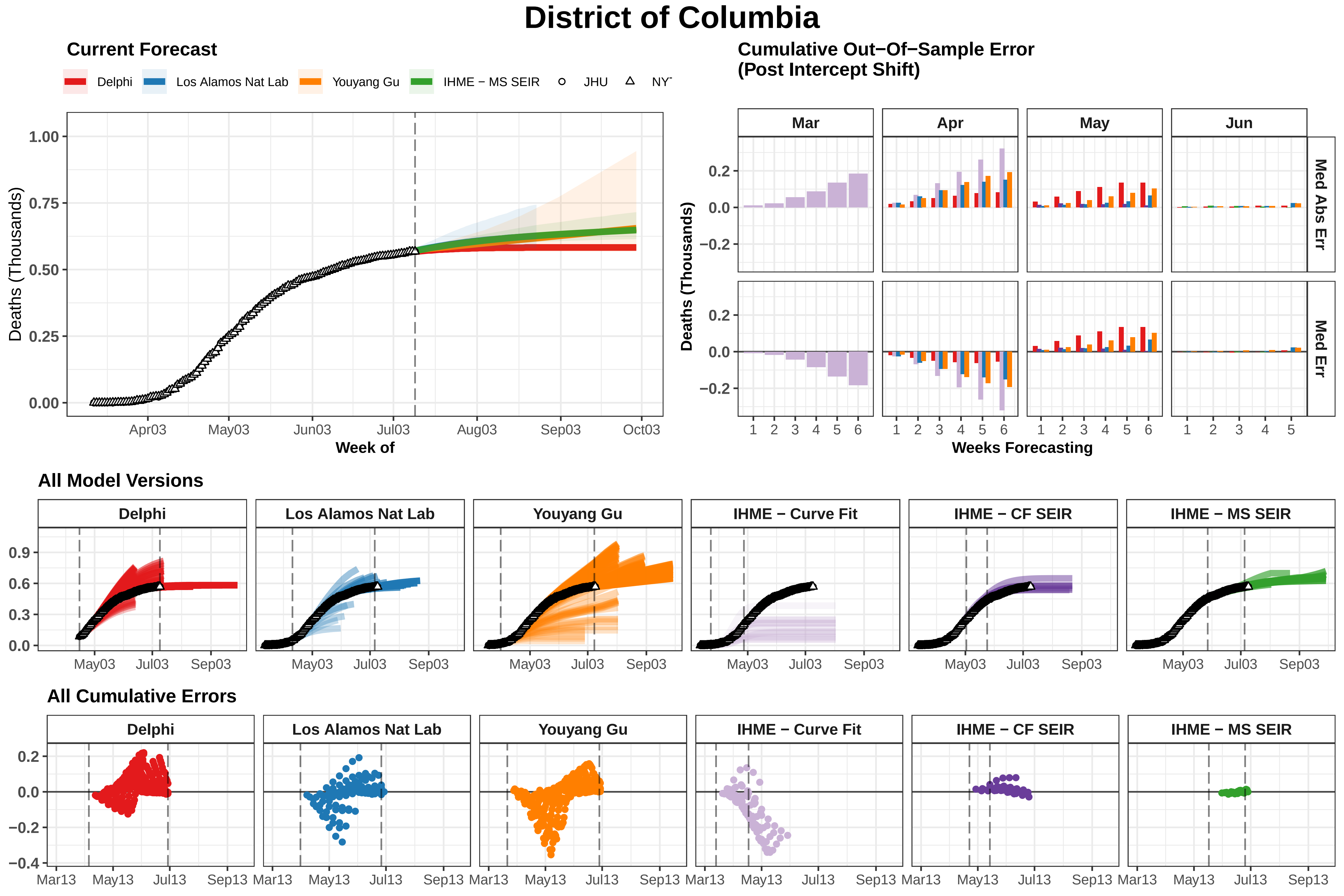

District of Columbia
Current Forecast
Delphi
Los Alamos Nat Lab
Youyang Gu
IHME − MS SEIR
JHU
NY
Deaths (Thousands)
Week of
Apr03
May03
Jun03
Jul03
Aug03
Sep03
Oct03
Cumulative Out−Of−Sample Error
(Post Intercept Shift)
Mar
Apr
May
Jun
Med Abs Err
Med Err
Weeks Forecasting
All Model Versions
Delphi
Los Alamos Nat Lab
Youyang Gu
IHME − Curve Fit
IHME − CF SEIR
IHME − MS SEIR
All Cumulative Errors
Mar13
May13
Jul13
Sep13

Armenia

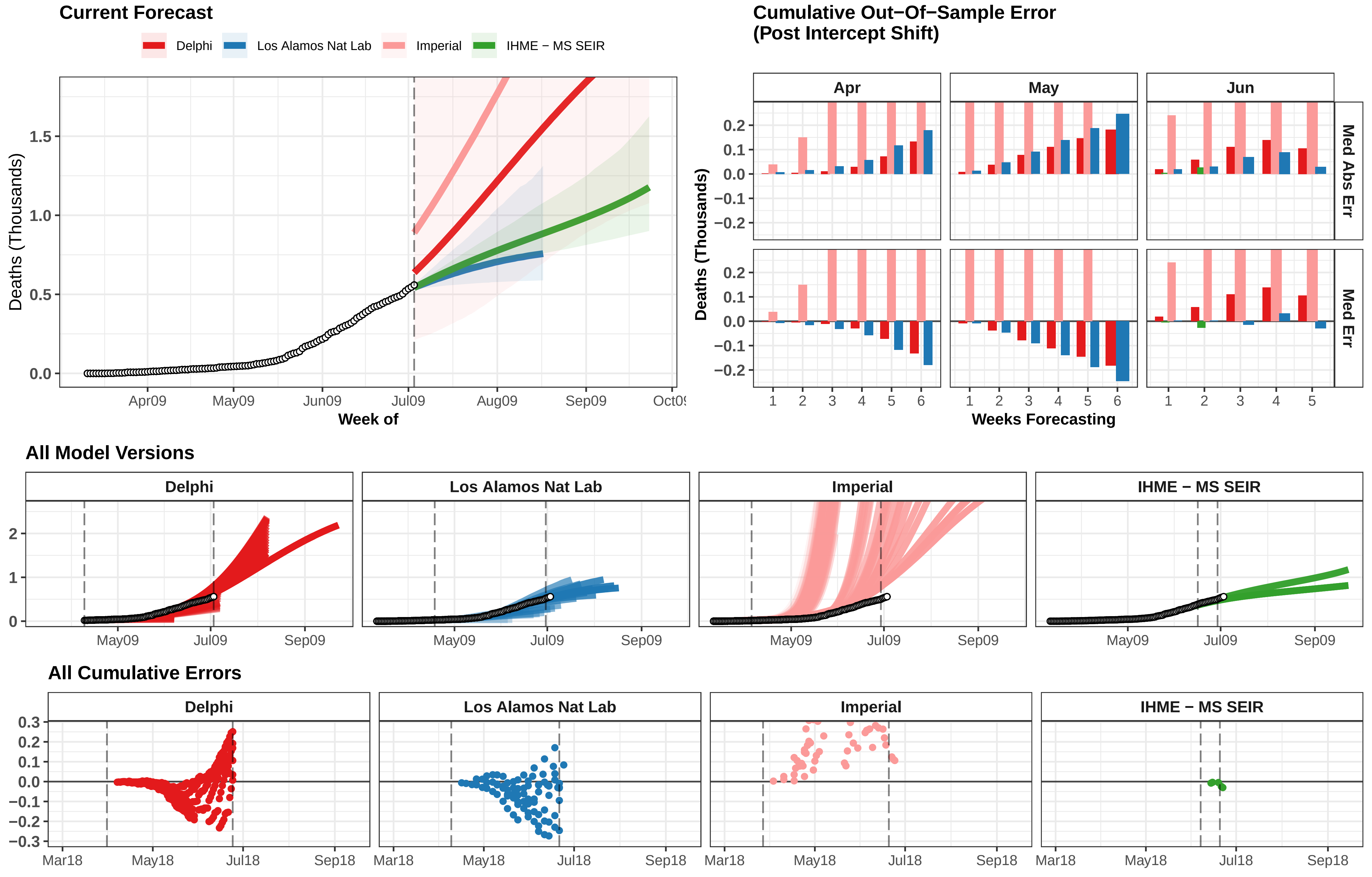
Current Forecast
Delphi
Los Alamos Nat Lab
Imperial
IHME − MS SEIR
Deaths (Thousands)
0.0
0.5
1.0
1.5
Apr09
May09
Jun09
Jul09
Aug09
Sep09
Week of
Cumulative Out−Of−Sample Error
(Post Intercept Shift)
Apr
May
Jun
Med Abs Err
Med Err
−0.2
−0.1
0.0
0.1
0.2
Weeks Forecasting
All Model Versions
Delphi
Los Alamos Nat Lab
Imperial
IHME − MS SEIR
0
1
2
May09
Jul09
Sep09
All Cumulative Errors
−0.3
0.3
Mar18
May18
Jul18
Sep18

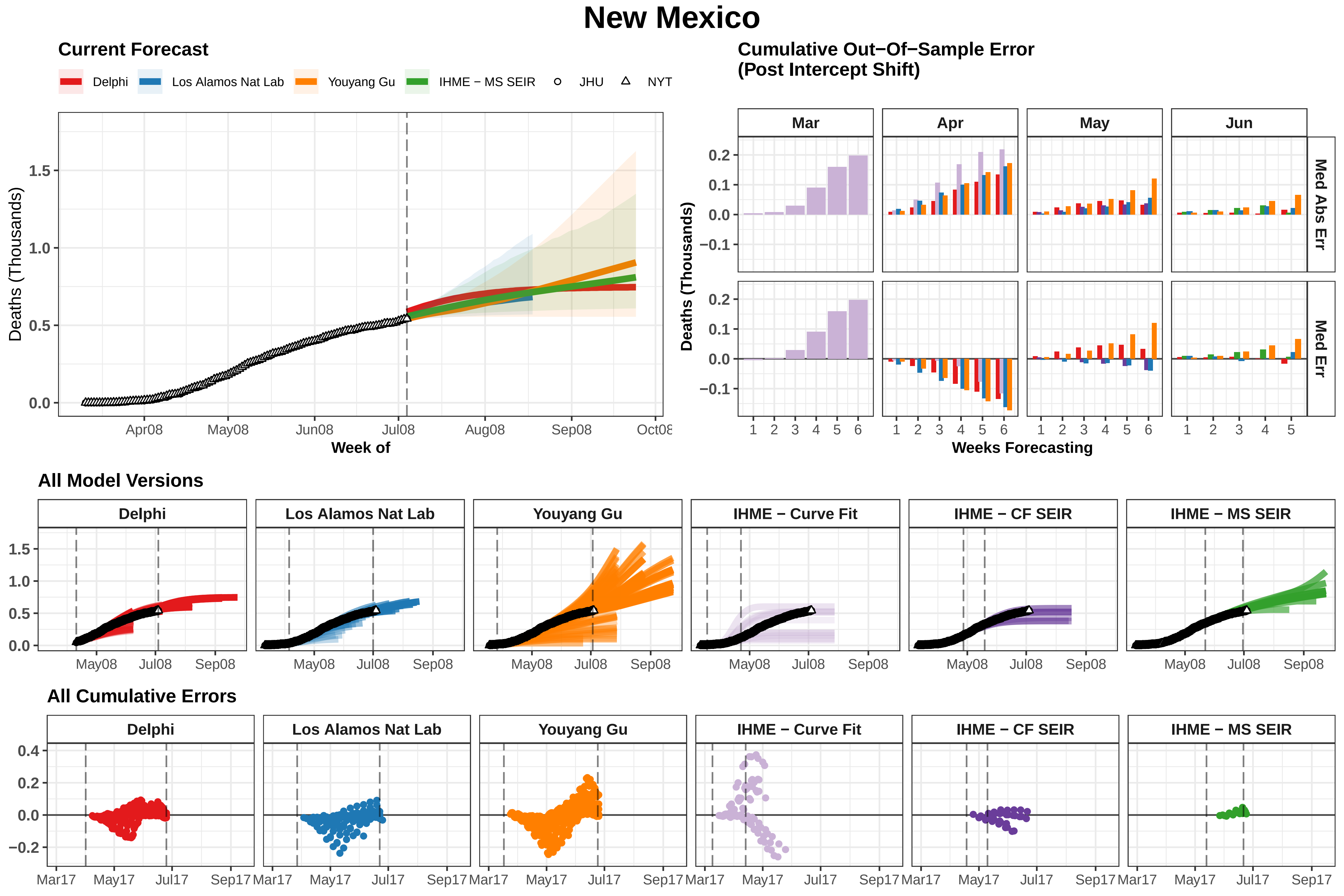
New Mexico
Current Forecast
Delphi
Los Alamos Nat Lab
Youyang Gu
IHME − MS SEIR
JHU
NYT
Deaths (Thousands)
0.0
0.5
1.0
1.5
Apr08
May08
Jun08
Jul08
Aug08
Sep08
Oct08
Week of
Cumulative Out−Of−Sample Error
(Post Intercept Shift)
Mar
Apr
May
Jun
Med Abs Err
Med Err
−0.1
0.0
0.1
0.2
Weeks Forecasting
All Model Versions
Delphi
Los Alamos Nat Lab
Youyang Gu
IHME − Curve Fit
IHME − CF SEIR
IHME − MS SEIR
All Cumulative Errors
−0.2
0.0
0.2
0.4
Mar17
May17
Jul17
Sep17

Delaware

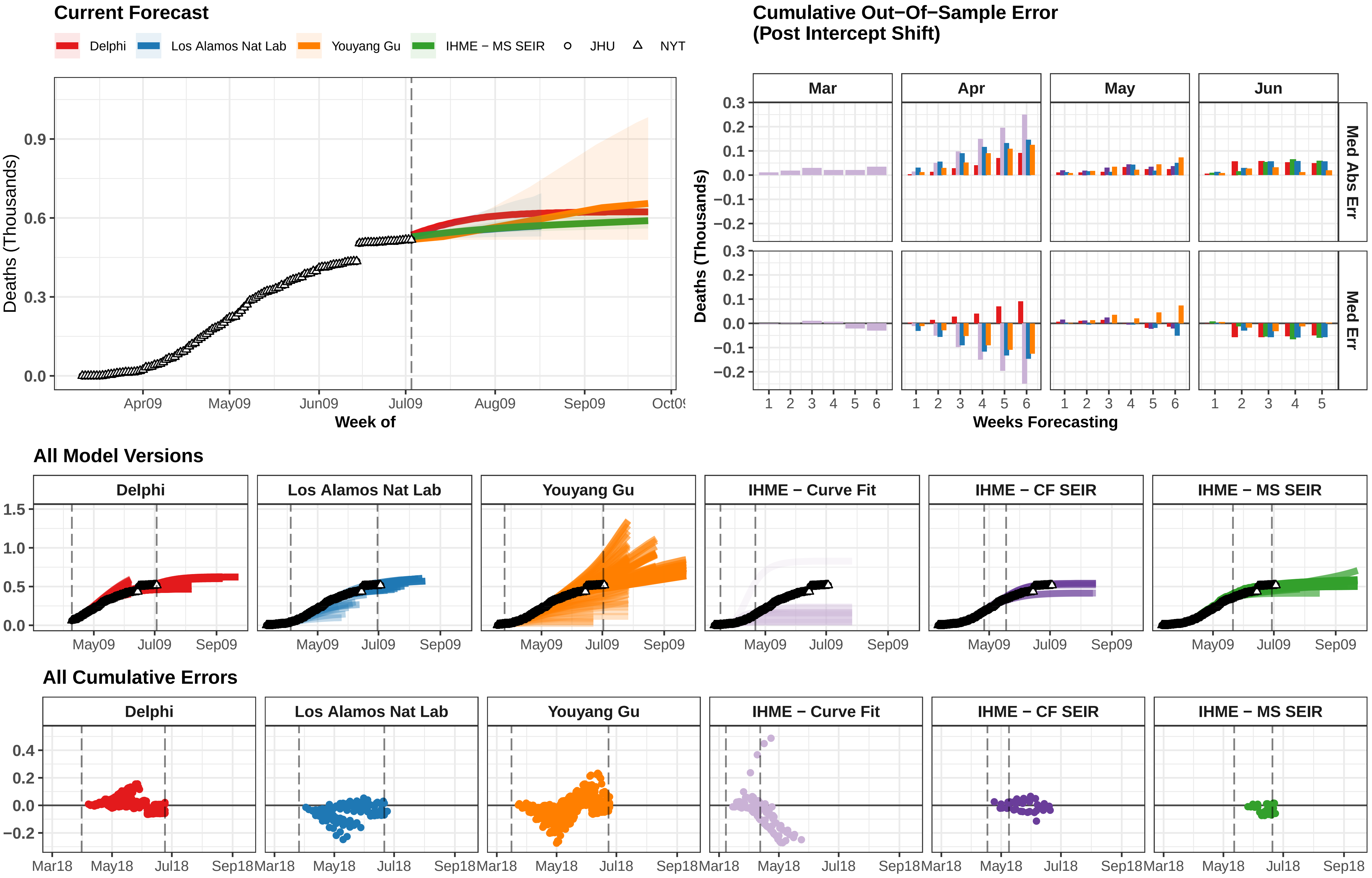

Current Forecast
Delphi
Los Alamos Nat Lab
Youyang Gu
IHME − MS SEIR
JHU
NYT
Deaths (Thousands)
0.0
0.3
0.6
0.9
Apr09
May09
Jun09
Jul09
Aug09
Sep09
Oct09
Week of
Cumulative Out−Of−Sample Error
(Post Intercept Shift)
Mar
Apr
May
Jun
Med Abs Err
Med Err
0.3
0.2
0.1
0.0
−0.1
−0.2
1 2 3 4 5 6
1 2 3 4 5
Weeks Forecasting
All Model Versions
Delphi
Los Alamos Nat Lab
Youyang Gu
IHME − Curve Fit
IHME − CF SEIR
IHME − MS SEIR
0.0
0.5
1.0
1.5
May09
Jul09
Sep09
All Cumulative Errors
−0.2
0.0
0.2
0.4
Mar18
May18
Jul18
Sep18

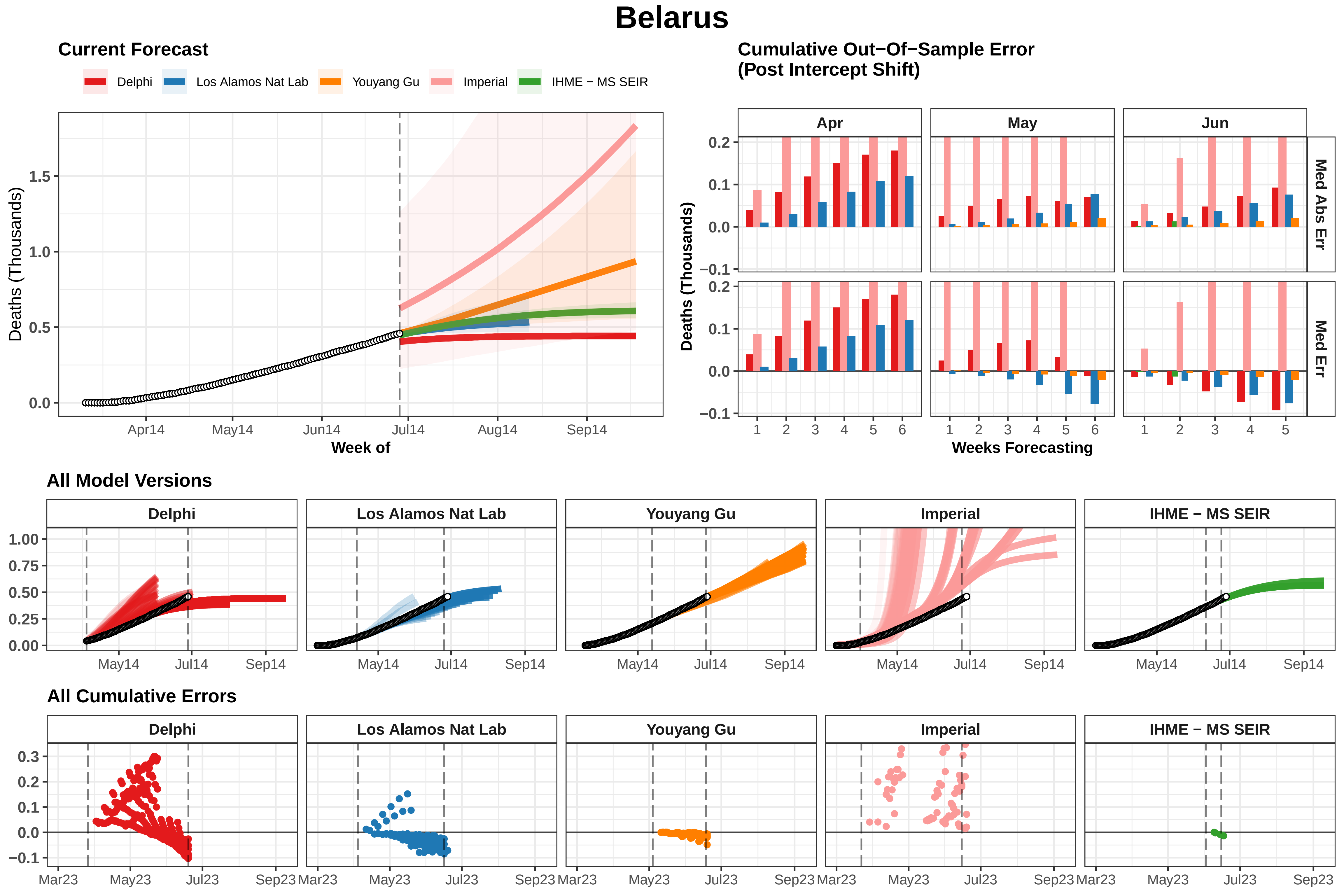

Belarus
Current Forecast
Delphi
Los Alamos Nat Lab
Youyang Gu
Imperial
IHME − MS SEIR
Deaths (Thousands)
1.5
1.0
0.5
0.0
Apr14
May14
Jun14
Jul14
Aug14
Sep14
Week of
Cumulative Out−Of−Sample Error
(Post Intercept Shift)
Apr
May
Jun
Med Abs Err
Med Err
0.2
0.1
0.0
−0.1
1
2
3
4
5
6
Weeks Forecasting
All Model Versions
1.00
0.75
0.50
0.25
0.00
All Cumulative Errors
0.3
Mar23
May23
Jul23
Sep23

Oklahoma

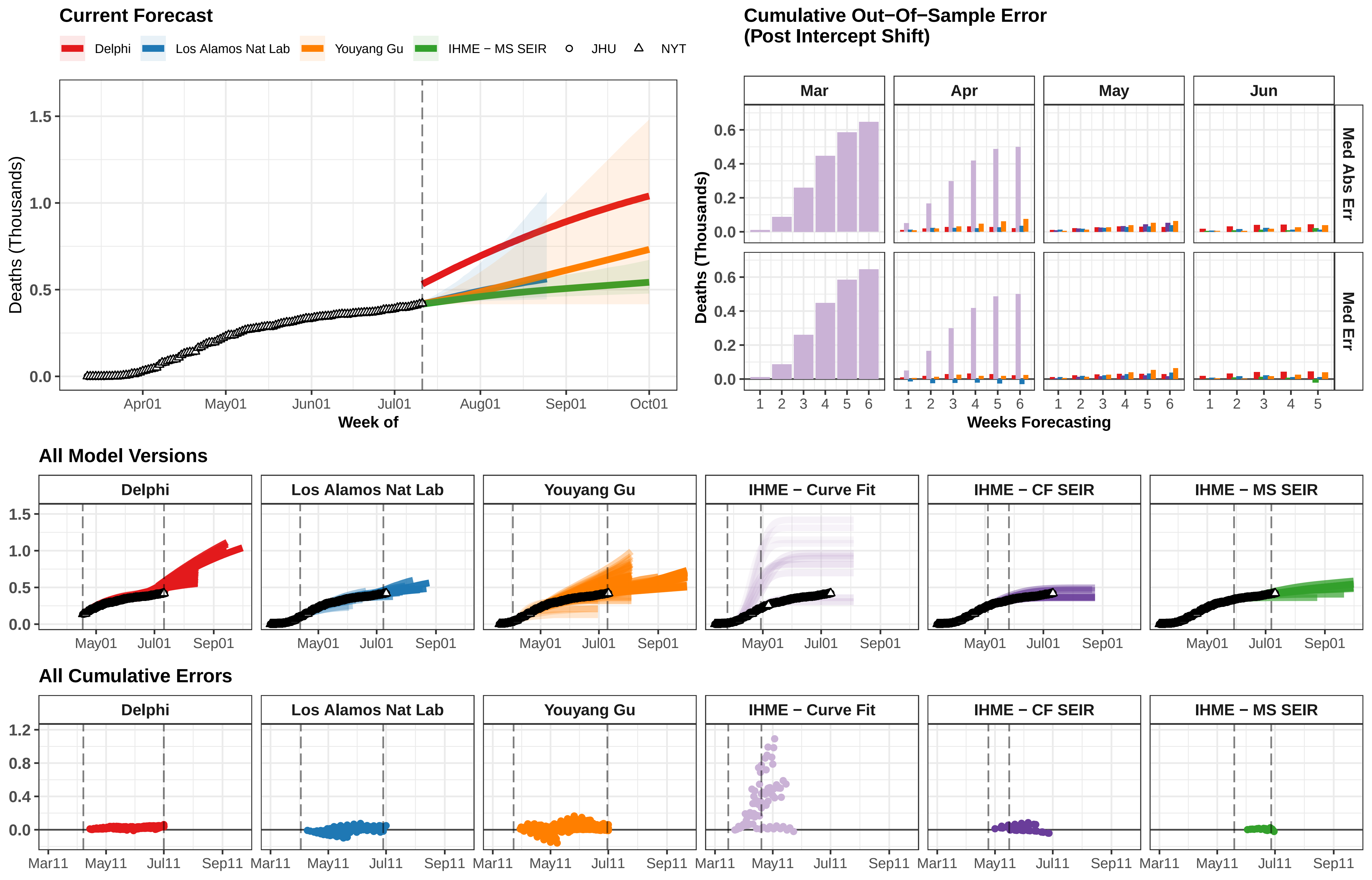
Current Forecast
Delphi
Los Alamos Nat Lab
Youyang Gu
IHME − MS SEIR
JHU
NYT
Deaths (Thousands)
Week of
Apr01
May01
Jun01
Jul01
Aug01
Sep01
Oct01
Cumulative Out−Of−Sample Error
(Post Intercept Shift)
Mar
Apr
May
Jun
Med Abs Err
Med Err
Weeks Forecasting
All Model Versions
Delphi
Los Alamos Nat Lab
Youyang Gu
IHME − Curve Fit
IHME − CF SEIR
IHME − MS SEIR
All Cumulative Errors
Mar11
May11
Jul11
Sep11

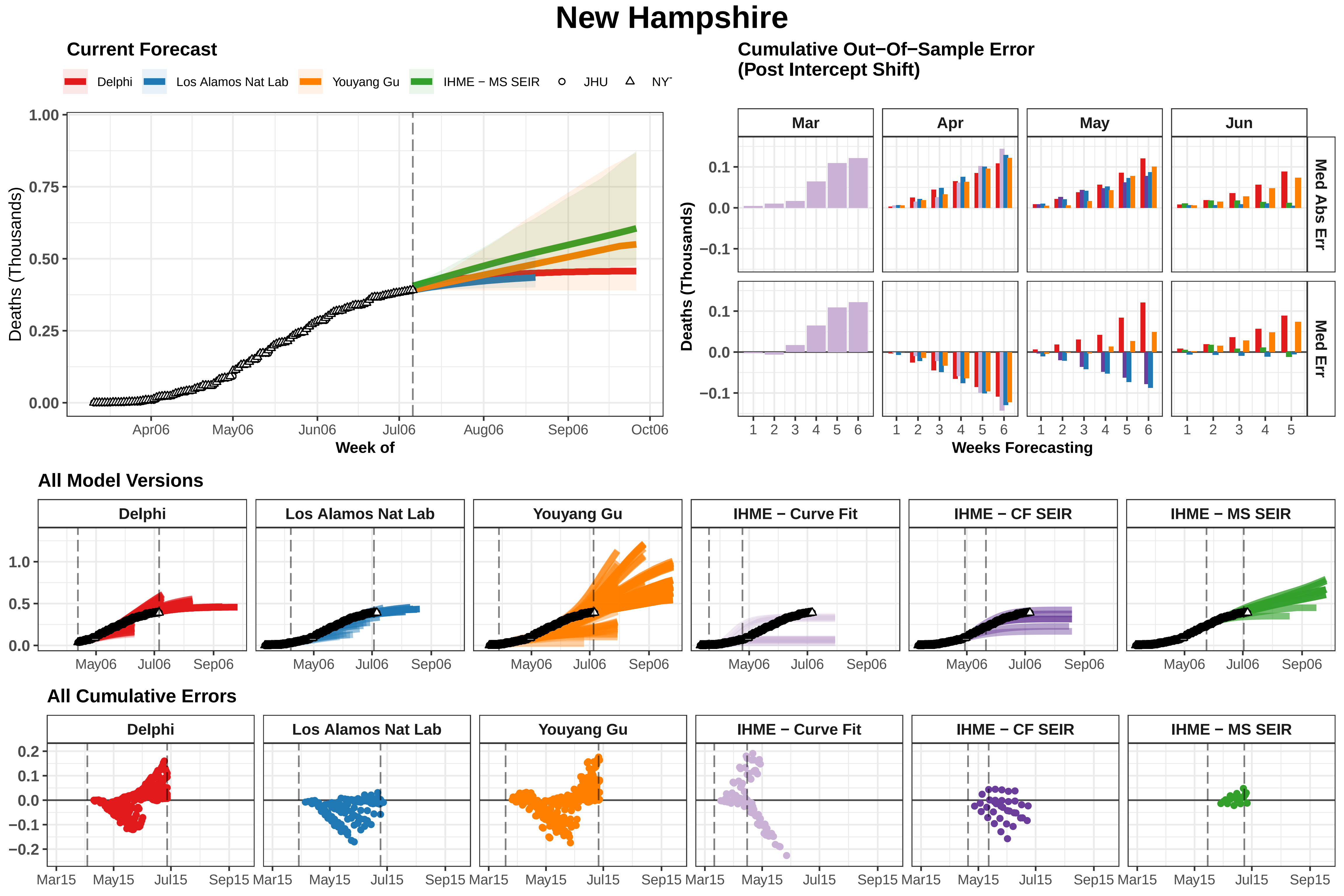
New Hampshire
Current Forecast
Delphi
Los Alamos Nat Lab
Youyang Gu
IHME − MS SEIR
JHU
NY
Deaths (Thousands)
Week of
Apr06
May06
Jun06
Jul06
Aug06
Sep06
Oct06
Cumulative Out−Of−Sample Error
(Post Intercept Shift)
Mar
Apr
May
Jun
Med Abs Err
Med Err
Weeks Forecasting
All Model Versions
IHME − Curve Fit
IHME − CF SEIR
All Cumulative Errors
Mar15
May15
Jul15
Sep15

Kuwait

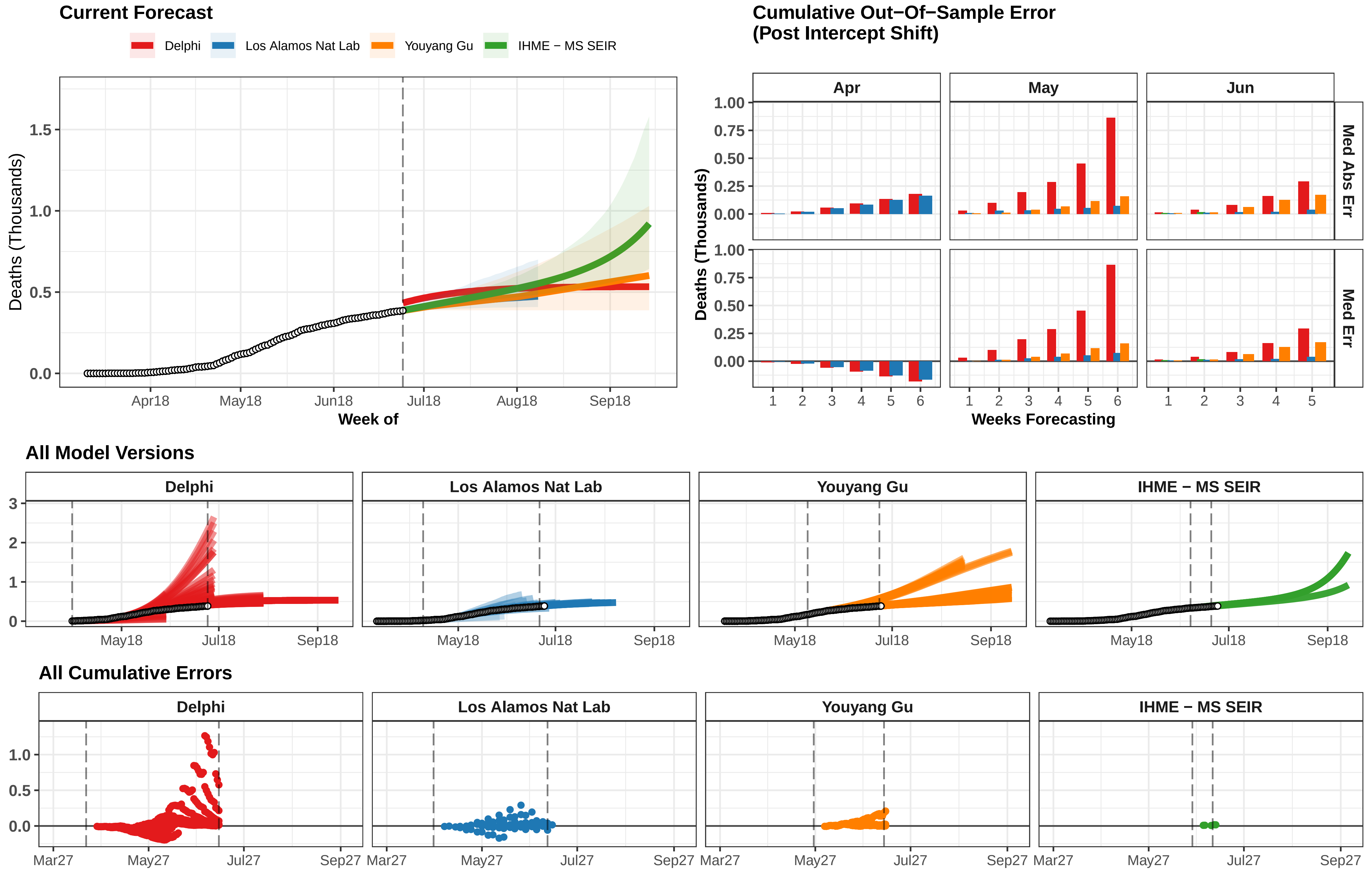
Current Forecast
Delphi
Los Alamos Nat Lab
Youyang Gu
IHME − MS SEIR
Deaths (Thousands)
0.0
0.5
1.0
1.5
Apr18
May18
Jun18
Jul18
Aug18
Sep18
Week of
Cumulative Out−Of−Sample Error
(Post Intercept Shift)
Apr
May
Jun
Med Abs Err
Med Err
Deaths (Thousands)
0.00
0.25
0.50
0.75
1.00
1
2
3
4
5
6
Weeks Forecasting
All Model Versions
Delphi
Los Alamos Nat Lab
Youyang Gu
IHME − MS SEIR
0
1
2
3
May18
Jul18
Sep18
All Cumulative Errors
Delphi
Los Alamos Nat Lab
Youyang Gu
IHME − MS SEIR
0.0
0.5
1.0
Mar27
May27
Jul27
Sep27

Serbia

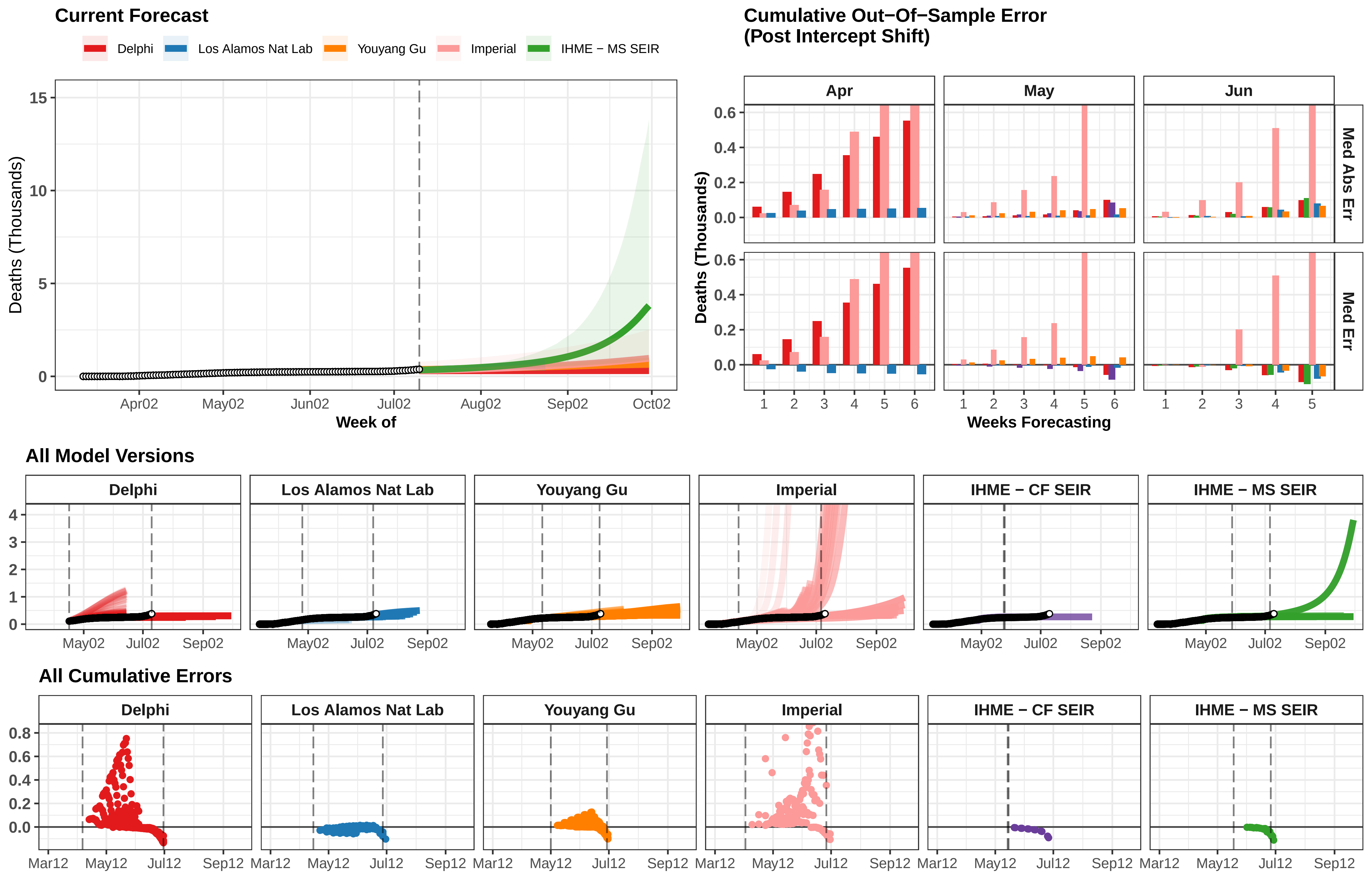

Current Forecast
Delphi
Los Alamos Nat Lab
Youyang Gu
Imperial
IHME − MS SEIR
Deaths (Thousands)
15
10
5
0
Apr02
May02
Jun02
Jul02
Aug02
Sep02
Oct02
Week of
Cumulative Out−Of−Sample Error
(Post Intercept Shift)
Apr
May
Jun
Med Abs Err
Med Err
Deaths (Thousands)
0.6
0.4
0.2
0.0
Weeks Forecasting
1
2
3
4
5
6
All Model Versions
Delphi
Los Alamos Nat Lab
Youyang Gu
Imperial
IHME − CF SEIR
IHME − MS SEIR
4
3
2
1
0
May02
Jul02
Sep02
All Cumulative Errors
Delphi
Los Alamos Nat Lab
Youyang Gu
Imperial
IHME − CF SEIR
IHME − MS SEIR
0.8
0.6
0.4
0.2
0.0
Mar12
May12
Jul12
Sep12

Macedonia

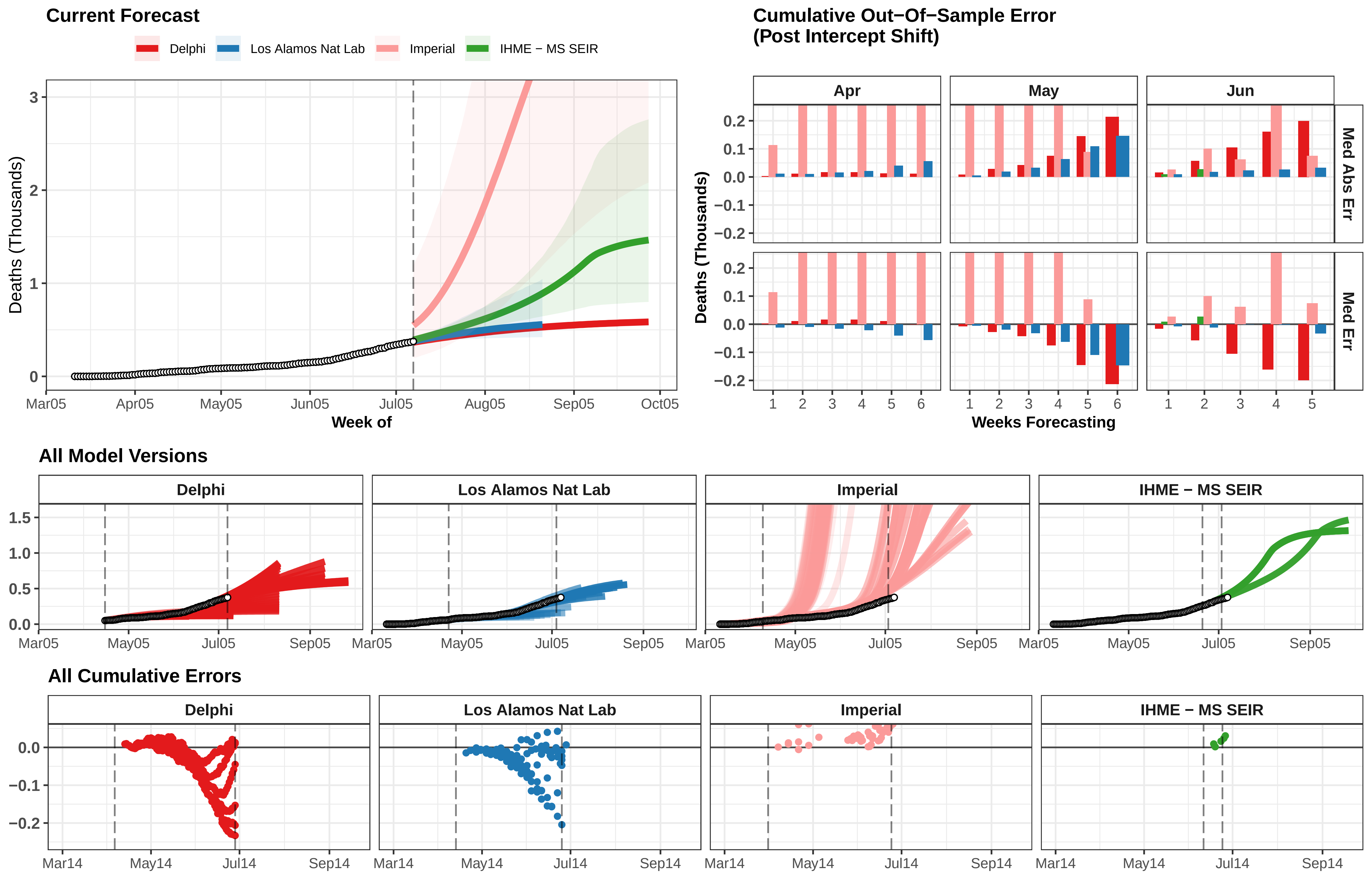
Current Forecast
Delphi
Los Alamos Nat Lab
Imperial
IHME − MS SEIR
Deaths (Thousands)
Week of
Mar05
Apr05
May05
Jun05
Jul05
Aug05
Sep05
Oct05
Cumulative Out−Of−Sample Error
(Post Intercept Shift)
Apr
May
Jun
Med Abs Err
Med Err
Weeks Forecasting
All Model Versions
All Cumulative Errors
Mar14
May14
Jul14
Sep14

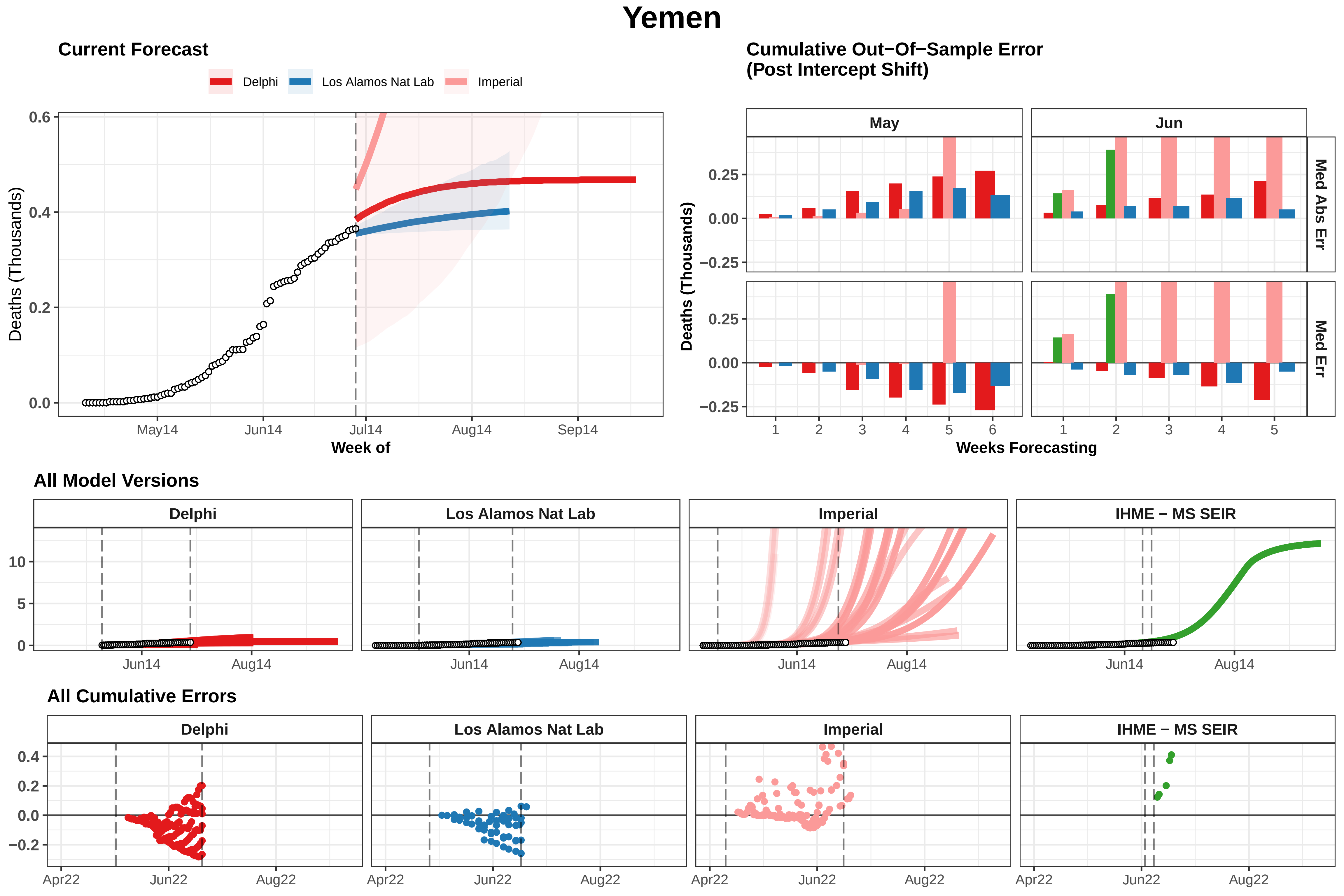

Yemen
Current Forecast
Delphi
Los Alamos Nat Lab
Imperial
Deaths (Thousands)
0.0
0.2
0.4
0.6
May14
Jun14
Jul14
Aug14
Sep14
Week of
Cumulative Out−Of−Sample Error
(Post Intercept Shift)
May
Jun
Med Abs Err
Med Err
0.25
0.00
−0.25
Weeks Forecasting
All Model Versions
Delphi
Los Alamos Nat Lab
Imperial
IHME − MS SEIR
0
5
10
Jun14
Aug14
All Cumulative Errors
0.4
0.2
0.0
−0.2
Apr22
Jun22
Aug22

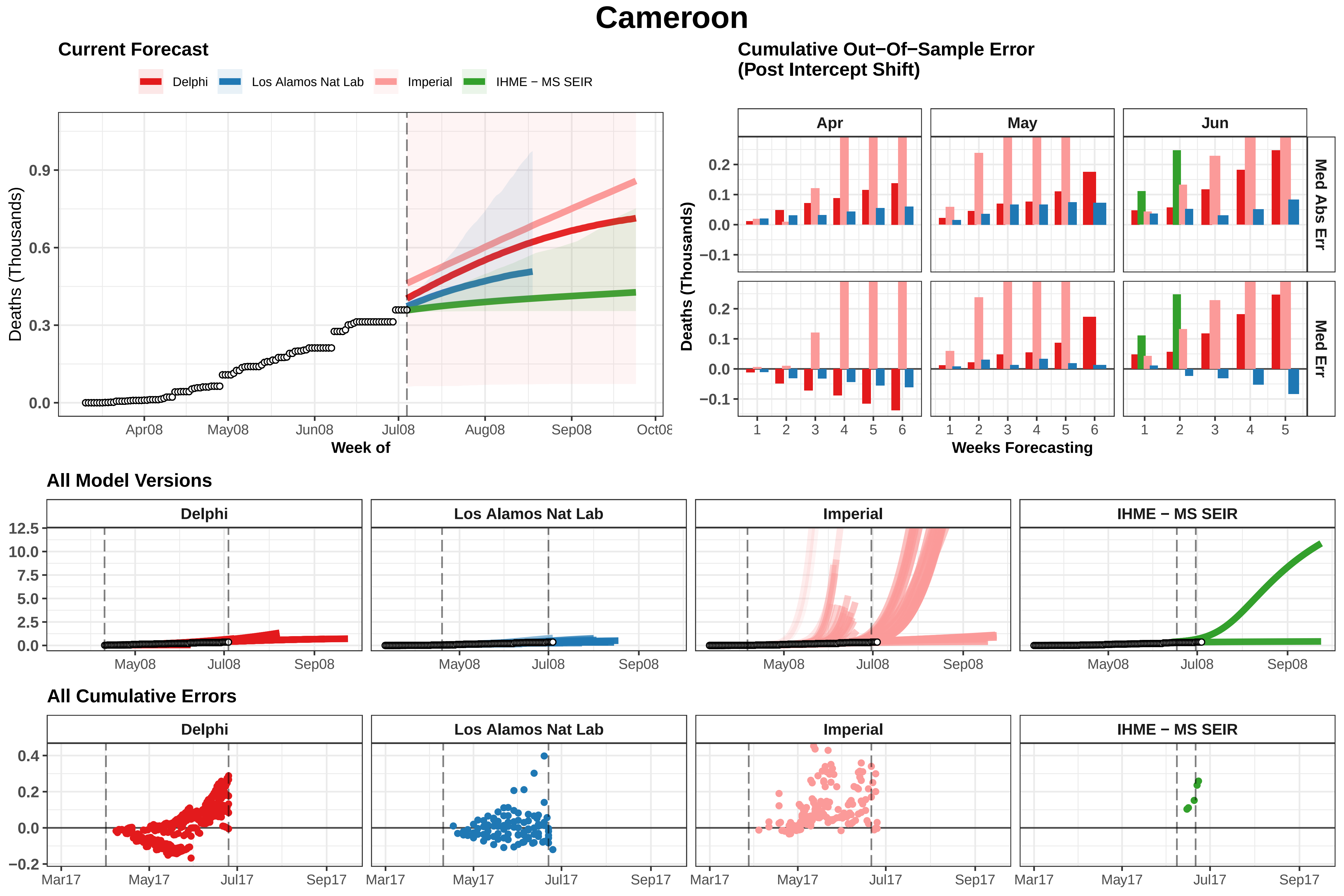

Cameroon
Current Forecast
Delphi
Los Alamos Nat Lab
Imperial
IHME − MS SEIR
Deaths (Thousands)
0.0
0.3
0.6
0.9
Apr08
May08
Jun08
Jul08
Aug08
Sep08
Oct08
Week of
Cumulative Out−Of−Sample Error
(Post Intercept Shift)
Apr
May
Jun
Med Abs Err
Med Err
0.2
0.1
0.0
−0.1
1
2
3
4
5
6
Weeks Forecasting
All Model Versions
12.5
10.0
7.5
5.0
2.5
0.0
All Cumulative Errors
0.4
0.2
0.0
−0.2
Mar17
May17
Jul17
Sep17

Israel

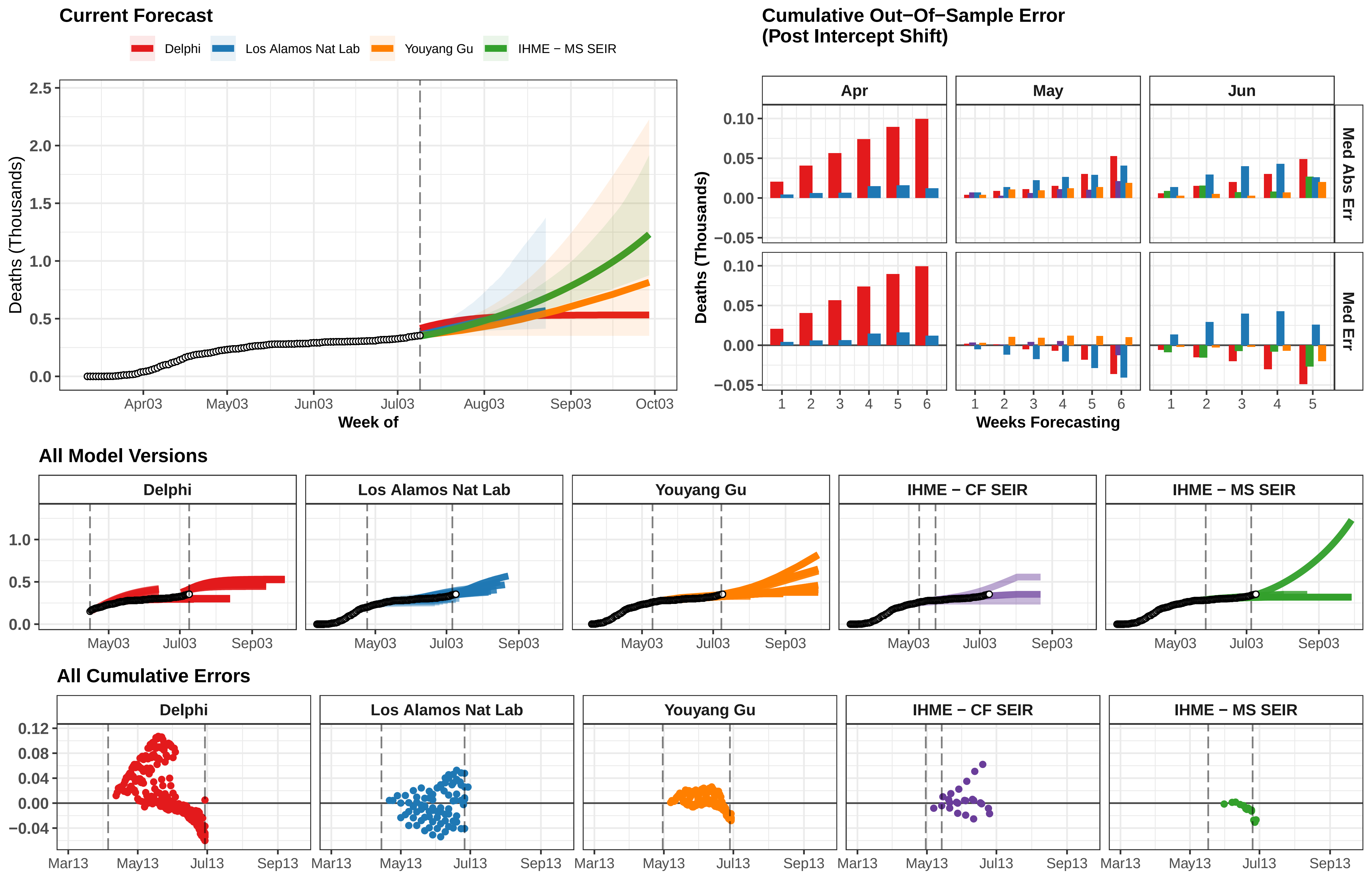

Current Forecast
Delphi
Los Alamos Nat Lab
Youyang Gu
IHME − MS SEIR
Deaths (Thousands)
Week of
Apr03
May03
Jun03
Jul03
Aug03
Sep03
Oct03
Cumulative Out−Of−Sample Error
(Post Intercept Shift)
Apr
May
Jun
Med Abs Err
Med Err
Weeks Forecasting
All Model Versions
Delphi
Los Alamos Nat Lab
Youyang Gu
IHME − CF SEIR
IHME − MS SEIR
All Cumulative Errors
Mar13
May13
Jul13
Sep13

Czech Republic

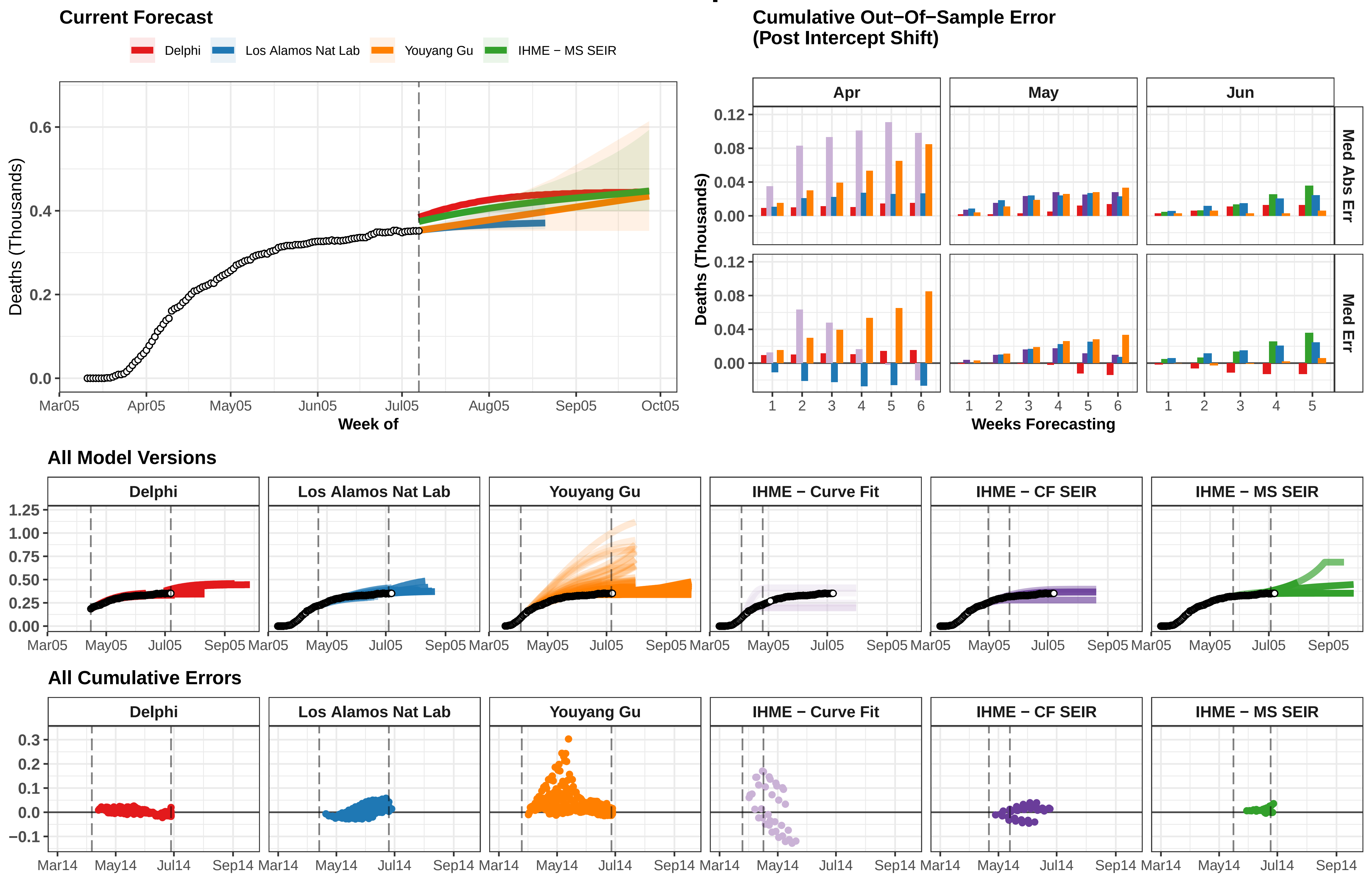

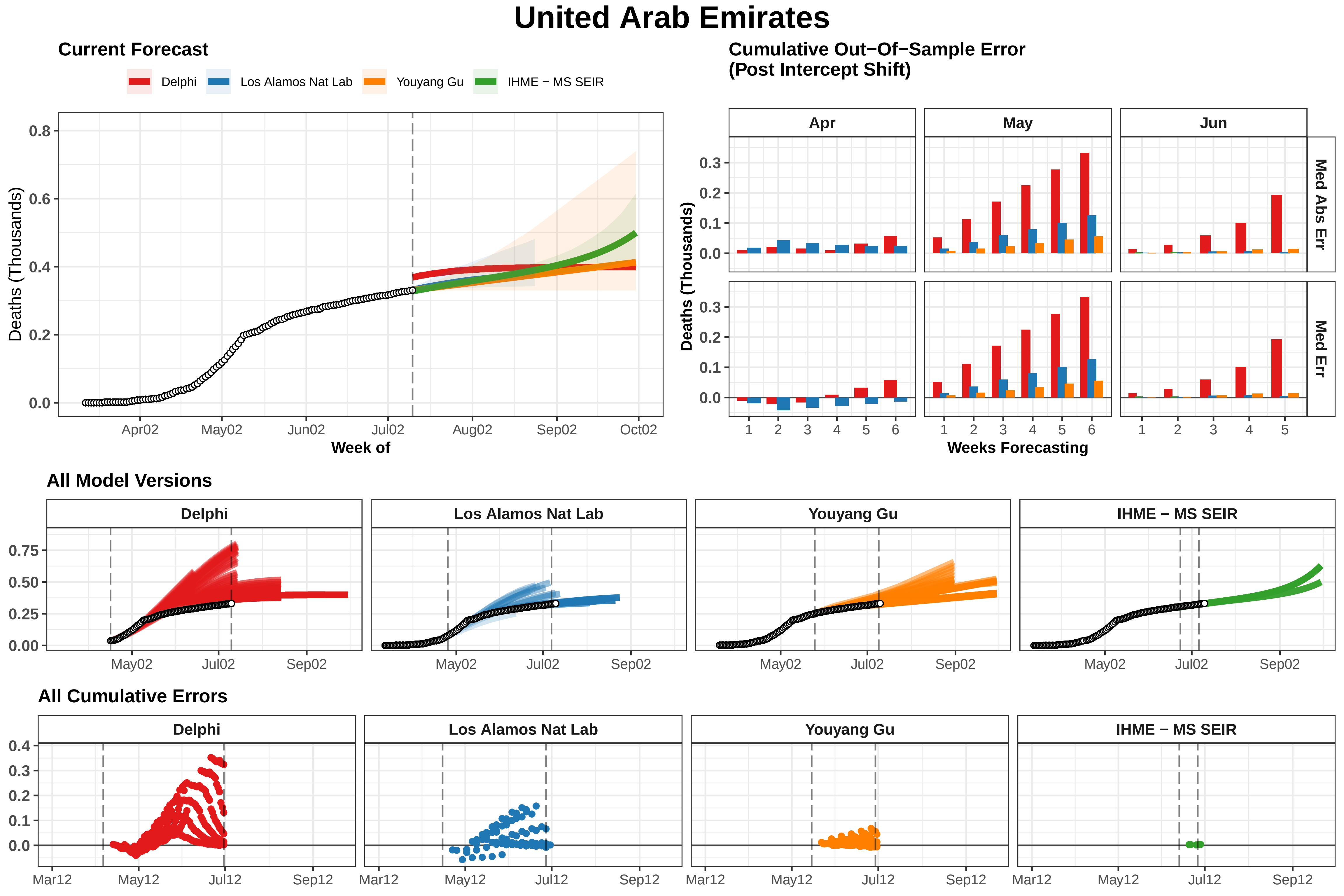

United Arab Emirates
Current Forecast
Delphi
Los Alamos Nat Lab
Youyang Gu
IHME − MS SEIR
Deaths (Thousands)
Week of
Apr02
May02
Jun02
Jul02
Aug02
Sep02
Oct02
Cumulative Out−Of−Sample Error
(Post Intercept Shift)
Apr
May
Jun
Med Abs Err
Med Err
Weeks Forecasting
All Model Versions
All Cumulative Errors
Mar12
May12
Jul12
Sep12

Finland

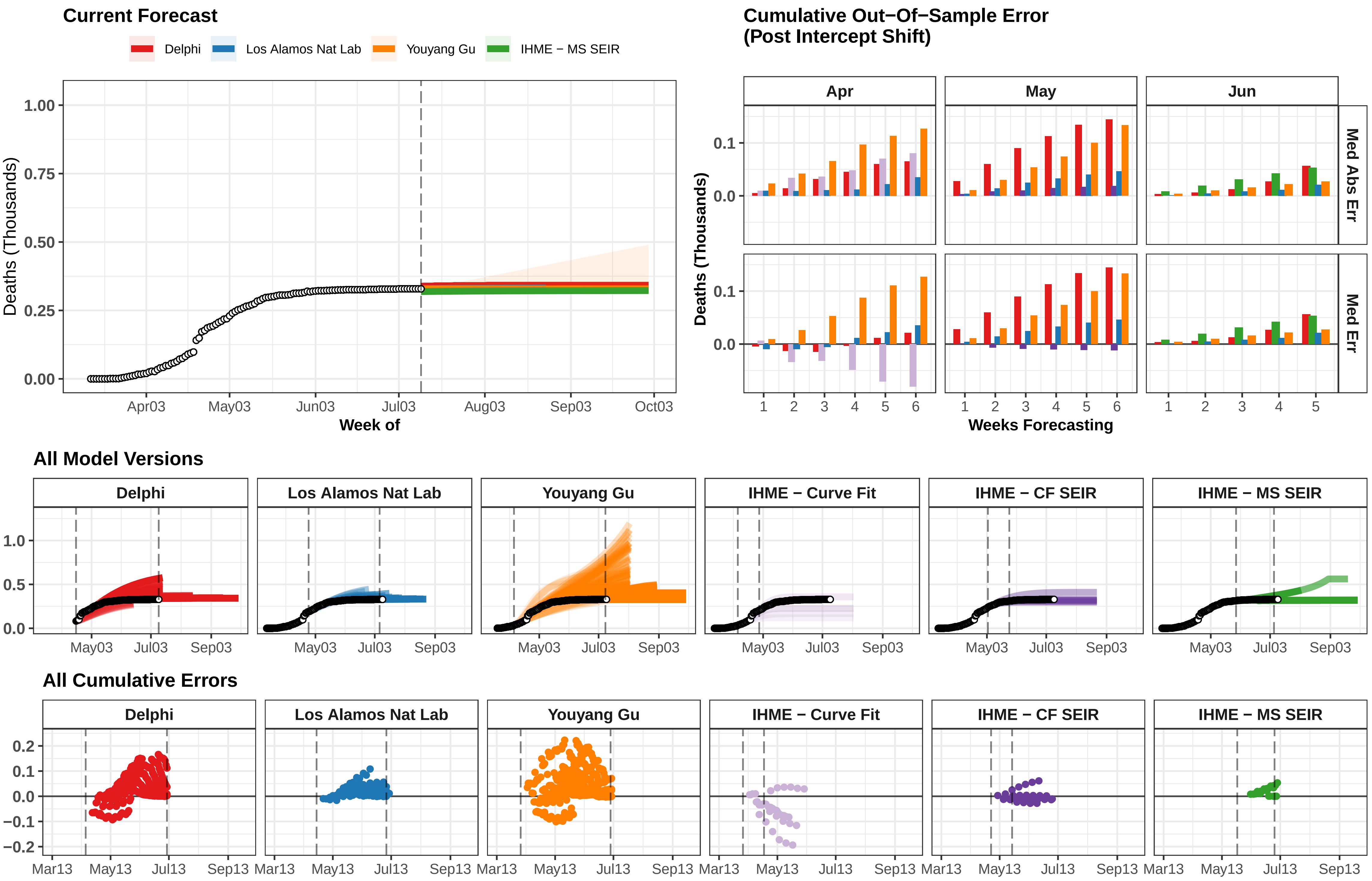

Current Forecast
Delphi
Los Alamos Nat Lab
Youyang Gu
IHME − MS SEIR
Deaths (Thousands)
1.00
0.75
0.50
0.25
0.00
Apr03
May03
Jun03
Jul03
Aug03
Sep03
Oct03
Week of
Cumulative Out−Of−Sample Error
(Post Intercept Shift)
Apr
May
Jun
Med Abs Err
Med Err
Deaths (Thousands)
0.1
0.0
1
2
3
4
5
6
Weeks Forecasting
All Model Versions
Delphi
Los Alamos Nat Lab
Youyang Gu
IHME − Curve Fit
IHME − CF SEIR
IHME − MS SEIR
1.0
0.5
0.0
May03
Jul03
Sep03
All Cumulative Errors
0.2
0.1
0.0
−0.1
−0.2
Mar13
May13
Jul13
Sep13

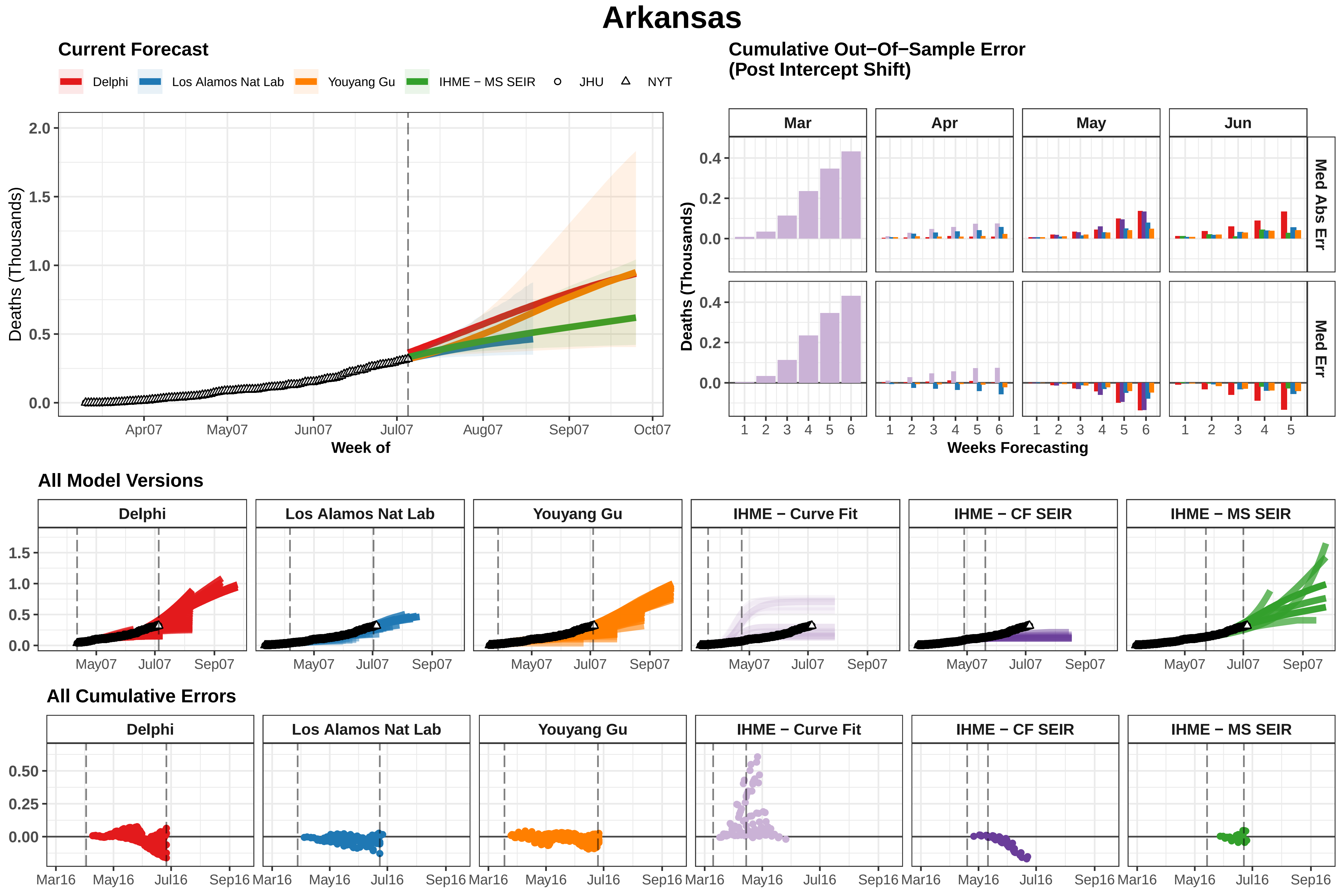
Arkansas
Current Forecast
Delphi
Los Alamos Nat Lab
Youyang Gu
IHME − MS SEIR
JHU
NYT
Deaths (Thousands)
Week of
Apr07
May07
Jun07
Jul07
Aug07
Sep07
Oct07
Cumulative Out−Of−Sample Error (Post Intercept Shift)
Mar
Apr
May
Jun
Med Abs Err
Med Err
Weeks Forecasting
All Model Versions
IHME − Curve Fit
IHME − CF SEIR
All Cumulative Errors
Mar16
May16
Jul16
Sep16

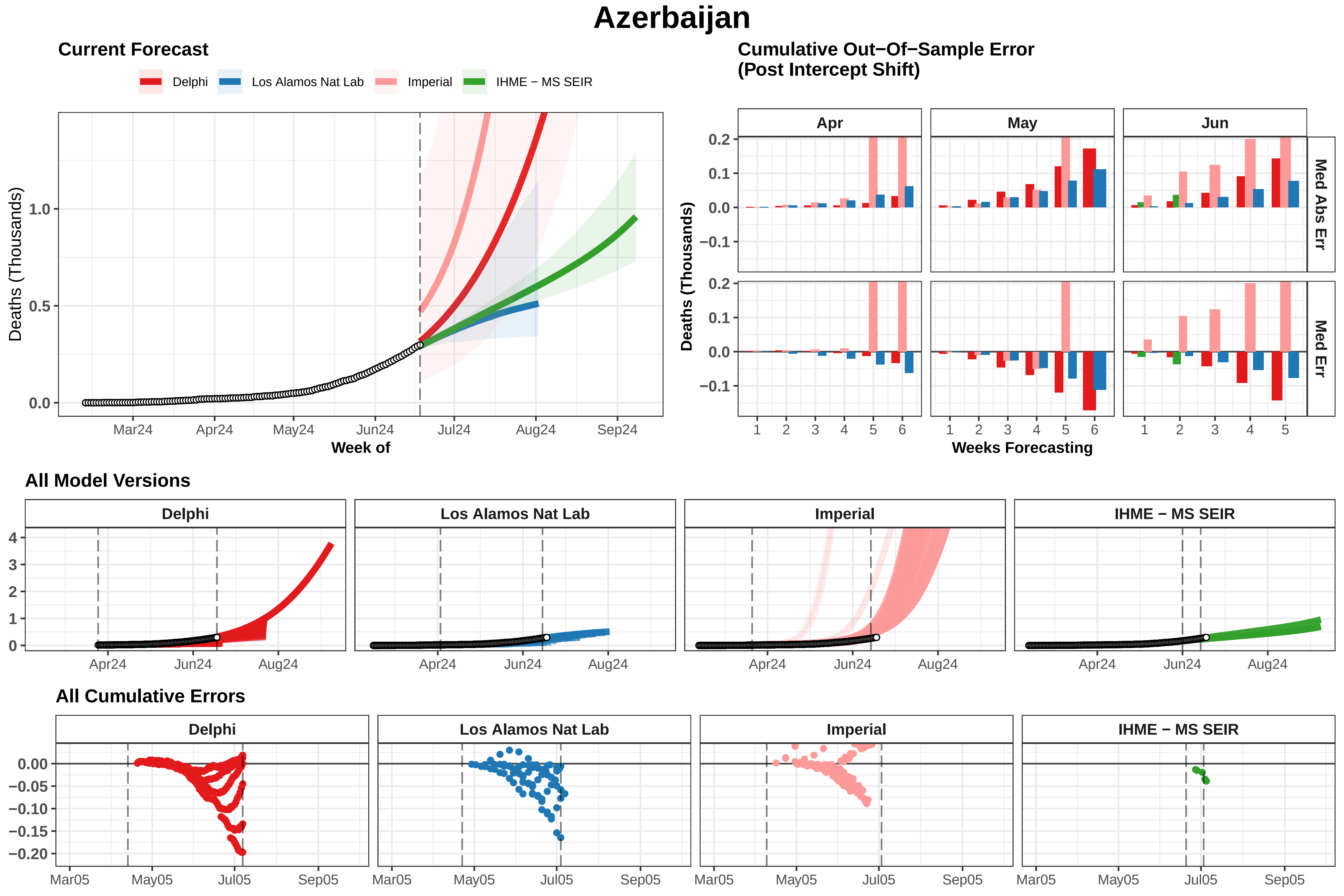

Azerbaijan
Current Forecast
Delphi
Los Alamos Nat Lab
Imperial
IHME − MS SEIR
Deaths (Thousands)
1.0
0.5
0.0
Mar24
Apr24
May24
Jun24
Jul24
Aug24
Sep24
Week of
Cumulative Out−Of−Sample Error
(Post Intercept Shift)
Apr
May
Jun
Med Abs Err
Med Err
0.2
0.1
0.0
−0.1
Weeks Forecasting
All Model Versions
Delphi
Los Alamos Nat Lab
Imperial
IHME − MS SEIR
4
3
2
1
0
Apr24
Jun24
Aug24
All Cumulative Errors
0.00
−0.05
−0.10
−0.15
−0.20
Mar05
May05
Jul05
Sep05

Nebraska

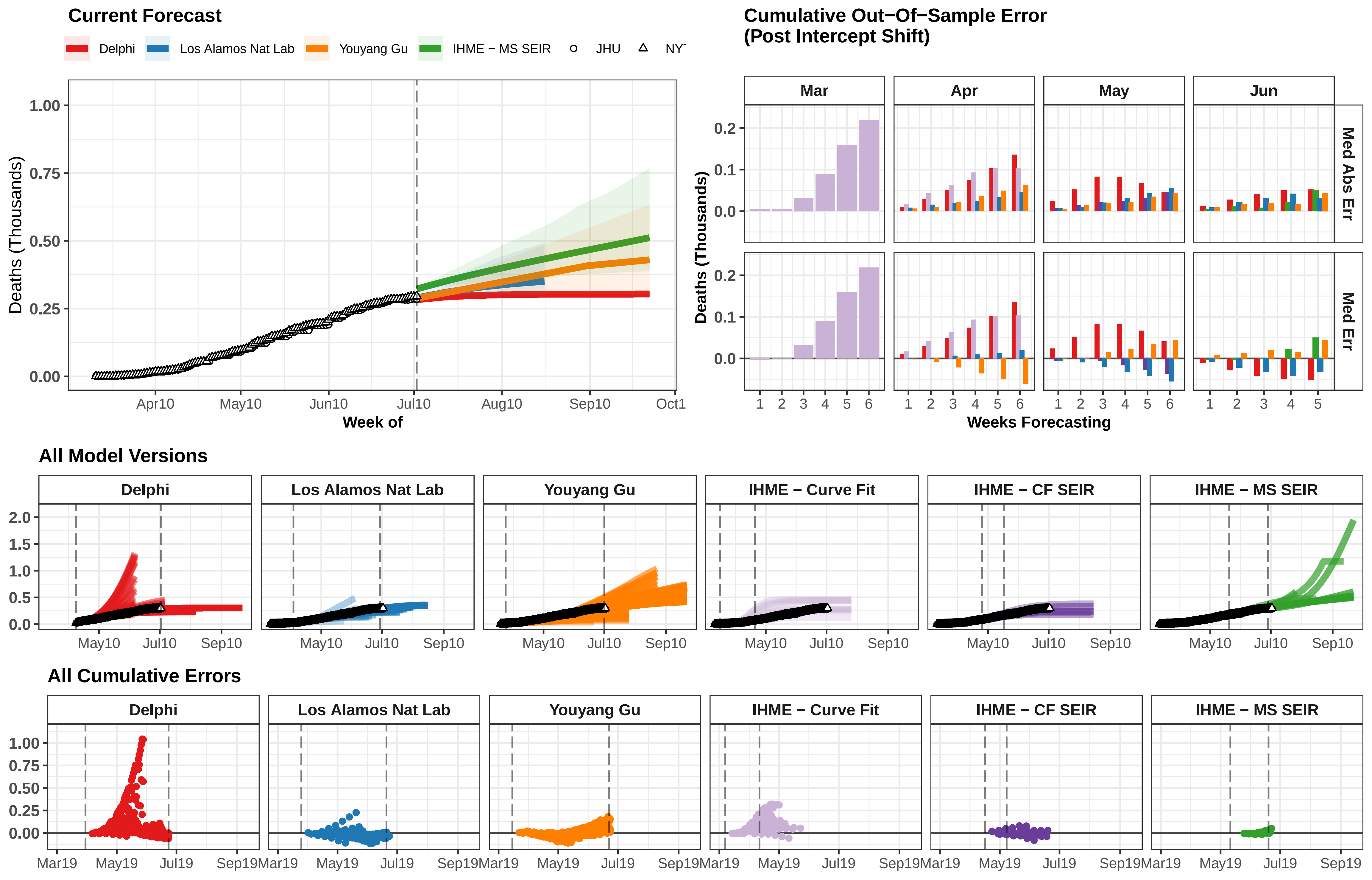

Current Forecast
Delphi
Los Alamos Nat Lab
Youyang Gu
IHME − MS SEIR
JHU
NY
Deaths (Thousands)
1.00
0.75
0.50
0.25
0.00
Apr10
May10
Jun10
Jul10
Aug10
Sep10
Oct1
Week of
Cumulative Out−Of−Sample Error
(Post Intercept Shift)
Mar
Apr
May
Jun
Med Abs Err
Med Err
0.2
0.1
0.0
Weeks Forecasting
All Model Versions
Delphi
Los Alamos Nat Lab
Youyang Gu
IHME − Curve Fit
IHME − CF SEIR
IHME − MS SEIR
2.0
1.5
1.0
0.5
0.0
May10
Jul10
Sep10
All Cumulative Errors
Mar19
May19
Jul19
Sep19

Kansas

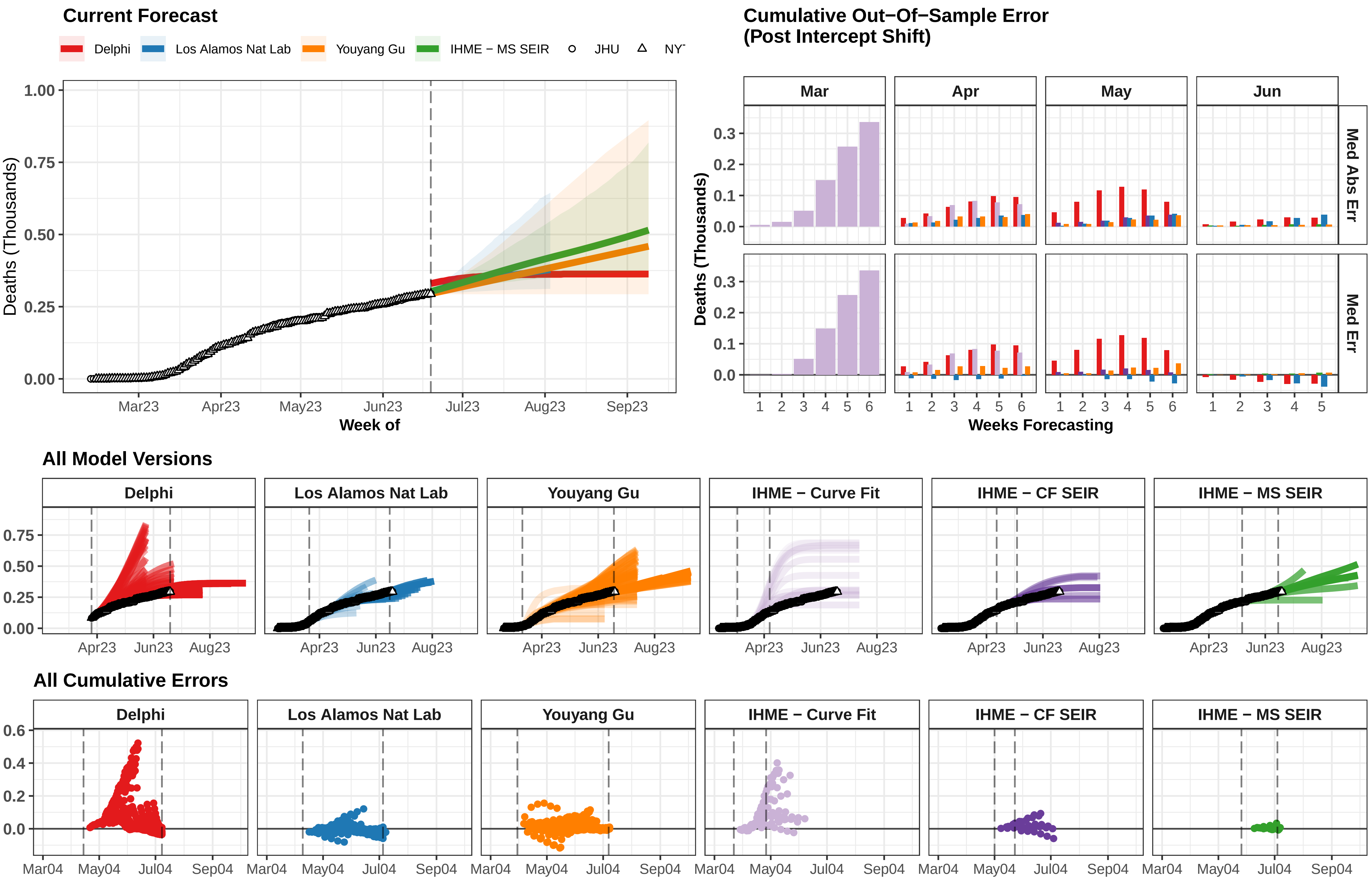

Current Forecast
Delphi
Los Alamos Nat Lab
Youyang Gu
IHME − MS SEIR
JHU
NY
Deaths (Thousands)
1.00
0.75
0.50
0.25
0.00
Mar23
Apr23
May23
Jun23
Jul23
Aug23
Sep23
Week of
Cumulative Out−Of−Sample Error
(Post Intercept Shift)
Mar
Apr
May
Jun
Med Abs Err
Med Err
0.3
0.2
0.1
0.0
Weeks Forecasting
All Model Versions
Delphi
Los Alamos Nat Lab
Youyang Gu
IHME − Curve Fit
IHME − CF SEIR
IHME − MS SEIR
Apr23
Jun23
Aug23
All Cumulative Errors
0.6
0.4
0.2
0.0
Mar04
May04
Jul04
Sep04

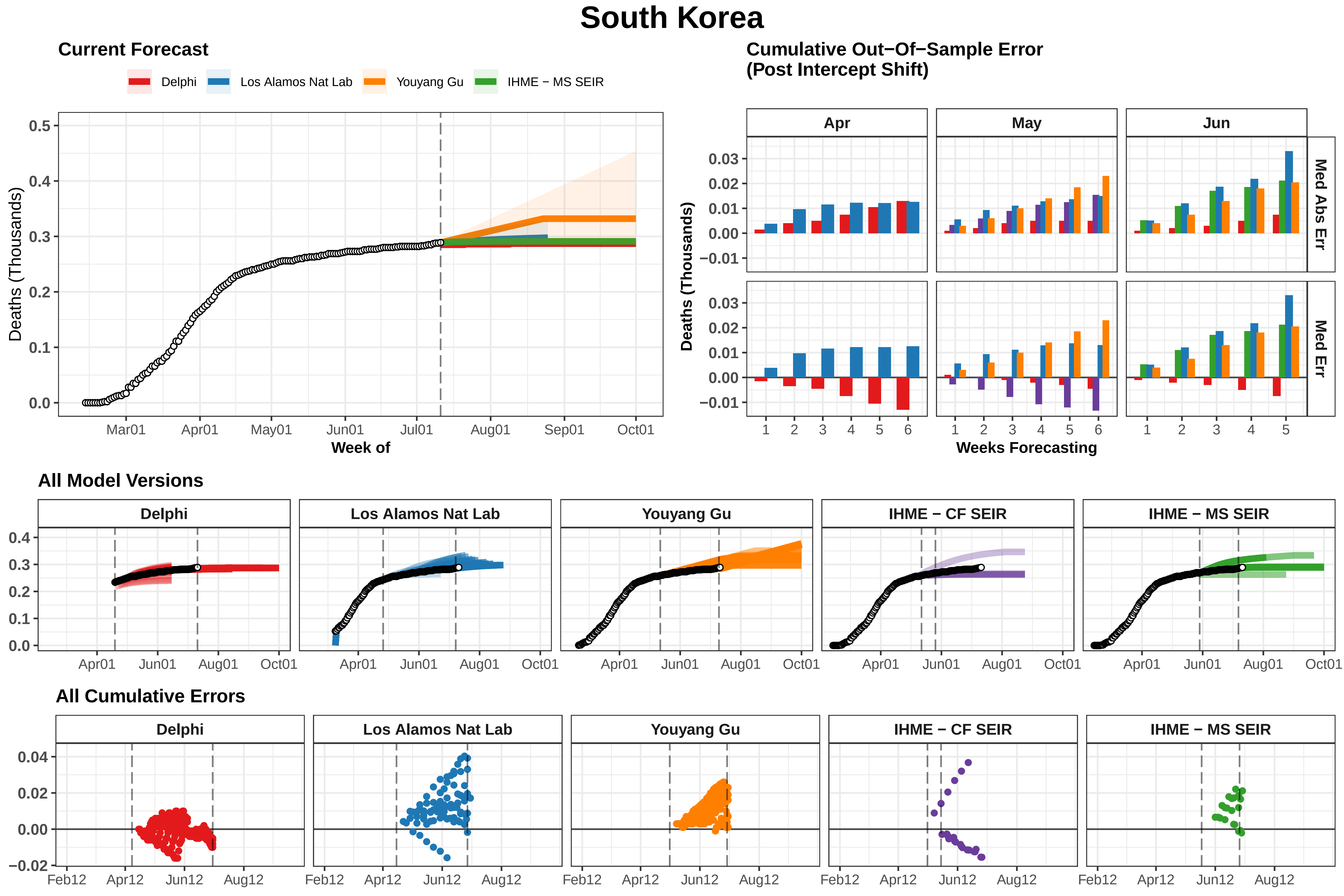

South Korea
Current Forecast
Delphi
Los Alamos Nat Lab
Youyang Gu
IHME − MS SEIR
Deaths (Thousands)
Week of
Mar01
Apr01
May01
Jun01
Jul01
Aug01
Sep01
Oct01
Cumulative Out−Of−Sample Error
(Post Intercept Shift)
Apr
May
Jun
Med Abs Err
Med Err
Weeks Forecasting
All Model Versions
Delphi
Los Alamos Nat Lab
Youyang Gu
IHME − CF SEIR
IHME − MS SEIR
All Cumulative Errors
Feb12
Apr12
Jun12
Aug12

Bulgaria

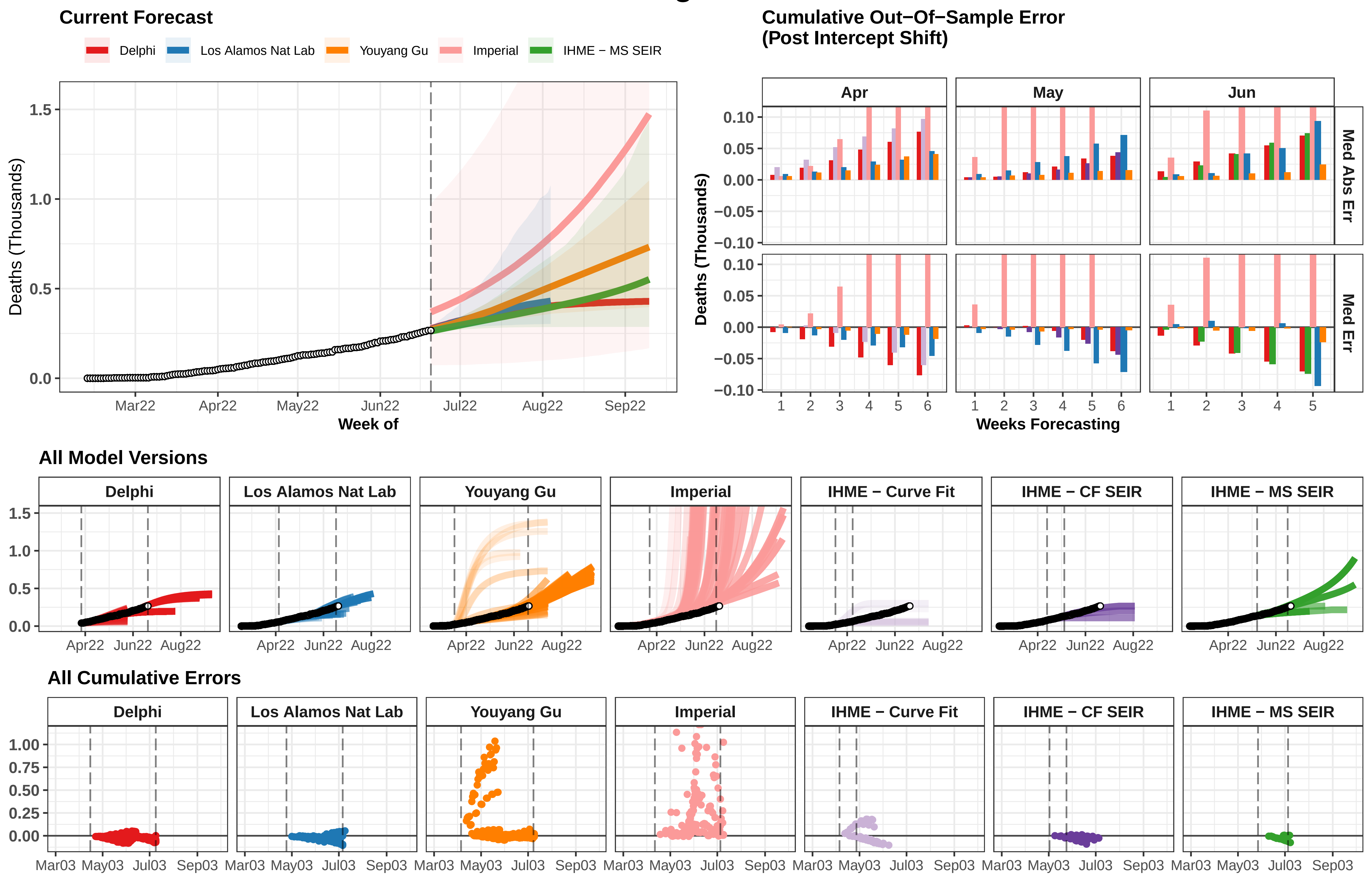

Current Forecast
Delphi
Los Alamos Nat Lab
Youyang Gu
Imperial
IHME − MS SEIR
Deaths (Thousands)
1.5
1.0
0.5
0.0
Mar22
Apr22
May22
Jun22
Jul22
Aug22
Sep22
Week of
Cumulative Out−Of−Sample Error
(Post Intercept Shift)
Apr
May
Jun
Med Abs Err
Med Err
0.10
0.05
0.00
−0.05
−0.10
1
2
3
4
5
6
Weeks Forecasting
All Model Versions
Delphi
Los Alamos Nat Lab
Youyang Gu
Imperial
IHME − Curve Fit
IHME − CF SEIR
IHME − MS SEIR
Apr22
Jun22
Aug22
All Cumulative Errors
1.00
0.75
0.50
0.25
0.00
Mar03
May03
Jul03
Sep03

Kazakhstan

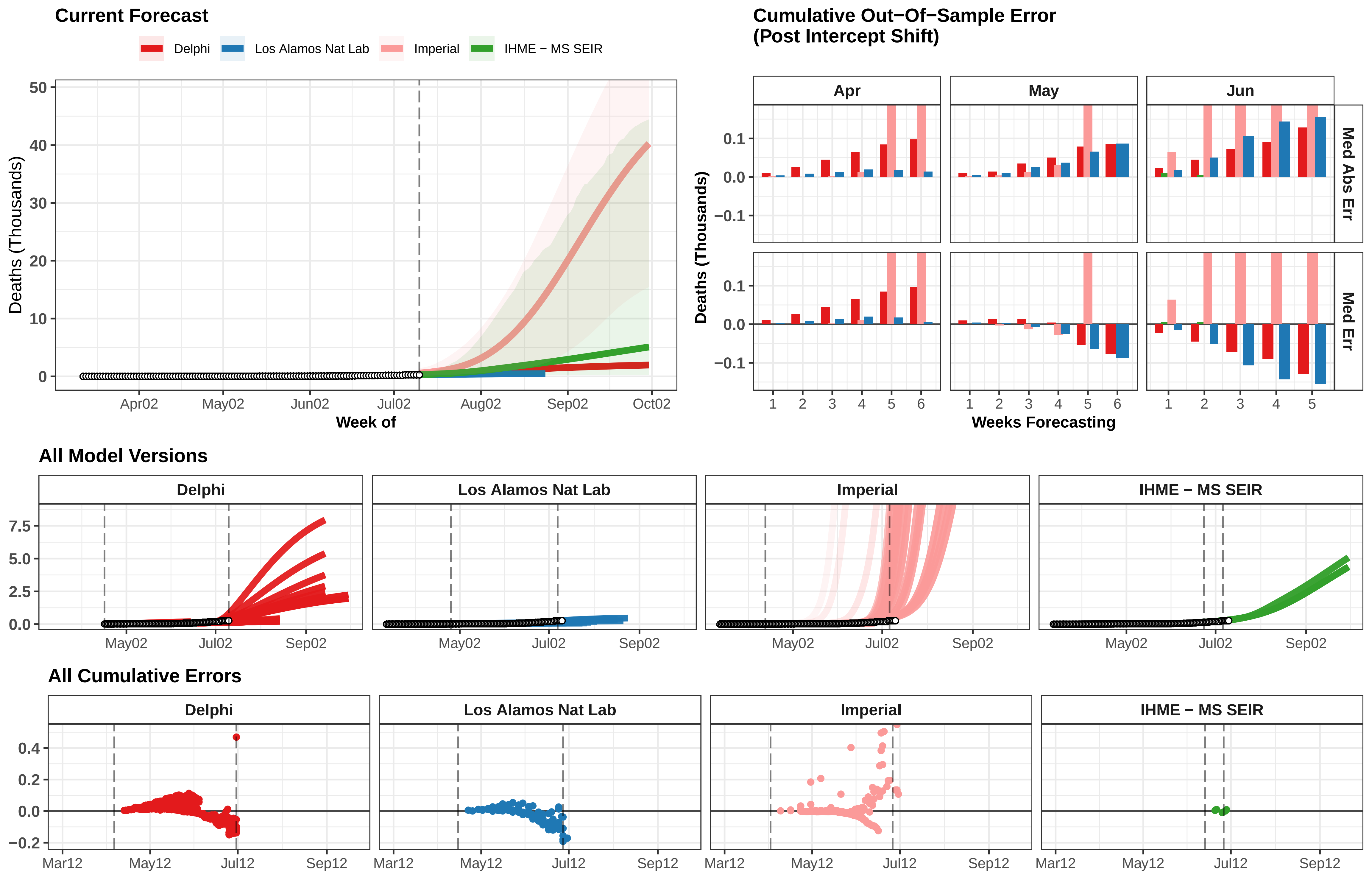

Current Forecast
Delphi
Los Alamos Nat Lab
Imperial
IHME − MS SEIR
Deaths (Thousands)
Week of
Apr02
May02
Jun02
Jul02
Aug02
Sep02
Oct02
Cumulative Out−Of−Sample Error
(Post Intercept Shift)
Apr
May
Jun
Med Abs Err
Med Err
Weeks Forecasting
All Model Versions
All Cumulative Errors
Mar12
May12
Jul12
Sep12

El Salvador

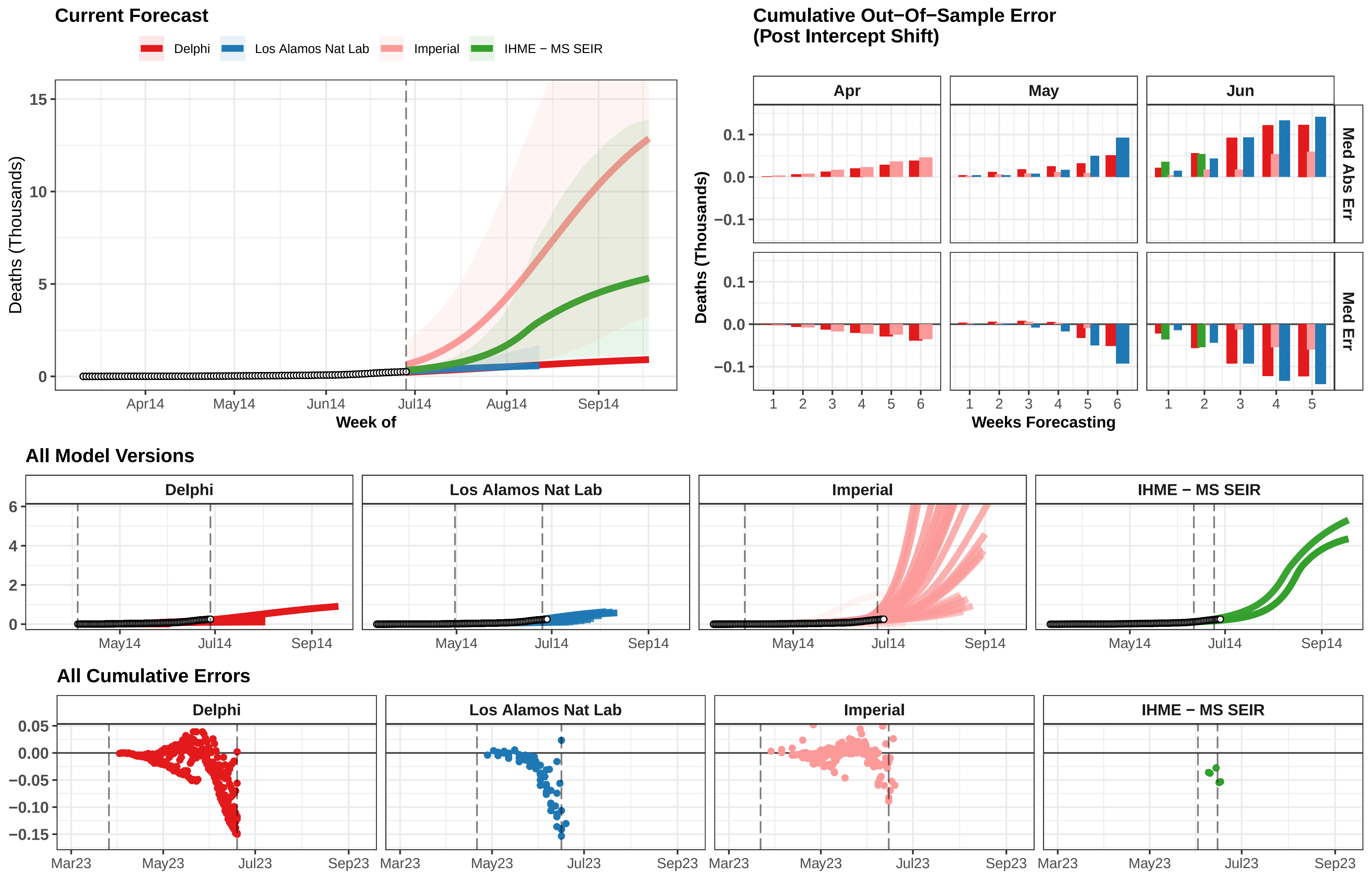
Current Forecast
Delphi
Los Alamos Nat Lab
Imperial
IHME − MS SEIR
Deaths (Thousands)
Week of
Apr14
May14
Jun14
Jul14
Aug14
Sep14
Cumulative Out−Of−Sample Error
(Post Intercept Shift)
Apr
May
Jun
Med Abs Err
Med Err
Weeks Forecasting
All Model Versions
All Cumulative Errors
Mar23
May23
Jul23
Sep23

Norway

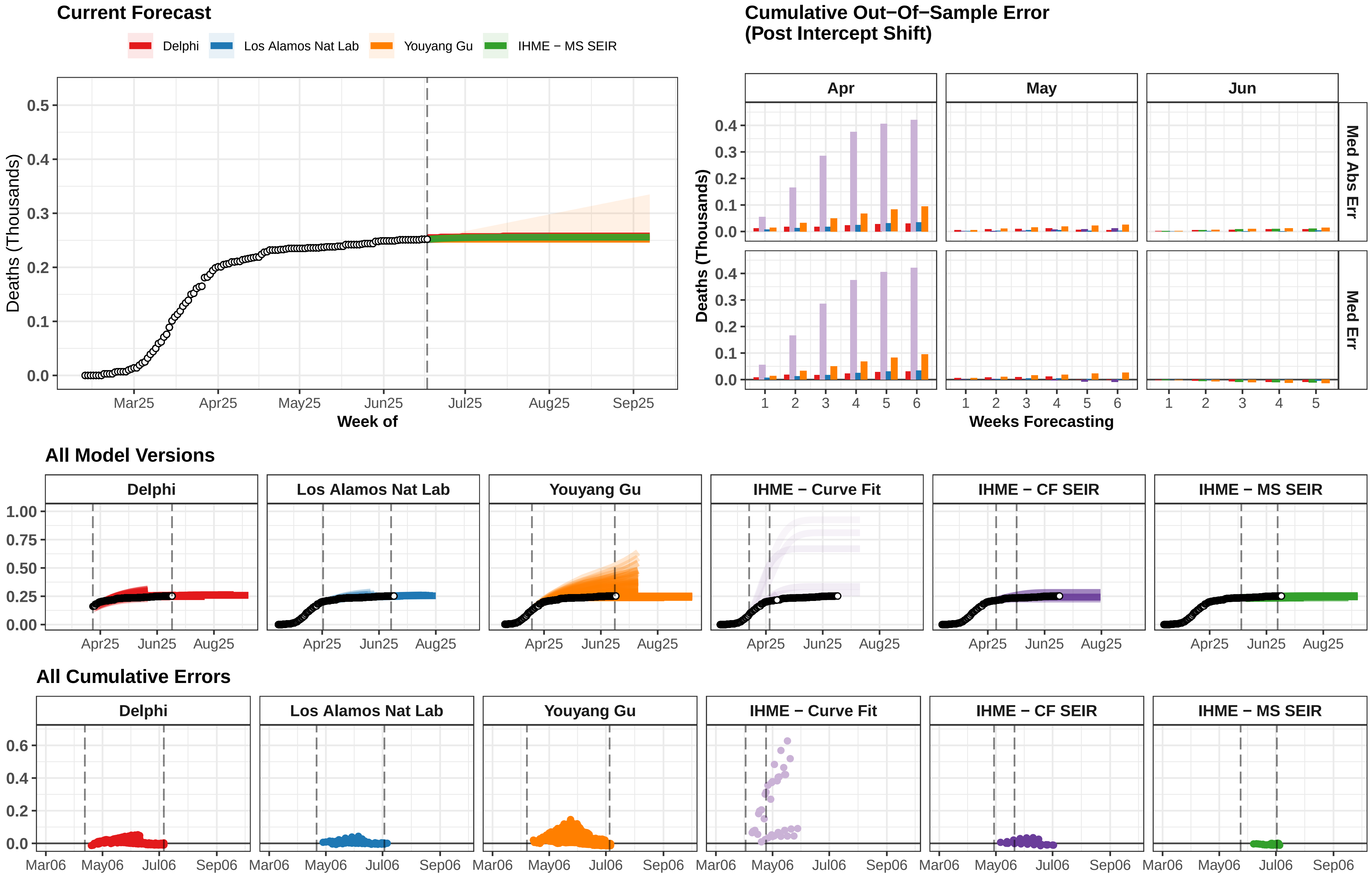

Current Forecast
Delphi
Los Alamos Nat Lab
Youyang Gu
IHME − MS SEIR
Deaths (Thousands)
Week of
Mar25
Apr25
May25
Jun25
Jul25
Aug25
Sep25
Cumulative Out−Of−Sample Error
(Post Intercept Shift)
Apr
May
Jun
Med Abs Err
Med Err
Weeks Forecasting
All Model Versions
Delphi
Los Alamos Nat Lab
Youyang Gu
IHME − Curve Fit
IHME − CF SEIR
IHME − MS SEIR
All Cumulative Errors
Mar06
May06
Jul06
Sep06

Oman

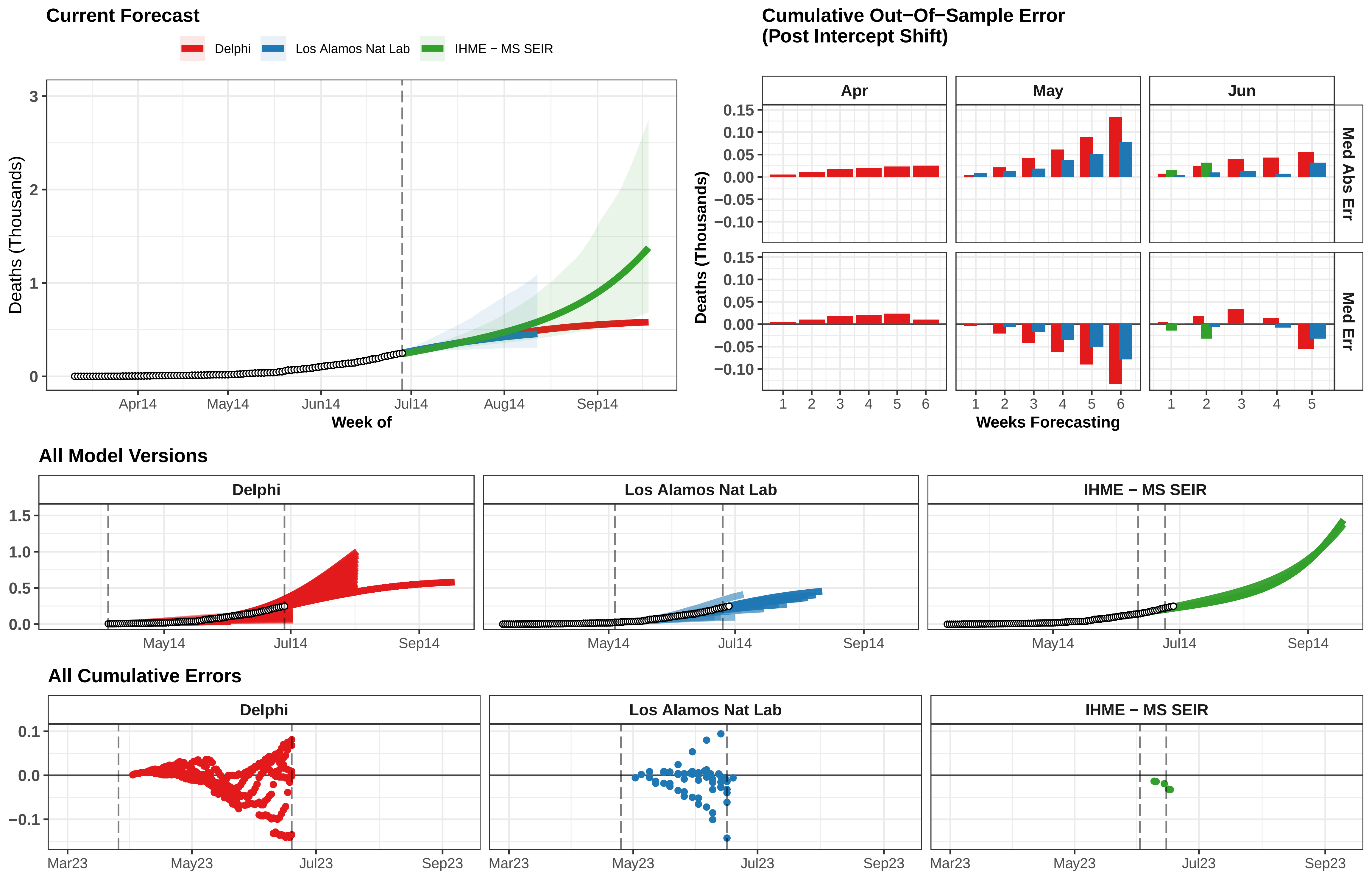

Current Forecast
Delphi
Los Alamos Nat Lab
IHME − MS SEIR
Deaths (Thousands)
Week of
Apr14
May14
Jun14
Jul14
Aug14
Sep14
Cumulative Out−Of−Sample Error
(Post Intercept Shift)
Apr
May
Jun
Med Abs Err
Med Err
Weeks Forecasting
All Model Versions
All Cumulative Errors
Mar23
May23
Jul23
Sep23

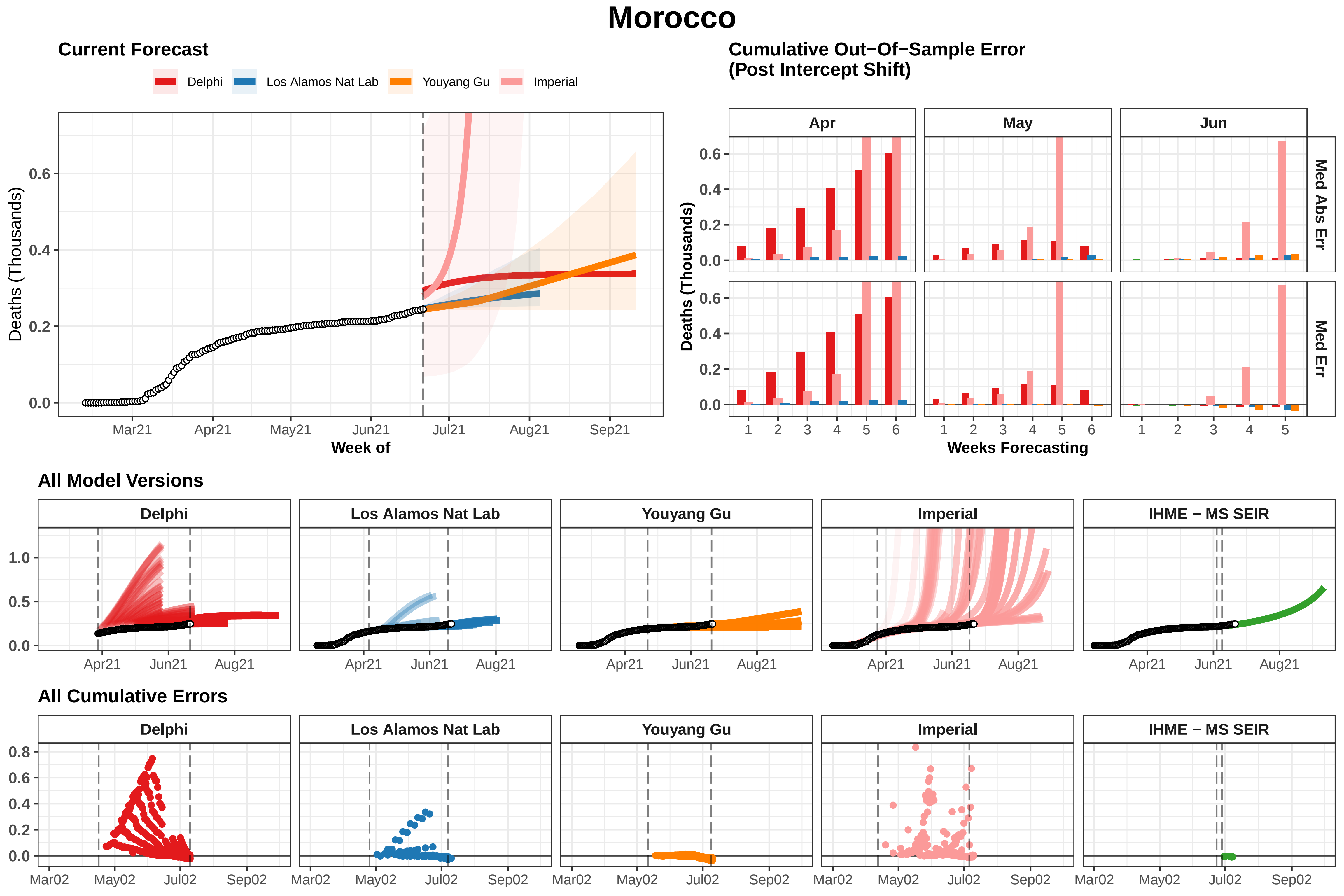

Morocco
Current Forecast
Delphi
Los Alamos Nat Lab
Youyang Gu
Imperial
Deaths (Thousands)
Week of
Mar21
Apr21
May21
Jun21
Jul21
Aug21
Sep21
Cumulative Out−Of−Sample Error
(Post Intercept Shift)
Apr
May
Jun
Med Abs Err
Med Err
Weeks Forecasting
All Model Versions
IHME − MS SEIR
All Cumulative Errors
Mar02
May02
Jul02
Sep02

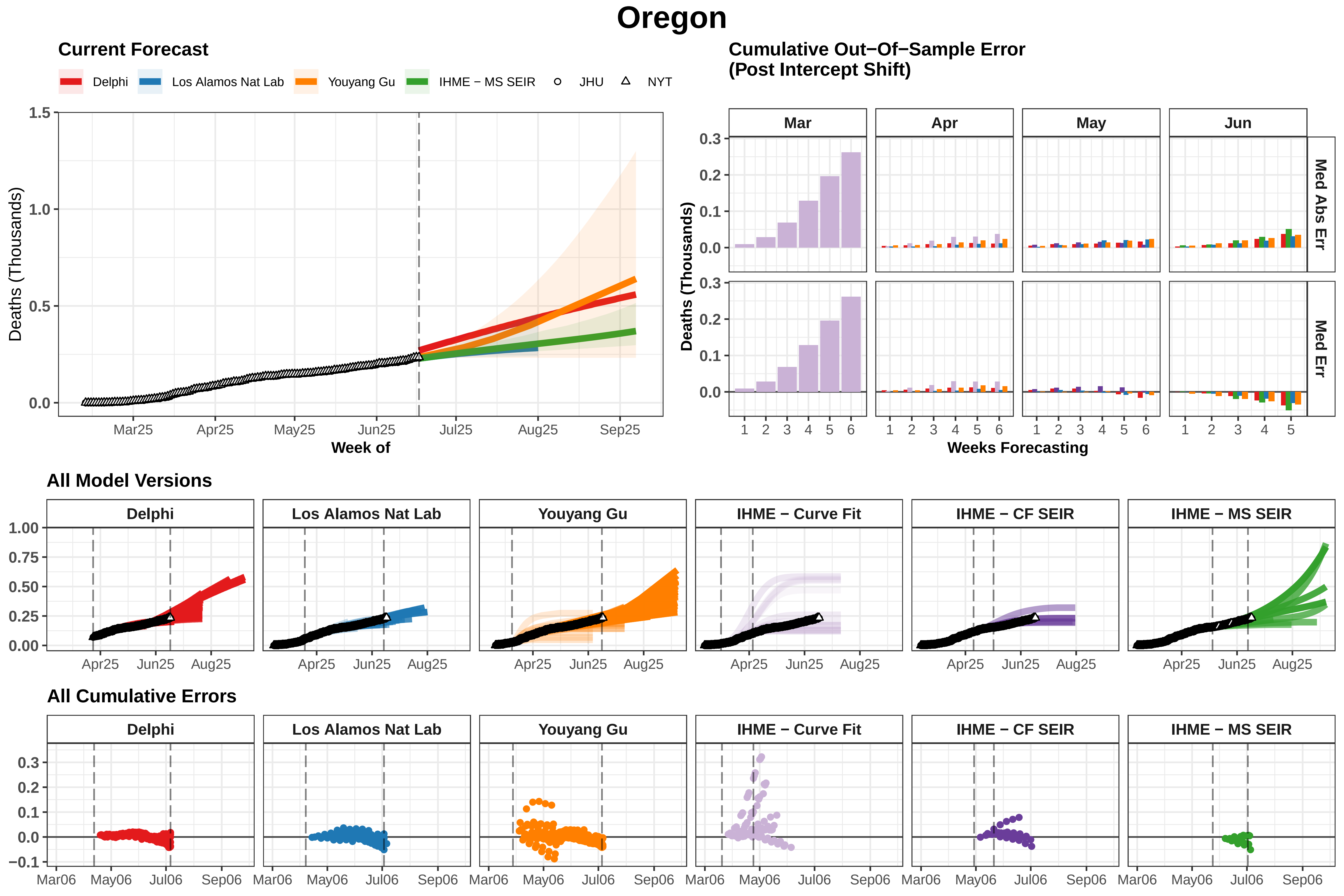
Oregon
Current Forecast
Delphi
Los Alamos Nat Lab
Youyang Gu
IHME − MS SEIR
JHU
NYT
Deaths (Thousands)
Week of
Mar25
Apr25
May25
Jun25
Jul25
Aug25
Sep25
Cumulative Out−Of−Sample Error
(Post Intercept Shift)
Mar
Apr
May
Jun
Med Abs Err
Med Err
Weeks Forecasting
All Model Versions
IHME − Curve Fit
IHME − CF SEIR
All Cumulative Errors
Mar06
May06
Jul06
Sep06

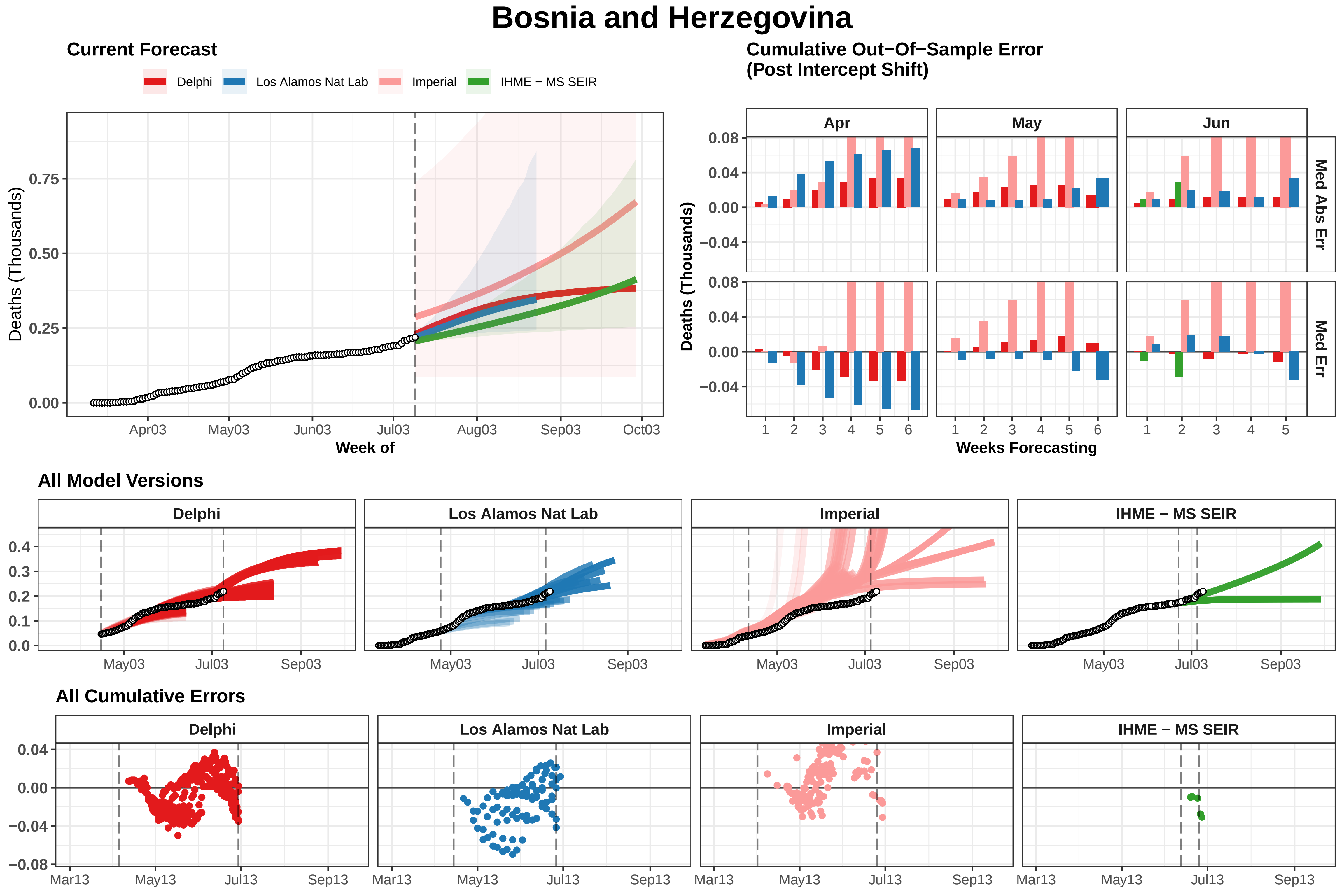
Bosnia and Herzegovina
Current Forecast
Delphi
Los Alamos Nat Lab
Imperial
IHME − MS SEIR
Deaths (Thousands)
Week of
Apr03
May03
Jun03
Jul03
Aug03
Sep03
Oct03
Cumulative Out−Of−Sample Error
(Post Intercept Shift)
Apr
May
Jun
Med Abs Err
Med Err
Weeks Forecasting
All Model Versions
All Cumulative Errors
Mar13
May13
Jul13
Sep13

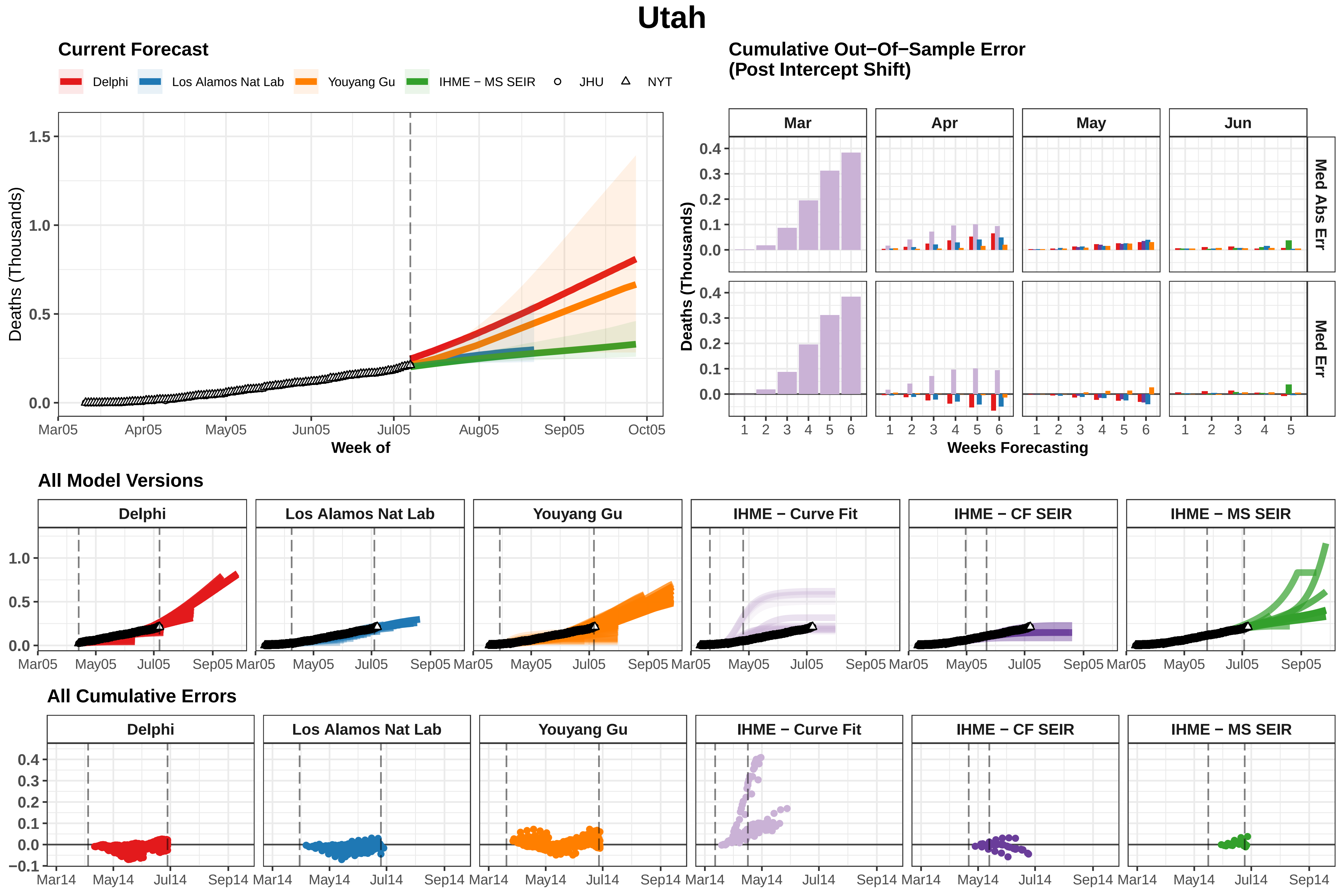
Utah
Current Forecast
Delphi
Los Alamos Nat Lab
Youyang Gu
IHME − MS SEIR
JHU
NYT
Deaths (Thousands)
Week of
Mar05
Apr05
May05
Jun05
Jul05
Aug05
Sep05
Oct05
Cumulative Out−Of−Sample Error
(Post Intercept Shift)
Mar
Apr
May
Jun
Med Abs Err
Med Err
Weeks Forecasting
All Model Versions
Delphi
Los Alamos Nat Lab
Youyang Gu
IHME − Curve Fit
IHME − CF SEIR
IHME − MS SEIR
All Cumulative Errors
Mar14
May14
Jul14
Sep14

Greece

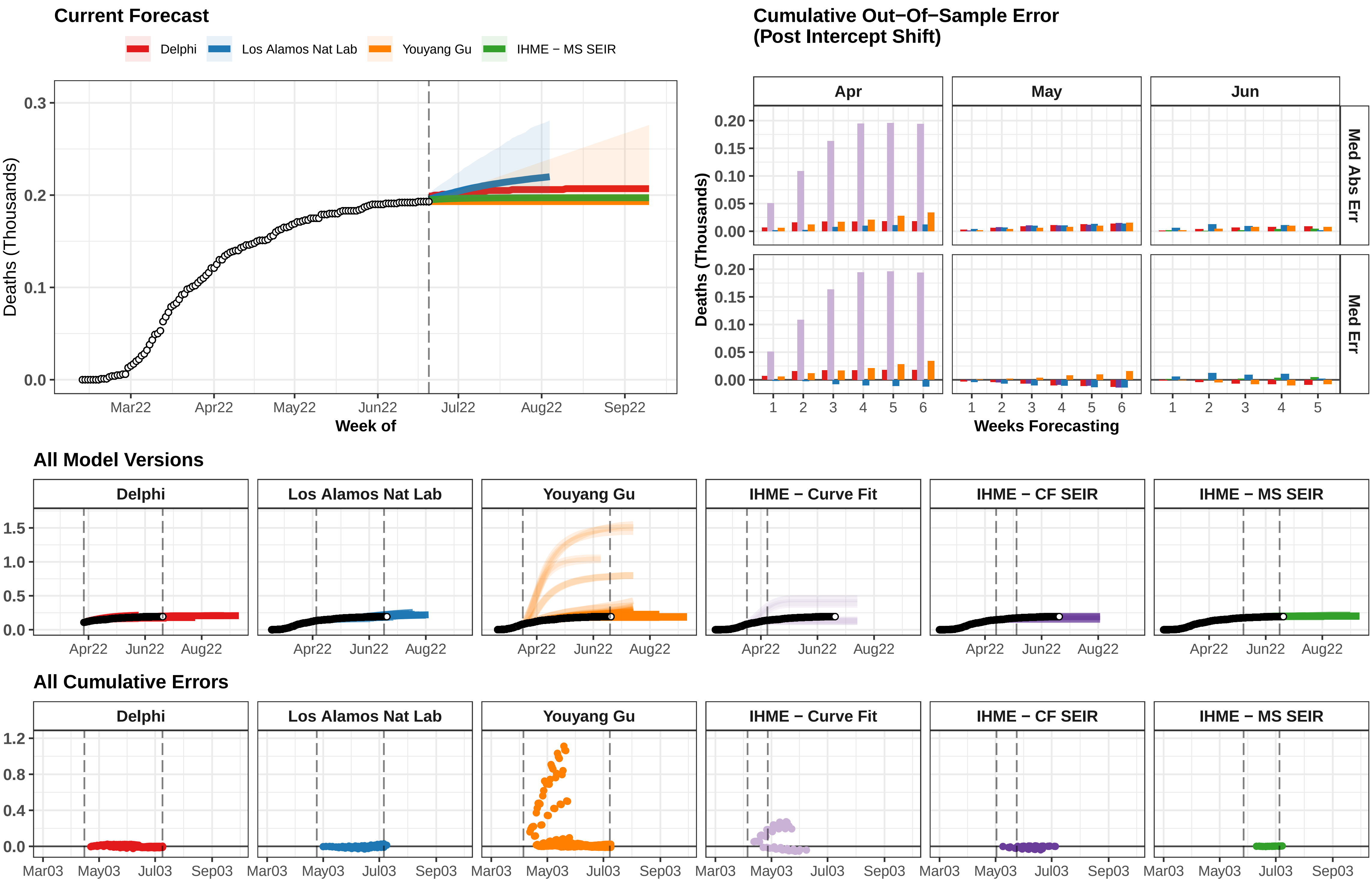
Current Forecast
Delphi
Los Alamos Nat Lab
Youyang Gu
IHME − MS SEIR
Deaths (Thousands)
0.0
0.1
0.2
0.3
Mar22
Apr22
May22
Jun22
Jul22
Aug22
Sep22
Week of
Cumulative Out−Of−Sample Error
(Post Intercept Shift)
Apr
May
Jun
Med Abs Err
Med Err
0.00
0.05
0.10
0.15
0.20
1
2
3
4
5
6
Weeks Forecasting
All Model Versions
Delphi
Los Alamos Nat Lab
Youyang Gu
IHME − Curve Fit
IHME − CF SEIR
IHME − MS SEIR
0.0
0.5
1.0
1.5
Apr22
Jun22
Aug22
All Cumulative Errors
0.0
0.4
0.8
1.2
Mar03
May03
Jul03
Sep03

Democratic Republic of the Congo

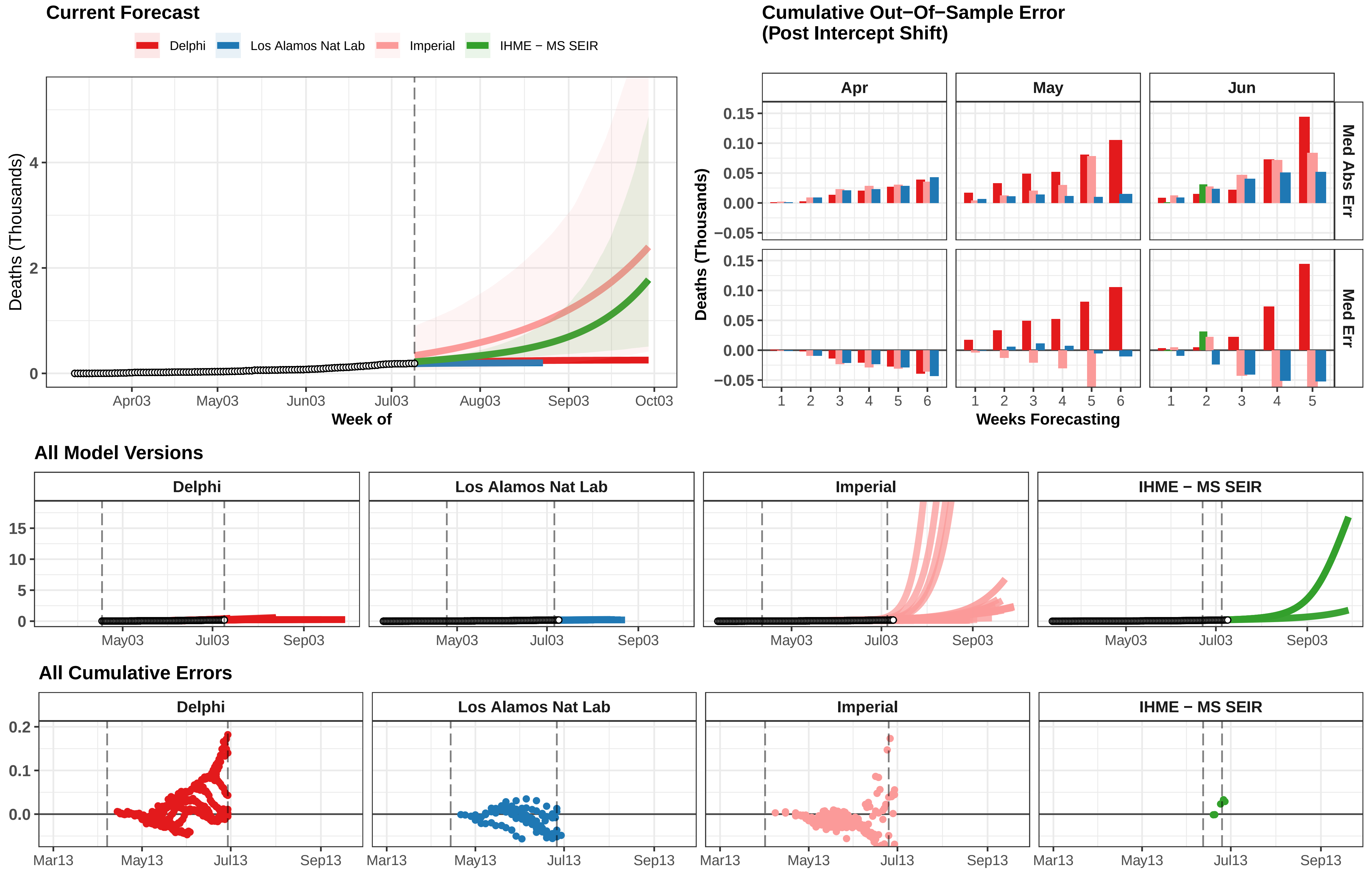

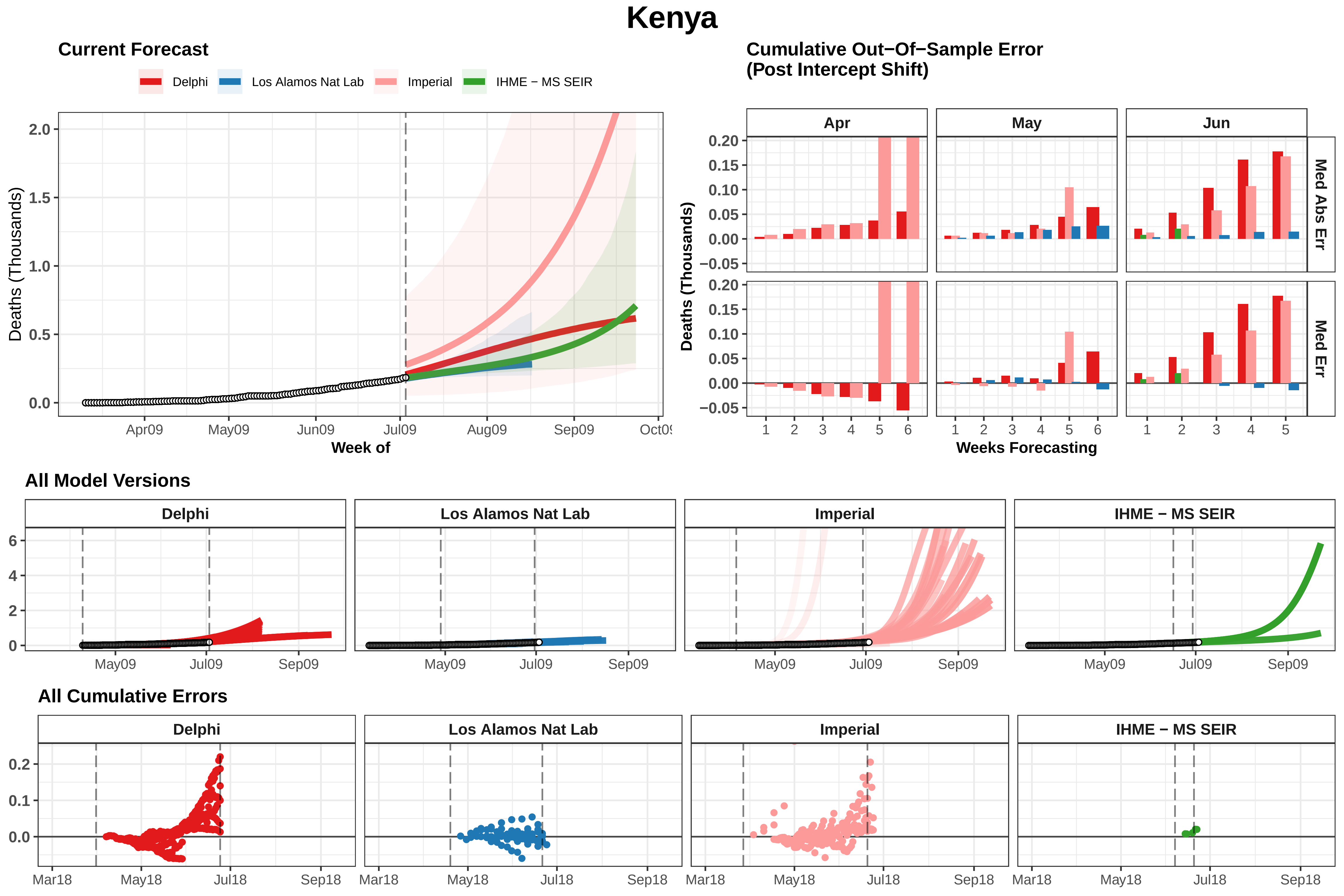
Kenya
Current Forecast
Delphi
Los Alamos Nat Lab
Imperial
IHME − MS SEIR
Deaths (Thousands)
2.0
1.5
1.0
0.5
0.0
Apr09
May09
Jun09
Jul09
Aug09
Sep09
Oct09
Week of
Cumulative Out−Of−Sample Error
(Post Intercept Shift)
Apr
May
Jun
Med Abs Err
Med Err
Deaths (Thousands)
0.20
0.15
0.10
0.05
0.00
−0.05
1
2
3
4
5
6
Weeks Forecasting
All Model Versions
Delphi
Los Alamos Nat Lab
Imperial
IHME − MS SEIR
6
4
2
0
May09
Jul09
Sep09
All Cumulative Errors
Delphi
Los Alamos Nat Lab
Imperial
IHME − MS SEIR
0.2
0.1
0.0
Mar18
May18
Jul18
Sep18

Puerto Rico

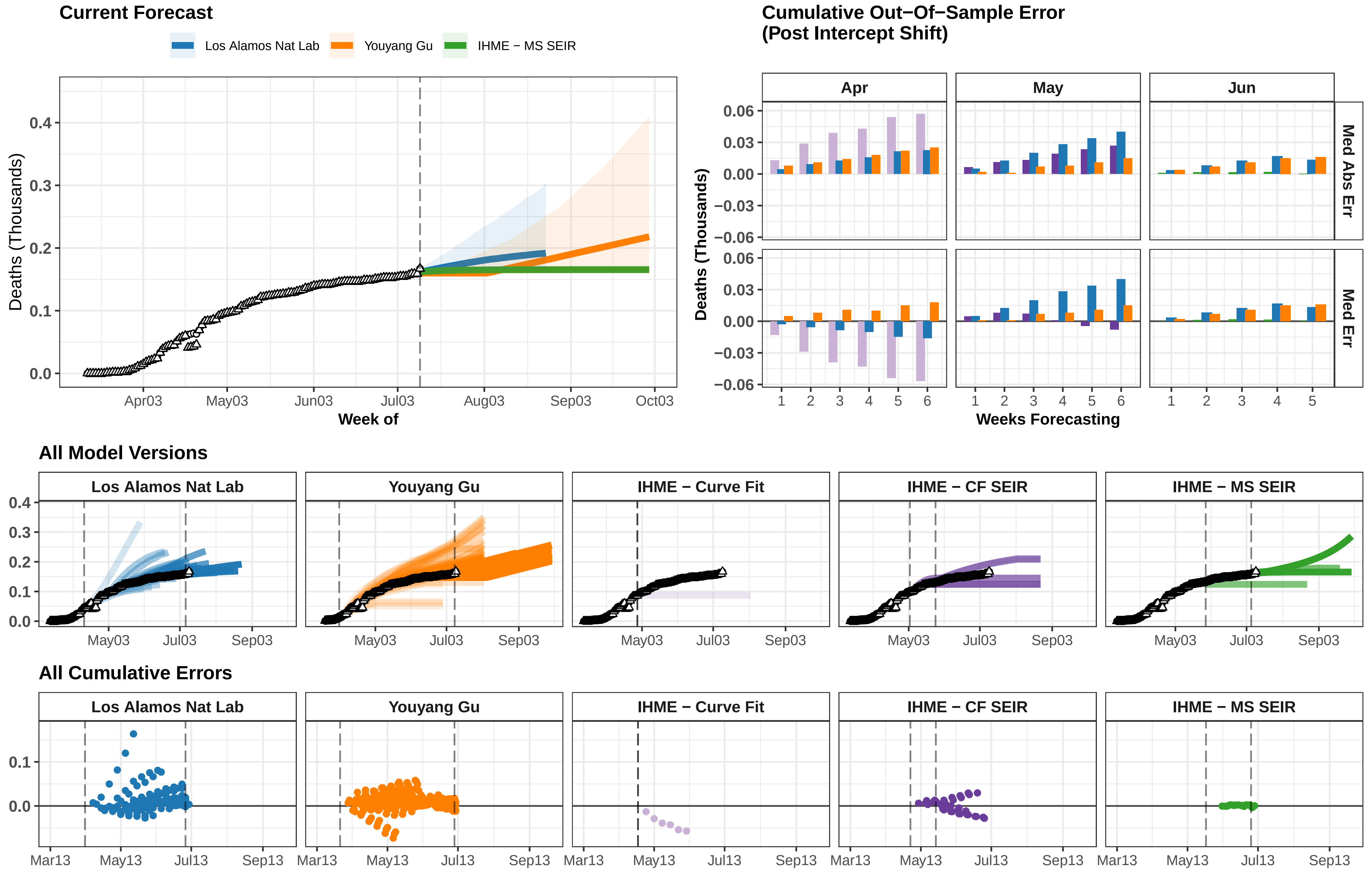

Current Forecast
Los Alamos Nat Lab
Youyang Gu
IHME − MS SEIR
Deaths (Thousands)
0.0
0.1
0.2
0.3
0.4
Apr03
May03
Jun03
Jul03
Aug03
Sep03
Oct03
Week of
Cumulative Out−Of−Sample Error
(Post Intercept Shift)
Apr
May
Jun
Med Abs Err
Med Err
0.06
0.03
0.00
−0.03
−0.06
1
2
3
4
5
6
Weeks Forecasting
All Model Versions
Los Alamos Nat Lab
Youyang Gu
IHME − Curve Fit
IHME − CF SEIR
IHME − MS SEIR
May03
Jul03
Sep03
All Cumulative Errors
0.1
0.0
Mar13
May13
Jul13
Sep13

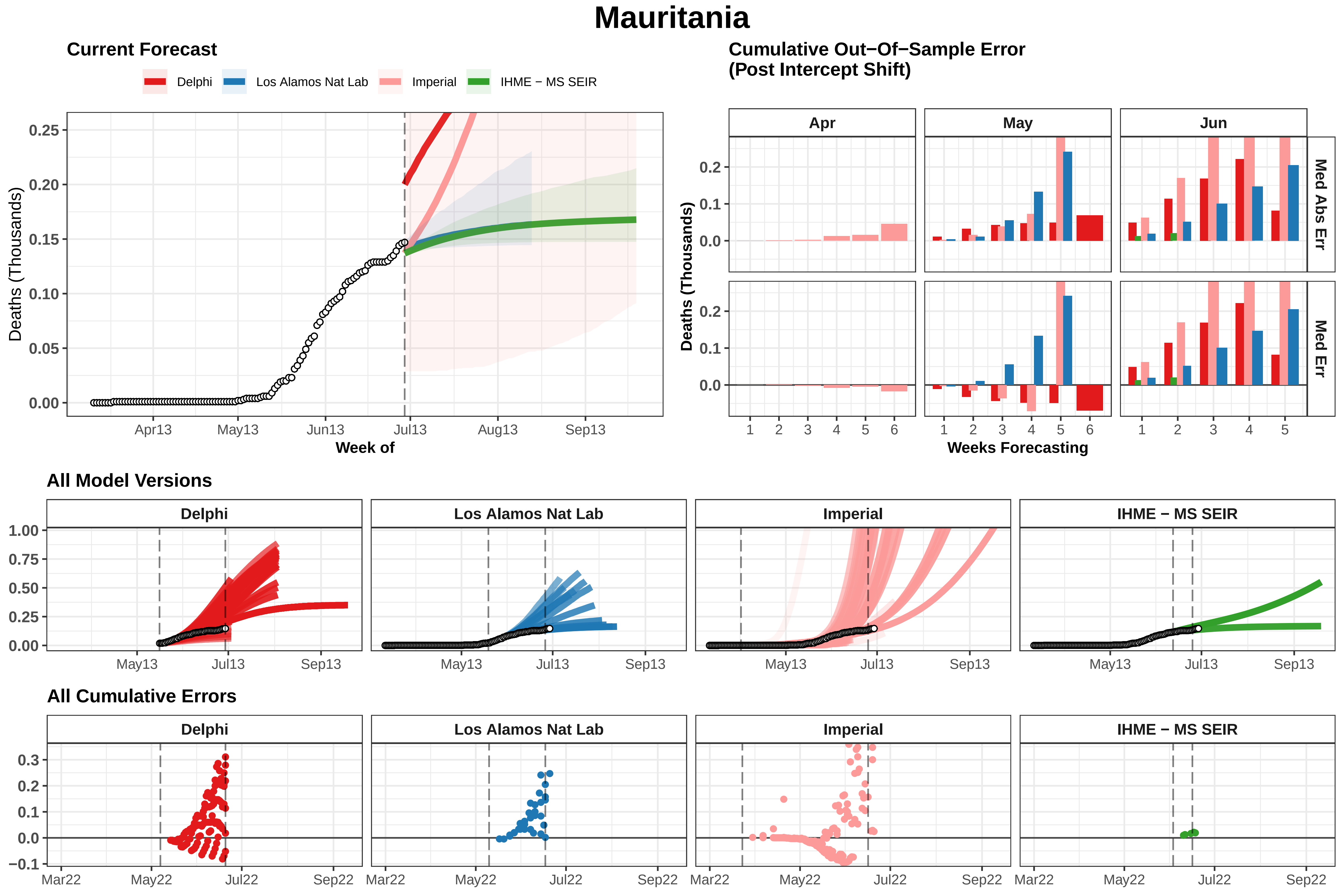
Mauritania
Current Forecast
Delphi
Los Alamos Nat Lab
Imperial
IHME − MS SEIR
Deaths (Thousands)
0.00
0.05
0.10
0.15
0.20
0.25
Apr13
May13
Jun13
Jul13
Aug13
Sep13
Week of
Cumulative Out−Of−Sample Error
(Post Intercept Shift)
Apr
May
Jun
Med Abs Err
Med Err
0.0
0.1
0.2
1
2
3
4
5
6
Weeks Forecasting
All Model Versions
Delphi
Los Alamos Nat Lab
Imperial
IHME − MS SEIR
0.00
0.25
0.50
0.75
1.00
May13
Jul13
Sep13
All Cumulative Errors
Delphi
Los Alamos Nat Lab
Imperial
IHME − MS SEIR
−0.1
0.0
0.1
0.2
0.3
Mar22
May22
Jul22
Sep22

Qatar

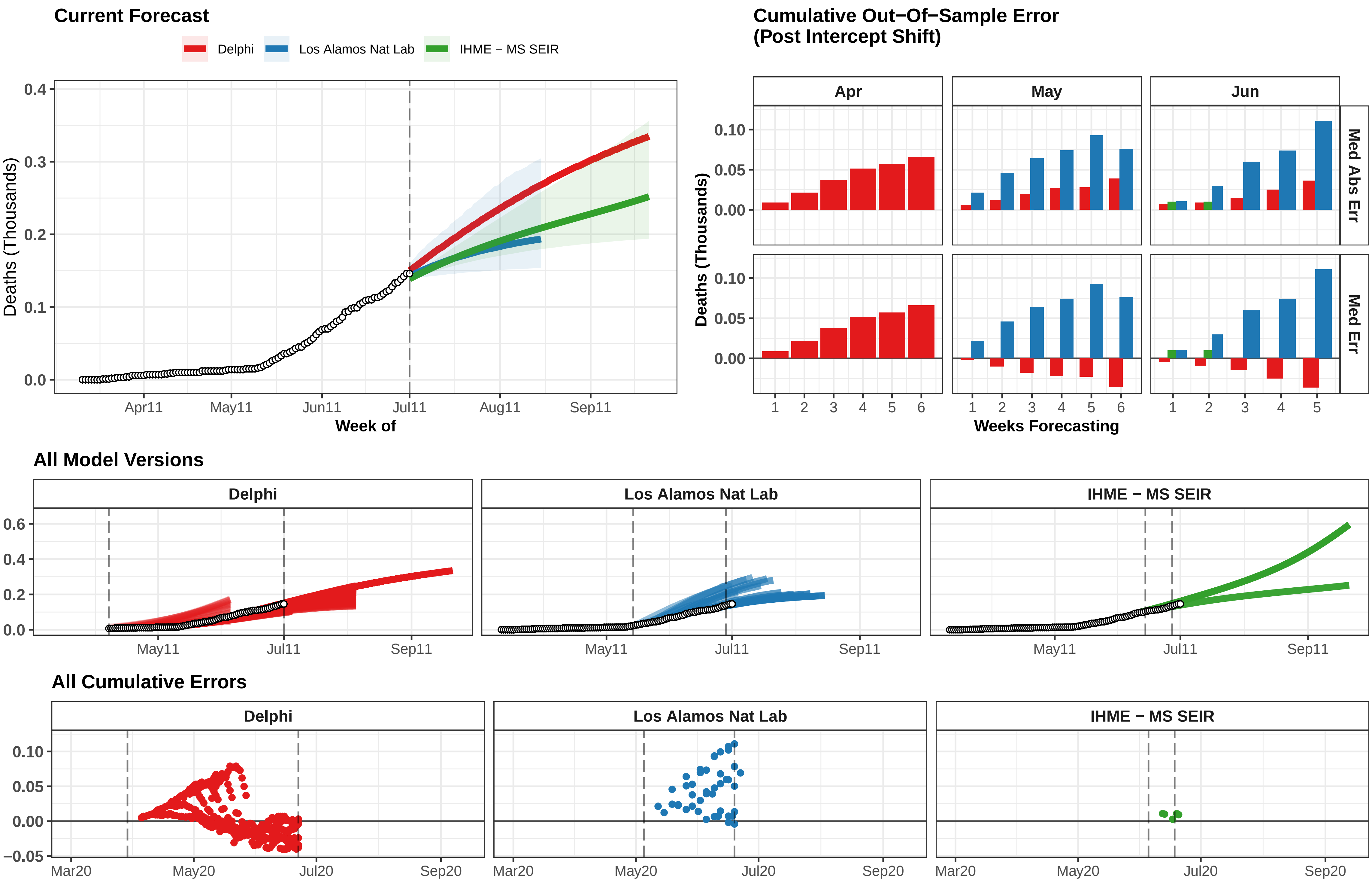
Current Forecast
Delphi
Los Alamos Nat Lab
IHME − MS SEIR
Deaths (Thousands)
0.0
0.1
0.2
0.3
0.4
Apr11
May11
Jun11
Jul11
Aug11
Sep11
Week of
Cumulative Out−Of−Sample Error
(Post Intercept Shift)
Apr
May
Jun
Med Abs Err
Med Err
Deaths (Thousands)
0.10
0.05
0.00
Weeks Forecasting
1
2
3
4
5
6
All Model Versions
Delphi
Los Alamos Nat Lab
IHME − MS SEIR
0.6
0.4
0.2
0.0
May11
Jul11
Sep11
All Cumulative Errors
Delphi
Los Alamos Nat Lab
IHME − MS SEIR
0.10
0.05
0.00
−0.05
Mar20
May20
Jul20
Sep20

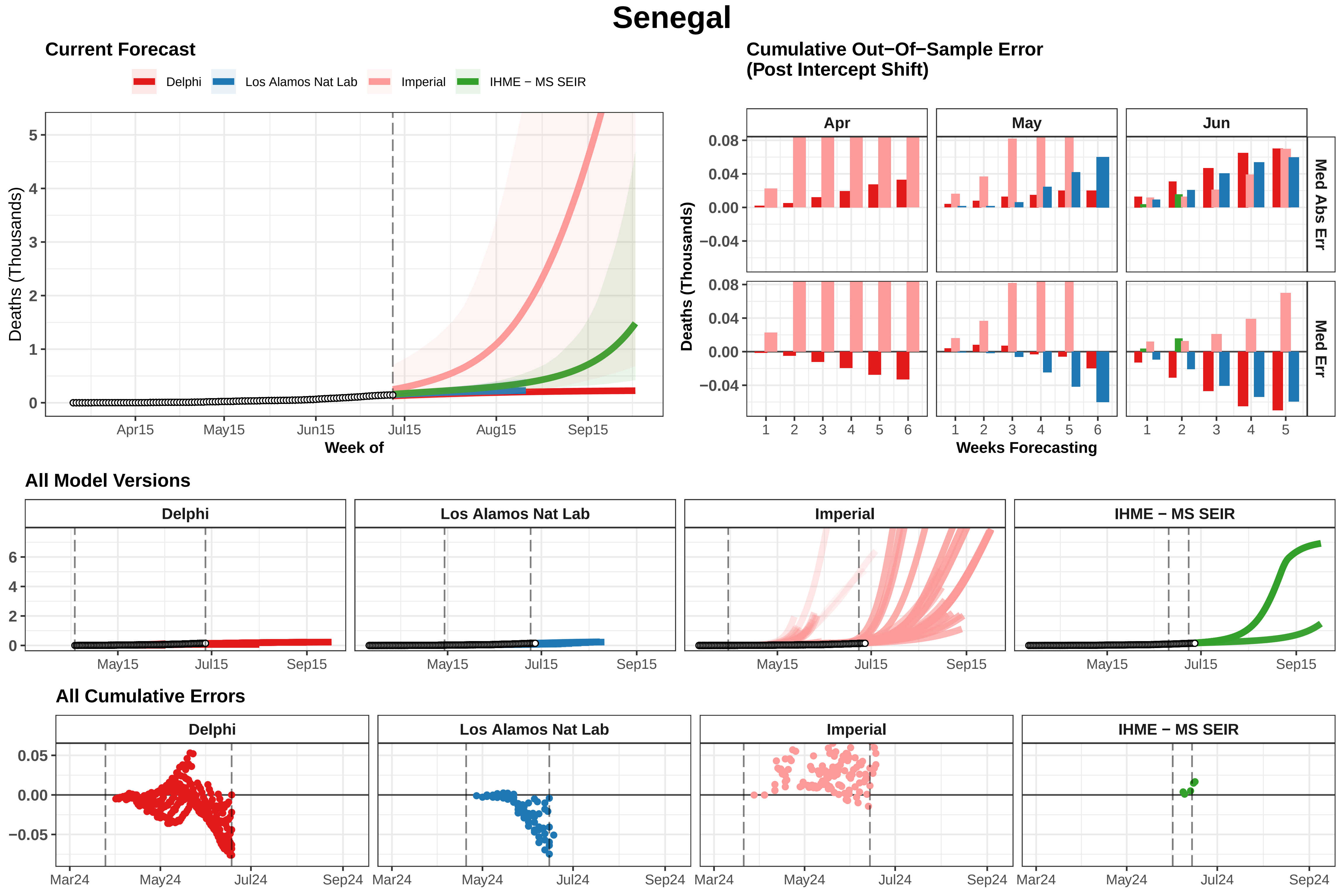
Senegal
Current Forecast
Delphi
Los Alamos Nat Lab
Imperial
IHME − MS SEIR
Deaths (Thousands)
Week of
Apr15
May15
Jun15
Jul15
Aug15
Sep15
Cumulative Out−Of−Sample Error
(Post Intercept Shift)
Apr
May
Jun
Med Abs Err
Med Err
Weeks Forecasting
All Model Versions
All Cumulative Errors
Mar24
May24
Jul24
Sep24

Haiti

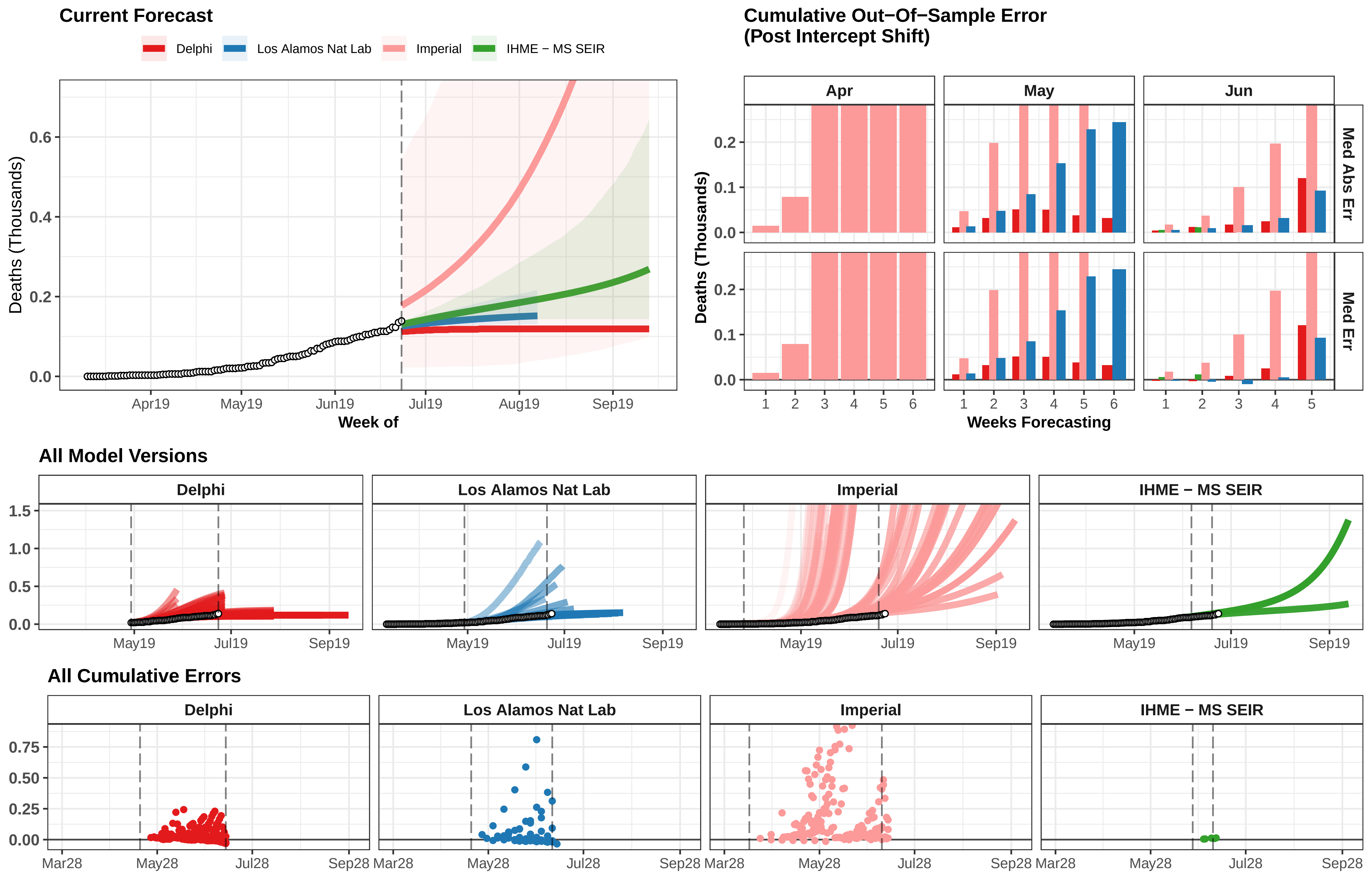
Current Forecast
Delphi
Los Alamos Nat Lab
Imperial
IHME − MS SEIR
Deaths (Thousands)
0.0
0.2
0.4
0.6
Apr19
May19
Jun19
Jul19
Aug19
Sep19
Week of
Cumulative Out−Of−Sample Error
(Post Intercept Shift)
Apr
May
Jun
Med Abs Err
Med Err
0.0
0.1
0.2
1
2
3
4
5
6
Weeks Forecasting
All Model Versions
Delphi
Los Alamos Nat Lab
Imperial
IHME − MS SEIR
0.0
0.5
1.0
1.5
May19
Jul19
Sep19
All Cumulative Errors
Delphi
Los Alamos Nat Lab
Imperial
IHME − MS SEIR
0.00
0.25
0.50
0.75
Mar28
May28
Jul28
Sep28

Ghana

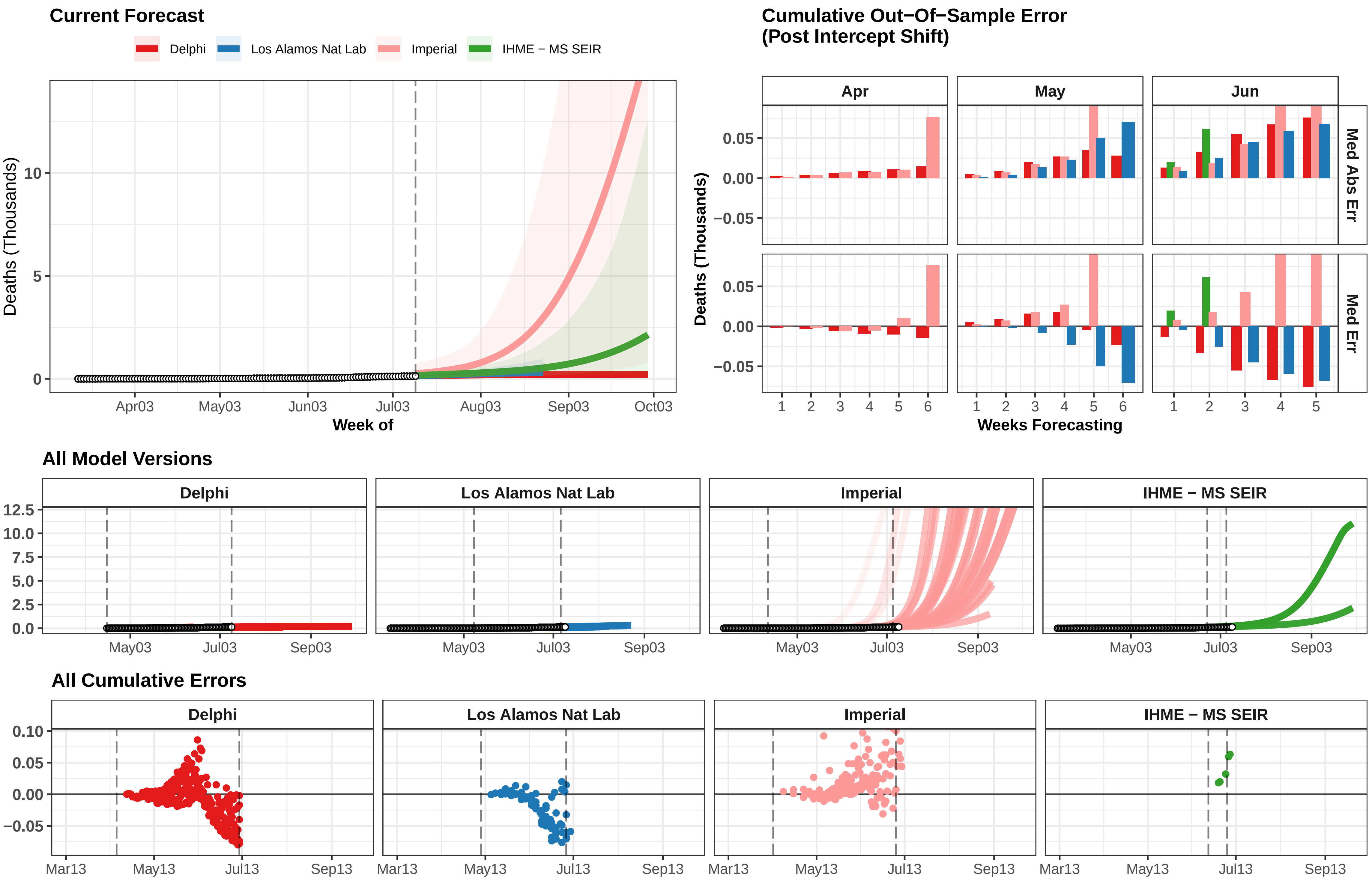

Current Forecast
Delphi
Los Alamos Nat Lab
Imperial
IHME − MS SEIR
Deaths (Thousands)
0
5
10
Apr03
May03
Jun03
Jul03
Aug03
Sep03
Oct03
Week of
Cumulative Out−Of−Sample Error
(Post Intercept Shift)
Apr
May
Jun
Med Abs Err
Med Err
0.05
0.00
−0.05
Weeks Forecasting
All Model Versions
12.5
10.0
7.5
5.0
2.5
0.0
May03
Jul03
Sep03
All Cumulative Errors
0.10
Mar13
May13
Jul13
Sep13

Kyrgyzstan

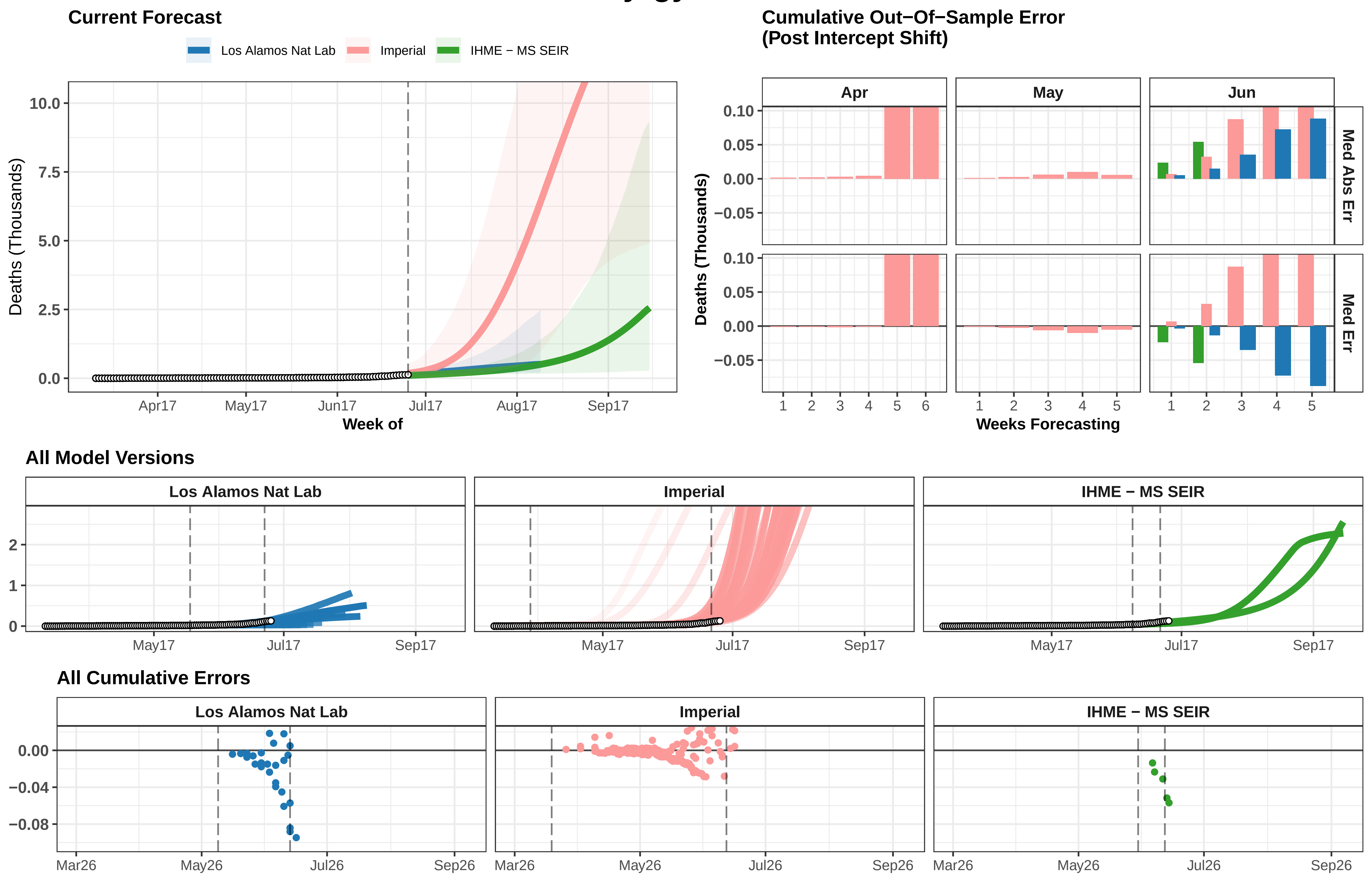

Current Forecast
Los Alamos Nat Lab
Imperial
IHME − MS SEIR
Deaths (Thousands)
10.0
7.5
5.0
2.5
0.0
Apr17
May17
Jun17
Jul17
Aug17
Sep17
Week of
Cumulative Out−Of−Sample Error
(Post Intercept Shift)
Apr
May
Jun
Med Abs Err
Med Err
0.10
0.05
0.00
−0.05
Weeks Forecasting
All Model Versions
2
1
0
All Cumulative Errors
0.00
−0.04
−0.08
Mar26
May26
Jul26
Sep26

Ethiopia

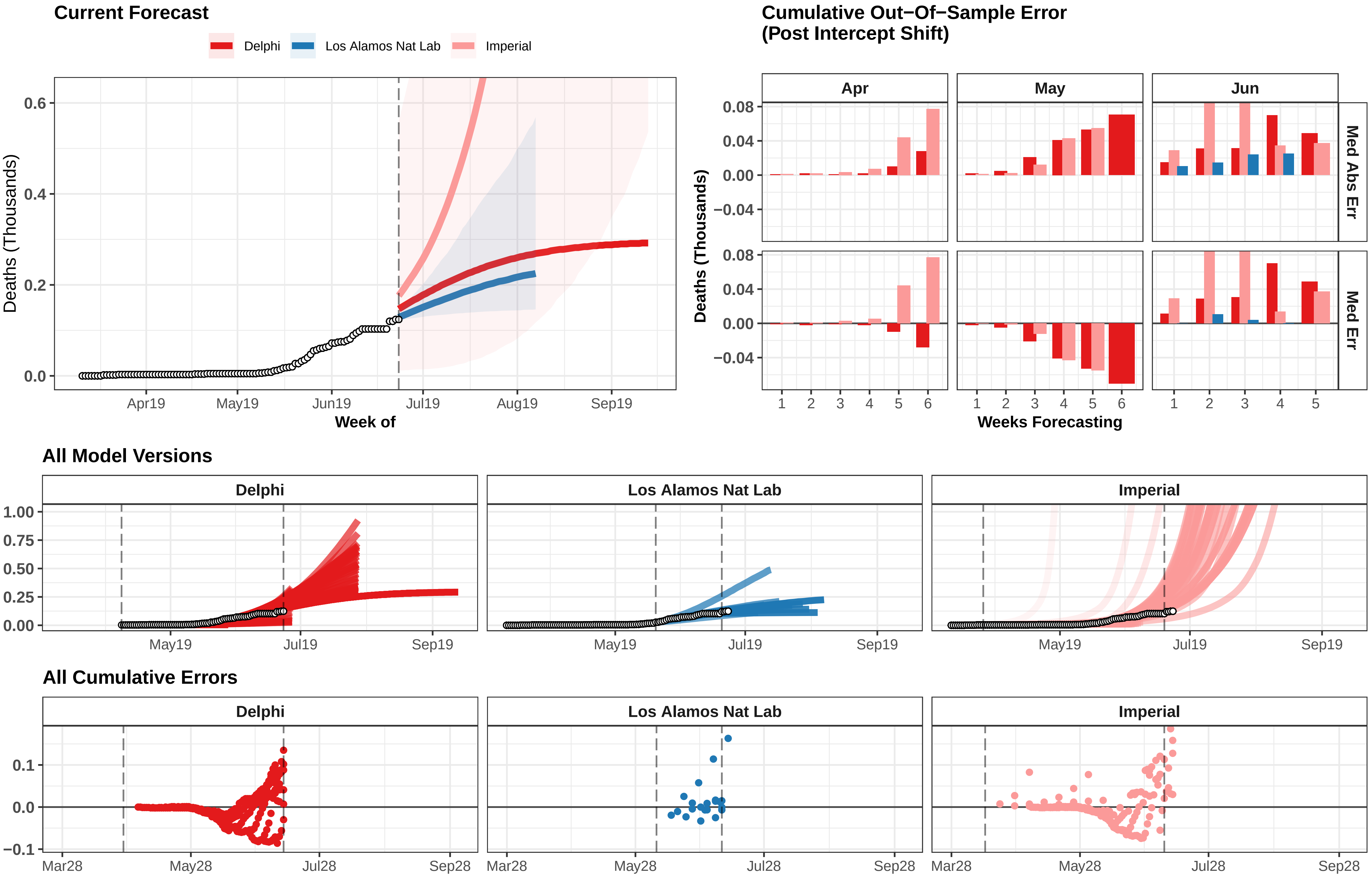

Current Forecast
Delphi
Los Alamos Nat Lab
Imperial
Deaths (Thousands)
0.6
0.4
0.2
0.0
Apr19
May19
Jun19
Jul19
Aug19
Sep19
Week of
Cumulative Out−Of−Sample Error
(Post Intercept Shift)
Apr
May
Jun
0.08
0.04
0.00
−0.04
Med Abs Err
Med Err
1
2
3
4
5
6
Weeks Forecasting
All Model Versions
Delphi
Los Alamos Nat Lab
Imperial
1.00
0.75
0.50
0.25
0.00
May19
Jul19
Sep19
All Cumulative Errors
0.1
0.0
−0.1
Mar28
May28
Jul28
Sep28

Malaysia

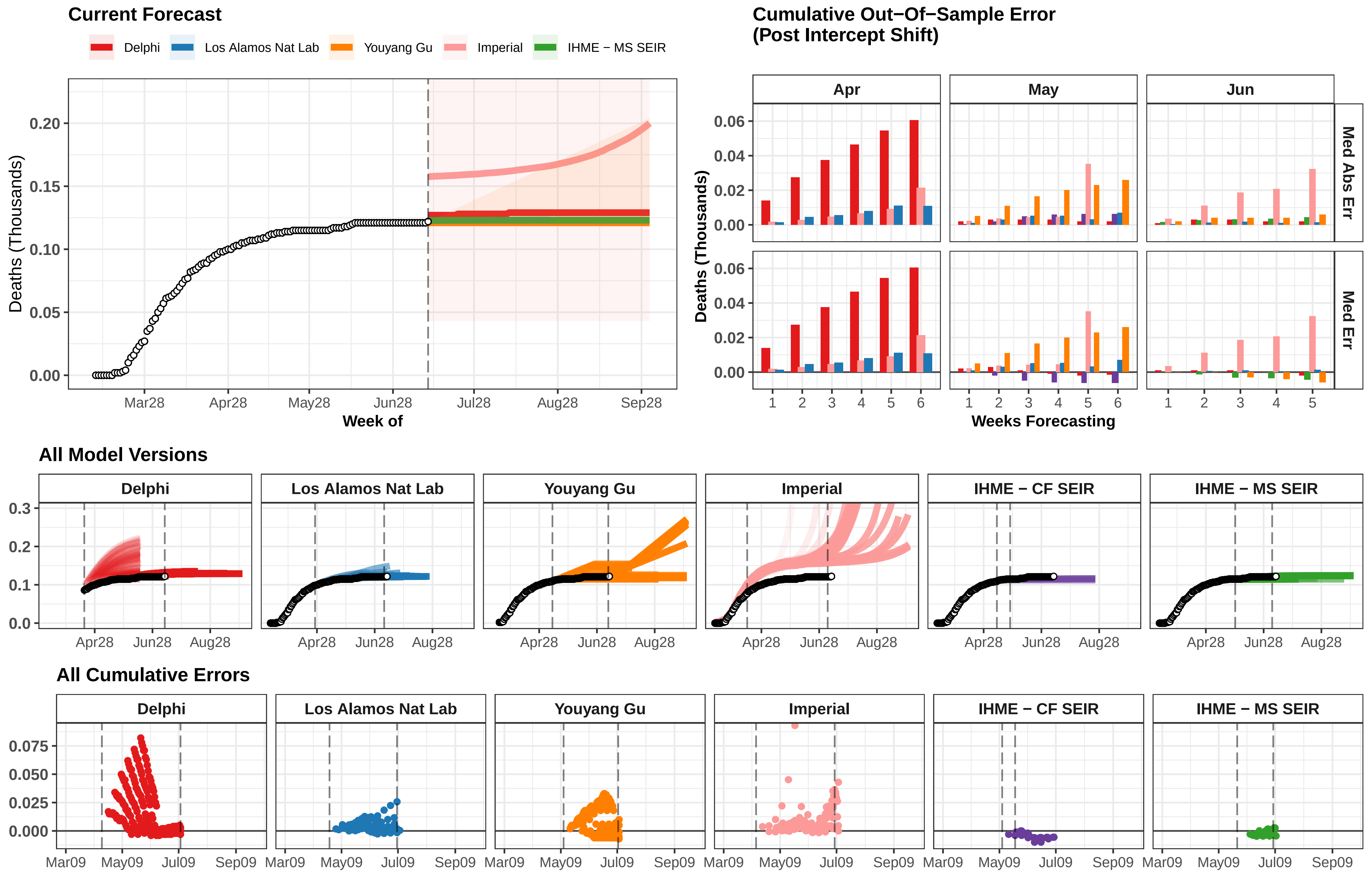

Current Forecast
Delphi
Los Alamos Nat Lab
Youyang Gu
Imperial
IHME − MS SEIR
Deaths (Thousands)
Week of
Mar28
Apr28
May28
Jun28
Jul28
Aug28
Sep28
Cumulative Out−Of−Sample Error
(Post Intercept Shift)
Apr
May
Jun
Med Abs Err
Med Err
Weeks Forecasting
All Model Versions
Delphi
Los Alamos Nat Lab
Youyang Gu
Imperial
IHME − CF SEIR
IHME − MS SEIR
All Cumulative Errors
Mar09
May09
Jul09
Sep09

Mali

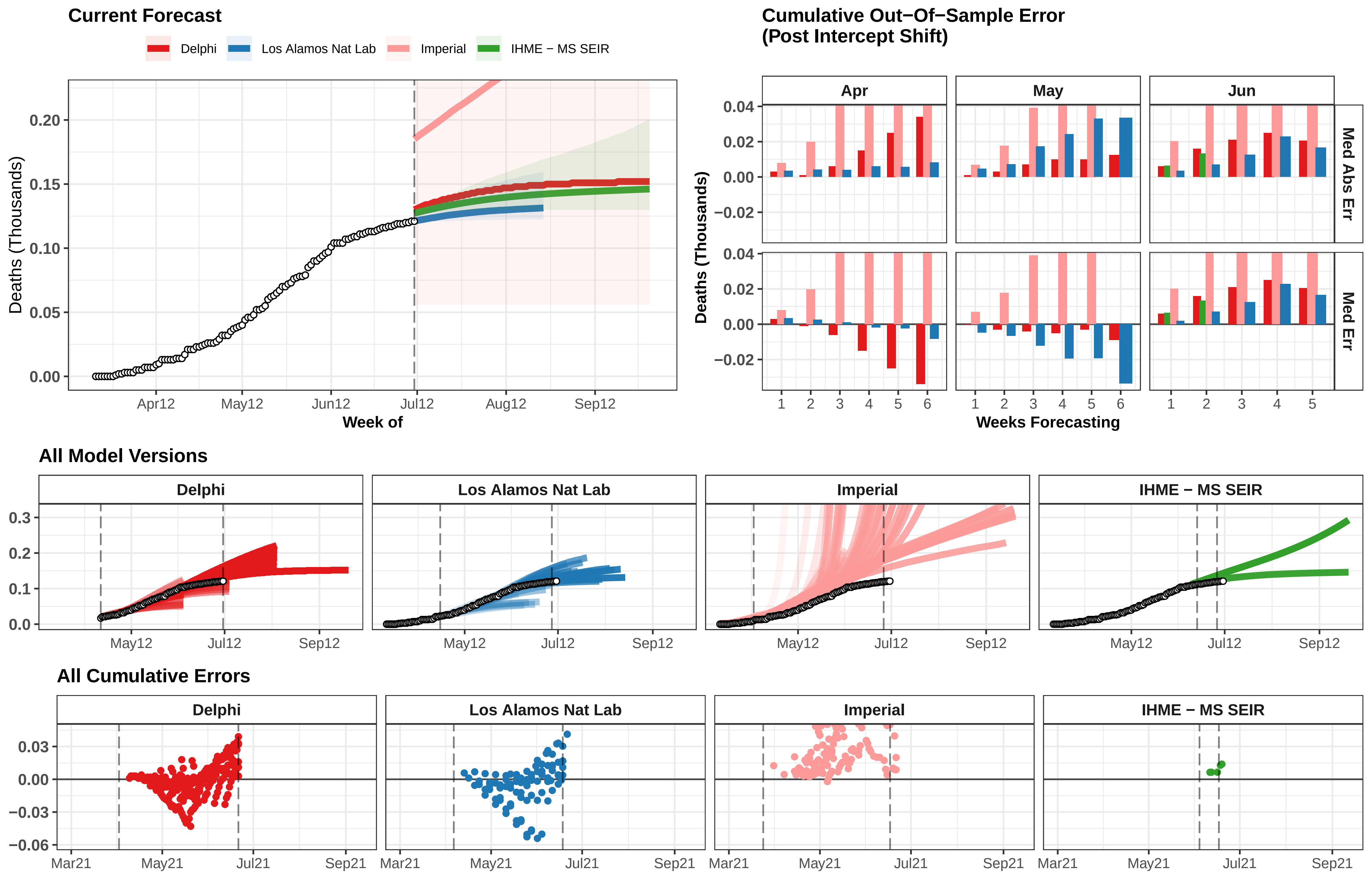

Current Forecast
Delphi
Los Alamos Nat Lab
Imperial
IHME − MS SEIR
Deaths (Thousands)
0.00
0.05
0.10
0.15
0.20
Apr12
May12
Jun12
Jul12
Aug12
Sep12
Week of
Cumulative Out−Of−Sample Error
(Post Intercept Shift)
Apr
May
Jun
Med Abs Err
Med Err
Deaths (Thousands)
0.04
0.02
0.00
−0.02
1
2
3
4
5
6
Weeks Forecasting
All Model Versions
Delphi
Los Alamos Nat Lab
Imperial
IHME − MS SEIR
0.3
0.2
0.1
0.0
May12
Jul12
Sep12
All Cumulative Errors
Delphi
Los Alamos Nat Lab
Imperial
IHME − MS SEIR
0.03
0.00
−0.03
−0.06
Mar21
May21
Jul21
Sep21

Croatia

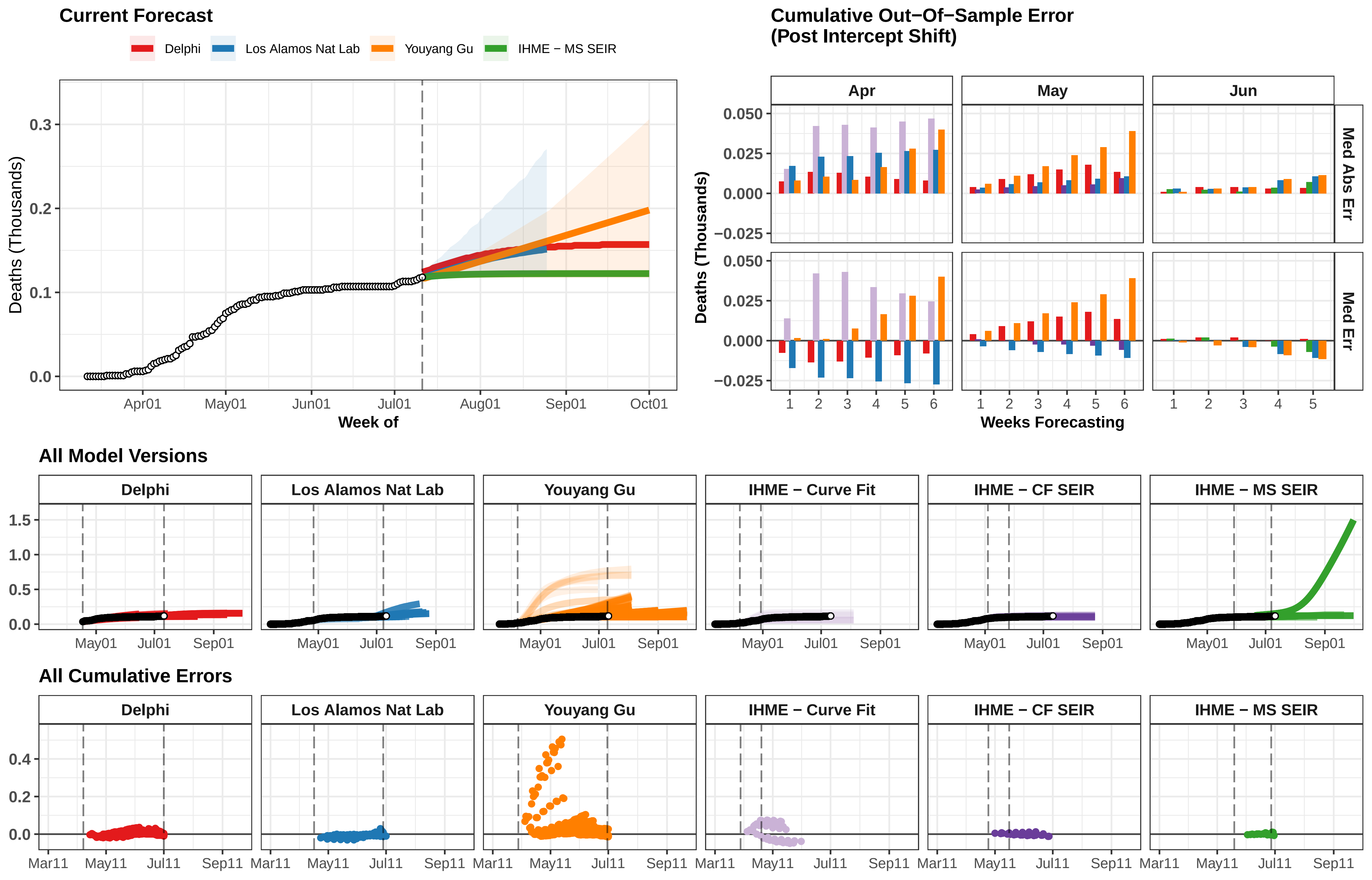

Current Forecast
Delphi
Los Alamos Nat Lab
Youyang Gu
IHME − MS SEIR
Deaths (Thousands)
Week of
Apr01
May01
Jun01
Jul01
Aug01
Sep01
Oct01
Cumulative Out−Of−Sample Error
(Post Intercept Shift)
Apr
May
Jun
Med Abs Err
Med Err
Weeks Forecasting
All Model Versions
IHME − Curve Fit
IHME − CF SEIR
All Cumulative Errors
Mar11
May11
Jul11
Sep11

Maine

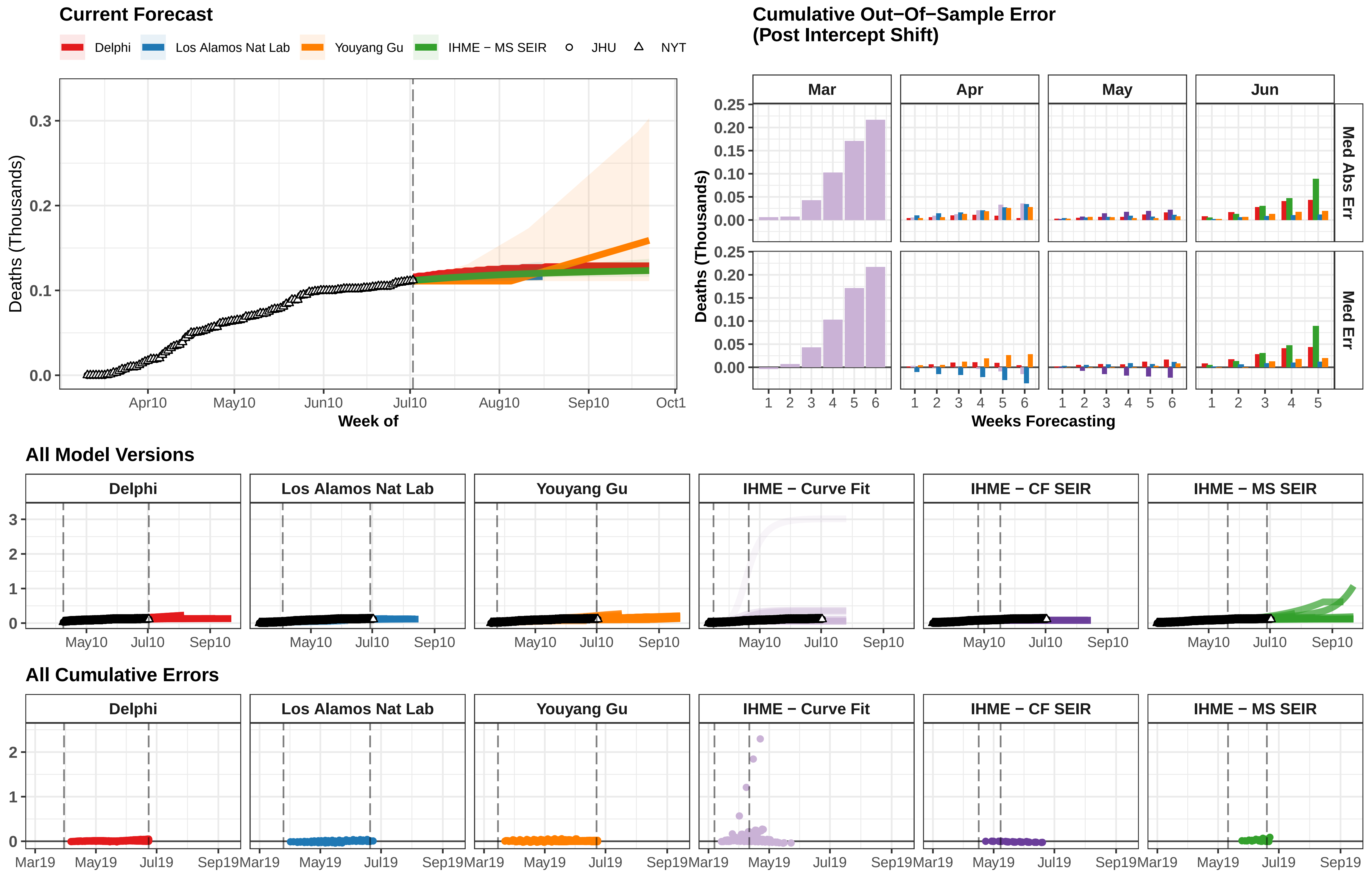
Current Forecast
Delphi
Los Alamos Nat Lab
Youyang Gu
IHME − MS SEIR
JHU
NYT
Deaths (Thousands)
0.0
0.1
0.2
0.3
Apr10
May10
Jun10
Jul10
Aug10
Sep10
Oct1
Week of
Cumulative Out−Of−Sample Error
(Post Intercept Shift)
Mar
Apr
May
Jun
Med Abs Err
Med Err
0.00
0.05
0.10
0.15
0.20
0.25
Weeks Forecasting
All Model Versions
Delphi
Los Alamos Nat Lab
Youyang Gu
IHME − Curve Fit
IHME − CF SEIR
IHME − MS SEIR
0
1
2
3
May10
Jul10
Sep10
All Cumulative Errors
Mar19
May19
Jul19
Sep19

Slovenia

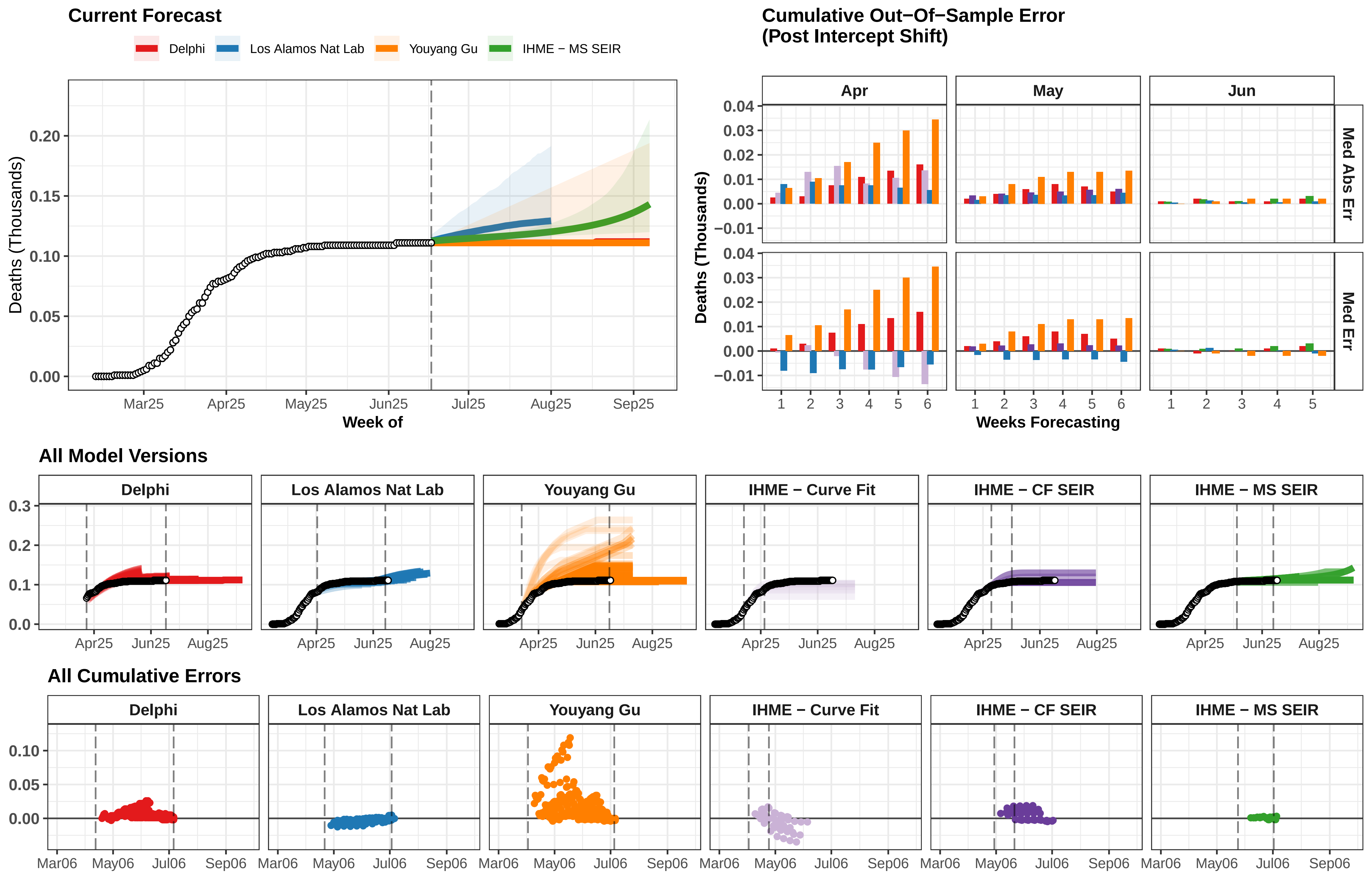

Current Forecast
Delphi
Los Alamos Nat Lab
Youyang Gu
IHME − MS SEIR
Deaths (Thousands)
0.00
0.05
0.10
0.15
0.20
Mar25
Apr25
May25
Jun25
Jul25
Aug25
Sep25
Week of
Cumulative Out−Of−Sample Error
(Post Intercept Shift)
Apr
May
Jun
Med Abs Err
Med Err
−0.01
0.00
0.01
0.02
0.03
0.04
1
2
3
4
5
6
Weeks Forecasting
All Model Versions
Delphi
Los Alamos Nat Lab
Youyang Gu
IHME − Curve Fit
IHME − CF SEIR
IHME − MS SEIR
0.0
0.1
0.2
0.3
Apr25
Jun25
Aug25
All Cumulative Errors
0.00
0.05
0.10
Mar06
May06
Jul06
Sep06

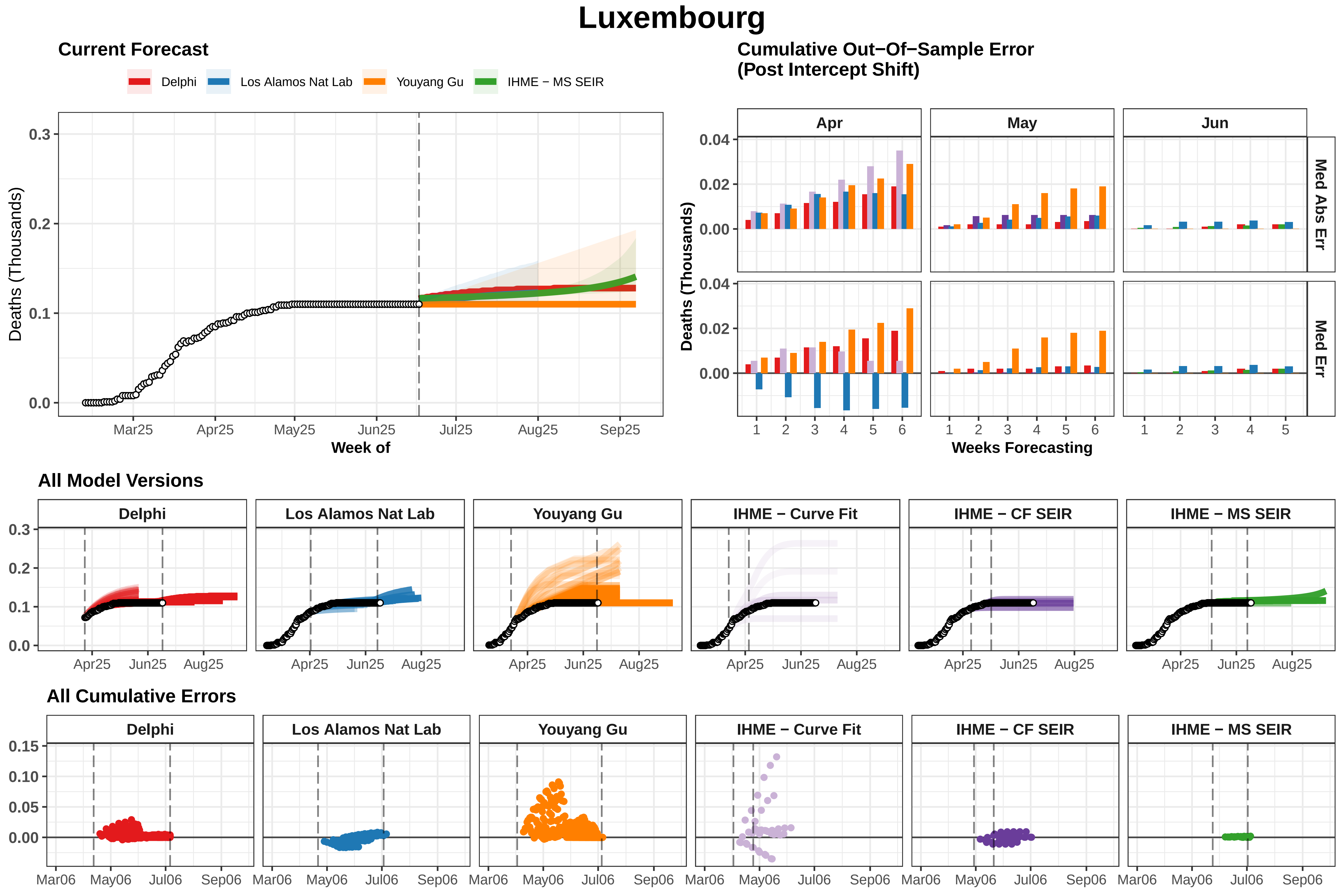
Luxembourg
Current Forecast
Delphi
Los Alamos Nat Lab
Youyang Gu
IHME − MS SEIR
Deaths (Thousands)
0.3
0.2
0.1
0.0
Mar25
Apr25
May25
Jun25
Jul25
Aug25
Sep25
Week of
Cumulative Out−Of−Sample Error
(Post Intercept Shift)
Apr
May
Jun
Med Abs Err
Med Err
0.04
0.02
0.00
Weeks Forecasting
All Model Versions
Delphi
Los Alamos Nat Lab
Youyang Gu
IHME − Curve Fit
IHME − CF SEIR
IHME − MS SEIR
All Cumulative Errors
0.15
0.10
0.05
0.00
Mar06
May06
Jul06
Sep06

South Dakota

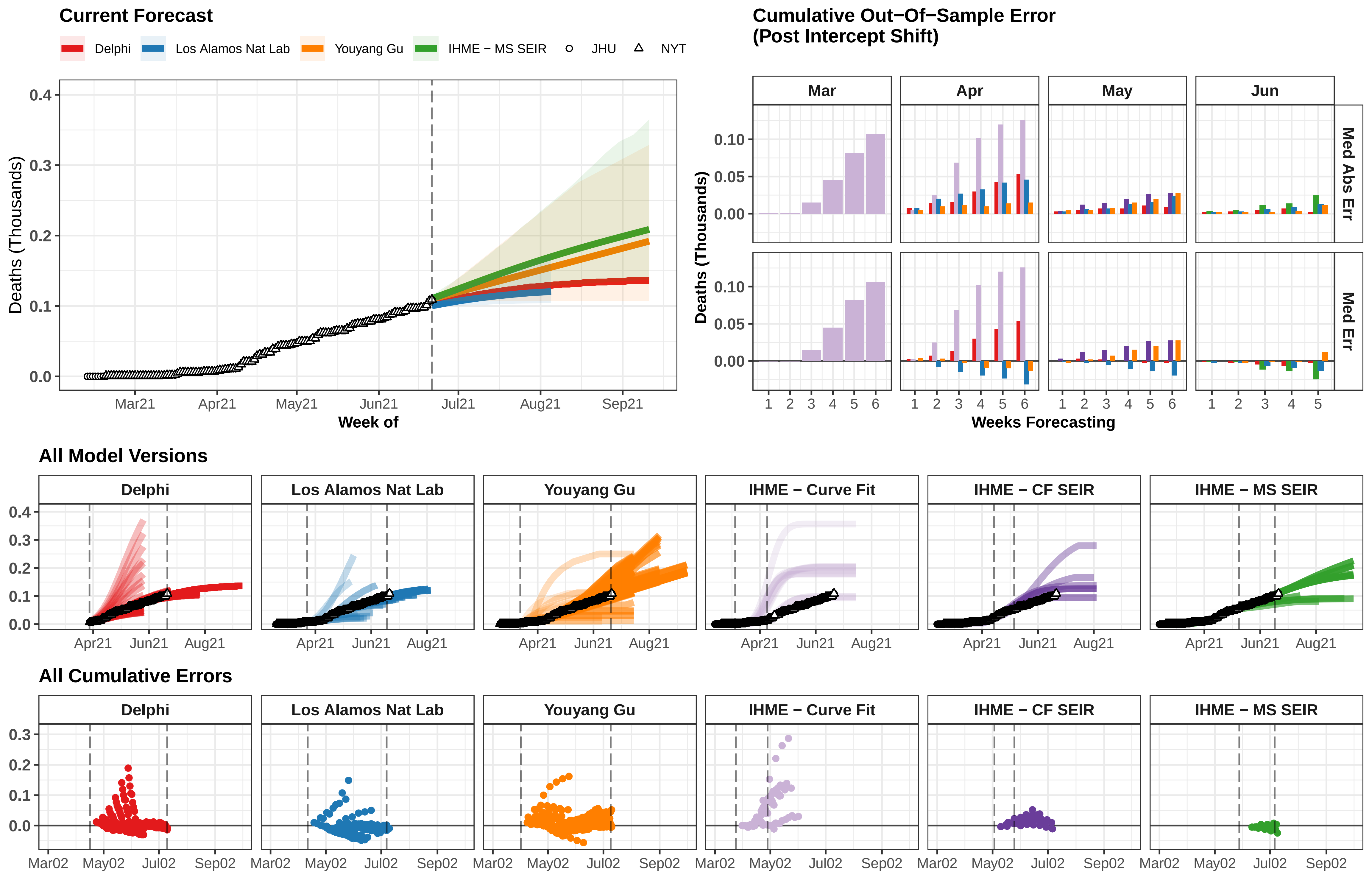

Australia

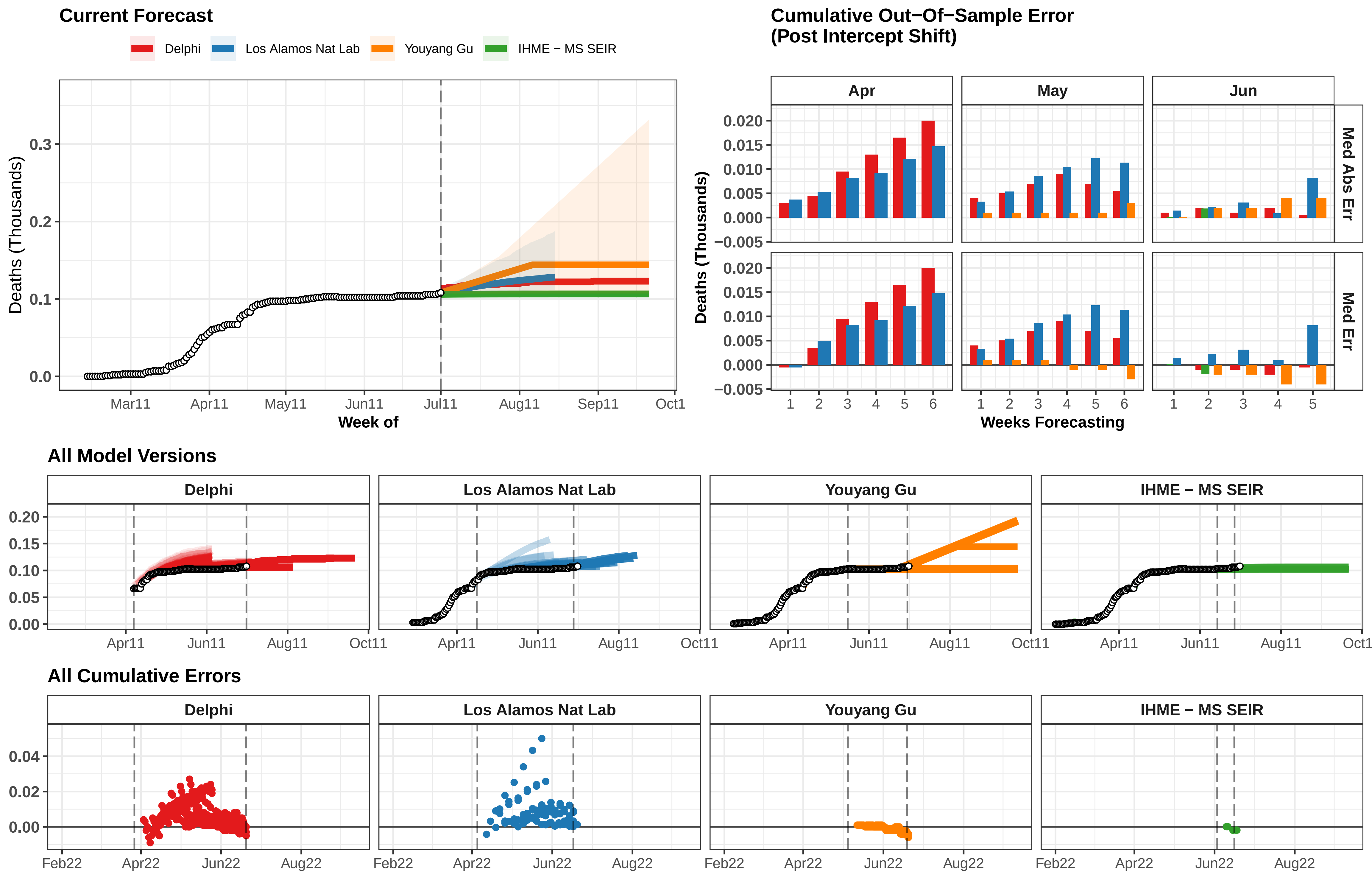
Current Forecast
Delphi
Los Alamos Nat Lab
Youyang Gu
IHME − MS SEIR
Deaths (Thousands)
Week of
Mar11
Apr11
May11
Jun11
Jul11
Aug11
Sep11
Oct1
Cumulative Out−Of−Sample Error
(Post Intercept Shift)
Apr
May
Jun
Med Abs Err
Med Err
Weeks Forecasting
All Model Versions
All Cumulative Errors
Feb22
Apr22
Jun22
Aug22

Bahrain

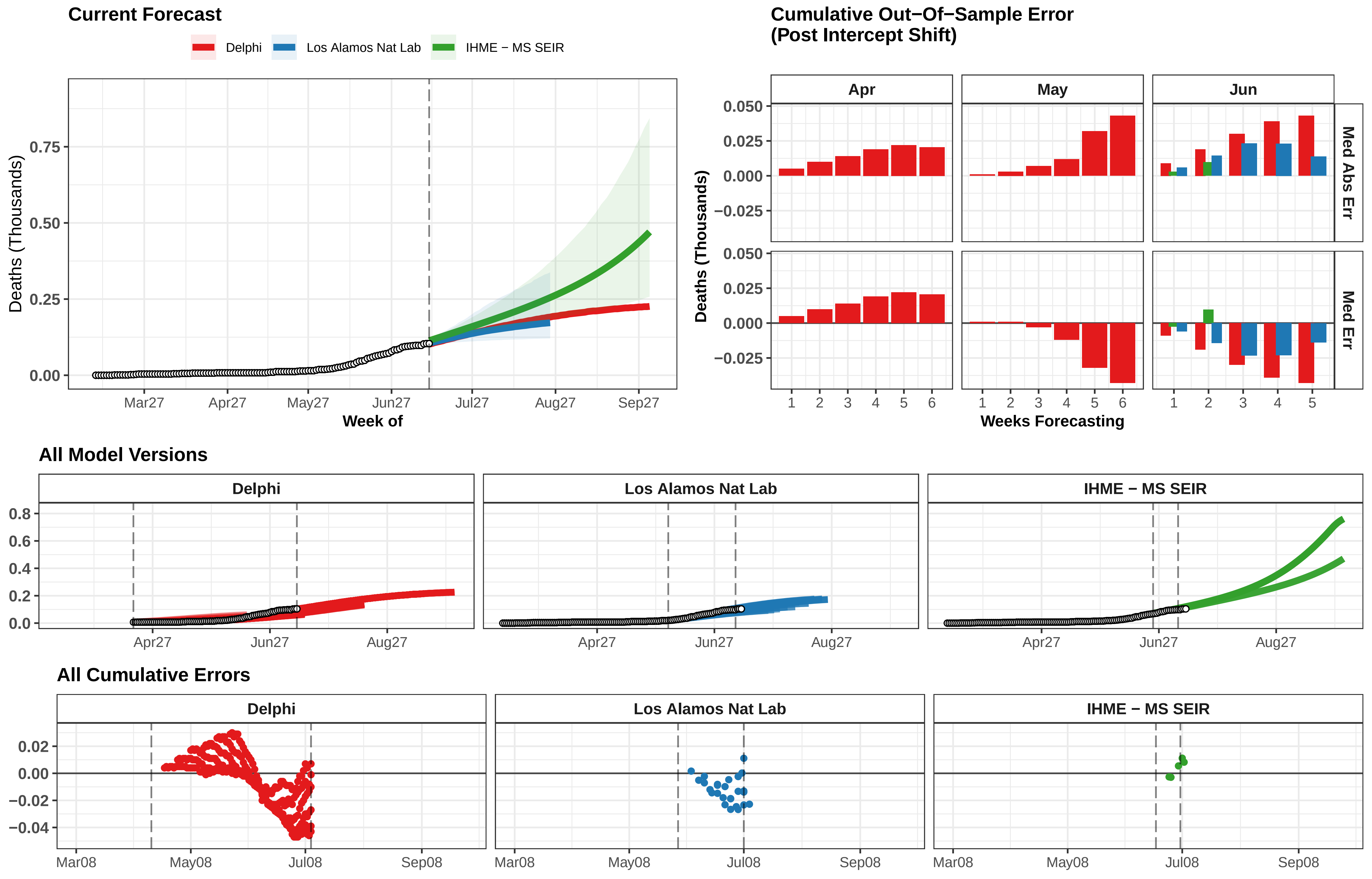

Current Forecast
Delphi
Los Alamos Nat Lab
IHME − MS SEIR
Deaths (Thousands)
0.00
0.25
0.50
0.75
Mar27
Apr27
May27
Jun27
Jul27
Aug27
Sep27
Week of
Cumulative Out−Of−Sample Error
(Post Intercept Shift)
Apr
May
Jun
Med Abs Err
Med Err
Deaths (Thousands)
0.050
0.025
0.000
−0.025
1
2
3
4
5
6
Weeks Forecasting
All Model Versions
Delphi
Los Alamos Nat Lab
IHME − MS SEIR
0.8
0.6
0.4
0.2
0.0
Apr27
Jun27
Aug27
All Cumulative Errors
Delphi
Los Alamos Nat Lab
IHME − MS SEIR
0.02
0.00
−0.02
−0.04
Mar08
May08
Jul08
Sep08

Idaho

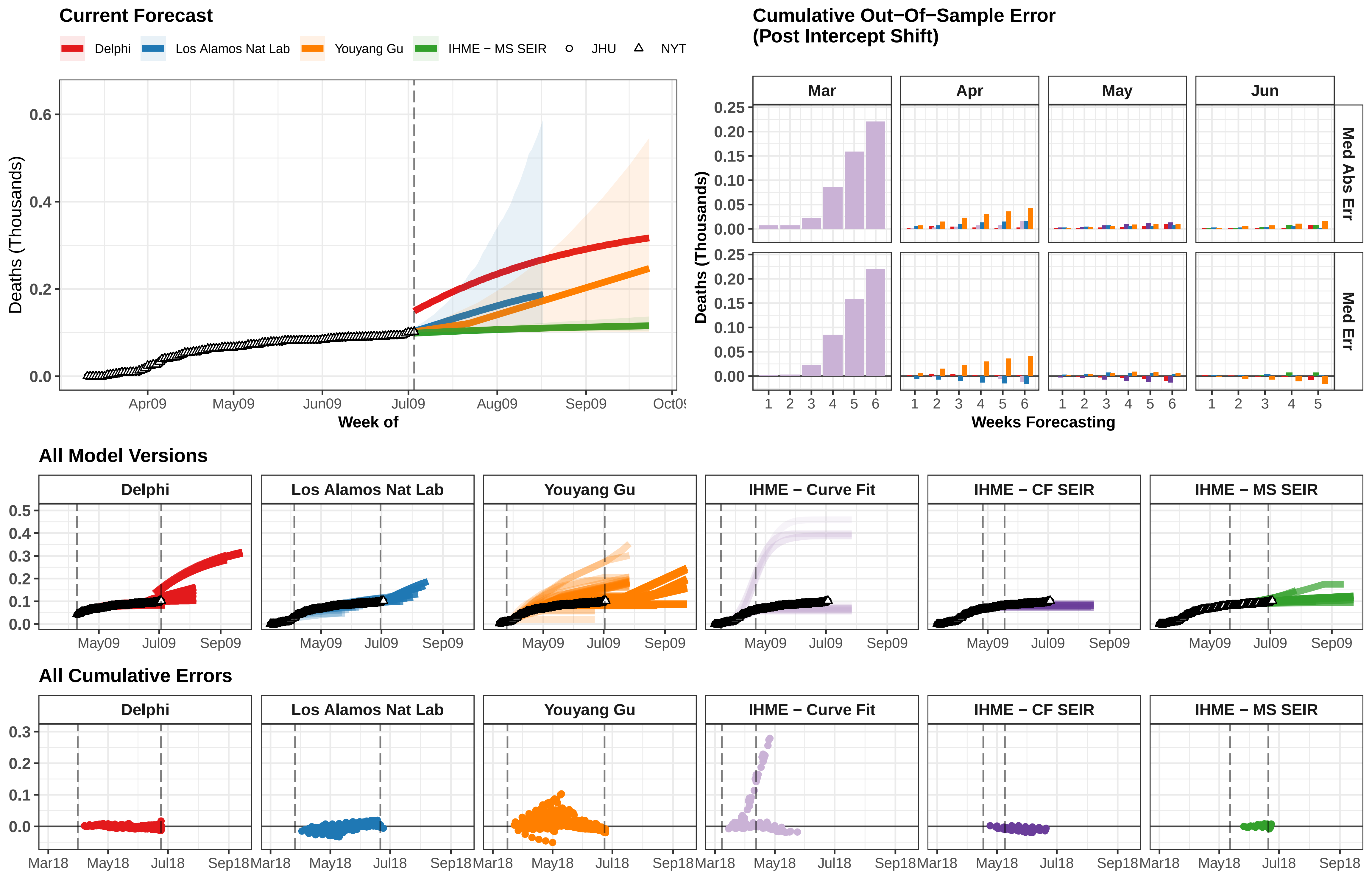
Current Forecast
Delphi
Los Alamos Nat Lab
Youyang Gu
IHME − MS SEIR
JHU
NYT
Deaths (Thousands)
Week of
Apr09
May09
Jun09
Jul09
Aug09
Sep09
Cumulative Out−Of−Sample Error (Post Intercept Shift)
Mar
Apr
May
Jun
Med Abs Err
Med Err
Weeks Forecasting
All Model Versions
Delphi
Los Alamos Nat Lab
Youyang Gu
IHME − Curve Fit
IHME − CF SEIR
IHME − MS SEIR
All Cumulative Errors
Mar18
May18
Jul18
Sep18

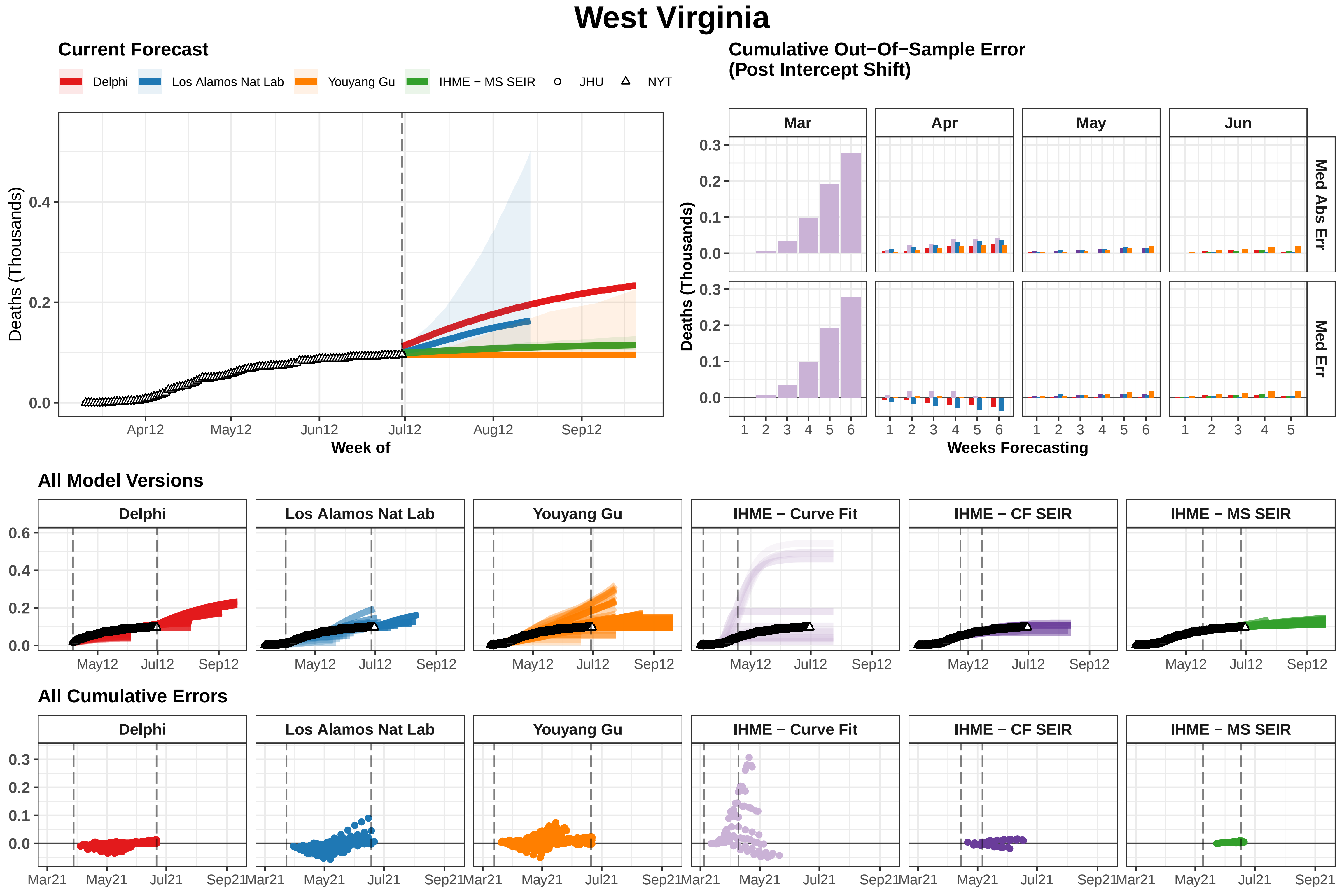
West Virginia
Current Forecast
Delphi
Los Alamos Nat Lab
Youyang Gu
IHME − MS SEIR
JHU
NYT
Deaths (Thousands)
0.0
0.2
0.4
Apr12
May12
Jun12
Jul12
Aug12
Sep12
Week of
Cumulative Out−Of−Sample Error (Post Intercept Shift)
Mar
Apr
May
Jun
Med Abs Err
Med Err
Deaths (Thousands)
0.0
0.1
0.2
0.3
1
2
3
4
5
6
Weeks Forecasting
All Model Versions
Delphi
Los Alamos Nat Lab
Youyang Gu
IHME − Curve Fit
IHME − CF SEIR
IHME − MS SEIR
0.0
0.2
0.4
0.6
May12
Jul12
Sep12
All Cumulative Errors
Delphi
Los Alamos Nat Lab
Youyang Gu
IHME − Curve Fit
IHME − CF SEIR
IHME − MS SEIR
0.0
0.1
0.2
0.3
Mar21
May21
Jul21
Sep21

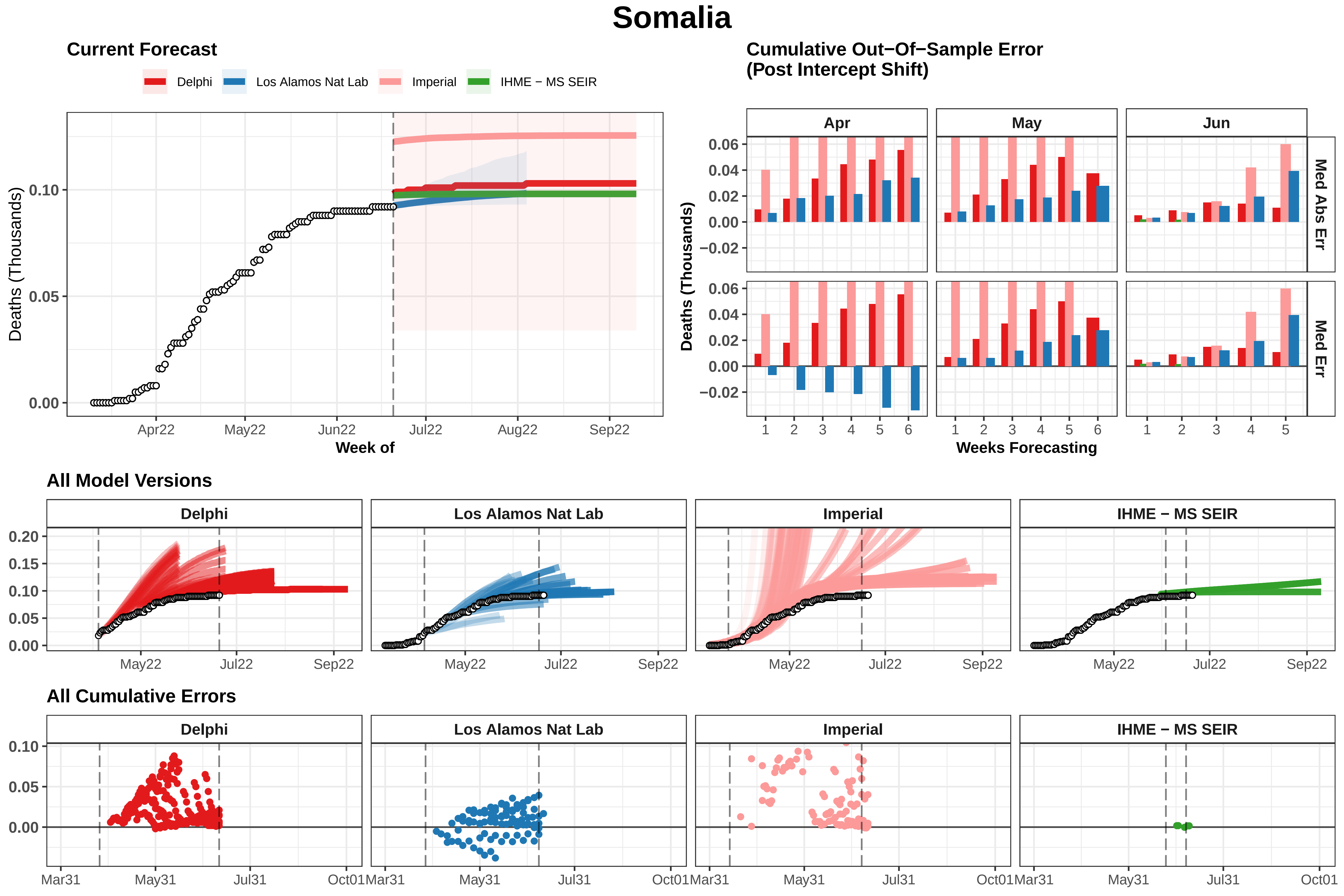
Somalia
Current Forecast
Delphi
Los Alamos Nat Lab
Imperial
IHME − MS SEIR
Deaths (Thousands)
Week of
Cumulative Out−Of−Sample Error
(Post Intercept Shift)
Apr
May
Jun
Med Abs Err
Med Err
Weeks Forecasting
All Model Versions
All Cumulative Errors

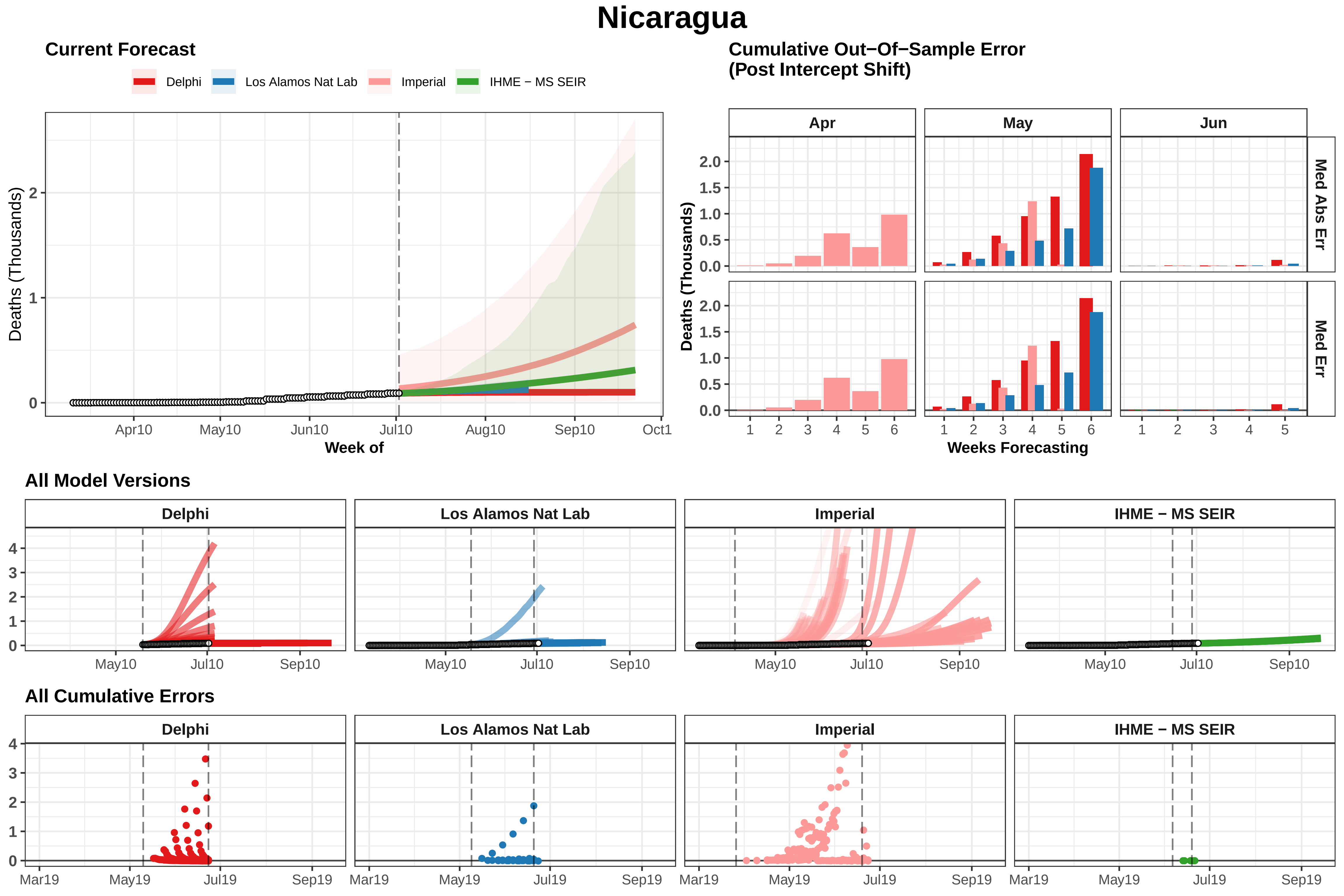
Nicaragua
Current Forecast
Delphi
Los Alamos Nat Lab
Imperial
IHME − MS SEIR
Deaths (Thousands)
0
1
2
Apr10
May10
Jun10
Jul10
Aug10
Sep10
Oct1
Week of
Cumulative Out−Of−Sample Error
(Post Intercept Shift)
Apr
May
Jun
Med Abs Err
Med Err
Deaths (Thousands)
0.0
0.5
1.0
1.5
2.0
1
2
3
4
5
6
Weeks Forecasting
All Model Versions
Delphi
Los Alamos Nat Lab
Imperial
IHME − MS SEIR
0
1
2
3
4
May10
Jul10
Sep10
All Cumulative Errors
Delphi
Los Alamos Nat Lab
Imperial
IHME − MS SEIR
0
1
2
3
4
Mar19
May19
Jul19
Sep19

North Dakota

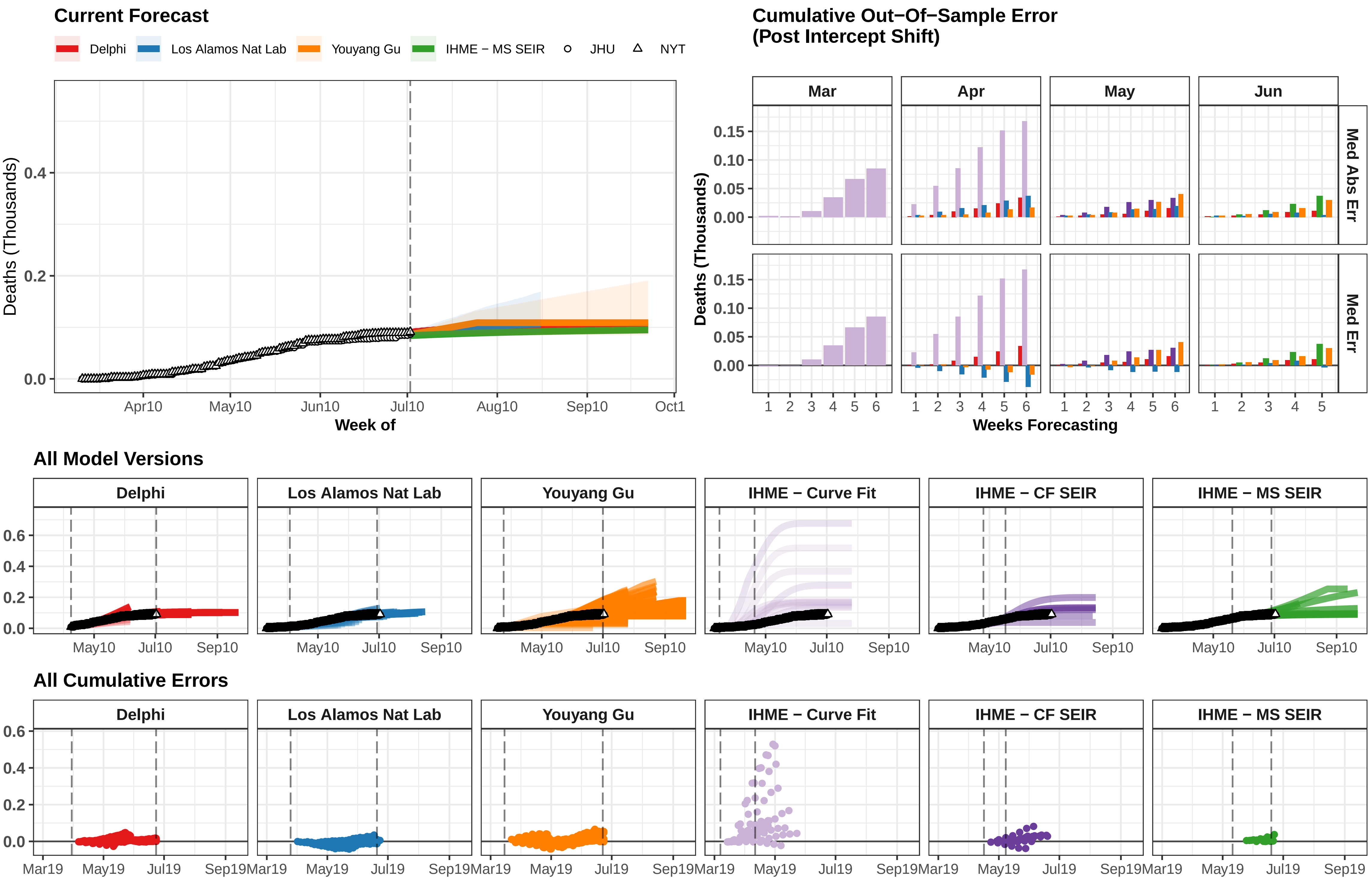

Albania

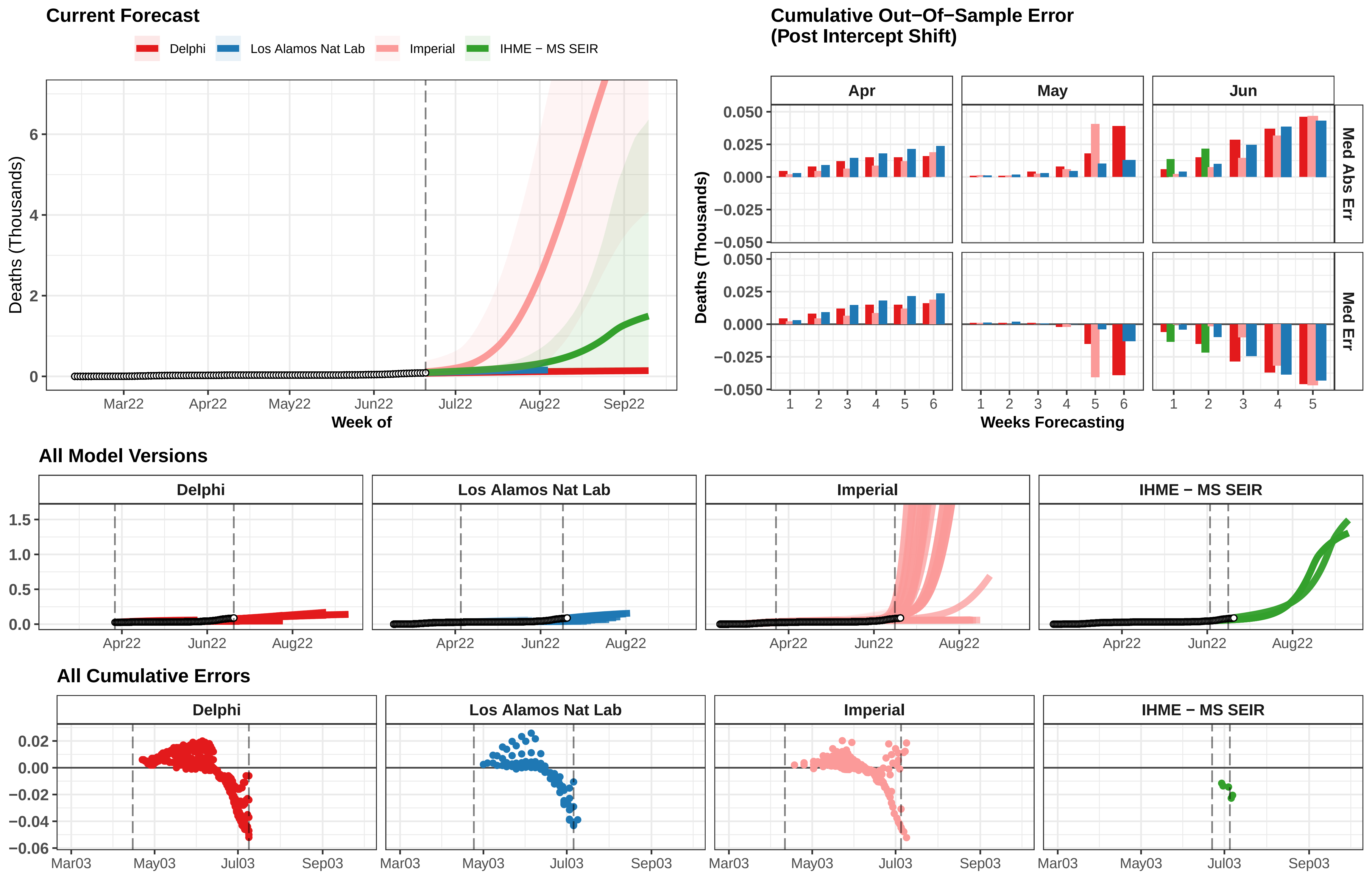
Current Forecast
Delphi
Los Alamos Nat Lab
Imperial
IHME − MS SEIR
Deaths (Thousands)
0
2
4
6
Mar22
Apr22
May22
Jun22
Jul22
Aug22
Sep22
Week of
Cumulative Out−Of−Sample Error
(Post Intercept Shift)
Apr
May
Jun
Med Abs Err
Med Err
0.050
0.025
0.000
−0.025
−0.050
1 2 3 4 5 6
1 2 3 4 5
Weeks Forecasting
All Model Versions
Delphi
Los Alamos Nat Lab
Imperial
IHME − MS SEIR
1.5
1.0
0.5
0.0
Apr22
Jun22
Aug22
All Cumulative Errors
0.02
0.00
−0.02
−0.04
−0.06
Mar03
May03
Jul03
Sep03

Cuba

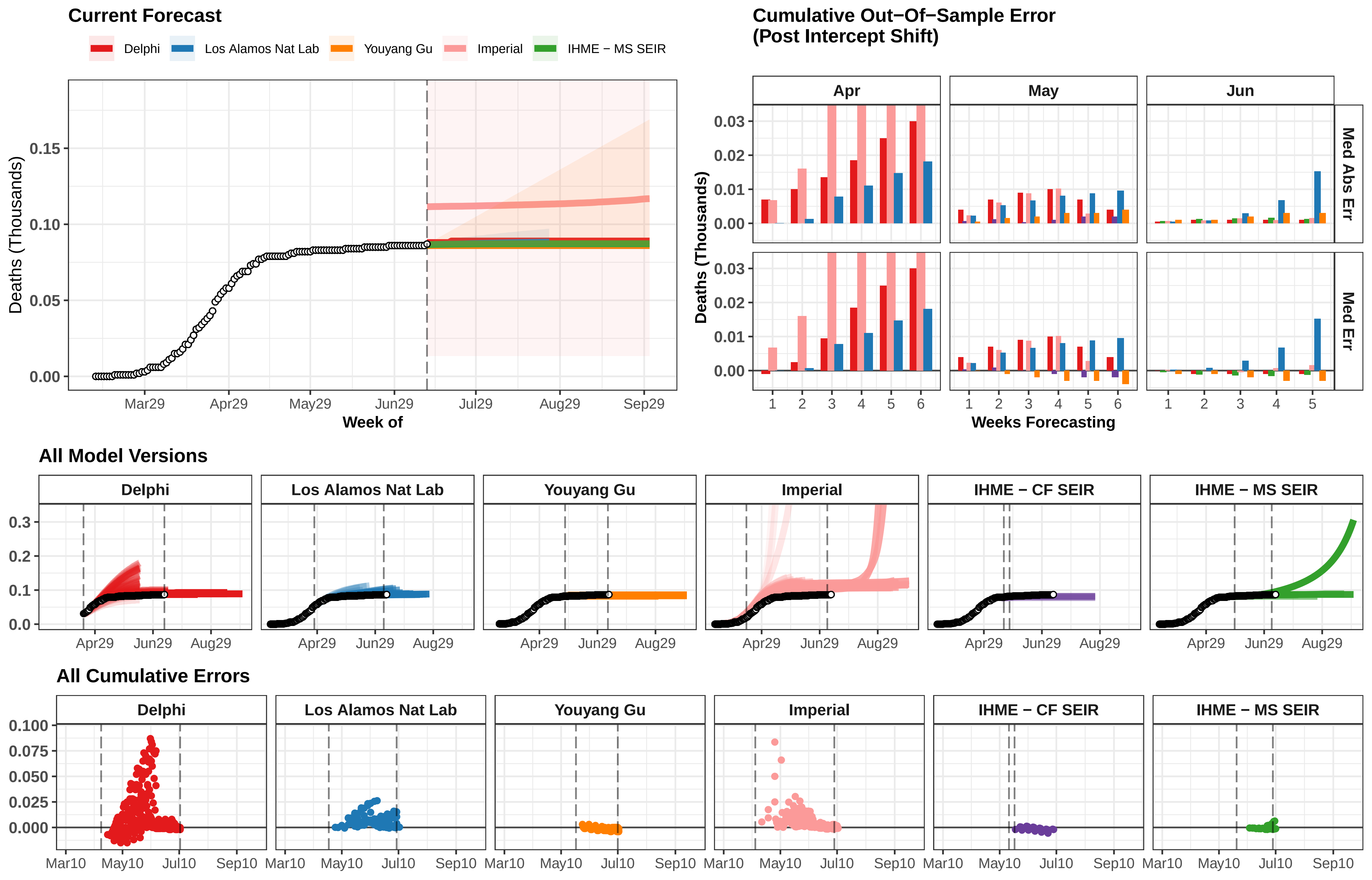

Current Forecast
Delphi
Los Alamos Nat Lab
Youyang Gu
Imperial
IHME − MS SEIR
Deaths (Thousands)
Week of
Mar29
Apr29
May29
Jun29
Jul29
Aug29
Sep29
Cumulative Out−Of−Sample Error
(Post Intercept Shift)
Apr
May
Jun
Med Abs Err
Med Err
Weeks Forecasting
All Model Versions
Delphi
Los Alamos Nat Lab
Youyang Gu
Imperial
IHME − CF SEIR
IHME − MS SEIR
All Cumulative Errors
Mar10
May10
Jul10
Sep10

Venezuela

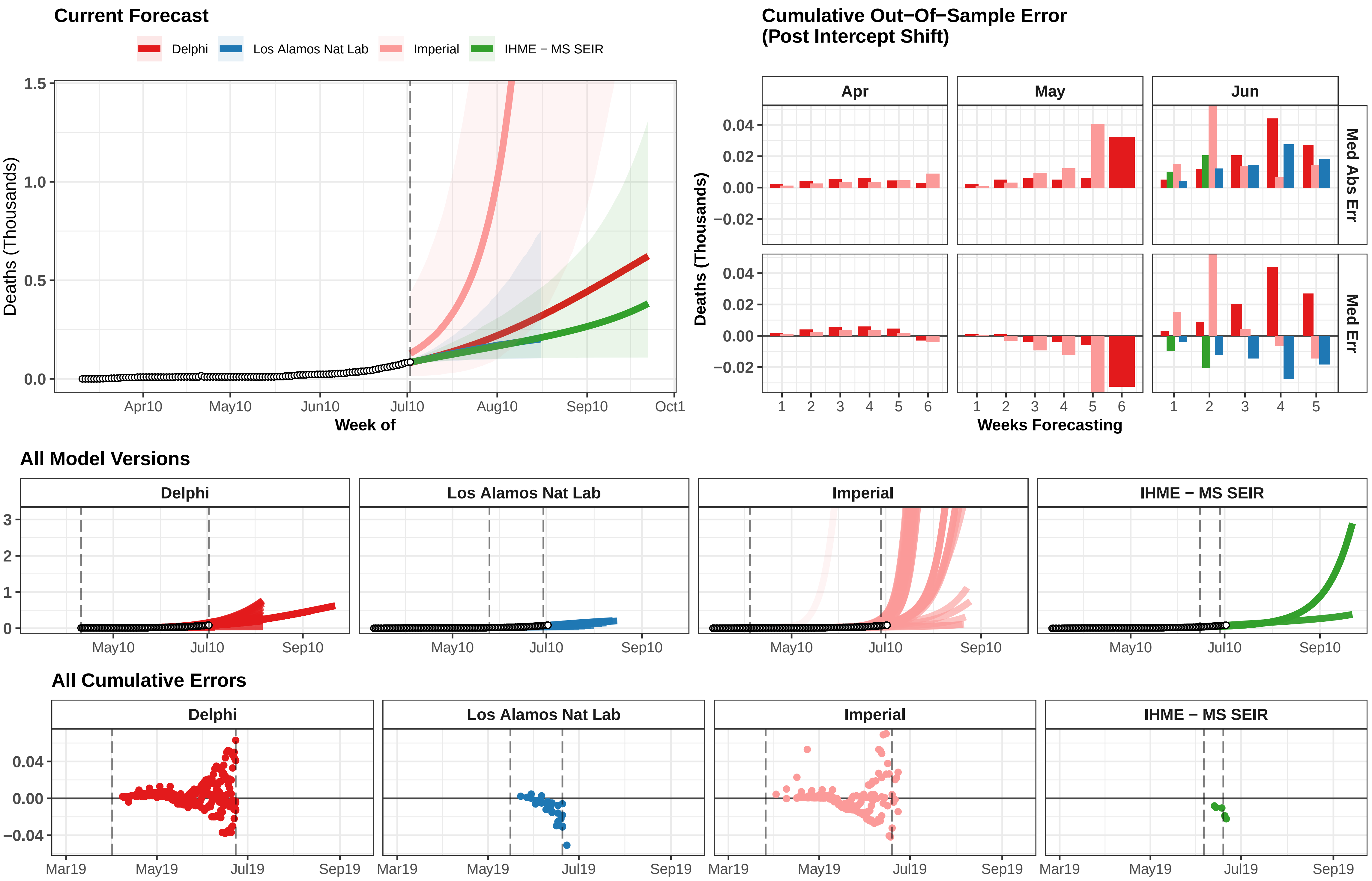

Current Forecast
Delphi
Los Alamos Nat Lab
Imperial
IHME − MS SEIR
Deaths (Thousands)
Week of
Apr10
May10
Jun10
Jul10
Aug10
Sep10
Oct1
Cumulative Out−Of−Sample Error
(Post Intercept Shift)
Apr
May
Jun
Med Abs Err
Med Err
Weeks Forecasting
All Model Versions
All Cumulative Errors
Mar19
May19
Jul19
Sep19

Cote d'Ivoire

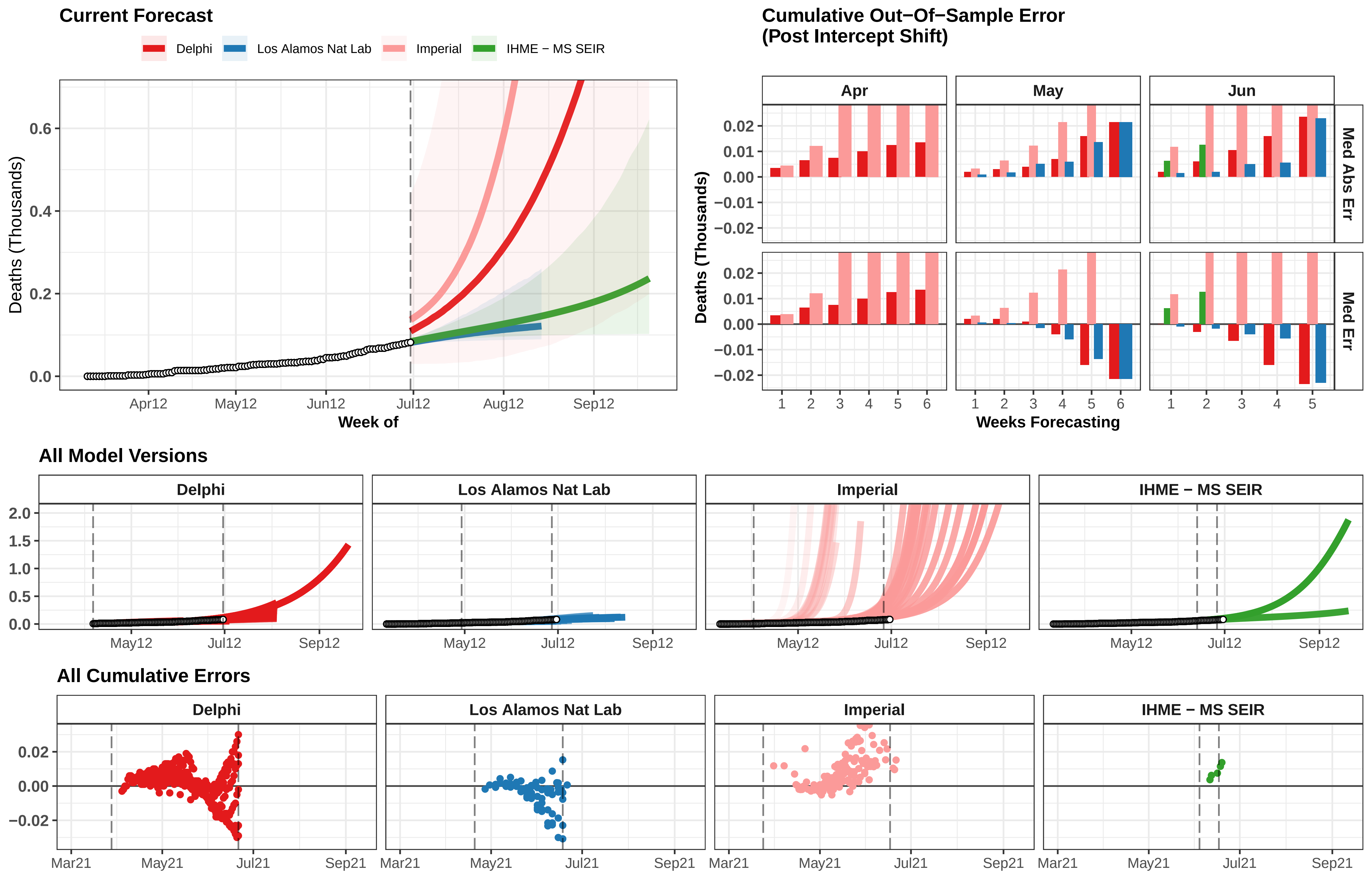

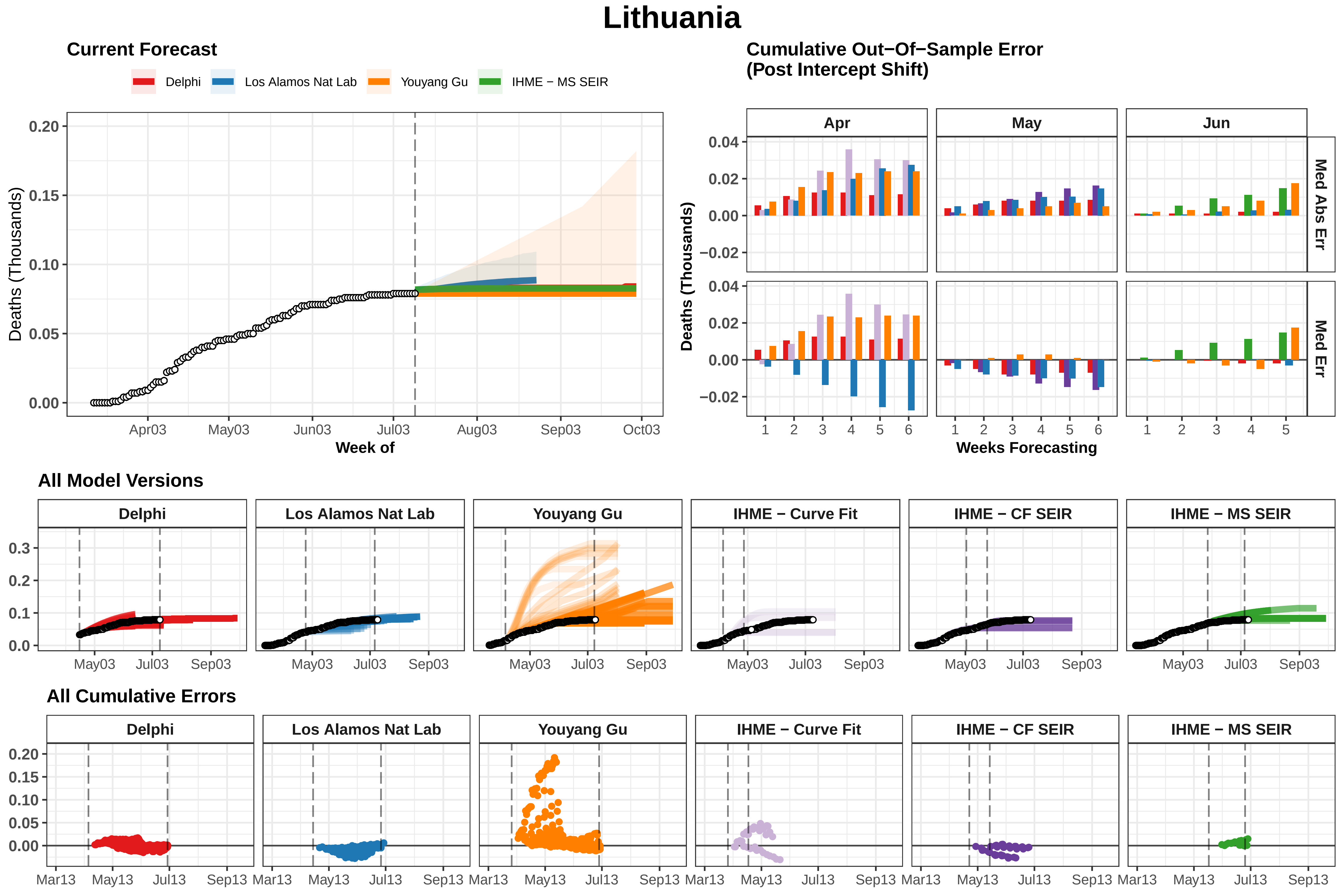
Lithuania
Current Forecast
Delphi
Los Alamos Nat Lab
Youyang Gu
IHME − MS SEIR
Deaths (Thousands)
Week of
Apr03
May03
Jun03
Jul03
Aug03
Sep03
Oct03
Cumulative Out−Of−Sample Error
(Post Intercept Shift)
Apr
May
Jun
Med Abs Err
Med Err
Weeks Forecasting
All Model Versions
Delphi
Los Alamos Nat Lab
Youyang Gu
IHME − Curve Fit
IHME − CF SEIR
IHME − MS SEIR
All Cumulative Errors
Mar13
May13
Jul13
Sep13

Chad

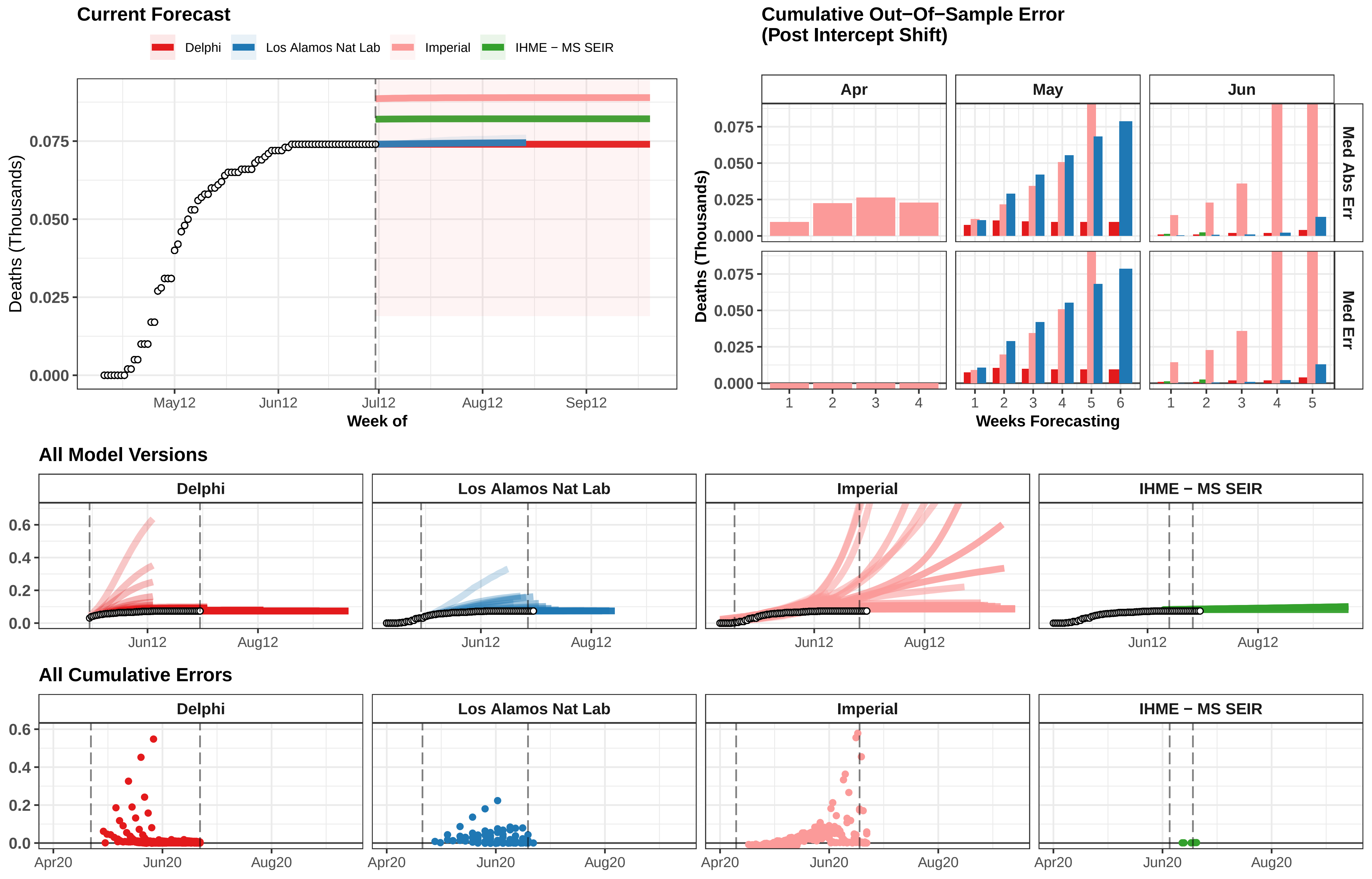
Current Forecast
Delphi
Los Alamos Nat Lab
Imperial
IHME − MS SEIR
Deaths (Thousands)
0.000
0.025
0.050
0.075
May12
Jun12
Jul12
Aug12
Sep12
Week of
Cumulative Out−Of−Sample Error
(Post Intercept Shift)
Apr
May
Jun
Med Abs Err
Med Err
Weeks Forecasting
All Model Versions
Delphi
Los Alamos Nat Lab
Imperial
IHME − MS SEIR
0.0
0.2
0.4
0.6
Jun12
Aug12
All Cumulative Errors
Apr20
Jun20
Aug20

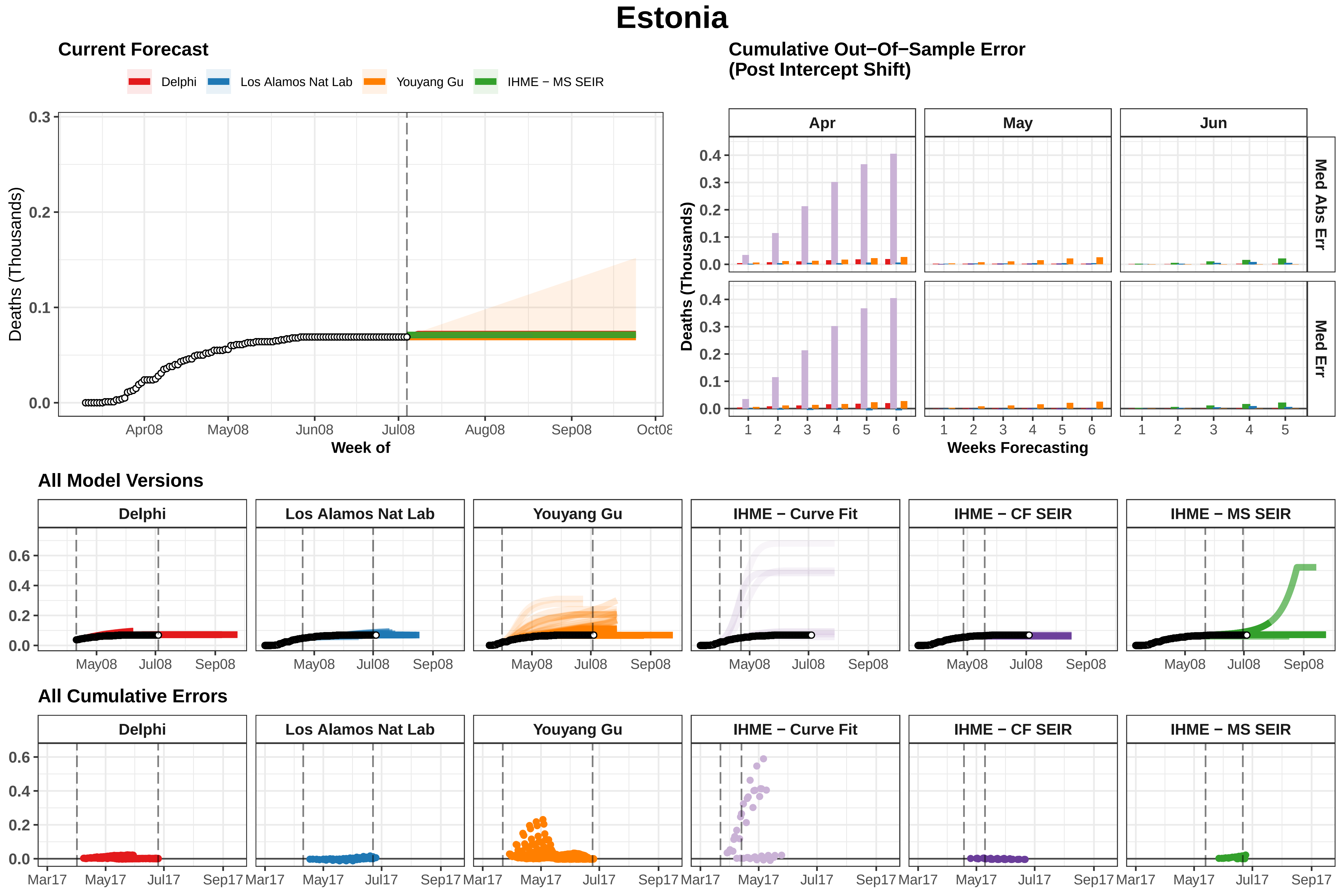

Estonia
Current Forecast
Delphi
Los Alamos Nat Lab
Youyang Gu
IHME − MS SEIR
Deaths (Thousands)
Week of
Cumulative Out−Of−Sample Error (Post Intercept Shift)
Apr
May
Jun
Med Abs Err
Med Err
Weeks Forecasting
All Model Versions
IHME − Curve Fit
IHME − CF SEIR
All Cumulative Errors

Niger

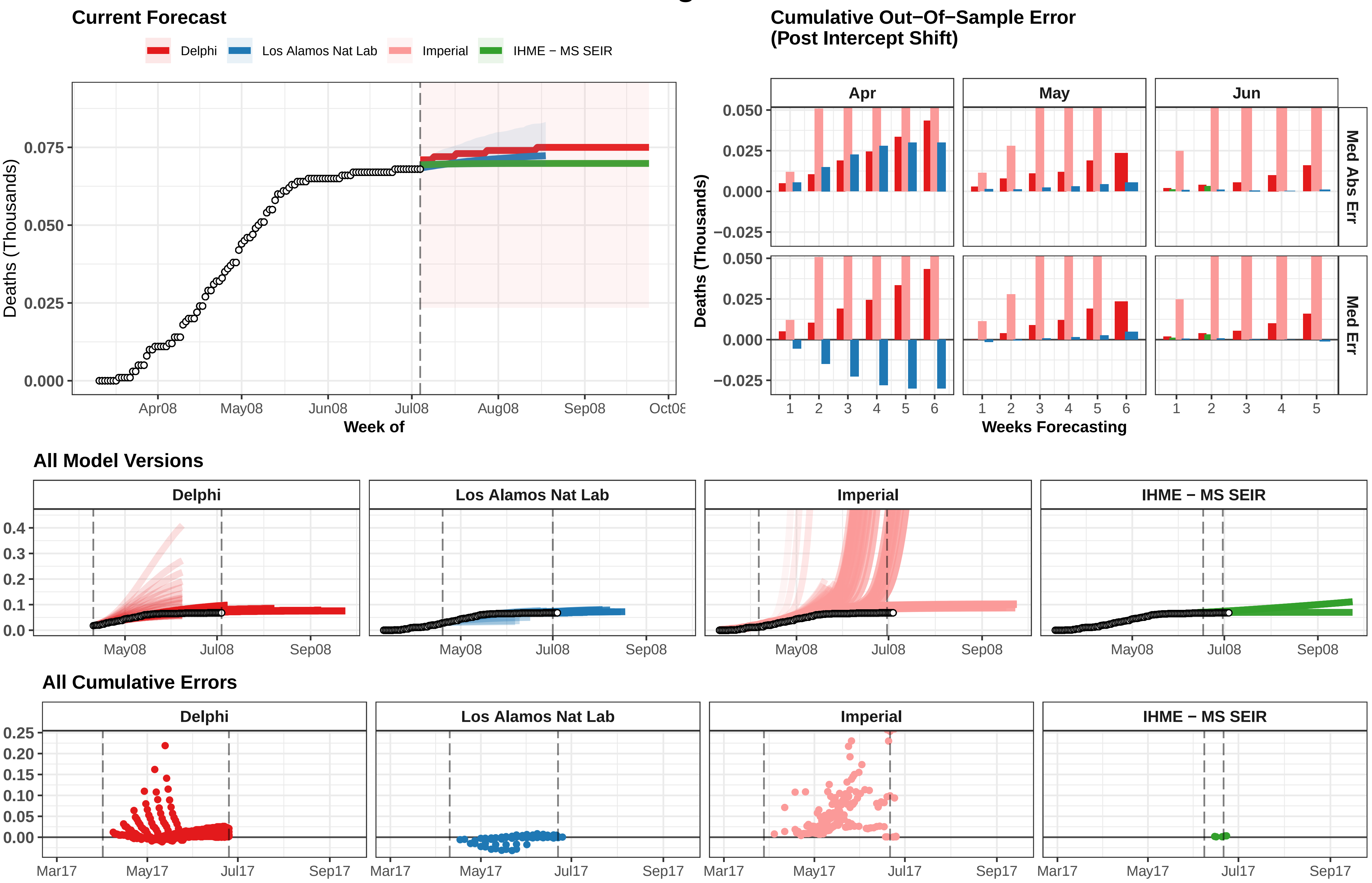

Current Forecast
Delphi
Los Alamos Nat Lab
Imperial
IHME − MS SEIR
Deaths (Thousands)
0.000
0.025
0.050
0.075
Apr08
May08
Jun08
Jul08
Aug08
Sep08
Week of
Cumulative Out−Of−Sample Error
(Post Intercept Shift)
Apr
May
Jun
Med Abs Err
Med Err
Weeks Forecasting
All Model Versions
All Cumulative Errors
Mar17
May17
Jul17
Sep17

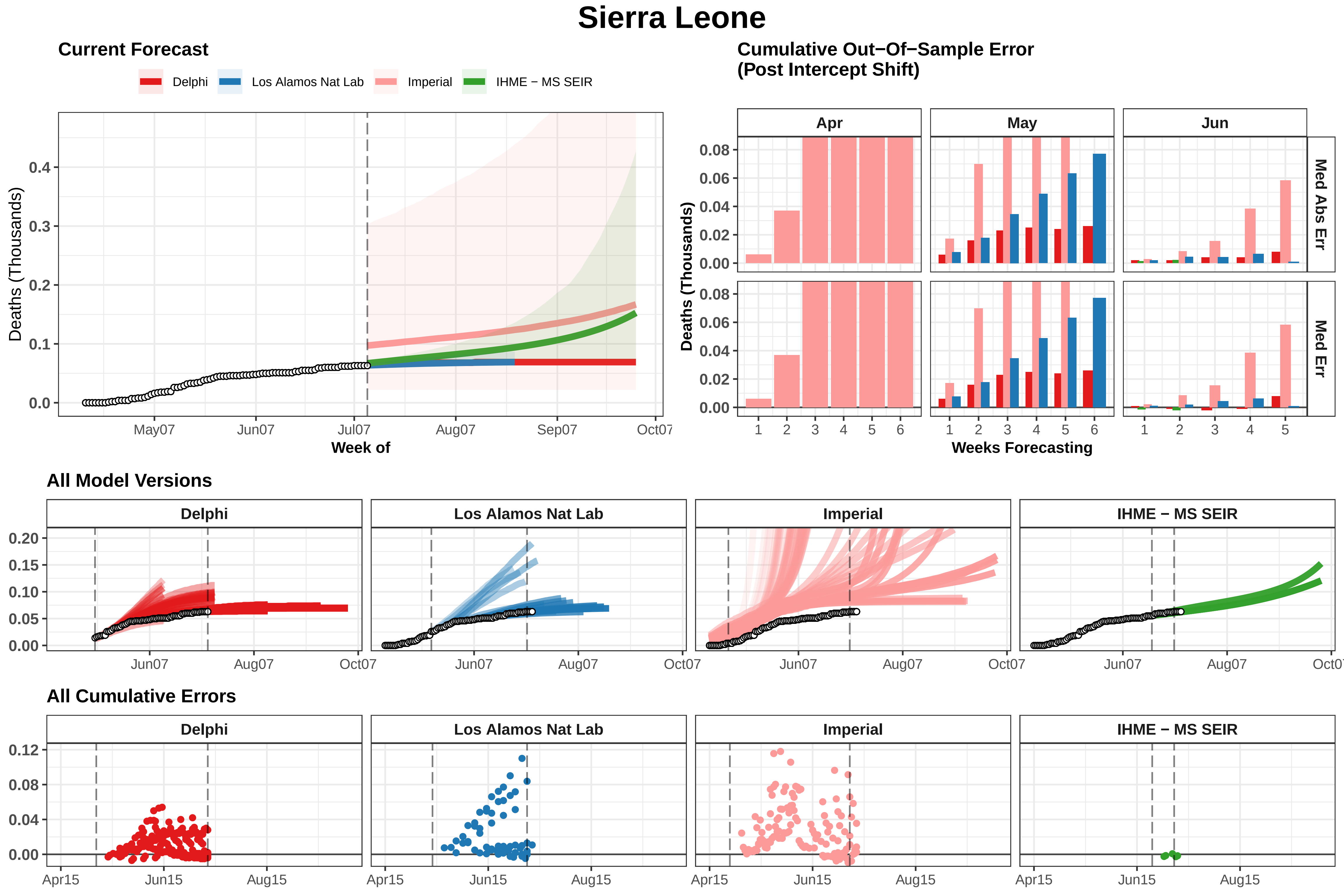
Sierra Leone
Current Forecast
Delphi
Los Alamos Nat Lab
Imperial
IHME − MS SEIR
Deaths (Thousands)
0.0
0.1
0.2
0.3
0.4
May07
Jun07
Jul07
Aug07
Sep07
Oct07
Week of
Cumulative Out−Of−Sample Error
(Post Intercept Shift)
Apr
May
Jun
Med Abs Err
Med Err
0.00
0.02
0.04
0.06
0.08
Weeks Forecasting
All Model Versions
0.00
0.05
0.10
0.15
0.20
All Cumulative Errors
0.00
0.04
0.08
0.12
Apr15
Jun15
Aug15

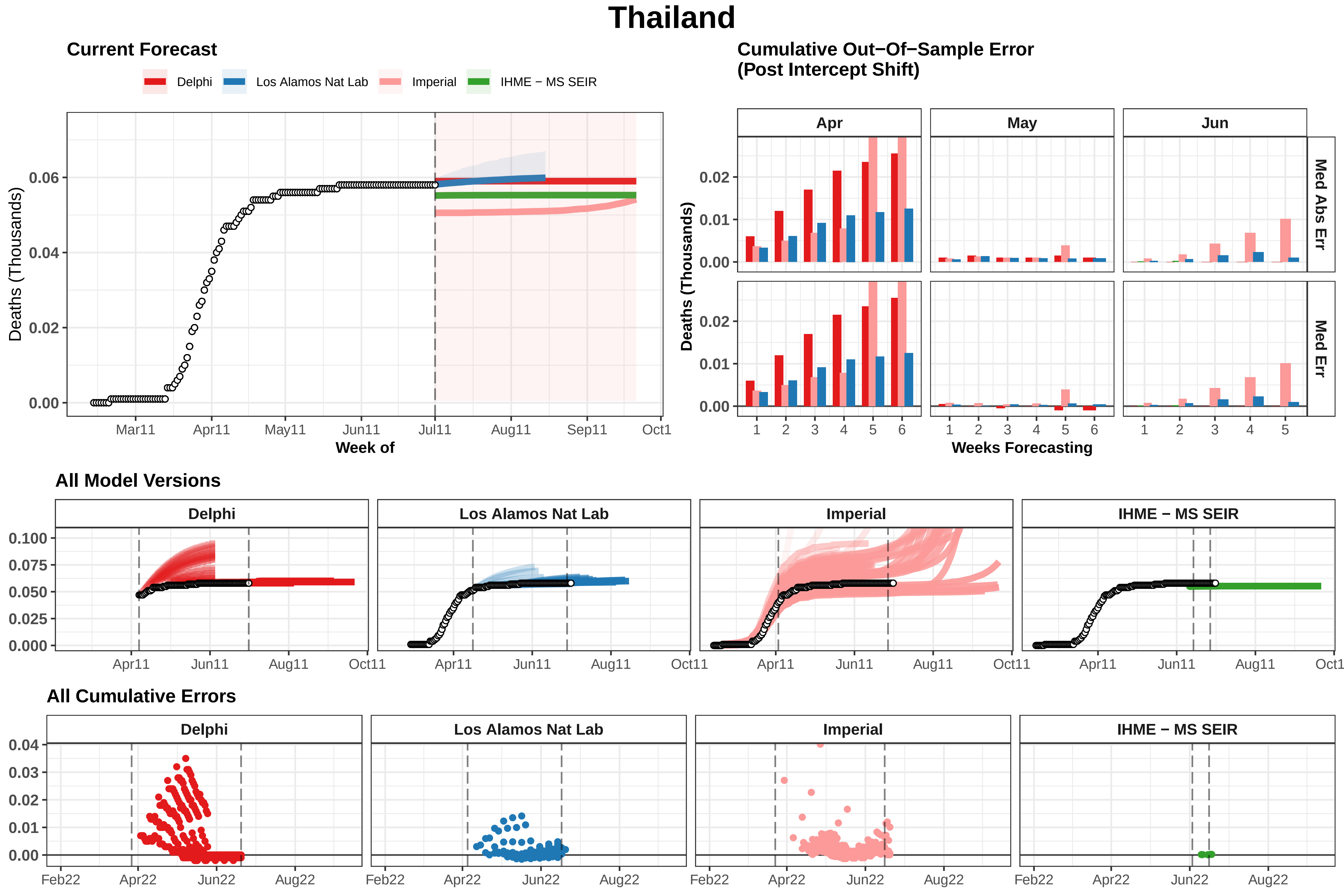
Thailand
Current Forecast
Delphi
Los Alamos Nat Lab
Imperial
IHME − MS SEIR
Deaths (Thousands)
0.00
0.02
0.04
0.06
Mar11
Apr11
May11
Jun11
Jul11
Aug11
Sep11
Oct1
Week of
Cumulative Out−Of−Sample Error
(Post Intercept Shift)
Apr
May
Jun
Med Abs Err
Med Err
Deaths (Thousands)
0.00
0.01
0.02
1
2
3
4
5
6
Weeks Forecasting
All Model Versions
Delphi
Los Alamos Nat Lab
Imperial
IHME − MS SEIR
0.000
0.025
0.050
0.075
0.100
Apr11
Jun11
Aug11
Oct11
All Cumulative Errors
Delphi
Los Alamos Nat Lab
Imperial
IHME − MS SEIR
0.00
0.01
0.02
0.03
0.04
Feb22
Apr22
Jun22
Aug22

Uzbekistan

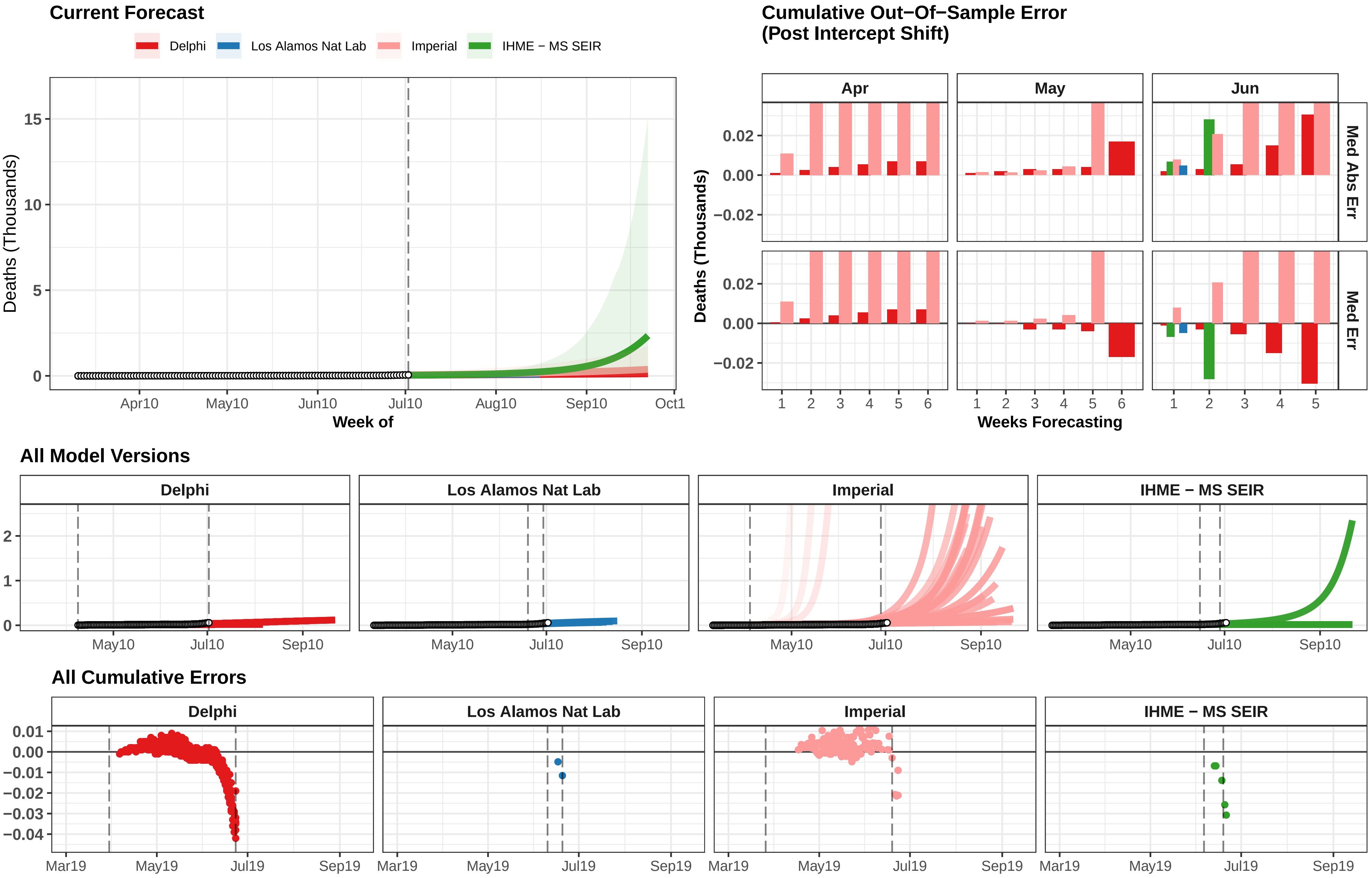

Current Forecast
Delphi
Los Alamos Nat Lab
Imperial
IHME − MS SEIR
Deaths (Thousands)
15
10
5
0
Apr10
May10
Jun10
Jul10
Aug10
Sep10
Oct1
Week of
Cumulative Out−Of−Sample Error
(Post Intercept Shift)
Apr
May
Jun
Med Abs Err
Med Err
0.02
0.00
−0.02
Weeks Forecasting
All Model Versions
Delphi
Los Alamos Nat Lab
Imperial
IHME − MS SEIR
2
1
0
May10
Jul10
Sep10
All Cumulative Errors
0.01
0.00
−0.01
−0.02
−0.03
−0.04
Mar19
May19
Jul19
Sep19

Djibouti

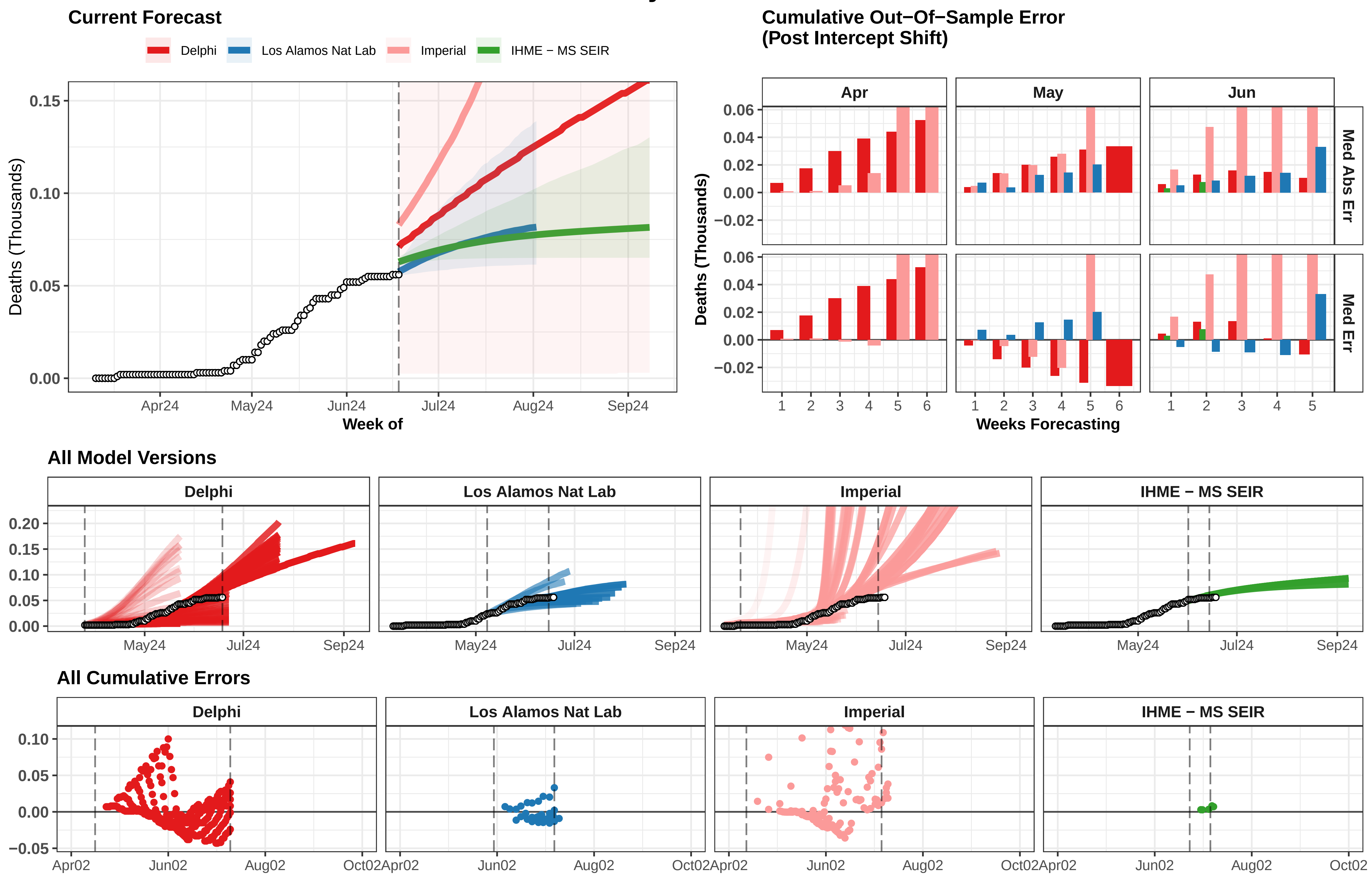

Current Forecast
Delphi
Los Alamos Nat Lab
Imperial
IHME − MS SEIR
Deaths (Thousands)
0.15
0.10
0.05
0.00
Apr24
May24
Jun24
Jul24
Aug24
Sep24
Week of
Cumulative Out−Of−Sample Error
(Post Intercept Shift)
Apr
May
Jun
Med Abs Err
Med Err
0.06
0.04
0.02
0.00
−0.02
1 2 3 4 5 6
1 2 3 4 5 6
1 2 3 4 5
Weeks Forecasting
All Model Versions
Delphi
Los Alamos Nat Lab
Imperial
IHME − MS SEIR
0.20
0.15
0.10
0.05
0.00
May24
Jul24
Sep24
All Cumulative Errors
Delphi
Los Alamos Nat Lab
Imperial
IHME − MS SEIR
0.10
0.05
0.00
−0.05
Apr02
Jun02
Aug02
Oct02

Vermont

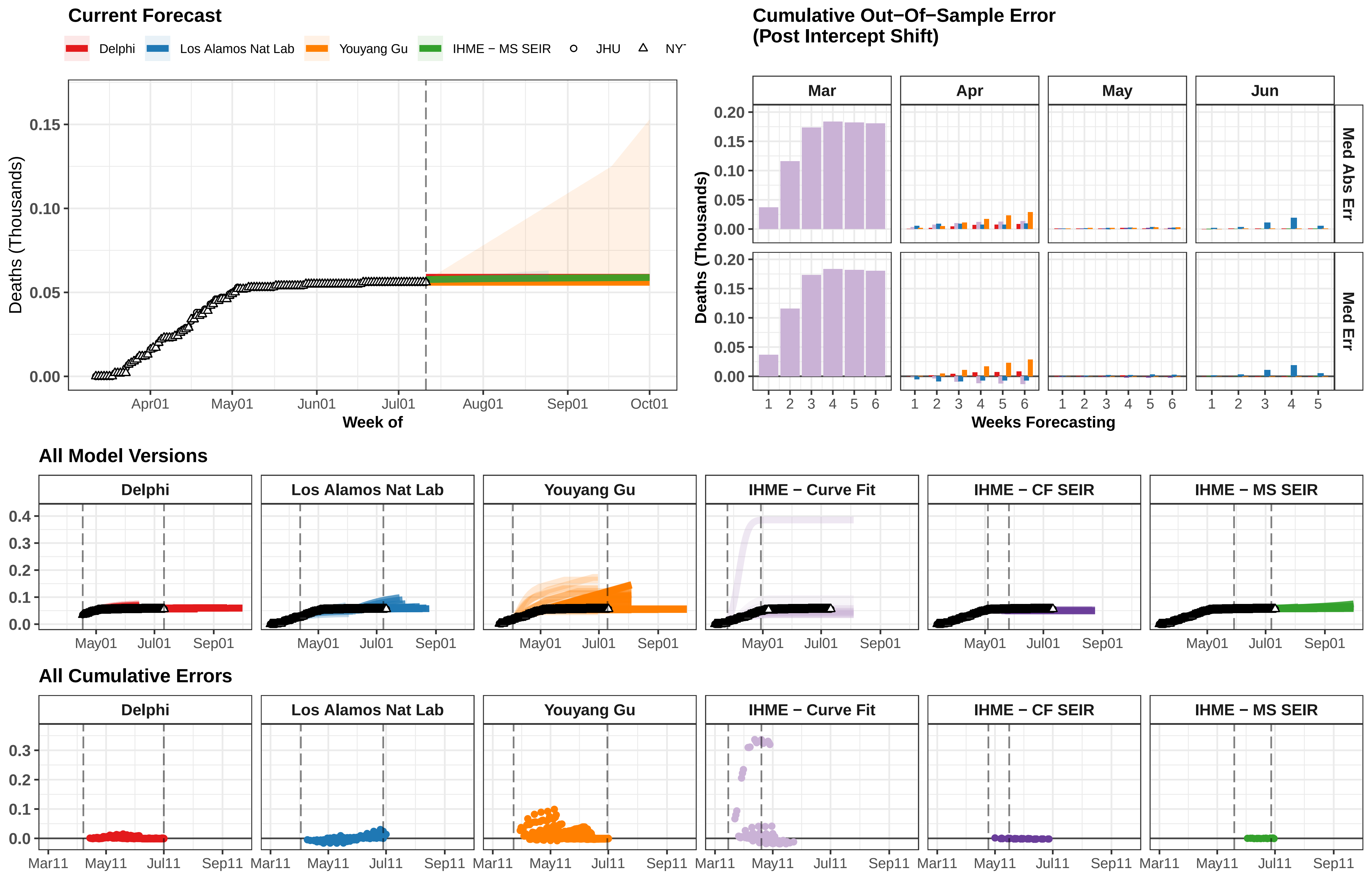

Current Forecast
Delphi
Los Alamos Nat Lab
Youyang Gu
IHME − MS SEIR
JHU
NY
Deaths (Thousands)
Week of
Apr01
May01
Jun01
Jul01
Aug01
Sep01
Oct01
Cumulative Out−Of−Sample Error (Post Intercept Shift)
Mar
Apr
May
Jun
Med Abs Err
Med Err
Weeks Forecasting
All Model Versions
Delphi
Los Alamos Nat Lab
Youyang Gu
IHME − Curve Fit
IHME − CF SEIR
IHME − MS SEIR
All Cumulative Errors
Mar11
May11
Jul11
Sep11

Tajikistan

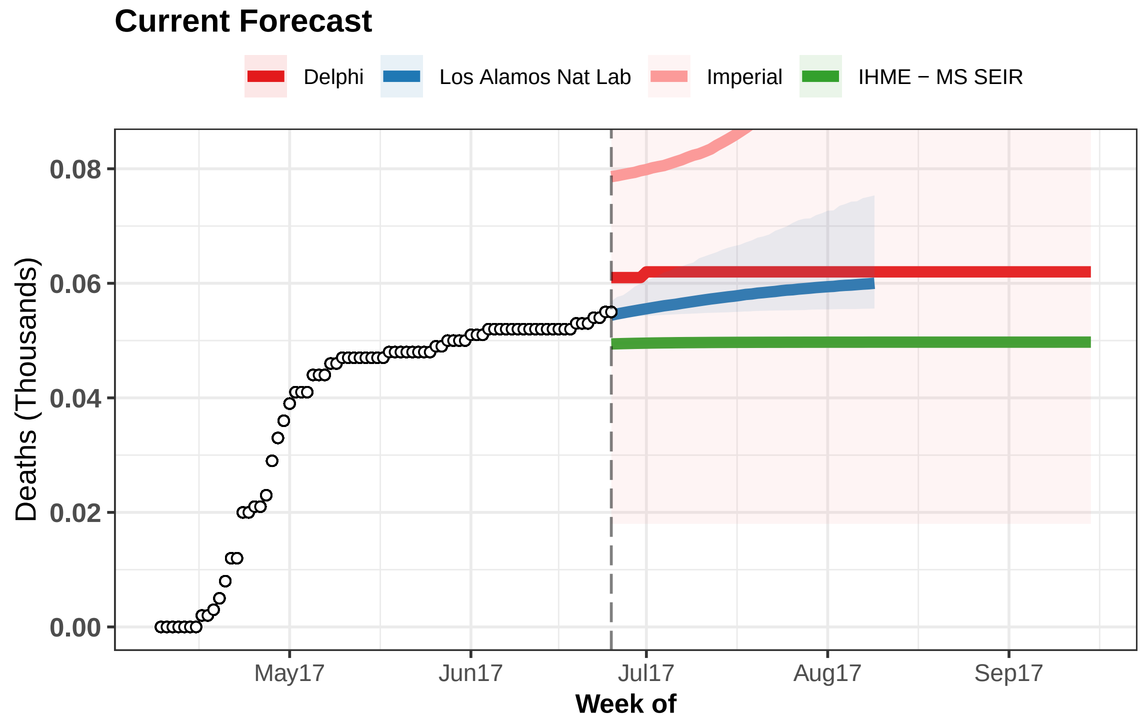

Current Forecast
Delphi
Los Alamos Nat Lab
Imperial
IHME − MS SEIR
Deaths (Thousands)
0.00
0.02
0.04
0.06
0.08
May17
Jun17
Jul17
Aug17
Sep17
Week of

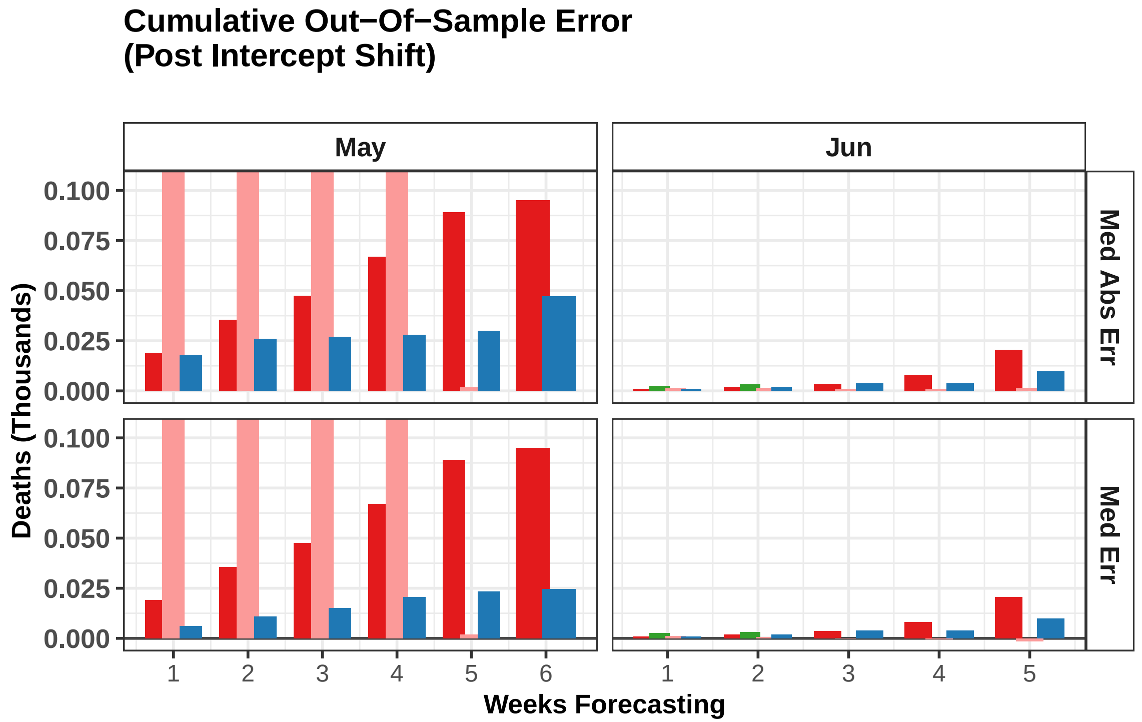

Cumulative Out−Of−Sample Error
(Post Intercept Shift)
May
Jun
Med Abs Err
Med Err
Deaths (Thousands)
0.000
0.025
0.050
0.075
0.100
1
2
3
4
5
6
Weeks Forecasting

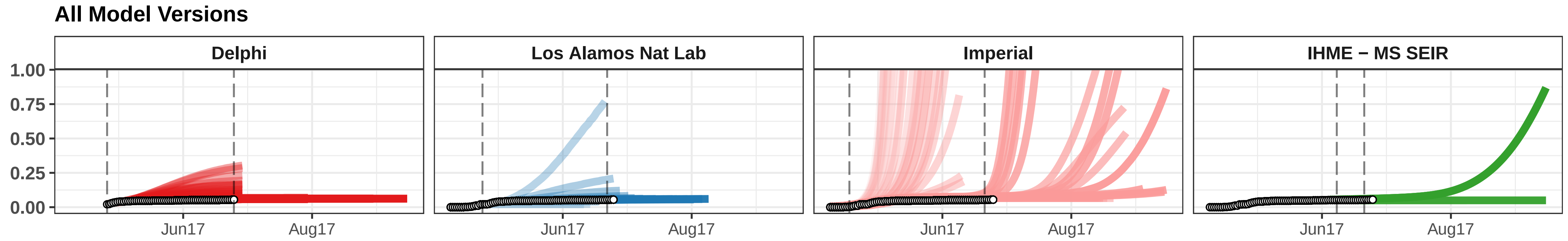

All Model Versions
Delphi
Los Alamos Nat Lab
Imperial
IHME − MS SEIR
0.00
0.25
0.50
0.75
1.00
Jun17
Aug17

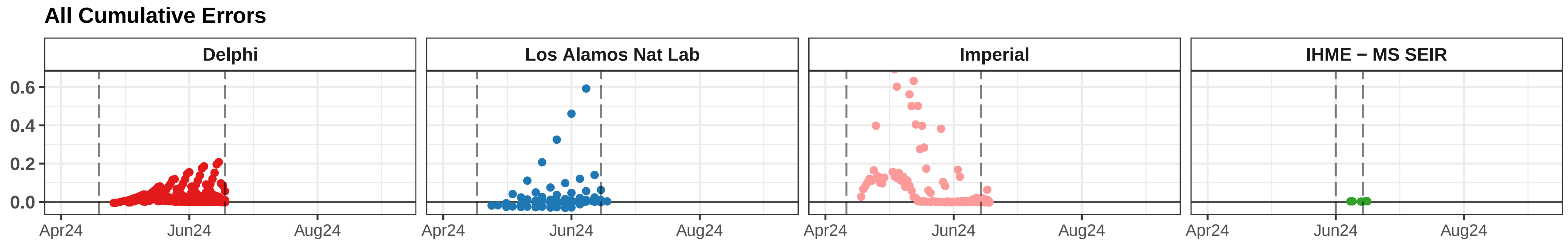

All Cumulative Errors
Delphi
Los Alamos Nat Lab
Imperial
IHME − MS SEIR
0.0
0.2
0.4
0.6
Apr24
Jun24
Aug24

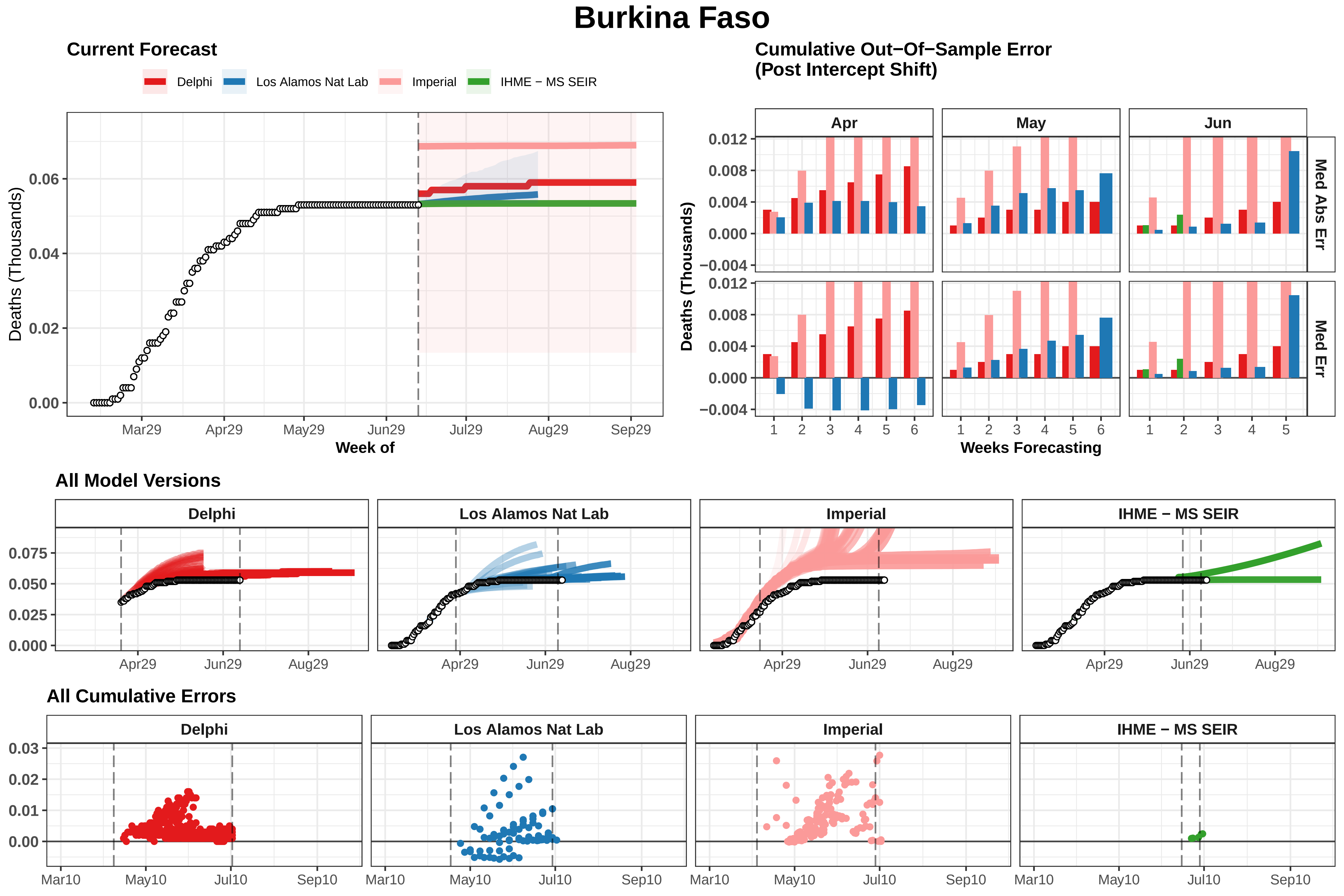
Burkina Faso
Current Forecast
Delphi
Los Alamos Nat Lab
Imperial
IHME − MS SEIR
Deaths (Thousands)
Week of
Mar29
Apr29
May29
Jun29
Jul29
Aug29
Sep29
Cumulative Out−Of−Sample Error
(Post Intercept Shift)
Apr
May
Jun
Med Abs Err
Med Err
Weeks Forecasting
All Model Versions
All Cumulative Errors
Mar10
May10
Jul10
Sep10

Central African Republic

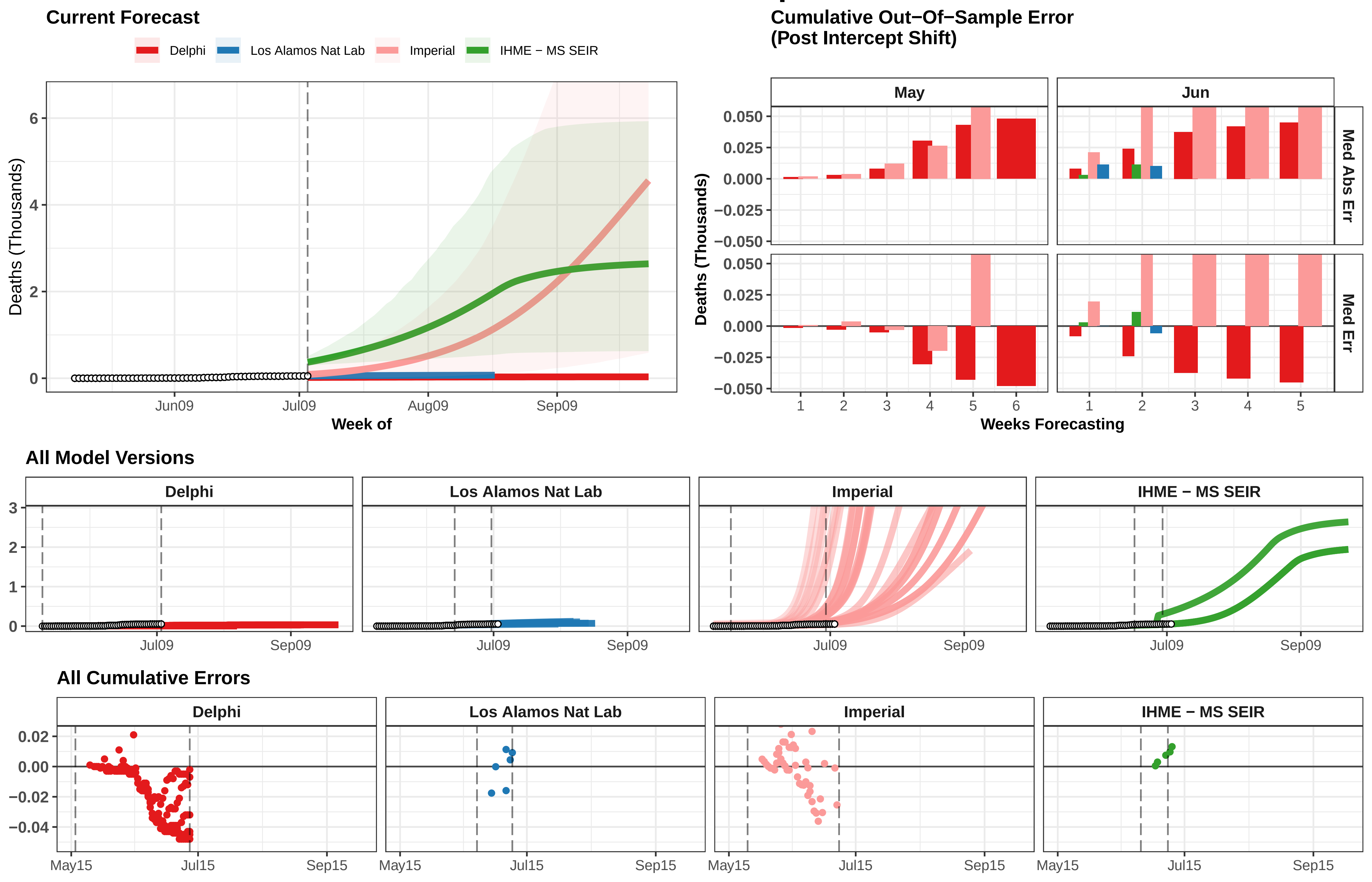

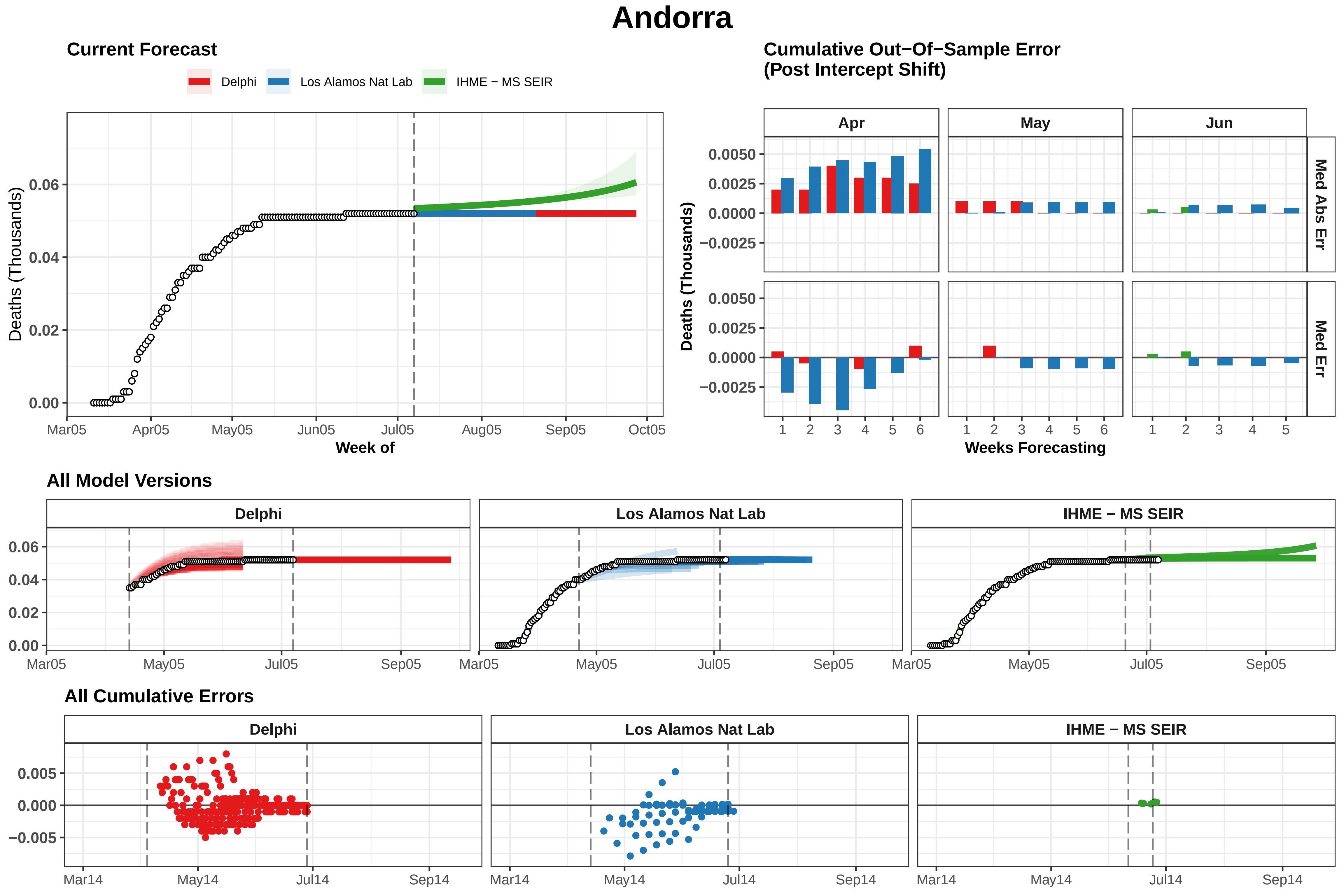

Andorra
Current Forecast
Delphi
Los Alamos Nat Lab
IHME − MS SEIR
Deaths (Thousands)
0.00
0.02
0.04
0.06
Mar05
Apr05
May05
Jun05
Jul05
Aug05
Sep05
Oct05
Week of
Cumulative Out−Of−Sample Error
(Post Intercept Shift)
Apr
May
Jun
Med Abs Err
Med Err
0.0050
0.0025
0.0000
−0.0025
Weeks Forecasting
All Model Versions
All Cumulative Errors
0.005
0.000
−0.005
Mar14
May14
Jul14
Sep14

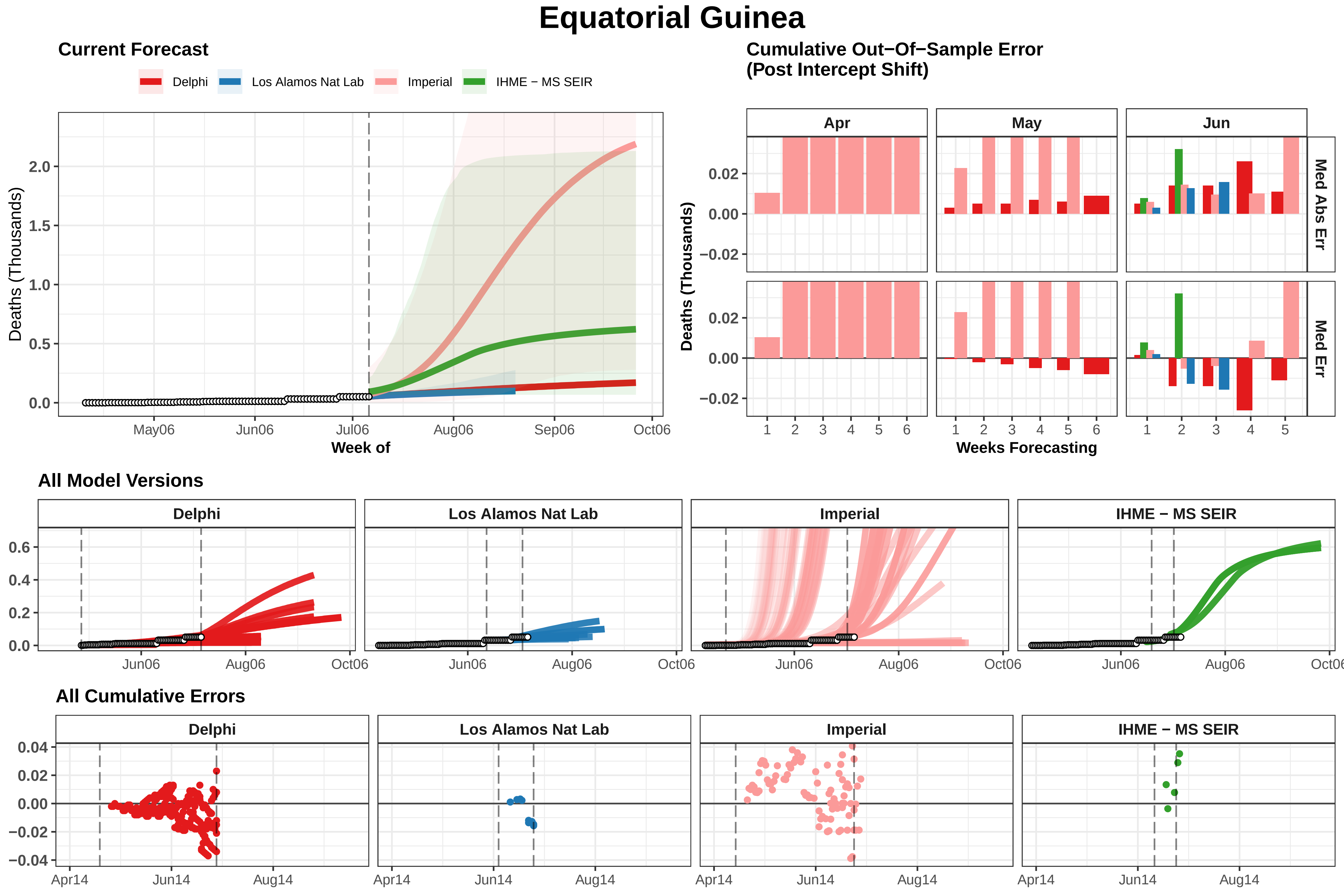
Equatorial Guinea
Current Forecast
Delphi
Los Alamos Nat Lab
Imperial
IHME − MS SEIR
Deaths (Thousands)
0.0
0.5
1.0
1.5
2.0
May06
Jun06
Jul06
Aug06
Sep06
Oct06
Week of
Cumulative Out−Of−Sample Error
(Post Intercept Shift)
Apr
May
Jun
Med Abs Err
Med Err
0.02
0.00
−0.02
Weeks Forecasting
All Model Versions
0.0
0.2
0.4
0.6
All Cumulative Errors
0.04
0.02
0.00
−0.02
−0.04
Apr14
Jun14
Aug14

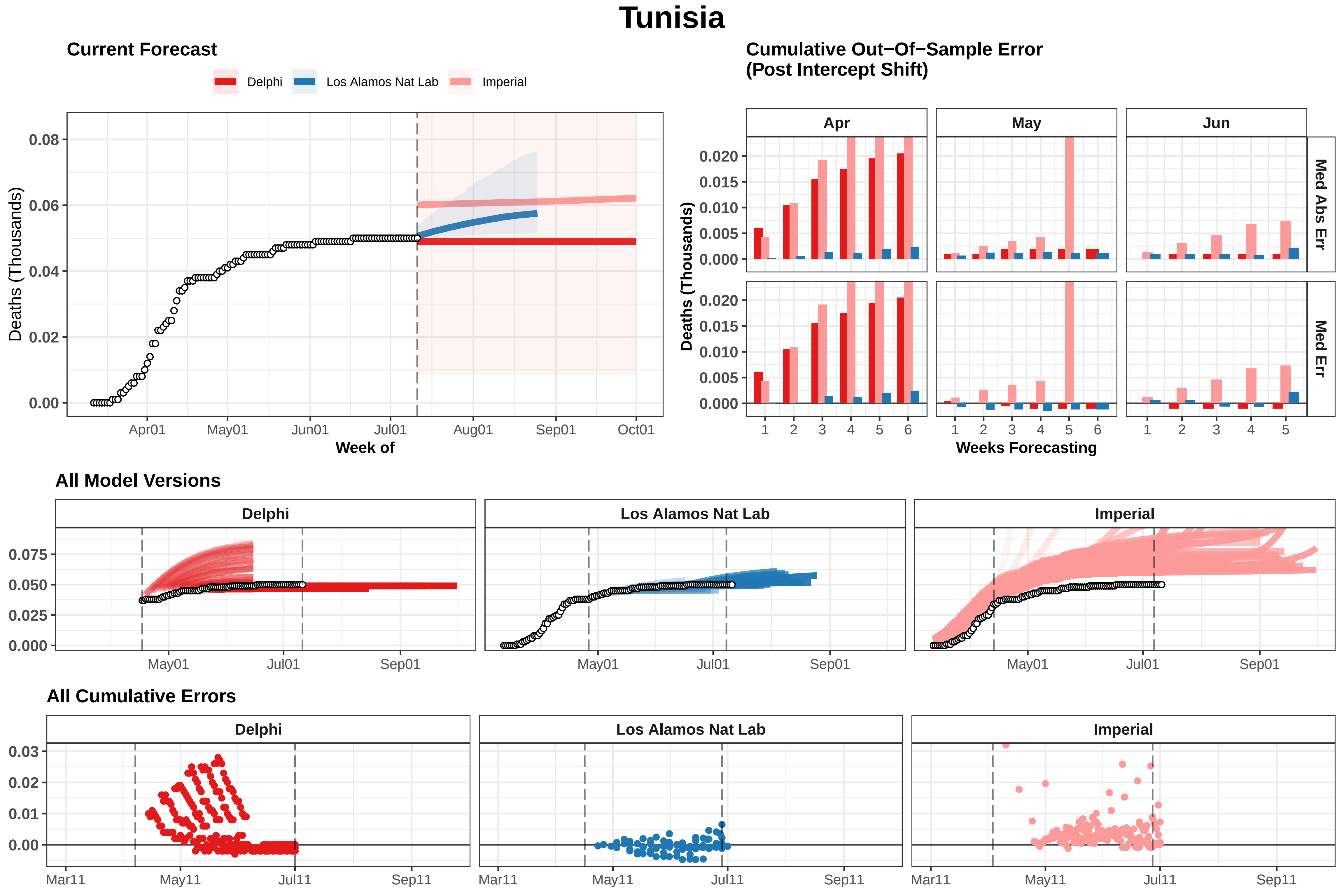
Tunisia
Current Forecast
Delphi
Los Alamos Nat Lab
Imperial
0.08
0.06
0.04
0.02
0.00
Deaths (Thousands)
Apr01
May01
Jun01
Jul01
Aug01
Sep01
Oct01
Week of
Cumulative Out−Of−Sample Error
(Post Intercept Shift)
Apr
May
Jun
0.020
0.015
0.010
0.005
0.000
Deaths (Thousands)
Med Abs Err
Med Err
1
2
3
4
5
6
Weeks Forecasting
All Model Versions
Delphi
Los Alamos Nat Lab
Imperial
0.075
0.050
0.025
0.000
May01
Jul01
Sep01
All Cumulative Errors
Delphi
Los Alamos Nat Lab
Imperial
0.03
0.02
0.01
0.00
Mar11
May11
Jul11
Sep11

Congo

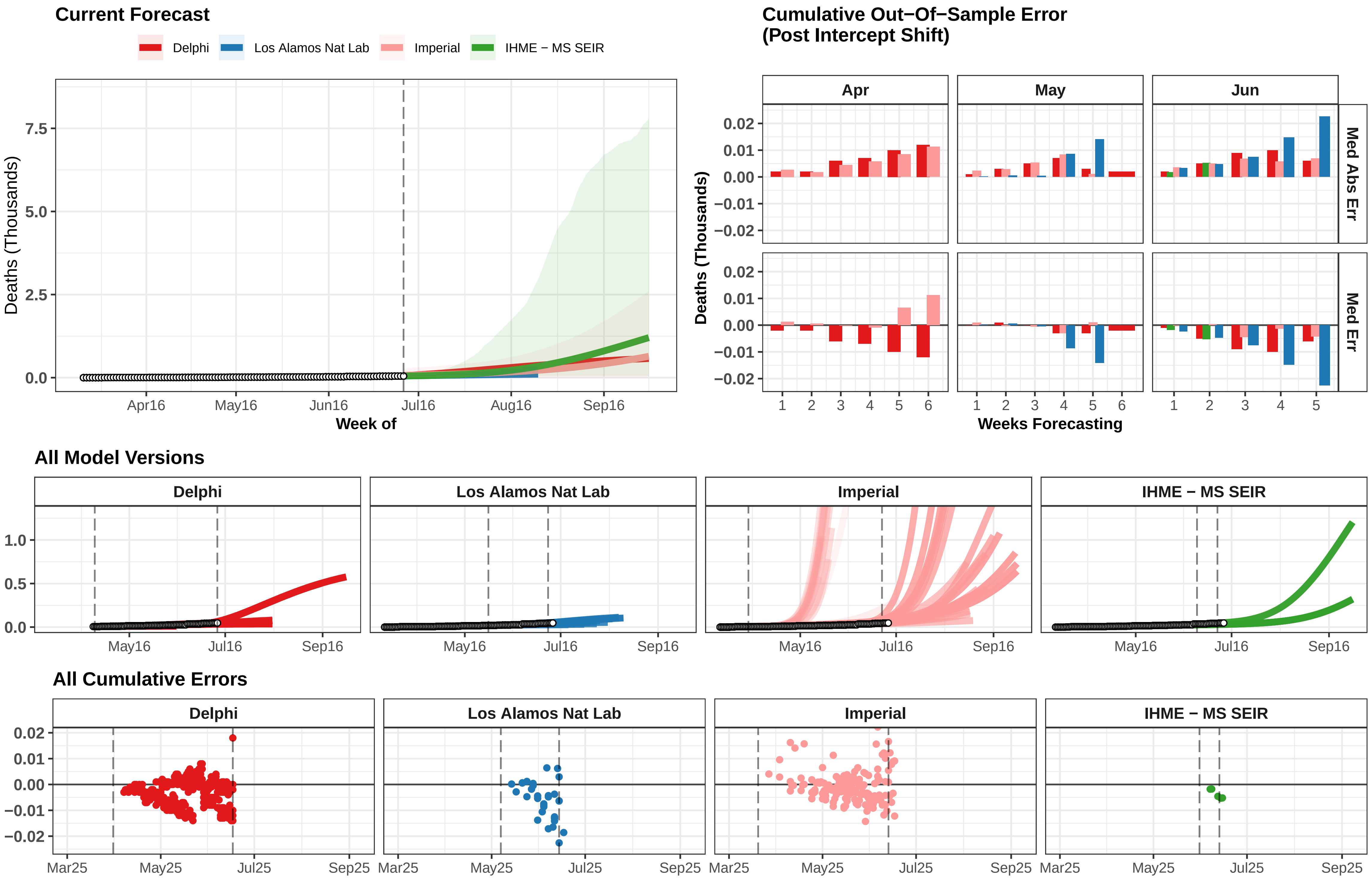
Current Forecast
Delphi
Los Alamos Nat Lab
Imperial
IHME − MS SEIR
Deaths (Thousands)
7.5
5.0
2.5
0.0
Apr16
May16
Jun16
Jul16
Aug16
Sep16
Week of
Cumulative Out−Of−Sample Error
(Post Intercept Shift)
Apr
May
Jun
Med Abs Err
Med Err
0.02
0.01
0.00
−0.01
−0.02
1
2
3
4
5
6
Weeks Forecasting
All Model Versions
Delphi
Los Alamos Nat Lab
Imperial
IHME − MS SEIR
1.0
0.5
0.0
May16
Jul16
Sep16
All Cumulative Errors
Mar25
May25
Jul25
Sep25

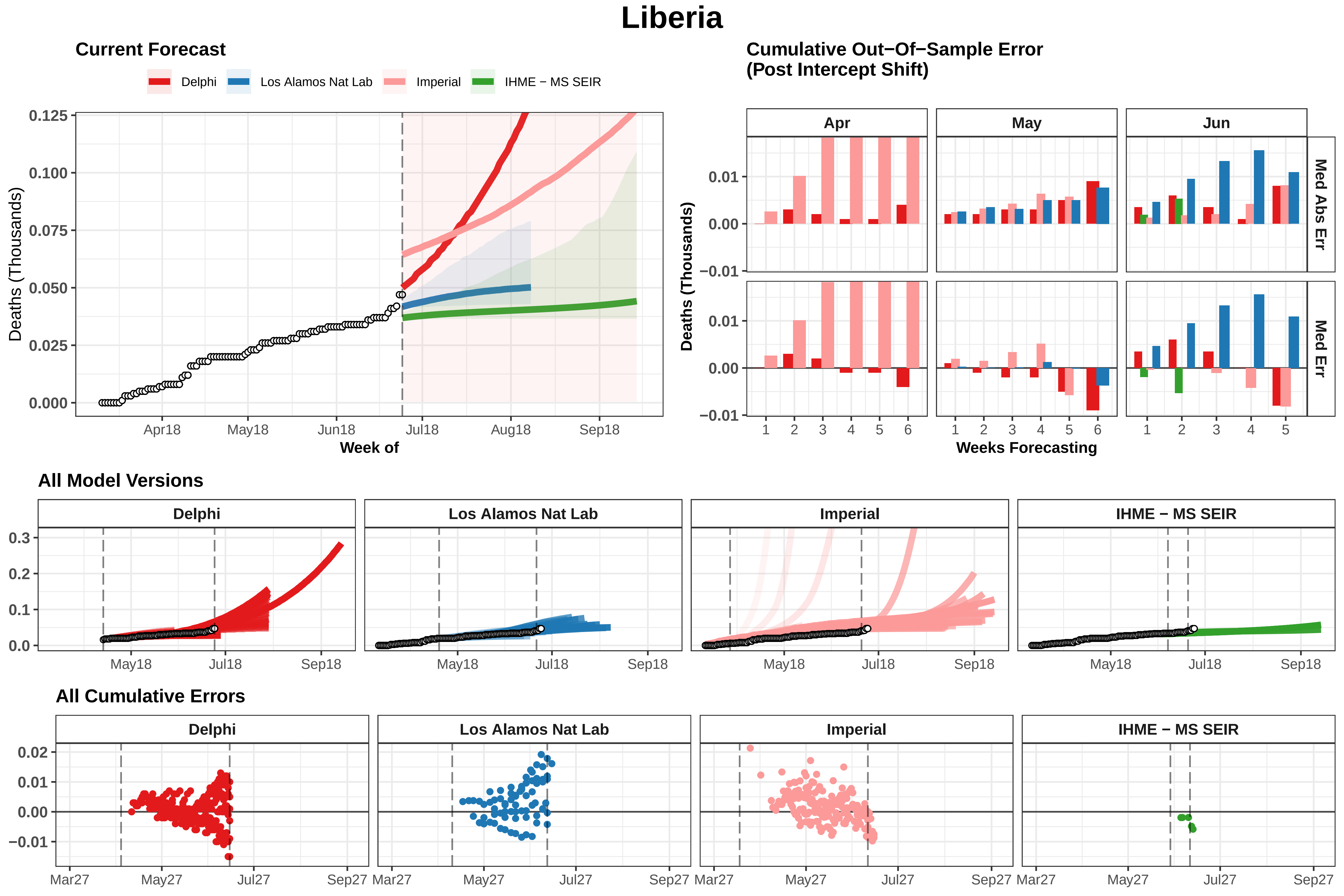

Liberia
Current Forecast
Delphi
Los Alamos Nat Lab
Imperial
IHME − MS SEIR
Deaths (Thousands)
0.125
0.100
0.075
0.050
0.025
0.000
Apr18
May18
Jun18
Jul18
Aug18
Sep18
Week of
Cumulative Out−Of−Sample Error
(Post Intercept Shift)
Apr
May
Jun
Med Abs Err
Med Err
0.01
0.00
−0.01
1
2
3
4
5
6
Weeks Forecasting
All Model Versions
0.3
0.2
0.1
0.0
All Cumulative Errors
0.02
Mar27
May27
Jul27
Sep27

Gabon

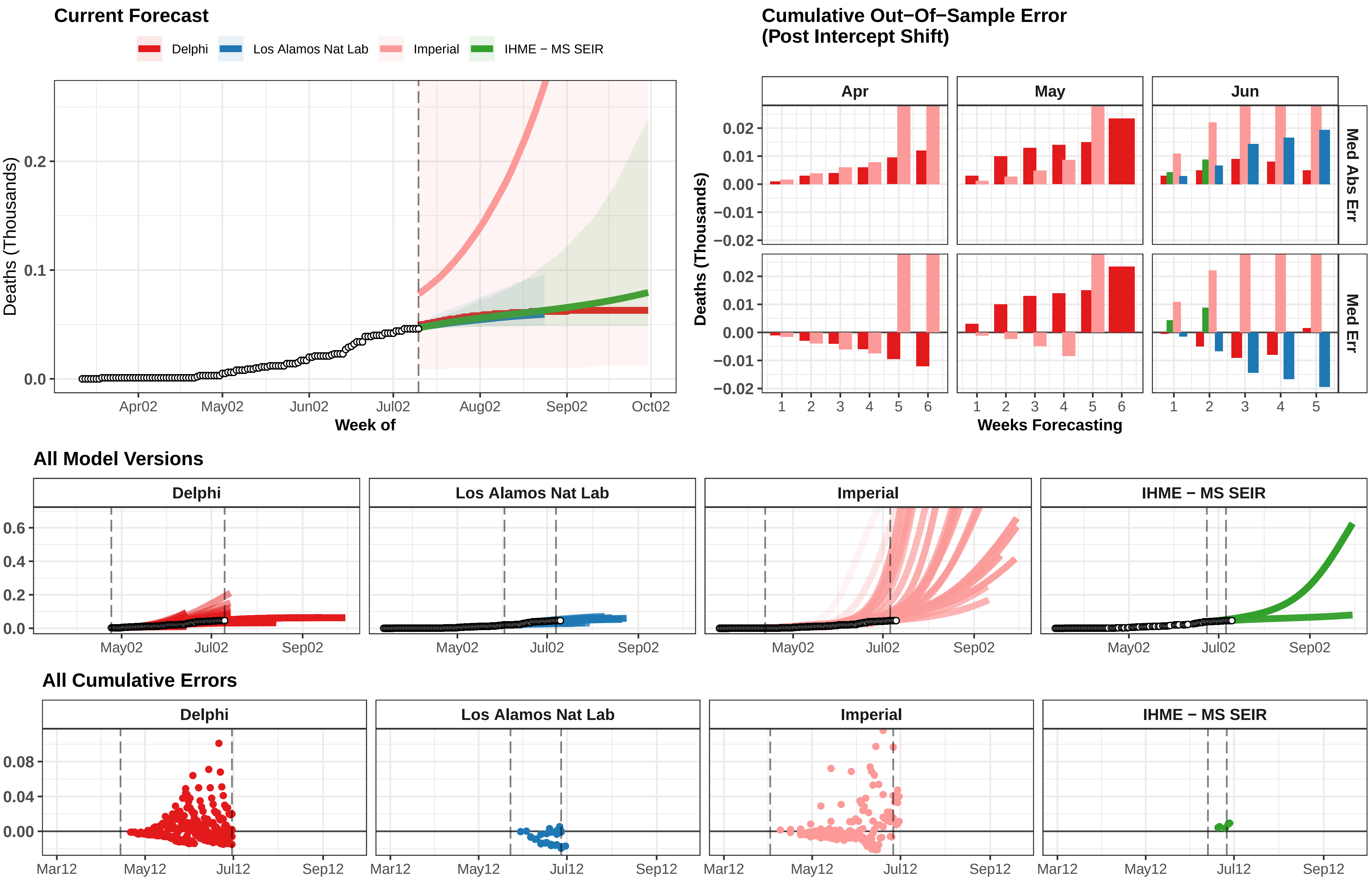

Current Forecast
Delphi
Los Alamos Nat Lab
Imperial
IHME − MS SEIR
Deaths (Thousands)
0.0
0.1
0.2
Apr02
May02
Jun02
Jul02
Aug02
Sep02
Oct02
Week of
Cumulative Out−Of−Sample Error
(Post Intercept Shift)
Apr
May
Jun
Med Abs Err
Med Err
Deaths (Thousands)
0.02
0.01
0.00
−0.01
−0.02
1
2
3
4
5
6
Weeks Forecasting
All Model Versions
Delphi
Los Alamos Nat Lab
Imperial
IHME − MS SEIR
0.0
0.2
0.4
0.6
May02
Jul02
Sep02
All Cumulative Errors
0.00
0.04
0.08
Mar12
May12
Jul12
Sep12

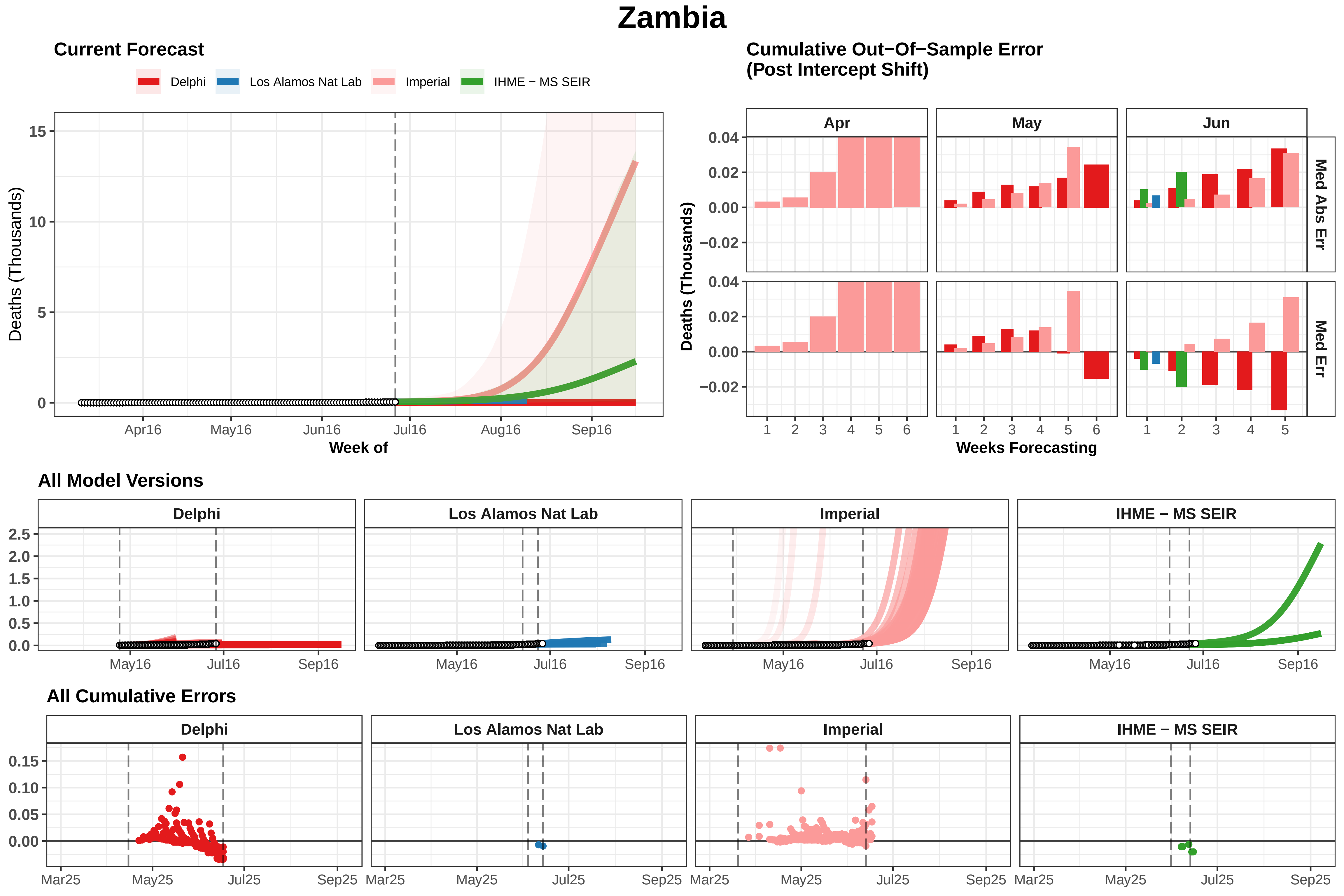

Zambia
Current Forecast
Delphi
Los Alamos Nat Lab
Imperial
IHME − MS SEIR
Deaths (Thousands)
Week of
Apr16
May16
Jun16
Jul16
Aug16
Sep16
Cumulative Out−Of−Sample Error
(Post Intercept Shift)
Apr
May
Jun
Med Abs Err
Med Err
Weeks Forecasting
All Model Versions
All Cumulative Errors
Mar25
May25
Jul25
Sep25

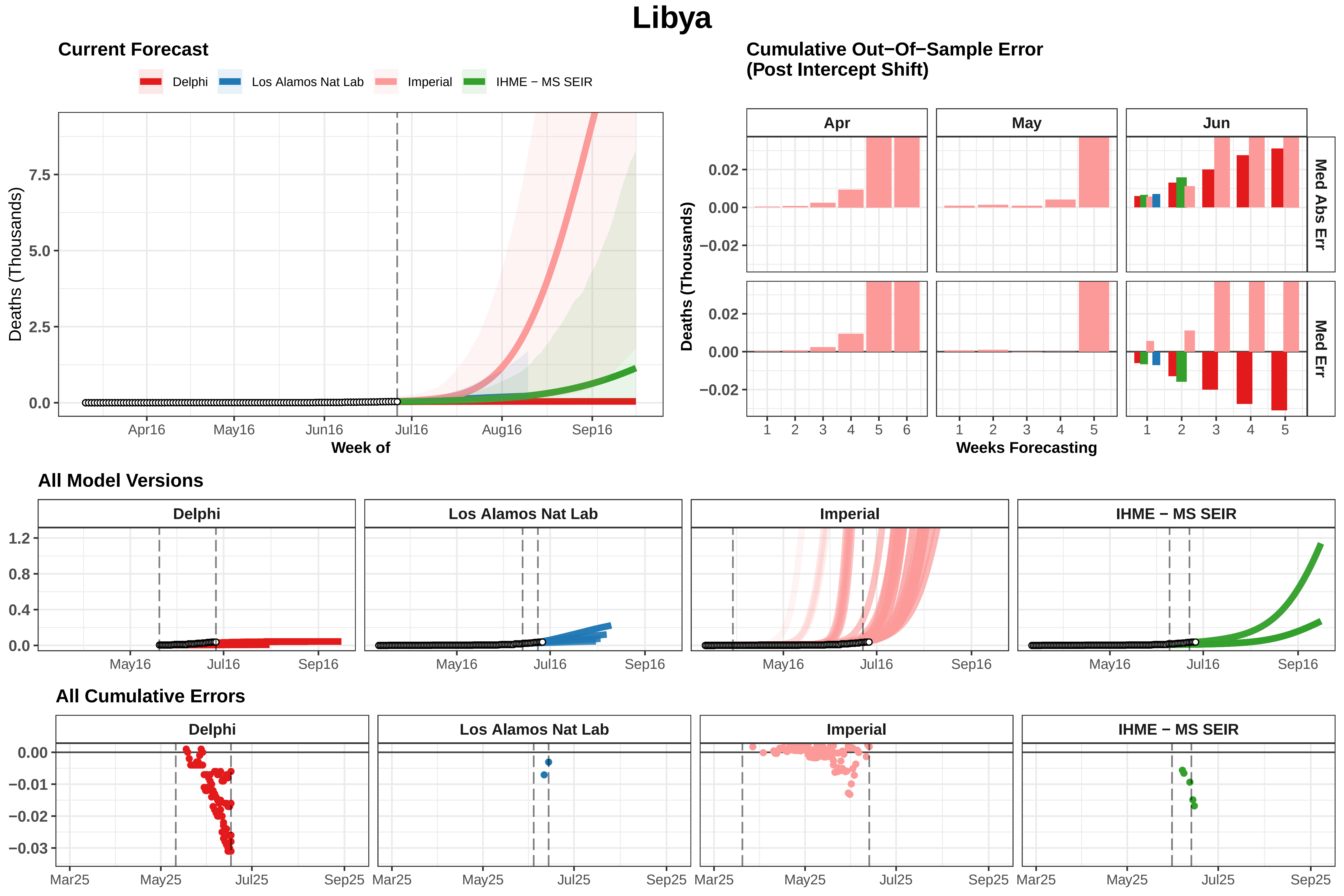

Libya
Current Forecast
Delphi
Los Alamos Nat Lab
Imperial
IHME − MS SEIR
Deaths (Thousands)
7.5
5.0
2.5
0.0
Apr16
May16
Jun16
Jul16
Aug16
Sep16
Week of
Cumulative Out−Of−Sample Error
(Post Intercept Shift)
Apr
May
Jun
Med Abs Err
Med Err
0.02
0.00
−0.02
Weeks Forecasting
All Model Versions
1.2
0.8
0.4
0.0
All Cumulative Errors
0.00
−0.01
−0.02
−0.03
Mar25
May25
Jul25
Sep25

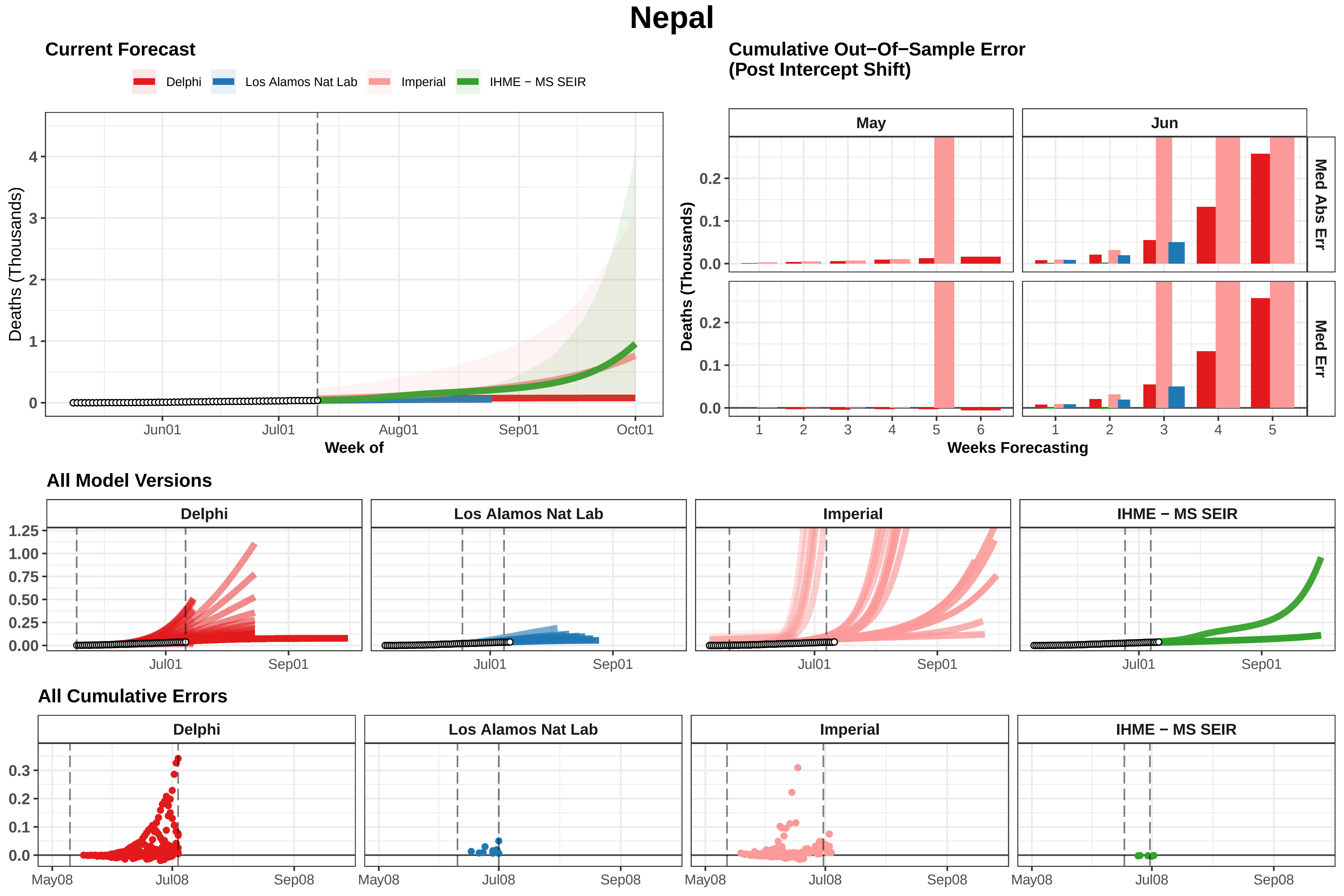

Nepal
Current Forecast
Delphi
Los Alamos Nat Lab
Imperial
IHME − MS SEIR
Deaths (Thousands)
Jun01
Jul01
Aug01
Sep01
Oct01
Week of
Cumulative Out−Of−Sample Error
(Post Intercept Shift)
May
Jun
Med Abs Err
Med Err
Weeks Forecasting
All Model Versions
All Cumulative Errors
May08
Jul08
Sep08

South Sudan

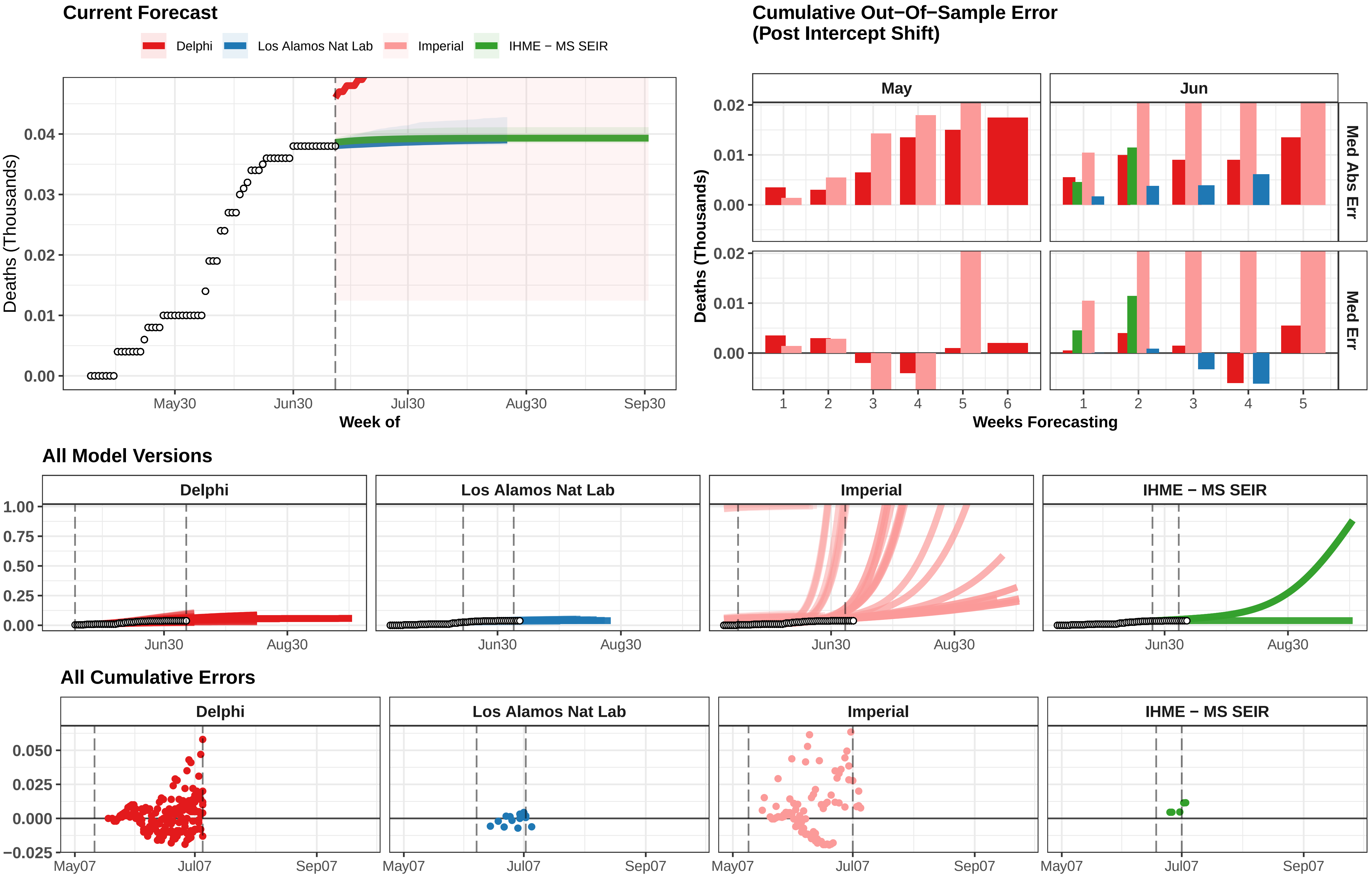

Current Forecast
Delphi
Los Alamos Nat Lab
Imperial
IHME − MS SEIR
Deaths (Thousands)
0.00
0.01
0.02
0.03
0.04
May30
Jun30
Jul30
Aug30
Sep30
Week of
Cumulative Out−Of−Sample Error
(Post Intercept Shift)
May
Jun
Med Abs Err
Med Err
Deaths (Thousands)
0.02
0.01
0.00
1
2
3
4
5
6
Weeks Forecasting
All Model Versions
Delphi
Los Alamos Nat Lab
Imperial
IHME − MS SEIR
1.00
0.75
0.50
0.25
0.00
Jun30
Aug30
All Cumulative Errors
Delphi
Los Alamos Nat Lab
Imperial
IHME − MS SEIR
0.050
0.025
0.000
−0.025
May07
Jul07
Sep07

Guinea

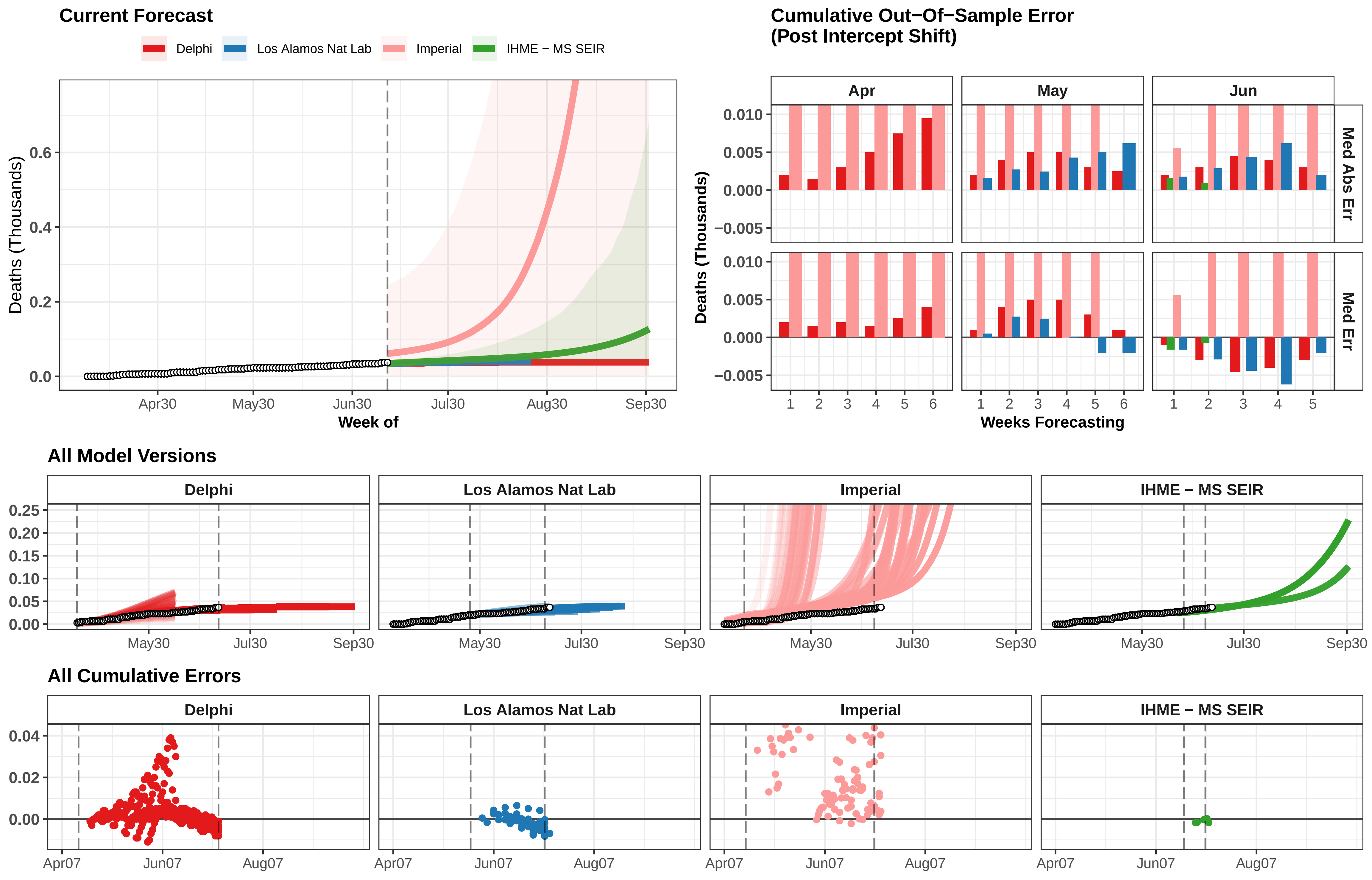

Current Forecast
Delphi
Los Alamos Nat Lab
Imperial
IHME − MS SEIR
Deaths (Thousands)
0.0
0.2
0.4
0.6
Apr30
May30
Jun30
Jul30
Aug30
Sep30
Week of
Cumulative Out−Of−Sample Error
(Post Intercept Shift)
Apr
May
Jun
Med Abs Err
Med Err
0.010
0.005
0.000
−0.005
Weeks Forecasting
All Model Versions
0.25
0.20
0.15
0.10
0.05
0.00
All Cumulative Errors
0.04
0.02
0.00
Apr07
Jun07
Aug07

Lebanon

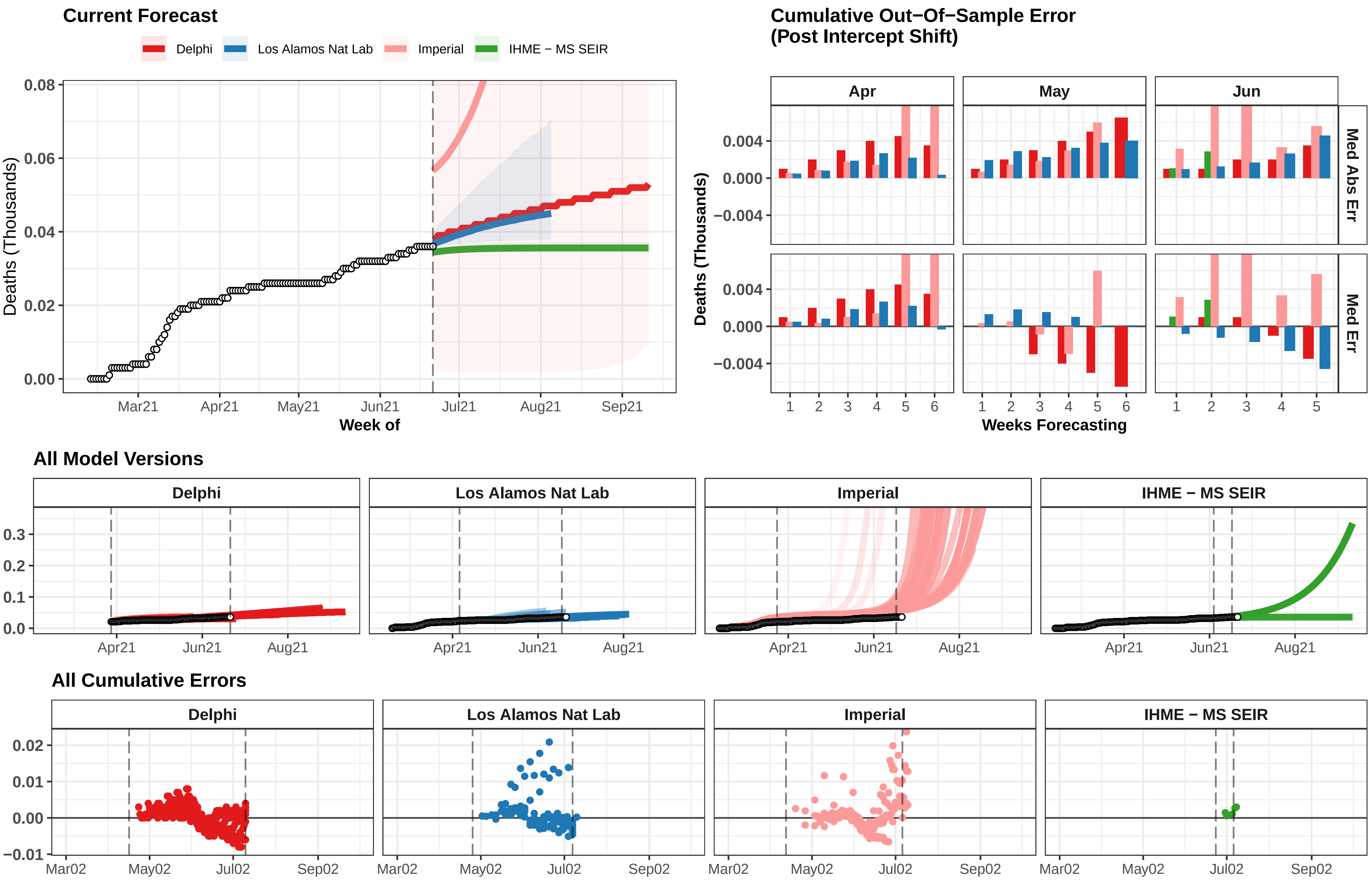
Current Forecast
Delphi
Los Alamos Nat Lab
Imperial
IHME − MS SEIR
Deaths (Thousands)
Week of
Cumulative Out−Of−Sample Error
(Post Intercept Shift)
Apr
May
Jun
Med Abs Err
Med Err
Weeks Forecasting
All Model Versions
All Cumulative Errors

Madagascar

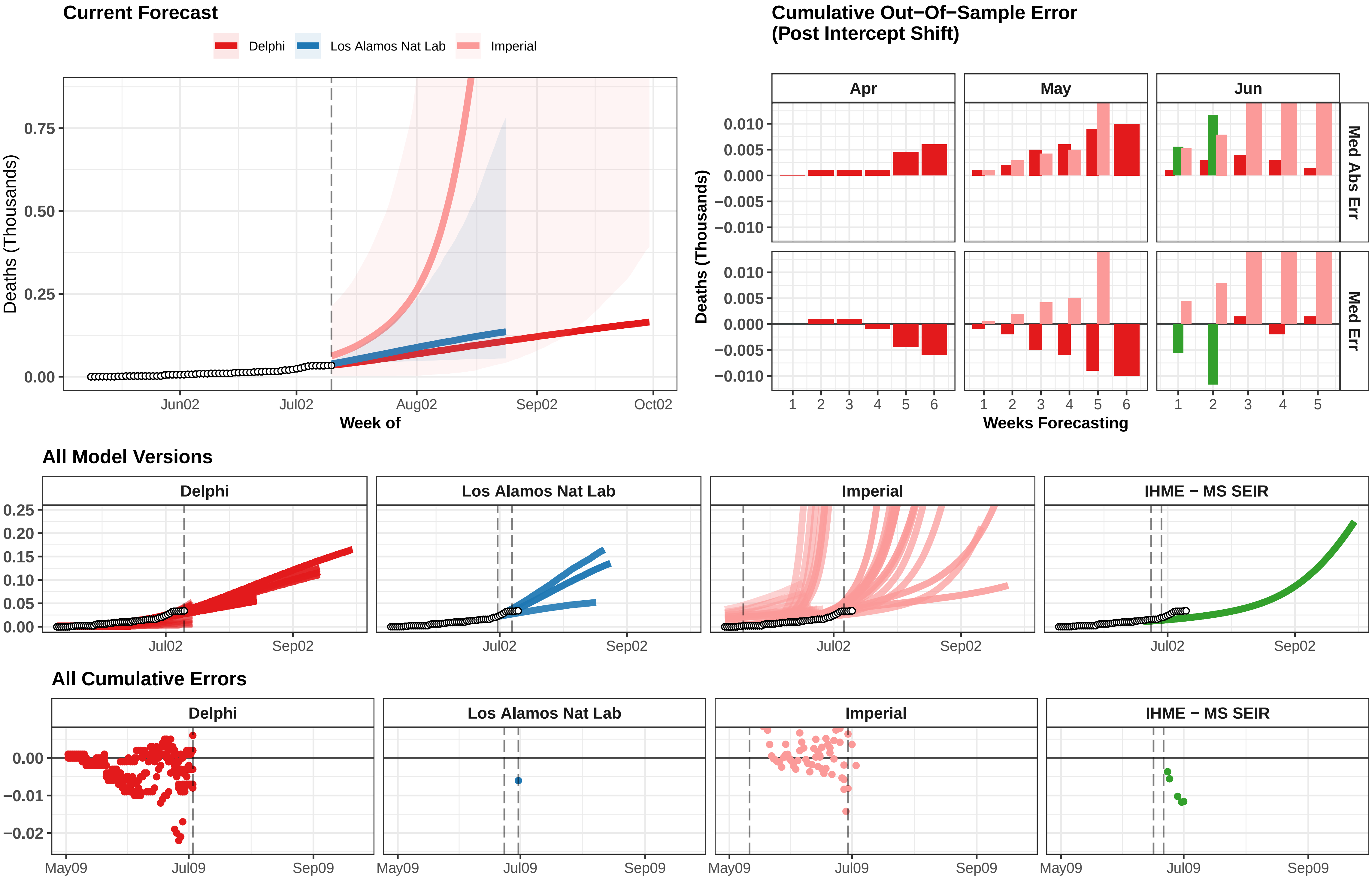

Current Forecast
Delphi
Los Alamos Nat Lab
Imperial
Deaths (Thousands)
0.75
0.50
0.25
0.00
Jun02
Jul02
Aug02
Sep02
Oct02
Week of
Cumulative Out−Of−Sample Error
(Post Intercept Shift)
Apr
May
Jun
Med Abs Err
Med Err
0.010
0.005
0.000
−0.005
−0.010
Weeks Forecasting
All Model Versions
Delphi
Los Alamos Nat Lab
Imperial
IHME − MS SEIR
0.25
0.20
0.15
0.10
0.05
0.00
Jul02
Sep02
All Cumulative Errors
0.00
−0.01
−0.02
May09
Jul09
Sep09

Malawi

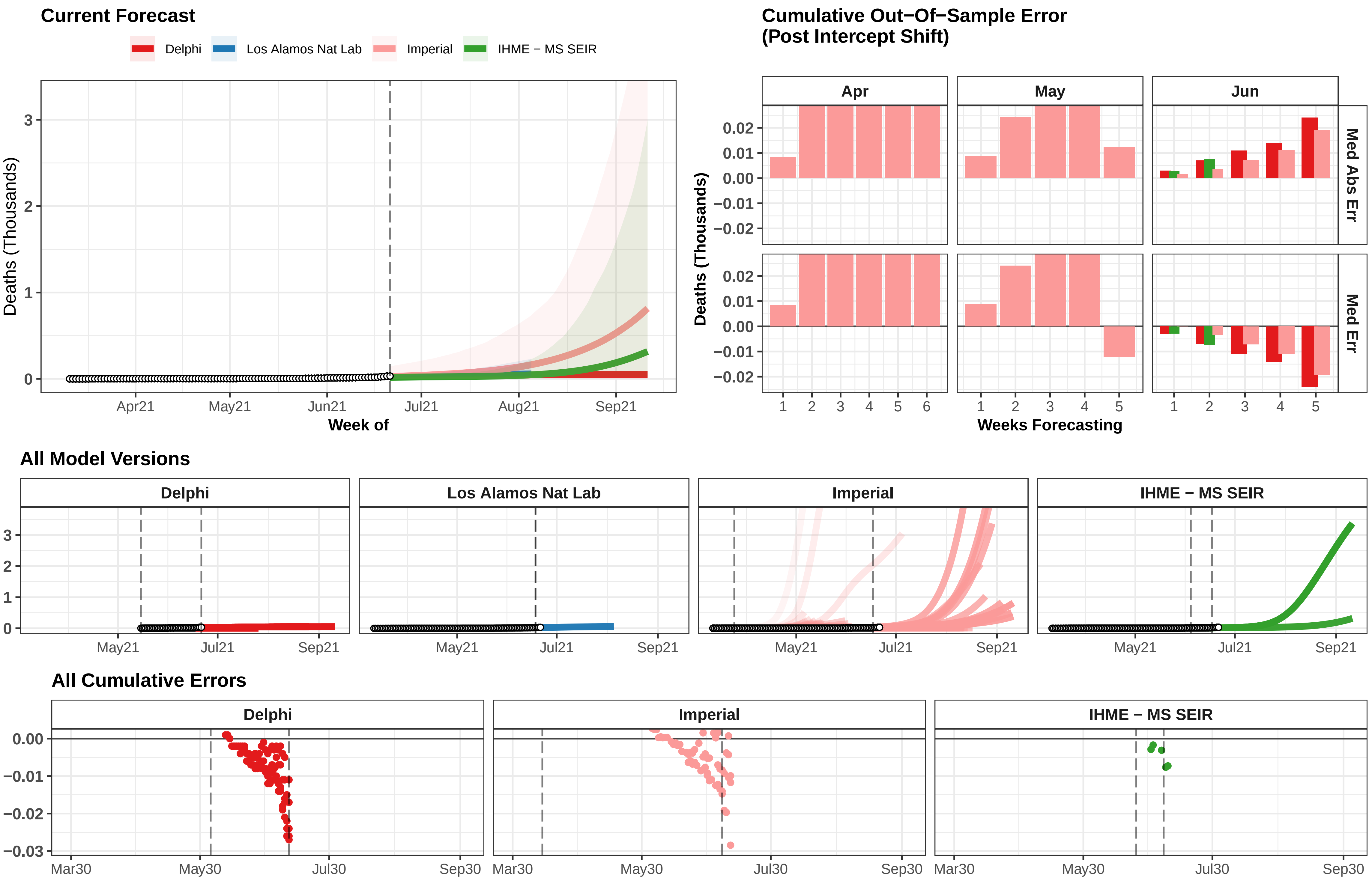
Current Forecast
Delphi
Los Alamos Nat Lab
Imperial
IHME − MS SEIR
Deaths (Thousands)
Week of
Apr21 May21 Jun21 Jul21 Aug21 Sep21
Cumulative Out−Of−Sample Error
(Post Intercept Shift)
Apr
May
Jun
Med Abs Err
Med Err
Weeks Forecasting
All Model Versions
Delphi
Los Alamos Nat Lab
Imperial
IHME − MS SEIR
May21 Jul21 Sep21
All Cumulative Errors
Delphi
Imperial
IHME − MS SEIR
Mar30 May30 Jul30 Sep30

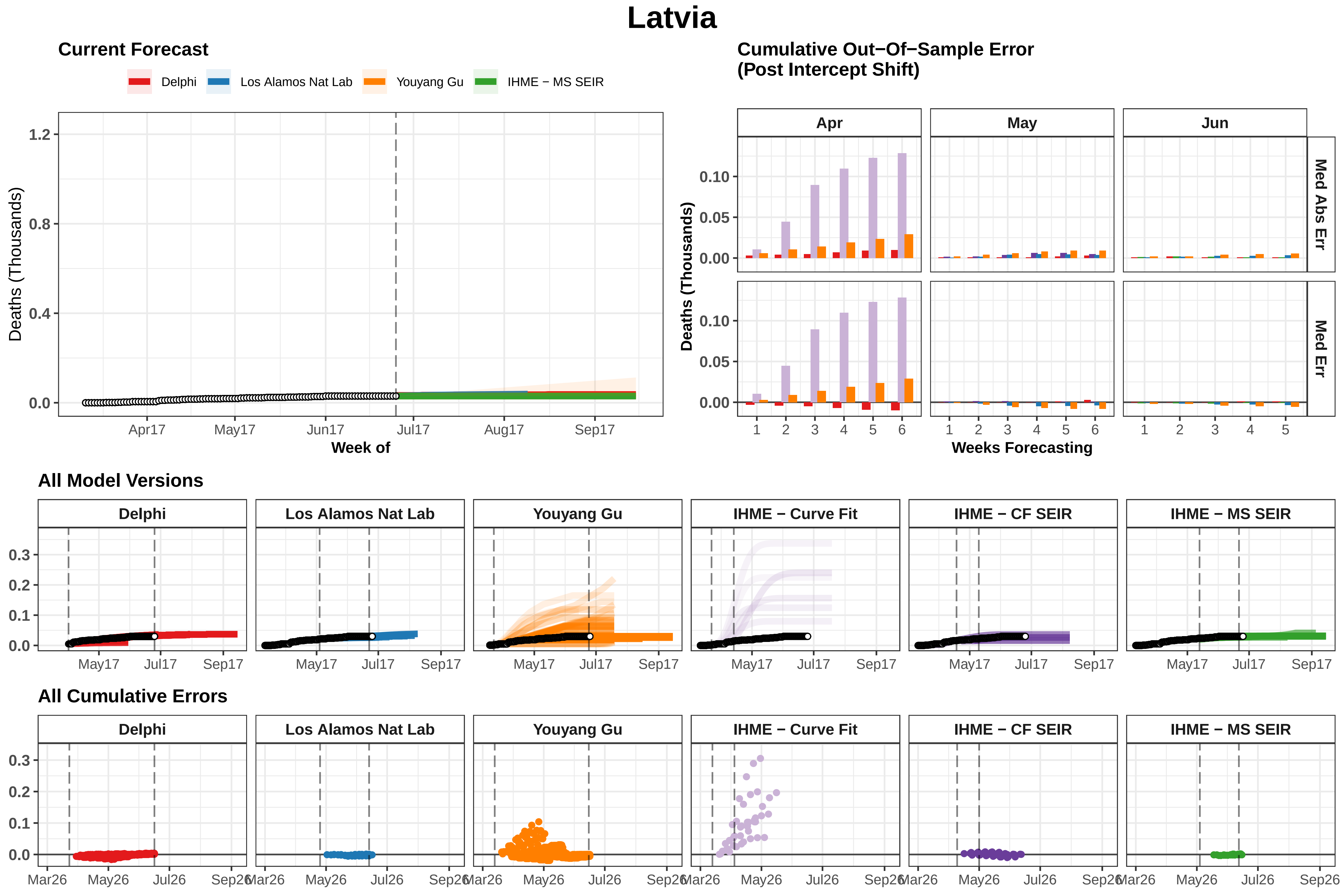

Latvia
Current Forecast
Delphi
Los Alamos Nat Lab
Youyang Gu
IHME − MS SEIR
Deaths (Thousands)
1.2
0.8
0.4
0.0
Apr17
May17
Jun17
Jul17
Aug17
Sep17
Week of
Cumulative Out−Of−Sample Error
(Post Intercept Shift)
Apr
May
Jun
Med Abs Err
Med Err
0.10
0.05
0.00
Weeks Forecasting
All Model Versions
Delphi
Los Alamos Nat Lab
Youyang Gu
IHME − Curve Fit
IHME − CF SEIR
IHME − MS SEIR
0.3
0.2
0.1
0.0
All Cumulative Errors
Mar26
May26
Jul26
Sep26

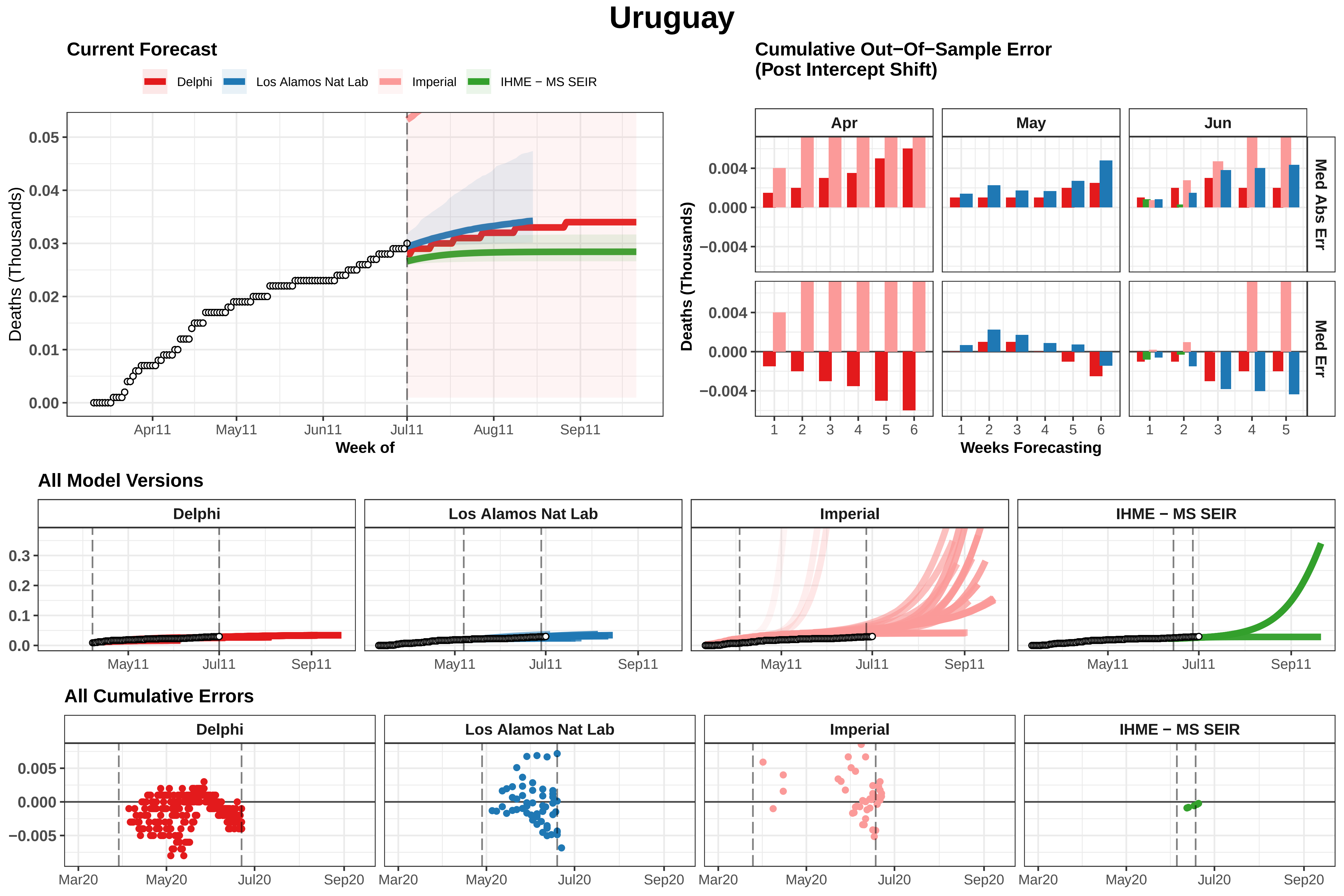
Uruguay
Current Forecast
Delphi
Los Alamos Nat Lab
Imperial
IHME − MS SEIR
Deaths (Thousands)
Week of
Apr11
May11
Jun11
Jul11
Aug11
Sep11
Cumulative Out−Of−Sample Error
(Post Intercept Shift)
Apr
May
Jun
Med Abs Err
Med Err
Weeks Forecasting
All Model Versions
All Cumulative Errors
Mar20
May20
Jul20
Sep20

Montana

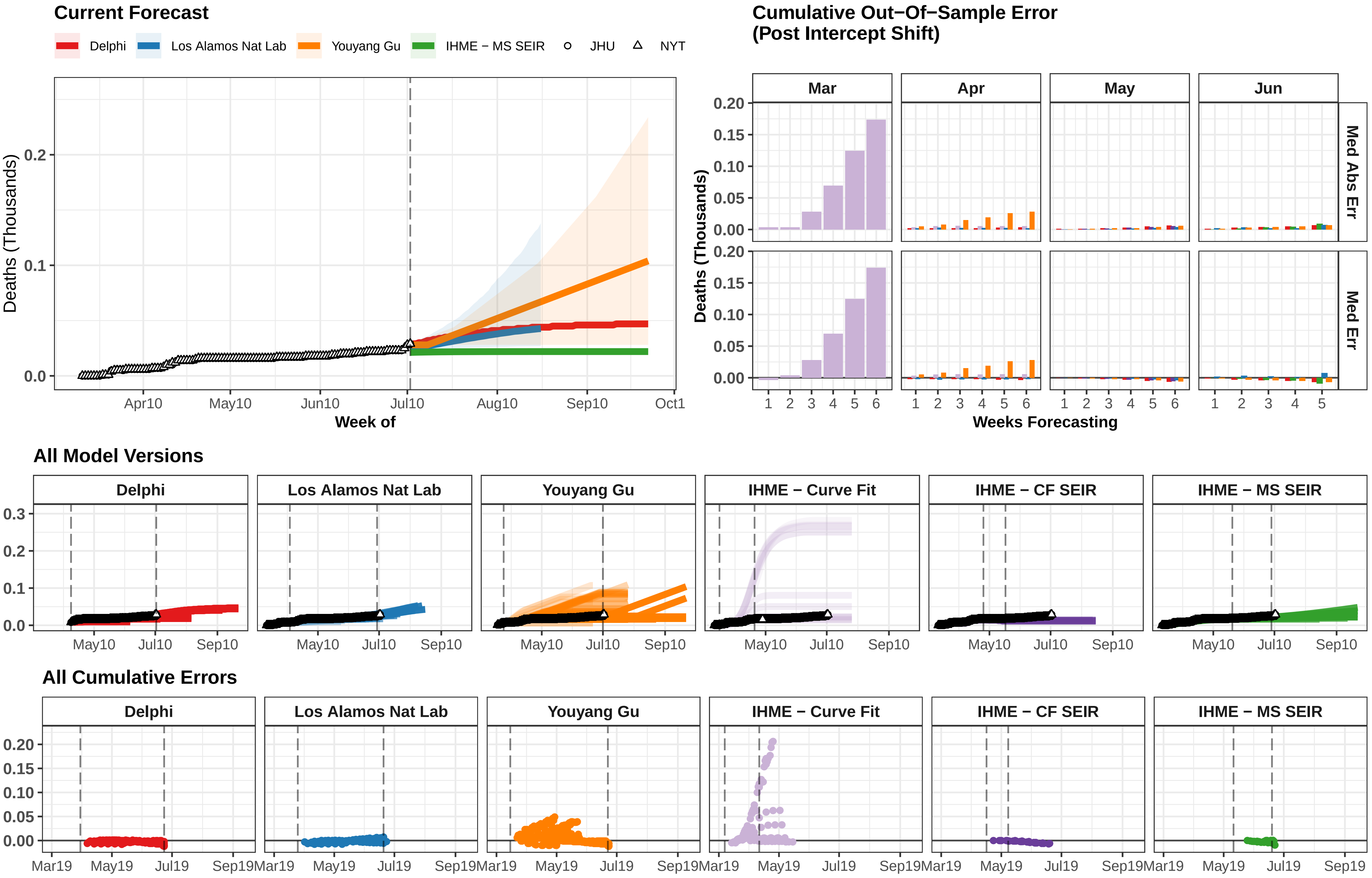
Current Forecast
Delphi
Los Alamos Nat Lab
Youyang Gu
IHME − MS SEIR
JHU
NYT
Deaths (Thousands)
0.0
0.1
0.2
Apr10
May10
Jun10
Jul10
Aug10
Sep10
Oct1
Week of
Cumulative Out−Of−Sample Error
(Post Intercept Shift)
Mar
Apr
May
Jun
Med Abs Err
Med Err
0.00
0.05
0.10
0.15
0.20
1
2
3
4
5
6
Weeks Forecasting
All Model Versions
Delphi
Los Alamos Nat Lab
Youyang Gu
IHME − Curve Fit
IHME − CF SEIR
IHME − MS SEIR
0.0
0.1
0.2
0.3
May10
Jul10
Sep10
All Cumulative Errors
Mar19
May19
Jul19
Sep19

Costa Rica

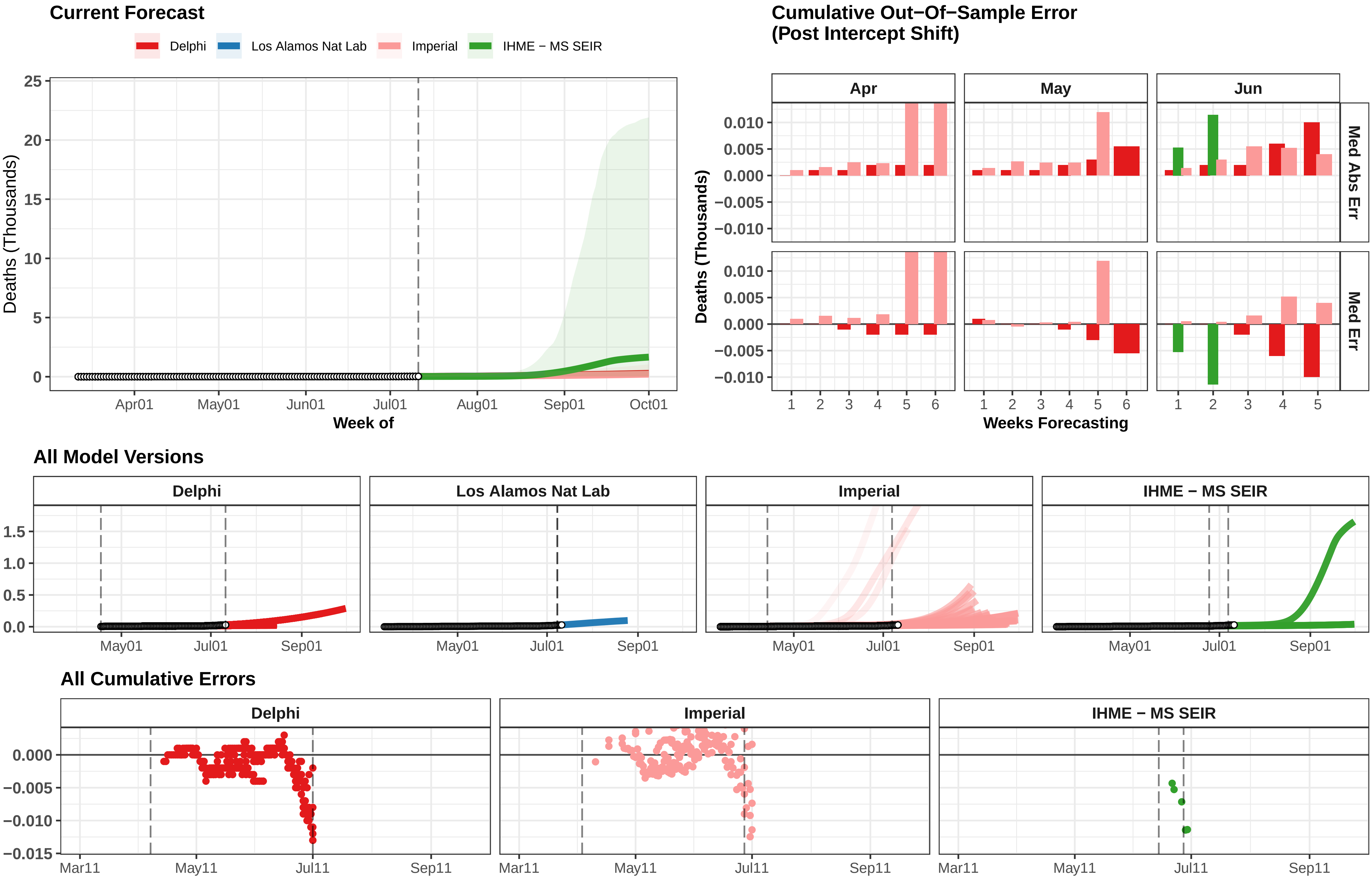
Current Forecast
Delphi
Los Alamos Nat Lab
Imperial
IHME − MS SEIR
Deaths (Thousands)
Week of
Apr01
May01
Jun01
Jul01
Aug01
Sep01
Oct01
Cumulative Out−Of−Sample Error
(Post Intercept Shift)
Apr
May
Jun
Med Abs Err
Med Err
Weeks Forecasting
All Model Versions
All Cumulative Errors
Mar11
May11
Jul11
Sep11

Slovakia

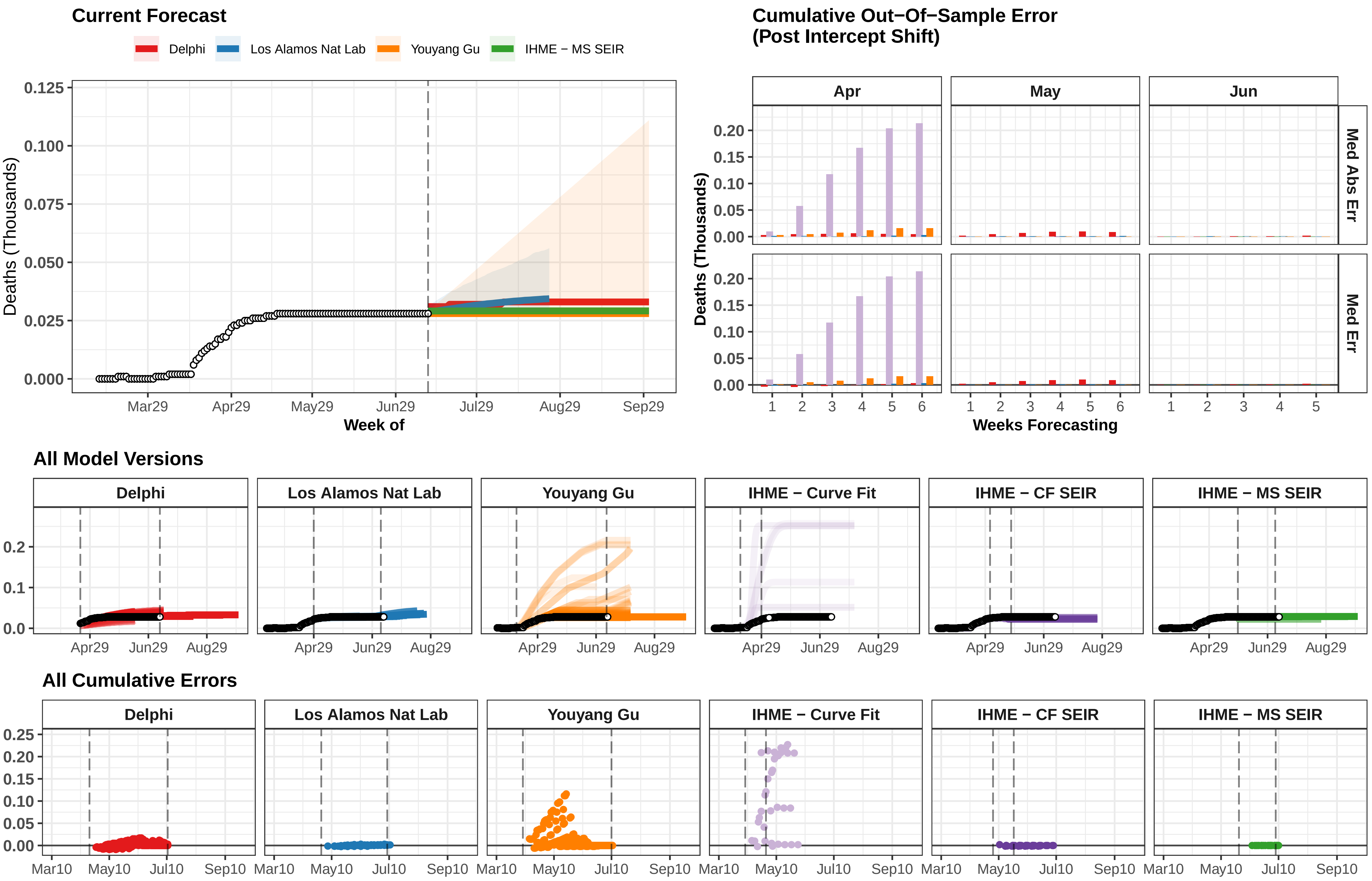

Current Forecast
Delphi
Los Alamos Nat Lab
Youyang Gu
IHME − MS SEIR
Deaths (Thousands)
0.125
0.100
0.075
0.050
0.025
0.000
Mar29
Apr29
May29
Jun29
Jul29
Aug29
Sep29
Week of
Cumulative Out−Of−Sample Error
(Post Intercept Shift)
Apr
May
Jun
Med Abs Err
Med Err
0.20
0.15
0.10
0.05
0.00
1
2
3
4
5
6
Weeks Forecasting
All Model Versions
Delphi
Los Alamos Nat Lab
Youyang Gu
IHME − Curve Fit
IHME − CF SEIR
IHME − MS SEIR
0.2
0.1
0.0
Apr29
Jun29
Aug29
All Cumulative Errors
0.25
0.20
0.15
0.10
0.05
0.00
Mar10
May10
Jul10
Sep10

Benin

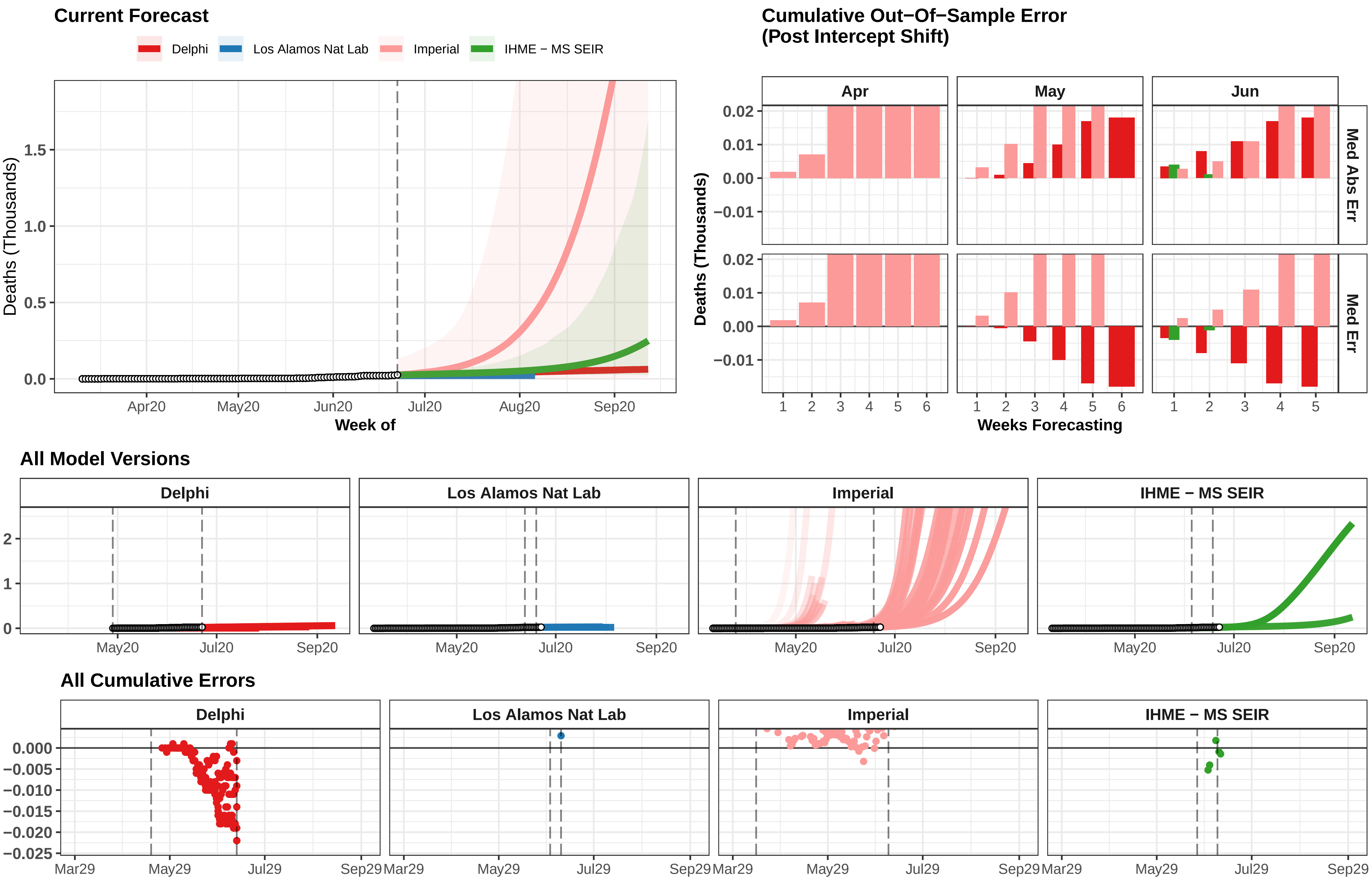

Current Forecast
Delphi
Los Alamos Nat Lab
Imperial
IHME − MS SEIR
Deaths (Thousands)
0.0
0.5
1.0
1.5
Apr20
May20
Jun20
Jul20
Aug20
Sep20
Week of
Cumulative Out−Of−Sample Error
(Post Intercept Shift)
Apr
May
Jun
Med Abs Err
Med Err
0.02
0.01
0.00
−0.01
Weeks Forecasting
All Model Versions
Delphi
Los Alamos Nat Lab
Imperial
IHME − MS SEIR
0
1
2
May20
Jul20
Sep20
All Cumulative Errors
0.000
−0.005
−0.010
−0.015
−0.020
−0.025
Mar29
May29
Jul29
Sep29

Guinea−Bissau

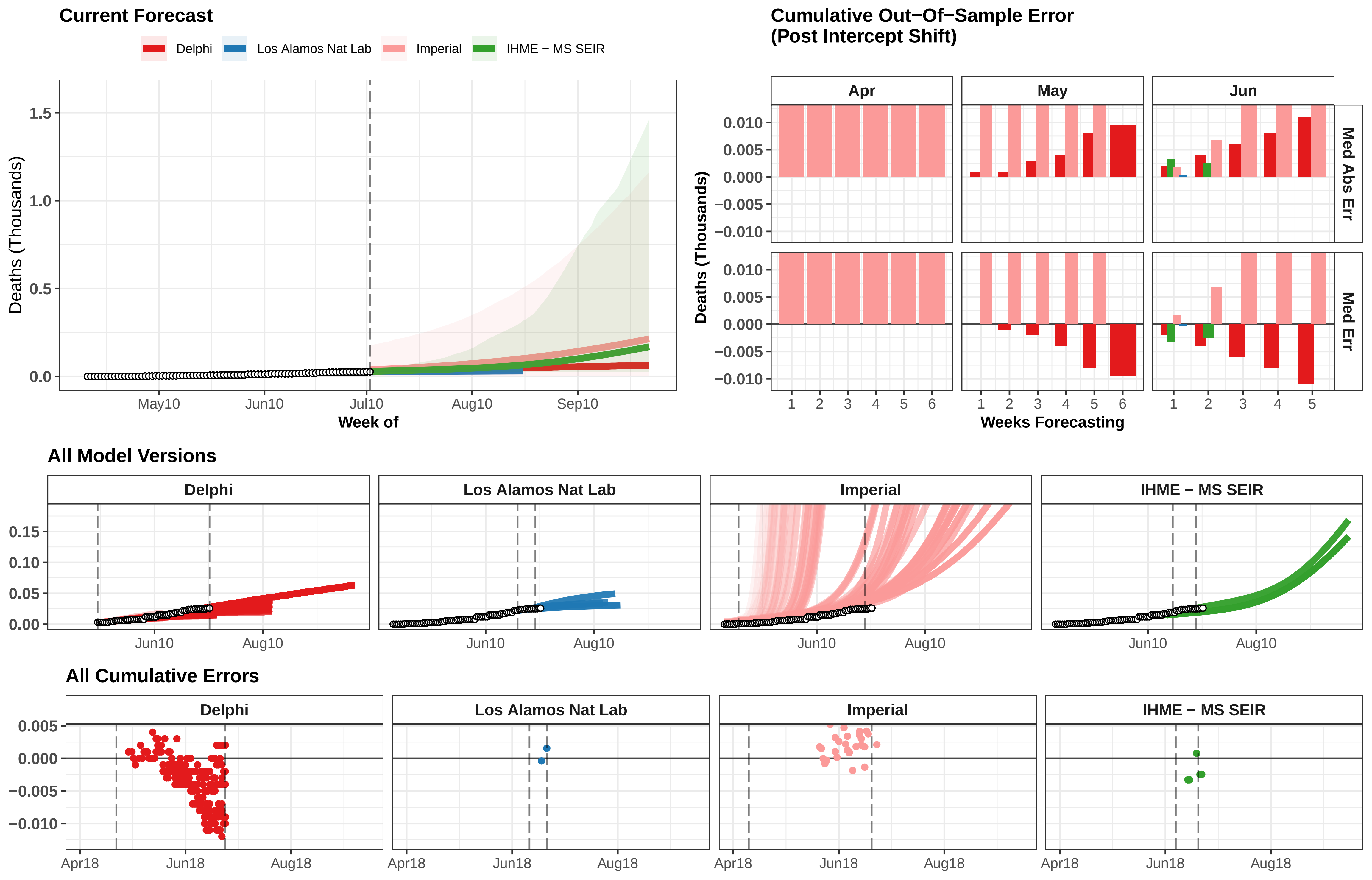

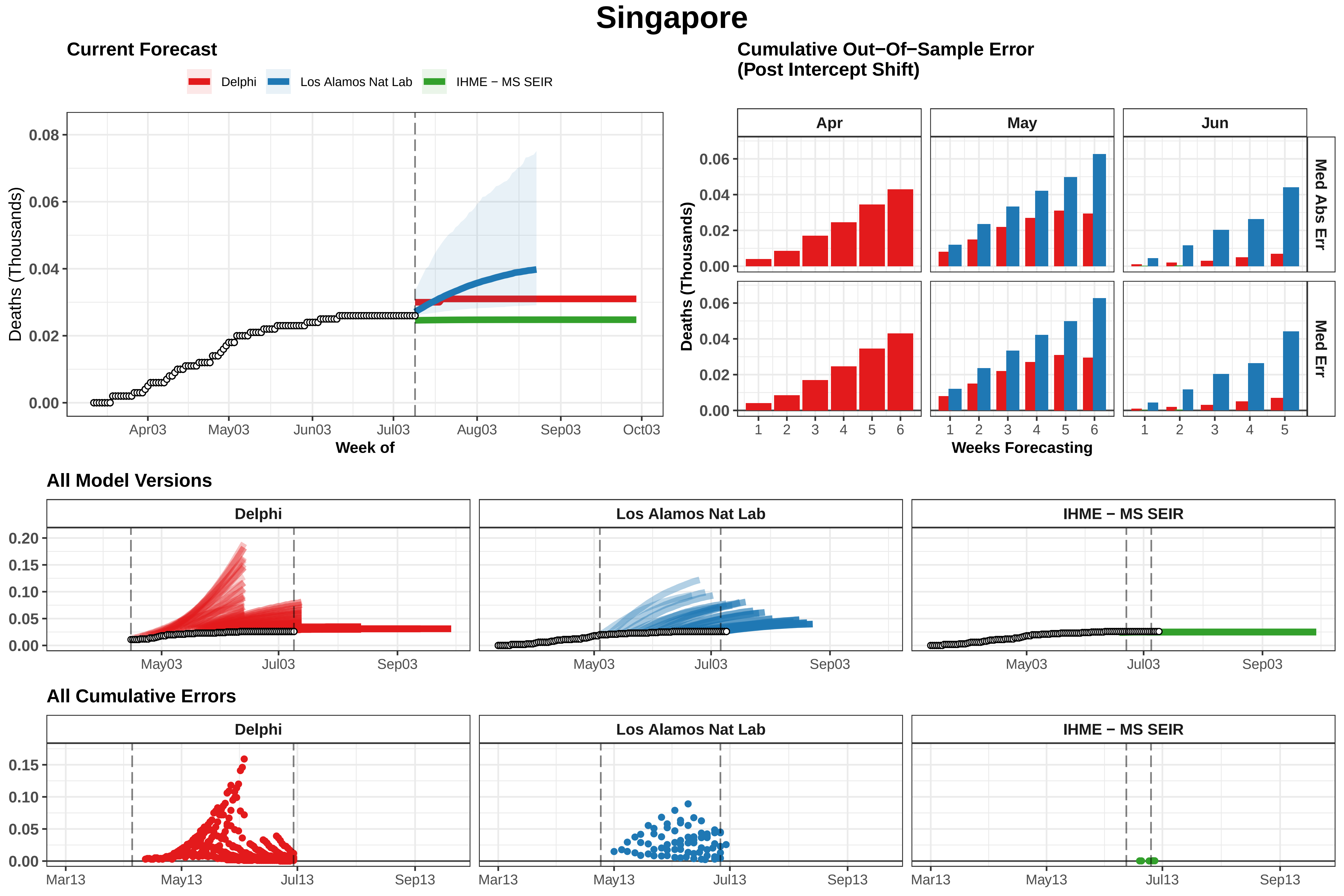
Singapore
Current Forecast
Delphi
Los Alamos Nat Lab
IHME − MS SEIR
Deaths (Thousands)
0.00
0.02
0.04
0.06
0.08
Apr03
May03
Jun03
Jul03
Aug03
Sep03
Oct03
Week of
Cumulative Out−Of−Sample Error
(Post Intercept Shift)
Apr
May
Jun
Med Abs Err
Med Err
Weeks Forecasting
All Model Versions
Delphi
Los Alamos Nat Lab
IHME − MS SEIR
0.00
0.05
0.10
0.15
0.20
May03
Jul03
Sep03
All Cumulative Errors
Mar13
May13
Jul13
Sep13

Angola

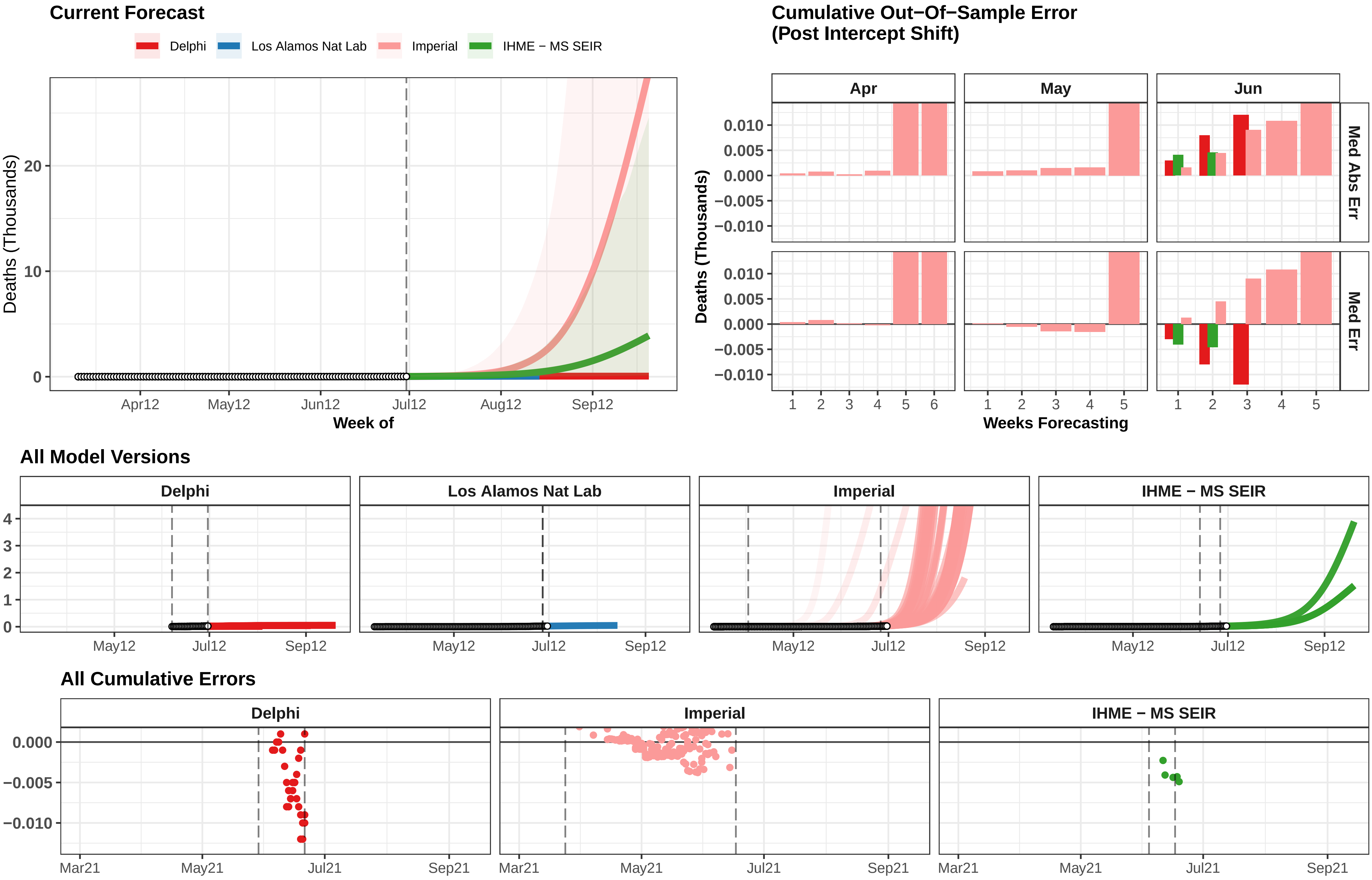
Current Forecast
Delphi
Los Alamos Nat Lab
Imperial
IHME − MS SEIR
Deaths (Thousands)
0
10
20
Apr12
May12
Jun12
Jul12
Aug12
Sep12
Week of
Cumulative Out−Of−Sample Error
(Post Intercept Shift)
Apr
May
Jun
Med Abs Err
Med Err
Deaths (Thousands)
0.010
0.005
0.000
−0.005
−0.010
1
2
3
4
5
6
Weeks Forecasting
All Model Versions
Delphi
Los Alamos Nat Lab
Imperial
IHME − MS SEIR
4
3
2
1
0
May12
Jul12
Sep12
All Cumulative Errors
Delphi
Imperial
IHME − MS SEIR
0.000
−0.005
−0.010
Mar21
May21
Jul21
Sep21

Montenegro

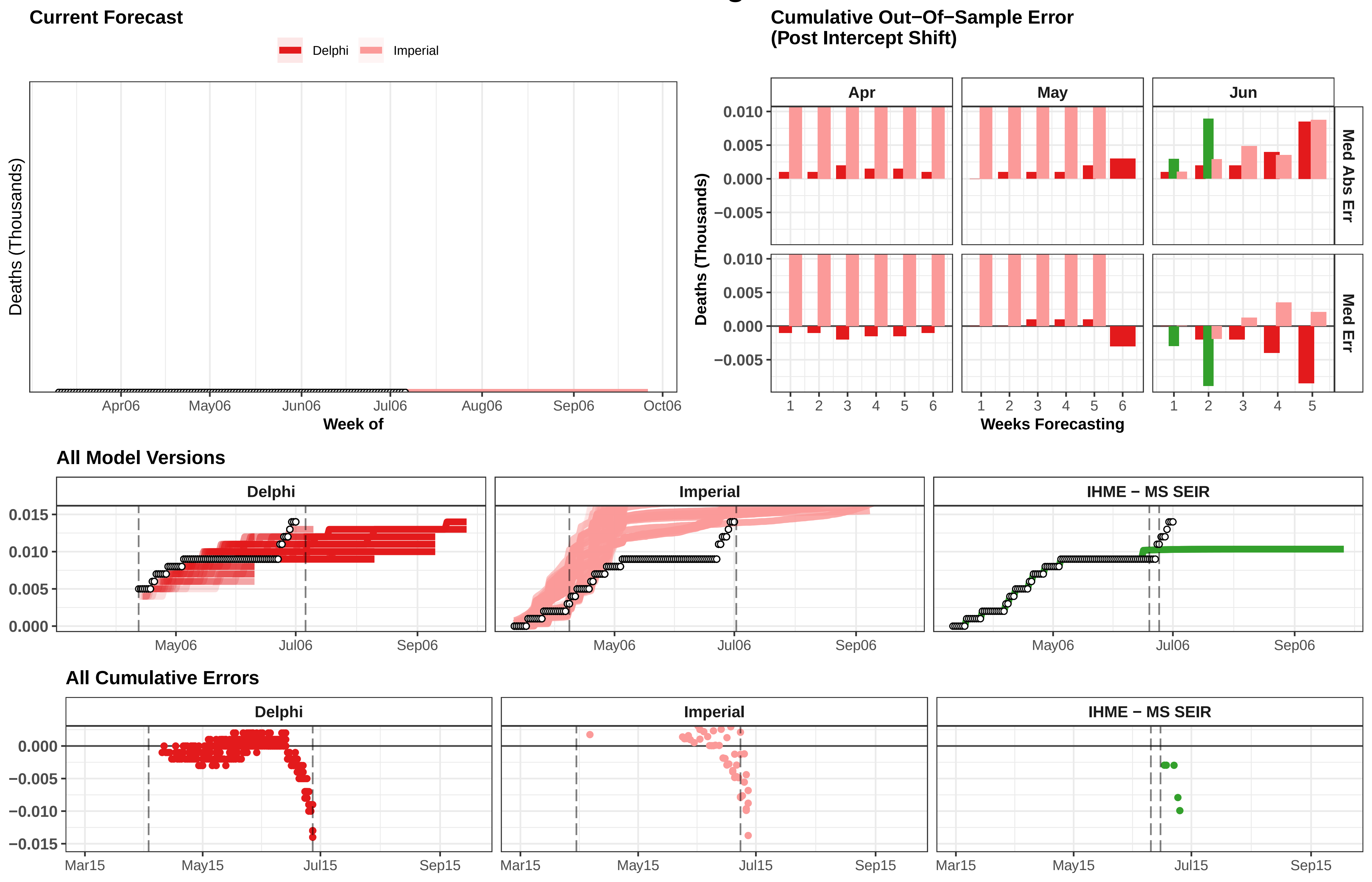
Current Forecast
Delphi
Imperial
Deaths (Thousands)
Apr06
May06
Jun06
Jul06
Aug06
Sep06
Oct06
Week of
Cumulative Out−Of−Sample Error
(Post Intercept Shift)
Apr
May
Jun
Med Abs Err
Med Err
0.010
0.005
0.000
−0.005
1 2 3 4 5 6
1 2 3 4 5
Weeks Forecasting
All Model Versions
Delphi
Imperial
IHME − MS SEIR
0.015
0.010
0.005
0.000
May06
Jul06
Sep06
All Cumulative Errors
−0.010
−0.015
Mar15
May15
Jul15
Sep15

New Zealand

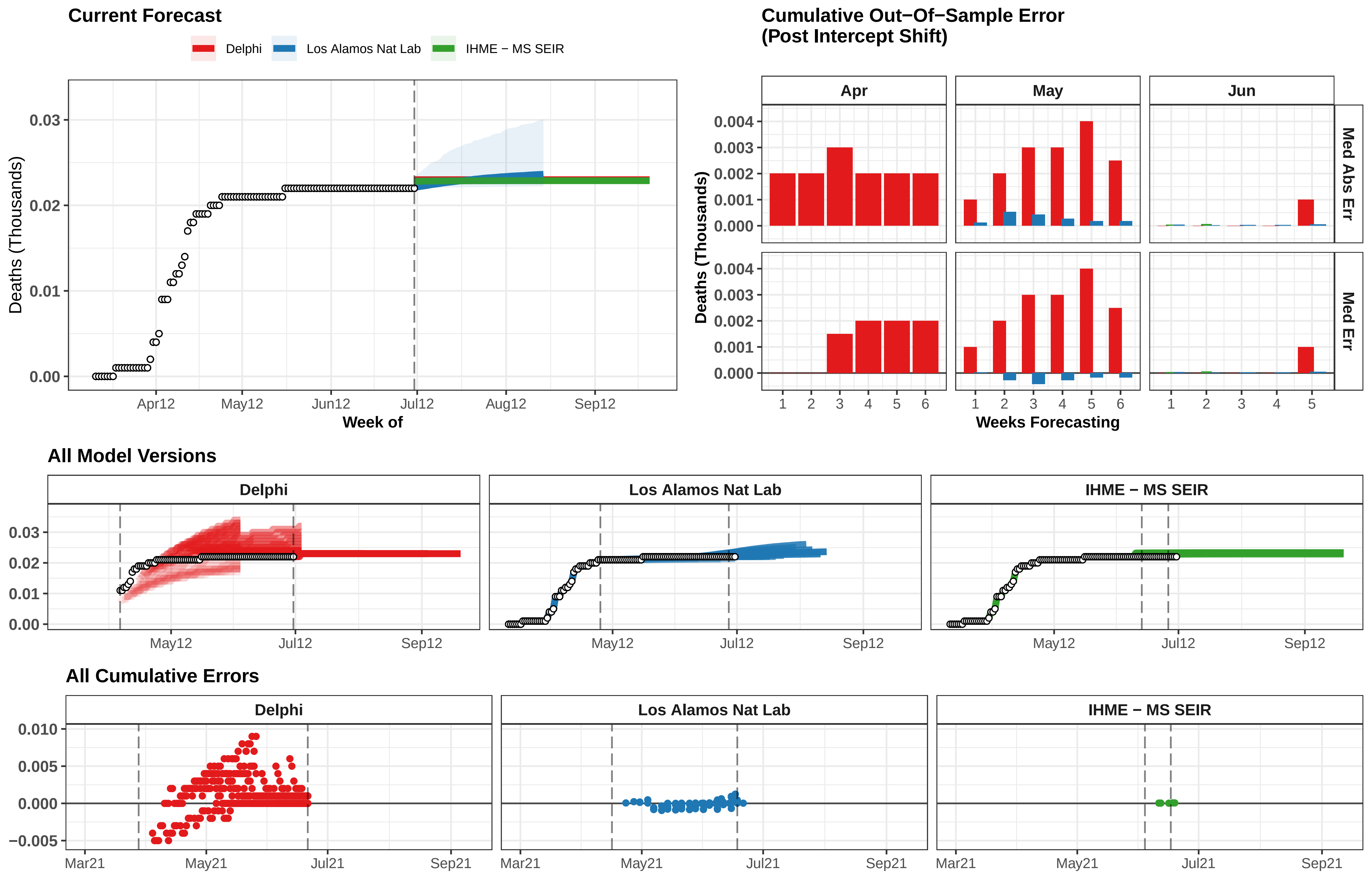

Current Forecast
Delphi
Los Alamos Nat Lab
IHME − MS SEIR
Deaths (Thousands)
0.00
0.01
0.02
0.03
Apr12
May12
Jun12
Jul12
Aug12
Sep12
Week of
Cumulative Out−Of−Sample Error
(Post Intercept Shift)
Apr
May
Jun
Med Abs Err
Med Err
0.000
0.001
0.002
0.003
0.004
Weeks Forecasting
All Model Versions
Delphi
Los Alamos Nat Lab
IHME − MS SEIR
All Cumulative Errors
−0.005
0.000
0.005
0.010
Mar21
May21
Jul21
Sep21

Paraguay

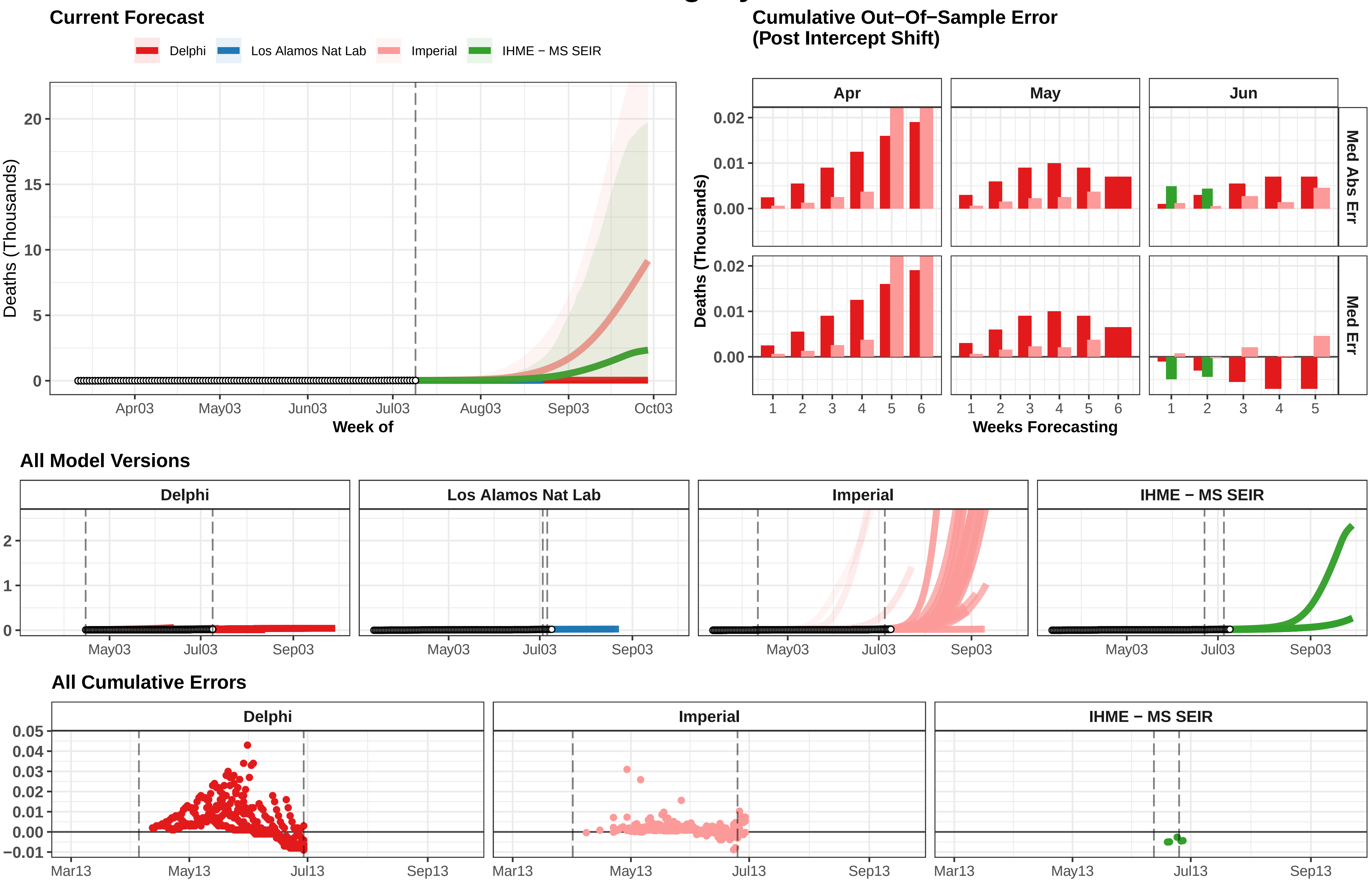

Current Forecast
Delphi
Los Alamos Nat Lab
Imperial
IHME − MS SEIR
Deaths (Thousands)
Week of
Apr03
May03
Jun03
Jul03
Aug03
Sep03
Oct03
Cumulative Out−Of−Sample Error
(Post Intercept Shift)
Apr
May
Jun
Med Abs Err
Med Err
Weeks Forecasting
All Model Versions
All Cumulative Errors
Mar13
May13
Jul13
Sep13

Tanzania

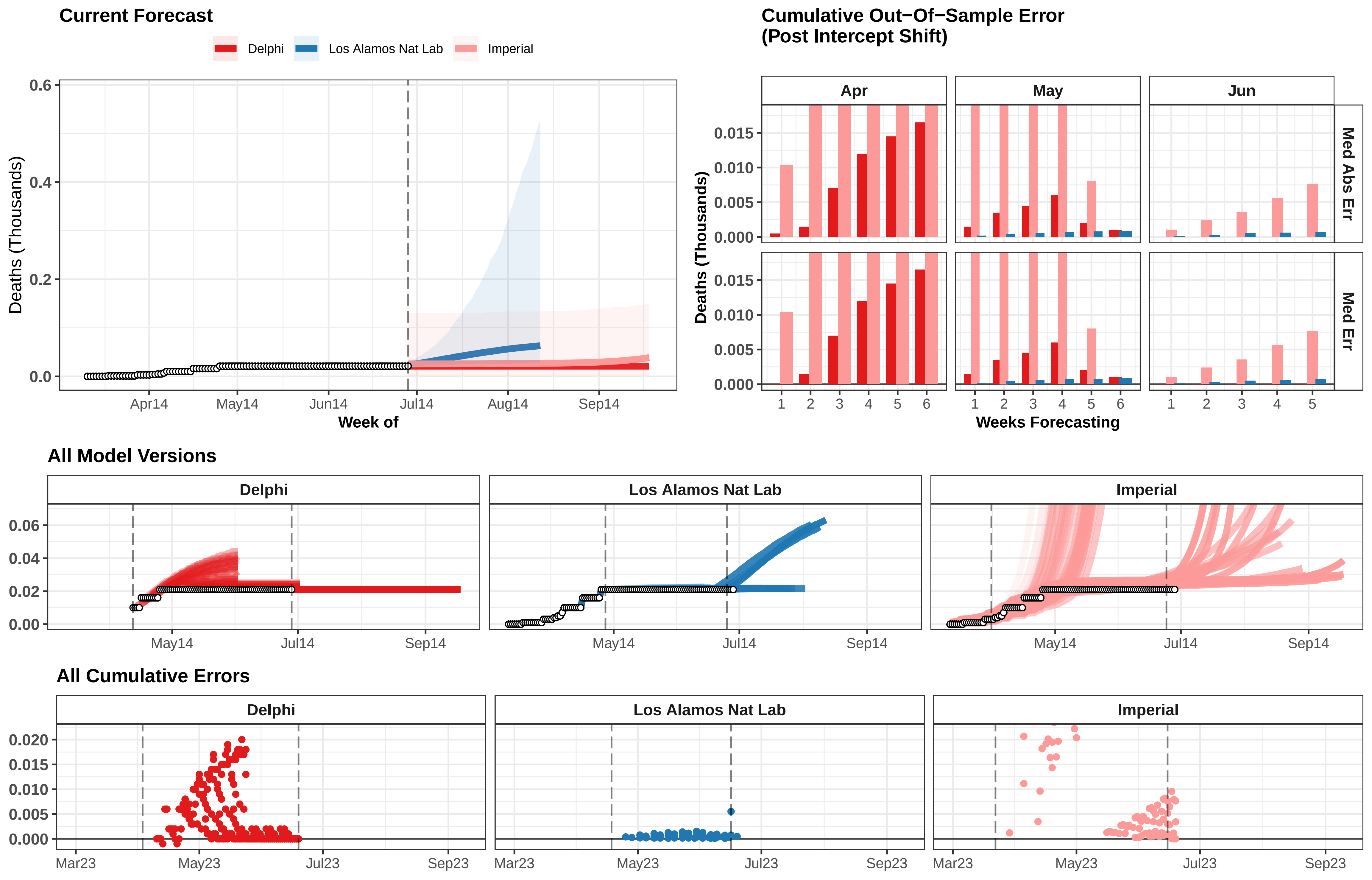

Current Forecast
Delphi
Los Alamos Nat Lab
Imperial
Deaths (Thousands)
0.6
0.4
0.2
0.0
Apr14
May14
Jun14
Jul14
Aug14
Sep14
Week of
Cumulative Out−Of−Sample Error
(Post Intercept Shift)
Apr
May
Jun
Med Abs Err
Med Err
0.015
0.010
0.005
0.000
1 2 3 4 5 6
Weeks Forecasting
All Model Versions
0.06
0.04
0.02
0.00
All Cumulative Errors
0.020
0.015
0.010
0.005
0.000
Mar23
May23
Jul23
Sep23

Wyoming

Current Forecast

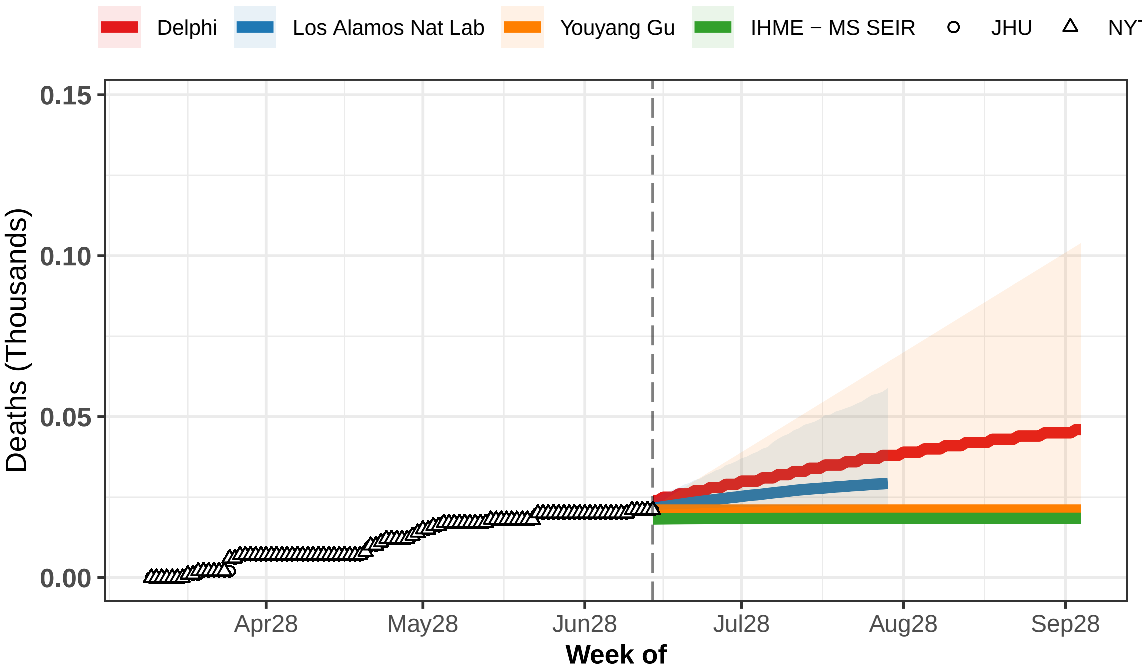

Cumulative Out−Of−Sample Error (Post Intercept Shift)

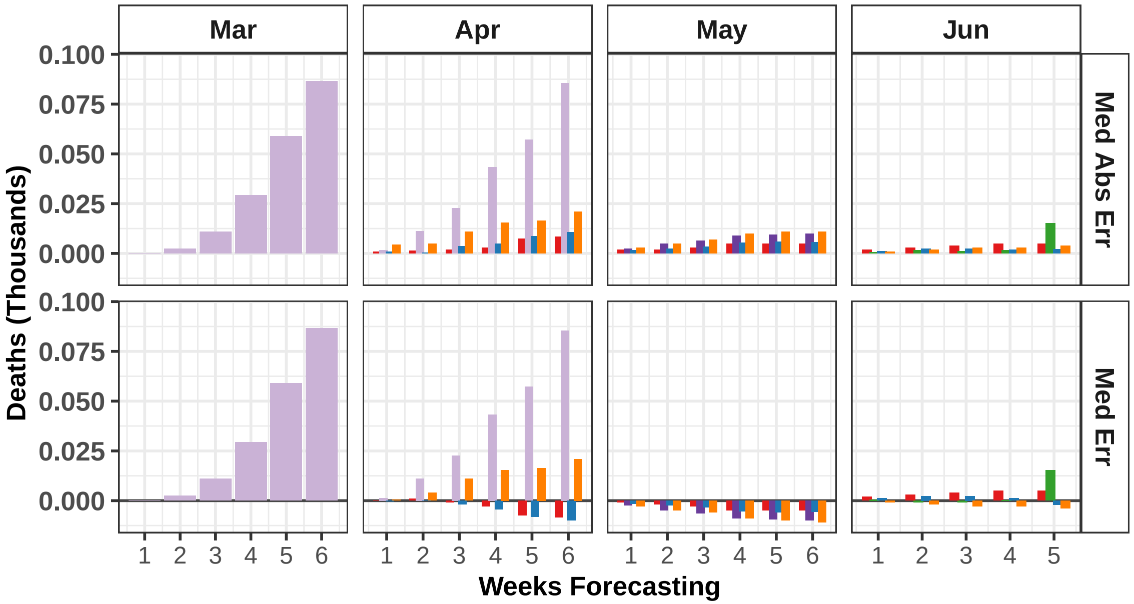

All Model Versions

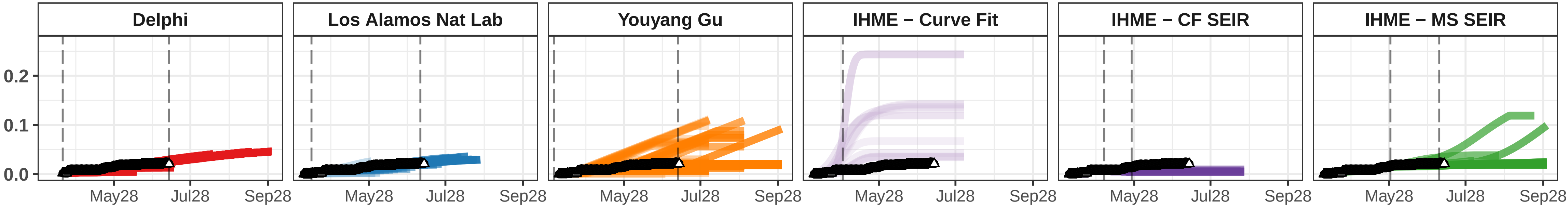

All Cumulative Errors

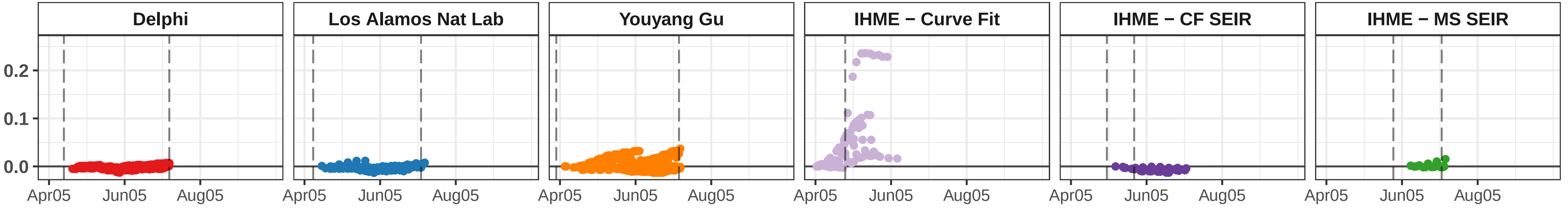

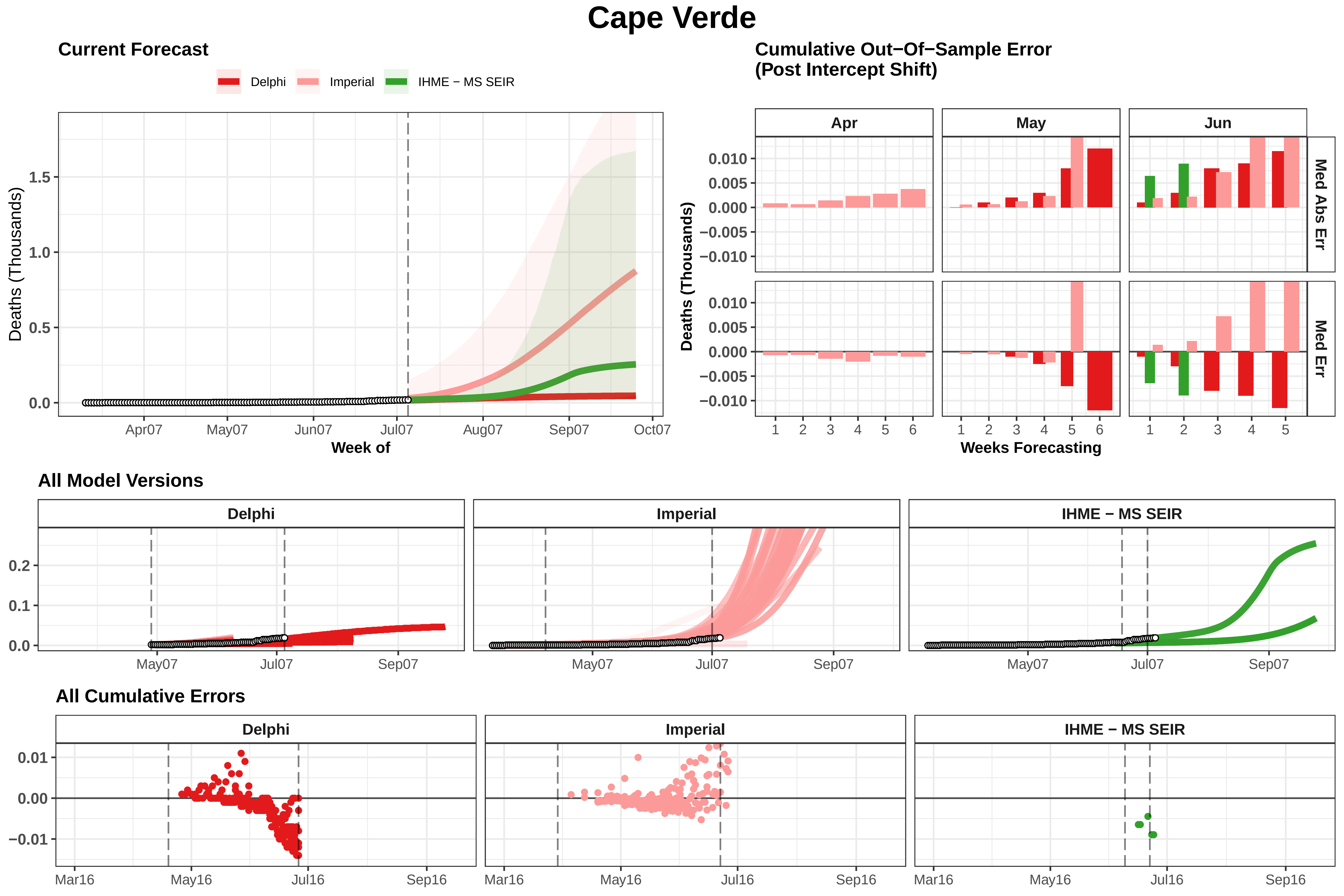
Cape Verde
Current Forecast
Delphi
Imperial
IHME − MS SEIR
Deaths (Thousands)
0.0
0.5
1.0
1.5
Apr07
May07
Jun07
Jul07
Aug07
Sep07
Oct07
Week of
Cumulative Out−Of−Sample Error
(Post Intercept Shift)
Apr
May
Jun
Med Abs Err
Med Err
0.010
0.005
0.000
−0.005
−0.010
Weeks Forecasting
All Model Versions
0.2
0.1
0.0
Sep07
All Cumulative Errors
0.01
0.00
−0.01
Mar16
May16
Jul16
Sep16

Cyprus

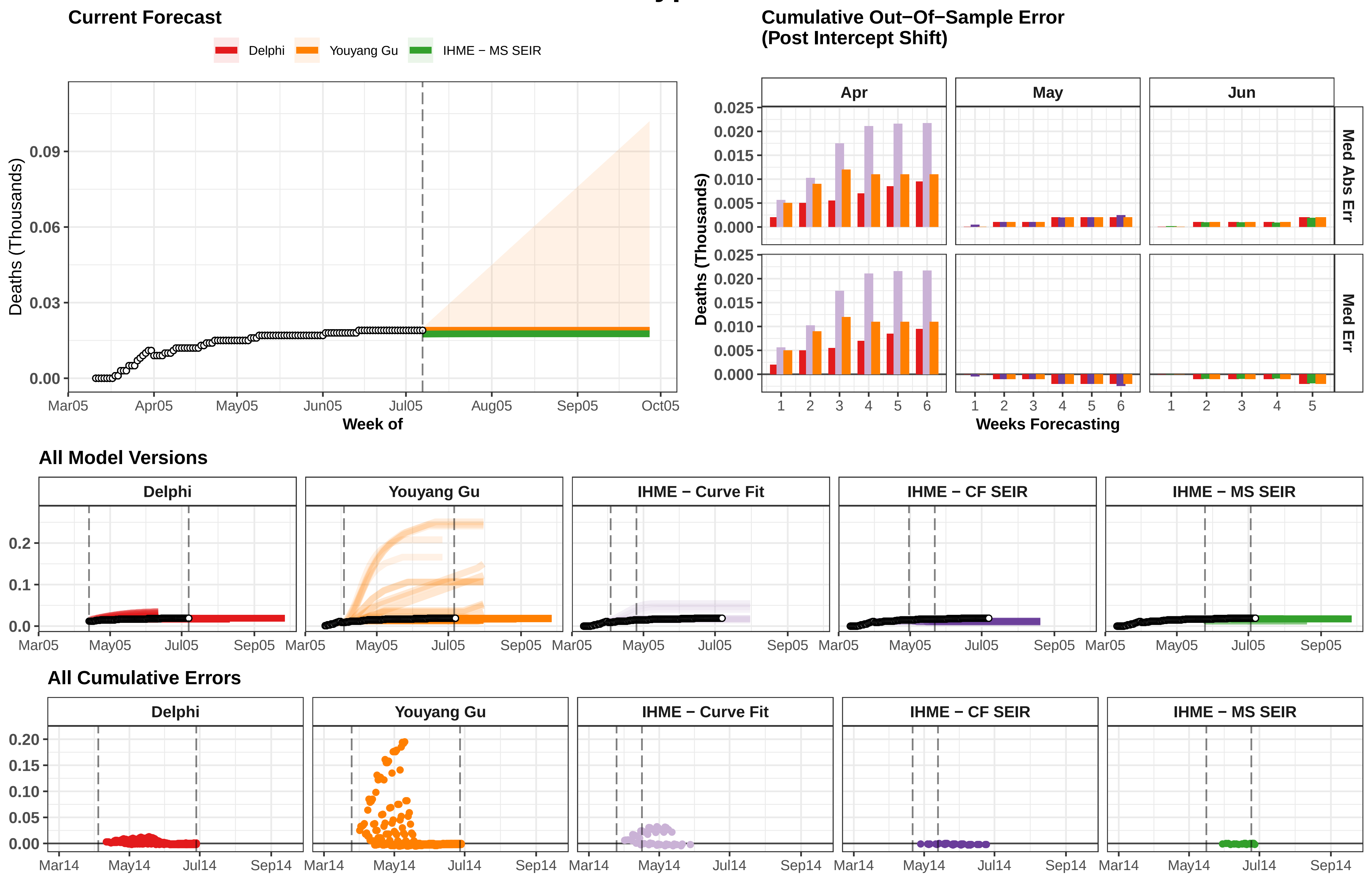

Current Forecast
Delphi
Youyang Gu
IHME − MS SEIR
Deaths (Thousands)
0.00
0.03
0.06
0.09
Mar05
Apr05
May05
Jun05
Jul05
Aug05
Sep05
Oct05
Week of
Cumulative Out−Of−Sample Error
(Post Intercept Shift)
Apr
May
Jun
Med Abs Err
Med Err
0.000
0.005
0.010
0.015
0.020
0.025
Weeks Forecasting
All Model Versions
Delphi
Youyang Gu
IHME − Curve Fit
IHME − CF SEIR
IHME − MS SEIR
0.0
0.1
0.2
May05
Jul05
Sep05
All Cumulative Errors
0.00
0.05
0.10
0.15
0.20
Mar14
May14
Jul14
Sep14

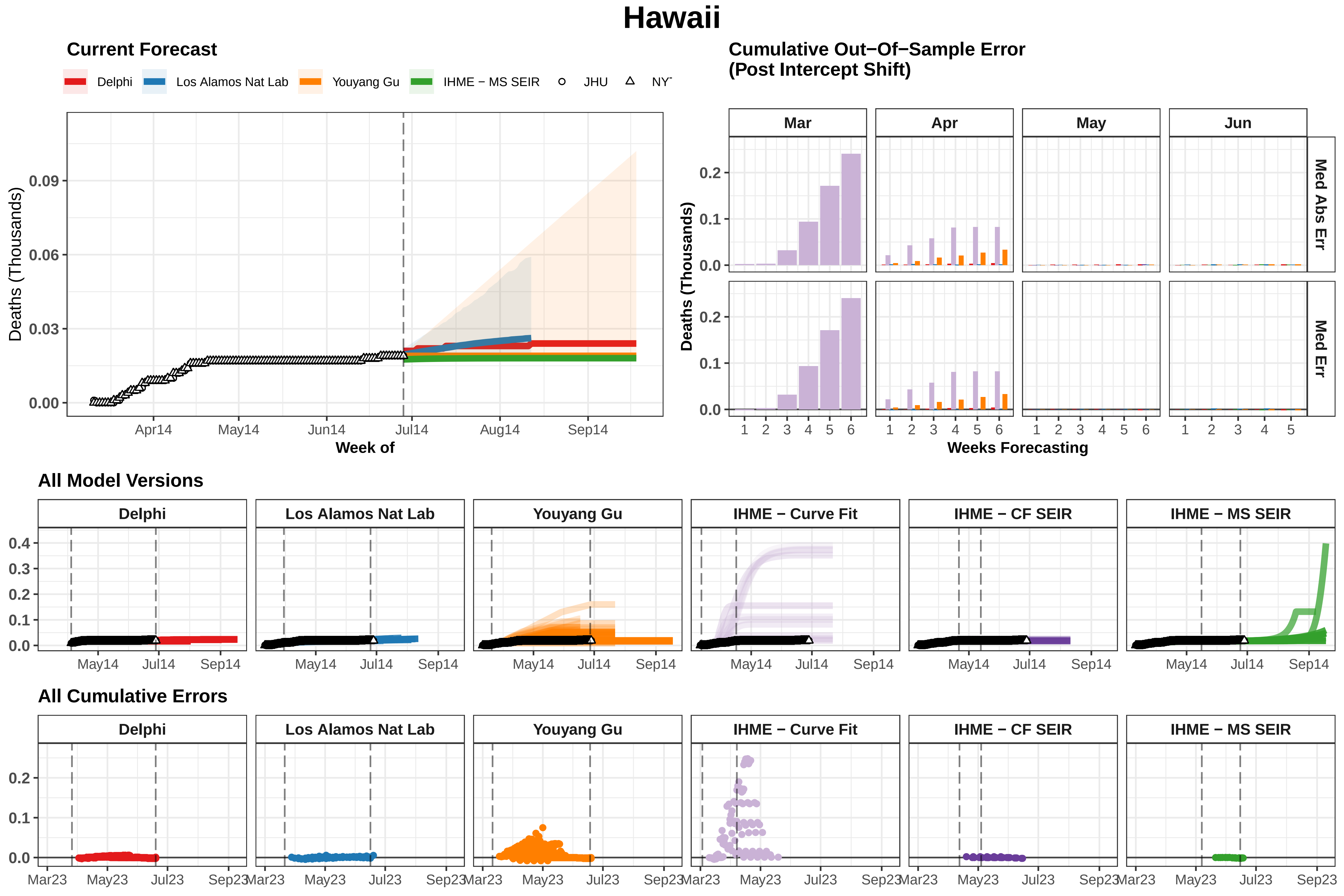

Hawaii
Current Forecast
Delphi
Los Alamos Nat Lab
Youyang Gu
IHME − MS SEIR
JHU
NY
Deaths (Thousands)
Week of
Apr14
May14
Jun14
Jul14
Aug14
Sep14
Cumulative Out−Of−Sample Error
(Post Intercept Shift)
Mar
Apr
May
Jun
Med Abs Err
Med Err
Weeks Forecasting
All Model Versions
Delphi
Los Alamos Nat Lab
Youyang Gu
IHME − Curve Fit
IHME − CF SEIR
IHME − MS SEIR
All Cumulative Errors
Mar23
May23
Jul23
Sep23

Suriname

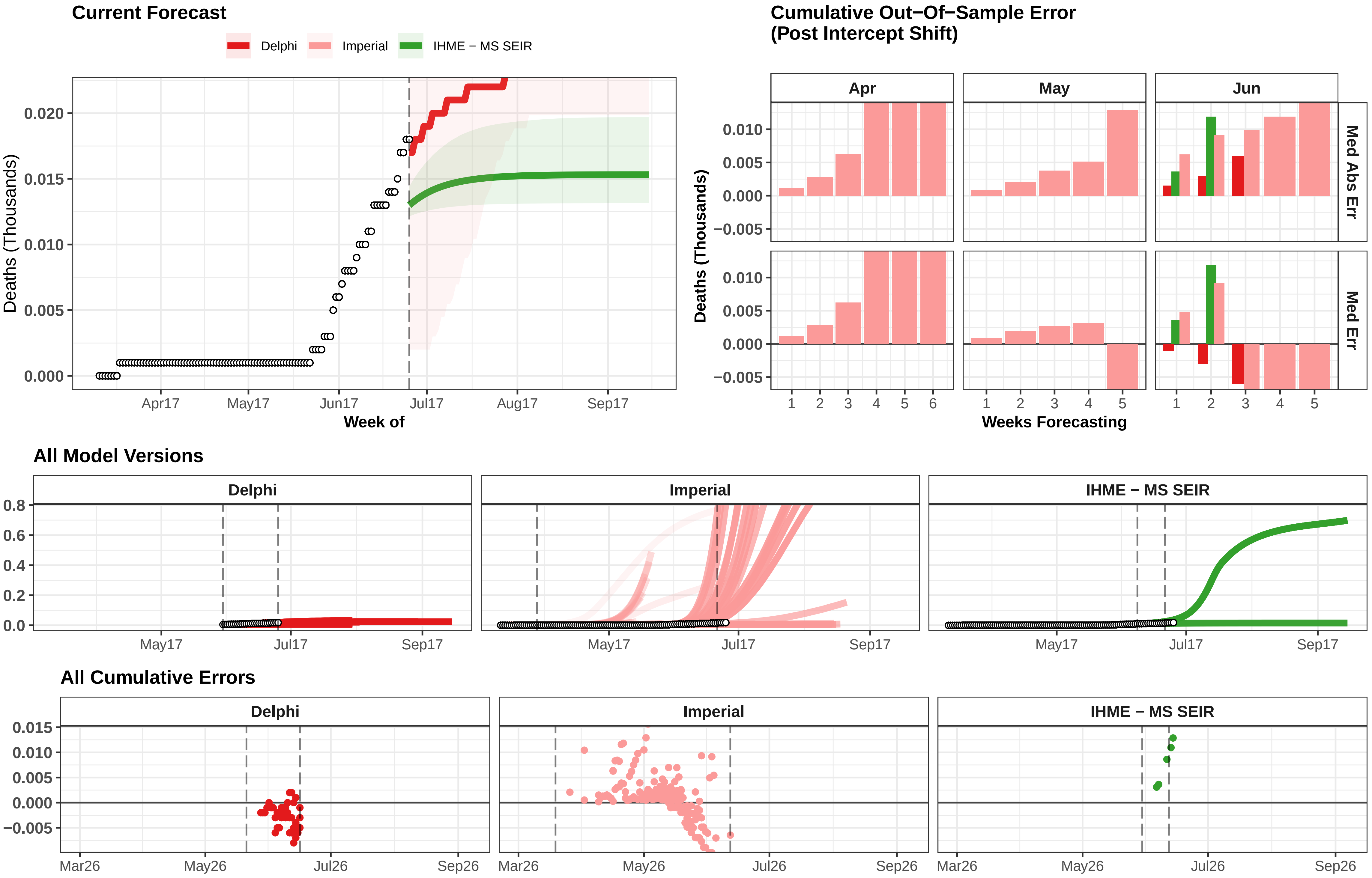

Current Forecast
Delphi
Imperial
IHME − MS SEIR
Deaths (Thousands)
0.000
0.005
0.010
0.015
0.020
Apr17
May17
Jun17
Jul17
Aug17
Sep17
Week of
Cumulative Out−Of−Sample Error
(Post Intercept Shift)
Apr
May
Jun
Med Abs Err
Med Err
−0.005
0.000
0.005
0.010
1
2
3
4
5
6
Weeks Forecasting
All Model Versions
Delphi
Imperial
IHME − MS SEIR
0.0
0.2
0.4
0.6
0.8
May17
Jul17
Sep17
All Cumulative Errors
−0.005
0.000
0.005
0.010
0.015
Mar26
May26
Jul26
Sep26

Swaziland

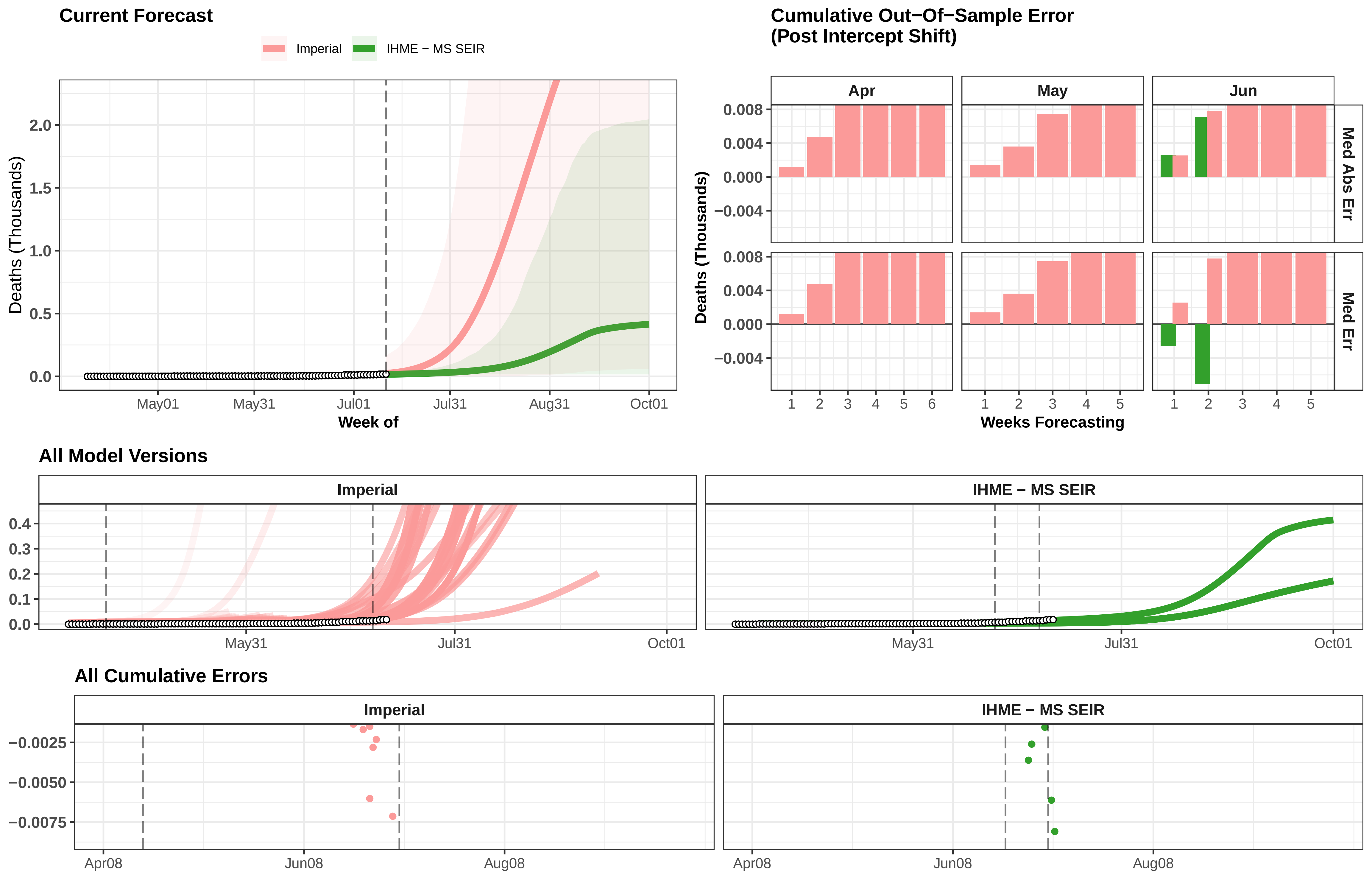
Current Forecast
Imperial
IHME − MS SEIR
Deaths (Thousands)
2.0
1.5
1.0
0.5
0.0
May01
May31
Jul01
Jul31
Aug31
Oct01
Week of
Cumulative Out−Of−Sample Error
(Post Intercept Shift)
Apr
May
Jun
Med Abs Err
Med Err
Deaths (Thousands)
0.008
0.004
0.000
−0.004
1
2
3
4
5
6
Weeks Forecasting
All Model Versions
Imperial
IHME − MS SEIR
0.4
0.3
0.2
0.1
0.0
May31
Jul31
Oct01
All Cumulative Errors
Imperial
IHME − MS SEIR
−0.0025
−0.0050
−0.0075
Apr08
Jun08
Aug08

Zimbabwe

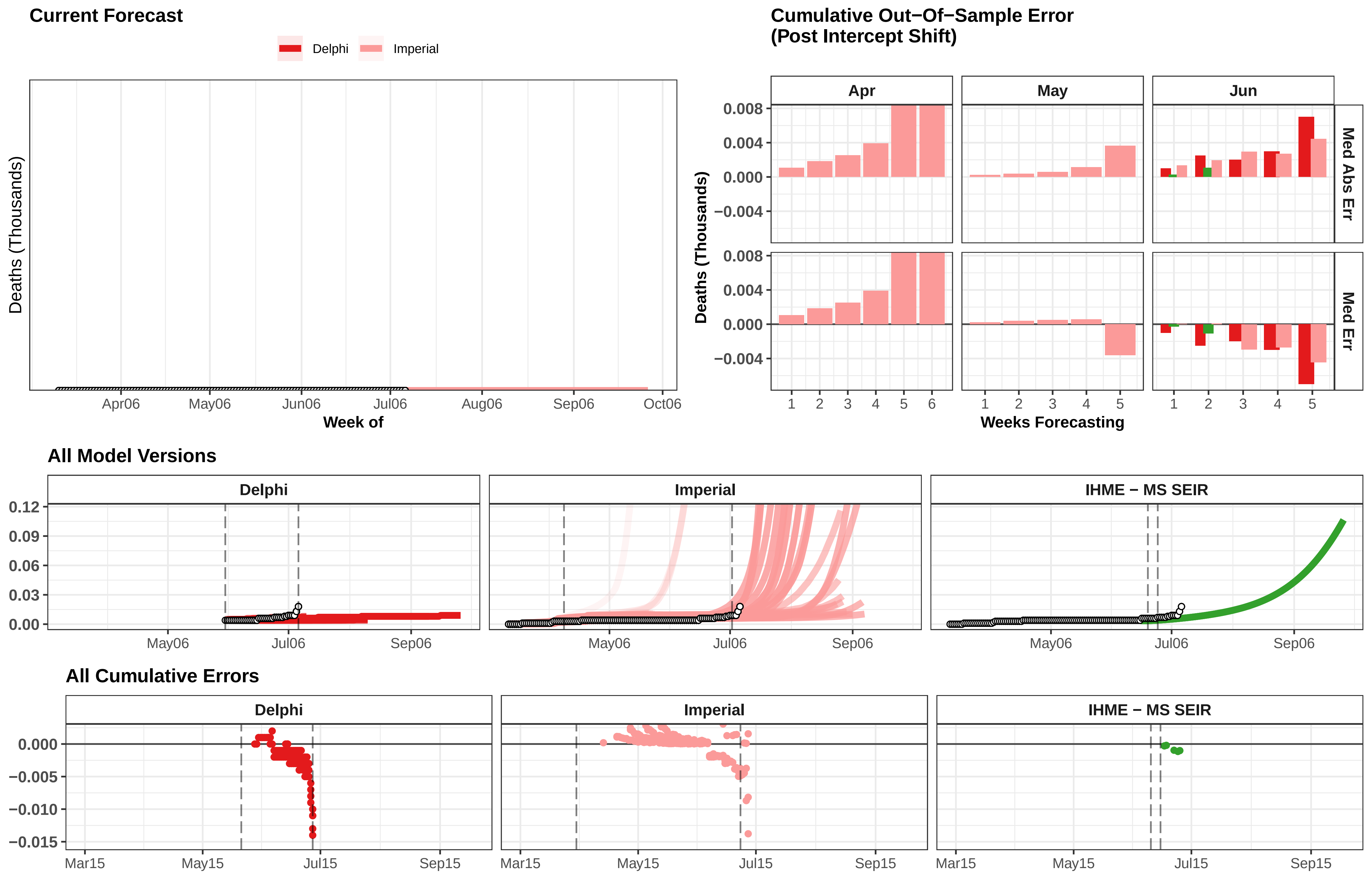

Current Forecast
Delphi
Imperial
Deaths (Thousands)
Apr06
May06
Jun06
Jul06
Aug06
Sep06
Oct06
Week of
Cumulative Out−Of−Sample Error
(Post Intercept Shift)
Apr
May
Jun
Med Abs Err
Med Err
0.008
0.004
0.000
−0.004
Weeks Forecasting
All Model Versions
Delphi
Imperial
IHME − MS SEIR
0.12
0.09
0.06
0.03
0.00
All Cumulative Errors
−0.005
−0.010
−0.015
Mar15
May15
Jul15
Sep15

Guyana

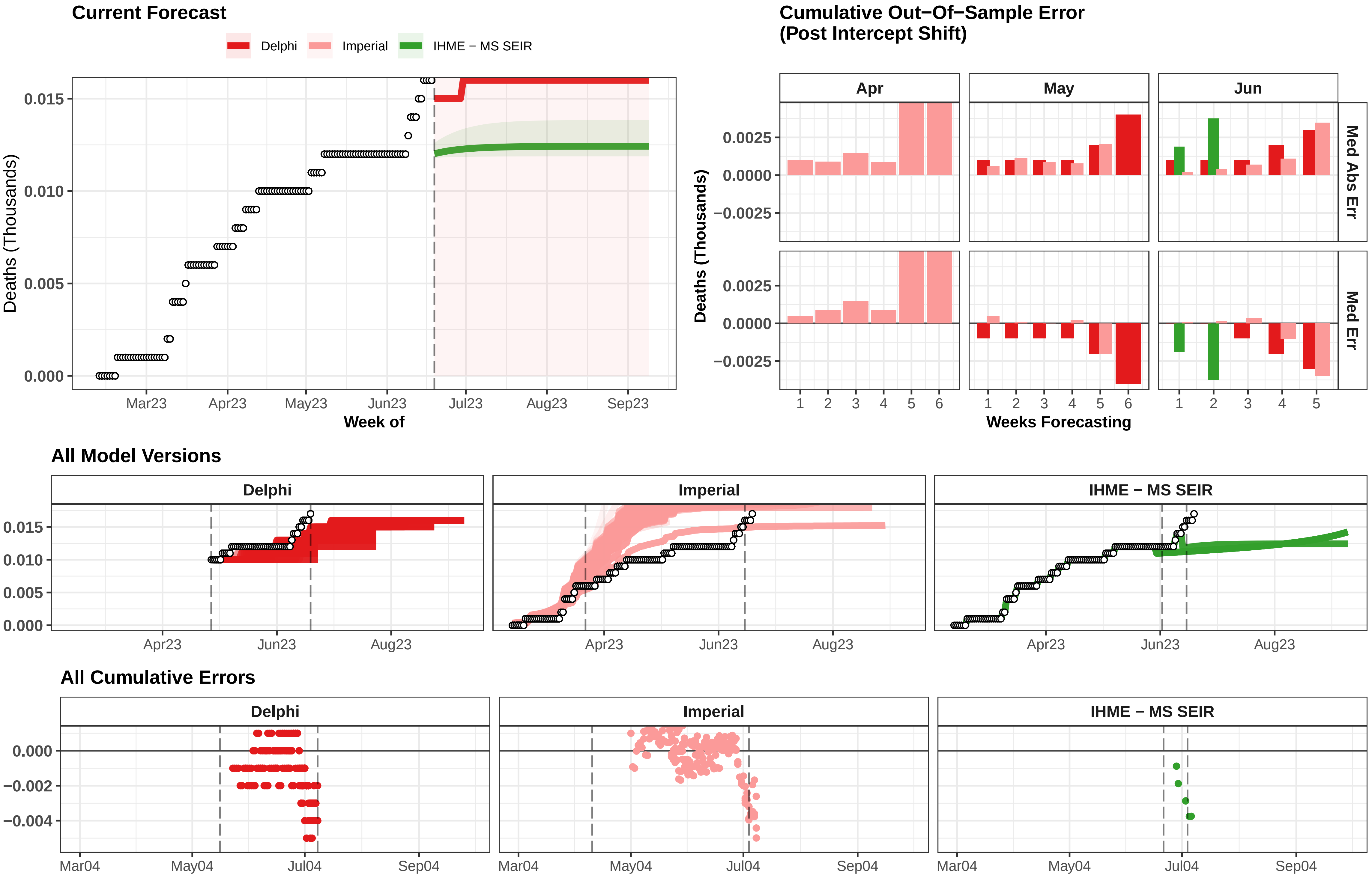
Current Forecast
Delphi
Imperial
IHME − MS SEIR
Deaths (Thousands)
0.015
0.010
0.005
0.000
Mar23
Apr23
May23
Jun23
Jul23
Aug23
Sep23
Week of
Cumulative Out−Of−Sample Error
(Post Intercept Shift)
Apr
May
Jun
Med Abs Err
Med Err
0.0025
0.0000
−0.0025
Weeks Forecasting
All Model Versions
Delphi
Imperial
IHME − MS SEIR
All Cumulative Errors
0.000
−0.002
−0.004
Mar04
May04
Jul04
Sep04

Syria

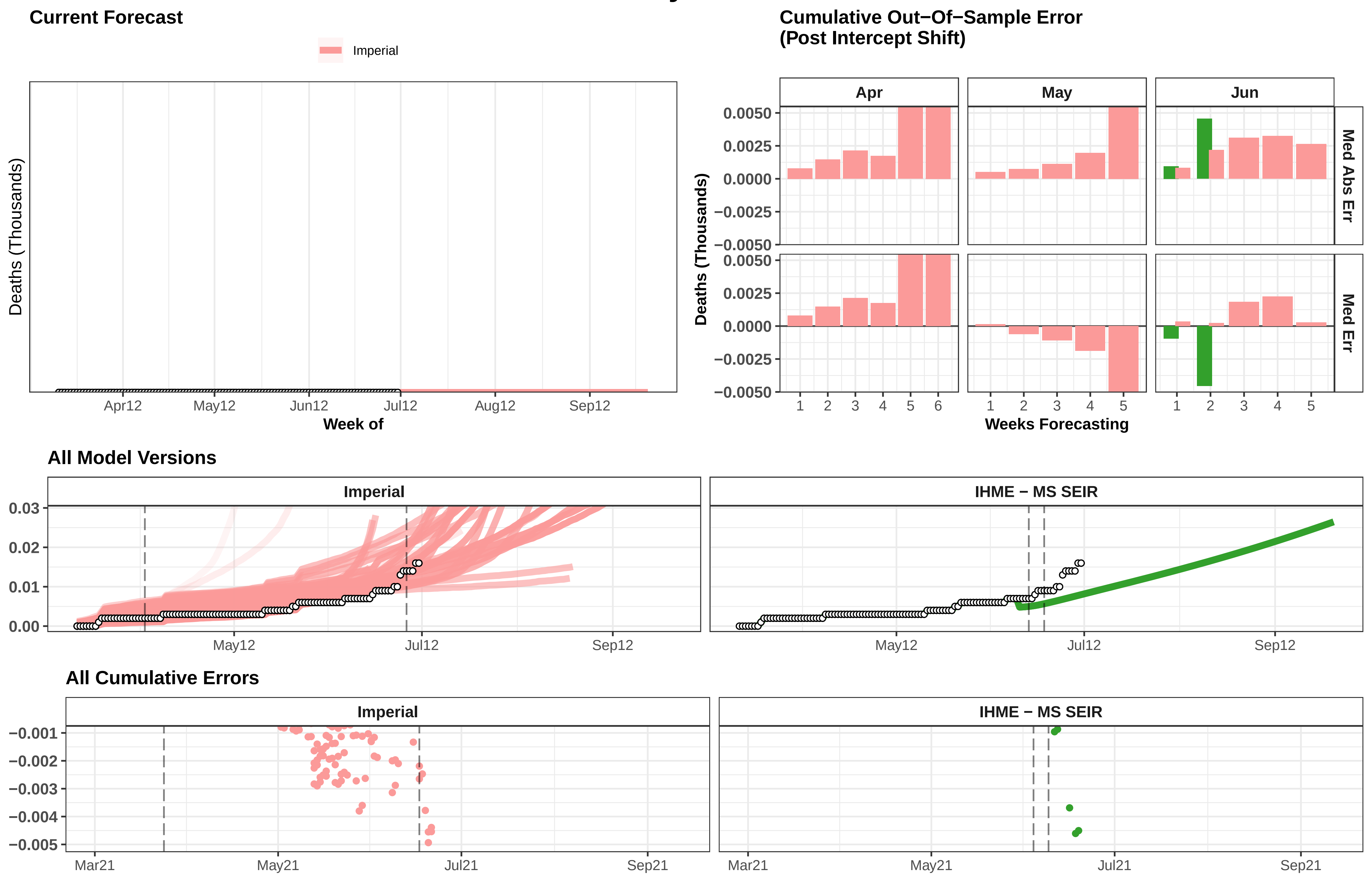
Current Forecast
Imperial
Deaths (Thousands)
Apr12
May12
Jun12
Jul12
Aug12
Sep12
Week of
Cumulative Out−Of−Sample Error
(Post Intercept Shift)
Apr
May
Jun
Med Abs Err
Med Err
0.0050
0.0025
0.0000
−0.0025
−0.0050
Weeks Forecasting
All Model Versions
IHME − MS SEIR
0.03
0.02
0.01
0.00
All Cumulative Errors
−0.001
−0.002
−0.003
−0.004
−0.005
Mar21
May21
Jul21
Sep21

Alaska

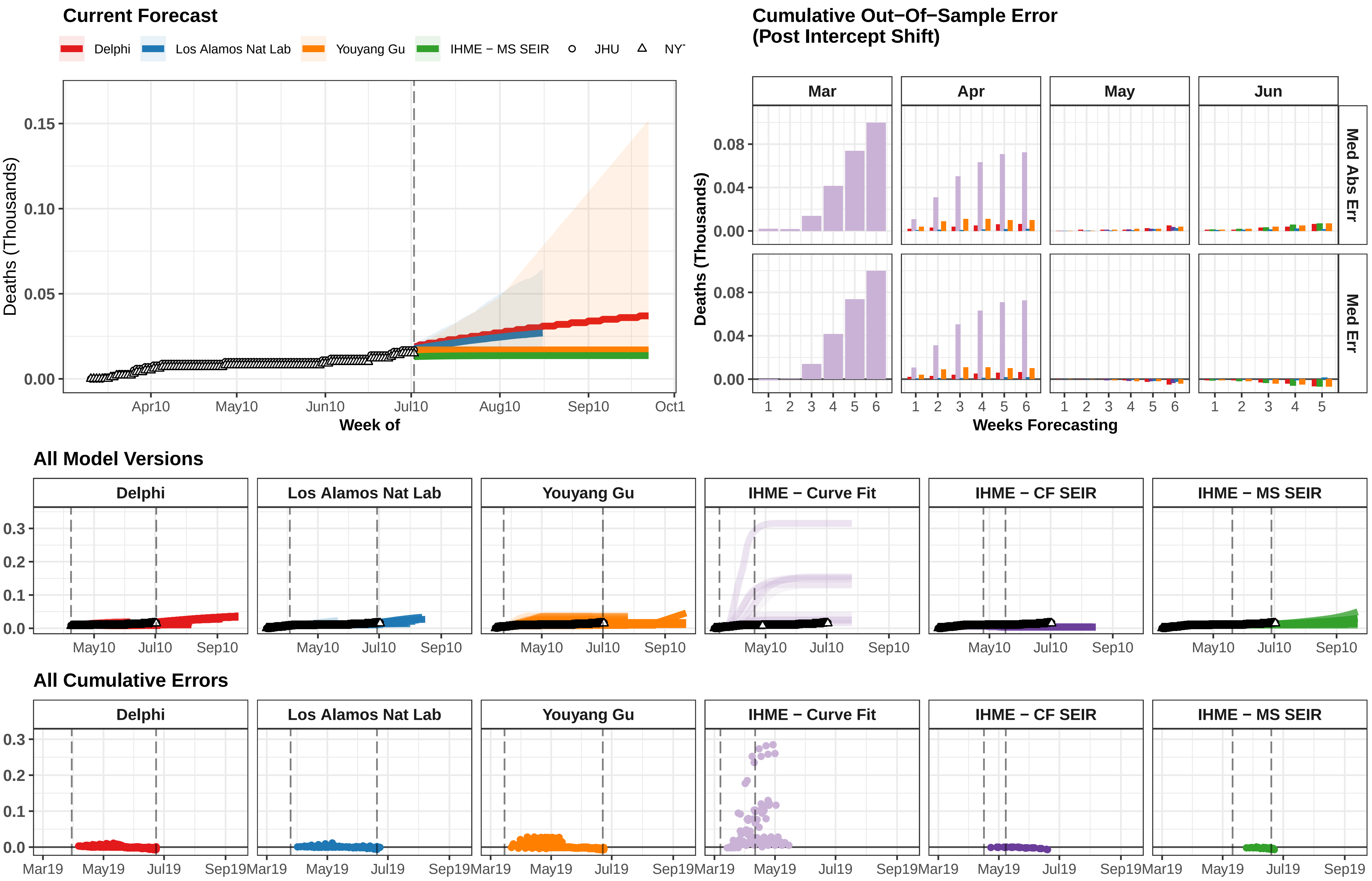

Current Forecast
Delphi
Los Alamos Nat Lab
Youyang Gu
IHME − MS SEIR
JHU
NY
0.15
0.10
0.05
0.00
Deaths (Thousands)
Apr10
May10
Jun10
Jul10
Aug10
Sep10
Oct1
Week of
Cumulative Out−Of−Sample Error
(Post Intercept Shift)
Mar
Apr
May
Jun
Med Abs Err
Med Err
0.08
0.04
0.00
Weeks Forecasting
All Model Versions
Delphi
Los Alamos Nat Lab
Youyang Gu
IHME − Curve Fit
IHME − CF SEIR
IHME − MS SEIR
0.3
0.2
0.1
0.0
May10
Jul10
Sep10
All Cumulative Errors
Mar19
May19
Jul19
Sep19

Georgia (Country)

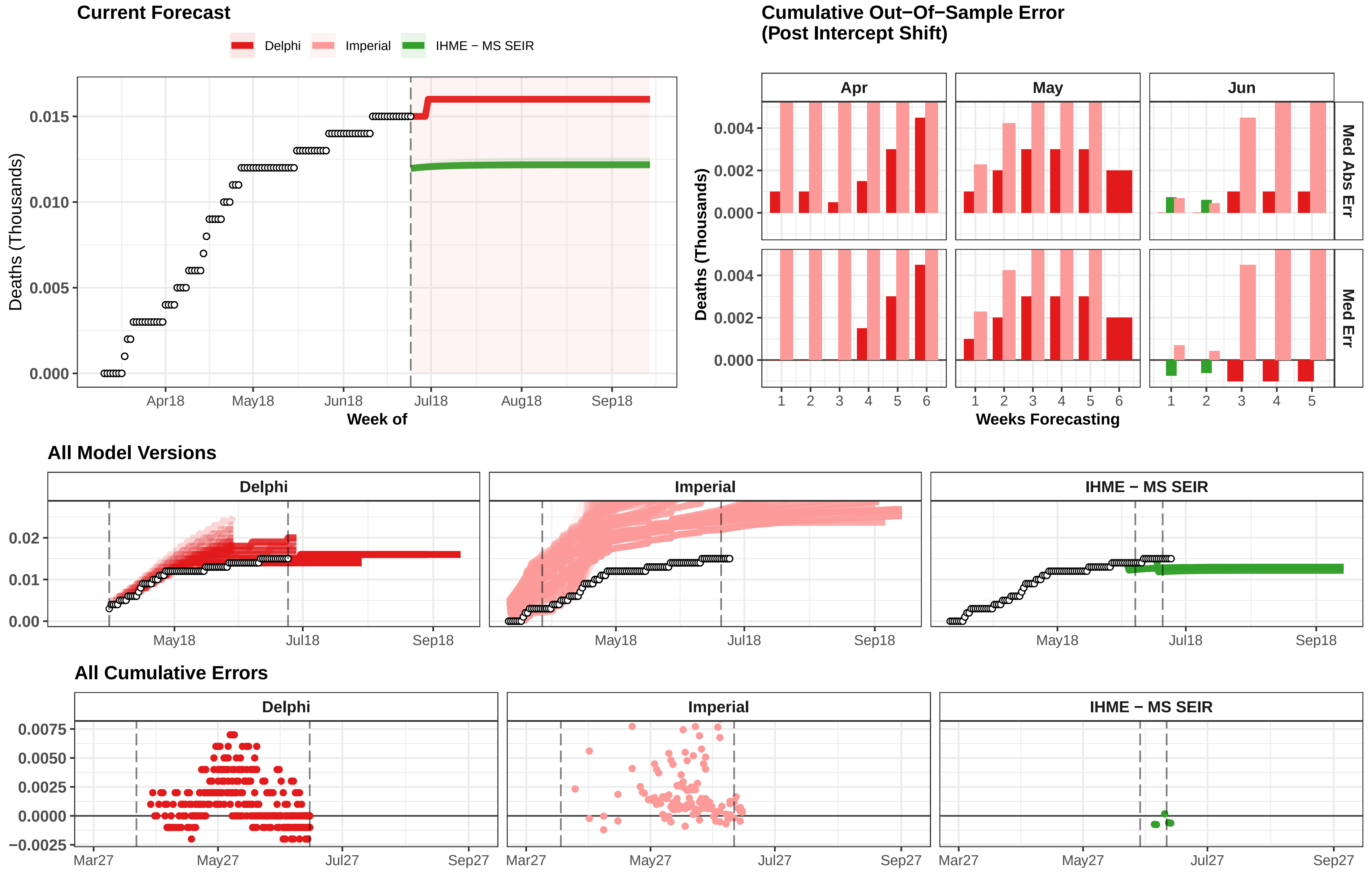

Togo

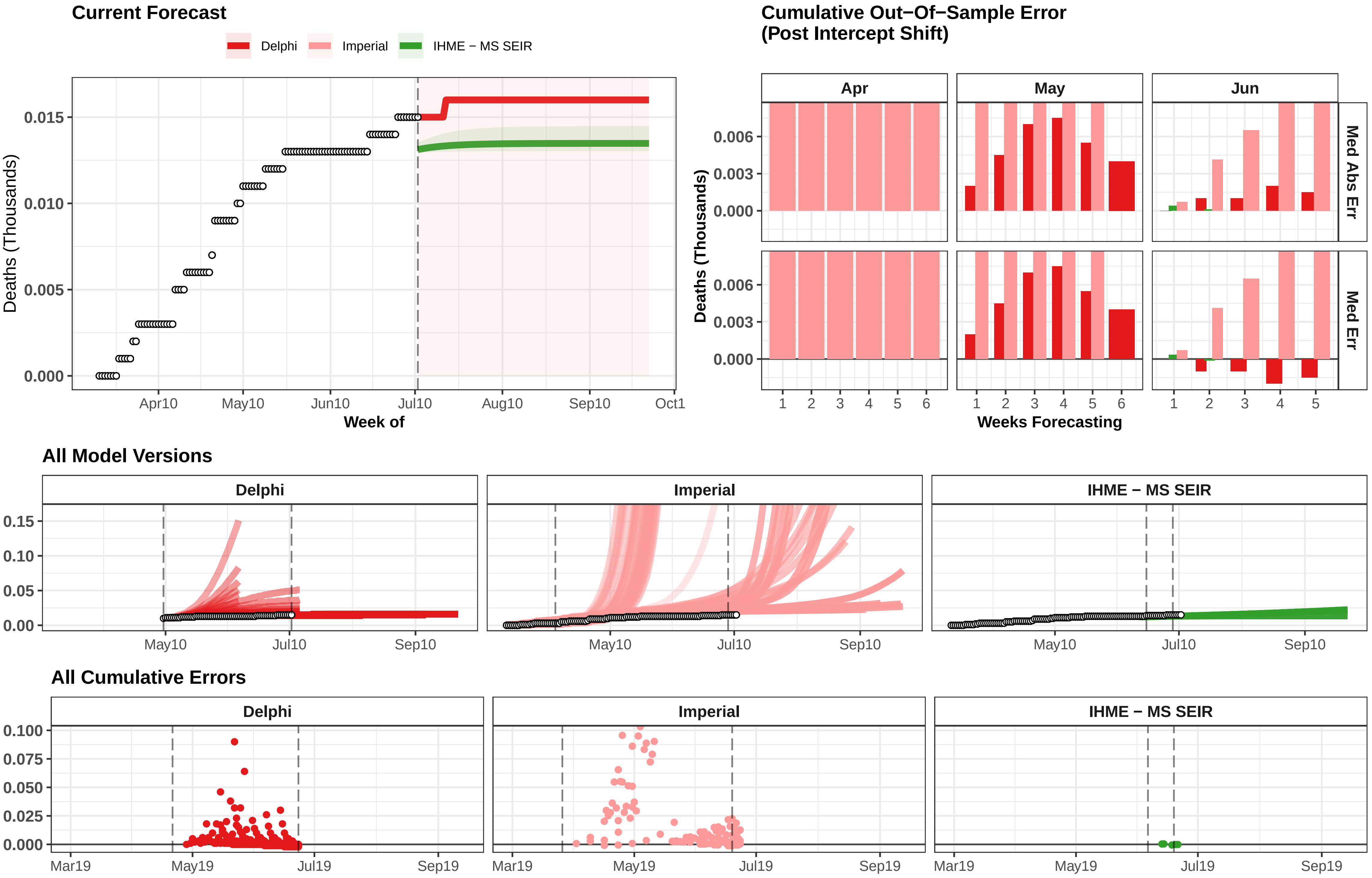

Current Forecast
Delphi
Imperial
IHME − MS SEIR
Deaths (Thousands)
0.000
0.005
0.010
0.015
Apr10
May10
Jun10
Jul10
Aug10
Sep10
Oct1
Week of
Cumulative Out−Of−Sample Error
(Post Intercept Shift)
Apr
May
Jun
Med Abs Err
Med Err
0.000
0.003
0.006
1
2
3
4
5
6
Weeks Forecasting
All Model Versions
Delphi
Imperial
IHME − MS SEIR
0.00
0.05
0.10
0.15
May10
Jul10
Sep10
All Cumulative Errors
0.000
0.025
0.050
0.075
0.100
Mar19
May19
Jul19
Sep19

Sao Tome and Principe

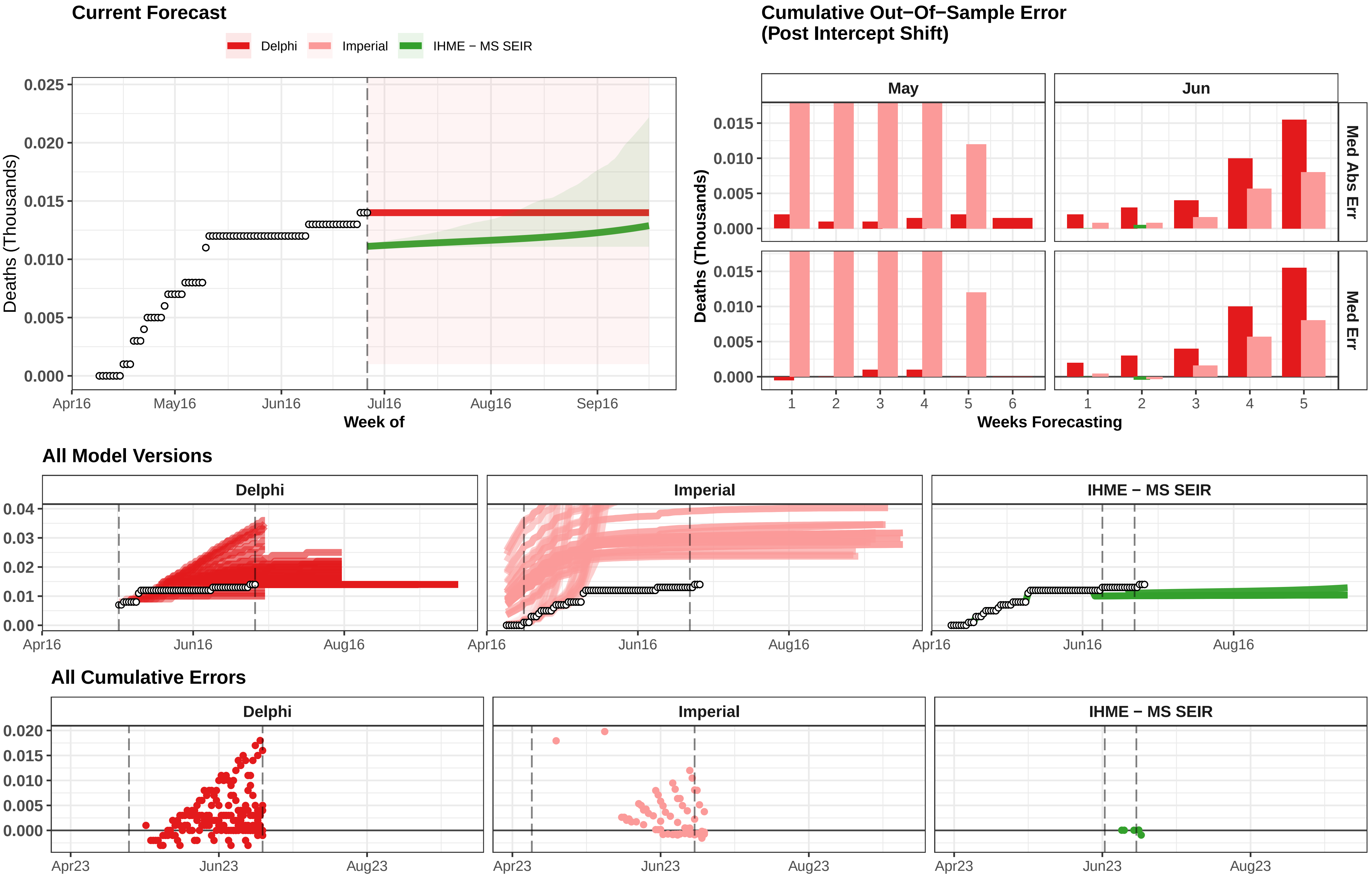

Maldives

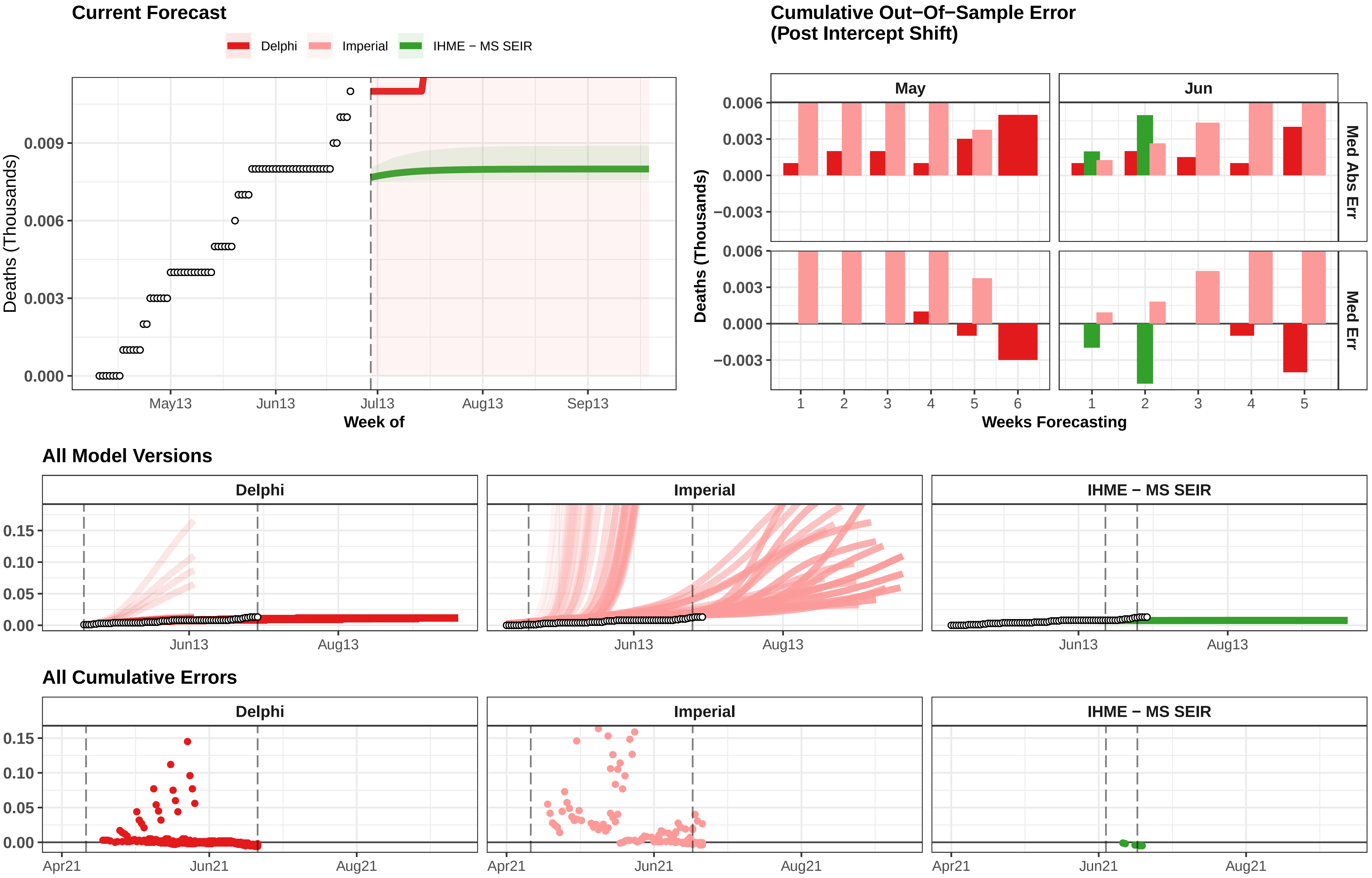

Current Forecast
Delphi
Imperial
IHME − MS SEIR
Deaths (Thousands)
0.000
0.003
0.006
0.009
May13
Jun13
Jul13
Aug13
Sep13
Week of
Cumulative Out−Of−Sample Error
(Post Intercept Shift)
May
Jun
Med Abs Err
Med Err
0.006
0.003
0.000
−0.003
1
2
3
4
5
6
Weeks Forecasting
All Model Versions
Delphi
Imperial
IHME − MS SEIR
0.15
0.10
0.05
0.00
Jun13
Aug13
All Cumulative Errors
Apr21
Jun21
Aug21

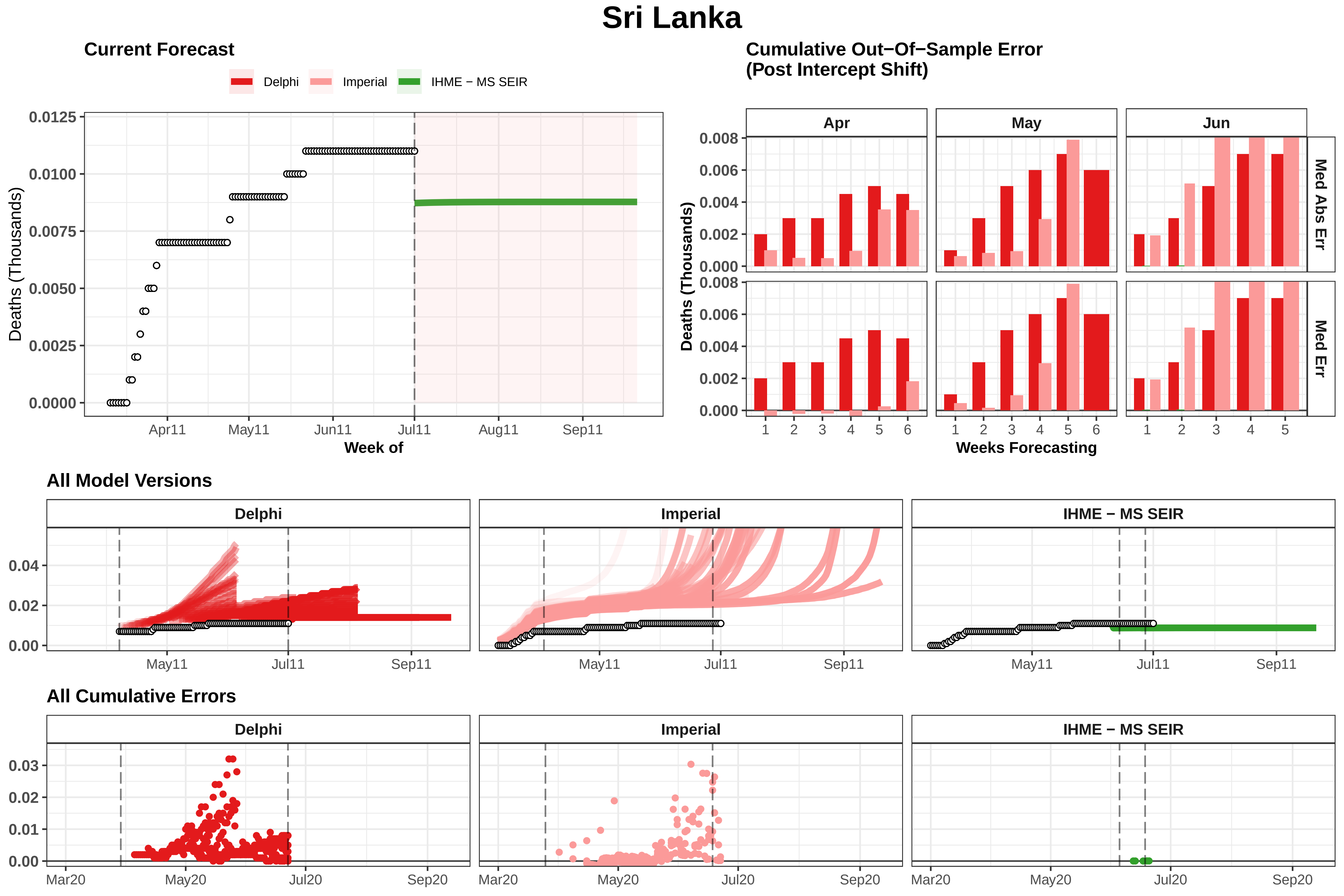

Sri Lanka
Current Forecast
Delphi
Imperial
IHME − MS SEIR
Deaths (Thousands)
0.0125
0.0100
0.0075
0.0050
0.0025
0.0000
Apr11
May11
Jun11
Jul11
Aug11
Sep11
Week of
Cumulative Out−Of−Sample Error
(Post Intercept Shift)
Apr
May
Jun
Med Abs Err
Med Err
0.008
0.006
0.004
0.002
0.000
Weeks Forecasting
All Model Versions
Delphi
Imperial
IHME − MS SEIR
0.04
0.02
0.00
All Cumulative Errors
0.03
0.02
0.01
0.00
Mar20
May20
Jul20
Sep20
