# Supplementary material for: Predictive performance of international COVID-19 mortality forecasting models
Source: medRxiv. 2020 Nov 19:2020.07.13.20151233. Preprint. [Version 5] doi: 10.1101/2020.07.13.20151233 (PMC7685335; doi:10.1101/2020.07.13.20151233)
Supplement: Supplement 2020 [file 89490-2020.07.13.20151233-3.pdf]

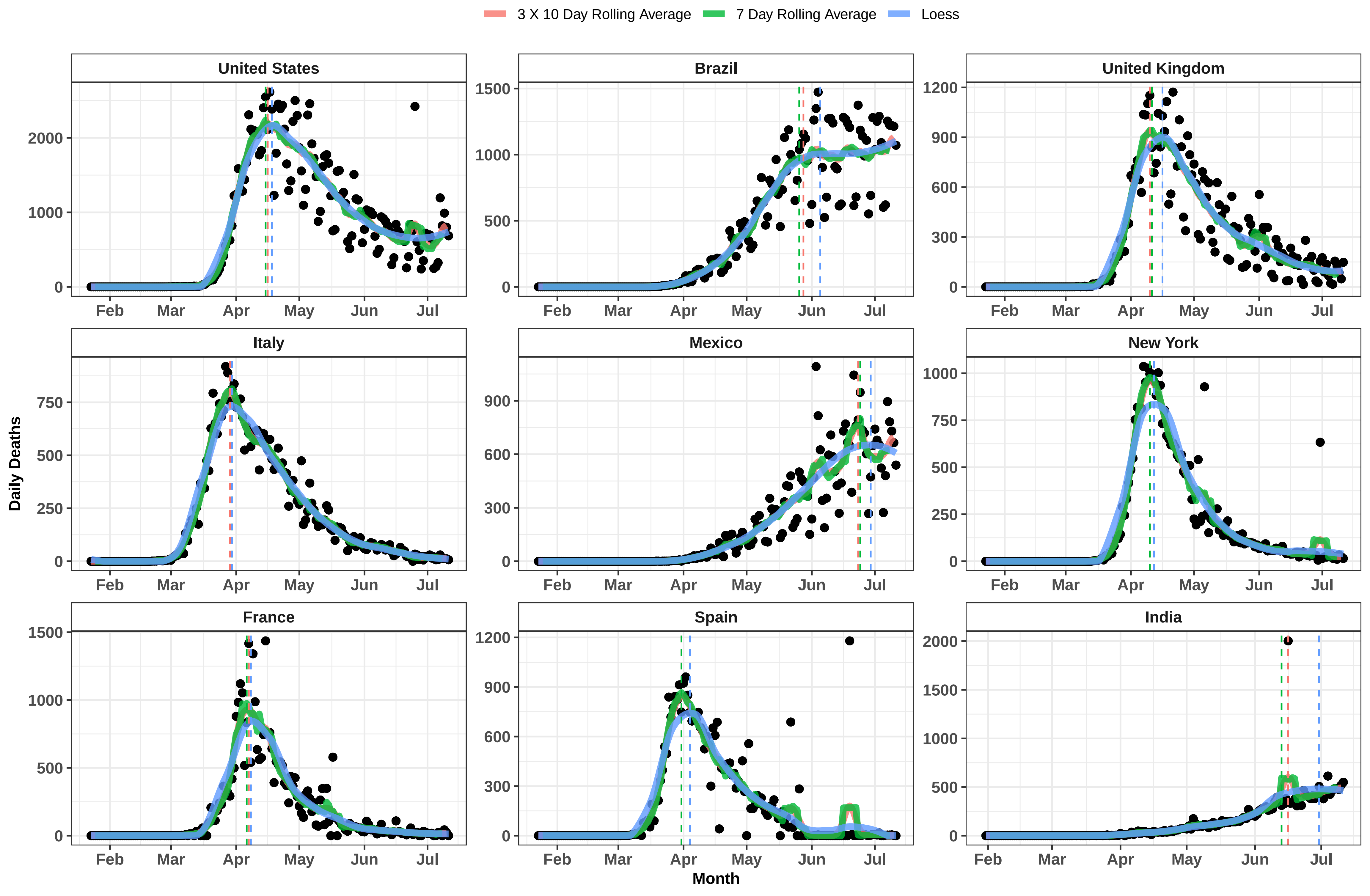

3 X 10 Day Rolling Average
7 Day Rolling Average
Loess
United States
Brazil
United Kingdom
Italy
Mexico
New York
France
Spain
India
Daily Deaths
Month
Feb
Mar
Apr
May
Jun
Jul

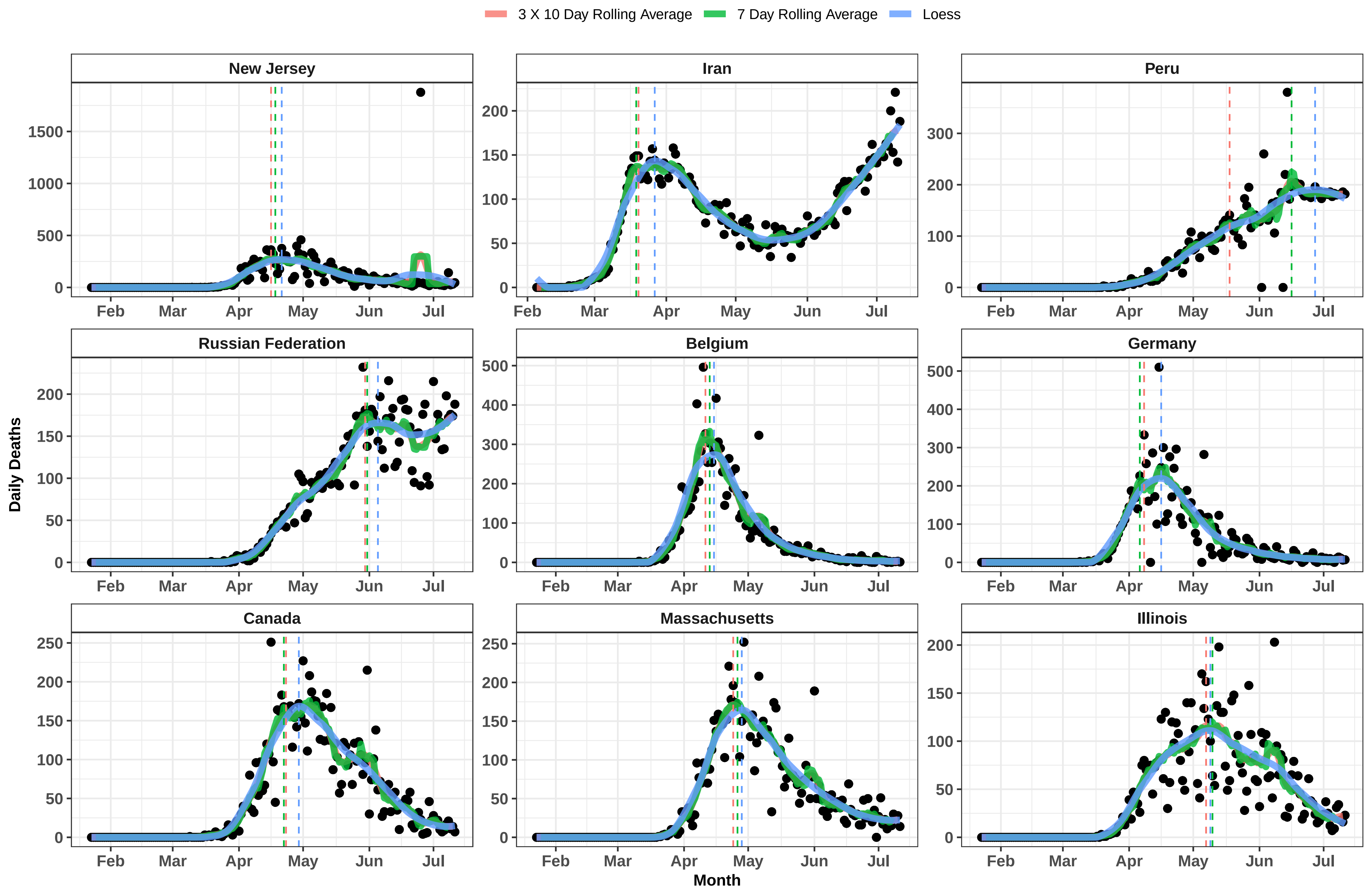

3 X 10 Day Rolling Average
7 Day Rolling Average
Loess
New Jersey
Iran
Peru
Russian Federation
Belgium
Germany
Canada
Massachusetts
Illinois
Daily Deaths
Month
Feb
Mar
Apr
May
Jun
Jul

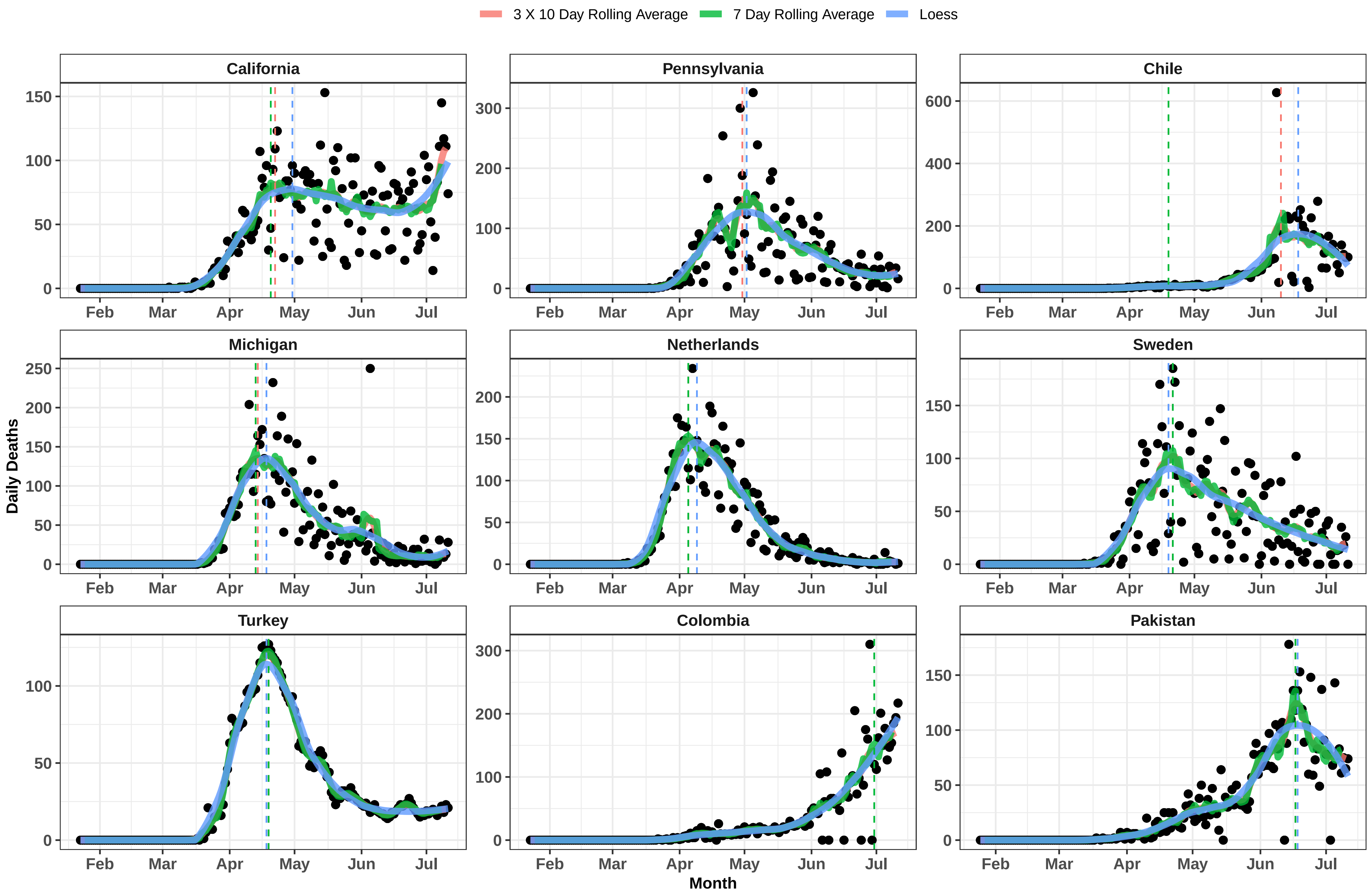

3 X 10 Day Rolling Average
7 Day Rolling Average
Loess
California
Pennsylvania
Chile
Michigan
Netherlands
Sweden
Turkey
Colombia
Pakistan
Daily Deaths
Month
Feb
Mar
Apr
May
Jun
Jul

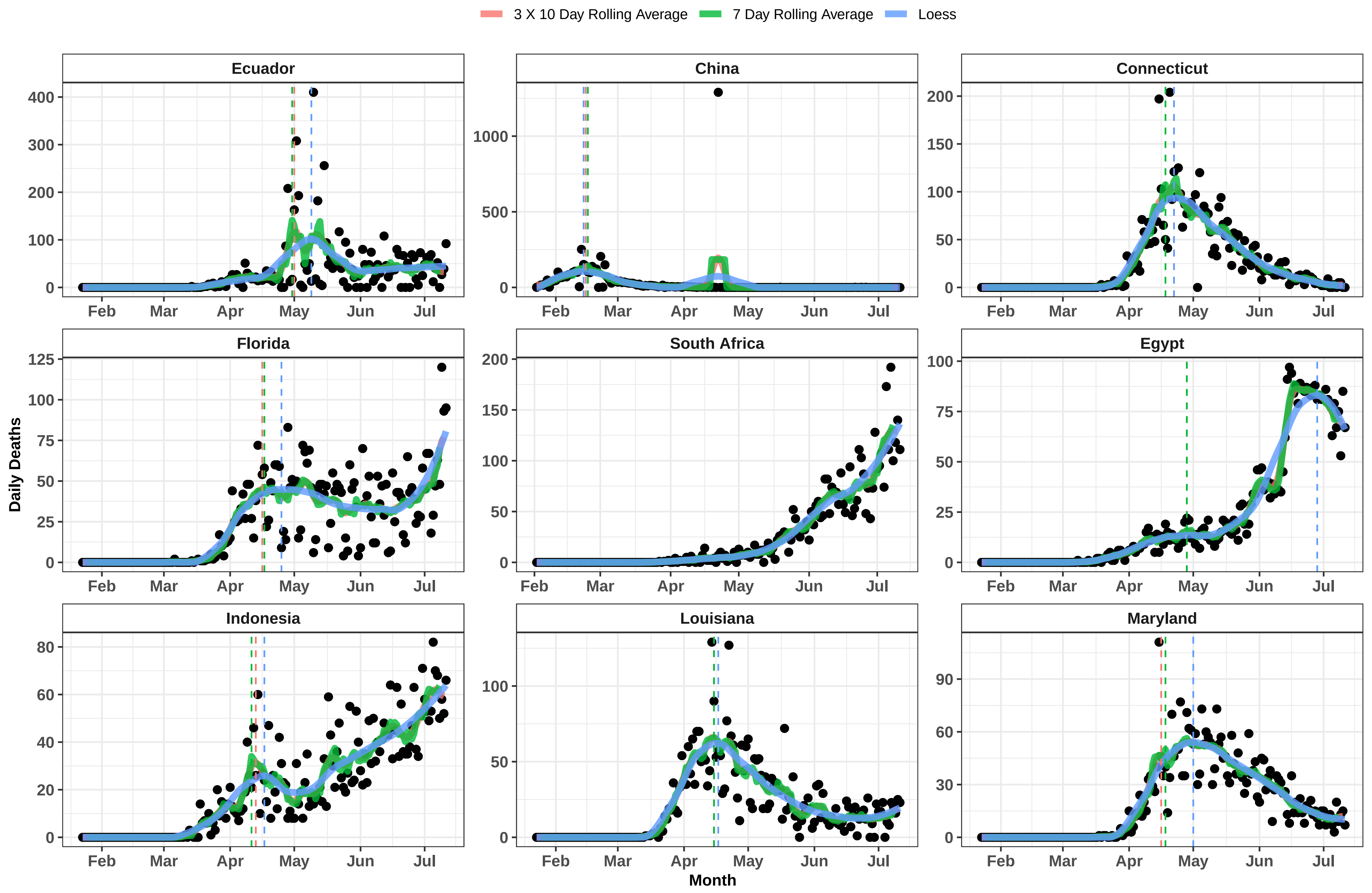

3 X 10 Day Rolling Average
7 Day Rolling Average
Loess
Ecuador
China
Connecticut
Florida
South Africa
Egypt
Indonesia
Louisiana
Maryland
Daily Deaths
Month
Feb
Mar
Apr
May
Jun
Jul

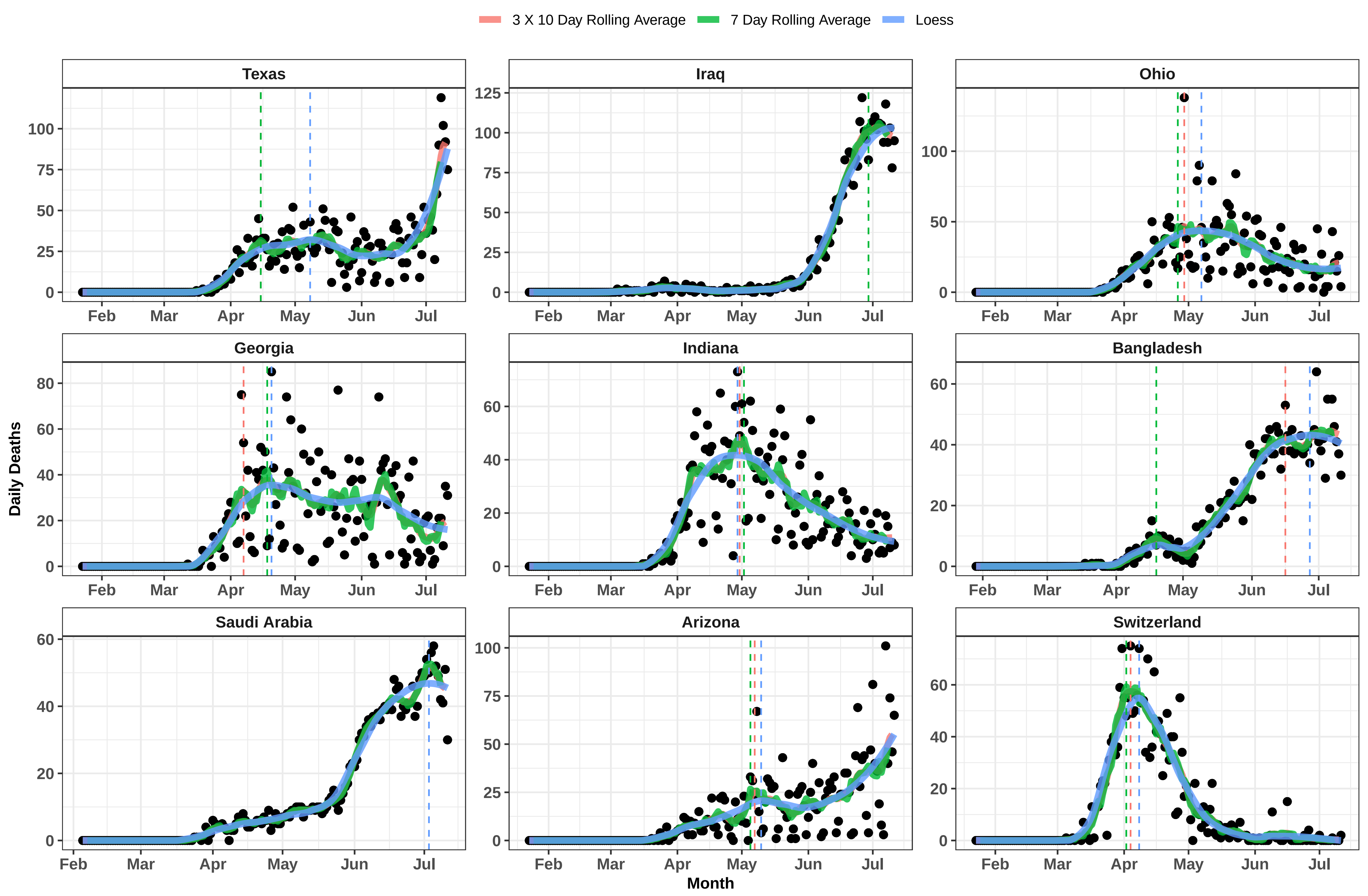

3 X 10 Day Rolling Average
7 Day Rolling Average
Loess
Texas
Iraq
Ohio
Georgia
Indiana
Bangladesh
Saudi Arabia
Arizona
Switzerland
Daily Deaths
Month
Feb
Mar
Apr
May
Jun
Jul

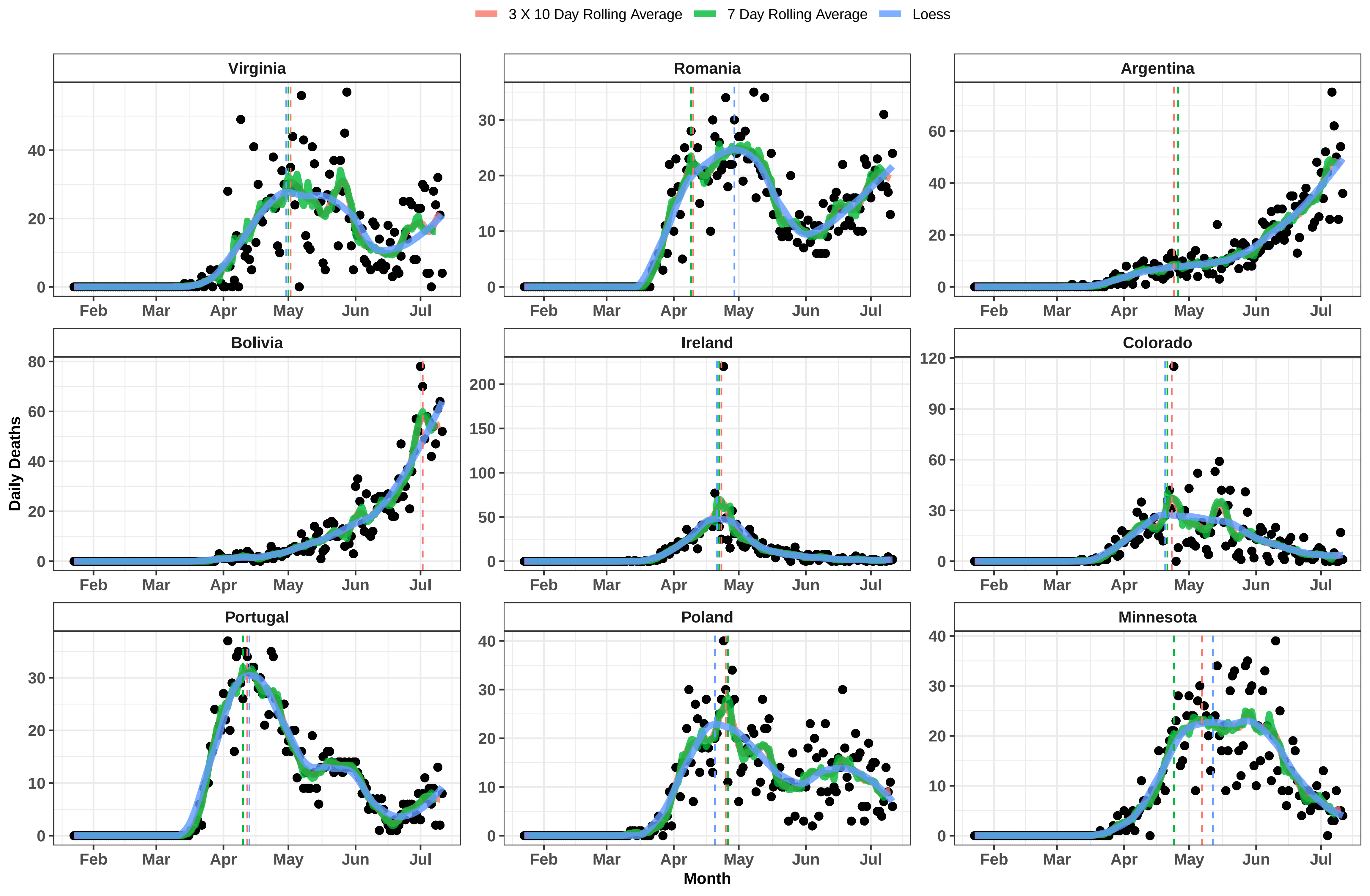

3 X 10 Day Rolling Average
7 Day Rolling Average
Loess
Virginia
Romania
Argentina
Bolivia
Ireland
Colorado
Portugal
Poland
Minnesota
Daily Deaths
Month
Feb
Mar
Apr
May
Jun
Jul

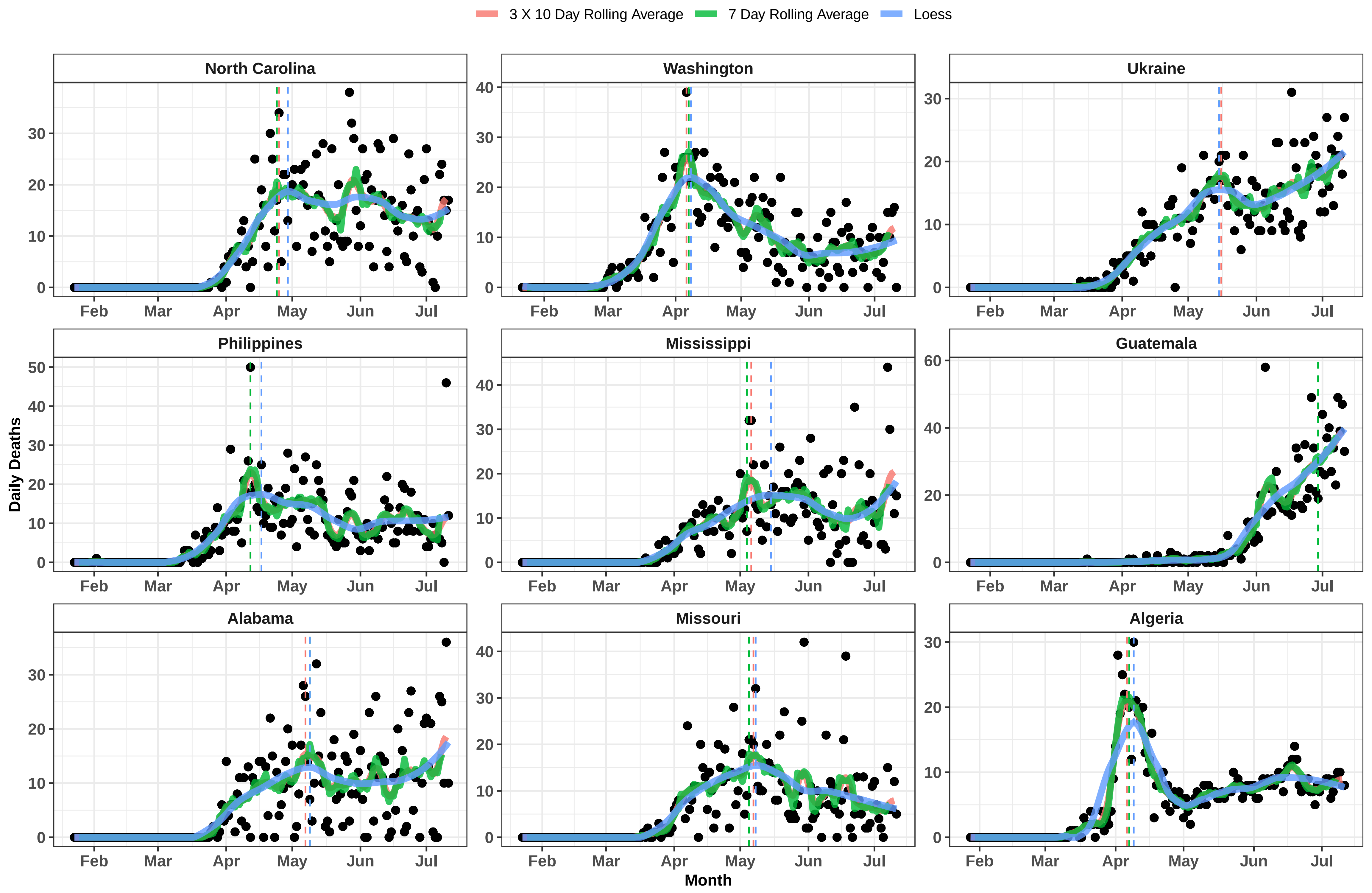
3 X 10 Day Rolling Average
7 Day Rolling Average
Loess
North Carolina
Washington
Ukraine
Philippines
Mississippi
Guatemala
Alabama
Missouri
Algeria
Daily Deaths
Month
Feb
Mar
Apr
May
Jun
Jul

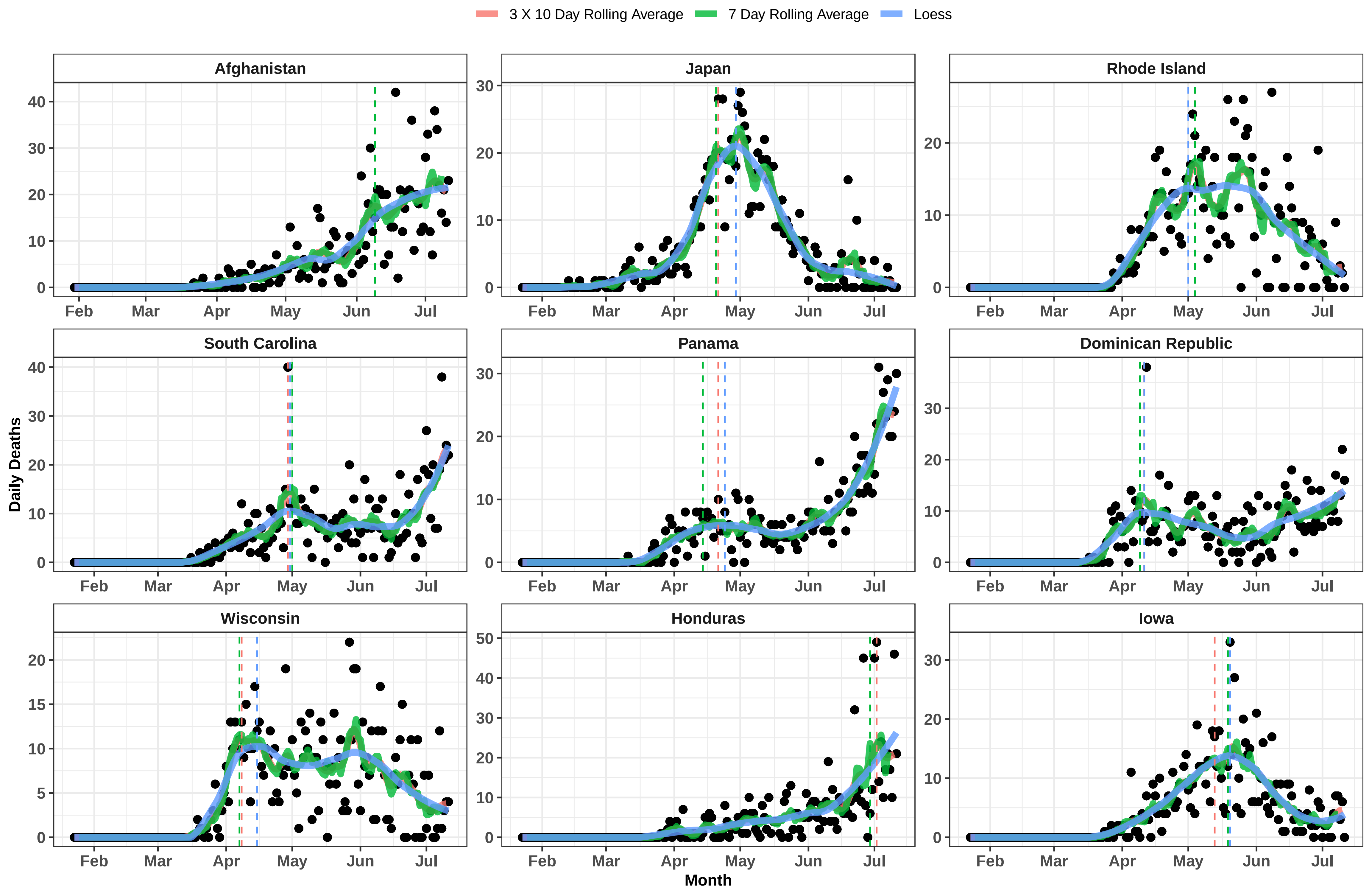

3 X 10 Day Rolling Average
7 Day Rolling Average
Loess
Afghanistan
Japan
Rhode Island
South Carolina
Panama
Dominican Republic
Wisconsin
Honduras
Iowa
Daily Deaths
Month
Feb
Mar
Apr
May
Jun
Jul

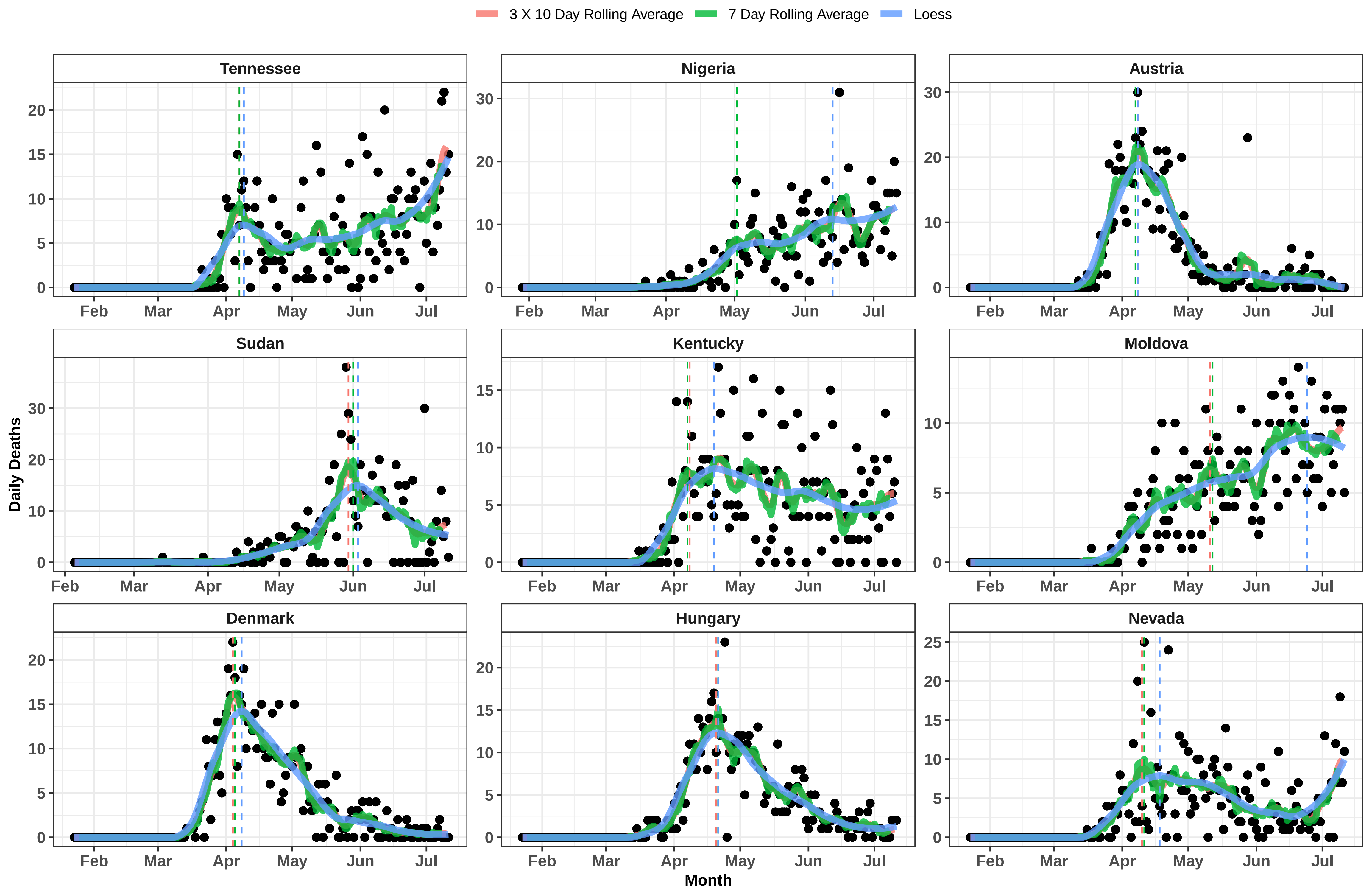

3 X 10 Day Rolling Average
7 Day Rolling Average
Loess
Tennessee
Nigeria
Austria
Sudan
Kentucky
Moldova
Denmark
Hungary
Nevada
Daily Deaths
Month
Feb
Mar
Apr
May
Jun
Jul

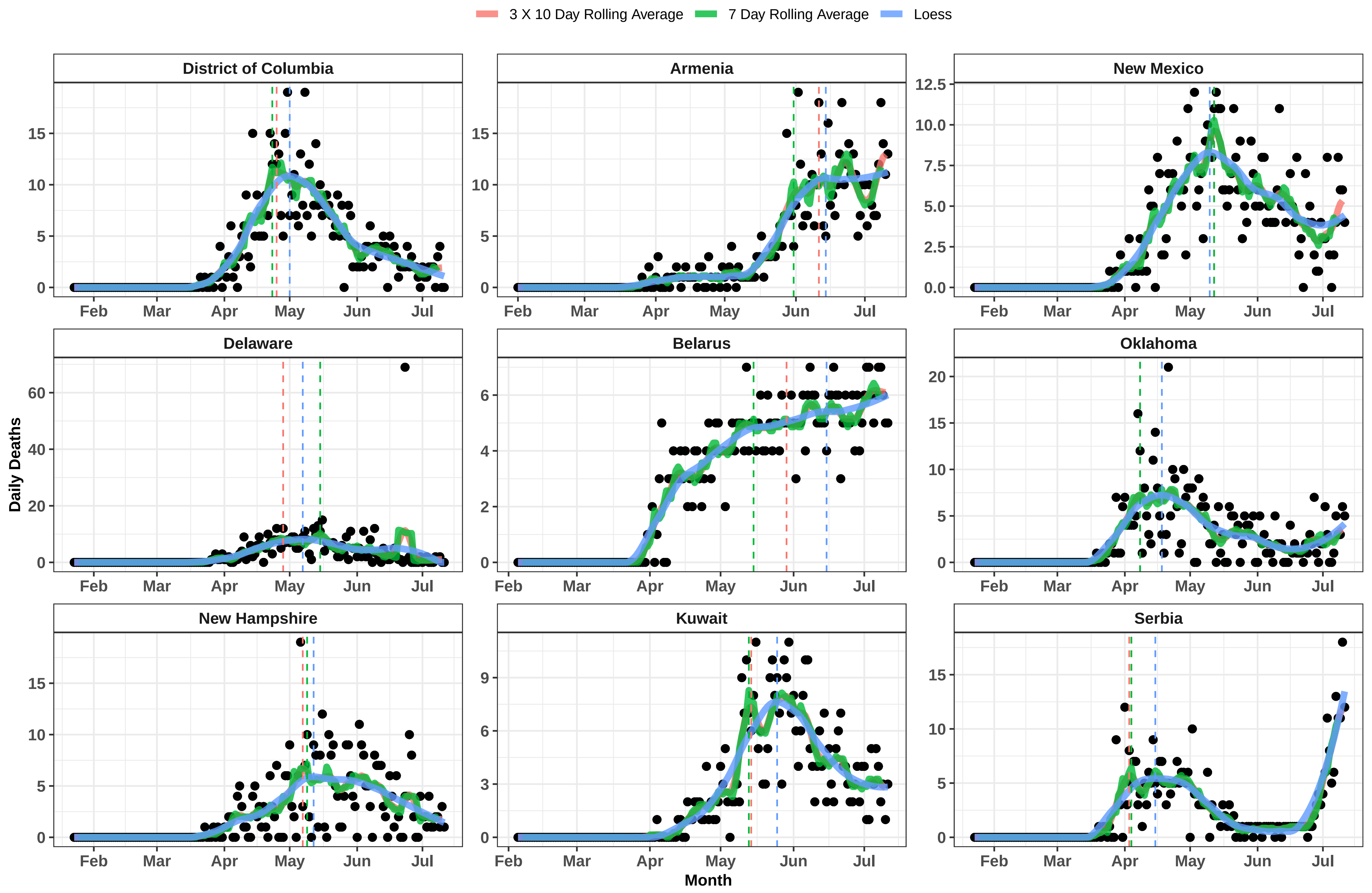

3 X 10 Day Rolling Average
7 Day Rolling Average
Loess
District of Columbia
Armenia
New Mexico
Delaware
Belarus
Oklahoma
New Hampshire
Kuwait
Serbia
Daily Deaths
Month
Feb
Mar
Apr
May
Jun
Jul

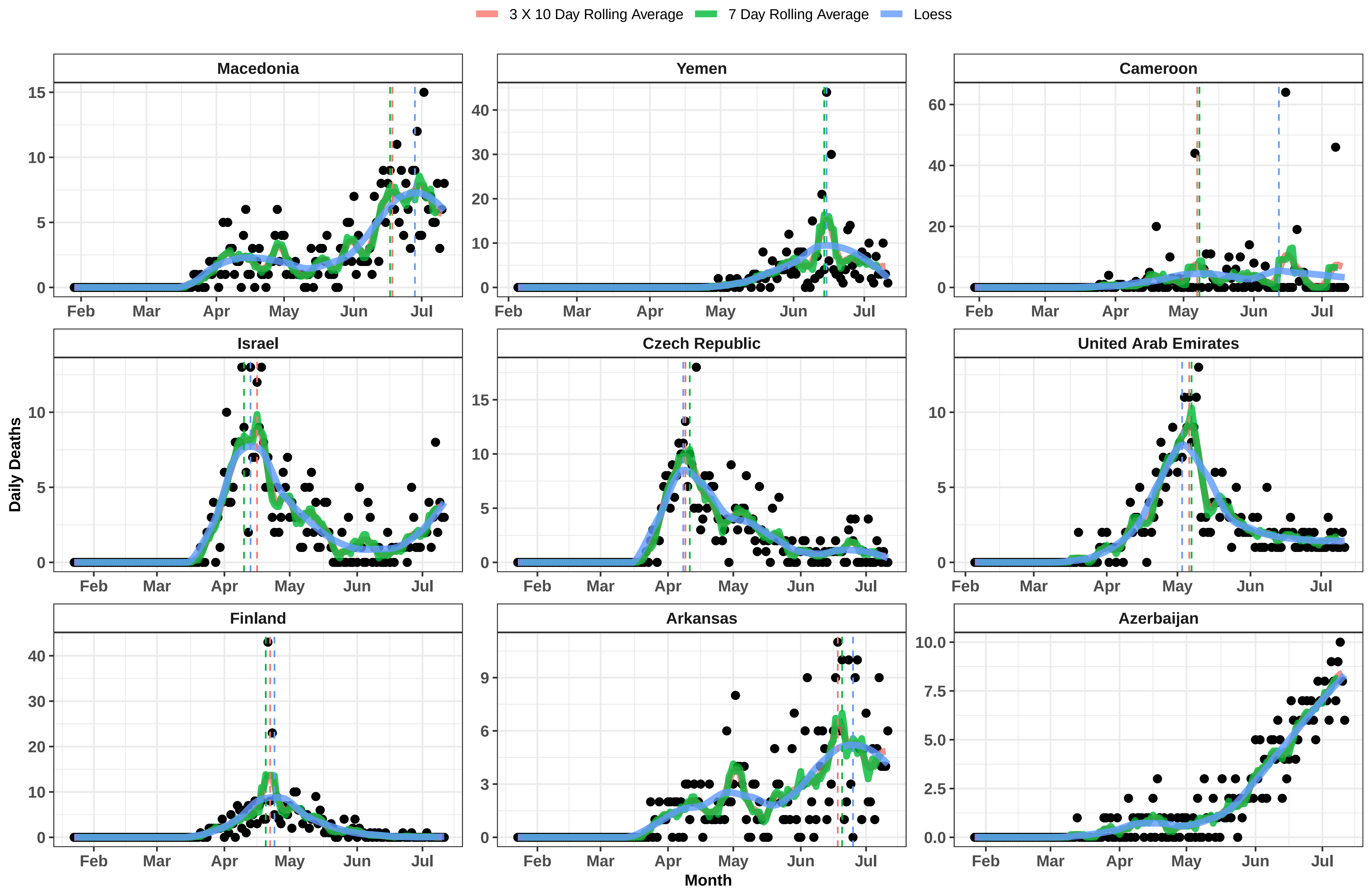

3 X 10 Day Rolling Average
7 Day Rolling Average
Loess
Macedonia
Yemen
Cameroon
Israel
Czech Republic
United Arab Emirates
Finland
Arkansas
Azerbaijan
Daily Deaths
Month
Feb
Mar
Apr
May
Jun
Jul

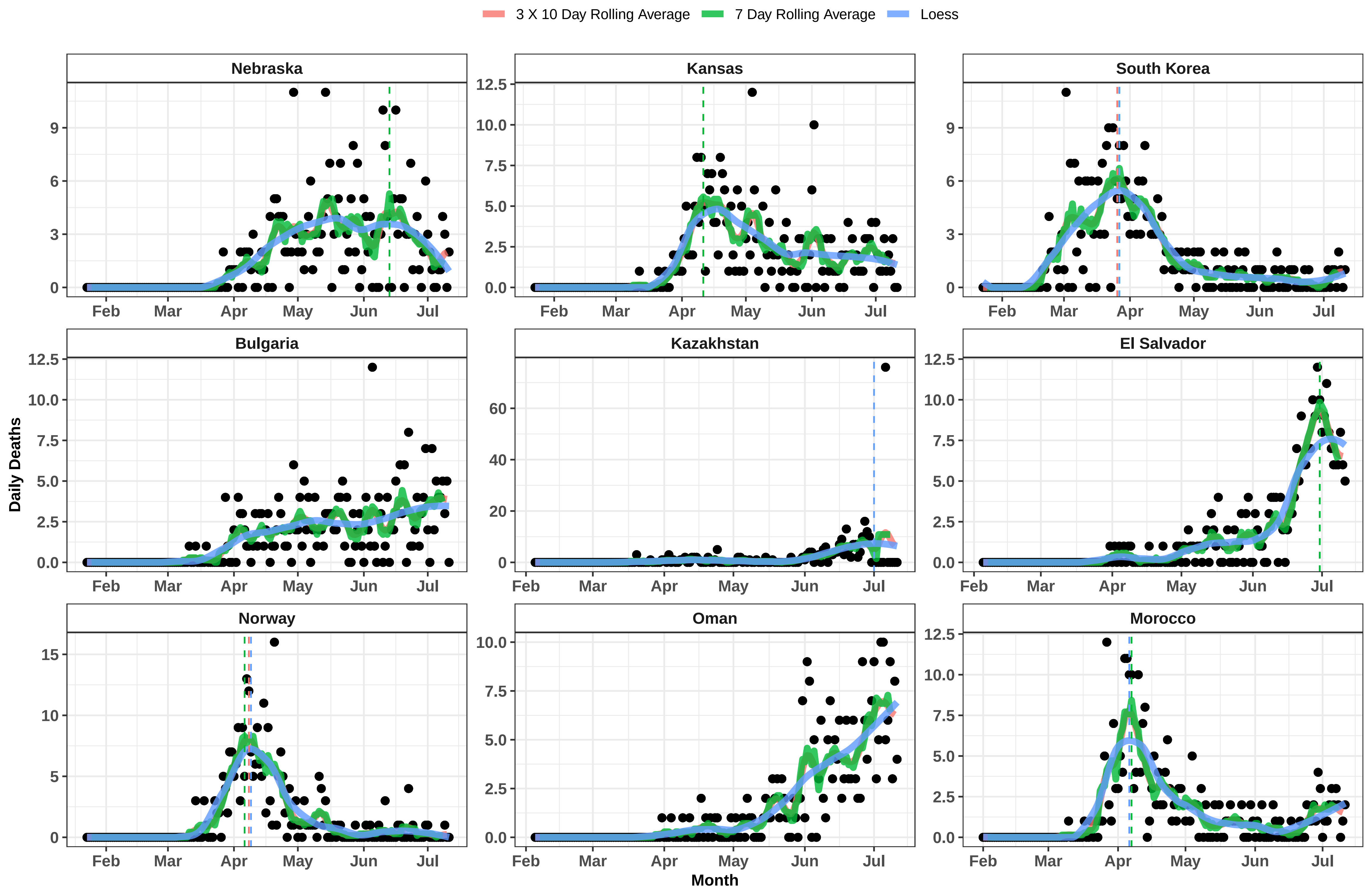

3 X 10 Day Rolling Average
7 Day Rolling Average
Loess
Nebraska
Kansas
South Korea
Bulgaria
Kazakhstan
El Salvador
Norway
Oman
Morocco
Daily Deaths
Month
Feb
Mar
Apr
May
Jun
Jul

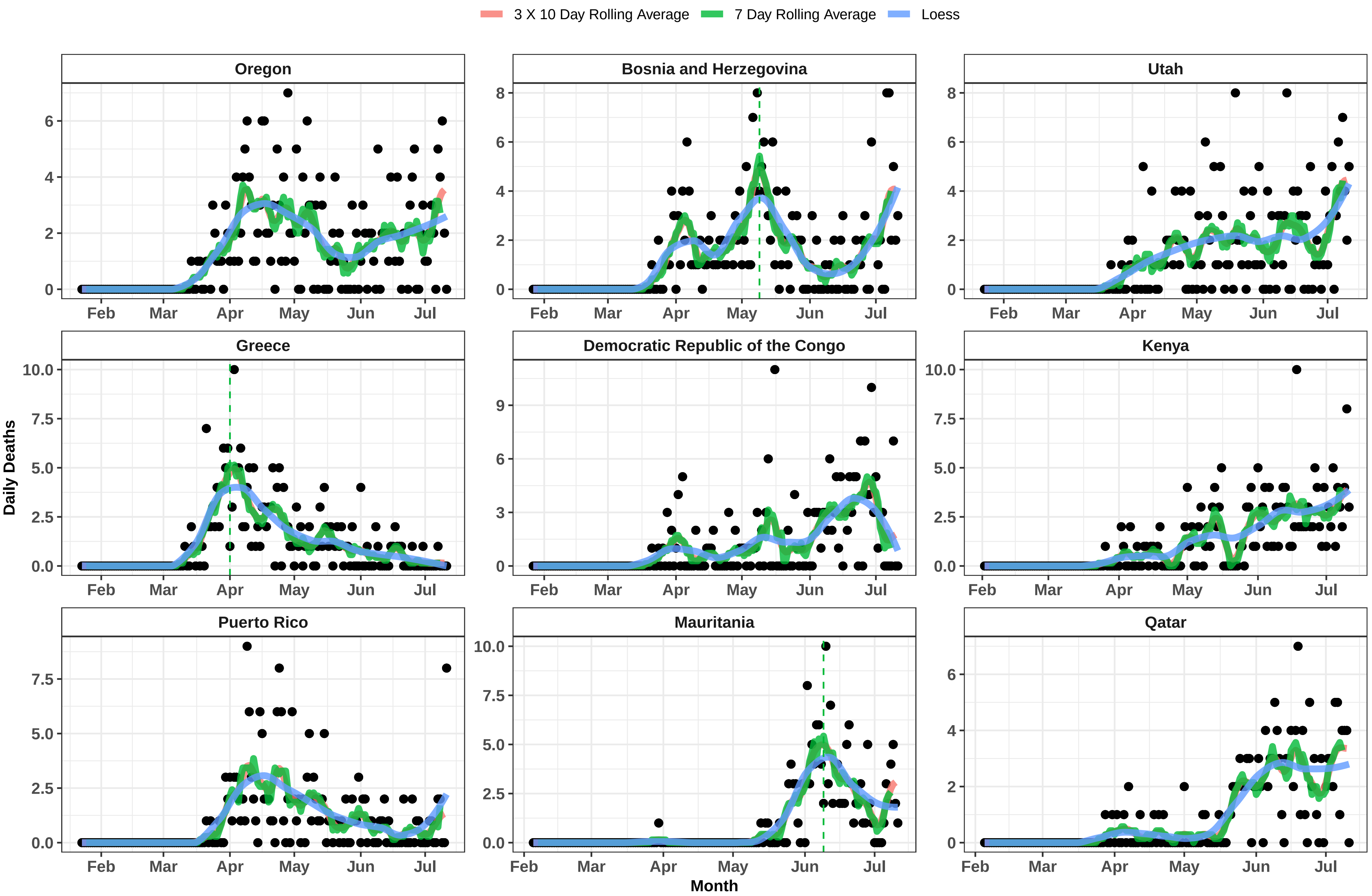

3 X 10 Day Rolling Average
7 Day Rolling Average
Loess
Oregon
Bosnia and Herzegovina
Utah
Greece
Democratic Republic of the Congo
Kenya
Puerto Rico
Mauritania
Qatar
Daily Deaths
Month
Feb
Mar
Apr
May
Jun
Jul

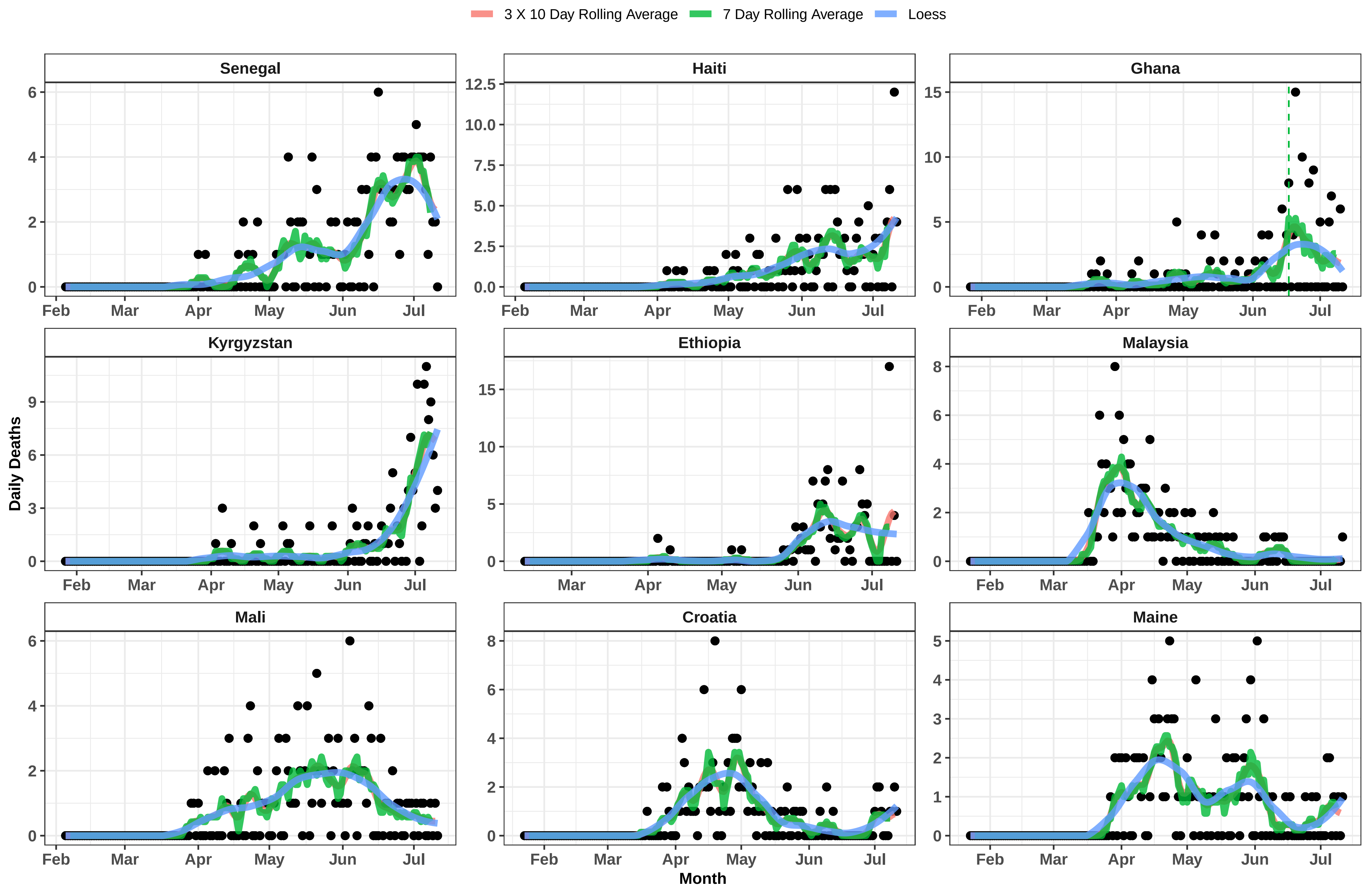
3 X 10 Day Rolling Average
7 Day Rolling Average
Loess
Senegal
Haiti
Ghana
Kyrgyzstan
Ethiopia
Malaysia
Mali
Croatia
Maine
Daily Deaths
Month
Feb
Mar
Apr
May
Jun
Jul

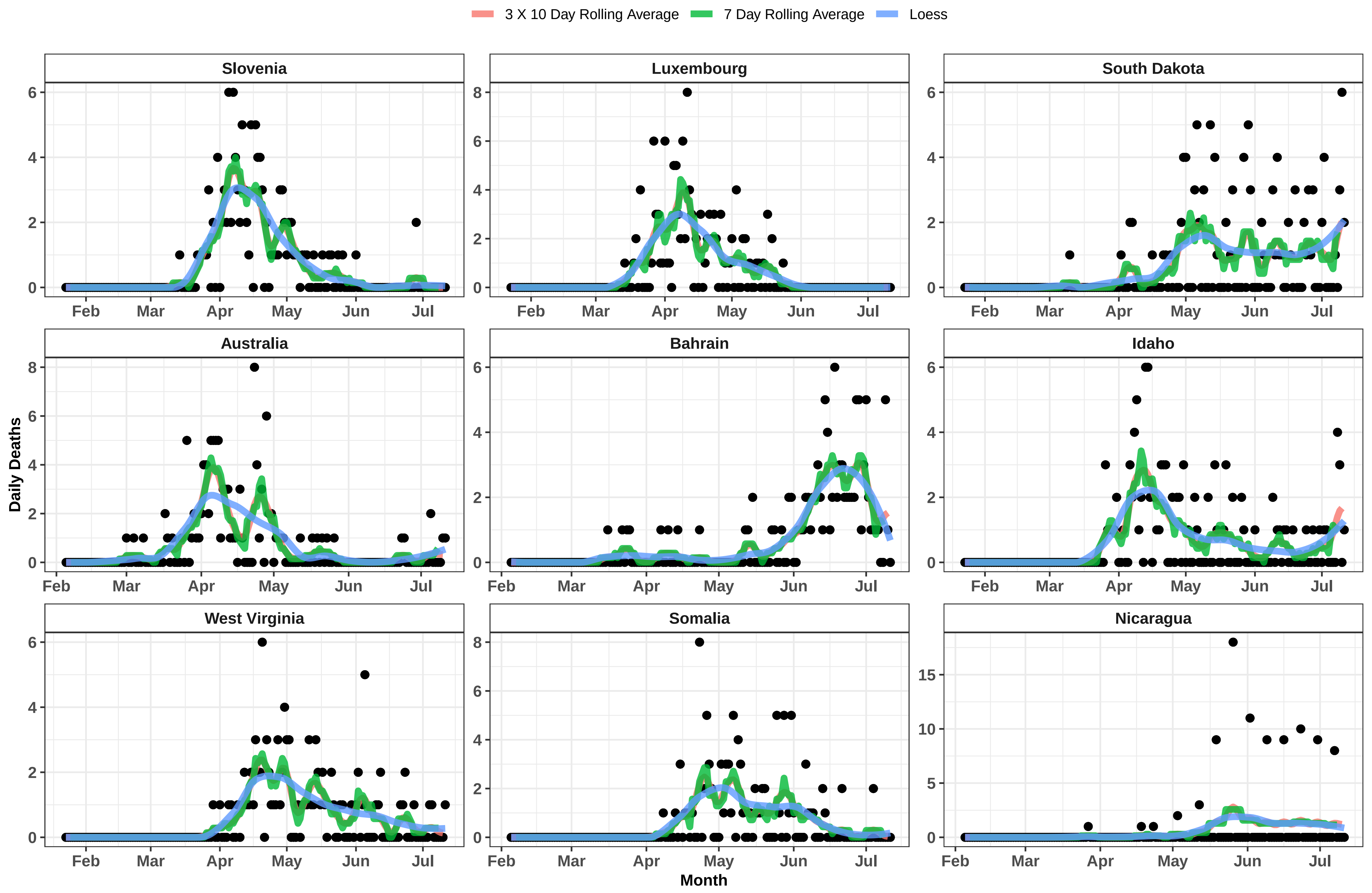

3 X 10 Day Rolling Average
7 Day Rolling Average
Loess
Slovenia
Luxembourg
South Dakota
Australia
Bahrain
Idaho
West Virginia
Somalia
Nicaragua
Daily Deaths
Month
Feb
Mar
Apr
May
Jun
Jul

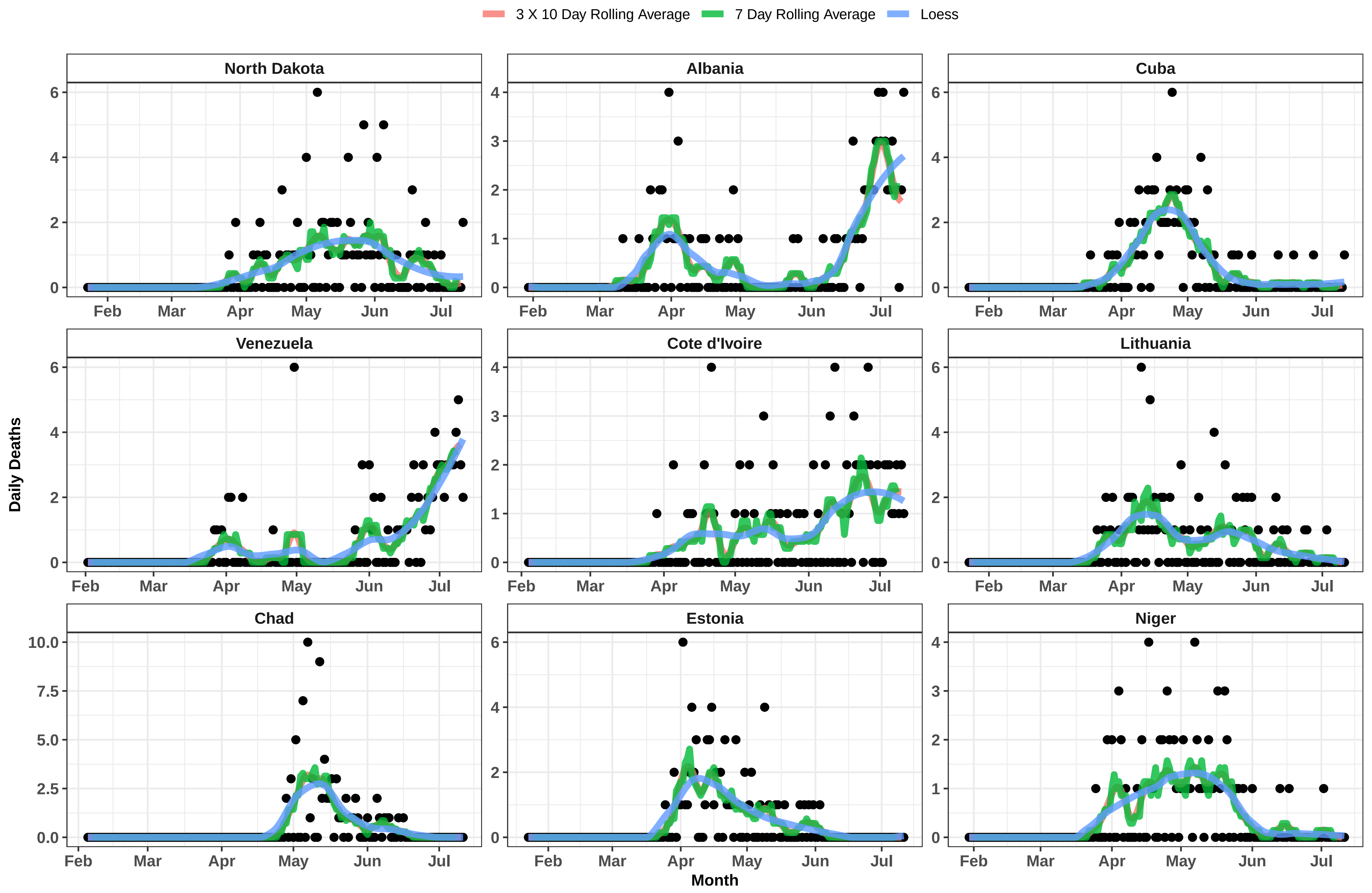

3 X 10 Day Rolling Average
7 Day Rolling Average
Loess
North Dakota
Albania
Cuba
Venezuela
Cote d'Ivoire
Lithuania
Chad
Estonia
Niger
Daily Deaths
Month
Feb
Mar
Apr
May
Jun
Jul

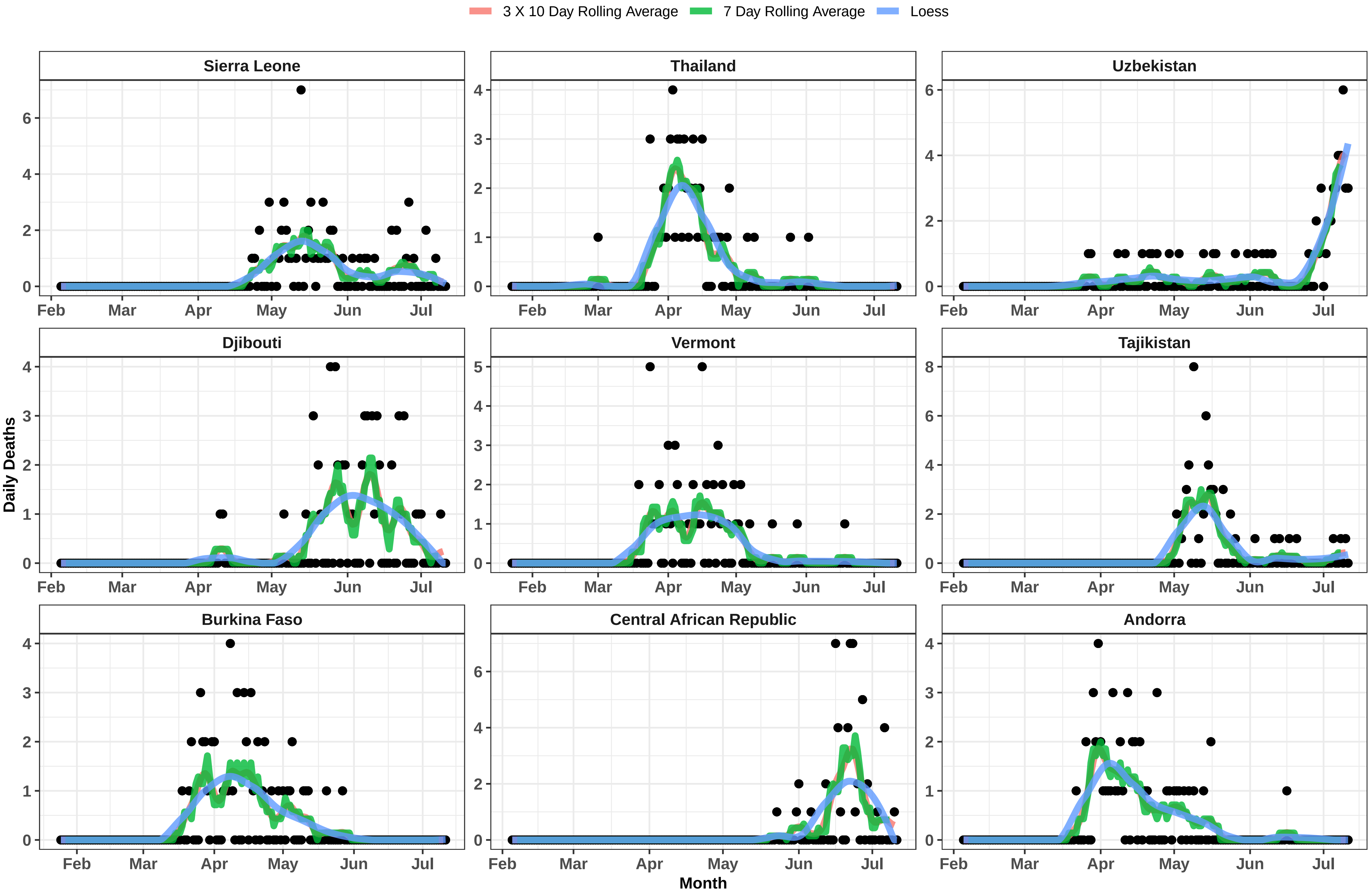

3 X 10 Day Rolling Average
7 Day Rolling Average
Loess
Sierra Leone
Thailand
Uzbekistan
Djibouti
Vermont
Tajikistan
Burkina Faso
Central African Republic
Andorra
Daily Deaths
Month
Feb
Mar
Apr
May
Jun
Jul

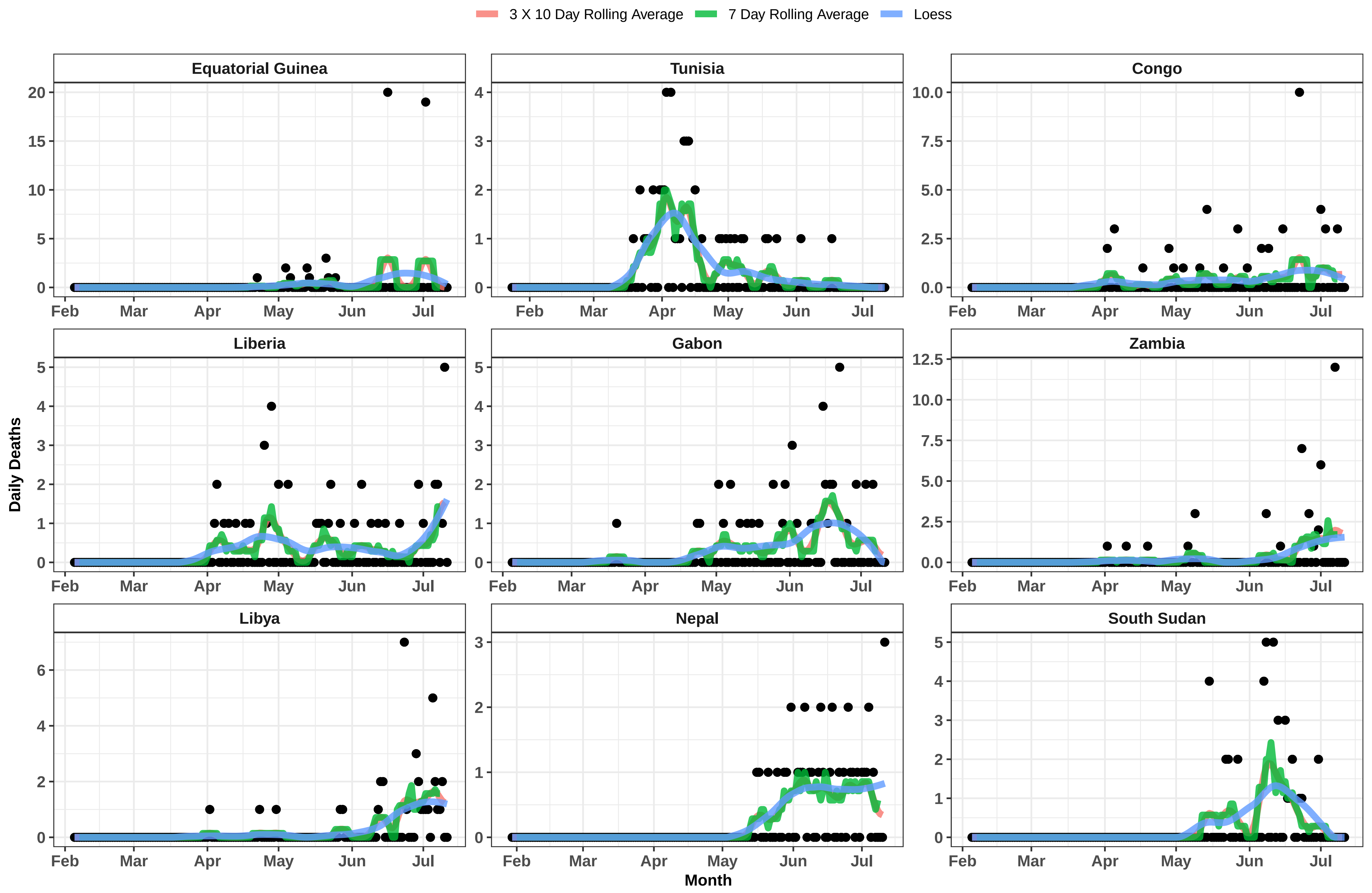

3 X 10 Day Rolling Average
7 Day Rolling Average
Loess
Equatorial Guinea
Tunisia
Congo
Liberia
Gabon
Zambia
Libya
Nepal
South Sudan
Daily Deaths
Month
Feb
Mar
Apr
May
Jun
Jul

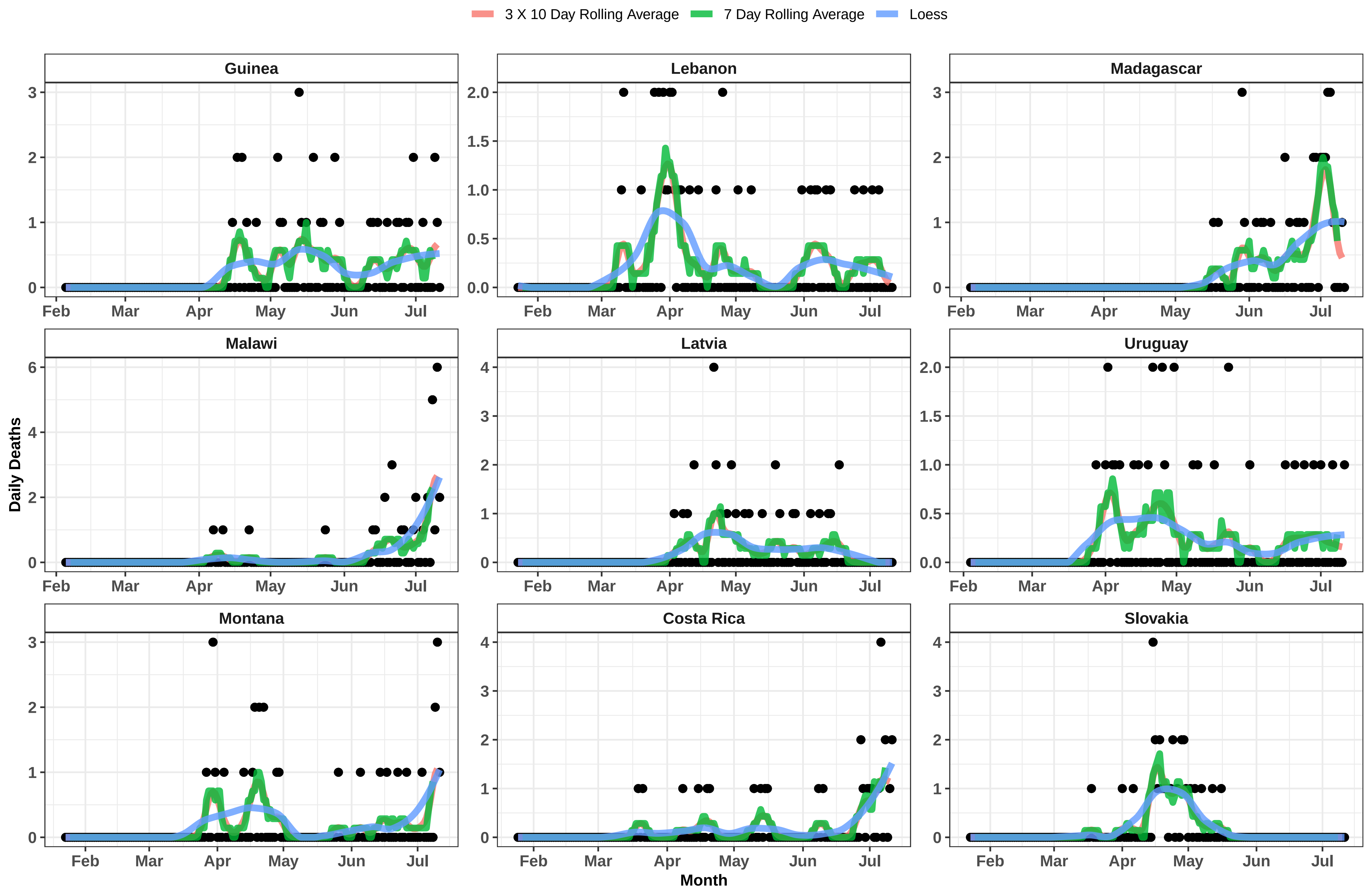
3 X 10 Day Rolling Average
7 Day Rolling Average
Loess
Guinea
Lebanon
Madagascar
Malawi
Latvia
Uruguay
Montana
Costa Rica
Slovakia
Daily Deaths
Month
Feb
Mar
Apr
May
Jun
Jul

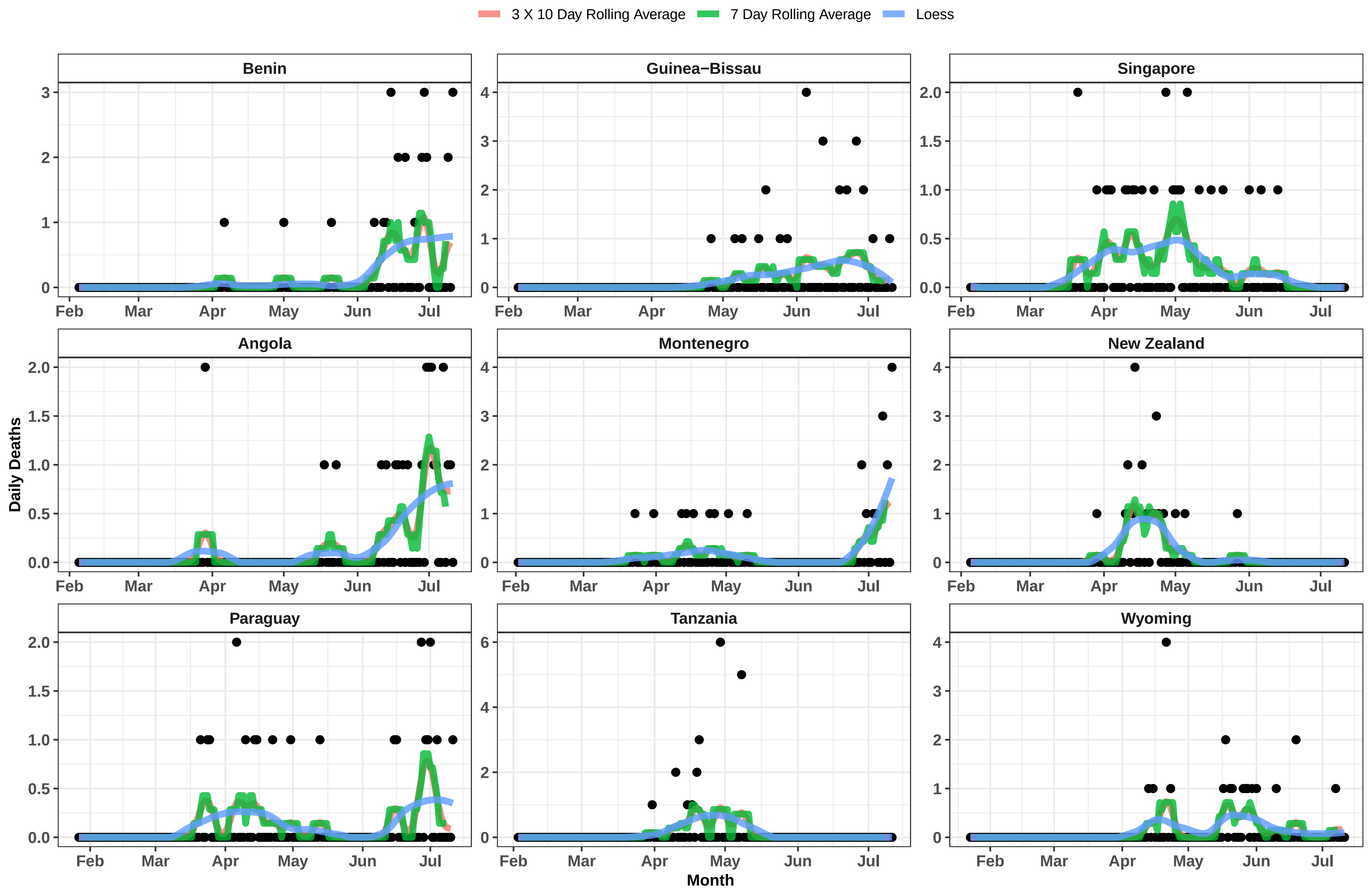

3 X 10 Day Rolling Average
7 Day Rolling Average
Loess
Benin
Guinea−Bissau
Singapore
Angola
Montenegro
New Zealand
Paraguay
Tanzania
Wyoming
Daily Deaths
Month
Feb
Mar
Apr
May
Jun
Jul

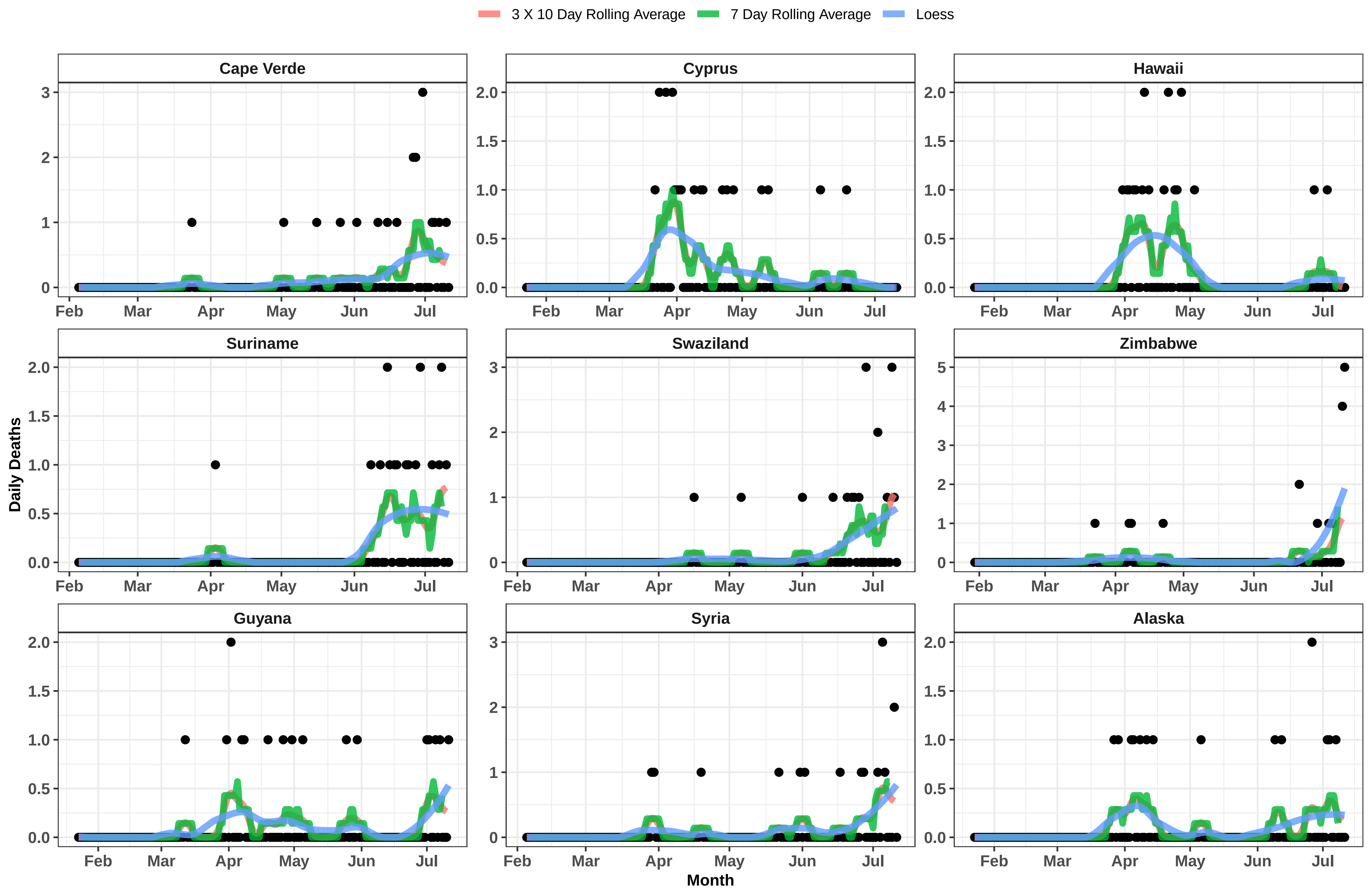

3 X 10 Day Rolling Average
7 Day Rolling Average
Loess
Cape Verde
Cyprus
Hawaii
Suriname
Swaziland
Zimbabwe
Guyana
Syria
Alaska
Daily Deaths
Month
Feb
Mar
Apr
May
Jun
Jul

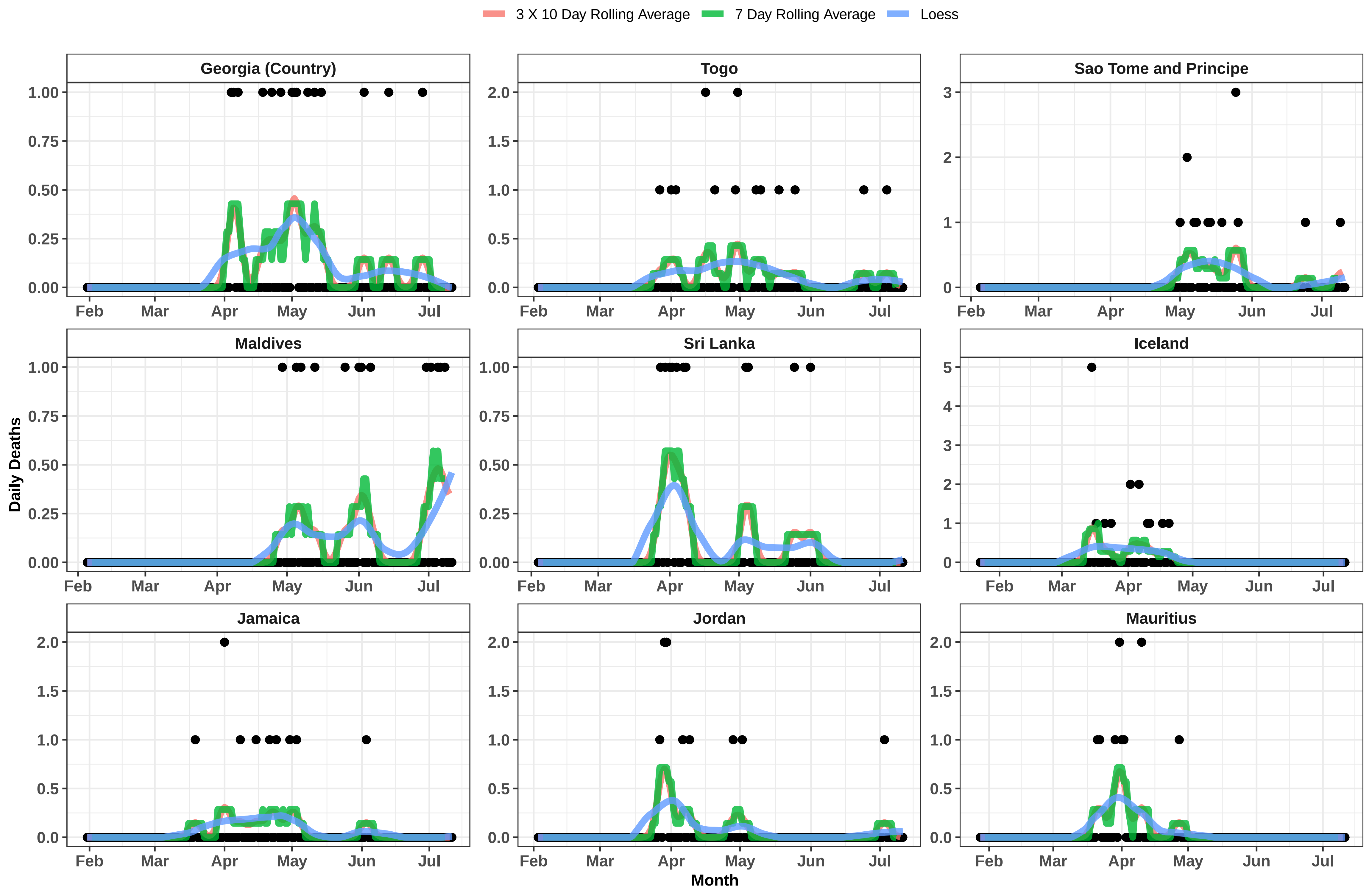

3 X 10 Day Rolling Average
7 Day Rolling Average
Loess
Georgia (Country)
Togo
Sao Tome and Principe
Maldives
Sri Lanka
Iceland
Jamaica
Jordan
Mauritius
Daily Deaths
Month
Feb Mar Apr May Jun Jul

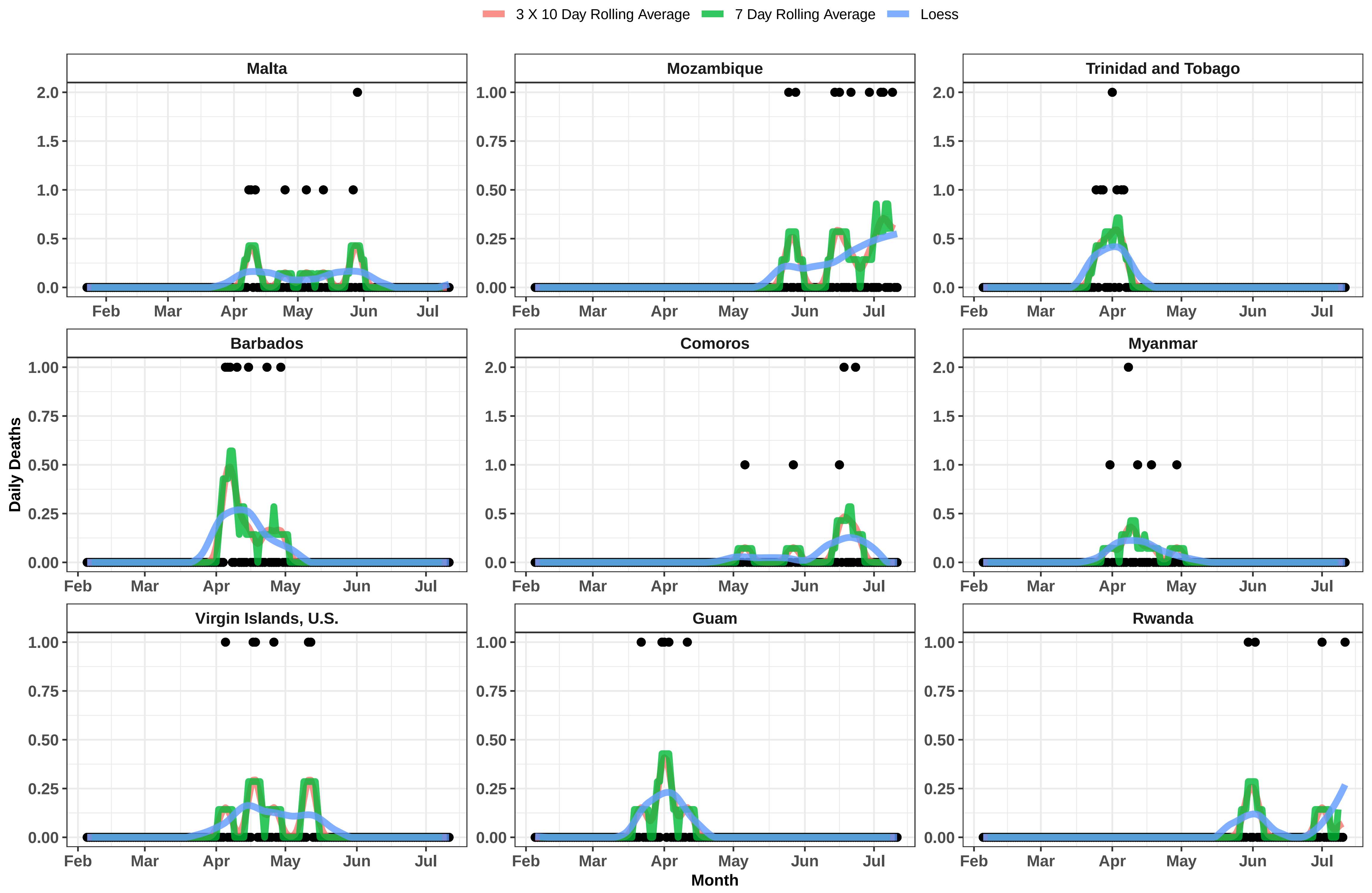

3 X 10 Day Rolling Average
7 Day Rolling Average
Loess
Malta
Mozambique
Trinidad and Tobago
Barbados
Comoros
Myanmar
Virgin Islands, U.S.
Guam
Rwanda
Daily Deaths
Month
Feb
Mar
Apr
May
Jun
Jul

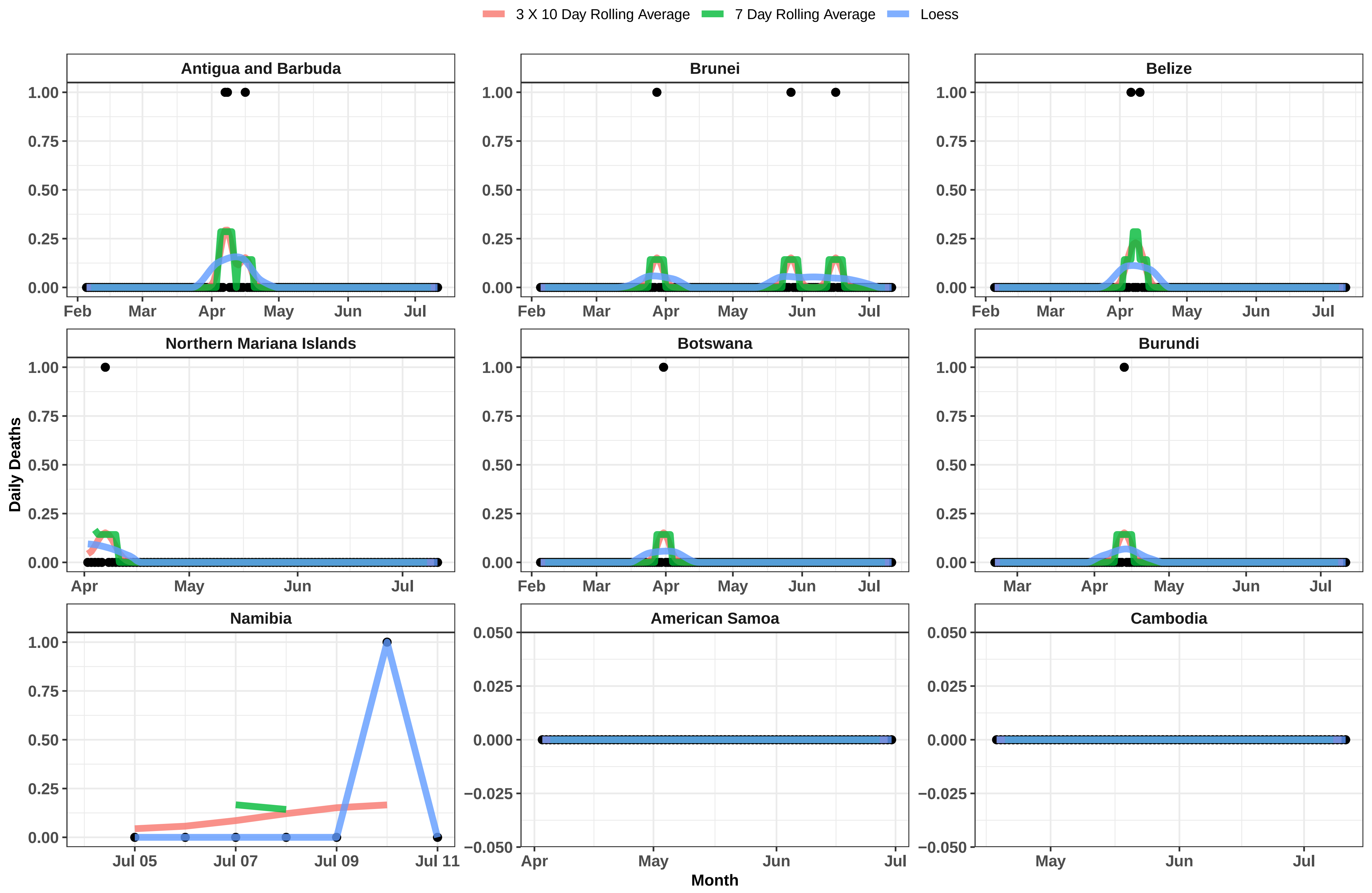

3 X 10 Day Rolling Average
7 Day Rolling Average
Loess
Antigua and Barbuda
Brunei
Belize
Northern Mariana Islands
Botswana
Burundi
Namibia
American Samoa
Cambodia
1.00
0.75
0.50
0.25
0.00
0.050
0.025
0.000
−0.025
−0.050
Feb
Mar
Apr
May
Jun
Jul
Jul 05
Jul 07
Jul 09
Jul 11
Daily Deaths
Month

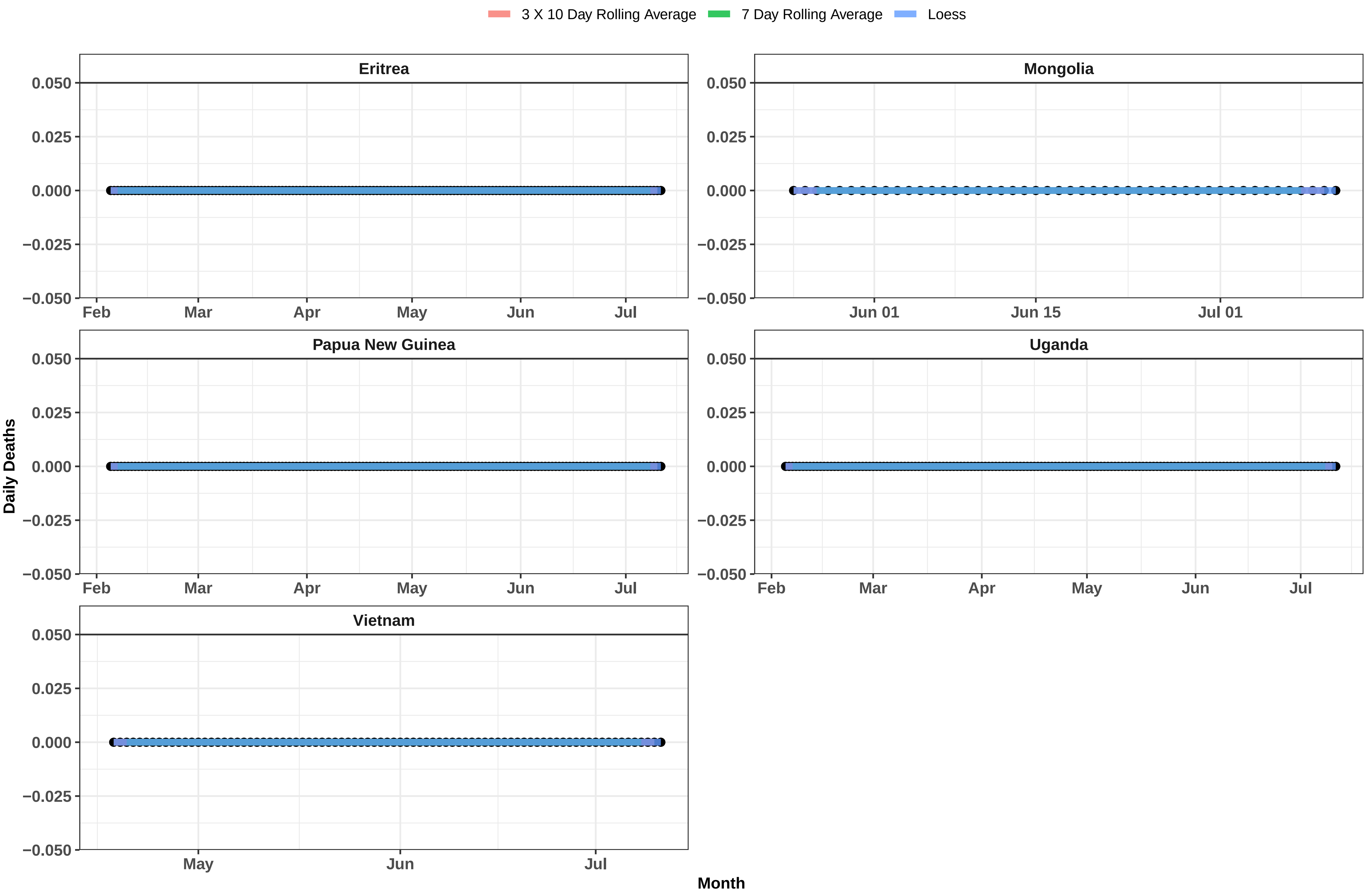

3 X 10 Day Rolling Average
7 Day Rolling Average
Loess
Eritrea
Mongolia
Papua New Guinea
Uganda
Vietnam
Daily Deaths
Month
0.050
0.025
0.000
−0.025
−0.050
Feb
Mar
Apr
May
Jun
Jul
Jun 01
Jun 15
Jul 01
