# Supplementary material for: Predictive performance of international COVID-19 mortality forecasting models
Source: medRxiv. 2020 Nov 19:2020.07.13.20151233. Preprint. [Version 5] doi: 10.1101/2020.07.13.20151233 (PMC7685335; doi:10.1101/2020.07.13.20151233)
Supplement: Supplement 2020 [file 90316-2020.07.13.20151233-1.pdf]

United States

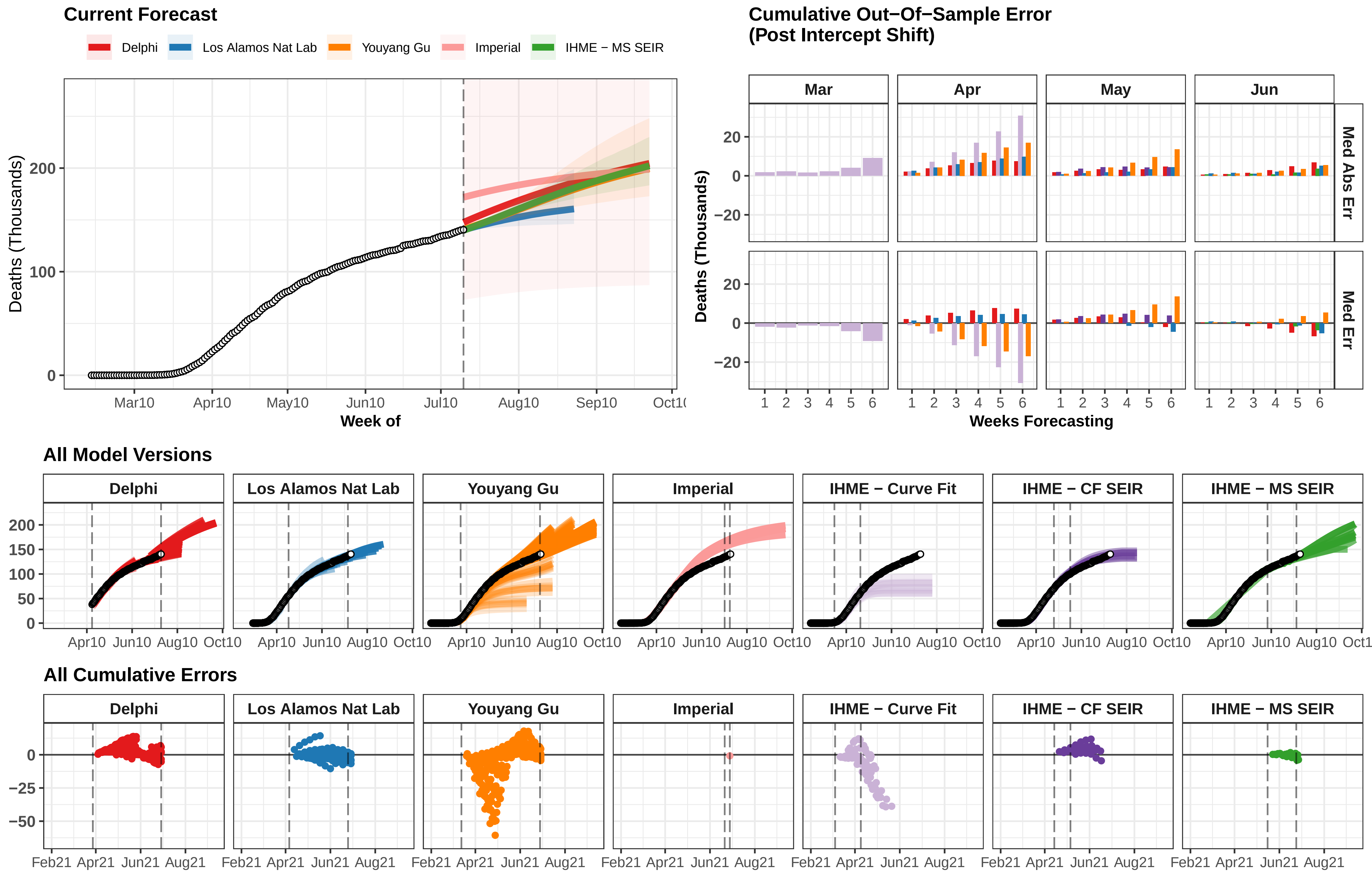

Brazil

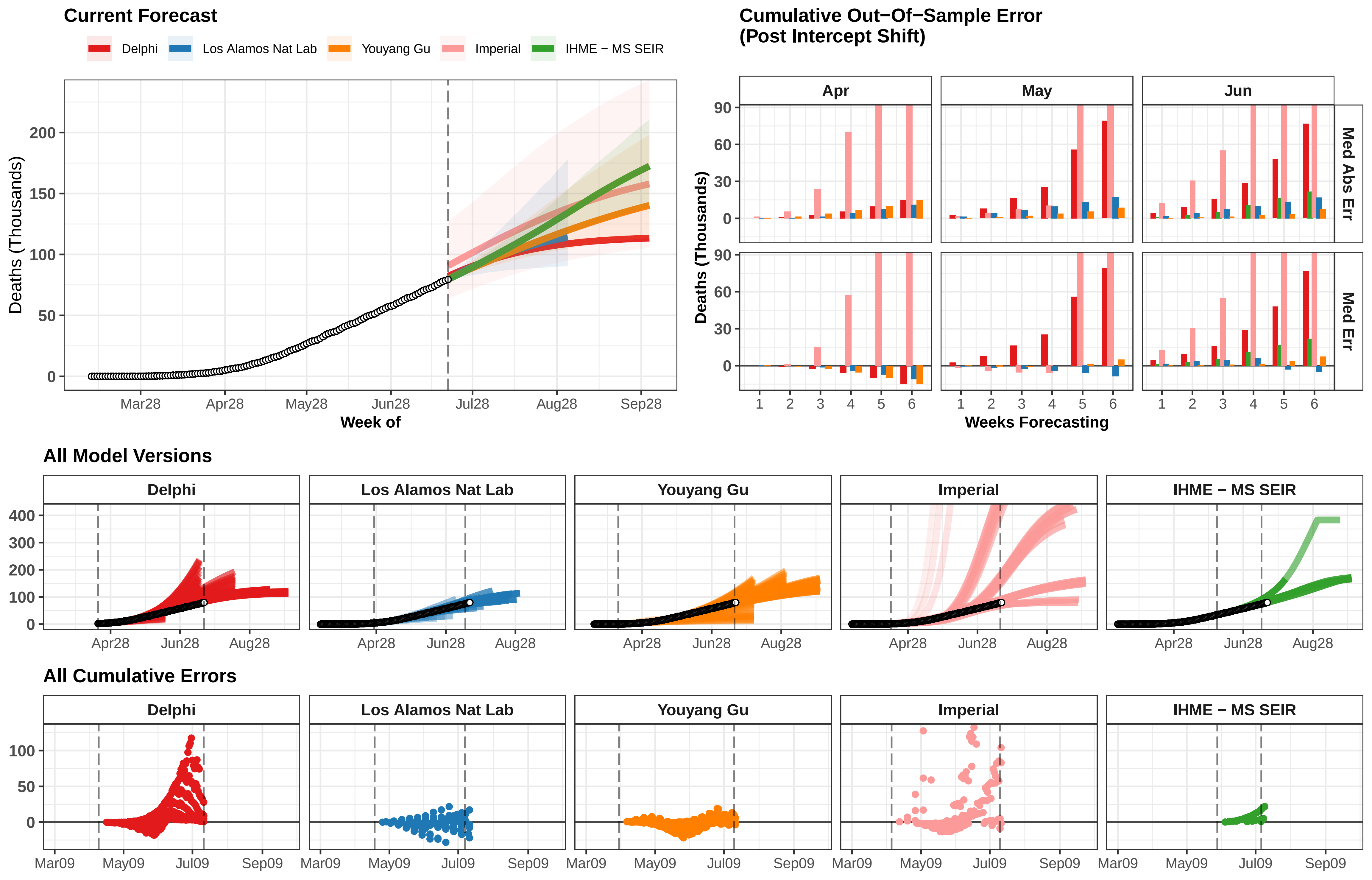
Current Forecast
Delphi
Los Alamos Nat Lab
Youyang Gu
Imperial
IHME − MS SEIR
Deaths (Thousands)
Week of
Mar28
Apr28
May28
Jun28
Jul28
Aug28
Sep28
Cumulative Out−Of−Sample Error
(Post Intercept Shift)
Apr
May
Jun
Med Abs Err
Med Err
Weeks Forecasting
All Model Versions
All Cumulative Errors
Mar09
May09
Jul09
Sep09

United Kingdom

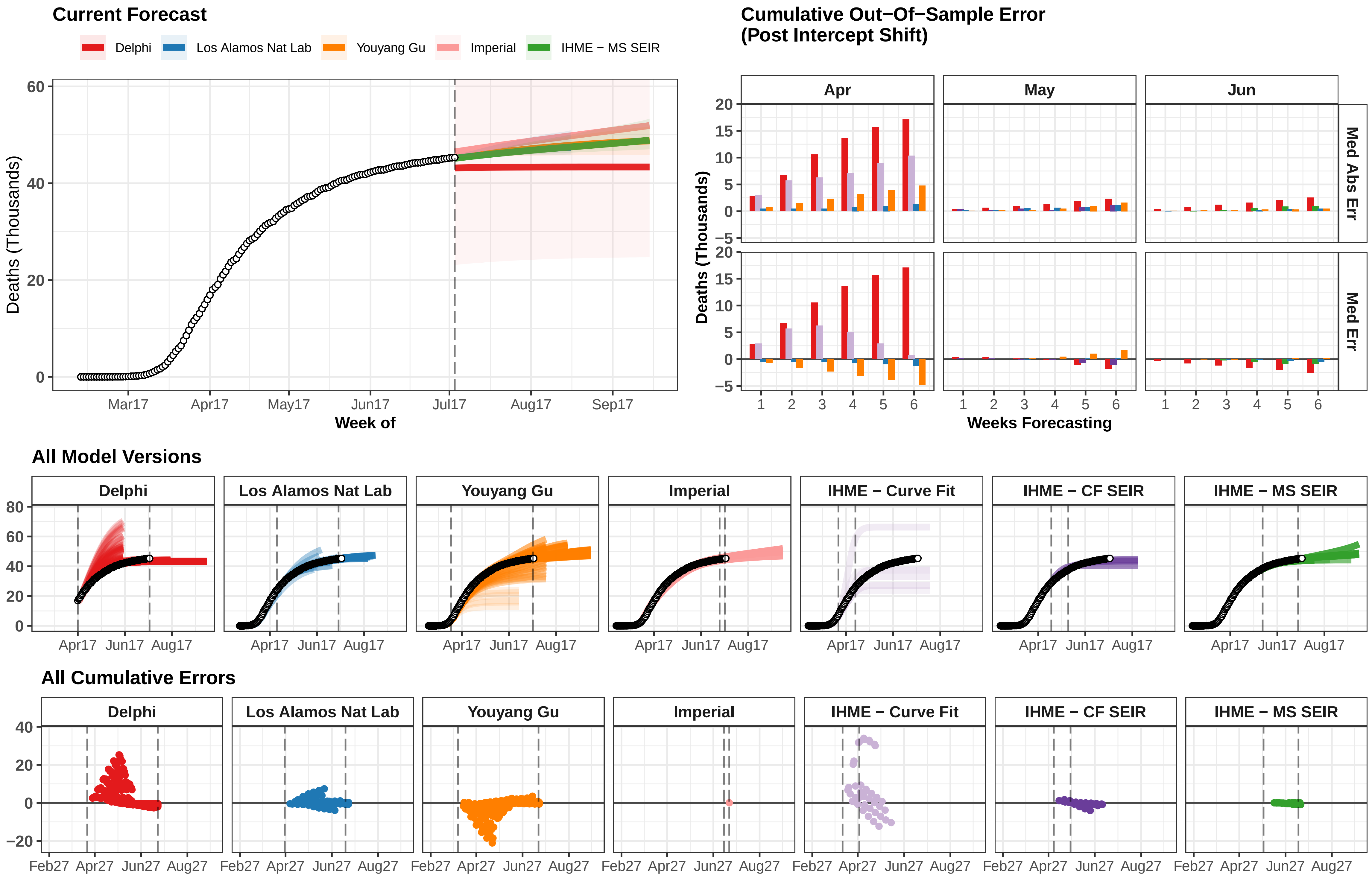

Mexico

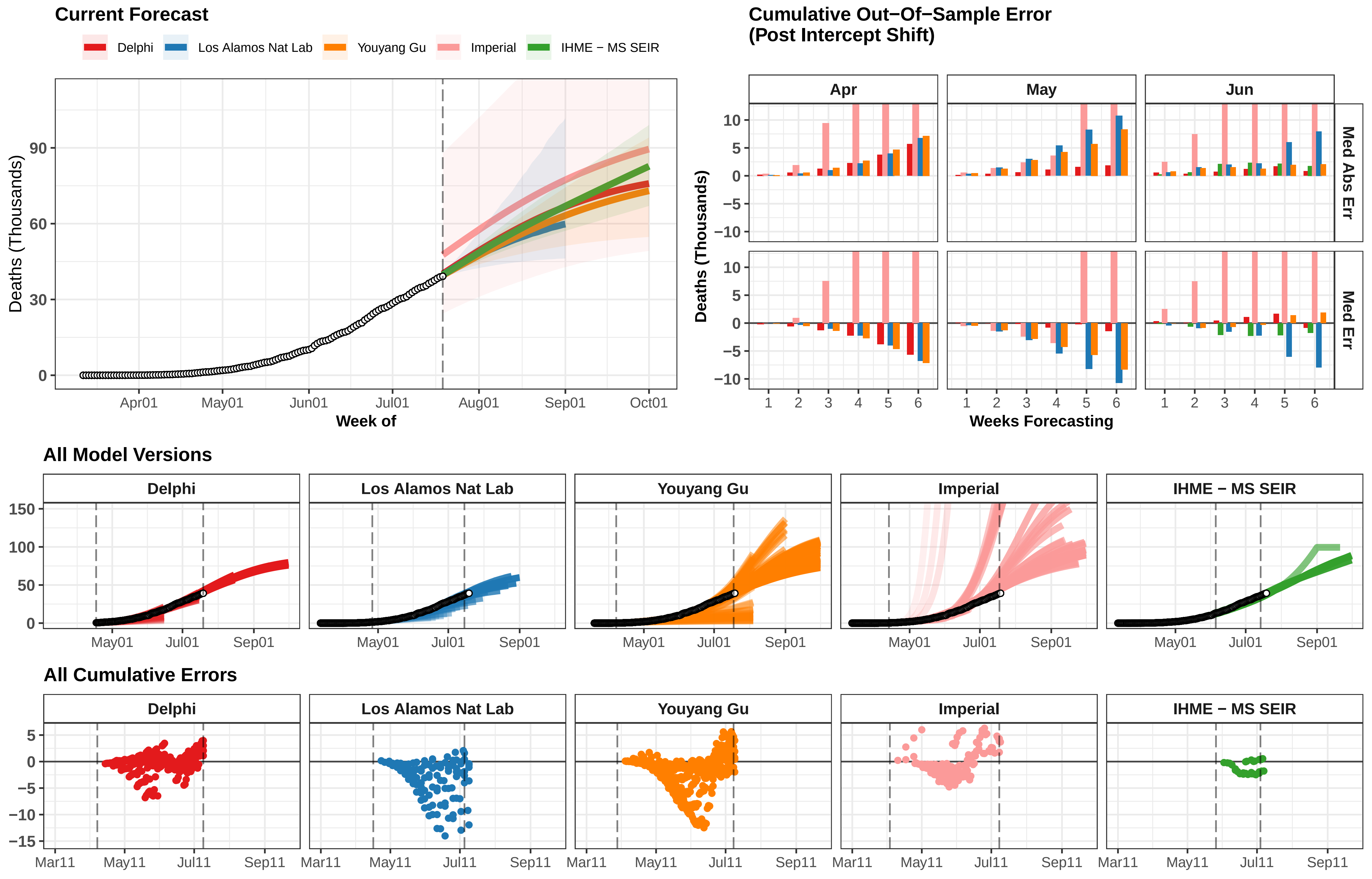
Current Forecast
Delphi
Los Alamos Nat Lab
Youyang Gu
Imperial
IHME − MS SEIR
Deaths (Thousands)
0
30
60
90
Apr01
May01
Jun01
Jul01
Aug01
Sep01
Oct01
Week of
Cumulative Out−Of−Sample Error
(Post Intercept Shift)
Apr
May
Jun
Med Abs Err
Med Err
Deaths (Thousands)
10
5
0
−5
−10
1
2
3
4
5
6
Weeks Forecasting
All Model Versions
Delphi
Los Alamos Nat Lab
Youyang Gu
Imperial
IHME − MS SEIR
150
100
50
0
May01
Jul01
Sep01
All Cumulative Errors
Delphi
Los Alamos Nat Lab
Youyang Gu
Imperial
IHME − MS SEIR
5
0
−5
−10
−15
Mar11
May11
Jul11
Sep11

Italy

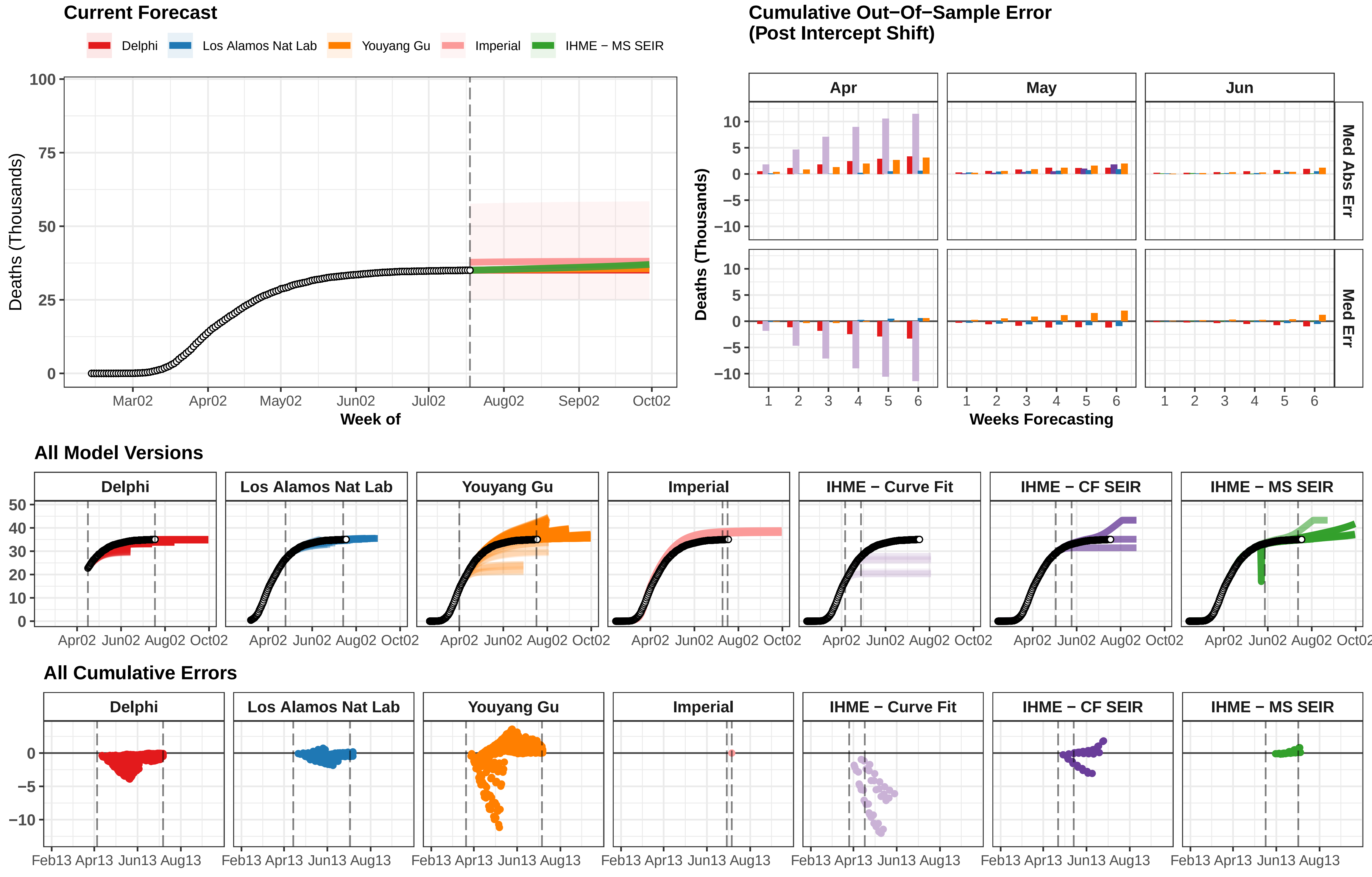
Current Forecast
Delphi
Los Alamos Nat Lab
Youyang Gu
Imperial
IHME − MS SEIR
Deaths (Thousands)
Week of
Mar02 Apr02 May02 Jun02 Jul02 Aug02 Sep02 Oct02
Cumulative Out−Of−Sample Error (Post Intercept Shift)
Apr
May
Jun
Med Abs Err
Med Err
Weeks Forecasting
All Model Versions
Delphi
Los Alamos Nat Lab
Youyang Gu
Imperial
IHME − Curve Fit
IHME − CF SEIR
IHME − MS SEIR
All Cumulative Errors
Feb13 Apr13 Jun13 Aug13

New York

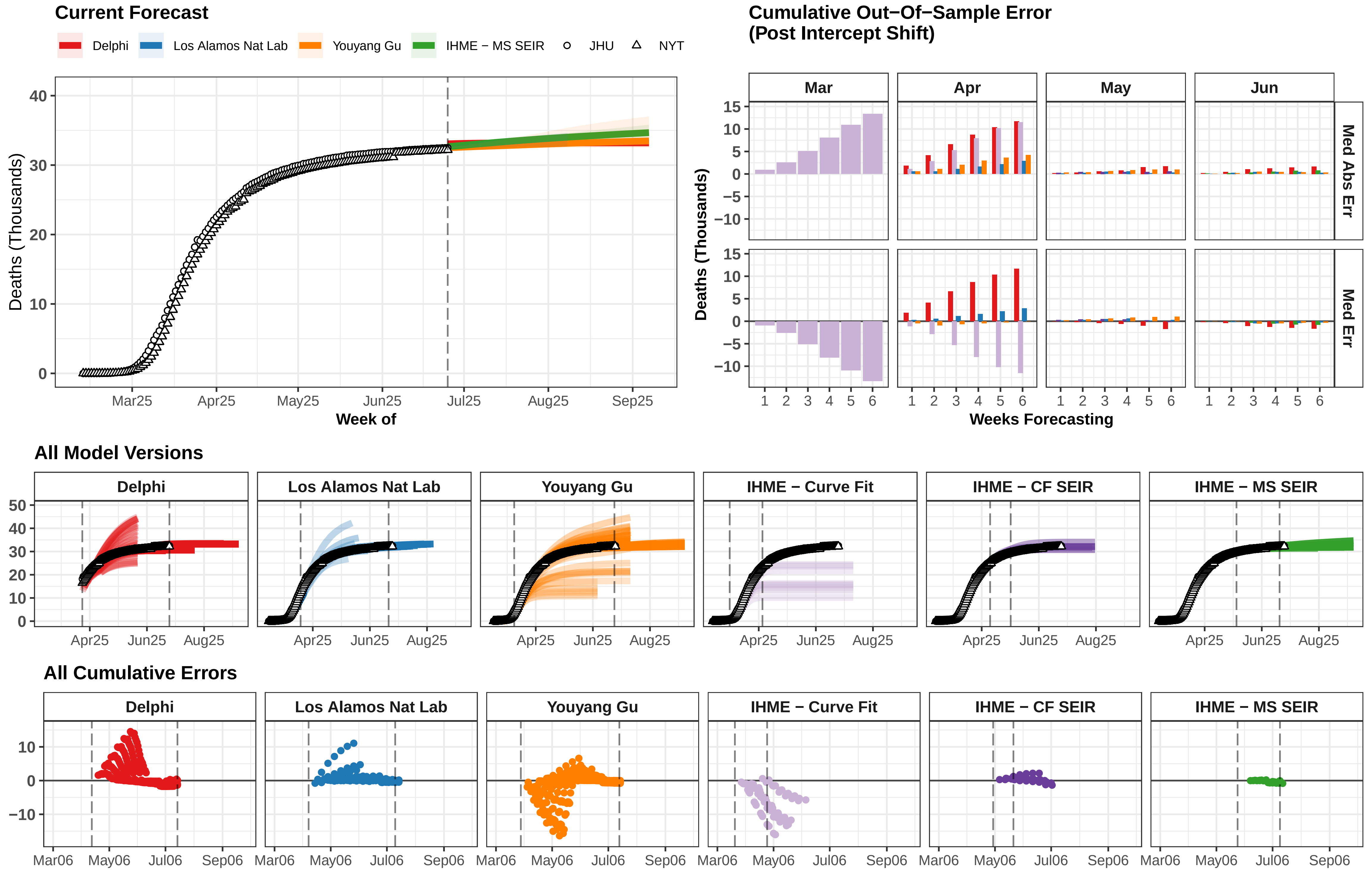
Current Forecast
Delphi
Los Alamos Nat Lab
Youyang Gu
IHME − MS SEIR
JHU
NYT
Deaths (Thousands)
Week of
Mar25
Apr25
May25
Jun25
Jul25
Aug25
Sep25
Cumulative Out−Of−Sample Error
(Post Intercept Shift)
Mar
Apr
May
Jun
Med Abs Err
Med Err
Weeks Forecasting
All Model Versions
Delphi
Los Alamos Nat Lab
Youyang Gu
IHME − Curve Fit
IHME − CF SEIR
IHME − MS SEIR
All Cumulative Errors
Mar06
May06
Jul06
Sep06

France

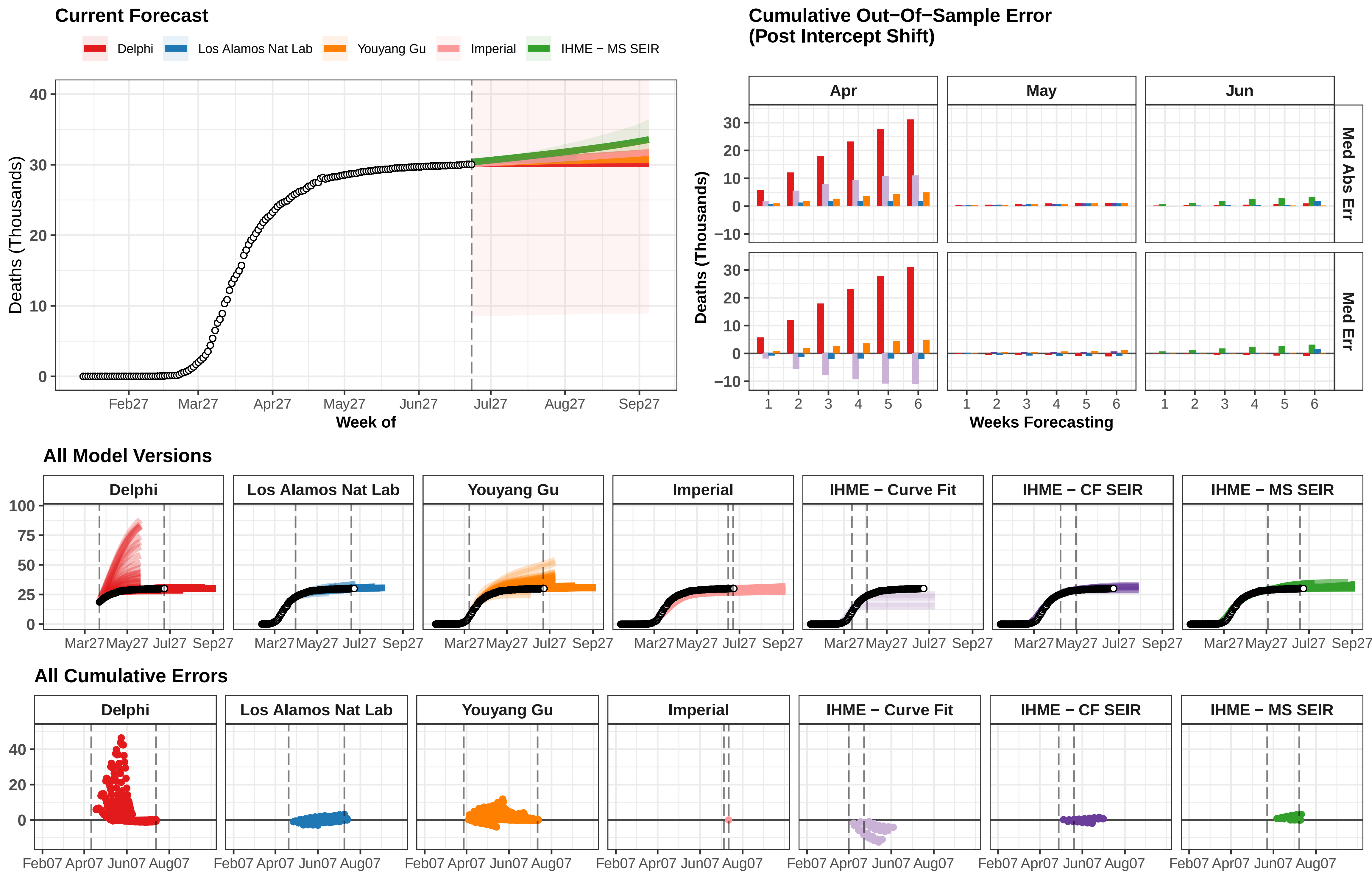

Current Forecast
Delphi
Los Alamos Nat Lab
Youyang Gu
Imperial
IHME − MS SEIR
Deaths (Thousands)
Week of
Feb27
Mar27
Apr27
May27
Jun27
Jul27
Aug27
Sep27
Cumulative Out−Of−Sample Error
(Post Intercept Shift)
Apr
May
Jun
Med Abs Err
Med Err
Weeks Forecasting
All Model Versions
Delphi
Los Alamos Nat Lab
Youyang Gu
Imperial
IHME − Curve Fit
IHME − CF SEIR
IHME − MS SEIR
All Cumulative Errors
Feb07
Apr07
Jun07
Aug07

Spain

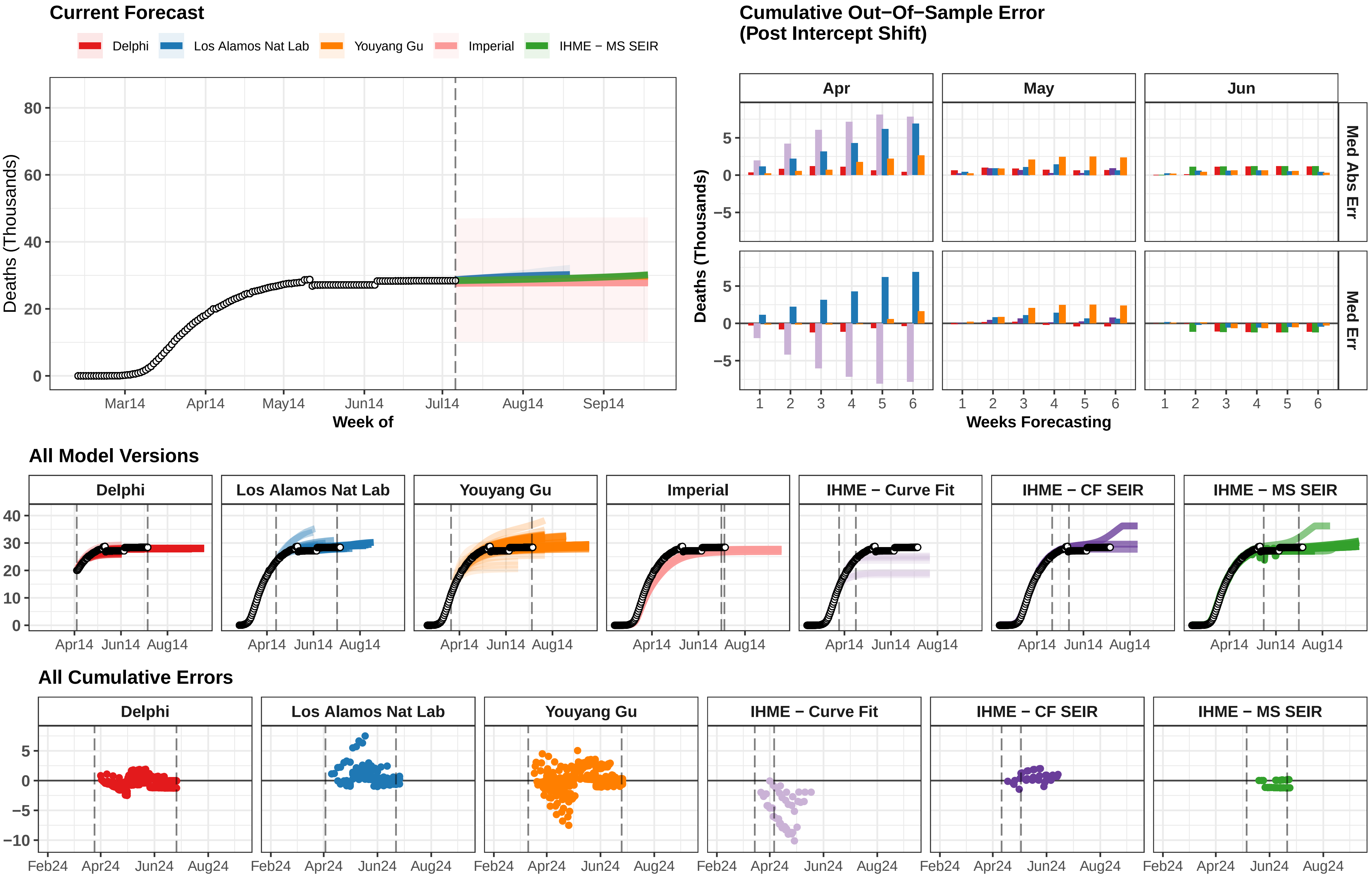

Current Forecast
Delphi
Los Alamos Nat Lab
Youyang Gu
Imperial
IHME − MS SEIR
Deaths (Thousands)
80
60
40
20
0
Mar14
Apr14
May14
Jun14
Jul14
Aug14
Sep14
Week of
Cumulative Out−Of−Sample Error
(Post Intercept Shift)
Apr
May
Jun
Med Abs Err
Med Err
Deaths (Thousands)
5
0
−5
1
2
3
4
5
6
Weeks Forecasting
All Model Versions
Delphi
Los Alamos Nat Lab
Youyang Gu
Imperial
IHME − Curve Fit
IHME − CF SEIR
IHME − MS SEIR
40
30
20
10
0
Apr14
Jun14
Aug14
All Cumulative Errors
Delphi
Los Alamos Nat Lab
Youyang Gu
IHME − Curve Fit
IHME − CF SEIR
IHME − MS SEIR
5
0
−5
−10
Feb24
Apr24
Jun24
Aug24

India

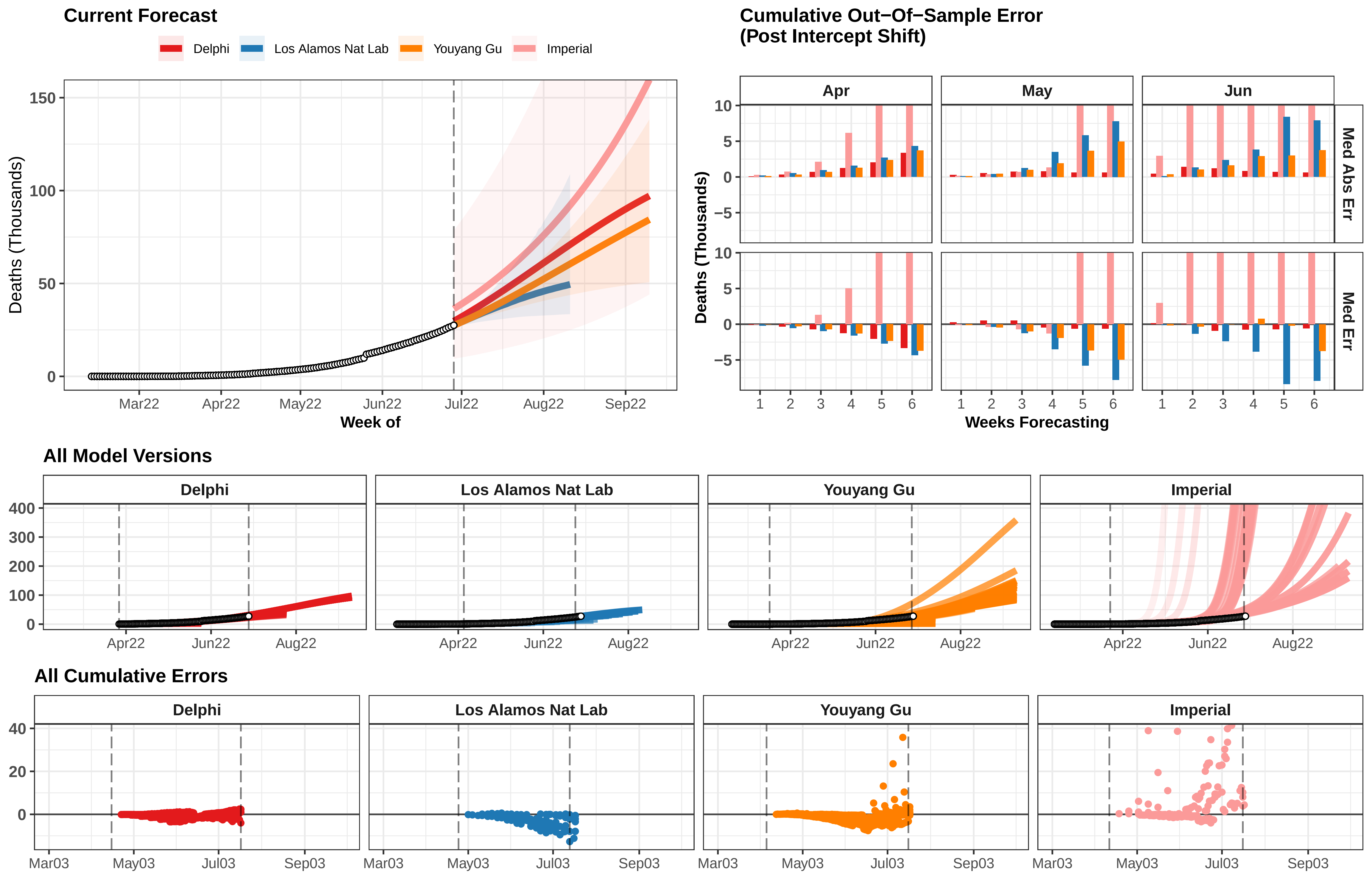
Current Forecast
Delphi
Los Alamos Nat Lab
Youyang Gu
Imperial
Deaths (Thousands)
Week of
Mar22
Apr22
May22
Jun22
Jul22
Aug22
Sep22
Cumulative Out−Of−Sample Error
(Post Intercept Shift)
Apr
May
Jun
Med Abs Err
Med Err
Weeks Forecasting
All Model Versions
All Cumulative Errors
Mar03
May03
Jul03
Sep03

New Jersey

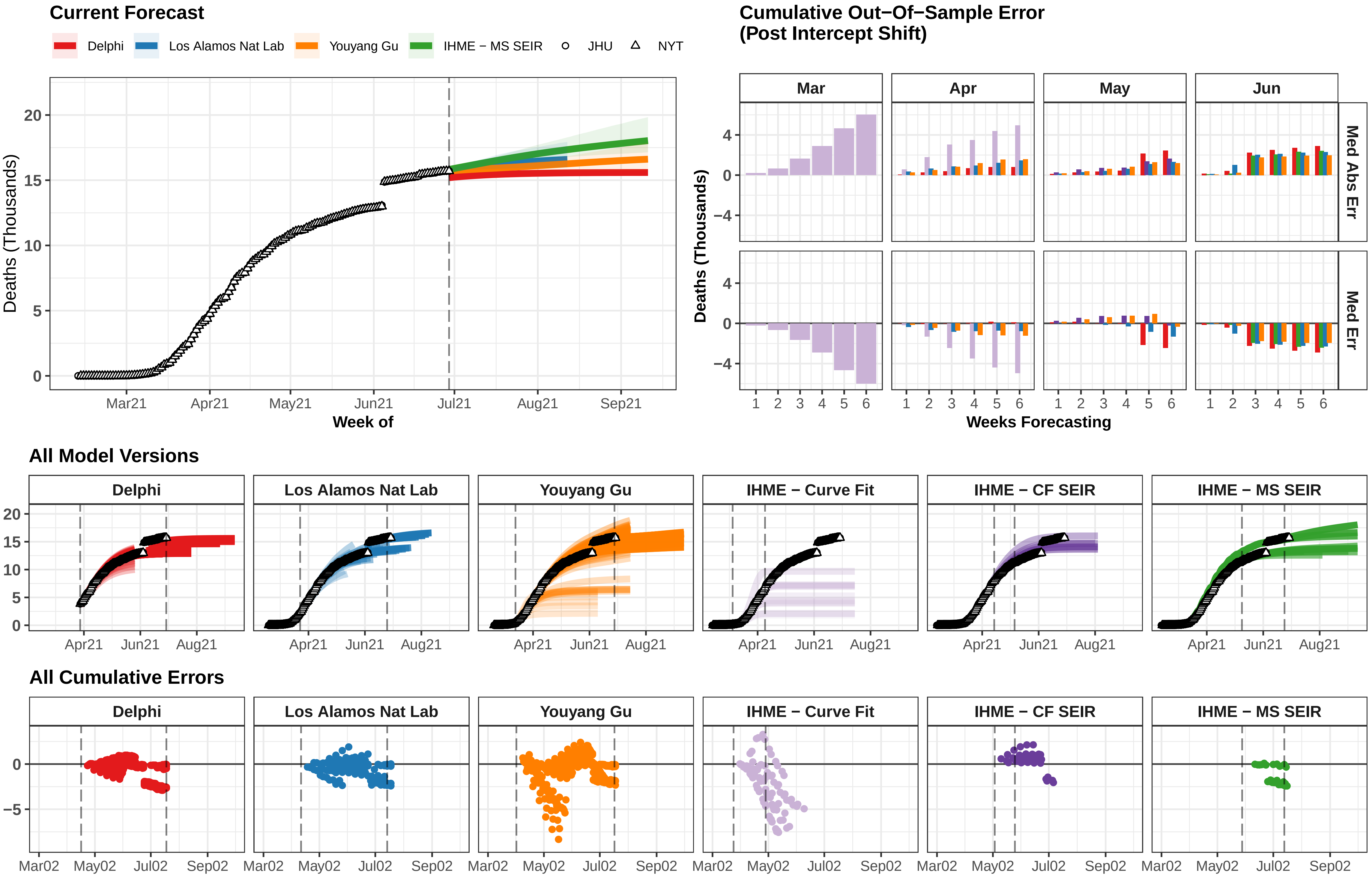

Current Forecast
Delphi
Los Alamos Nat Lab
Youyang Gu
IHME − MS SEIR
JHU
NYT
Deaths (Thousands)
Week of
Mar21
Apr21
May21
Jun21
Jul21
Aug21
Sep21
Cumulative Out−Of−Sample Error
(Post Intercept Shift)
Mar
Apr
May
Jun
Med Abs Err
Med Err
Weeks Forecasting
All Model Versions
Delphi
Los Alamos Nat Lab
Youyang Gu
IHME − Curve Fit
IHME − CF SEIR
IHME − MS SEIR
All Cumulative Errors
Mar02
May02
Jul02
Sep02

Iran

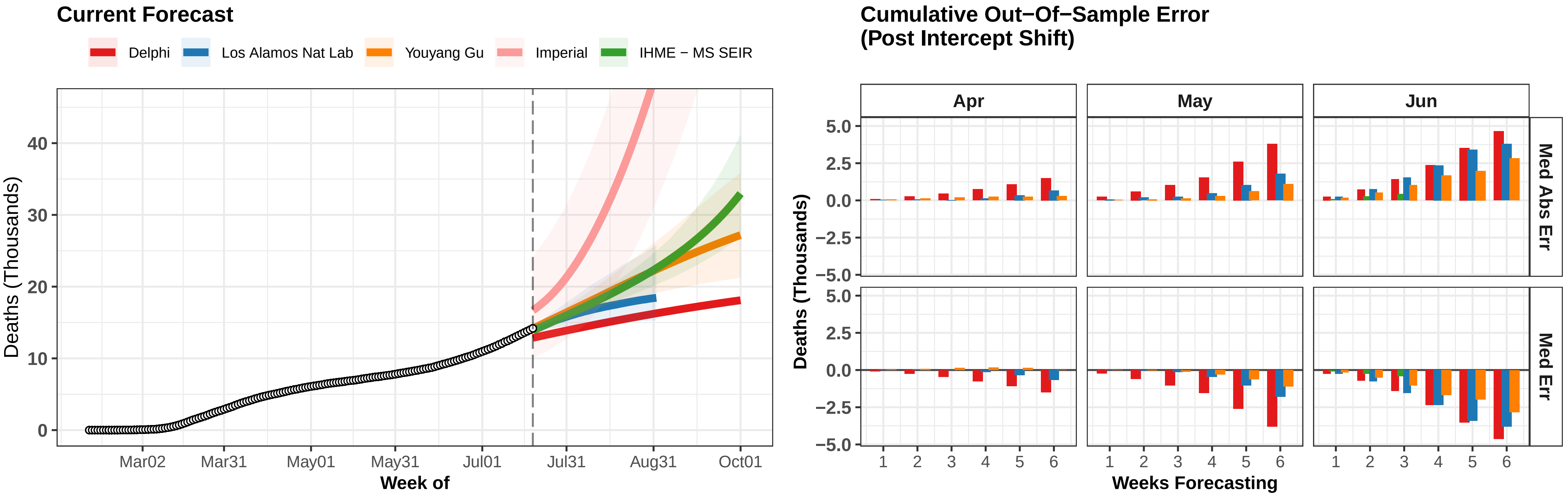

Current Forecast
Delphi
Los Alamos Nat Lab
Youyang Gu
Imperial
IHME − MS SEIR
Deaths (Thousands)
Week of
Mar02
Mar31
May01
May31
Jul01
Jul31
Aug31
Oct01
Cumulative Out−Of−Sample Error
(Post Intercept Shift)
Apr
May
Jun
Med Abs Err
Med Err
Weeks Forecasting

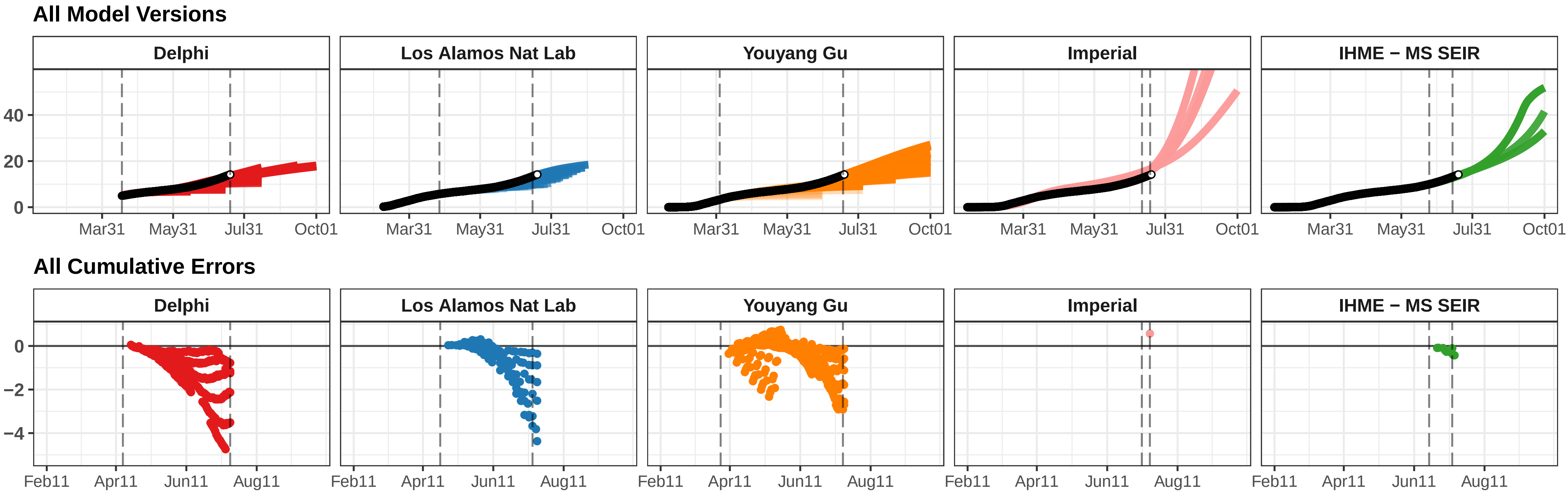

All Model Versions
Delphi
Los Alamos Nat Lab
Youyang Gu
Imperial
IHME − MS SEIR
Mar31
May31
Jul31
Oct01
All Cumulative Errors
Feb11
Apr11
Jun11
Aug11

Russian Federation

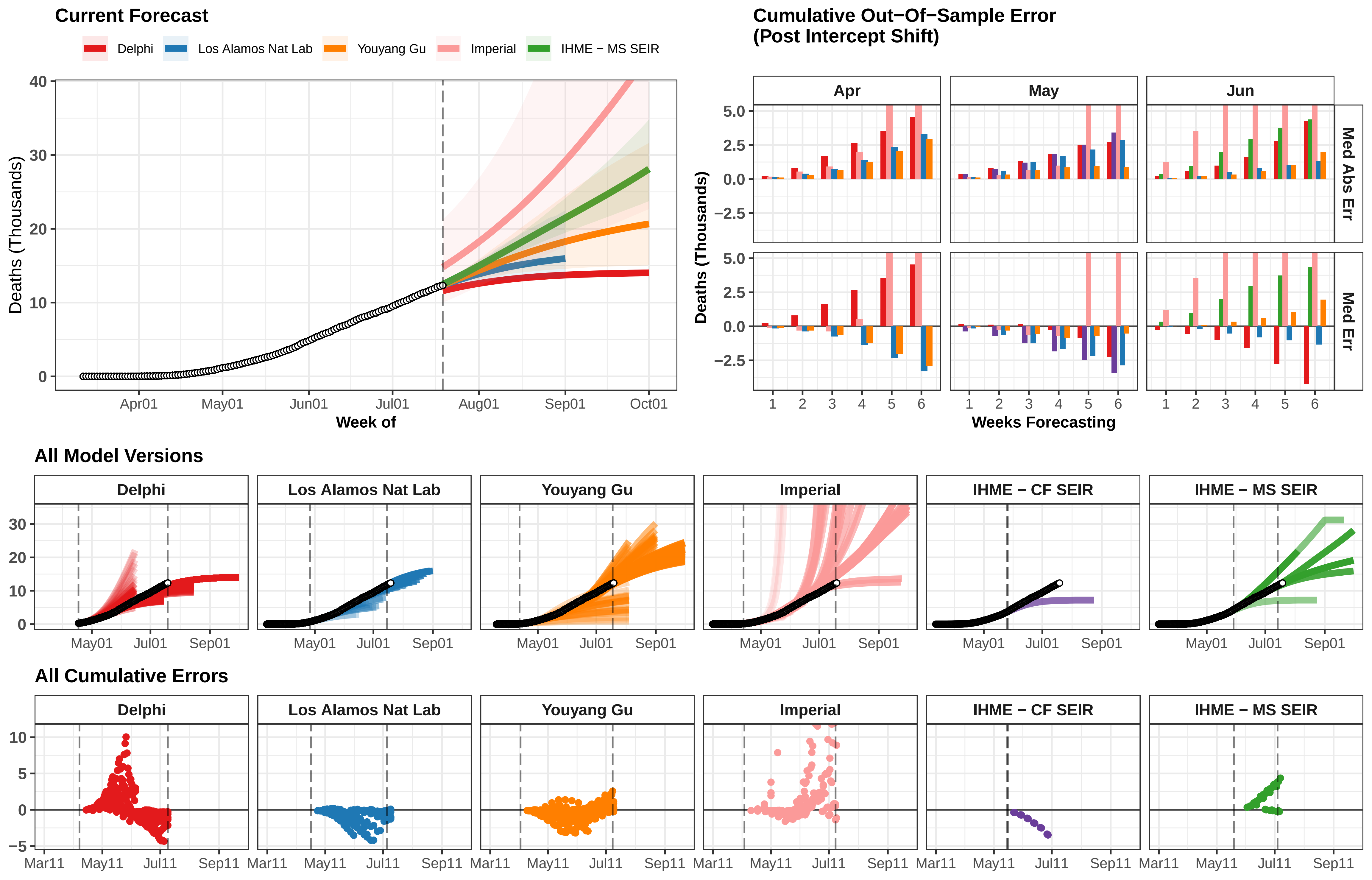

Belgium

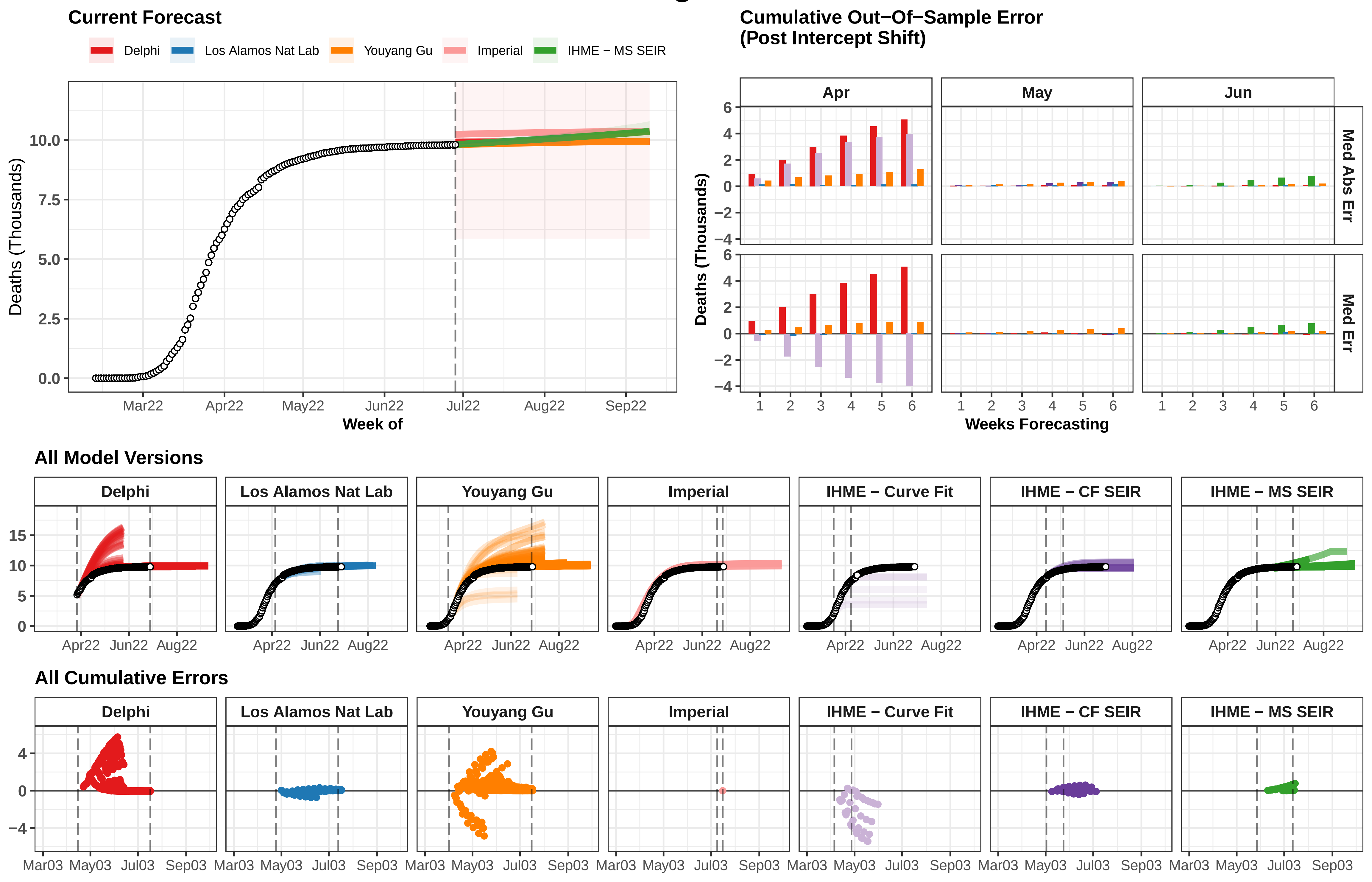
Current Forecast
Delphi
Los Alamos Nat Lab
Youyang Gu
Imperial
IHME − MS SEIR
Deaths (Thousands)
Week of
Mar22
Apr22
May22
Jun22
Jul22
Aug22
Sep22
Cumulative Out−Of−Sample Error
(Post Intercept Shift)
Apr
May
Jun
Med Abs Err
Med Err
Weeks Forecasting
All Model Versions
Delphi
Los Alamos Nat Lab
Youyang Gu
Imperial
IHME − Curve Fit
IHME − CF SEIR
IHME − MS SEIR
All Cumulative Errors
Mar03
May03
Jul03
Sep03

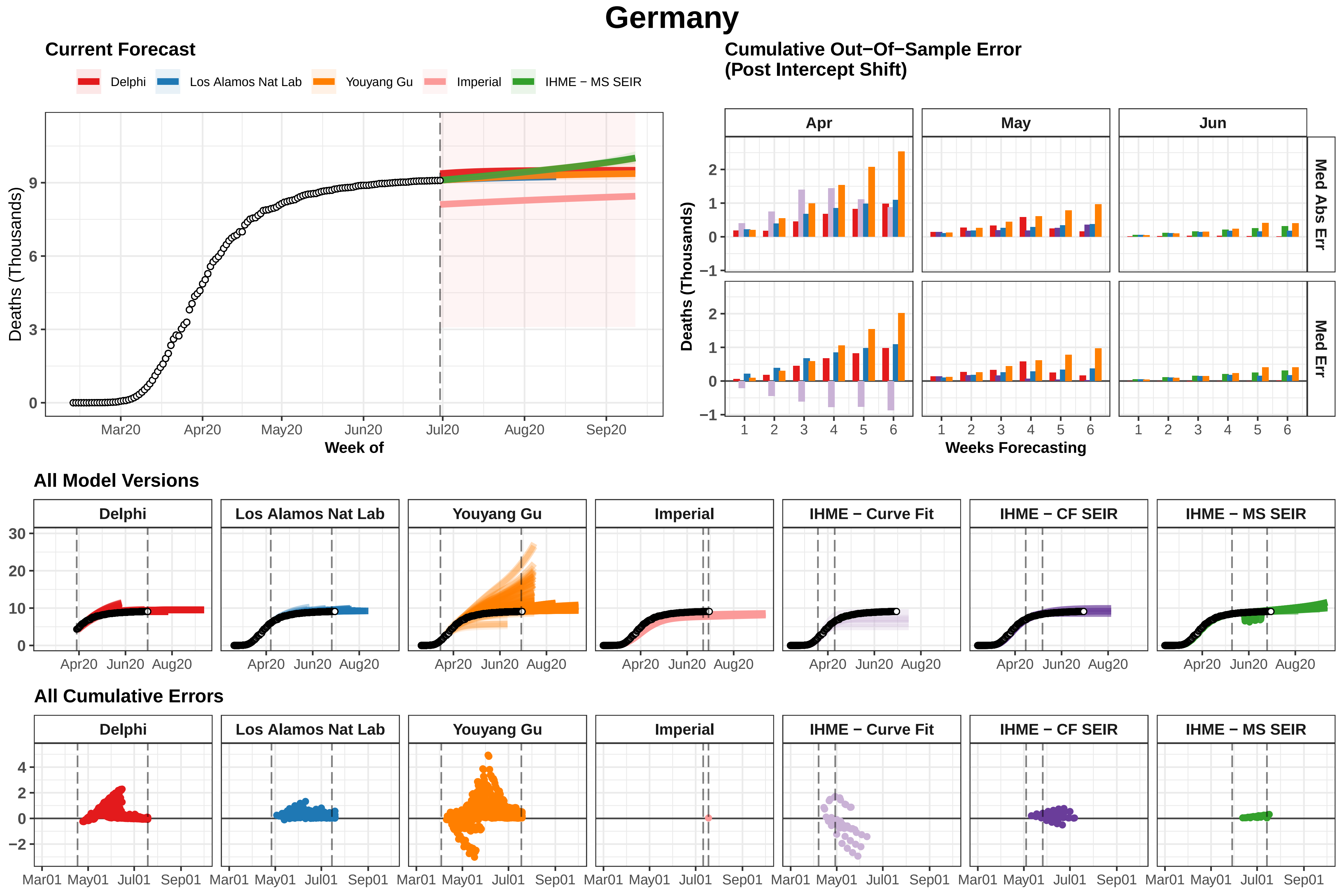

Germany
Current Forecast
Delphi
Los Alamos Nat Lab
Youyang Gu
Imperial
IHME − MS SEIR
Deaths (Thousands)
Week of
Mar20
Apr20
May20
Jun20
Jul20
Aug20
Sep20
Cumulative Out−Of−Sample Error
(Post Intercept Shift)
Apr
May
Jun
Med Abs Err
Med Err
Weeks Forecasting
All Model Versions
Delphi
Los Alamos Nat Lab
Youyang Gu
Imperial
IHME − Curve Fit
IHME − CF SEIR
IHME − MS SEIR
All Cumulative Errors
Mar01
May01
Jul01
Sep01

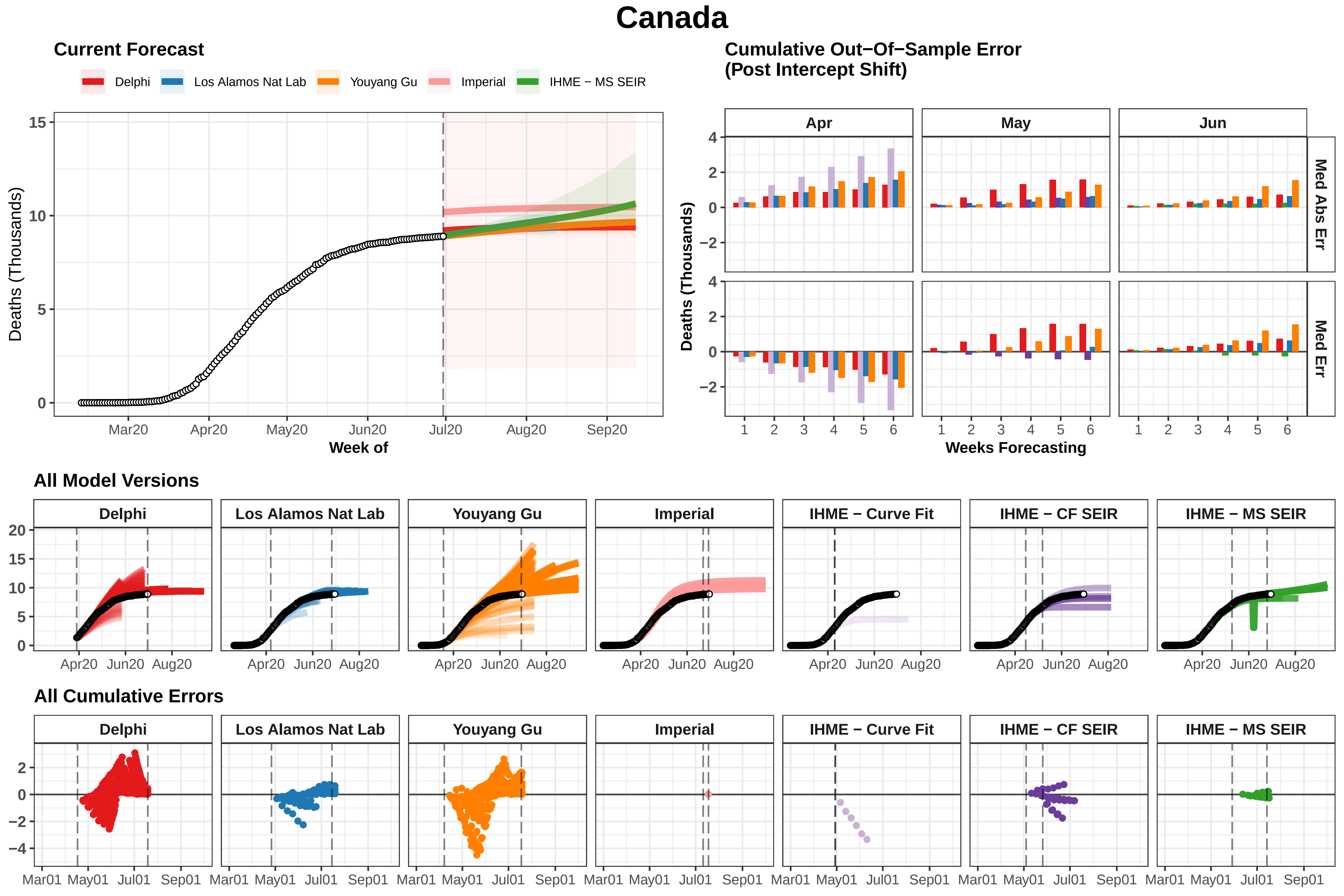

Canada
Current Forecast
Delphi
Los Alamos Nat Lab
Youyang Gu
Imperial
IHME − MS SEIR
Deaths (Thousands)
Week of
Mar20
Apr20
May20
Jun20
Jul20
Aug20
Sep20
Cumulative Out−Of−Sample Error
(Post Intercept Shift)
Apr
May
Jun
Med Abs Err
Med Err
Weeks Forecasting
All Model Versions
IHME − Curve Fit
IHME − CF SEIR
All Cumulative Errors
Mar01
May01
Jul01
Sep01

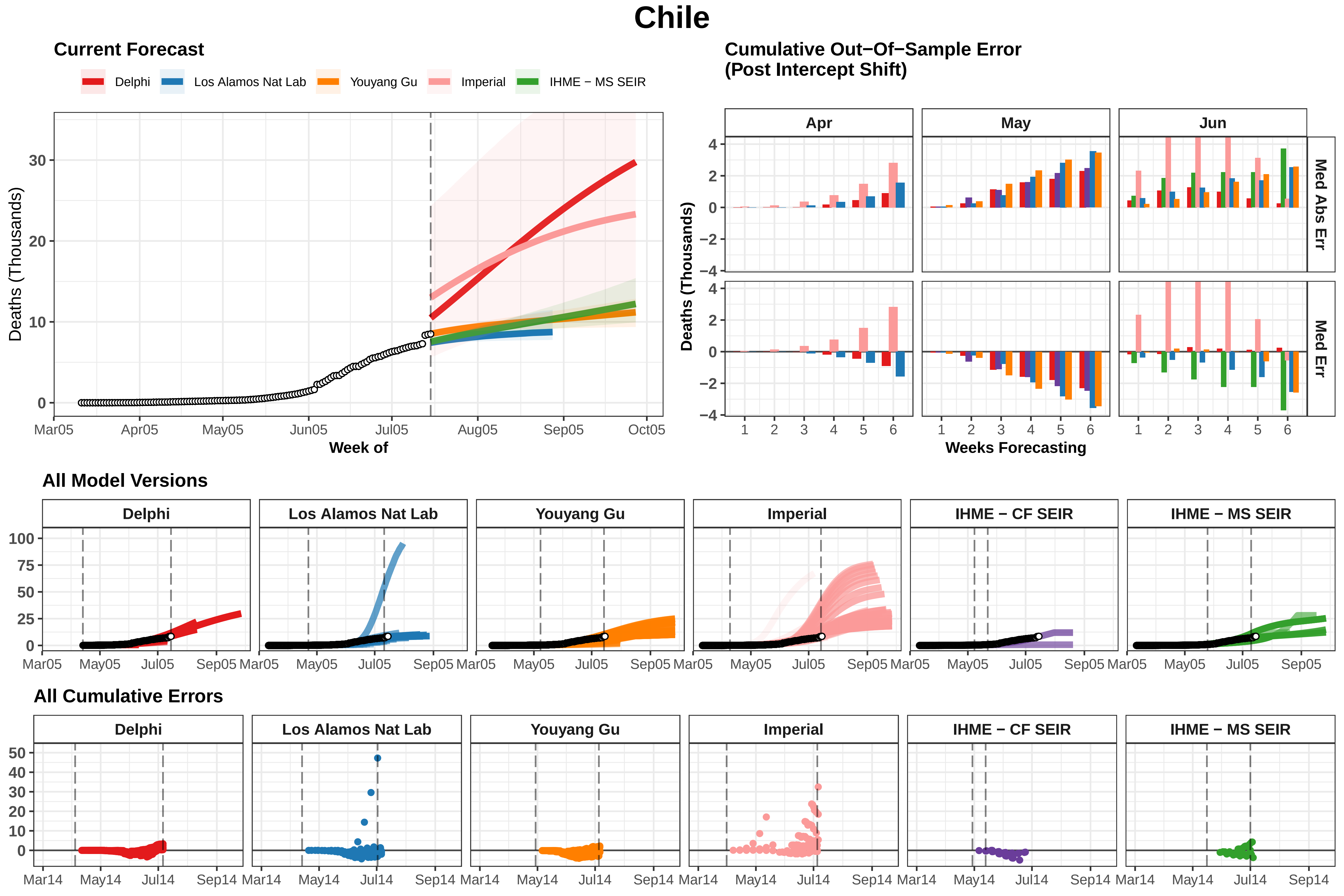
Chile
Current Forecast
Delphi
Los Alamos Nat Lab
Youyang Gu
Imperial
IHME − MS SEIR
Deaths (Thousands)
Week of
Mar05
Apr05
May05
Jun05
Jul05
Aug05
Sep05
Oct05
Cumulative Out−Of−Sample Error
(Post Intercept Shift)
Apr
May
Jun
Med Abs Err
Med Err
Weeks Forecasting
All Model Versions
IHME − CF SEIR
All Cumulative Errors
Mar14
May14
Jul14
Sep14

Massachusetts

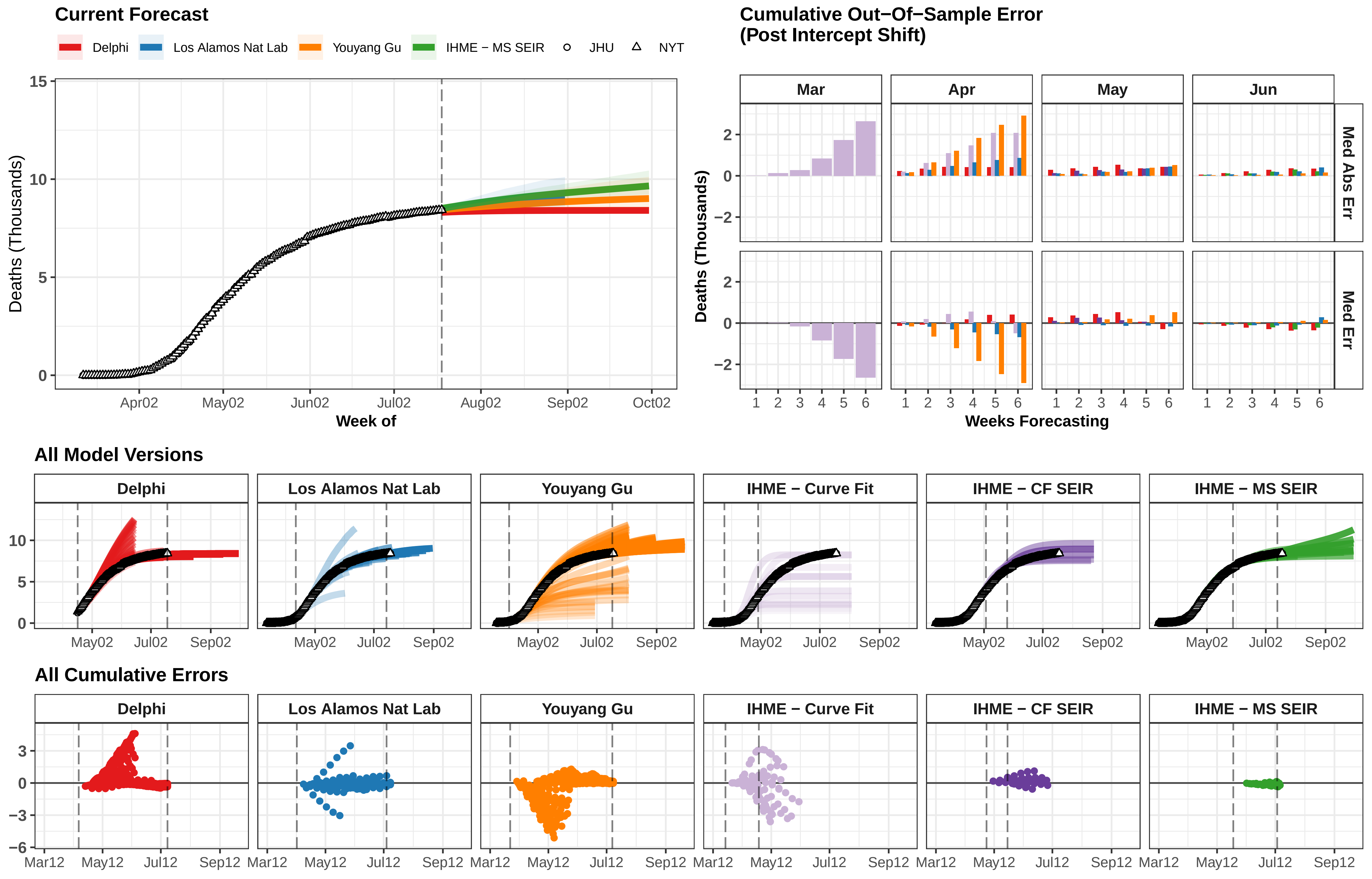

California

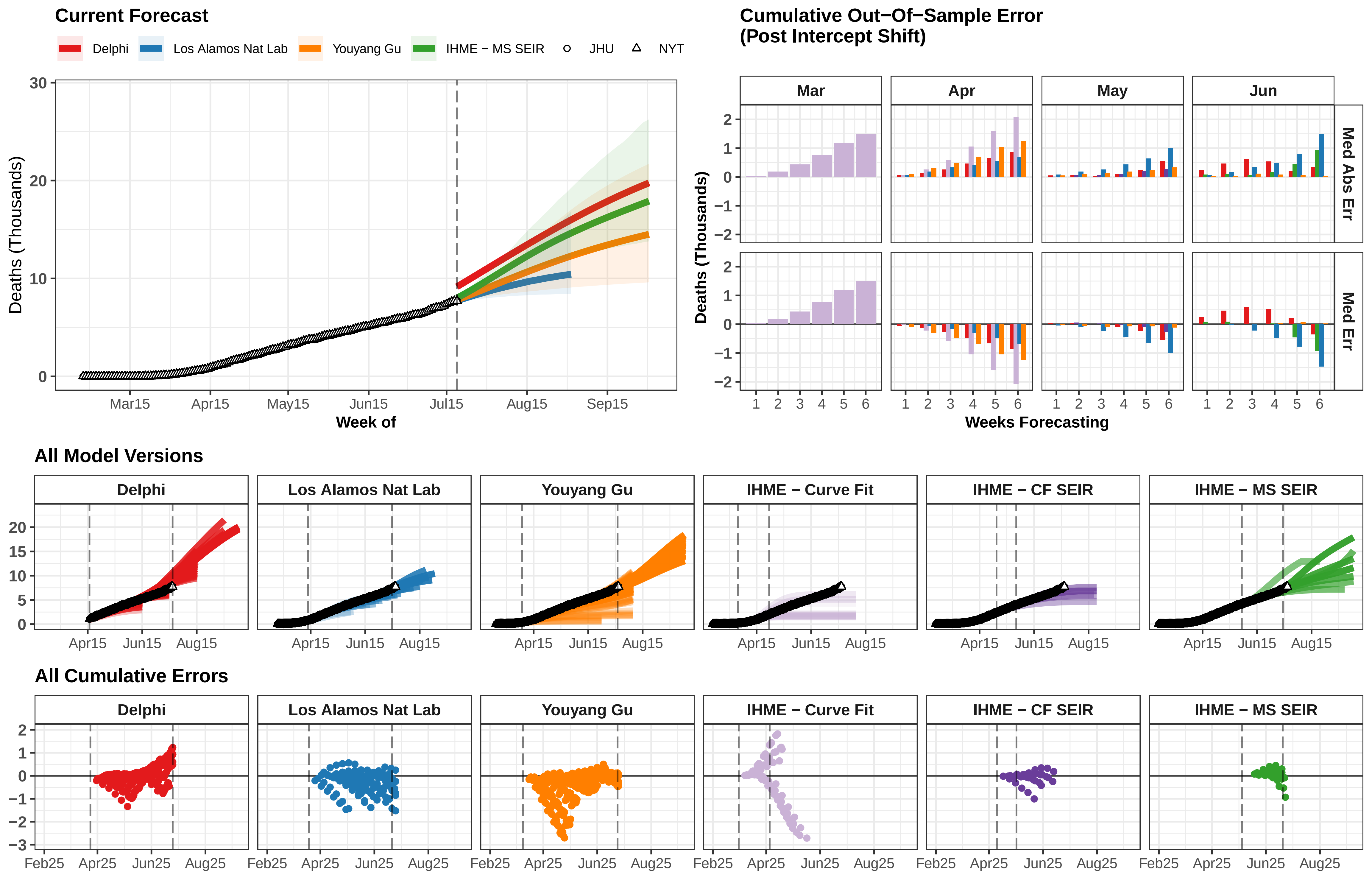
Current Forecast
Delphi
Los Alamos Nat Lab
Youyang Gu
IHME − MS SEIR
JHU
NYT
Deaths (Thousands)
Week of
Mar15
Apr15
May15
Jun15
Jul15
Aug15
Sep15
Cumulative Out−Of−Sample Error (Post Intercept Shift)
Mar
Apr
May
Jun
Med Abs Err
Med Err
Weeks Forecasting
All Model Versions
Delphi
Los Alamos Nat Lab
Youyang Gu
IHME − Curve Fit
IHME − CF SEIR
IHME − MS SEIR
All Cumulative Errors
Feb25
Apr25
Jun25
Aug25

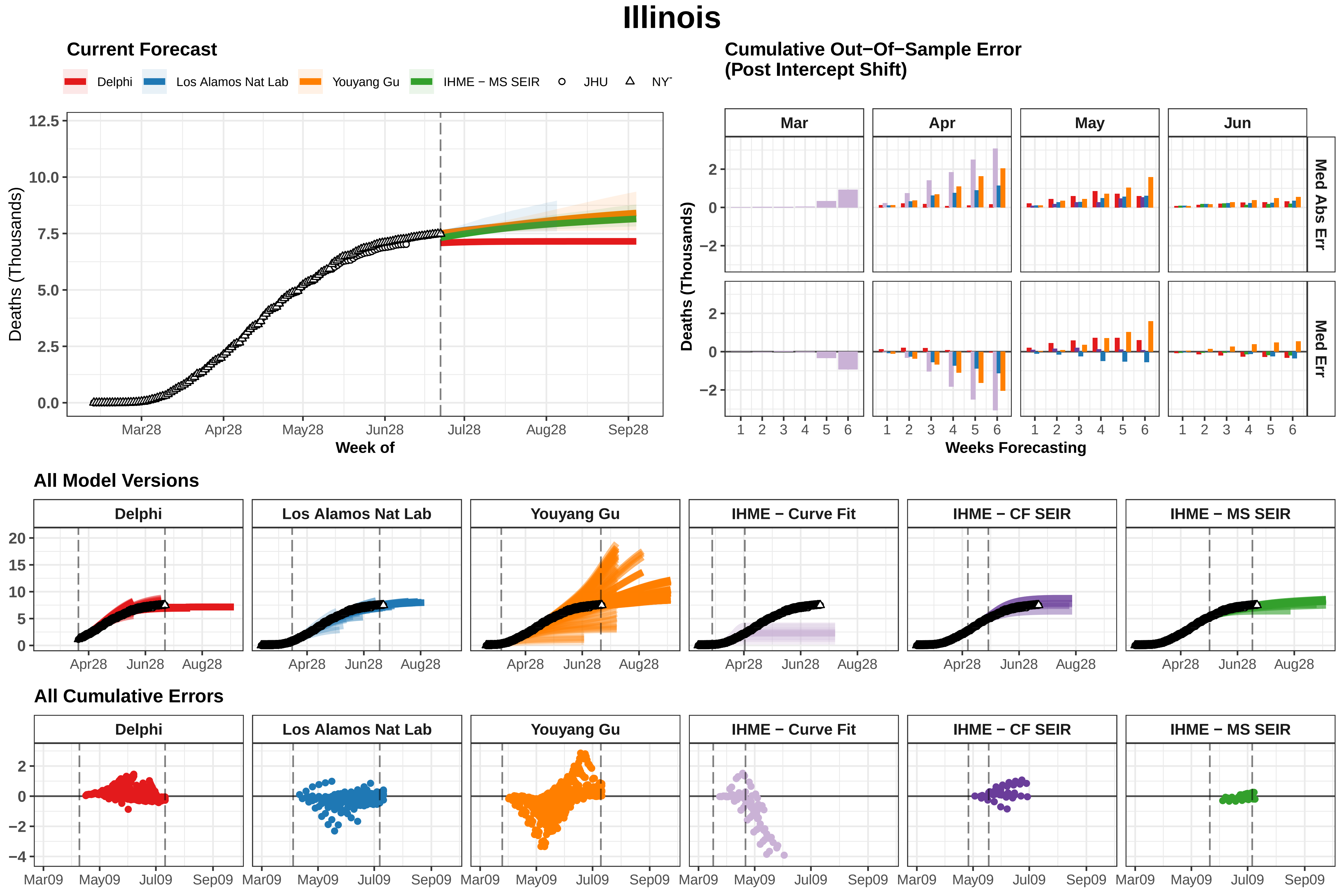

Illinois
Current Forecast
Delphi
Los Alamos Nat Lab
Youyang Gu
IHME − MS SEIR
JHU
NY
Deaths (Thousands)
12.5
10.0
7.5
5.0
2.5
0.0
Mar28
Apr28
May28
Jun28
Jul28
Aug28
Sep28
Week of
Cumulative Out−Of−Sample Error
(Post Intercept Shift)
Mar
Apr
May
Jun
Med Abs Err
Med Err
Weeks Forecasting
All Model Versions
IHME − Curve Fit
IHME − CF SEIR
20
15
10
5
0
All Cumulative Errors
2
0
−2
−4
Mar09
May09
Jul09
Sep09

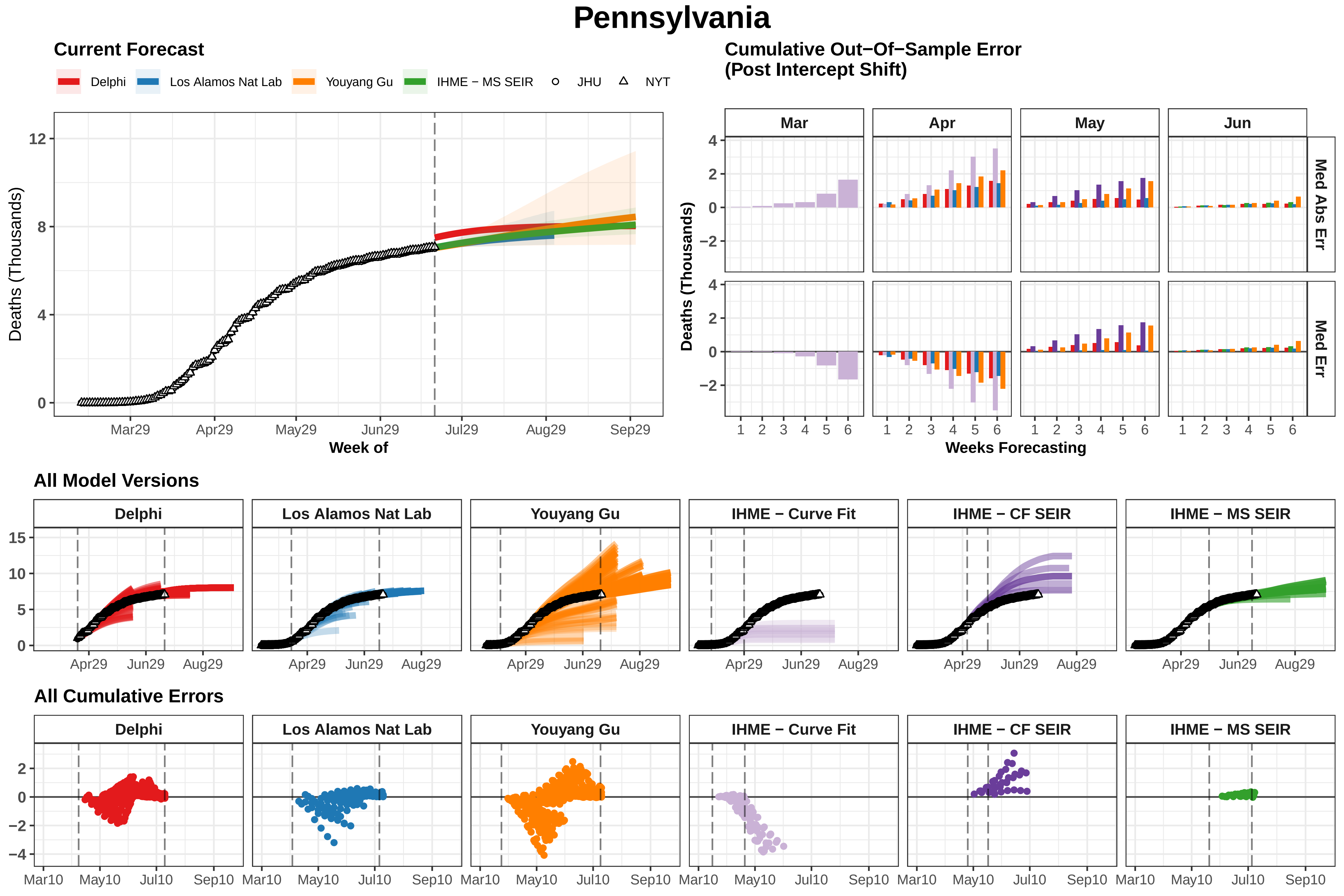
Pennsylvania
Current Forecast
Delphi
Los Alamos Nat Lab
Youyang Gu
IHME − MS SEIR
JHU
NYT
Deaths (Thousands)
Week of
Mar29
Apr29
May29
Jun29
Jul29
Aug29
Sep29
Cumulative Out−Of−Sample Error
(Post Intercept Shift)
Mar
Apr
May
Jun
Med Abs Err
Med Err
Weeks Forecasting
All Model Versions
IHME − Curve Fit
IHME − CF SEIR
All Cumulative Errors
Mar10
May10
Jul10
Sep10

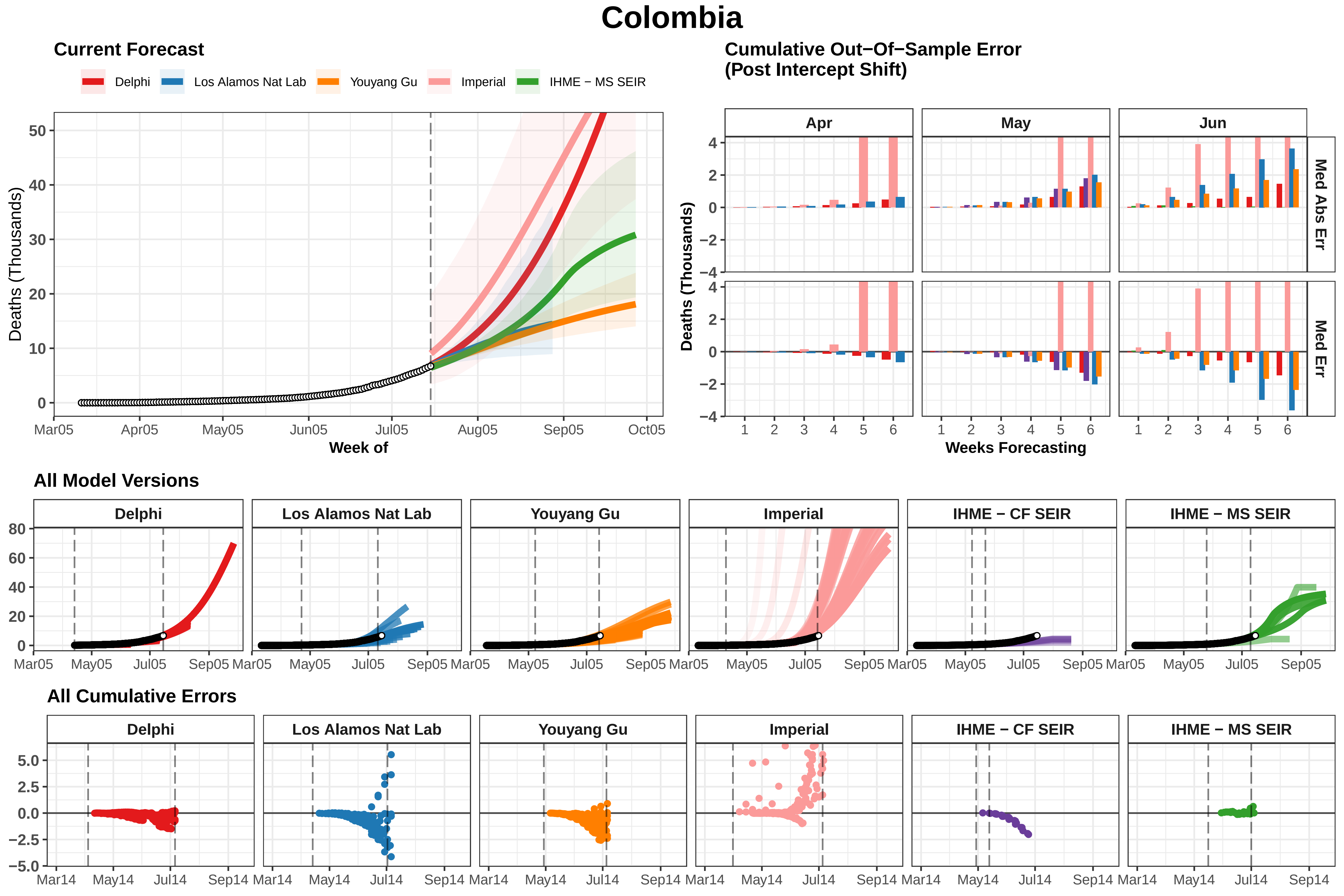

Colombia
Current Forecast
Delphi
Los Alamos Nat Lab
Youyang Gu
Imperial
IHME − MS SEIR
Deaths (Thousands)
Week of
Mar05
Apr05
May05
Jun05
Jul05
Aug05
Sep05
Oct05
Cumulative Out−Of−Sample Error
(Post Intercept Shift)
Apr
May
Jun
Med Abs Err
Med Err
Weeks Forecasting
All Model Versions
IHME − CF SEIR
All Cumulative Errors
Mar14
May14
Jul14
Sep14

Michigan

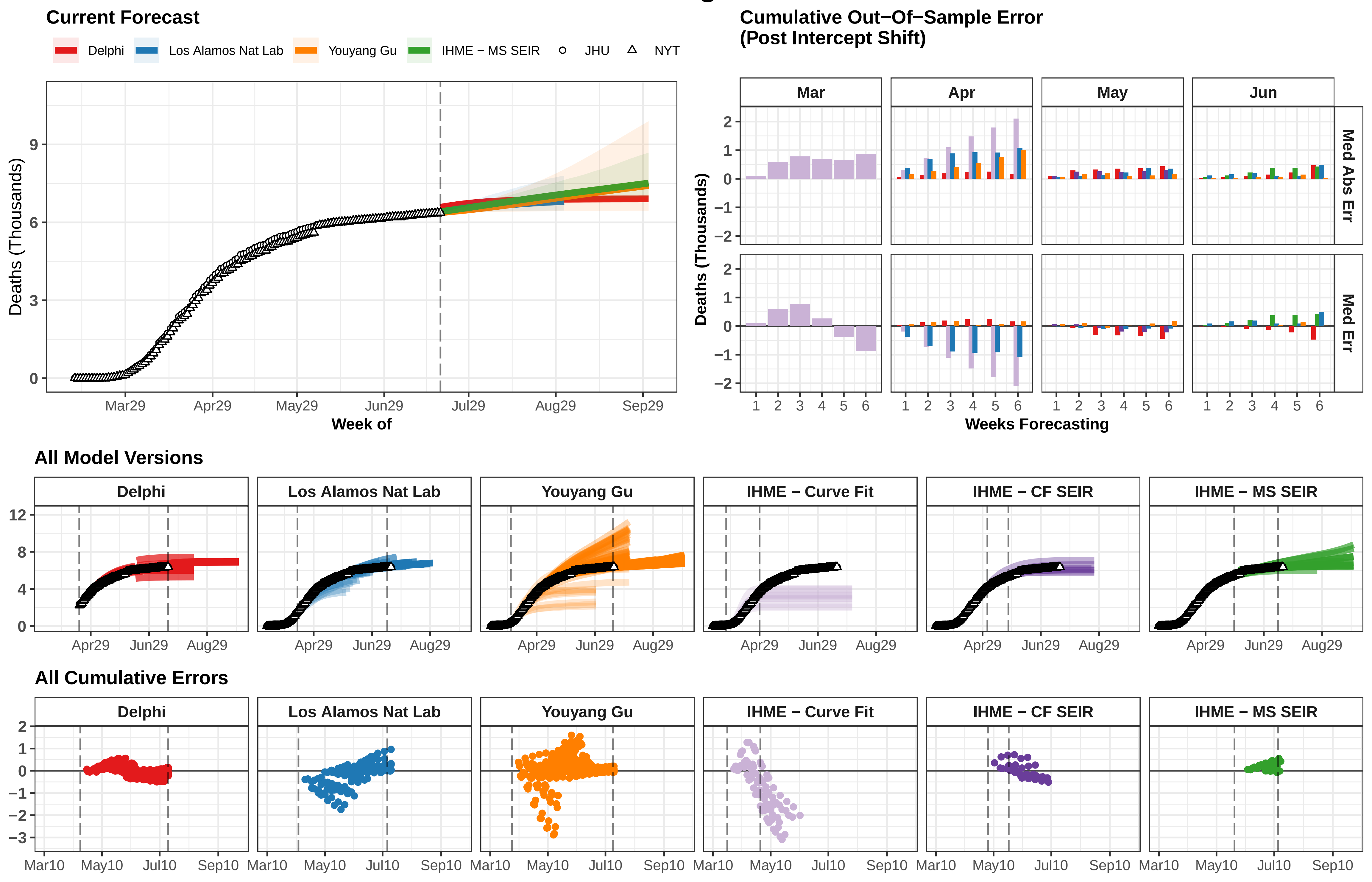
Current Forecast
Delphi
Los Alamos Nat Lab
Youyang Gu
IHME − MS SEIR
JHU
NYT
Deaths (Thousands)
Week of
Mar29
Apr29
May29
Jun29
Jul29
Aug29
Sep29
Cumulative Out−Of−Sample Error
(Post Intercept Shift)
Mar
Apr
May
Jun
Med Abs Err
Med Err
Weeks Forecasting
All Model Versions
Delphi
Los Alamos Nat Lab
Youyang Gu
IHME − Curve Fit
IHME − CF SEIR
IHME − MS SEIR
All Cumulative Errors
Mar10
May10
Jul10
Sep10

Netherlands

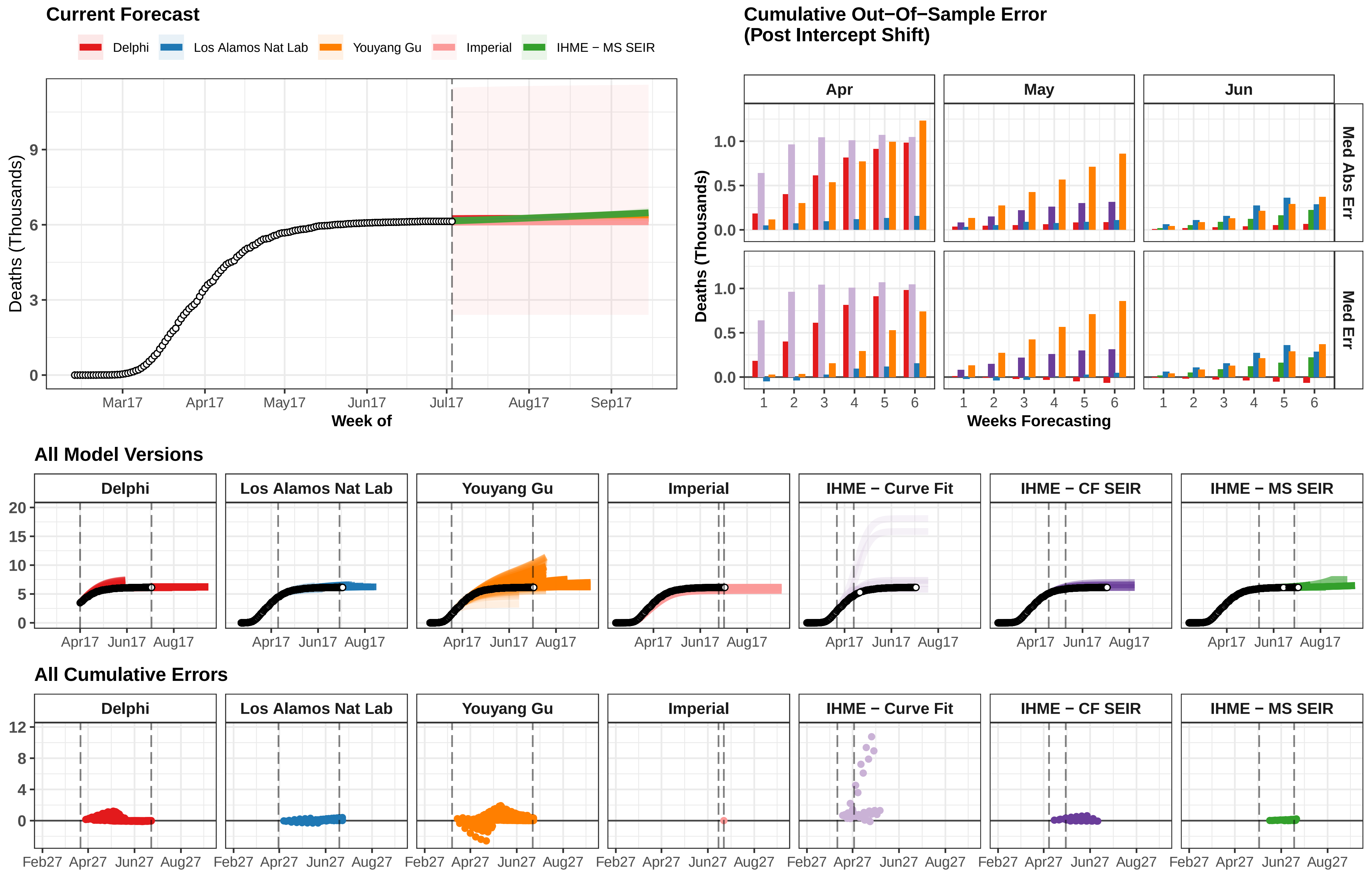

Sweden

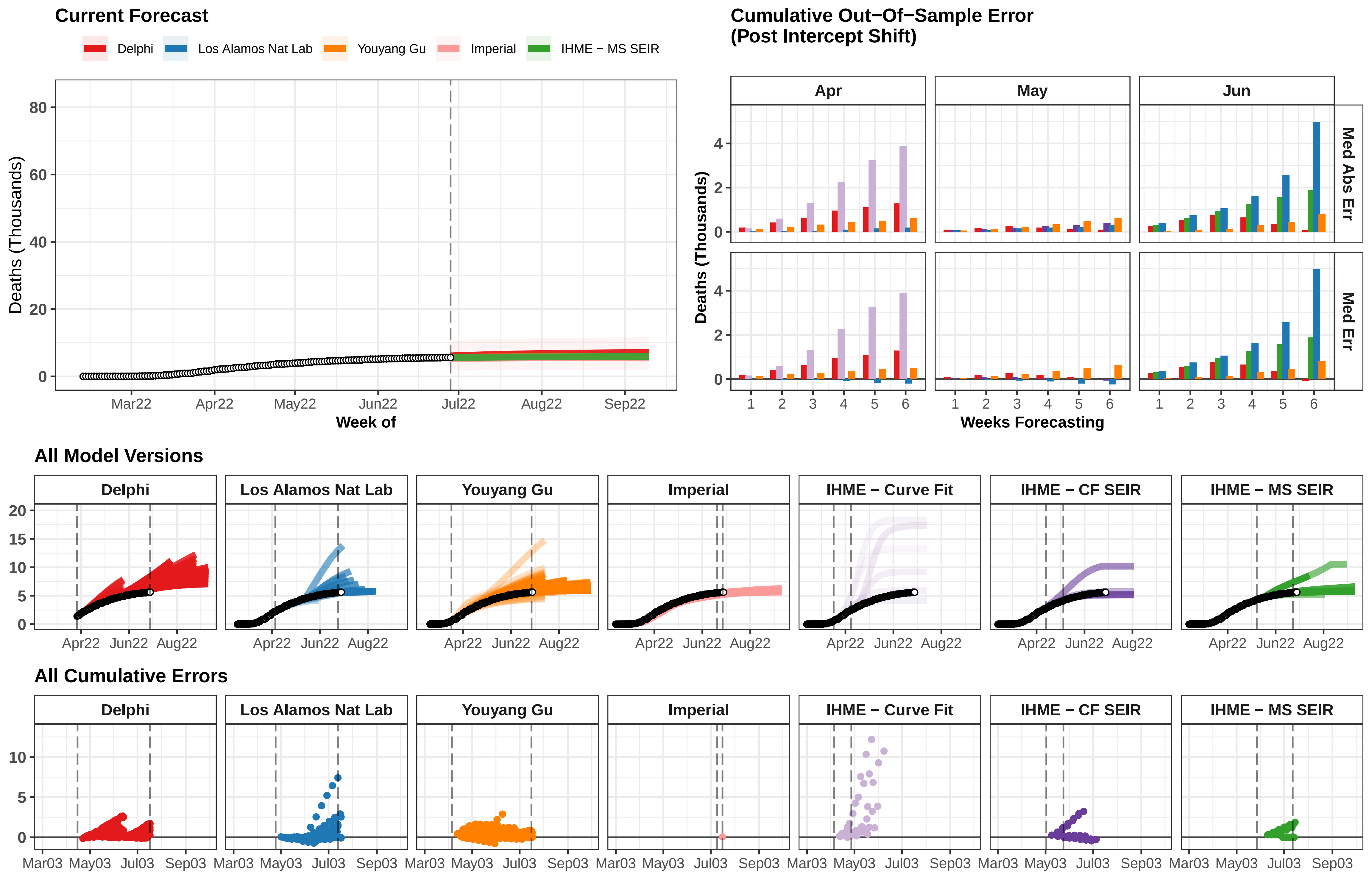

Current Forecast
Delphi
Los Alamos Nat Lab
Youyang Gu
Imperial
IHME − MS SEIR
Deaths (Thousands)
Week of
Mar22
Apr22
May22
Jun22
Jul22
Aug22
Sep22
Cumulative Out−Of−Sample Error (Post Intercept Shift)
Apr
May
Jun
Med Abs Err
Med Err
Weeks Forecasting
All Model Versions
Delphi
Los Alamos Nat Lab
Youyang Gu
Imperial
IHME − Curve Fit
IHME − CF SEIR
IHME − MS SEIR
All Cumulative Errors
Mar03
May03
Jul03
Sep03

Pakistan

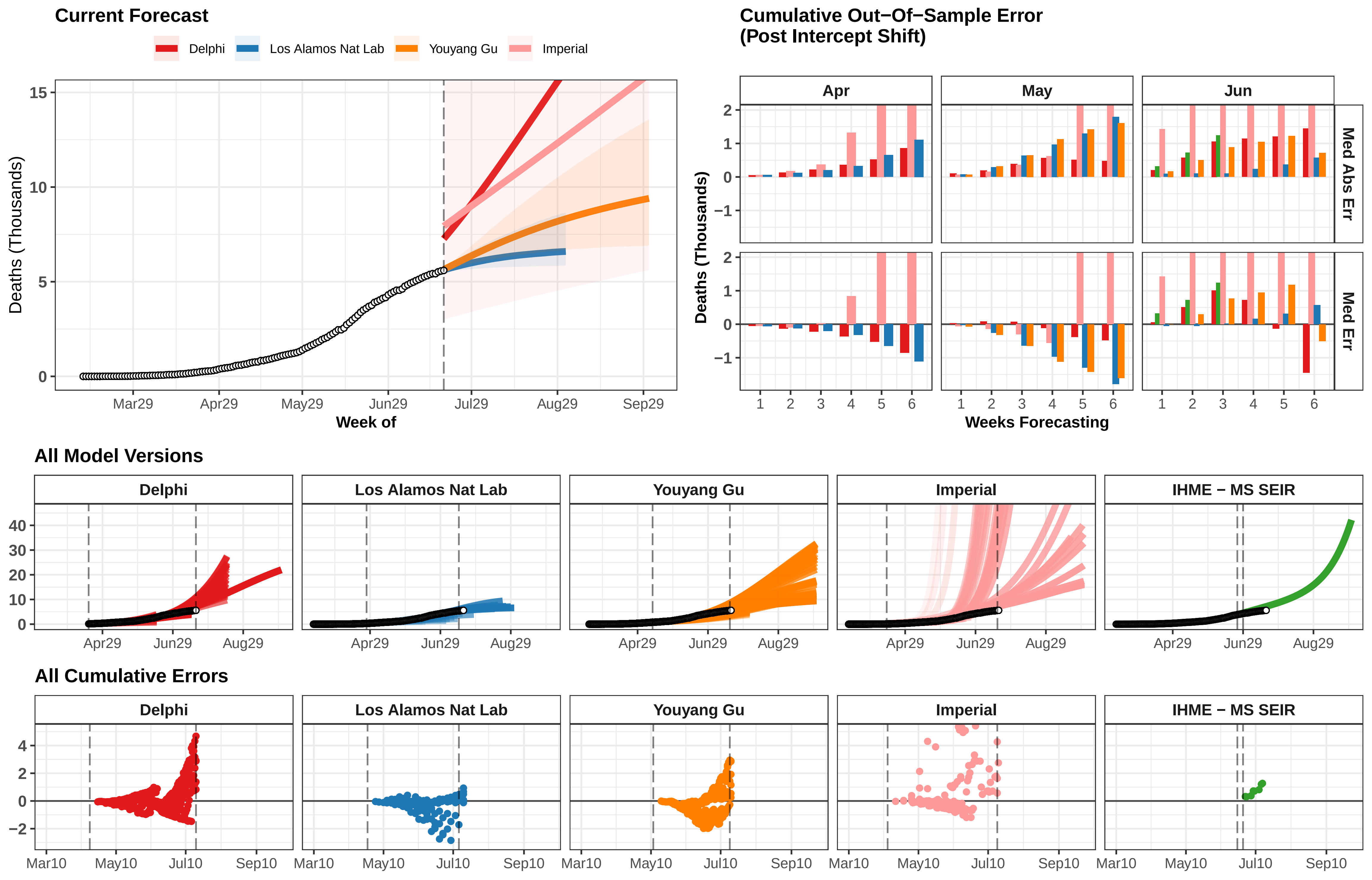

Current Forecast
Delphi
Los Alamos Nat Lab
Youyang Gu
Imperial
Deaths (Thousands)
Week of
Mar29
Apr29
May29
Jun29
Jul29
Aug29
Sep29
Cumulative Out−Of−Sample Error
(Post Intercept Shift)
Apr
May
Jun
Med Abs Err
Med Err
Weeks Forecasting
All Model Versions
IHME − MS SEIR
All Cumulative Errors
Mar10
May10
Jul10
Sep10

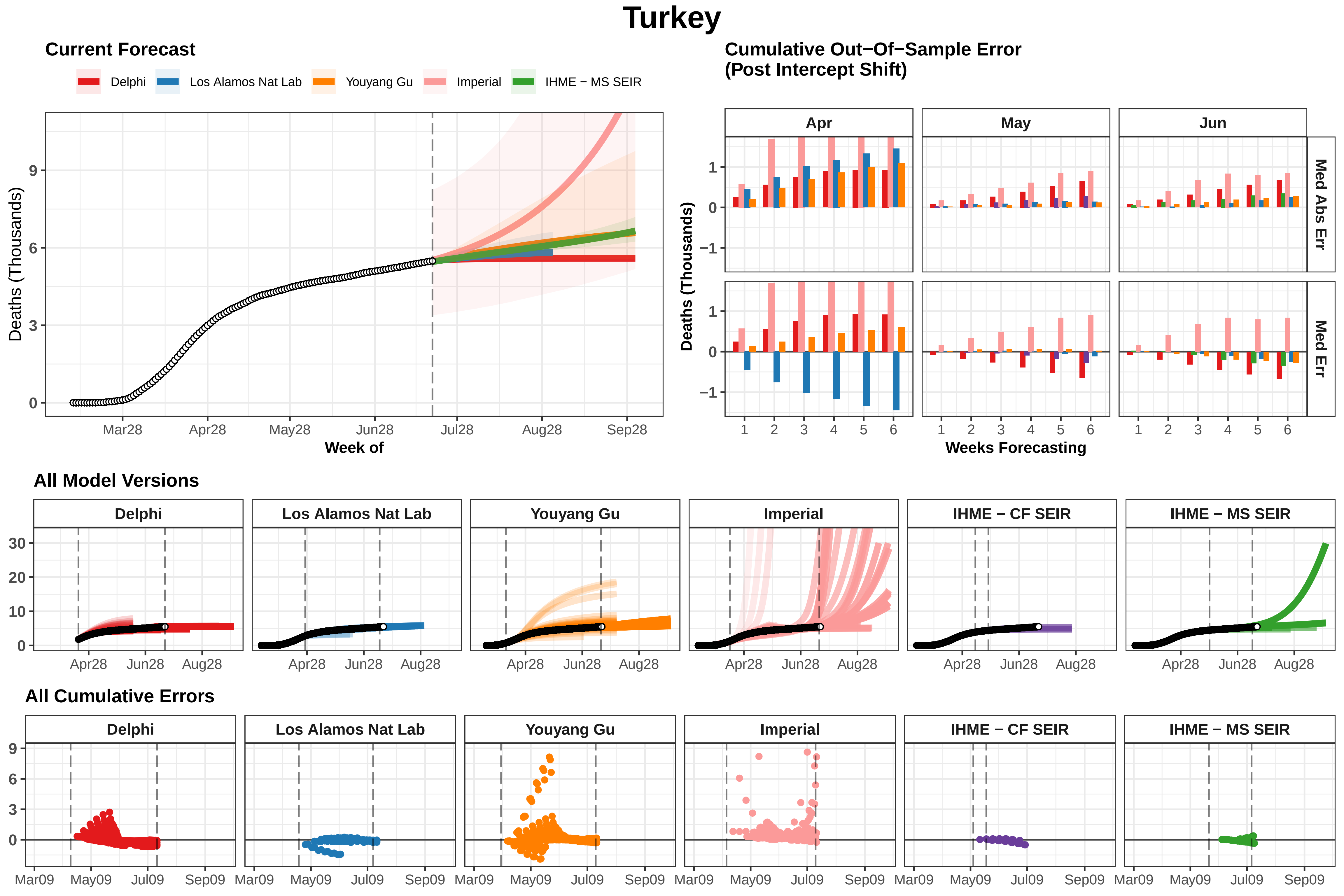
Turkey
Current Forecast
Delphi
Los Alamos Nat Lab
Youyang Gu
Imperial
IHME − MS SEIR
Deaths (Thousands)
Week of
Mar28
Apr28
May28
Jun28
Jul28
Aug28
Sep28
Cumulative Out−Of−Sample Error
(Post Intercept Shift)
Apr
May
Jun
Med Abs Err
Med Err
Weeks Forecasting
All Model Versions
IHME − CF SEIR
All Cumulative Errors
Mar09
May09
Jul09
Sep09

South Africa

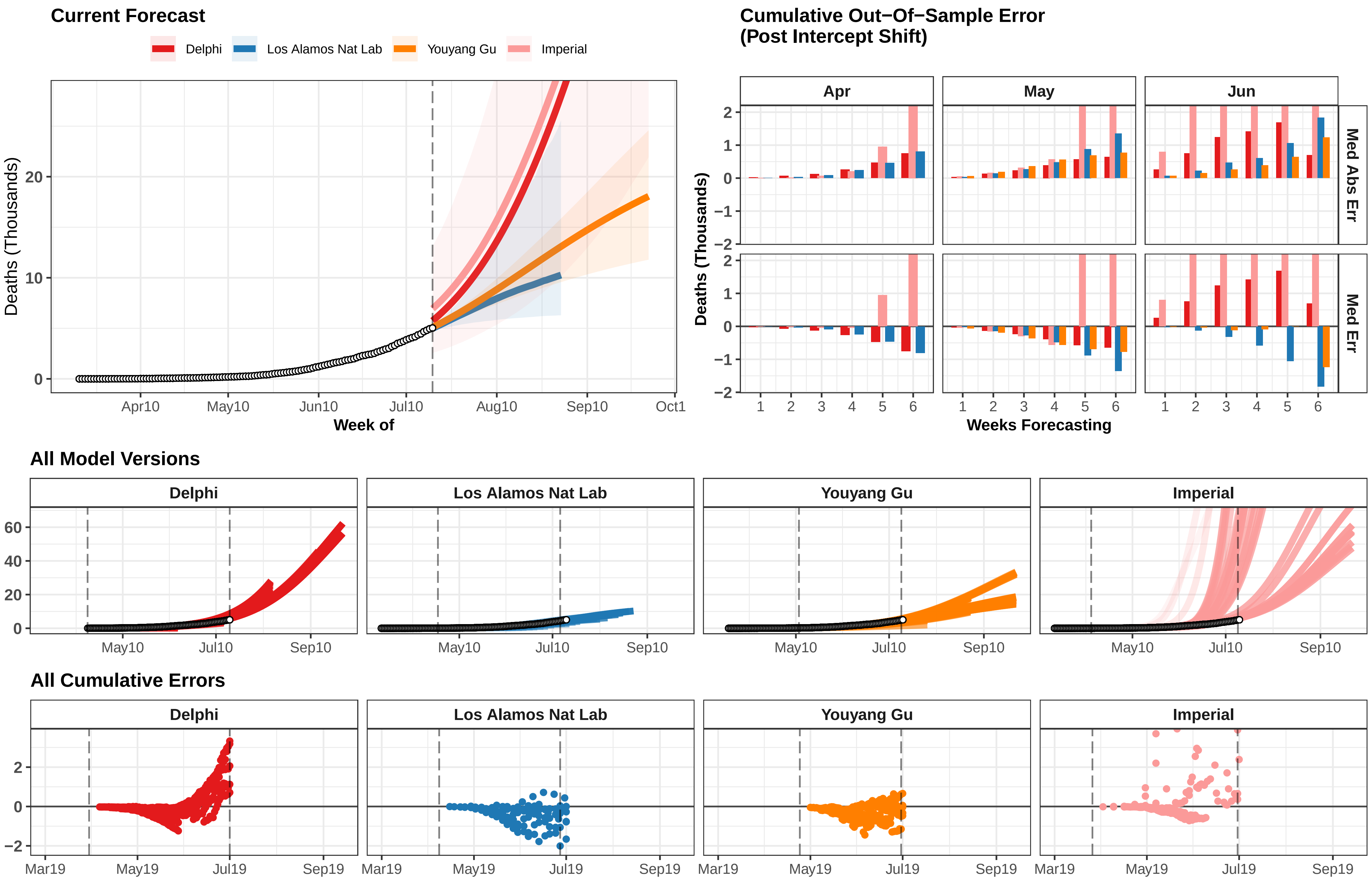

Florida

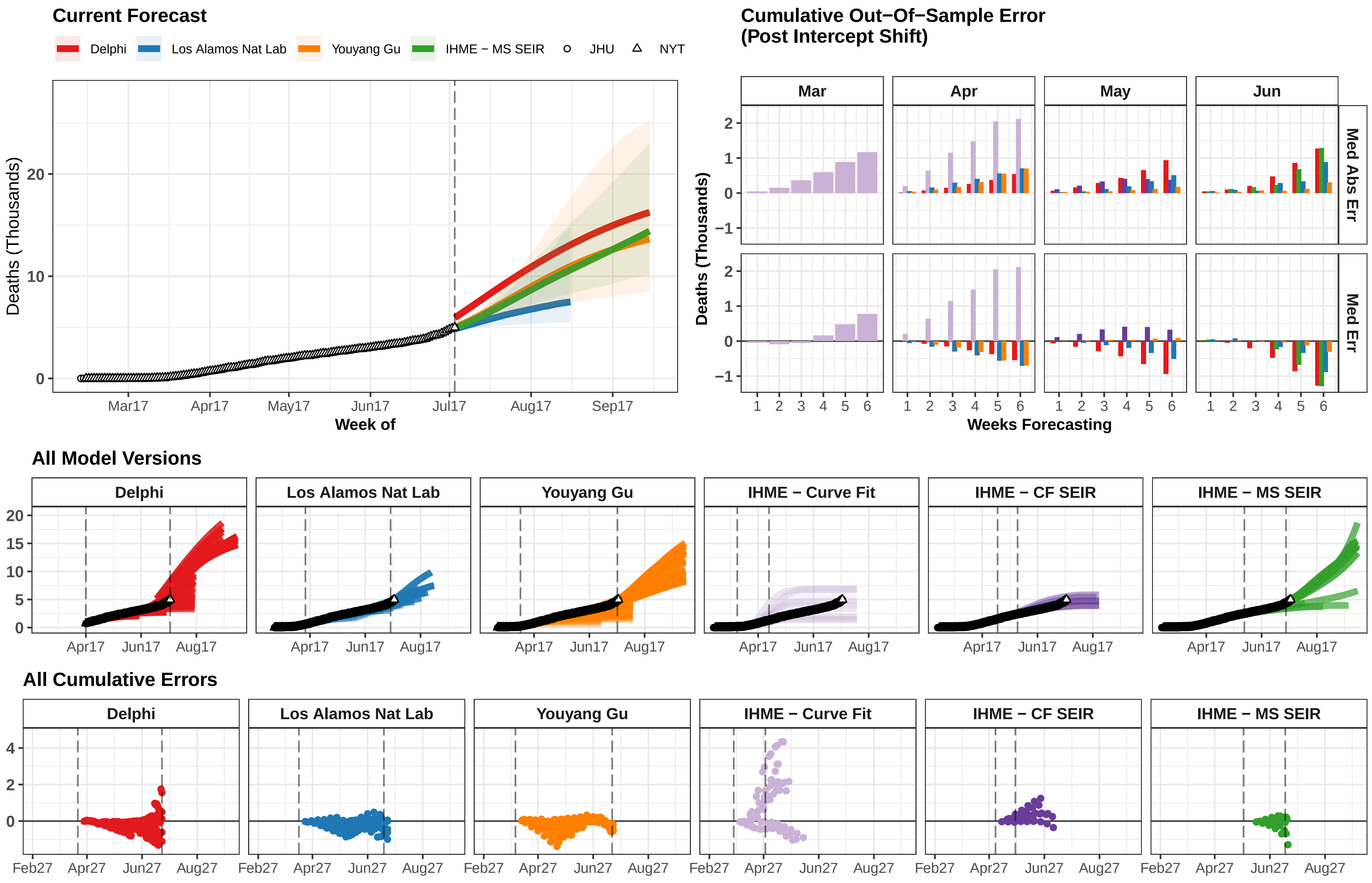
Current Forecast
Delphi
Los Alamos Nat Lab
Youyang Gu
IHME − MS SEIR
JHU
NYT
Deaths (Thousands)
20
10
0
Mar17
Apr17
May17
Jun17
Jul17
Aug17
Sep17
Week of
Cumulative Out−Of−Sample Error
(Post Intercept Shift)
Mar
Apr
May
Jun
Med Abs Err
Med Err
Deaths (Thousands)
2
1
0
−1
1 2 3 4 5 6
Weeks Forecasting
All Model Versions
Delphi
Los Alamos Nat Lab
Youyang Gu
IHME − Curve Fit
IHME − CF SEIR
IHME − MS SEIR
20
15
10
5
0
Apr17
Jun17
Aug17
All Cumulative Errors
Delphi
Los Alamos Nat Lab
Youyang Gu
IHME − Curve Fit
IHME − CF SEIR
IHME − MS SEIR
4
2
0
Feb27
Apr27
Jun27
Aug27

China

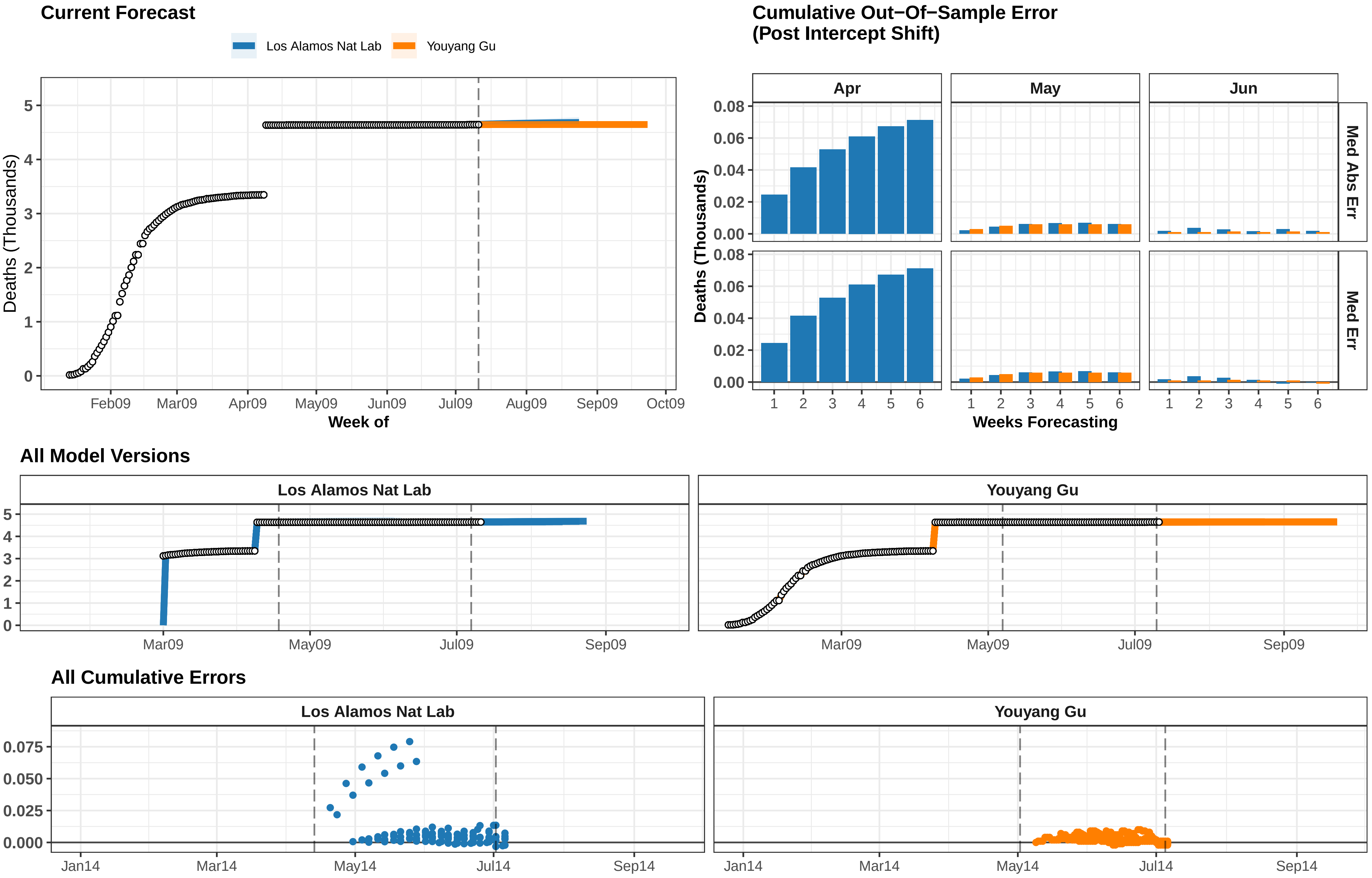

Current Forecast
Los Alamos Nat Lab
Youyang Gu
Deaths (Thousands)
Week of
Feb09
Mar09
Apr09
May09
Jun09
Jul09
Aug09
Sep09
Oct09
Cumulative Out−Of−Sample Error
(Post Intercept Shift)
Apr
May
Jun
Med Abs Err
Med Err
Weeks Forecasting
All Model Versions
Los Alamos Nat Lab
Youyang Gu
All Cumulative Errors
Jan14
Mar14
May14
Jul14
Sep14

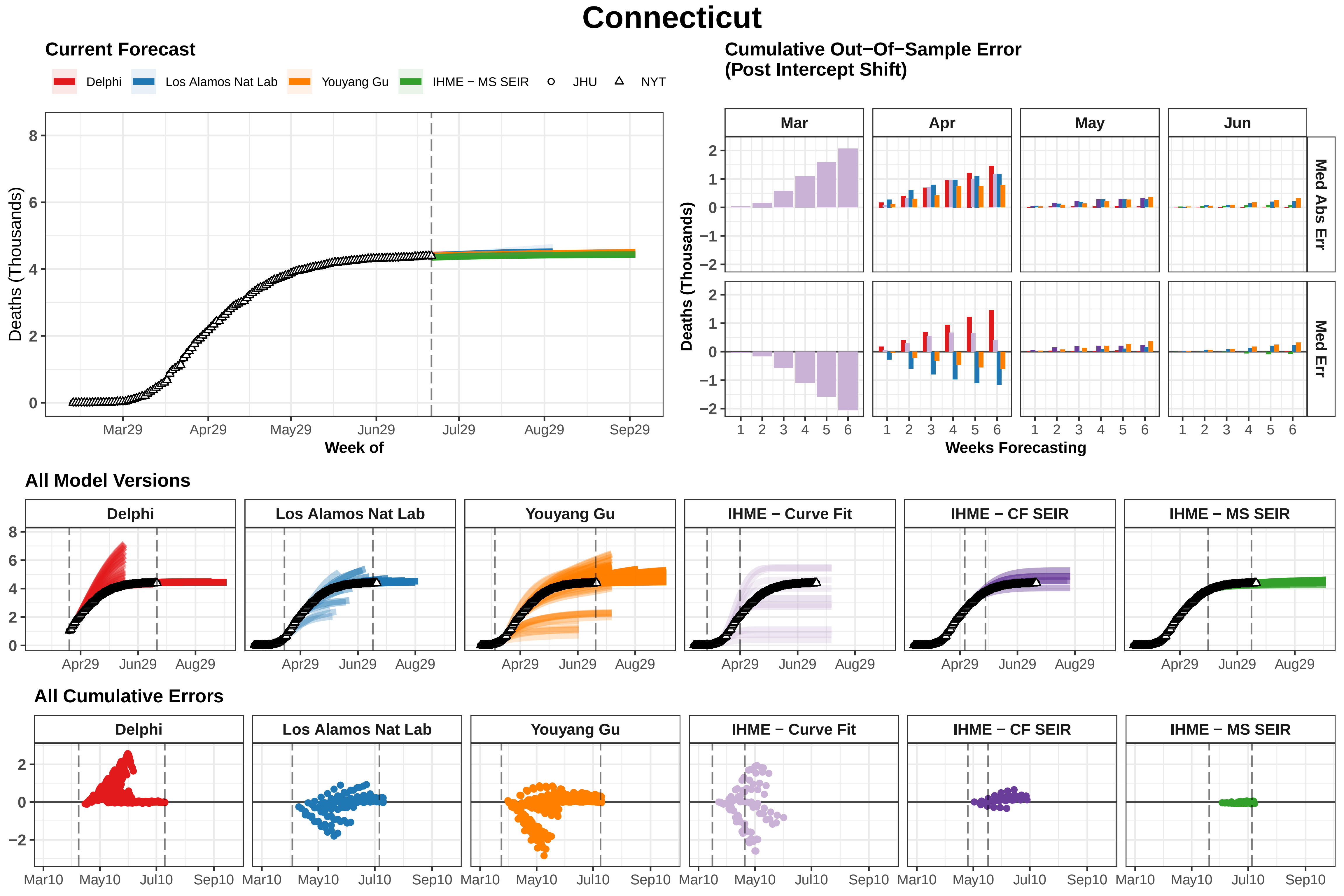
Connecticut
Current Forecast
Delphi
Los Alamos Nat Lab
Youyang Gu
IHME − MS SEIR
JHU
NYT
Deaths (Thousands)
Week of
Mar29
Apr29
May29
Jun29
Jul29
Aug29
Sep29
Cumulative Out−Of−Sample Error
(Post Intercept Shift)
Mar
Apr
May
Jun
Med Abs Err
Med Err
Weeks Forecasting
All Model Versions
Delphi
Los Alamos Nat Lab
Youyang Gu
IHME − Curve Fit
IHME − CF SEIR
IHME − MS SEIR
All Cumulative Errors
Mar10
May10
Jul10
Sep10

Egypt

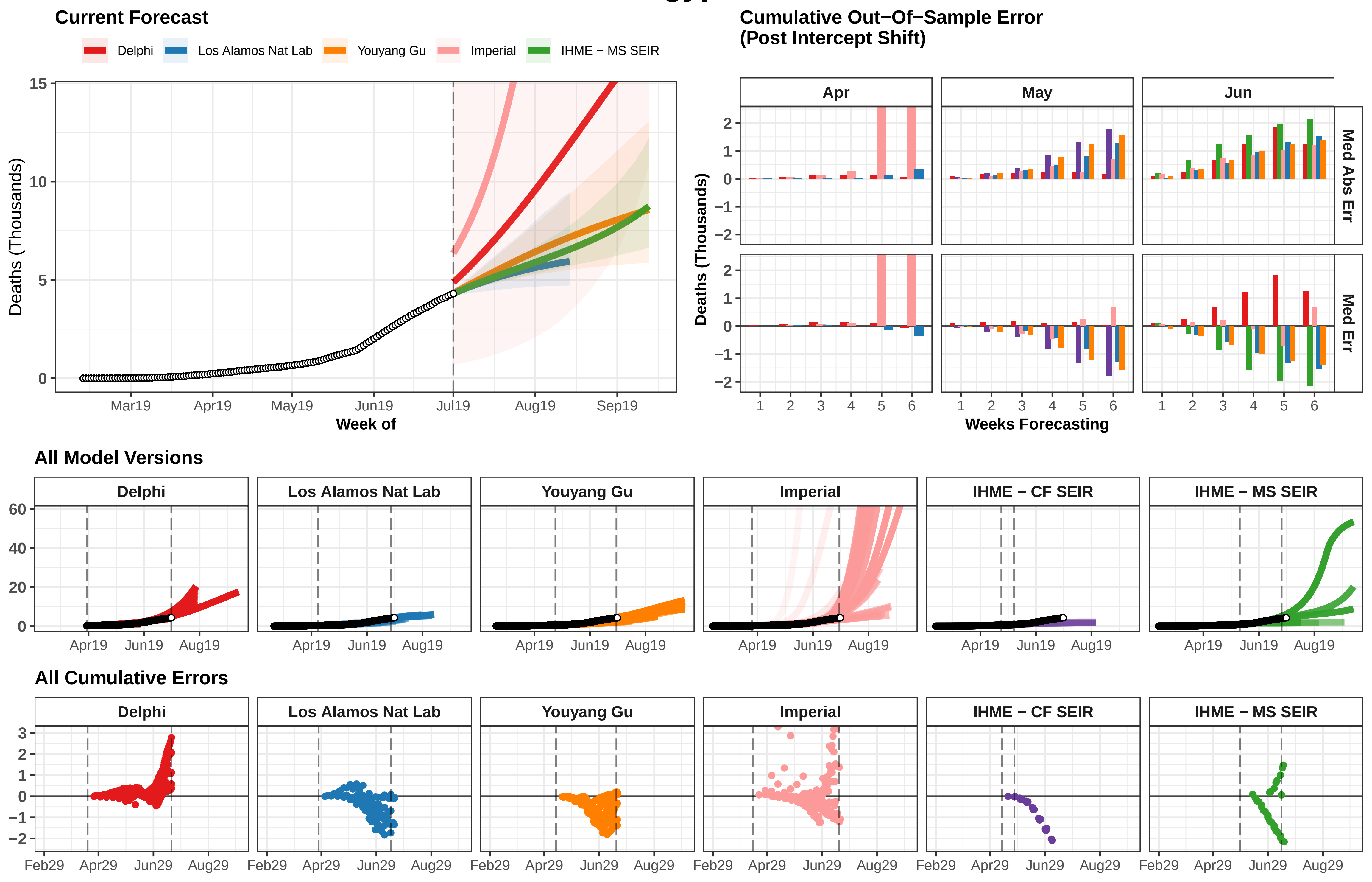

Current Forecast
Delphi
Los Alamos Nat Lab
Youyang Gu
Imperial
IHME − MS SEIR
Deaths (Thousands)
Week of
Mar19
Apr19
May19
Jun19
Jul19
Aug19
Sep19
Cumulative Out−Of−Sample Error
(Post Intercept Shift)
Apr
May
Jun
Med Abs Err
Med Err
Weeks Forecasting
All Model Versions
IHME − CF SEIR
All Cumulative Errors
Feb29
Apr29
Jun29
Aug29

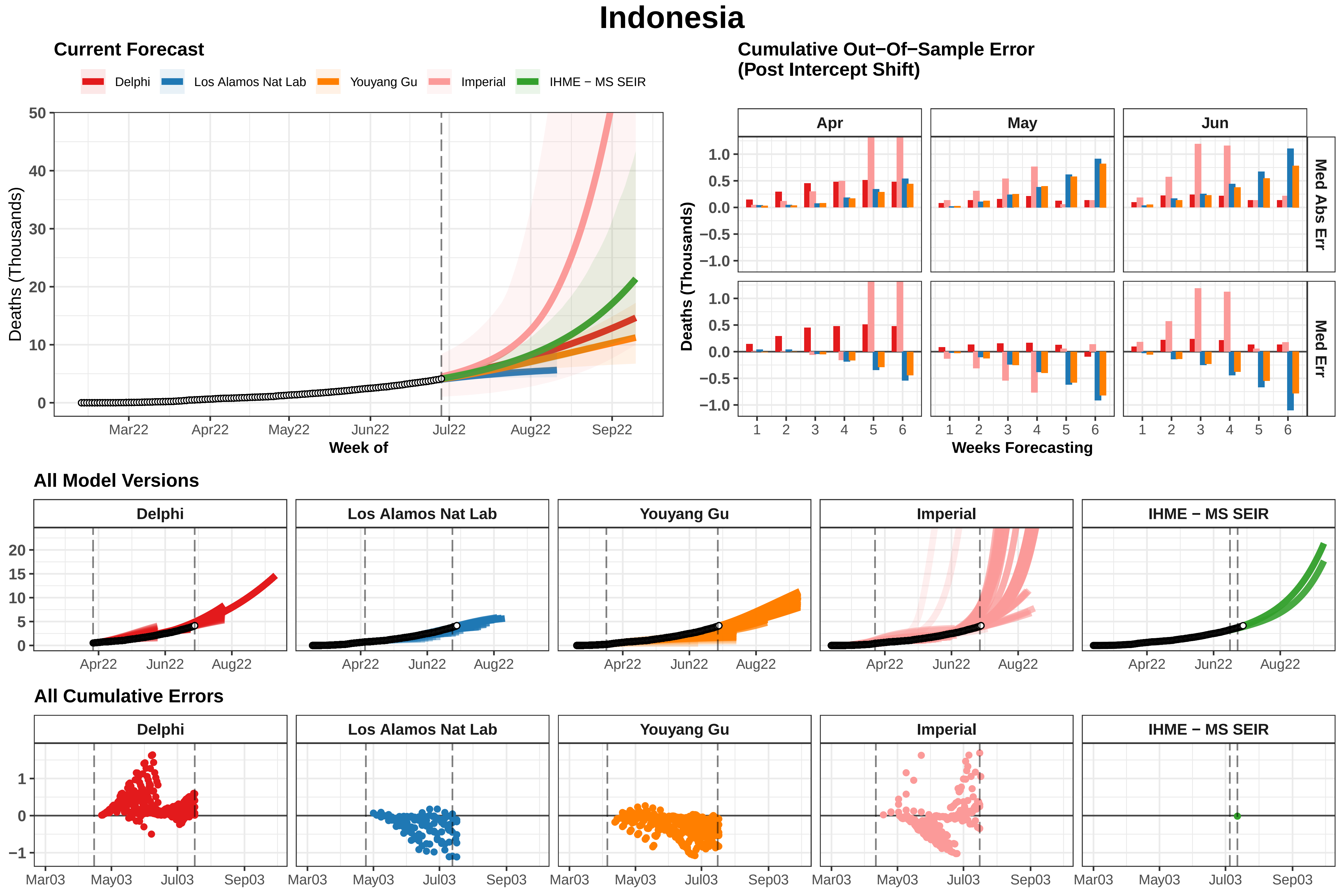
Indonesia
Current Forecast
Delphi
Los Alamos Nat Lab
Youyang Gu
Imperial
IHME − MS SEIR
Deaths (Thousands)
Week of
Mar22
Apr22
May22
Jun22
Jul22
Aug22
Sep22
Cumulative Out−Of−Sample Error
(Post Intercept Shift)
Apr
May
Jun
Med Abs Err
Med Err
Weeks Forecasting
All Model Versions
All Cumulative Errors
Mar03
May03
Jul03
Sep03

Texas

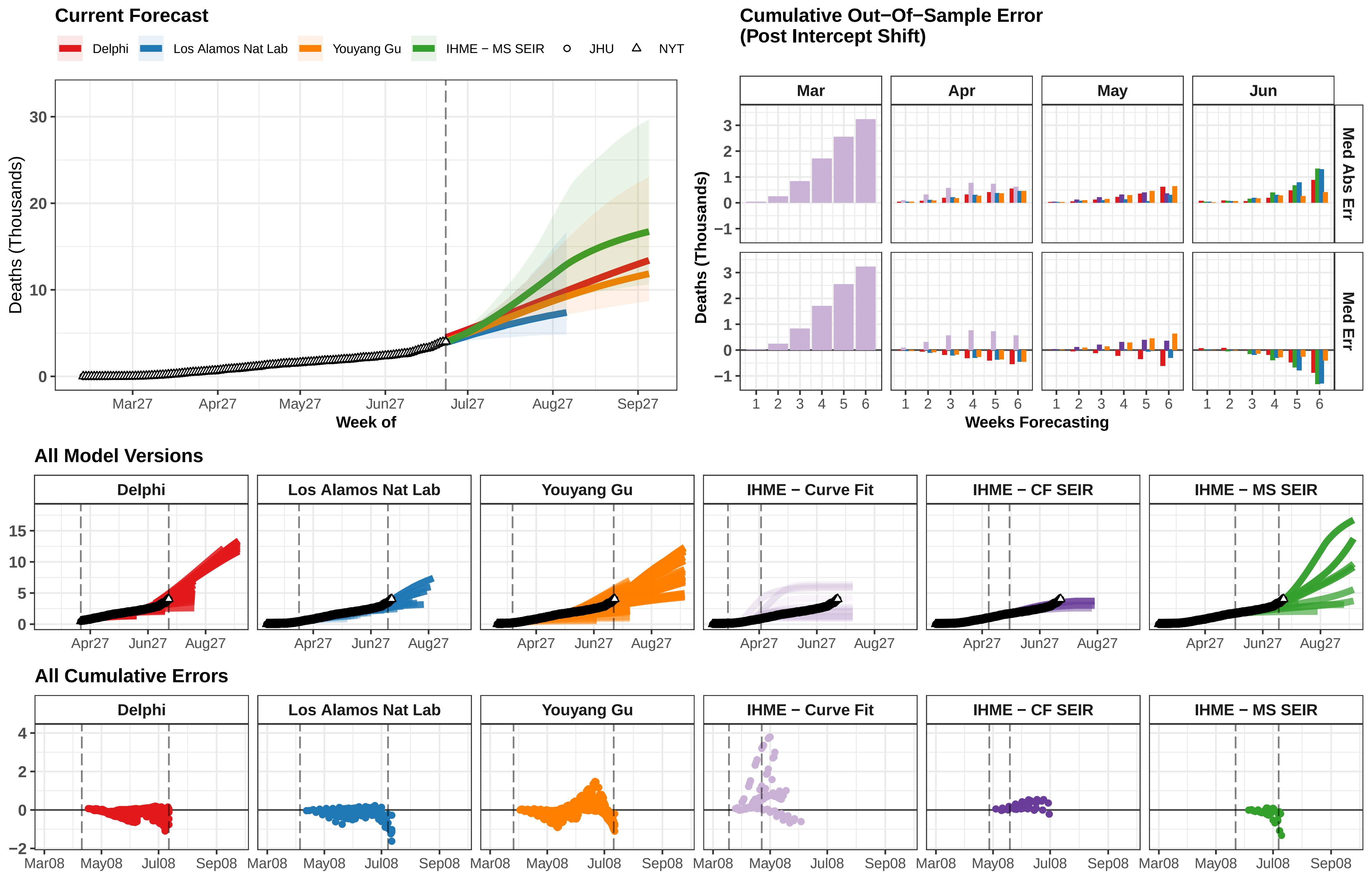

Current Forecast
Delphi
Los Alamos Nat Lab
Youyang Gu
IHME − MS SEIR
JHU
NYT
Deaths (Thousands)
Week of
Mar27
Apr27
May27
Jun27
Jul27
Aug27
Sep27
Cumulative Out−Of−Sample Error
(Post Intercept Shift)
Mar
Apr
May
Jun
Med Abs Err
Med Err
Weeks Forecasting
All Model Versions
Delphi
Los Alamos Nat Lab
Youyang Gu
IHME − Curve Fit
IHME − CF SEIR
IHME − MS SEIR
All Cumulative Errors
Mar08
May08
Jul08
Sep08

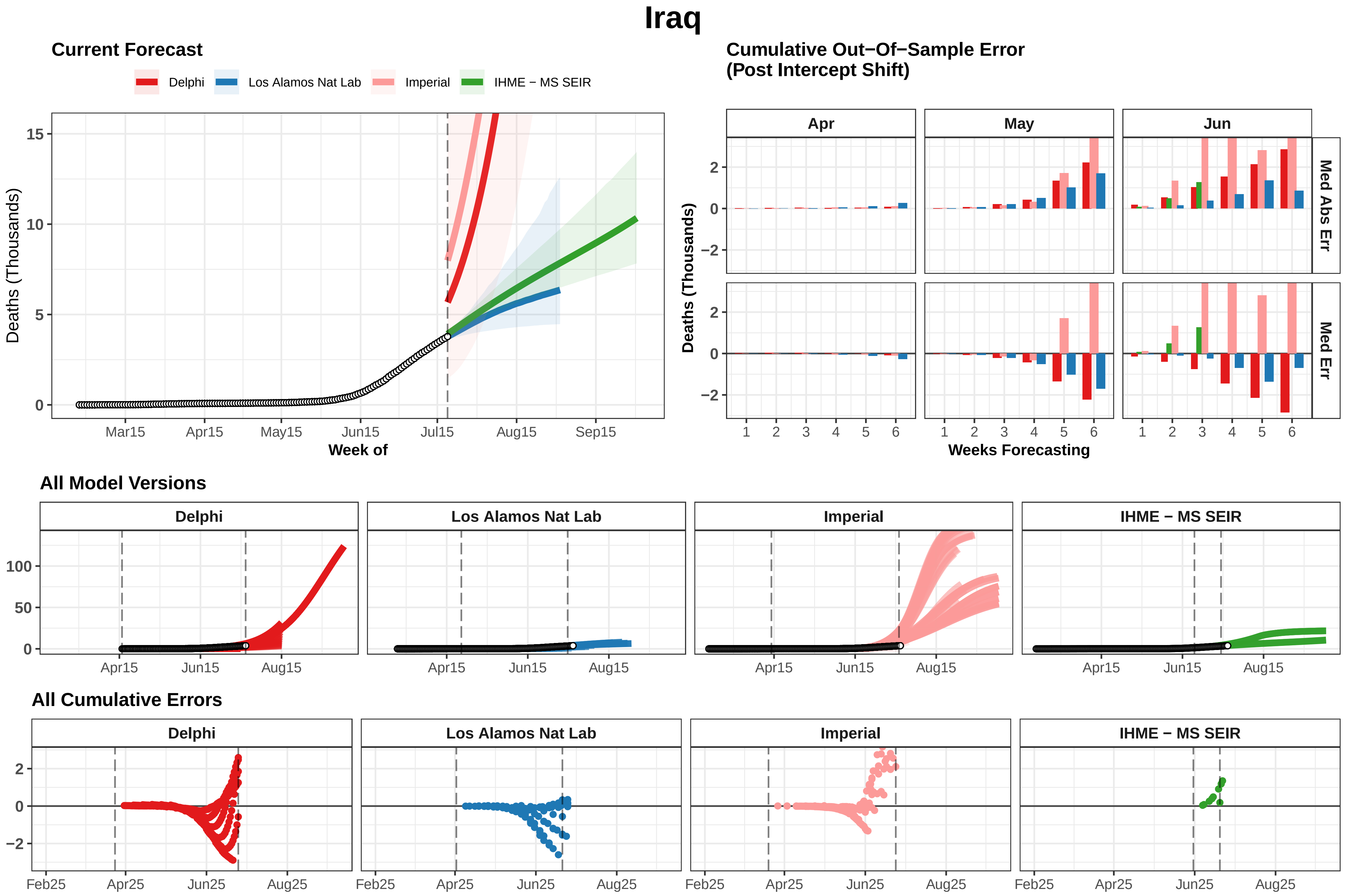
Iraq
Current Forecast
Delphi
Los Alamos Nat Lab
Imperial
IHME − MS SEIR
Deaths (Thousands)
15
10
5
0
Mar15
Apr15
May15
Jun15
Jul15
Aug15
Sep15
Week of
Cumulative Out−Of−Sample Error
(Post Intercept Shift)
Apr
May
Jun
Med Abs Err
Med Err
2
0
−2
1
2
3
4
5
6
Weeks Forecasting
All Model Versions
100
50
Jun15
All Cumulative Errors
Feb25
Apr25
Jun25
Aug25

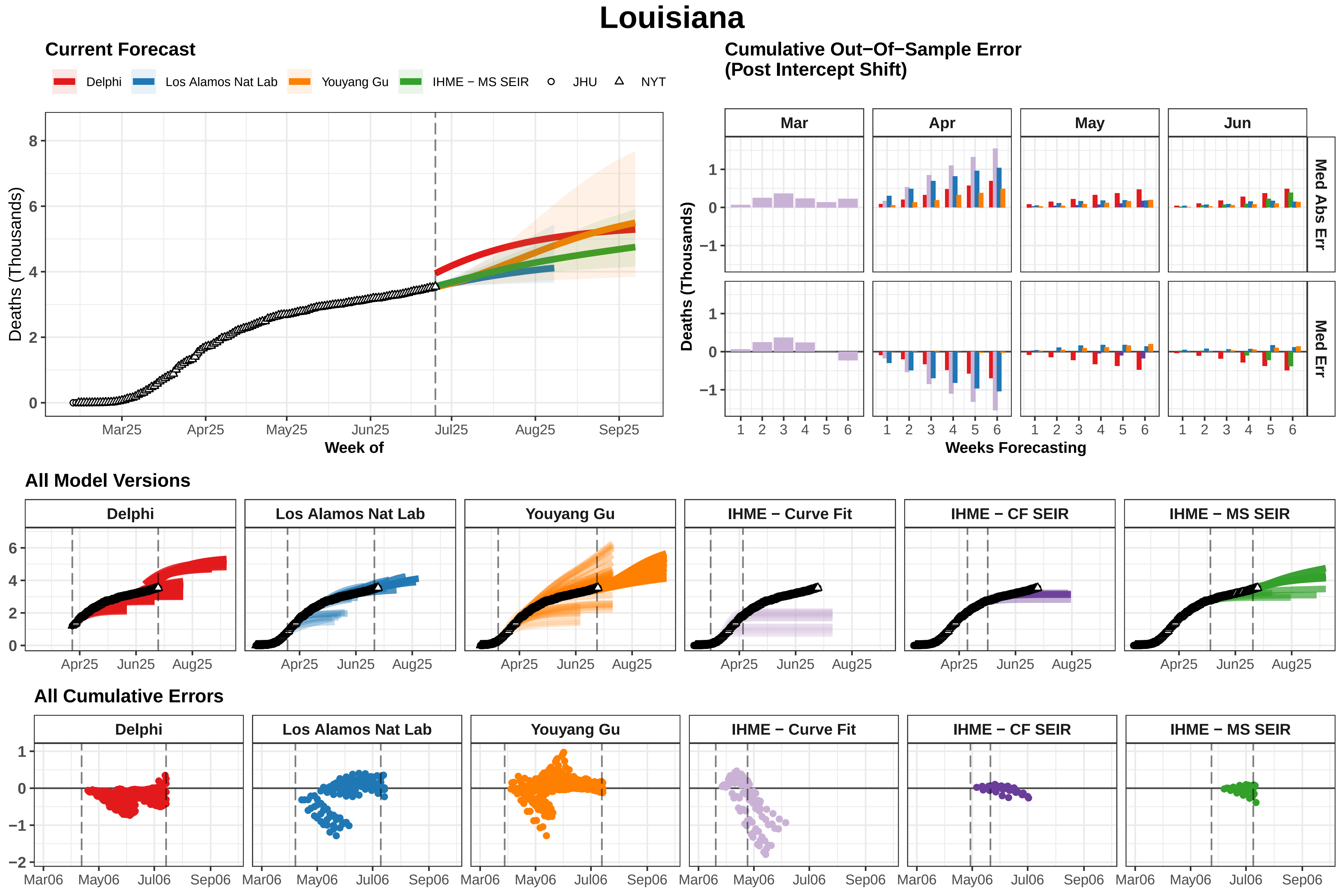

Louisiana
Current Forecast
Delphi
Los Alamos Nat Lab
Youyang Gu
IHME − MS SEIR
JHU
NYT
Deaths (Thousands)
Week of
Mar25
Apr25
May25
Jun25
Jul25
Aug25
Sep25
Cumulative Out−Of−Sample Error
(Post Intercept Shift)
Mar
Apr
May
Jun
Med Abs Err
Med Err
Weeks Forecasting
All Model Versions
Delphi
Los Alamos Nat Lab
Youyang Gu
IHME − Curve Fit
IHME − CF SEIR
IHME − MS SEIR
All Cumulative Errors
Mar06
May06
Jul06
Sep06

Maryland

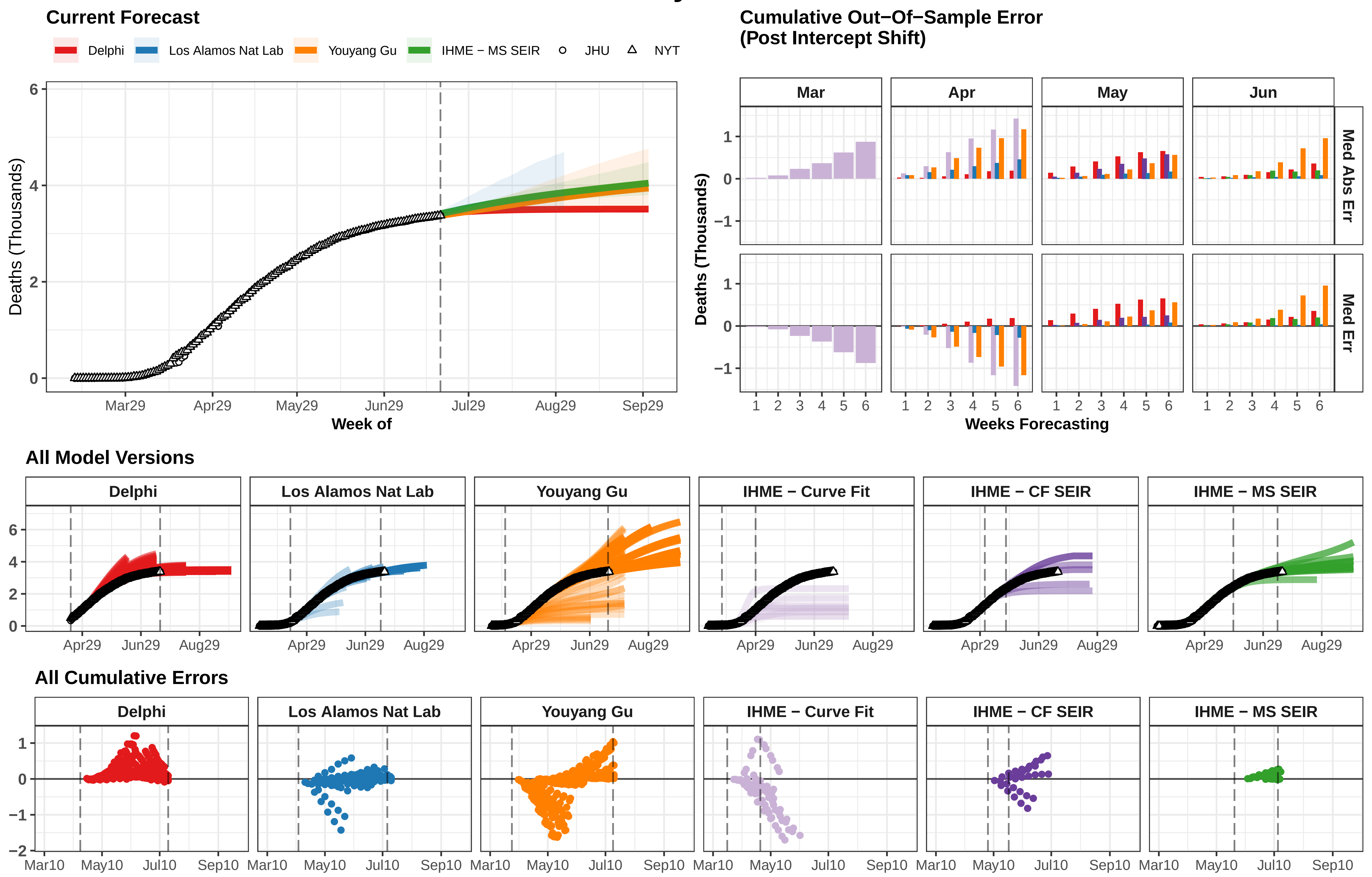

Current Forecast
Delphi
Los Alamos Nat Lab
Youyang Gu
IHME − MS SEIR
JHU
NYT
Deaths (Thousands)
Week of
Mar29
Apr29
May29
Jun29
Jul29
Aug29
Sep29
Cumulative Out−Of−Sample Error
(Post Intercept Shift)
Mar
Apr
May
Jun
Med Abs Err
Med Err
Weeks Forecasting
All Model Versions
IHME − Curve Fit
IHME − CF SEIR
All Cumulative Errors
Mar10
May10
Jul10
Sep10

Ohio

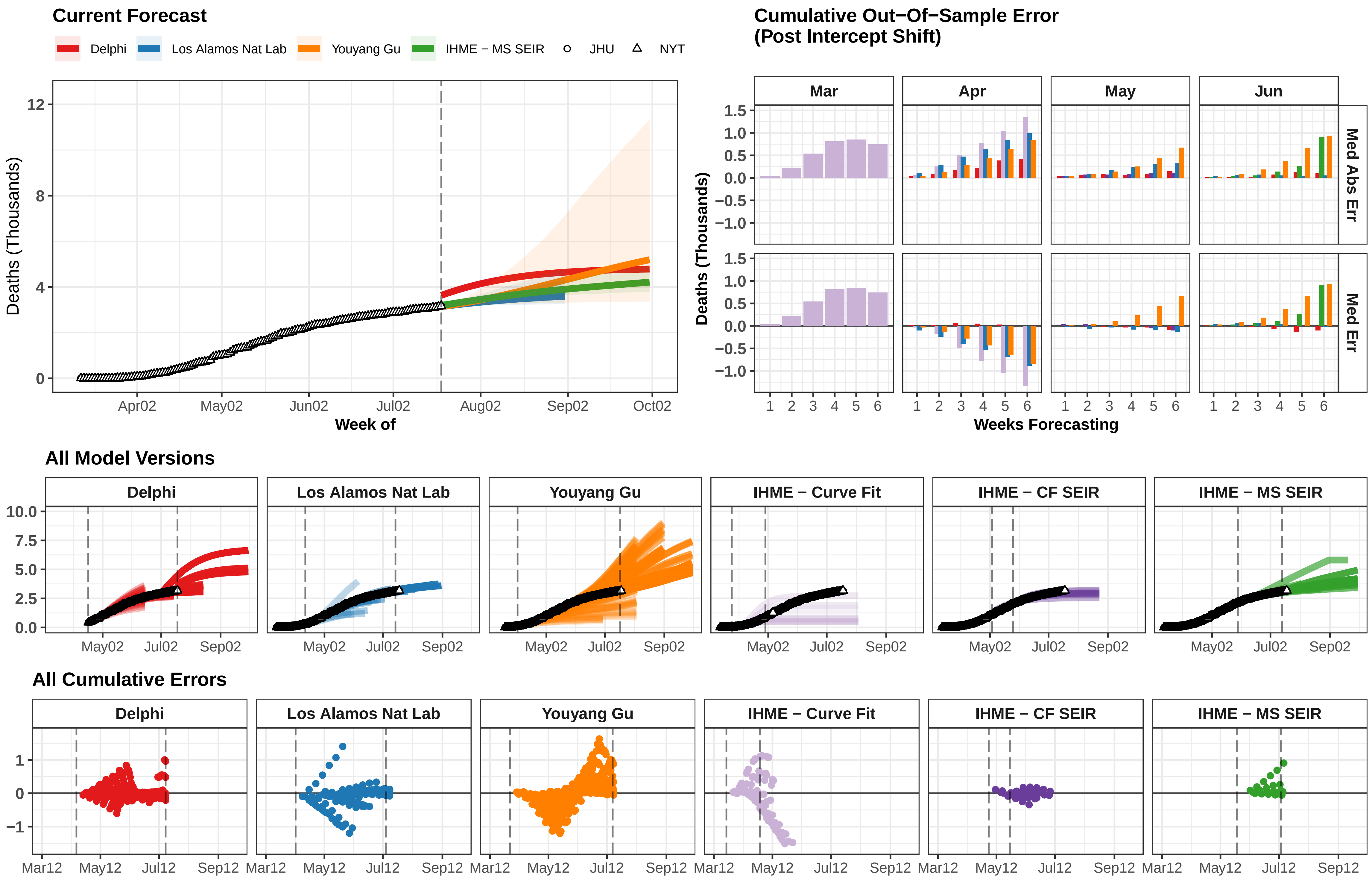
Current Forecast
Delphi
Los Alamos Nat Lab
Youyang Gu
IHME − MS SEIR
JHU
NYT
Deaths (Thousands)
Week of
Apr02
May02
Jun02
Jul02
Aug02
Sep02
Oct02
Cumulative Out−Of−Sample Error (Post Intercept Shift)
Mar
Apr
May
Jun
Med Abs Err
Med Err
Weeks Forecasting
All Model Versions
IHME − Curve Fit
IHME − CF SEIR
All Cumulative Errors
Mar12
May12
Jul12
Sep12

Georgia

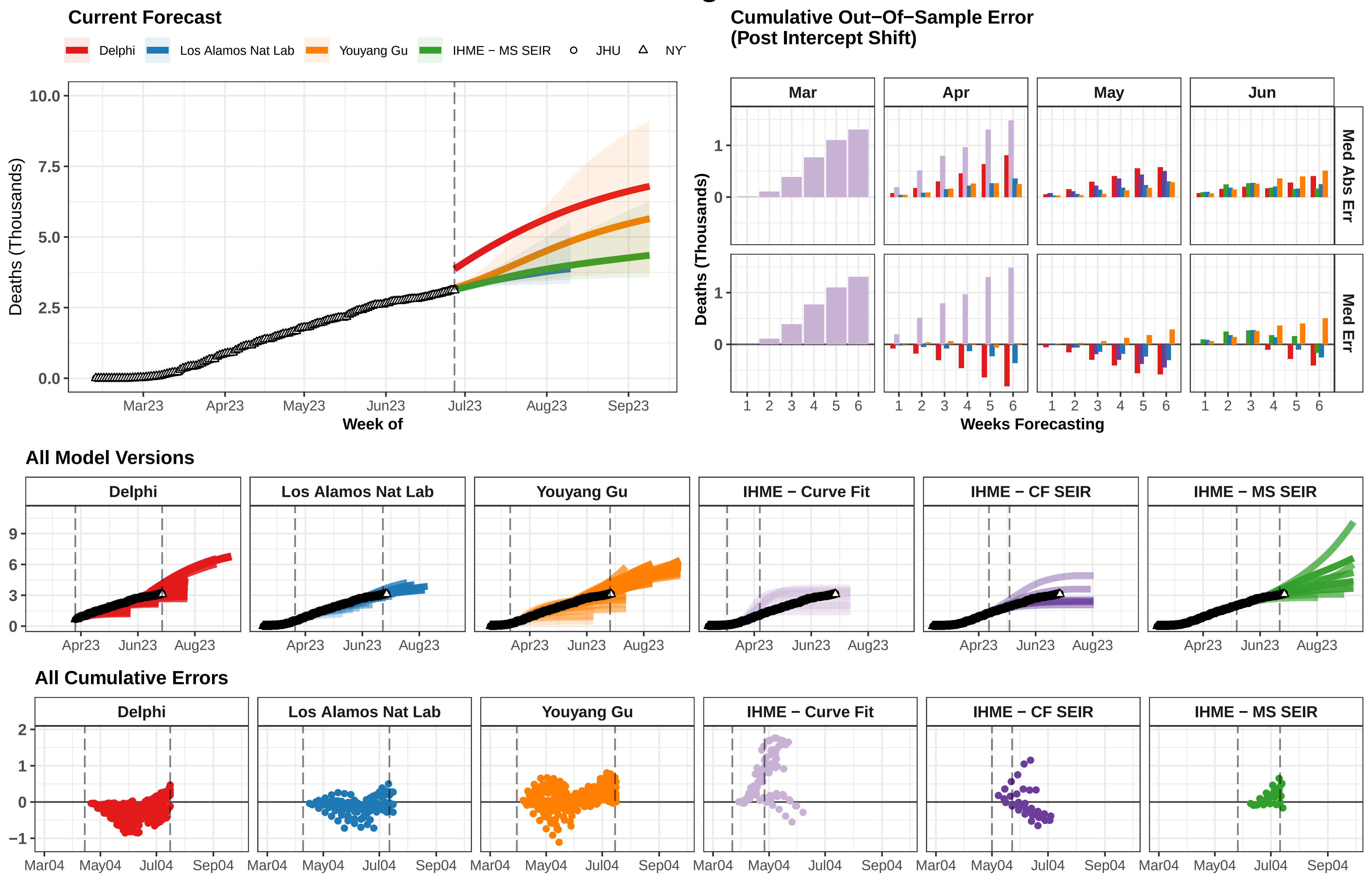
Current Forecast
Delphi
Los Alamos Nat Lab
Youyang Gu
IHME − MS SEIR
JHU
NY
Deaths (Thousands)
10.0
7.5
5.0
2.5
0.0
Mar23
Apr23
May23
Jun23
Jul23
Aug23
Sep23
Week of
Cumulative Out−Of−Sample Error
(Post Intercept Shift)
Mar
Apr
May
Jun
Med Abs Err
Med Err
Weeks Forecasting
All Model Versions
Delphi
Los Alamos Nat Lab
Youyang Gu
IHME − Curve Fit
IHME − CF SEIR
IHME − MS SEIR
All Cumulative Errors
Mar04
May04
Jul04
Sep04

Indiana

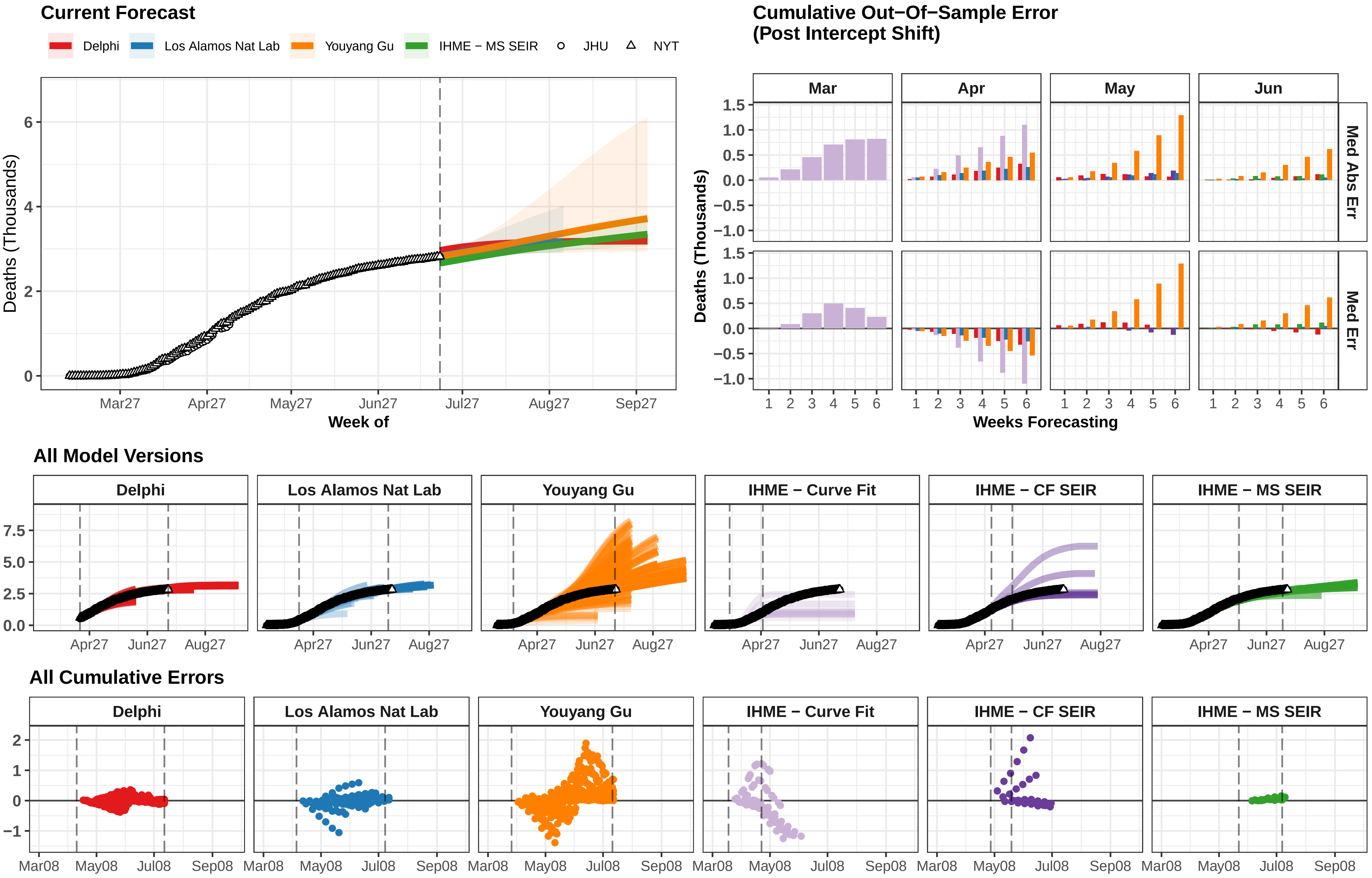

Current Forecast
Delphi
Los Alamos Nat Lab
Youyang Gu
IHME − MS SEIR
JHU
NYT
Deaths (Thousands)
Week of
Mar27
Apr27
May27
Jun27
Jul27
Aug27
Sep27
Cumulative Out−Of−Sample Error
(Post Intercept Shift)
Mar
Apr
May
Jun
Med Abs Err
Med Err
Weeks Forecasting
All Model Versions
Delphi
Los Alamos Nat Lab
Youyang Gu
IHME − Curve Fit
IHME − CF SEIR
IHME − MS SEIR
All Cumulative Errors
Mar08
May08
Jul08
Sep08

Arizona

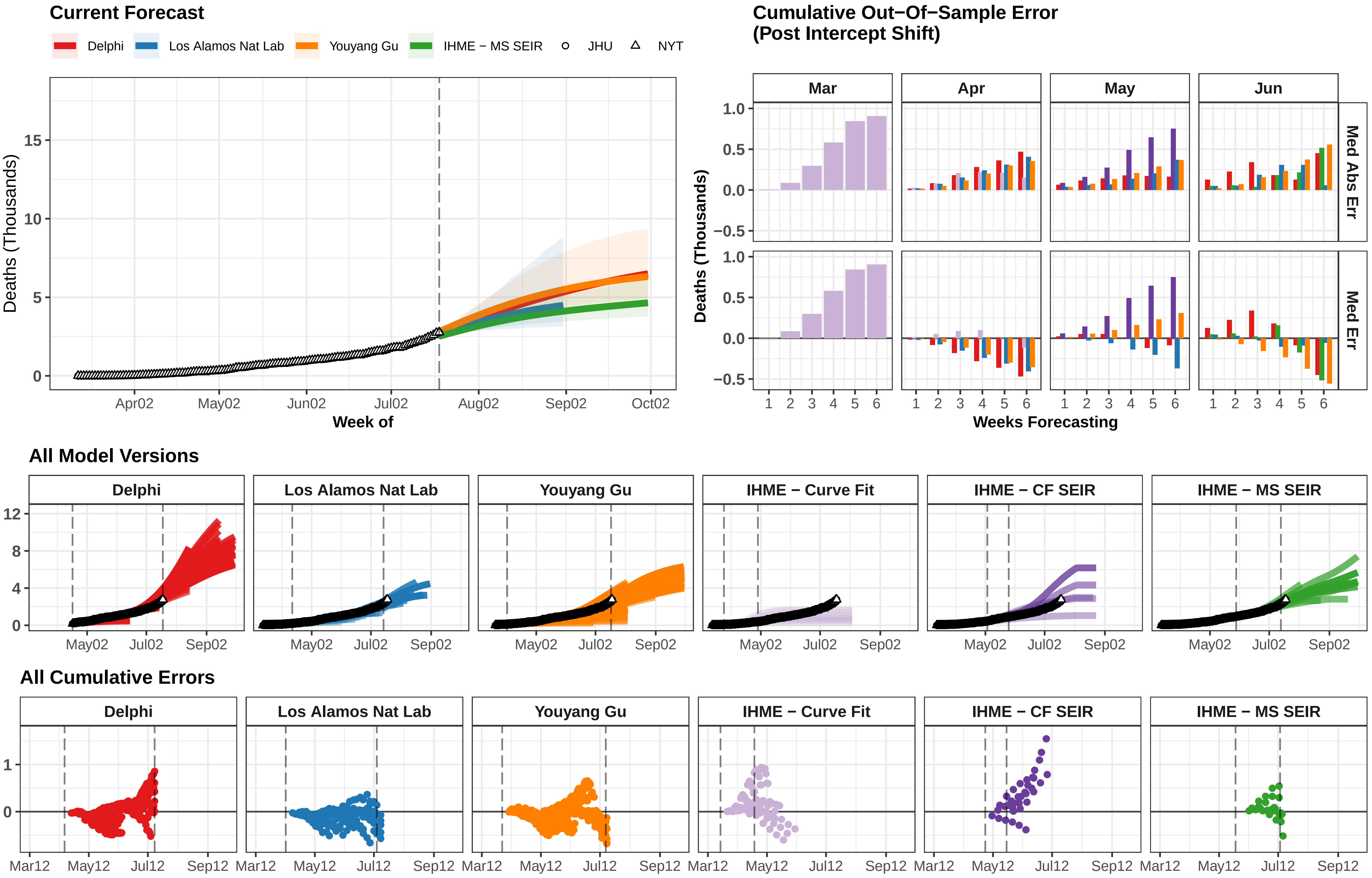
Current Forecast
Delphi
Los Alamos Nat Lab
Youyang Gu
IHME − MS SEIR
JHU
NYT
Deaths (Thousands)
Week of
Apr02
May02
Jun02
Jul02
Aug02
Sep02
Oct02
Cumulative Out−Of−Sample Error (Post Intercept Shift)
Mar
Apr
May
Jun
Med Abs Err
Med Err
Weeks Forecasting
All Model Versions
Delphi
Los Alamos Nat Lab
Youyang Gu
IHME − Curve Fit
IHME − CF SEIR
IHME − MS SEIR
All Cumulative Errors
Mar12
May12
Jul12
Sep12

Bangladesh

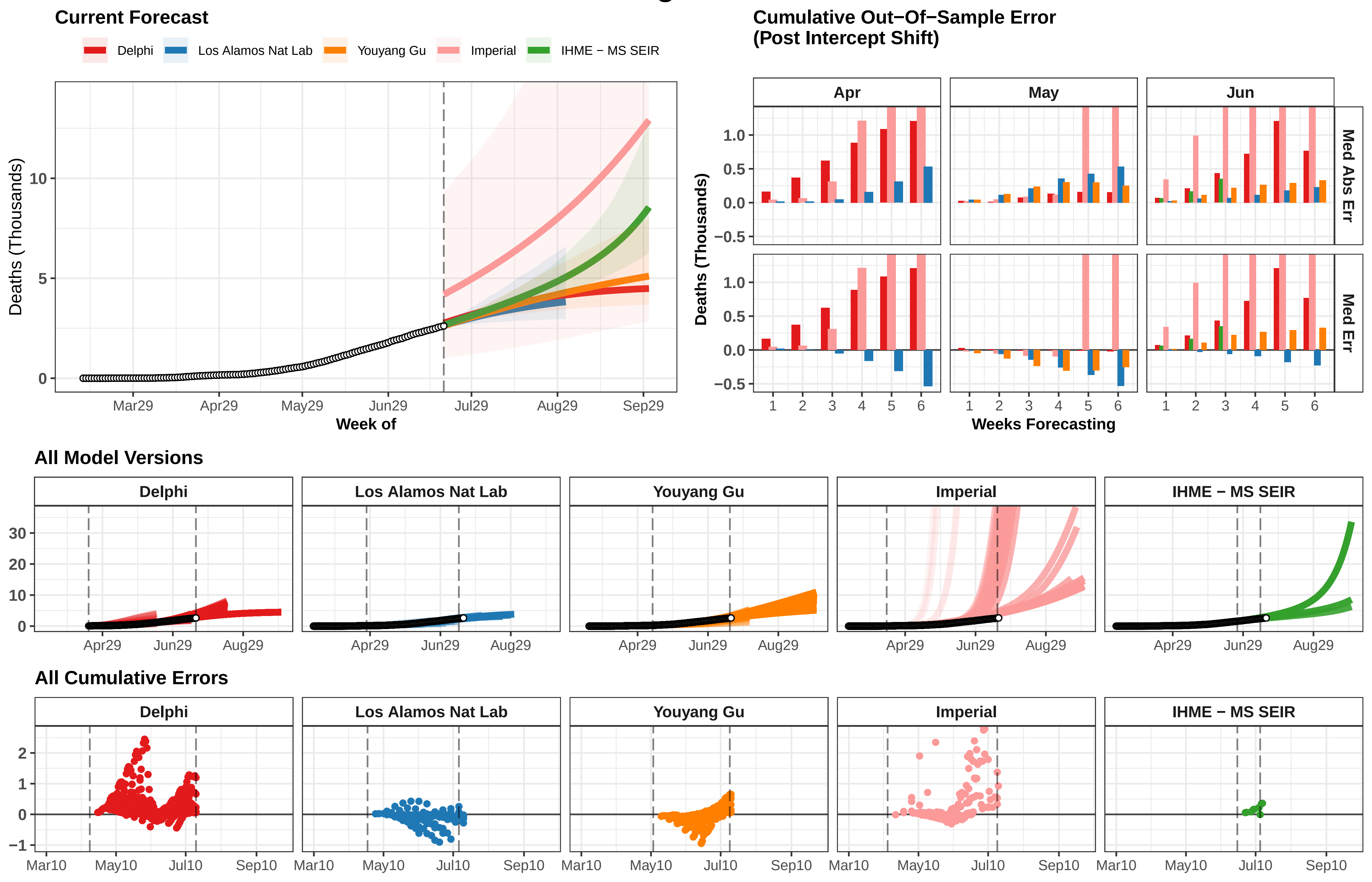

Current Forecast
Delphi
Los Alamos Nat Lab
Youyang Gu
Imperial
IHME − MS SEIR
Deaths (Thousands)
Week of
Mar29
Apr29
May29
Jun29
Jul29
Aug29
Sep29
Cumulative Out−Of−Sample Error
(Post Intercept Shift)
Apr
May
Jun
Med Abs Err
Med Err
Weeks Forecasting
All Model Versions
All Cumulative Errors
Mar10
May10
Jul10
Sep10

Saudi Arabia

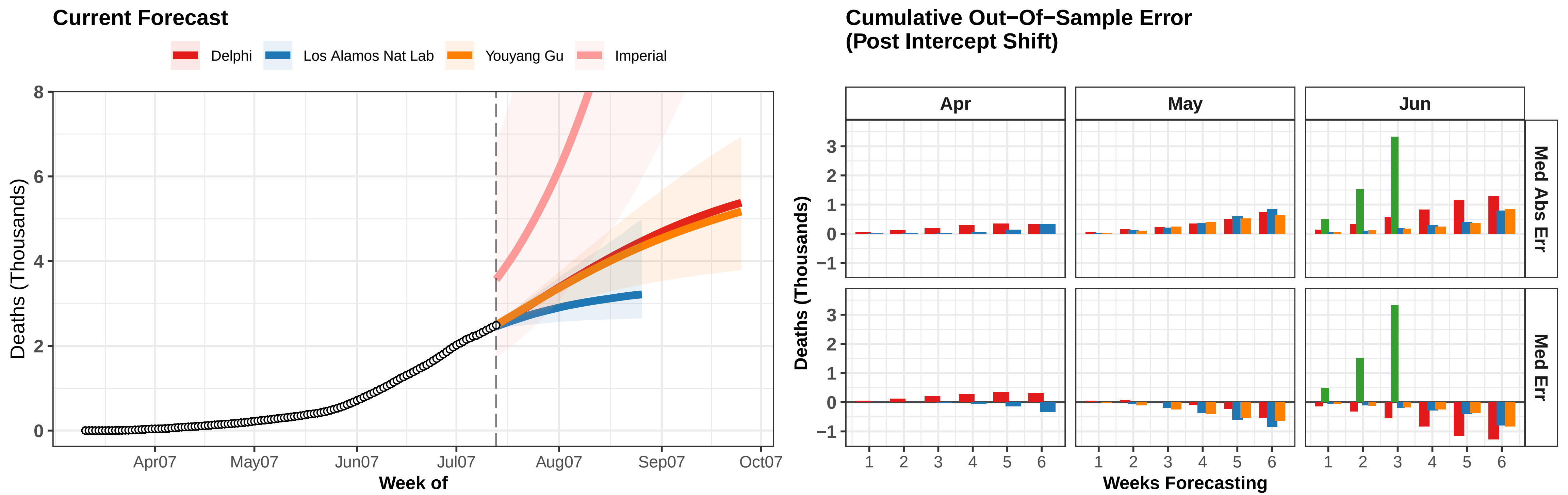

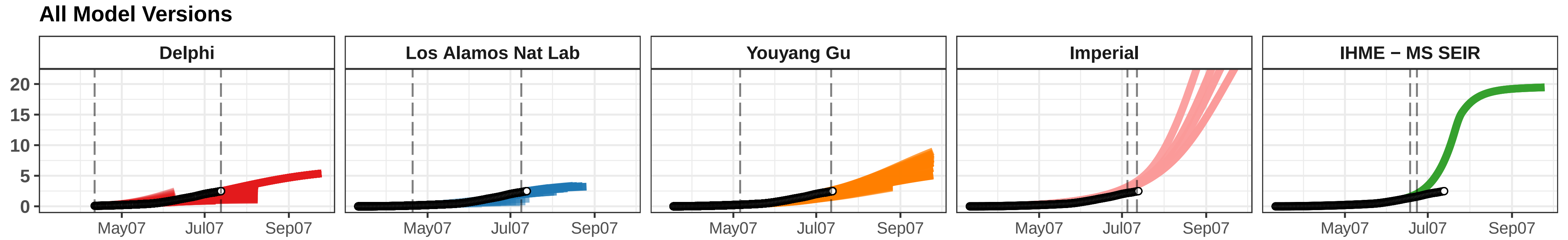

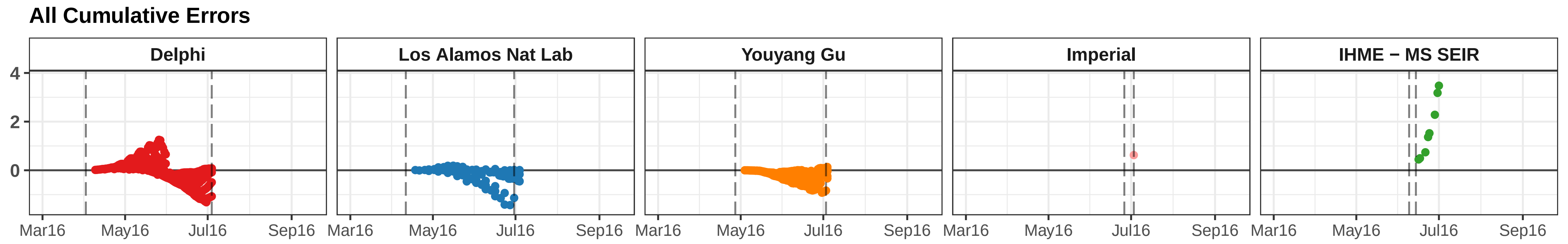

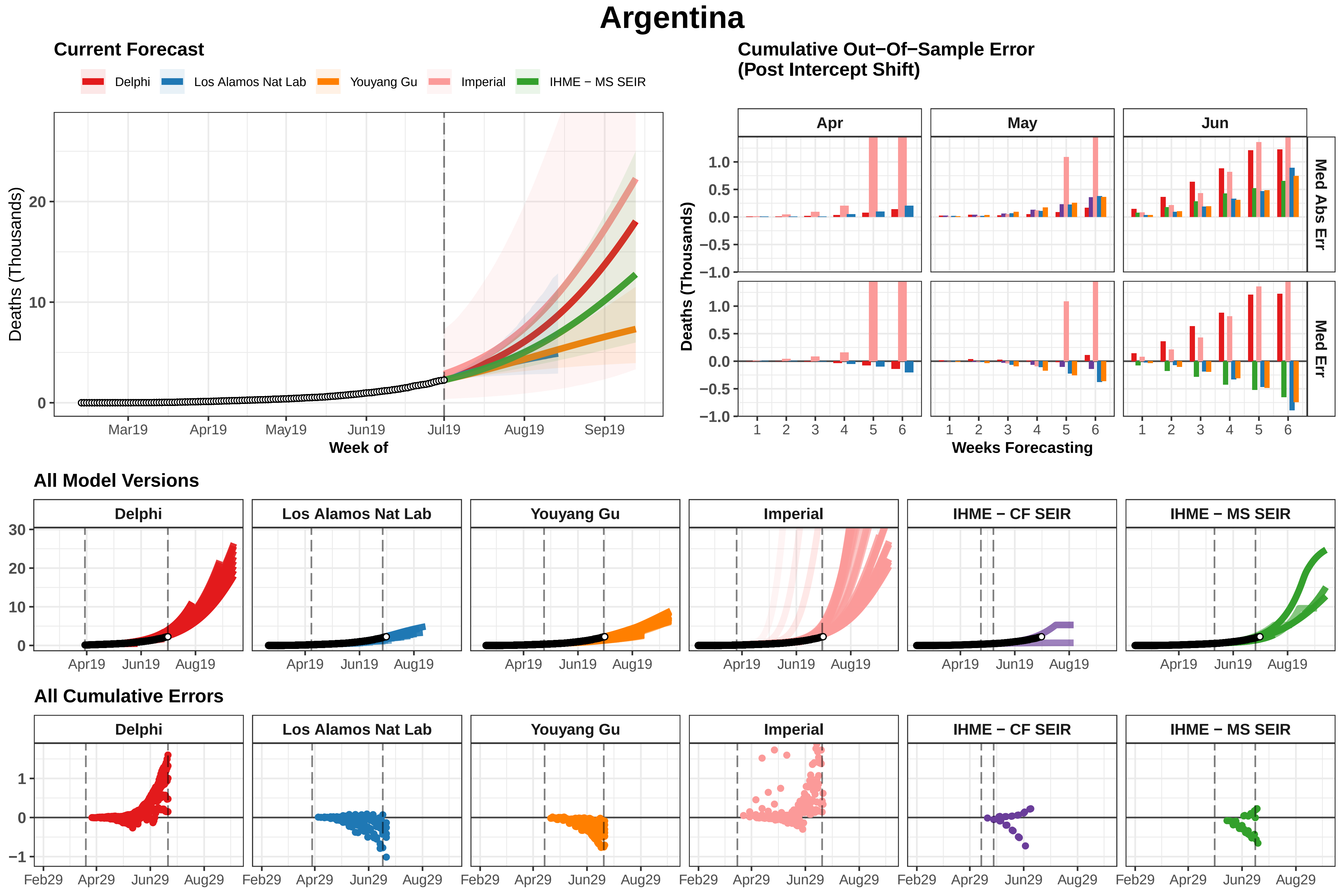

Argentina
Current Forecast
Delphi
Los Alamos Nat Lab
Youyang Gu
Imperial
IHME − MS SEIR
Deaths (Thousands)
0
10
20
Mar19
Apr19
May19
Jun19
Jul19
Aug19
Sep19
Week of
Cumulative Out−Of−Sample Error
(Post Intercept Shift)
Apr
May
Jun
Med Abs Err
Med Err
1.0
0.5
0.0
−0.5
−1.0
1
2
3
4
5
6
Weeks Forecasting
All Model Versions
Delphi
Los Alamos Nat Lab
Youyang Gu
Imperial
IHME − CF SEIR
IHME − MS SEIR
30
20
10
0
Apr19
Jun19
Aug19
All Cumulative Errors
1
0
−1
Feb29
Apr29
Jun29
Aug29

Bolivia

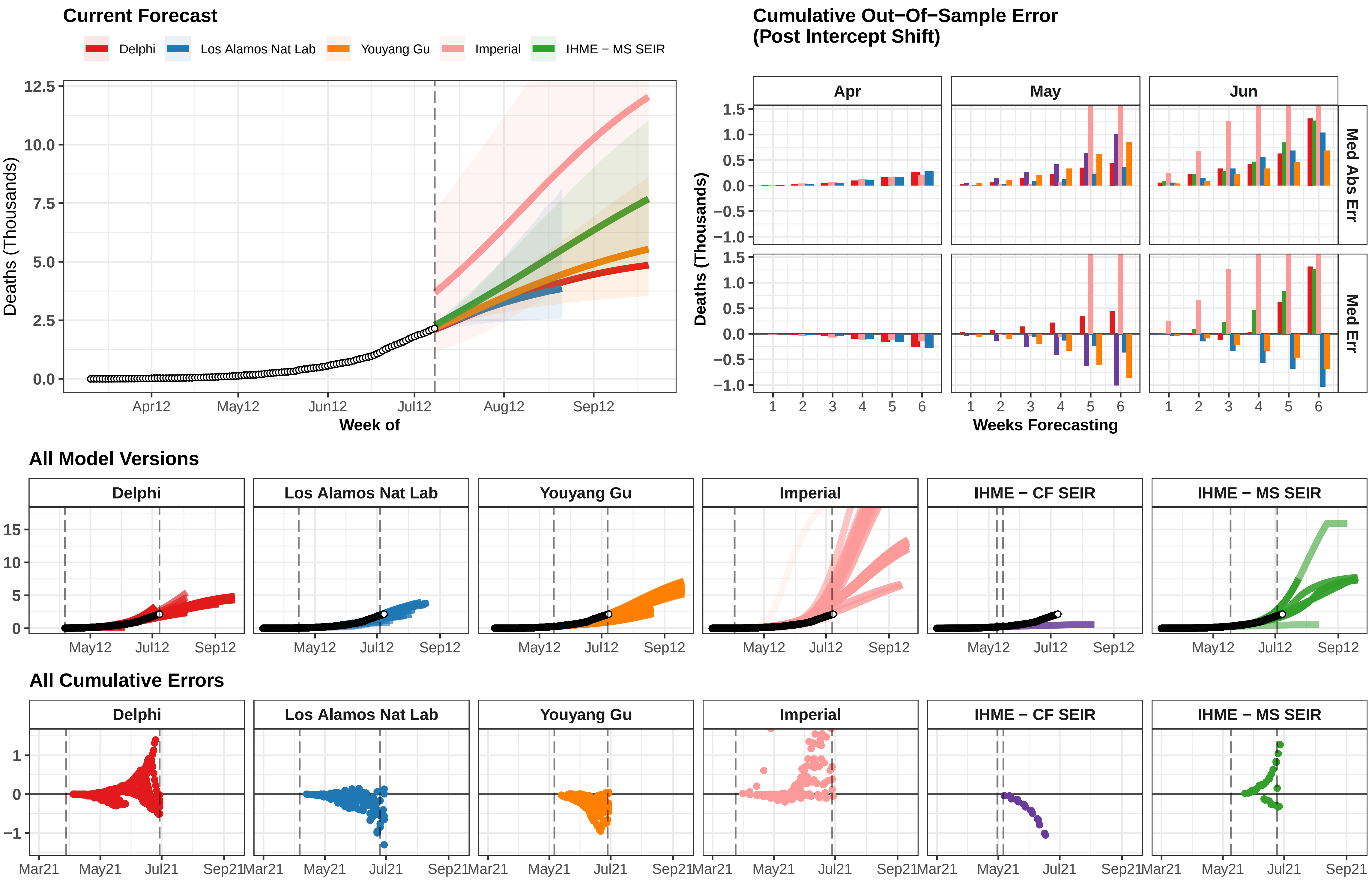

Current Forecast
Delphi
Los Alamos Nat Lab
Youyang Gu
Imperial
IHME − MS SEIR
Deaths (Thousands)
Week of
Apr12
May12
Jun12
Jul12
Aug12
Sep12
Cumulative Out−Of−Sample Error
(Post Intercept Shift)
Apr
May
Jun
Med Abs Err
Med Err
Weeks Forecasting
All Model Versions
Delphi
Los Alamos Nat Lab
Youyang Gu
Imperial
IHME − CF SEIR
IHME − MS SEIR
All Cumulative Errors
Mar21
May21
Jul21
Sep21

Virginia

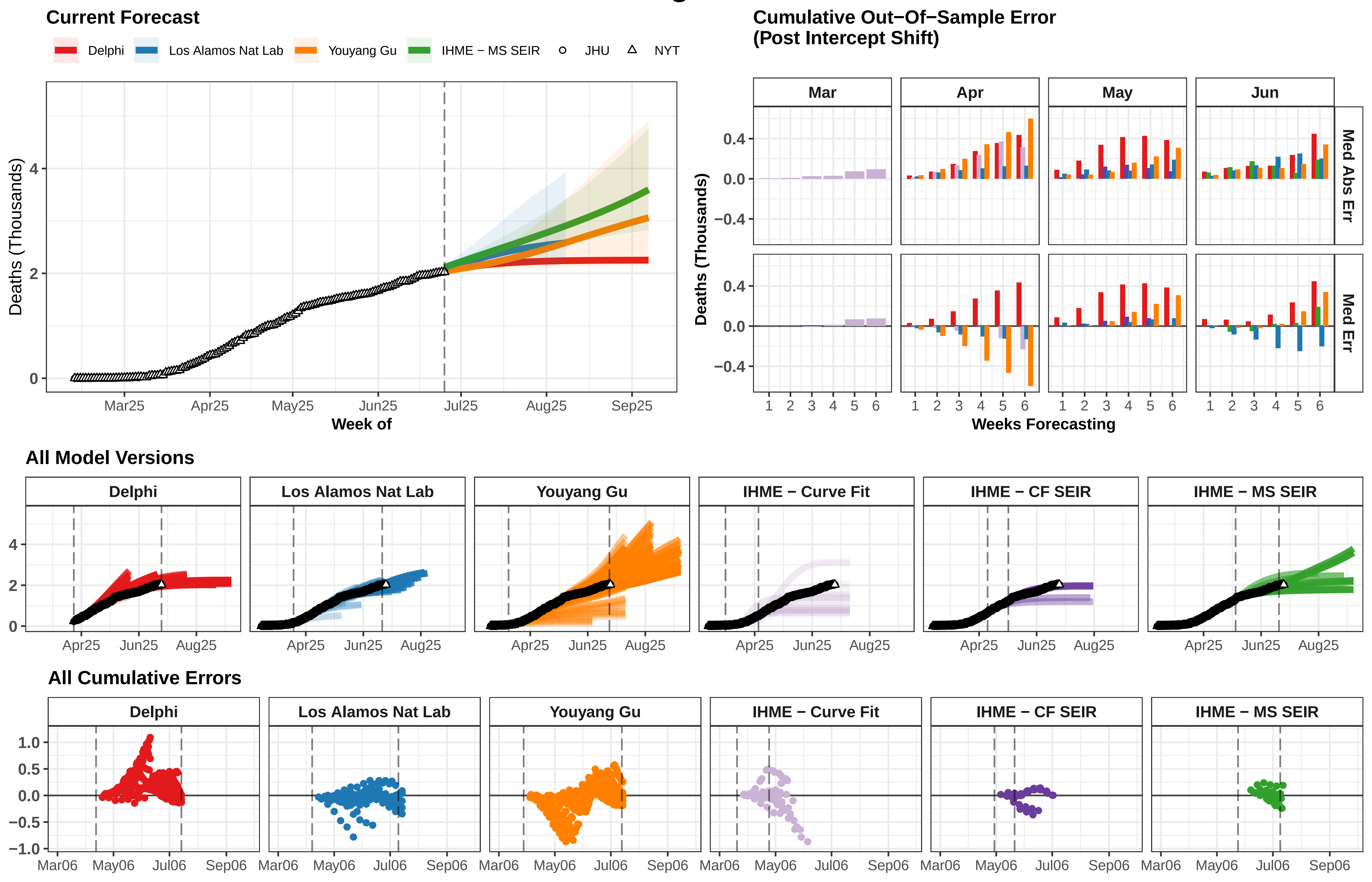
Current Forecast
Delphi
Los Alamos Nat Lab
Youyang Gu
IHME − MS SEIR
JHU
NYT
Deaths (Thousands)
Week of
Mar25
Apr25
May25
Jun25
Jul25
Aug25
Sep25
Cumulative Out−Of−Sample Error
(Post Intercept Shift)
Mar
Apr
May
Jun
Med Abs Err
Med Err
Weeks Forecasting
All Model Versions
Delphi
Los Alamos Nat Lab
Youyang Gu
IHME − Curve Fit
IHME − CF SEIR
IHME − MS SEIR
All Cumulative Errors
Mar06
May06
Jul06
Sep06

Romania

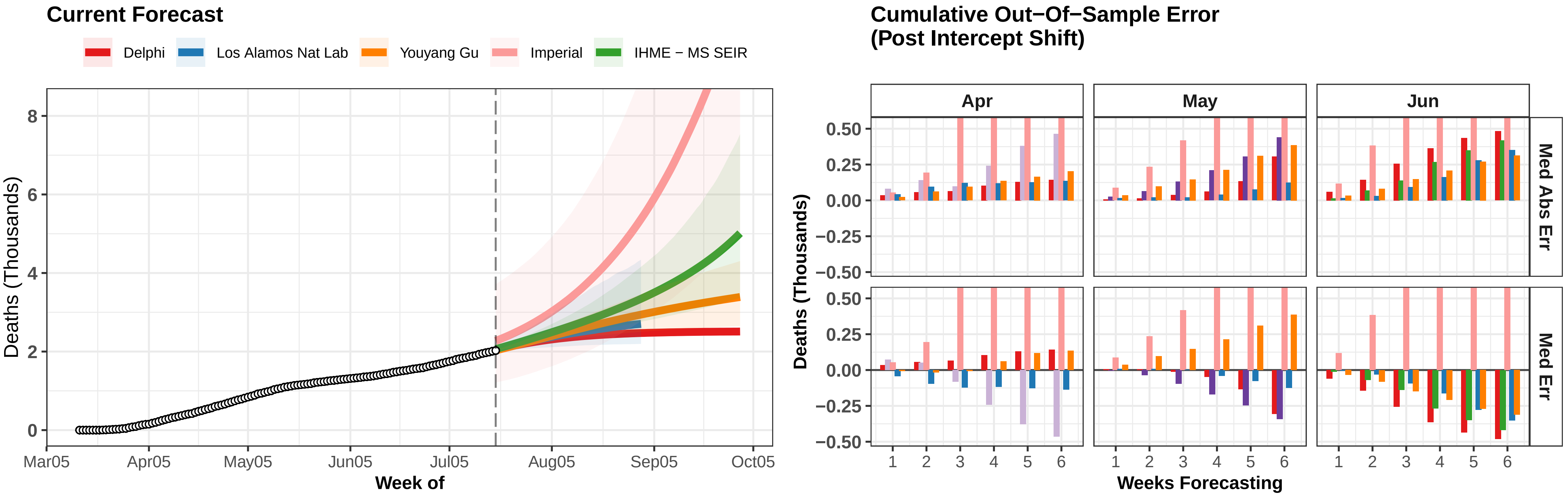
Current Forecast
Delphi
Los Alamos Nat Lab
Youyang Gu
Imperial
IHME − MS SEIR
Deaths (Thousands)
Week of
Mar05
Apr05
May05
Jun05
Jul05
Aug05
Sep05
Oct05
Cumulative Out−Of−Sample Error
(Post Intercept Shift)
Apr
May
Jun
Med Abs Err
Med Err
Weeks Forecasting

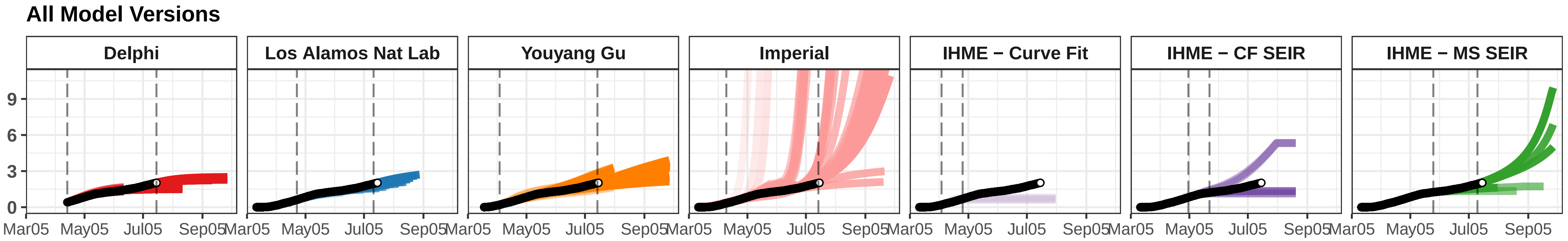
All Model Versions
Delphi
Los Alamos Nat Lab
Youyang Gu
Imperial
IHME − Curve Fit
IHME − CF SEIR
IHME − MS SEIR

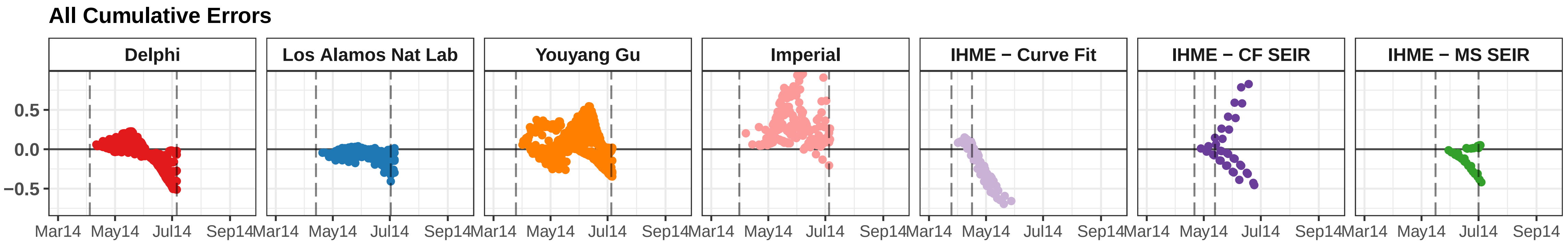
All Cumulative Errors
Delphi
Los Alamos Nat Lab
Youyang Gu
Imperial
IHME − Curve Fit
IHME − CF SEIR
IHME − MS SEIR
Mar14
May14
Jul14
Sep14

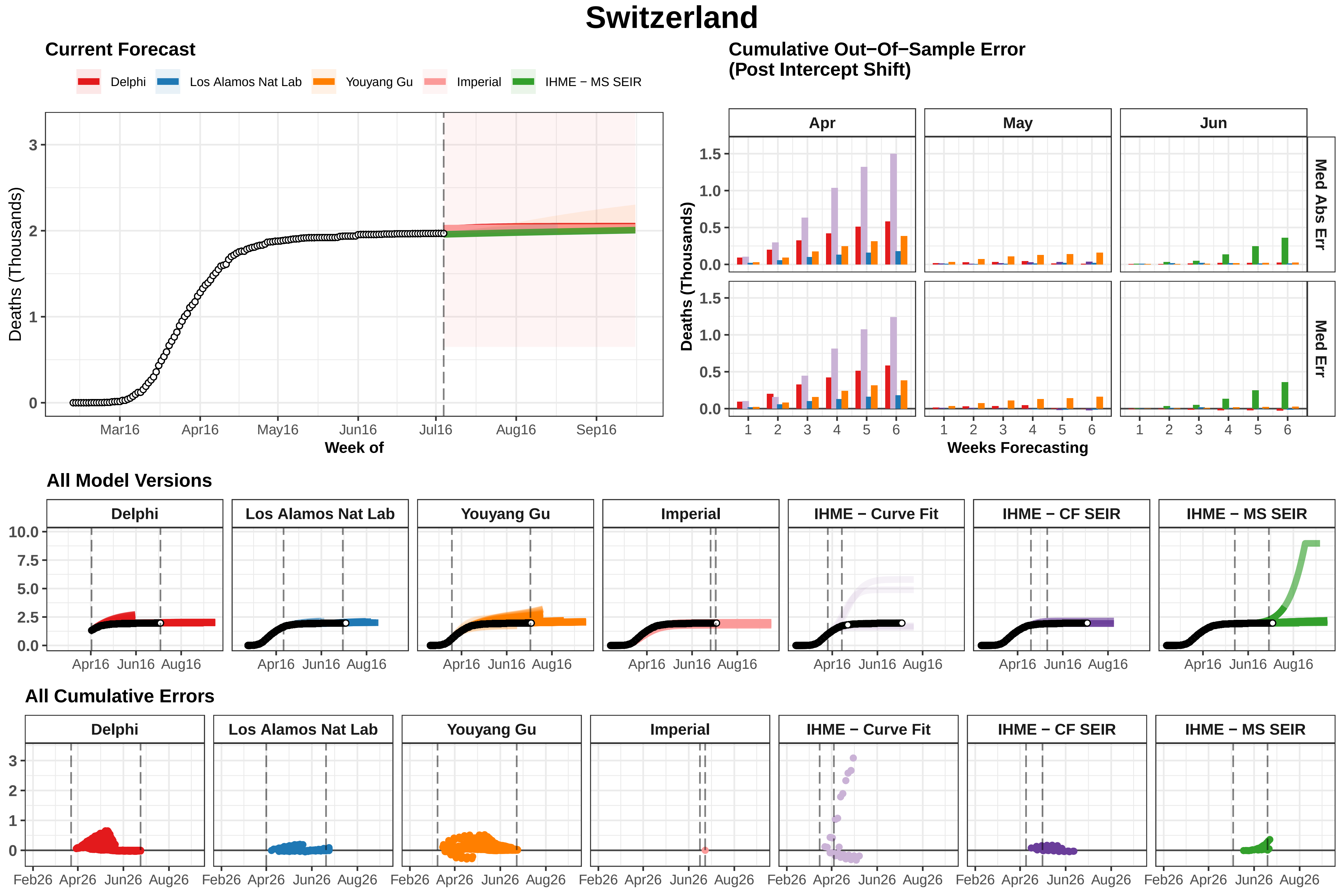

Switzerland
Current Forecast
Delphi
Los Alamos Nat Lab
Youyang Gu
Imperial
IHME − MS SEIR
Deaths (Thousands)
Week of
Mar16
Apr16
May16
Jun16
Jul16
Aug16
Sep16
Cumulative Out−Of−Sample Error
(Post Intercept Shift)
Apr
May
Jun
Med Abs Err
Med Err
Weeks Forecasting
All Model Versions
IHME − Curve Fit
IHME − CF SEIR
All Cumulative Errors
Feb26
Apr26
Jun26
Aug26

Philippines

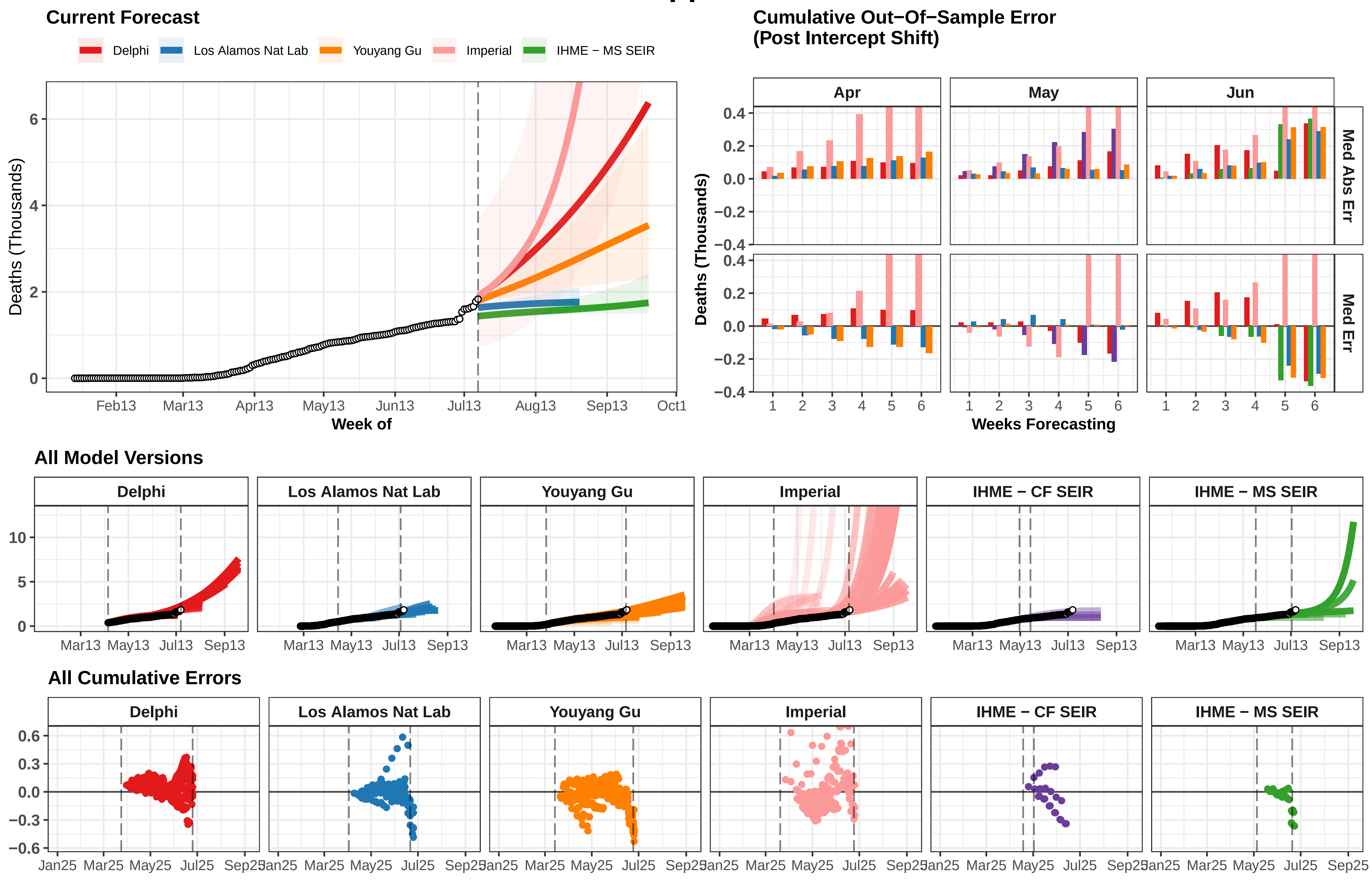

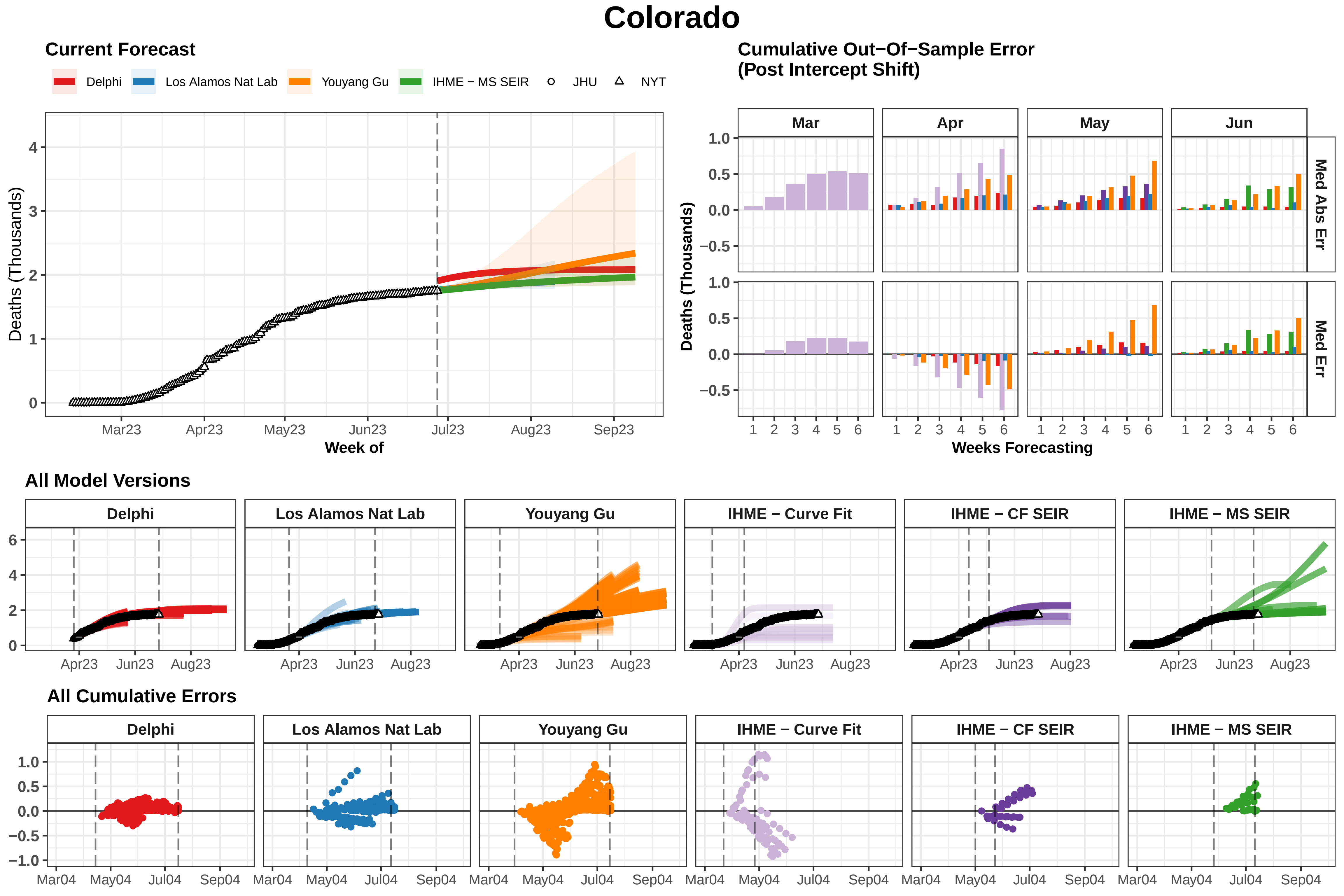
Colorado
Current Forecast
Delphi
Los Alamos Nat Lab
Youyang Gu
IHME − MS SEIR
JHU
NYT
Deaths (Thousands)
Week of
Mar23
Apr23
May23
Jun23
Jul23
Aug23
Sep23
Cumulative Out−Of−Sample Error
(Post Intercept Shift)
Mar
Apr
May
Jun
Med Abs Err
Med Err
Weeks Forecasting
All Model Versions
Delphi
Los Alamos Nat Lab
Youyang Gu
IHME − Curve Fit
IHME − CF SEIR
IHME − MS SEIR
All Cumulative Errors
Mar04
May04
Jul04
Sep04

Ireland

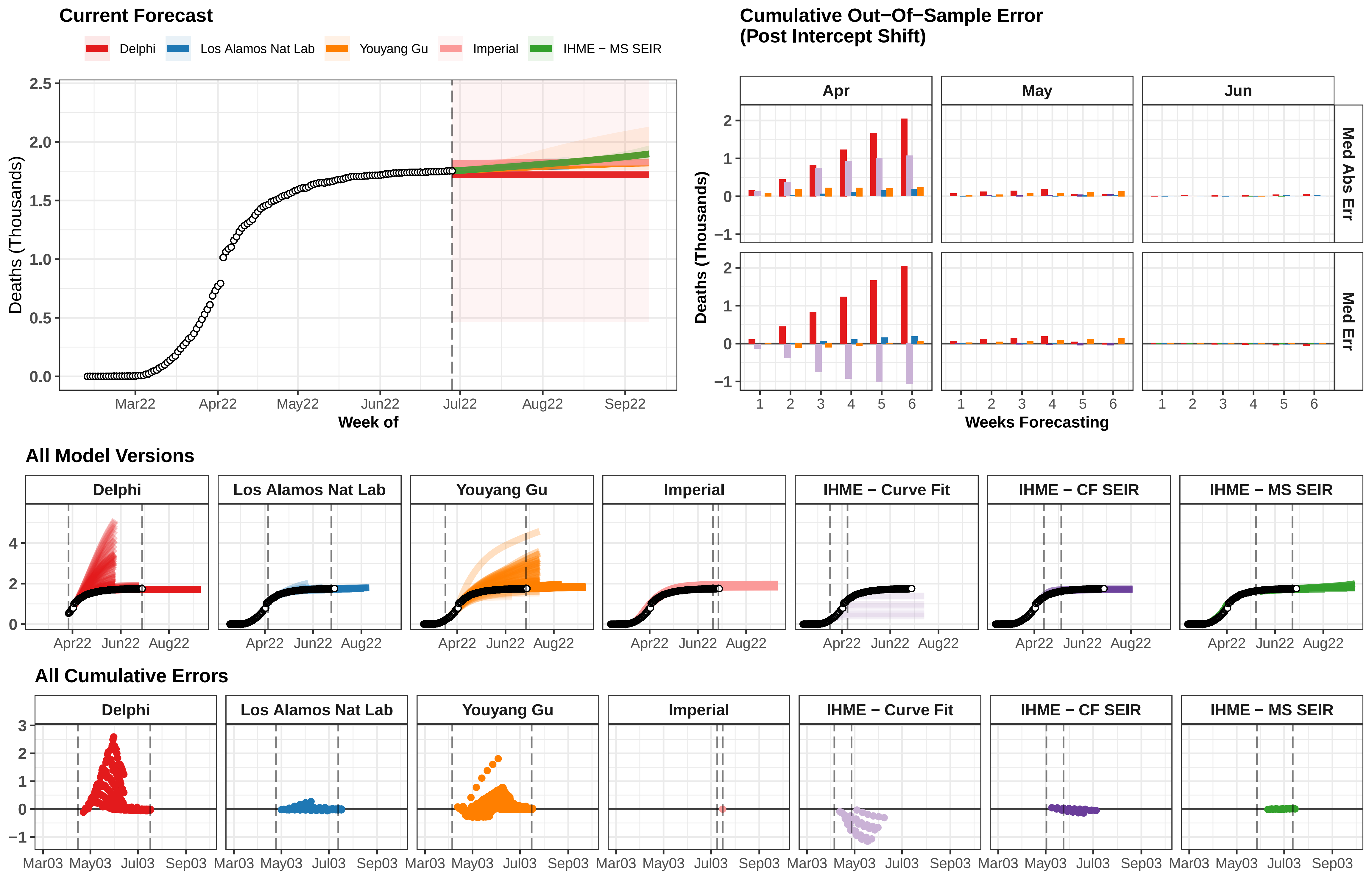

Ireland
Current Forecast
Delphi
Los Alamos Nat Lab
Youyang Gu
Imperial
IHME − MS SEIR
Deaths (Thousands)
Week of
Mar22 Apr22 May22 Jun22 Jul22 Aug22 Sep22
Cumulative Out−Of−Sample Error
(Post Intercept Shift)
Apr
May
Jun
Med Abs Err
Med Err
Weeks Forecasting
All Model Versions
Delphi
Los Alamos Nat Lab
Youyang Gu
Imperial
IHME − Curve Fit
IHME − CF SEIR
IHME − MS SEIR
Apr22 Jun22 Aug22
All Cumulative Errors
Mar03 May03 Jul03 Sep03

Portugal

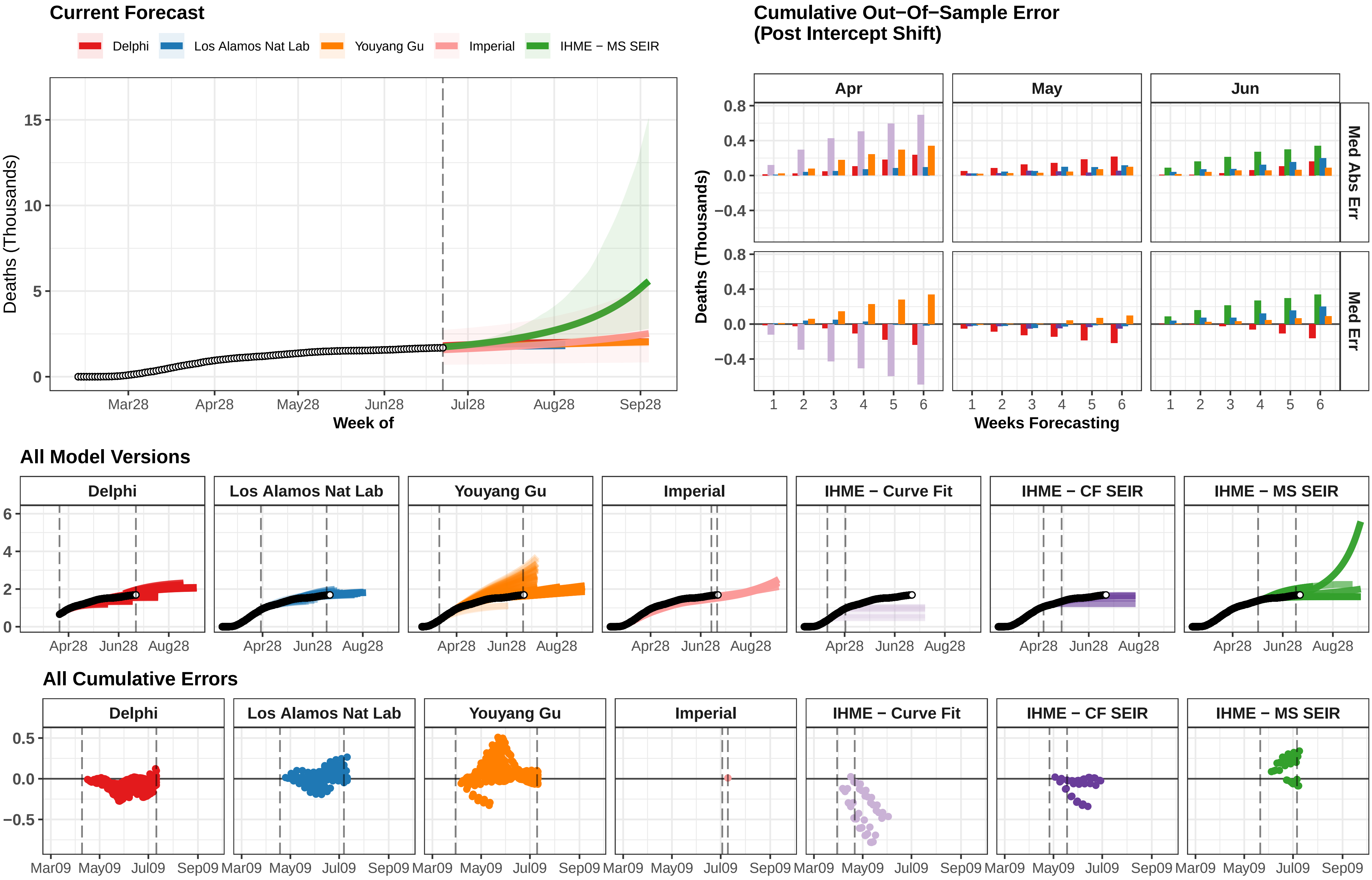

Current Forecast
Delphi
Los Alamos Nat Lab
Youyang Gu
Imperial
IHME − MS SEIR
Deaths (Thousands)
Week of
Mar28
Apr28
May28
Jun28
Jul28
Aug28
Sep28
Cumulative Out−Of−Sample Error
(Post Intercept Shift)
Apr
May
Jun
Med Abs Err
Med Err
Weeks Forecasting
All Model Versions
Delphi
Los Alamos Nat Lab
Youyang Gu
Imperial
IHME − Curve Fit
IHME − CF SEIR
IHME − MS SEIR
All Cumulative Errors
Mar09
May09
Jul09
Sep09

North Carolina

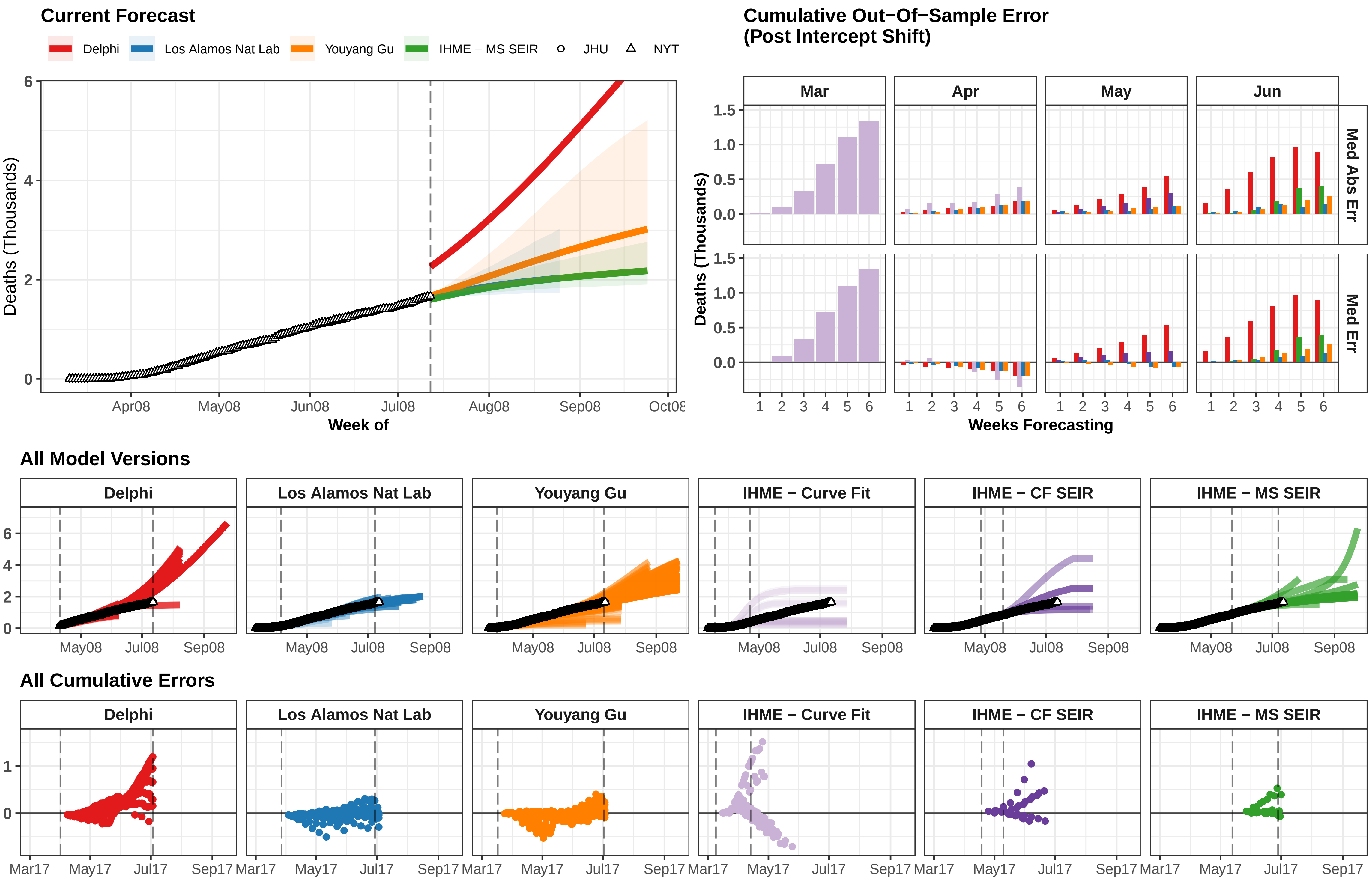

Poland

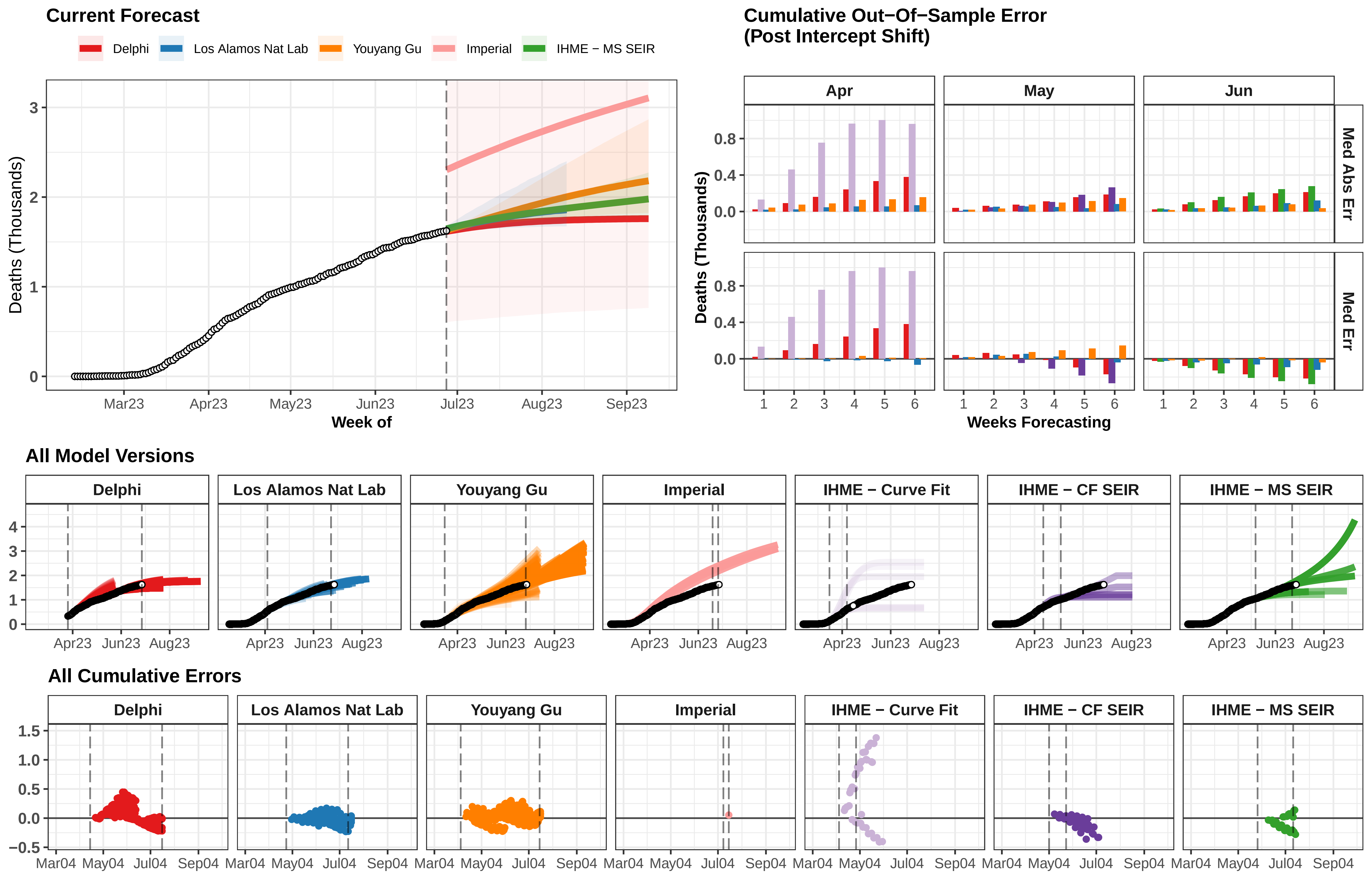

Current Forecast
Delphi
Los Alamos Nat Lab
Youyang Gu
Imperial
IHME − MS SEIR
Deaths (Thousands)
Week of
Mar23
Apr23
May23
Jun23
Jul23
Aug23
Sep23
Cumulative Out−Of−Sample Error (Post Intercept Shift)
Apr
May
Jun
Med Abs Err
Med Err
Weeks Forecasting
All Model Versions
Delphi
Los Alamos Nat Lab
Youyang Gu
Imperial
IHME − Curve Fit
IHME − CF SEIR
IHME − MS SEIR
All Cumulative Errors
Mar04
May04
Jul04
Sep04

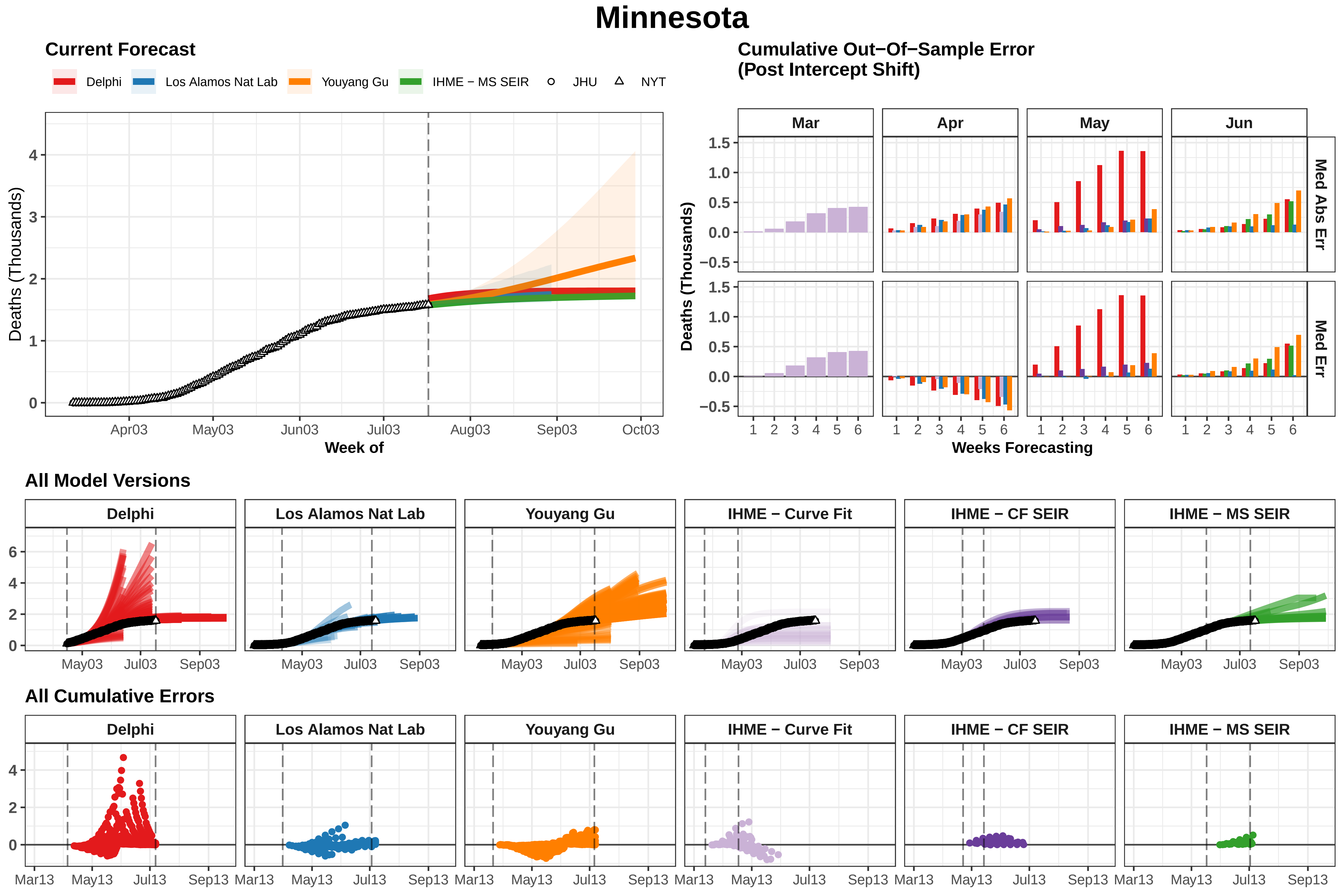

Minnesota
Current Forecast
Delphi
Los Alamos Nat Lab
Youyang Gu
IHME − MS SEIR
JHU
NYT
Deaths (Thousands)
Week of
Apr03
May03
Jun03
Jul03
Aug03
Sep03
Oct03
Cumulative Out−Of−Sample Error
(Post Intercept Shift)
Mar
Apr
May
Jun
Med Abs Err
Med Err
Weeks Forecasting
All Model Versions
Delphi
Los Alamos Nat Lab
Youyang Gu
IHME − Curve Fit
IHME − CF SEIR
IHME − MS SEIR
All Cumulative Errors
Mar13
May13
Jul13
Sep13

Washington

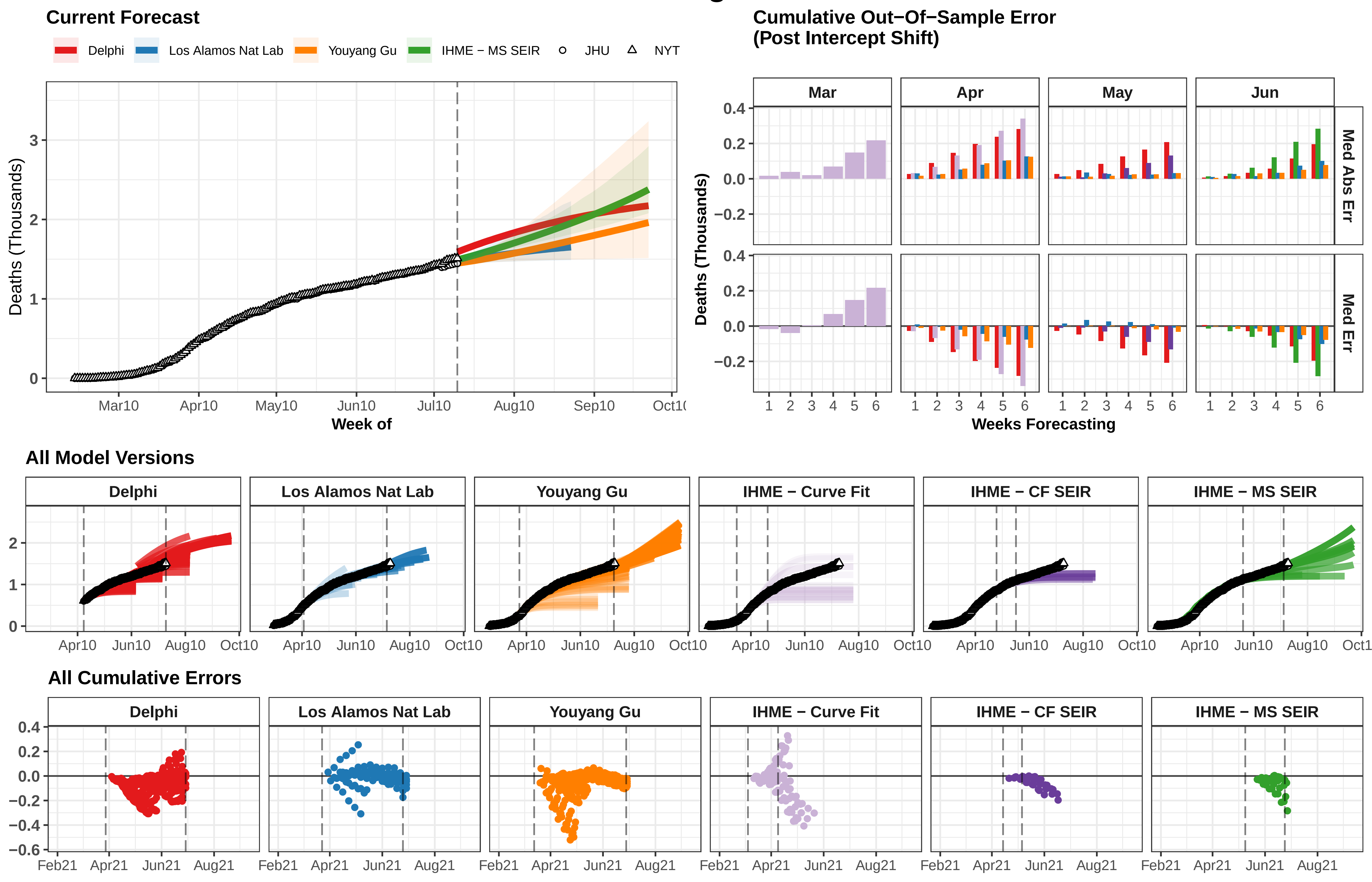
Current Forecast
Delphi
Los Alamos Nat Lab
Youyang Gu
IHME − MS SEIR
JHU
NYT
Deaths (Thousands)
Week of
Mar10
Apr10
May10
Jun10
Jul10
Aug10
Sep10
Cumulative Out−Of−Sample Error
(Post Intercept Shift)
Mar
Apr
May
Jun
Med Abs Err
Med Err
Weeks Forecasting
All Model Versions
Delphi
Los Alamos Nat Lab
Youyang Gu
IHME − Curve Fit
IHME − CF SEIR
IHME − MS SEIR
Apr10
Jun10
Aug10
Oct10
All Cumulative Errors
Feb21
Apr21
Jun21
Aug21

Ukraine

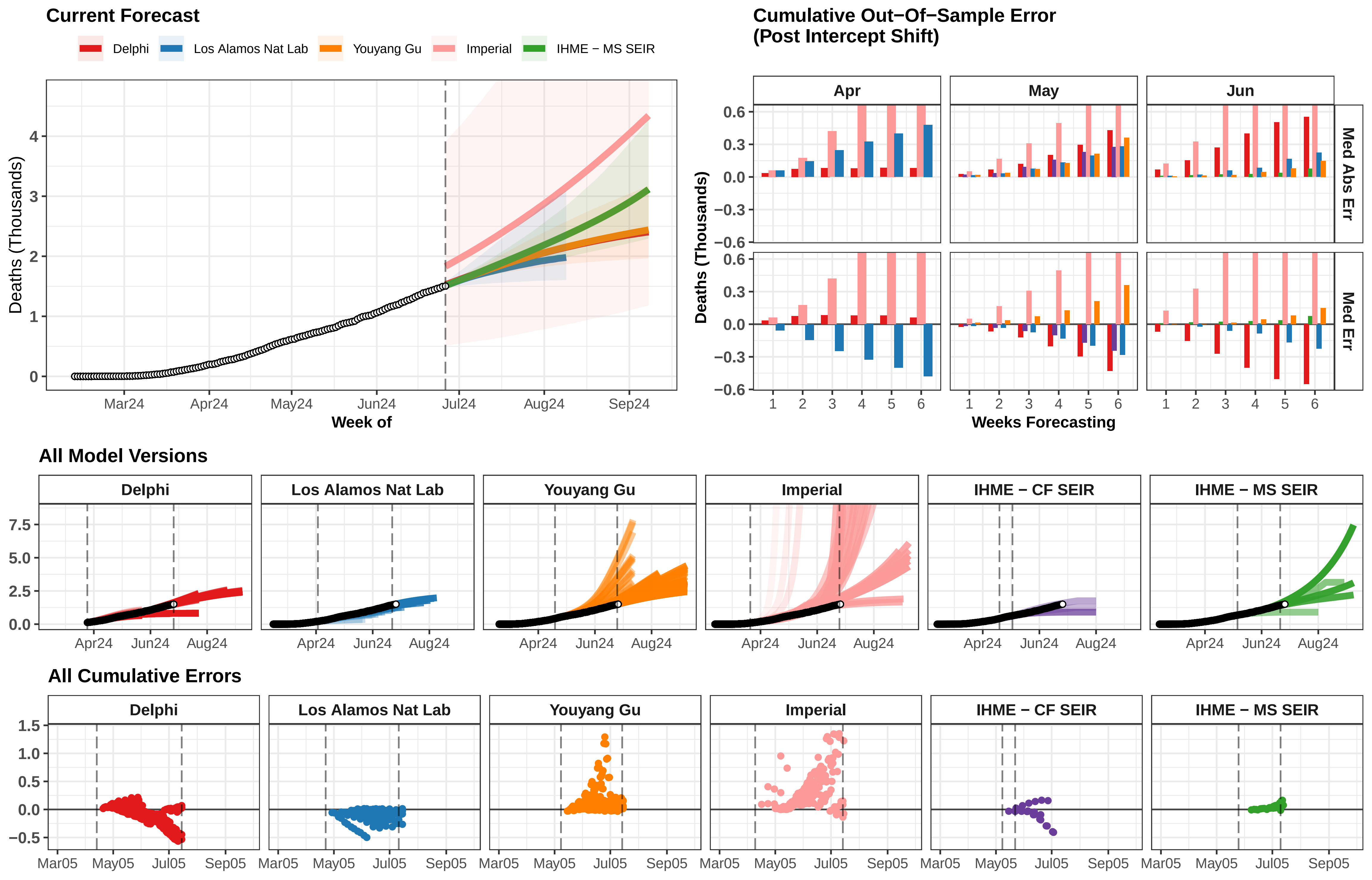

Current Forecast
Delphi
Los Alamos Nat Lab
Youyang Gu
Imperial
IHME − MS SEIR
Deaths (Thousands)
Week of
Mar24
Apr24
May24
Jun24
Jul24
Aug24
Sep24
Cumulative Out−Of−Sample Error
(Post Intercept Shift)
Apr
May
Jun
Med Abs Err
Med Err
Weeks Forecasting
All Model Versions
IHME − CF SEIR
All Cumulative Errors
Mar05
May05
Jul05
Sep05

Guatemala

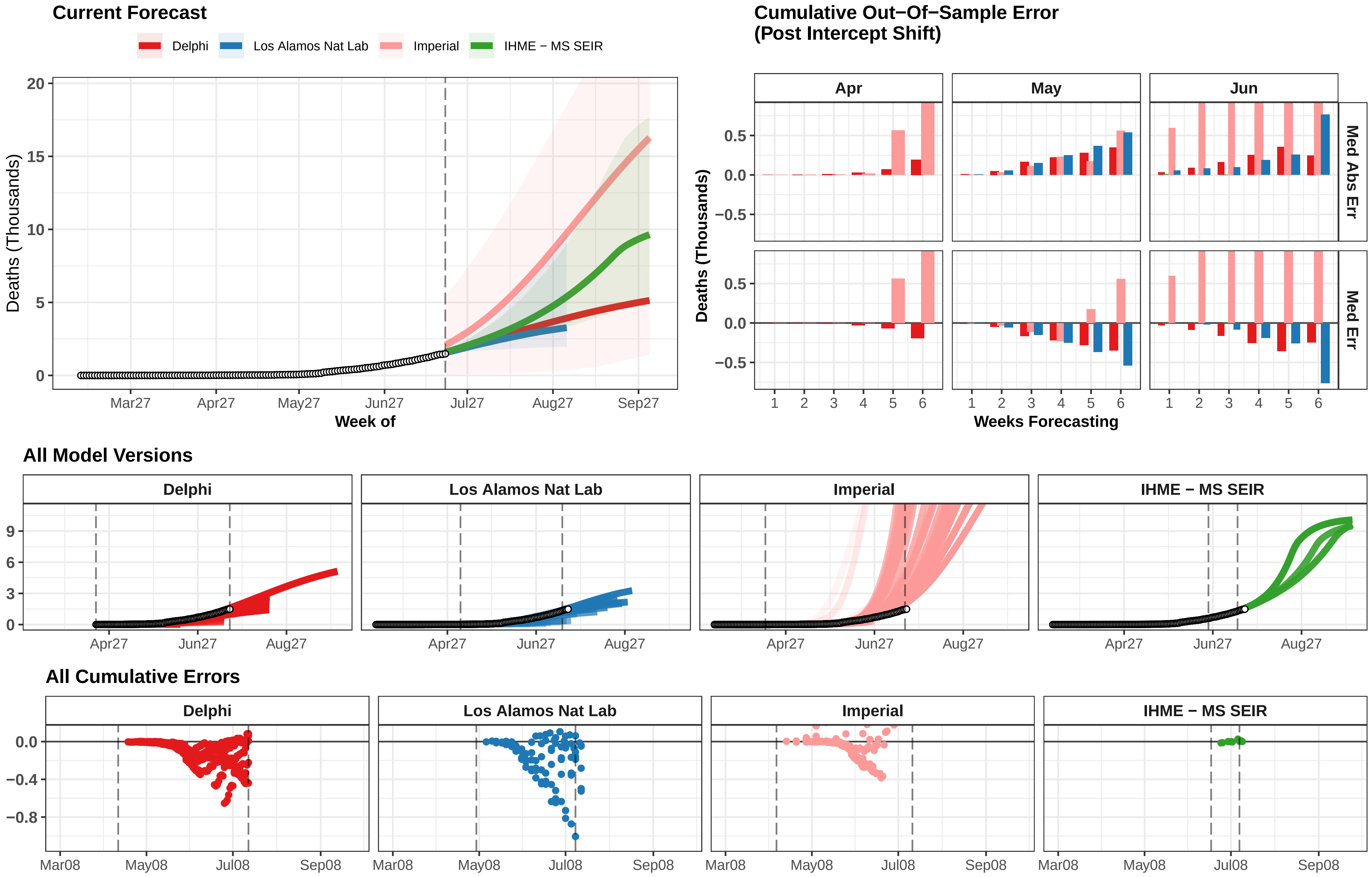

Current Forecast
Delphi
Los Alamos Nat Lab
Imperial
IHME − MS SEIR
Deaths (Thousands)
Week of
Mar27
Apr27
May27
Jun27
Jul27
Aug27
Sep27
Cumulative Out−Of−Sample Error
(Post Intercept Shift)
Apr
May
Jun
Med Abs Err
Med Err
Weeks Forecasting
All Model Versions
All Cumulative Errors
Mar08
May08
Jul08
Sep08

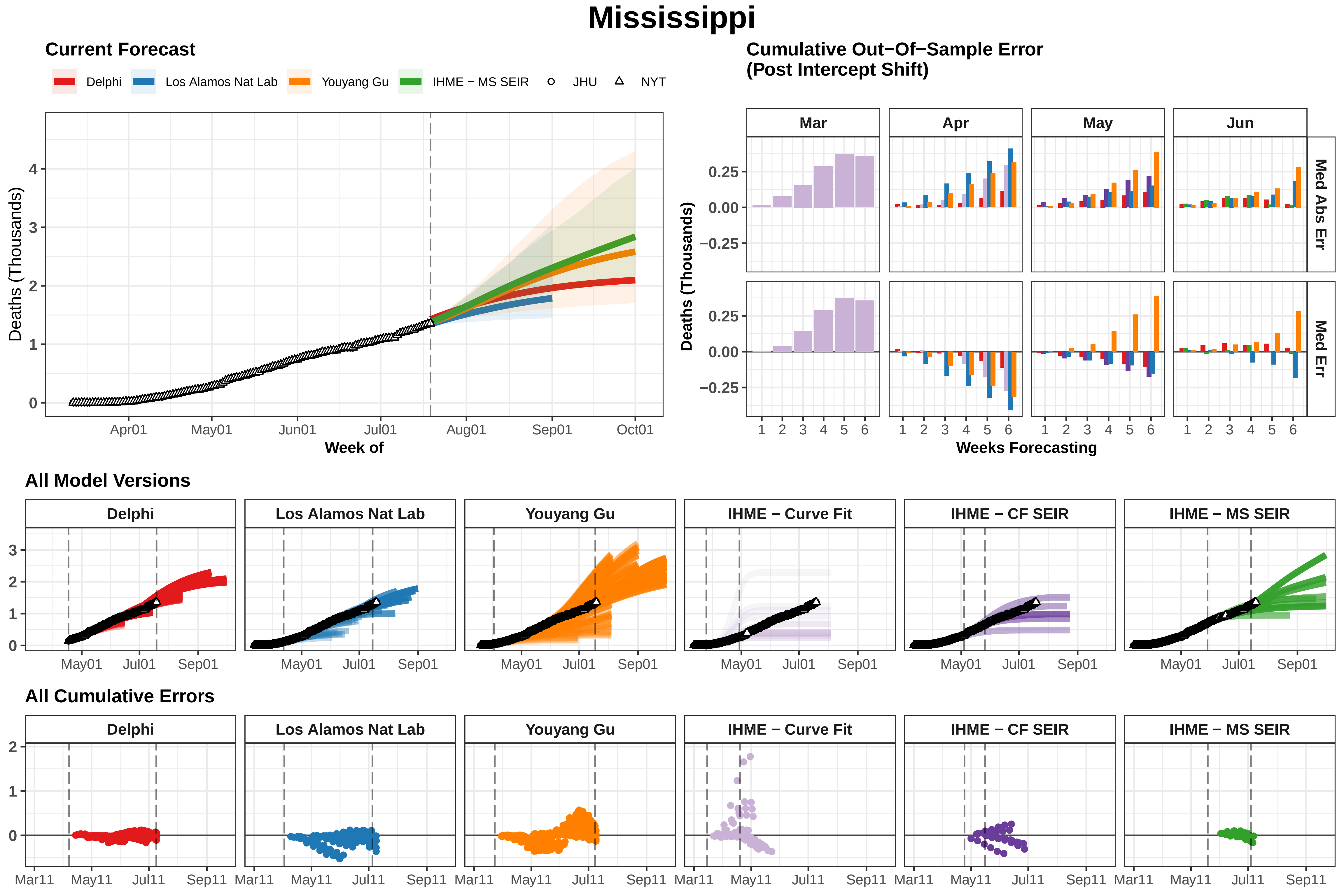

Mississippi
Current Forecast
Delphi
Los Alamos Nat Lab
Youyang Gu
IHME − MS SEIR
JHU
NYT
Deaths (Thousands)
Week of
Apr01
May01
Jun01
Jul01
Aug01
Sep01
Oct01
Cumulative Out−Of−Sample Error
(Post Intercept Shift)
Mar
Apr
May
Jun
Med Abs Err
Med Err
Weeks Forecasting
All Model Versions
Delphi
Los Alamos Nat Lab
Youyang Gu
IHME − Curve Fit
IHME − CF SEIR
IHME − MS SEIR
All Cumulative Errors
Mar11
May11
Jul11
Sep11

Alabama

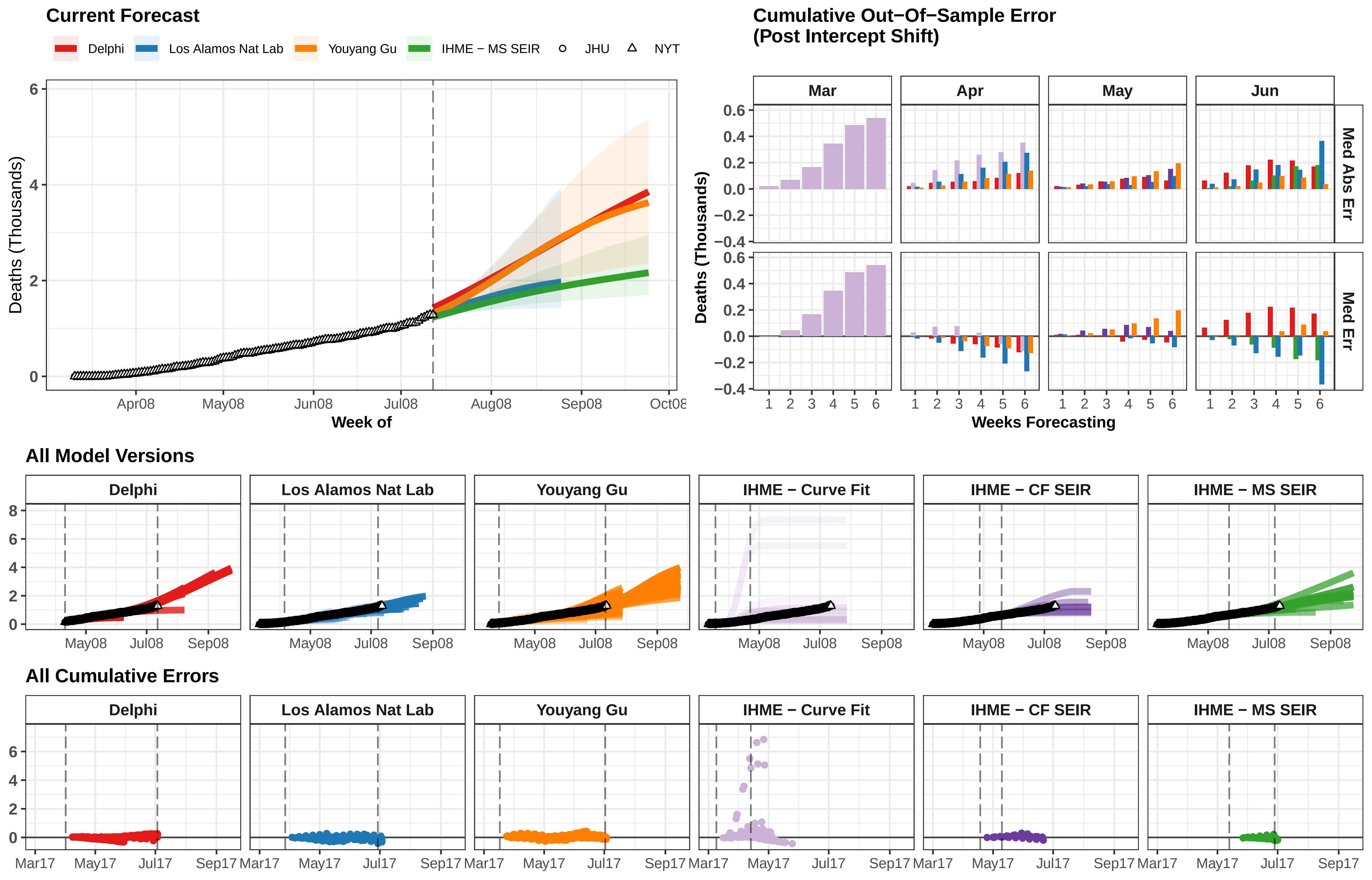
Current Forecast
Delphi
Los Alamos Nat Lab
Youyang Gu
IHME − MS SEIR
JHU
NYT
Deaths (Thousands)
Week of
Apr08
May08
Jun08
Jul08
Aug08
Sep08
Oct08
Cumulative Out−Of−Sample Error
(Post Intercept Shift)
Mar
Apr
May
Jun
Med Abs Err
Med Err
Weeks Forecasting
All Model Versions
Delphi
Los Alamos Nat Lab
Youyang Gu
IHME − Curve Fit
IHME − CF SEIR
IHME − MS SEIR
All Cumulative Errors
Mar17
May17
Jul17
Sep17

Afghanistan

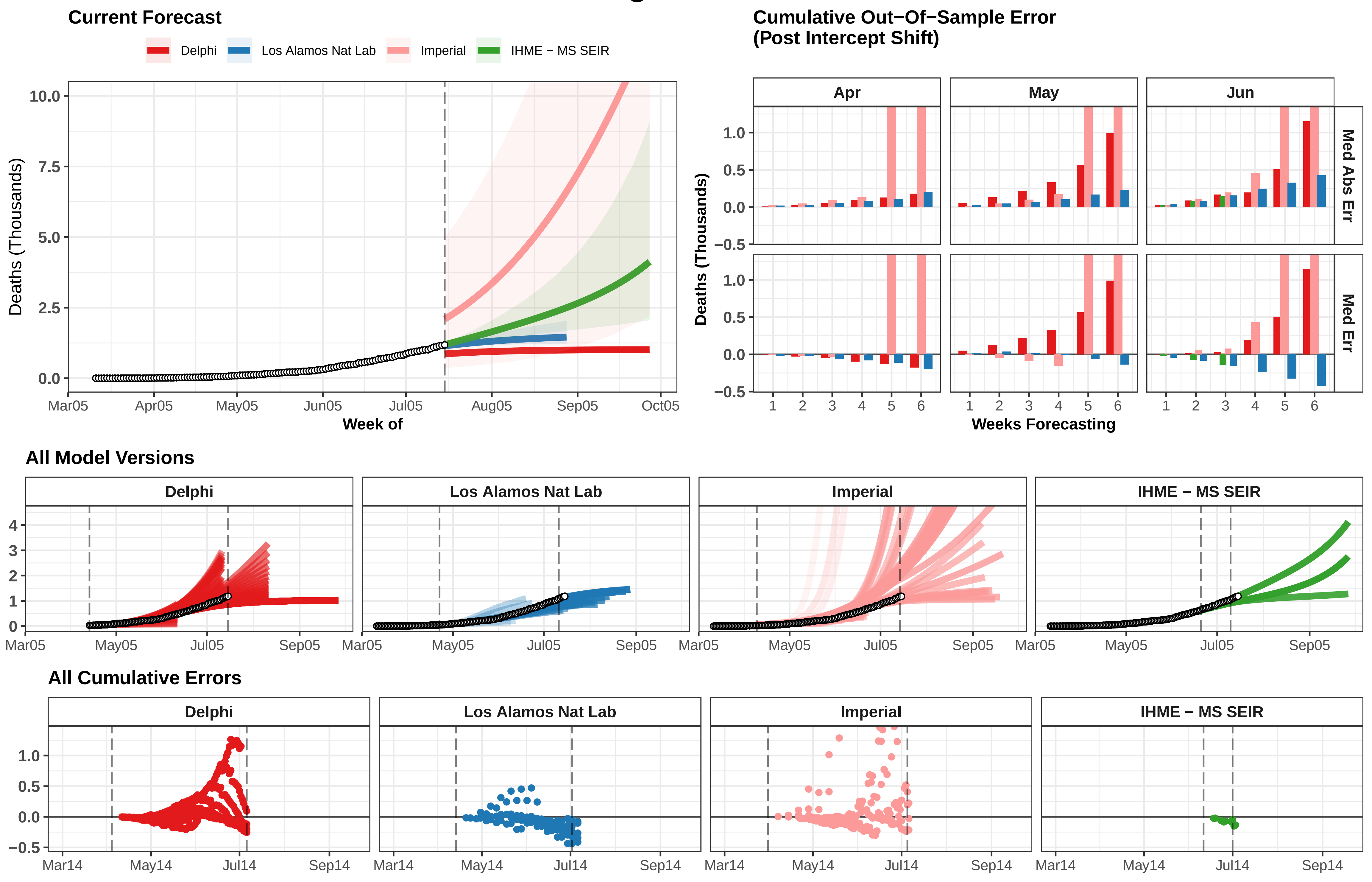

Missouri

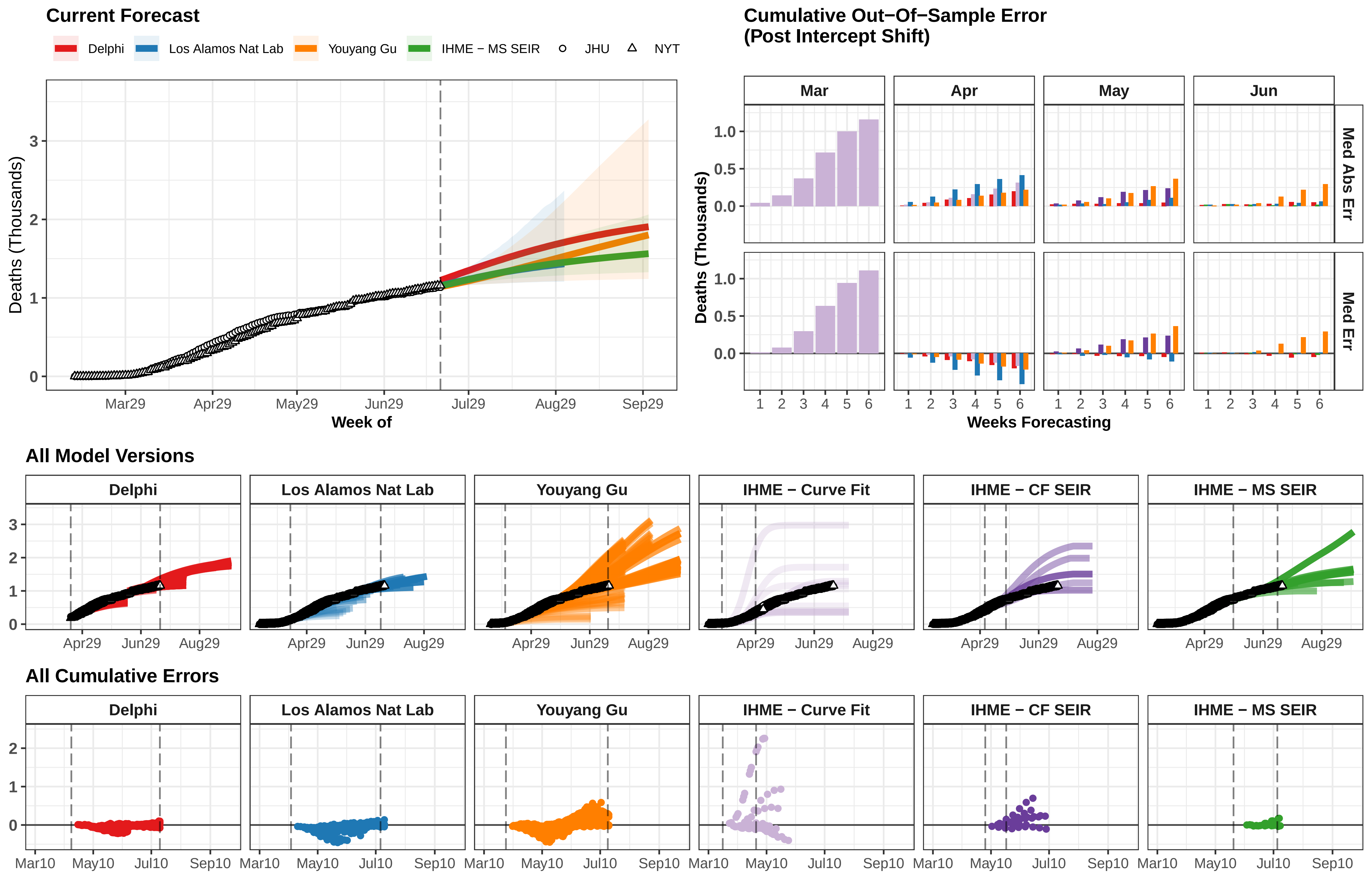

Current Forecast
Delphi
Los Alamos Nat Lab
Youyang Gu
IHME − MS SEIR
JHU
NYT
Deaths (Thousands)
Week of
Mar29
Apr29
May29
Jun29
Jul29
Aug29
Sep29
Cumulative Out−Of−Sample Error
(Post Intercept Shift)
Mar
Apr
May
Jun
Med Abs Err
Med Err
Weeks Forecasting
All Model Versions
Delphi
Los Alamos Nat Lab
Youyang Gu
IHME − Curve Fit
IHME − CF SEIR
IHME − MS SEIR
All Cumulative Errors
Mar10
May10
Jul10
Sep10

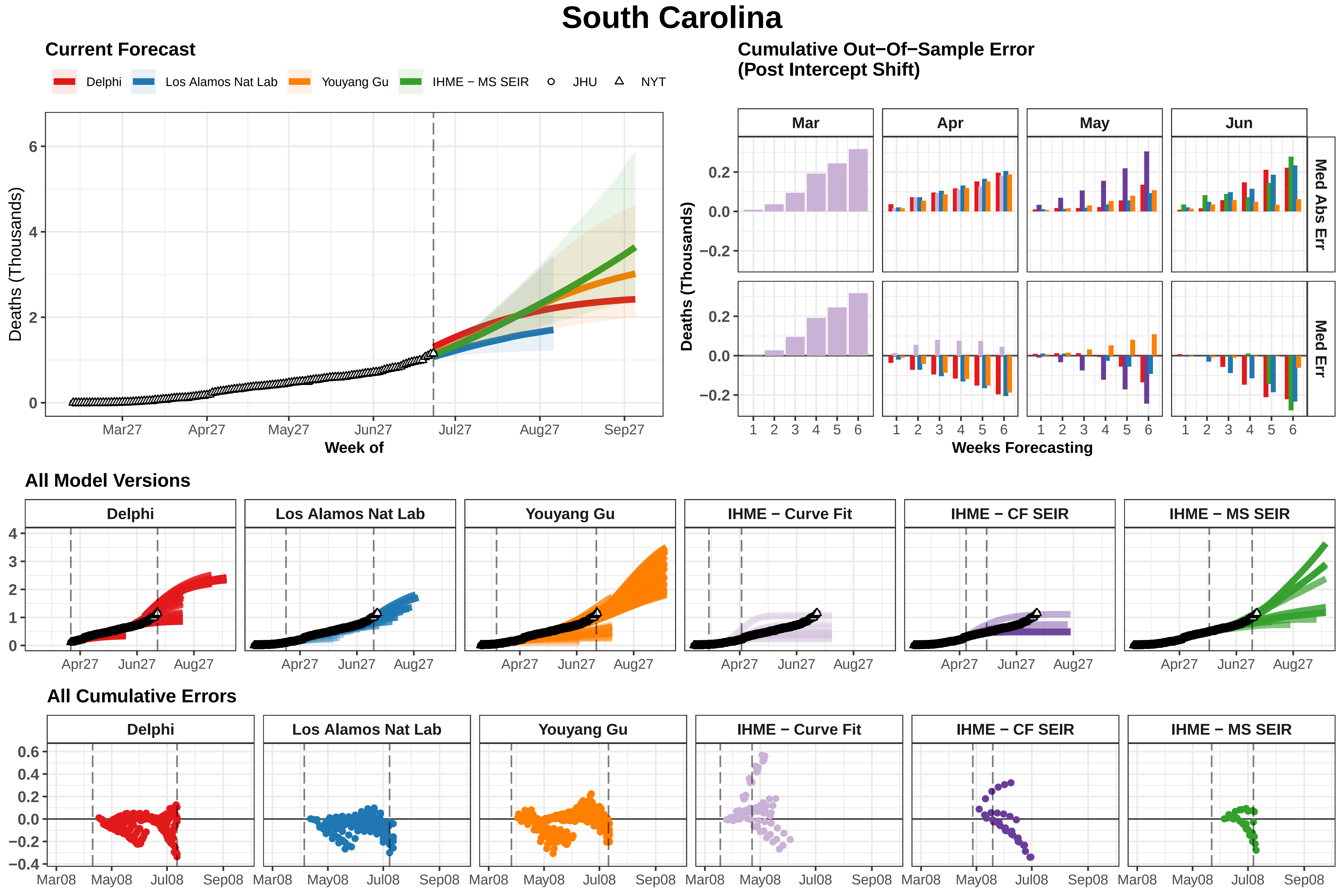

South Carolina
Current Forecast
Delphi
Los Alamos Nat Lab
Youyang Gu
IHME − MS SEIR
JHU
NYT
Deaths (Thousands)
Week of
Mar27
Apr27
May27
Jun27
Jul27
Aug27
Sep27
Cumulative Out−Of−Sample Error
(Post Intercept Shift)
Mar
Apr
May
Jun
Med Abs Err
Med Err
Weeks Forecasting
All Model Versions
Delphi
Los Alamos Nat Lab
Youyang Gu
IHME − Curve Fit
IHME − CF SEIR
IHME − MS SEIR
All Cumulative Errors
Mar08
May08
Jul08
Sep08

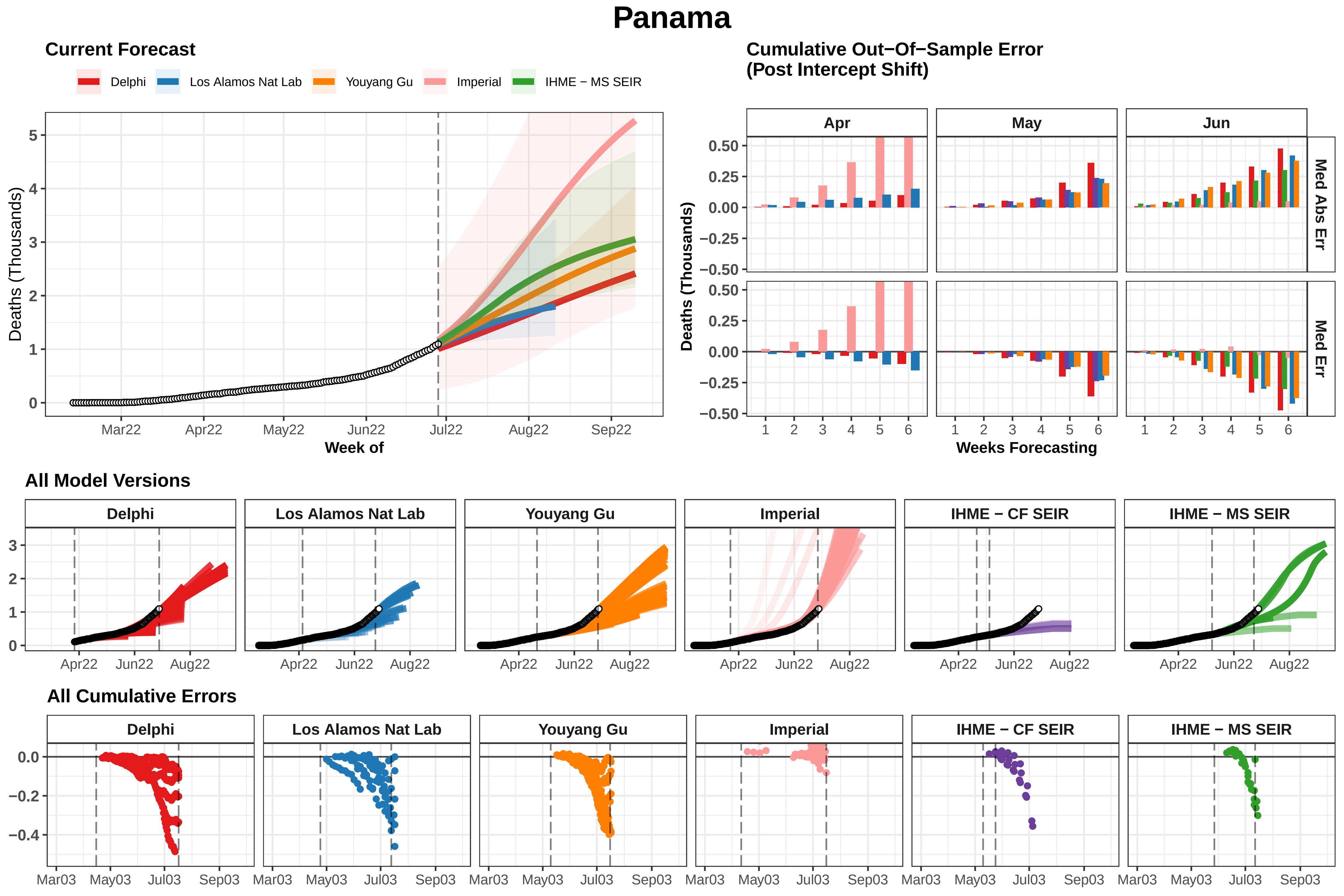
Panama
Current Forecast
Delphi
Los Alamos Nat Lab
Youyang Gu
Imperial
IHME − MS SEIR
Deaths (Thousands)
Week of
Mar22
Apr22
May22
Jun22
Jul22
Aug22
Sep22
Cumulative Out−Of−Sample Error
(Post Intercept Shift)
Apr
May
Jun
Med Abs Err
Med Err
Weeks Forecasting
All Model Versions
IHME − CF SEIR
All Cumulative Errors
Mar03
May03
Jul03
Sep03

Algeria

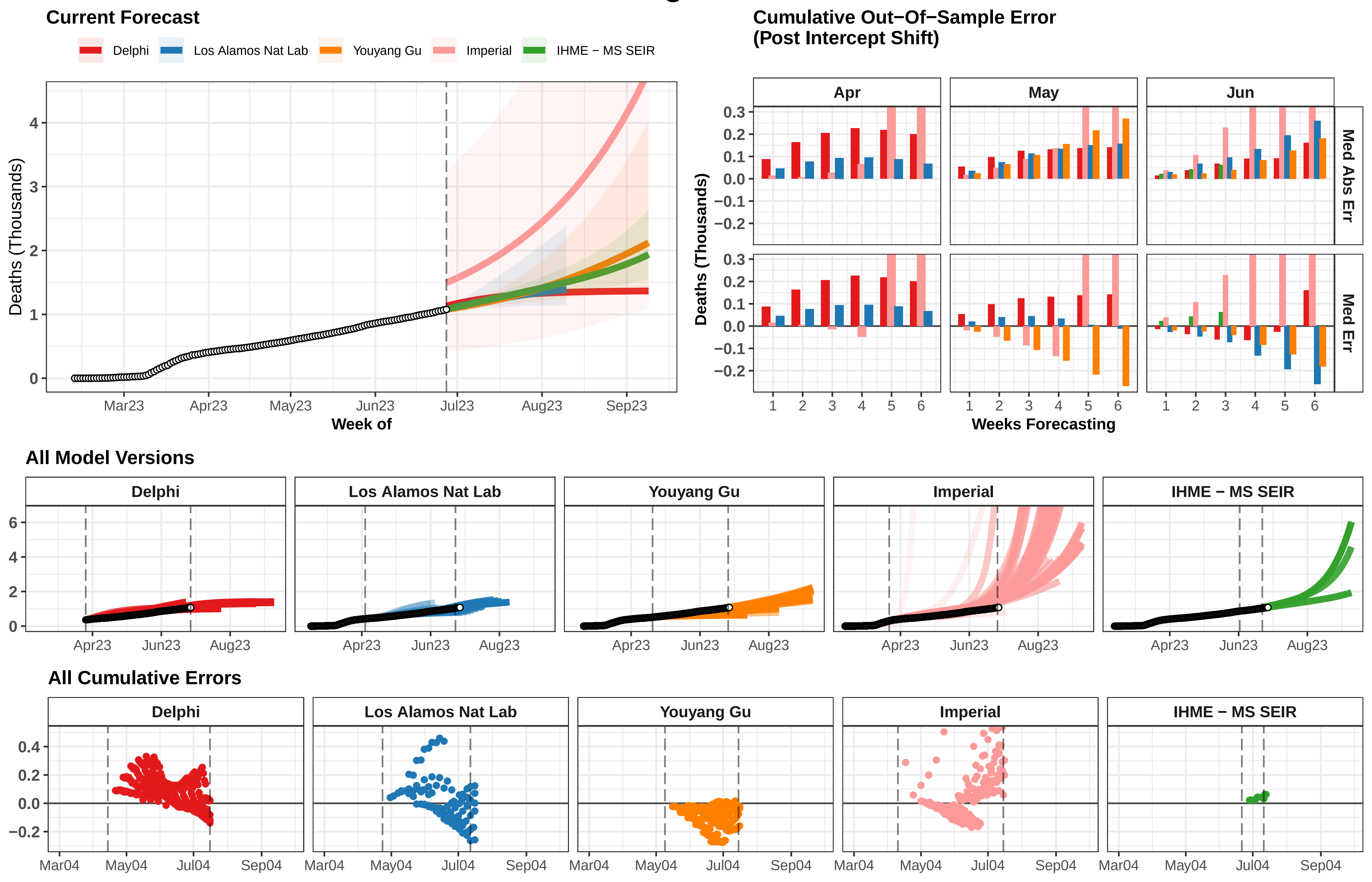

Current Forecast
Delphi
Los Alamos Nat Lab
Youyang Gu
Imperial
IHME − MS SEIR
Deaths (Thousands)
Week of
Mar23
Apr23
May23
Jun23
Jul23
Aug23
Sep23
Cumulative Out−Of−Sample Error
(Post Intercept Shift)
Apr
May
Jun
Med Abs Err
Med Err
Weeks Forecasting
All Model Versions
All Cumulative Errors
Mar04
May04
Jul04
Sep04

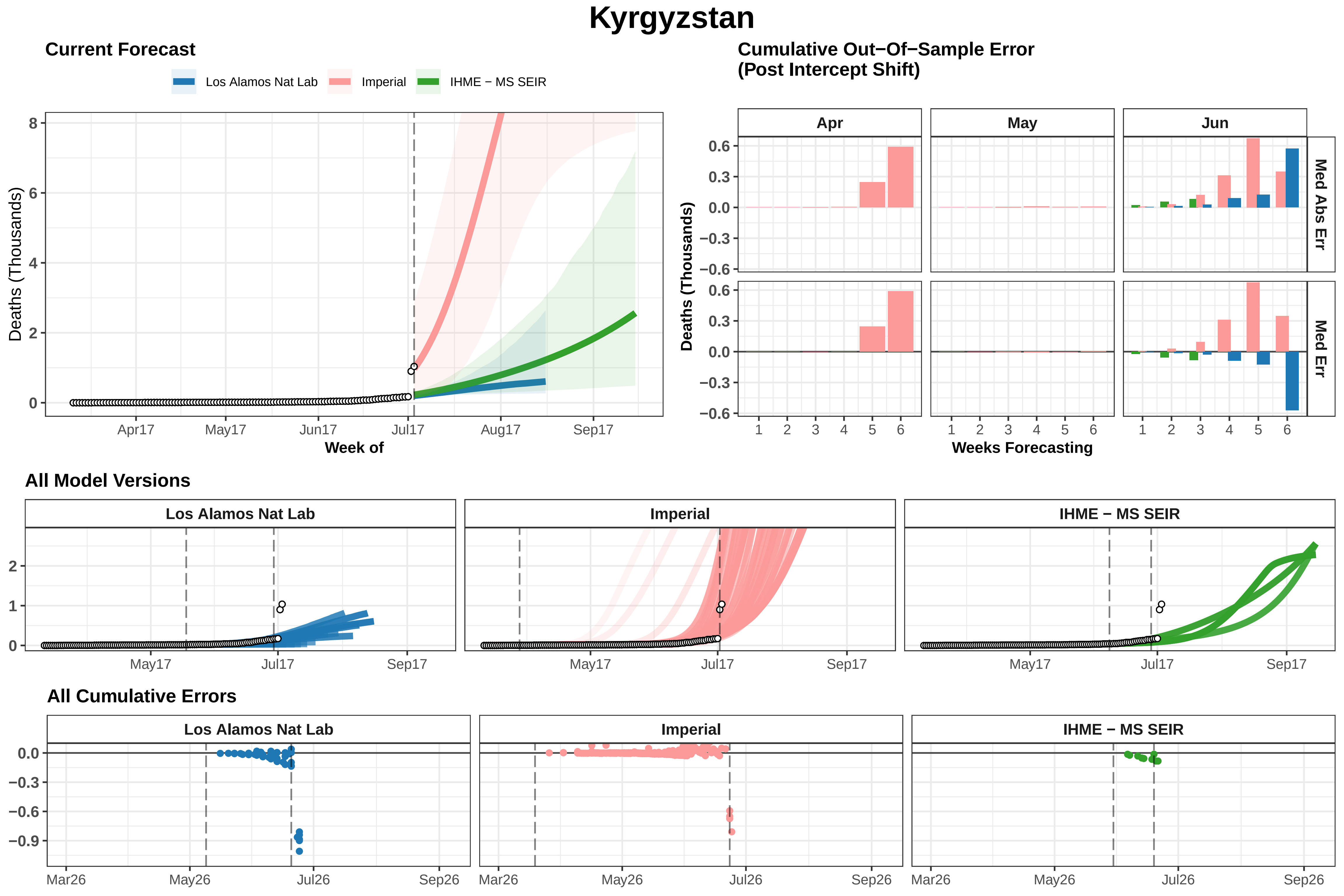
Kyrgyzstan
Current Forecast
Los Alamos Nat Lab
Imperial
IHME − MS SEIR
Deaths (Thousands)
Week of
Apr17
May17
Jun17
Jul17
Aug17
Sep17
Cumulative Out−Of−Sample Error
(Post Intercept Shift)
Apr
May
Jun
Med Abs Err
Med Err
Weeks Forecasting
All Model Versions
Los Alamos Nat Lab
Imperial
IHME − MS SEIR
All Cumulative Errors
Los Alamos Nat Lab
Imperial
IHME − MS SEIR
Mar26
May26
Jul26
Sep26

Rhode Island

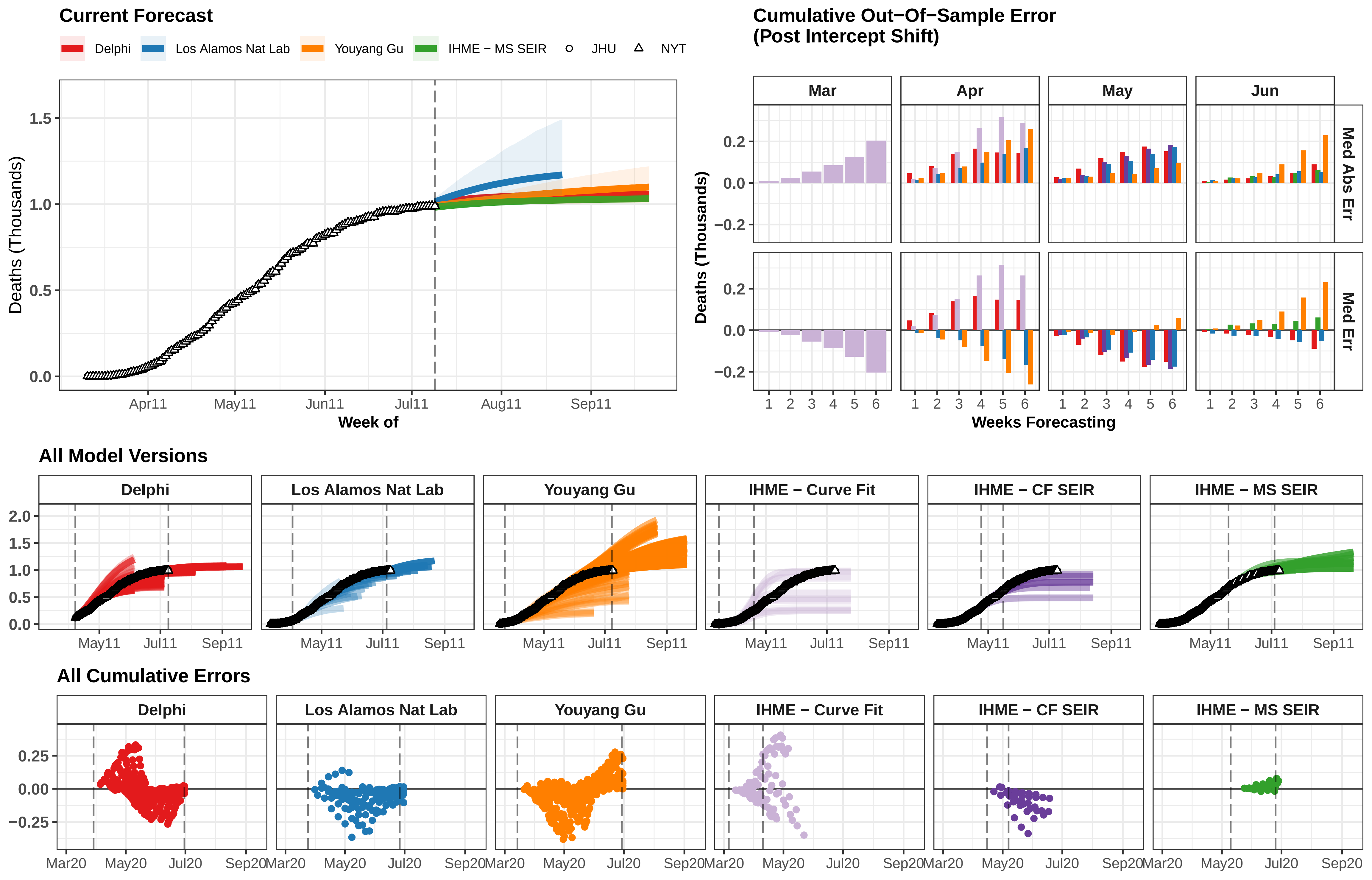

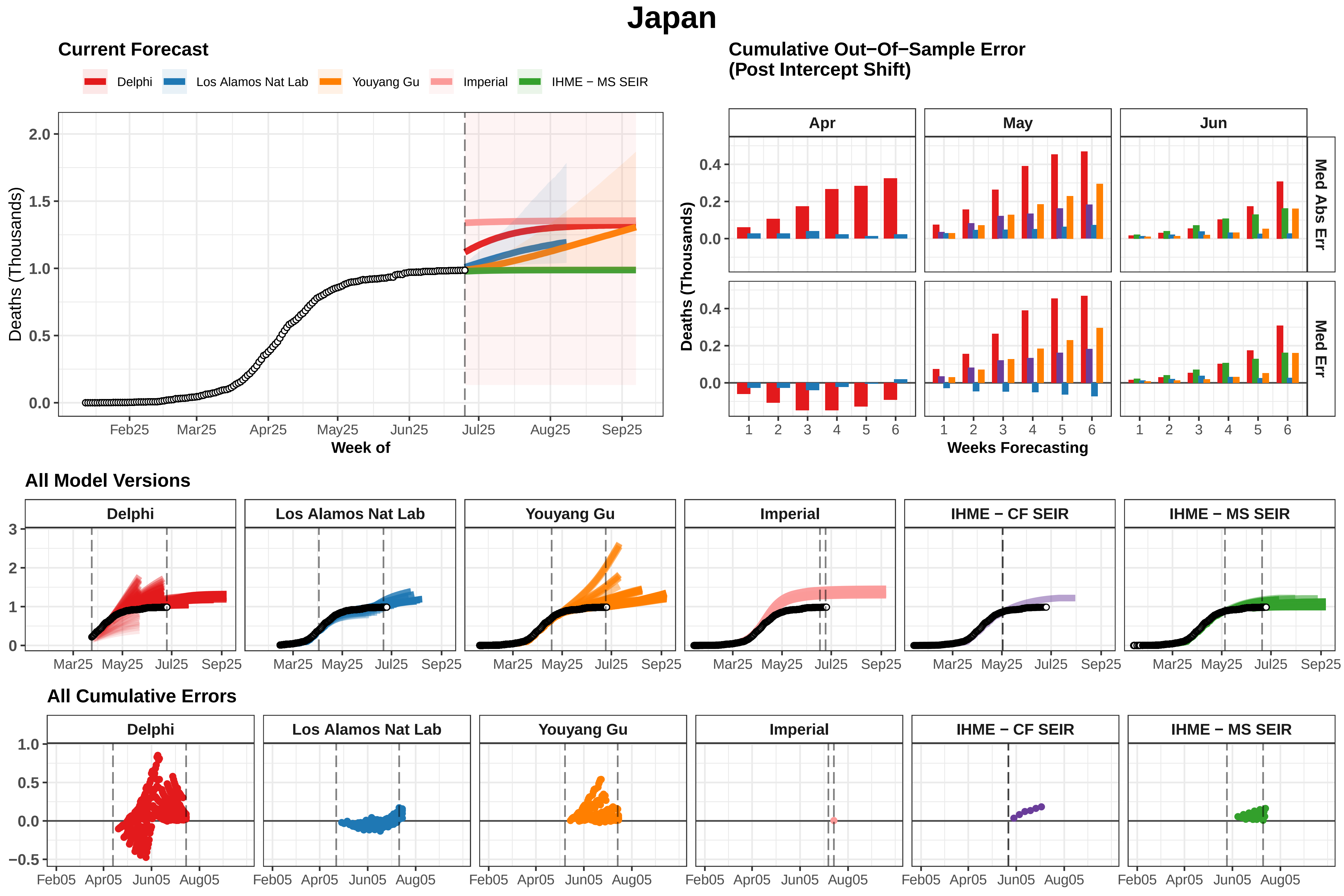
Japan
Current Forecast
Delphi
Los Alamos Nat Lab
Youyang Gu
Imperial
IHME − MS SEIR
Deaths (Thousands)
Week of
Feb25
Mar25
Apr25
May25
Jun25
Jul25
Aug25
Sep25
Cumulative Out−Of−Sample Error
(Post Intercept Shift)
Apr
May
Jun
Med Abs Err
Med Err
Weeks Forecasting
All Model Versions
IHME − CF SEIR
All Cumulative Errors
Feb05
Apr05
Jun05
Aug05

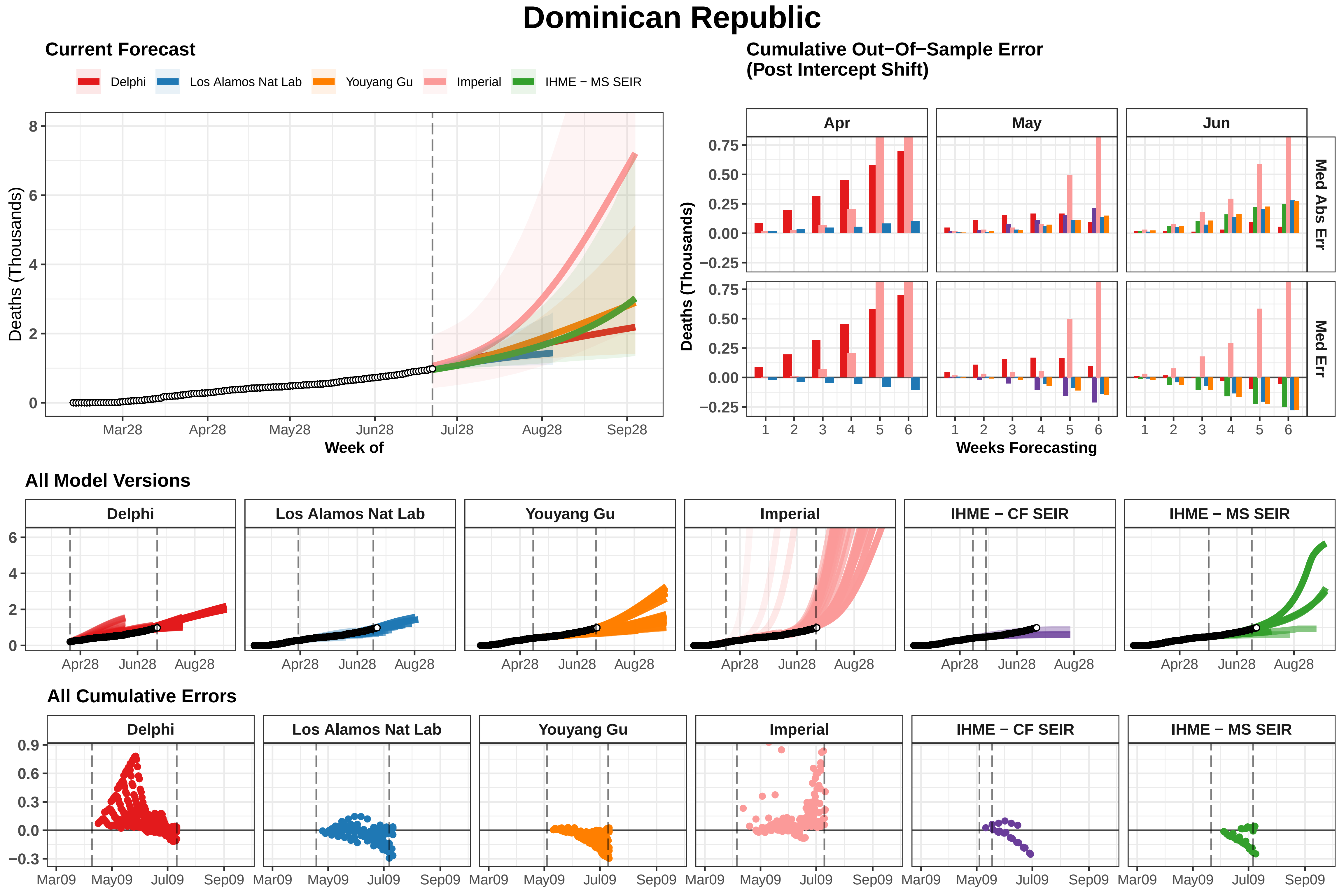

Dominican Republic
Current Forecast
Delphi
Los Alamos Nat Lab
Youyang Gu
Imperial
IHME − MS SEIR
Deaths (Thousands)
Week of
Mar28
Apr28
May28
Jun28
Jul28
Aug28
Sep28
Cumulative Out−Of−Sample Error
(Post Intercept Shift)
Apr
May
Jun
Med Abs Err
Med Err
Weeks Forecasting
All Model Versions
IHME − CF SEIR
All Cumulative Errors
Mar09
May09
Jul09
Sep09

Honduras

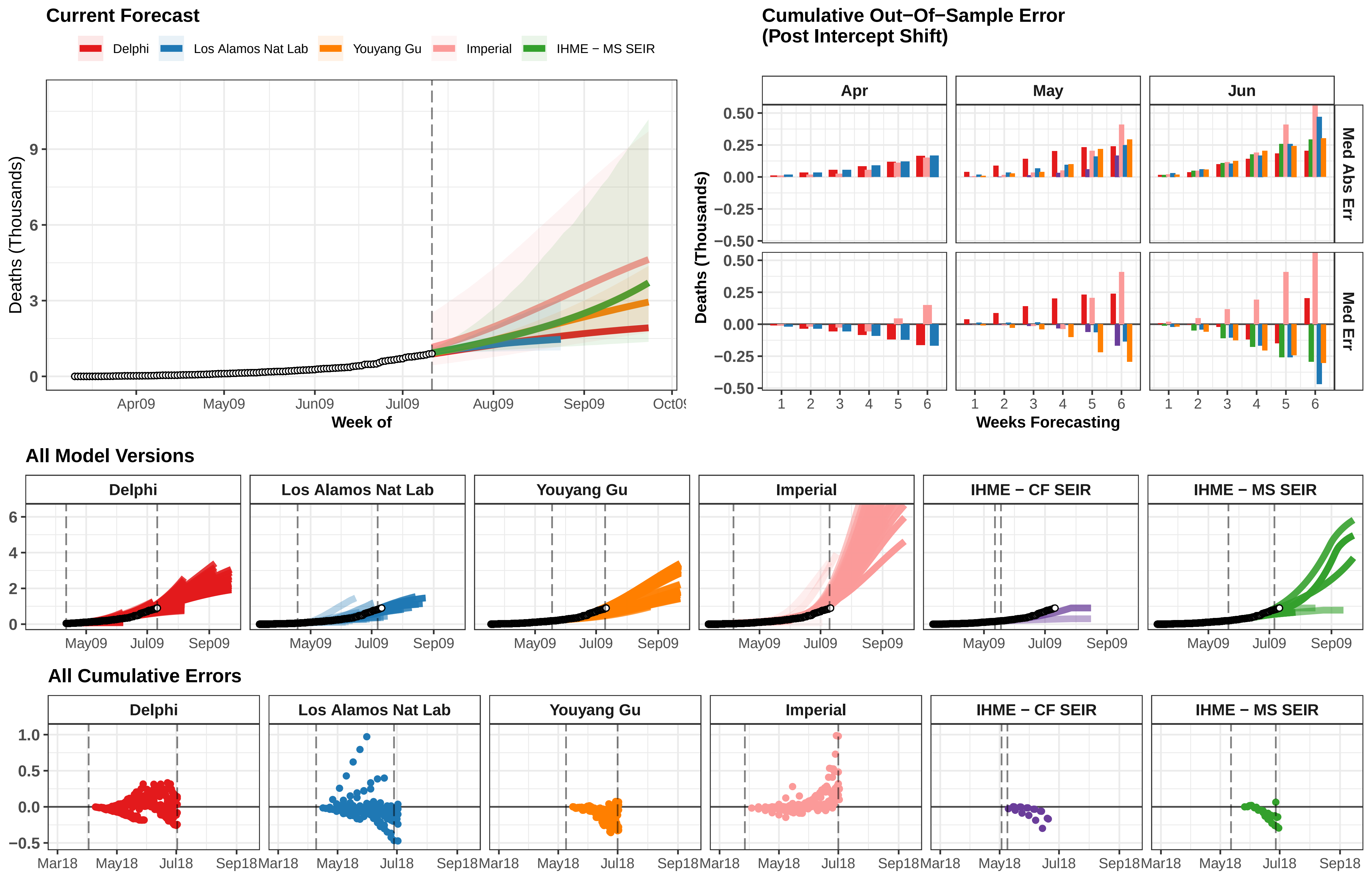

Current Forecast
Delphi
Los Alamos Nat Lab
Youyang Gu
Imperial
IHME − MS SEIR
Deaths (Thousands)
0
3
6
9
Apr09
May09
Jun09
Jul09
Aug09
Sep09
Week of
Cumulative Out−Of−Sample Error
(Post Intercept Shift)
Apr
May
Jun
Med Abs Err
Med Err
0.50
0.25
0.00
−0.25
−0.50
1
2
3
4
5
6
Weeks Forecasting
All Model Versions
Delphi
Los Alamos Nat Lab
Youyang Gu
Imperial
IHME − CF SEIR
IHME − MS SEIR
0
2
4
6
May09
Jul09
Sep09
All Cumulative Errors
1.0
0.5
0.0
−0.5
Mar18
May18
Jul18
Sep18

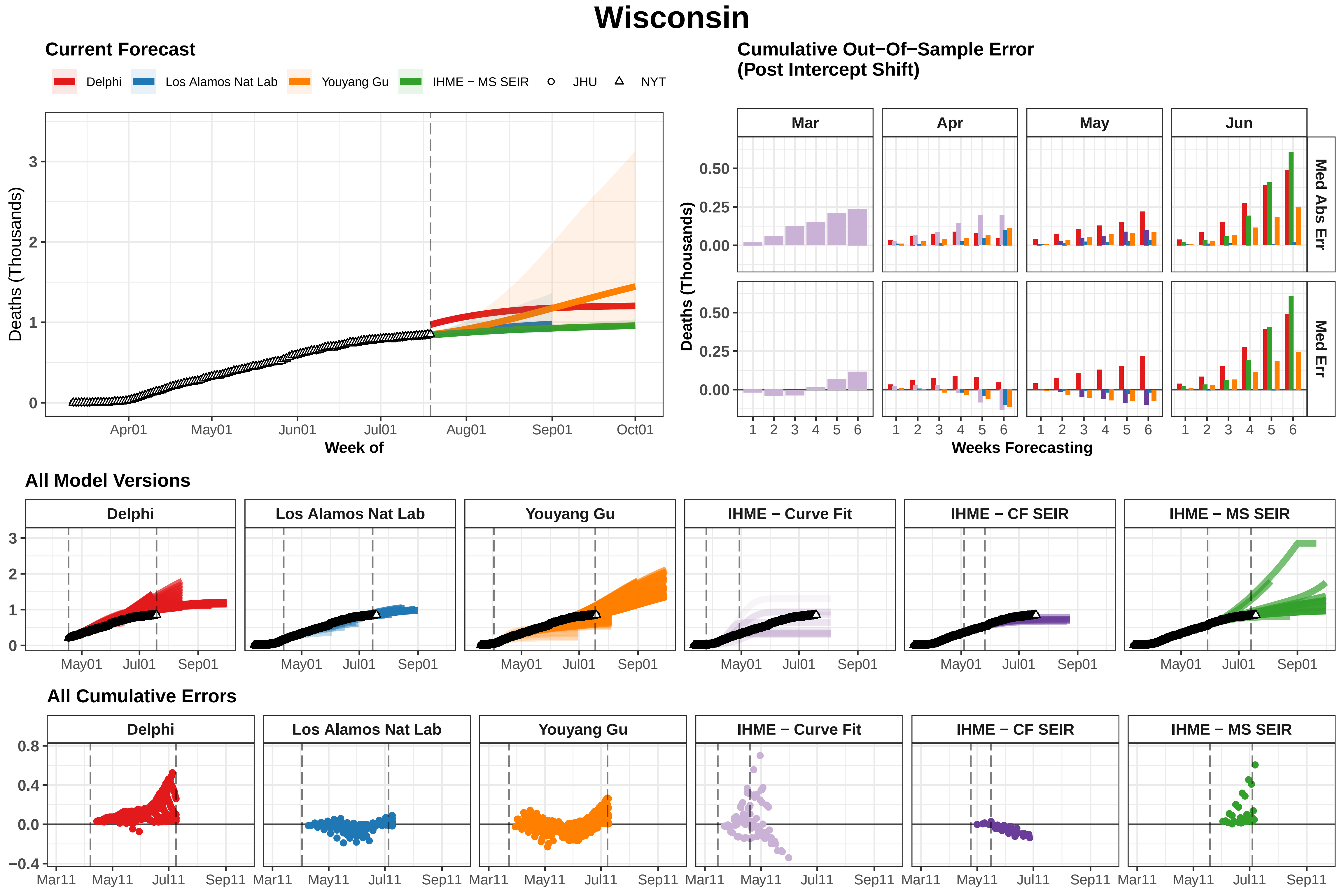
Wisconsin
Current Forecast
Delphi
Los Alamos Nat Lab
Youyang Gu
IHME − MS SEIR
JHU
NYT
Deaths (Thousands)
Week of
Apr01
May01
Jun01
Jul01
Aug01
Sep01
Oct01
Cumulative Out−Of−Sample Error
(Post Intercept Shift)
Mar
Apr
May
Jun
Med Abs Err
Med Err
Weeks Forecasting
All Model Versions
Delphi
Los Alamos Nat Lab
Youyang Gu
IHME − Curve Fit
IHME − CF SEIR
IHME − MS SEIR
All Cumulative Errors
Mar11
May11
Jul11
Sep11

Tennessee

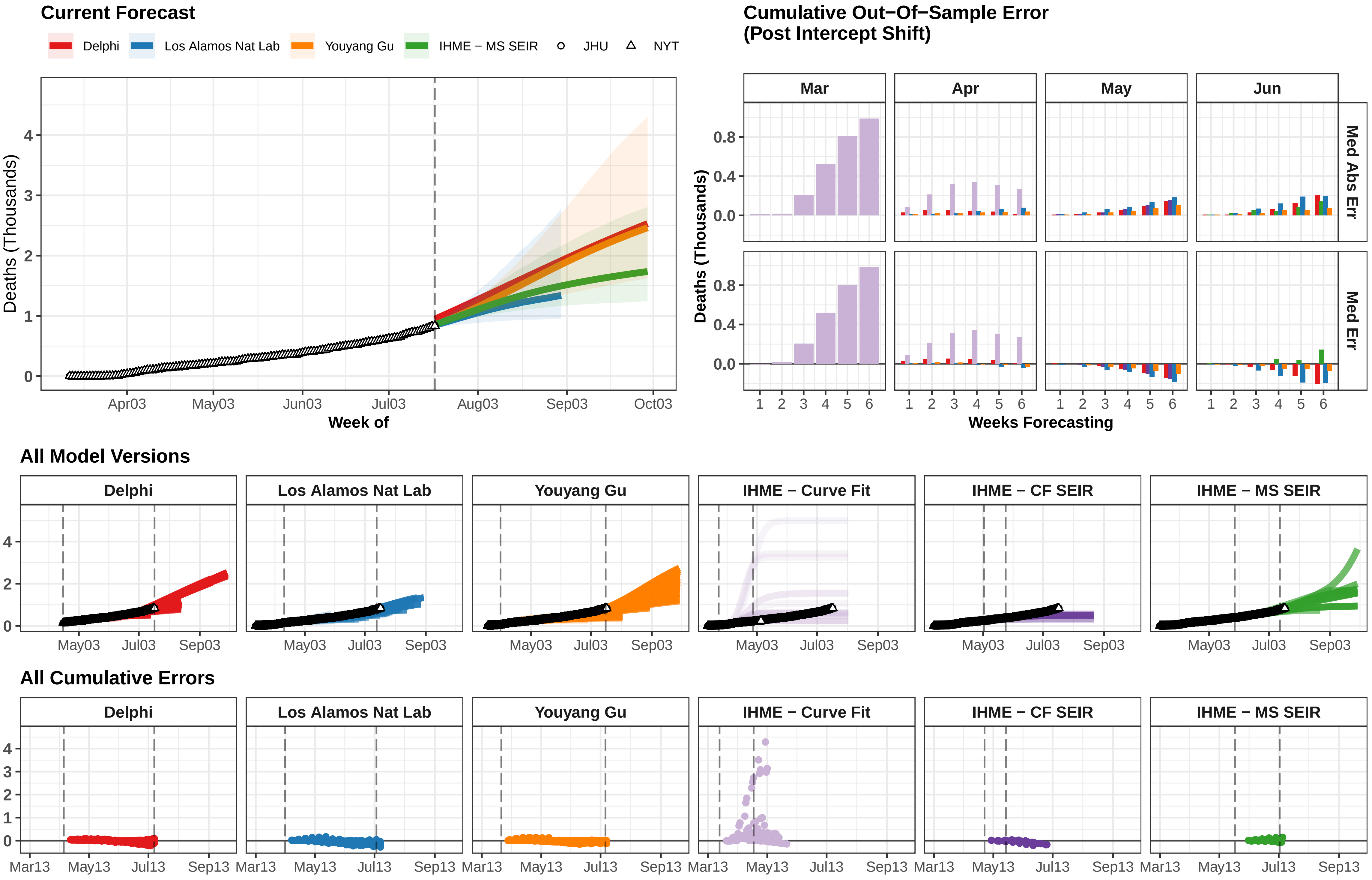
Current Forecast
Delphi
Los Alamos Nat Lab
Youyang Gu
IHME − MS SEIR
JHU
NYT
Deaths (Thousands)
Week of
Apr03
May03
Jun03
Jul03
Aug03
Sep03
Oct03
Cumulative Out−Of−Sample Error
(Post Intercept Shift)
Mar
Apr
May
Jun
Med Abs Err
Med Err
Deaths (Thousands)
Weeks Forecasting
All Model Versions
Delphi
Los Alamos Nat Lab
Youyang Gu
IHME − Curve Fit
IHME − CF SEIR
IHME − MS SEIR
May03
Jul03
Sep03
All Cumulative Errors
Delphi
Los Alamos Nat Lab
Youyang Gu
IHME − Curve Fit
IHME − CF SEIR
IHME − MS SEIR
Mar13
May13
Jul13
Sep13

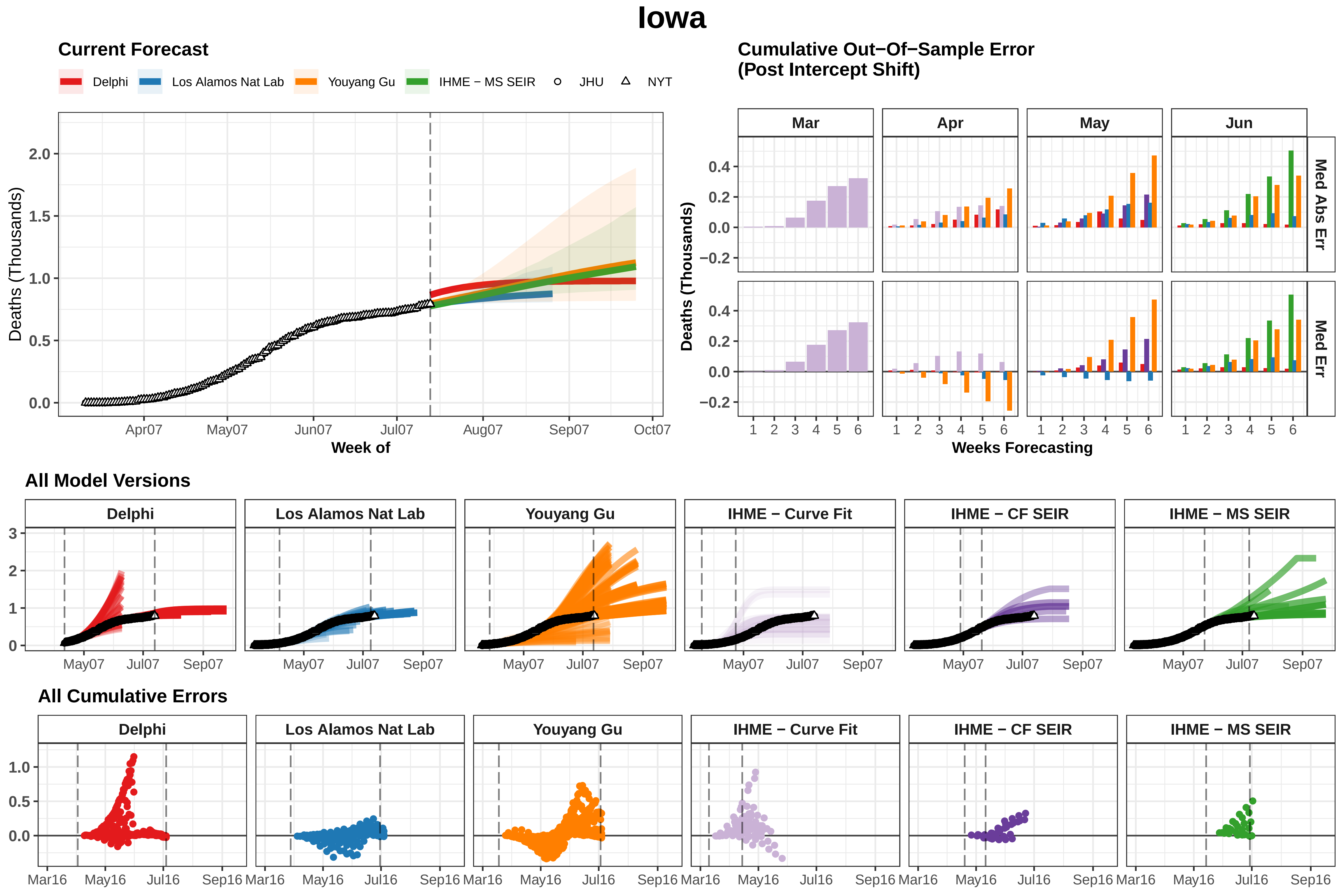

Iowa
Current Forecast
Delphi
Los Alamos Nat Lab
Youyang Gu
IHME − MS SEIR
JHU
NYT
Deaths (Thousands)
Week of
Apr07
May07
Jun07
Jul07
Aug07
Sep07
Oct07
Cumulative Out−Of−Sample Error (Post Intercept Shift)
Mar
Apr
May
Jun
Med Abs Err
Med Err
Weeks Forecasting
All Model Versions
Delphi
Los Alamos Nat Lab
Youyang Gu
IHME − Curve Fit
IHME − CF SEIR
IHME − MS SEIR
All Cumulative Errors
Mar16
May16
Jul16
Sep16

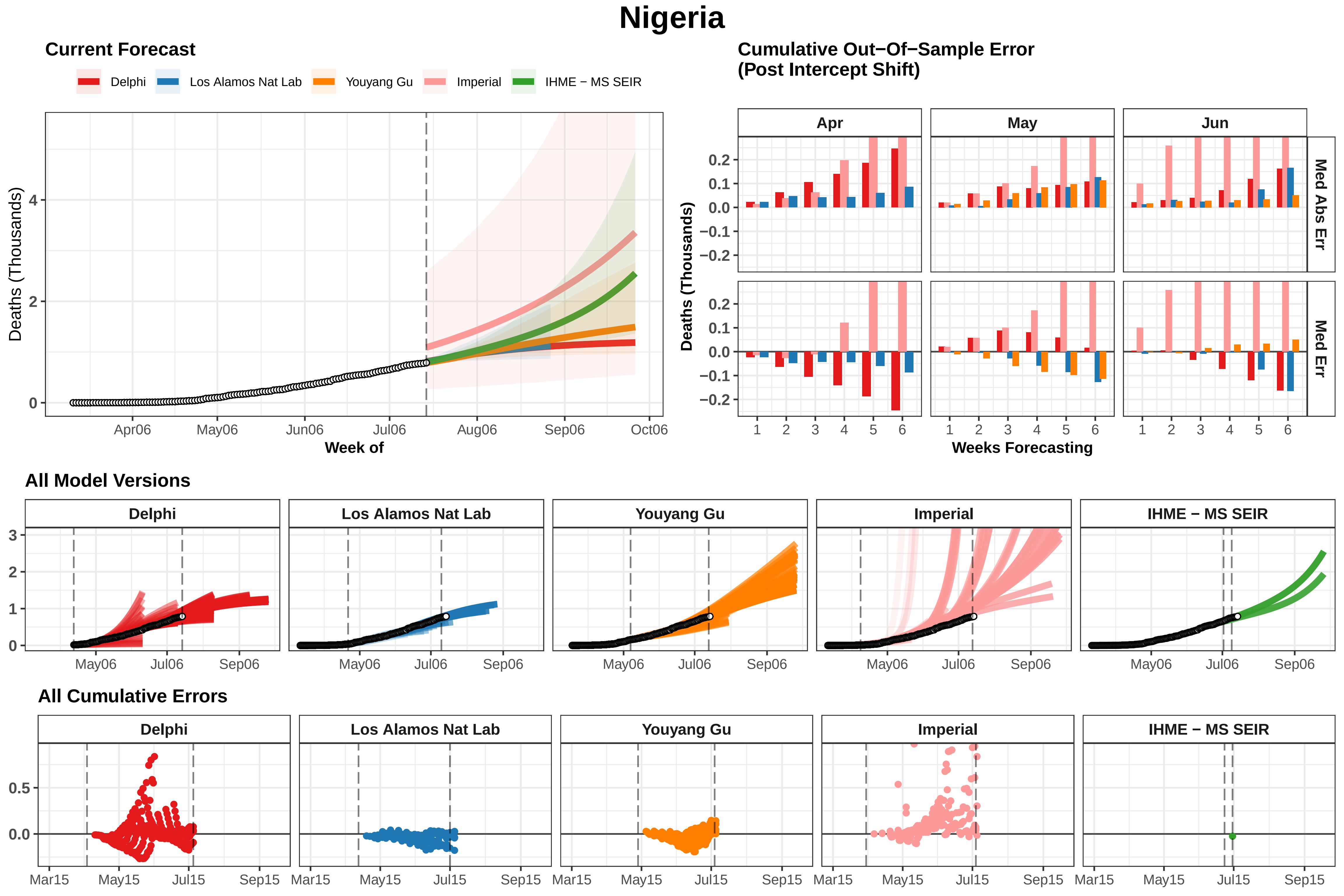

Nigeria
Current Forecast
Delphi
Los Alamos Nat Lab
Youyang Gu
Imperial
IHME − MS SEIR
Deaths (Thousands)
Week of
Apr06
May06
Jun06
Jul06
Aug06
Sep06
Oct06
Cumulative Out−Of−Sample Error
(Post Intercept Shift)
Apr
May
Jun
Med Abs Err
Med Err
Weeks Forecasting
All Model Versions
All Cumulative Errors
Mar15
May15
Jul15
Sep15

Austria

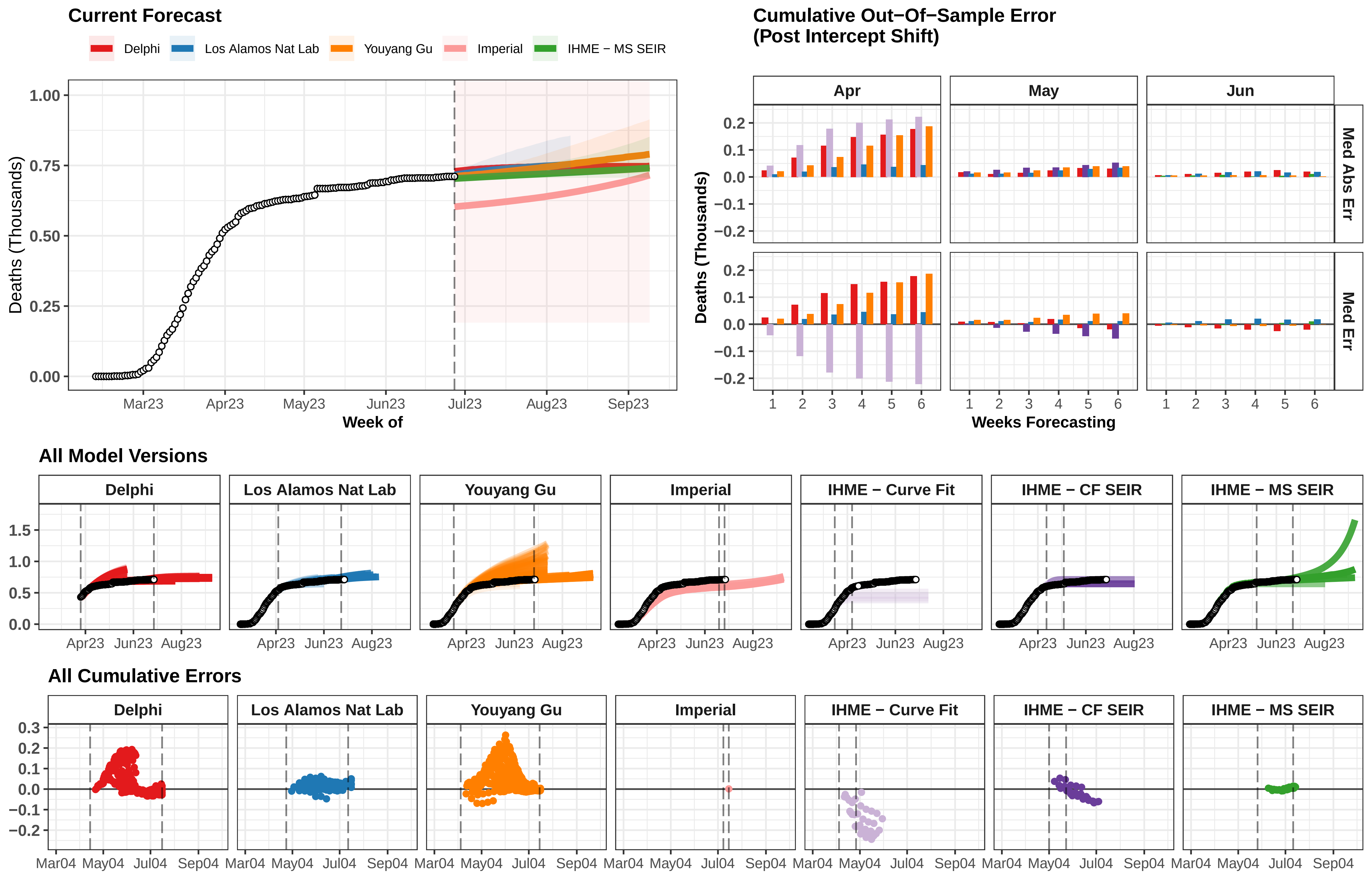
Current Forecast
Delphi
Los Alamos Nat Lab
Youyang Gu
Imperial
IHME − MS SEIR
Deaths (Thousands)
1.00
0.75
0.50
0.25
0.00
Mar23
Apr23
May23
Jun23
Jul23
Aug23
Sep23
Week of
Cumulative Out−Of−Sample Error
(Post Intercept Shift)
Apr
May
Jun
Med Abs Err
Med Err
0.2
0.1
0.0
−0.1
−0.2
Weeks Forecasting
All Model Versions
Delphi
Los Alamos Nat Lab
Youyang Gu
Imperial
IHME − Curve Fit
IHME − CF SEIR
IHME − MS SEIR
1.5
1.0
0.5
0.0
Apr23
Jun23
Aug23
All Cumulative Errors
0.3
0.2
0.1
0.0
−0.1
−0.2
Mar04
May04
Jul04
Sep04

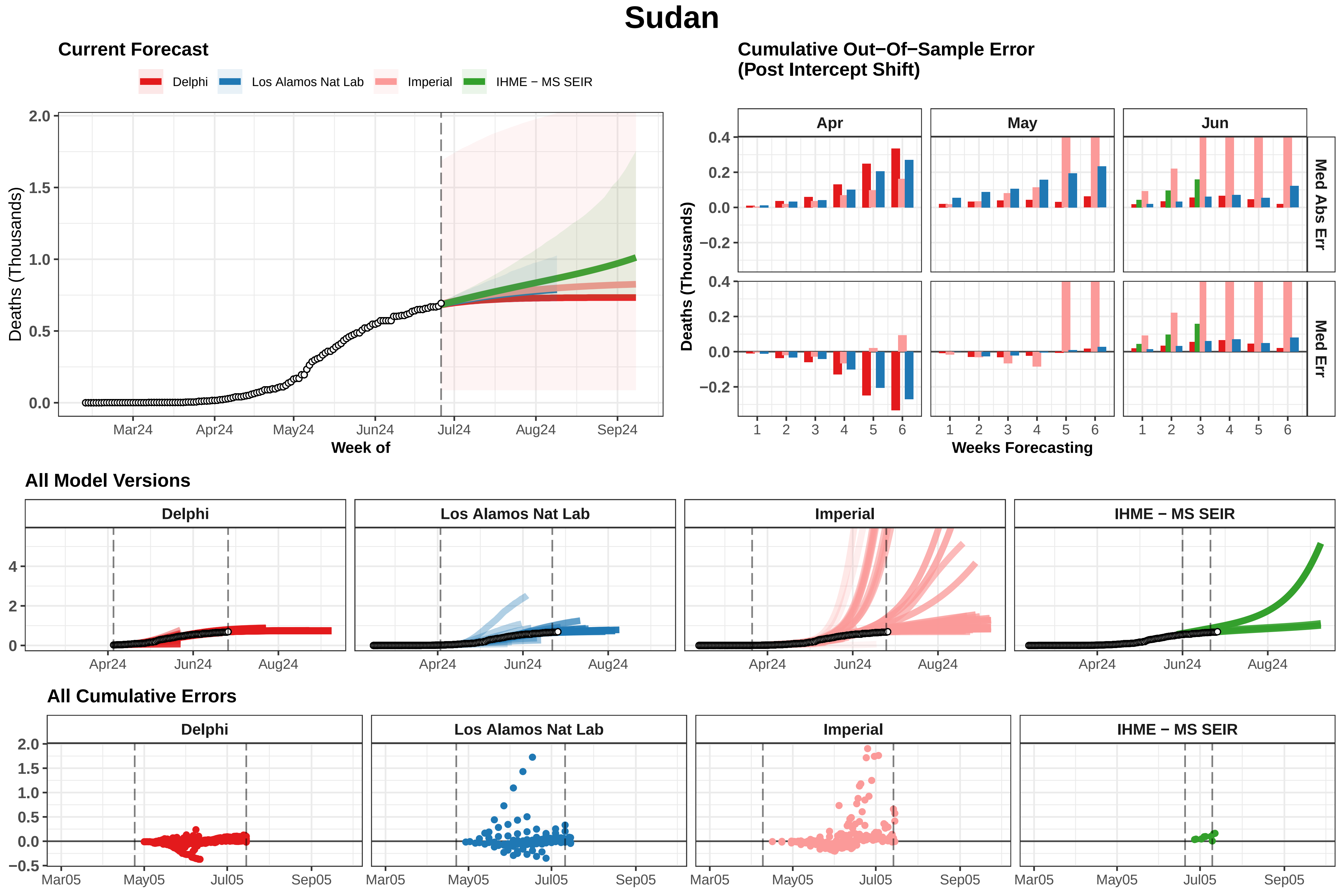
Sudan
Current Forecast
Delphi
Los Alamos Nat Lab
Imperial
IHME − MS SEIR
Deaths (Thousands)
Week of
Mar24
Apr24
May24
Jun24
Jul24
Aug24
Sep24
Cumulative Out−Of−Sample Error
(Post Intercept Shift)
Apr
May
Jun
Med Abs Err
Med Err
Weeks Forecasting
All Model Versions
All Cumulative Errors
Mar05
May05
Jul05
Sep05

Kentucky

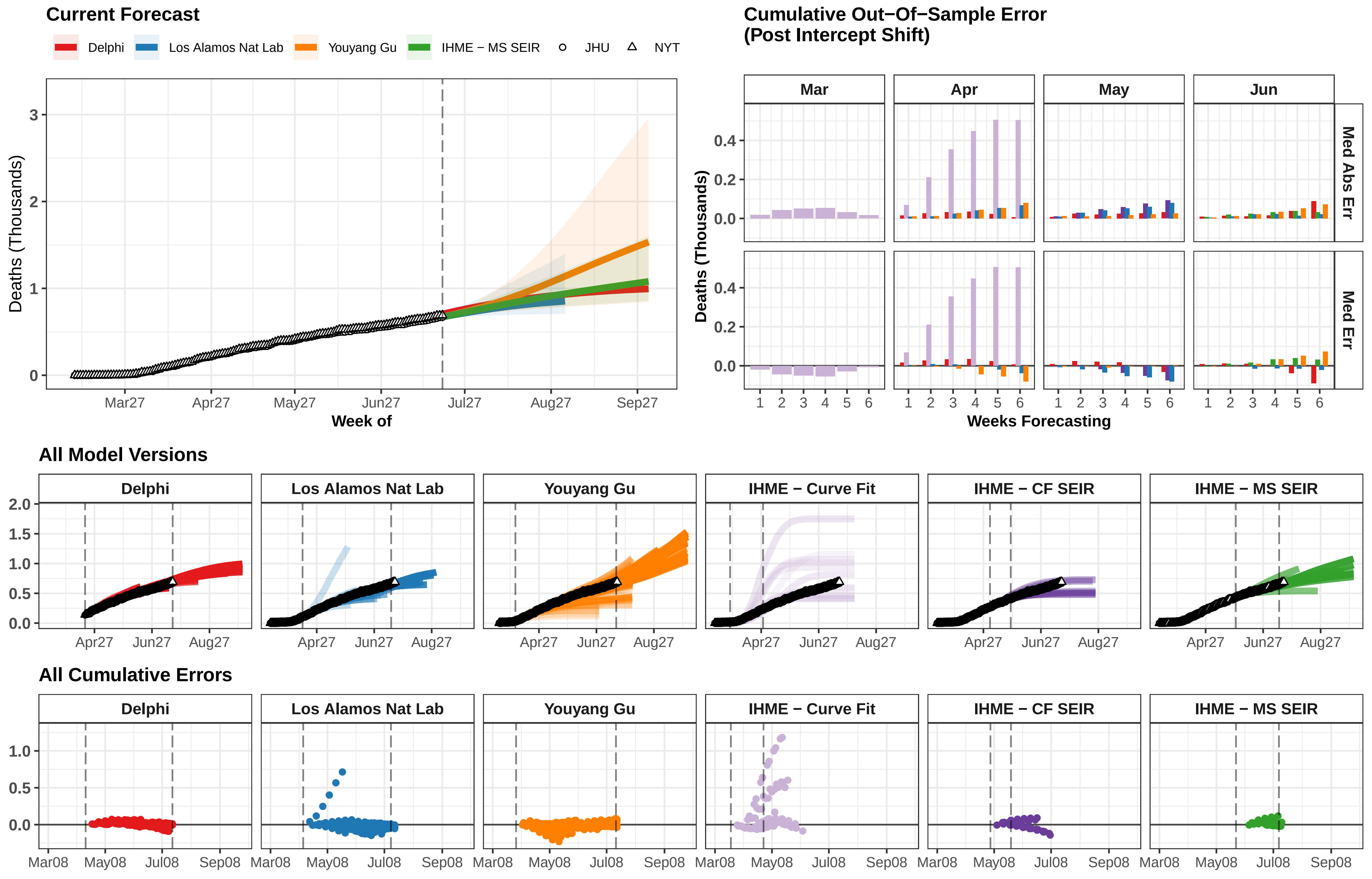
Current Forecast
Delphi
Los Alamos Nat Lab
Youyang Gu
IHME − MS SEIR
JHU
NYT
Deaths (Thousands)
Week of
Mar27
Apr27
May27
Jun27
Jul27
Aug27
Sep27
Cumulative Out−Of−Sample Error
(Post Intercept Shift)
Mar
Apr
May
Jun
Med Abs Err
Med Err
Weeks Forecasting
All Model Versions
Delphi
Los Alamos Nat Lab
Youyang Gu
IHME − Curve Fit
IHME − CF SEIR
IHME − MS SEIR
All Cumulative Errors
Mar08
May08
Jul08
Sep08

Moldova

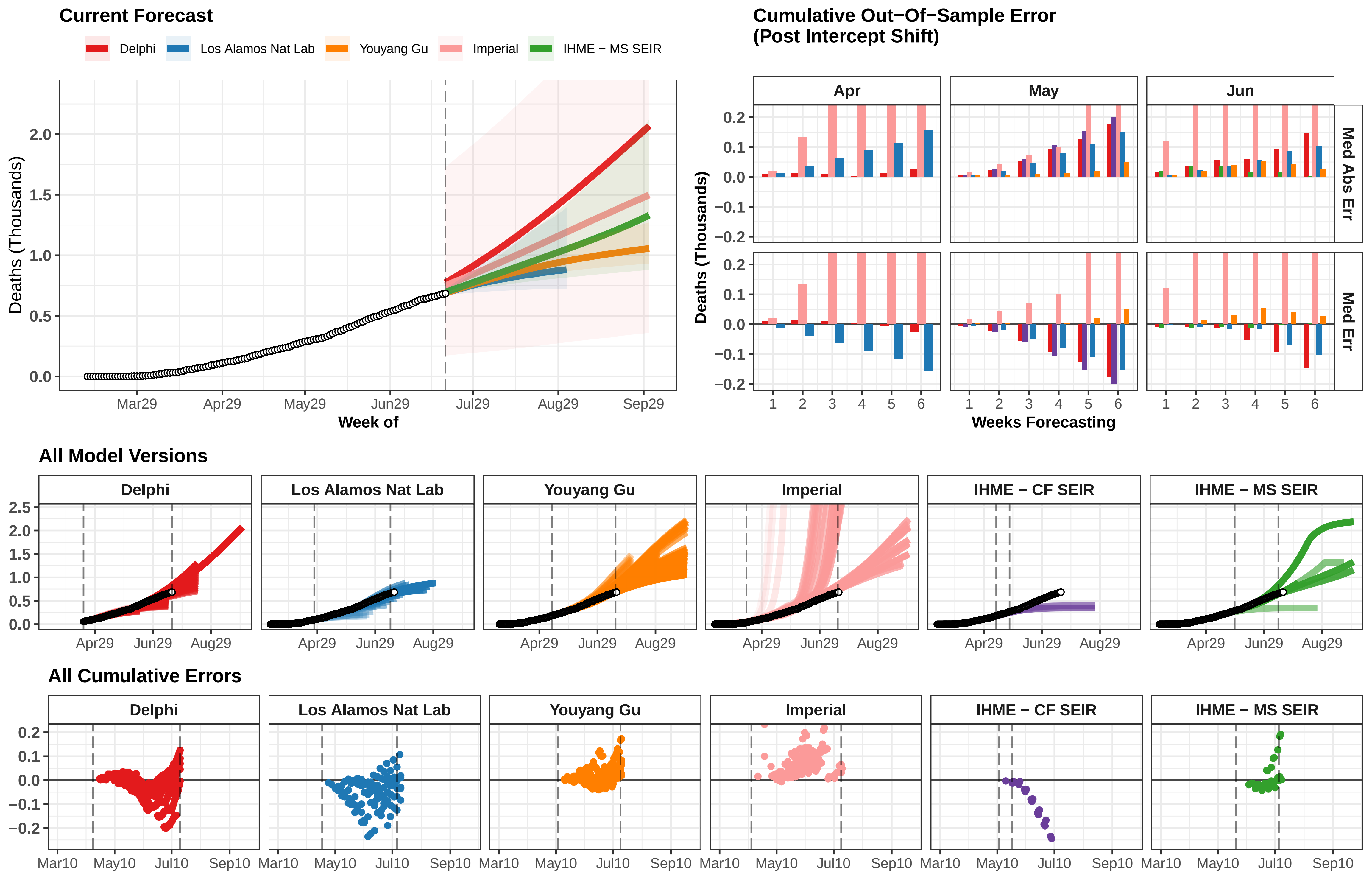

Current Forecast
Delphi
Los Alamos Nat Lab
Youyang Gu
Imperial
IHME − MS SEIR
Deaths (Thousands)
Week of
Mar29
Apr29
May29
Jun29
Jul29
Aug29
Sep29
Cumulative Out−Of−Sample Error (Post Intercept Shift)
Apr
May
Jun
Med Abs Err
Med Err
Weeks Forecasting
All Model Versions
Delphi
Los Alamos Nat Lab
Youyang Gu
Imperial
IHME − CF SEIR
IHME − MS SEIR
All Cumulative Errors
Mar10
May10
Jul10
Sep10

Nevada

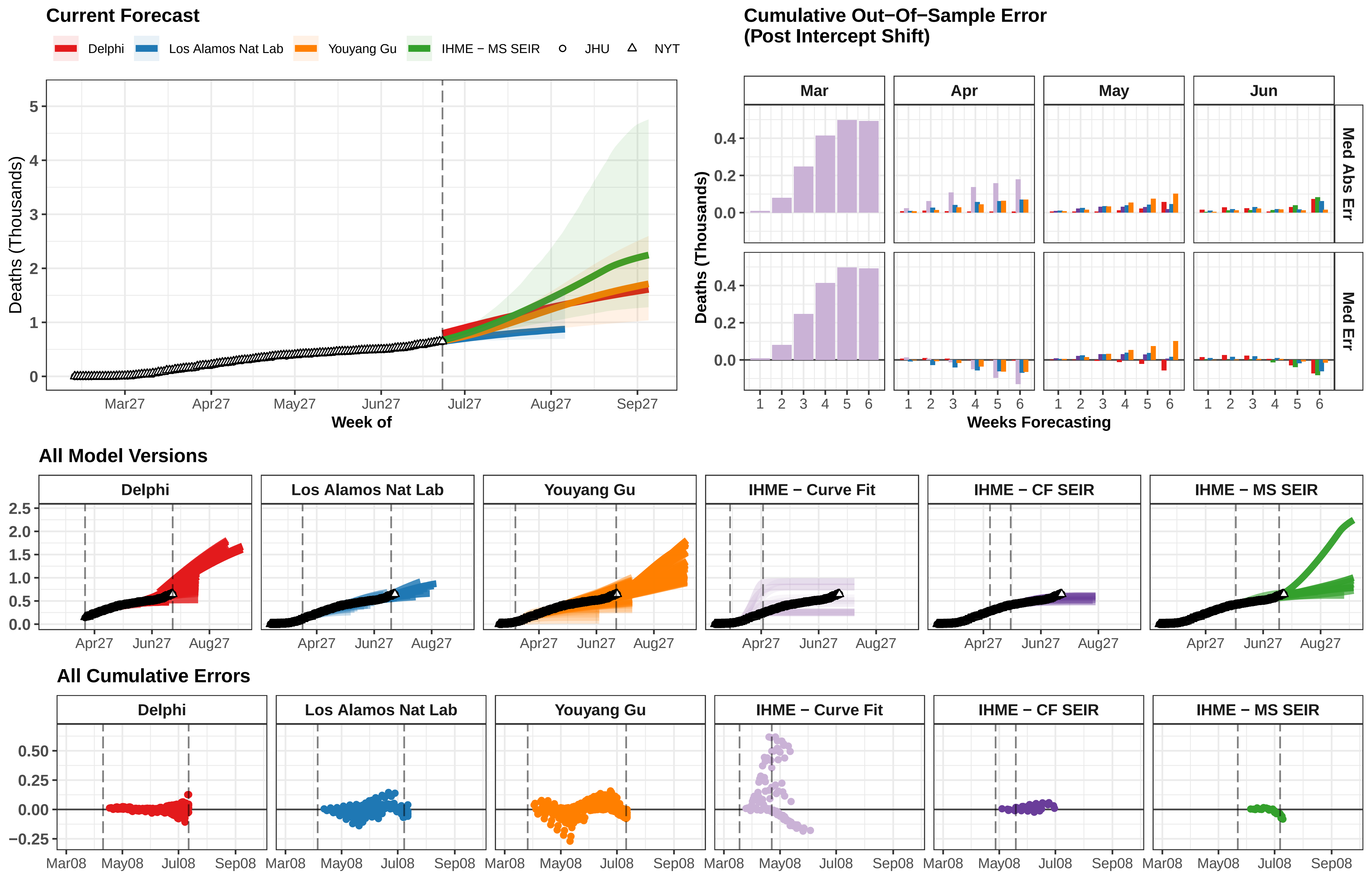
Current Forecast
Delphi
Los Alamos Nat Lab
Youyang Gu
IHME − MS SEIR
JHU
NYT
Deaths (Thousands)
Week of
Mar27
Apr27
May27
Jun27
Jul27
Aug27
Sep27
Cumulative Out−Of−Sample Error
(Post Intercept Shift)
Mar
Apr
May
Jun
Med Abs Err
Med Err
Weeks Forecasting
All Model Versions
Delphi
Los Alamos Nat Lab
Youyang Gu
IHME − Curve Fit
IHME − CF SEIR
IHME − MS SEIR
All Cumulative Errors
Mar08
May08
Jul08
Sep08

Armenia

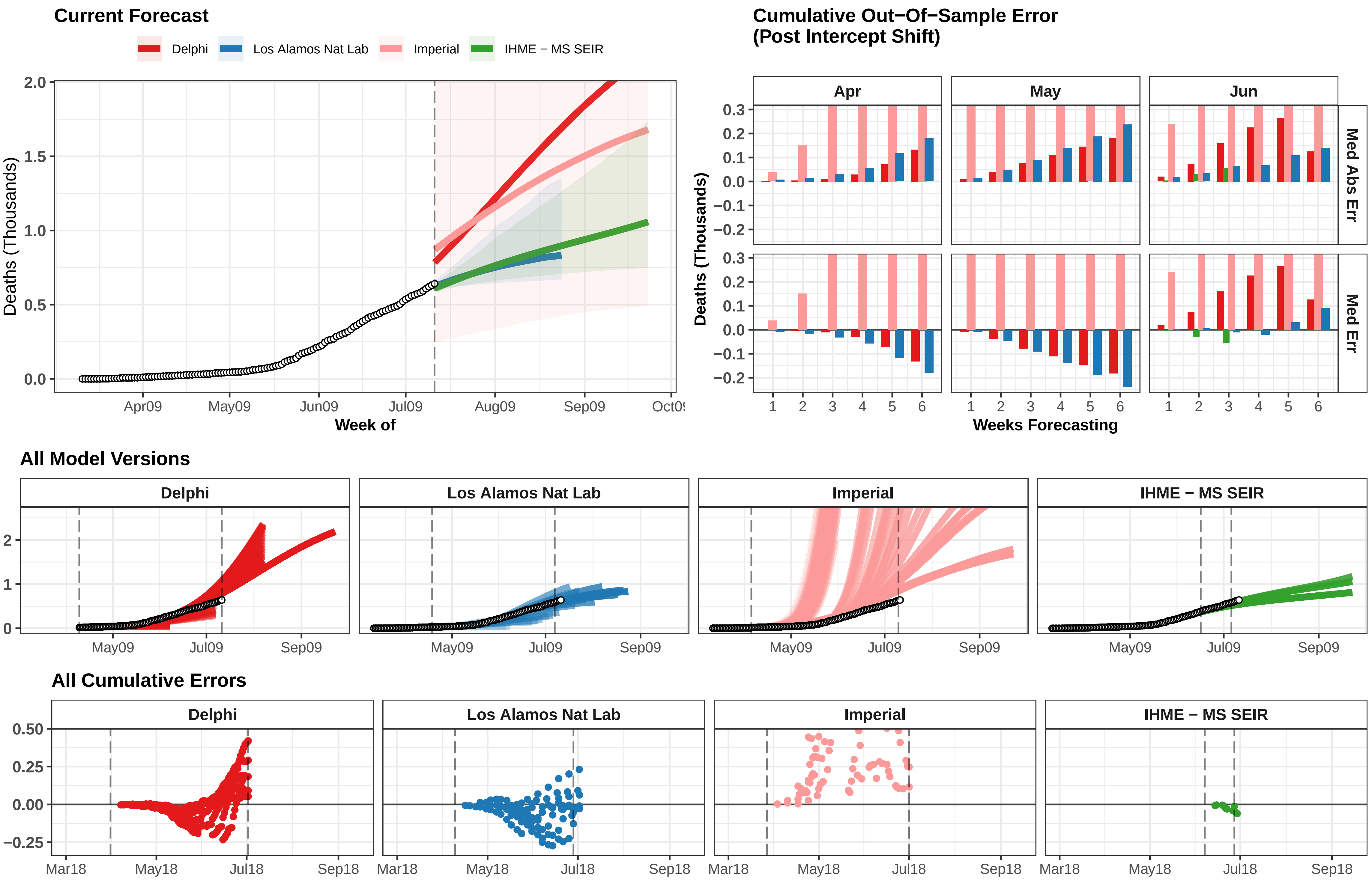

Current Forecast
Delphi
Los Alamos Nat Lab
Imperial
IHME − MS SEIR
Deaths (Thousands)
2.0
1.5
1.0
0.5
0.0
Apr09
May09
Jun09
Jul09
Aug09
Sep09
Oct09
Week of
Cumulative Out−Of−Sample Error
(Post Intercept Shift)
Apr
May
Jun
Med Abs Err
Med Err
Deaths (Thousands)
0.3
0.2
0.1
0.0
−0.1
−0.2
1
2
3
4
5
6
Weeks Forecasting
All Model Versions
Delphi
Los Alamos Nat Lab
Imperial
IHME − MS SEIR
2
1
0
May09
Jul09
Sep09
All Cumulative Errors
Delphi
Los Alamos Nat Lab
Imperial
IHME − MS SEIR
0.50
0.25
0.00
−0.25
Mar18
May18
Jul18
Sep18

Denmark

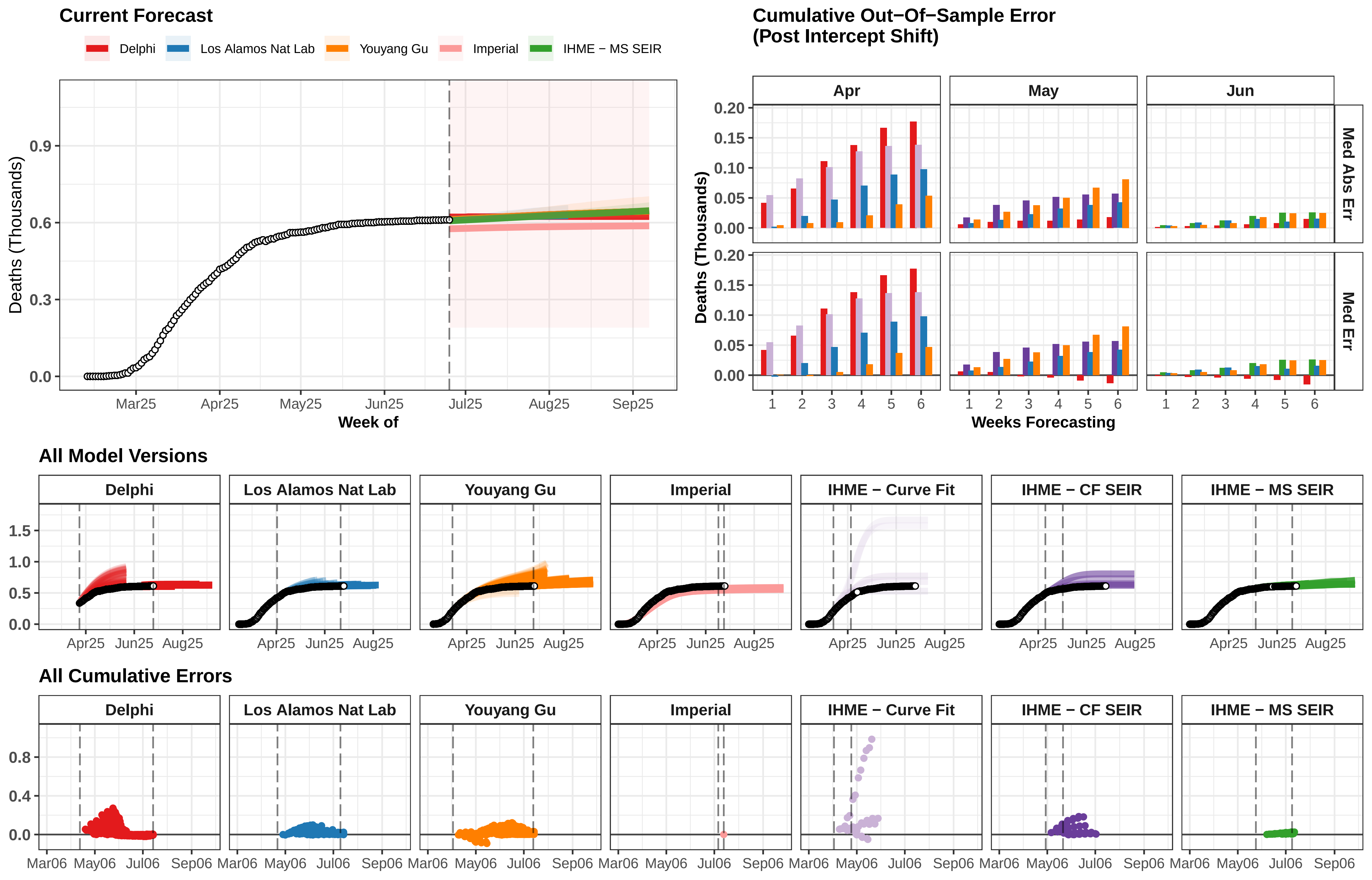

Current Forecast
Delphi
Los Alamos Nat Lab
Youyang Gu
Imperial
IHME − MS SEIR
Deaths (Thousands)
Week of
Cumulative Out−Of−Sample Error (Post Intercept Shift)
Apr
May
Jun
Med Abs Err
Med Err
Weeks Forecasting
All Model Versions
IHME − Curve Fit
IHME − CF SEIR
All Cumulative Errors

Hungary

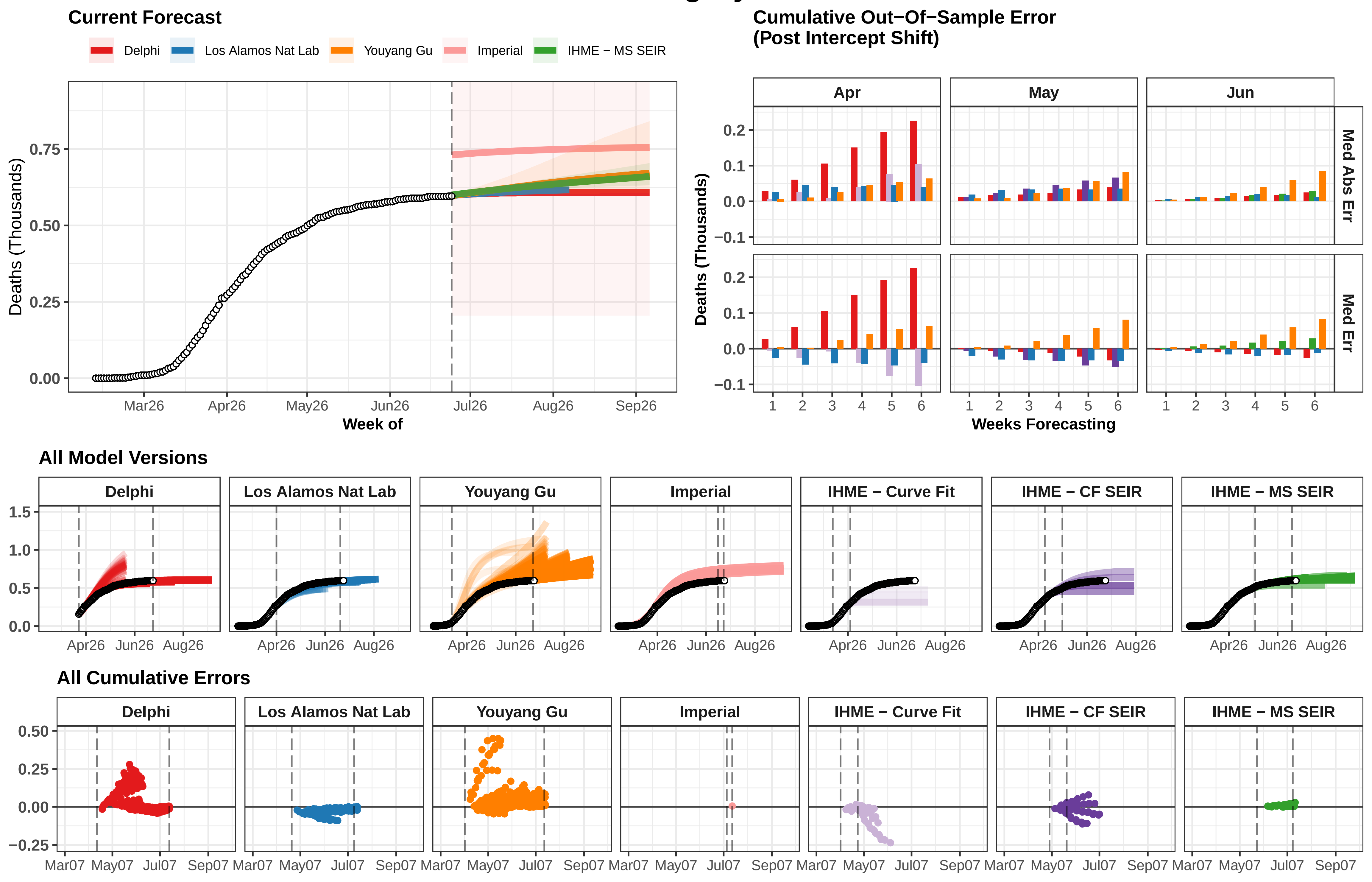

Current Forecast
Delphi
Los Alamos Nat Lab
Youyang Gu
Imperial
IHME − MS SEIR
Deaths (Thousands)
Week of
Mar26
Apr26
May26
Jun26
Jul26
Aug26
Sep26
Cumulative Out−Of−Sample Error
(Post Intercept Shift)
Apr
May
Jun
Med Abs Err
Med Err
Weeks Forecasting
All Model Versions
IHME − Curve Fit
IHME − CF SEIR
All Cumulative Errors
Mar07
May07
Jul07
Sep07

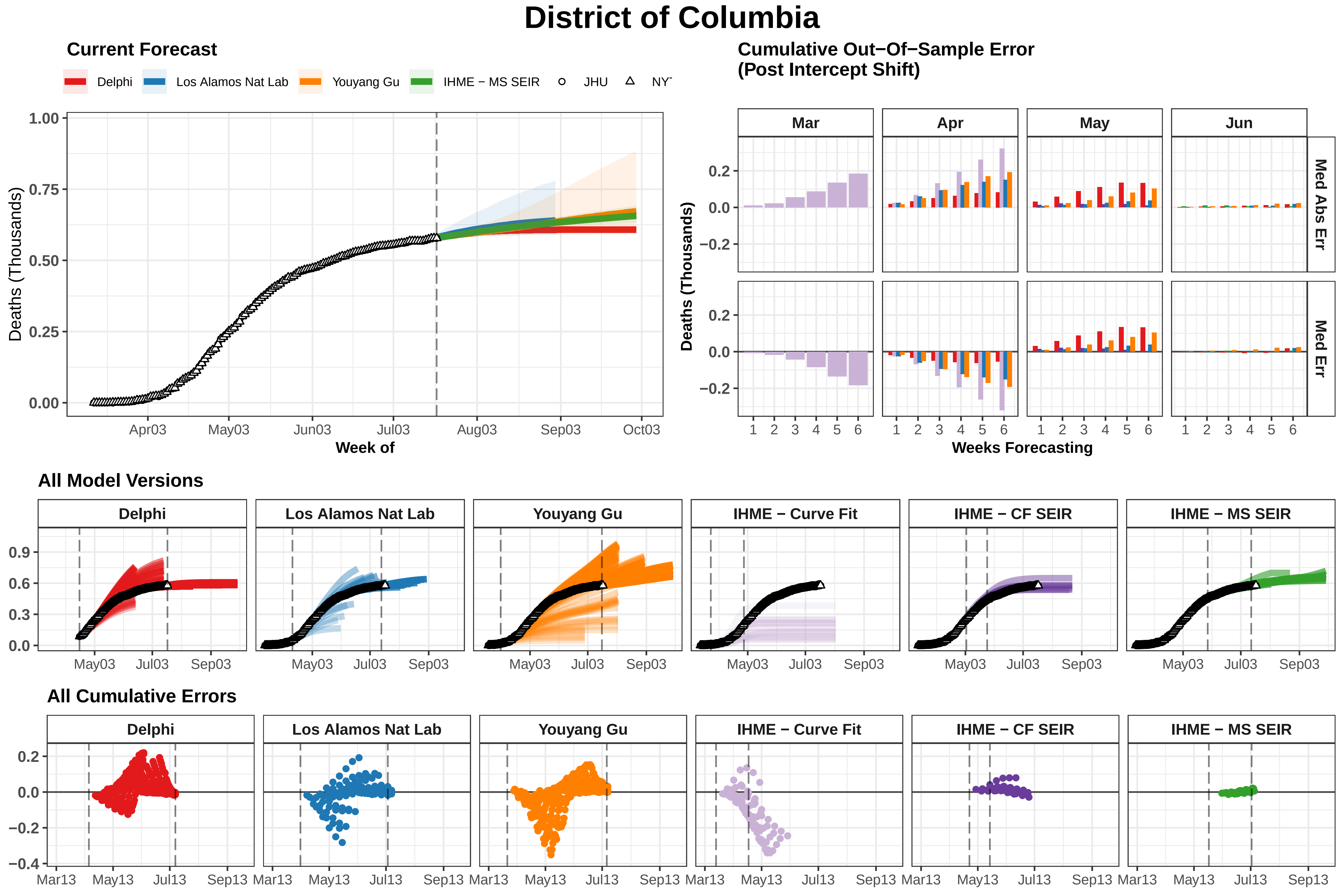

District of Columbia
Current Forecast
Delphi
Los Alamos Nat Lab
Youyang Gu
IHME − MS SEIR
JHU
NY
Deaths (Thousands)
Week of
Apr03
May03
Jun03
Jul03
Aug03
Sep03
Oct03
Cumulative Out−Of−Sample Error (Post Intercept Shift)
Mar
Apr
May
Jun
Med Abs Err
Med Err
Weeks Forecasting
All Model Versions
Delphi
Los Alamos Nat Lab
Youyang Gu
IHME − Curve Fit
IHME − CF SEIR
IHME − MS SEIR
All Cumulative Errors
Mar13
May13
Jul13
Sep13

New Mexico

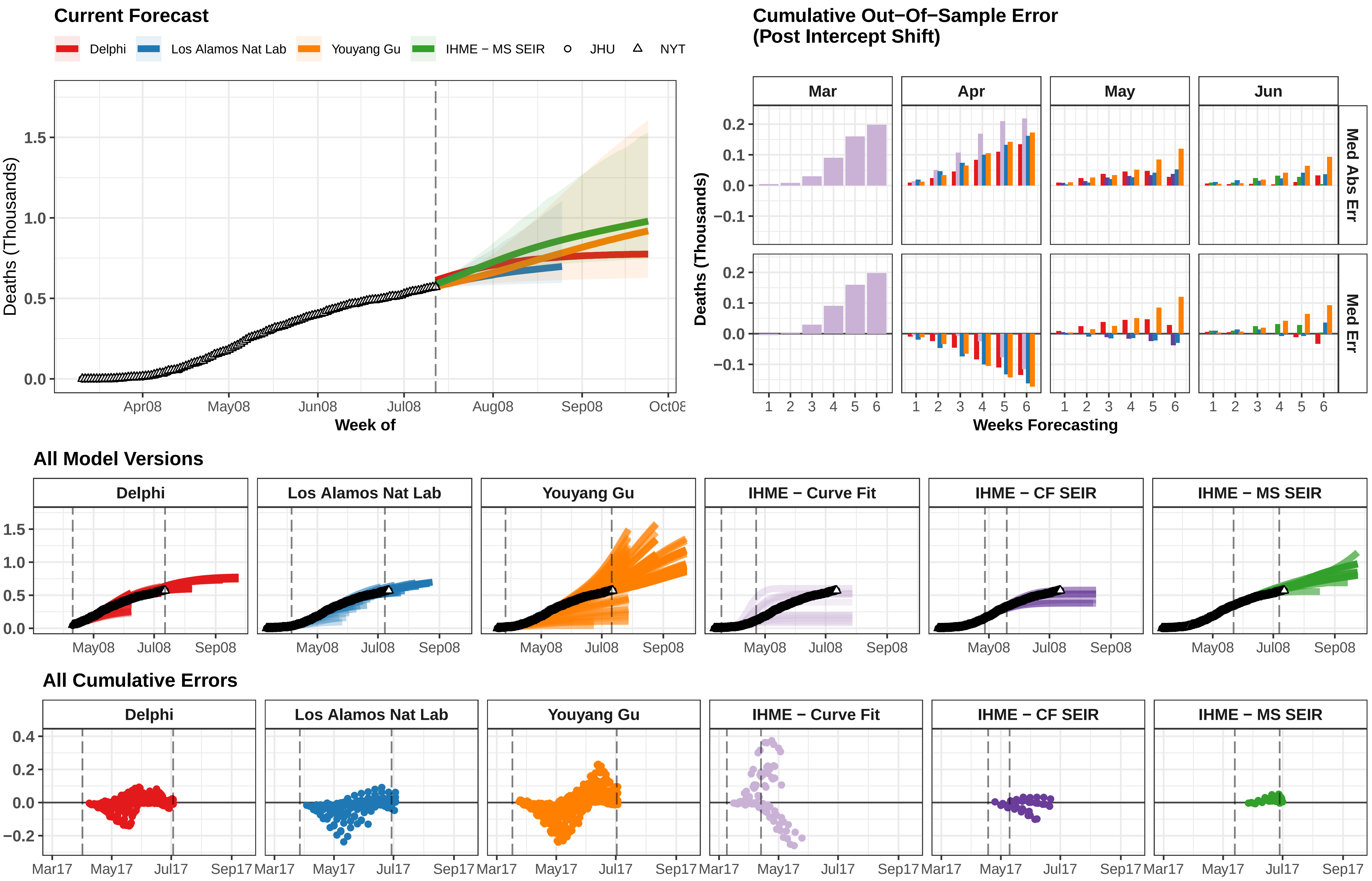

Current Forecast
Delphi
Los Alamos Nat Lab
Youyang Gu
IHME − MS SEIR
JHU
NYT
Deaths (Thousands)
Week of
Apr08
May08
Jun08
Jul08
Aug08
Sep08
Oct08
Cumulative Out−Of−Sample Error
(Post Intercept Shift)
Mar
Apr
May
Jun
Med Abs Err
Med Err
Weeks Forecasting
All Model Versions
Delphi
Los Alamos Nat Lab
Youyang Gu
IHME − Curve Fit
IHME − CF SEIR
IHME − MS SEIR
All Cumulative Errors
Mar17
May17
Jul17
Sep17

Delaware

Current Forecast

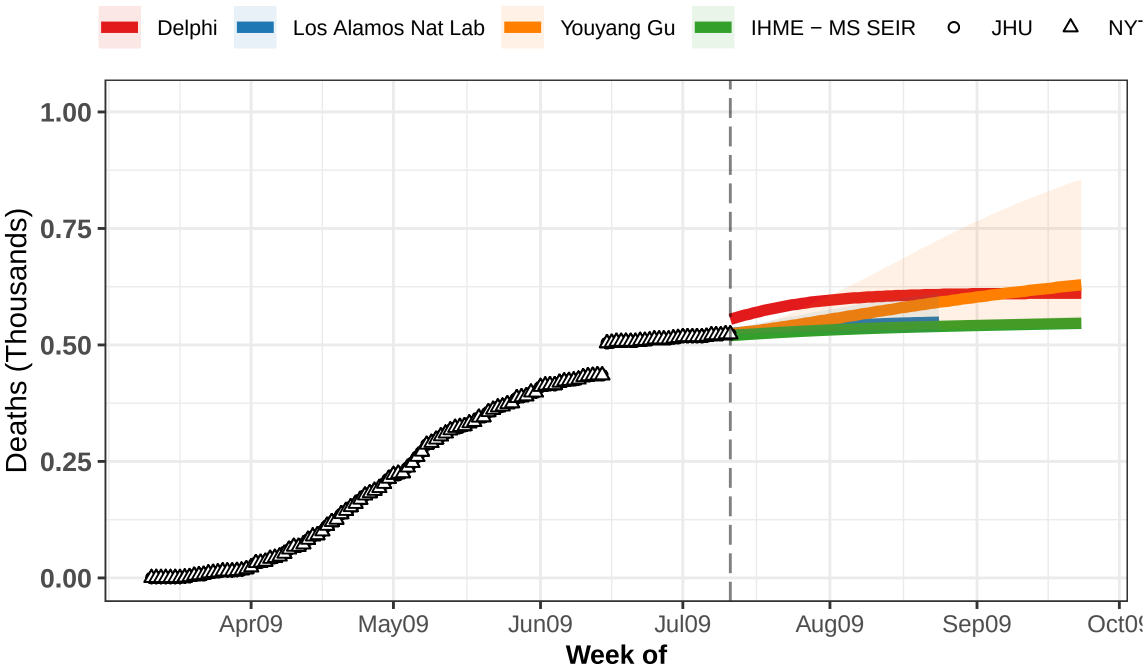

Cumulative Out−Of−Sample Error (Post Intercept Shift)

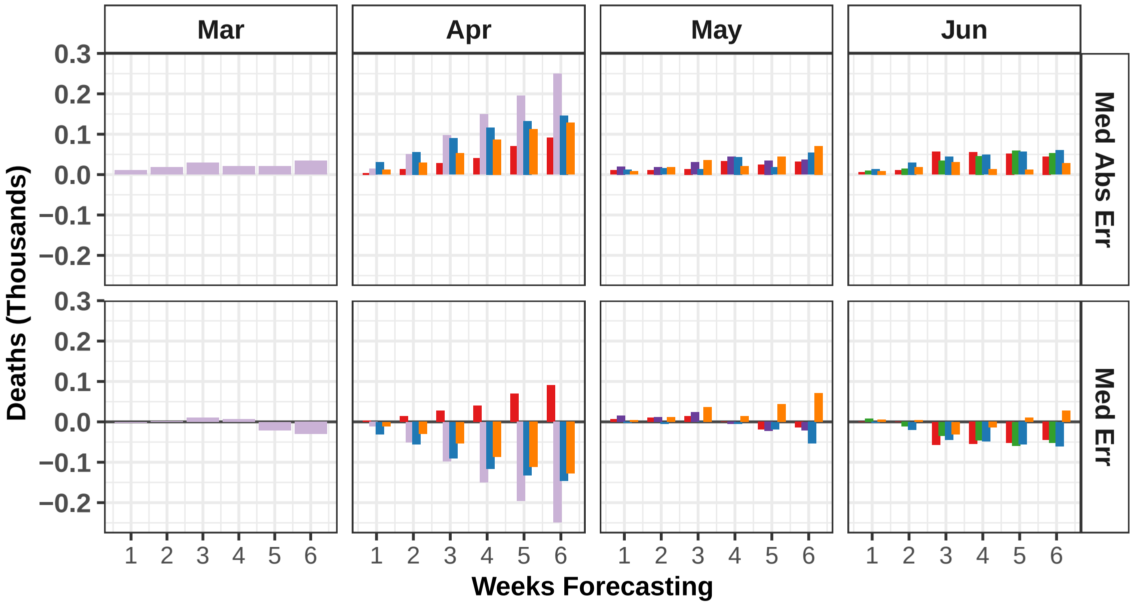

All Model Versions

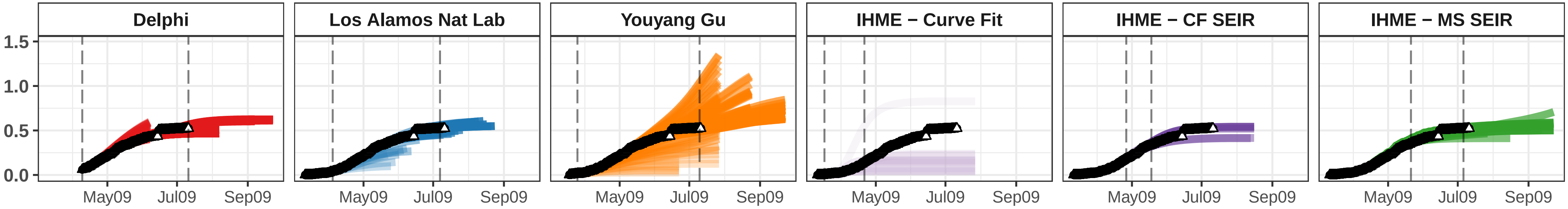

All Cumulative Errors

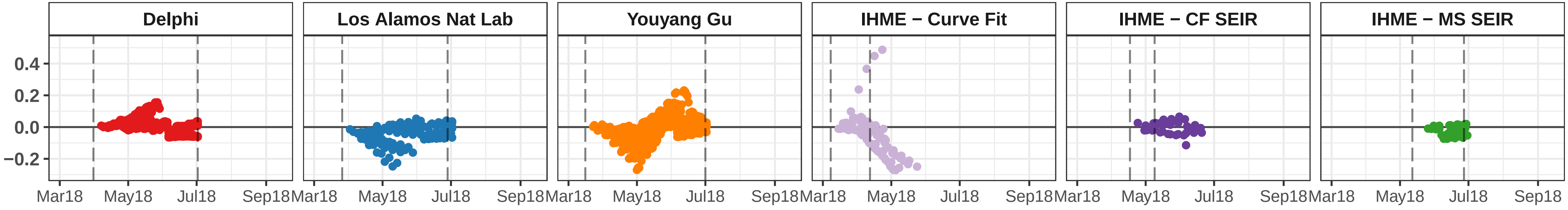

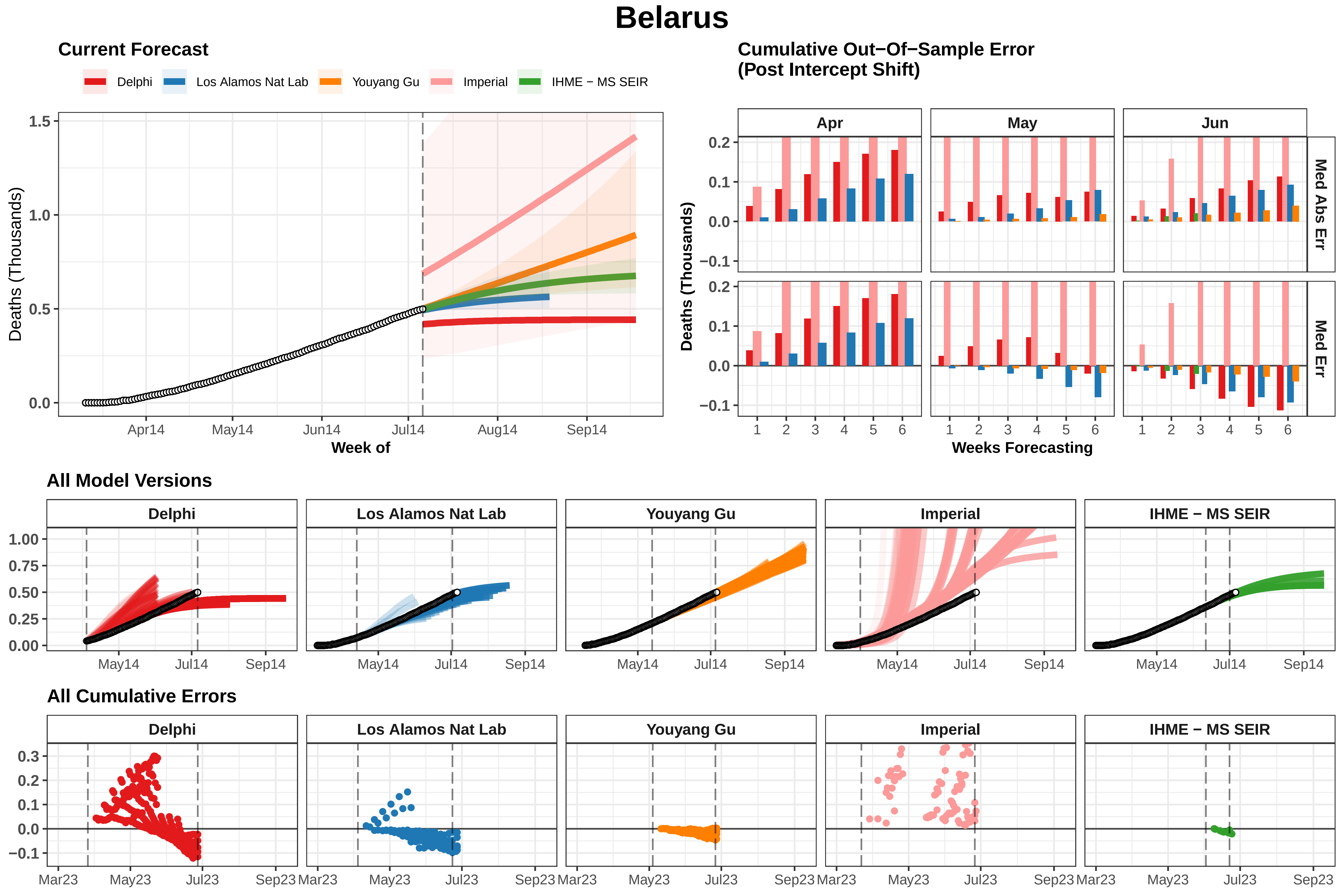
Belarus
Current Forecast
Delphi
Los Alamos Nat Lab
Youyang Gu
Imperial
IHME − MS SEIR
Deaths (Thousands)
Week of
Apr14
May14
Jun14
Jul14
Aug14
Sep14
Cumulative Out−Of−Sample Error
(Post Intercept Shift)
Apr
May
Jun
Med Abs Err
Med Err
Weeks Forecasting
All Model Versions
All Cumulative Errors
Mar23
May23
Jul23
Sep23

Serbia

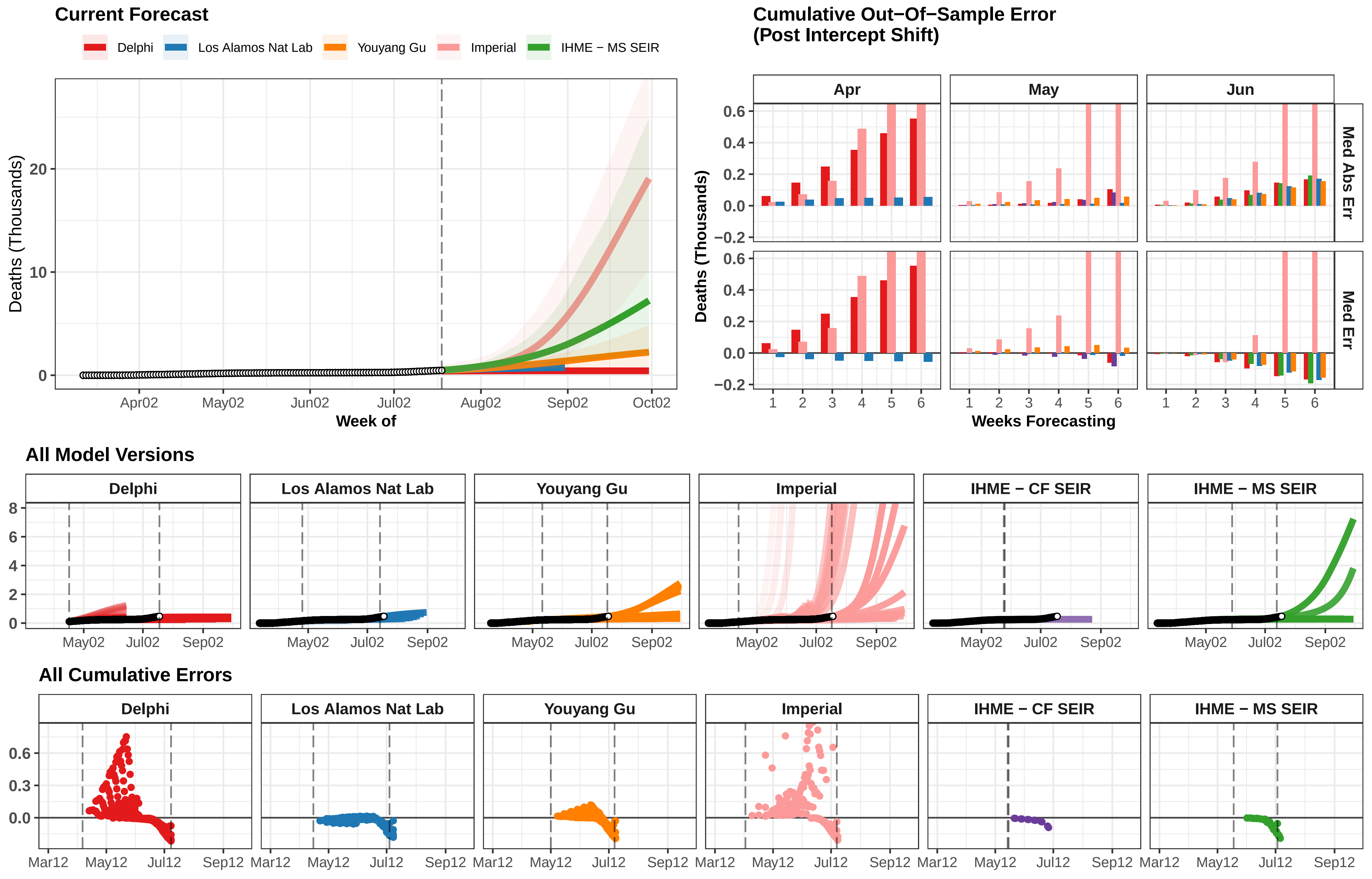
Current Forecast
Delphi
Los Alamos Nat Lab
Youyang Gu
Imperial
IHME − MS SEIR
Deaths (Thousands)
Week of
Apr02
May02
Jun02
Jul02
Aug02
Sep02
Oct02
Cumulative Out−Of−Sample Error
(Post Intercept Shift)
Apr
May
Jun
Med Abs Err
Med Err
Weeks Forecasting
All Model Versions
Delphi
Los Alamos Nat Lab
Youyang Gu
Imperial
IHME − CF SEIR
IHME − MS SEIR
All Cumulative Errors
Mar12
May12
Jul12
Sep12

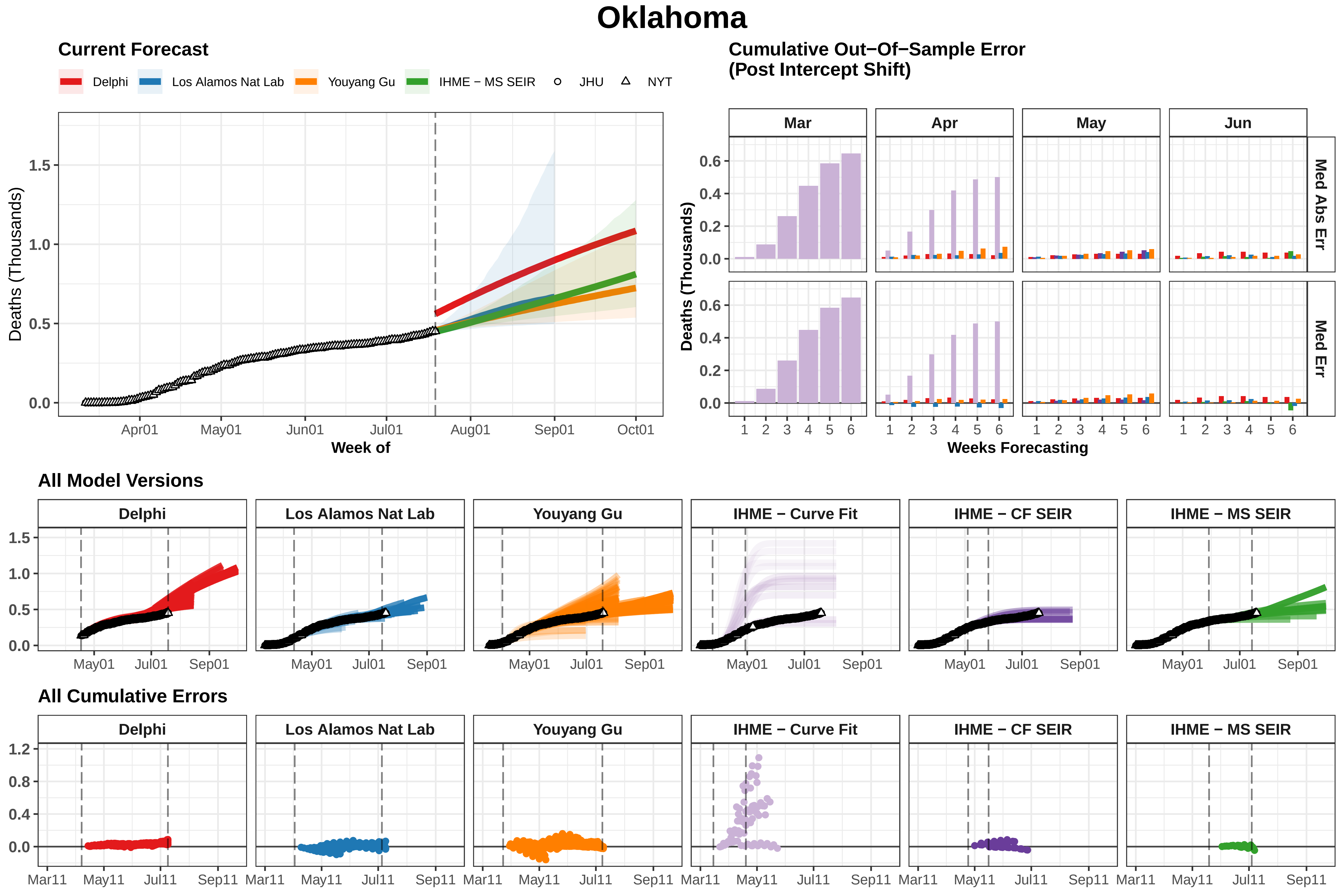
Oklahoma
Current Forecast
Delphi
Los Alamos Nat Lab
Youyang Gu
IHME − MS SEIR
JHU
NYT
Deaths (Thousands)
0.0
0.5
1.0
1.5
Apr01
May01
Jun01
Jul01
Aug01
Sep01
Oct01
Week of
Cumulative Out−Of−Sample Error
(Post Intercept Shift)
Mar
Apr
May
Jun
Med Abs Err
Med Err
Deaths (Thousands)
0.0
0.2
0.4
0.6
1 2 3 4 5 6
Weeks Forecasting
All Model Versions
Delphi
Los Alamos Nat Lab
Youyang Gu
IHME − Curve Fit
IHME − CF SEIR
IHME − MS SEIR
0.0
0.5
1.0
1.5
May01
Jul01
Sep01
All Cumulative Errors
Delphi
Los Alamos Nat Lab
Youyang Gu
IHME − Curve Fit
IHME − CF SEIR
IHME − MS SEIR
0.0
0.4
0.8
1.2
Mar11
May11
Jul11
Sep11

Yemen

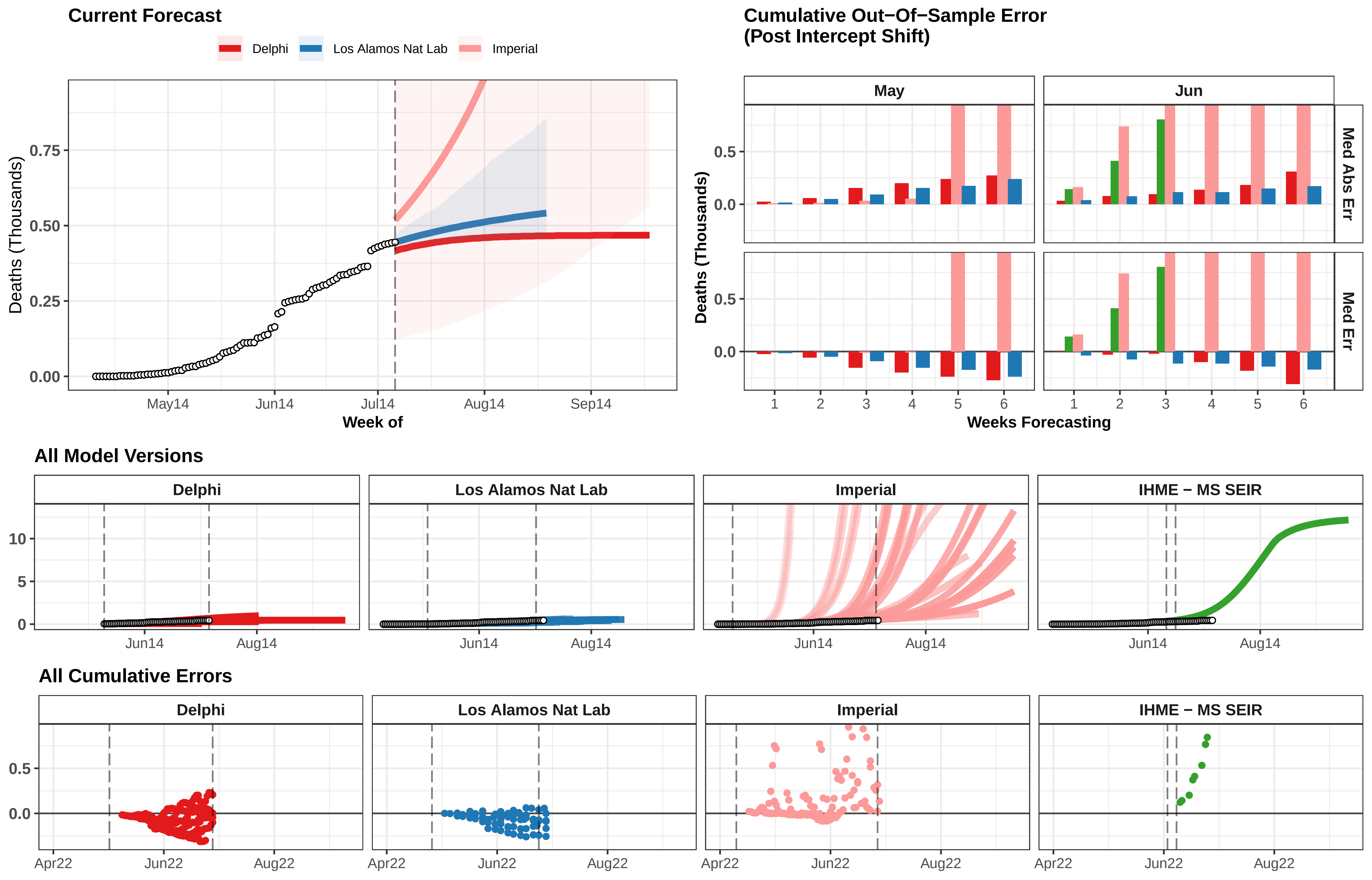

Current Forecast
Delphi
Los Alamos Nat Lab
Imperial
Deaths (Thousands)
0.00
0.25
0.50
0.75
May14
Jun14
Jul14
Aug14
Sep14
Week of
Cumulative Out−Of−Sample Error
(Post Intercept Shift)
May
Jun
Med Abs Err
Med Err
Deaths (Thousands)
0.0
0.5
1
2
3
4
5
6
Weeks Forecasting
All Model Versions
Delphi
Los Alamos Nat Lab
Imperial
IHME − MS SEIR
0
5
10
Jun14
Aug14
All Cumulative Errors
Delphi
Los Alamos Nat Lab
Imperial
IHME − MS SEIR
0.0
0.5
Apr22
Jun22
Aug22

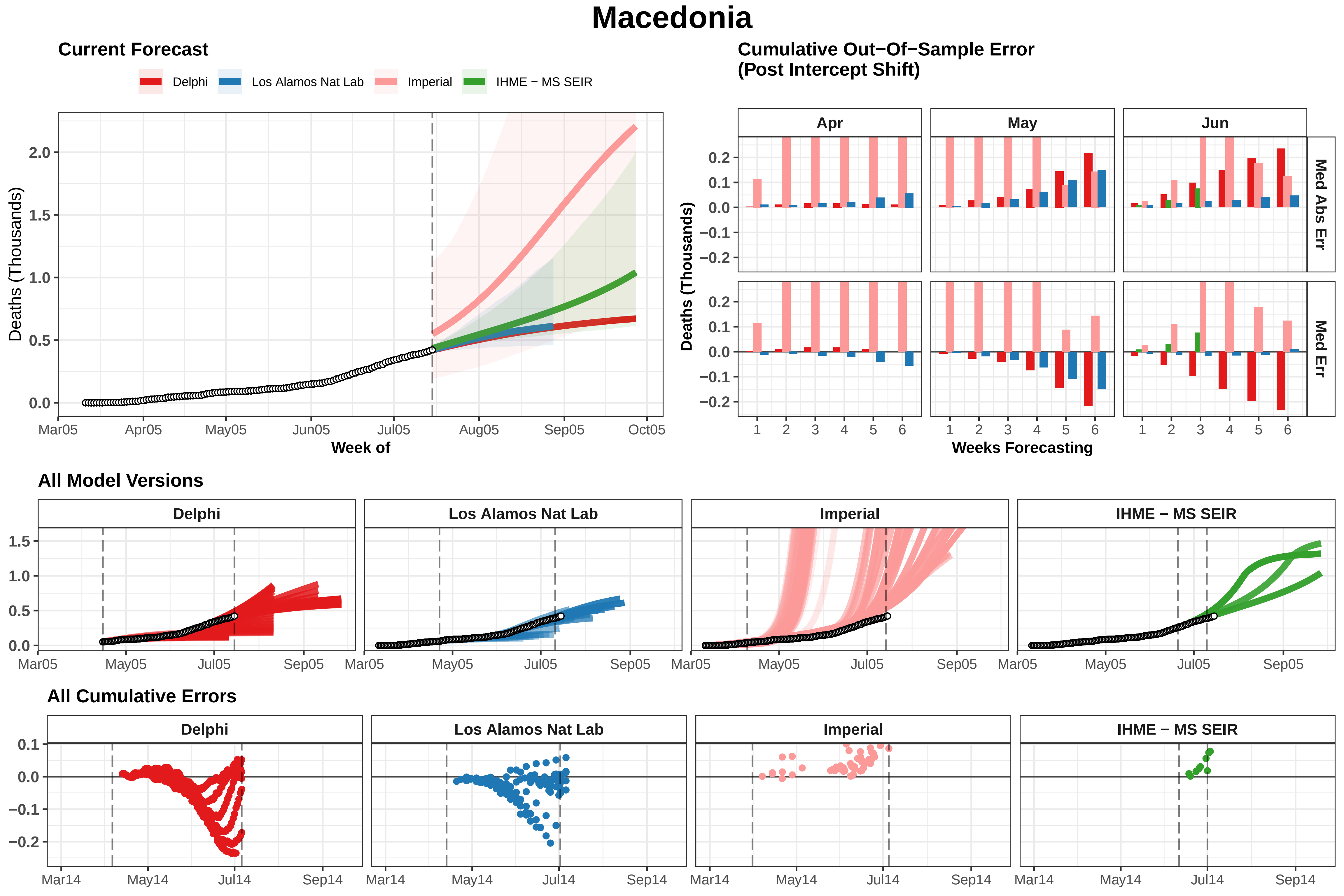

Macedonia
Current Forecast
Delphi
Los Alamos Nat Lab
Imperial
IHME − MS SEIR
Deaths (Thousands)
Week of
Mar05
Apr05
May05
Jun05
Jul05
Aug05
Sep05
Oct05
Cumulative Out−Of−Sample Error
(Post Intercept Shift)
Apr
May
Jun
Med Abs Err
Med Err
Weeks Forecasting
All Model Versions
All Cumulative Errors
Mar14
May14
Jul14
Sep14

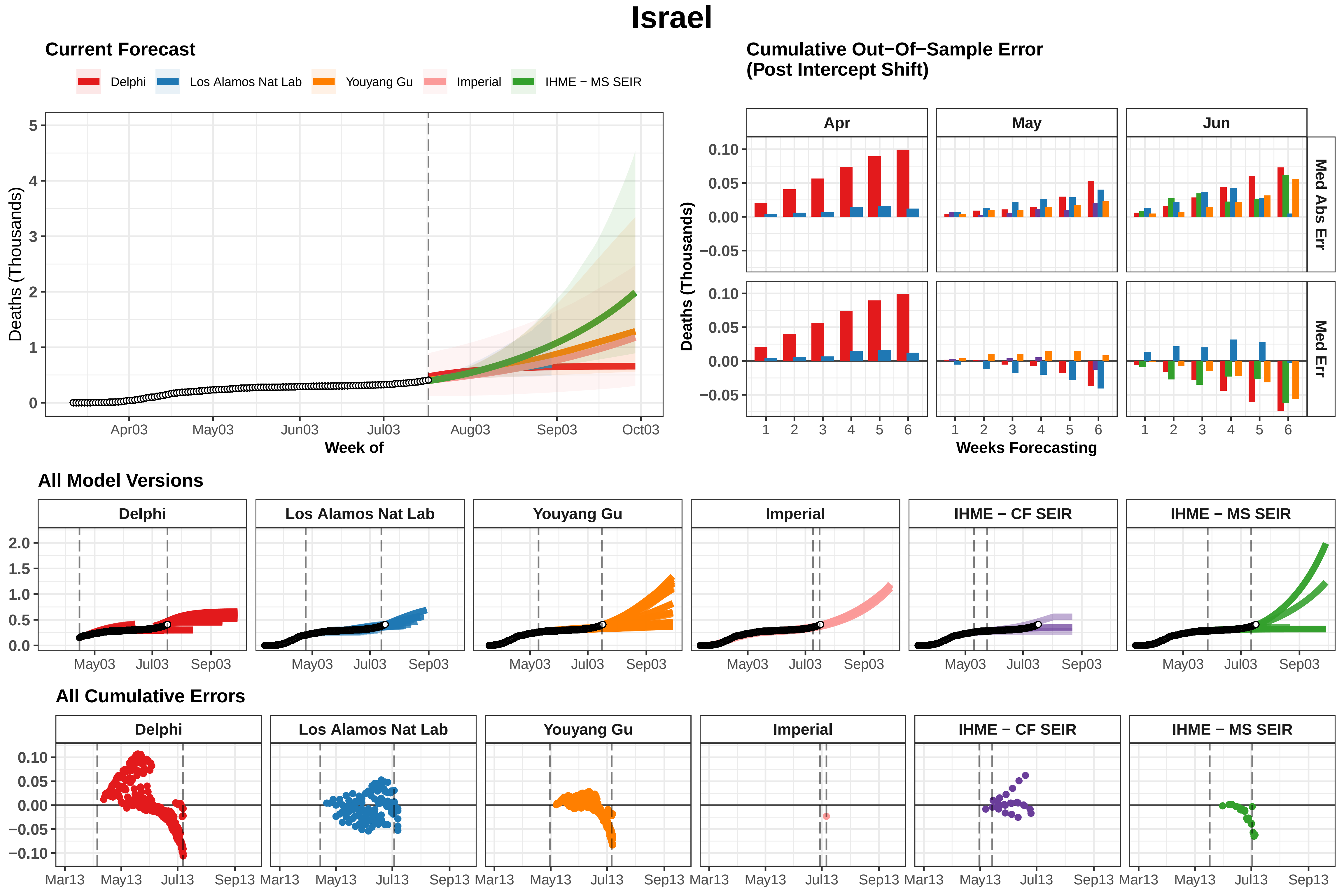
Israel
Current Forecast
Delphi
Los Alamos Nat Lab
Youyang Gu
Imperial
IHME − MS SEIR
Deaths (Thousands)
Week of
Apr03
May03
Jun03
Jul03
Aug03
Sep03
Oct03
Cumulative Out−Of−Sample Error
(Post Intercept Shift)
Apr
May
Jun
Med Abs Err
Med Err
Weeks Forecasting
All Model Versions
Delphi
Los Alamos Nat Lab
Youyang Gu
Imperial
IHME − CF SEIR
IHME − MS SEIR
All Cumulative Errors
Mar13
May13
Jul13
Sep13

Kuwait

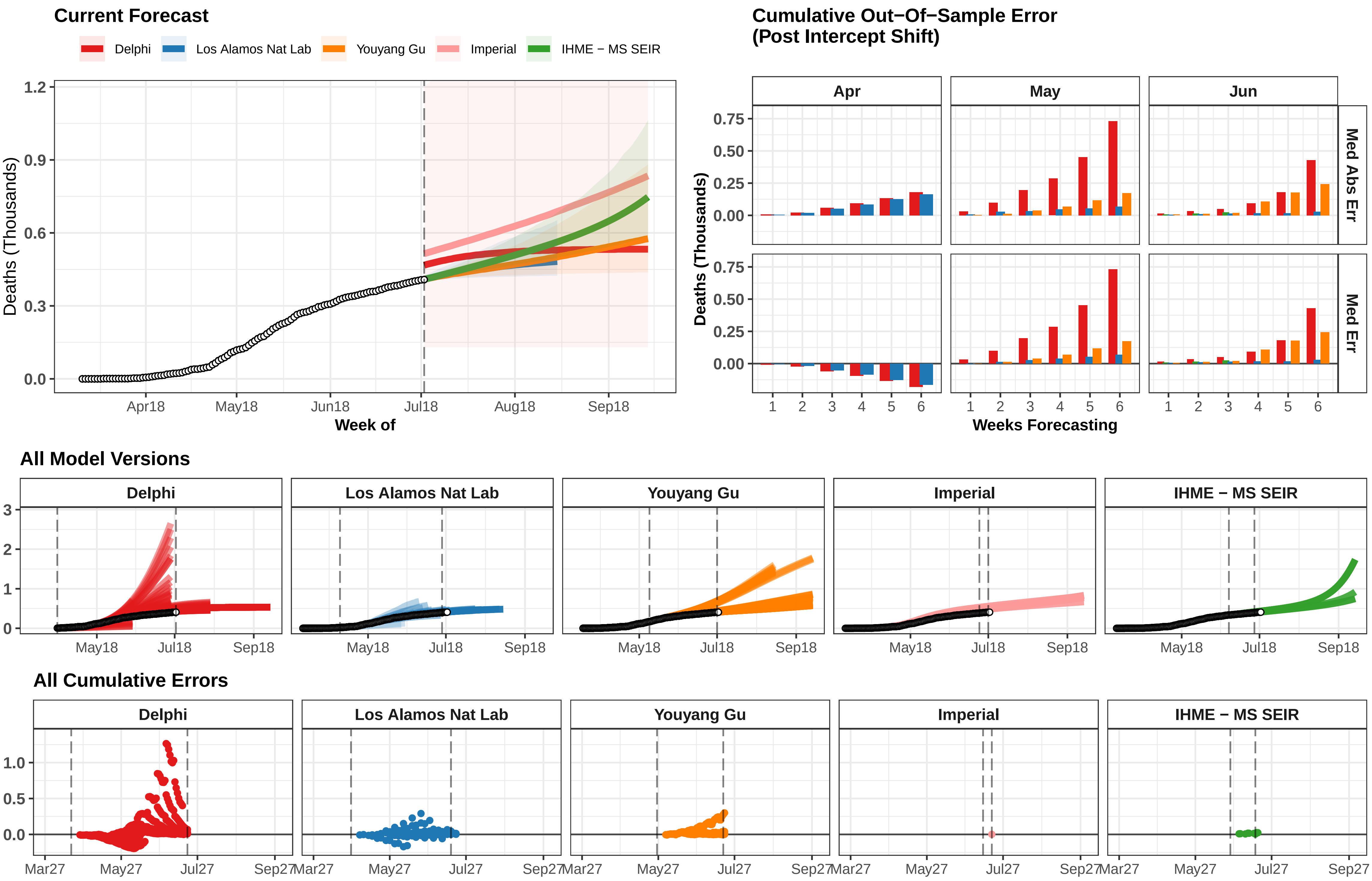

Current Forecast
Delphi
Los Alamos Nat Lab
Youyang Gu
Imperial
IHME − MS SEIR
Deaths (Thousands)
Week of
Apr18
May18
Jun18
Jul18
Aug18
Sep18
Cumulative Out−Of−Sample Error
(Post Intercept Shift)
Apr
May
Jun
Med Abs Err
Med Err
Weeks Forecasting
All Model Versions
All Cumulative Errors
Mar27
May27
Jul27
Sep27

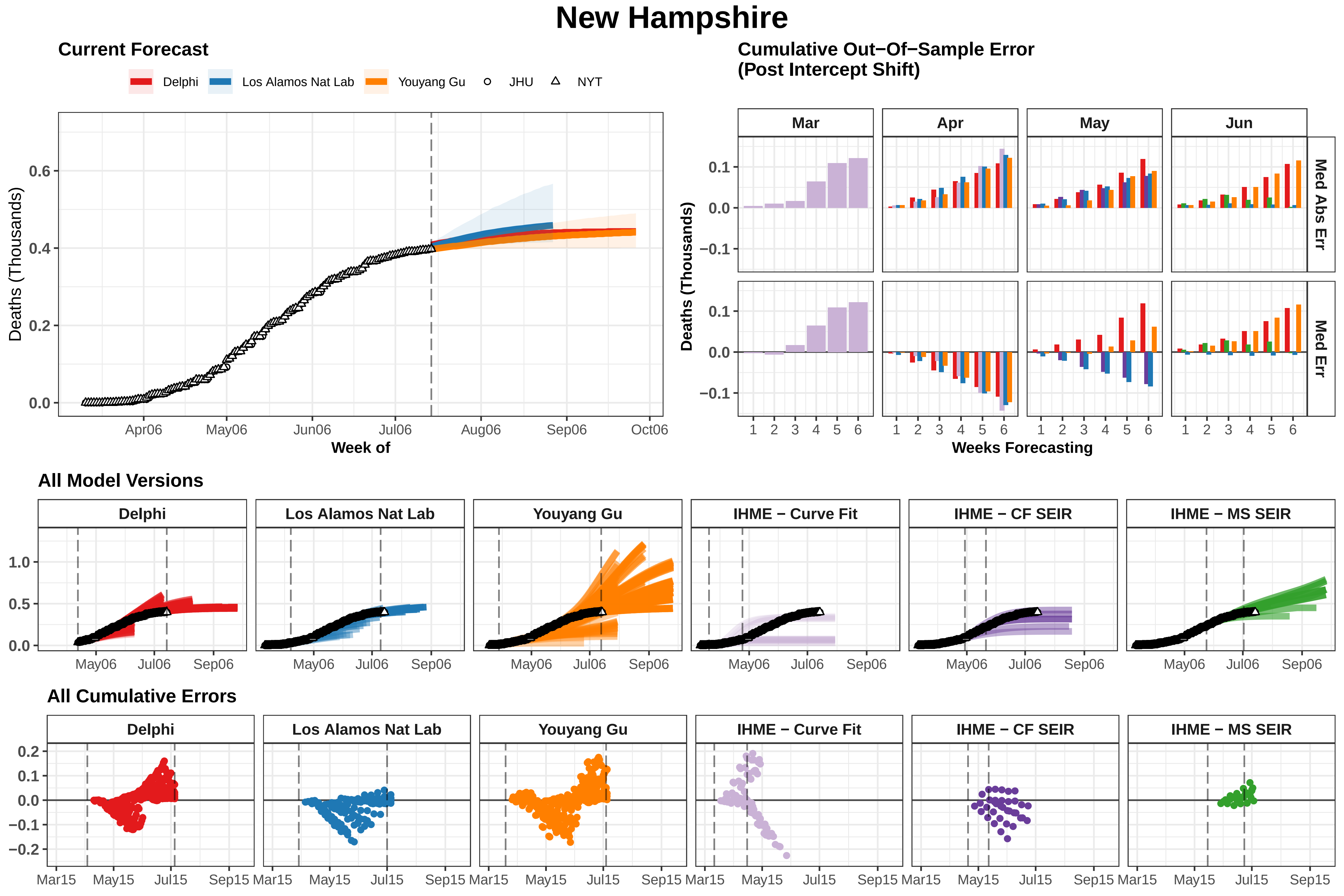

New Hampshire
Current Forecast
Delphi
Los Alamos Nat Lab
Youyang Gu
JHU
NYT
Deaths (Thousands)
Week of
Apr06
May06
Jun06
Jul06
Aug06
Sep06
Oct06
Cumulative Out−Of−Sample Error
(Post Intercept Shift)
Mar
Apr
May
Jun
Med Abs Err
Med Err
Weeks Forecasting
All Model Versions
Delphi
Los Alamos Nat Lab
Youyang Gu
IHME − Curve Fit
IHME − CF SEIR
IHME − MS SEIR
All Cumulative Errors
Mar15
May15
Jul15
Sep15

Kazakhstan

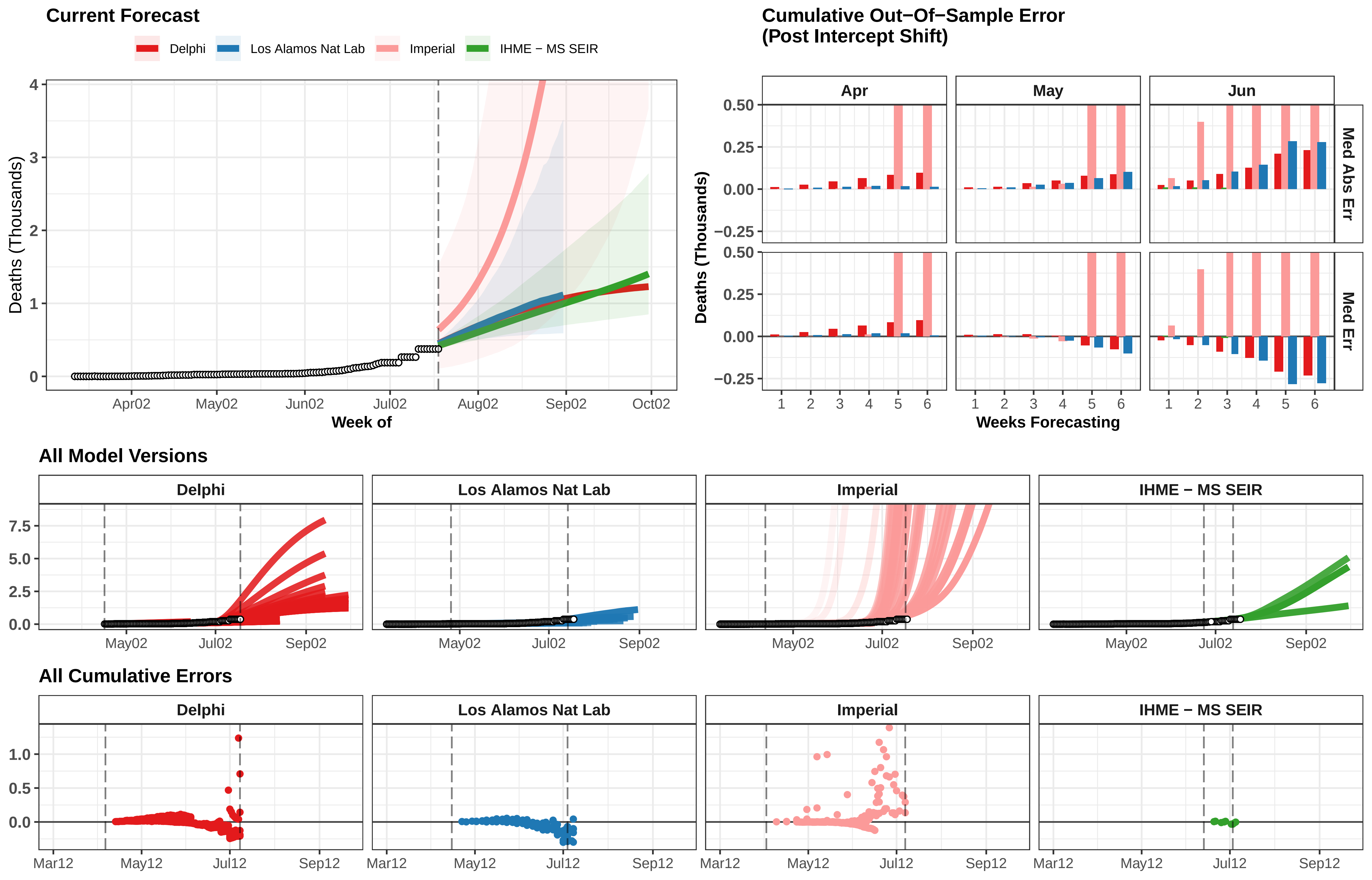
Current Forecast
Delphi
Los Alamos Nat Lab
Imperial
IHME − MS SEIR
Deaths (Thousands)
Week of
Apr02
May02
Jun02
Jul02
Aug02
Sep02
Oct02
Cumulative Out−Of−Sample Error
(Post Intercept Shift)
Apr
May
Jun
Med Abs Err
Med Err
Weeks Forecasting
All Model Versions
All Cumulative Errors
Mar12
May12
Jul12
Sep12

Cameroon

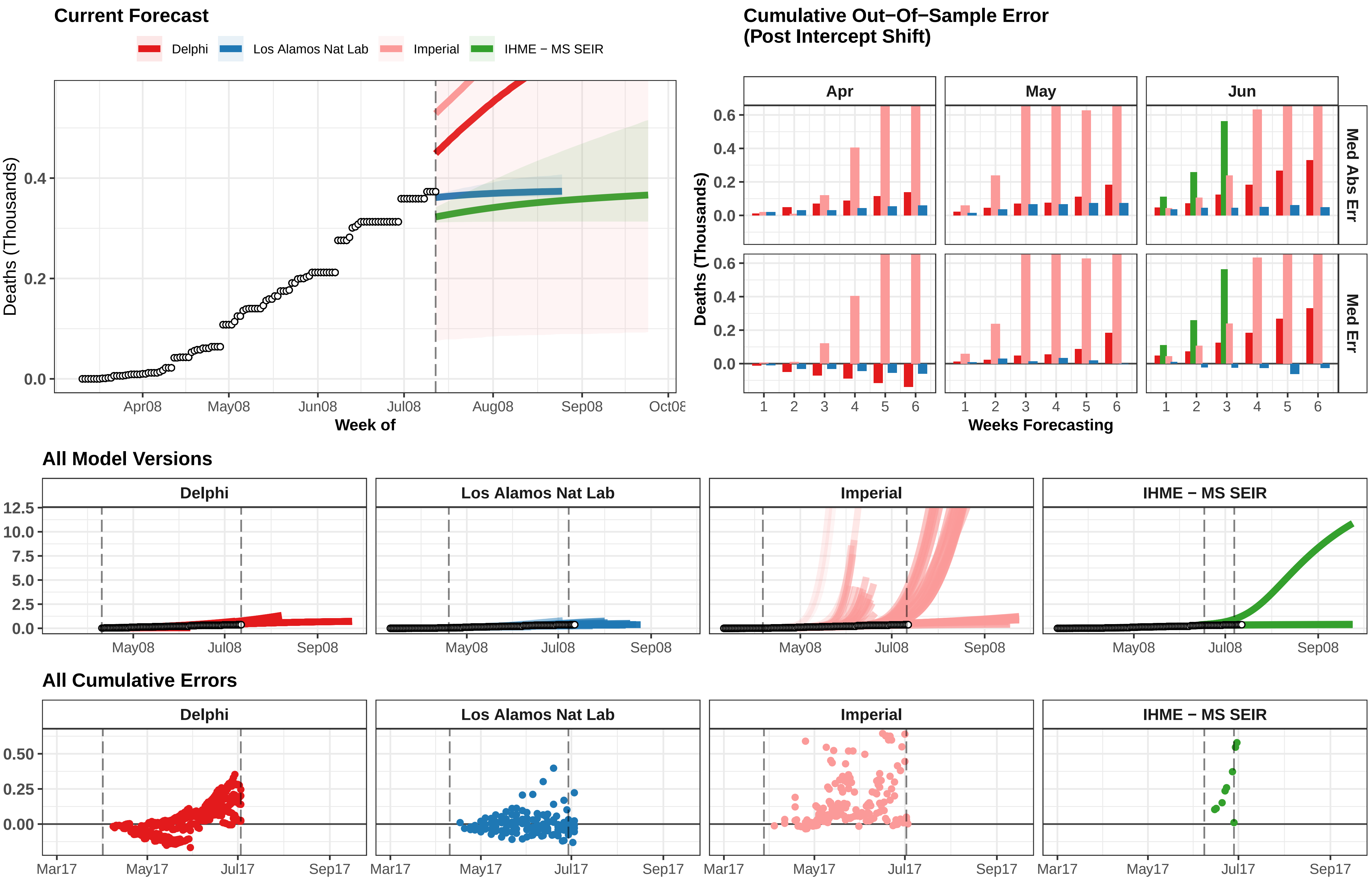

Current Forecast
Delphi
Los Alamos Nat Lab
Imperial
IHME − MS SEIR
Deaths (Thousands)
0.0
0.2
0.4
Apr08
May08
Jun08
Jul08
Aug08
Sep08
Oct08
Week of
Cumulative Out−Of−Sample Error
(Post Intercept Shift)
Apr
May
Jun
Med Abs Err
Med Err
0.6
0.4
0.2
0.0
1
2
3
4
5
6
Weeks Forecasting
All Model Versions
12.5
10.0
7.5
5.0
2.5
0.0
Sep08
All Cumulative Errors
0.50
0.25
0.00
Mar17
May17
Jul17
Sep17

Czech Republic

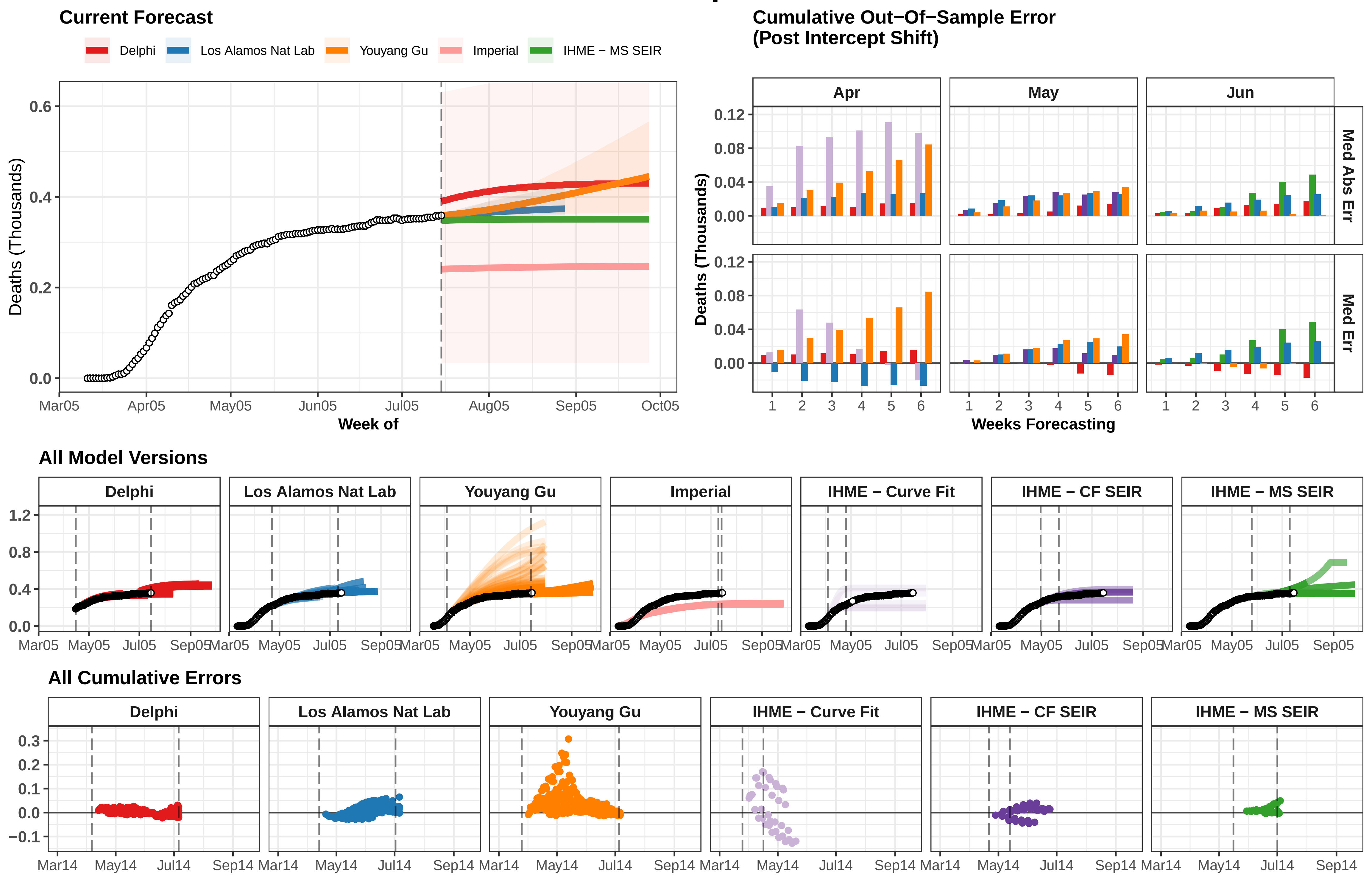

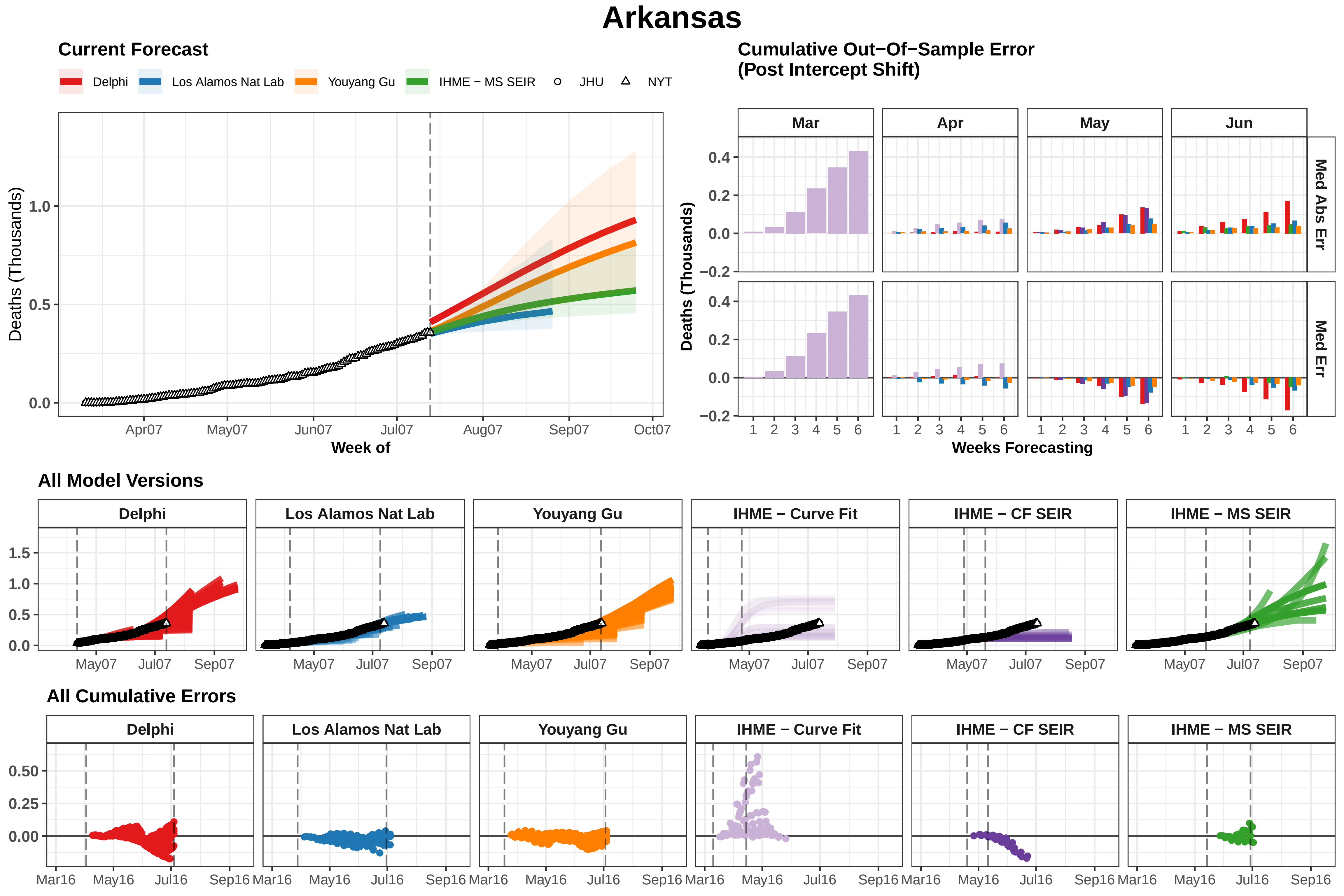
Arkansas
Current Forecast
Delphi
Los Alamos Nat Lab
Youyang Gu
IHME − MS SEIR
JHU
NYT
Deaths (Thousands)
Week of
Apr07
May07
Jun07
Jul07
Aug07
Sep07
Oct07
Cumulative Out−Of−Sample Error
(Post Intercept Shift)
Mar
Apr
May
Jun
Med Abs Err
Med Err
Weeks Forecasting
All Model Versions
Delphi
Los Alamos Nat Lab
Youyang Gu
IHME − Curve Fit
IHME − CF SEIR
IHME − MS SEIR
All Cumulative Errors
Mar16
May16
Jul16
Sep16

Azerbaijan

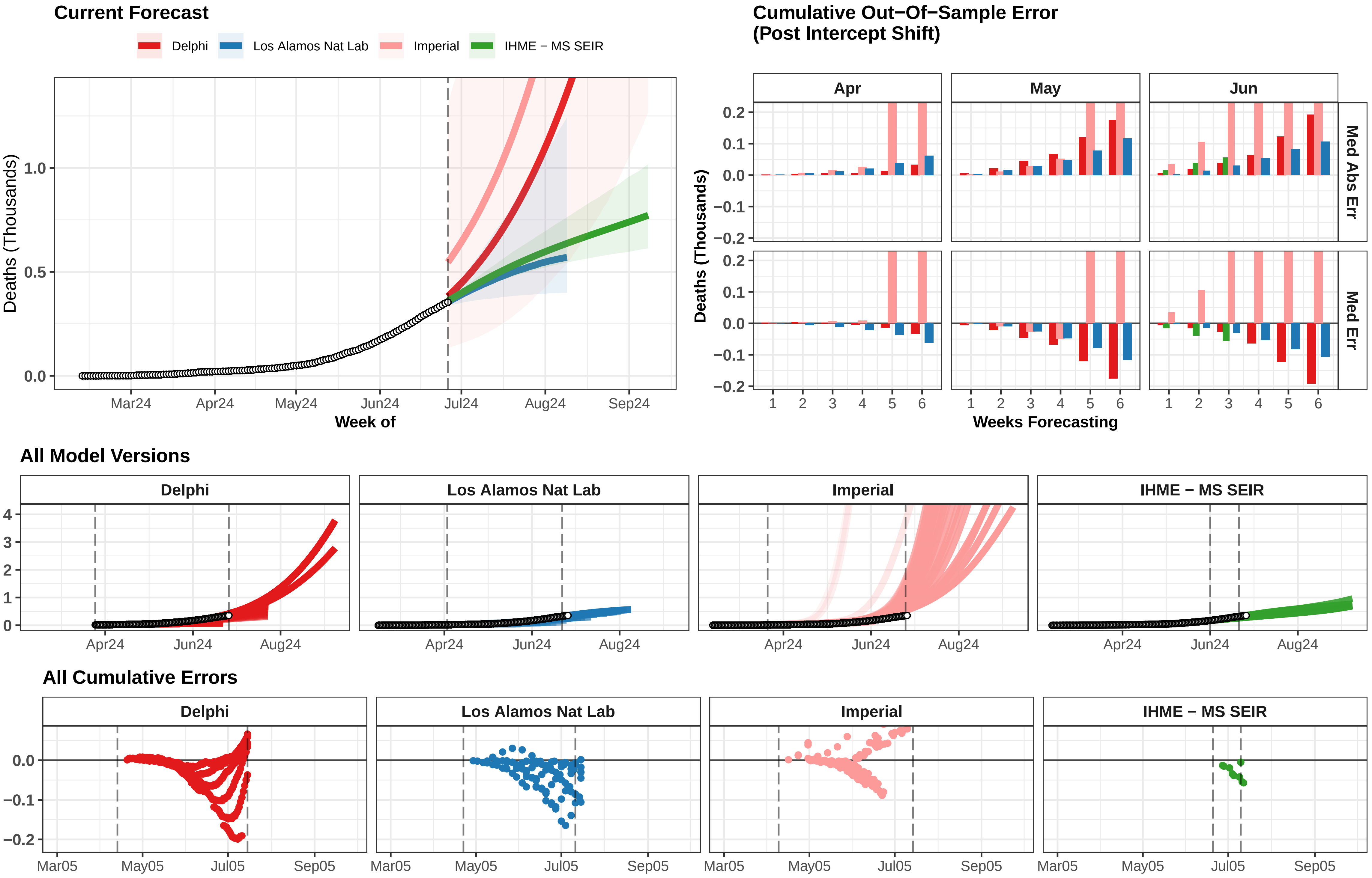

Current Forecast
Delphi
Los Alamos Nat Lab
Imperial
IHME − MS SEIR
Deaths (Thousands)
Week of
Mar24
Apr24
May24
Jun24
Jul24
Aug24
Sep24
Cumulative Out−Of−Sample Error
(Post Intercept Shift)
Apr
May
Jun
Med Abs Err
Med Err
Weeks Forecasting
All Model Versions
All Cumulative Errors
Mar05
May05
Jul05
Sep05

United Arab Emirates

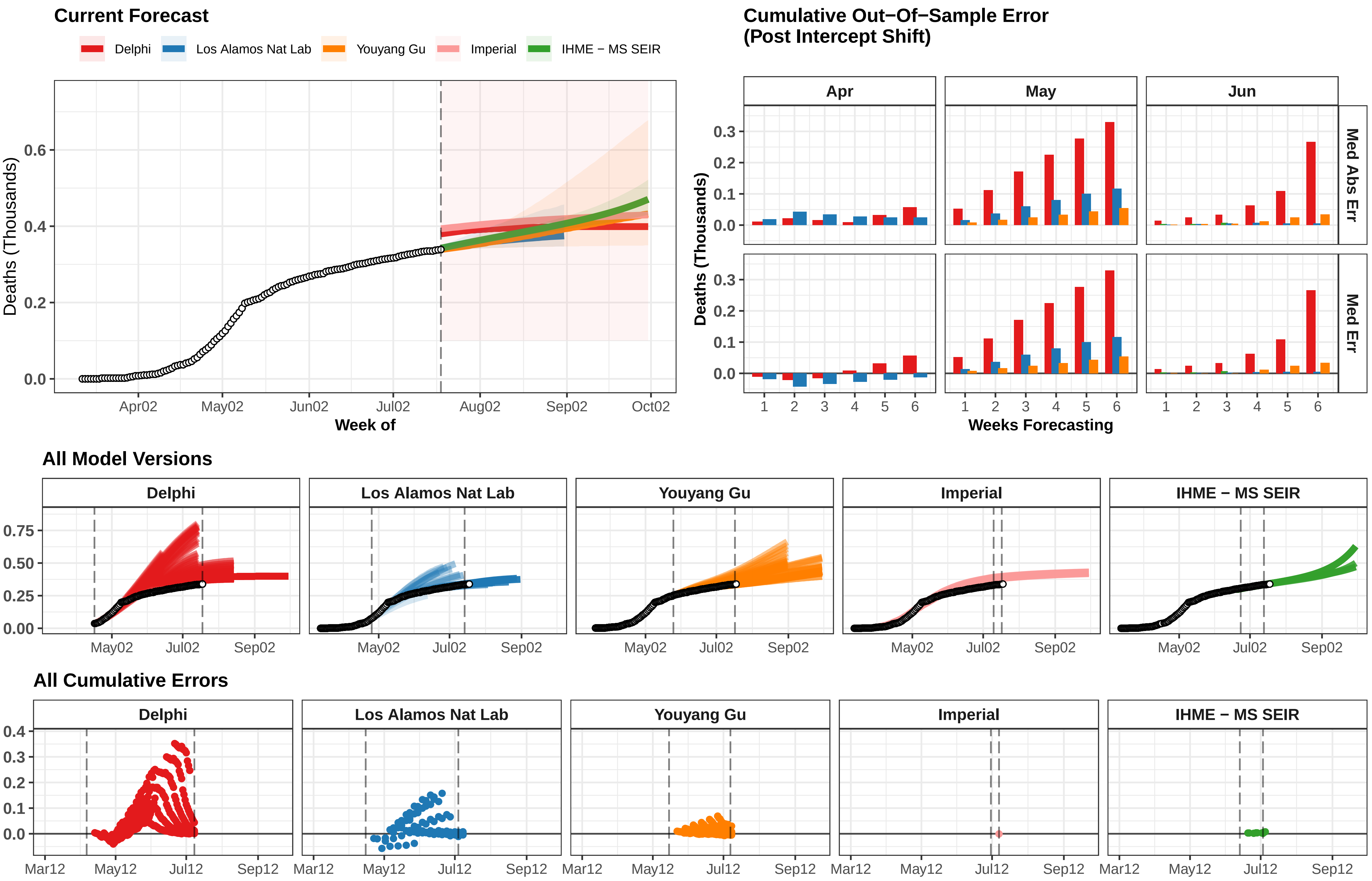

El Salvador

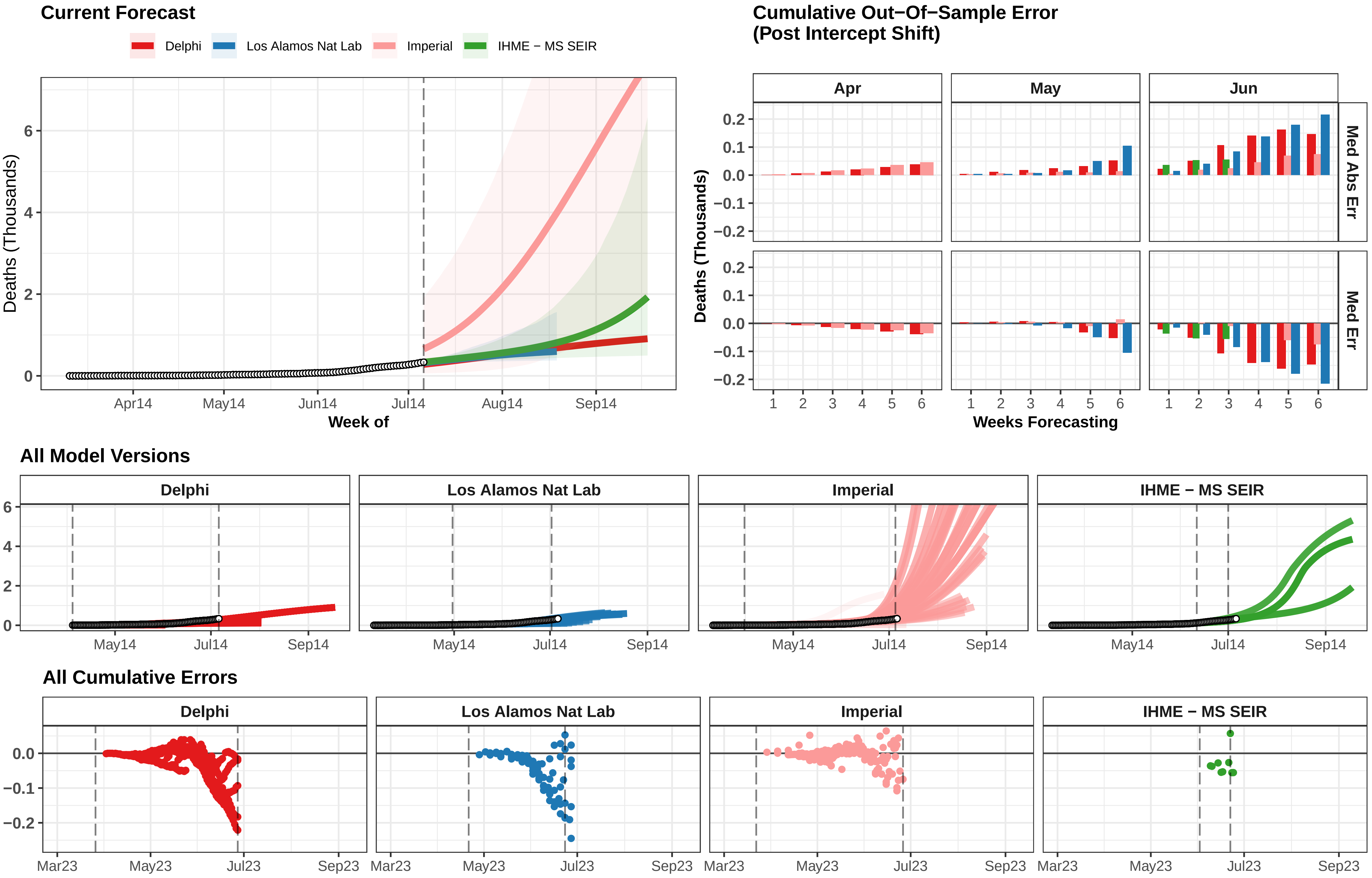

Current Forecast
Delphi
Los Alamos Nat Lab
Imperial
IHME − MS SEIR
Deaths (Thousands)
Week of
Apr14
May14
Jun14
Jul14
Aug14
Sep14
Cumulative Out−Of−Sample Error
(Post Intercept Shift)
Apr
May
Jun
Med Abs Err
Med Err
Weeks Forecasting
All Model Versions
All Cumulative Errors
Mar23
May23
Jul23
Sep23

Finland

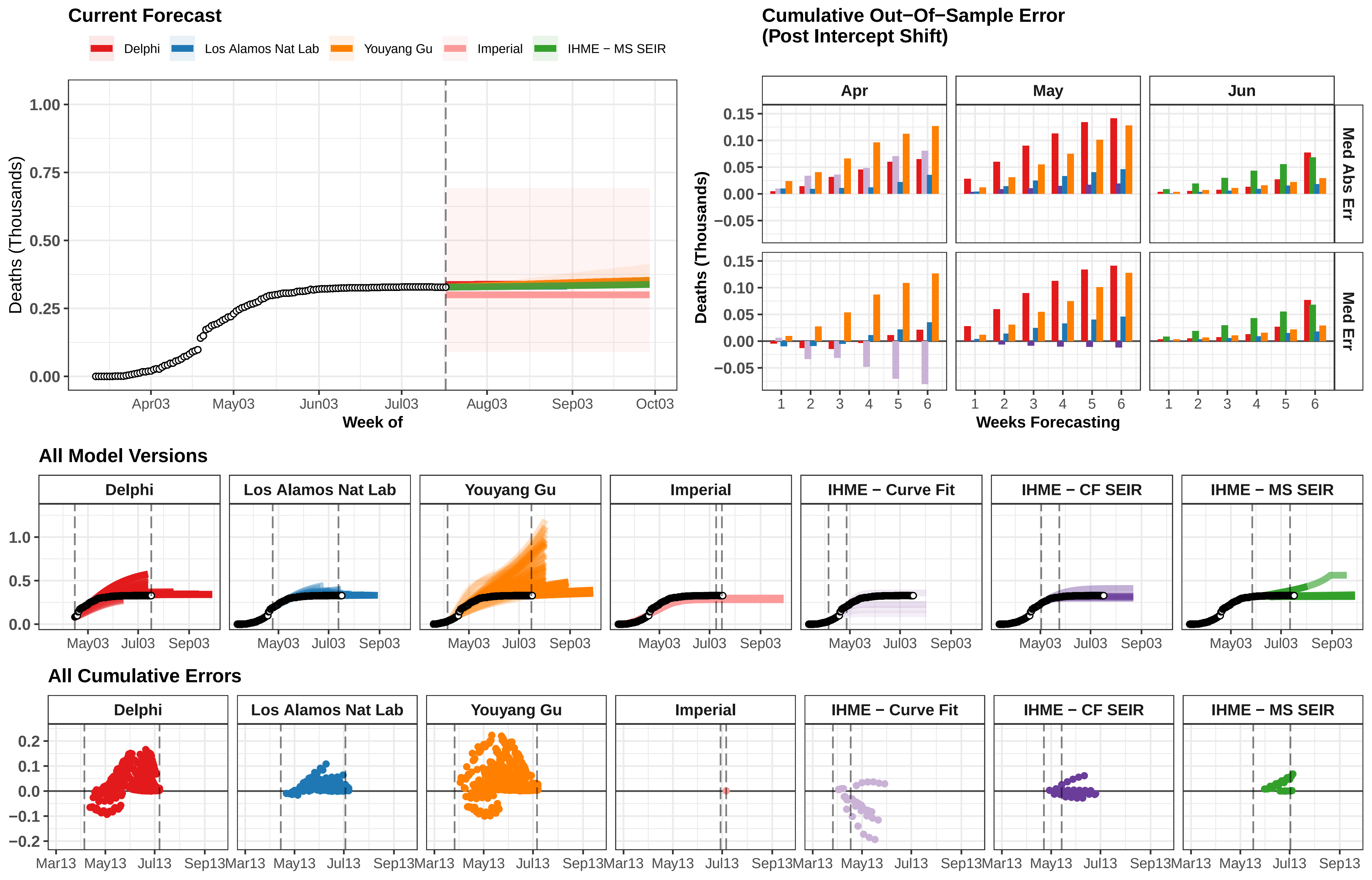
Current Forecast
Delphi
Los Alamos Nat Lab
Youyang Gu
Imperial
IHME − MS SEIR
Deaths (Thousands)
1.00
0.75
0.50
0.25
0.00
Apr03
May03
Jun03
Jul03
Aug03
Sep03
Oct03
Week of
Cumulative Out−Of−Sample Error
(Post Intercept Shift)
Apr
May
Jun
Med Abs Err
Med Err
0.15
0.10
0.05
0.00
−0.05
1
2
3
4
5
6
Weeks Forecasting
All Model Versions
Delphi
Los Alamos Nat Lab
Youyang Gu
Imperial
IHME − Curve Fit
IHME − CF SEIR
IHME − MS SEIR
1.0
0.5
0.0
May03
Jul03
Sep03
All Cumulative Errors
0.2
0.1
0.0
−0.1
−0.2
Mar13
May13
Jul13
Sep13

Oman

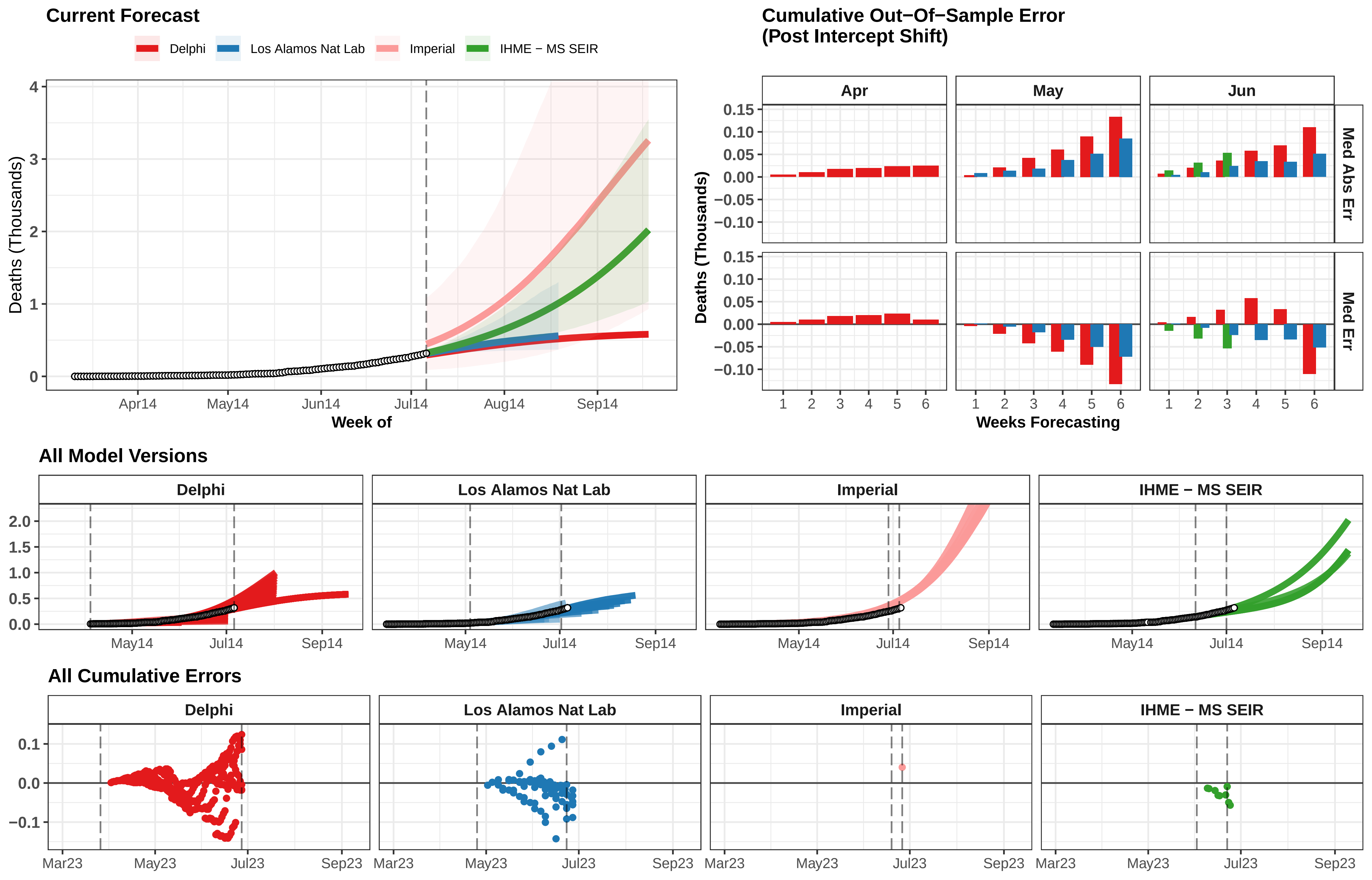
Current Forecast
Delphi
Los Alamos Nat Lab
Imperial
IHME − MS SEIR
Deaths (Thousands)
Week of
Apr14
May14
Jun14
Jul14
Aug14
Sep14
Cumulative Out−Of−Sample Error
(Post Intercept Shift)
Apr
May
Jun
Med Abs Err
Med Err
Weeks Forecasting
All Model Versions
All Cumulative Errors
Mar23
May23
Jul23
Sep23

Kansas

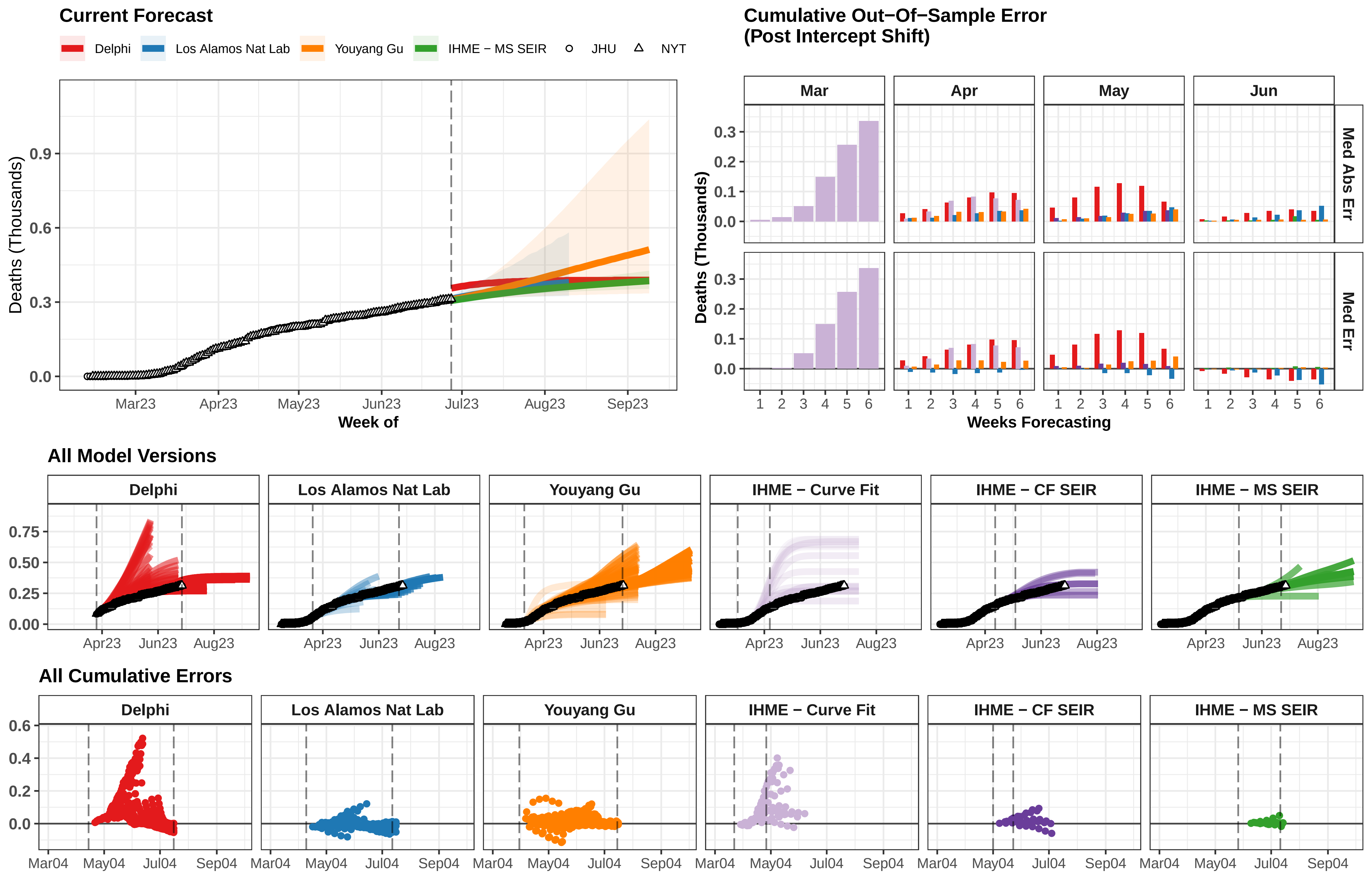

Current Forecast
Delphi
Los Alamos Nat Lab
Youyang Gu
IHME − MS SEIR
JHU
NYT
Deaths (Thousands)
0.0
0.3
0.6
0.9
Mar23
Apr23
May23
Jun23
Jul23
Aug23
Sep23
Week of
Cumulative Out−Of−Sample Error
(Post Intercept Shift)
Mar
Apr
May
Jun
Med Abs Err
Med Err
Deaths (Thousands)
0.0
0.1
0.2
0.3
1
2
3
4
5
6
Weeks Forecasting
All Model Versions
Delphi
Los Alamos Nat Lab
Youyang Gu
IHME − Curve Fit
IHME − CF SEIR
IHME − MS SEIR
0.00
0.25
0.50
0.75
Apr23
Jun23
Aug23
All Cumulative Errors
Delphi
Los Alamos Nat Lab
Youyang Gu
IHME − Curve Fit
IHME − CF SEIR
IHME − MS SEIR
0.0
0.2
0.4
0.6
Mar04
May04
Jul04
Sep04

Nebraska

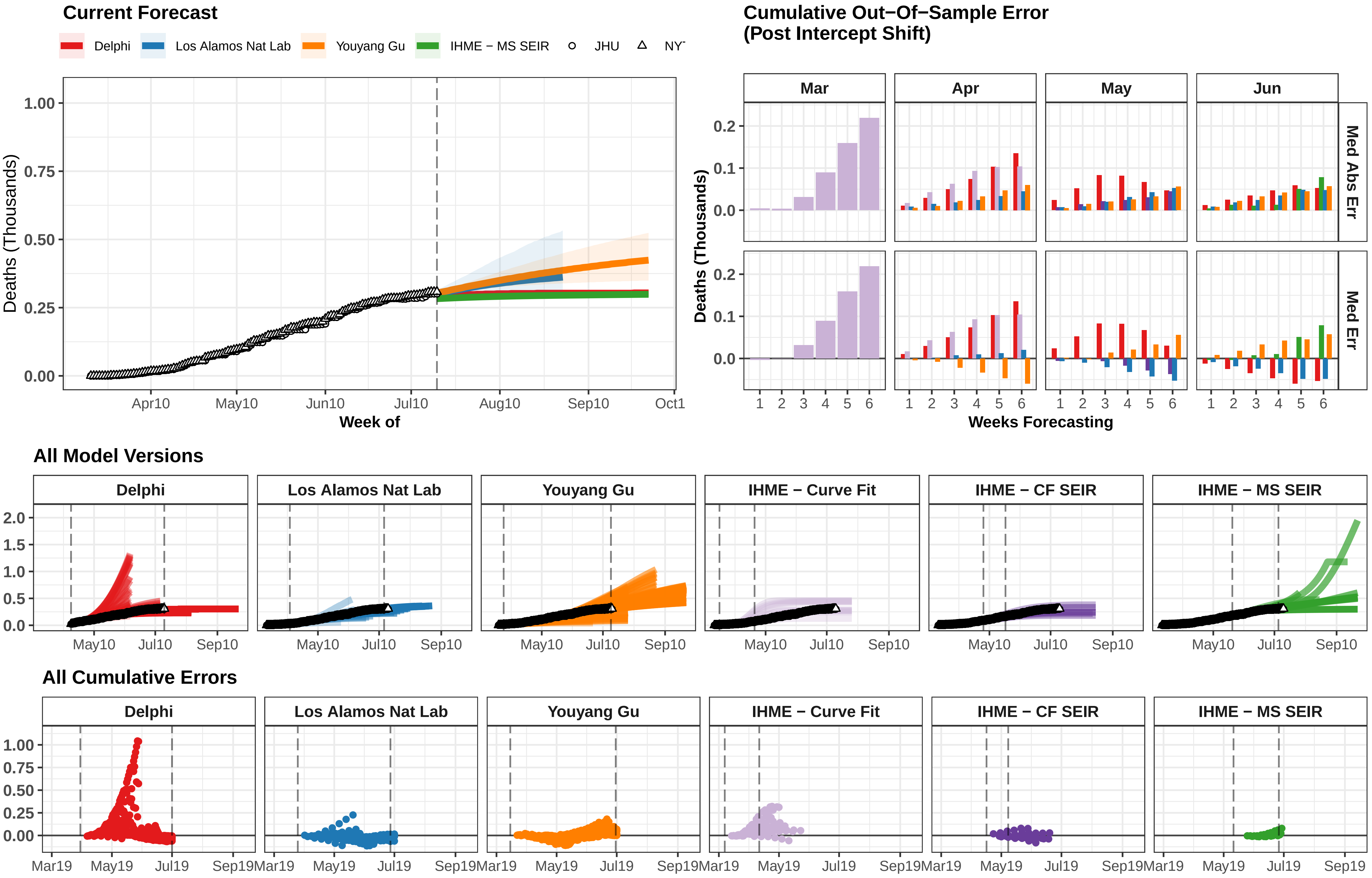

Current Forecast
Delphi
Los Alamos Nat Lab
Youyang Gu
IHME − MS SEIR
JHU
NY
Deaths (Thousands)
1.00
0.75
0.50
0.25
0.00
Apr10
May10
Jun10
Jul10
Aug10
Sep10
Oct1
Week of
Cumulative Out−Of−Sample Error
(Post Intercept Shift)
Mar
Apr
May
Jun
Med Abs Err
Med Err
0.2
0.1
0.0
1
2
3
4
5
6
Weeks Forecasting
All Model Versions
Delphi
Los Alamos Nat Lab
Youyang Gu
IHME − Curve Fit
IHME − CF SEIR
IHME − MS SEIR
2.0
1.5
1.0
0.5
0.0
May10
Jul10
Sep10
All Cumulative Errors
1.00
0.75
0.50
0.25
0.00
Mar19
May19
Jul19
Sep19

Bulgaria

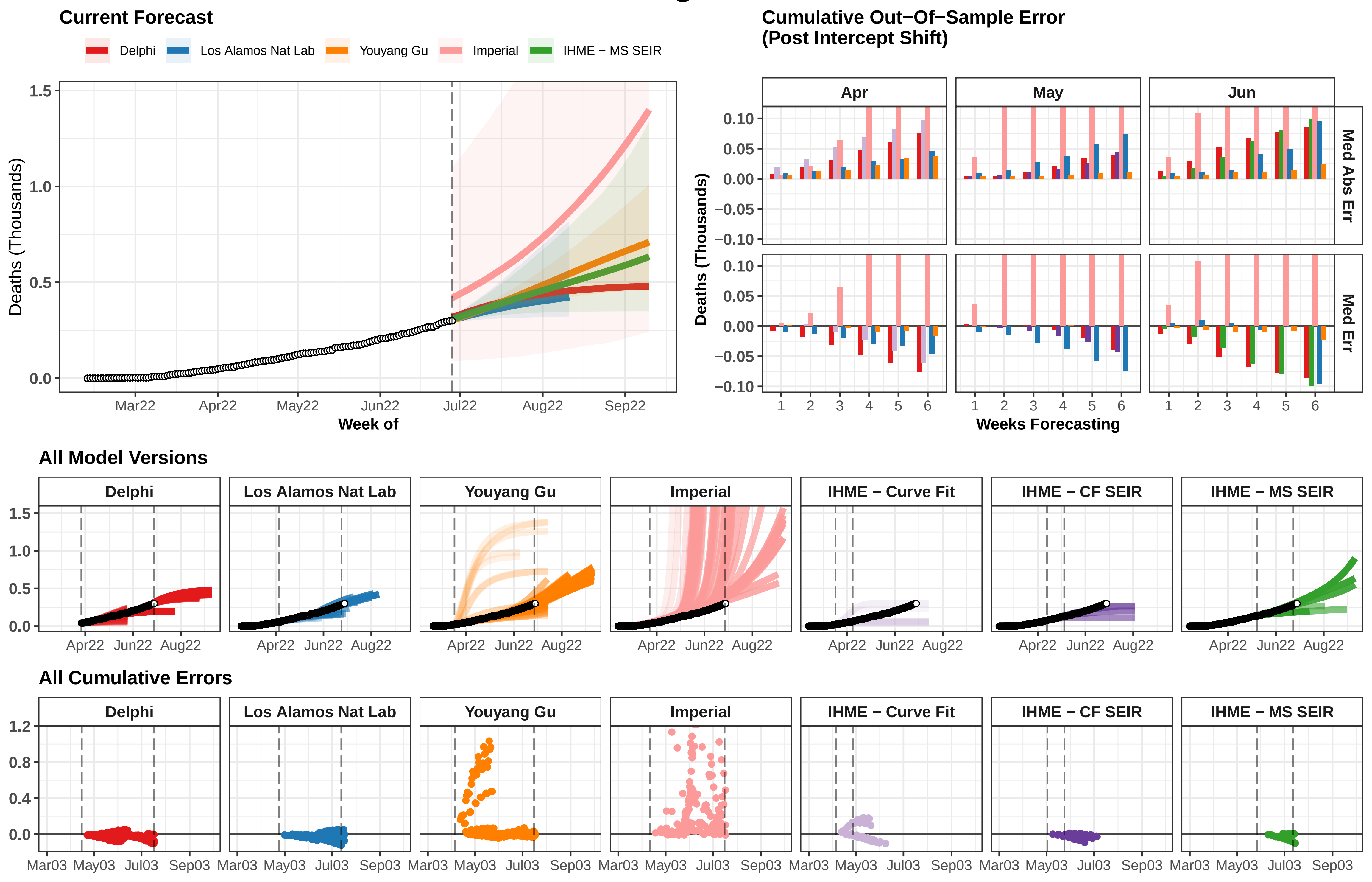
Current Forecast
Delphi
Los Alamos Nat Lab
Youyang Gu
Imperial
IHME − MS SEIR
Deaths (Thousands)
0.0
0.5
1.0
1.5
Mar22
Apr22
May22
Jun22
Jul22
Aug22
Sep22
Week of
Cumulative Out−Of−Sample Error
(Post Intercept Shift)
Apr
May
Jun
Med Abs Err
Med Err
Deaths (Thousands)
0.10
0.05
0.00
−0.05
−0.10
1
2
3
4
5
6
Weeks Forecasting
All Model Versions
Delphi
Los Alamos Nat Lab
Youyang Gu
Imperial
IHME − Curve Fit
IHME − CF SEIR
IHME − MS SEIR
0.0
0.5
1.0
1.5
Apr22
Jun22
Aug22
All Cumulative Errors
Delphi
Los Alamos Nat Lab
Youyang Gu
Imperial
IHME − Curve Fit
IHME − CF SEIR
IHME − MS SEIR
0.0
0.4
0.8
1.2
Mar03
May03
Jul03
Sep03

South Korea

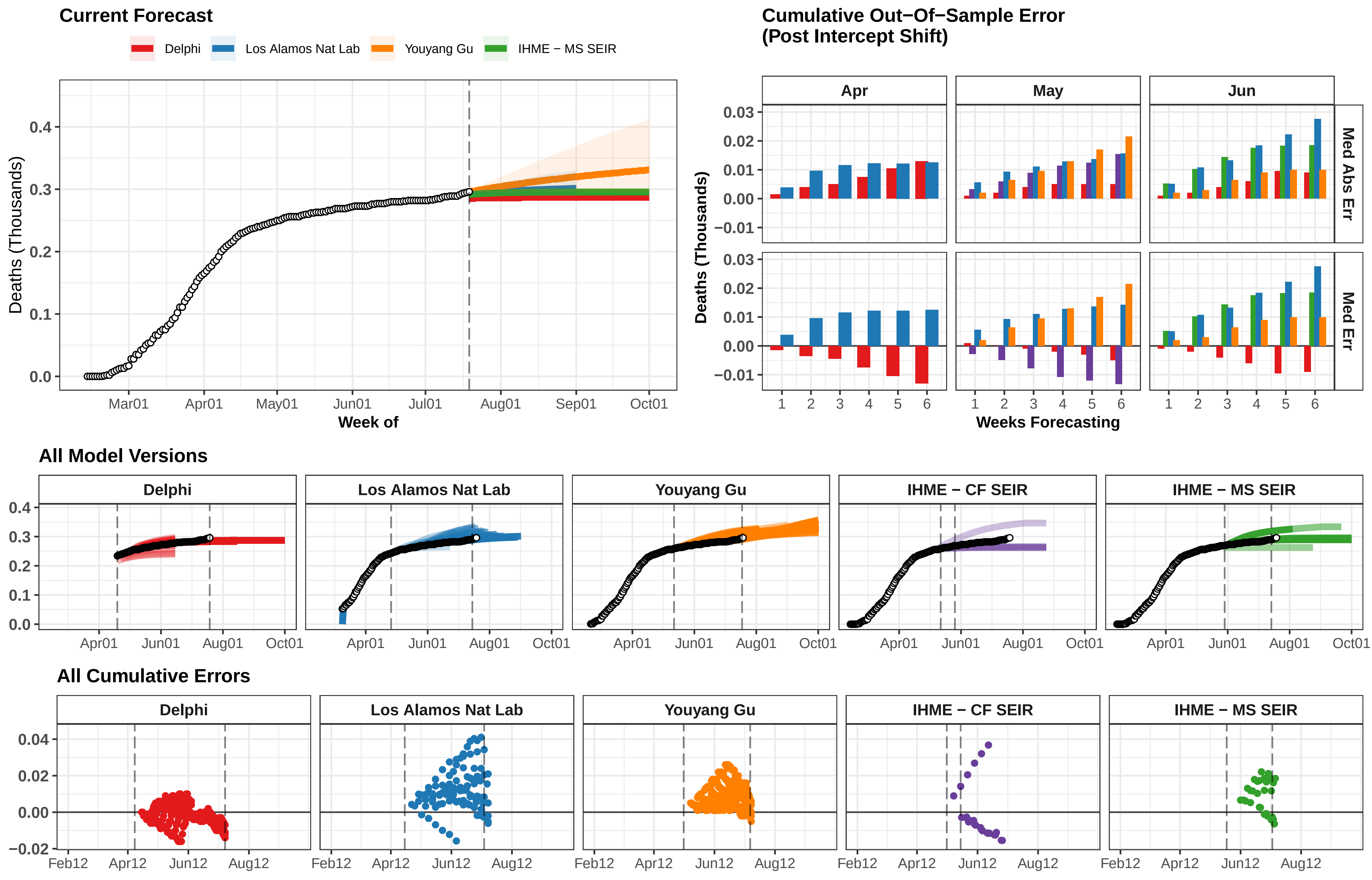

Current Forecast
Delphi
Los Alamos Nat Lab
Youyang Gu
IHME − MS SEIR
Deaths (Thousands)
0.0
0.1
0.2
0.3
0.4
Mar01
Apr01
May01
Jun01
Jul01
Aug01
Sep01
Oct01
Week of
Cumulative Out−Of−Sample Error
(Post Intercept Shift)
Apr
May
Jun
Med Abs Err
Med Err
−0.01
0.00
0.01
0.02
0.03
1
2
3
4
5
6
Weeks Forecasting
All Model Versions
Delphi
Los Alamos Nat Lab
Youyang Gu
IHME − CF SEIR
IHME − MS SEIR
Apr01
Jun01
Aug01
Oct01
All Cumulative Errors
−0.02
0.00
0.02
0.04
Feb12
Apr12
Jun12
Aug12

Morocco

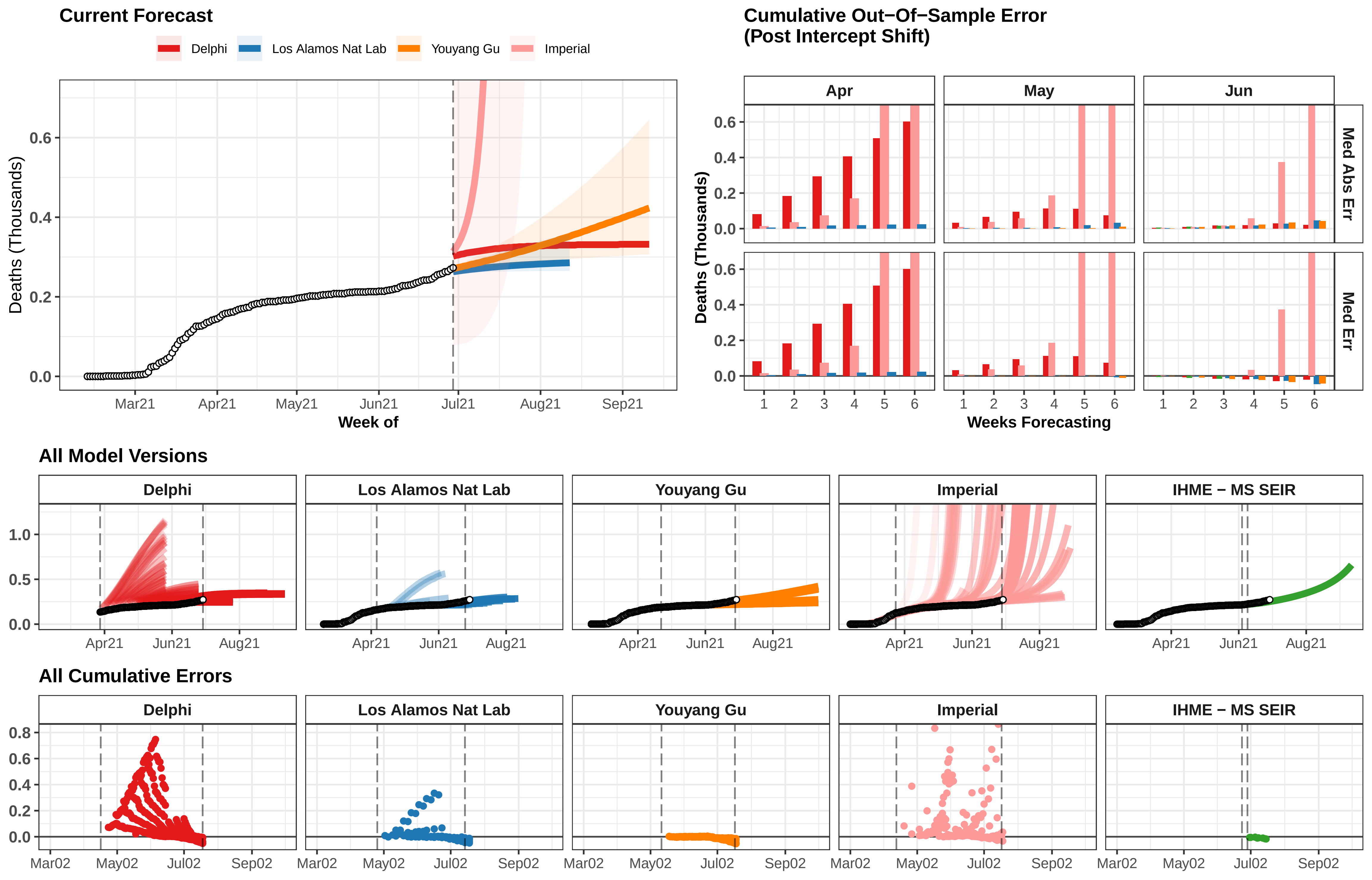

Current Forecast
Delphi
Los Alamos Nat Lab
Youyang Gu
Imperial
Deaths (Thousands)
0.0
0.2
0.4
0.6
Mar21
Apr21
May21
Jun21
Jul21
Aug21
Sep21
Week of
Cumulative Out−Of−Sample Error
(Post Intercept Shift)
Apr
May
Jun
Med Abs Err
Med Err
Weeks Forecasting
1
2
3
4
5
6
All Model Versions
Delphi
Los Alamos Nat Lab
Youyang Gu
Imperial
IHME − MS SEIR
0.0
0.5
1.0
Apr21
Jun21
Aug21
All Cumulative Errors
0.8
Mar02
May02
Jul02
Sep02

Oregon

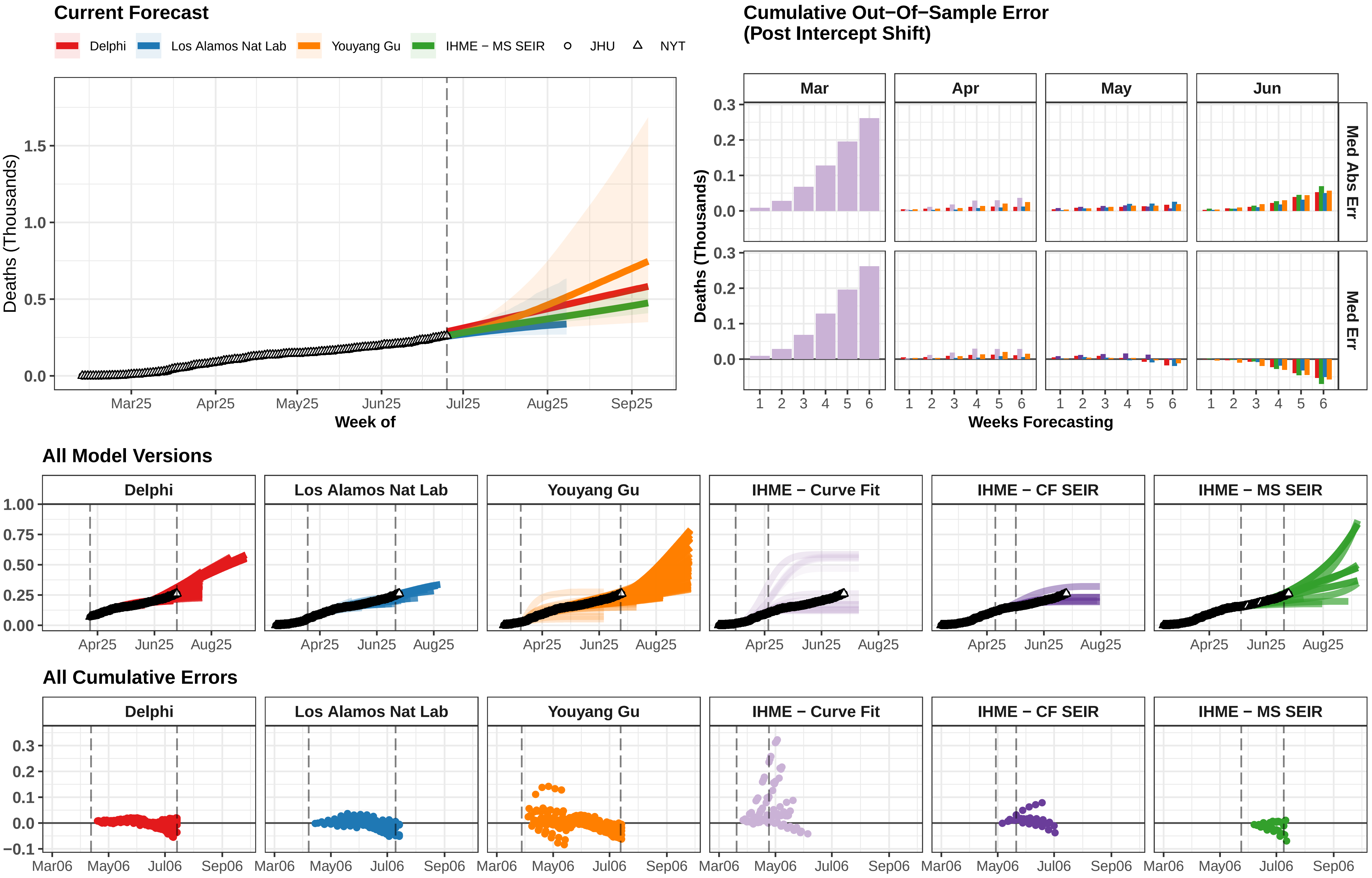

Current Forecast
Delphi
Los Alamos Nat Lab
Youyang Gu
IHME − MS SEIR
JHU
NYT
Deaths (Thousands)
0.0
0.5
1.0
1.5
Mar25
Apr25
May25
Jun25
Jul25
Aug25
Sep25
Week of
Cumulative Out−Of−Sample Error
(Post Intercept Shift)
Mar
Apr
May
Jun
Med Abs Err
Med Err
Deaths (Thousands)
0.3
0.2
0.1
0.0
1
2
3
4
5
6
Weeks Forecasting
All Model Versions
Delphi
Los Alamos Nat Lab
Youyang Gu
IHME − Curve Fit
IHME − CF SEIR
IHME − MS SEIR
1.00
0.75
0.50
0.25
0.00
Apr25
Jun25
Aug25
All Cumulative Errors
0.3
0.2
0.1
0.0
−0.1
Mar06
May06
Jul06
Sep06

Norway

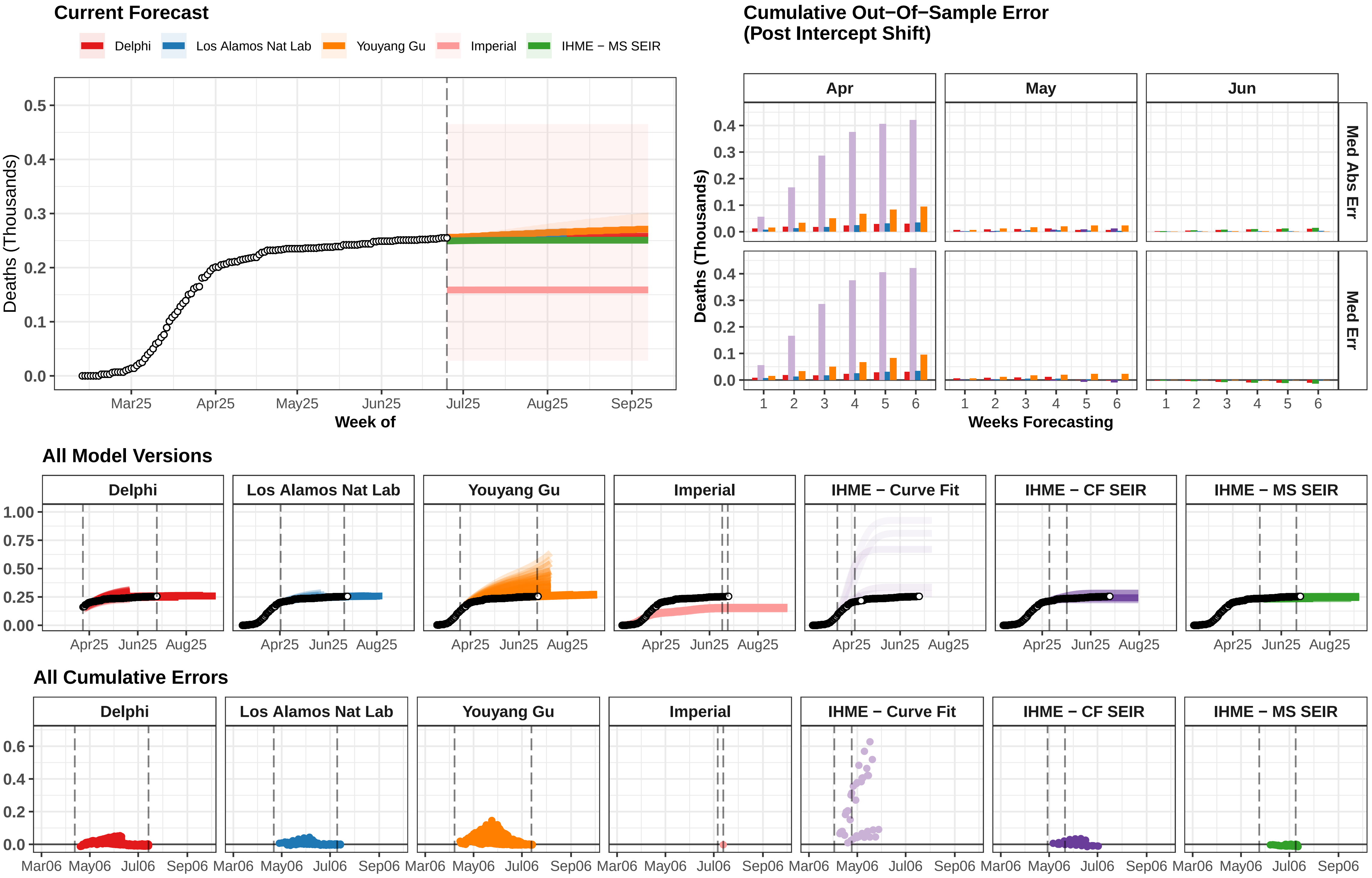

Current Forecast
Delphi
Los Alamos Nat Lab
Youyang Gu
Imperial
IHME − MS SEIR
Deaths (Thousands)
Week of
Mar25
Apr25
May25
Jun25
Jul25
Aug25
Sep25
Cumulative Out−Of−Sample Error
(Post Intercept Shift)
Apr
May
Jun
Med Abs Err
Med Err
Weeks Forecasting
All Model Versions
Delphi
Los Alamos Nat Lab
Youyang Gu
Imperial
IHME − Curve Fit
IHME − CF SEIR
IHME − MS SEIR
All Cumulative Errors
Mar06
May06
Jul06
Sep06

Bosnia and Herzegovina

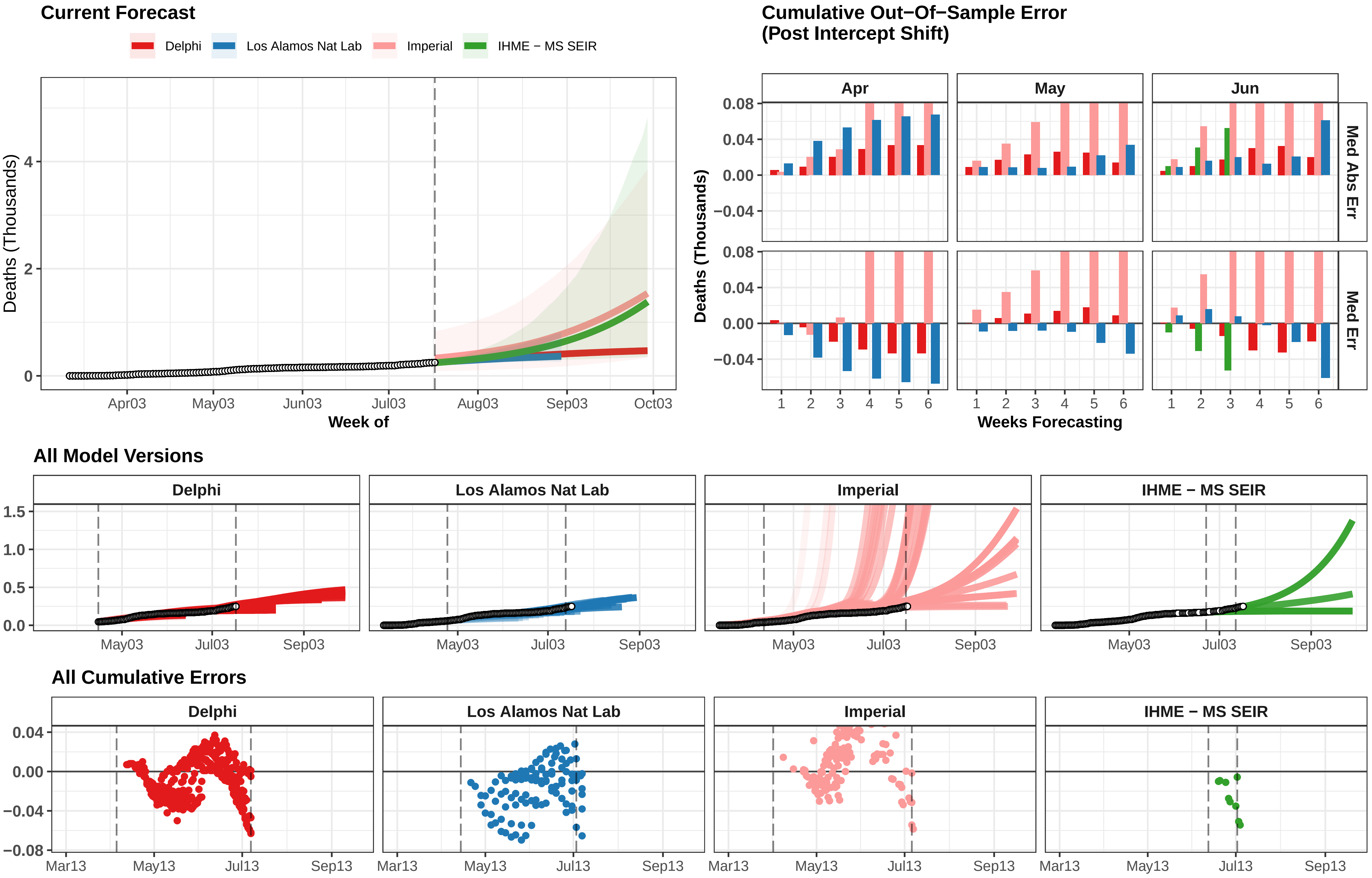

Utah

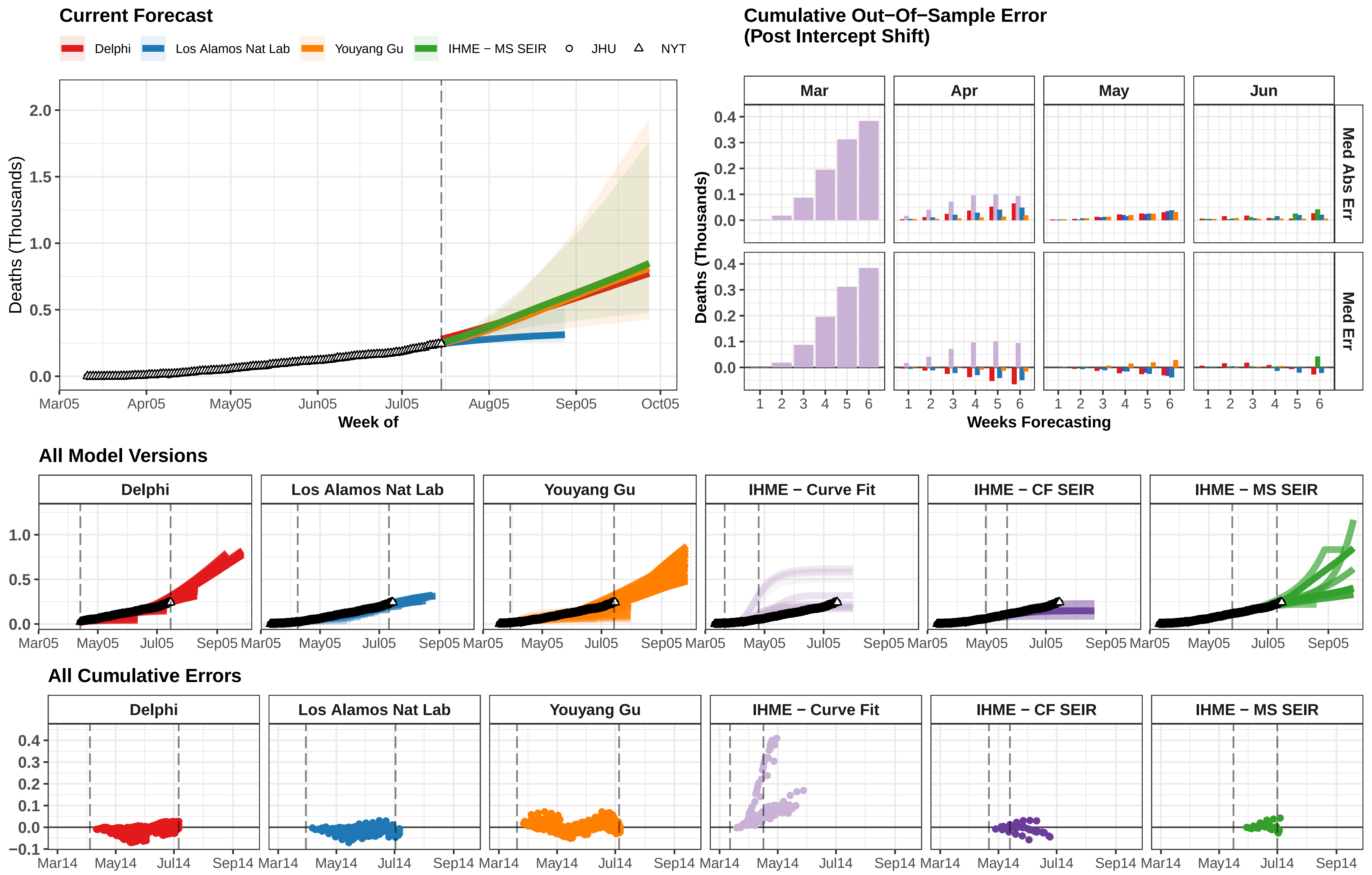

Current Forecast
Delphi
Los Alamos Nat Lab
Youyang Gu
IHME − MS SEIR
JHU
NYT
Deaths (Thousands)
2.0
1.5
1.0
0.5
0.0
Mar05
Apr05
May05
Jun05
Jul05
Aug05
Sep05
Oct05
Week of
Cumulative Out−Of−Sample Error
(Post Intercept Shift)
Mar
Apr
May
Jun
Med Abs Err
Med Err
0.4
0.3
0.2
0.1
0.0
1 2 3 4 5 6
Weeks Forecasting
All Model Versions
Delphi
Los Alamos Nat Lab
Youyang Gu
IHME − Curve Fit
IHME − CF SEIR
IHME − MS SEIR
1.0
0.5
0.0
Mar05
May05
Jul05
Sep05
All Cumulative Errors
0.4
0.3
0.2
0.1
0.0
−0.1
Mar14
May14
Jul14
Sep14

Kenya

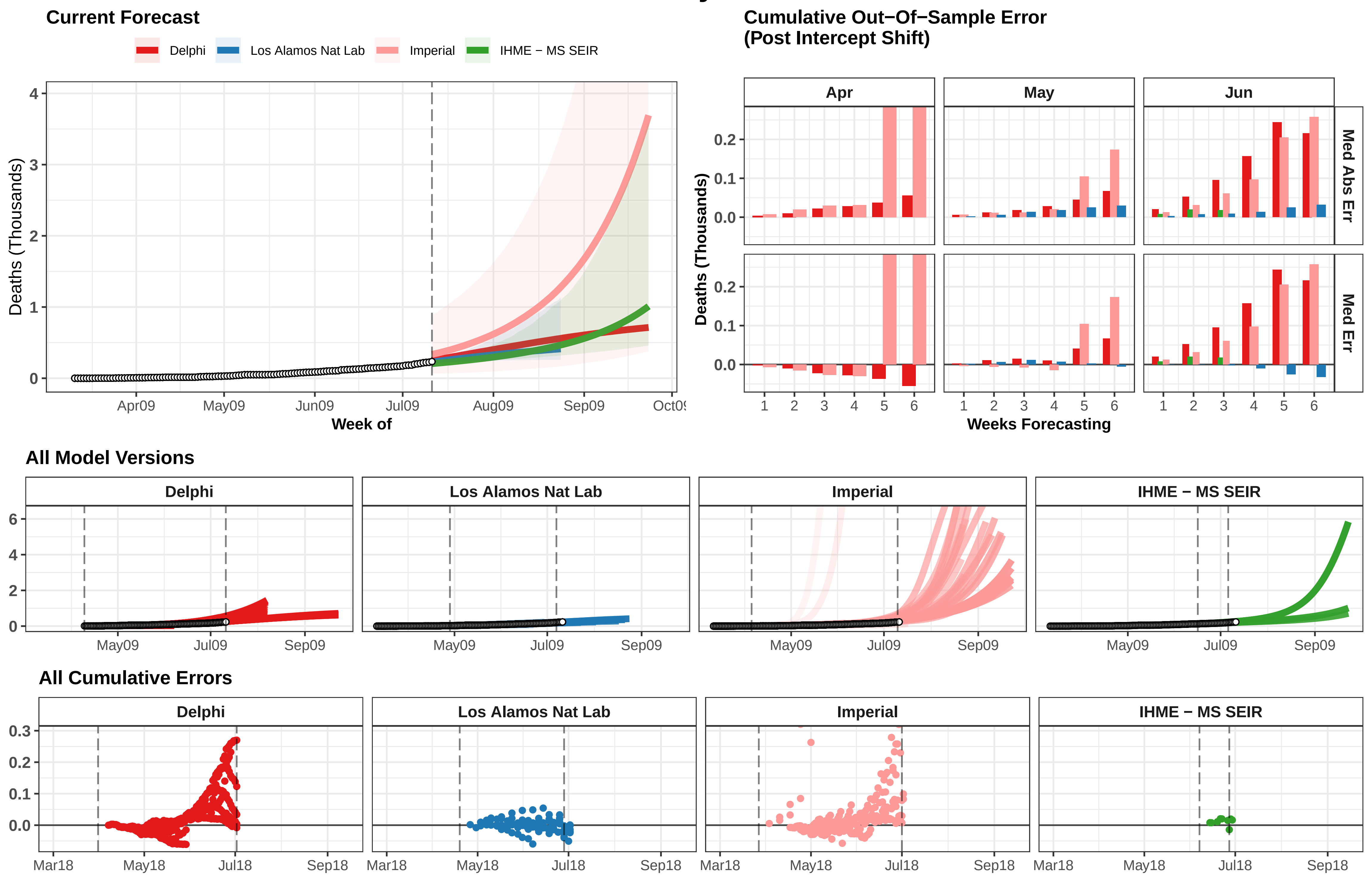

Current Forecast
Delphi
Los Alamos Nat Lab
Imperial
IHME − MS SEIR
Deaths (Thousands)
4
3
2
1
0
Apr09
May09
Jun09
Jul09
Aug09
Sep09
Oct09
Week of
Cumulative Out−Of−Sample Error
(Post Intercept Shift)
Apr
May
Jun
Med Abs Err
Med Err
0.2
0.1
0.0
1 2 3 4 5 6
Weeks Forecasting
All Model Versions
Delphi
Los Alamos Nat Lab
Imperial
IHME − MS SEIR
6
4
2
0
May09
Jul09
Sep09
All Cumulative Errors
0.3
0.2
0.1
0.0
Mar18
May18
Jul18
Sep18

Democratic Republic of the Congo

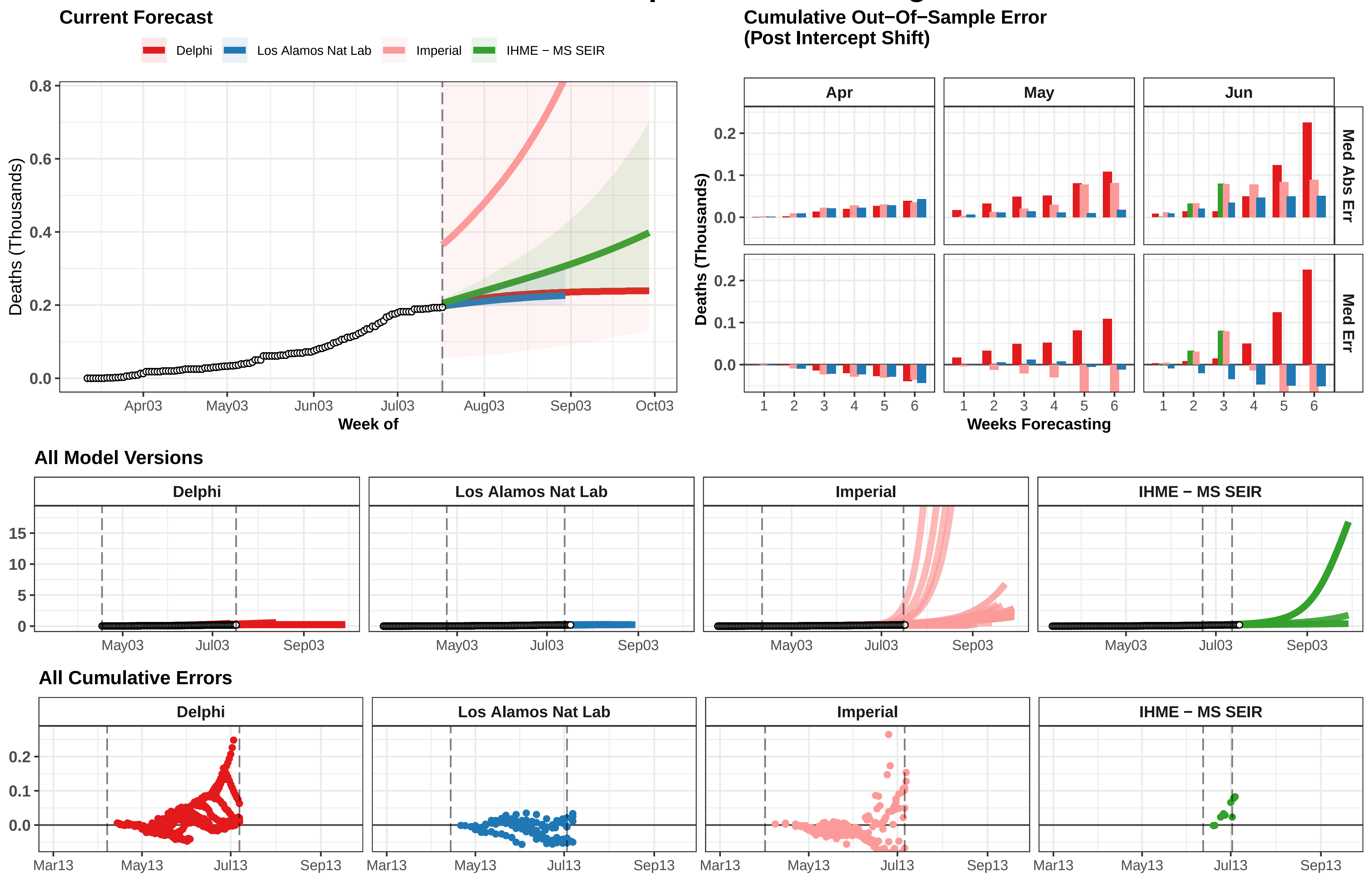

Greece

Current Forecast

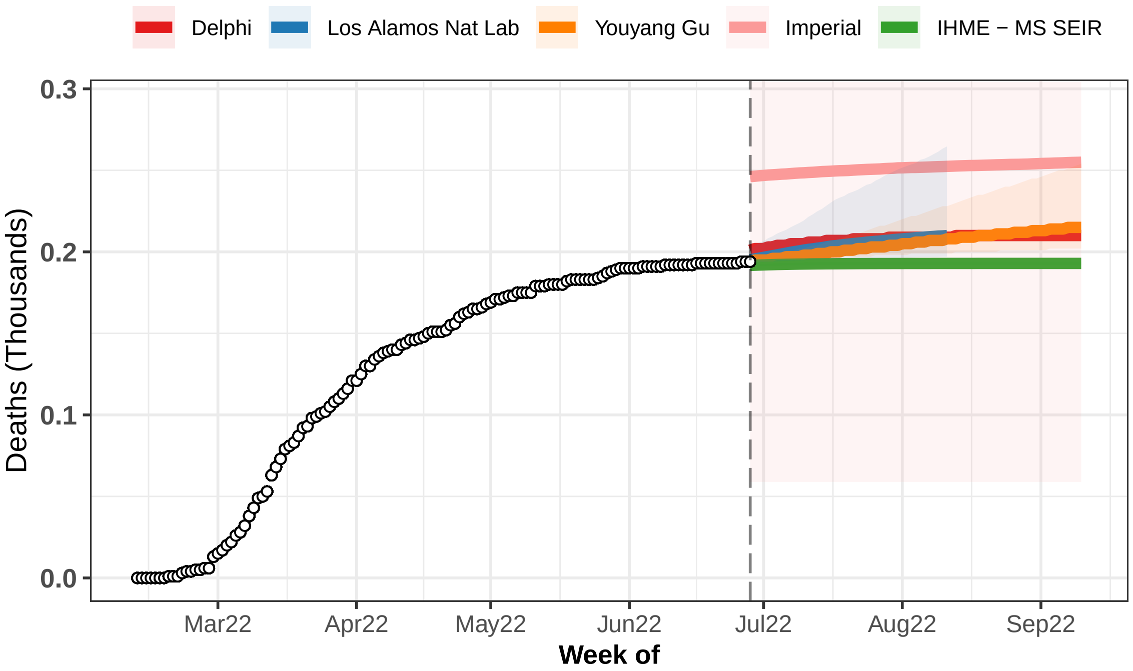

Cumulative Out−Of−Sample Error (Post Intercept Shift)

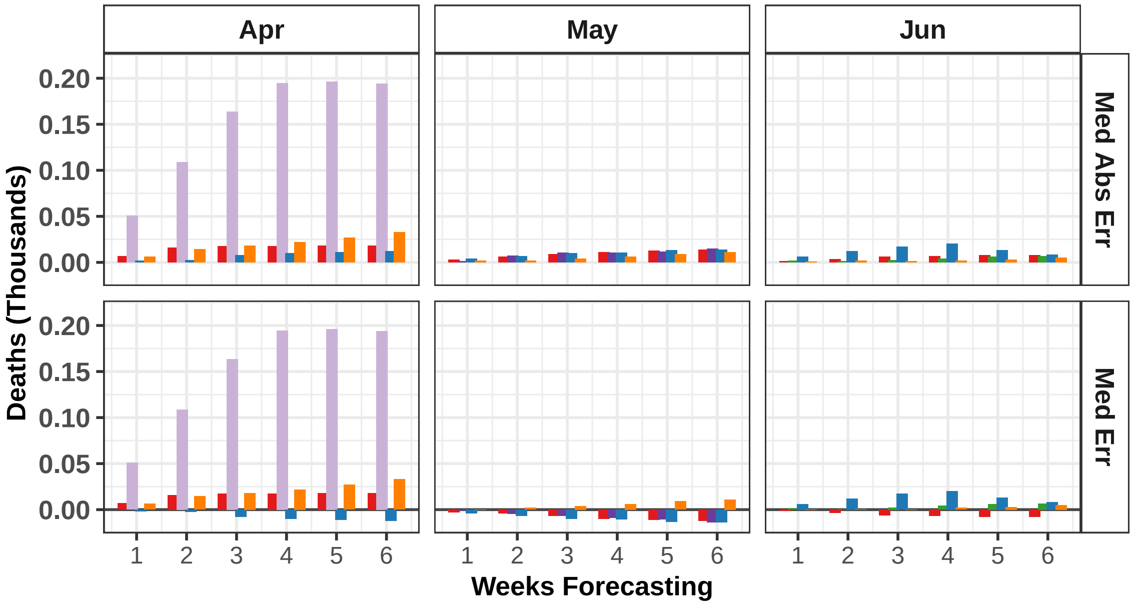

All Model Versions

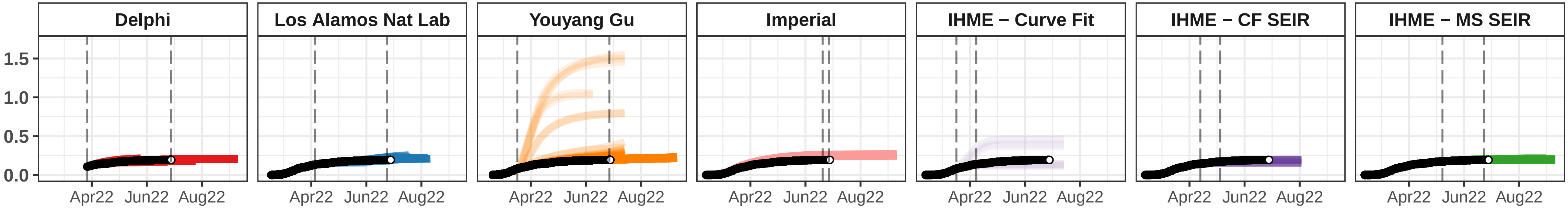

All Cumulative Errors

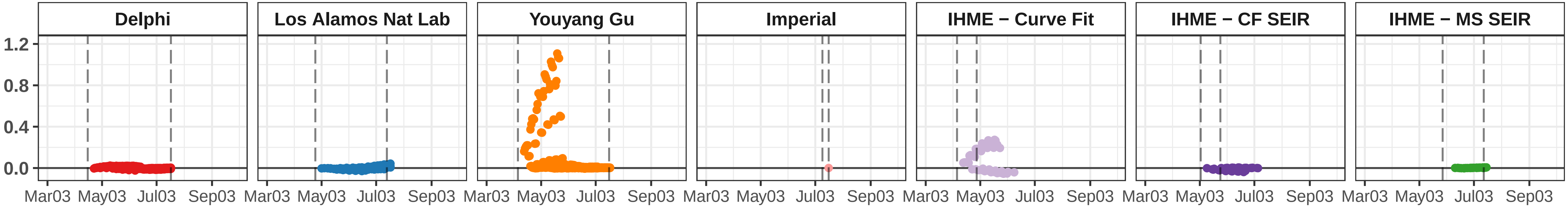

Puerto Rico

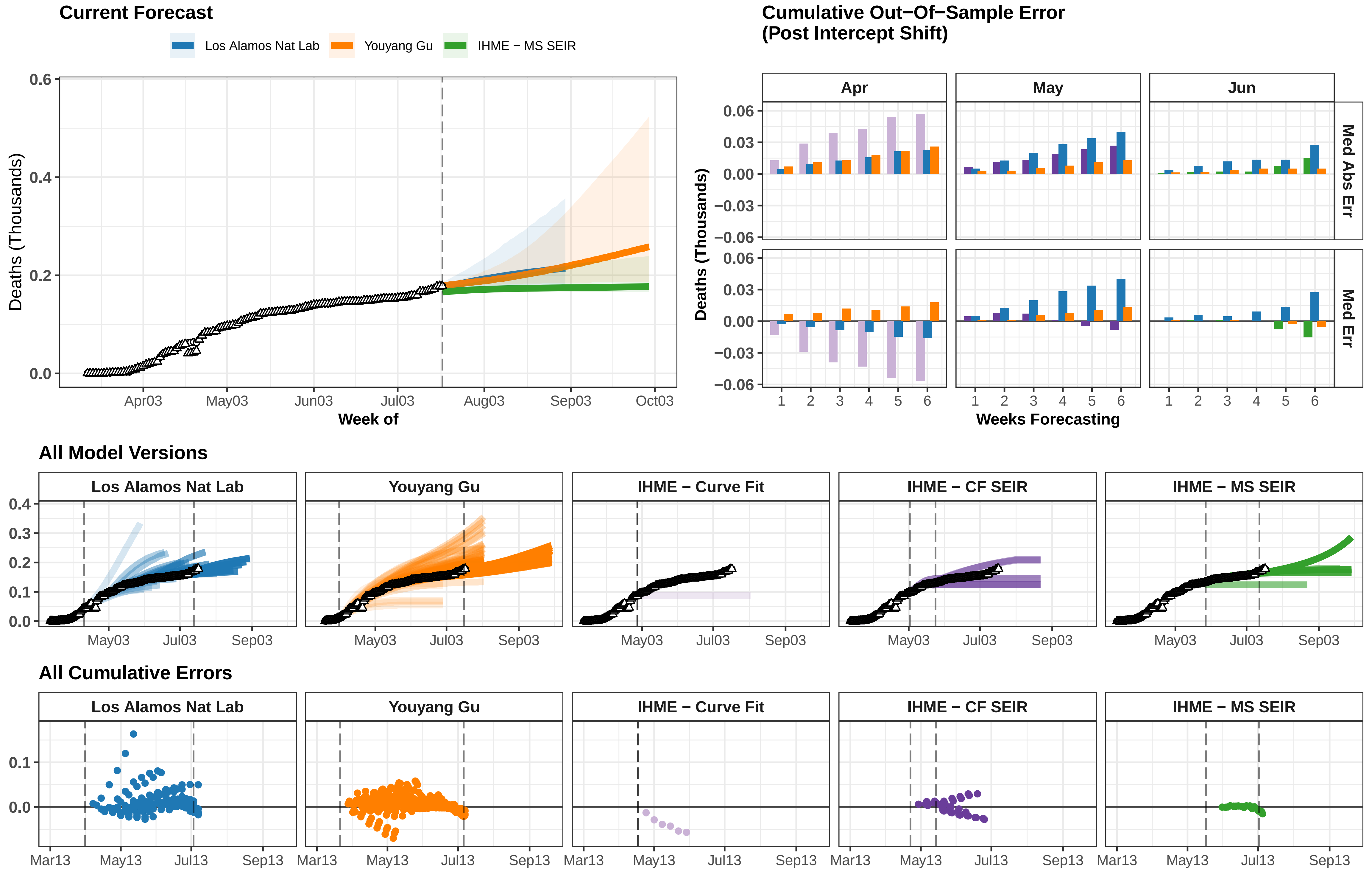

Current Forecast
Los Alamos Nat Lab
Youyang Gu
IHME − MS SEIR
Deaths (Thousands)
0.0
0.2
0.4
0.6
Apr03
May03
Jun03
Jul03
Aug03
Sep03
Oct03
Week of
Cumulative Out−Of−Sample Error
(Post Intercept Shift)
Apr
May
Jun
Med Abs Err
Med Err
Deaths (Thousands)
0.06
0.03
0.00
−0.03
−0.06
1
2
3
4
5
6
Weeks Forecasting
All Model Versions
Los Alamos Nat Lab
Youyang Gu
IHME − Curve Fit
IHME − CF SEIR
IHME − MS SEIR
0.4
0.3
0.2
0.1
0.0
May03
Jul03
Sep03
All Cumulative Errors
Los Alamos Nat Lab
Youyang Gu
IHME − Curve Fit
IHME − CF SEIR
IHME − MS SEIR
0.1
0.0
Mar13
May13
Jul13
Sep13

Ethiopia

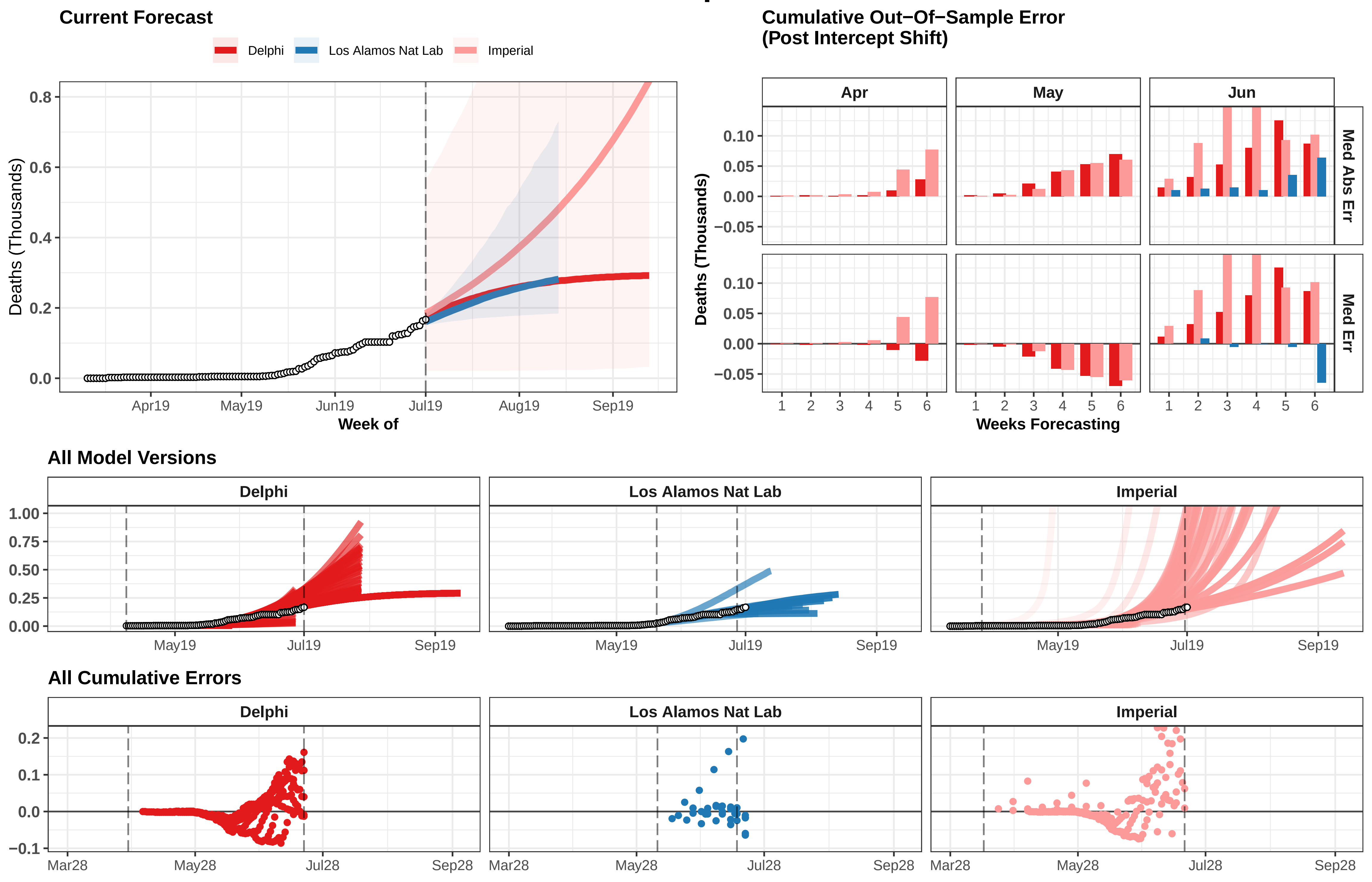
Current Forecast
Delphi
Los Alamos Nat Lab
Imperial
Deaths (Thousands)
Week of
Apr19
May19
Jun19
Jul19
Aug19
Sep19
Cumulative Out−Of−Sample Error
(Post Intercept Shift)
Apr
May
Jun
Med Abs Err
Med Err
Weeks Forecasting
All Model Versions
All Cumulative Errors
Mar28
May28
Jul28
Sep28

Senegal

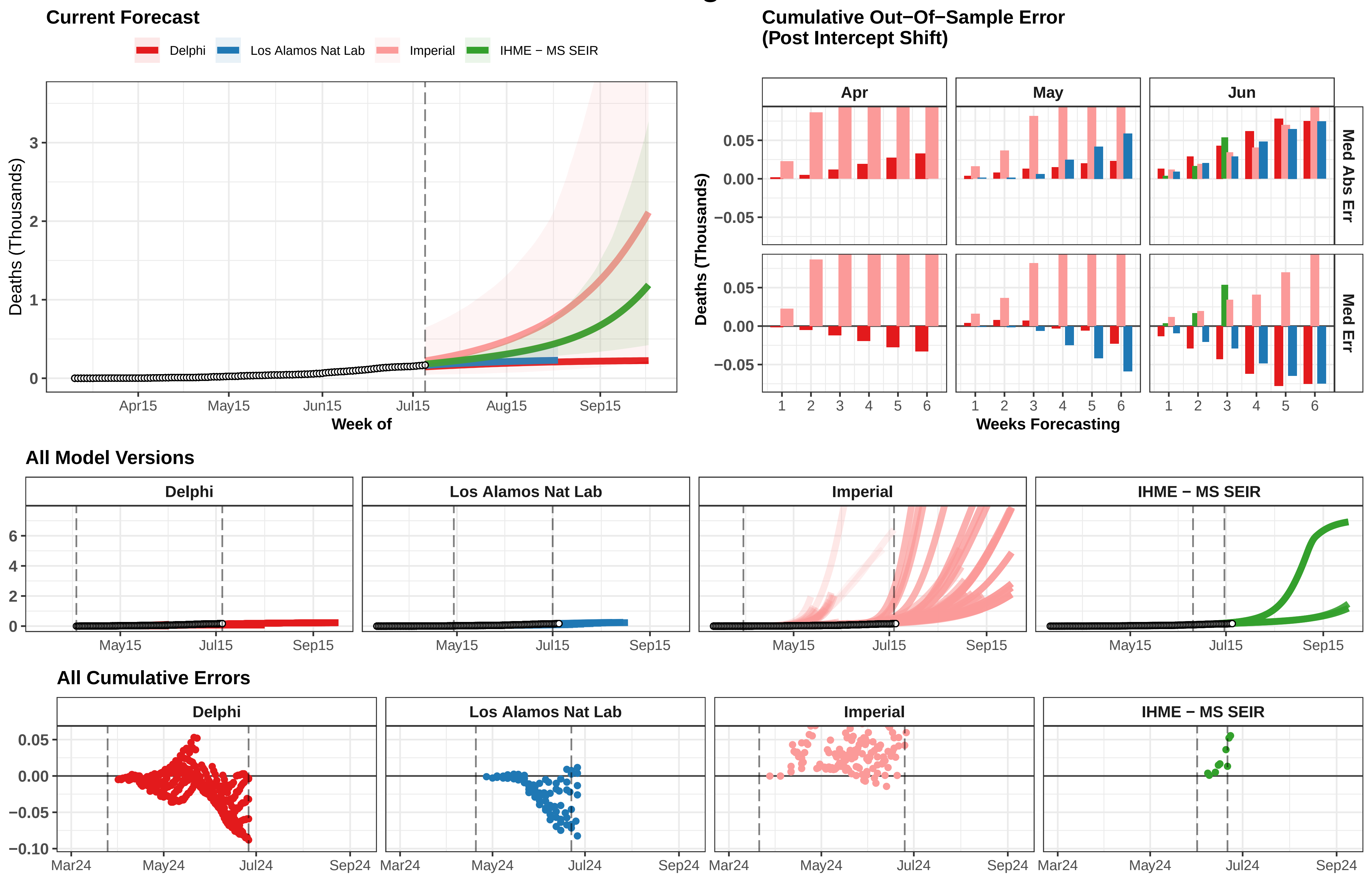

Current Forecast
Delphi
Los Alamos Nat Lab
Imperial
IHME − MS SEIR
Deaths (Thousands)
0
1
2
3
Apr15
May15
Jun15
Jul15
Aug15
Sep15
Week of
Cumulative Out−Of−Sample Error
(Post Intercept Shift)
Apr
May
Jun
Med Abs Err
Med Err
0.05
0.00
−0.05
1
2
3
4
5
6
Weeks Forecasting
All Model Versions
Delphi
Los Alamos Nat Lab
Imperial
IHME − MS SEIR
0
2
4
6
May15
Jul15
Sep15
All Cumulative Errors
Delphi
Los Alamos Nat Lab
Imperial
IHME − MS SEIR
0.05
0.00
−0.05
−0.10
Mar24
May24
Jul24
Sep24

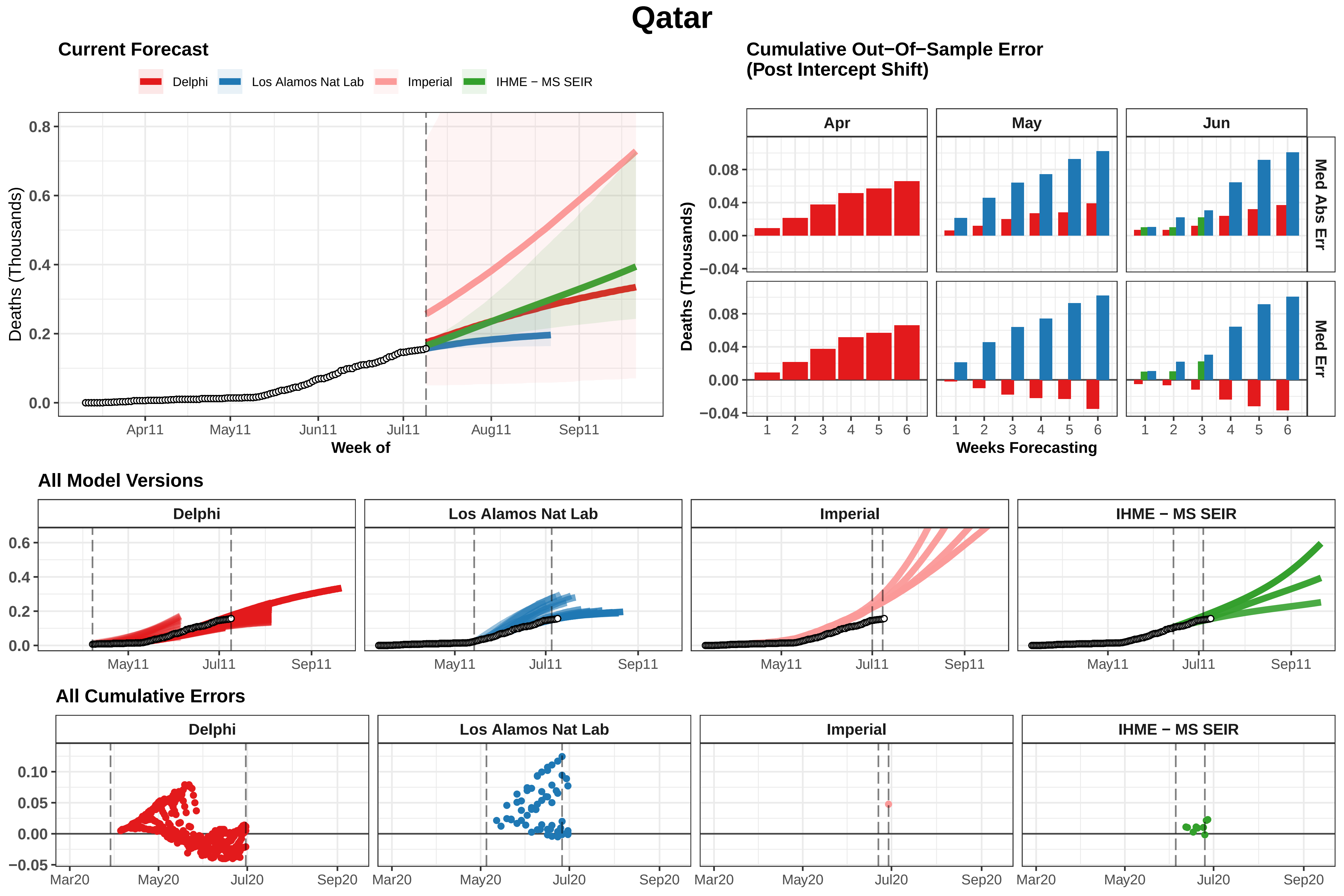
Qatar
Current Forecast
Delphi
Los Alamos Nat Lab
Imperial
IHME − MS SEIR
Deaths (Thousands)
0.0
0.2
0.4
0.6
0.8
Apr11
May11
Jun11
Jul11
Aug11
Sep11
Week of
Cumulative Out−Of−Sample Error
(Post Intercept Shift)
Apr
May
Jun
Med Abs Err
Med Err
Deaths (Thousands)
0.08
0.04
0.00
−0.04
1
2
3
4
5
6
Weeks Forecasting
All Model Versions
Delphi
Los Alamos Nat Lab
Imperial
IHME − MS SEIR
0.0
0.2
0.4
0.6
May11
Jul11
Sep11
All Cumulative Errors
Delphi
Los Alamos Nat Lab
Imperial
IHME − MS SEIR
0.10
0.05
0.00
−0.05
Mar20
May20
Jul20
Sep20

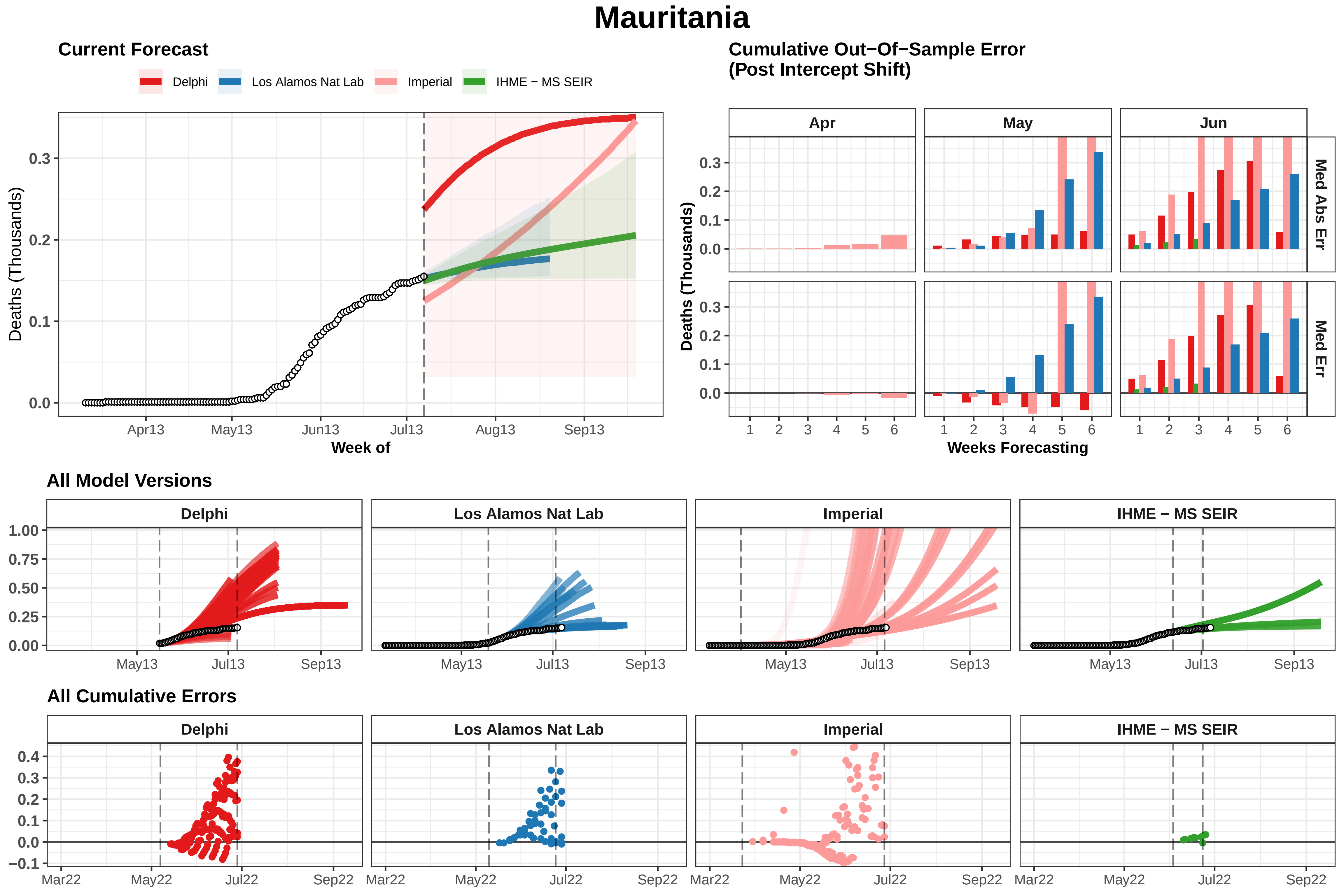

Mauritania
Current Forecast
Delphi
Los Alamos Nat Lab
Imperial
IHME − MS SEIR
Deaths (Thousands)
Week of
Apr13
May13
Jun13
Jul13
Aug13
Sep13
Cumulative Out−Of−Sample Error
(Post Intercept Shift)
Apr
May
Jun
Med Abs Err
Med Err
Weeks Forecasting
All Model Versions
All Cumulative Errors
Mar22
May22
Jul22
Sep22

Ghana

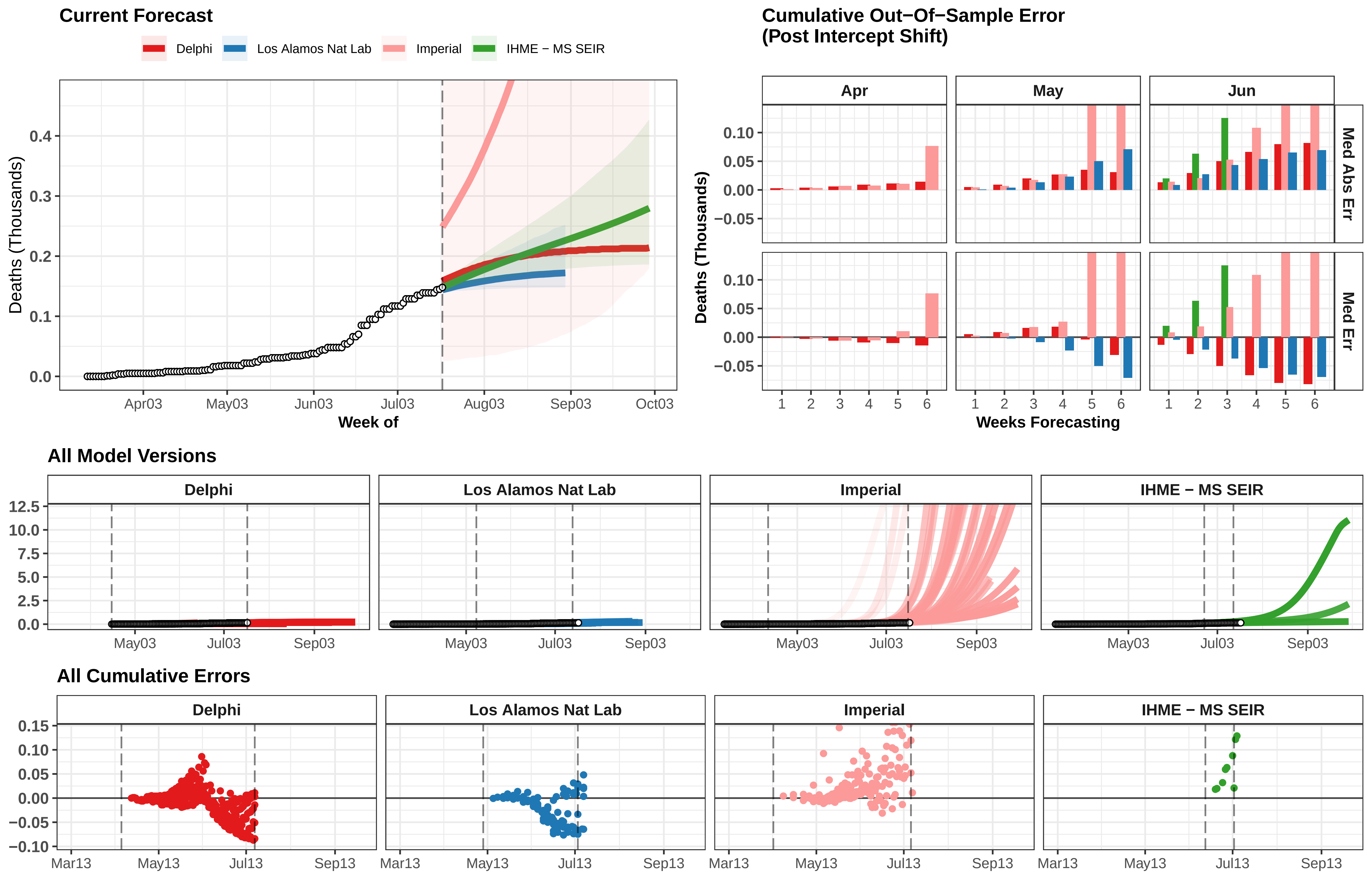
Current Forecast
Delphi
Los Alamos Nat Lab
Imperial
IHME − MS SEIR
Deaths (Thousands)
Week of
Apr03
May03
Jun03
Jul03
Aug03
Sep03
Oct03
Cumulative Out−Of−Sample Error (Post Intercept Shift)
Apr
May
Jun
Med Abs Err
Med Err
Weeks Forecasting
All Model Versions
All Cumulative Errors
Mar13
May13
Jul13
Sep13

Haiti

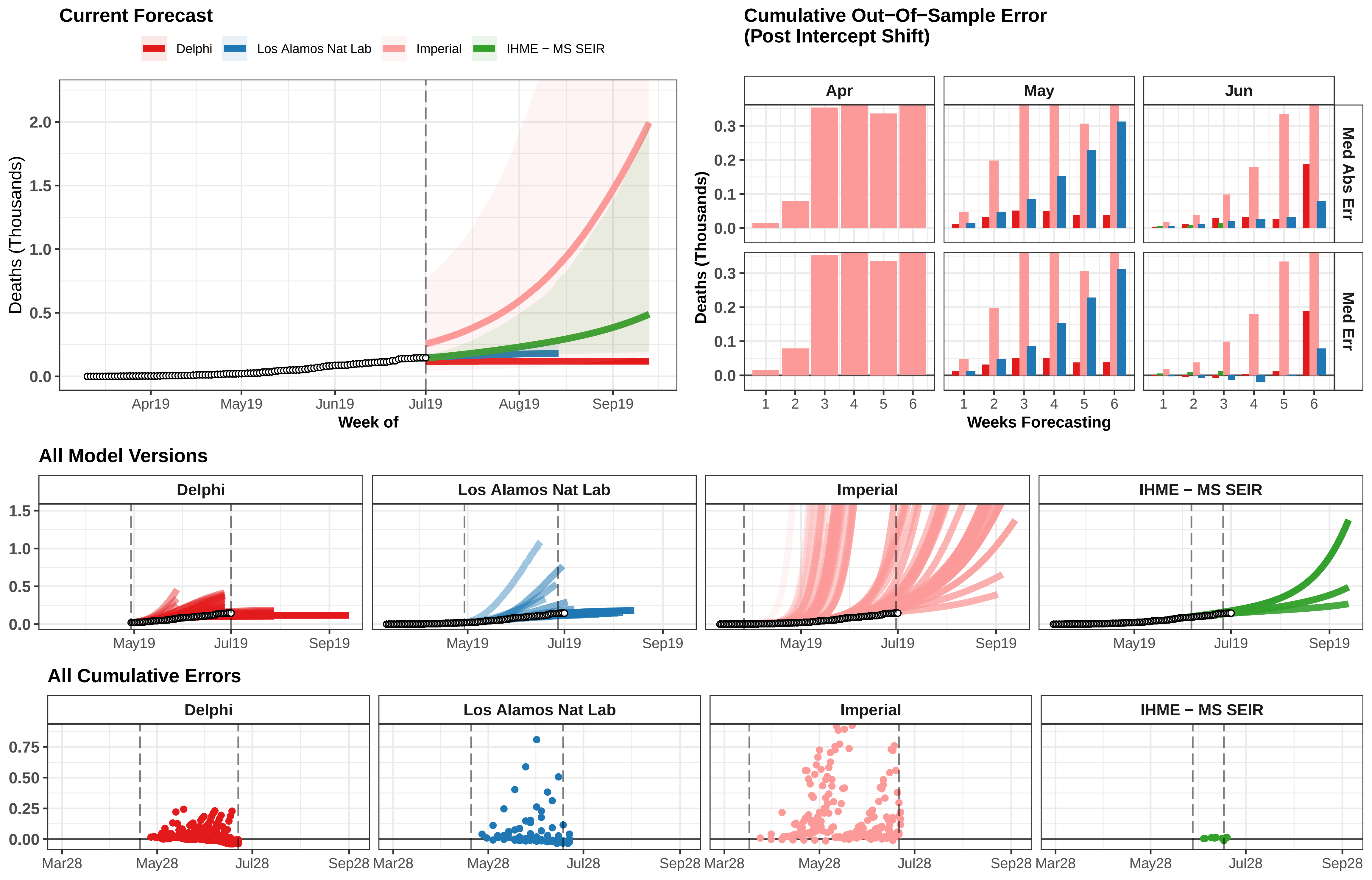

Current Forecast
Delphi
Los Alamos Nat Lab
Imperial
IHME − MS SEIR
Deaths (Thousands)
Week of
Apr19
May19
Jun19
Jul19
Aug19
Sep19
Cumulative Out−Of−Sample Error
(Post Intercept Shift)
Apr
May
Jun
Med Abs Err
Med Err
Weeks Forecasting
All Model Versions
All Cumulative Errors
Mar28
May28
Jul28
Sep28

Bahrain

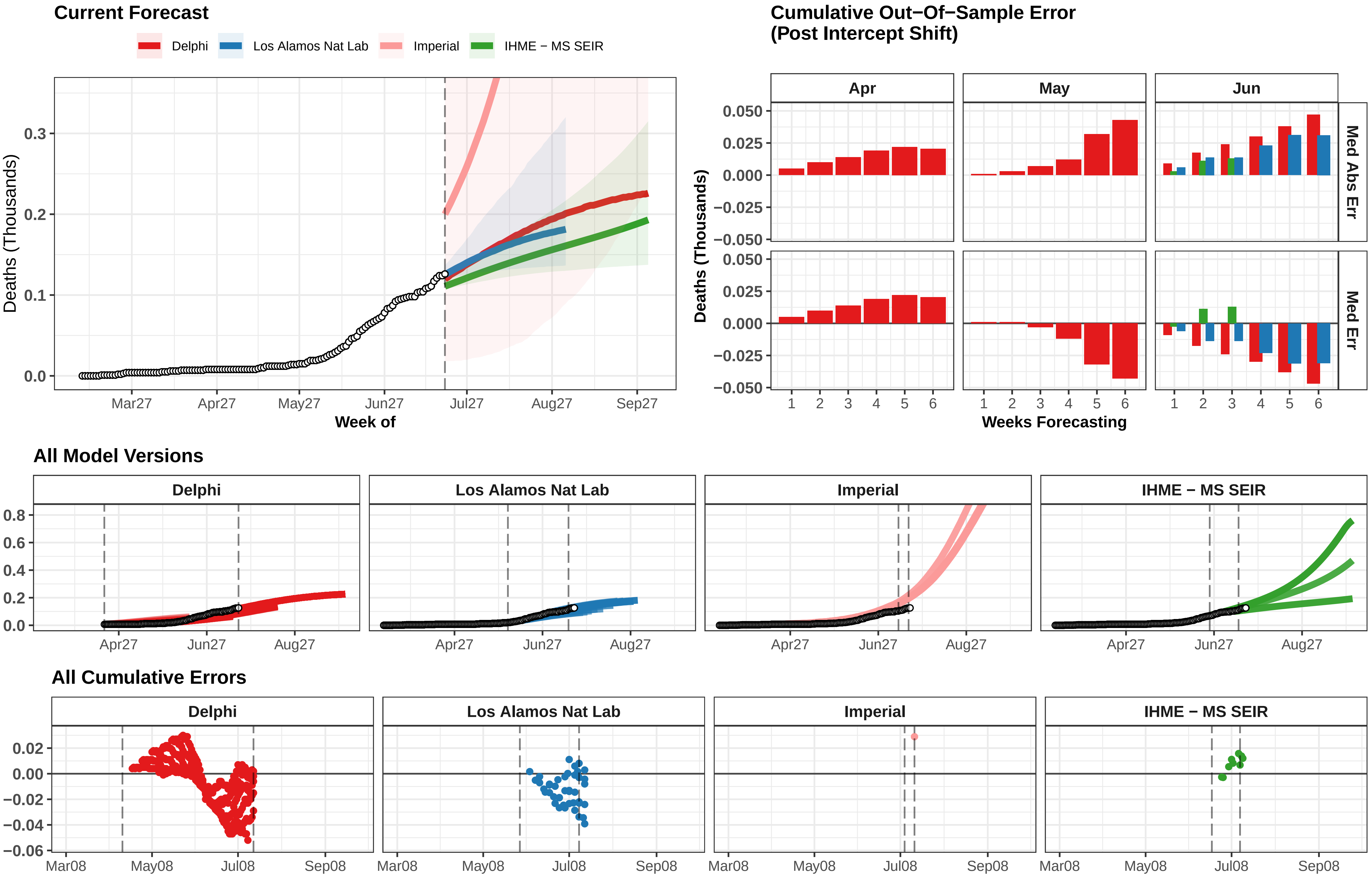
Current Forecast
Delphi
Los Alamos Nat Lab
Imperial
IHME − MS SEIR
Deaths (Thousands)
0.0
0.1
0.2
0.3
Mar27
Apr27
May27
Jun27
Jul27
Aug27
Sep27
Week of
Cumulative Out−Of−Sample Error
(Post Intercept Shift)
Apr
May
Jun
Med Abs Err
Med Err
0.050
0.025
0.000
−0.025
−0.050
1
2
3
4
5
6
Weeks Forecasting
All Model Versions
Delphi
Los Alamos Nat Lab
Imperial
IHME − MS SEIR
0.0
0.2
0.4
0.6
0.8
Apr27
Jun27
Aug27
All Cumulative Errors
Delphi
Los Alamos Nat Lab
Imperial
IHME − MS SEIR
0.02
0.00
−0.02
−0.04
−0.06
Mar08
May08
Jul08
Sep08

Australia

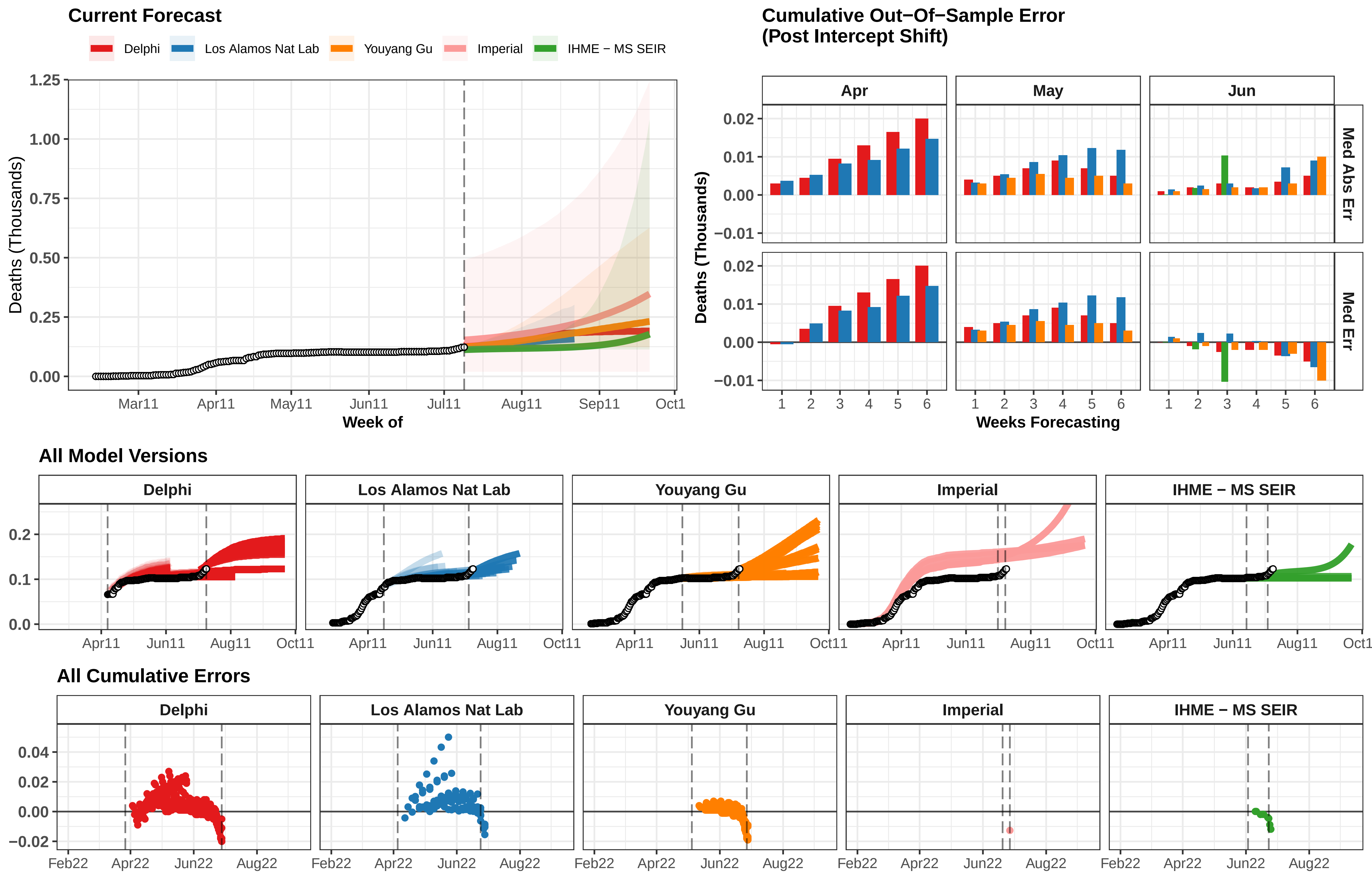
Current Forecast
Delphi
Los Alamos Nat Lab
Youyang Gu
Imperial
IHME − MS SEIR
Deaths (Thousands)
1.25
1.00
0.75
0.50
0.25
0.00
Mar11
Apr11
May11
Jun11
Jul11
Aug11
Sep11
Oct1
Week of
Cumulative Out−Of−Sample Error
(Post Intercept Shift)
Apr
May
Jun
Med Abs Err
Med Err
Deaths (Thousands)
0.02
0.01
0.00
−0.01
1
2
3
4
5
6
Weeks Forecasting
All Model Versions
Delphi
Los Alamos Nat Lab
Youyang Gu
Imperial
IHME − MS SEIR
0.2
0.1
0.0
Apr11
Jun11
Aug11
Oct11
All Cumulative Errors
Delphi
Los Alamos Nat Lab
Youyang Gu
Imperial
IHME − MS SEIR
0.04
0.02
0.00
−0.02
Feb22
Apr22
Jun22
Aug22

Malaysia

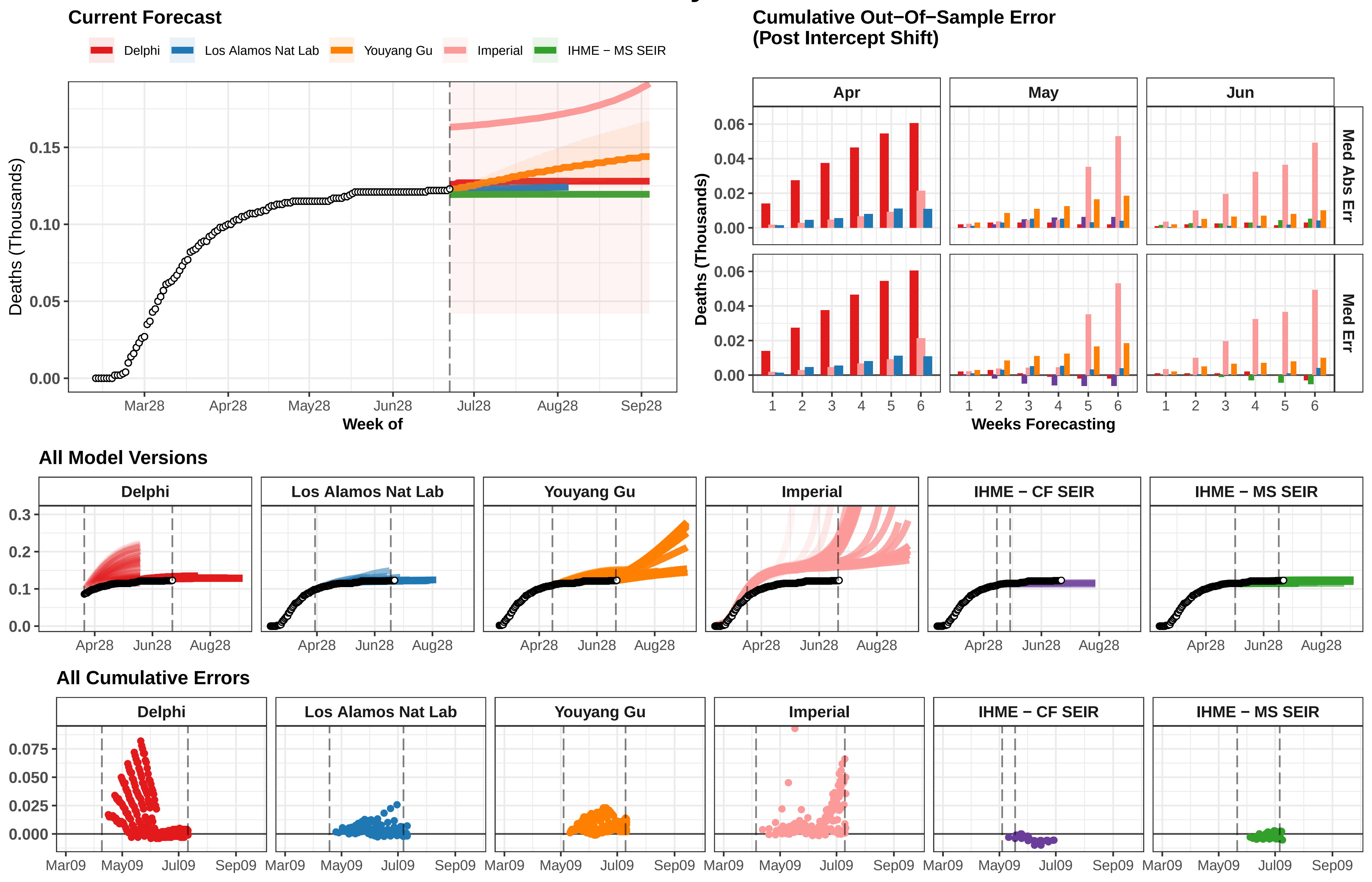
Current Forecast
Delphi
Los Alamos Nat Lab
Youyang Gu
Imperial
IHME − MS SEIR
Deaths (Thousands)
0.00
0.05
0.10
0.15
Mar28
Apr28
May28
Jun28
Jul28
Aug28
Sep28
Week of
Cumulative Out−Of−Sample Error
(Post Intercept Shift)
Apr
May
Jun
Med Abs Err
Med Err
0.00
0.02
0.04
0.06
1
2
3
4
5
6
Weeks Forecasting
All Model Versions
Delphi
Los Alamos Nat Lab
Youyang Gu
Imperial
IHME − CF SEIR
IHME − MS SEIR
0.0
0.1
0.2
0.3
Apr28
Jun28
Aug28
All Cumulative Errors
0.000
0.025
0.050
0.075
Mar09
May09
Jul09
Sep09

Mali

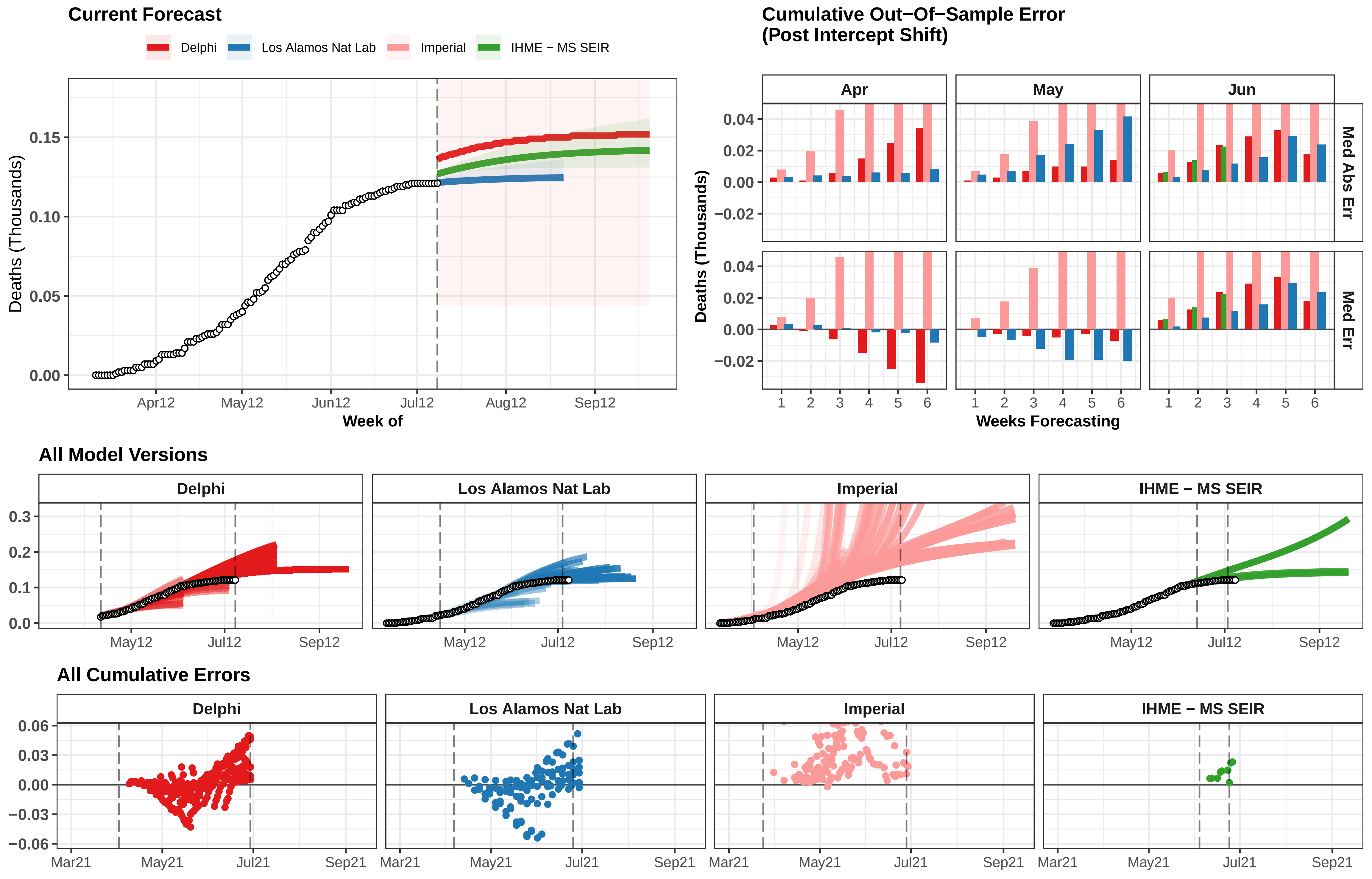

Current Forecast
Delphi
Los Alamos Nat Lab
Imperial
IHME − MS SEIR
Deaths (Thousands)
0.15
0.10
0.05
0.00
Apr12
May12
Jun12
Jul12
Aug12
Sep12
Week of
Cumulative Out−Of−Sample Error
(Post Intercept Shift)
Apr
May
Jun
Med Abs Err
Med Err
0.04
0.02
0.00
−0.02
1
2
3
4
5
6
Weeks Forecasting
All Model Versions
Delphi
Los Alamos Nat Lab
Imperial
IHME − MS SEIR
0.3
0.2
0.1
0.0
May12
Jul12
Sep12
All Cumulative Errors
Delphi
Los Alamos Nat Lab
Imperial
IHME − MS SEIR
0.06
0.03
0.00
−0.03
−0.06
Mar21
May21
Jul21
Sep21

Croatia

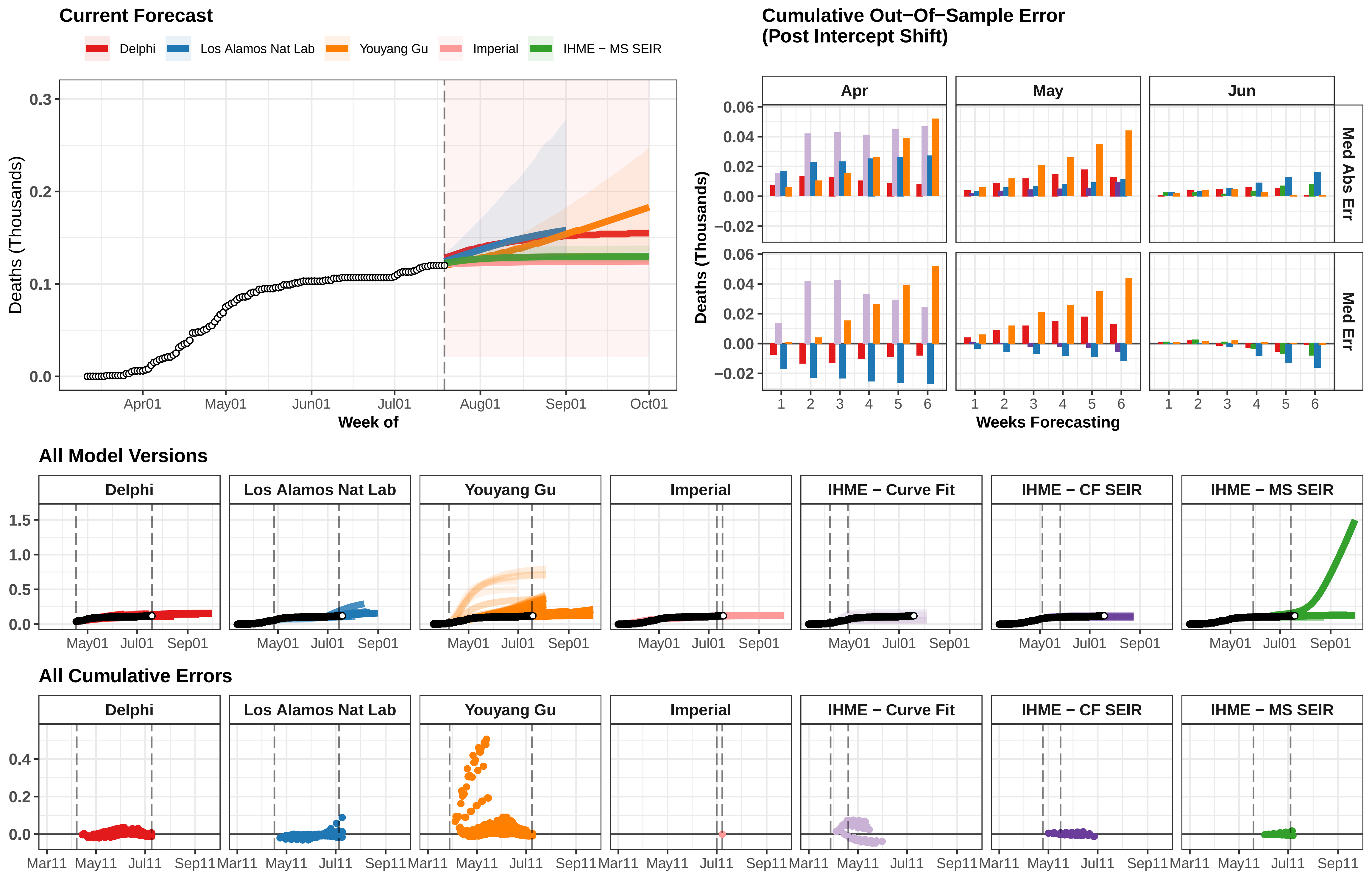

Current Forecast
Delphi
Los Alamos Nat Lab
Youyang Gu
Imperial
IHME − MS SEIR
Deaths (Thousands)
Week of
Apr01
May01
Jun01
Jul01
Aug01
Sep01
Oct01
Cumulative Out−Of−Sample Error (Post Intercept Shift)
Apr
May
Jun
Med Abs Err
Med Err
Weeks Forecasting
All Model Versions
Delphi
Los Alamos Nat Lab
Youyang Gu
Imperial
IHME − Curve Fit
IHME − CF SEIR
IHME − MS SEIR
All Cumulative Errors
Mar11
May11
Jul11
Sep11

Idaho

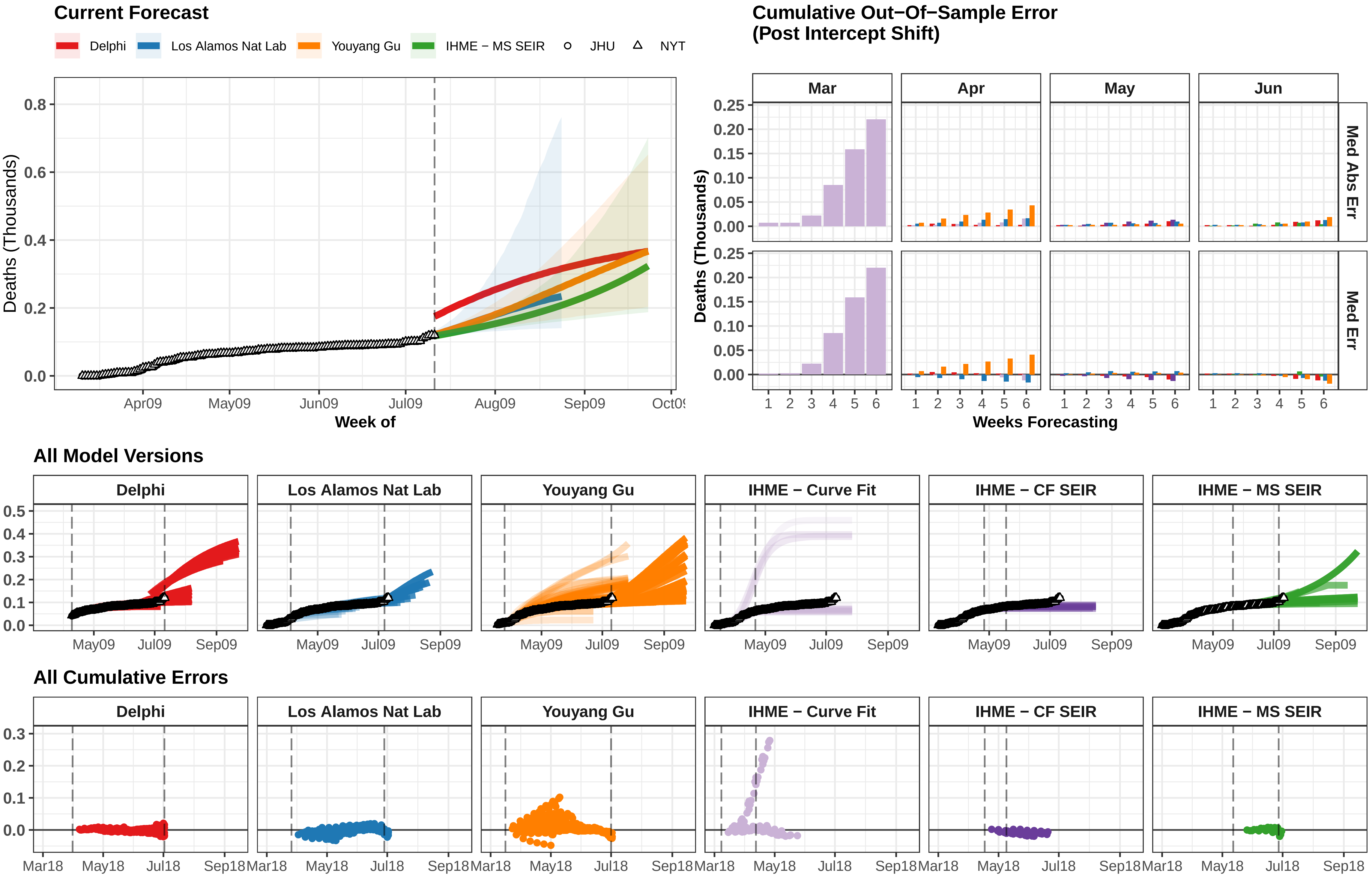

Current Forecast
Delphi
Los Alamos Nat Lab
Youyang Gu
IHME − MS SEIR
JHU
NYT
Deaths (Thousands)
Week of
Cumulative Out−Of−Sample Error
(Post Intercept Shift)
Mar
Apr
May
Jun
Med Abs Err
Med Err
Weeks Forecasting
All Model Versions
Delphi
Los Alamos Nat Lab
Youyang Gu
IHME − Curve Fit
IHME − CF SEIR
IHME − MS SEIR
All Cumulative Errors

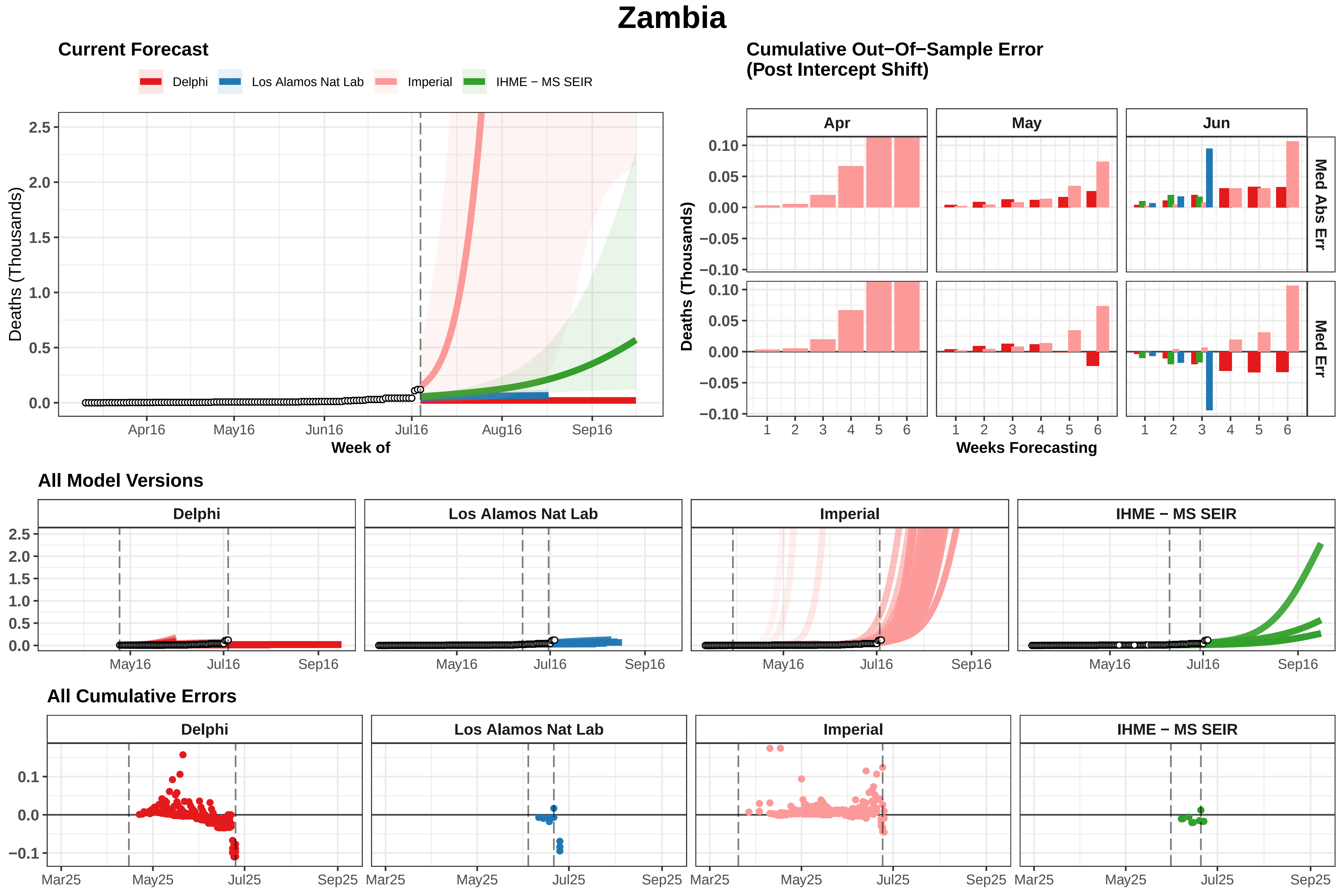
Zambia
Current Forecast
Delphi
Los Alamos Nat Lab
Imperial
IHME − MS SEIR
Deaths (Thousands)
Week of
Apr16
May16
Jun16
Jul16
Aug16
Sep16
Cumulative Out−Of−Sample Error
(Post Intercept Shift)
Apr
May
Jun
Med Abs Err
Med Err
Weeks Forecasting
All Model Versions
All Cumulative Errors
Mar25
May25
Jul25
Sep25

South Dakota

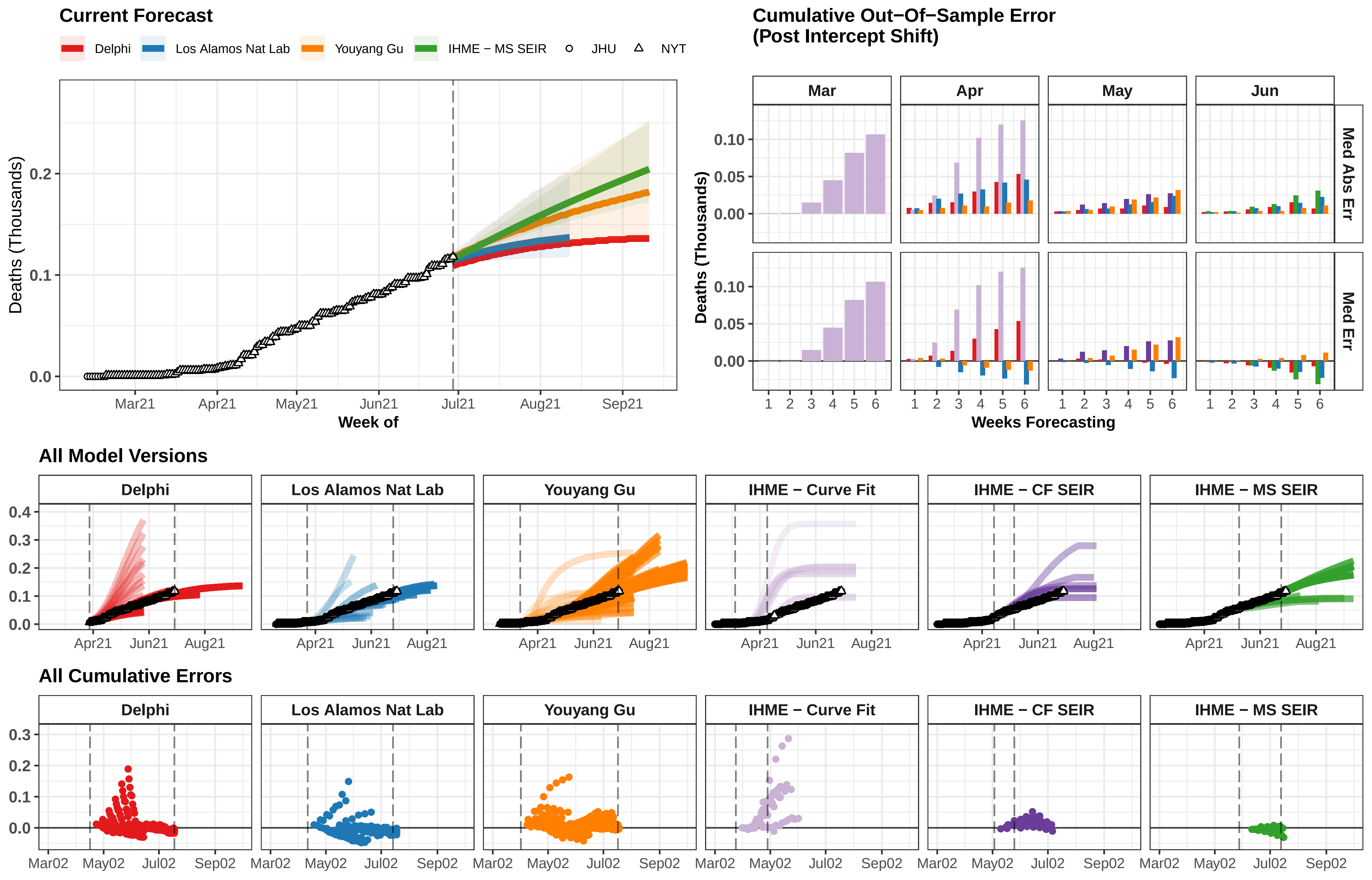

Maine

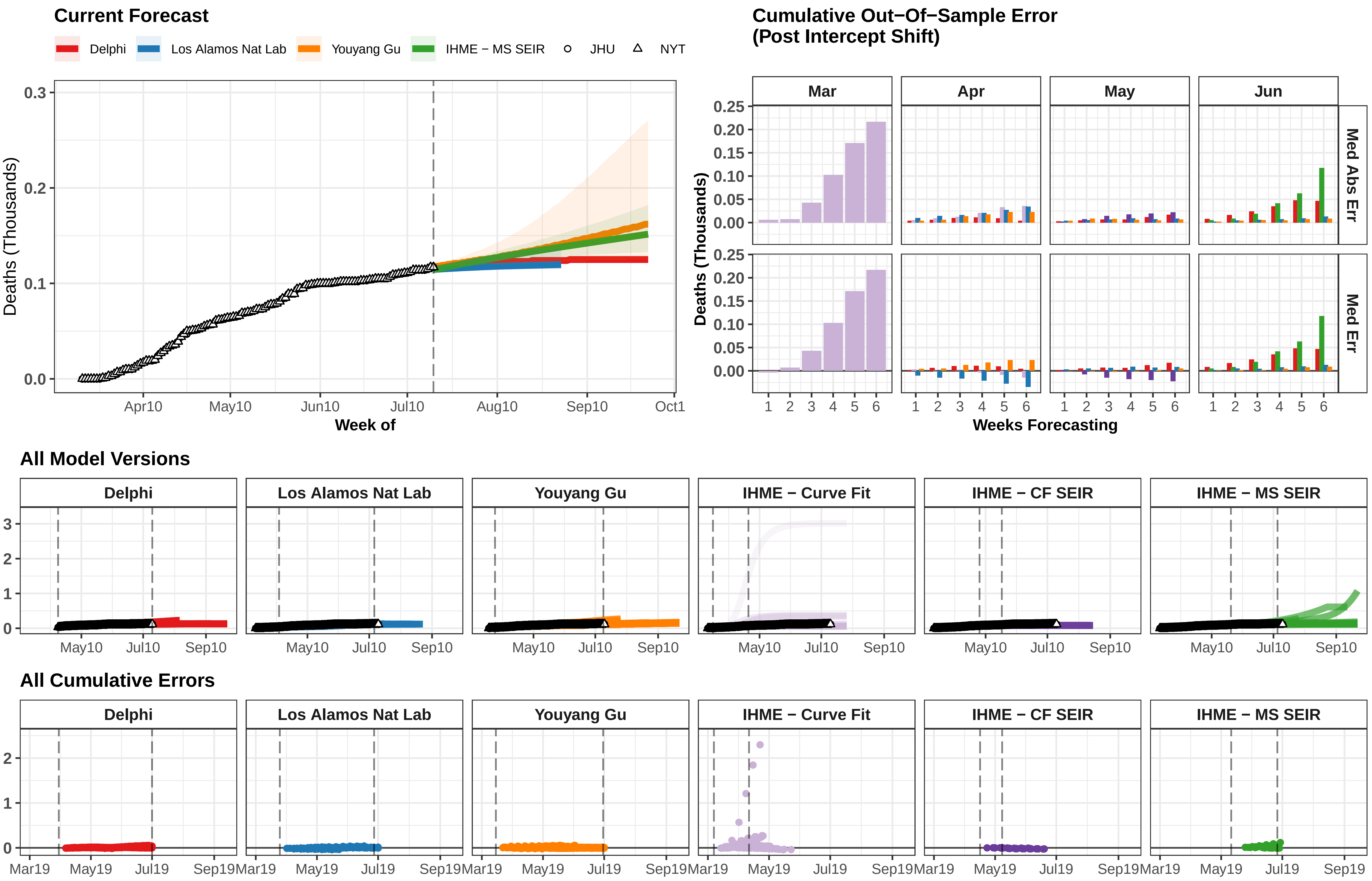

Current Forecast
Delphi
Los Alamos Nat Lab
Youyang Gu
IHME − MS SEIR
JHU
NYT
Deaths (Thousands)
0.0
0.1
0.2
0.3
Apr10
May10
Jun10
Jul10
Aug10
Sep10
Oct1
Week of
Cumulative Out−Of−Sample Error
(Post Intercept Shift)
Mar
Apr
May
Jun
Med Abs Err
Med Err
Deaths (Thousands)
0.00
0.05
0.10
0.15
0.20
0.25
1 2 3 4 5 6
Weeks Forecasting
All Model Versions
Delphi
Los Alamos Nat Lab
Youyang Gu
IHME − Curve Fit
IHME − CF SEIR
IHME − MS SEIR
0
1
2
3
May10
Jul10
Sep10
All Cumulative Errors
Delphi
Los Alamos Nat Lab
Youyang Gu
IHME − Curve Fit
IHME − CF SEIR
IHME − MS SEIR
0
1
2
Mar19
May19
Jul19
Sep19

Albania

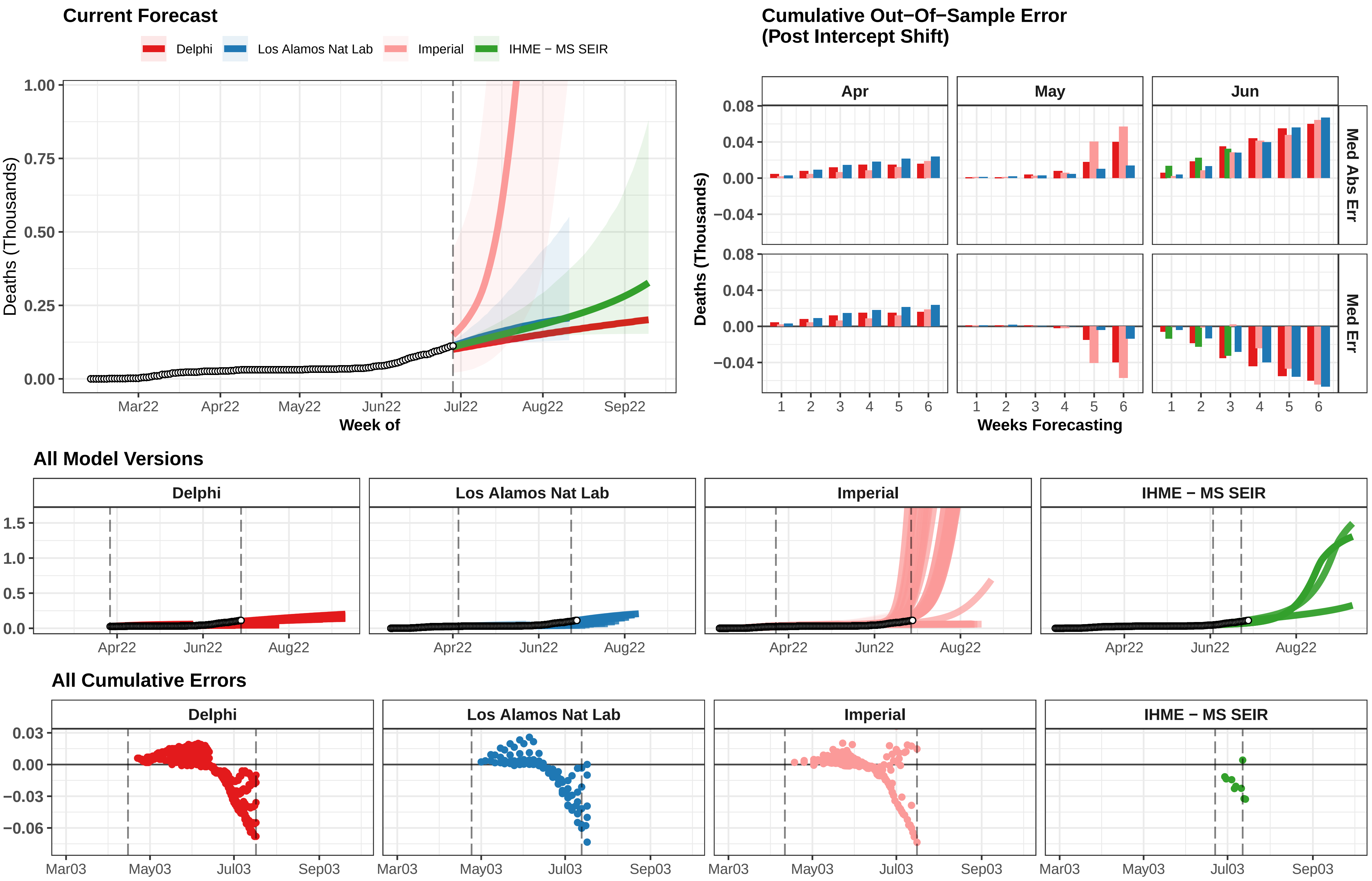

Current Forecast
Delphi
Los Alamos Nat Lab
Imperial
IHME − MS SEIR
Deaths (Thousands)
1.00
0.75
0.50
0.25
0.00
Mar22
Apr22
May22
Jun22
Jul22
Aug22
Sep22
Week of
Cumulative Out−Of−Sample Error
(Post Intercept Shift)
Apr
May
Jun
Med Abs Err
Med Err
0.08
0.04
0.00
−0.04
Weeks Forecasting
All Model Versions
1.5
1.0
0.5
0.0
All Cumulative Errors
0.03
0.00
−0.03
−0.06
Mar03
May03
Jul03
Sep03

Slovenia

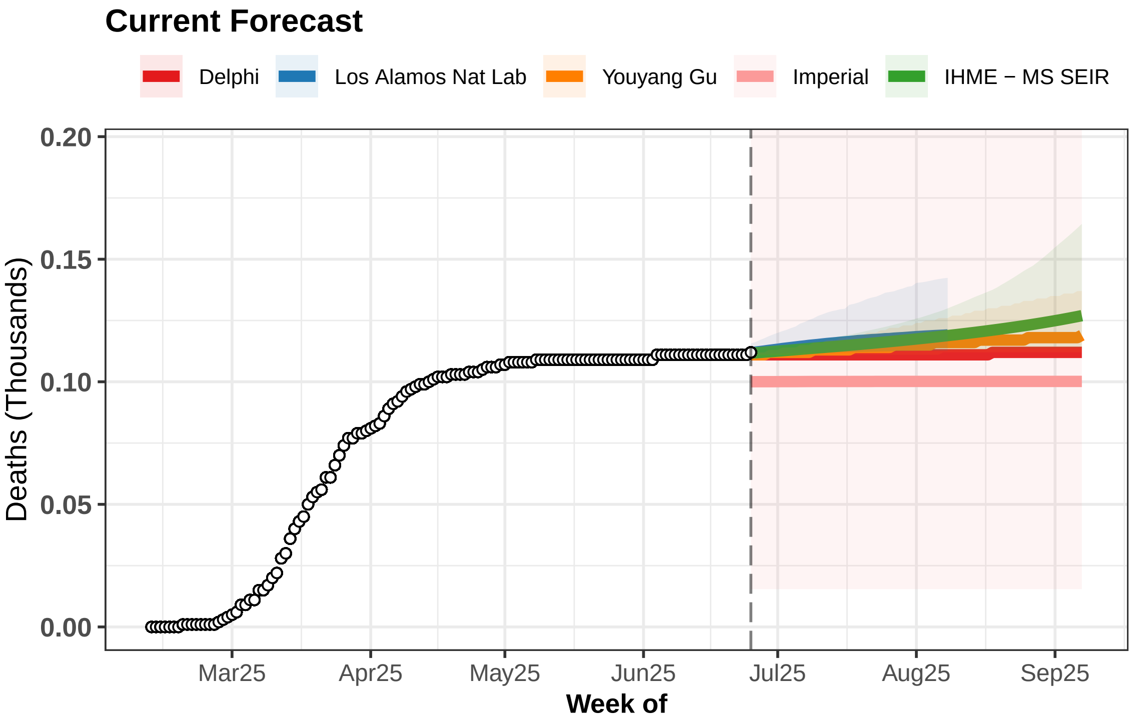

Current Forecast
Delphi
Los Alamos Nat Lab
Youyang Gu
Imperial
IHME − MS SEIR
Deaths (Thousands)
0.20
0.15
0.10
0.05
0.00
Mar25
Apr25
May25
Jun25
Jul25
Aug25
Sep25
Week of

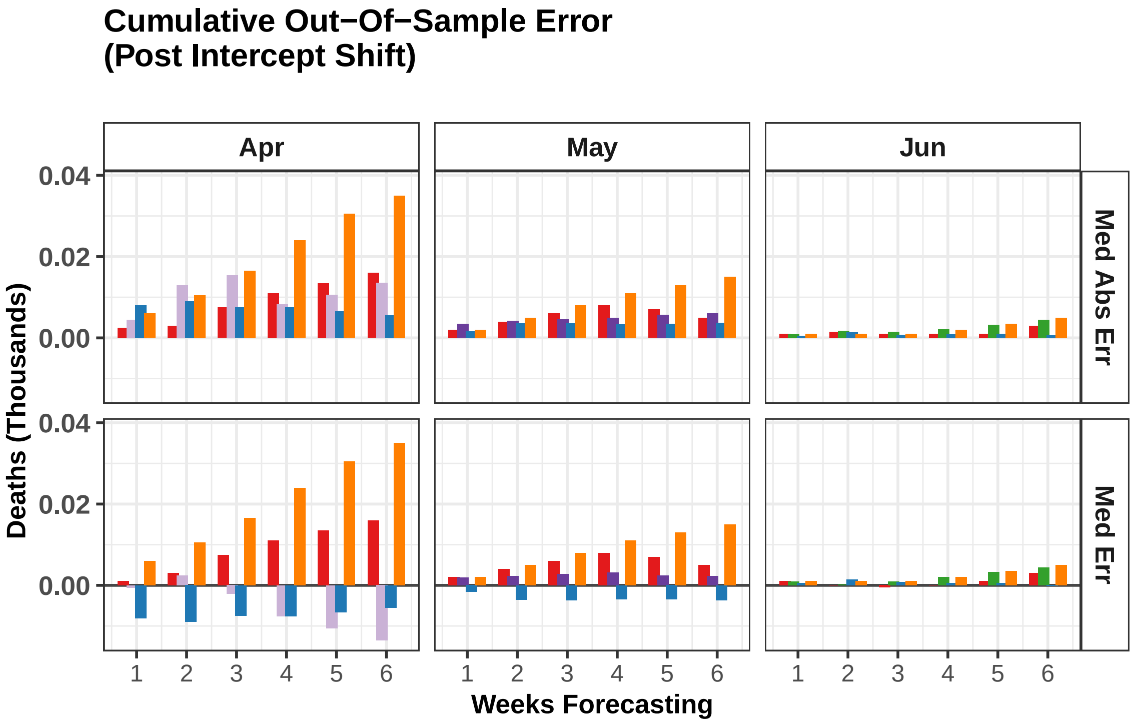

Cumulative Out−Of−Sample Error
(Post Intercept Shift)
Apr
May
Jun
Med Abs Err
Med Err
Deaths (Thousands)
0.04
0.02
0.00
1
2
3
4
5
6
Weeks Forecasting

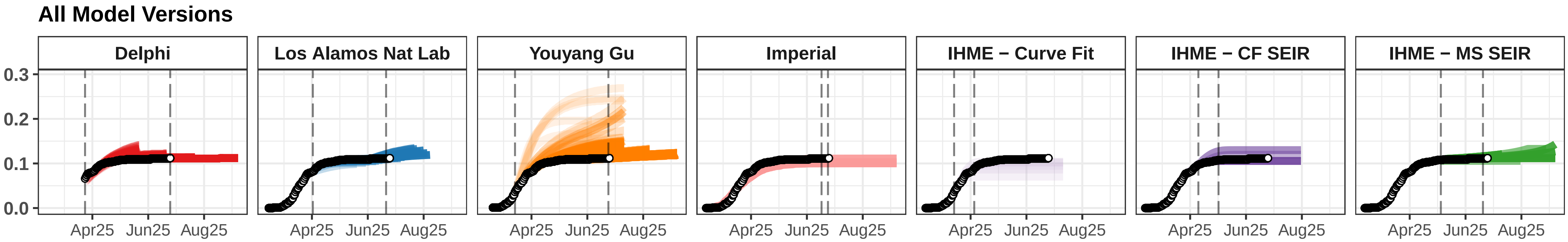

All Model Versions
Delphi
Los Alamos Nat Lab
Youyang Gu
Imperial
IHME − Curve Fit
IHME − CF SEIR
IHME − MS SEIR
0.3
0.2
0.1
0.0
Apr25
Jun25
Aug25

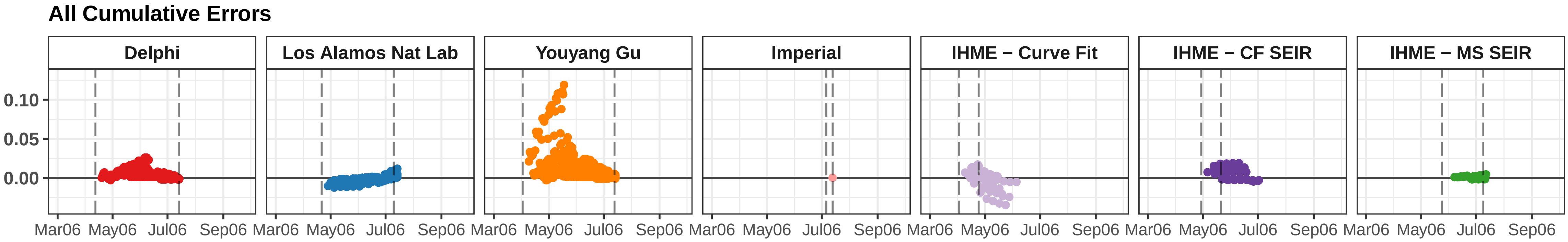

All Cumulative Errors
Delphi
Los Alamos Nat Lab
Youyang Gu
Imperial
IHME − Curve Fit
IHME − CF SEIR
IHME − MS SEIR
0.10
0.05
0.00
Mar06
May06
Jul06
Sep06

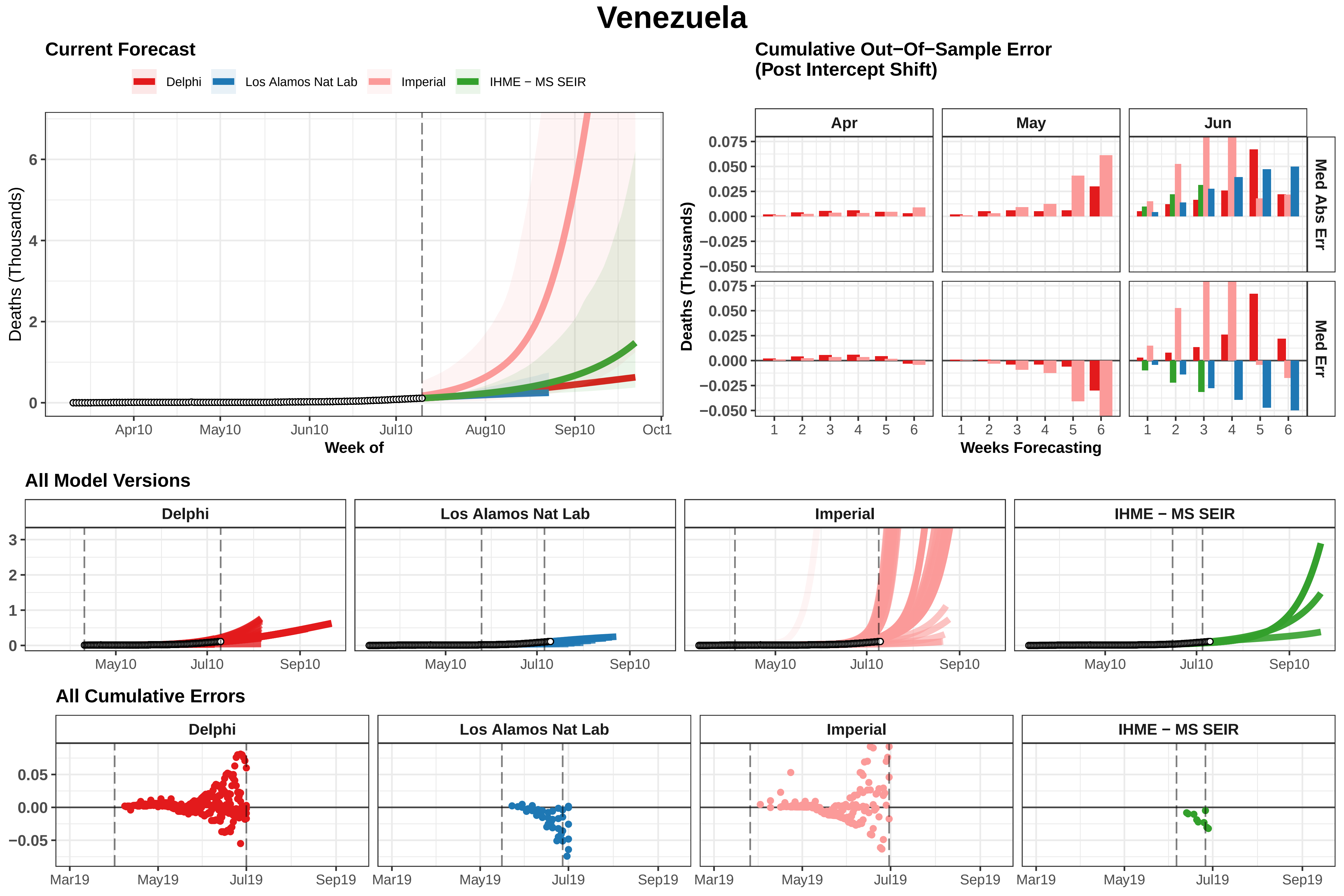
Venezuela
Current Forecast
Delphi
Los Alamos Nat Lab
Imperial
IHME − MS SEIR
Deaths (Thousands)
Week of
Apr10
May10
Jun10
Jul10
Aug10
Sep10
Oct1
Cumulative Out−Of−Sample Error
(Post Intercept Shift)
Apr
May
Jun
Med Abs Err
Med Err
Weeks Forecasting
All Model Versions
All Cumulative Errors
Mar19
May19
Jul19
Sep19

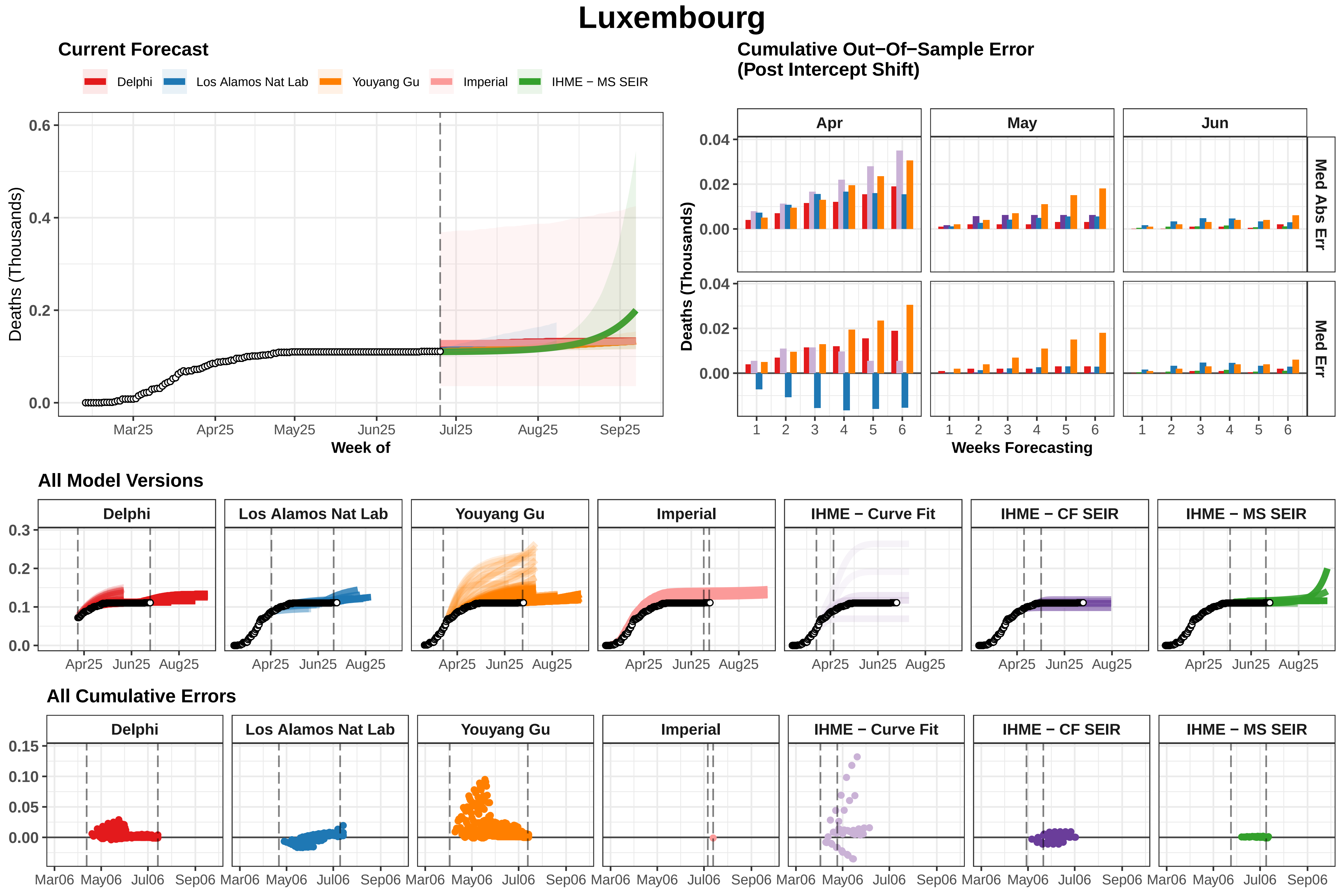
Luxembourg
Current Forecast
Delphi
Los Alamos Nat Lab
Youyang Gu
Imperial
IHME − MS SEIR
Deaths (Thousands)
Week of
Mar25
Apr25
May25
Jun25
Jul25
Aug25
Sep25
Cumulative Out−Of−Sample Error
(Post Intercept Shift)
Apr
May
Jun
Med Abs Err
Med Err
Weeks Forecasting
All Model Versions
IHME − Curve Fit
IHME − CF SEIR
All Cumulative Errors
Mar06
May06
Jul06
Sep06

West Virginia

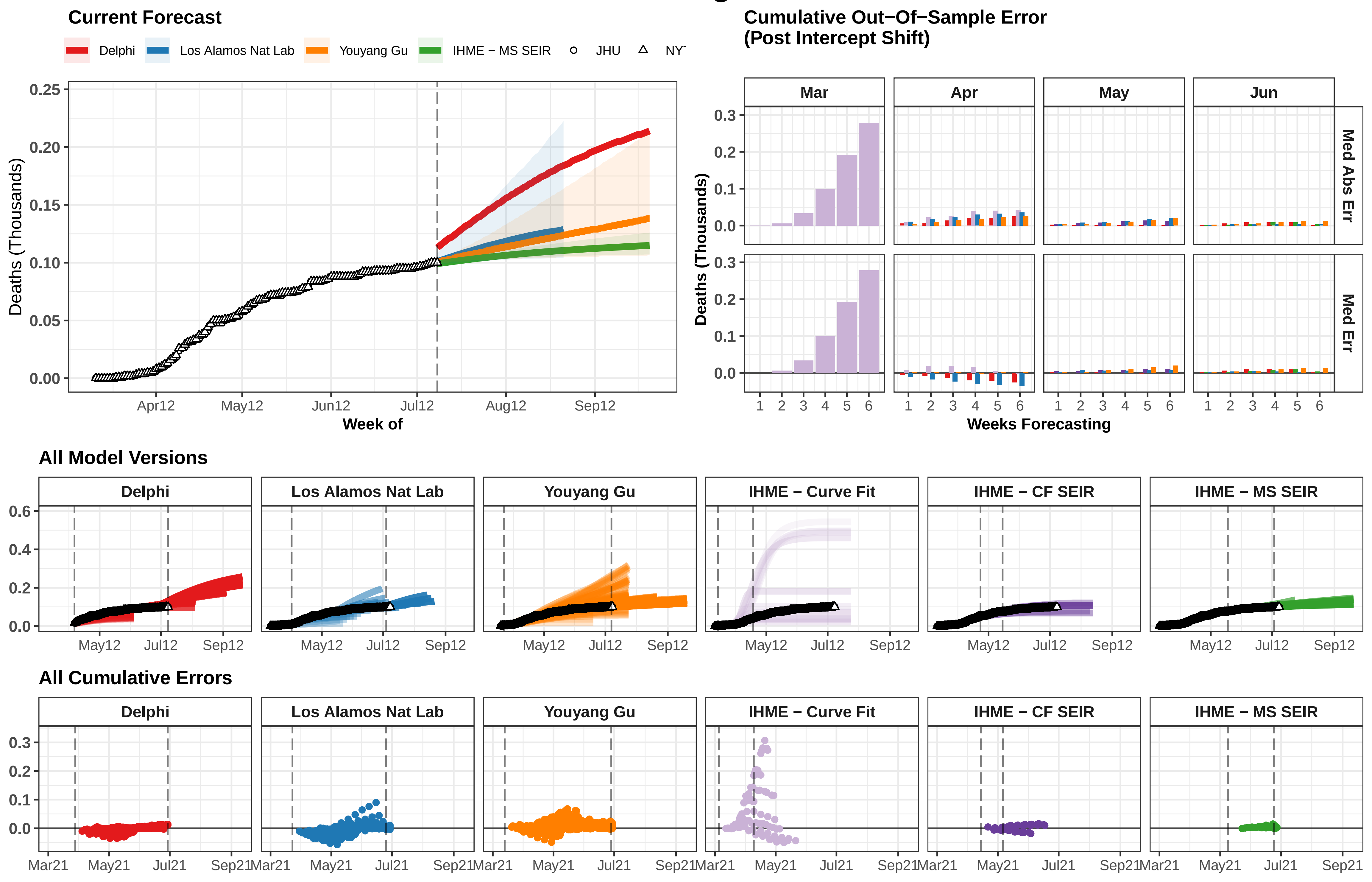

Nicaragua

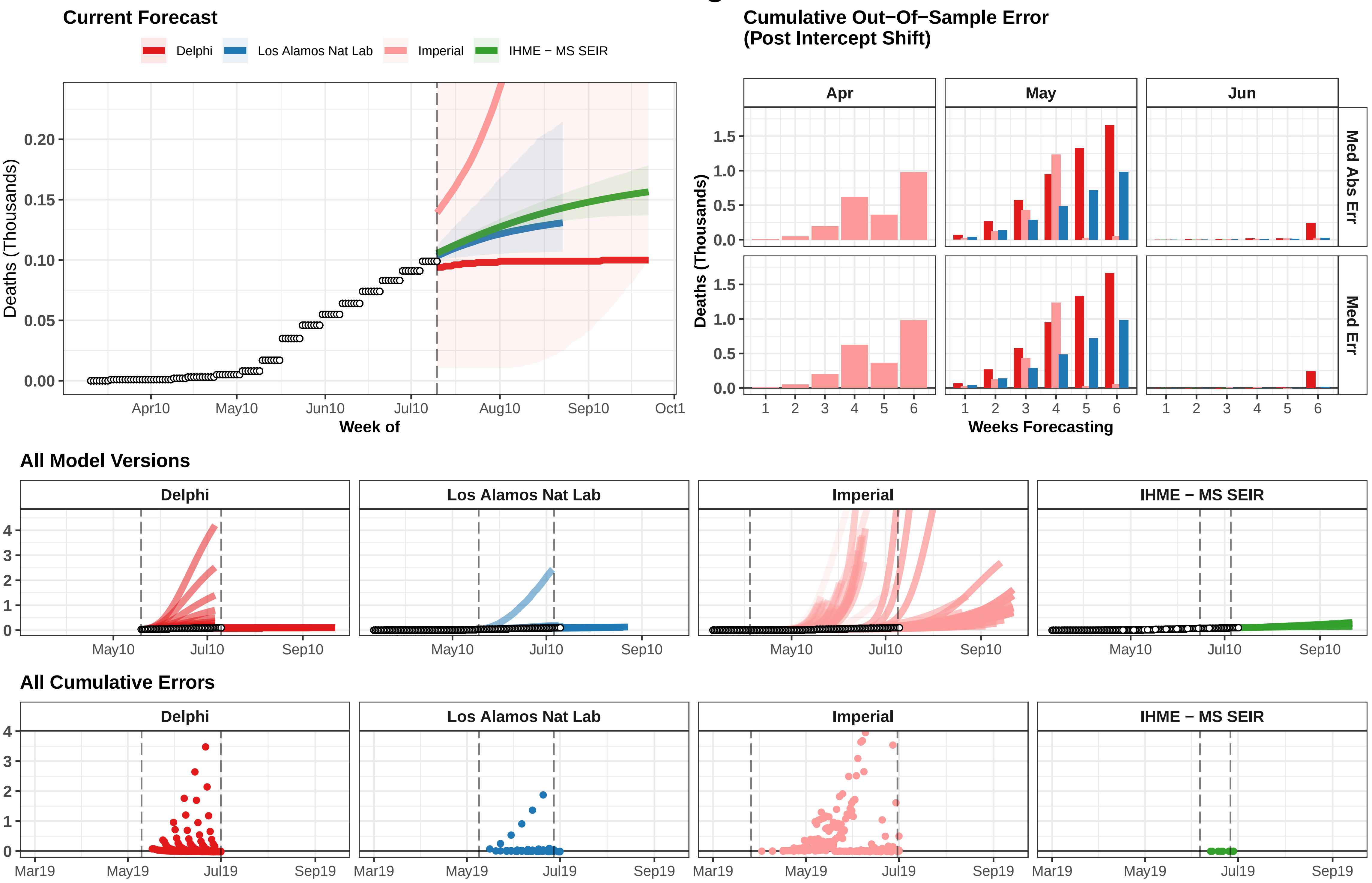

Current Forecast
Delphi
Los Alamos Nat Lab
Imperial
IHME − MS SEIR
Deaths (Thousands)
0.00
0.05
0.10
0.15
0.20
Apr10
May10
Jun10
Jul10
Aug10
Sep10
Oct1
Week of
Cumulative Out−Of−Sample Error
(Post Intercept Shift)
Apr
May
Jun
Med Abs Err
Med Err
Deaths (Thousands)
0.0
0.5
1.0
1.5
1
2
3
4
5
6
Weeks Forecasting
All Model Versions
Delphi
Los Alamos Nat Lab
Imperial
IHME − MS SEIR
0
1
2
3
4
May10
Jul10
Sep10
All Cumulative Errors
Delphi
Los Alamos Nat Lab
Imperial
IHME − MS SEIR
0
1
2
3
4
Mar19
May19
Jul19
Sep19

North Dakota

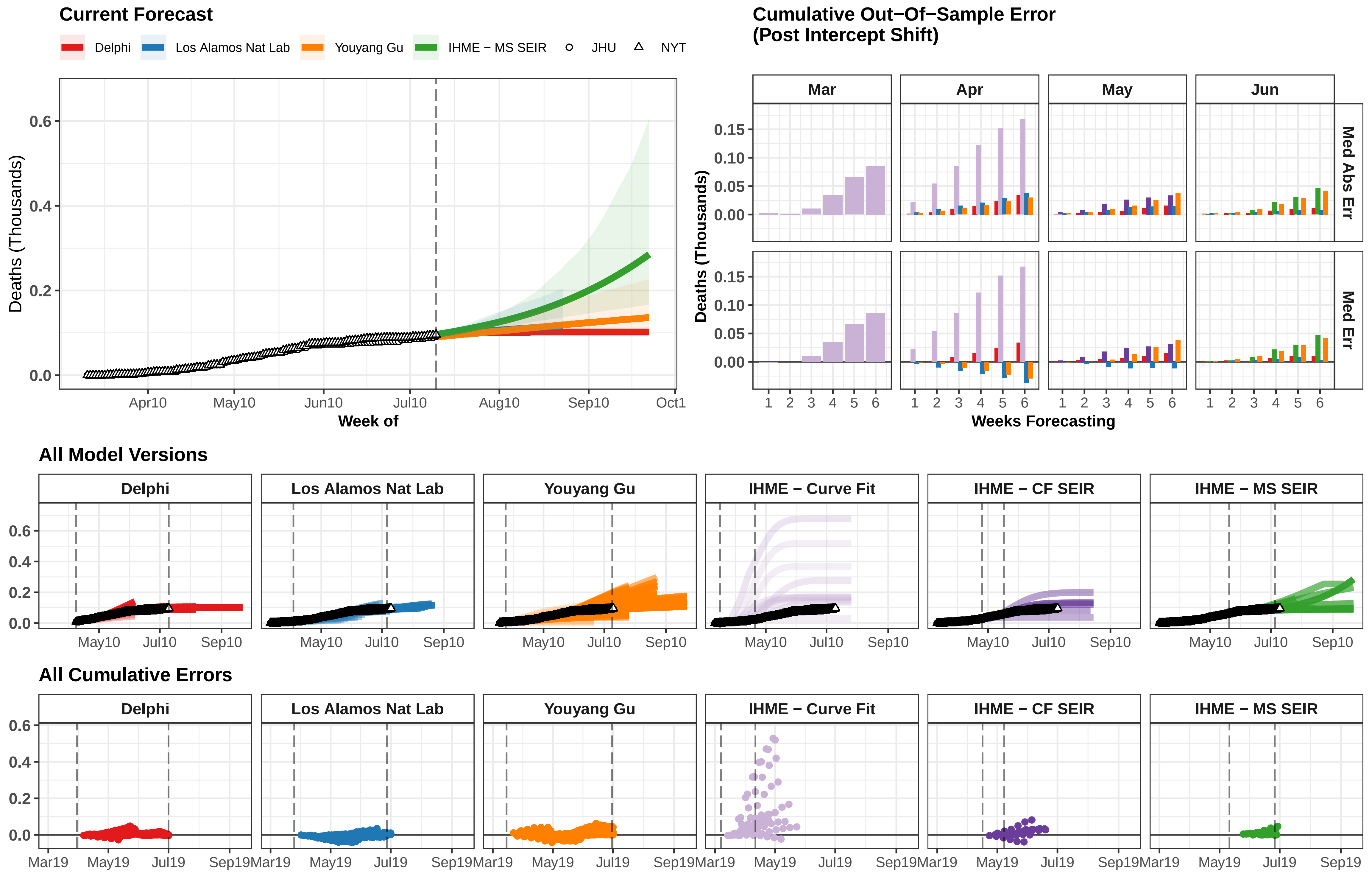

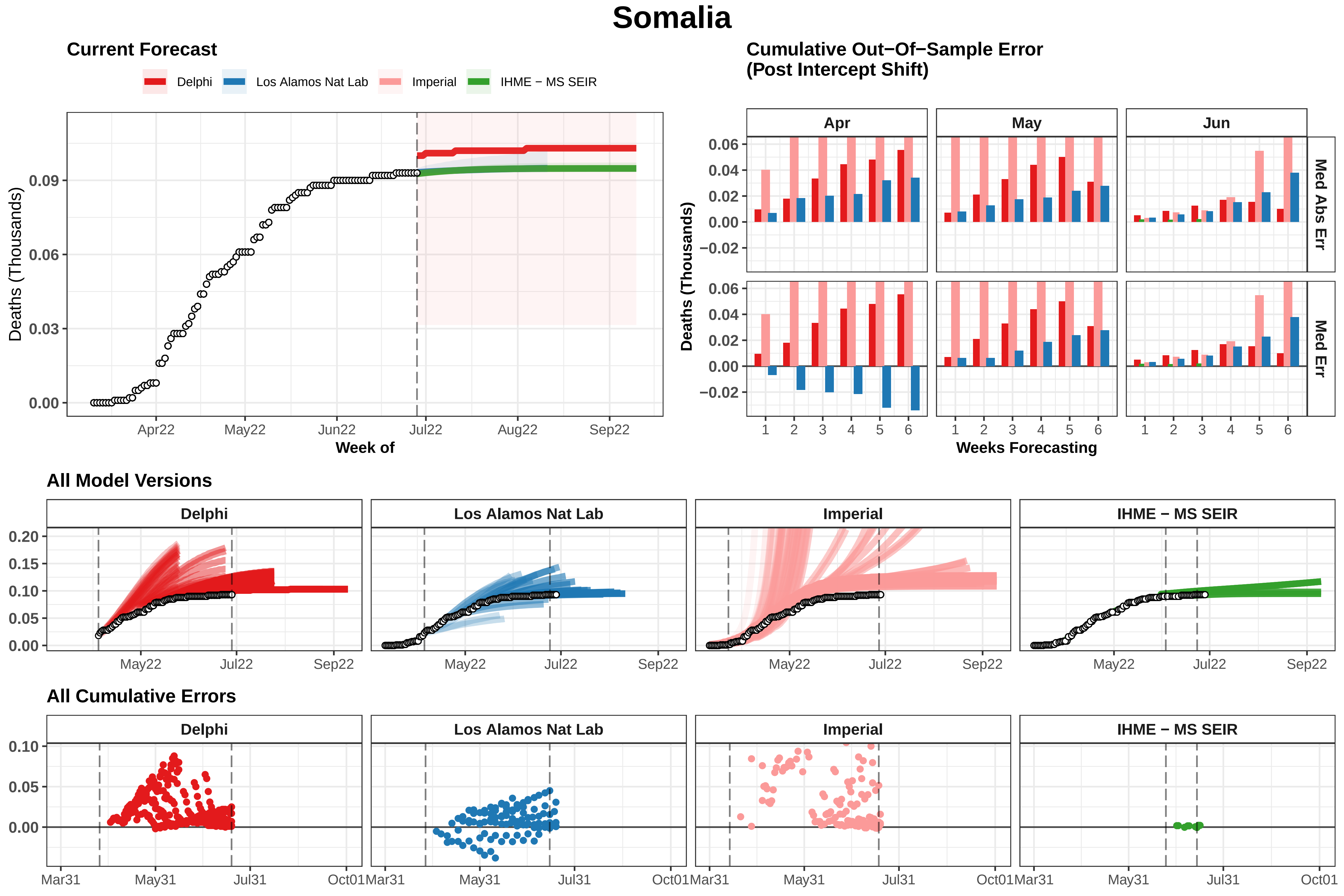
Somalia
Current Forecast
Delphi
Los Alamos Nat Lab
Imperial
IHME − MS SEIR
Deaths (Thousands)
0.00
0.03
0.06
0.09
Apr22
May22
Jun22
Jul22
Aug22
Sep22
Week of
Cumulative Out−Of−Sample Error
(Post Intercept Shift)
Apr
May
Jun
Med Abs Err
Med Err
Weeks Forecasting
All Model Versions
All Cumulative Errors
Mar31
May31
Jul31
Oct01

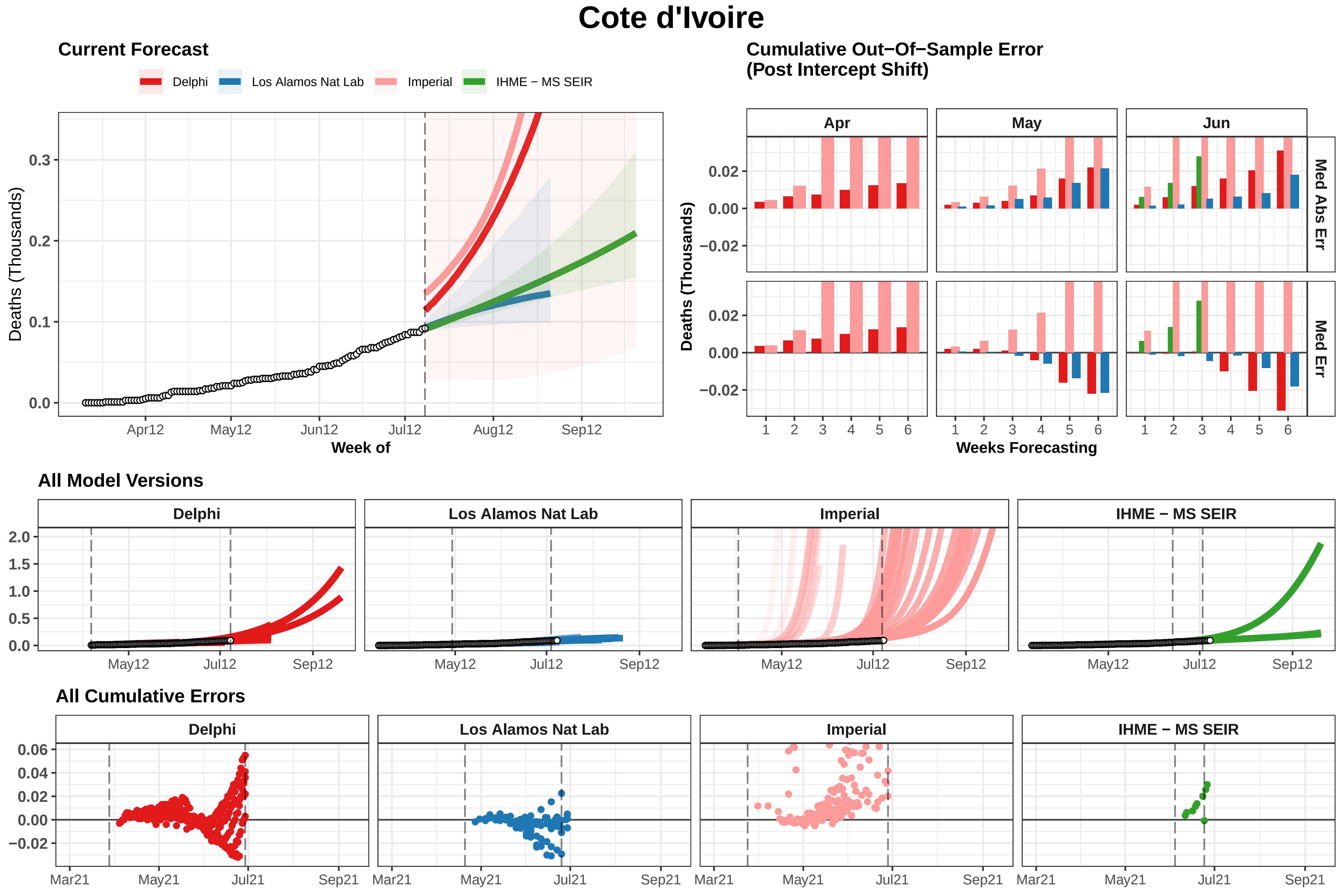
Cote d'Ivoire
Current Forecast
Delphi
Los Alamos Nat Lab
Imperial
IHME − MS SEIR
Deaths (Thousands)
0.0
0.1
0.2
0.3
Apr12
May12
Jun12
Jul12
Aug12
Sep12
Week of
Cumulative Out−Of−Sample Error
(Post Intercept Shift)
Apr
May
Jun
Med Abs Err
Med Err
0.02
0.00
−0.02
1 2 3 4 5 6
Weeks Forecasting
All Model Versions
Delphi
Los Alamos Nat Lab
Imperial
IHME − MS SEIR
2.0
1.5
1.0
0.5
0.0
May12
Jul12
Sep12
All Cumulative Errors
0.06
0.04
0.02
0.00
−0.02
Mar21
May21
Jul21
Sep21

Cuba

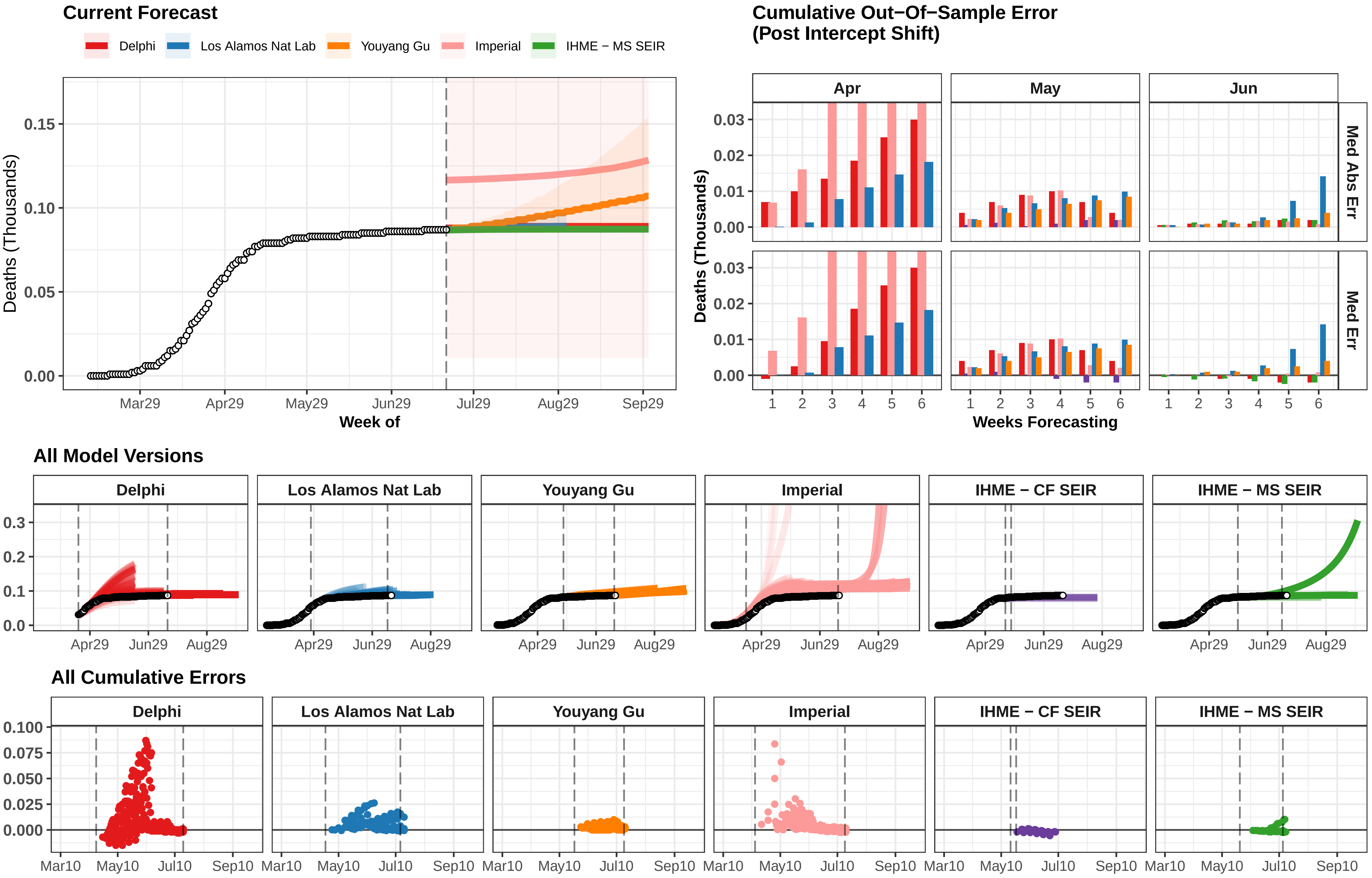

Current Forecast
Delphi
Los Alamos Nat Lab
Youyang Gu
Imperial
IHME − MS SEIR
Deaths (Thousands)
0.00
0.05
0.10
0.15
Mar29
Apr29
May29
Jun29
Jul29
Aug29
Sep29
Week of
Cumulative Out−Of−Sample Error
(Post Intercept Shift)
Apr
May
Jun
Med Abs Err
Med Err
0.00
0.01
0.02
0.03
1
2
3
4
5
6
Weeks Forecasting
All Model Versions
Delphi
Los Alamos Nat Lab
Youyang Gu
Imperial
IHME − CF SEIR
IHME − MS SEIR
0.0
0.1
0.2
0.3
Apr29
Jun29
Aug29
All Cumulative Errors
0.000
0.025
0.050
0.075
0.100
Mar10
May10
Jul10
Sep10

Uzbekistan

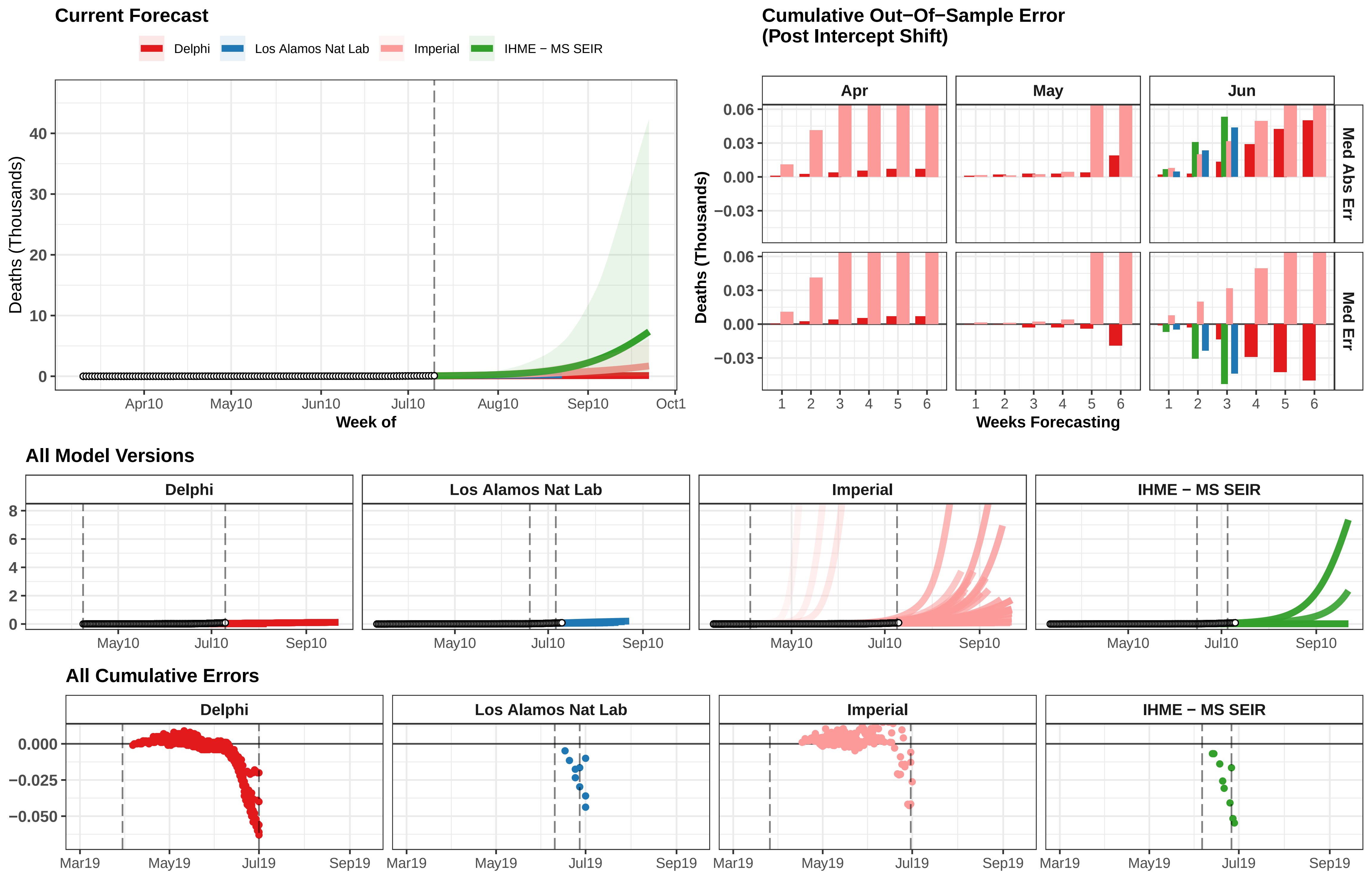

Current Forecast
Delphi
Los Alamos Nat Lab
Imperial
IHME − MS SEIR
Deaths (Thousands)
Week of
Apr10
May10
Jun10
Jul10
Aug10
Sep10
Oct1
Cumulative Out−Of−Sample Error
(Post Intercept Shift)
Apr
May
Jun
Med Abs Err
Med Err
Weeks Forecasting
All Model Versions
All Cumulative Errors
Mar19
May19
Jul19
Sep19

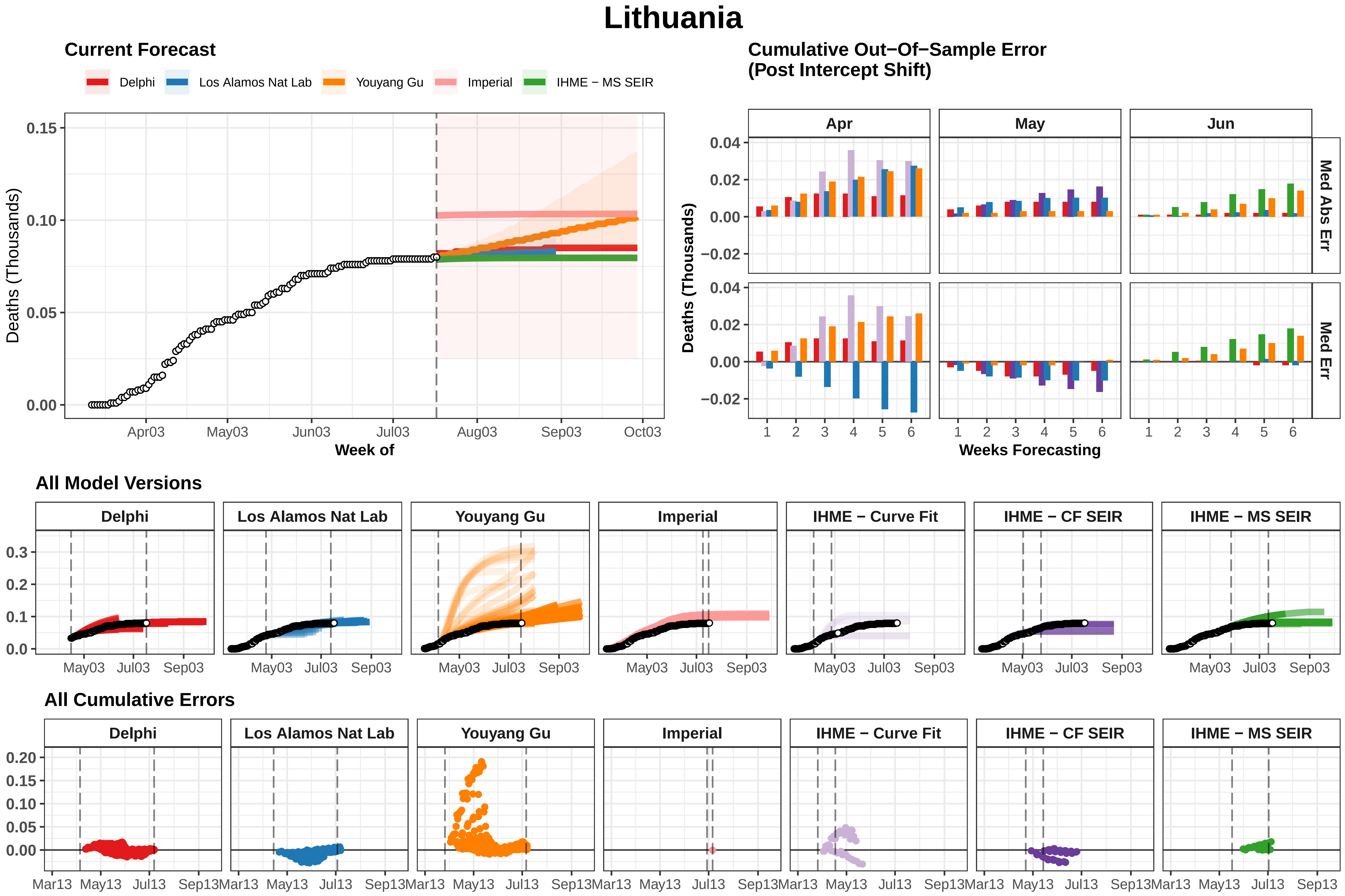
Lithuania
Current Forecast
Delphi
Los Alamos Nat Lab
Youyang Gu
Imperial
IHME − MS SEIR
Deaths (Thousands)
0.15
0.10
0.05
0.00
Apr03
May03
Jun03
Jul03
Aug03
Sep03
Oct03
Week of
Cumulative Out−Of−Sample Error
(Post Intercept Shift)
Apr
May
Jun
Med Abs Err
Med Err
0.04
0.02
0.00
−0.02
1 2 3 4 5 6
Weeks Forecasting
All Model Versions
Delphi
Los Alamos Nat Lab
Youyang Gu
Imperial
IHME − Curve Fit
IHME − CF SEIR
IHME − MS SEIR
0.3
0.2
0.1
0.0
May03 Jul03 Sep03
All Cumulative Errors
0.20
0.15
0.10
0.05
0.00
Mar13 May13 Jul13 Sep13

Chad

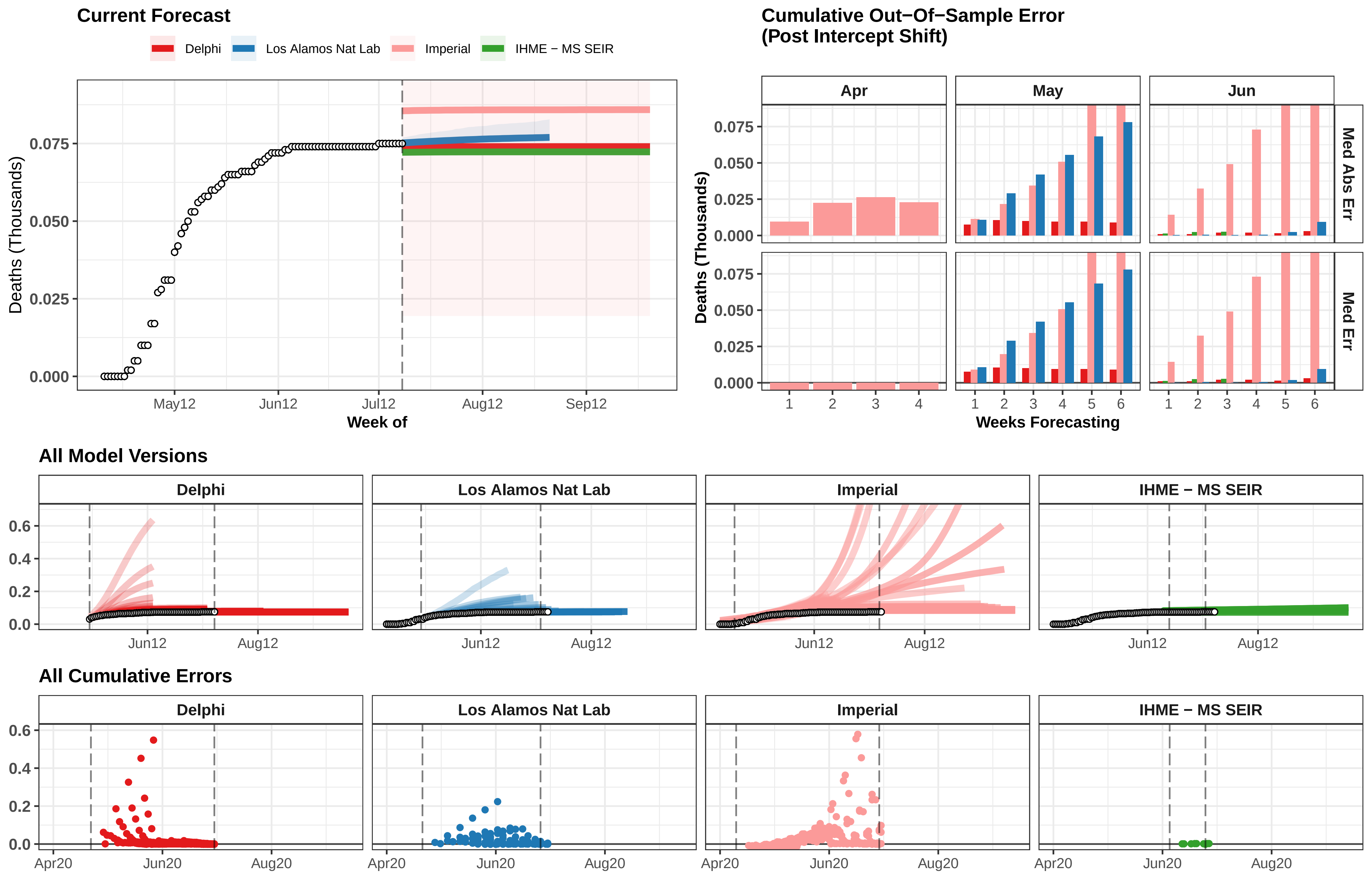

Current Forecast
Delphi
Los Alamos Nat Lab
Imperial
IHME − MS SEIR
Deaths (Thousands)
0.075
0.050
0.025
0.000
May12
Jun12
Jul12
Aug12
Sep12
Week of
Cumulative Out−Of−Sample Error
(Post Intercept Shift)
Apr
May
Jun
Med Abs Err
Med Err
Weeks Forecasting
All Model Versions
Delphi
Los Alamos Nat Lab
Imperial
IHME − MS SEIR
0.6
0.4
0.2
0.0
Jun12
Aug12
All Cumulative Errors
Apr20
Jun20
Aug20

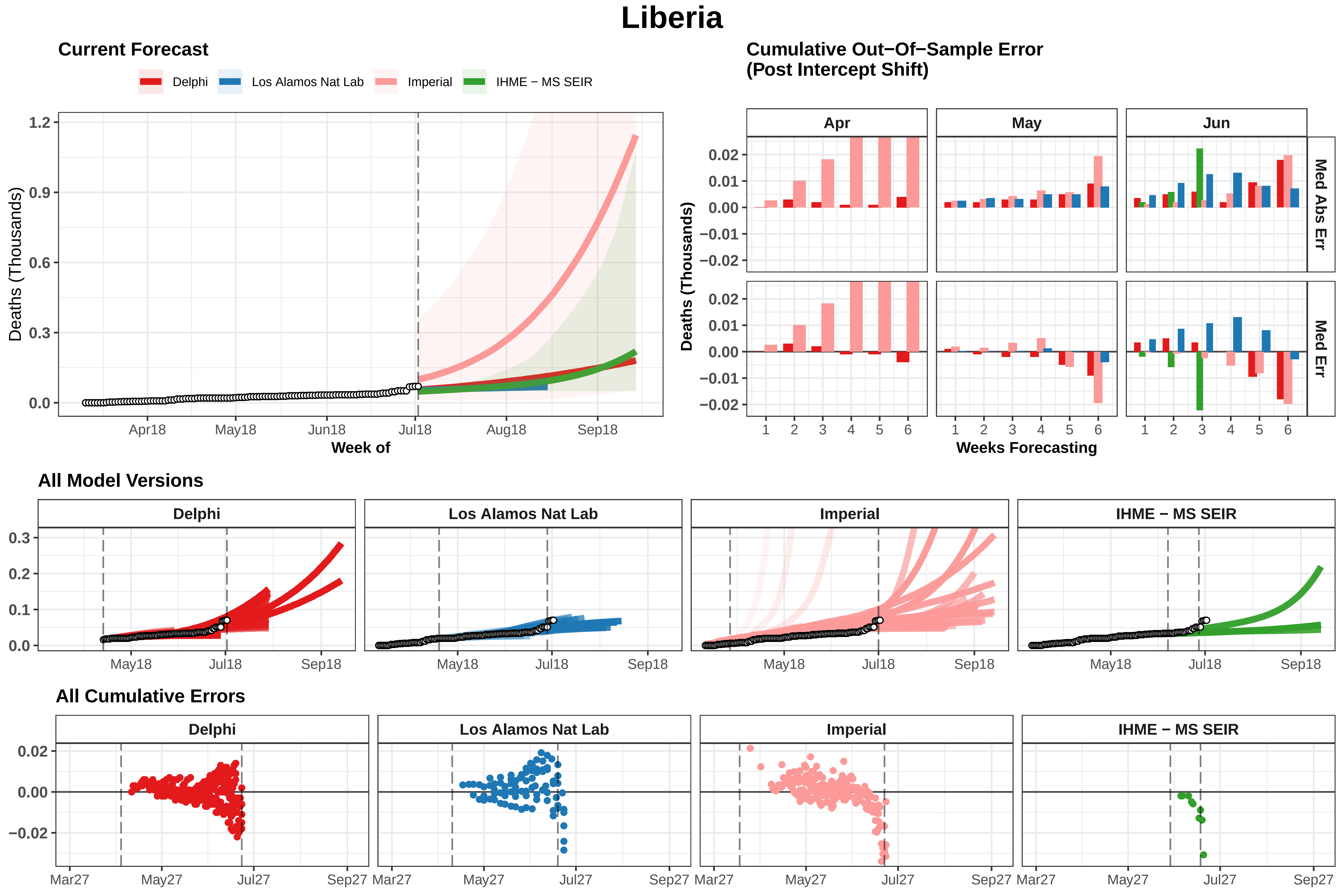
Liberia
Current Forecast
Delphi
Los Alamos Nat Lab
Imperial
IHME − MS SEIR
Deaths (Thousands)
Week of
Apr18
May18
Jun18
Jul18
Aug18
Sep18
Cumulative Out−Of−Sample Error (Post Intercept Shift)
Apr
May
Jun
Med Abs Err
Med Err
Weeks Forecasting
All Model Versions
All Cumulative Errors
Mar27
May27
Jul27
Sep27

Estonia

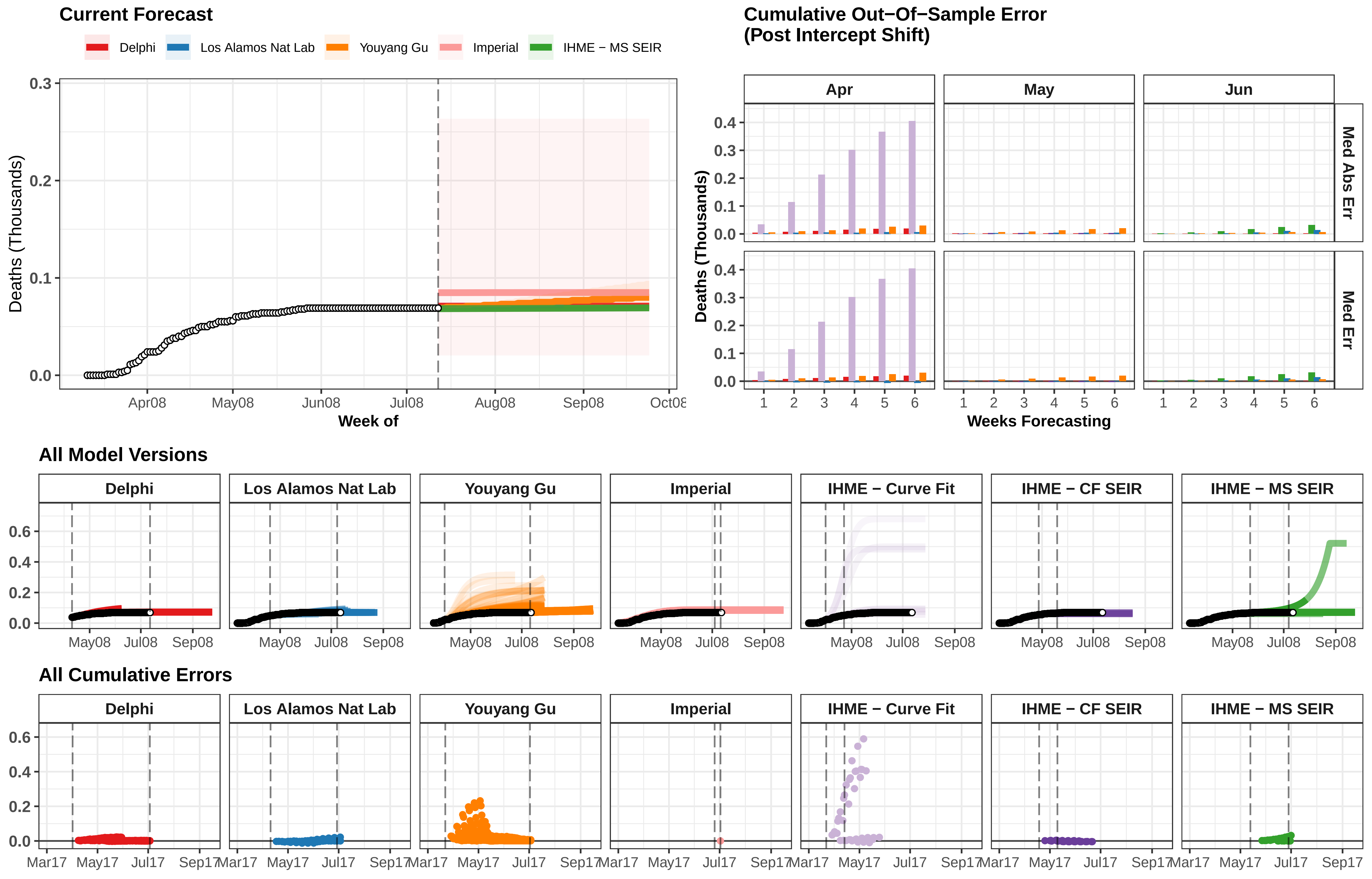

Current Forecast
Delphi
Los Alamos Nat Lab
Youyang Gu
Imperial
IHME − MS SEIR
Deaths (Thousands)
Week of
Apr08
May08
Jun08
Jul08
Aug08
Sep08
Oct08
Cumulative Out−Of−Sample Error
(Post Intercept Shift)
Apr
May
Jun
Med Abs Err
Med Err
Weeks Forecasting
All Model Versions
Delphi
Los Alamos Nat Lab
Youyang Gu
Imperial
IHME − Curve Fit
IHME − CF SEIR
IHME − MS SEIR
All Cumulative Errors
Mar17
May17
Jul17
Sep17

Niger

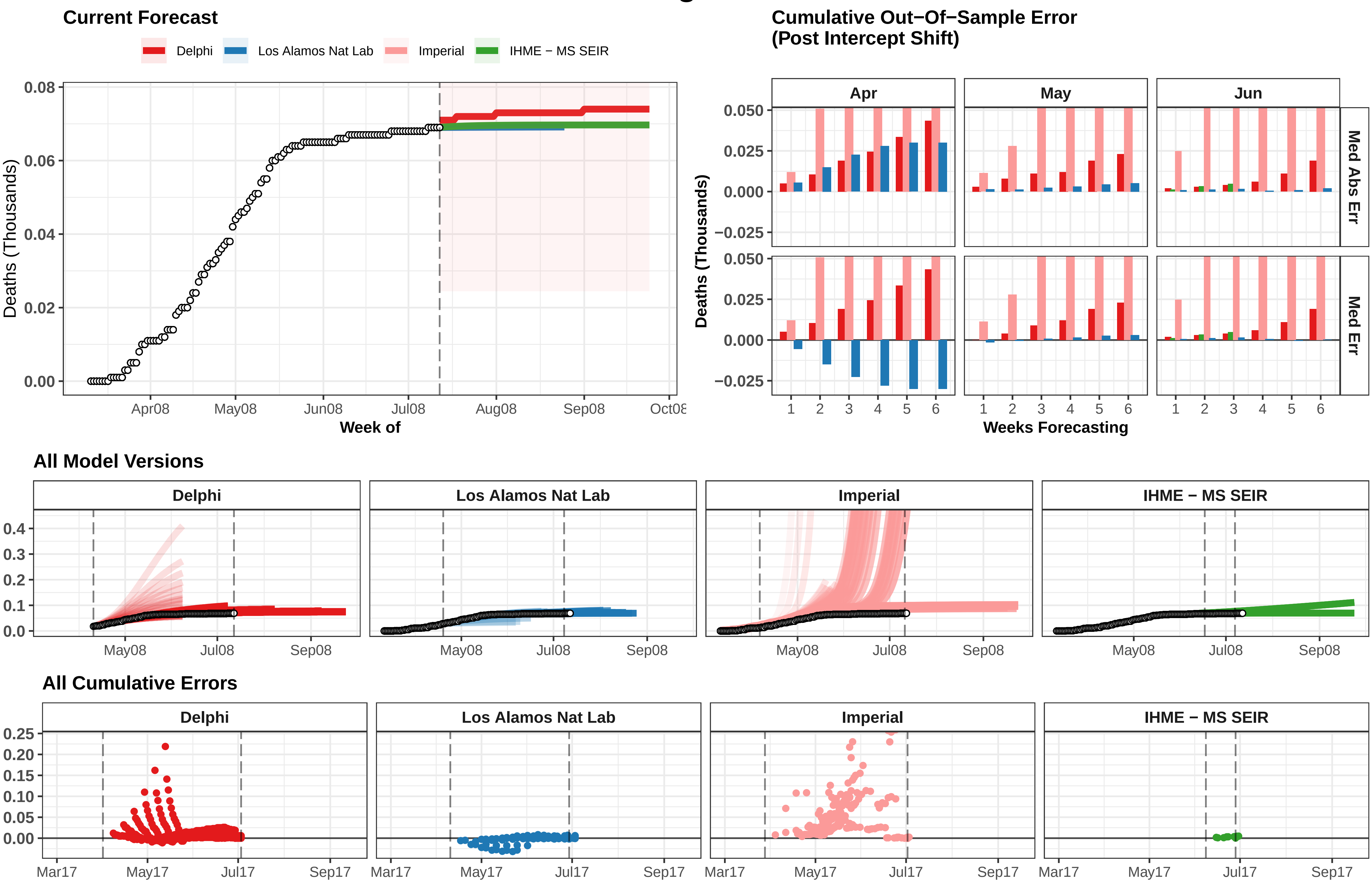

Current Forecast
Delphi
Los Alamos Nat Lab
Imperial
IHME − MS SEIR
Deaths (Thousands)
0.08
0.06
0.04
0.02
0.00
Apr08
May08
Jun08
Jul08
Aug08
Sep08
Week of
Cumulative Out−Of−Sample Error
(Post Intercept Shift)
Apr
May
Jun
Med Abs Err
Med Err
0.050
0.025
0.000
−0.025
1
2
3
4
5
6
Weeks Forecasting
All Model Versions
0.4
0.3
0.2
0.1
0.0
All Cumulative Errors
0.25
0.20
0.15
0.10
0.05
0.00
Mar17
May17
Jul17
Sep17

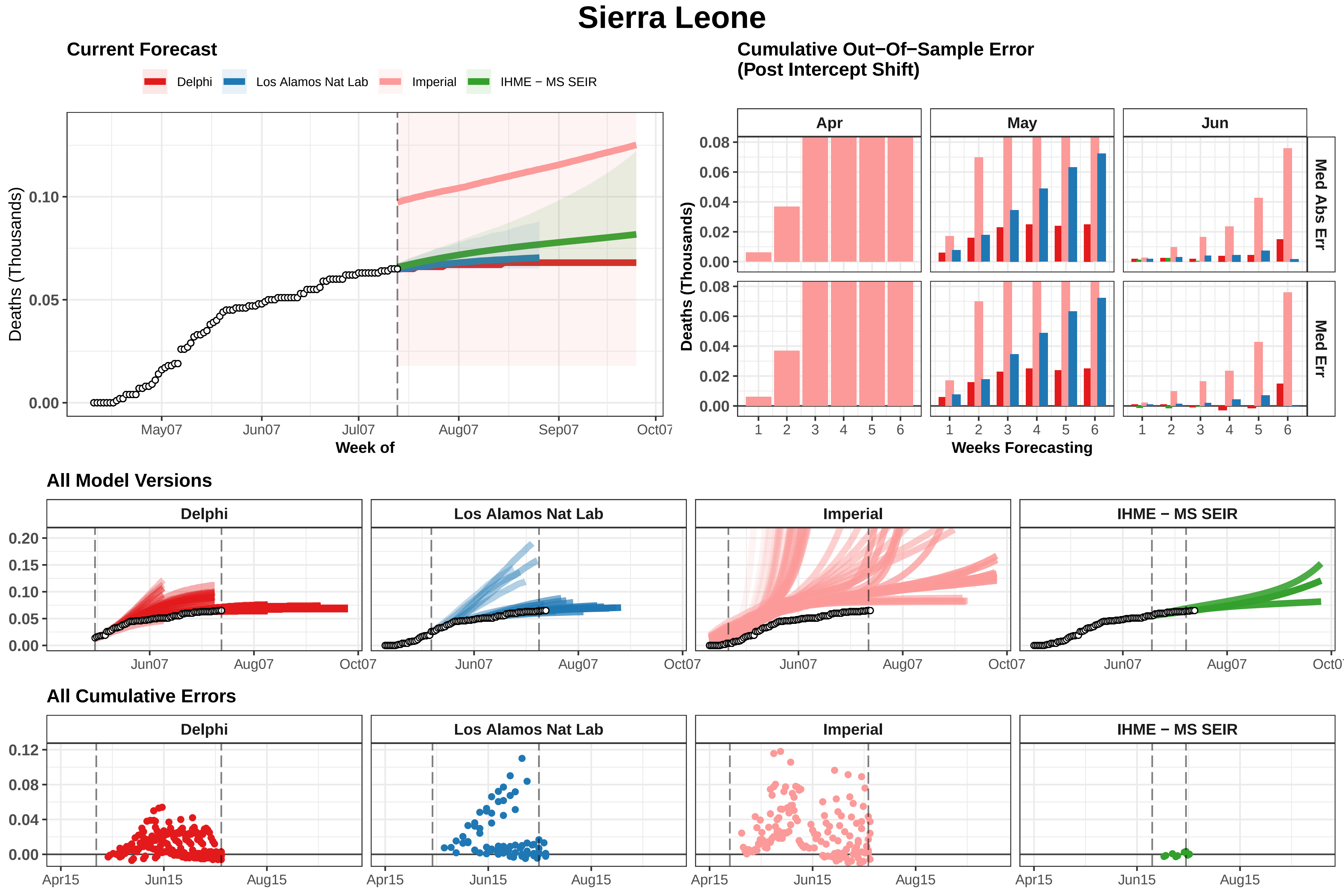

Sierra Leone
Current Forecast
Delphi
Los Alamos Nat Lab
Imperial
IHME − MS SEIR
Deaths (Thousands)
Week of
Cumulative Out−Of−Sample Error
(Post Intercept Shift)
Apr
May
Jun
Med Abs Err
Med Err
Weeks Forecasting
All Model Versions
All Cumulative Errors

Costa Rica

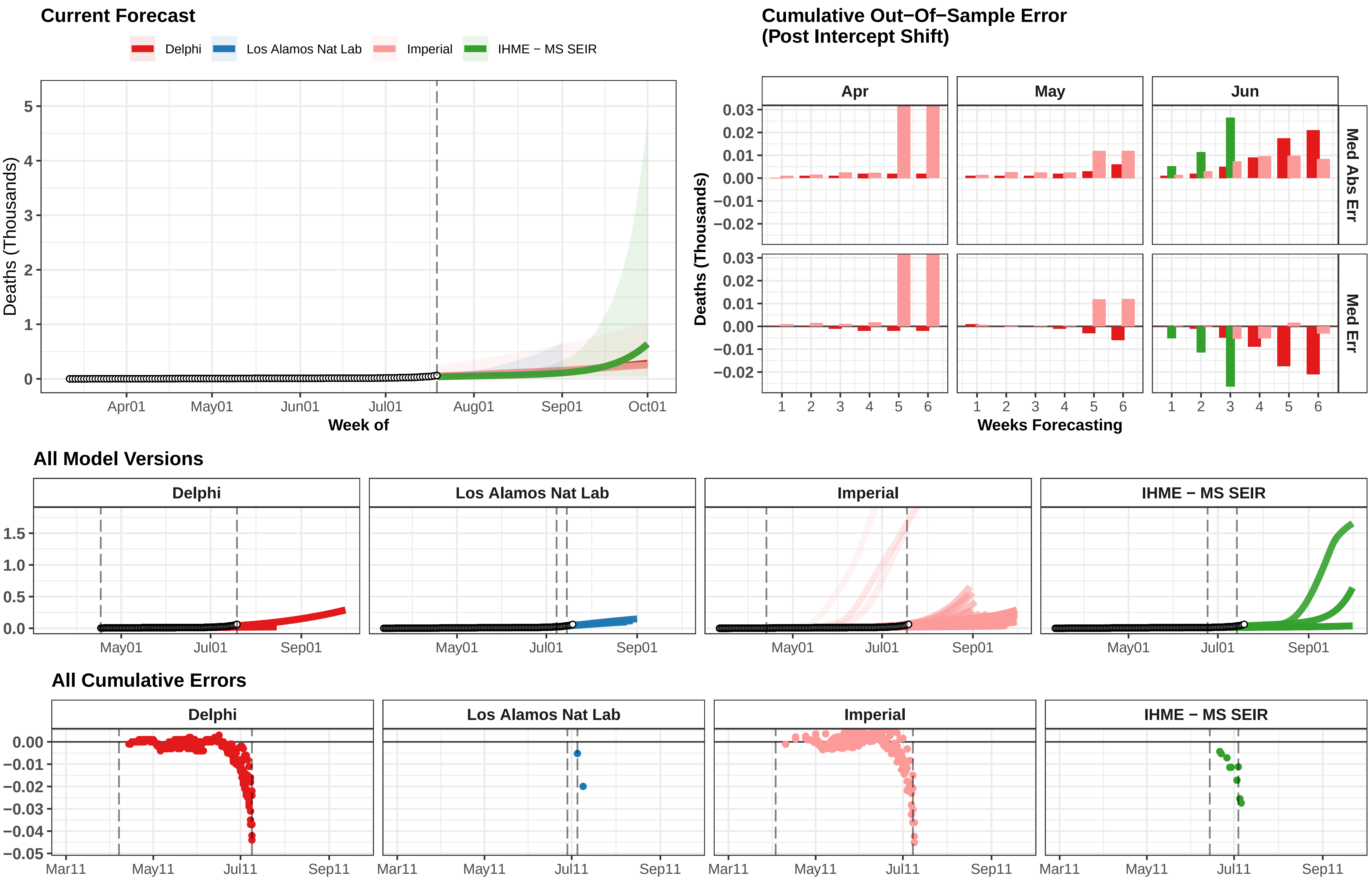

Current Forecast
Delphi
Los Alamos Nat Lab
Imperial
IHME − MS SEIR
Deaths (Thousands)
Week of
Apr01
May01
Jun01
Jul01
Aug01
Sep01
Oct01
Cumulative Out−Of−Sample Error
(Post Intercept Shift)
Apr
May
Jun
Med Abs Err
Med Err
Weeks Forecasting
All Model Versions
All Cumulative Errors
Mar11
May11
Jul11
Sep11

Madagascar

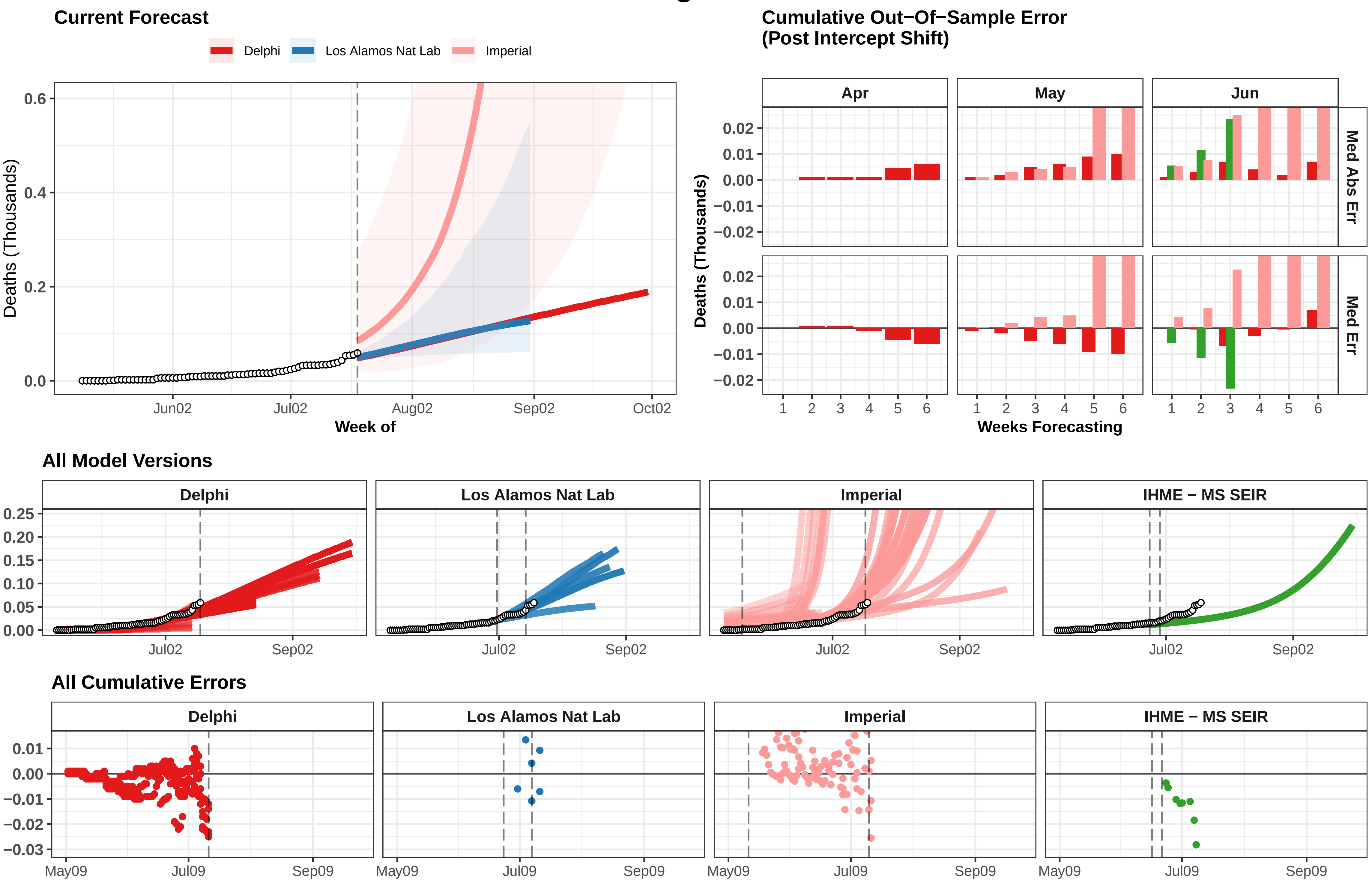
Current Forecast
Delphi
Los Alamos Nat Lab
Imperial
Deaths (Thousands)
Week of
Jun02
Jul02
Aug02
Sep02
Oct02
Cumulative Out−Of−Sample Error
(Post Intercept Shift)
Apr
May
Jun
Med Abs Err
Med Err
Weeks Forecasting
All Model Versions
Delphi
Los Alamos Nat Lab
Imperial
IHME − MS SEIR
All Cumulative Errors
May09
Jul09
Sep09

Malawi

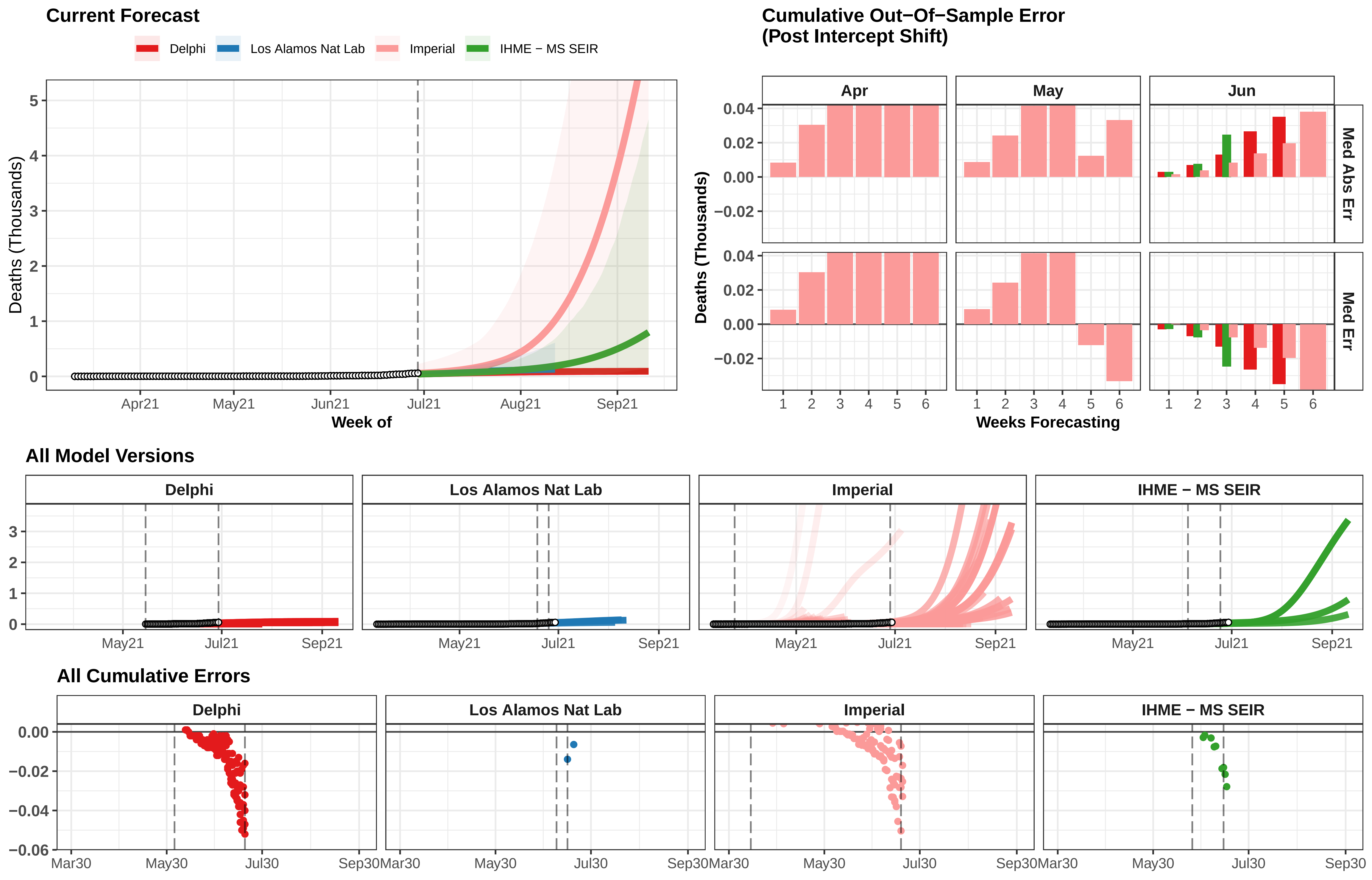

Current Forecast
Delphi
Los Alamos Nat Lab
Imperial
IHME − MS SEIR
Deaths (Thousands)
Week of
Apr21
May21
Jun21
Jul21
Aug21
Sep21
Cumulative Out−Of−Sample Error
(Post Intercept Shift)
Apr
May
Jun
Med Abs Err
Med Err
Weeks Forecasting
All Model Versions
All Cumulative Errors
Mar30
May30
Jul30
Sep30

Thailand

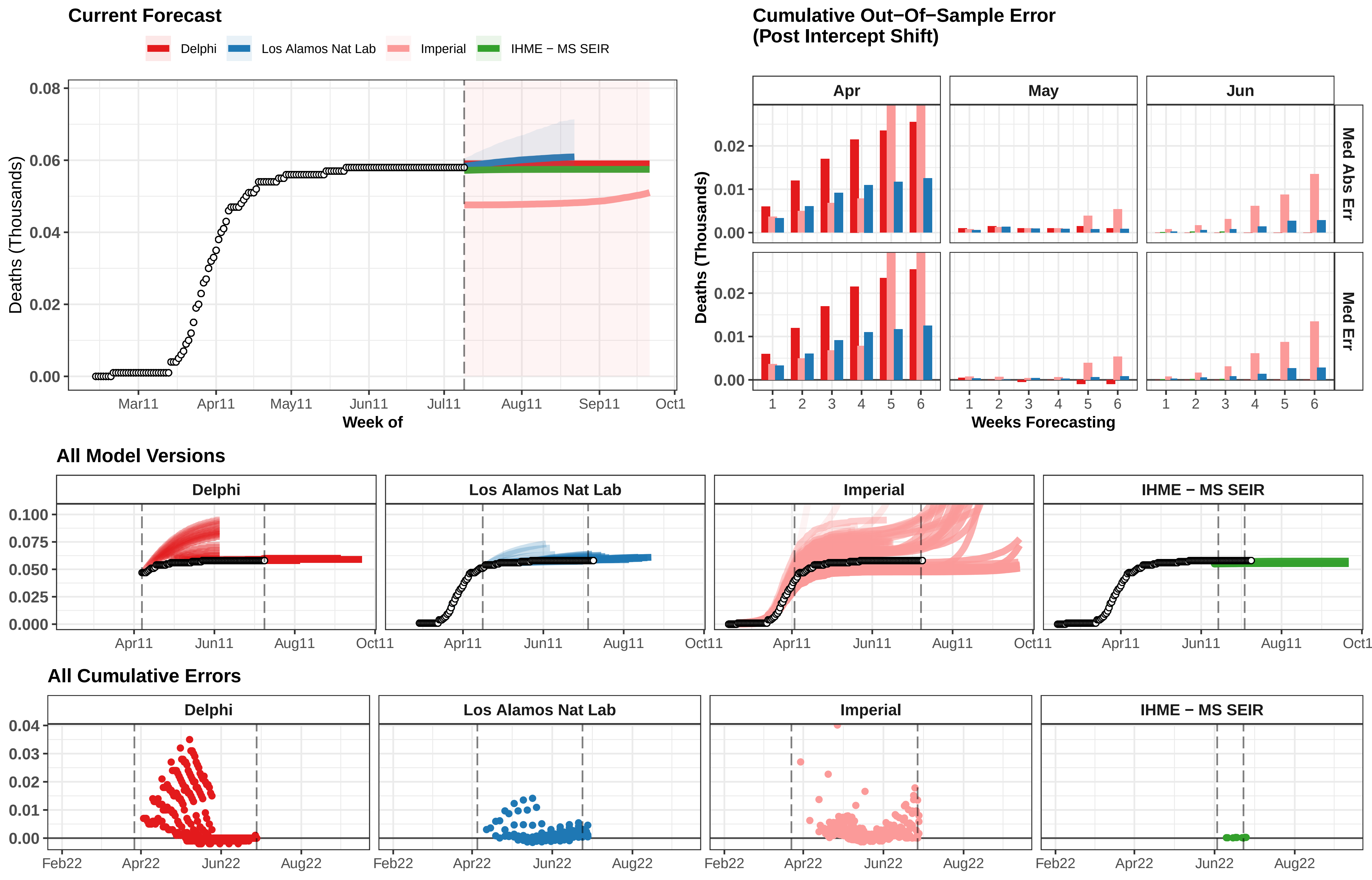
Current Forecast
Delphi
Los Alamos Nat Lab
Imperial
IHME − MS SEIR
Deaths (Thousands)
0.08
0.06
0.04
0.02
0.00
Mar11
Apr11
May11
Jun11
Jul11
Aug11
Sep11
Oct1
Week of
Cumulative Out−Of−Sample Error
(Post Intercept Shift)
Apr
May
Jun
Med Abs Err
Med Err
Deaths (Thousands)
0.02
0.01
0.00
1
2
3
4
5
6
Weeks Forecasting
All Model Versions
Delphi
Los Alamos Nat Lab
Imperial
IHME − MS SEIR
0.100
0.075
0.050
0.025
0.000
Apr11
Jun11
Aug11
Oct11
All Cumulative Errors
Delphi
Los Alamos Nat Lab
Imperial
IHME − MS SEIR
0.04
0.03
0.02
0.01
0.00
Feb22
Apr22
Jun22
Aug22

Tajikistan

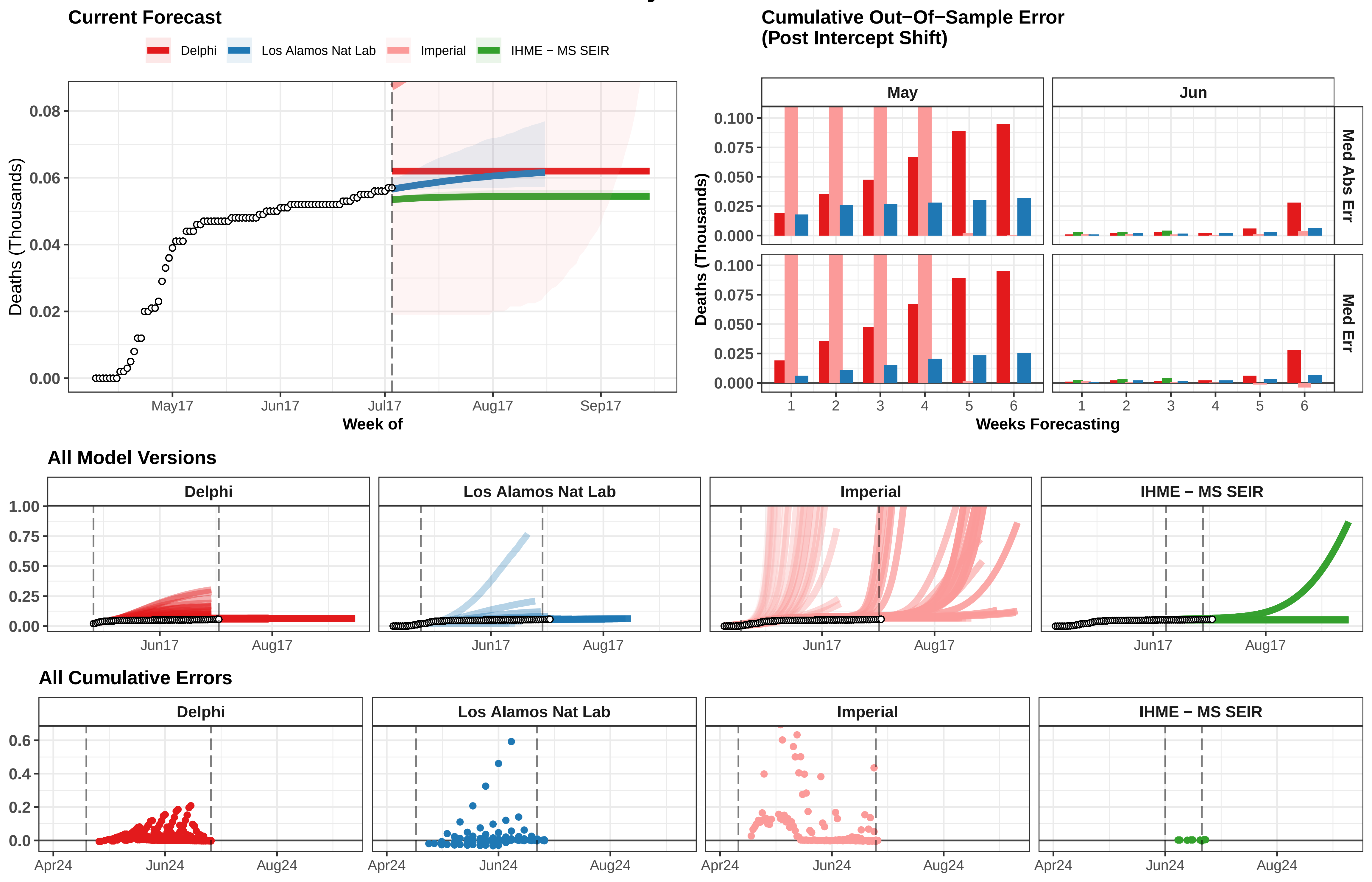

Current Forecast
Delphi
Los Alamos Nat Lab
Imperial
IHME − MS SEIR
Deaths (Thousands)
0.08
0.06
0.04
0.02
0.00
May17
Jun17
Jul17
Aug17
Sep17
Week of
Cumulative Out−Of−Sample Error
(Post Intercept Shift)
May
Jun
Med Abs Err
Med Err
0.100
0.075
0.050
0.025
0.000
Weeks Forecasting
All Model Versions
Delphi
Los Alamos Nat Lab
Imperial
IHME − MS SEIR
1.00
0.75
0.50
0.25
0.00
Jun17
Aug17
All Cumulative Errors
0.6
0.4
0.2
0.0
Apr24
Jun24
Aug24

Djibouti

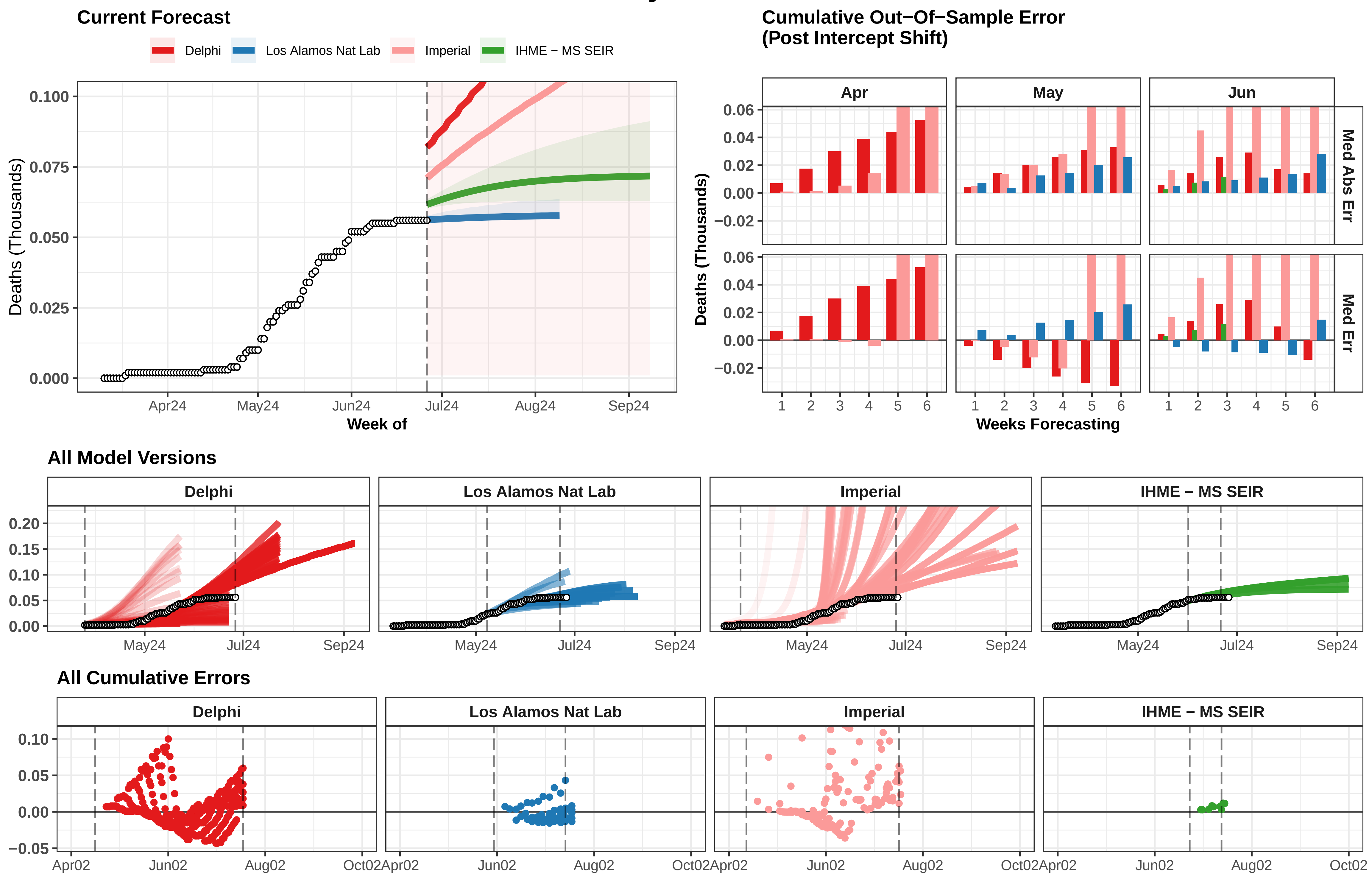
Current Forecast
Delphi
Los Alamos Nat Lab
Imperial
IHME − MS SEIR
Deaths (Thousands)
Week of
Apr24
May24
Jun24
Jul24
Aug24
Sep24
Cumulative Out−Of−Sample Error
(Post Intercept Shift)
Apr
May
Jun
Med Abs Err
Med Err
Weeks Forecasting
All Model Versions
All Cumulative Errors
Apr02
Jun02
Aug02
Oct02

Vermont

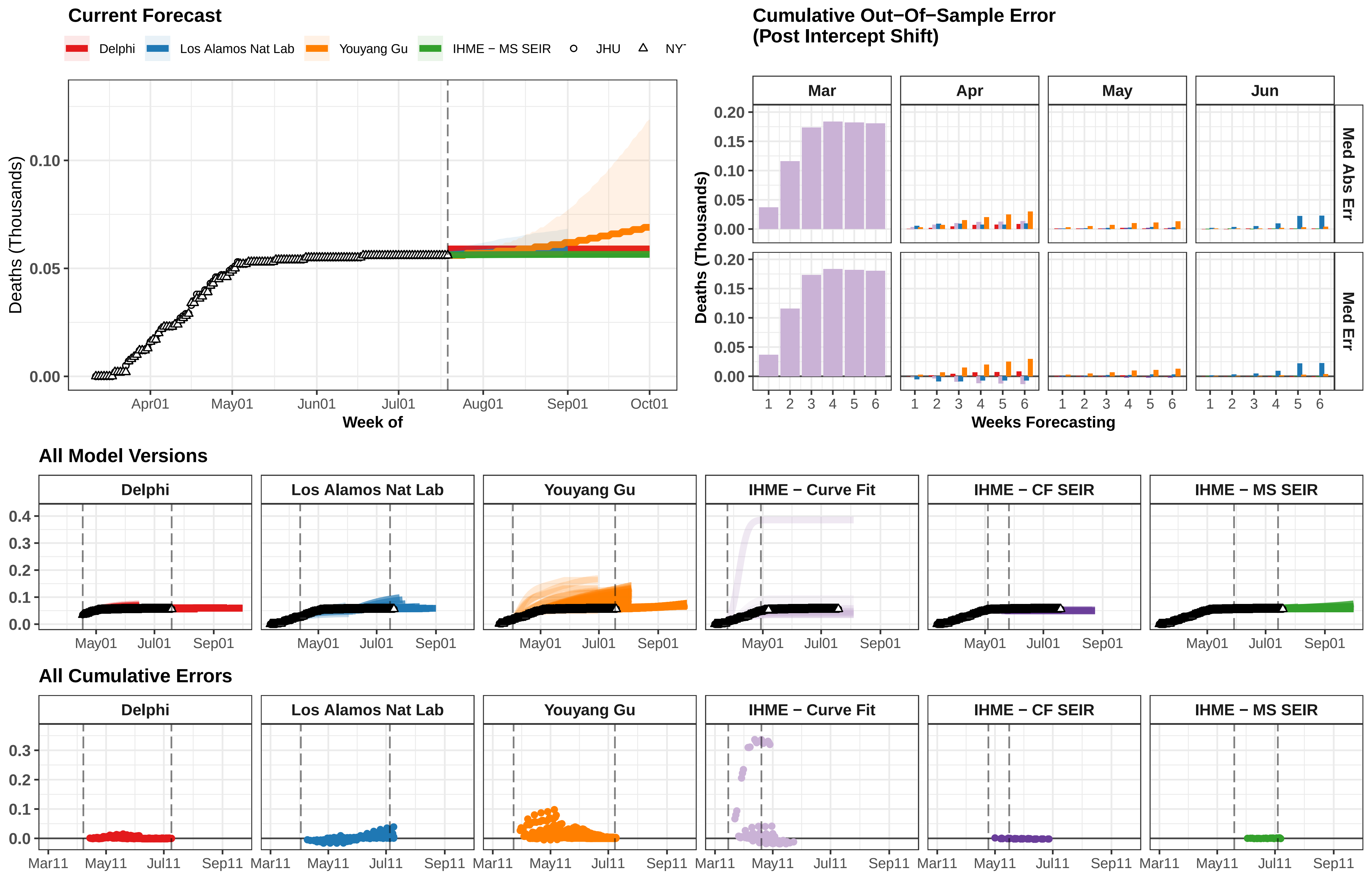

Current Forecast
Delphi
Los Alamos Nat Lab
Youyang Gu
IHME − MS SEIR
JHU
NY
Deaths (Thousands)
0.00
0.05
0.10
Apr01
May01
Jun01
Jul01
Aug01
Sep01
Oct01
Week of
Cumulative Out−Of−Sample Error
(Post Intercept Shift)
Mar
Apr
May
Jun
Med Abs Err
Med Err
0.20
0.15
0.10
0.05
0.00
1
2
3
4
5
6
Weeks Forecasting
All Model Versions
Delphi
Los Alamos Nat Lab
Youyang Gu
IHME − Curve Fit
IHME − CF SEIR
IHME − MS SEIR
0.4
0.3
0.2
0.1
0.0
May01
Jul01
Sep01
All Cumulative Errors
Mar11
May11
Jul11
Sep11

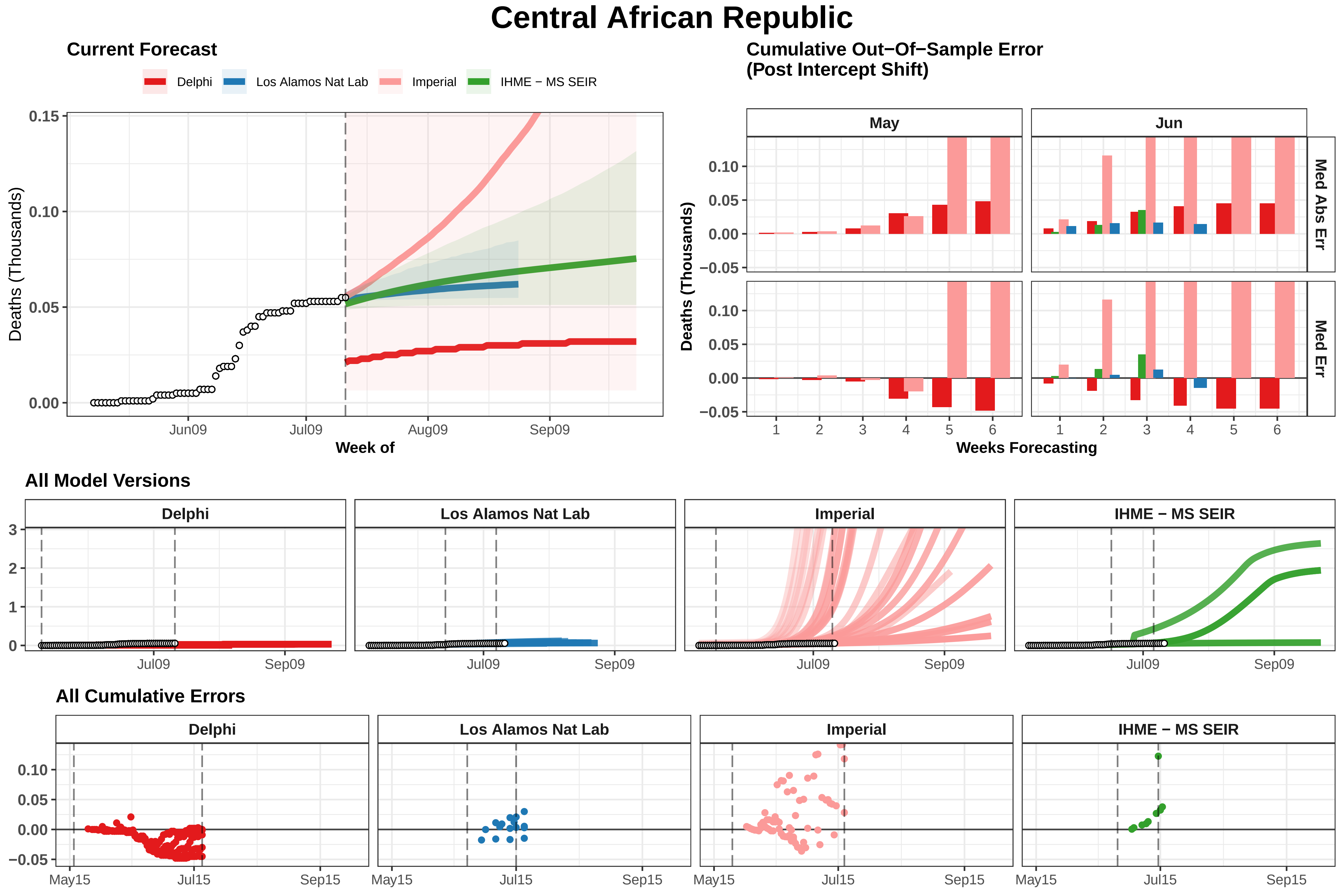

Central African Republic
Current Forecast
Delphi
Los Alamos Nat Lab
Imperial
IHME − MS SEIR
Deaths (Thousands)
0.15
0.10
0.05
0.00
Jun09
Jul09
Aug09
Sep09
Week of
Cumulative Out−Of−Sample Error (Post Intercept Shift)
May
Jun
Med Abs Err
Med Err
Deaths (Thousands)
0.10
0.05
0.00
−0.05
1
2
3
4
5
6
Weeks Forecasting
All Model Versions
Delphi
Los Alamos Nat Lab
Imperial
IHME − MS SEIR
3
2
1
0
Jul09
Sep09
All Cumulative Errors
Delphi
Los Alamos Nat Lab
Imperial
IHME − MS SEIR
0.10
0.05
0.00
−0.05
May15
Jul15
Sep15

Burkina Faso

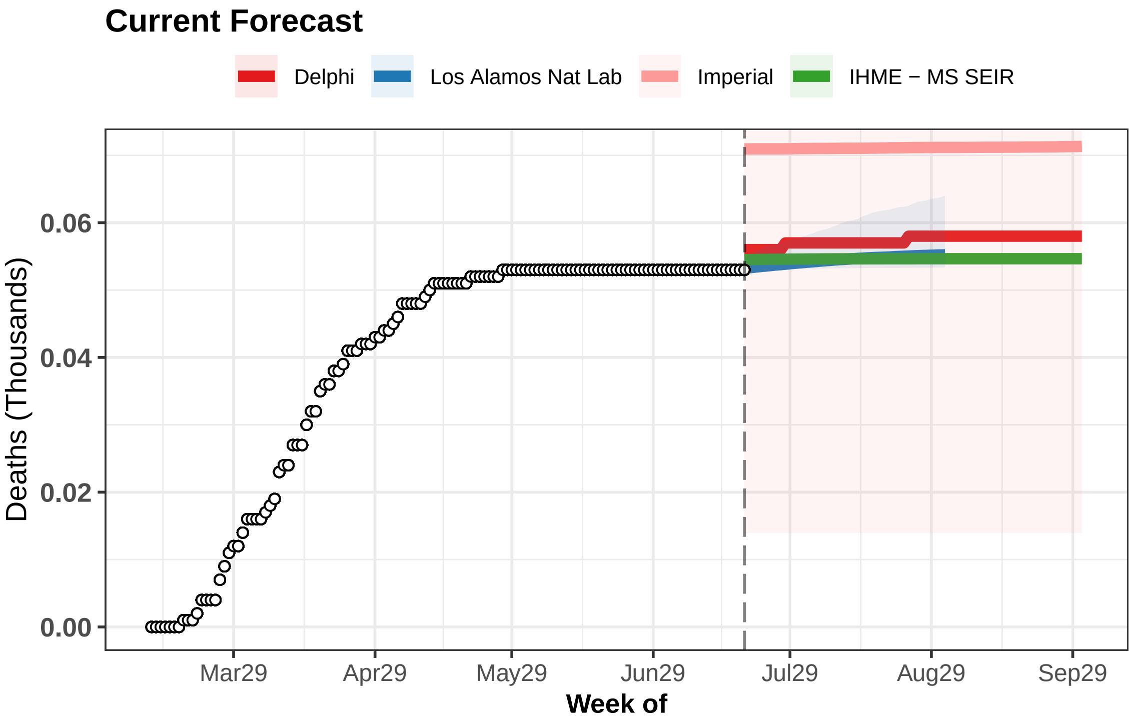

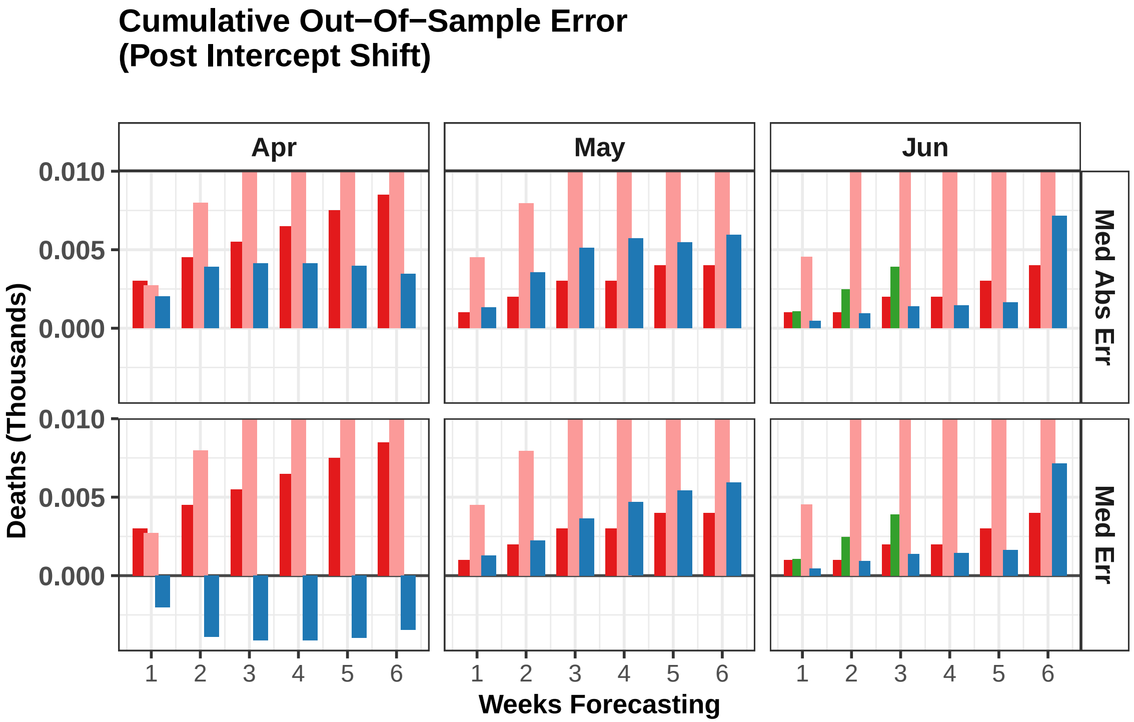

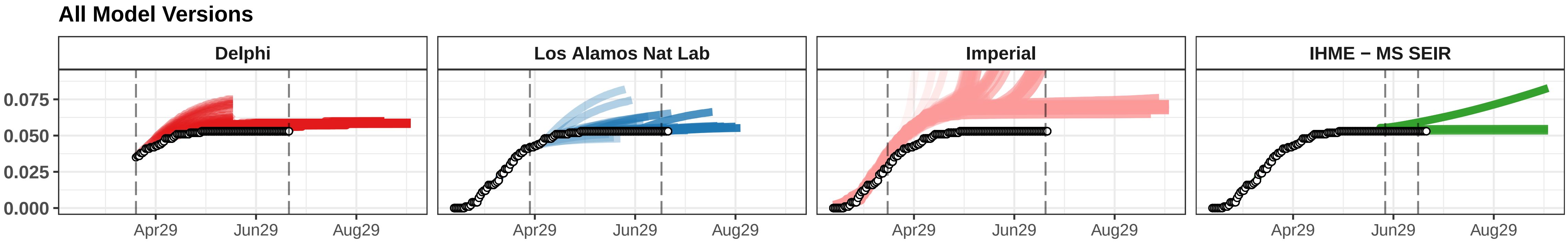

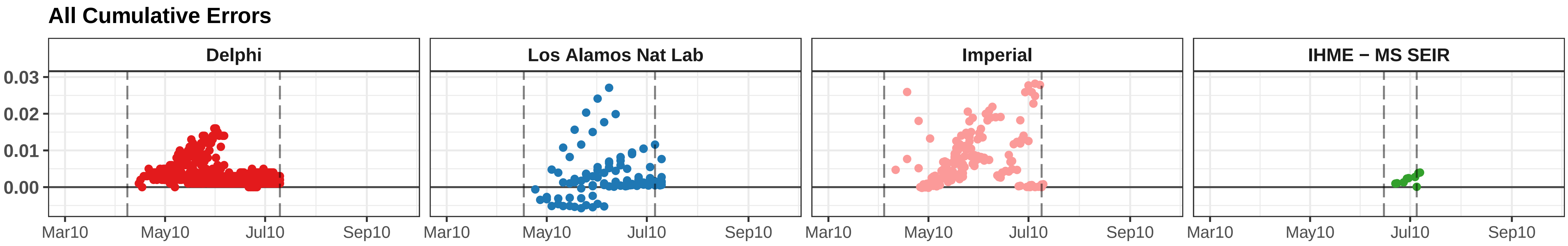

Andorra

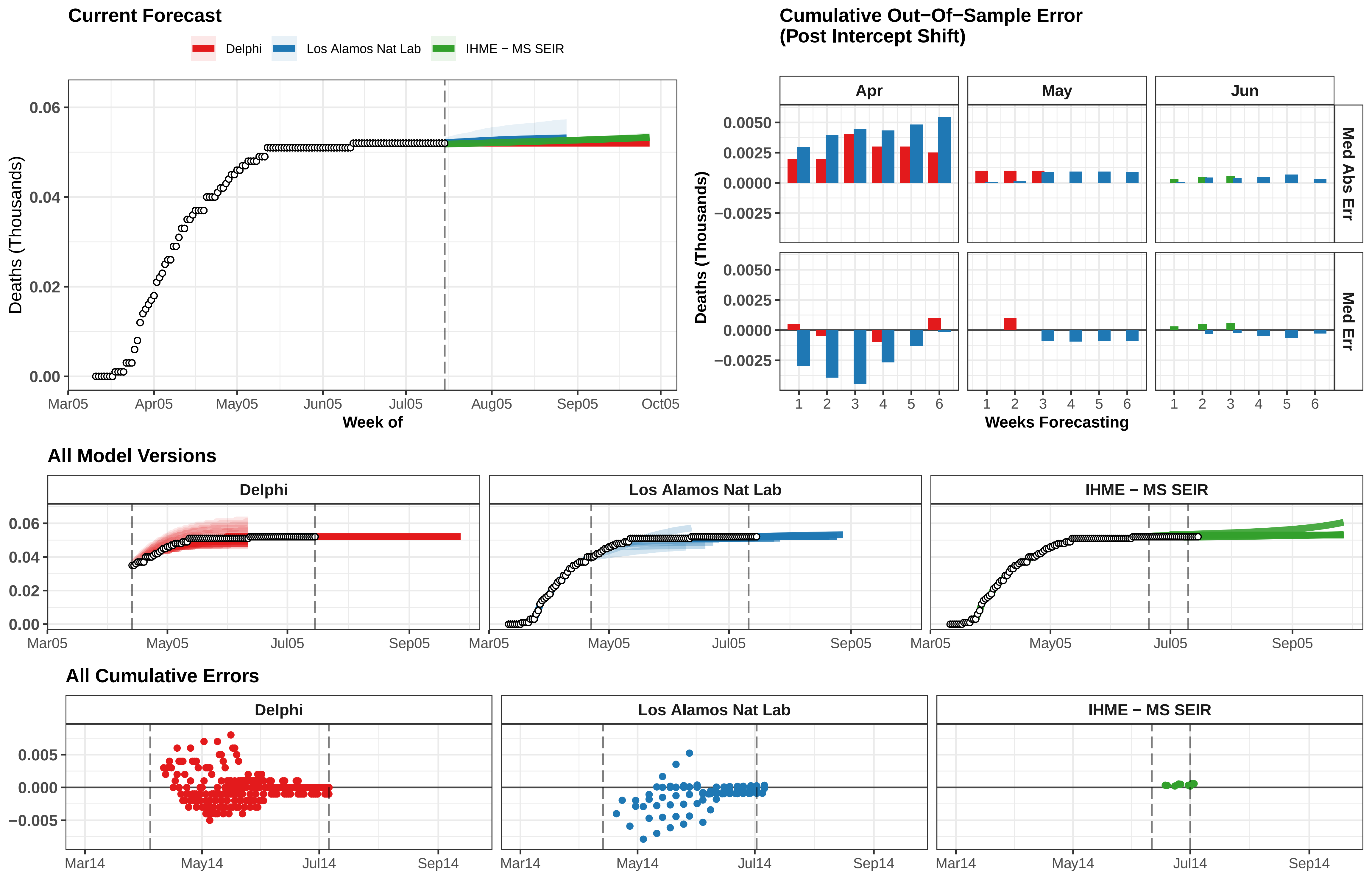

Current Forecast
Delphi
Los Alamos Nat Lab
IHME − MS SEIR
Deaths (Thousands)
0.06
0.04
0.02
0.00
Mar05
Apr05
May05
Jun05
Jul05
Aug05
Sep05
Oct05
Week of
Cumulative Out−Of−Sample Error
(Post Intercept Shift)
Apr
May
Jun
Med Abs Err
Med Err
0.0050
0.0025
0.0000
−0.0025
1
2
3
4
5
6
Weeks Forecasting
All Model Versions
Delphi
Los Alamos Nat Lab
IHME − MS SEIR
0.06
0.04
0.02
0.00
Mar05
May05
Jul05
Sep05
All Cumulative Errors
Delphi
Los Alamos Nat Lab
IHME − MS SEIR
0.005
0.000
−0.005
Mar14
May14
Jul14
Sep14

Equatorial Guinea

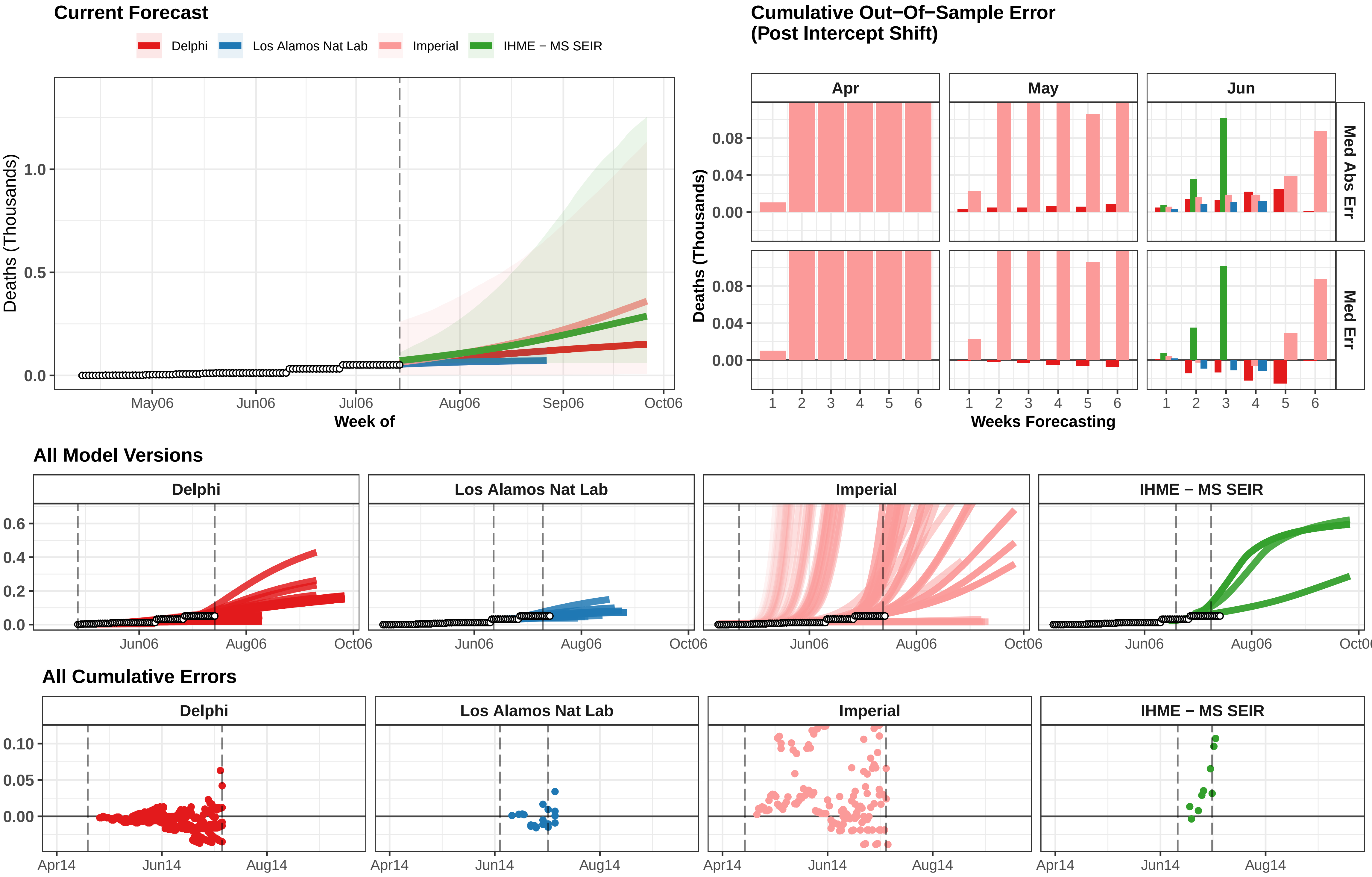

Tunisia

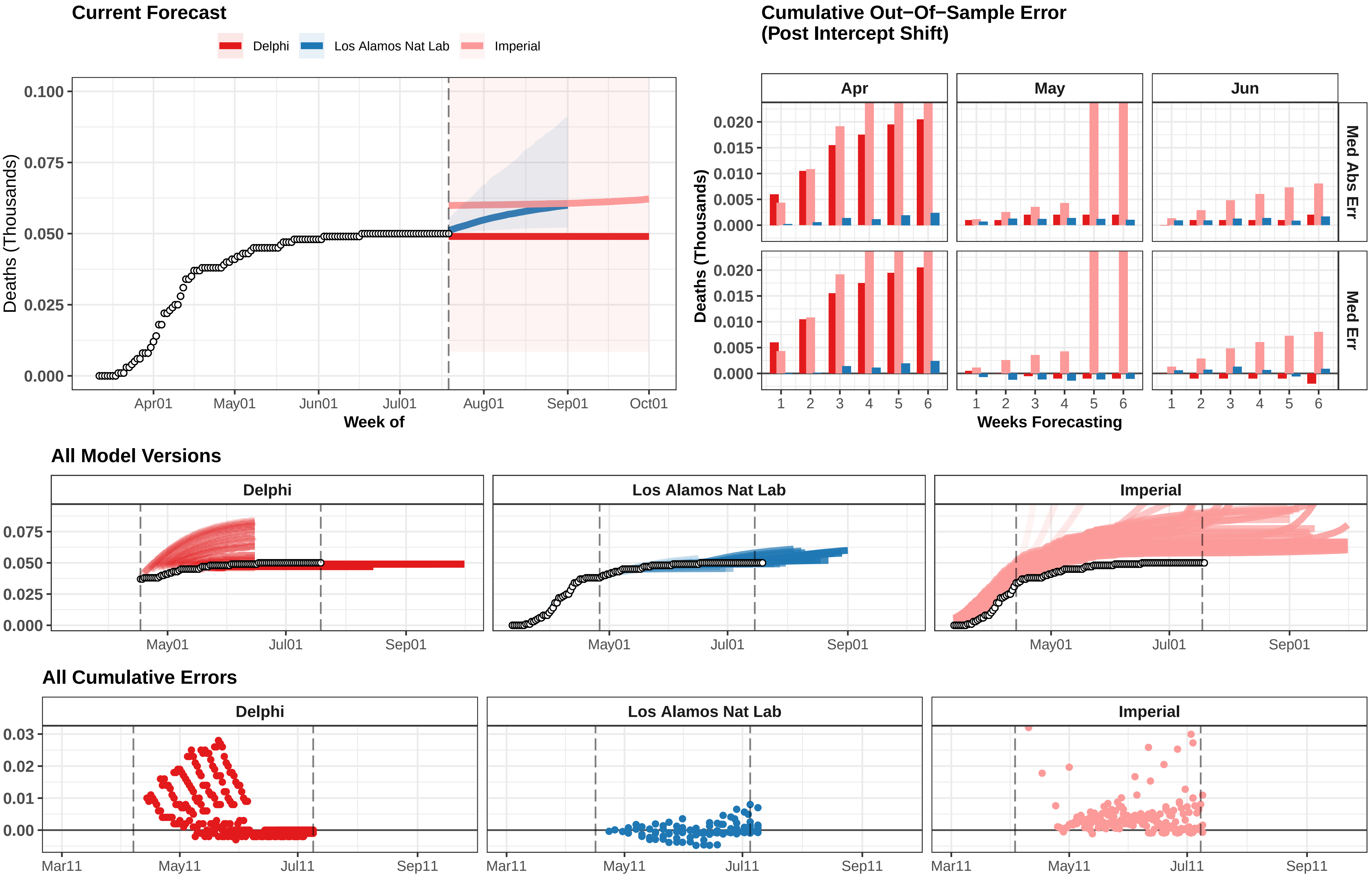

Current Forecast
Delphi
Los Alamos Nat Lab
Imperial
Deaths (Thousands)
0.100
0.075
0.050
0.025
0.000
Apr01
May01
Jun01
Jul01
Aug01
Sep01
Oct01
Week of
Cumulative Out−Of−Sample Error
(Post Intercept Shift)
Apr
May
Jun
Med Abs Err
Med Err
0.020
0.015
0.010
0.005
0.000
1
2
3
4
5
6
Weeks Forecasting
All Model Versions
Delphi
Los Alamos Nat Lab
Imperial
0.075
0.050
0.025
0.000
May01
Jul01
Sep01
All Cumulative Errors
0.03
0.02
0.01
0.00
Mar11
May11
Jul11
Sep11

Congo

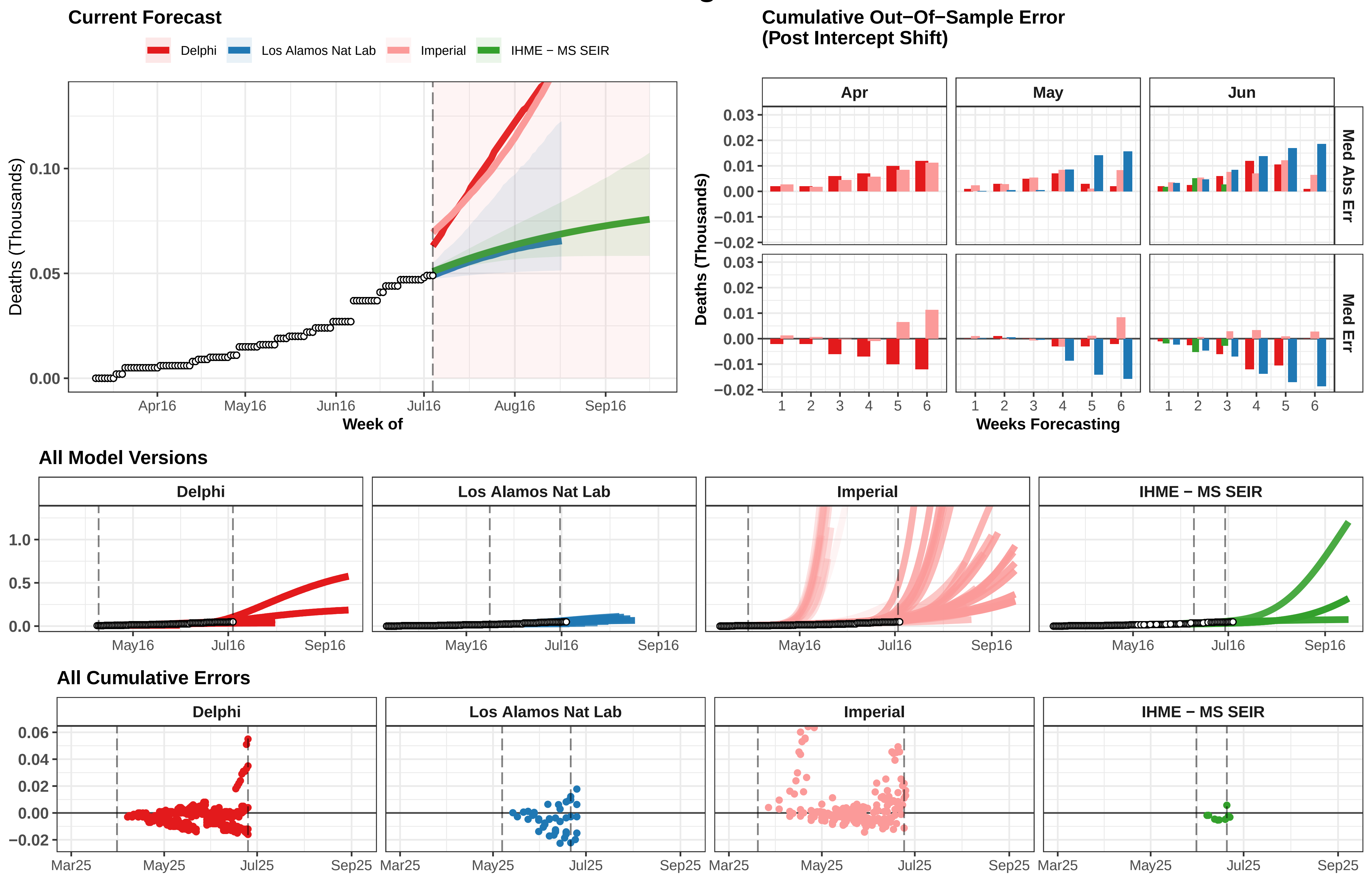

Current Forecast
Delphi
Los Alamos Nat Lab
Imperial
IHME − MS SEIR
Deaths (Thousands)
0.00
0.05
0.10
Apr16
May16
Jun16
Jul16
Aug16
Sep16
Week of
Cumulative Out−Of−Sample Error
(Post Intercept Shift)
Apr
May
Jun
Med Abs Err
Med Err
0.03
0.02
0.01
0.00
−0.01
−0.02
1
2
3
4
5
6
Weeks Forecasting
All Model Versions
1.0
0.5
0.0
All Cumulative Errors
0.06
0.04
0.02
Mar25
May25
Jul25
Sep25

Libya

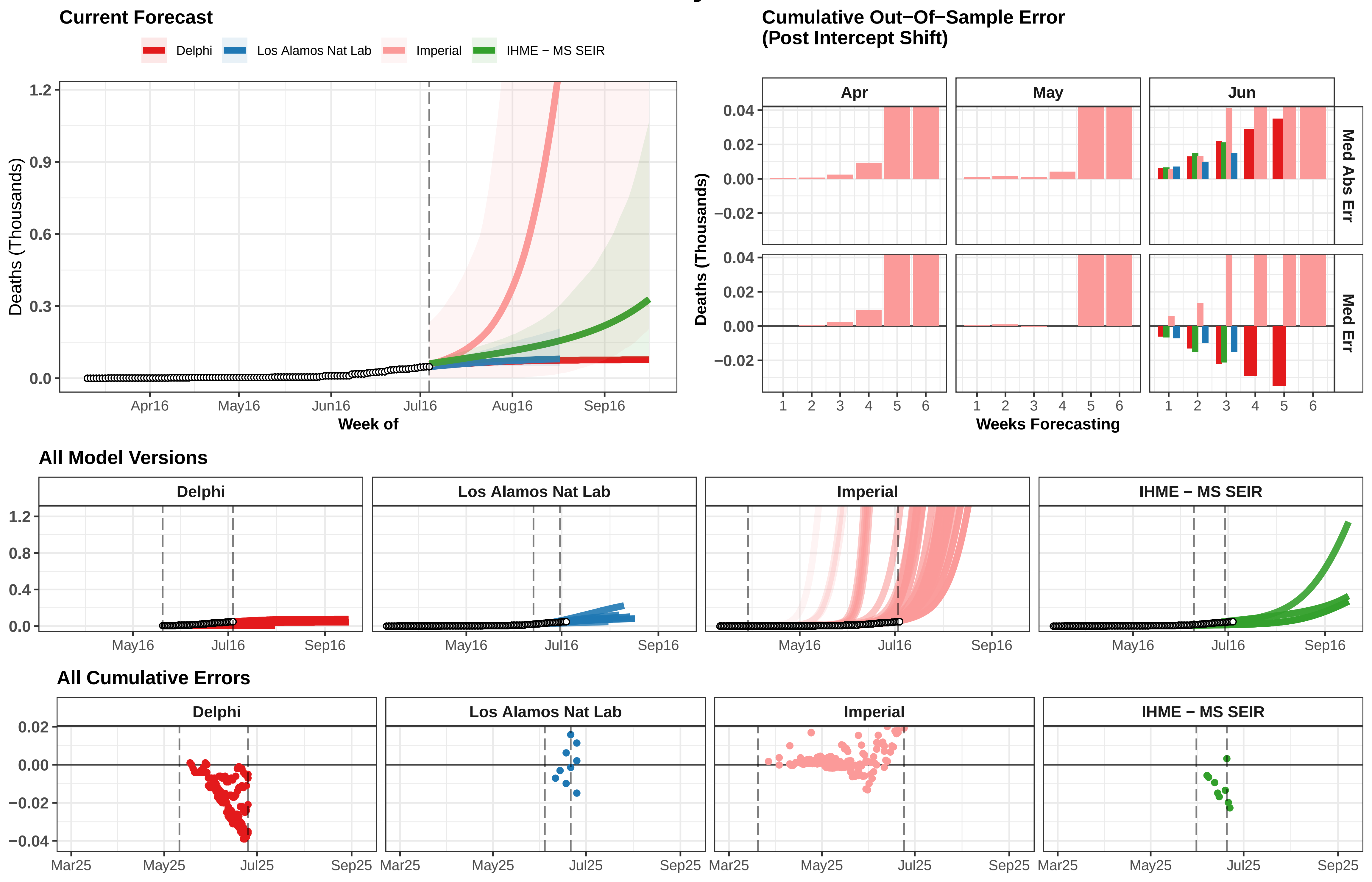

Current Forecast
Delphi
Los Alamos Nat Lab
Imperial
IHME − MS SEIR
Deaths (Thousands)
1.2
0.9
0.6
0.3
0.0
Apr16
May16
Jun16
Jul16
Aug16
Sep16
Week of
Cumulative Out−Of−Sample Error
(Post Intercept Shift)
Apr
May
Jun
Med Abs Err
Med Err
0.04
0.02
0.00
−0.02
1
2
3
4
5
6
Weeks Forecasting
All Model Versions
1.2
0.8
0.4
0.0
May16
Jul16
Sep16
All Cumulative Errors
0.02
0.00
−0.02
−0.04
Mar25
May25
Jul25
Sep25

Gabon

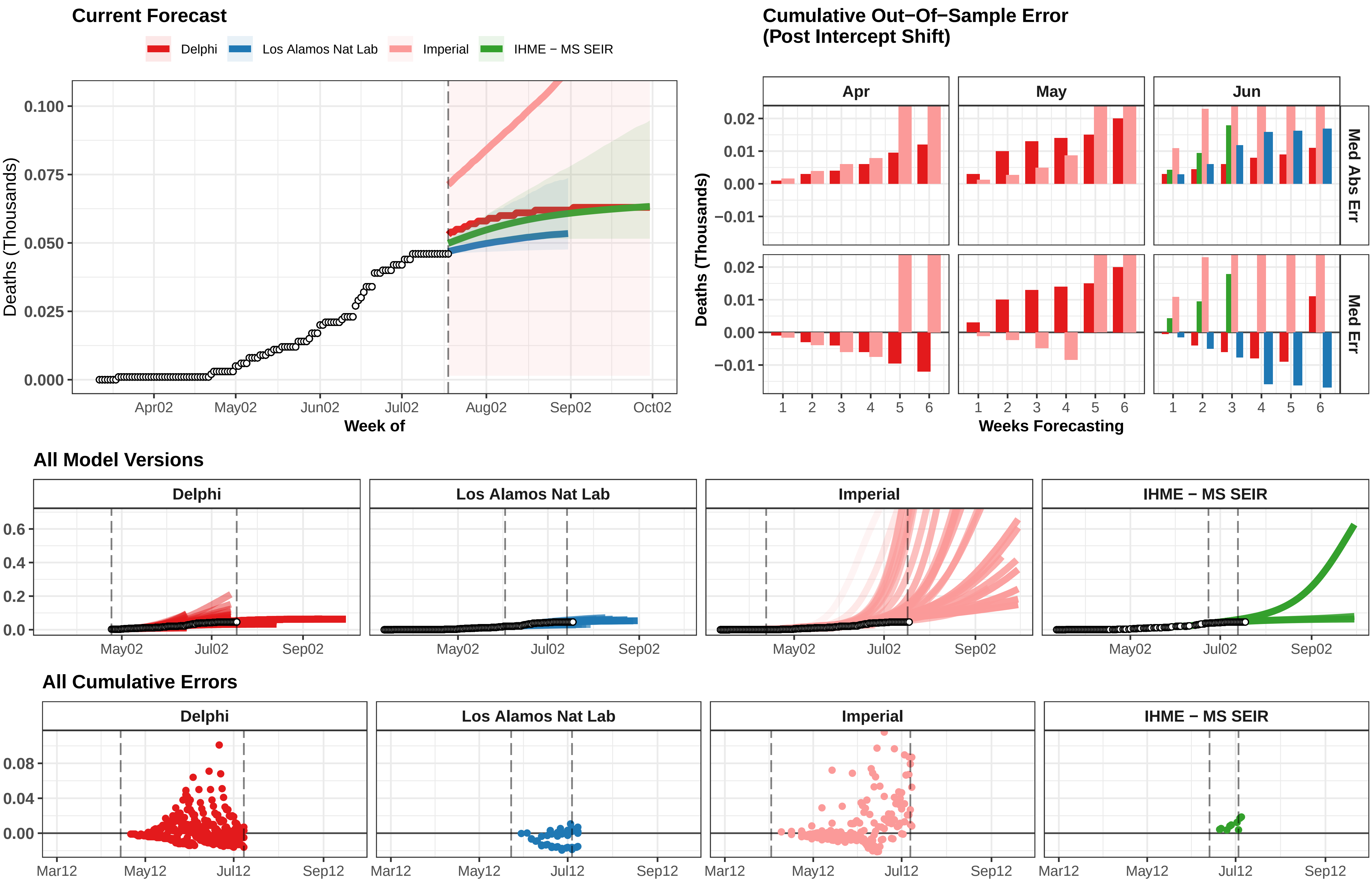

Current Forecast
Delphi
Los Alamos Nat Lab
Imperial
IHME − MS SEIR
Deaths (Thousands)
0.100
0.075
0.050
0.025
0.000
Apr02
May02
Jun02
Jul02
Aug02
Sep02
Oct02
Week of
Cumulative Out−Of−Sample Error
(Post Intercept Shift)
Apr
May
Jun
Med Abs Err
Med Err
0.02
0.01
0.00
−0.01
1
2
3
4
5
6
Weeks Forecasting
All Model Versions
Delphi
Los Alamos Nat Lab
Imperial
IHME − MS SEIR
0.6
0.4
0.2
0.0
May02
Jul02
Sep02
All Cumulative Errors
0.08
0.04
0.00
Mar12
May12
Jul12
Sep12

South Sudan

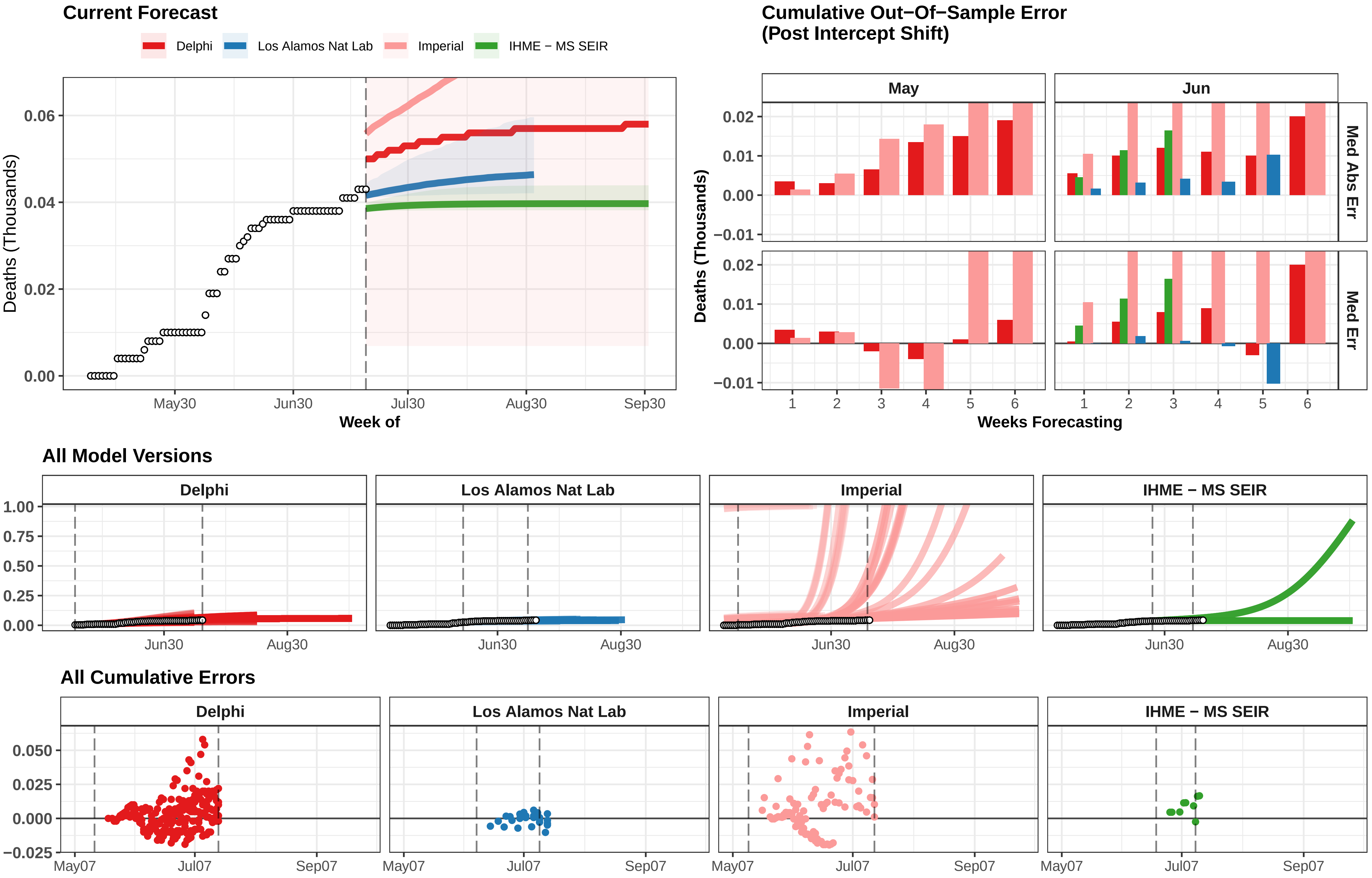

Current Forecast
Delphi
Los Alamos Nat Lab
Imperial
IHME − MS SEIR
Deaths (Thousands)
Week of
May30
Jun30
Jul30
Aug30
Sep30
Cumulative Out−Of−Sample Error
(Post Intercept Shift)
May
Jun
Med Abs Err
Med Err
Weeks Forecasting
All Model Versions
All Cumulative Errors
May07
Jul07
Sep07

Lebanon

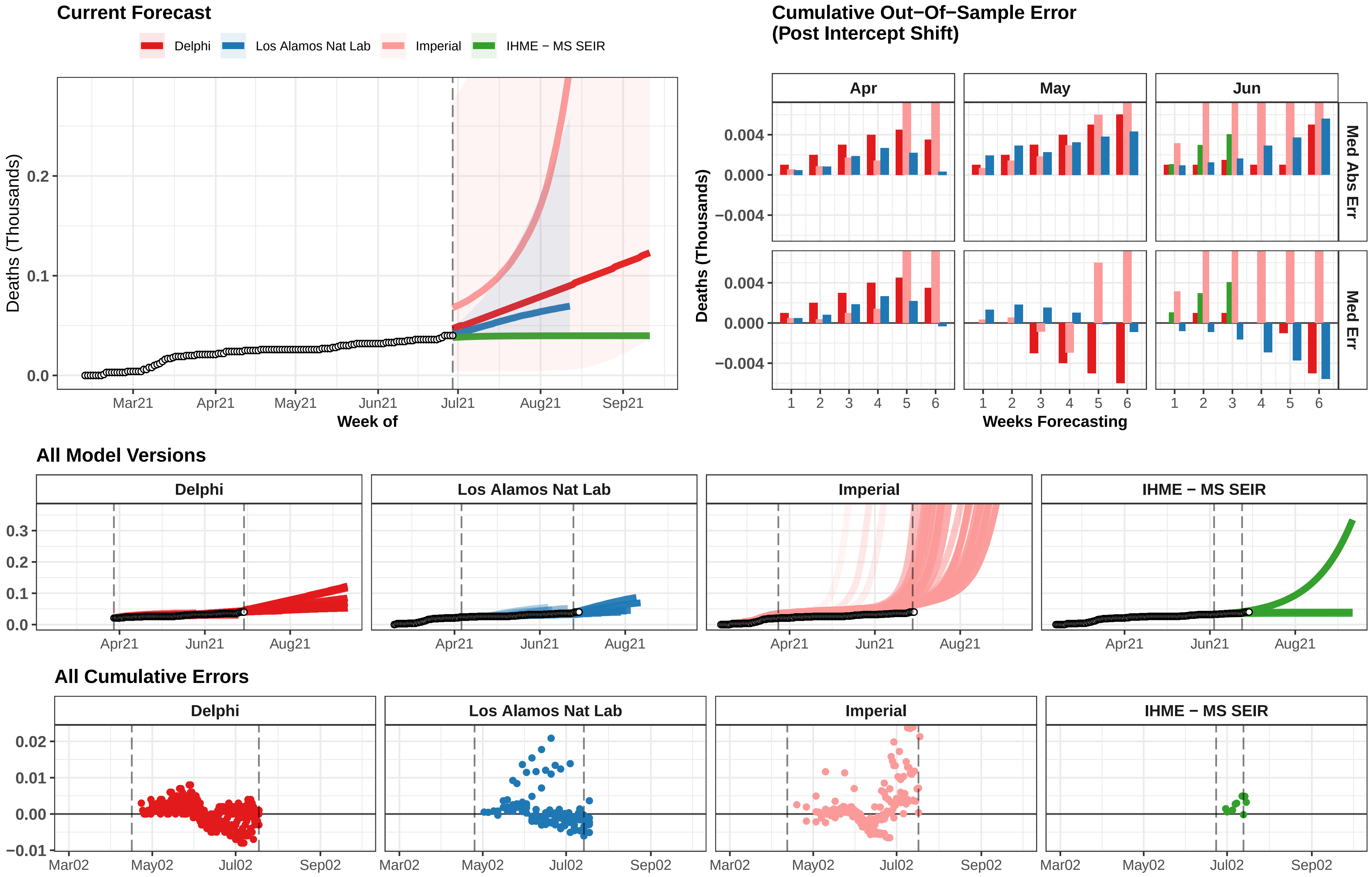
Current Forecast
Delphi
Los Alamos Nat Lab
Imperial
IHME − MS SEIR
Deaths (Thousands)
0.0
0.1
0.2
Mar21
Apr21
May21
Jun21
Jul21
Aug21
Sep21
Week of
Cumulative Out−Of−Sample Error
(Post Intercept Shift)
Apr
May
Jun
Med Abs Err
Med Err
Deaths (Thousands)
0.004
0.000
−0.004
1
2
3
4
5
6
Weeks Forecasting
All Model Versions
Delphi
Los Alamos Nat Lab
Imperial
IHME − MS SEIR
0.0
0.1
0.2
0.3
Apr21
Jun21
Aug21
All Cumulative Errors
Delphi
Los Alamos Nat Lab
Imperial
IHME − MS SEIR
−0.01
0.00
0.01
0.02
Mar02
May02
Jul02
Sep02

Nepal

Current Forecast

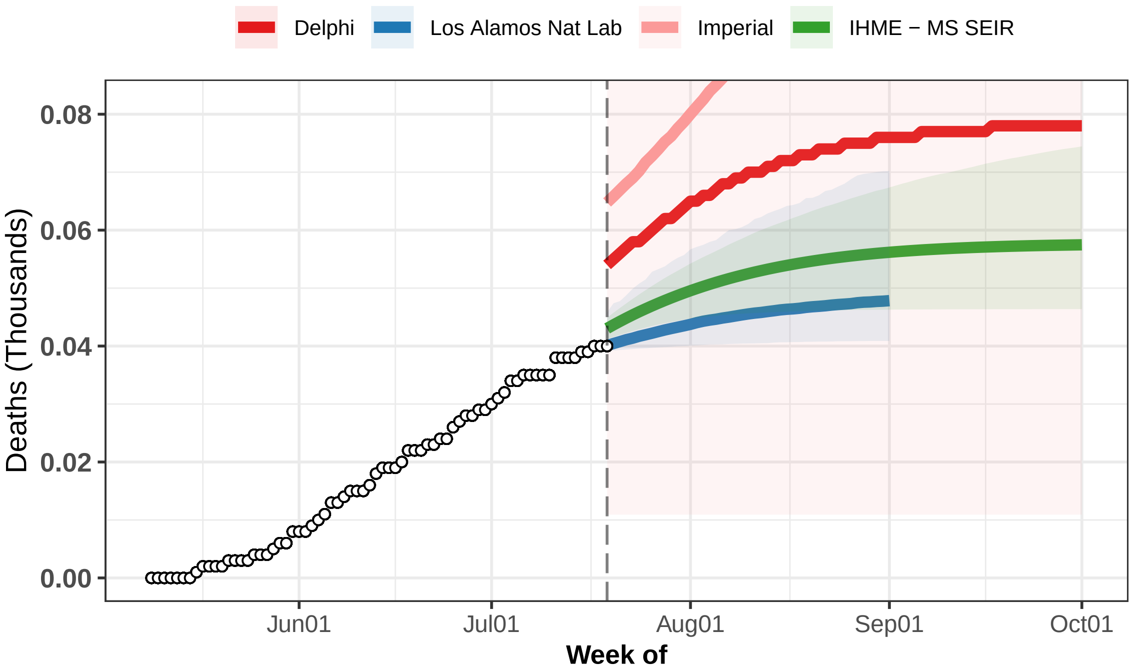

Cumulative Out−Of−Sample Error (Post Intercept Shift)

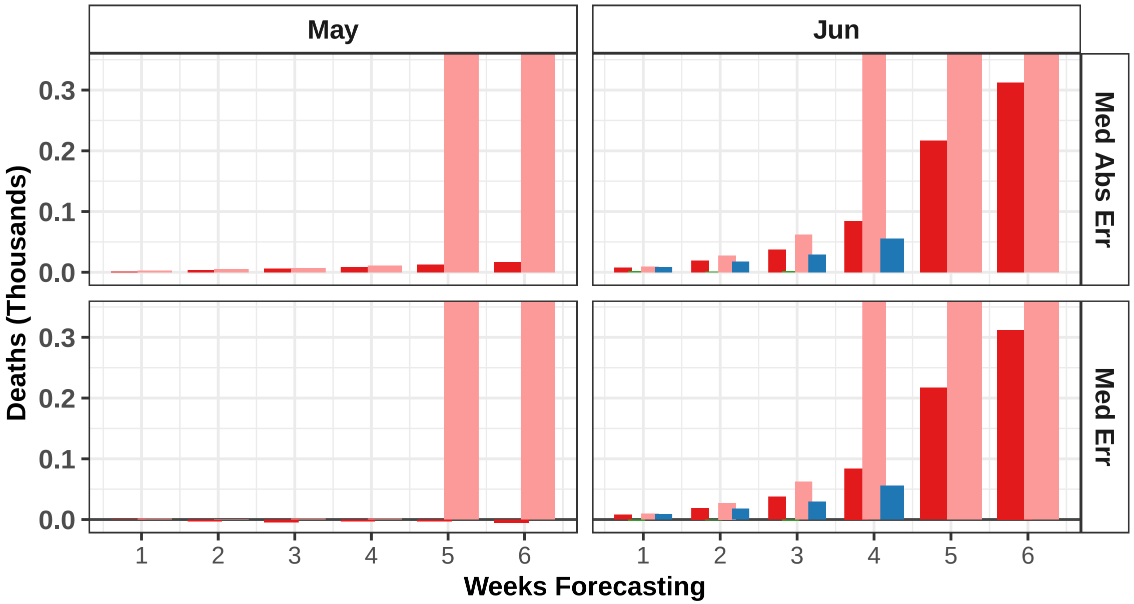

All Model Versions

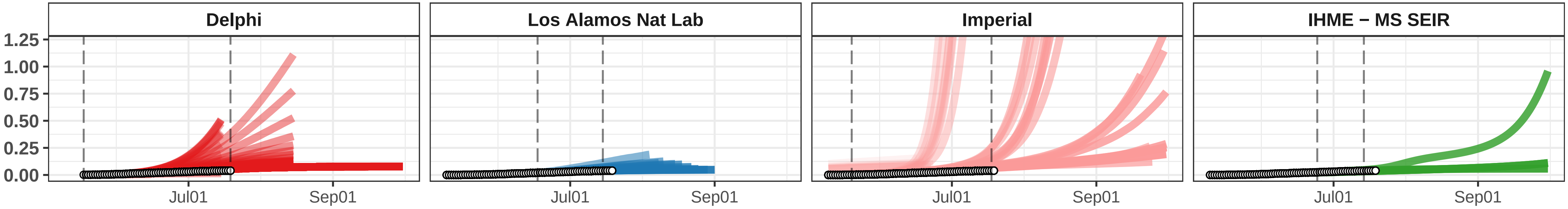

All Cumulative Errors

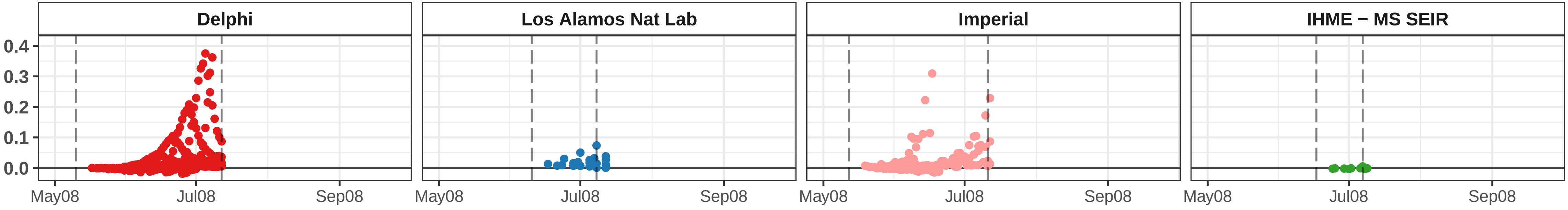

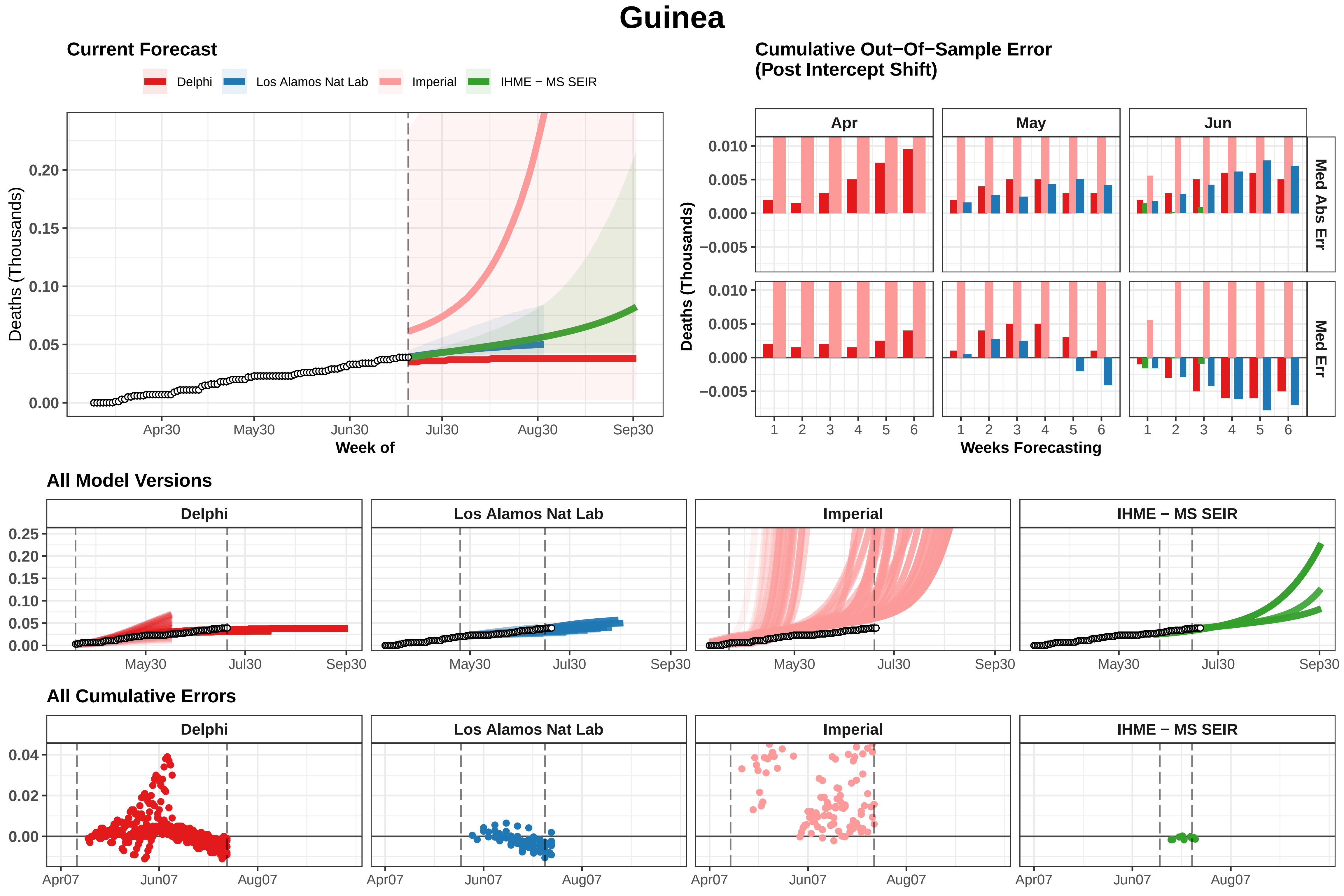
Guinea
Current Forecast
Delphi
Los Alamos Nat Lab
Imperial
IHME − MS SEIR
Deaths (Thousands)
0.00
0.05
0.10
0.15
0.20
Apr30
May30
Jun30
Jul30
Aug30
Sep30
Week of
Cumulative Out−Of−Sample Error
(Post Intercept Shift)
Apr
May
Jun
0.010
0.005
0.000
−0.005
Med Abs Err
Med Err
1
2
3
4
5
6
Weeks Forecasting
All Model Versions
0.25
0.20
0.15
0.10
0.05
0.00
All Cumulative Errors
0.04
0.02
0.00
Apr07
Jun07
Aug07

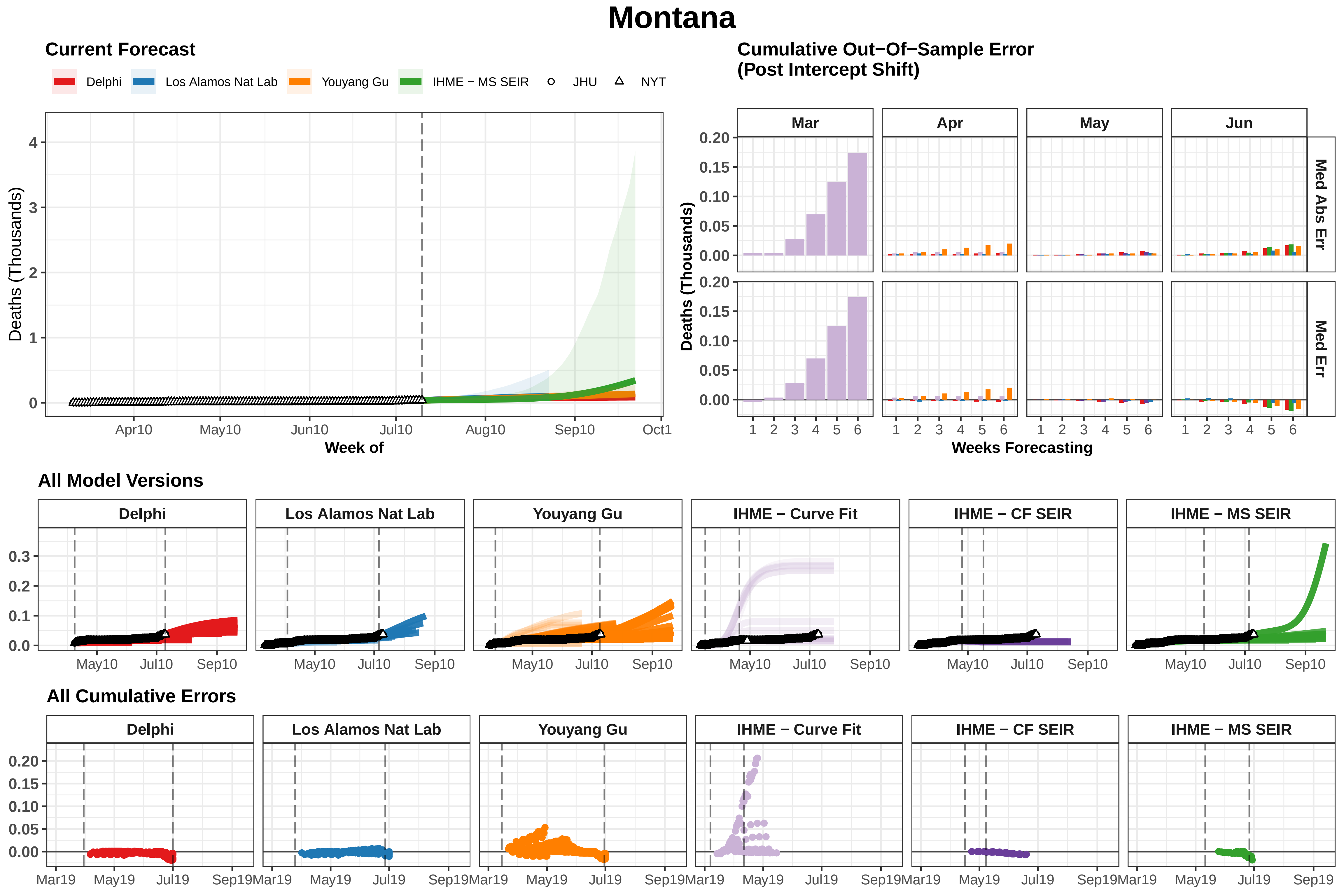
Montana
Current Forecast
Delphi
Los Alamos Nat Lab
Youyang Gu
IHME − MS SEIR
JHU
NYT
Deaths (Thousands)
Week of
Apr10
May10
Jun10
Jul10
Aug10
Sep10
Oct1
Cumulative Out−Of−Sample Error
(Post Intercept Shift)
Mar
Apr
May
Jun
Med Abs Err
Med Err
Weeks Forecasting
All Model Versions
IHME − Curve Fit
IHME − CF SEIR
All Cumulative Errors
Mar19
May19
Jul19
Sep19

Uruguay

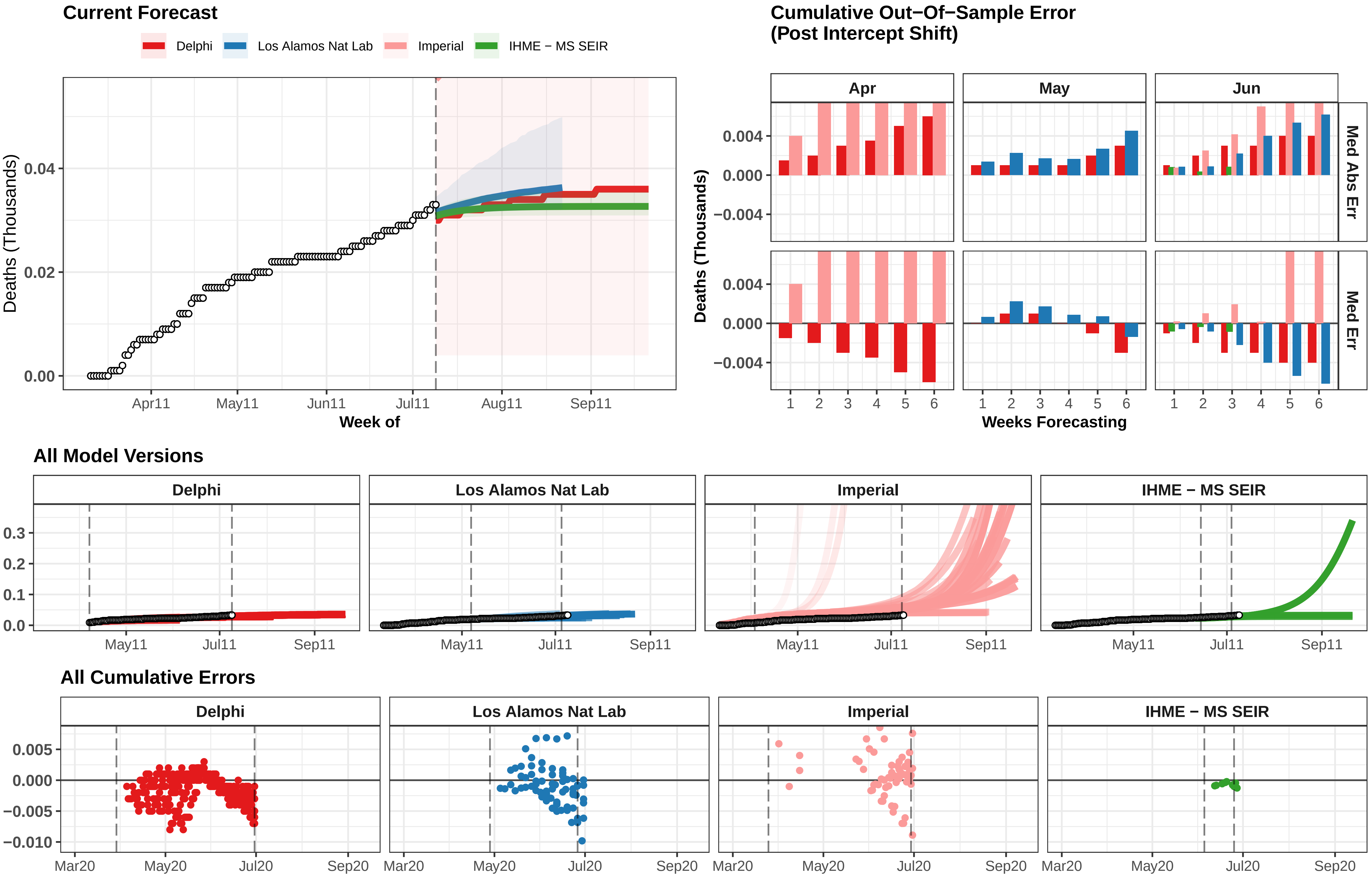

Current Forecast
Delphi
Los Alamos Nat Lab
Imperial
IHME − MS SEIR
Deaths (Thousands)
0.00
0.02
0.04
Apr11
May11
Jun11
Jul11
Aug11
Sep11
Week of
Cumulative Out−Of−Sample Error
(Post Intercept Shift)
Apr
May
Jun
Med Abs Err
Med Err
0.004
0.000
−0.004
Weeks Forecasting
All Model Versions
0.0
0.1
0.2
0.3
All Cumulative Errors
0.005
0.000
−0.005
−0.010
Mar20
May20
Jul20
Sep20

Montenegro

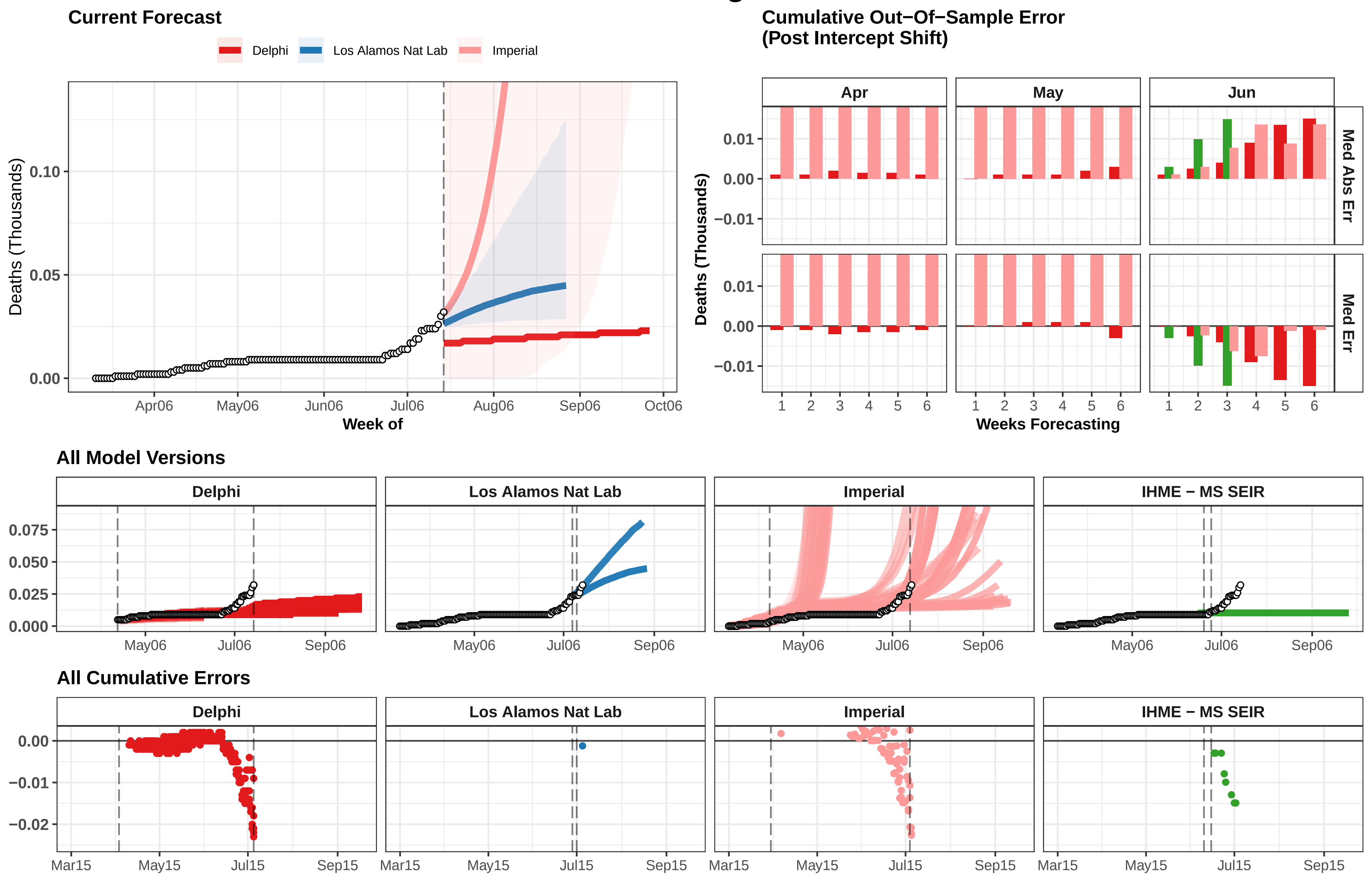

Current Forecast
Delphi
Los Alamos Nat Lab
Imperial
Deaths (Thousands)
0.10
0.05
0.00
Apr06
May06
Jun06
Jul06
Aug06
Sep06
Oct06
Week of
Cumulative Out−Of−Sample Error
(Post Intercept Shift)
Apr
May
Jun
Med Abs Err
Med Err
0.01
0.00
−0.01
Weeks Forecasting
All Model Versions
Delphi
Los Alamos Nat Lab
Imperial
IHME − MS SEIR
0.075
0.050
0.025
0.000
All Cumulative Errors
−0.02
Mar15
May15
Jul15
Sep15

Benin

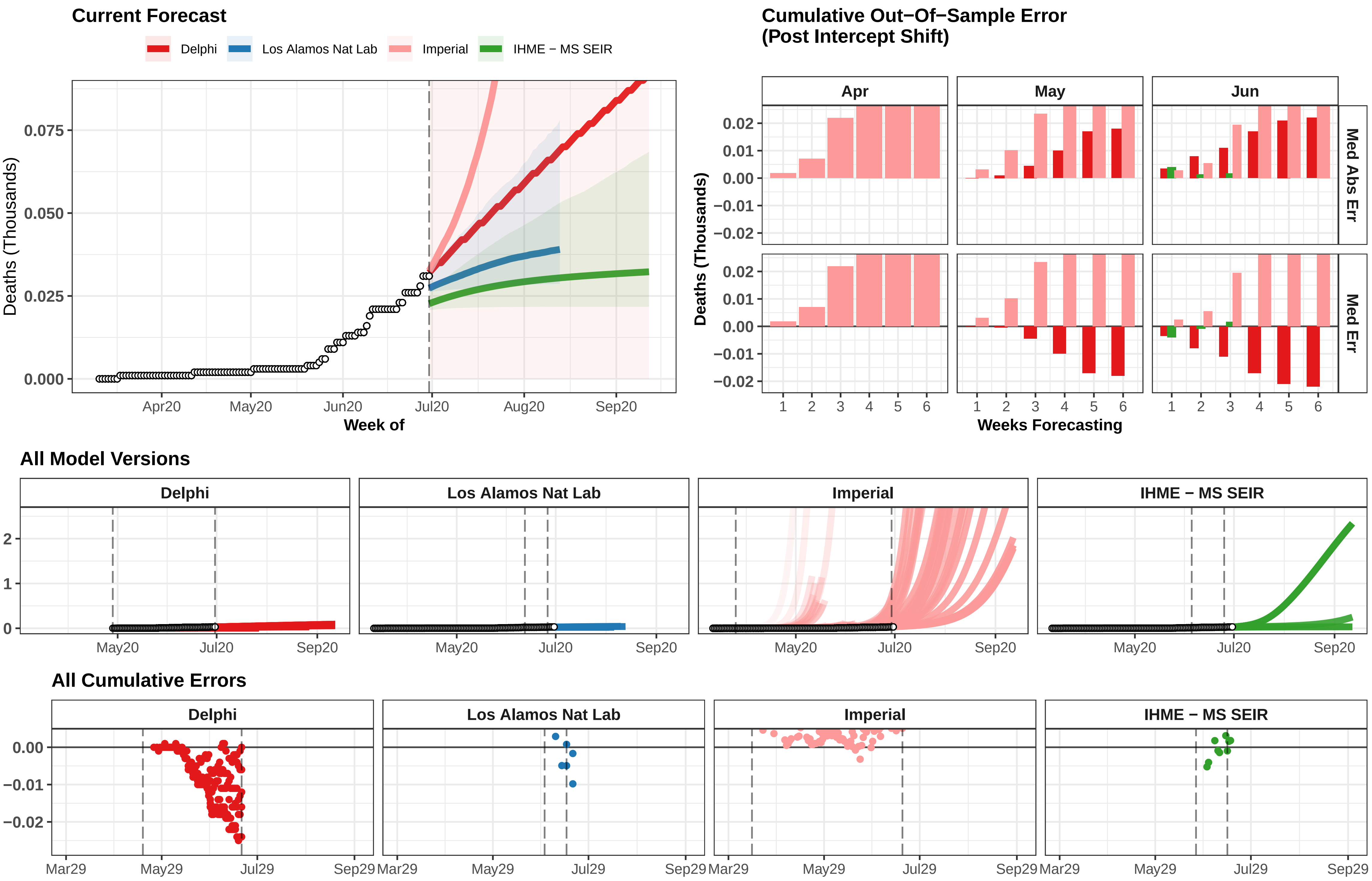

Current Forecast
Delphi
Los Alamos Nat Lab
Imperial
IHME − MS SEIR
Deaths (Thousands)
0.000
0.025
0.050
0.075
Apr20
May20
Jun20
Jul20
Aug20
Sep20
Week of
Cumulative Out−Of−Sample Error
(Post Intercept Shift)
Apr
May
Jun
Med Abs Err
Med Err
Deaths (Thousands)
0.02
0.01
0.00
−0.01
−0.02
1
2
3
4
5
6
Weeks Forecasting
All Model Versions
Delphi
Los Alamos Nat Lab
Imperial
IHME − MS SEIR
0
1
2
May20
Jul20
Sep20
All Cumulative Errors
Delphi
Los Alamos Nat Lab
Imperial
IHME − MS SEIR
0.00
−0.01
−0.02
Mar29
May29
Jul29
Sep29

Latvia

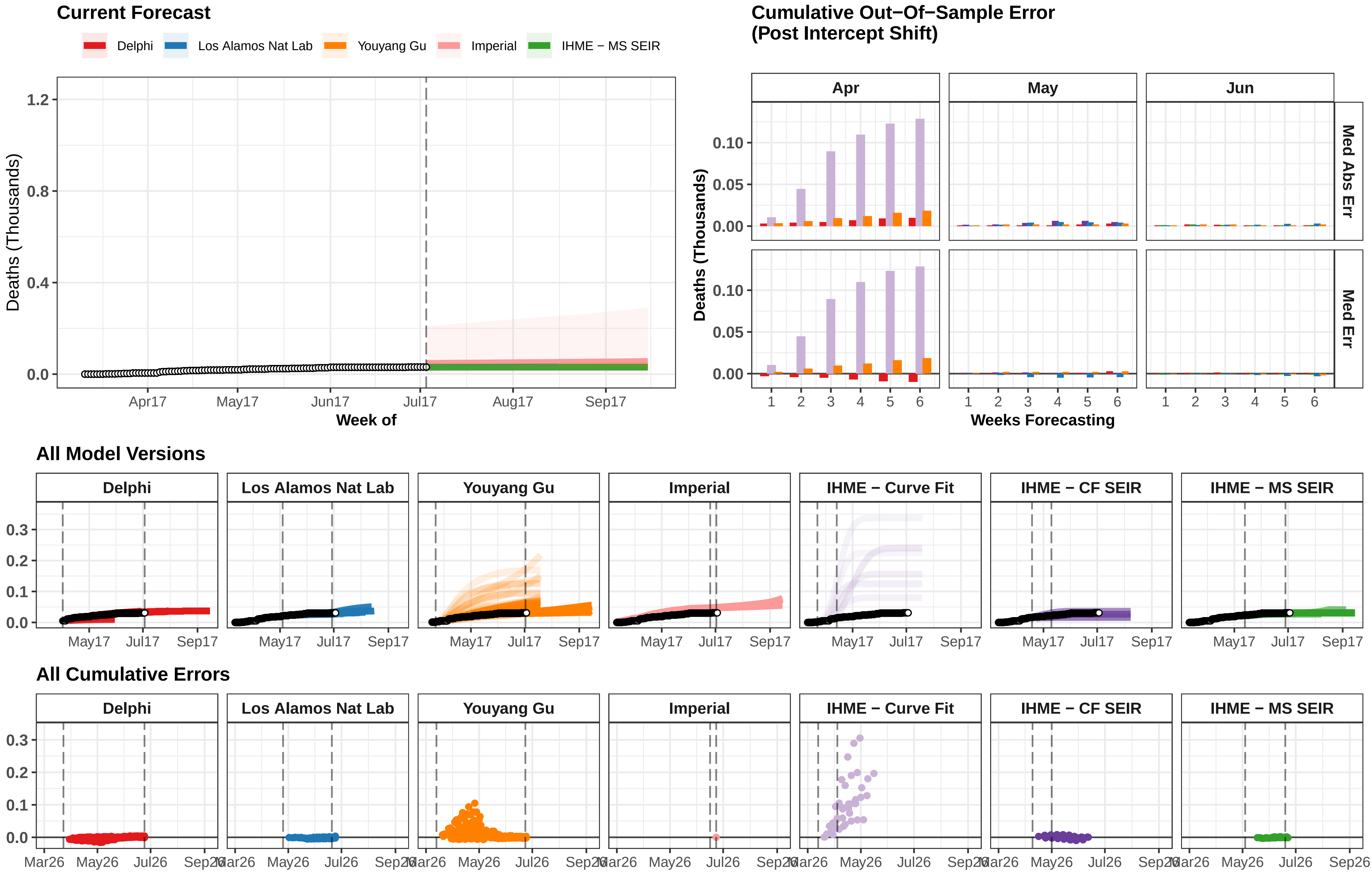
Current Forecast
Delphi
Los Alamos Nat Lab
Youyang Gu
Imperial
IHME − MS SEIR
Deaths (Thousands)
1.2
0.8
0.4
0.0
Apr17
May17
Jun17
Jul17
Aug17
Sep17
Week of
Cumulative Out−Of−Sample Error
(Post Intercept Shift)
Apr
May
Jun
Med Abs Err
Med Err
0.10
0.05
0.00
Weeks Forecasting
All Model Versions
Delphi
Los Alamos Nat Lab
Youyang Gu
Imperial
IHME − Curve Fit
IHME − CF SEIR
IHME − MS SEIR
0.3
0.2
0.1
0.0
May17
Jul17
Sep17
All Cumulative Errors
Mar26
May26
Jul26
Sep26

Paraguay

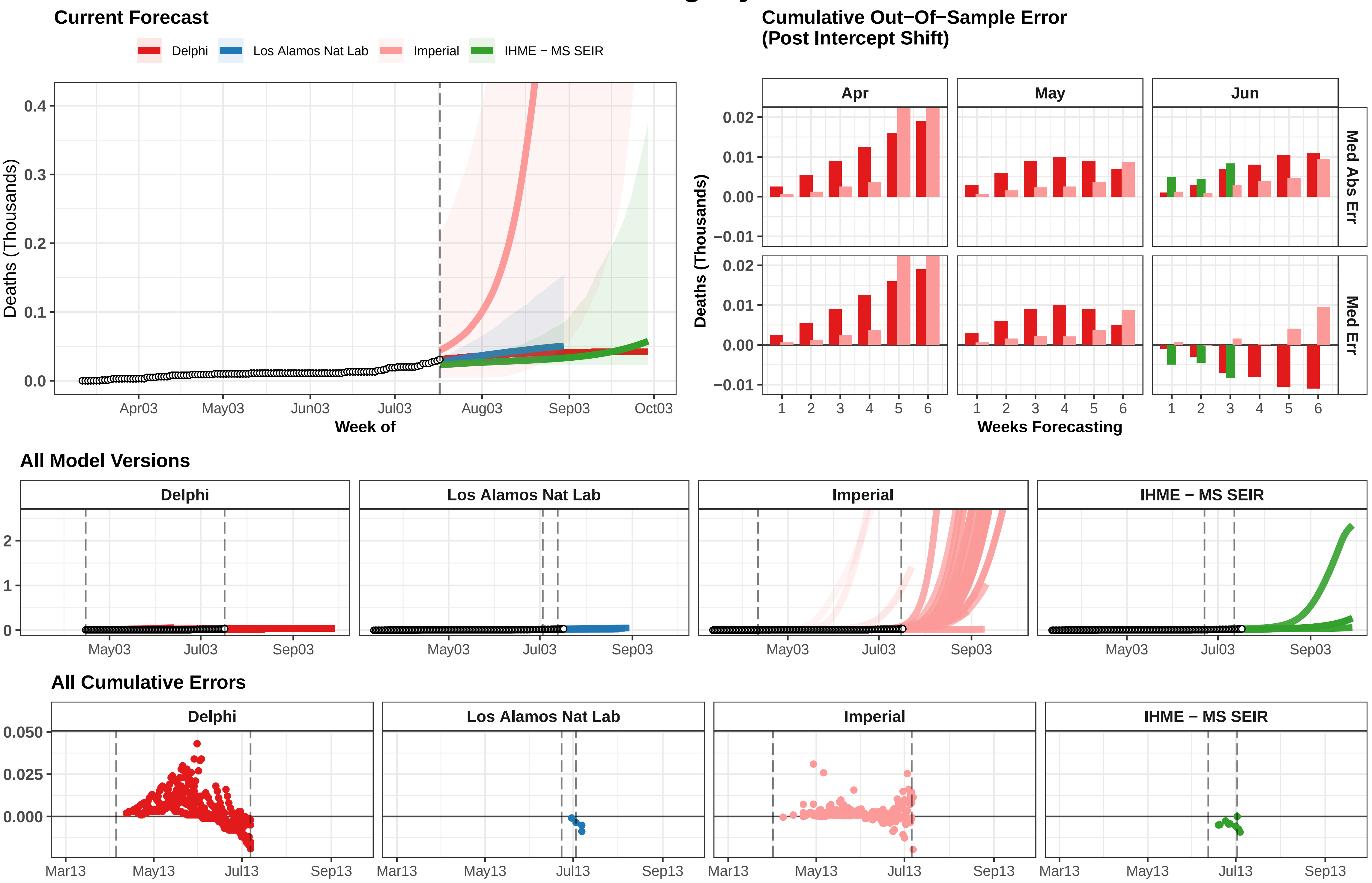

Current Forecast
Delphi
Los Alamos Nat Lab
Imperial
IHME − MS SEIR
Deaths (Thousands)
0.0
0.1
0.2
0.3
0.4
Apr03
May03
Jun03
Jul03
Aug03
Sep03
Oct03
Week of
Cumulative Out−Of−Sample Error
(Post Intercept Shift)
Apr
May
Jun
Med Abs Err
Med Err
0.02
0.01
0.00
−0.01
1
2
3
4
5
6
Weeks Forecasting
All Model Versions
Delphi
Los Alamos Nat Lab
Imperial
IHME − MS SEIR
0
1
2
May03
Jul03
Sep03
All Cumulative Errors
0.050
0.025
0.000
Mar13
May13
Jul13
Sep13

Angola

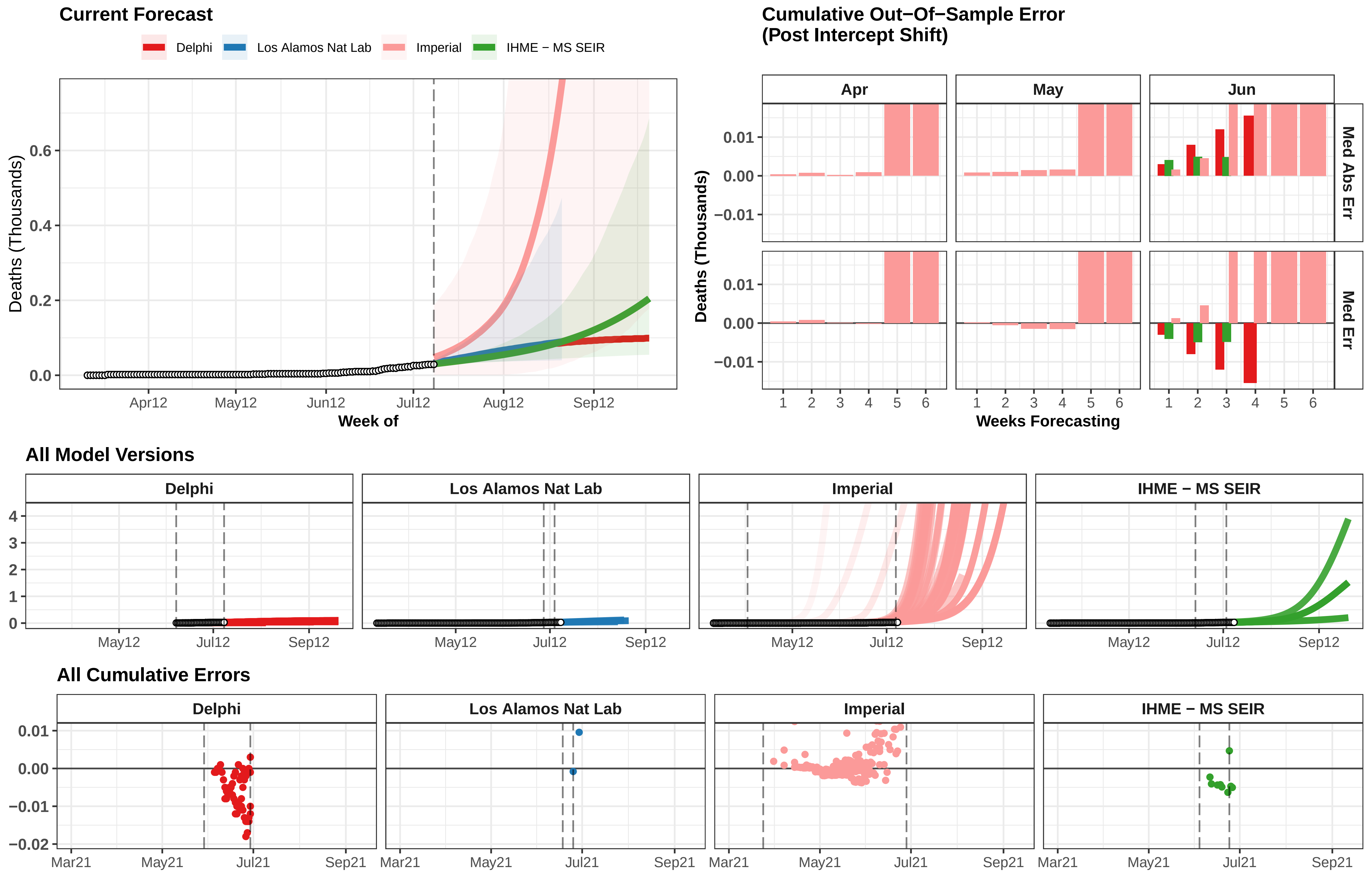
Current Forecast
Delphi
Los Alamos Nat Lab
Imperial
IHME − MS SEIR
Deaths (Thousands)
0.0
0.2
0.4
0.6
Apr12
May12
Jun12
Jul12
Aug12
Sep12
Week of
Cumulative Out−Of−Sample Error
(Post Intercept Shift)
Apr
May
Jun
Med Abs Err
Med Err
0.01
0.00
−0.01
1
2
3
4
5
6
Weeks Forecasting
All Model Versions
Delphi
Los Alamos Nat Lab
Imperial
IHME − MS SEIR
0
1
2
3
4
May12
Jul12
Sep12
All Cumulative Errors
0.01
0.00
−0.01
−0.02
Mar21
May21
Jul21
Sep21

Slovakia

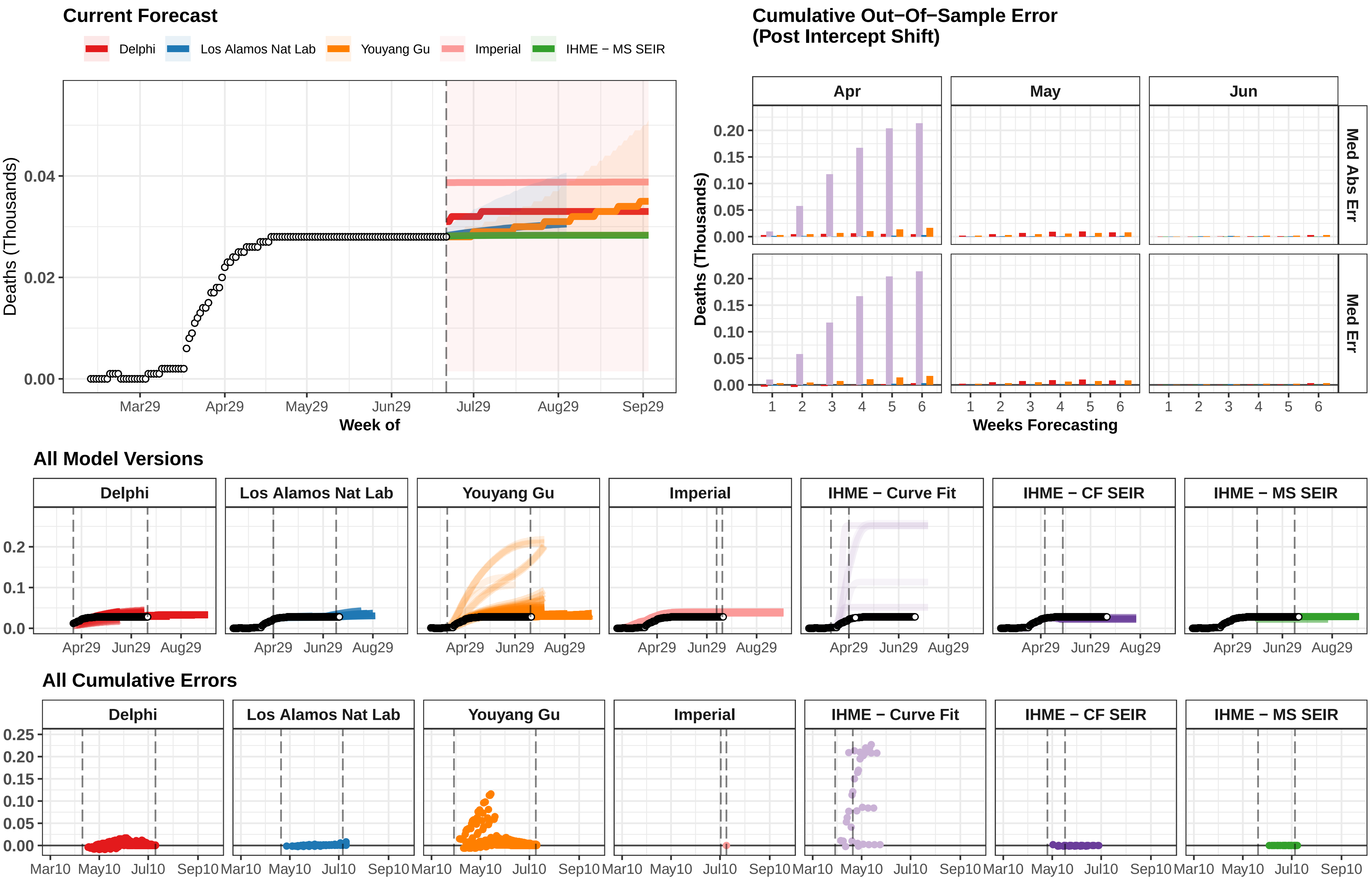
Current Forecast
Delphi
Los Alamos Nat Lab
Youyang Gu
Imperial
IHME − MS SEIR
Deaths (Thousands)
0.04
0.02
0.00
Mar29
Apr29
May29
Jun29
Jul29
Aug29
Sep29
Week of
Cumulative Out−Of−Sample Error
(Post Intercept Shift)
Apr
May
Jun
Med Abs Err
Med Err
0.20
0.15
0.10
0.05
0.00
1
2
3
4
5
6
Weeks Forecasting
All Model Versions
Delphi
Los Alamos Nat Lab
Youyang Gu
Imperial
IHME − Curve Fit
IHME − CF SEIR
IHME − MS SEIR
0.2
0.1
0.0
All Cumulative Errors
0.25
Mar10
May10
Jul10
Sep10

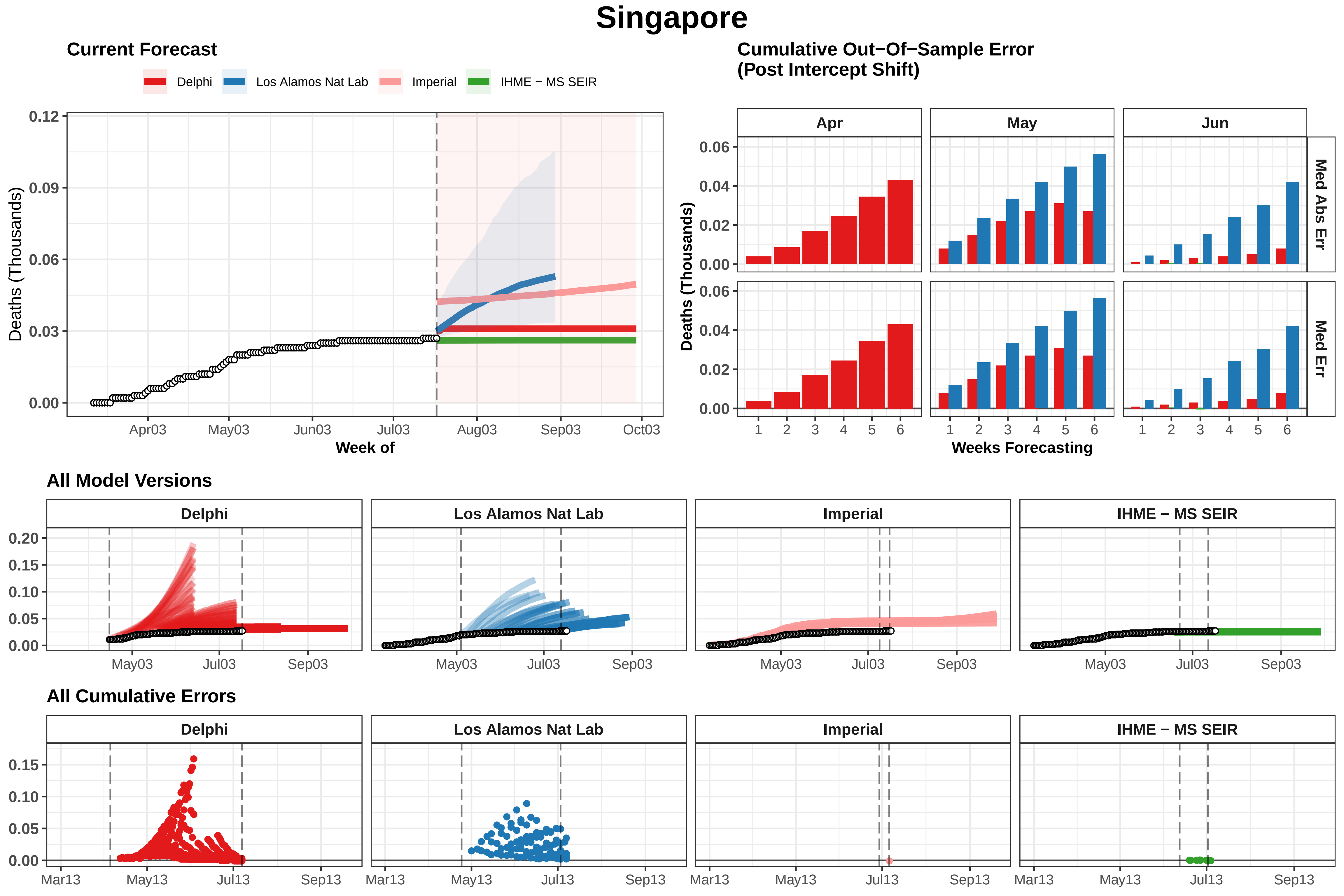

Singapore
Current Forecast
Delphi
Los Alamos Nat Lab
Imperial
IHME − MS SEIR
Deaths (Thousands)
Week of
Cumulative Out−Of−Sample Error
(Post Intercept Shift)
Apr
May
Jun
Med Abs Err
Med Err
Weeks Forecasting
All Model Versions
All Cumulative Errors

Guinea−Bissau

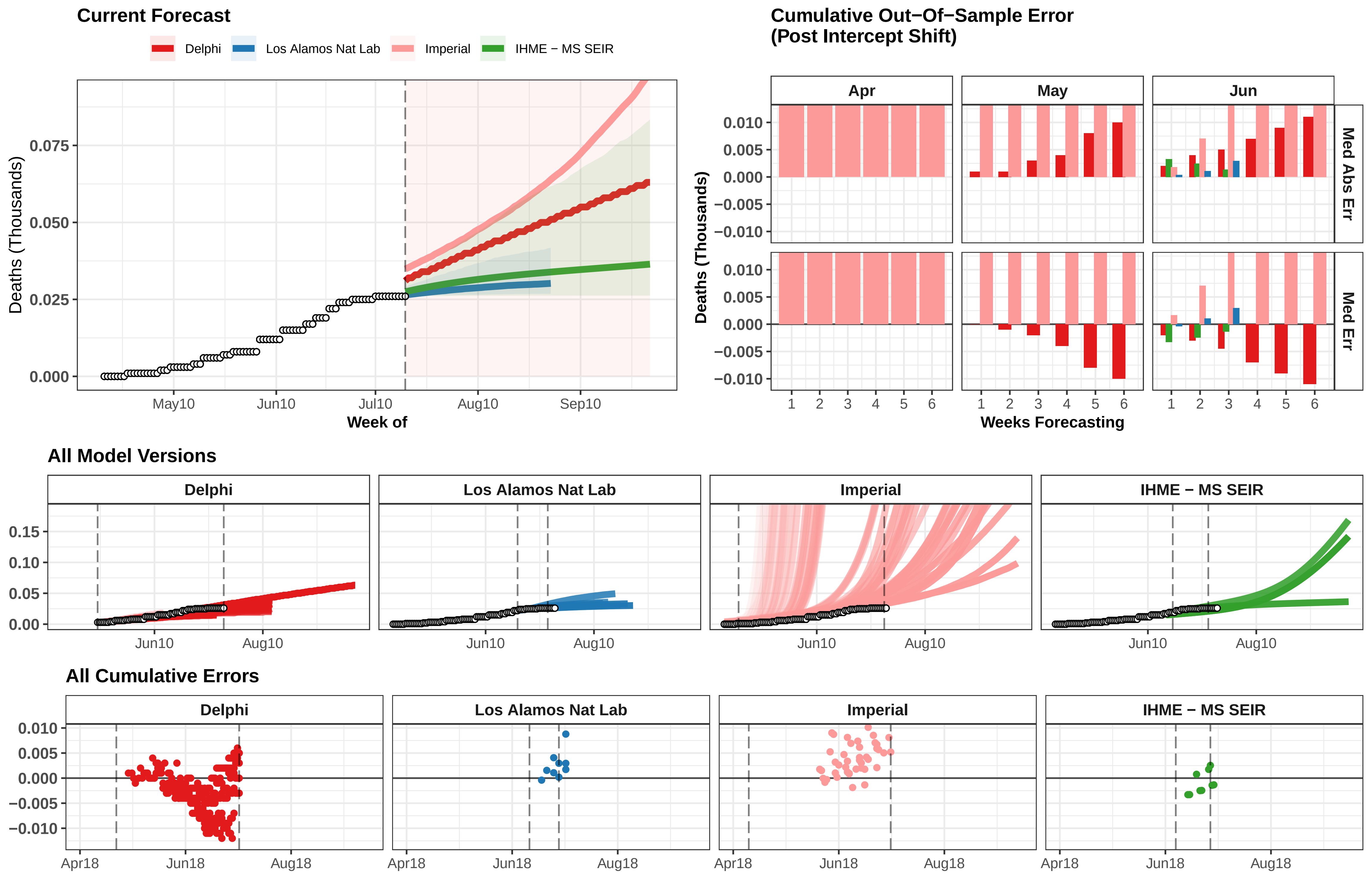

Syria

Current Forecast

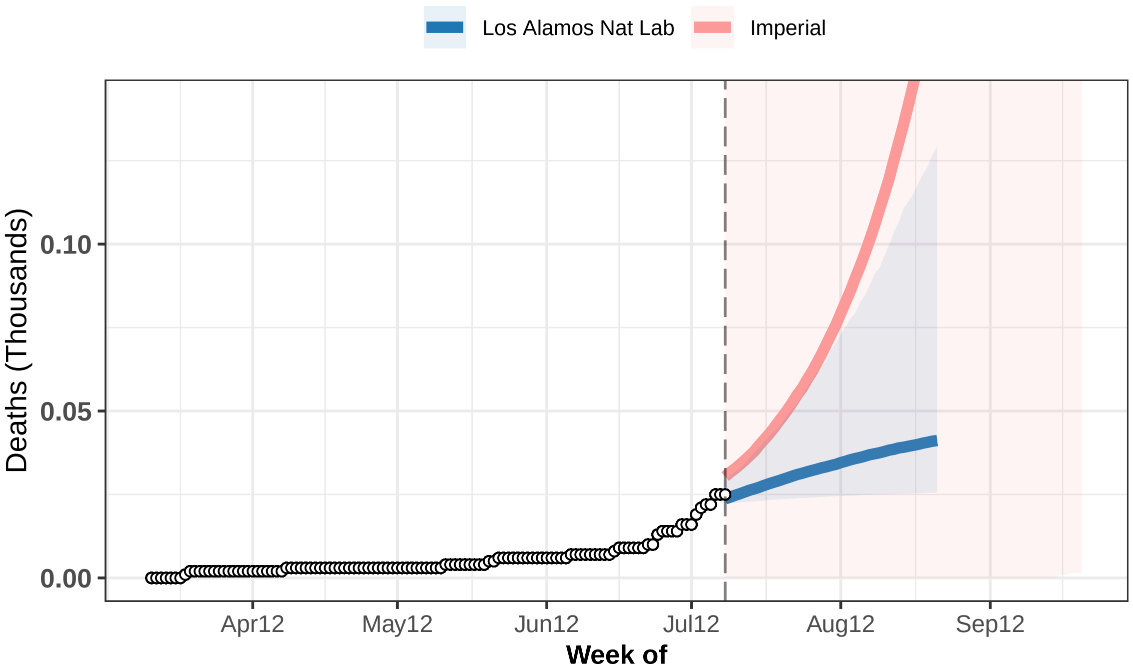

Cumulative Out−Of−Sample Error (Post Intercept Shift)

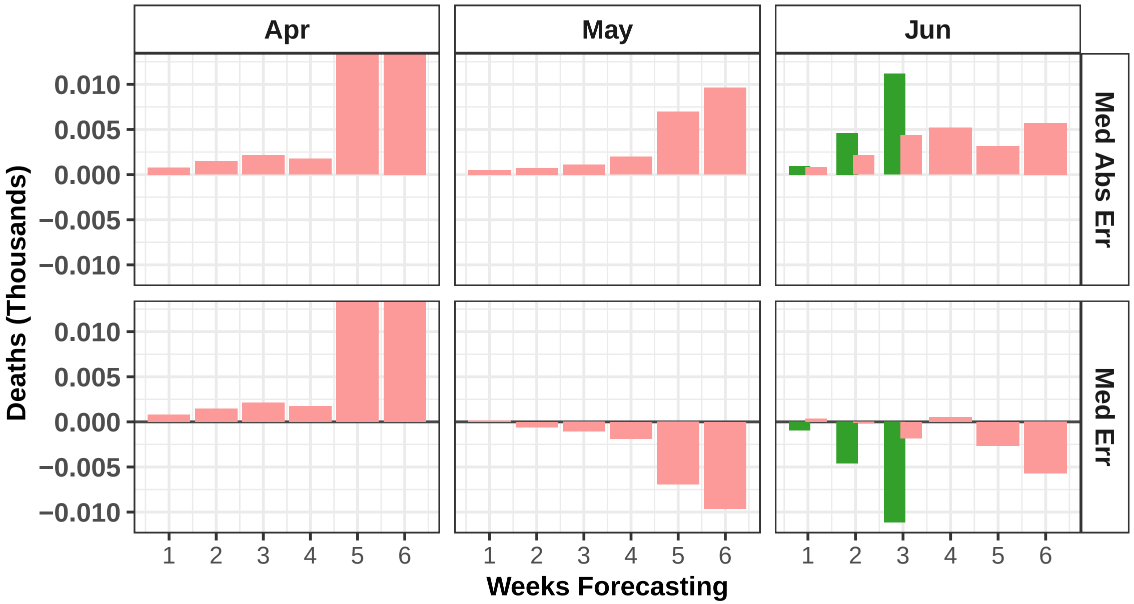

All Model Versions

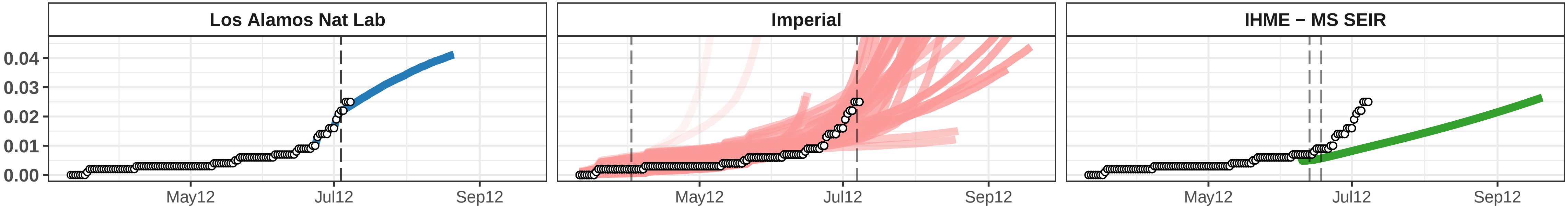

All Cumulative Errors

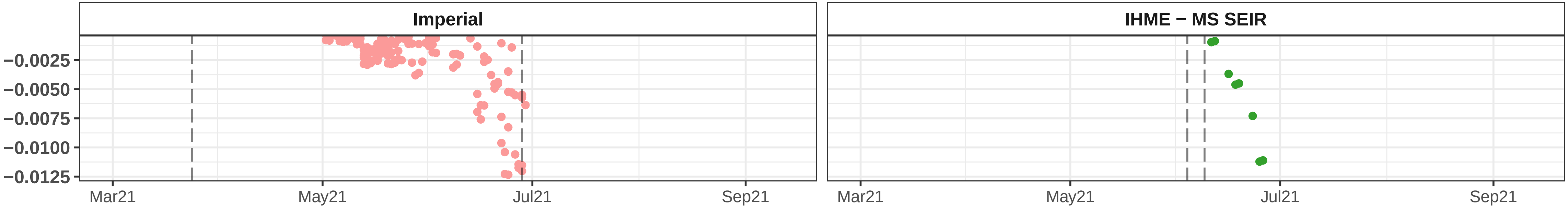

Zimbabwe

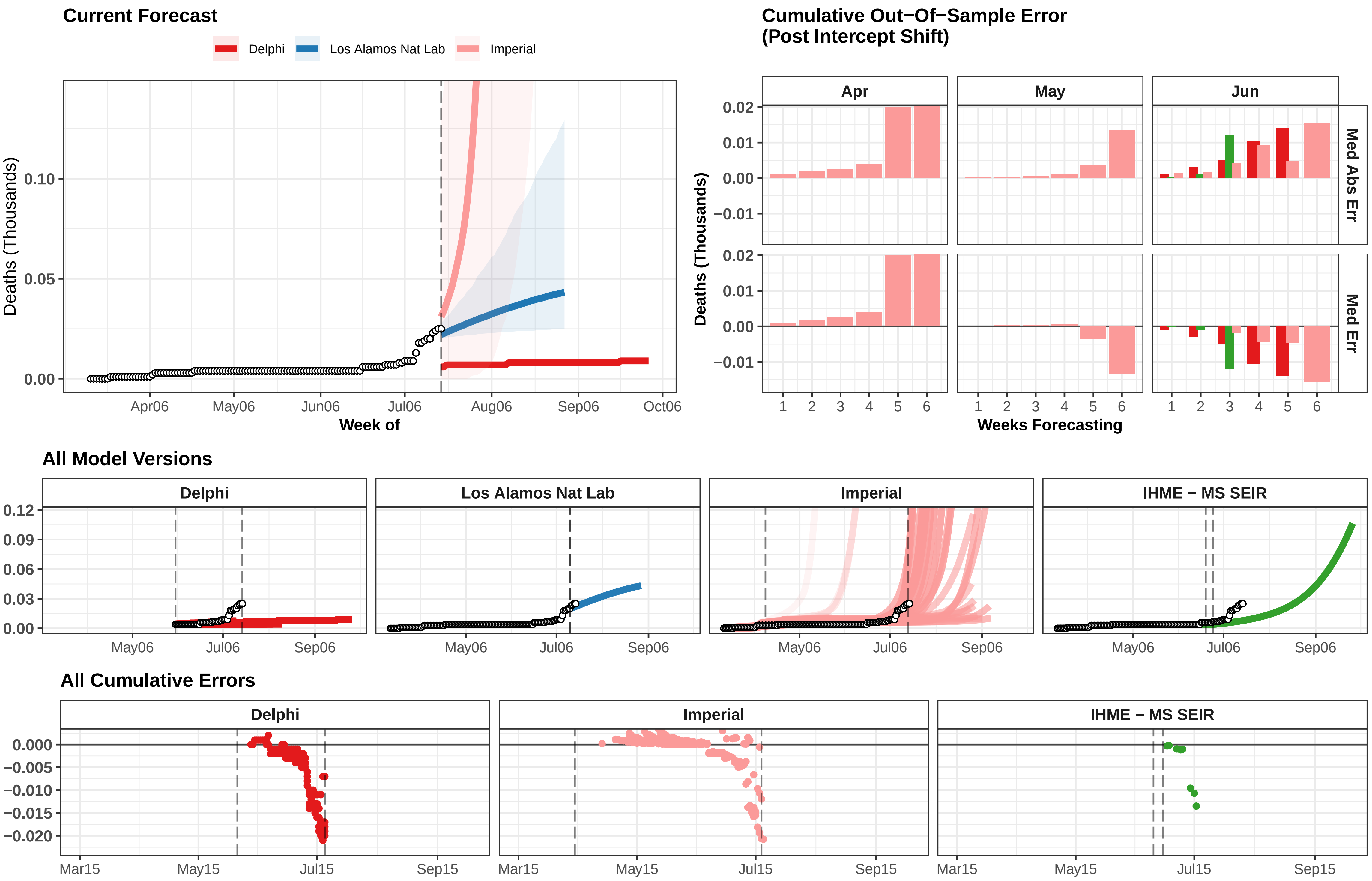

Current Forecast
Delphi
Los Alamos Nat Lab
Imperial
Deaths (Thousands)
0.10
0.05
0.00
Apr06
May06
Jun06
Jul06
Aug06
Sep06
Oct06
Week of
Cumulative Out−Of−Sample Error
(Post Intercept Shift)
Apr
May
Jun
Med Abs Err
Med Err
Deaths (Thousands)
0.02
0.01
0.00
−0.01
1
2
3
4
5
6
Weeks Forecasting
All Model Versions
Delphi
Los Alamos Nat Lab
Imperial
IHME − MS SEIR
0.12
0.09
0.06
0.03
0.00
May06
Jul06
Sep06
All Cumulative Errors
Delphi
Imperial
IHME − MS SEIR
0.000
−0.005
−0.010
−0.015
−0.020
Mar15
May15
Jul15
Sep15

Wyoming

Current Forecast

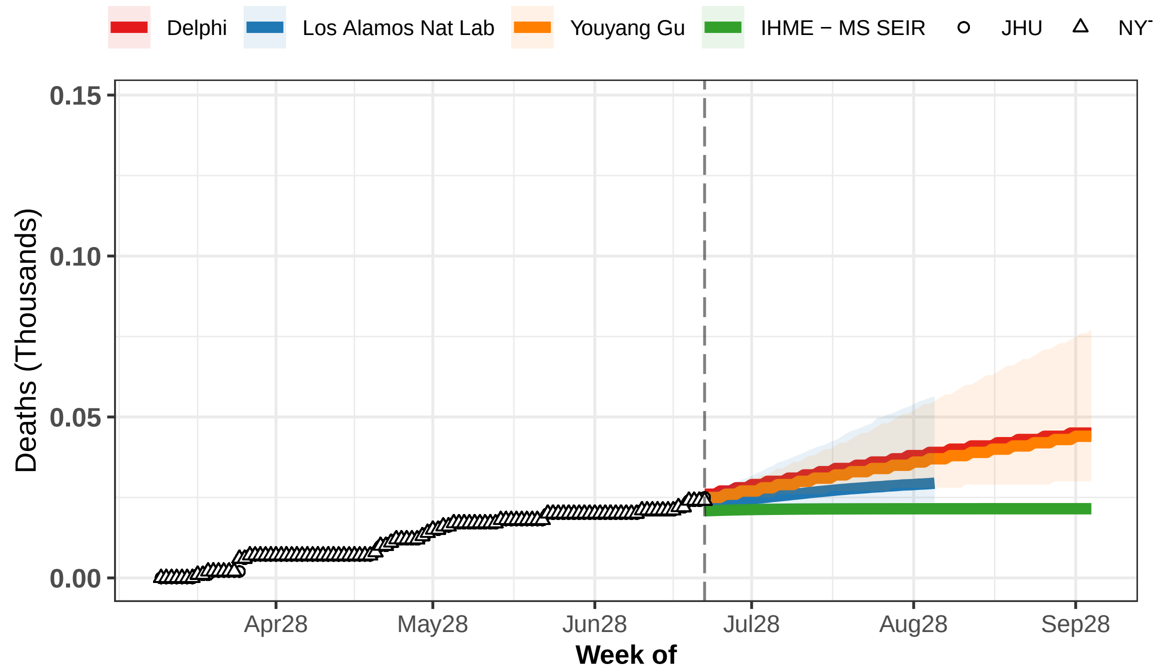

Cumulative Out−Of−Sample Error (Post Intercept Shift)

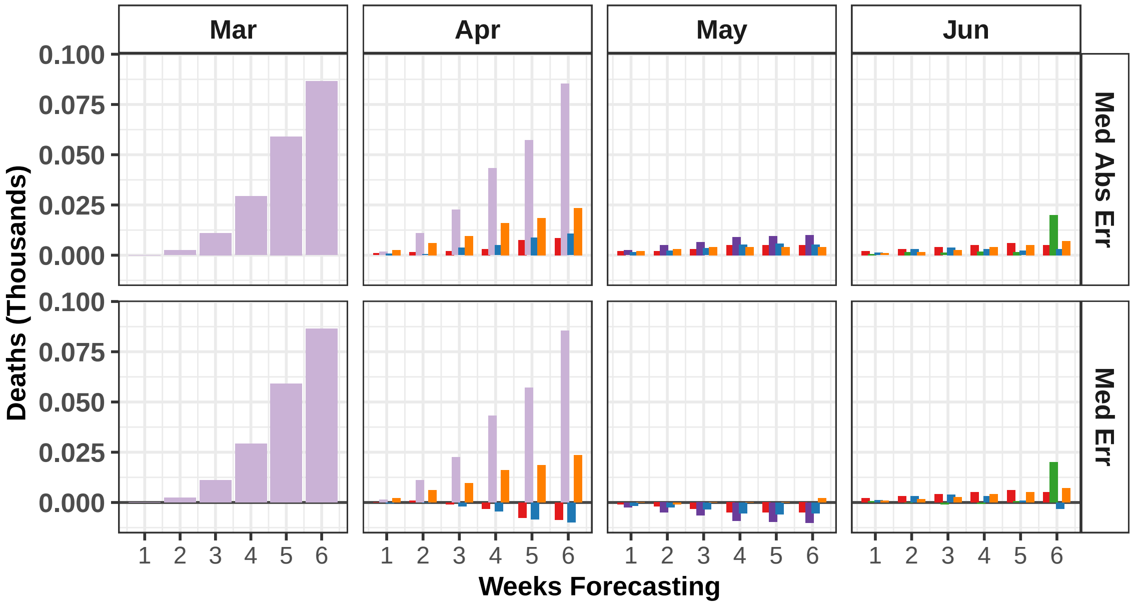

All Model Versions

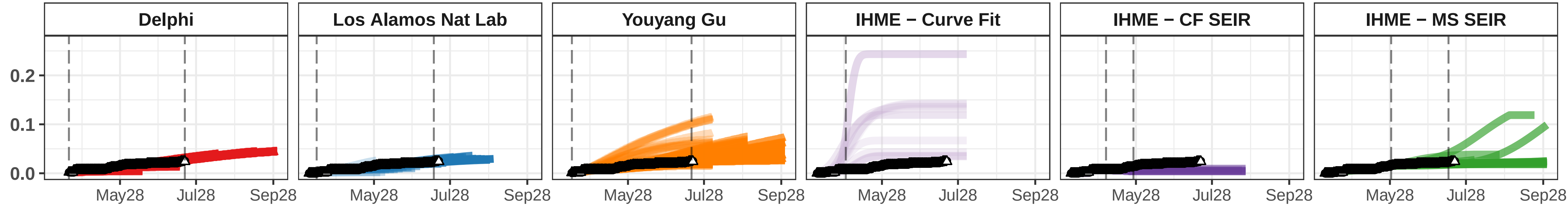

All Cumulative Errors

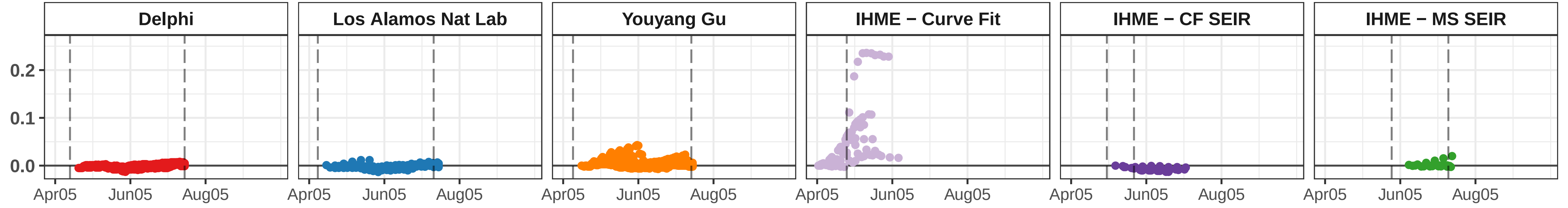

Hawaii

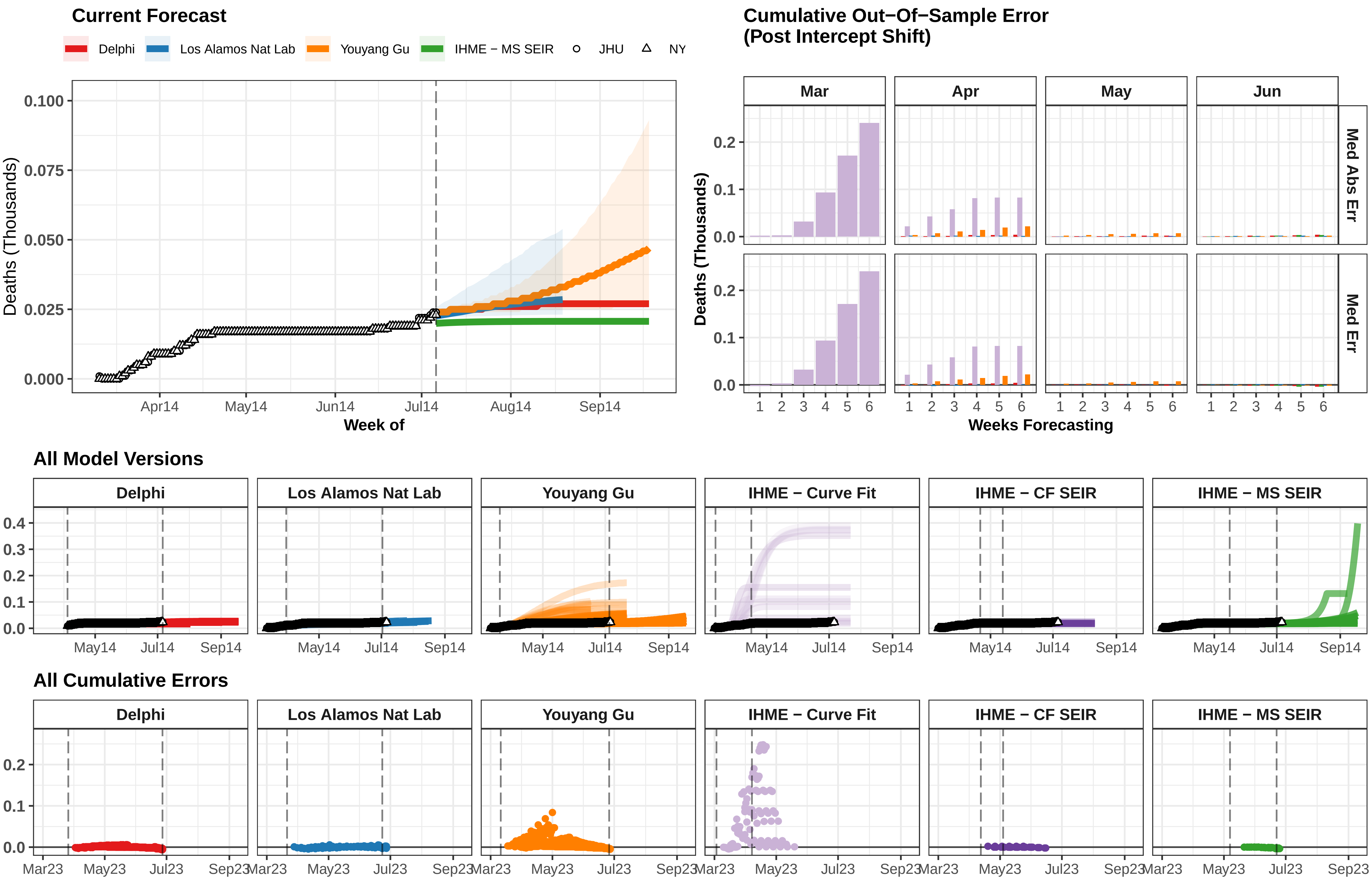

Current Forecast
Delphi
Los Alamos Nat Lab
Youyang Gu
IHME − MS SEIR
JHU
NY
Deaths (Thousands)
0.100
0.075
0.050
0.025
0.000
Apr14
May14
Jun14
Jul14
Aug14
Sep14
Week of
Cumulative Out−Of−Sample Error
(Post Intercept Shift)
Mar
Apr
May
Jun
Med Abs Err
Med Err
Deaths (Thousands)
0.2
0.1
0.0
1 2 3 4 5 6
Weeks Forecasting
All Model Versions
Delphi
Los Alamos Nat Lab
Youyang Gu
IHME − Curve Fit
IHME − CF SEIR
IHME − MS SEIR
0.4
0.3
0.2
0.1
0.0
May14
Jul14
Sep14
All Cumulative Errors
Delphi
Los Alamos Nat Lab
Youyang Gu
IHME − Curve Fit
IHME − CF SEIR
IHME − MS SEIR
0.2
0.1
0.0
Mar23
May23
Jul23
Sep23

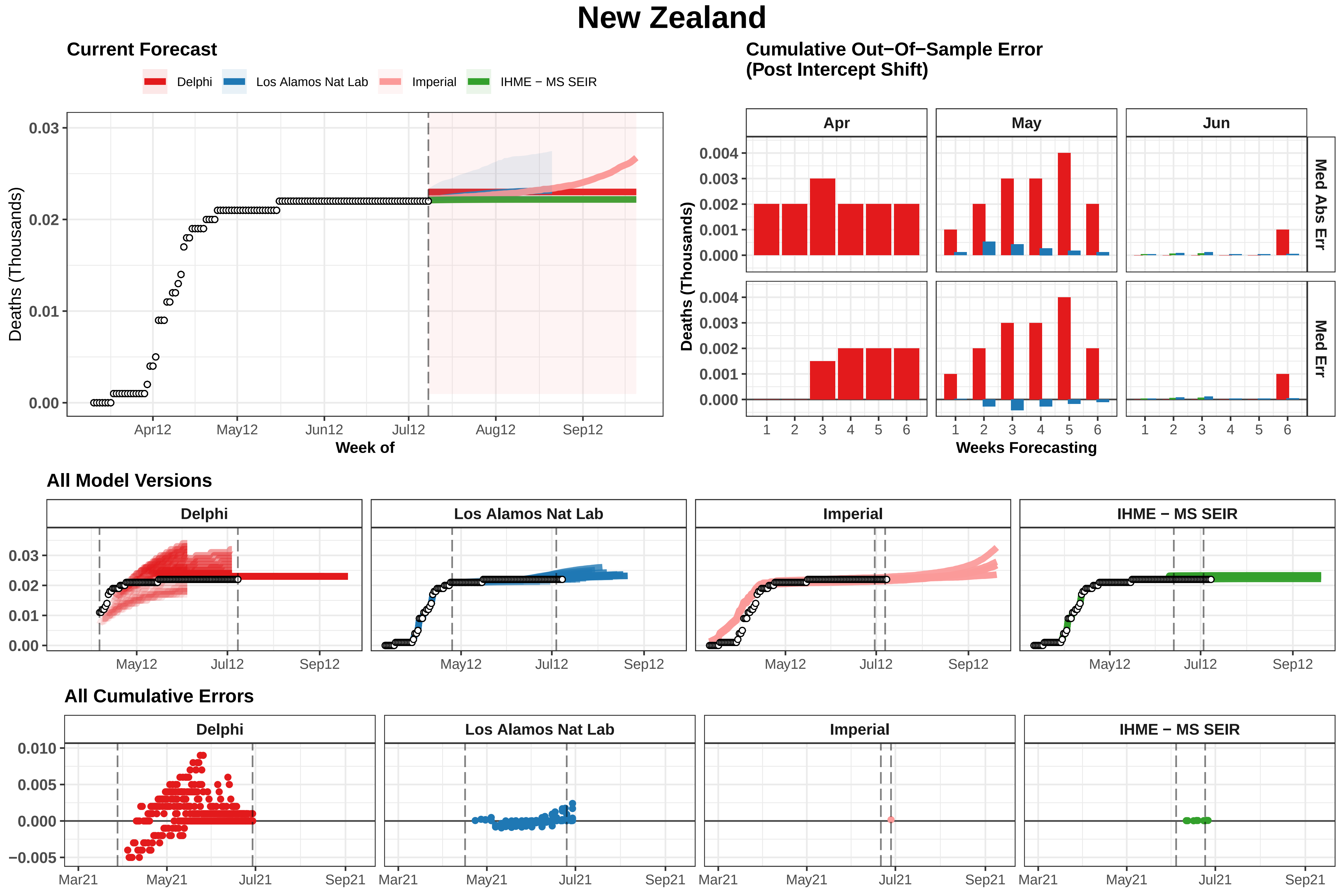
New Zealand
Current Forecast
Delphi
Los Alamos Nat Lab
Imperial
IHME − MS SEIR
Deaths (Thousands)
0.00
0.01
0.02
0.03
Apr12
May12
Jun12
Jul12
Aug12
Sep12
Week of
Cumulative Out−Of−Sample Error
(Post Intercept Shift)
Apr
May
Jun
Med Abs Err
Med Err
0.000
0.001
0.002
0.003
0.004
1
2
3
4
5
6
Weeks Forecasting
All Model Versions
Delphi
Los Alamos Nat Lab
Imperial
IHME − MS SEIR
All Cumulative Errors
0.010
0.005
0.000
−0.005
Mar21
May21
Jul21
Sep21

Cape Verde

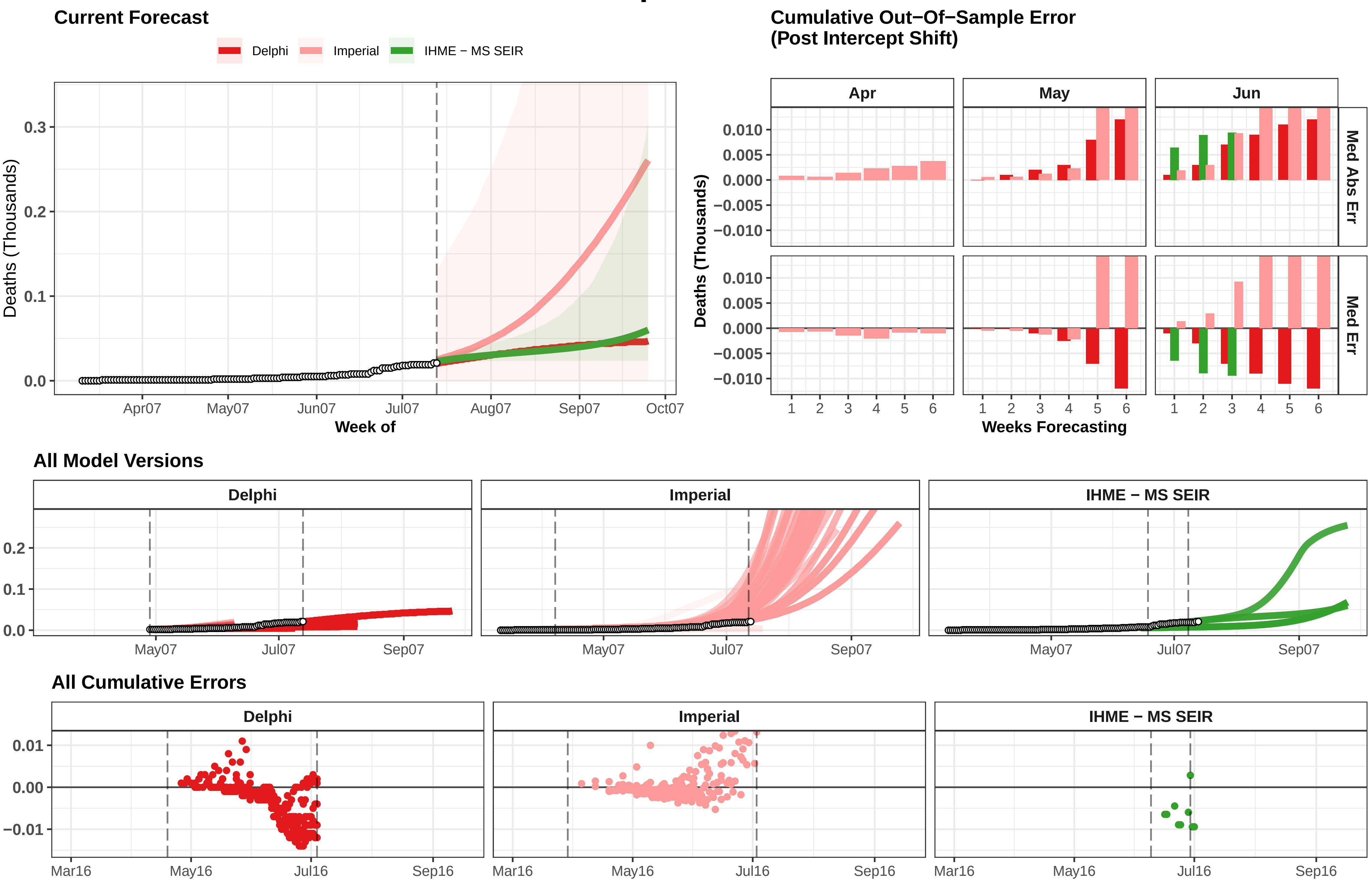

Current Forecast
Delphi
Imperial
IHME − MS SEIR
Deaths (Thousands)
0.0
0.1
0.2
0.3
Apr07
May07
Jun07
Jul07
Aug07
Sep07
Oct07
Week of
Cumulative Out−Of−Sample Error
(Post Intercept Shift)
Apr
May
Jun
Med Abs Err
Med Err
Deaths (Thousands)
0.010
0.005
0.000
−0.005
−0.010
1
2
3
4
5
6
Weeks Forecasting
All Model Versions
Delphi
Imperial
IHME − MS SEIR
0.0
0.1
0.2
May07
Jul07
Sep07
All Cumulative Errors
Delphi
Imperial
IHME − MS SEIR
0.01
0.00
−0.01
Mar16
May16
Jul16
Sep16

Suriname

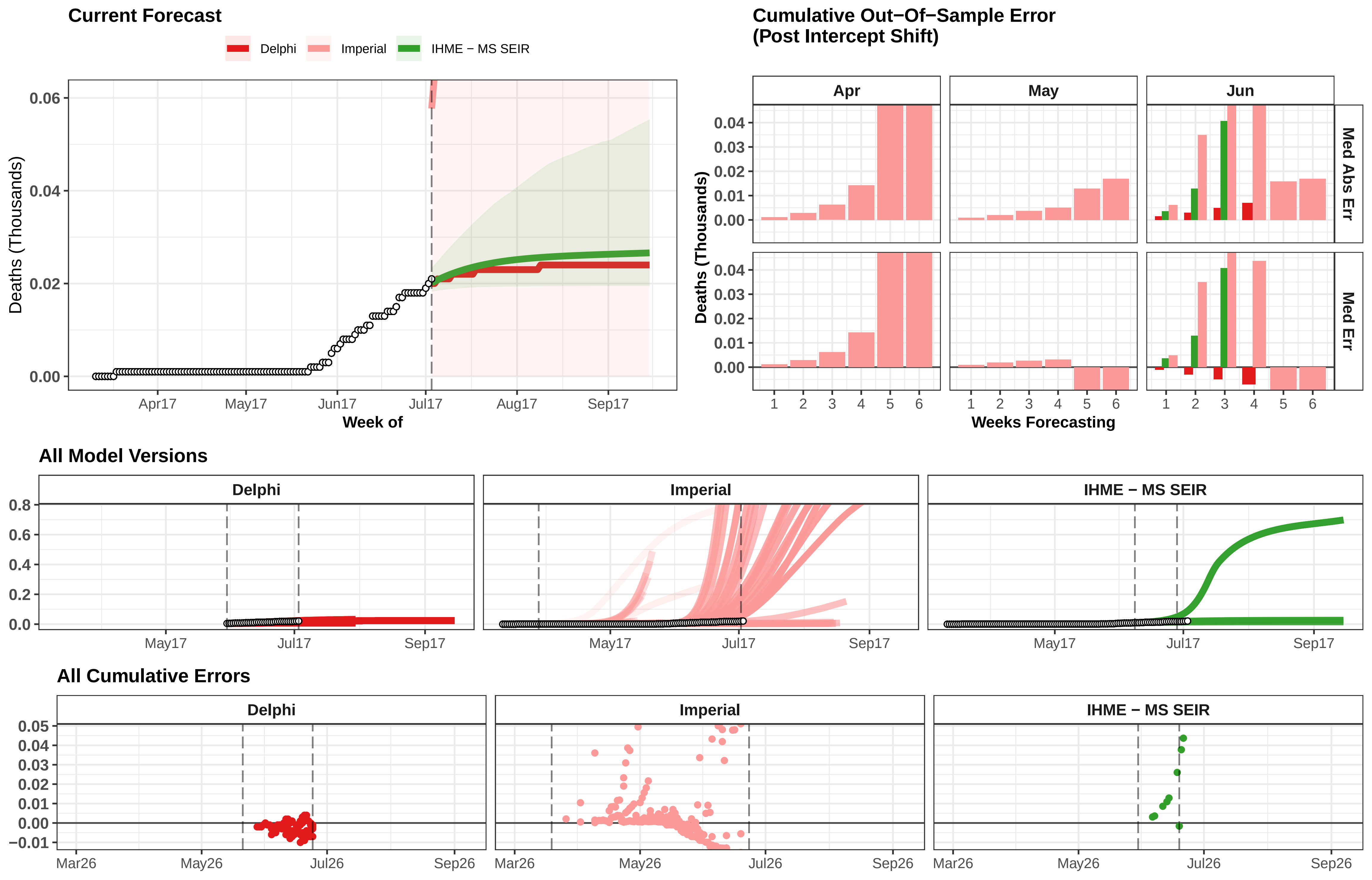
Current Forecast
Delphi
Imperial
IHME − MS SEIR
Deaths (Thousands)
0.06
0.04
0.02
0.00
Apr17
May17
Jun17
Jul17
Aug17
Sep17
Week of
Cumulative Out−Of−Sample Error
(Post Intercept Shift)
Apr
May
Jun
Med Abs Err
Med Err
0.04
0.03
0.02
0.01
0.00
1
2
3
4
5
6
Weeks Forecasting
All Model Versions
Delphi
Imperial
IHME − MS SEIR
0.8
0.6
0.4
0.2
0.0
May17
Jul17
Sep17
All Cumulative Errors
0.05
0.04
0.03
0.02
0.01
0.00
−0.01
Mar26
May26
Jul26
Sep26

Swaziland

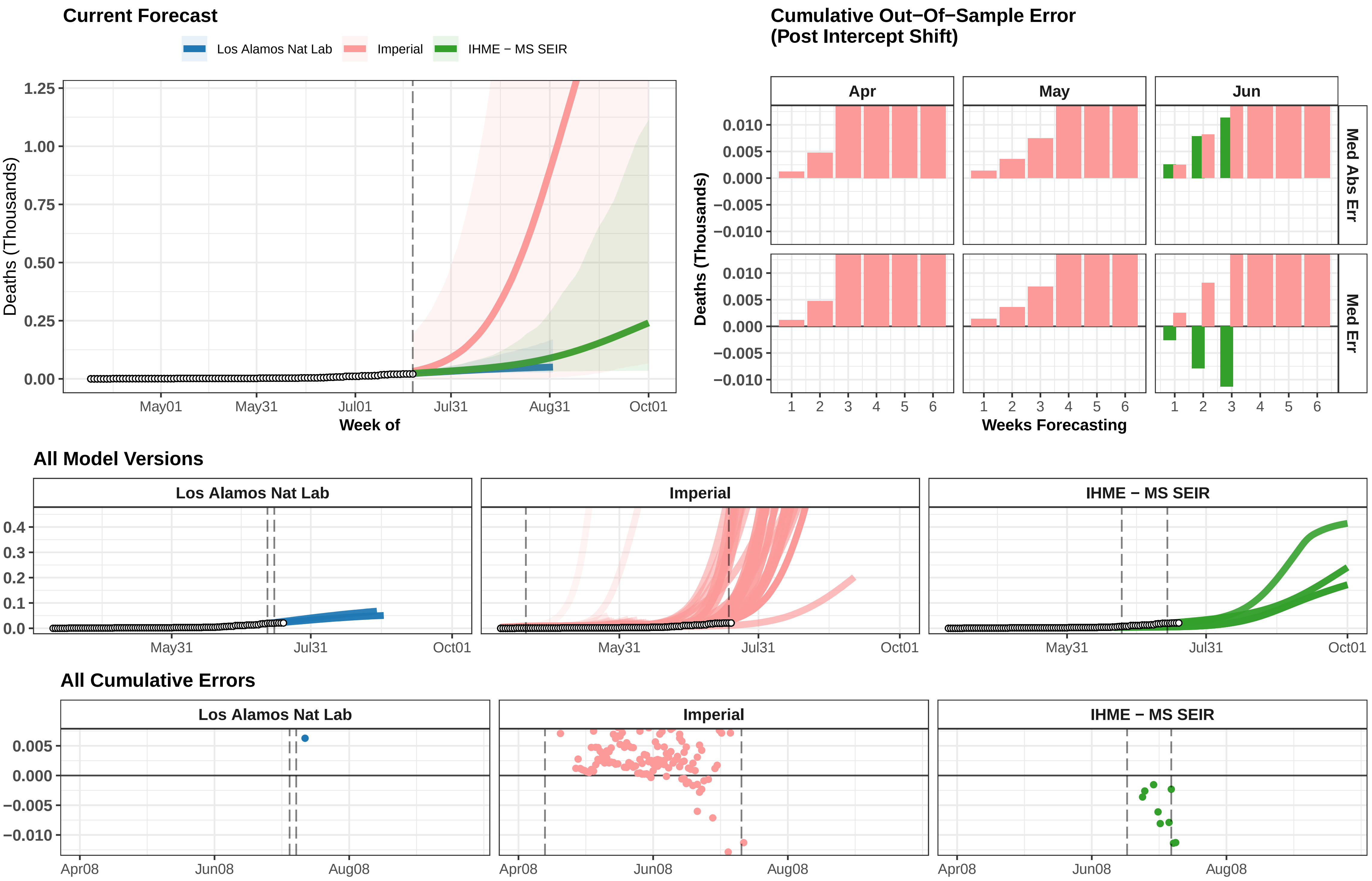
Current Forecast
Los Alamos Nat Lab
Imperial
IHME − MS SEIR
Deaths (Thousands)
Week of
May01
May31
Jul01
Jul31
Aug31
Oct01
Cumulative Out−Of−Sample Error
(Post Intercept Shift)
Apr
May
Jun
Med Abs Err
Med Err
Weeks Forecasting
All Model Versions
All Cumulative Errors
Apr08
Jun08
Aug08

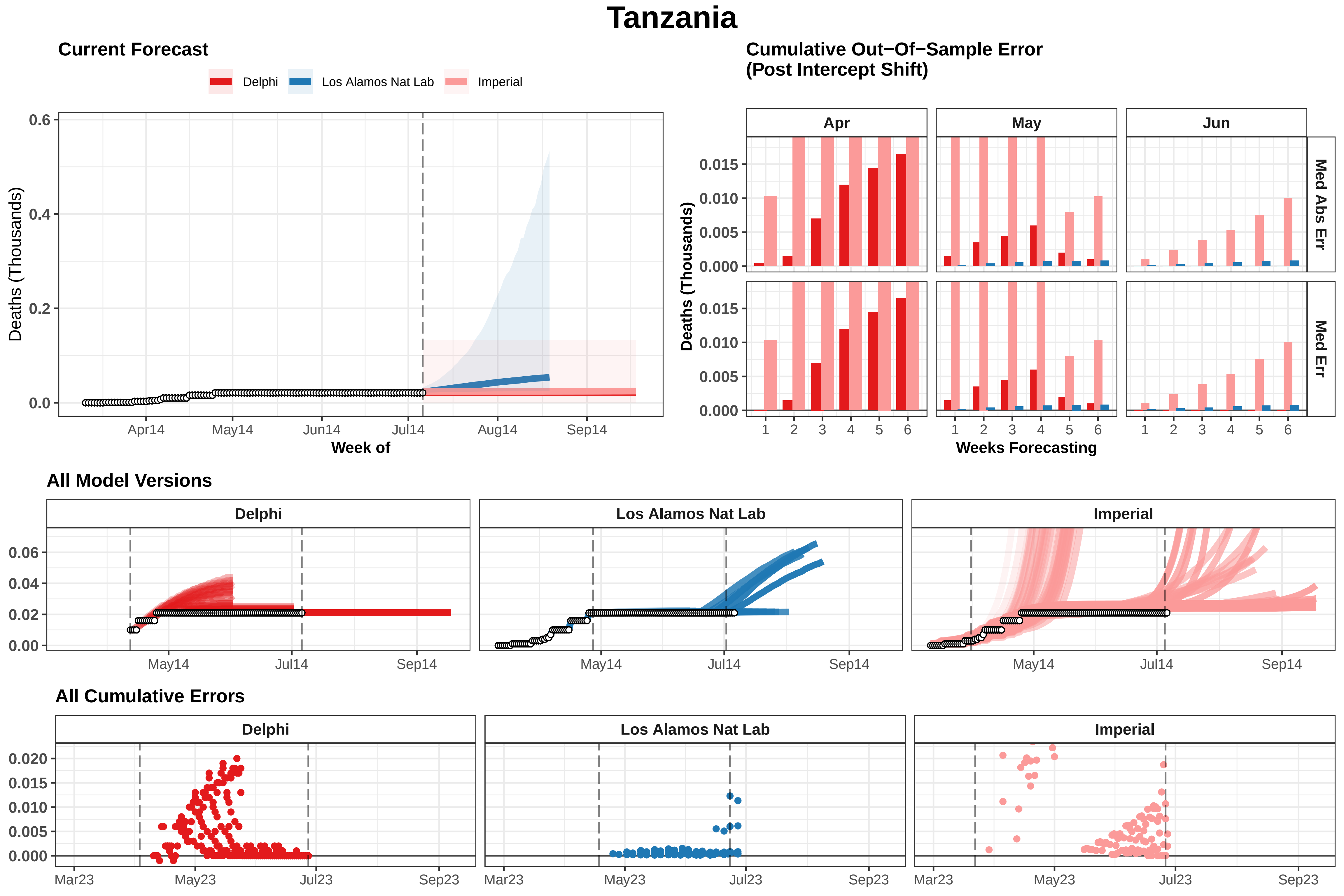

Tanzania
Current Forecast
Delphi
Los Alamos Nat Lab
Imperial
Deaths (Thousands)
0.6
0.4
0.2
0.0
Apr14
May14
Jun14
Jul14
Aug14
Sep14
Week of
Cumulative Out−Of−Sample Error
(Post Intercept Shift)
Apr
May
Jun
Med Abs Err
Med Err
0.015
0.010
0.005
0.000
1 2 3 4 5 6
Weeks Forecasting
All Model Versions
Delphi
Los Alamos Nat Lab
Imperial
0.06
0.04
0.02
0.00
May14
Jul14
Sep14
All Cumulative Errors
Delphi
Los Alamos Nat Lab
Imperial
0.020
0.015
0.010
0.005
0.000
Mar23
May23
Jul23
Sep23

Cyprus

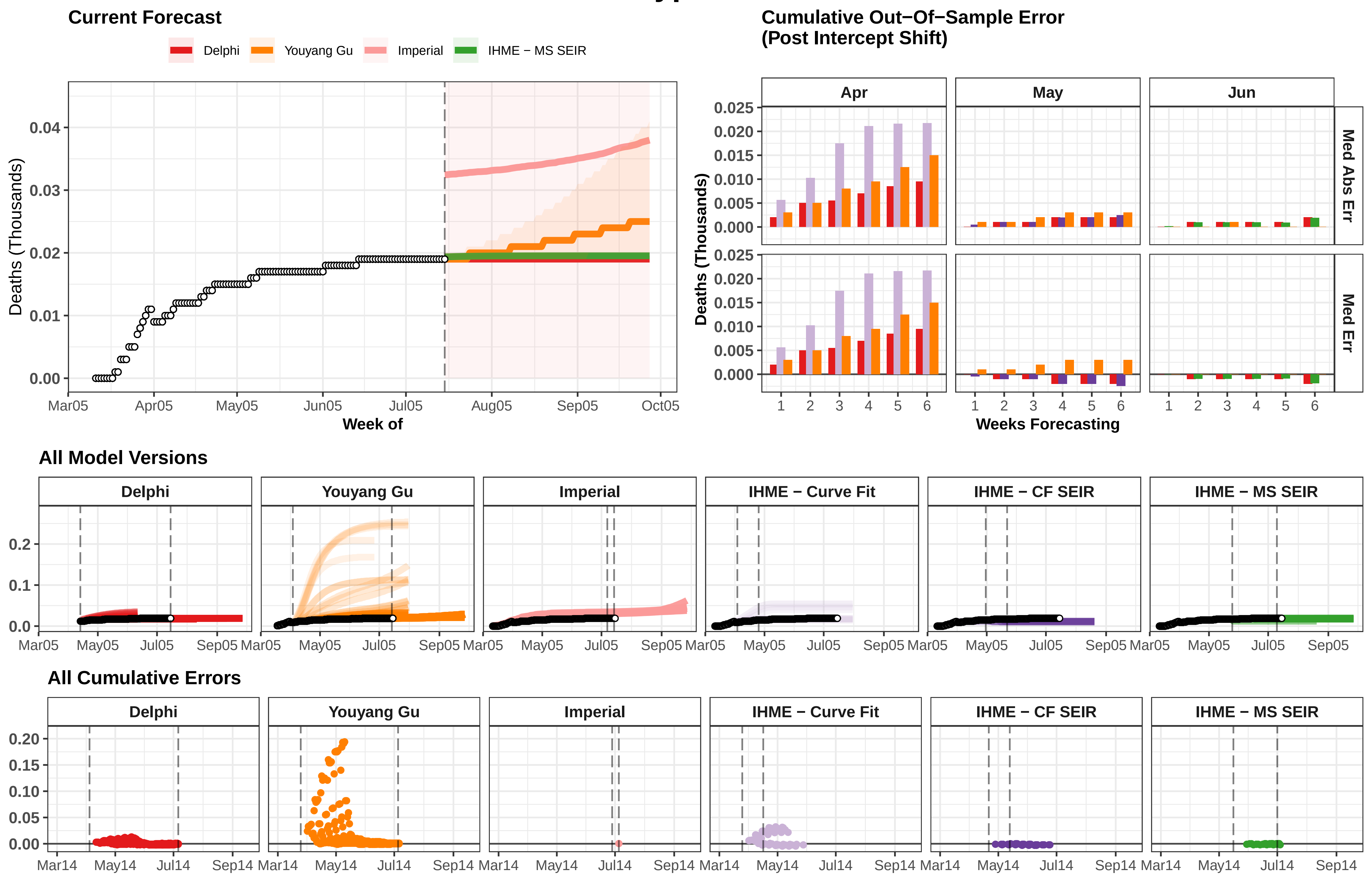
Current Forecast
Delphi
Youyang Gu
Imperial
IHME − MS SEIR
Deaths (Thousands)
Week of
Cumulative Out−Of−Sample Error
(Post Intercept Shift)
Apr
May
Jun
Med Abs Err
Med Err
Weeks Forecasting
All Model Versions
IHME − Curve Fit
IHME − CF SEIR
All Cumulative Errors

Guyana

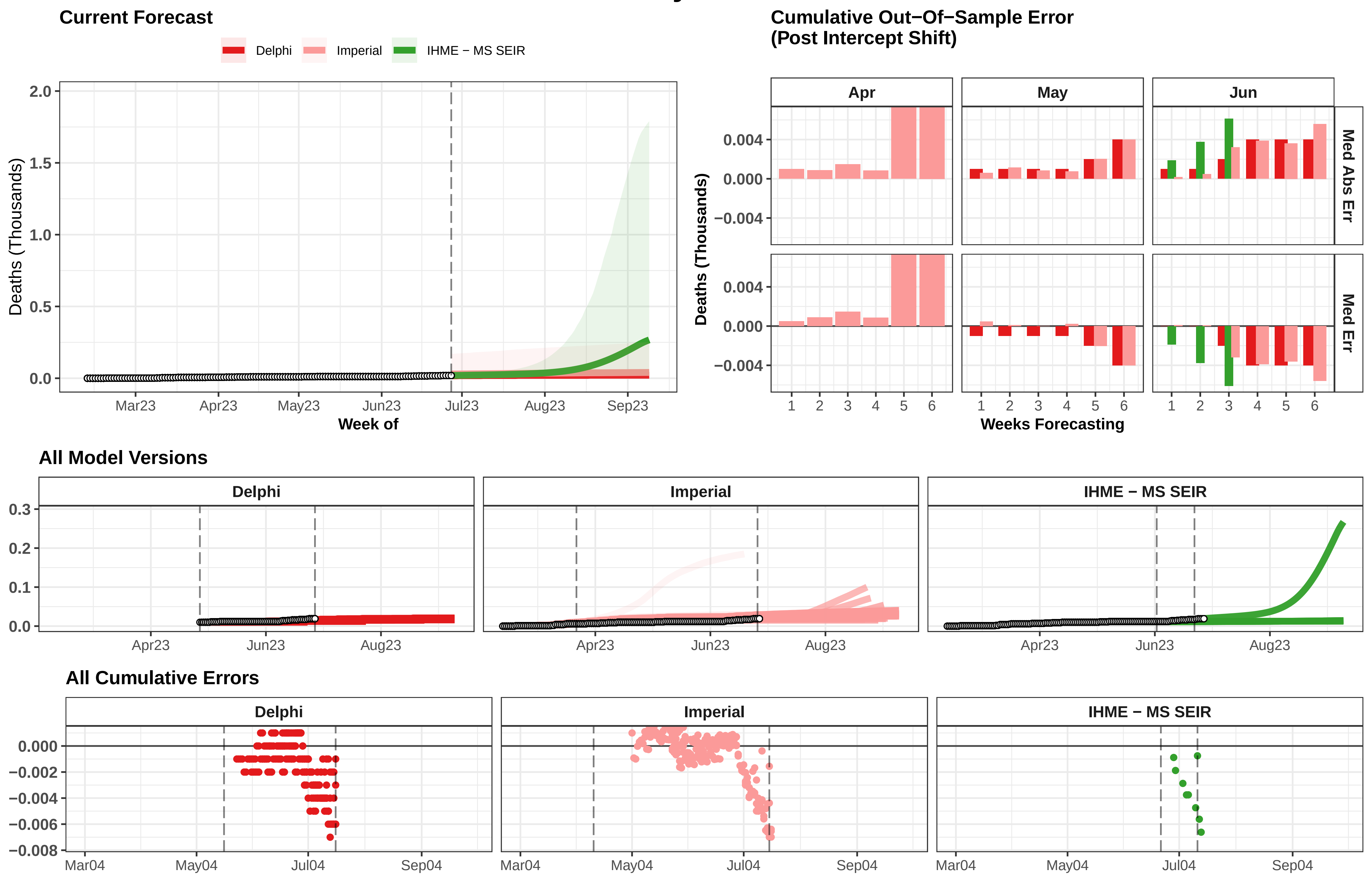
Current Forecast
Delphi
Imperial
IHME − MS SEIR
Deaths (Thousands)
Week of
Mar23
Apr23
May23
Jun23
Jul23
Aug23
Sep23
Cumulative Out−Of−Sample Error
(Post Intercept Shift)
Apr
May
Jun
Med Abs Err
Med Err
Weeks Forecasting
All Model Versions
All Cumulative Errors
Mar04
May04
Jul04
Sep04

Alaska

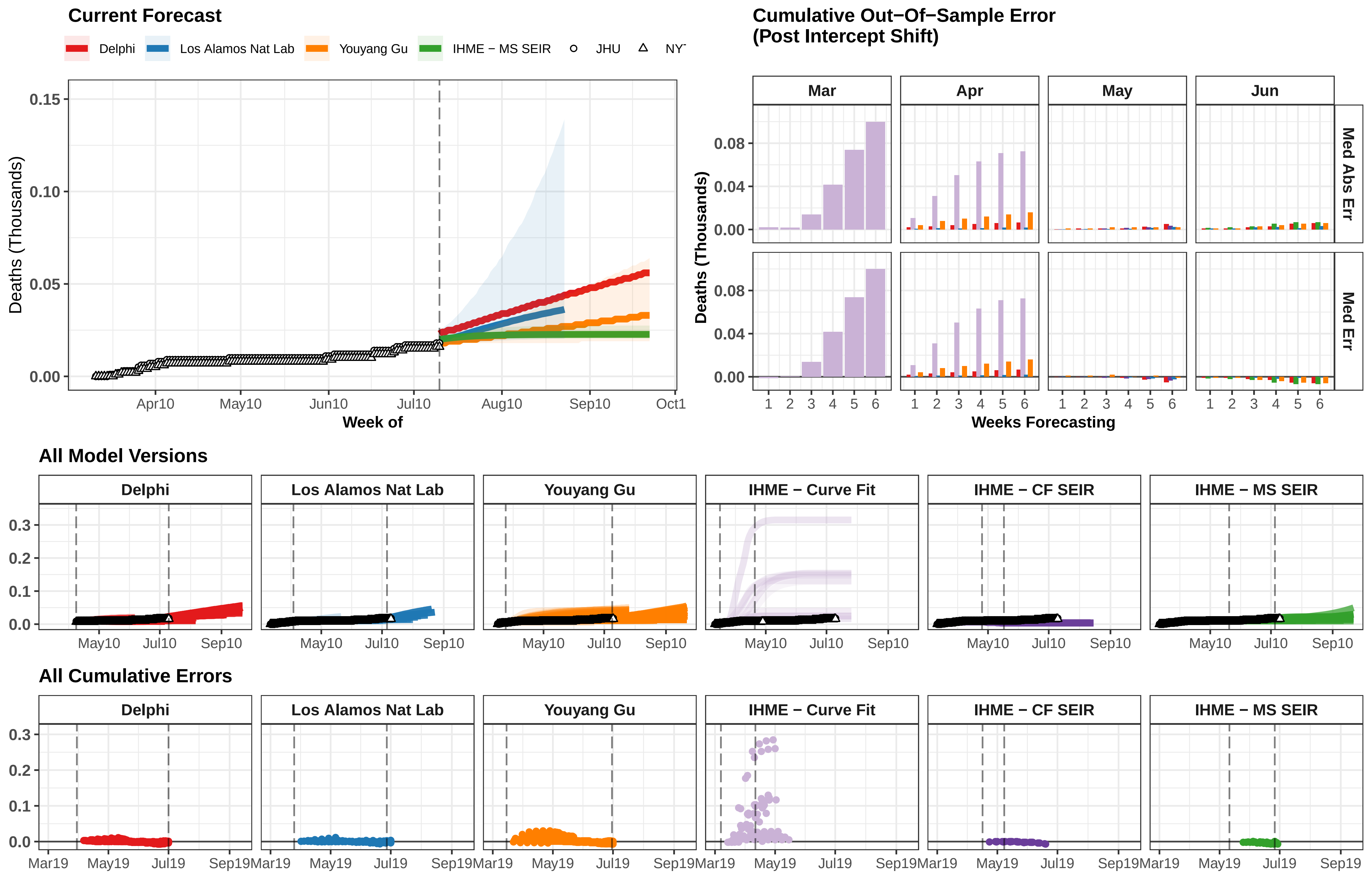

Current Forecast
Delphi
Los Alamos Nat Lab
Youyang Gu
IHME − MS SEIR
JHU
NY
0.15
0.10
0.05
0.00
Deaths (Thousands)
Apr10
May10
Jun10
Jul10
Aug10
Sep10
Oct1
Week of
Cumulative Out−Of−Sample Error
(Post Intercept Shift)
Mar
Apr
May
Jun
Med Abs Err
Med Err
0.08
0.04
0.00
Weeks Forecasting
All Model Versions
Delphi
Los Alamos Nat Lab
Youyang Gu
IHME − Curve Fit
IHME − CF SEIR
IHME − MS SEIR
0.3
0.2
0.1
0.0
May10
Jul10
Sep10
All Cumulative Errors
Mar19
May19
Jul19
Sep19

Georgia (Country)

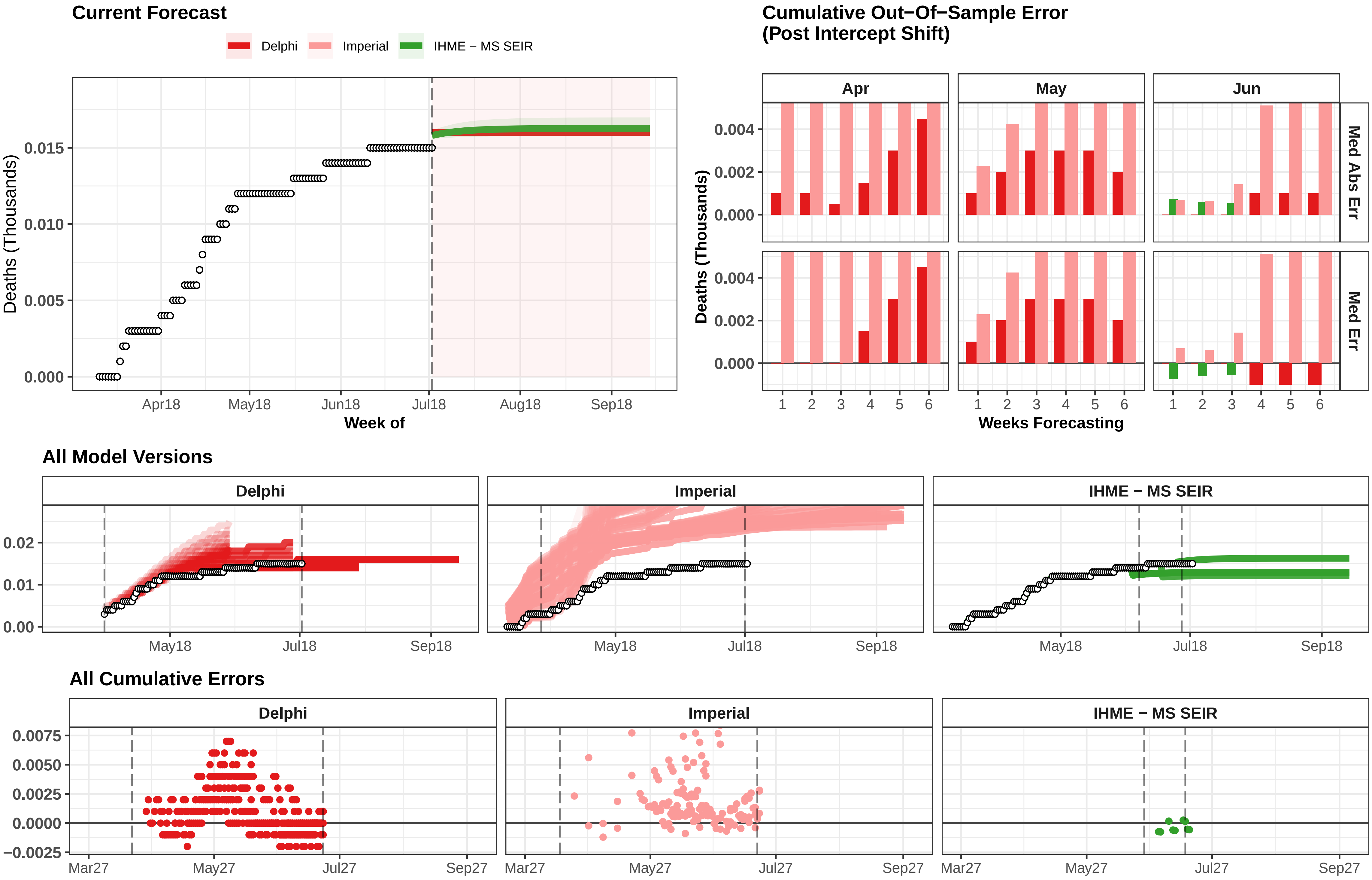

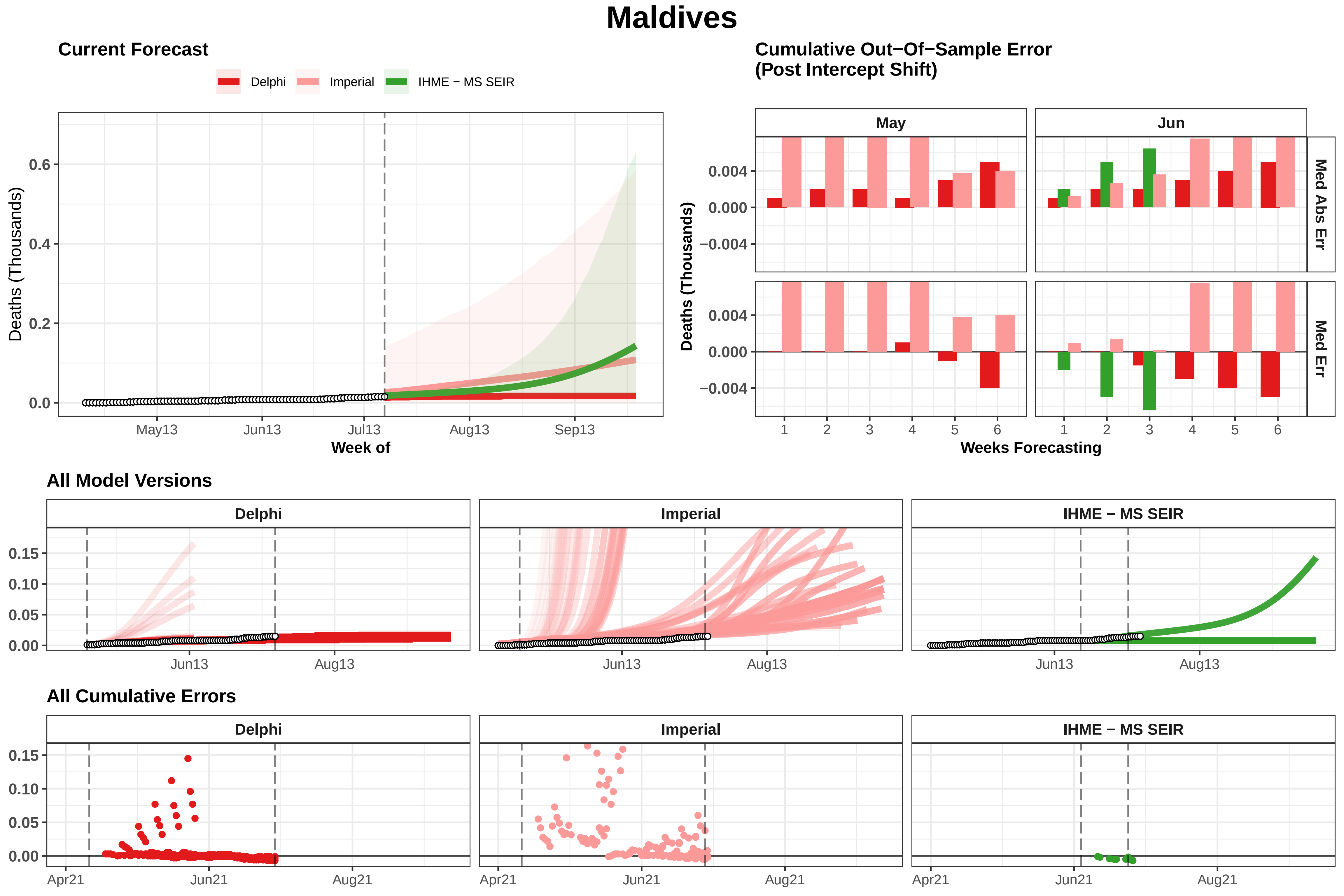

Maldives
Current Forecast
Delphi
Imperial
IHME − MS SEIR
Deaths (Thousands)
0.0
0.2
0.4
0.6
May13
Jun13
Jul13
Aug13
Sep13
Week of
Cumulative Out−Of−Sample Error
(Post Intercept Shift)
May
Jun
Med Abs Err
Med Err
0.004
0.000
−0.004
1
2
3
4
5
6
Weeks Forecasting
All Model Versions
0.15
0.10
0.05
0.00
Aug13
All Cumulative Errors
Apr21
Jun21
Aug21

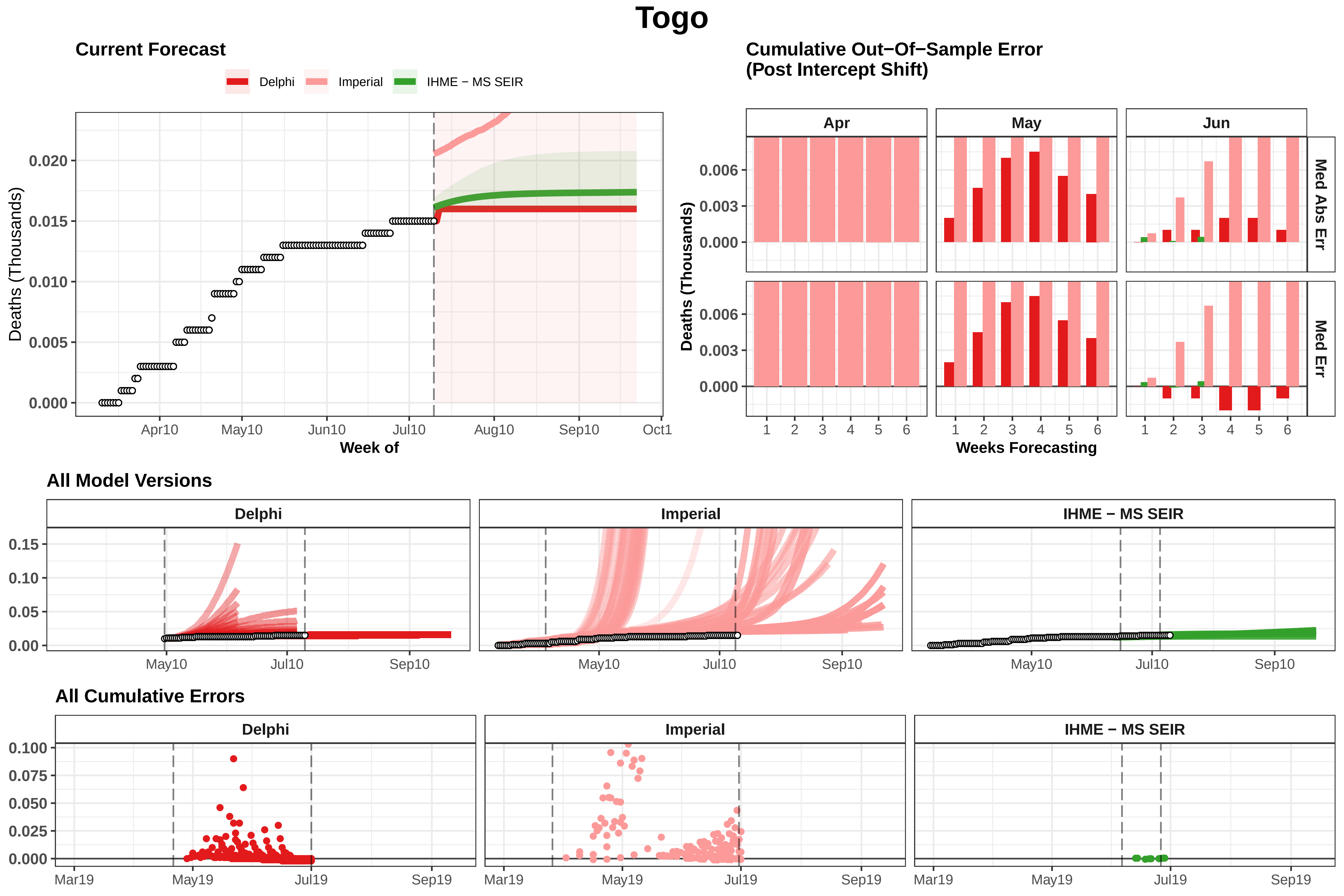

Togo
Current Forecast
Delphi
Imperial
IHME − MS SEIR
Deaths (Thousands)
0.020
0.015
0.010
0.005
0.000
Apr10
May10
Jun10
Jul10
Aug10
Sep10
Oct1
Week of
Cumulative Out−Of−Sample Error
(Post Intercept Shift)
Apr
May
Jun
Med Abs Err
Med Err
0.006
0.003
0.000
1
2
3
4
5
6
Weeks Forecasting
All Model Versions
Delphi
Imperial
IHME − MS SEIR
0.15
0.10
0.05
0.00
May10
Jul10
Sep10
All Cumulative Errors
0.100
0.075
0.050
0.025
0.000
Mar19
May19
Jul19
Sep19

Sao Tome and Principe

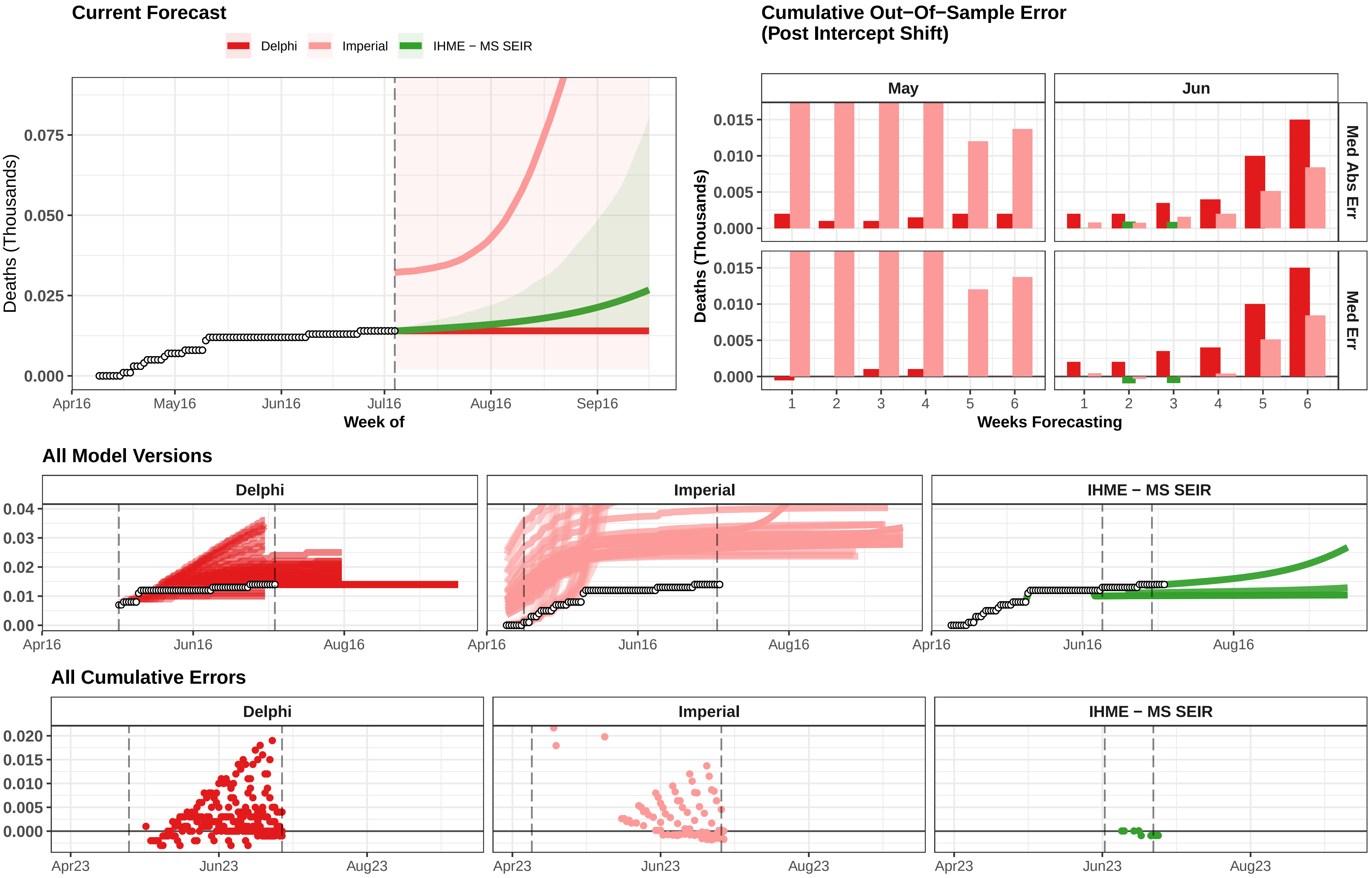

Jordan

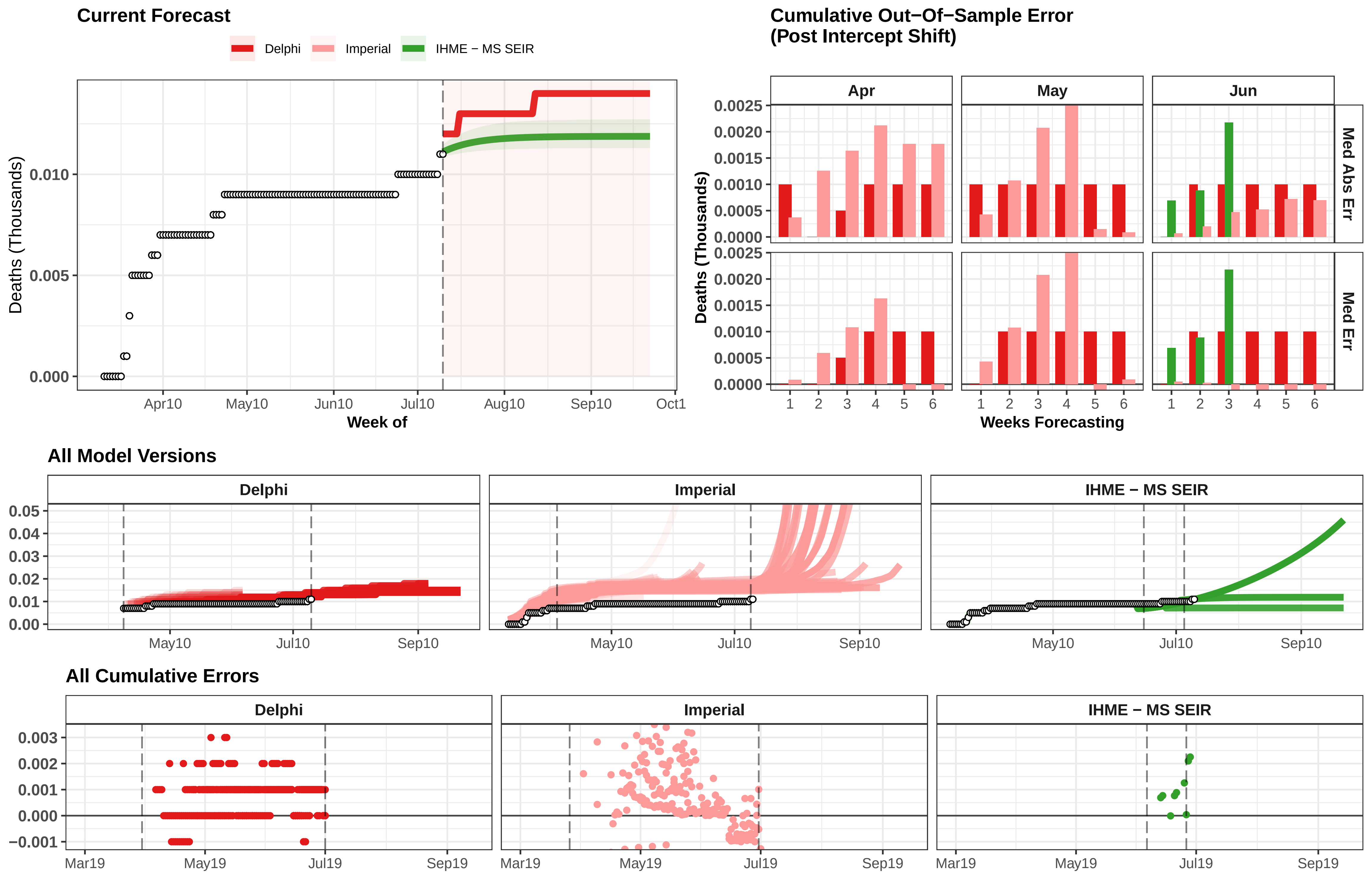

Current Forecast
Delphi
Imperial
IHME − MS SEIR
Deaths (Thousands)
0.010
0.005
0.000
Apr10
May10
Jun10
Jul10
Aug10
Sep10
Oct1
Week of
Cumulative Out−Of−Sample Error
(Post Intercept Shift)
Apr
May
Jun
Med Abs Err
Med Err
0.0025
0.0020
0.0015
0.0010
0.0005
0.0000
1
2
3
4
5
6
Weeks Forecasting
All Model Versions
Delphi
Imperial
IHME − MS SEIR
0.05
0.04
0.03
0.02
0.01
0.00
May10
Jul10
Sep10
All Cumulative Errors
0.003
0.002
0.001
0.000
−0.001
Mar19
May19
Jul19
Sep19

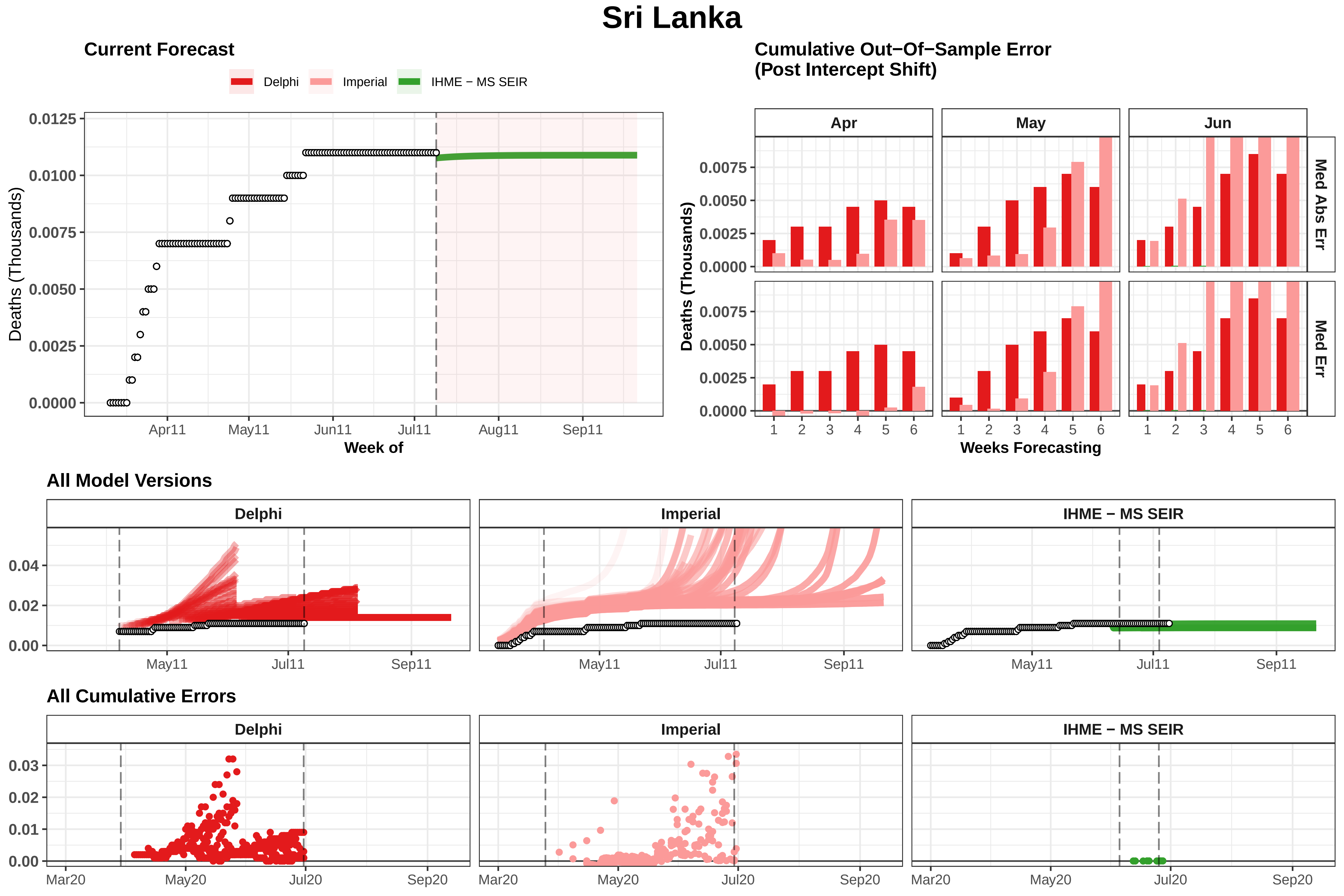

Sri Lanka
Current Forecast
Delphi
Imperial
IHME − MS SEIR
Deaths (Thousands)
0.0125
0.0100
0.0075
0.0050
0.0025
0.0000
Apr11
May11
Jun11
Jul11
Aug11
Sep11
Week of
Cumulative Out−Of−Sample Error
(Post Intercept Shift)
Apr
May
Jun
Med Abs Err
Med Err
Weeks Forecasting
All Model Versions
Delphi
Imperial
IHME − MS SEIR
0.04
0.02
0.00
All Cumulative Errors
0.03
0.02
0.01
0.00
Mar20
May20
Jul20
Sep20
