# Supplementary material for: Predictive performance of international COVID-19 mortality forecasting models
Source: medRxiv. 2020 Nov 19:2020.07.13.20151233. Preprint. [Version 5] doi: 10.1101/2020.07.13.20151233 (PMC7685335; doi:10.1101/2020.07.13.20151233)
Supplement: Supplement 2020 [file 90316-2020.07.13.20151233-3.pdf]

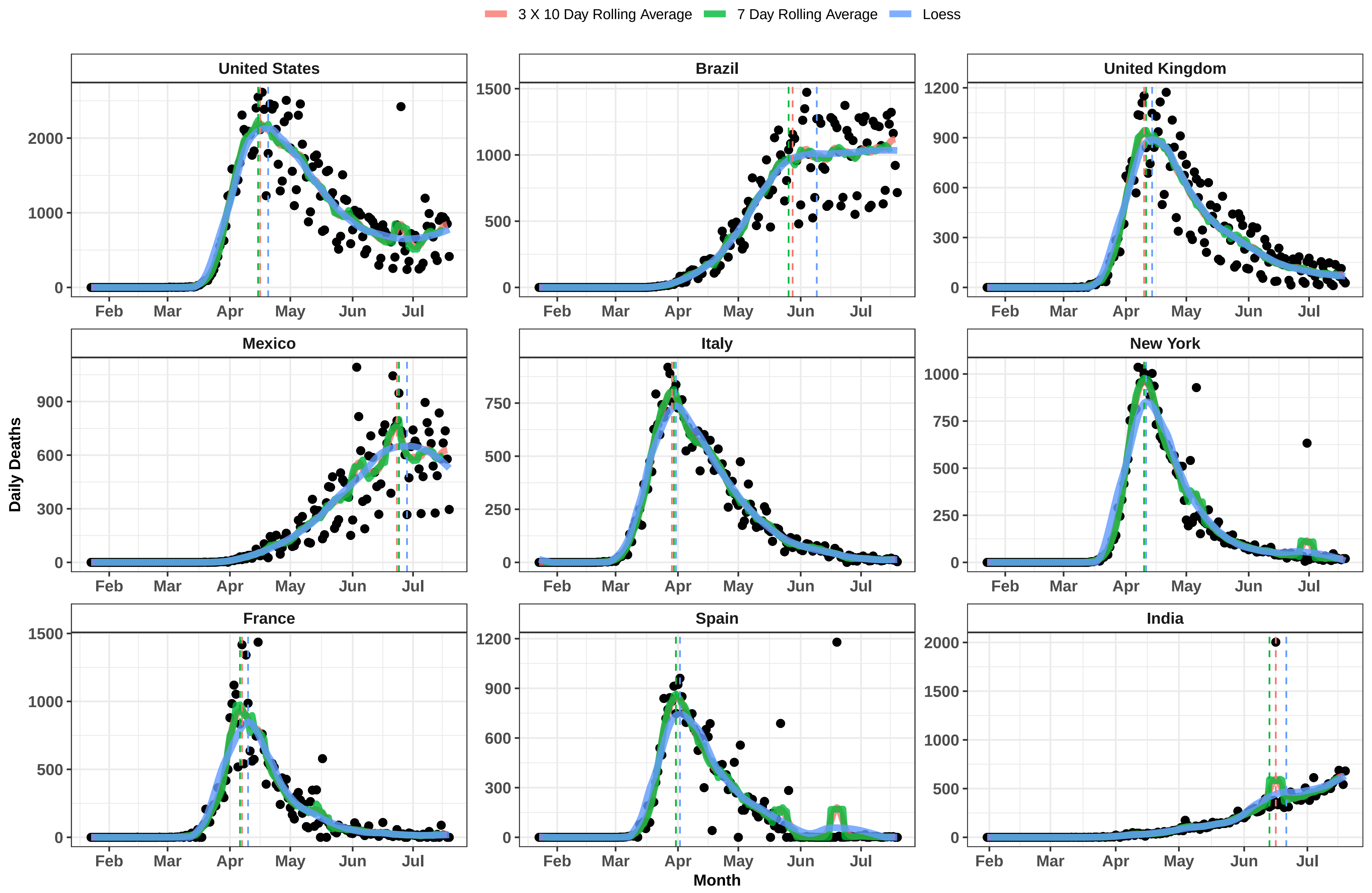

3 X 10 Day Rolling Average
7 Day Rolling Average
Loess
United States
Brazil
United Kingdom
Mexico
Italy
New York
France
Spain
India
Daily Deaths
Month
Feb
Mar
Apr
May
Jun
Jul

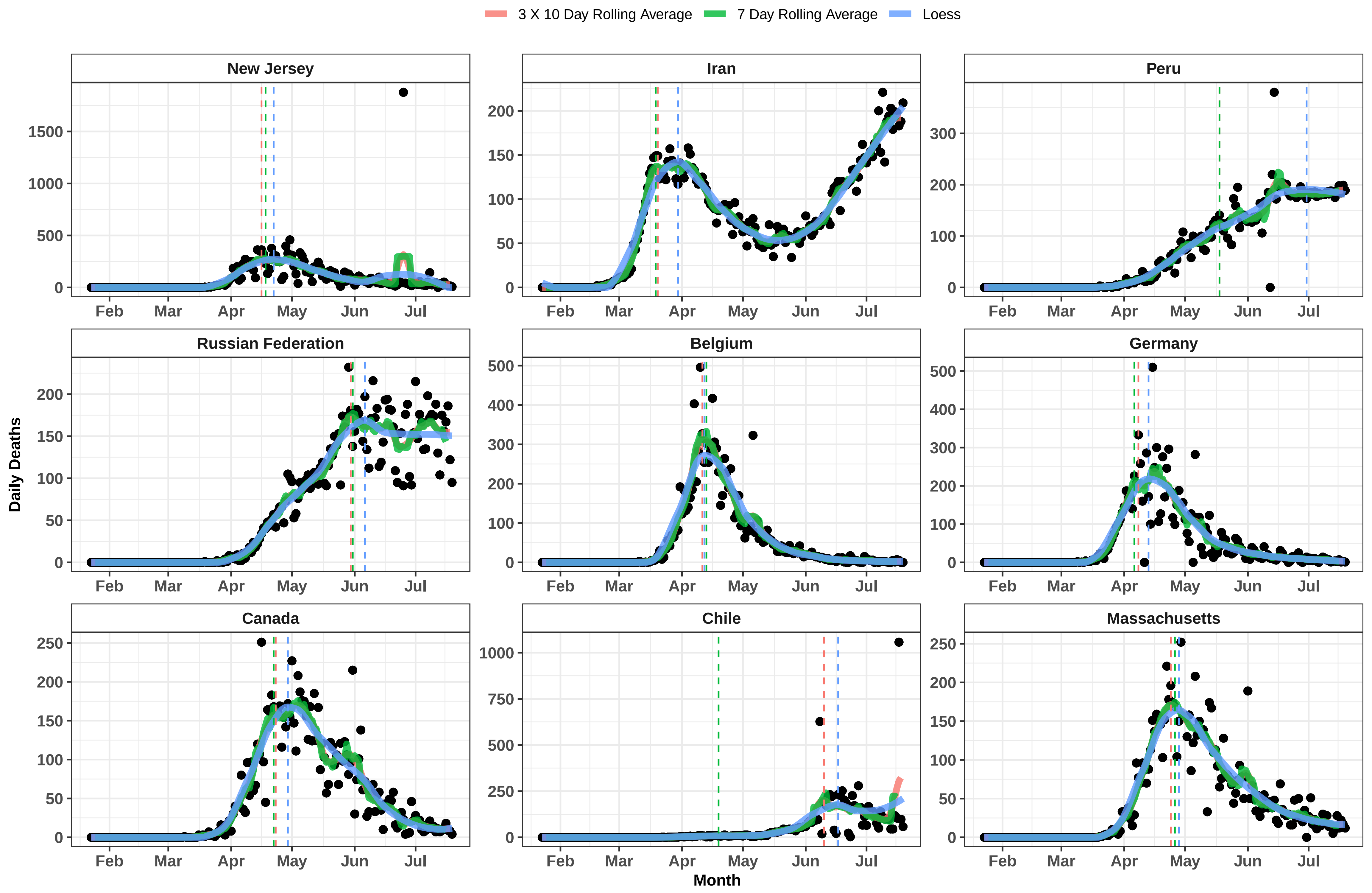
3 X 10 Day Rolling Average
7 Day Rolling Average
Loess
New Jersey
Iran
Peru
Russian Federation
Belgium
Germany
Canada
Chile
Massachusetts
Daily Deaths
Month
Feb
Mar
Apr
May
Jun
Jul

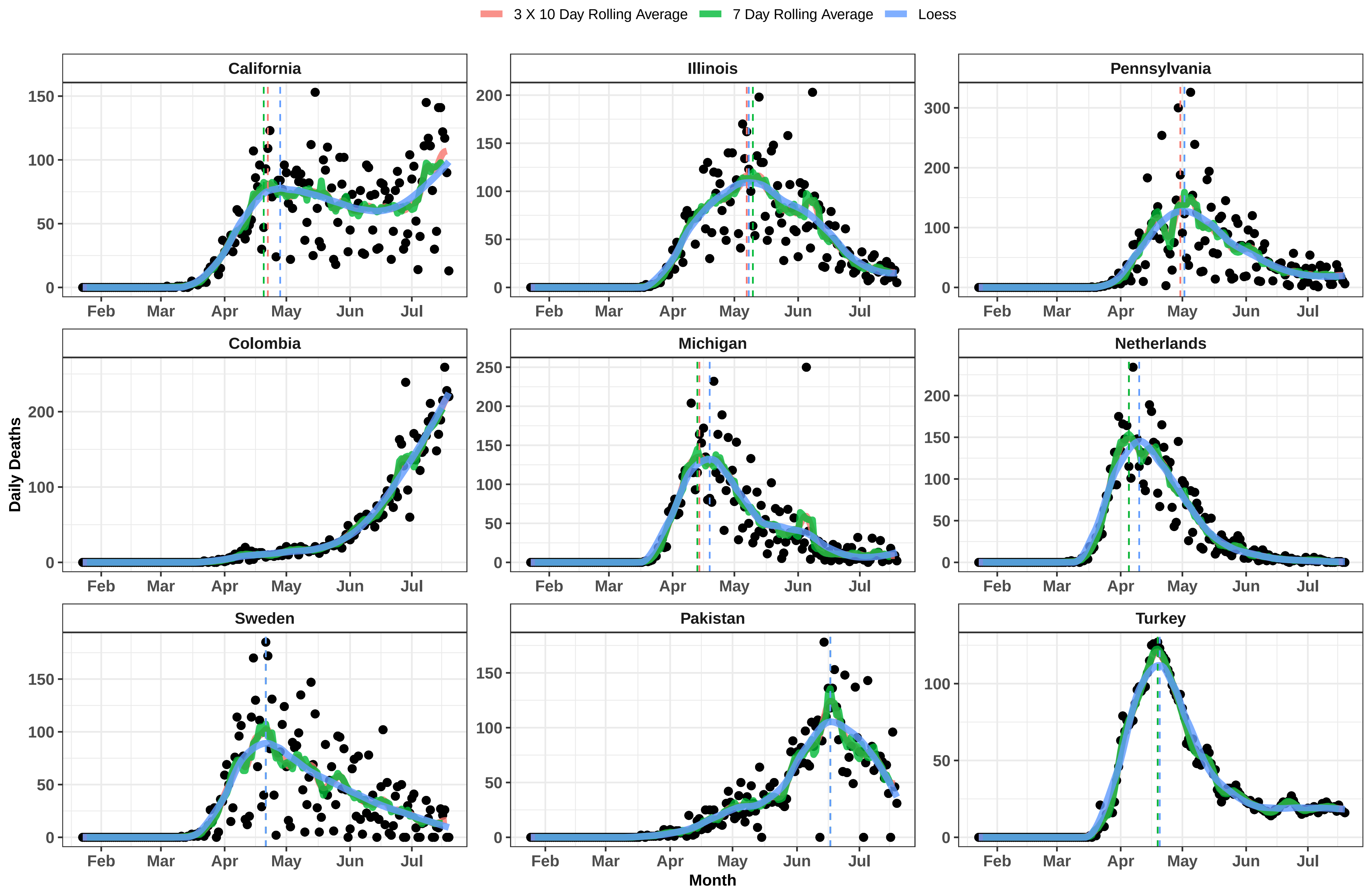

3 X 10 Day Rolling Average
7 Day Rolling Average
Loess
California
Illinois
Pennsylvania
Colombia
Michigan
Netherlands
Sweden
Pakistan
Turkey
Daily Deaths
Month
Feb
Mar
Apr
May
Jun
Jul

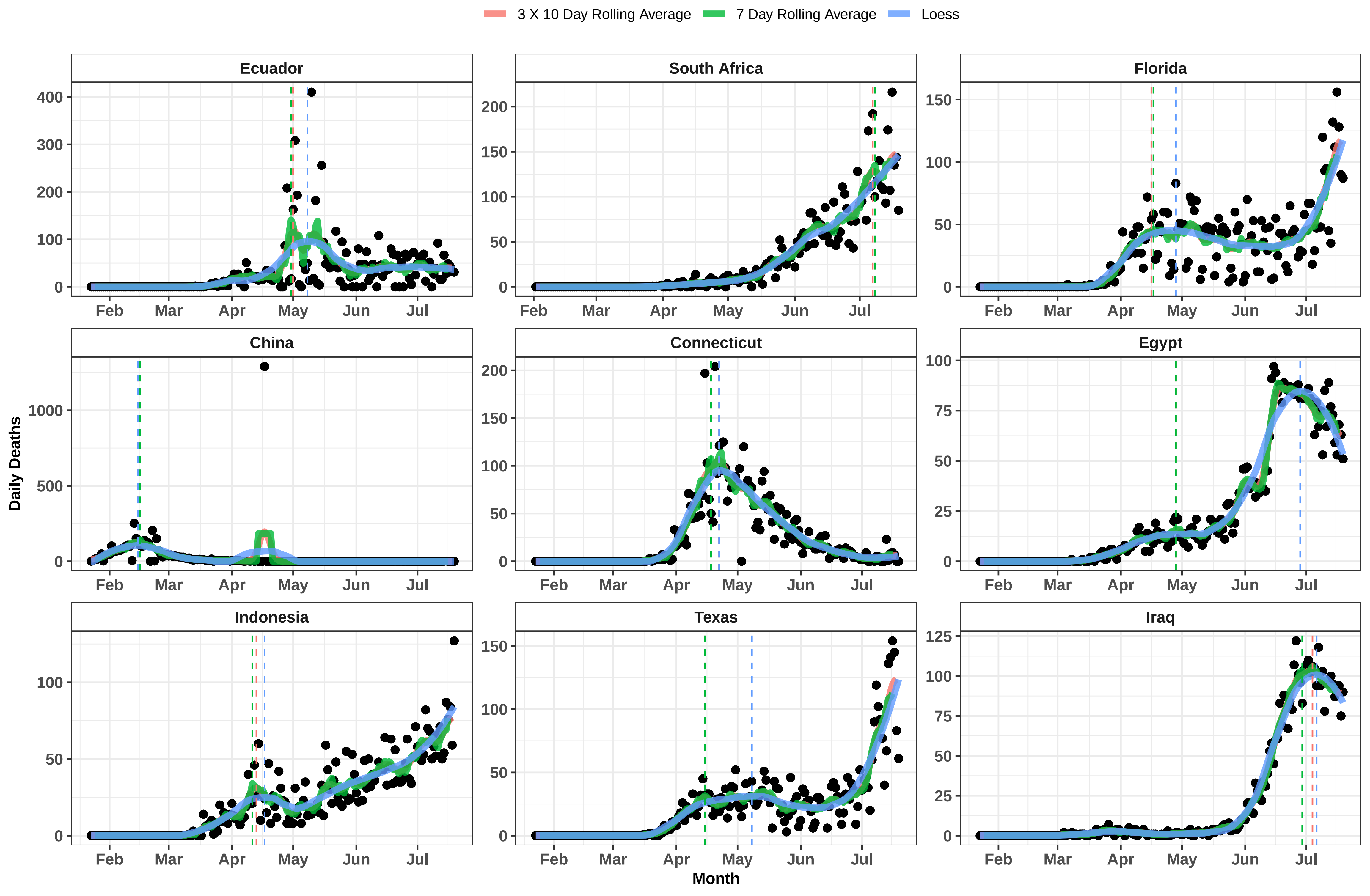

3 X 10 Day Rolling Average
7 Day Rolling Average
Loess
Ecuador
South Africa
Florida
China
Connecticut
Egypt
Indonesia
Texas
Iraq
Daily Deaths
Month
Feb
Mar
Apr
May
Jun
Jul

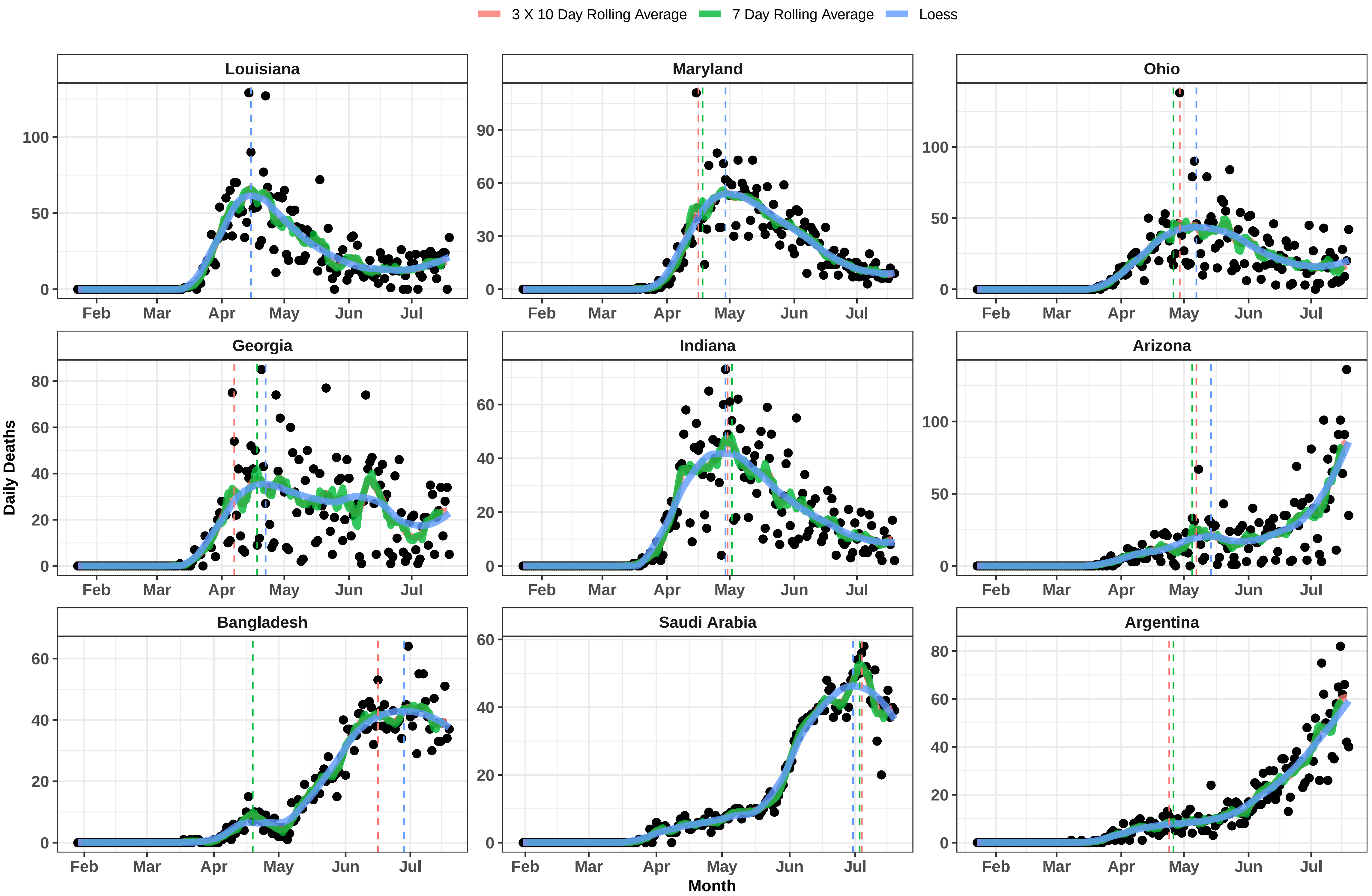

3 X 10 Day Rolling Average
7 Day Rolling Average
Loess
Louisiana
Maryland
Ohio
Georgia
Indiana
Arizona
Bangladesh
Saudi Arabia
Argentina
Daily Deaths
Month
Feb
Mar
Apr
May
Jun
Jul

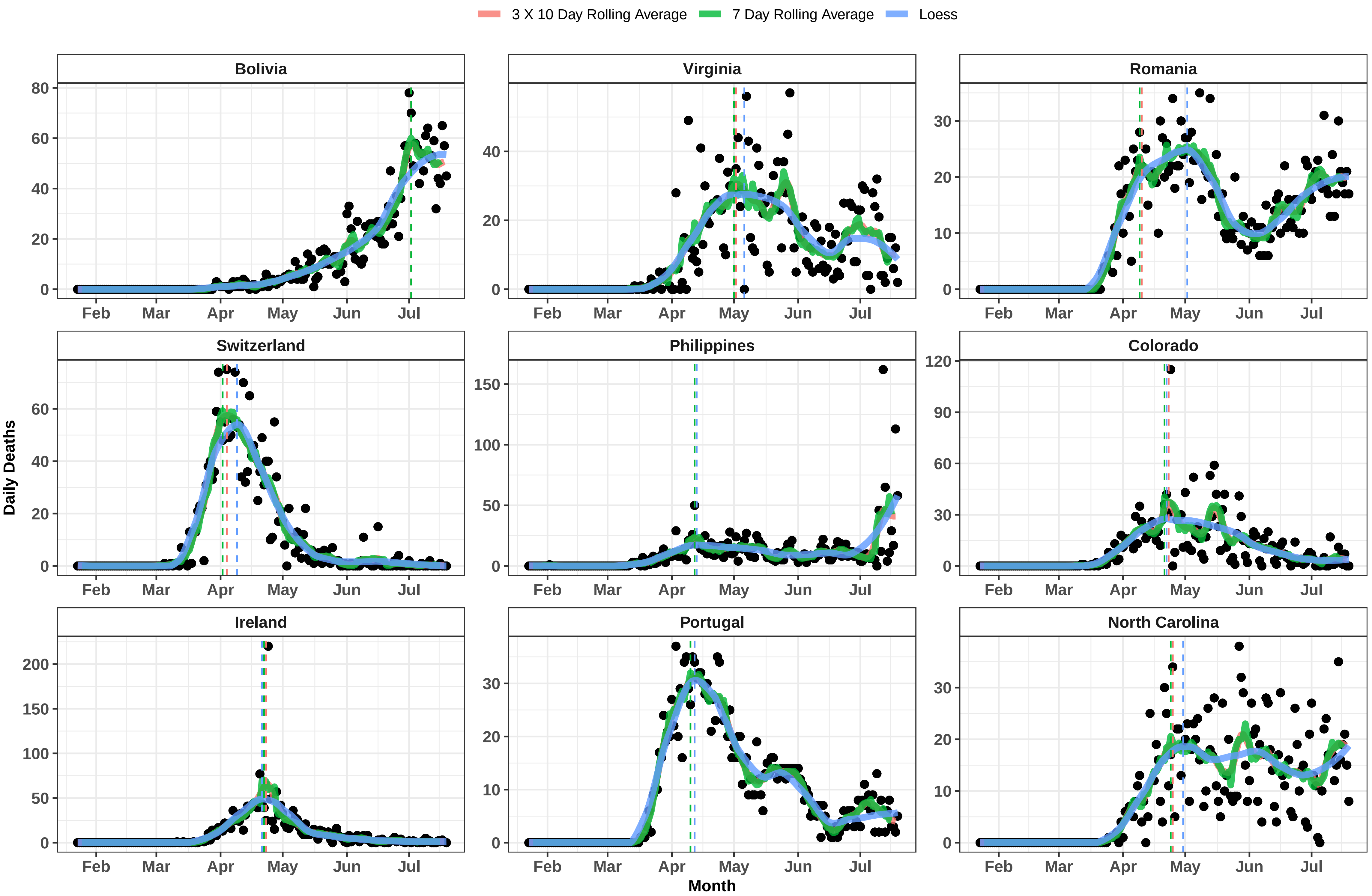

3 X 10 Day Rolling Average
7 Day Rolling Average
Loess
Bolivia
Virginia
Romania
Switzerland
Philippines
Colorado
Ireland
Portugal
North Carolina
Daily Deaths
Month
Feb
Mar
Apr
May
Jun
Jul

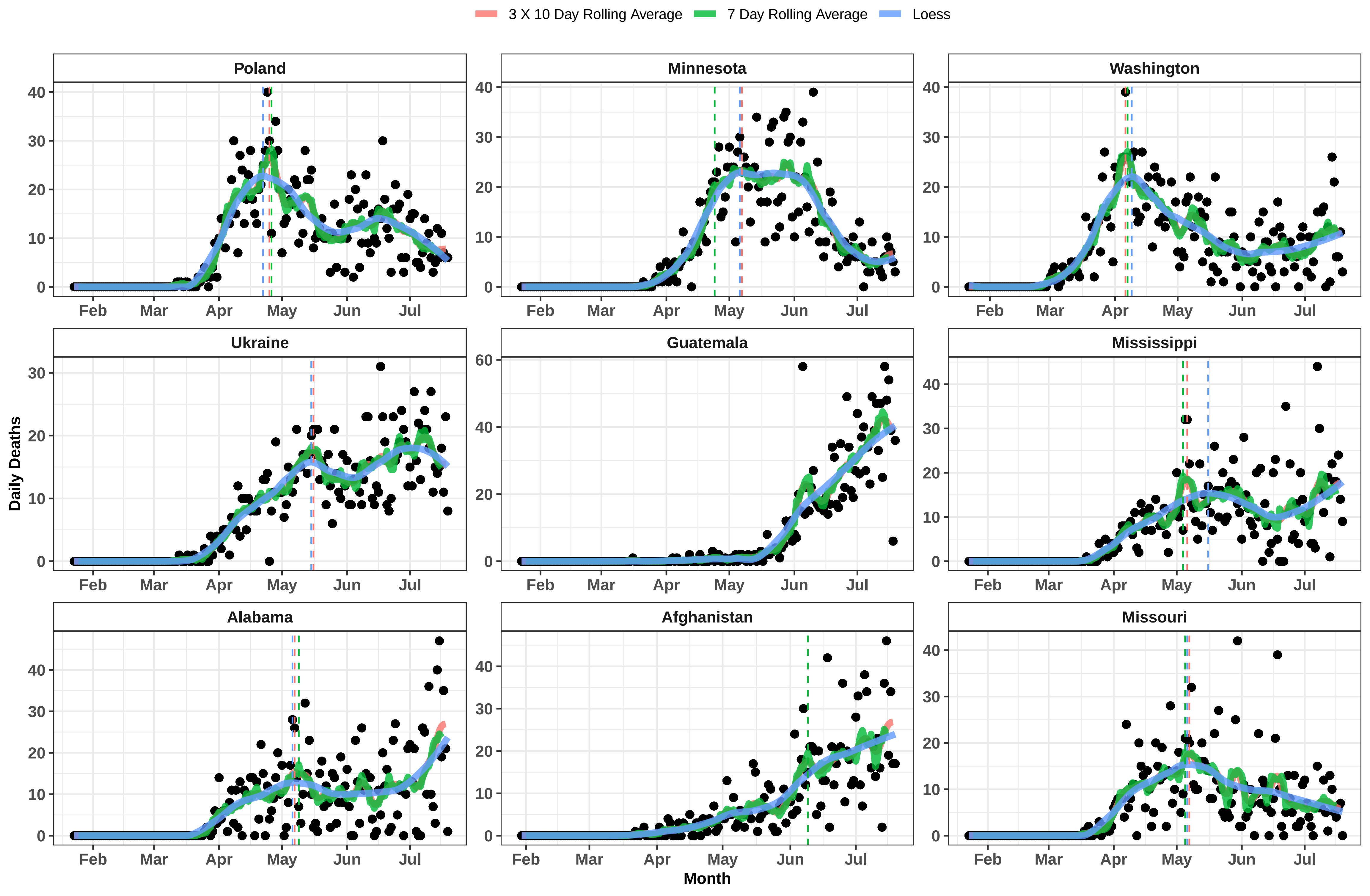
3 X 10 Day Rolling Average
7 Day Rolling Average
Loess
Poland
Minnesota
Washington
Ukraine
Guatemala
Mississippi
Alabama
Afghanistan
Missouri
Daily Deaths
Month
Feb
Mar
Apr
May
Jun
Jul

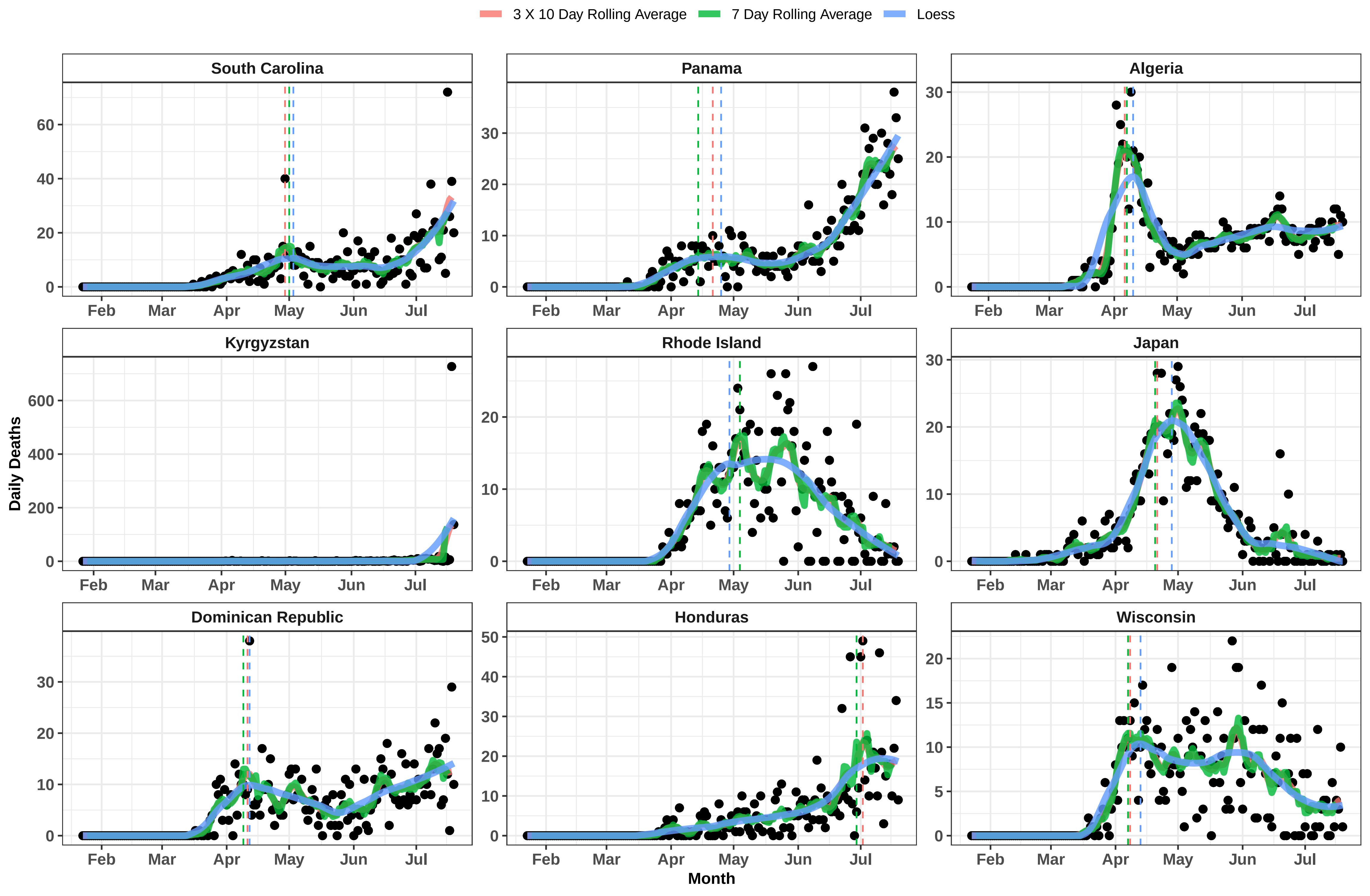

3 X 10 Day Rolling Average
7 Day Rolling Average
Loess
South Carolina
Panama
Algeria
Kyrgyzstan
Rhode Island
Japan
Dominican Republic
Honduras
Wisconsin
Daily Deaths
Month
Feb
Mar
Apr
May
Jun
Jul

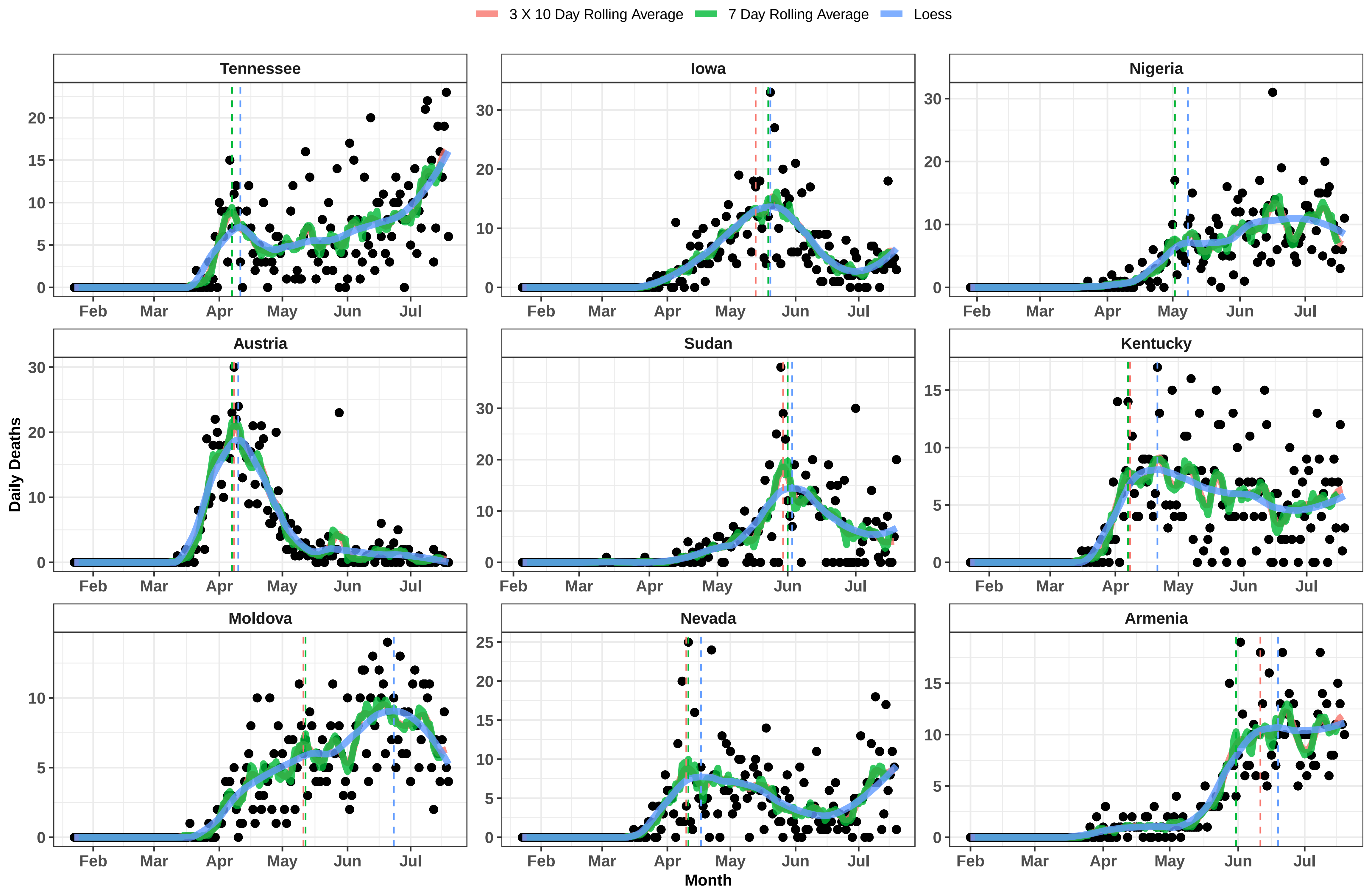

3 X 10 Day Rolling Average
7 Day Rolling Average
Loess
Tennessee
Iowa
Nigeria
Austria
Sudan
Kentucky
Moldova
Nevada
Armenia
Daily Deaths
Month
Feb
Mar
Apr
May
Jun
Jul

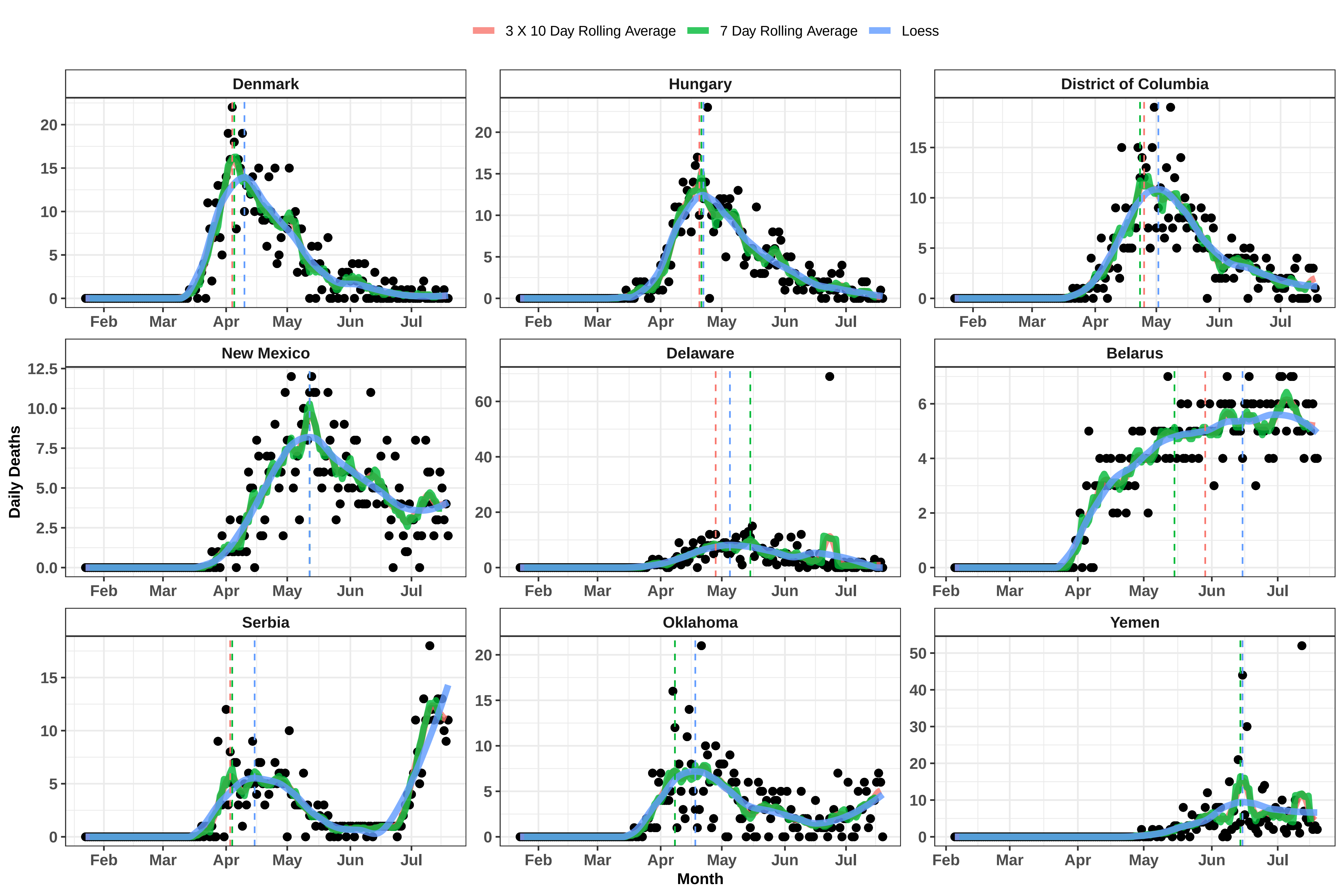

3 X 10 Day Rolling Average
7 Day Rolling Average
Loess
Denmark
Hungary
District of Columbia
New Mexico
Delaware
Belarus
Serbia
Oklahoma
Yemen
Daily Deaths
Month
Feb
Mar
Apr
May
Jun
Jul

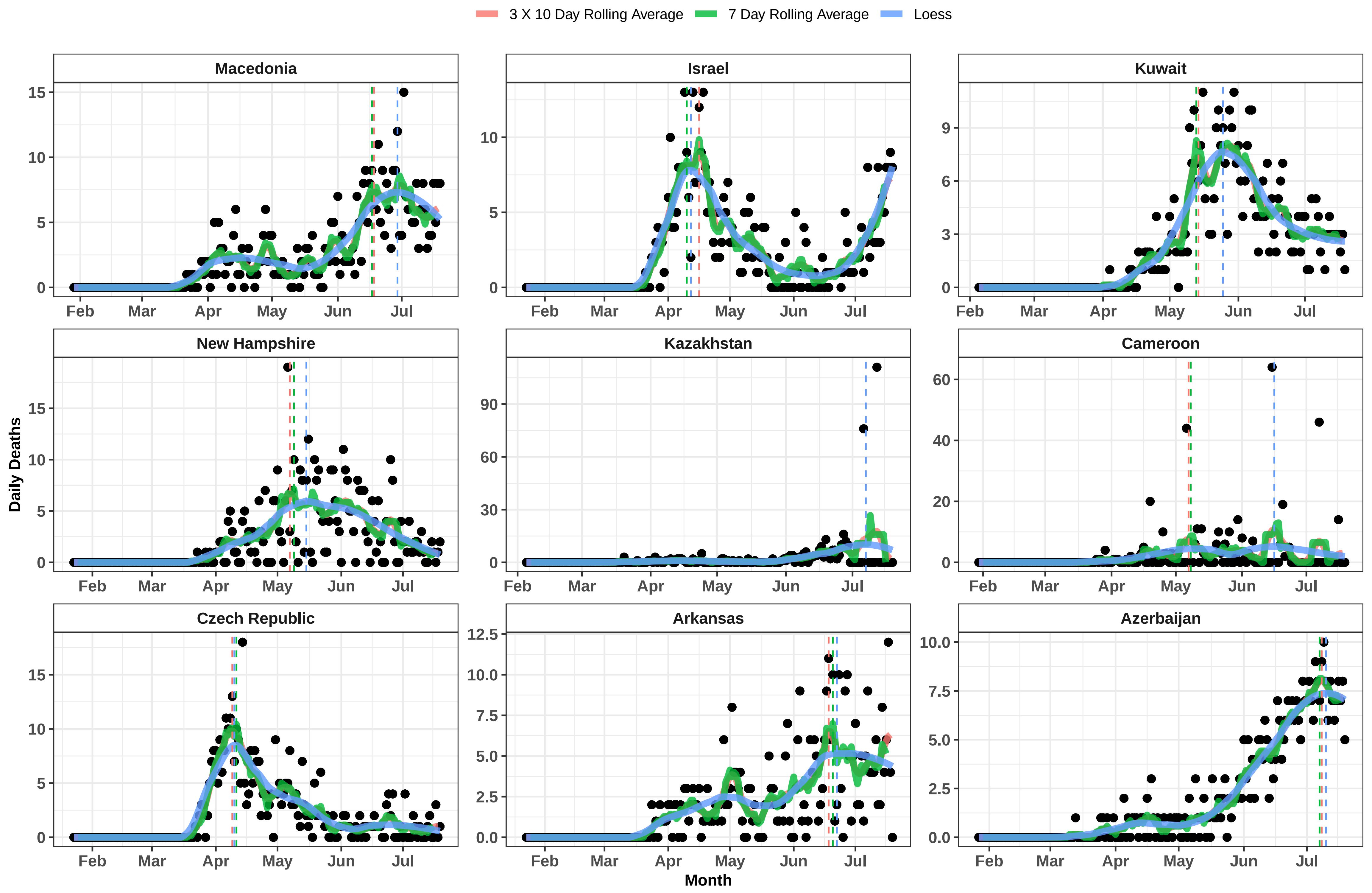

3 X 10 Day Rolling Average
7 Day Rolling Average
Loess
Macedonia
Israel
Kuwait
New Hampshire
Kazakhstan
Cameroon
Czech Republic
Arkansas
Azerbaijan
Daily Deaths
Month
Feb
Mar
Apr
May
Jun
Jul

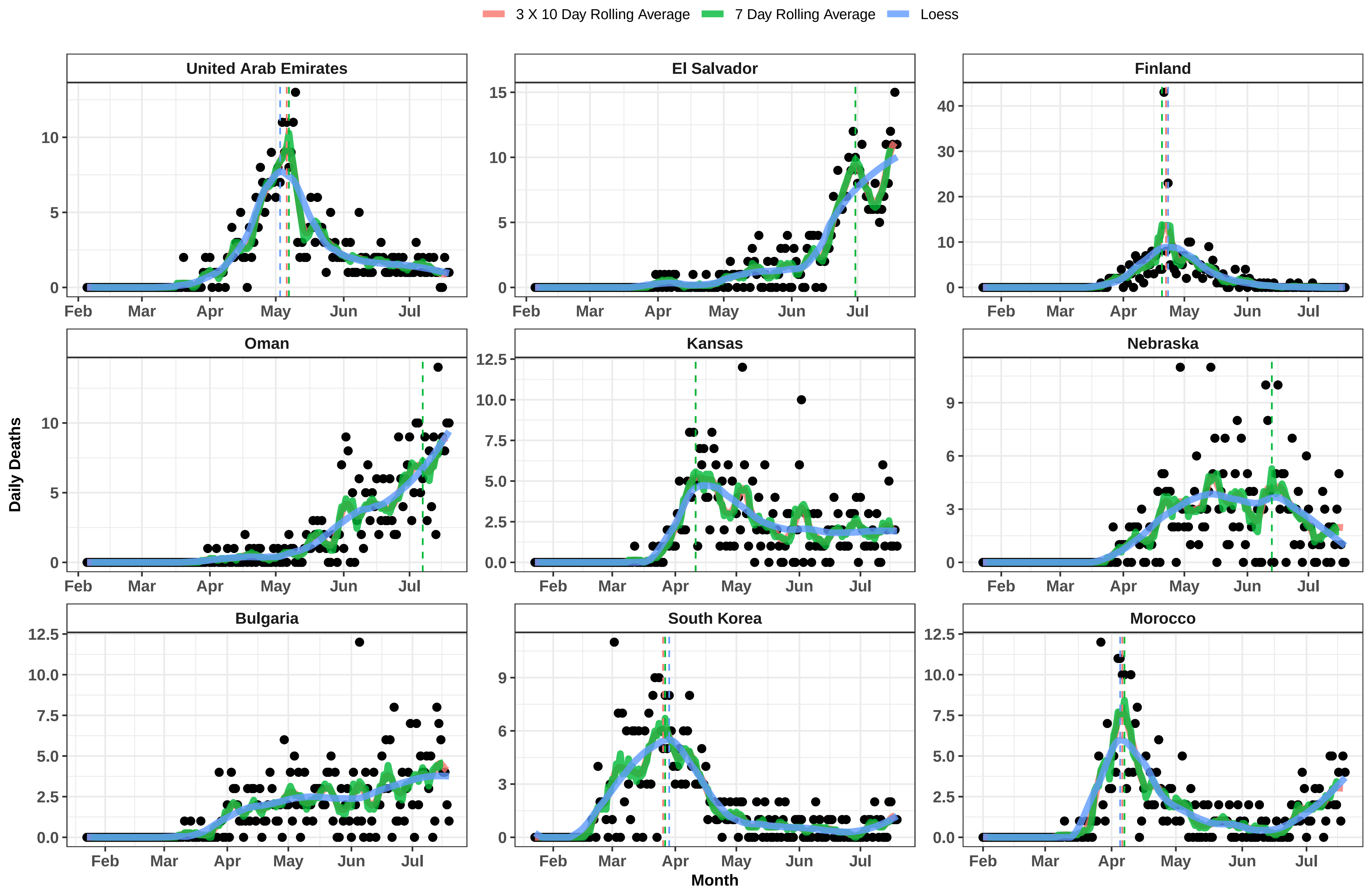

3 X 10 Day Rolling Average
7 Day Rolling Average
Loess
United Arab Emirates
El Salvador
Finland
Oman
Kansas
Nebraska
Bulgaria
South Korea
Morocco
Daily Deaths
Month
Feb
Mar
Apr
May
Jun
Jul

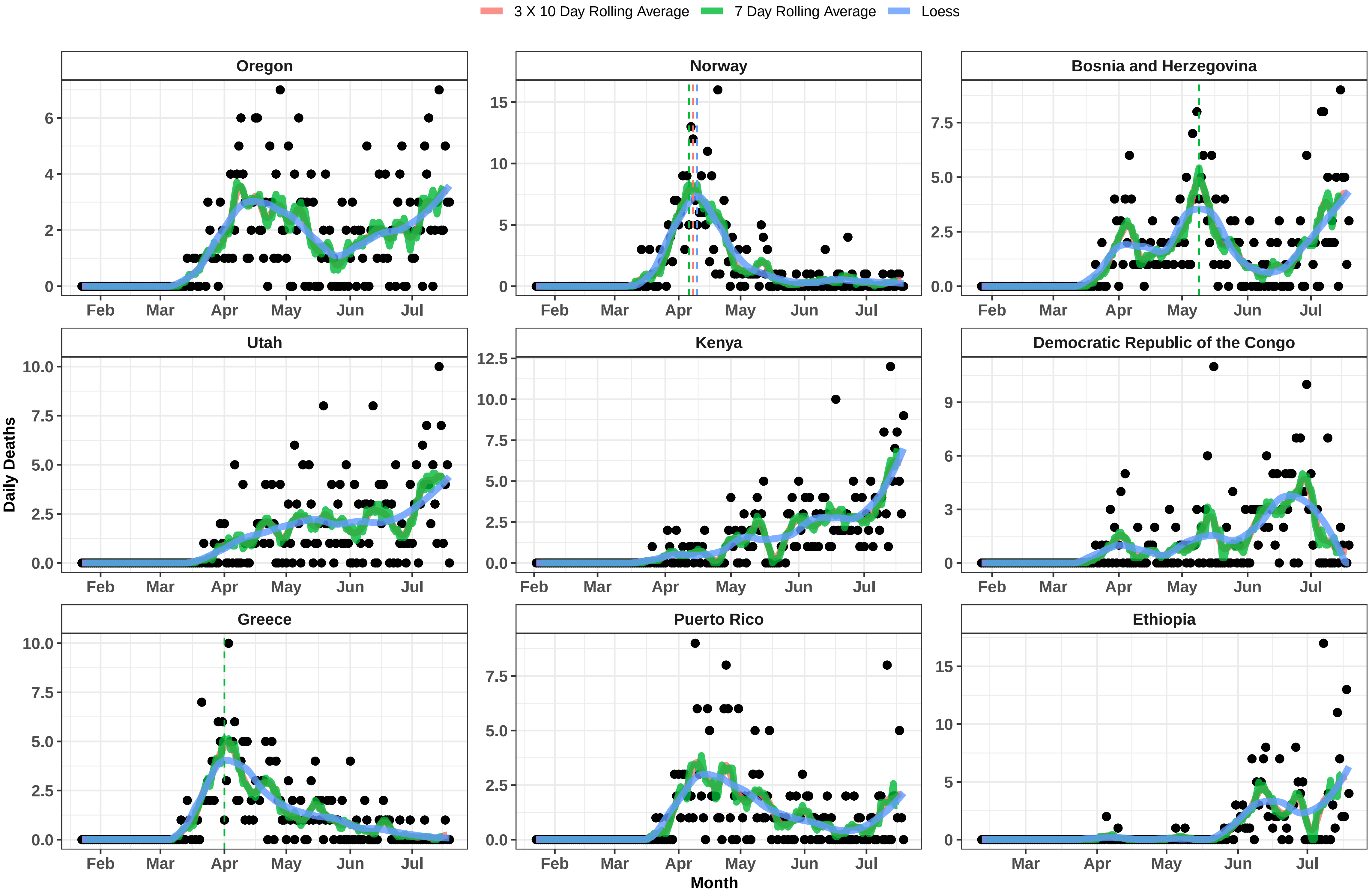

3 X 10 Day Rolling Average
7 Day Rolling Average
Loess
Oregon
Norway
Bosnia and Herzegovina
Utah
Kenya
Democratic Republic of the Congo
Greece
Puerto Rico
Ethiopia
Daily Deaths
Month
Feb
Mar
Apr
May
Jun
Jul

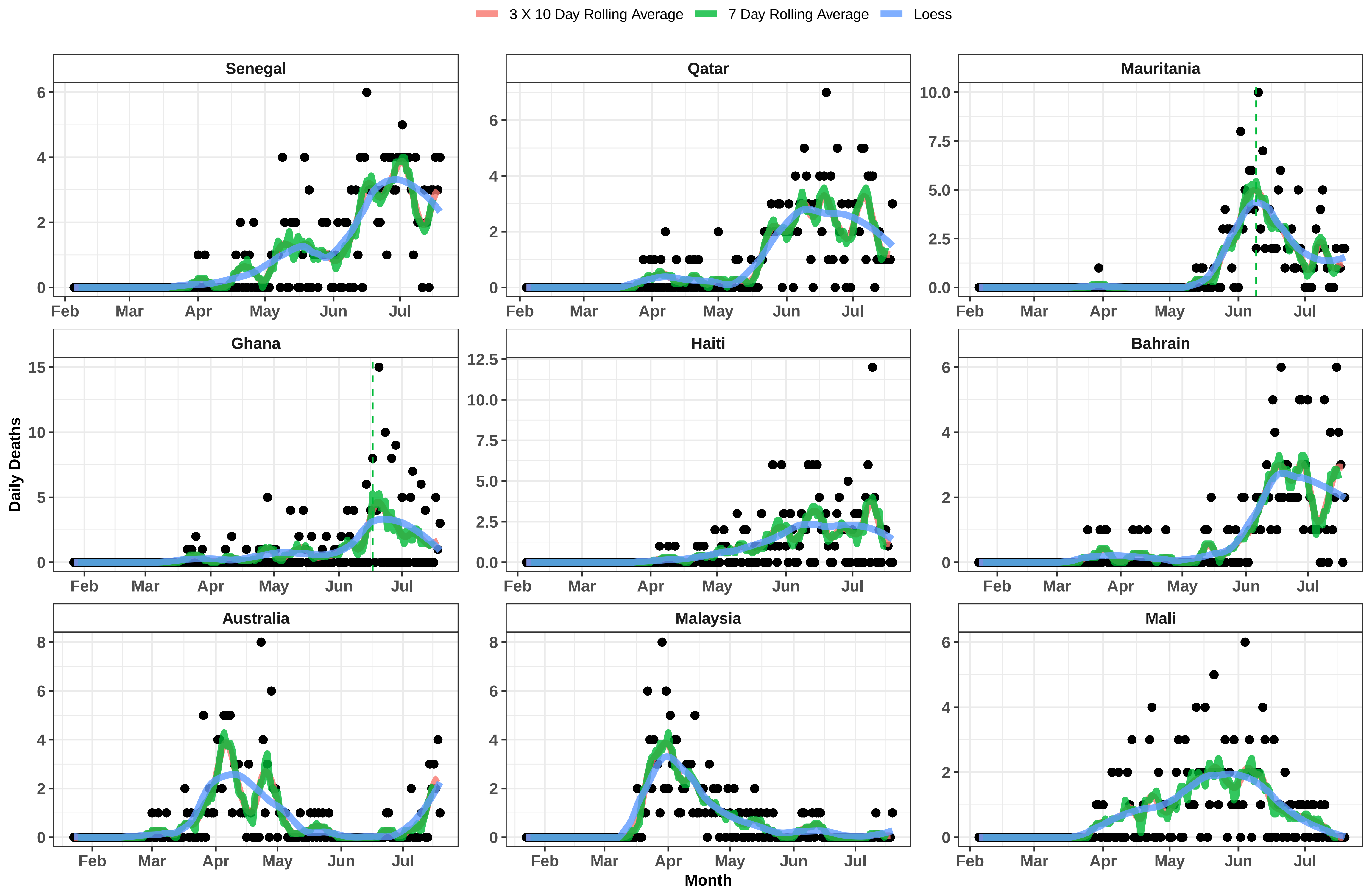

3 X 10 Day Rolling Average
7 Day Rolling Average
Loess
Senegal
Qatar
Mauritania
Ghana
Haiti
Bahrain
Australia
Malaysia
Mali
Daily Deaths
Month
Feb
Mar
Apr
May
Jun
Jul

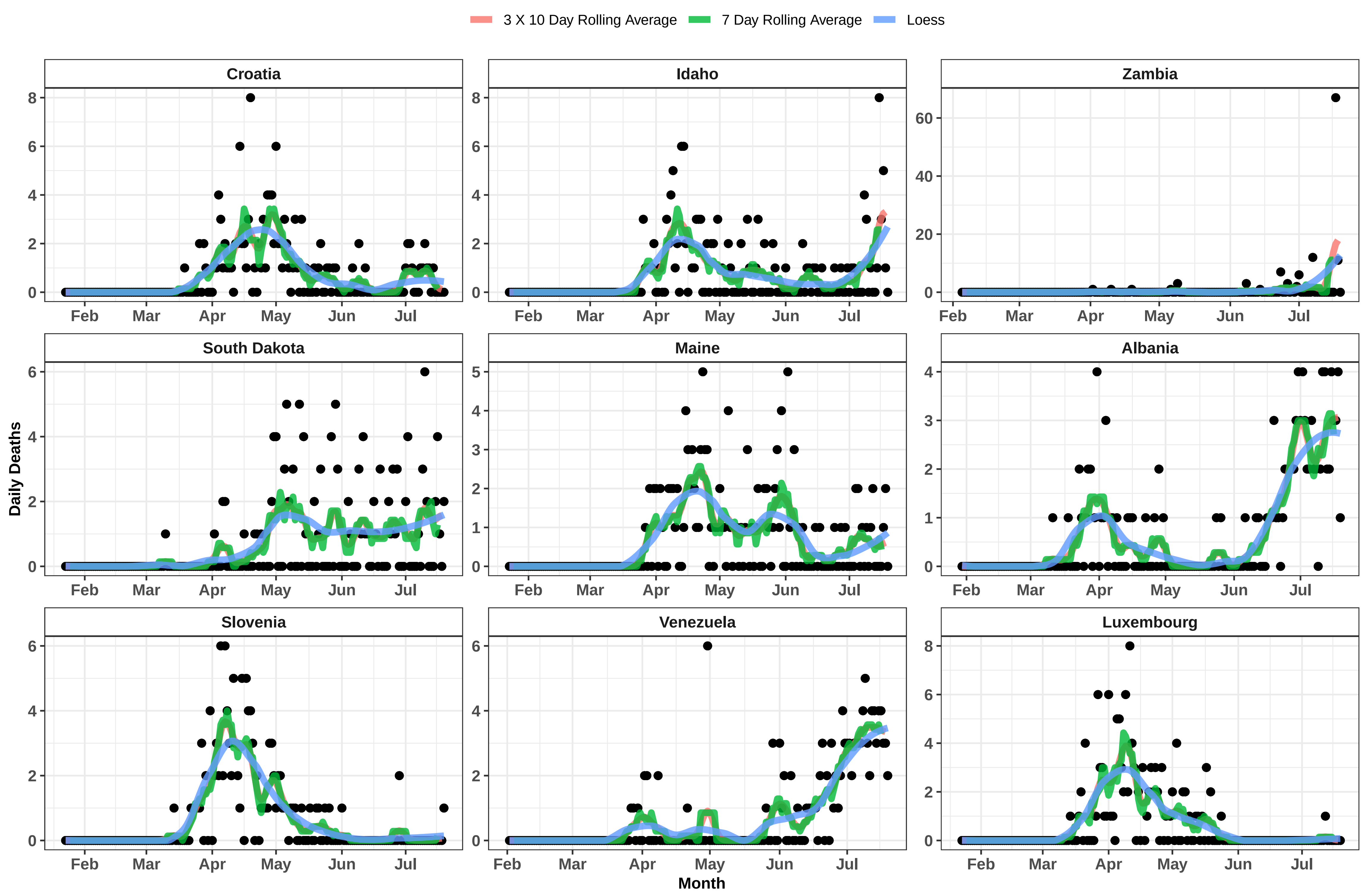

3 X 10 Day Rolling Average
7 Day Rolling Average
Loess
Croatia
Idaho
Zambia
South Dakota
Maine
Albania
Slovenia
Venezuela
Luxembourg
Daily Deaths
Month
Feb
Mar
Apr
May
Jun
Jul

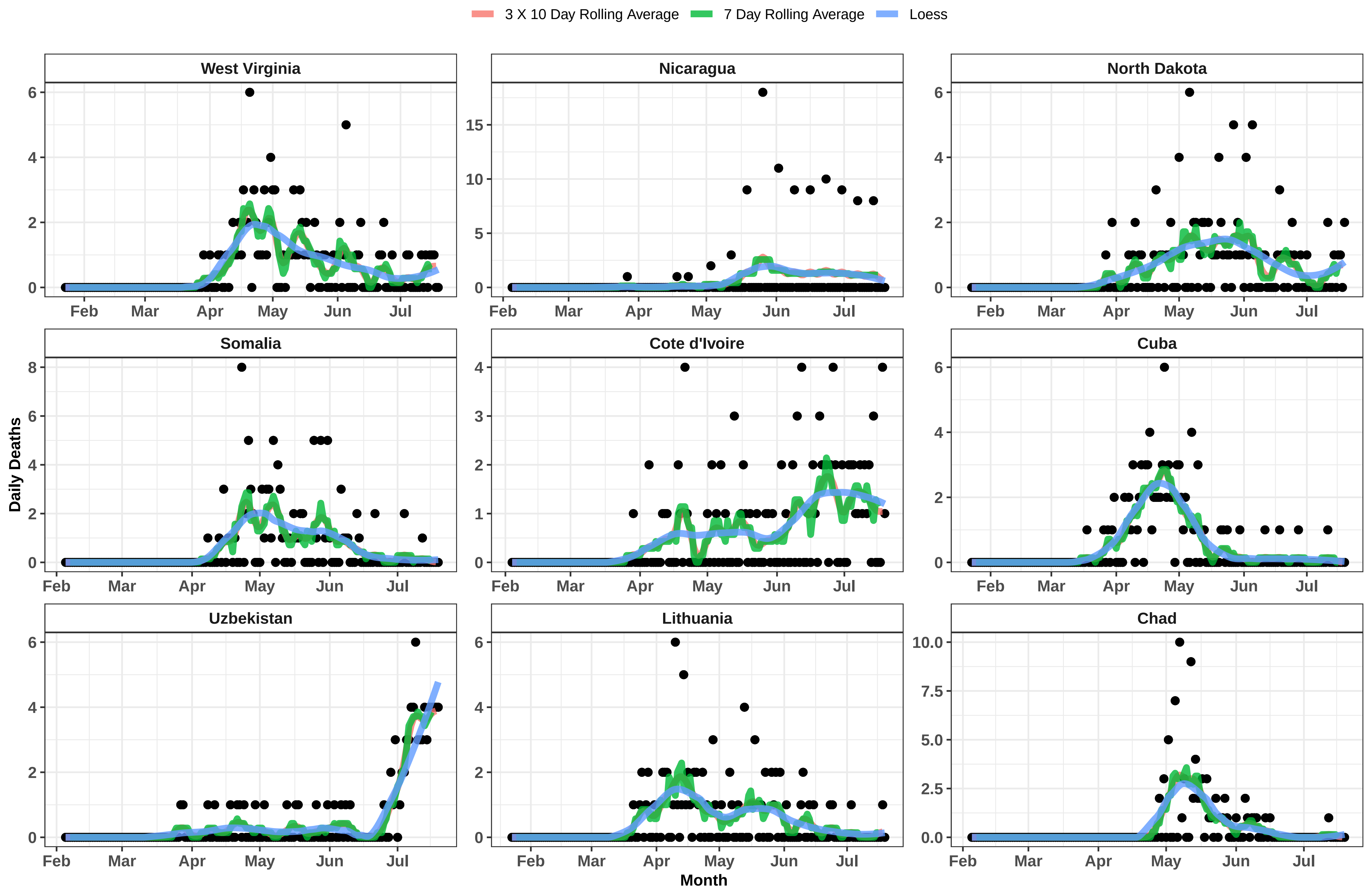

3 X 10 Day Rolling Average
7 Day Rolling Average
Loess
West Virginia
Nicaragua
North Dakota
Somalia
Cote d'Ivoire
Cuba
Uzbekistan
Lithuania
Chad
Daily Deaths
Month
Feb
Mar
Apr
May
Jun
Jul

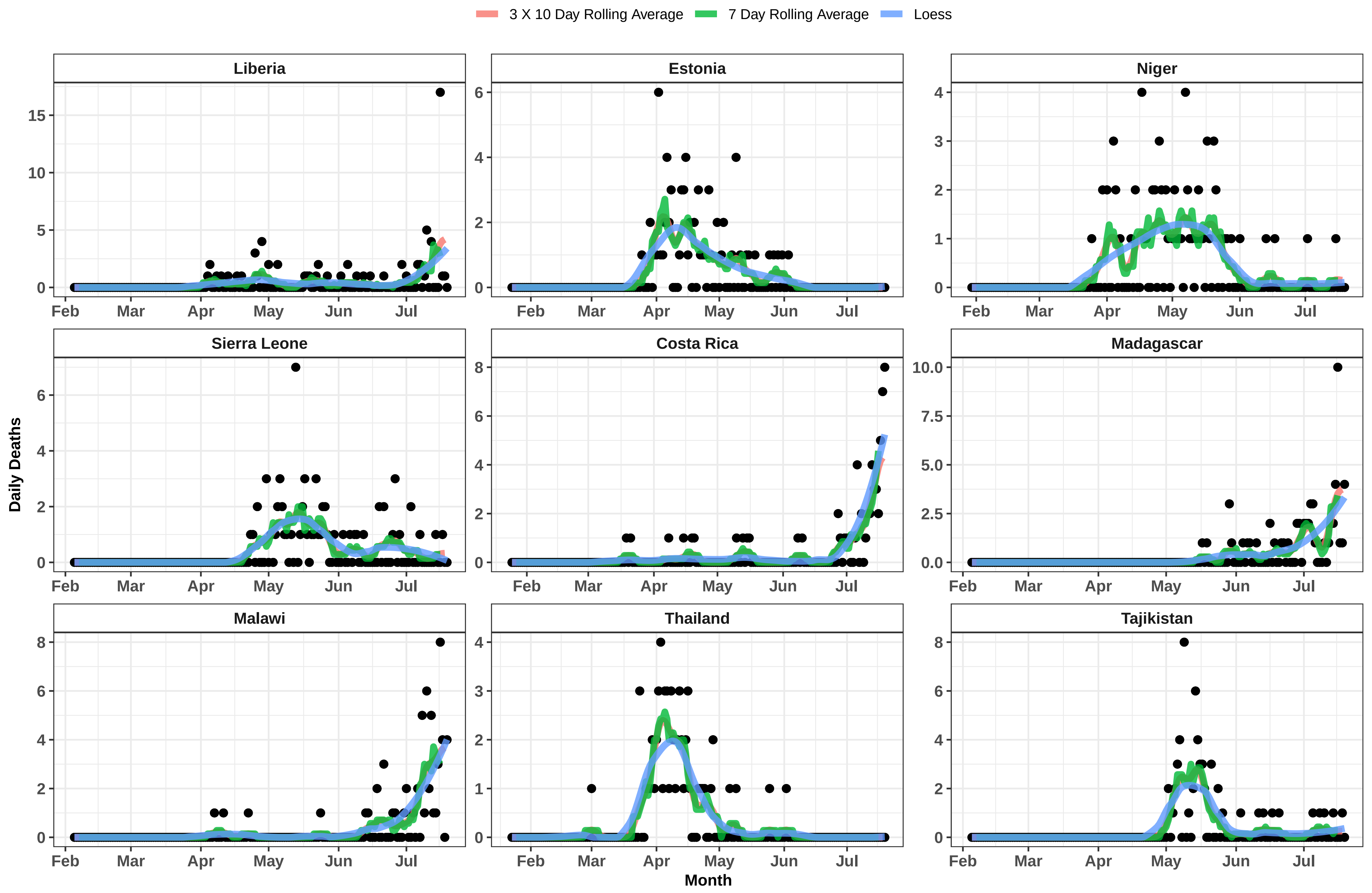

3 X 10 Day Rolling Average
7 Day Rolling Average
Loess
Liberia
Estonia
Niger
Sierra Leone
Costa Rica
Madagascar
Malawi
Thailand
Tajikistan
Daily Deaths
Month
Feb
Mar
Apr
May
Jun
Jul

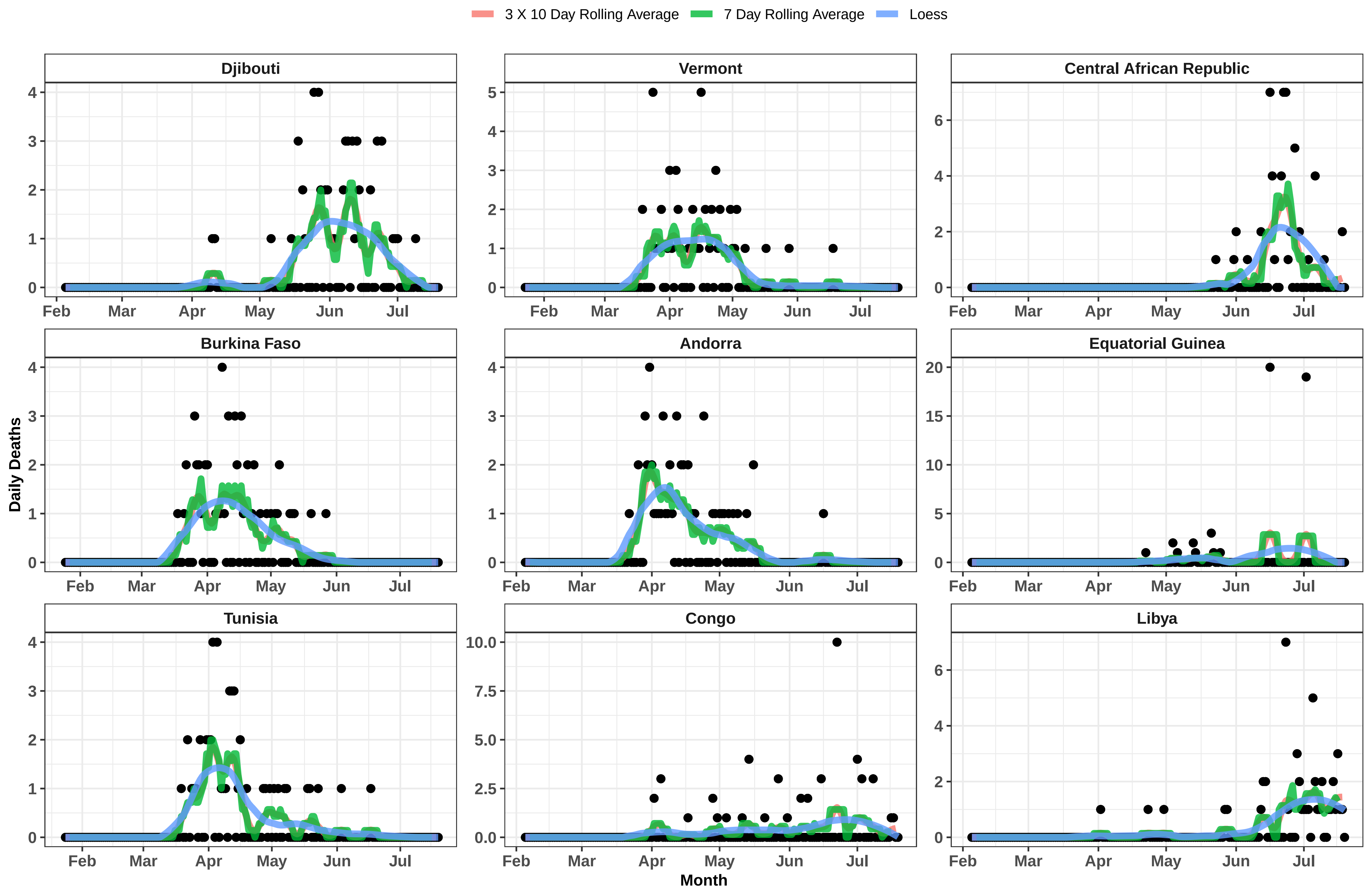

3 X 10 Day Rolling Average
7 Day Rolling Average
Loess
Djibouti
Vermont
Central African Republic
Burkina Faso
Andorra
Equatorial Guinea
Tunisia
Congo
Libya
Daily Deaths
Month
Feb
Mar
Apr
May
Jun
Jul

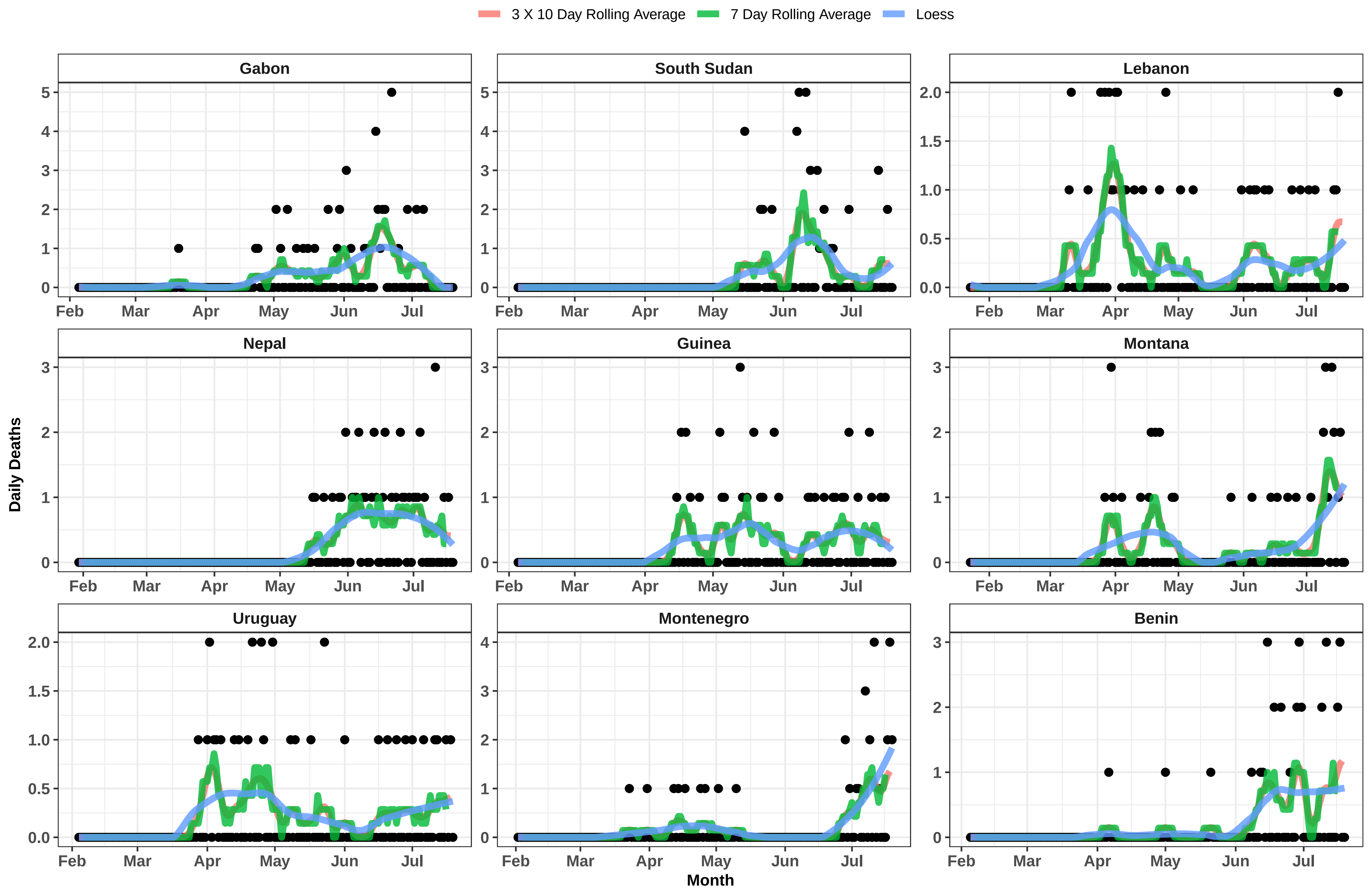

3 X 10 Day Rolling Average
7 Day Rolling Average
Loess
Gabon
South Sudan
Lebanon
Nepal
Guinea
Montana
Uruguay
Montenegro
Benin
Daily Deaths
Month
Feb
Mar
Apr
May
Jun
Jul

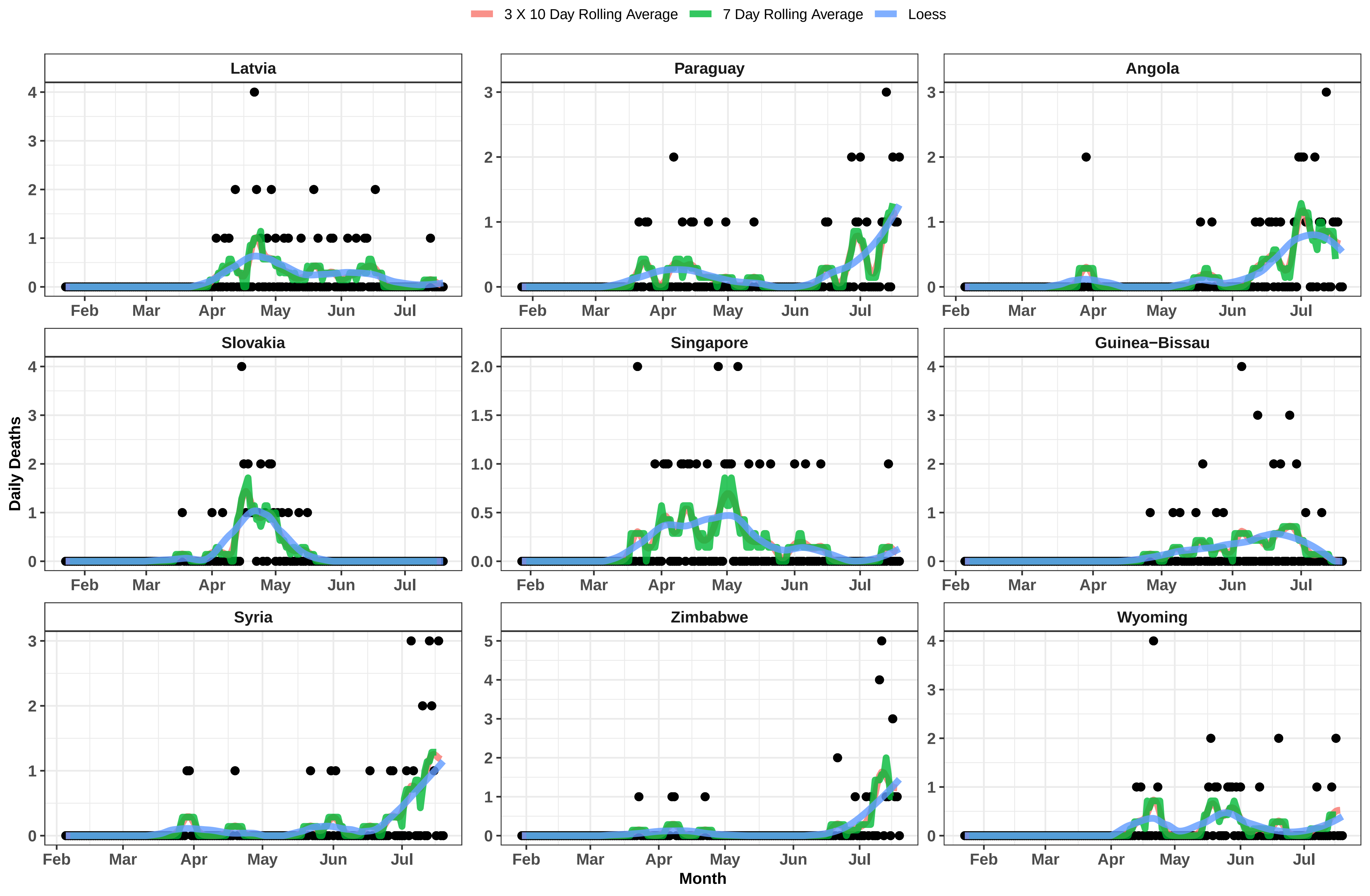

3 X 10 Day Rolling Average
7 Day Rolling Average
Loess
Latvia
Paraguay
Angola
Slovakia
Singapore
Guinea−Bissau
Syria
Zimbabwe
Wyoming
Daily Deaths
Month
Feb
Mar
Apr
May
Jun
Jul

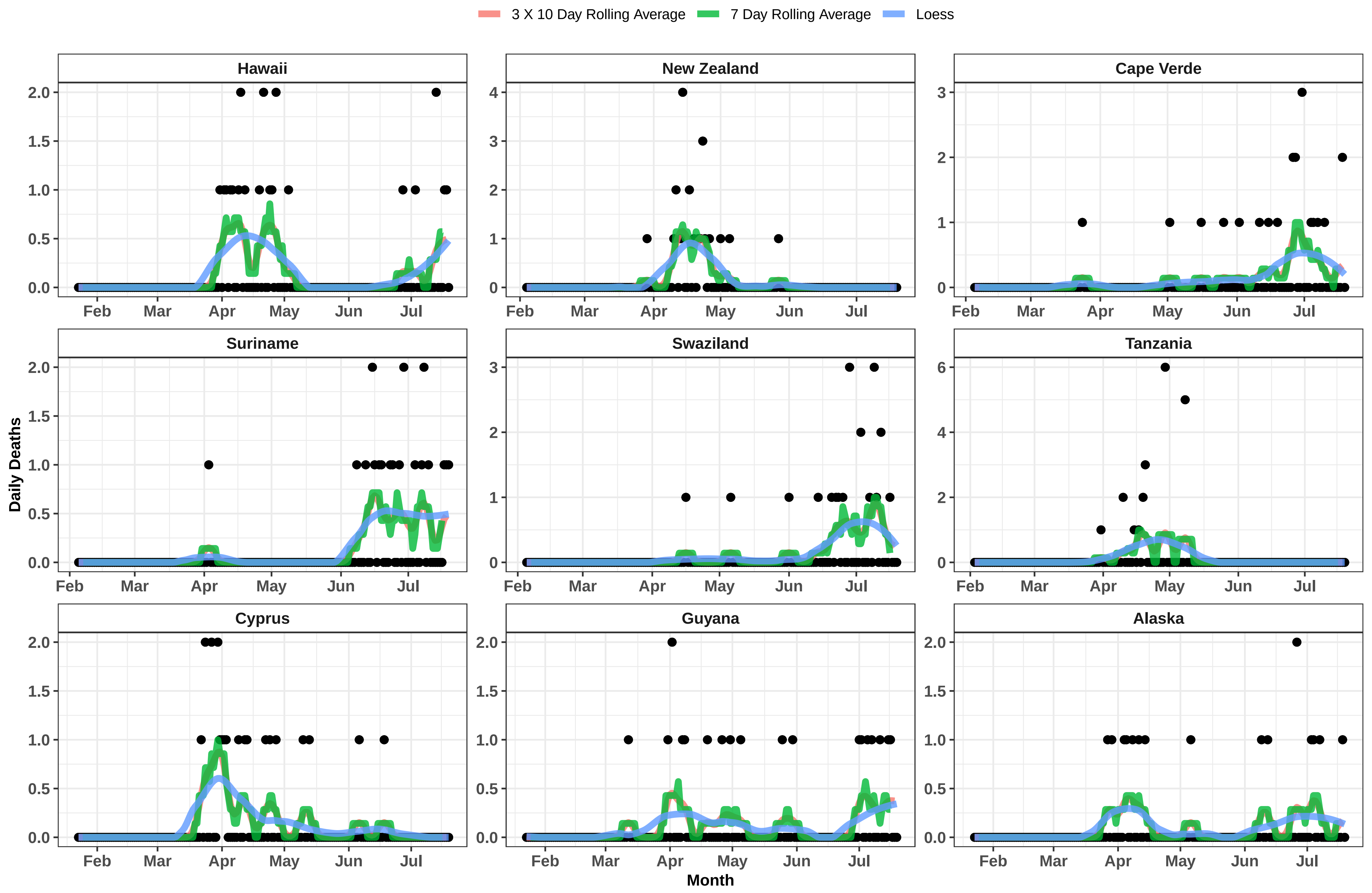
3 X 10 Day Rolling Average
7 Day Rolling Average
Loess
Hawaii
New Zealand
Cape Verde
Suriname
Swaziland
Tanzania
Cyprus
Guyana
Alaska
Daily Deaths
Month
Feb
Mar
Apr
May
Jun
Jul

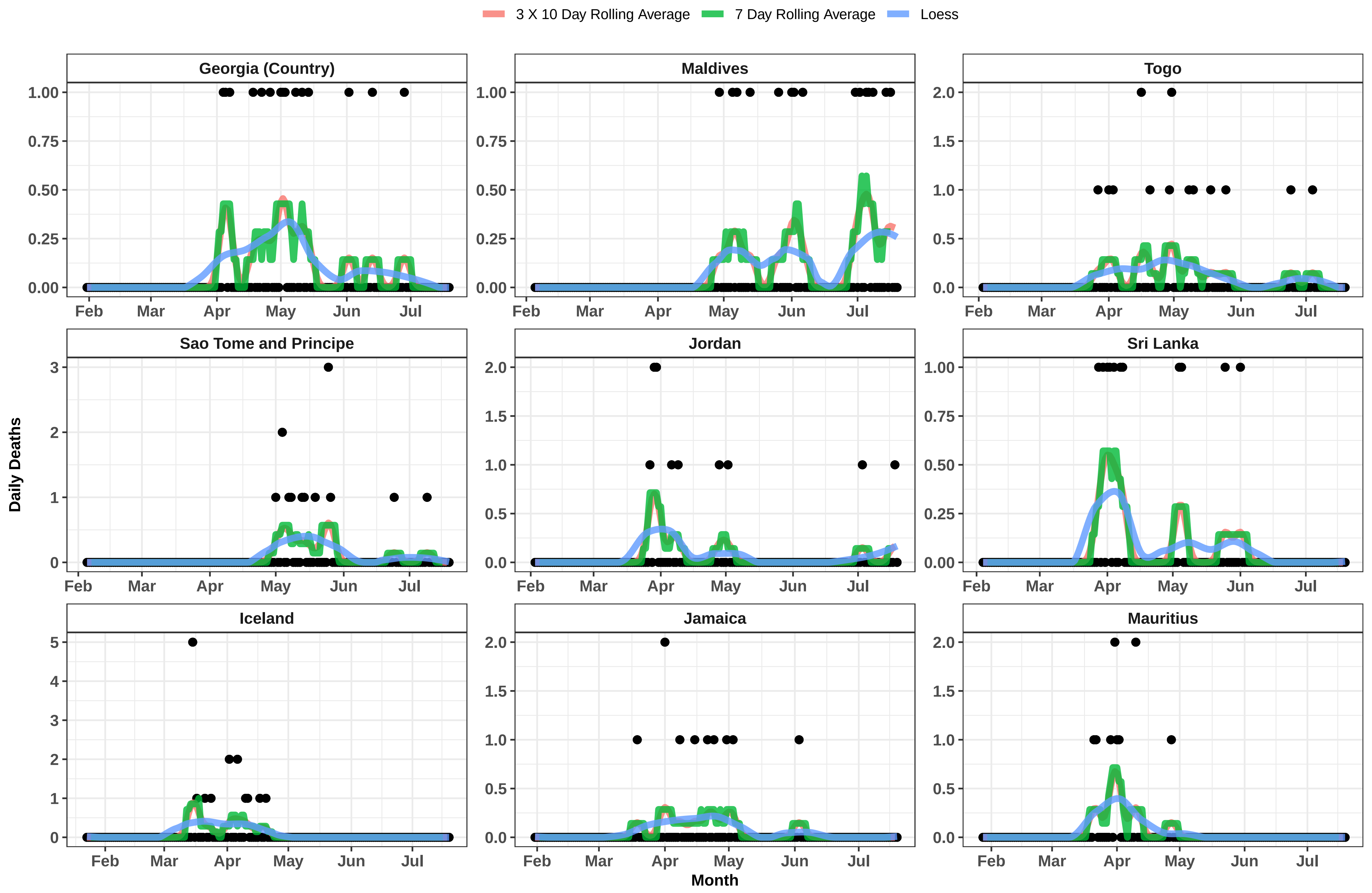

3 X 10 Day Rolling Average
7 Day Rolling Average
Loess
Georgia (Country)
Maldives
Togo
Sao Tome and Principe
Jordan
Sri Lanka
Iceland
Jamaica
Mauritius
Daily Deaths
Month
Feb
Mar
Apr
May
Jun
Jul

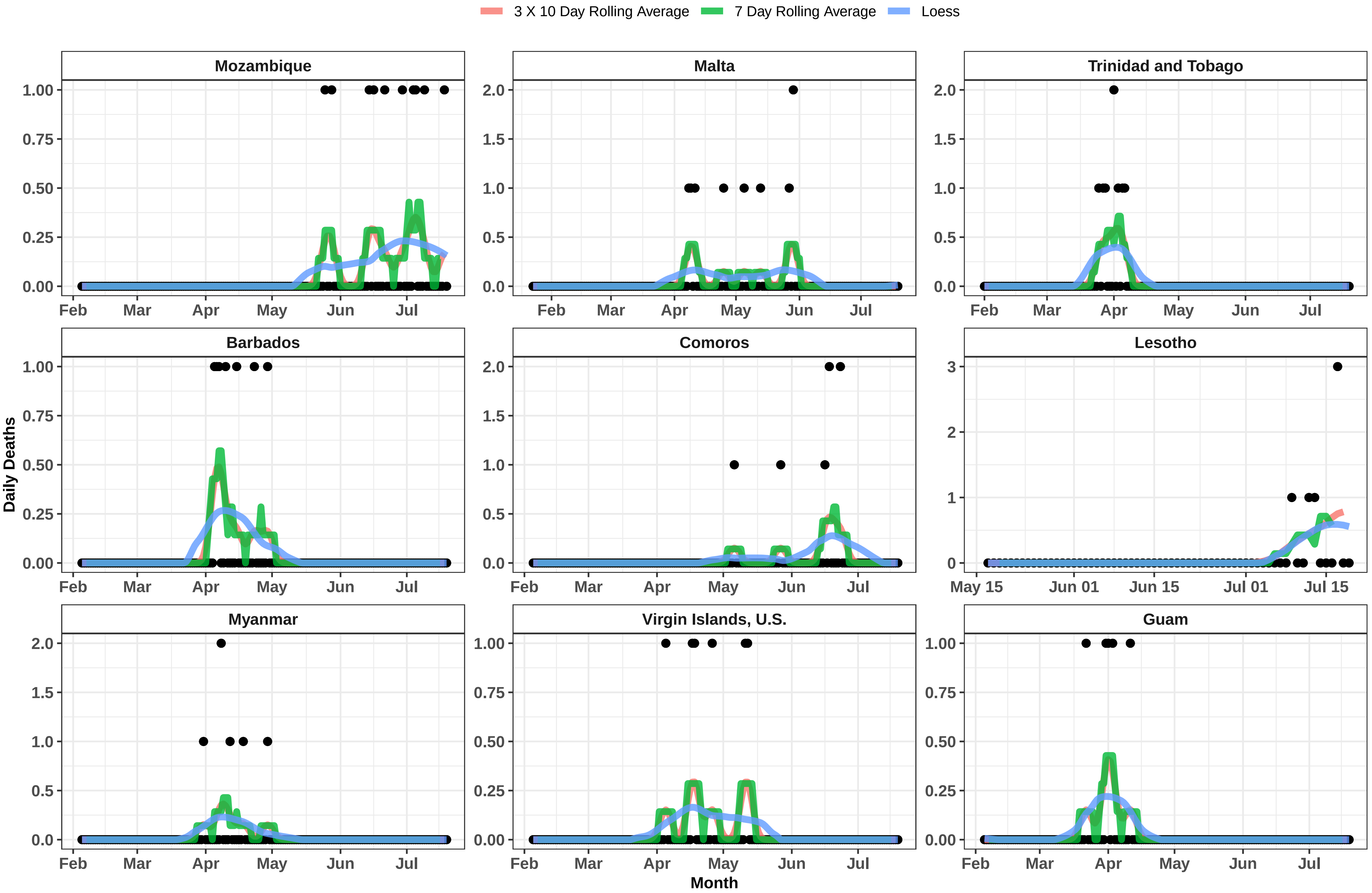

3 X 10 Day Rolling Average
7 Day Rolling Average
Loess
Mozambique
Malta
Trinidad and Tobago
Barbados
Comoros
Lesotho
Myanmar
Virgin Islands, U.S.
Guam
Daily Deaths
Month
Feb
Mar
Apr
May
Jun
Jul
May 15
Jun 01
Jun 15
Jul 01
Jul 15

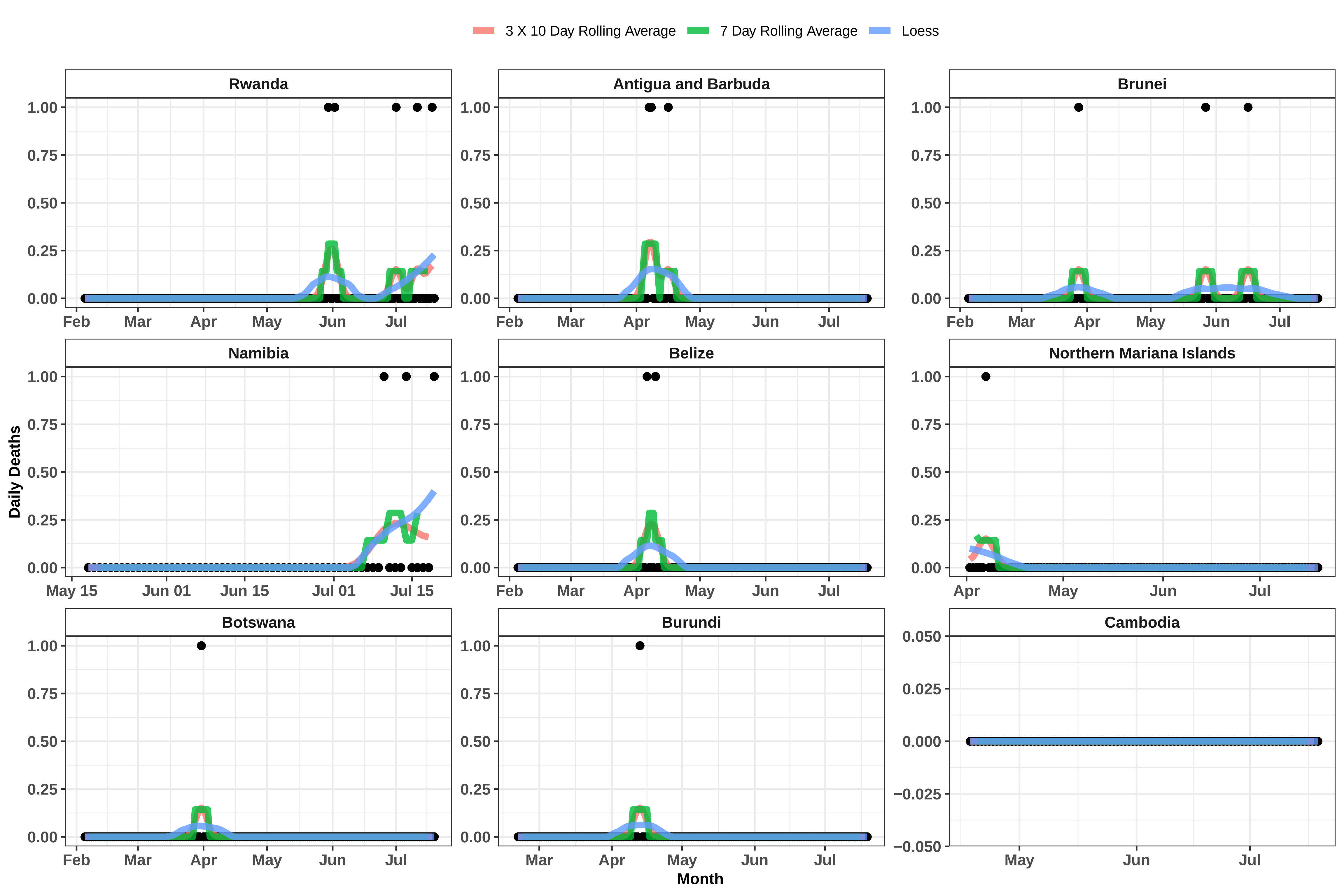

3 X 10 Day Rolling Average
7 Day Rolling Average
Loess
Rwanda
Antigua and Barbuda
Brunei
Namibia
Belize
Northern Mariana Islands
Botswana
Burundi
Cambodia
Daily Deaths
Month
1.00
0.75
0.50
0.25
0.00
0.050
0.025
0.000
−0.025
−0.050
Feb
Mar
Apr
May
Jun
Jul
May 15
Jun 01
Jun 15
Jul 01
Jul 15

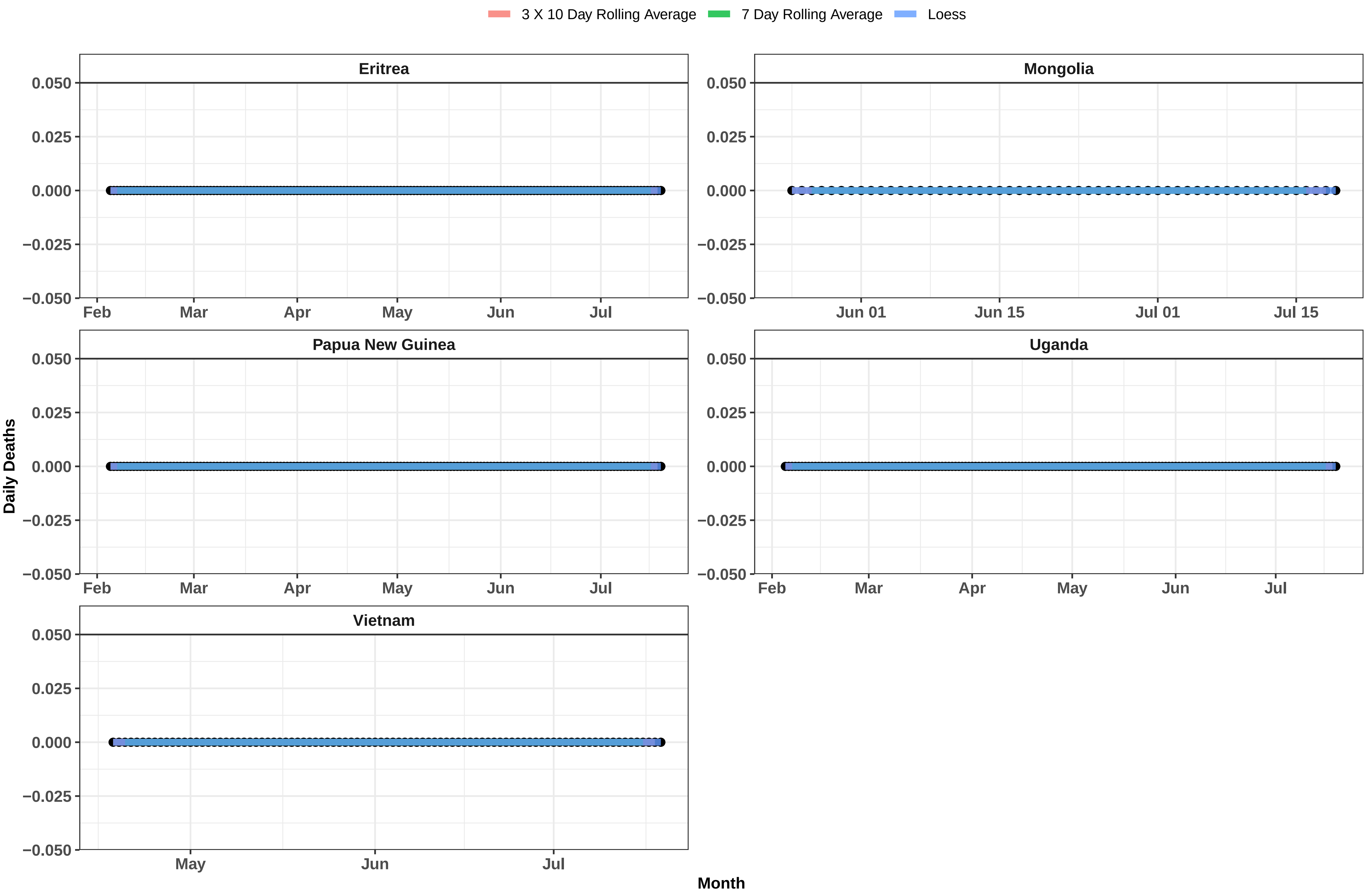

3 X 10 Day Rolling Average
7 Day Rolling Average
Loess
Eritrea
Mongolia
Papua New Guinea
Uganda
Vietnam
Daily Deaths
Month
0.050
0.025
0.000
−0.025
−0.050
Feb
Mar
Apr
May
Jun
Jul
Jun 01
Jun 15
Jul 01
Jul 15
