# Supplementary material for: Predictive performance of international COVID-19 mortality forecasting models
Source: medRxiv. 2020 Nov 19:2020.07.13.20151233. Preprint. [Version 5] doi: 10.1101/2020.07.13.20151233 (PMC7685335; doi:10.1101/2020.07.13.20151233)
Supplement: Supplement 2020 [file 94423-2020.07.13.20151233-3.pdf]

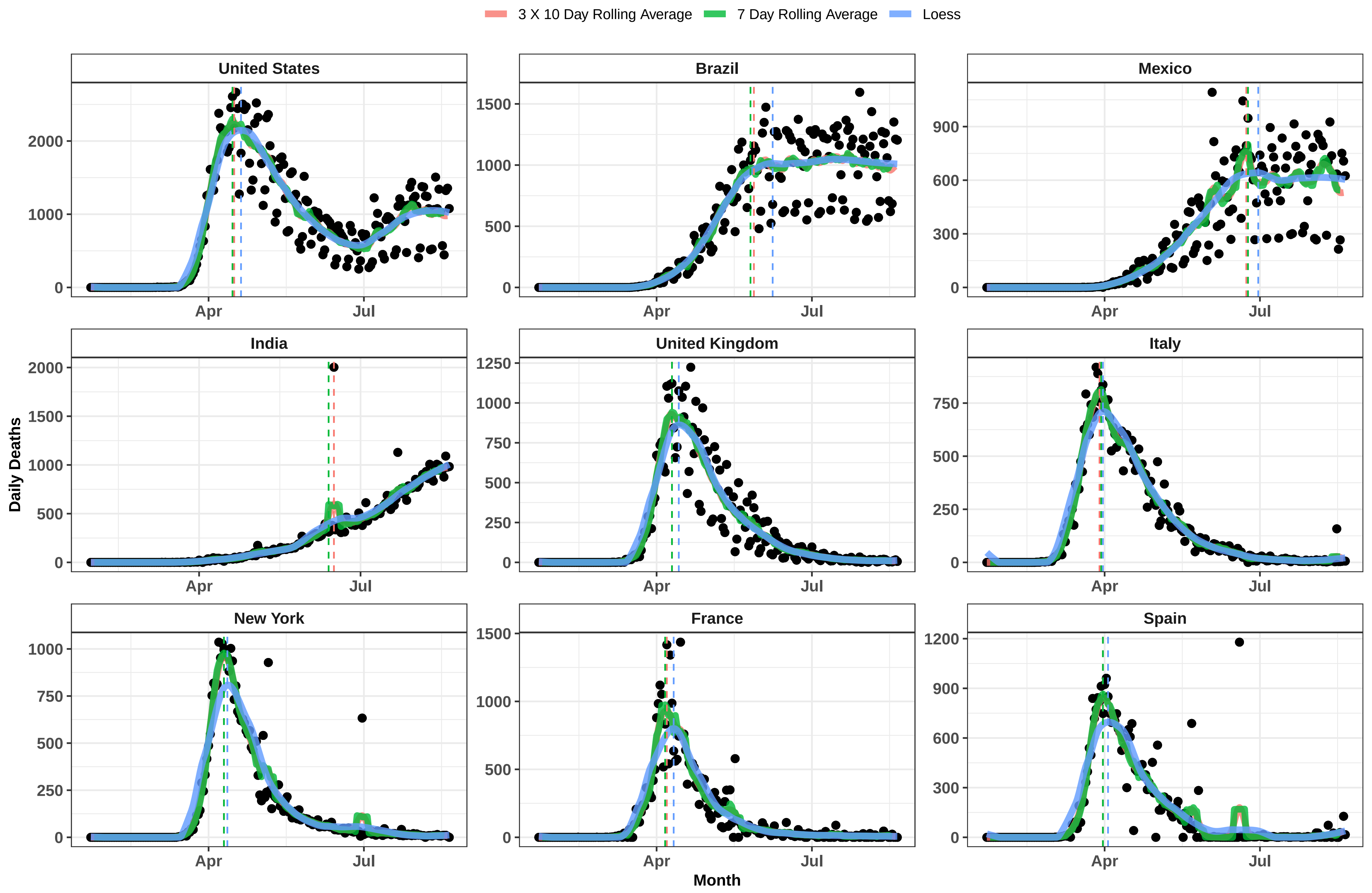

3 X 10 Day Rolling Average
7 Day Rolling Average
Loess
United States
Brazil
Mexico
India
United Kingdom
Italy
New York
France
Spain
Daily Deaths
Month
Apr
Jul

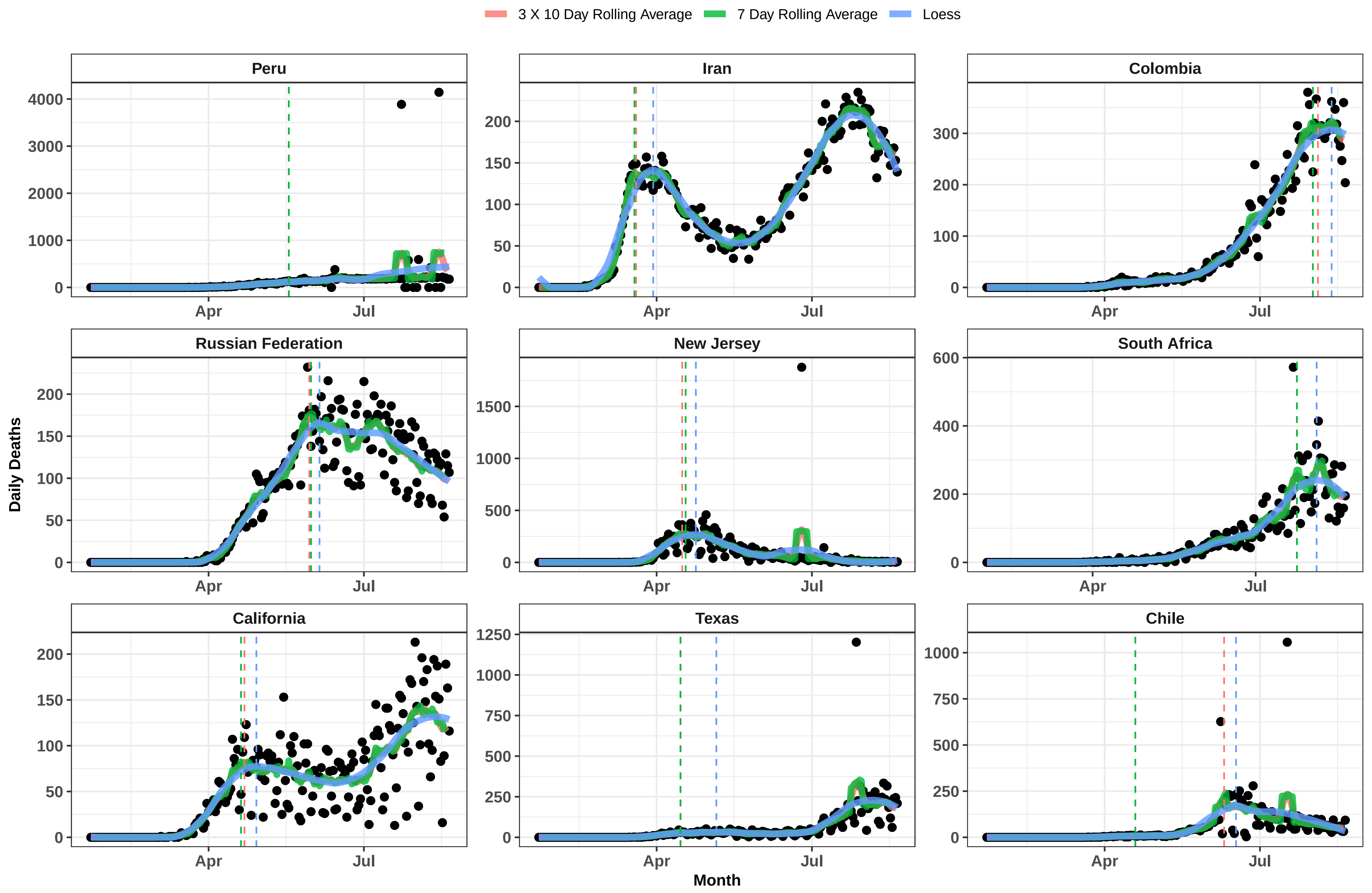

3 X 10 Day Rolling Average
7 Day Rolling Average
Loess
Peru
Iran
Colombia
Russian Federation
New Jersey
South Africa
California
Texas
Chile
Daily Deaths
Month
Apr
Jul

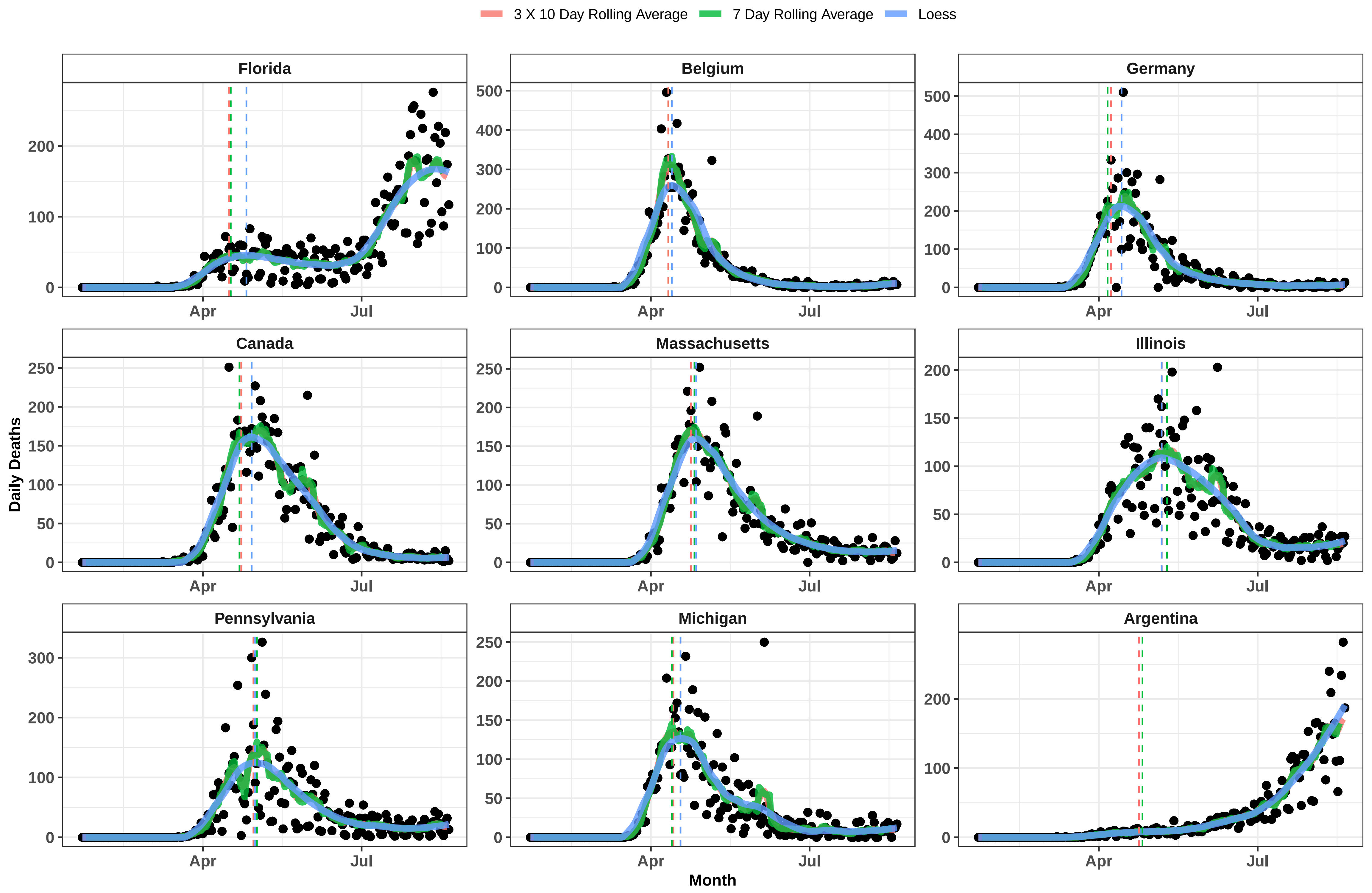

3 X 10 Day Rolling Average
7 Day Rolling Average
Loess
Florida
Belgium
Germany
Canada
Massachusetts
Illinois
Pennsylvania
Michigan
Argentina
Daily Deaths
Month
Apr
Jul

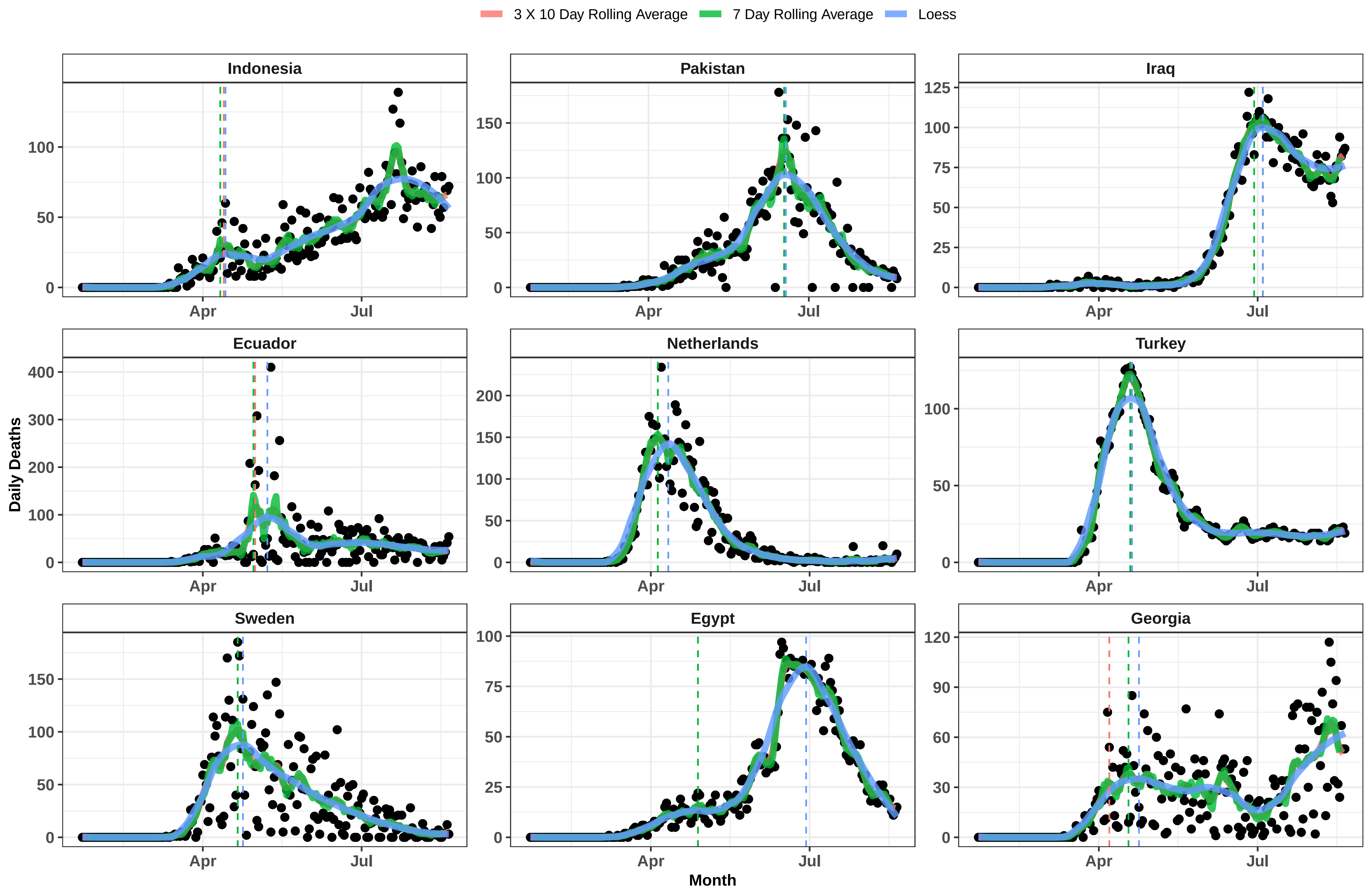

3 X 10 Day Rolling Average
7 Day Rolling Average
Loess
Indonesia
Pakistan
Iraq
Ecuador
Netherlands
Turkey
Sweden
Egypt
Georgia
Daily Deaths
Month
Apr
Jul

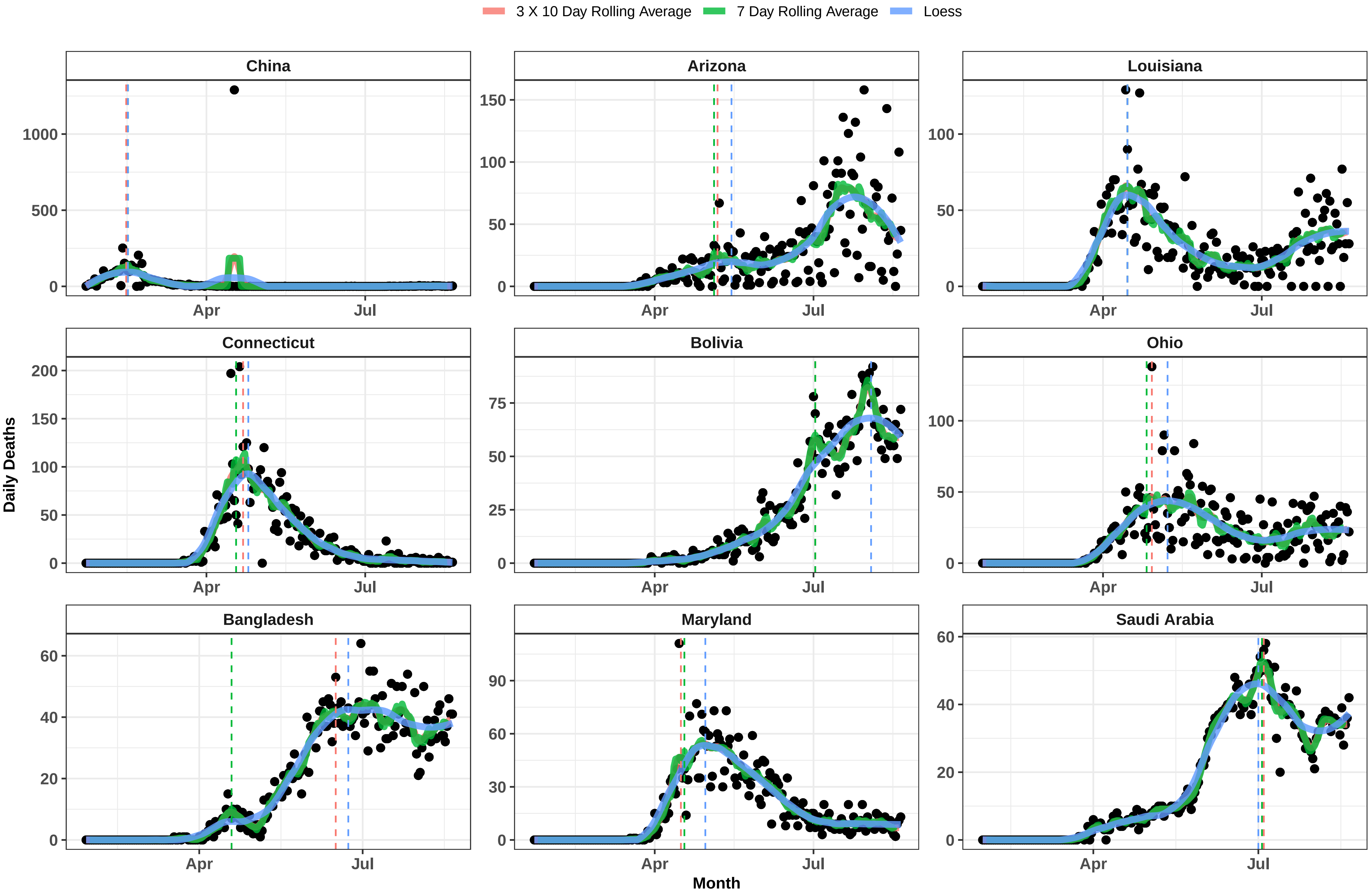

3 X 10 Day Rolling Average
7 Day Rolling Average
Loess
China
Arizona
Louisiana
Connecticut
Bolivia
Ohio
Bangladesh
Maryland
Saudi Arabia
Daily Deaths
Month
Apr
Jul

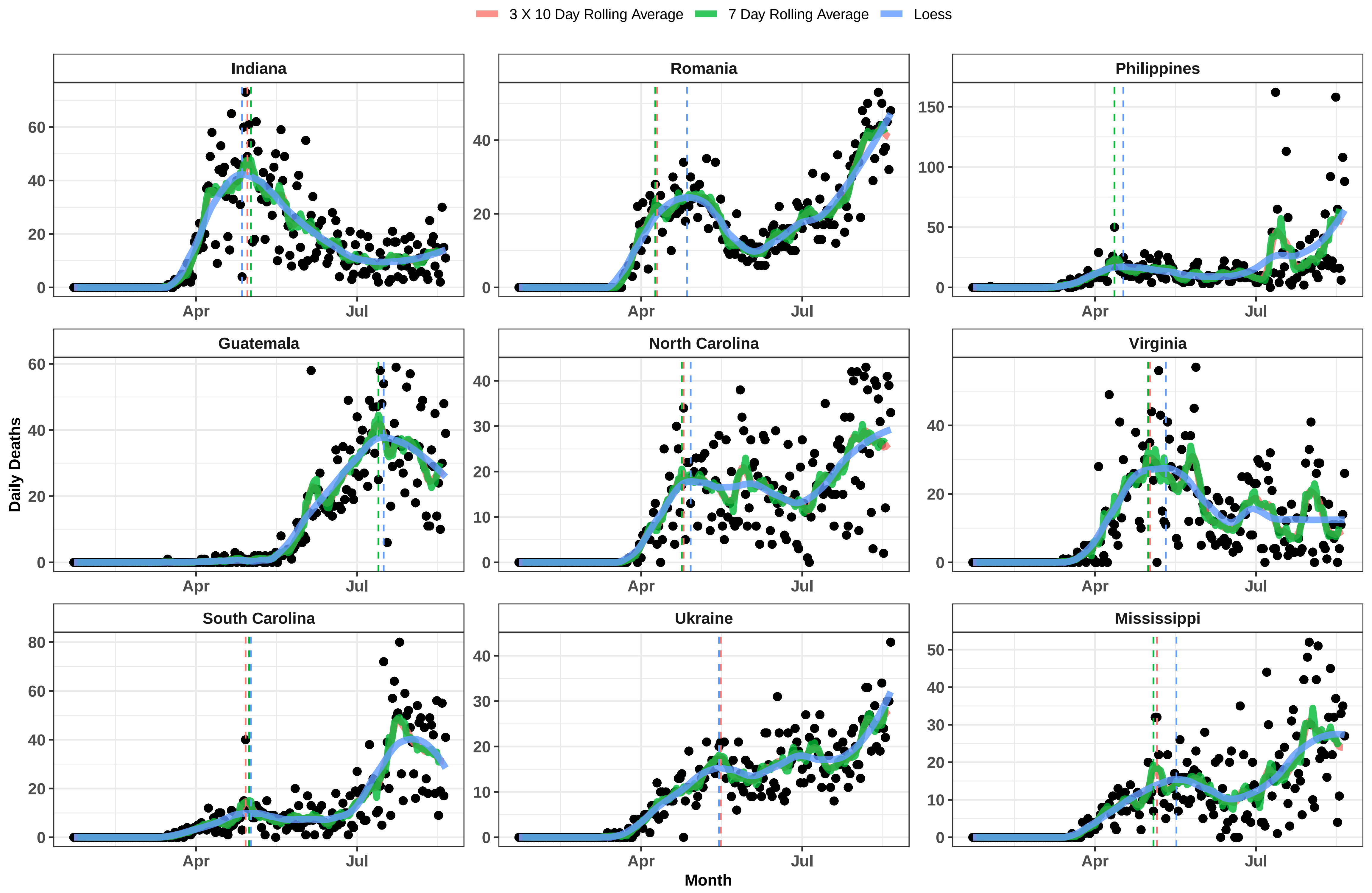
3 X 10 Day Rolling Average
7 Day Rolling Average
Loess
Indiana
Romania
Philippines
Guatemala
North Carolina
Virginia
South Carolina
Ukraine
Mississippi
Daily Deaths
Month
Apr
Jul

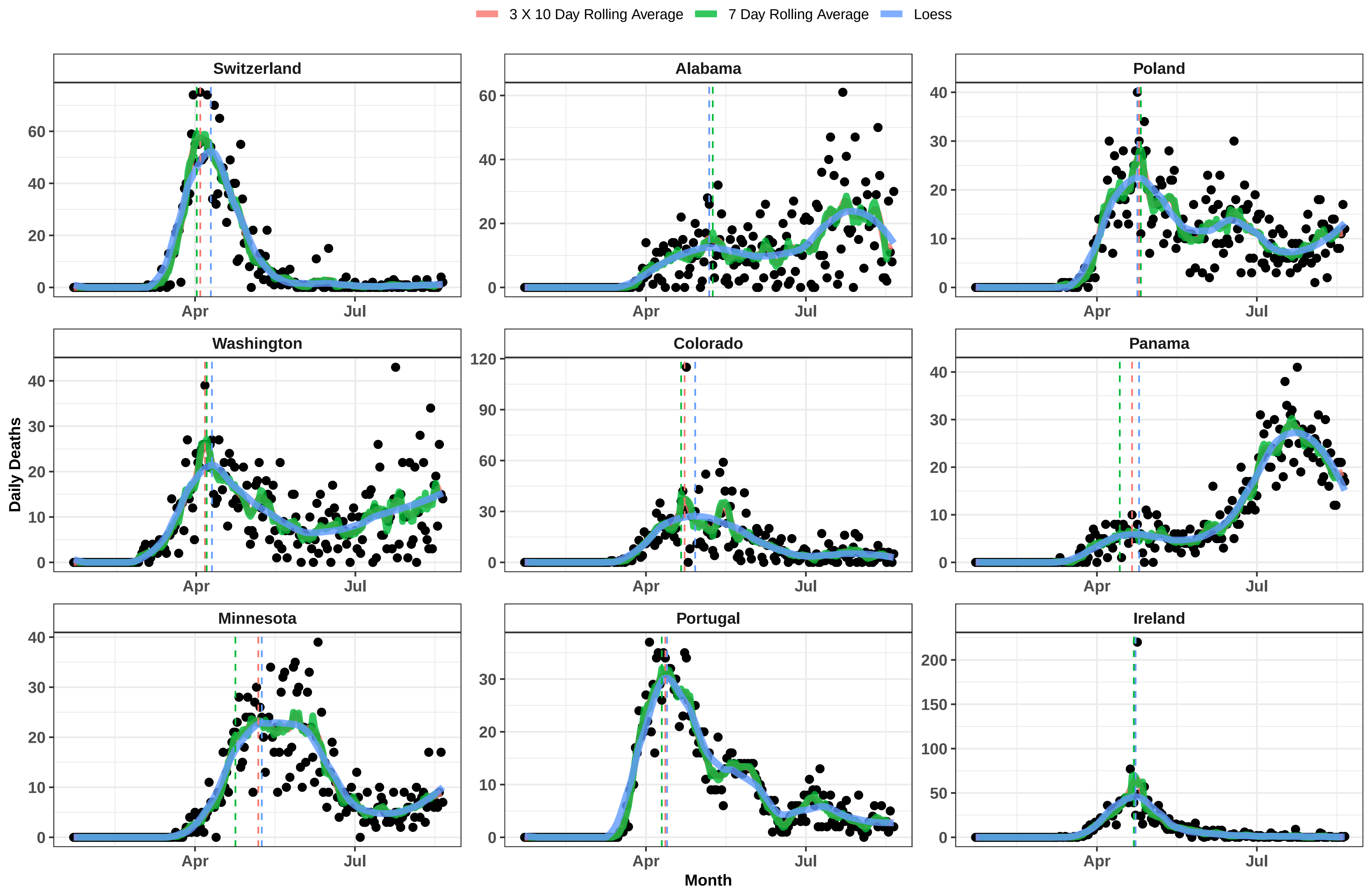
3 X 10 Day Rolling Average
7 Day Rolling Average
Loess
Switzerland
Alabama
Poland
Washington
Colorado
Panama
Minnesota
Portugal
Ireland
Daily Deaths
Month
Apr
Jul

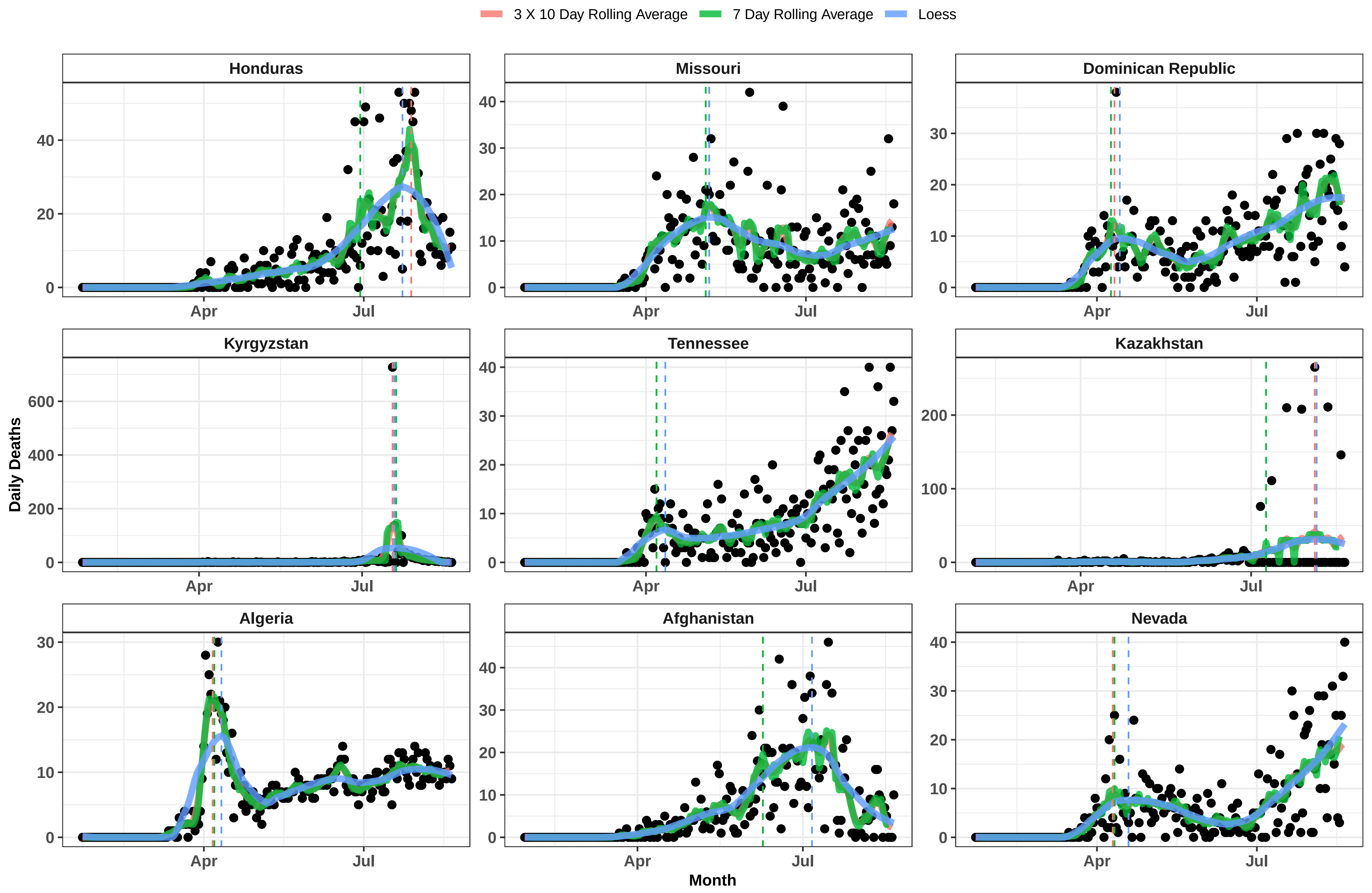

3 X 10 Day Rolling Average
7 Day Rolling Average
Loess
Honduras
Missouri
Dominican Republic
Kyrgyzstan
Tennessee
Kazakhstan
Algeria
Afghanistan
Nevada
Daily Deaths
Month
Apr
Jul

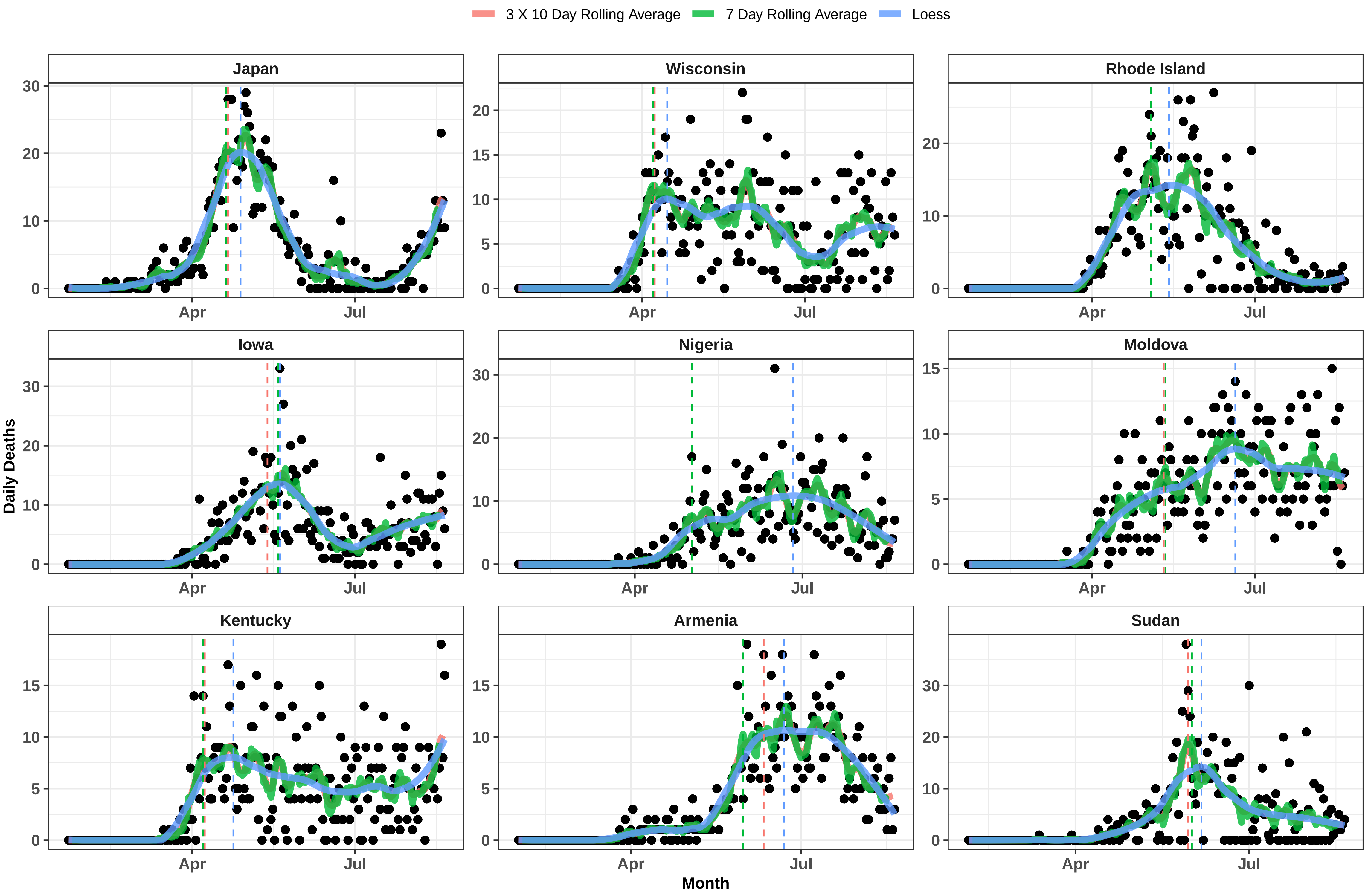

3 X 10 Day Rolling Average
7 Day Rolling Average
Loess
Japan
Wisconsin
Rhode Island
Iowa
Nigeria
Moldova
Kentucky
Armenia
Sudan
Daily Deaths
Month
Apr
Jul

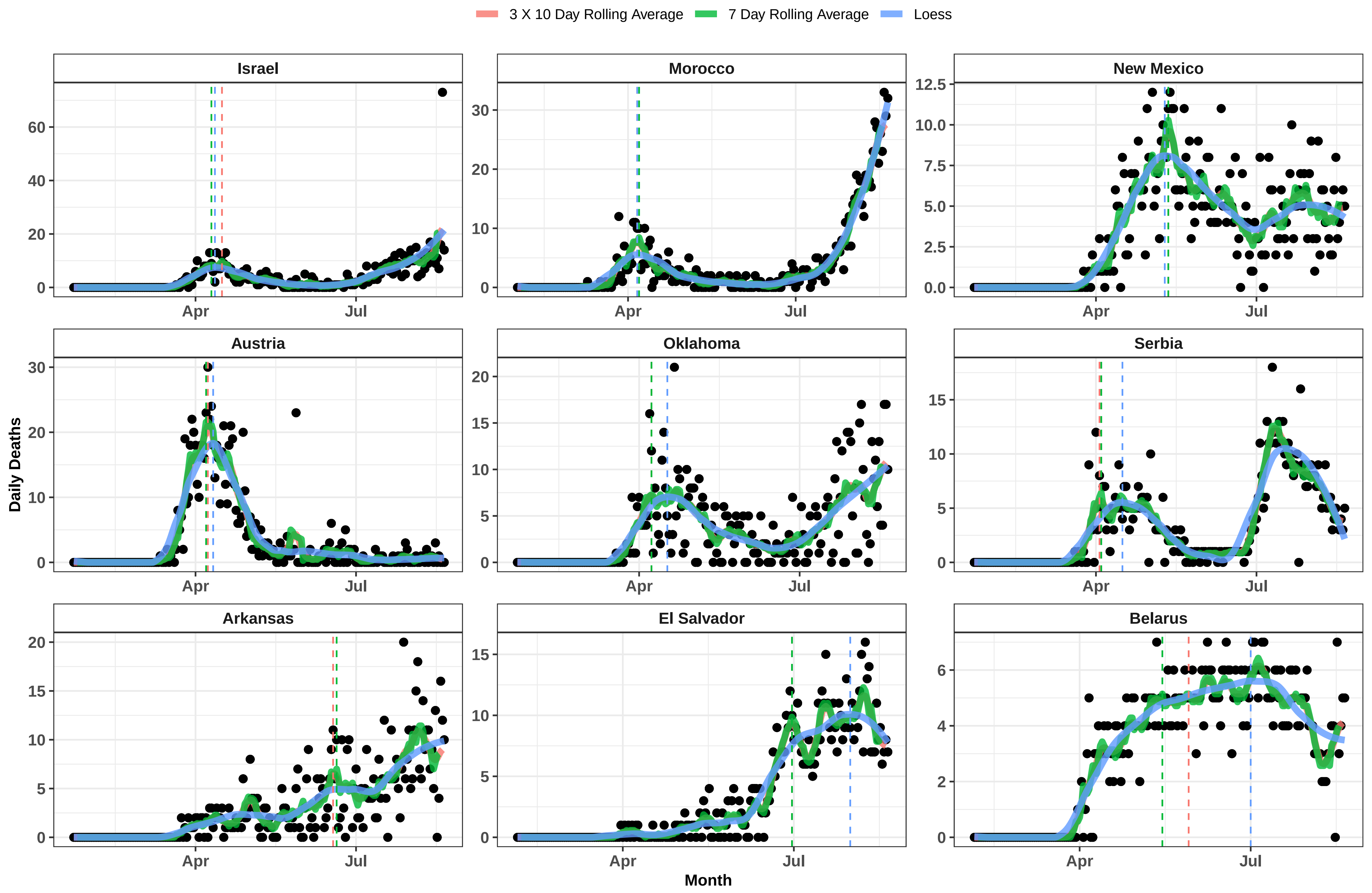

3 X 10 Day Rolling Average
7 Day Rolling Average
Loess
Israel
Morocco
New Mexico
Austria
Oklahoma
Serbia
Arkansas
El Salvador
Belarus
Daily Deaths
Month
Apr
Jul

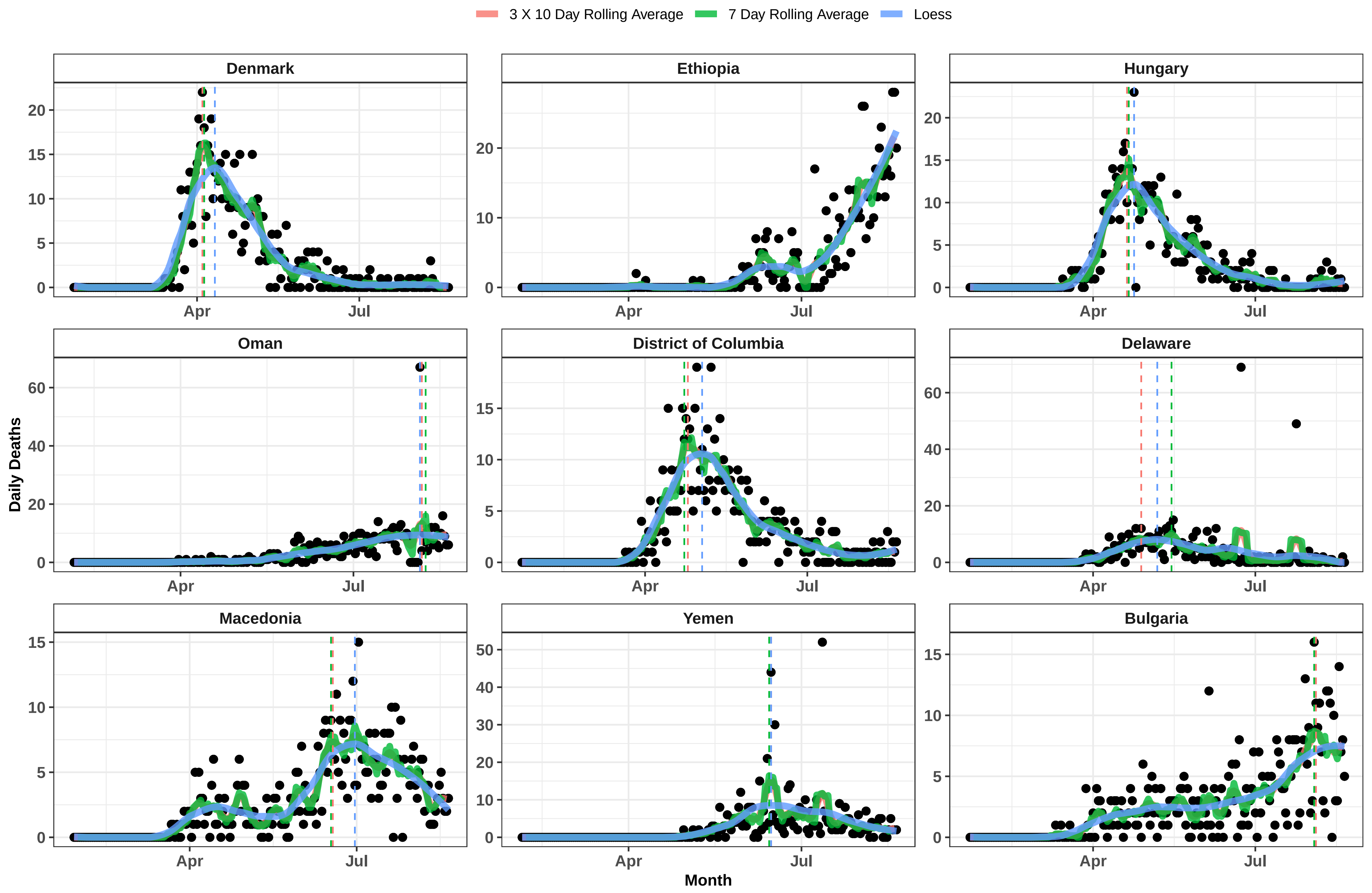
3 X 10 Day Rolling Average
7 Day Rolling Average
Loess
Denmark
Ethiopia
Hungary
Oman
District of Columbia
Delaware
Macedonia
Yemen
Bulgaria
Daily Deaths
Month
Apr
Jul

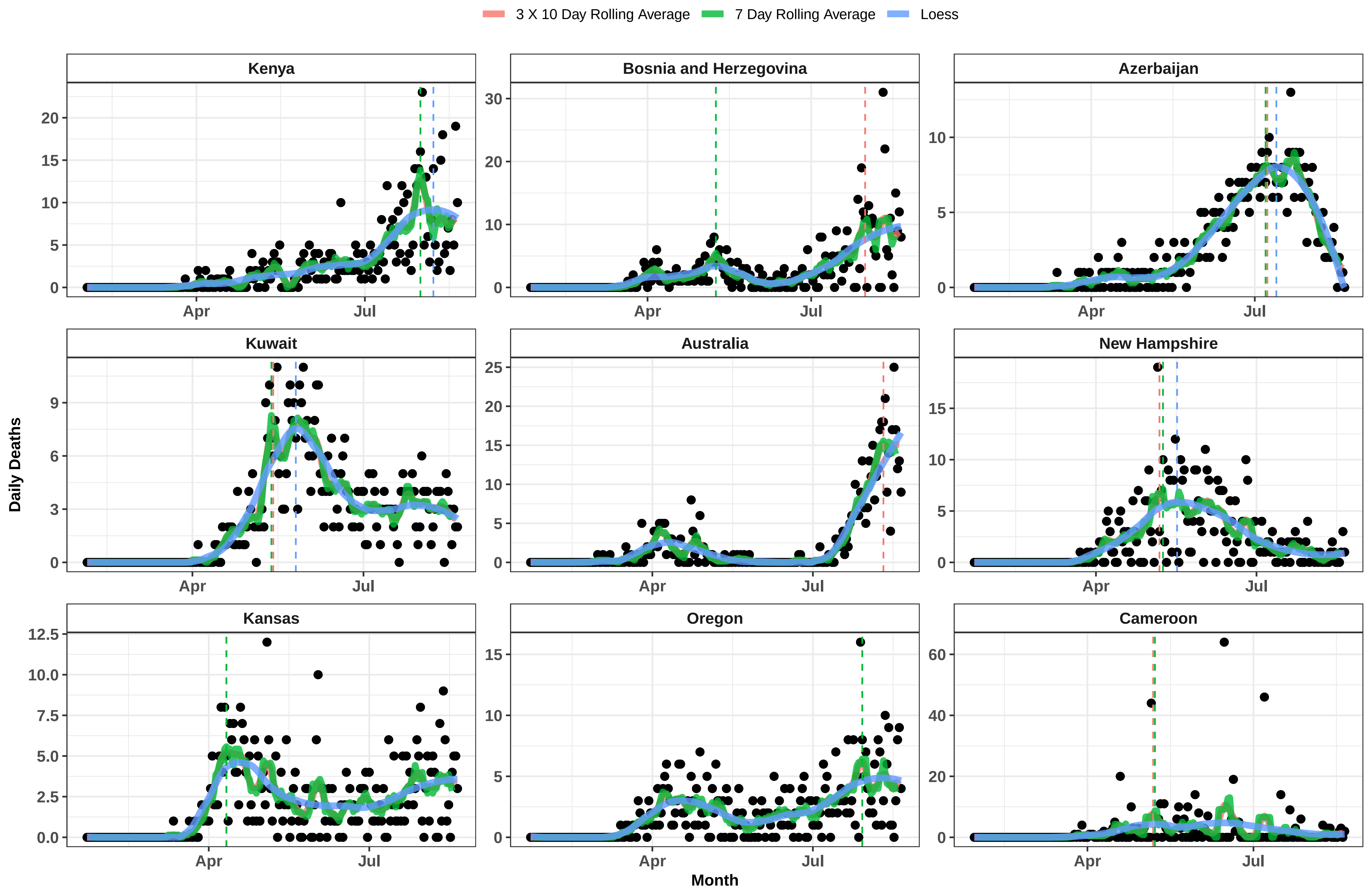

3 X 10 Day Rolling Average
7 Day Rolling Average
Loess
Kenya
Bosnia and Herzegovina
Azerbaijan
Kuwait
Australia
New Hampshire
Kansas
Oregon
Cameroon
Daily Deaths
Month
Apr
Jul

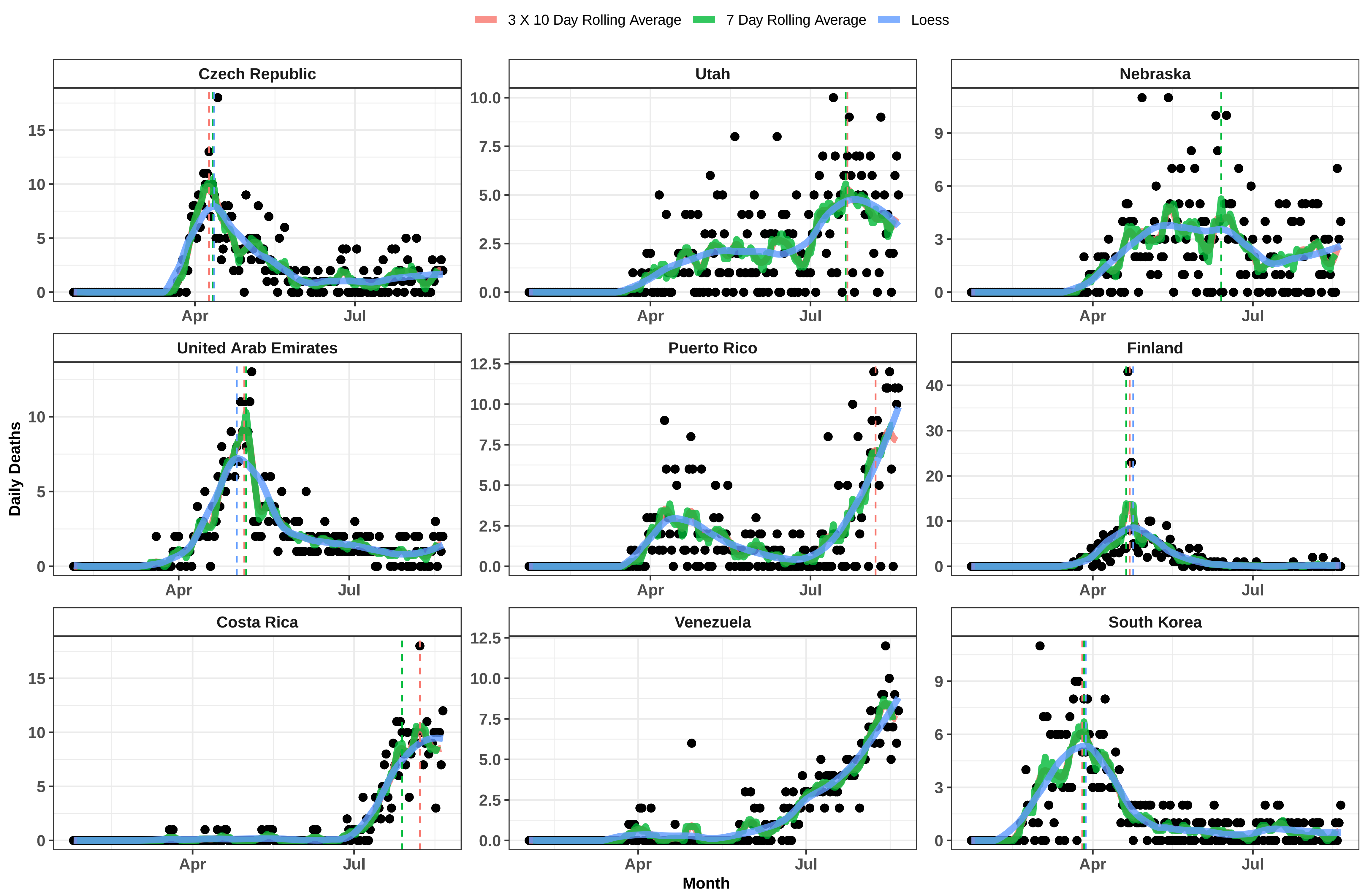

3 X 10 Day Rolling Average
7 Day Rolling Average
Loess
Czech Republic
Utah
Nebraska
United Arab Emirates
Puerto Rico
Finland
Costa Rica
Venezuela
South Korea
Daily Deaths
Month
Apr
Jul

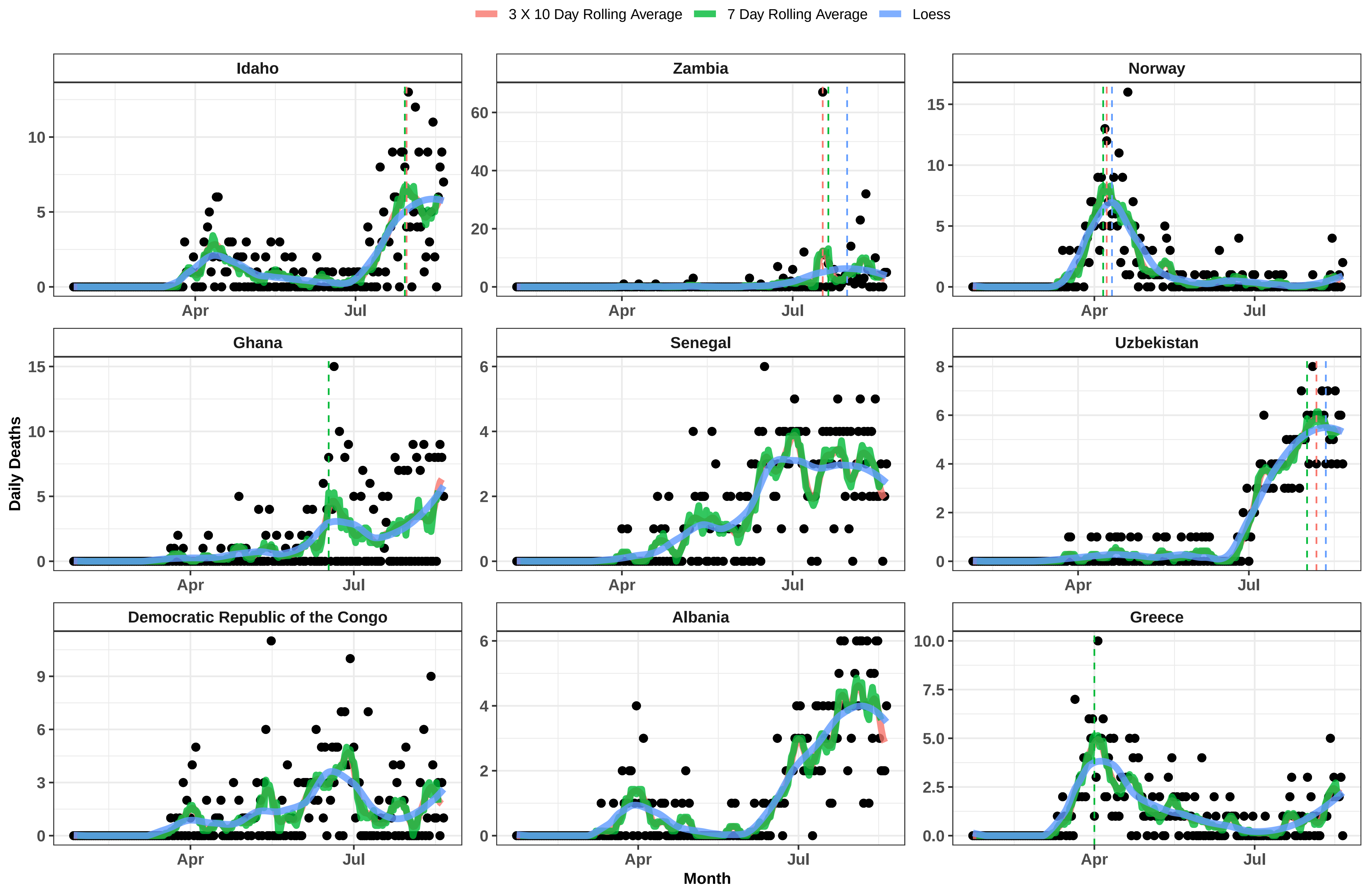

3 X 10 Day Rolling Average
7 Day Rolling Average
Loess
Idaho
Zambia
Norway
Ghana
Senegal
Uzbekistan
Democratic Republic of the Congo
Albania
Greece
Daily Deaths
Month
Apr
Jul

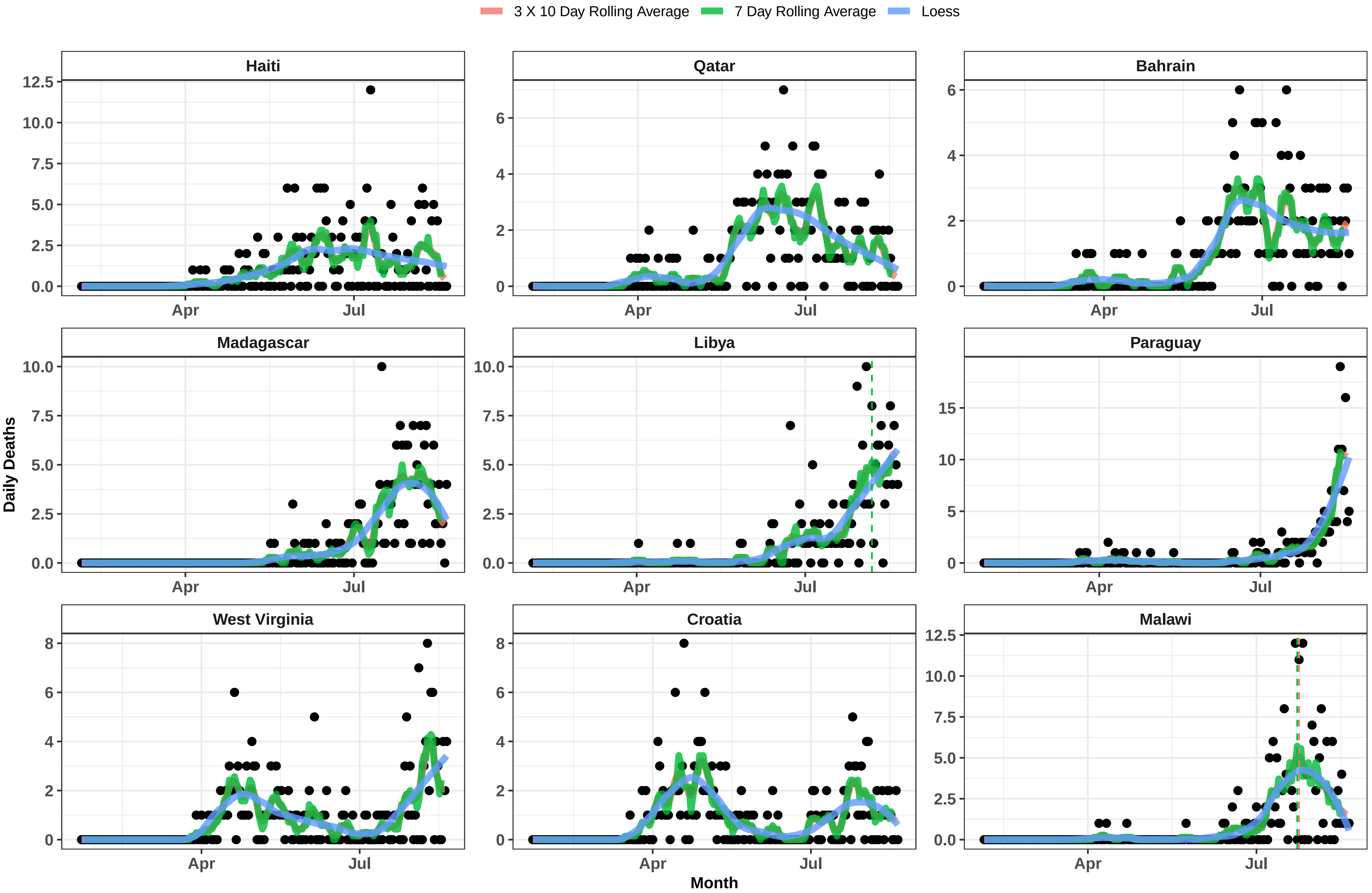

3 X 10 Day Rolling Average
7 Day Rolling Average
Loess
Haiti
Qatar
Bahrain
Madagascar
Libya
Paraguay
West Virginia
Croatia
Malawi
Daily Deaths
Month
Apr
Jul

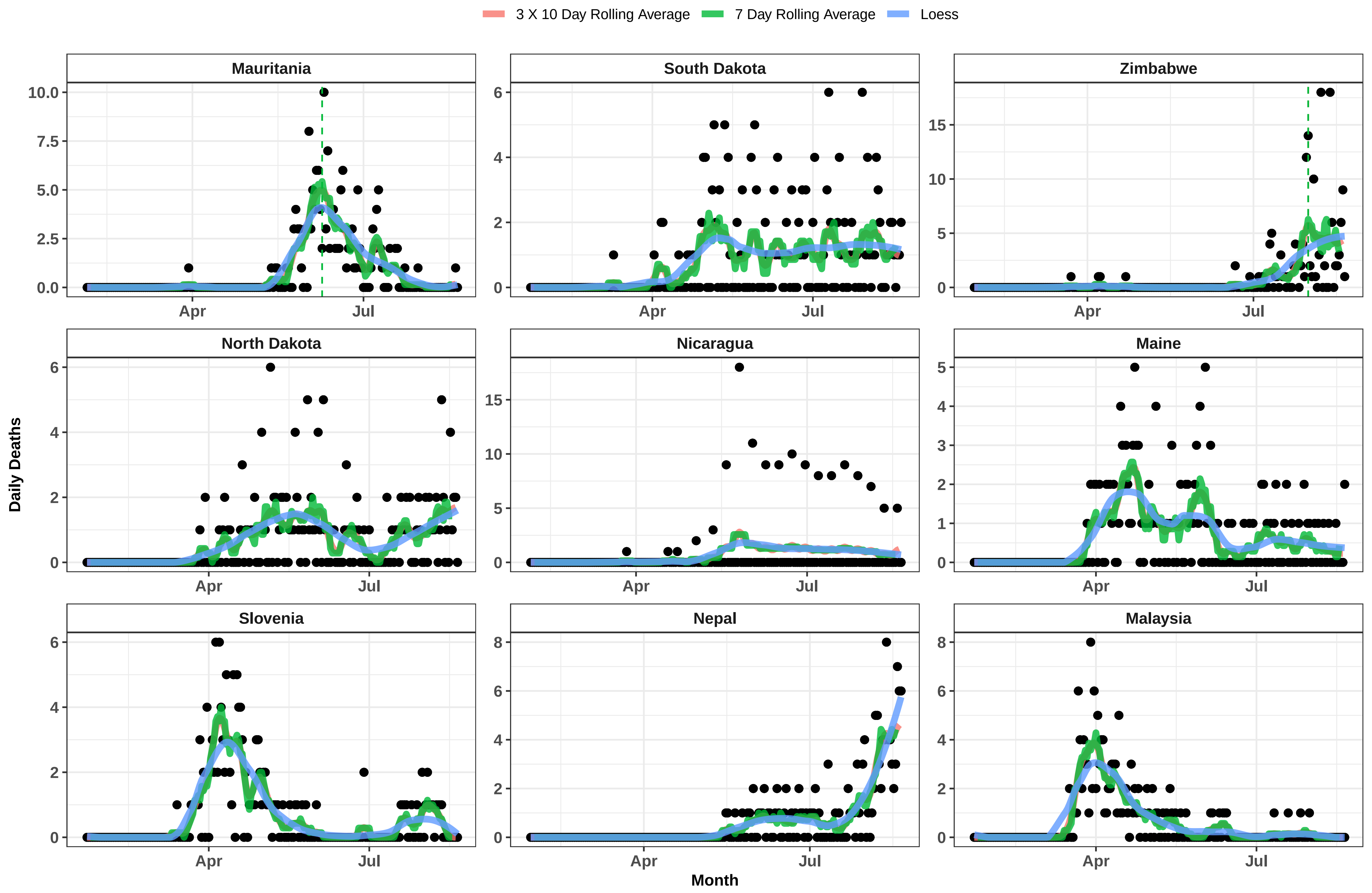

3 X 10 Day Rolling Average
7 Day Rolling Average
Loess
Mauritania
South Dakota
Zimbabwe
North Dakota
Nicaragua
Maine
Slovenia
Nepal
Malaysia
Daily Deaths
Month
Apr
Jul

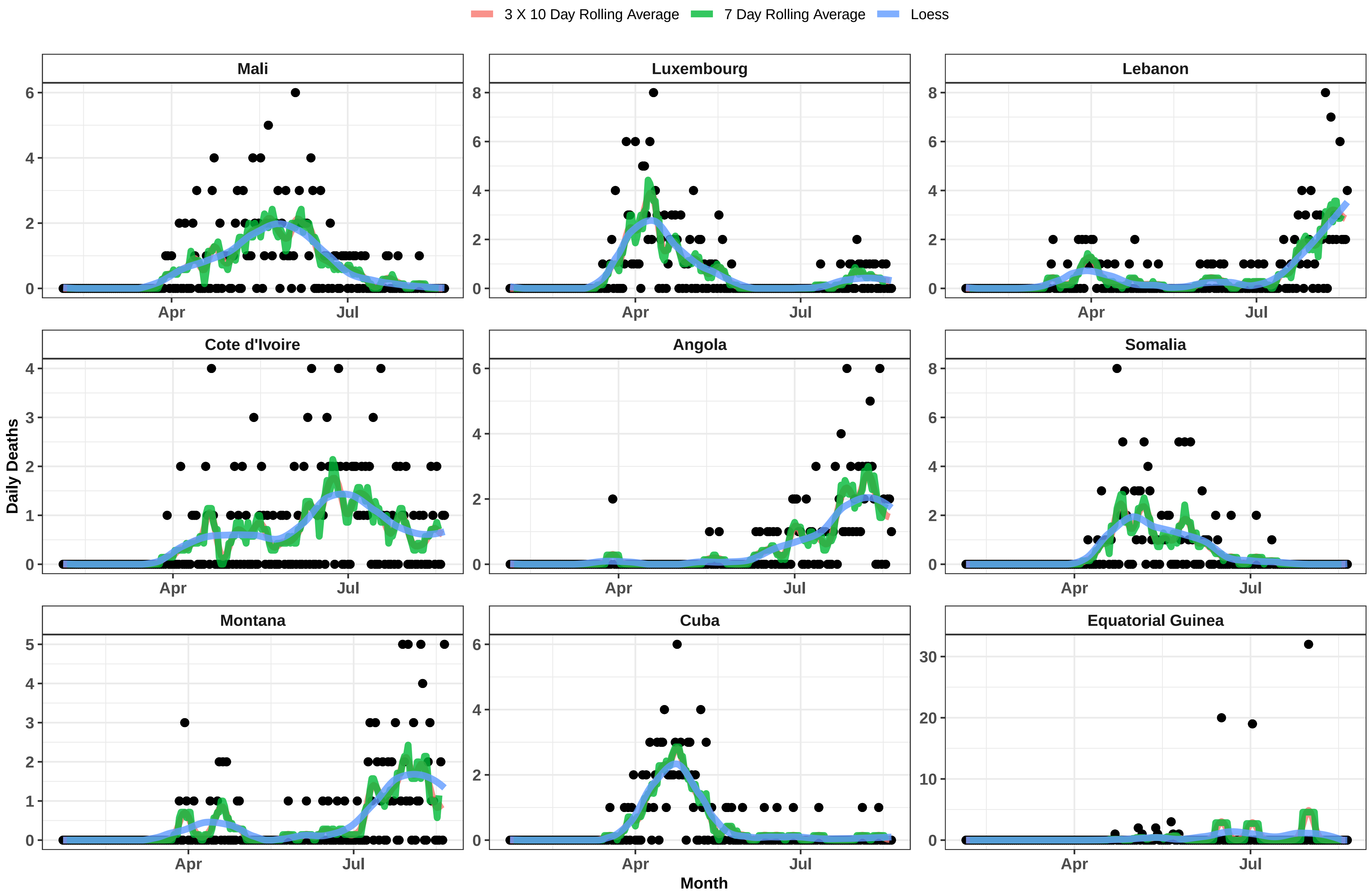

3 X 10 Day Rolling Average
7 Day Rolling Average
Loess
Mali
Luxembourg
Lebanon
Cote d'Ivoire
Angola
Somalia
Montana
Cuba
Equatorial Guinea
Daily Deaths
Month
Apr
Jul

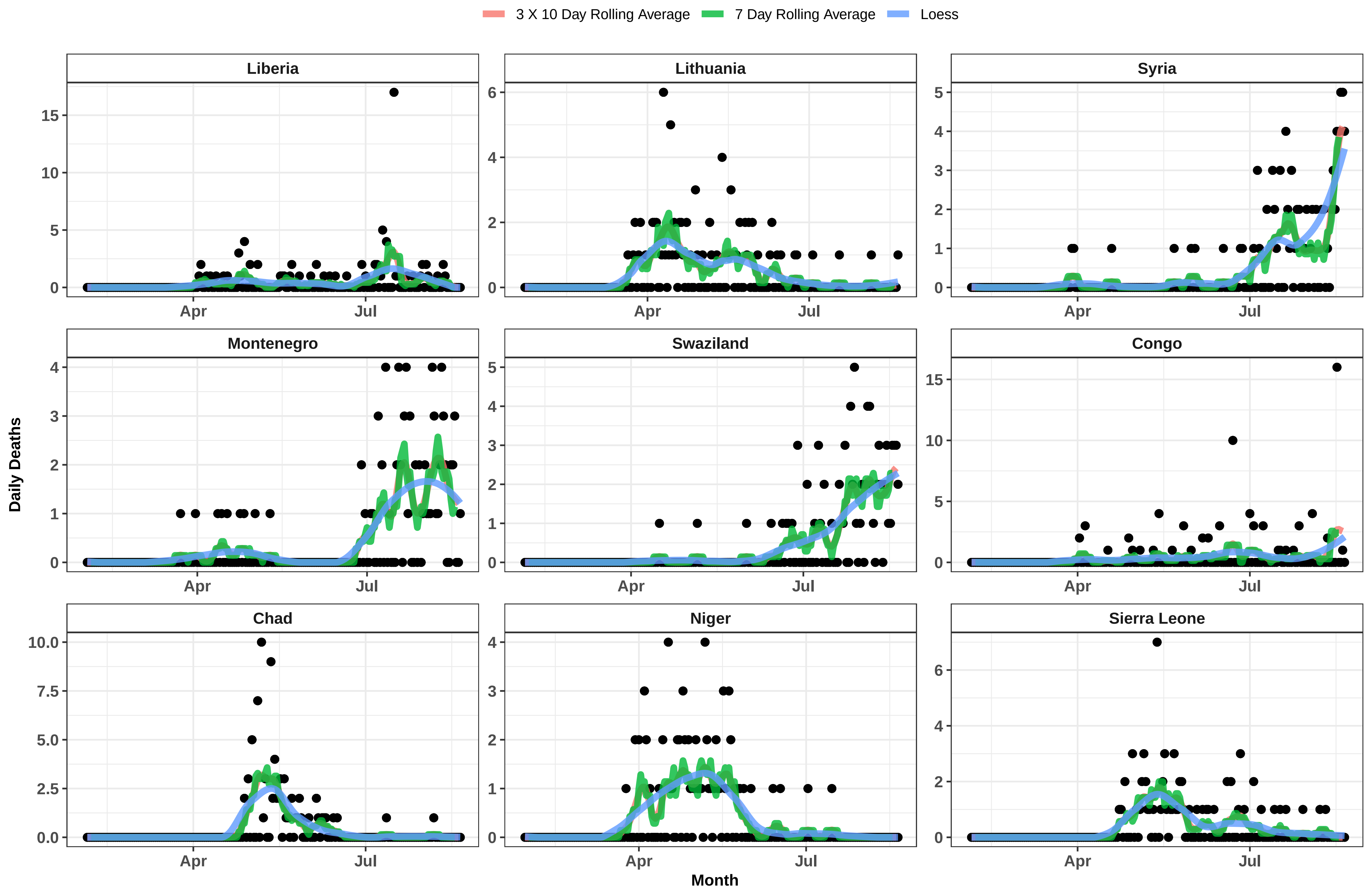

3 X 10 Day Rolling Average
7 Day Rolling Average
Loess
Liberia
Lithuania
Syria
Montenegro
Swaziland
Congo
Chad
Niger
Sierra Leone
Daily Deaths
Month
Apr
Jul

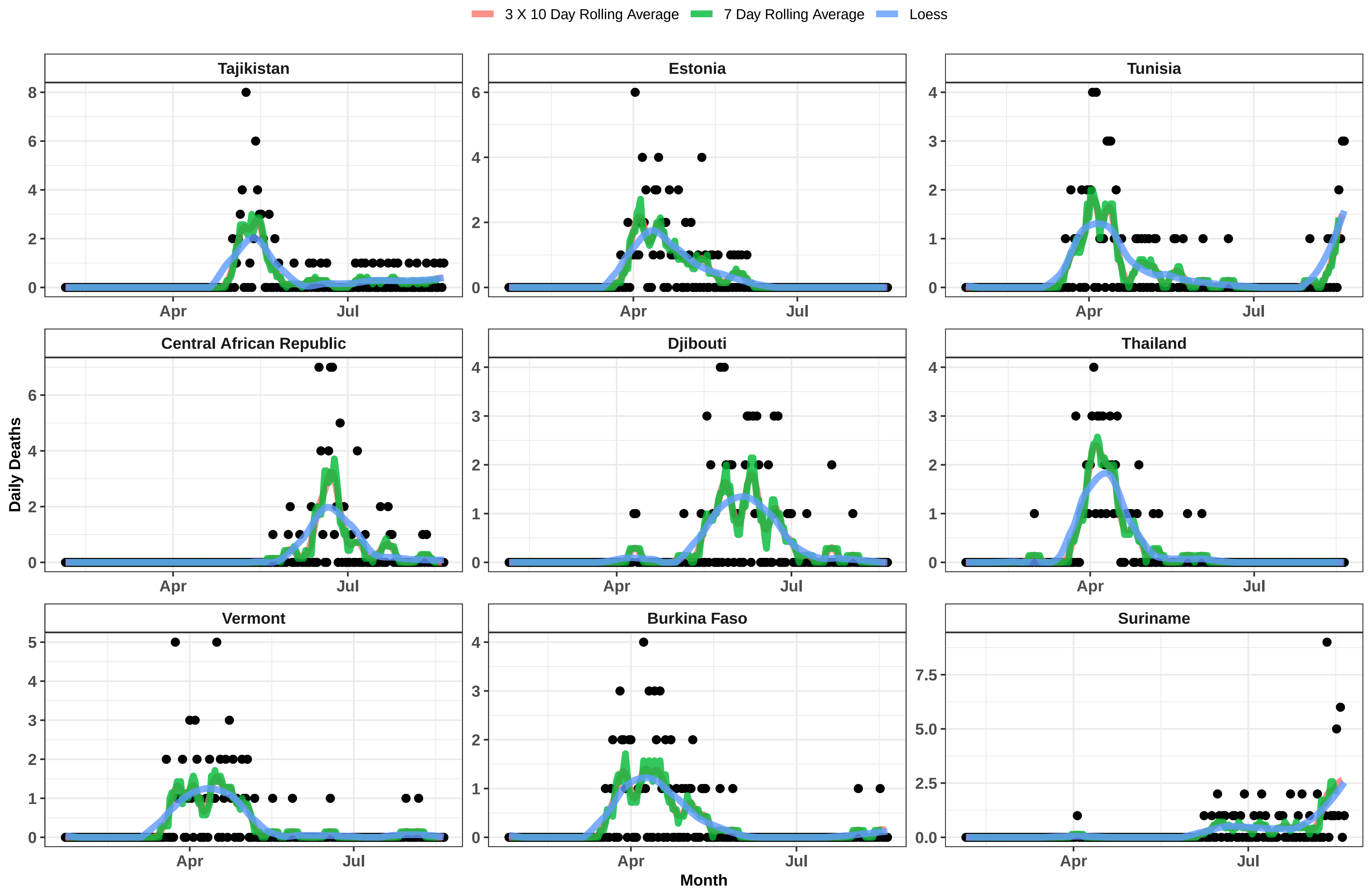

3 X 10 Day Rolling Average
7 Day Rolling Average
Loess
Tajikistan
Estonia
Tunisia
Central African Republic
Djibouti
Thailand
Vermont
Burkina Faso
Suriname
Daily Deaths
Month
Apr
Jul

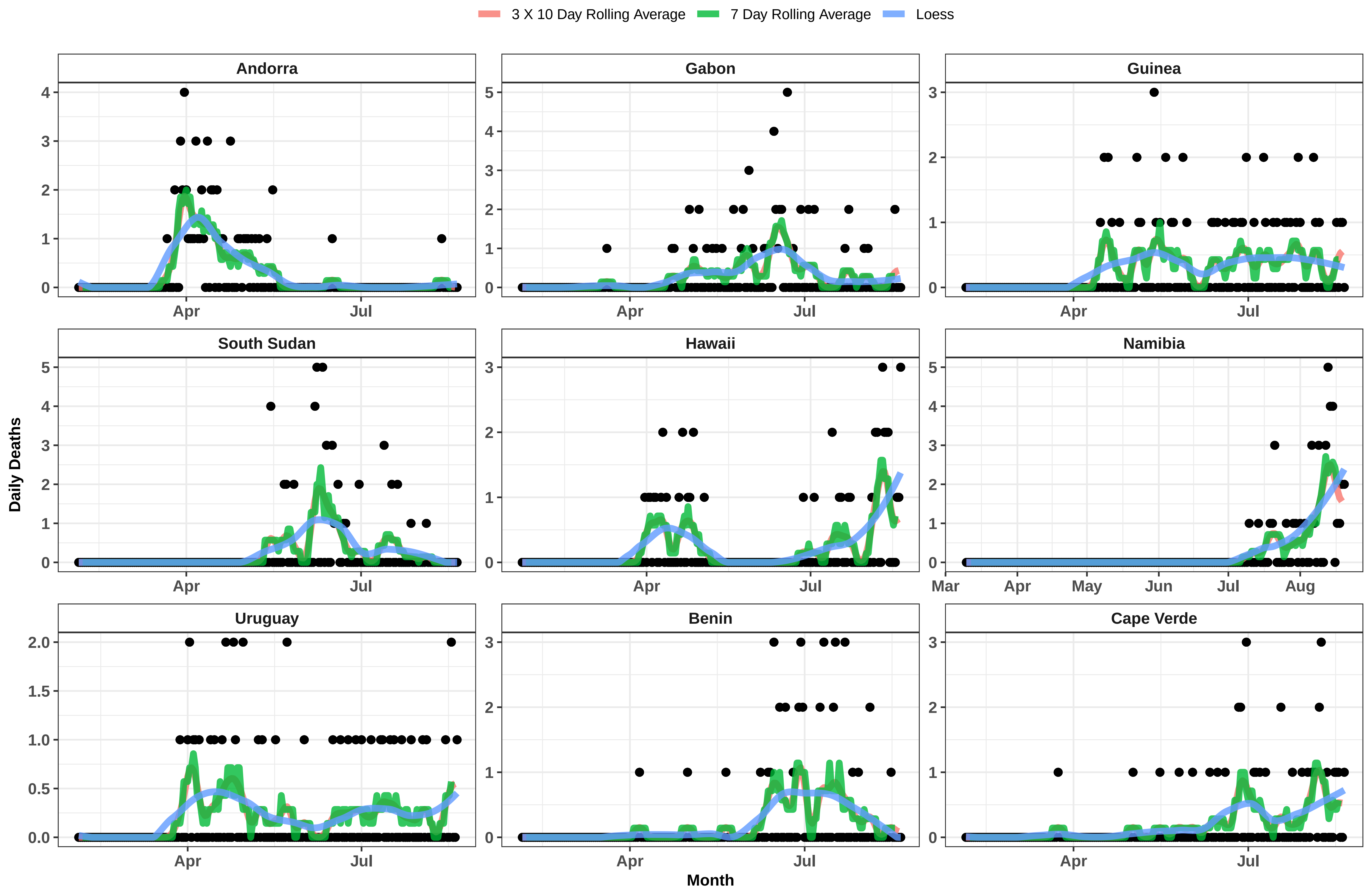
3 X 10 Day Rolling Average
7 Day Rolling Average
Loess
Andorra
Gabon
Guinea
South Sudan
Hawaii
Namibia
Uruguay
Benin
Cape Verde
Daily Deaths
Month
Apr
Jul
Mar
May
Jun
Aug

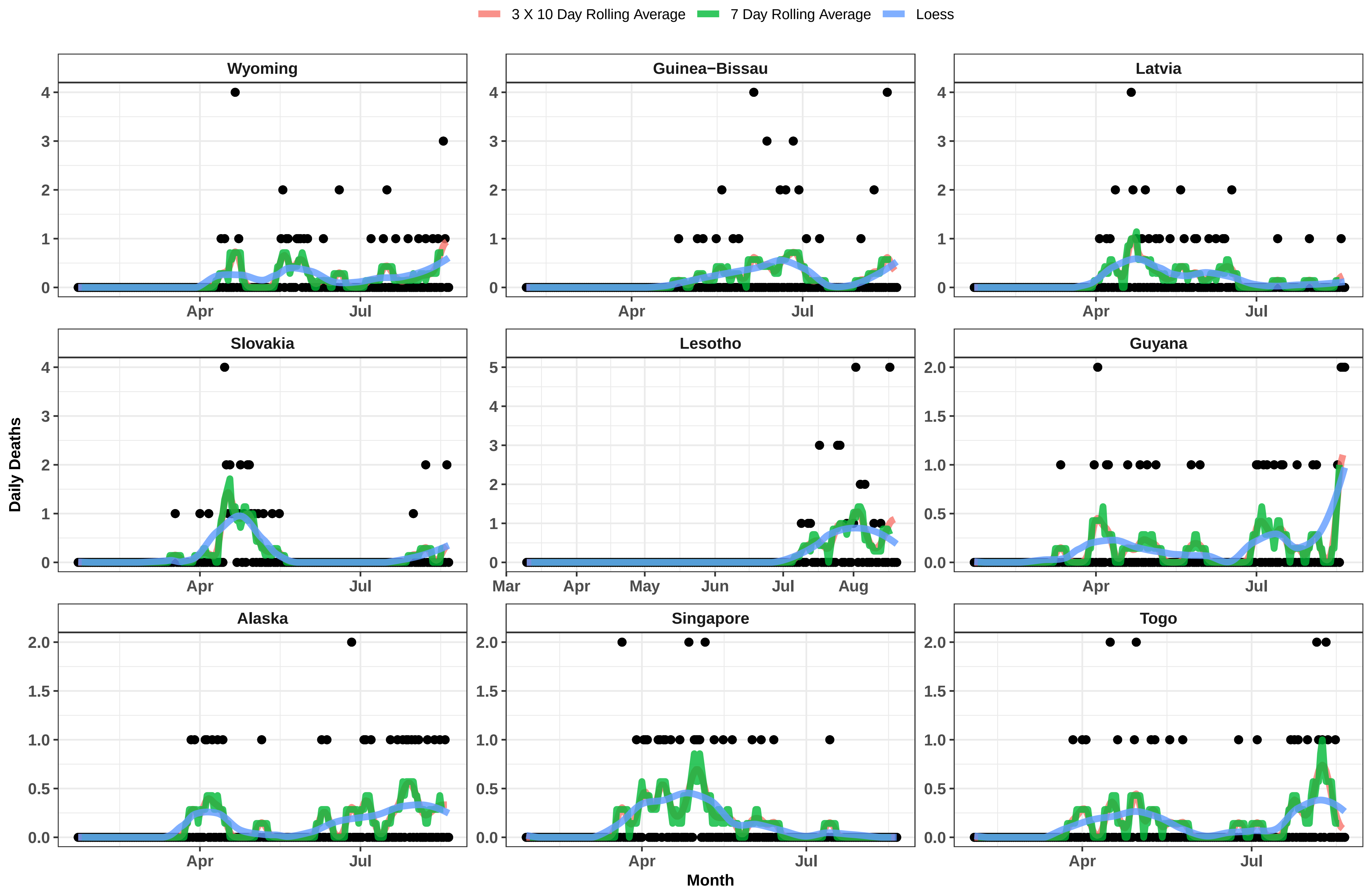
3 X 10 Day Rolling Average
7 Day Rolling Average
Loess
Wyoming
Guinea−Bissau
Latvia
Slovakia
Lesotho
Guyana
Alaska
Singapore
Togo
Daily Deaths
Month
Mar
Apr
May
Jun
Jul
Aug

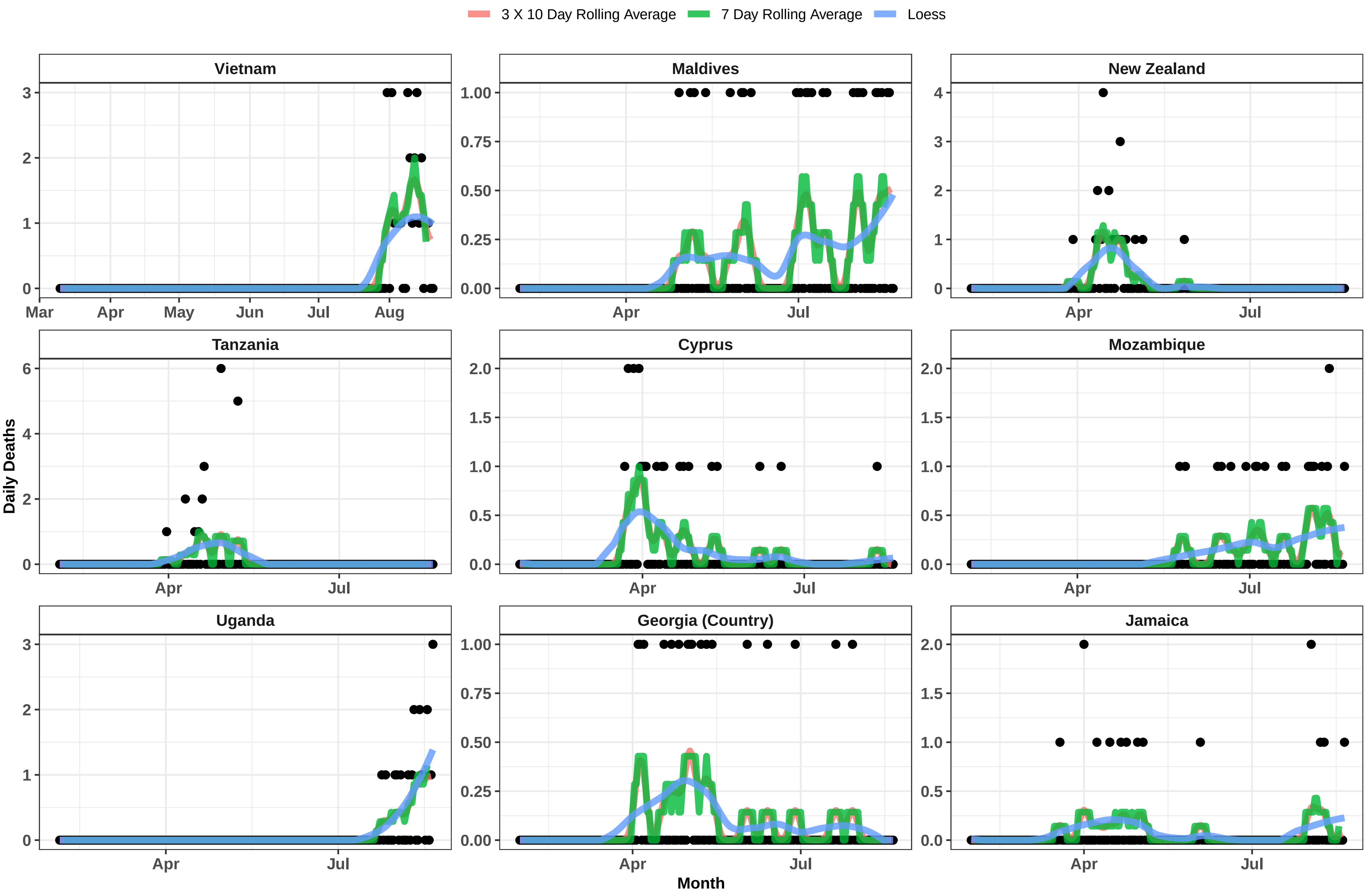

3 X 10 Day Rolling Average
7 Day Rolling Average
Loess
Vietnam
Maldives
New Zealand
Tanzania
Cyprus
Mozambique
Uganda
Georgia (Country)
Jamaica
Daily Deaths
Month
Mar
Apr
May
Jun
Jul
Aug

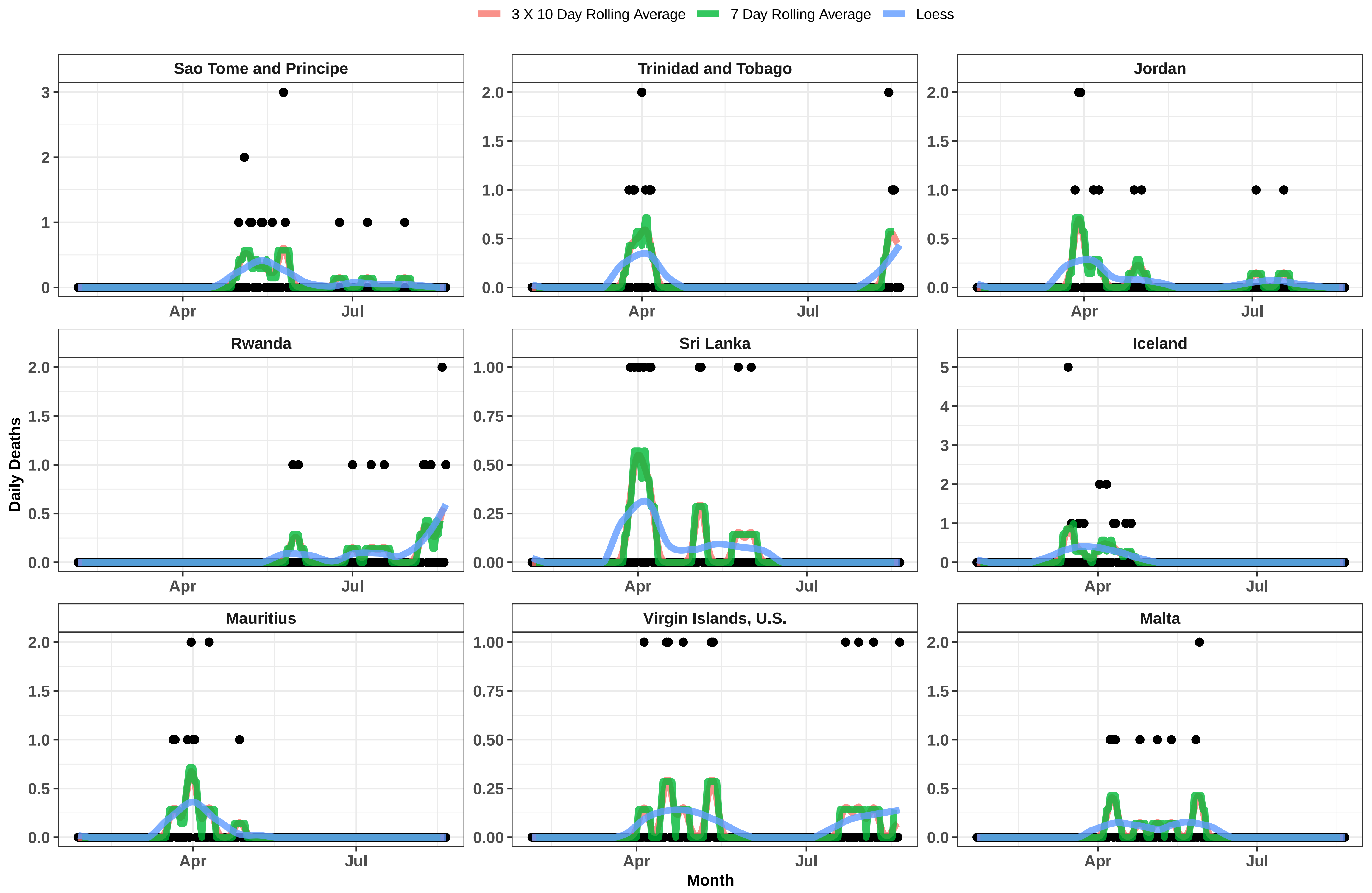

3 X 10 Day Rolling Average
7 Day Rolling Average
Loess
Sao Tome and Principe
Trinidad and Tobago
Jordan
Rwanda
Sri Lanka
Iceland
Mauritius
Virgin Islands, U.S.
Malta
Daily Deaths
Month
Apr
Jul

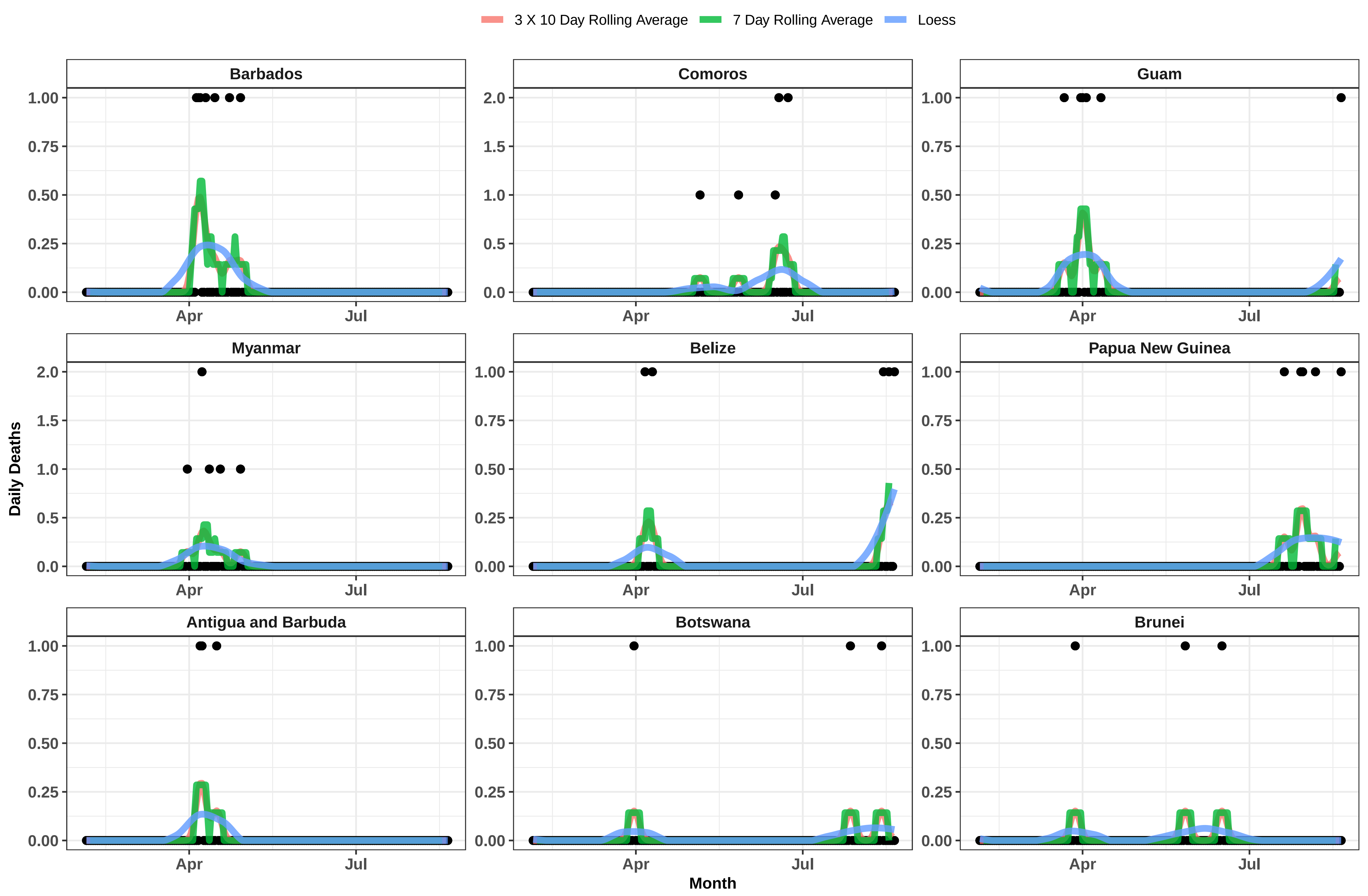

3 X 10 Day Rolling Average
7 Day Rolling Average
Loess
Barbados
Comoros
Guam
Myanmar
Belize
Papua New Guinea
Antigua and Barbuda
Botswana
Brunei
Daily Deaths
Month
Apr
Jul
0.00
0.25
0.50
0.75
1.00
0.0
0.5
1.0
1.5
2.0

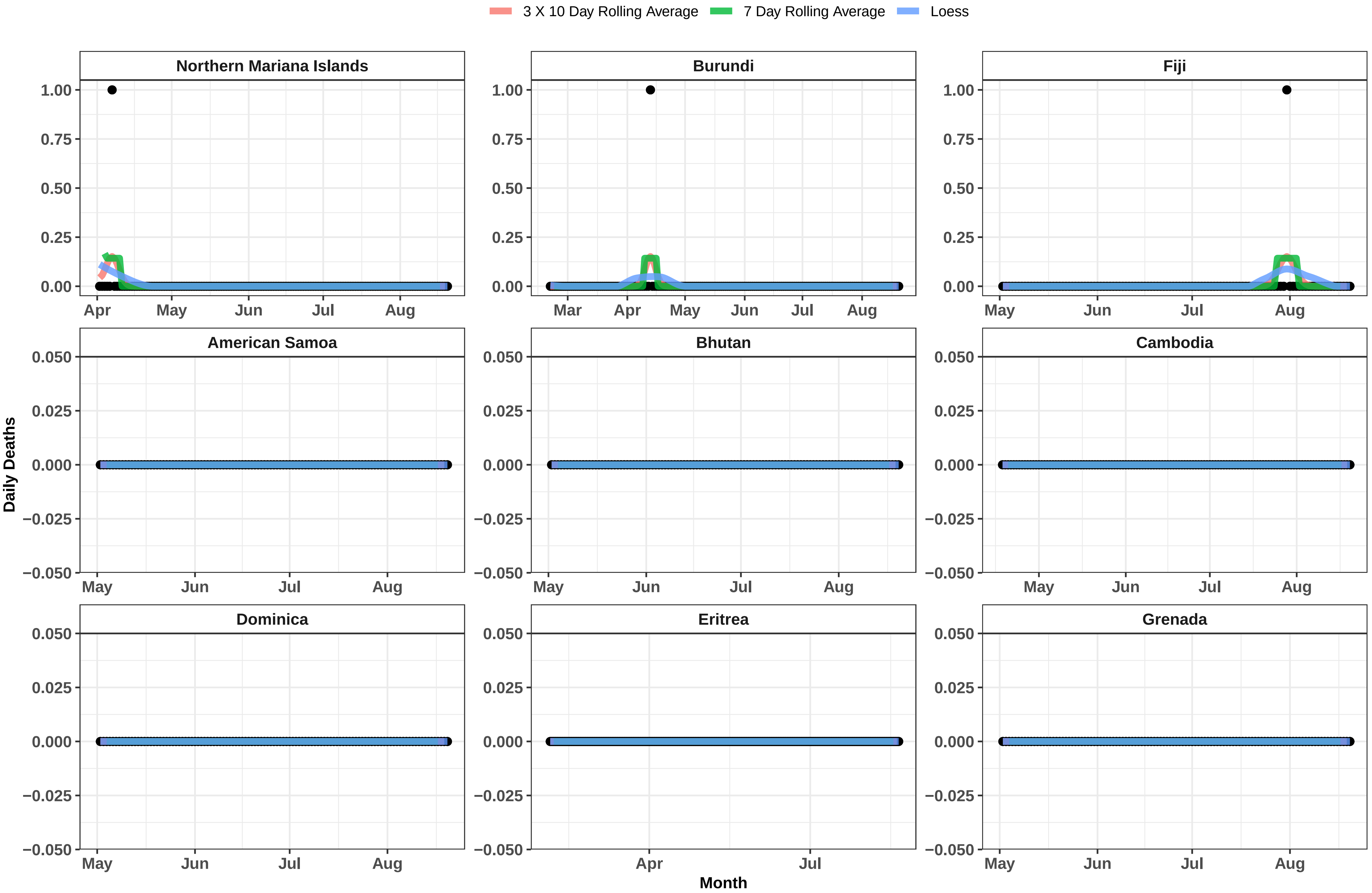

3 X 10 Day Rolling Average
7 Day Rolling Average
Loess
Northern Mariana Islands
Burundi
Fiji
American Samoa
Bhutan
Cambodia
Dominica
Eritrea
Grenada
Daily Deaths
Month

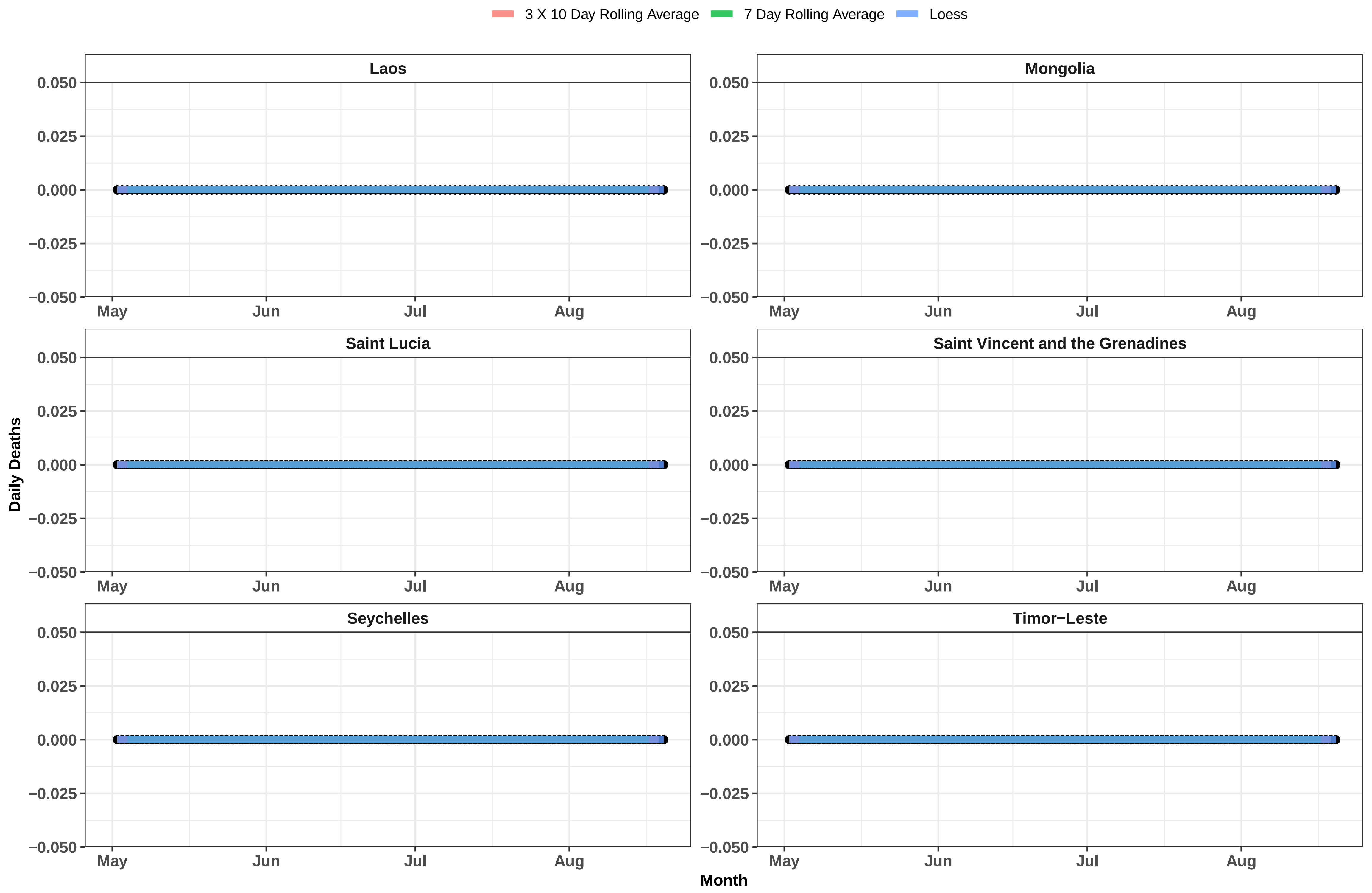
3 X 10 Day Rolling Average
7 Day Rolling Average
Loess
Laos
Mongolia
Saint Lucia
Saint Vincent and the Grenadines
Seychelles
Timor−Leste
0.050
0.025
0.000
−0.025
−0.050
May
Jun
Jul
Aug
Daily Deaths
Month
