# Supplementary material for: Predictive performance of international COVID-19 mortality forecasting models
Source: medRxiv. 2020 Nov 19:2020.07.13.20151233. Preprint. [Version 5] doi: 10.1101/2020.07.13.20151233 (PMC7685335; doi:10.1101/2020.07.13.20151233)
Supplement: Supplement 2020 [file 94423-2020.07.13.20151233-4.pdf]

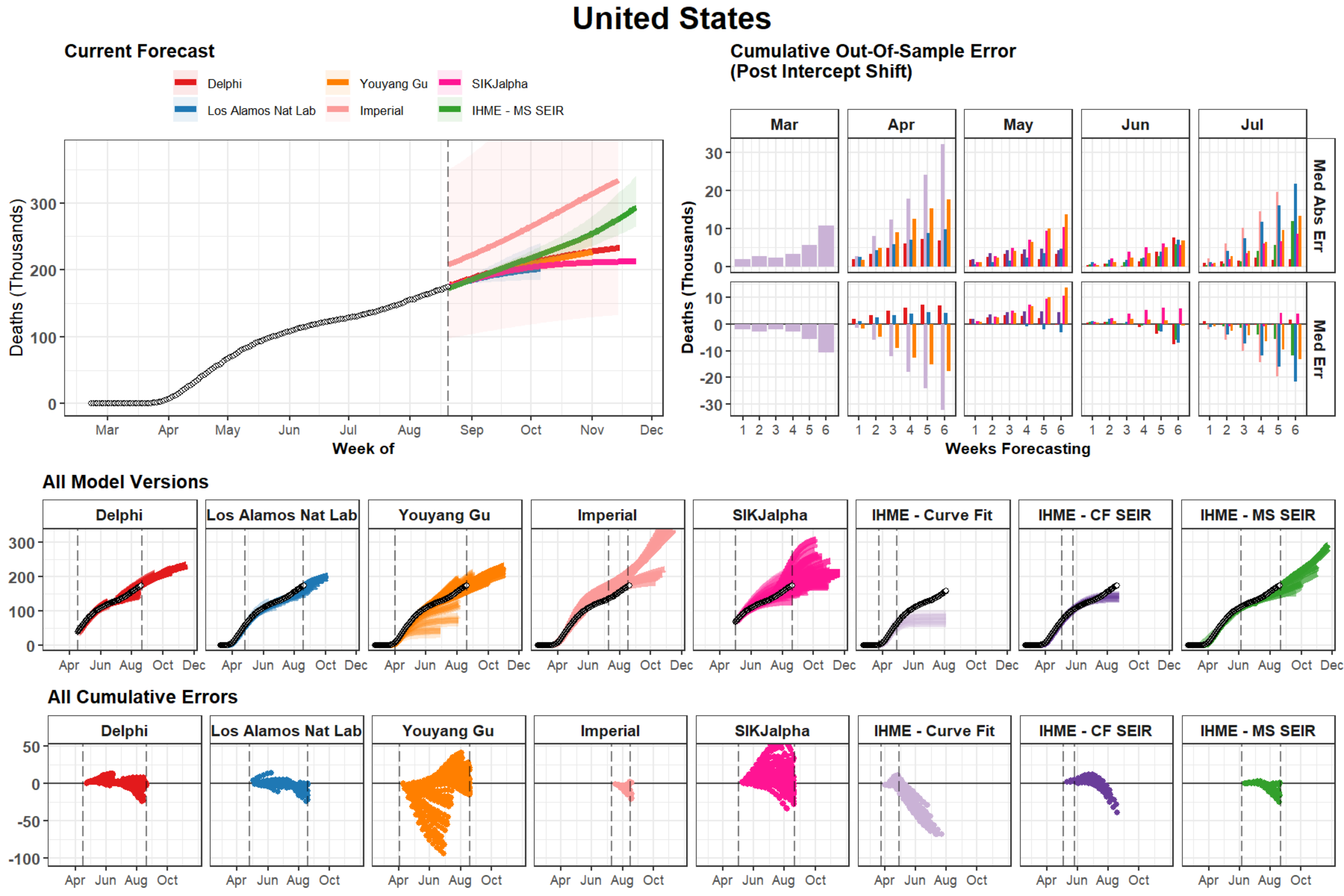
United States
Current Forecast
Delphi
Youyang Gu
SIKJalpha
Los Alamos Nat Lab
Imperial
IHME - MS SEIR
Deaths (Thousands)
300
200
100
0
Mar
Apr
May
Jun
Jul
Aug
Sep
Oct
Nov
Dec
Week of
Cumulative Out-Of-Sample Error
(Post Intercept Shift)
Mar
Apr
May
Jun
Jul
Med Abs Err
Med Err
30
20
10
0
-10
-20
-30
1 2 3 4 5 6
Weeks Forecasting
All Model Versions
Delphi
Los Alamos Nat Lab
Youyang Gu
Imperial
SIKJalpha
IHME - Curve Fit
IHME - CF SEIR
IHME - MS SEIR
Apr Jun Aug Oct Dec
All Cumulative Errors
50
0
-50
-100
Apr Jun Aug Oct

Brazil

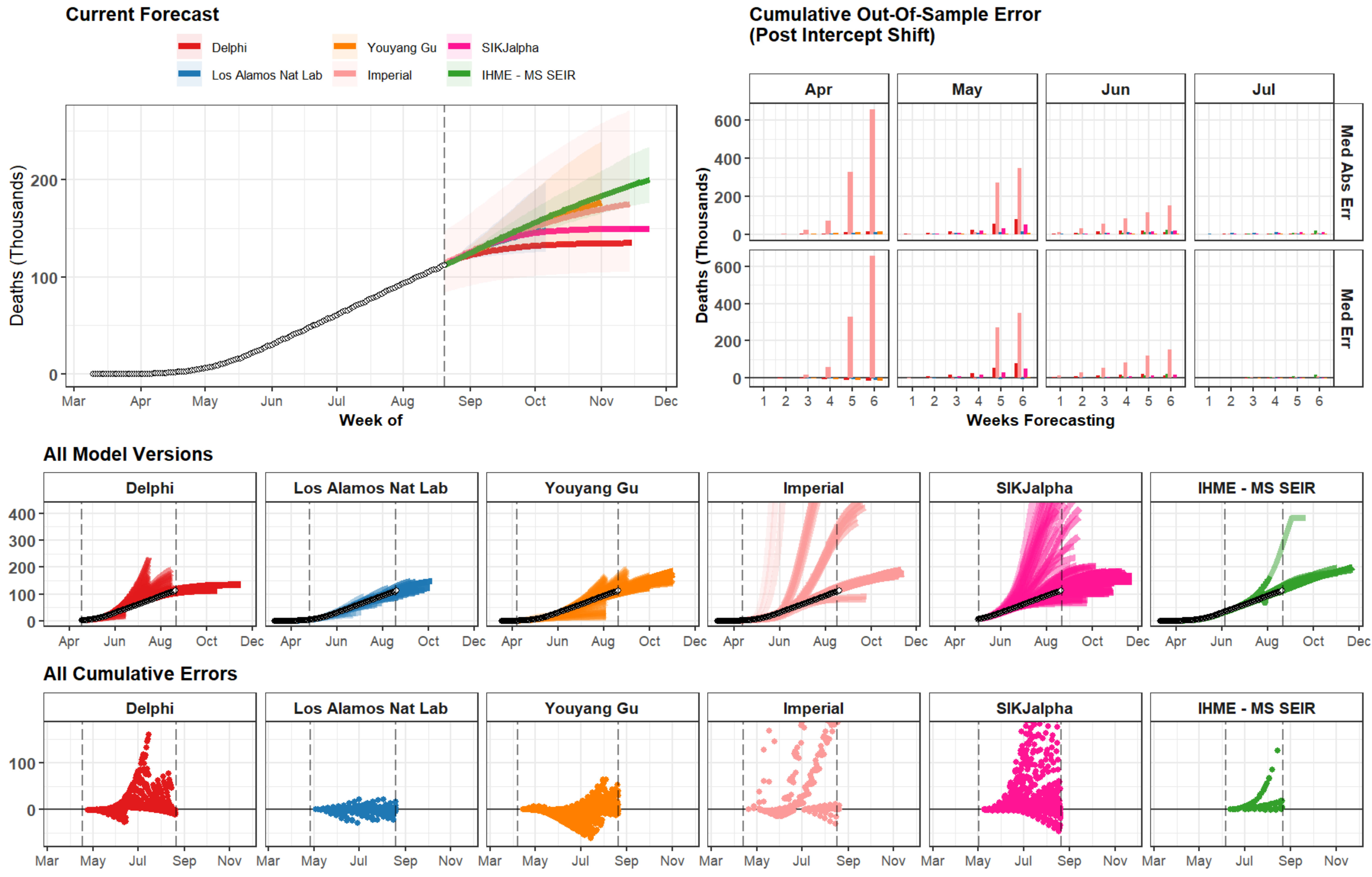
Current Forecast
Delphi
Youyang Gu
SIKJalpha
Los Alamos Nat Lab
Imperial
IHME - MS SEIR
Deaths (Thousands)
0
100
200
Mar
Apr
May
Jun
Jul
Aug
Sep
Oct
Nov
Dec
Week of
Cumulative Out-Of-Sample Error
(Post Intercept Shift)
Apr
May
Jun
Jul
Med Abs Err
Med Err
0
200
400
600
1
2
3
4
5
6
Weeks Forecasting
All Model Versions
Delphi
Los Alamos Nat Lab
Youyang Gu
Imperial
SIKJalpha
IHME - MS SEIR
0
100
200
300
400
Apr
Jun
Aug
Oct
Dec
All Cumulative Errors
Delphi
Los Alamos Nat Lab
Youyang Gu
Imperial
SIKJalpha
IHME - MS SEIR
0
100
Mar
May
Jul
Sep
Nov

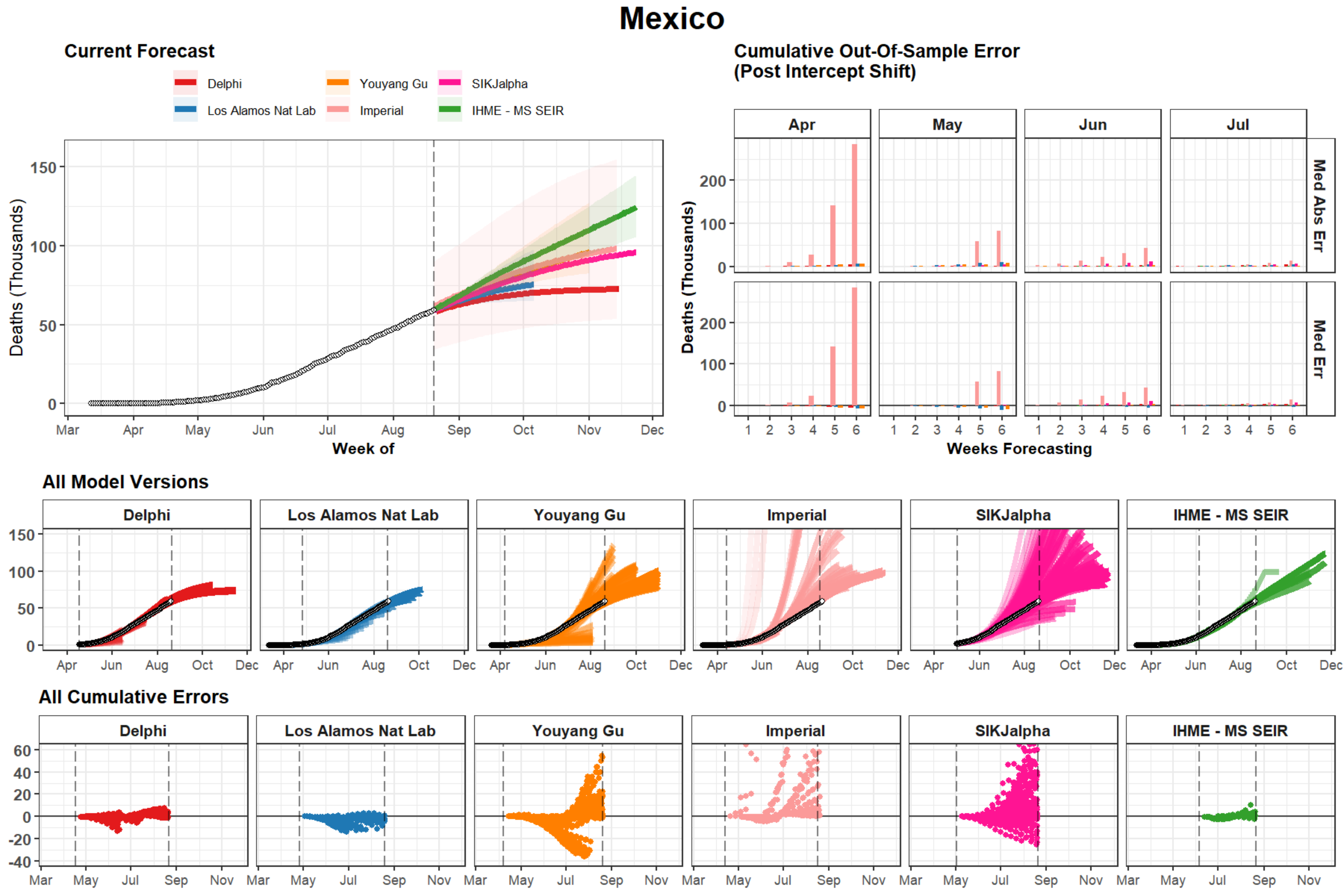

Mexico
Current Forecast
Delphi
Los Alamos Nat Lab
Youyang Gu
Imperial
SIKJalpha
IHME - MS SEIR
Deaths (Thousands)
Week of
Cumulative Out-Of-Sample Error
(Post Intercept Shift)
Apr
May
Jun
Jul
Med Abs Err
Med Err
Weeks Forecasting
All Model Versions
All Cumulative Errors

India

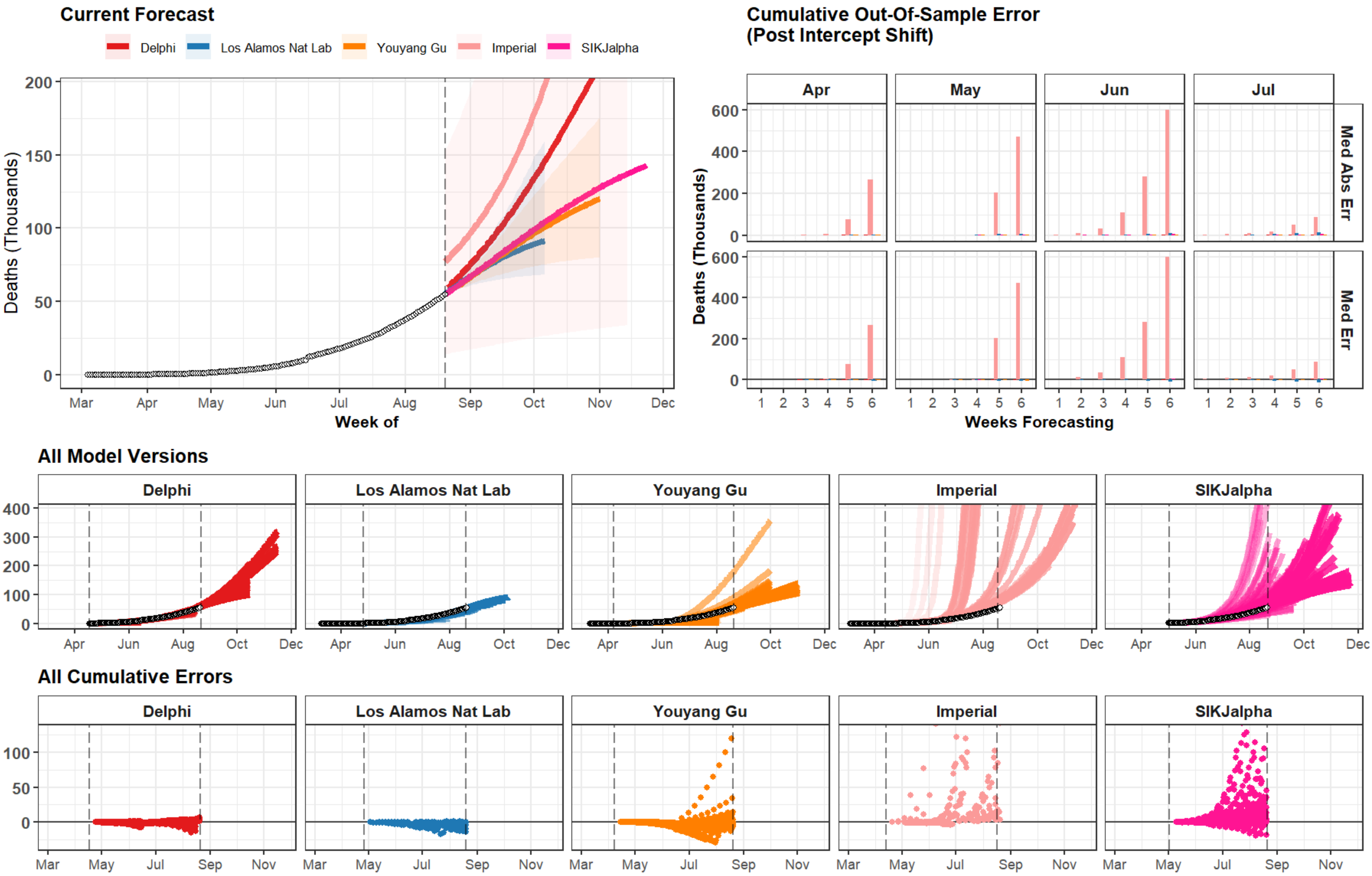
Current Forecast
Delphi
Los Alamos Nat Lab
Youyang Gu
Imperial
SIKJalpha
Deaths (Thousands)
Week of
Mar
Apr
May
Jun
Jul
Aug
Sep
Oct
Nov
Dec
Cumulative Out-Of-Sample Error
(Post Intercept Shift)
Med Abs Err
Med Err
Weeks Forecasting
All Model Versions
All Cumulative Errors

United Kingdom

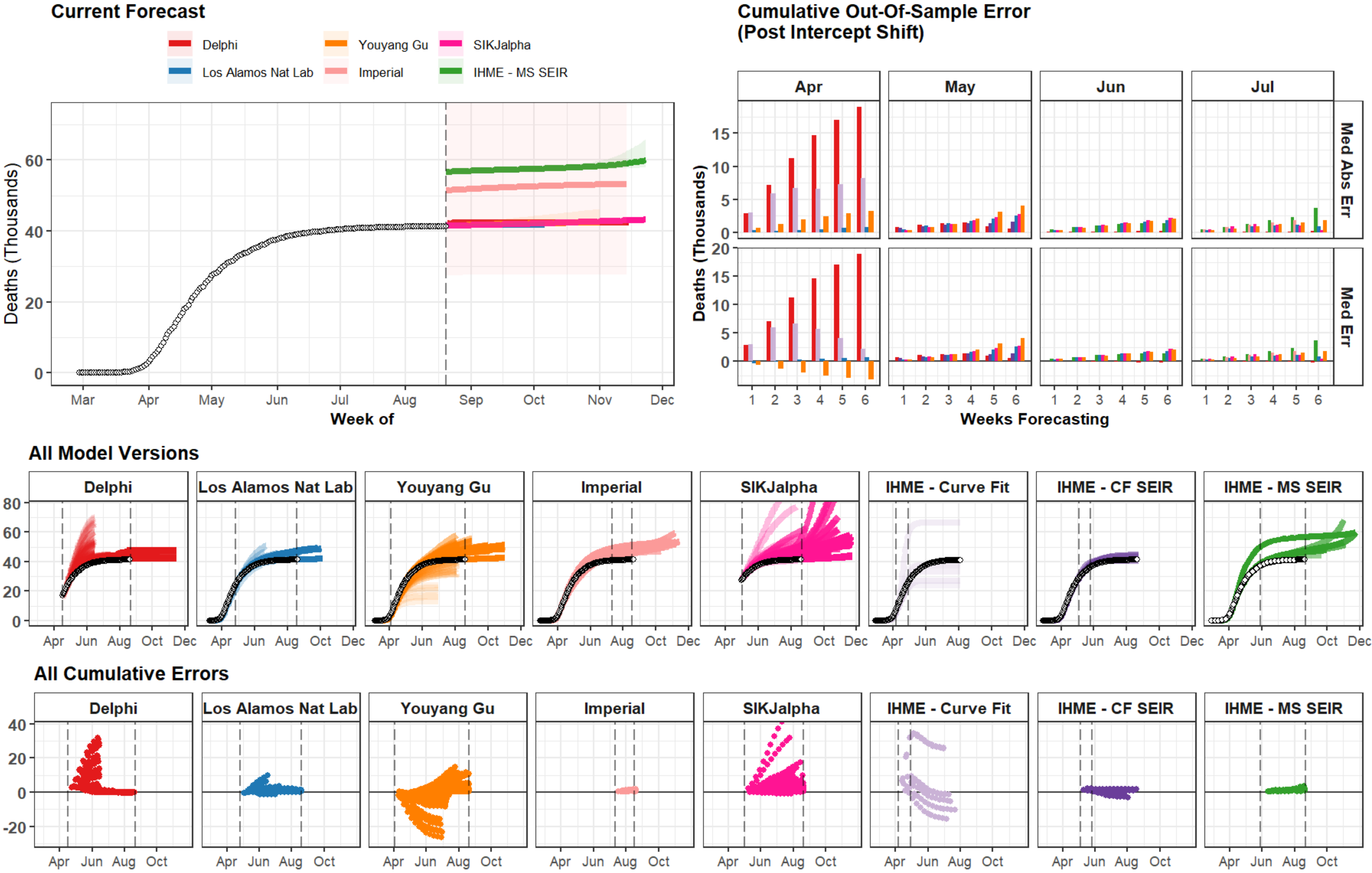

Italy

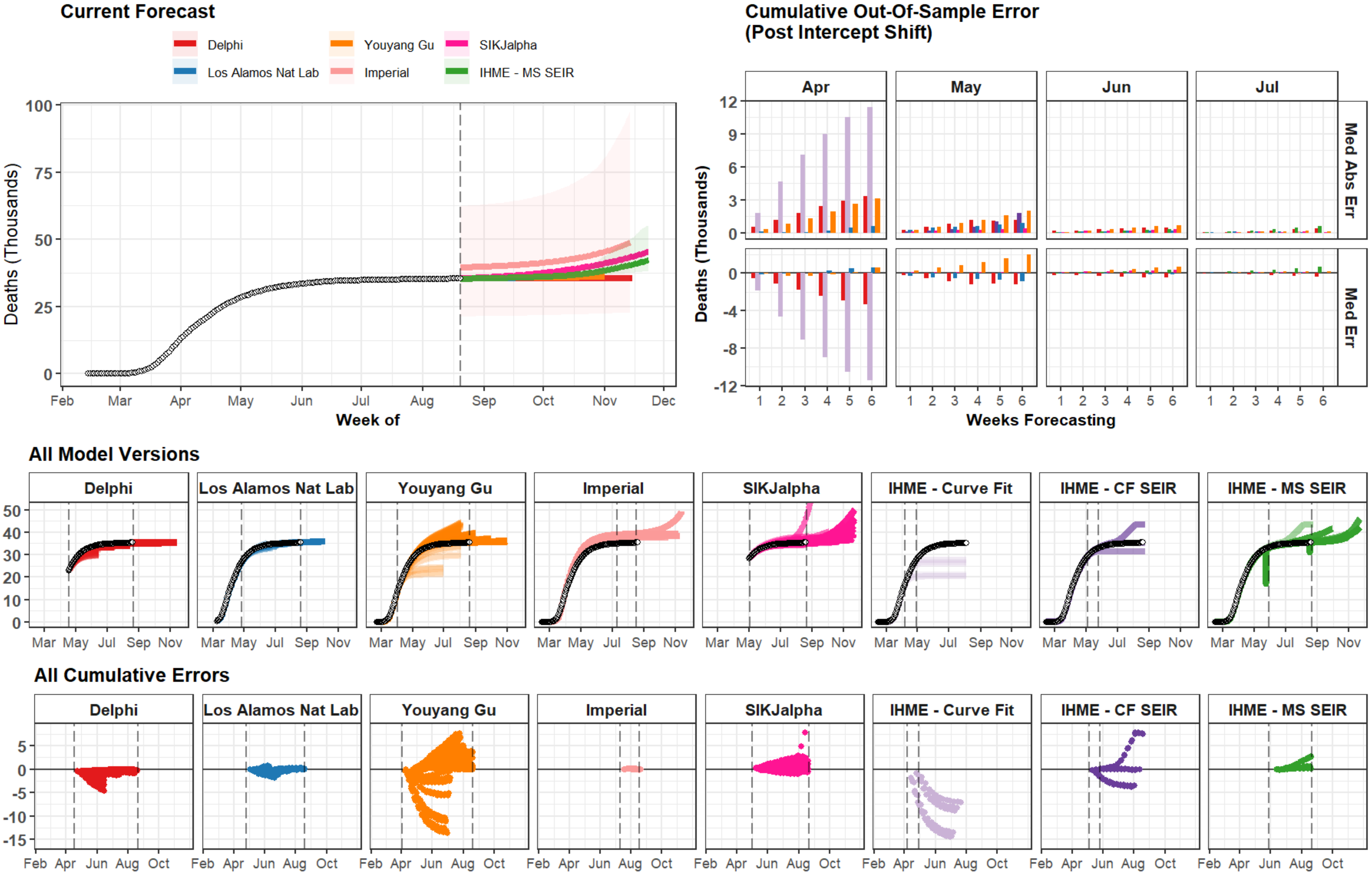
Current Forecast
Delphi
Youyang Gu
SIKJalpha
Los Alamos Nat Lab
Imperial
IHME - MS SEIR
Deaths (Thousands)
Week of
Feb Mar Apr May Jun Jul Aug Sep Oct Nov Dec
Cumulative Out-Of-Sample Error
(Post Intercept Shift)
Apr May Jun Jul
Med Abs Err
Med Err
Weeks Forecasting
All Model Versions
Delphi
Los Alamos Nat Lab
Youyang Gu
Imperial
SIKJalpha
IHME - Curve Fit
IHME - CF SEIR
IHME - MS SEIR
All Cumulative Errors

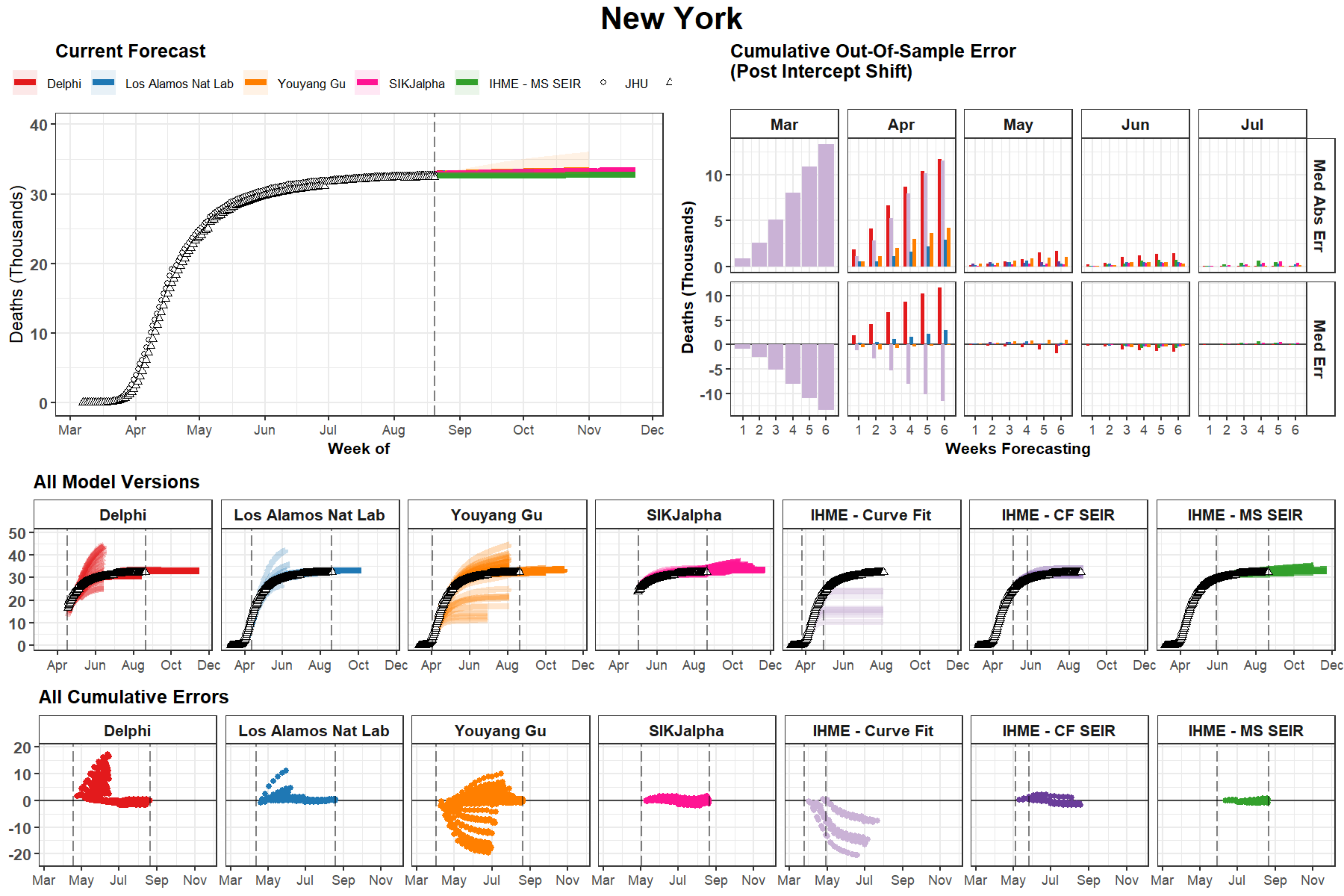
New York
Current Forecast
Delphi
Los Alamos Nat Lab
Youyang Gu
SIKJalpha
IHME - MS SEIR
JHU
Deaths (Thousands)
Week of
Mar
Apr
May
Jun
Jul
Aug
Sep
Oct
Nov
Dec
Cumulative Out-Of-Sample Error
(Post Intercept Shift)
Med Abs Err
Med Err
Weeks Forecasting
All Model Versions
IHME - Curve Fit
IHME - CF SEIR
All Cumulative Errors

France

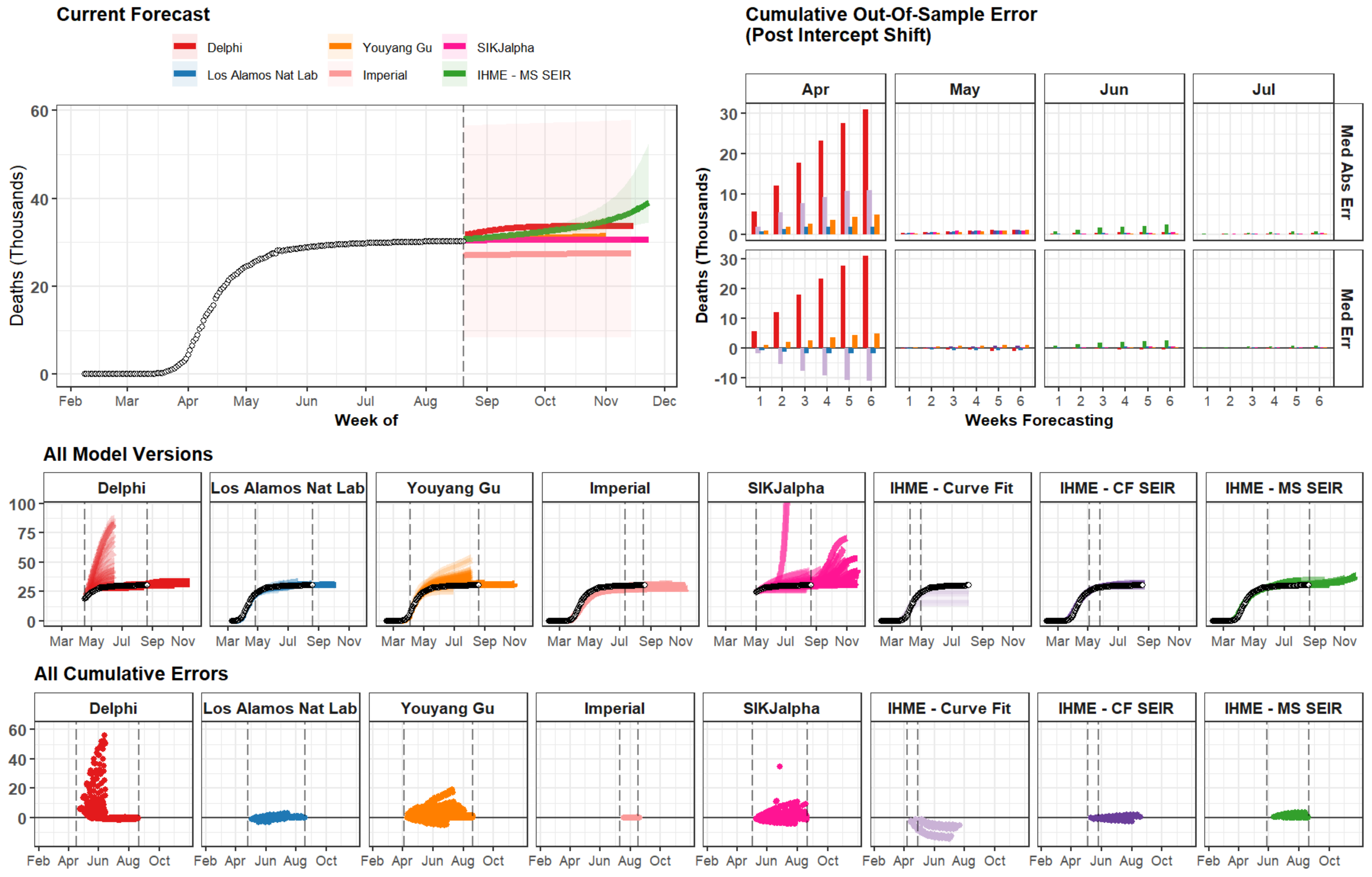
Current Forecast
Delphi
Los Alamos Nat Lab
Youyang Gu
Imperial
SIKJalpha
IHME - MS SEIR
Deaths (Thousands)
Week of
Feb Mar Apr May Jun Jul Aug Sep Oct Nov Dec
Cumulative Out-Of-Sample Error
(Post Intercept Shift)
Apr
May
Jun
Jul
Med Abs Err
Med Err
Weeks Forecasting
All Model Versions
Delphi
Los Alamos Nat Lab
Youyang Gu
Imperial
SIKJalpha
IHME - Curve Fit
IHME - CF SEIR
IHME - MS SEIR
Mar May Jul Sep Nov
All Cumulative Errors
Feb Apr Jun Aug Oct

Spain

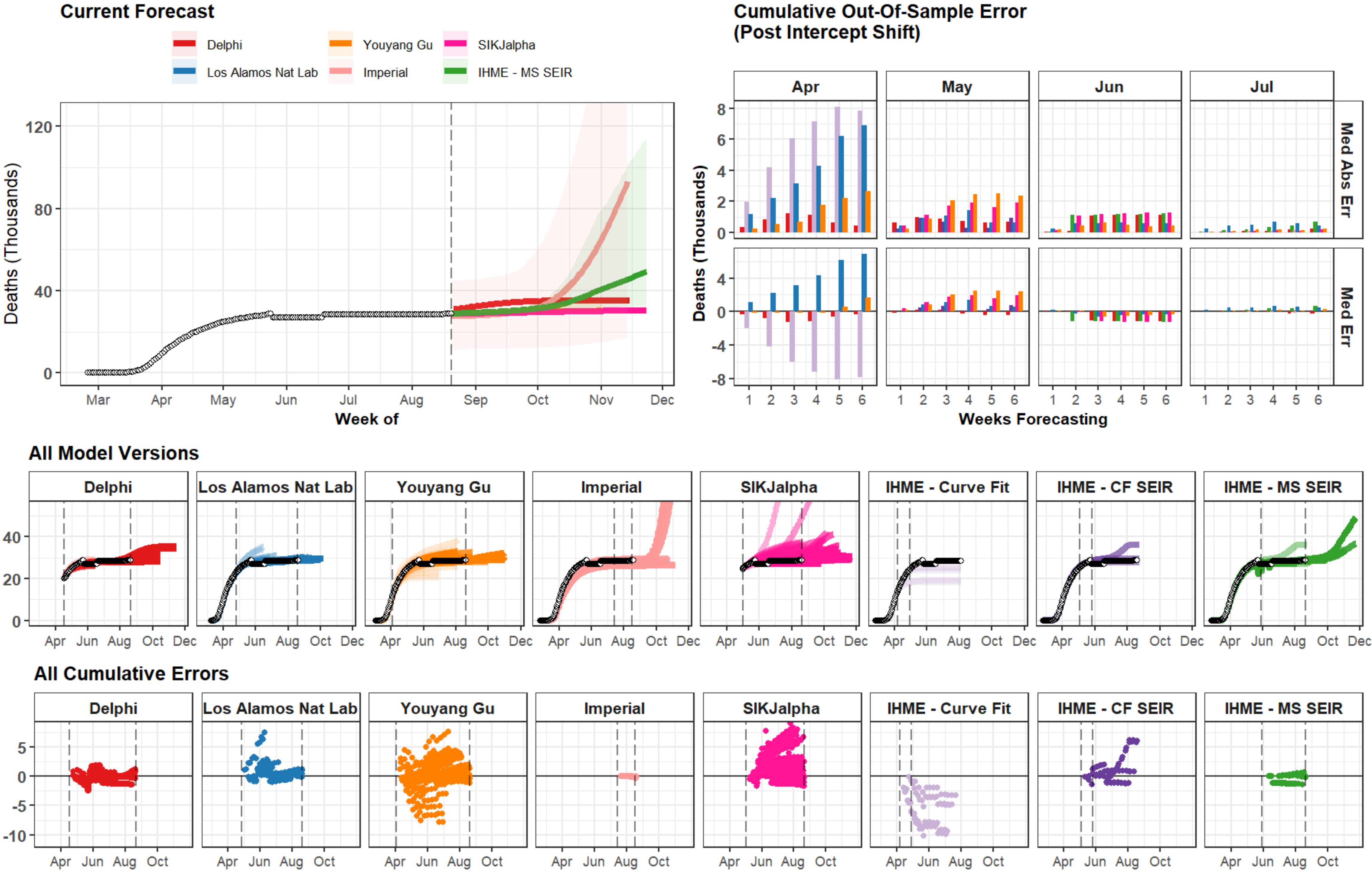

Current Forecast
Delphi
Los Alamos Nat Lab
Youyang Gu
Imperial
SIKJalpha
IHME - MS SEIR
Deaths (Thousands)
Week of
Cumulative Out-Of-Sample Error
(Post Intercept Shift)
Apr
May
Jun
Jul
Med Abs Err
Med Err
Weeks Forecasting
All Model Versions
Delphi
Los Alamos Nat Lab
Youyang Gu
Imperial
SIKJalpha
IHME - Curve Fit
IHME - CF SEIR
IHME - MS SEIR
All Cumulative Errors

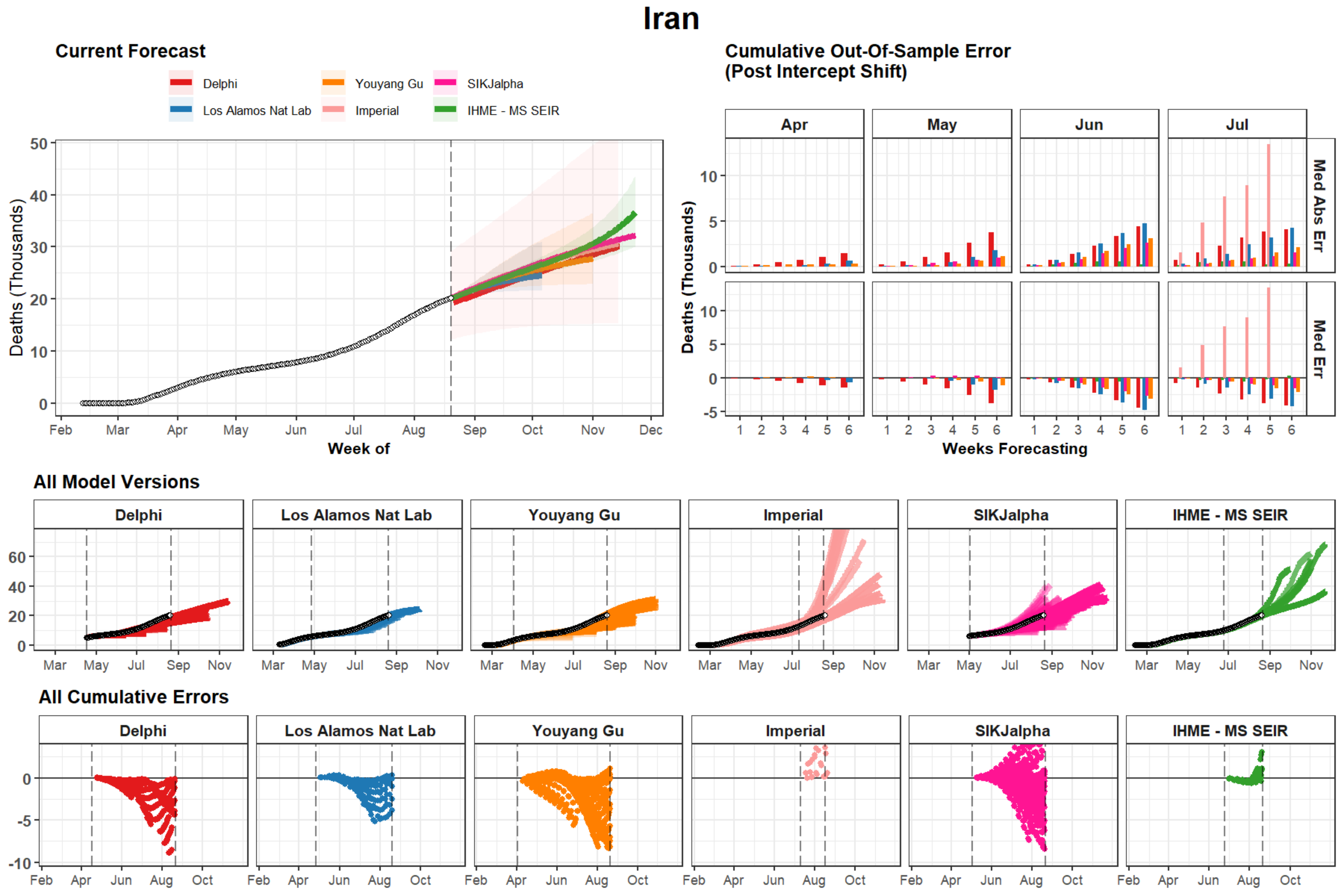

Iran
Current Forecast
Delphi
Los Alamos Nat Lab
Youyang Gu
Imperial
SIKJalpha
IHME - MS SEIR
Deaths (Thousands)
Week of
Feb
Mar
Apr
May
Jun
Jul
Aug
Sep
Oct
Nov
Dec
Cumulative Out-Of-Sample Error
(Post Intercept Shift)
Apr
May
Jun
Jul
Med Abs Err
Med Err
Weeks Forecasting
All Model Versions
All Cumulative Errors

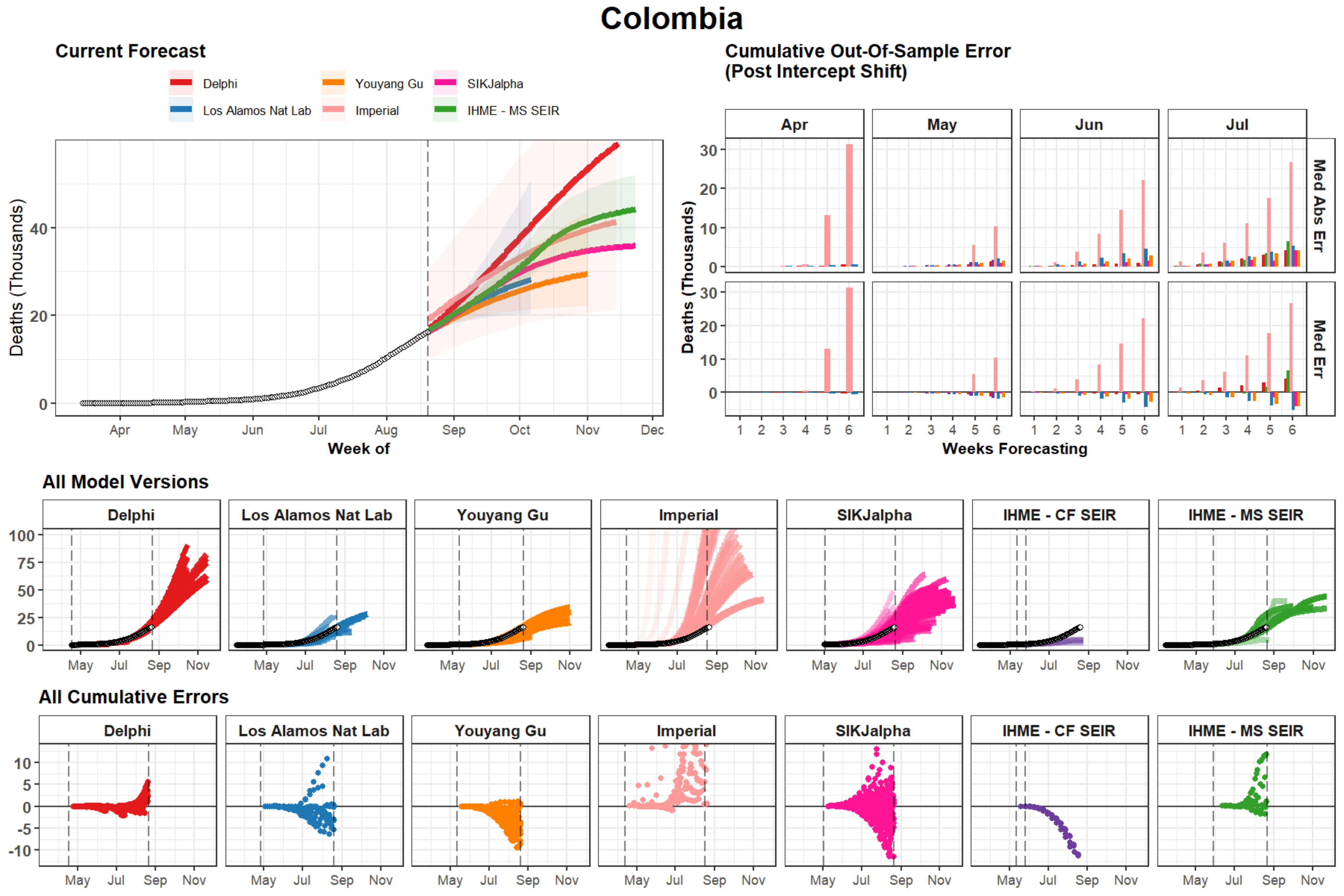
Colombia
Current Forecast
Delphi
Los Alamos Nat Lab
Youyang Gu
Imperial
SIKJalpha
IHME - MS SEIR
Deaths (Thousands)
Week of
Apr
May
Jun
Jul
Aug
Sep
Oct
Nov
Dec
Cumulative Out-Of-Sample Error
(Post Intercept Shift)
Med Abs Err
Med Err
Weeks Forecasting
All Model Versions
IHME - CF SEIR
All Cumulative Errors

Russian Federation

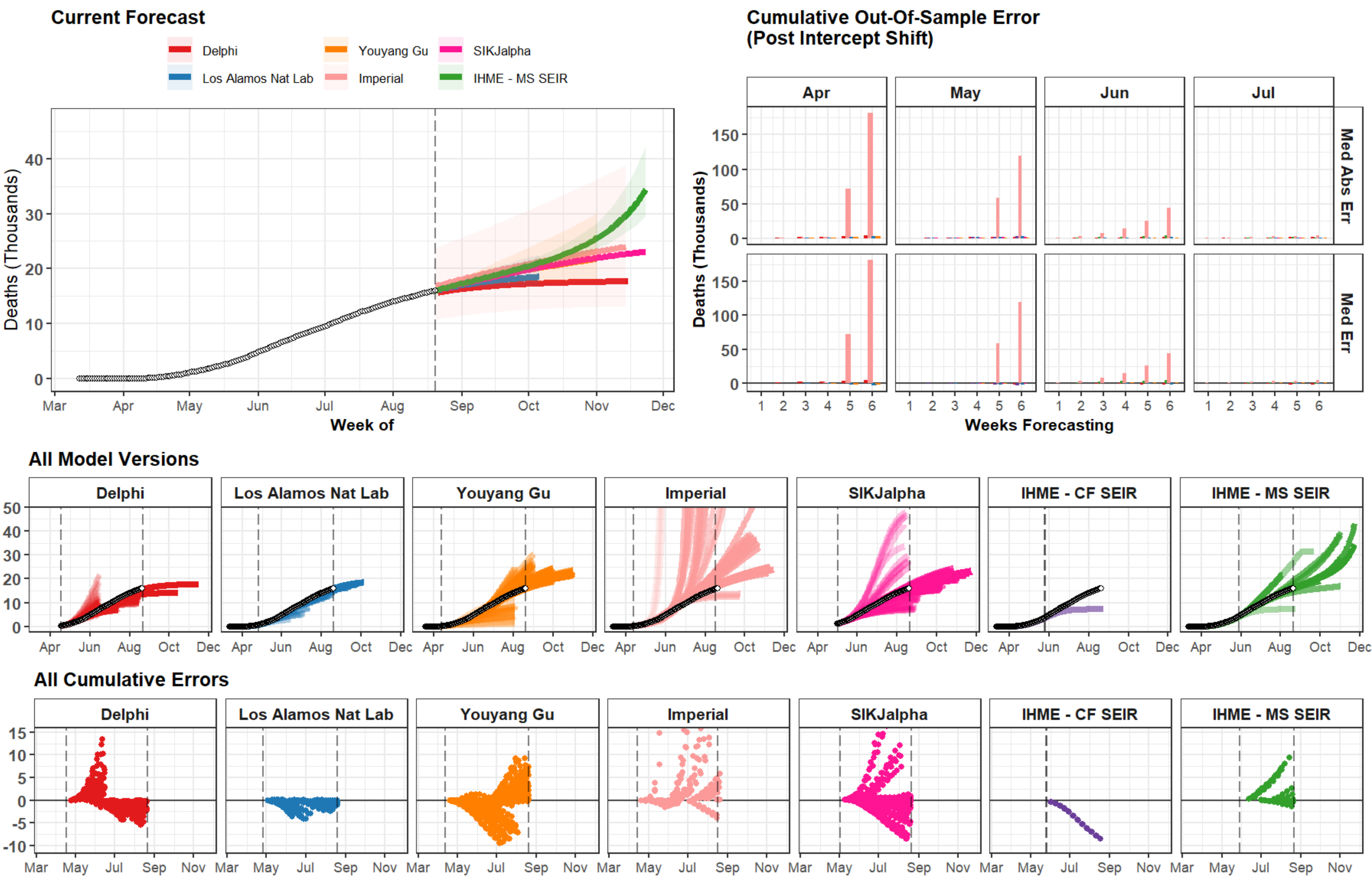

New Jersey

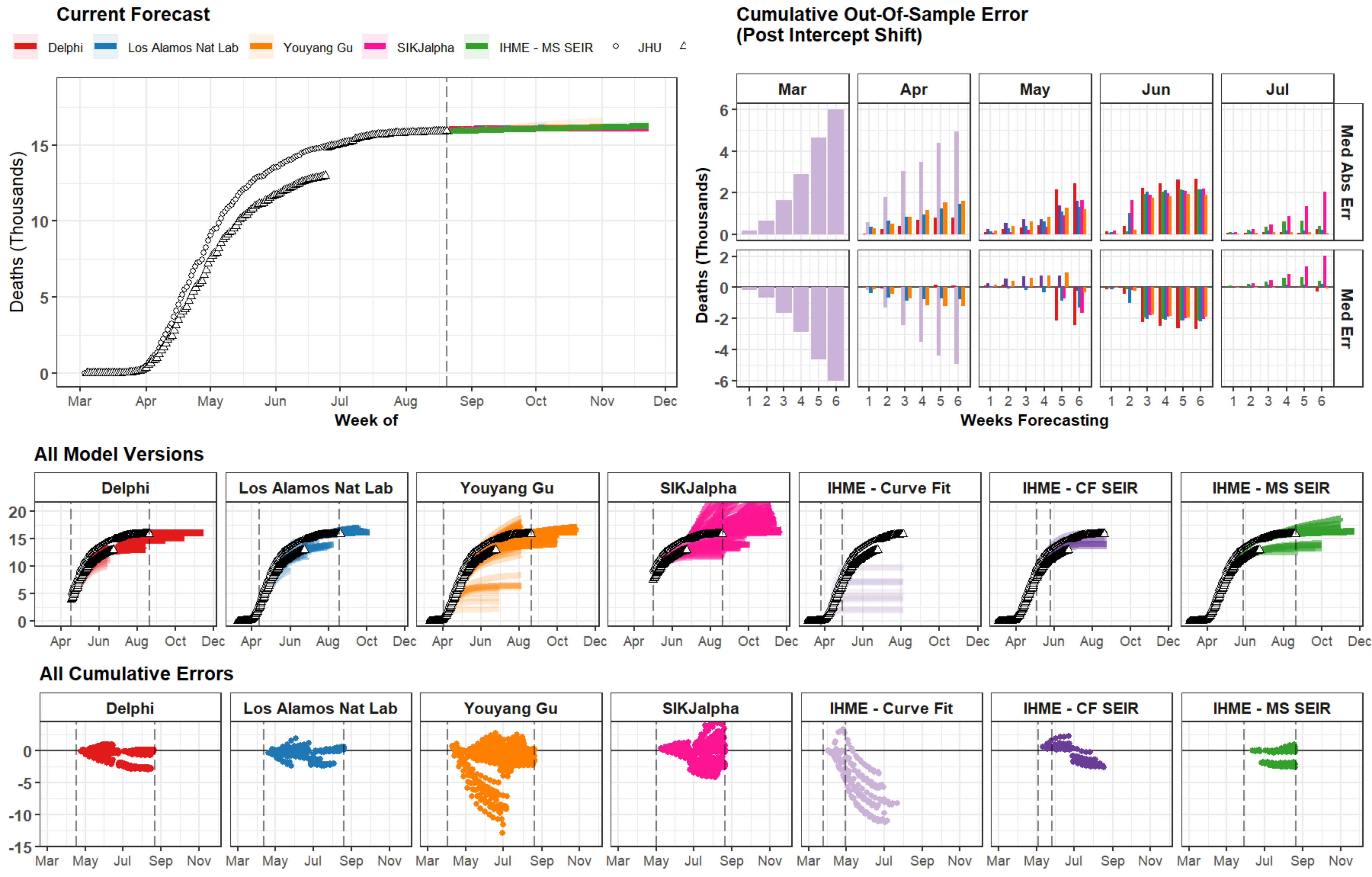
Current Forecast
Delphi
Los Alamos Nat Lab
Youyang Gu
SIKJalpha
IHME - MS SEIR
JHU
Deaths (Thousands)
Week of
Mar
Apr
May
Jun
Jul
Aug
Sep
Oct
Nov
Dec
Cumulative Out-Of-Sample Error
(Post Intercept Shift)
Med Abs Err
Med Err
Weeks Forecasting
All Model Versions
IHME - Curve Fit
IHME - CF SEIR
All Cumulative Errors

South Africa

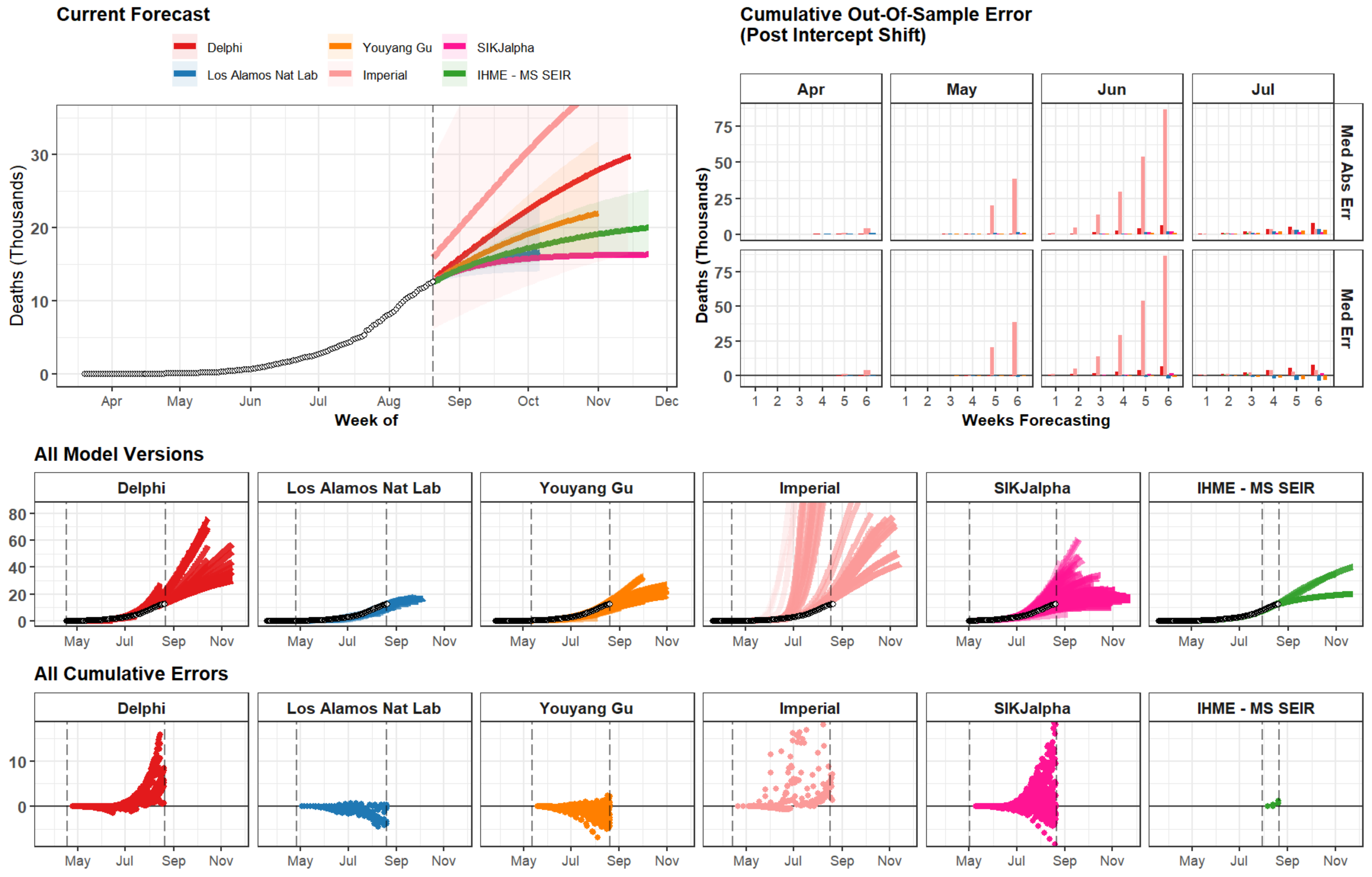

California

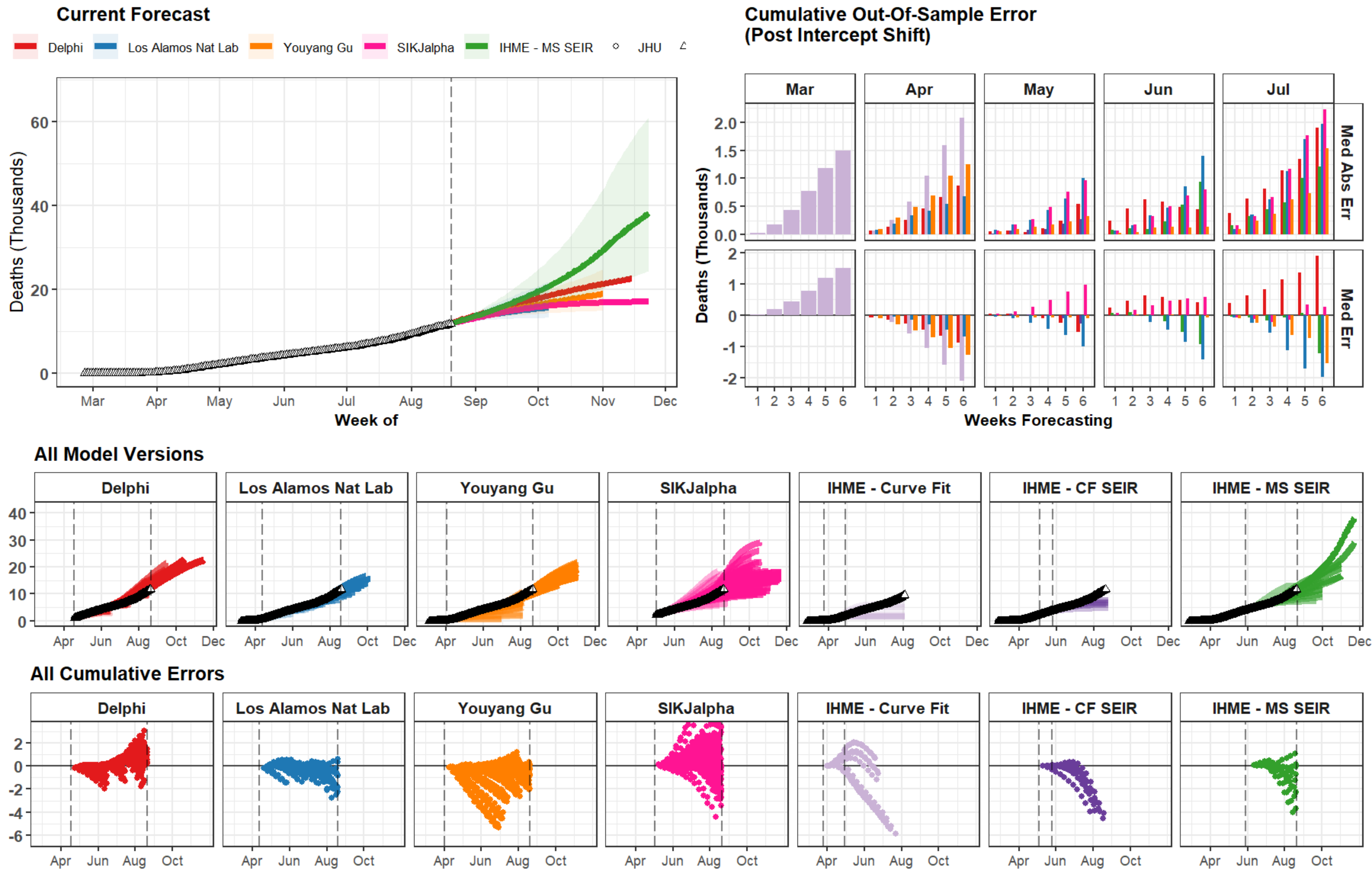
Current Forecast
Delphi
Los Alamos Nat Lab
Youyang Gu
SIKJalpha
IHME - MS SEIR
JHU
Deaths (Thousands)
0
20
40
60
Mar
Apr
May
Jun
Jul
Aug
Sep
Oct
Nov
Dec
Week of
Cumulative Out-Of-Sample Error
(Post Intercept Shift)
Mar
Apr
May
Jun
Jul
Med Abs Err
Med Err
Deaths (Thousands)
0.0
0.5
1.0
1.5
2.0
-2
-1
0
1
2
1 2 3 4 5 6
Weeks Forecasting
All Model Versions
Delphi
Los Alamos Nat Lab
Youyang Gu
SIKJalpha
IHME - Curve Fit
IHME - CF SEIR
IHME - MS SEIR
0
10
20
30
40
Apr
Jun
Aug
Oct
Dec
All Cumulative Errors
Delphi
Los Alamos Nat Lab
Youyang Gu
SIKJalpha
IHME - Curve Fit
IHME - CF SEIR
IHME - MS SEIR
-6
-4
-2
0
2
Apr
Jun
Aug
Oct

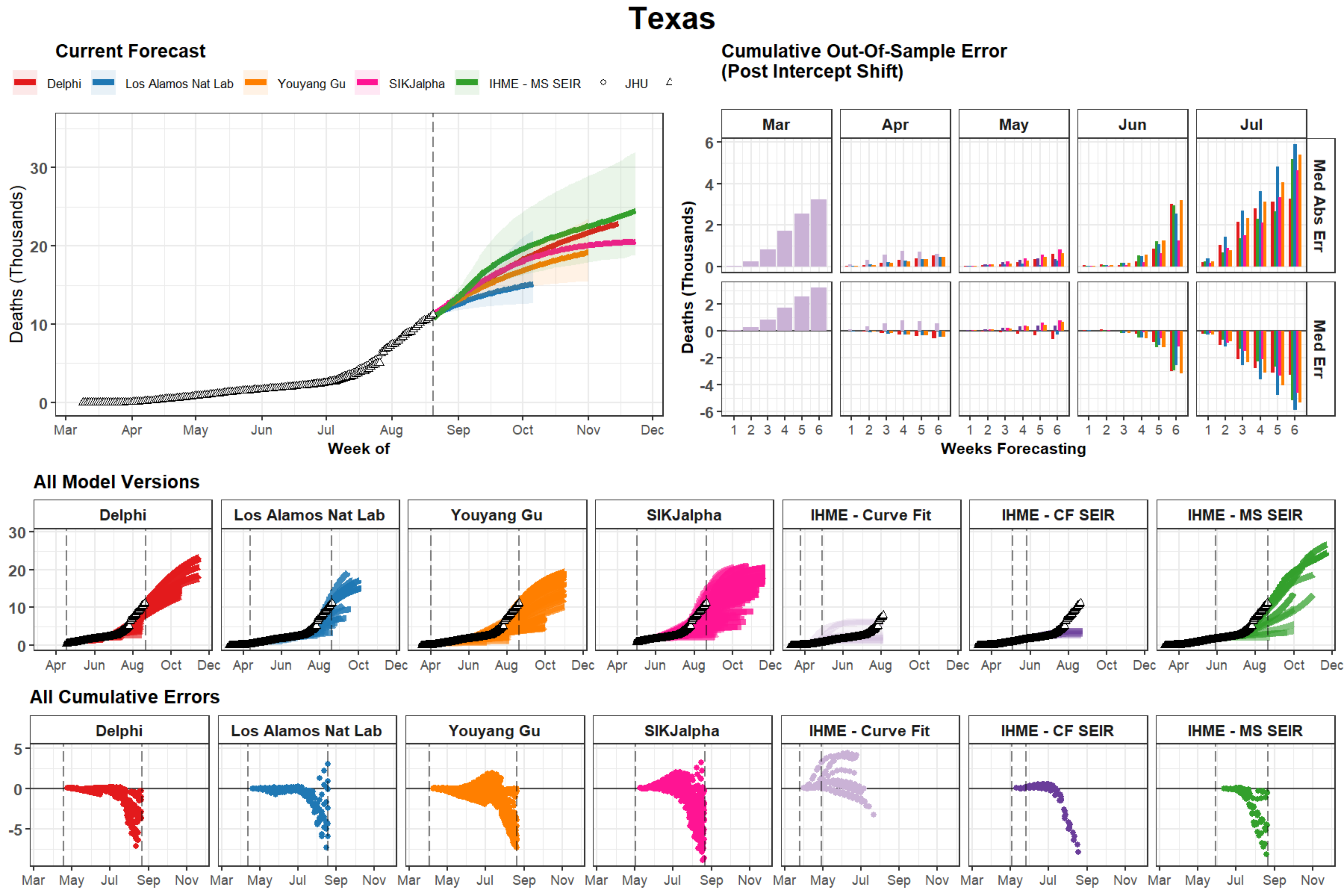

Texas
Current Forecast
Delphi
Los Alamos Nat Lab
Youyang Gu
SIKJalpha
IHME - MS SEIR
JHU
Deaths (Thousands)
Week of
Mar
Apr
May
Jun
Jul
Aug
Sep
Oct
Nov
Dec
Cumulative Out-Of-Sample Error
(Post Intercept Shift)
Med Abs Err
Med Err
Weeks Forecasting
All Model Versions
IHME - Curve Fit
IHME - CF SEIR
All Cumulative Errors

Chile

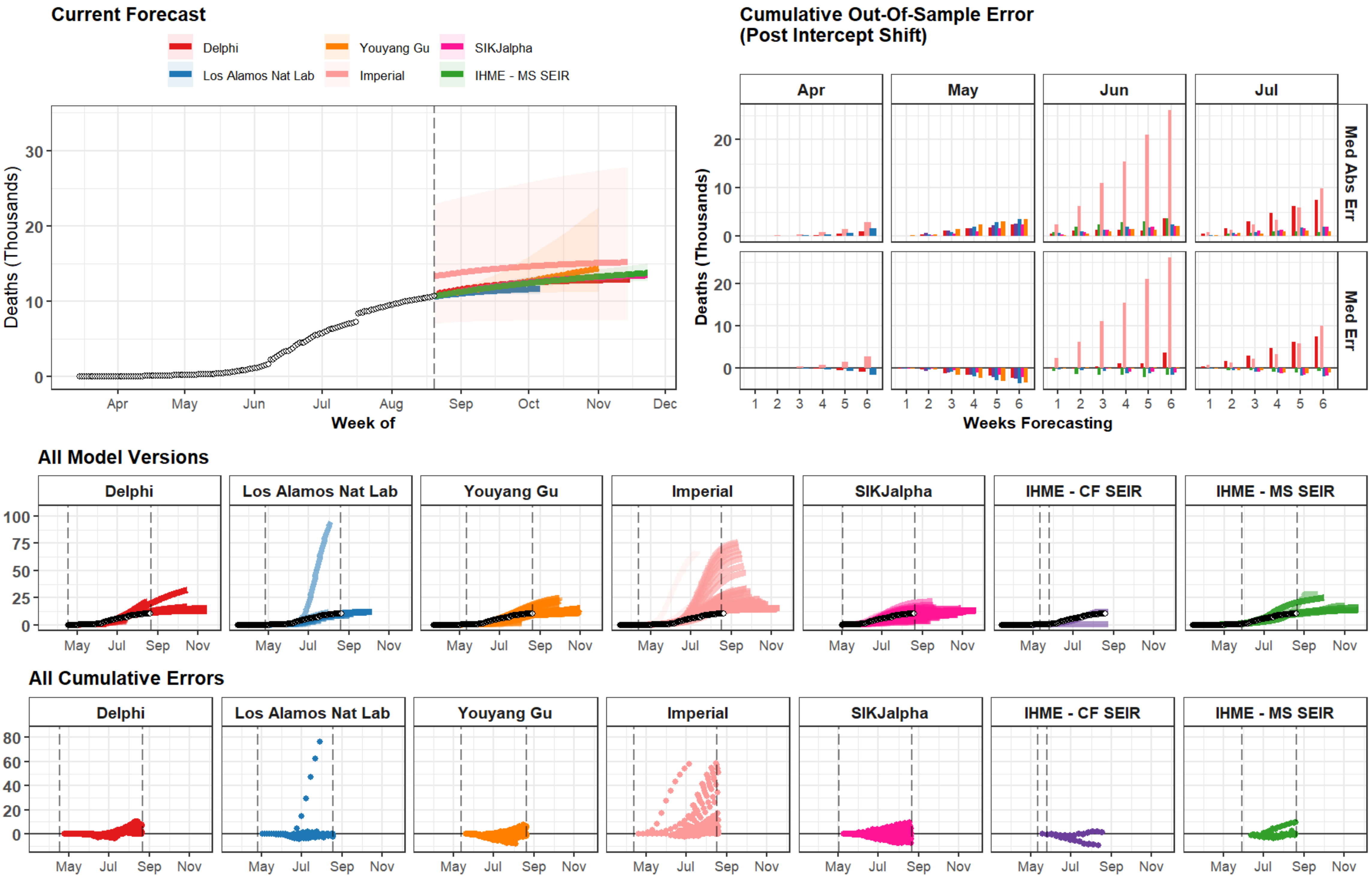

Current Forecast
Delphi
Los Alamos Nat Lab
Youyang Gu
Imperial
SIKJalpha
IHME - MS SEIR
Deaths (Thousands)
Week of
Apr
May
Jun
Jul
Aug
Sep
Oct
Nov
Dec
Cumulative Out-Of-Sample Error
(Post Intercept Shift)
Med Abs Err
Med Err
Weeks Forecasting
All Model Versions
IHME - CF SEIR
All Cumulative Errors

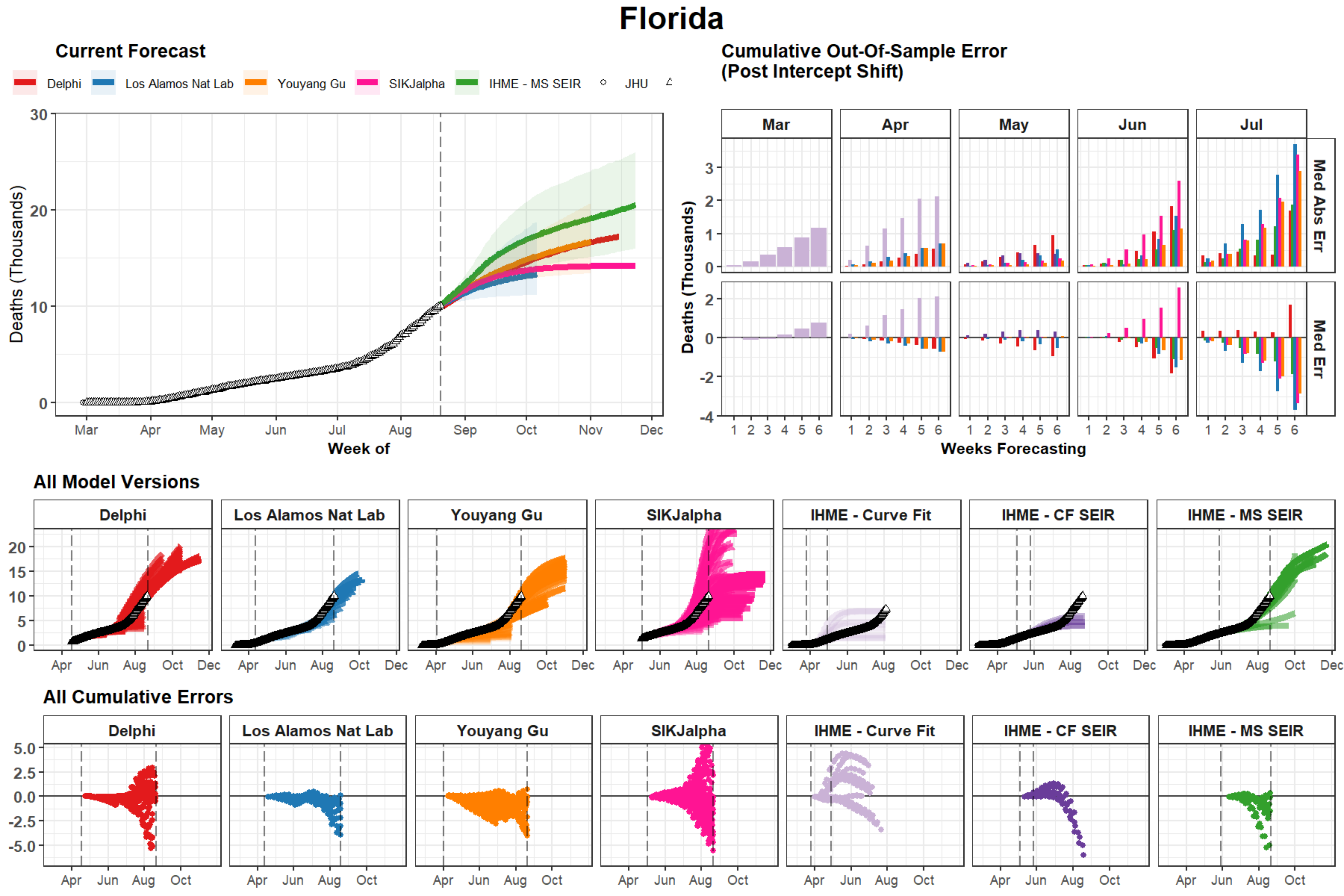
Florida
Current Forecast
Delphi
Los Alamos Nat Lab
Youyang Gu
SIKJalpha
IHME - MS SEIR
JHU
Deaths (Thousands)
Week of
Mar
Apr
May
Jun
Jul
Aug
Sep
Oct
Nov
Dec
Cumulative Out-Of-Sample Error
(Post Intercept Shift)
Med Abs Err
Med Err
Weeks Forecasting
All Model Versions
IHME - Curve Fit
IHME - CF SEIR
All Cumulative Errors

Belgium

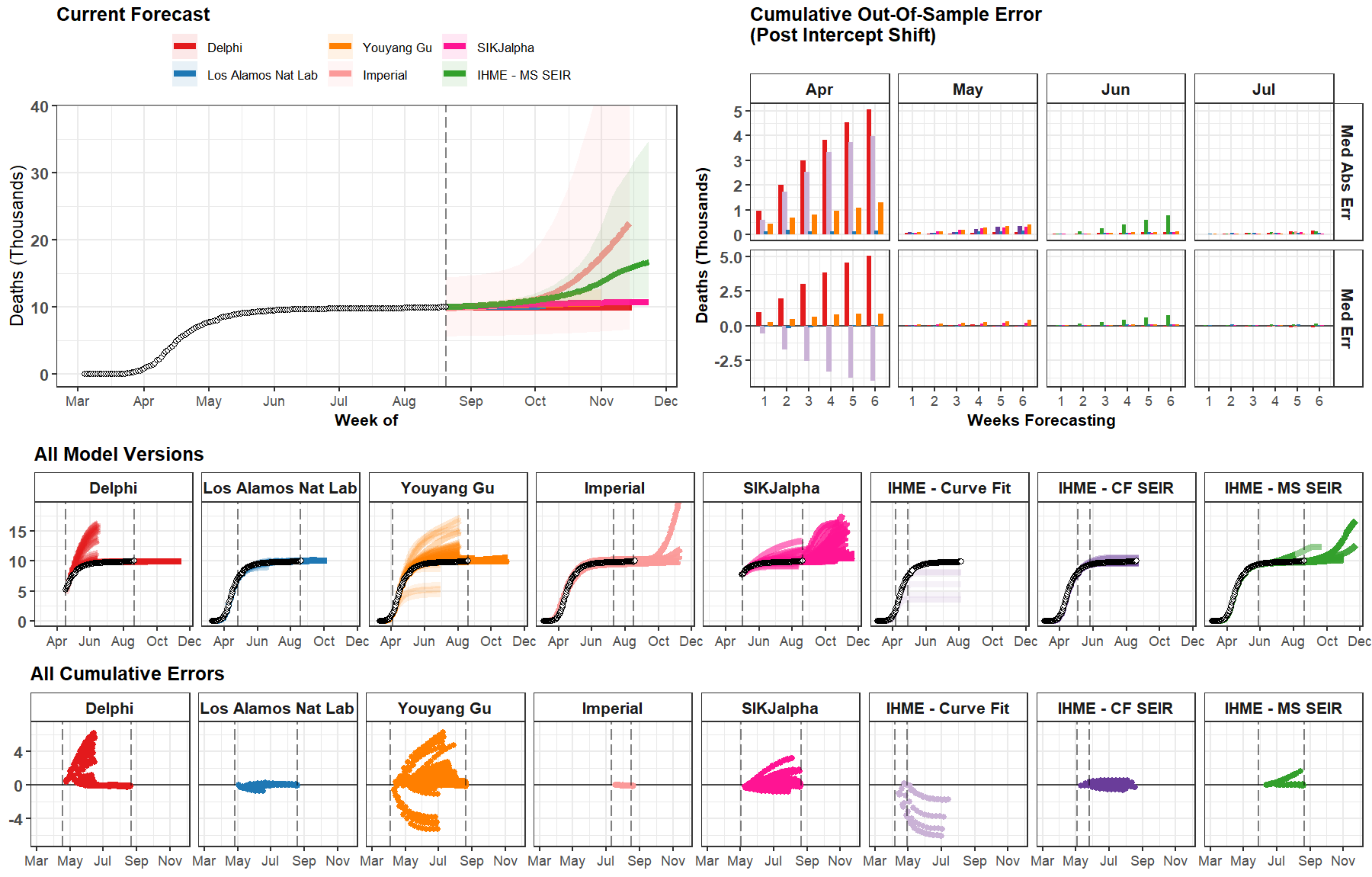
Current Forecast
Delphi
Los Alamos Nat Lab
Youyang Gu
Imperial
SIKJalpha
IHME - MS SEIR
Deaths (Thousands)
Week of
Mar Apr May Jun Jul Aug Sep Oct Nov Dec
Cumulative Out-Of-Sample Error
(Post Intercept Shift)
Apr May Jun Jul
Med Abs Err
Med Err
Weeks Forecasting
All Model Versions
Delphi
Los Alamos Nat Lab
Youyang Gu
Imperial
SIKJalpha
IHME - Curve Fit
IHME - CF SEIR
IHME - MS SEIR
All Cumulative Errors

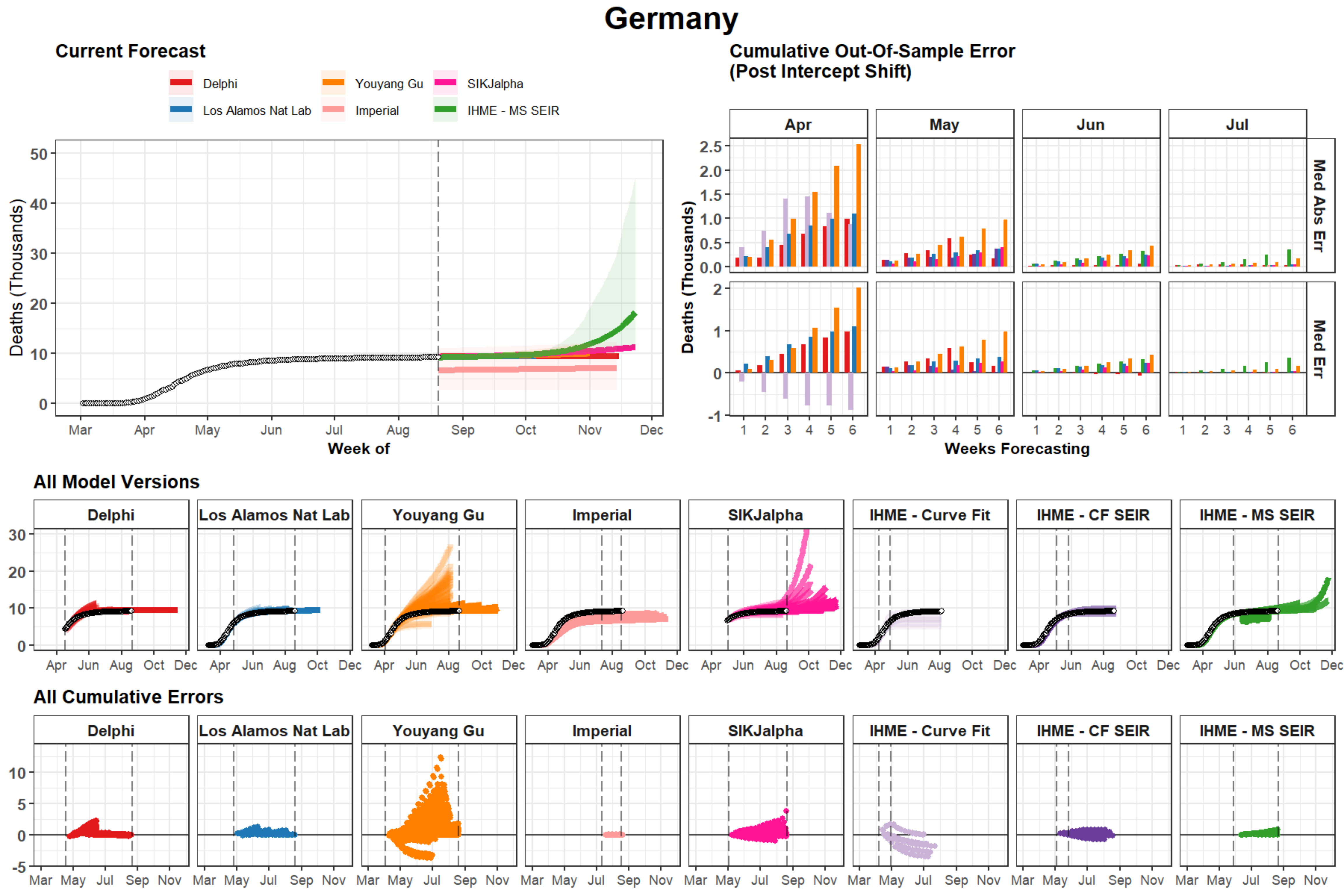

Germany
Current Forecast
Delphi
Los Alamos Nat Lab
Youyang Gu
Imperial
SIKJalpha
IHME - MS SEIR
Deaths (Thousands)
Week of
Mar Apr May Jun Jul Aug Sep Oct Nov Dec
Cumulative Out-Of-Sample Error
(Post Intercept Shift)
Apr May Jun Jul
Med Abs Err
Med Err
Weeks Forecasting
All Model Versions
Delphi
Los Alamos Nat Lab
Youyang Gu
Imperial
SIKJalpha
IHME - Curve Fit
IHME - CF SEIR
IHME - MS SEIR
All Cumulative Errors

Canada

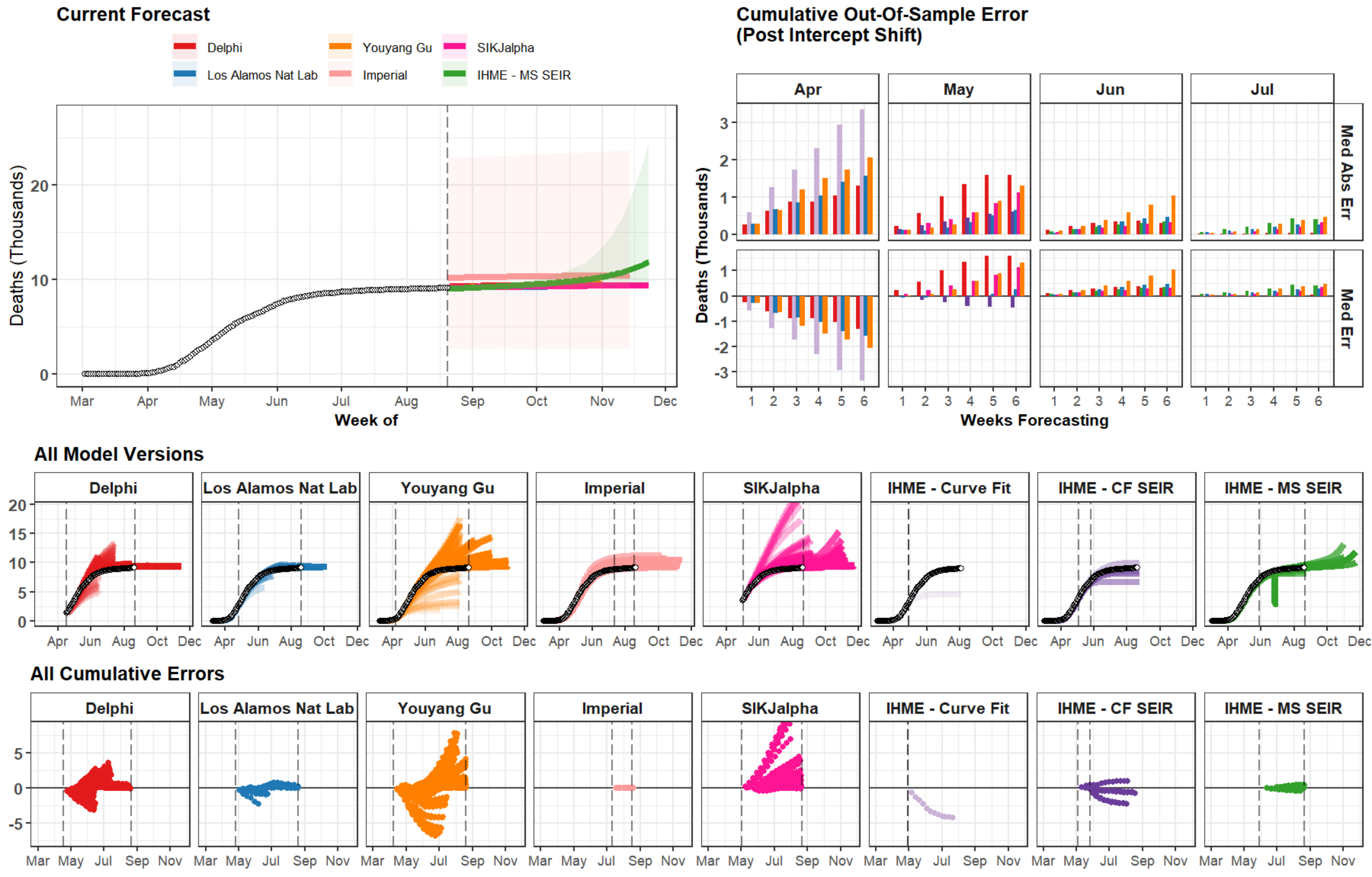

Current Forecast
Delphi
Youyang Gu
SIKJalpha
Los Alamos Nat Lab
Imperial
IHME - MS SEIR
Deaths (Thousands)
Week of
Mar
Apr
May
Jun
Jul
Aug
Sep
Oct
Nov
Dec
Cumulative Out-Of-Sample Error
(Post Intercept Shift)
Apr
May
Jun
Jul
Med Abs Err
Med Err
Weeks Forecasting
All Model Versions
Delphi
Los Alamos Nat Lab
Youyang Gu
Imperial
SIKJalpha
IHME - Curve Fit
IHME - CF SEIR
IHME - MS SEIR
All Cumulative Errors

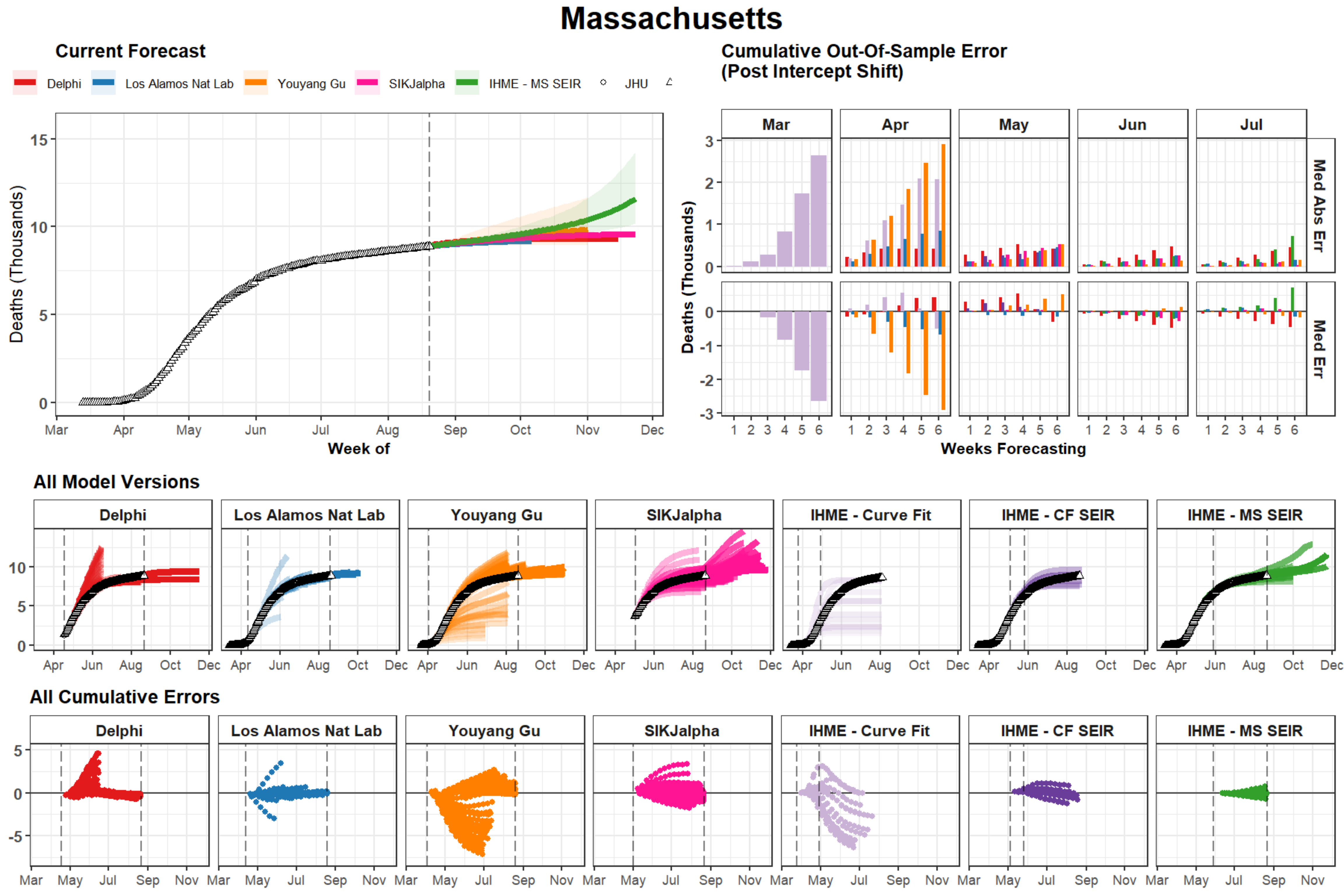

Massachusetts
Current Forecast
Delphi
Los Alamos Nat Lab
Youyang Gu
SIKJalpha
IHME - MS SEIR
JHU
Deaths (Thousands)
Week of
Cumulative Out-Of-Sample Error
(Post Intercept Shift)
Mar
Apr
May
Jun
Jul
Med Abs Err
Med Err
Weeks Forecasting
All Model Versions
IHME - Curve Fit
IHME - CF SEIR
All Cumulative Errors

Illinois

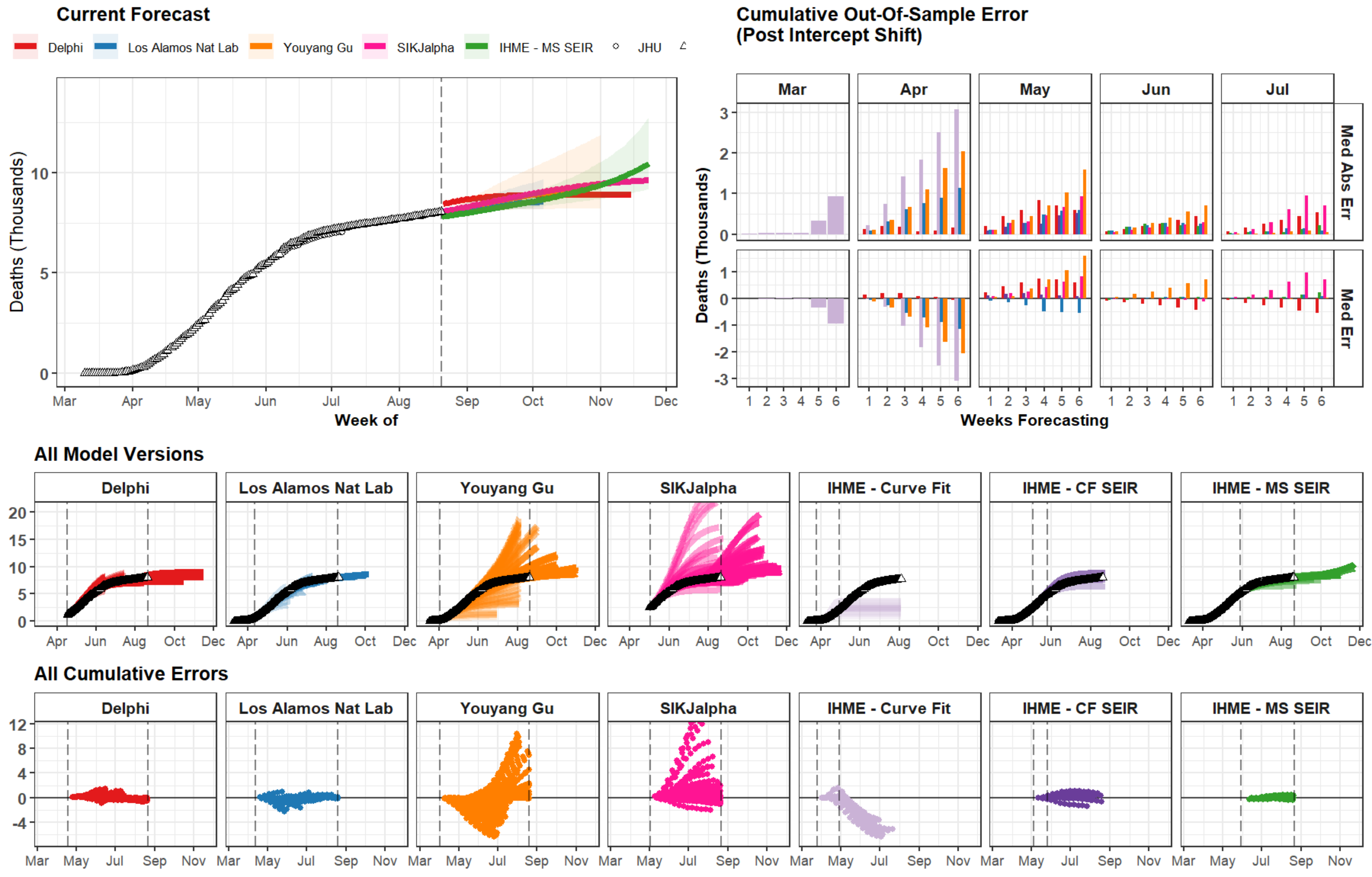
Current Forecast
Delphi
Los Alamos Nat Lab
Youyang Gu
SIKJalpha
IHME - MS SEIR
JHU
Deaths (Thousands)
Week of
Mar
Apr
May
Jun
Jul
Aug
Sep
Oct
Nov
Dec
Cumulative Out-Of-Sample Error
(Post Intercept Shift)
Med Abs Err
Med Err
Weeks Forecasting
All Model Versions
IHME - Curve Fit
IHME - CF SEIR
All Cumulative Errors

Pennsylvania

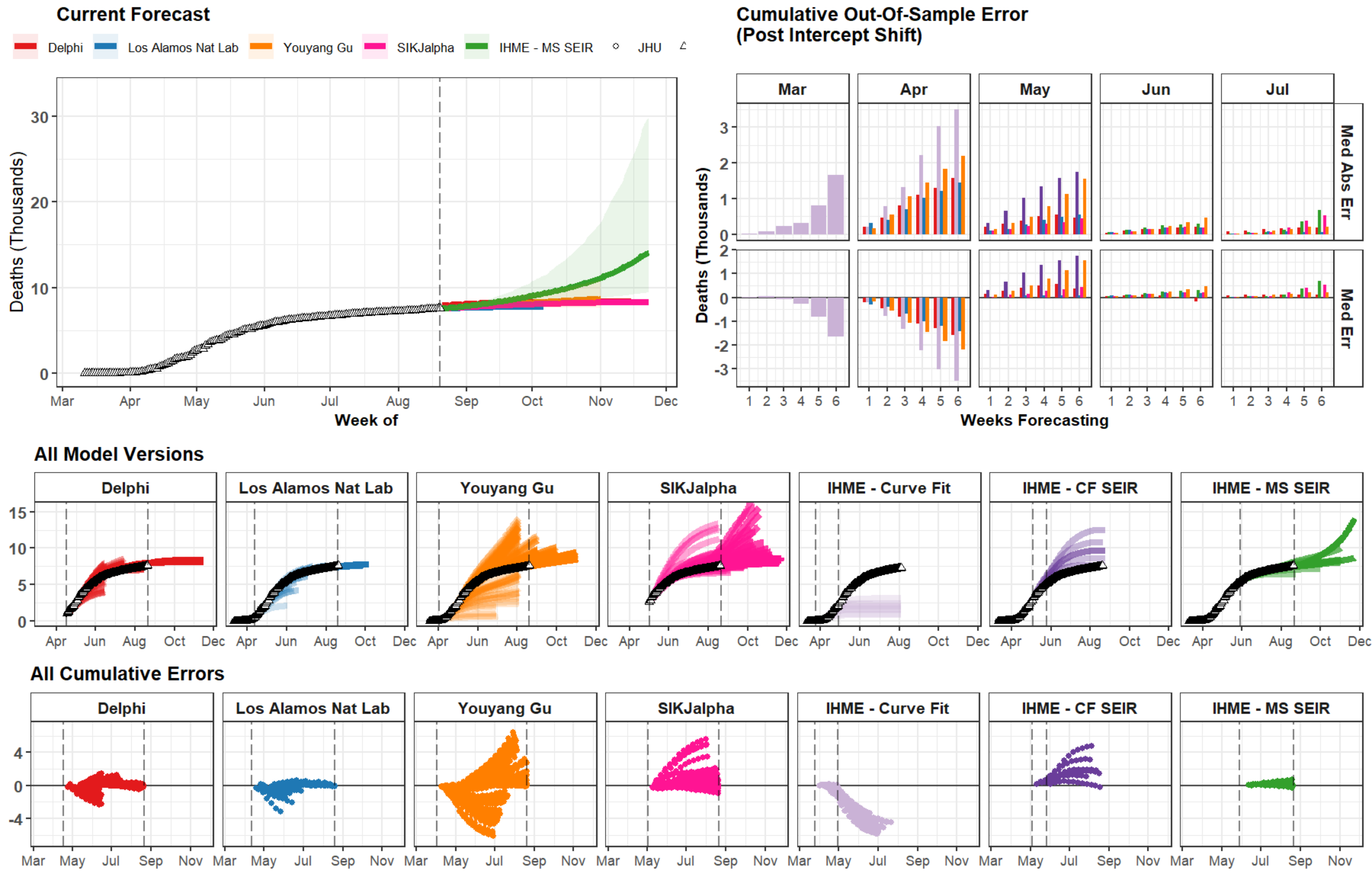

Michigan

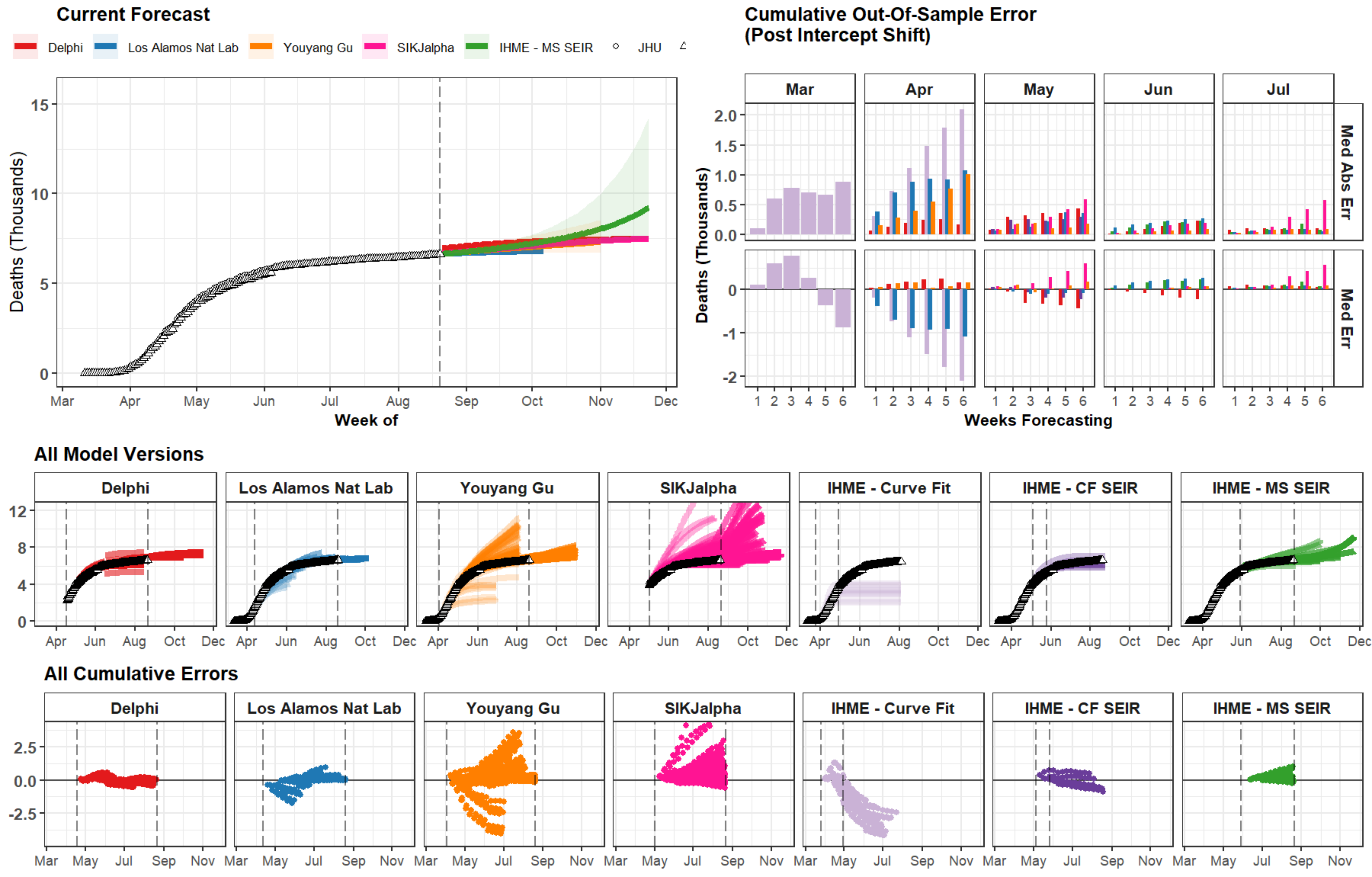
Current Forecast
Delphi
Los Alamos Nat Lab
Youyang Gu
SIKJalpha
IHME - MS SEIR
JHU
Deaths (Thousands)
Week of
Mar Apr May Jun Jul Aug Sep Oct Nov Dec
Cumulative Out-Of-Sample Error
(Post Intercept Shift)
Mar Apr May Jun Jul
Med Abs Err
Med Err
Weeks Forecasting
All Model Versions
Delphi
Los Alamos Nat Lab
Youyang Gu
SIKJalpha
IHME - Curve Fit
IHME - CF SEIR
IHME - MS SEIR
All Cumulative Errors

Argentina

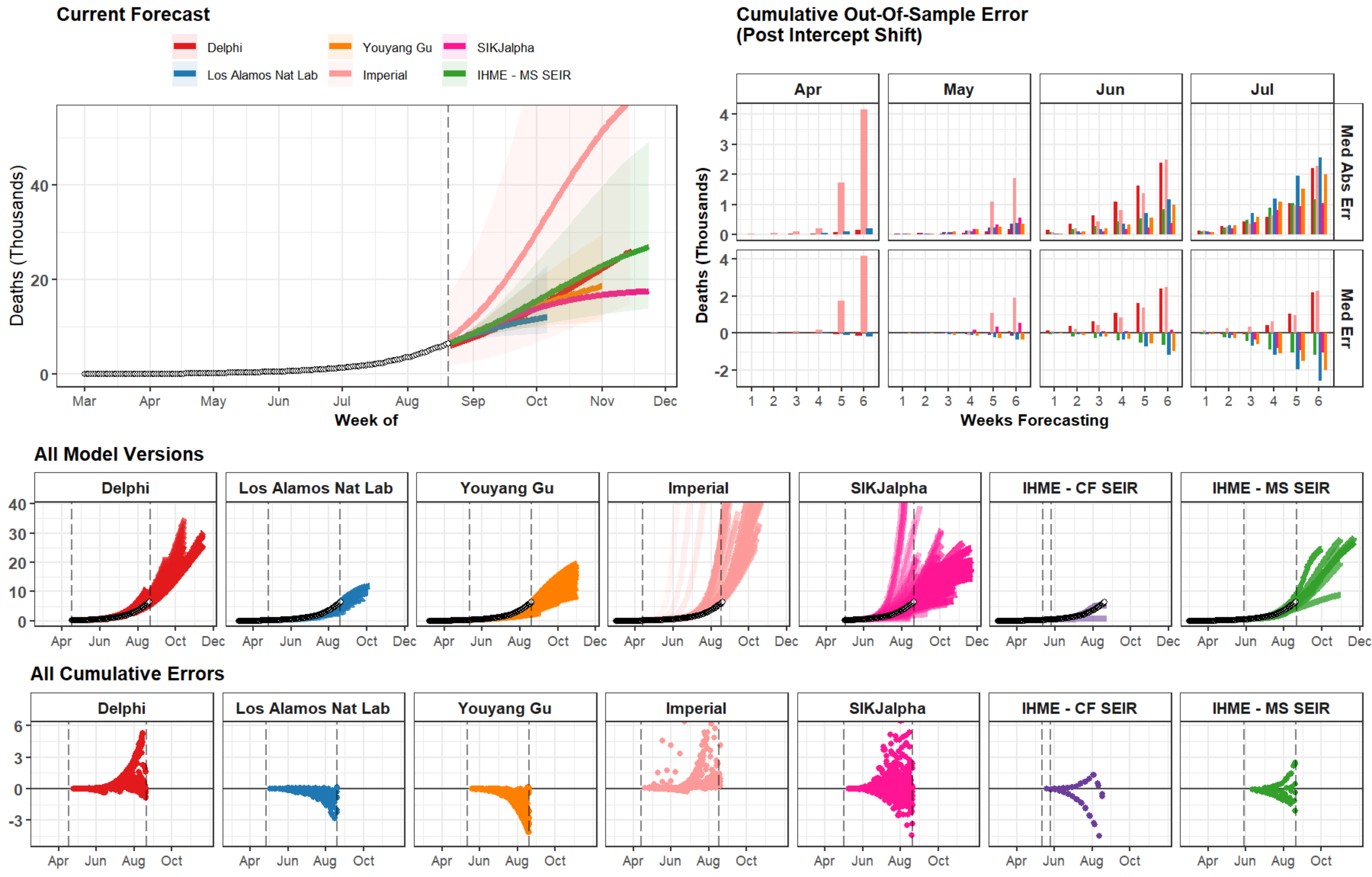
Current Forecast
Delphi
Los Alamos Nat Lab
Youyang Gu
Imperial
SIKJalpha
IHME - MS SEIR
Deaths (Thousands)
Week of
Mar
Apr
May
Jun
Jul
Aug
Sep
Oct
Nov
Dec
Cumulative Out-Of-Sample Error
(Post Intercept Shift)
Apr
May
Jun
Jul
Med Abs Err
Med Err
Weeks Forecasting
All Model Versions
Delphi
Los Alamos Nat Lab
Youyang Gu
Imperial
SIKJalpha
IHME - CF SEIR
IHME - MS SEIR
All Cumulative Errors

Indonesia

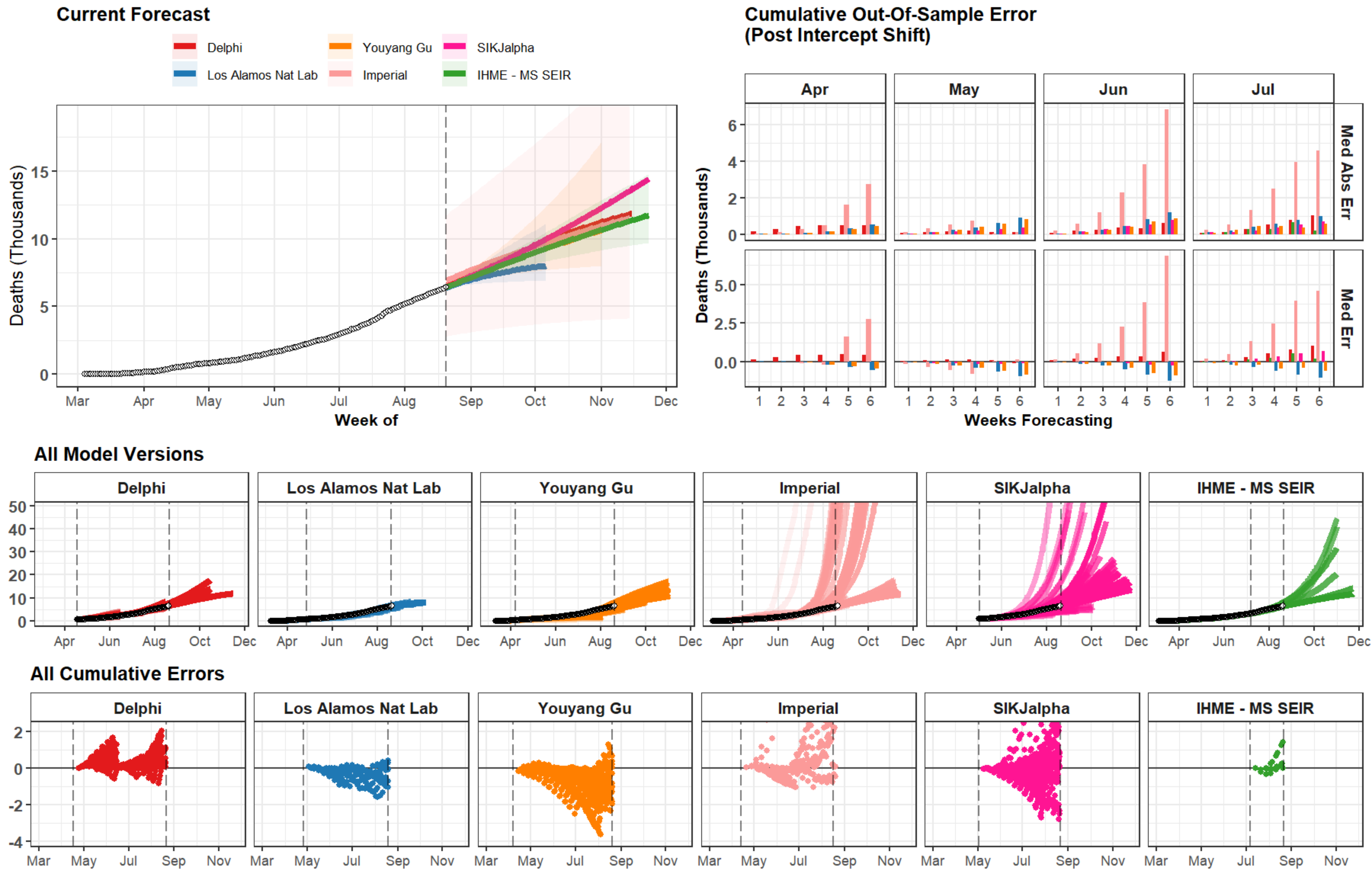
Current Forecast
Delphi
Los Alamos Nat Lab
Youyang Gu
Imperial
SIKJalpha
IHME - MS SEIR
Deaths (Thousands)
Week of
Mar
Apr
May
Jun
Jul
Aug
Sep
Oct
Nov
Dec
Cumulative Out-Of-Sample Error
(Post Intercept Shift)
Apr
May
Jun
Jul
Med Abs Err
Med Err
Weeks Forecasting
All Model Versions
All Cumulative Errors

Pakistan

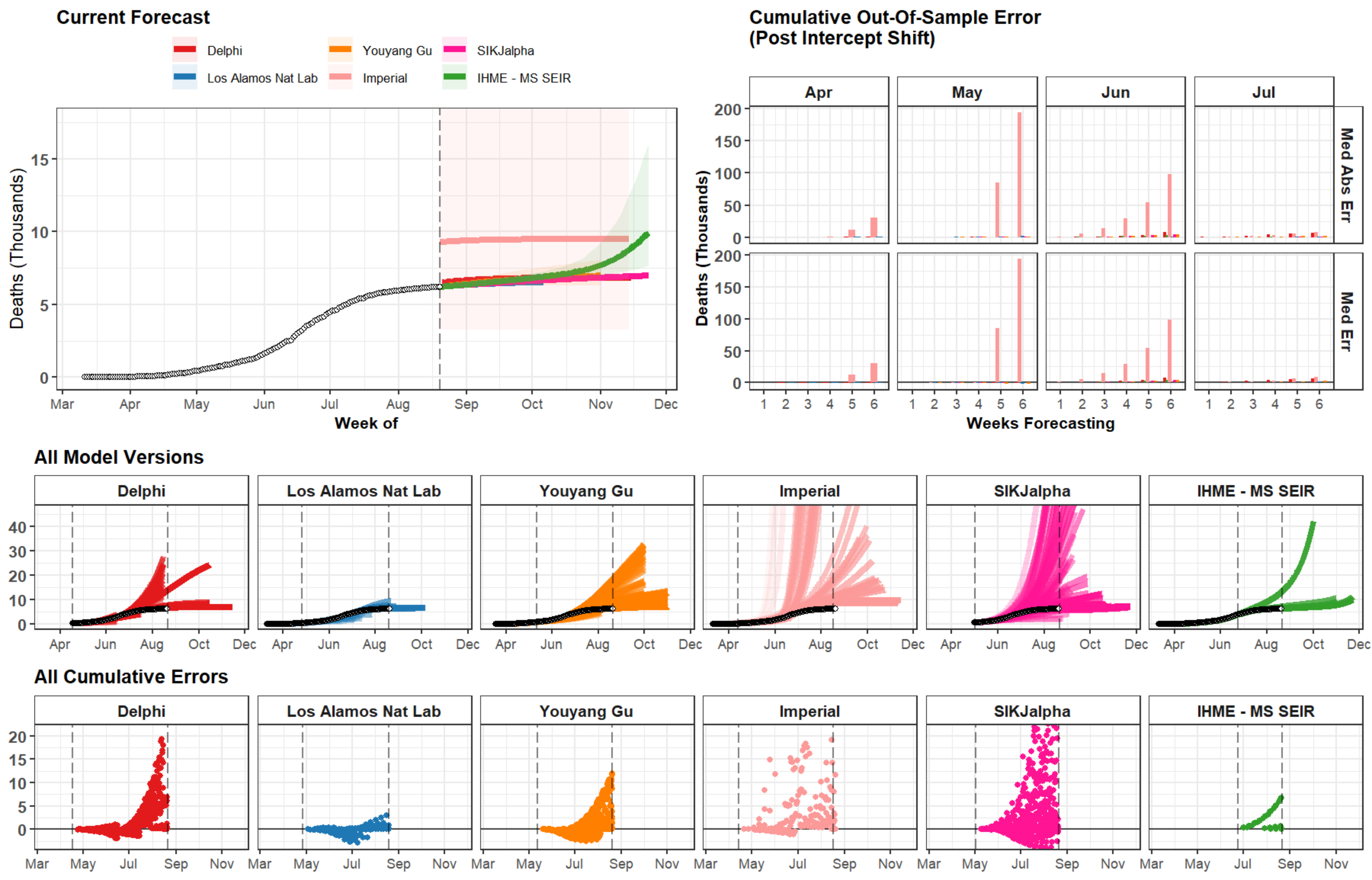
Current Forecast
Delphi
Los Alamos Nat Lab
Youyang Gu
Imperial
SIKJalpha
IHME - MS SEIR
Deaths (Thousands)
Week of
Cumulative Out-Of-Sample Error
(Post Intercept Shift)
Apr
May
Jun
Jul
Med Abs Err
Med Err
Weeks Forecasting
All Model Versions
All Cumulative Errors

Iraq

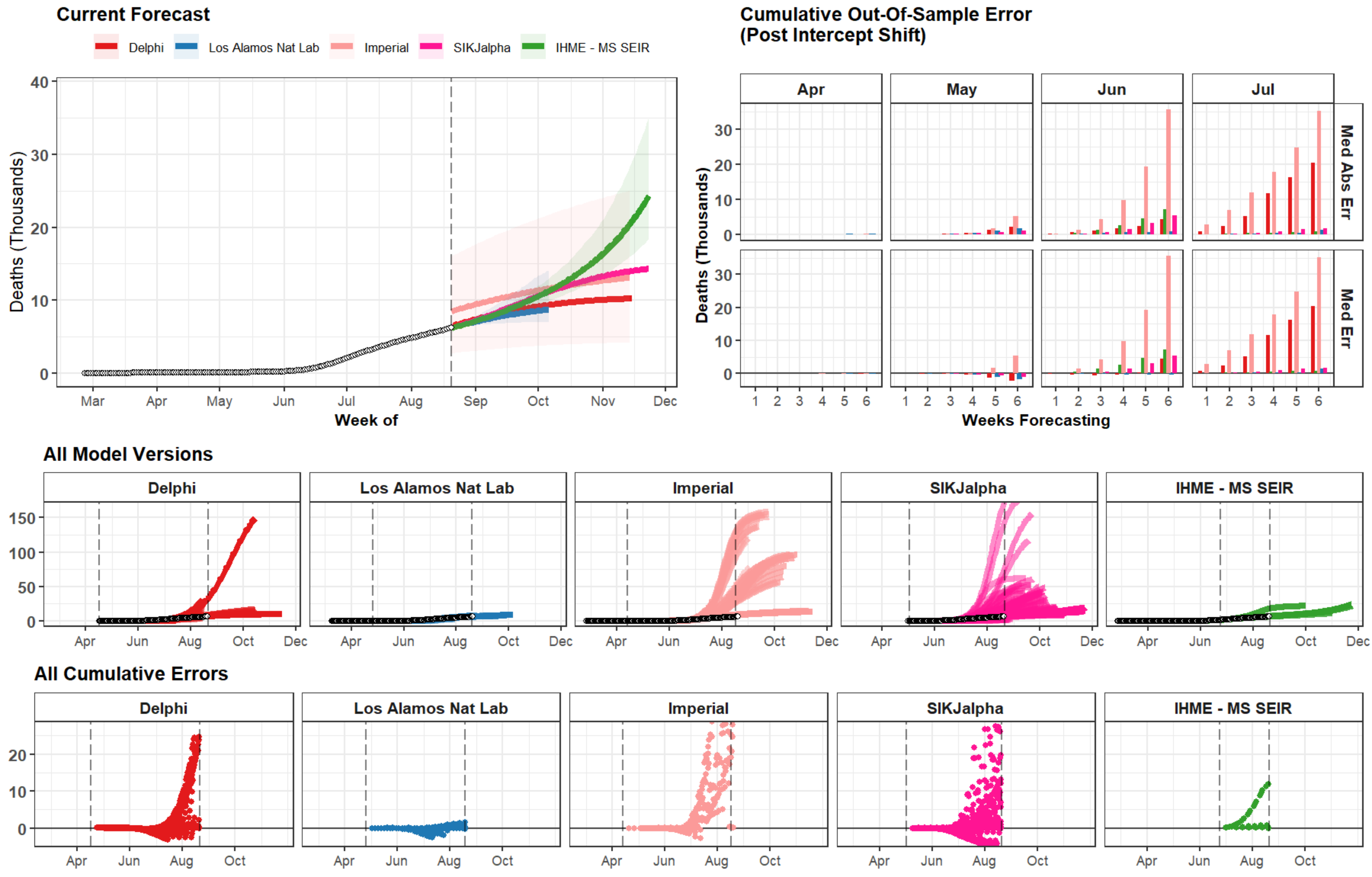

Current Forecast
Delphi
Los Alamos Nat Lab
Imperial
SIKJalpha
IHME - MS SEIR
Deaths (Thousands)
Week of
Cumulative Out-Of-Sample Error
(Post Intercept Shift)
Apr
May
Jun
Jul
Med Abs Err
Med Err
Weeks Forecasting
All Model Versions
All Cumulative Errors

Netherlands

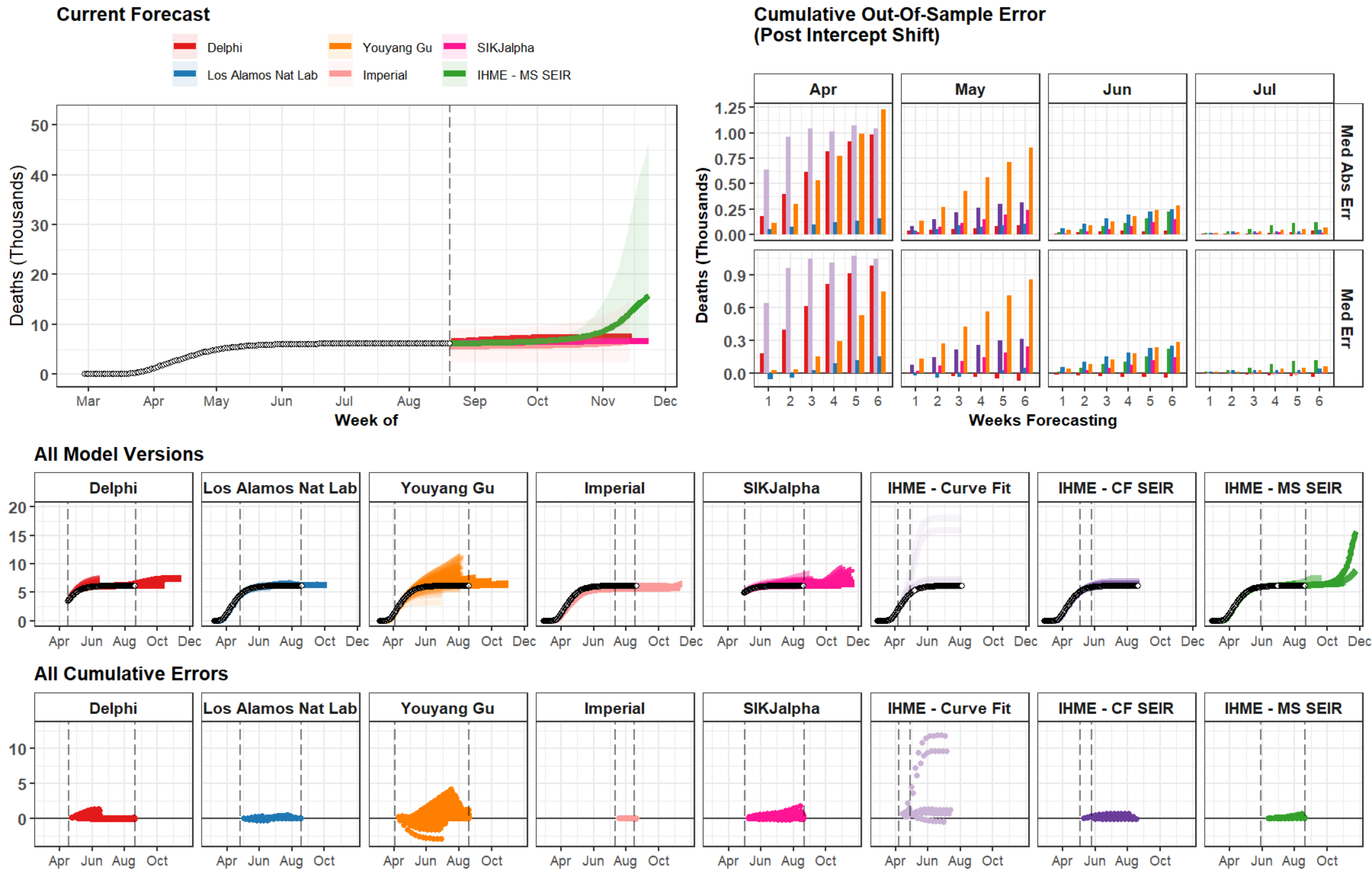

Turkey

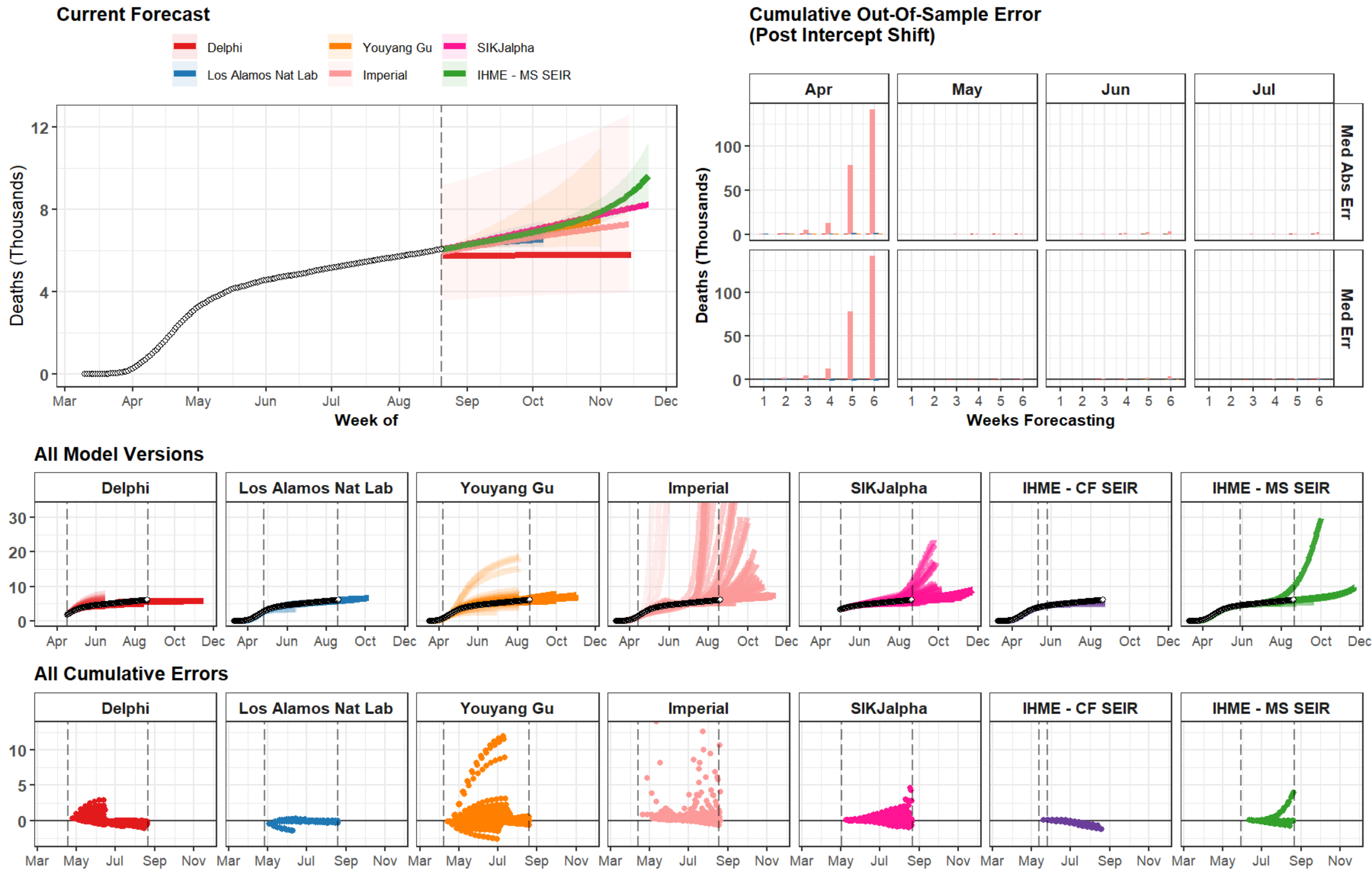

Current Forecast
Delphi
Youyang Gu
SIKJalpha
Los Alamos Nat Lab
Imperial
IHME - MS SEIR
Deaths (Thousands)
Week of
Cumulative Out-Of-Sample Error
(Post Intercept Shift)
Apr
May
Jun
Jul
Med Abs Err
Med Err
Weeks Forecasting
All Model Versions
Delphi
Los Alamos Nat Lab
Youyang Gu
Imperial
SIKJalpha
IHME - CF SEIR
IHME - MS SEIR
All Cumulative Errors

Sweden

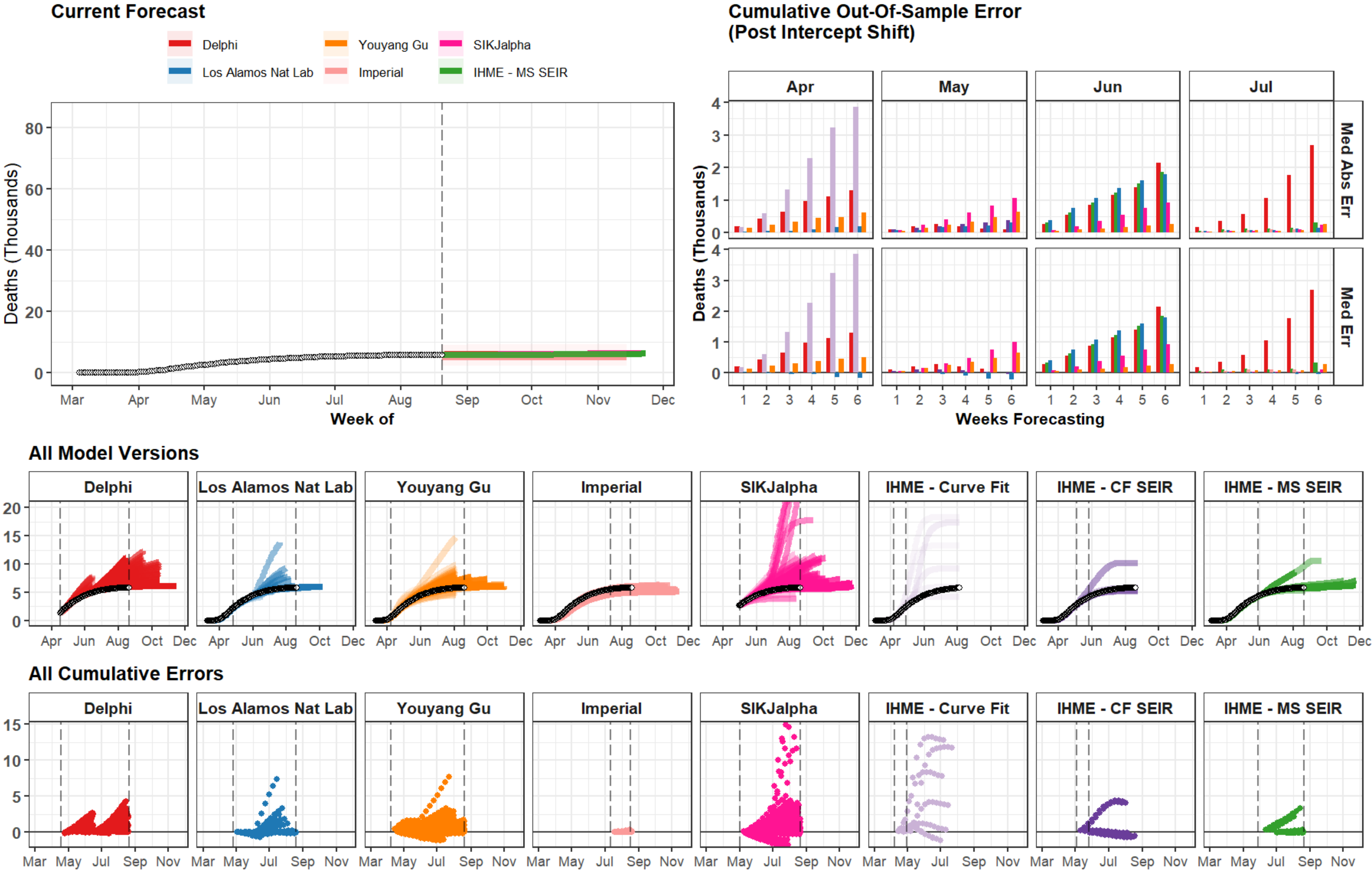

Current Forecast
Delphi
Los Alamos Nat Lab
Youyang Gu
Imperial
SIKJalpha
IHME - MS SEIR
Deaths (Thousands)
Week of
Mar Apr May Jun Jul Aug Sep Oct Nov Dec
Cumulative Out-Of-Sample Error
(Post Intercept Shift)
Apr
May
Jun
Jul
Med Abs Err
Med Err
Weeks Forecasting
All Model Versions
Delphi
Los Alamos Nat Lab
Youyang Gu
Imperial
SIKJalpha
IHME - Curve Fit
IHME - CF SEIR
IHME - MS SEIR
All Cumulative Errors

Egypt

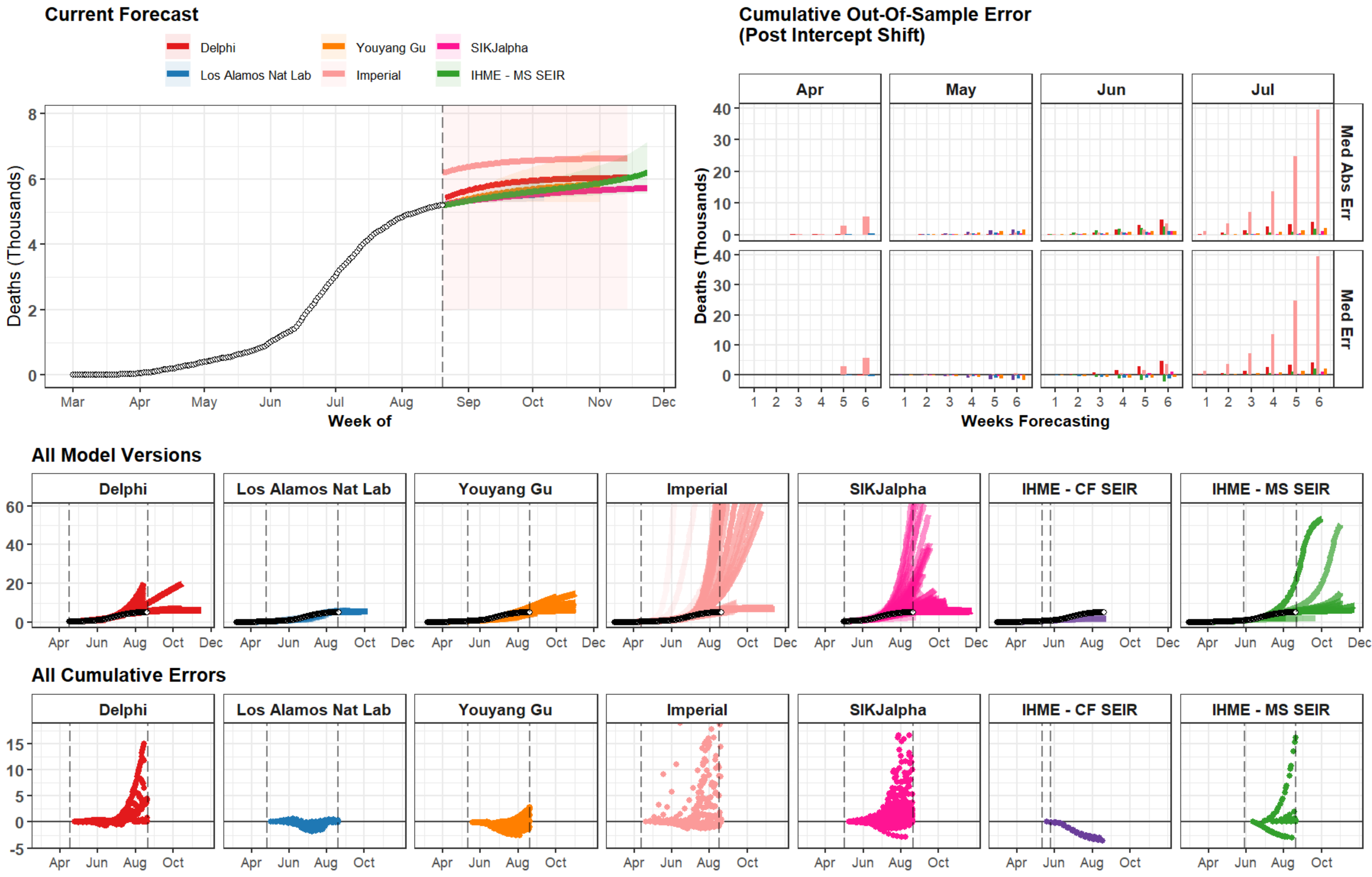

Current Forecast
Delphi
Youyang Gu
SIKJalpha
Los Alamos Nat Lab
Imperial
IHME - MS SEIR
Deaths (Thousands)
Week of
Mar
Apr
May
Jun
Jul
Aug
Sep
Oct
Nov
Dec
Cumulative Out-Of-Sample Error
(Post Intercept Shift)
Apr
May
Jun
Jul
Med Abs Err
Med Err
Weeks Forecasting
All Model Versions
Delphi
Los Alamos Nat Lab
Youyang Gu
Imperial
SIKJalpha
IHME - CF SEIR
IHME - MS SEIR
All Cumulative Errors

Georgia

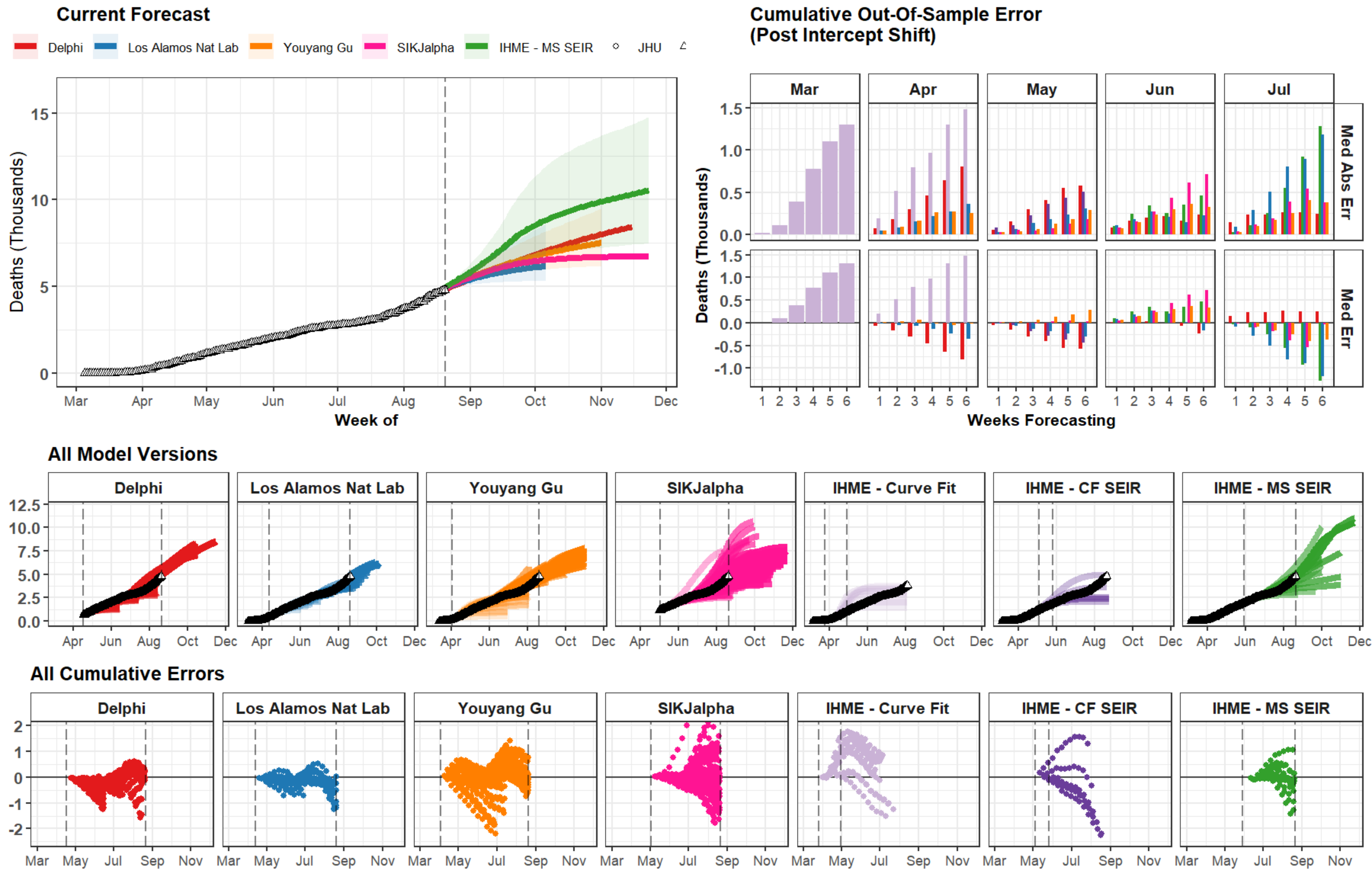

Current Forecast
Delphi
Los Alamos Nat Lab
Youyang Gu
SIKJalpha
IHME - MS SEIR
JHU
Deaths (Thousands)
Week of
Cumulative Out-Of-Sample Error
(Post Intercept Shift)
Mar
Apr
May
Jun
Jul
Med Abs Err
Med Err
Deaths (Thousands)
Weeks Forecasting
All Model Versions
Delphi
Los Alamos Nat Lab
Youyang Gu
SIKJalpha
IHME - Curve Fit
IHME - CF SEIR
IHME - MS SEIR
All Cumulative Errors
Delphi
Los Alamos Nat Lab
Youyang Gu
SIKJalpha
IHME - Curve Fit
IHME - CF SEIR
IHME - MS SEIR

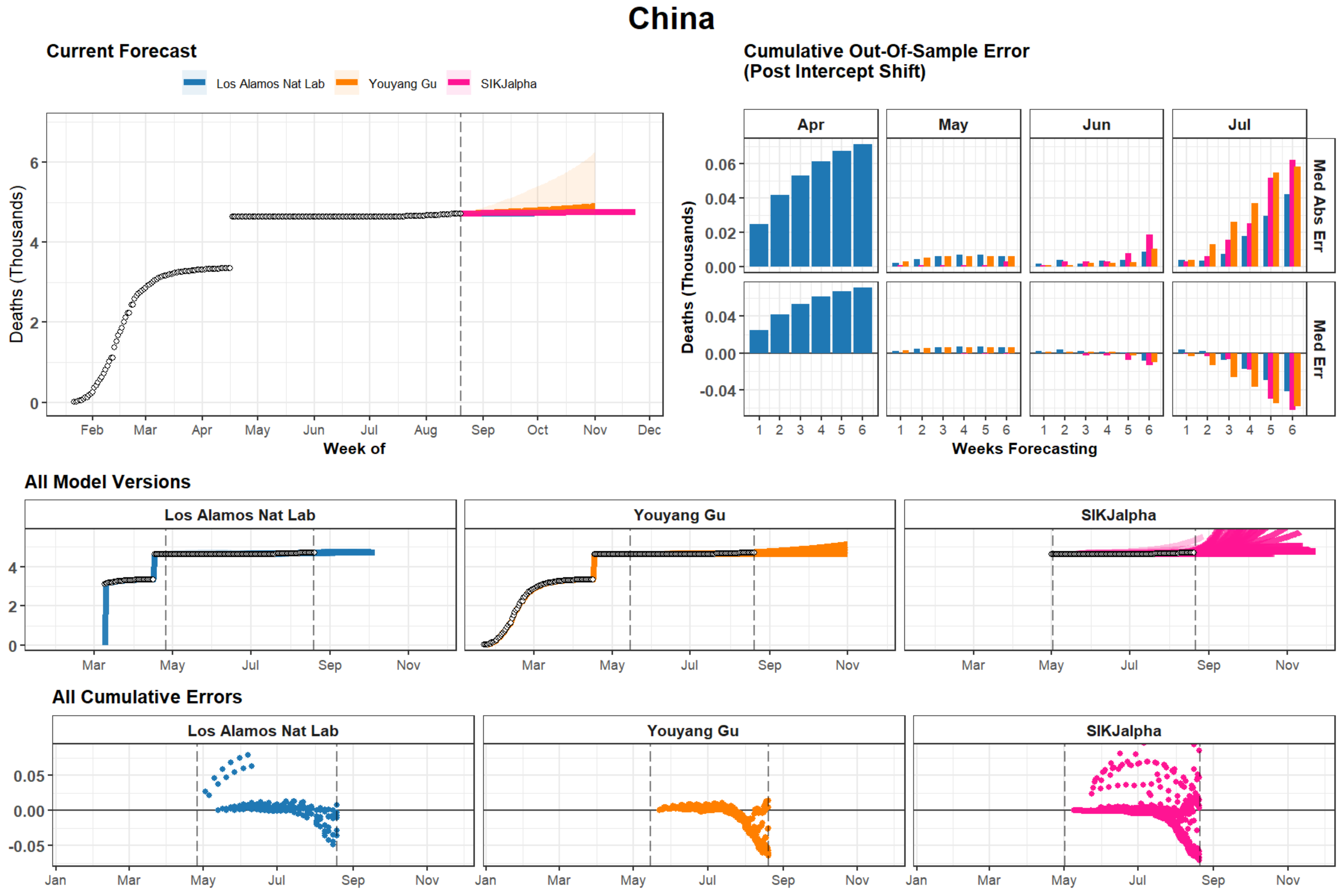
China
Current Forecast
Los Alamos Nat Lab
Youyang Gu
SIKJalpha
Deaths (Thousands)
0
2
4
6
Feb
Mar
Apr
May
Jun
Jul
Aug
Sep
Oct
Nov
Dec
Week of
Cumulative Out-Of-Sample Error
(Post Intercept Shift)
Apr
May
Jun
Jul
Med Abs Err
Med Err
0.00
0.02
0.04
0.06
-0.04
1 2 3 4 5 6
Weeks Forecasting
All Model Versions
Los Alamos Nat Lab
Youyang Gu
SIKJalpha
Mar
May
Jul
Sep
Nov
All Cumulative Errors
Los Alamos Nat Lab
Youyang Gu
SIKJalpha
0.05
0.00
-0.05
Jan
Mar
May
Jul
Sep
Nov

Arizona

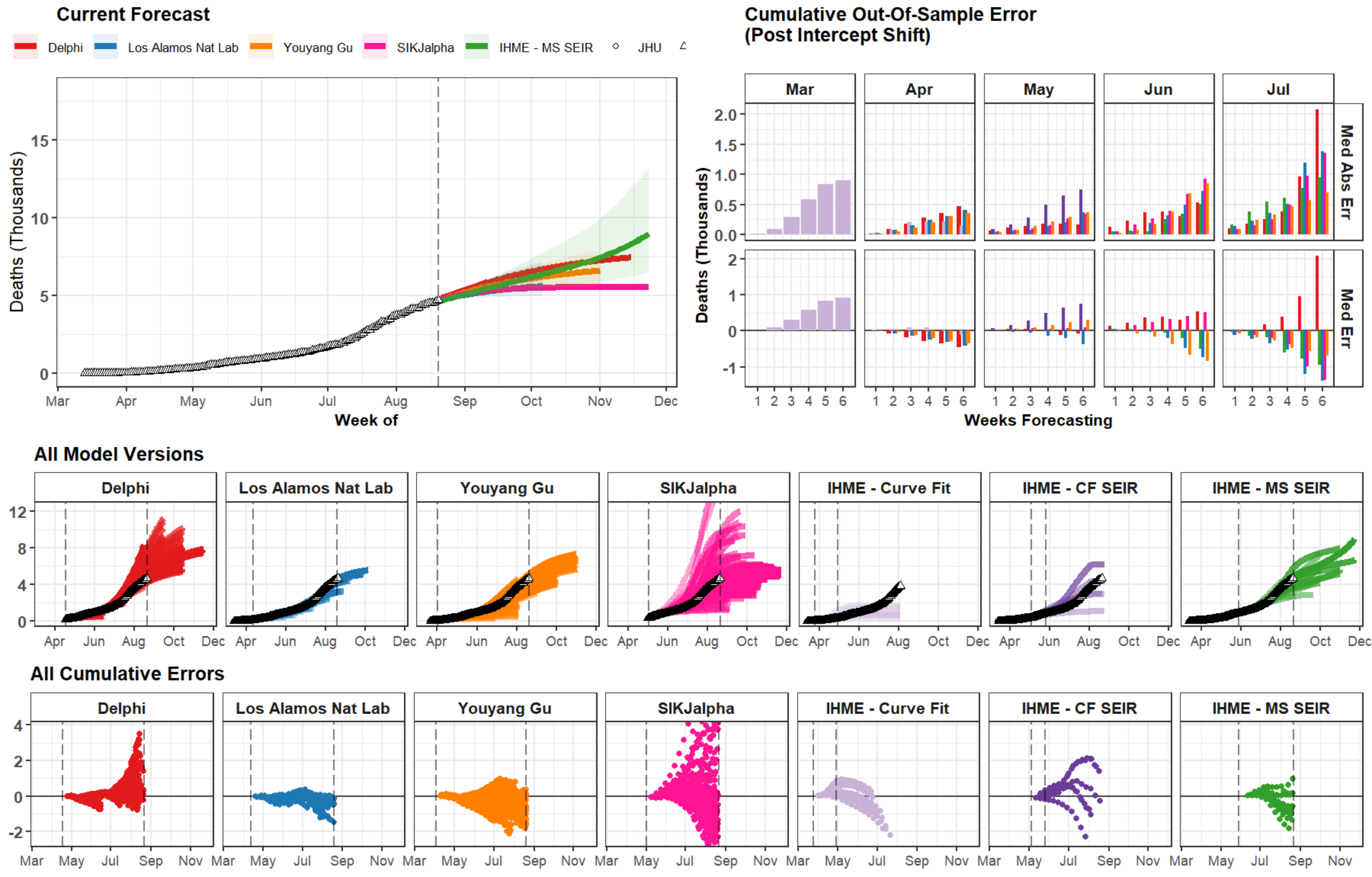
Current Forecast
Delphi
Los Alamos Nat Lab
Youyang Gu
SIKJalpha
IHME - MS SEIR
JHU
Deaths (Thousands)
Week of
Mar
Apr
May
Jun
Jul
Aug
Sep
Oct
Nov
Dec
Cumulative Out-Of-Sample Error
(Post Intercept Shift)
Med Abs Err
Med Err
Weeks Forecasting
All Model Versions
IHME - Curve Fit
IHME - CF SEIR
All Cumulative Errors

Louisiana

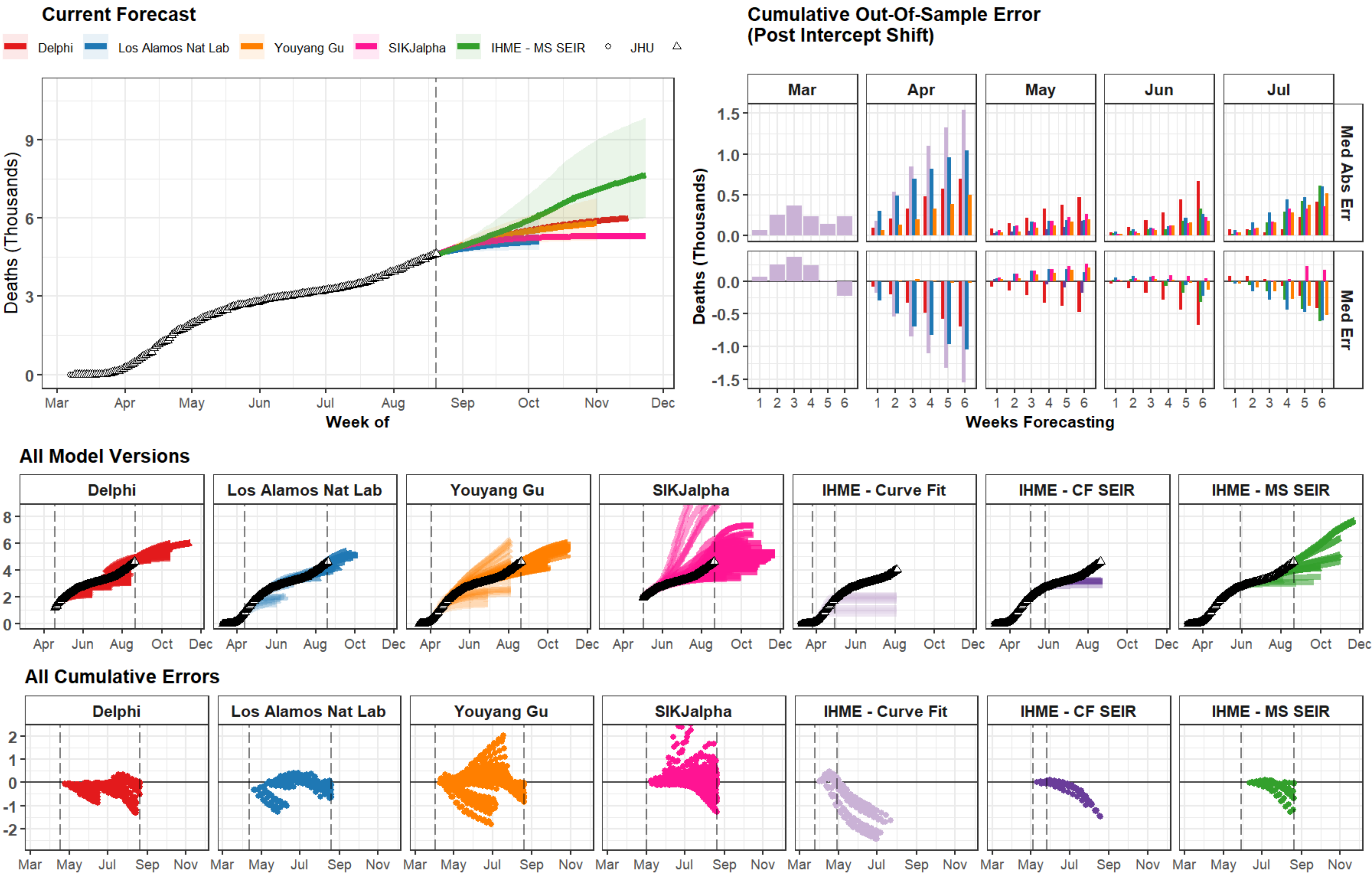

Current Forecast
Delphi
Los Alamos Nat Lab
Youyang Gu
SIKJalpha
IHME - MS SEIR
JHU
Deaths (Thousands)
Week of
Mar
Apr
May
Jun
Jul
Aug
Sep
Oct
Nov
Dec
Cumulative Out-Of-Sample Error
(Post Intercept Shift)
Med Abs Err
Med Err
Weeks Forecasting
All Model Versions
IHME - Curve Fit
IHME - CF SEIR
All Cumulative Errors

Connecticut

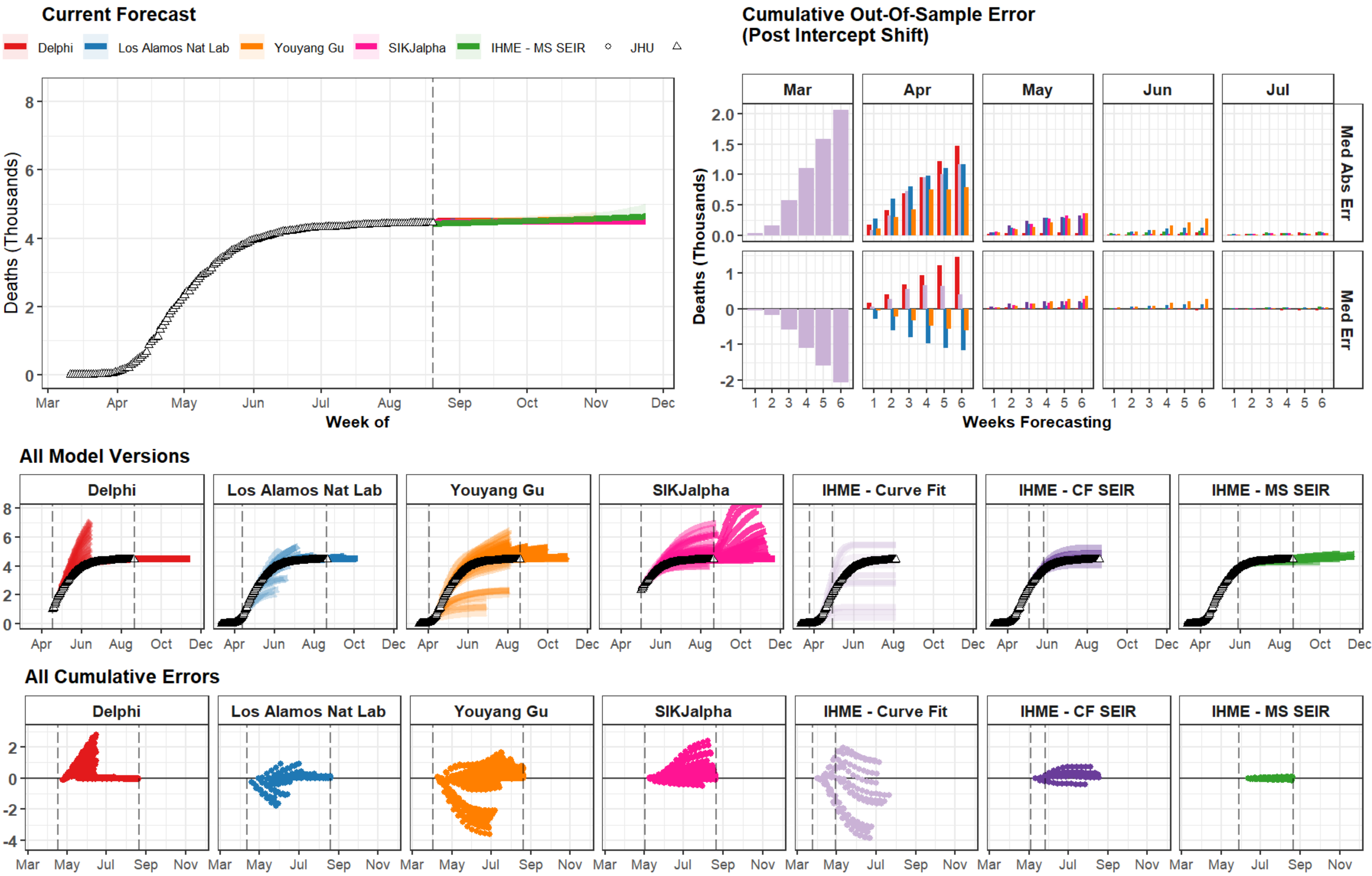

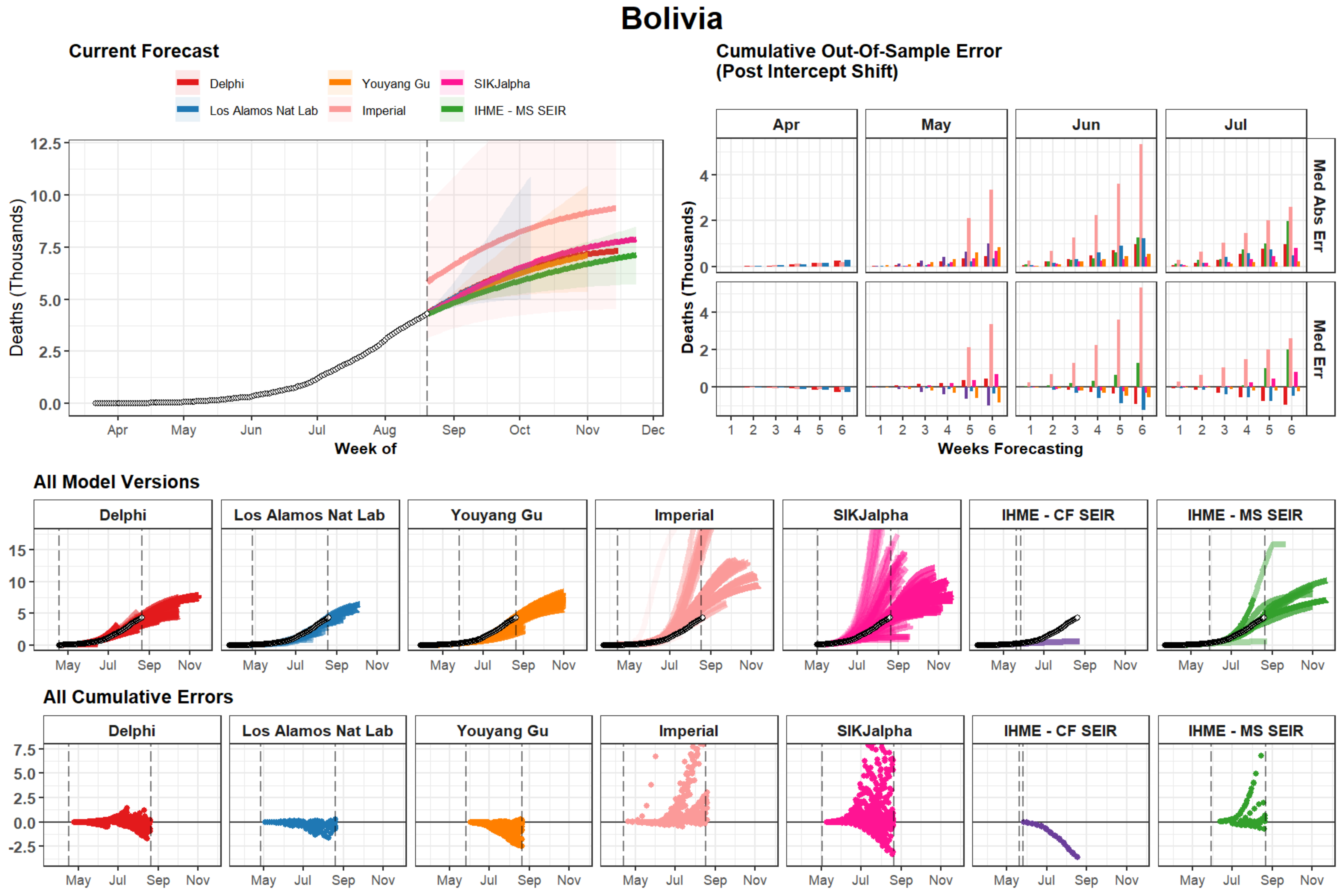
Bolivia
Current Forecast
Delphi
Los Alamos Nat Lab
Youyang Gu
Imperial
SIKJalpha
IHME - MS SEIR
Deaths (Thousands)
12.5
10.0
7.5
5.0
2.5
0.0
Apr
May
Jun
Jul
Aug
Sep
Oct
Nov
Dec
Week of
Cumulative Out-Of-Sample Error
(Post Intercept Shift)
Apr
May
Jun
Jul
Med Abs Err
Med Err
Deaths (Thousands)
1 2 3 4 5 6
Weeks Forecasting
All Model Versions
Delphi
Los Alamos Nat Lab
Youyang Gu
Imperial
SIKJalpha
IHME - CF SEIR
IHME - MS SEIR
15
10
5
0
May
Jul
Sep
Nov
All Cumulative Errors
Delphi
Los Alamos Nat Lab
Youyang Gu
Imperial
SIKJalpha
IHME - CF SEIR
IHME - MS SEIR
7.5
5.0
2.5
0.0
-2.5
May
Jul
Sep
Nov

Ohio

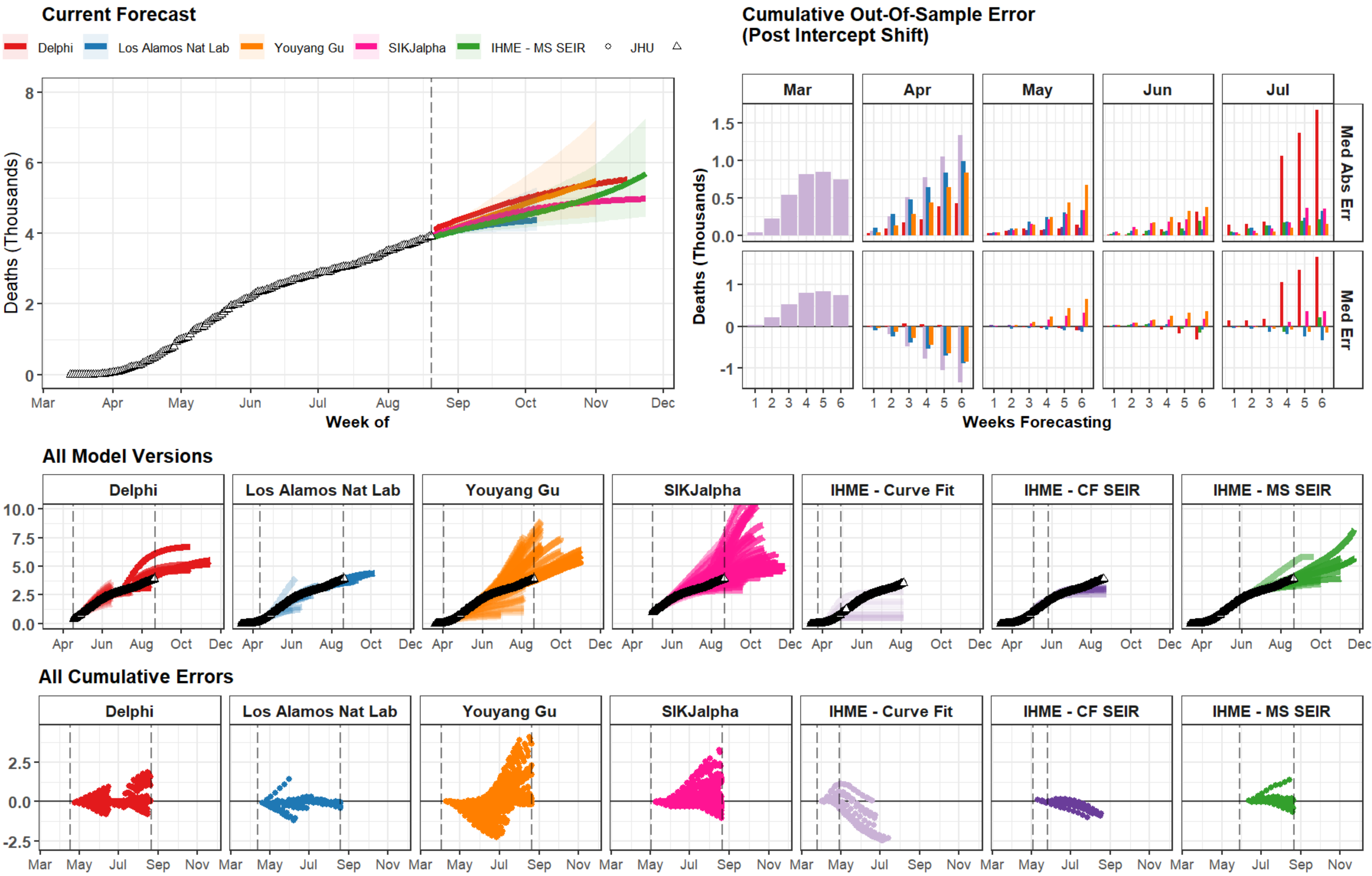

Current Forecast
Delphi
Los Alamos Nat Lab
Youyang Gu
SIKJalpha
IHME - MS SEIR
JHU
Deaths (Thousands)
Week of
Mar
Apr
May
Jun
Jul
Aug
Sep
Oct
Nov
Dec
Cumulative Out-Of-Sample Error
(Post Intercept Shift)
Med Abs Err
Med Err
Weeks Forecasting
All Model Versions
IHME - Curve Fit
IHME - CF SEIR
All Cumulative Errors

Bangladesh

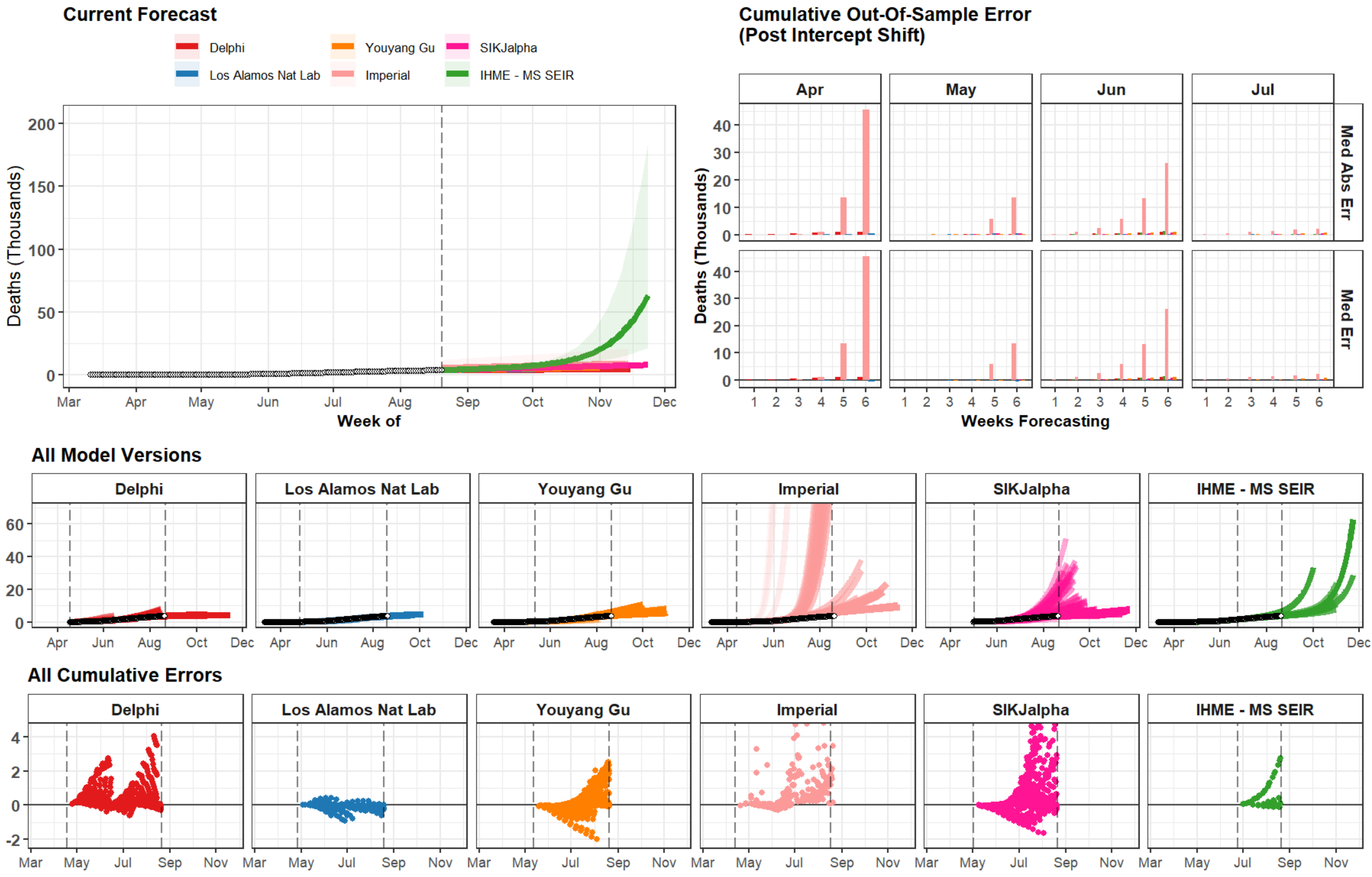

Current Forecast
Delphi
Youyang Gu
SIKJalpha
Los Alamos Nat Lab
Imperial
IHME - MS SEIR
Deaths (Thousands)
200
150
100
50
0
Mar
Apr
May
Jun
Jul
Aug
Sep
Oct
Nov
Dec
Week of
Cumulative Out-Of-Sample Error
(Post Intercept Shift)
Apr
May
Jun
Jul
Med Abs Err
Med Err
Deaths (Thousands)
40
30
20
10
0
1
2
3
4
5
6
Weeks Forecasting
All Model Versions
Delphi
Los Alamos Nat Lab
Youyang Gu
Imperial
SIKJalpha
IHME - MS SEIR
60
40
20
0
Apr
Jun
Aug
Oct
Dec
All Cumulative Errors
Delphi
Los Alamos Nat Lab
Youyang Gu
Imperial
SIKJalpha
IHME - MS SEIR
4
2
0
-2
Mar
May
Jul
Sep
Nov

Maryland

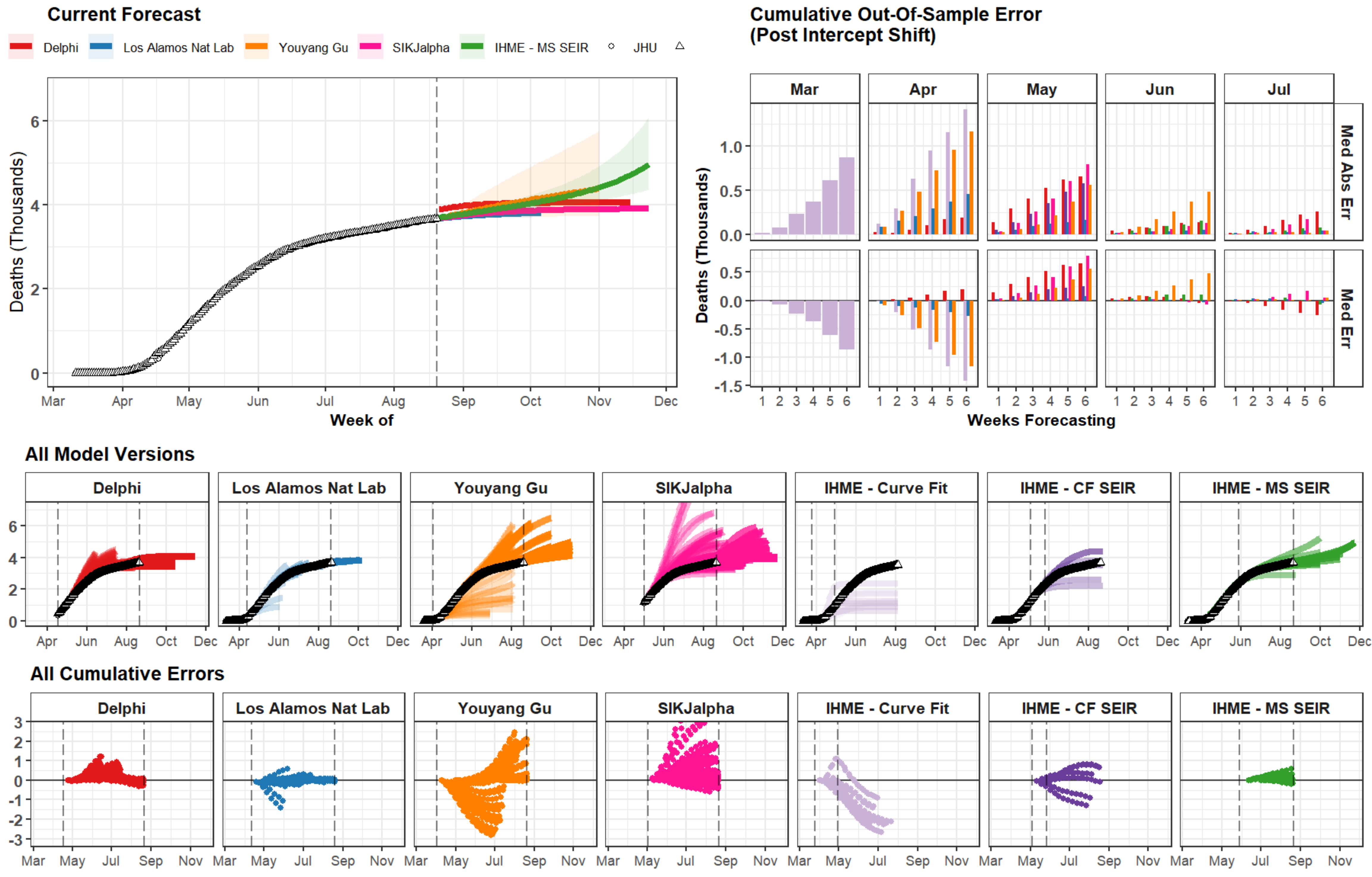

Current Forecast
Delphi
Los Alamos Nat Lab
Youyang Gu
SIKJalpha
IHME - MS SEIR
JHU
Deaths (Thousands)
Week of
Cumulative Out-Of-Sample Error
(Post Intercept Shift)
Mar
Apr
May
Jun
Jul
Med Abs Err
Med Err
Weeks Forecasting
All Model Versions
Delphi
Los Alamos Nat Lab
Youyang Gu
SIKJalpha
IHME - Curve Fit
IHME - CF SEIR
IHME - MS SEIR
All Cumulative Errors

Saudi Arabia

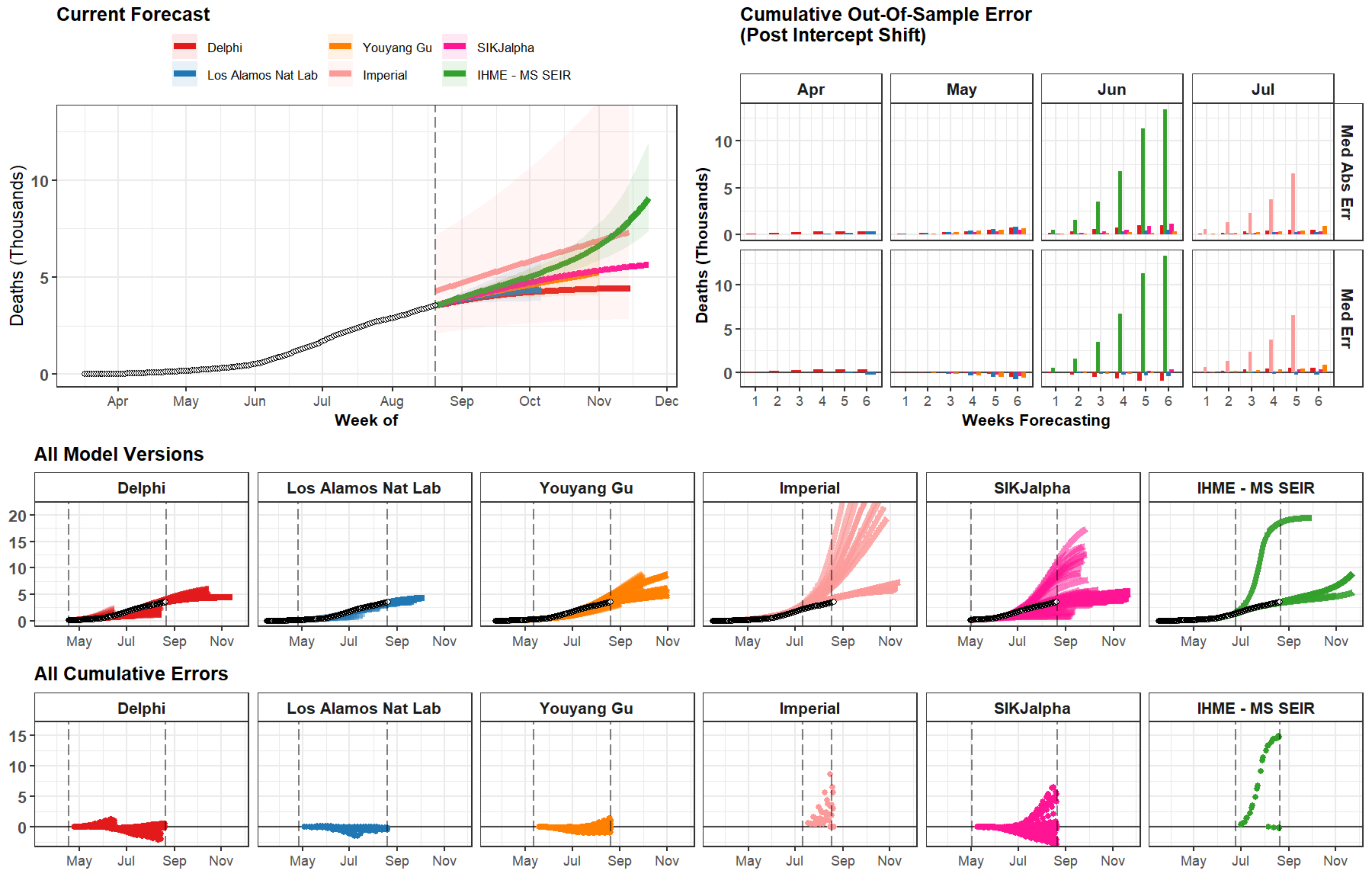

Indiana

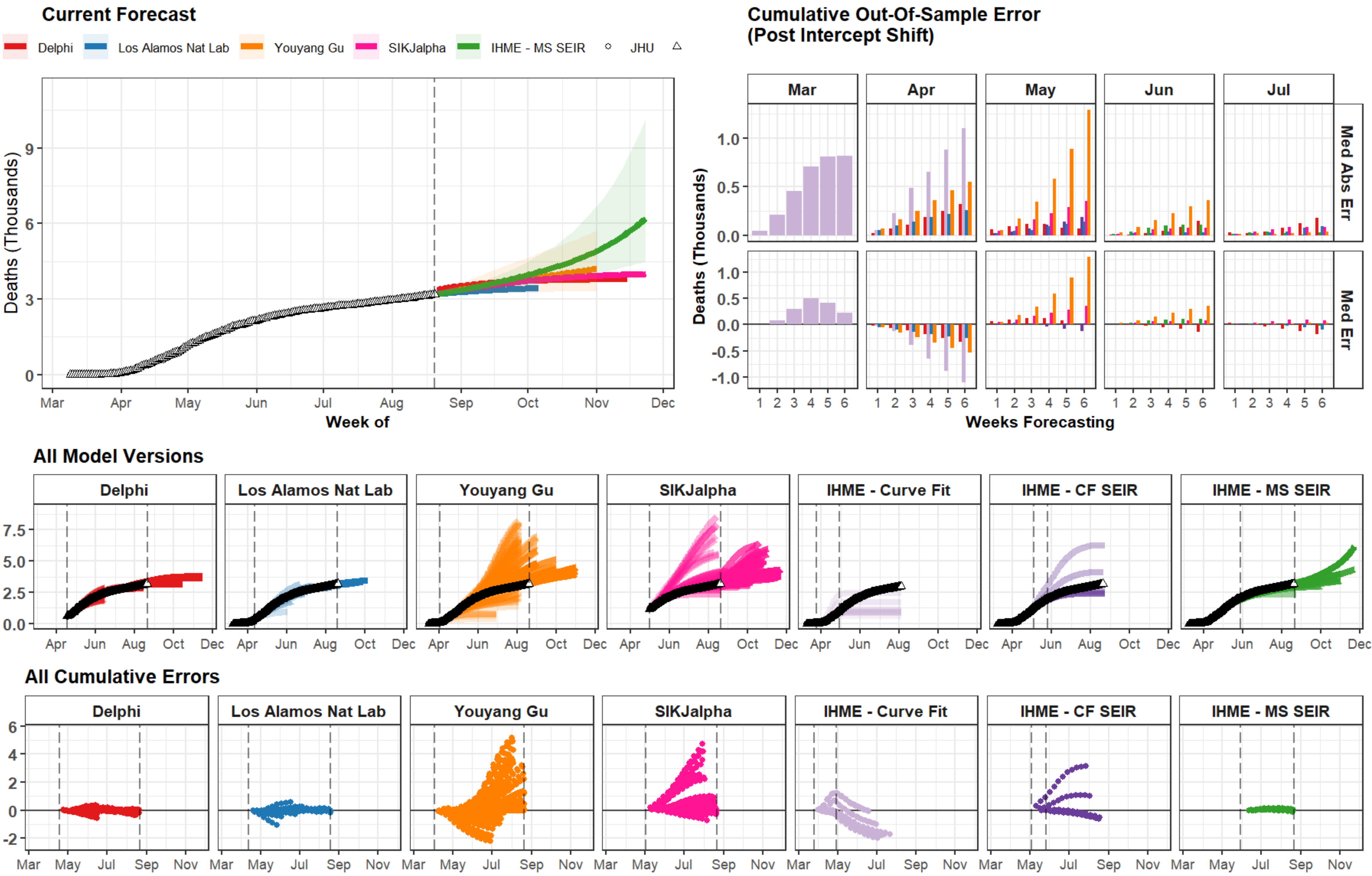

Current Forecast
Delphi
Los Alamos Nat Lab
Youyang Gu
SIKJalpha
IHME - MS SEIR
JHU
Deaths (Thousands)
Week of
Mar
Apr
May
Jun
Jul
Aug
Sep
Oct
Nov
Dec
Cumulative Out-Of-Sample Error
(Post Intercept Shift)
Med Abs Err
Med Err
Weeks Forecasting
All Model Versions
IHME - Curve Fit
IHME - CF SEIR
All Cumulative Errors

Romania

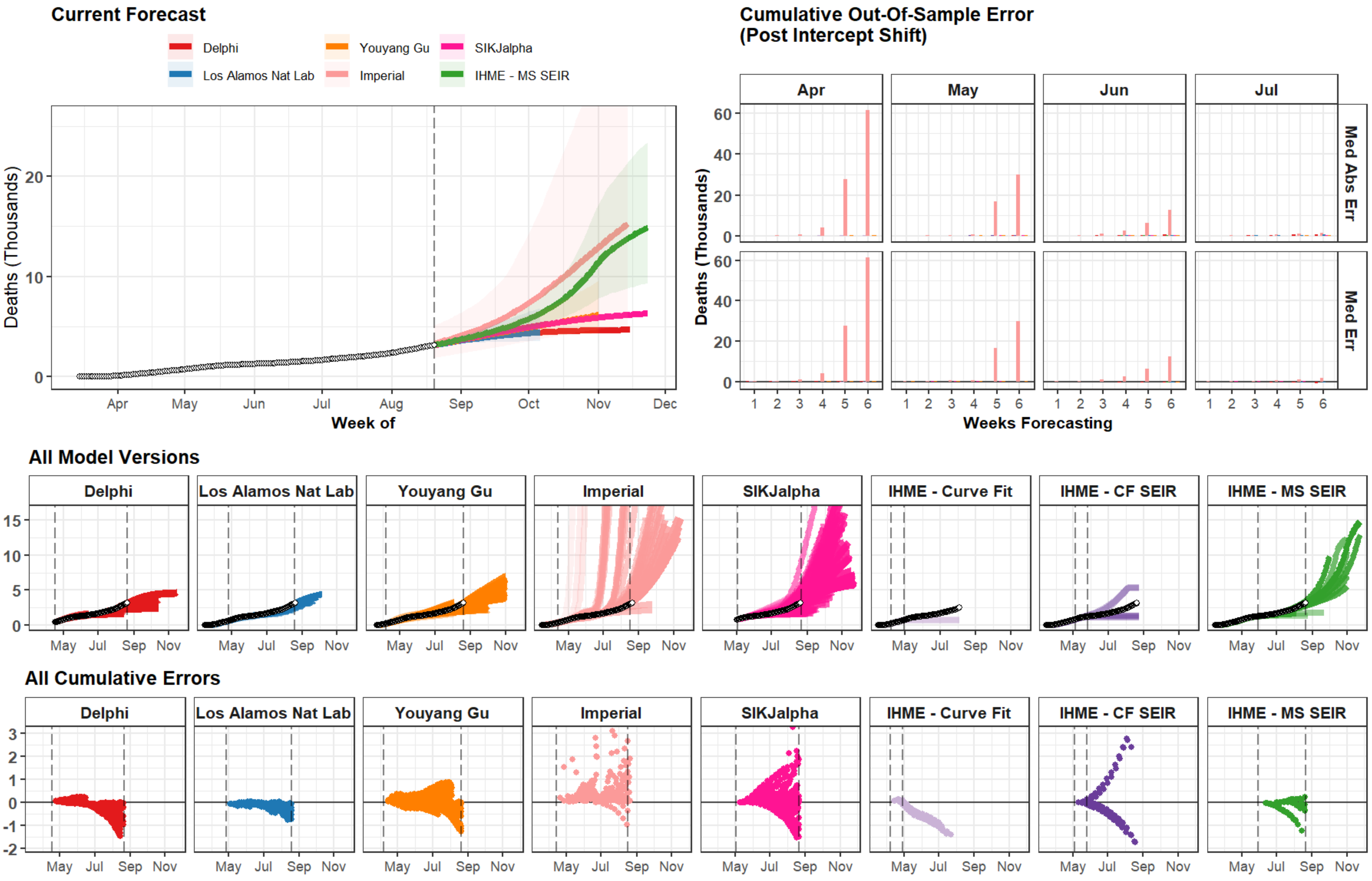
Current Forecast
Delphi
Los Alamos Nat Lab
Youyang Gu
Imperial
SIKJalpha
IHME - MS SEIR
Deaths (Thousands)
0
10
20
Apr
May
Jun
Jul
Aug
Sep
Oct
Nov
Dec
Week of
Cumulative Out-Of-Sample Error
(Post Intercept Shift)
Apr
May
Jun
Jul
Med Abs Err
Med Err
Deaths (Thousands)
0
20
40
60
1
2
3
4
5
6
Weeks Forecasting
All Model Versions
Delphi
Los Alamos Nat Lab
Youyang Gu
Imperial
SIKJalpha
IHME - Curve Fit
IHME - CF SEIR
IHME - MS SEIR
0
5
10
15
May
Jul
Sep
Nov
All Cumulative Errors
Delphi
Los Alamos Nat Lab
Youyang Gu
Imperial
SIKJalpha
IHME - Curve Fit
IHME - CF SEIR
IHME - MS SEIR
-2
-1
0
1
2
3
May
Jul
Sep
Nov

Philippines

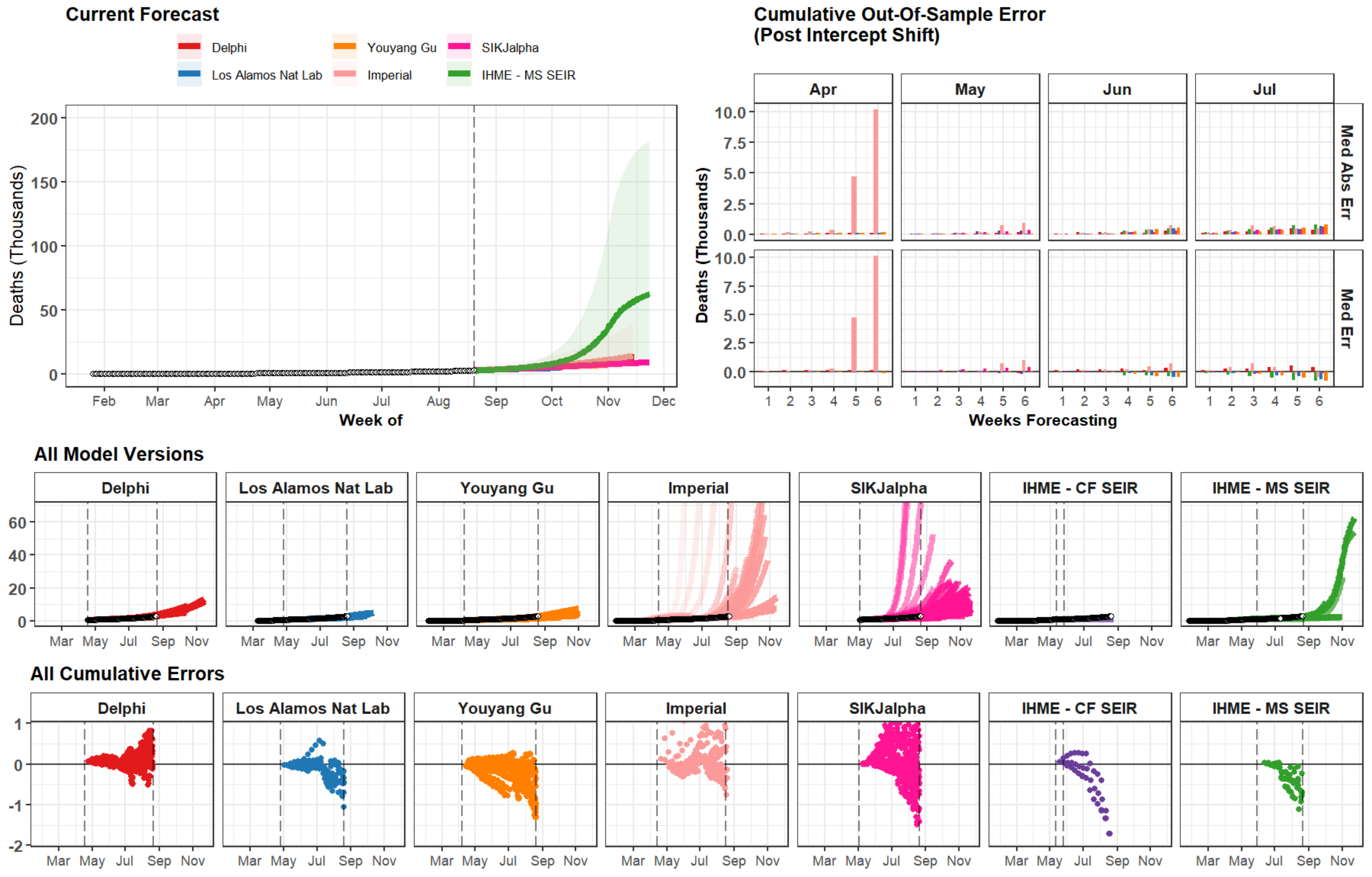

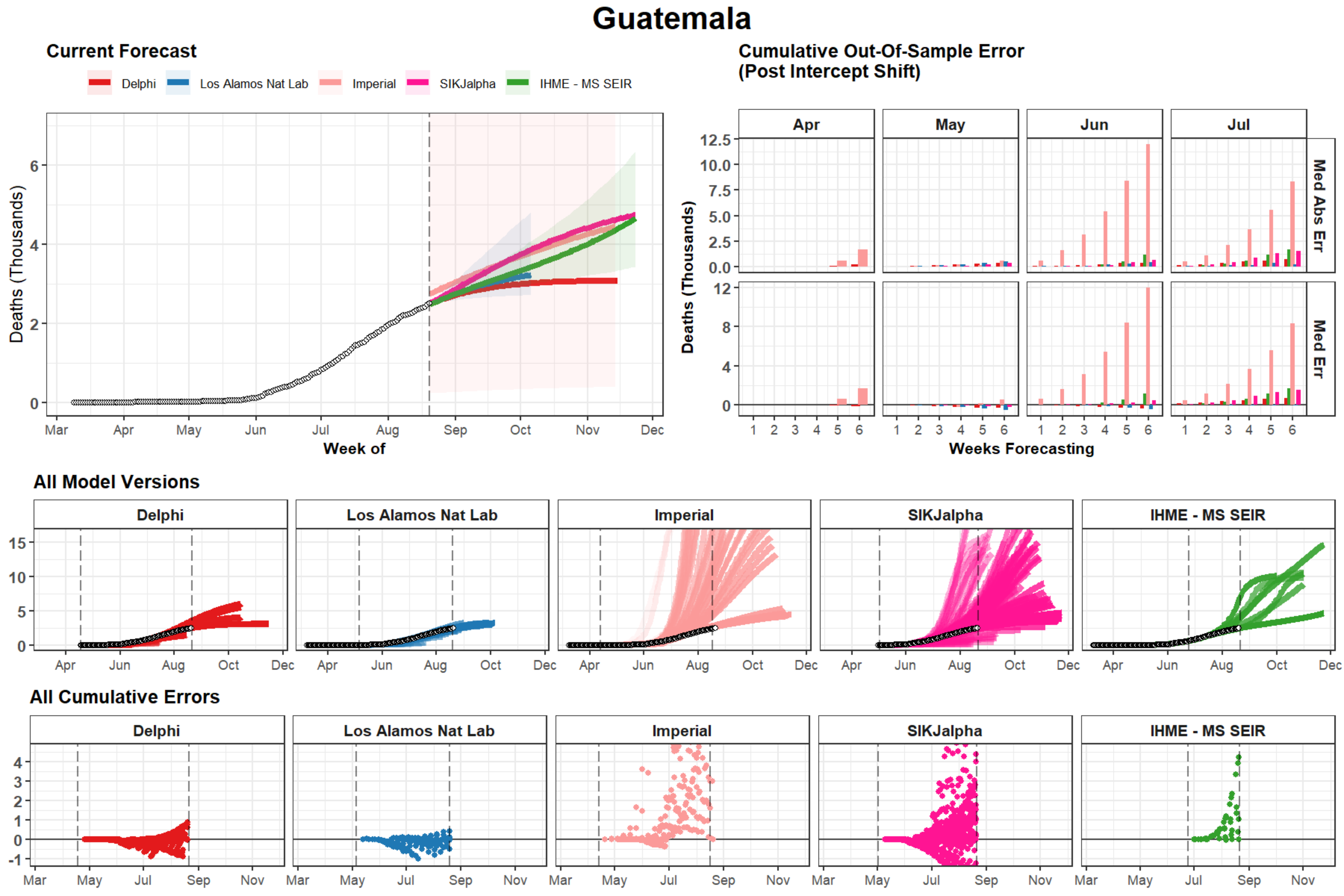
Guatemala
Current Forecast
Delphi
Los Alamos Nat Lab
Imperial
SIKJalpha
IHME - MS SEIR
Deaths (Thousands)
Week of
Mar Apr May Jun Jul Aug Sep Oct Nov Dec
0 2 4 6
Cumulative Out-Of-Sample Error
(Post Intercept Shift)
Apr May Jun Jul
Med Abs Err
Med Err
12.5 10.0 7.5 5.0 2.5 0.0
12 8 4 0
1 2 3 4 5 6
Weeks Forecasting
All Model Versions
0 5 10 15
Apr Jun Aug Oct Dec
All Cumulative Errors
4 3 2 1 0 -1
Mar May Jul Sep Nov

North Carolina

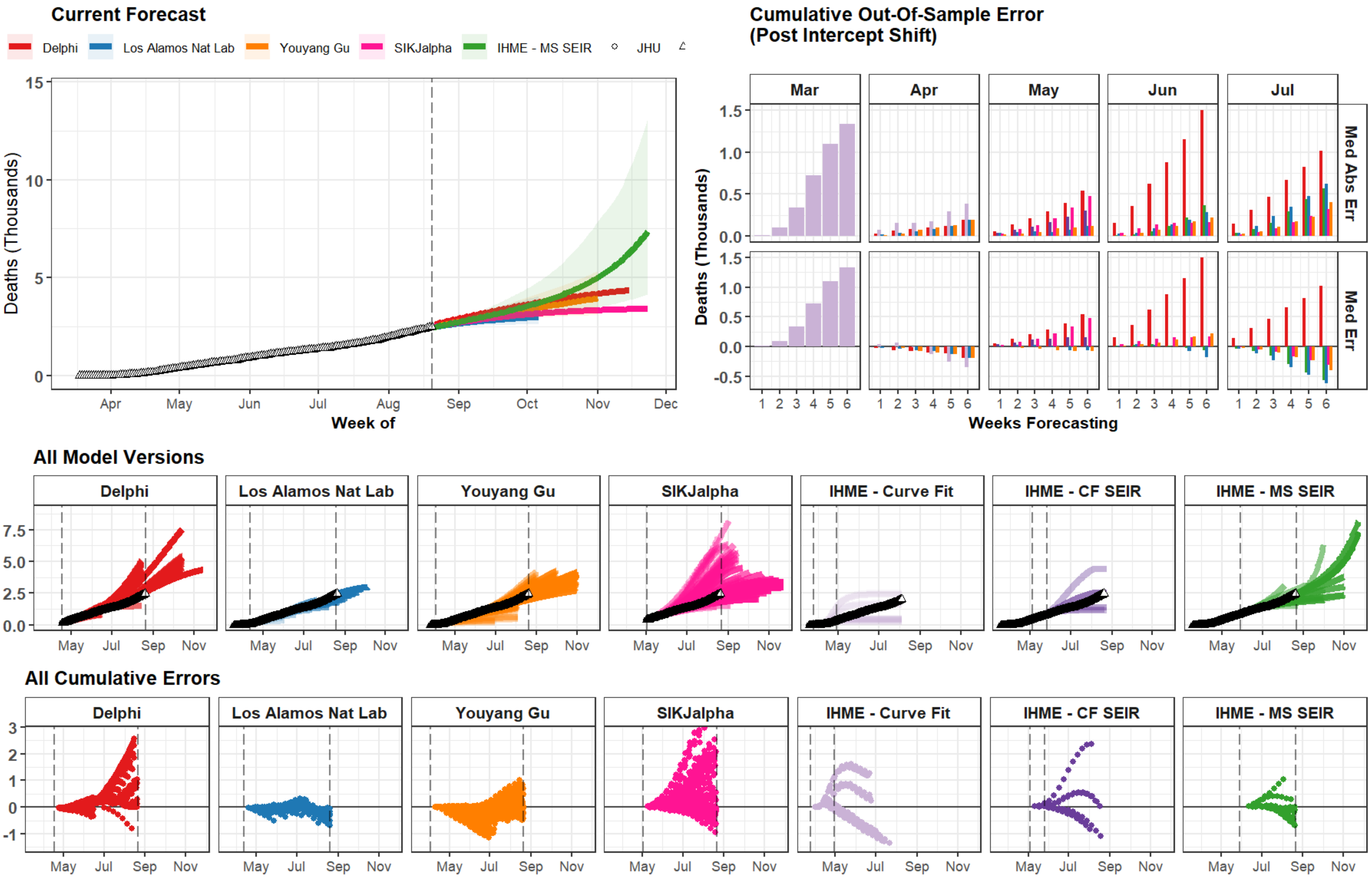

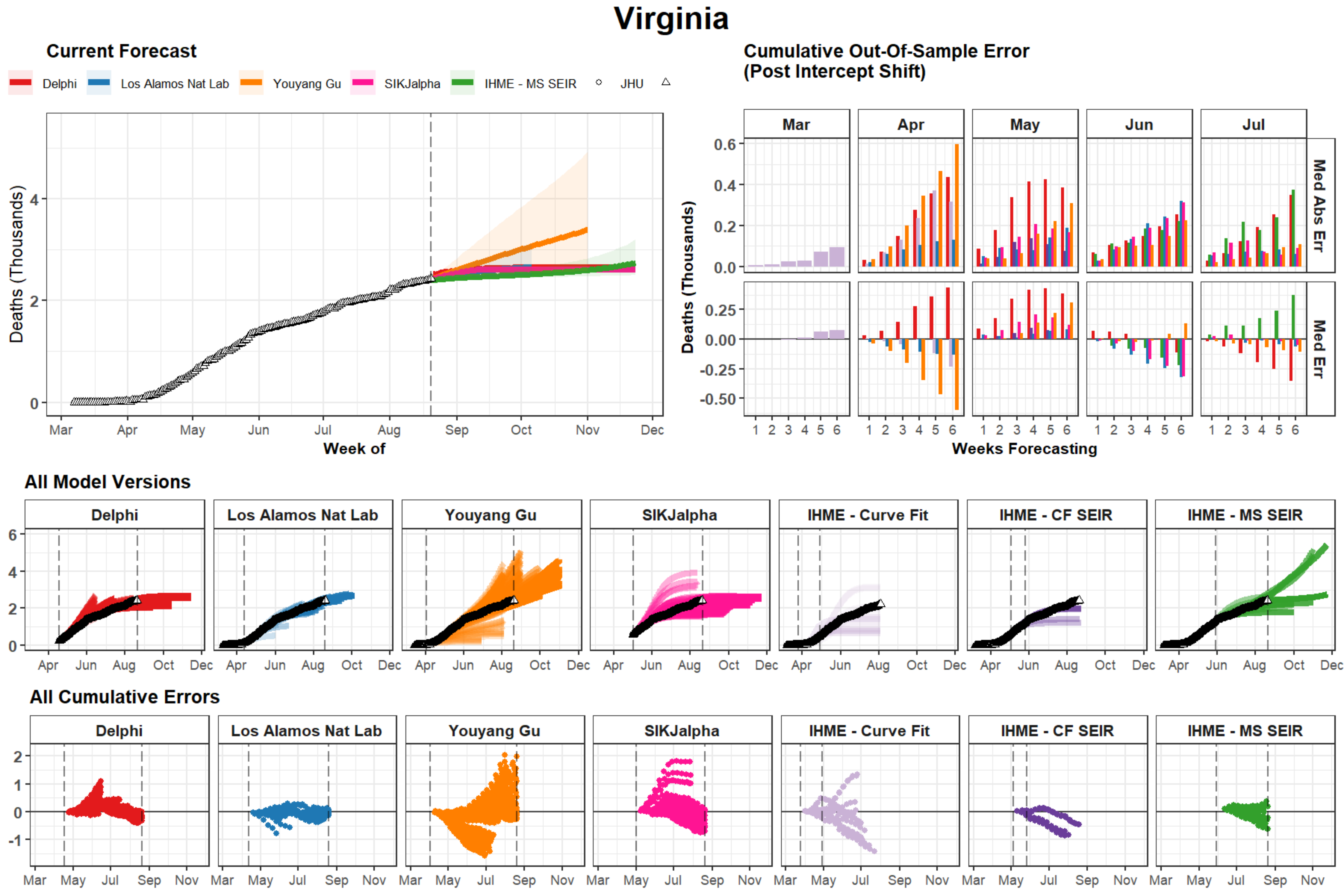

Virginia
Current Forecast
Delphi
Los Alamos Nat Lab
Youyang Gu
SIKJalpha
IHME - MS SEIR
JHU
Deaths (Thousands)
Week of
Mar Apr May Jun Jul Aug Sep Oct Nov Dec
Cumulative Out-Of-Sample Error
(Post Intercept Shift)
Mar Apr May Jun Jul
Med Abs Err
Med Err
Weeks Forecasting
All Model Versions
Delphi
Los Alamos Nat Lab
Youyang Gu
SIKJalpha
IHME - Curve Fit
IHME - CF SEIR
IHME - MS SEIR
All Cumulative Errors

South Carolina

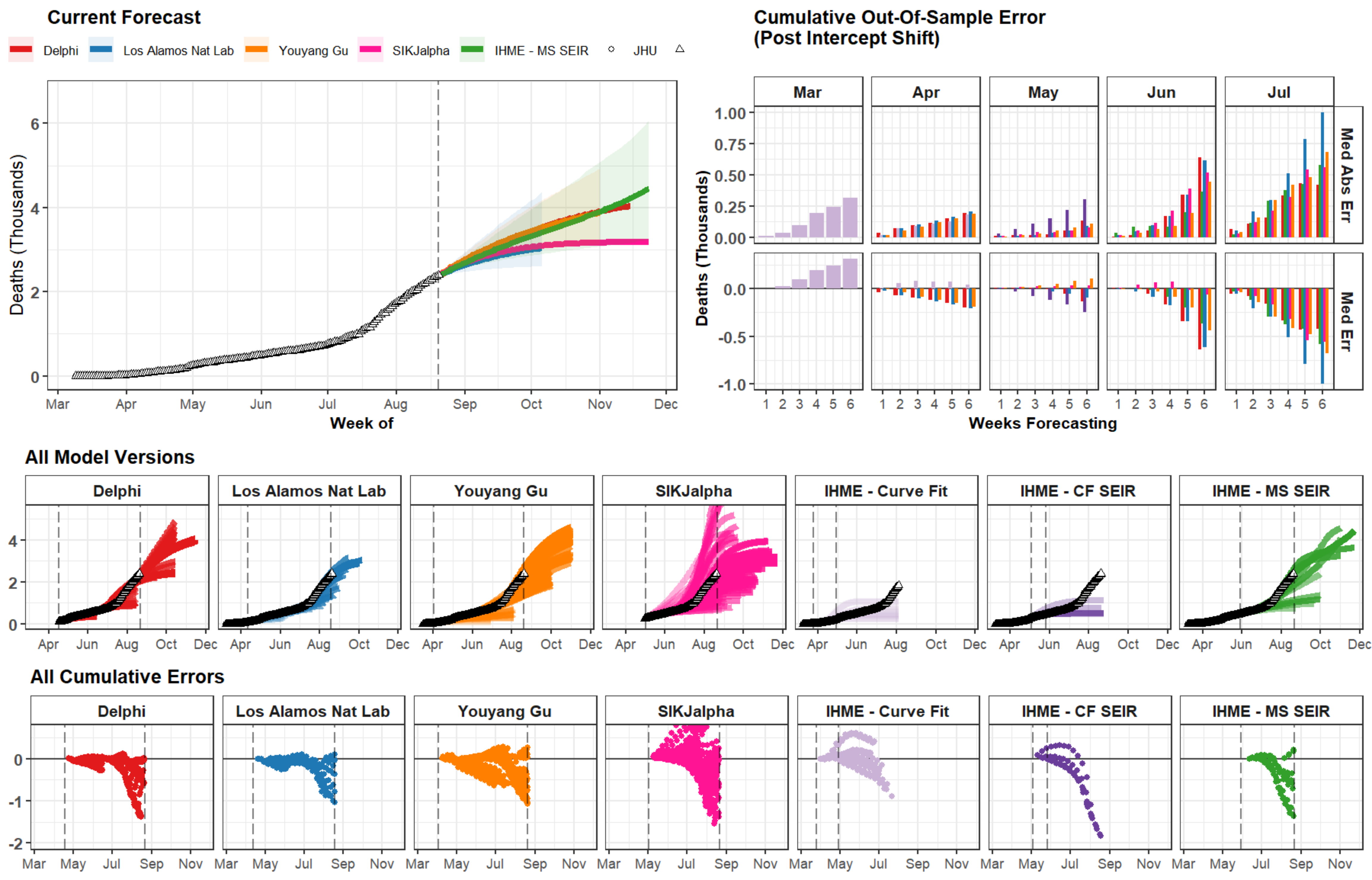

Ukraine

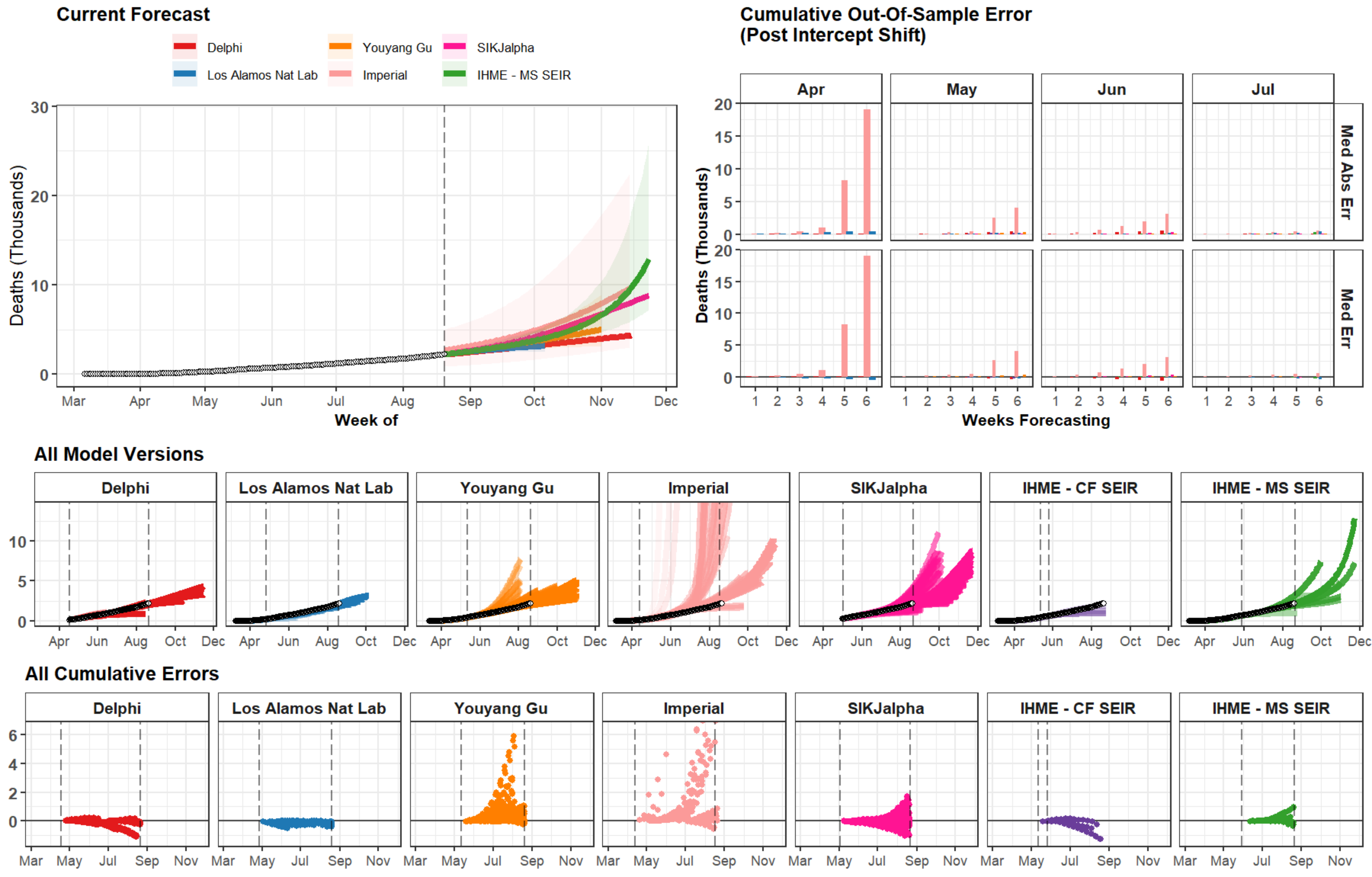
Current Forecast
Delphi
Los Alamos Nat Lab
Youyang Gu
Imperial
SIKJalpha
IHME - MS SEIR
Deaths (Thousands)
Week of
Cumulative Out-Of-Sample Error
(Post Intercept Shift)
Apr
May
Jun
Jul
Med Abs Err
Med Err
Weeks Forecasting
All Model Versions
Delphi
Los Alamos Nat Lab
Youyang Gu
Imperial
SIKJalpha
IHME - CF SEIR
IHME - MS SEIR
All Cumulative Errors

Mississippi

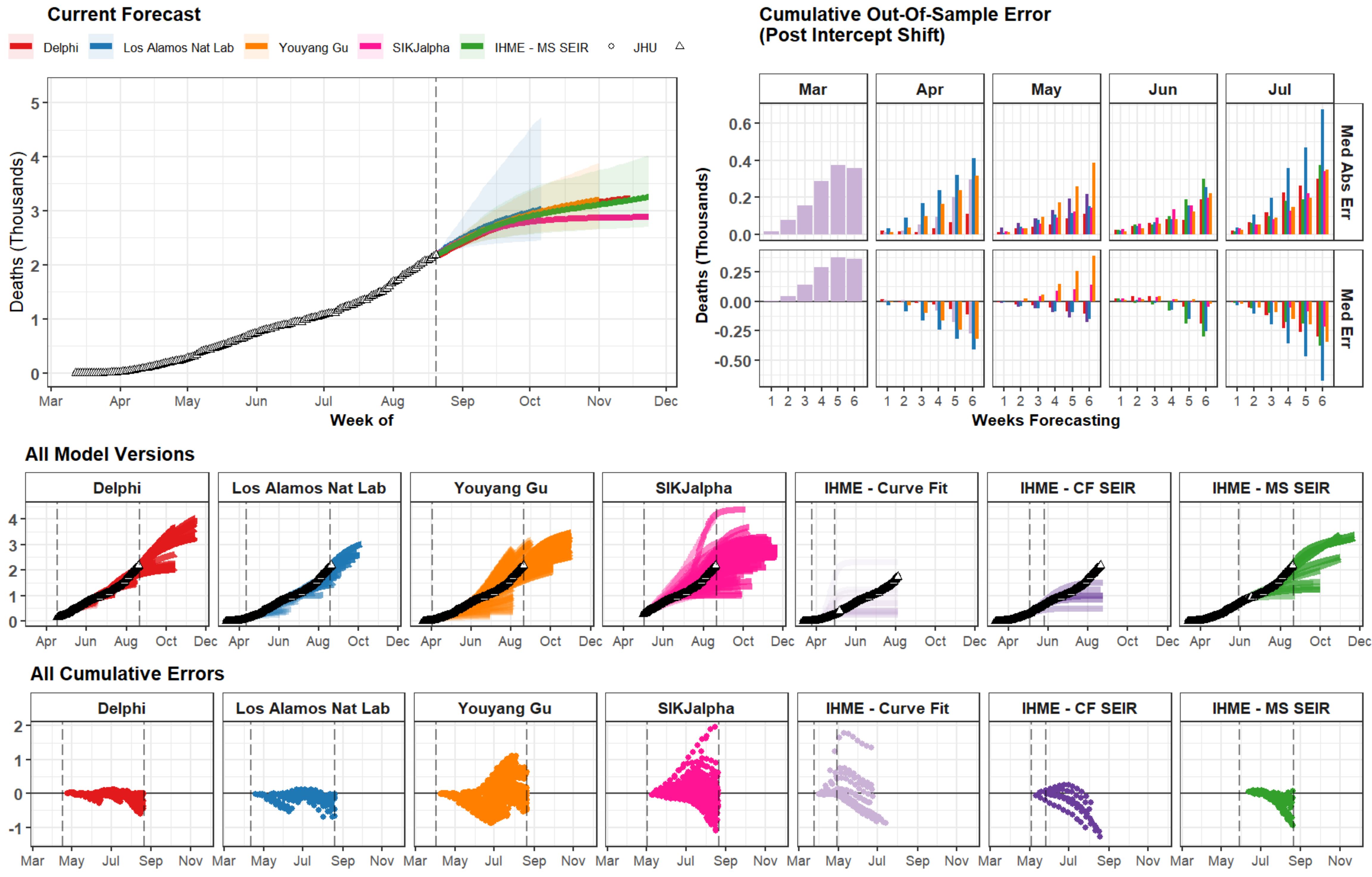

Switzerland

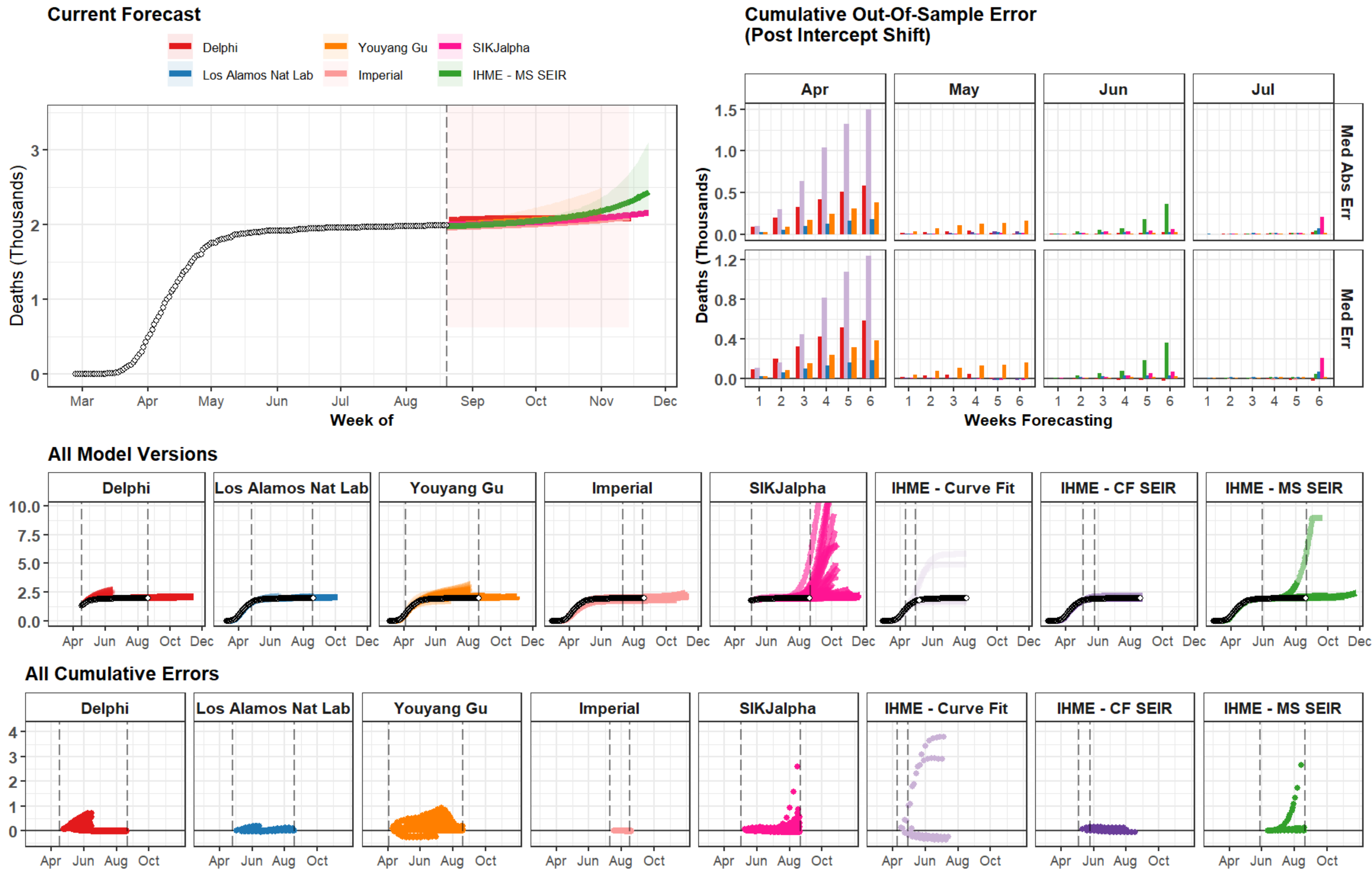

Alabama

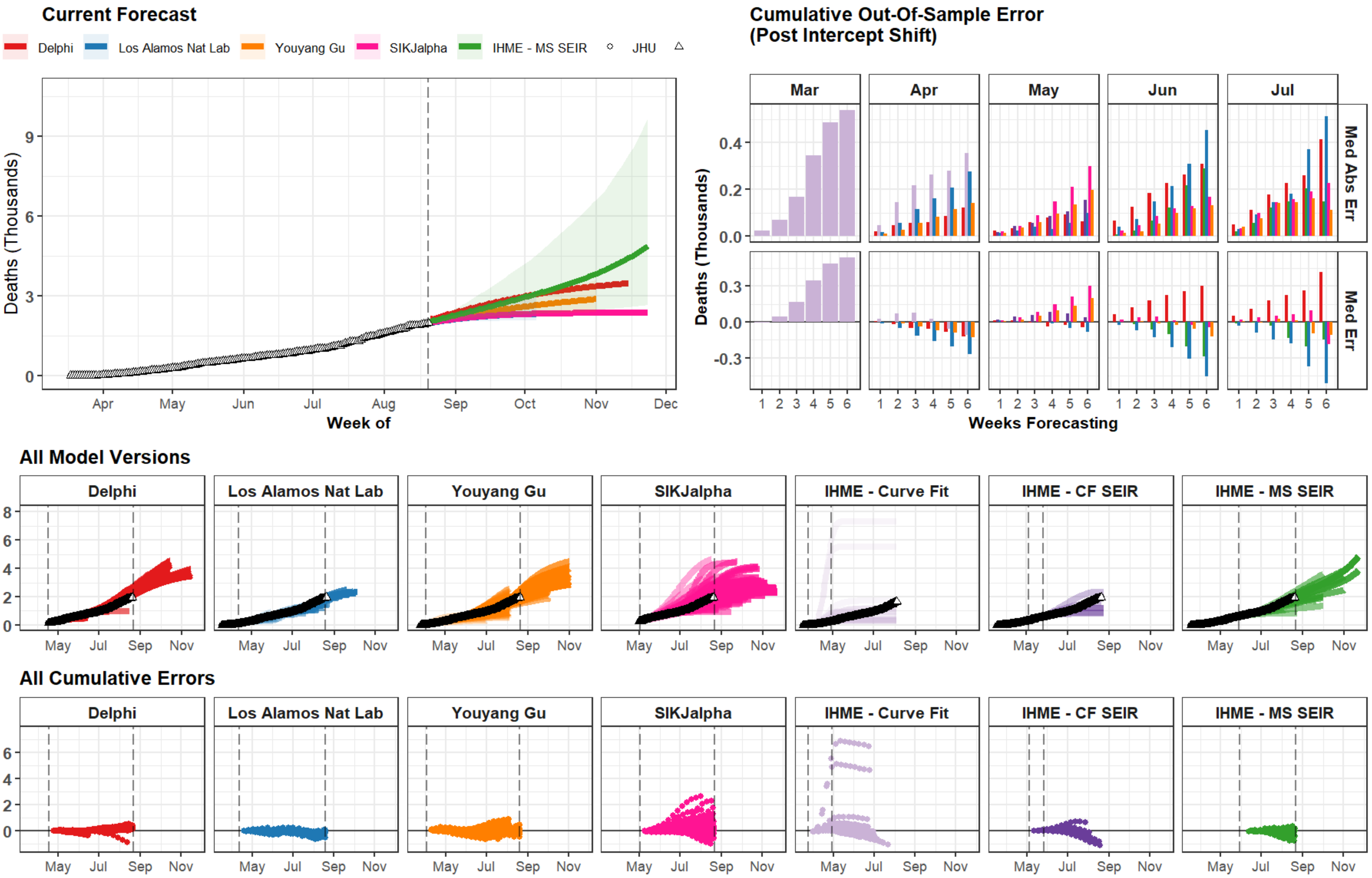
Current Forecast
Delphi
Los Alamos Nat Lab
Youyang Gu
SIKJalpha
IHME - MS SEIR
JHU
Deaths (Thousands)
Week of
Apr May Jun Jul Aug Sep Oct Nov Dec
Cumulative Out-Of-Sample Error
(Post Intercept Shift)
Mar Apr May Jun Jul
Med Abs Err
Med Err
Weeks Forecasting
All Model Versions
Delphi
Los Alamos Nat Lab
Youyang Gu
SIKJalpha
IHME - Curve Fit
IHME - CF SEIR
IHME - MS SEIR
All Cumulative Errors

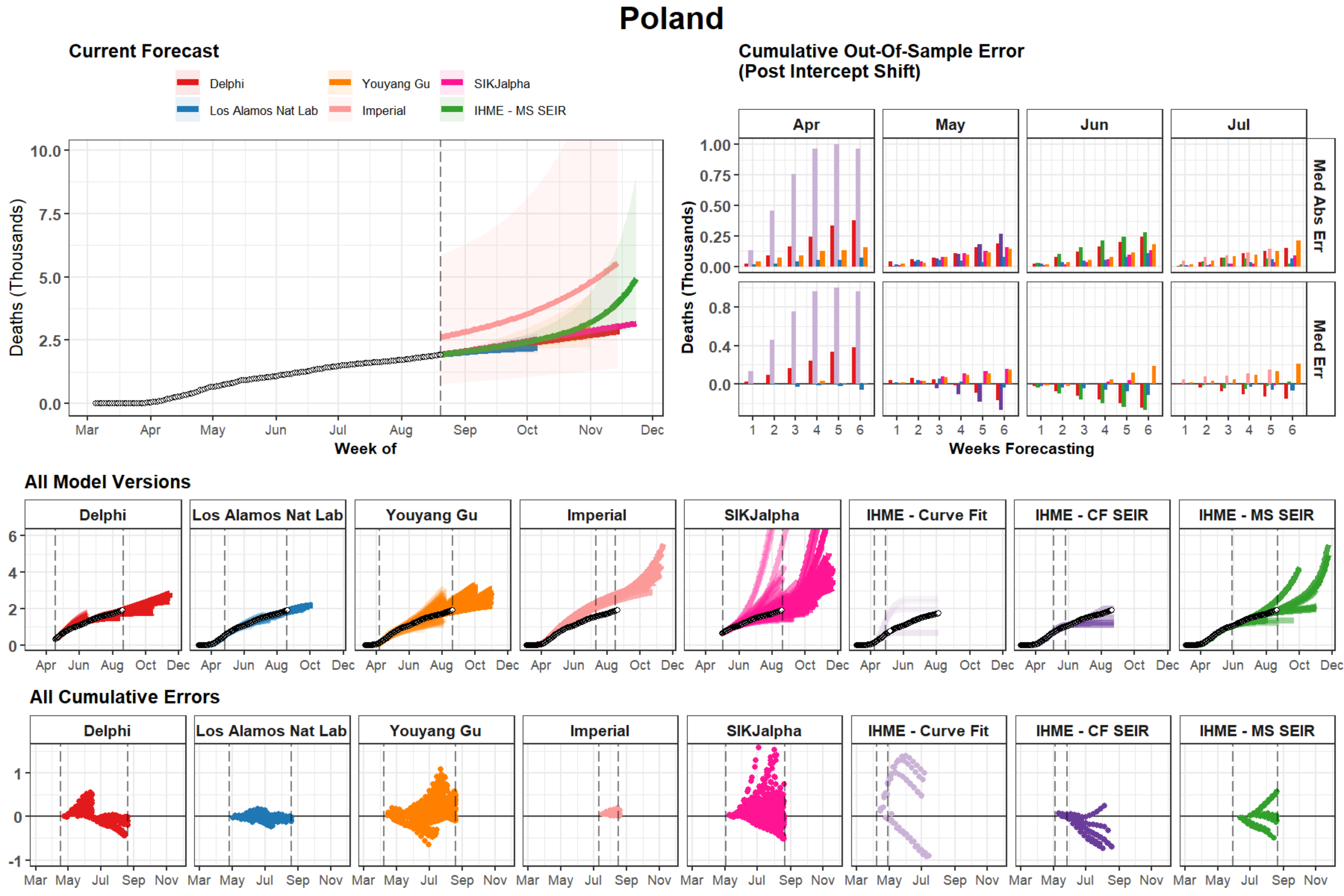

Poland
Current Forecast
Delphi
Youyang Gu
SIKJalpha
Los Alamos Nat Lab
Imperial
IHME - MS SEIR
Deaths (Thousands)
Week of
Cumulative Out-Of-Sample Error
(Post Intercept Shift)
Apr
May
Jun
Jul
Med Abs Err
Med Err
Weeks Forecasting
All Model Versions
Delphi
Los Alamos Nat Lab
Youyang Gu
Imperial
SIKJalpha
IHME - Curve Fit
IHME - CF SEIR
IHME - MS SEIR
All Cumulative Errors

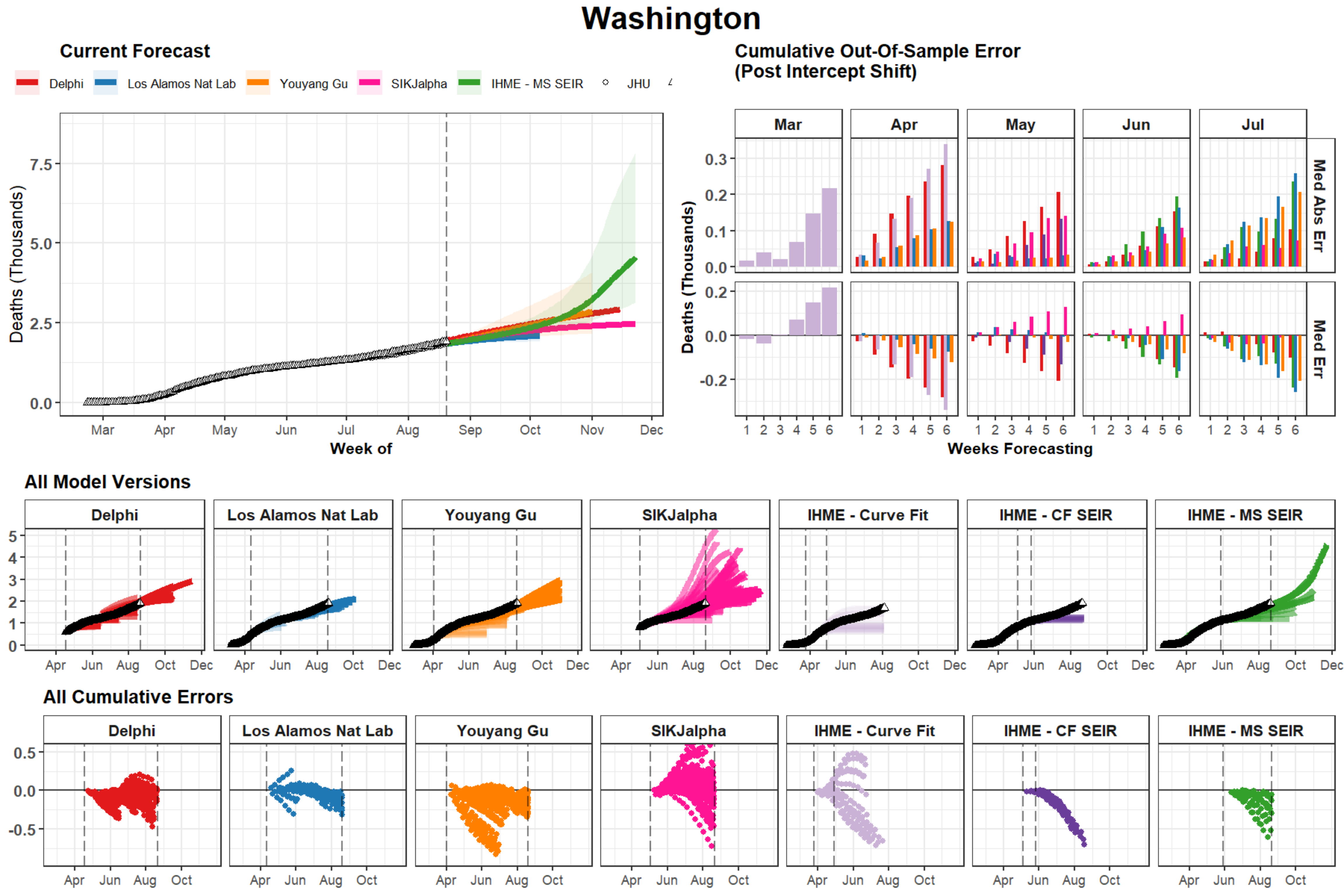

Washington
Current Forecast
Delphi
Los Alamos Nat Lab
Youyang Gu
SIKJalpha
IHME - MS SEIR
JHU
Deaths (Thousands)
7.5
5.0
2.5
0.0
Mar
Apr
May
Jun
Jul
Aug
Sep
Oct
Nov
Dec
Week of
Cumulative Out-Of-Sample Error
(Post Intercept Shift)
Mar
Apr
May
Jun
Jul
Med Abs Err
Med Err
Deaths (Thousands)
0.3
0.2
0.1
0.0
-0.2
1 2 3 4 5 6
Weeks Forecasting
All Model Versions
Delphi
Los Alamos Nat Lab
Youyang Gu
SIKJalpha
IHME - Curve Fit
IHME - CF SEIR
IHME - MS SEIR
5
4
3
2
1
0
Apr
Jun
Aug
Oct
Dec
All Cumulative Errors
Delphi
Los Alamos Nat Lab
Youyang Gu
SIKJalpha
IHME - Curve Fit
IHME - CF SEIR
IHME - MS SEIR
0.5
0.0
-0.5
Apr
Jun
Aug
Oct

Colorado

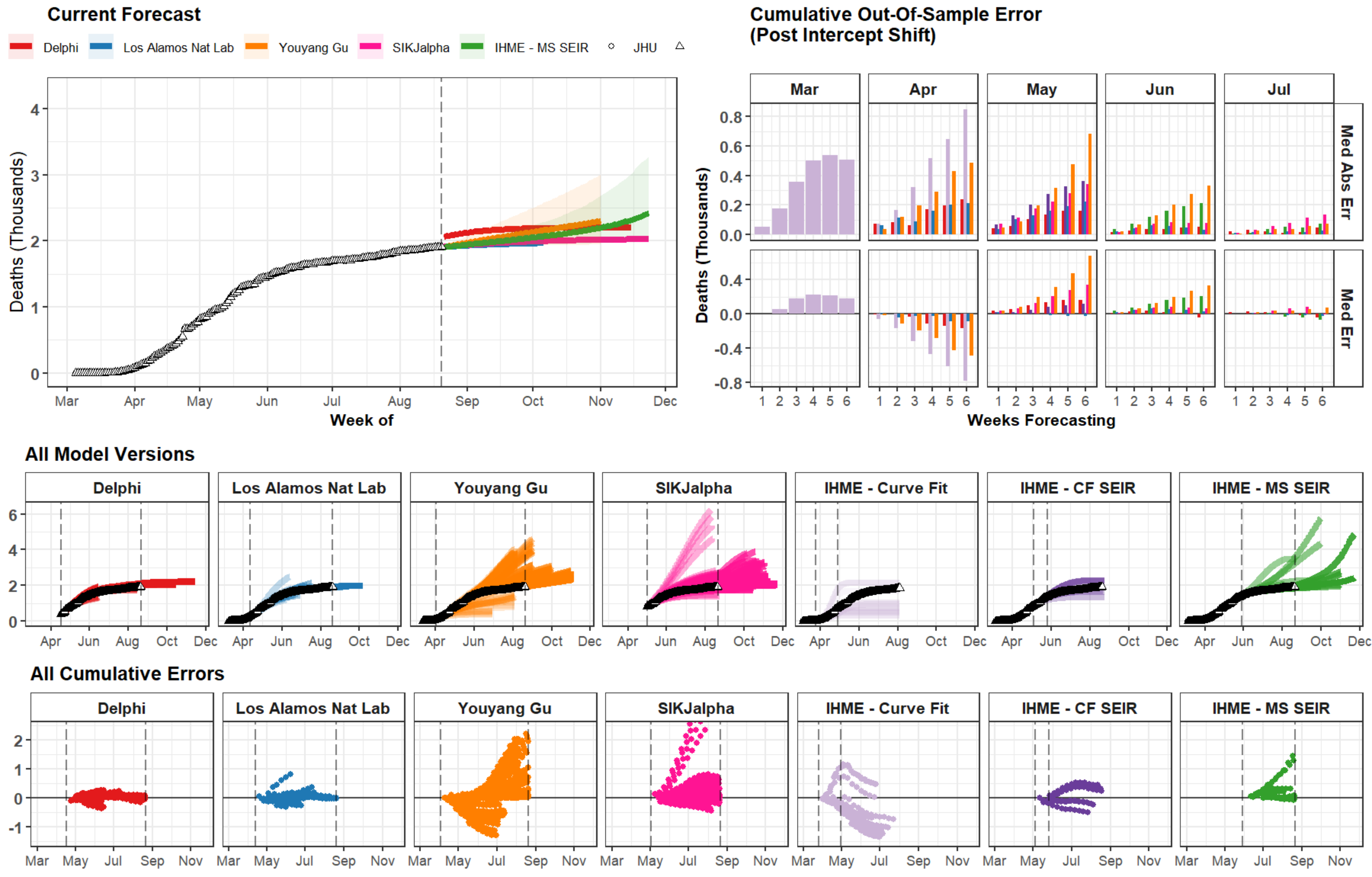
Current Forecast
Delphi
Los Alamos Nat Lab
Youyang Gu
SIKJalpha
IHME - MS SEIR
JHU
Deaths (Thousands)
Week of
Mar Apr May Jun Jul Aug Sep Oct Nov Dec
Cumulative Out-Of-Sample Error
(Post Intercept Shift)
Mar
Apr
May
Jun
Jul
Med Abs Err
Med Err
Deaths (Thousands)
Weeks Forecasting
All Model Versions
Delphi
Los Alamos Nat Lab
Youyang Gu
SIKJalpha
IHME - Curve Fit
IHME - CF SEIR
IHME - MS SEIR
All Cumulative Errors
Delphi
Los Alamos Nat Lab
Youyang Gu
SIKJalpha
IHME - Curve Fit
IHME - CF SEIR
IHME - MS SEIR

Panama

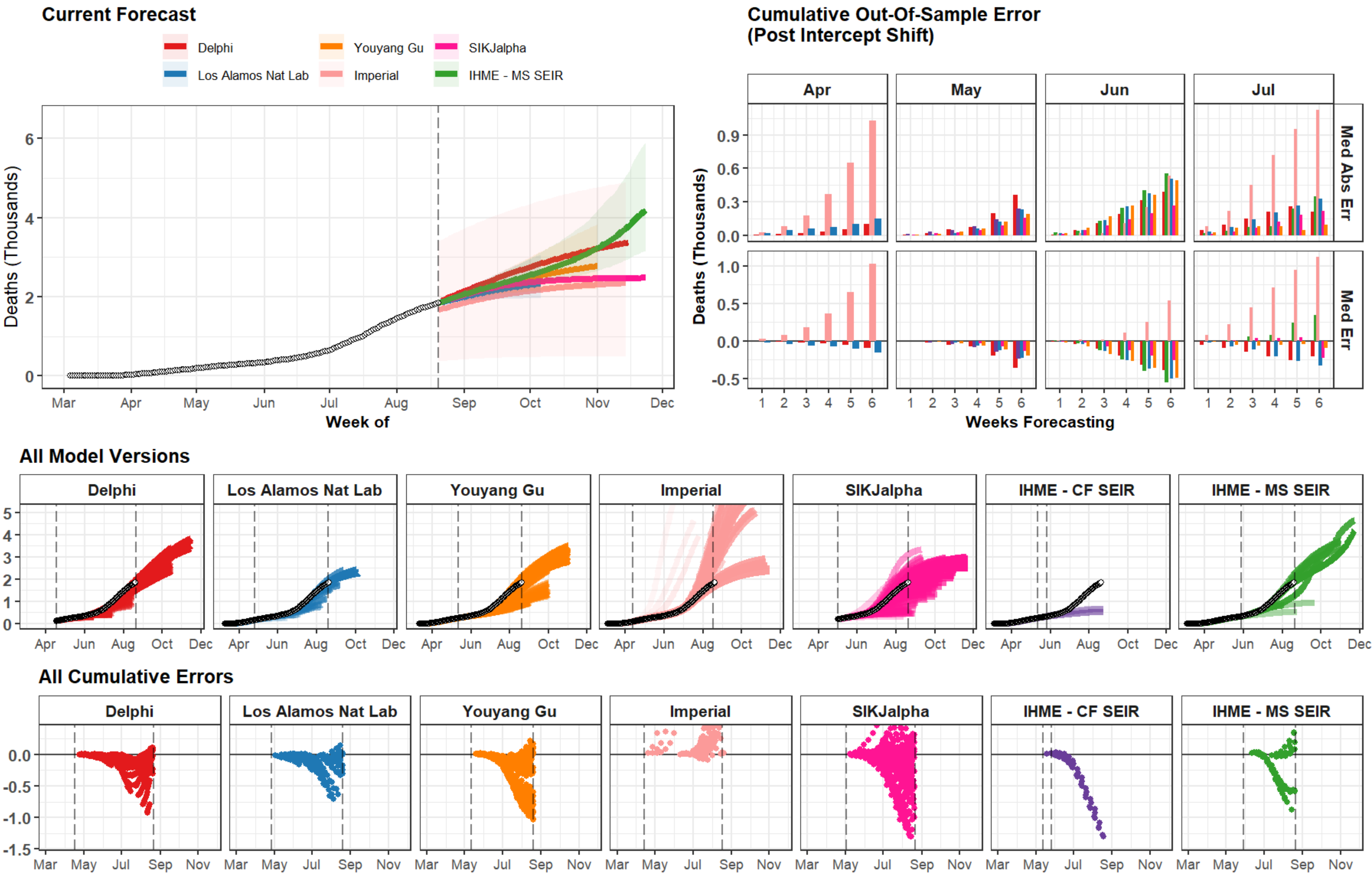

Current Forecast
Delphi
Los Alamos Nat Lab
Youyang Gu
Imperial
SIKJalpha
IHME - MS SEIR
Deaths (Thousands)
Week of
Mar
Apr
May
Jun
Jul
Aug
Sep
Oct
Nov
Dec
Cumulative Out-Of-Sample Error
(Post Intercept Shift)
Apr
May
Jun
Jul
Med Abs Err
Med Err
Weeks Forecasting
All Model Versions
Delphi
Los Alamos Nat Lab
Youyang Gu
Imperial
SIKJalpha
IHME - CF SEIR
IHME - MS SEIR
All Cumulative Errors

Minnesota

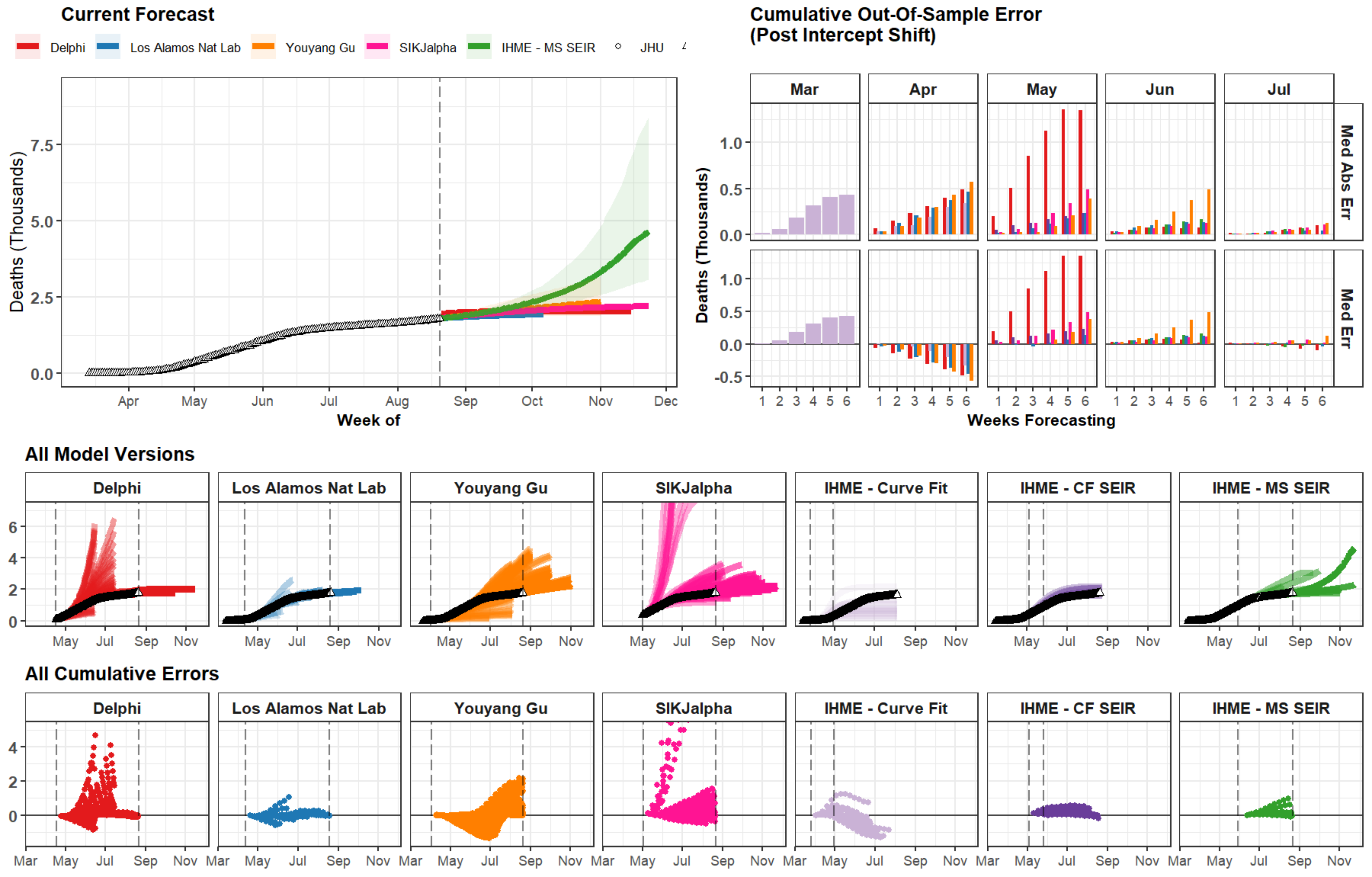
Current Forecast
Delphi
Los Alamos Nat Lab
Youyang Gu
SIKJalpha
IHME - MS SEIR
JHU
Deaths (Thousands)
7.5
5.0
2.5
0.0
Apr
May
Jun
Jul
Aug
Sep
Oct
Nov
Dec
Week of
Cumulative Out-Of-Sample Error
(Post Intercept Shift)
Mar
Apr
May
Jun
Jul
Med Abs Err
Med Err
Deaths (Thousands)
1.0
0.5
0.0
-0.5
1 2 3 4 5 6
Weeks Forecasting
All Model Versions
Delphi
Los Alamos Nat Lab
Youyang Gu
SIKJalpha
IHME - Curve Fit
IHME - CF SEIR
IHME - MS SEIR
6
4
2
0
May
Jul
Sep
Nov
All Cumulative Errors
Delphi
Los Alamos Nat Lab
Youyang Gu
SIKJalpha
IHME - Curve Fit
IHME - CF SEIR
IHME - MS SEIR
4
2
0
Mar
May
Jul
Sep
Nov

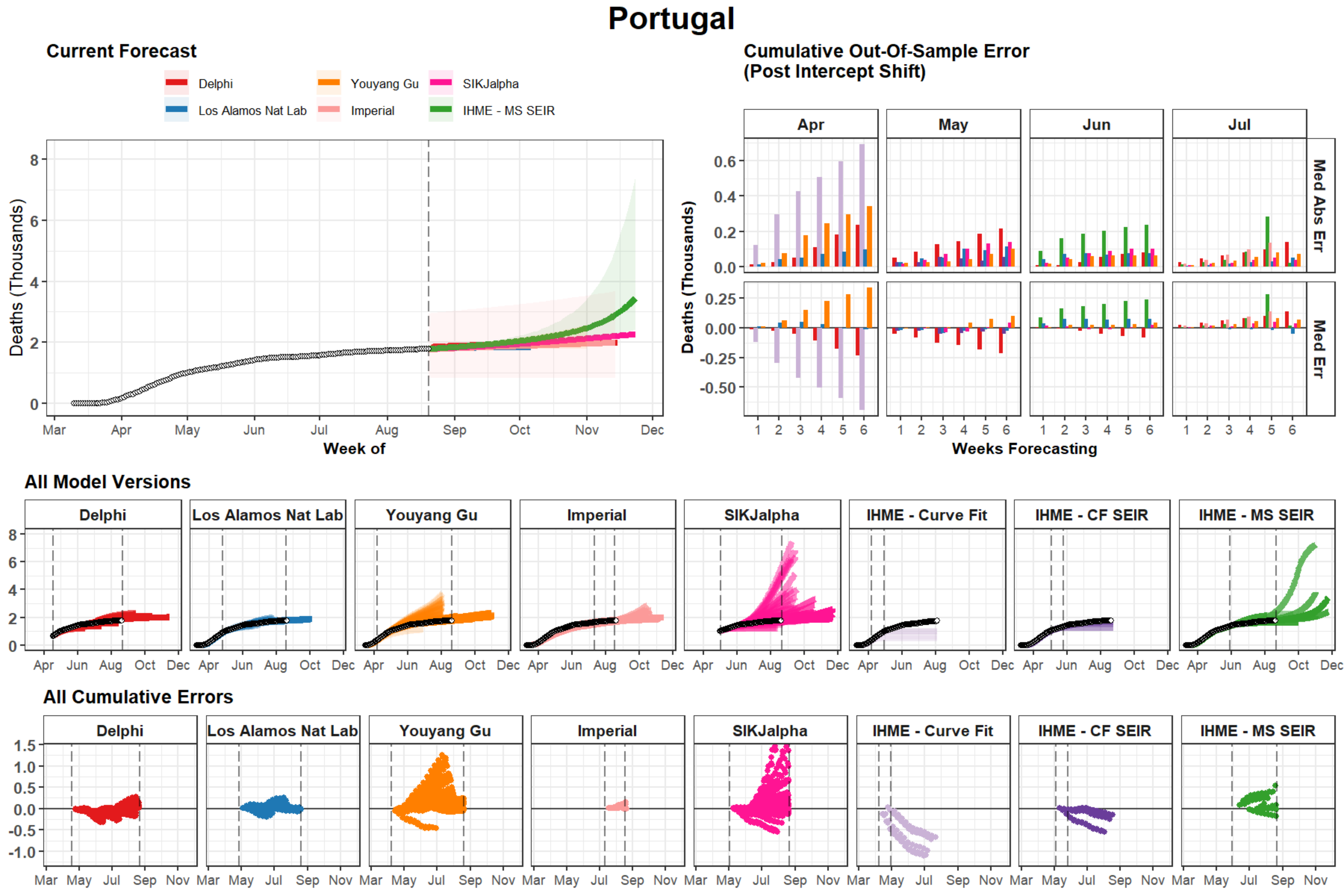

Portugal
Current Forecast
Delphi
Youyang Gu
SIKJalpha
Los Alamos Nat Lab
Imperial
IHME - MS SEIR
Deaths (Thousands)
Week of
Cumulative Out-Of-Sample Error
(Post Intercept Shift)
Apr
May
Jun
Jul
Med Abs Err
Med Err
Weeks Forecasting
All Model Versions
Delphi
Los Alamos Nat Lab
Youyang Gu
Imperial
SIKJalpha
IHME - Curve Fit
IHME - CF SEIR
IHME - MS SEIR
All Cumulative Errors

Ireland

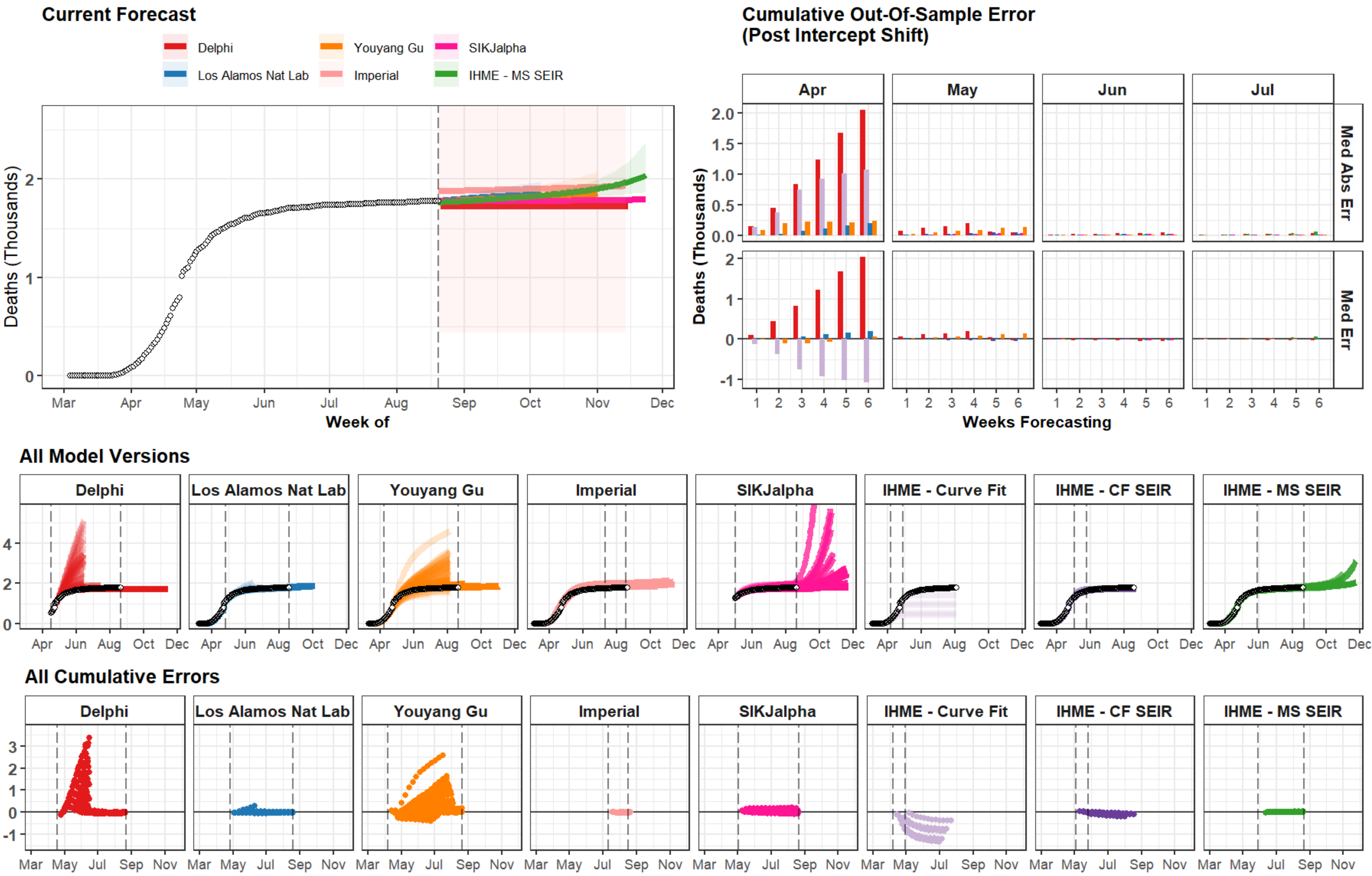
Current Forecast
Delphi
Los Alamos Nat Lab
Youyang Gu
Imperial
SIKJalpha
IHME - MS SEIR
Deaths (Thousands)
Week of
Mar Apr May Jun Jul Aug Sep Oct Nov Dec
Cumulative Out-Of-Sample Error
(Post Intercept Shift)
Apr
May
Jun
Jul
Med Abs Err
Med Err
Weeks Forecasting
All Model Versions
Delphi
Los Alamos Nat Lab
Youyang Gu
Imperial
SIKJalpha
IHME - Curve Fit
IHME - CF SEIR
IHME - MS SEIR
All Cumulative Errors

Honduras

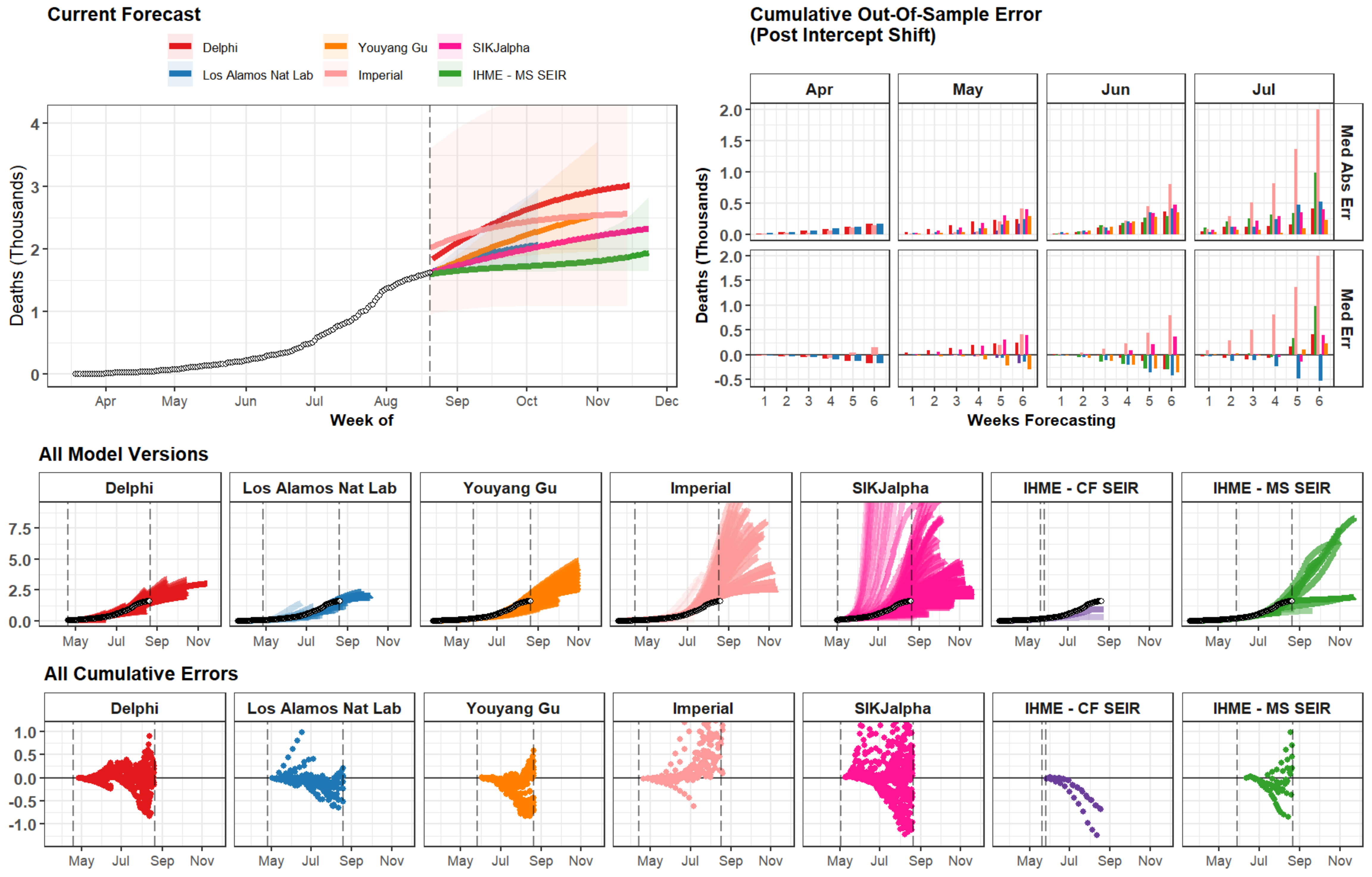

Current Forecast
Delphi
Los Alamos Nat Lab
Youyang Gu
Imperial
SIKJalpha
IHME - MS SEIR
Deaths (Thousands)
Week of
Apr
May
Jun
Jul
Aug
Sep
Oct
Nov
Dec
Cumulative Out-Of-Sample Error
(Post Intercept Shift)
Med Abs Err
Med Err
Weeks Forecasting
All Model Versions
IHME - CF SEIR
All Cumulative Errors

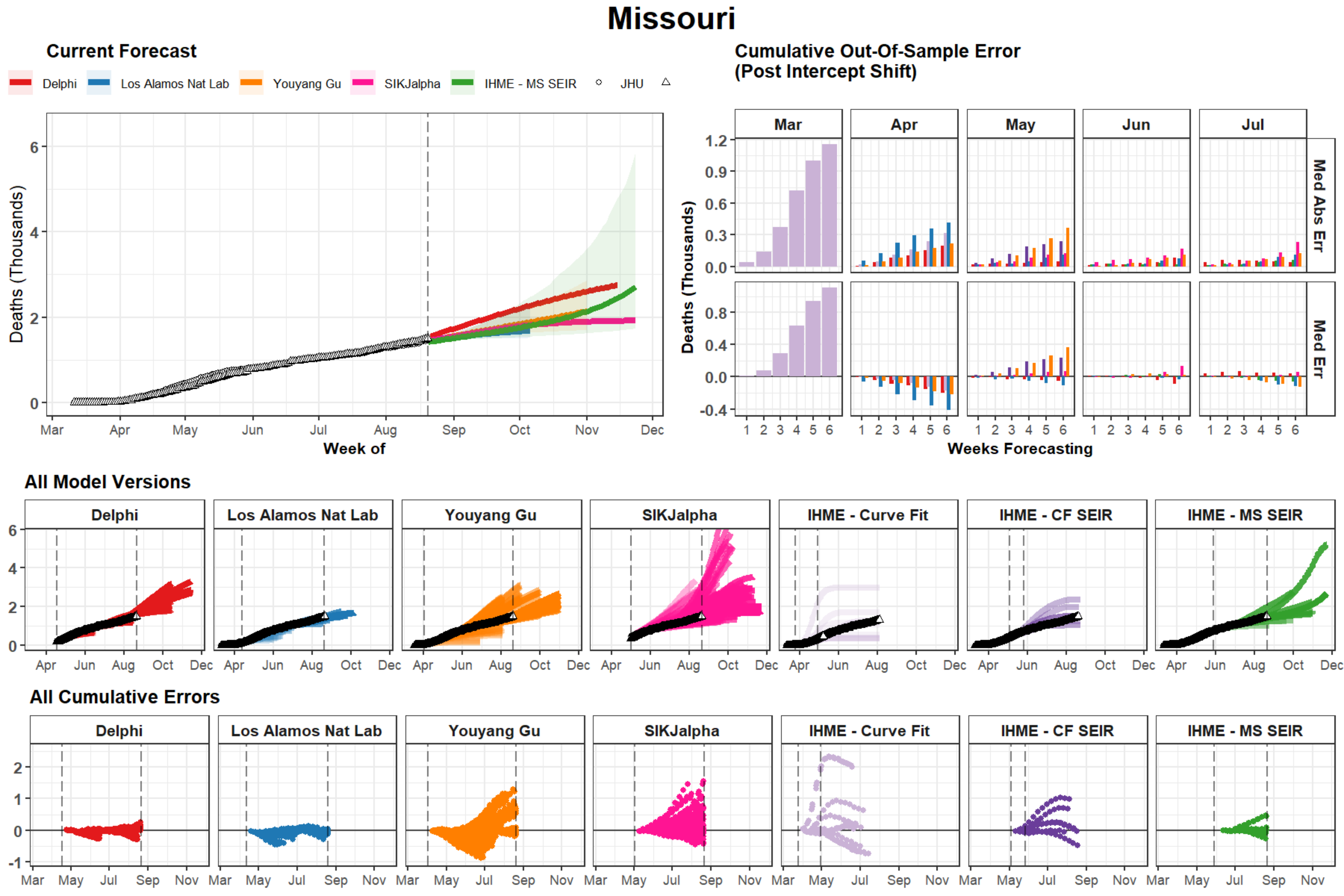

Missouri
Current Forecast
Delphi
Los Alamos Nat Lab
Youyang Gu
SIKJalpha
IHME - MS SEIR
JHU
Deaths (Thousands)
Week of
Cumulative Out-Of-Sample Error
(Post Intercept Shift)
Mar
Apr
May
Jun
Jul
Med Abs Err
Med Err
Weeks Forecasting
All Model Versions
Delphi
Los Alamos Nat Lab
Youyang Gu
SIKJalpha
IHME - Curve Fit
IHME - CF SEIR
IHME - MS SEIR
All Cumulative Errors
Delphi
Los Alamos Nat Lab
Youyang Gu
SIKJalpha
IHME - Curve Fit
IHME - CF SEIR
IHME - MS SEIR

Dominican Republic

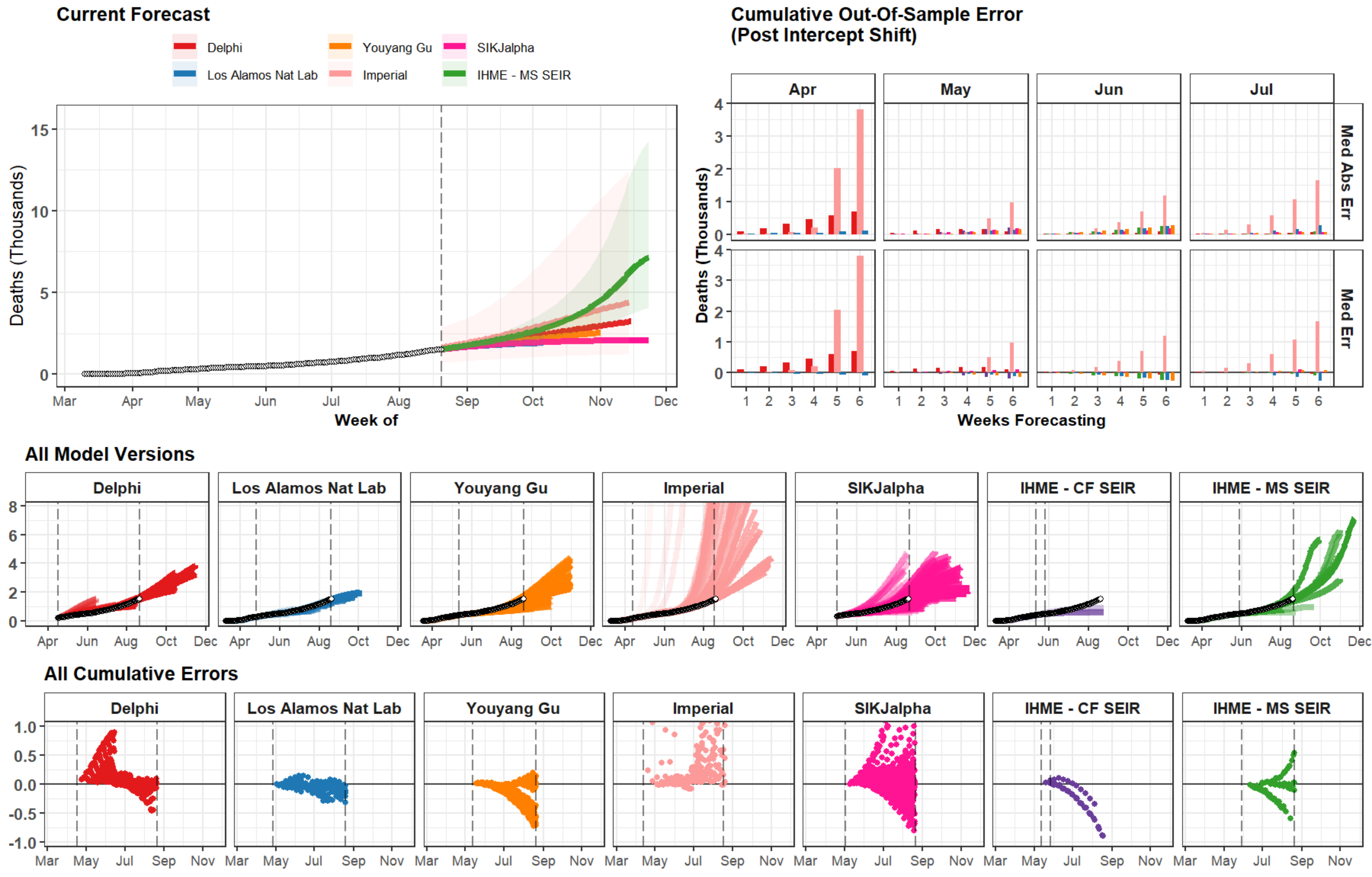

Kyrgyzstan

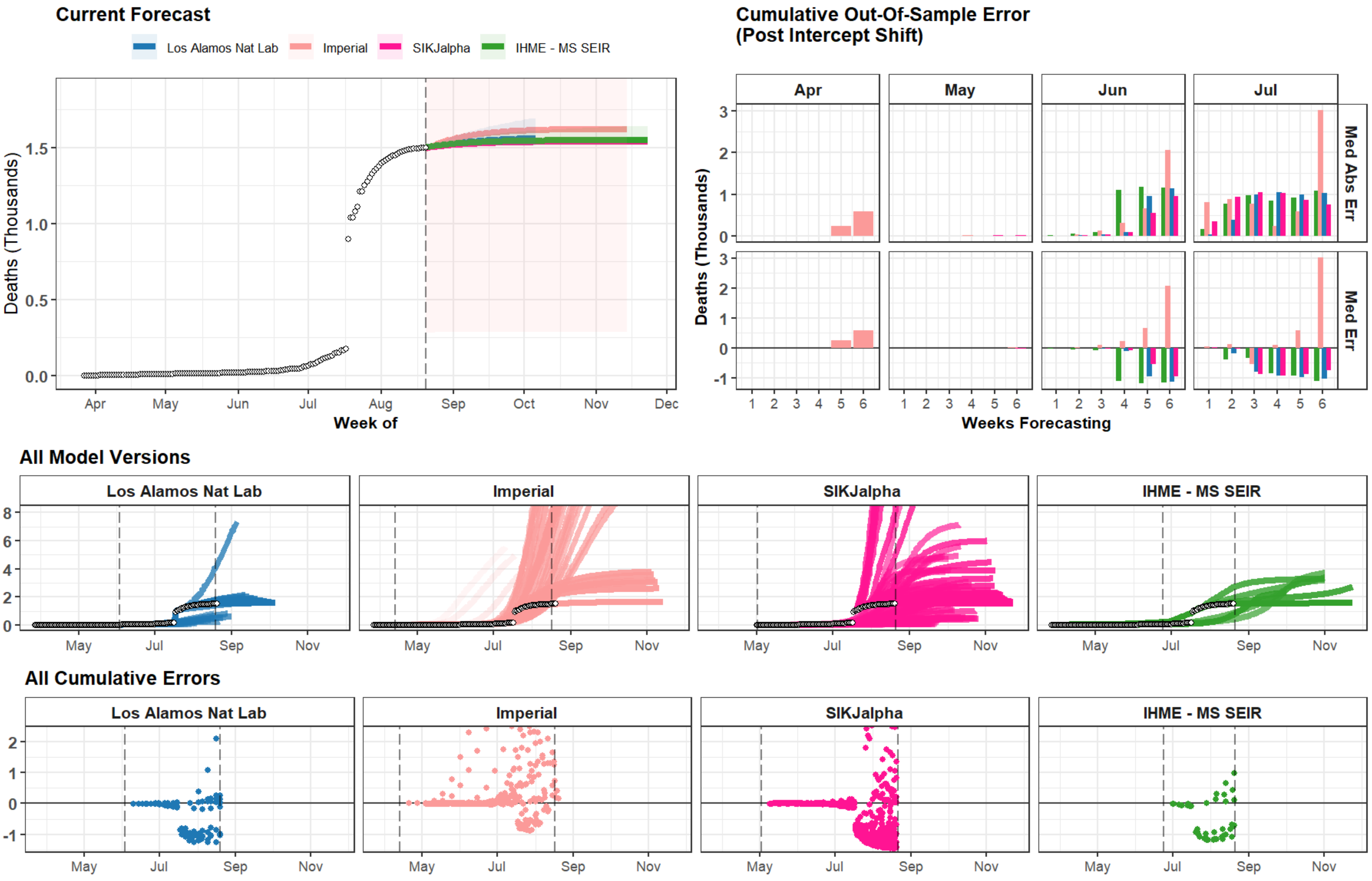

Current Forecast
Los Alamos Nat Lab
Imperial
SIKJalpha
IHME - MS SEIR
Deaths (Thousands)
1.5
1.0
0.5
0.0
Apr
May
Jun
Jul
Aug
Sep
Oct
Nov
Dec
Week of
Cumulative Out-Of-Sample Error
(Post Intercept Shift)
Apr
May
Jun
Jul
Med Abs Err
Med Err
Deaths (Thousands)
Weeks Forecasting
All Model Versions
Los Alamos Nat Lab
Imperial
SIKJalpha
IHME - MS SEIR
8
6
4
2
0
May
Jul
Sep
Nov
All Cumulative Errors
Los Alamos Nat Lab
Imperial
SIKJalpha
IHME - MS SEIR
2
1
0
-1
May
Jul
Sep
Nov

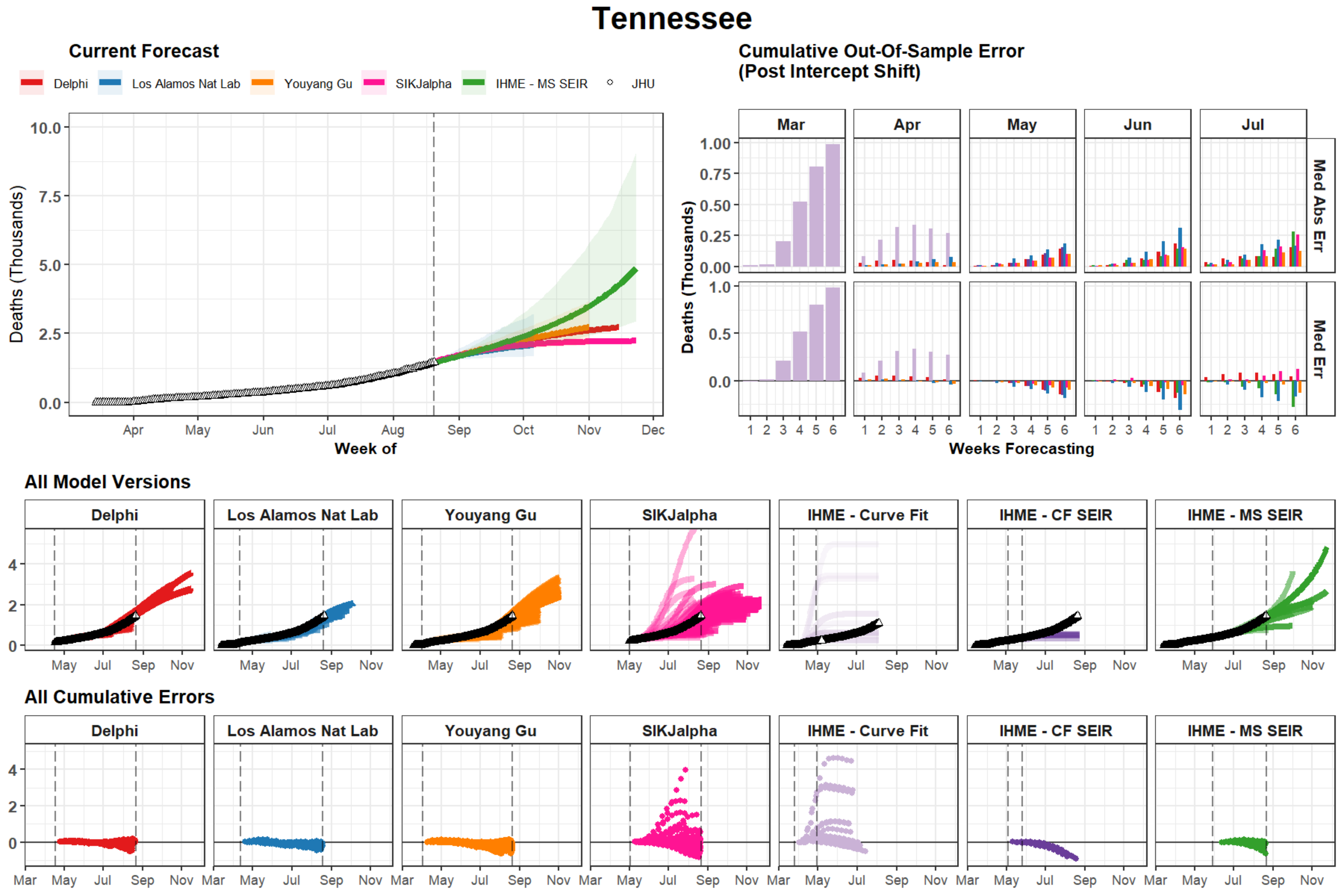
Tennessee
Current Forecast
Delphi
Los Alamos Nat Lab
Youyang Gu
SIKJalpha
IHME - MS SEIR
JHU
Deaths (Thousands)
Week of
Apr
May
Jun
Jul
Aug
Sep
Oct
Nov
Dec
Cumulative Out-Of-Sample Error
(Post Intercept Shift)
Mar
Apr
May
Jun
Jul
Med Abs Err
Med Err
Weeks Forecasting
All Model Versions
IHME - Curve Fit
IHME - CF SEIR
All Cumulative Errors

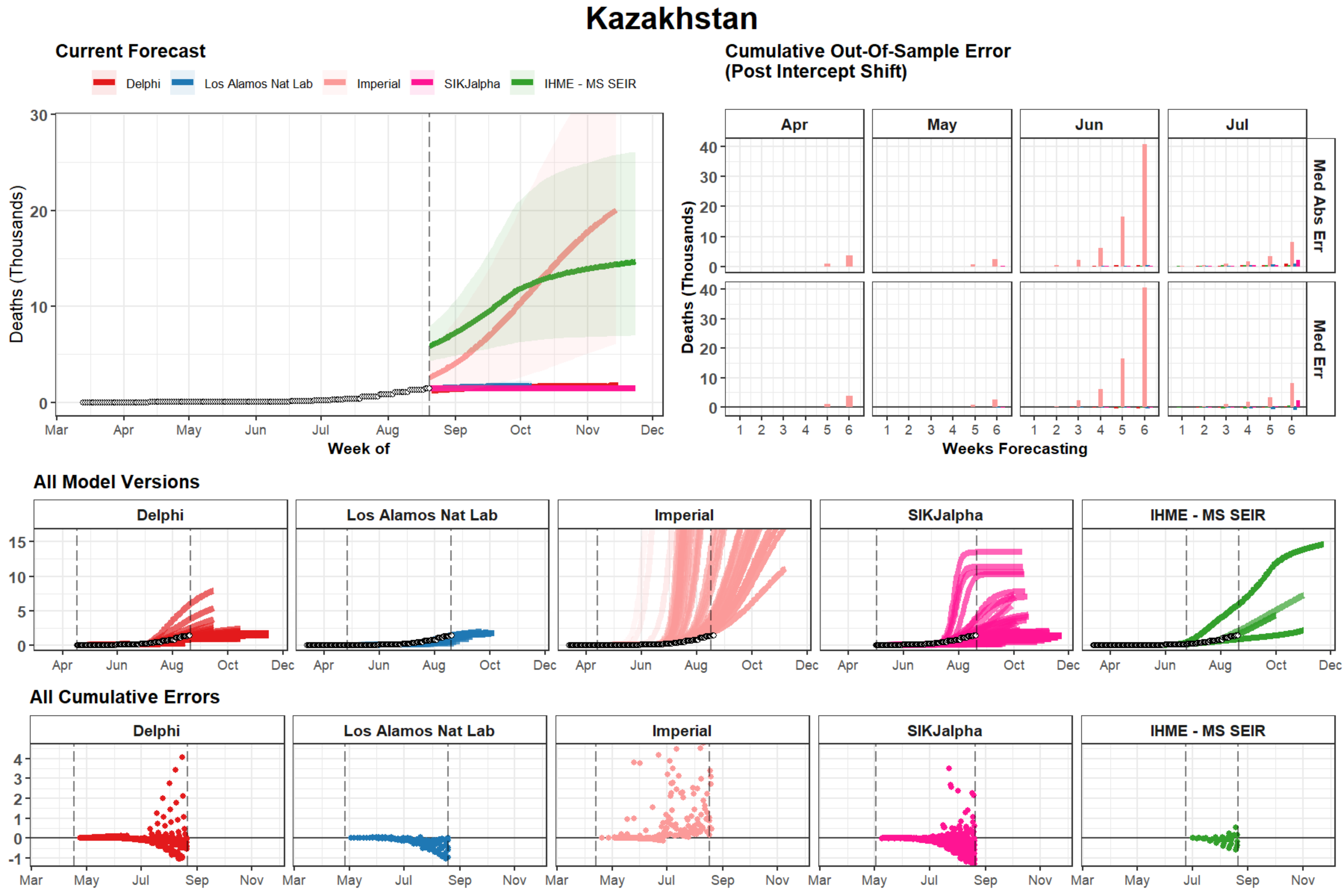

Kazakhstan
Current Forecast
Delphi
Los Alamos Nat Lab
Imperial
SIKJalpha
IHME - MS SEIR
Deaths (Thousands)
Week of
Cumulative Out-Of-Sample Error
(Post Intercept Shift)
Apr
May
Jun
Jul
Med Abs Err
Med Err
Weeks Forecasting
All Model Versions
All Cumulative Errors

Algeria

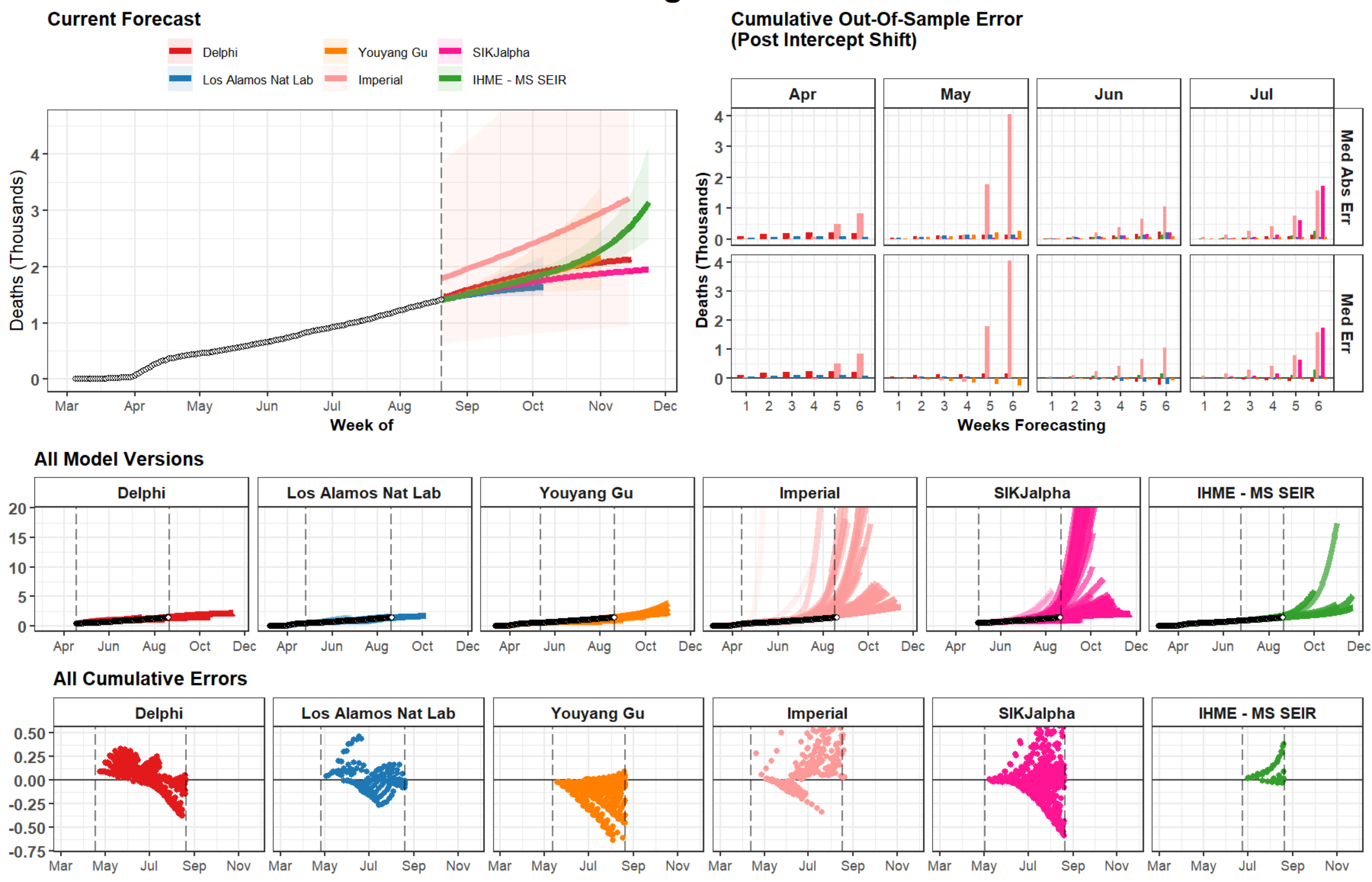

Current Forecast
Delphi
Los Alamos Nat Lab
Youyang Gu
Imperial
SIKJalpha
IHME - MS SEIR
Deaths (Thousands)
Week of
Mar
Apr
May
Jun
Jul
Aug
Sep
Oct
Nov
Dec
Cumulative Out-Of-Sample Error
(Post Intercept Shift)
Apr
May
Jun
Jul
Med Abs Err
Med Err
Weeks Forecasting
All Model Versions
All Cumulative Errors

Afghanistan

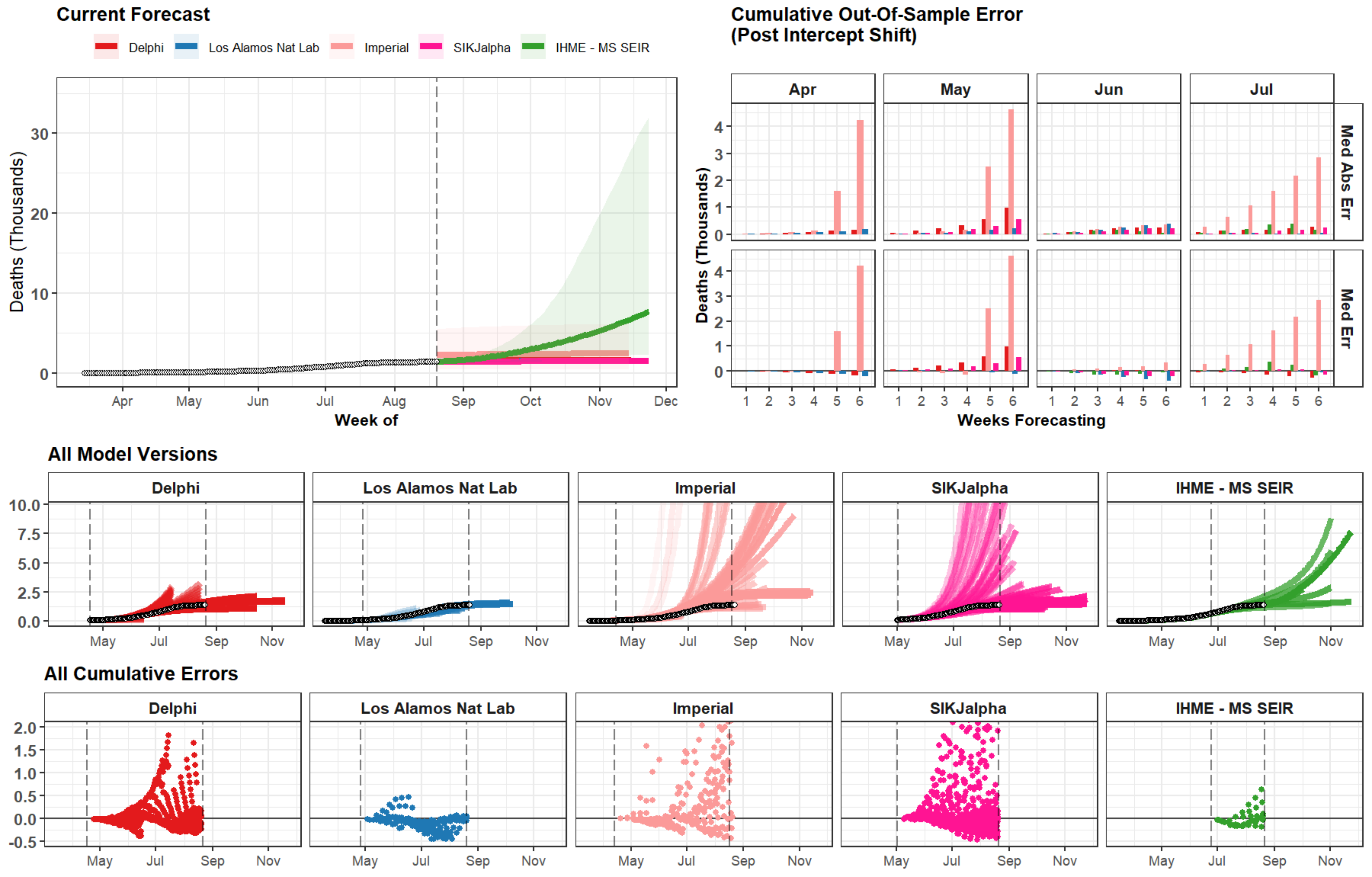

Nevada

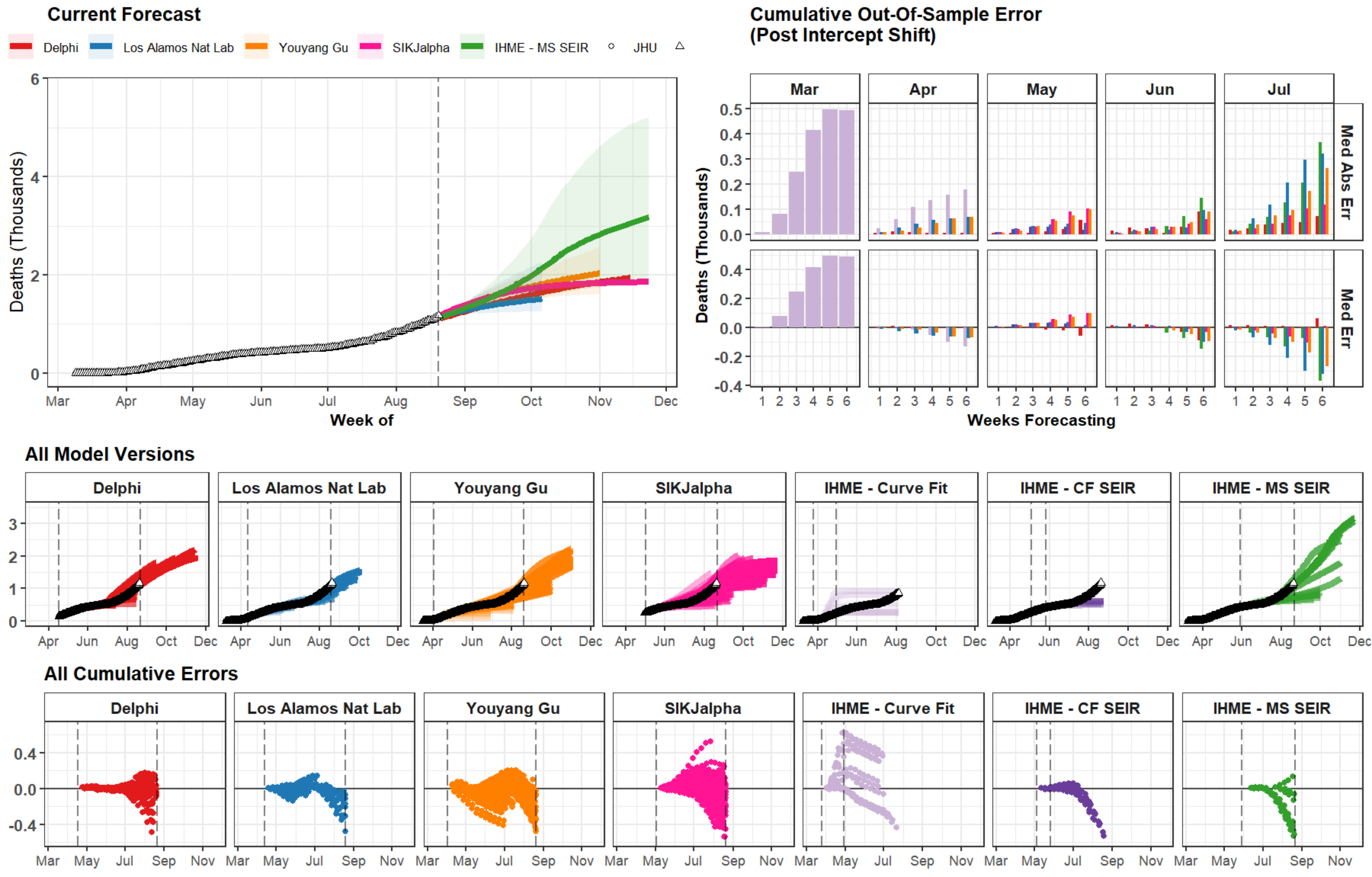

Current Forecast
Delphi
Los Alamos Nat Lab
Youyang Gu
SIKJalpha
IHME - MS SEIR
JHU
Deaths (Thousands)
Week of
Mar
Apr
May
Jun
Jul
Aug
Sep
Oct
Nov
Dec
Cumulative Out-Of-Sample Error
(Post Intercept Shift)
Med Abs Err
Med Err
Weeks Forecasting
All Model Versions
IHME - Curve Fit
IHME - CF SEIR
All Cumulative Errors

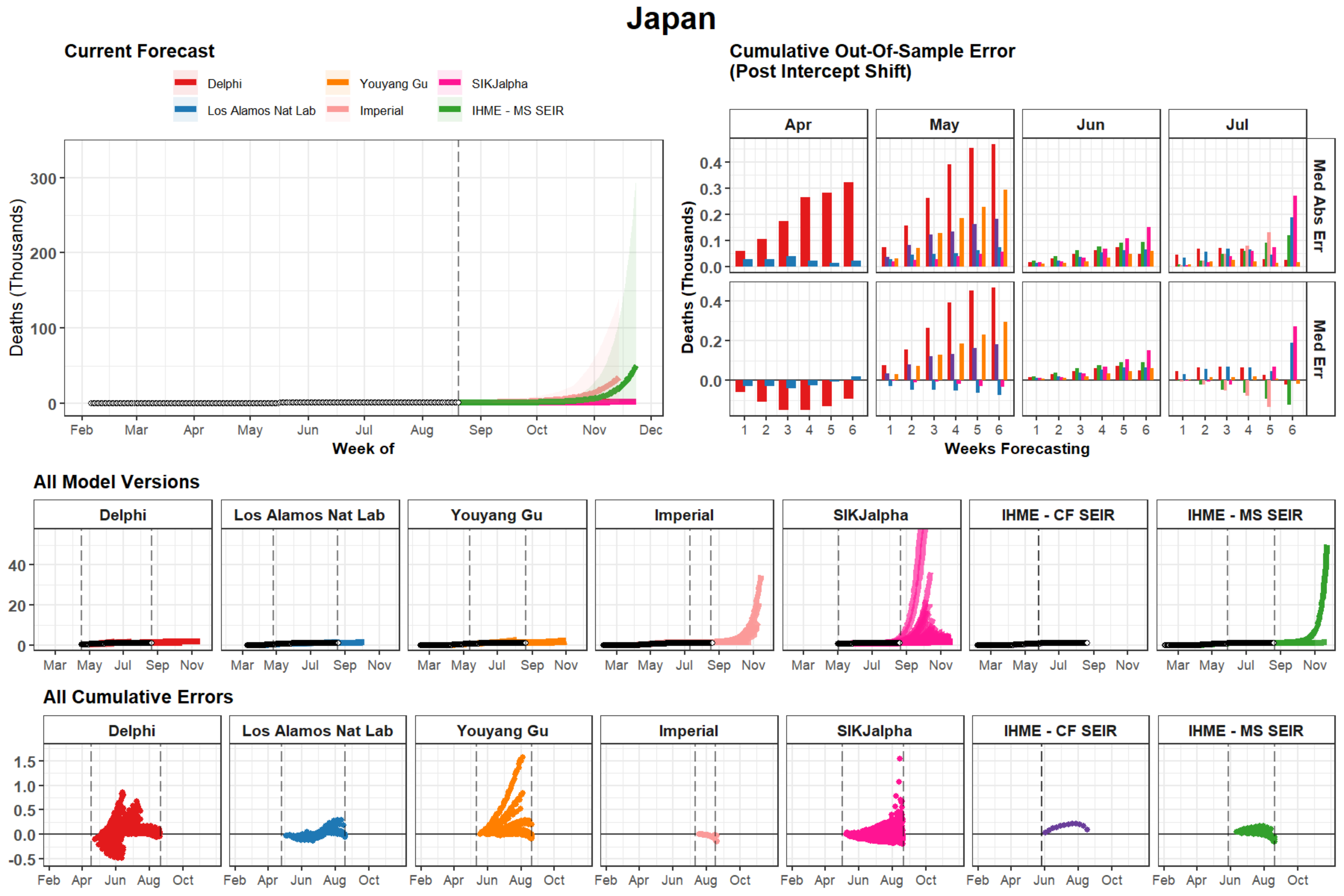
Japan
Current Forecast
Delphi
Youyang Gu
SIKJalpha
Los Alamos Nat Lab
Imperial
IHME - MS SEIR
Deaths (Thousands)
300
200
100
0
Feb
Mar
Apr
May
Jun
Jul
Aug
Sep
Oct
Nov
Dec
Week of
Cumulative Out-Of-Sample Error
(Post Intercept Shift)
Apr
May
Jun
Jul
Med Abs Err
Med Err
0.4
0.3
0.2
0.1
0.0
1
2
3
4
5
6
Weeks Forecasting
All Model Versions
Delphi
Los Alamos Nat Lab
Youyang Gu
Imperial
SIKJalpha
IHME - CF SEIR
IHME - MS SEIR
40
20
0
All Cumulative Errors
1.5
1.0
0.5
0.0
-0.5

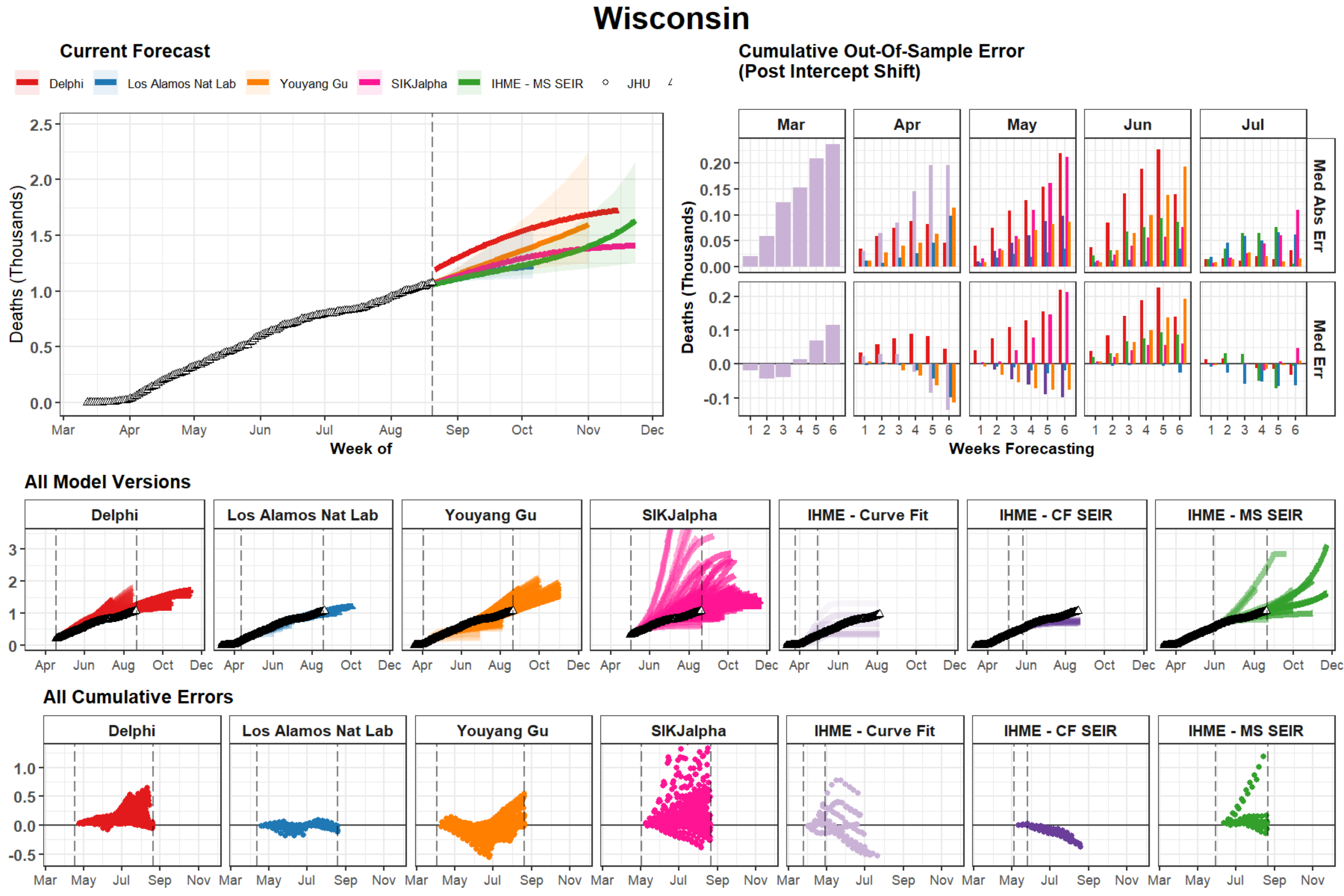
Wisconsin
Current Forecast
Delphi
Los Alamos Nat Lab
Youyang Gu
SIKJalpha
IHME - MS SEIR
JHU
Deaths (Thousands)
Week of
Mar
Apr
May
Jun
Jul
Aug
Sep
Oct
Nov
Dec
Cumulative Out-Of-Sample Error
(Post Intercept Shift)
Med Abs Err
Med Err
Weeks Forecasting
All Model Versions
IHME - Curve Fit
IHME - CF SEIR
All Cumulative Errors

Rhode Island

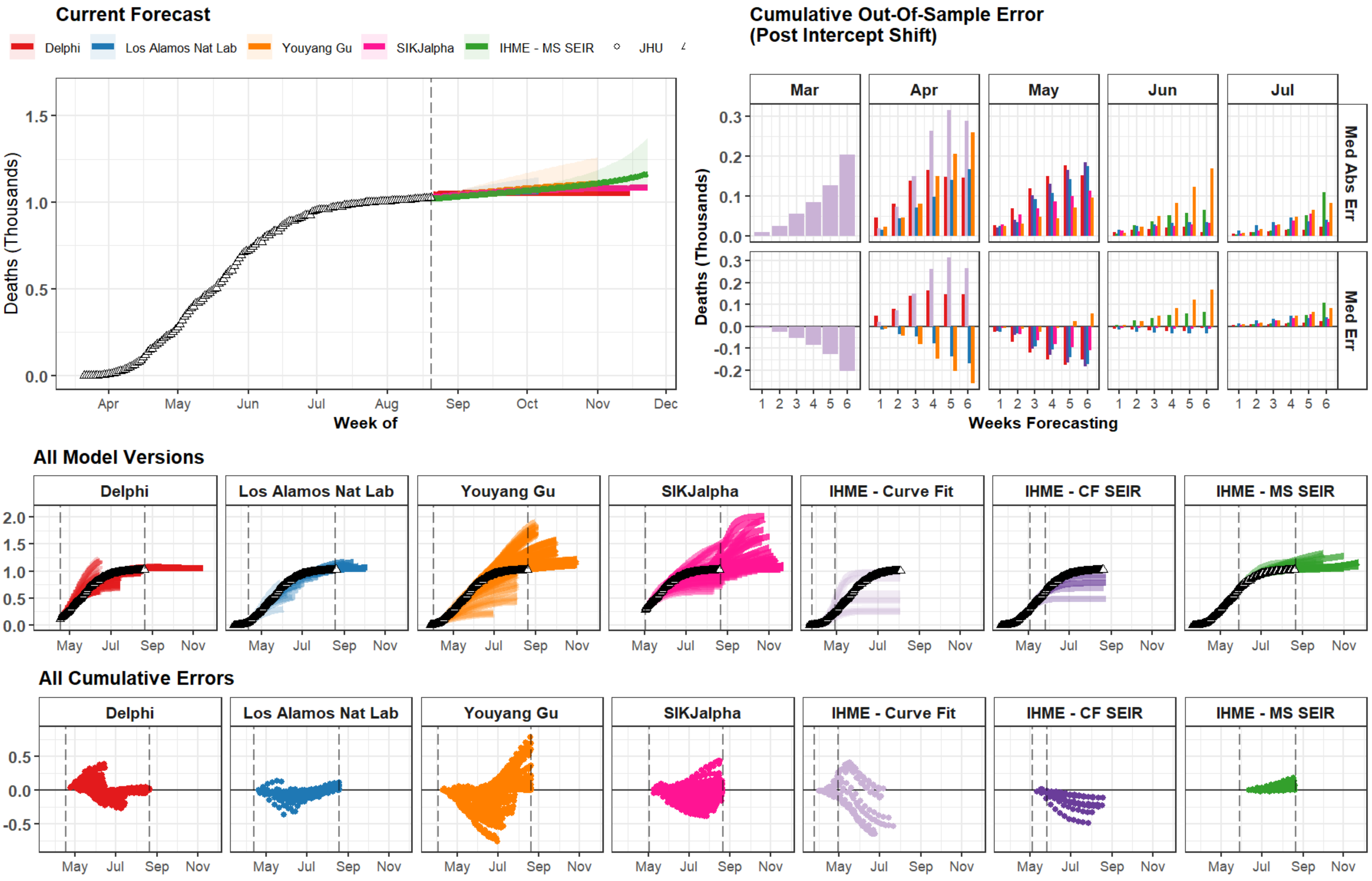

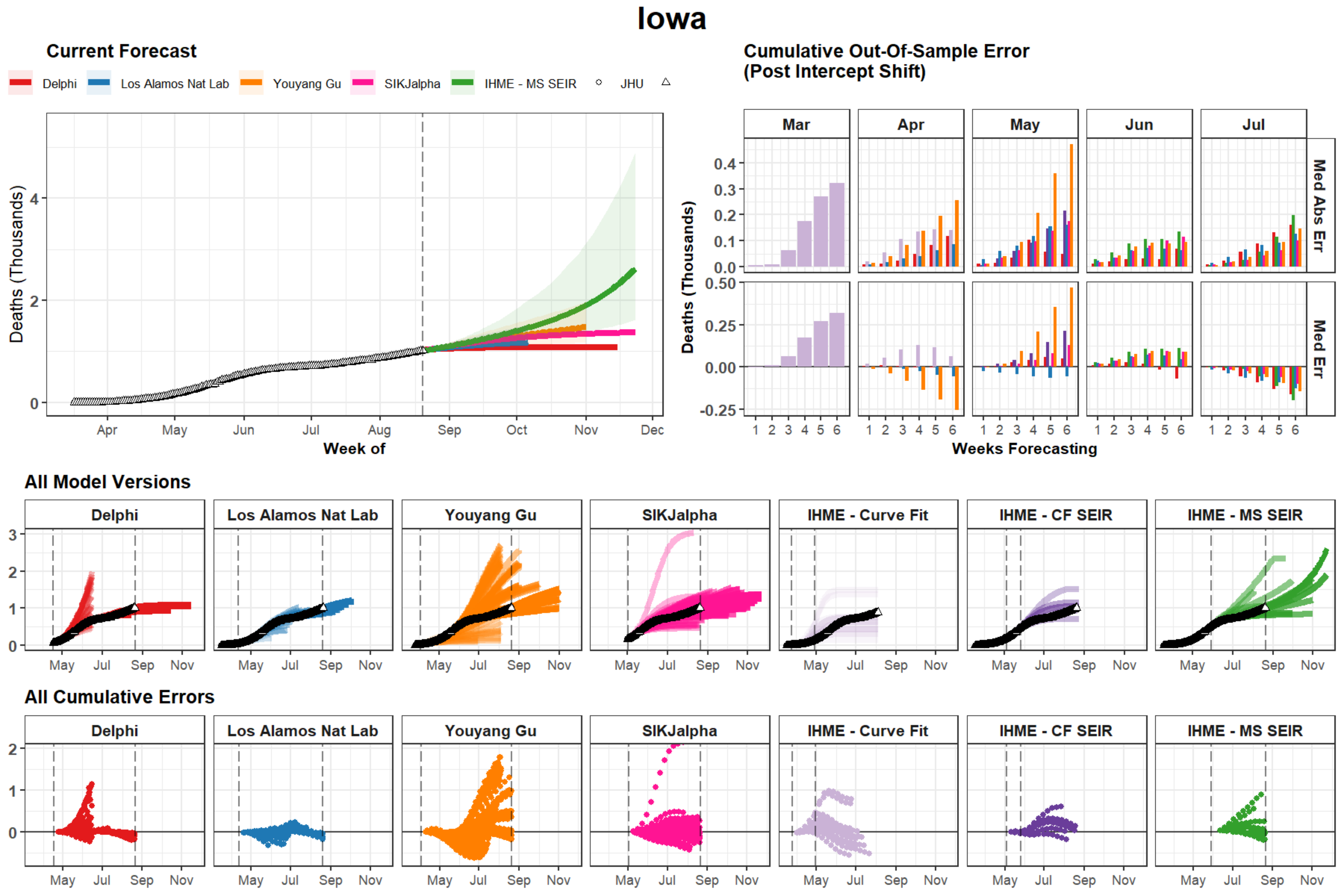
Iowa
Current Forecast
Delphi
Los Alamos Nat Lab
Youyang Gu
SIKJalpha
IHME - MS SEIR
JHU
Deaths (Thousands)
Week of
Apr
May
Jun
Jul
Aug
Sep
Oct
Nov
Dec
Cumulative Out-Of-Sample Error
(Post Intercept Shift)
Mar
Apr
May
Jun
Jul
Med Abs Err
Med Err
Weeks Forecasting
All Model Versions
Delphi
Los Alamos Nat Lab
Youyang Gu
SIKJalpha
IHME - Curve Fit
IHME - CF SEIR
IHME - MS SEIR
All Cumulative Errors

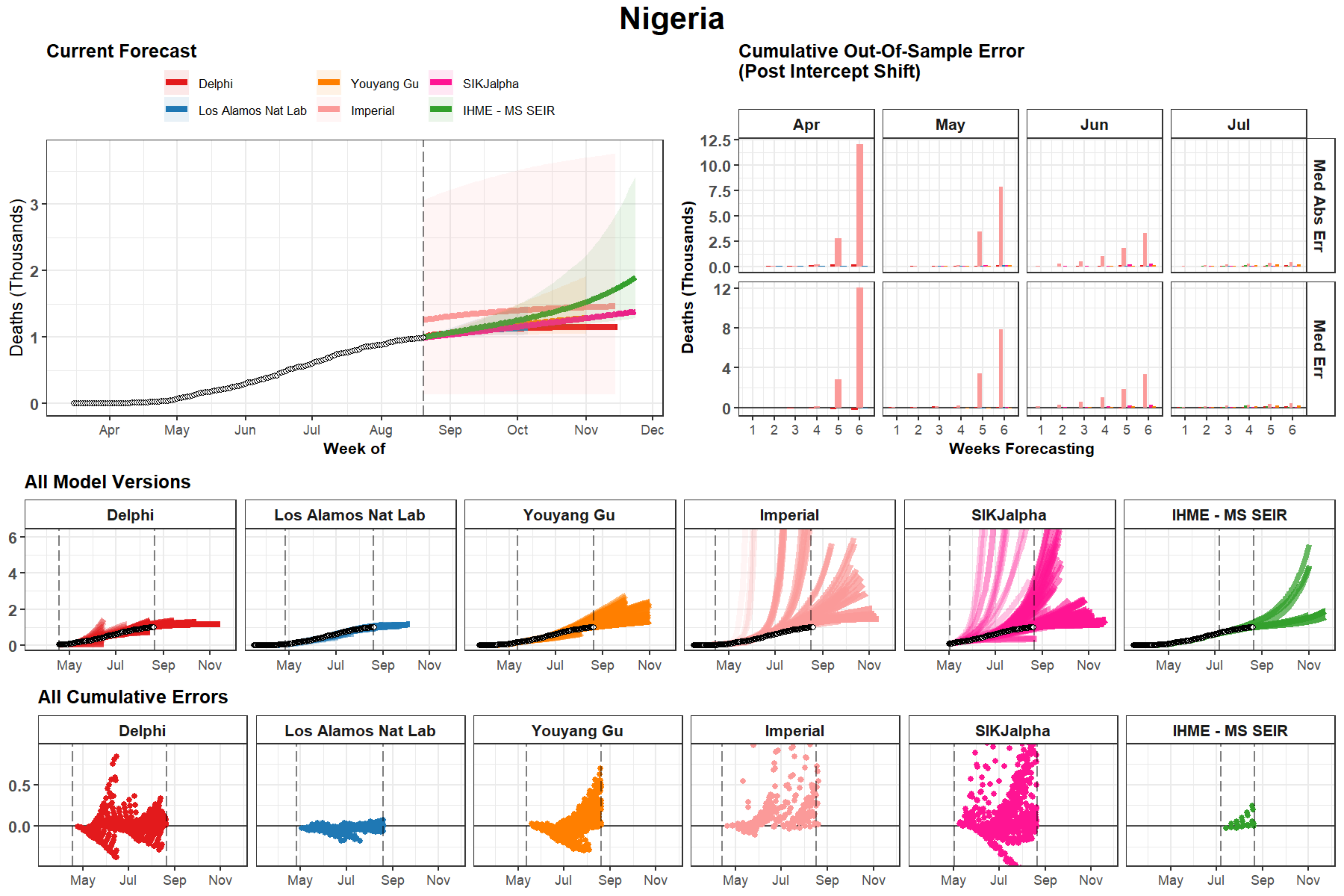
Nigeria
Current Forecast
Delphi
Los Alamos Nat Lab
Youyang Gu
Imperial
SIKJalpha
IHME - MS SEIR
Deaths (Thousands)
Week of
Apr
May
Jun
Jul
Aug
Sep
Oct
Nov
Dec
Cumulative Out-Of-Sample Error
(Post Intercept Shift)
Med Abs Err
Med Err
Weeks Forecasting
All Model Versions
All Cumulative Errors

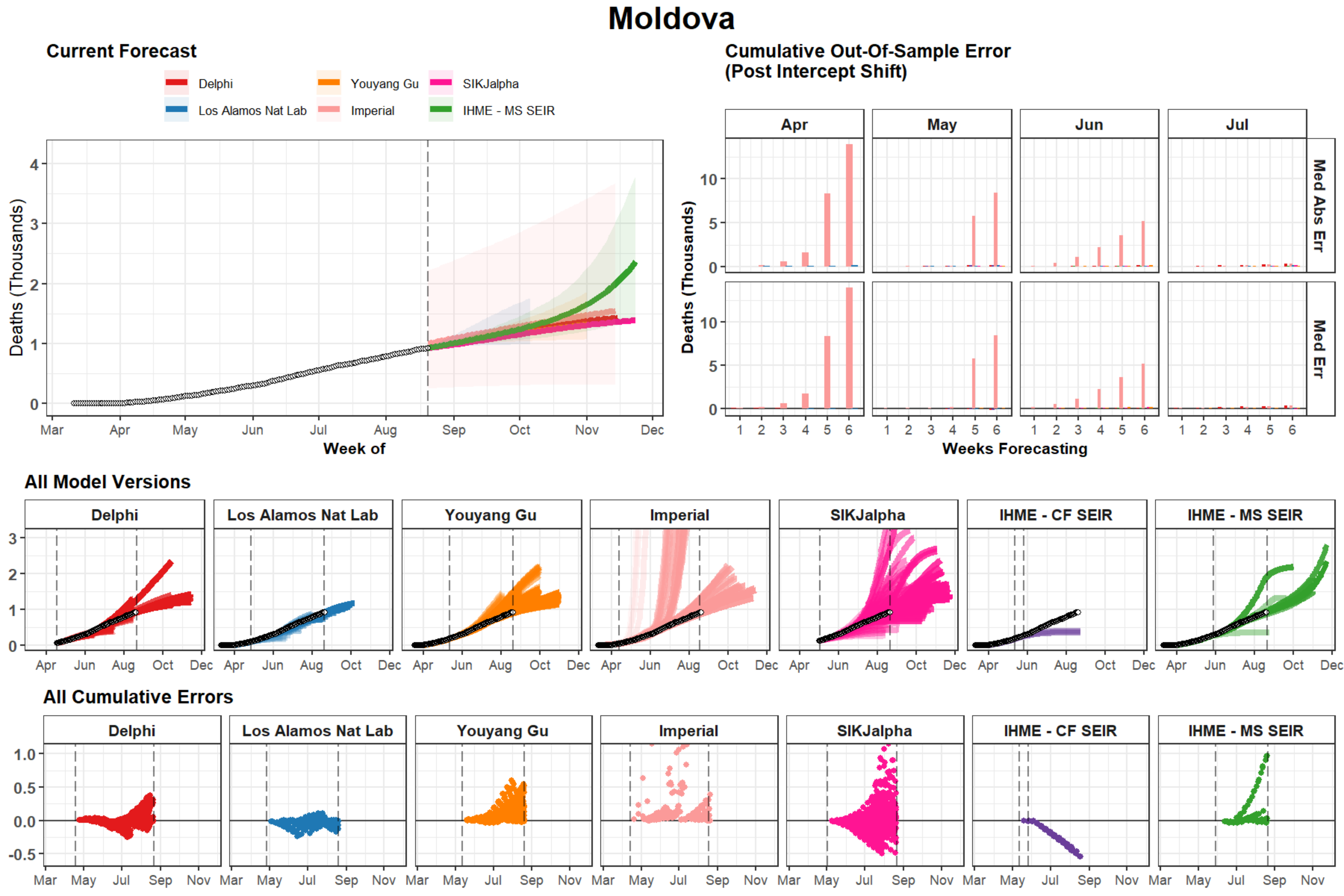
Moldova
Current Forecast
Delphi
Los Alamos Nat Lab
Youyang Gu
Imperial
SIKJalpha
IHME - MS SEIR
Deaths (Thousands)
Week of
Mar
Apr
May
Jun
Jul
Aug
Sep
Oct
Nov
Dec
Cumulative Out-Of-Sample Error
(Post Intercept Shift)
Apr
May
Jun
Jul
Med Abs Err
Med Err
Weeks Forecasting
All Model Versions
Delphi
Los Alamos Nat Lab
Youyang Gu
Imperial
SIKJalpha
IHME - CF SEIR
IHME - MS SEIR
All Cumulative Errors

Kentucky

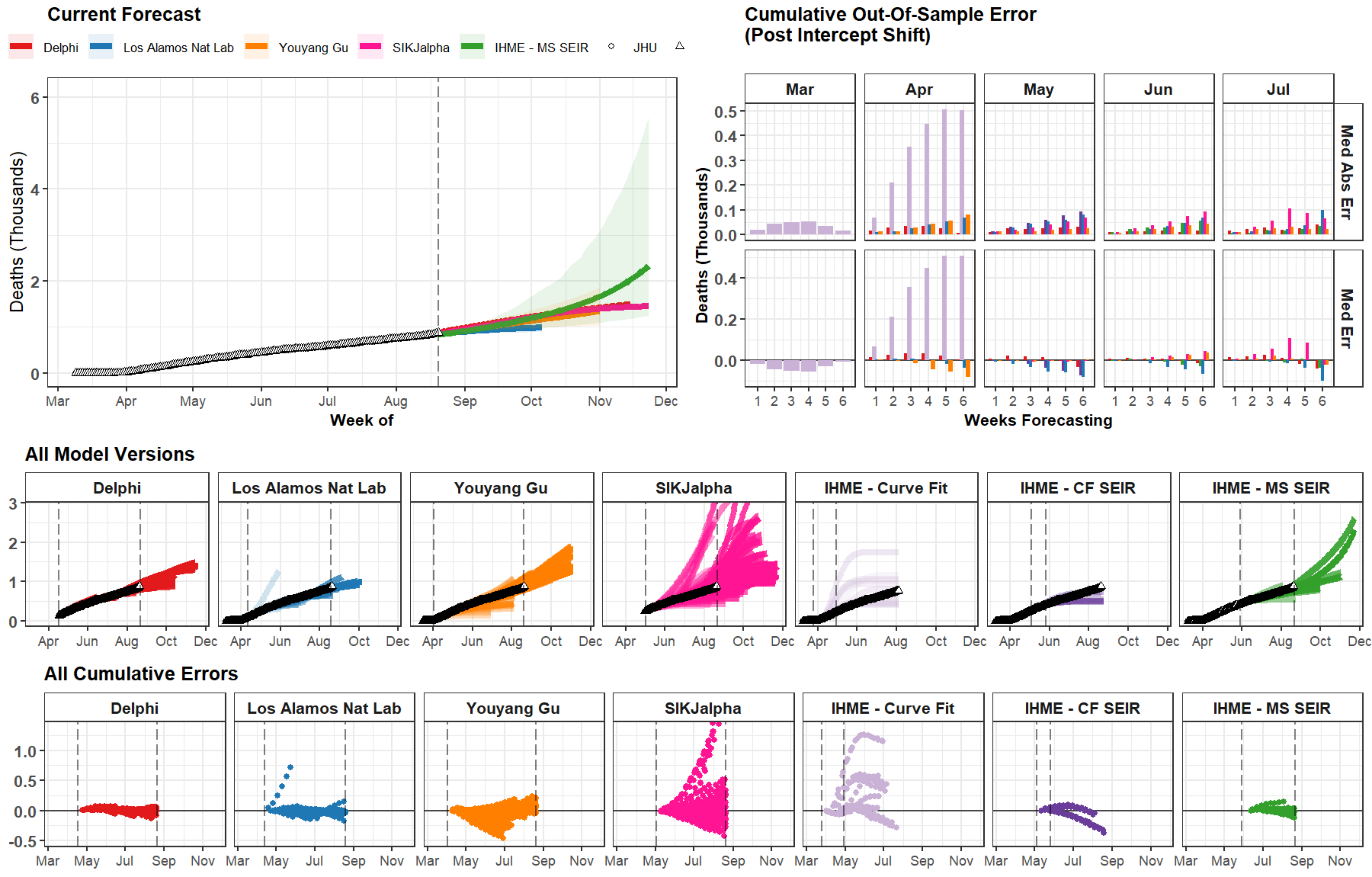
Current Forecast
Delphi
Los Alamos Nat Lab
Youyang Gu
SIKJalpha
IHME - MS SEIR
JHU
Deaths (Thousands)
Week of
Mar
Apr
May
Jun
Jul
Aug
Sep
Oct
Nov
Dec
Cumulative Out-Of-Sample Error
(Post Intercept Shift)
Med Abs Err
Med Err
Weeks Forecasting
All Model Versions
IHME - Curve Fit
IHME - CF SEIR
All Cumulative Errors

Armenia

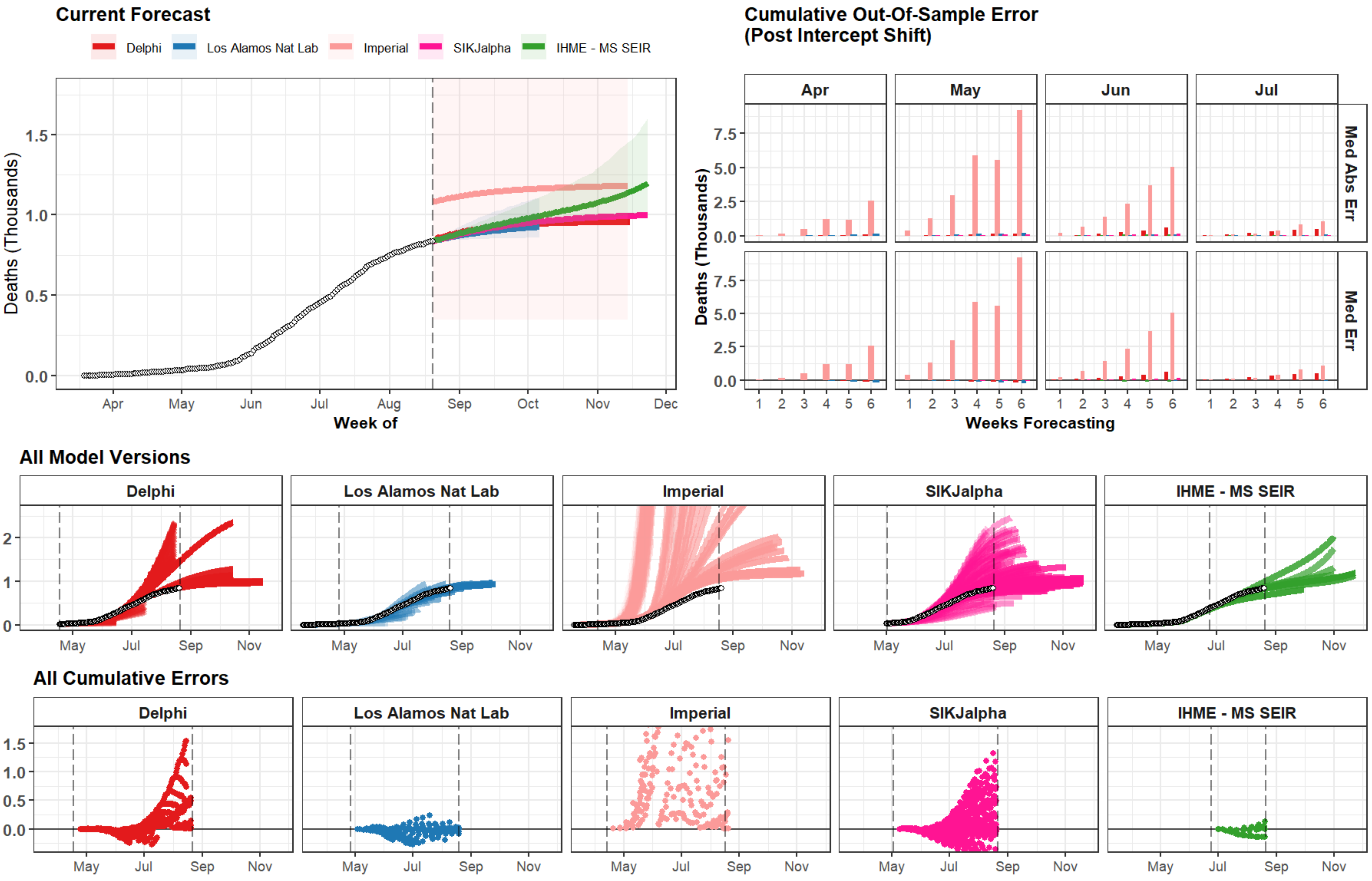
Current Forecast
Delphi
Los Alamos Nat Lab
Imperial
SIKJalpha
IHME - MS SEIR
Deaths (Thousands)
Week of
Cumulative Out-Of-Sample Error
(Post Intercept Shift)
Apr
May
Jun
Jul
Med Abs Err
Med Err
Weeks Forecasting
All Model Versions
All Cumulative Errors

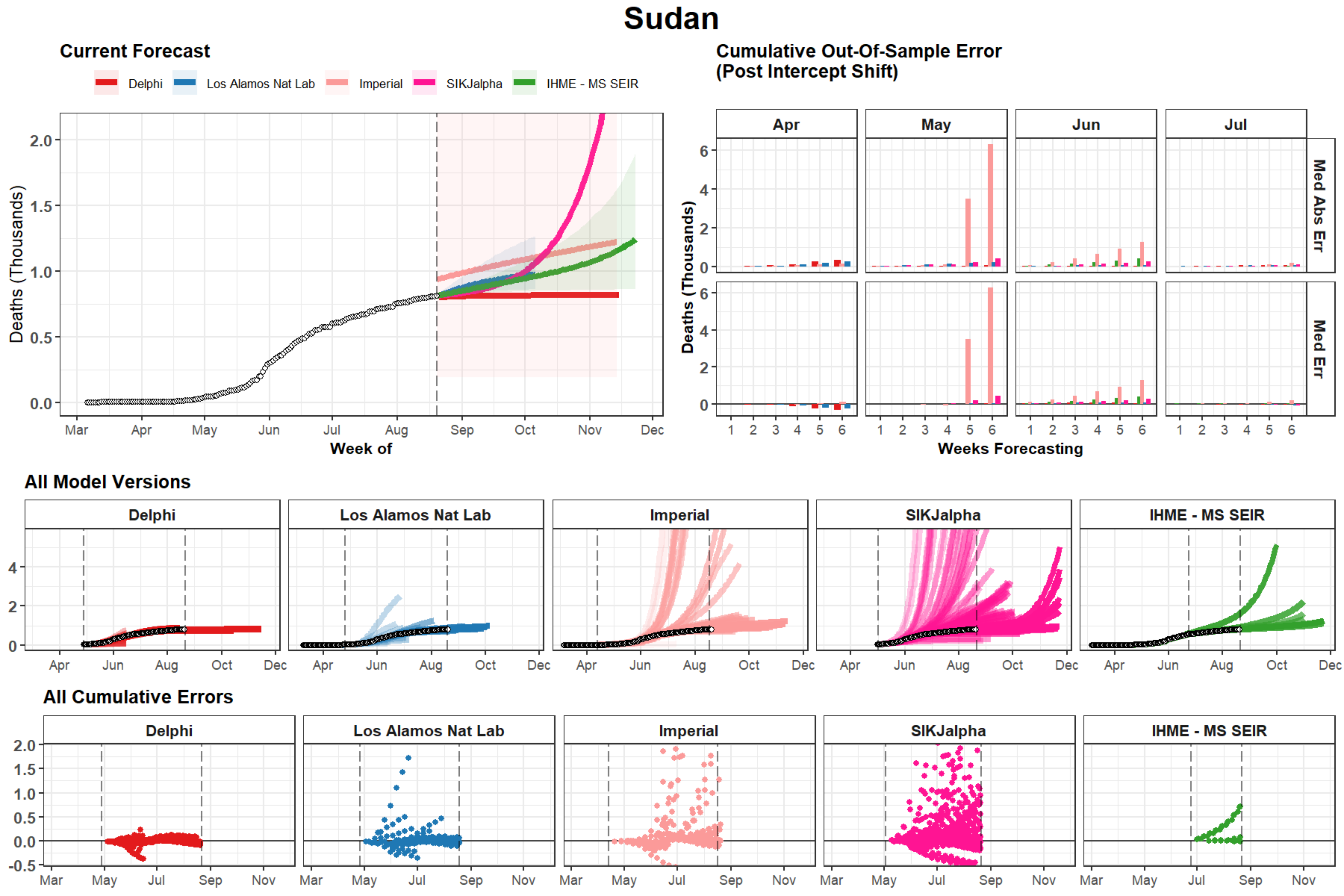
Sudan
Current Forecast
Delphi
Los Alamos Nat Lab
Imperial
SIKJalpha
IHME - MS SEIR
Deaths (Thousands)
Week of
Cumulative Out-Of-Sample Error
(Post Intercept Shift)
Apr
May
Jun
Jul
Med Abs Err
Med Err
Weeks Forecasting
All Model Versions
All Cumulative Errors

Israel

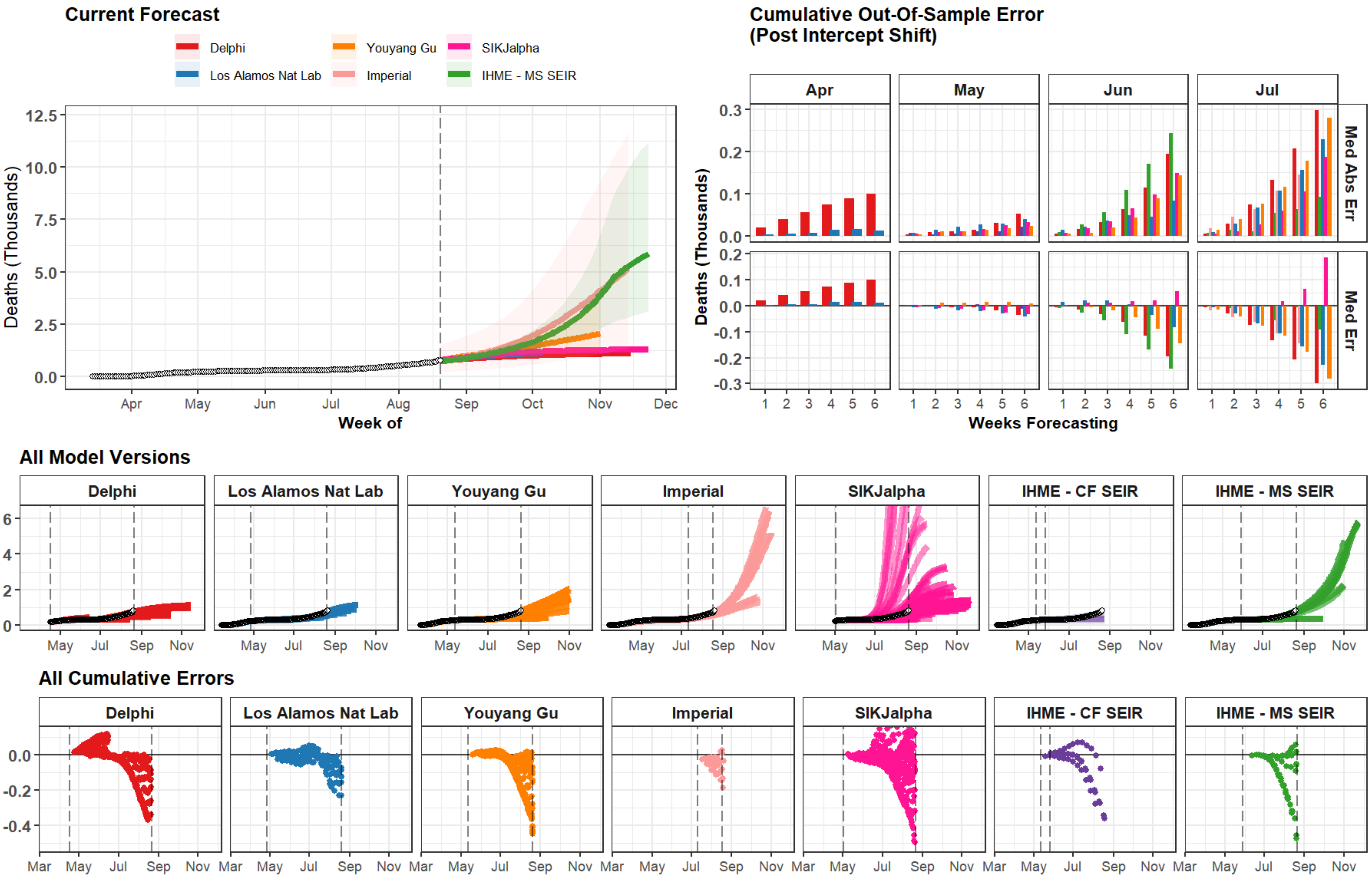

Current Forecast
Delphi
Los Alamos Nat Lab
Youyang Gu
Imperial
SIKJalpha
IHME - MS SEIR
Deaths (Thousands)
12.5
10.0
7.5
5.0
2.5
0.0
Apr
May
Jun
Jul
Aug
Sep
Oct
Nov
Dec
Week of
Cumulative Out-Of-Sample Error
(Post Intercept Shift)
Apr
May
Jun
Jul
Med Abs Err
Med Err
Deaths (Thousands)
Weeks Forecasting
All Model Versions
Delphi
Los Alamos Nat Lab
Youyang Gu
Imperial
SIKJalpha
IHME - CF SEIR
IHME - MS SEIR
All Cumulative Errors
Mar
May
Jul
Sep
Nov

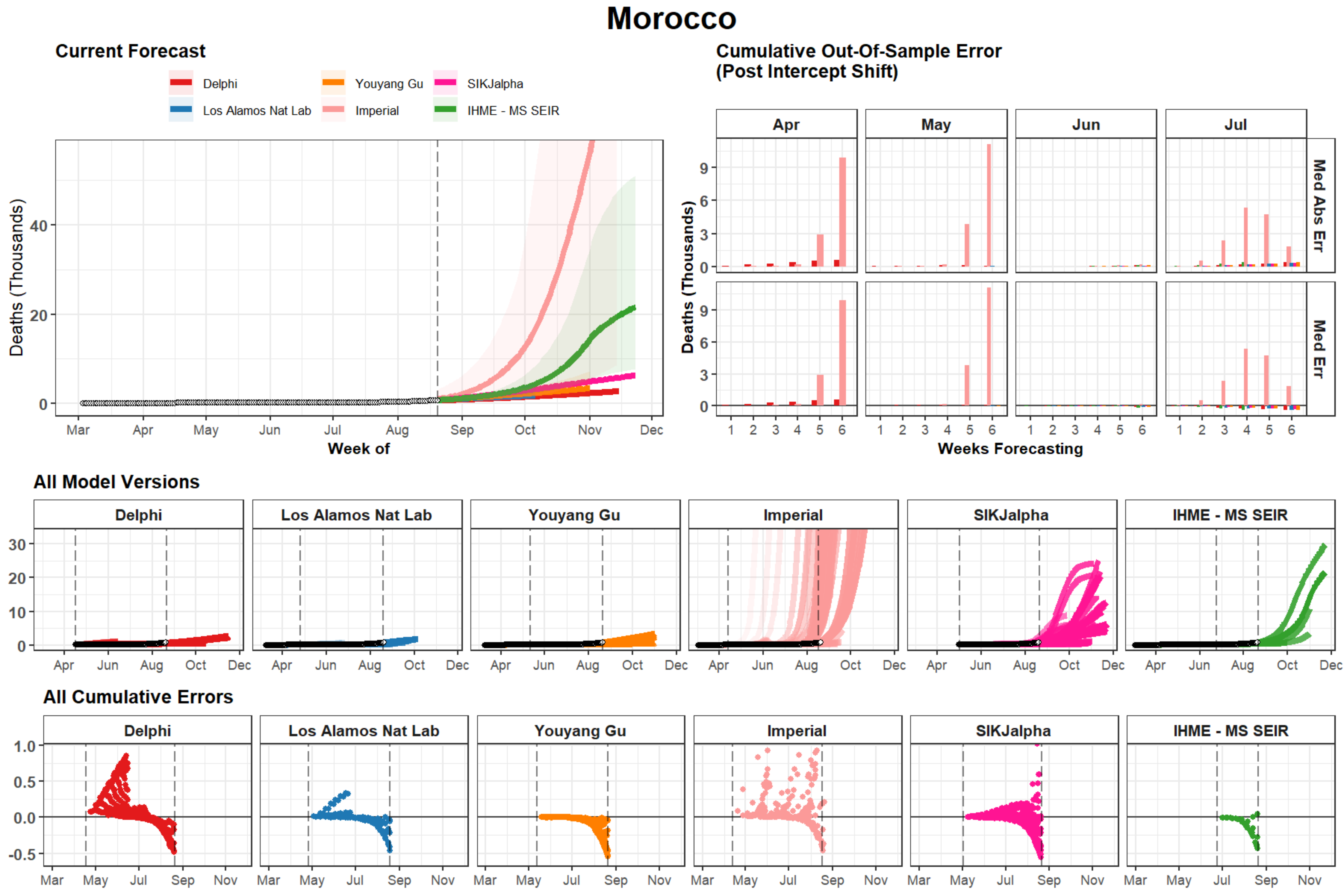

Morocco
Current Forecast
Delphi
Youyang Gu
SIKJalpha
Los Alamos Nat Lab
Imperial
IHME - MS SEIR
Deaths (Thousands)
Week of
Cumulative Out-Of-Sample Error
(Post Intercept Shift)
Apr
May
Jun
Jul
Med Abs Err
Med Err
Weeks Forecasting
All Model Versions
All Cumulative Errors

New Mexico

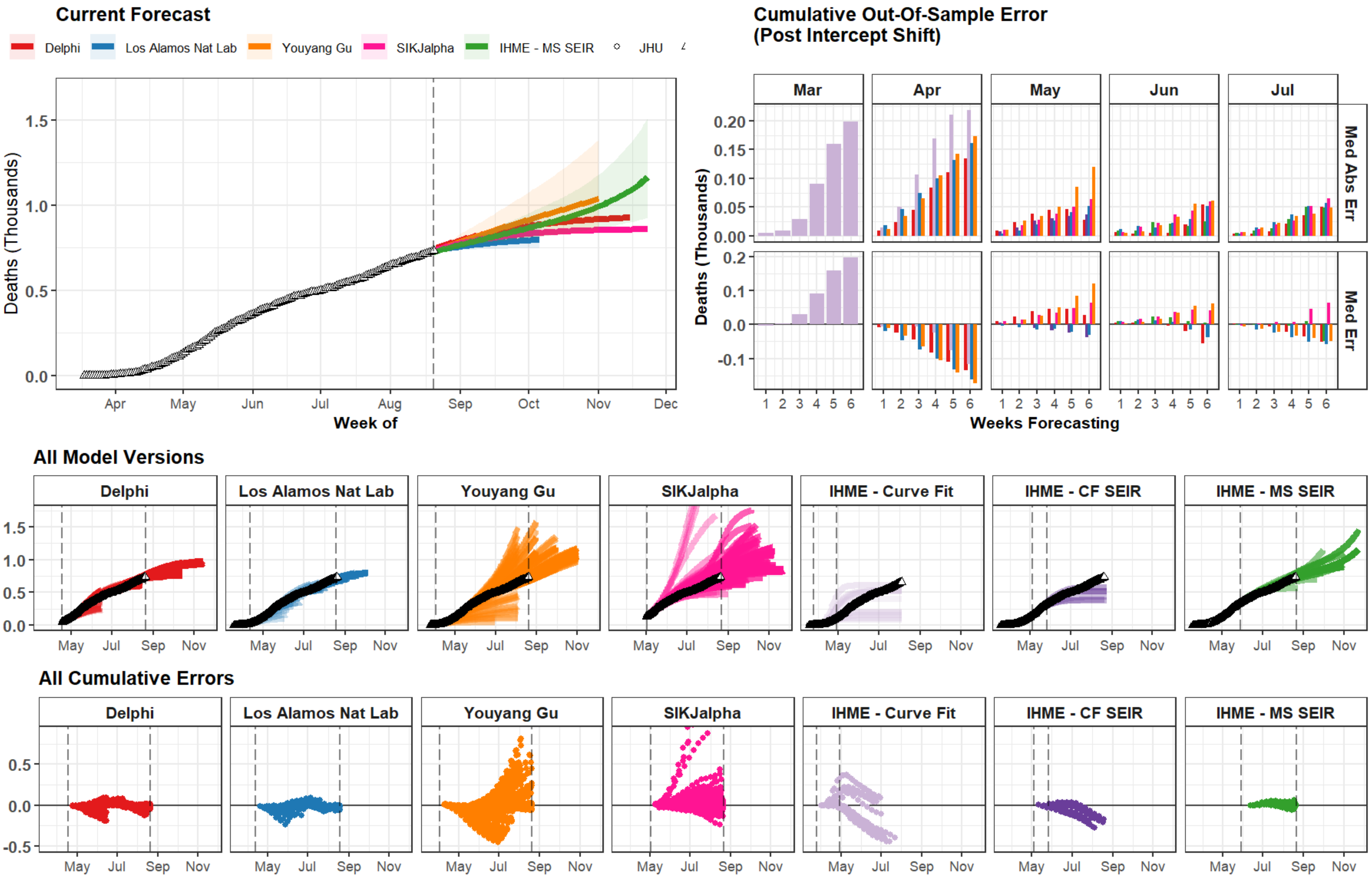

Current Forecast
Delphi
Los Alamos Nat Lab
Youyang Gu
SIKJalpha
IHME - MS SEIR
JHU
Deaths (Thousands)
Week of
Apr
May
Jun
Jul
Aug
Sep
Oct
Nov
Dec
Cumulative Out-Of-Sample Error
(Post Intercept Shift)
Mar
Apr
May
Jun
Jul
Med Abs Err
Med Err
Weeks Forecasting
All Model Versions
Delphi
Los Alamos Nat Lab
Youyang Gu
SIKJalpha
IHME - Curve Fit
IHME - CF SEIR
IHME - MS SEIR
All Cumulative Errors

Austria

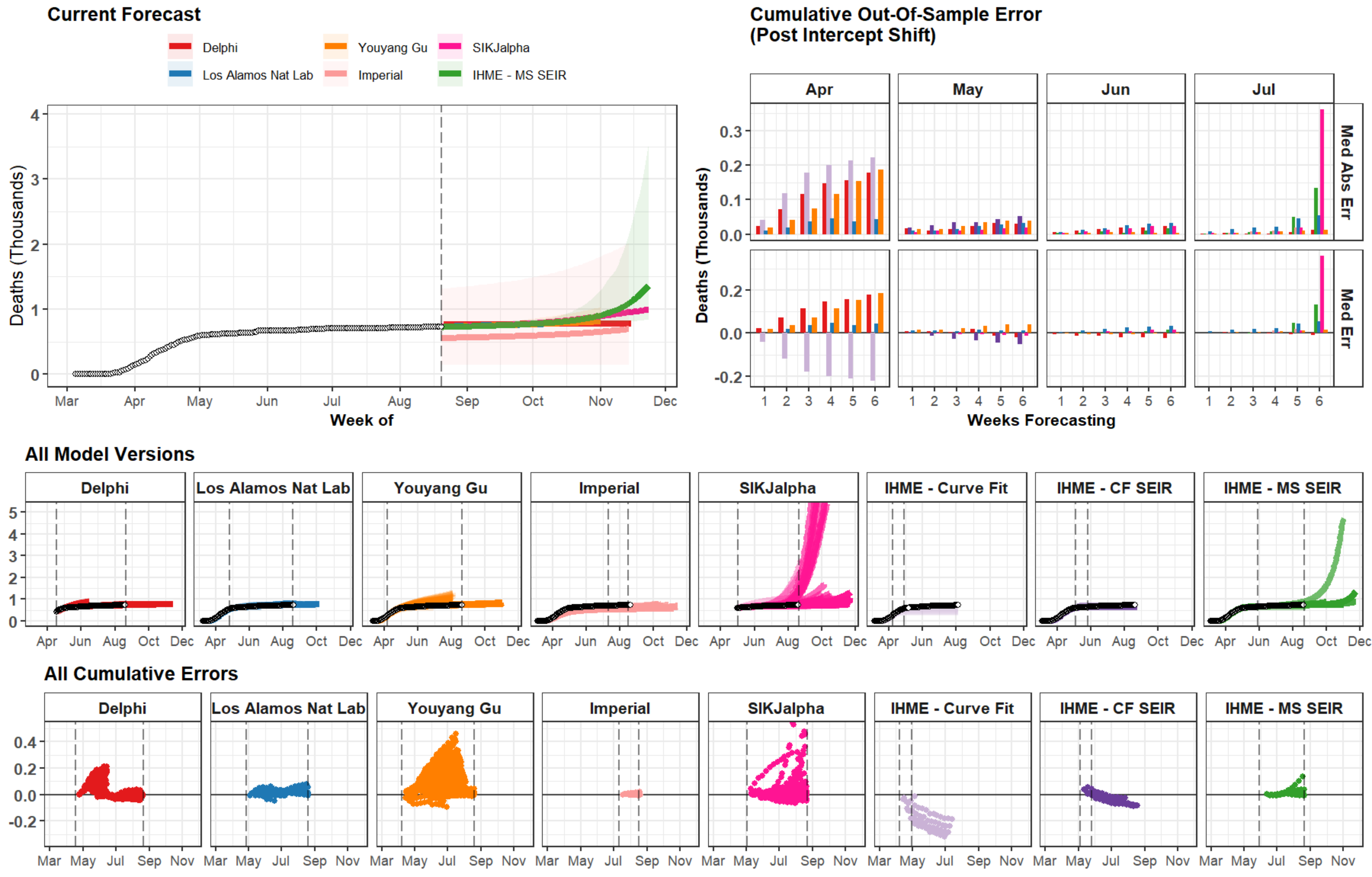

Current Forecast
Delphi
Los Alamos Nat Lab
Youyang Gu
Imperial
SIKJalpha
IHME - MS SEIR
Deaths (Thousands)
Week of
Mar Apr May Jun Jul Aug Sep Oct Nov Dec
Cumulative Out-Of-Sample Error
(Post Intercept Shift)
Apr
May
Jun
Jul
Med Abs Err
Med Err
Weeks Forecasting
All Model Versions
Delphi
Los Alamos Nat Lab
Youyang Gu
Imperial
SIKJalpha
IHME - Curve Fit
IHME - CF SEIR
IHME - MS SEIR
All Cumulative Errors

Oklahoma

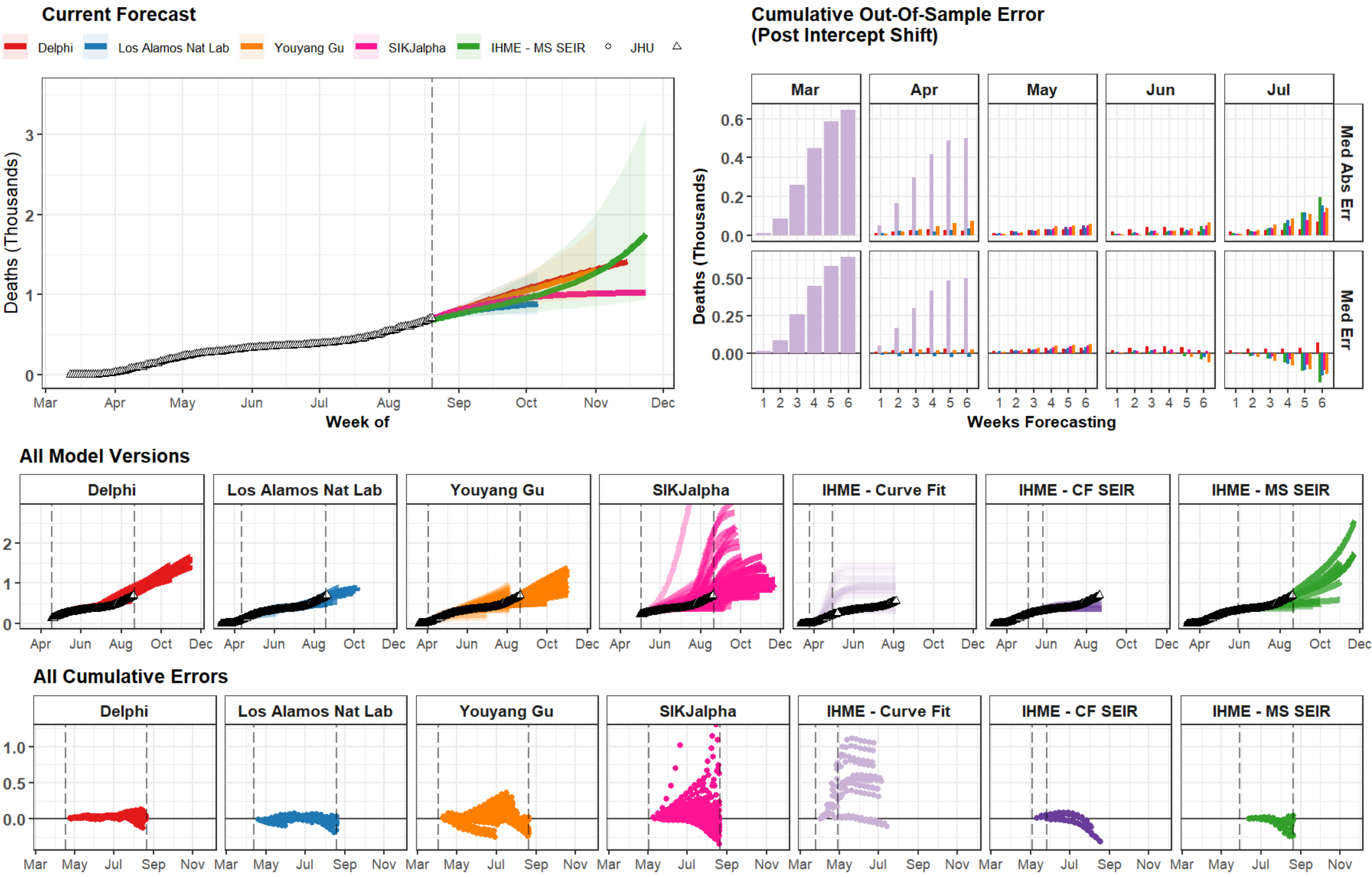
Current Forecast
Delphi
Los Alamos Nat Lab
Youyang Gu
SIKJalpha
IHME - MS SEIR
JHU
Deaths (Thousands)
Week of
Mar
Apr
May
Jun
Jul
Aug
Sep
Oct
Nov
Dec
Cumulative Out-Of-Sample Error
(Post Intercept Shift)
Med Abs Err
Med Err
Weeks Forecasting
All Model Versions
IHME - Curve Fit
IHME - CF SEIR
All Cumulative Errors

Serbia

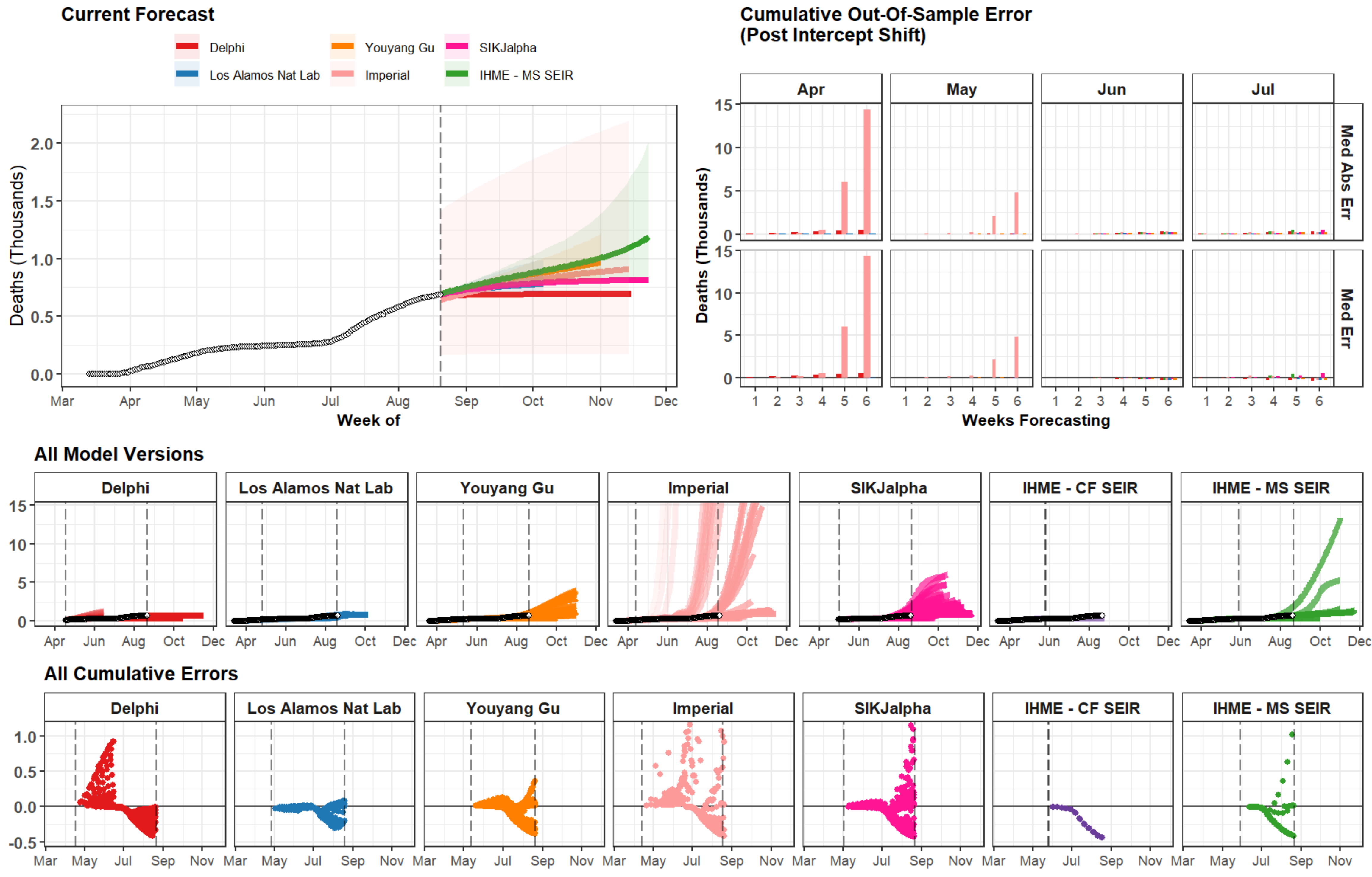

Current Forecast
Delphi
Youyang Gu
SIKJalpha
Los Alamos Nat Lab
Imperial
IHME - MS SEIR
Deaths (Thousands)
Week of
Mar
Apr
May
Jun
Jul
Aug
Sep
Oct
Nov
Dec
Cumulative Out-Of-Sample Error
(Post Intercept Shift)
Med Abs Err
Med Err
Weeks Forecasting
All Model Versions
IHME - CF SEIR
All Cumulative Errors

Arkansas

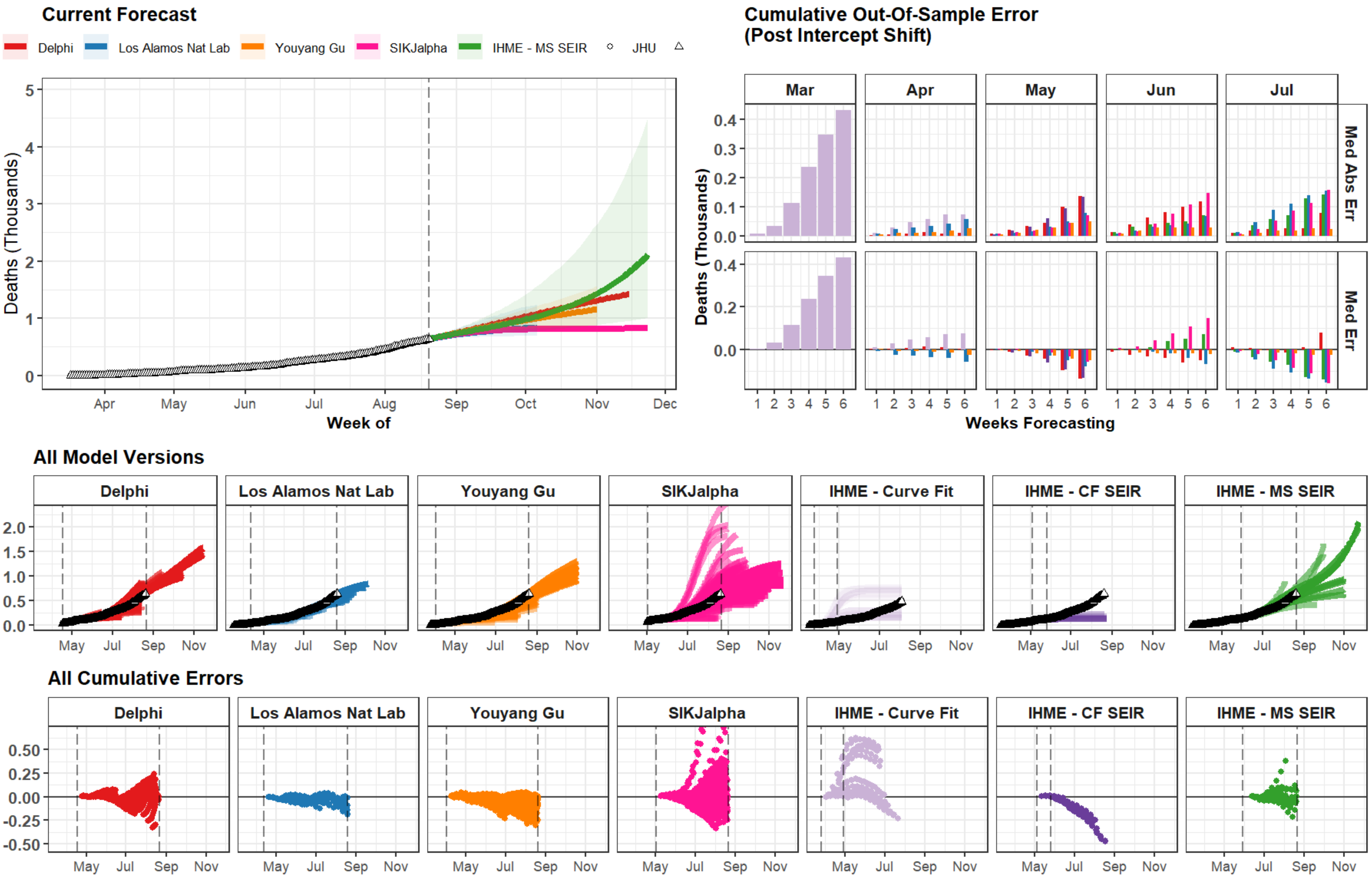

Current Forecast
Delphi
Los Alamos Nat Lab
Youyang Gu
SIKJalpha
IHME - MS SEIR
JHU
Deaths (Thousands)
Week of
Apr
May
Jun
Jul
Aug
Sep
Oct
Nov
Dec
Cumulative Out-Of-Sample Error
(Post Intercept Shift)
Mar
Apr
May
Jun
Jul
Med Abs Err
Med Err
Weeks Forecasting
All Model Versions
Delphi
Los Alamos Nat Lab
Youyang Gu
SIKJalpha
IHME - Curve Fit
IHME - CF SEIR
IHME - MS SEIR
All Cumulative Errors

El Salvador

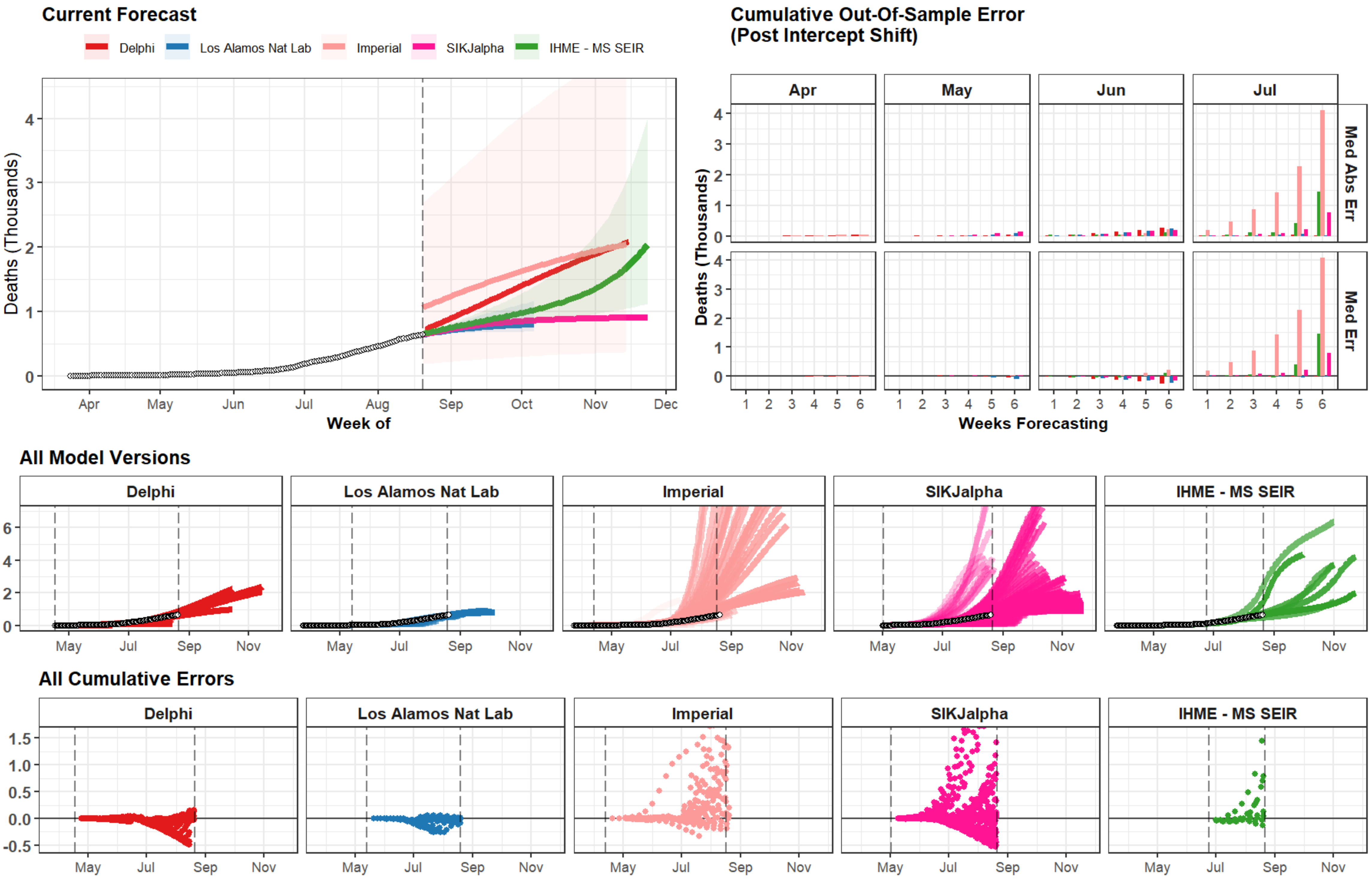
Current Forecast
Delphi
Los Alamos Nat Lab
Imperial
SIKJalpha
IHME - MS SEIR
Deaths (Thousands)
Week of
Apr
May
Jun
Jul
Aug
Sep
Oct
Nov
Dec
Cumulative Out-Of-Sample Error
(Post Intercept Shift)
Med Abs Err
Med Err
Weeks Forecasting
All Model Versions
All Cumulative Errors

Belarus

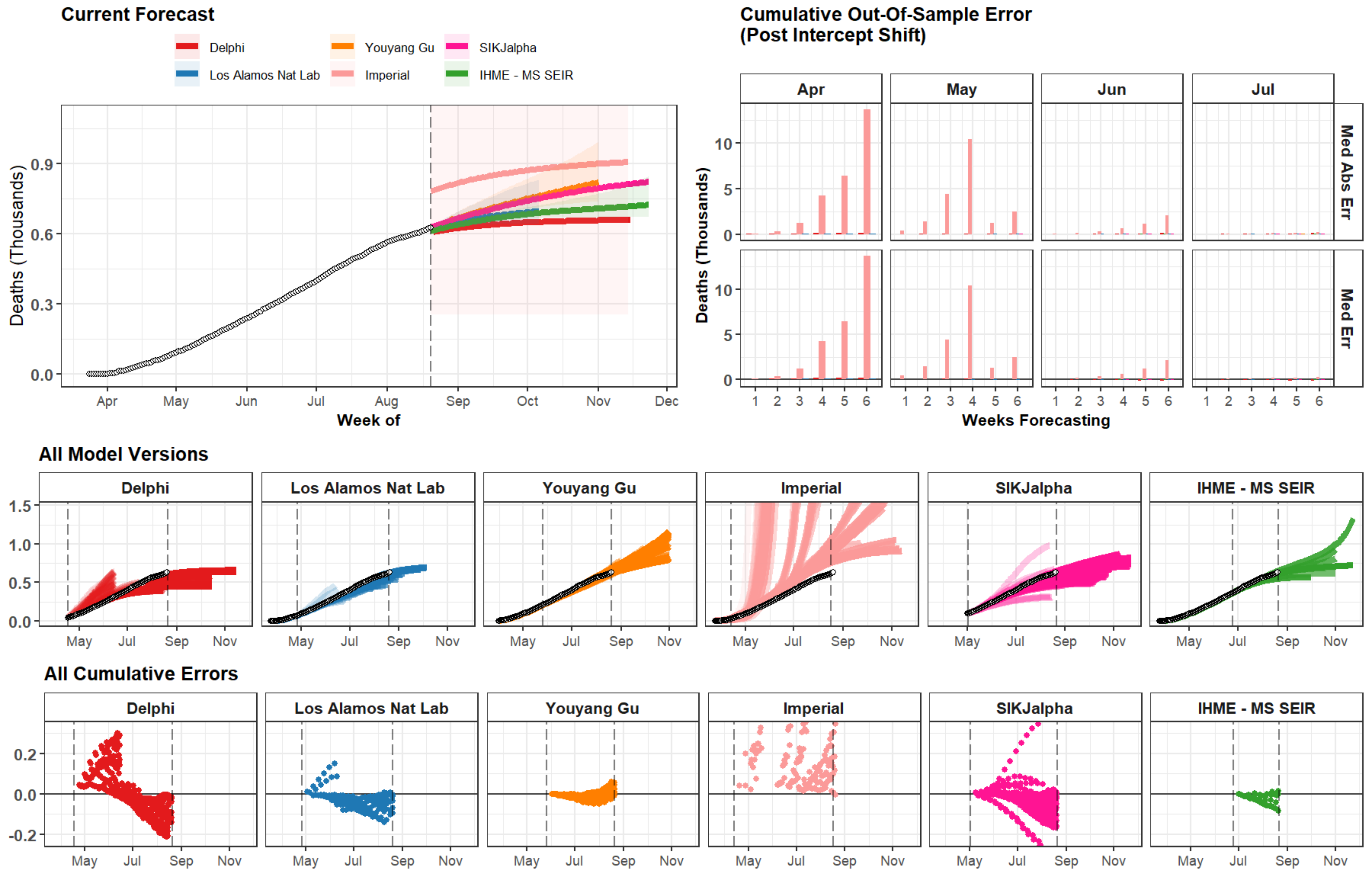

Current Forecast
Delphi
Los Alamos Nat Lab
Youyang Gu
Imperial
SIKJalpha
IHME - MS SEIR
Deaths (Thousands)
0.0
0.3
0.6
0.9
Apr
May
Jun
Jul
Aug
Sep
Oct
Nov
Dec
Week of
Cumulative Out-Of-Sample Error
(Post Intercept Shift)
Apr
May
Jun
Jul
Med Abs Err
Med Err
Deaths (Thousands)
0
5
10
1 2 3 4 5 6
Weeks Forecasting
All Model Versions
Delphi
Los Alamos Nat Lab
Youyang Gu
Imperial
SIKJalpha
IHME - MS SEIR
0.0
0.5
1.0
1.5
May
Jul
Sep
Nov
All Cumulative Errors
Delphi
Los Alamos Nat Lab
Youyang Gu
Imperial
SIKJalpha
IHME - MS SEIR
-0.2
0.0
0.2
May
Jul
Sep
Nov

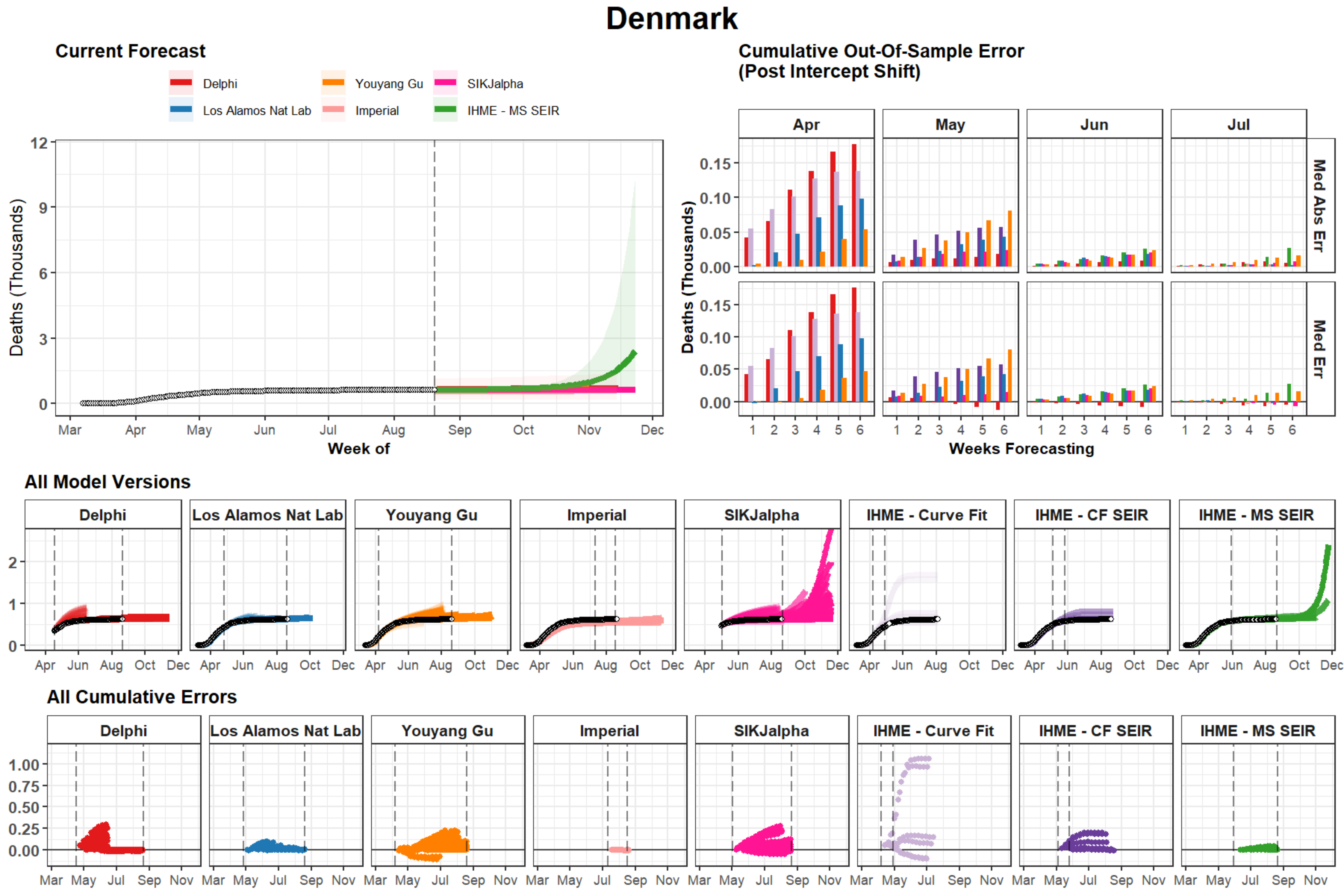

Denmark
Current Forecast
Delphi
Los Alamos Nat Lab
Youyang Gu
Imperial
SIKJalpha
IHME - MS SEIR
Deaths (Thousands)
Week of
Cumulative Out-Of-Sample Error
(Post Intercept Shift)
Apr
May
Jun
Jul
Med Abs Err
Med Err
Weeks Forecasting
All Model Versions
Delphi
Los Alamos Nat Lab
Youyang Gu
Imperial
SIKJalpha
IHME - Curve Fit
IHME - CF SEIR
IHME - MS SEIR
All Cumulative Errors

Ethiopia

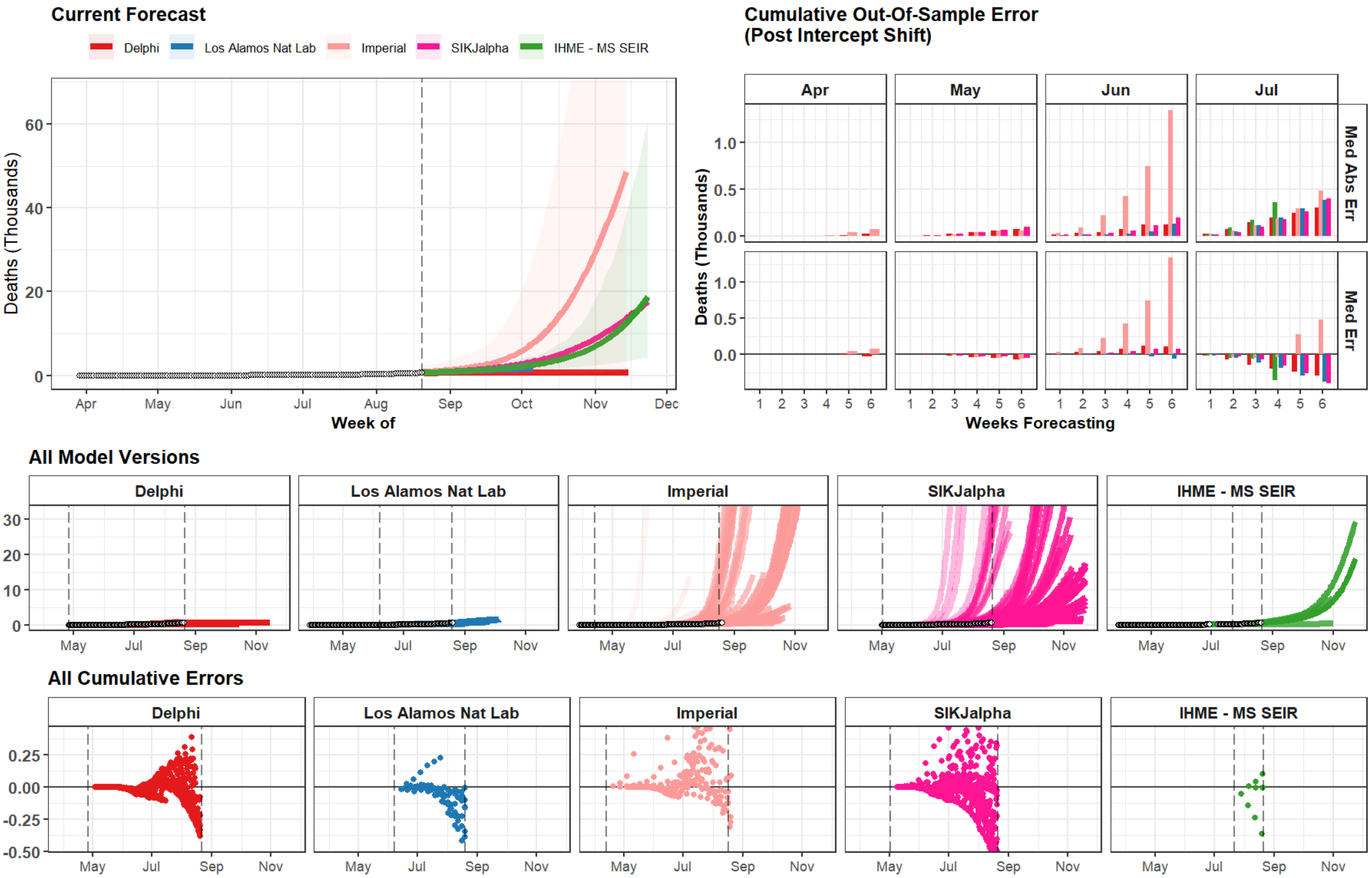

Current Forecast
Delphi
Los Alamos Nat Lab
Imperial
SIKJalpha
IHME - MS SEIR
Deaths (Thousands)
0
20
40
60
Apr
May
Jun
Jul
Aug
Sep
Oct
Nov
Dec
Week of
Cumulative Out-Of-Sample Error
(Post Intercept Shift)
Apr
May
Jun
Jul
Med Abs Err
Med Err
Deaths (Thousands)
0.0
0.5
1.0
1
2
3
4
5
6
Weeks Forecasting
All Model Versions
Delphi
Los Alamos Nat Lab
Imperial
SIKJalpha
IHME - MS SEIR
0
10
20
30
May
Jul
Sep
Nov
All Cumulative Errors
Delphi
Los Alamos Nat Lab
Imperial
SIKJalpha
IHME - MS SEIR
-0.50
-0.25
0.00
0.25
May
Jul
Sep
Nov

Hungary

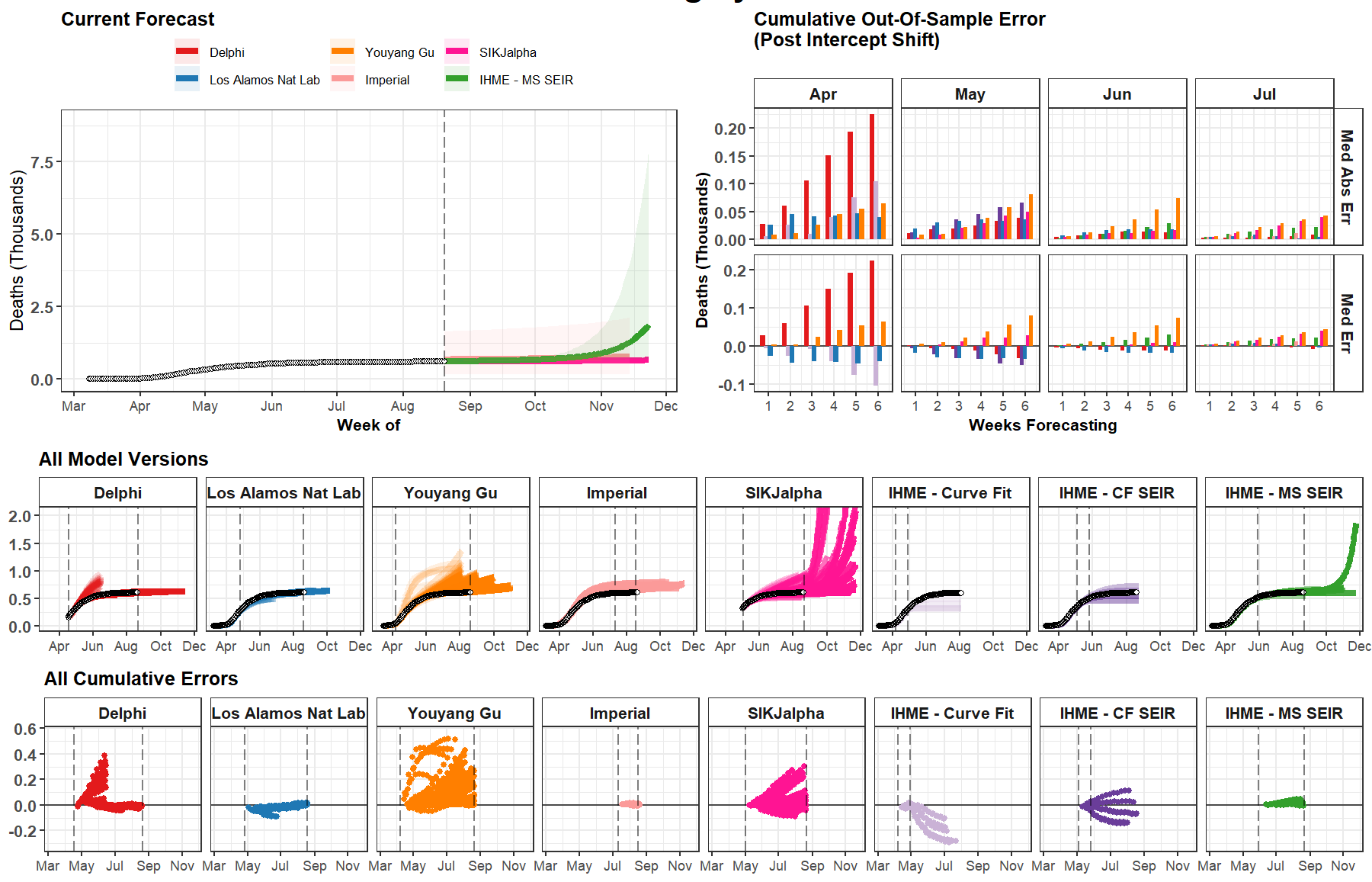
Current Forecast
Delphi
Youyang Gu
SIKJalpha
Los Alamos Nat Lab
Imperial
IHME - MS SEIR
Deaths (Thousands)
Week of
Cumulative Out-Of-Sample Error
(Post Intercept Shift)
Apr
May
Jun
Jul
Med Abs Err
Med Err
Weeks Forecasting
All Model Versions
Delphi
Los Alamos Nat Lab
Youyang Gu
Imperial
SIKJalpha
IHME - Curve Fit
IHME - CF SEIR
IHME - MS SEIR
All Cumulative Errors

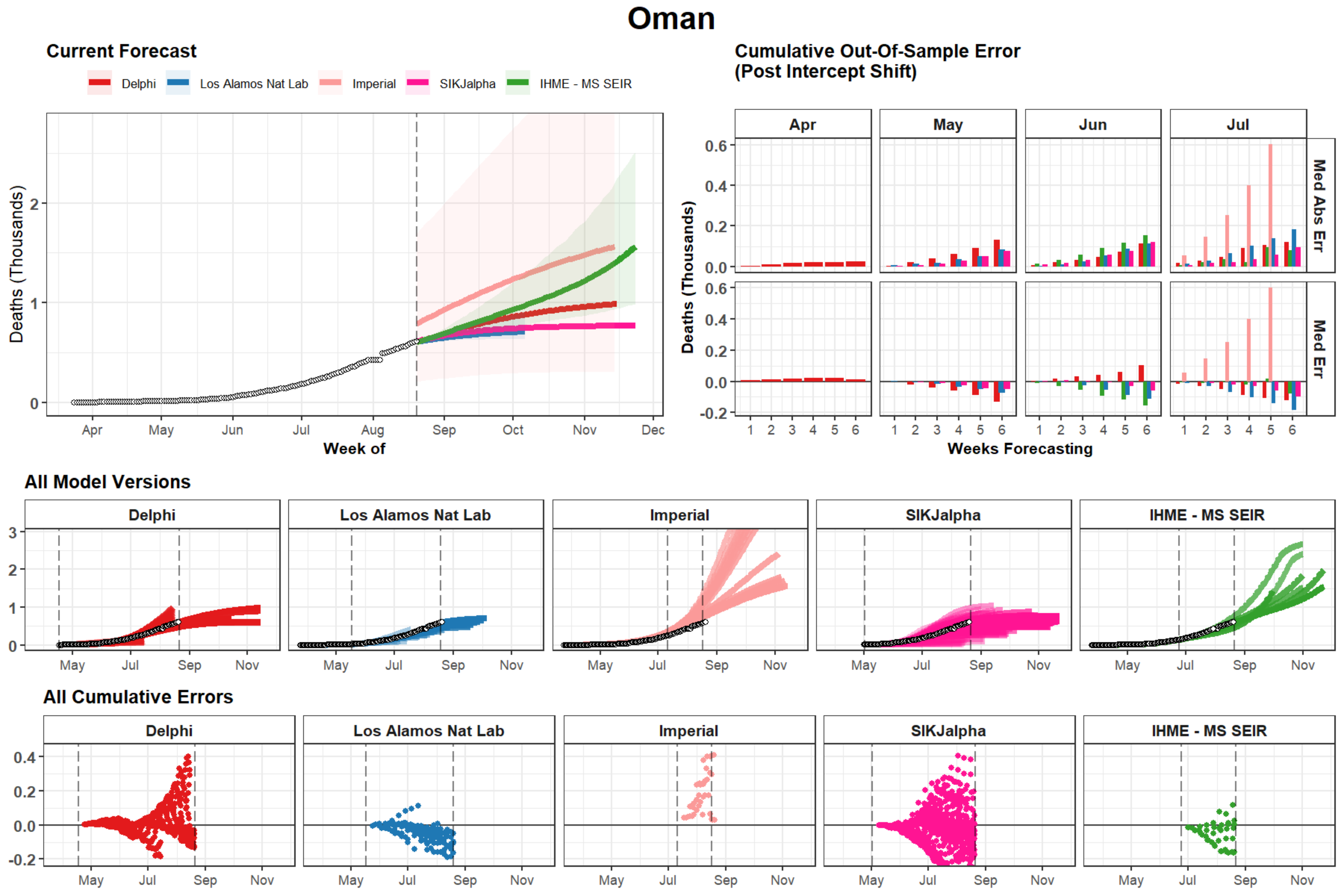

Oman
Current Forecast
Delphi
Los Alamos Nat Lab
Imperial
SIKJalpha
IHME - MS SEIR
Deaths (Thousands)
0
1
2
Apr
May
Jun
Jul
Aug
Sep
Oct
Nov
Dec
Week of
Cumulative Out-Of-Sample Error
(Post Intercept Shift)
Apr
May
Jun
Jul
Med Abs Err
Med Err
0.6
0.4
0.2
0.0
-0.2
1 2 3 4 5 6
Weeks Forecasting
All Model Versions
Delphi
Los Alamos Nat Lab
Imperial
SIKJalpha
IHME - MS SEIR
0
1
2
3
May
Jul
Sep
Nov
All Cumulative Errors
Delphi
Los Alamos Nat Lab
Imperial
SIKJalpha
IHME - MS SEIR
0.4
0.2
0.0
-0.2
May
Jul
Sep
Nov

District of Columbia

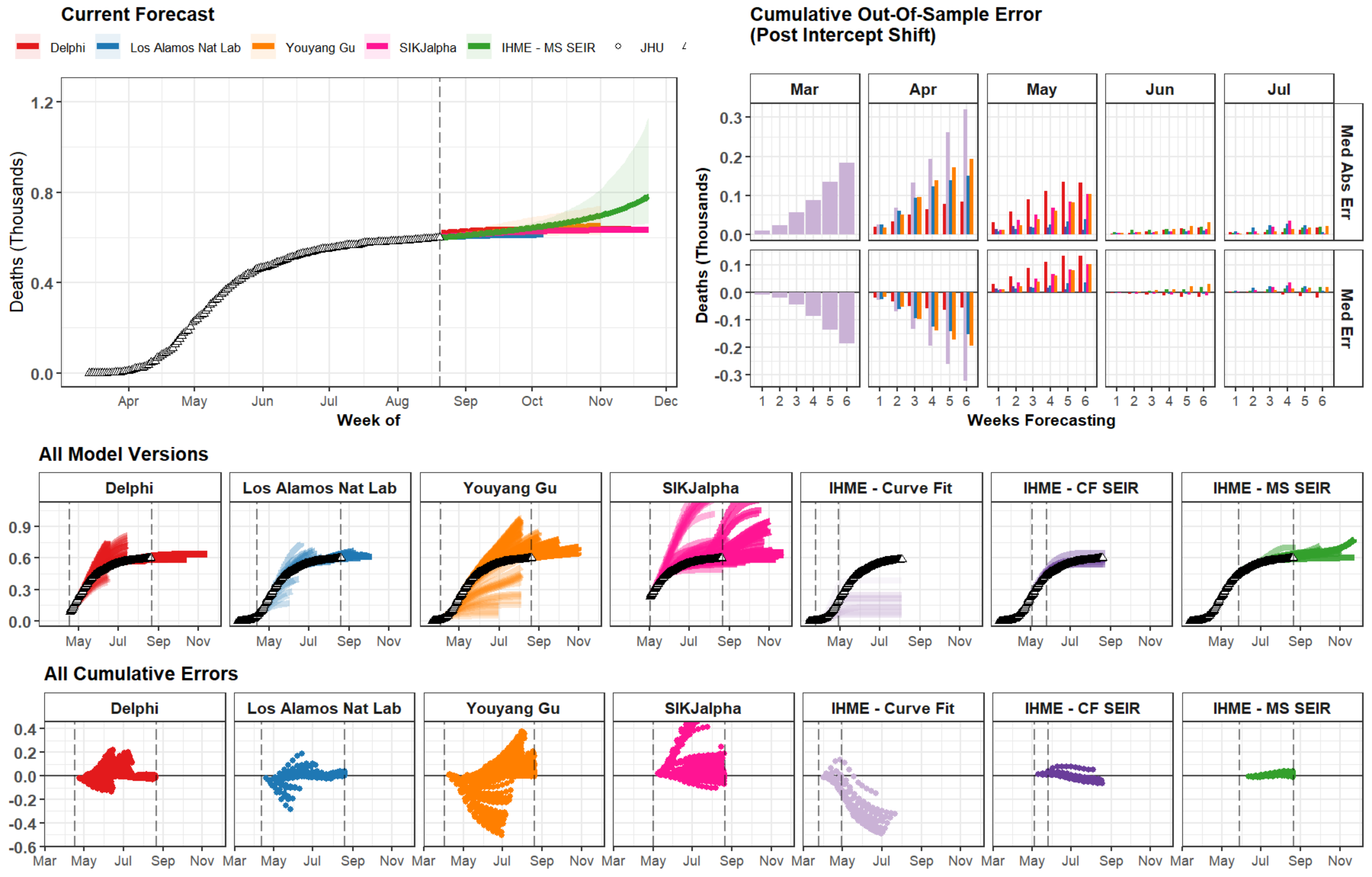

Delaware

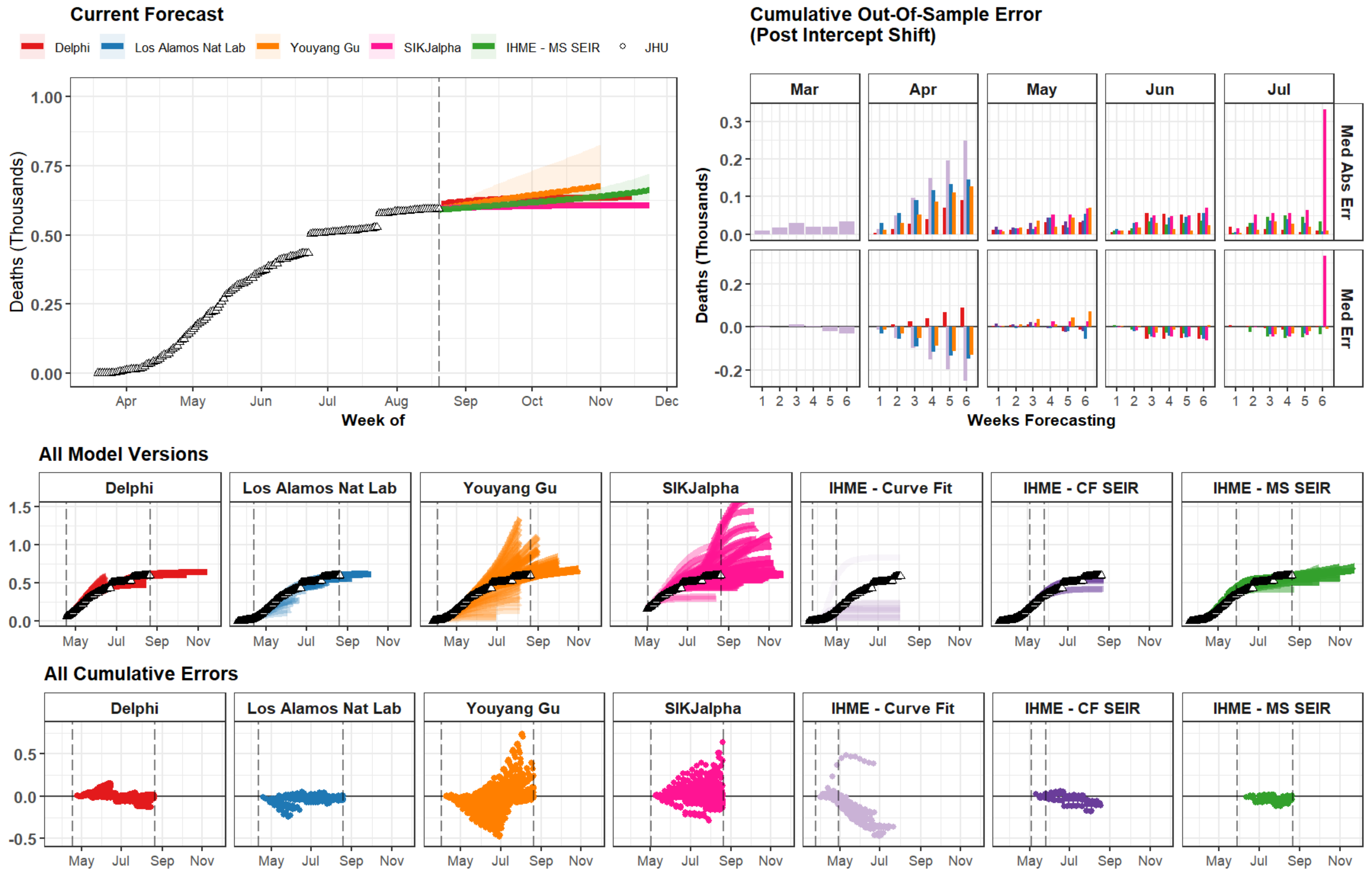

Current Forecast
Delphi
Los Alamos Nat Lab
Youyang Gu
SIKJalpha
IHME - MS SEIR
JHU
Deaths (Thousands)
1.00
0.75
0.50
0.25
0.00
Apr
May
Jun
Jul
Aug
Sep
Oct
Nov
Dec
Week of
Cumulative Out-Of-Sample Error
(Post Intercept Shift)
Mar
Apr
May
Jun
Jul
Med Abs Err
Med Err
0.3
0.2
0.1
0.0
-0.2
1 2 3 4 5 6
Weeks Forecasting
All Model Versions
Delphi
Los Alamos Nat Lab
Youyang Gu
SIKJalpha
IHME - Curve Fit
IHME - CF SEIR
IHME - MS SEIR
1.5
1.0
0.5
0.0
May
Jul
Sep
Nov
All Cumulative Errors
Delphi
Los Alamos Nat Lab
Youyang Gu
SIKJalpha
IHME - Curve Fit
IHME - CF SEIR
IHME - MS SEIR
0.5
0.0
-0.5
May
Jul
Sep
Nov

Macedonia

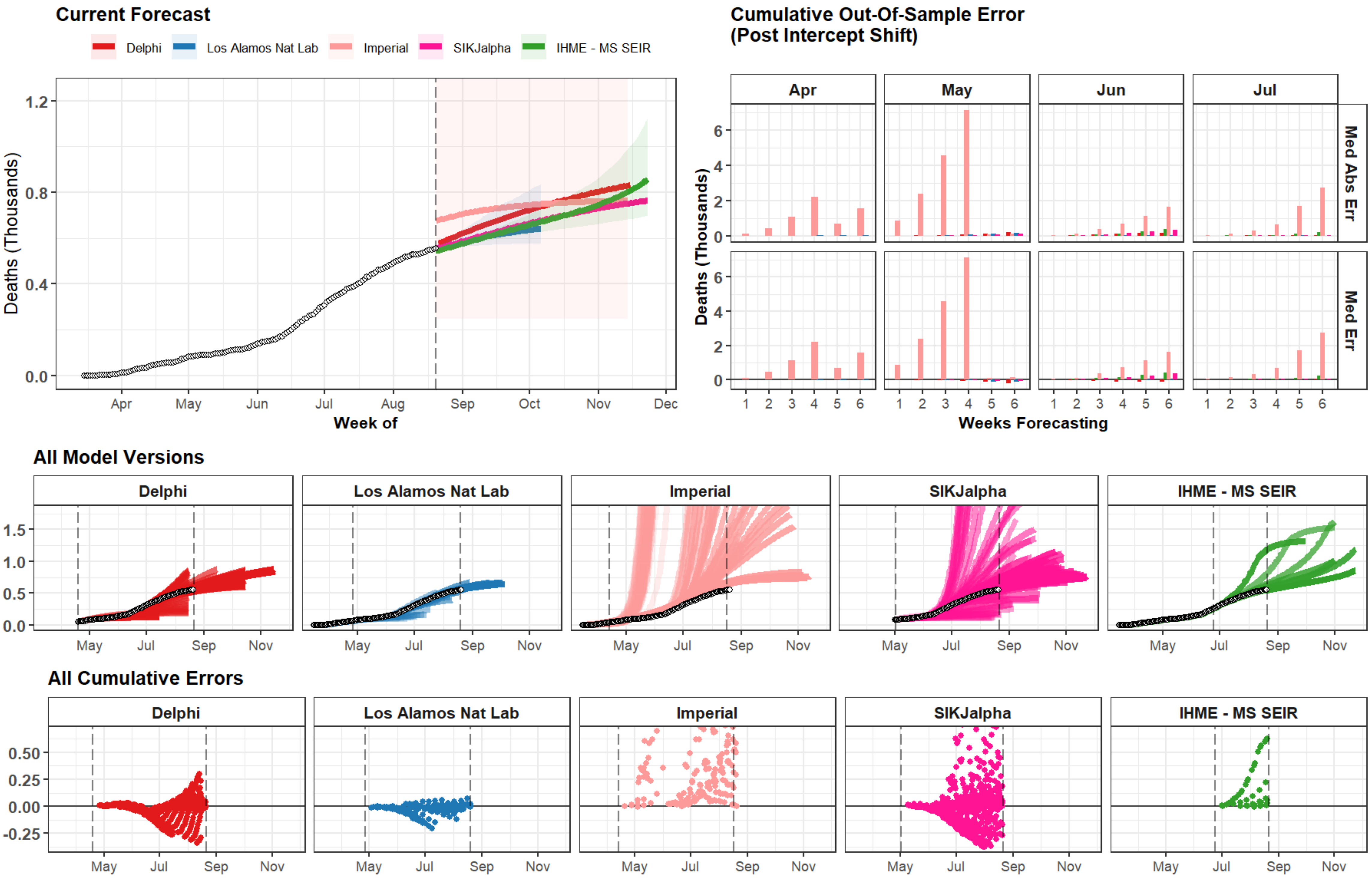
Current Forecast
Delphi
Los Alamos Nat Lab
Imperial
SIKJalpha
IHME - MS SEIR
Deaths (Thousands)
1.2
0.8
0.4
0.0
Apr
May
Jun
Jul
Aug
Sep
Oct
Nov
Dec
Week of
Cumulative Out-Of-Sample Error
(Post Intercept Shift)
Apr
May
Jun
Jul
Med Abs Err
Med Err
Deaths (Thousands)
Weeks Forecasting
All Model Versions
Delphi
Los Alamos Nat Lab
Imperial
SIKJalpha
IHME - MS SEIR
1.5
1.0
0.5
0.0
May
Jul
Sep
Nov
All Cumulative Errors
Delphi
Los Alamos Nat Lab
Imperial
SIKJalpha
IHME - MS SEIR
0.50
0.25
0.00
-0.25
May
Jul
Sep
Nov

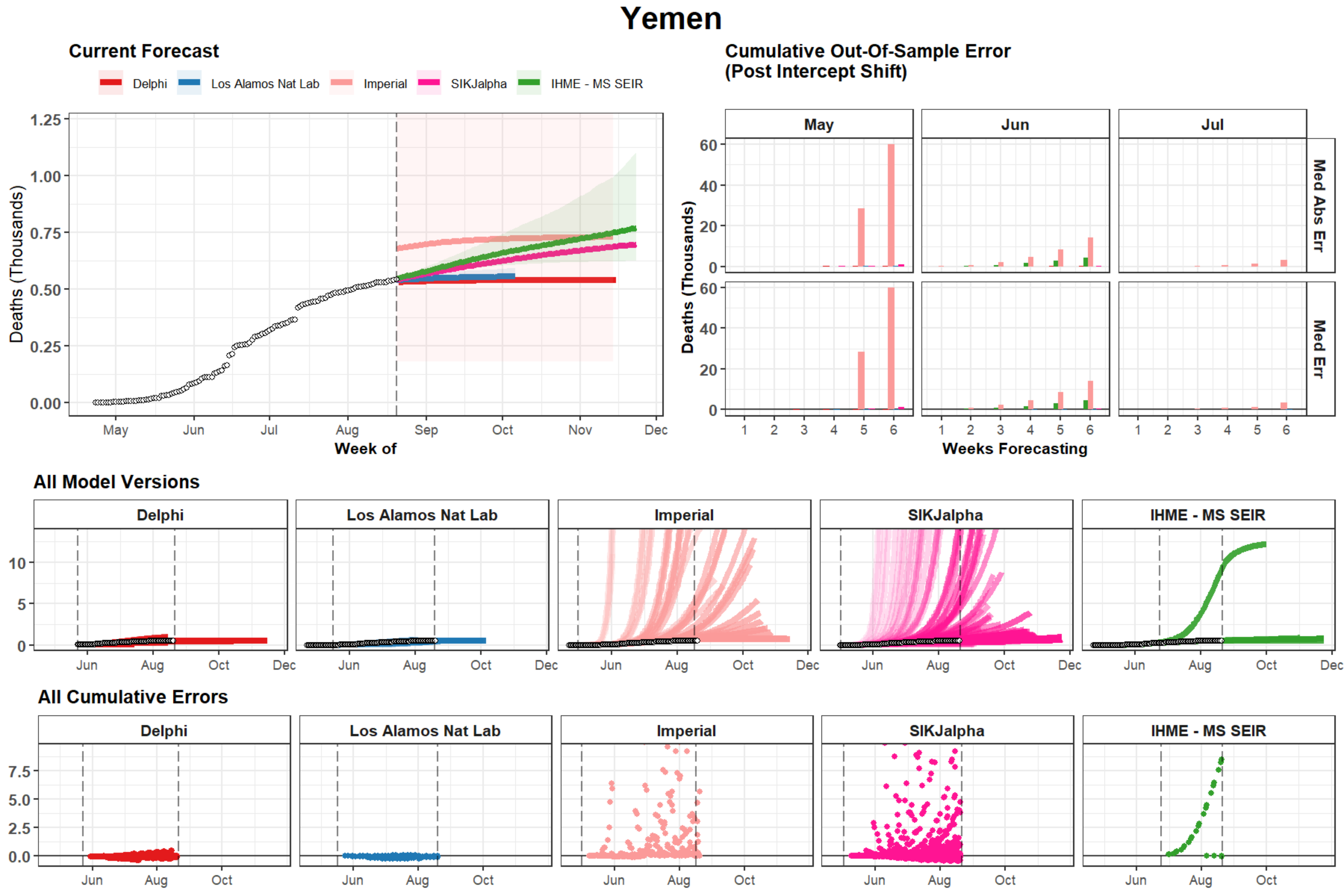
Yemen
Current Forecast
Delphi
Los Alamos Nat Lab
Imperial
SIKJalpha
IHME - MS SEIR
Deaths (Thousands)
Week of
Cumulative Out-Of-Sample Error
(Post Intercept Shift)
May
Jun
Jul
Med Abs Err
Med Err
Weeks Forecasting
All Model Versions
All Cumulative Errors

Bulgaria

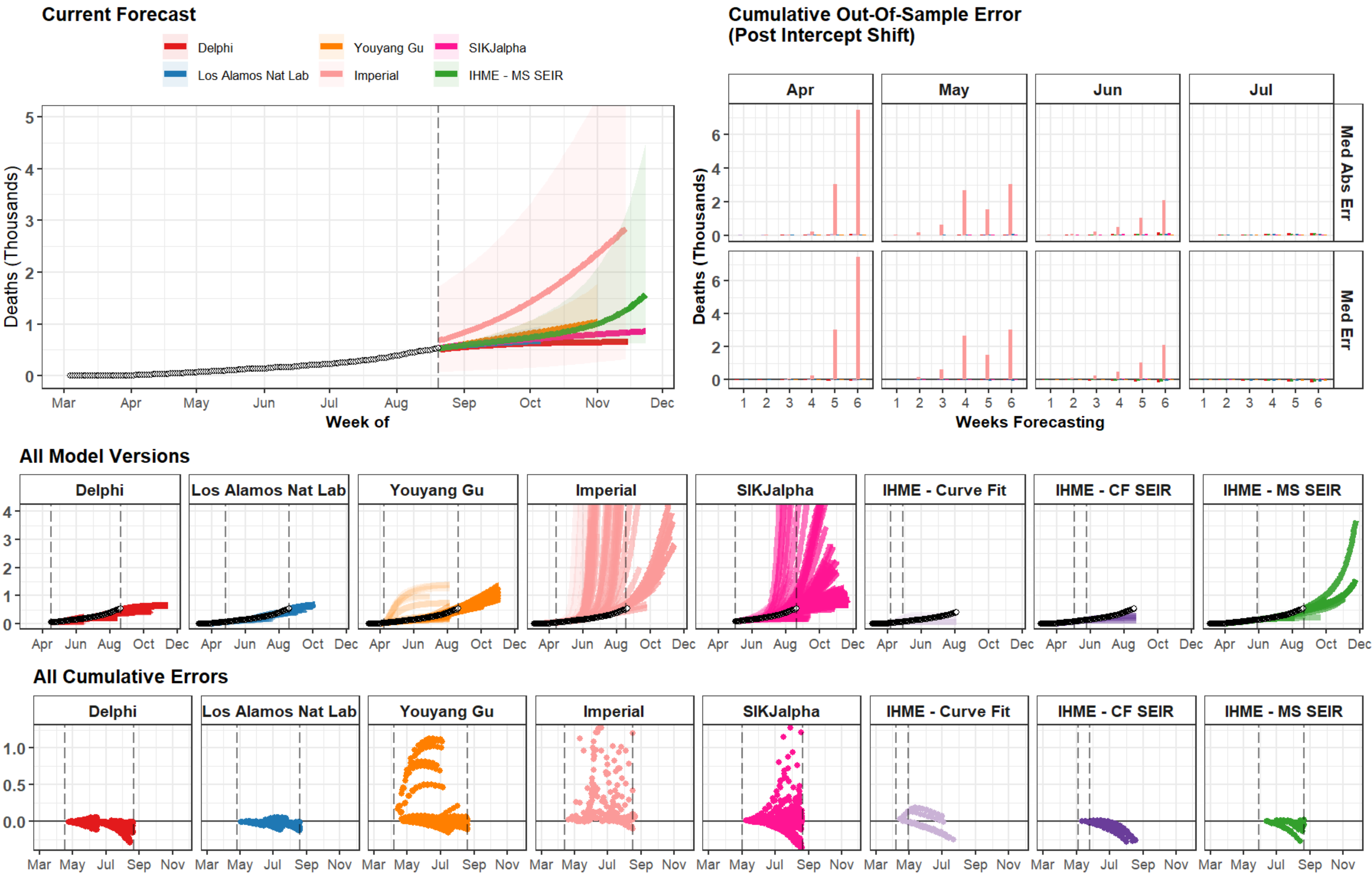

Current Forecast
Delphi
Los Alamos Nat Lab
Youyang Gu
Imperial
SIKJalpha
IHME - MS SEIR
Deaths (Thousands)
Week of
Cumulative Out-Of-Sample Error
(Post Intercept Shift)
Apr
May
Jun
Jul
Med Abs Err
Med Err
Weeks Forecasting
All Model Versions
Delphi
Los Alamos Nat Lab
Youyang Gu
Imperial
SIKJalpha
IHME - Curve Fit
IHME - CF SEIR
IHME - MS SEIR
All Cumulative Errors

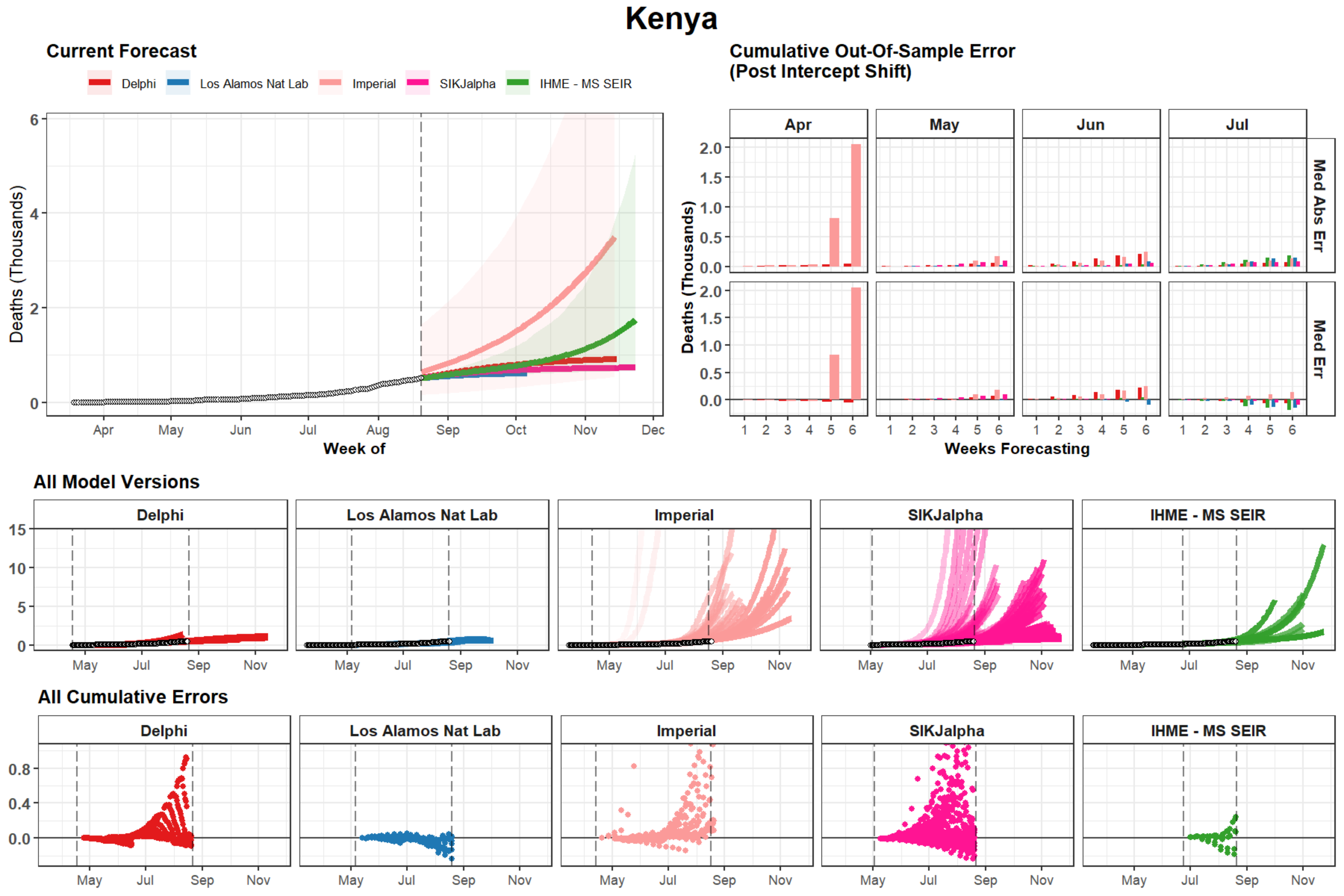

Kenya
Current Forecast
Delphi
Los Alamos Nat Lab
Imperial
SIKJalpha
IHME - MS SEIR
Deaths (Thousands)
Week of
Apr
May
Jun
Jul
Aug
Sep
Oct
Nov
Dec
Cumulative Out-Of-Sample Error
(Post Intercept Shift)
Med Abs Err
Med Err
Weeks Forecasting
All Model Versions
All Cumulative Errors

Bosnia and Herzegovina

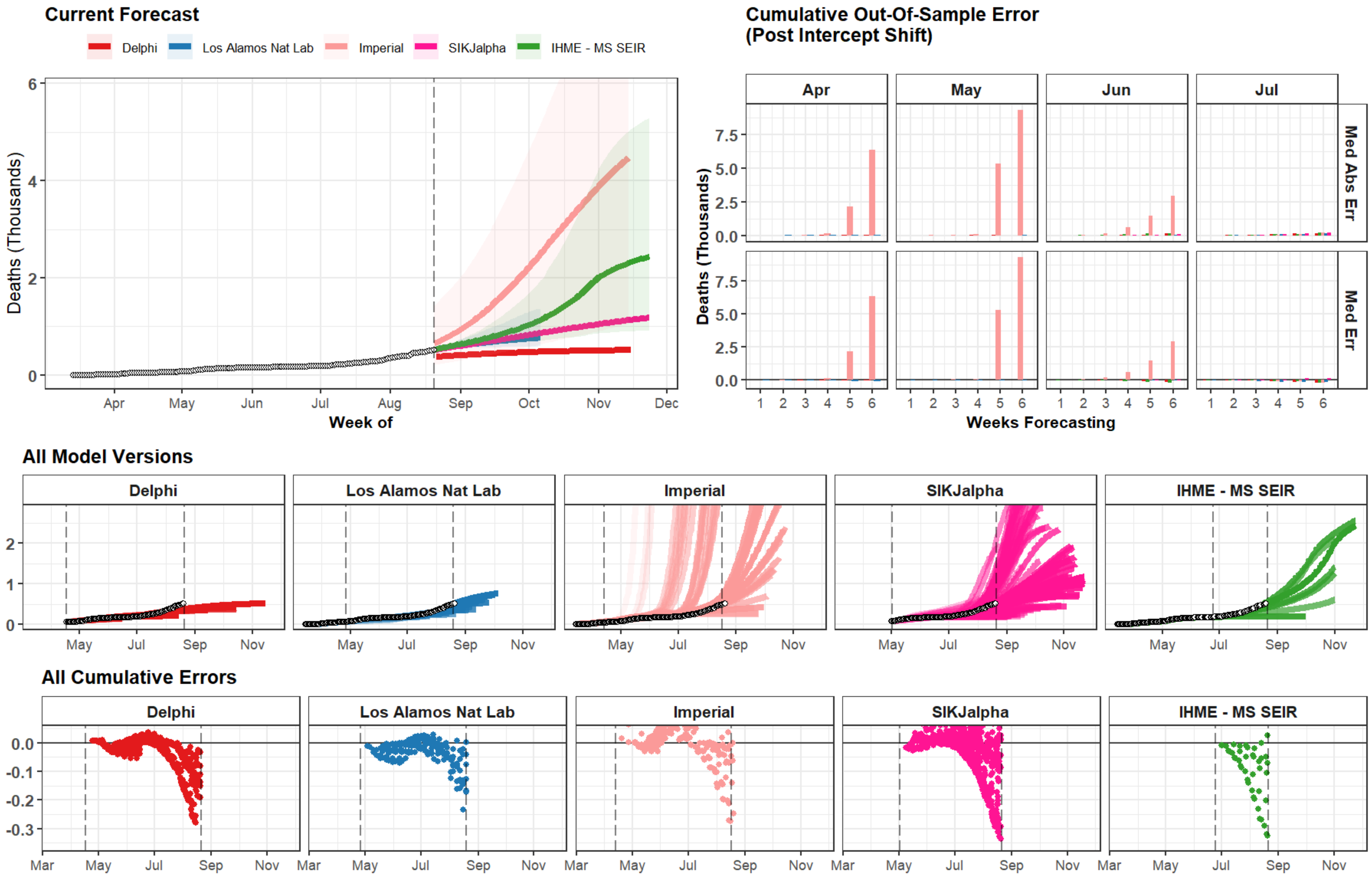

Azerbaijan

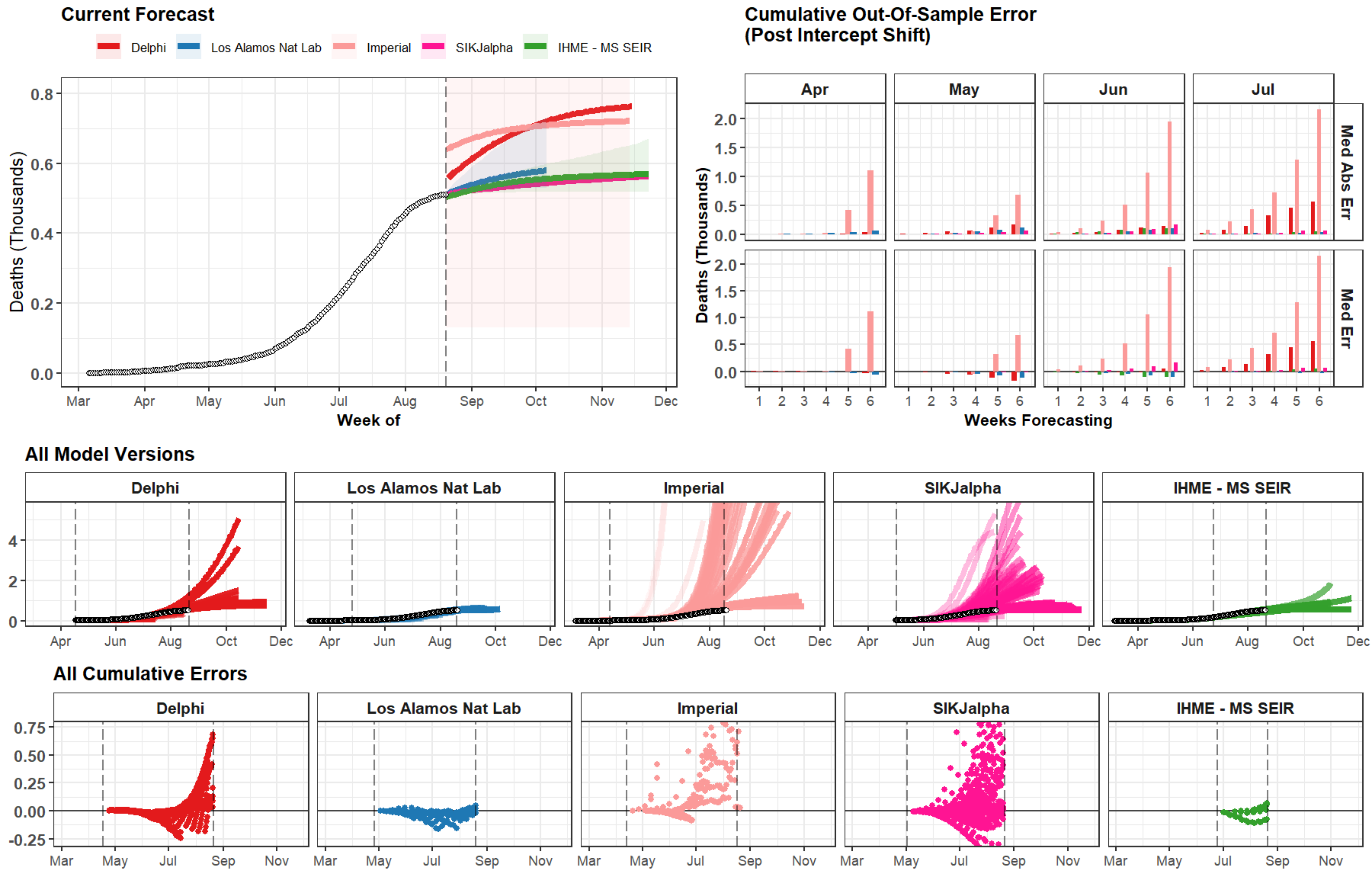
Current Forecast
Delphi
Los Alamos Nat Lab
Imperial
SIKJalpha
IHME - MS SEIR
Deaths (Thousands)
0.0
0.2
0.4
0.6
0.8
Mar
Apr
May
Jun
Jul
Aug
Sep
Oct
Nov
Dec
Week of
Cumulative Out-Of-Sample Error
(Post Intercept Shift)
Apr
May
Jun
Jul
Med Abs Err
Med Err
Deaths (Thousands)
0.0
0.5
1.0
1.5
2.0
1
2
3
4
5
6
Weeks Forecasting
All Model Versions
Delphi
Los Alamos Nat Lab
Imperial
SIKJalpha
IHME - MS SEIR
0
2
4
Apr
Jun
Aug
Oct
Dec
All Cumulative Errors
Delphi
Los Alamos Nat Lab
Imperial
SIKJalpha
IHME - MS SEIR
0.75
0.50
0.25
0.00
-0.25
Mar
May
Jul
Sep
Nov

Kuwait

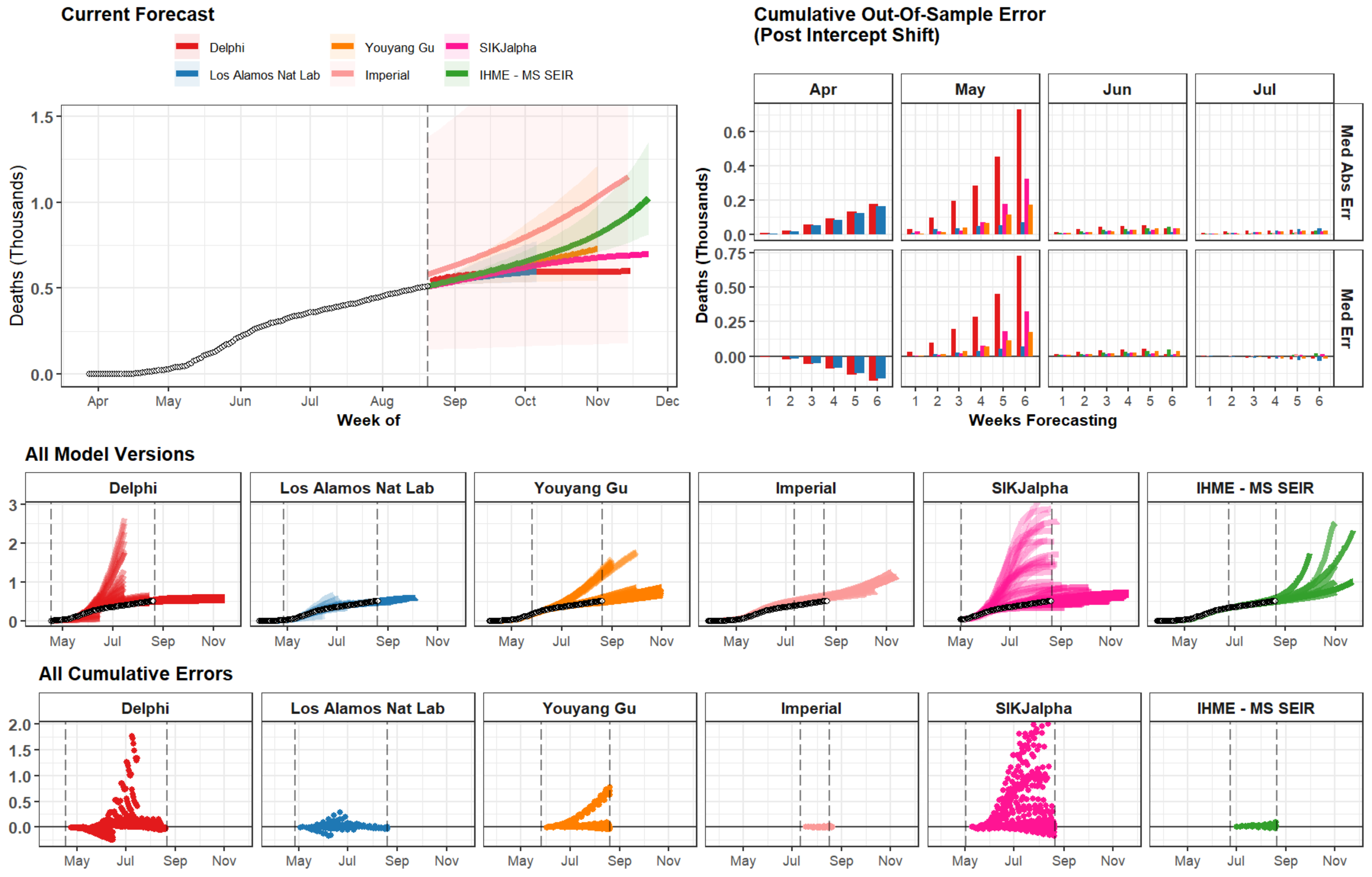
Current Forecast
Delphi
Los Alamos Nat Lab
Youyang Gu
Imperial
SIKJalpha
IHME - MS SEIR
Deaths (Thousands)
1.5
1.0
0.5
0.0
Apr
May
Jun
Jul
Aug
Sep
Oct
Nov
Dec
Week of
Cumulative Out-Of-Sample Error
(Post Intercept Shift)
Apr
May
Jun
Jul
Med Abs Err
Med Err
0.6
0.4
0.2
0.0
0.75
0.50
0.25
0.00
1
2
3
4
5
6
Weeks Forecasting
All Model Versions
All Cumulative Errors
3
2
1
0
2.0
1.5
1.0
0.5
0.0
May
Jul
Sep
Nov

Australia

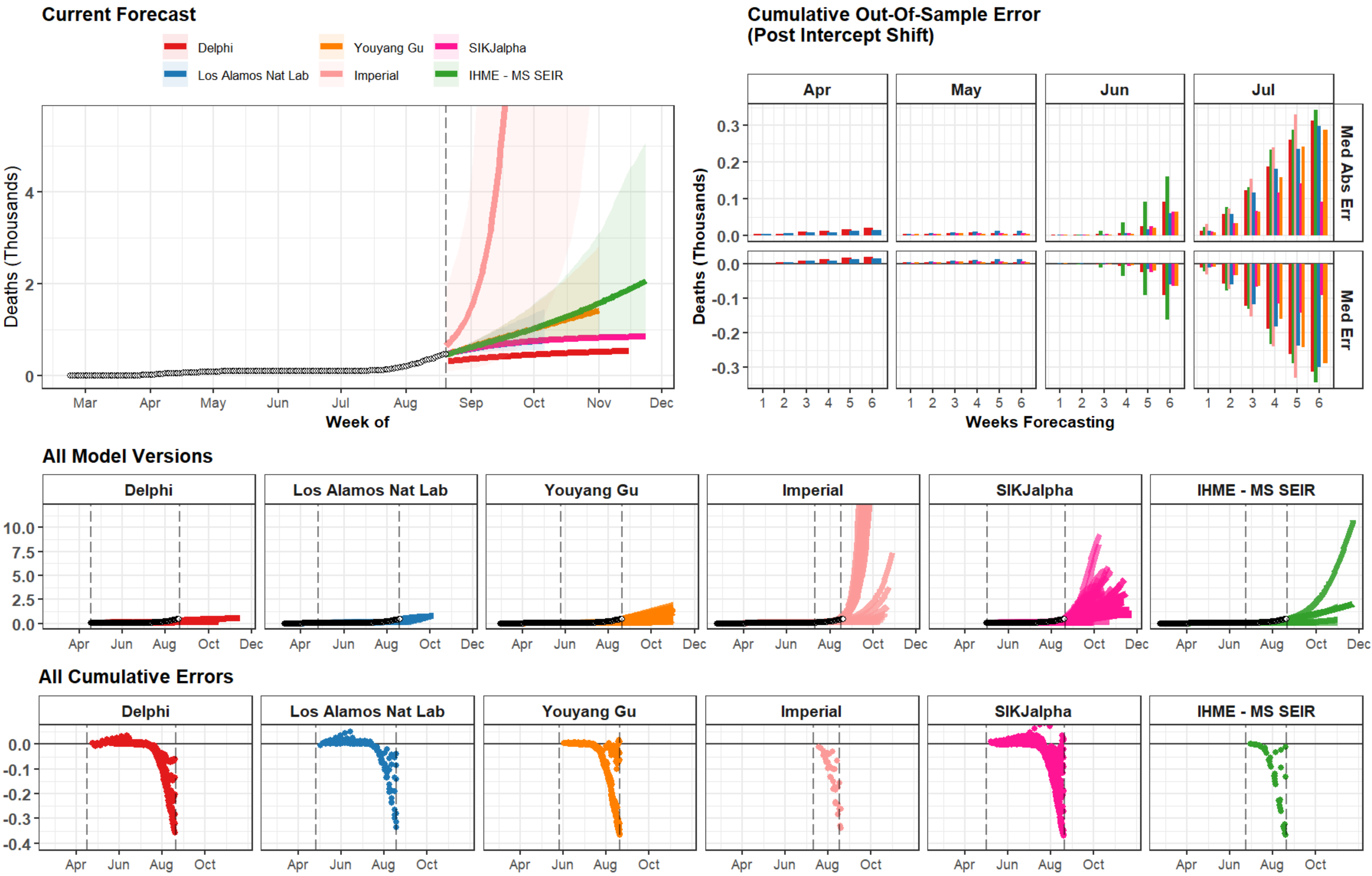

Current Forecast
Delphi
Youyang Gu
SIKJalpha
Los Alamos Nat Lab
Imperial
IHME - MS SEIR
Deaths (Thousands)
Week of
Mar Apr May Jun Jul Aug Sep Oct Nov Dec
Cumulative Out-Of-Sample Error
(Post Intercept Shift)
Apr May Jun Jul
Med Abs Err
Med Err
Weeks Forecasting
All Model Versions
All Cumulative Errors

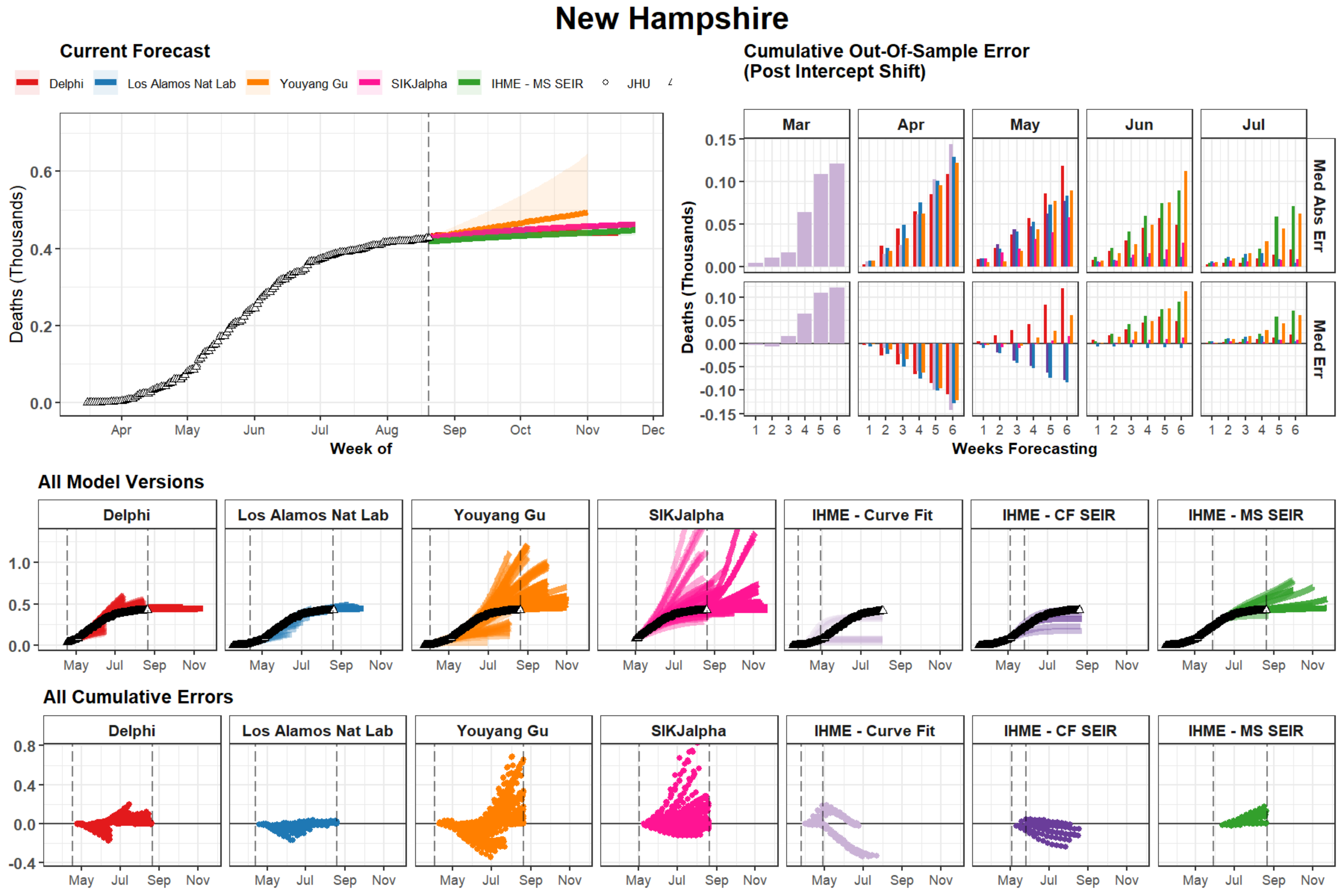
New Hampshire
Current Forecast
Delphi
Los Alamos Nat Lab
Youyang Gu
SIKJalpha
IHME - MS SEIR
JHU
Deaths (Thousands)
Week of
Apr
May
Jun
Jul
Aug
Sep
Oct
Nov
Dec
Cumulative Out-Of-Sample Error
(Post Intercept Shift)
Mar
Apr
May
Jun
Jul
Med Abs Err
Med Err
Weeks Forecasting
All Model Versions
Delphi
Los Alamos Nat Lab
Youyang Gu
SIKJalpha
IHME - Curve Fit
IHME - CF SEIR
IHME - MS SEIR
All Cumulative Errors

Kansas

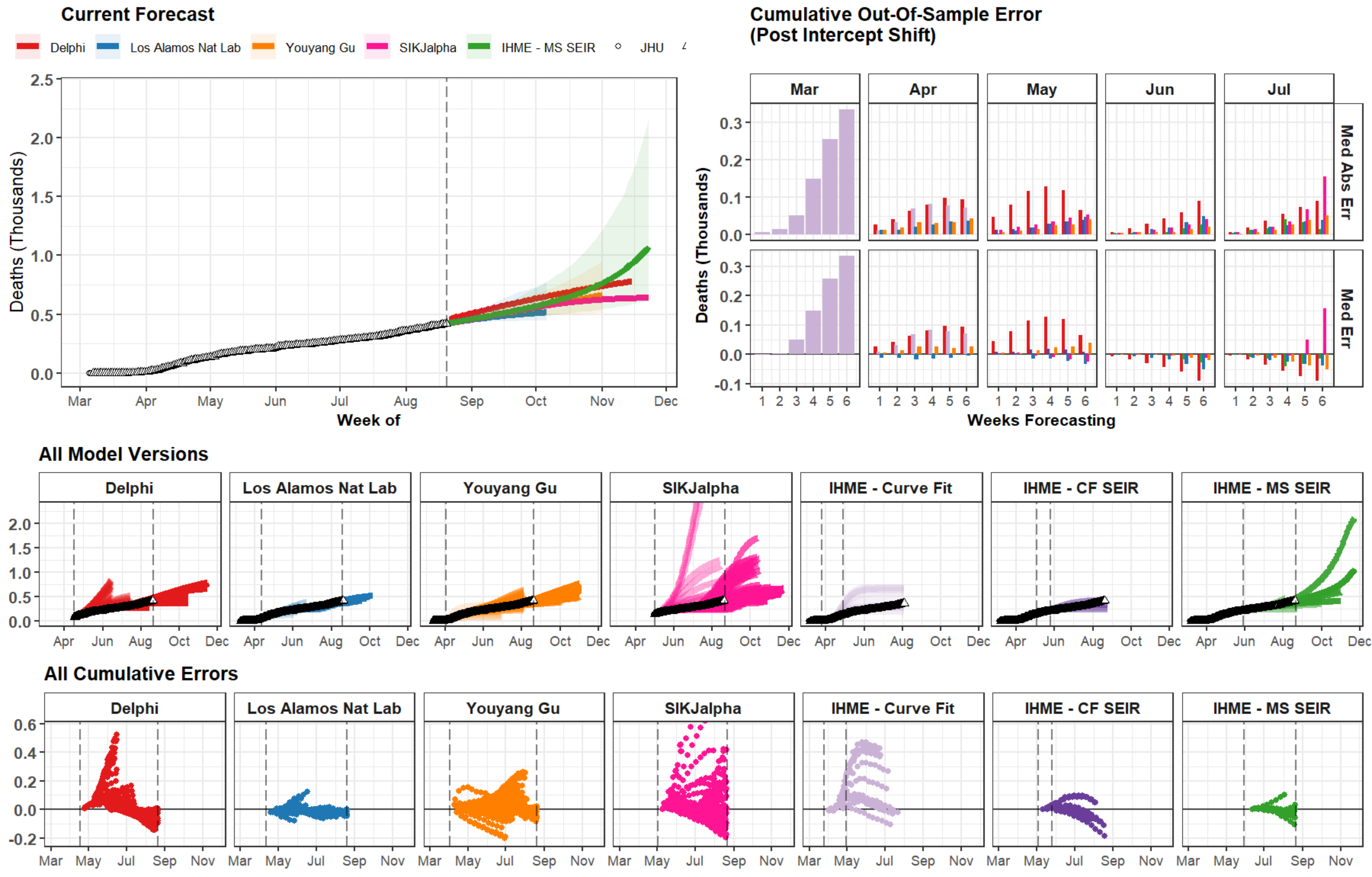
Current Forecast
Delphi
Los Alamos Nat Lab
Youyang Gu
SIKJalpha
IHME - MS SEIR
JHU
Deaths (Thousands)
Week of
Cumulative Out-Of-Sample Error
(Post Intercept Shift)
Mar
Apr
May
Jun
Jul
Med Abs Err
Med Err
Weeks Forecasting
All Model Versions
Delphi
Los Alamos Nat Lab
Youyang Gu
SIKJalpha
IHME - Curve Fit
IHME - CF SEIR
IHME - MS SEIR
All Cumulative Errors

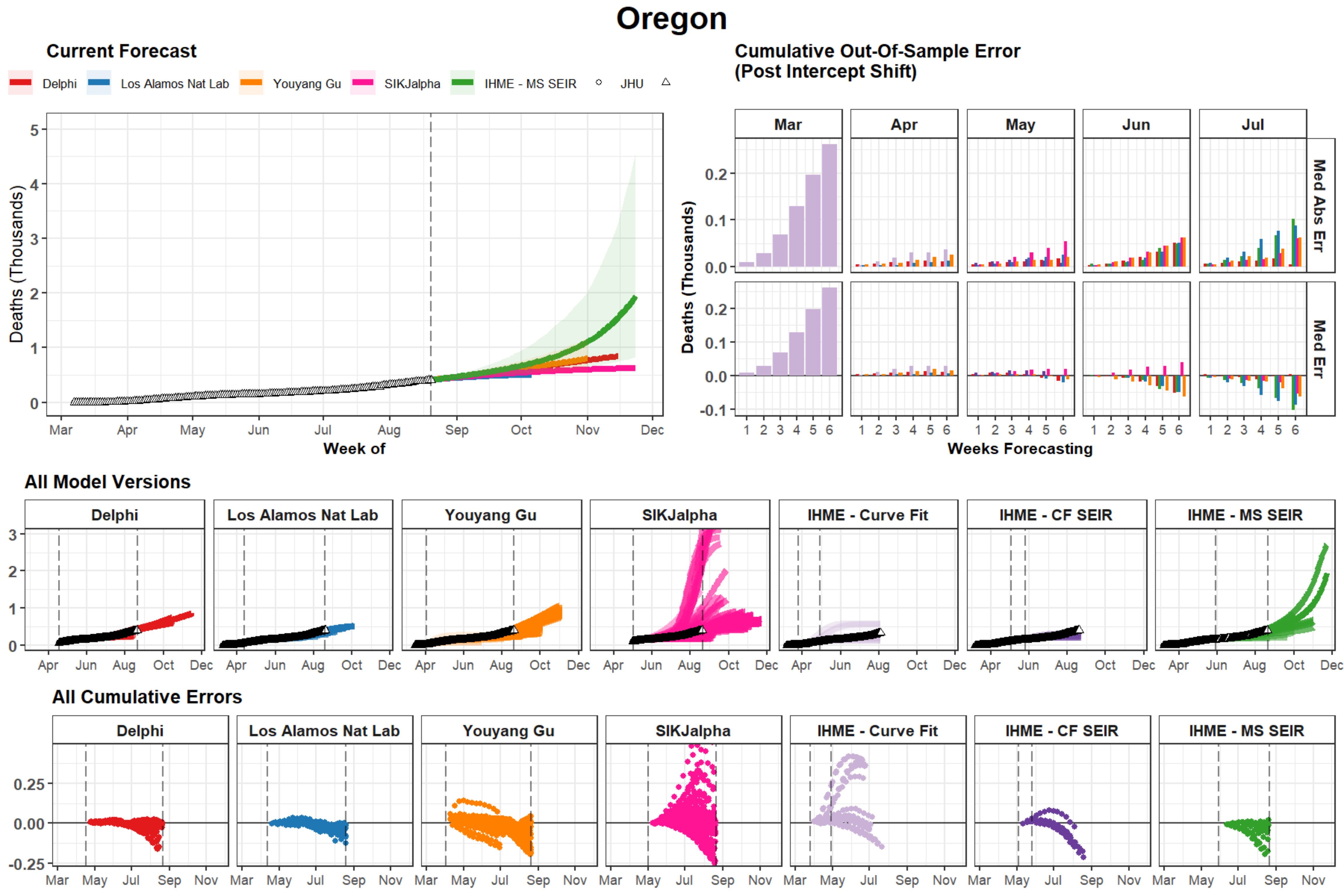
Oregon
Current Forecast
Delphi
Los Alamos Nat Lab
Youyang Gu
SIKJalpha
IHME - MS SEIR
JHU
Deaths (Thousands)
Week of
Mar
Apr
May
Jun
Jul
Aug
Sep
Oct
Nov
Dec
Cumulative Out-Of-Sample Error
(Post Intercept Shift)
Med Abs Err
Med Err
Weeks Forecasting
All Model Versions
IHME - Curve Fit
IHME - CF SEIR
All Cumulative Errors

Cameroon

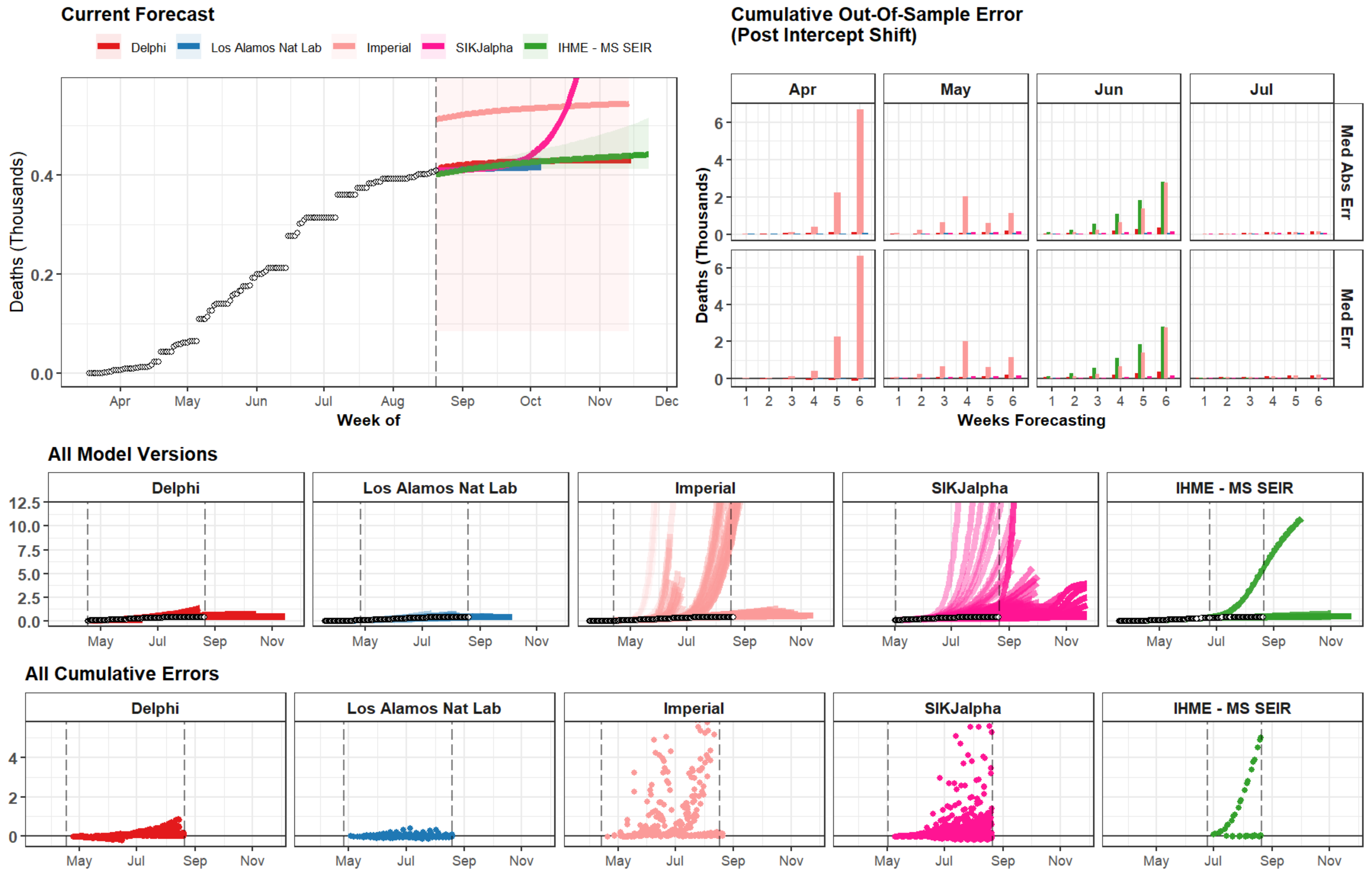

Current Forecast
Delphi
Los Alamos Nat Lab
Imperial
SIKJalpha
IHME - MS SEIR
Deaths (Thousands)
0.0
0.2
0.4
Apr
May
Jun
Jul
Aug
Sep
Oct
Nov
Dec
Week of
Cumulative Out-Of-Sample Error
(Post Intercept Shift)
Apr
May
Jun
Jul
Med Abs Err
Med Err
Deaths (Thousands)
0
2
4
6
1
2
3
4
5
6
Weeks Forecasting
All Model Versions
Delphi
Los Alamos Nat Lab
Imperial
SIKJalpha
IHME - MS SEIR
0.0
2.5
5.0
7.5
10.0
12.5
May
Jul
Sep
Nov
All Cumulative Errors
Delphi
Los Alamos Nat Lab
Imperial
SIKJalpha
IHME - MS SEIR
0
2
4
May
Jul
Sep
Nov

Czech Republic

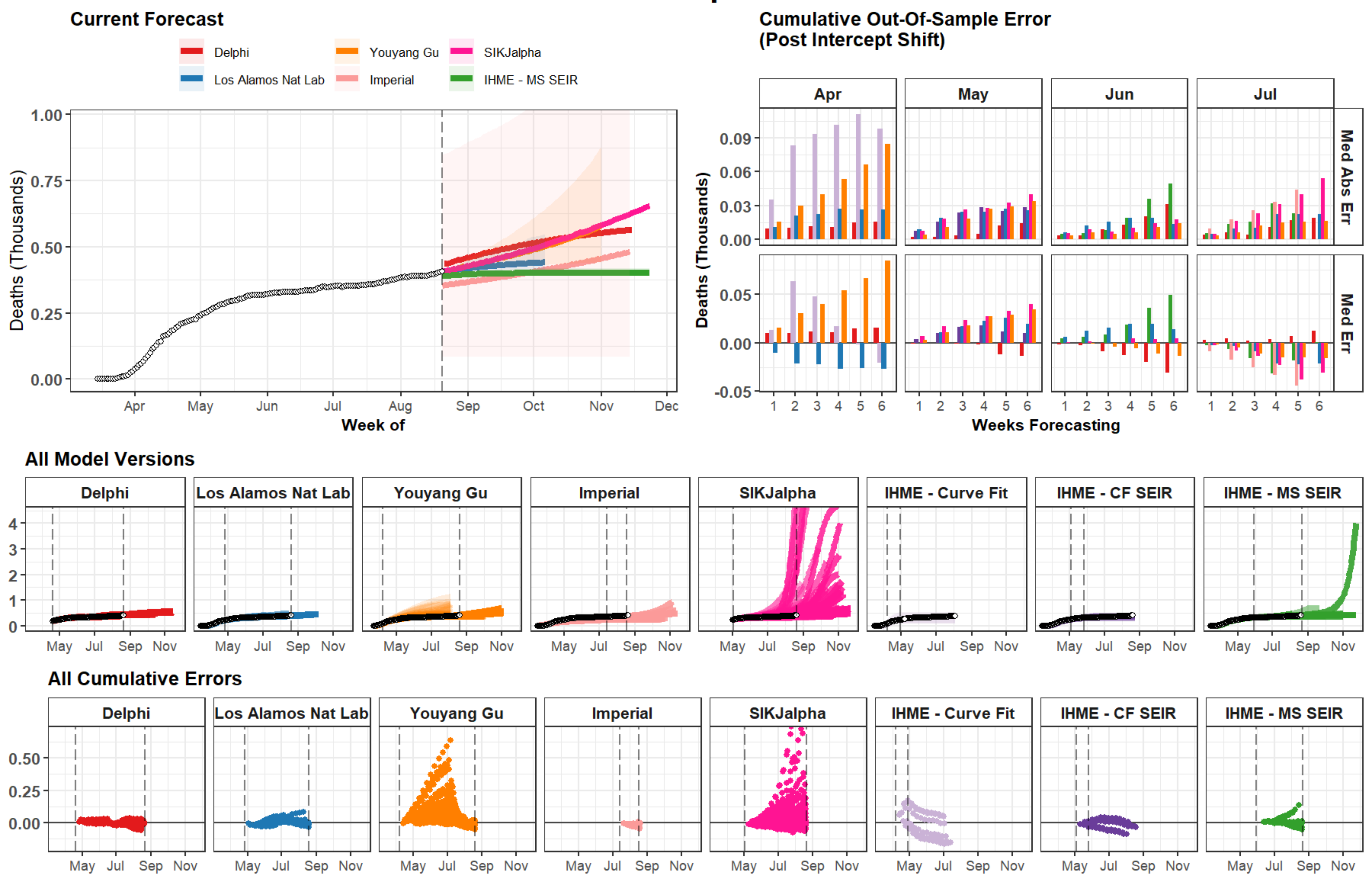

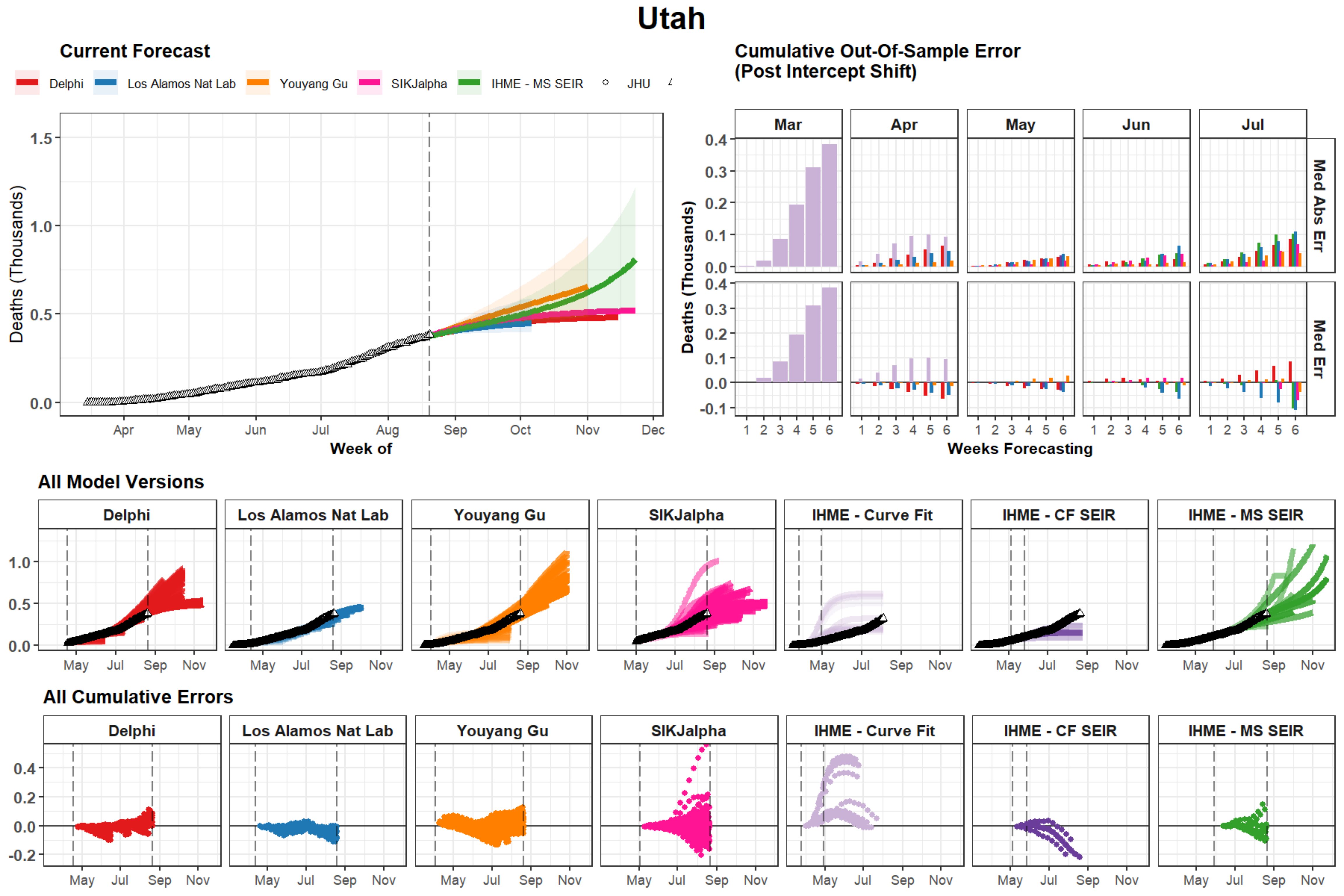

Utah
Current Forecast
Delphi
Los Alamos Nat Lab
Youyang Gu
SIKJalpha
IHME - MS SEIR
JHU
Deaths (Thousands)
Week of
Apr
May
Jun
Jul
Aug
Sep
Oct
Nov
Dec
Cumulative Out-Of-Sample Error
(Post Intercept Shift)
Mar
Apr
May
Jun
Jul
Med Abs Err
Med Err
Weeks Forecasting
All Model Versions
IHME - Curve Fit
IHME - CF SEIR
All Cumulative Errors

Nebraska

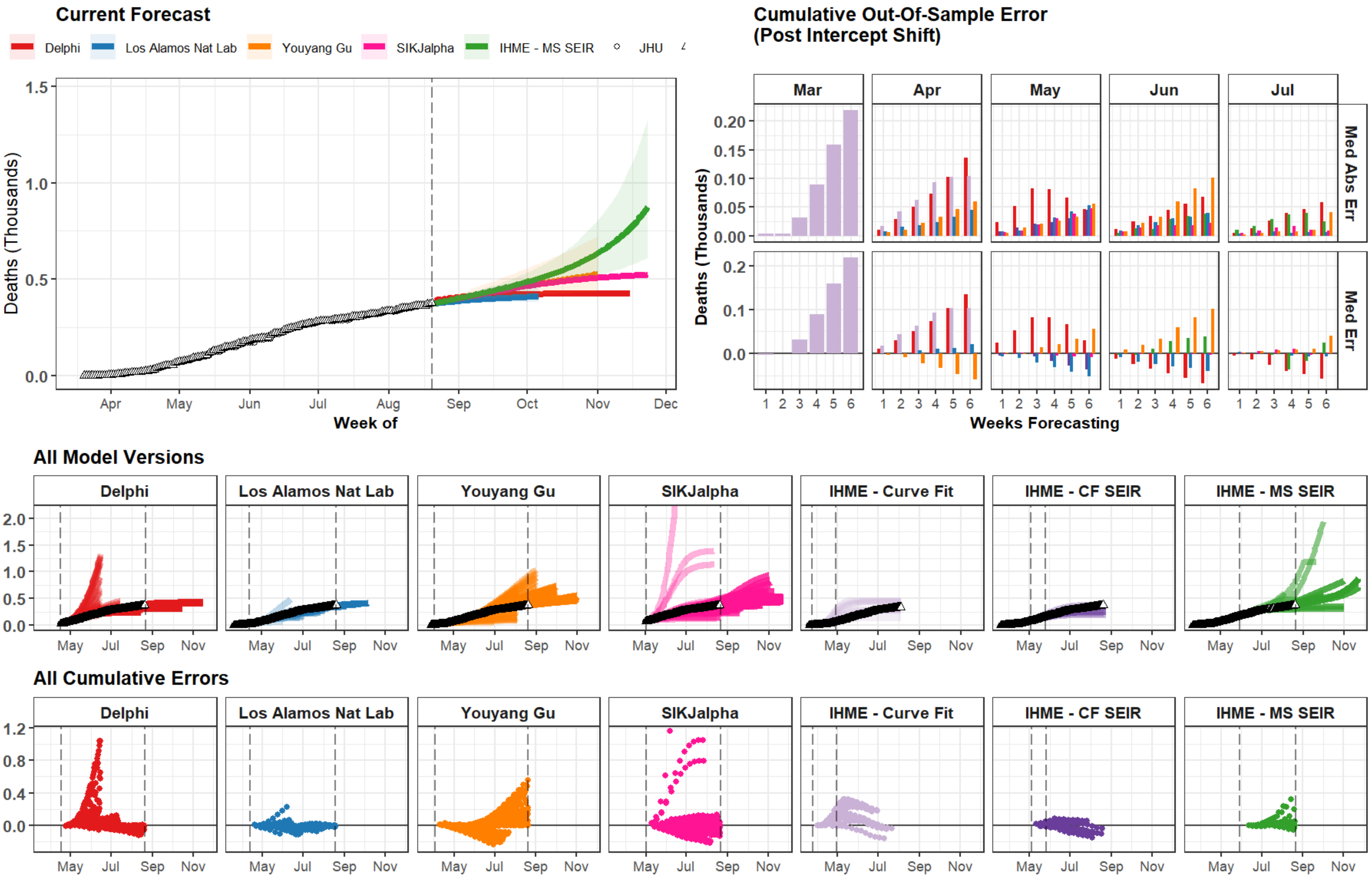
Current Forecast
Delphi
Los Alamos Nat Lab
Youyang Gu
SIKJalpha
IHME - MS SEIR
JHU
Deaths (Thousands)
Week of
Apr
May
Jun
Jul
Aug
Sep
Oct
Nov
Dec
Cumulative Out-Of-Sample Error
(Post Intercept Shift)
Mar
Apr
May
Jun
Jul
Med Abs Err
Med Err
Weeks Forecasting
All Model Versions
Delphi
Los Alamos Nat Lab
Youyang Gu
SIKJalpha
IHME - Curve Fit
IHME - CF SEIR
IHME - MS SEIR
All Cumulative Errors

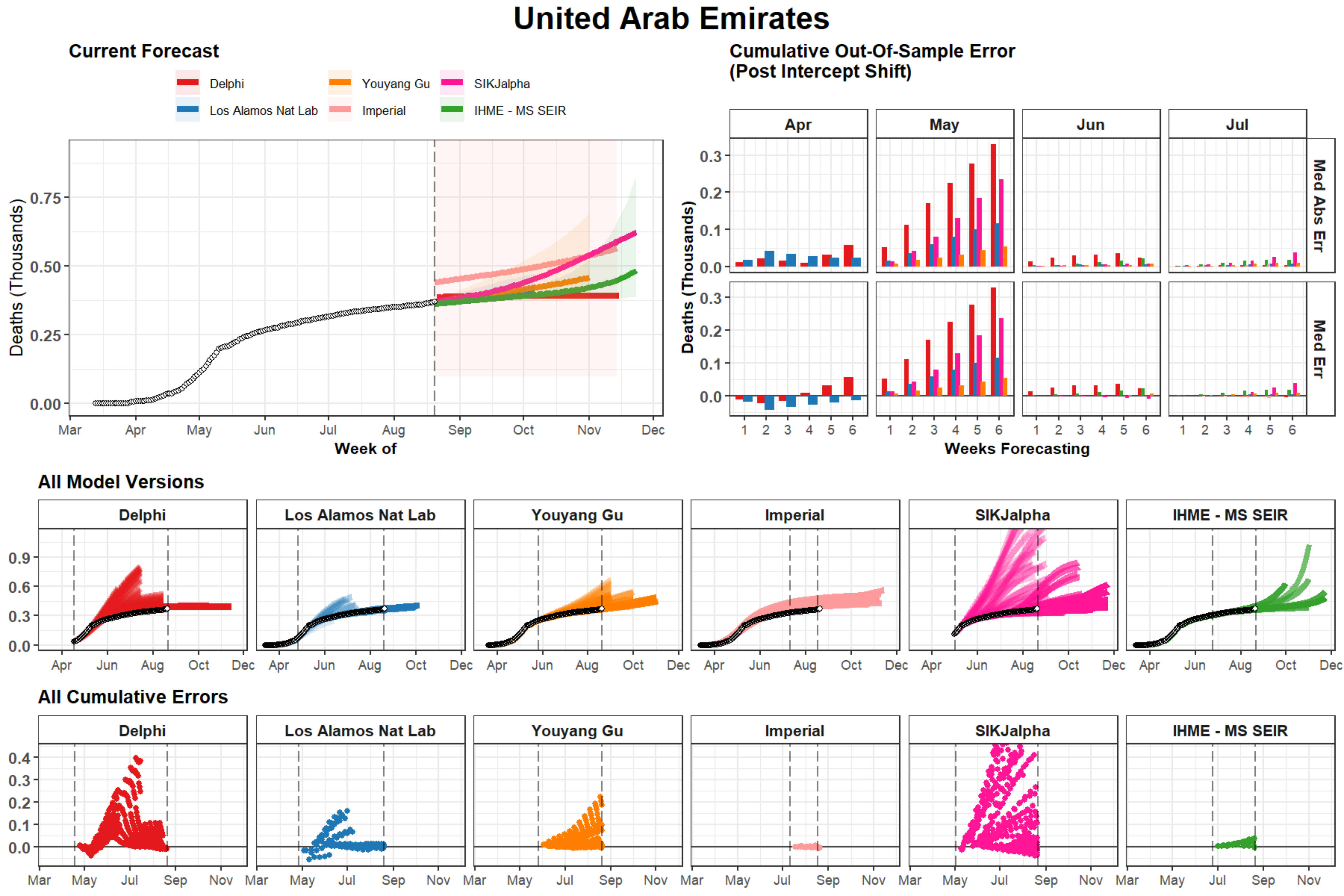
United Arab Emirates
Current Forecast
Delphi
Youyang Gu
SIKJalpha
Los Alamos Nat Lab
Imperial
IHME - MS SEIR
Deaths (Thousands)
0.00
0.25
0.50
0.75
Mar
Apr
May
Jun
Jul
Aug
Sep
Oct
Nov
Dec
Week of
Cumulative Out-Of-Sample Error
(Post Intercept Shift)
Apr
May
Jun
Jul
Med Abs Err
Med Err
0.0
0.1
0.2
0.3
1
2
3
4
5
6
Weeks Forecasting
All Model Versions
Delphi
Los Alamos Nat Lab
Youyang Gu
Imperial
SIKJalpha
IHME - MS SEIR
0.0
0.3
0.6
0.9
Apr
Jun
Aug
Oct
Dec
All Cumulative Errors
0.0
0.1
0.2
0.3
0.4
Mar
May
Jul
Sep
Nov

Puerto Rico

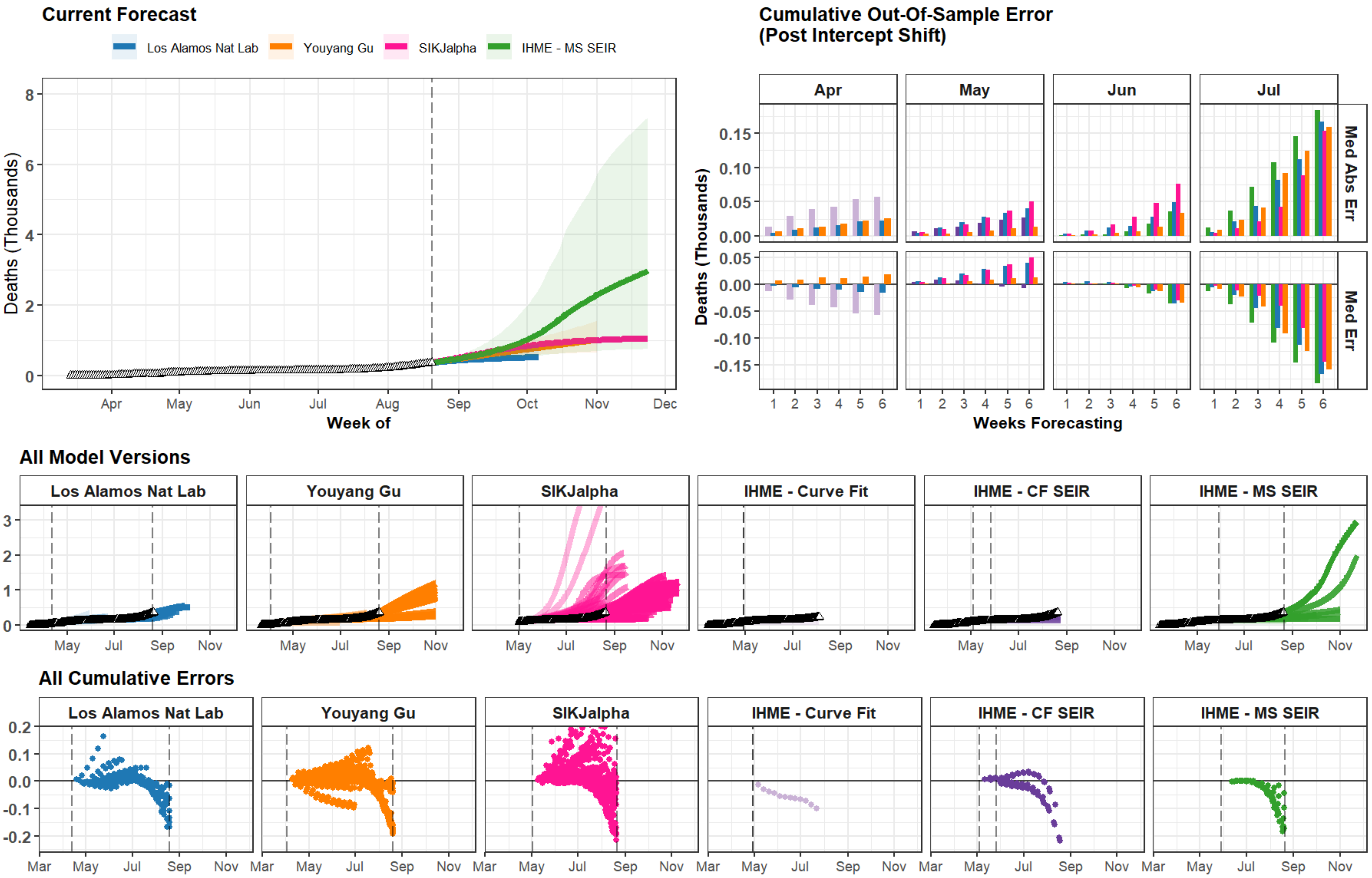
Current Forecast
Los Alamos Nat Lab
Youyang Gu
SIKJalpha
IHME - MS SEIR
Deaths (Thousands)
Week of
Apr May Jun Jul Aug Sep Oct Nov Dec
Cumulative Out-Of-Sample Error
(Post Intercept Shift)
Apr May Jun Jul
Med Abs Err
Med Err
Deaths (Thousands)
Weeks Forecasting
All Model Versions
Los Alamos Nat Lab
Youyang Gu
SIKJalpha
IHME - Curve Fit
IHME - CF SEIR
IHME - MS SEIR
All Cumulative Errors
Los Alamos Nat Lab
Youyang Gu
SIKJalpha
IHME - Curve Fit
IHME - CF SEIR
IHME - MS SEIR
Mar May Jul Sep Nov

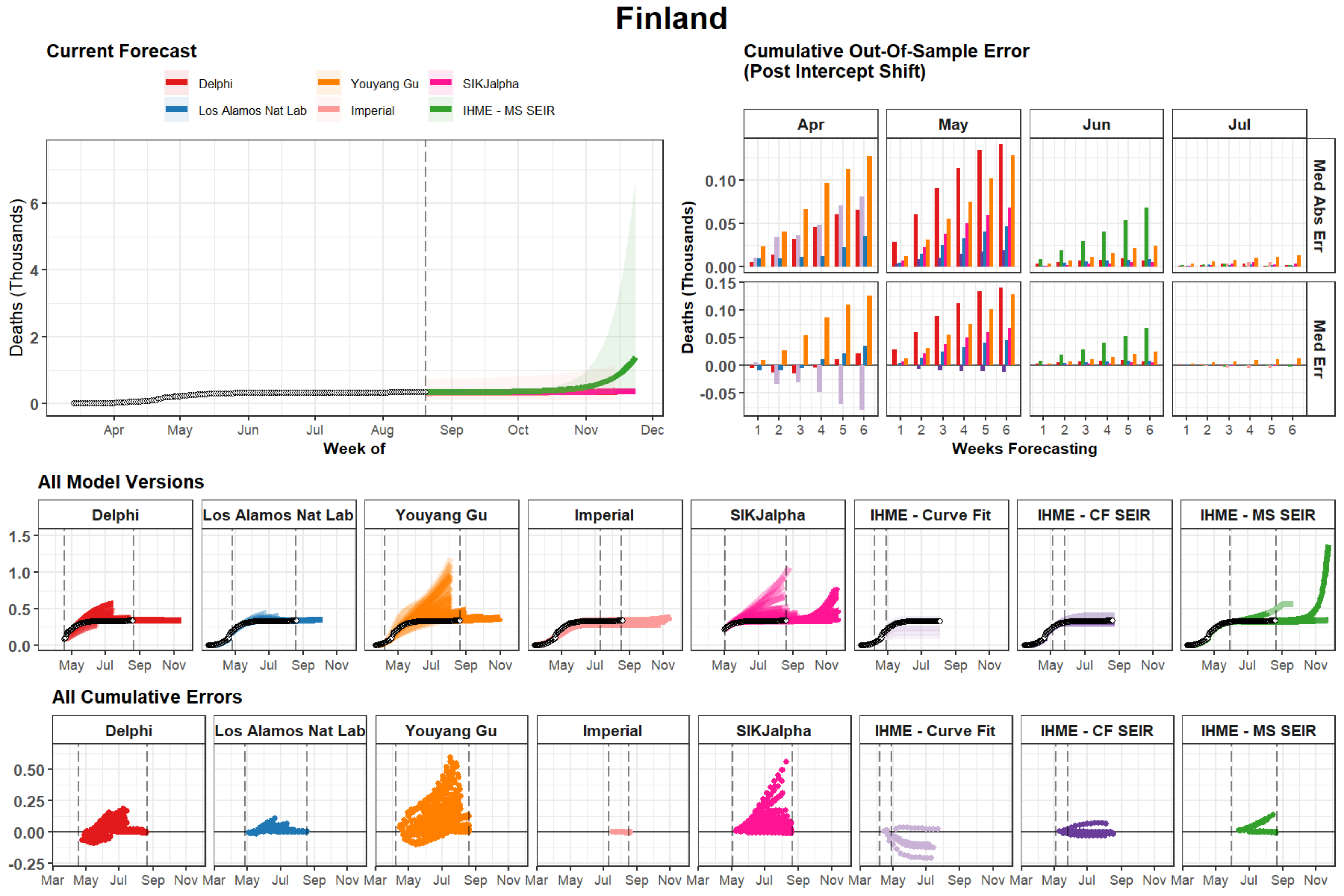

Finland
Current Forecast
Delphi
Los Alamos Nat Lab
Youyang Gu
Imperial
SIKJalpha
IHME - MS SEIR
Deaths (Thousands)
Week of
Apr
May
Jun
Jul
Aug
Sep
Oct
Nov
Dec
Cumulative Out-Of-Sample Error
(Post Intercept Shift)
Med Abs Err
Med Err
Weeks Forecasting
All Model Versions
Delphi
Los Alamos Nat Lab
Youyang Gu
Imperial
SIKJalpha
IHME - Curve Fit
IHME - CF SEIR
IHME - MS SEIR
All Cumulative Errors

Costa Rica

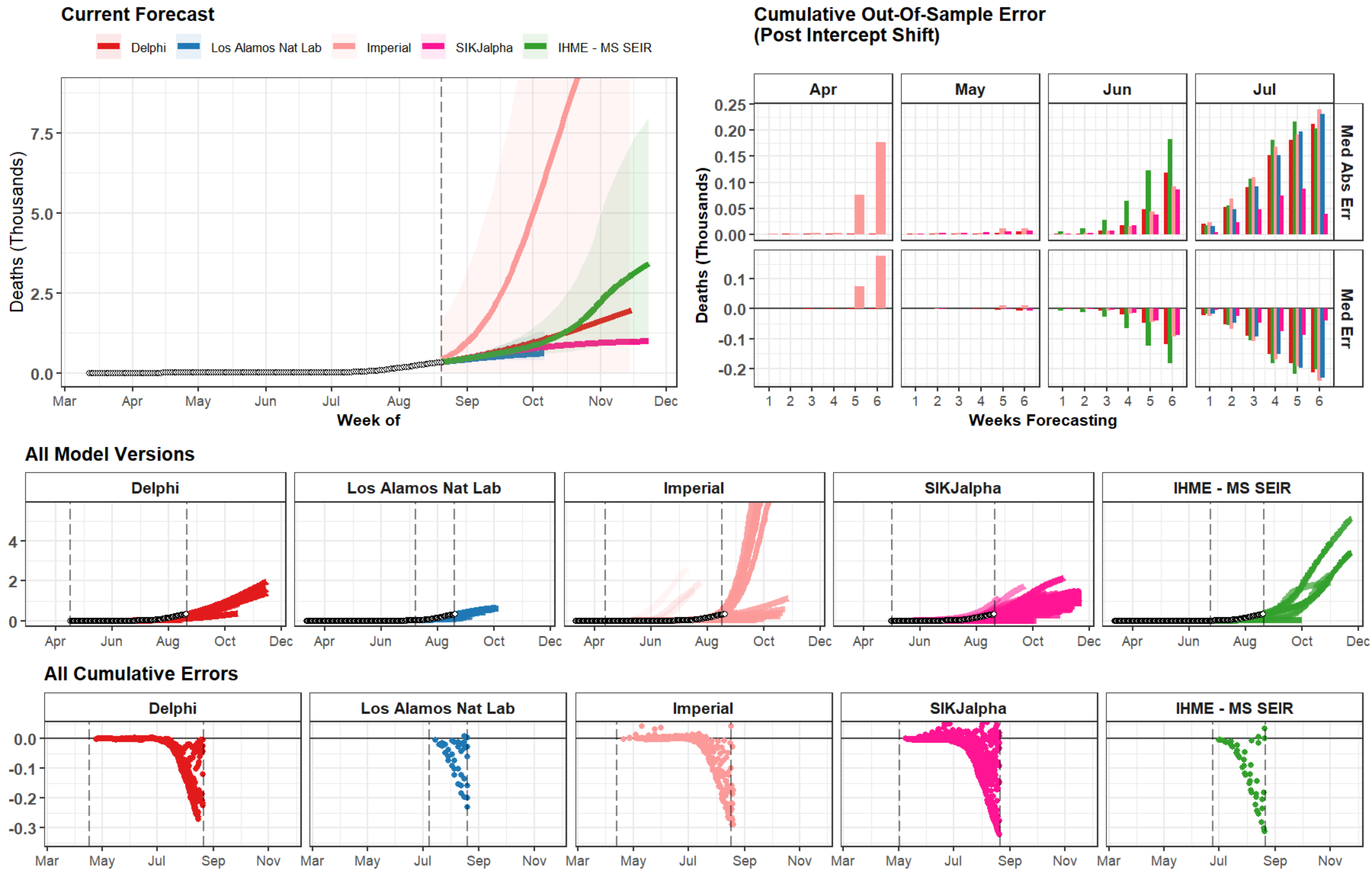

Current Forecast
Delphi
Los Alamos Nat Lab
Imperial
SIKJalpha
IHME - MS SEIR
Deaths (Thousands)
Week of
Cumulative Out-Of-Sample Error
(Post Intercept Shift)
Apr
May
Jun
Jul
Med Abs Err
Med Err
Weeks Forecasting
All Model Versions
All Cumulative Errors

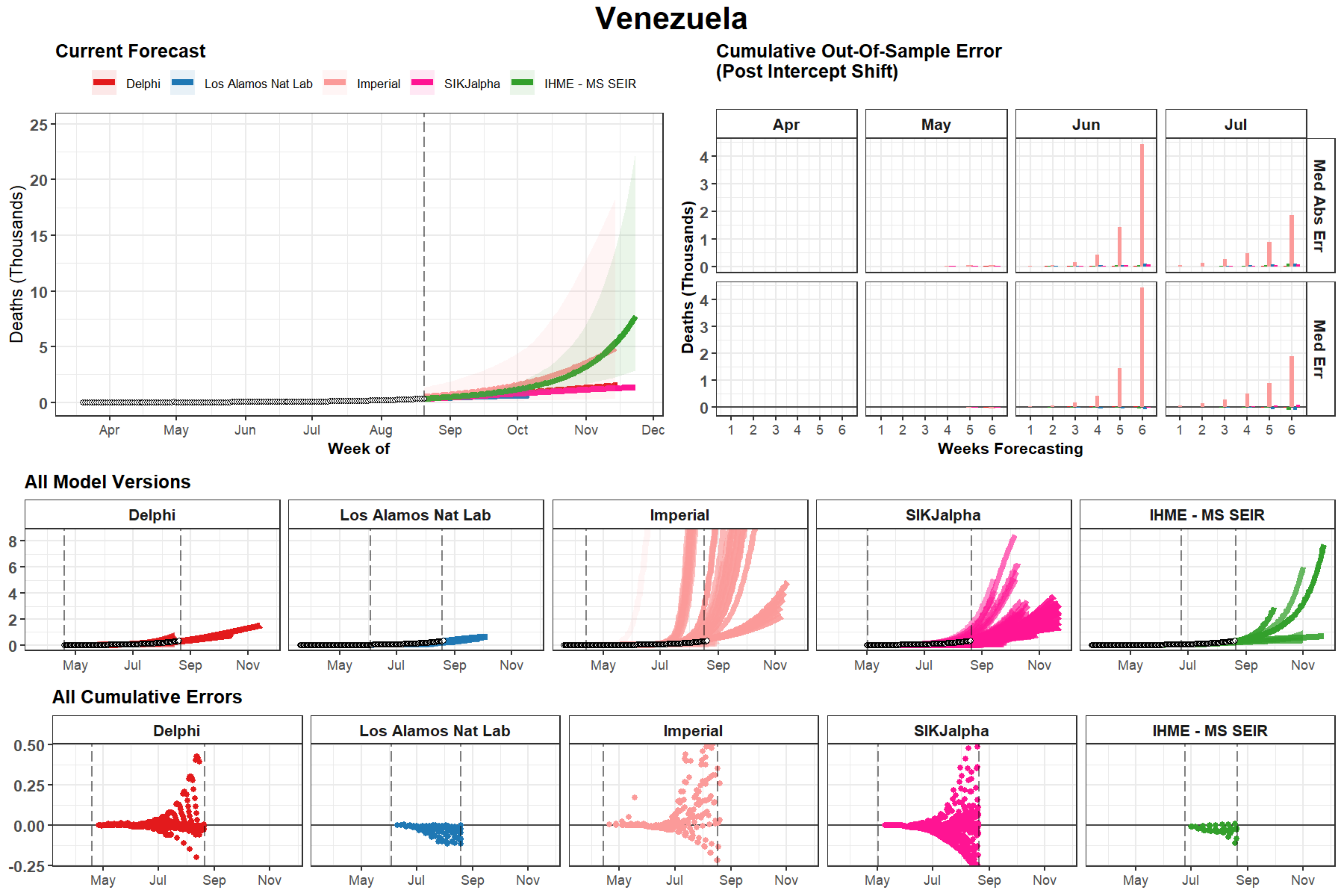

Venezuela
Current Forecast
Delphi
Los Alamos Nat Lab
Imperial
SIKJalpha
IHME - MS SEIR
Deaths (Thousands)
Week of
Apr
May
Jun
Jul
Aug
Sep
Oct
Nov
Dec
Cumulative Out-Of-Sample Error
(Post Intercept Shift)
Med Abs Err
Med Err
Weeks Forecasting
All Model Versions
All Cumulative Errors

South Korea

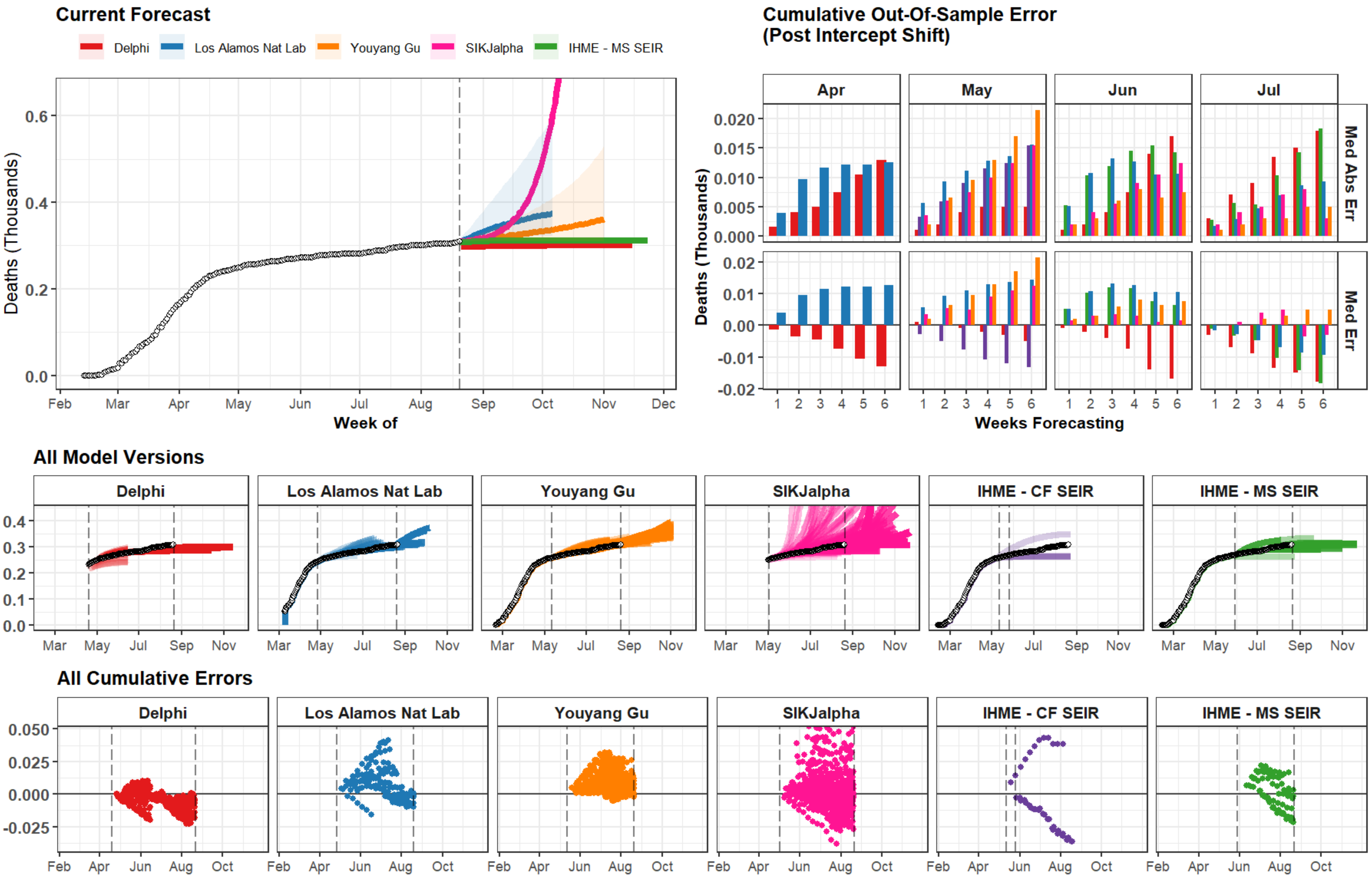

Current Forecast
Delphi
Los Alamos Nat Lab
Youyang Gu
SIKJalpha
IHME - MS SEIR
Deaths (Thousands)
Week of
Cumulative Out-Of-Sample Error
(Post Intercept Shift)
Apr
May
Jun
Jul
Med Abs Err
Med Err
Weeks Forecasting
All Model Versions
Delphi
Los Alamos Nat Lab
Youyang Gu
SIKJalpha
IHME - CF SEIR
IHME - MS SEIR
All Cumulative Errors

Idaho

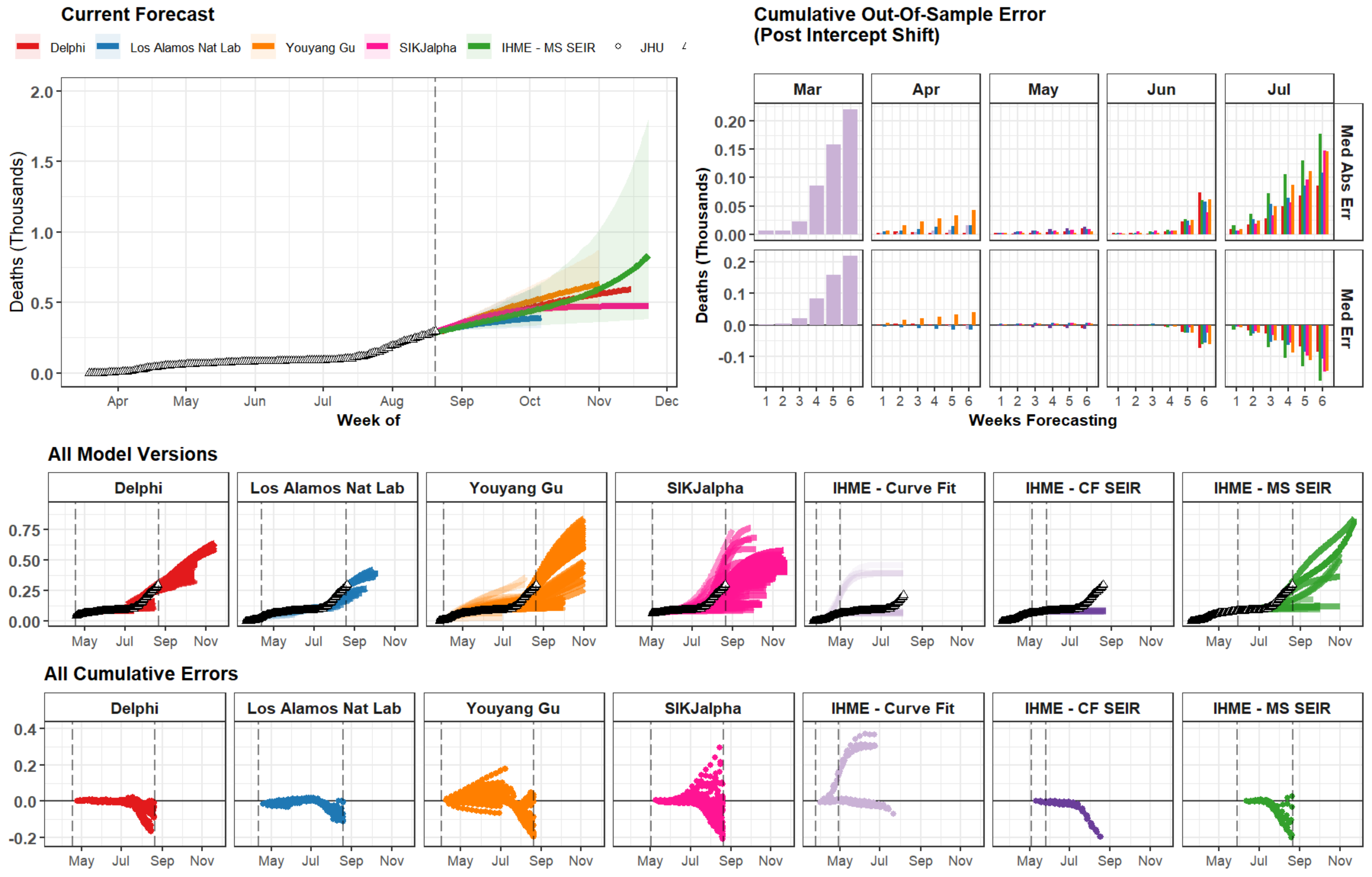

Current Forecast
Delphi
Los Alamos Nat Lab
Youyang Gu
SIKJalpha
IHME - MS SEIR
JHU
Deaths (Thousands)
2.0
1.5
1.0
0.5
0.0
Apr
May
Jun
Jul
Aug
Sep
Oct
Nov
Dec
Week of
Cumulative Out-Of-Sample Error
(Post Intercept Shift)
Mar
Apr
May
Jun
Jul
Med Abs Err
Med Err
Deaths (Thousands)
0.20
0.15
0.10
0.05
0.00
0.2
0.1
0.0
-0.1
1 2 3 4 5 6
Weeks Forecasting
All Model Versions
Delphi
Los Alamos Nat Lab
Youyang Gu
SIKJalpha
IHME - Curve Fit
IHME - CF SEIR
IHME - MS SEIR
0.75
0.50
0.25
0.00
May
Jul
Sep
Nov
All Cumulative Errors
Delphi
Los Alamos Nat Lab
Youyang Gu
SIKJalpha
IHME - Curve Fit
IHME - CF SEIR
IHME - MS SEIR
0.4
0.2
0.0
-0.2
May
Jul
Sep
Nov

Zambia

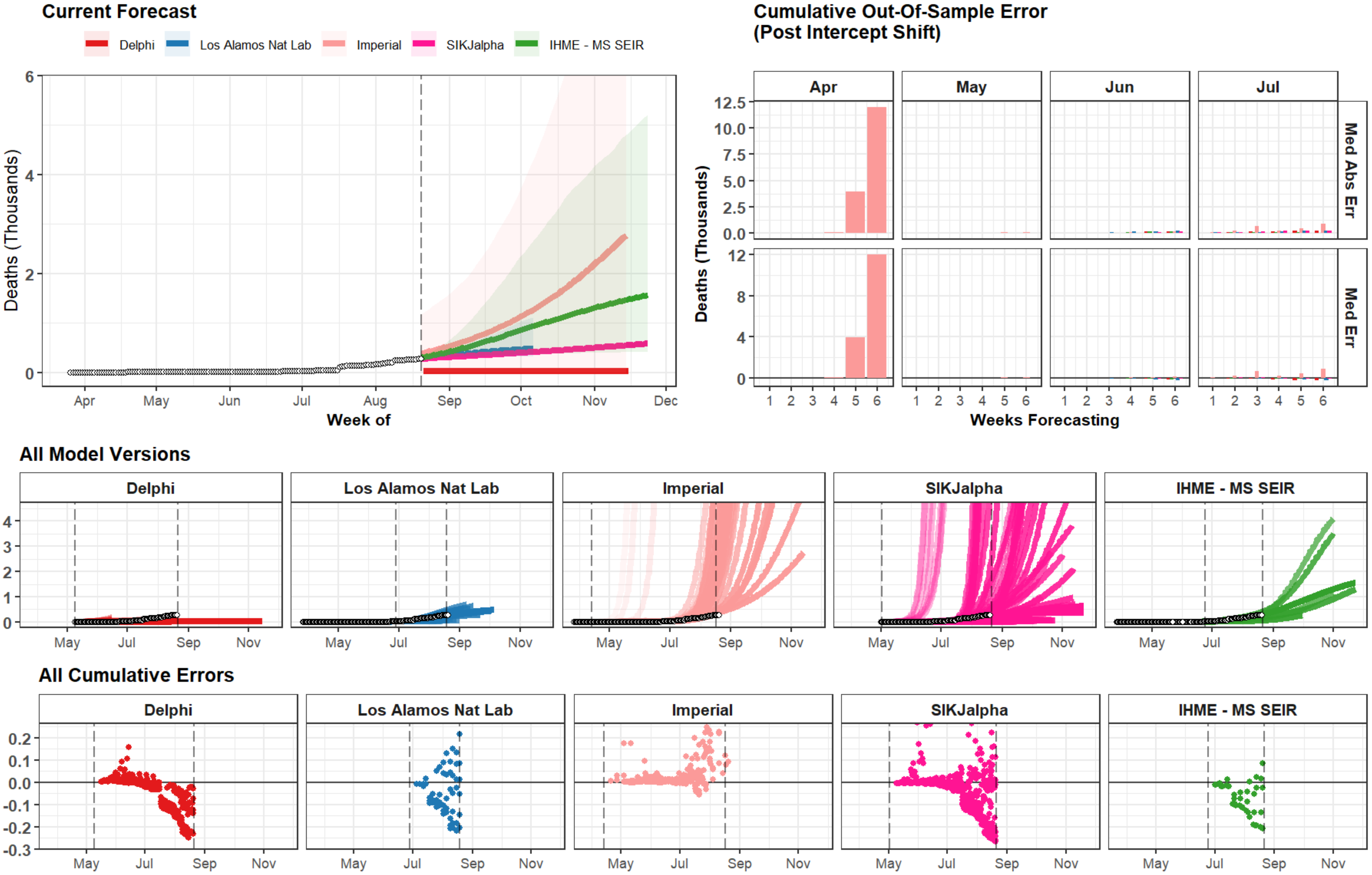

Current Forecast
Delphi
Los Alamos Nat Lab
Imperial
SIKJalpha
IHME - MS SEIR
Deaths (Thousands)
Week of
Apr
May
Jun
Jul
Aug
Sep
Oct
Nov
Dec
Cumulative Out-Of-Sample Error
(Post Intercept Shift)
Med Abs Err
Med Err
Weeks Forecasting
All Model Versions
All Cumulative Errors

Norway

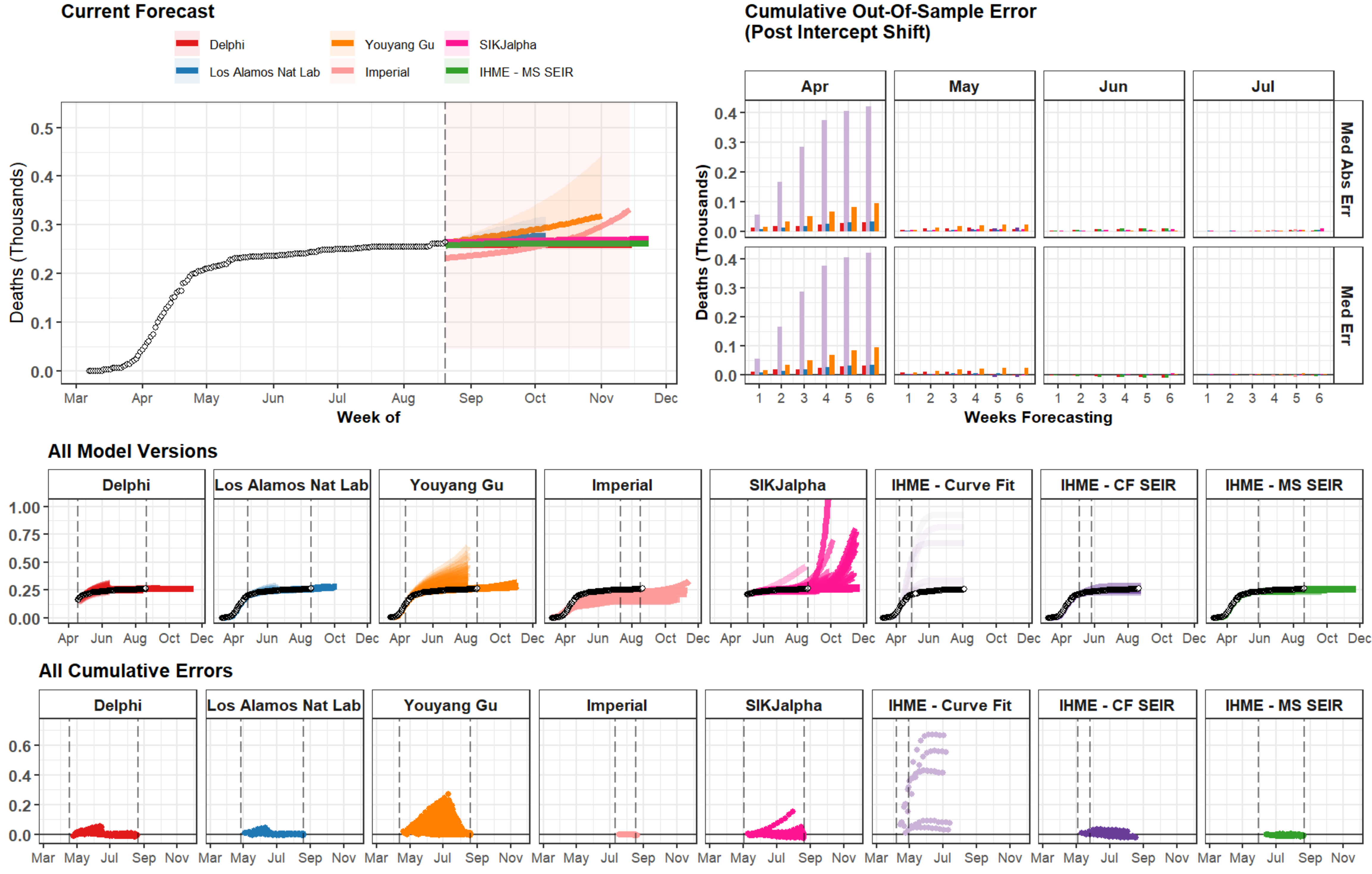
Current Forecast
Delphi
Los Alamos Nat Lab
Youyang Gu
Imperial
SIKJalpha
IHME - MS SEIR
Deaths (Thousands)
Week of
Cumulative Out-Of-Sample Error (Post Intercept Shift)
Apr
May
Jun
Jul
Med Abs Err
Med Err
Weeks Forecasting
All Model Versions
Delphi
Los Alamos Nat Lab
Youyang Gu
Imperial
SIKJalpha
IHME - Curve Fit
IHME - CF SEIR
IHME - MS SEIR
All Cumulative Errors

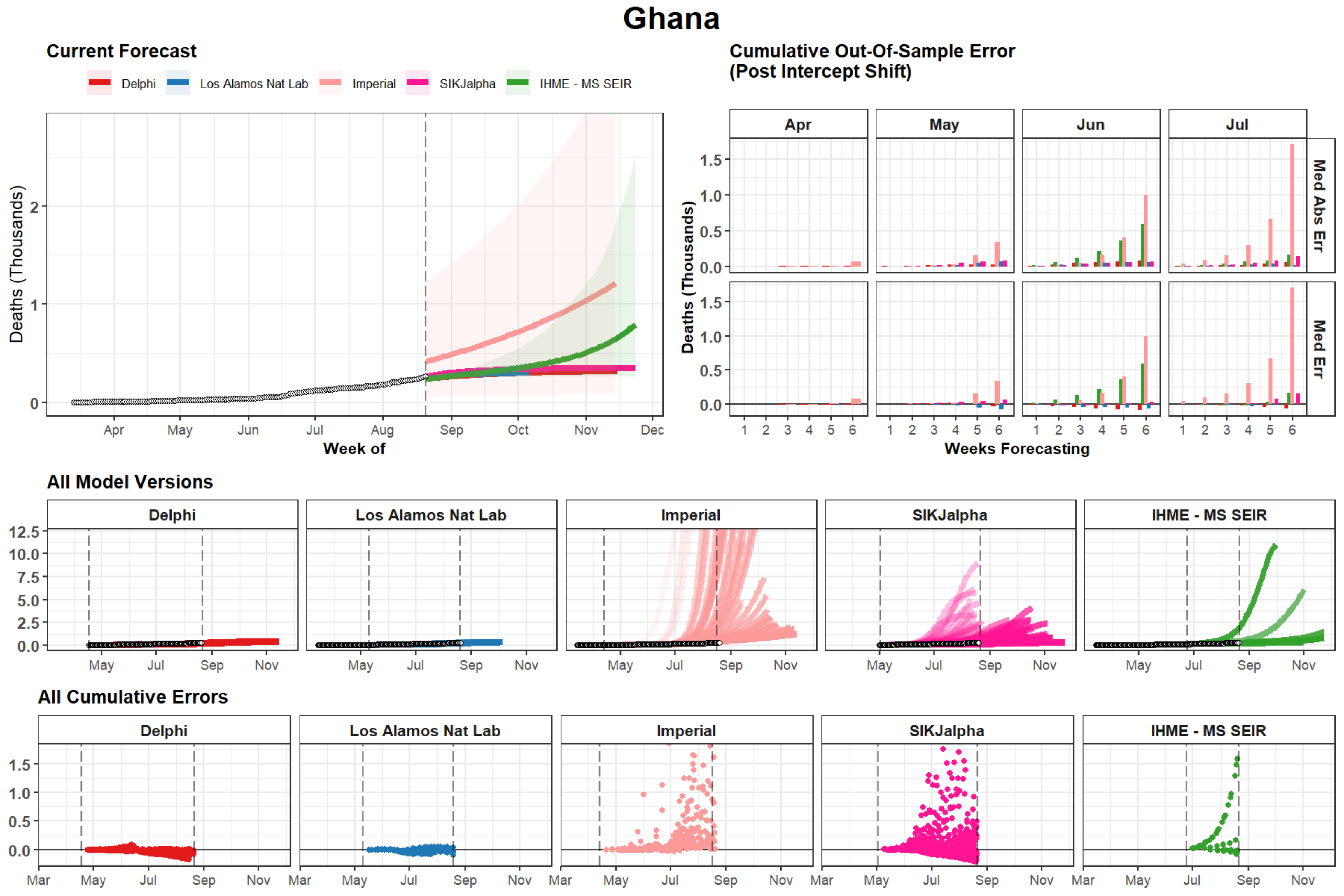
Ghana
Current Forecast
Delphi
Los Alamos Nat Lab
Imperial
SIKJalpha
IHME - MS SEIR
Deaths (Thousands)
Week of
Apr
May
Jun
Jul
Aug
Sep
Oct
Nov
Dec
Cumulative Out-Of-Sample Error
(Post Intercept Shift)
Med Abs Err
Med Err
Weeks Forecasting
All Model Versions
All Cumulative Errors
Mar

Senegal

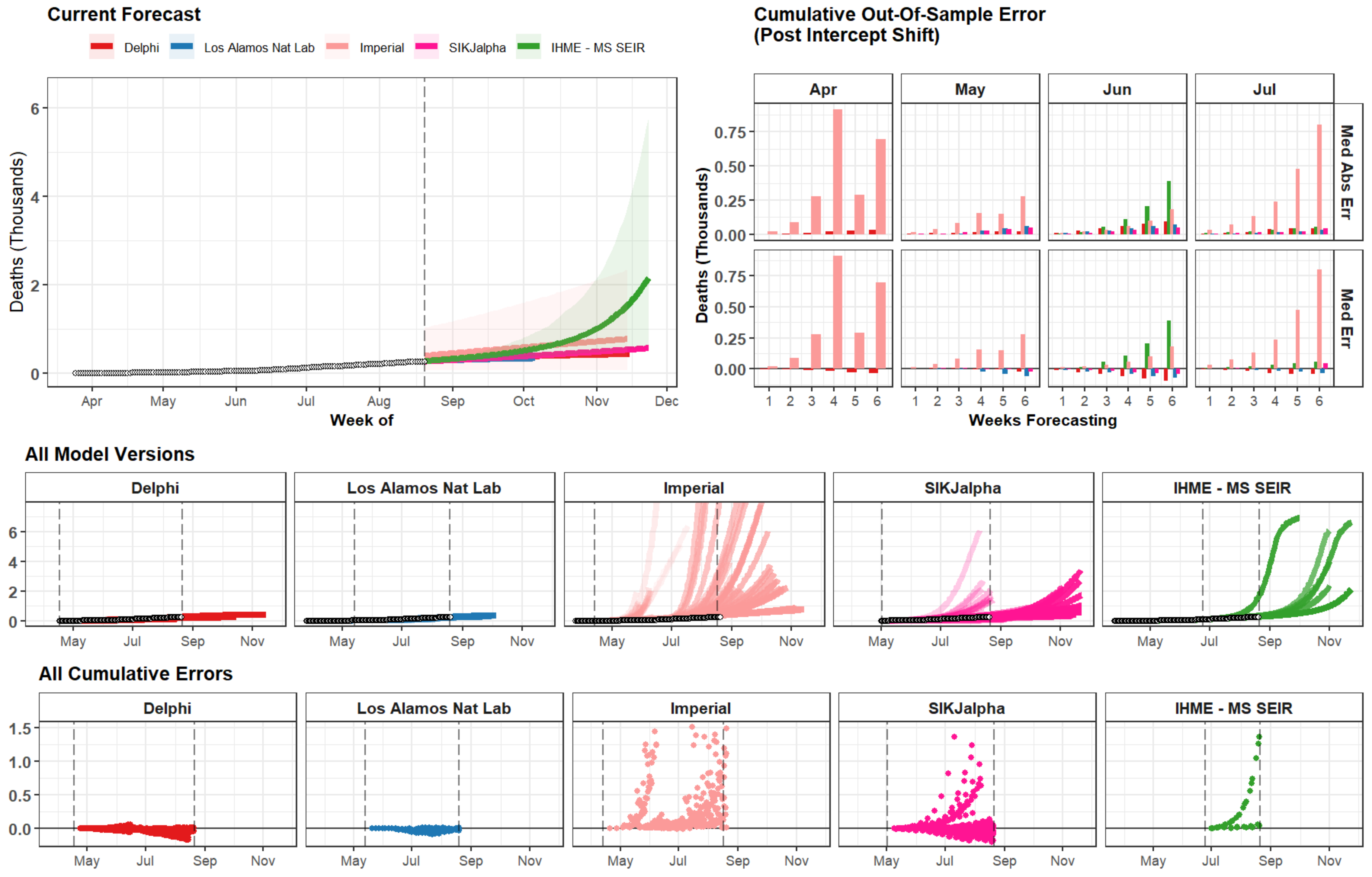
Current Forecast
Delphi
Los Alamos Nat Lab
Imperial
SIKJalpha
IHME - MS SEIR
Deaths (Thousands)
Week of
Apr
May
Jun
Jul
Aug
Sep
Oct
Nov
Dec
Cumulative Out-Of-Sample Error
(Post Intercept Shift)
Med Abs Err
Med Err
Weeks Forecasting
All Model Versions
All Cumulative Errors

Uzbekistan

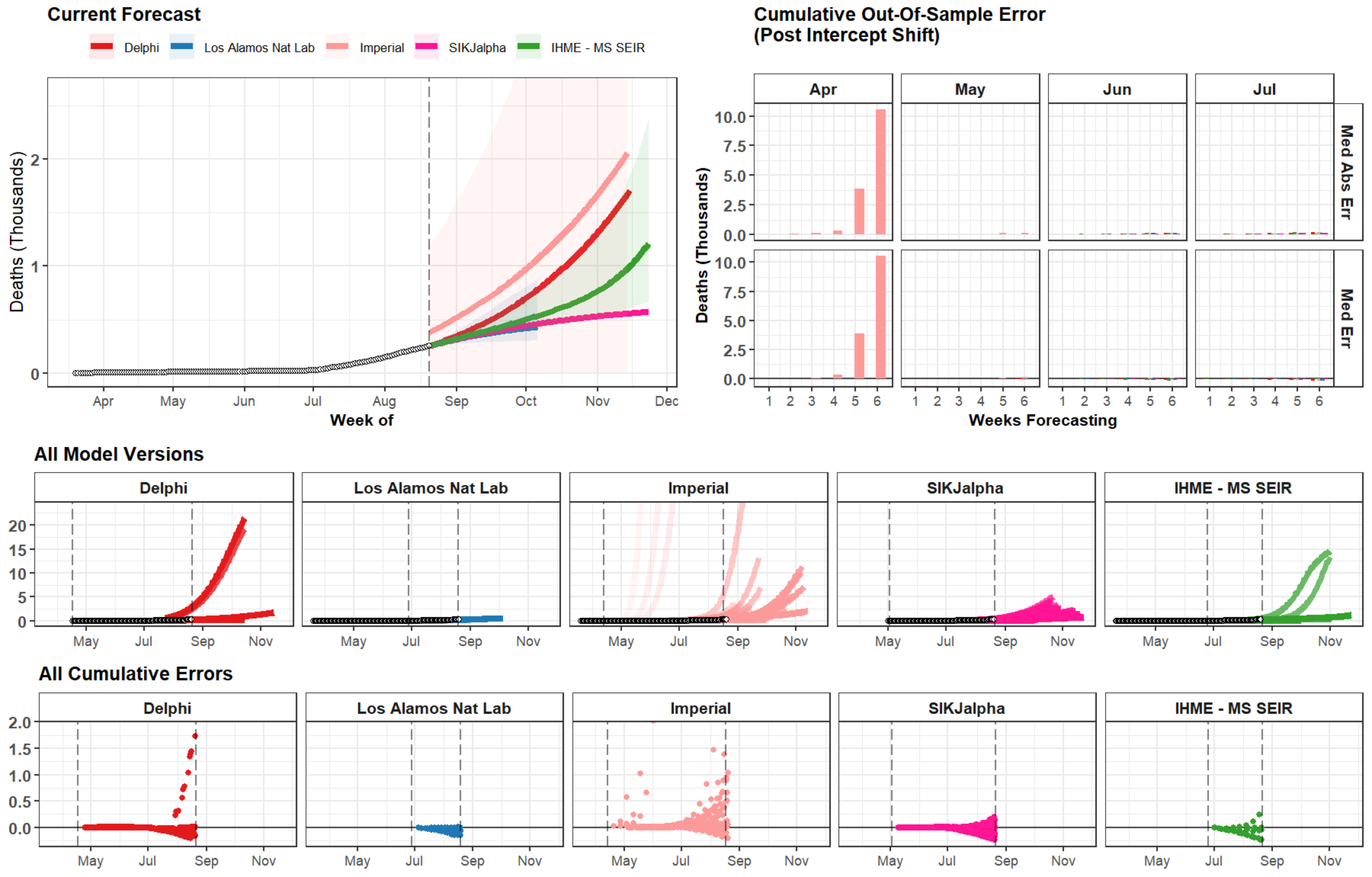

Current Forecast
Delphi
Los Alamos Nat Lab
Imperial
SIKJalpha
IHME - MS SEIR
Deaths (Thousands)
Week of
Apr
May
Jun
Jul
Aug
Sep
Oct
Nov
Dec
Cumulative Out-Of-Sample Error
(Post Intercept Shift)
Med Abs Err
Med Err
Weeks Forecasting
All Model Versions
All Cumulative Errors

Democratic Republic of the Congo

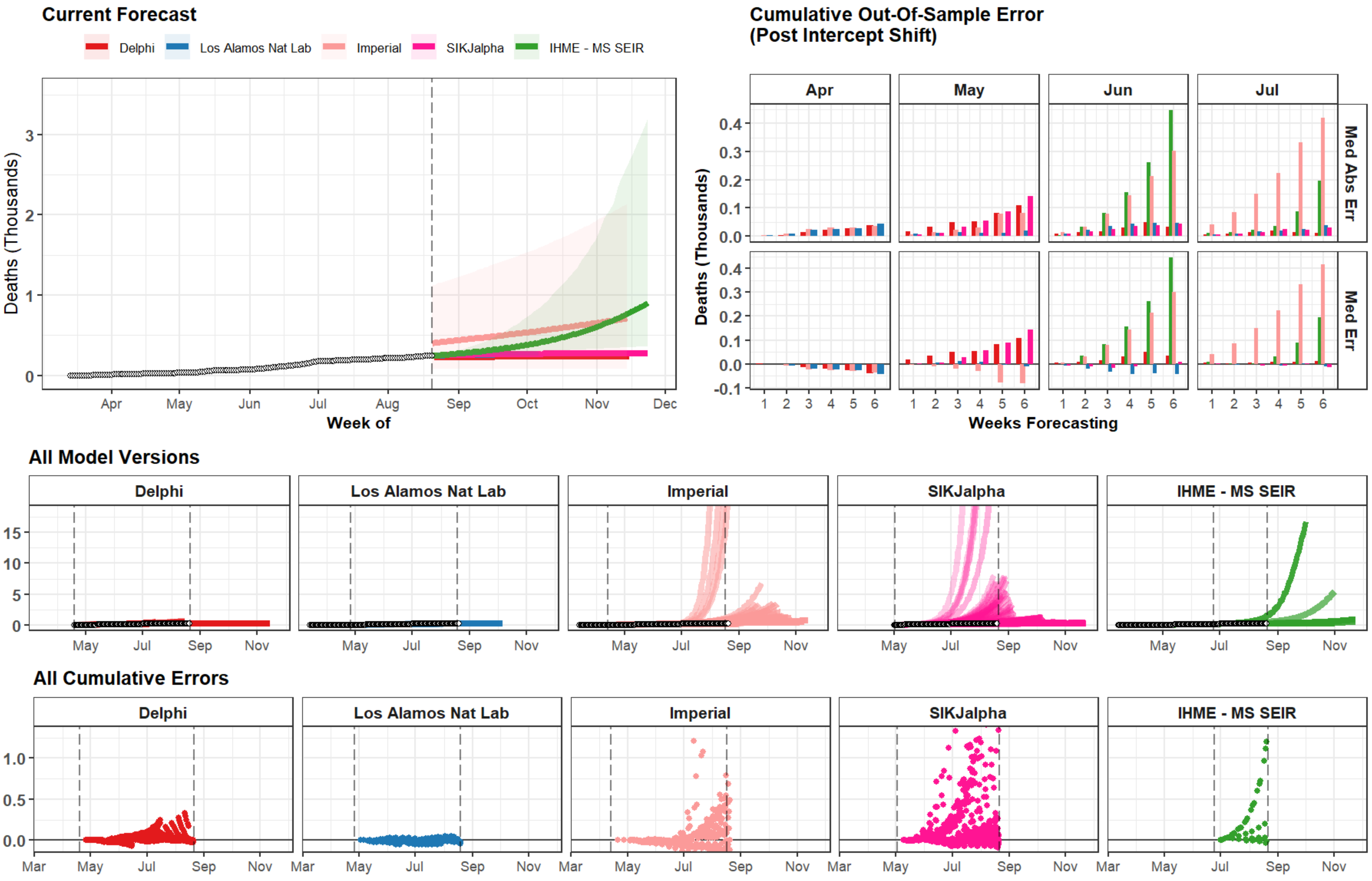

Albania

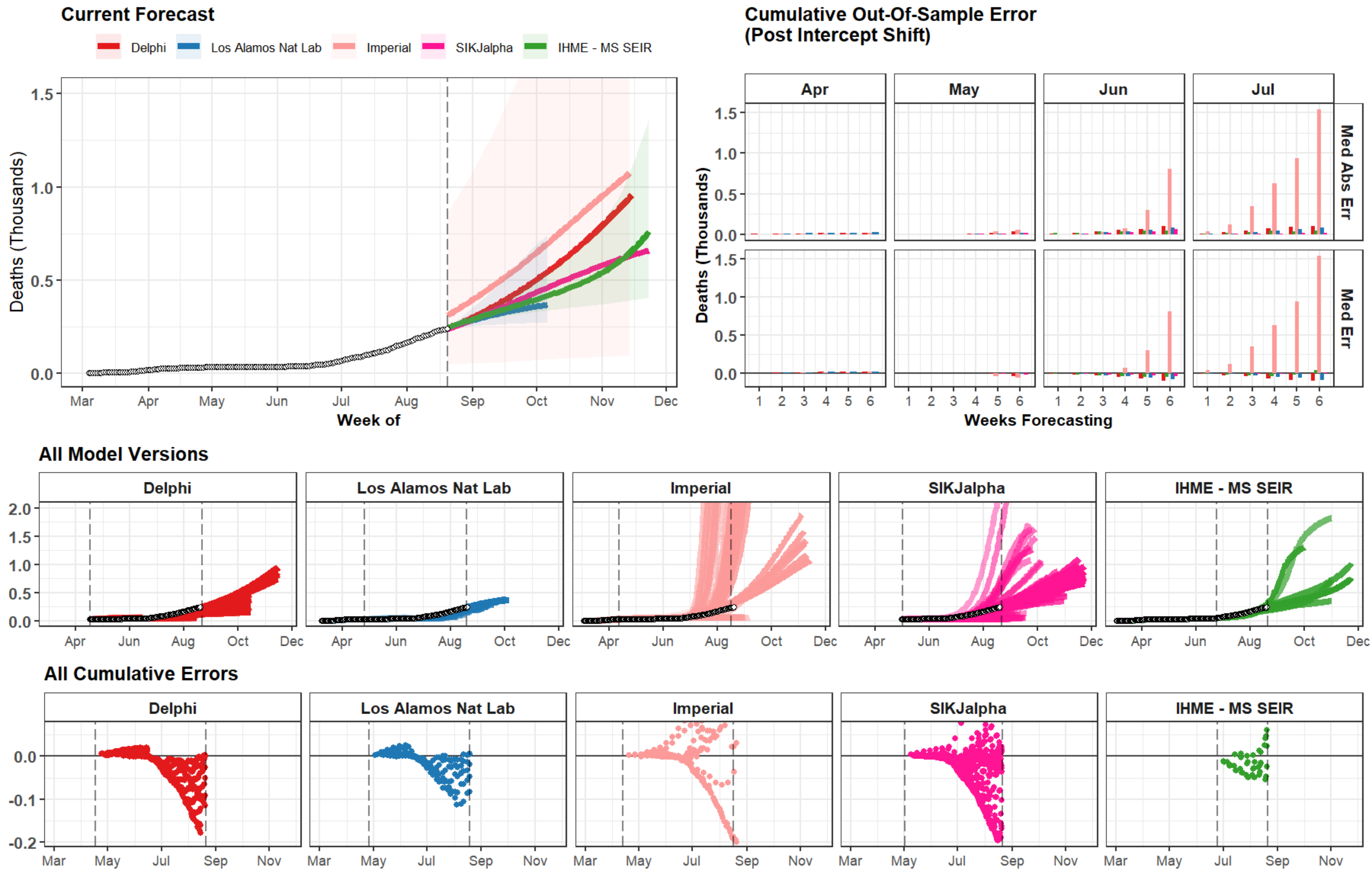
Current Forecast
Delphi
Los Alamos Nat Lab
Imperial
SIKJalpha
IHME - MS SEIR
Deaths (Thousands)
1.5
1.0
0.5
0.0
Mar
Apr
May
Jun
Jul
Aug
Sep
Oct
Nov
Dec
Week of
Cumulative Out-Of-Sample Error
(Post Intercept Shift)
Apr
May
Jun
Jul
Med Abs Err
Med Err
Deaths (Thousands)
1 2 3 4 5 6
Weeks Forecasting
All Model Versions
Delphi
Los Alamos Nat Lab
Imperial
SIKJalpha
IHME - MS SEIR
2.0
1.5
1.0
0.5
0.0
Apr
Jun
Aug
Oct
Dec
All Cumulative Errors
Delphi
Los Alamos Nat Lab
Imperial
SIKJalpha
IHME - MS SEIR
0.0
-0.1
-0.2
Mar
May
Jul
Sep
Nov

Greece

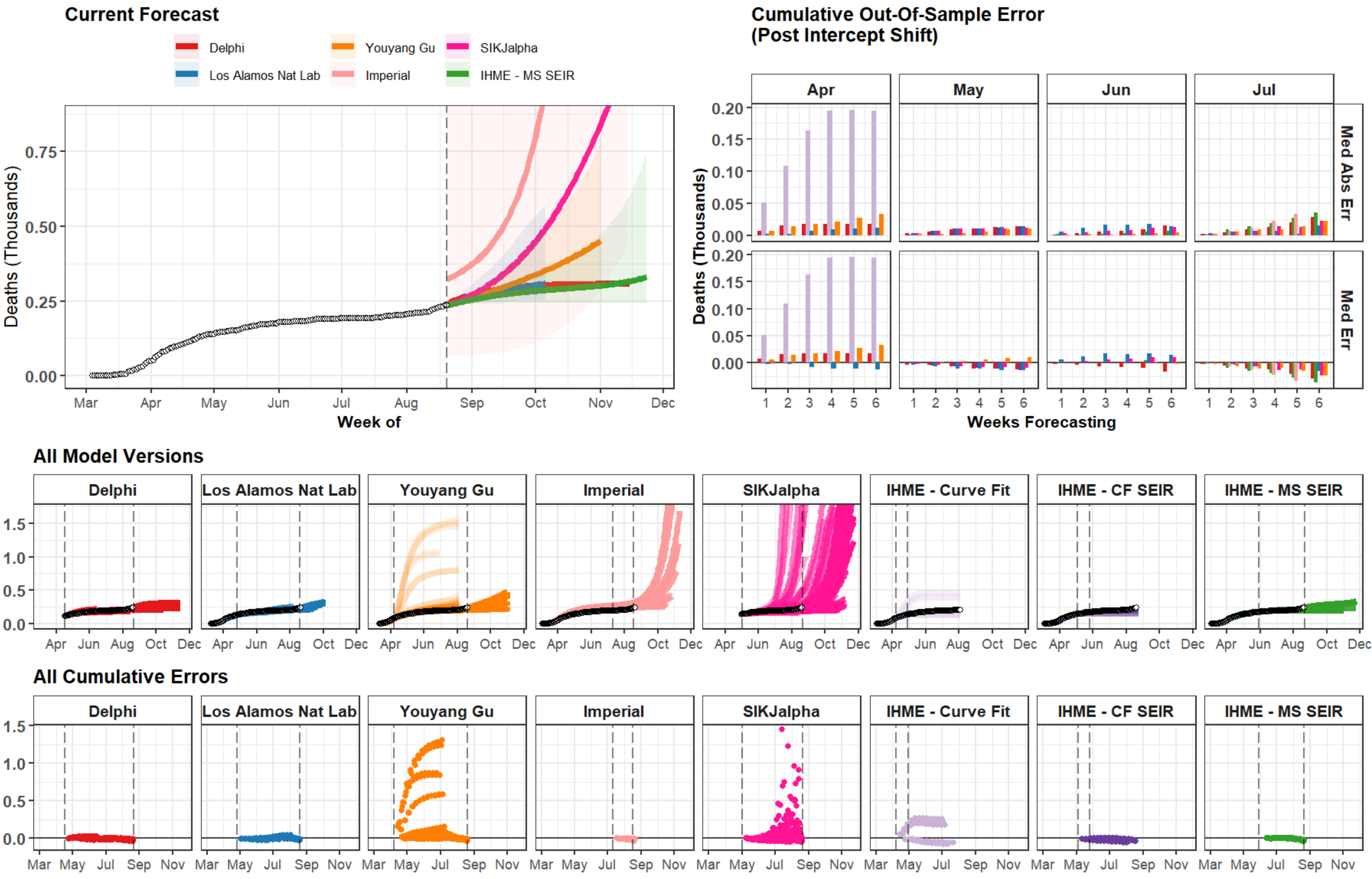

Current Forecast
Delphi
Los Alamos Nat Lab
Youyang Gu
Imperial
SIKJalpha
IHME - MS SEIR
Deaths (Thousands)
Week of
Cumulative Out-Of-Sample Error
(Post Intercept Shift)
Apr
May
Jun
Jul
Med Abs Err
Med Err
Weeks Forecasting
All Model Versions
Delphi
Los Alamos Nat Lab
Youyang Gu
Imperial
SIKJalpha
IHME - Curve Fit
IHME - CF SEIR
IHME - MS SEIR
All Cumulative Errors

Haiti

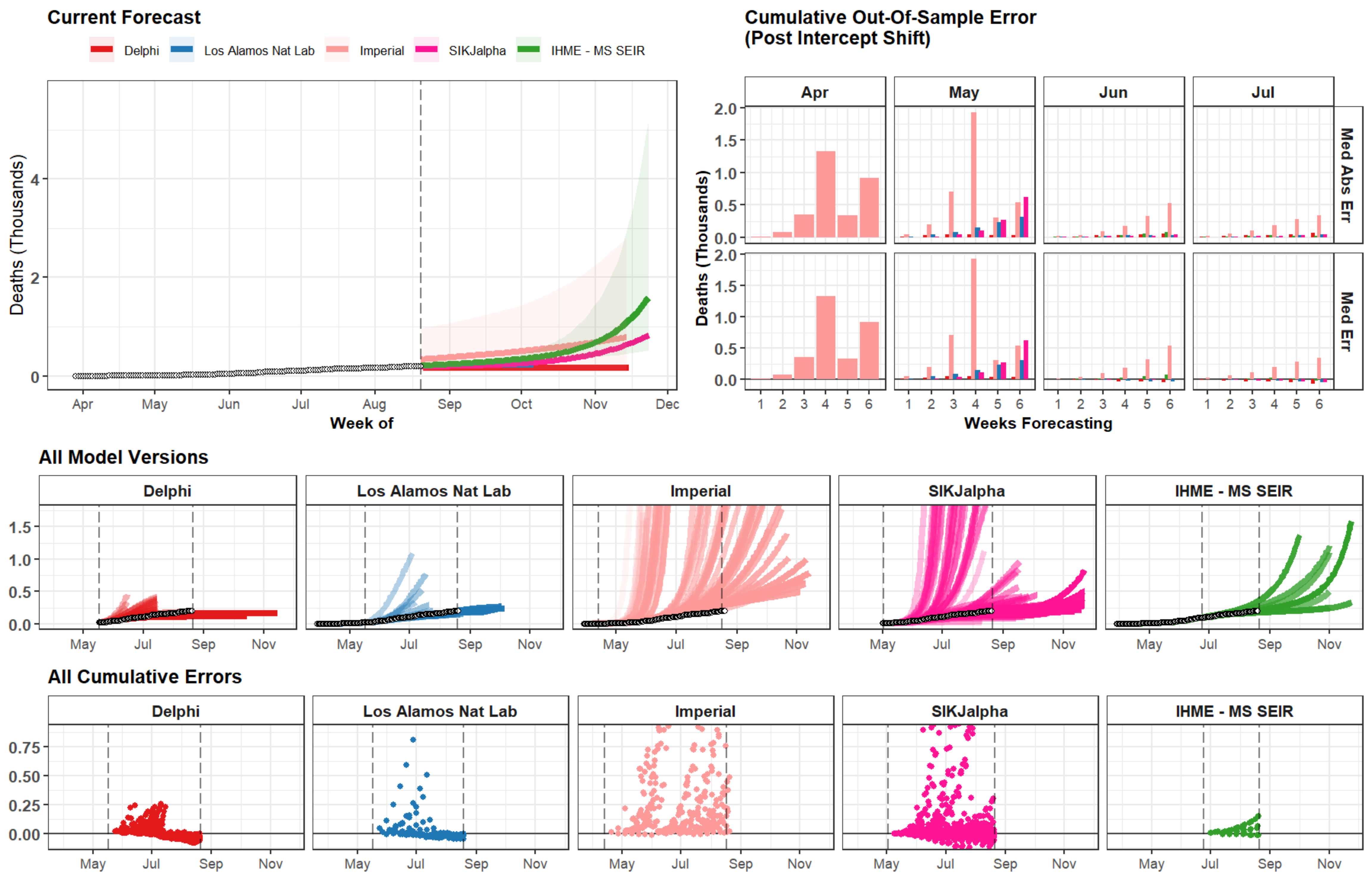

Current Forecast
Delphi
Los Alamos Nat Lab
Imperial
SIKJalpha
IHME - MS SEIR
Deaths (Thousands)
Week of
Apr
May
Jun
Jul
Aug
Sep
Oct
Nov
Dec
Cumulative Out-Of-Sample Error
(Post Intercept Shift)
Med Abs Err
Med Err
Weeks Forecasting
All Model Versions
All Cumulative Errors

Qatar

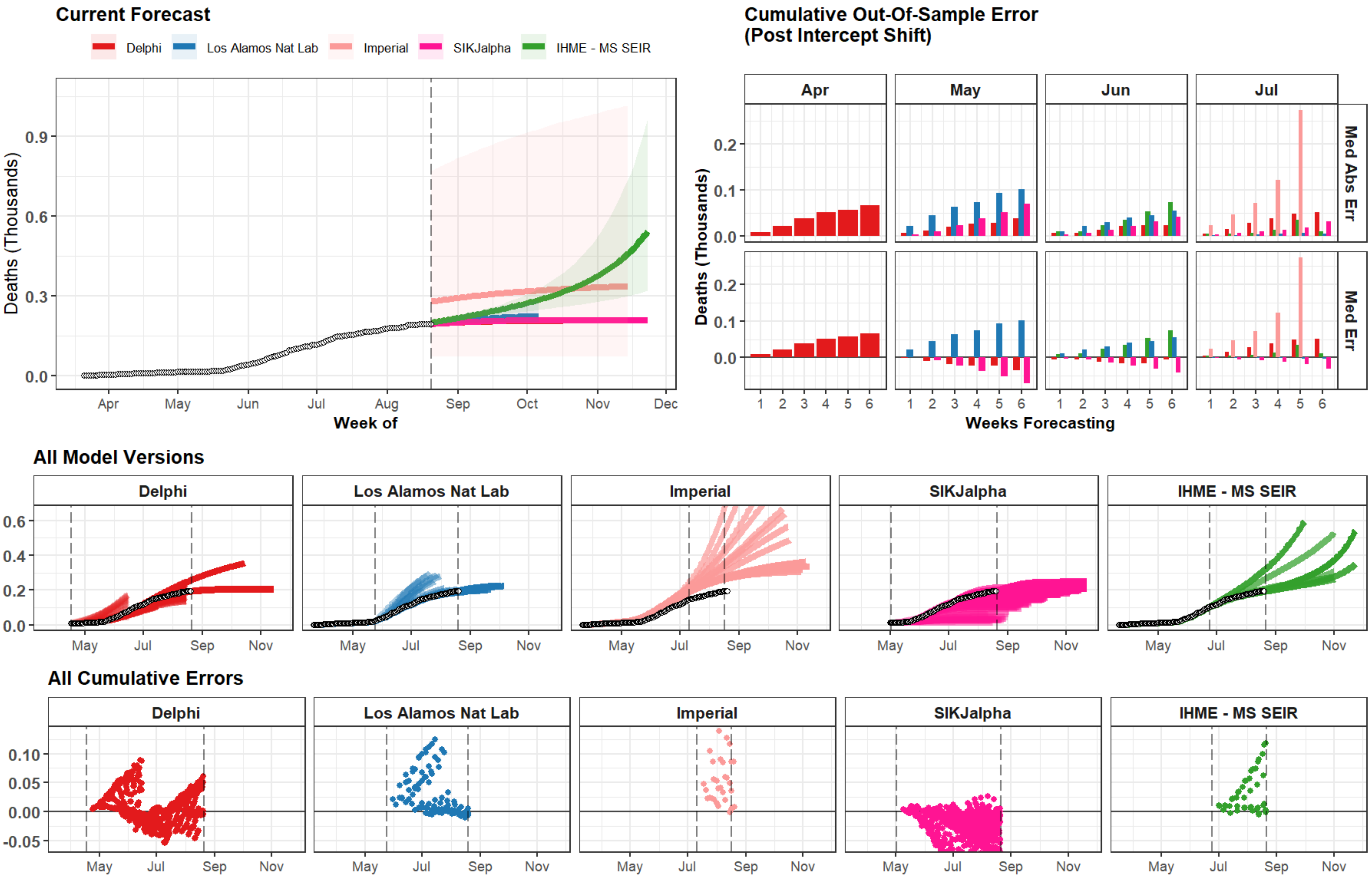
Current Forecast
Delphi
Los Alamos Nat Lab
Imperial
SIKJalpha
IHME - MS SEIR
Deaths (Thousands)
0.0
0.3
0.6
0.9
Apr
May
Jun
Jul
Aug
Sep
Oct
Nov
Dec
Week of
Cumulative Out-Of-Sample Error
(Post Intercept Shift)
Apr
May
Jun
Jul
Med Abs Err
Med Err
Deaths (Thousands)
0.0
0.1
0.2
1
2
3
4
5
6
Weeks Forecasting
All Model Versions
Delphi
Los Alamos Nat Lab
Imperial
SIKJalpha
IHME - MS SEIR
0.0
0.2
0.4
0.6
May
Jul
Sep
Nov
All Cumulative Errors
Delphi
Los Alamos Nat Lab
Imperial
SIKJalpha
IHME - MS SEIR
-0.05
0.00
0.05
0.10
May
Jul
Sep
Nov

Bahrain

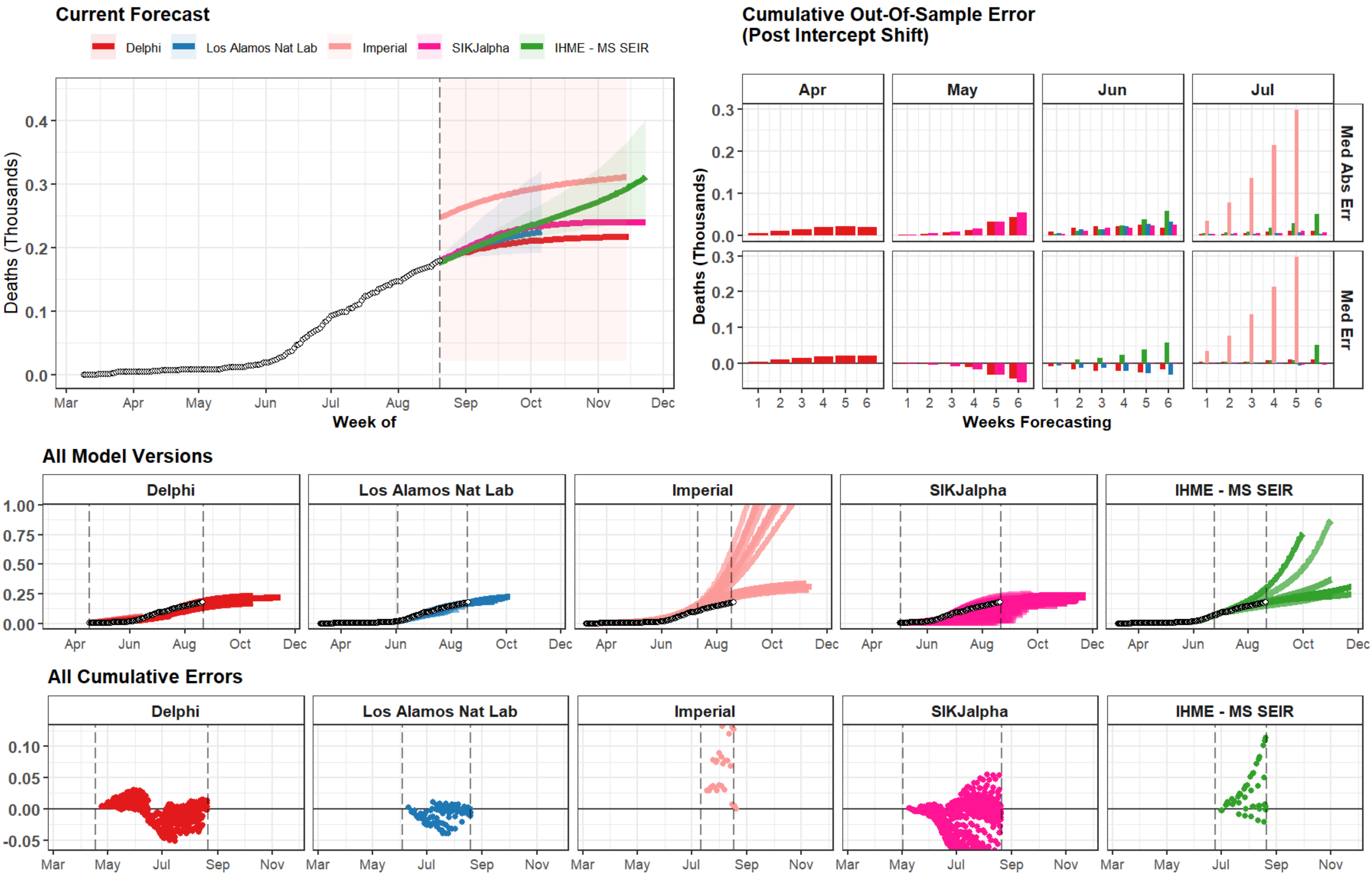

Current Forecast
Delphi
Los Alamos Nat Lab
Imperial
SIKJalpha
IHME - MS SEIR
Deaths (Thousands)
Week of
Cumulative Out-Of-Sample Error
(Post Intercept Shift)
Apr
May
Jun
Jul
Med Abs Err
Med Err
Weeks Forecasting
All Model Versions
All Cumulative Errors

Madagascar

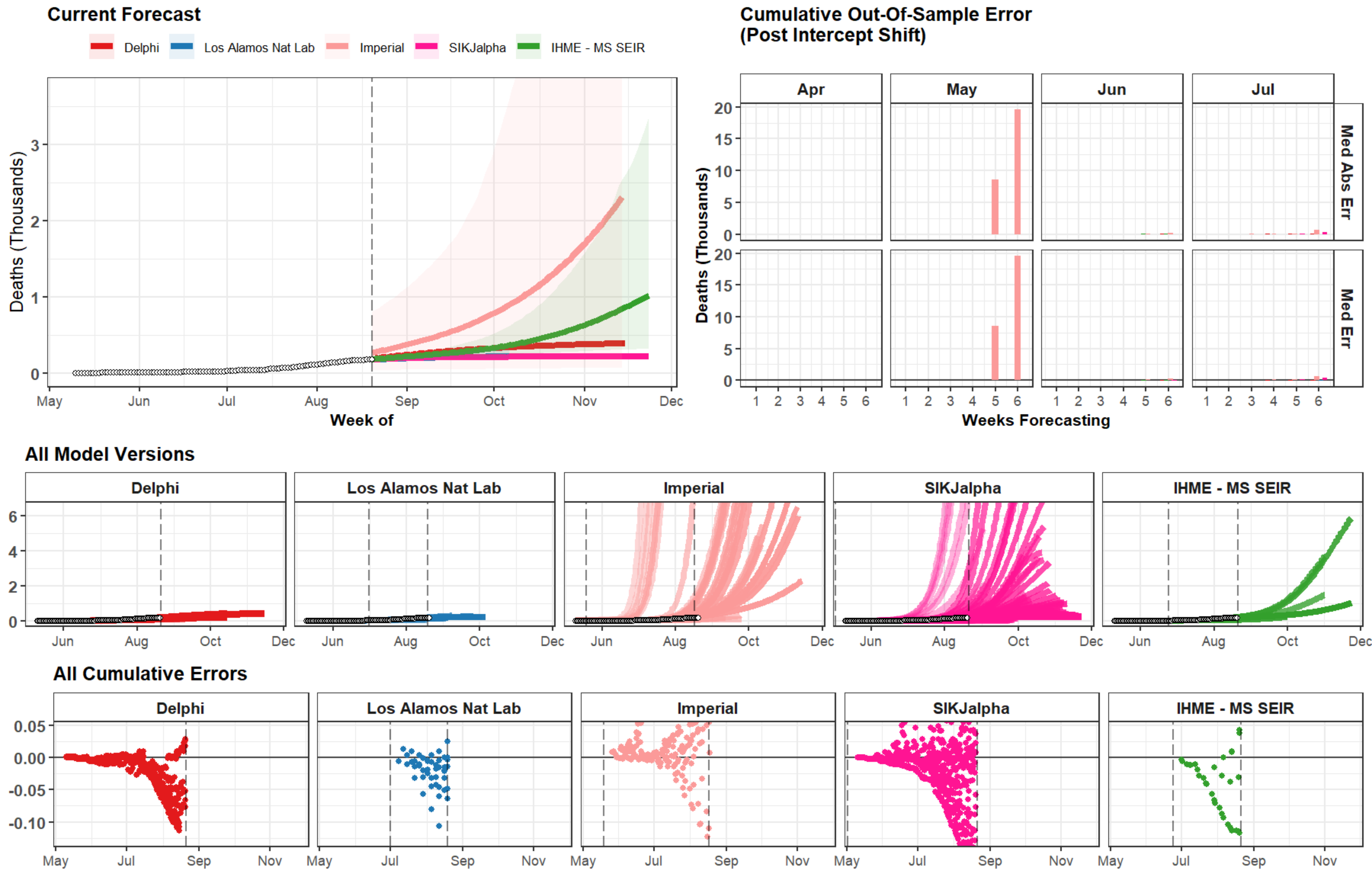
Current Forecast
Delphi
Los Alamos Nat Lab
Imperial
SIKJalpha
IHME - MS SEIR
Deaths (Thousands)
Week of
Cumulative Out-Of-Sample Error
(Post Intercept Shift)
Apr
May
Jun
Jul
Med Abs Err
Med Err
Weeks Forecasting
All Model Versions
All Cumulative Errors

Libya

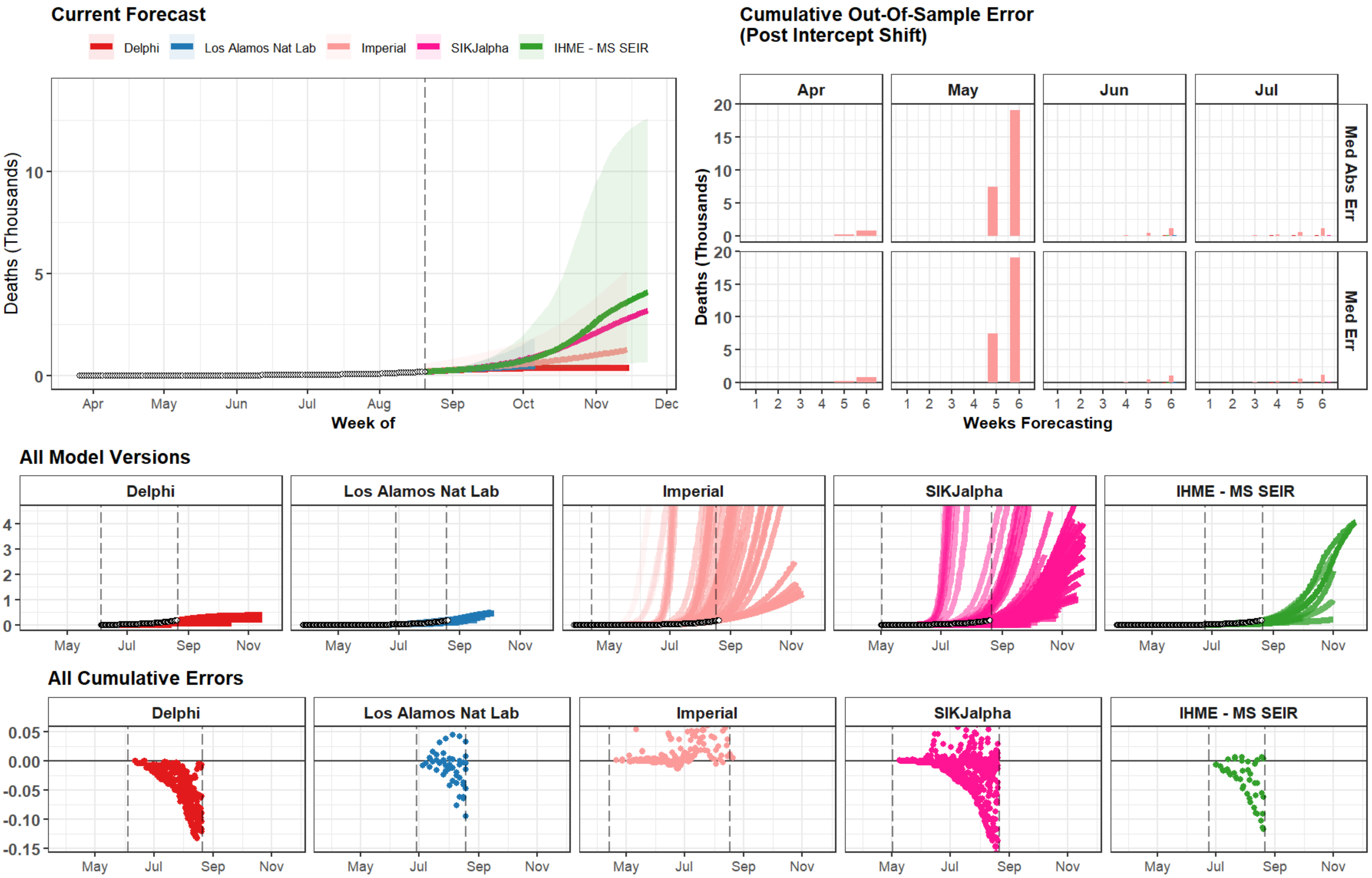
Current Forecast
Delphi
Los Alamos Nat Lab
Imperial
SIKJalpha
IHME - MS SEIR
Deaths (Thousands)
Week of
Apr
May
Jun
Jul
Aug
Sep
Oct
Nov
Dec
Cumulative Out-Of-Sample Error
(Post Intercept Shift)
Med Abs Err
Med Err
Weeks Forecasting
All Model Versions
All Cumulative Errors

Paraguay

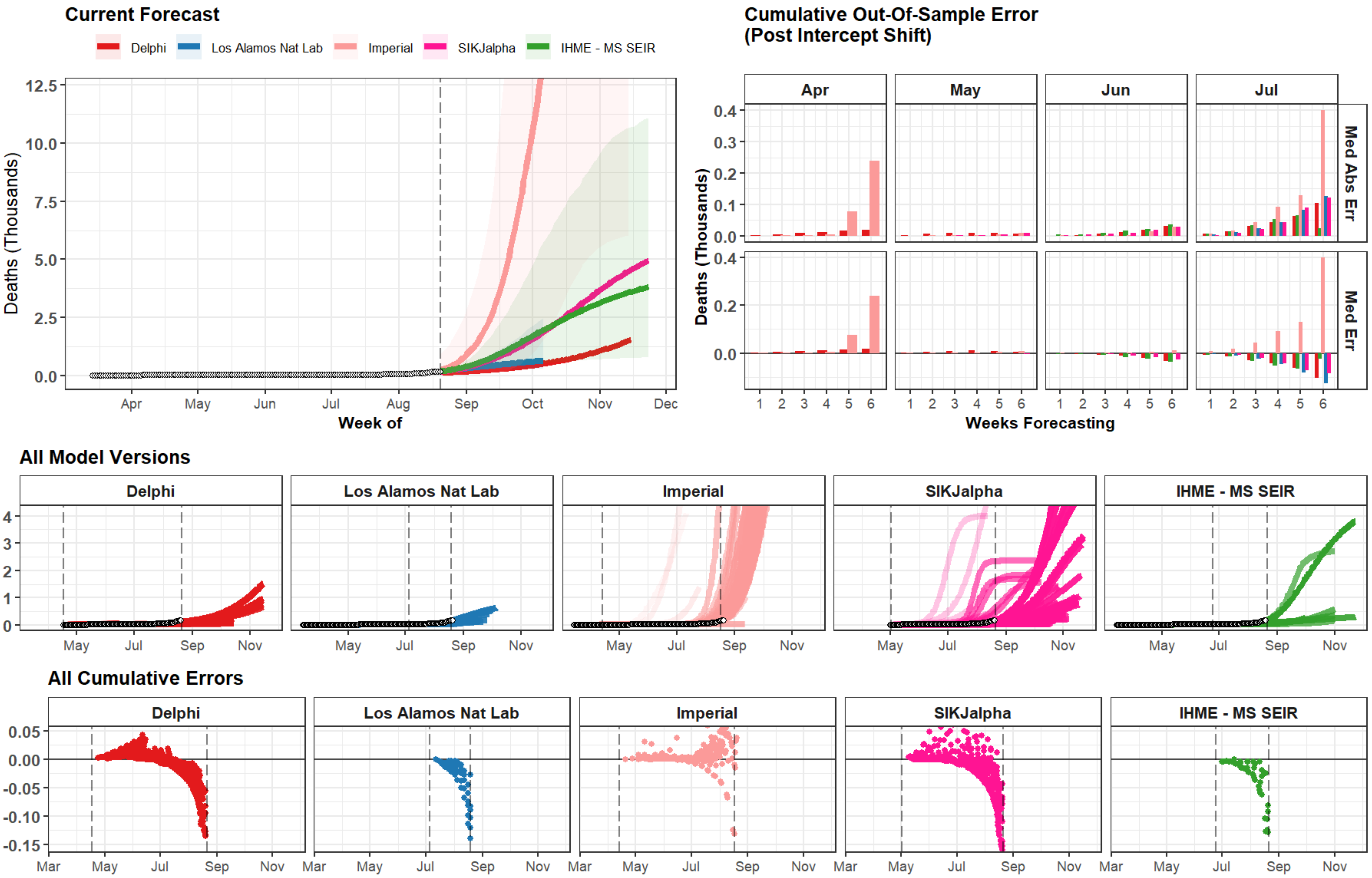

Current Forecast
Delphi
Los Alamos Nat Lab
Imperial
SIKJalpha
IHME - MS SEIR
Deaths (Thousands)
Week of
Apr
May
Jun
Jul
Aug
Sep
Oct
Nov
Dec
Cumulative Out-Of-Sample Error
(Post Intercept Shift)
Med Abs Err
Med Err
Weeks Forecasting
All Model Versions
All Cumulative Errors
Mar

West Virginia

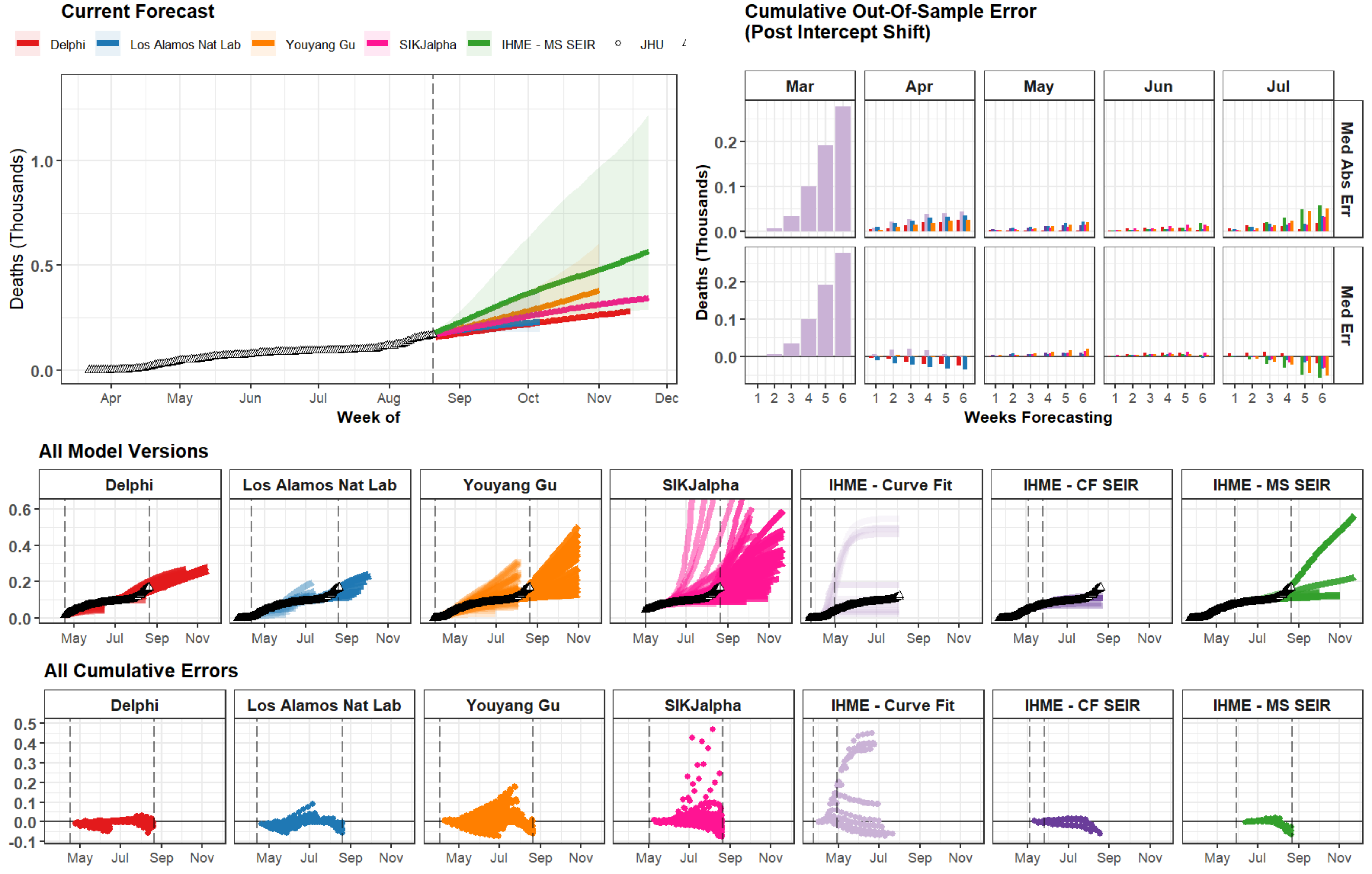

Croatia

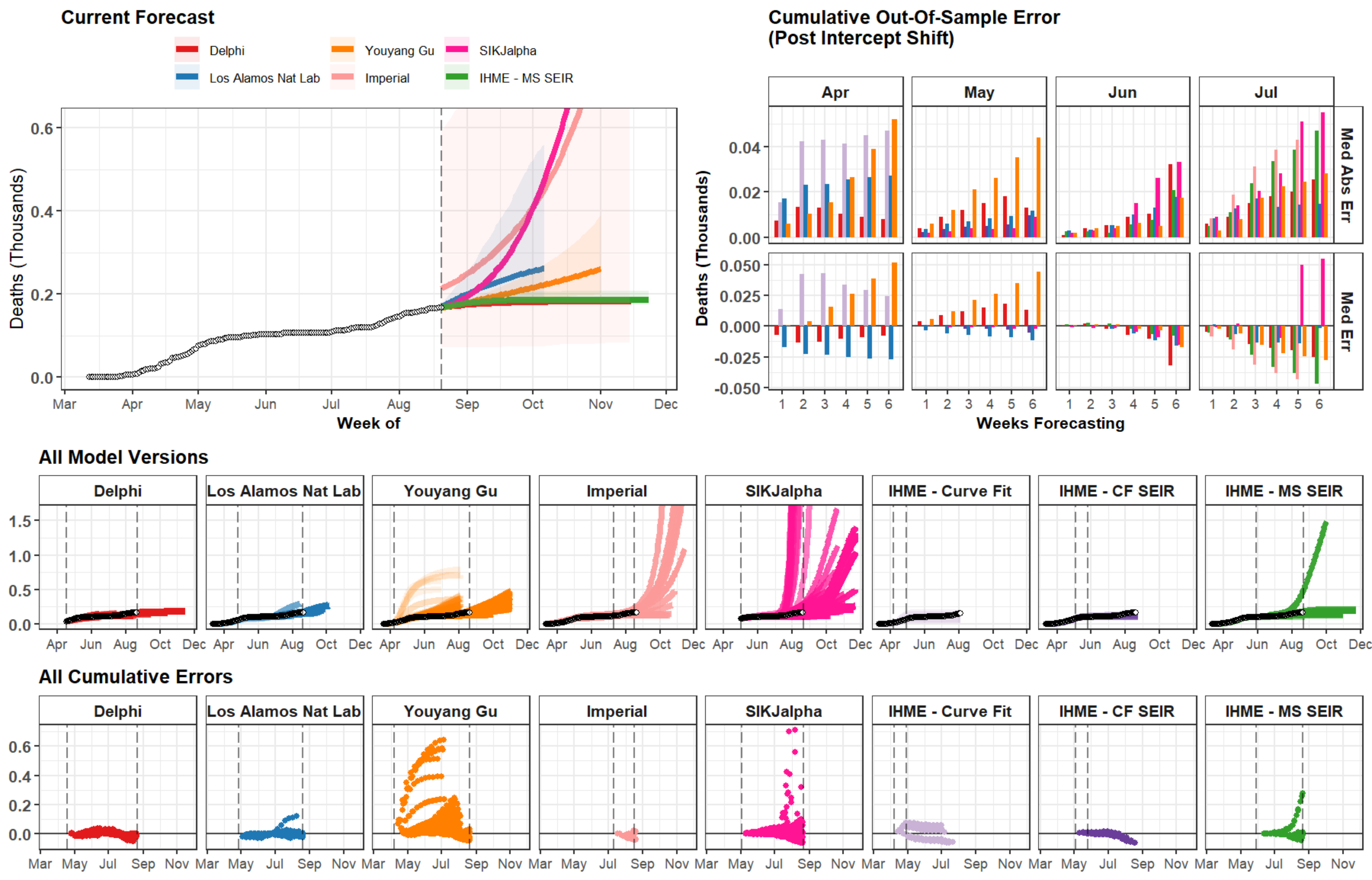

Current Forecast
Delphi
Youyang Gu
SIKJalpha
Los Alamos Nat Lab
Imperial
IHME - MS SEIR
Deaths (Thousands)
Week of
Cumulative Out-Of-Sample Error
(Post Intercept Shift)
Apr
May
Jun
Jul
Med Abs Err
Med Err
Weeks Forecasting
All Model Versions
Delphi
Los Alamos Nat Lab
Youyang Gu
Imperial
SIKJalpha
IHME - Curve Fit
IHME - CF SEIR
IHME - MS SEIR
All Cumulative Errors

Malawi

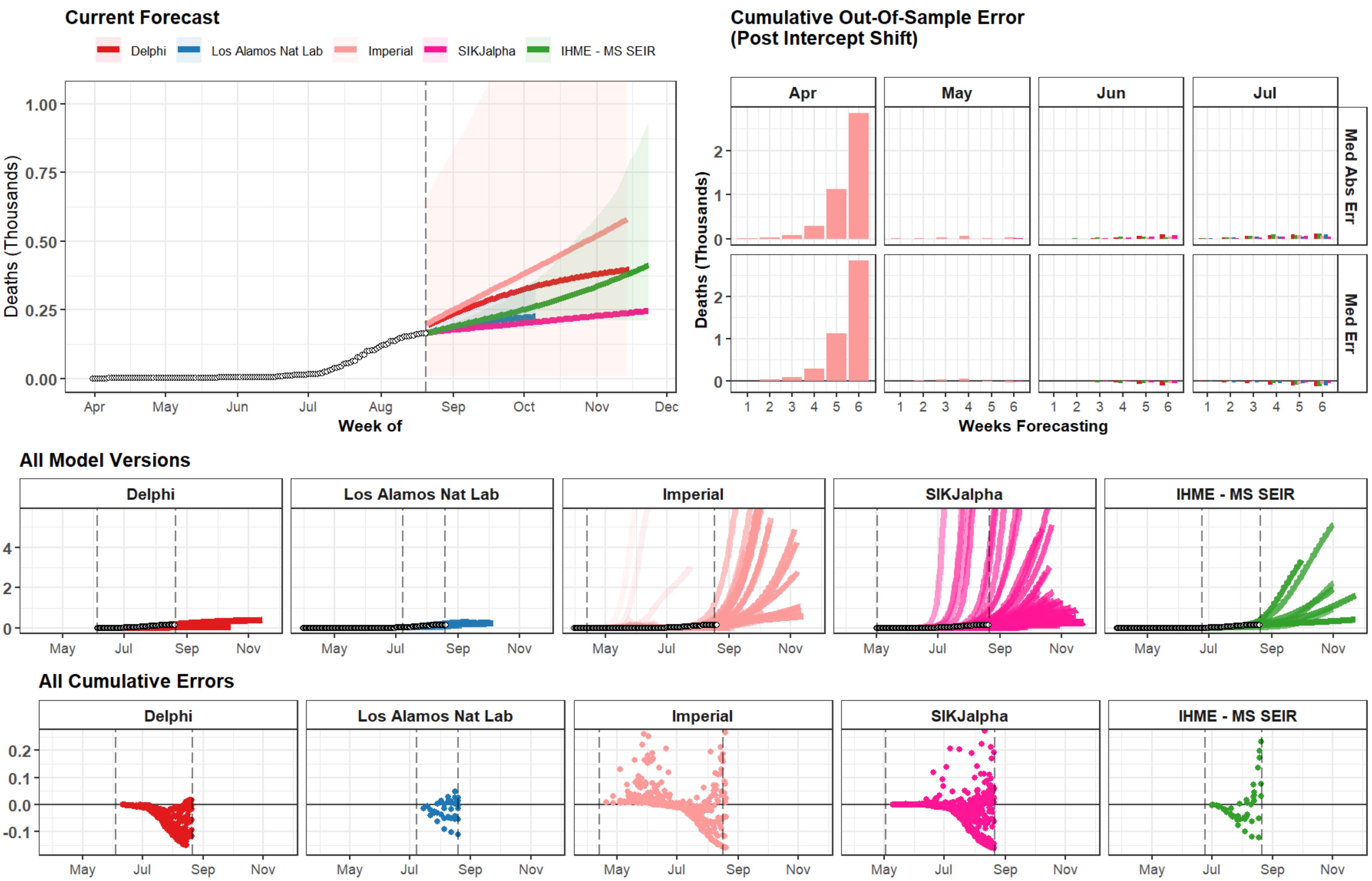
Current Forecast
Delphi
Los Alamos Nat Lab
Imperial
SIKJalpha
IHME - MS SEIR
Deaths (Thousands)
Week of
Cumulative Out-Of-Sample Error
(Post Intercept Shift)
Apr
May
Jun
Jul
Med Abs Err
Med Err
Weeks Forecasting
All Model Versions
All Cumulative Errors

Mauritania

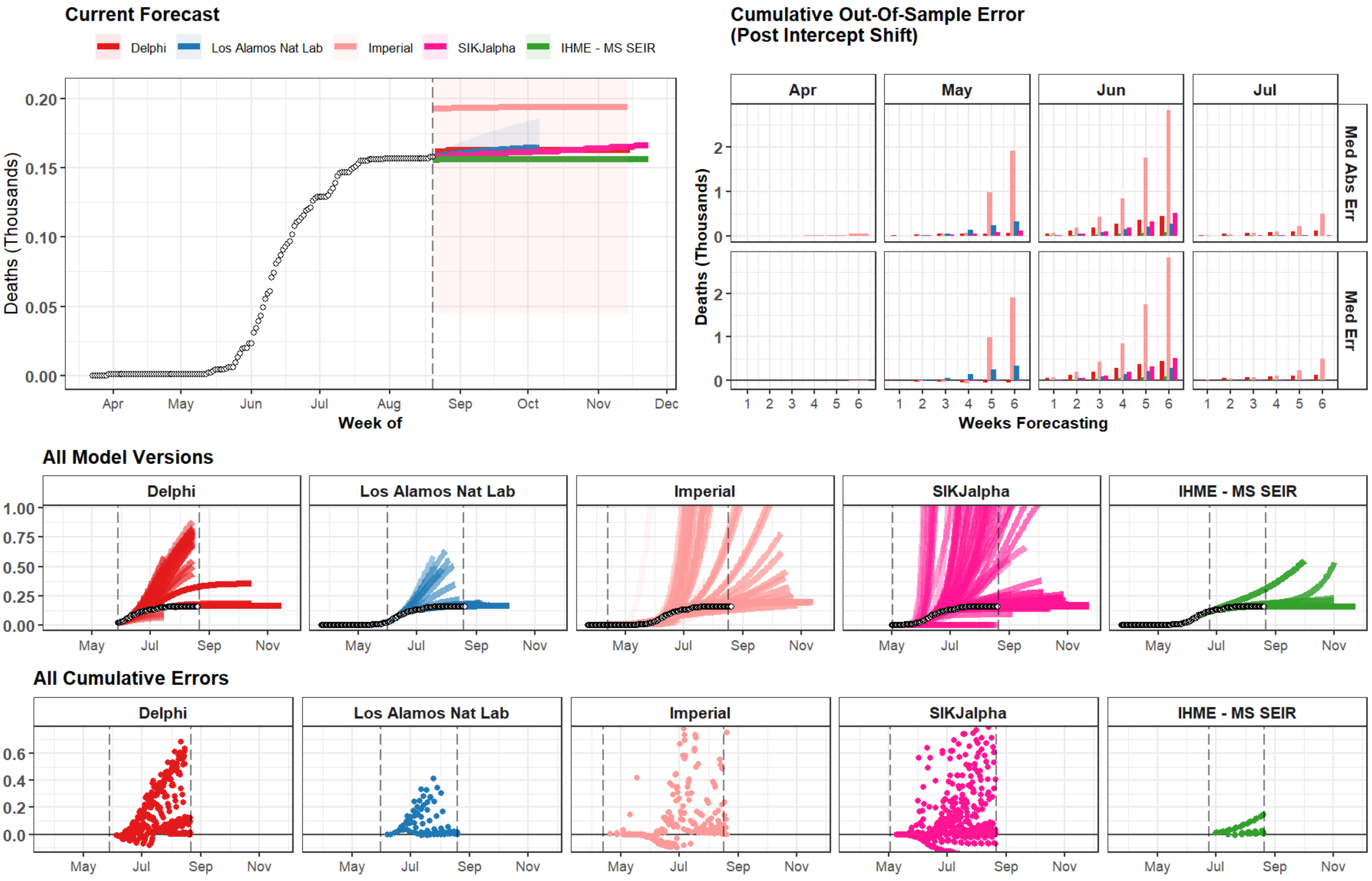

Current Forecast
Delphi
Los Alamos Nat Lab
Imperial
SIKJalpha
IHME - MS SEIR
Deaths (Thousands)
0.20
0.15
0.10
0.05
0.00
Apr
May
Jun
Jul
Aug
Sep
Oct
Nov
Dec
Week of
Cumulative Out-Of-Sample Error
(Post Intercept Shift)
Apr
May
Jun
Jul
Med Abs Err
Med Err
Deaths (Thousands)
2
1
0
1
2
3
4
5
6
Weeks Forecasting
All Model Versions
Delphi
Los Alamos Nat Lab
Imperial
SIKJalpha
IHME - MS SEIR
1.00
0.75
0.50
0.25
0.00
May
Jul
Sep
Nov
All Cumulative Errors
Delphi
Los Alamos Nat Lab
Imperial
SIKJalpha
IHME - MS SEIR
0.6
0.4
0.2
0.0
May
Jul
Sep
Nov

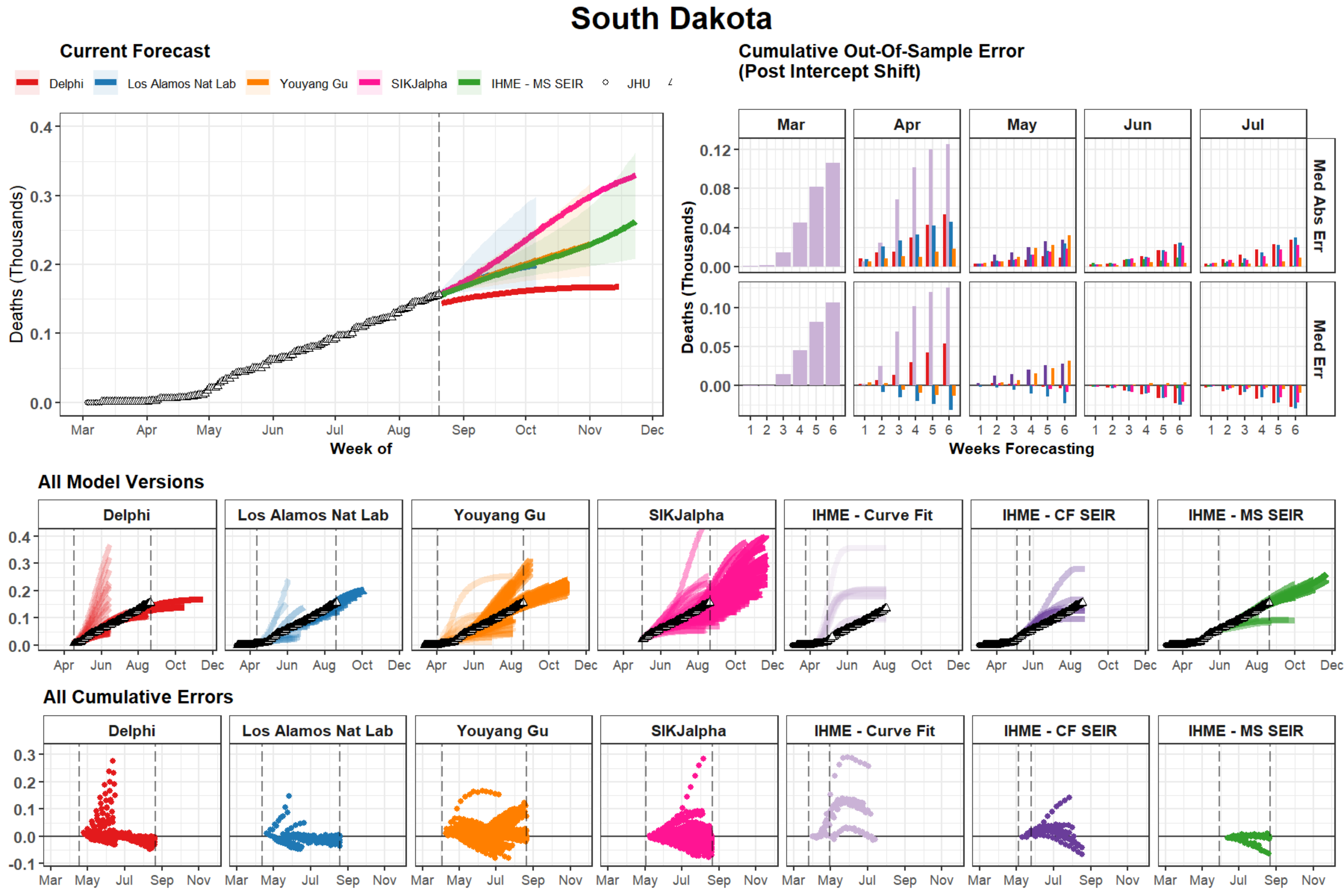
South Dakota
Current Forecast
Delphi
Los Alamos Nat Lab
Youyang Gu
SIKJalpha
IHME - MS SEIR
JHU
Deaths (Thousands)
Week of
Mar
Apr
May
Jun
Jul
Aug
Sep
Oct
Nov
Dec
Cumulative Out-Of-Sample Error
(Post Intercept Shift)
Med Abs Err
Med Err
Weeks Forecasting
All Model Versions
IHME - Curve Fit
IHME - CF SEIR
All Cumulative Errors

Zimbabwe

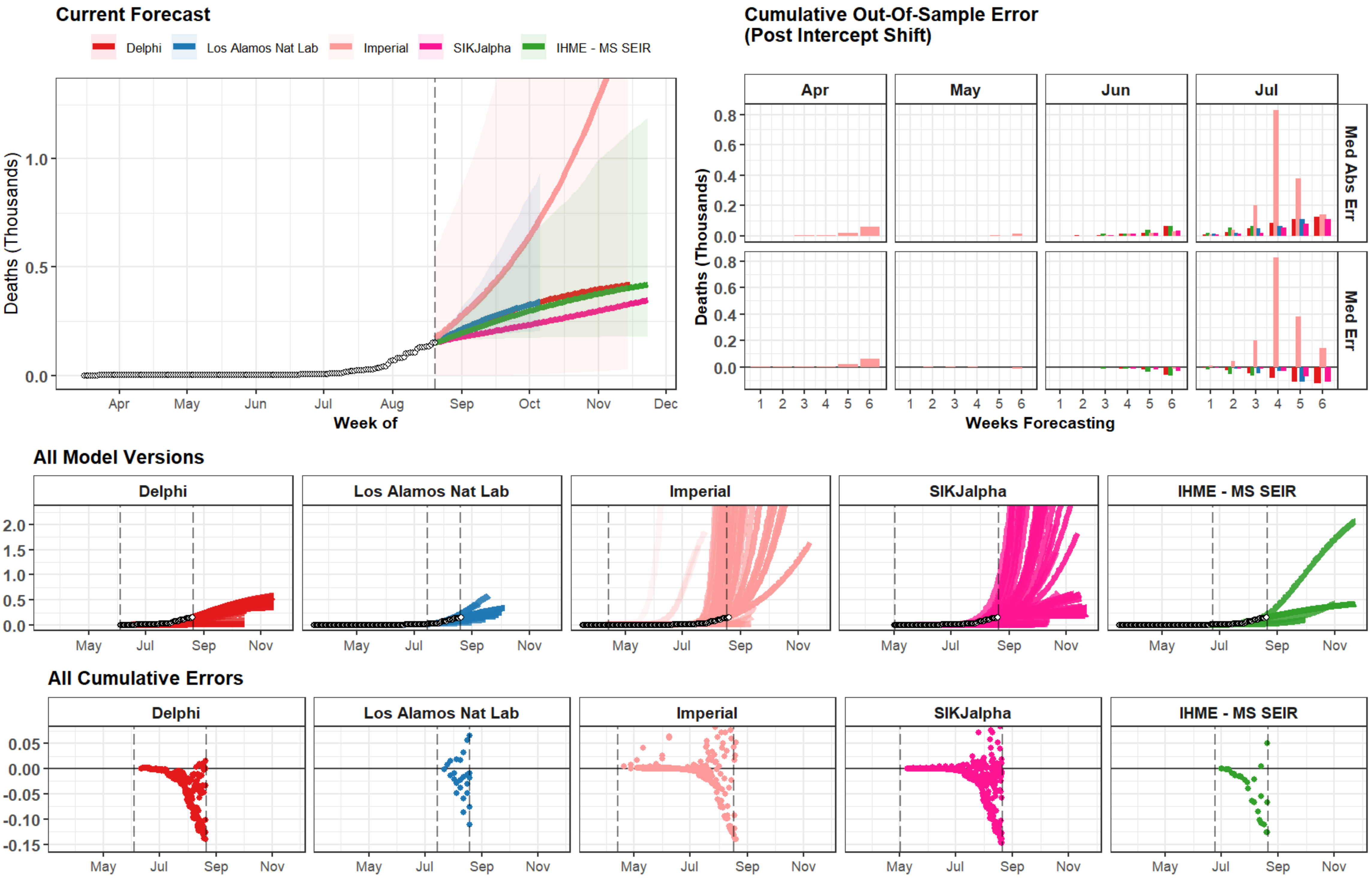

Current Forecast
Delphi
Los Alamos Nat Lab
Imperial
SIKJalpha
IHME - MS SEIR
Deaths (Thousands)
Week of
Cumulative Out-Of-Sample Error
(Post Intercept Shift)
Apr
May
Jun
Jul
Med Abs Err
Med Err
Weeks Forecasting
All Model Versions
All Cumulative Errors

North Dakota

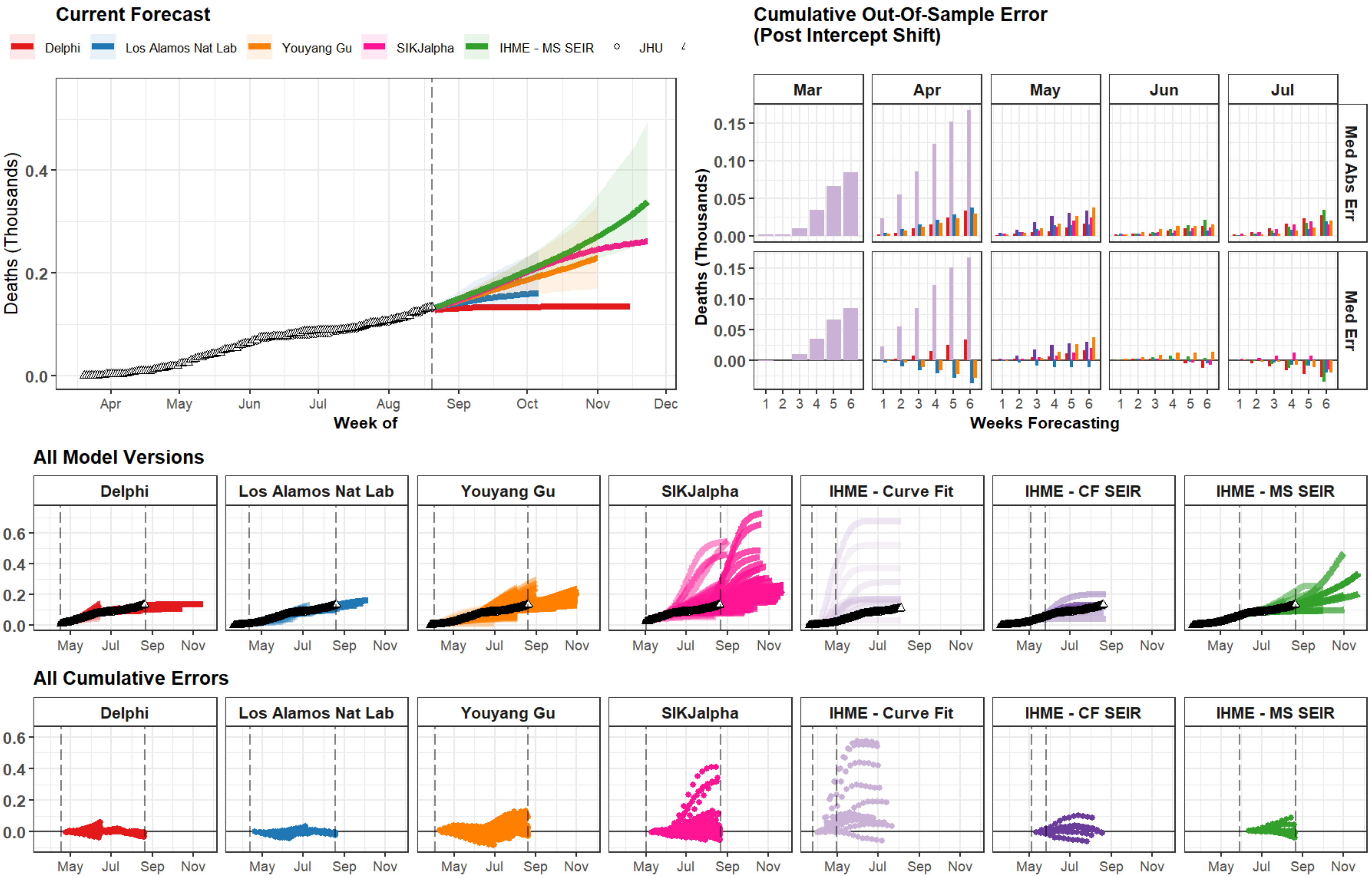

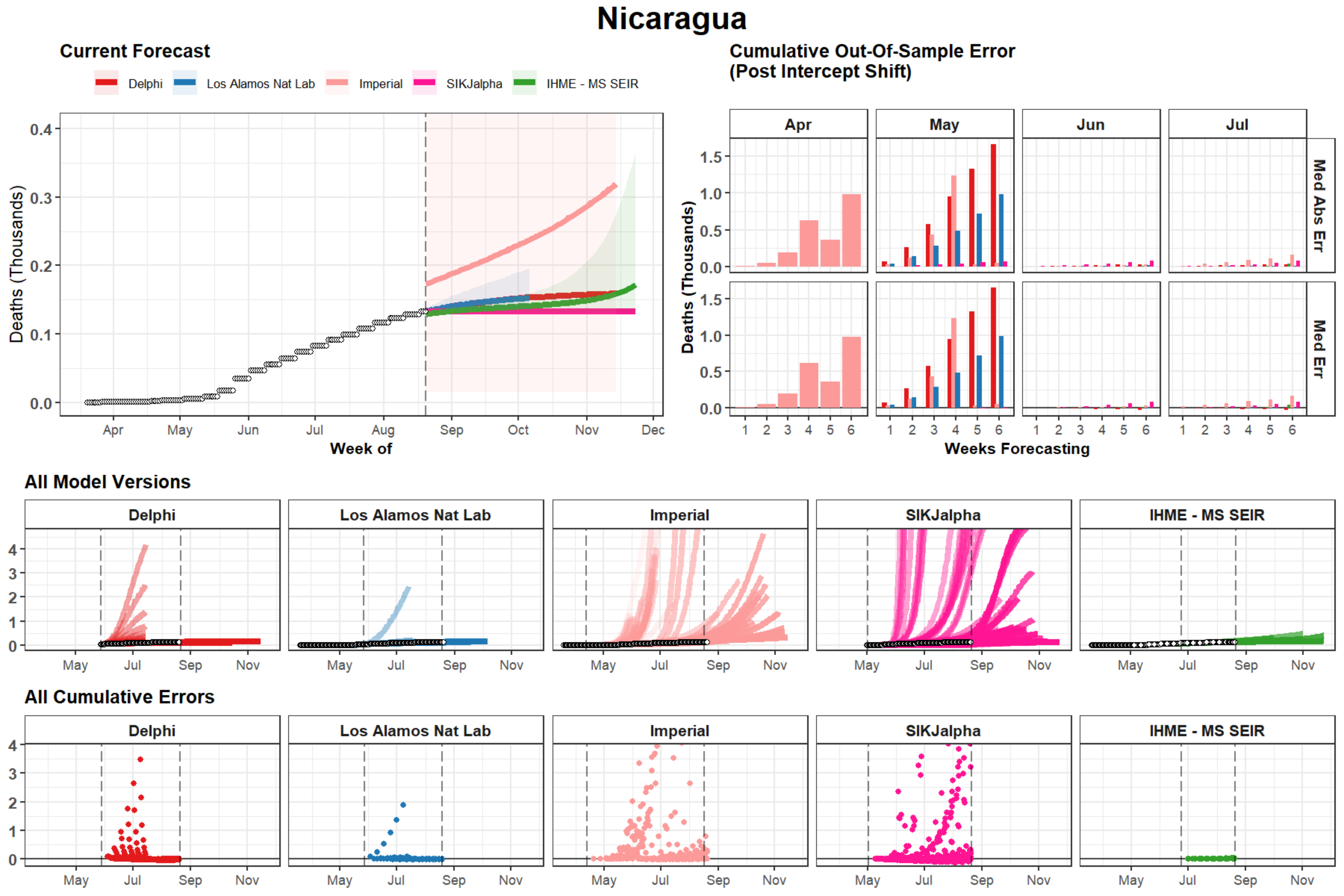

Nicaragua
Current Forecast
Delphi
Los Alamos Nat Lab
Imperial
SIKJalpha
IHME - MS SEIR
Deaths (Thousands)
Week of
Cumulative Out-Of-Sample Error
(Post Intercept Shift)
Apr
May
Jun
Jul
Med Abs Err
Med Err
Weeks Forecasting
All Model Versions
All Cumulative Errors

Maine

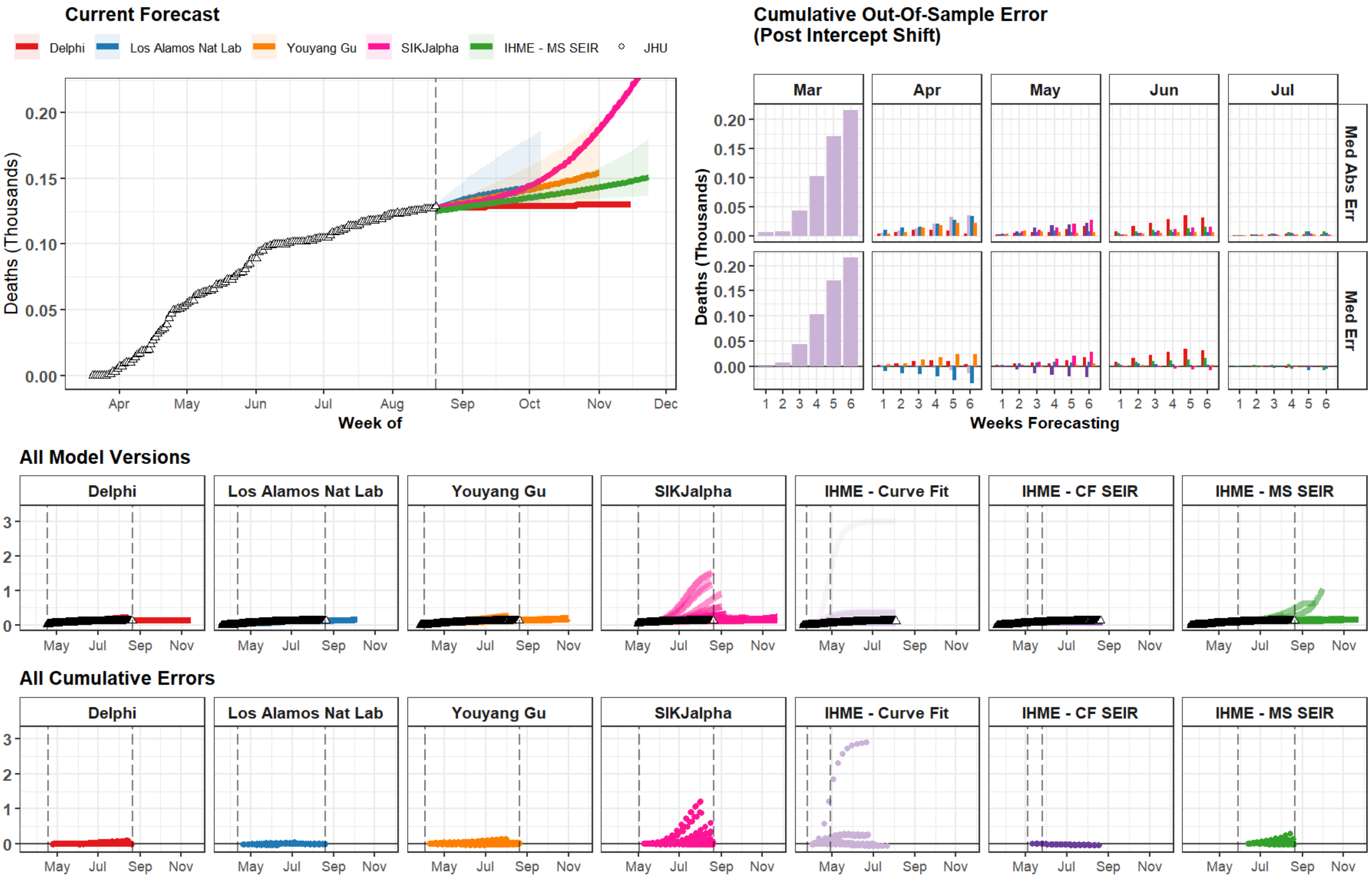

Current Forecast
Delphi
Los Alamos Nat Lab
Youyang Gu
SIKJalpha
IHME - MS SEIR
JHU
Deaths (Thousands)
0.00
0.05
0.10
0.15
0.20
Apr
May
Jun
Jul
Aug
Sep
Oct
Nov
Dec
Week of
Cumulative Out-Of-Sample Error
(Post Intercept Shift)
Mar
Apr
May
Jun
Jul
Med Abs Err
Med Err
Weeks Forecasting
1
2
3
4
5
6
All Model Versions
Delphi
Los Alamos Nat Lab
Youyang Gu
SIKJalpha
IHME - Curve Fit
IHME - CF SEIR
IHME - MS SEIR
0
1
2
3
May
Jul
Sep
Nov
All Cumulative Errors

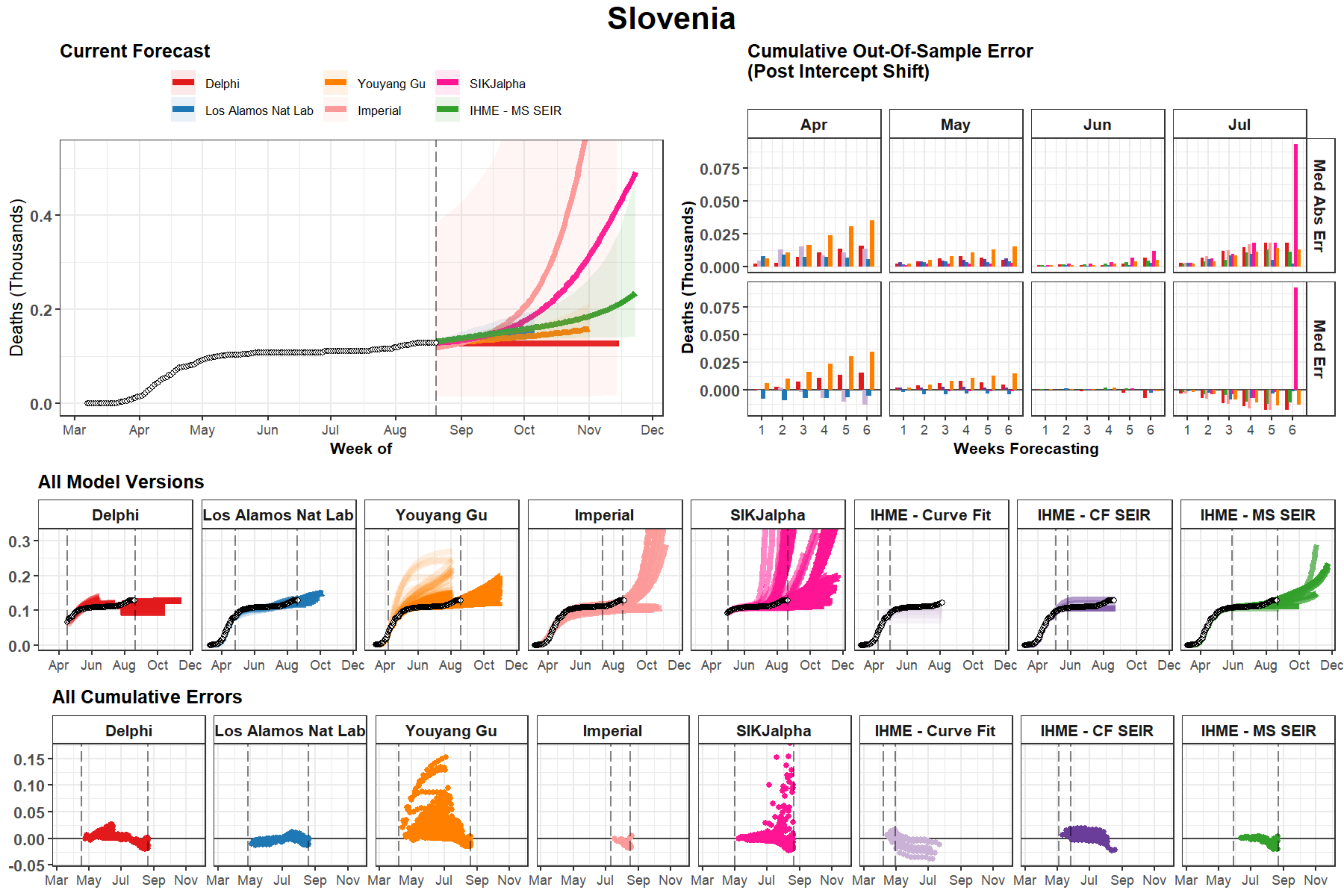

Slovenia
Current Forecast
Delphi
Youyang Gu
SIKJalpha
Los Alamos Nat Lab
Imperial
IHME - MS SEIR
Deaths (Thousands)
Week of
Cumulative Out-Of-Sample Error
(Post Intercept Shift)
Apr
May
Jun
Jul
Med Abs Err
Med Err
Weeks Forecasting
All Model Versions
Delphi
Los Alamos Nat Lab
Youyang Gu
Imperial
SIKJalpha
IHME - Curve Fit
IHME - CF SEIR
IHME - MS SEIR
All Cumulative Errors

Nepal

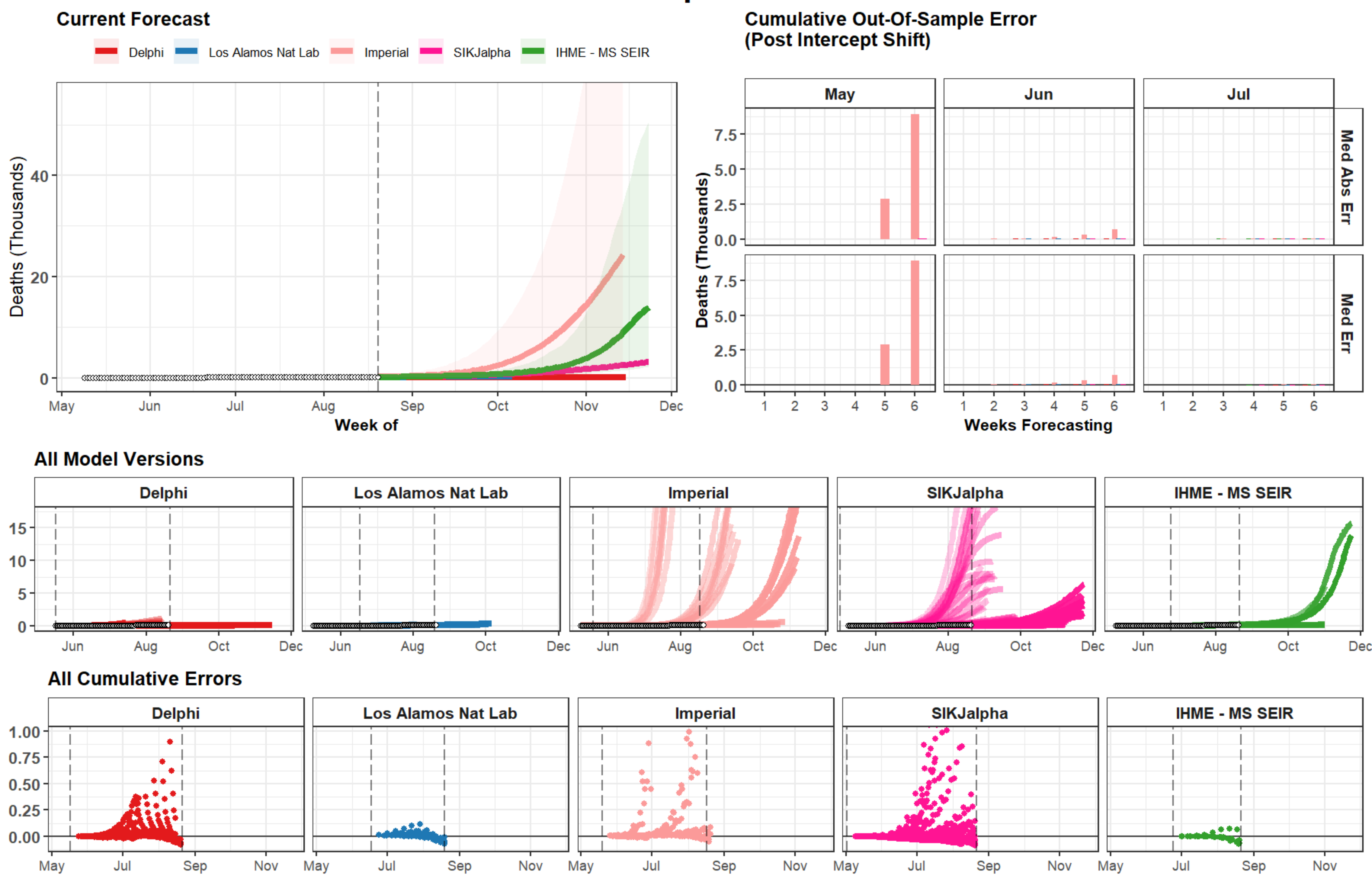

Current Forecast
Delphi
Los Alamos Nat Lab
Imperial
SIKJalpha
IHME - MS SEIR
Deaths (Thousands)
40
20
0
May
Jun
Jul
Aug
Sep
Oct
Nov
Dec
Week of
Cumulative Out-Of-Sample Error
(Post Intercept Shift)
May
Jun
Jul
Med Abs Err
Med Err
7.5
5.0
2.5
0.0
1 2 3 4 5 6
Weeks Forecasting
All Model Versions
15
10
5
0
All Cumulative Errors
1.00
0.75
0.50
0.25
0.00

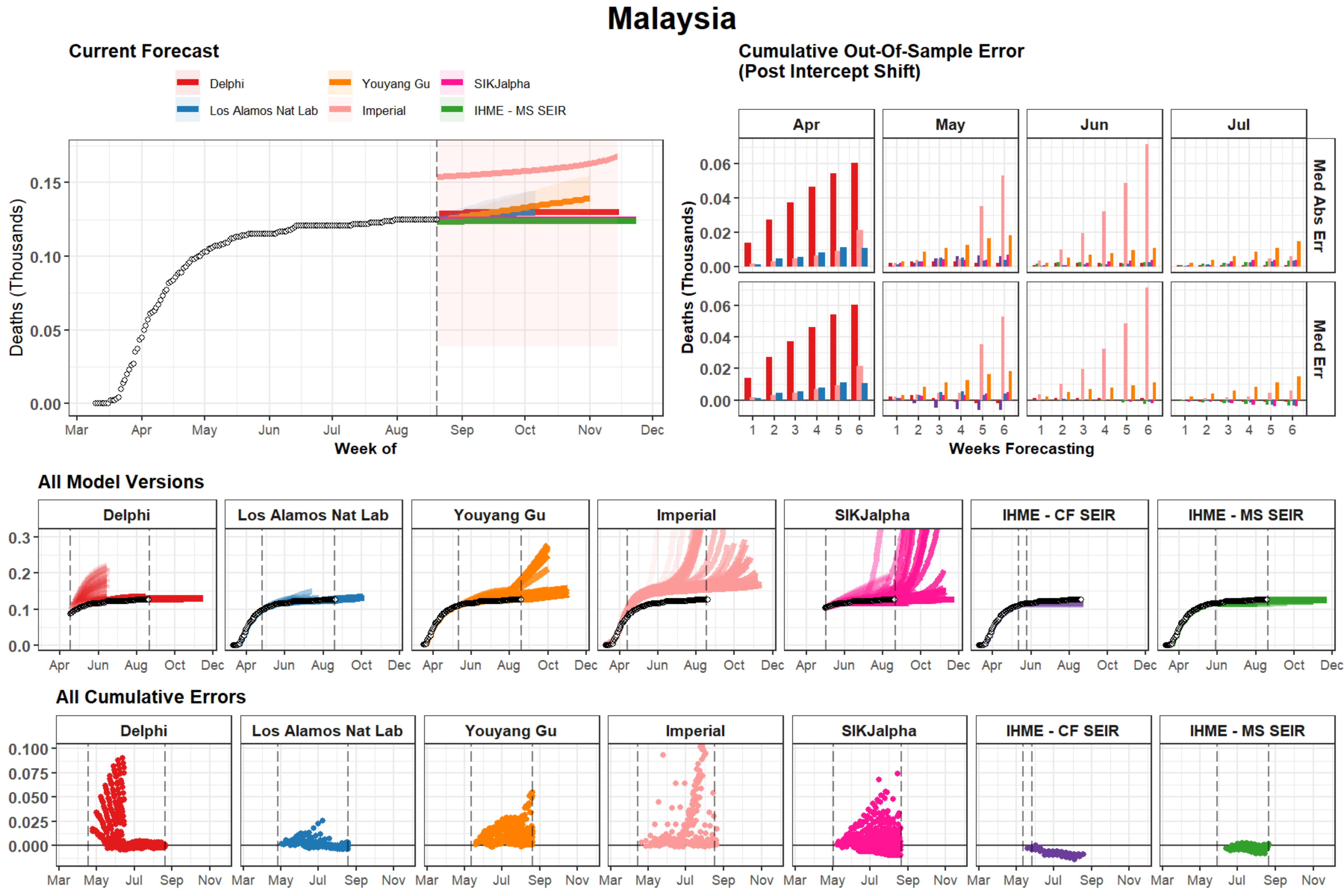

Malaysia
Current Forecast
Delphi
Youyang Gu
SIKJalpha
Los Alamos Nat Lab
Imperial
IHME - MS SEIR
Deaths (Thousands)
0.15
0.10
0.05
0.00
Mar
Apr
May
Jun
Jul
Aug
Sep
Oct
Nov
Dec
Week of
Cumulative Out-Of-Sample Error
(Post Intercept Shift)
Apr
May
Jun
Jul
Med Abs Err
Med Err
0.06
0.04
0.02
0.00
Weeks Forecasting
All Model Versions
Delphi
Los Alamos Nat Lab
Youyang Gu
Imperial
SIKJalpha
IHME - CF SEIR
IHME - MS SEIR
0.3
0.2
0.1
0.0
All Cumulative Errors
0.100
0.075
0.050
0.025
0.000

Mali

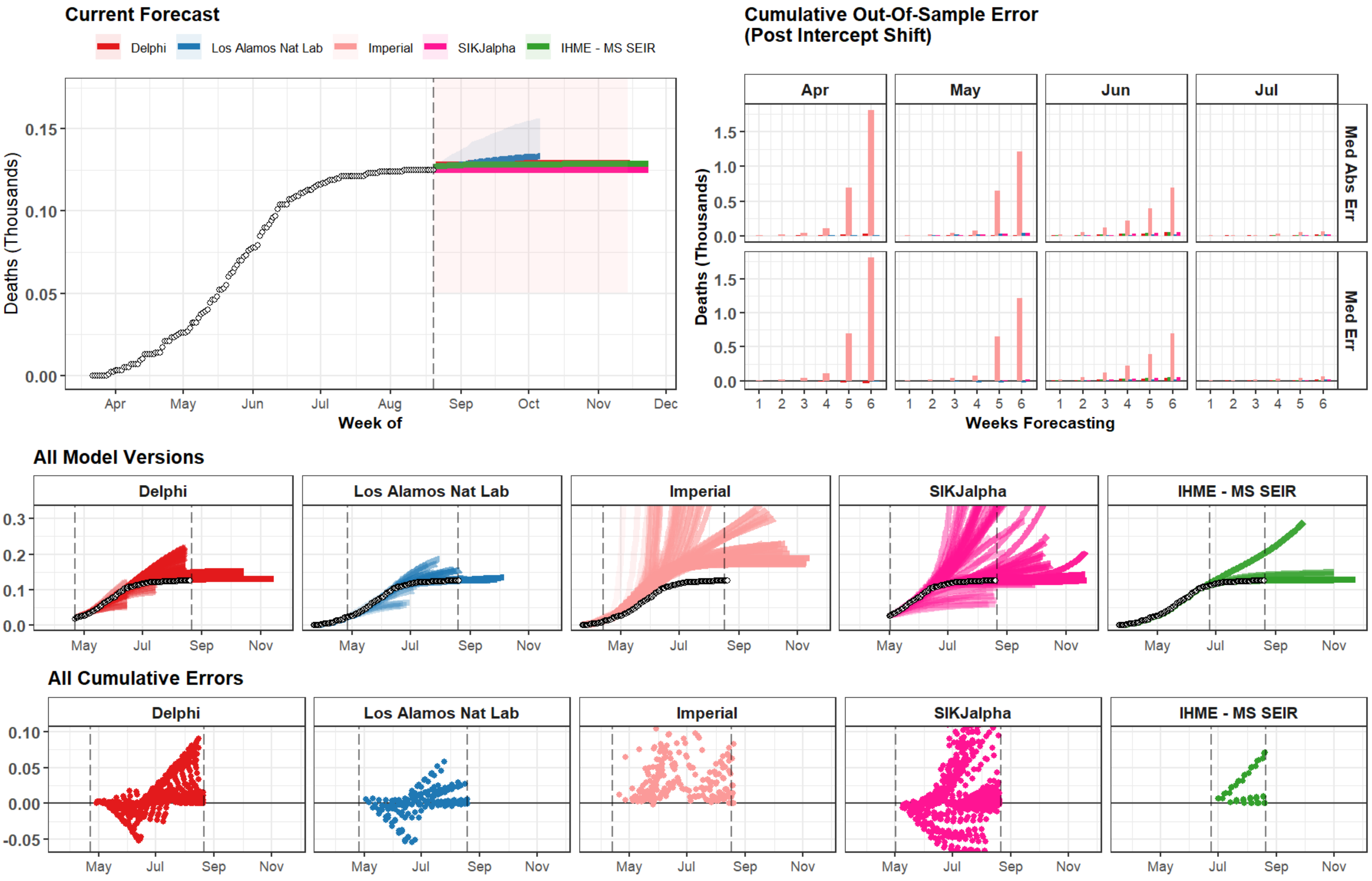

Current Forecast
Delphi
Los Alamos Nat Lab
Imperial
SIKJalpha
IHME - MS SEIR
Deaths (Thousands)
0.00
0.05
0.10
0.15
Apr
May
Jun
Jul
Aug
Sep
Oct
Nov
Dec
Week of
Cumulative Out-Of-Sample Error
(Post Intercept Shift)
Apr
May
Jun
Jul
Med Abs Err
Med Err
Deaths (Thousands)
0.0
0.5
1.0
1.5
1
2
3
4
5
6
Weeks Forecasting
All Model Versions
Delphi
Los Alamos Nat Lab
Imperial
SIKJalpha
IHME - MS SEIR
0.0
0.1
0.2
0.3
May
Jul
Sep
Nov
All Cumulative Errors
Delphi
Los Alamos Nat Lab
Imperial
SIKJalpha
IHME - MS SEIR
-0.05
0.00
0.05
0.10
May
Jul
Sep
Nov

Luxembourg

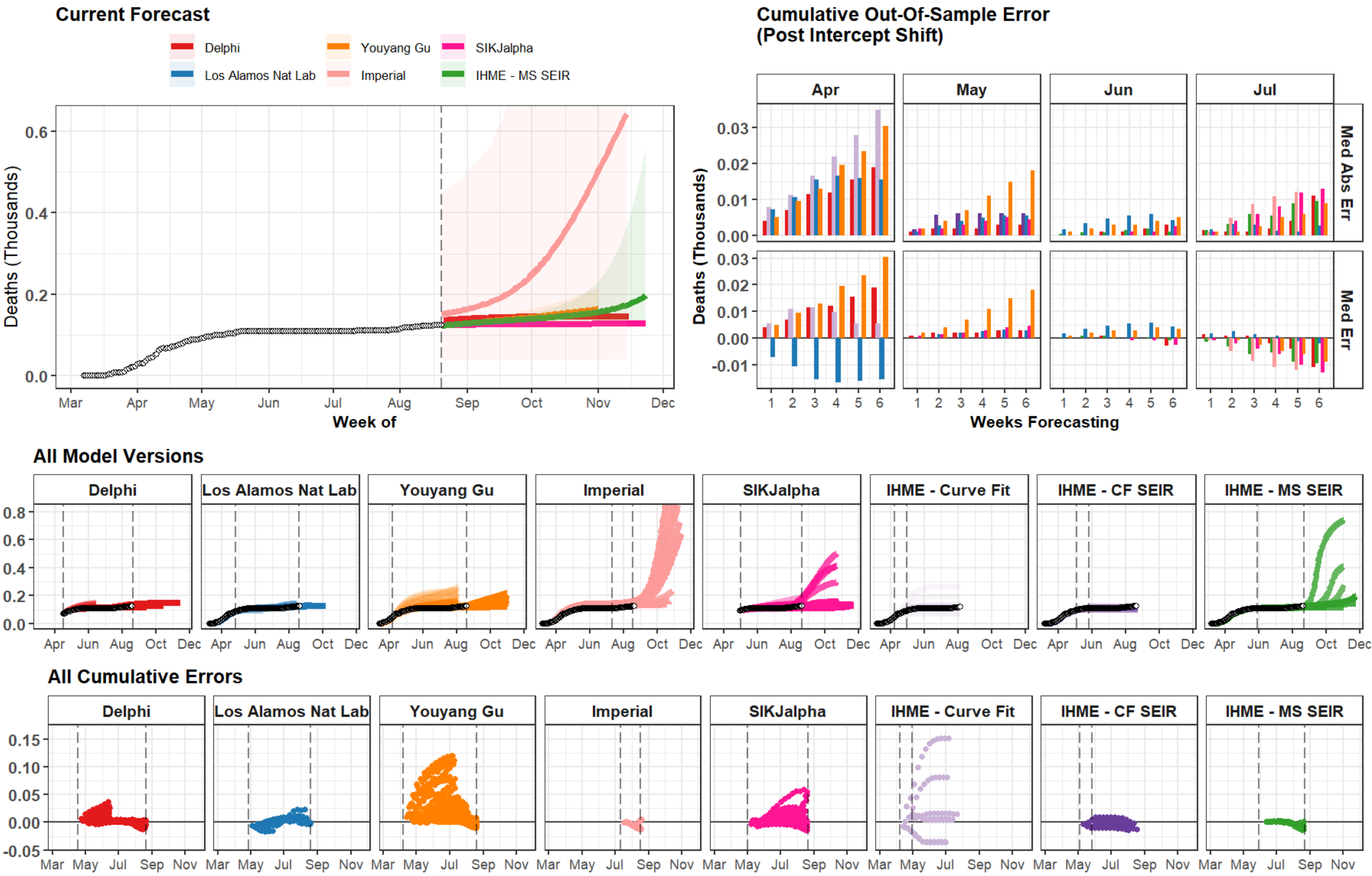
Current Forecast
Delphi
Los Alamos Nat Lab
Youyang Gu
Imperial
SIKJalpha
IHME - MS SEIR
Deaths (Thousands)
Week of
Mar
Apr
May
Jun
Jul
Aug
Sep
Oct
Nov
Dec
Cumulative Out-Of-Sample Error
(Post Intercept Shift)
Med Abs Err
Med Err
Weeks Forecasting
All Model Versions
IHME - Curve Fit
IHME - CF SEIR
All Cumulative Errors

Lebanon

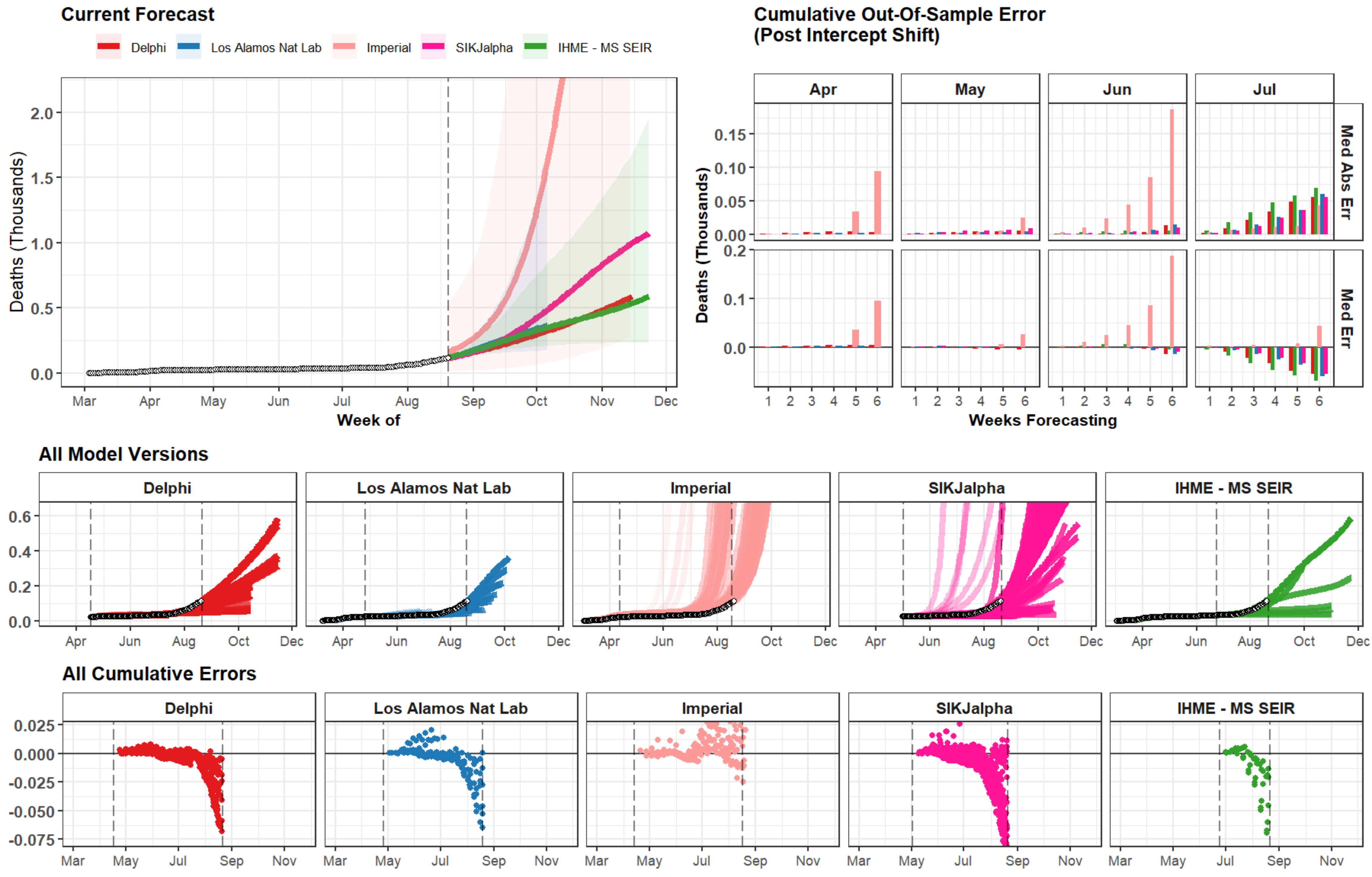

Current Forecast
Delphi
Los Alamos Nat Lab
Imperial
SIKJalpha
IHME - MS SEIR
Deaths (Thousands)
0.0
0.5
1.0
1.5
2.0
Mar
Apr
May
Jun
Jul
Aug
Sep
Oct
Nov
Dec
Week of
Cumulative Out-Of-Sample Error
(Post Intercept Shift)
Apr
May
Jun
Jul
Med Abs Err
Med Err
0.00
0.05
0.10
0.15
0.0
0.1
0.2
1
2
3
4
5
6
Weeks Forecasting
All Model Versions
Delphi
Los Alamos Nat Lab
Imperial
SIKJalpha
IHME - MS SEIR
0.0
0.2
0.4
0.6
Apr
Jun
Aug
Oct
Dec
All Cumulative Errors
Delphi
Los Alamos Nat Lab
Imperial
SIKJalpha
IHME - MS SEIR
0.025
0.000
-0.025
-0.050
-0.075
Mar
May
Jul
Sep
Nov

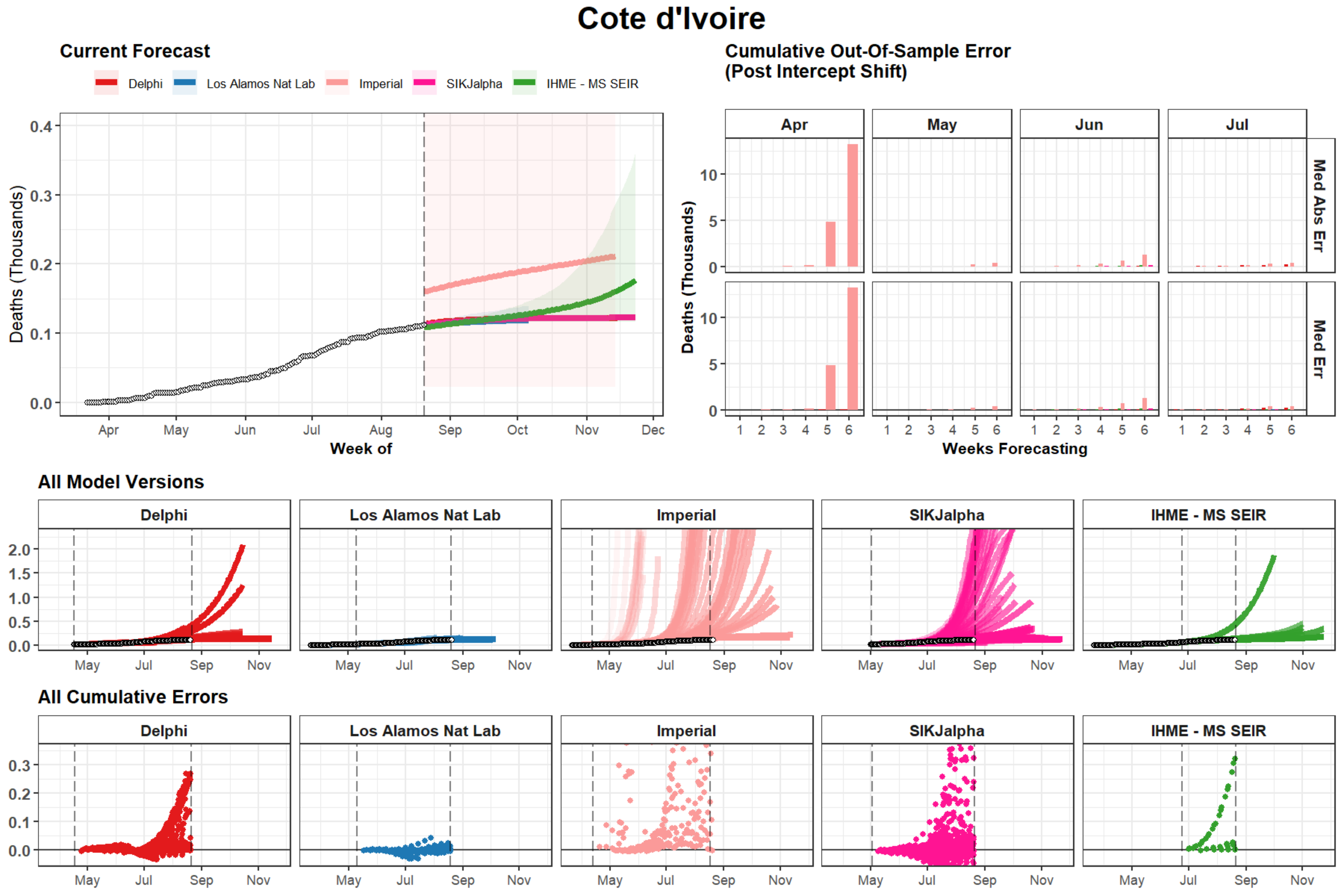
Cote d'Ivoire
Current Forecast
Delphi
Los Alamos Nat Lab
Imperial
SIKJalpha
IHME - MS SEIR
Deaths (Thousands)
Week of
Apr
May
Jun
Jul
Aug
Sep
Oct
Nov
Dec
Cumulative Out-Of-Sample Error
(Post Intercept Shift)
Med Abs Err
Med Err
Weeks Forecasting
All Model Versions
All Cumulative Errors

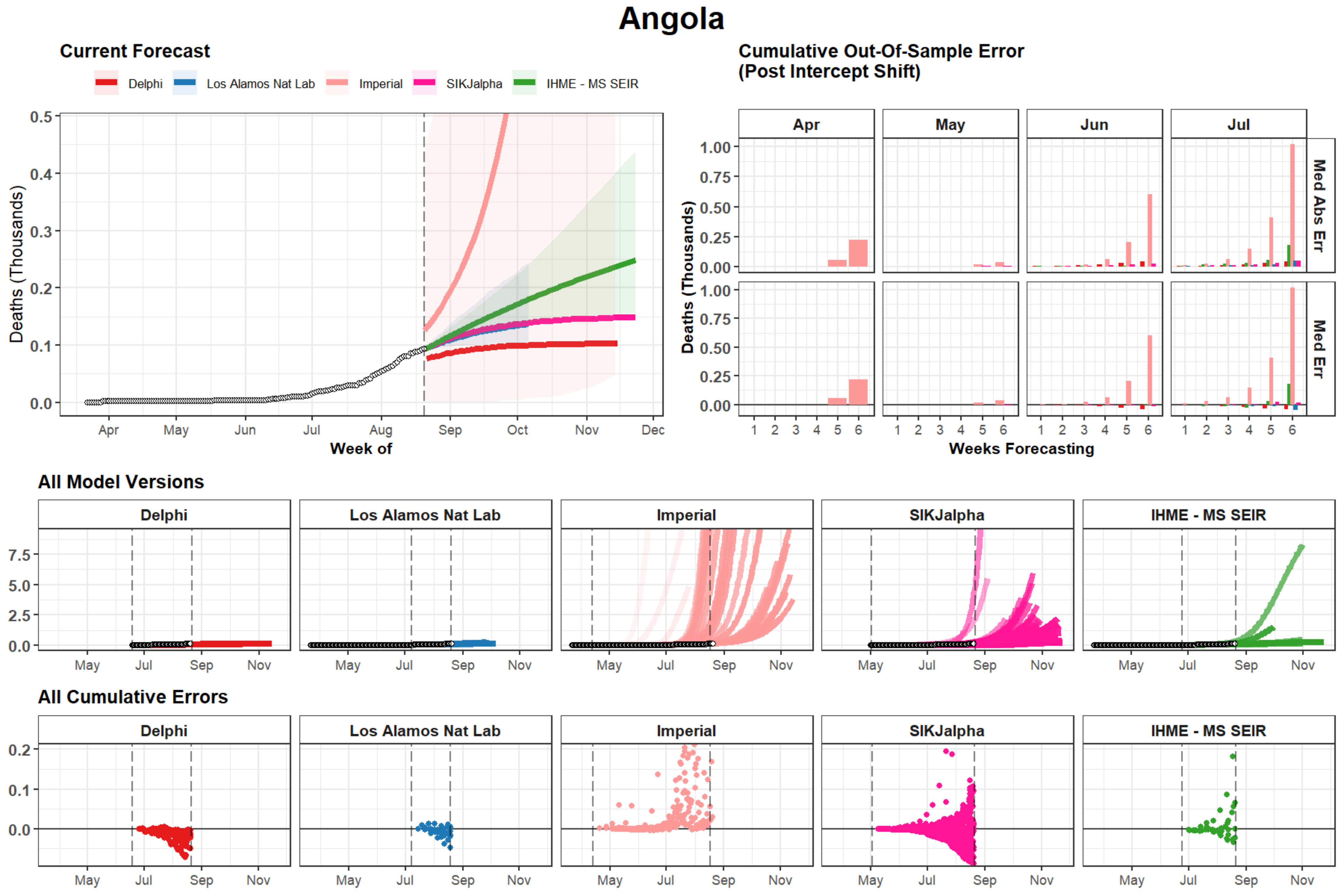
Angola
Current Forecast
Delphi
Los Alamos Nat Lab
Imperial
SIKJalpha
IHME - MS SEIR
Deaths (Thousands)
Week of
Cumulative Out-Of-Sample Error
(Post Intercept Shift)
Apr
May
Jun
Jul
Med Abs Err
Med Err
Weeks Forecasting
All Model Versions
All Cumulative Errors

Somalia

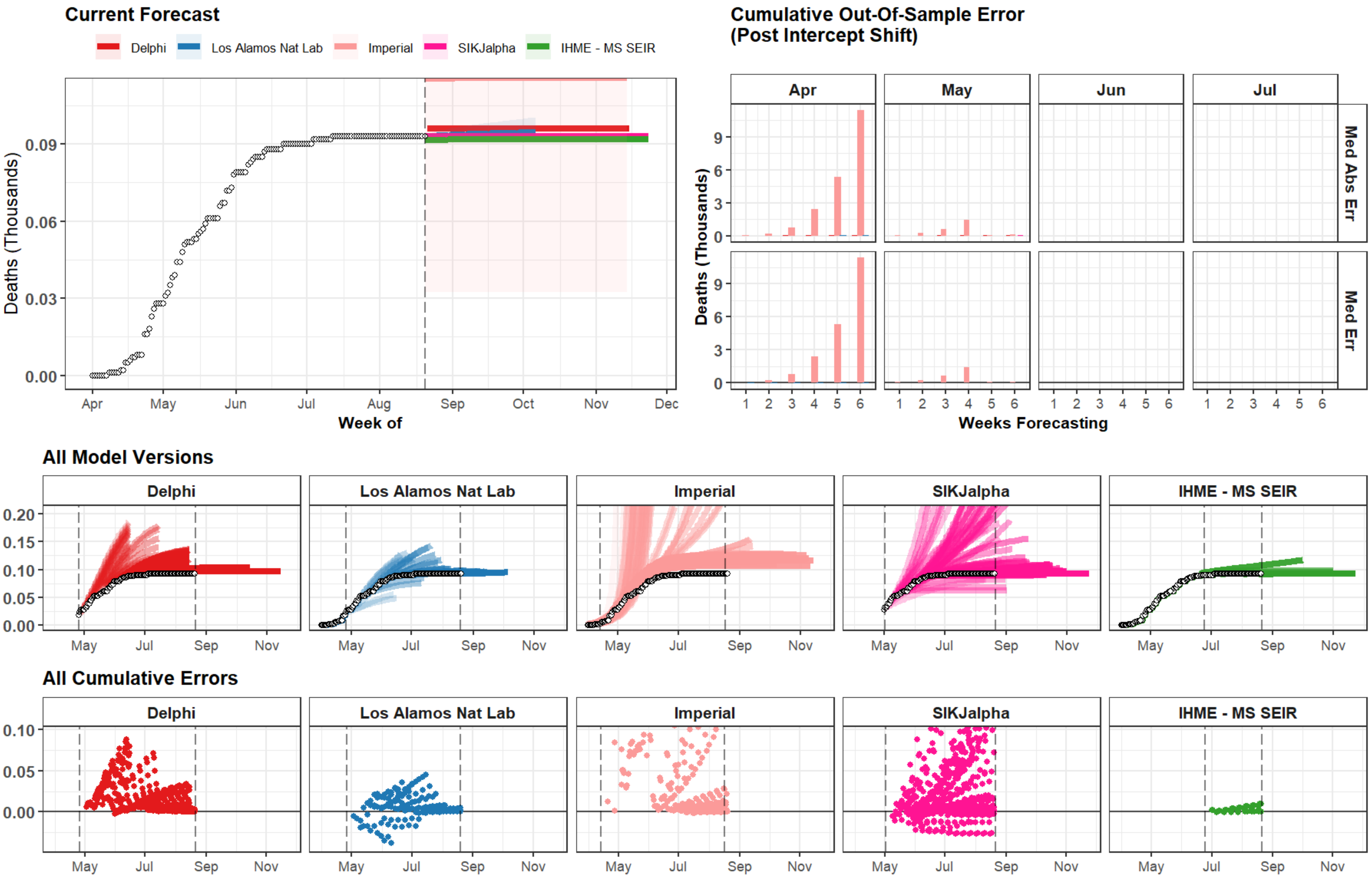
Current Forecast
Delphi
Los Alamos Nat Lab
Imperial
SIKJalpha
IHME - MS SEIR
Deaths (Thousands)
Week of
Cumulative Out-Of-Sample Error
(Post Intercept Shift)
Apr
May
Jun
Jul
Med Abs Err
Med Err
Weeks Forecasting
All Model Versions
All Cumulative Errors

Montana

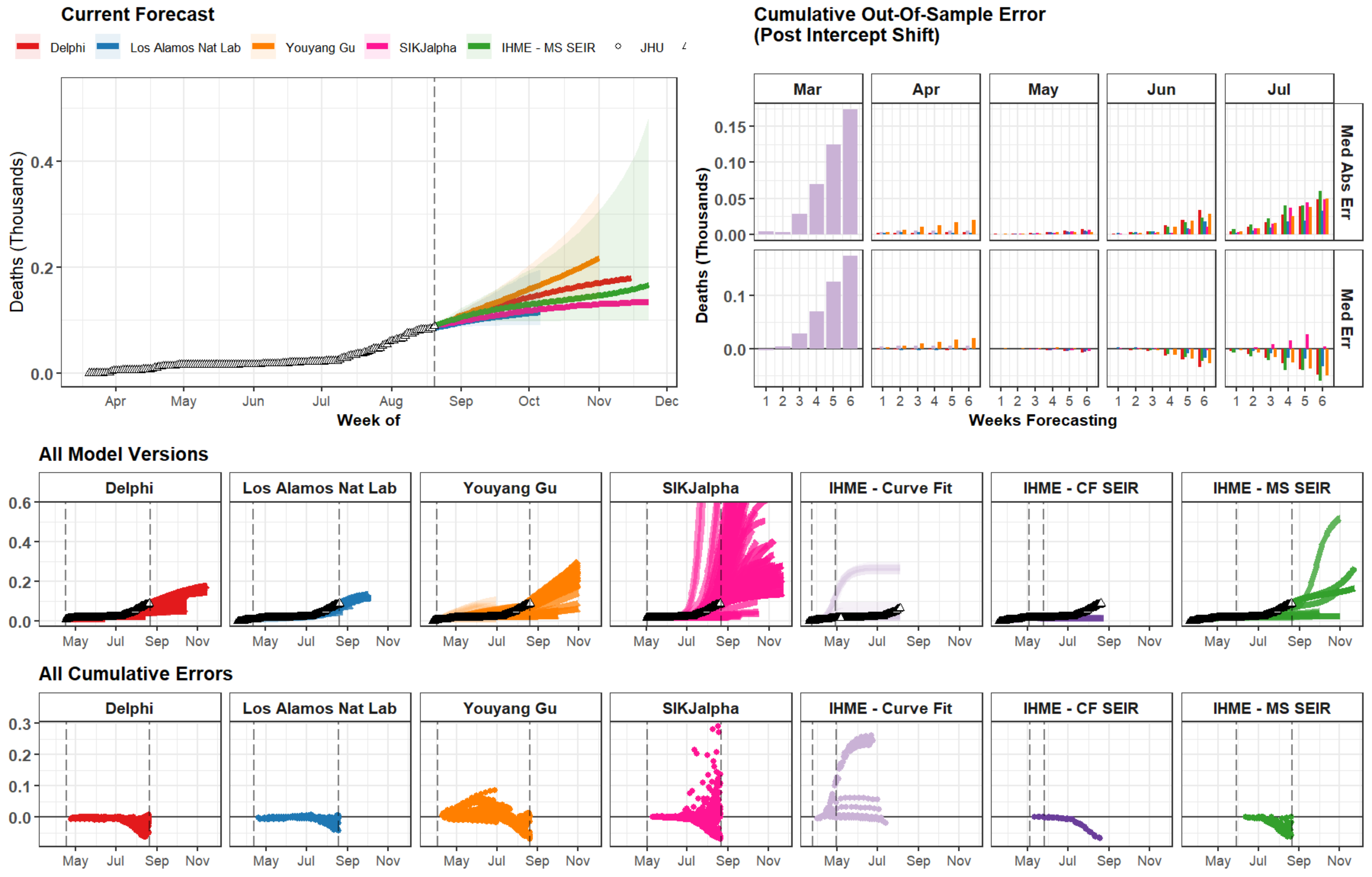

Current Forecast
Delphi
Los Alamos Nat Lab
Youyang Gu
SIKJalpha
IHME - MS SEIR
JHU
Deaths (Thousands)
0.0
0.2
0.4
Apr
May
Jun
Jul
Aug
Sep
Oct
Nov
Dec
Week of
Cumulative Out-Of-Sample Error
(Post Intercept Shift)
Mar
Apr
May
Jun
Jul
Med Abs Err
Med Err
0.00
0.05
0.10
0.15
0.0
0.1
1 2 3 4 5 6
Weeks Forecasting
All Model Versions
Delphi
Los Alamos Nat Lab
Youyang Gu
SIKJalpha
IHME - Curve Fit
IHME - CF SEIR
IHME - MS SEIR
0.0
0.2
0.4
0.6
May
Jul
Sep
Nov
All Cumulative Errors
0.0
0.1
0.2
0.3

Cuba

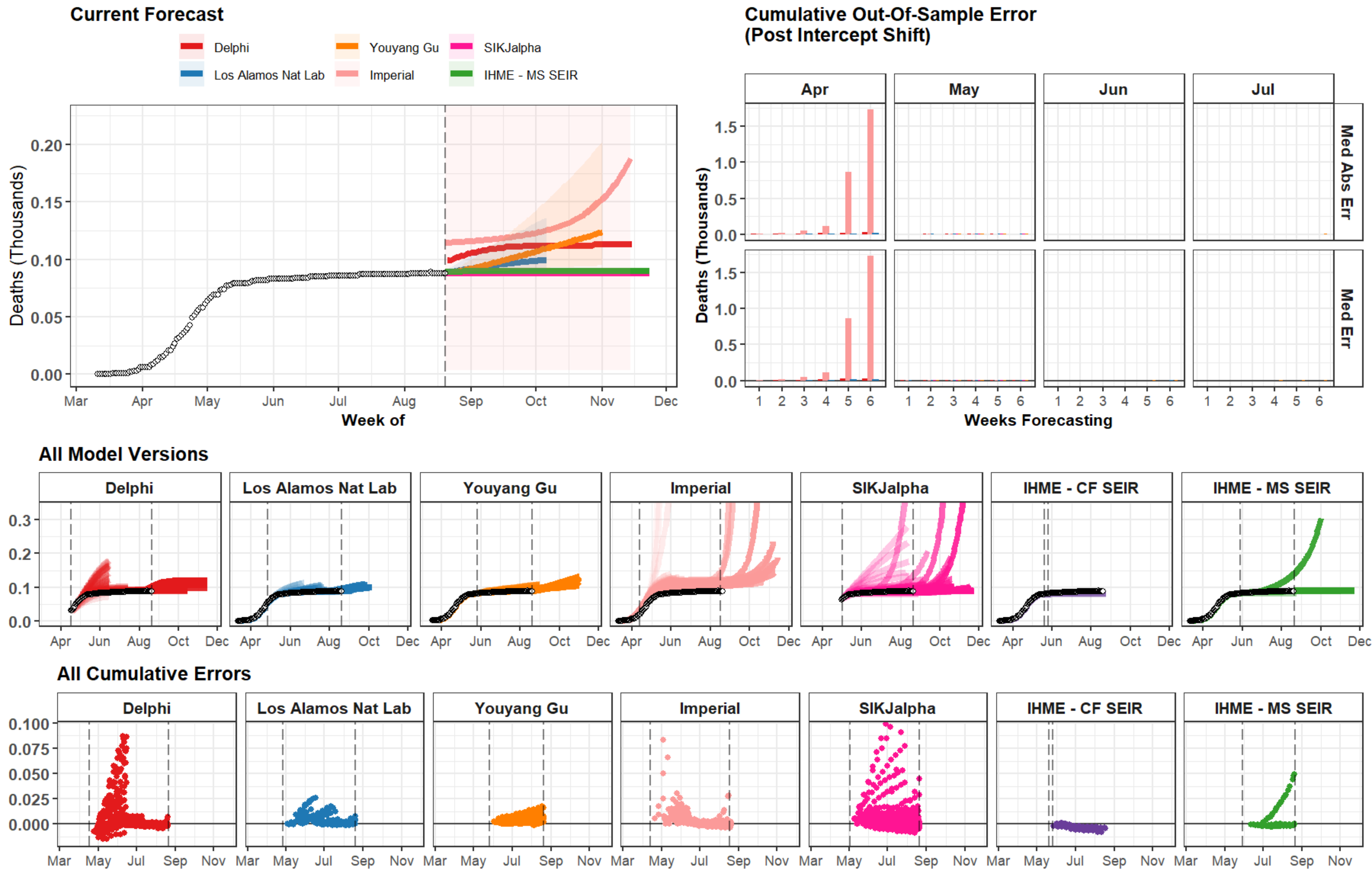
Current Forecast
Delphi
Los Alamos Nat Lab
Youyang Gu
Imperial
SIKJalpha
IHME - MS SEIR
Deaths (Thousands)
0.00
0.05
0.10
0.15
0.20
Mar
Apr
May
Jun
Jul
Aug
Sep
Oct
Nov
Dec
Week of
Cumulative Out-Of-Sample Error
(Post Intercept Shift)
Apr
May
Jun
Jul
Med Abs Err
Med Err
Deaths (Thousands)
0.0
0.5
1.0
1.5
1
2
3
4
5
6
Weeks Forecasting
All Model Versions
Delphi
Los Alamos Nat Lab
Youyang Gu
Imperial
SIKJalpha
IHME - CF SEIR
IHME - MS SEIR
0.0
0.1
0.2
0.3
Apr
Jun
Aug
Oct
Dec
All Cumulative Errors
Delphi
Los Alamos Nat Lab
Youyang Gu
Imperial
SIKJalpha
IHME - CF SEIR
IHME - MS SEIR
0.000
0.025
0.050
0.075
0.100
Mar
May
Jul
Sep
Nov

Equatorial Guinea

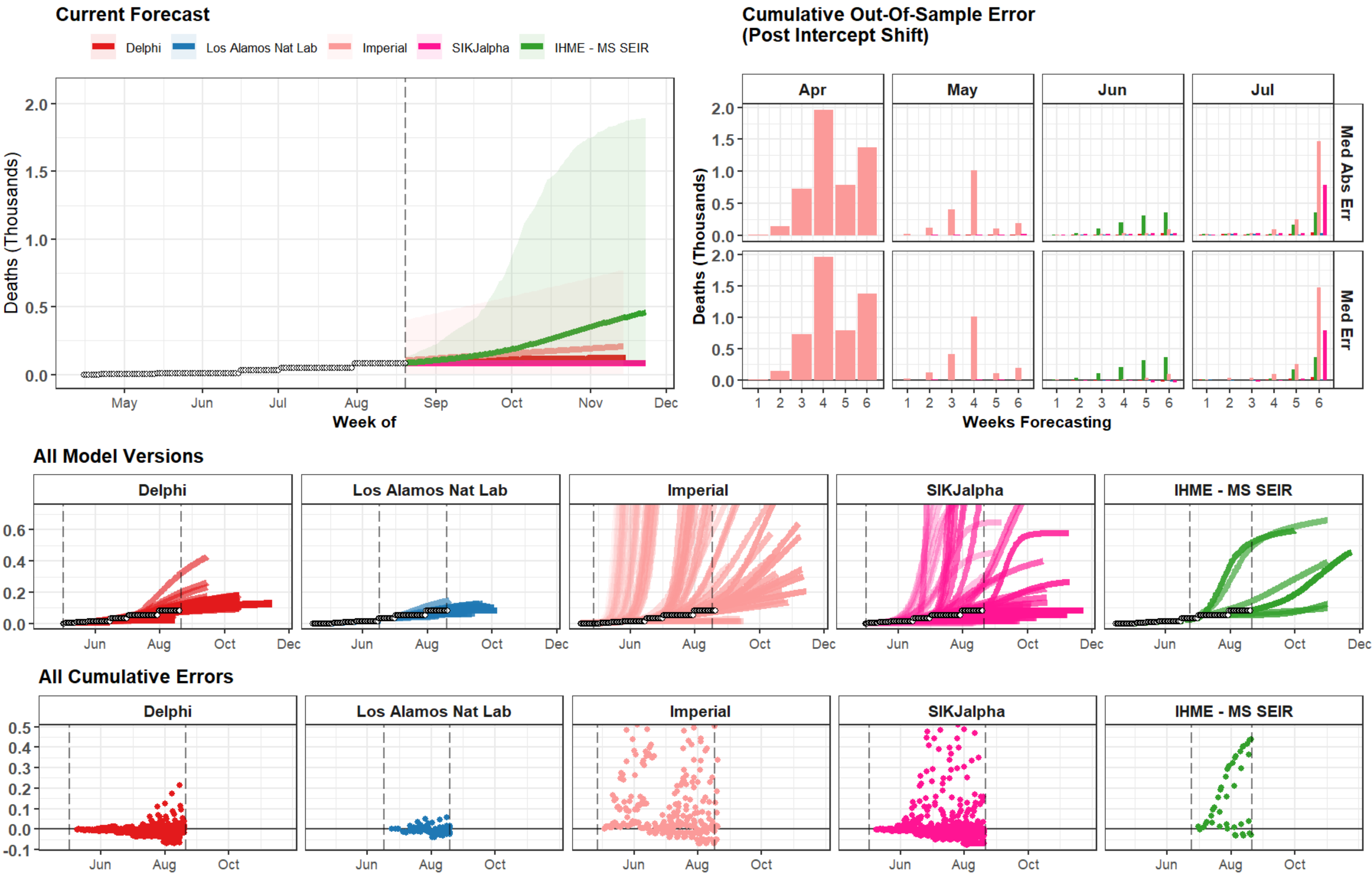

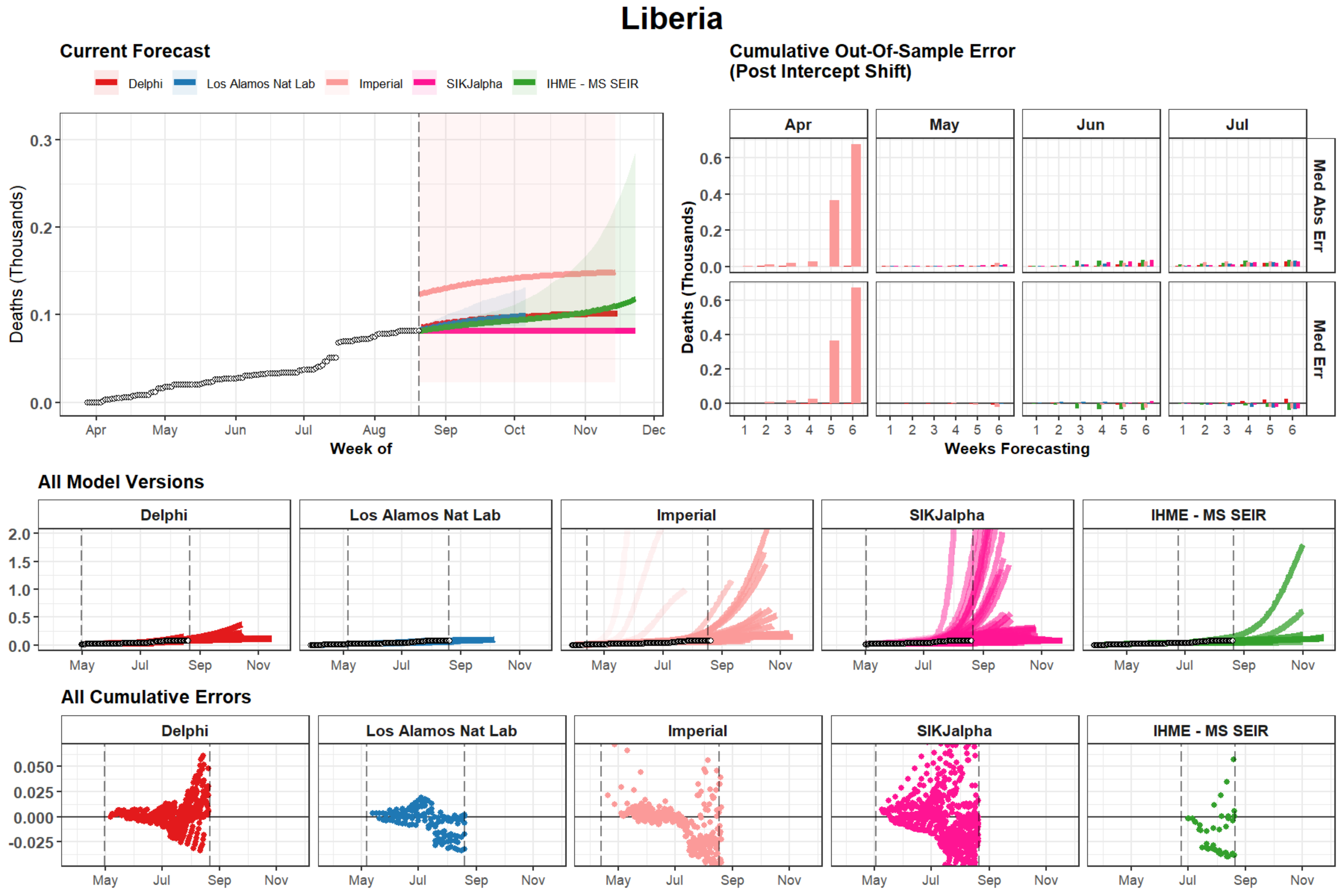

Liberia
Current Forecast
Delphi
Los Alamos Nat Lab
Imperial
SIKJalpha
IHME - MS SEIR
Deaths (Thousands)
Week of
Apr
May
Jun
Jul
Aug
Sep
Oct
Nov
Dec
Cumulative Out-Of-Sample Error
(Post Intercept Shift)
Med Abs Err
Med Err
Weeks Forecasting
All Model Versions
All Cumulative Errors

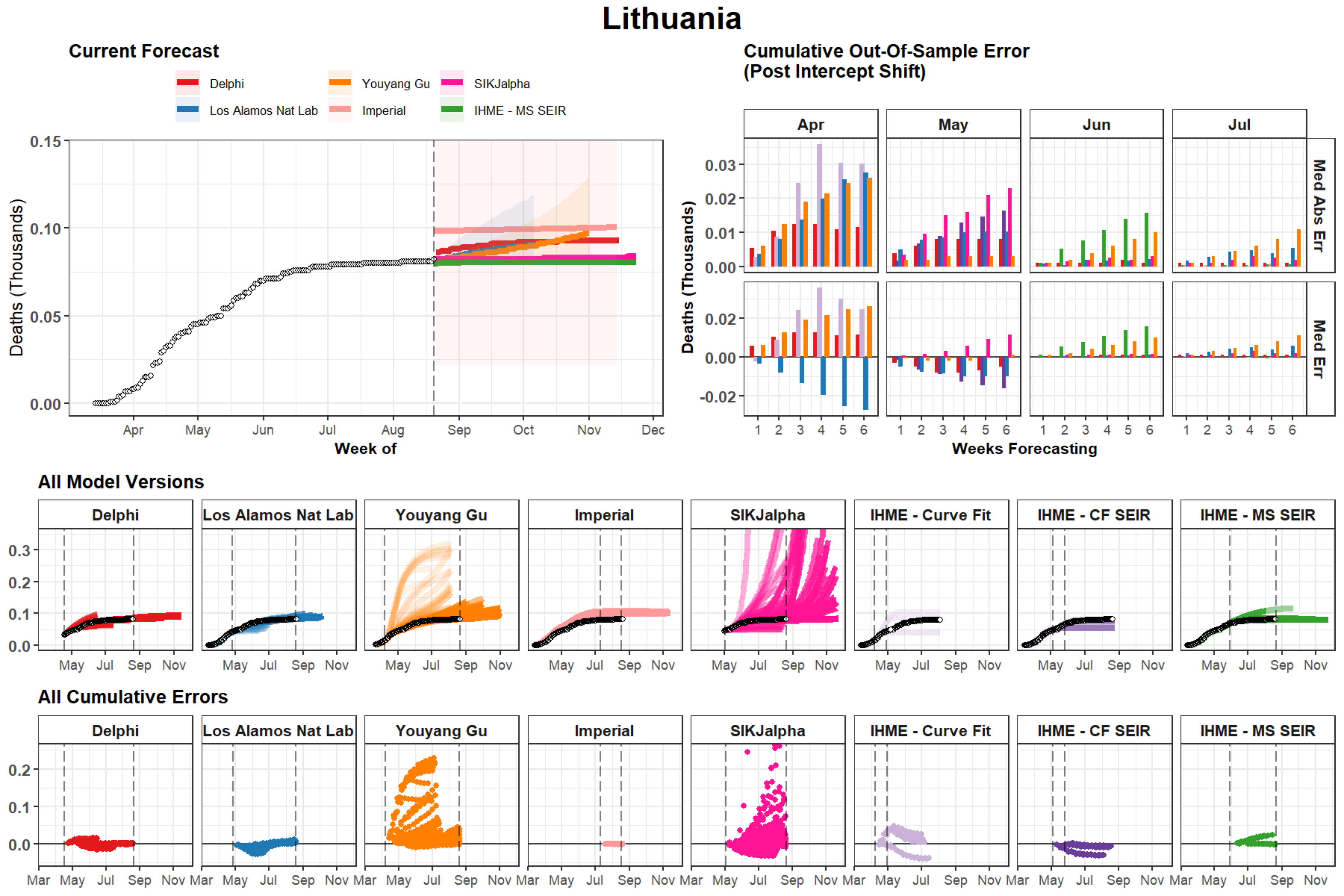

Lithuania
Current Forecast
Delphi
Youyang Gu
SIKJalpha
Los Alamos Nat Lab
Imperial
IHME - MS SEIR
Deaths (Thousands)
Week of
Apr May Jun Jul Aug Sep Oct Nov Dec
Cumulative Out-Of-Sample Error
(Post Intercept Shift)
Apr May Jun Jul
Med Abs Err
Med Err
Weeks Forecasting
All Model Versions
Delphi
Los Alamos Nat Lab
Youyang Gu
Imperial
SIKJalpha
IHME - Curve Fit
IHME - CF SEIR
IHME - MS SEIR
All Cumulative Errors
Mar May Jul Sep Nov

Syria

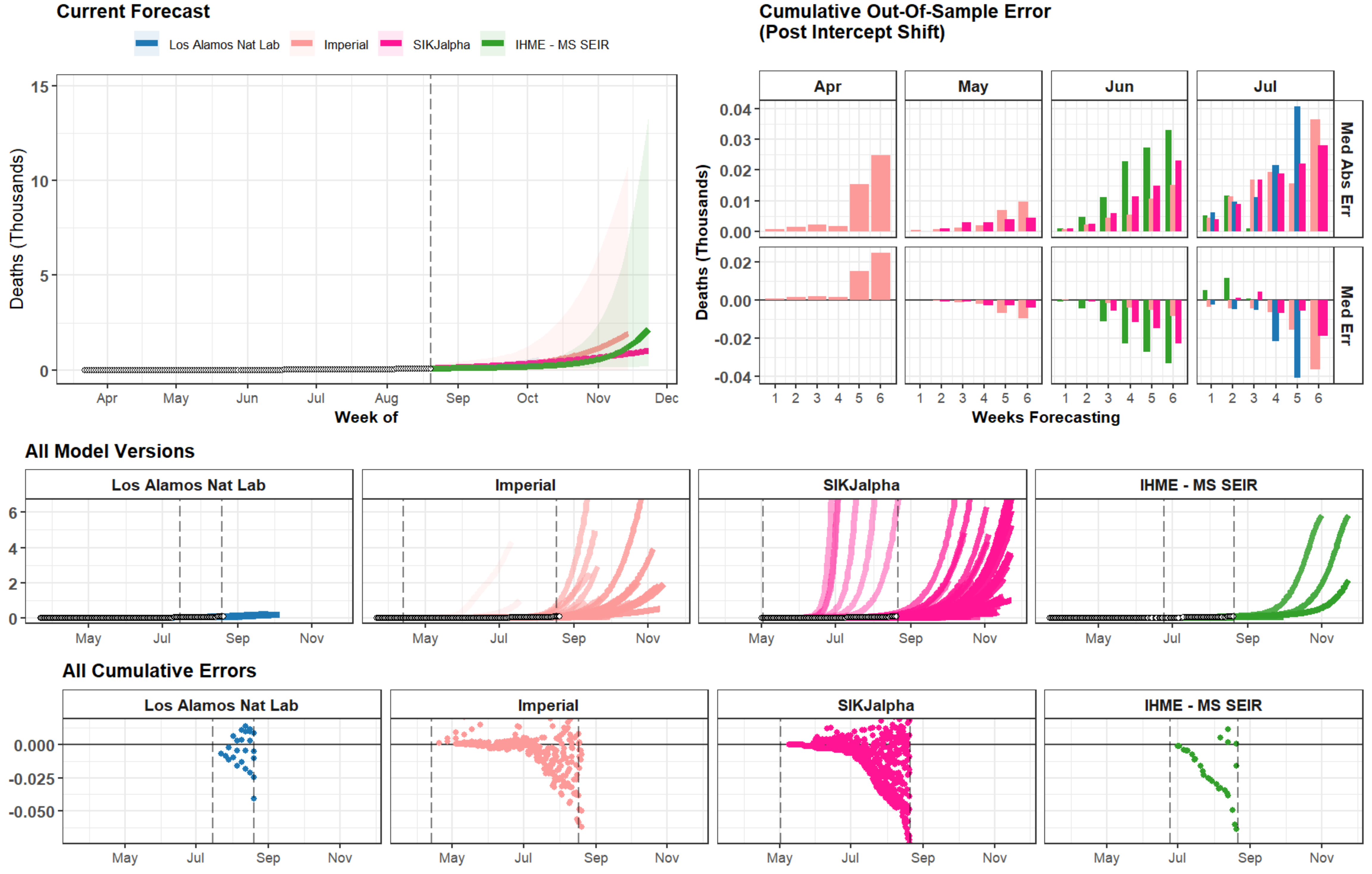

Current Forecast
Los Alamos Nat Lab
Imperial
SIKJalpha
IHME - MS SEIR
Deaths (Thousands)
15
10
5
0
Apr
May
Jun
Jul
Aug
Sep
Oct
Nov
Dec
Week of
Cumulative Out-Of-Sample Error
(Post Intercept Shift)
Apr
May
Jun
Jul
Med Abs Err
Med Err
0.04
0.03
0.02
0.01
0.00
-0.02
-0.04
1 2 3 4 5 6
Weeks Forecasting
All Model Versions
Los Alamos Nat Lab
Imperial
SIKJalpha
IHME - MS SEIR
6
4
2
0
All Cumulative Errors
0.000
-0.025
-0.050

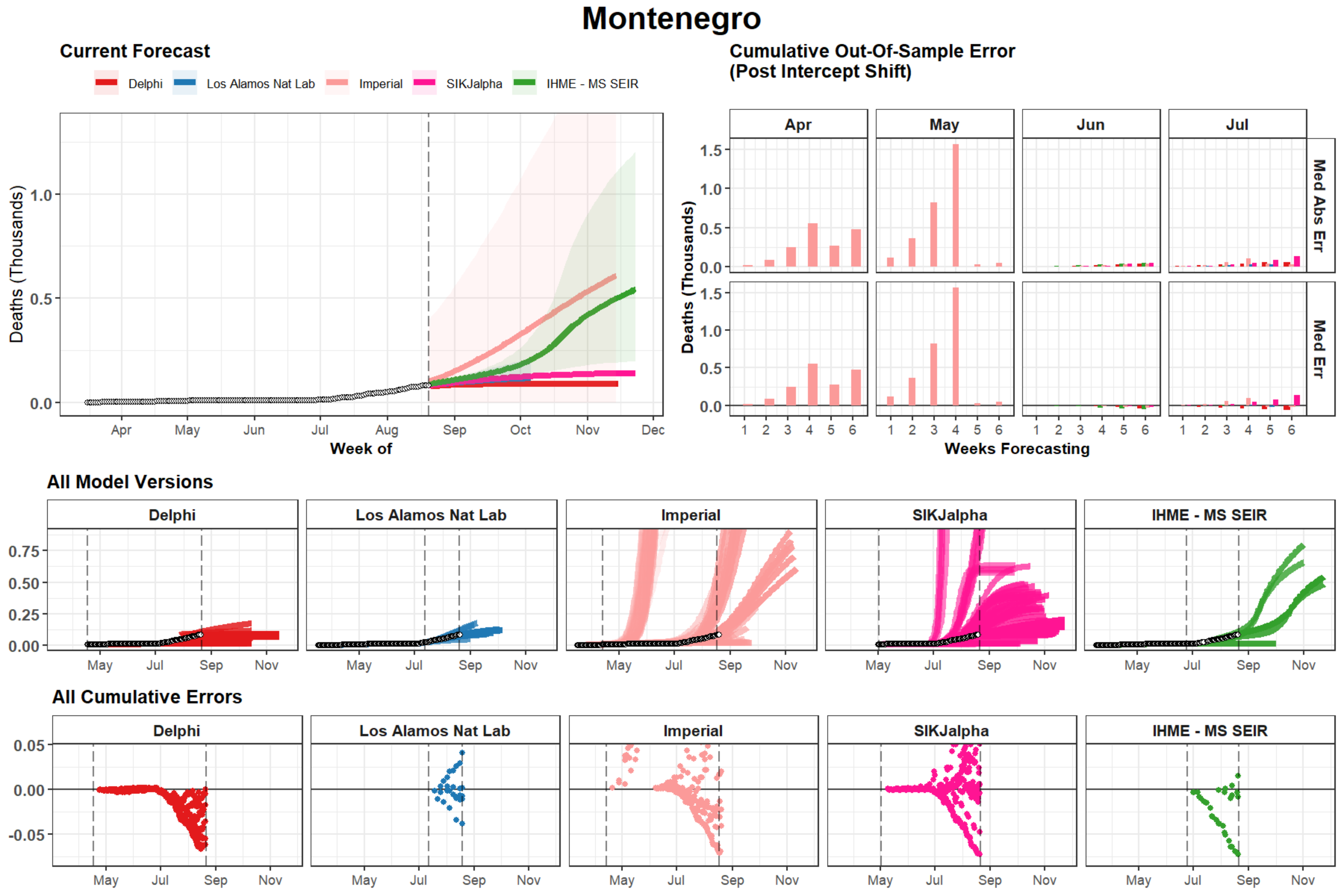

Montenegro
Current Forecast
Delphi
Los Alamos Nat Lab
Imperial
SIKJalpha
IHME - MS SEIR
Deaths (Thousands)
Week of
Cumulative Out-Of-Sample Error (Post Intercept Shift)
Apr
May
Jun
Jul
Med Abs Err
Med Err
Weeks Forecasting
All Model Versions
All Cumulative Errors

Swaziland

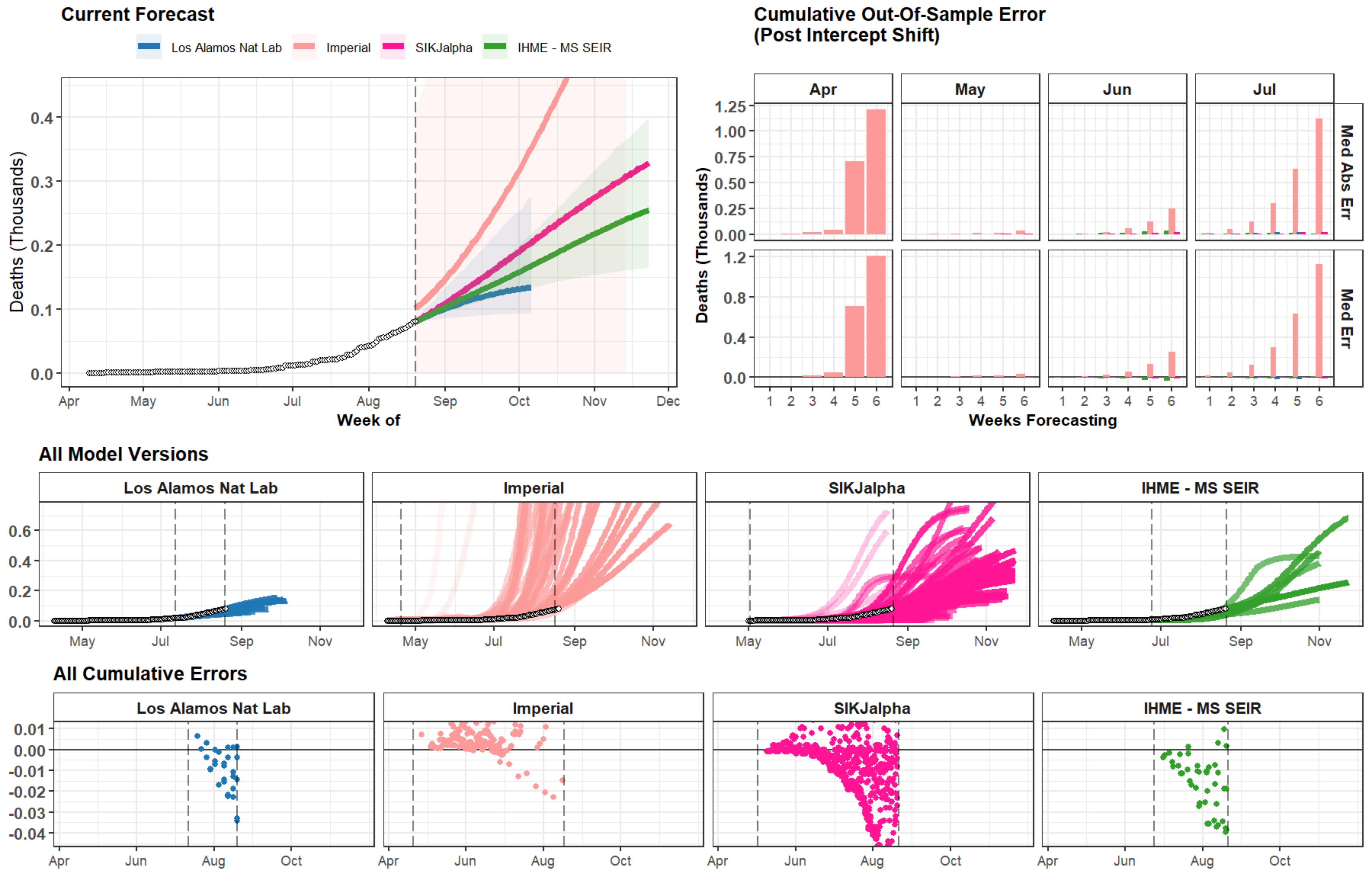

Current Forecast
Los Alamos Nat Lab
Imperial
SIKJalpha
IHME - MS SEIR
Deaths (Thousands)
Week of
Apr
May
Jun
Jul
Aug
Sep
Oct
Nov
Dec
Cumulative Out-Of-Sample Error
(Post Intercept Shift)
Apr
May
Jun
Jul
Med Abs Err
Med Err
Deaths (Thousands)
Weeks Forecasting
All Model Versions
Los Alamos Nat Lab
Imperial
SIKJalpha
IHME - MS SEIR
All Cumulative Errors
Los Alamos Nat Lab
Imperial
SIKJalpha
IHME - MS SEIR

Congo

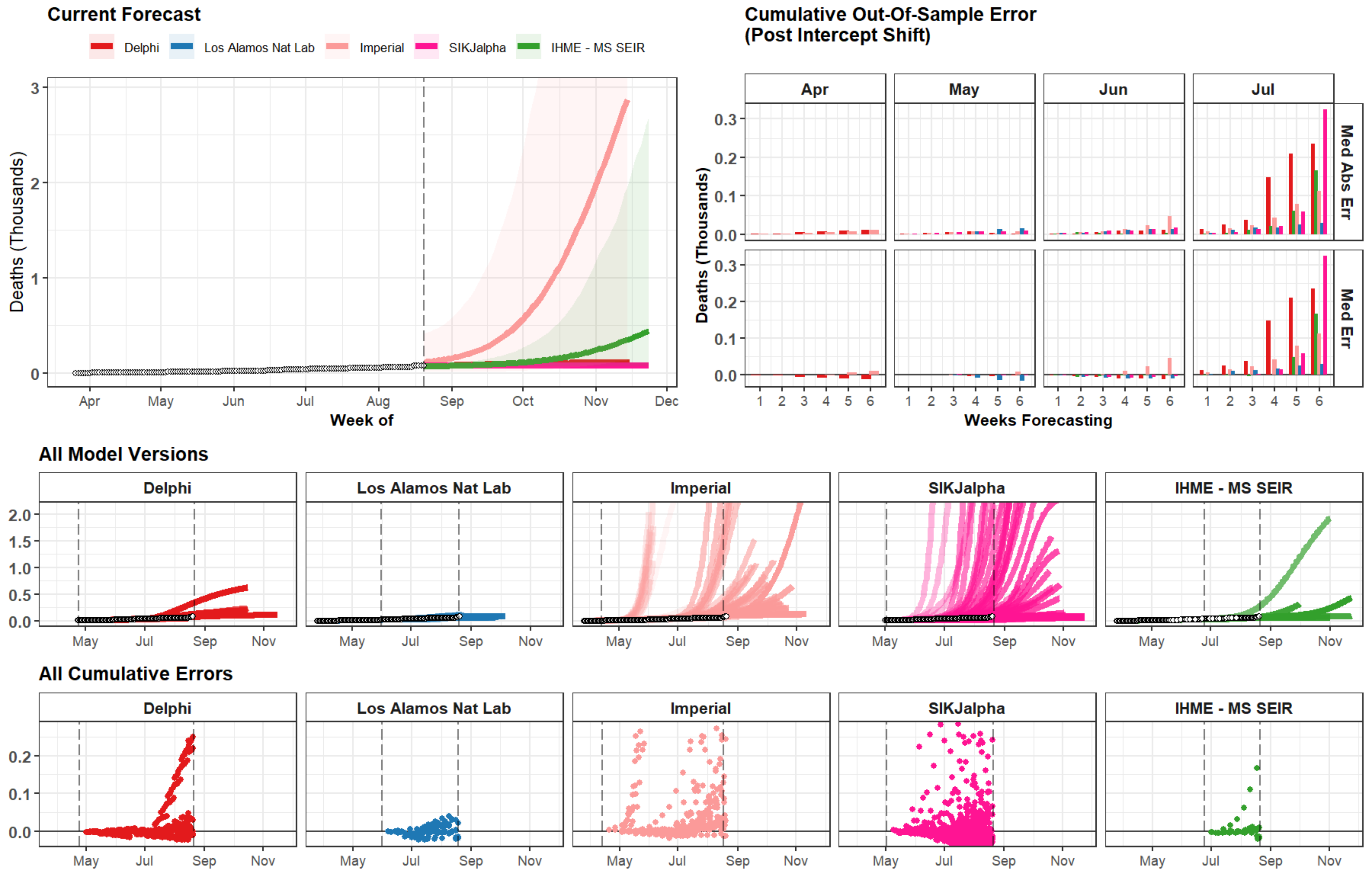

Current Forecast
Delphi
Los Alamos Nat Lab
Imperial
SIKJalpha
IHME - MS SEIR
Deaths (Thousands)
3
2
1
0
Apr
May
Jun
Jul
Aug
Sep
Oct
Nov
Dec
Week of
Cumulative Out-Of-Sample Error
(Post Intercept Shift)
Apr
May
Jun
Jul
Med Abs Err
Med Err
0.3
0.2
0.1
0.0
1
2
3
4
5
6
Weeks Forecasting
All Model Versions
Delphi
Los Alamos Nat Lab
Imperial
SIKJalpha
IHME - MS SEIR
2.0
1.5
1.0
0.5
0.0
May
Jul
Sep
Nov
All Cumulative Errors
Delphi
Los Alamos Nat Lab
Imperial
SIKJalpha
IHME - MS SEIR
0.2
0.1
0.0
May
Jul
Sep
Nov

Chad

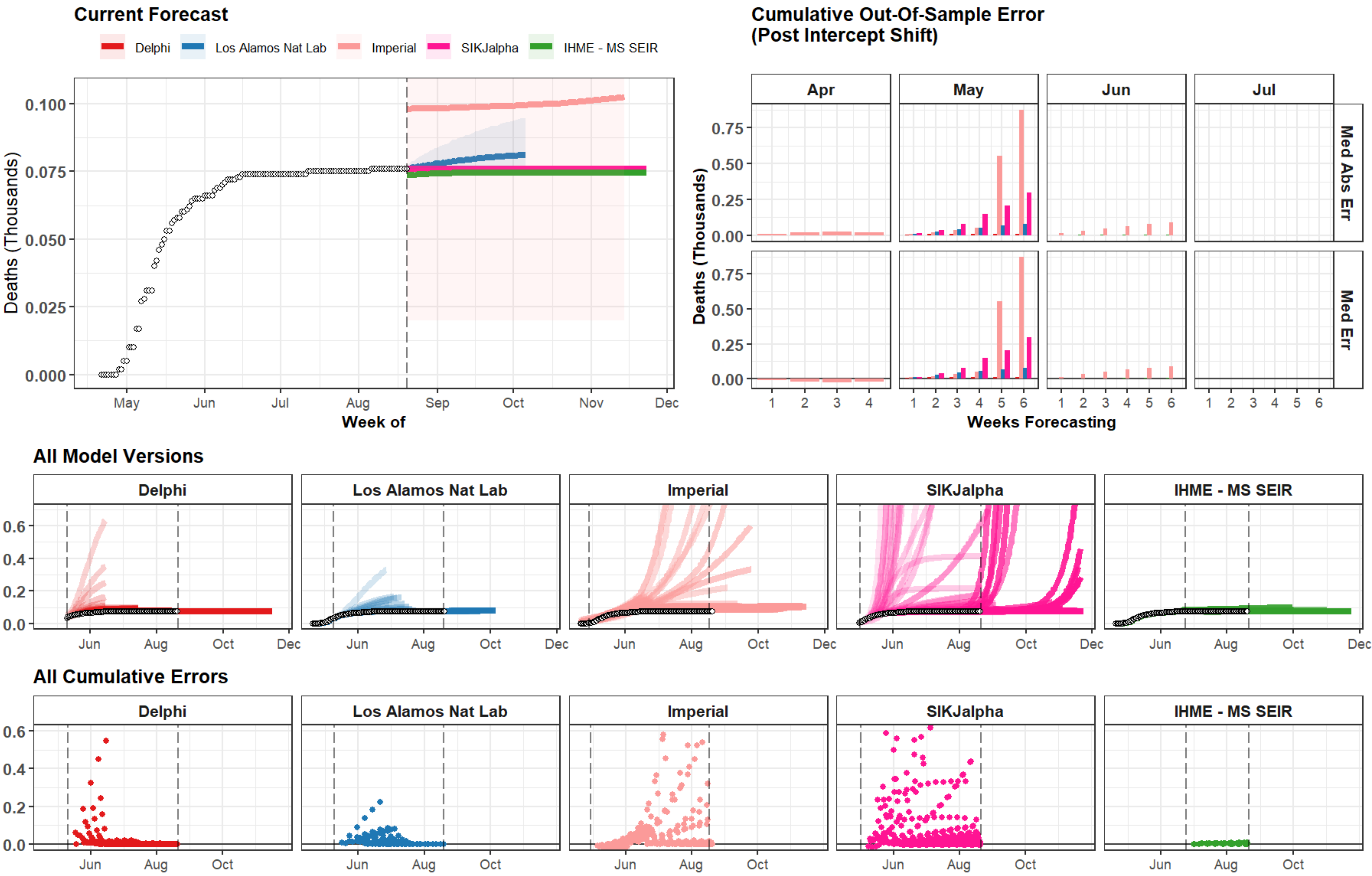
Current Forecast
Delphi
Los Alamos Nat Lab
Imperial
SIKJalpha
IHME - MS SEIR
Deaths (Thousands)
0.100
0.075
0.050
0.025
0.000
May
Jun
Jul
Aug
Sep
Oct
Nov
Dec
Week of
Cumulative Out-Of-Sample Error
(Post Intercept Shift)
Apr
May
Jun
Jul
Med Abs Err
Med Err
0.75
0.50
0.25
0.00
Weeks Forecasting
All Model Versions
Delphi
Los Alamos Nat Lab
Imperial
SIKJalpha
IHME - MS SEIR
0.6
0.4
0.2
0.0
All Cumulative Errors

Niger

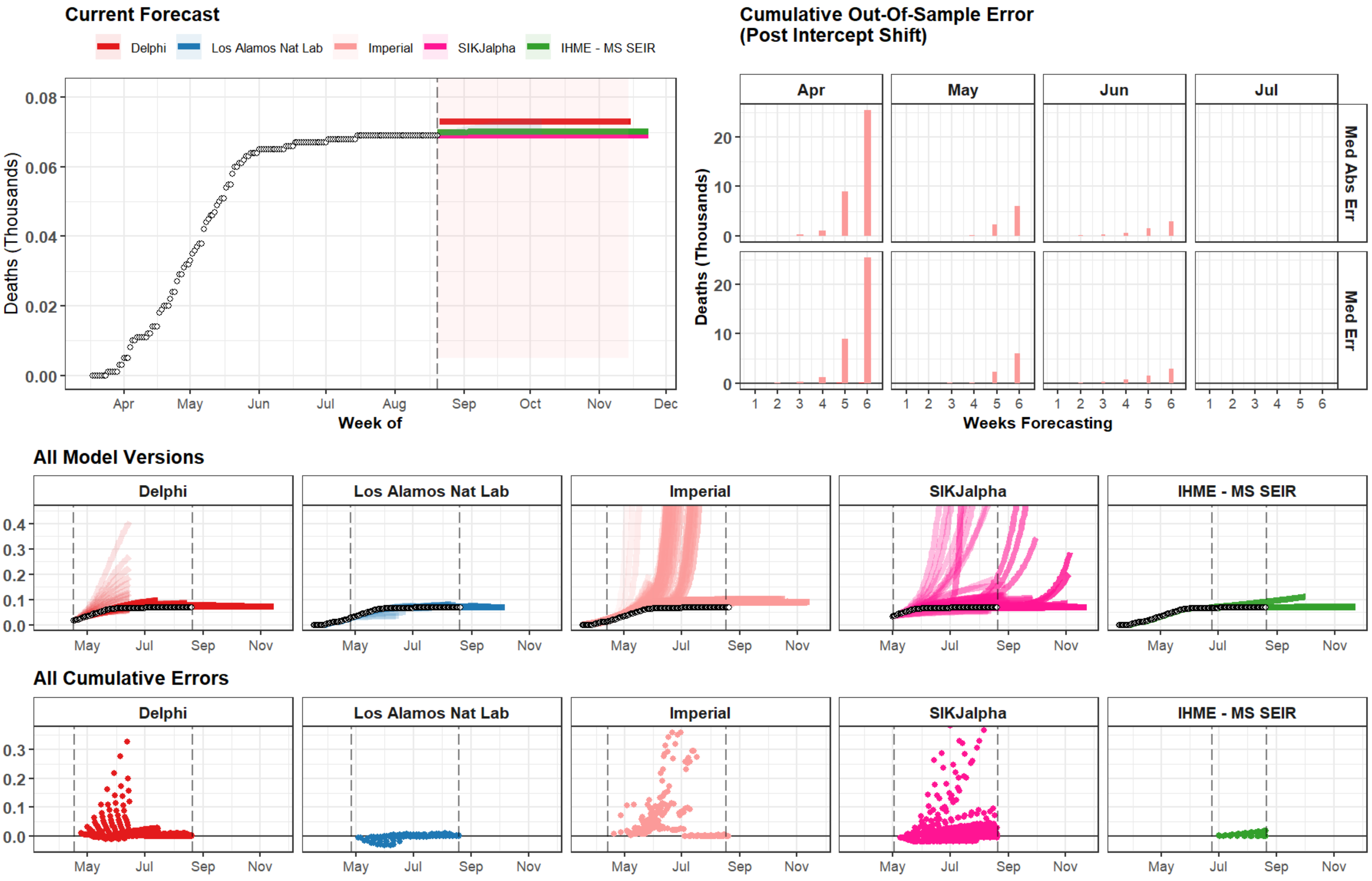

Current Forecast
Delphi
Los Alamos Nat Lab
Imperial
SIKJalpha
IHME - MS SEIR
Deaths (Thousands)
0.08
0.06
0.04
0.02
0.00
Apr
May
Jun
Jul
Aug
Sep
Oct
Nov
Dec
Week of
Cumulative Out-Of-Sample Error
(Post Intercept Shift)
Apr
May
Jun
Jul
Med Abs Err
Med Err
Deaths (Thousands)
20
10
0
1
2
3
4
5
6
Weeks Forecasting
All Model Versions
Delphi
Los Alamos Nat Lab
Imperial
SIKJalpha
IHME - MS SEIR
0.4
0.3
0.2
0.1
0.0
May
Jul
Sep
Nov
All Cumulative Errors
Delphi
Los Alamos Nat Lab
Imperial
SIKJalpha
IHME - MS SEIR
0.3
0.2
0.1
0.0
May
Jul
Sep
Nov

Sierra Leone

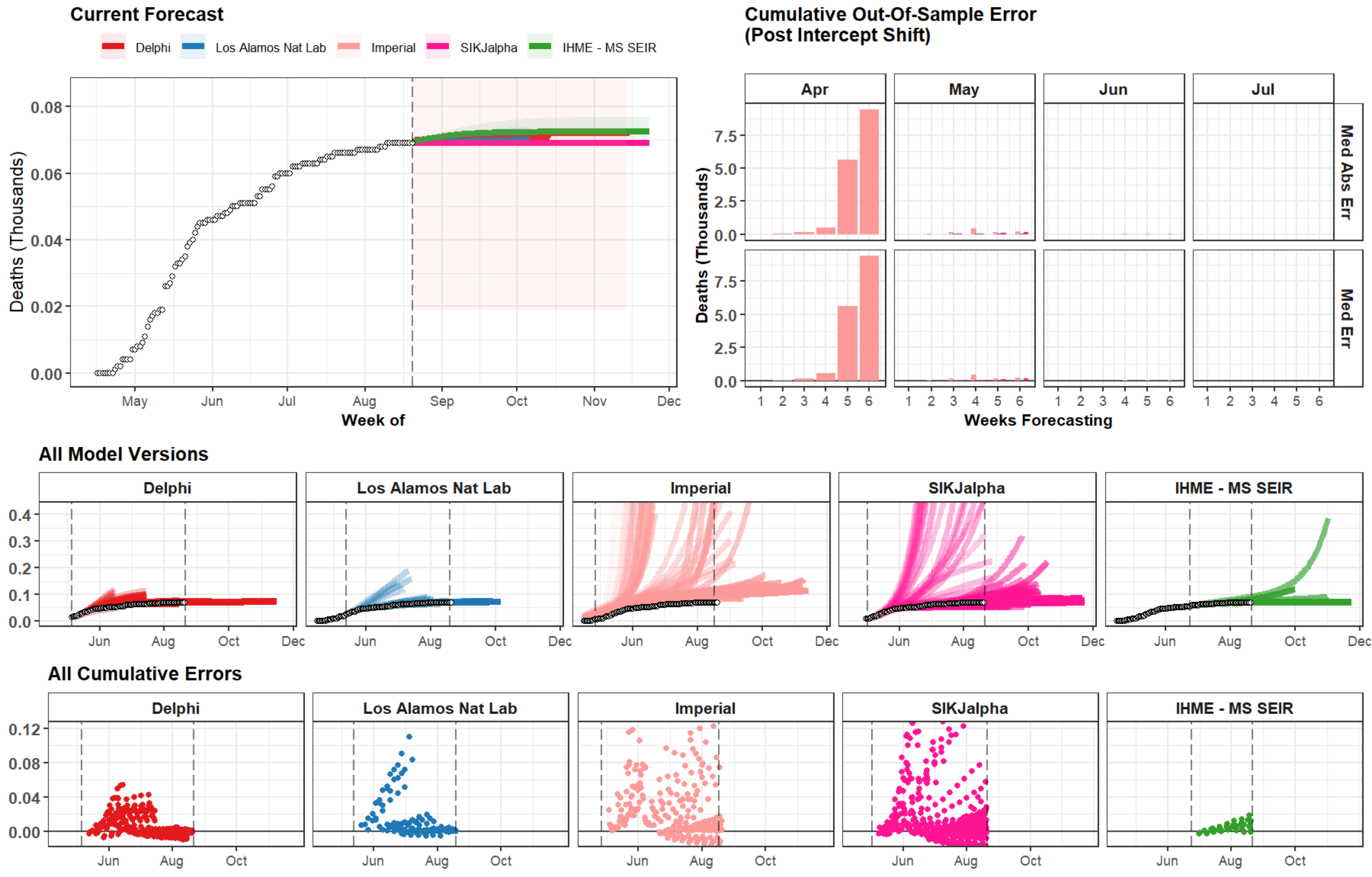

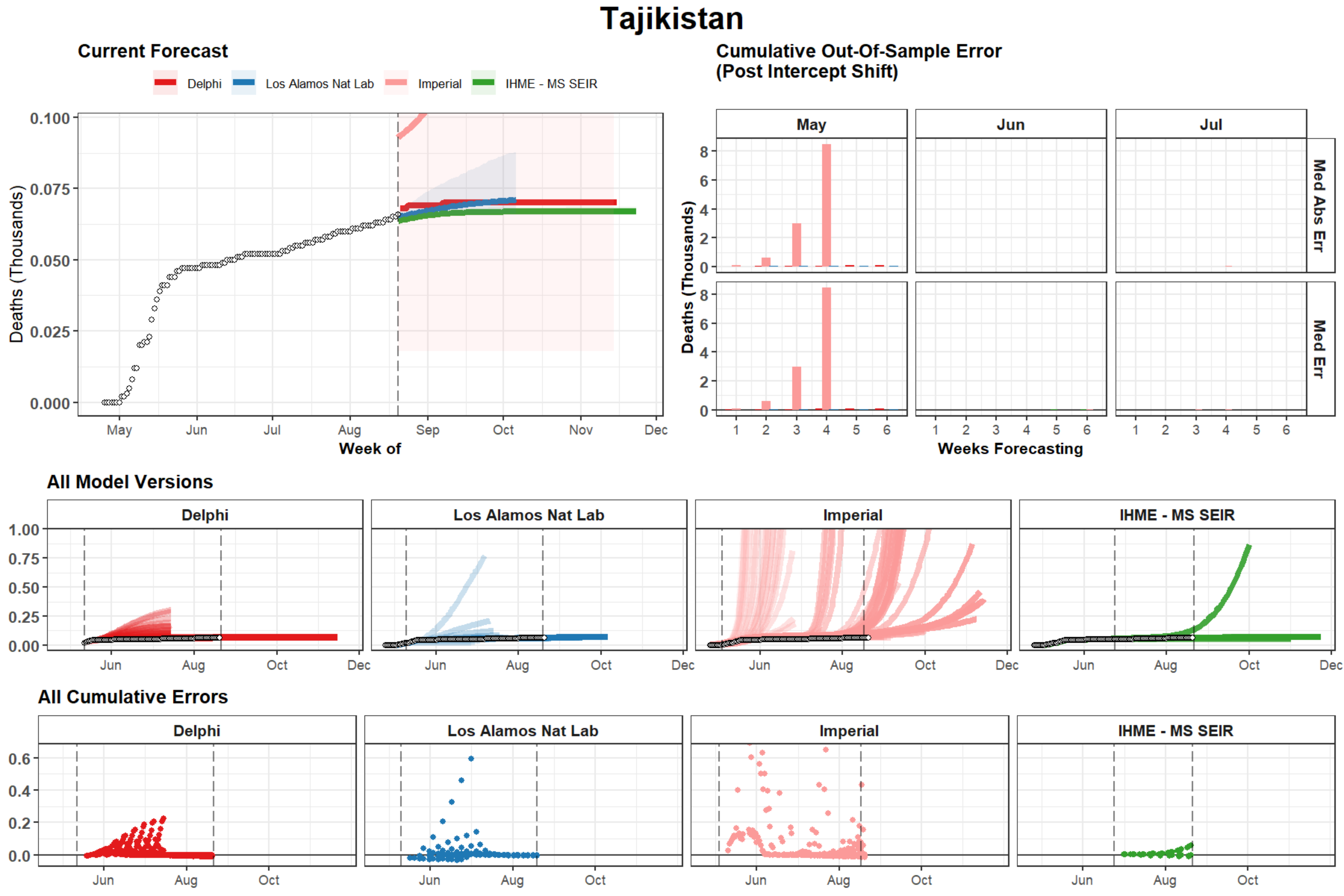
Tajikistan
Current Forecast
Delphi
Los Alamos Nat Lab
Imperial
IHME - MS SEIR
Deaths (Thousands)
0.100
0.075
0.050
0.025
0.000
May
Jun
Jul
Aug
Sep
Oct
Nov
Dec
Week of
Cumulative Out-Of-Sample Error
(Post Intercept Shift)
May
Jun
Jul
Med Abs Err
Med Err
Weeks Forecasting
All Model Versions
1.00
0.75
0.50
0.25
0.00
All Cumulative Errors
0.6
0.4
0.2
0.0

Estonia

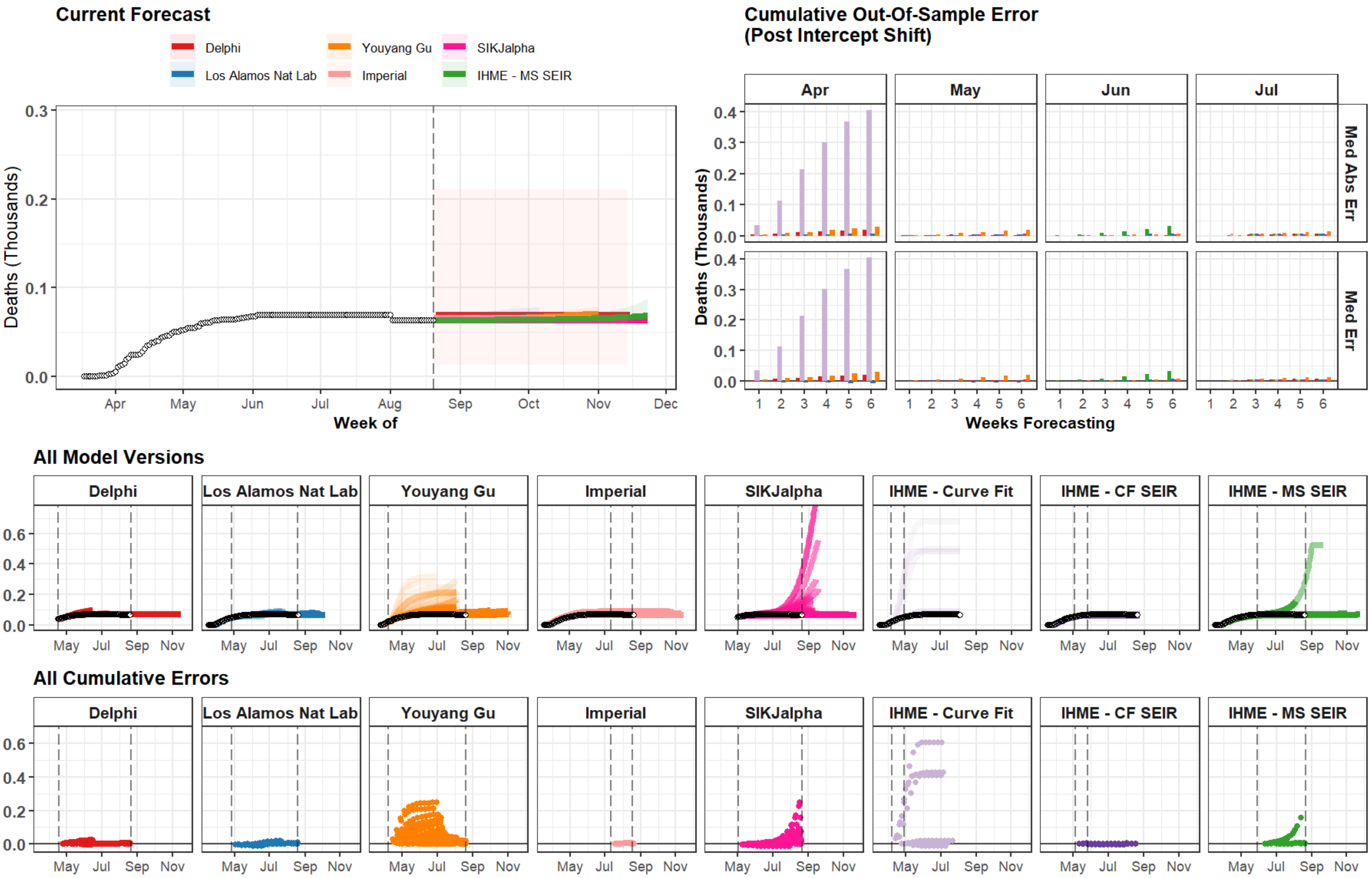

Current Forecast
Delphi
Youyang Gu
SIKJalpha
Los Alamos Nat Lab
Imperial
IHME - MS SEIR
Deaths (Thousands)
Week of
Apr May Jun Jul Aug Sep Oct Nov Dec
Cumulative Out-Of-Sample Error
(Post Intercept Shift)
Apr
May
Jun
Jul
Med Abs Err
Med Err
Weeks Forecasting
All Model Versions
Delphi
Los Alamos Nat Lab
Youyang Gu
Imperial
SIKJalpha
IHME - Curve Fit
IHME - CF SEIR
IHME - MS SEIR
All Cumulative Errors

Tunisia

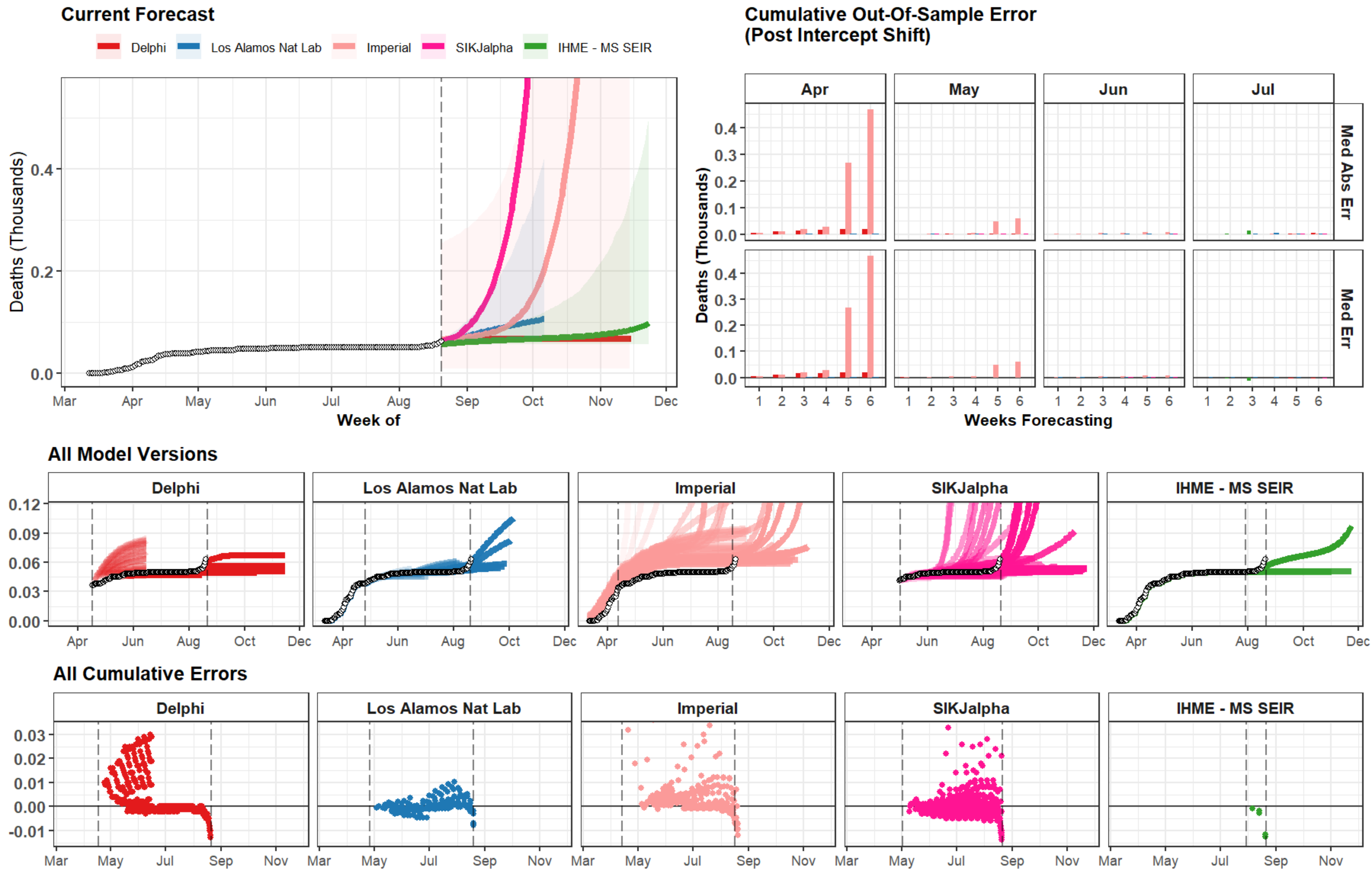

Current Forecast
Delphi
Los Alamos Nat Lab
Imperial
SIKJalpha
IHME - MS SEIR
Deaths (Thousands)
0.0
0.2
0.4
Mar
Apr
May
Jun
Jul
Aug
Sep
Oct
Nov
Dec
Week of
Cumulative Out-Of-Sample Error
(Post Intercept Shift)
Apr
May
Jun
Jul
Med Abs Err
Med Err
0.0
0.1
0.2
0.3
0.4
1
2
3
4
5
6
Weeks Forecasting
All Model Versions
Delphi
Los Alamos Nat Lab
Imperial
SIKJalpha
IHME - MS SEIR
0.00
0.03
0.06
0.09
0.12
Apr
Jun
Aug
Oct
Dec
All Cumulative Errors
Delphi
Los Alamos Nat Lab
Imperial
SIKJalpha
IHME - MS SEIR
-0.01
0.00
0.01
0.02
0.03
Mar
May
Jul
Sep
Nov

Central African Republic

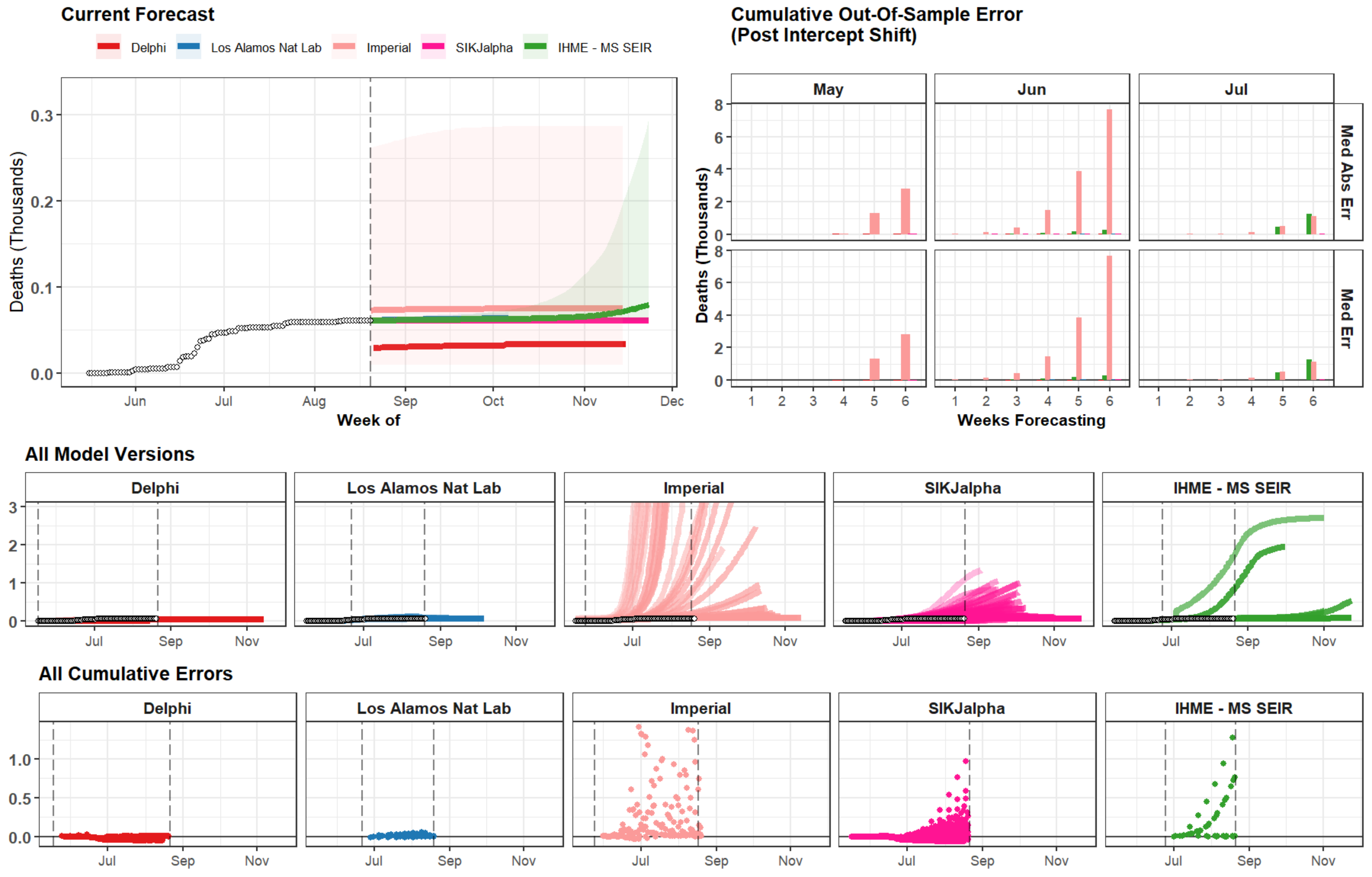

Djibouti

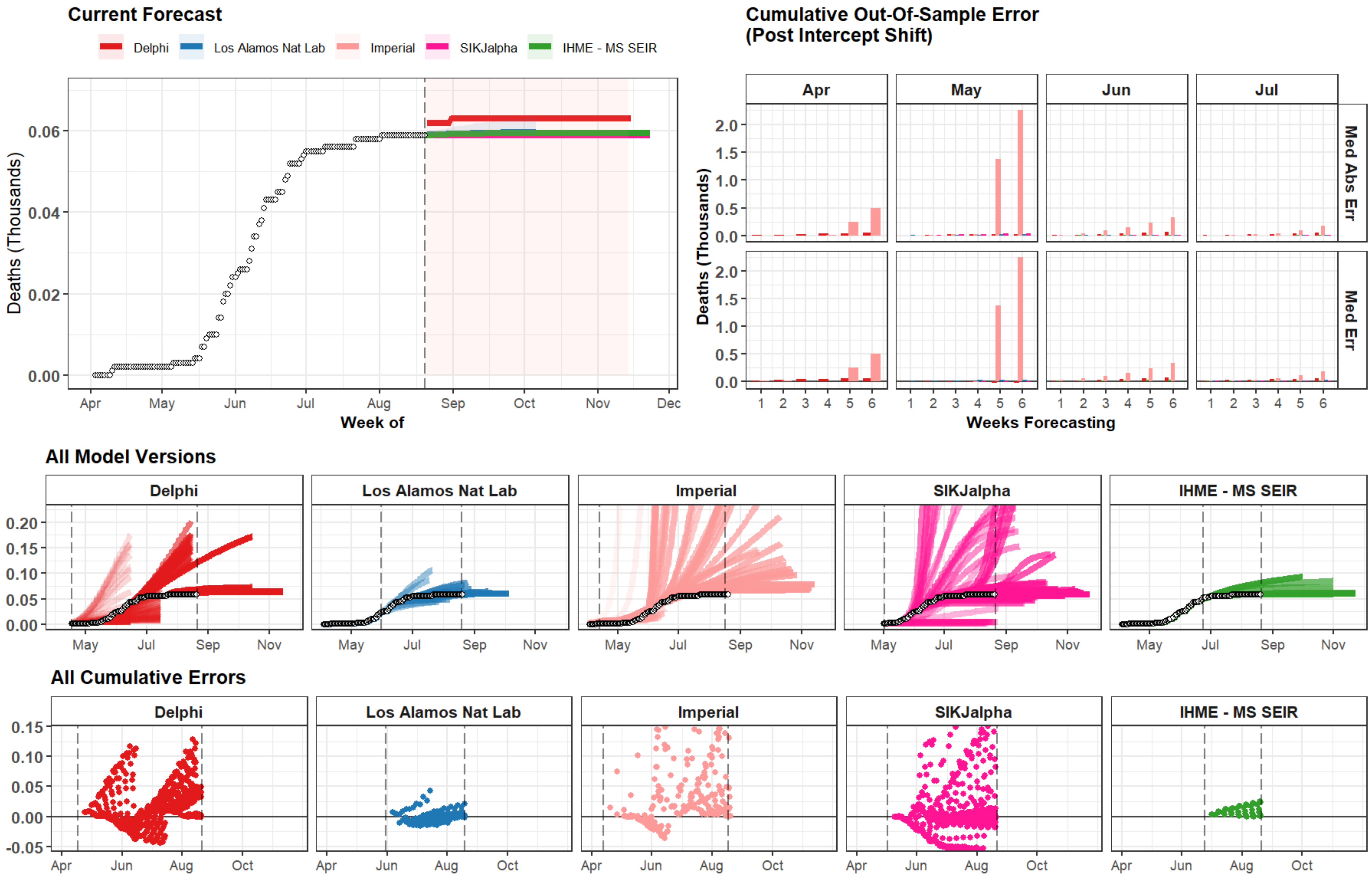
Current Forecast
Delphi
Los Alamos Nat Lab
Imperial
SIKJalpha
IHME - MS SEIR
Deaths (Thousands)
0.00
0.02
0.04
0.06
Apr
May
Jun
Jul
Aug
Sep
Oct
Nov
Dec
Week of
Cumulative Out-Of-Sample Error
(Post Intercept Shift)
Apr
May
Jun
Jul
Med Abs Err
Med Err
0.0
0.5
1.0
1.5
2.0
1
2
3
4
5
6
Weeks Forecasting
All Model Versions
Delphi
Los Alamos Nat Lab
Imperial
SIKJalpha
IHME - MS SEIR
0.00
0.05
0.10
0.15
0.20
May
Jul
Sep
Nov
All Cumulative Errors
Delphi
Los Alamos Nat Lab
Imperial
SIKJalpha
IHME - MS SEIR
-0.05
0.00
0.05
0.10
0.15
Apr
Jun
Aug
Oct

Thailand

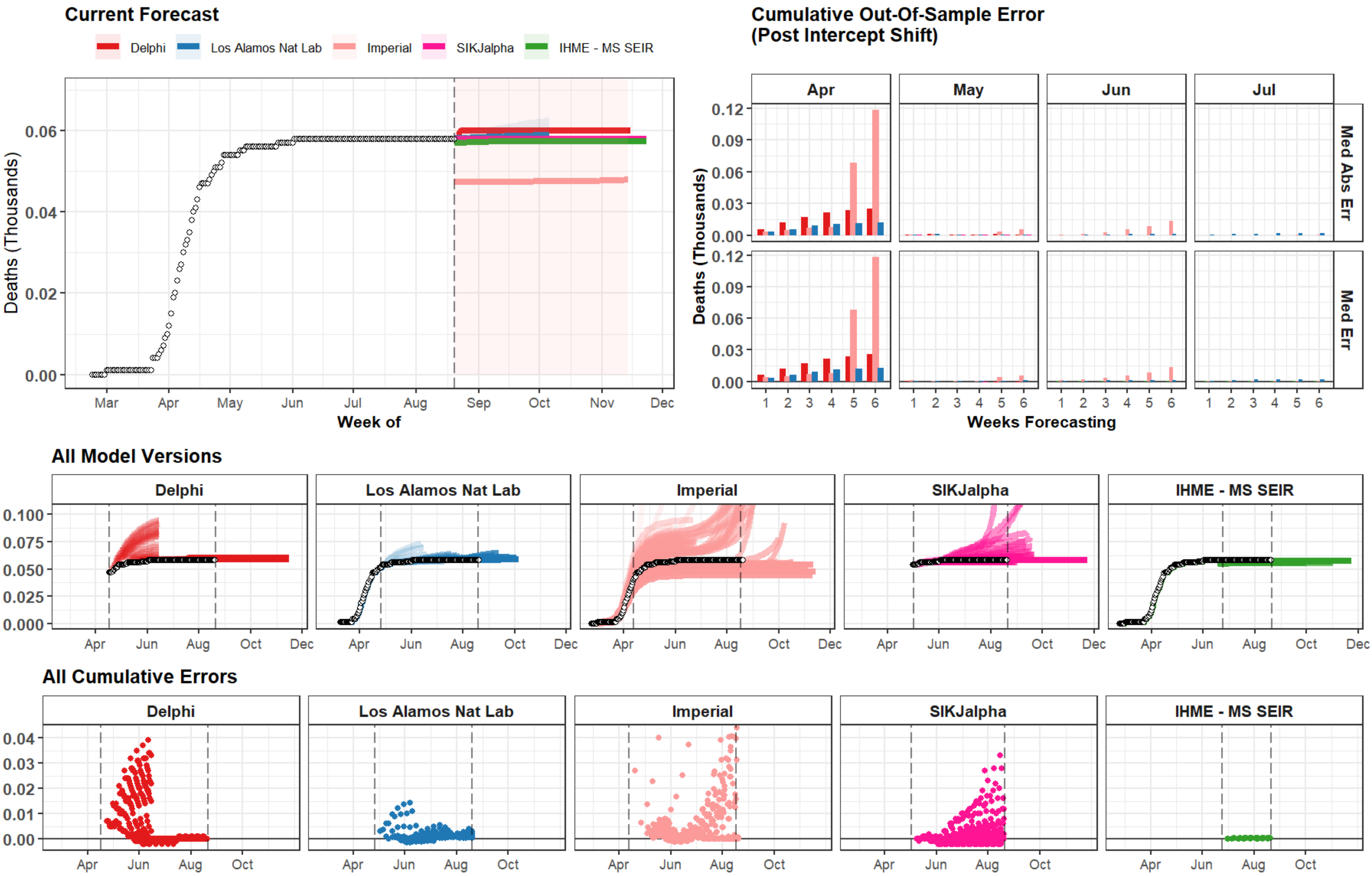
Current Forecast
Delphi
Los Alamos Nat Lab
Imperial
SIKJalpha
IHME - MS SEIR
Deaths (Thousands)
Week of
Cumulative Out-Of-Sample Error
(Post Intercept Shift)
Apr
May
Jun
Jul
Med Abs Err
Med Err
Weeks Forecasting
All Model Versions
All Cumulative Errors

Vermont

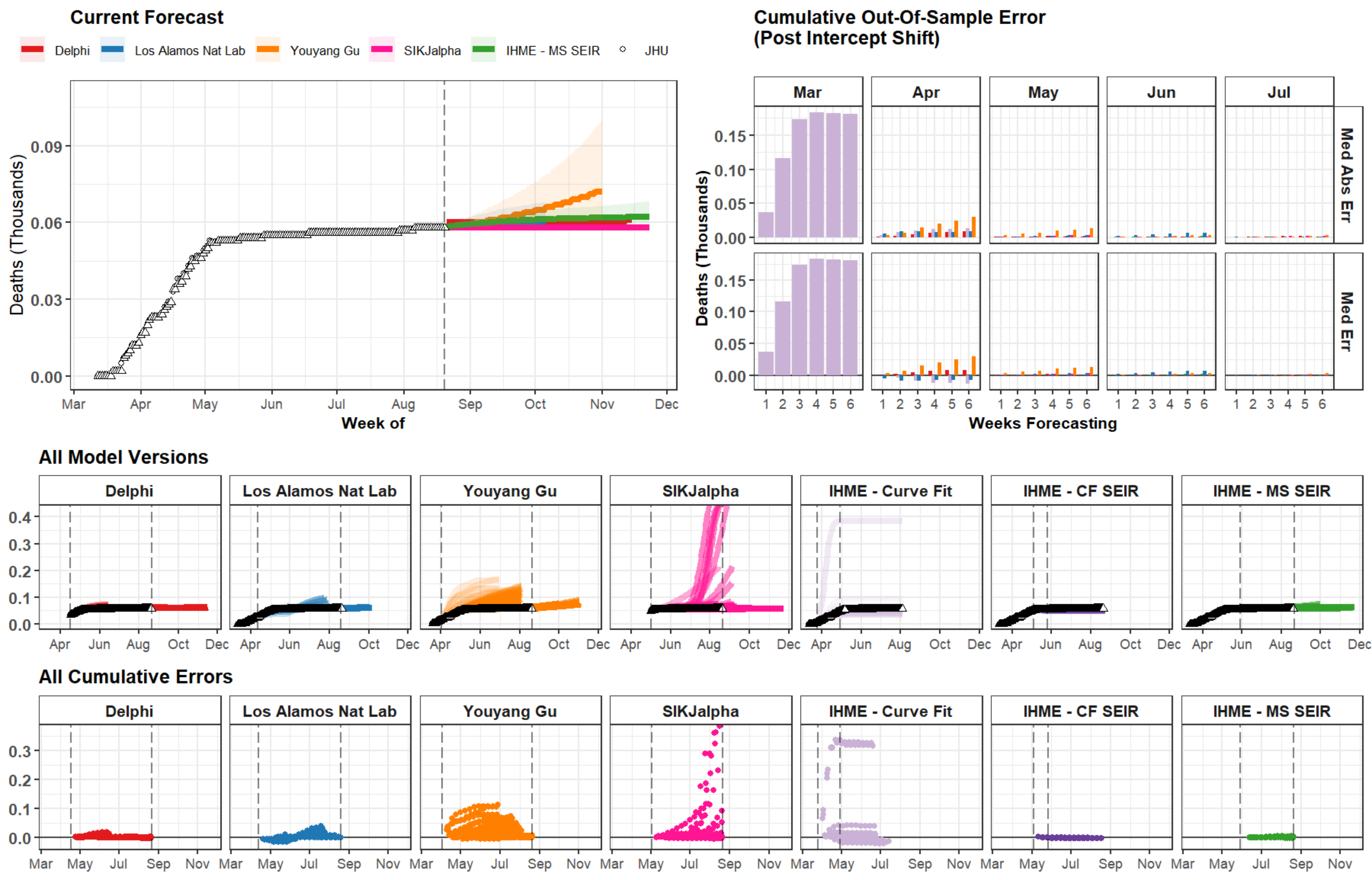

Current Forecast
Delphi
Los Alamos Nat Lab
Youyang Gu
SIKJalpha
IHME - MS SEIR
JHU
Deaths (Thousands)
0.09
0.06
0.03
0.00
Mar
Apr
May
Jun
Jul
Aug
Sep
Oct
Nov
Dec
Week of
Cumulative Out-Of-Sample Error
(Post Intercept Shift)
Mar
Apr
May
Jun
Jul
0.15
0.10
0.05
0.00
Med Abs Err
Med Err
1 2 3 4 5 6
Weeks Forecasting
All Model Versions
Delphi
Los Alamos Nat Lab
Youyang Gu
SIKJalpha
IHME - Curve Fit
IHME - CF SEIR
IHME - MS SEIR
0.4
0.3
0.2
0.1
0.0
Apr
Jun
Aug
Oct
Dec
All Cumulative Errors
0.3
0.2
0.1
0.0
Mar
May
Jul
Sep
Nov

Burkina Faso

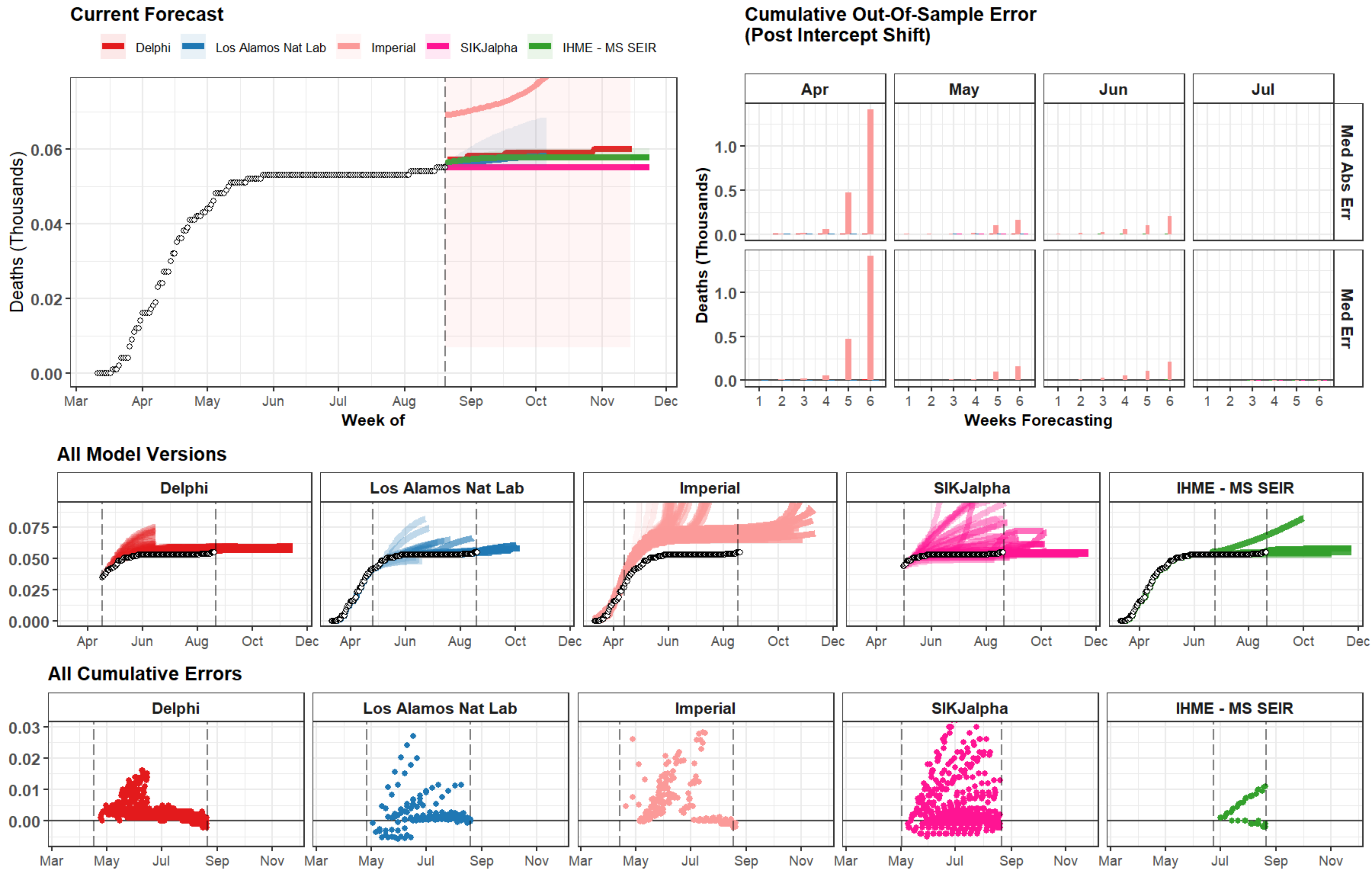

Suriname

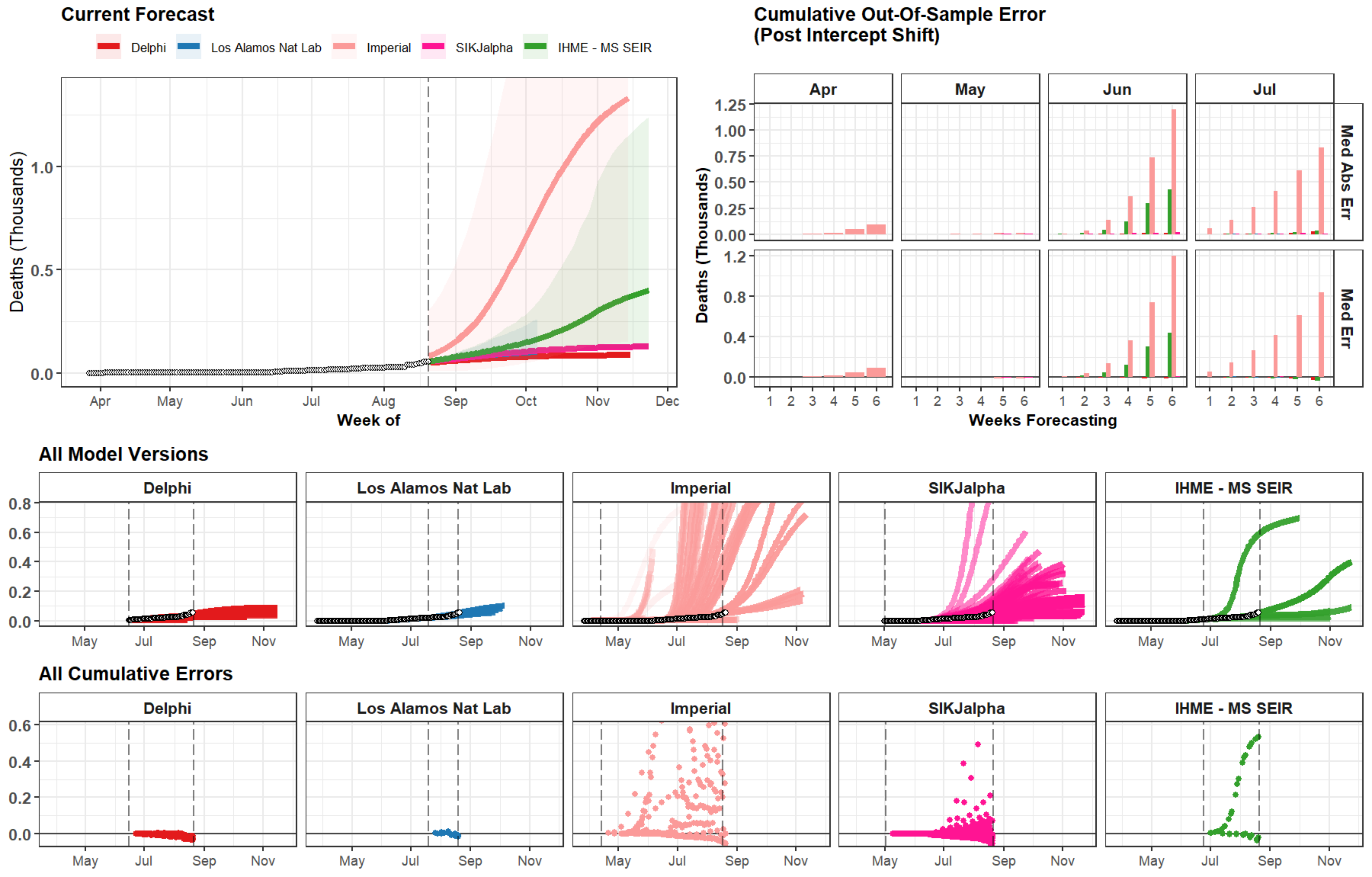
Current Forecast
Delphi
Los Alamos Nat Lab
Imperial
SIKJalpha
IHME - MS SEIR
Deaths (Thousands)
Week of
Apr
May
Jun
Jul
Aug
Sep
Oct
Nov
Dec
Cumulative Out-Of-Sample Error
(Post Intercept Shift)
Med Abs Err
Med Err
Weeks Forecasting
All Model Versions
All Cumulative Errors

Andorra

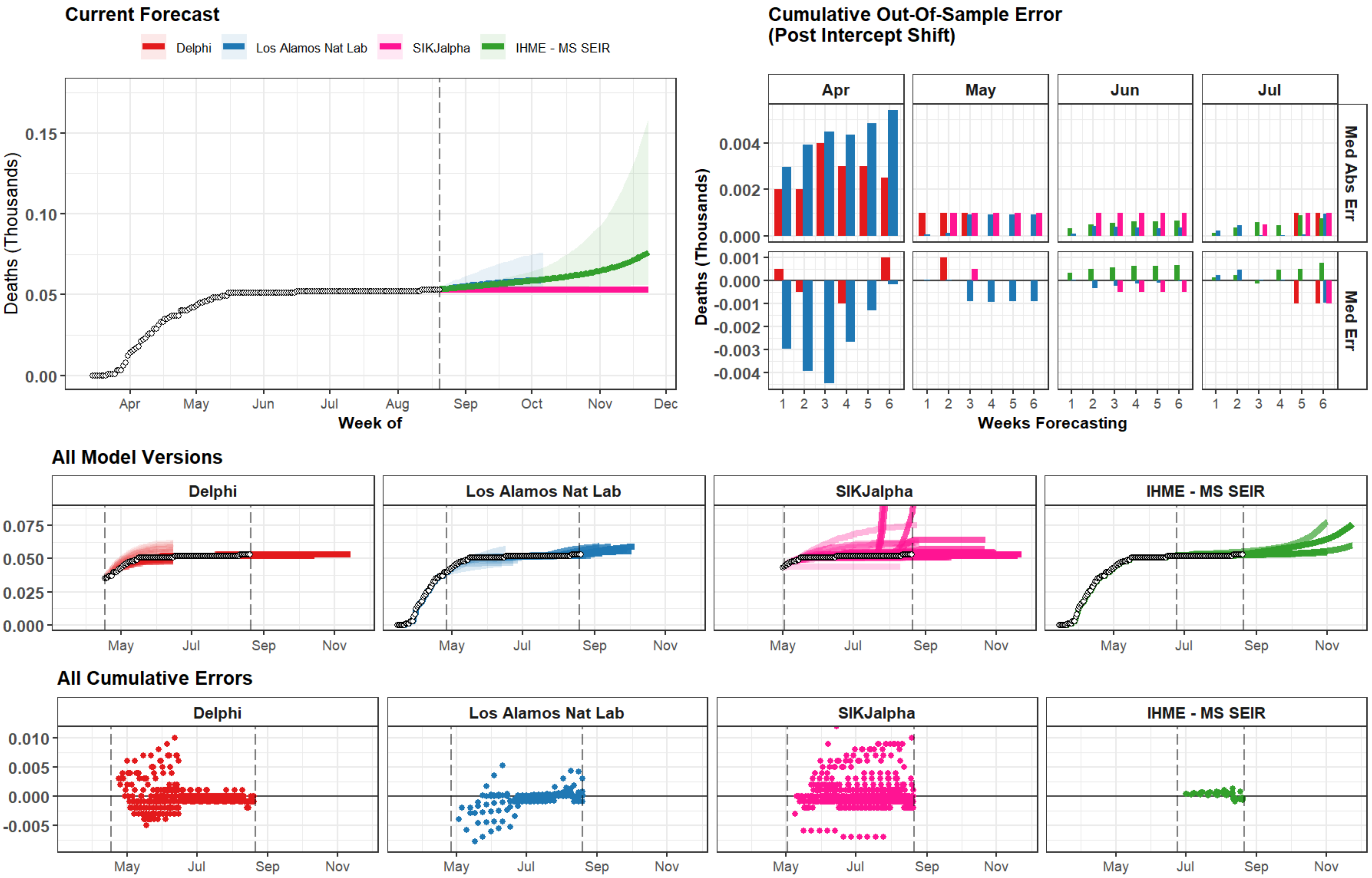

Current Forecast
Delphi
Los Alamos Nat Lab
SIKJalpha
IHME - MS SEIR
Deaths (Thousands)
0.15
0.10
0.05
0.00
Apr
May
Jun
Jul
Aug
Sep
Oct
Nov
Dec
Week of
Cumulative Out-Of-Sample Error
(Post Intercept Shift)
Apr
May
Jun
Jul
Med Abs Err
Med Err
0.004
0.002
0.000
0.001
0.000
-0.001
-0.002
-0.003
-0.004
1 2 3 4 5 6
Weeks Forecasting
All Model Versions
Delphi
Los Alamos Nat Lab
SIKJalpha
IHME - MS SEIR
0.075
0.050
0.025
0.000
May
Jul
Sep
Nov
All Cumulative Errors
Delphi
Los Alamos Nat Lab
SIKJalpha
IHME - MS SEIR
0.010
0.005
0.000
-0.005
May
Jul
Sep
Nov

Gabon

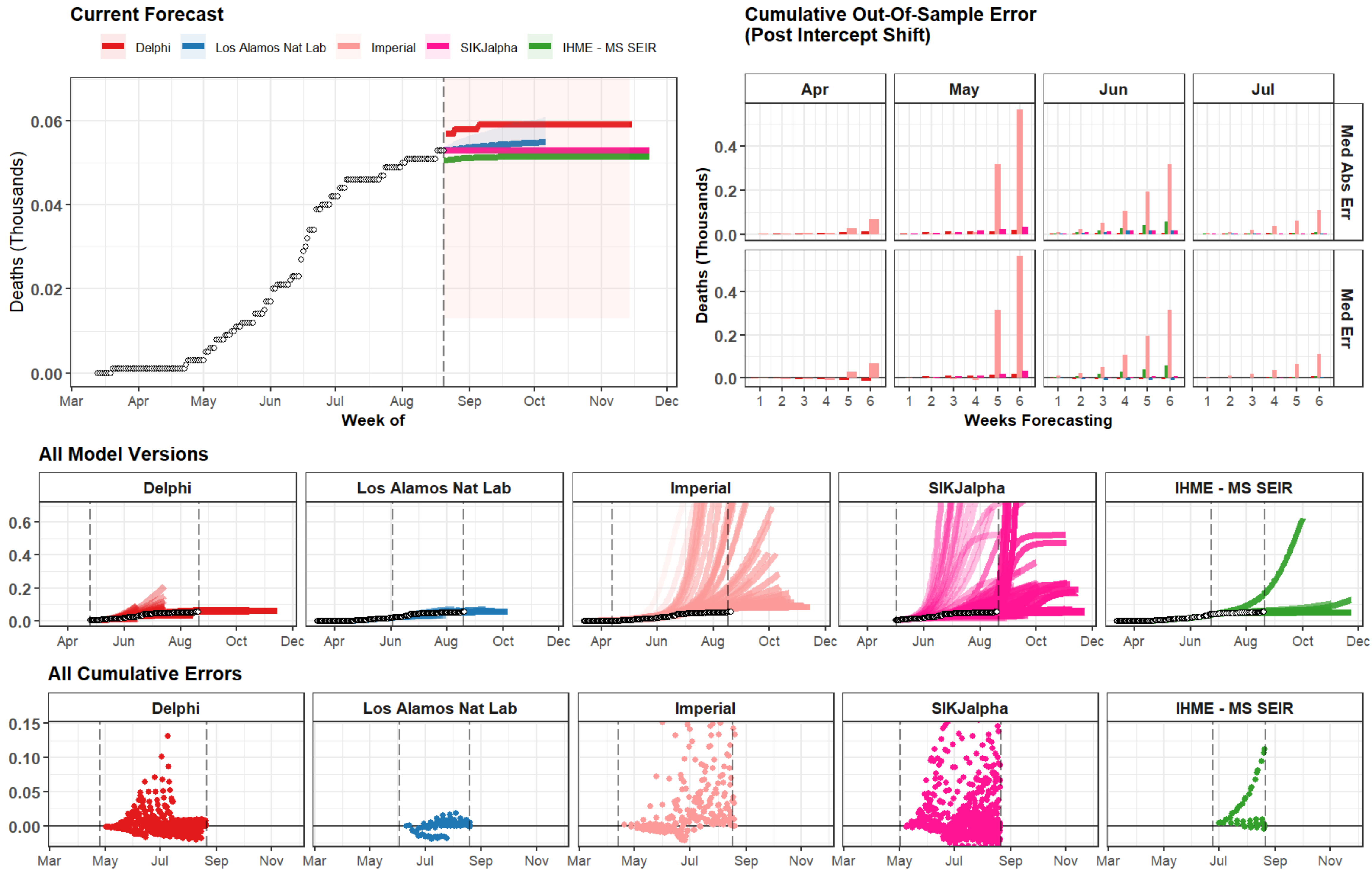

Current Forecast
Delphi
Los Alamos Nat Lab
Imperial
SIKJalpha
IHME - MS SEIR
Deaths (Thousands)
0.06
0.04
0.02
0.00
Mar
Apr
May
Jun
Jul
Aug
Sep
Oct
Nov
Dec
Week of
Cumulative Out-Of-Sample Error
(Post Intercept Shift)
Apr
May
Jun
Jul
Med Abs Err
Med Err
Deaths (Thousands)
0.4
0.2
0.0
1
2
3
4
5
6
Weeks Forecasting
All Model Versions
Delphi
Los Alamos Nat Lab
Imperial
SIKJalpha
IHME - MS SEIR
0.6
0.4
0.2
0.0
Apr
Jun
Aug
Oct
Dec
All Cumulative Errors
Delphi
Los Alamos Nat Lab
Imperial
SIKJalpha
IHME - MS SEIR
0.15
0.10
0.05
0.00
Mar
May
Jul
Sep
Nov

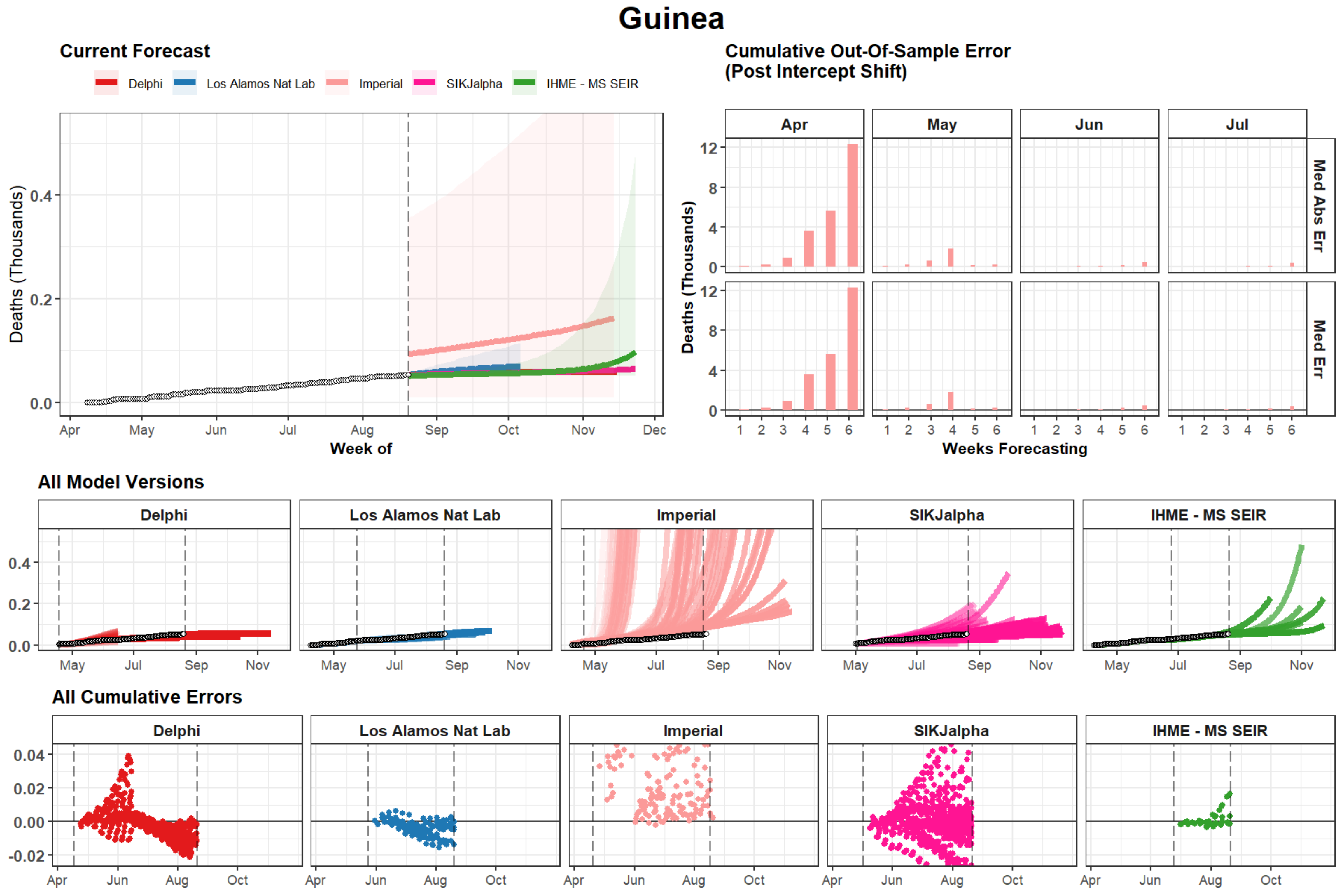
Guinea
Current Forecast
Delphi
Los Alamos Nat Lab
Imperial
SIKJalpha
IHME - MS SEIR
Deaths (Thousands)
Week of
Cumulative Out-Of-Sample Error
(Post Intercept Shift)
Apr
May
Jun
Jul
Med Abs Err
Med Err
Weeks Forecasting
All Model Versions
All Cumulative Errors

South Sudan

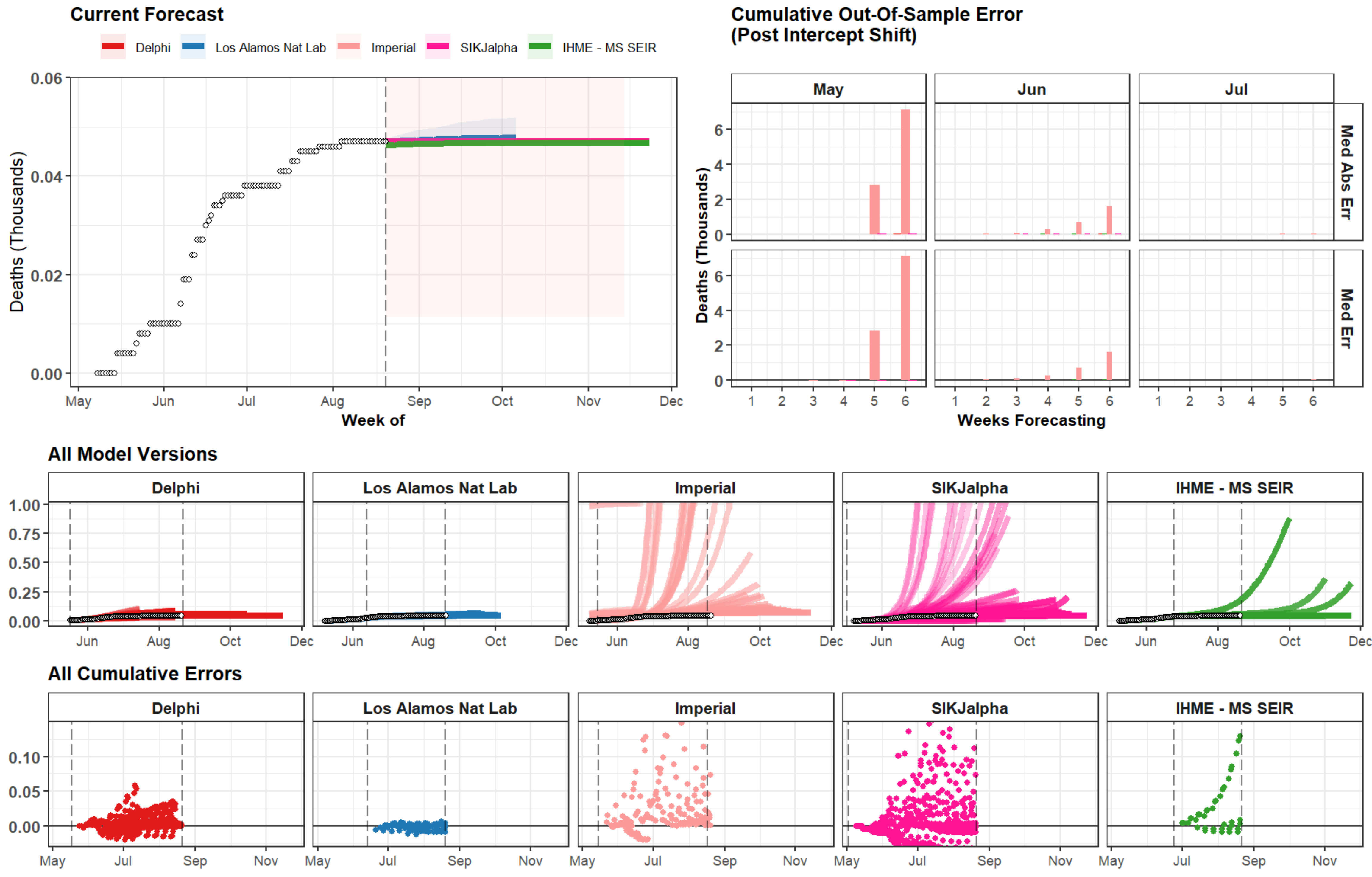
Current Forecast
Delphi
Los Alamos Nat Lab
Imperial
SIKJalpha
IHME - MS SEIR
Deaths (Thousands)
0.06
0.04
0.02
0.00
May
Jun
Jul
Aug
Sep
Oct
Nov
Dec
Week of
Cumulative Out-Of-Sample Error
(Post Intercept Shift)
May
Jun
Jul
Med Abs Err
Med Err
6
4
2
0
1
2
3
4
5
6
Weeks Forecasting
All Model Versions
1.00
0.75
0.50
0.25
0.00
All Cumulative Errors
0.10
0.05
0.00

Hawaii

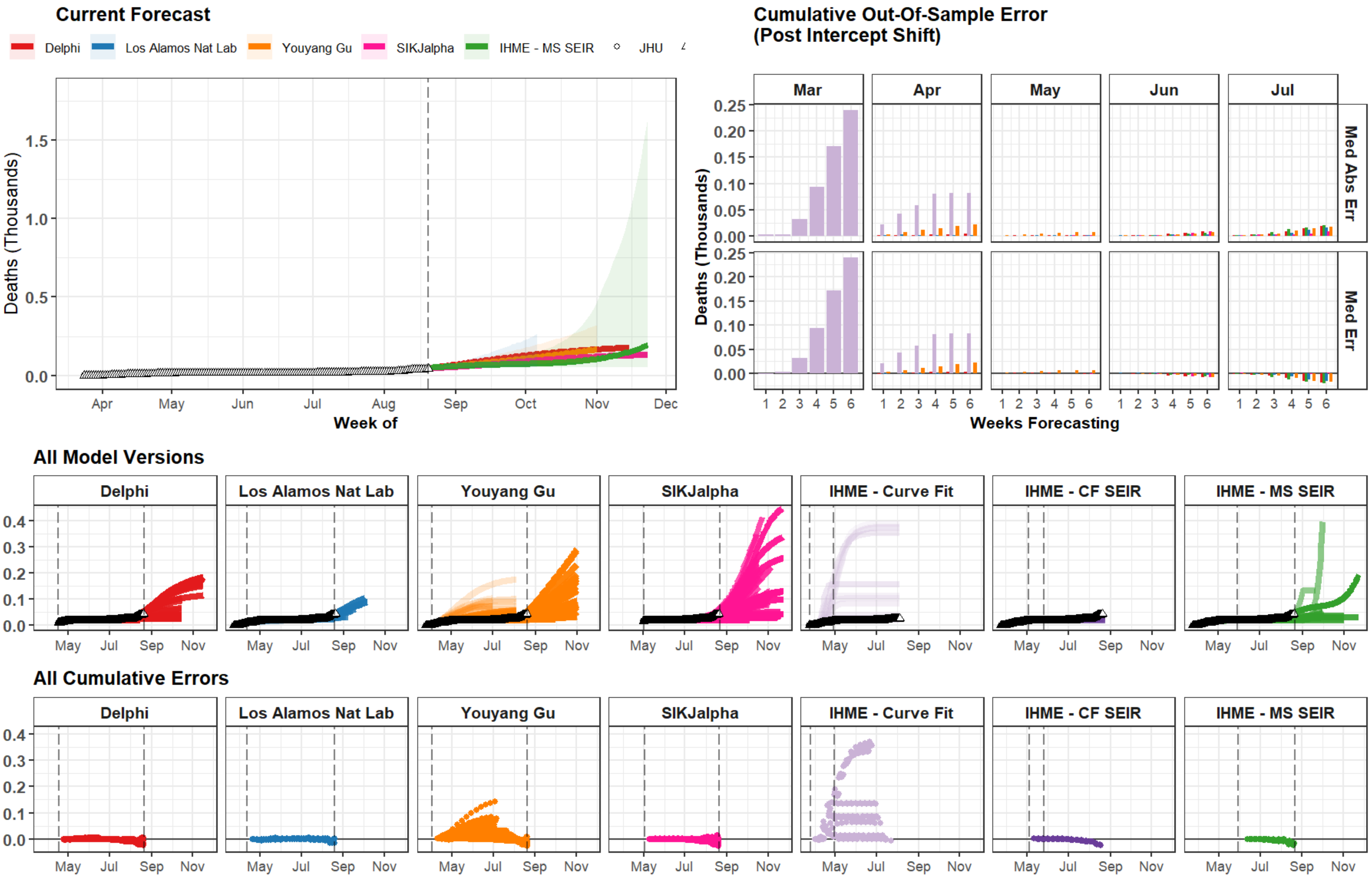
Current Forecast
Delphi
Los Alamos Nat Lab
Youyang Gu
SIKJalpha
IHME - MS SEIR
JHU
Deaths (Thousands)
Week of
Cumulative Out-Of-Sample Error
(Post Intercept Shift)
Mar
Apr
May
Jun
Jul
Med Abs Err
Med Err
Weeks Forecasting
All Model Versions
IHME - Curve Fit
IHME - CF SEIR
All Cumulative Errors

Namibia

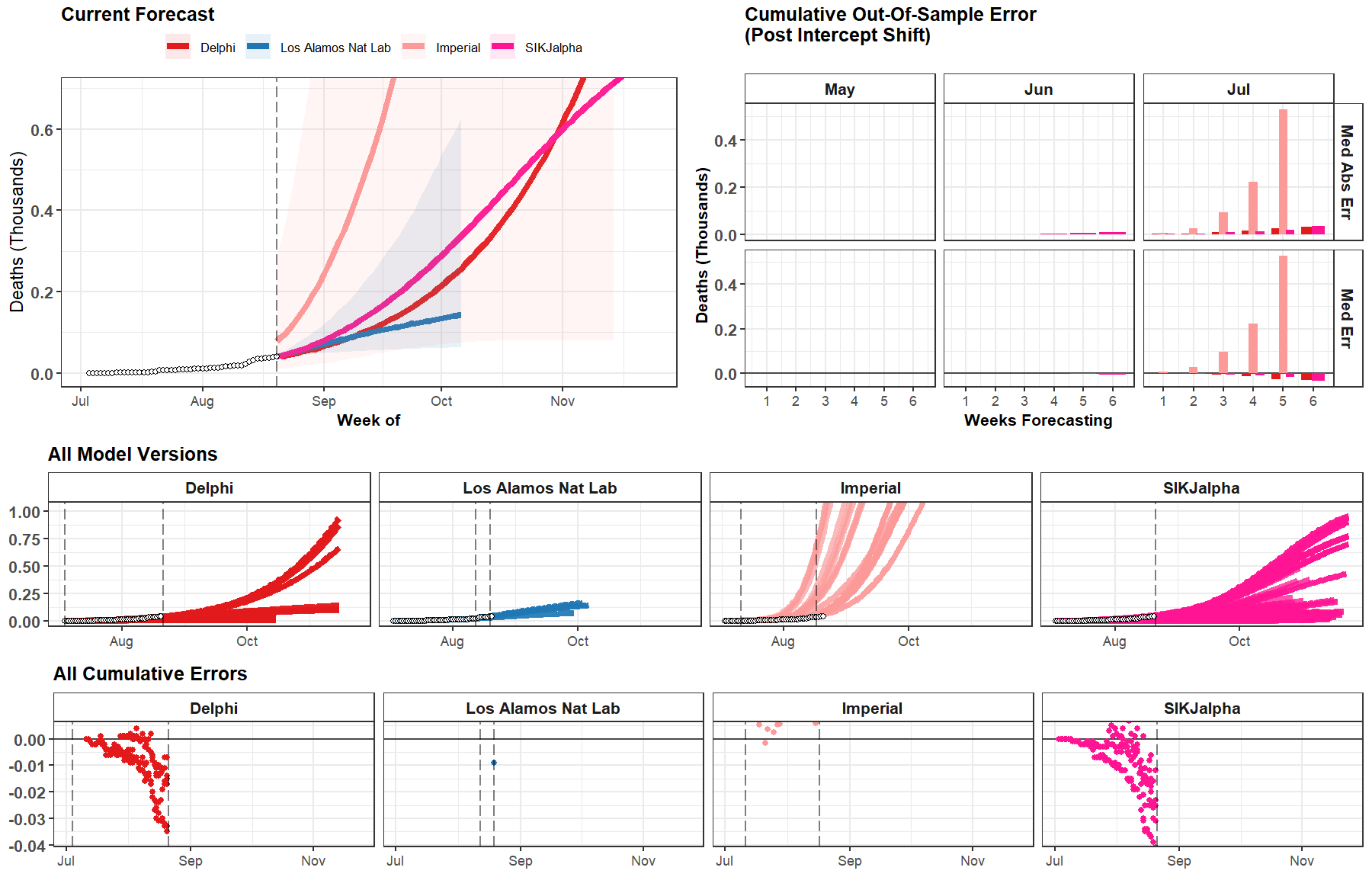

Current Forecast
Delphi
Los Alamos Nat Lab
Imperial
SIKJalpha
Deaths (Thousands)
0.0
0.2
0.4
0.6
Jul
Aug
Sep
Oct
Nov
Week of
Cumulative Out-Of-Sample Error
(Post Intercept Shift)
May
Jun
Jul
Med Abs Err
Med Err
Deaths (Thousands)
0.0
0.2
0.4
1
2
3
4
5
6
Weeks Forecasting
All Model Versions
Delphi
Los Alamos Nat Lab
Imperial
SIKJalpha
0.00
0.25
0.50
0.75
1.00
Aug
Oct
All Cumulative Errors
Delphi
Los Alamos Nat Lab
Imperial
SIKJalpha
0.00
-0.01
-0.02
-0.03
-0.04
Jul
Sep
Nov

Uruguay

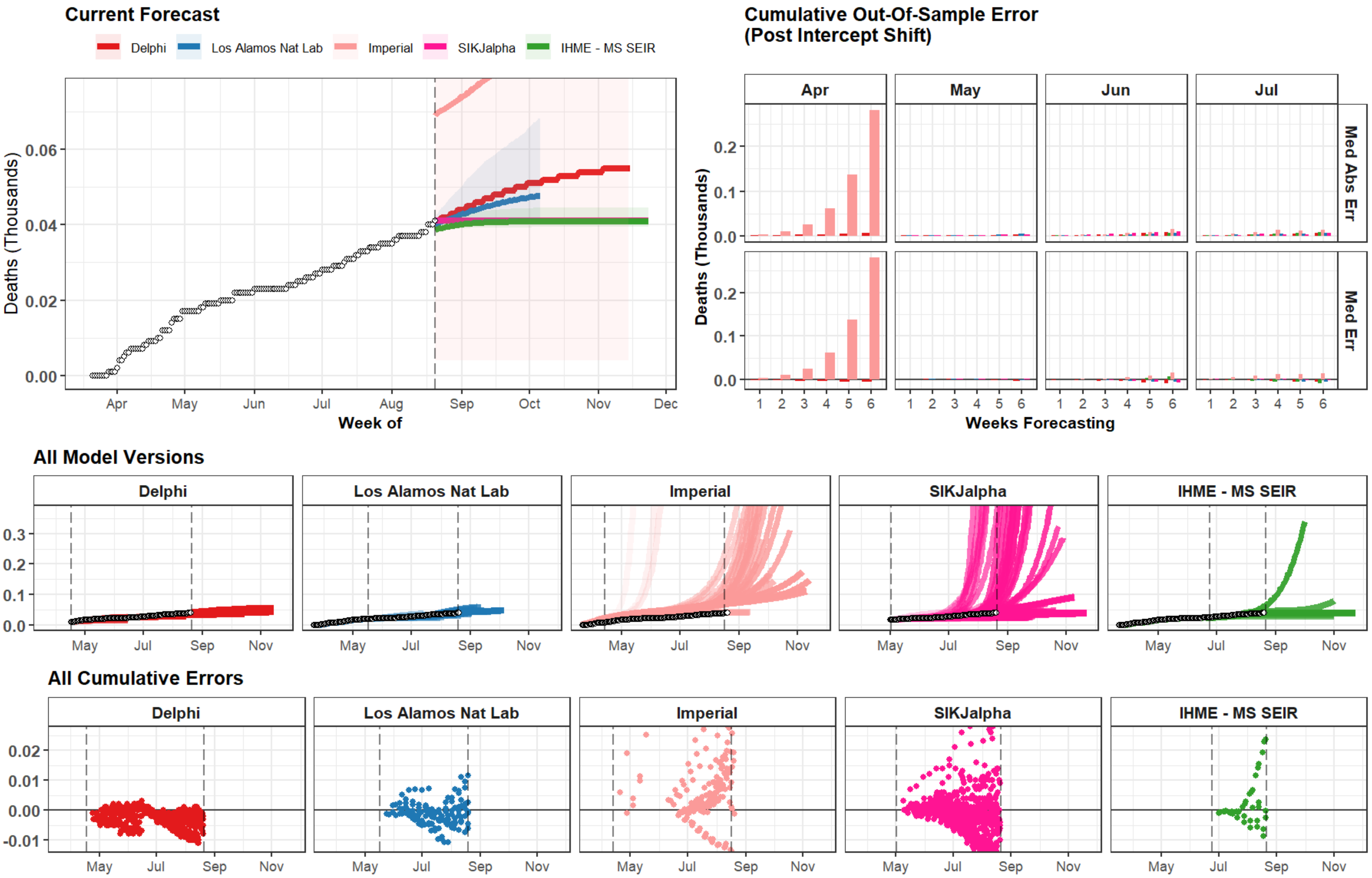
Current Forecast
Delphi
Los Alamos Nat Lab
Imperial
SIKJalpha
IHME - MS SEIR
Deaths (Thousands)
0.06
0.04
0.02
0.00
Apr
May
Jun
Jul
Aug
Sep
Oct
Nov
Dec
Week of
Cumulative Out-Of-Sample Error
(Post Intercept Shift)
Apr
May
Jun
Jul
Med Abs Err
Med Err
Deaths (Thousands)
0.2
0.1
0.0
1 2 3 4 5 6
Weeks Forecasting
All Model Versions
Delphi
Los Alamos Nat Lab
Imperial
SIKJalpha
IHME - MS SEIR
0.3
0.2
0.1
0.0
May
Jul
Sep
Nov
All Cumulative Errors
Delphi
Los Alamos Nat Lab
Imperial
SIKJalpha
IHME - MS SEIR
0.02
0.01
0.00
-0.01
May
Jul
Sep
Nov

Benin

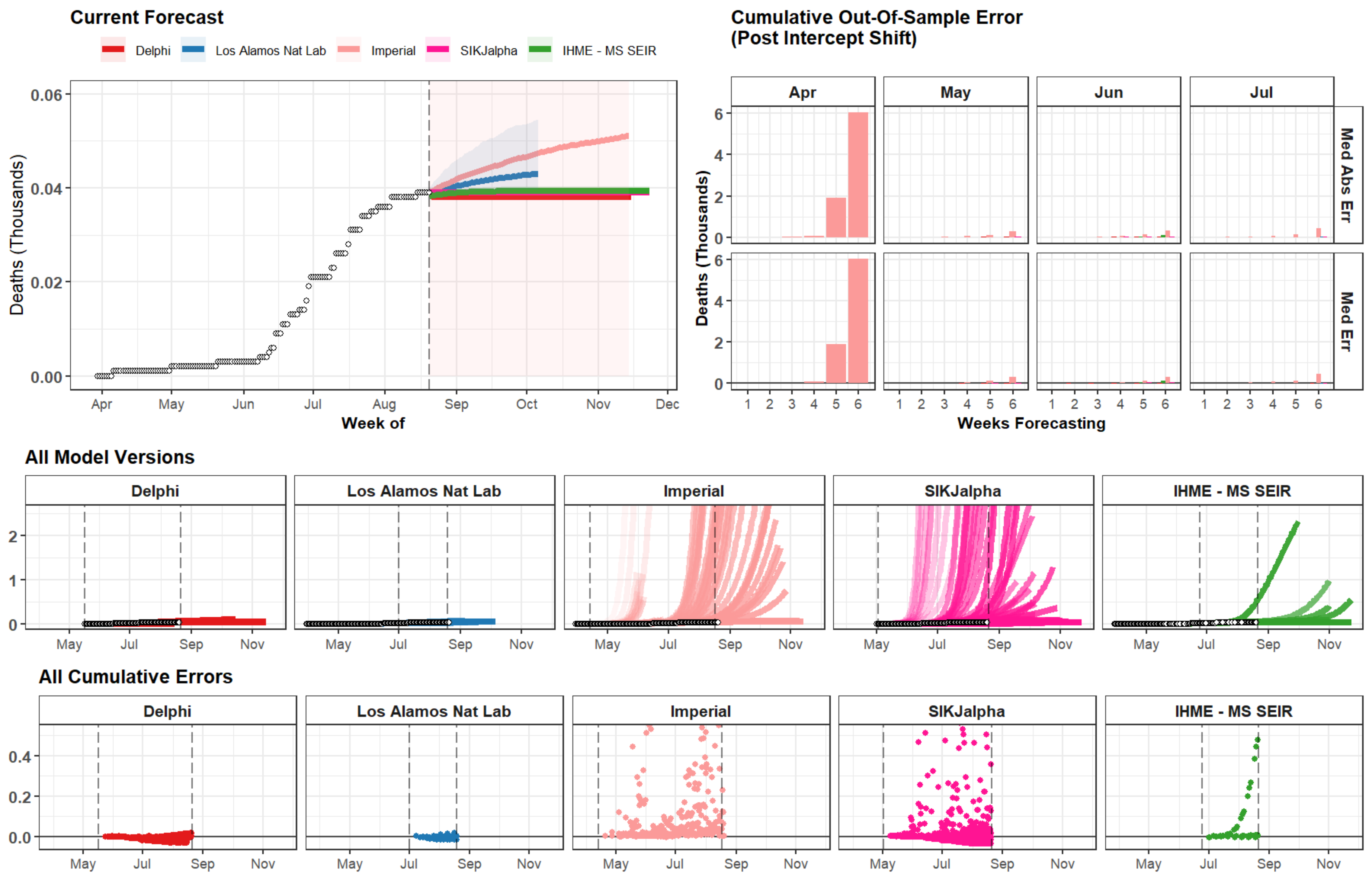
Current Forecast
Delphi
Los Alamos Nat Lab
Imperial
SIKJalpha
IHME - MS SEIR
0.06
0.04
0.02
0.00
Deaths (Thousands)
Apr
May
Jun
Jul
Aug
Sep
Oct
Nov
Dec
Week of
Cumulative Out-Of-Sample Error
(Post Intercept Shift)
Apr
May
Jun
Jul
Med Abs Err
Med Err
6
4
2
0
Deaths (Thousands)
1
2
3
4
5
6
Weeks Forecasting
All Model Versions
Delphi
Los Alamos Nat Lab
Imperial
SIKJalpha
IHME - MS SEIR
2
1
0
May
Jul
Sep
Nov
All Cumulative Errors
Delphi
Los Alamos Nat Lab
Imperial
SIKJalpha
IHME - MS SEIR
0.4
0.2
0.0
May
Jul
Sep
Nov

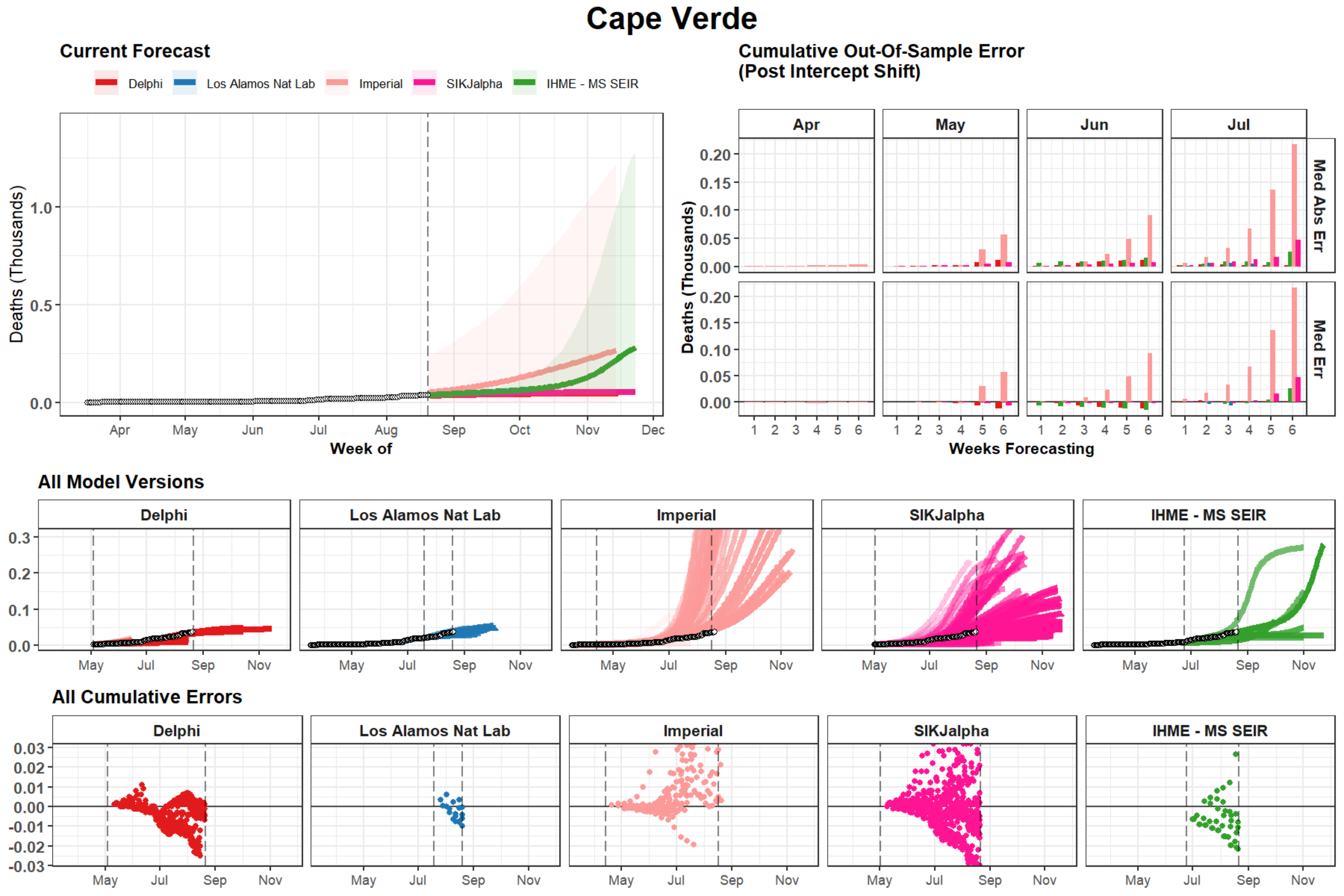

Cape Verde
Current Forecast
Delphi
Los Alamos Nat Lab
Imperial
SIKJalpha
IHME - MS SEIR
Deaths (Thousands)
Week of
Apr
May
Jun
Jul
Aug
Sep
Oct
Nov
Dec
Cumulative Out-Of-Sample Error
(Post Intercept Shift)
Med Abs Err
Med Err
Weeks Forecasting
All Model Versions
All Cumulative Errors

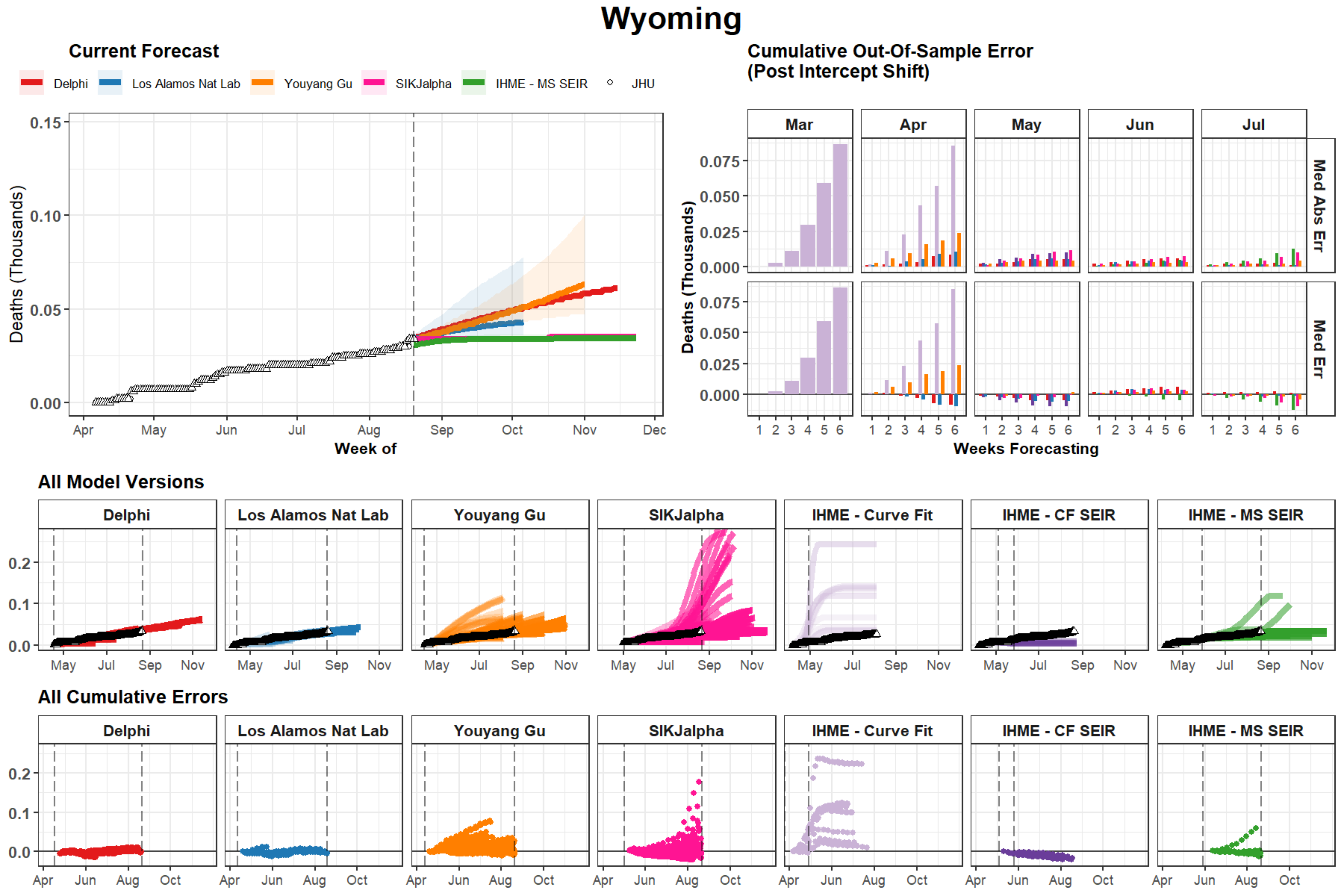
Wyoming
Current Forecast
Delphi
Los Alamos Nat Lab
Youyang Gu
SIKJalpha
IHME - MS SEIR
JHU
Deaths (Thousands)
0.15
0.10
0.05
0.00
Apr
May
Jun
Jul
Aug
Sep
Oct
Nov
Dec
Week of
Cumulative Out-Of-Sample Error
(Post Intercept Shift)
Mar
Apr
May
Jun
Jul
Med Abs Err
Med Err
0.075
0.050
0.025
0.000
1 2 3 4 5 6
Weeks Forecasting
All Model Versions
Delphi
Los Alamos Nat Lab
Youyang Gu
SIKJalpha
IHME - Curve Fit
IHME - CF SEIR
IHME - MS SEIR
0.2
0.1
0.0
May
Jul
Sep
Nov
All Cumulative Errors
Apr
Jun
Aug
Oct

Guinea-Bissau

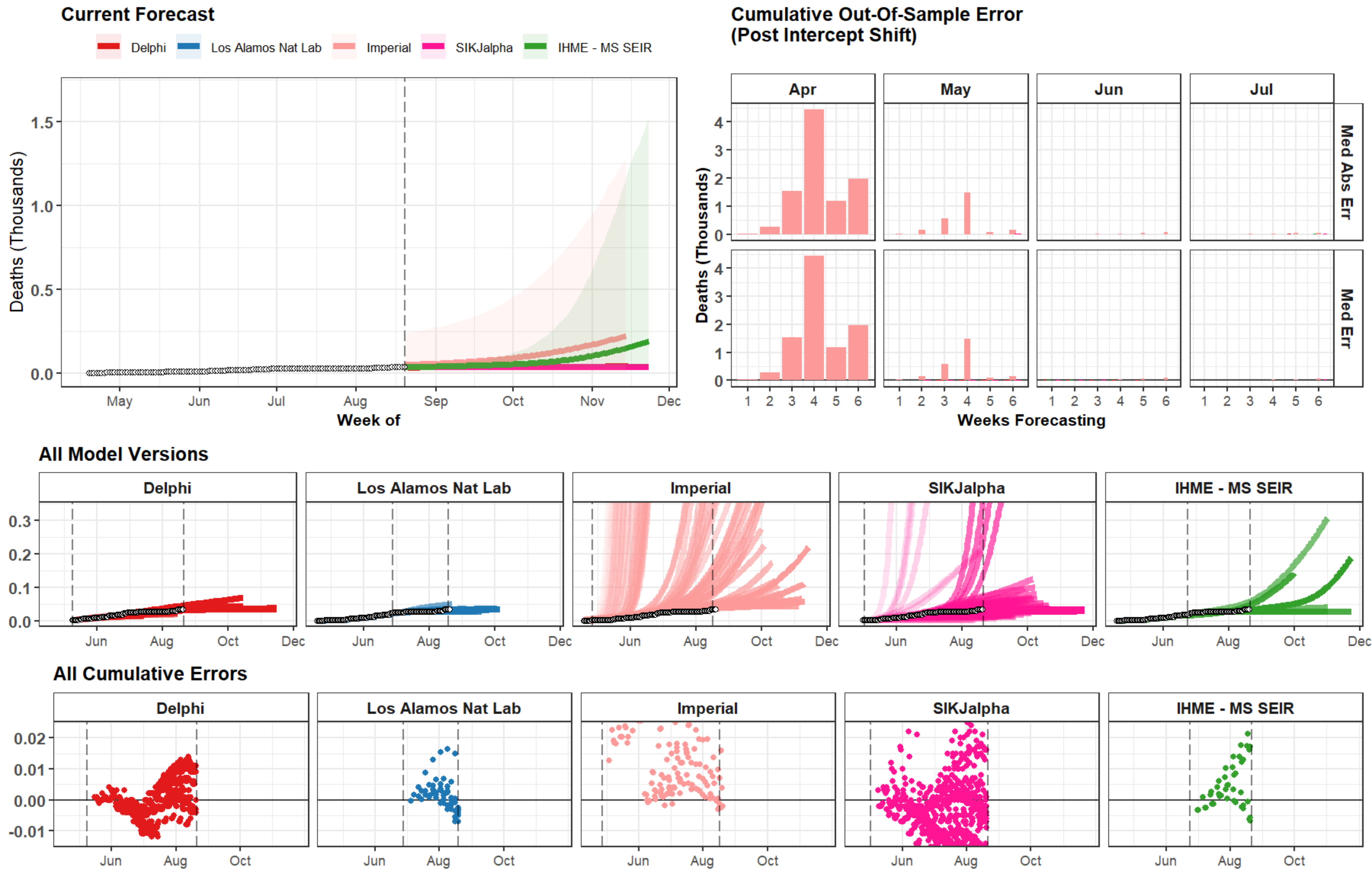

Latvia

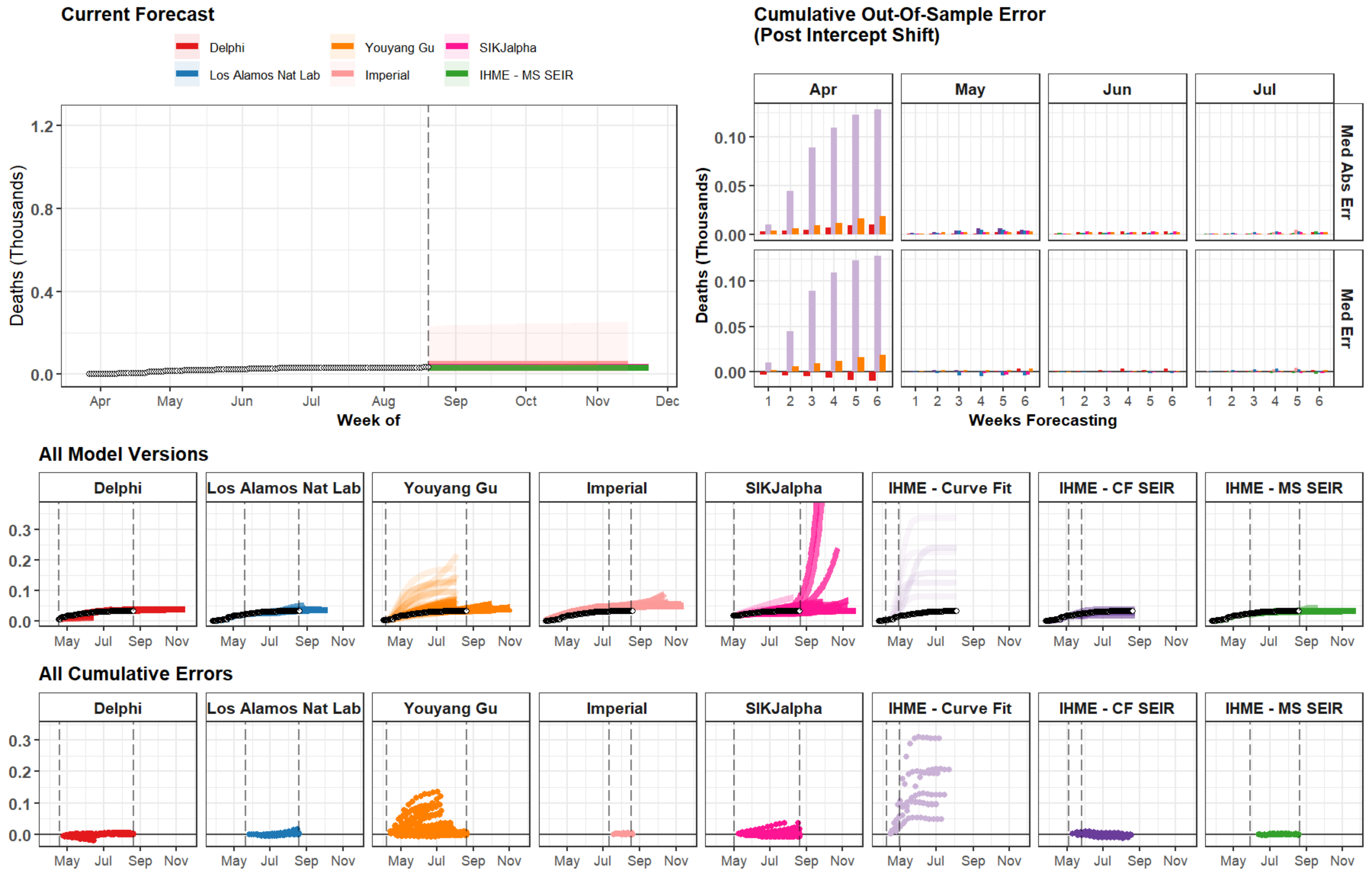
Current Forecast
Delphi
Los Alamos Nat Lab
Youyang Gu
Imperial
SIKJalpha
IHME - MS SEIR
Deaths (Thousands)
Week of
Cumulative Out-Of-Sample Error
(Post Intercept Shift)
Apr
May
Jun
Jul
Med Abs Err
Med Err
Weeks Forecasting
All Model Versions
Delphi
Los Alamos Nat Lab
Youyang Gu
Imperial
SIKJalpha
IHME - Curve Fit
IHME - CF SEIR
IHME - MS SEIR
All Cumulative Errors

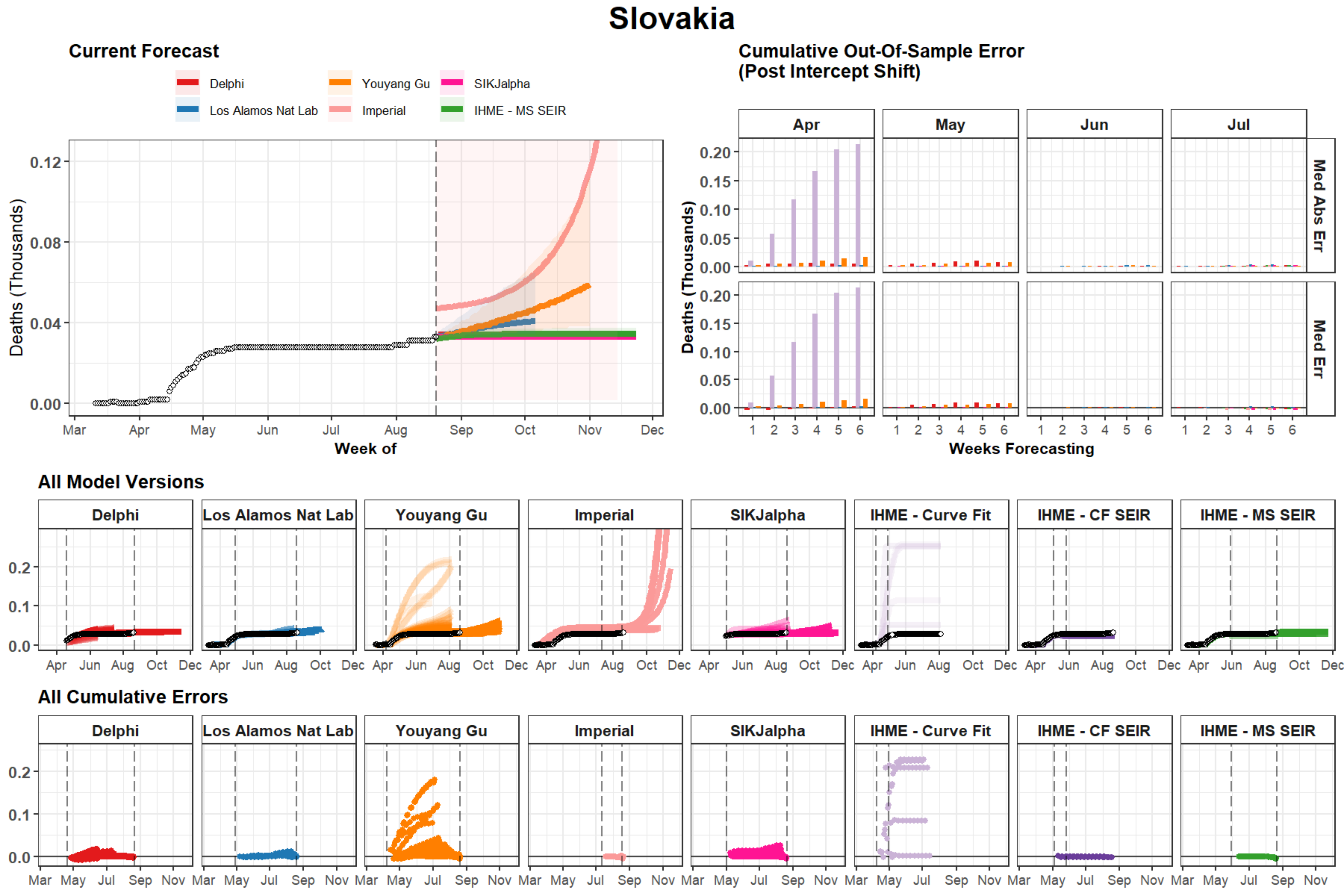
Slovakia
Current Forecast
Delphi
Los Alamos Nat Lab
Youyang Gu
Imperial
SIKJalpha
IHME - MS SEIR
Deaths (Thousands)
Week of
Cumulative Out-Of-Sample Error
(Post Intercept Shift)
Apr
May
Jun
Jul
Med Abs Err
Med Err
Weeks Forecasting
All Model Versions
Delphi
Los Alamos Nat Lab
Youyang Gu
Imperial
SIKJalpha
IHME - Curve Fit
IHME - CF SEIR
IHME - MS SEIR
All Cumulative Errors

Lesotho

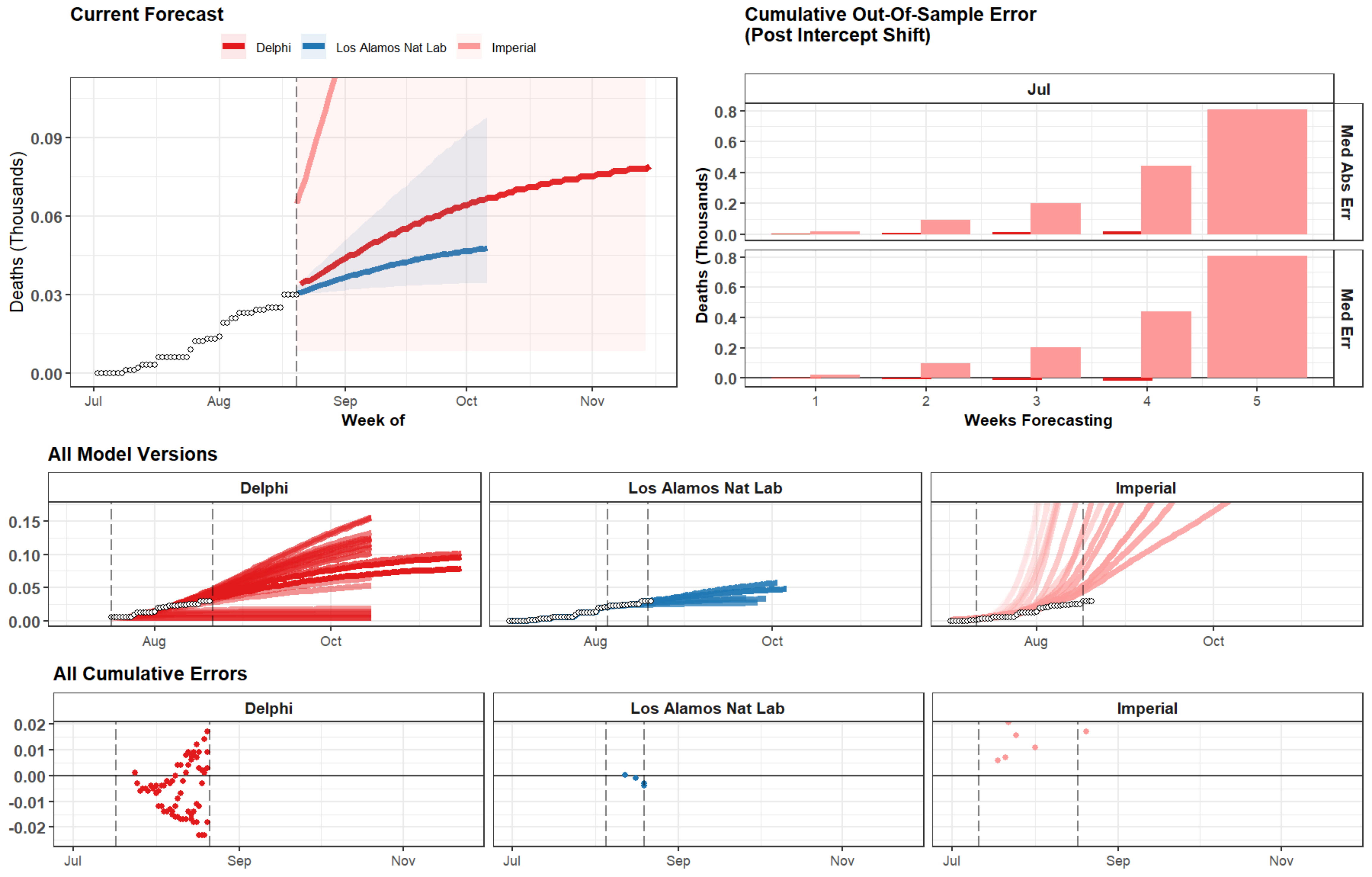

Current Forecast
Delphi
Los Alamos Nat Lab
Imperial
Deaths (Thousands)
0.00
0.03
0.06
0.09
Jul
Aug
Sep
Oct
Nov
Week of
Cumulative Out-Of-Sample Error
(Post Intercept Shift)
Jul
Med Abs Err
Med Err
0.0
0.2
0.4
0.6
0.8
1
2
3
4
5
Weeks Forecasting
All Model Versions
Delphi
Los Alamos Nat Lab
Imperial
0.00
0.05
0.10
0.15
Aug
Oct
All Cumulative Errors
Delphi
Los Alamos Nat Lab
Imperial
0.02
0.01
0.00
-0.01
-0.02
Jul
Sep
Nov

Guyana

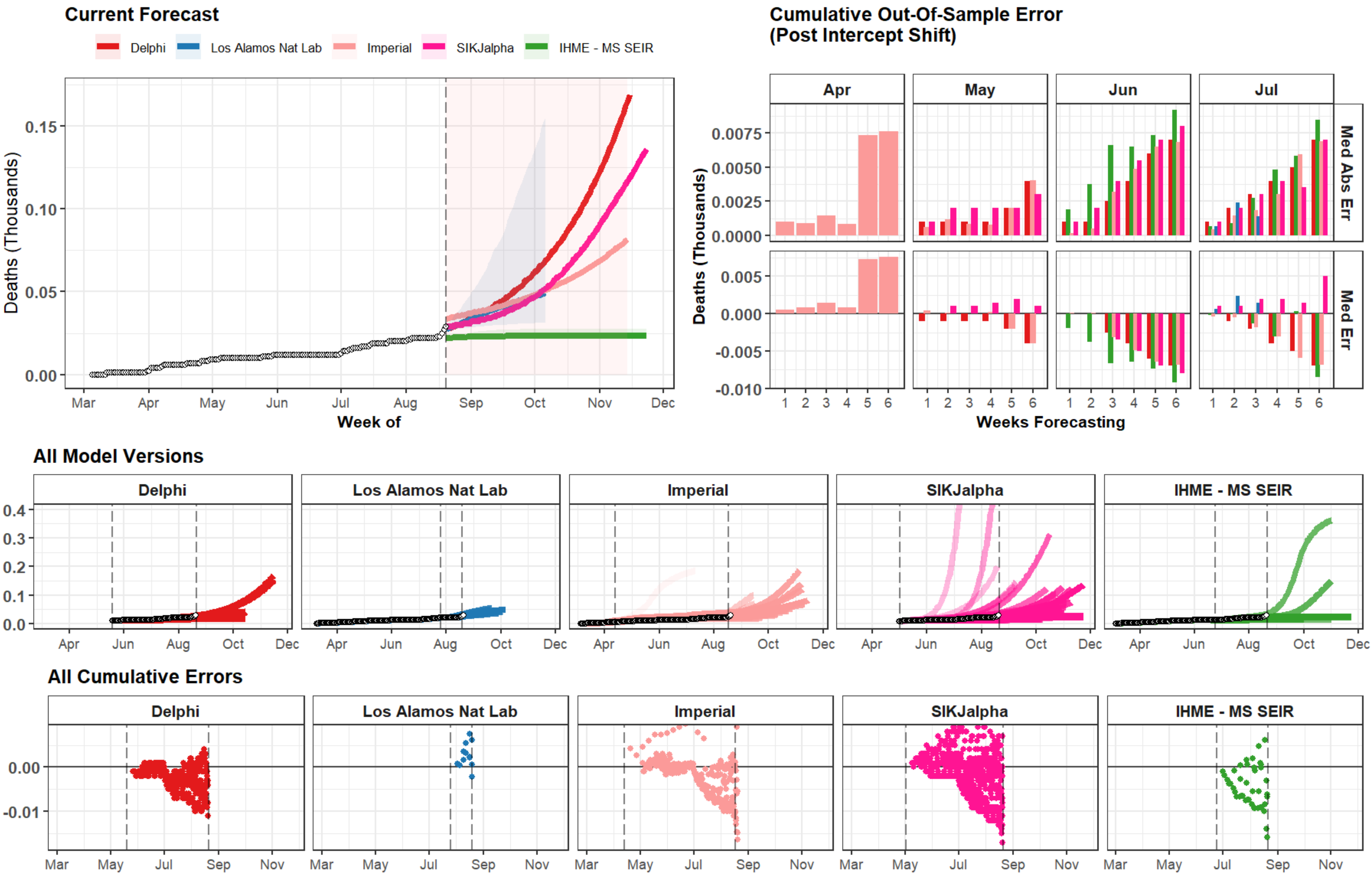

Current Forecast
Delphi
Los Alamos Nat Lab
Imperial
SIKJalpha
IHME - MS SEIR
Deaths (Thousands)
0.15
0.10
0.05
0.00
Mar
Apr
May
Jun
Jul
Aug
Sep
Oct
Nov
Dec
Week of
Cumulative Out-Of-Sample Error
(Post Intercept Shift)
Apr
May
Jun
Jul
Med Abs Err
Med Err
0.0075
0.0050
0.0025
0.0000
0.005
0.000
-0.005
-0.010
1
2
3
4
5
6
Weeks Forecasting
All Model Versions
0.4
0.3
0.2
0.1
0.0
All Cumulative Errors
0.00
-0.01

Alaska

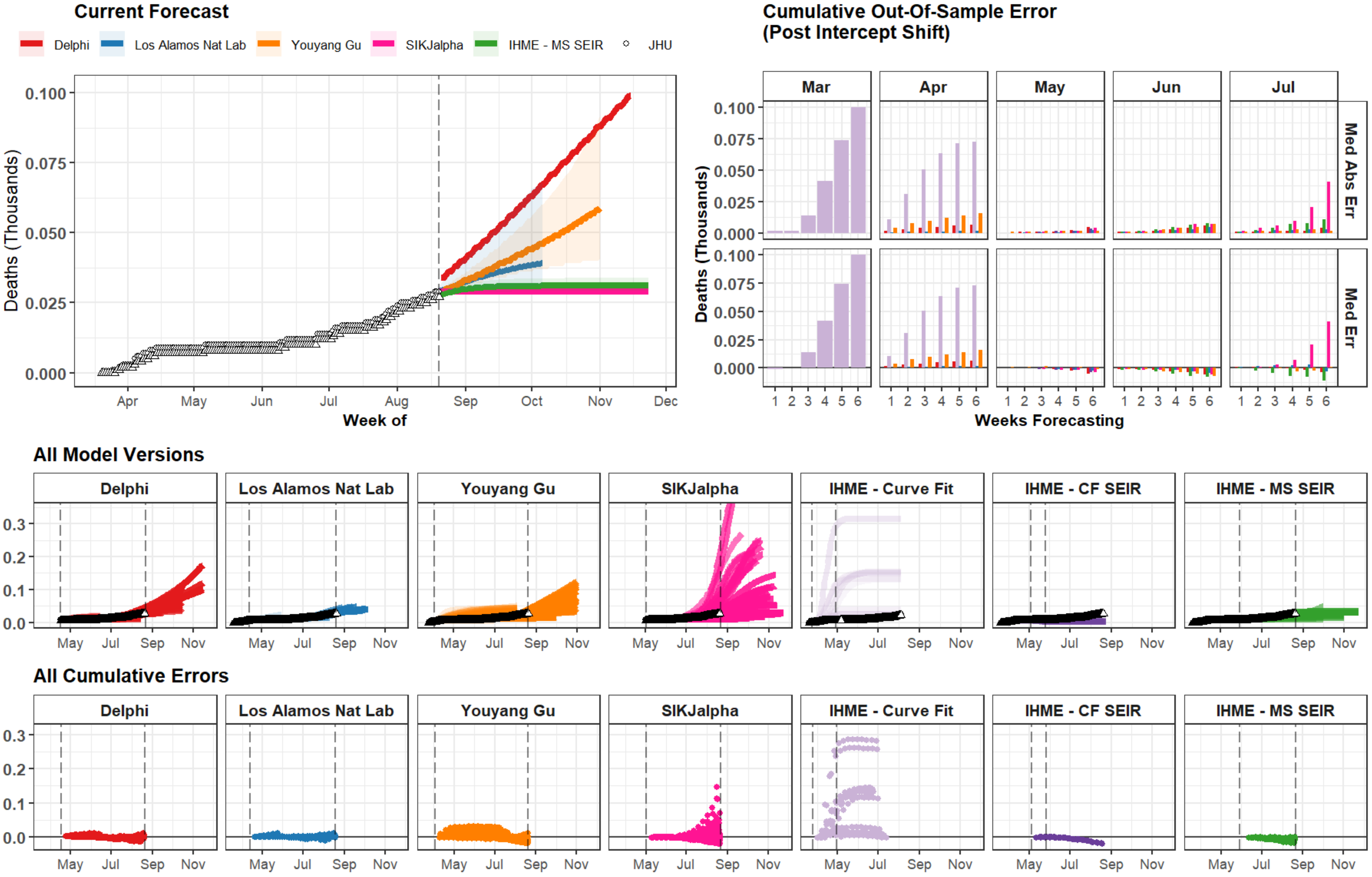

Current Forecast
Delphi
Los Alamos Nat Lab
Youyang Gu
SIKJalpha
IHME - MS SEIR
JHU
Deaths (Thousands)
0.100
0.075
0.050
0.025
0.000
Apr
May
Jun
Jul
Aug
Sep
Oct
Nov
Dec
Week of
Cumulative Out-Of-Sample Error
(Post Intercept Shift)
Mar
Apr
May
Jun
Jul
Med Abs Err
Med Err
Weeks Forecasting
All Model Versions
Delphi
Los Alamos Nat Lab
Youyang Gu
SIKJalpha
IHME - Curve Fit
IHME - CF SEIR
IHME - MS SEIR
0.3
0.2
0.1
0.0
All Cumulative Errors

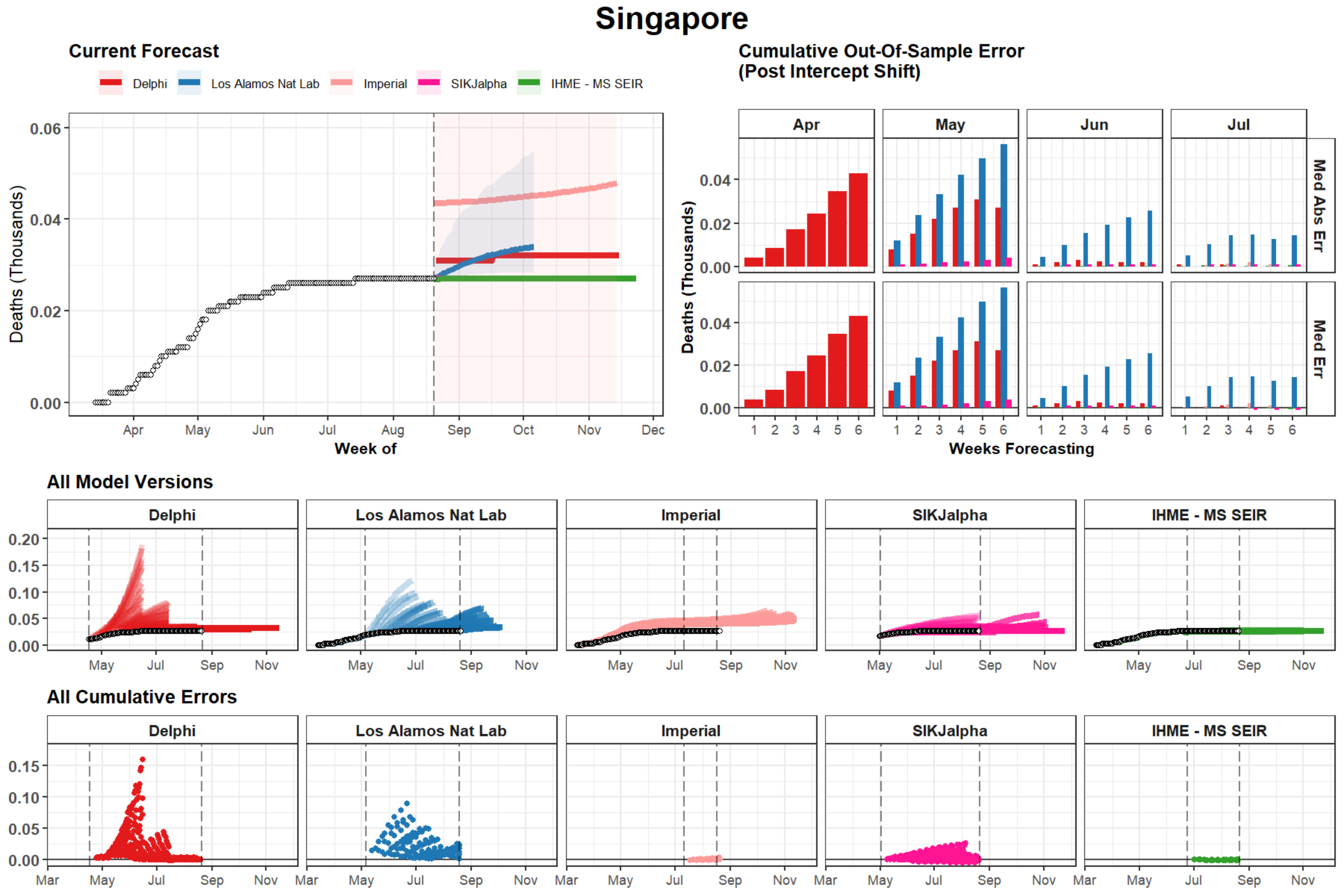

Singapore
Current Forecast
Delphi
Los Alamos Nat Lab
Imperial
SIKJalpha
IHME - MS SEIR
Deaths (Thousands)
Week of
Cumulative Out-Of-Sample Error
(Post Intercept Shift)
Apr
May
Jun
Jul
Med Abs Err
Med Err
Weeks Forecasting
All Model Versions
All Cumulative Errors

Togo

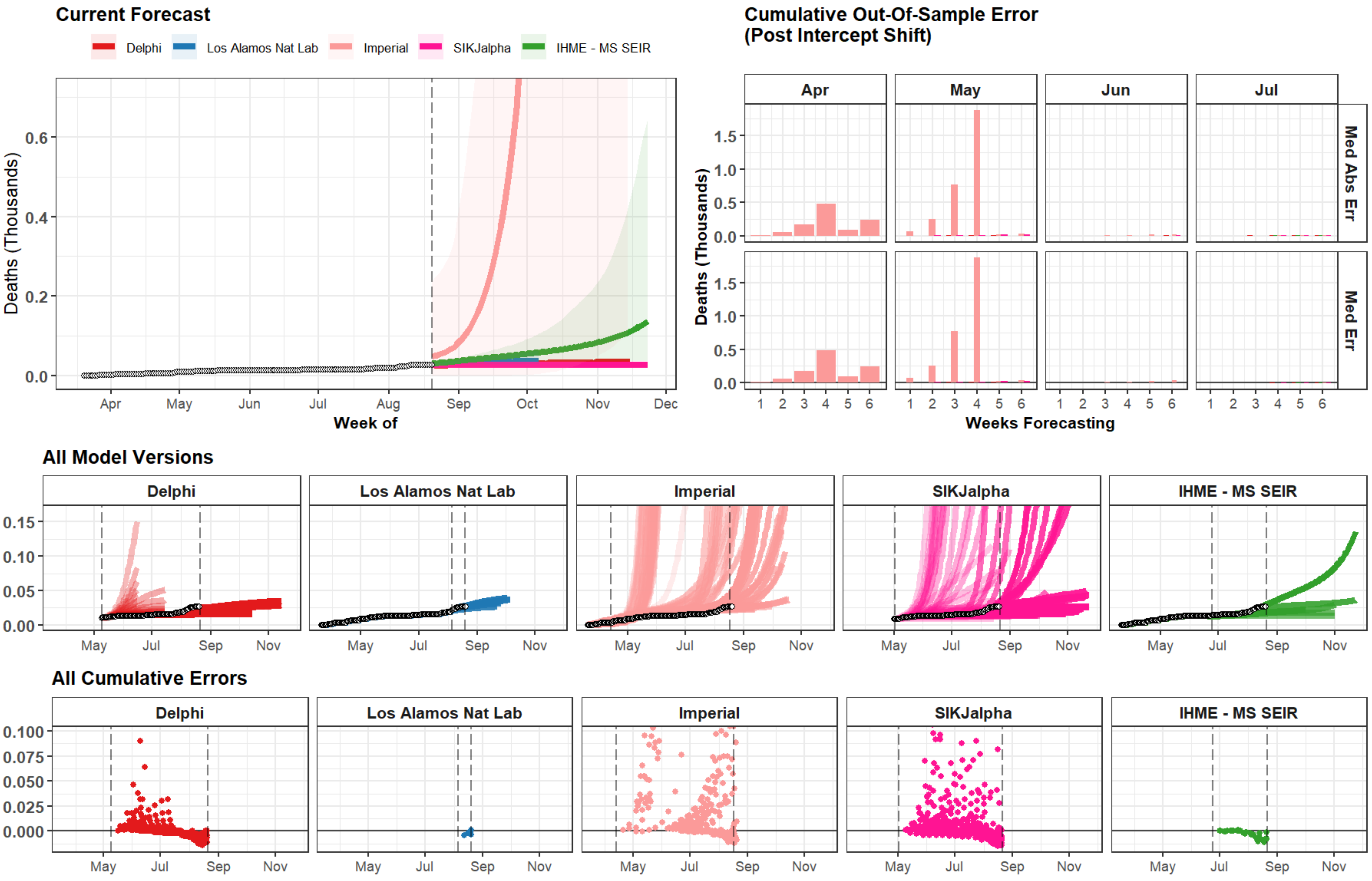

Current Forecast
Delphi
Los Alamos Nat Lab
Imperial
SIKJalpha
IHME - MS SEIR
Deaths (Thousands)
Week of
Cumulative Out-Of-Sample Error
(Post Intercept Shift)
Apr
May
Jun
Jul
Med Abs Err
Med Err
Weeks Forecasting
All Model Versions
All Cumulative Errors

Vietnam

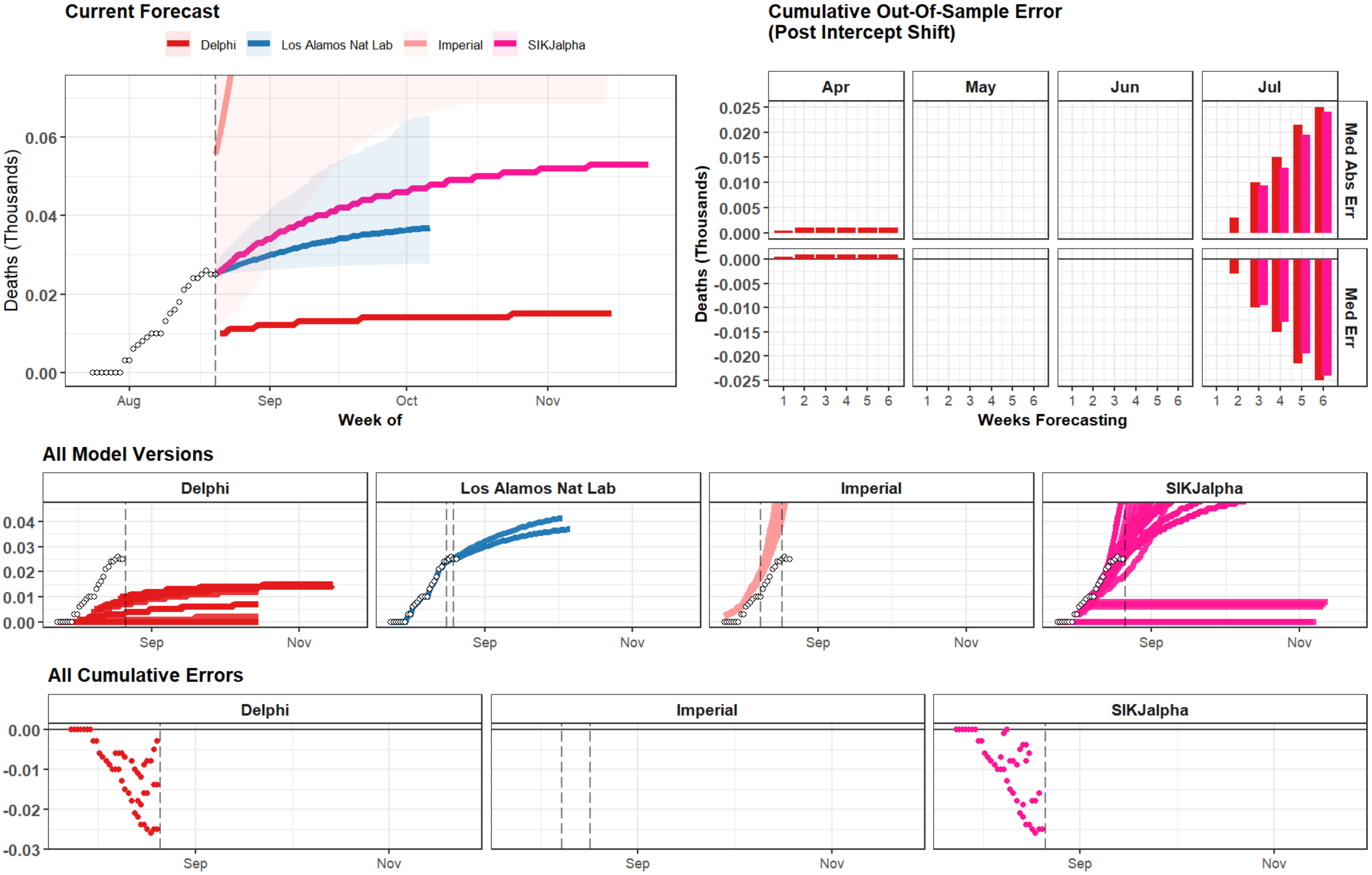

Current Forecast
Delphi
Los Alamos Nat Lab
Imperial
SIKJalpha
0.06
0.04
0.02
0.00
Deaths (Thousands)
Aug
Sep
Oct
Nov
Week of
Cumulative Out-Of-Sample Error
(Post Intercept Shift)
Apr
May
Jun
Jul
0.025
0.020
0.015
0.010
0.005
0.000
-0.005
-0.010
-0.015
-0.020
-0.025
Deaths (Thousands)
Med Abs Err
Med Err
1 2 3 4 5 6
Weeks Forecasting
All Model Versions
Delphi
Los Alamos Nat Lab
Imperial
SIKJalpha
0.04
0.03
0.02
0.01
0.00
Sep
Nov
All Cumulative Errors
Delphi
Imperial
SIKJalpha
0.00
-0.01
-0.02
-0.03
Sep
Nov

Maldives

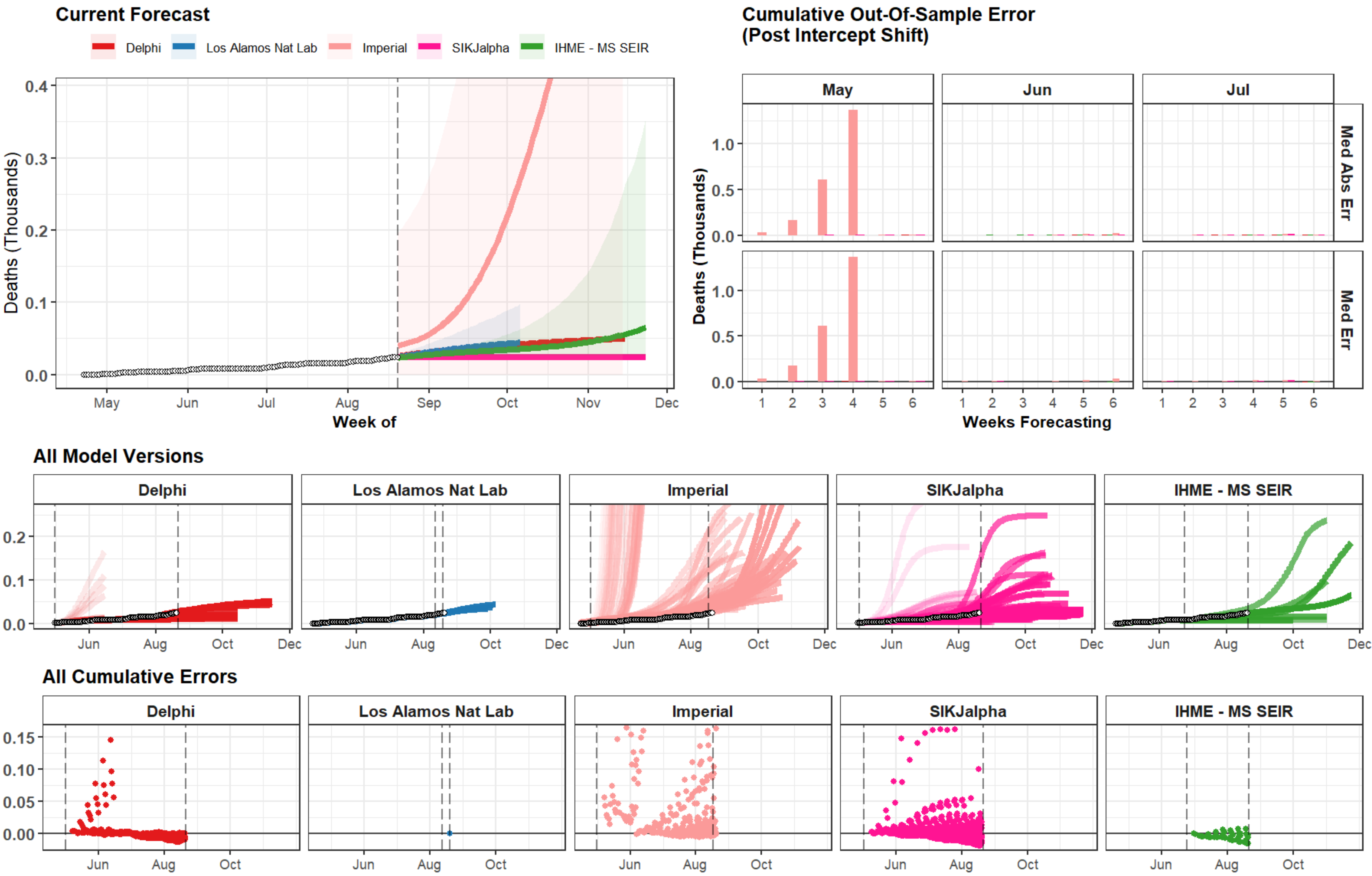

Current Forecast
Delphi
Los Alamos Nat Lab
Imperial
SIKJalpha
IHME - MS SEIR
Deaths (Thousands)
Week of
Cumulative Out-Of-Sample Error
(Post Intercept Shift)
May
Jun
Jul
Med Abs Err
Med Err
Weeks Forecasting
All Model Versions
All Cumulative Errors

New Zealand

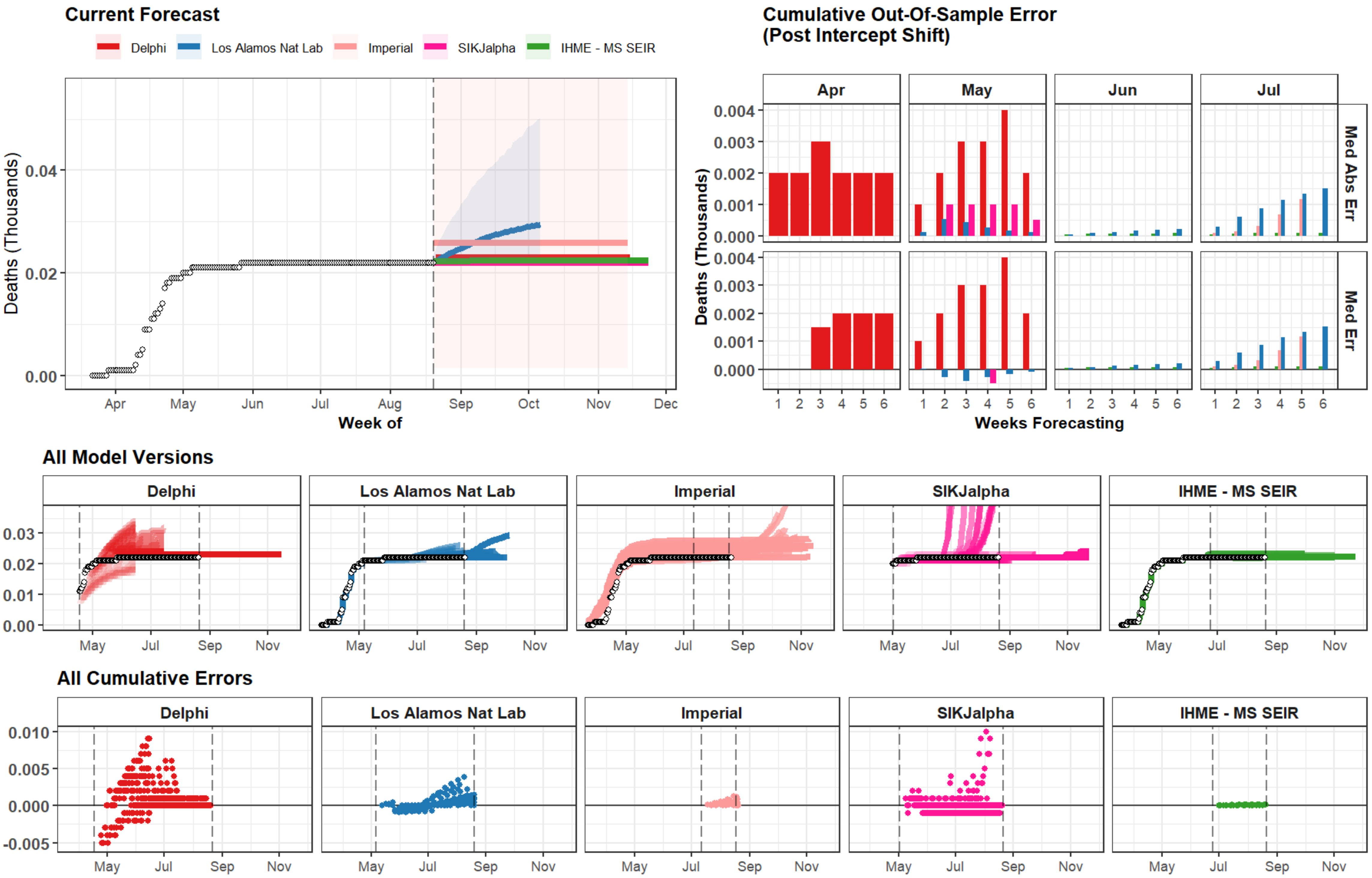

Current Forecast
Delphi
Los Alamos Nat Lab
Imperial
SIKJalpha
IHME - MS SEIR
Deaths (Thousands)
0.00
0.02
0.04
Apr
May
Jun
Jul
Aug
Sep
Oct
Nov
Dec
Week of
Cumulative Out-Of-Sample Error
(Post Intercept Shift)
Apr
May
Jun
Jul
Med Abs Err
Med Err
0.000
0.001
0.002
0.003
0.004
1 2 3 4 5 6
Weeks Forecasting
All Model Versions
Delphi
Los Alamos Nat Lab
Imperial
SIKJalpha
IHME - MS SEIR
0.00
0.01
0.02
0.03
May
Jul
Sep
Nov
All Cumulative Errors
Delphi
Los Alamos Nat Lab
Imperial
SIKJalpha
IHME - MS SEIR
-0.005
0.000
0.005
0.010
May
Jul
Sep
Nov

Tanzania

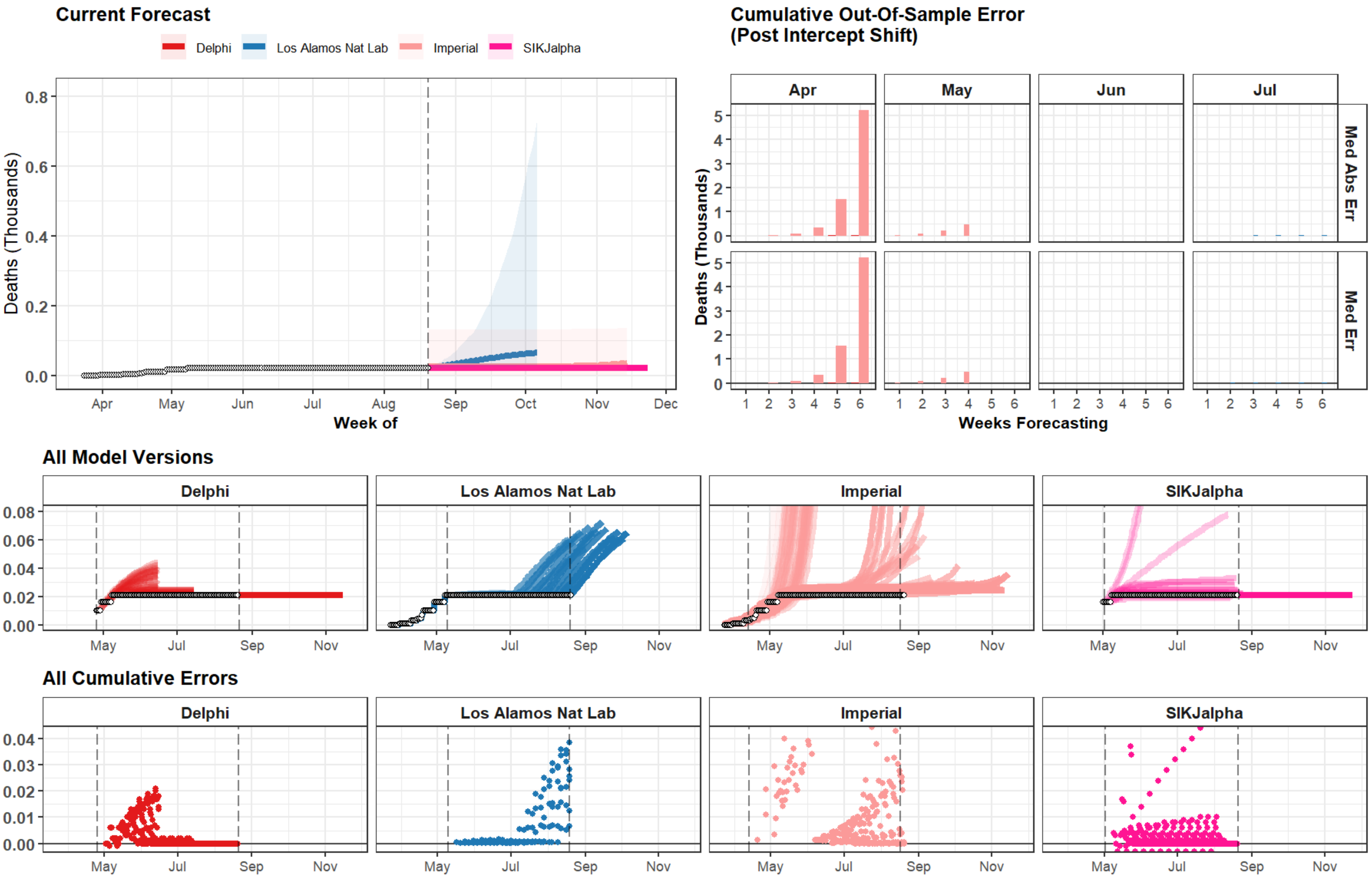
Current Forecast
Delphi
Los Alamos Nat Lab
Imperial
SIKJalpha
Deaths (Thousands)
0.0
0.2
0.4
0.6
0.8
Apr
May
Jun
Jul
Aug
Sep
Oct
Nov
Dec
Week of
Cumulative Out-Of-Sample Error
(Post Intercept Shift)
Apr
May
Jun
Jul
Med Abs Err
Med Err
Deaths (Thousands)
Weeks Forecasting
All Model Versions
Delphi
Los Alamos Nat Lab
Imperial
SIKJalpha
0.00
0.02
0.04
0.06
0.08
May
Jul
Sep
Nov
All Cumulative Errors
Delphi
Los Alamos Nat Lab
Imperial
SIKJalpha
0.00
0.01
0.02
0.03
0.04

Cyprus

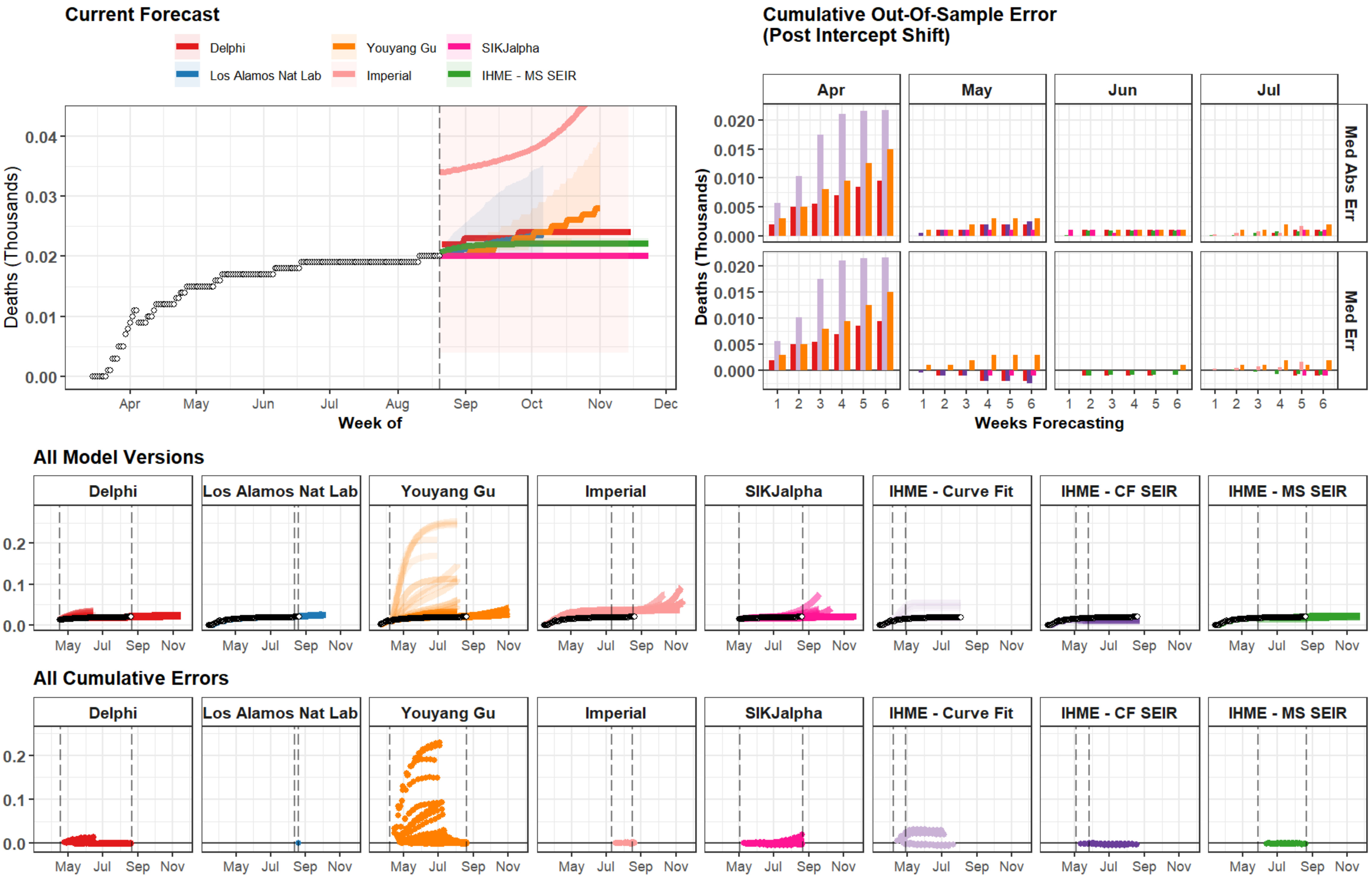

Current Forecast
Delphi
Youyang Gu
SIKJalpha
Los Alamos Nat Lab
Imperial
IHME - MS SEIR
Deaths (Thousands)
0.04
0.03
0.02
0.01
0.00
Apr
May
Jun
Jul
Aug
Sep
Oct
Nov
Dec
Week of
Cumulative Out-Of-Sample Error
(Post Intercept Shift)
Apr
May
Jun
Jul
Med Abs Err
Med Err
0.020
0.015
0.010
0.005
0.000
1
2
3
4
5
6
Weeks Forecasting
All Model Versions
Delphi
Los Alamos Nat Lab
Youyang Gu
Imperial
SIKJalpha
IHME - Curve Fit
IHME - CF SEIR
IHME - MS SEIR
0.2
0.1
0.0
May
Jul
Sep
Nov
All Cumulative Errors

Mozambique

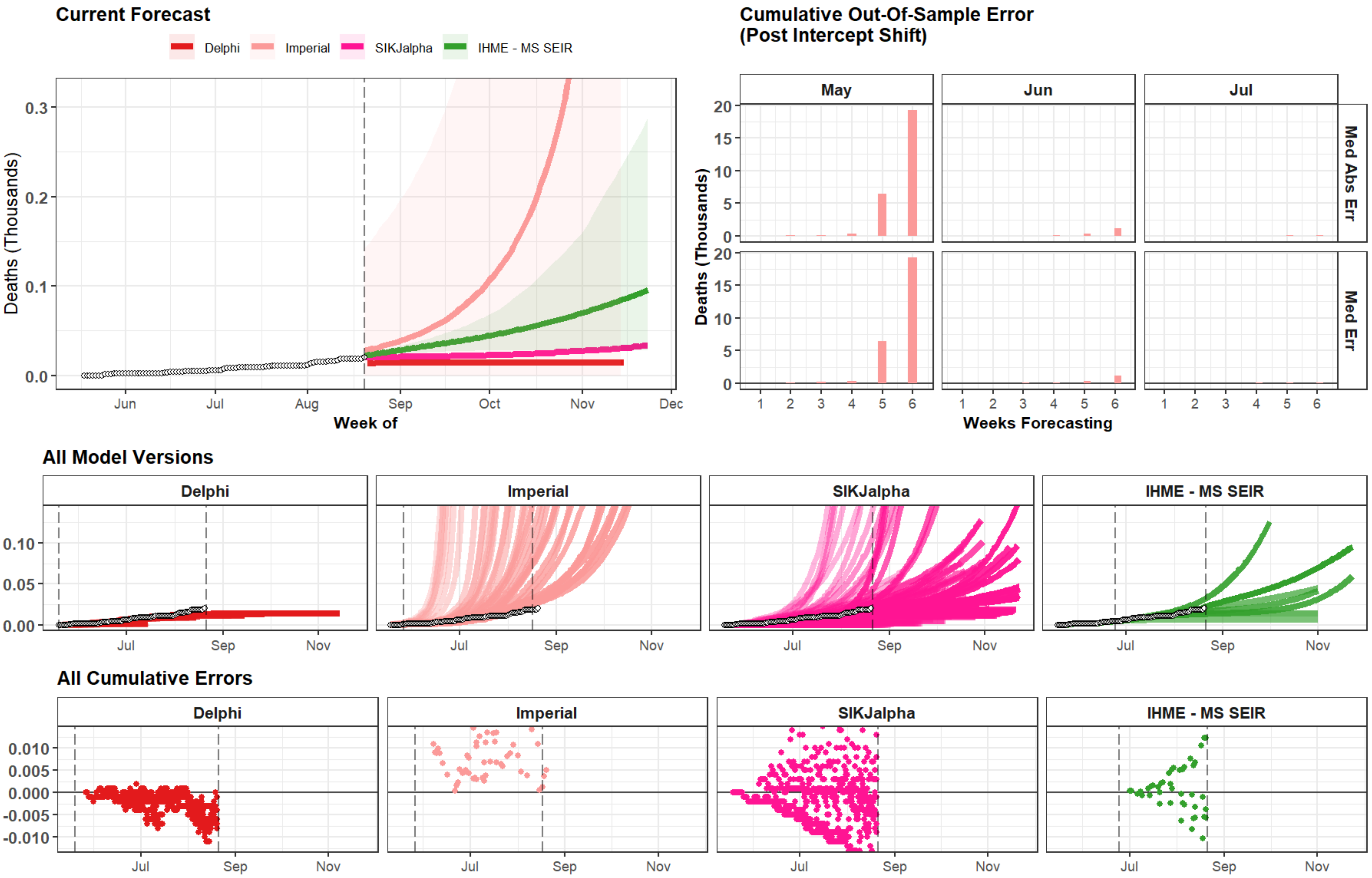

Current Forecast
Delphi
Imperial
SIKJalpha
IHME - MS SEIR
Deaths (Thousands)
0.3
0.2
0.1
0.0
Jun
Jul
Aug
Sep
Oct
Nov
Dec
Week of
Cumulative Out-Of-Sample Error
(Post Intercept Shift)
May
Jun
Jul
Med Abs Err
Med Err
20
15
10
5
0
1
2
3
4
5
6
Weeks Forecasting
All Model Versions
0.10
0.05
0.00
All Cumulative Errors
0.010
0.005
0.000
-0.005
-0.010

Uganda

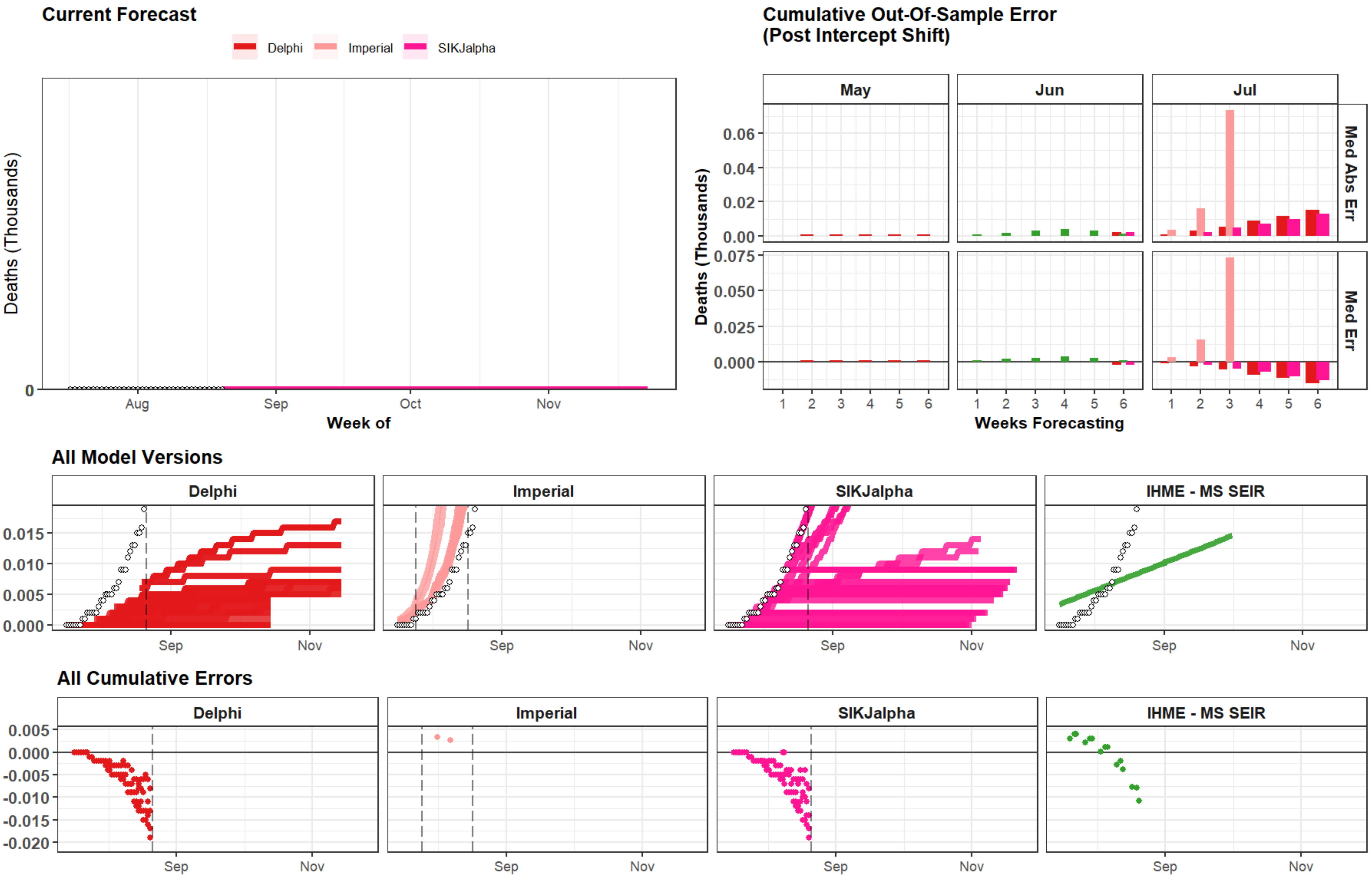

Current Forecast
Delphi
Imperial
SIKJalpha
Deaths (Thousands)
0
Aug
Sep
Oct
Nov
Week of
Cumulative Out-Of-Sample Error
(Post Intercept Shift)
May
Jun
Jul
Med Abs Err
Med Err
0.06
0.04
0.02
0.00
0.075
0.050
0.025
0.000
Weeks Forecasting
All Model Versions
Delphi
Imperial
SIKJalpha
IHME - MS SEIR
0.015
0.010
0.005
0.000
All Cumulative Errors
0.005
0.000
-0.005
-0.010
-0.015
-0.020

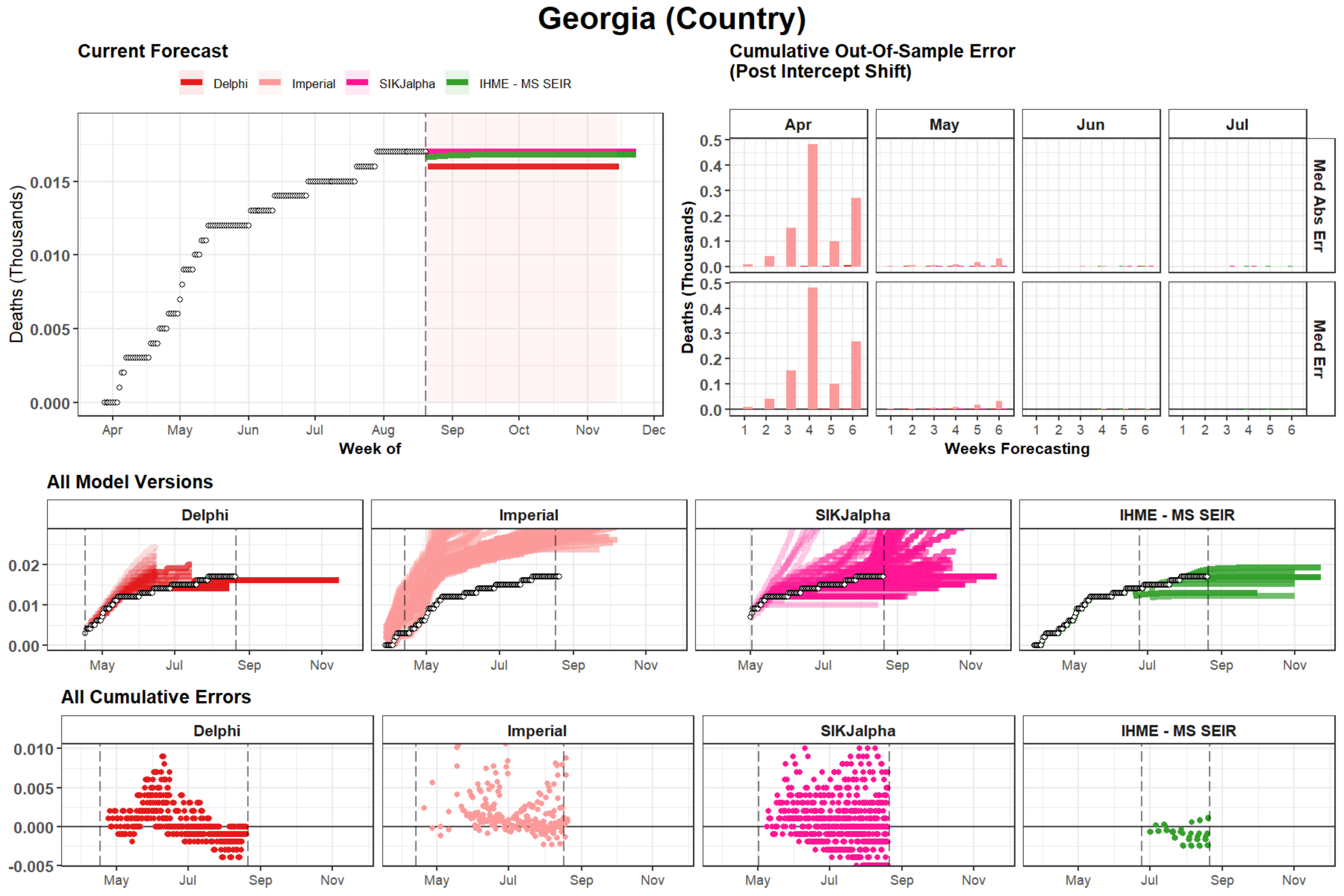

Georgia (Country)
Current Forecast
Delphi
Imperial
SIKJalpha
IHME - MS SEIR
Deaths (Thousands)
Week of
Cumulative Out-Of-Sample Error
(Post Intercept Shift)
Apr
May
Jun
Jul
Med Abs Err
Med Err
Weeks Forecasting
All Model Versions
All Cumulative Errors

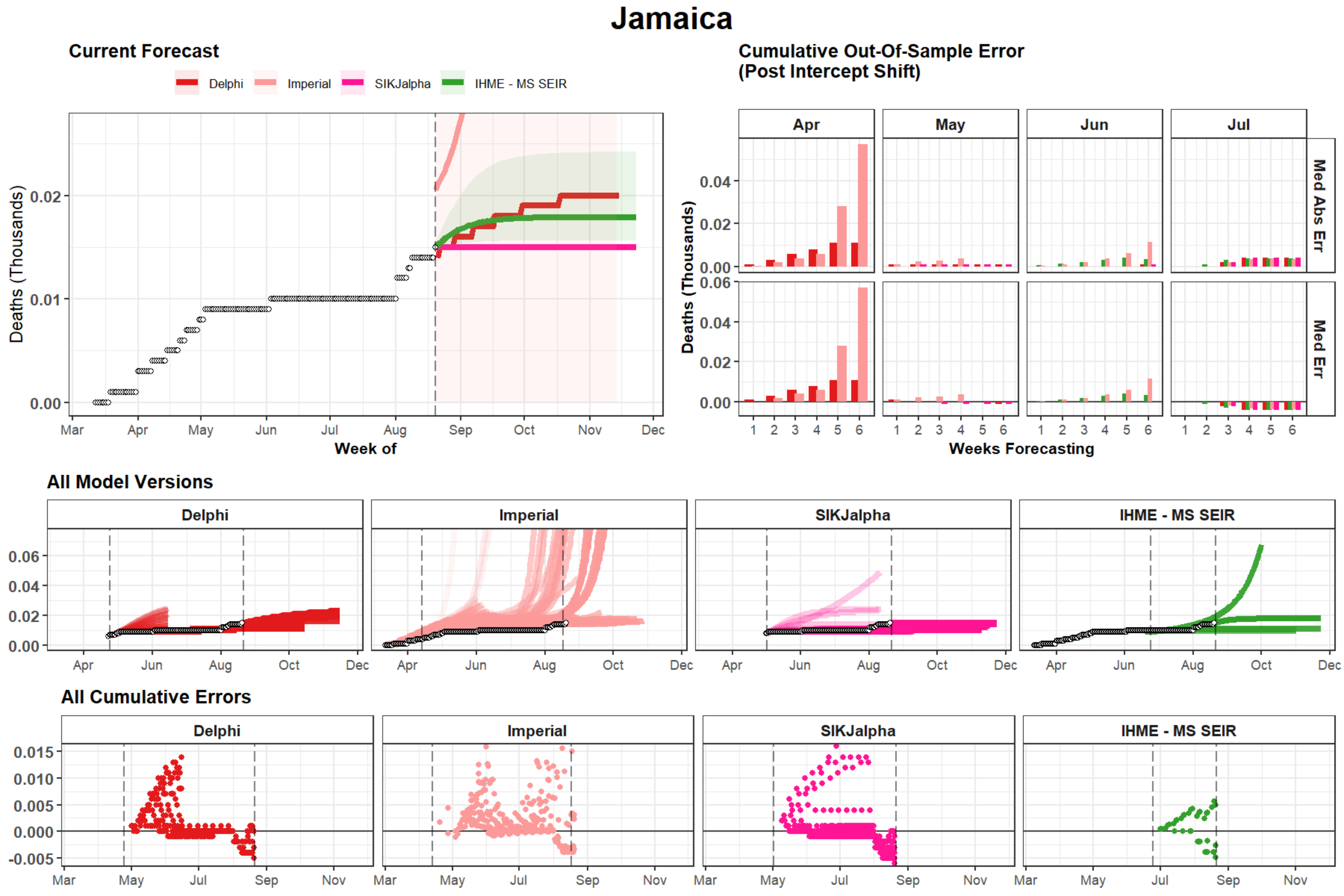
Jamaica
Current Forecast
Delphi
Imperial
SIKJalpha
IHME - MS SEIR
Deaths (Thousands)
0.00
0.01
0.02
Mar
Apr
May
Jun
Jul
Aug
Sep
Oct
Nov
Dec
Week of
Cumulative Out-Of-Sample Error
(Post Intercept Shift)
Apr
May
Jun
Jul
Med Abs Err
Med Err
0.00
0.02
0.04
0.06
1 2 3 4 5 6
Weeks Forecasting
All Model Versions
Delphi
Imperial
SIKJalpha
IHME - MS SEIR
0.00
0.02
0.04
0.06
Apr
Jun
Aug
Oct
Dec
All Cumulative Errors
Delphi
Imperial
SIKJalpha
IHME - MS SEIR
-0.005
0.000
0.005
0.010
0.015
Mar
May
Jul
Sep
Nov

Sao Tome and Principe

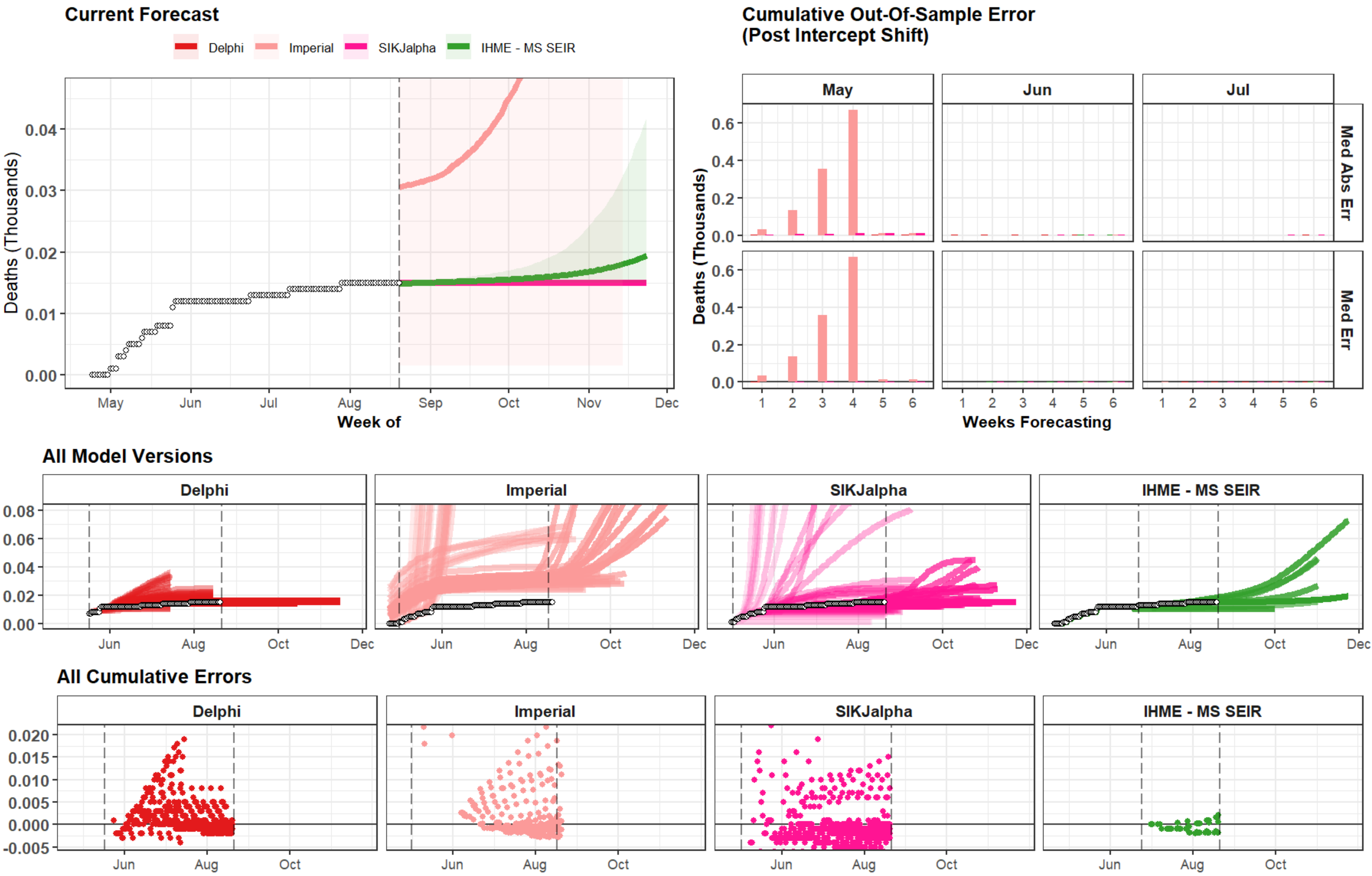

Trinidad and Tobago

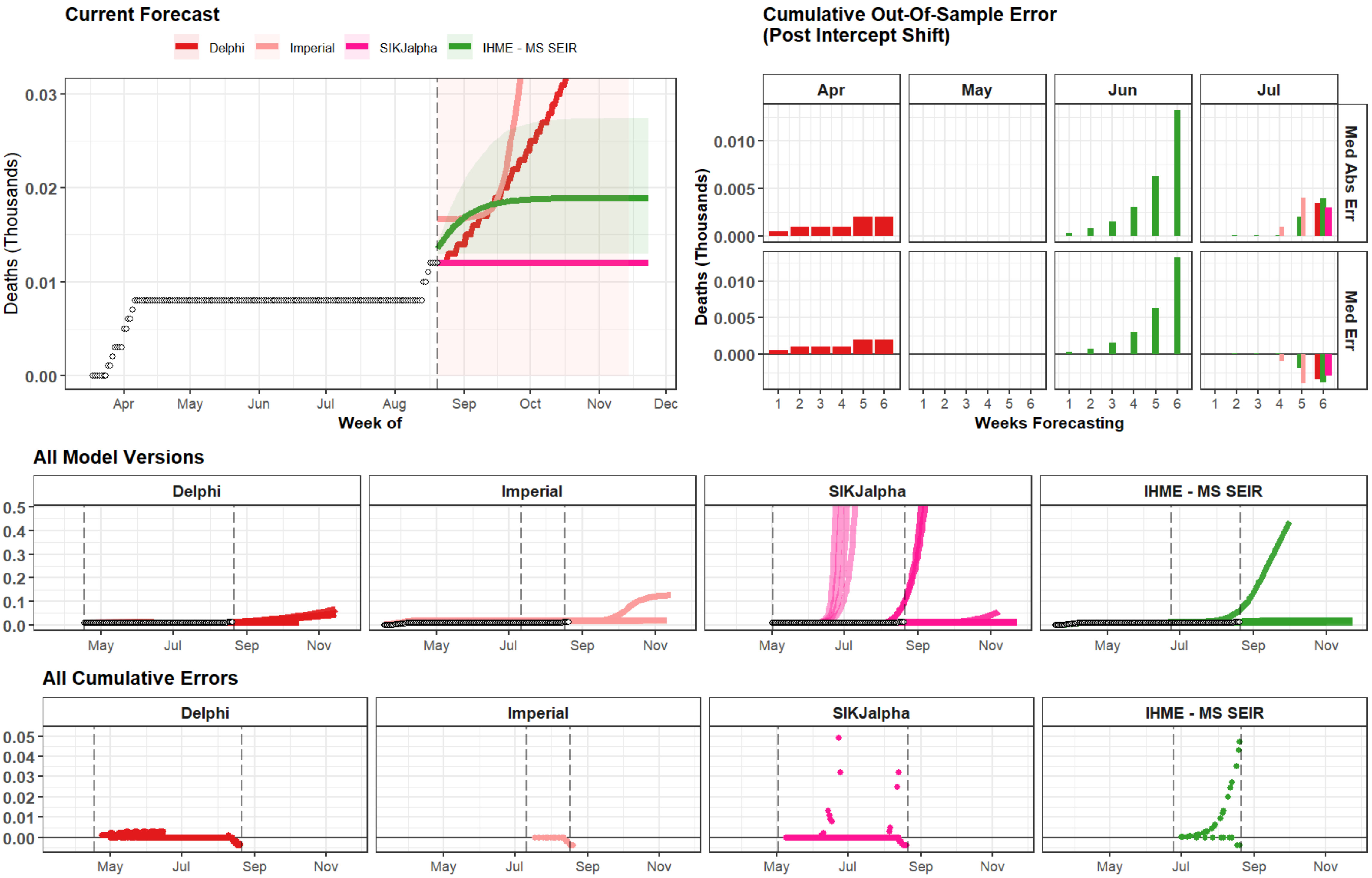

Jordan

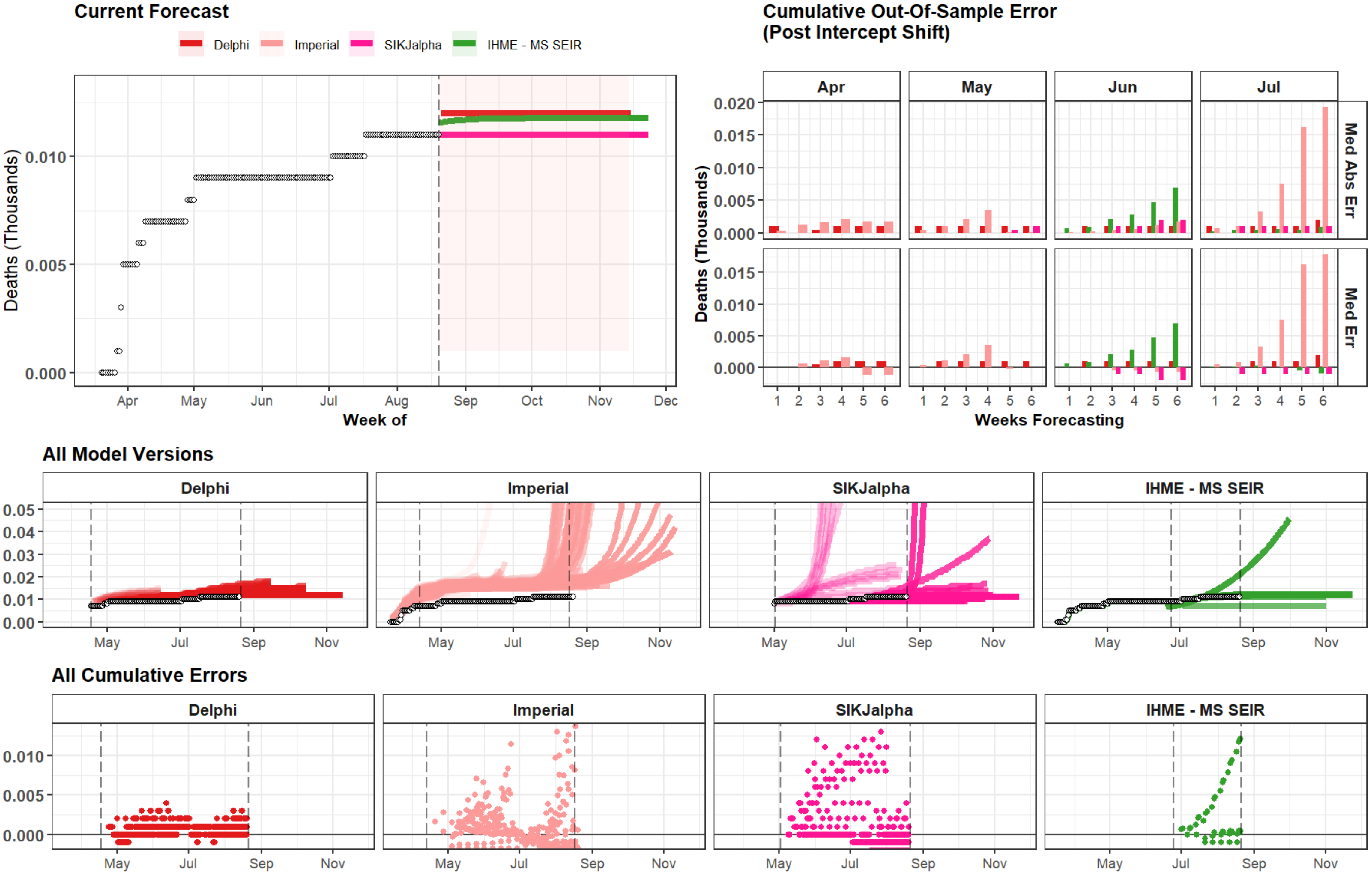

Current Forecast
Delphi
Imperial
SIKJalpha
IHME - MS SEIR
Deaths (Thousands)
0.000
0.005
0.010
Apr
May
Jun
Jul
Aug
Sep
Oct
Nov
Dec
Week of
Cumulative Out-Of-Sample Error
(Post Intercept Shift)
Apr
May
Jun
Jul
Med Abs Err
Med Err
0.000
0.005
0.010
0.015
0.020
1
2
3
4
5
6
Weeks Forecasting
All Model Versions
Delphi
Imperial
SIKJalpha
IHME - MS SEIR
0.00
0.01
0.02
0.03
0.04
0.05
May
Jul
Sep
Nov
All Cumulative Errors
Delphi
Imperial
SIKJalpha
IHME - MS SEIR
0.000
0.005
0.010
May
Jul
Sep
Nov

Rwanda

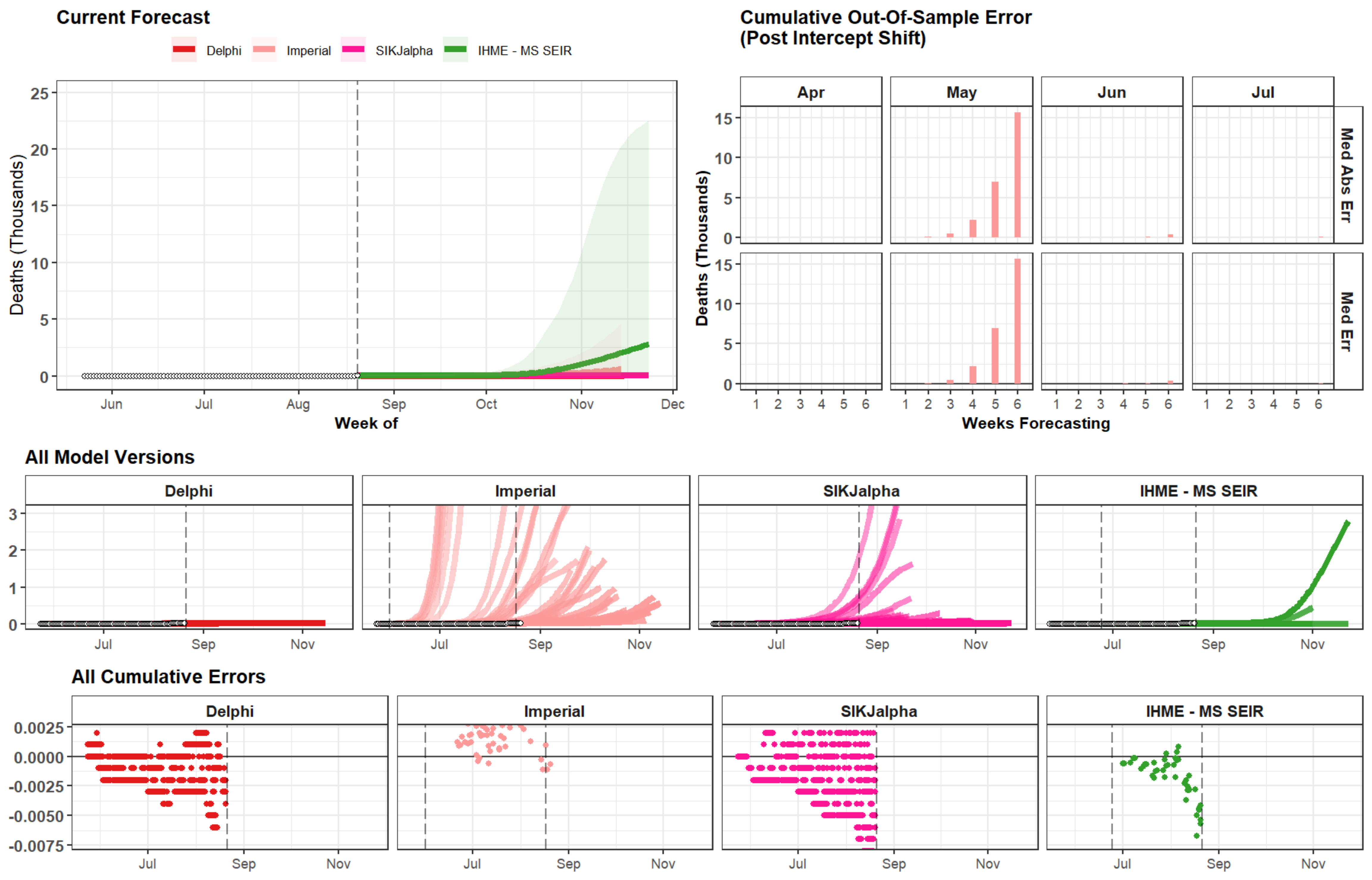

Current Forecast
Delphi
Imperial
SIKJalpha
IHME - MS SEIR
Deaths (Thousands)
25
20
15
10
5
0
Jun
Jul
Aug
Sep
Oct
Nov
Dec
Week of
Cumulative Out-Of-Sample Error
(Post Intercept Shift)
Apr
May
Jun
Jul
Med Abs Err
Med Err
Deaths (Thousands)
Weeks Forecasting
All Model Versions
Delphi
Imperial
SIKJalpha
IHME - MS SEIR
All Cumulative Errors
0.0025
0.0000
-0.0025
-0.0050
-0.0075

Sri Lanka

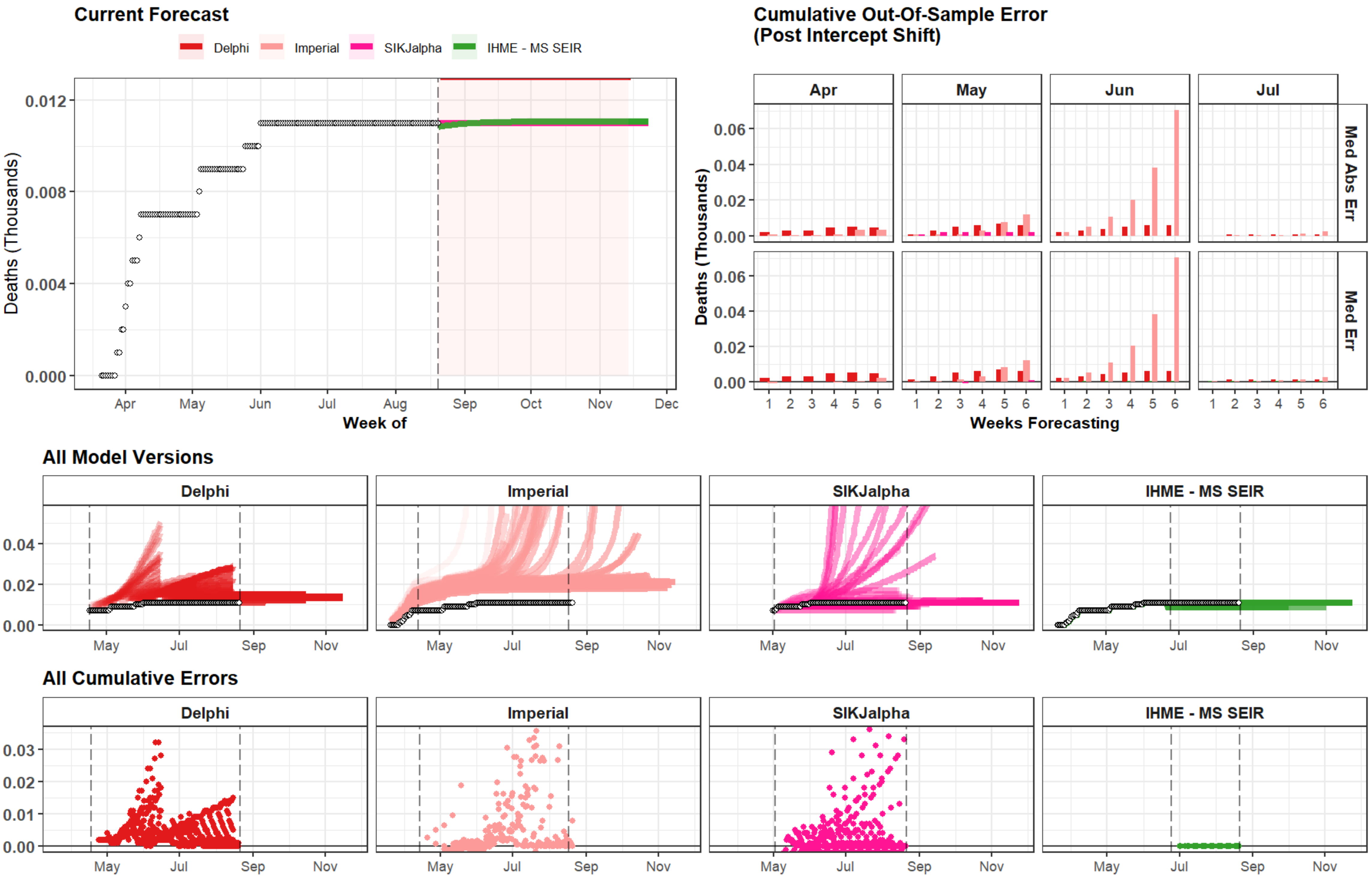

Current Forecast
Delphi
Imperial
SIKJalpha
IHME - MS SEIR
0.012
0.008
0.004
0.000
Deaths (Thousands)
Apr
May
Jun
Jul
Aug
Sep
Oct
Nov
Dec
Week of
Cumulative Out-Of-Sample Error
(Post Intercept Shift)
Apr
May
Jun
Jul
0.06
0.04
0.02
0.00
Med Abs Err
Med Err
Deaths (Thousands)
1
2
3
4
5
6
Weeks Forecasting
All Model Versions
Delphi
Imperial
SIKJalpha
IHME - MS SEIR
0.04
0.02
0.00
May
Jul
Sep
Nov
All Cumulative Errors
Delphi
Imperial
SIKJalpha
IHME - MS SEIR
0.03
0.02
0.01
0.00
May
Jul
Sep
Nov
