# Supplementary material for: Predictive performance of international COVID-19 mortality forecasting models
Source: medRxiv. 2020 Nov 19:2020.07.13.20151233. Preprint. [Version 5] doi: 10.1101/2020.07.13.20151233 (PMC7685335; doi:10.1101/2020.07.13.20151233)
Supplement: Supplement 2 [file media-2.pdf]

United States

Current Forecast

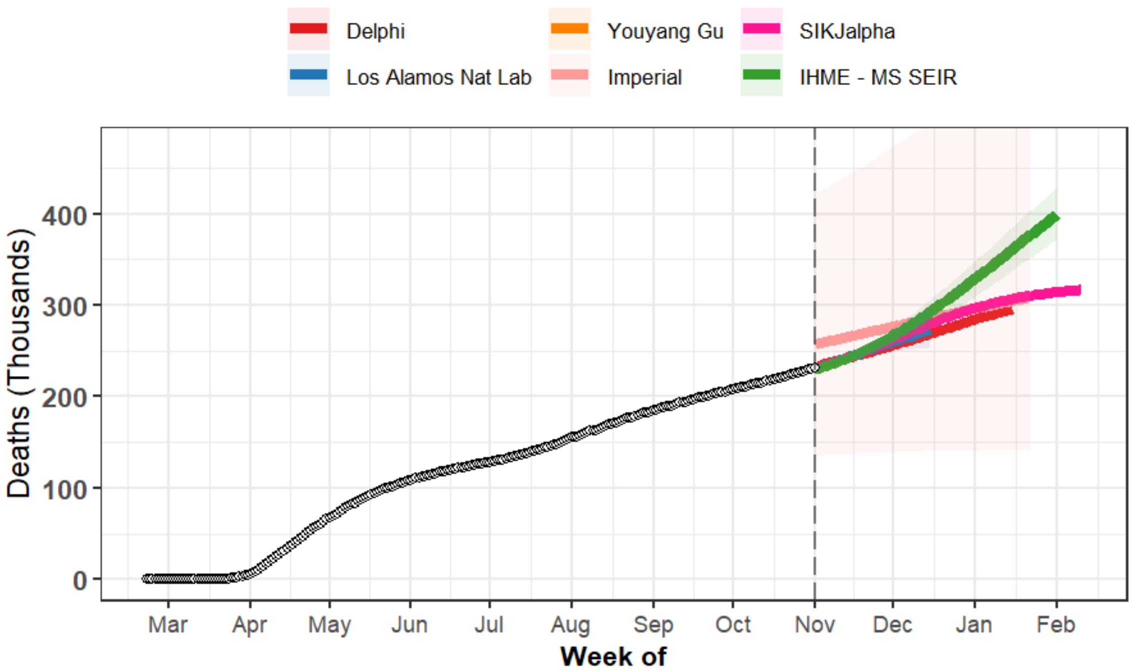

Cumulative Out-Of-Sample Error (Post Intercept Shift)

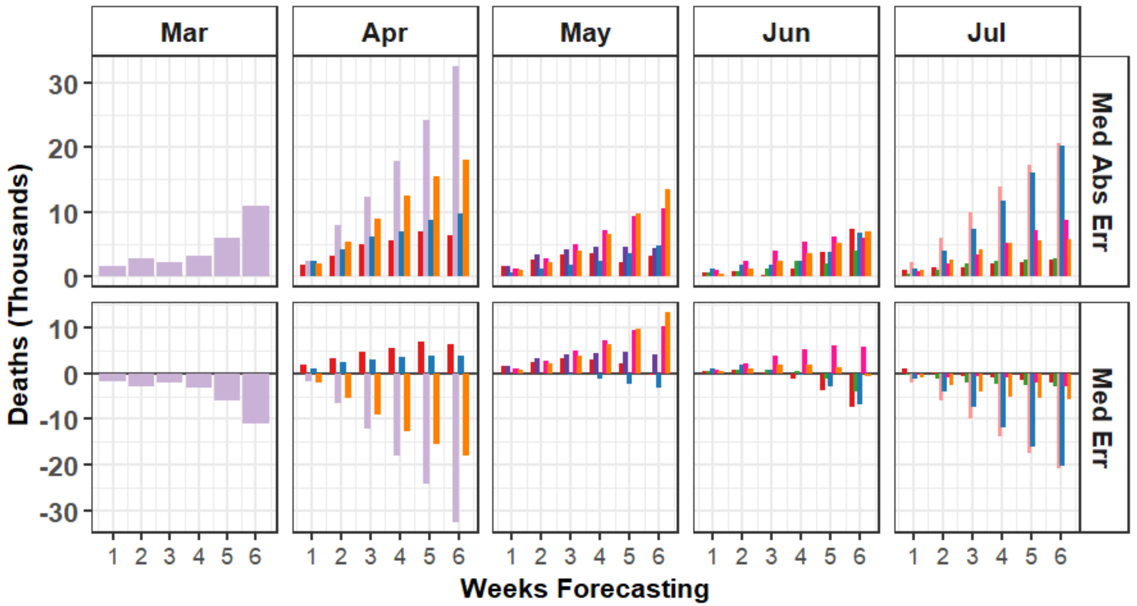

All Model Versions

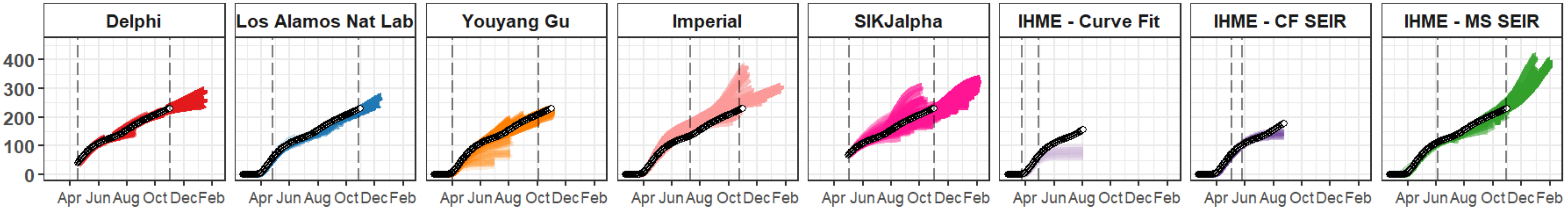

All Cumulative Errors

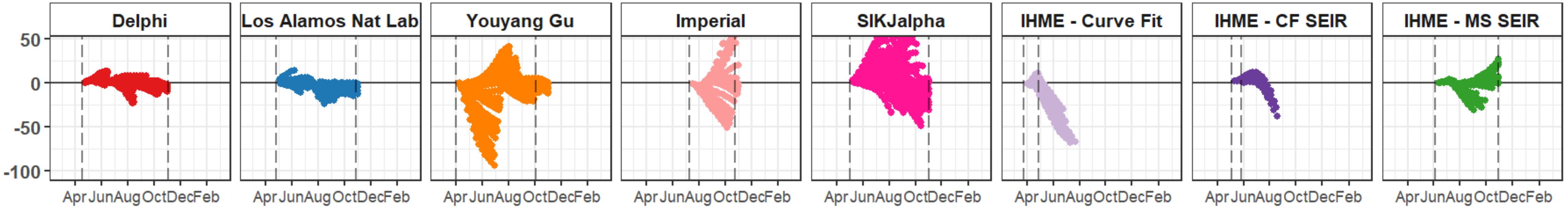

Brazil

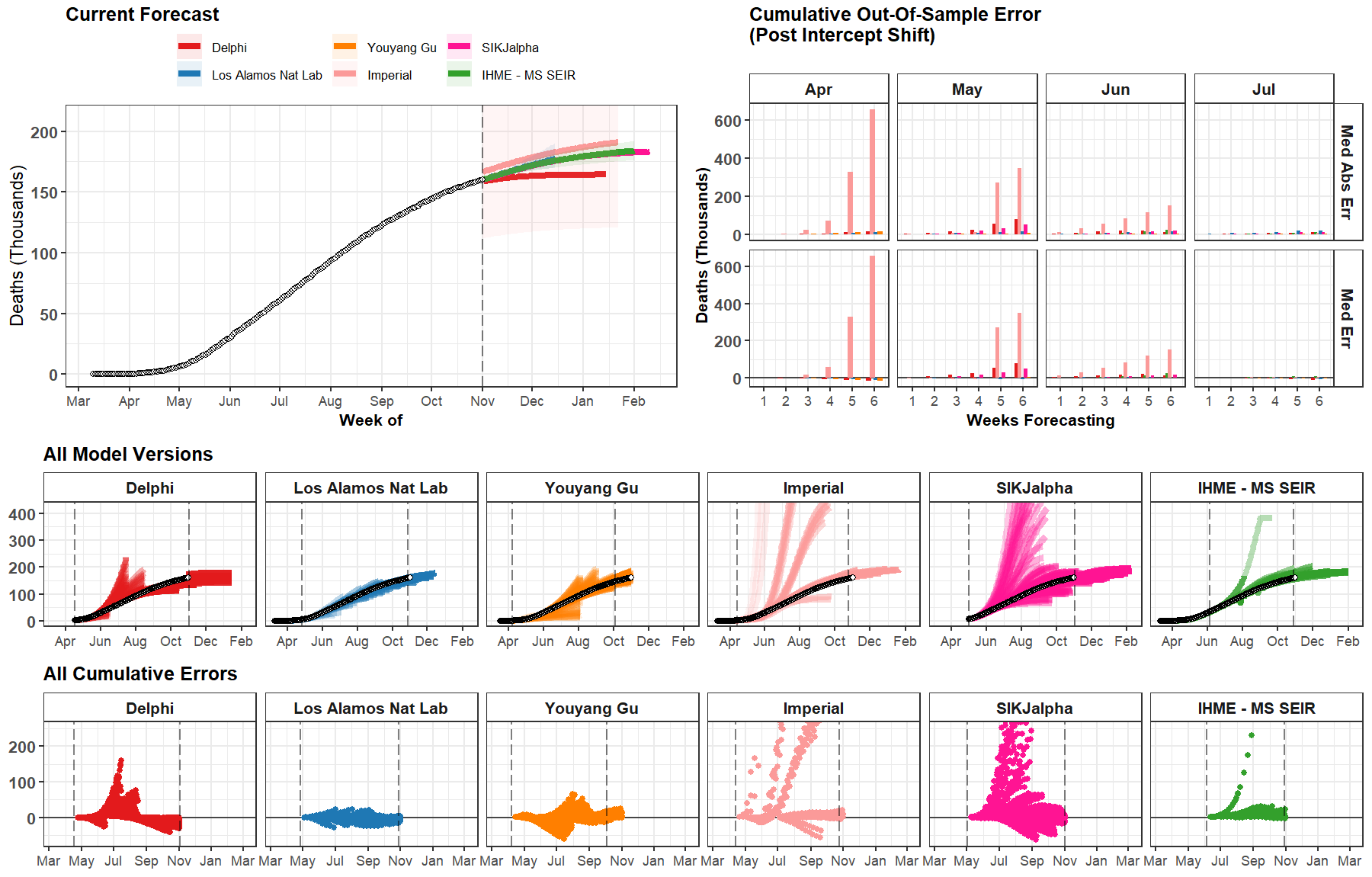
Current Forecast
Delphi
Youyang Gu
SIKJalpha
Los Alamos Nat Lab
Imperial
IHME - MS SEIR
Deaths (Thousands)
Week of
Mar
Apr
May
Jun
Jul
Aug
Sep
Oct
Nov
Dec
Jan
Feb
Cumulative Out-Of-Sample Error
(Post Intercept Shift)
Med Abs Err
Med Err
Weeks Forecasting
All Model Versions
All Cumulative Errors

India

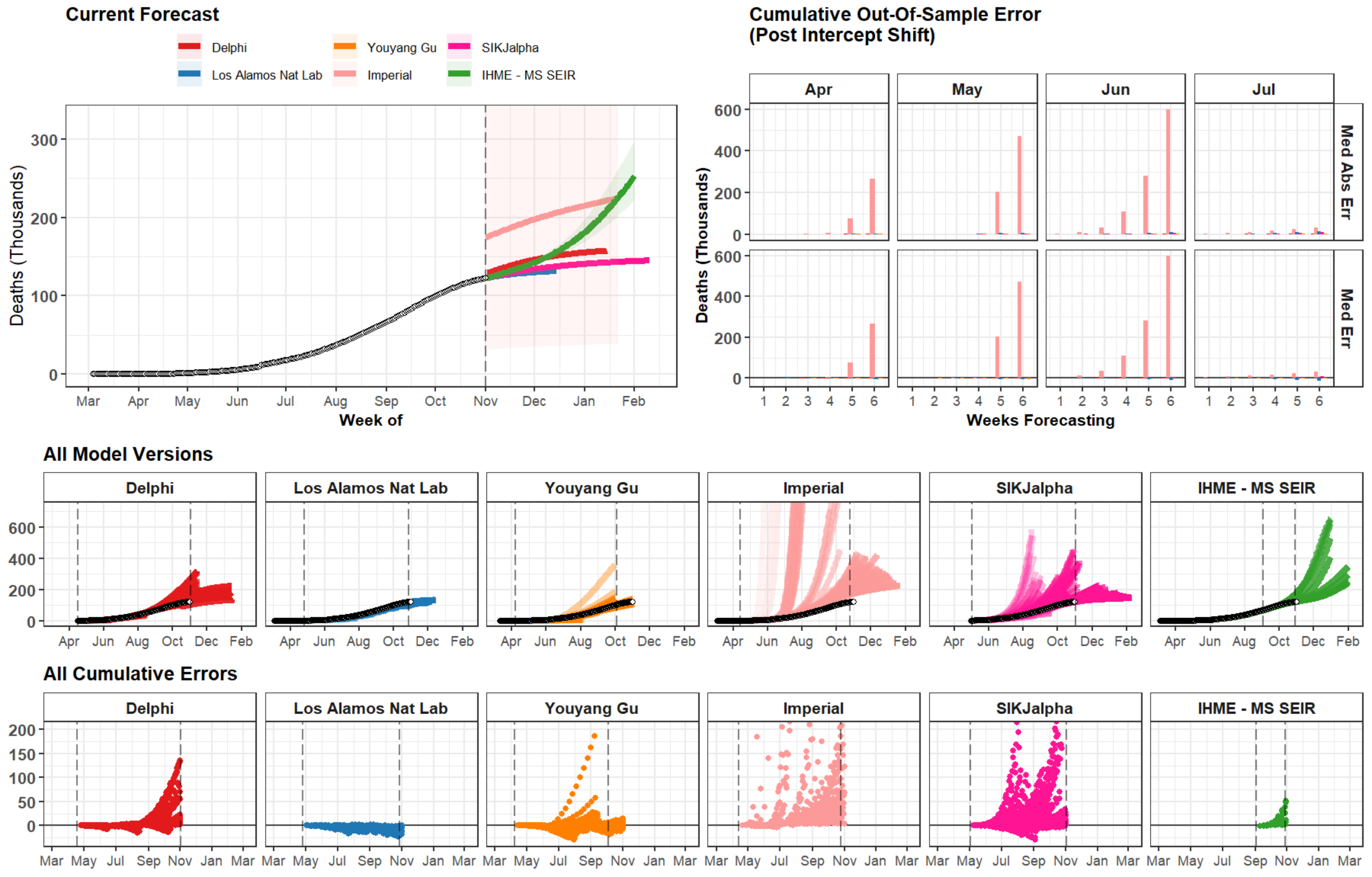
Current Forecast
Delphi
Youyang Gu
SIKJalpha
Los Alamos Nat Lab
Imperial
IHME - MS SEIR
Deaths (Thousands)
300
200
100
0
Mar
Apr
May
Jun
Jul
Aug
Sep
Oct
Nov
Dec
Jan
Feb
Week of
Cumulative Out-Of-Sample Error
(Post Intercept Shift)
Apr
May
Jun
Jul
Med Abs Err
Med Err
600
400
200
0
1
2
3
4
5
6
Weeks Forecasting
All Model Versions
Delphi
Los Alamos Nat Lab
Youyang Gu
Imperial
SIKJalpha
IHME - MS SEIR
600
400
200
0
Apr
Jun
Aug
Oct
Dec
Feb
All Cumulative Errors
Delphi
Los Alamos Nat Lab
Youyang Gu
Imperial
SIKJalpha
IHME - MS SEIR
200
150
100
50
0
Mar
May
Jul
Sep
Nov
Jan
Mar

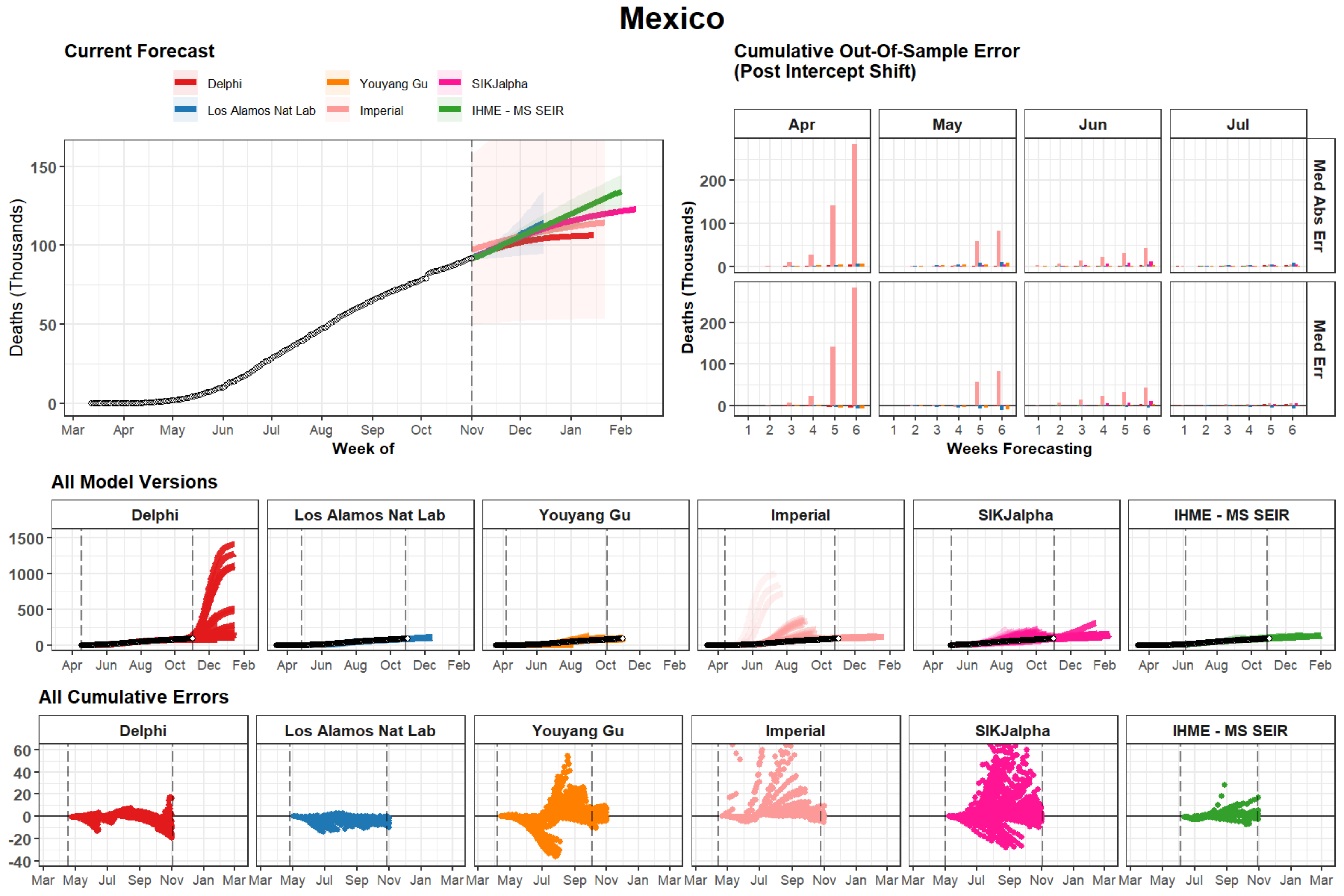
Mexico
Current Forecast
Delphi
Los Alamos Nat Lab
Youyang Gu
Imperial
SIKJalpha
IHME - MS SEIR
Deaths (Thousands)
150
100
50
0
Mar
Apr
May
Jun
Jul
Aug
Sep
Oct
Nov
Dec
Jan
Feb
Week of
Cumulative Out-Of-Sample Error
(Post Intercept Shift)
Apr
May
Jun
Jul
Med Abs Err
Med Err
200
100
0
1
2
3
4
5
6
Weeks Forecasting
All Model Versions
1500
1000
500
All Cumulative Errors
60
40
20
-20
-40

United Kingdom

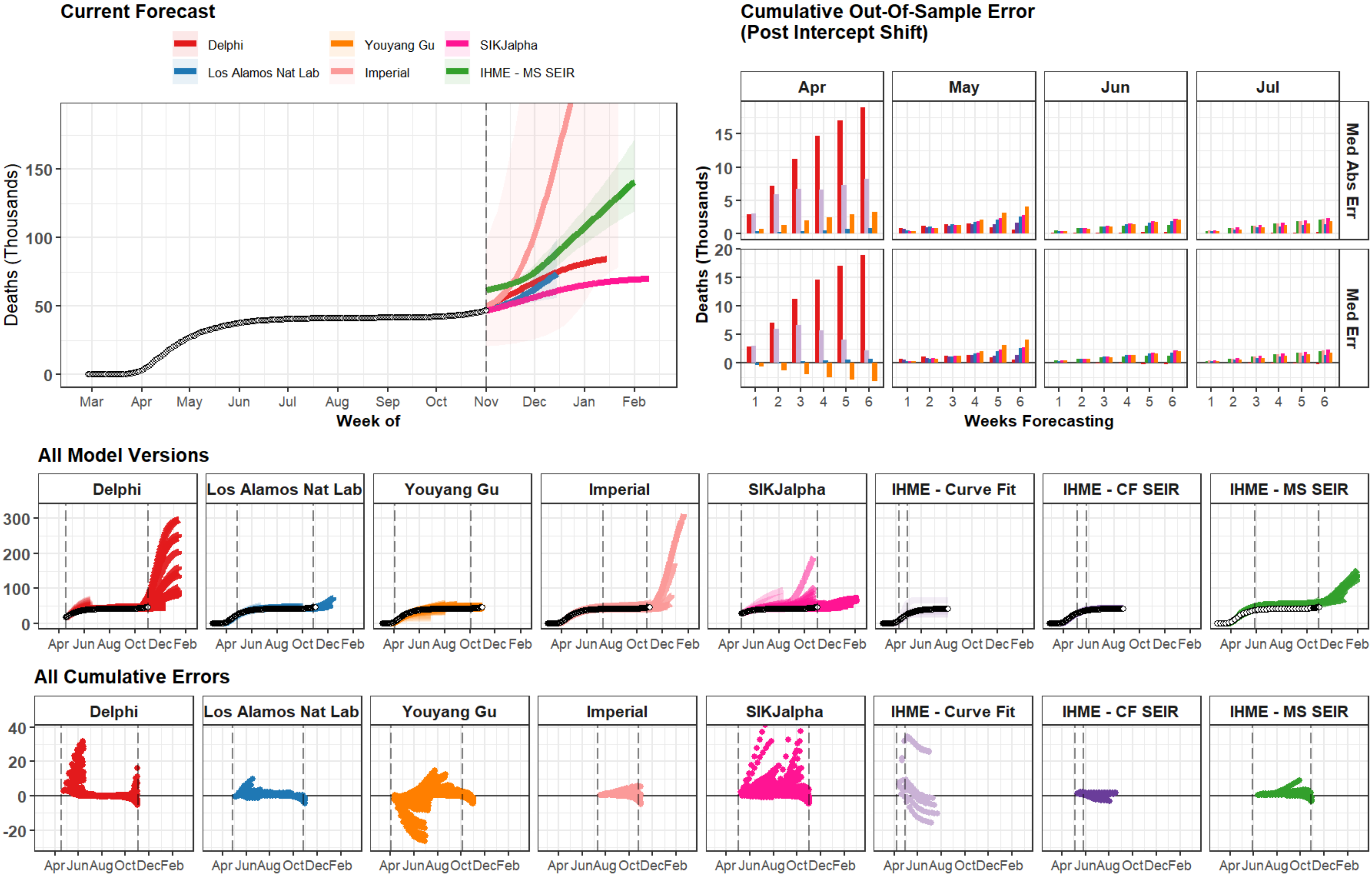

Italy

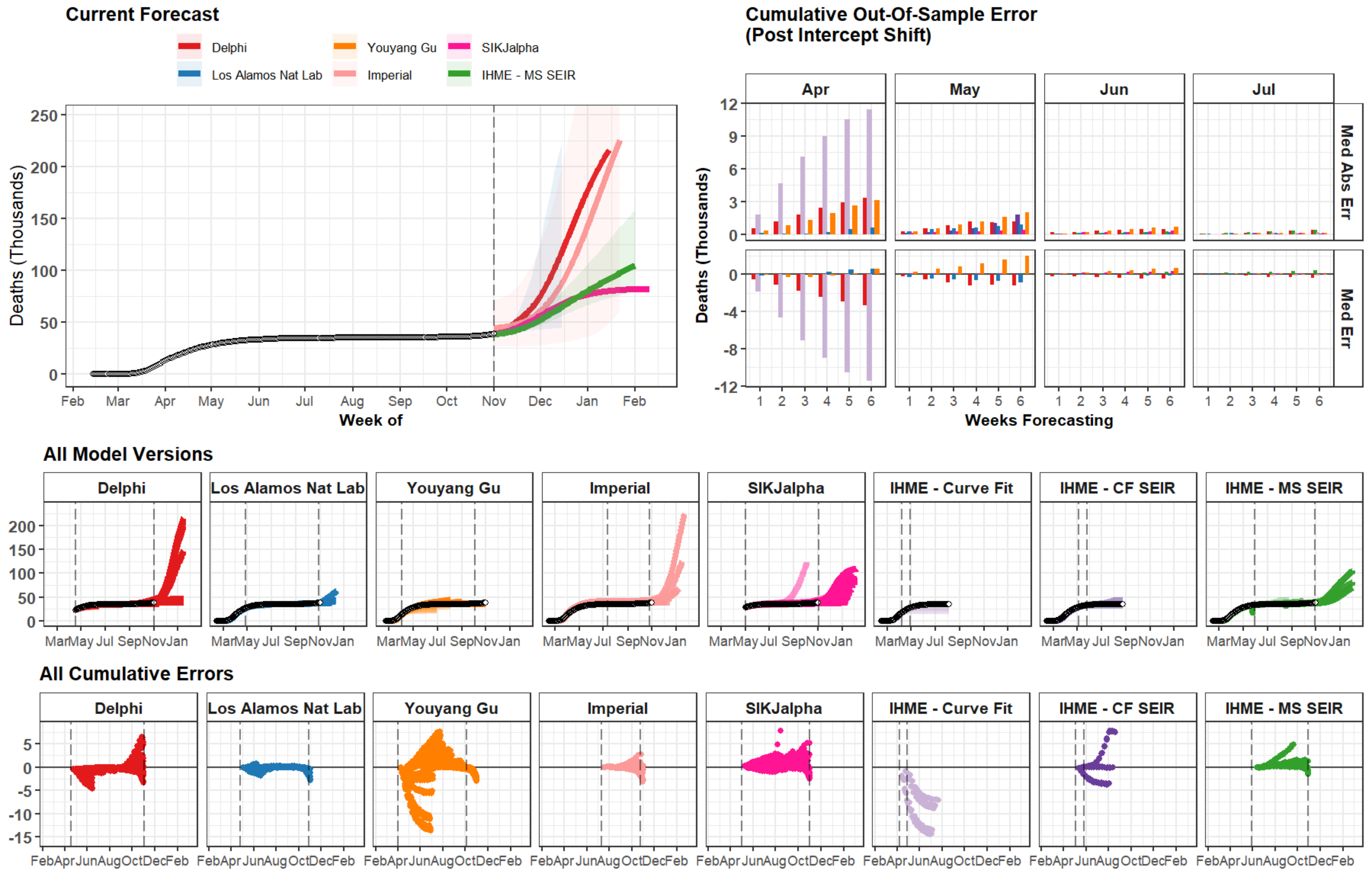
Current Forecast
Delphi
Los Alamos Nat Lab
Youyang Gu
Imperial
SIKJalpha
IHME - MS SEIR
Deaths (Thousands)
Week of
Cumulative Out-Of-Sample Error
(Post Intercept Shift)
Apr
May
Jun
Jul
Med Abs Err
Med Err
Weeks Forecasting
All Model Versions
Delphi
Los Alamos Nat Lab
Youyang Gu
Imperial
SIKJalpha
IHME - Curve Fit
IHME - CF SEIR
IHME - MS SEIR
All Cumulative Errors

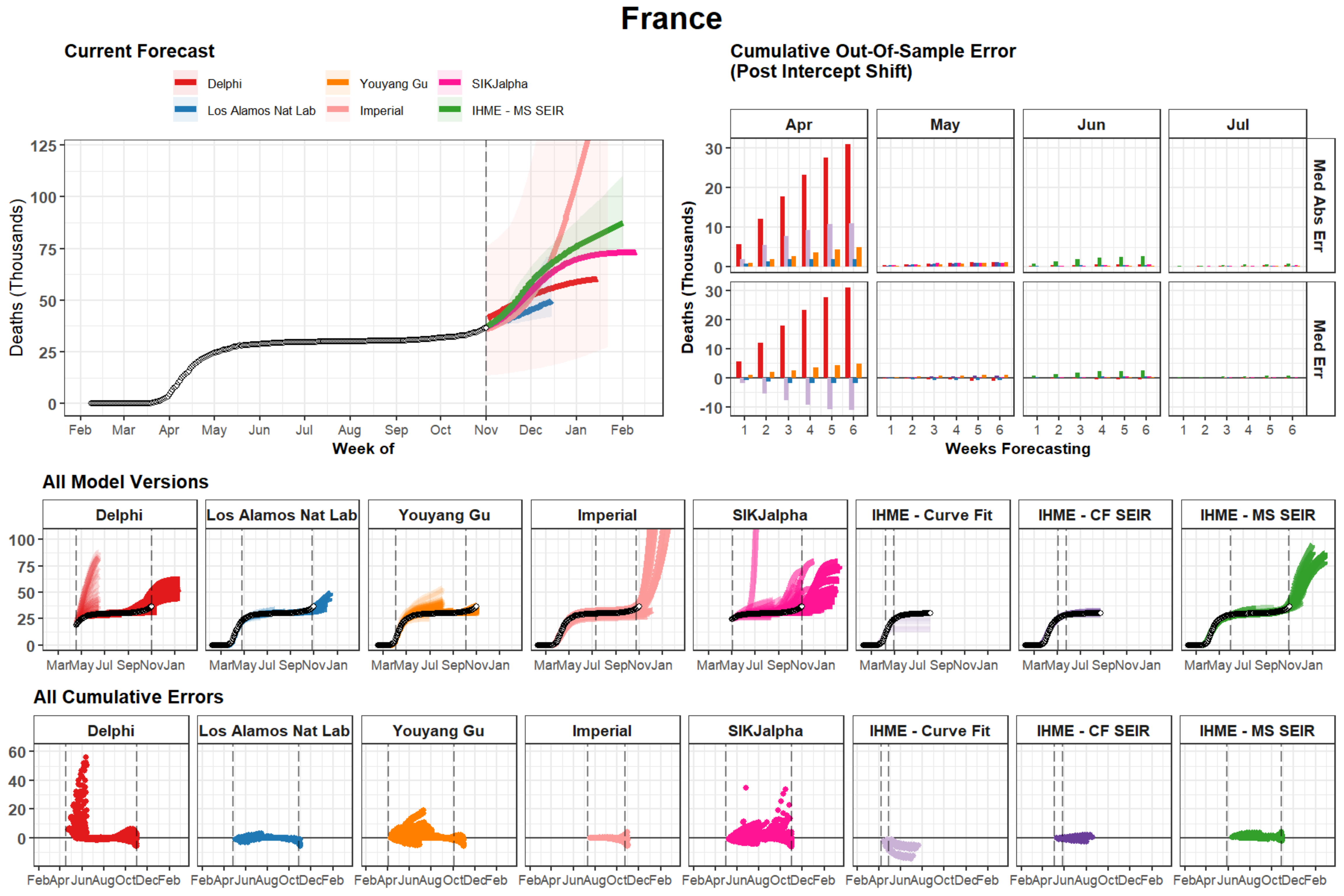
France
Current Forecast
Delphi
Youyang Gu
SIKJalpha
Los Alamos Nat Lab
Imperial
IHME - MS SEIR
Deaths (Thousands)
Week of
Cumulative Out-Of-Sample Error
(Post Intercept Shift)
Apr
May
Jun
Jul
Med Abs Err
Med Err
Weeks Forecasting
All Model Versions
IHME - Curve Fit
IHME - CF SEIR
All Cumulative Errors

Spain

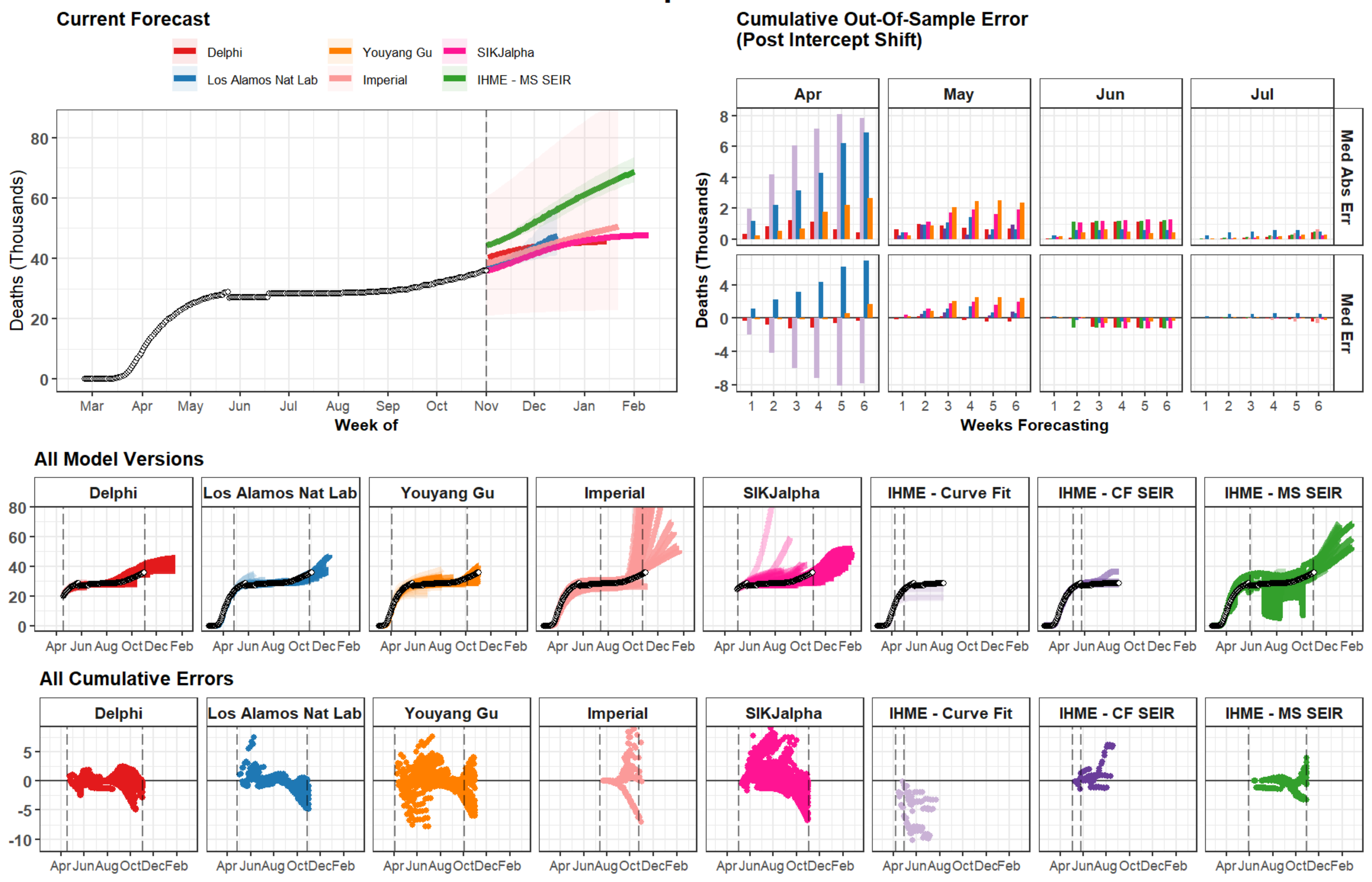
Current Forecast
Delphi
Los Alamos Nat Lab
Youyang Gu
Imperial
SIKJalpha
IHME - MS SEIR
Deaths (Thousands)
Week of
Mar Apr May Jun Jul Aug Sep Oct Nov Dec Jan Feb
Cumulative Out-Of-Sample Error
(Post Intercept Shift)
Apr May Jun Jul
Med Abs Err
Med Err
Weeks Forecasting
All Model Versions
Delphi
Los Alamos Nat Lab
Youyang Gu
Imperial
SIKJalpha
IHME - Curve Fit
IHME - CF SEIR
IHME - MS SEIR
Apr Jun Aug Oct Dec Feb
All Cumulative Errors

Iran

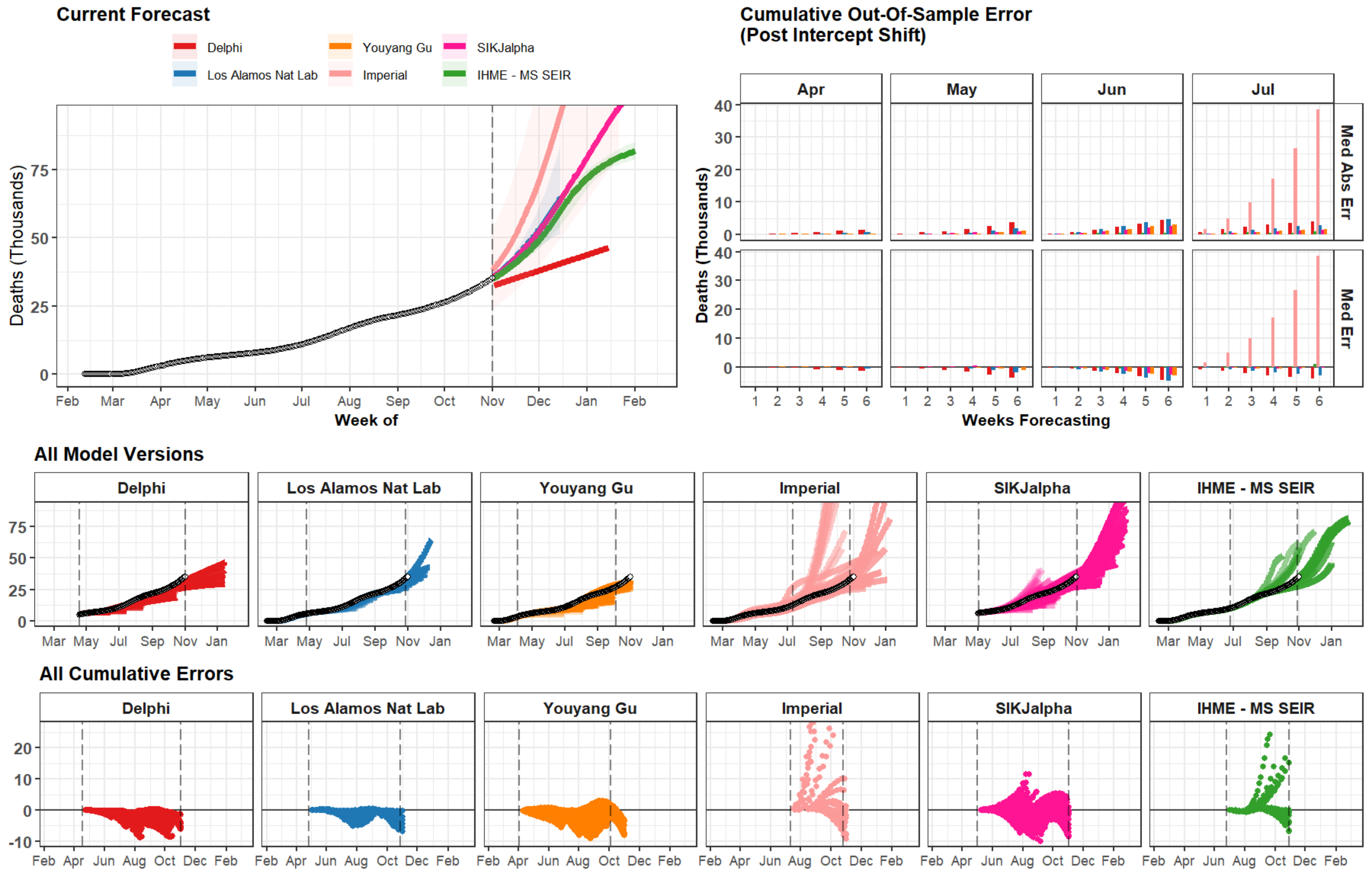
Current Forecast
Delphi
Los Alamos Nat Lab
Youyang Gu
Imperial
SIKJalpha
IHME - MS SEIR
Deaths (Thousands)
0
25
50
75
Feb
Mar
Apr
May
Jun
Jul
Aug
Sep
Oct
Nov
Dec
Jan
Feb
Week of
Cumulative Out-Of-Sample Error
(Post Intercept Shift)
Apr
May
Jun
Jul
Med Abs Err
Med Err
Deaths (Thousands)
0
10
20
30
40
1
2
3
4
5
6
Weeks Forecasting
All Model Versions
Delphi
Los Alamos Nat Lab
Youyang Gu
Imperial
SIKJalpha
IHME - MS SEIR
0
25
50
75
Mar
May
Jul
Sep
Nov
Jan
All Cumulative Errors
Delphi
Los Alamos Nat Lab
Youyang Gu
Imperial
SIKJalpha
IHME - MS SEIR
-10
0
10
20
Feb
Apr
Jun
Aug
Oct
Dec
Feb

New York

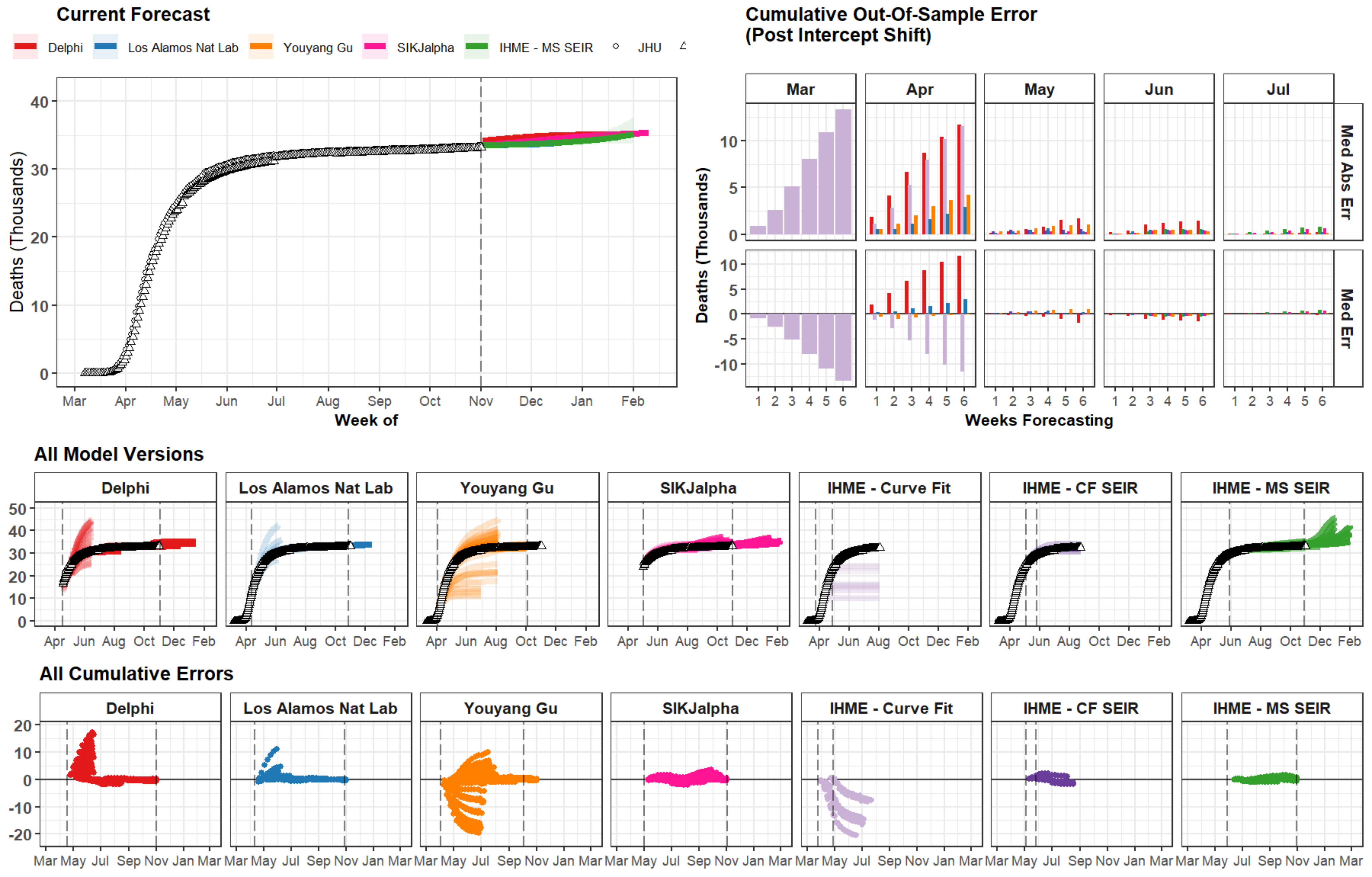
Current Forecast
Delphi
Los Alamos Nat Lab
Youyang Gu
SIKJalpha
IHME - MS SEIR
JHU
Deaths (Thousands)
Week of
Mar Apr May Jun Jul Aug Sep Oct Nov Dec Jan Feb
Cumulative Out-Of-Sample Error
(Post Intercept Shift)
Mar Apr May Jun Jul
Med Abs Err
Med Err
Deaths (Thousands)
Weeks Forecasting
All Model Versions
Delphi
Los Alamos Nat Lab
Youyang Gu
SIKJalpha
IHME - Curve Fit
IHME - CF SEIR
IHME - MS SEIR
Apr Jun Aug Oct Dec Feb
All Cumulative Errors
Delphi
Los Alamos Nat Lab
Youyang Gu
SIKJalpha
IHME - Curve Fit
IHME - CF SEIR
IHME - MS SEIR
Mar May Jul Sep Nov Jan Mar

Colombia

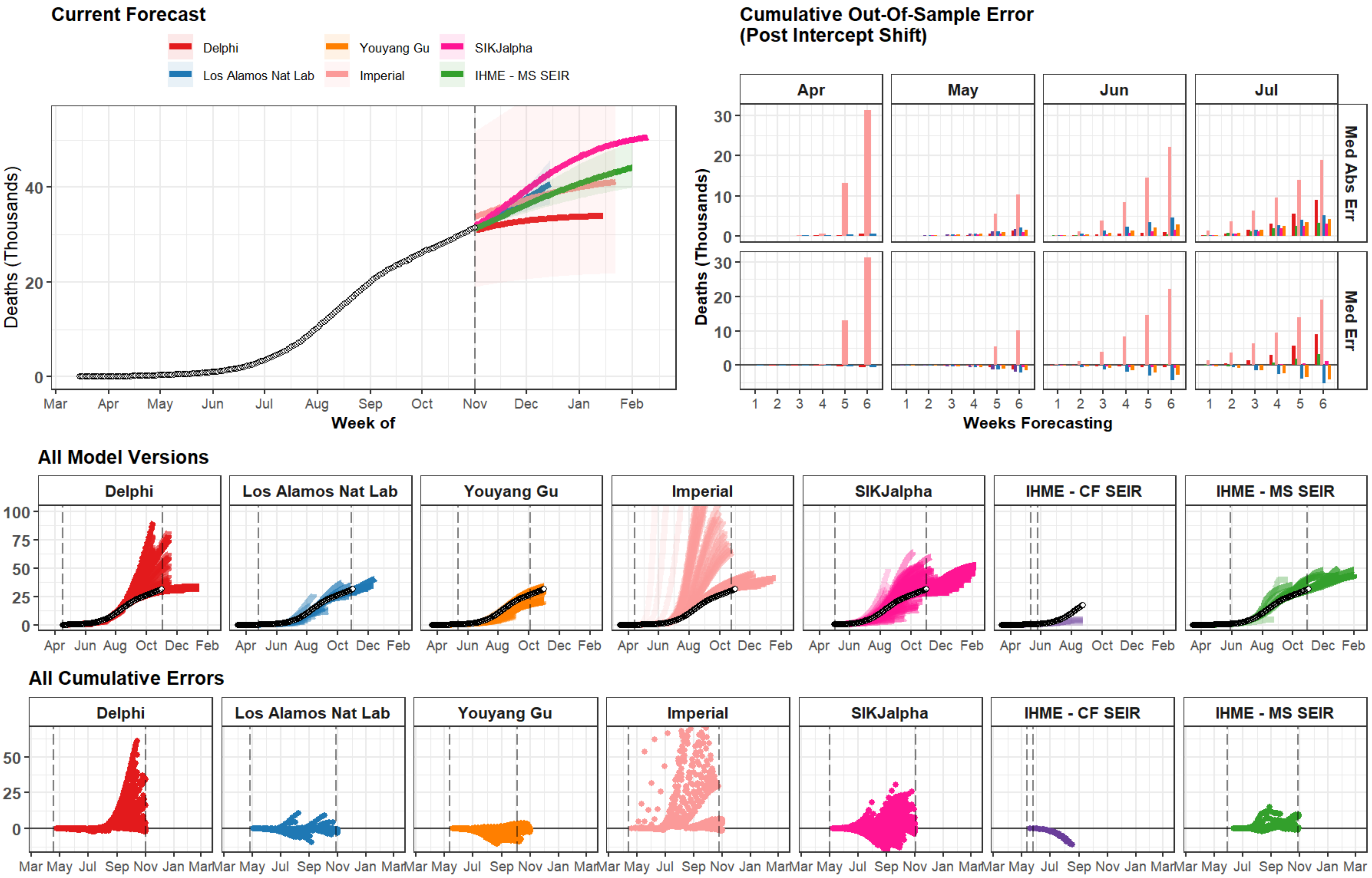

Current Forecast
Delphi
Los Alamos Nat Lab
Youyang Gu
Imperial
SIKJalpha
IHME - MS SEIR
Deaths (Thousands)
Week of
Mar Apr May Jun Jul Aug Sep Oct Nov Dec Jan Feb
Cumulative Out-Of-Sample Error
(Post Intercept Shift)
Apr May Jun Jul
Med Abs Err
Med Err
Weeks Forecasting
All Model Versions
Delphi
Los Alamos Nat Lab
Youyang Gu
Imperial
SIKJalpha
IHME - CF SEIR
IHME - MS SEIR
All Cumulative Errors

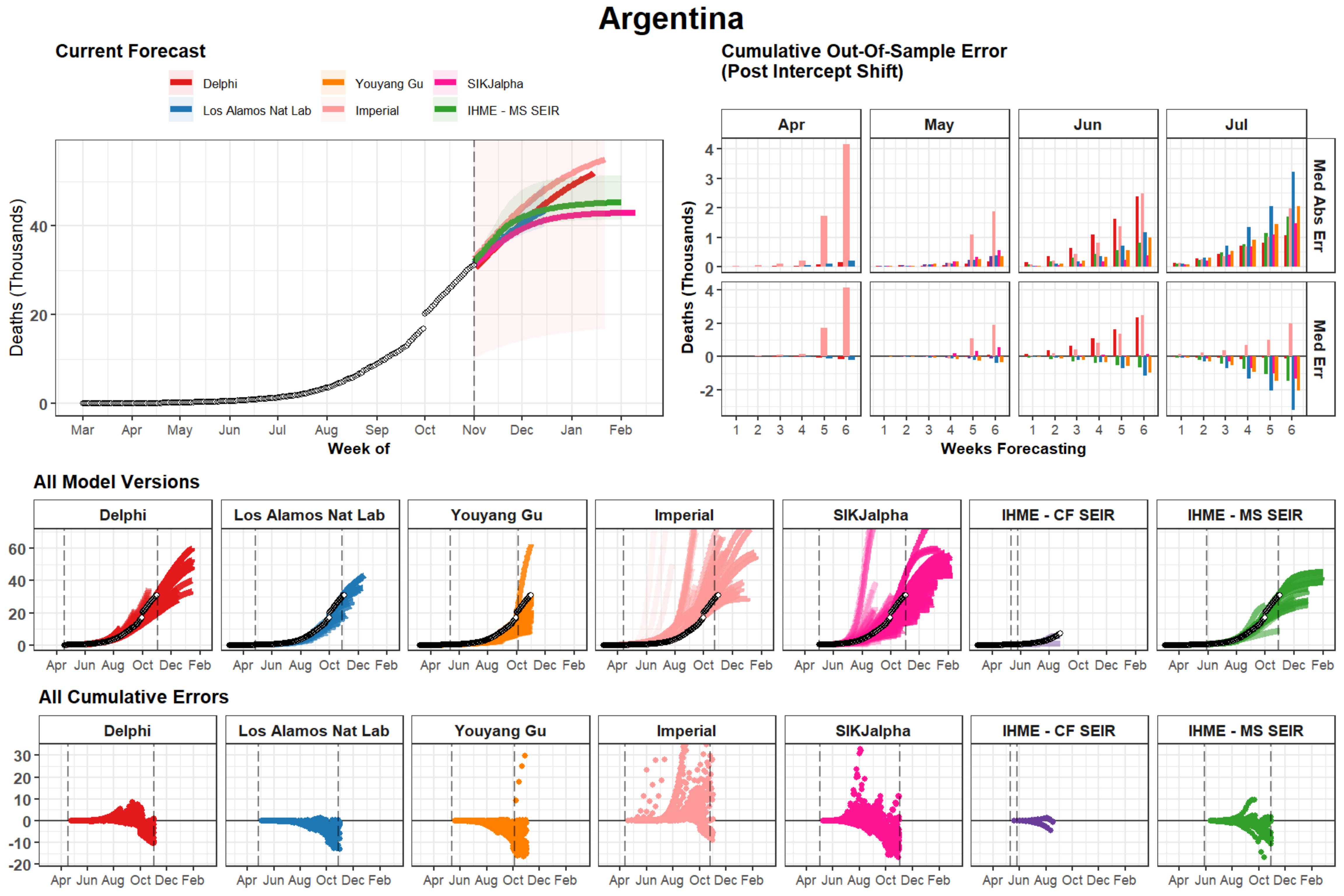
Argentina
Current Forecast
Delphi
Youyang Gu
SIKJalpha
Los Alamos Nat Lab
Imperial
IHME - MS SEIR
Deaths (Thousands)
Week of
Mar Apr May Jun Jul Aug Sep Oct Nov Dec Jan Feb
Cumulative Out-Of-Sample Error
(Post Intercept Shift)
Apr May Jun Jul
Med Abs Err
Med Err
Weeks Forecasting
All Model Versions
Delphi Los Alamos Nat Lab Youyang Gu Imperial SIKJalpha IHME - CF SEIR IHME - MS SEIR
All Cumulative Errors
Delphi Los Alamos Nat Lab Youyang Gu Imperial SIKJalpha IHME - CF SEIR IHME - MS SEIR

Russian Federation

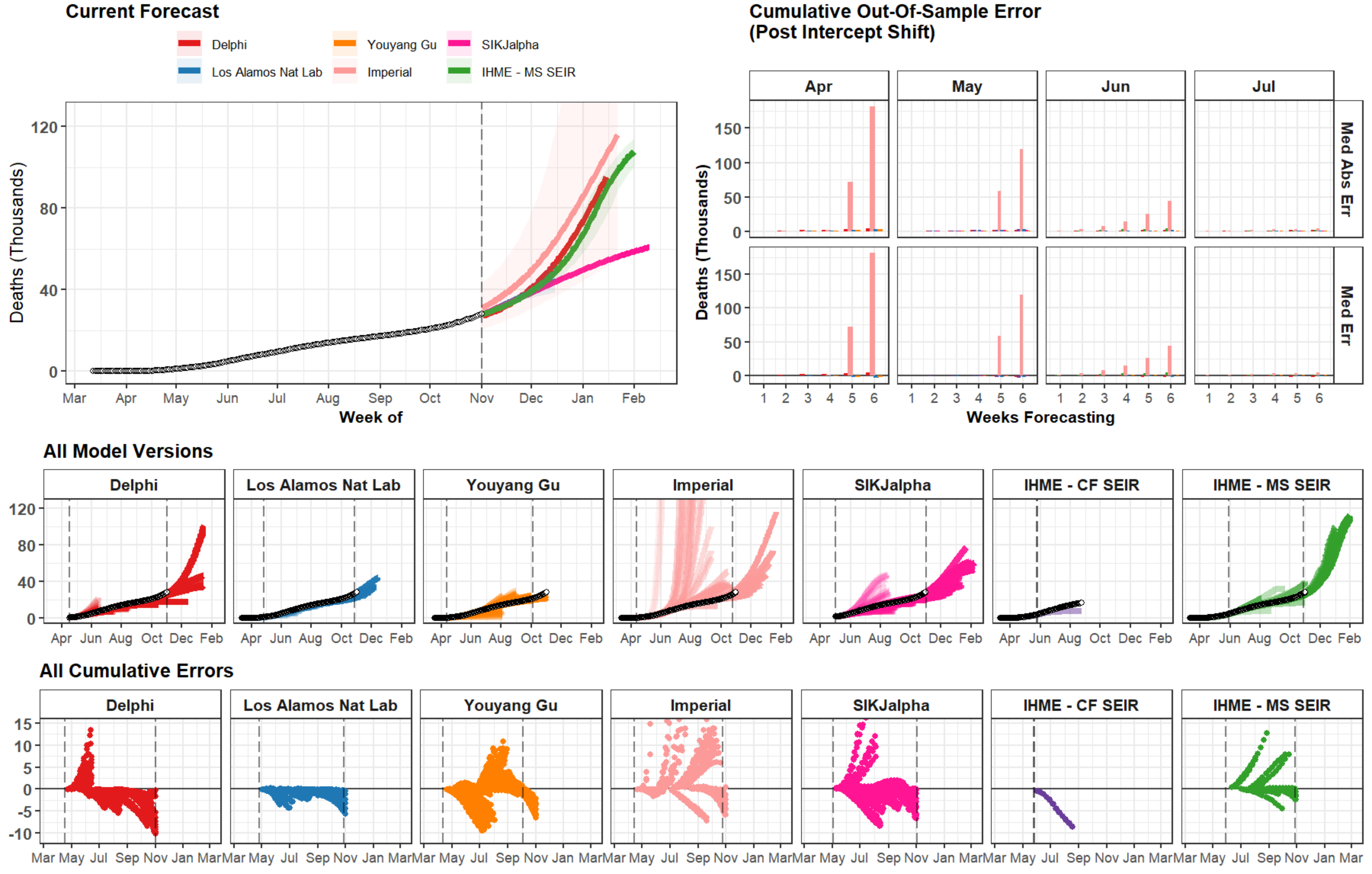

South Africa

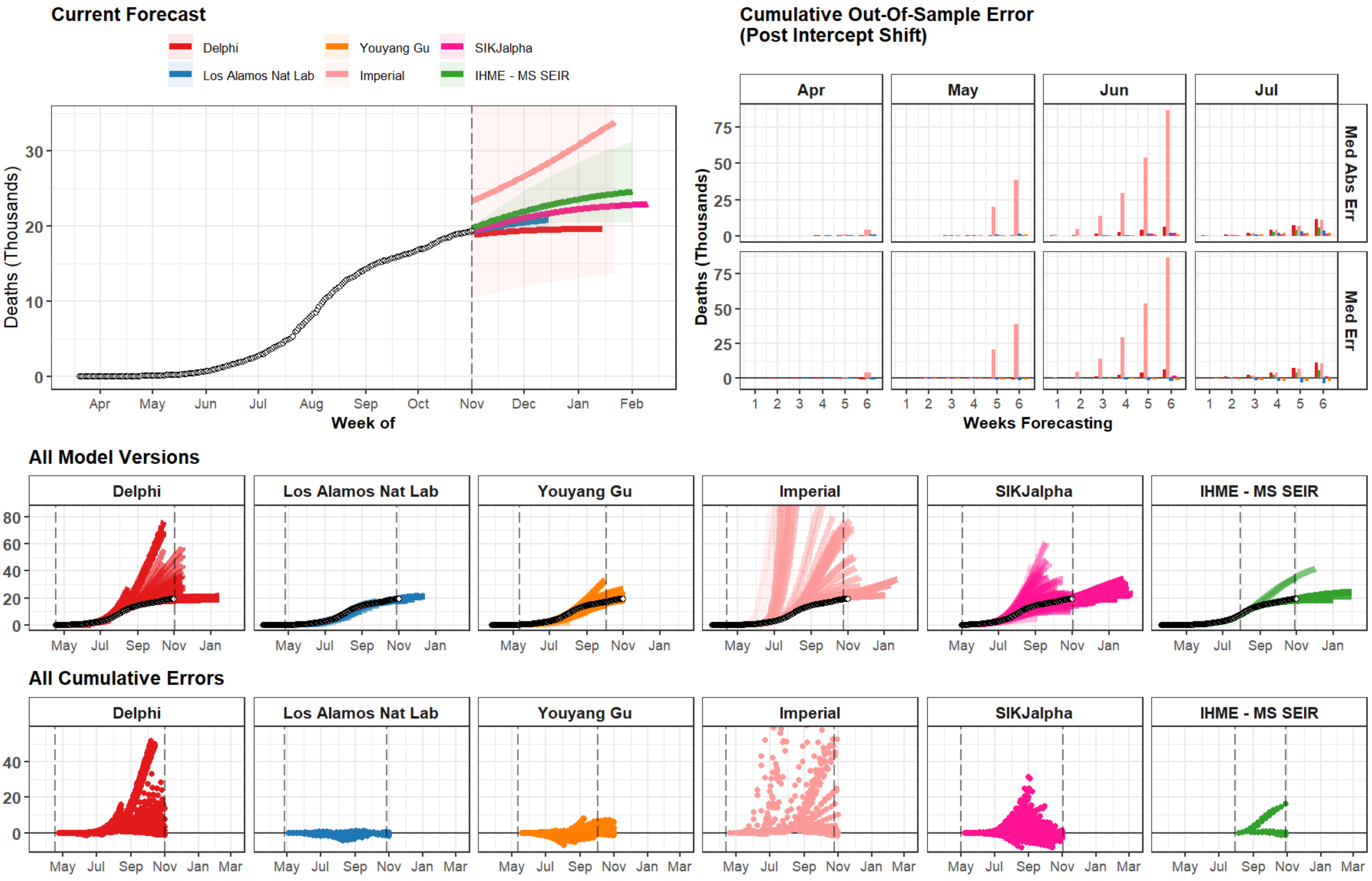

Texas

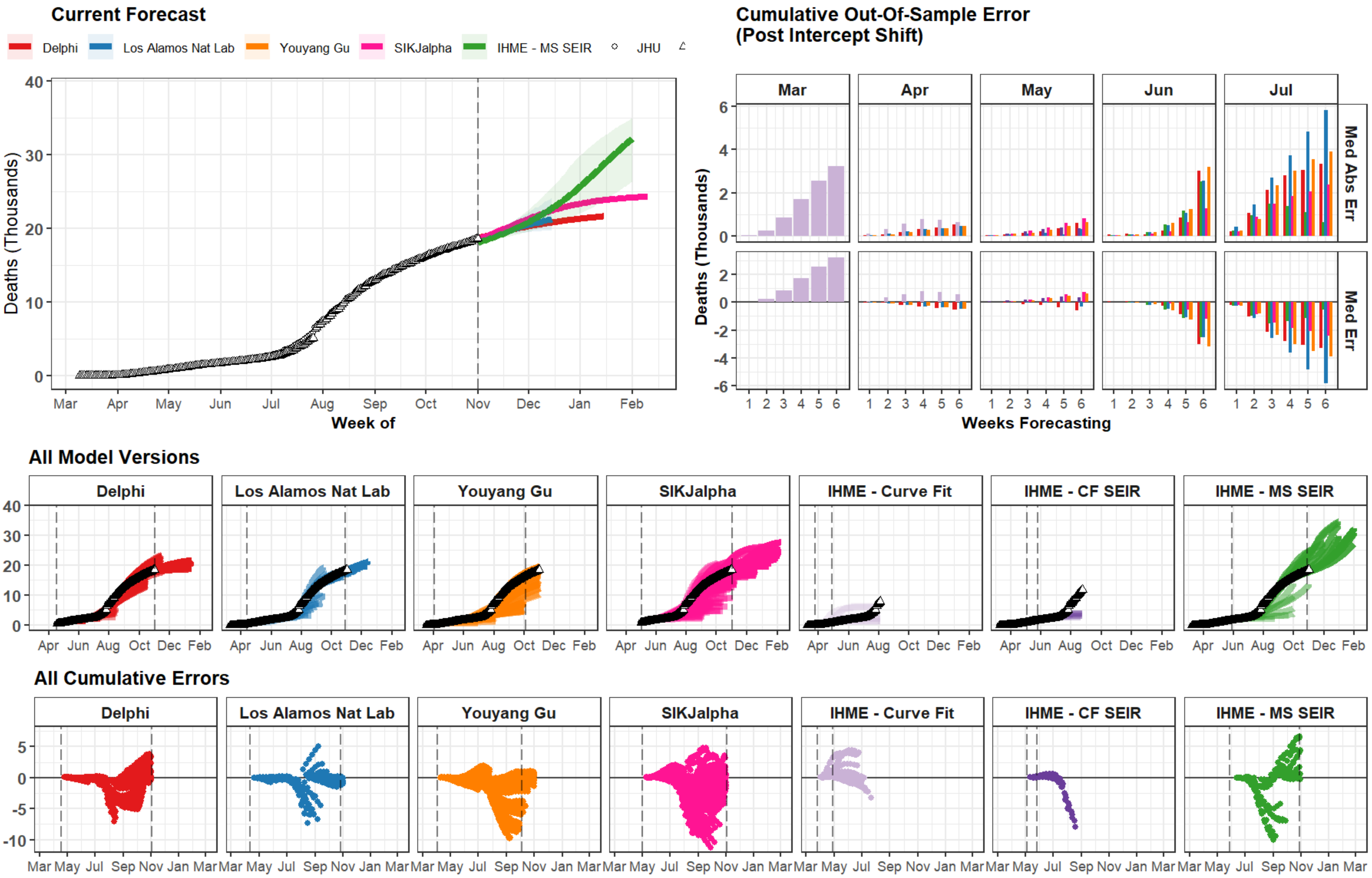

Current Forecast
Delphi
Los Alamos Nat Lab
Youyang Gu
SIKJalpha
IHME - MS SEIR
JHU
Deaths (Thousands)
Week of
Mar
Apr
May
Jun
Jul
Aug
Sep
Oct
Nov
Dec
Jan
Feb
Cumulative Out-Of-Sample Error
(Post Intercept Shift)
Med Abs Err
Med Err
Weeks Forecasting
All Model Versions
IHME - Curve Fit
IHME - CF SEIR
All Cumulative Errors

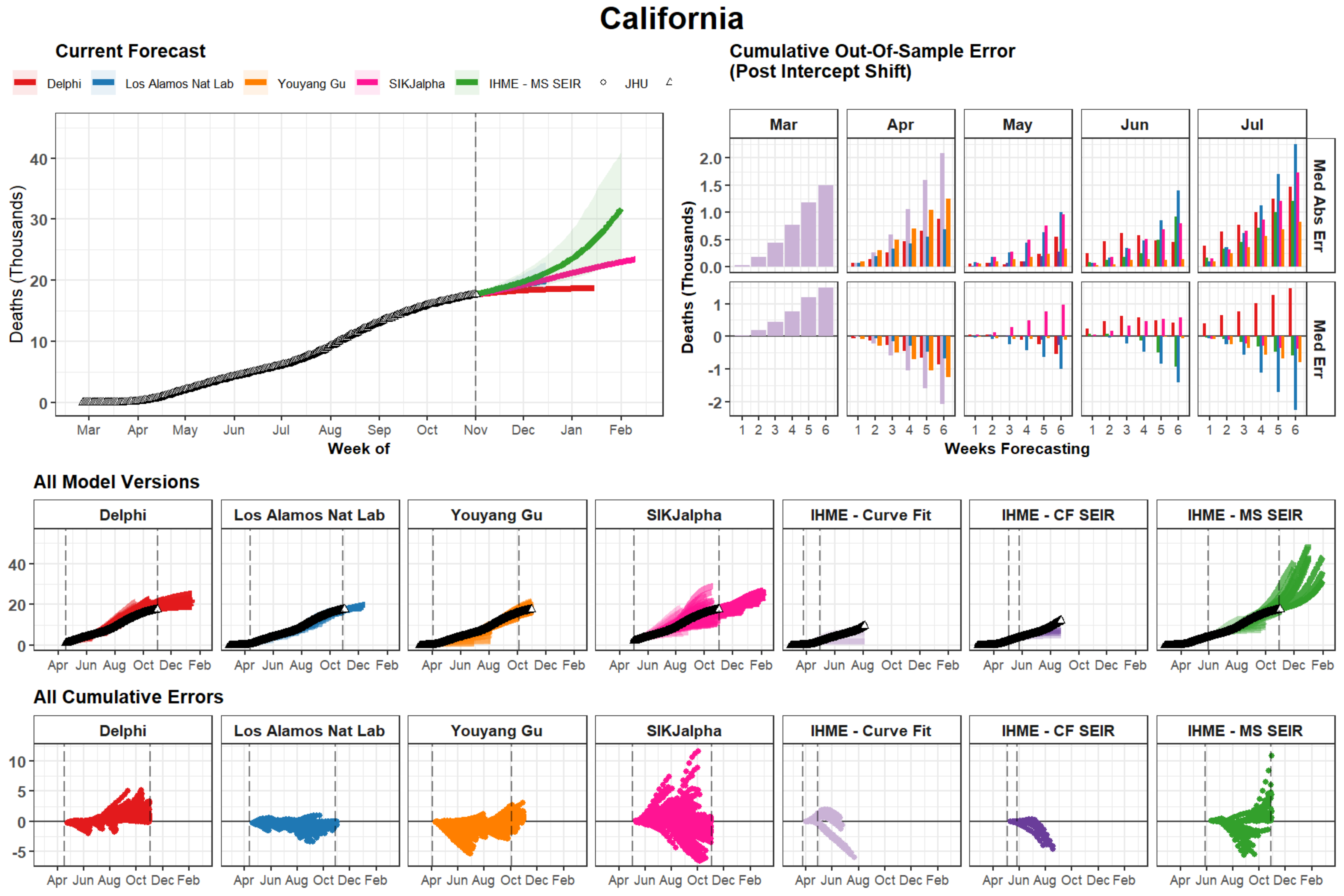

California
Current Forecast
Delphi
Los Alamos Nat Lab
Youyang Gu
SIKJalpha
IHME - MS SEIR
JHU
Deaths (Thousands)
Week of
Mar
Apr
May
Jun
Jul
Aug
Sep
Oct
Nov
Dec
Jan
Feb
Cumulative Out-Of-Sample Error
(Post Intercept Shift)
Med Abs Err
Med Err
Weeks Forecasting
All Model Versions
IHME - Curve Fit
IHME - CF SEIR
All Cumulative Errors

Florida

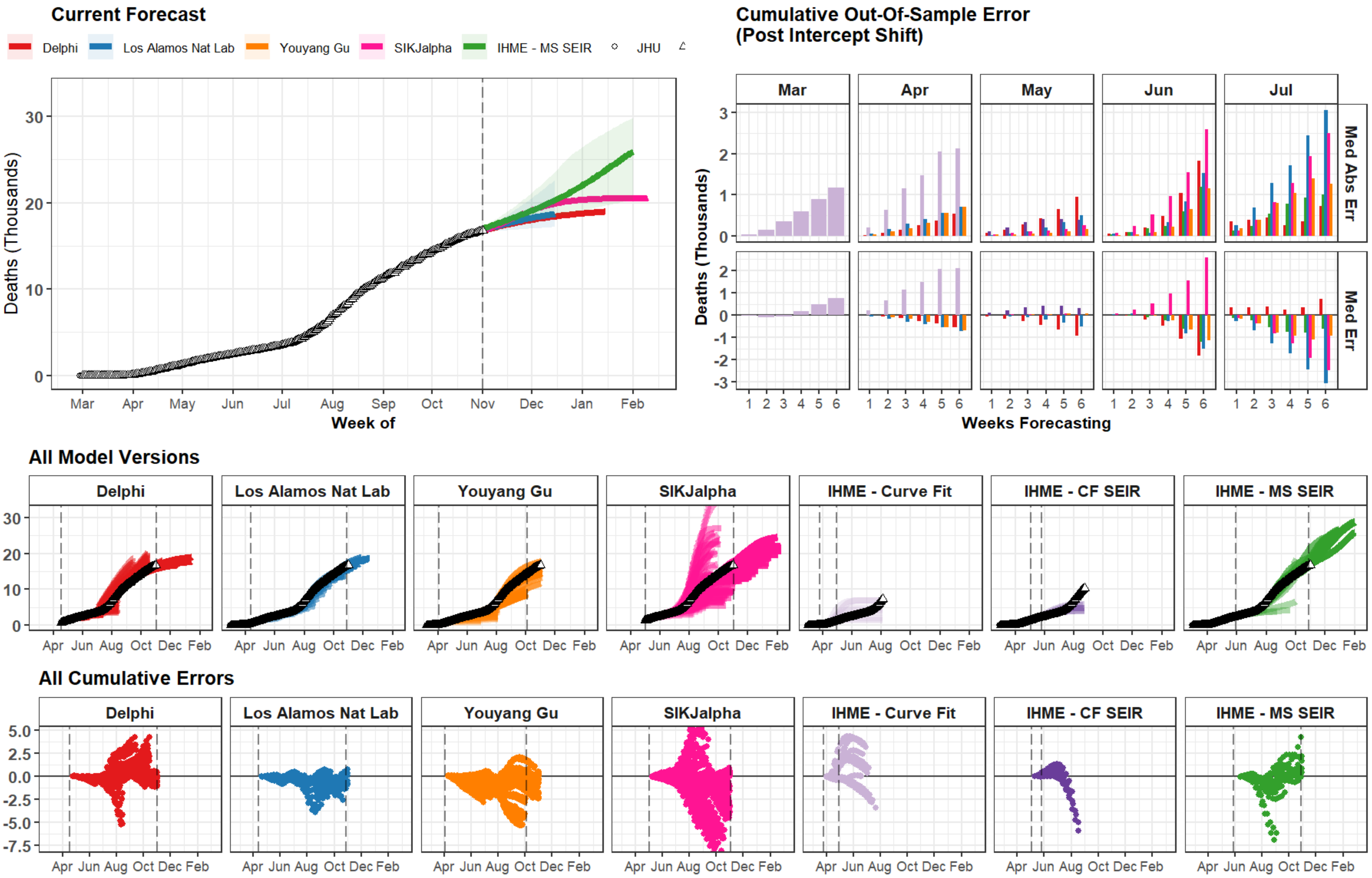
Current Forecast
Delphi
Los Alamos Nat Lab
Youyang Gu
SIKJalpha
IHME - MS SEIR
JHU
Deaths (Thousands)
Week of
Mar Apr May Jun Jul Aug Sep Oct Nov Dec Jan Feb
Cumulative Out-Of-Sample Error
(Post Intercept Shift)
Mar Apr May Jun Jul
Med Abs Err
Med Err
Weeks Forecasting
All Model Versions
Delphi
Los Alamos Nat Lab
Youyang Gu
SIKJalpha
IHME - Curve Fit
IHME - CF SEIR
IHME - MS SEIR
All Cumulative Errors

New Jersey

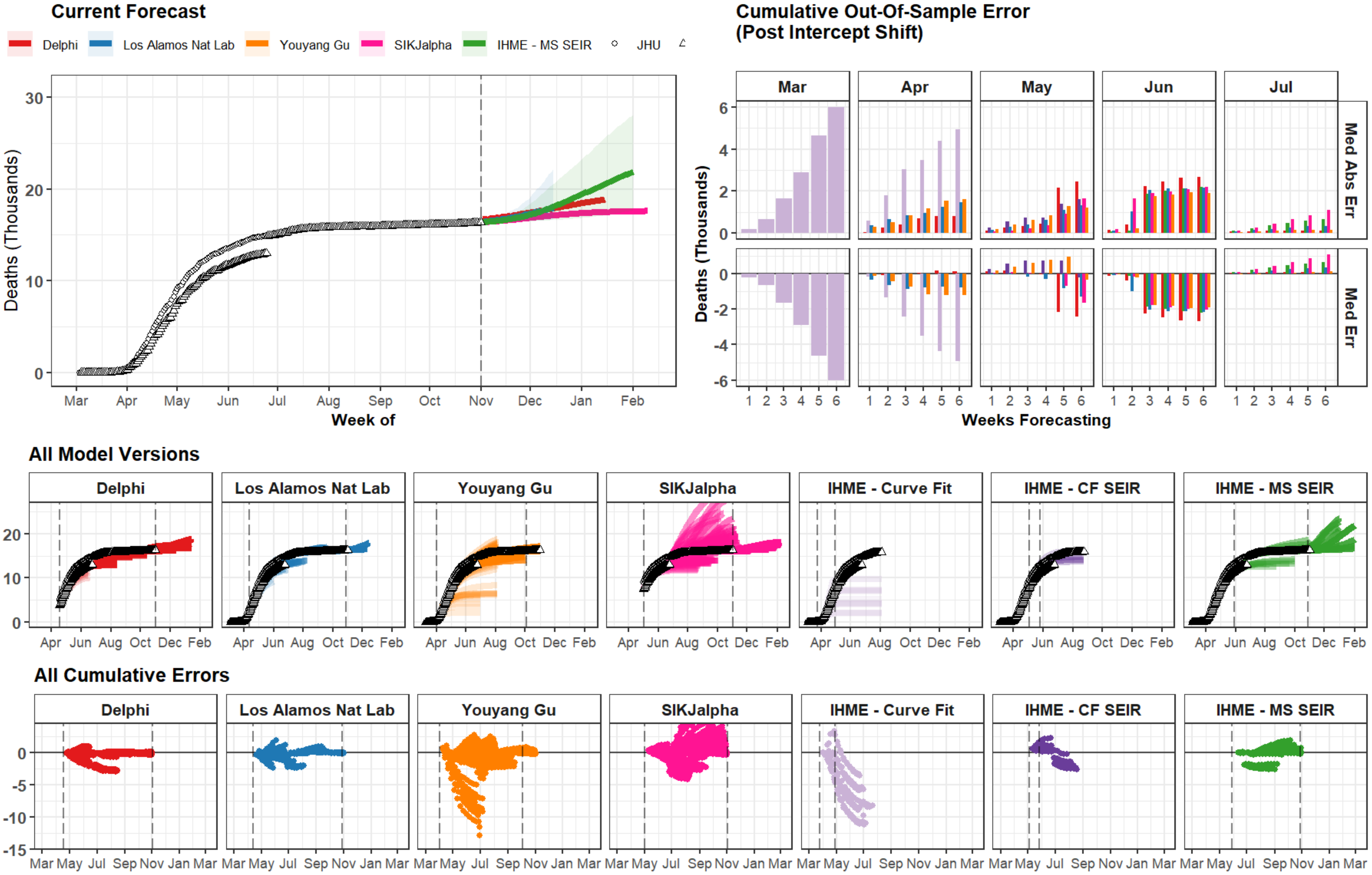
Current Forecast
Delphi
Los Alamos Nat Lab
Youyang Gu
SIKJalpha
IHME - MS SEIR
JHU
Deaths (Thousands)
0
10
20
30
Mar Apr May Jun Jul Aug Sep Oct Nov Dec Jan Feb
Week of
Cumulative Out-Of-Sample Error
(Post Intercept Shift)
Mar
Apr
May
Jun
Jul
Med Abs Err
Med Err
6
4
2
0
-2
-4
-6
1 2 3 4 5 6
Weeks Forecasting
All Model Versions
Delphi
Los Alamos Nat Lab
Youyang Gu
SIKJalpha
IHME - Curve Fit
IHME - CF SEIR
IHME - MS SEIR
Apr Jun Aug Oct Dec Feb
All Cumulative Errors
0
-5
-10
-15
Mar May Jul Sep Nov Jan Mar

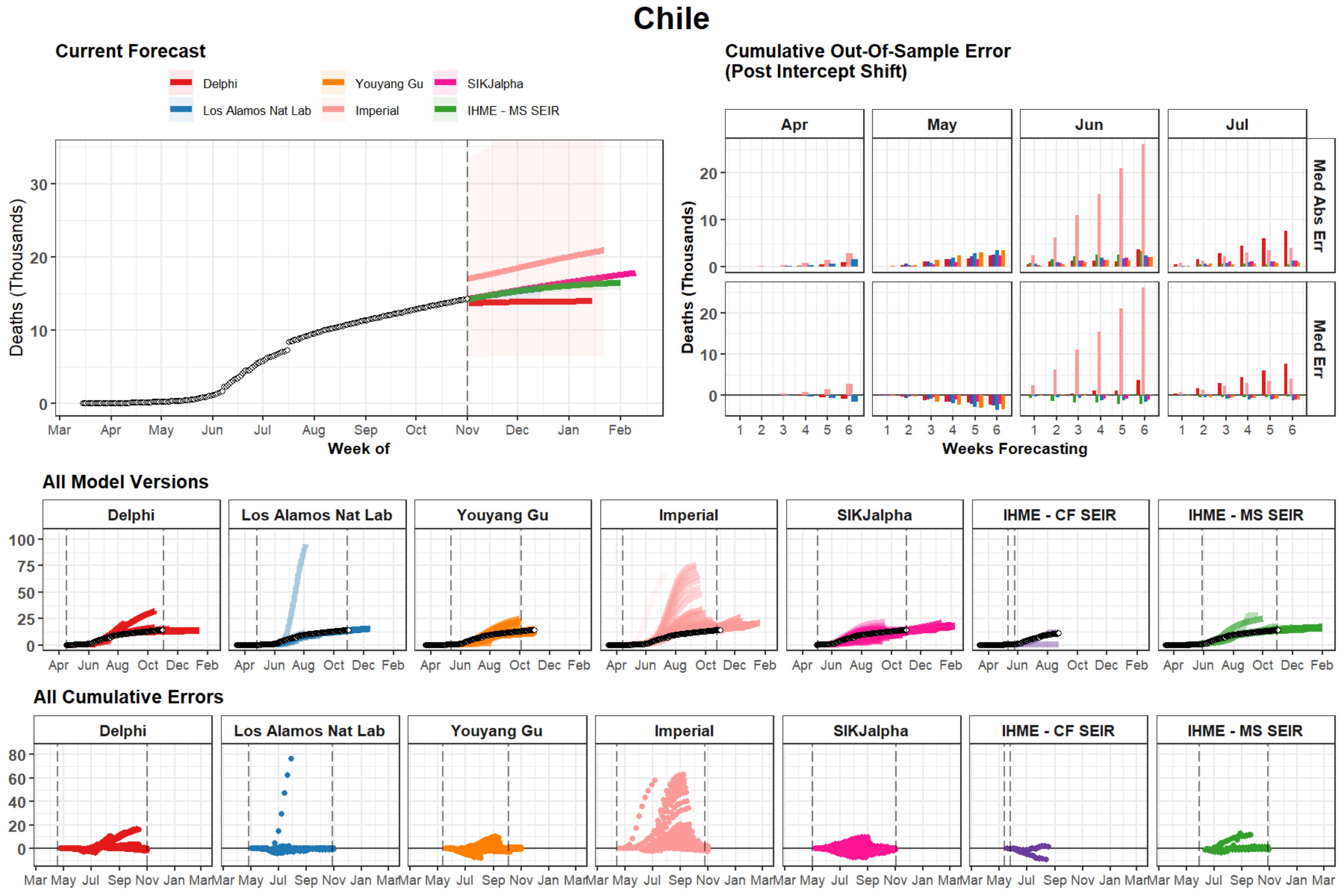
Chile
Current Forecast
Delphi
Los Alamos Nat Lab
Youyang Gu
Imperial
SIKJalpha
IHME - MS SEIR
Deaths (Thousands)
Week of
Mar Apr May Jun Jul Aug Sep Oct Nov Dec Jan Feb
Cumulative Out-Of-Sample Error
(Post Intercept Shift)
Apr
May
Jun
Jul
Med Abs Err
Med Err
Weeks Forecasting
All Model Versions
Delphi
Los Alamos Nat Lab
Youyang Gu
Imperial
SIKJalpha
IHME - CF SEIR
IHME - MS SEIR
All Cumulative Errors

Indonesia

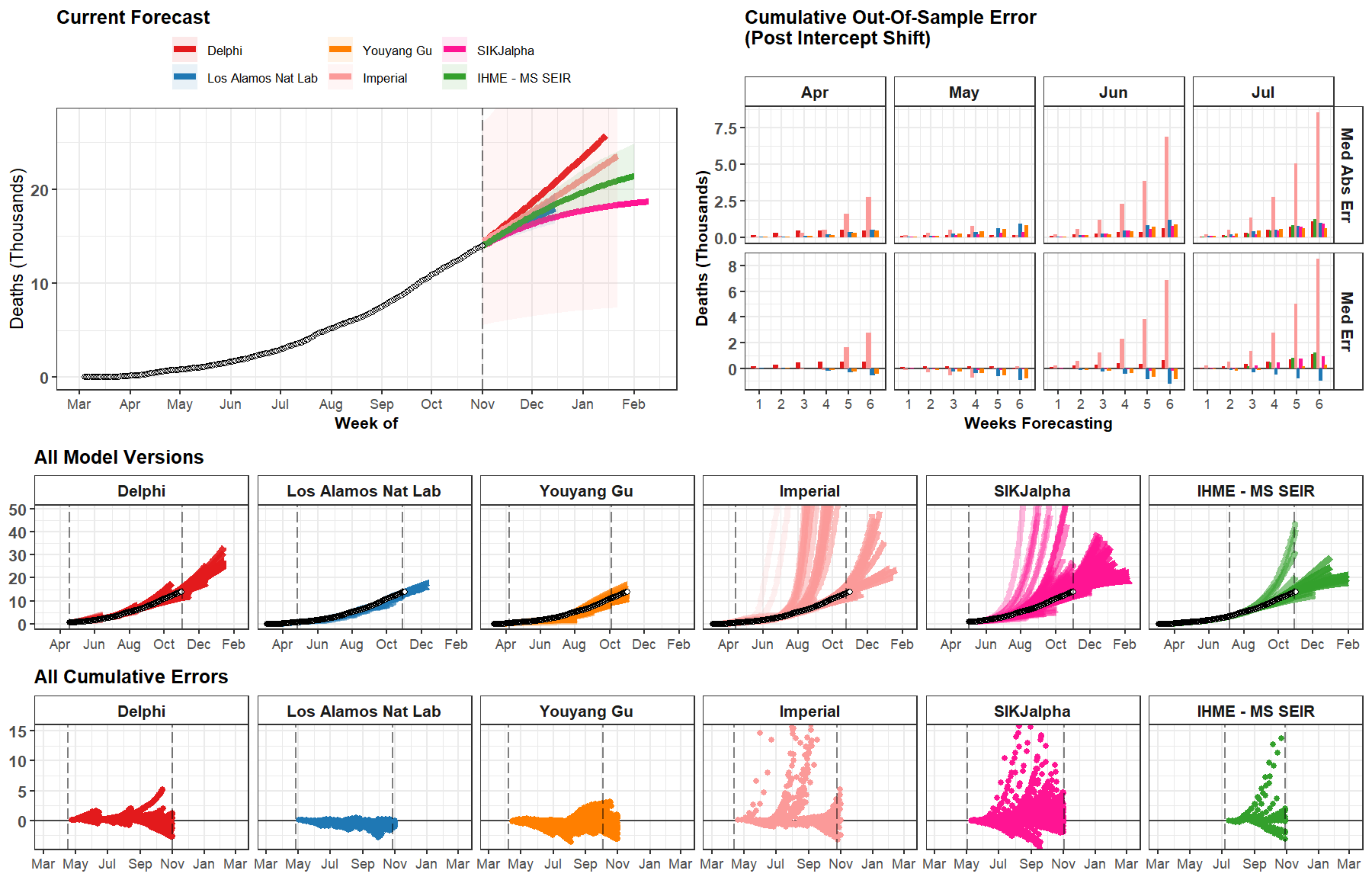
Current Forecast
Delphi
Youyang Gu
SIKJalpha
Los Alamos Nat Lab
Imperial
IHME - MS SEIR
Deaths (Thousands)
Week of
Mar Apr May Jun Jul Aug Sep Oct Nov Dec Jan Feb
Cumulative Out-Of-Sample Error
(Post Intercept Shift)
Apr May Jun Jul
Med Abs Err
Med Err
Weeks Forecasting
All Model Versions
All Cumulative Errors

Belgium

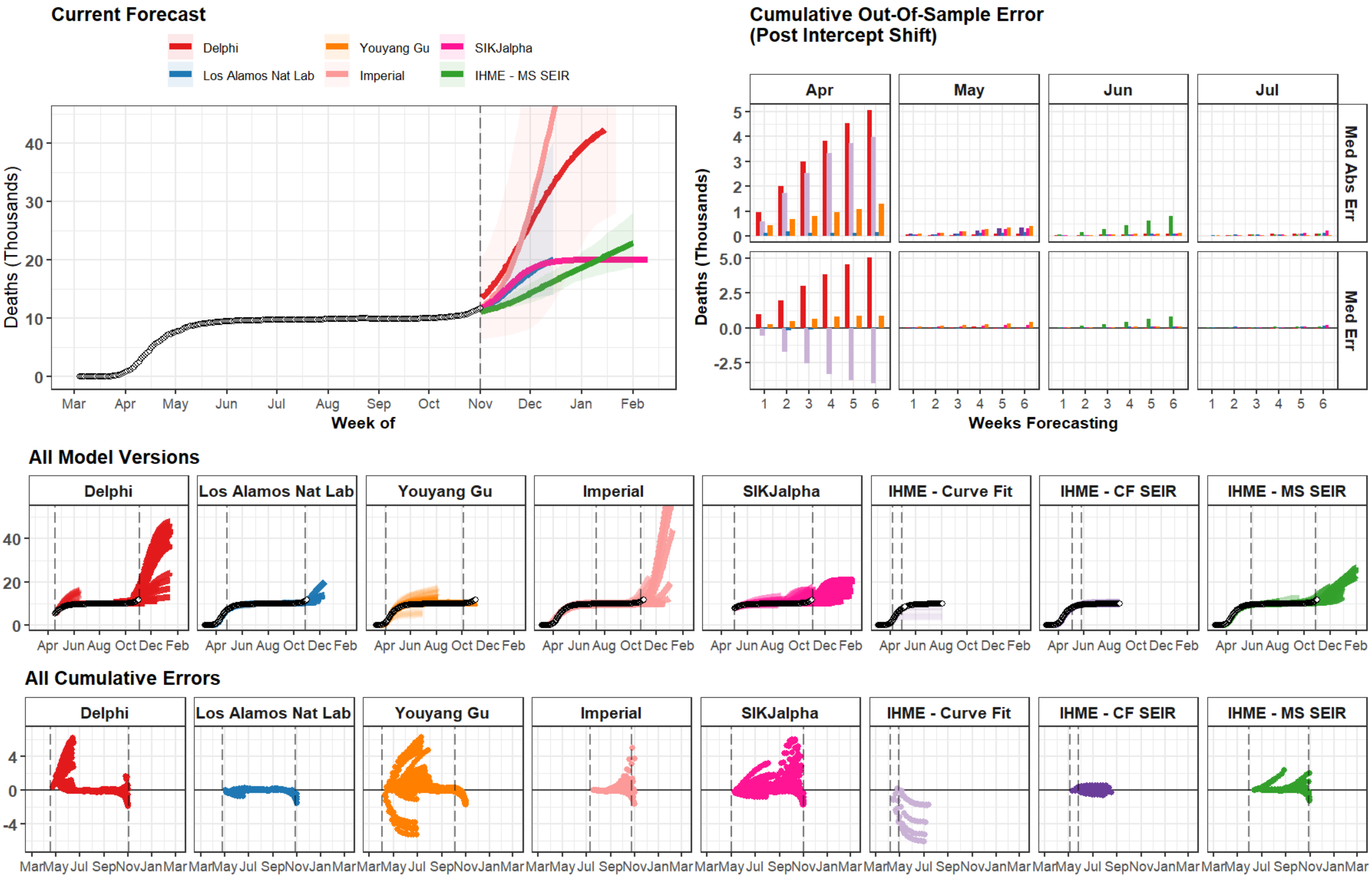
Current Forecast
Delphi
Youyang Gu
SIKJalpha
Los Alamos Nat Lab
Imperial
IHME - MS SEIR
Deaths (Thousands)
Week of
Mar Apr May Jun Jul Aug Sep Oct Nov Dec Jan Feb
Cumulative Out-Of-Sample Error
(Post Intercept Shift)
Apr
May
Jun
Jul
Med Abs Err
Med Err
Weeks Forecasting
All Model Versions
Delphi
Los Alamos Nat Lab
Youyang Gu
Imperial
SIKJalpha
IHME - Curve Fit
IHME - CF SEIR
IHME - MS SEIR
Apr Jun Aug Oct Dec Feb
All Cumulative Errors
MarMay Jul SepNovJanMar

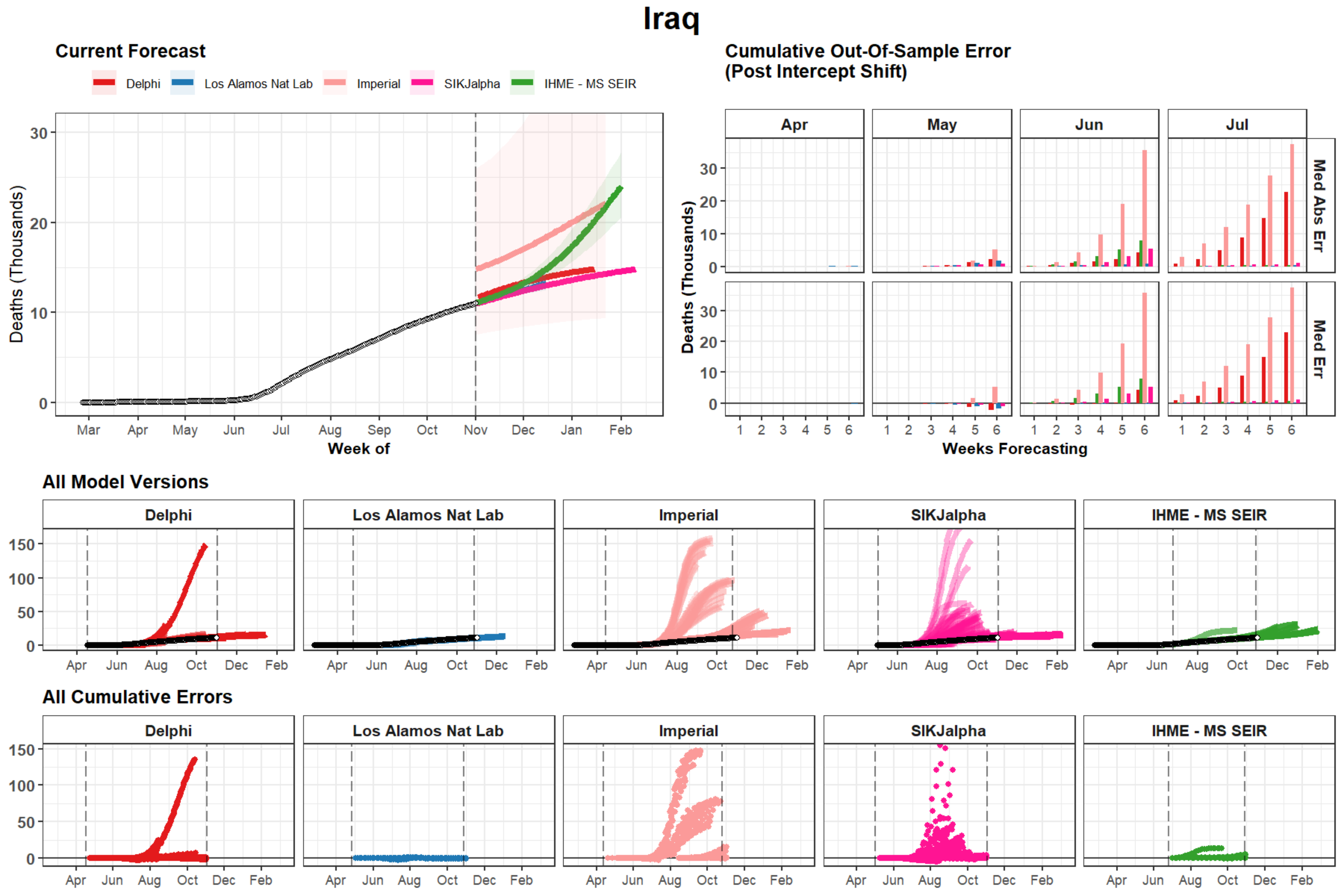
Iraq
Current Forecast
Delphi
Los Alamos Nat Lab
Imperial
SIKJalpha
IHME - MS SEIR
Deaths (Thousands)
Week of
Cumulative Out-Of-Sample Error
(Post Intercept Shift)
Apr
May
Jun
Jul
Med Abs Err
Med Err
Weeks Forecasting
All Model Versions
All Cumulative Errors

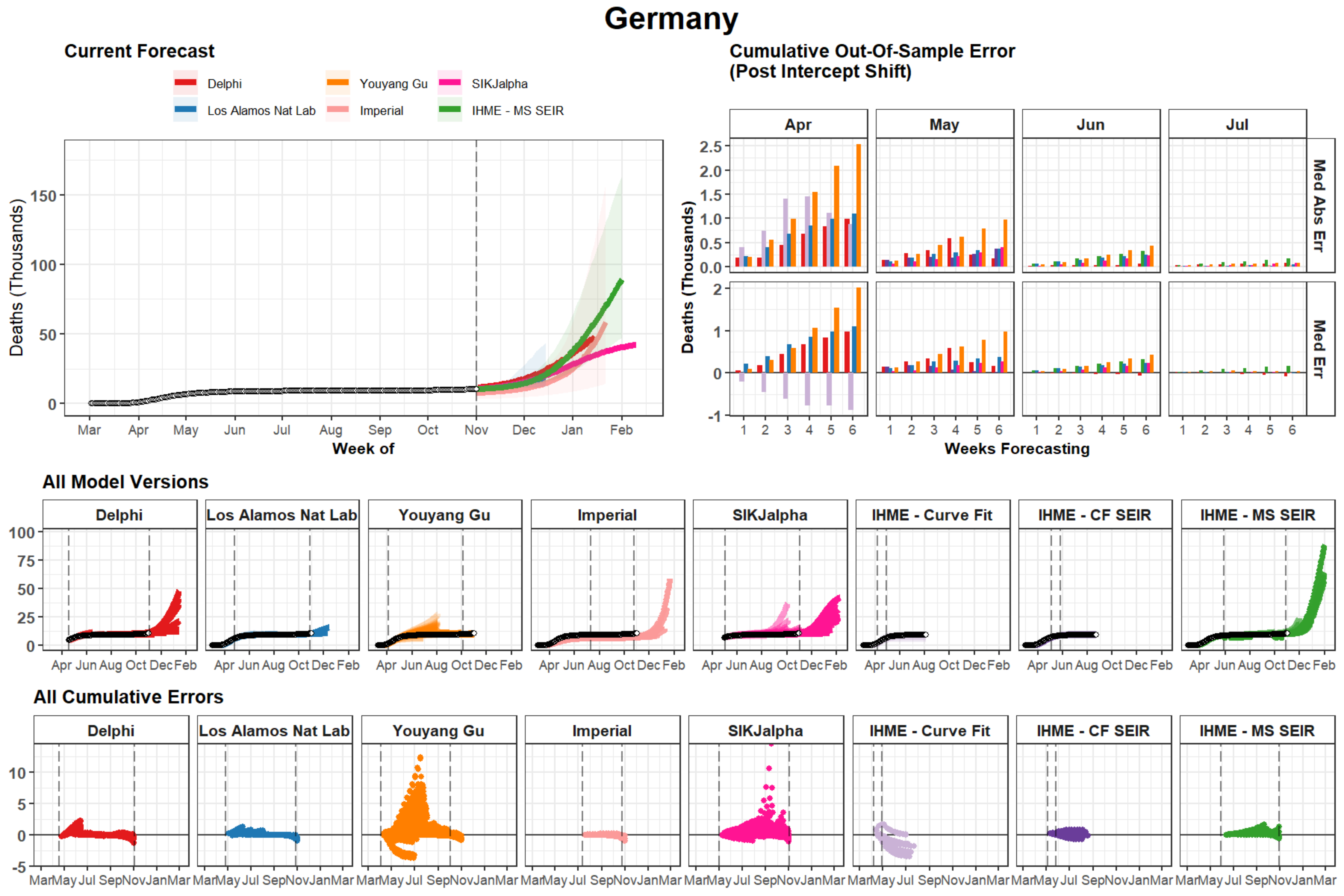
Germany
Current Forecast
Delphi
Los Alamos Nat Lab
Youyang Gu
Imperial
SIKJalpha
IHME - MS SEIR
Deaths (Thousands)
Week of
Cumulative Out-Of-Sample Error
(Post Intercept Shift)
Apr
May
Jun
Jul
Med Abs Err
Med Err
Weeks Forecasting
All Model Versions
Delphi
Los Alamos Nat Lab
Youyang Gu
Imperial
SIKJalpha
IHME - Curve Fit
IHME - CF SEIR
IHME - MS SEIR
All Cumulative Errors

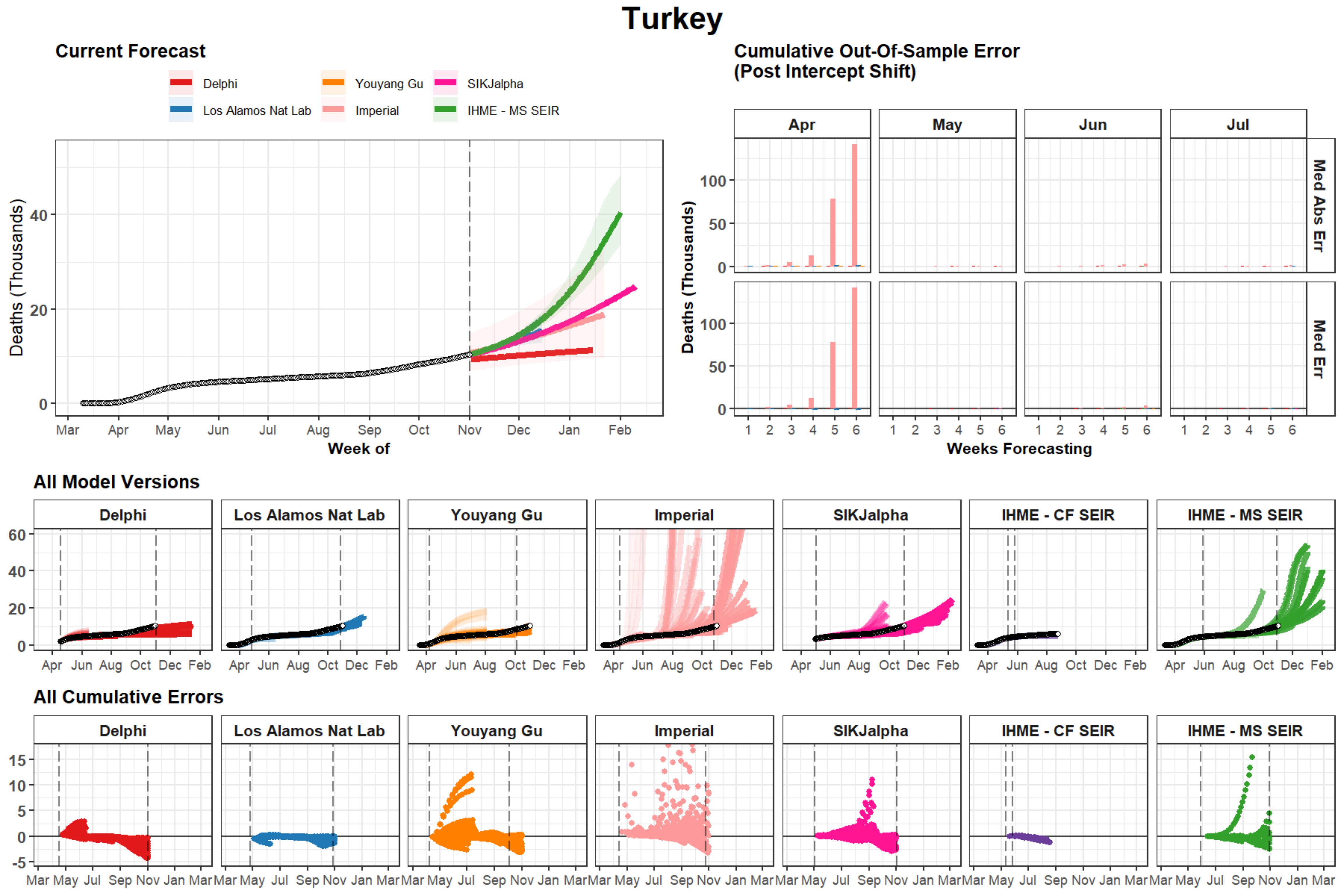

Turkey
Current Forecast
Delphi
Los Alamos Nat Lab
Youyang Gu
Imperial
SIKJalpha
IHME - MS SEIR
Deaths (Thousands)
Week of
Cumulative Out-Of-Sample Error
(Post Intercept Shift)
Apr
May
Jun
Jul
Med Abs Err
Med Err
Weeks Forecasting
All Model Versions
Delphi
Los Alamos Nat Lab
Youyang Gu
Imperial
SIKJalpha
IHME - CF SEIR
IHME - MS SEIR
All Cumulative Errors

Canada

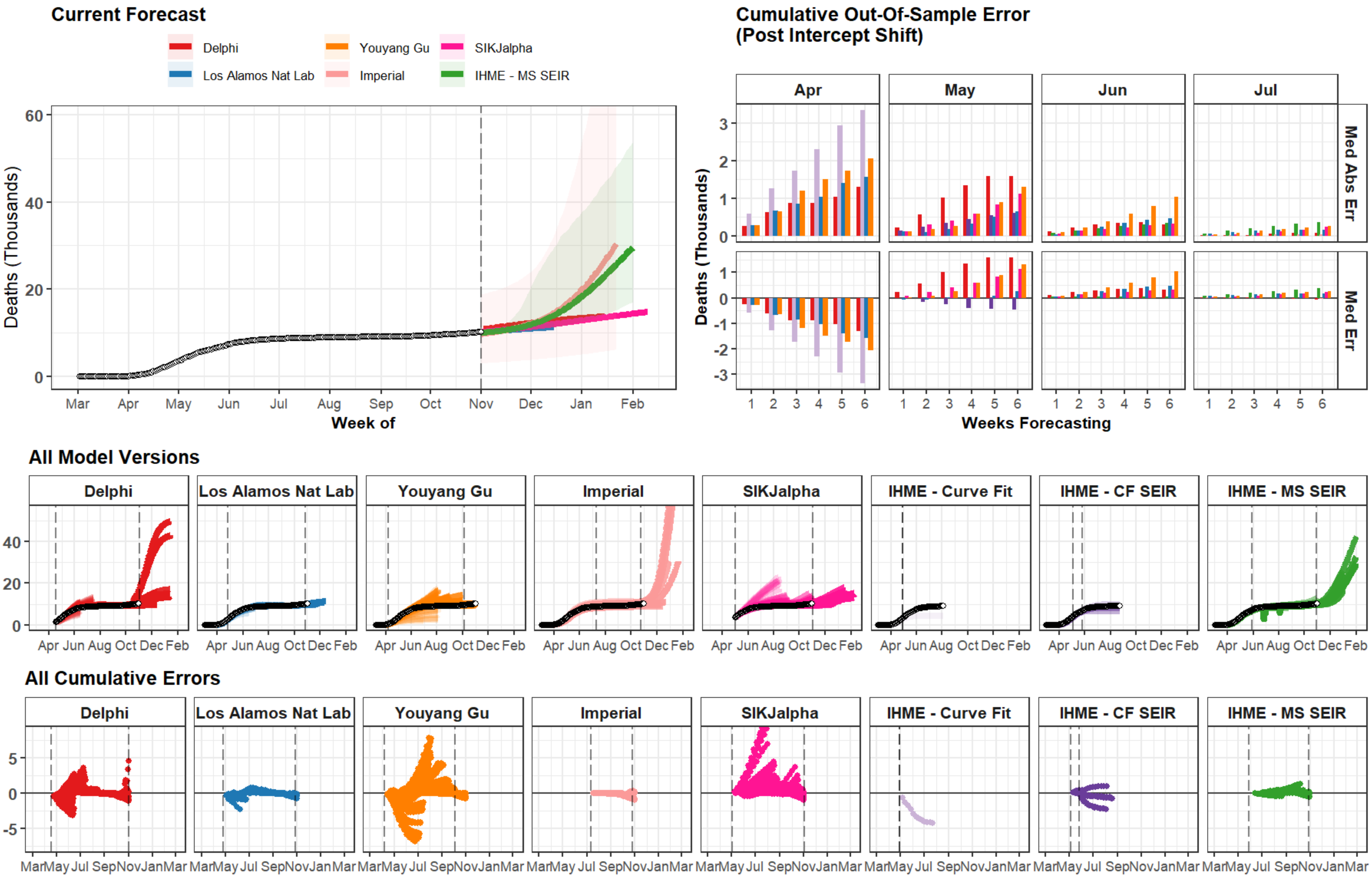

Current Forecast
Delphi
Youyang Gu
SIKJalpha
Los Alamos Nat Lab
Imperial
IHME - MS SEIR
Deaths (Thousands)
0
20
40
60
Mar
Apr
May
Jun
Jul
Aug
Sep
Oct
Nov
Dec
Jan
Feb
Week of
Cumulative Out-Of-Sample Error
(Post Intercept Shift)
Apr
May
Jun
Jul
Med Abs Err
Med Err
Weeks Forecasting
All Model Versions
Delphi
Los Alamos Nat Lab
Youyang Gu
Imperial
SIKJalpha
IHME - Curve Fit
IHME - CF SEIR
IHME - MS SEIR
Apr Jun Aug Oct Dec Feb
All Cumulative Errors
MarMay Jul SepNovJanMar

Illinois

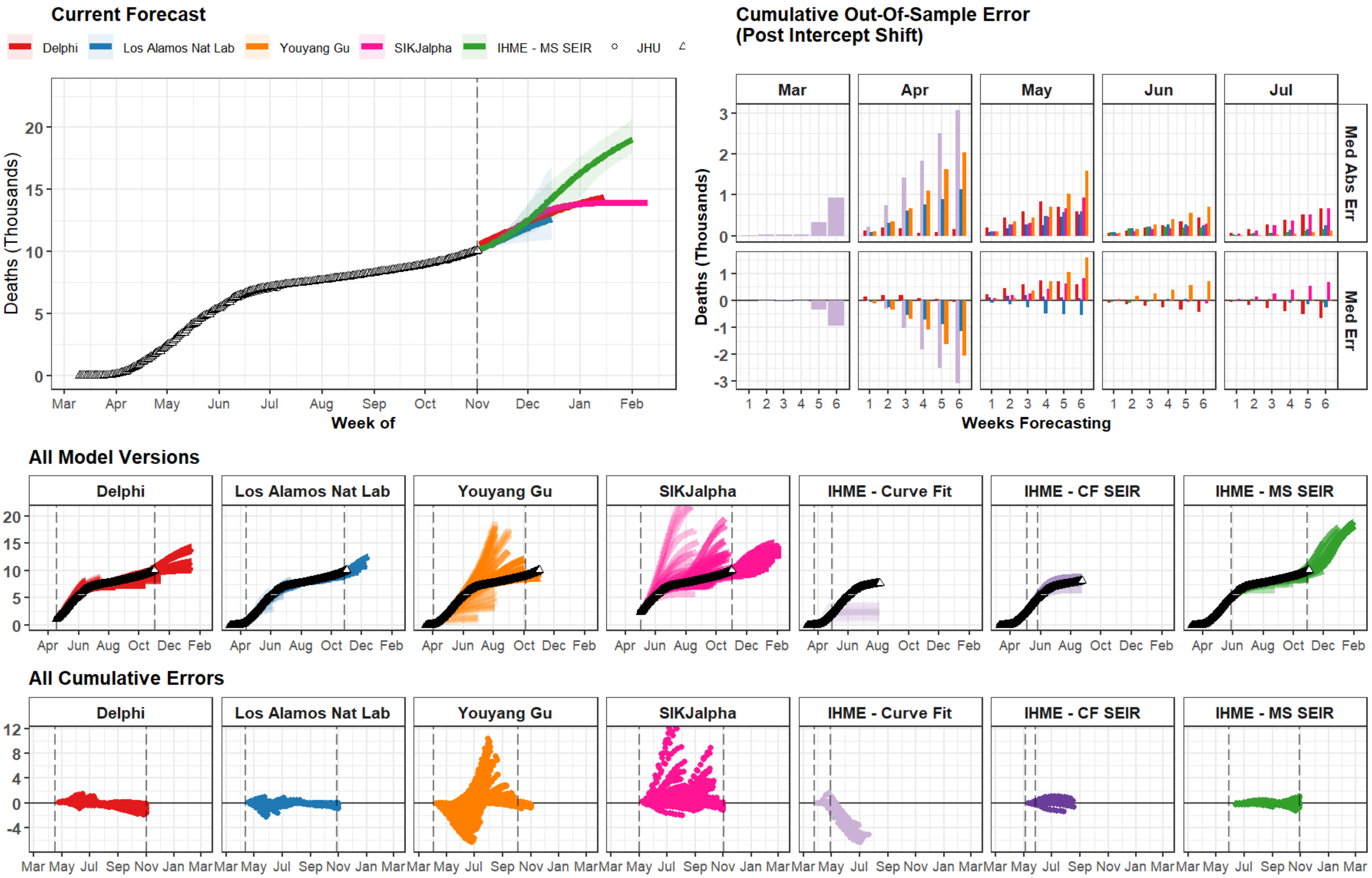

Current Forecast
Delphi
Los Alamos Nat Lab
Youyang Gu
SIKJalpha
IHME - MS SEIR
JHU
Deaths (Thousands)
Week of
Mar Apr May Jun Jul Aug Sep Oct Nov Dec Jan Feb
Cumulative Out-Of-Sample Error
(Post Intercept Shift)
Mar
Apr
May
Jun
Jul
Med Abs Err
Med Err
Weeks Forecasting
All Model Versions
Delphi
Los Alamos Nat Lab
Youyang Gu
SIKJalpha
IHME - Curve Fit
IHME - CF SEIR
IHME - MS SEIR
All Cumulative Errors

Massachusetts

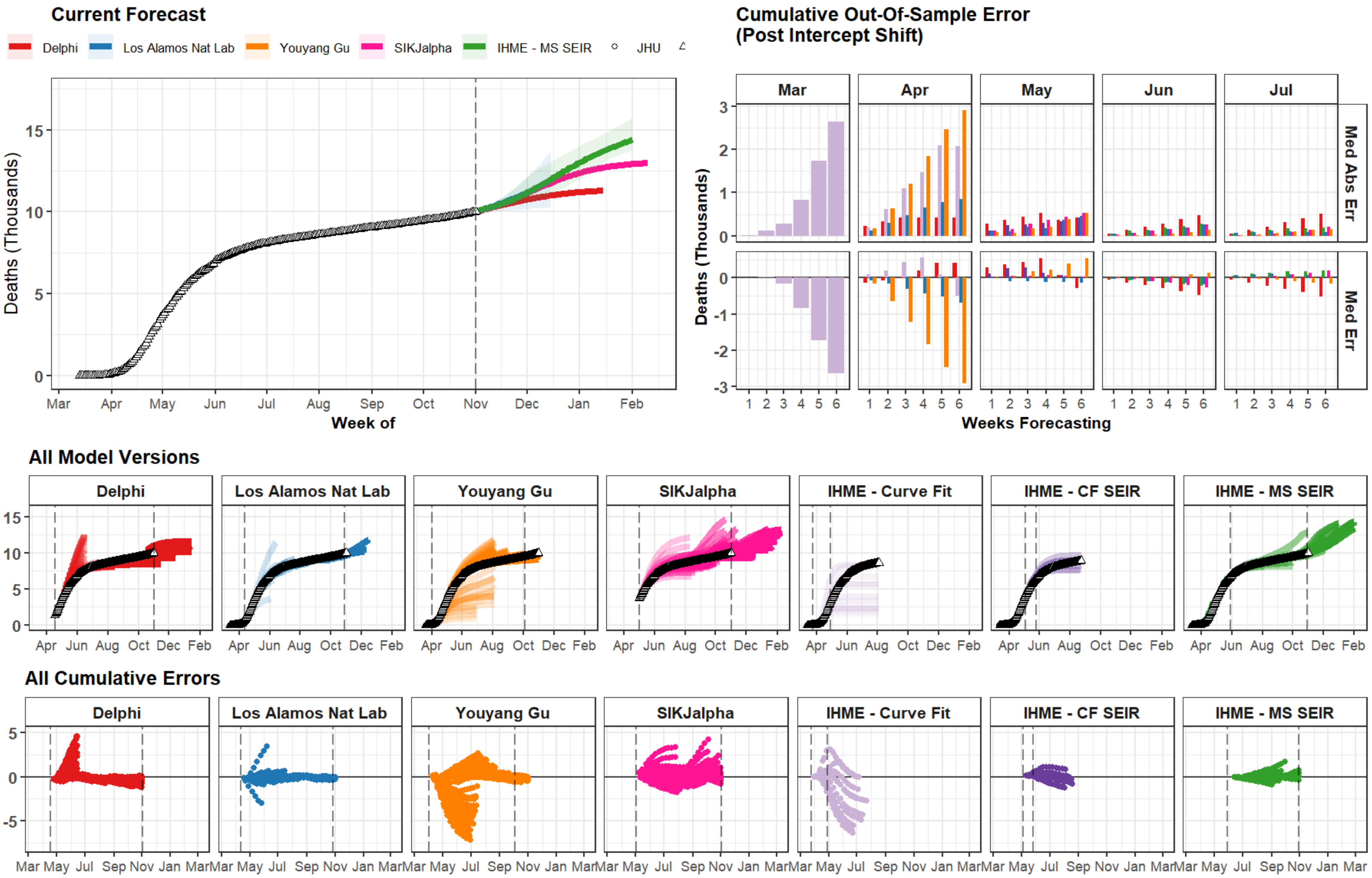

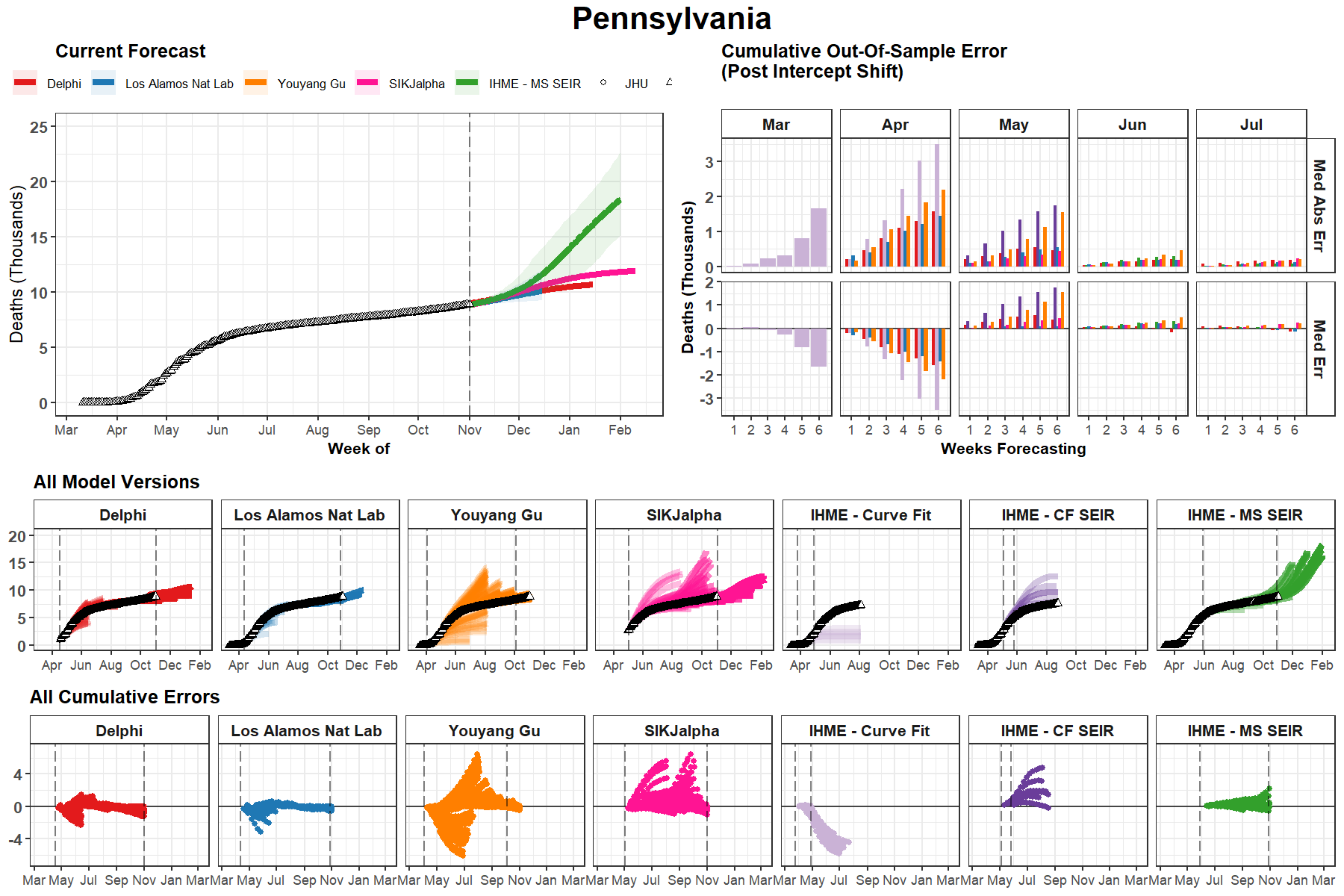

Pennsylvania
Current Forecast
Delphi
Los Alamos Nat Lab
Youyang Gu
SIKJalpha
IHME - MS SEIR
JHU
Deaths (Thousands)
Week of
Mar
Apr
May
Jun
Jul
Aug
Sep
Oct
Nov
Dec
Jan
Feb
Cumulative Out-Of-Sample Error
(Post Intercept Shift)
Med Abs Err
Med Err
Weeks Forecasting
All Model Versions
IHME - Curve Fit
IHME - CF SEIR
All Cumulative Errors

Bolivia

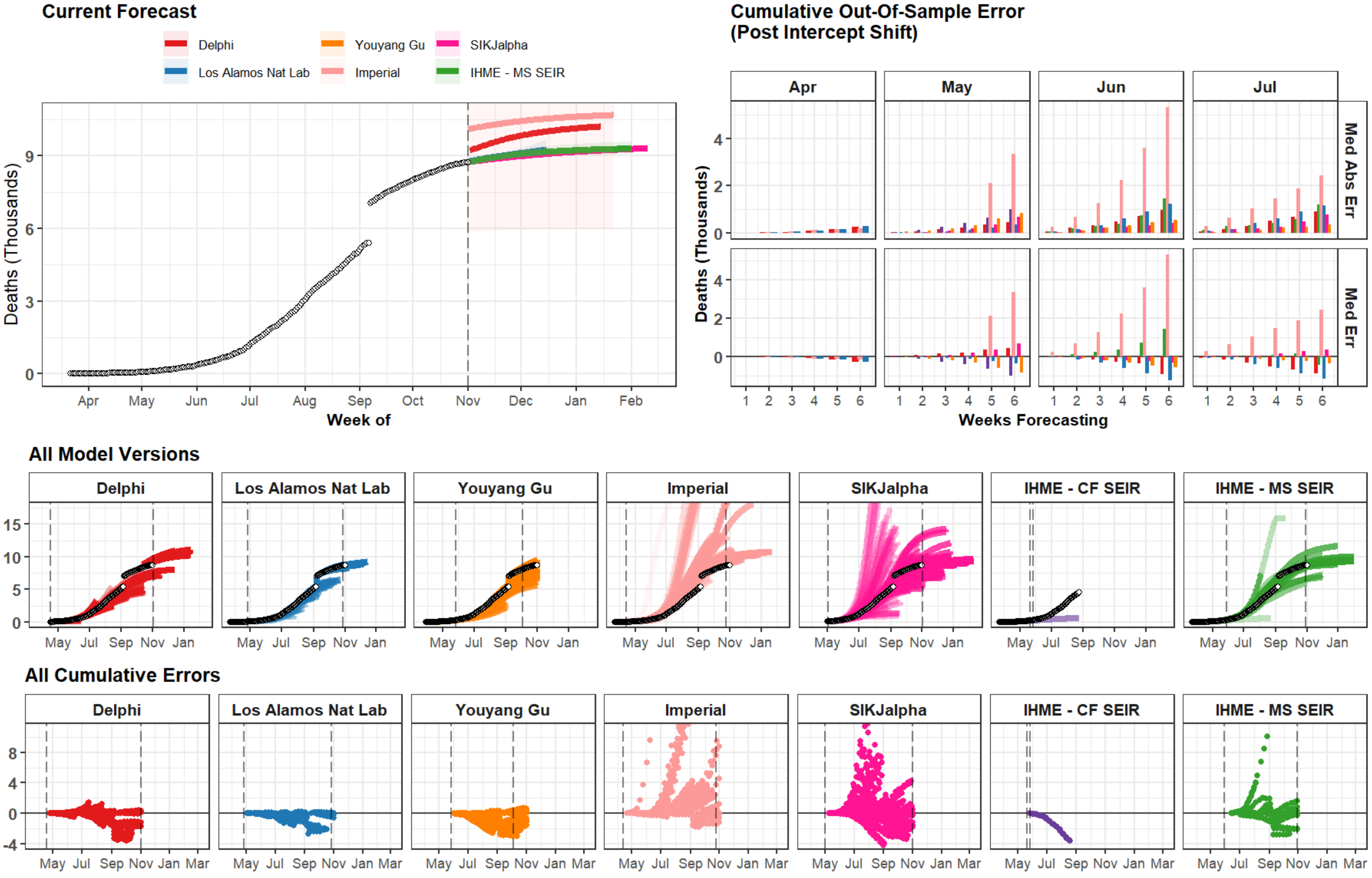
Current Forecast
Delphi
Youyang Gu
SIKJalpha
Los Alamos Nat Lab
Imperial
IHME - MS SEIR
Deaths (Thousands)
Week of
Apr May Jun Jul Aug Sep Oct Nov Dec Jan Feb
Cumulative Out-Of-Sample Error
(Post Intercept Shift)
Apr May Jun Jul
Med Abs Err
Med Err
Weeks Forecasting
All Model Versions
Delphi
Los Alamos Nat Lab
Youyang Gu
Imperial
SIKJalpha
IHME - CF SEIR
IHME - MS SEIR
All Cumulative Errors

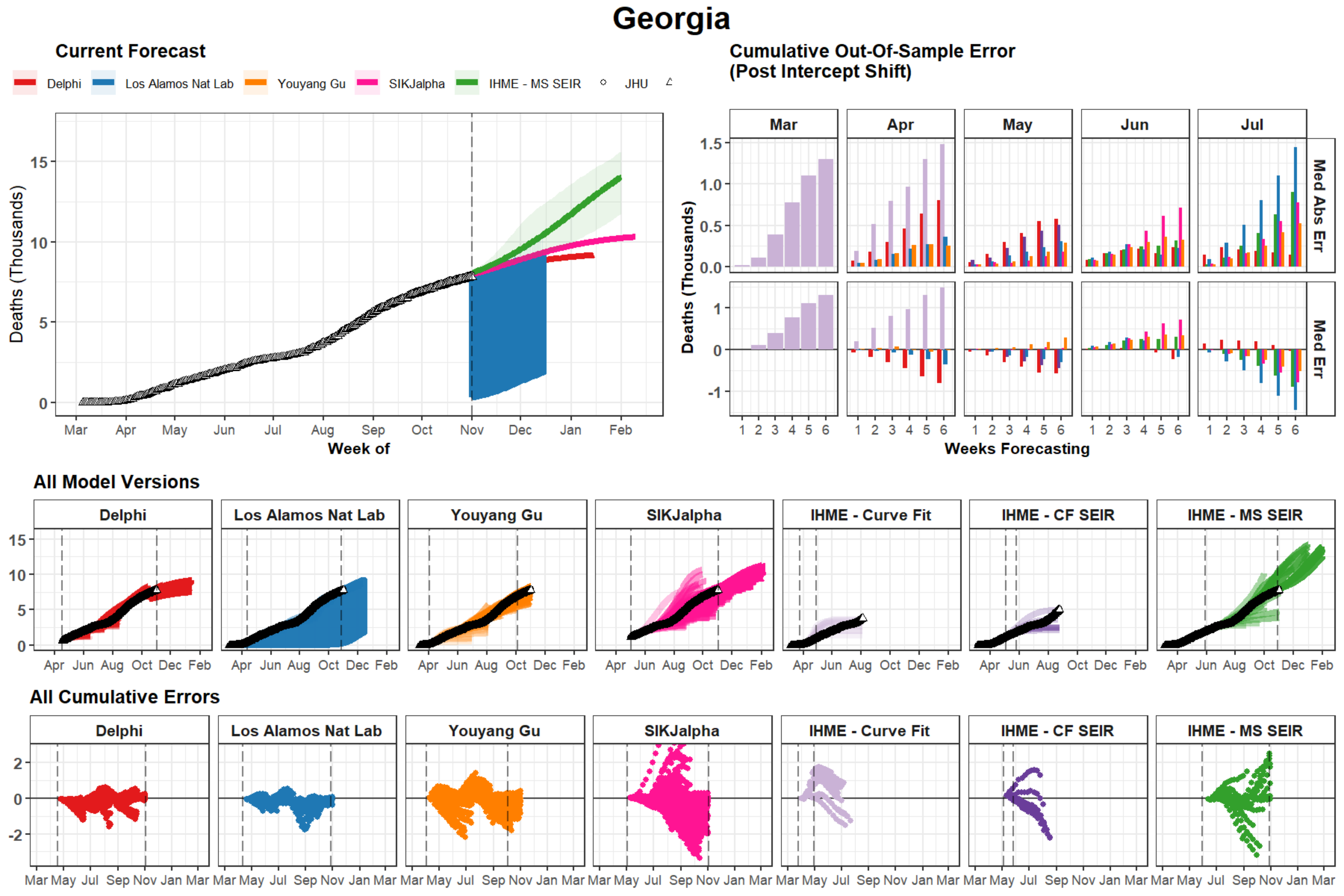
Georgia
Current Forecast
Delphi
Los Alamos Nat Lab
Youyang Gu
SIKJalpha
IHME - MS SEIR
JHU
Deaths (Thousands)
Week of
Mar Apr May Jun Jul Aug Sep Oct Nov Dec Jan Feb
Cumulative Out-Of-Sample Error
(Post Intercept Shift)
Mar Apr May Jun Jul
Med Abs Err
Med Err
Weeks Forecasting
All Model Versions
Delphi
Los Alamos Nat Lab
Youyang Gu
SIKJalpha
IHME - Curve Fit
IHME - CF SEIR
IHME - MS SEIR
All Cumulative Errors
Delphi
Los Alamos Nat Lab
Youyang Gu
SIKJalpha
IHME - Curve Fit
IHME - CF SEIR
IHME - MS SEIR

Michigan

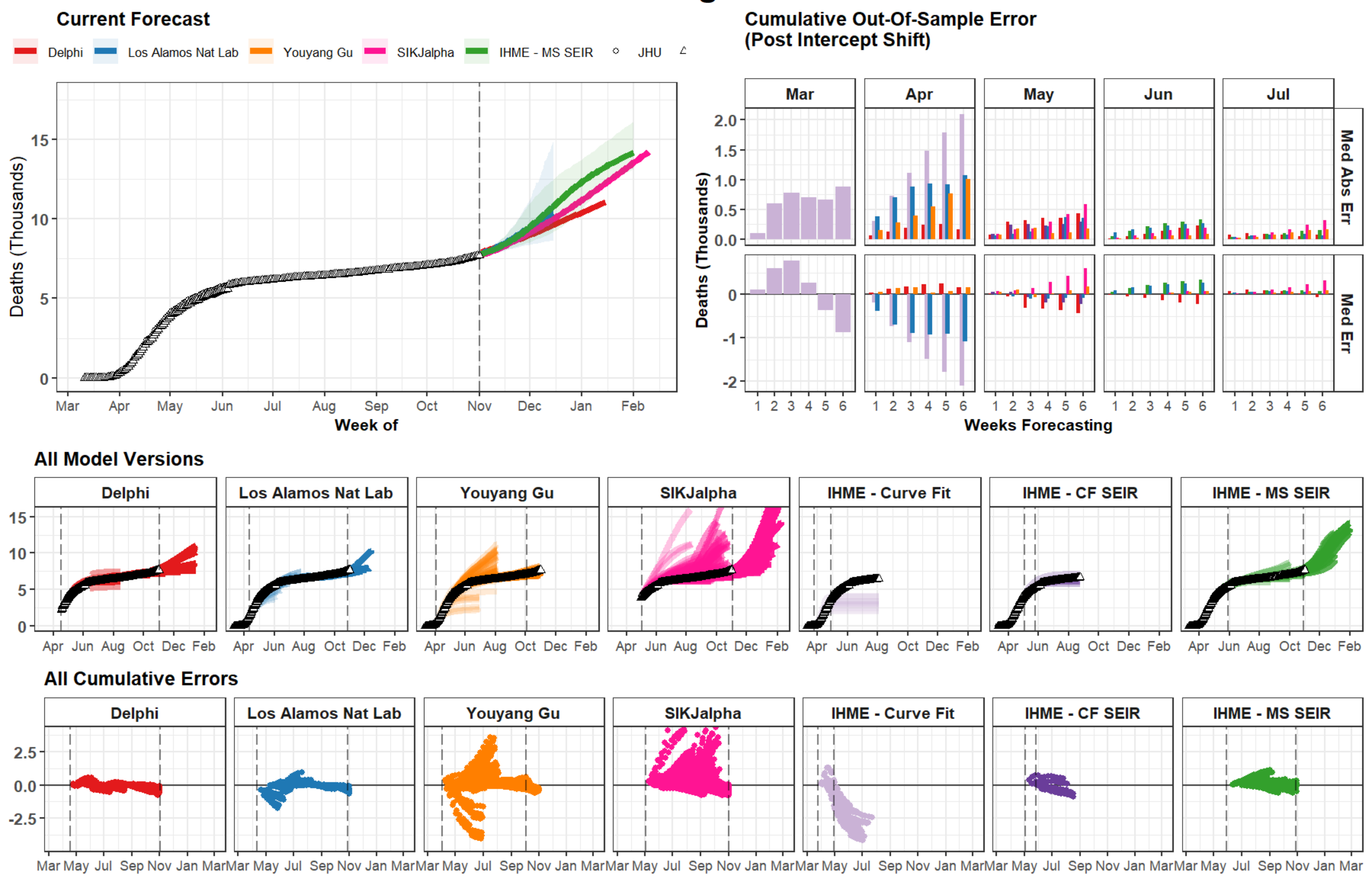
Current Forecast
Delphi
Los Alamos Nat Lab
Youyang Gu
SIKJalpha
IHME - MS SEIR
JHU
Deaths (Thousands)
Week of
Cumulative Out-Of-Sample Error
(Post Intercept Shift)
Mar
Apr
May
Jun
Jul
Med Abs Err
Med Err
Weeks Forecasting
All Model Versions
Delphi
Los Alamos Nat Lab
Youyang Gu
SIKJalpha
IHME - Curve Fit
IHME - CF SEIR
IHME - MS SEIR
All Cumulative Errors

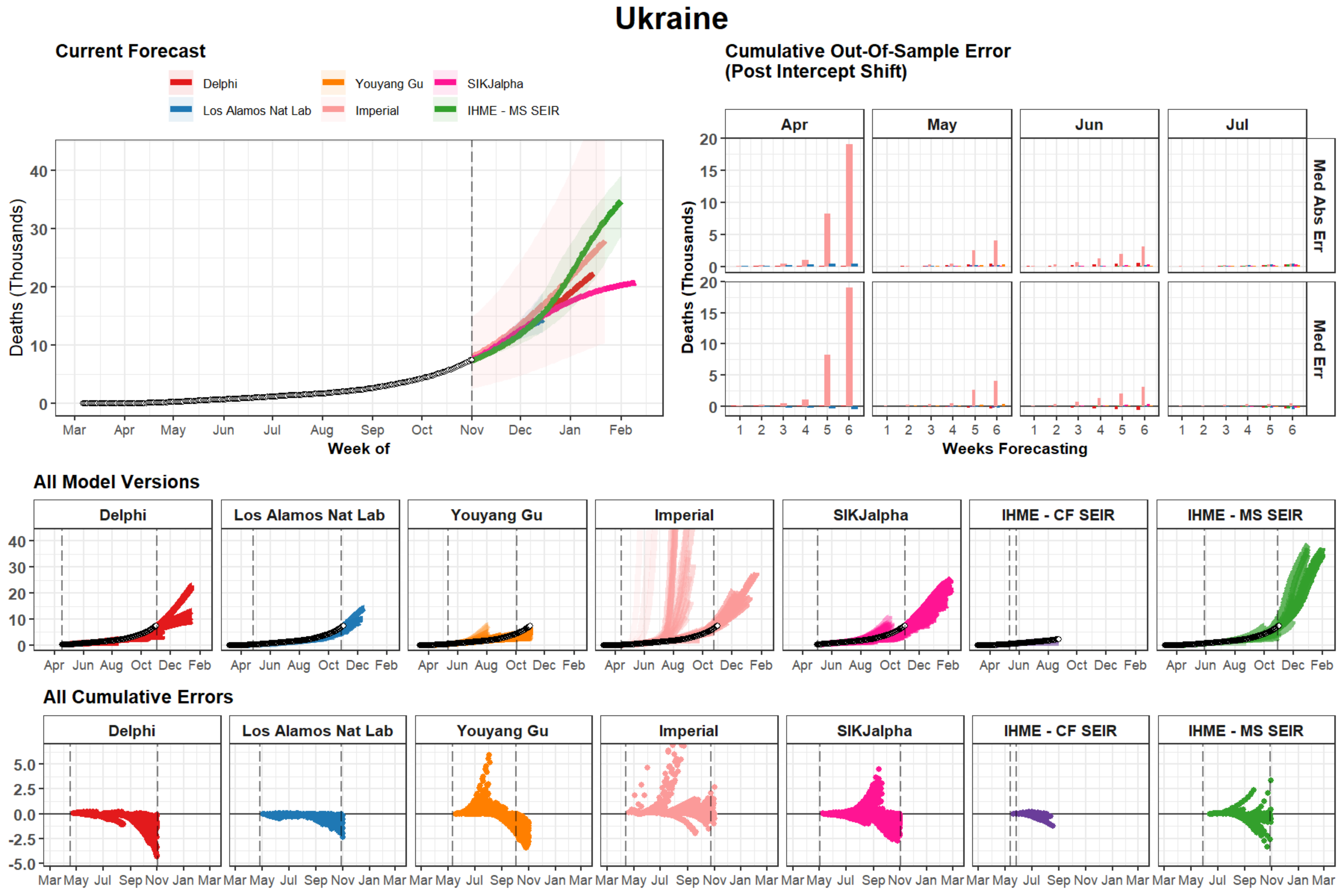
Ukraine
Current Forecast
Delphi
Los Alamos Nat Lab
Youyang Gu
Imperial
SIKJalpha
IHME - MS SEIR
Deaths (Thousands)
Week of
Cumulative Out-Of-Sample Error
(Post Intercept Shift)
Apr
May
Jun
Jul
Med Abs Err
Med Err
Weeks Forecasting
All Model Versions
IHME - CF SEIR
All Cumulative Errors

Netherlands

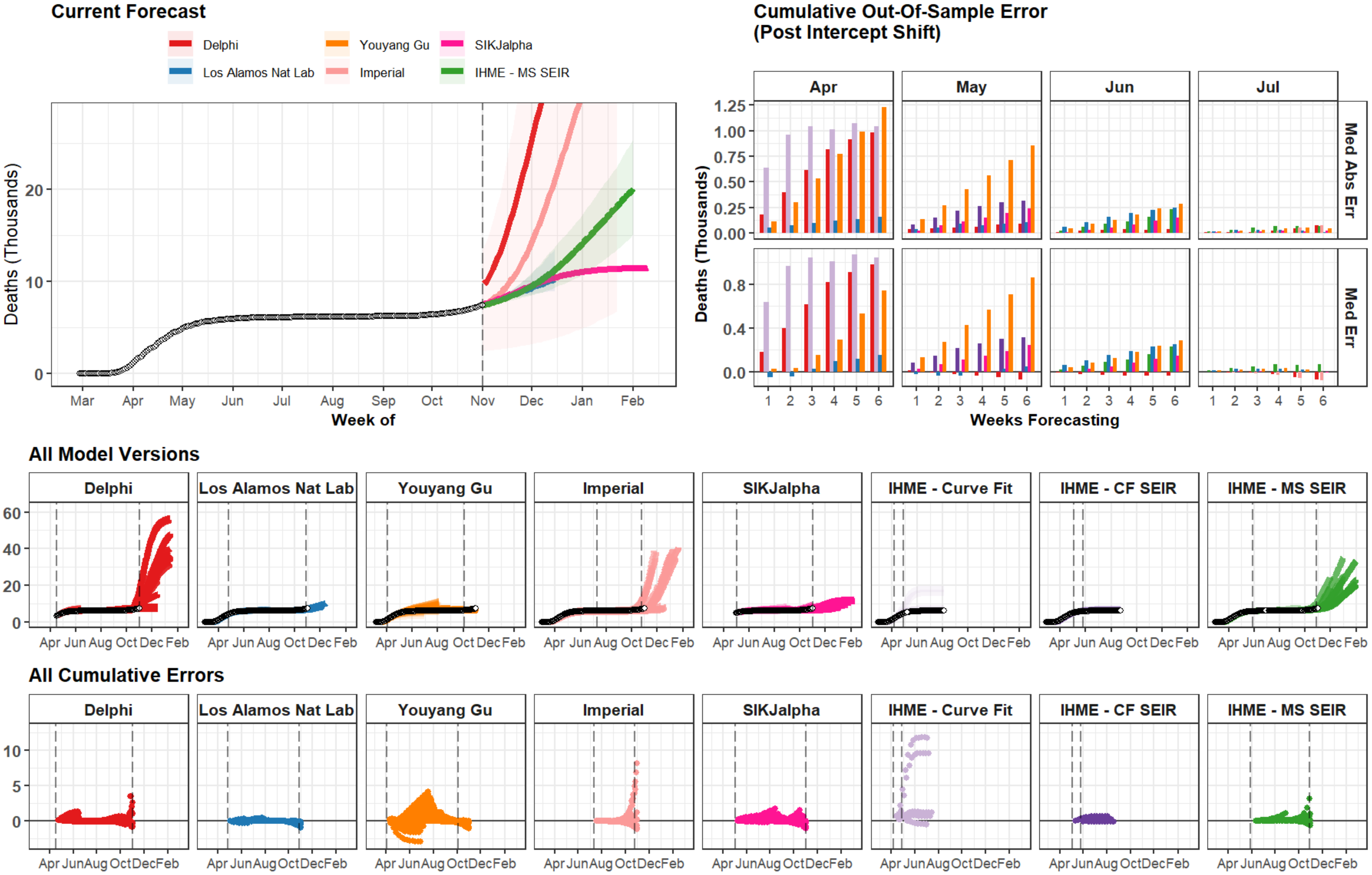

Philippines

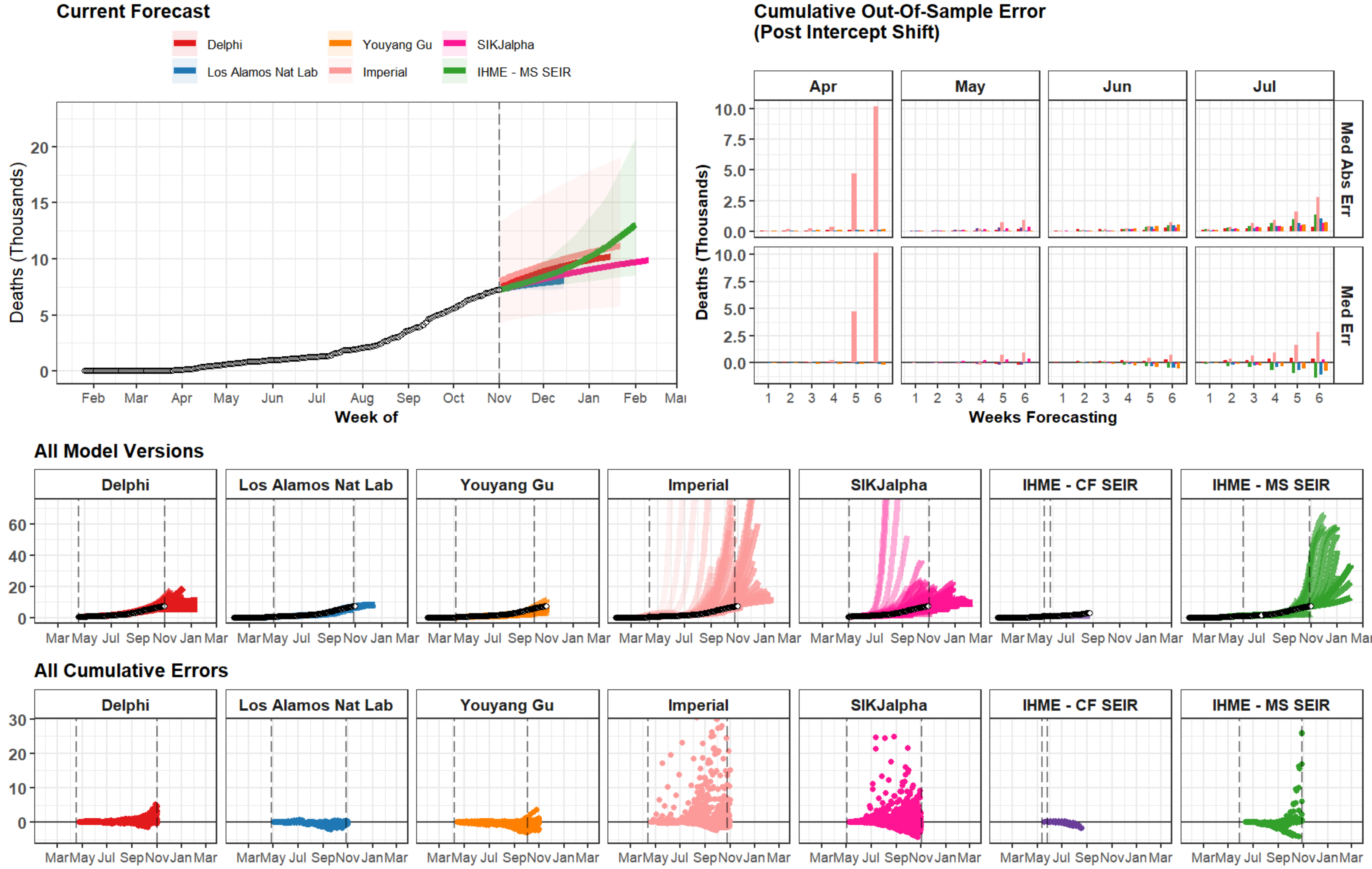

Romania

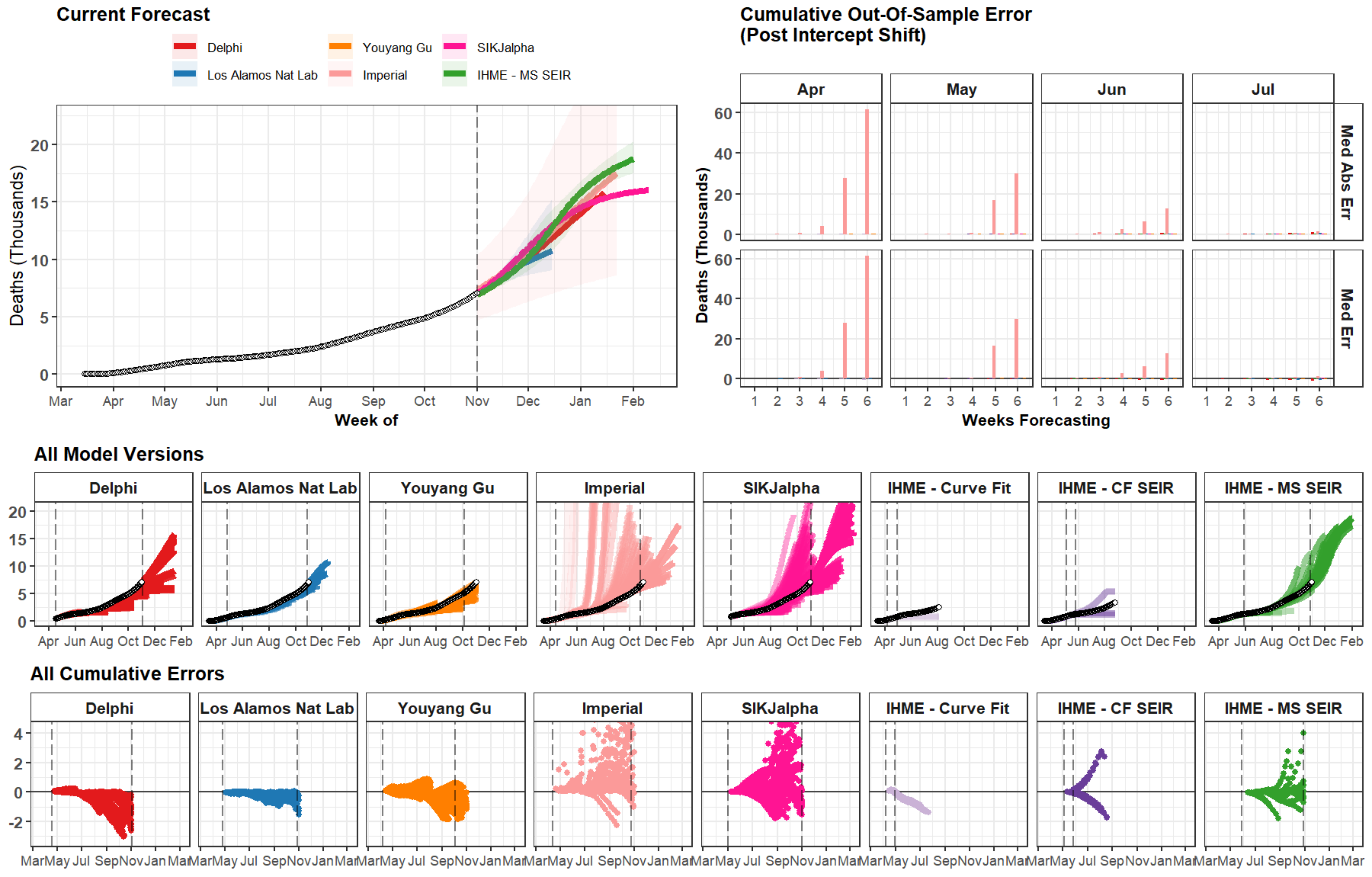

Current Forecast
Delphi
Los Alamos Nat Lab
Youyang Gu
Imperial
SIKJalpha
IHME - MS SEIR
Deaths (Thousands)
Week of
Mar Apr May Jun Jul Aug Sep Oct Nov Dec Jan Feb
Cumulative Out-Of-Sample Error
(Post Intercept Shift)
Apr
May
Jun
Jul
Med Abs Err
Med Err
Weeks Forecasting
All Model Versions
Delphi
Los Alamos Nat Lab
Youyang Gu
Imperial
SIKJalpha
IHME - Curve Fit
IHME - CF SEIR
IHME - MS SEIR
Apr Jun Aug Oct Dec Feb
All Cumulative Errors
MarMay Jul SepNovJanMar

Pakistan

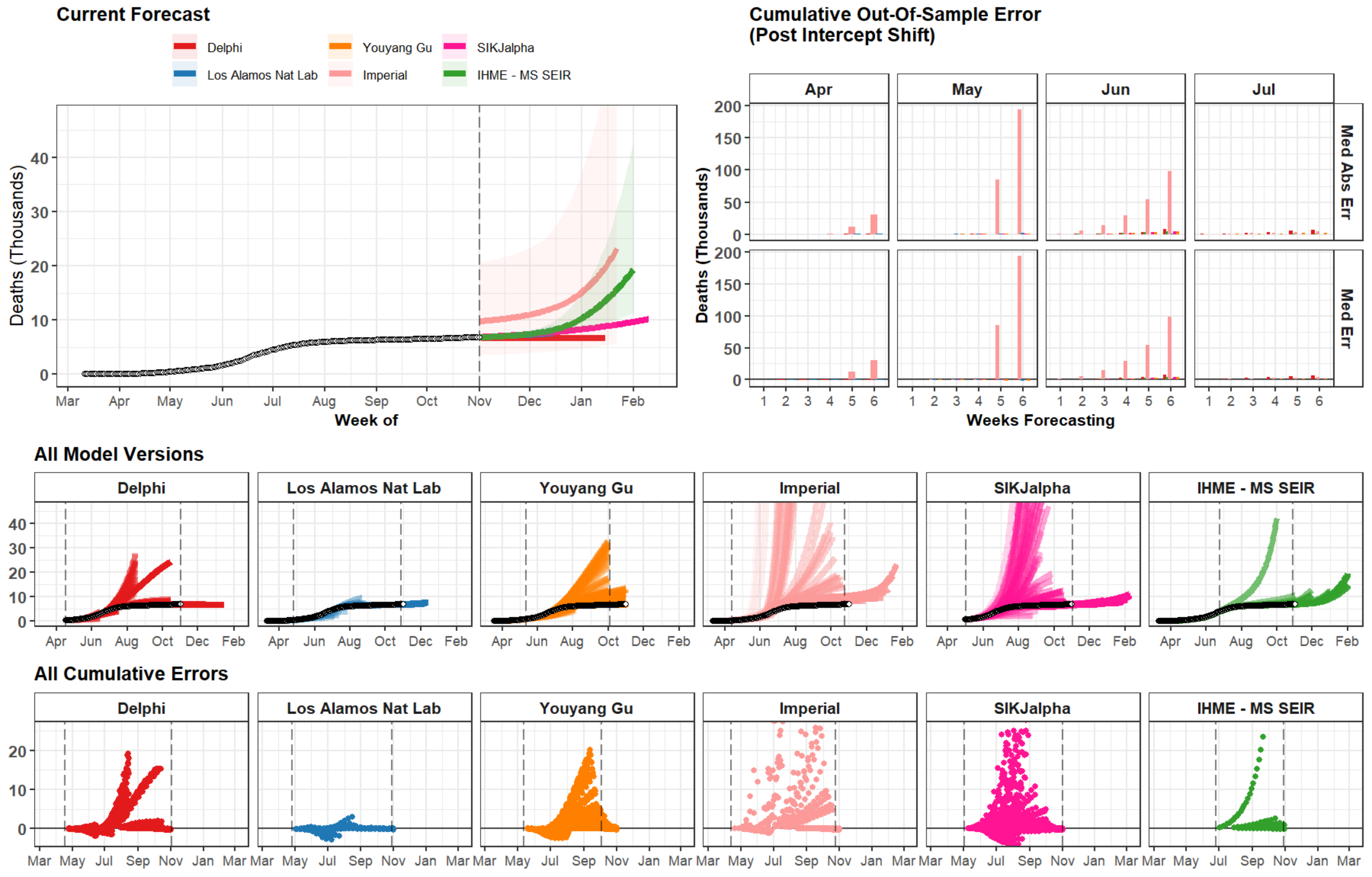

Current Forecast
Delphi
Los Alamos Nat Lab
Youyang Gu
Imperial
SIKJalpha
IHME - MS SEIR
Deaths (Thousands)
Week of
Mar Apr May Jun Jul Aug Sep Oct Nov Dec Jan Feb
Cumulative Out-Of-Sample Error
(Post Intercept Shift)
Apr May Jun Jul
Med Abs Err
Med Err
Weeks Forecasting
All Model Versions
All Cumulative Errors

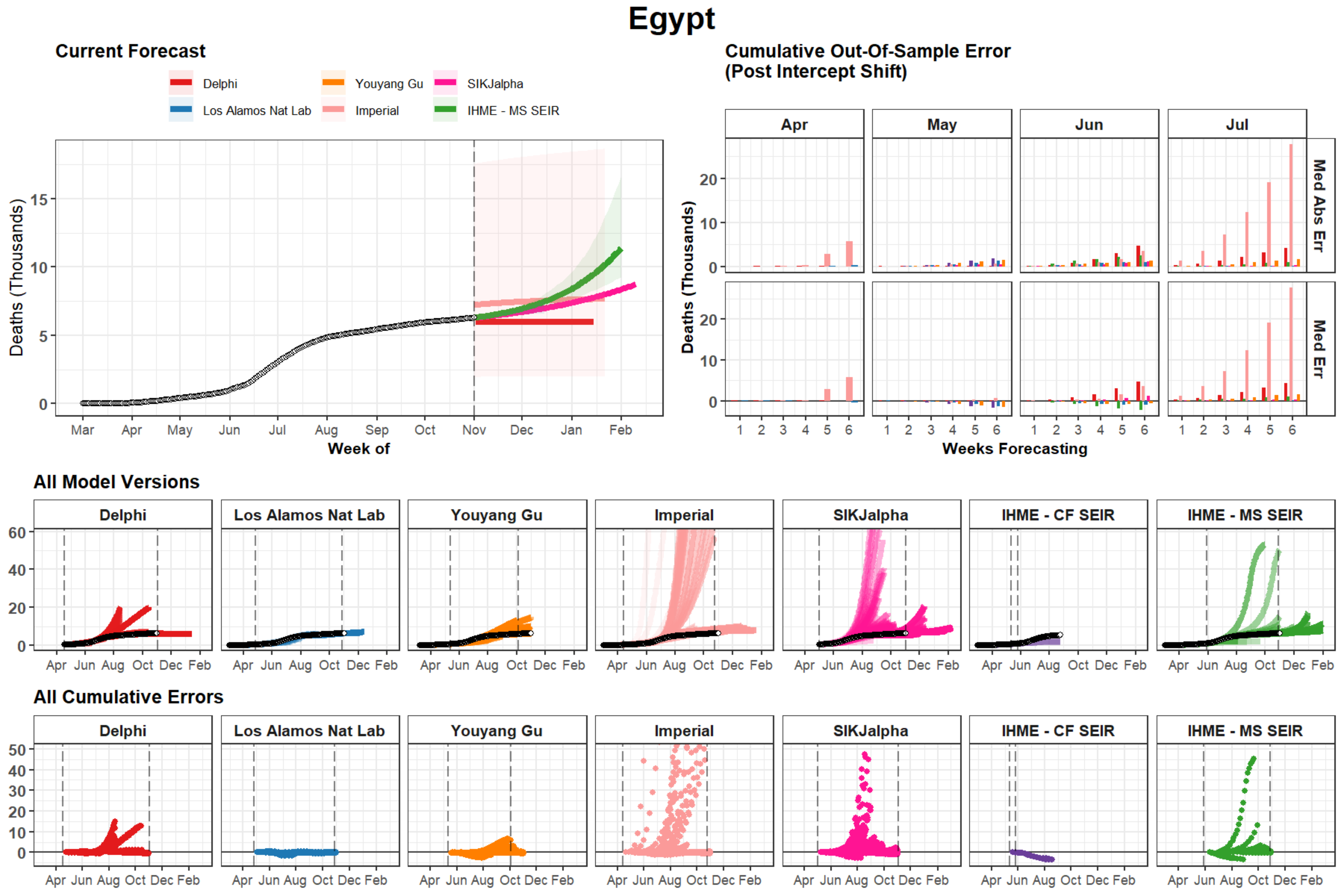
Egypt
Current Forecast
Delphi
Los Alamos Nat Lab
Youyang Gu
Imperial
SIKJalpha
IHME - MS SEIR
Deaths (Thousands)
Week of
Mar Apr May Jun Jul Aug Sep Oct Nov Dec Jan Feb
Cumulative Out-Of-Sample Error
(Post Intercept Shift)
Apr May Jun Jul
Med Abs Err
Med Err
Weeks Forecasting
All Model Versions
Delphi
Los Alamos Nat Lab
Youyang Gu
Imperial
SIKJalpha
IHME - CF SEIR
IHME - MS SEIR
All Cumulative Errors
Apr Jun Aug Oct Dec Feb

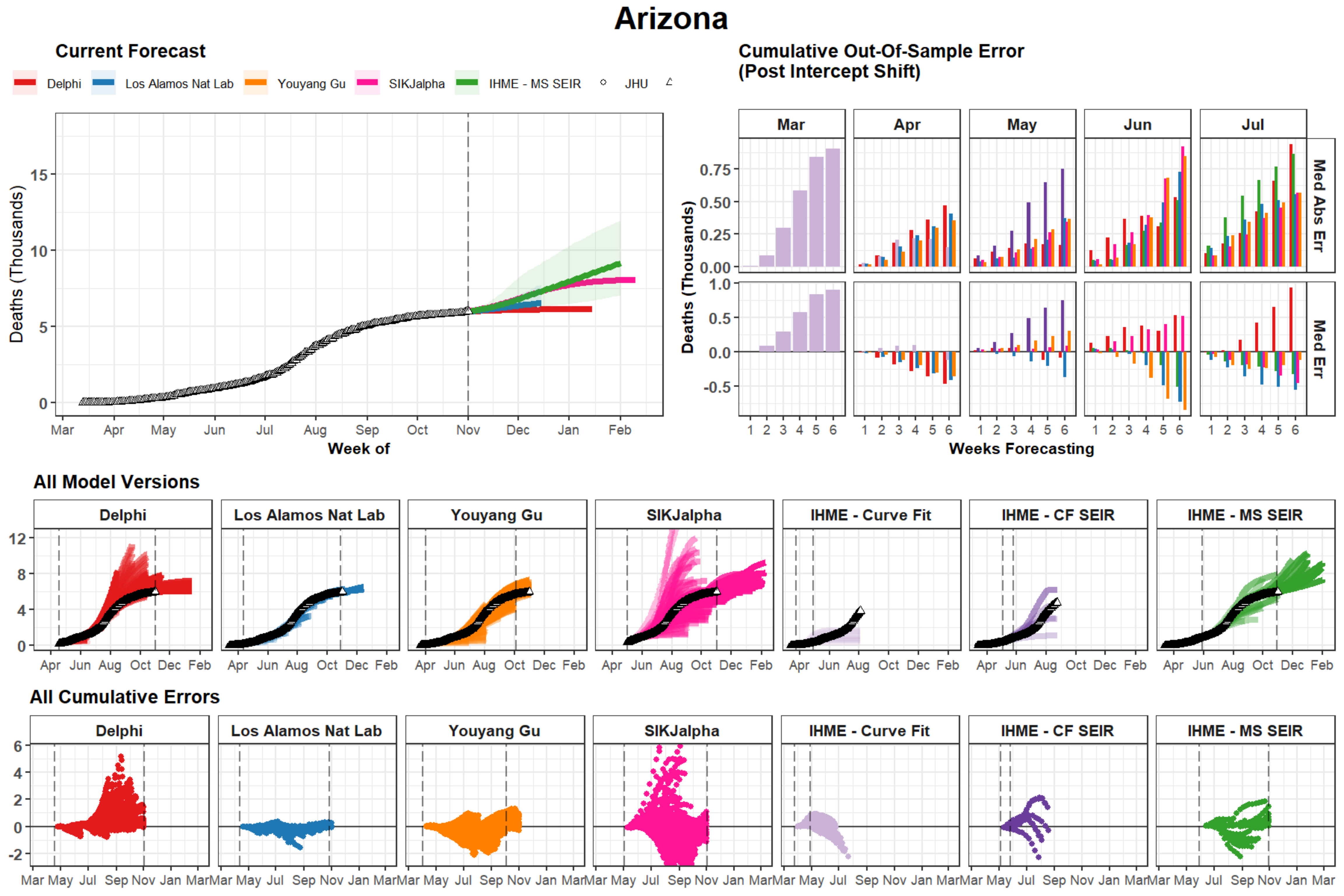
Arizona
Current Forecast
Delphi
Los Alamos Nat Lab
Youyang Gu
SIKJalpha
IHME - MS SEIR
JHU
Deaths (Thousands)
15
10
5
0
Mar Apr May Jun Jul Aug Sep Oct Nov Dec Jan Feb
Week of
Cumulative Out-Of-Sample Error
(Post Intercept Shift)
Mar
Apr
May
Jun
Jul
Med Abs Err
Med Err
0.75
0.50
0.25
0.00
1.0
0.5
0.0
-0.5
1 2 3 4 5 6
Weeks Forecasting
All Model Versions
Delphi
Los Alamos Nat Lab
Youyang Gu
SIKJalpha
IHME - Curve Fit
IHME - CF SEIR
IHME - MS SEIR
12
8
4
0
Apr Jun Aug Oct Dec Feb
All Cumulative Errors
6
4
2
0
-2
Mar May Jul Sep Nov Jan Mar

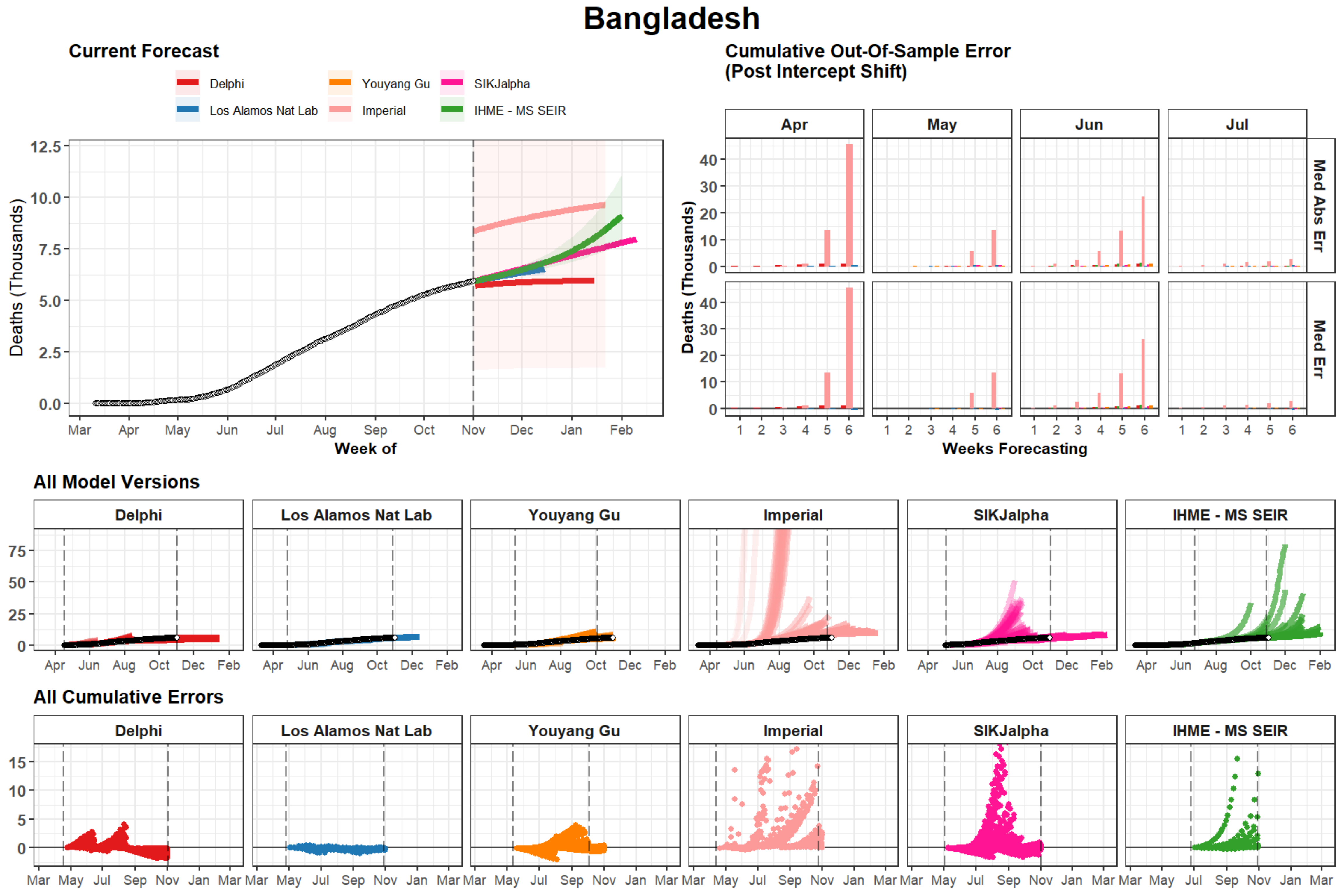
Bangladesh
Current Forecast
Delphi
Youyang Gu
SIKJalpha
Los Alamos Nat Lab
Imperial
IHME - MS SEIR
Deaths (Thousands)
Week of
Cumulative Out-Of-Sample Error
(Post Intercept Shift)
Apr
May
Jun
Jul
Med Abs Err
Med Err
Weeks Forecasting
All Model Versions
All Cumulative Errors

Sweden

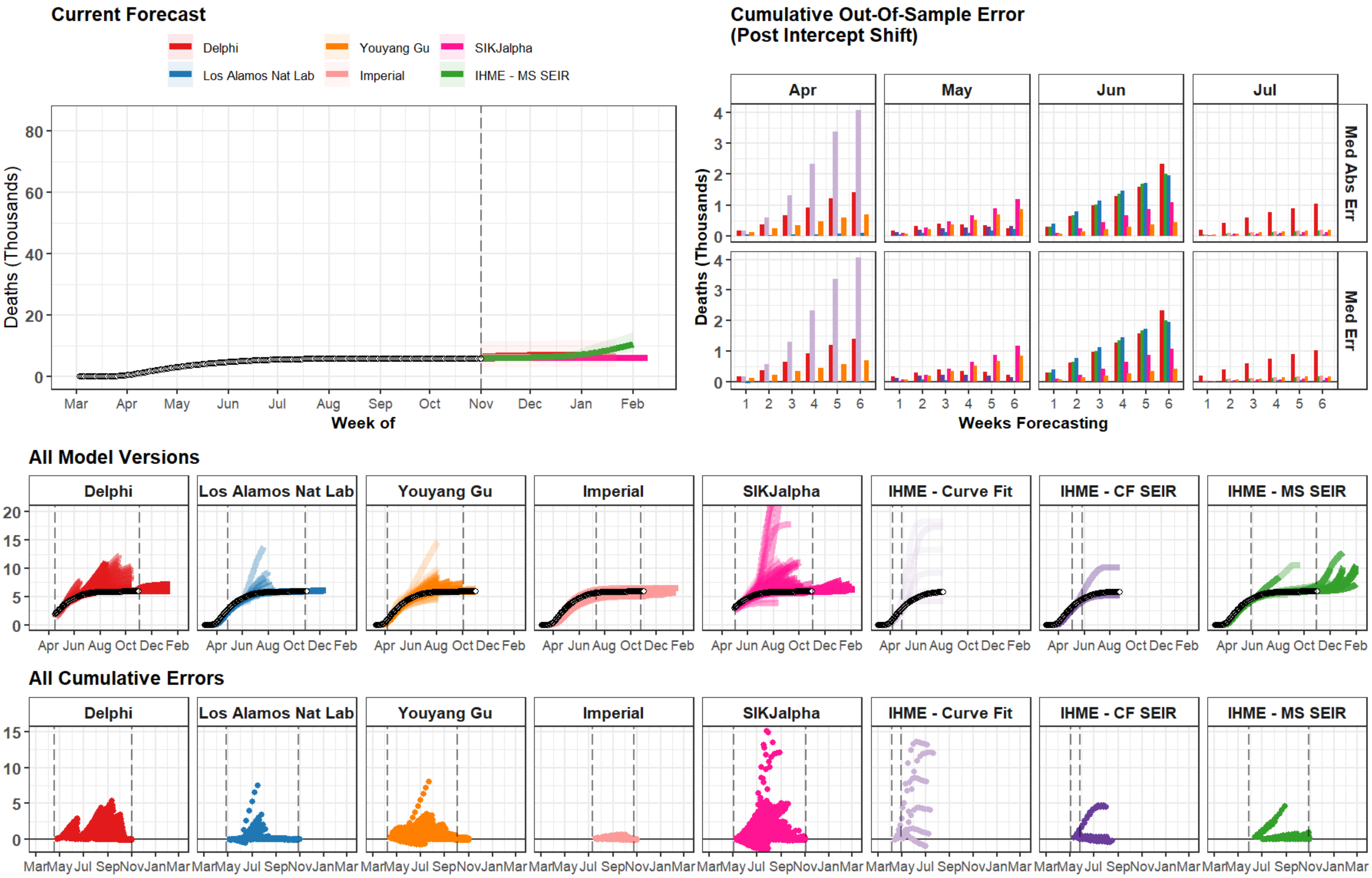
Current Forecast
Delphi
Youyang Gu
SIKJalpha
Los Alamos Nat Lab
Imperial
IHME - MS SEIR
Deaths (Thousands)
Week of
Mar Apr May Jun Jul Aug Sep Oct Nov Dec Jan Feb
Cumulative Out-Of-Sample Error
(Post Intercept Shift)
Apr
May
Jun
Jul
Med Abs Err
Med Err
Weeks Forecasting
All Model Versions
Delphi
Los Alamos Nat Lab
Youyang Gu
Imperial
SIKJalpha
IHME - Curve Fit
IHME - CF SEIR
IHME - MS SEIR
All Cumulative Errors

Louisiana

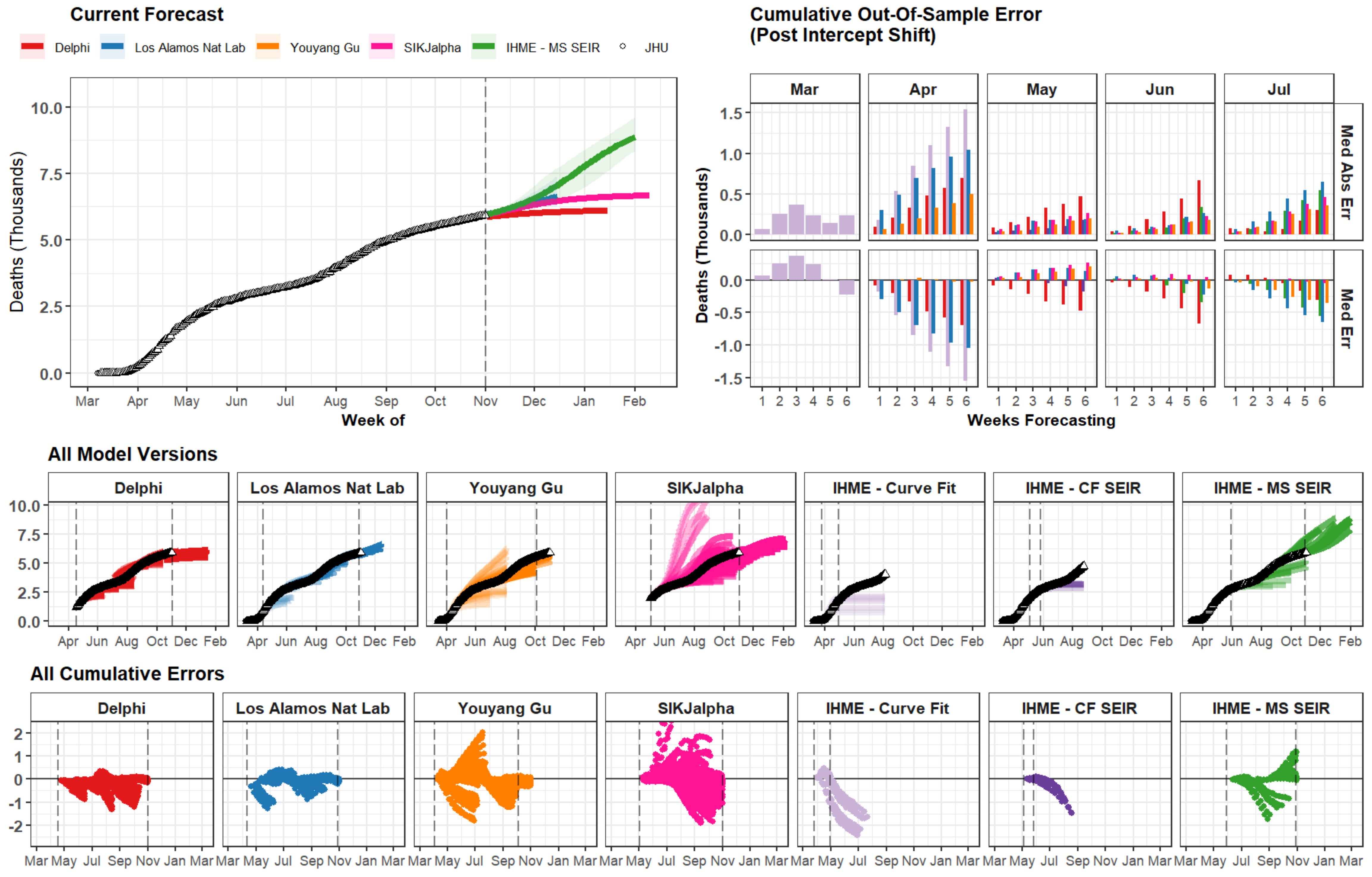
Current Forecast
Delphi
Los Alamos Nat Lab
Youyang Gu
SIKJalpha
IHME - MS SEIR
JHU
Deaths (Thousands)
10.0
7.5
5.0
2.5
0.0
Mar Apr May Jun Jul Aug Sep Oct Nov Dec Jan Feb
Week of
Cumulative Out-Of-Sample Error
(Post Intercept Shift)
Mar
Apr
May
Jun
Jul
Med Abs Err
Med Err
1.5
1.0
0.5
0.0
-0.5
-1.0
-1.5
1 2 3 4 5 6
Weeks Forecasting
All Model Versions
Delphi
Los Alamos Nat Lab
Youyang Gu
SIKJalpha
IHME - Curve Fit
IHME - CF SEIR
IHME - MS SEIR
Apr Jun Aug Oct Dec Feb
All Cumulative Errors
Delphi
Los Alamos Nat Lab
Youyang Gu
SIKJalpha
IHME - Curve Fit
IHME - CF SEIR
IHME - MS SEIR
2
1
0
-1
-2
Mar May Jul Sep Nov Jan Mar

Poland

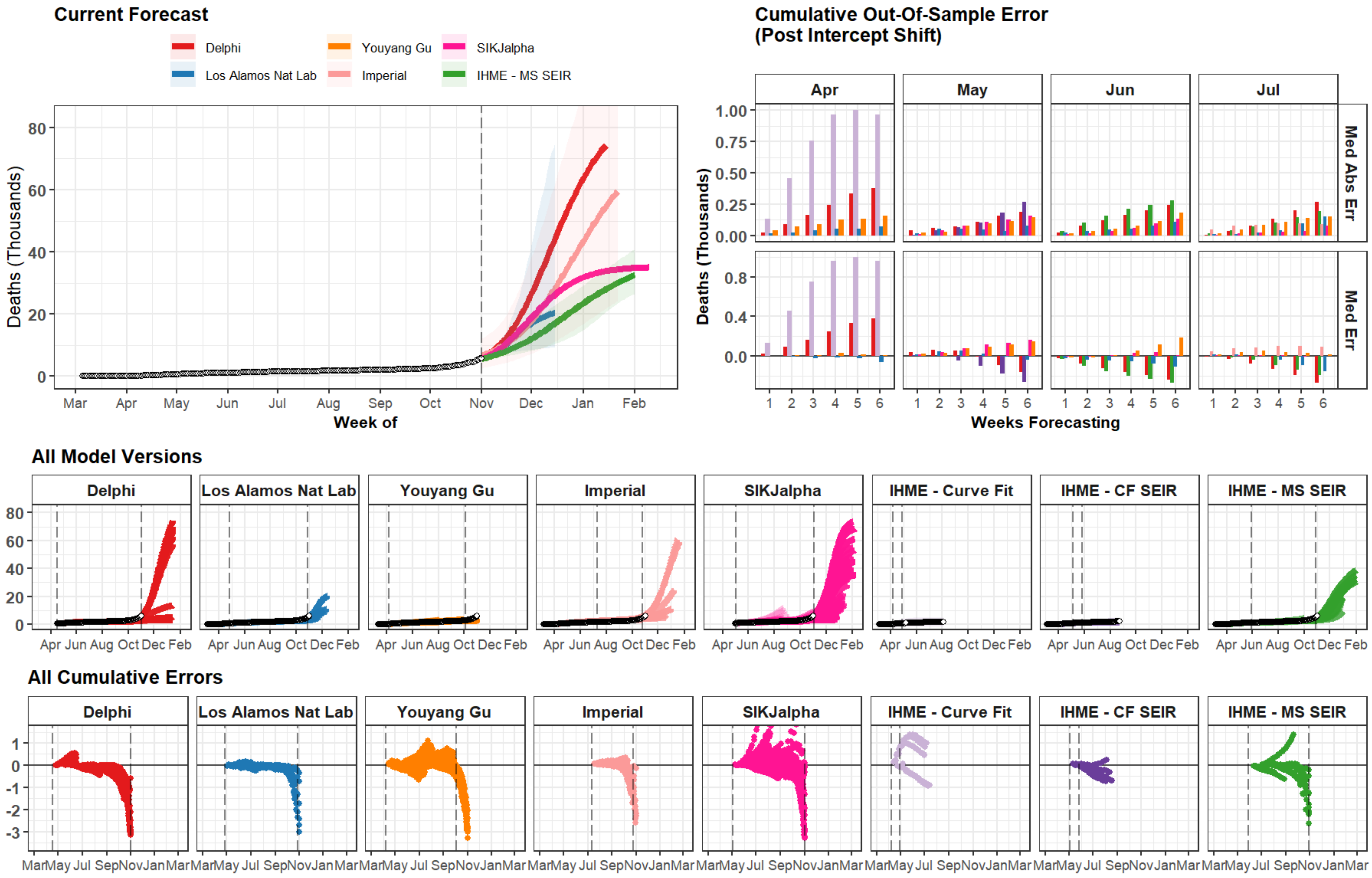

Current Forecast
Delphi
Youyang Gu
SIKJalpha
Los Alamos Nat Lab
Imperial
IHME - MS SEIR
Deaths (Thousands)
Week of
Mar Apr May Jun Jul Aug Sep Oct Nov Dec Jan Feb
Cumulative Out-Of-Sample Error
(Post Intercept Shift)
Apr May Jun Jul
Med Abs Err
Med Err
Weeks Forecasting
All Model Versions
Delphi
Los Alamos Nat Lab
Youyang Gu
Imperial
SIKJalpha
IHME - Curve Fit
IHME - CF SEIR
IHME - MS SEIR
All Cumulative Errors

Saudi Arabia

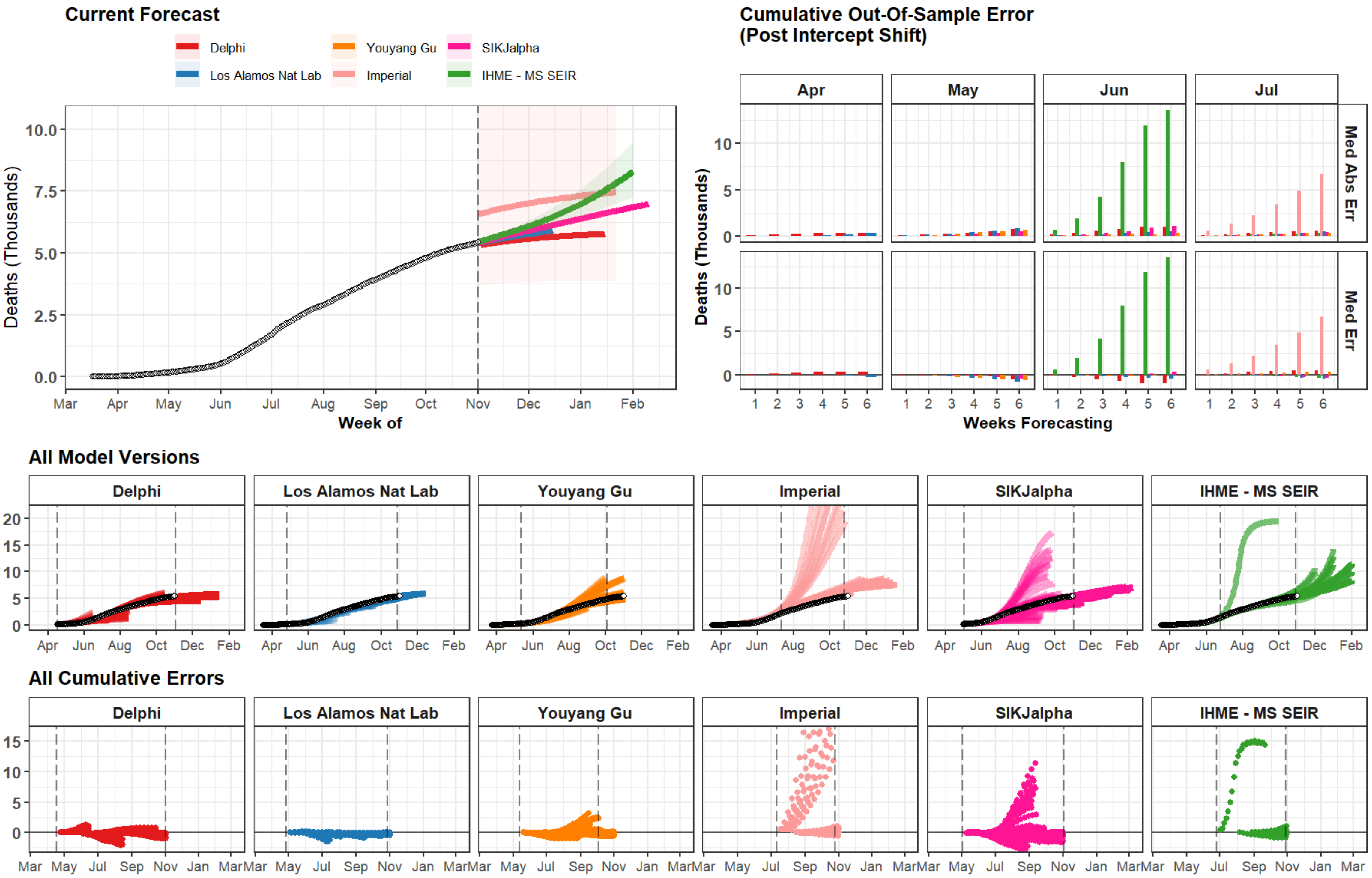

Ohio

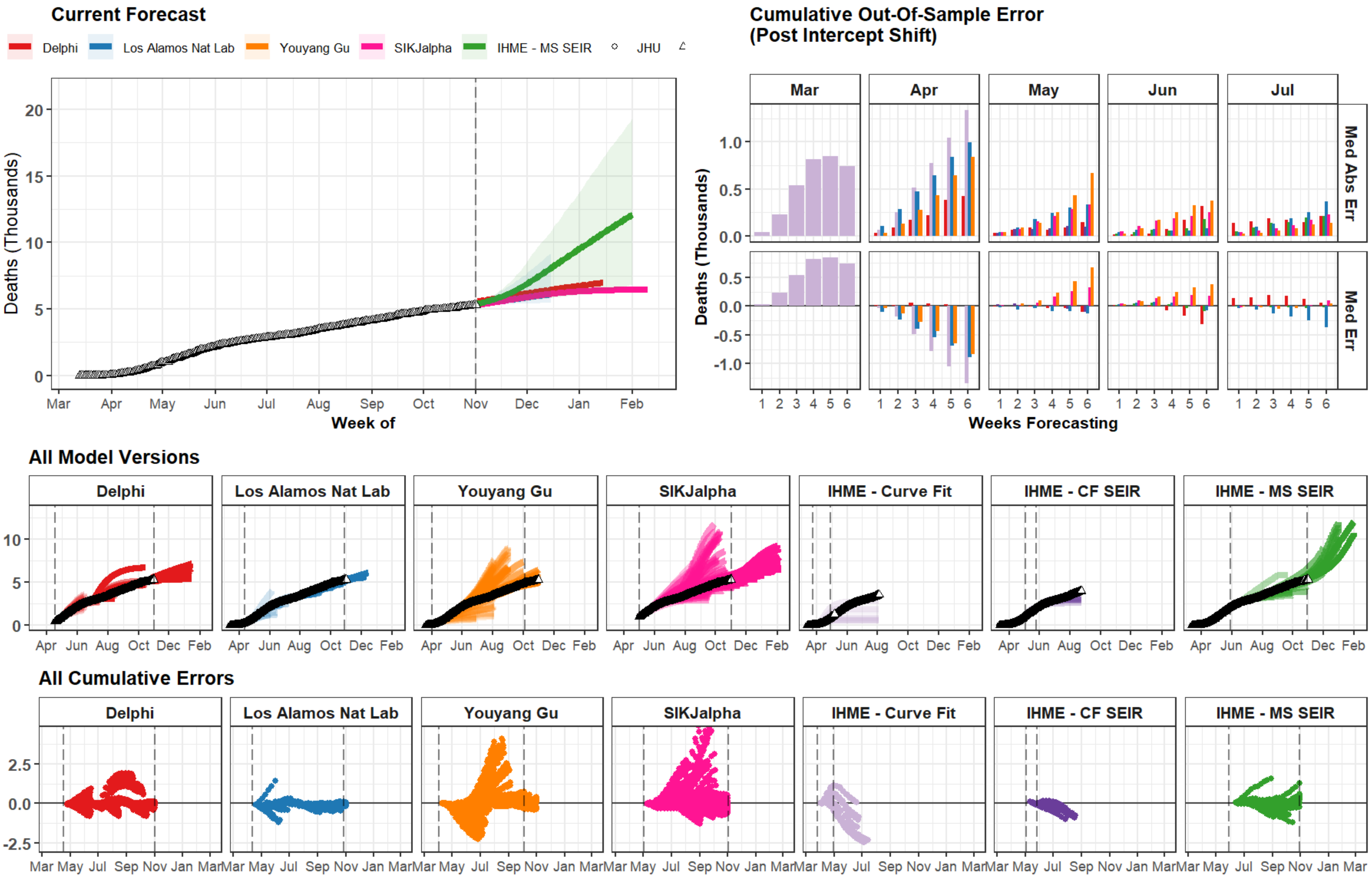

Current Forecast
Delphi
Los Alamos Nat Lab
Youyang Gu
SIKJalpha
IHME - MS SEIR
JHU
Deaths (Thousands)
Week of
Mar Apr May Jun Jul Aug Sep Oct Nov Dec Jan Feb
Cumulative Out-Of-Sample Error
(Post Intercept Shift)
Mar
Apr
May
Jun
Jul
Med Abs Err
Med Err
Weeks Forecasting
All Model Versions
Delphi
Los Alamos Nat Lab
Youyang Gu
SIKJalpha
IHME - Curve Fit
IHME - CF SEIR
IHME - MS SEIR
Apr Jun Aug Oct Dec Feb
All Cumulative Errors
Mar May Jul Sep Nov Jan Mar

China

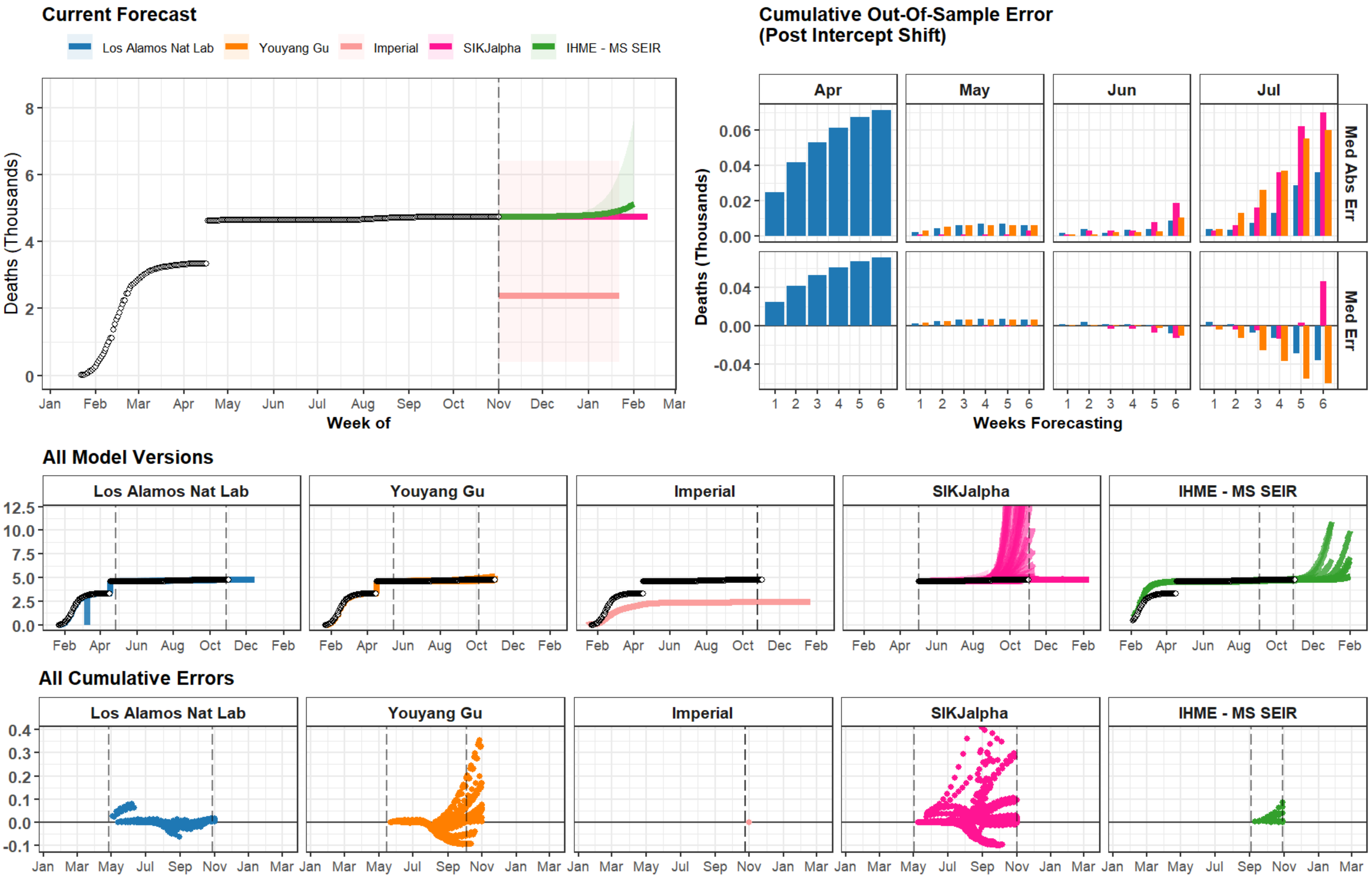
Current Forecast
Los Alamos Nat Lab
Youyang Gu
Imperial
SIKJalpha
IHME - MS SEIR
Deaths (Thousands)
Week of
Jan
Feb
Mar
Apr
May
Jun
Jul
Aug
Sep
Oct
Nov
Dec
Cumulative Out-Of-Sample Error
(Post Intercept Shift)
Apr
May
Jun
Jul
Med Abs Err
Med Err
Weeks Forecasting
All Model Versions
All Cumulative Errors

Connecticut

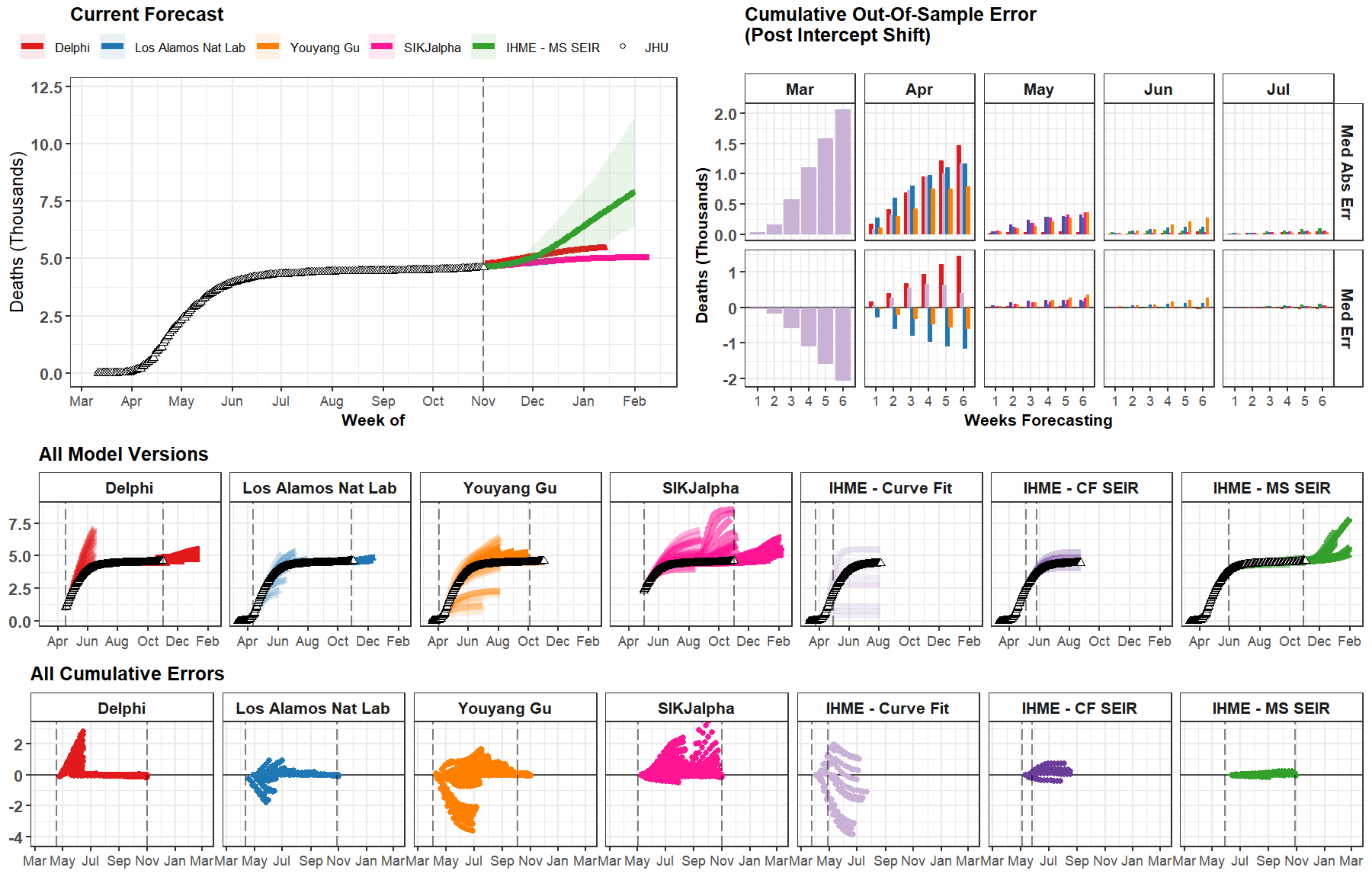

North Carolina

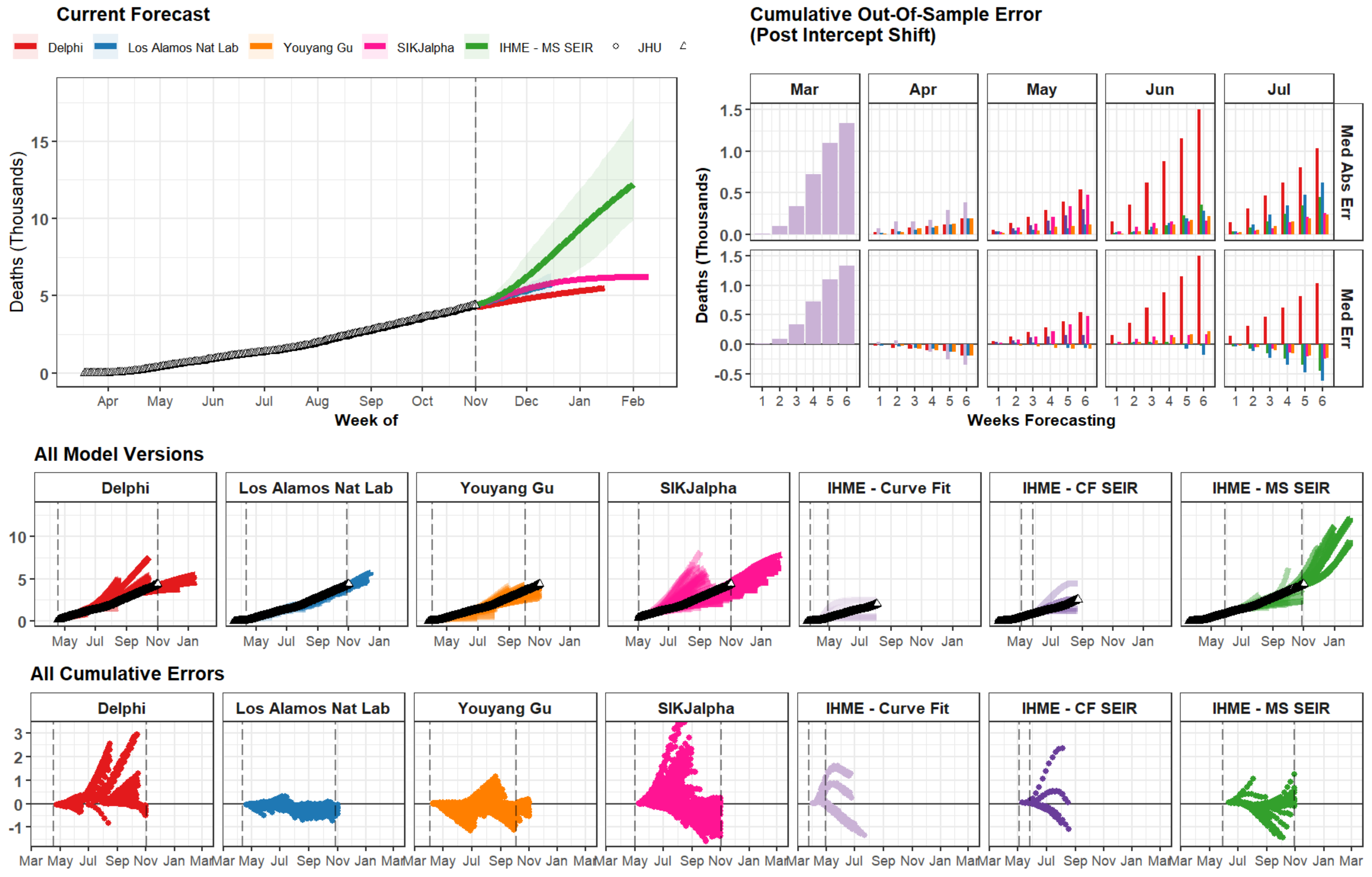

Indiana

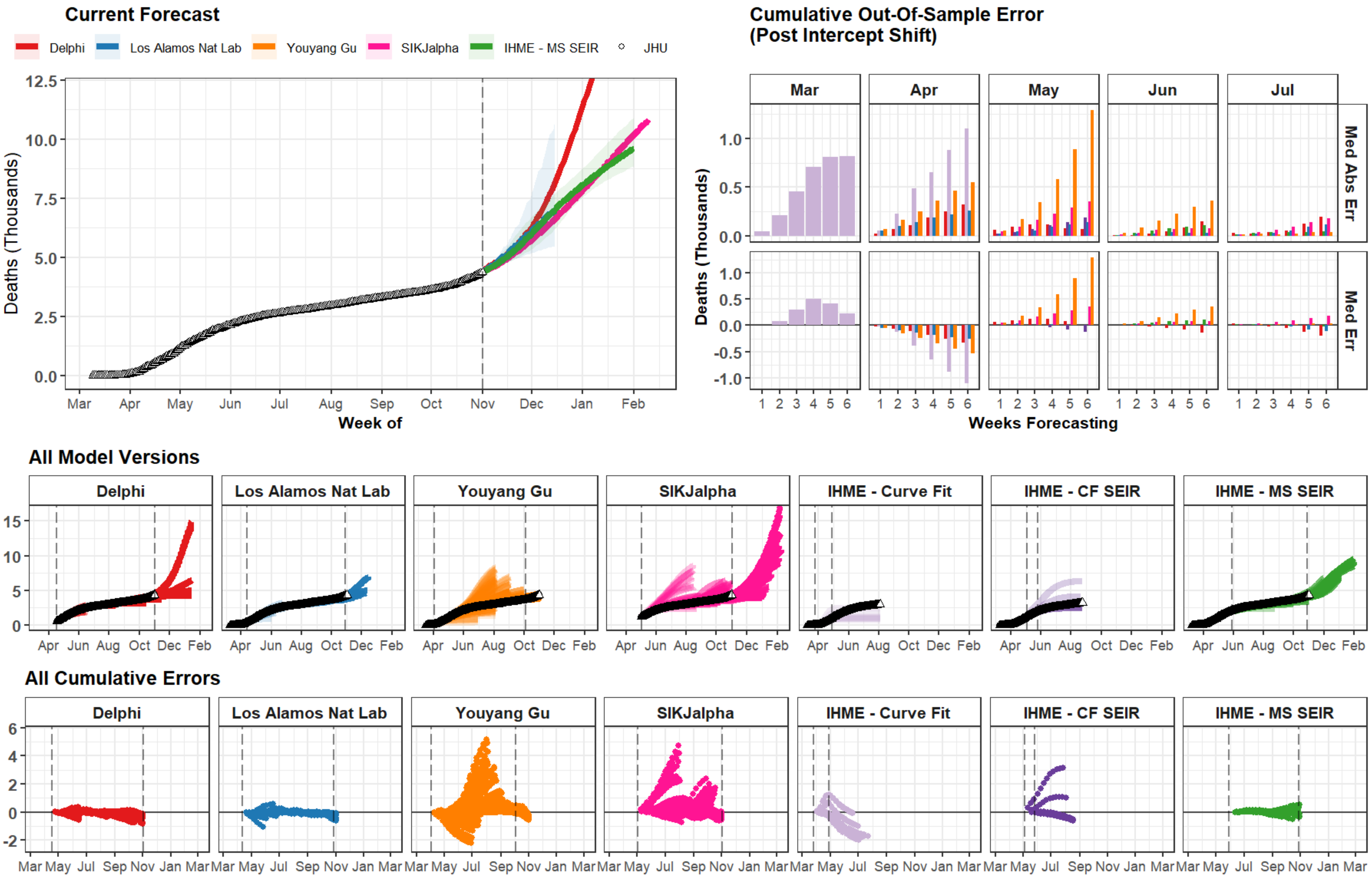

Current Forecast
Delphi
Los Alamos Nat Lab
Youyang Gu
SIKJalpha
IHME - MS SEIR
JHU
Deaths (Thousands)
Week of
Cumulative Out-Of-Sample Error
(Post Intercept Shift)
Mar
Apr
May
Jun
Jul
Med Abs Err
Med Err
Weeks Forecasting
All Model Versions
Delphi
Los Alamos Nat Lab
Youyang Gu
SIKJalpha
IHME - Curve Fit
IHME - CF SEIR
IHME - MS SEIR
All Cumulative Errors

Maryland

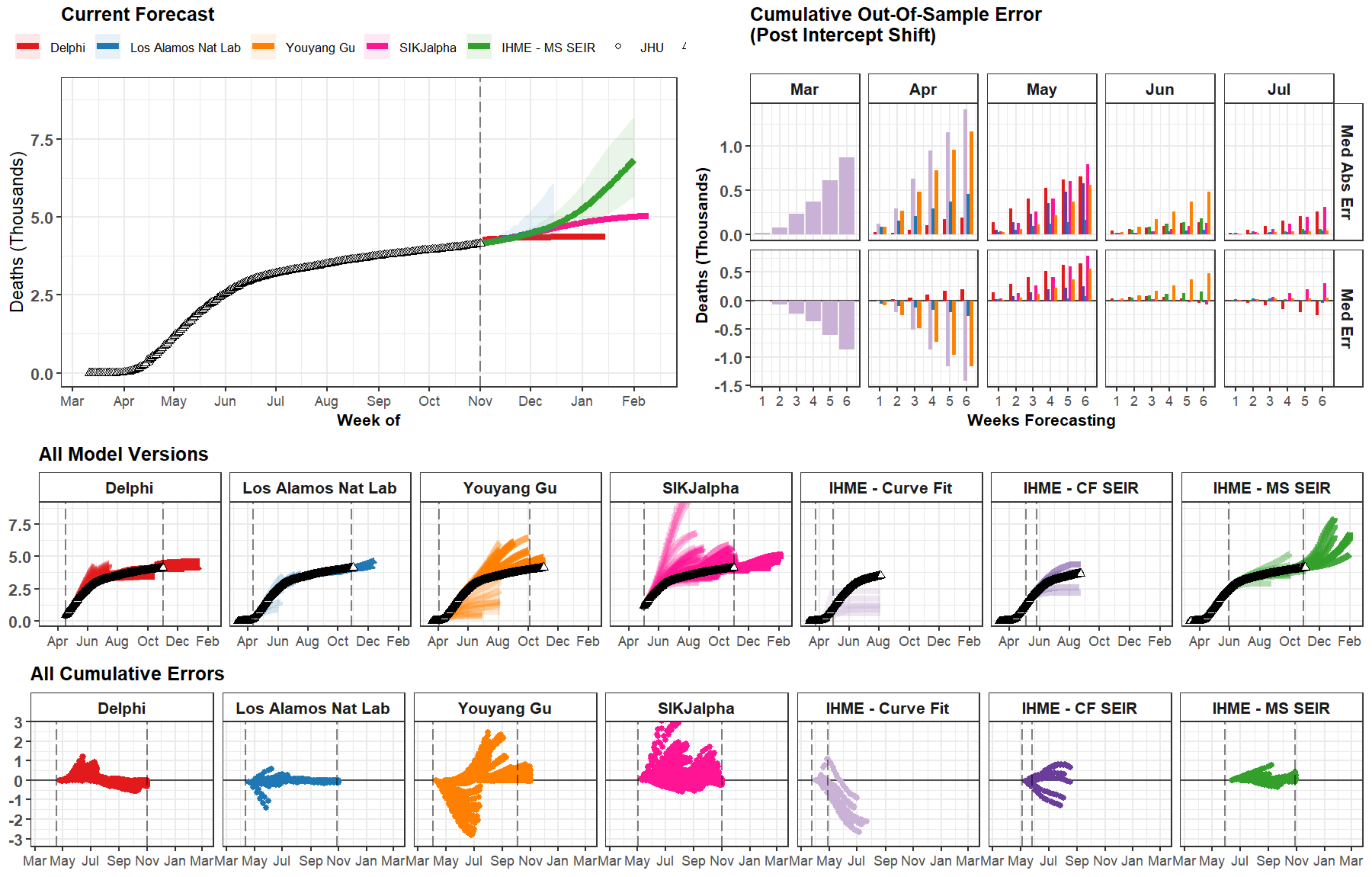
Current Forecast
Delphi
Los Alamos Nat Lab
Youyang Gu
SIKJalpha
IHME - MS SEIR
JHU
Deaths (Thousands)
Week of
Mar Apr May Jun Jul Aug Sep Oct Nov Dec Jan Feb
Cumulative Out-Of-Sample Error
(Post Intercept Shift)
Mar Apr May Jun Jul
Med Abs Err
Med Err
Weeks Forecasting
All Model Versions
Delphi
Los Alamos Nat Lab
Youyang Gu
SIKJalpha
IHME - Curve Fit
IHME - CF SEIR
IHME - MS SEIR
All Cumulative Errors

South Carolina

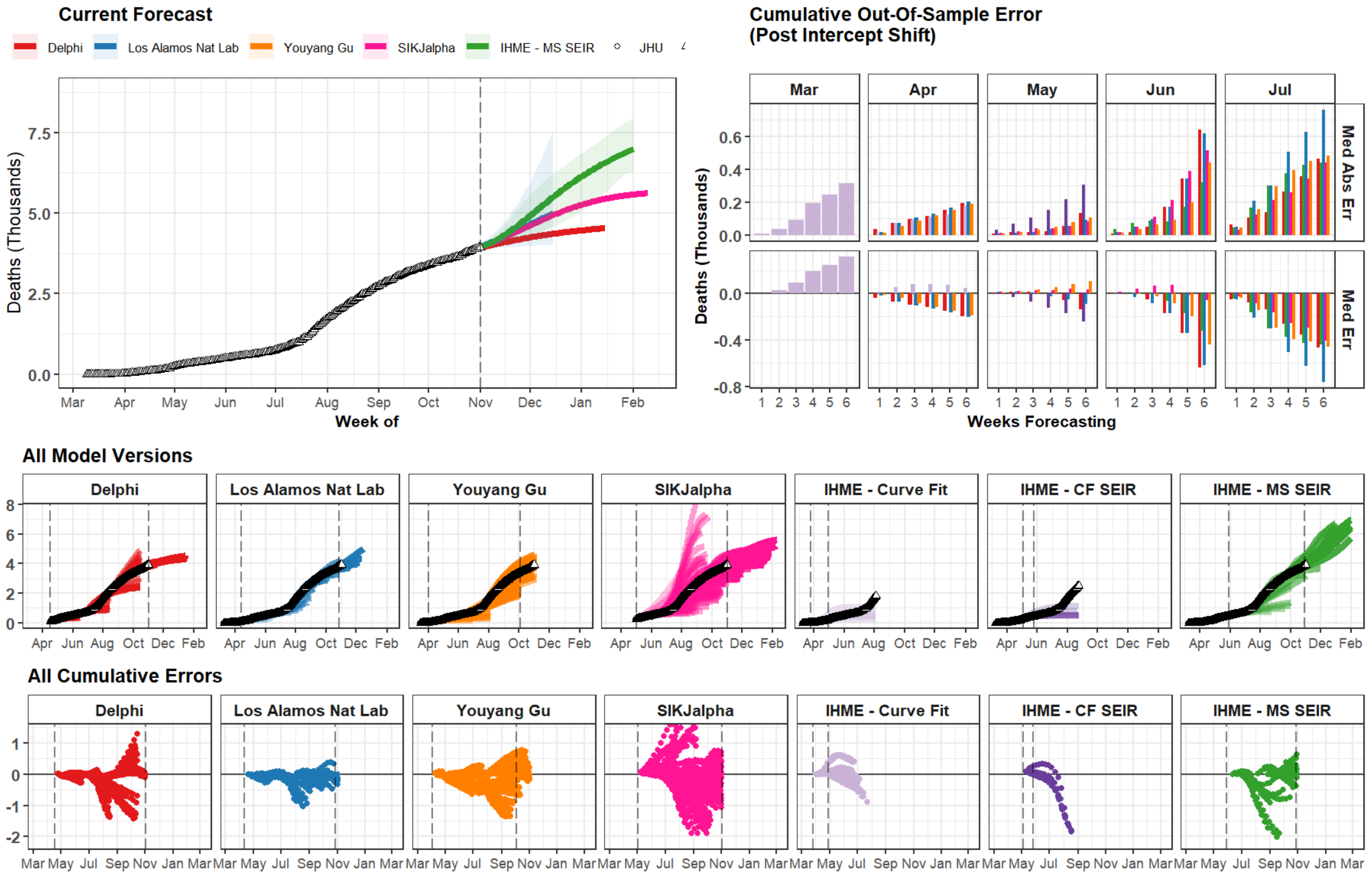

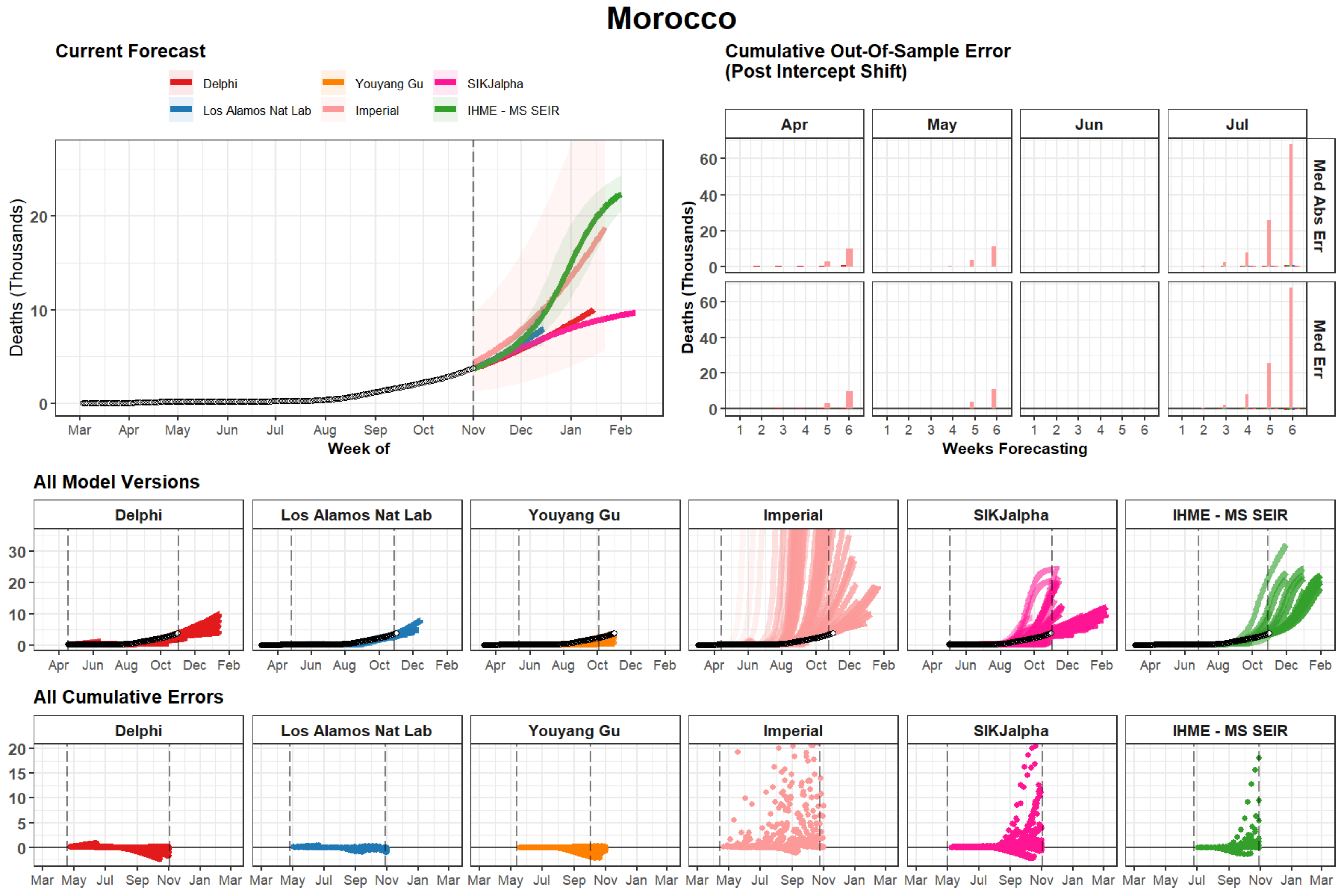
Morocco
Current Forecast
Delphi
Los Alamos Nat Lab
Youyang Gu
Imperial
SIKJalpha
IHME - MS SEIR
Deaths (Thousands)
Week of
Mar
Apr
May
Jun
Jul
Aug
Sep
Oct
Nov
Dec
Jan
Feb
Cumulative Out-Of-Sample Error
(Post Intercept Shift)
Med Abs Err
Med Err
Weeks Forecasting
All Model Versions
All Cumulative Errors

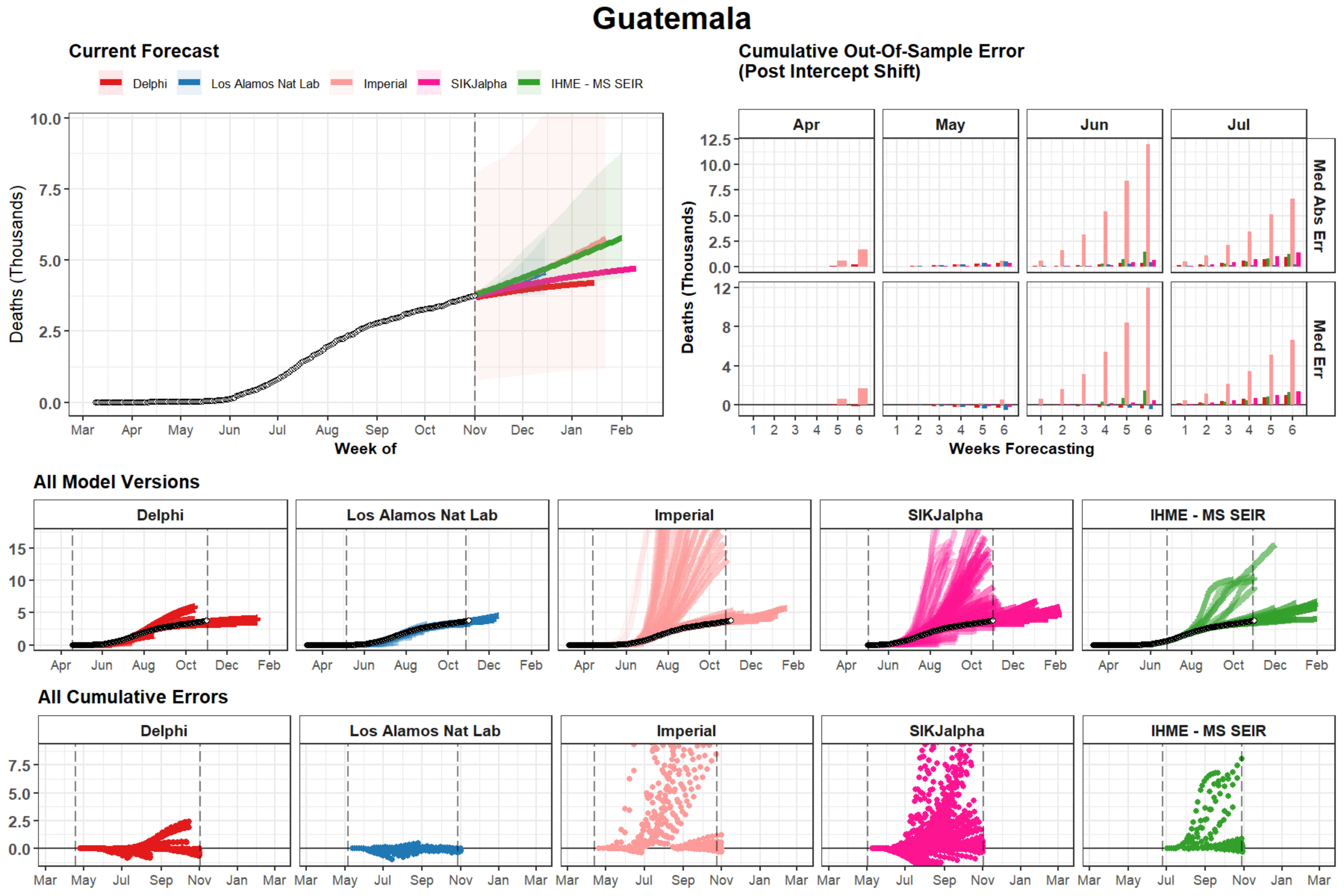

Guatemala
Current Forecast
Delphi
Los Alamos Nat Lab
Imperial
SIKJalpha
IHME - MS SEIR
Deaths (Thousands)
Week of
Cumulative Out-Of-Sample Error
(Post Intercept Shift)
Apr
May
Jun
Jul
Med Abs Err
Med Err
Weeks Forecasting
All Model Versions
All Cumulative Errors

Virginia

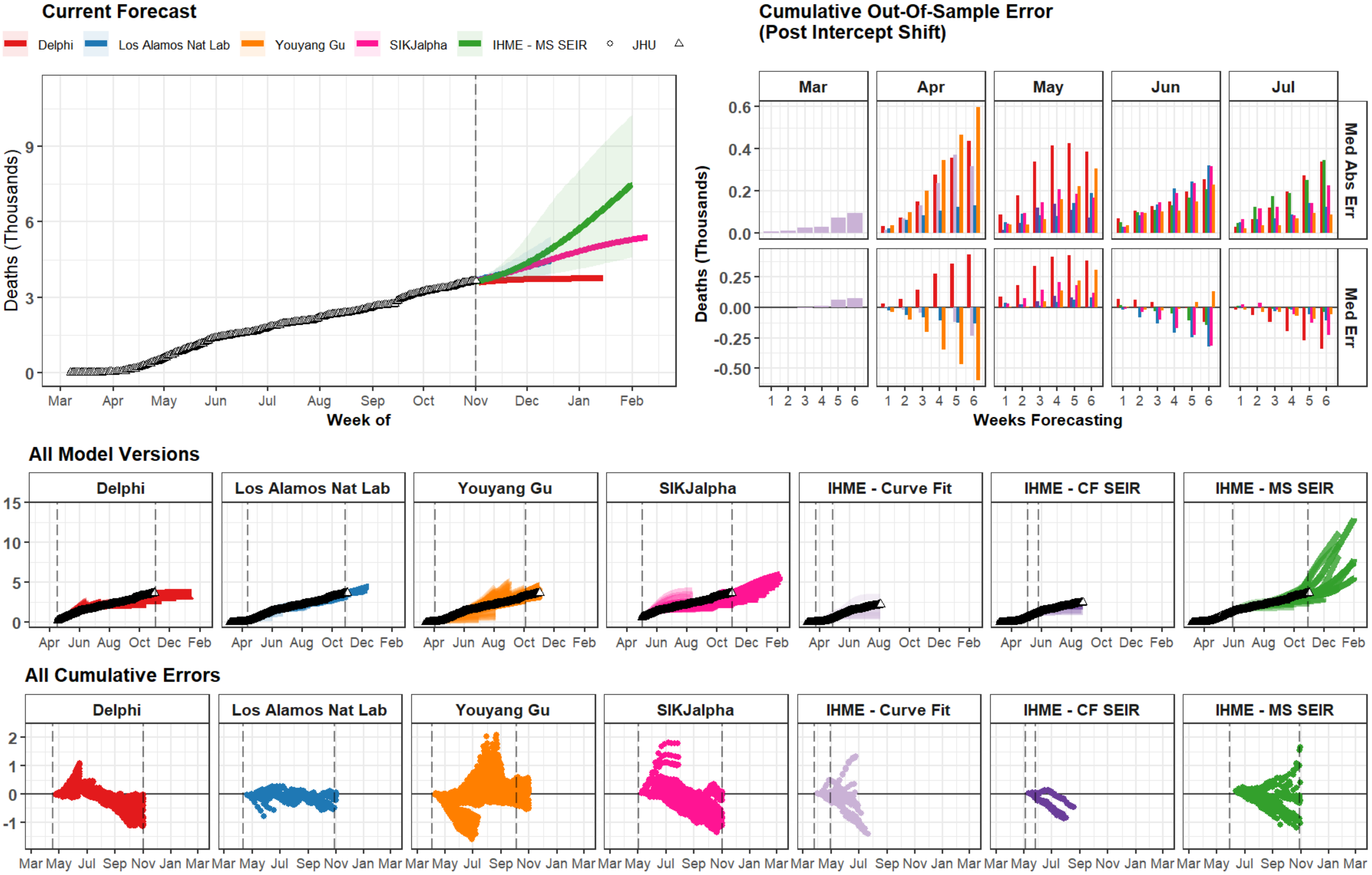
Current Forecast
Delphi
Los Alamos Nat Lab
Youyang Gu
SIKJalpha
IHME - MS SEIR
JHU
Deaths (Thousands)
Week of
Mar Apr May Jun Jul Aug Sep Oct Nov Dec Jan Feb
Cumulative Out-Of-Sample Error
(Post Intercept Shift)
Mar Apr May Jun Jul
Med Abs Err
Med Err
Weeks Forecasting
All Model Versions
Delphi
Los Alamos Nat Lab
Youyang Gu
SIKJalpha
IHME - Curve Fit
IHME - CF SEIR
IHME - MS SEIR
All Cumulative Errors

Czech Republic

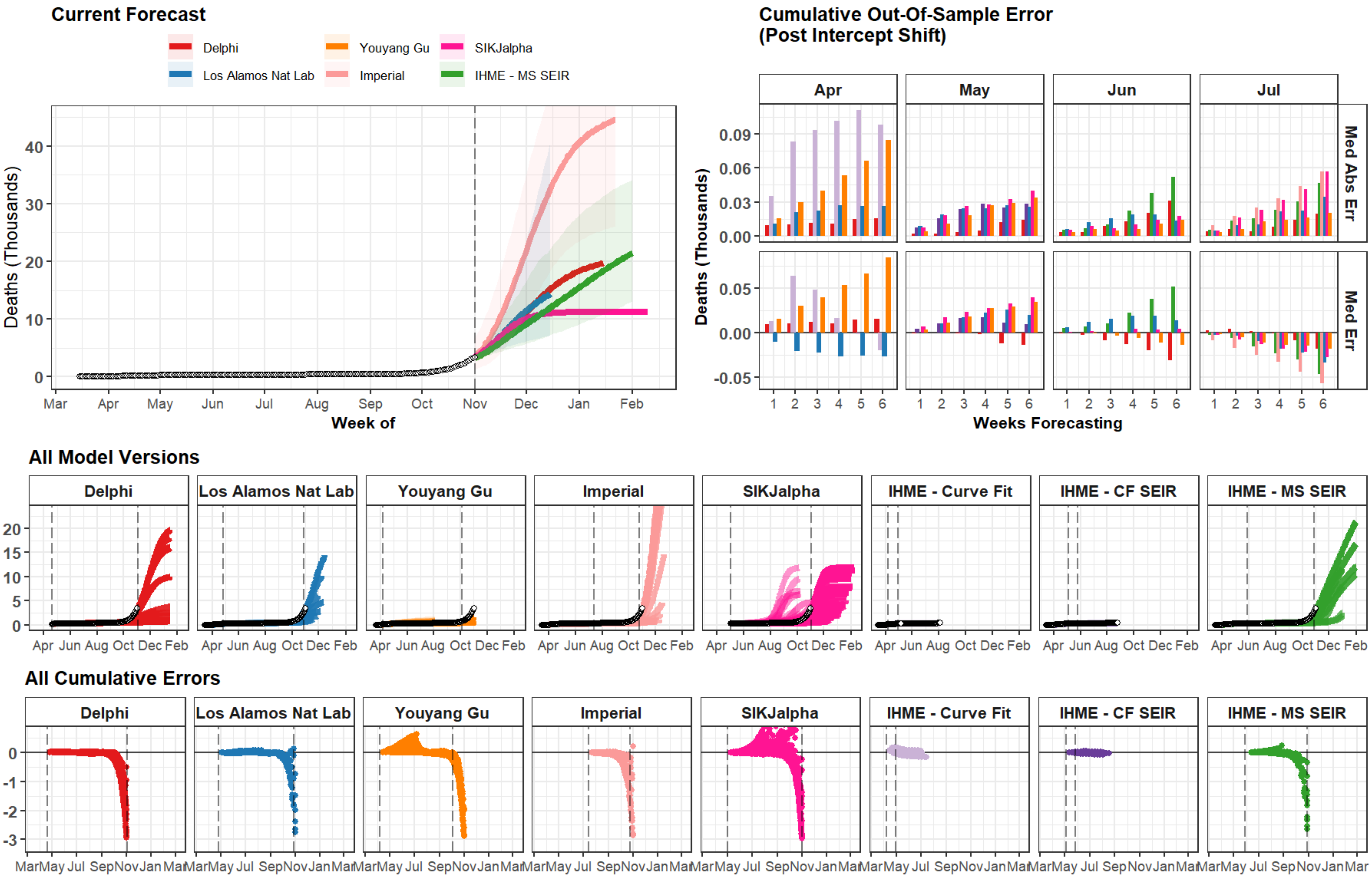

Mississippi

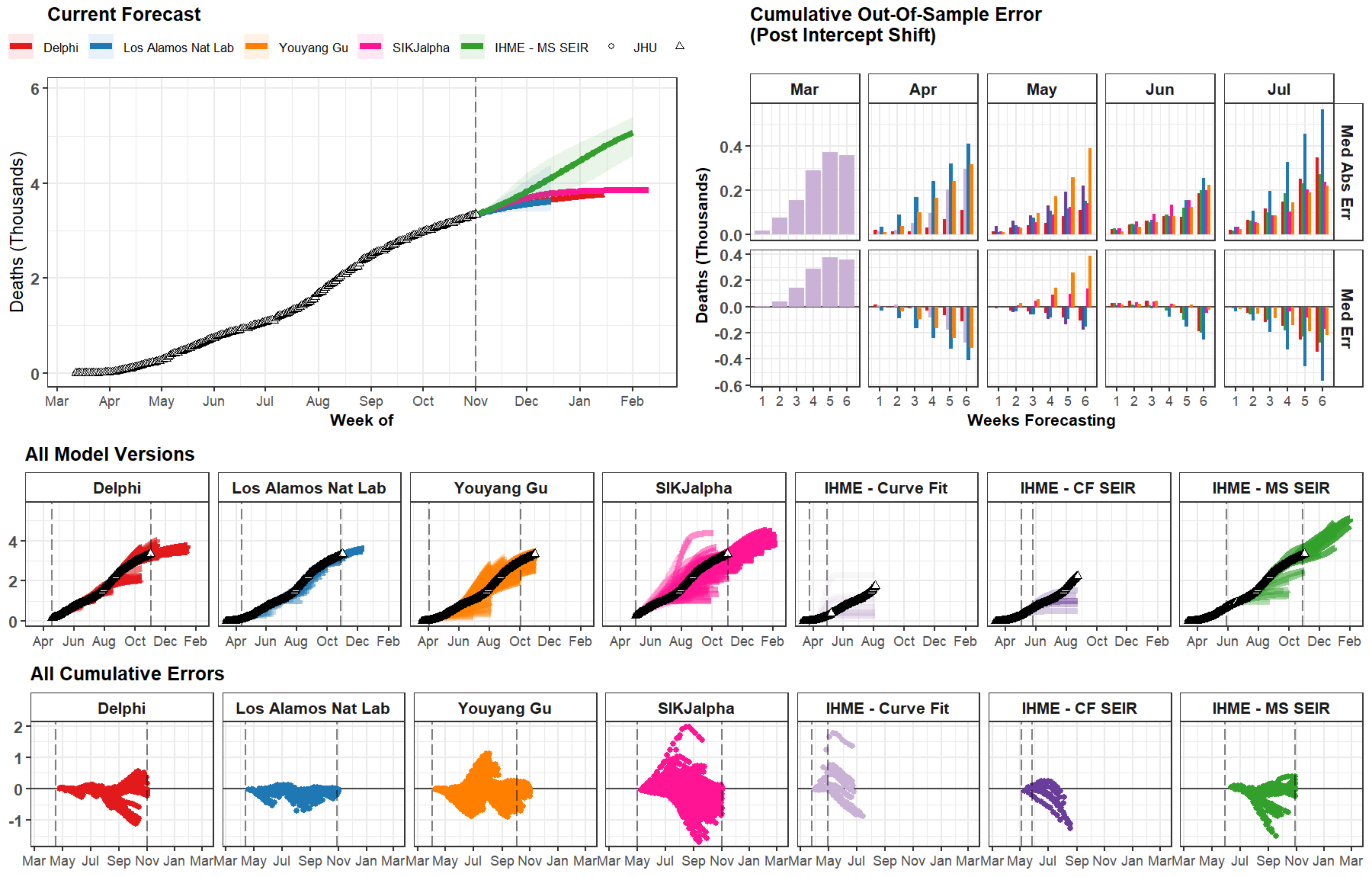

Tennessee

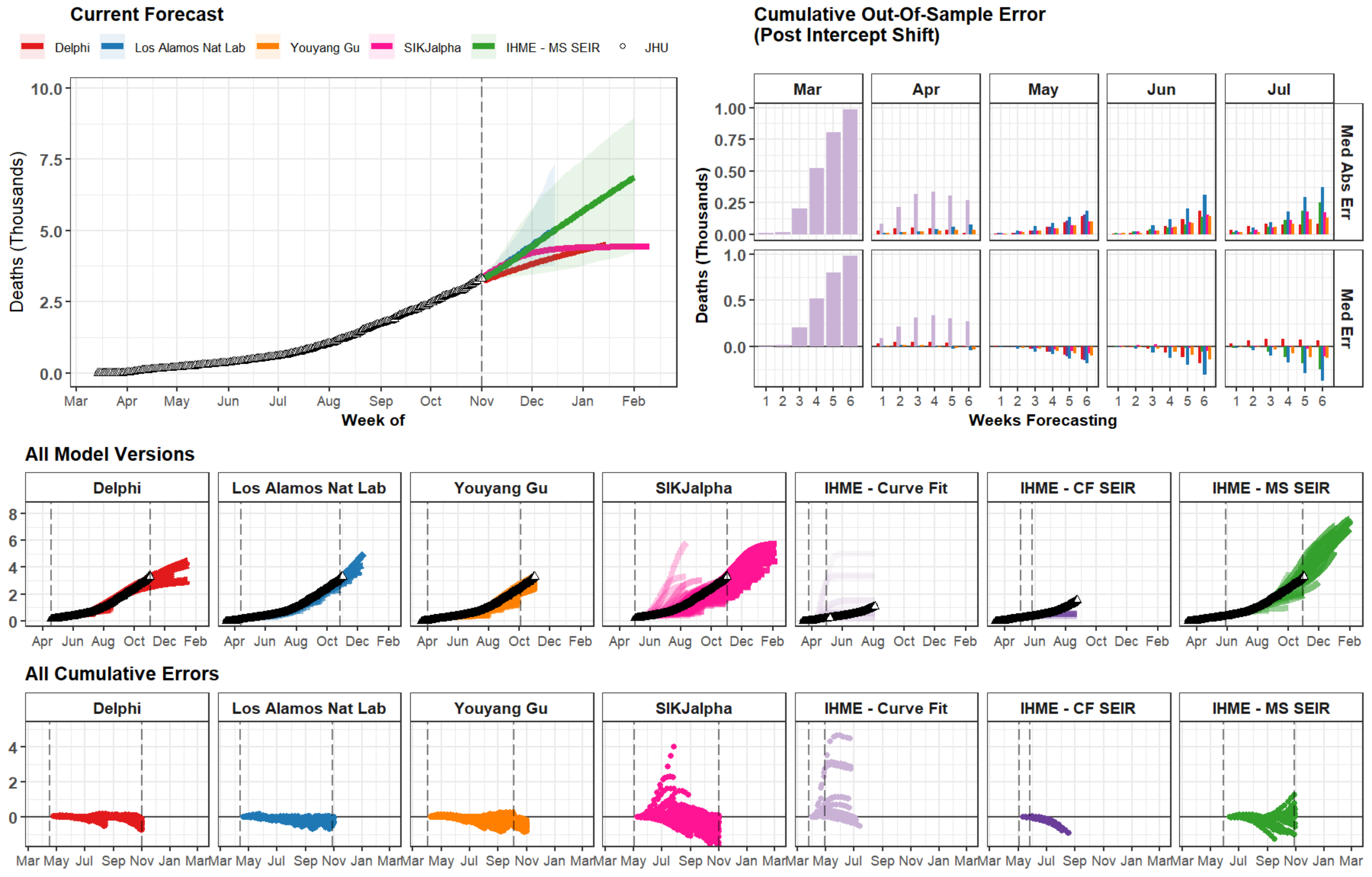

Current Forecast
Delphi
Los Alamos Nat Lab
Youyang Gu
SIKJalpha
IHME - MS SEIR
JHU
Deaths (Thousands)
10.0
7.5
5.0
2.5
0.0
Mar Apr May Jun Jul Aug Sep Oct Nov Dec Jan Feb
Week of
Cumulative Out-Of-Sample Error
(Post Intercept Shift)
Mar
Apr
May
Jun
Jul
Med Abs Err
Med Err
1.00
0.75
0.50
0.25
0.00
1.0
0.5
0.0
1 2 3 4 5 6
Weeks Forecasting
All Model Versions
Delphi
Los Alamos Nat Lab
Youyang Gu
SIKJalpha
IHME - Curve Fit
IHME - CF SEIR
IHME - MS SEIR
8
6
4
2
0
Apr Jun Aug Oct Dec Feb
All Cumulative Errors
4
2
0
Mar May Jul Sep Nov Jan Mar

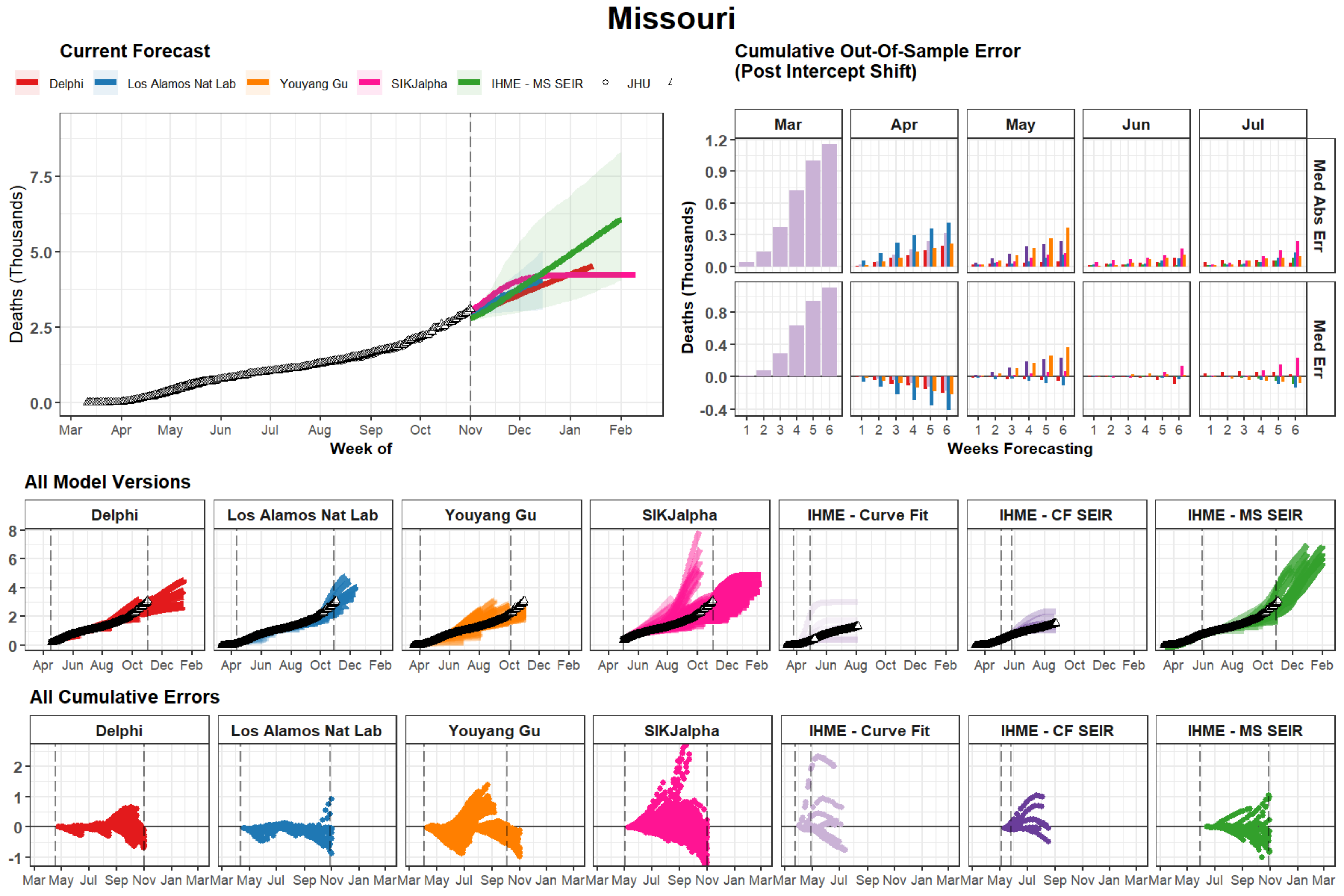

Missouri
Current Forecast
Delphi
Los Alamos Nat Lab
Youyang Gu
SIKJalpha
IHME - MS SEIR
JHU
Deaths (Thousands)
Week of
Cumulative Out-Of-Sample Error
(Post Intercept Shift)
Mar
Apr
May
Jun
Jul
Med Abs Err
Med Err
Weeks Forecasting
All Model Versions
IHME - Curve Fit
IHME - CF SEIR
All Cumulative Errors

Alabama

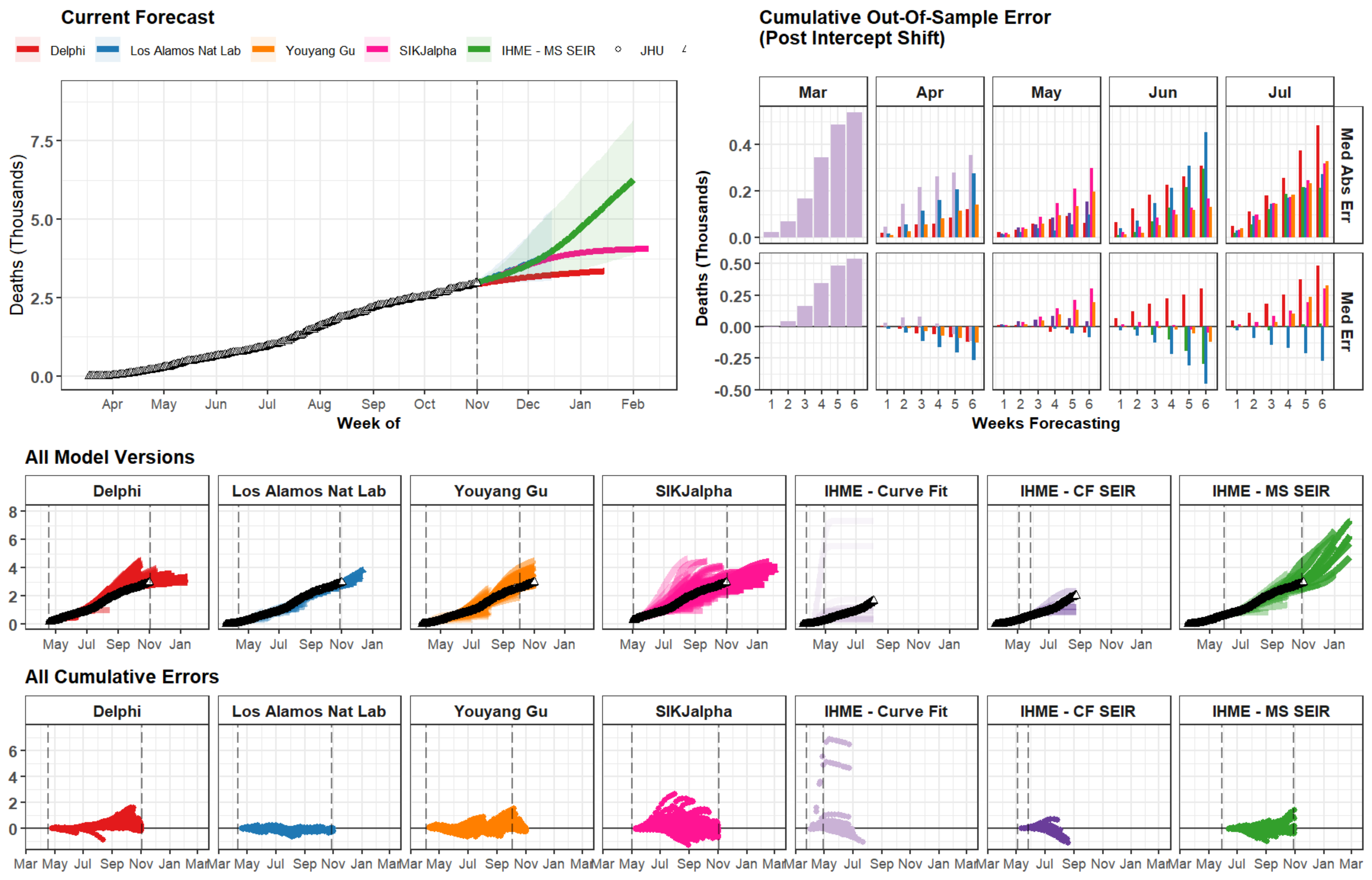
Current Forecast
Delphi
Los Alamos Nat Lab
Youyang Gu
SIKJalpha
IHME - MS SEIR
JHU
Deaths (Thousands)
Week of
Apr May Jun Jul Aug Sep Oct Nov Dec Jan Feb
Cumulative Out-Of-Sample Error
(Post Intercept Shift)
Mar Apr May Jun Jul
Med Abs Err
Med Err
Weeks Forecasting
All Model Versions
Delphi
Los Alamos Nat Lab
Youyang Gu
SIKJalpha
IHME - Curve Fit
IHME - CF SEIR
IHME - MS SEIR
All Cumulative Errors

Panama

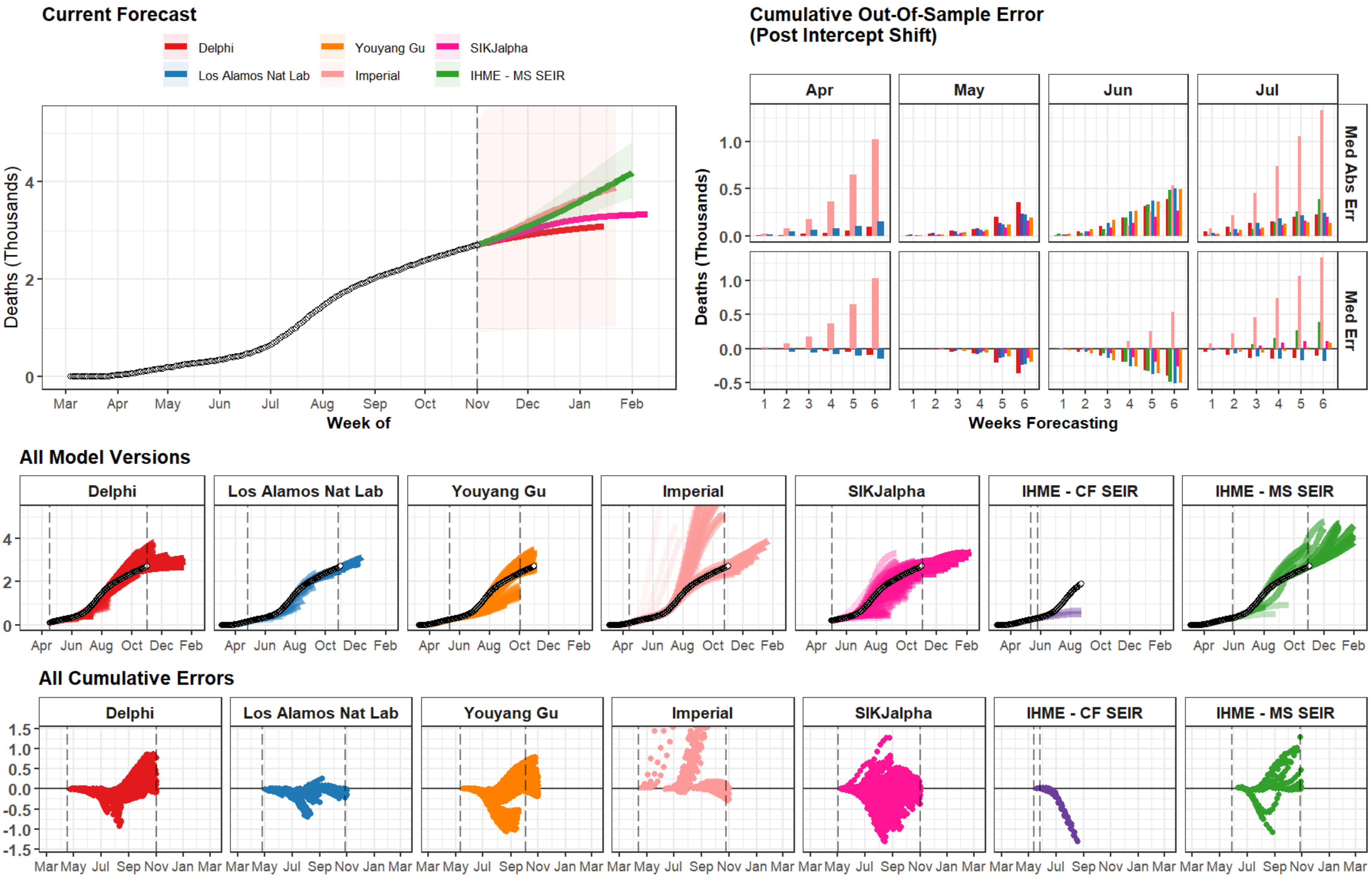

Current Forecast
Delphi
Youyang Gu
SIKJalpha
Los Alamos Nat Lab
Imperial
IHME - MS SEIR
Deaths (Thousands)
Week of
Cumulative Out-Of-Sample Error
(Post Intercept Shift)
Apr
May
Jun
Jul
Med Abs Err
Med Err
Weeks Forecasting
All Model Versions
Delphi
Los Alamos Nat Lab
Youyang Gu
Imperial
SIKJalpha
IHME - CF SEIR
IHME - MS SEIR
All Cumulative Errors

Honduras

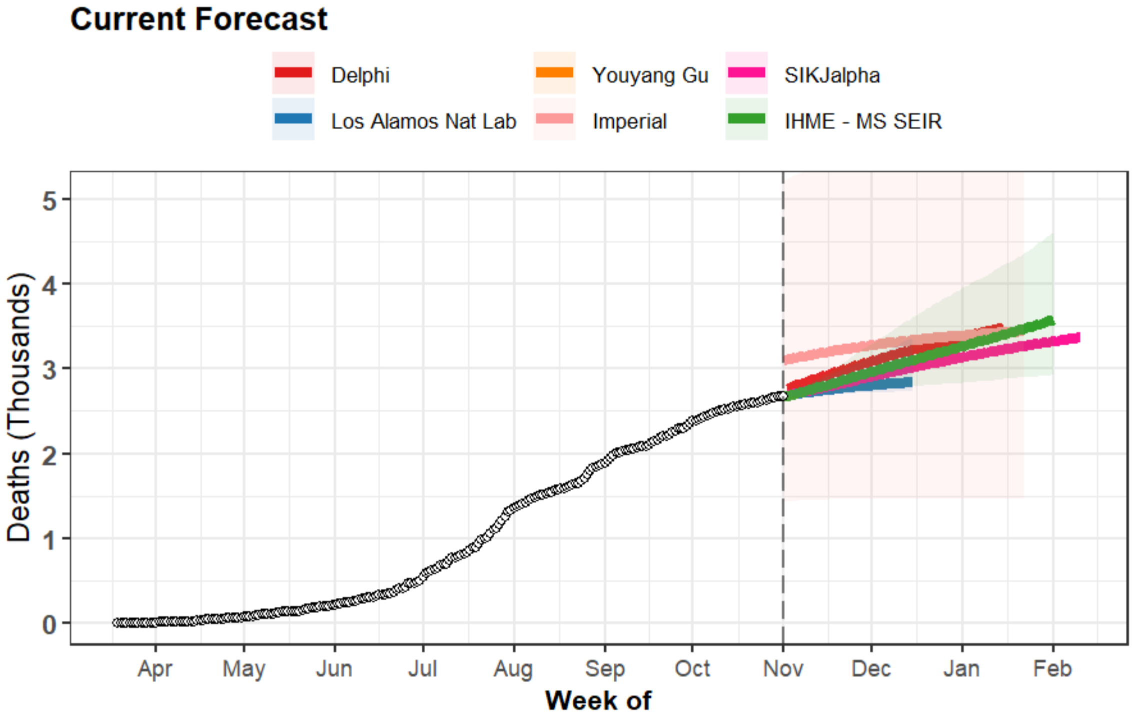

Current Forecast
Delphi
Los Alamos Nat Lab
Youyang Gu
Imperial
SIKJalpha
IHME - MS SEIR
Deaths (Thousands)
0
1
2
3
4
5
Apr
May
Jun
Jul
Aug
Sep
Oct
Nov
Dec
Jan
Feb
Week of

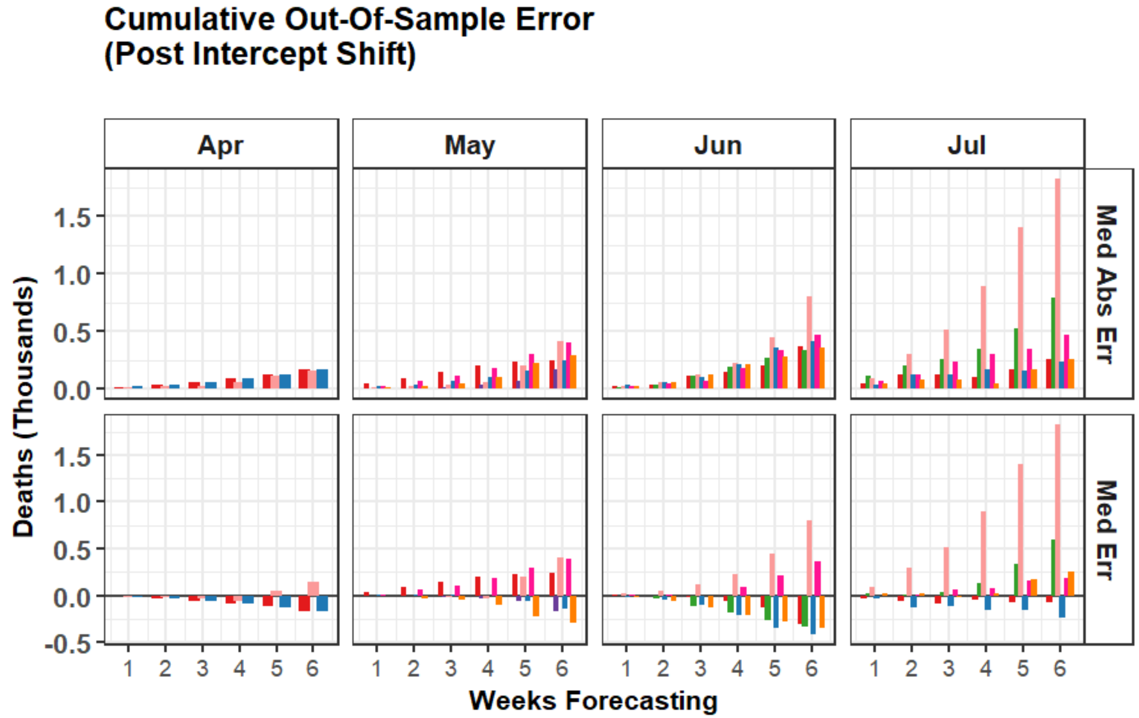

Cumulative Out-Of-Sample Error
(Post Intercept Shift)
Apr
May
Jun
Jul
Med Abs Err
Med Err
Deaths (Thousands)
1.5
1.0
0.5
0.0
-0.5
1 2 3 4 5 6
Weeks Forecasting

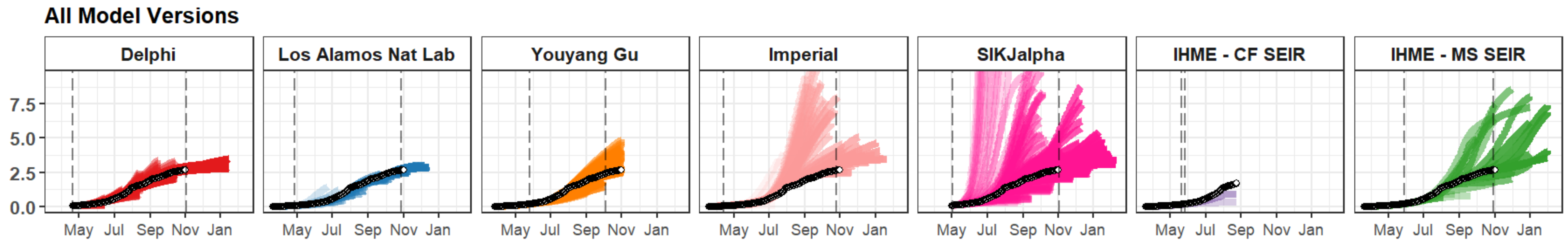

All Model Versions
Delphi
Los Alamos Nat Lab
Youyang Gu
Imperial
SIKJalpha
IHME - CF SEIR
IHME - MS SEIR
7.5
5.0
2.5
0.0
May Jul Sep Nov Jan

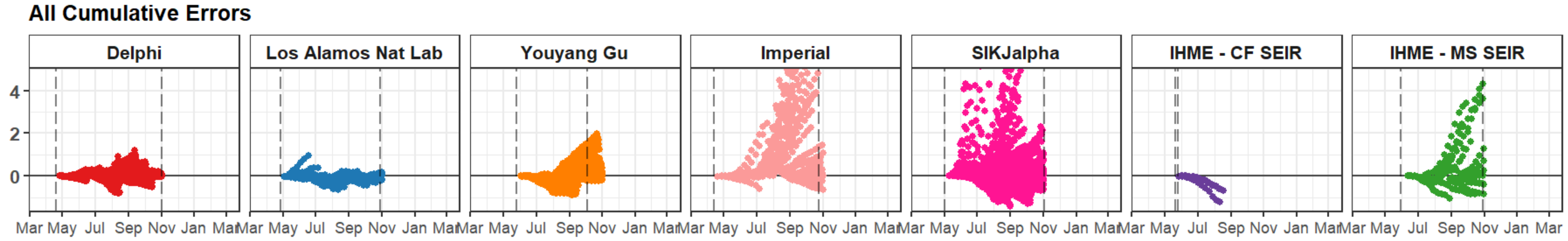

All Cumulative Errors
Delphi
Los Alamos Nat Lab
Youyang Gu
Imperial
SIKJalpha
IHME - CF SEIR
IHME - MS SEIR
4
2
0
Mar May Jul Sep Nov Jan Mar

Israel

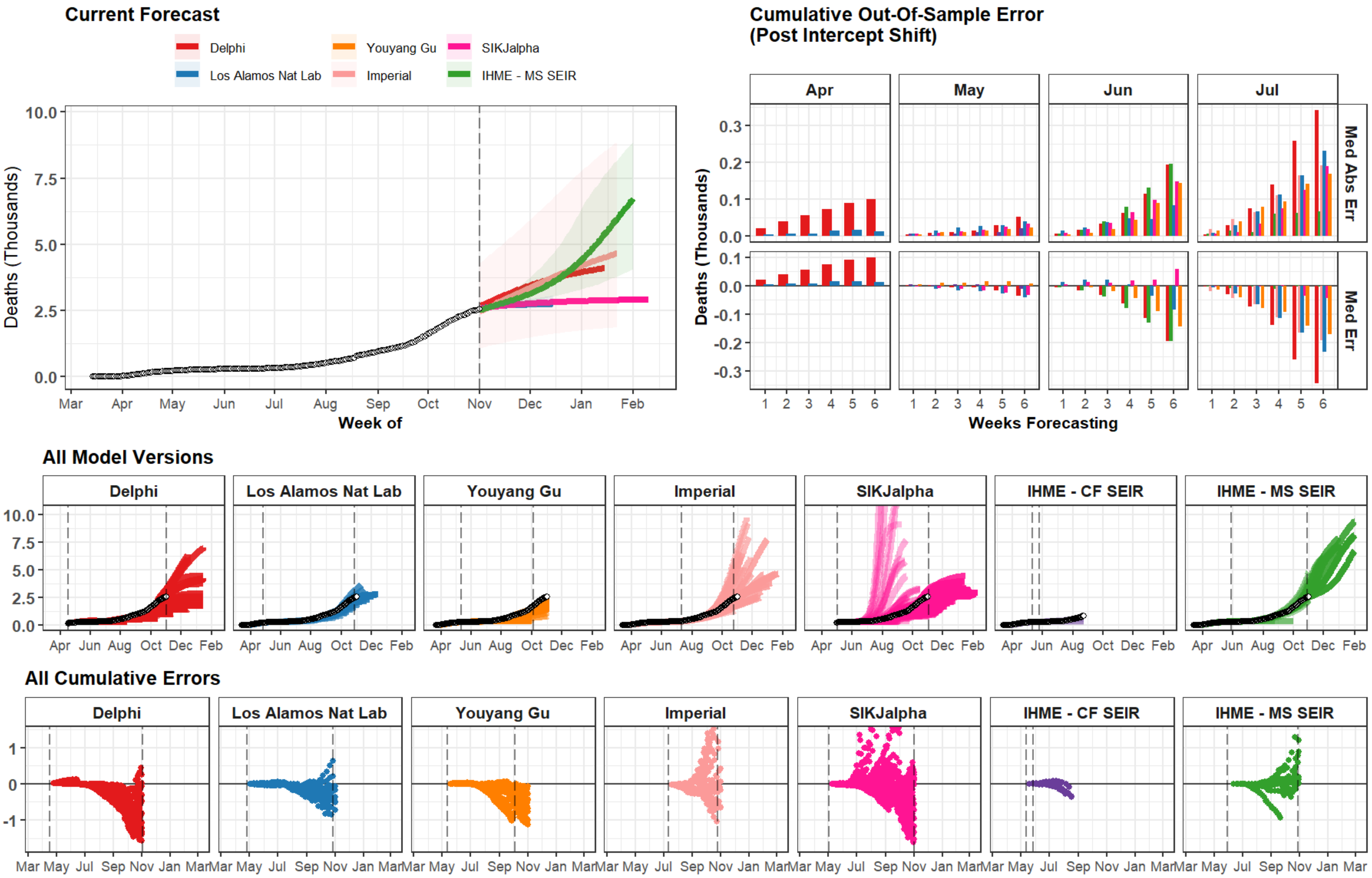
Current Forecast
Delphi
Los Alamos Nat Lab
Youyang Gu
Imperial
SIKJalpha
IHME - MS SEIR
Deaths (Thousands)
Week of
Cumulative Out-Of-Sample Error
(Post Intercept Shift)
Apr
May
Jun
Jul
Med Abs Err
Med Err
Weeks Forecasting
All Model Versions
Delphi
Los Alamos Nat Lab
Youyang Gu
Imperial
SIKJalpha
IHME - CF SEIR
IHME - MS SEIR
All Cumulative Errors

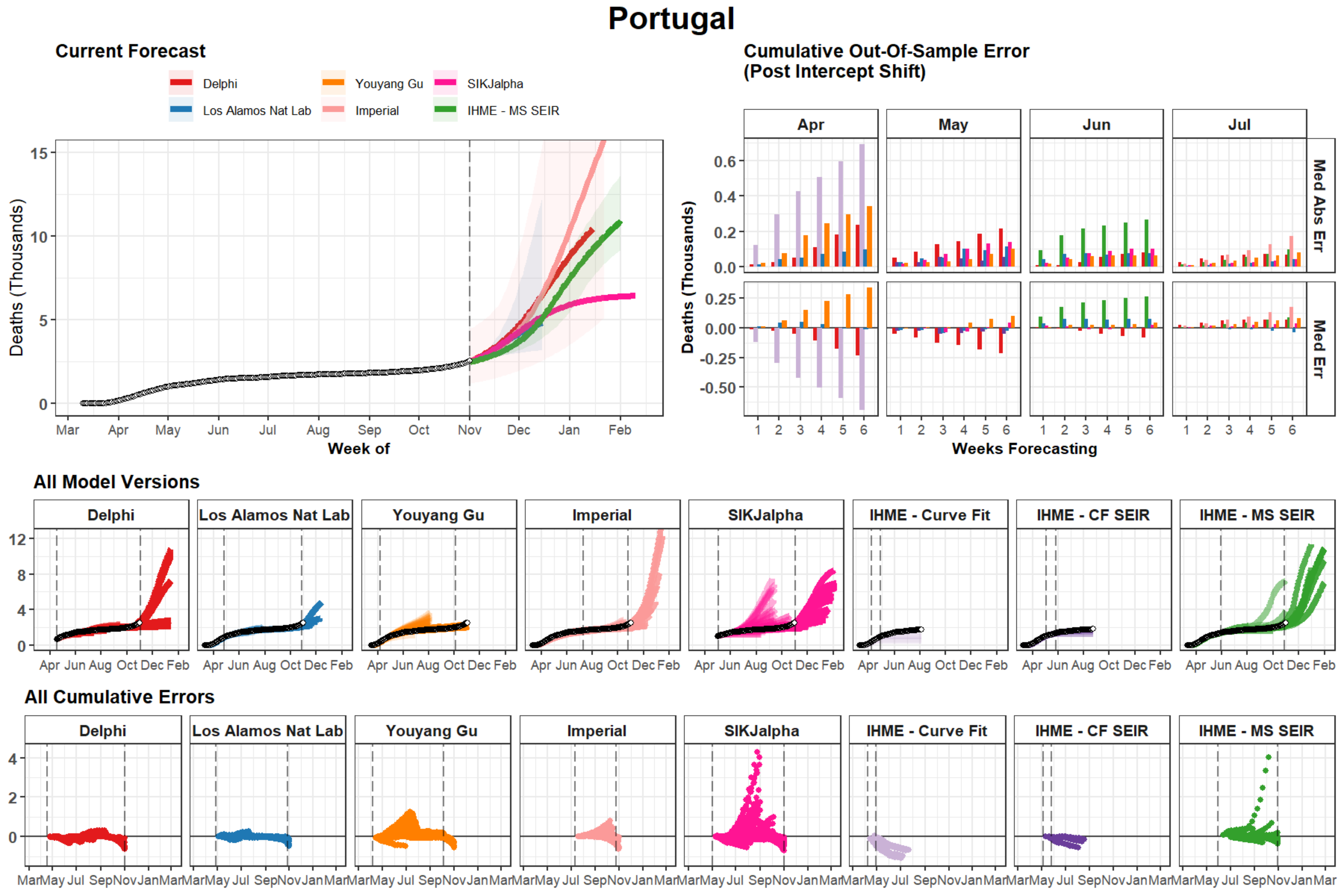
Portugal
Current Forecast
Delphi
Los Alamos Nat Lab
Youyang Gu
Imperial
SIKJalpha
IHME - MS SEIR
Deaths (Thousands)
15
10
5
0
Mar Apr May Jun Jul Aug Sep Oct Nov Dec Jan Feb
Week of
Cumulative Out-Of-Sample Error
(Post Intercept Shift)
Apr
May
Jun
Jul
Med Abs Err
Med Err
0.6
0.4
0.2
0.0
0.25
0.00
-0.25
-0.50
1 2 3 4 5 6
Weeks Forecasting
All Model Versions
Delphi
Los Alamos Nat Lab
Youyang Gu
Imperial
SIKJalpha
IHME - Curve Fit
IHME - CF SEIR
IHME - MS SEIR
12
8
4
0
Apr Jun Aug Oct Dec Feb
All Cumulative Errors
4
2
0
Mar May Jul Sep Nov Jan Mar

Minnesota

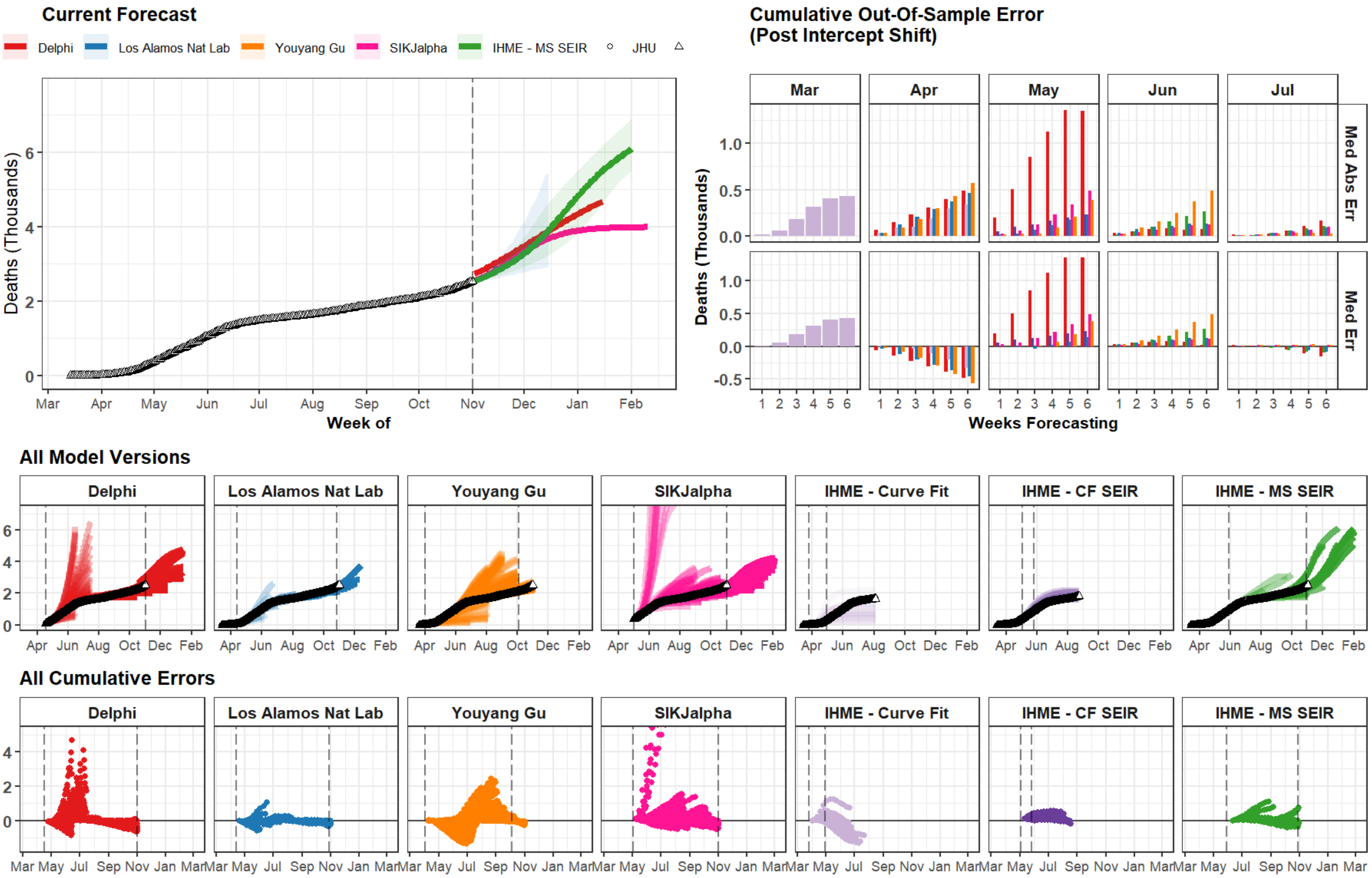
Current Forecast
Delphi
Los Alamos Nat Lab
Youyang Gu
SIKJalpha
IHME - MS SEIR
JHU
Deaths (Thousands)
Week of
Mar Apr May Jun Jul Aug Sep Oct Nov Dec Jan Feb
Cumulative Out-Of-Sample Error
(Post Intercept Shift)
Mar Apr May Jun Jul
Med Abs Err
Med Err
Weeks Forecasting
All Model Versions
Delphi
Los Alamos Nat Lab
Youyang Gu
SIKJalpha
IHME - Curve Fit
IHME - CF SEIR
IHME - MS SEIR
All Cumulative Errors

Washington

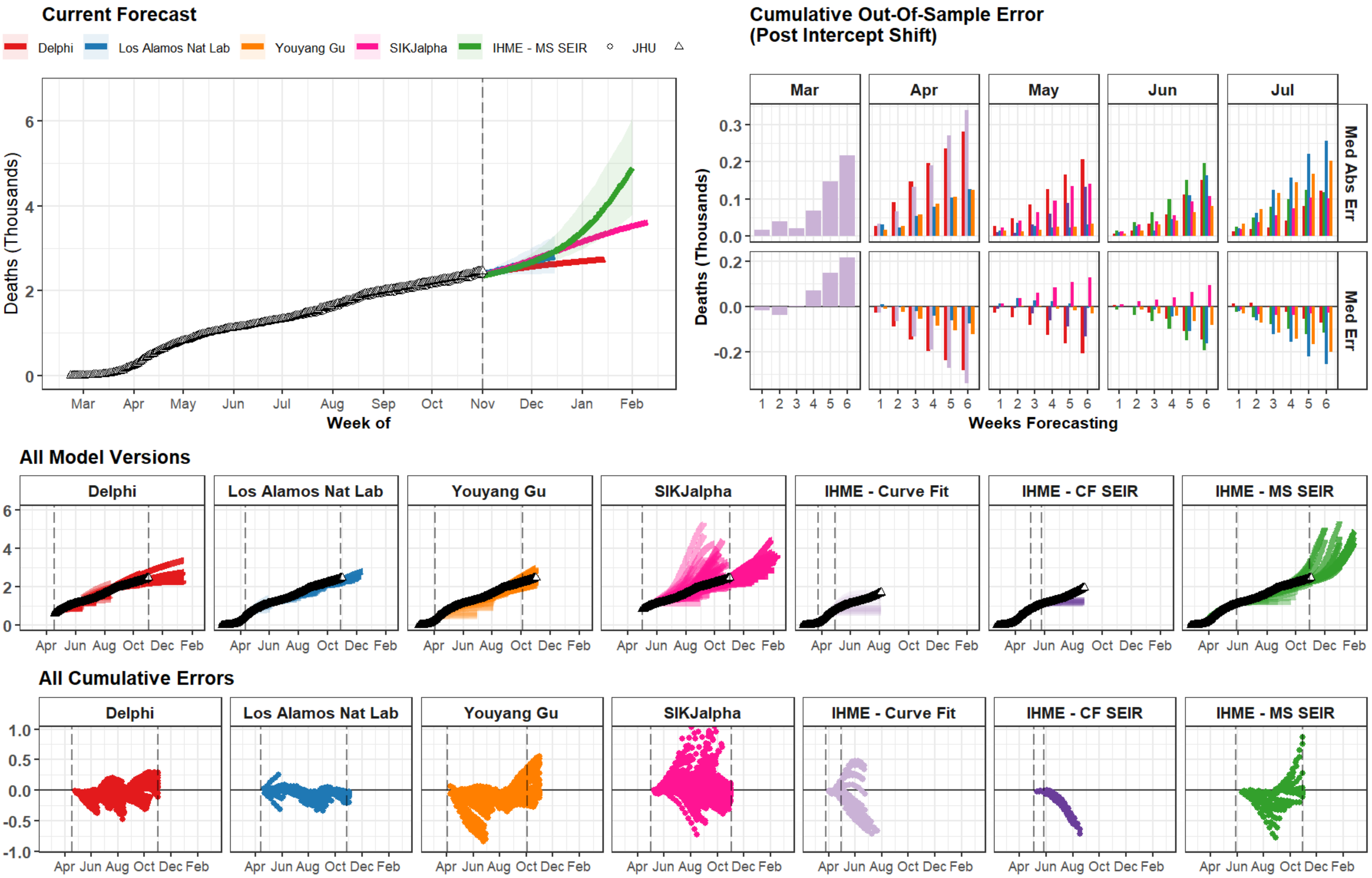
Current Forecast
Delphi
Los Alamos Nat Lab
Youyang Gu
SIKJalpha
IHME - MS SEIR
JHU
Deaths (Thousands)
Week of
Mar Apr May Jun Jul Aug Sep Oct Nov Dec Jan Feb
Cumulative Out-Of-Sample Error
(Post Intercept Shift)
Mar
Apr
May
Jun
Jul
Med Abs Err
Med Err
Deaths (Thousands)
Weeks Forecasting
All Model Versions
Delphi
Los Alamos Nat Lab
Youyang Gu
SIKJalpha
IHME - Curve Fit
IHME - CF SEIR
IHME - MS SEIR
Apr Jun Aug Oct Dec Feb
All Cumulative Errors
Delphi
Los Alamos Nat Lab
Youyang Gu
SIKJalpha
IHME - Curve Fit
IHME - CF SEIR
IHME - MS SEIR
Apr Jun Aug Oct Dec Feb

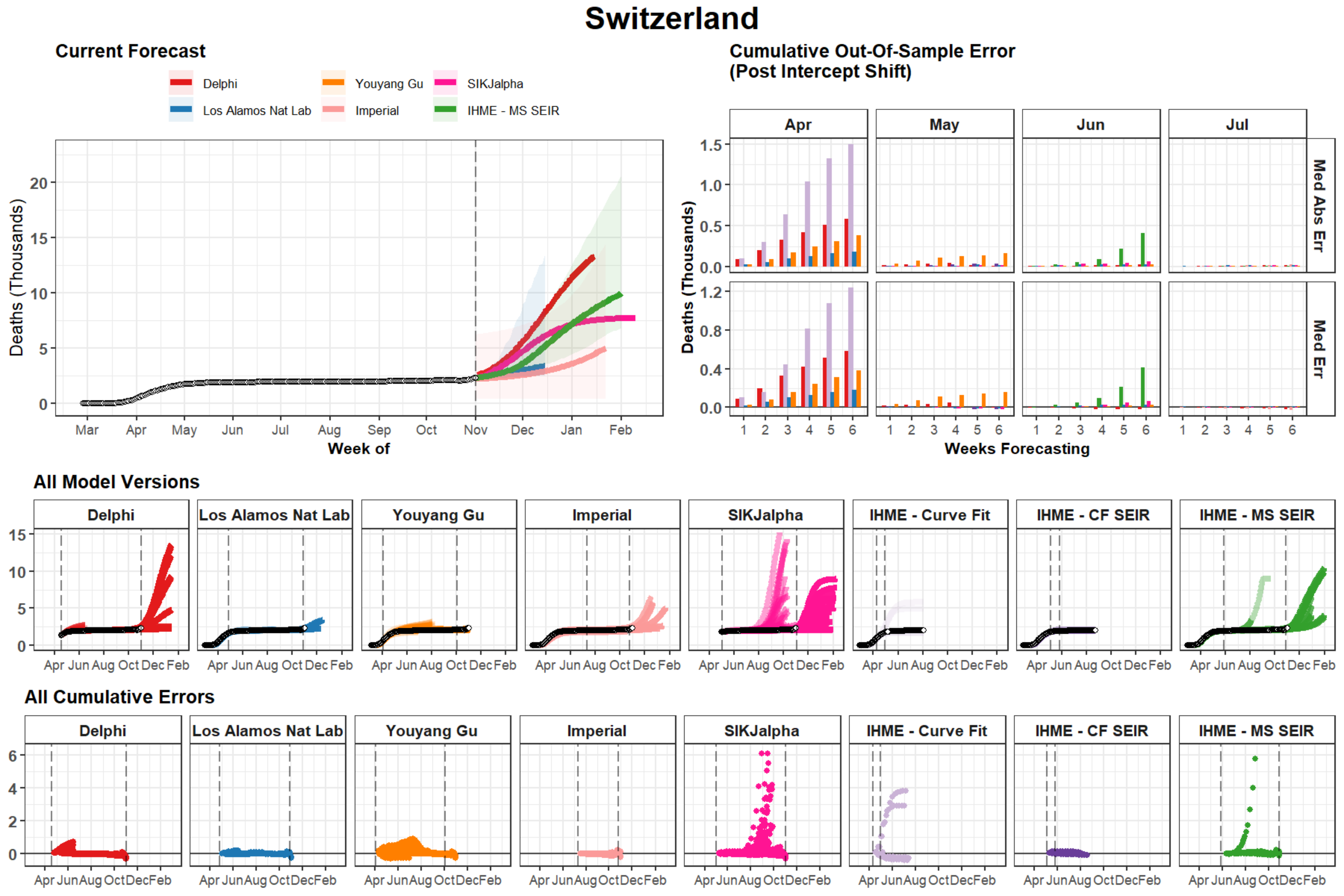
Switzerland
Current Forecast
Delphi
Los Alamos Nat Lab
Youyang Gu
Imperial
SIKJalpha
IHME - MS SEIR
Deaths (Thousands)
Week of
Mar Apr May Jun Jul Aug Sep Oct Nov Dec Jan Feb
Cumulative Out-Of-Sample Error
(Post Intercept Shift)
Apr May Jun Jul
Med Abs Err
Med Err
Weeks Forecasting
All Model Versions
Delphi Los Alamos Nat Lab Youyang Gu Imperial SIKJalpha IHME - Curve Fit IHME - CF SEIR IHME - MS SEIR
Apr Jun Aug Oct Dec Feb
All Cumulative Errors
Delphi Los Alamos Nat Lab Youyang Gu Imperial SIKJalpha IHME - Curve Fit IHME - CF SEIR IHME - MS SEIR

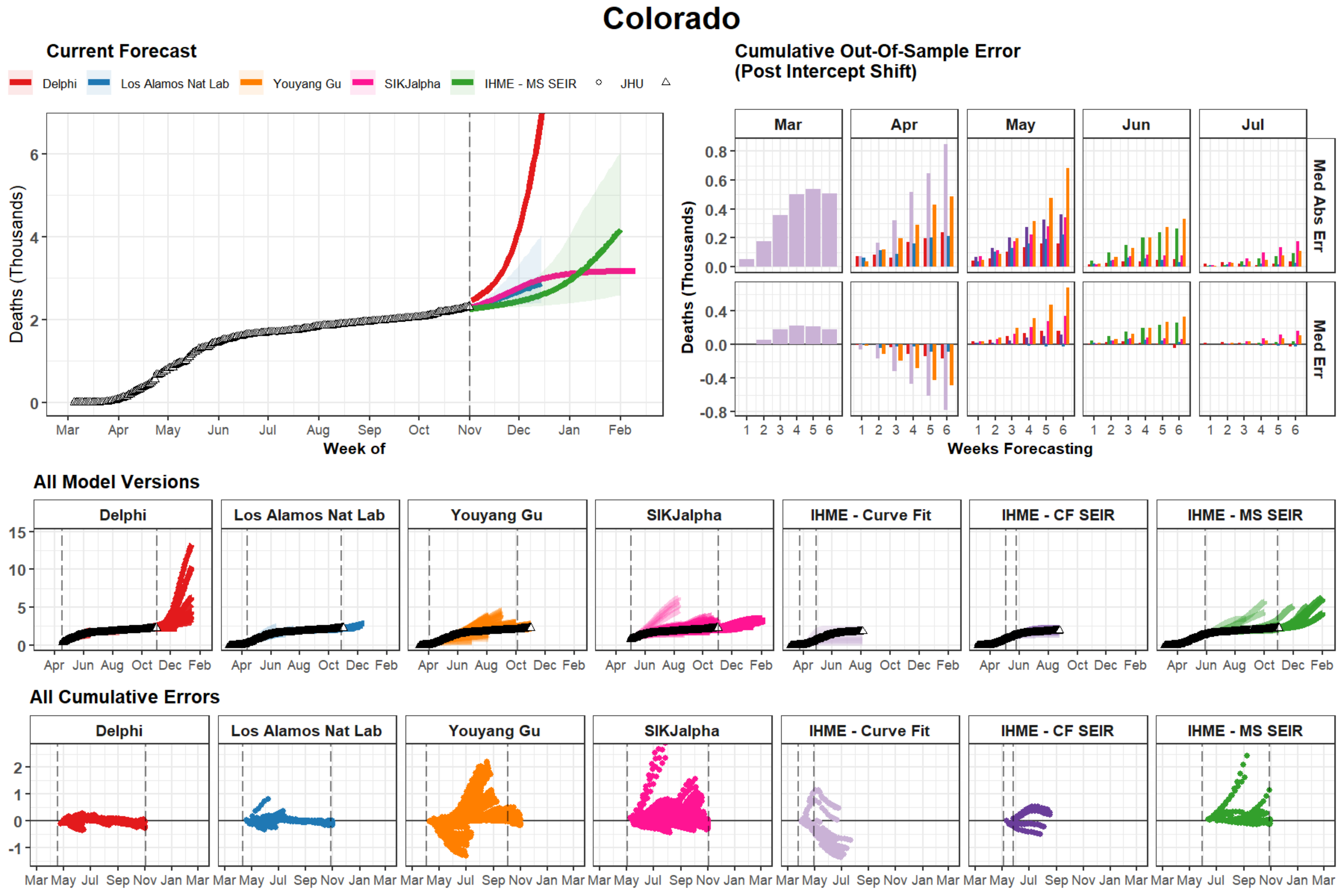
Colorado
Current Forecast
Delphi
Los Alamos Nat Lab
Youyang Gu
SIKJalpha
IHME - MS SEIR
JHU
Deaths (Thousands)
Week of
Cumulative Out-Of-Sample Error
(Post Intercept Shift)
Mar
Apr
May
Jun
Jul
Med Abs Err
Med Err
Weeks Forecasting
All Model Versions
Delphi
Los Alamos Nat Lab
Youyang Gu
SIKJalpha
IHME - Curve Fit
IHME - CF SEIR
IHME - MS SEIR
All Cumulative Errors

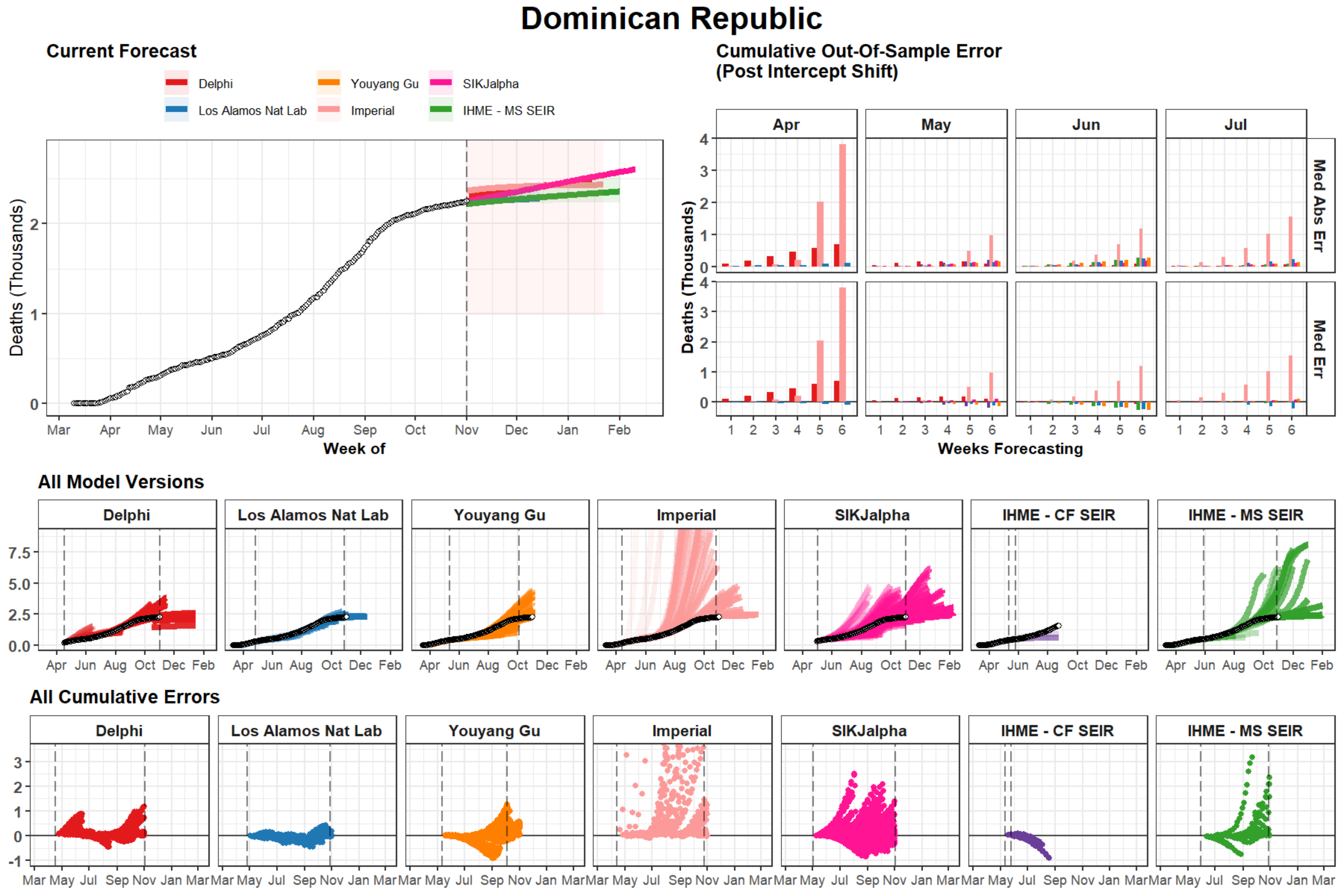

Dominican Republic
Current Forecast
Delphi
Los Alamos Nat Lab
Youyang Gu
Imperial
SIKJalpha
IHME - MS SEIR
Deaths (Thousands)
Week of
Cumulative Out-Of-Sample Error
(Post Intercept Shift)
Apr
May
Jun
Jul
Med Abs Err
Med Err
Weeks Forecasting
All Model Versions
Delphi
Los Alamos Nat Lab
Youyang Gu
Imperial
SIKJalpha
IHME - CF SEIR
IHME - MS SEIR
All Cumulative Errors

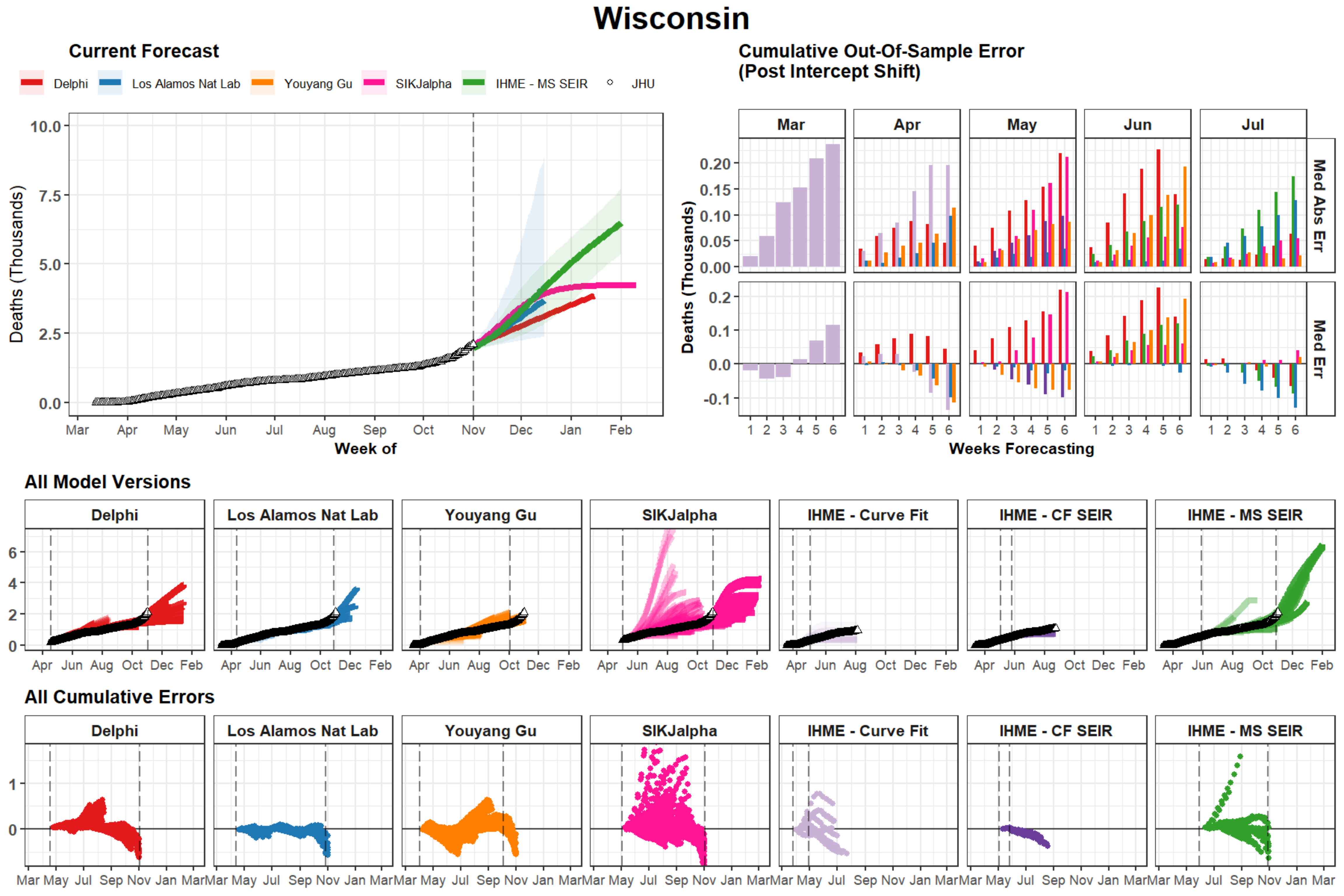
Wisconsin
Current Forecast
Delphi
Los Alamos Nat Lab
Youyang Gu
SIKJalpha
IHME - MS SEIR
JHU
Deaths (Thousands)
Week of
Mar
Apr
May
Jun
Jul
Aug
Sep
Oct
Nov
Dec
Jan
Feb
Cumulative Out-Of-Sample Error
(Post Intercept Shift)
Med Abs Err
Med Err
Weeks Forecasting
All Model Versions
IHME - Curve Fit
IHME - CF SEIR
All Cumulative Errors

Algeria

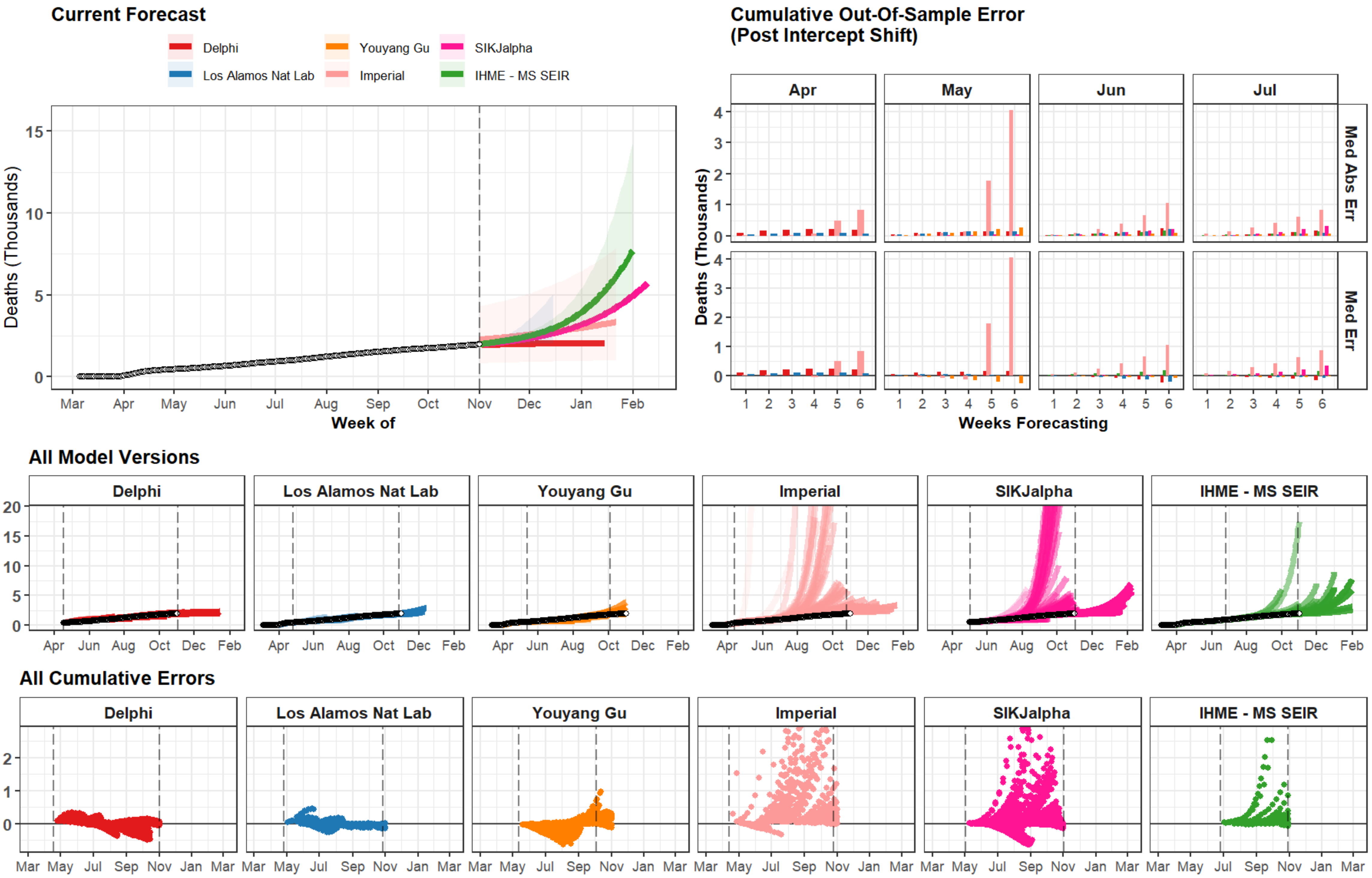
Current Forecast
Delphi
Los Alamos Nat Lab
Youyang Gu
Imperial
SIKJalpha
IHME - MS SEIR
Deaths (Thousands)
0
5
10
15
Mar
Apr
May
Jun
Jul
Aug
Sep
Oct
Nov
Dec
Jan
Feb
Week of
Cumulative Out-Of-Sample Error
(Post Intercept Shift)
Apr
May
Jun
Jul
Med Abs Err
Med Err
Deaths (Thousands)
Weeks Forecasting
All Model Versions
Delphi
Los Alamos Nat Lab
Youyang Gu
Imperial
SIKJalpha
IHME - MS SEIR
0
5
10
15
20
Apr
Jun
Aug
Oct
Dec
Feb
All Cumulative Errors
Delphi
Los Alamos Nat Lab
Youyang Gu
Imperial
SIKJalpha
IHME - MS SEIR
0
1
2
Mar
May
Jul
Sep
Nov
Jan
Mar

Arkansas

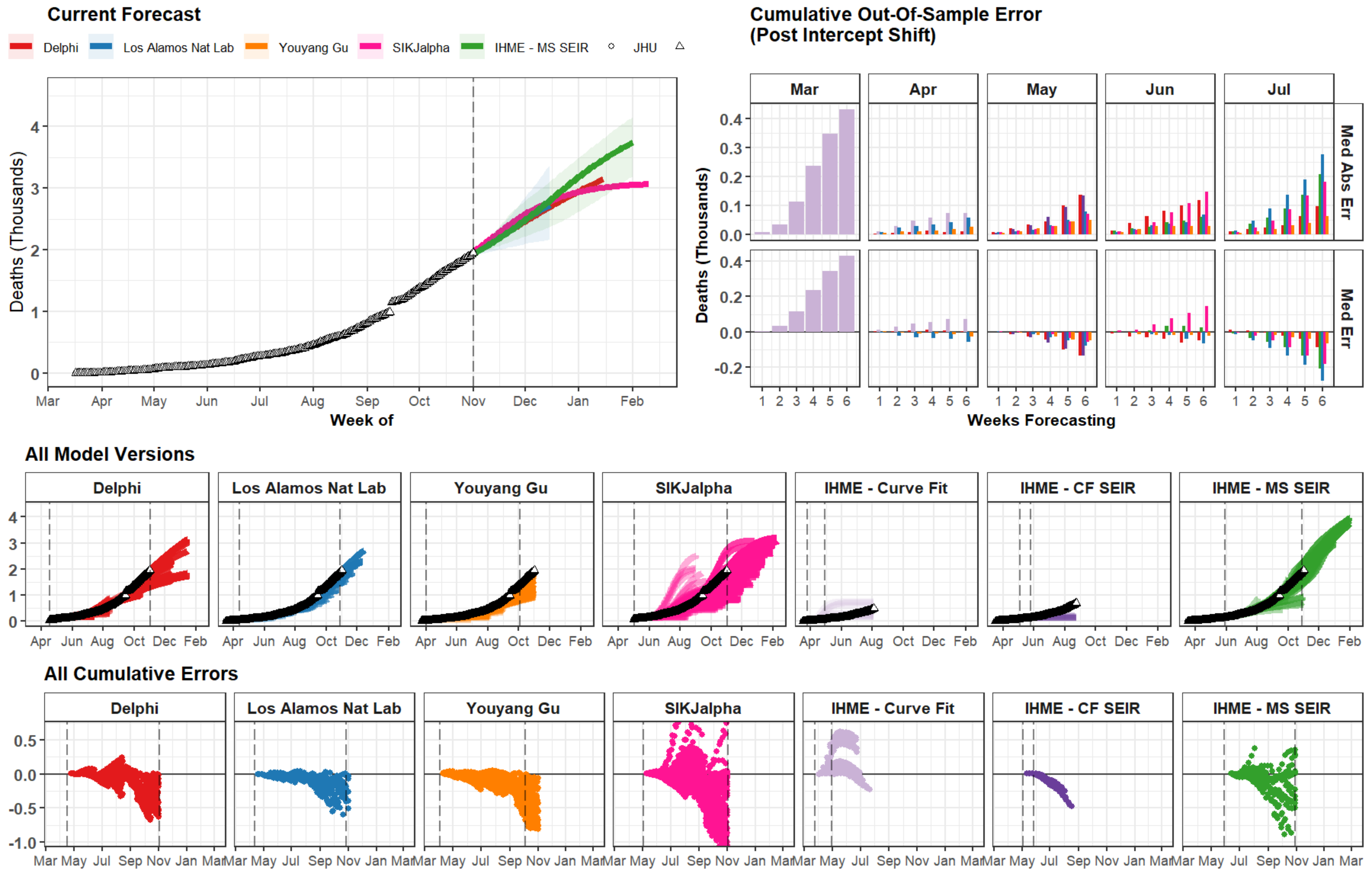
Current Forecast
Delphi
Los Alamos Nat Lab
Youyang Gu
SIKJalpha
IHME - MS SEIR
JHU
Deaths (Thousands)
Week of
Mar Apr May Jun Jul Aug Sep Oct Nov Dec Jan Feb
Cumulative Out-Of-Sample Error
(Post Intercept Shift)
Mar
Apr
May
Jun
Jul
Med Abs Err
Med Err
Weeks Forecasting
All Model Versions
IHME - Curve Fit
IHME - CF SEIR
All Cumulative Errors

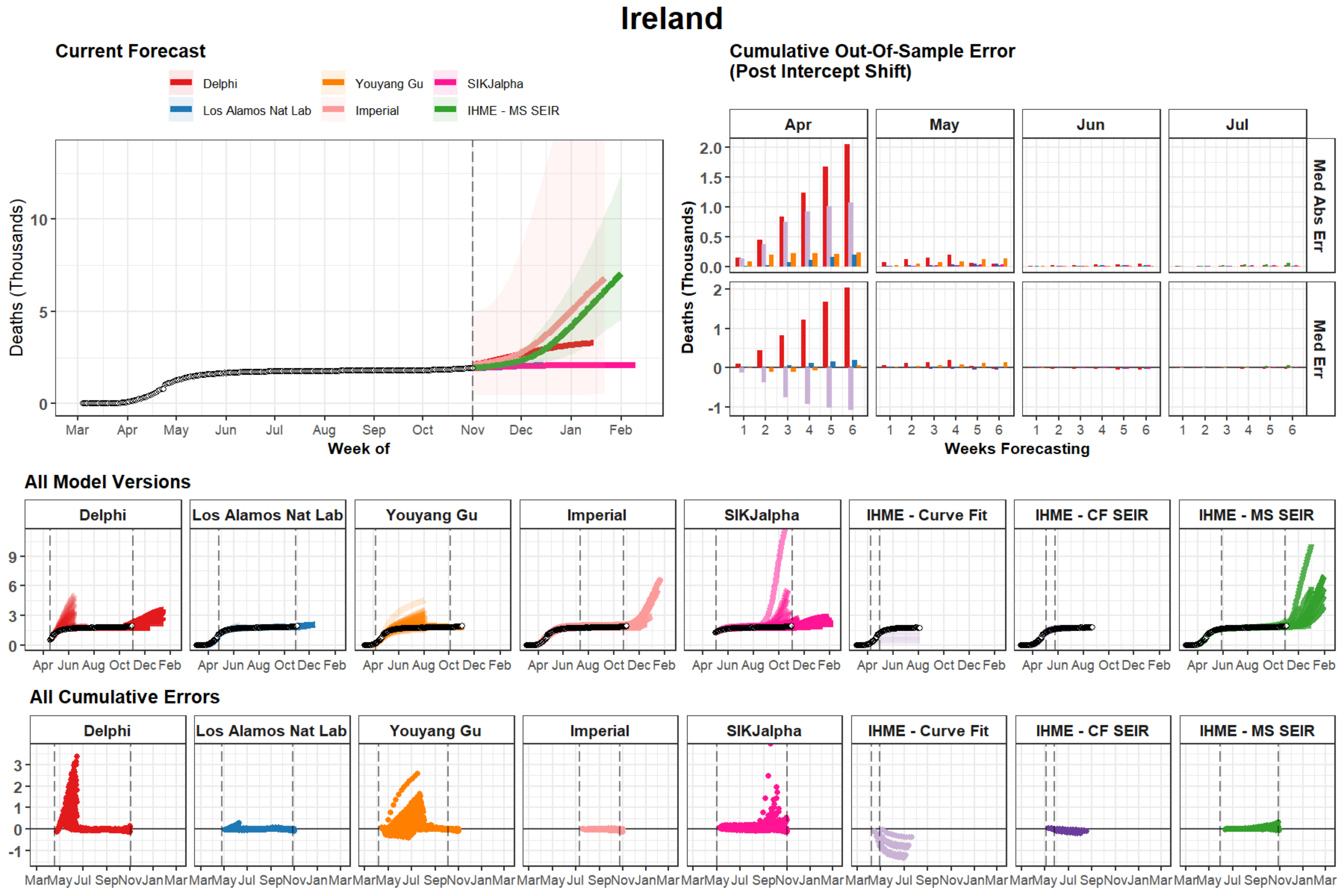

Ireland
Current Forecast
Delphi
Los Alamos Nat Lab
Youyang Gu
Imperial
SIKJalpha
IHME - MS SEIR
Deaths (Thousands)
Week of
Mar Apr May Jun Jul Aug Sep Oct Nov Dec Jan Feb
Cumulative Out-Of-Sample Error
(Post Intercept Shift)
Apr May Jun Jul
Med Abs Err
Med Err
Weeks Forecasting
All Model Versions
Delphi
Los Alamos Nat Lab
Youyang Gu
Imperial
SIKJalpha
IHME - Curve Fit
IHME - CF SEIR
IHME - MS SEIR
All Cumulative Errors

Kazakhstan

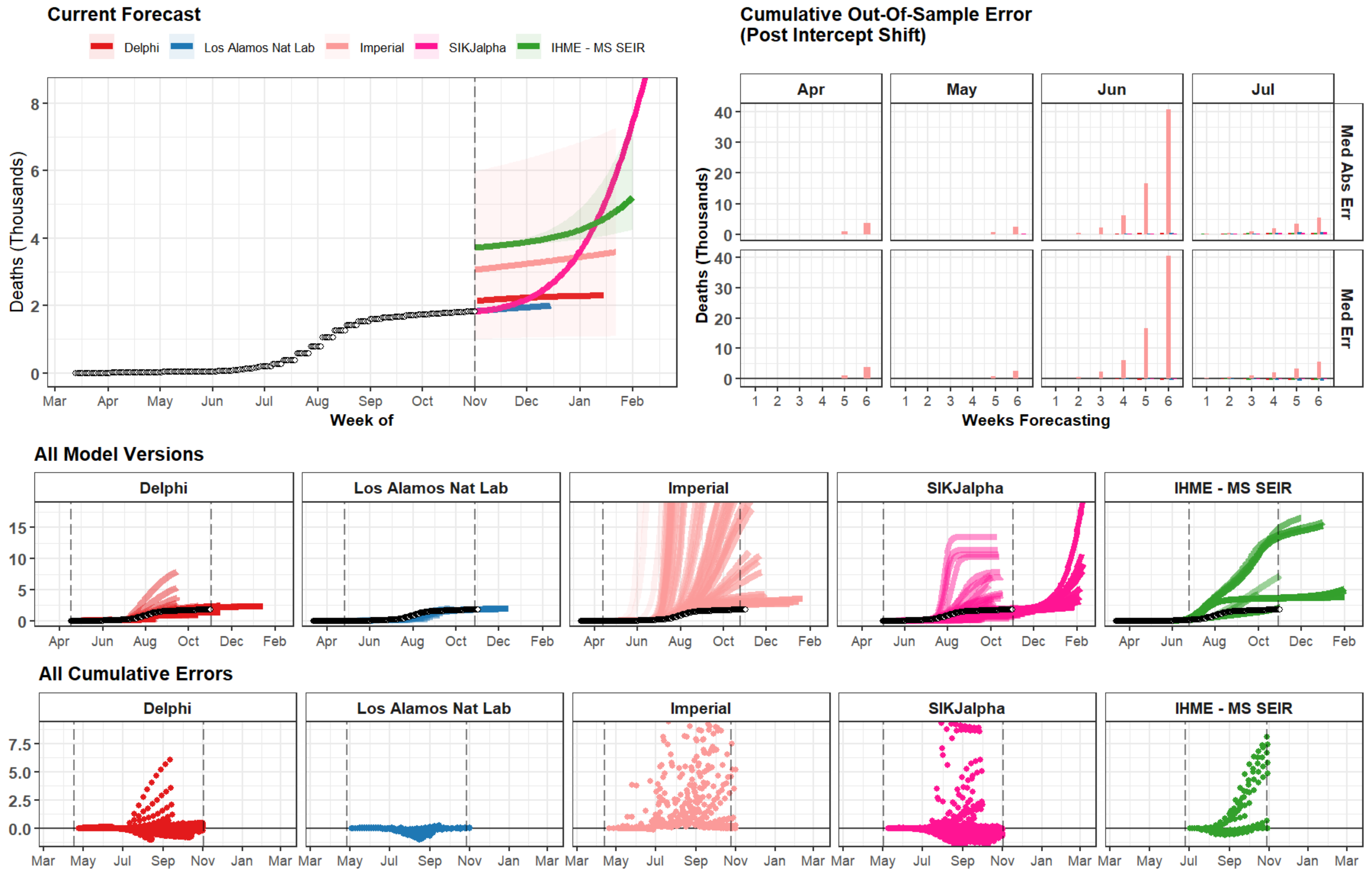
Current Forecast
Delphi
Los Alamos Nat Lab
Imperial
SIKJalpha
IHME - MS SEIR
Deaths (Thousands)
0
2
4
6
8
Mar
Apr
May
Jun
Jul
Aug
Sep
Oct
Nov
Dec
Jan
Feb
Week of
Cumulative Out-Of-Sample Error
(Post Intercept Shift)
Apr
May
Jun
Jul
Med Abs Err
Med Err
Deaths (Thousands)
0
10
20
30
40
1
2
3
4
5
6
Weeks Forecasting
All Model Versions
Delphi
Los Alamos Nat Lab
Imperial
SIKJalpha
IHME - MS SEIR
0
5
10
15
Apr
Jun
Aug
Oct
Dec
Feb
All Cumulative Errors
Delphi
Los Alamos Nat Lab
Imperial
SIKJalpha
IHME - MS SEIR
0.0
2.5
5.0
7.5
Mar
May
Jul
Sep
Nov
Jan
Mar

Hungary

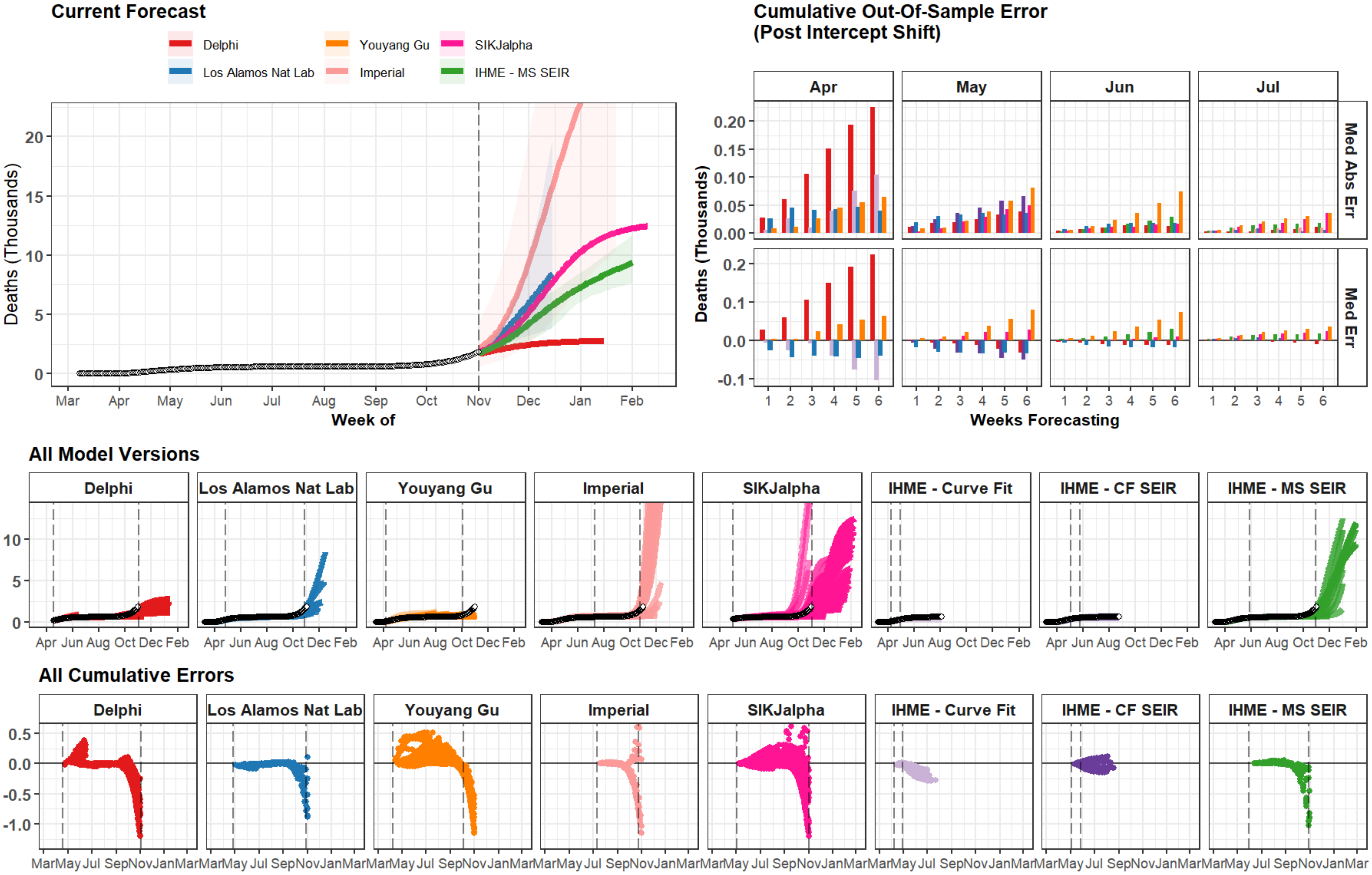
Current Forecast
Delphi
Los Alamos Nat Lab
Youyang Gu
Imperial
SIKJalpha
IHME - MS SEIR
Deaths (Thousands)
Week of
Cumulative Out-Of-Sample Error
(Post Intercept Shift)
Apr
May
Jun
Jul
Med Abs Err
Med Err
Weeks Forecasting
All Model Versions
Delphi
Los Alamos Nat Lab
Youyang Gu
Imperial
SIKJalpha
IHME - Curve Fit
IHME - CF SEIR
IHME - MS SEIR
All Cumulative Errors

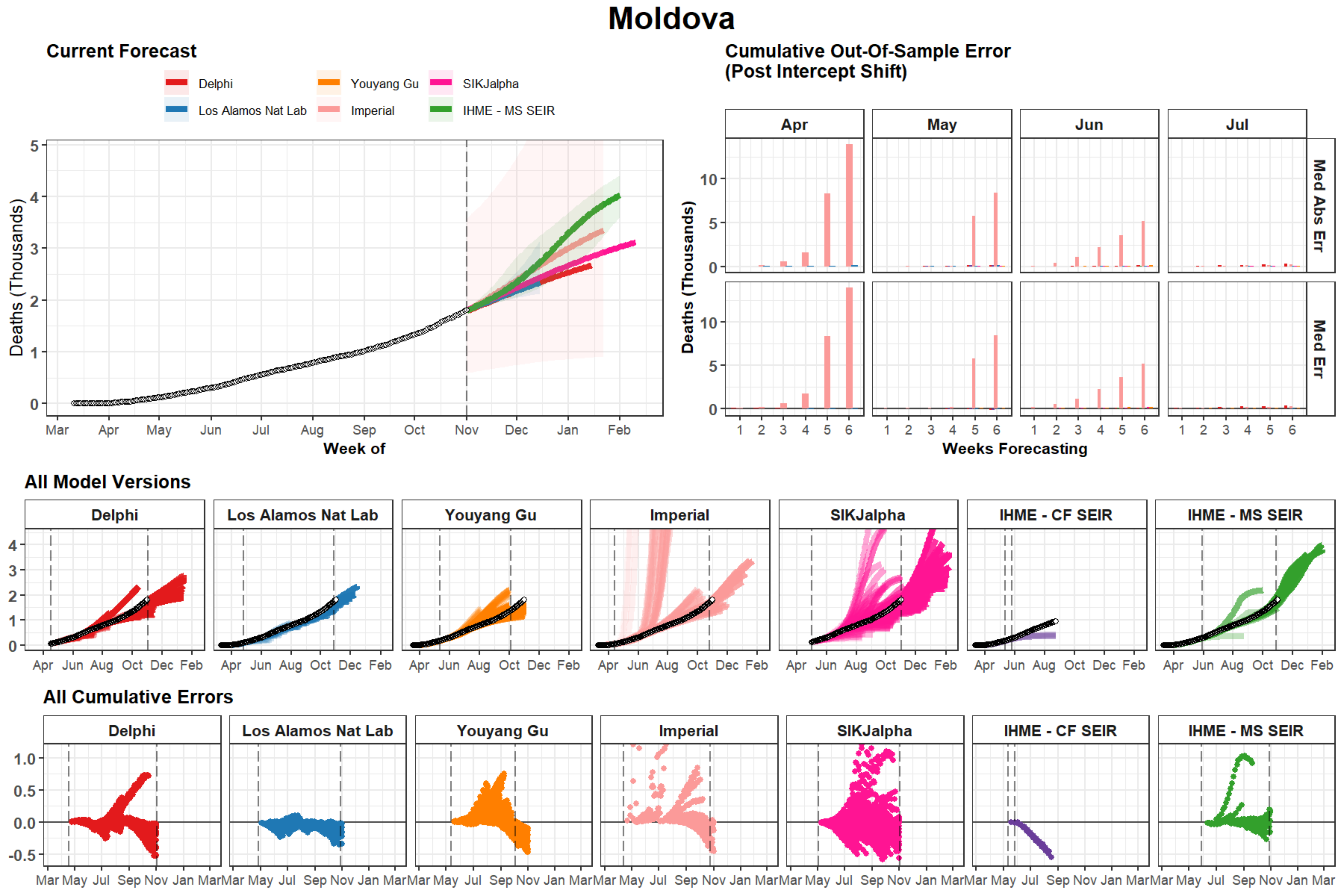
Moldova
Current Forecast
Delphi
Youyang Gu
SIKJalpha
Los Alamos Nat Lab
Imperial
IHME - MS SEIR
Deaths (Thousands)
Week of
Mar Apr May Jun Jul Aug Sep Oct Nov Dec Jan Feb
Cumulative Out-Of-Sample Error
(Post Intercept Shift)
Apr May Jun Jul
Med Abs Err
Med Err
Deaths (Thousands)
Weeks Forecasting
All Model Versions
Delphi
Los Alamos Nat Lab
Youyang Gu
Imperial
SIKJalpha
IHME - CF SEIR
IHME - MS SEIR
Apr Jun Aug Oct Dec Feb
All Cumulative Errors
Delphi
Los Alamos Nat Lab
Youyang Gu
Imperial
SIKJalpha
IHME - CF SEIR
IHME - MS SEIR
Mar May Jul Sep Nov Jan Mar

Nevada

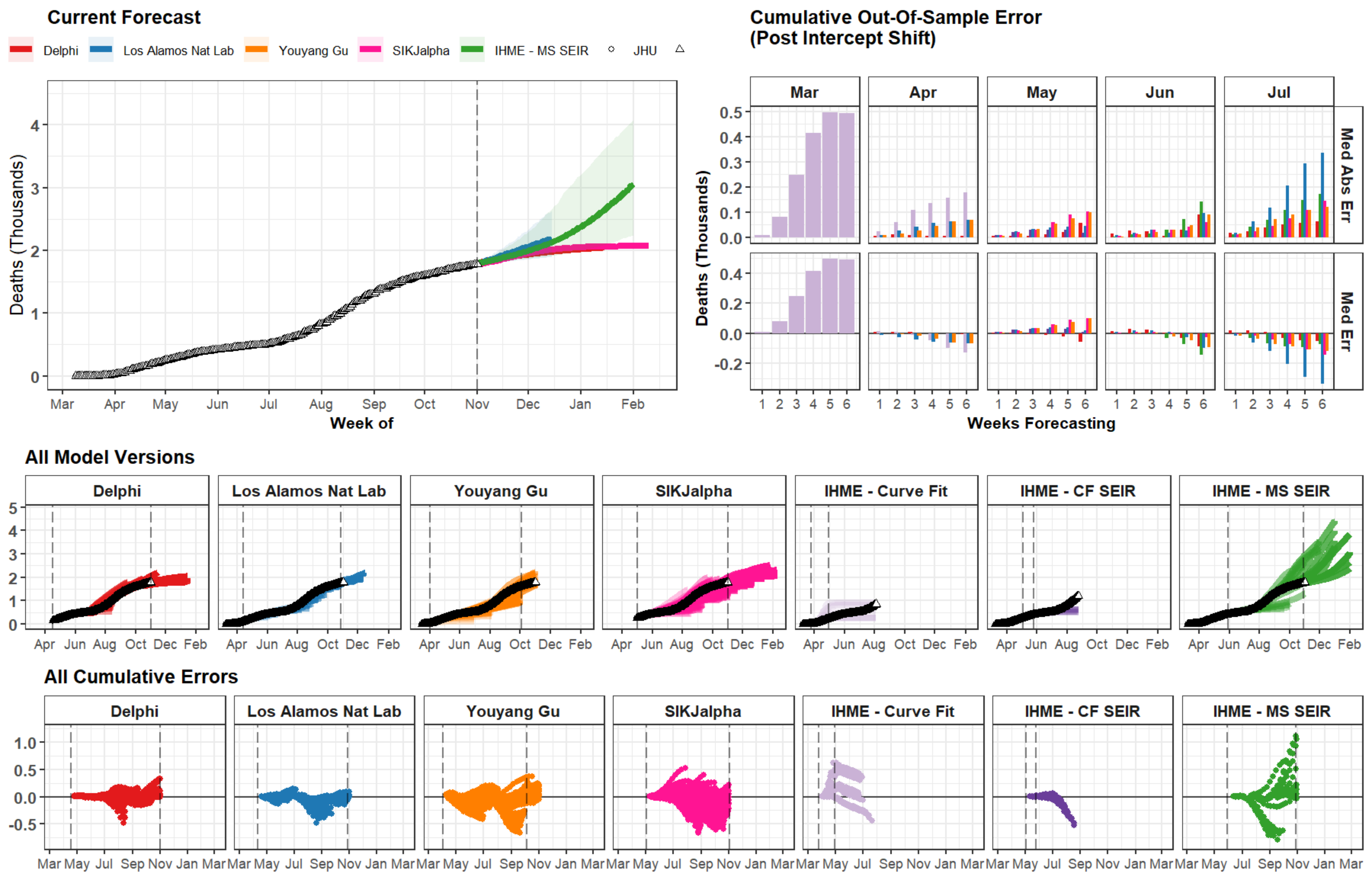

Current Forecast
Delphi
Los Alamos Nat Lab
Youyang Gu
SIKJalpha
IHME - MS SEIR
JHU
Deaths (Thousands)
Week of
Mar Apr May Jun Jul Aug Sep Oct Nov Dec Jan Feb
Cumulative Out-Of-Sample Error
(Post Intercept Shift)
Mar Apr May Jun Jul
Med Abs Err
Med Err
Weeks Forecasting
All Model Versions
Delphi
Los Alamos Nat Lab
Youyang Gu
SIKJalpha
IHME - Curve Fit
IHME - CF SEIR
IHME - MS SEIR
Apr Jun Aug Oct Dec Feb
All Cumulative Errors
Mar May Jul Sep Nov Jan Mar

Japan

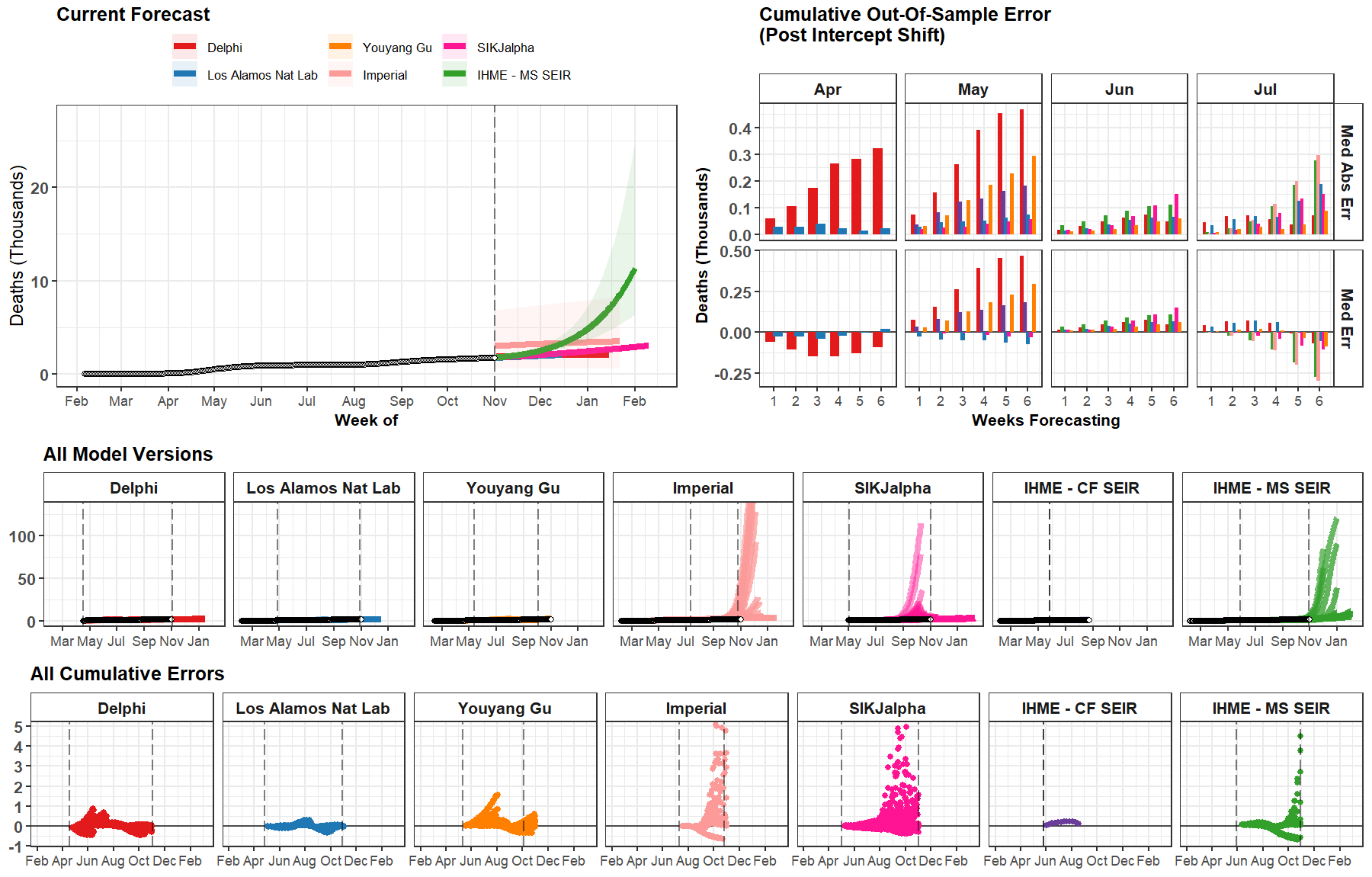
Current Forecast
Delphi
Los Alamos Nat Lab
Youyang Gu
Imperial
SIKJalpha
IHME - MS SEIR
Deaths (Thousands)
Week of
Feb
Mar
Apr
May
Jun
Jul
Aug
Sep
Oct
Nov
Dec
Jan
Cumulative Out-Of-Sample Error
(Post Intercept Shift)
Apr
May
Jun
Jul
Med Abs Err
Med Err
Weeks Forecasting
All Model Versions
IHME - CF SEIR
All Cumulative Errors

Iowa

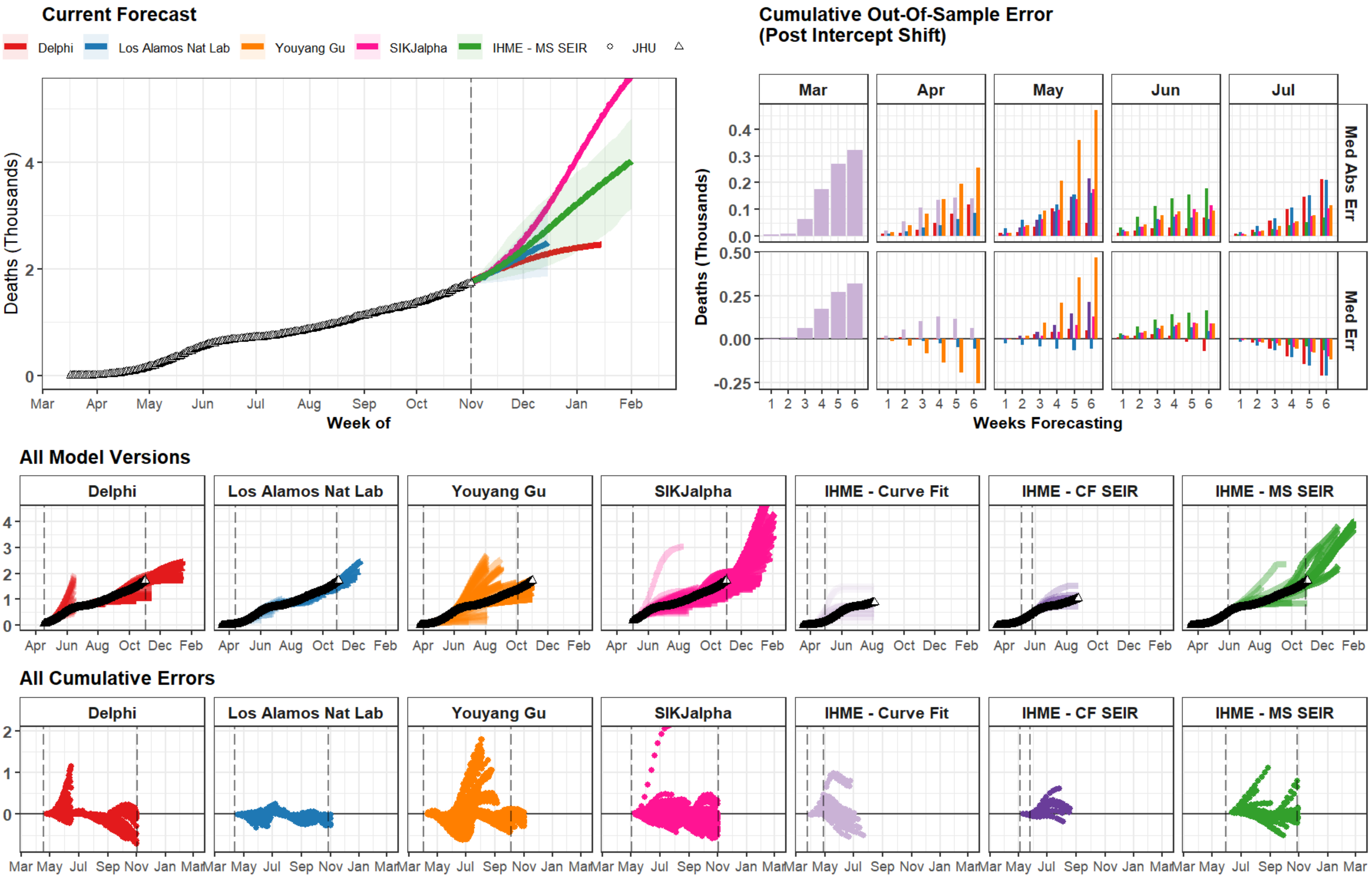

Current Forecast
Delphi
Los Alamos Nat Lab
Youyang Gu
SIKJalpha
IHME - MS SEIR
JHU
Deaths (Thousands)
Week of
Mar
Apr
May
Jun
Jul
Aug
Sep
Oct
Nov
Dec
Jan
Feb
Cumulative Out-Of-Sample Error
(Post Intercept Shift)
Med Abs Err
Med Err
Weeks Forecasting
All Model Versions
IHME - Curve Fit
IHME - CF SEIR
All Cumulative Errors

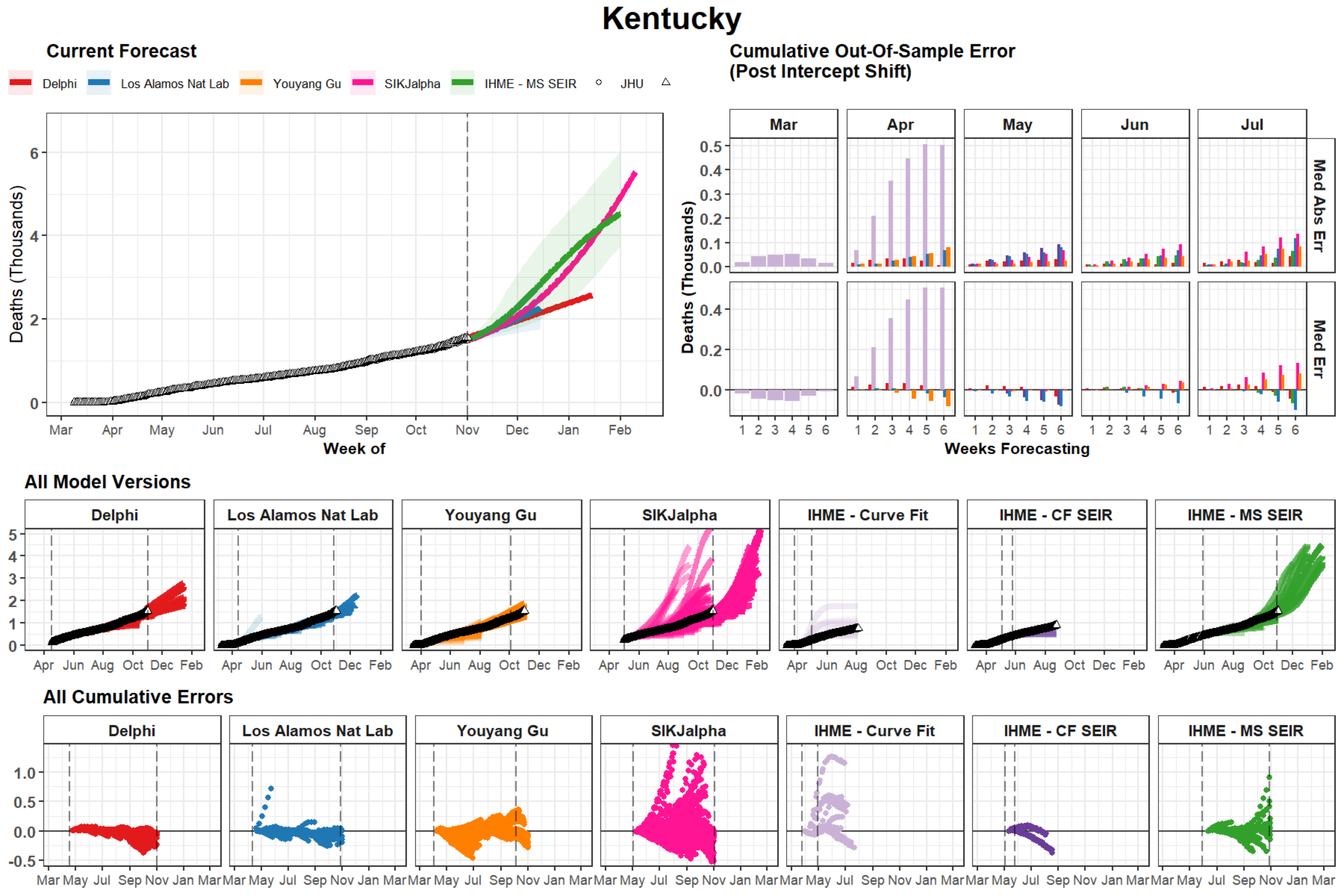
Kentucky
Current Forecast
Delphi
Los Alamos Nat Lab
Youyang Gu
SIKJalpha
IHME - MS SEIR
JHU
Deaths (Thousands)
Week of
Mar
Apr
May
Jun
Jul
Aug
Sep
Oct
Nov
Dec
Jan
Feb
Cumulative Out-Of-Sample Error
(Post Intercept Shift)
Med Abs Err
Med Err
Weeks Forecasting
All Model Versions
IHME - Curve Fit
IHME - CF SEIR
All Cumulative Errors

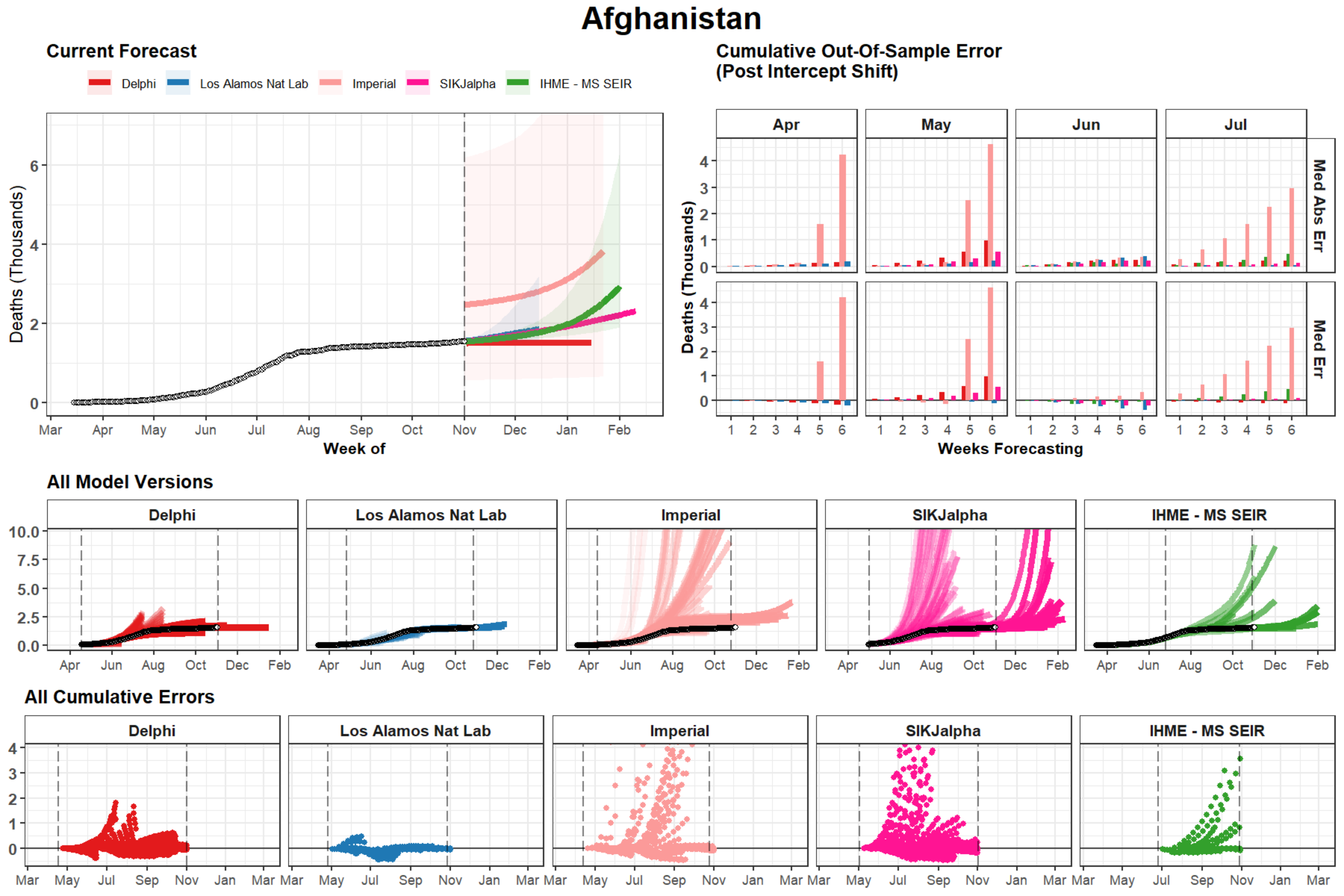

Afghanistan
Current Forecast
Delphi
Los Alamos Nat Lab
Imperial
SIKJalpha
IHME - MS SEIR
Deaths (Thousands)
Week of
Cumulative Out-Of-Sample Error
(Post Intercept Shift)
Apr
May
Jun
Jul
Med Abs Err
Med Err
Weeks Forecasting
All Model Versions
All Cumulative Errors

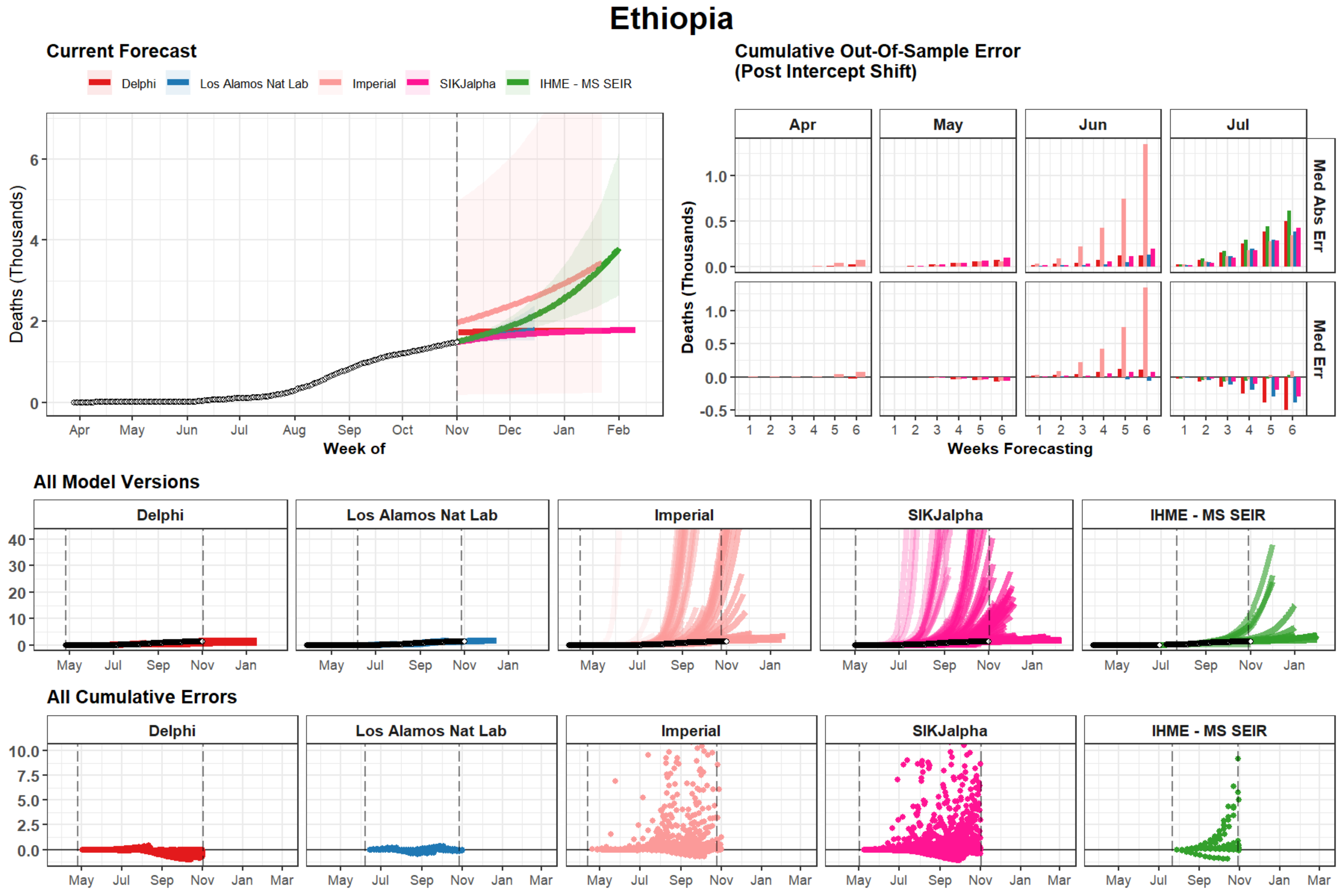

Ethiopia
Current Forecast
Delphi
Los Alamos Nat Lab
Imperial
SIKJalpha
IHME - MS SEIR
Deaths (Thousands)
Week of
Apr
May
Jun
Jul
Aug
Sep
Oct
Nov
Dec
Jan
Feb
Cumulative Out-Of-Sample Error
(Post Intercept Shift)
Med Abs Err
Med Err
Weeks Forecasting
All Model Versions
All Cumulative Errors
Mar

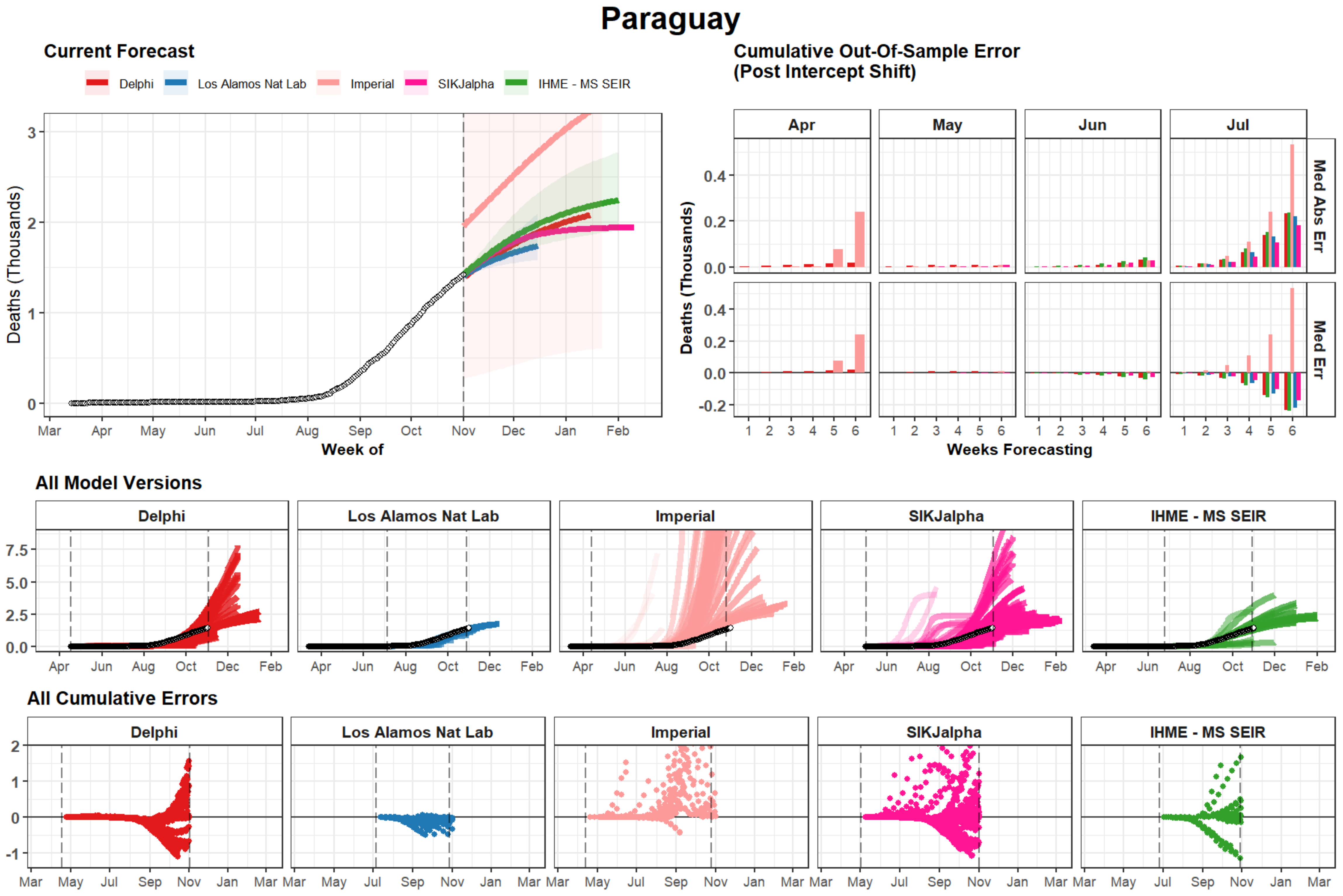

Paraguay
Current Forecast
Delphi
Los Alamos Nat Lab
Imperial
SIKJalpha
IHME - MS SEIR
Deaths (Thousands)
Week of
Mar Apr May Jun Jul Aug Sep Oct Nov Dec Jan Feb
Cumulative Out-Of-Sample Error
(Post Intercept Shift)
Apr May Jun Jul
Med Abs Err
Med Err
Weeks Forecasting
All Model Versions
All Cumulative Errors

Costa Rica

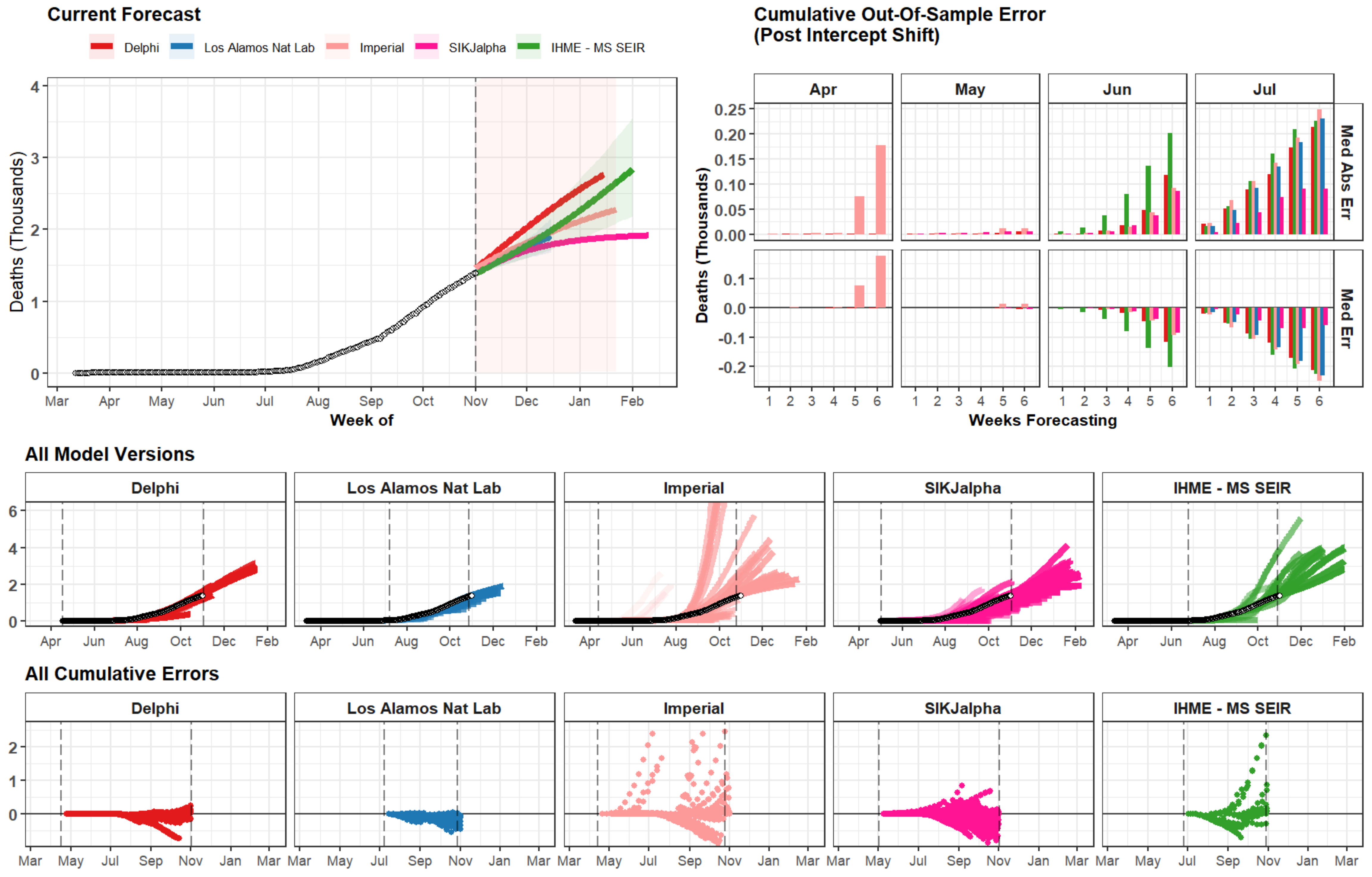

Current Forecast
Delphi
Los Alamos Nat Lab
Imperial
SIKJalpha
IHME - MS SEIR
Deaths (Thousands)
Week of
Cumulative Out-Of-Sample Error
(Post Intercept Shift)
Apr
May
Jun
Jul
Med Abs Err
Med Err
Weeks Forecasting
All Model Versions
All Cumulative Errors

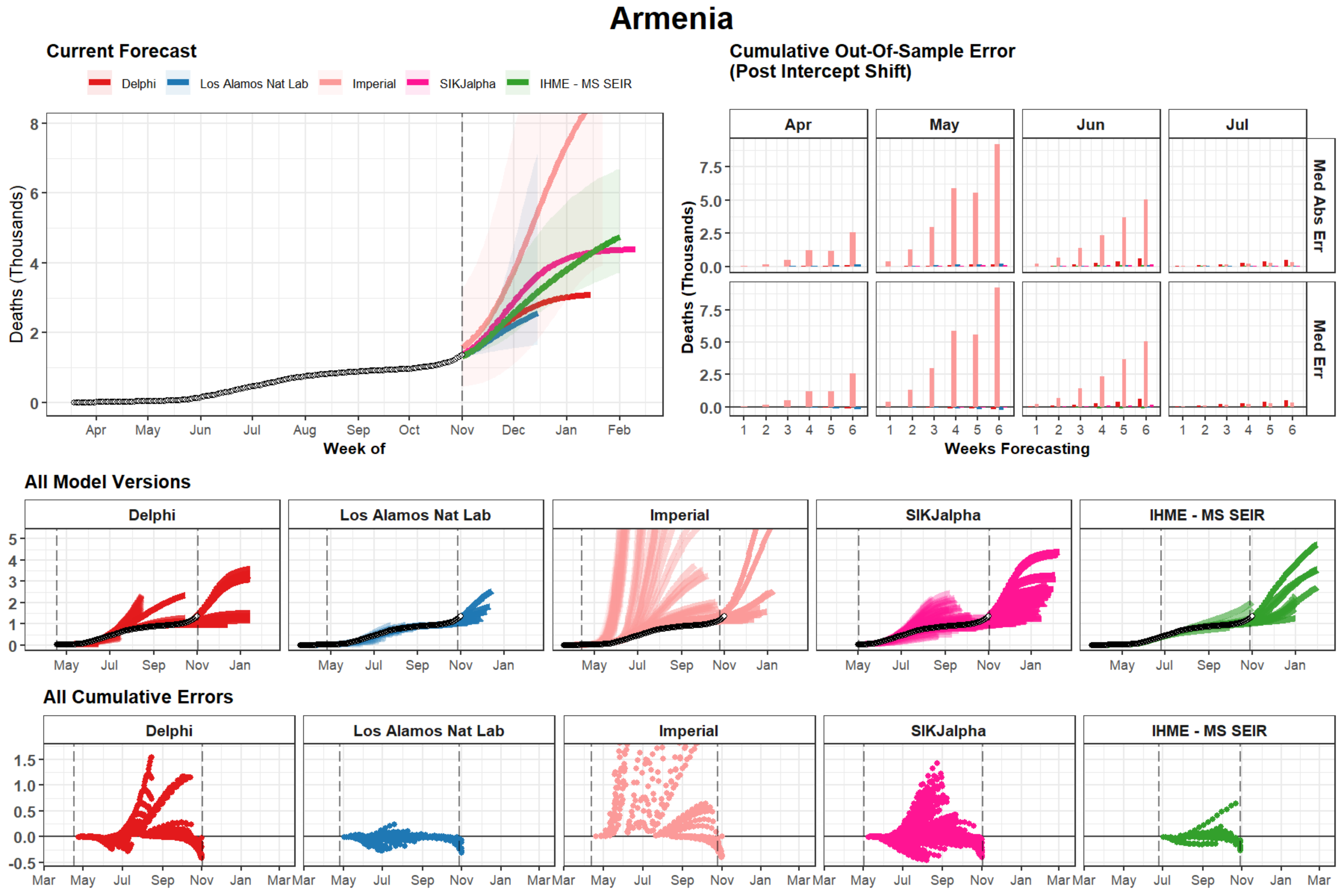
Armenia
Current Forecast
Delphi
Los Alamos Nat Lab
Imperial
SIKJalpha
IHME - MS SEIR
Deaths (Thousands)
Week of
Cumulative Out-Of-Sample Error
(Post Intercept Shift)
Apr
May
Jun
Jul
Med Abs Err
Med Err
Weeks Forecasting
All Model Versions
All Cumulative Errors

Tunisia

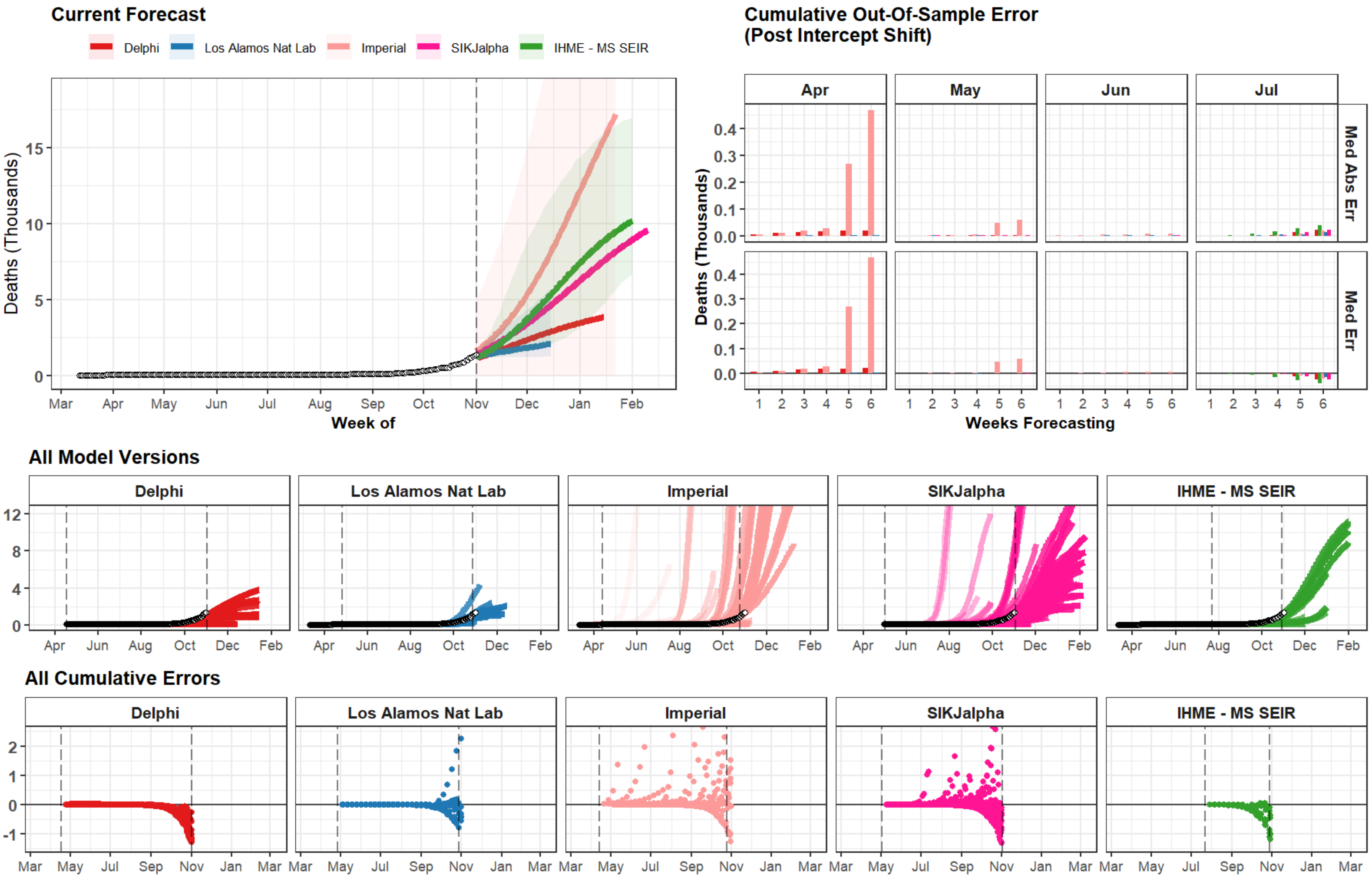
Current Forecast
Delphi
Los Alamos Nat Lab
Imperial
SIKJalpha
IHME - MS SEIR
Deaths (Thousands)
Week of
Cumulative Out-Of-Sample Error
(Post Intercept Shift)
Apr
May
Jun
Jul
Med Abs Err
Med Err
Weeks Forecasting
All Model Versions
All Cumulative Errors

Oklahoma

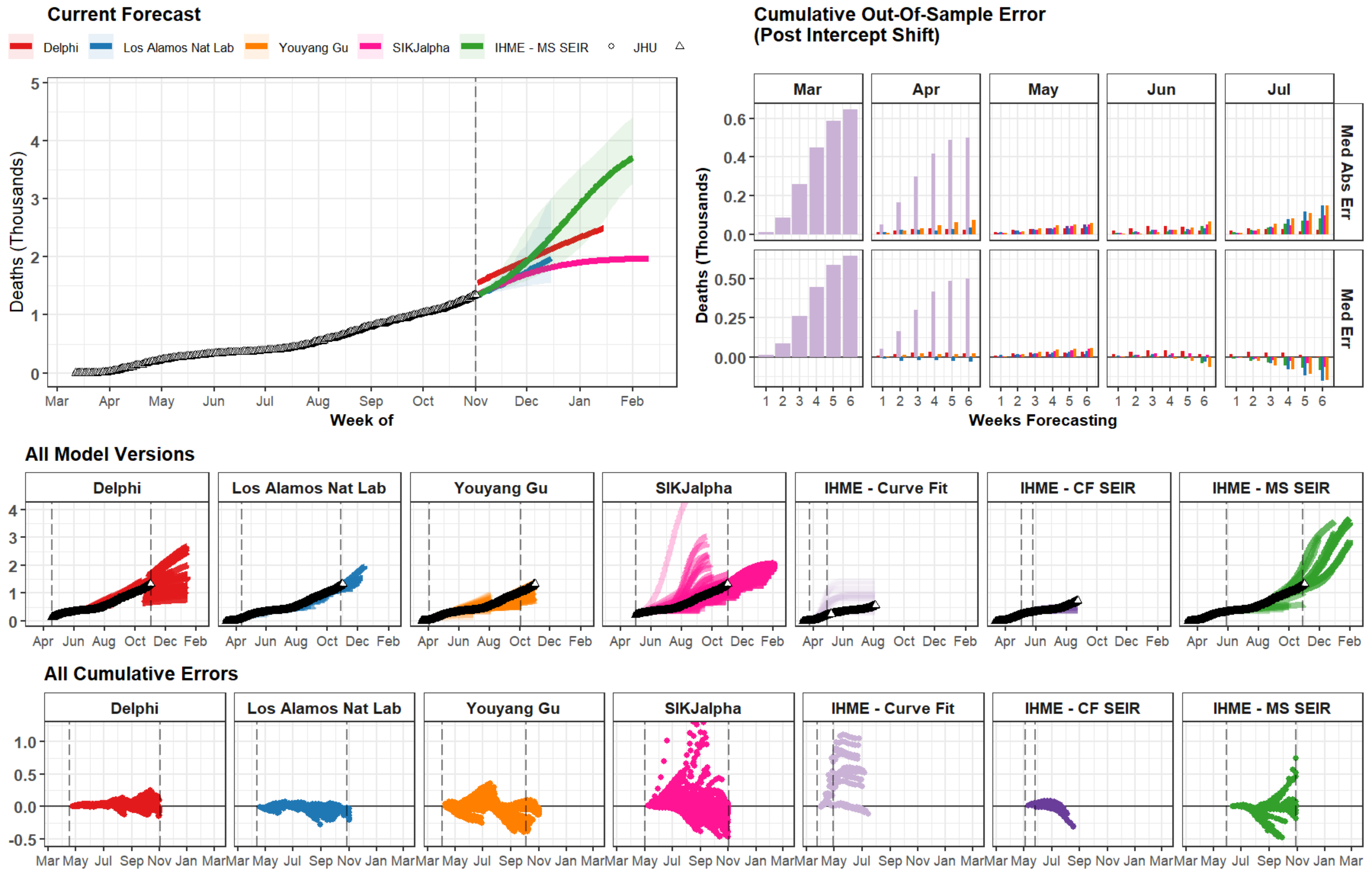

Current Forecast
Delphi
Los Alamos Nat Lab
Youyang Gu
SIKJalpha
IHME - MS SEIR
JHU
Deaths (Thousands)
Week of
Mar Apr May Jun Jul Aug Sep Oct Nov Dec Jan Feb
Cumulative Out-Of-Sample Error
(Post Intercept Shift)
Mar
Apr
May
Jun
Jul
Med Abs Err
Med Err
Deaths (Thousands)
Weeks Forecasting
All Model Versions
Delphi
Los Alamos Nat Lab
Youyang Gu
SIKJalpha
IHME - Curve Fit
IHME - CF SEIR
IHME - MS SEIR
All Cumulative Errors

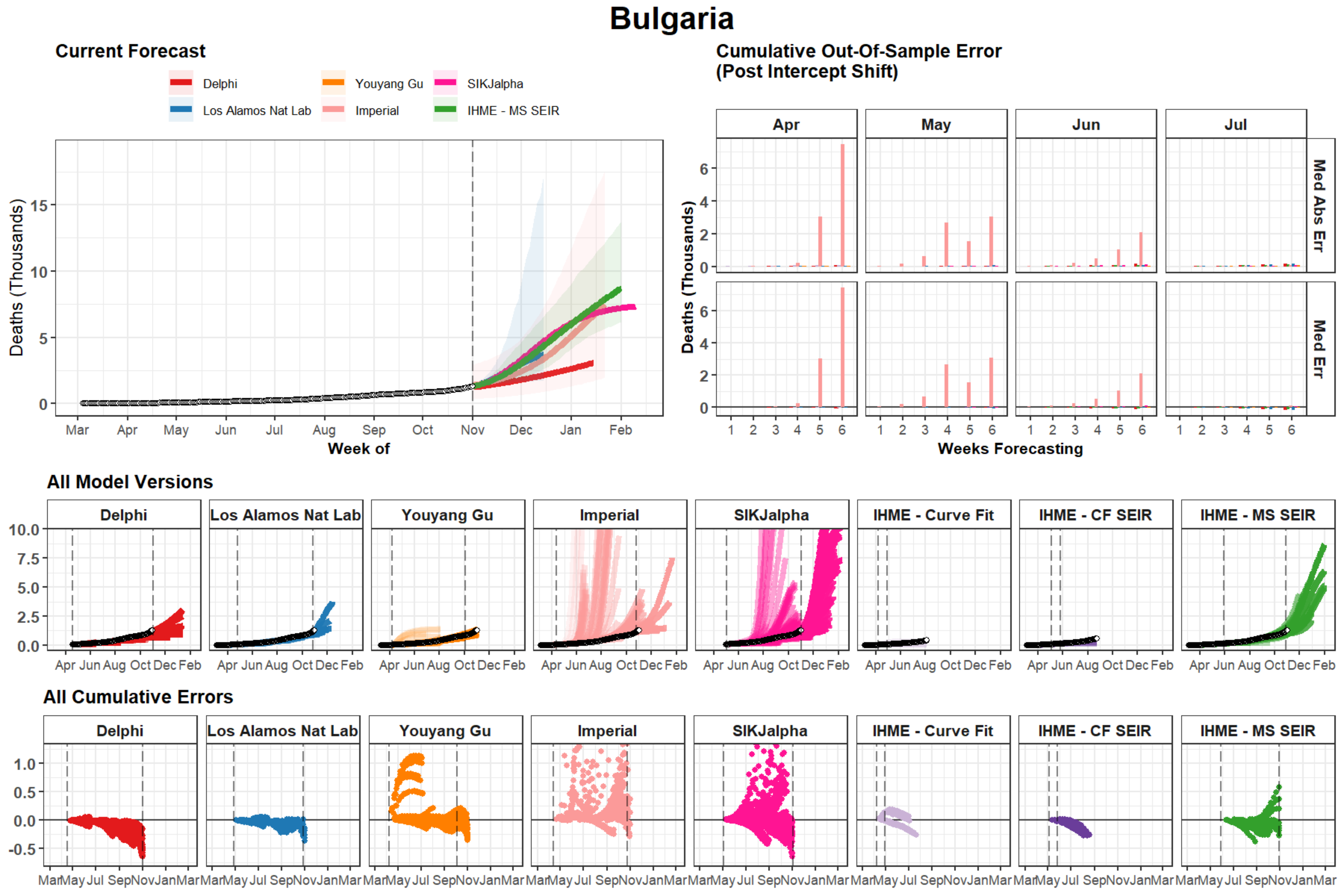
Bulgaria
Current Forecast
Delphi
Los Alamos Nat Lab
Youyang Gu
Imperial
SIKJalpha
IHME - MS SEIR
Deaths (Thousands)
Week of
Mar Apr May Jun Jul Aug Sep Oct Nov Dec Jan Feb
Cumulative Out-Of-Sample Error
(Post Intercept Shift)
Apr May Jun Jul
Med Abs Err
Med Err
Weeks Forecasting
All Model Versions
Delphi Los Alamos Nat Lab Youyang Gu Imperial SIKJalpha IHME - Curve Fit IHME - CF SEIR IHME - MS SEIR
All Cumulative Errors

Myanmar

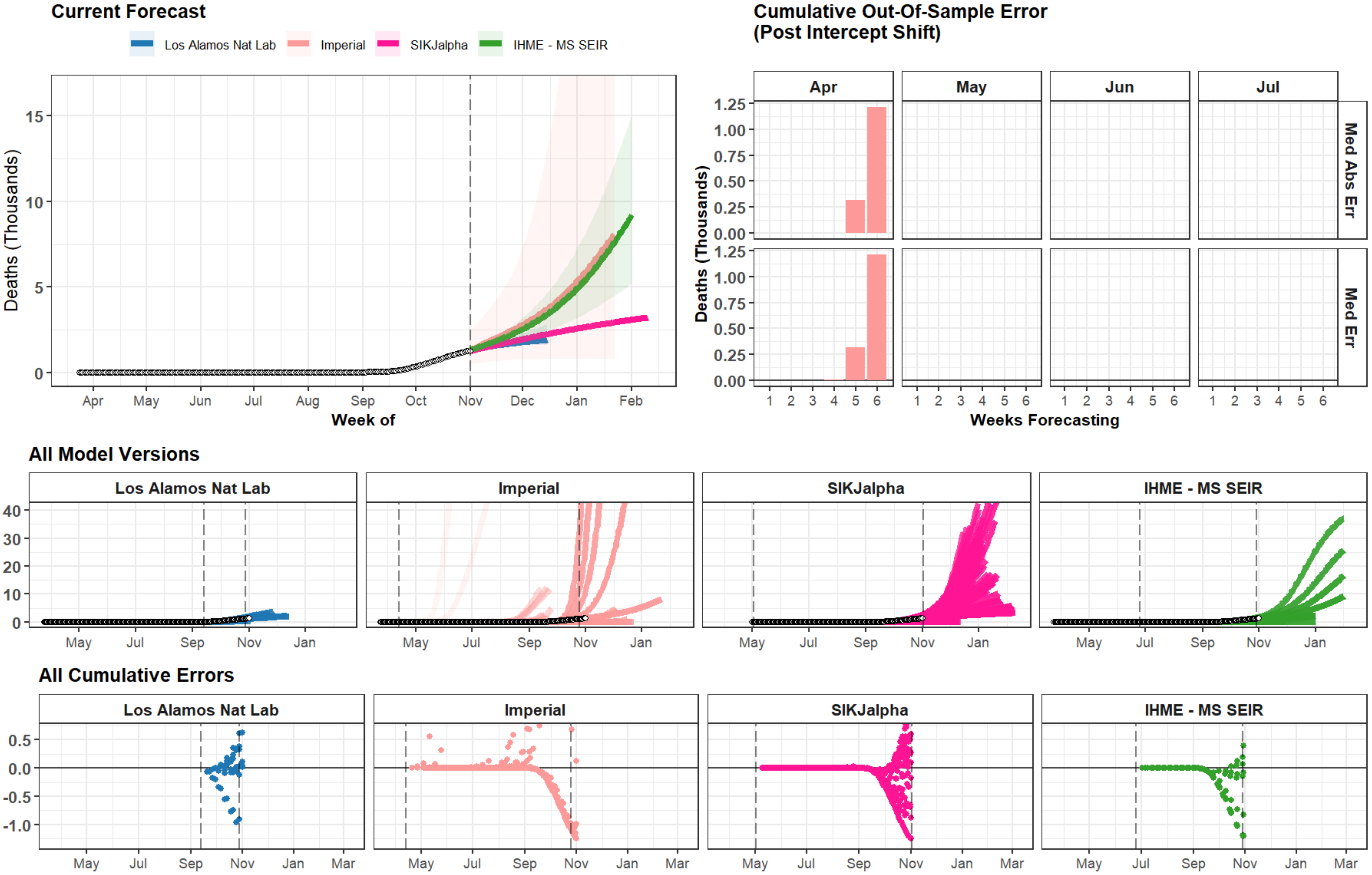
Current Forecast
Los Alamos Nat Lab
Imperial
SIKJalpha
IHME - MS SEIR
Deaths (Thousands)
Week of
Apr
May
Jun
Jul
Aug
Sep
Oct
Nov
Dec
Jan
Feb
Cumulative Out-Of-Sample Error
(Post Intercept Shift)
Apr
May
Jun
Jul
Med Abs Err
Med Err
Weeks Forecasting
All Model Versions
All Cumulative Errors
Mar

Bosnia and Herzegovina

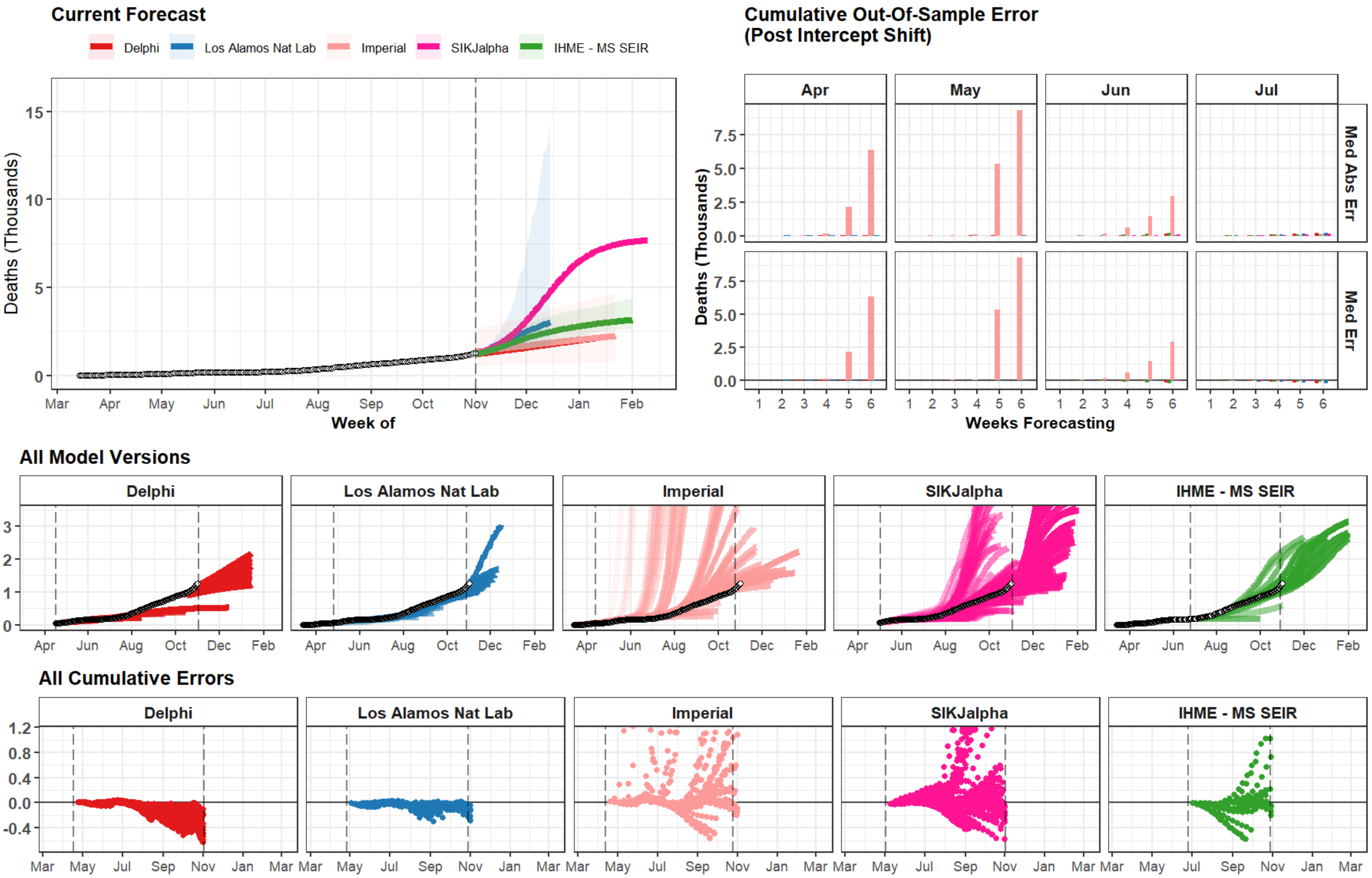

Oman

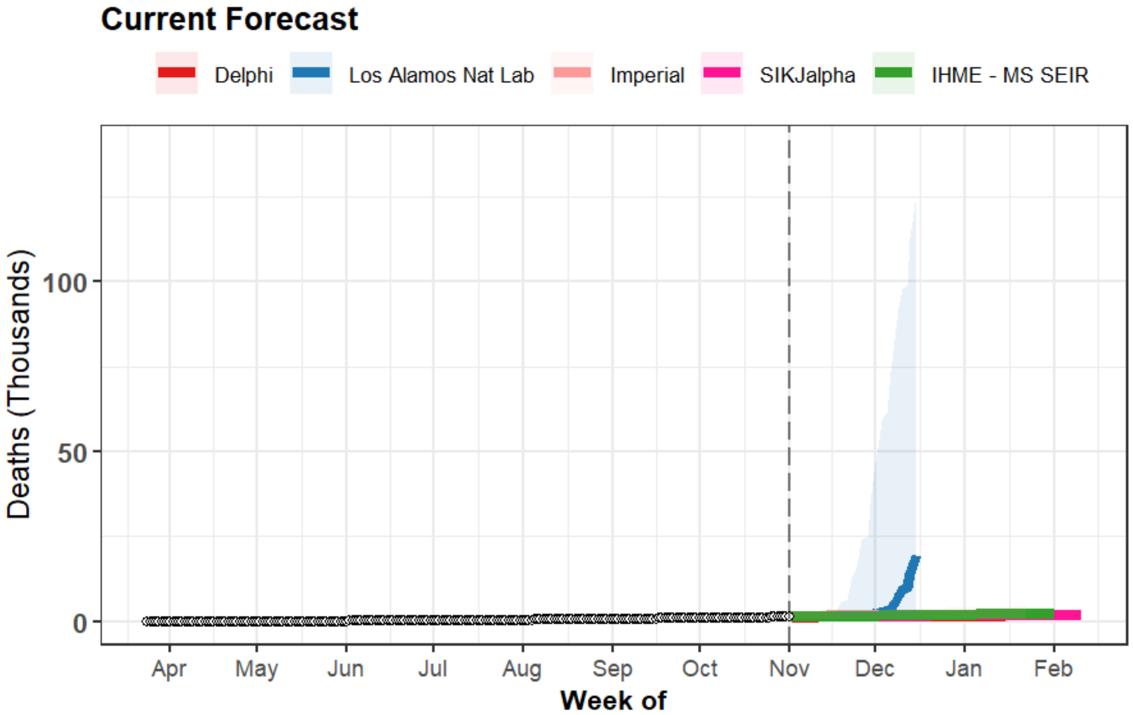

Current Forecast
Delphi
Los Alamos Nat Lab
Imperial
SIKJalpha
IHME - MS SEIR
Deaths (Thousands)
100
50
0
Apr
May
Jun
Jul
Aug
Sep
Oct
Nov
Dec
Jan
Feb
Week of

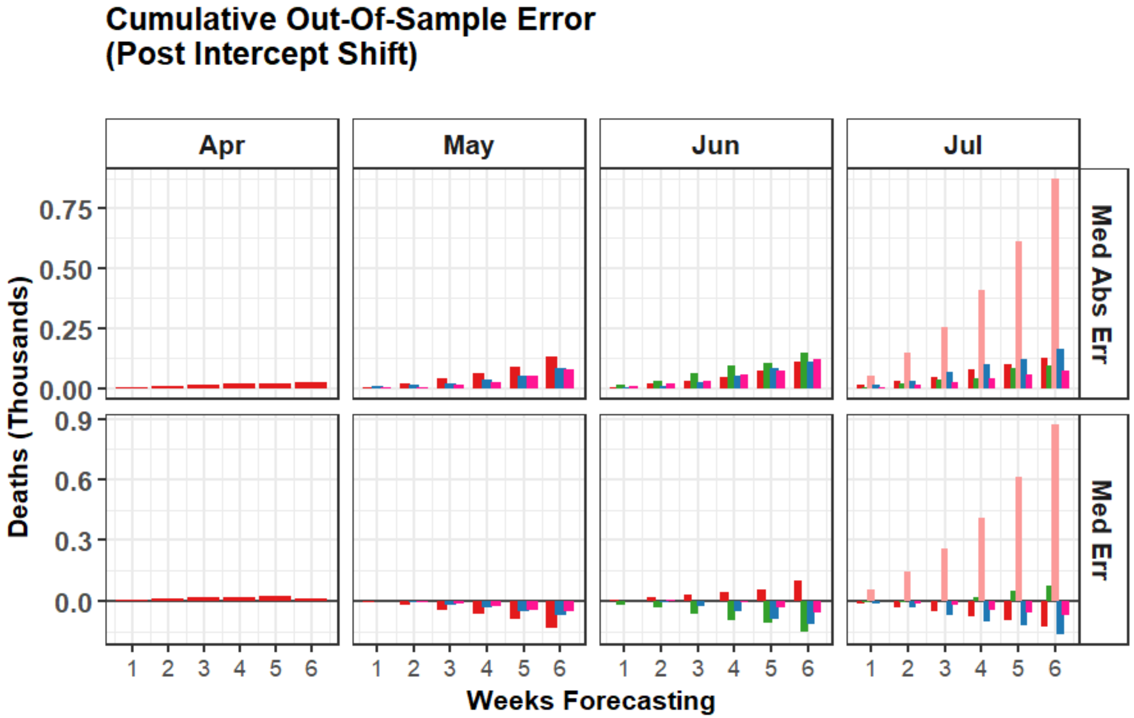

Cumulative Out-Of-Sample Error
(Post Intercept Shift)
Apr
May
Jun
Jul
Med Abs Err
Med Err
Deaths (Thousands)
0.75
0.50
0.25
0.00
0.9
0.6
0.3
0.0
1
2
3
4
5
6
Weeks Forecasting

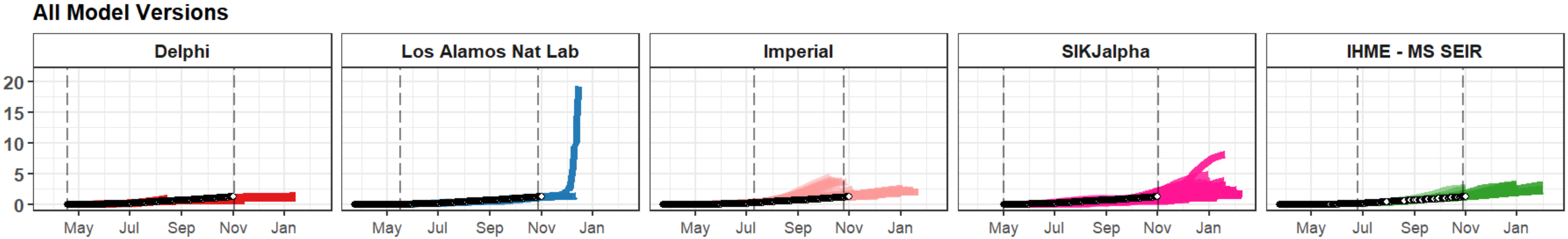

All Model Versions
Delphi
Los Alamos Nat Lab
Imperial
SIKJalpha
IHME - MS SEIR
20
15
10
5
0
May
Jul
Sep
Nov
Jan

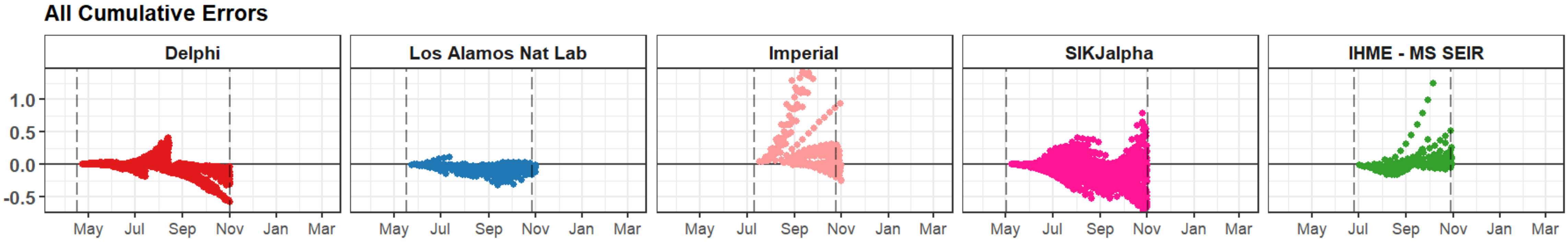

All Cumulative Errors
Delphi
Los Alamos Nat Lab
Imperial
SIKJalpha
IHME - MS SEIR
1.0
0.5
0.0
-0.5
May
Jul
Sep
Nov
Jan
Mar

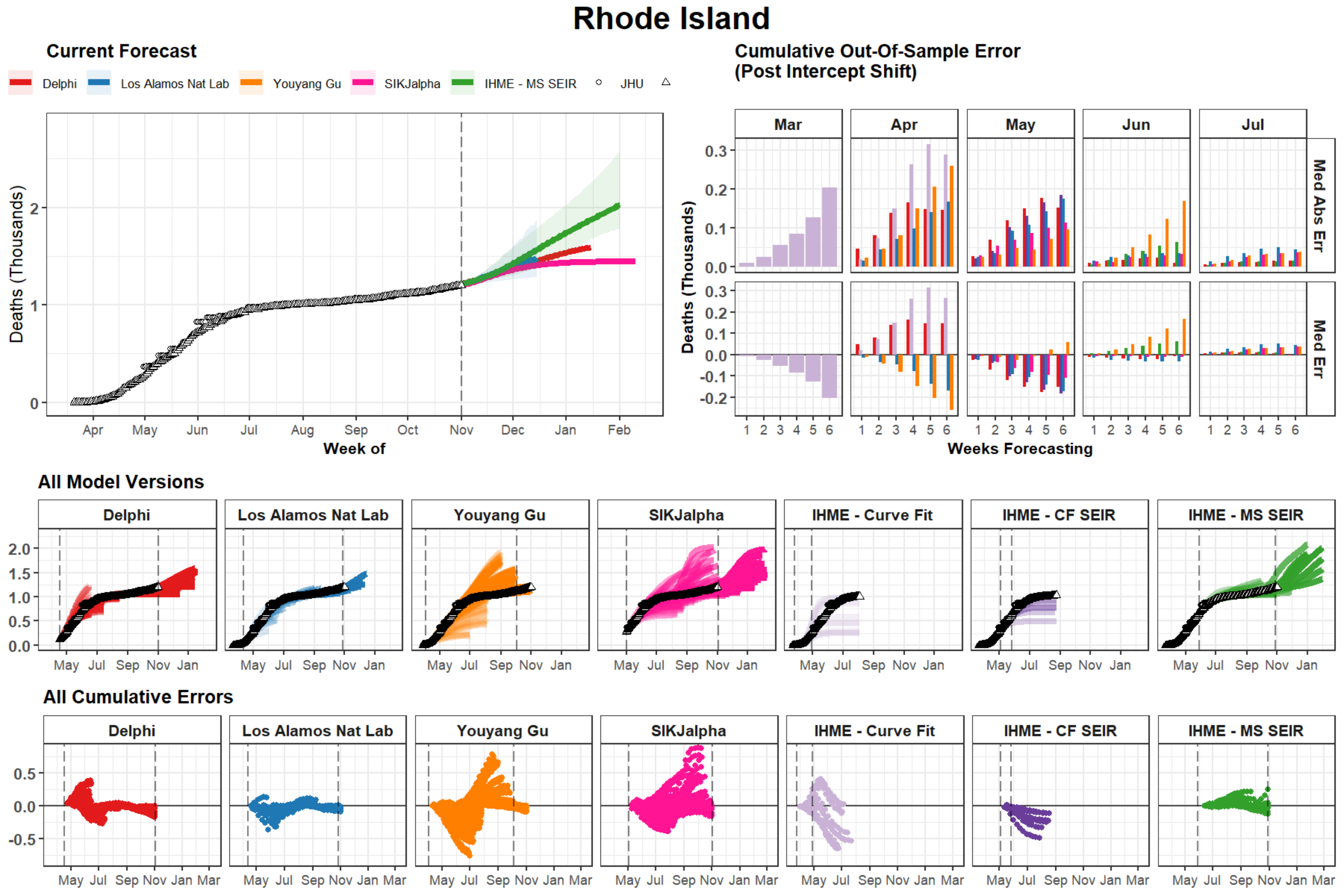
Rhode Island
Current Forecast
Delphi
Los Alamos Nat Lab
Youyang Gu
SIKJalpha
IHME - MS SEIR
JHU
Deaths (Thousands)
Week of
Apr May Jun Jul Aug Sep Oct Nov Dec Jan Feb
Cumulative Out-Of-Sample Error
(Post Intercept Shift)
Mar
Apr
May
Jun
Jul
Med Abs Err
Med Err
Deaths (Thousands)
Weeks Forecasting
All Model Versions
Delphi
Los Alamos Nat Lab
Youyang Gu
SIKJalpha
IHME - Curve Fit
IHME - CF SEIR
IHME - MS SEIR
May Jul Sep Nov Jan
All Cumulative Errors
Delphi
Los Alamos Nat Lab
Youyang Gu
SIKJalpha
IHME - Curve Fit
IHME - CF SEIR
IHME - MS SEIR
May Jul Sep Nov Jan Mar

Nigeria

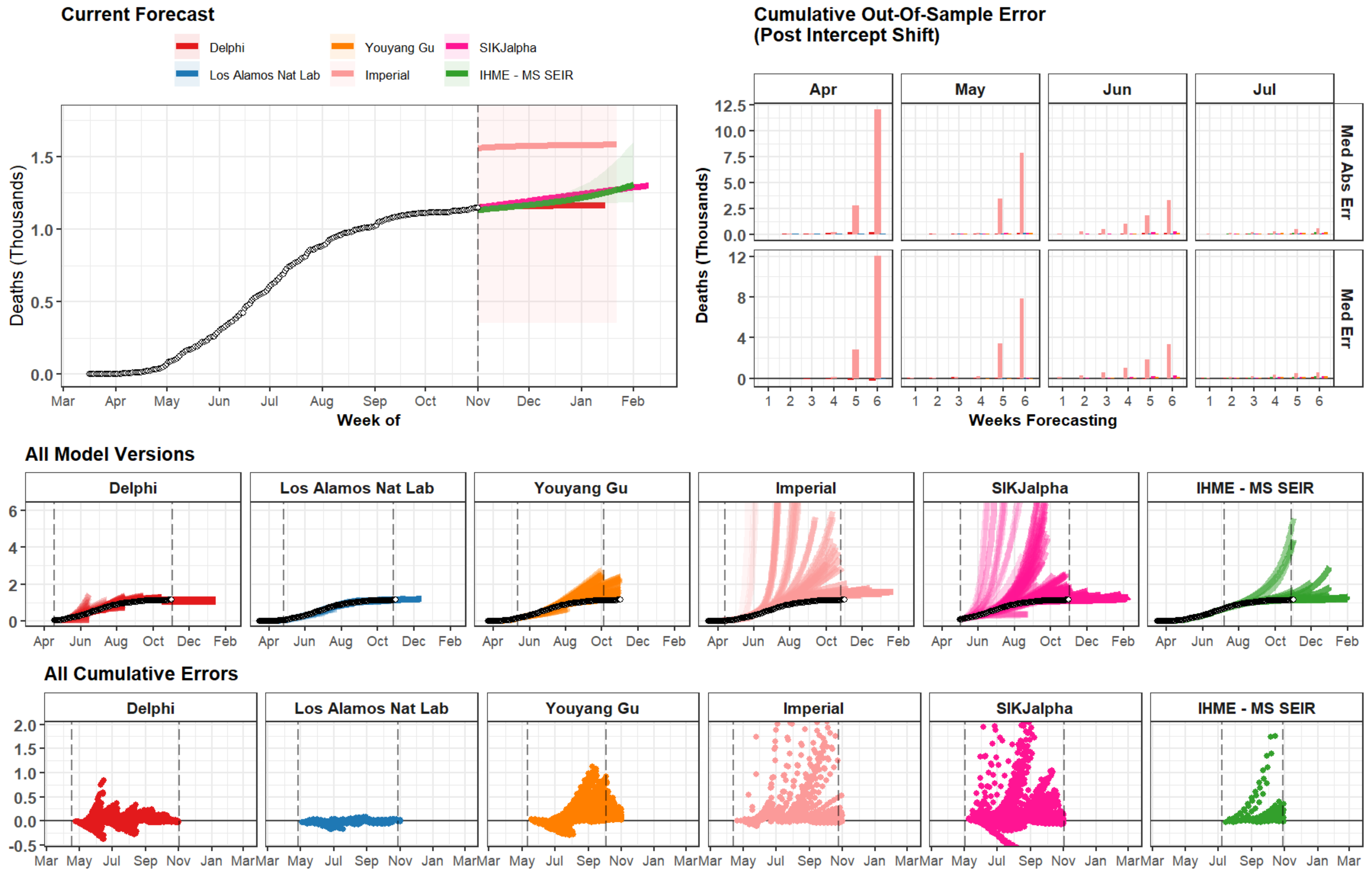

Current Forecast
Delphi
Youyang Gu
SIKJalpha
Los Alamos Nat Lab
Imperial
IHME - MS SEIR
Deaths (Thousands)
Week of
Cumulative Out-Of-Sample Error
(Post Intercept Shift)
Apr
May
Jun
Jul
Med Abs Err
Med Err
Weeks Forecasting
All Model Versions
All Cumulative Errors

Kyrgyzstan

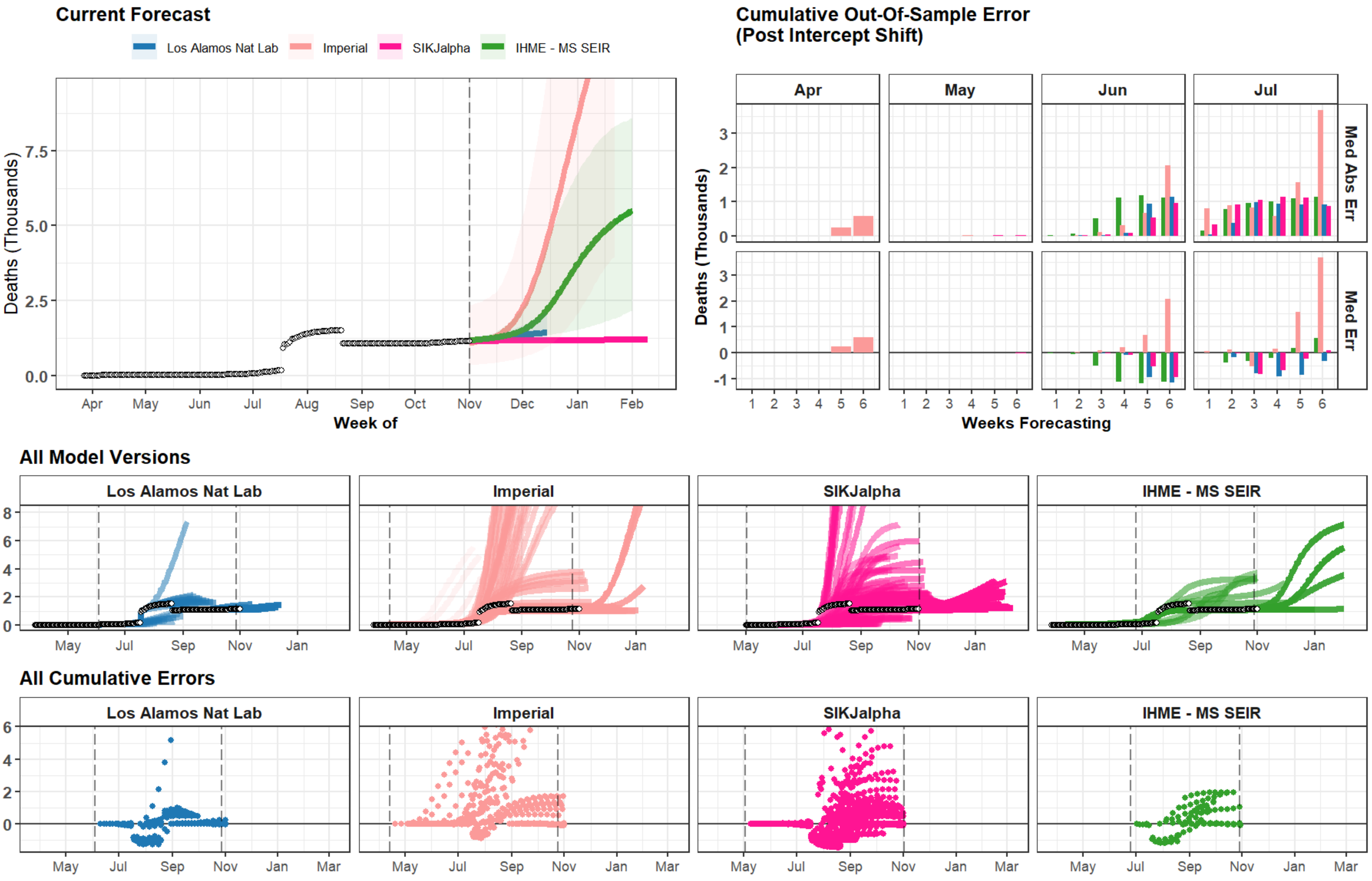

Current Forecast
Los Alamos Nat Lab
Imperial
SIKJalpha
IHME - MS SEIR
Deaths (Thousands)
7.5
5.0
2.5
0.0
Apr
May
Jun
Jul
Aug
Sep
Oct
Nov
Dec
Jan
Feb
Week of
Cumulative Out-Of-Sample Error
(Post Intercept Shift)
Apr
May
Jun
Jul
Med Abs Err
Med Err
Weeks Forecasting
All Model Versions
All Cumulative Errors

Austria

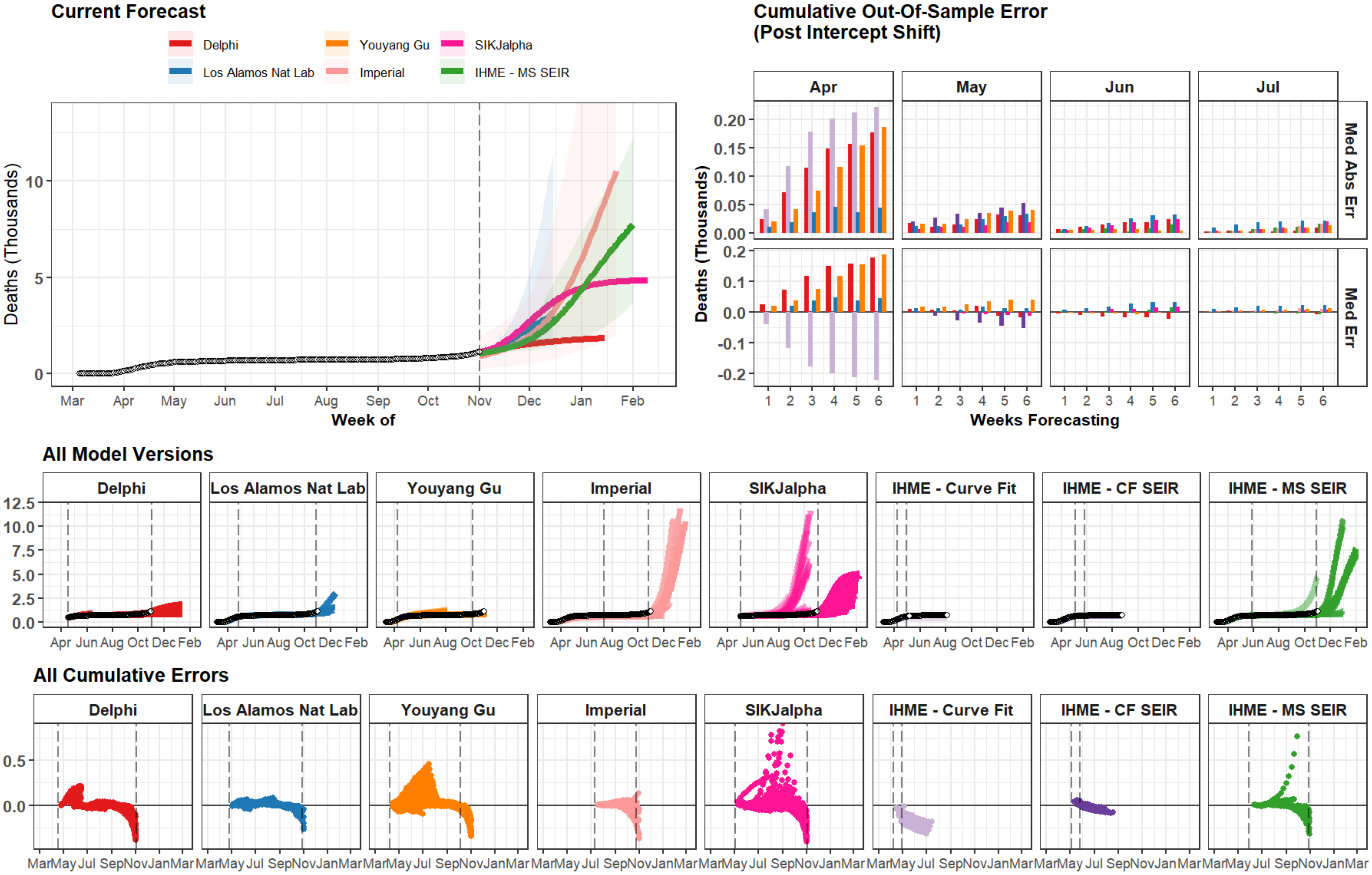
Current Forecast
Delphi
Los Alamos Nat Lab
Youyang Gu
Imperial
SIKJalpha
IHME - MS SEIR
Deaths (Thousands)
Week of
Mar Apr May Jun Jul Aug Sep Oct Nov Dec Jan Feb
Cumulative Out-Of-Sample Error
(Post Intercept Shift)
Apr
May
Jun
Jul
Med Abs Err
Med Err
Deaths (Thousands)
Weeks Forecasting
All Model Versions
Delphi
Los Alamos Nat Lab
Youyang Gu
Imperial
SIKJalpha
IHME - Curve Fit
IHME - CF SEIR
IHME - MS SEIR
Apr Jun Aug Oct Dec Feb
All Cumulative Errors
Delphi
Los Alamos Nat Lab
Youyang Gu
Imperial
SIKJalpha
IHME - Curve Fit
IHME - CF SEIR
IHME - MS SEIR
Mar May Jul Sep Nov Jan Mar

Kansas

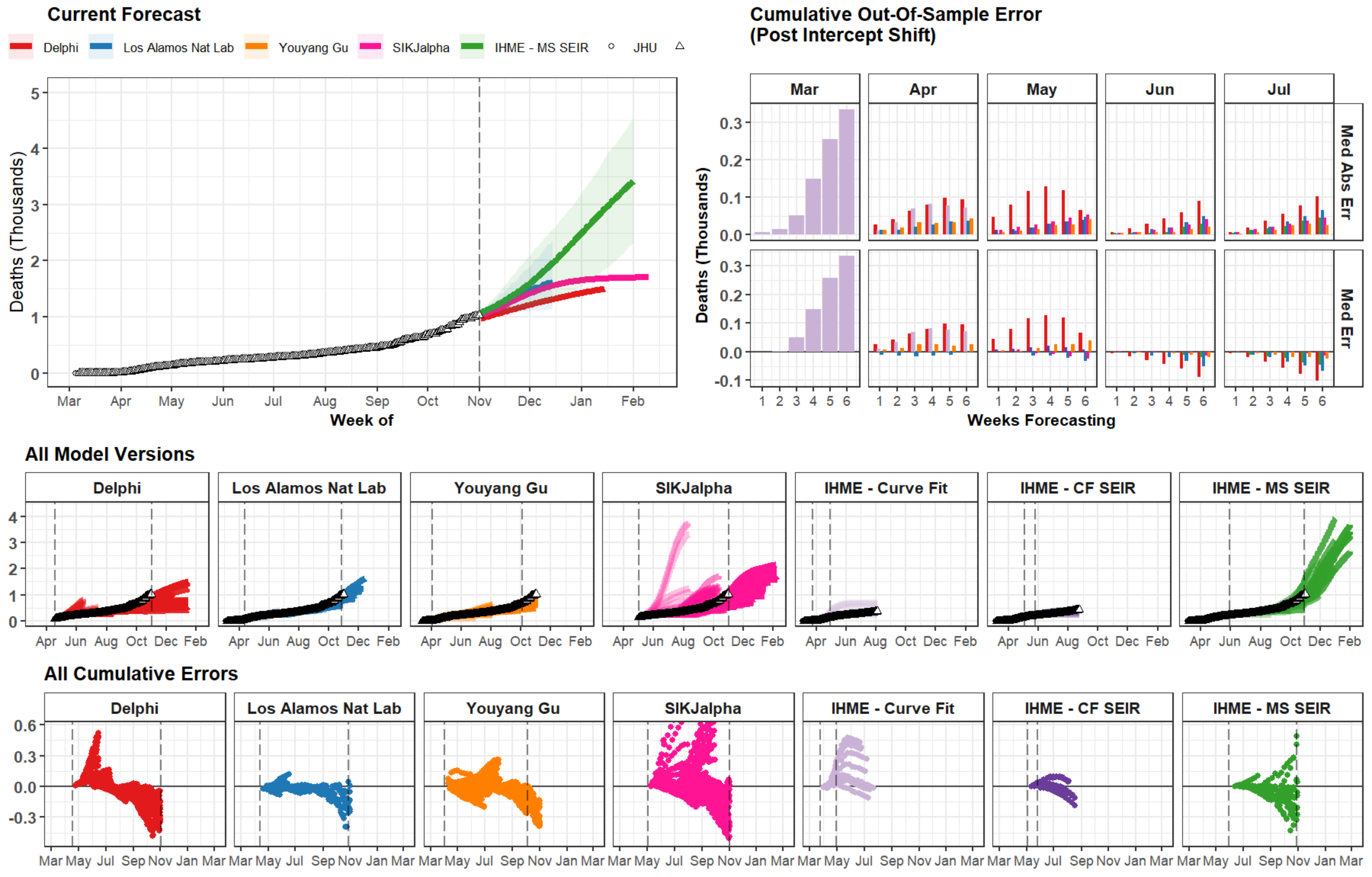
Current Forecast
Delphi
Los Alamos Nat Lab
Youyang Gu
SIKJalpha
IHME - MS SEIR
JHU
Deaths (Thousands)
Week of
Mar
Apr
May
Jun
Jul
Aug
Sep
Oct
Nov
Dec
Jan
Feb
Cumulative Out-Of-Sample Error
(Post Intercept Shift)
Med Abs Err
Med Err
Weeks Forecasting
All Model Versions
IHME - Curve Fit
IHME - CF SEIR
All Cumulative Errors

New Mexico

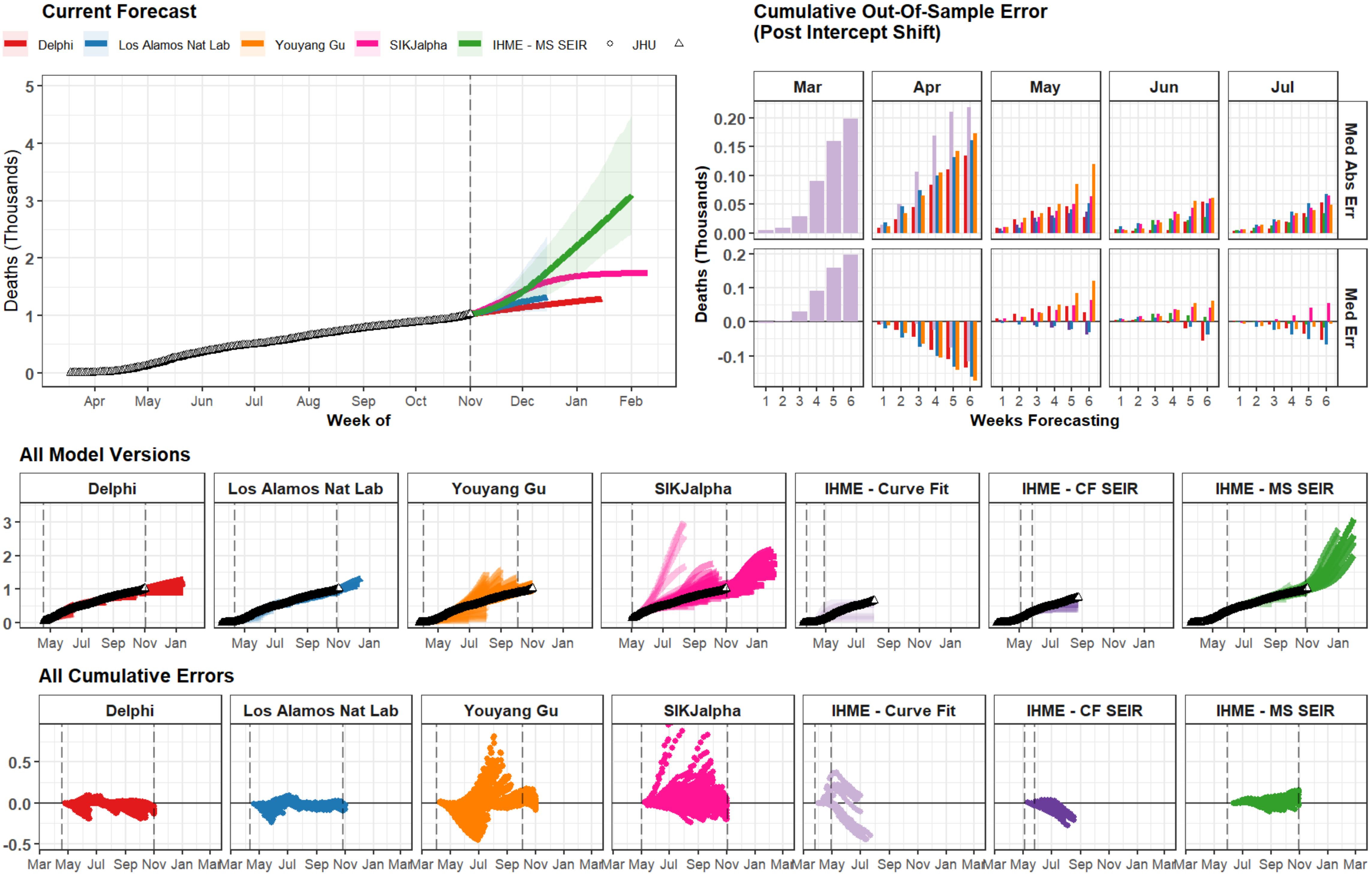
Current Forecast
Delphi
Los Alamos Nat Lab
Youyang Gu
SIKJalpha
IHME - MS SEIR
JHU
Deaths (Thousands)
0
1
2
3
4
5
Apr
May
Jun
Jul
Aug
Sep
Oct
Nov
Dec
Jan
Feb
Week of
Cumulative Out-Of-Sample Error
(Post Intercept Shift)
Mar
Apr
May
Jun
Jul
Med Abs Err
Med Err
0.00
0.05
0.10
0.15
0.20
-0.1
0.0
0.1
0.2
1 2 3 4 5 6
Weeks Forecasting
All Model Versions
Delphi
Los Alamos Nat Lab
Youyang Gu
SIKJalpha
IHME - Curve Fit
IHME - CF SEIR
IHME - MS SEIR
0
1
2
3
May Jul Sep Nov Jan
All Cumulative Errors
Delphi
Los Alamos Nat Lab
Youyang Gu
SIKJalpha
IHME - Curve Fit
IHME - CF SEIR
IHME - MS SEIR
-0.5
0.0
0.5
Mar May Jul Sep Nov Jan Mar

Kenya

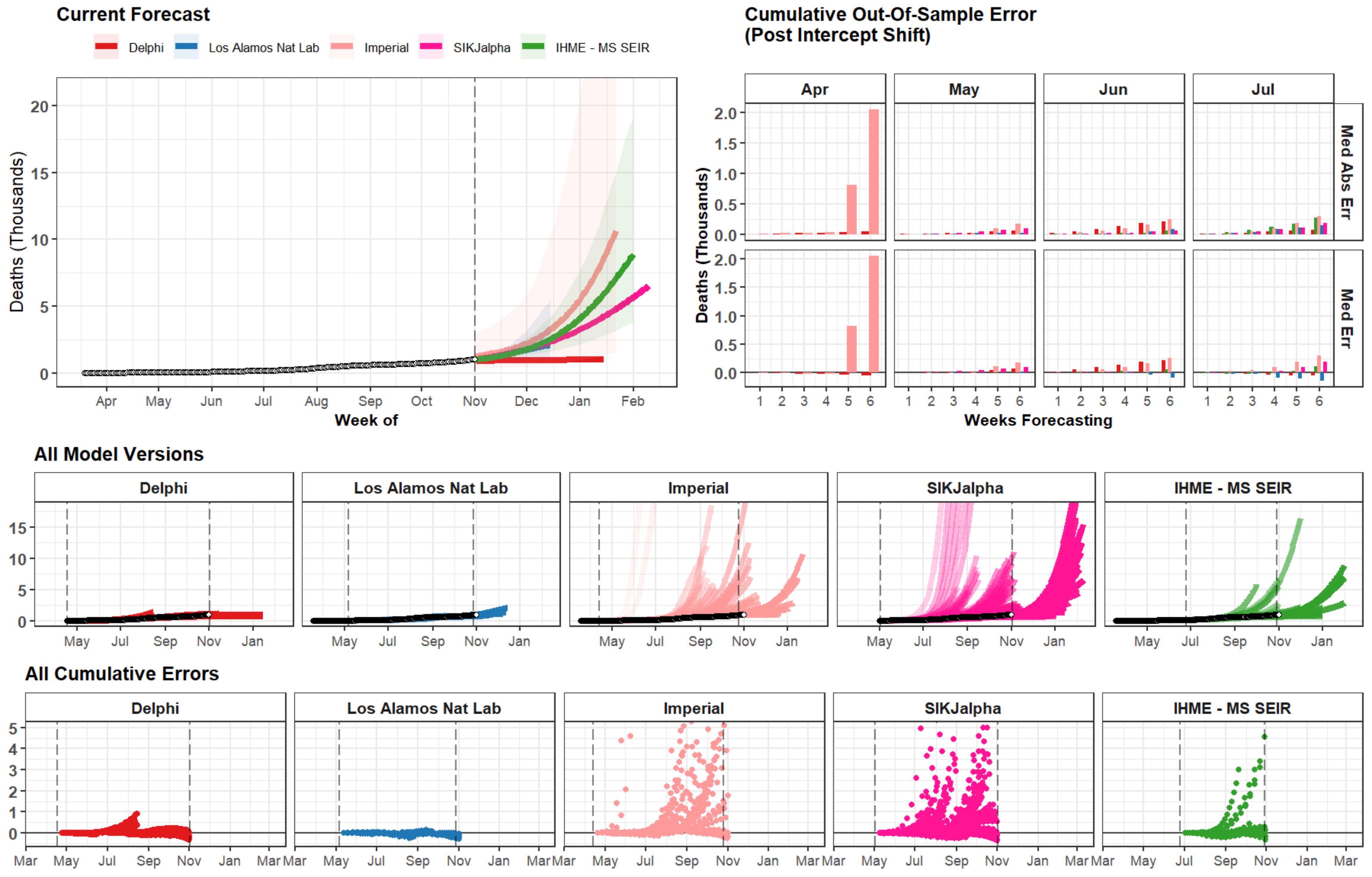

Current Forecast
Delphi
Los Alamos Nat Lab
Imperial
SIKJalpha
IHME - MS SEIR
Deaths (Thousands)
Week of
Apr May Jun Jul Aug Sep Oct Nov Dec Jan Feb
Cumulative Out-Of-Sample Error
(Post Intercept Shift)
Apr
May
Jun
Jul
Med Abs Err
Med Err
Deaths (Thousands)
Weeks Forecasting
All Model Versions
Delphi
Los Alamos Nat Lab
Imperial
SIKJalpha
IHME - MS SEIR
All Cumulative Errors
Delphi
Los Alamos Nat Lab
Imperial
SIKJalpha
IHME - MS SEIR
Mar May Jul Sep Nov Jan Mar

Macedonia

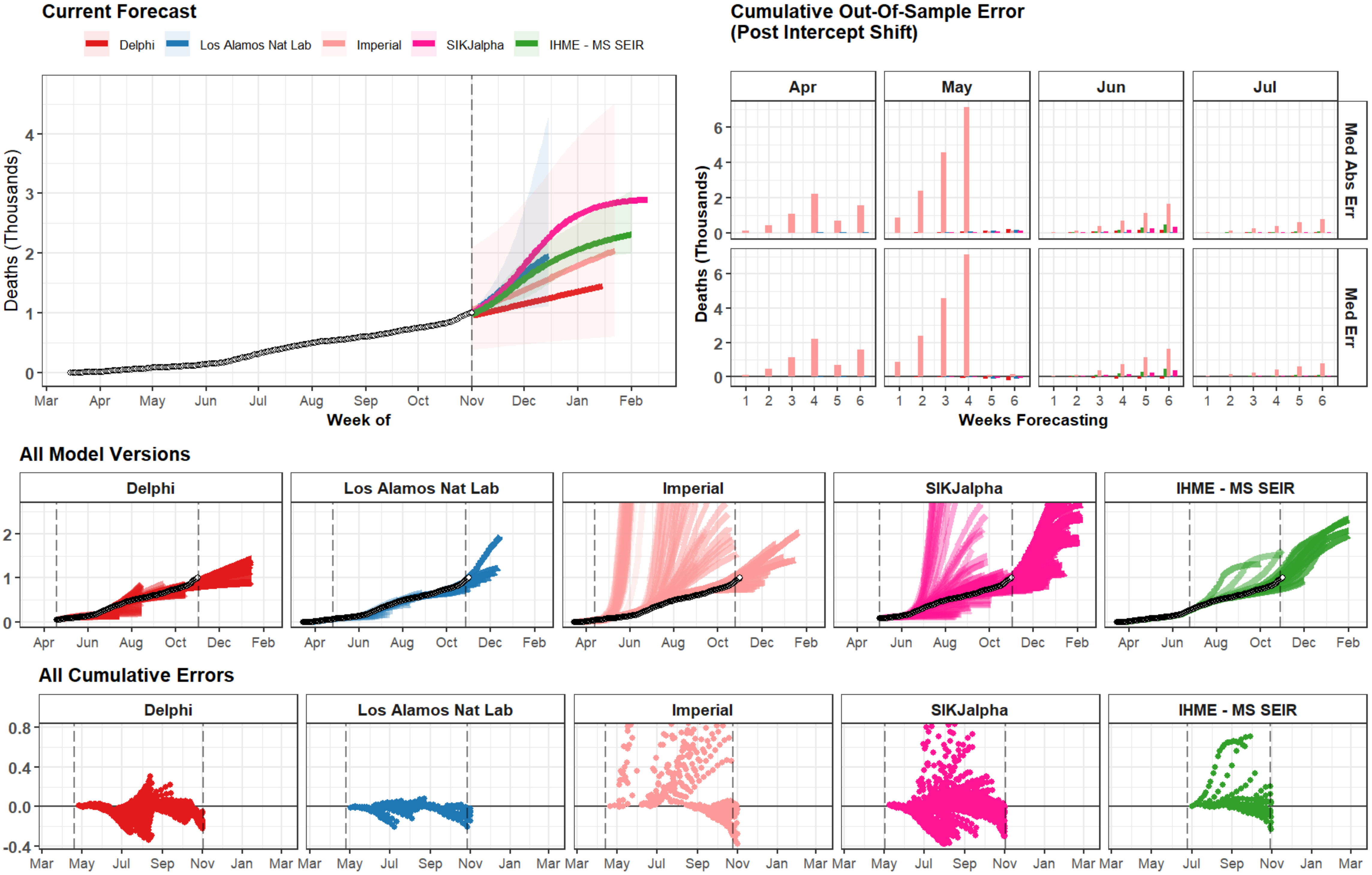

Current Forecast
Delphi
Los Alamos Nat Lab
Imperial
SIKJalpha
IHME - MS SEIR
Deaths (Thousands)
Week of
Mar
Apr
May
Jun
Jul
Aug
Sep
Oct
Nov
Dec
Jan
Feb
Cumulative Out-Of-Sample Error
(Post Intercept Shift)
Apr
May
Jun
Jul
Med Abs Err
Med Err
Weeks Forecasting
All Model Versions
All Cumulative Errors

Belarus

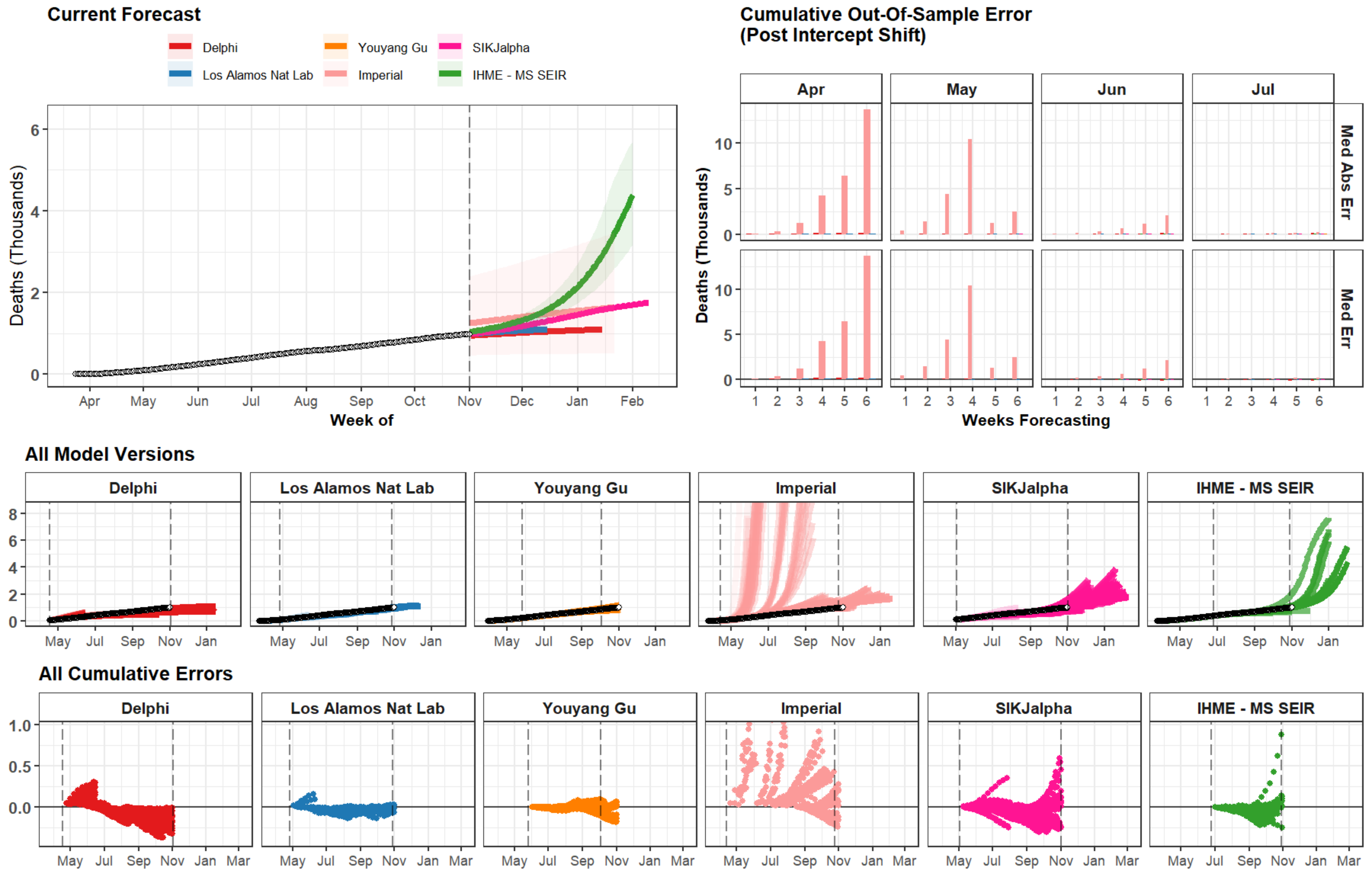

Current Forecast
Delphi
Los Alamos Nat Lab
Youyang Gu
Imperial
SIKJalpha
IHME - MS SEIR
Deaths (Thousands)
Week of
Apr May Jun Jul Aug Sep Oct Nov Dec Jan Feb
Cumulative Out-Of-Sample Error
(Post Intercept Shift)
Apr May Jun Jul
Med Abs Err
Med Err
Weeks Forecasting
All Model Versions
All Cumulative Errors
May Jul Sep Nov Jan Mar

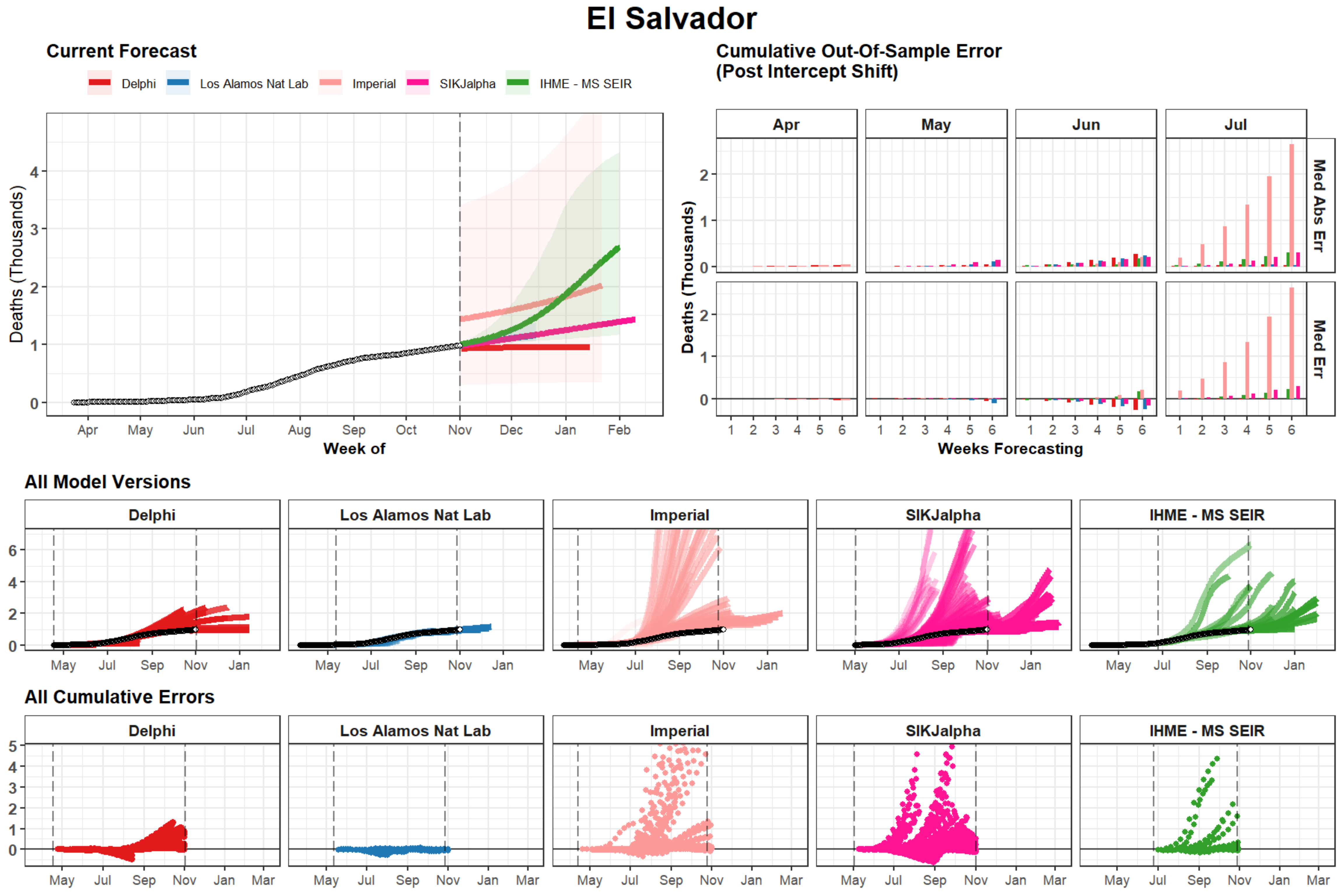

El Salvador
Current Forecast
Delphi
Los Alamos Nat Lab
Imperial
SIKJalpha
IHME - MS SEIR
Deaths (Thousands)
Week of
Apr
May
Jun
Jul
Aug
Sep
Oct
Nov
Dec
Jan
Feb
Cumulative Out-Of-Sample Error
(Post Intercept Shift)
Apr
May
Jun
Jul
Med Abs Err
Med Err
Weeks Forecasting
All Model Versions
Delphi
Los Alamos Nat Lab
Imperial
SIKJalpha
IHME - MS SEIR
All Cumulative Errors
Delphi
Los Alamos Nat Lab
Imperial
SIKJalpha
IHME - MS SEIR

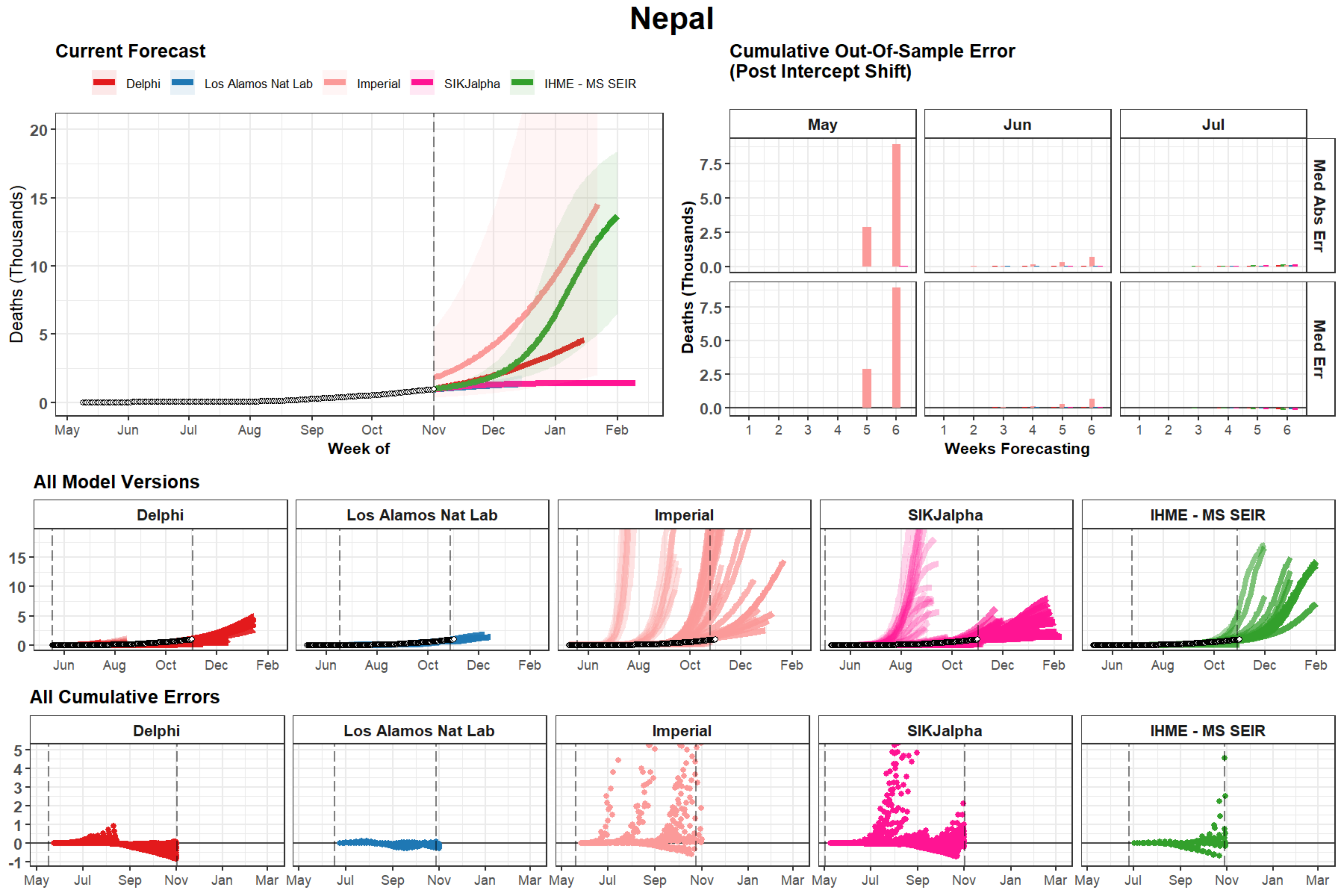
Nepal
Current Forecast
Delphi
Los Alamos Nat Lab
Imperial
SIKJalpha
IHME - MS SEIR
Deaths (Thousands)
Week of
Cumulative Out-Of-Sample Error
(Post Intercept Shift)
May
Jun
Jul
Med Abs Err
Med Err
Weeks Forecasting
All Model Versions
All Cumulative Errors

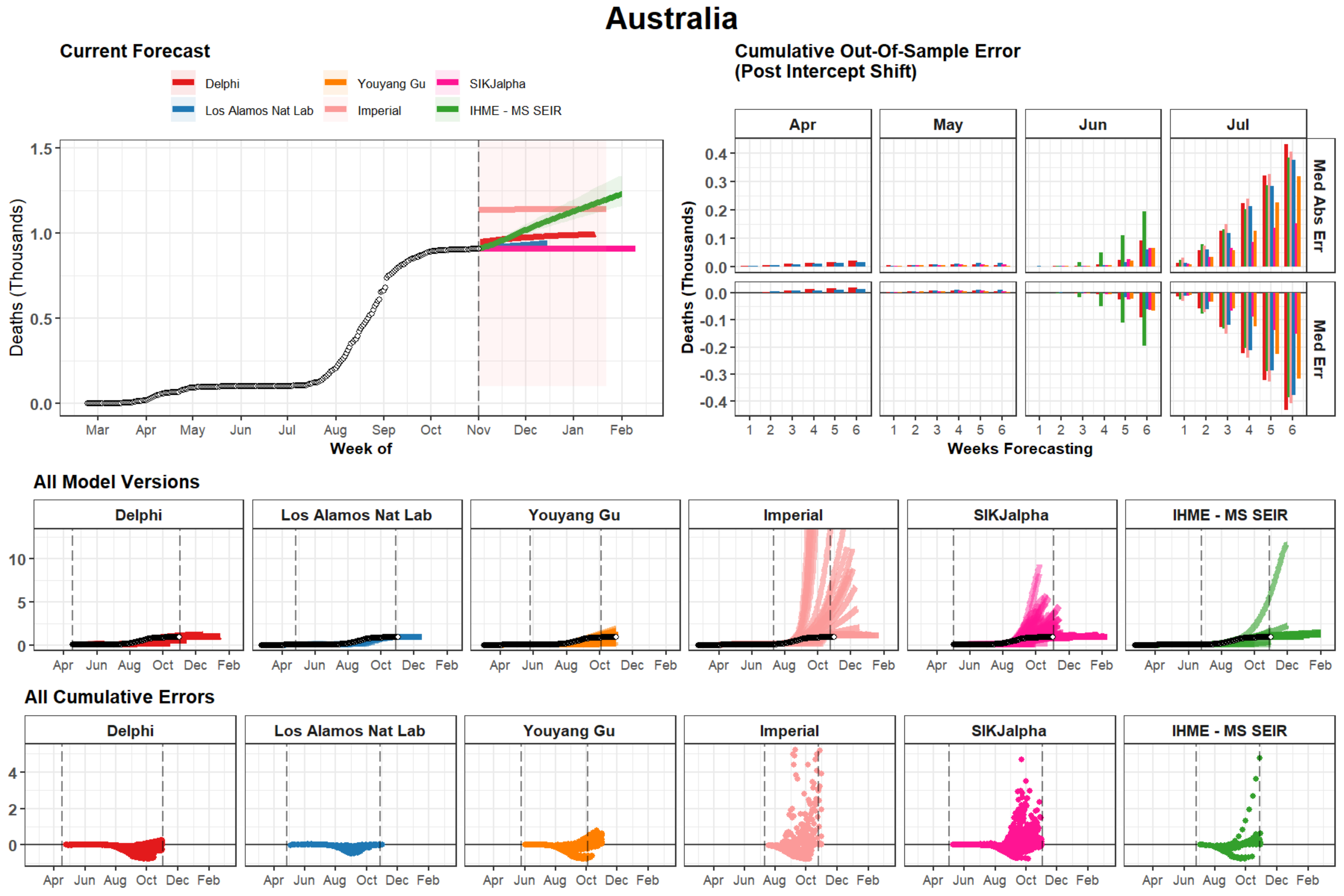

Australia
Current Forecast
Delphi
Los Alamos Nat Lab
Youyang Gu
Imperial
SIKJalpha
IHME - MS SEIR
Deaths (Thousands)
Week of
Cumulative Out-Of-Sample Error
(Post Intercept Shift)
Apr
May
Jun
Jul
Med Abs Err
Med Err
Weeks Forecasting
All Model Versions
All Cumulative Errors

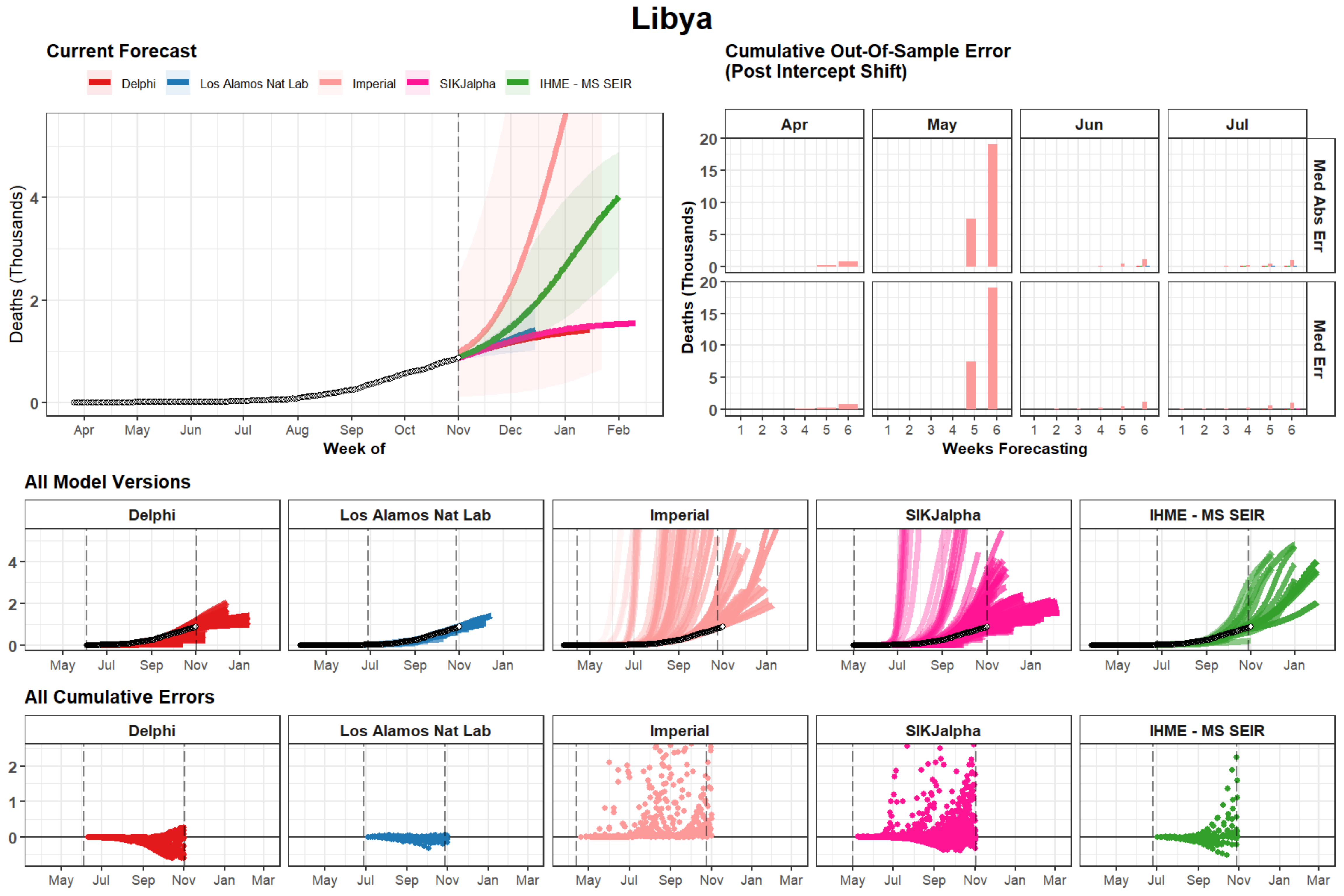
Libya
Current Forecast
Delphi
Los Alamos Nat Lab
Imperial
SIKJalpha
IHME - MS SEIR
Deaths (Thousands)
Week of
Apr May Jun Jul Aug Sep Oct Nov Dec Jan Feb
Cumulative Out-Of-Sample Error
(Post Intercept Shift)
Apr
May
Jun
Jul
Med Abs Err
Med Err
Weeks Forecasting
All Model Versions
Delphi
Los Alamos Nat Lab
Imperial
SIKJalpha
IHME - MS SEIR
May Jul Sep Nov Jan
All Cumulative Errors
May Jul Sep Nov Jan Mar

Jordan

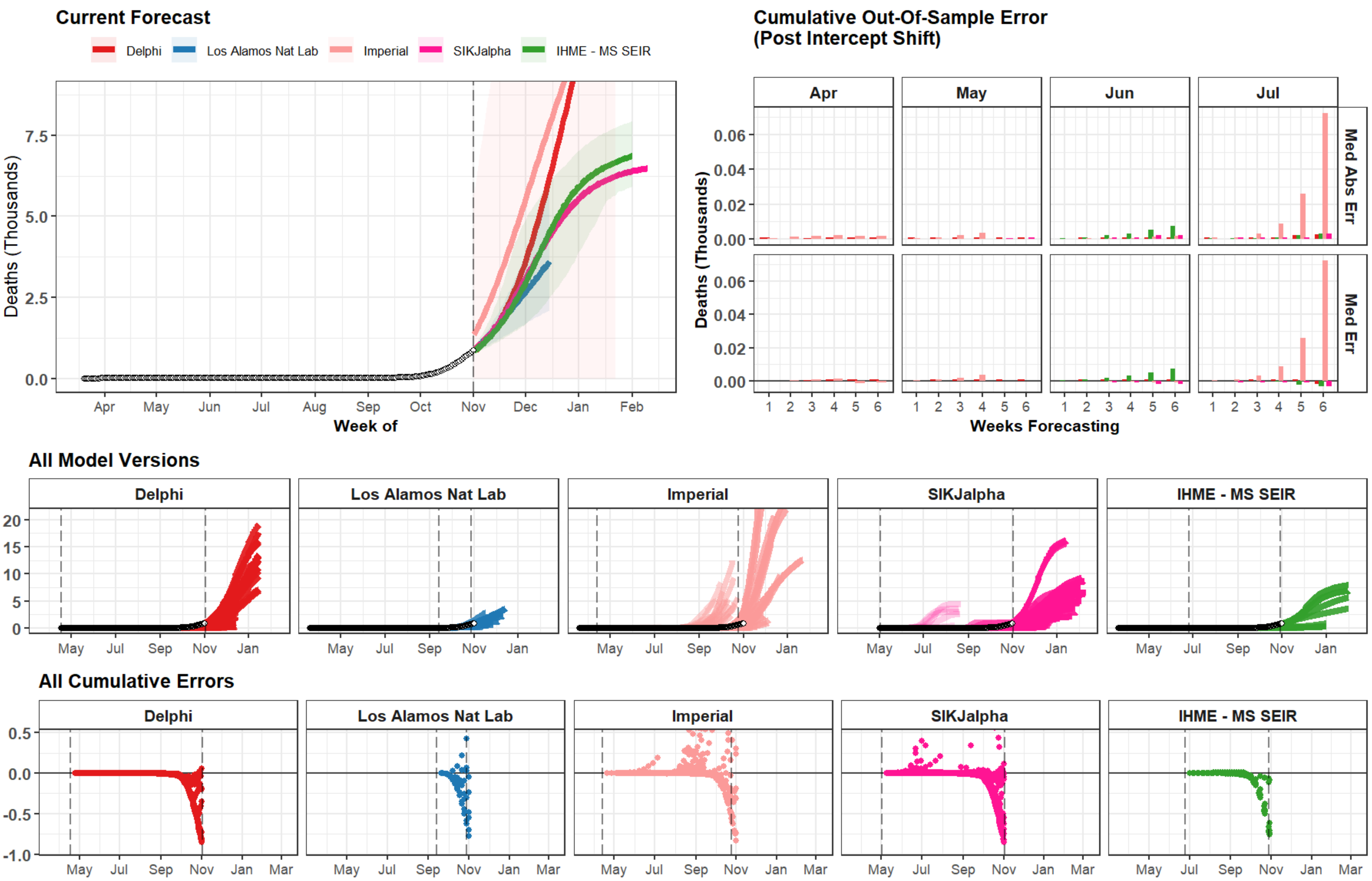
Current Forecast
Delphi
Los Alamos Nat Lab
Imperial
SIKJalpha
IHME - MS SEIR
Deaths (Thousands)
Week of
Apr
May
Jun
Jul
Aug
Sep
Oct
Nov
Dec
Jan
Feb
Cumulative Out-Of-Sample Error
(Post Intercept Shift)
Apr
May
Jun
Jul
Med Abs Err
Med Err
Weeks Forecasting
All Model Versions
All Cumulative Errors

Sudan

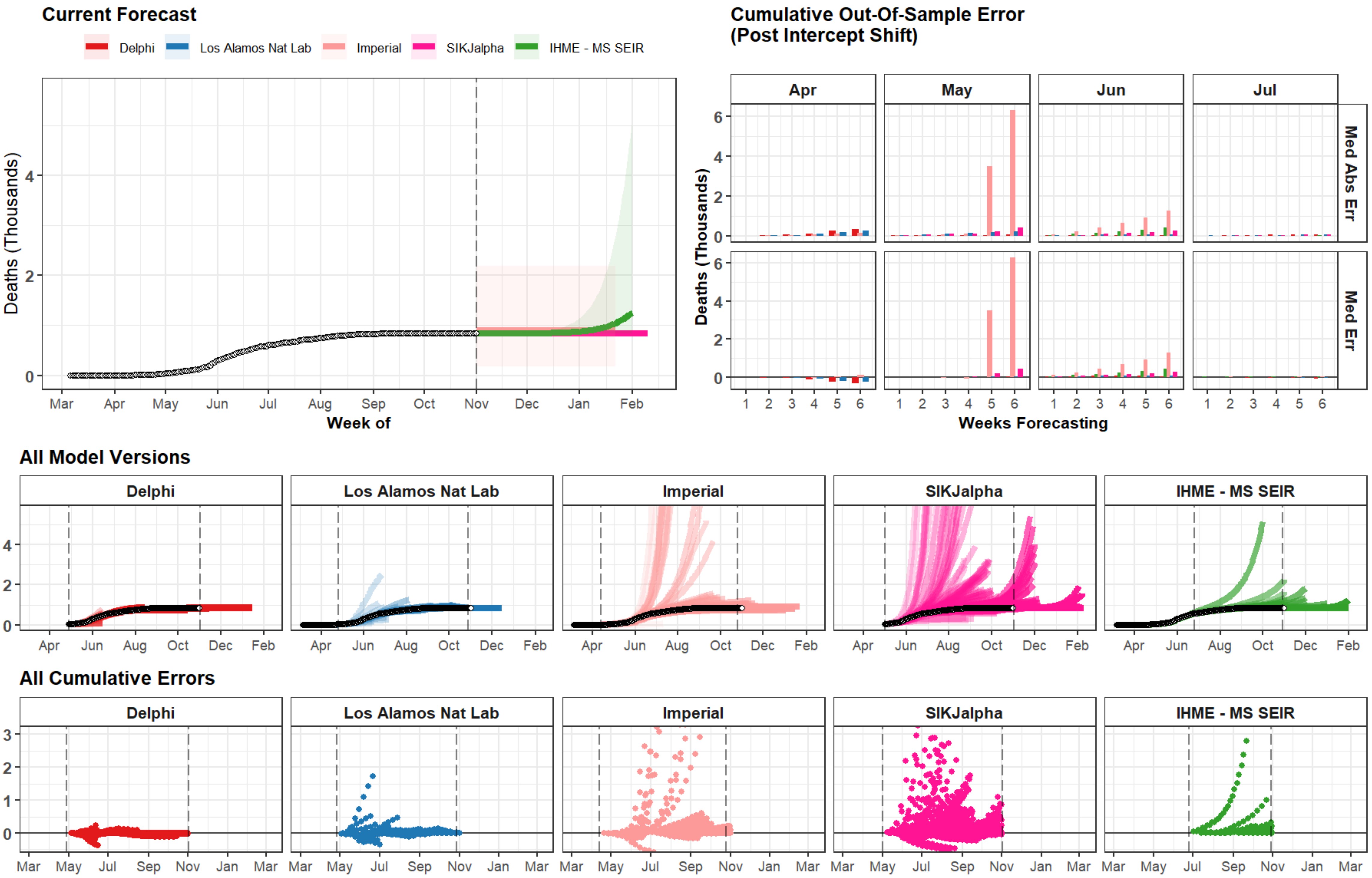

Current Forecast
Delphi
Los Alamos Nat Lab
Imperial
SIKJalpha
IHME - MS SEIR
Deaths (Thousands)
Week of
Cumulative Out-Of-Sample Error
(Post Intercept Shift)
Apr
May
Jun
Jul
Med Abs Err
Med Err
Weeks Forecasting
All Model Versions
All Cumulative Errors

Puerto Rico

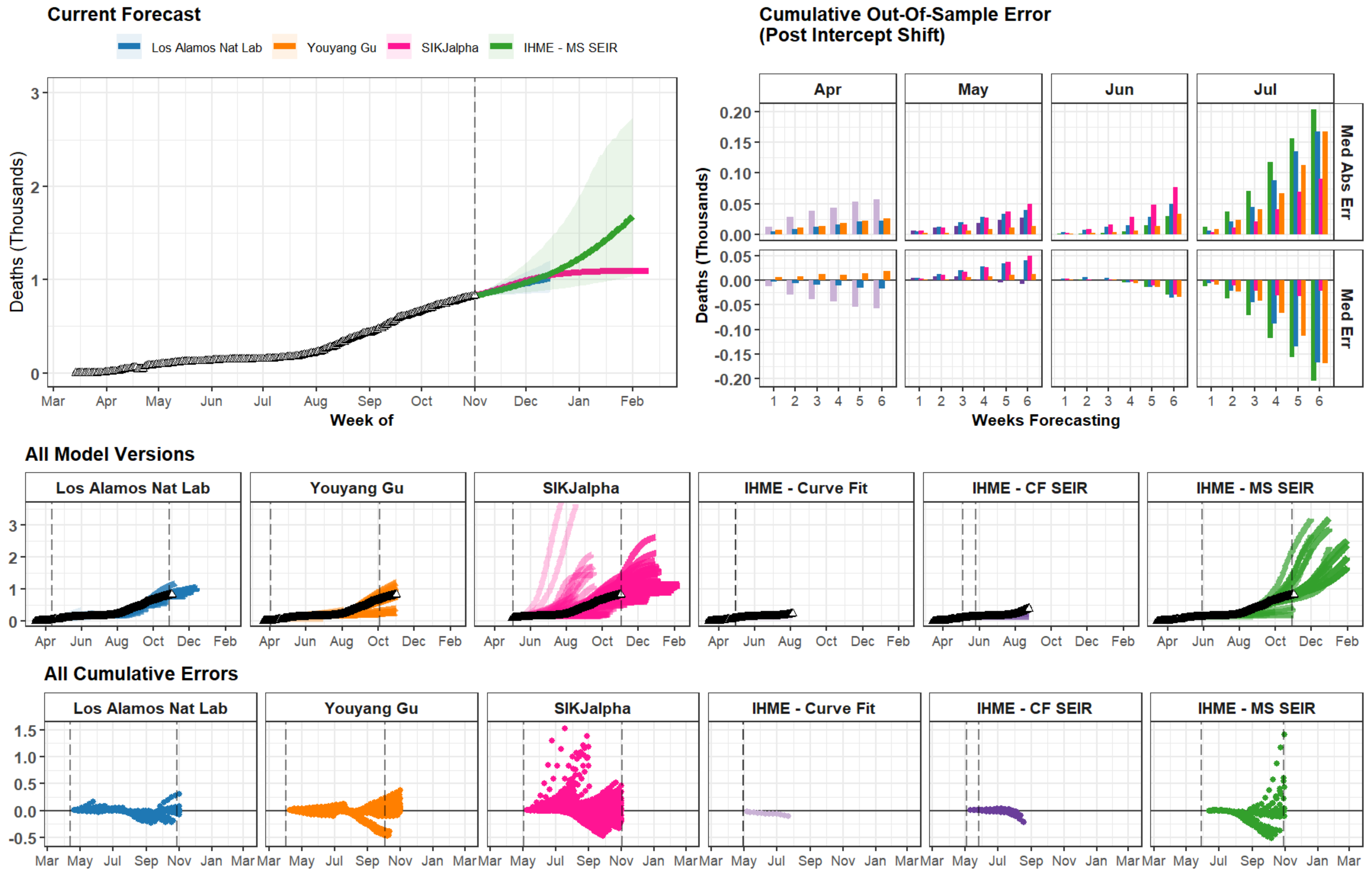

Current Forecast
Los Alamos Nat Lab
Youyang Gu
SIKJalpha
IHME - MS SEIR
Deaths (Thousands)
Week of
Mar Apr May Jun Jul Aug Sep Oct Nov Dec Jan Feb
Cumulative Out-Of-Sample Error
(Post Intercept Shift)
Apr
May
Jun
Jul
Med Abs Err
Med Err
Weeks Forecasting
All Model Versions
Los Alamos Nat Lab
Youyang Gu
SIKJalpha
IHME - Curve Fit
IHME - CF SEIR
IHME - MS SEIR
All Cumulative Errors
Los Alamos Nat Lab
Youyang Gu
SIKJalpha
IHME - Curve Fit
IHME - CF SEIR
IHME - MS SEIR

Serbia

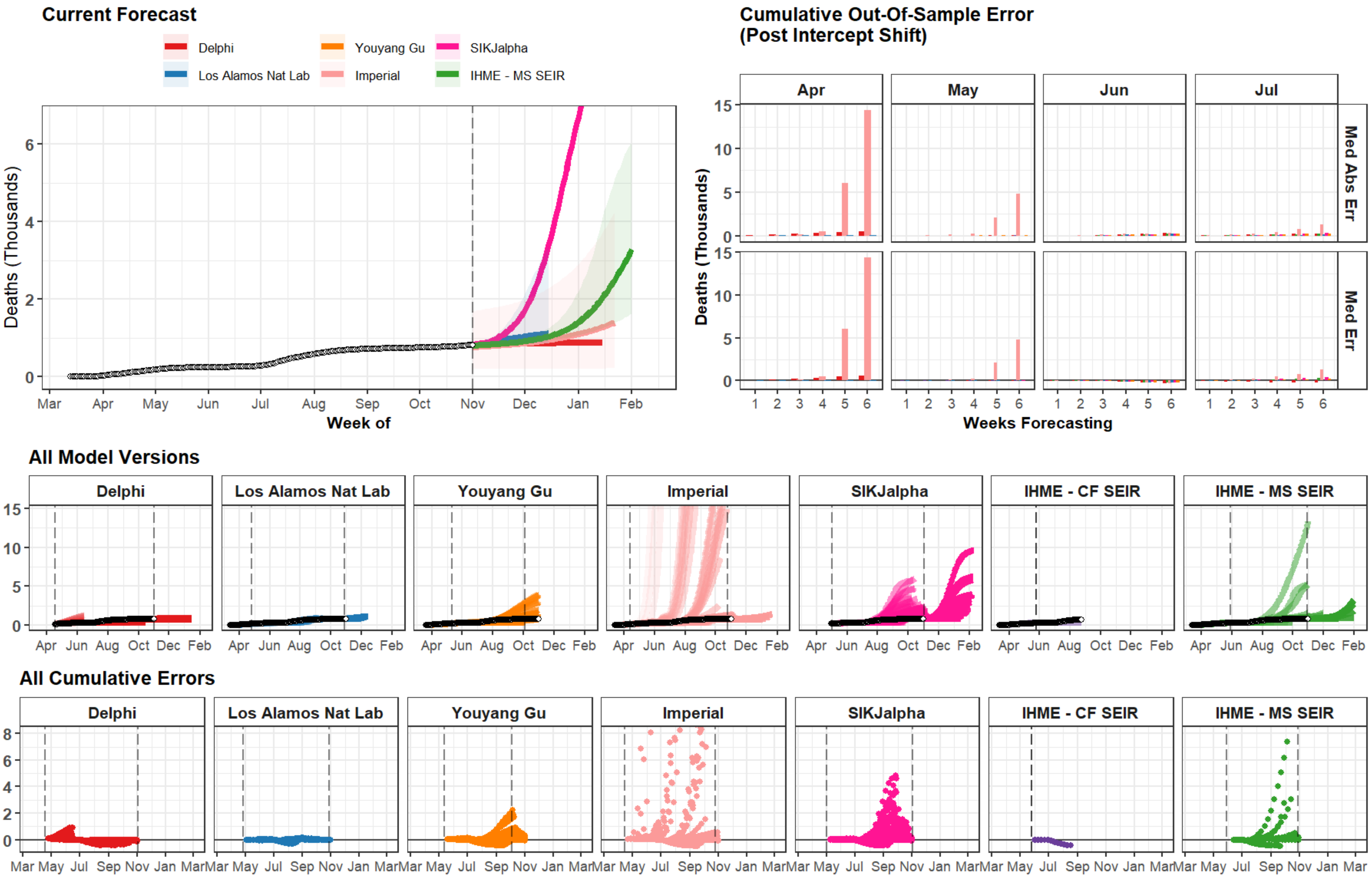
Current Forecast
Delphi
Los Alamos Nat Lab
Youyang Gu
Imperial
SIKJalpha
IHME - MS SEIR
Deaths (Thousands)
Week of
Mar Apr May Jun Jul Aug Sep Oct Nov Dec Jan Feb
Cumulative Out-Of-Sample Error
(Post Intercept Shift)
Apr
May
Jun
Jul
Med Abs Err
Med Err
Deaths (Thousands)
Weeks Forecasting
All Model Versions
Delphi
Los Alamos Nat Lab
Youyang Gu
Imperial
SIKJalpha
IHME - CF SEIR
IHME - MS SEIR
Apr Jun Aug Oct Dec Feb
All Cumulative Errors
Delphi
Los Alamos Nat Lab
Youyang Gu
Imperial
SIKJalpha
IHME - CF SEIR
IHME - MS SEIR
Mar May Jul Sep Nov Jan Mar

Venezuela

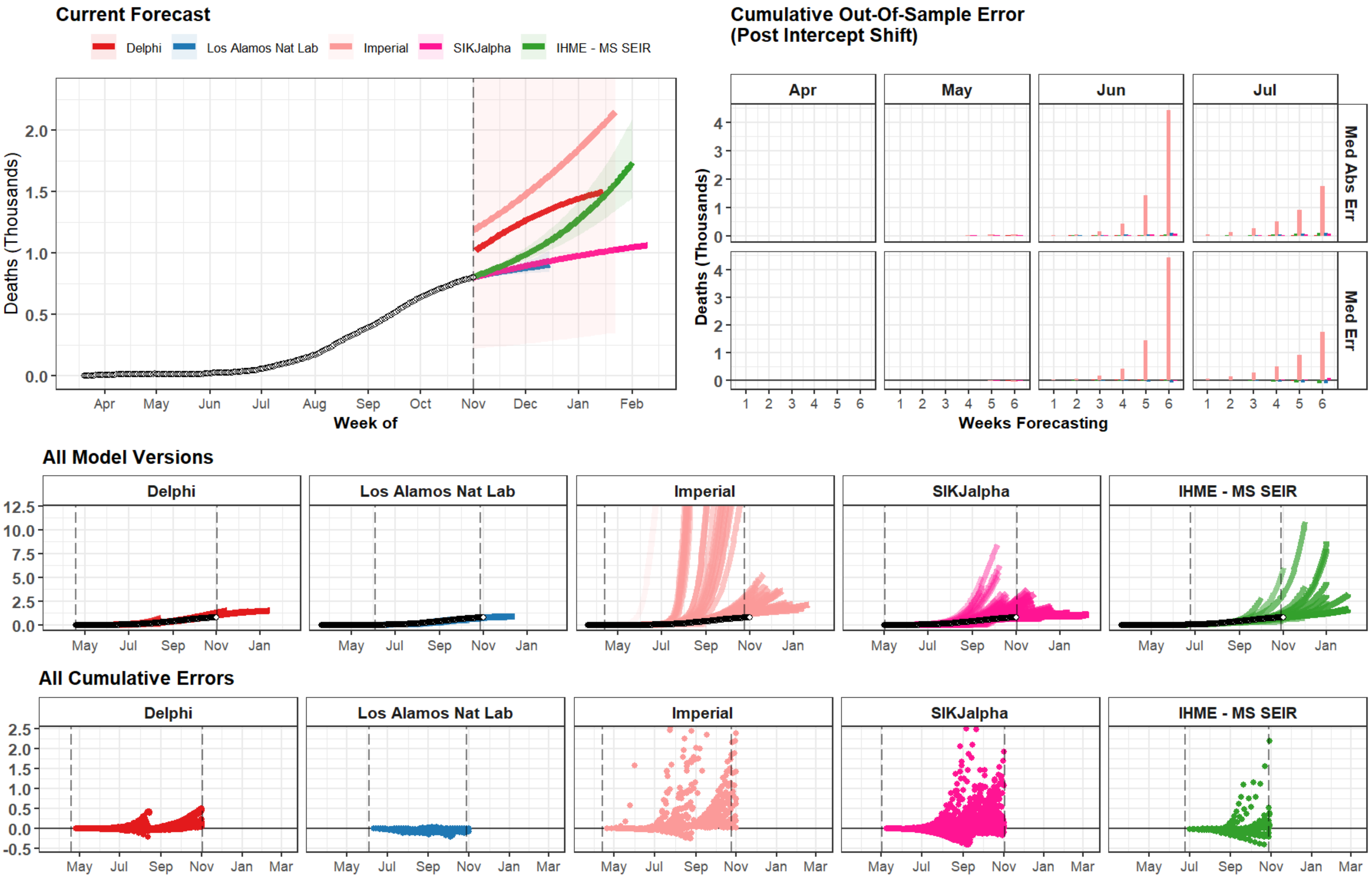
Current Forecast
Delphi
Los Alamos Nat Lab
Imperial
SIKJalpha
IHME - MS SEIR
Deaths (Thousands)
0.0
0.5
1.0
1.5
2.0
Apr
May
Jun
Jul
Aug
Sep
Oct
Nov
Dec
Jan
Feb
Week of
Cumulative Out-Of-Sample Error
(Post Intercept Shift)
Apr
May
Jun
Jul
Med Abs Err
Med Err
Deaths (Thousands)
0
1
2
3
4
1
2
3
4
5
6
Weeks Forecasting
All Model Versions
Delphi
Los Alamos Nat Lab
Imperial
SIKJalpha
IHME - MS SEIR
0.0
2.5
5.0
7.5
10.0
12.5
May
Jul
Sep
Nov
Jan
All Cumulative Errors
Delphi
Los Alamos Nat Lab
Imperial
SIKJalpha
IHME - MS SEIR
-0.5
0.0
0.5
1.0
1.5
2.0
2.5
May
Jul
Sep
Nov
Jan
Mar

Kuwait

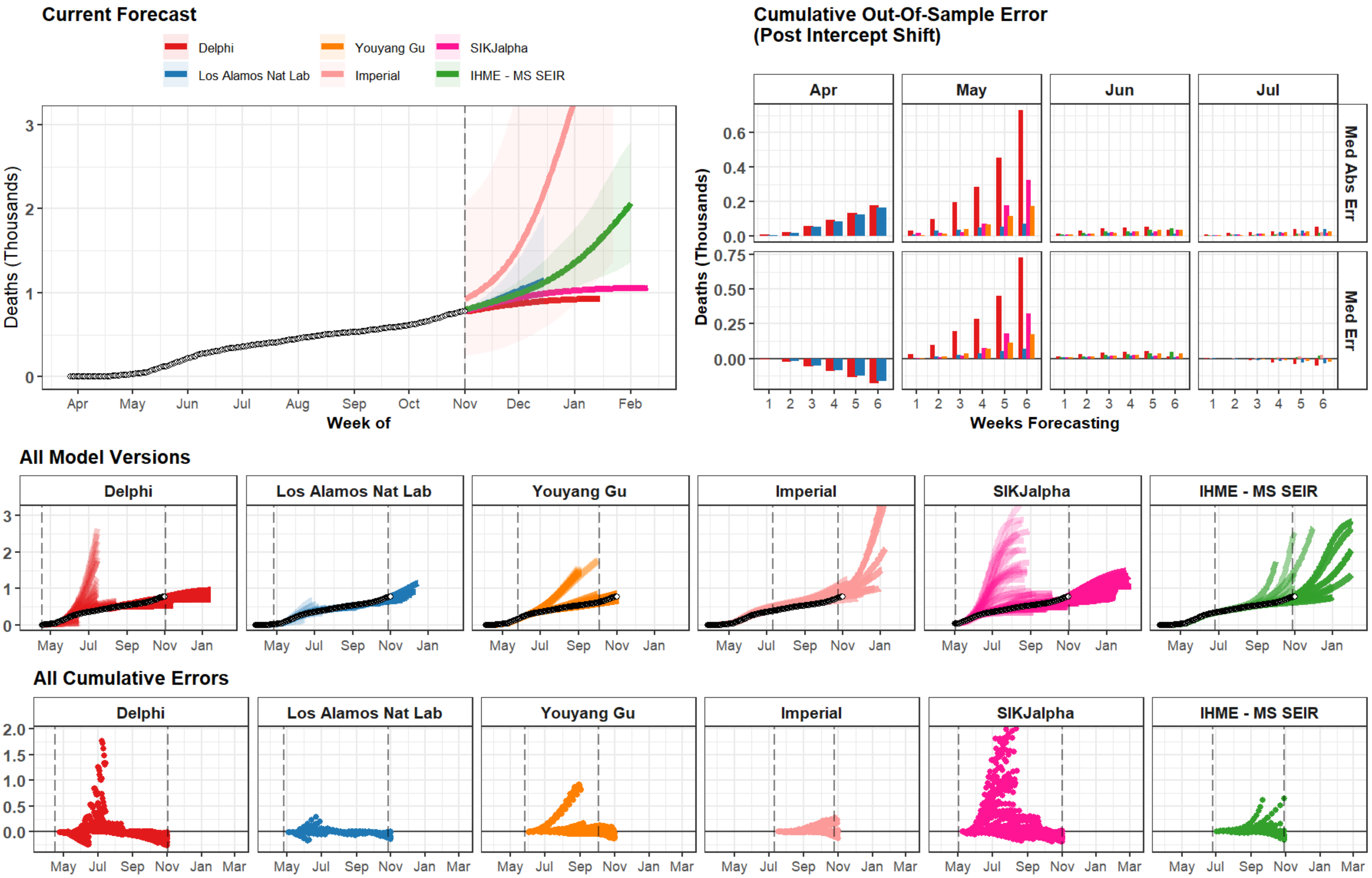
Current Forecast
Delphi
Los Alamos Nat Lab
Youyang Gu
Imperial
SIKJalpha
IHME - MS SEIR
Deaths (Thousands)
Week of
Apr
May
Jun
Jul
Aug
Sep
Oct
Nov
Dec
Jan
Feb
Cumulative Out-Of-Sample Error
(Post Intercept Shift)
Med Abs Err
Med Err
Weeks Forecasting
All Model Versions
All Cumulative Errors
Mar

Azerbaijan

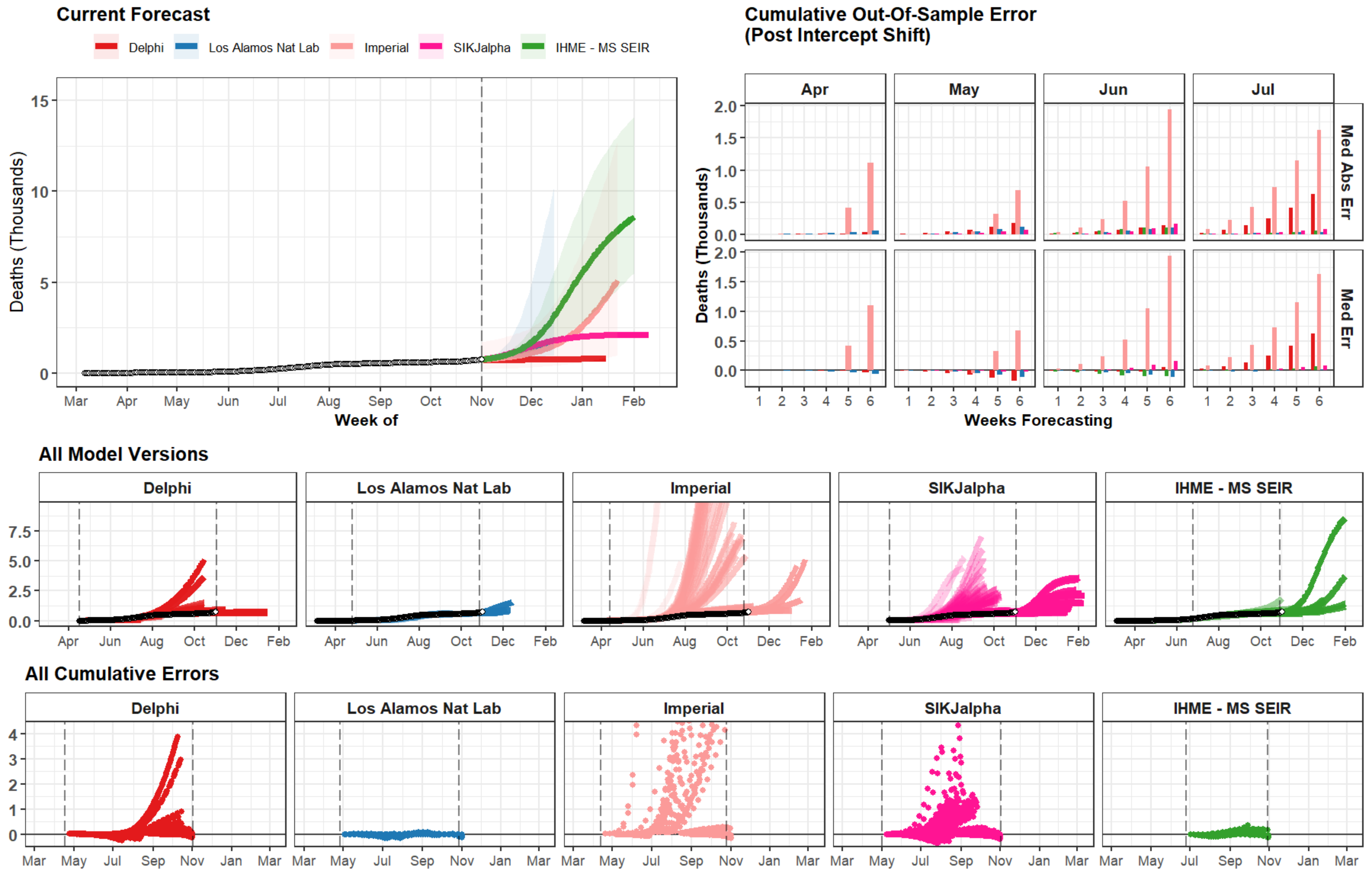

Current Forecast
Delphi
Los Alamos Nat Lab
Imperial
SIKJalpha
IHME - MS SEIR
Deaths (Thousands)
Week of
Cumulative Out-Of-Sample Error (Post Intercept Shift)
Apr
May
Jun
Jul
Med Abs Err
Med Err
Weeks Forecasting
All Model Versions
All Cumulative Errors

Denmark

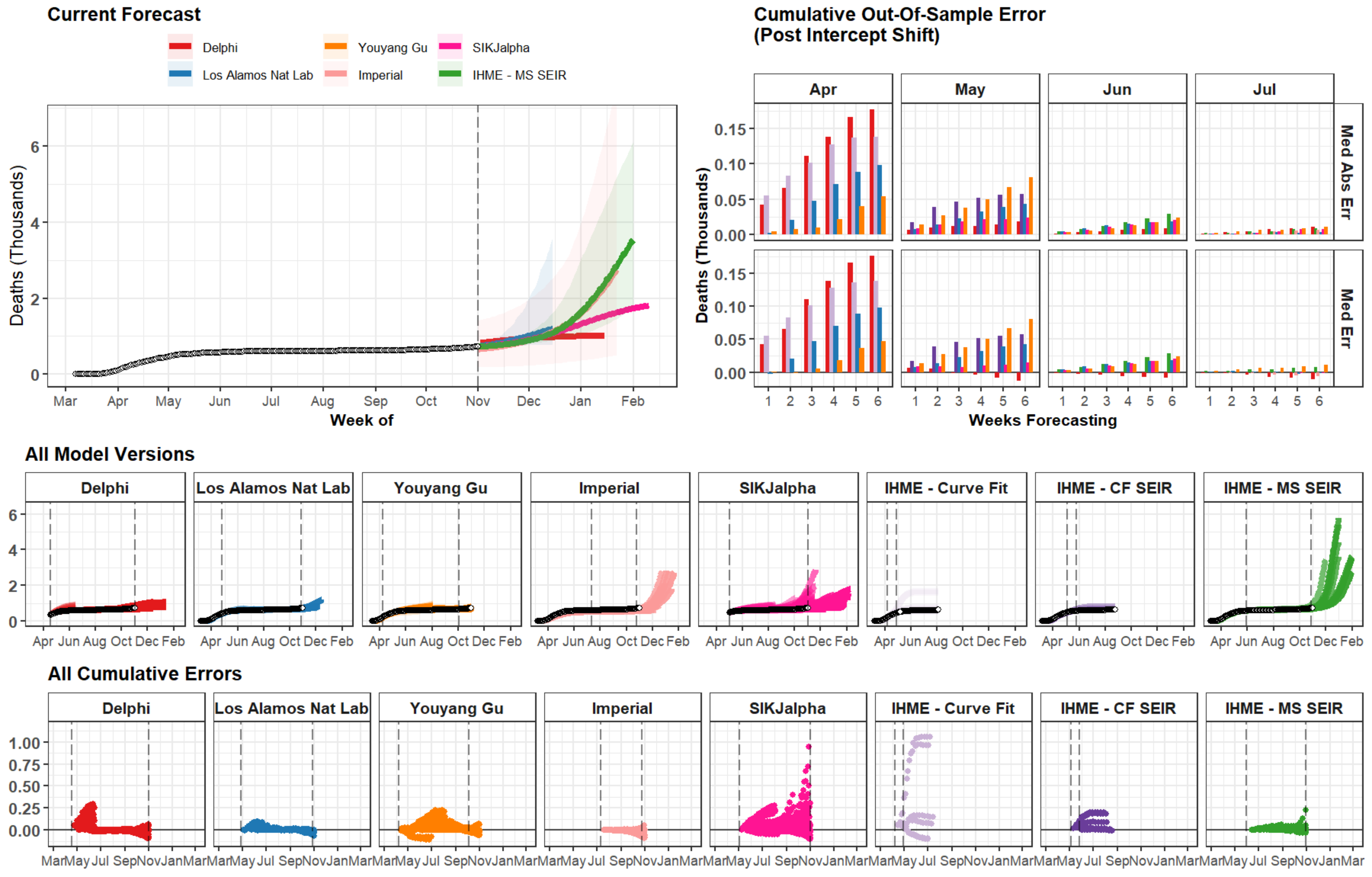

Current Forecast
Delphi
Youyang Gu
SIKJalpha
Los Alamos Nat Lab
Imperial
IHME - MS SEIR
Deaths (Thousands)
Week of
Mar Apr May Jun Jul Aug Sep Oct Nov Dec Jan Feb
Cumulative Out-Of-Sample Error
(Post Intercept Shift)
Apr
May
Jun
Jul
Med Abs Err
Med Err
Weeks Forecasting
All Model Versions
Delphi
Los Alamos Nat Lab
Youyang Gu
Imperial
SIKJalpha
IHME - Curve Fit
IHME - CF SEIR
IHME - MS SEIR
Apr Jun Aug Oct Dec Feb
All Cumulative Errors
MarMay Jul SepNovJanMar

Delaware

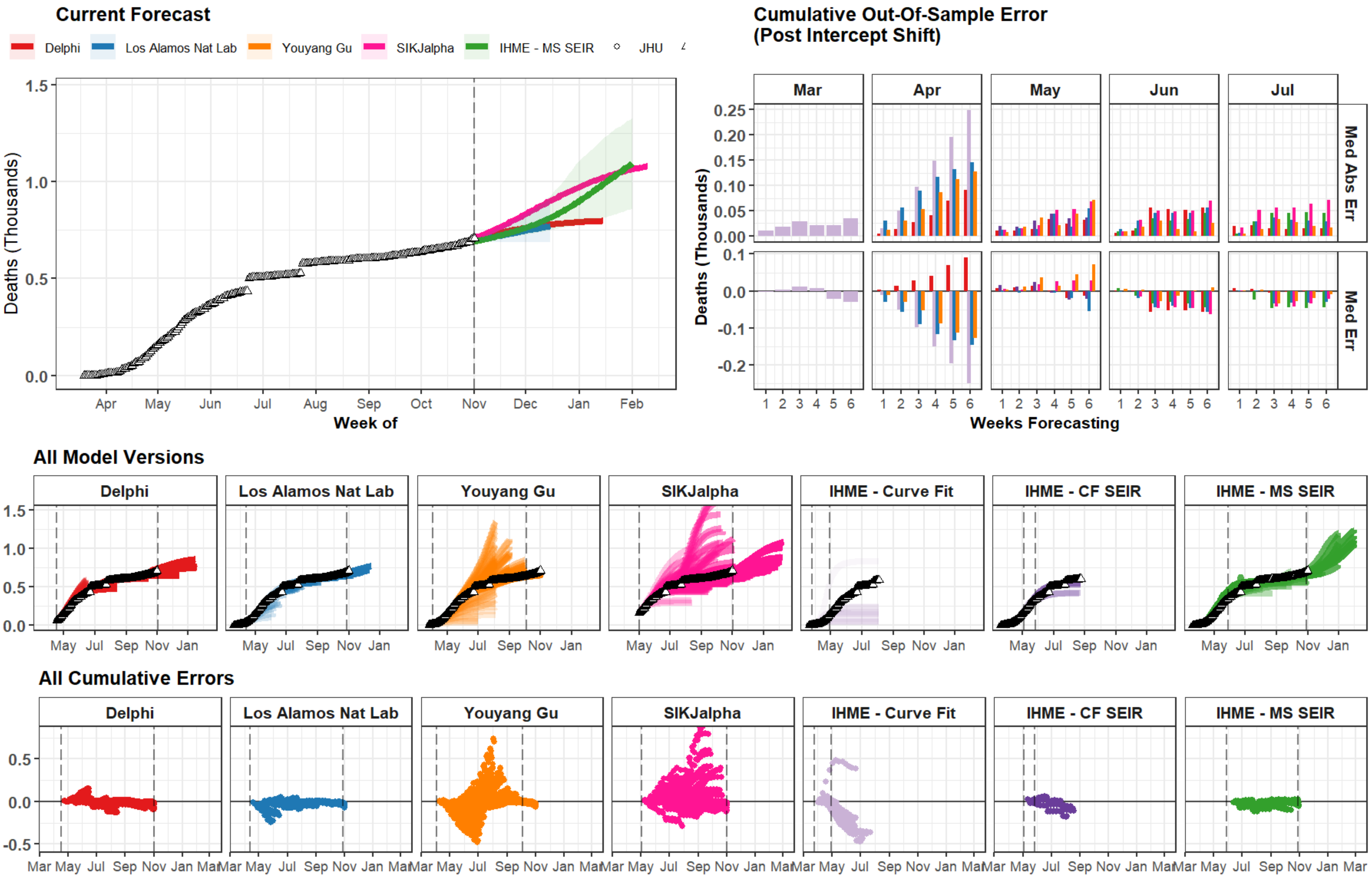
Current Forecast
Delphi
Los Alamos Nat Lab
Youyang Gu
SIKJalpha
IHME - MS SEIR
JHU
Deaths (Thousands)
Week of
Cumulative Out-Of-Sample Error
(Post Intercept Shift)
Mar
Apr
May
Jun
Jul
Med Abs Err
Med Err
Weeks Forecasting
All Model Versions
Delphi
Los Alamos Nat Lab
Youyang Gu
SIKJalpha
IHME - Curve Fit
IHME - CF SEIR
IHME - MS SEIR
All Cumulative Errors

Oregon

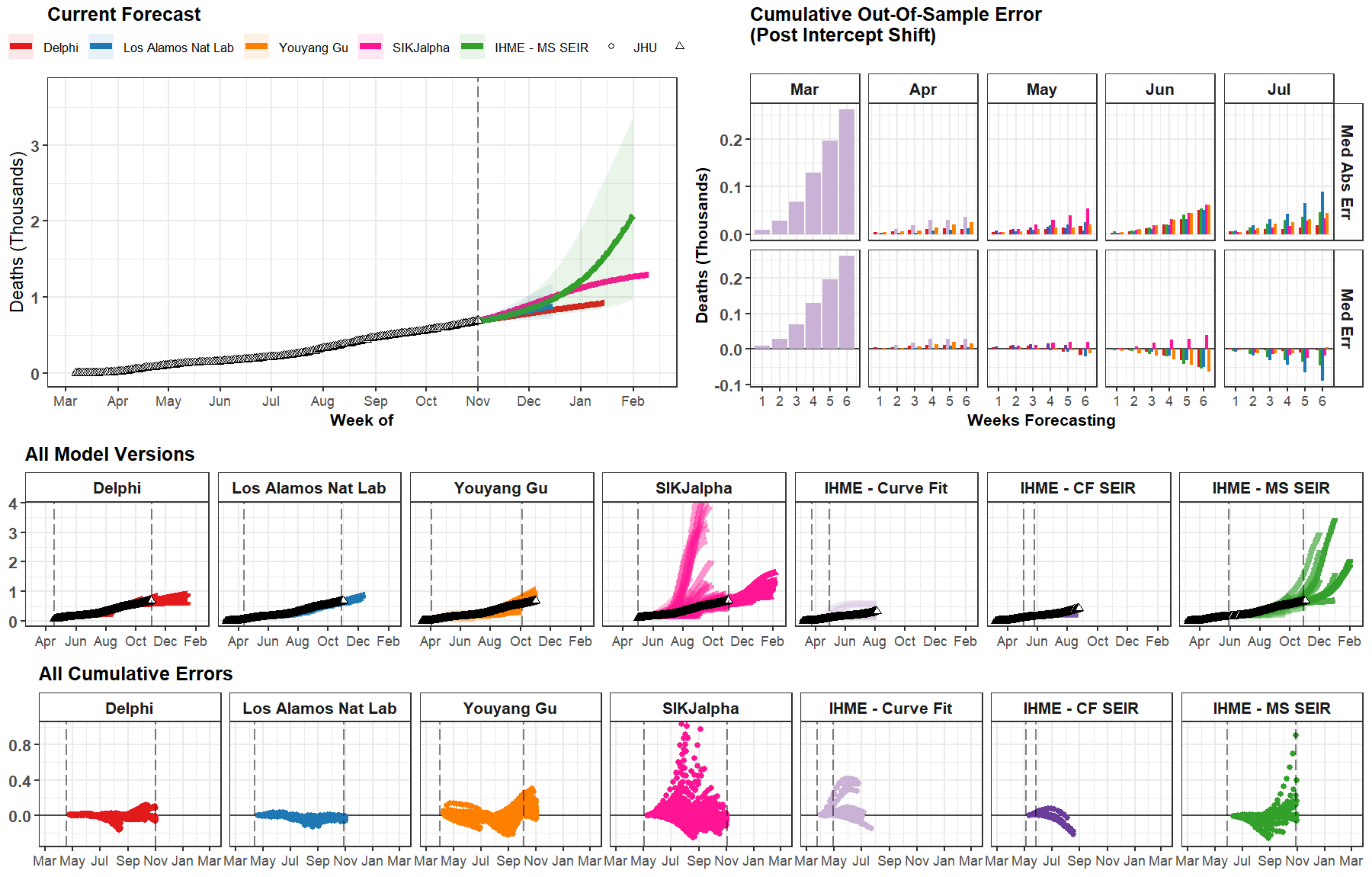
Current Forecast
Delphi
Los Alamos Nat Lab
Youyang Gu
SIKJalpha
IHME - MS SEIR
JHU
Deaths (Thousands)
Week of
Mar
Apr
May
Jun
Jul
Aug
Sep
Oct
Nov
Dec
Jan
Feb
Cumulative Out-Of-Sample Error
(Post Intercept Shift)
Med Abs Err
Med Err
Weeks Forecasting
All Model Versions
IHME - Curve Fit
IHME - CF SEIR
All Cumulative Errors

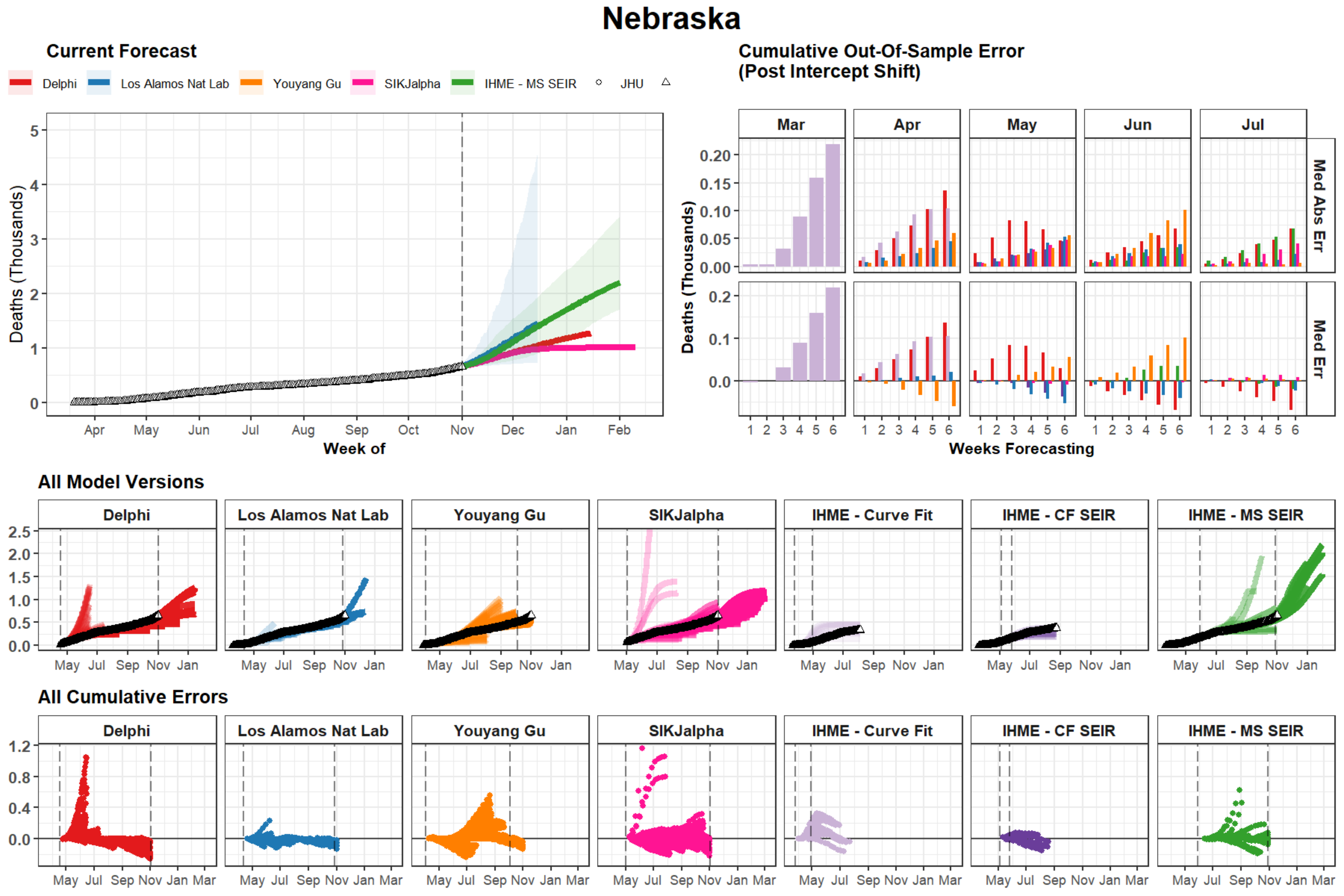
Nebraska
Current Forecast
Delphi
Los Alamos Nat Lab
Youyang Gu
SIKJalpha
IHME - MS SEIR
JHU
Deaths (Thousands)
Week of
Apr May Jun Jul Aug Sep Oct Nov Dec Jan Feb
Cumulative Out-Of-Sample Error
(Post Intercept Shift)
Mar Apr May Jun Jul
Med Abs Err
Med Err
Weeks Forecasting
All Model Versions
Delphi
Los Alamos Nat Lab
Youyang Gu
SIKJalpha
IHME - Curve Fit
IHME - CF SEIR
IHME - MS SEIR
All Cumulative Errors

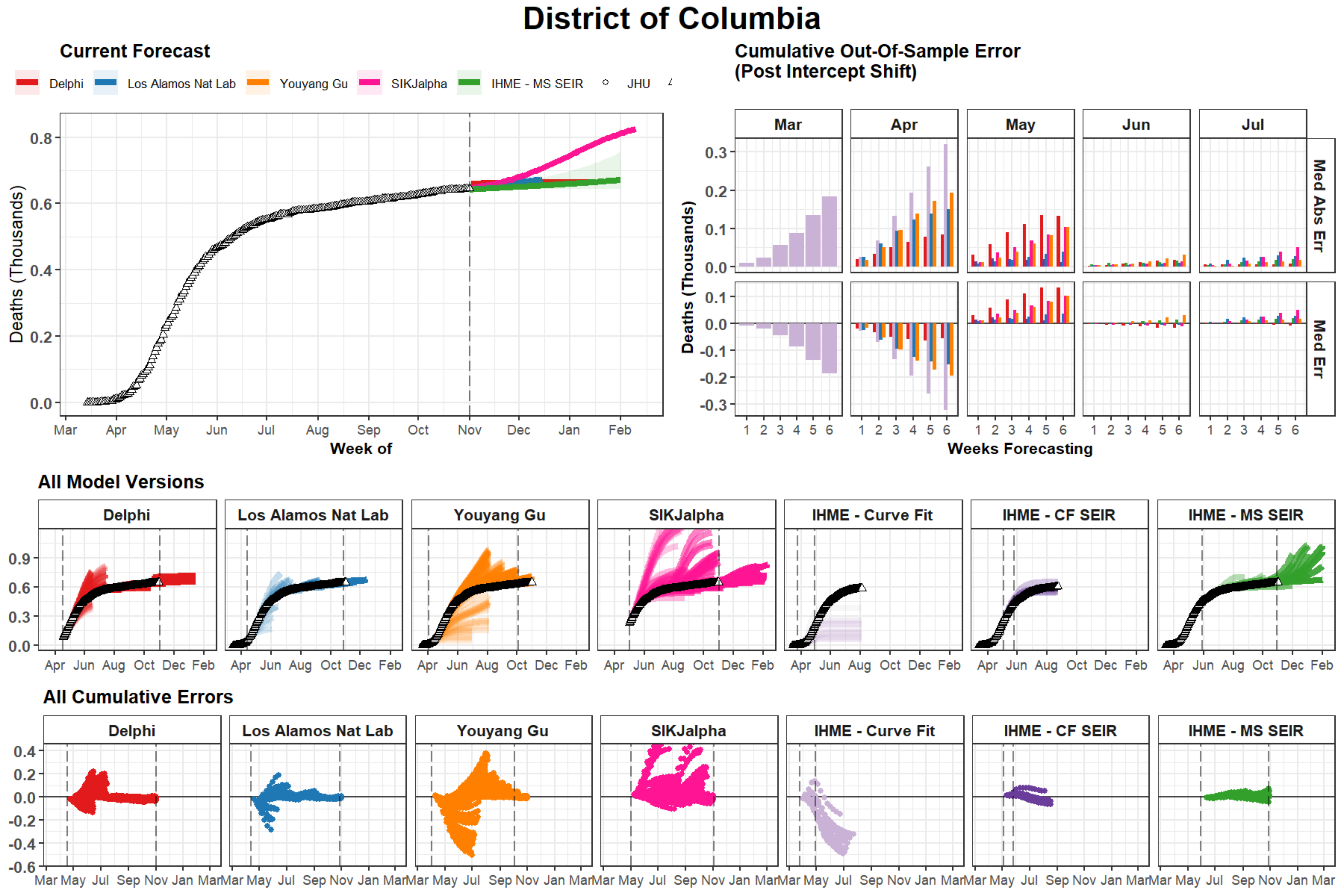
District of Columbia
Current Forecast
Delphi
Los Alamos Nat Lab
Youyang Gu
SIKJalpha
IHME - MS SEIR
JHU
Deaths (Thousands)
Week of
Cumulative Out-Of-Sample Error
(Post Intercept Shift)
Mar
Apr
May
Jun
Jul
Med Abs Err
Med Err
Weeks Forecasting
All Model Versions
Delphi
Los Alamos Nat Lab
Youyang Gu
SIKJalpha
IHME - Curve Fit
IHME - CF SEIR
IHME - MS SEIR
All Cumulative Errors

Lebanon

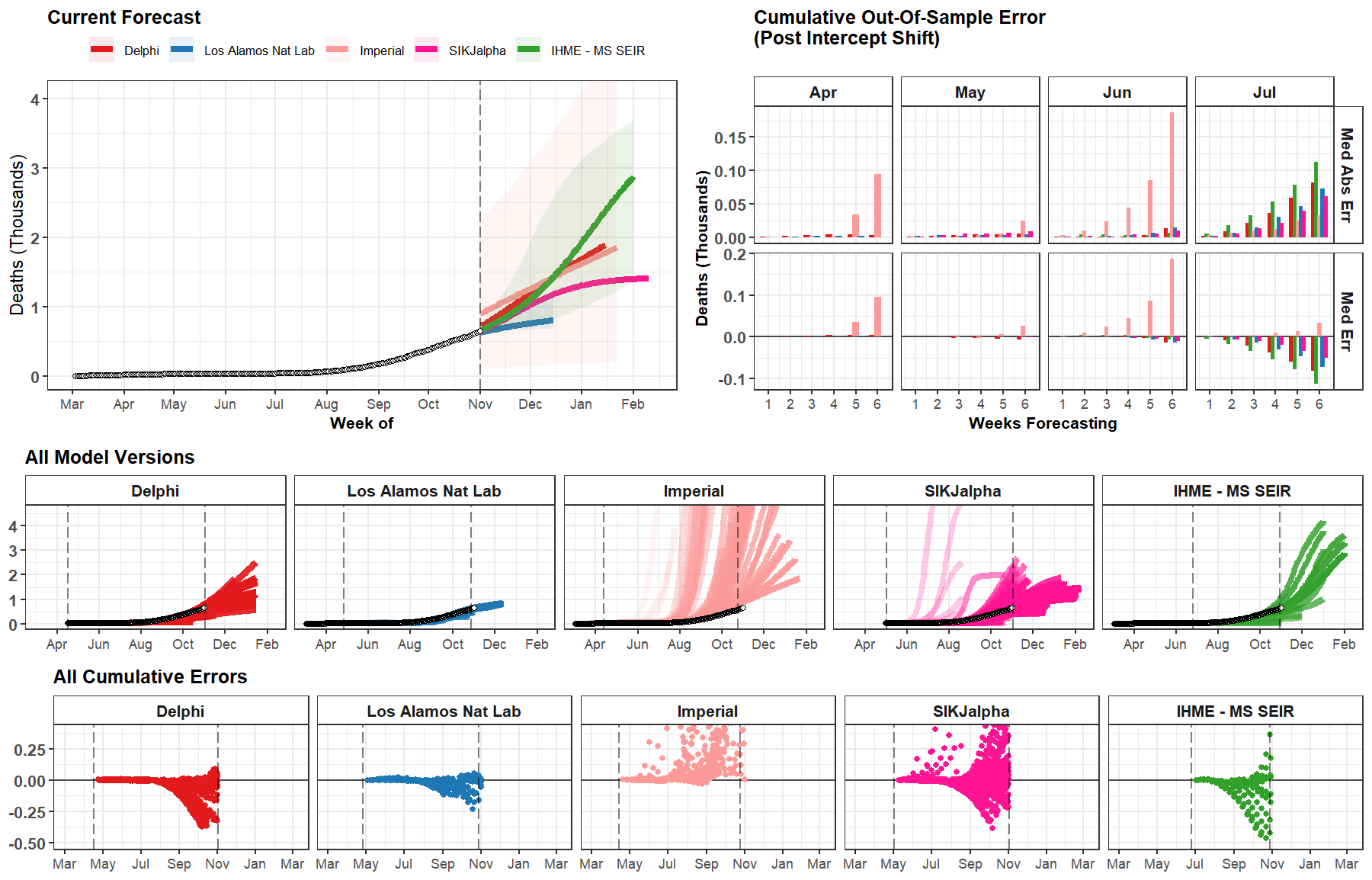

Current Forecast
Delphi
Los Alamos Nat Lab
Imperial
SIKJalpha
IHME - MS SEIR
Deaths (Thousands)
Week of
Mar
Apr
May
Jun
Jul
Aug
Sep
Oct
Nov
Dec
Jan
Feb
Cumulative Out-Of-Sample Error
(Post Intercept Shift)
Apr
May
Jun
Jul
Med Abs Err
Med Err
Weeks Forecasting
All Model Versions
All Cumulative Errors

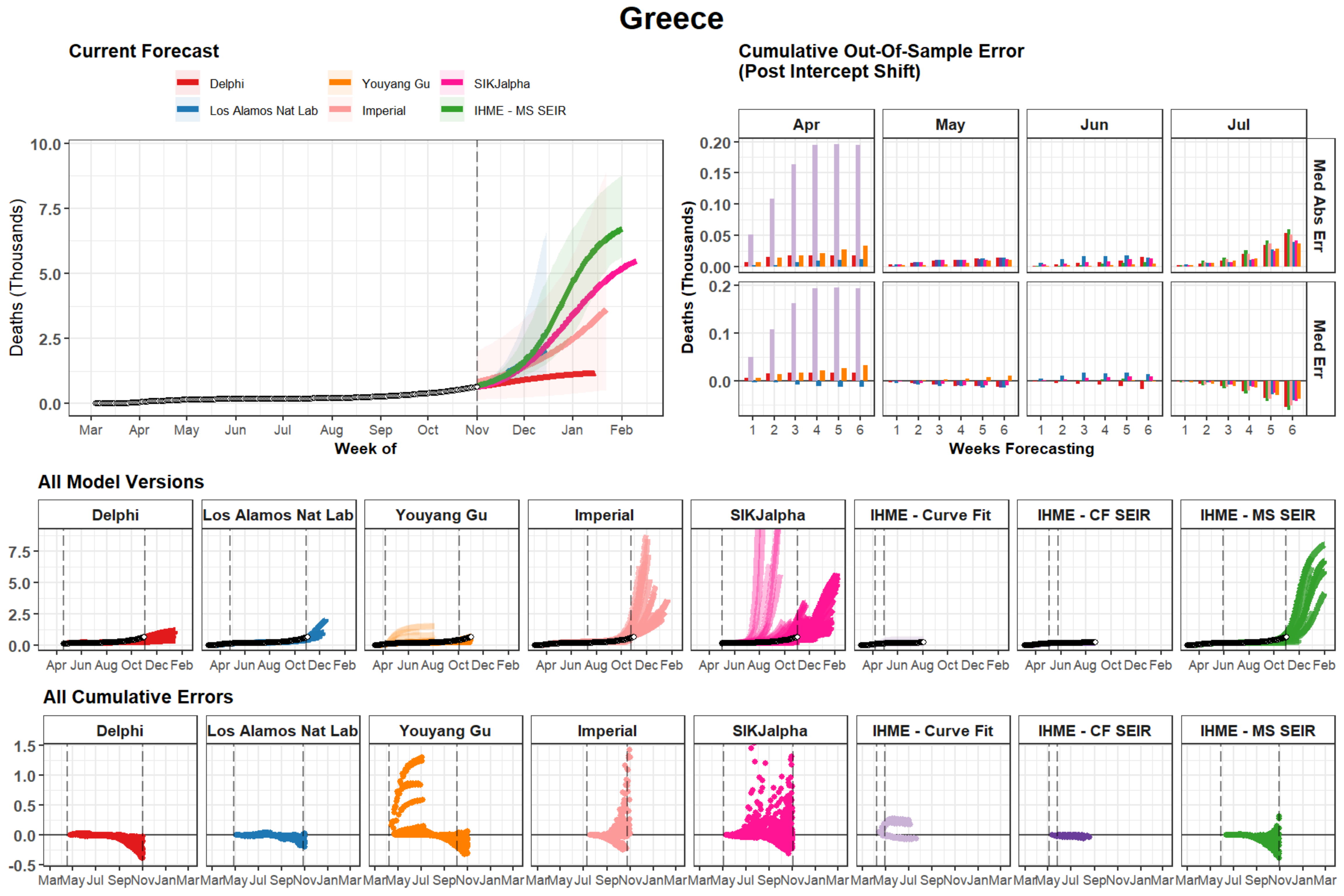

Greece
Current Forecast
Delphi
Los Alamos Nat Lab
Youyang Gu
Imperial
SIKJalpha
IHME - MS SEIR
Deaths (Thousands)
Week of
Cumulative Out-Of-Sample Error
(Post Intercept Shift)
Apr
May
Jun
Jul
Med Abs Err
Med Err
Weeks Forecasting
All Model Versions
Delphi
Los Alamos Nat Lab
Youyang Gu
Imperial
SIKJalpha
IHME - Curve Fit
IHME - CF SEIR
IHME - MS SEIR
All Cumulative Errors

Idaho

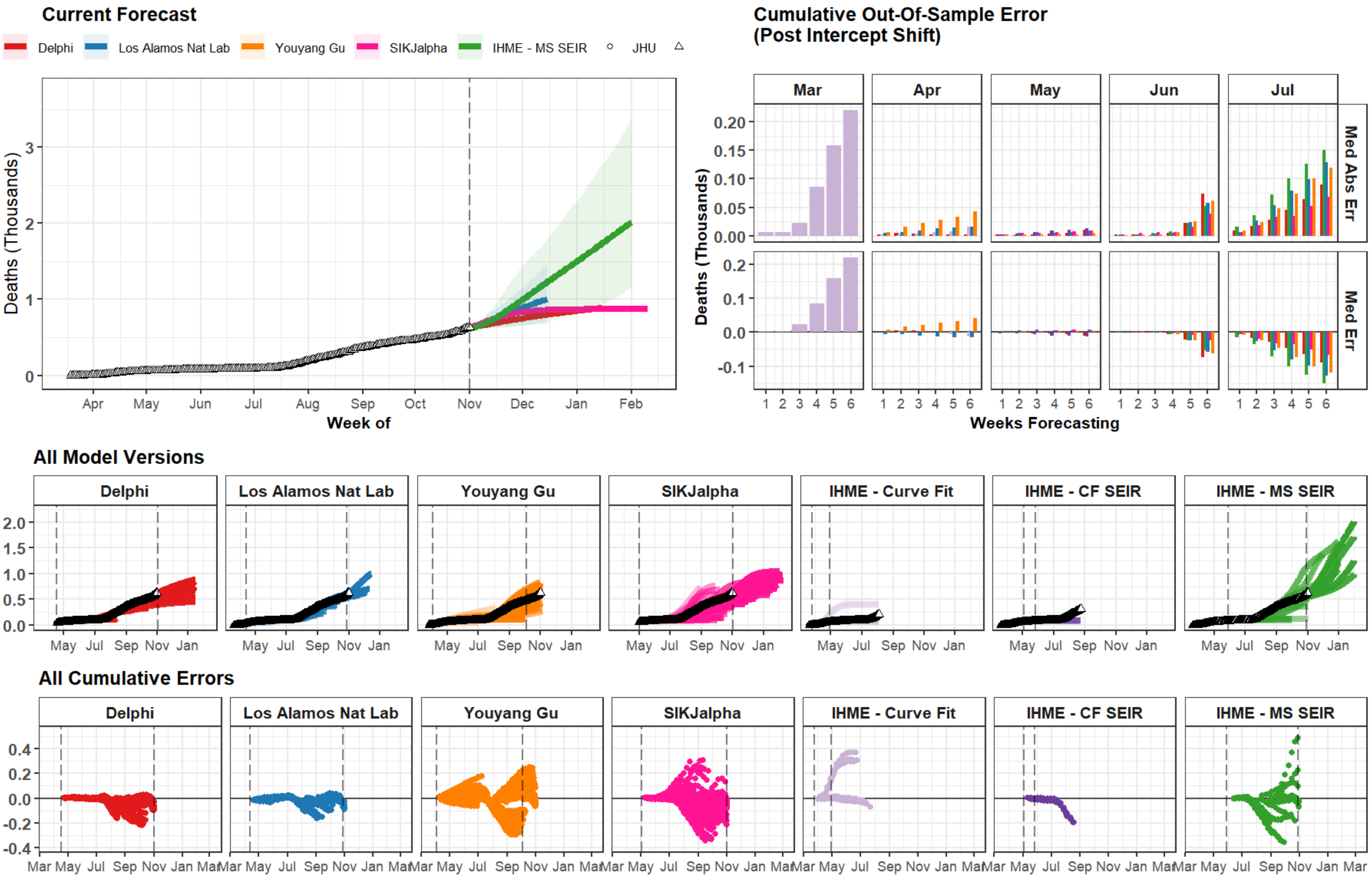

Current Forecast
Delphi
Los Alamos Nat Lab
Youyang Gu
SIKJalpha
IHME - MS SEIR
JHU
Deaths (Thousands)
Week of
Apr May Jun Jul Aug Sep Oct Nov Dec Jan Feb
Cumulative Out-Of-Sample Error
(Post Intercept Shift)
Mar
Apr
May
Jun
Jul
Med Abs Err
Med Err
Weeks Forecasting
All Model Versions
Delphi
Los Alamos Nat Lab
Youyang Gu
SIKJalpha
IHME - Curve Fit
IHME - CF SEIR
IHME - MS SEIR
All Cumulative Errors

Utah

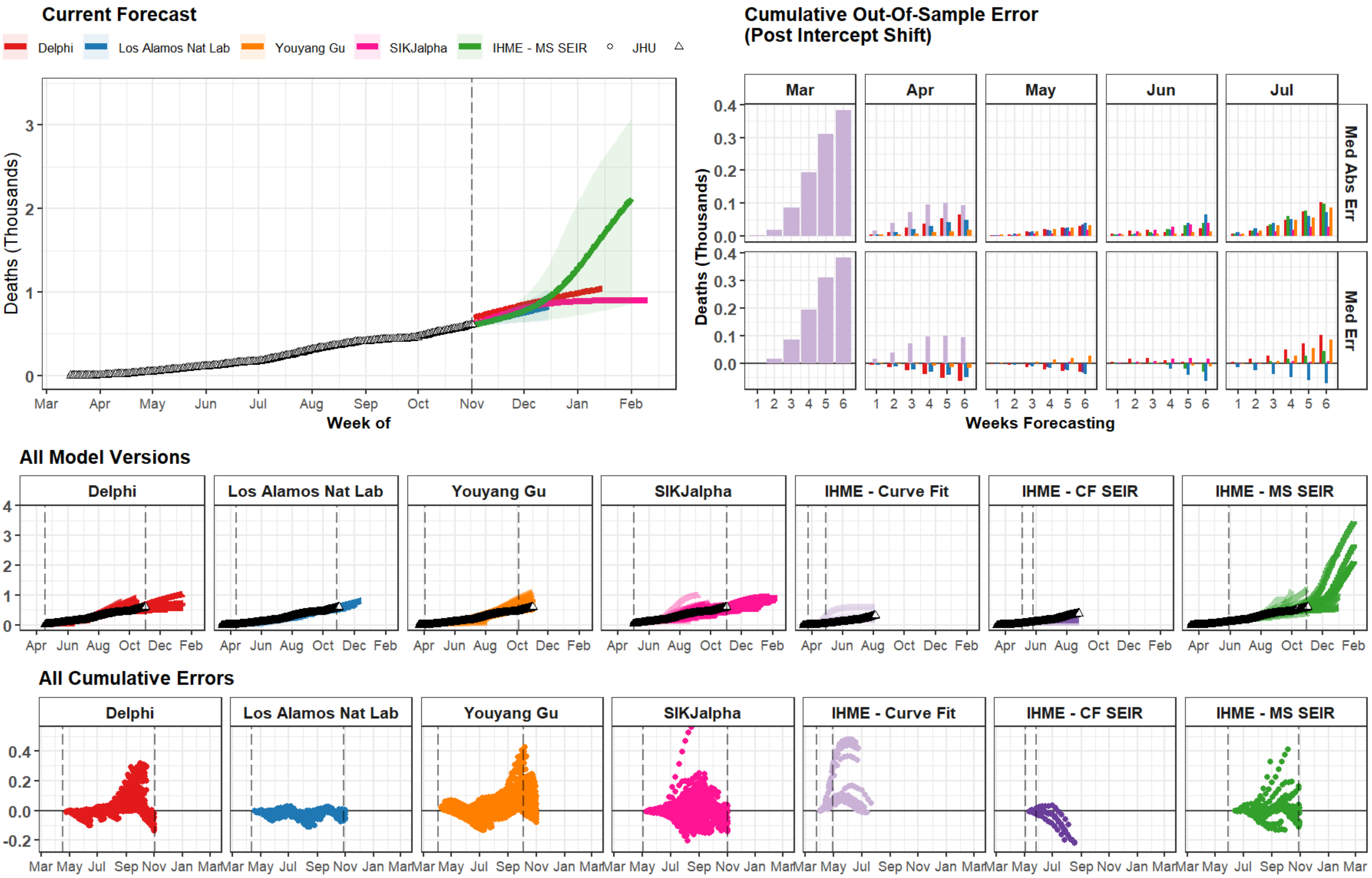

Current Forecast
Delphi
Los Alamos Nat Lab
Youyang Gu
SIKJalpha
IHME - MS SEIR
JHU
Deaths (Thousands)
Week of
Mar
Apr
May
Jun
Jul
Aug
Sep
Oct
Nov
Dec
Jan
Feb
Cumulative Out-Of-Sample Error
(Post Intercept Shift)
Mar
Apr
May
Jun
Jul
Med Abs Err
Med Err
Deaths (Thousands)
Weeks Forecasting
All Model Versions
Delphi
Los Alamos Nat Lab
Youyang Gu
SIKJalpha
IHME - Curve Fit
IHME - CF SEIR
IHME - MS SEIR
All Cumulative Errors
Delphi
Los Alamos Nat Lab
Youyang Gu
SIKJalpha
IHME - Curve Fit
IHME - CF SEIR
IHME - MS SEIR

Yemen

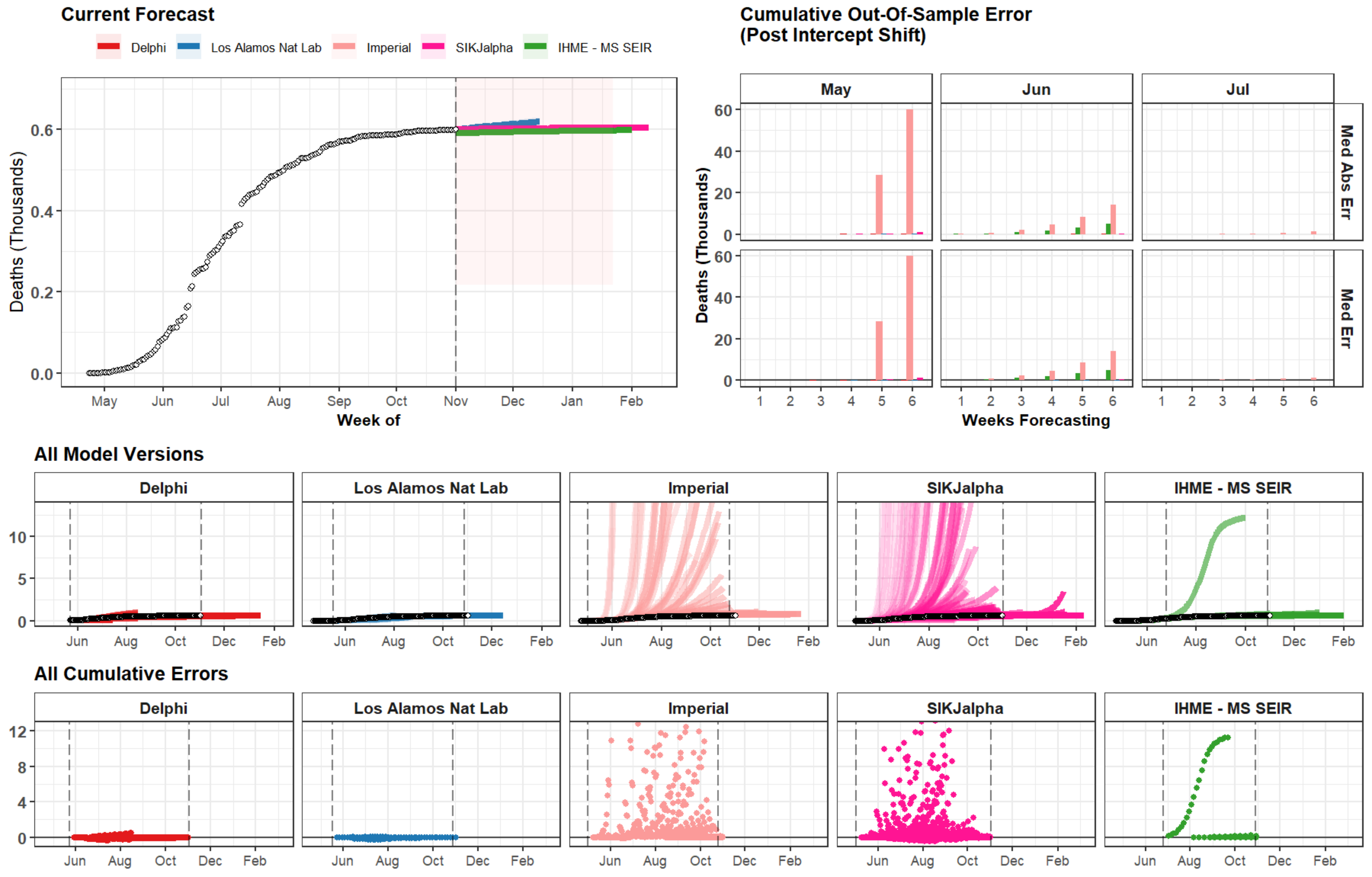

Current Forecast
Delphi
Los Alamos Nat Lab
Imperial
SIKJalpha
IHME - MS SEIR
Deaths (Thousands)
0.0
0.2
0.4
0.6
May
Jun
Jul
Aug
Sep
Oct
Nov
Dec
Jan
Feb
Week of
Cumulative Out-Of-Sample Error
(Post Intercept Shift)
May
Jun
Jul
Med Abs Err
Med Err
Deaths (Thousands)
0
20
40
60
1
2
3
4
5
6
Weeks Forecasting
All Model Versions
Delphi
Los Alamos Nat Lab
Imperial
SIKJalpha
IHME - MS SEIR
0
5
10
Jun
Aug
Oct
Dec
Feb
All Cumulative Errors
Delphi
Los Alamos Nat Lab
Imperial
SIKJalpha
IHME - MS SEIR
0
4
8
12
Jun
Aug
Oct
Dec
Feb

Uzbekistan

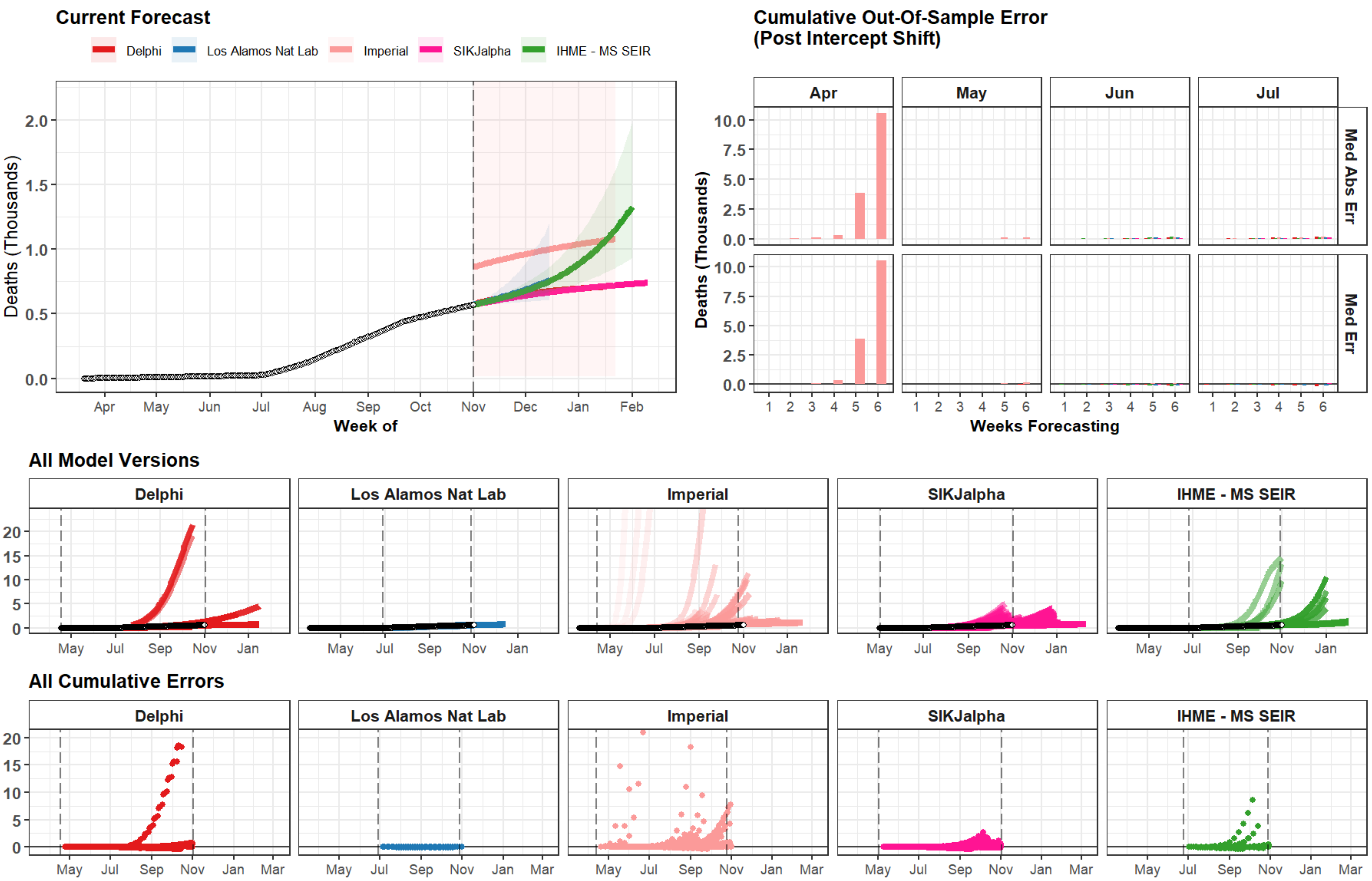

Current Forecast
Delphi
Los Alamos Nat Lab
Imperial
SIKJalpha
IHME - MS SEIR
Deaths (Thousands)
Week of
Apr
May
Jun
Jul
Aug
Sep
Oct
Nov
Dec
Jan
Feb
Cumulative Out-Of-Sample Error
(Post Intercept Shift)
Med Abs Err
Med Err
Weeks Forecasting
All Model Versions
All Cumulative Errors
Mar

Croatia

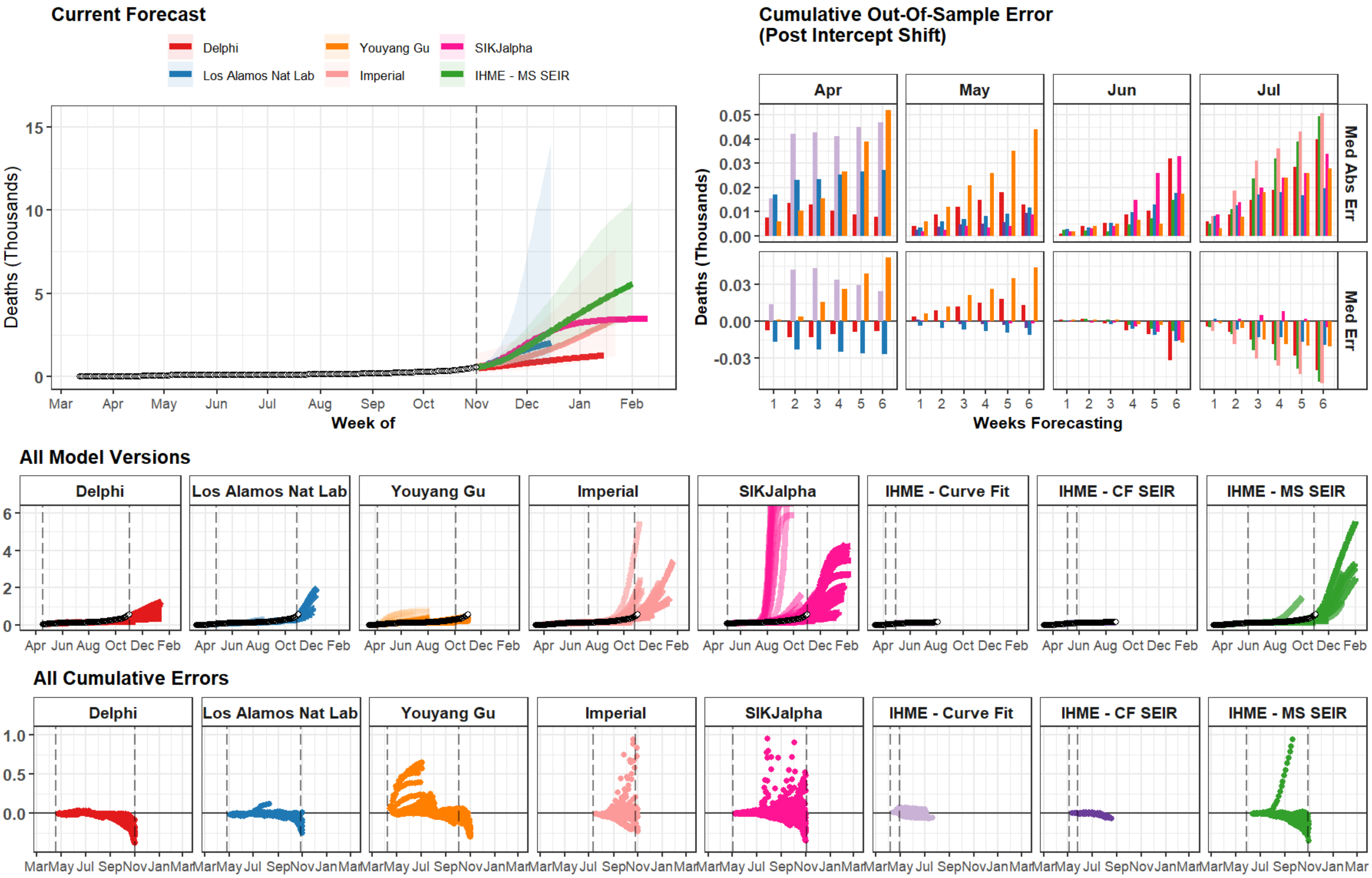
Current Forecast
Delphi
Los Alamos Nat Lab
Youyang Gu
Imperial
SIKJalpha
IHME - MS SEIR
Deaths (Thousands)
Week of
Mar Apr May Jun Jul Aug Sep Oct Nov Dec Jan Feb
Cumulative Out-Of-Sample Error
(Post Intercept Shift)
Apr
May
Jun
Jul
Med Abs Err
Med Err
Weeks Forecasting
All Model Versions
Delphi
Los Alamos Nat Lab
Youyang Gu
Imperial
SIKJalpha
IHME - Curve Fit
IHME - CF SEIR
IHME - MS SEIR
All Cumulative Errors

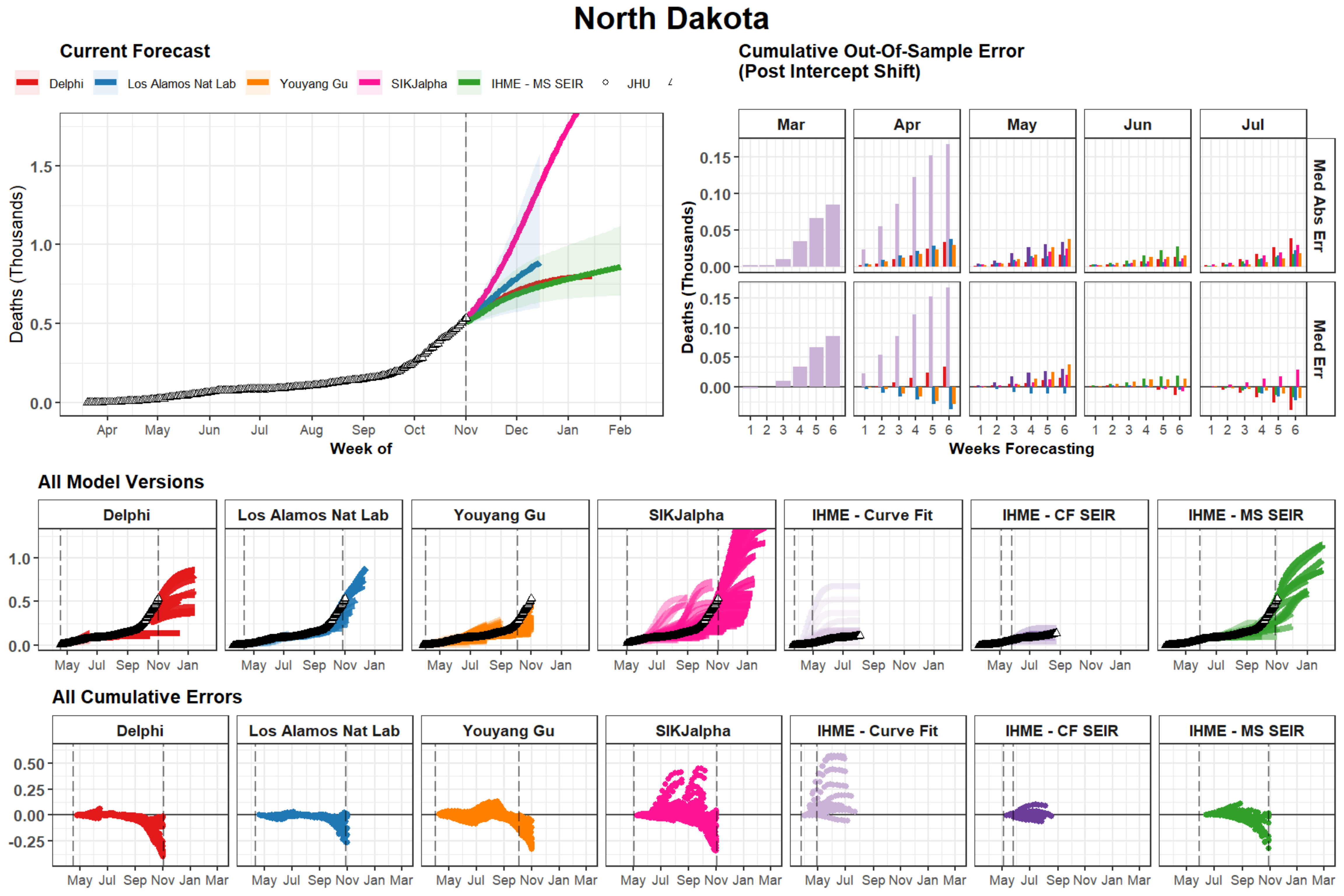
North Dakota
Current Forecast
Delphi
Los Alamos Nat Lab
Youyang Gu
SIKJalpha
IHME - MS SEIR
JHU
Deaths (Thousands)
Week of
Apr May Jun Jul Aug Sep Oct Nov Dec Jan Feb
Cumulative Out-Of-Sample Error
(Post Intercept Shift)
Mar Apr May Jun Jul
Med Abs Err
Med Err
Weeks Forecasting
All Model Versions
Delphi
Los Alamos Nat Lab
Youyang Gu
SIKJalpha
IHME - Curve Fit
IHME - CF SEIR
IHME - MS SEIR
All Cumulative Errors

Albania

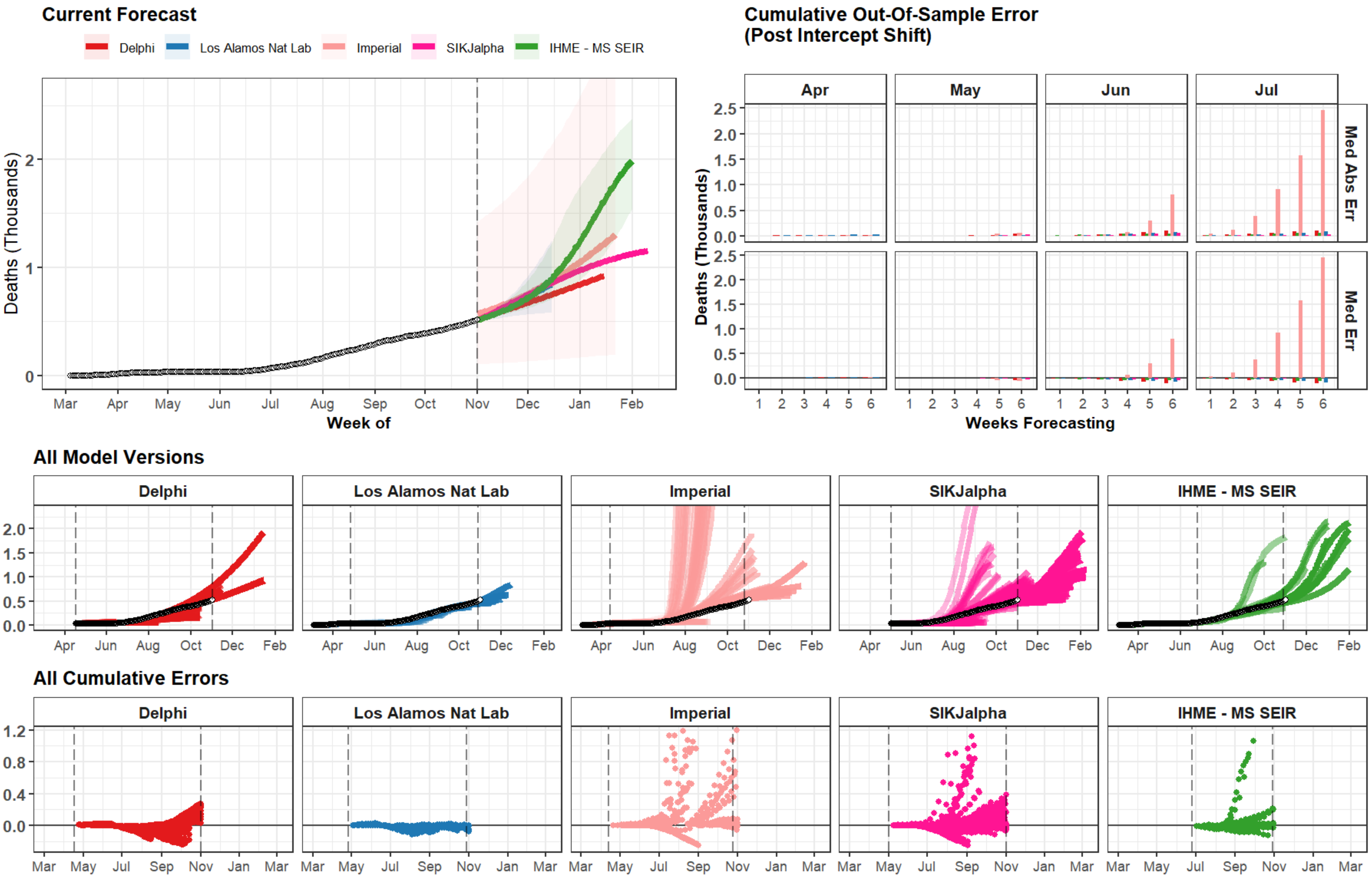

Current Forecast
Delphi
Los Alamos Nat Lab
Imperial
SIKJalpha
IHME - MS SEIR
Deaths (Thousands)
Week of
Mar Apr May Jun Jul Aug Sep Oct Nov Dec Jan Feb
Cumulative Out-Of-Sample Error
(Post Intercept Shift)
Apr May Jun Jul
Med Abs Err
Med Err
Weeks Forecasting
All Model Versions
All Cumulative Errors
Delphi
Los Alamos Nat Lab
Imperial
SIKJalpha
IHME - MS SEIR

United Arab Emirates

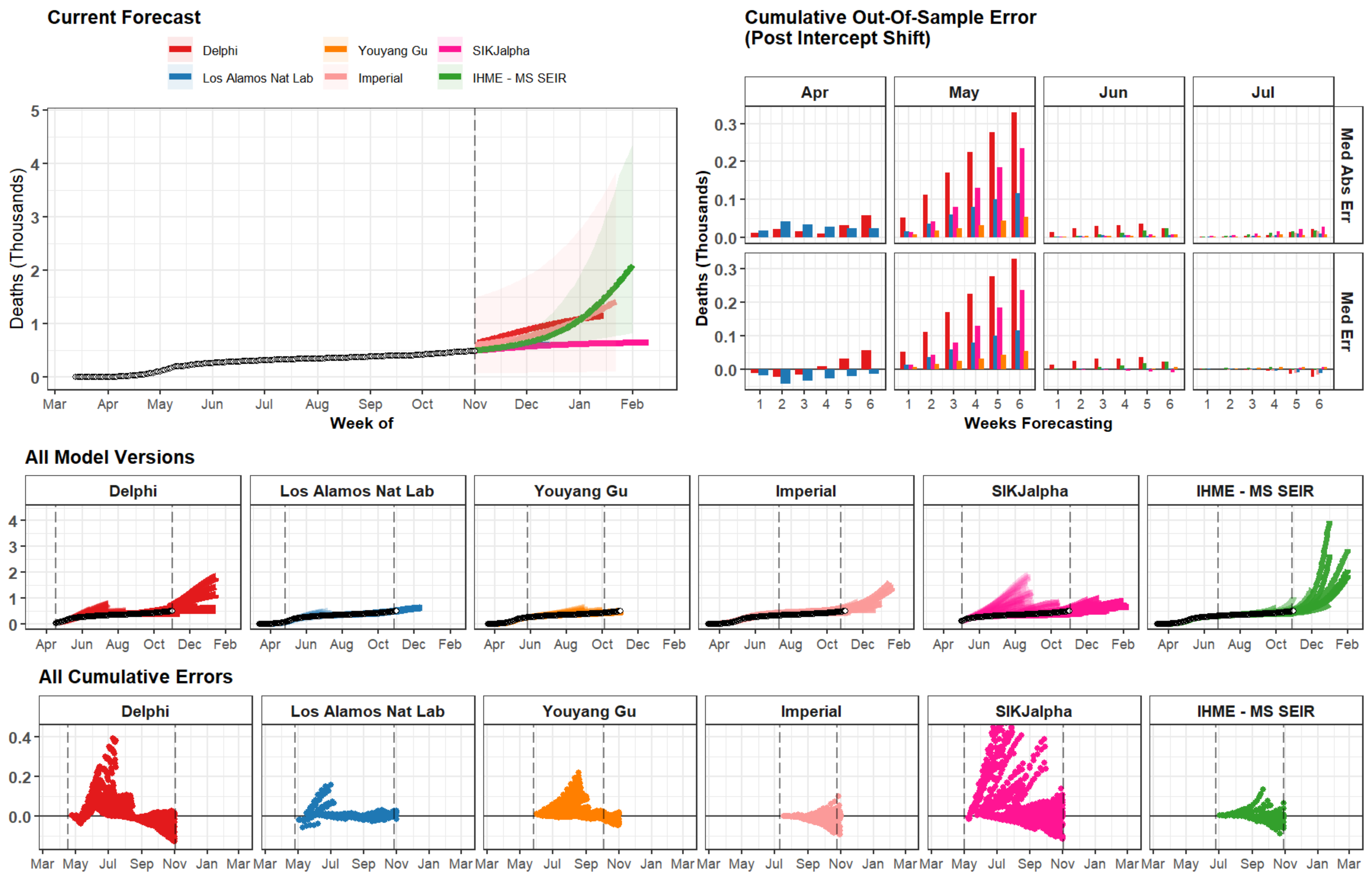

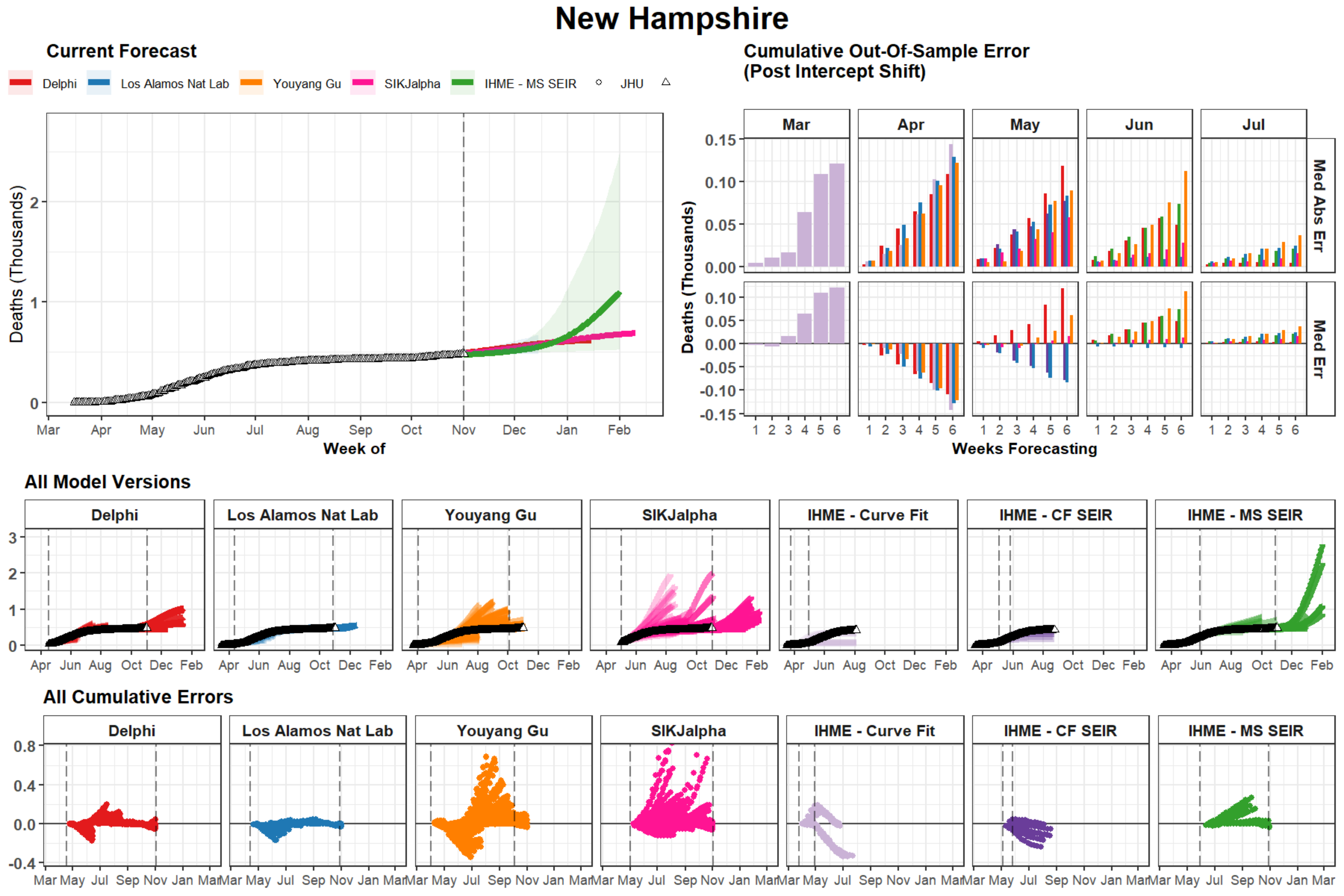
New Hampshire
Current Forecast
Delphi
Los Alamos Nat Lab
Youyang Gu
SIKJalpha
IHME - MS SEIR
JHU
Deaths (Thousands)
Week of
Mar Apr May Jun Jul Aug Sep Oct Nov Dec Jan Feb
Cumulative Out-Of-Sample Error
(Post Intercept Shift)
Mar Apr May Jun Jul
Med Abs Err
Med Err
Weeks Forecasting
All Model Versions
Delphi
Los Alamos Nat Lab
Youyang Gu
SIKJalpha
IHME - Curve Fit
IHME - CF SEIR
IHME - MS SEIR
All Cumulative Errors
Delphi
Los Alamos Nat Lab
Youyang Gu
SIKJalpha
IHME - Curve Fit
IHME - CF SEIR
IHME - MS SEIR

South Korea

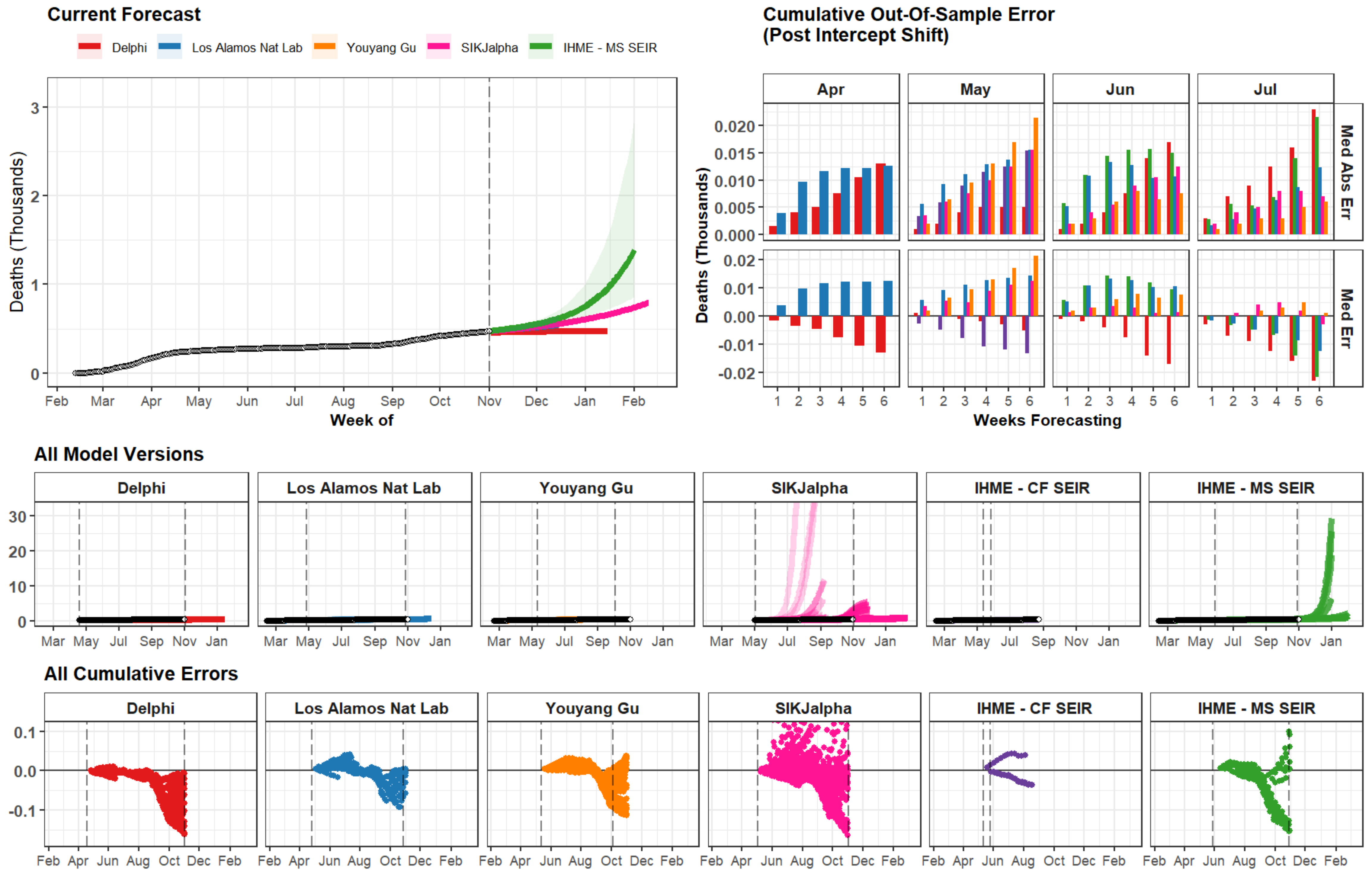

Current Forecast
Delphi
Los Alamos Nat Lab
Youyang Gu
SIKJalpha
IHME - MS SEIR
Deaths (Thousands)
3
2
1
0
Feb Mar Apr May Jun Jul Aug Sep Oct Nov Dec Jan Feb
Week of
Cumulative Out-Of-Sample Error
(Post Intercept Shift)
Apr
May
Jun
Jul
Med Abs Err
Med Err
0.020
0.015
0.010
0.005
0.000
0.02
0.01
0.00
-0.01
-0.02
1 2 3 4 5 6
Weeks Forecasting
All Model Versions
Delphi
Los Alamos Nat Lab
Youyang Gu
SIKJalpha
IHME - CF SEIR
IHME - MS SEIR
30
20
10
0
Mar May Jul Sep Nov Jan
All Cumulative Errors
Delphi
Los Alamos Nat Lab
Youyang Gu
SIKJalpha
IHME - CF SEIR
IHME - MS SEIR
0.1
0.0
-0.1
Feb Apr Jun Aug Oct Dec Feb

West Virginia

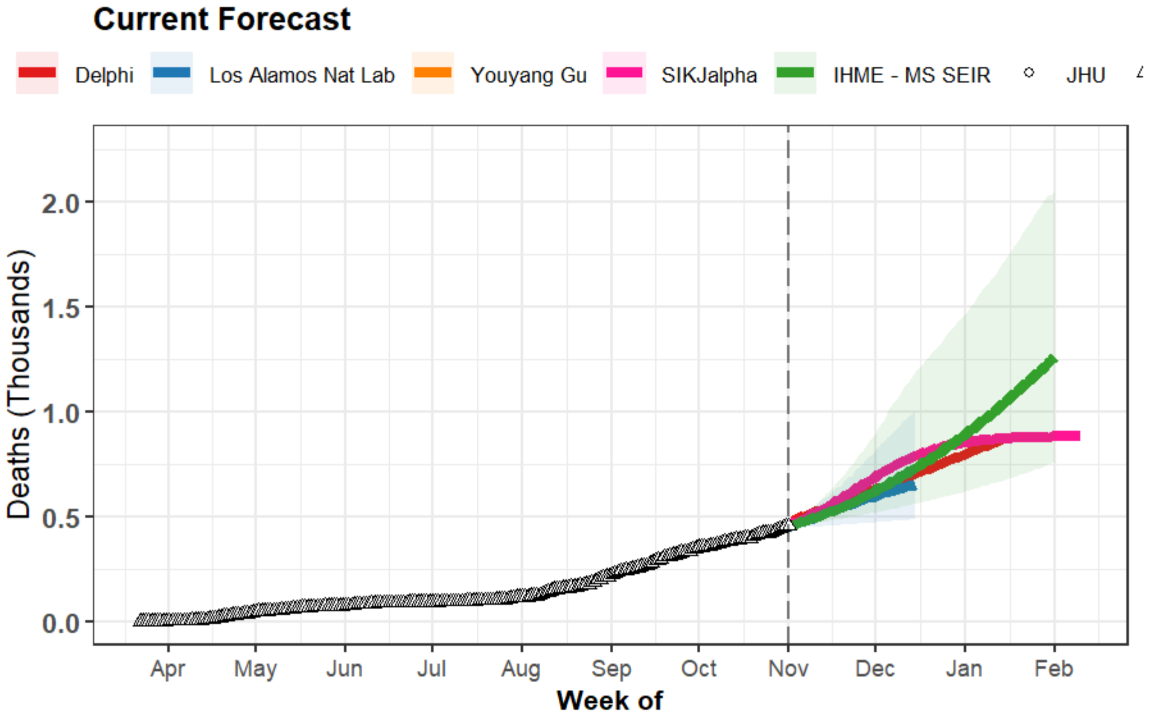

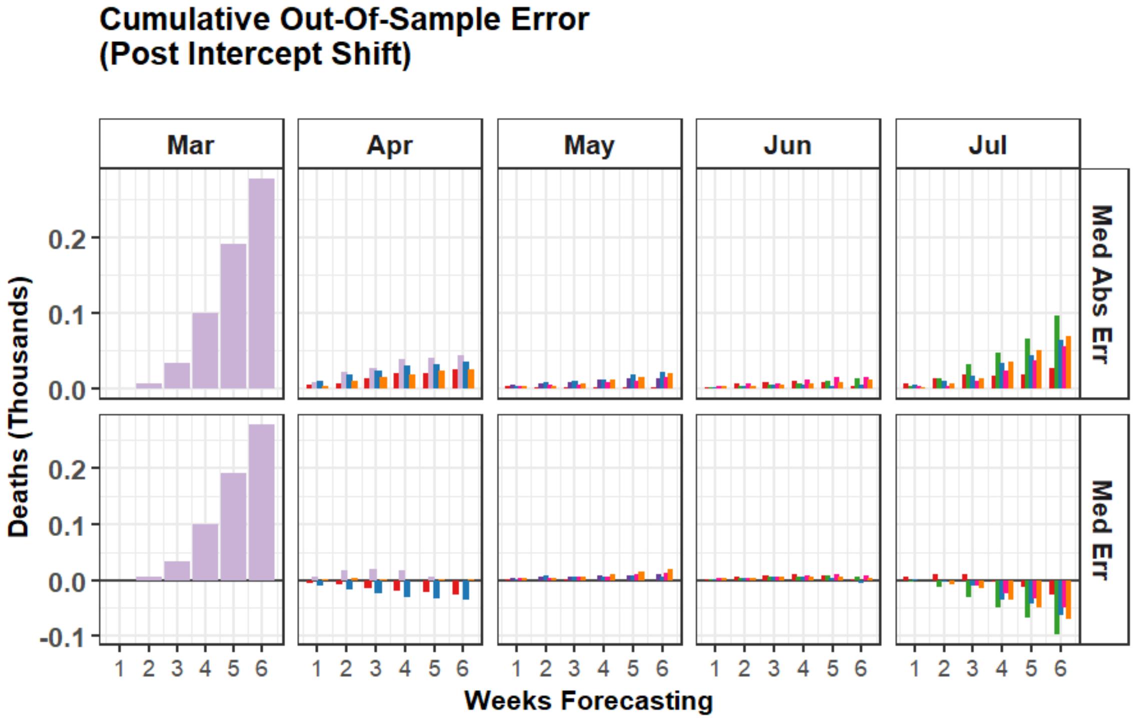

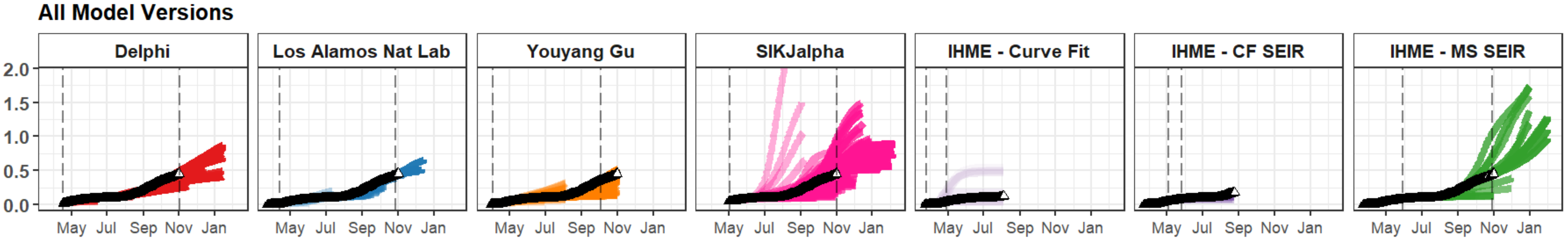

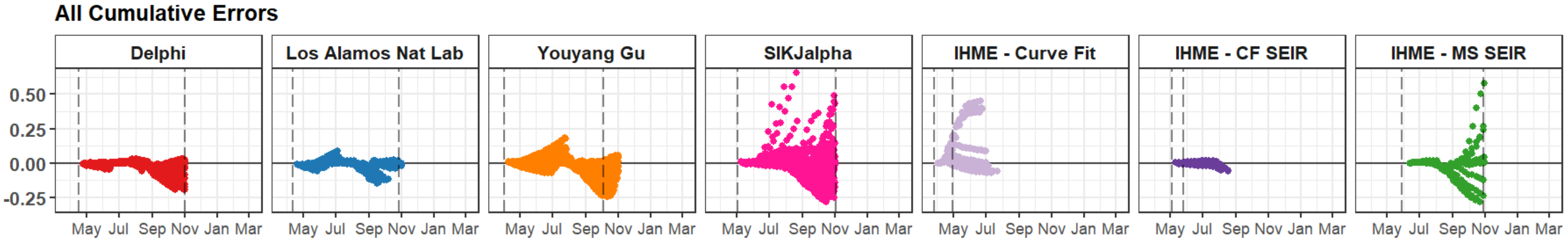

South Dakota

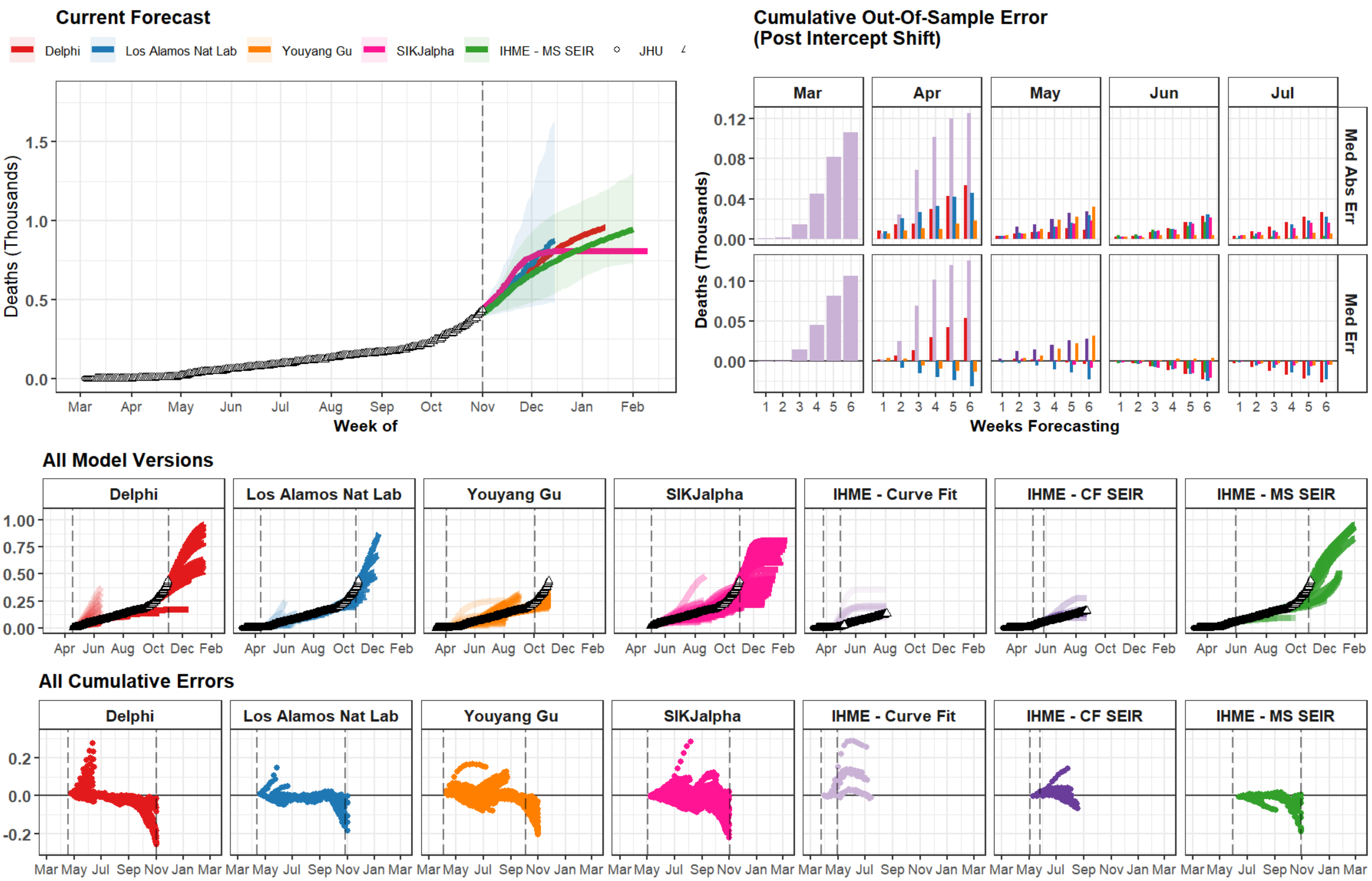

Cameroon

Current Forecast

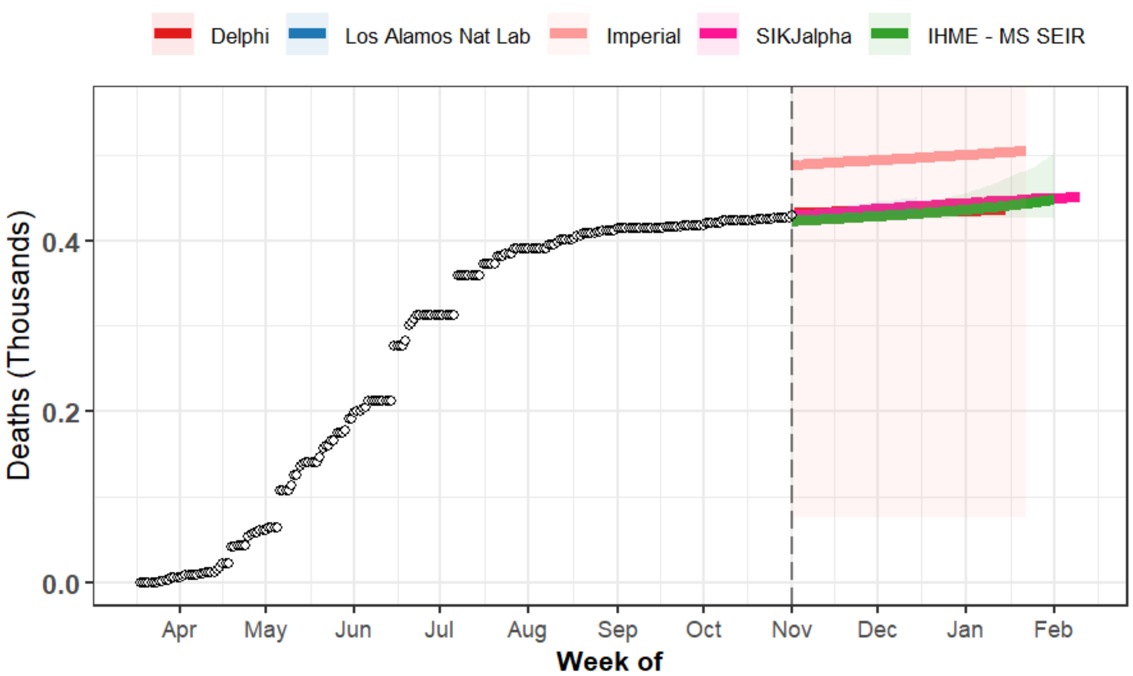

Cumulative Out-Of-Sample Error (Post Intercept Shift)

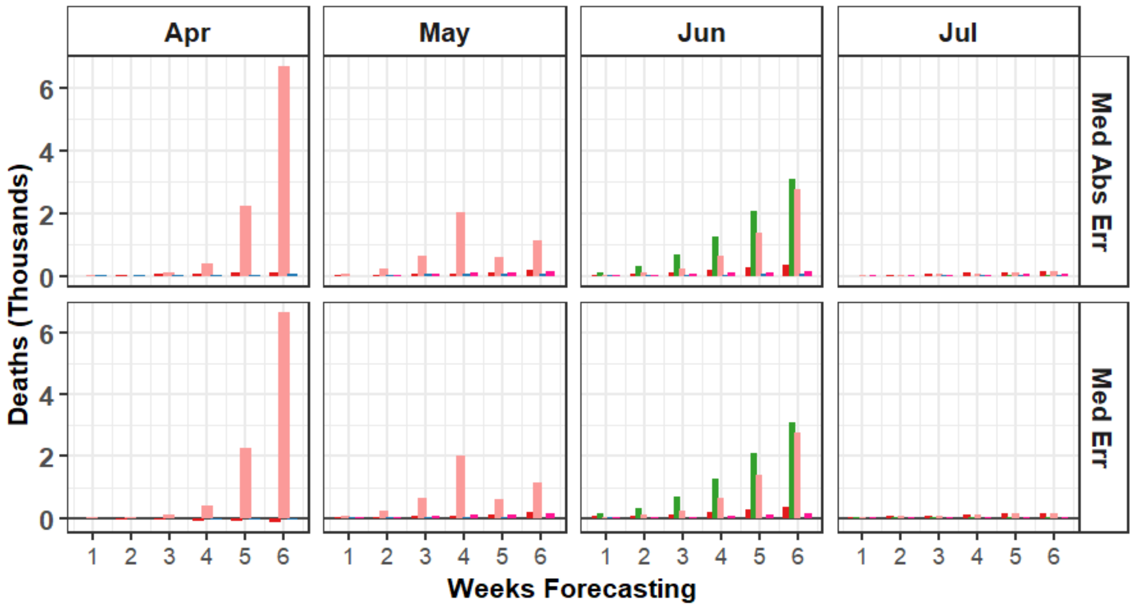

All Model Versions

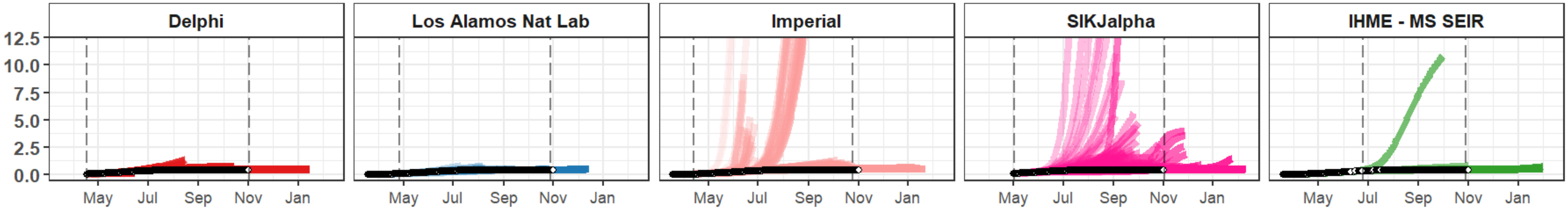

All Cumulative Errors

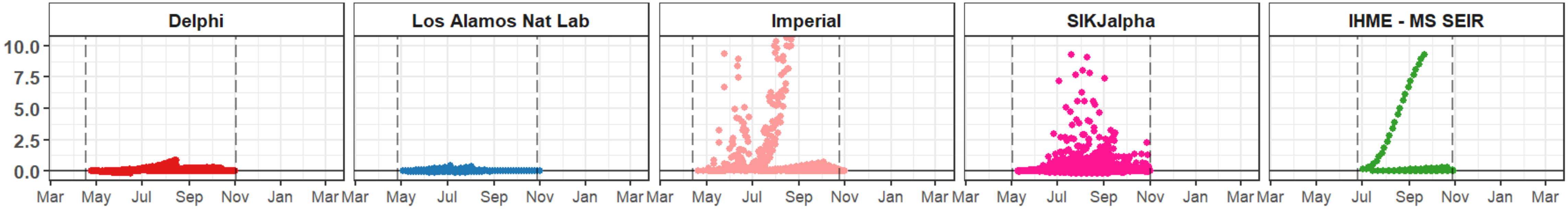

Montana

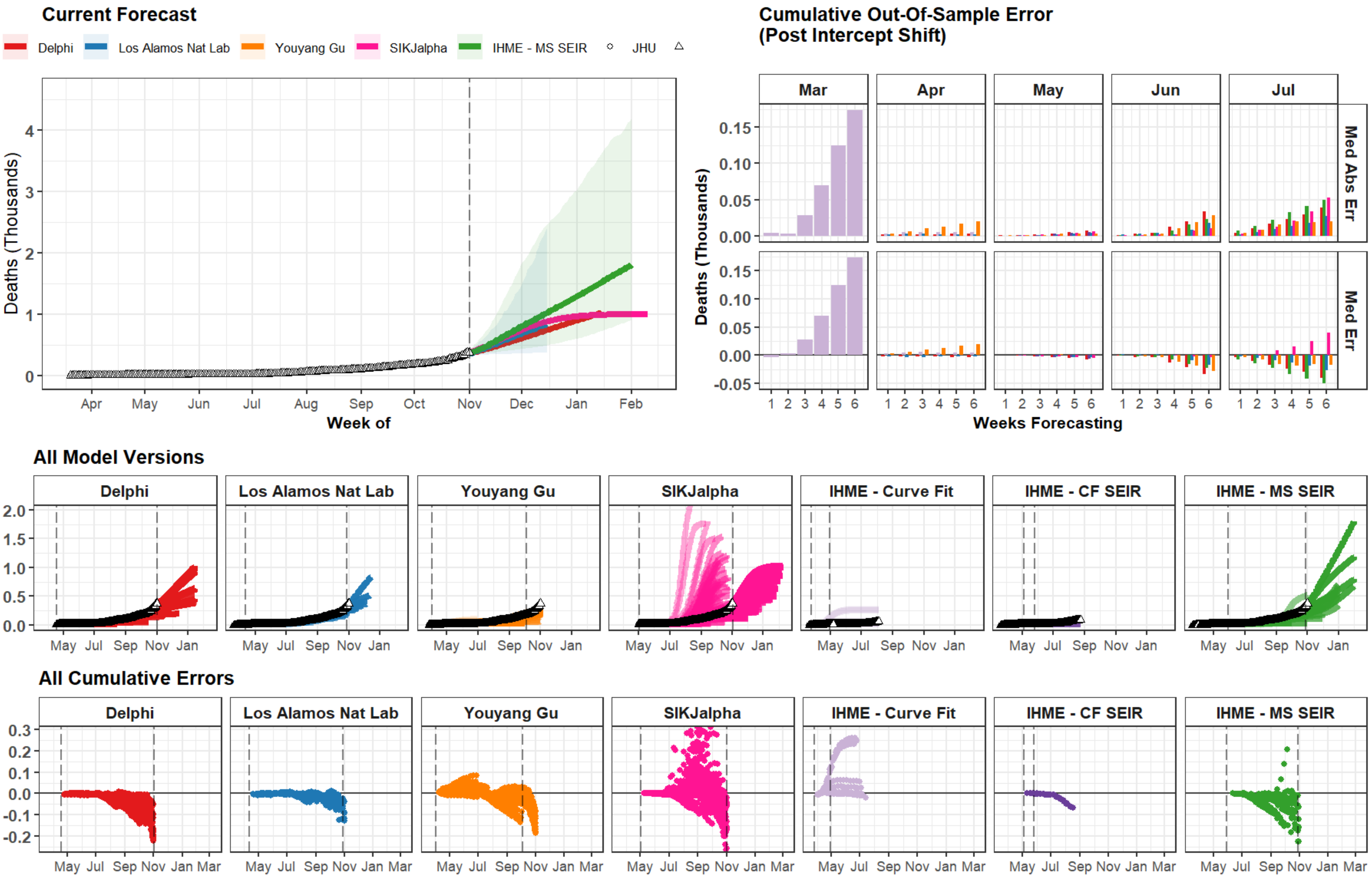
Current Forecast
Delphi
Los Alamos Nat Lab
Youyang Gu
SIKJalpha
IHME - MS SEIR
JHU
Deaths (Thousands)
Week of
Apr May Jun Jul Aug Sep Oct Nov Dec Jan Feb
Cumulative Out-Of-Sample Error (Post Intercept Shift)
Mar Apr May Jun Jul
Med Abs Err
Med Err
Weeks Forecasting
All Model Versions
Delphi
Los Alamos Nat Lab
Youyang Gu
SIKJalpha
IHME - Curve Fit
IHME - CF SEIR
IHME - MS SEIR
May Jul Sep Nov Jan
All Cumulative Errors
May Jul Sep Nov Jan Mar

Slovenia

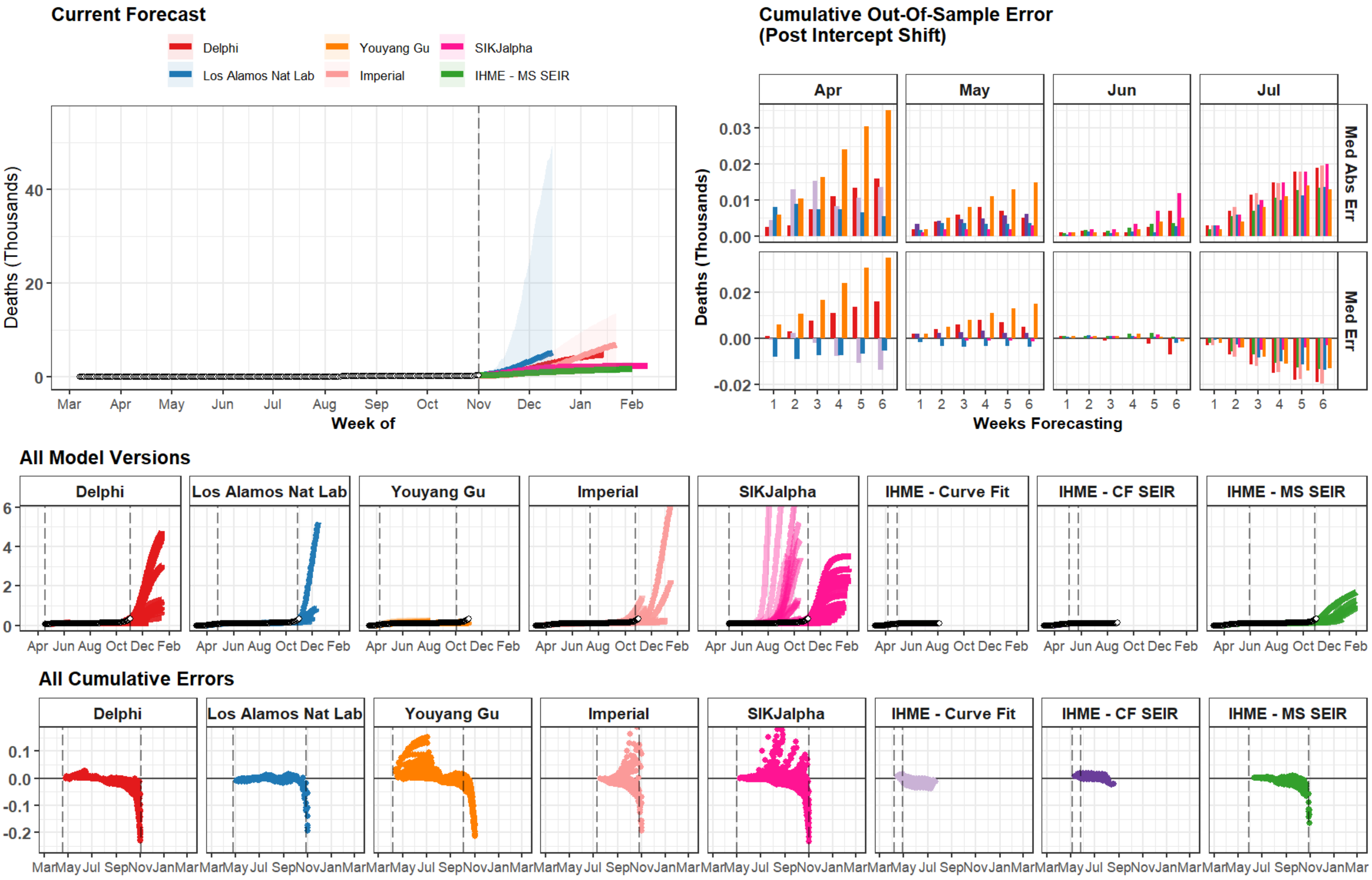

Current Forecast
Delphi
Los Alamos Nat Lab
Youyang Gu
Imperial
SIKJalpha
IHME - MS SEIR
Deaths (Thousands)
0
20
40
Mar
Apr
May
Jun
Jul
Aug
Sep
Oct
Nov
Dec
Jan
Feb
Week of
Cumulative Out-Of-Sample Error
(Post Intercept Shift)
Apr
May
Jun
Jul
Med Abs Err
Med Err
0.00
0.01
0.02
0.03
-0.02
1
2
3
4
5
6
Weeks Forecasting
All Model Versions
Delphi
Los Alamos Nat Lab
Youyang Gu
Imperial
SIKJalpha
IHME - Curve Fit
IHME - CF SEIR
IHME - MS SEIR
0
2
4
6
Apr Jun Aug Oct Dec Feb
All Cumulative Errors
0.1
0.0
-0.1
-0.2
Mar May Jul Sep Nov Jan Mar

Finland

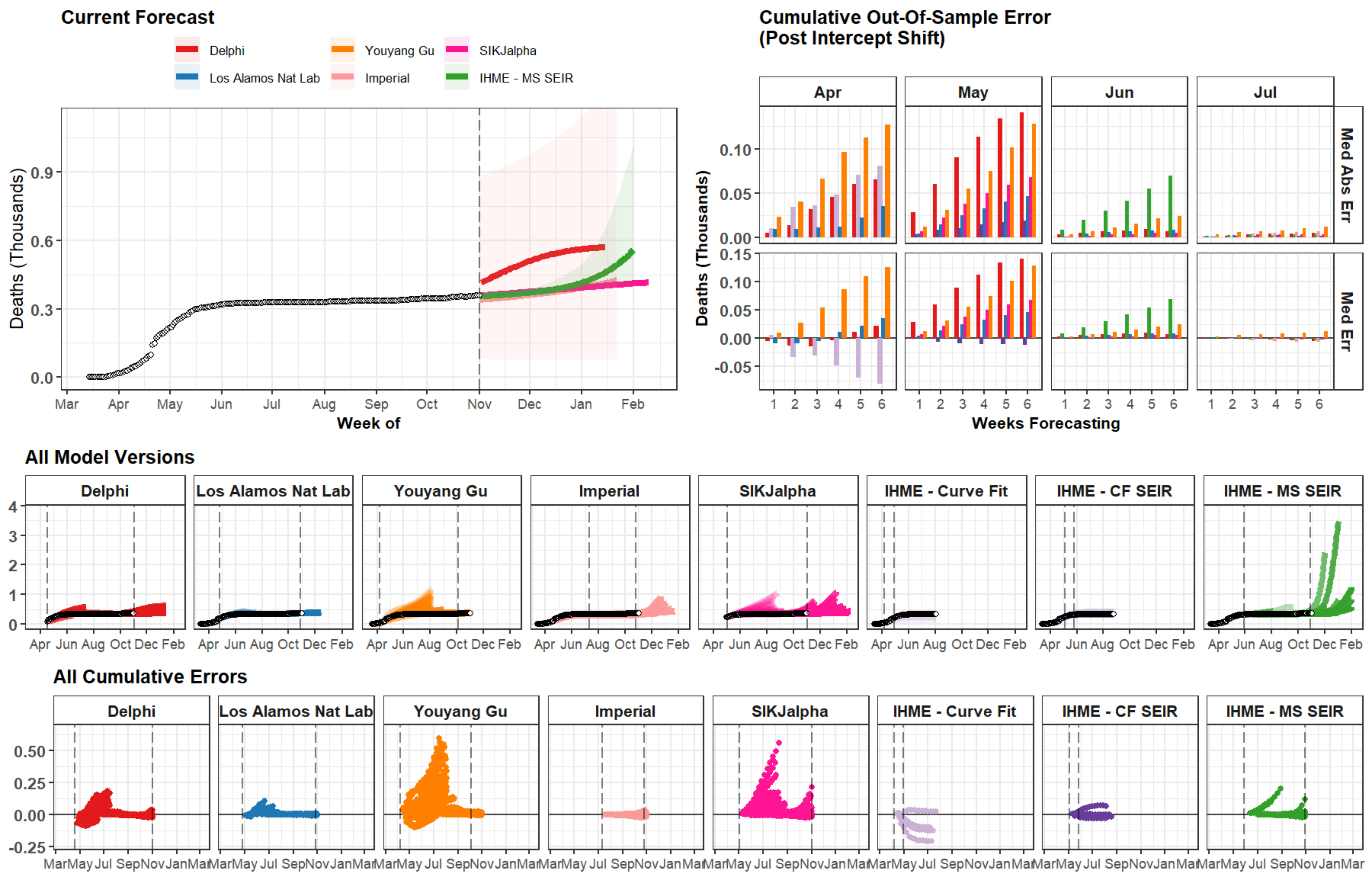
Current Forecast
Delphi
Youyang Gu
SIKJalpha
Los Alamos Nat Lab
Imperial
IHME - MS SEIR
Deaths (Thousands)
Week of
Cumulative Out-Of-Sample Error
(Post Intercept Shift)
Apr
May
Jun
Jul
Med Abs Err
Med Err
Weeks Forecasting
All Model Versions
Delphi
Los Alamos Nat Lab
Youyang Gu
Imperial
SIKJalpha
IHME - Curve Fit
IHME - CF SEIR
IHME - MS SEIR
All Cumulative Errors

Zambia

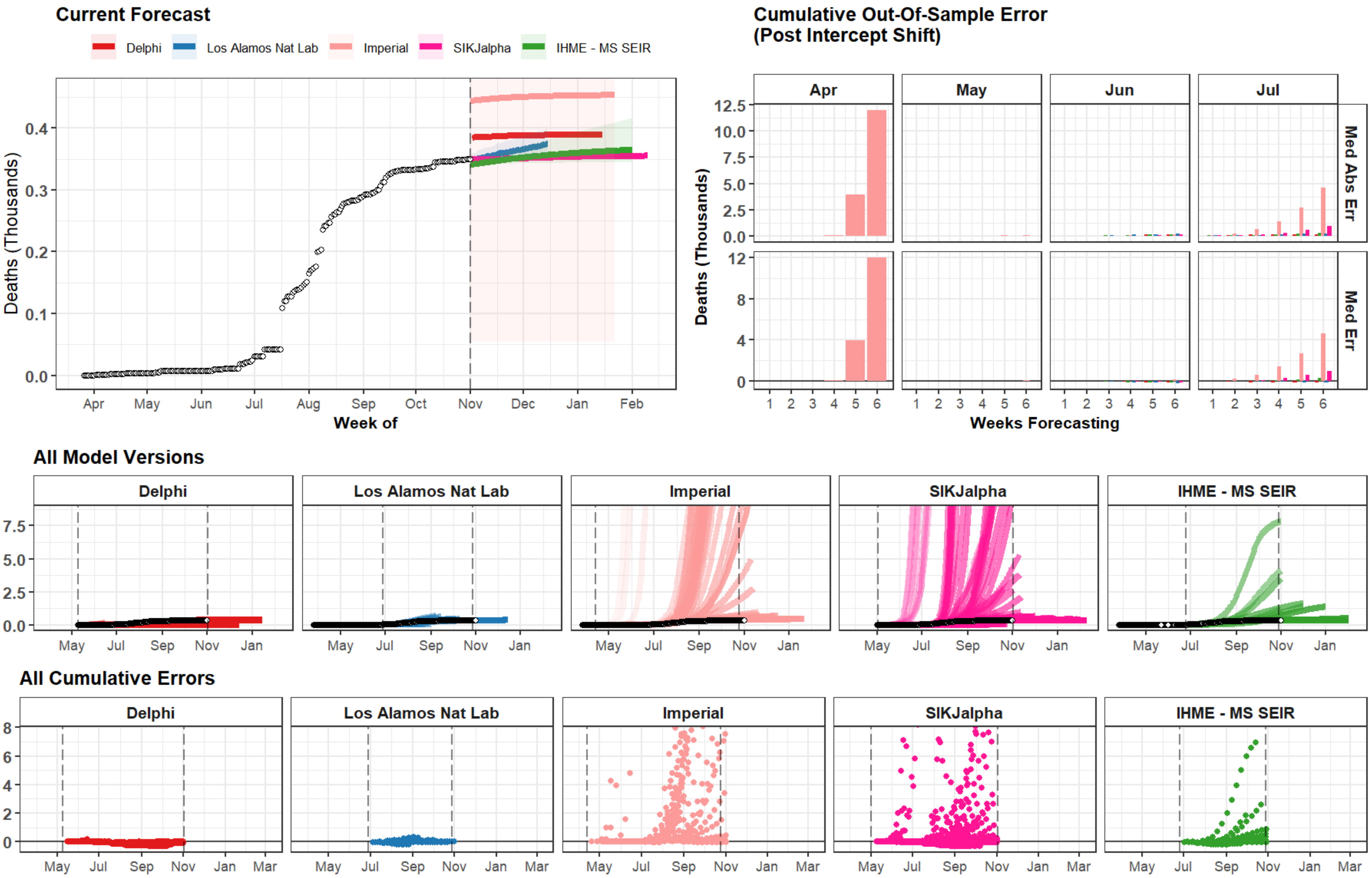

Current Forecast
Delphi
Los Alamos Nat Lab
Imperial
SIKJalpha
IHME - MS SEIR
Deaths (Thousands)
0.0
0.1
0.2
0.3
0.4
Apr
May
Jun
Jul
Aug
Sep
Oct
Nov
Dec
Jan
Feb
Week of
Cumulative Out-Of-Sample Error
(Post Intercept Shift)
Apr
May
Jun
Jul
Med Abs Err
Med Err
Deaths (Thousands)
0.0
2.5
5.0
7.5
10.0
12.5
0
4
8
12
1
2
3
4
5
6
Weeks Forecasting
All Model Versions
Delphi
Los Alamos Nat Lab
Imperial
SIKJalpha
IHME - MS SEIR
0.0
2.5
5.0
7.5
May
Jul
Sep
Nov
Jan
All Cumulative Errors
Delphi
Los Alamos Nat Lab
Imperial
SIKJalpha
IHME - MS SEIR
0
2
4
6
8
May
Jul
Sep
Nov
Jan
Mar

Georgia (Country)

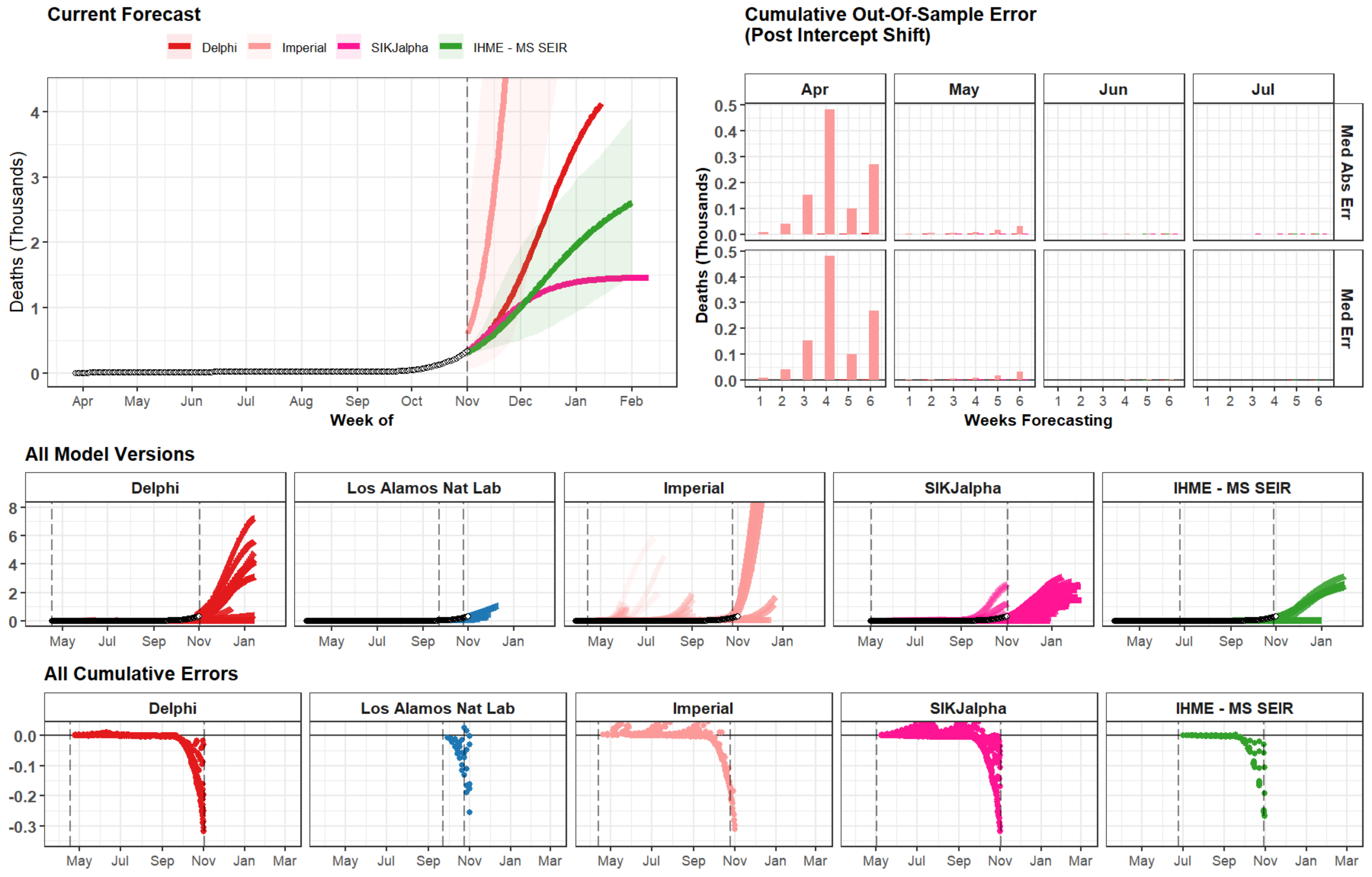

Senegal

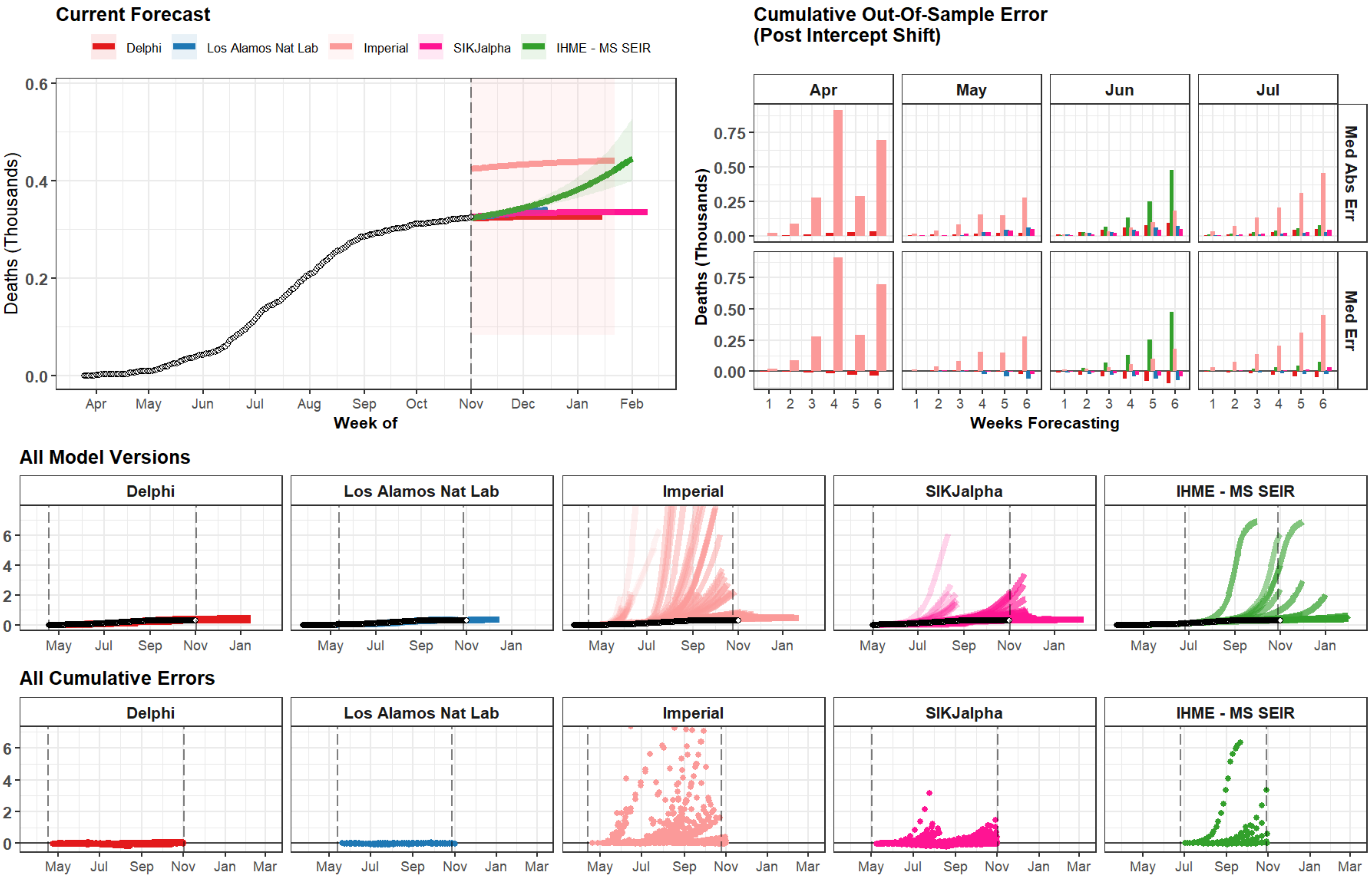
Current Forecast
Delphi
Los Alamos Nat Lab
Imperial
SIKJalpha
IHME - MS SEIR
Deaths (Thousands)
Week of
Cumulative Out-Of-Sample Error
(Post Intercept Shift)
Apr
May
Jun
Jul
Med Abs Err
Med Err
Weeks Forecasting
All Model Versions
All Cumulative Errors

Bahrain

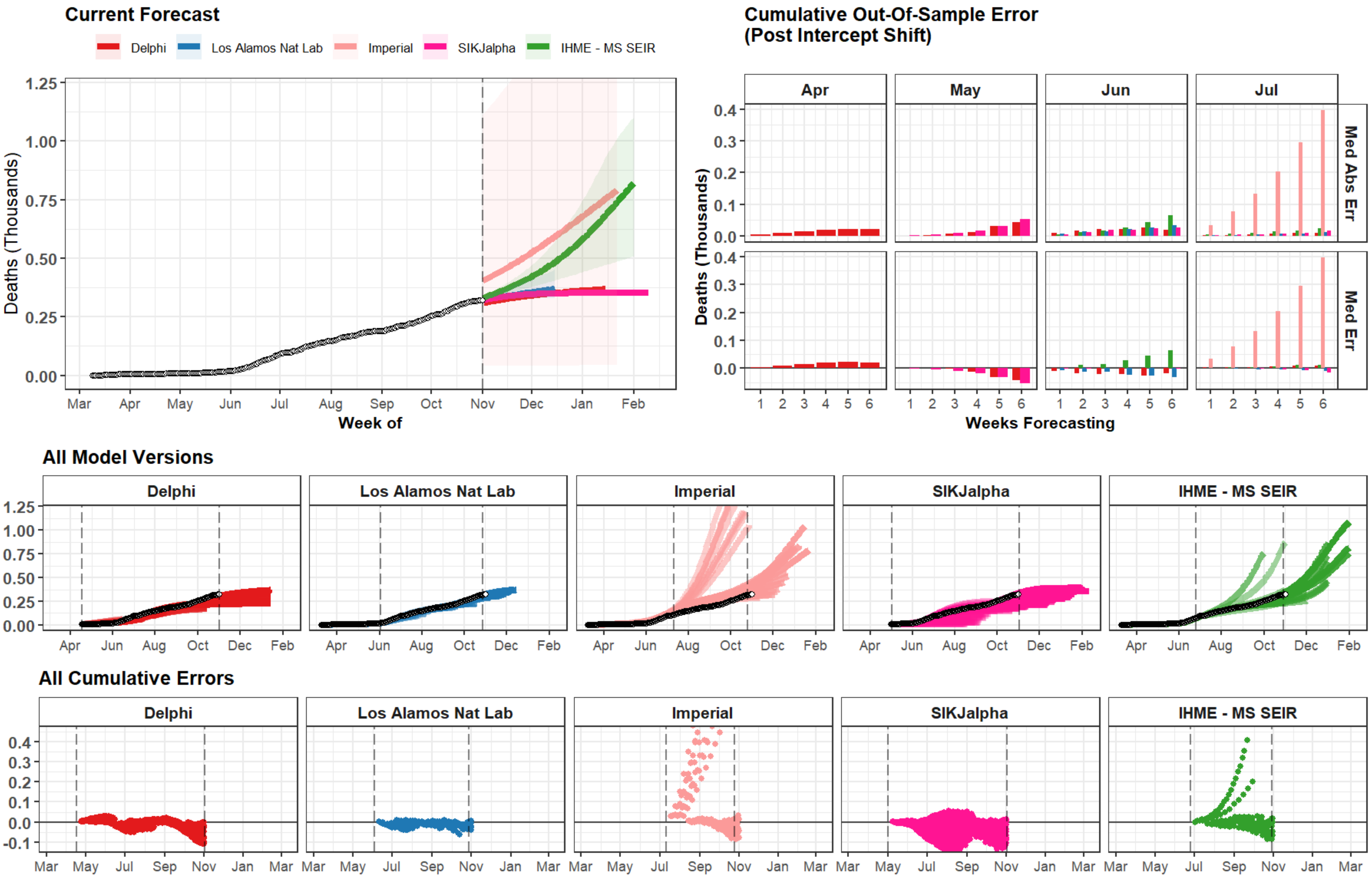

Current Forecast
Delphi
Los Alamos Nat Lab
Imperial
SIKJalpha
IHME - MS SEIR
Deaths (Thousands)
1.25
1.00
0.75
0.50
0.25
0.00
Mar
Apr
May
Jun
Jul
Aug
Sep
Oct
Nov
Dec
Jan
Feb
Week of
Cumulative Out-Of-Sample Error
(Post Intercept Shift)
Apr
May
Jun
Jul
Med Abs Err
Med Err
Deaths (Thousands)
0.4
0.3
0.2
0.1
0.0
1
2
3
4
5
6
Weeks Forecasting
All Model Versions
Delphi
Los Alamos Nat Lab
Imperial
SIKJalpha
IHME - MS SEIR
1.25
1.00
0.75
0.50
0.25
0.00
Apr
Jun
Aug
Oct
Dec
Feb
All Cumulative Errors
Delphi
Los Alamos Nat Lab
Imperial
SIKJalpha
IHME - MS SEIR
0.4
0.3
0.2
0.1
0.0
-0.1
Mar
May
Jul
Sep
Nov
Jan
Mar

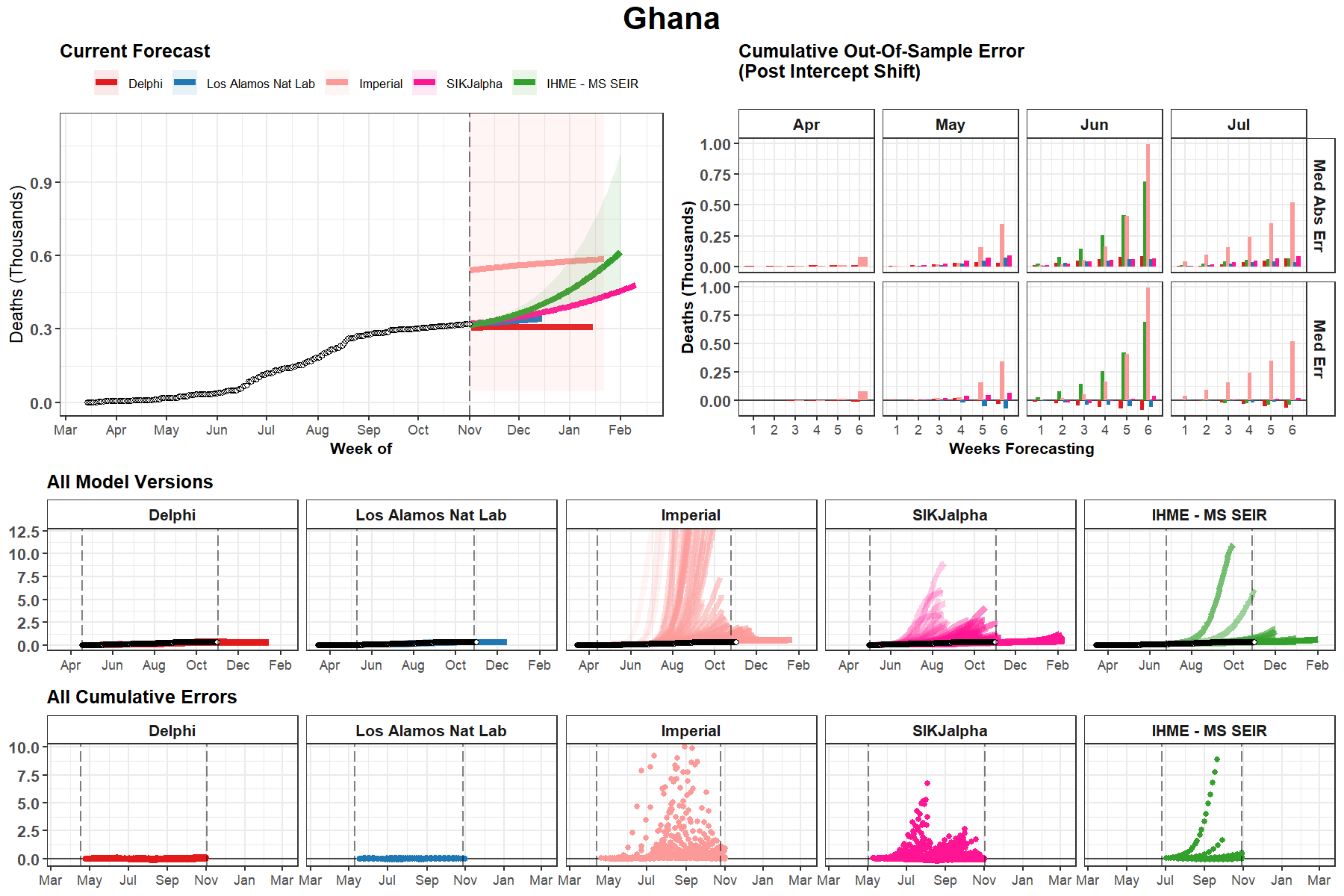
Ghana
Current Forecast
Delphi
Los Alamos Nat Lab
Imperial
SIKJalpha
IHME - MS SEIR
Deaths (Thousands)
Week of
Cumulative Out-Of-Sample Error
(Post Intercept Shift)
Apr
May
Jun
Jul
Med Abs Err
Med Err
Weeks Forecasting
All Model Versions
All Cumulative Errors

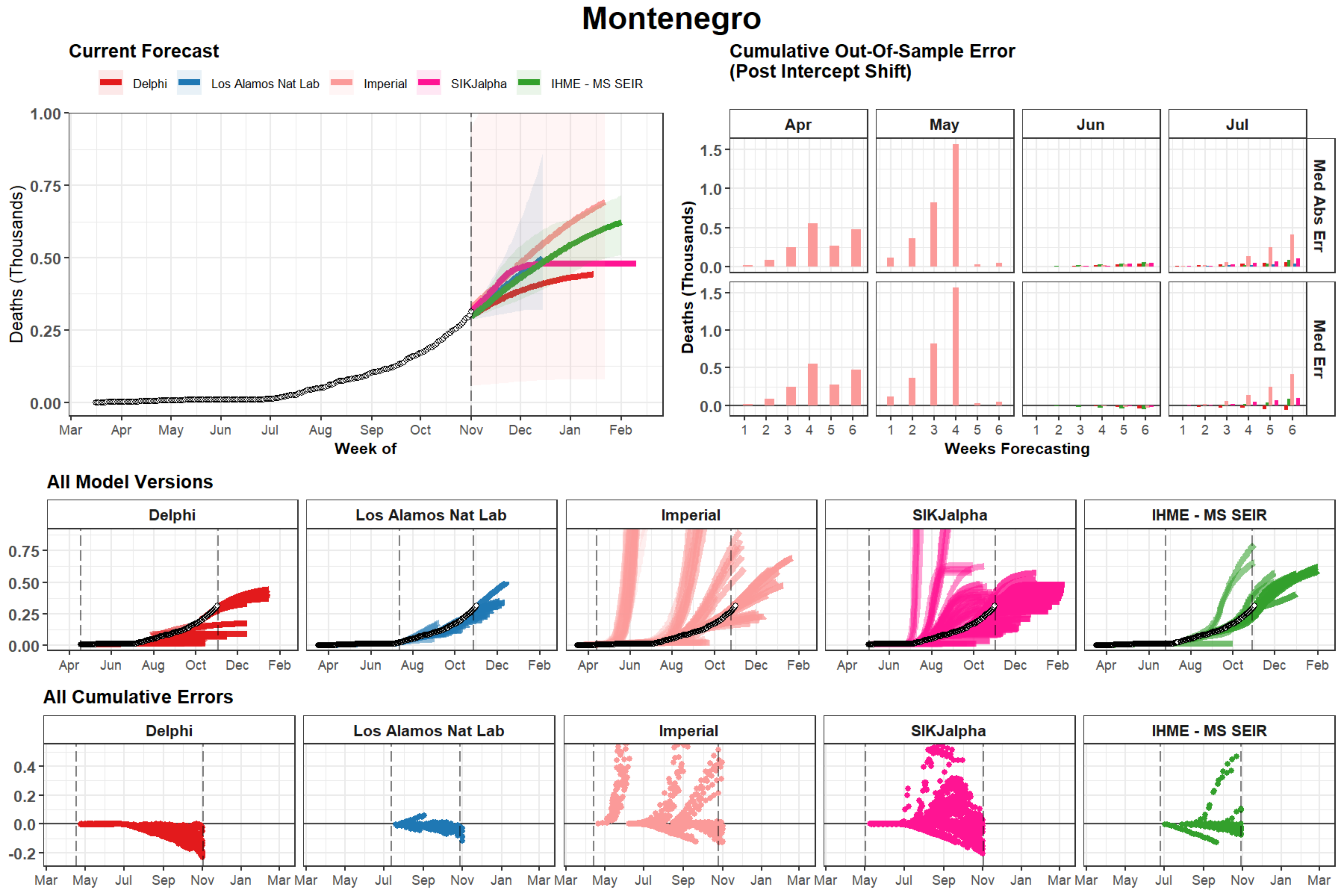
Montenegro
Current Forecast
Delphi
Los Alamos Nat Lab
Imperial
SIKJalpha
IHME - MS SEIR
Deaths (Thousands)
Week of
Cumulative Out-Of-Sample Error
(Post Intercept Shift)
Apr
May
Jun
Jul
Med Abs Err
Med Err
Weeks Forecasting
All Model Versions
All Cumulative Errors

Democratic Republic of the Congo

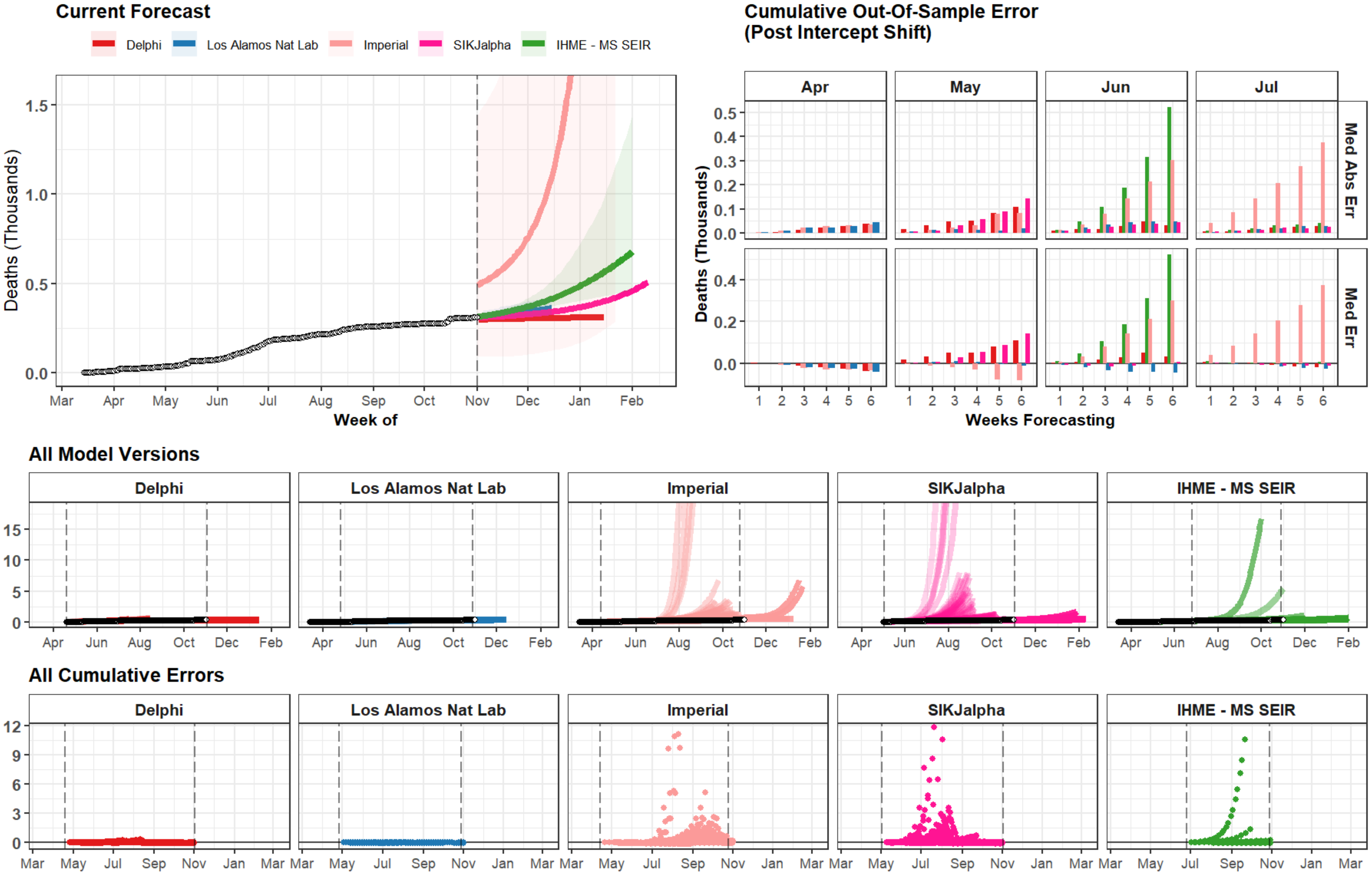

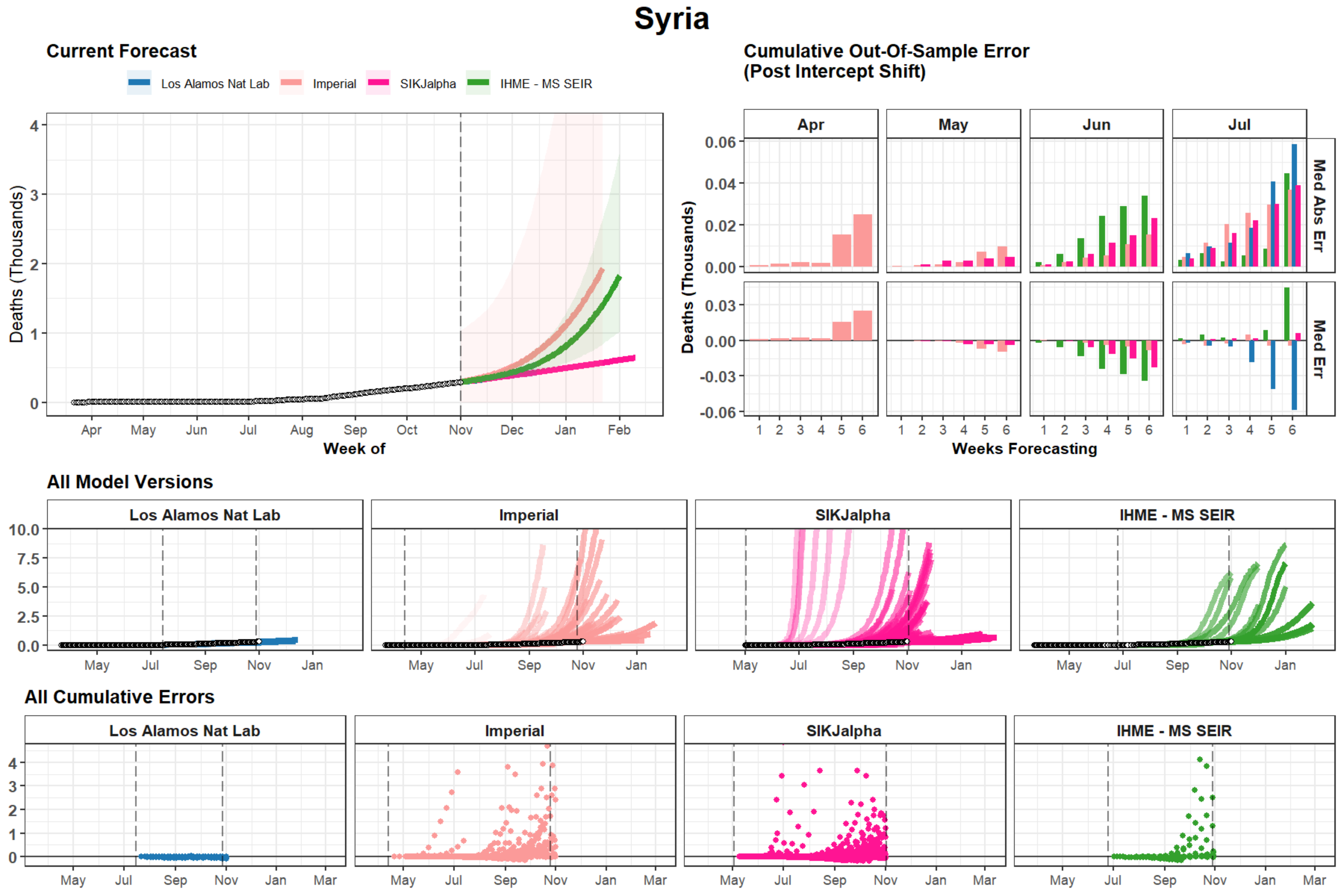

Syria
Current Forecast
Los Alamos Nat Lab
Imperial
SIKJalpha
IHME - MS SEIR
Deaths (Thousands)
Week of
Apr May Jun Jul Aug Sep Oct Nov Dec Jan Feb
Cumulative Out-Of-Sample Error
(Post Intercept Shift)
Apr May Jun Jul
Med Abs Err
Med Err
Deaths (Thousands)
Weeks Forecasting
All Model Versions
Los Alamos Nat Lab
Imperial
SIKJalpha
IHME - MS SEIR
All Cumulative Errors
Los Alamos Nat Lab
Imperial
SIKJalpha
IHME - MS SEIR

Angola

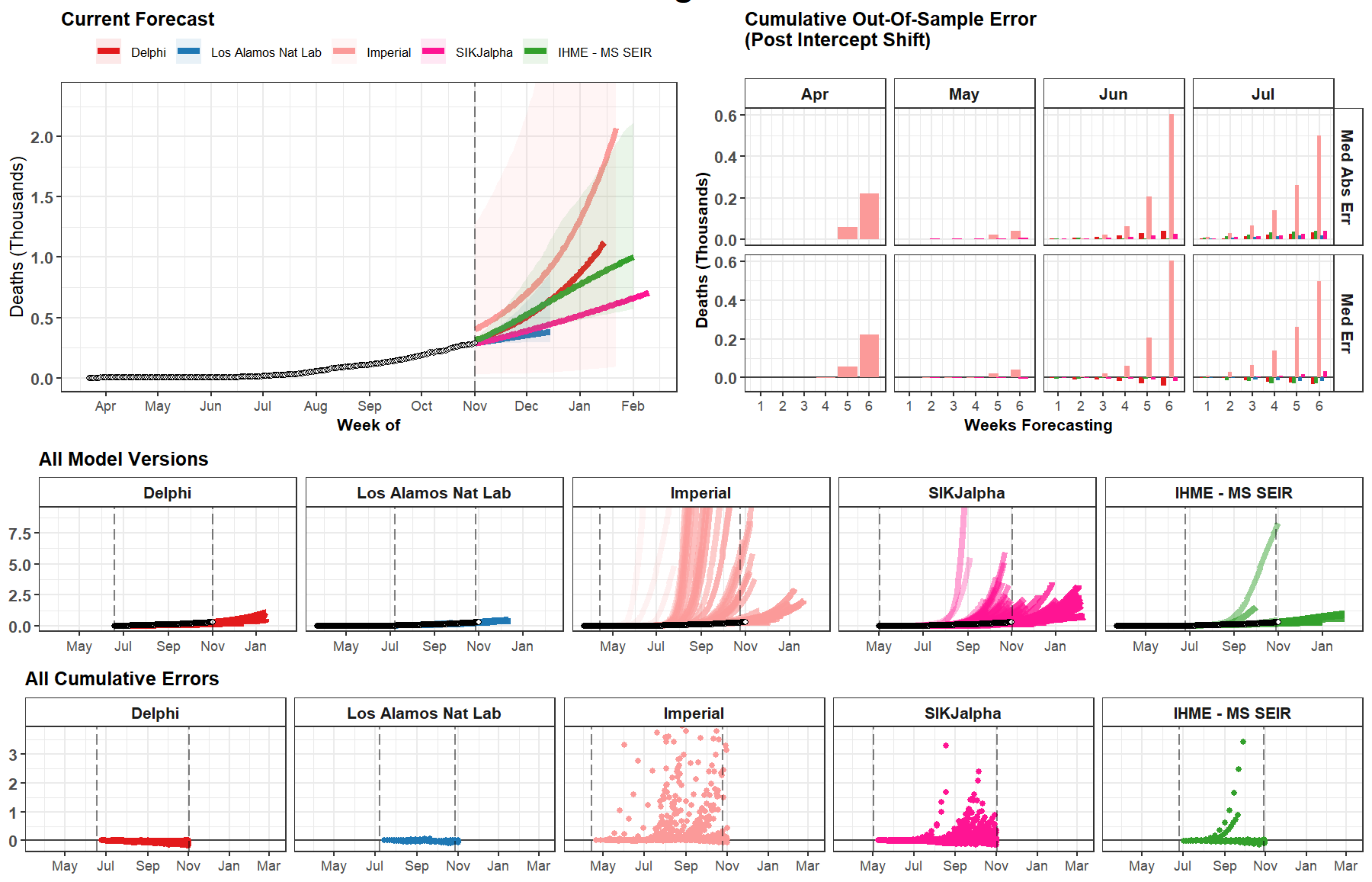

Current Forecast
Delphi
Los Alamos Nat Lab
Imperial
SIKJalpha
IHME - MS SEIR
Deaths (Thousands)
Week of
Apr May Jun Jul Aug Sep Oct Nov Dec Jan Feb
Cumulative Out-Of-Sample Error (Post Intercept Shift)
Apr May Jun Jul
Med Abs Err
Med Err
Weeks Forecasting
All Model Versions
All Cumulative Errors

Norway

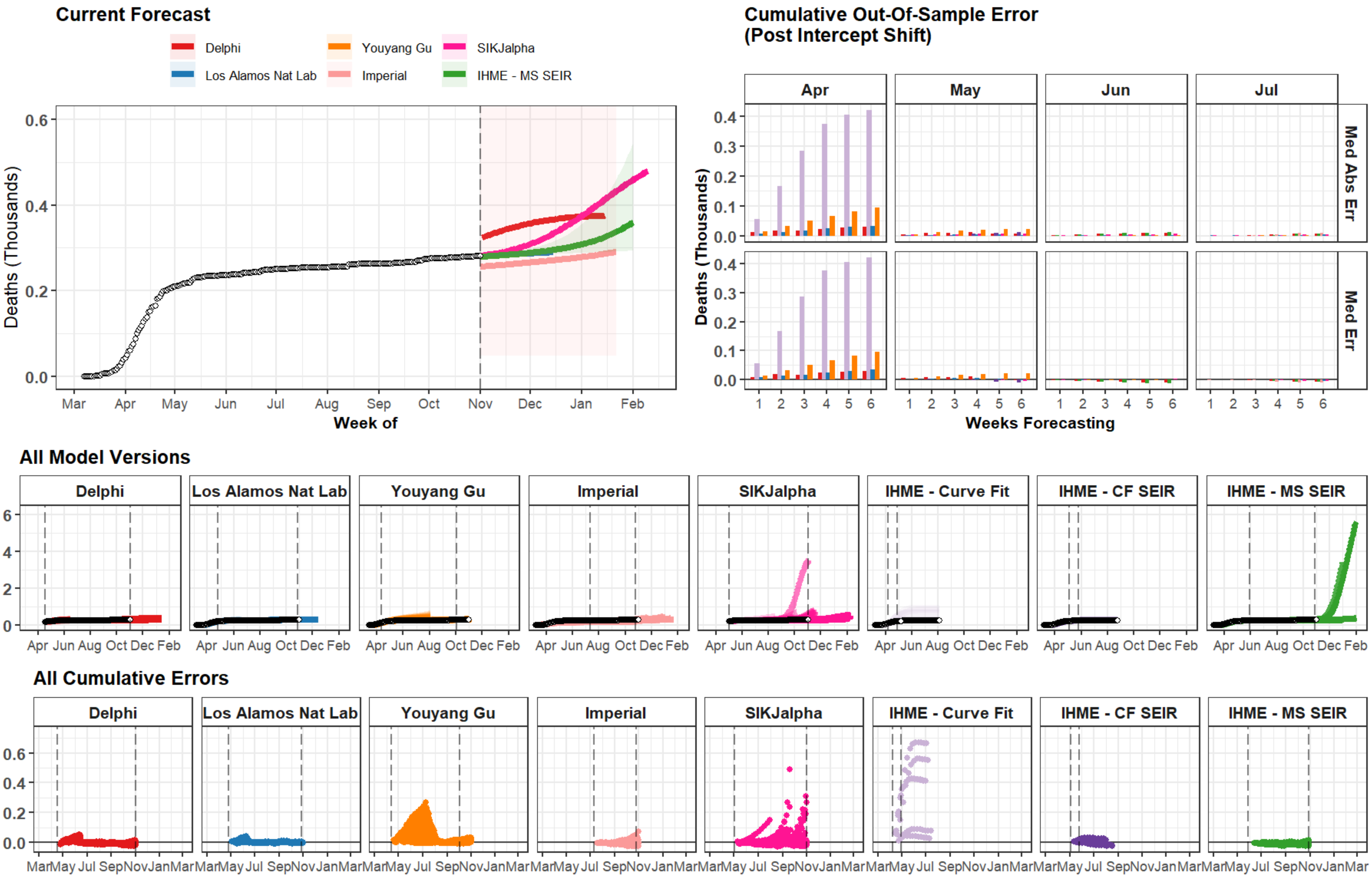

Current Forecast
Delphi
Los Alamos Nat Lab
Youyang Gu
Imperial
SIKJalpha
IHME - MS SEIR
Deaths (Thousands)
Week of
Mar Apr May Jun Jul Aug Sep Oct Nov Dec Jan Feb
Cumulative Out-Of-Sample Error
(Post Intercept Shift)
Apr
May
Jun
Jul
Med Abs Err
Med Err
Weeks Forecasting
All Model Versions
Delphi
Los Alamos Nat Lab
Youyang Gu
Imperial
SIKJalpha
IHME - Curve Fit
IHME - CF SEIR
IHME - MS SEIR
All Cumulative Errors

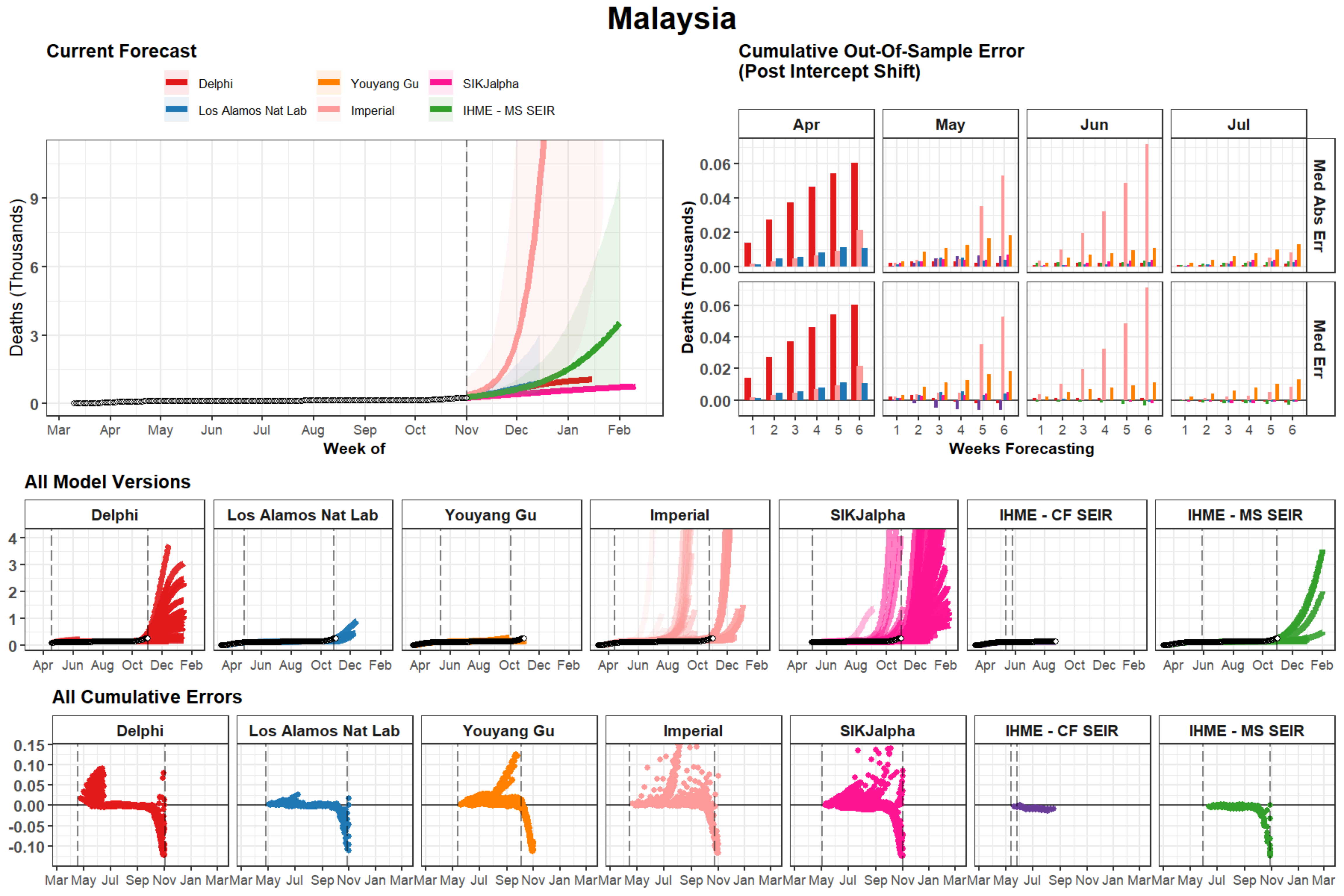
Malaysia
Current Forecast
Delphi
Los Alamos Nat Lab
Youyang Gu
Imperial
SIKJalpha
IHME - MS SEIR
Deaths (Thousands)
Week of
Mar Apr May Jun Jul Aug Sep Oct Nov Dec Jan Feb
Cumulative Out-Of-Sample Error (Post Intercept Shift)
Apr May Jun Jul
Med Abs Err
Med Err
Weeks Forecasting
All Model Versions
Delphi
Los Alamos Nat Lab
Youyang Gu
Imperial
SIKJalpha
IHME - CF SEIR
IHME - MS SEIR
All Cumulative Errors

Madagascar

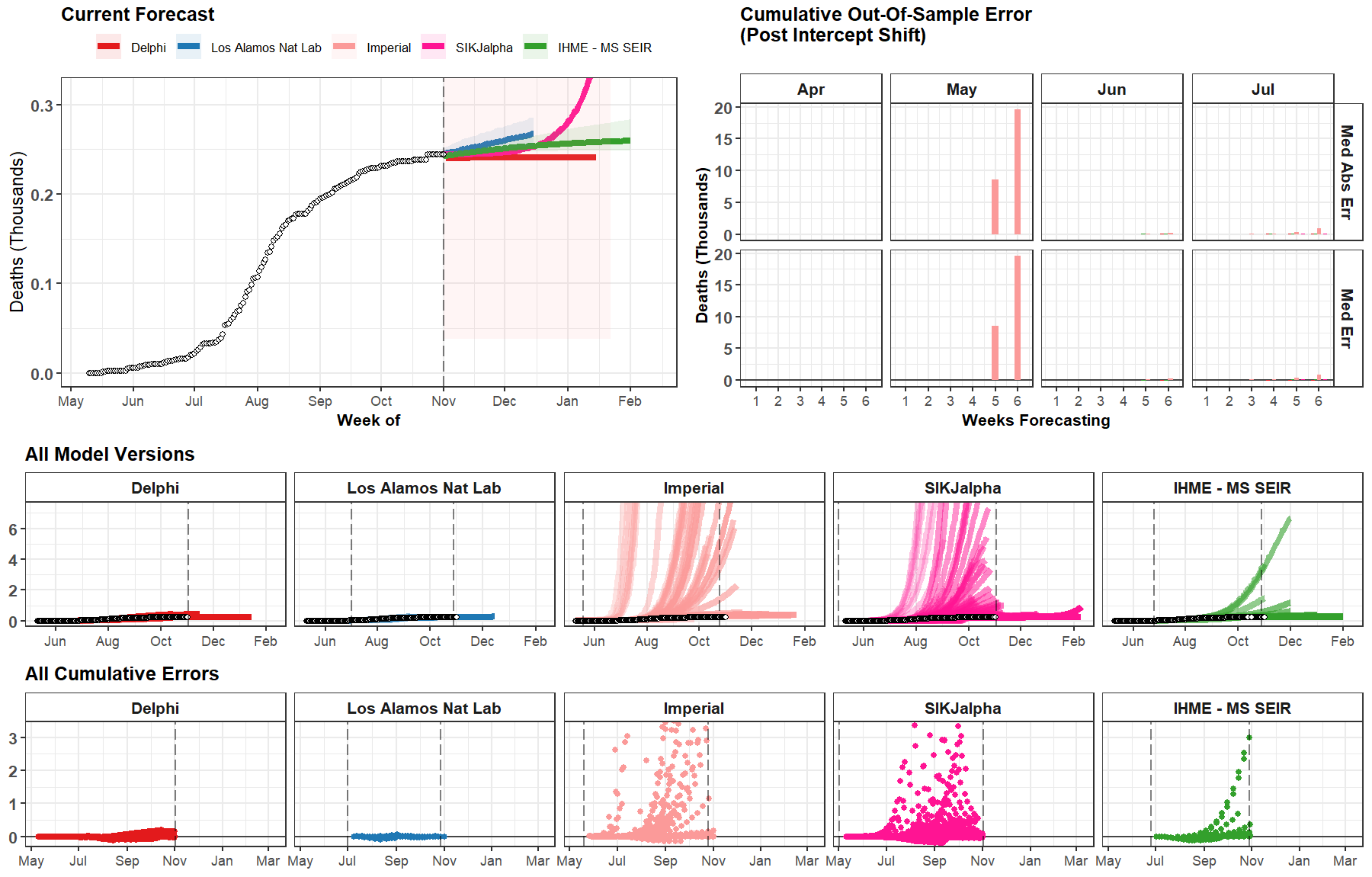

Current Forecast
Delphi
Los Alamos Nat Lab
Imperial
SIKJalpha
IHME - MS SEIR
Deaths (Thousands)
0.0
0.1
0.2
0.3
May
Jun
Jul
Aug
Sep
Oct
Nov
Dec
Jan
Feb
Week of
Cumulative Out-Of-Sample Error
(Post Intercept Shift)
Apr
May
Jun
Jul
Med Abs Err
Med Err
Deaths (Thousands)
0
5
10
15
20
1
2
3
4
5
6
Weeks Forecasting
All Model Versions
Delphi
Los Alamos Nat Lab
Imperial
SIKJalpha
IHME - MS SEIR
0
2
4
6
Jun
Aug
Oct
Dec
Feb
All Cumulative Errors
Delphi
Los Alamos Nat Lab
Imperial
SIKJalpha
IHME - MS SEIR
0
1
2
3
May
Jul
Sep
Nov
Jan
Mar

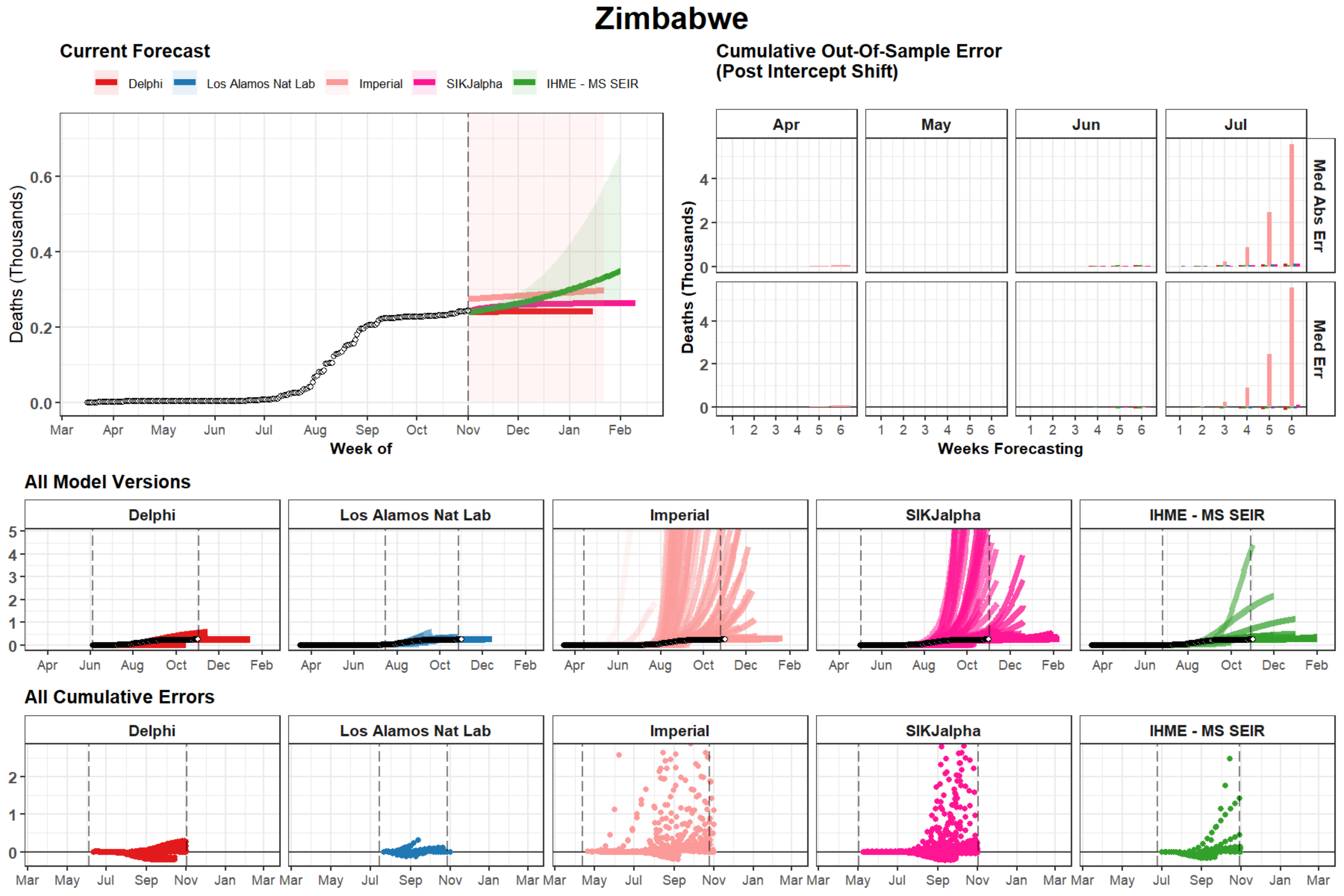

Zimbabwe
Current Forecast
Delphi
Los Alamos Nat Lab
Imperial
SIKJalpha
IHME - MS SEIR
Deaths (Thousands)
Week of
Cumulative Out-Of-Sample Error
(Post Intercept Shift)
Apr
May
Jun
Jul
Med Abs Err
Med Err
Weeks Forecasting
All Model Versions
All Cumulative Errors

Haiti

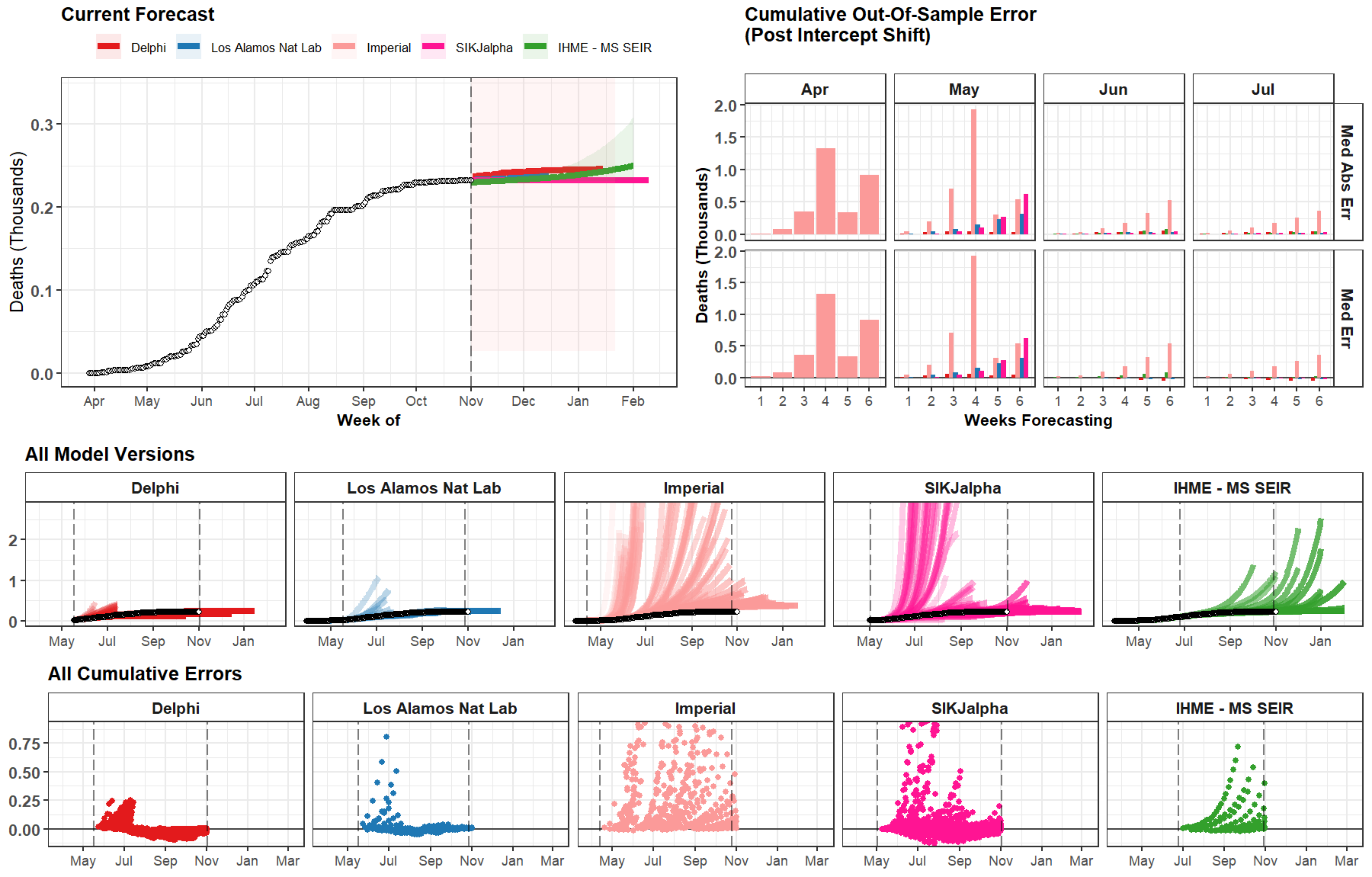

Current Forecast
Delphi
Los Alamos Nat Lab
Imperial
SIKJalpha
IHME - MS SEIR
Deaths (Thousands)
Week of
Apr
May
Jun
Jul
Aug
Sep
Oct
Nov
Dec
Jan
Feb
Cumulative Out-Of-Sample Error
(Post Intercept Shift)
Med Abs Err
Med Err
Weeks Forecasting
All Model Versions
All Cumulative Errors
Mar

Qatar

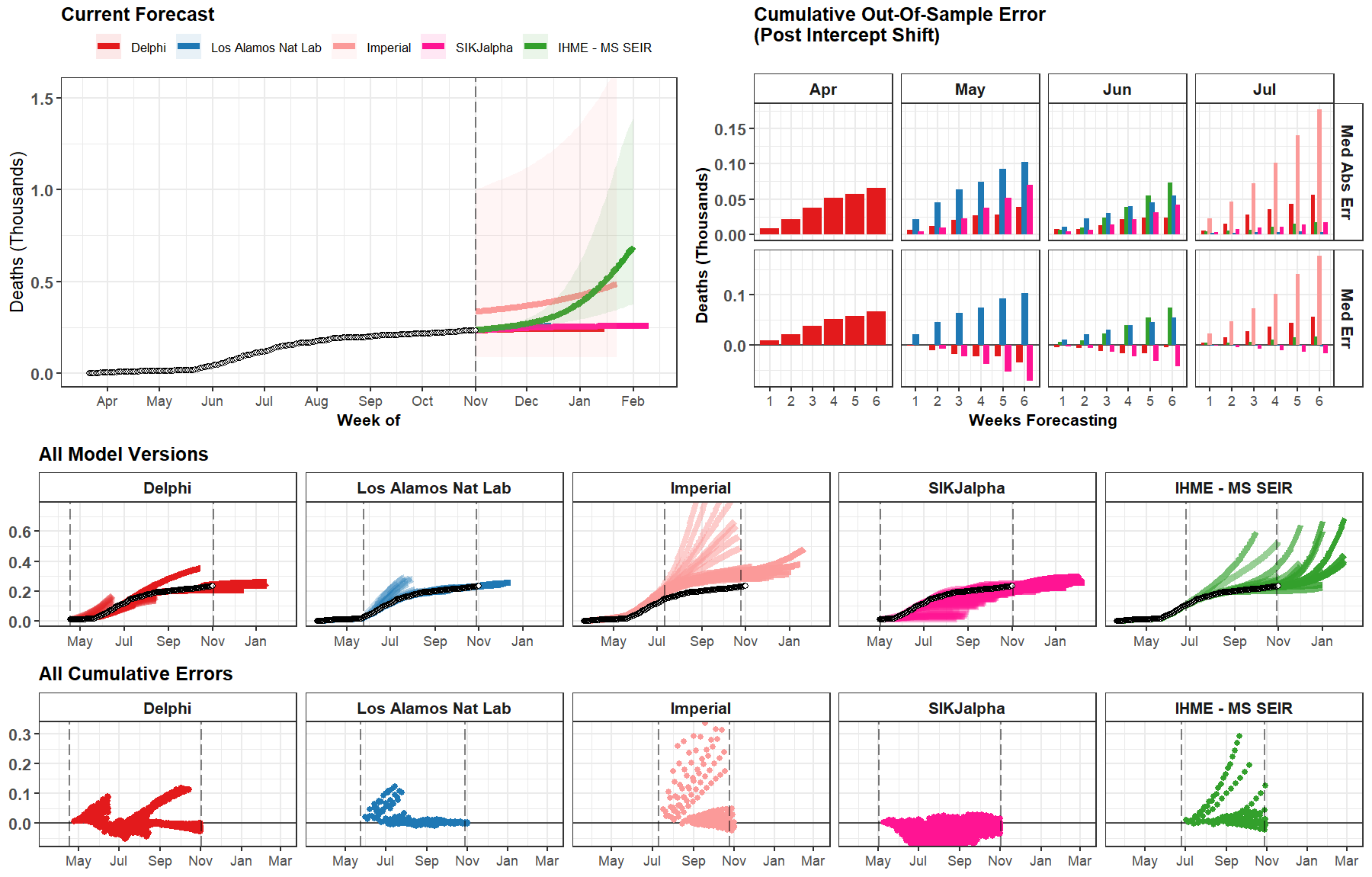
Current Forecast
Delphi
Los Alamos Nat Lab
Imperial
SIKJalpha
IHME - MS SEIR
Deaths (Thousands)
Week of
Cumulative Out-Of-Sample Error
(Post Intercept Shift)
Apr
May
Jun
Jul
Med Abs Err
Med Err
Weeks Forecasting
All Model Versions
All Cumulative Errors

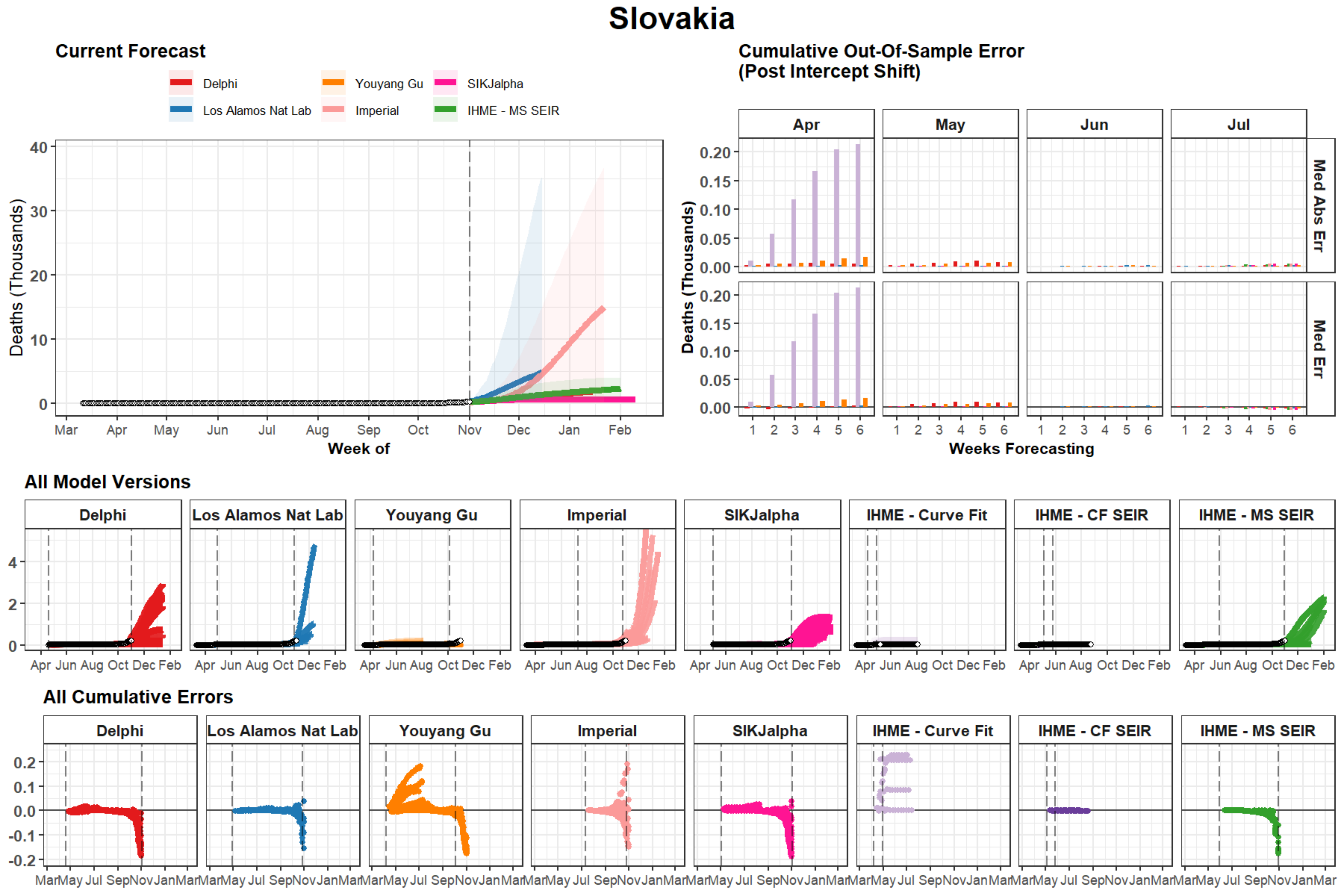
Slovakia
Current Forecast
Delphi
Los Alamos Nat Lab
Youyang Gu
Imperial
SIKJalpha
IHME - MS SEIR
Deaths (Thousands)
Week of
Mar Apr May Jun Jul Aug Sep Oct Nov Dec Jan Feb
Cumulative Out-Of-Sample Error
(Post Intercept Shift)
Apr
May
Jun
Jul
Med Abs Err
Med Err
Weeks Forecasting
All Model Versions
Delphi
Los Alamos Nat Lab
Youyang Gu
Imperial
SIKJalpha
IHME - Curve Fit
IHME - CF SEIR
IHME - MS SEIR
All Cumulative Errors

Hawaii

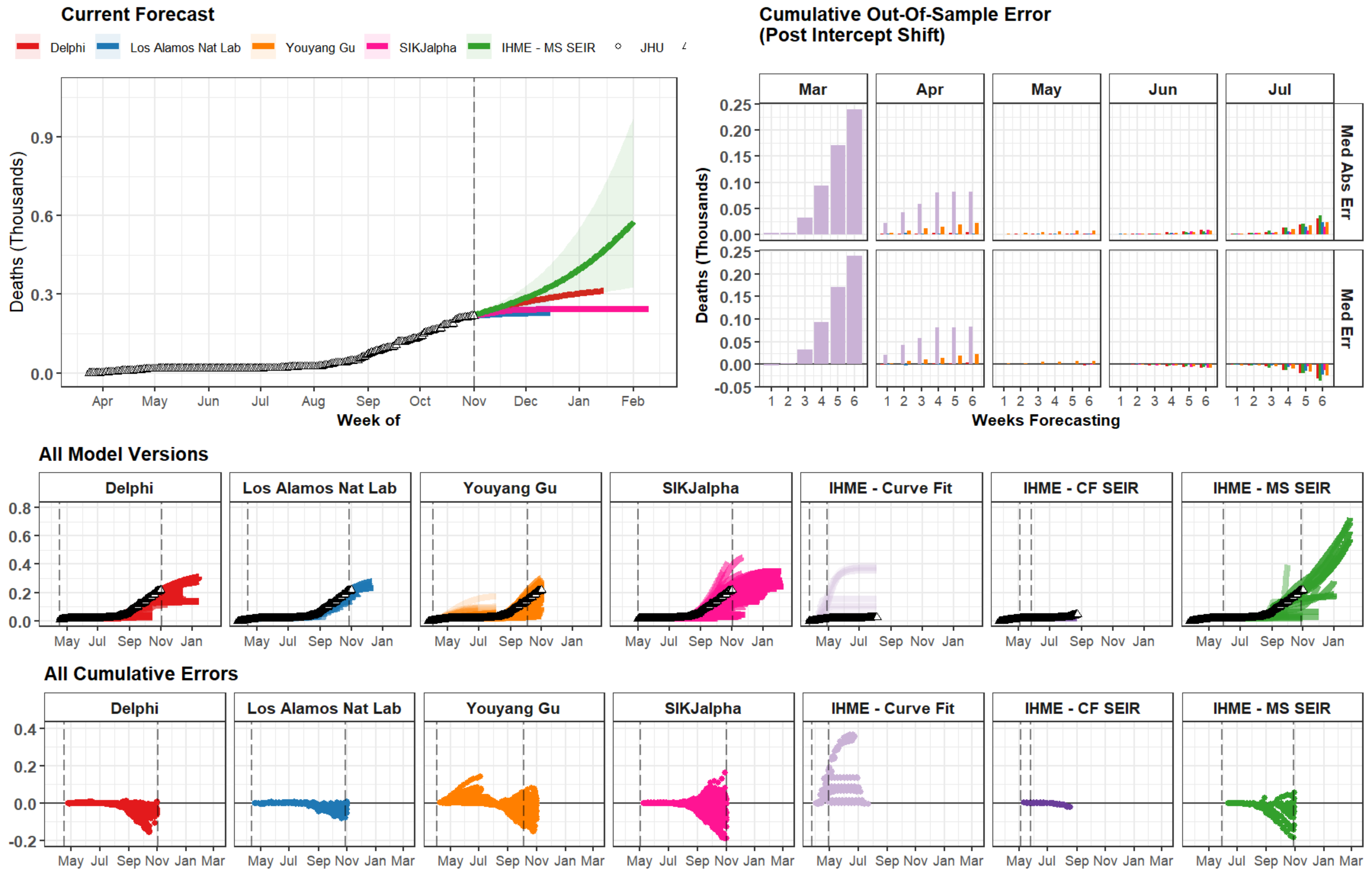
Current Forecast
Delphi
Los Alamos Nat Lab
Youyang Gu
SIKJalpha
IHME - MS SEIR
JHU
Deaths (Thousands)
0.0
0.3
0.6
0.9
Apr May Jun Jul Aug Sep Oct Nov Dec Jan Feb
Week of
Cumulative Out-Of-Sample Error
(Post Intercept Shift)
Mar
Apr
May
Jun
Jul
Med Abs Err
Med Err
Deaths (Thousands)
0.25 0.20 0.15 0.10 0.05 0.00 -0.05
1 2 3 4 5 6
Weeks Forecasting
All Model Versions
Delphi
Los Alamos Nat Lab
Youyang Gu
SIKJalpha
IHME - Curve Fit
IHME - CF SEIR
IHME - MS SEIR
0.8 0.6 0.4 0.2 0.0
May Jul Sep Nov Jan
All Cumulative Errors
Delphi
Los Alamos Nat Lab
Youyang Gu
SIKJalpha
IHME - Curve Fit
IHME - CF SEIR
IHME - MS SEIR
0.4 0.2 0.0 -0.2
May Jul Sep Nov Jan Mar

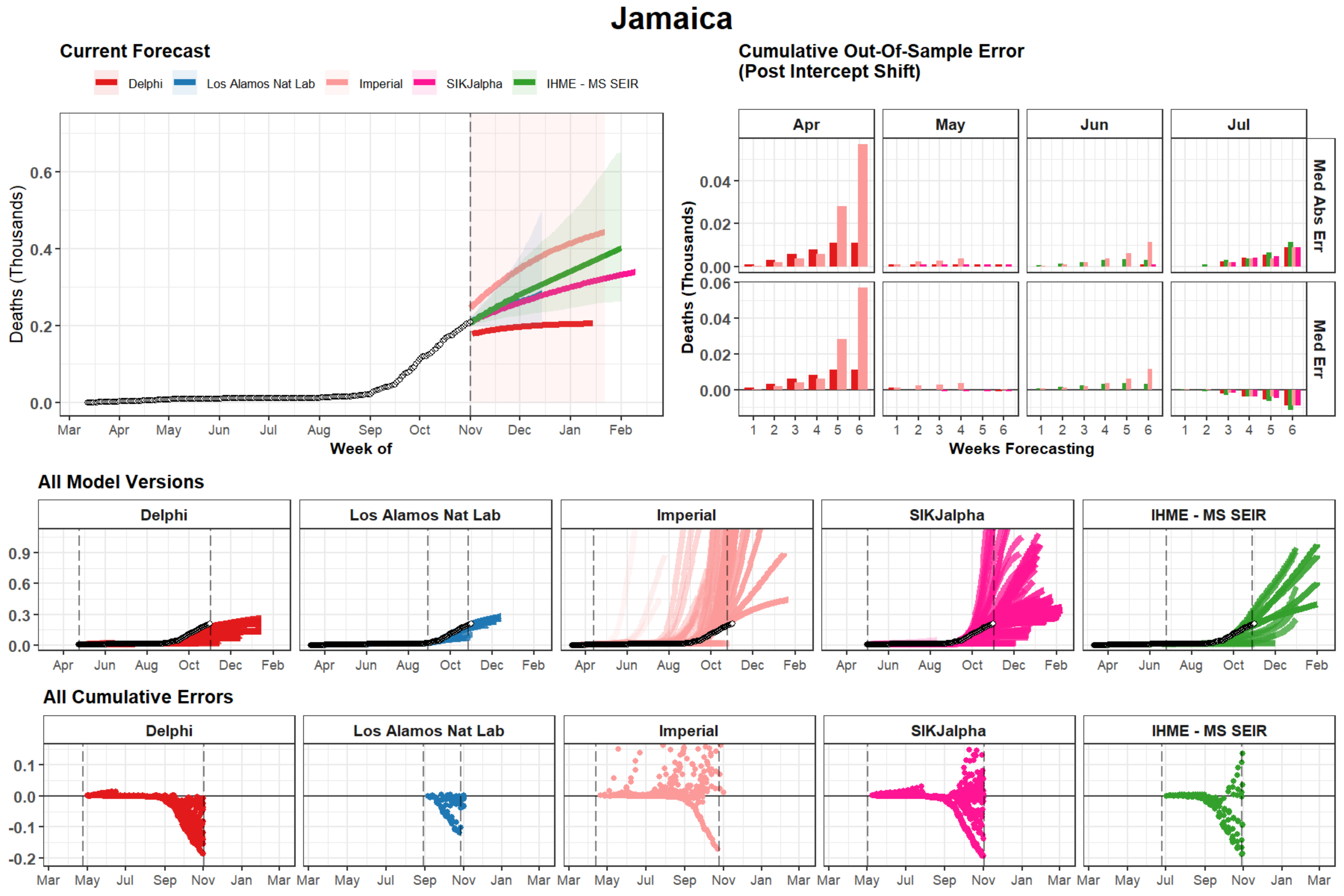
Jamaica
Current Forecast
Delphi
Los Alamos Nat Lab
Imperial
SIKJalpha
IHME - MS SEIR
Deaths (Thousands)
Week of
Cumulative Out-Of-Sample Error
(Post Intercept Shift)
Apr
May
Jun
Jul
Med Abs Err
Med Err
Weeks Forecasting
All Model Versions
All Cumulative Errors

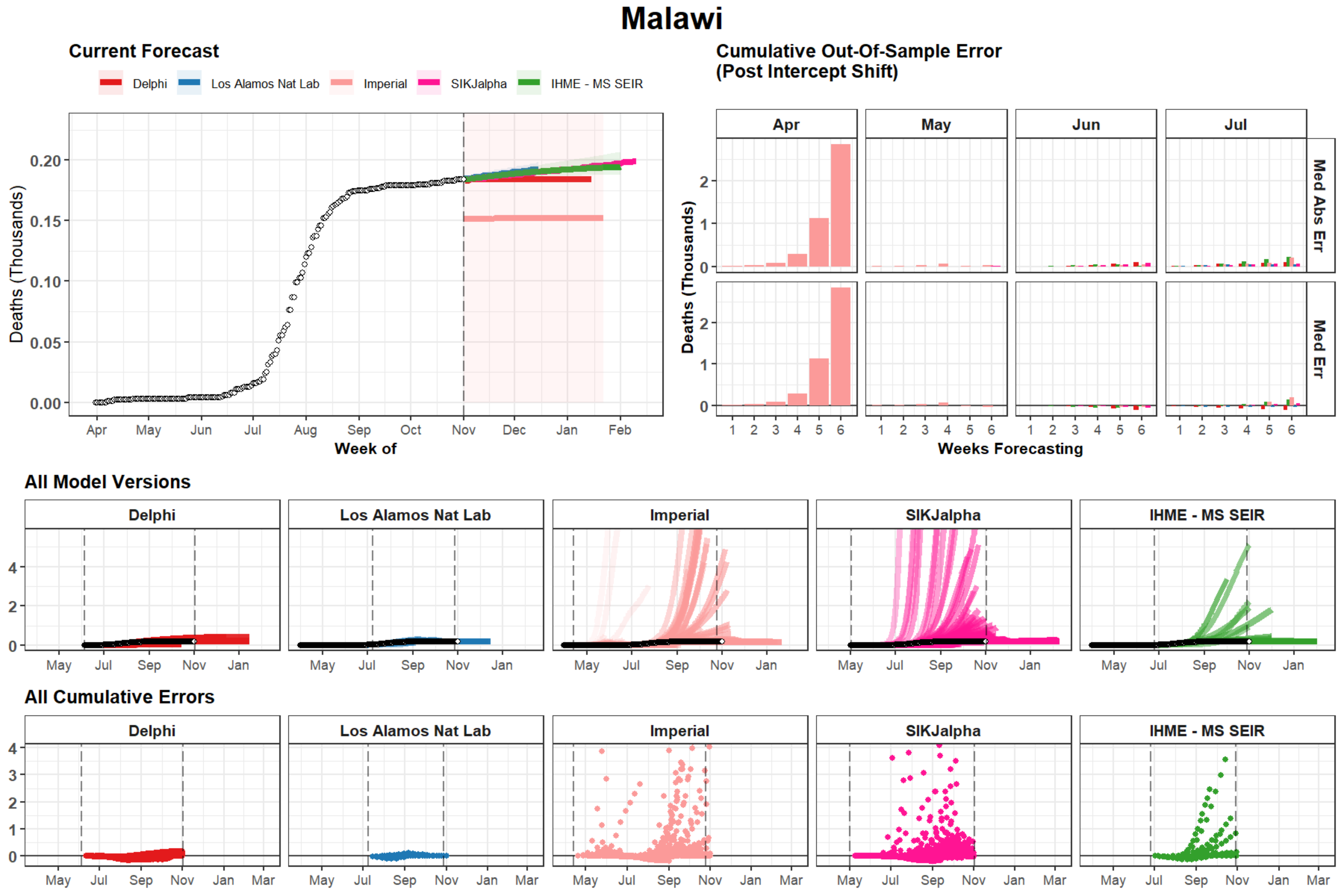
Malawi
Current Forecast
Delphi
Los Alamos Nat Lab
Imperial
SIKJalpha
IHME - MS SEIR
Deaths (Thousands)
0.00
0.05
0.10
0.15
0.20
Apr
May
Jun
Jul
Aug
Sep
Oct
Nov
Dec
Jan
Feb
Week of
Cumulative Out-Of-Sample Error
(Post Intercept Shift)
Apr
May
Jun
Jul
Med Abs Err
Med Err
Deaths (Thousands)
Weeks Forecasting
All Model Versions
All Cumulative Errors
May
Jul
Sep
Nov
Jan
Mar

Lithuania

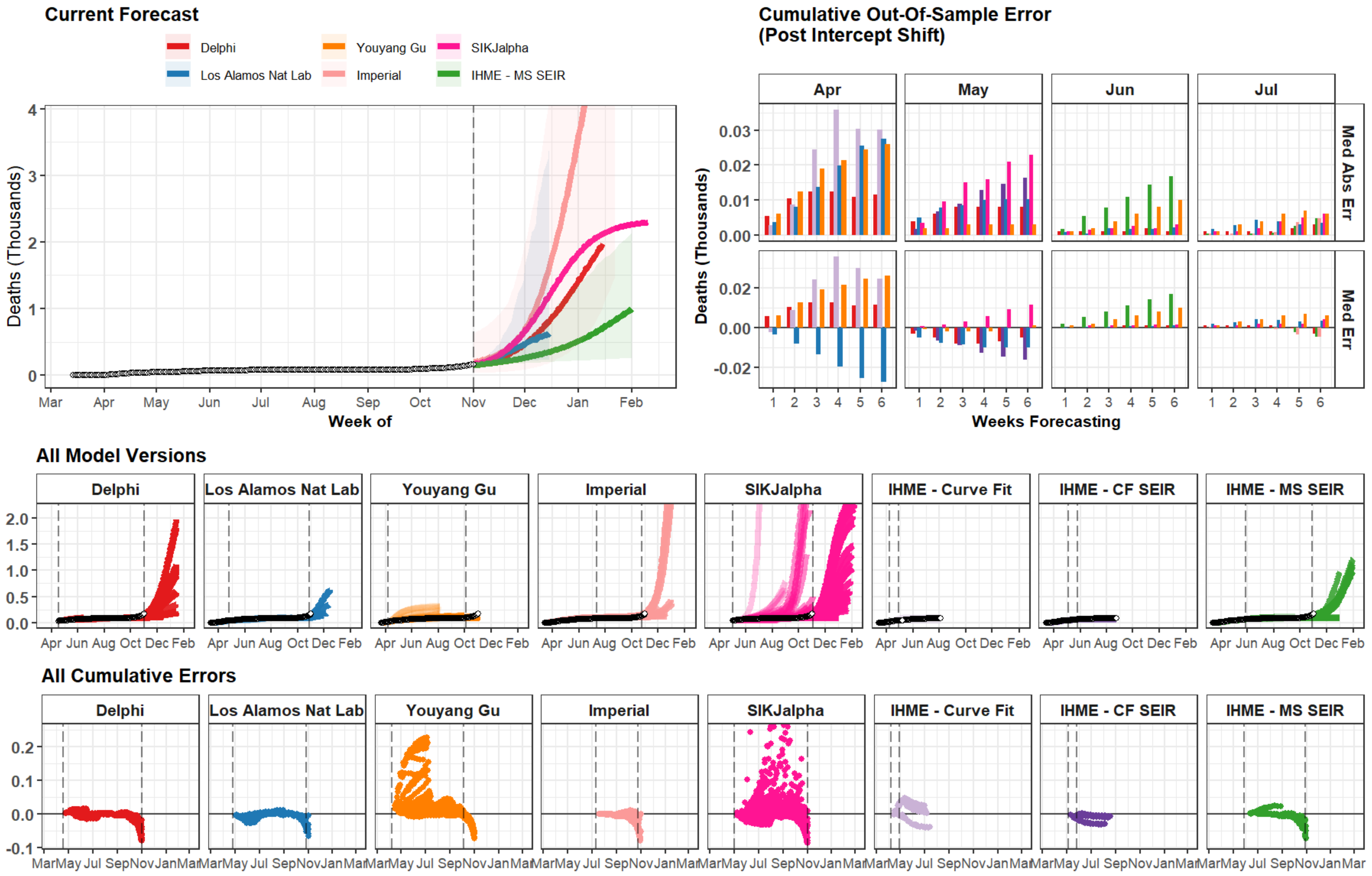
Current Forecast
Delphi
Los Alamos Nat Lab
Youyang Gu
Imperial
SIKJalpha
IHME - MS SEIR
Deaths (Thousands)
0
1
2
3
4
Mar Apr May Jun Jul Aug Sep Oct Nov Dec Jan Feb
Week of
Cumulative Out-Of-Sample Error
(Post Intercept Shift)
Apr
May
Jun
Jul
Med Abs Err
Med Err
Deaths (Thousands)
0.00 0.01 0.02 0.03
-0.02 0.00 0.02
1 2 3 4 5 6
Weeks Forecasting
All Model Versions
Delphi
Los Alamos Nat Lab
Youyang Gu
Imperial
SIKJalpha
IHME - Curve Fit
IHME - CF SEIR
IHME - MS SEIR
0.0 0.5 1.0 1.5 2.0
Apr Jun Aug Oct Dec Feb
All Cumulative Errors
Delphi
Los Alamos Nat Lab
Youyang Gu
Imperial
SIKJalpha
IHME - Curve Fit
IHME - CF SEIR
IHME - MS SEIR
-0.1 0.0 0.1 0.2
Mar May Jul Sep Nov Jan Mar

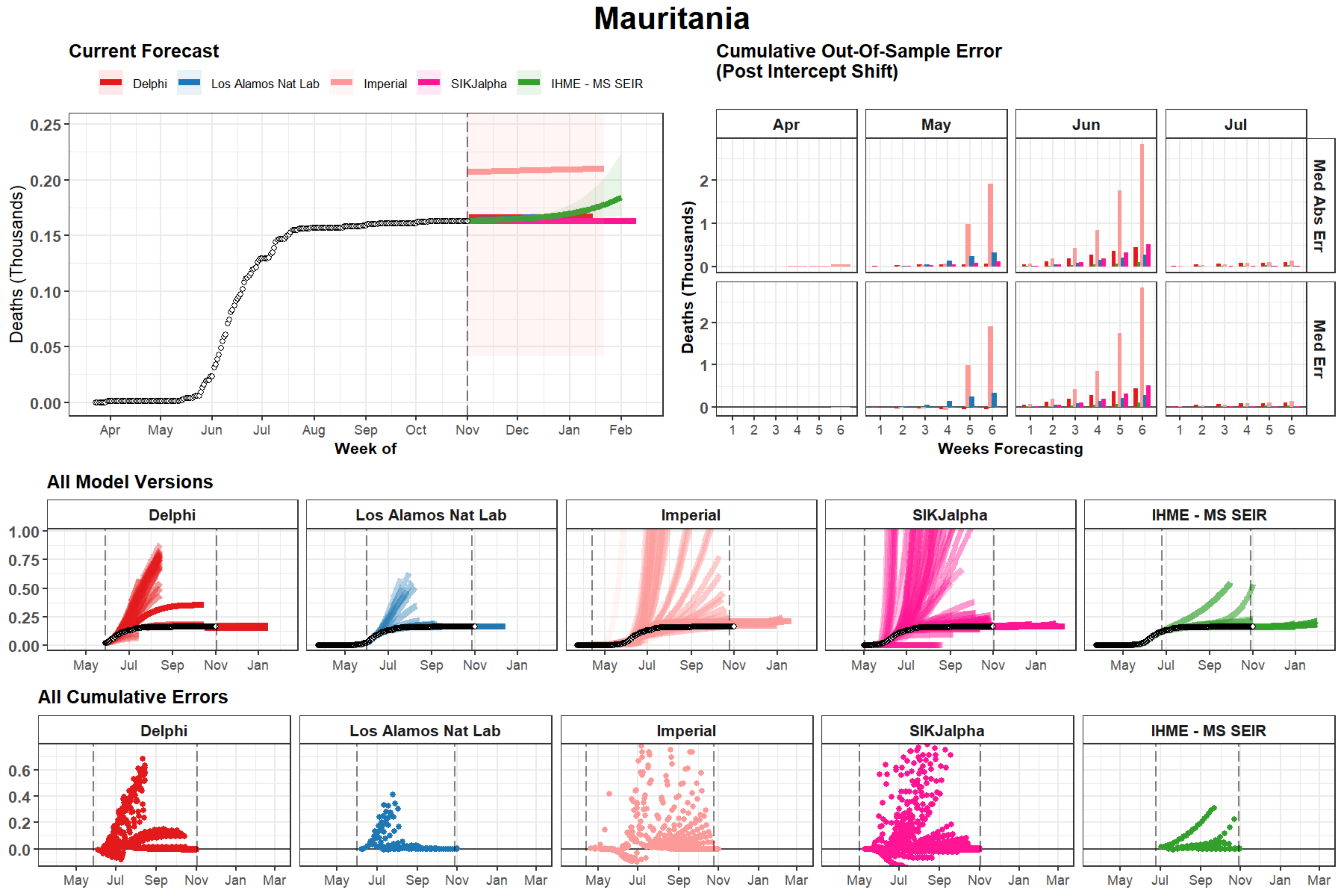

Mauritania
Current Forecast
Delphi
Los Alamos Nat Lab
Imperial
SIKJalpha
IHME - MS SEIR
Deaths (Thousands)
Week of
Cumulative Out-Of-Sample Error
(Post Intercept Shift)
Apr
May
Jun
Jul
Med Abs Err
Med Err
Weeks Forecasting
All Model Versions
All Cumulative Errors

Nicaragua

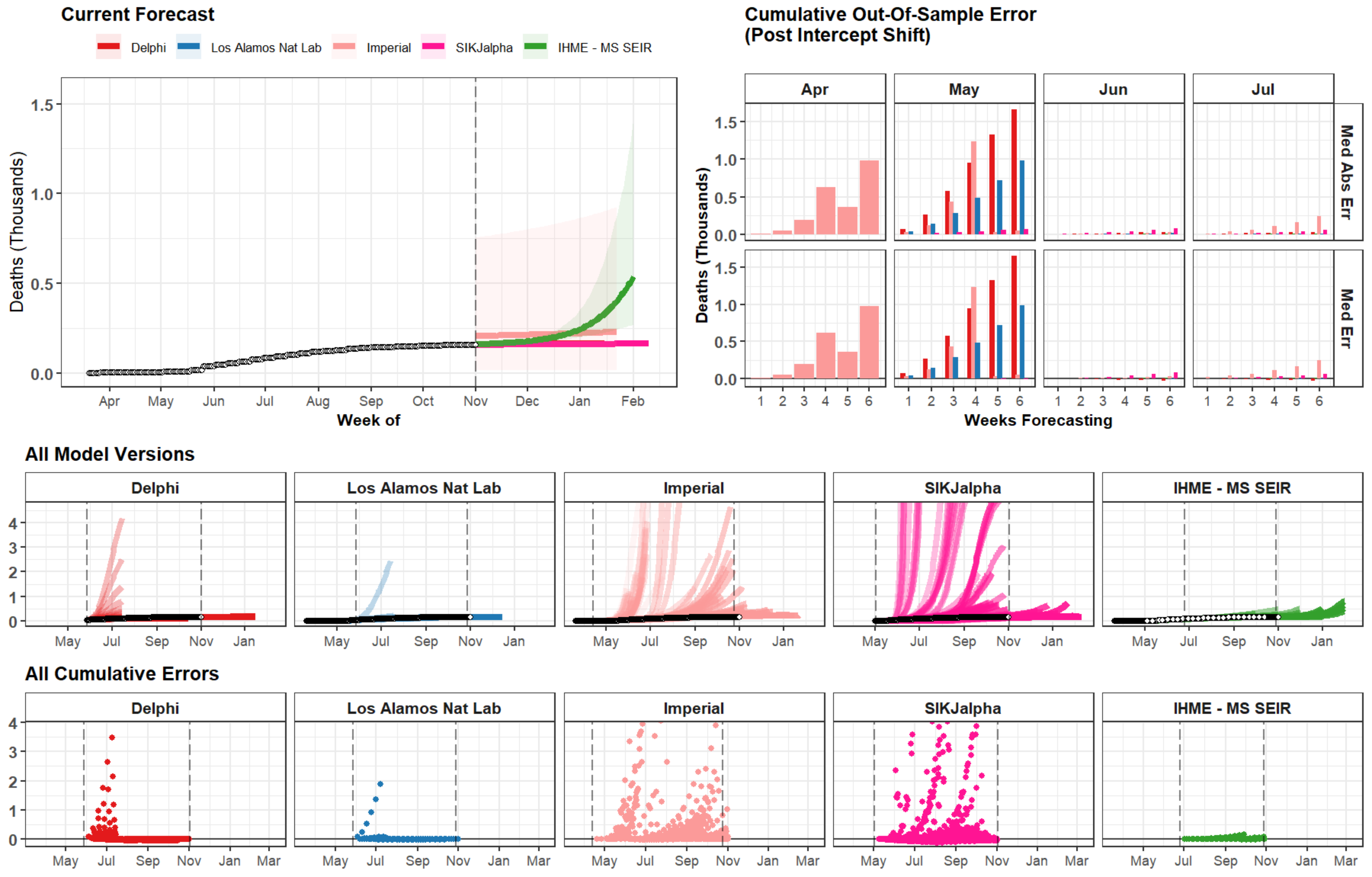
Current Forecast
Delphi
Los Alamos Nat Lab
Imperial
SIKJalpha
IHME - MS SEIR
Deaths (Thousands)
1.5
1.0
0.5
0.0
Apr
May
Jun
Jul
Aug
Sep
Oct
Nov
Dec
Jan
Feb
Week of
Cumulative Out-Of-Sample Error
(Post Intercept Shift)
Apr
May
Jun
Jul
Med Abs Err
Med Err
Weeks Forecasting
All Model Versions
All Cumulative Errors
Mar

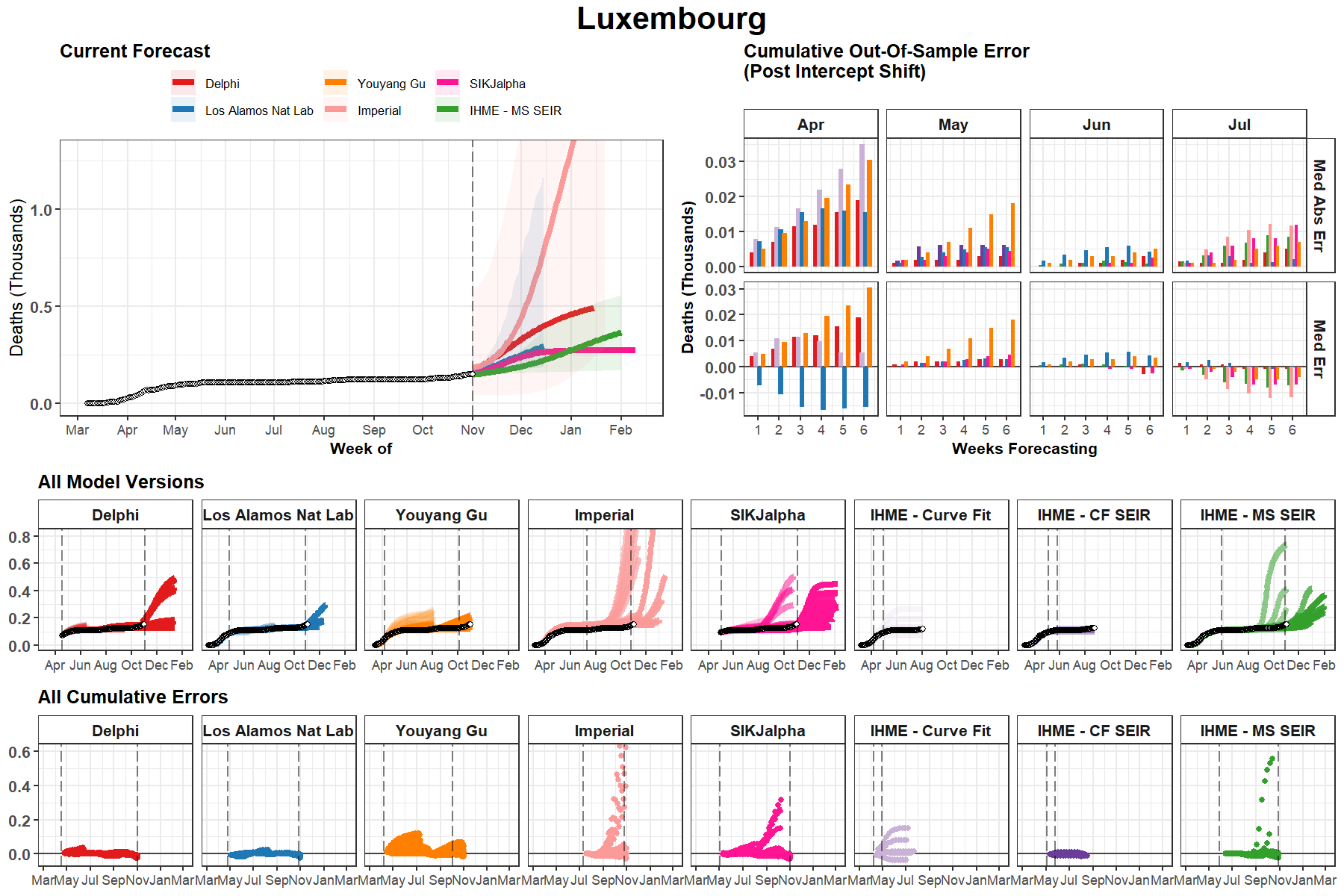

Luxembourg
Current Forecast
Delphi
Los Alamos Nat Lab
Youyang Gu
Imperial
SIKJalpha
IHME - MS SEIR
Deaths (Thousands)
Week of
Mar Apr May Jun Jul Aug Sep Oct Nov Dec Jan Feb
Cumulative Out-Of-Sample Error (Post Intercept Shift)
Apr May Jun Jul
Med Abs Err
Med Err
Weeks Forecasting
All Model Versions
Delphi Los Alamos Nat Lab Youyang Gu Imperial SIKJalpha IHME - Curve Fit IHME - CF SEIR IHME - MS SEIR
All Cumulative Errors

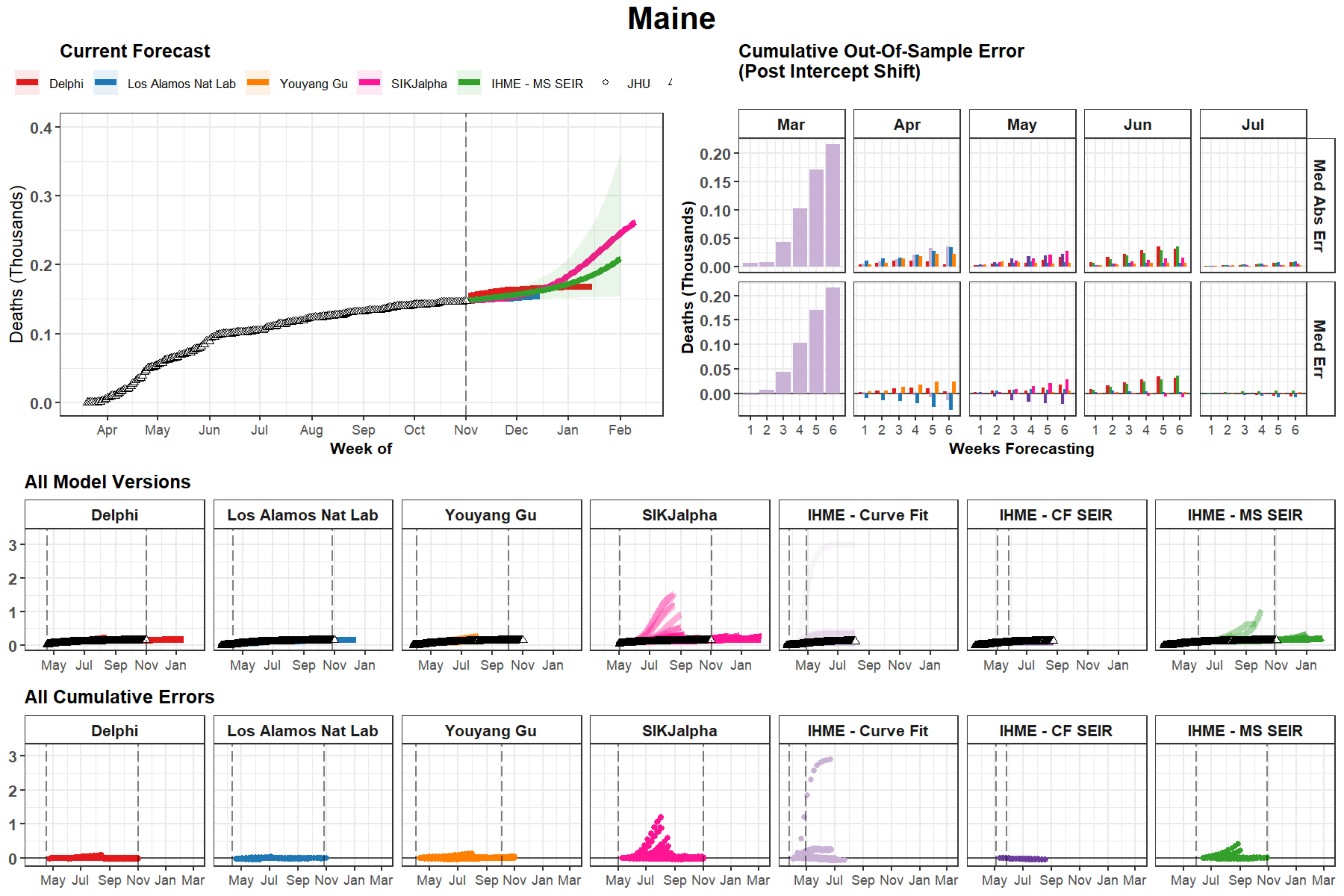
Maine
Current Forecast
Delphi
Los Alamos Nat Lab
Youyang Gu
SIKJalpha
IHME - MS SEIR
JHU
Deaths (Thousands)
Week of
Cumulative Out-Of-Sample Error
(Post Intercept Shift)
Mar
Apr
May
Jun
Jul
Med Abs Err
Med Err
Weeks Forecasting
All Model Versions
IHME - Curve Fit
IHME - CF SEIR
All Cumulative Errors

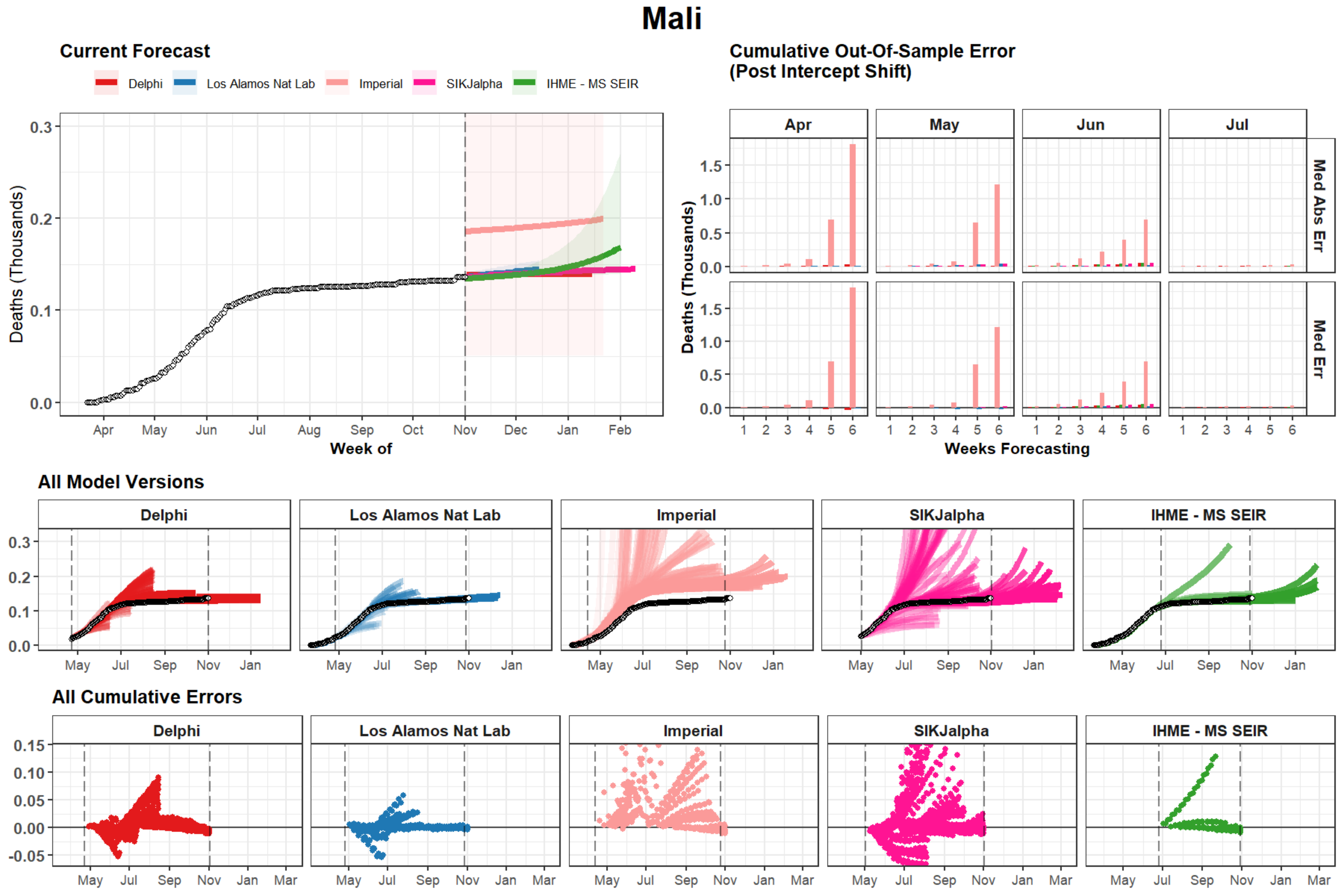
Mali
Current Forecast
Delphi
Los Alamos Nat Lab
Imperial
SIKJalpha
IHME - MS SEIR
Deaths (Thousands)
Week of
Cumulative Out-Of-Sample Error
(Post Intercept Shift)
Apr
May
Jun
Jul
Med Abs Err
Med Err
Weeks Forecasting
All Model Versions
All Cumulative Errors

Namibia

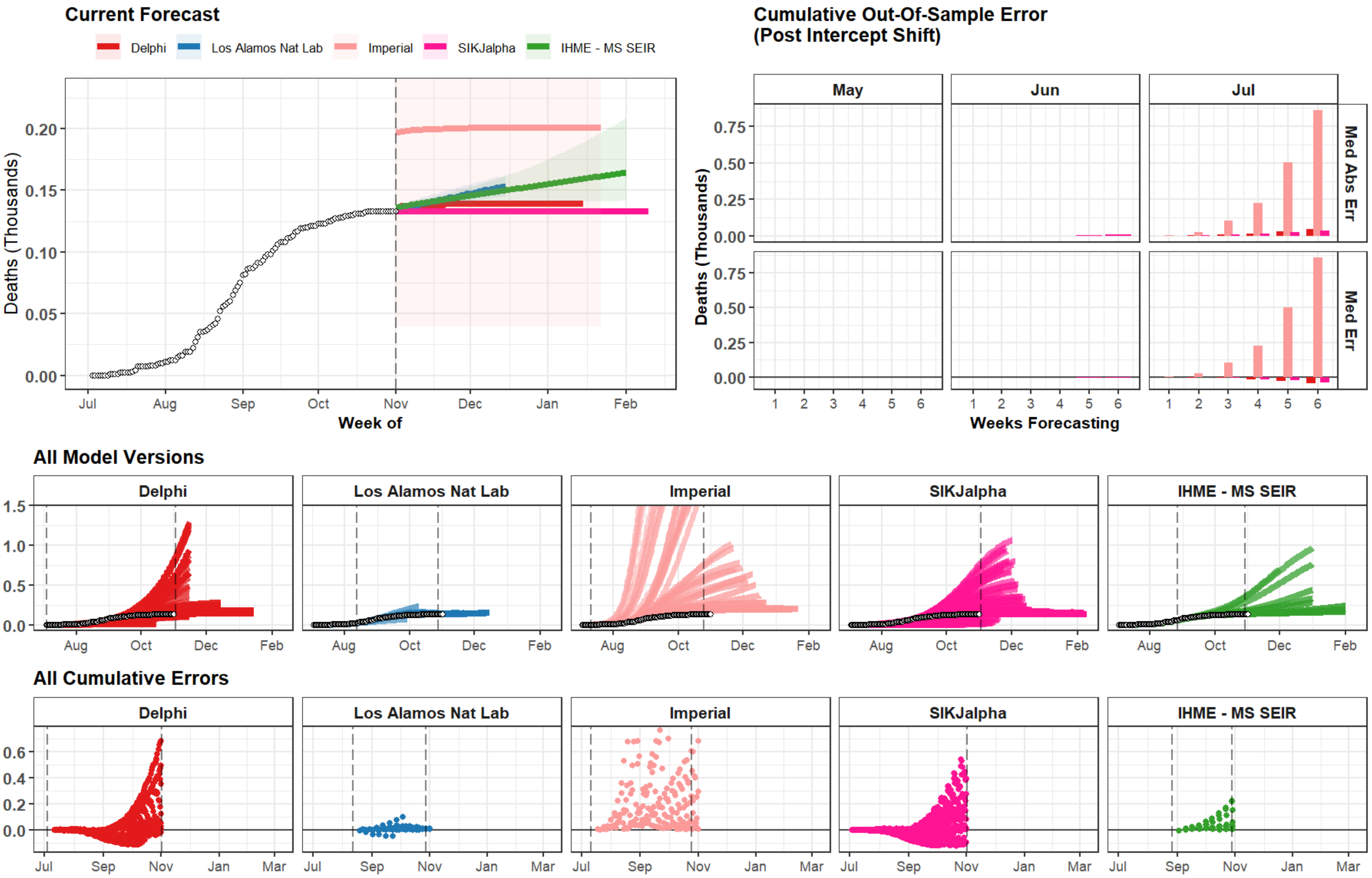
Current Forecast
Delphi
Los Alamos Nat Lab
Imperial
SIKJalpha
IHME - MS SEIR
Deaths (Thousands)
Week of
Cumulative Out-Of-Sample Error
(Post Intercept Shift)
May
Jun
Jul
Med Abs Err
Med Err
Weeks Forecasting
All Model Versions
All Cumulative Errors

Cuba

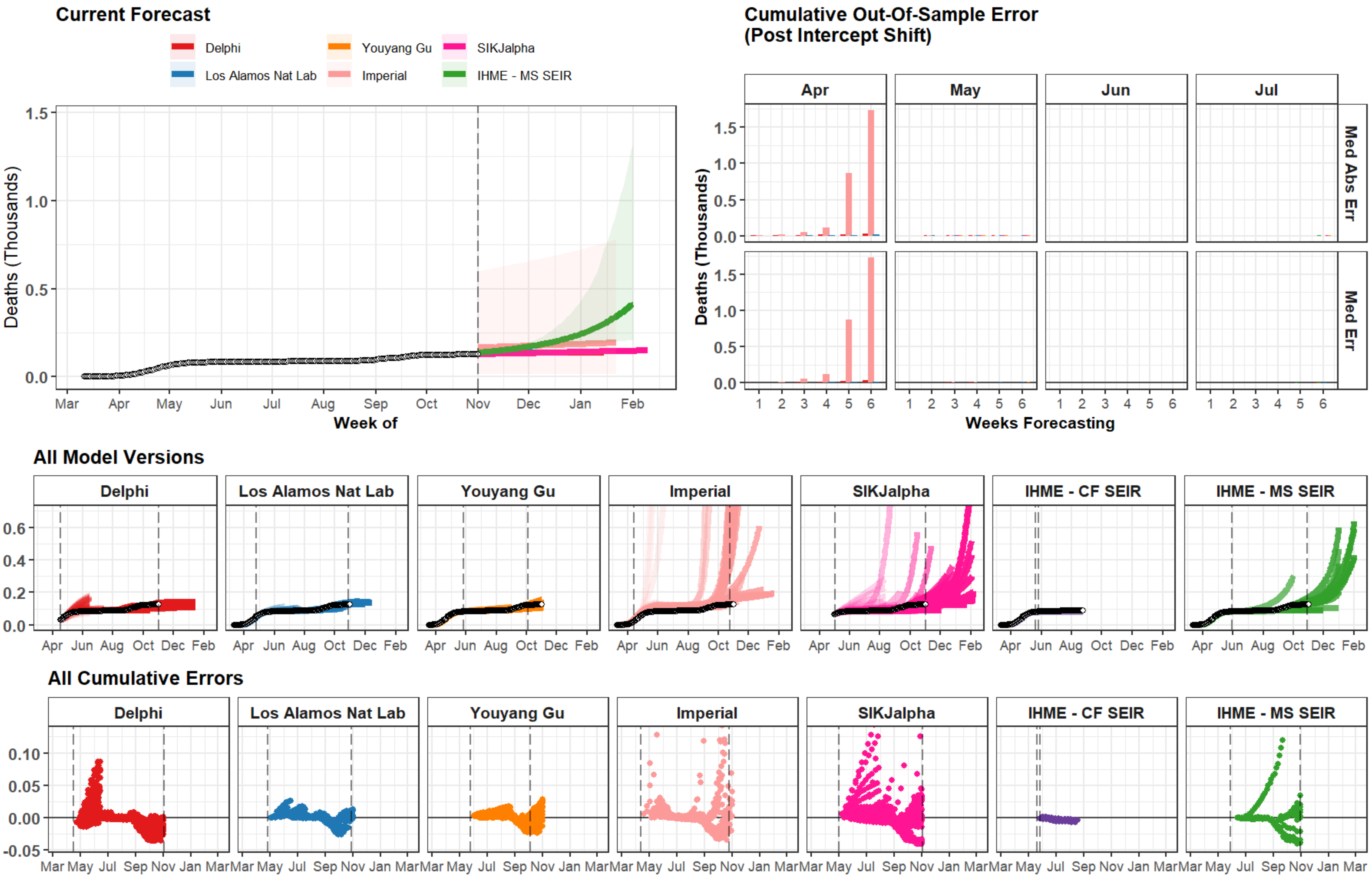
Current Forecast
Delphi
Los Alamos Nat Lab
Youyang Gu
Imperial
SIKJalpha
IHME - MS SEIR
Deaths (Thousands)
1.5
1.0
0.5
0.0
Mar
Apr
May
Jun
Jul
Aug
Sep
Oct
Nov
Dec
Jan
Feb
Week of
Cumulative Out-Of-Sample Error
(Post Intercept Shift)
Apr
May
Jun
Jul
Med Abs Err
Med Err
Deaths (Thousands)
1 2 3 4 5 6
Weeks Forecasting
All Model Versions
Delphi
Los Alamos Nat Lab
Youyang Gu
Imperial
SIKJalpha
IHME - CF SEIR
IHME - MS SEIR
0.6
0.4
0.2
0.0
Apr Jun Aug Oct Dec Feb
All Cumulative Errors
Delphi
Los Alamos Nat Lab
Youyang Gu
Imperial
SIKJalpha
IHME - CF SEIR
IHME - MS SEIR
0.10
0.05
0.00
-0.05
Mar May Jul Sep Nov Jan Mar

Cote d'Ivoire

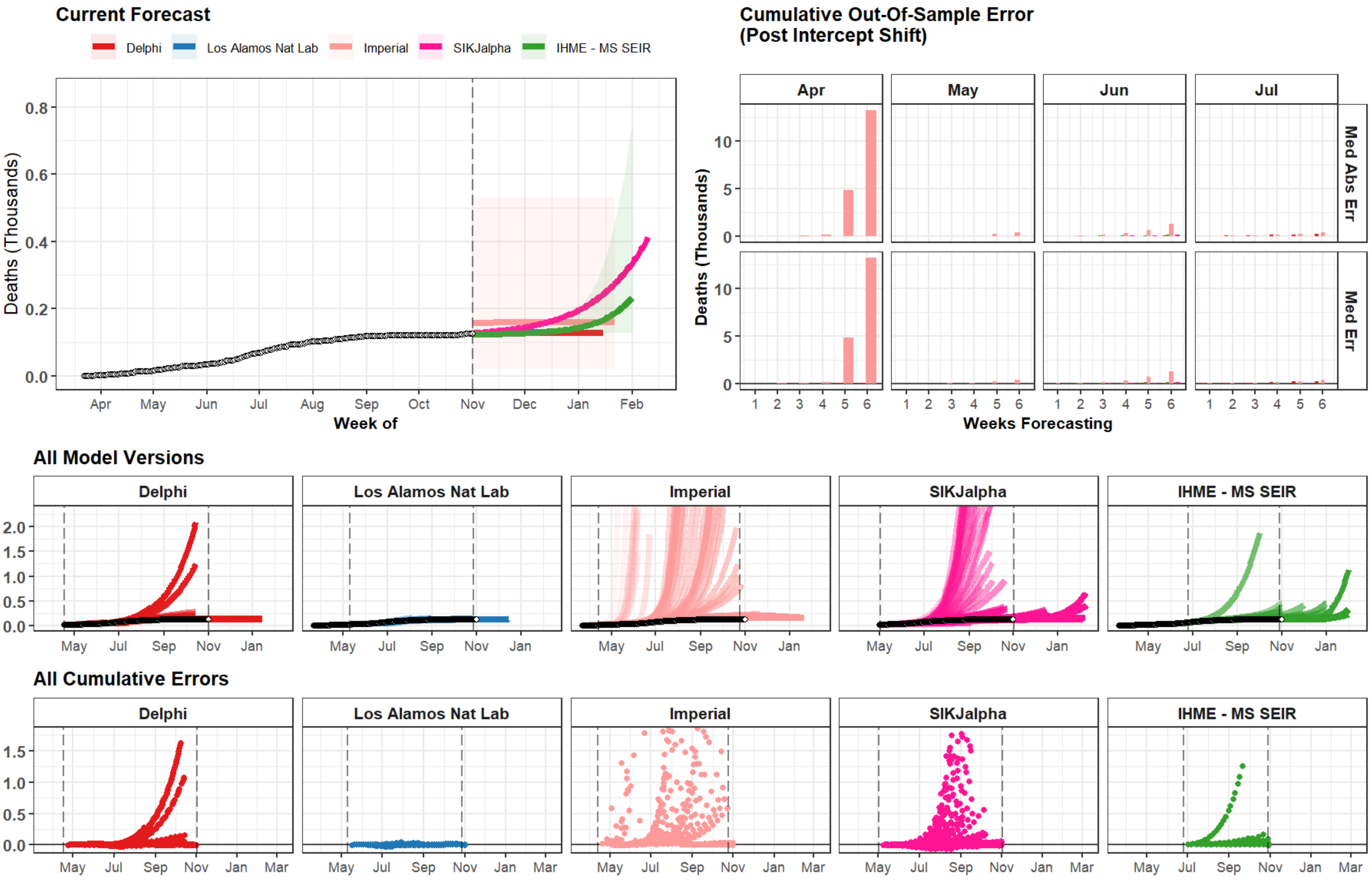

Guyana

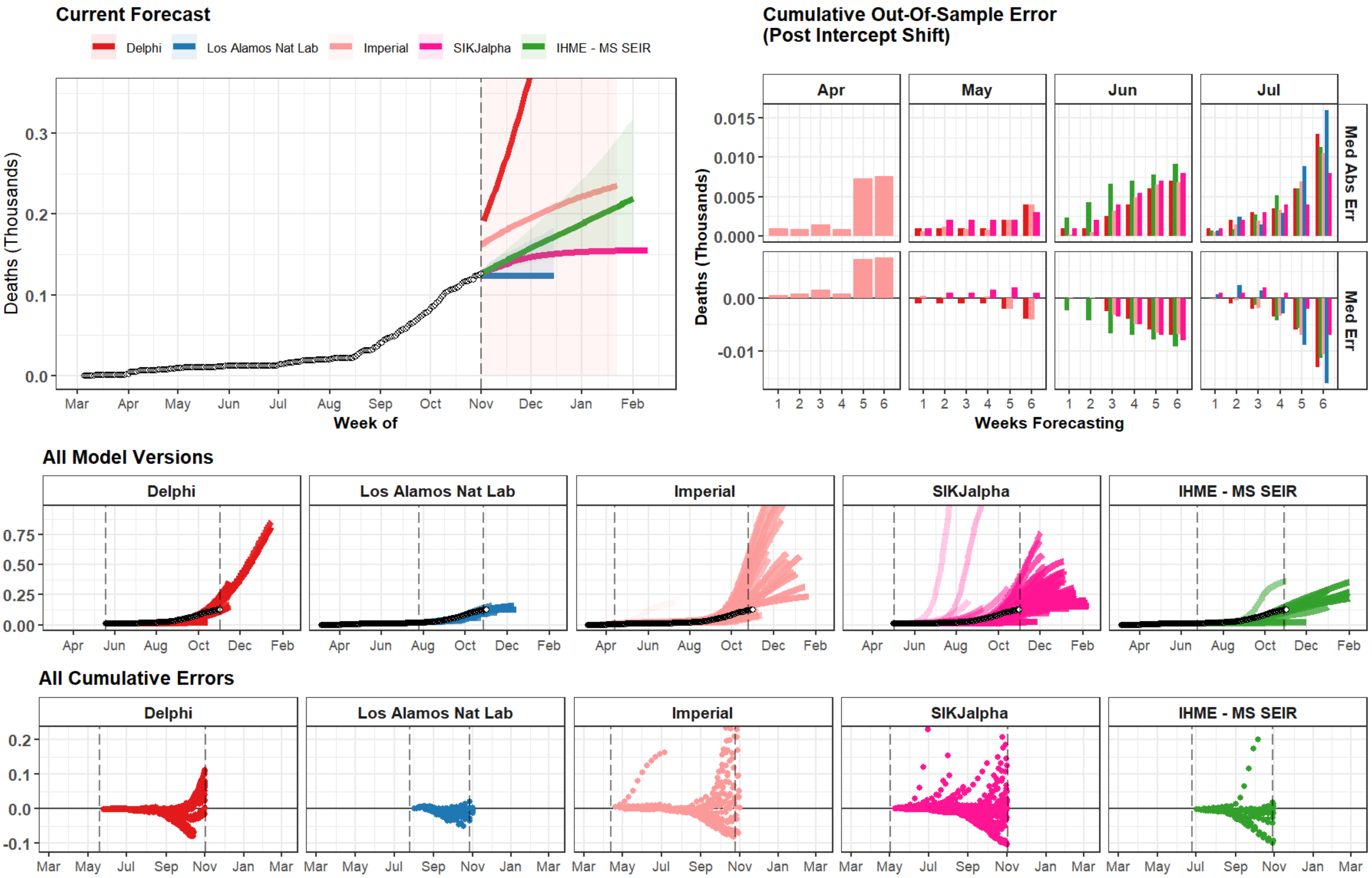

Current Forecast
Delphi
Los Alamos Nat Lab
Imperial
SIKJalpha
IHME - MS SEIR
Deaths (Thousands)
Week of
Cumulative Out-Of-Sample Error
(Post Intercept Shift)
Apr
May
Jun
Jul
Med Abs Err
Med Err
Weeks Forecasting
All Model Versions
All Cumulative Errors

Swaziland

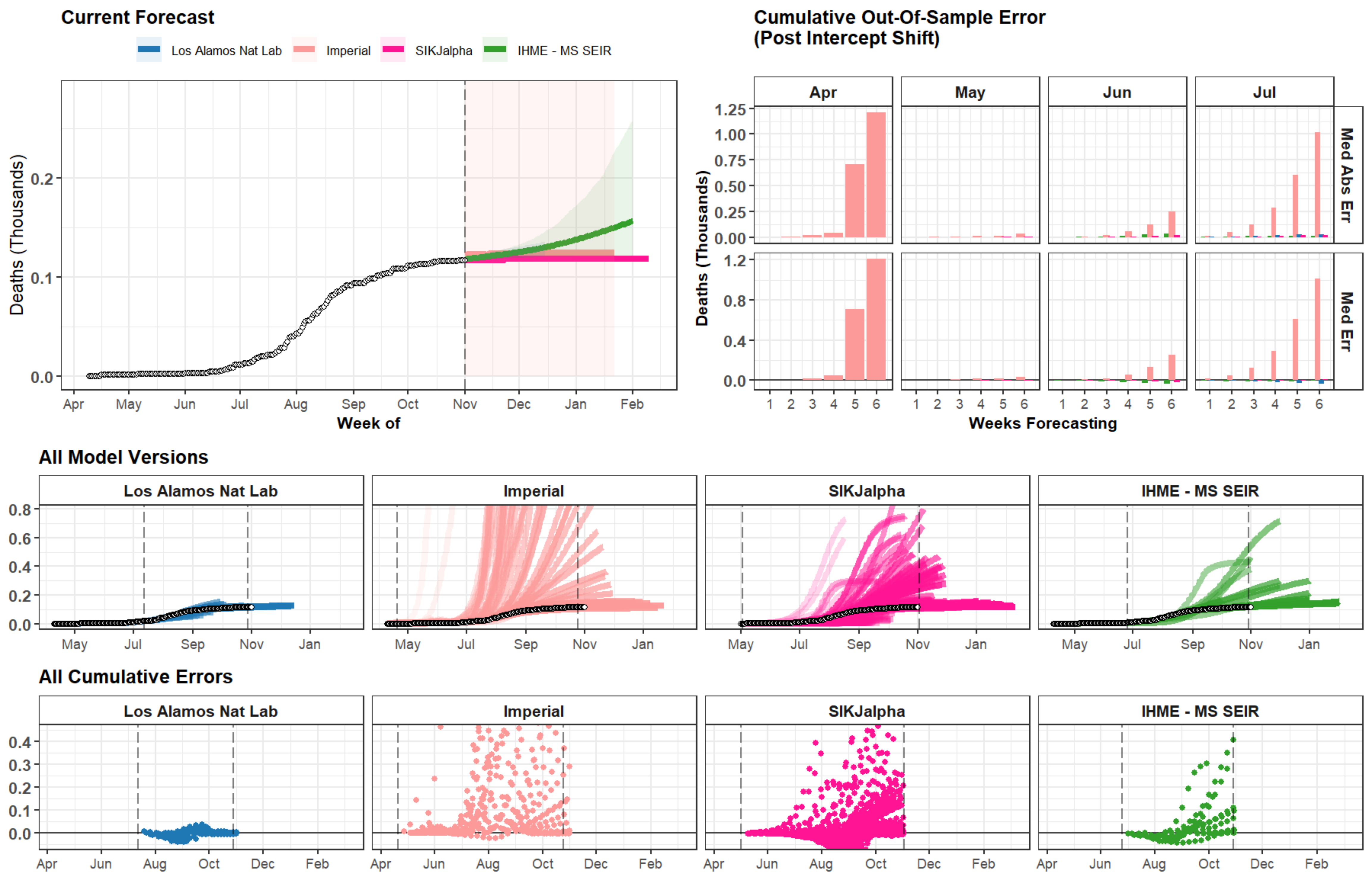
Current Forecast
Los Alamos Nat Lab
Imperial
SIKJalpha
IHME - MS SEIR
Deaths (Thousands)
Week of
Cumulative Out-Of-Sample Error
(Post Intercept Shift)
Apr
May
Jun
Jul
Med Abs Err
Med Err
Weeks Forecasting
All Model Versions
All Cumulative Errors

Uganda

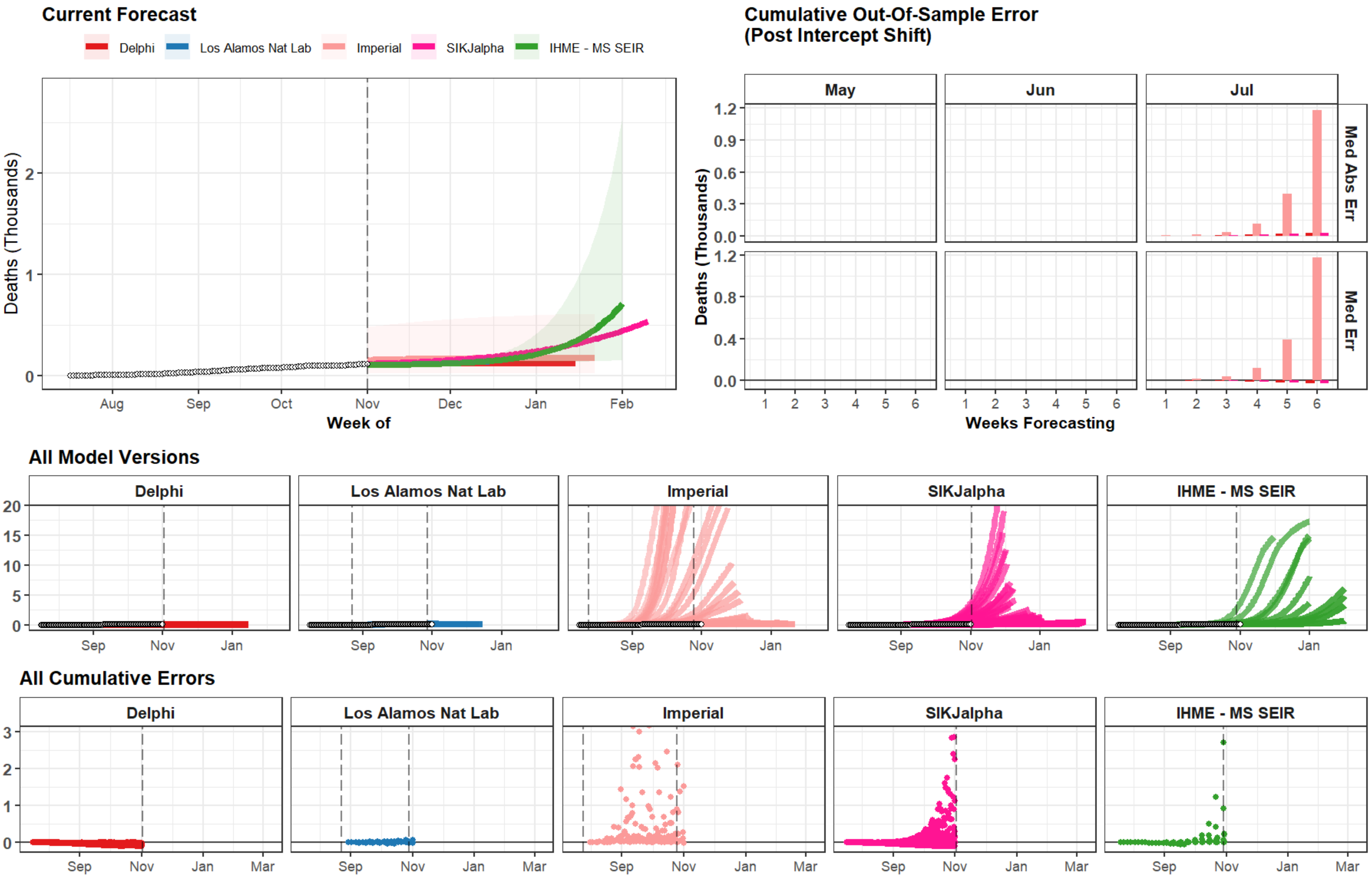
Current Forecast
Delphi
Los Alamos Nat Lab
Imperial
SIKJalpha
IHME - MS SEIR
Deaths (Thousands)
Week of
Aug
Sep
Oct
Nov
Dec
Jan
Feb
Cumulative Out-Of-Sample Error
(Post Intercept Shift)
May
Jun
Jul
Med Abs Err
Med Err
Weeks Forecasting
All Model Versions
All Cumulative Errors
Mar

Suriname

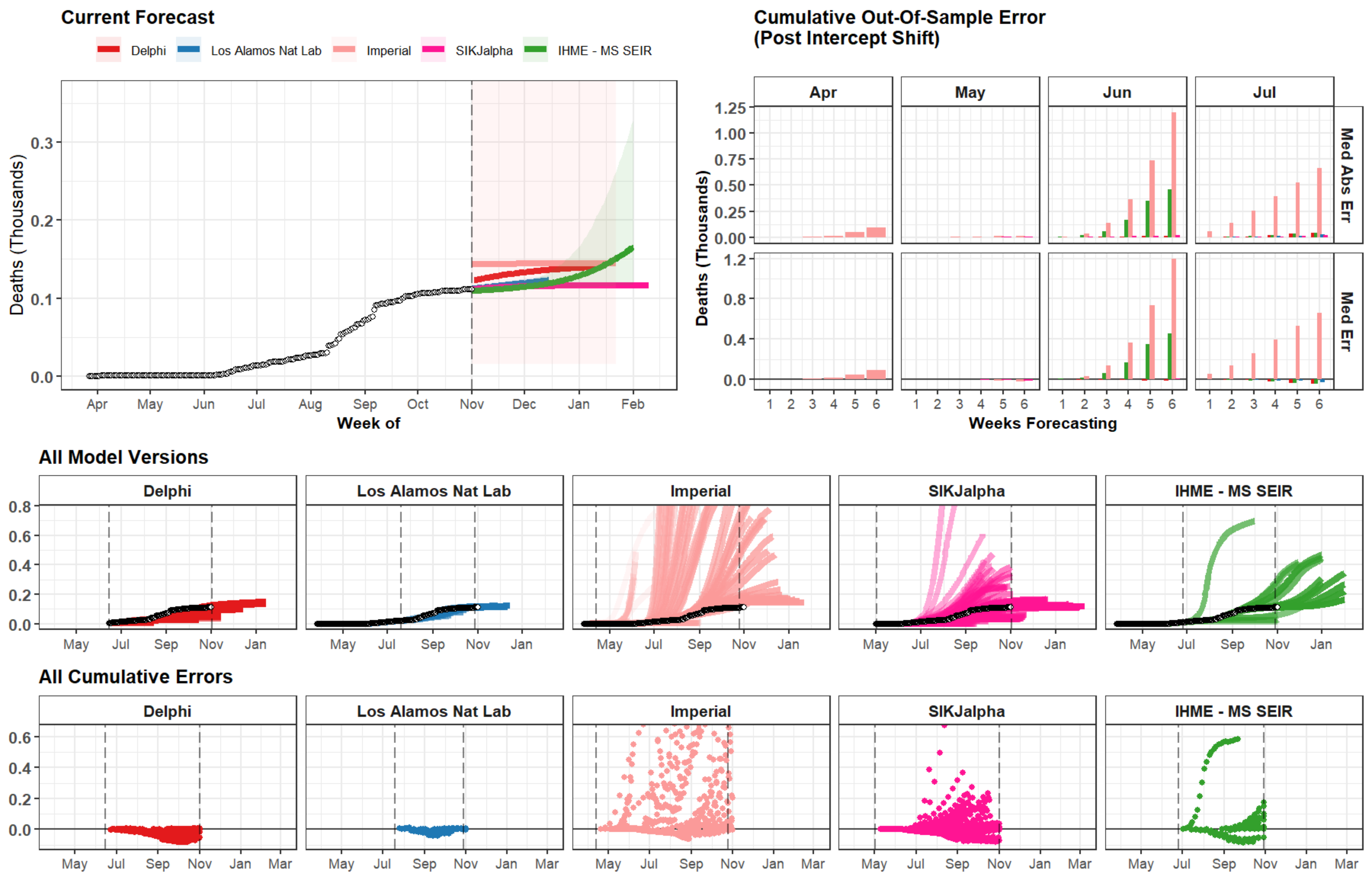

Current Forecast
Delphi
Los Alamos Nat Lab
Imperial
SIKJalpha
IHME - MS SEIR
Deaths (Thousands)
Week of
Apr
May
Jun
Jul
Aug
Sep
Oct
Nov
Dec
Jan
Feb
Cumulative Out-Of-Sample Error
(Post Intercept Shift)
Med Abs Err
Med Err
Weeks Forecasting
All Model Versions
All Cumulative Errors
Mar

Trinidad and Tobago

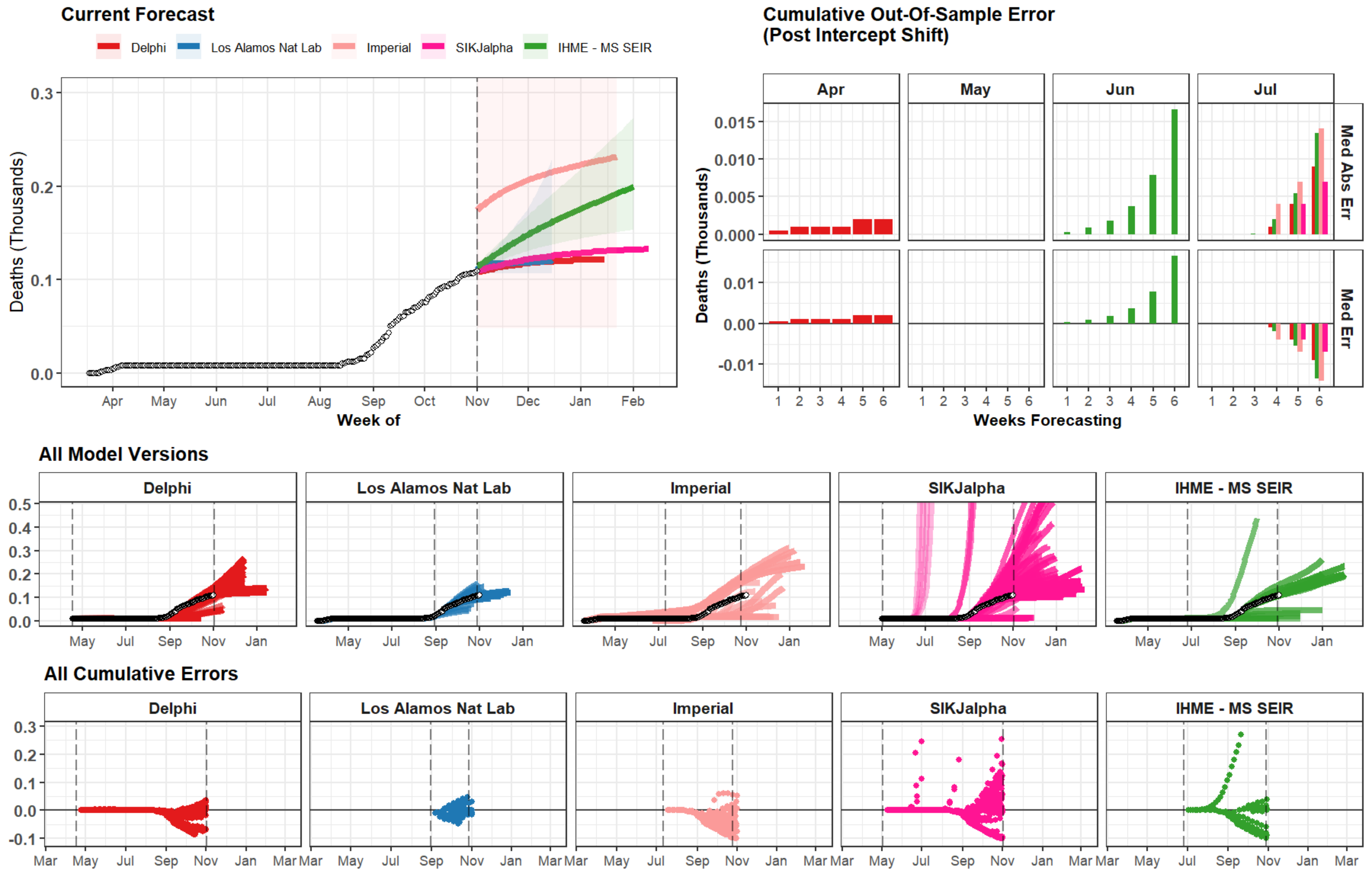

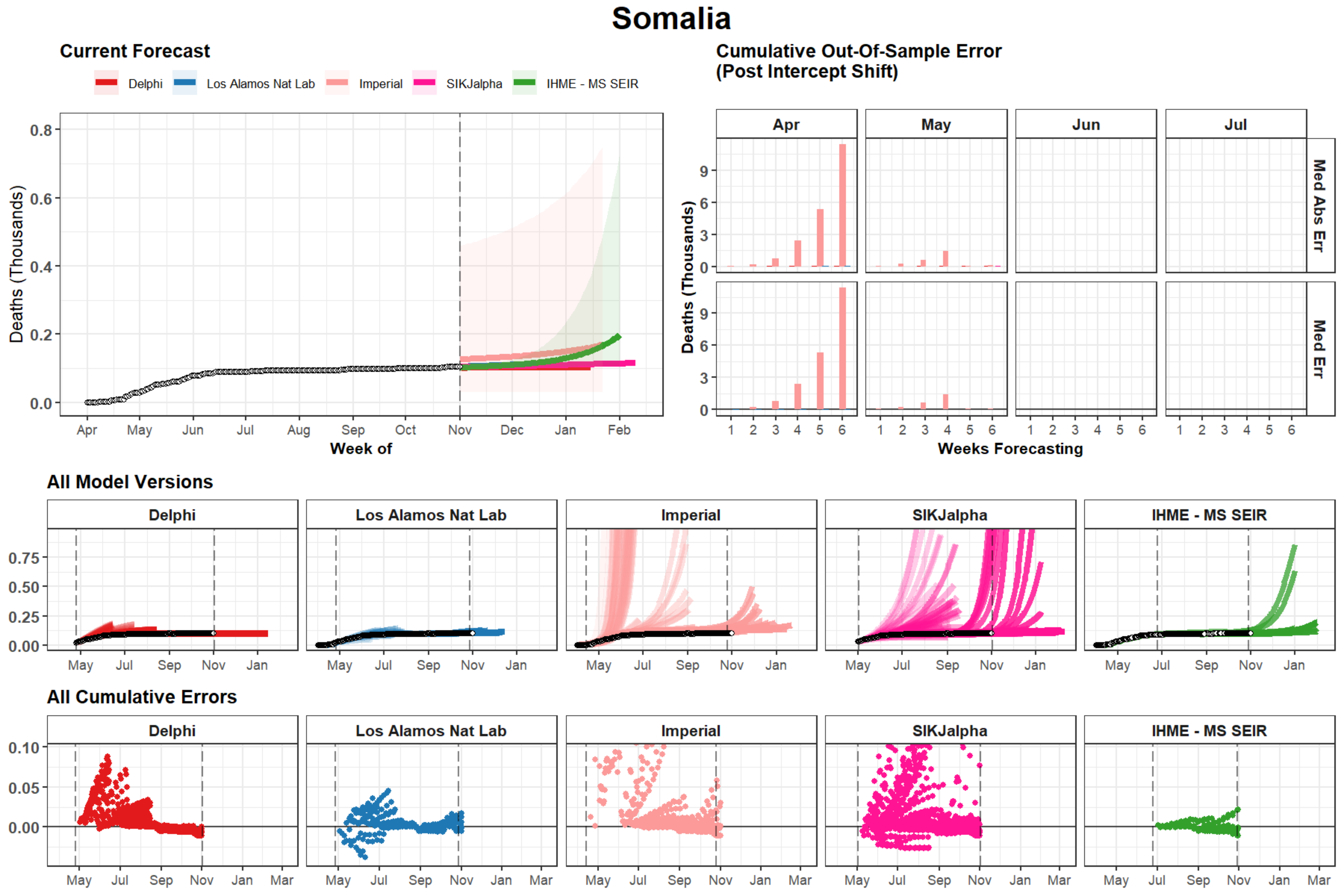
Somalia
Current Forecast
Delphi
Los Alamos Nat Lab
Imperial
SIKJalpha
IHME - MS SEIR
Deaths (Thousands)
0.0
0.2
0.4
0.6
0.8
Apr
May
Jun
Jul
Aug
Sep
Oct
Nov
Dec
Jan
Feb
Week of
Cumulative Out-Of-Sample Error
(Post Intercept Shift)
Apr
May
Jun
Jul
Med Abs Err
Med Err
0
3
6
9
1
2
3
4
5
6
Weeks Forecasting
All Model Versions
0.00
0.25
0.50
0.75
Jan
All Cumulative Errors
0.00
0.05
0.10
Mar

Chad

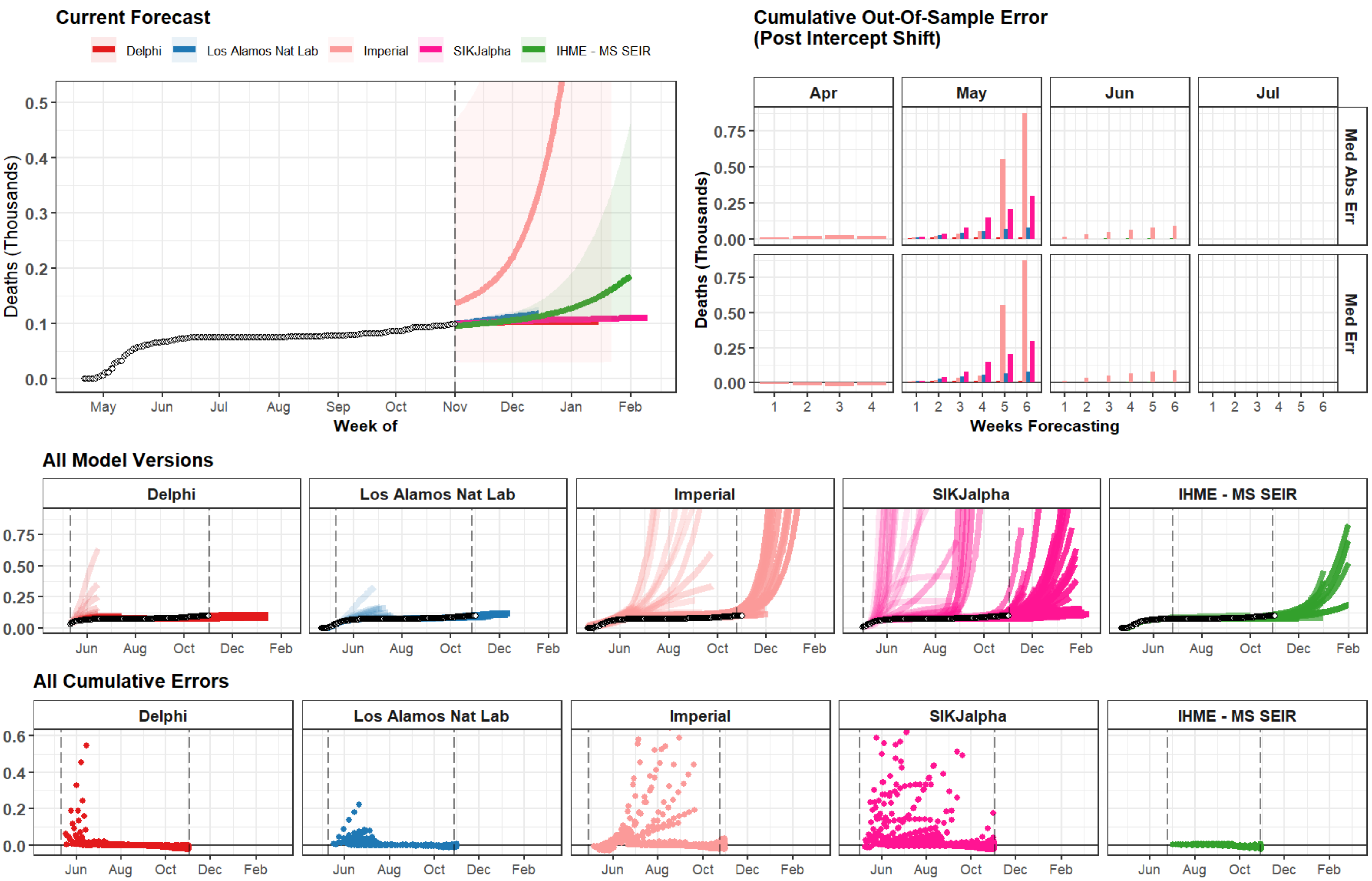

Current Forecast
Delphi
Los Alamos Nat Lab
Imperial
SIKJalpha
IHME - MS SEIR
Deaths (Thousands)
Week of
May
Jun
Jul
Aug
Sep
Oct
Nov
Dec
Jan
Feb
Cumulative Out-Of-Sample Error (Post Intercept Shift)
Apr
May
Jun
Jul
Med Abs Err
Med Err
Weeks Forecasting
All Model Versions
All Cumulative Errors

Cape Verde

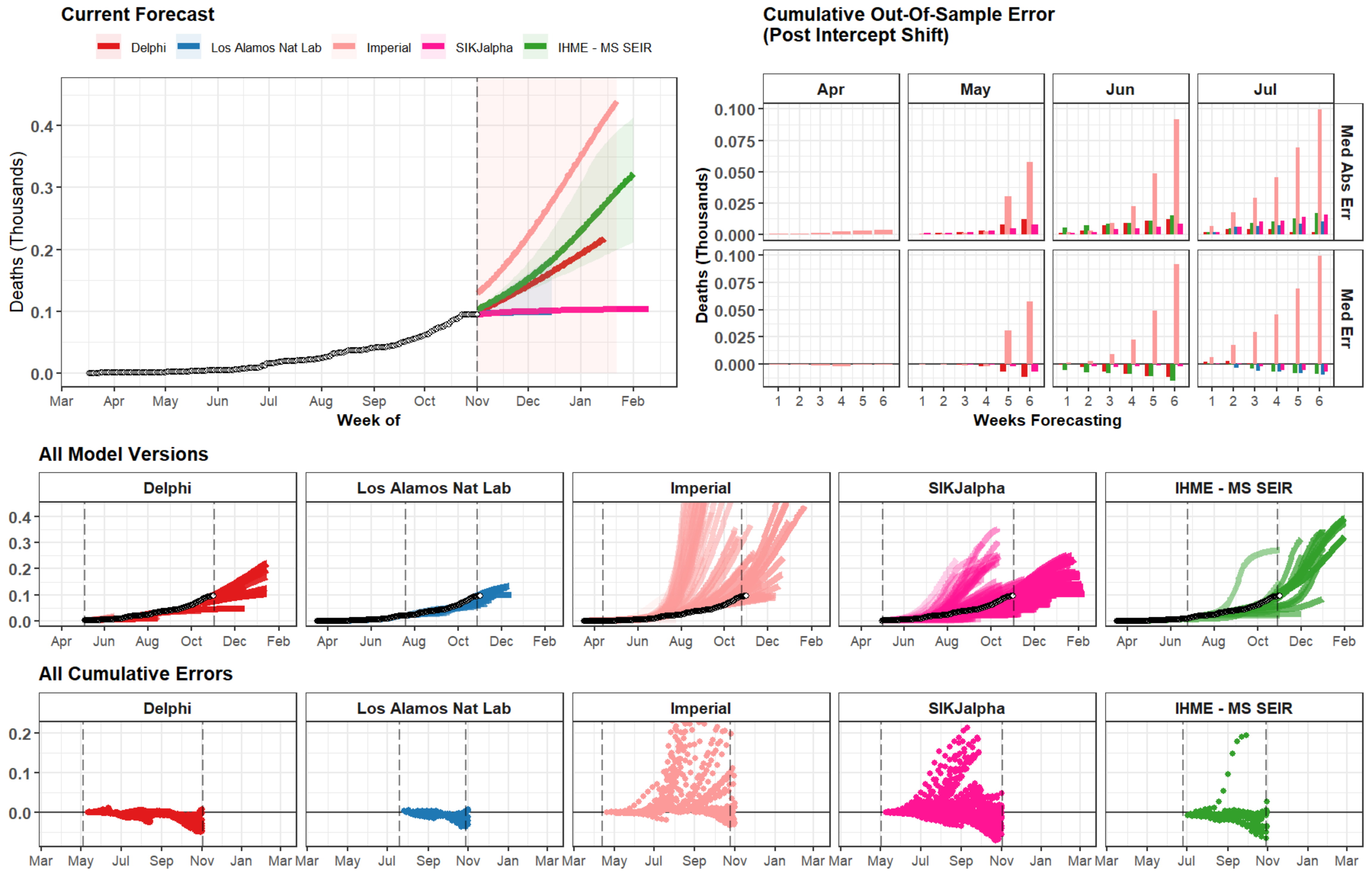

Current Forecast
Delphi
Los Alamos Nat Lab
Imperial
SIKJalpha
IHME - MS SEIR
Deaths (Thousands)
Week of
Cumulative Out-Of-Sample Error
(Post Intercept Shift)
Apr
May
Jun
Jul
Med Abs Err
Med Err
Weeks Forecasting
All Model Versions
All Cumulative Errors

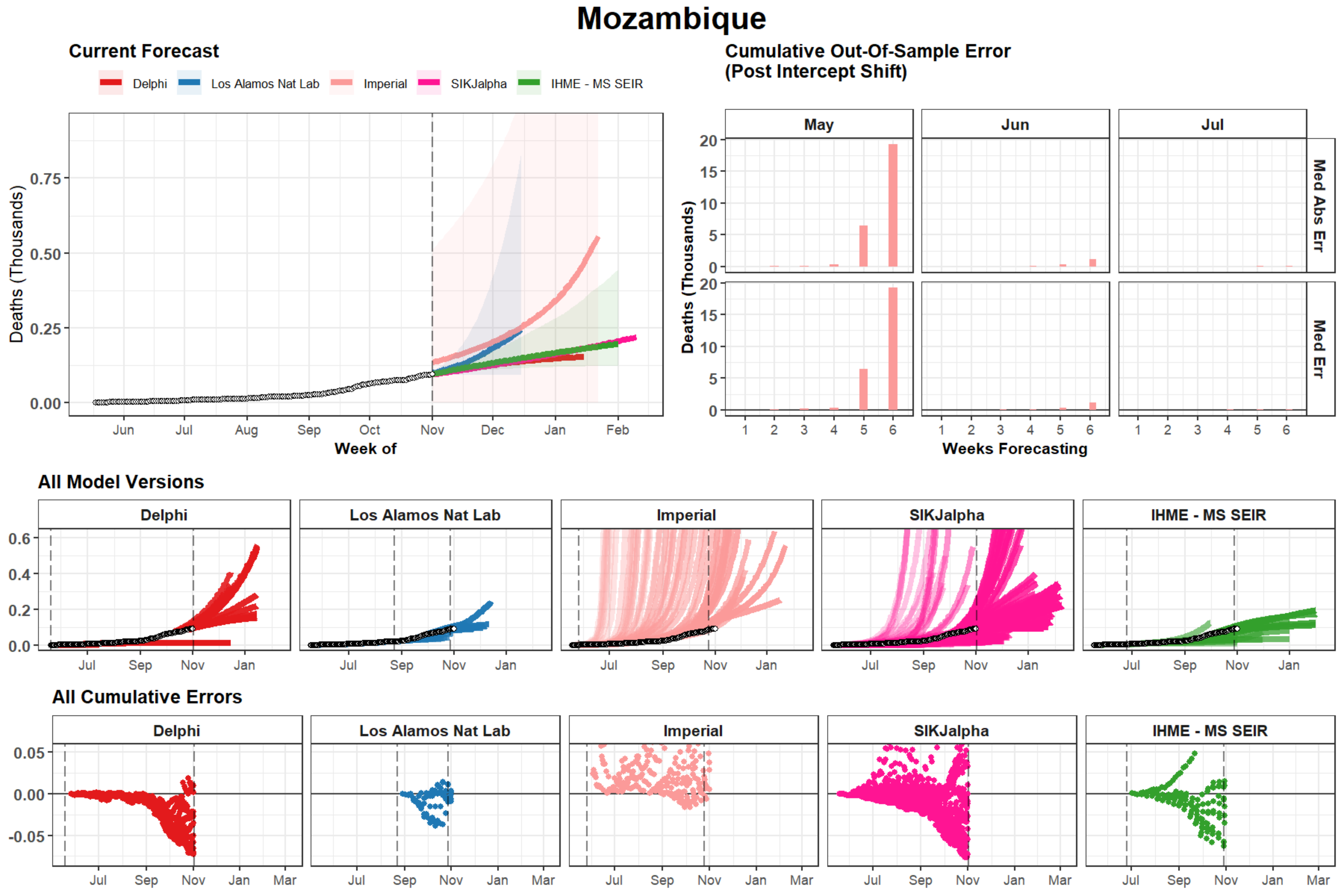

Mozambique
Current Forecast
Delphi
Los Alamos Nat Lab
Imperial
SIKJalpha
IHME - MS SEIR
Deaths (Thousands)
0.75
0.50
0.25
0.00
Jun
Jul
Aug
Sep
Oct
Nov
Dec
Jan
Feb
Week of
Cumulative Out-Of-Sample Error
(Post Intercept Shift)
May
Jun
Jul
Med Abs Err
Med Err
Weeks Forecasting
All Model Versions
All Cumulative Errors
Mar

Congo

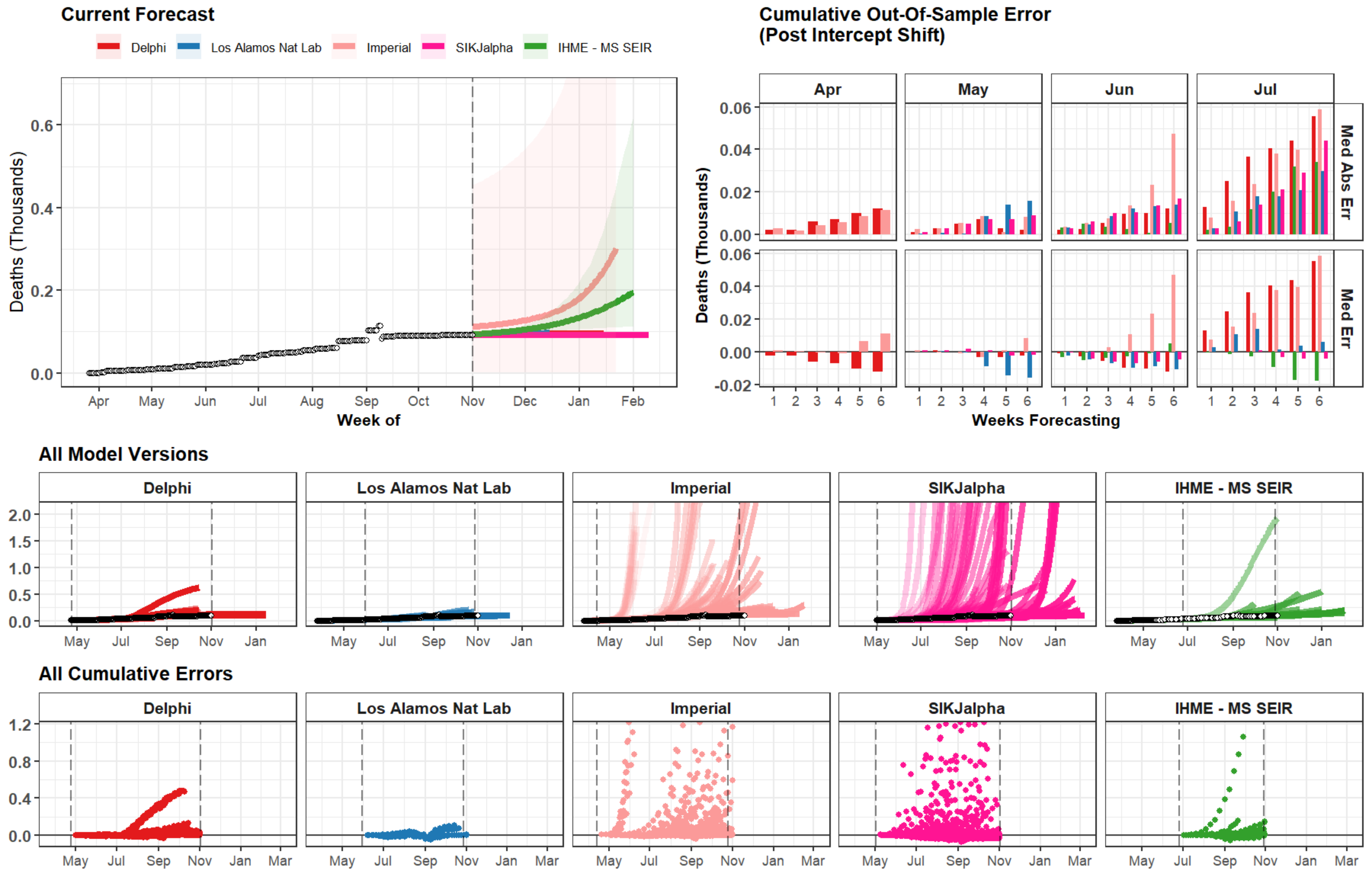
Current Forecast
Delphi
Los Alamos Nat Lab
Imperial
SIKJalpha
IHME - MS SEIR
Deaths (Thousands)
Week of
Apr
May
Jun
Jul
Aug
Sep
Oct
Nov
Dec
Jan
Feb
Cumulative Out-Of-Sample Error
(Post Intercept Shift)
Med Abs Err
Med Err
Weeks Forecasting
All Model Versions
All Cumulative Errors
Mar

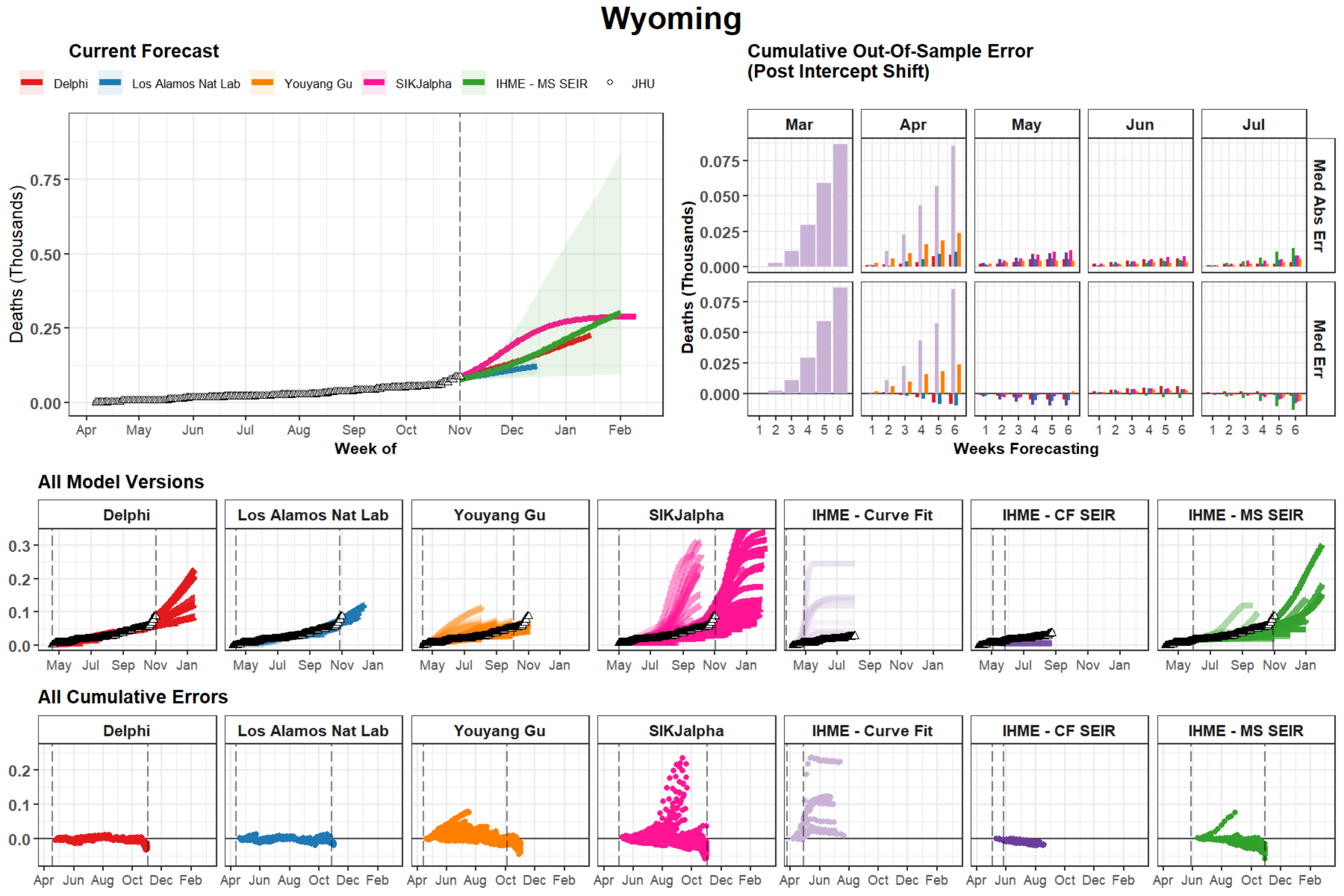
Wyoming
Current Forecast
Delphi
Los Alamos Nat Lab
Youyang Gu
SIKJalpha
IHME - MS SEIR
JHU
Deaths (Thousands)
0.00
0.25
0.50
0.75
Apr May Jun Jul Aug Sep Oct Nov Dec Jan Feb
Week of
Cumulative Out-Of-Sample Error
(Post Intercept Shift)
Mar
Apr
May
Jun
Jul
Med Abs Err
Med Err
0.000
0.025
0.050
0.075
1 2 3 4 5 6
Weeks Forecasting
All Model Versions
Delphi
Los Alamos Nat Lab
Youyang Gu
SIKJalpha
IHME - Curve Fit
IHME - CF SEIR
IHME - MS SEIR
0.0
0.1
0.2
0.3
May Jul Sep Nov Jan
All Cumulative Errors
Delphi
Los Alamos Nat Lab
Youyang Gu
SIKJalpha
IHME - Curve Fit
IHME - CF SEIR
IHME - MS SEIR
0.0
0.1
0.2
Apr Jun Aug Oct Dec Feb

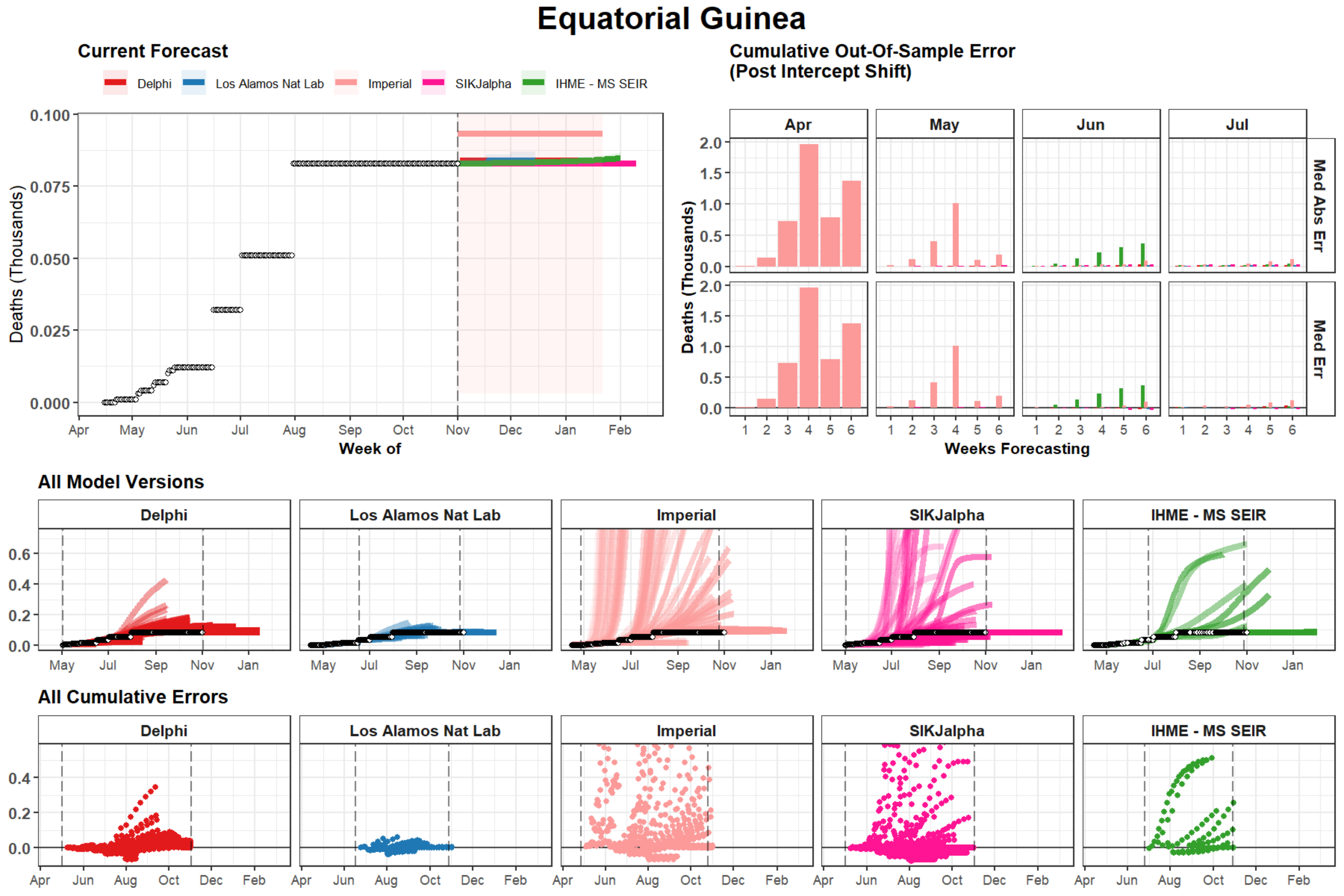
Equatorial Guinea
Current Forecast
Delphi
Los Alamos Nat Lab
Imperial
SIKJalpha
IHME - MS SEIR
Deaths (Thousands)
0.100
0.075
0.050
0.025
0.000
Apr May Jun Jul Aug Sep Oct Nov Dec Jan Feb
Week of
Cumulative Out-Of-Sample Error
(Post Intercept Shift)
Apr
May
Jun
Jul
Med Abs Err
Med Err
2.0
1.5
1.0
0.5
0.0
1 2 3 4 5 6
Weeks Forecasting
All Model Versions
Delphi
Los Alamos Nat Lab
Imperial
SIKJalpha
IHME - MS SEIR
0.6
0.4
0.2
0.0
May Jul Sep Nov Jan
All Cumulative Errors
Delphi
Los Alamos Nat Lab
Imperial
SIKJalpha
IHME - MS SEIR
0.4
0.2
0.0
Apr Jun Aug Oct Dec Feb

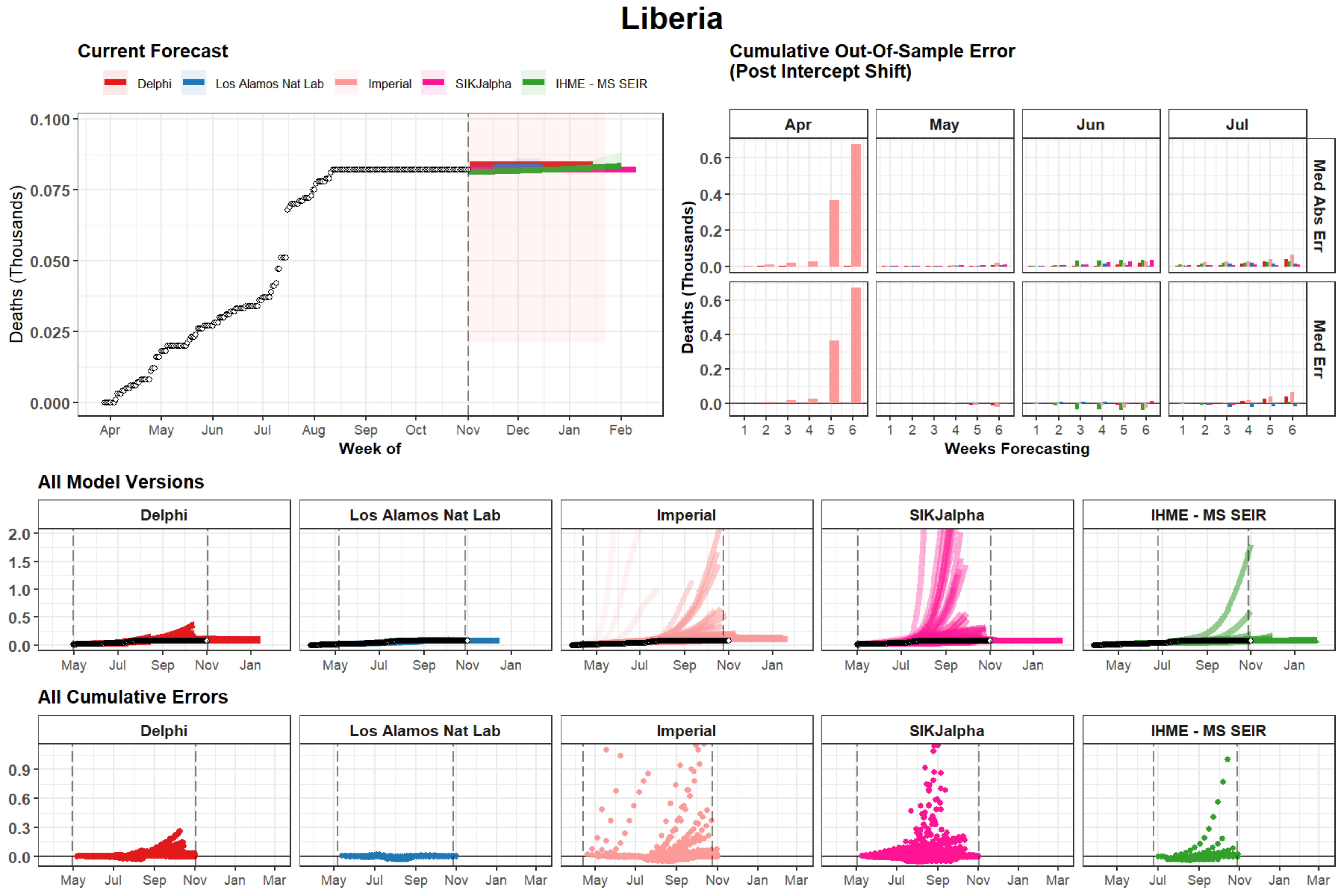
Liberia
Current Forecast
Delphi
Los Alamos Nat Lab
Imperial
SIKJalpha
IHME - MS SEIR
Deaths (Thousands)
Week of
Cumulative Out-Of-Sample Error
(Post Intercept Shift)
Apr
May
Jun
Jul
Med Abs Err
Med Err
Weeks Forecasting
All Model Versions
All Cumulative Errors

Tajikistan

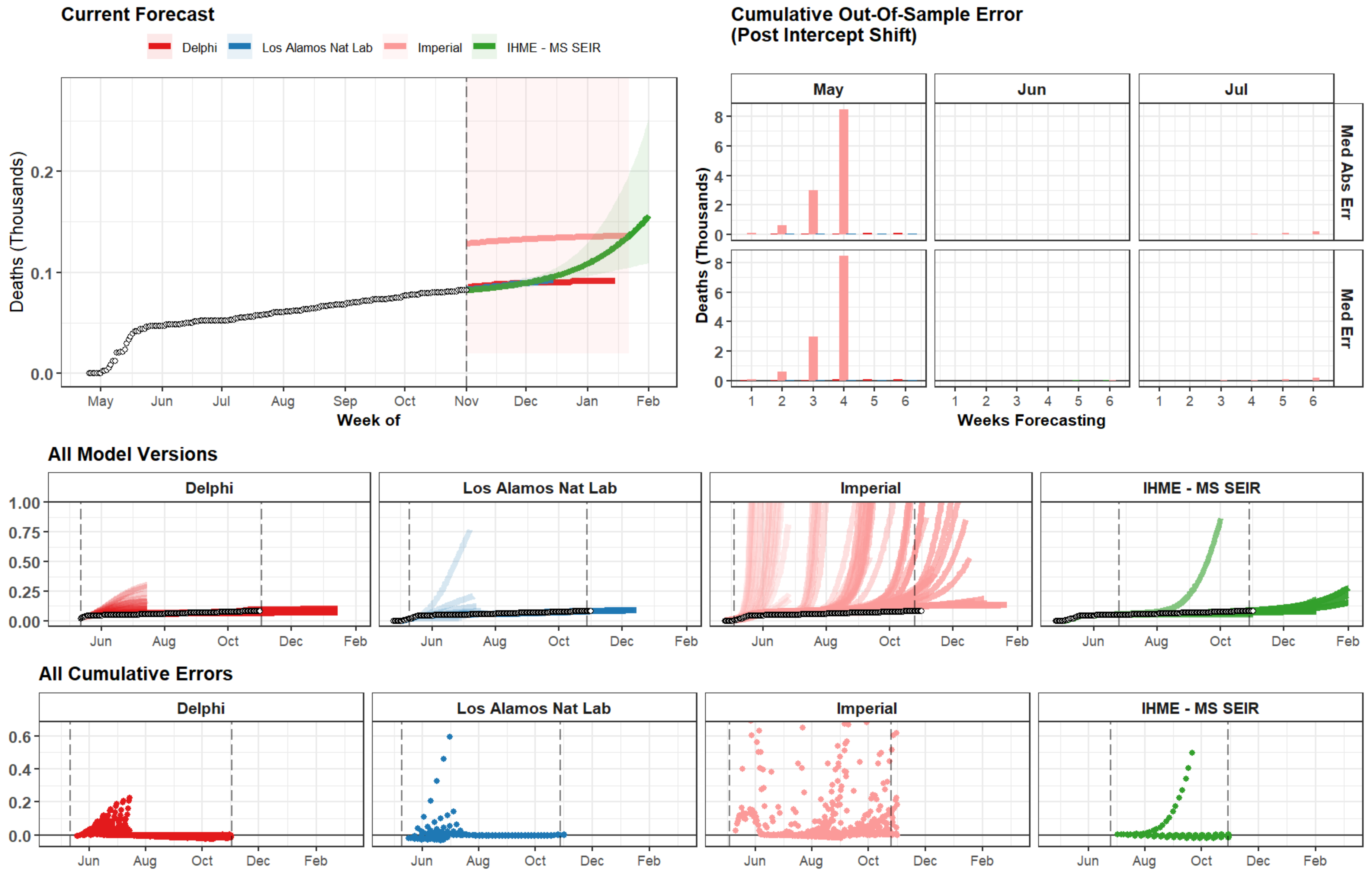
Current Forecast
Delphi
Los Alamos Nat Lab
Imperial
IHME - MS SEIR
Deaths (Thousands)
0.0
0.1
0.2
May
Jun
Jul
Aug
Sep
Oct
Nov
Dec
Jan
Feb
Week of
Cumulative Out-Of-Sample Error
(Post Intercept Shift)
May
Jun
Jul
Med Abs Err
Med Err
Deaths (Thousands)
0
2
4
6
8
1
2
3
4
5
6
Weeks Forecasting
All Model Versions
Delphi
Los Alamos Nat Lab
Imperial
IHME - MS SEIR
0.00
0.25
0.50
0.75
1.00
Jun
Aug
Oct
Dec
Feb
All Cumulative Errors
Delphi
Los Alamos Nat Lab
Imperial
IHME - MS SEIR
0.0
0.2
0.4
0.6
Jun
Aug
Oct
Dec
Feb

Guam

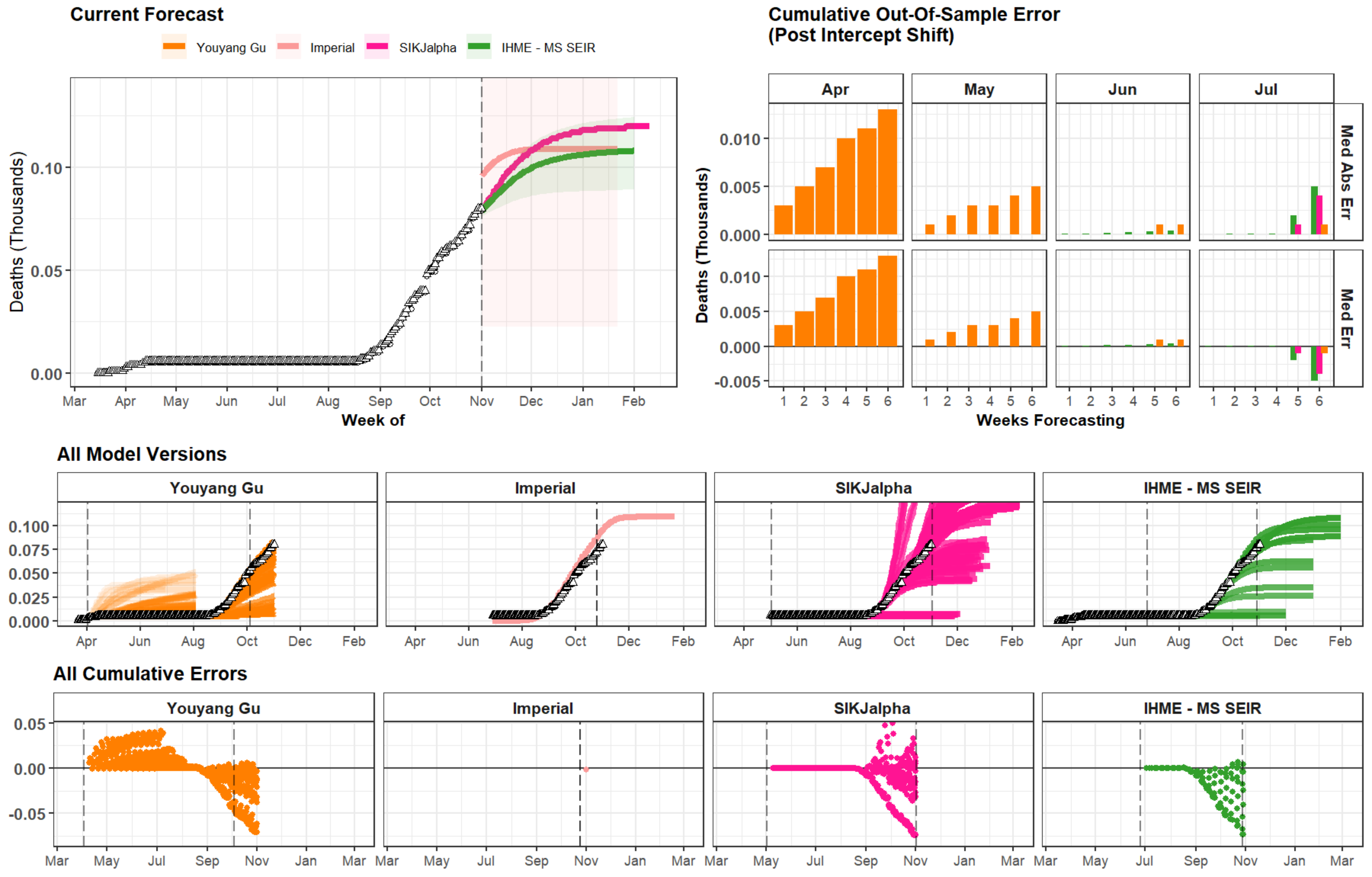

Current Forecast
Youyang Gu
Imperial
SIKJalpha
IHME - MS SEIR
Deaths (Thousands)
0.10
0.05
0.00
Mar
Apr
May
Jun
Jul
Aug
Sep
Oct
Nov
Dec
Jan
Feb
Week of
Cumulative Out-Of-Sample Error
(Post Intercept Shift)
Apr
May
Jun
Jul
Med Abs Err
Med Err
0.010
0.005
0.000
-0.005
1 2 3 4 5 6
Weeks Forecasting
All Model Versions
Youyang Gu
Imperial
SIKJalpha
IHME - MS SEIR
0.100
0.075
0.050
0.025
0.000
Apr
Jun
Aug
Oct
Dec
Feb
All Cumulative Errors
0.05
0.00
-0.05
Mar
May
Jul
Sep
Nov
Jan
Mar

Alaska

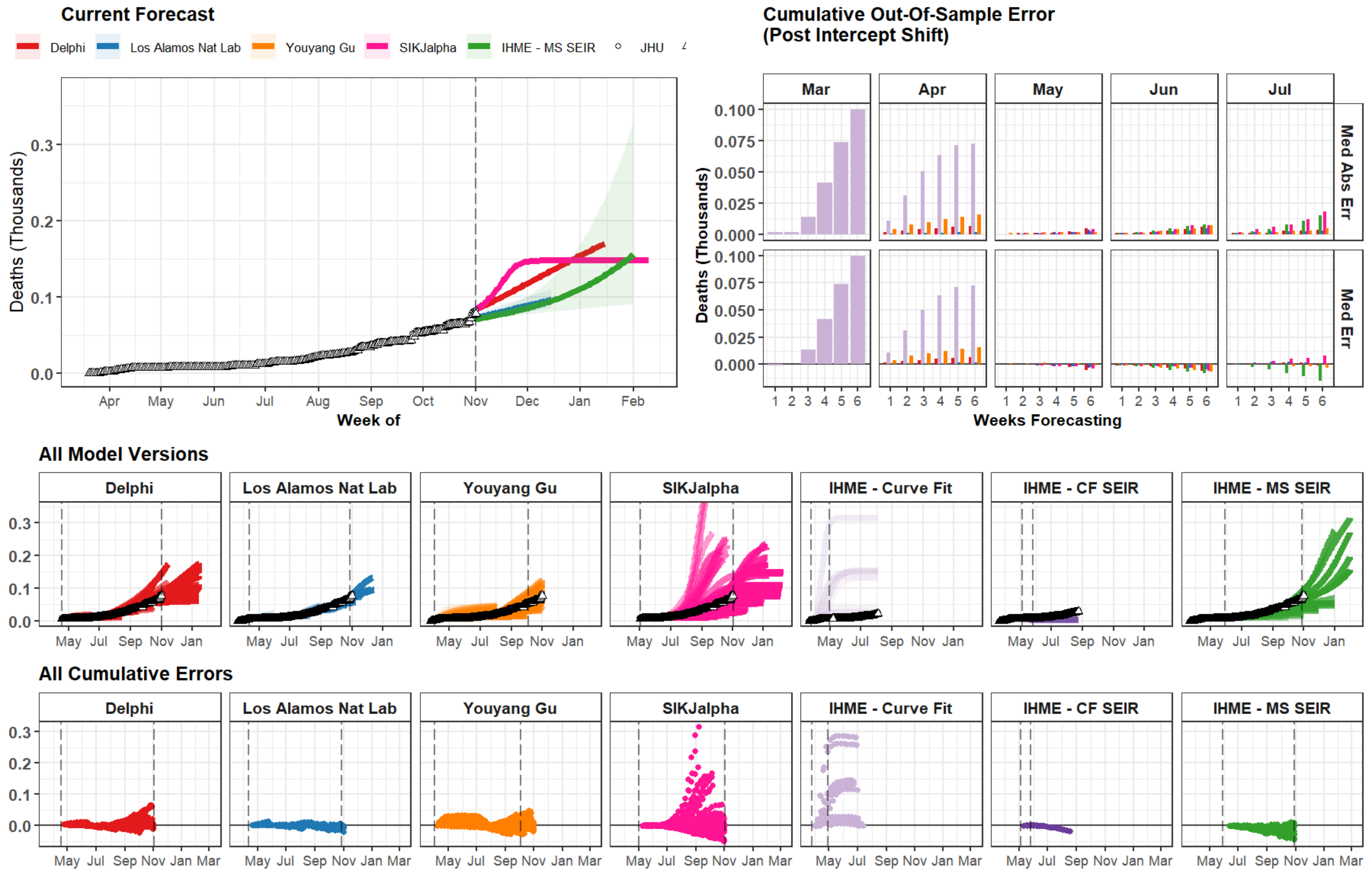
Current Forecast
Delphi
Los Alamos Nat Lab
Youyang Gu
SIKJalpha
IHME - MS SEIR
JHU
Deaths (Thousands)
Week of
Apr May Jun Jul Aug Sep Oct Nov Dec Jan Feb
Cumulative Out-Of-Sample Error
(Post Intercept Shift)
Mar Apr May Jun Jul
Med Abs Err
Med Err
Weeks Forecasting
All Model Versions
Delphi
Los Alamos Nat Lab
Youyang Gu
SIKJalpha
IHME - Curve Fit
IHME - CF SEIR
IHME - MS SEIR
May Jul Sep Nov Jan
All Cumulative Errors
May Jul Sep Nov Jan Mar

Andorra

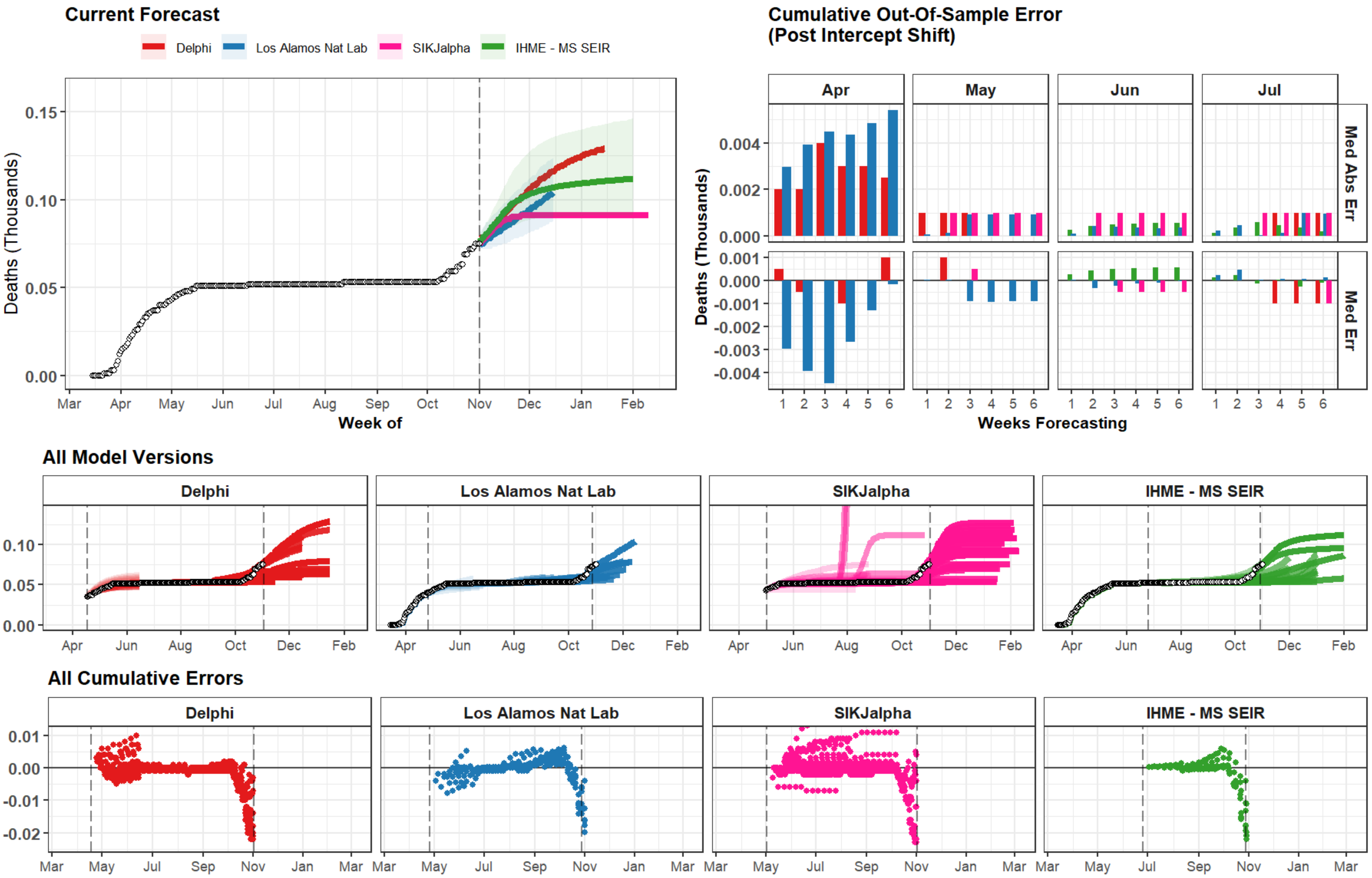
Current Forecast
Delphi
Los Alamos Nat Lab
SIKJalpha
IHME - MS SEIR
Deaths (Thousands)
Week of
Cumulative Out-Of-Sample Error
(Post Intercept Shift)
Apr
May
Jun
Jul
Med Abs Err
Med Err
Weeks Forecasting
All Model Versions
All Cumulative Errors

Latvia

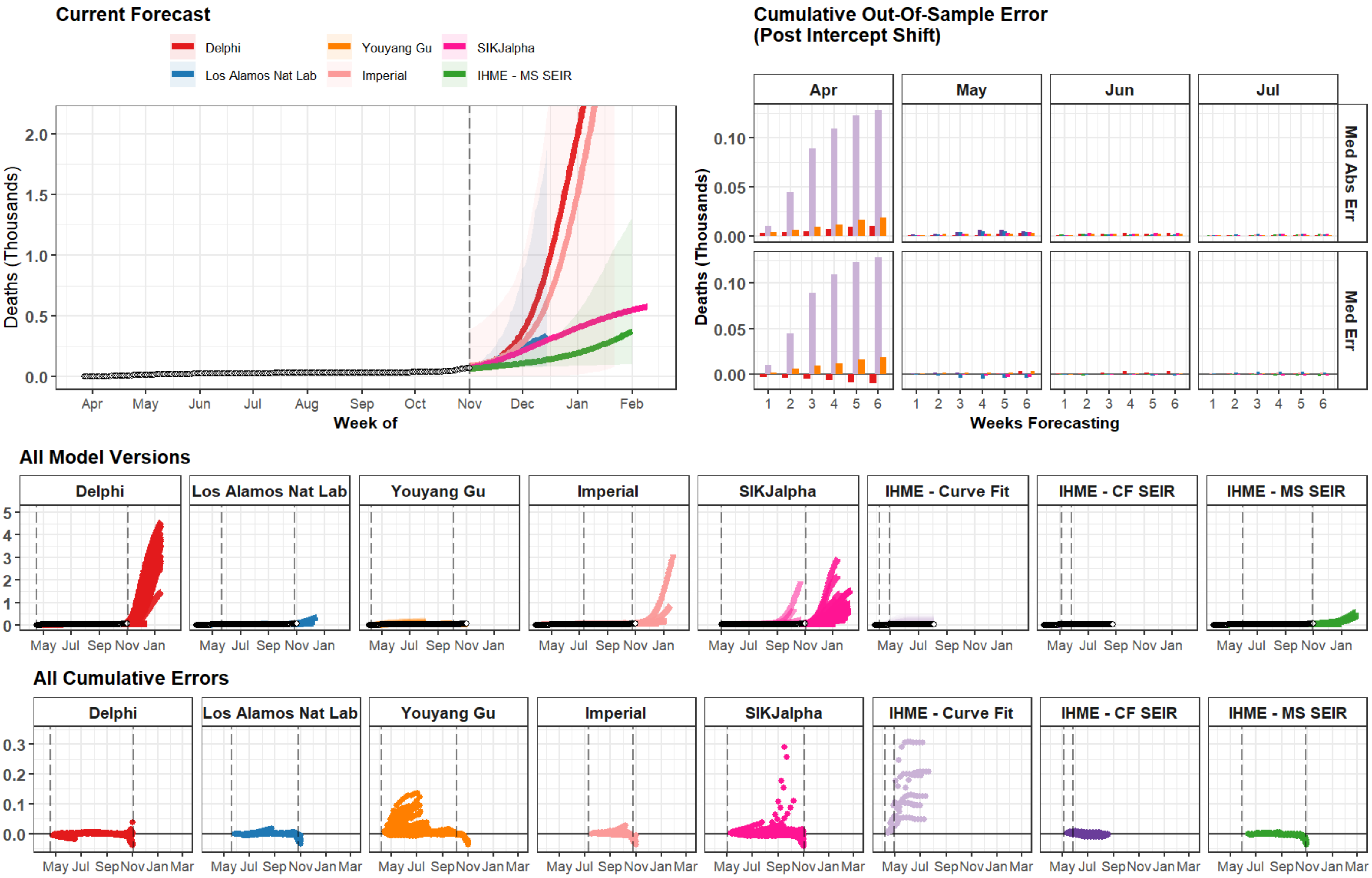
Current Forecast
Delphi
Los Alamos Nat Lab
Youyang Gu
Imperial
SIKJalpha
IHME - MS SEIR
Deaths (Thousands)
2.0
1.5
1.0
0.5
0.0
Apr
May
Jun
Jul
Aug
Sep
Oct
Nov
Dec
Jan
Feb
Week of
Cumulative Out-Of-Sample Error
(Post Intercept Shift)
Apr
May
Jun
Jul
Med Abs Err
Med Err
0.10
0.05
0.00
1 2 3 4 5 6
Weeks Forecasting
All Model Versions
Delphi
Los Alamos Nat Lab
Youyang Gu
Imperial
SIKJalpha
IHME - Curve Fit
IHME - CF SEIR
IHME - MS SEIR
5
4
3
2
1
0
May Jul Sep Nov Jan
All Cumulative Errors
0.3
0.2
0.1
0.0
May Jul Sep Nov Jan Mar

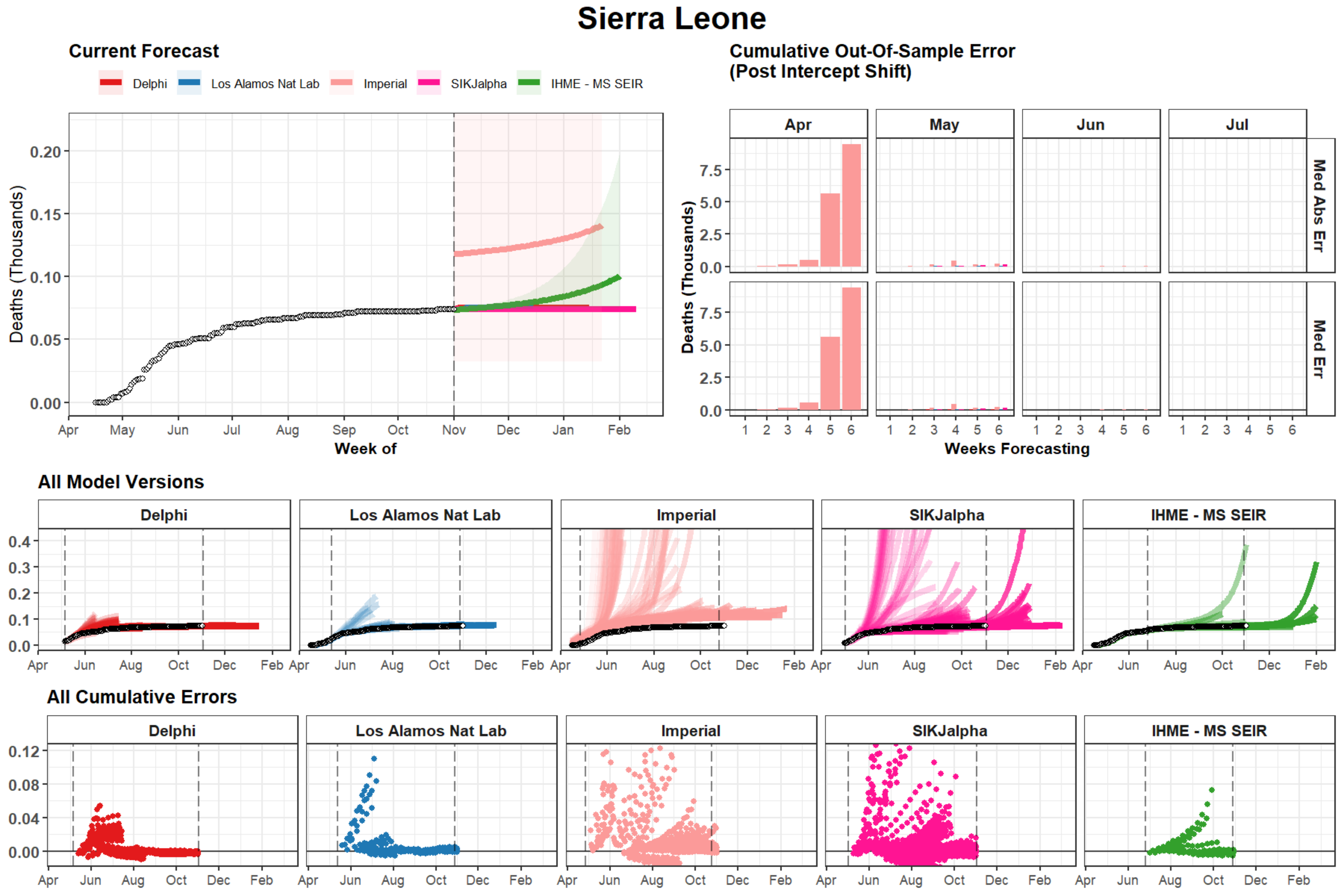

Sierra Leone
Current Forecast
Delphi
Los Alamos Nat Lab
Imperial
SIKJalpha
IHME - MS SEIR
Deaths (Thousands)
0.00
0.05
0.10
0.15
0.20
Apr
May
Jun
Jul
Aug
Sep
Oct
Nov
Dec
Jan
Feb
Week of
Cumulative Out-Of-Sample Error
(Post Intercept Shift)
Apr
May
Jun
Jul
Med Abs Err
Med Err
0.0
2.5
5.0
7.5
1
2
3
4
5
6
Weeks Forecasting
All Model Versions
0.0
0.1
0.2
0.3
0.4
All Cumulative Errors
0.00
0.04
0.08
0.12

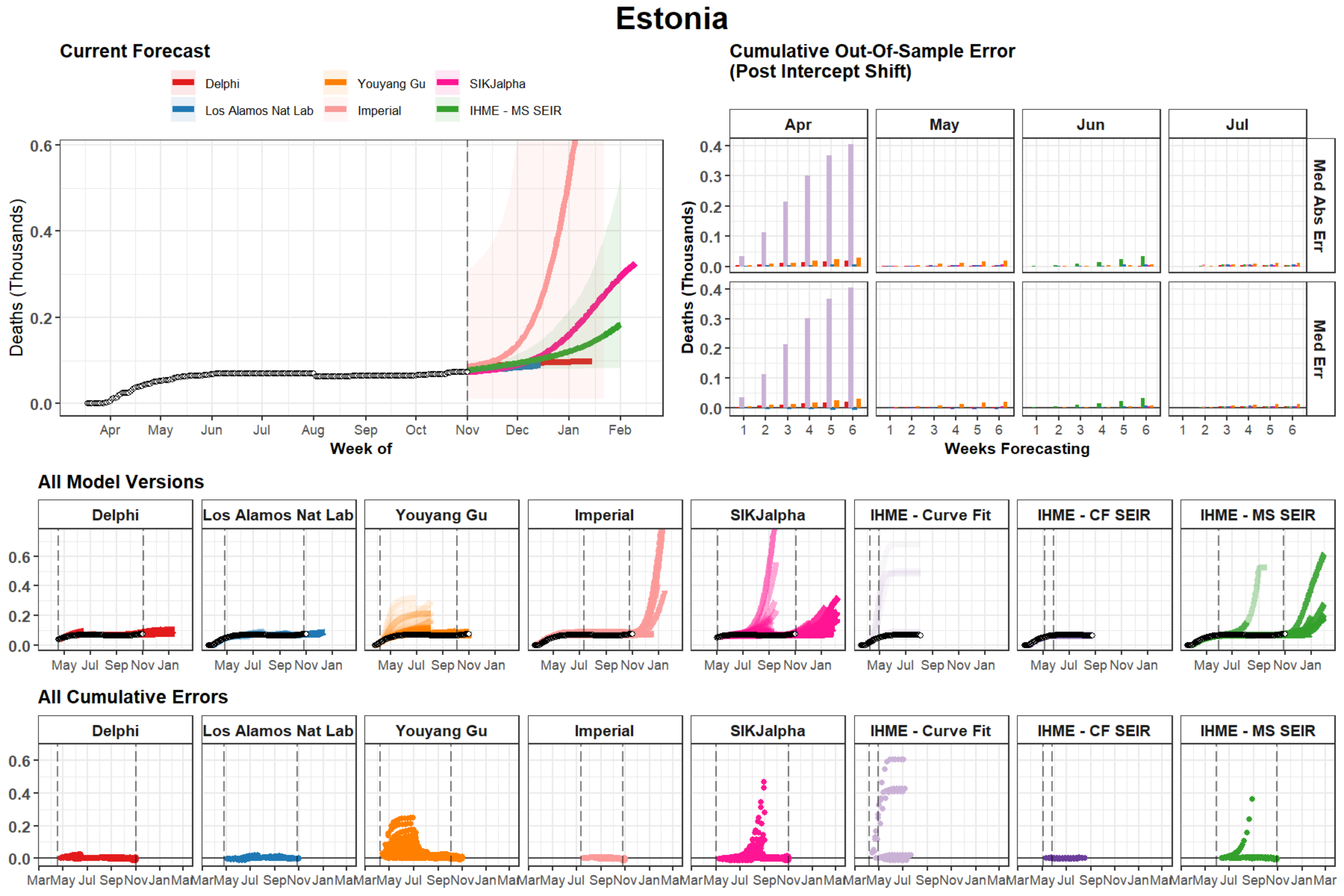
Estonia
Current Forecast
Delphi
Youyang Gu
SIKJalpha
Los Alamos Nat Lab
Imperial
IHME - MS SEIR
Deaths (Thousands)
Week of
Apr May Jun Jul Aug Sep Oct Nov Dec Jan Feb
Cumulative Out-Of-Sample Error
(Post Intercept Shift)
Apr May Jun Jul
Med Abs Err
Med Err
Weeks Forecasting
All Model Versions
Delphi
Los Alamos Nat Lab
Youyang Gu
Imperial
SIKJalpha
IHME - Curve Fit
IHME - CF SEIR
IHME - MS SEIR
All Cumulative Errors

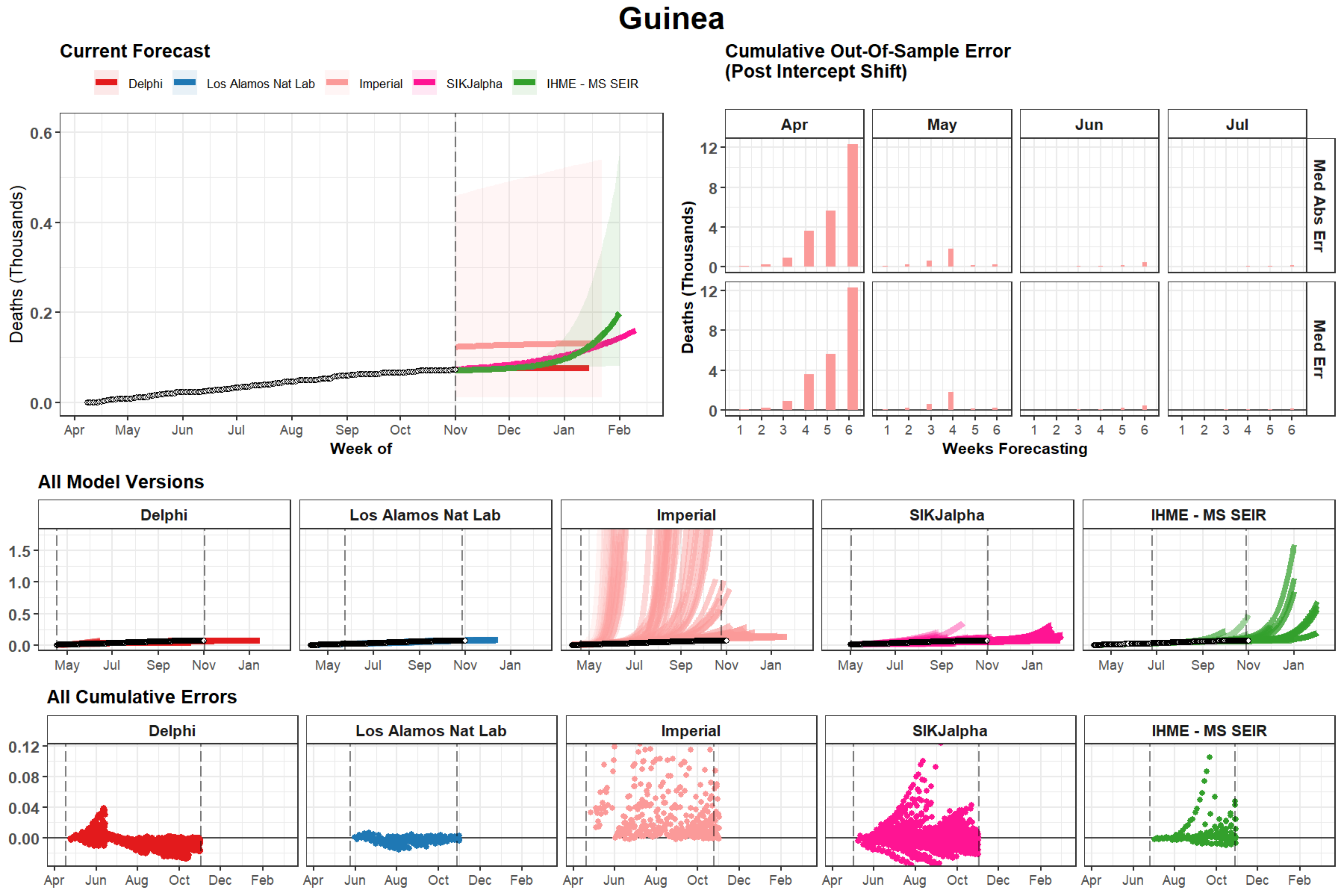
Guinea
Current Forecast
Delphi
Los Alamos Nat Lab
Imperial
SIKJalpha
IHME - MS SEIR
Deaths (Thousands)
0.0
0.2
0.4
0.6
Apr
May
Jun
Jul
Aug
Sep
Oct
Nov
Dec
Jan
Feb
Week of
Cumulative Out-Of-Sample Error
(Post Intercept Shift)
Apr
May
Jun
Jul
Med Abs Err
Med Err
0
4
8
12
1 2 3 4 5 6
Weeks Forecasting
All Model Versions
0.0
0.5
1.0
1.5
May Jul Sep Nov Jan
All Cumulative Errors
0.00
0.04
0.08
0.12
Apr Jun Aug Oct Dec Feb

Niger

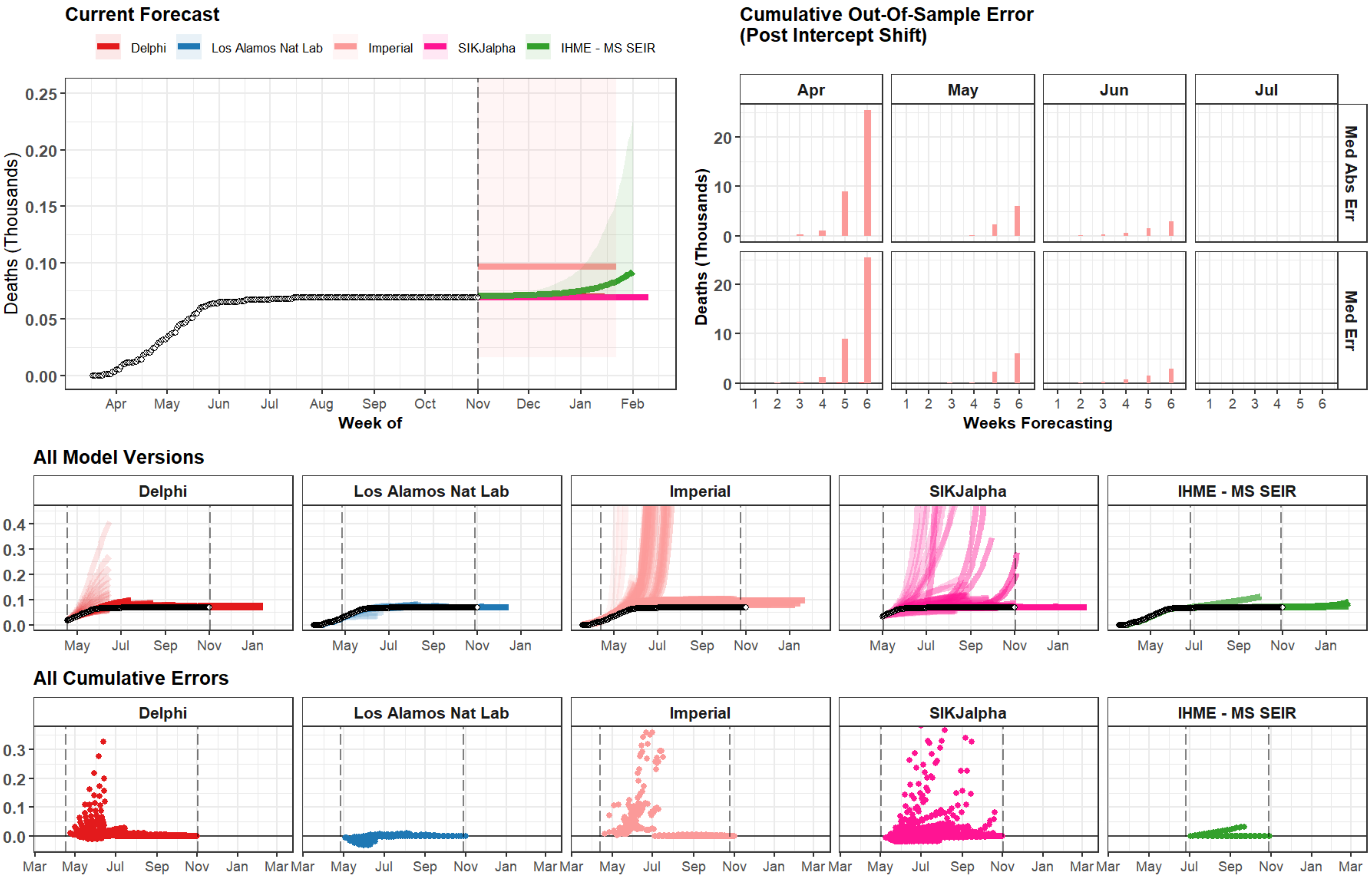
Current Forecast
Delphi
Los Alamos Nat Lab
Imperial
SIKJalpha
IHME - MS SEIR
Deaths (Thousands)
0.25
0.20
0.15
0.10
0.05
0.00
Apr
May
Jun
Jul
Aug
Sep
Oct
Nov
Dec
Jan
Feb
Week of
Cumulative Out-Of-Sample Error
(Post Intercept Shift)
Apr
May
Jun
Jul
Med Abs Err
Med Err
20
10
0
1
2
3
4
5
6
Weeks Forecasting
All Model Versions
Delphi
Los Alamos Nat Lab
Imperial
SIKJalpha
IHME - MS SEIR
0.4
0.3
0.2
0.1
0.0
May
Jul
Sep
Nov
Jan
All Cumulative Errors
0.3
0.2
0.1
0.0
Mar
May
Jul
Sep
Nov
Jan
Mar

Burkina Faso

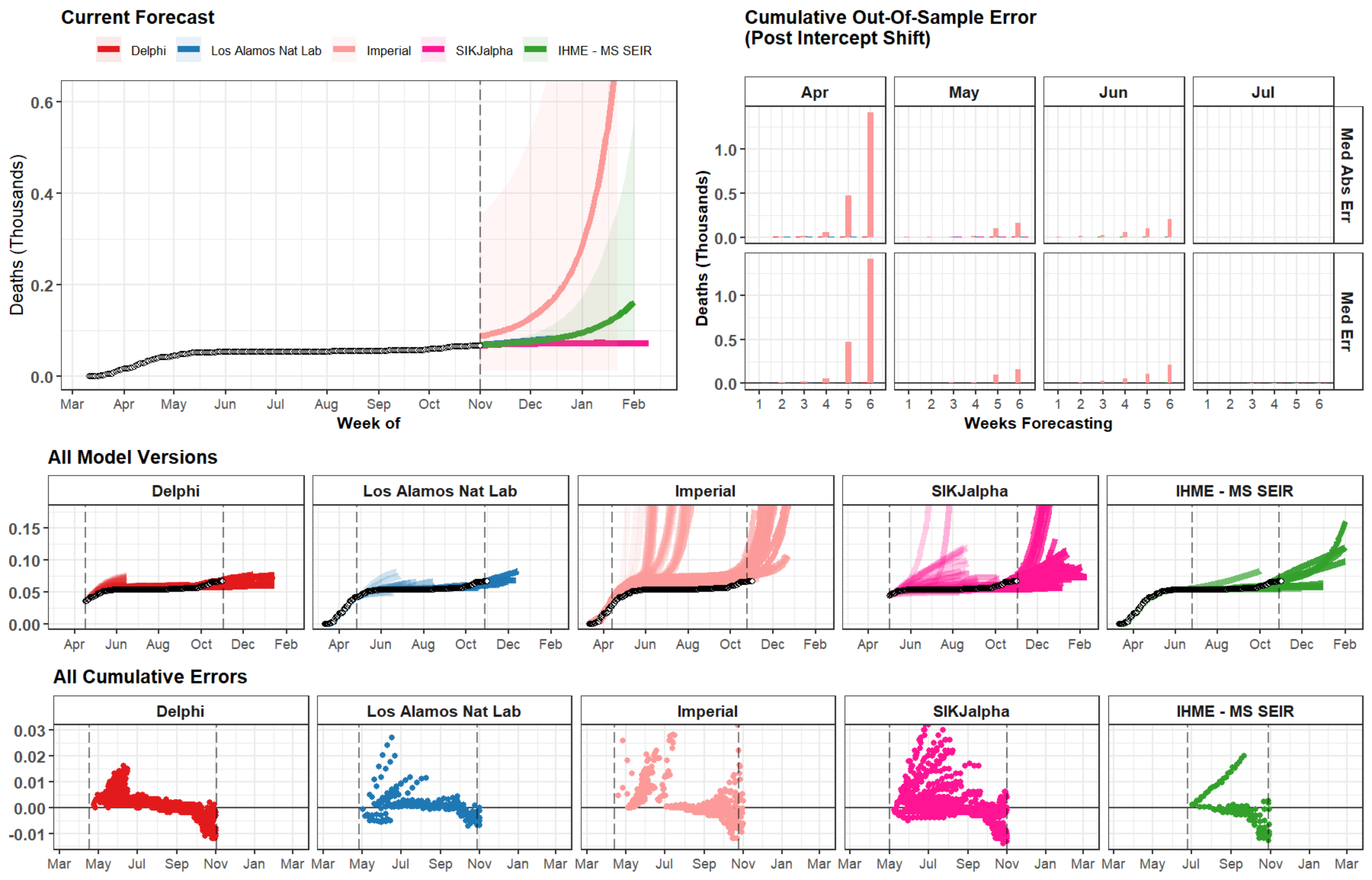

Malta

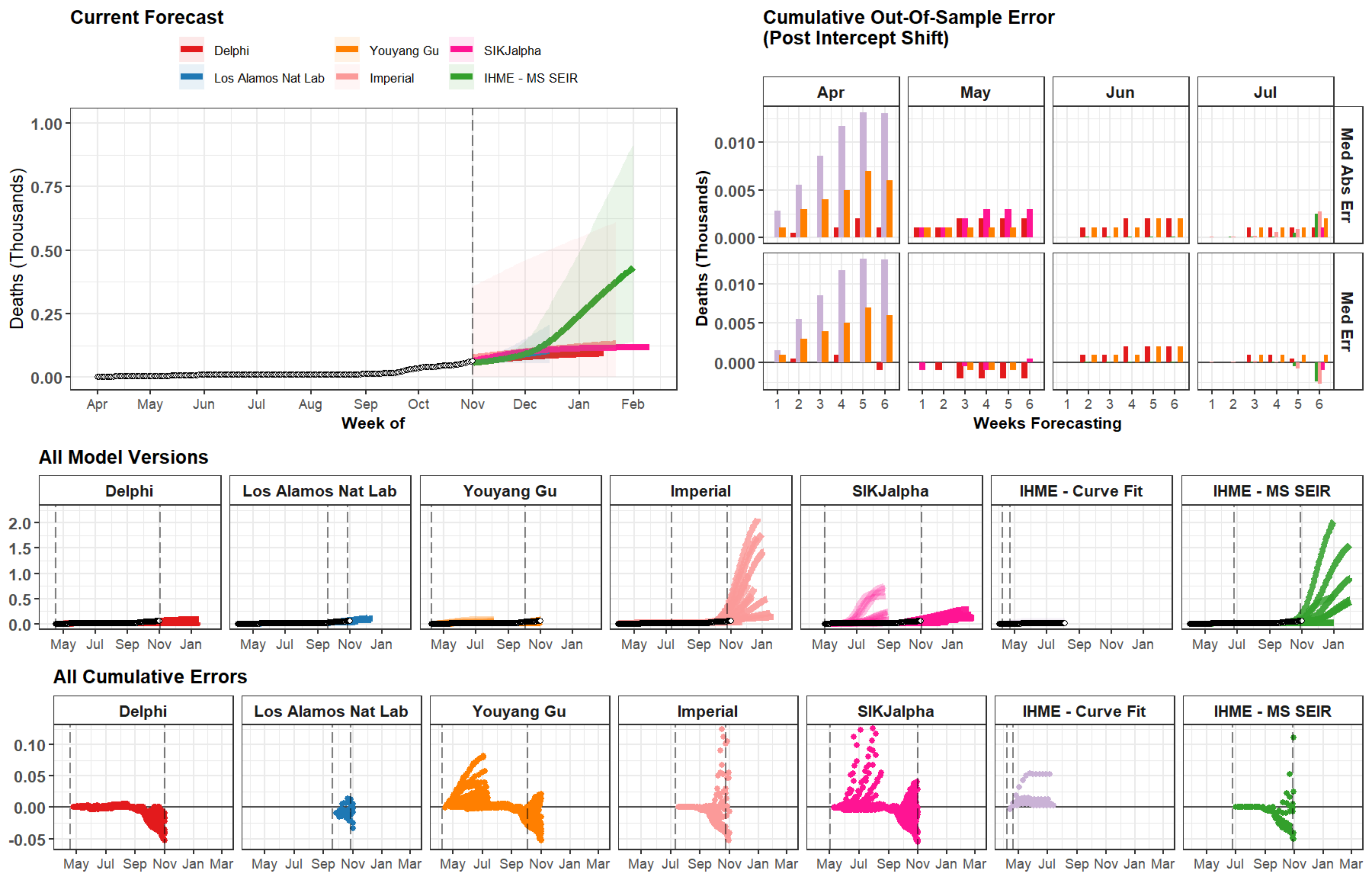

Current Forecast
Delphi
Youyang Gu
SIKJalpha
Los Alamos Nat Lab
Imperial
IHME - MS SEIR
Deaths (Thousands)
1.00
0.75
0.50
0.25
0.00
Apr May Jun Jul Aug Sep Oct Nov Dec Jan Feb
Week of
Cumulative Out-Of-Sample Error
(Post Intercept Shift)
Apr May Jun Jul
Med Abs Err
Med Err
0.010
0.005
0.000
1 2 3 4 5 6
Weeks Forecasting
All Model Versions
Delphi
Los Alamos Nat Lab
Youyang Gu
Imperial
SIKJalpha
IHME - Curve Fit
IHME - MS SEIR
2.0
1.5
1.0
0.5
0.0
May Jul Sep Nov Jan
All Cumulative Errors
0.10
0.05
0.00
-0.05
May Jul Sep Nov Jan Mar

Central African Republic

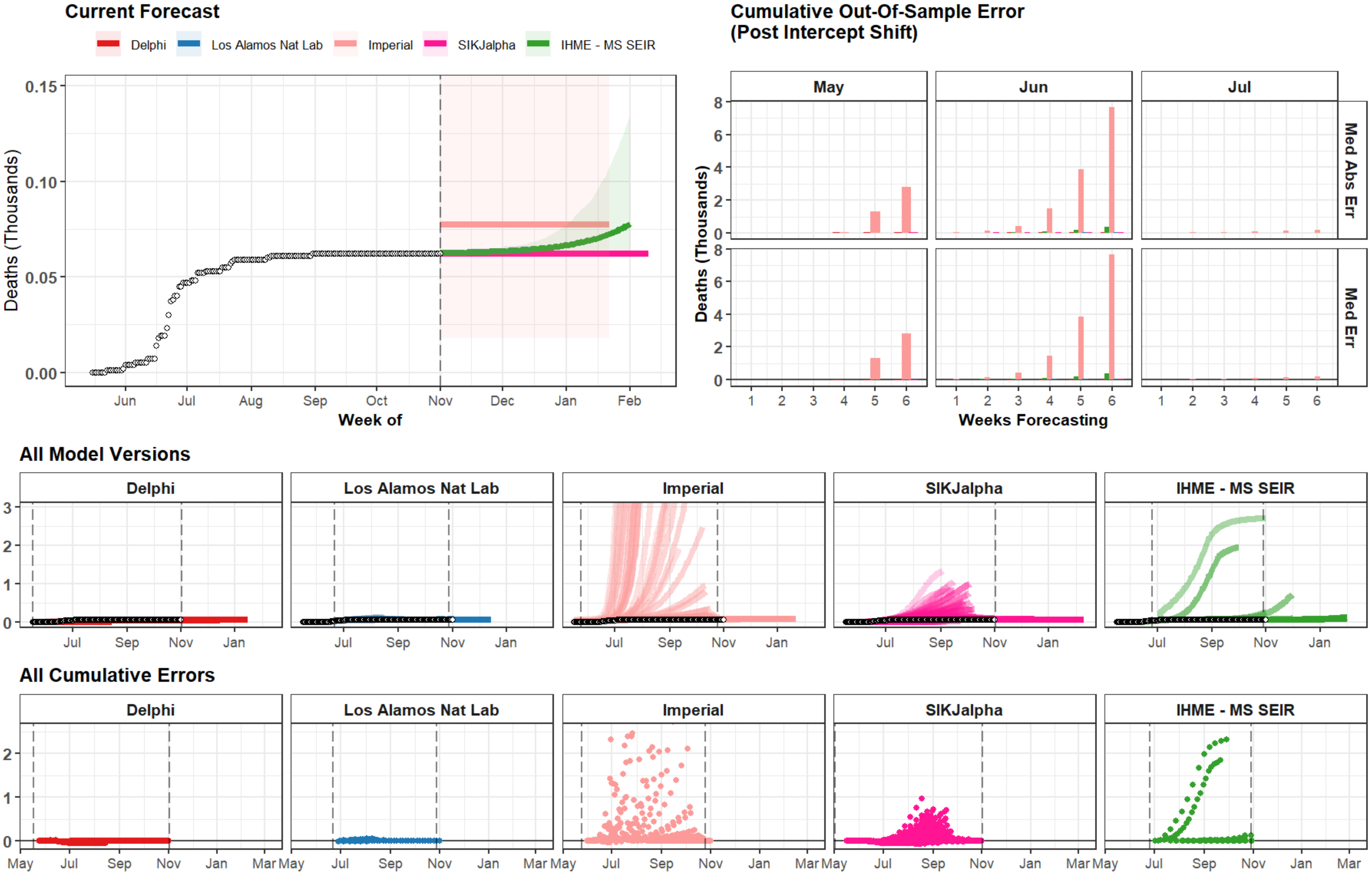

Djibouti

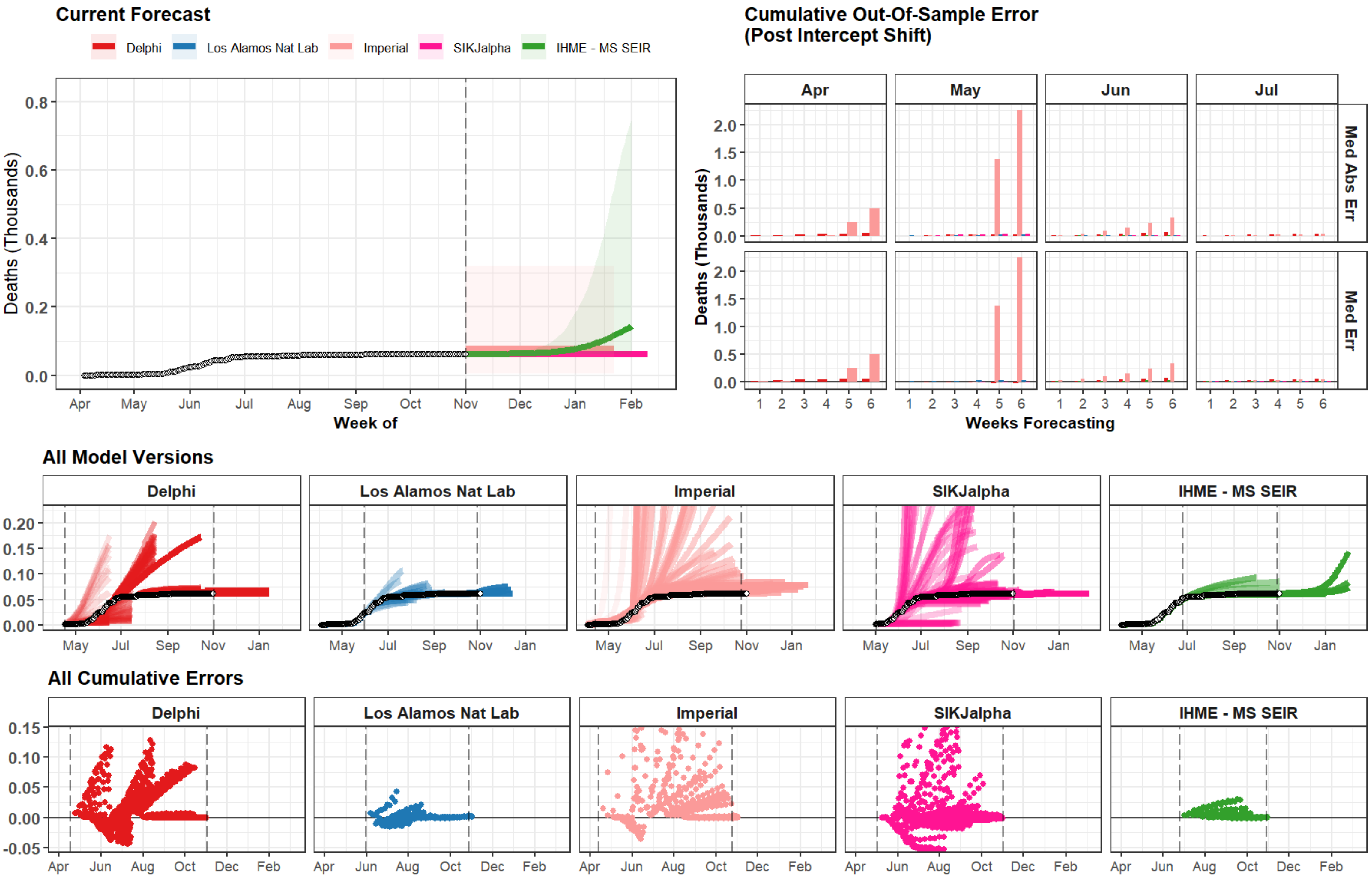

Current Forecast
Delphi
Los Alamos Nat Lab
Imperial
SIKJalpha
IHME - MS SEIR
Deaths (Thousands)
0.0
0.2
0.4
0.6
0.8
Apr
May
Jun
Jul
Aug
Sep
Oct
Nov
Dec
Jan
Feb
Week of
Cumulative Out-Of-Sample Error
(Post Intercept Shift)
Apr
May
Jun
Jul
Med Abs Err
Med Err
0.0
0.5
1.0
1.5
2.0
1
2
3
4
5
6
Weeks Forecasting
All Model Versions
Delphi
Los Alamos Nat Lab
Imperial
SIKJalpha
IHME - MS SEIR
0.00
0.05
0.10
0.15
0.20
May
Jul
Sep
Nov
Jan
All Cumulative Errors
Delphi
Los Alamos Nat Lab
Imperial
SIKJalpha
IHME - MS SEIR
-0.05
0.00
0.05
0.10
0.15
Apr
Jun
Aug
Oct
Dec
Feb

Belize

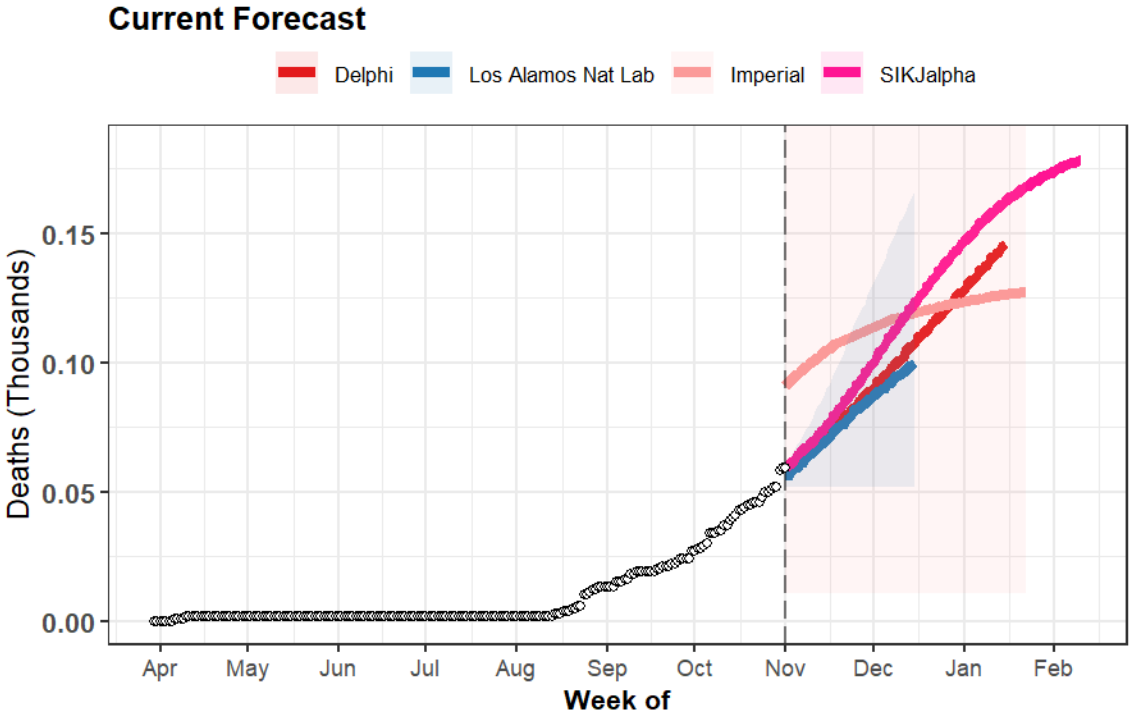

Current Forecast
Delphi
Los Alamos Nat Lab
Imperial
SIKJalpha
Deaths (Thousands)
0.15
0.10
0.05
0.00
Apr
May
Jun
Jul
Aug
Sep
Oct
Nov
Dec
Jan
Feb
Week of

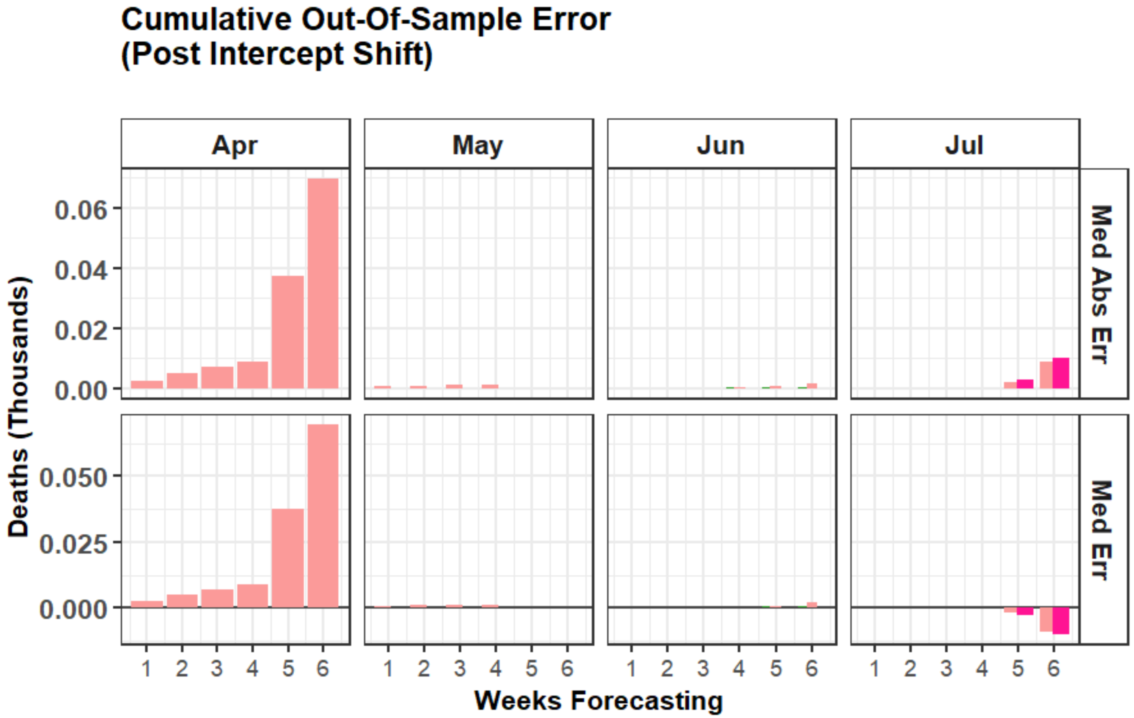

Cumulative Out-Of-Sample Error
(Post Intercept Shift)
Apr
May
Jun
Jul
Med Abs Err
Med Err
Deaths (Thousands)
0.06
0.04
0.02
0.00
0.050
0.025
0.000
1 2 3 4 5 6
Weeks Forecasting

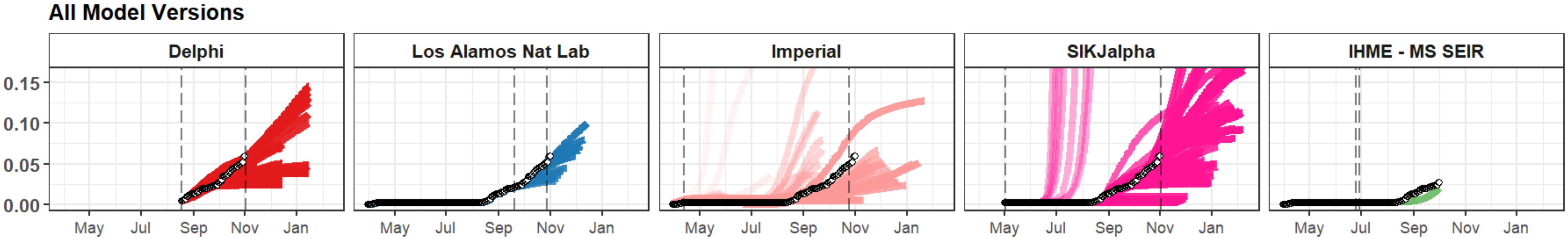

All Model Versions
Delphi
Los Alamos Nat Lab
Imperial
SIKJalpha
IHME - MS SEIR
0.15
0.10
0.05
0.00
May
Jul
Sep
Nov
Jan

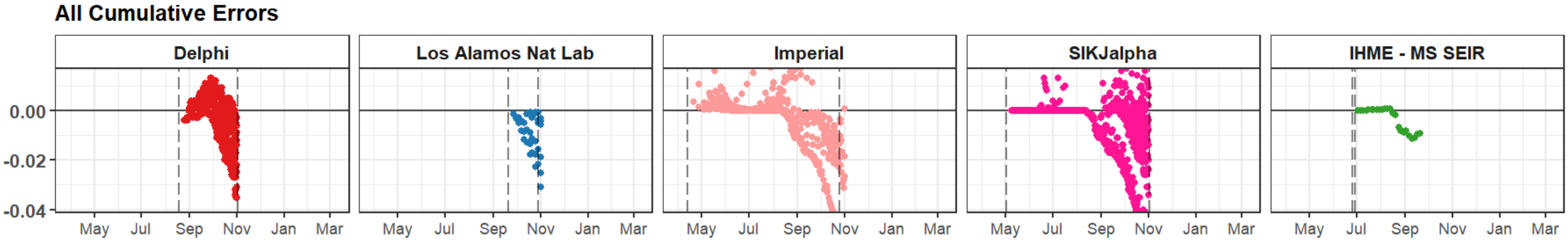

All Cumulative Errors
Delphi
Los Alamos Nat Lab
Imperial
SIKJalpha
IHME - MS SEIR
0.00
-0.02
-0.04
May
Jul
Sep
Nov
Jan
Mar

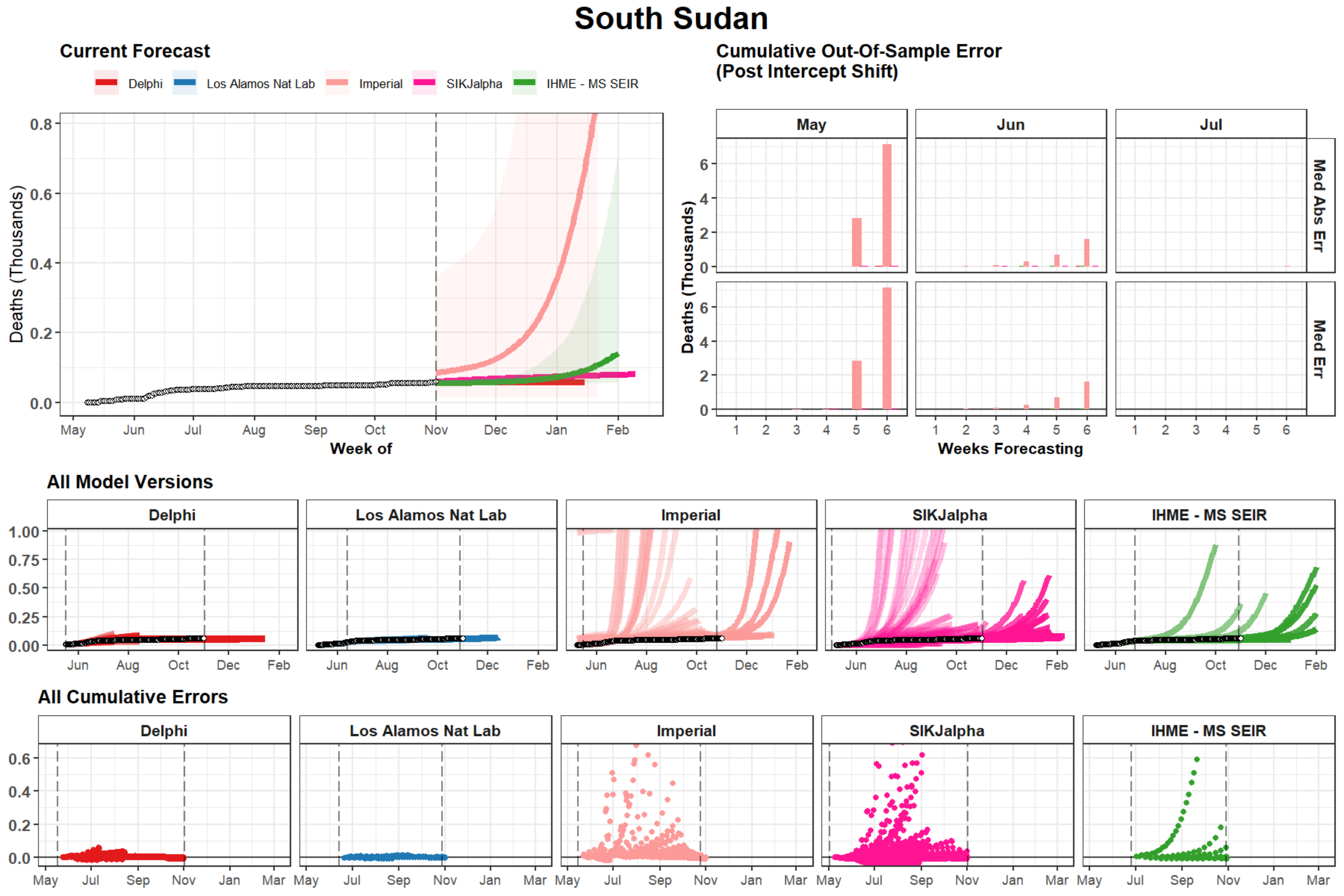

South Sudan
Current Forecast
Delphi
Los Alamos Nat Lab
Imperial
SIKJalpha
IHME - MS SEIR
Deaths (Thousands)
Week of
Cumulative Out-Of-Sample Error
(Post Intercept Shift)
May
Jun
Jul
Med Abs Err
Med Err
Weeks Forecasting
All Model Versions
All Cumulative Errors

Thailand

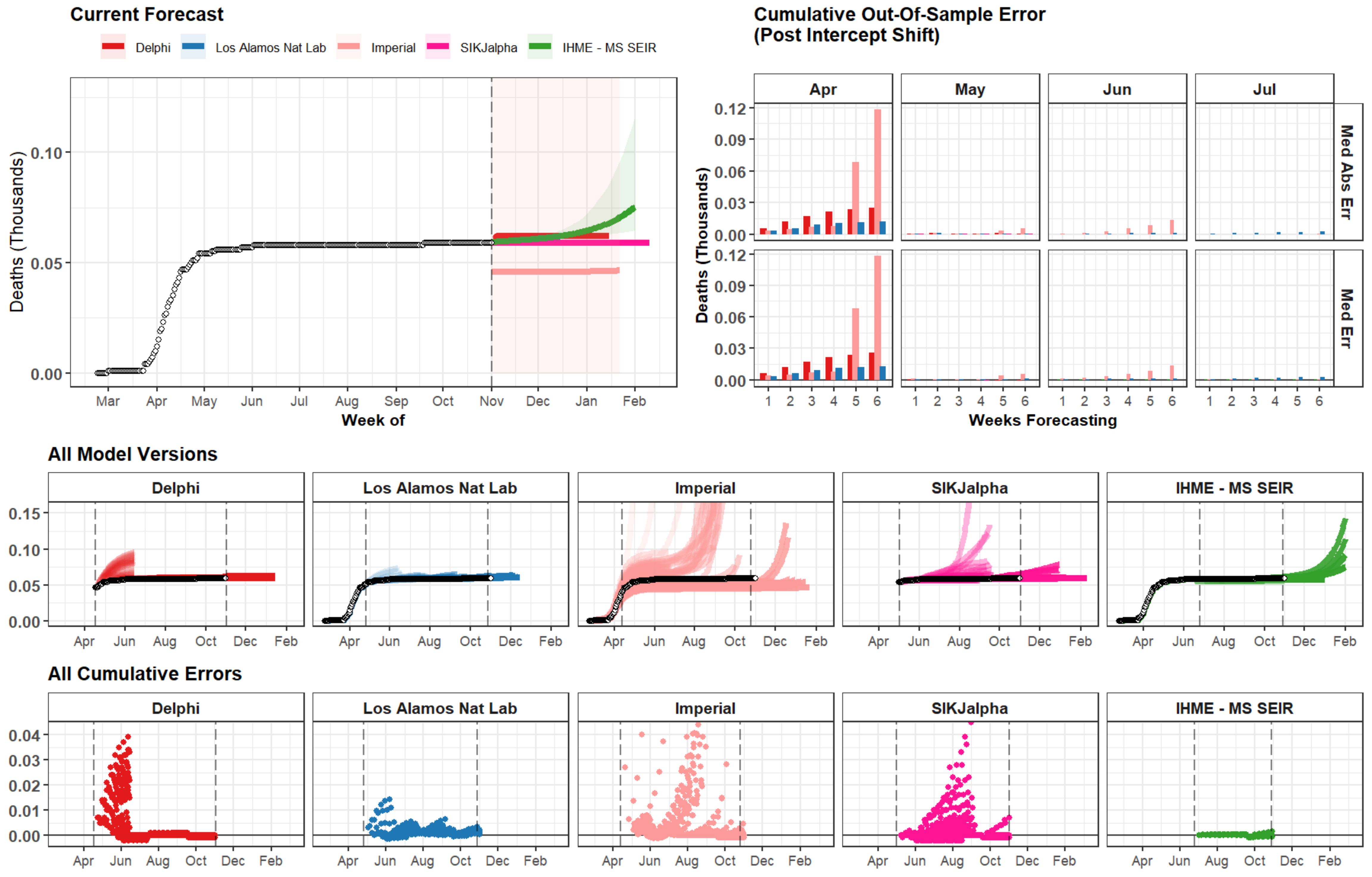

Current Forecast
Delphi
Los Alamos Nat Lab
Imperial
SIKJalpha
IHME - MS SEIR
Deaths (Thousands)
0.10
0.05
0.00
Mar
Apr
May
Jun
Jul
Aug
Sep
Oct
Nov
Dec
Jan
Feb
Week of
Cumulative Out-Of-Sample Error
(Post Intercept Shift)
Apr
May
Jun
Jul
Med Abs Err
Med Err
0.12
0.09
0.06
0.03
0.00
1
2
3
4
5
6
Weeks Forecasting
All Model Versions
Delphi
Los Alamos Nat Lab
Imperial
SIKJalpha
IHME - MS SEIR
0.15
0.10
0.05
0.00
Apr
Jun
Aug
Oct
Dec
Feb
All Cumulative Errors
Delphi
Los Alamos Nat Lab
Imperial
SIKJalpha
IHME - MS SEIR
0.04
0.03
0.02
0.01
0.00

Uruguay

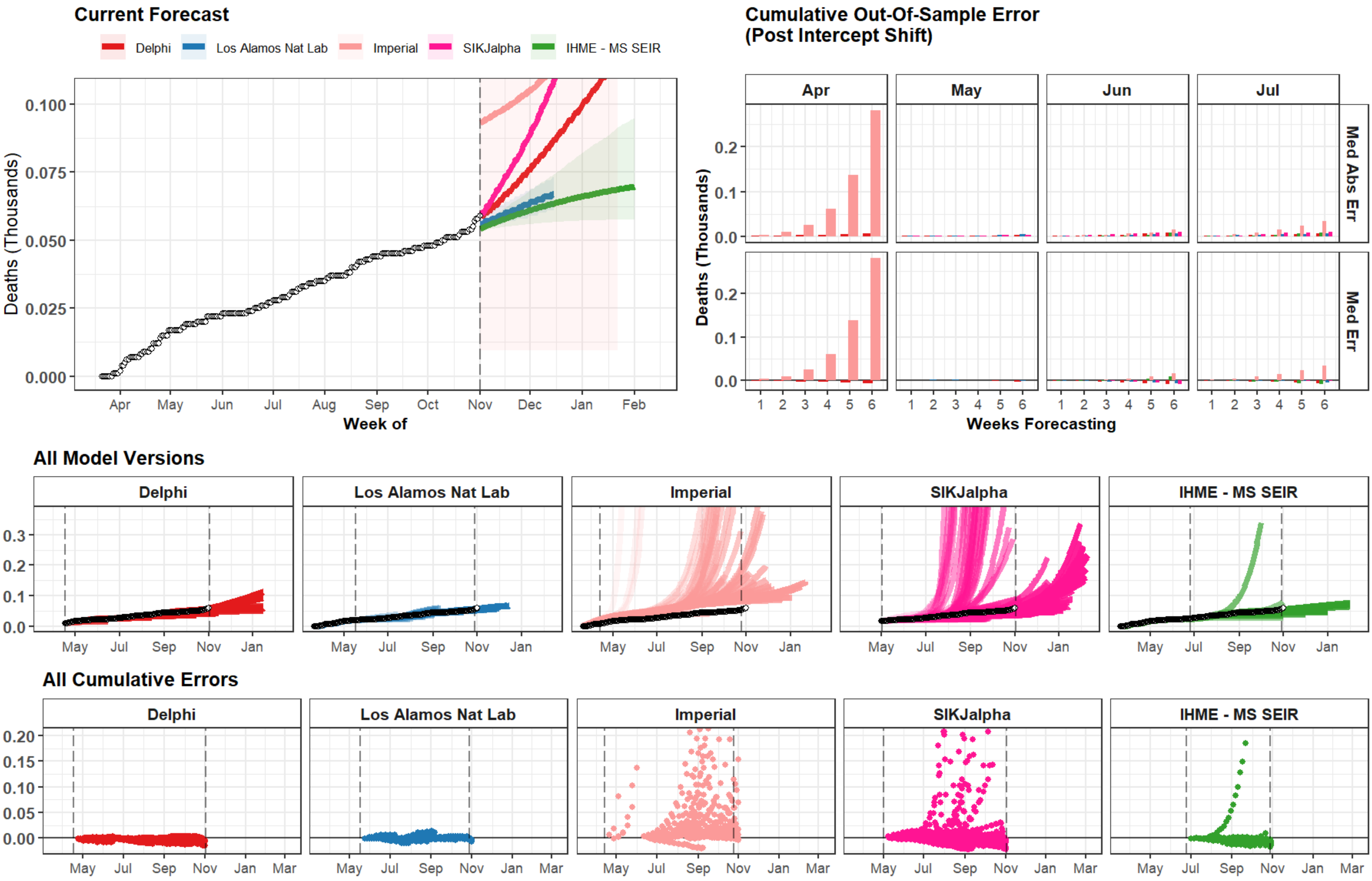

Current Forecast
Delphi
Los Alamos Nat Lab
Imperial
SIKJalpha
IHME - MS SEIR
Deaths (Thousands)
Week of
Cumulative Out-Of-Sample Error
(Post Intercept Shift)
Apr
May
Jun
Jul
Med Abs Err
Med Err
Weeks Forecasting
All Model Versions
All Cumulative Errors

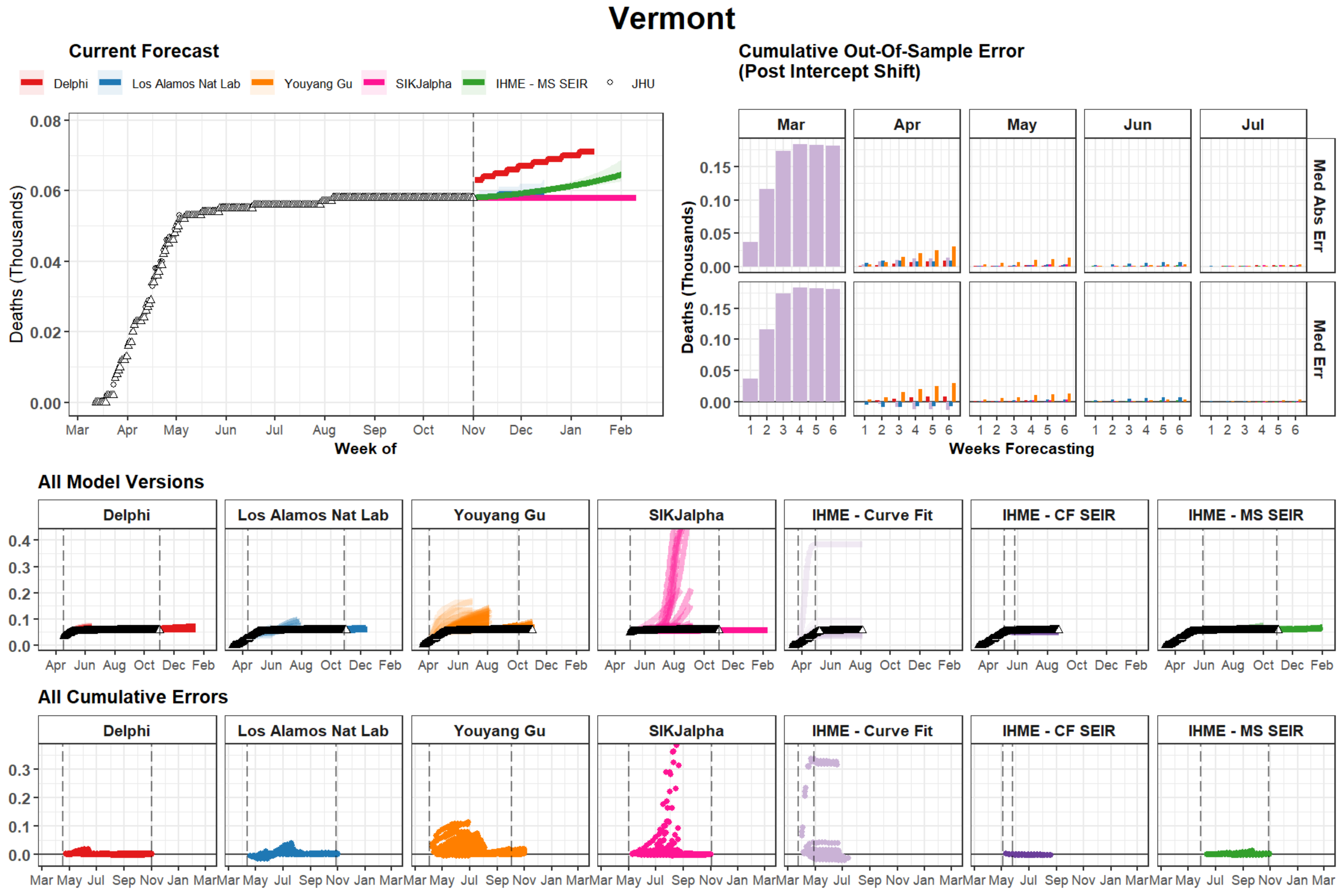
Vermont
Current Forecast
Delphi
Los Alamos Nat Lab
Youyang Gu
SIKJalpha
IHME - MS SEIR
JHU
Deaths (Thousands)
Week of
Cumulative Out-Of-Sample Error
(Post Intercept Shift)
Mar
Apr
May
Jun
Jul
Med Abs Err
Med Err
Weeks Forecasting
All Model Versions
IHME - Curve Fit
IHME - CF SEIR
All Cumulative Errors

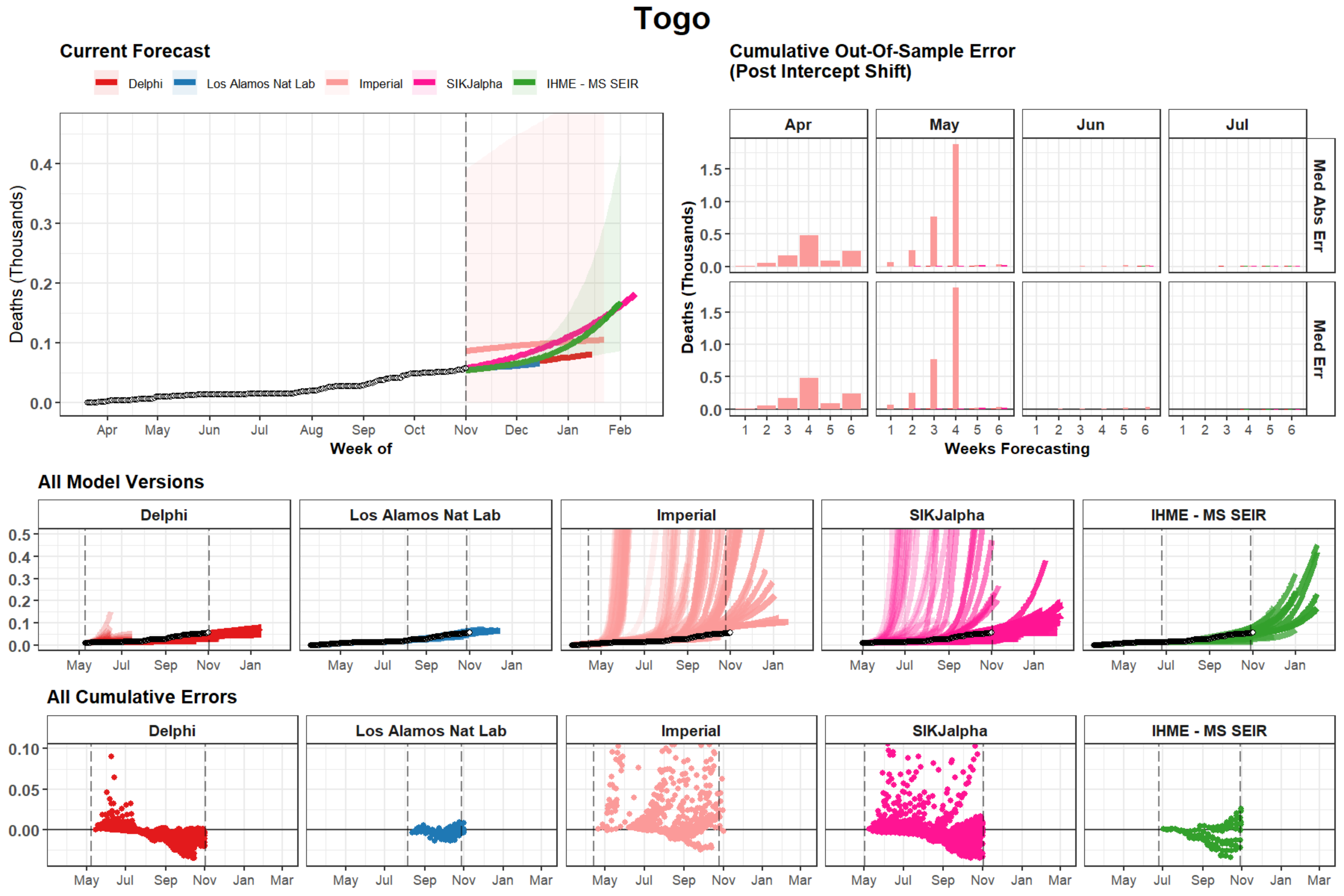
Togo
Current Forecast
Delphi
Los Alamos Nat Lab
Imperial
SIKJalpha
IHME - MS SEIR
Deaths (Thousands)
0.0
0.1
0.2
0.3
0.4
Apr
May
Jun
Jul
Aug
Sep
Oct
Nov
Dec
Jan
Feb
Week of
Cumulative Out-Of-Sample Error
(Post Intercept Shift)
Apr
May
Jun
Jul
Med Abs Err
Med Err
0.0
0.5
1.0
1.5
1
2
3
4
5
6
Weeks Forecasting
All Model Versions
0.0
0.1
0.2
0.3
0.4
0.5
May
Jul
Sep
Nov
Jan
All Cumulative Errors
0.00
0.05
0.10
Mar

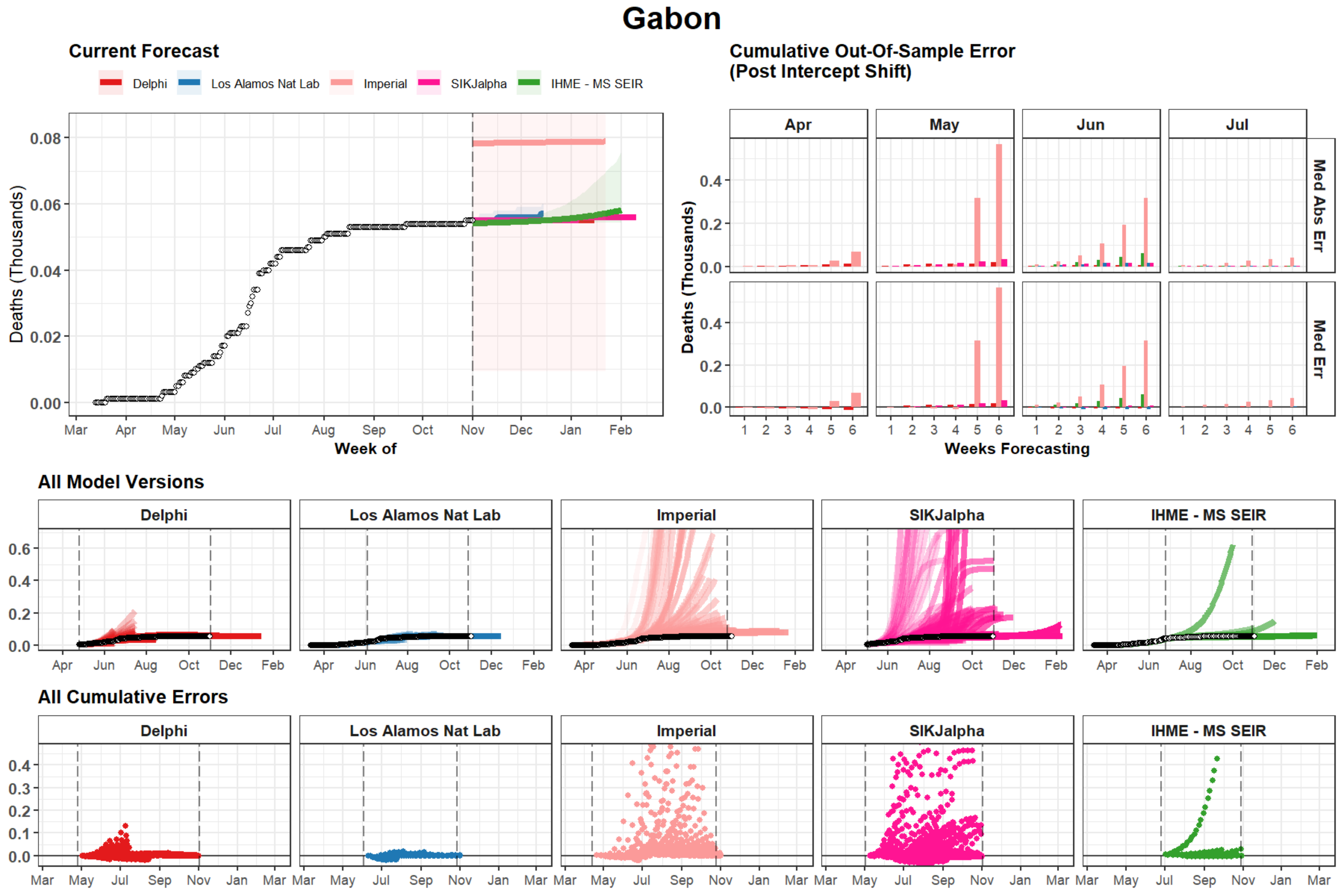

Gabon
Current Forecast
Delphi
Los Alamos Nat Lab
Imperial
SIKJalpha
IHME - MS SEIR
Deaths (Thousands)
Week of
Cumulative Out-Of-Sample Error
(Post Intercept Shift)
Apr
May
Jun
Jul
Med Abs Err
Med Err
Weeks Forecasting
All Model Versions
All Cumulative Errors

Lesotho

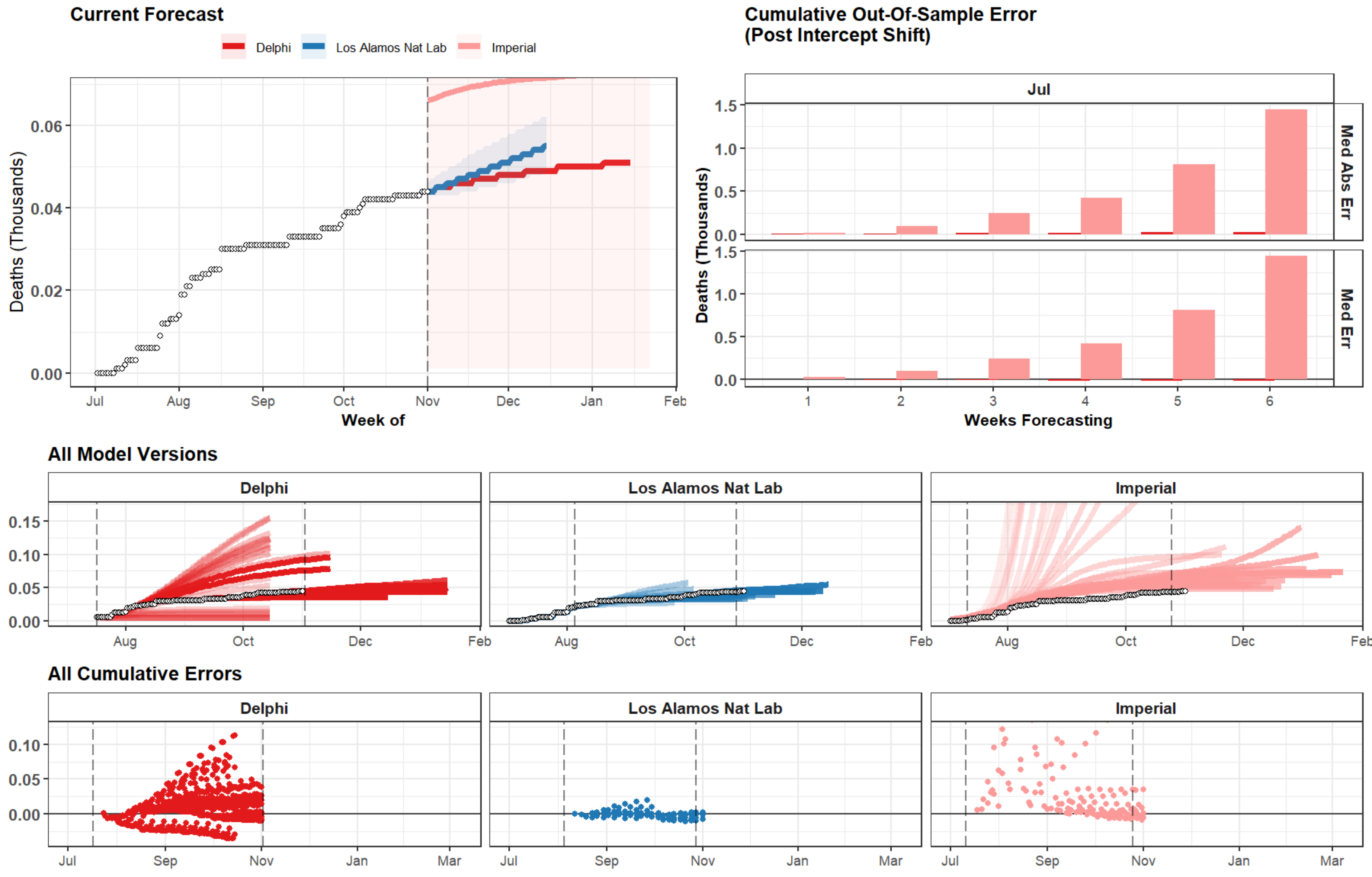
Current Forecast
Delphi
Los Alamos Nat Lab
Imperial
Deaths (Thousands)
0.00
0.02
0.04
0.06
Jul
Aug
Sep
Oct
Nov
Dec
Jan
Feb
Week of
Cumulative Out-Of-Sample Error
(Post Intercept Shift)
Jul
Med Abs Err
Med Err
Deaths (Thousands)
0.0
0.5
1.0
1.5
1
2
3
4
5
6
Weeks Forecasting
All Model Versions
Delphi
Los Alamos Nat Lab
Imperial
0.00
0.05
0.10
0.15
Aug
Oct
Dec
Feb
All Cumulative Errors
Delphi
Los Alamos Nat Lab
Imperial
0.00
0.05
0.10
Jul
Sep
Nov
Jan
Mar

Benin

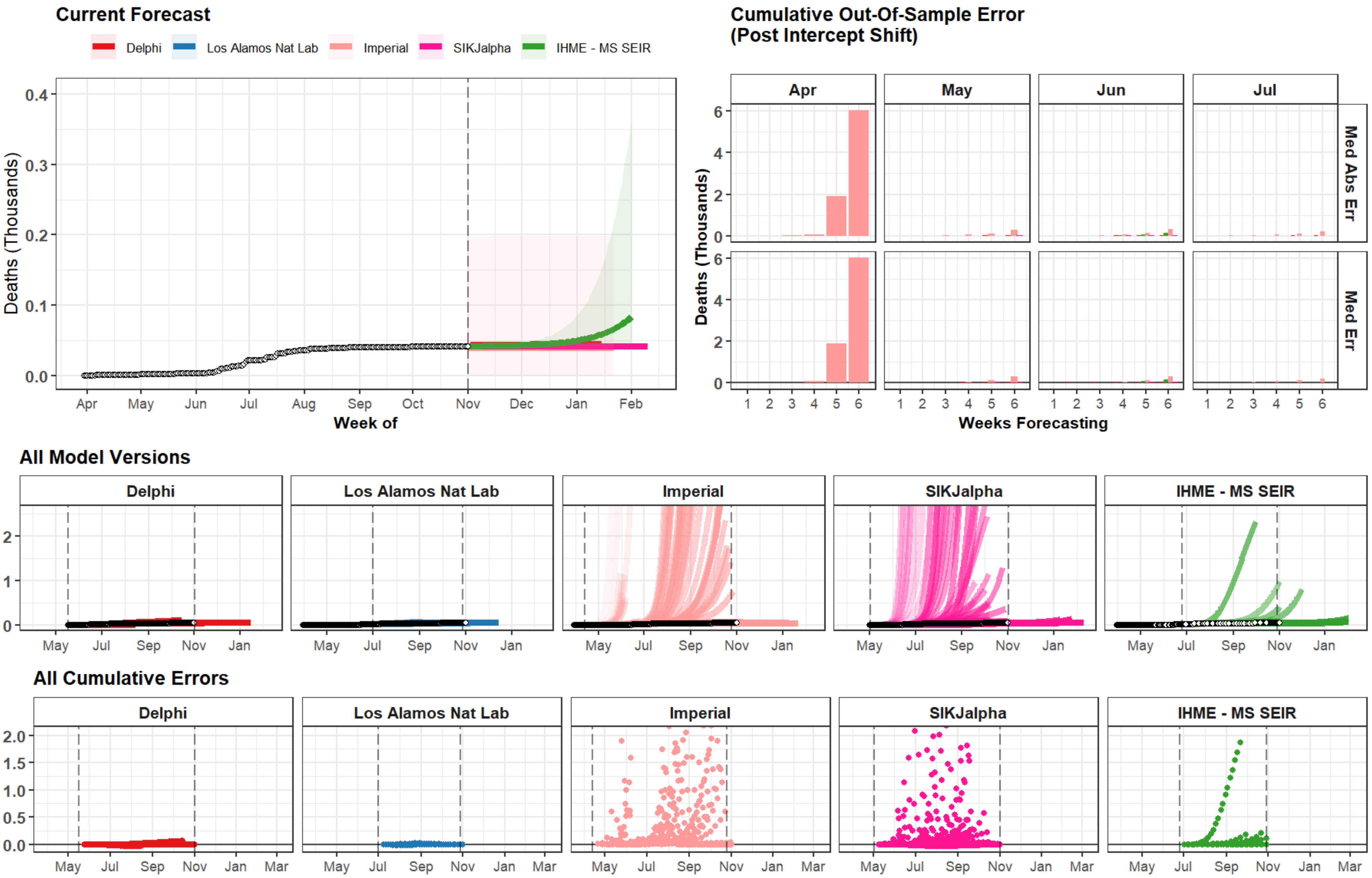
Current Forecast
Delphi
Los Alamos Nat Lab
Imperial
SIKJalpha
IHME - MS SEIR
Deaths (Thousands)
Week of
Apr May Jun Jul Aug Sep Oct Nov Dec Jan Feb
Cumulative Out-Of-Sample Error
(Post Intercept Shift)
Apr
May
Jun
Jul
Med Abs Err
Med Err
Weeks Forecasting
All Model Versions
Delphi
Los Alamos Nat Lab
Imperial
SIKJalpha
IHME - MS SEIR
All Cumulative Errors
Delphi
Los Alamos Nat Lab
Imperial
SIKJalpha
IHME - MS SEIR

Guinea-Bissau

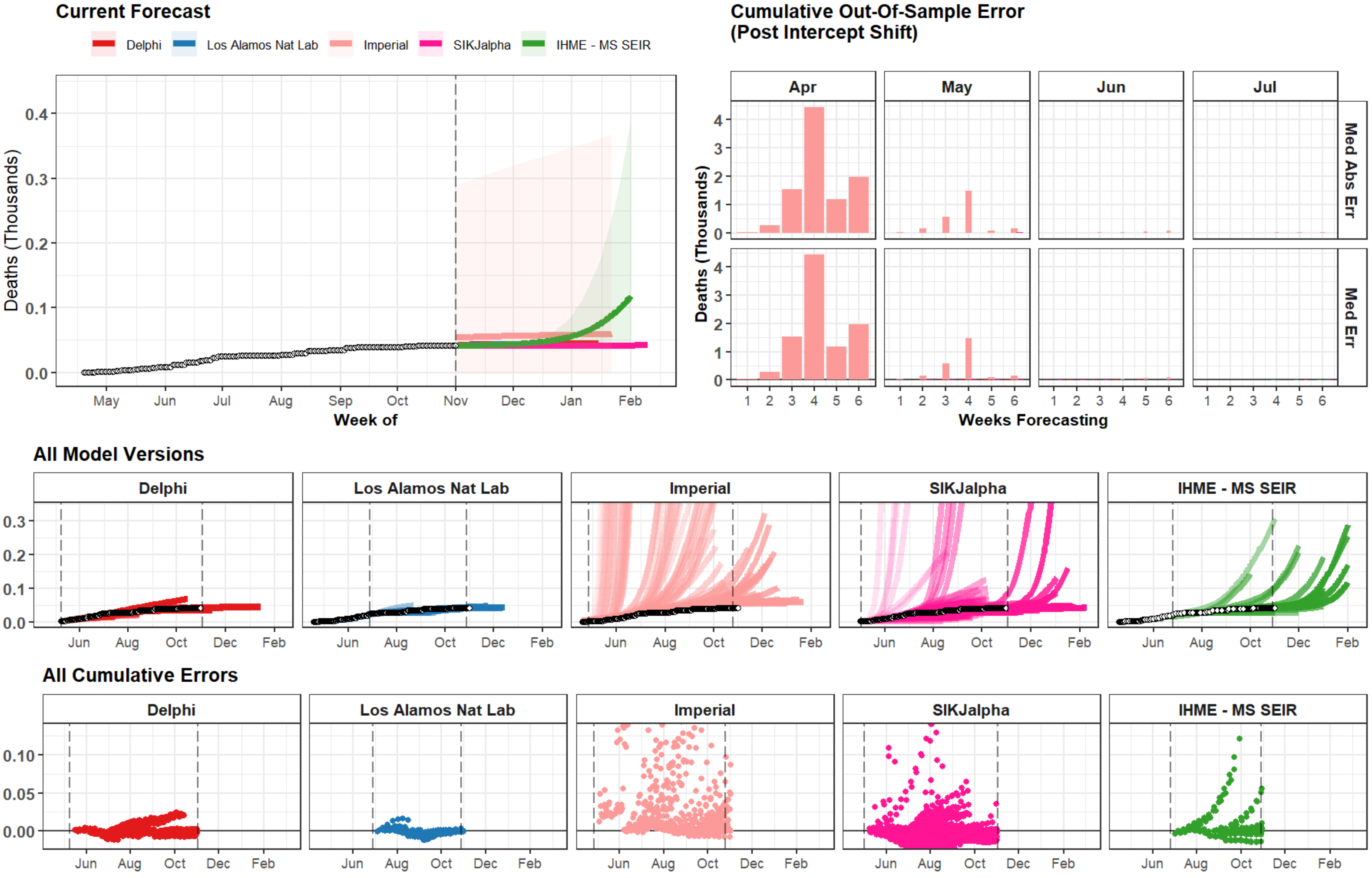

Maldives

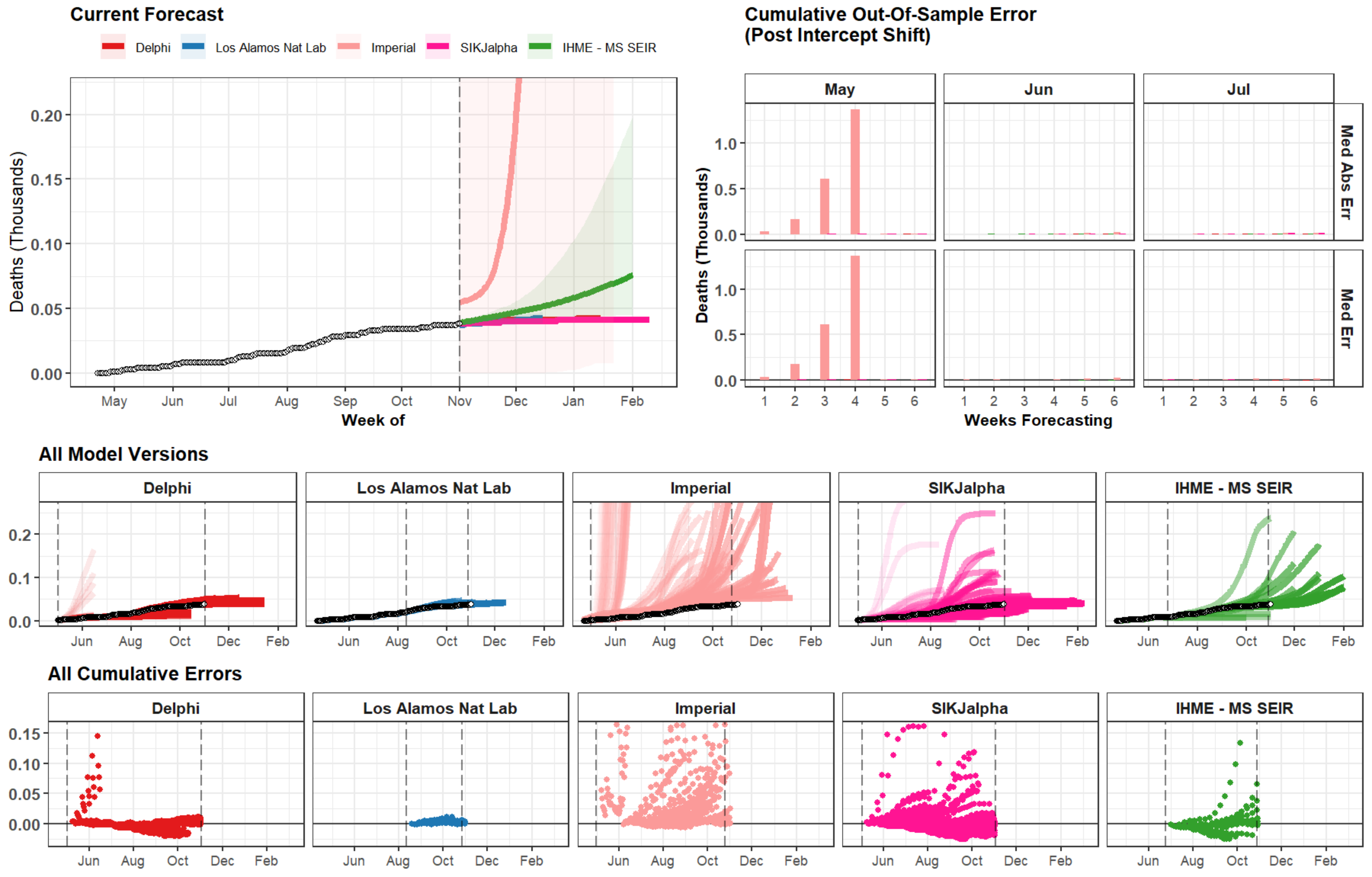

Current Forecast
Delphi
Los Alamos Nat Lab
Imperial
SIKJalpha
IHME - MS SEIR
Deaths (Thousands)
0.00
0.05
0.10
0.15
0.20
May
Jun
Jul
Aug
Sep
Oct
Nov
Dec
Jan
Feb
Week of
Cumulative Out-Of-Sample Error
(Post Intercept Shift)
May
Jun
Jul
Med Abs Err
Med Err
0.0
0.5
1.0
1
2
3
4
5
6
Weeks Forecasting
All Model Versions
0.0
0.1
0.2
Jun
Aug
Oct
Dec
Feb
All Cumulative Errors
0.00
0.05
0.10
0.15

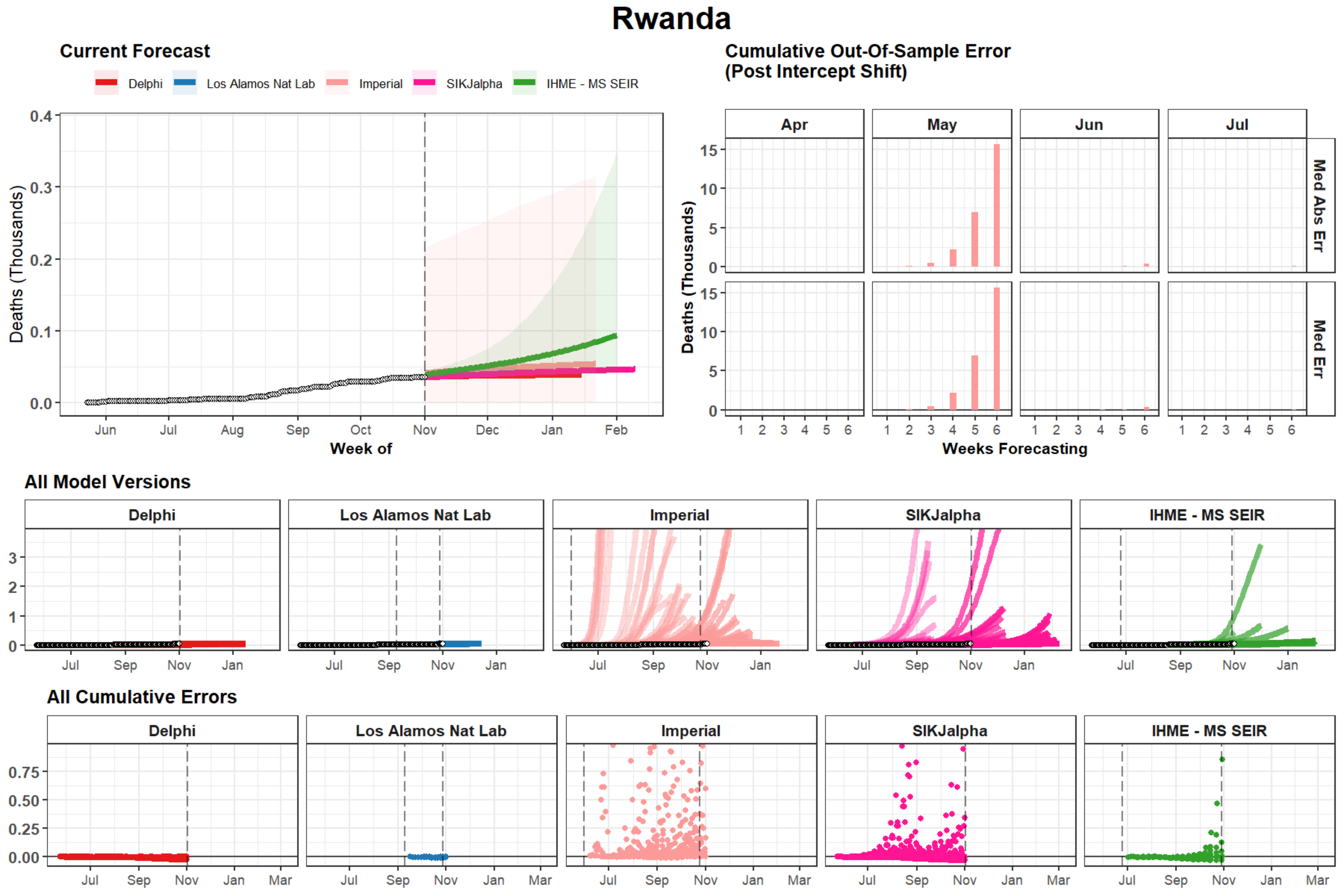

Rwanda
Current Forecast
Delphi
Los Alamos Nat Lab
Imperial
SIKJalpha
IHME - MS SEIR
Deaths (Thousands)
Week of
Cumulative Out-Of-Sample Error
(Post Intercept Shift)
Apr
May
Jun
Jul
Med Abs Err
Med Err
Weeks Forecasting
All Model Versions
All Cumulative Errors

Vietnam

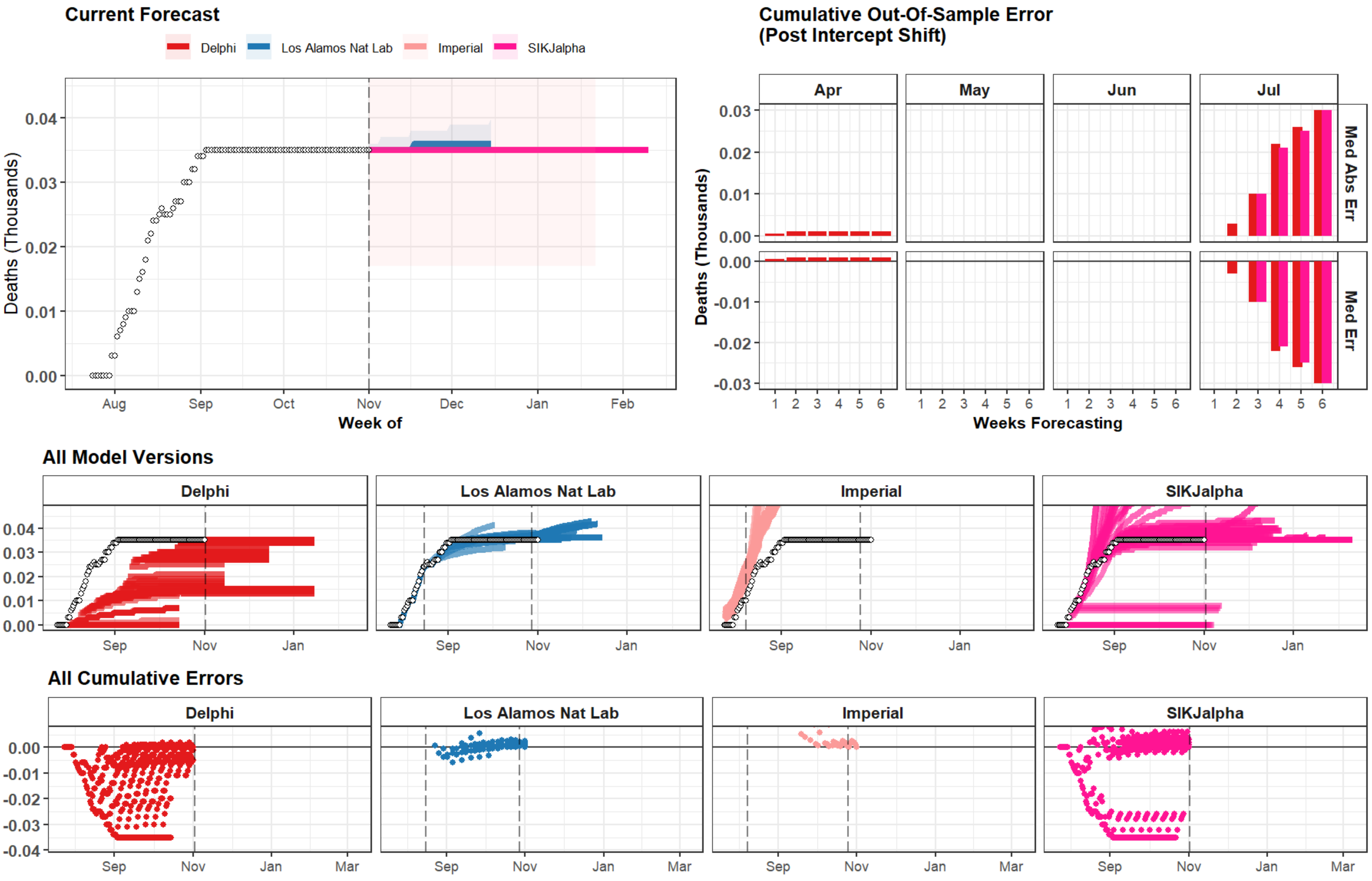

Current Forecast
Delphi
Los Alamos Nat Lab
Imperial
SIKJalpha
Deaths (Thousands)
Week of
Aug
Sep
Oct
Nov
Dec
Jan
Feb
Cumulative Out-Of-Sample Error
(Post Intercept Shift)
Apr
May
Jun
Jul
Med Abs Err
Med Err
Weeks Forecasting
All Model Versions
All Cumulative Errors
Mar

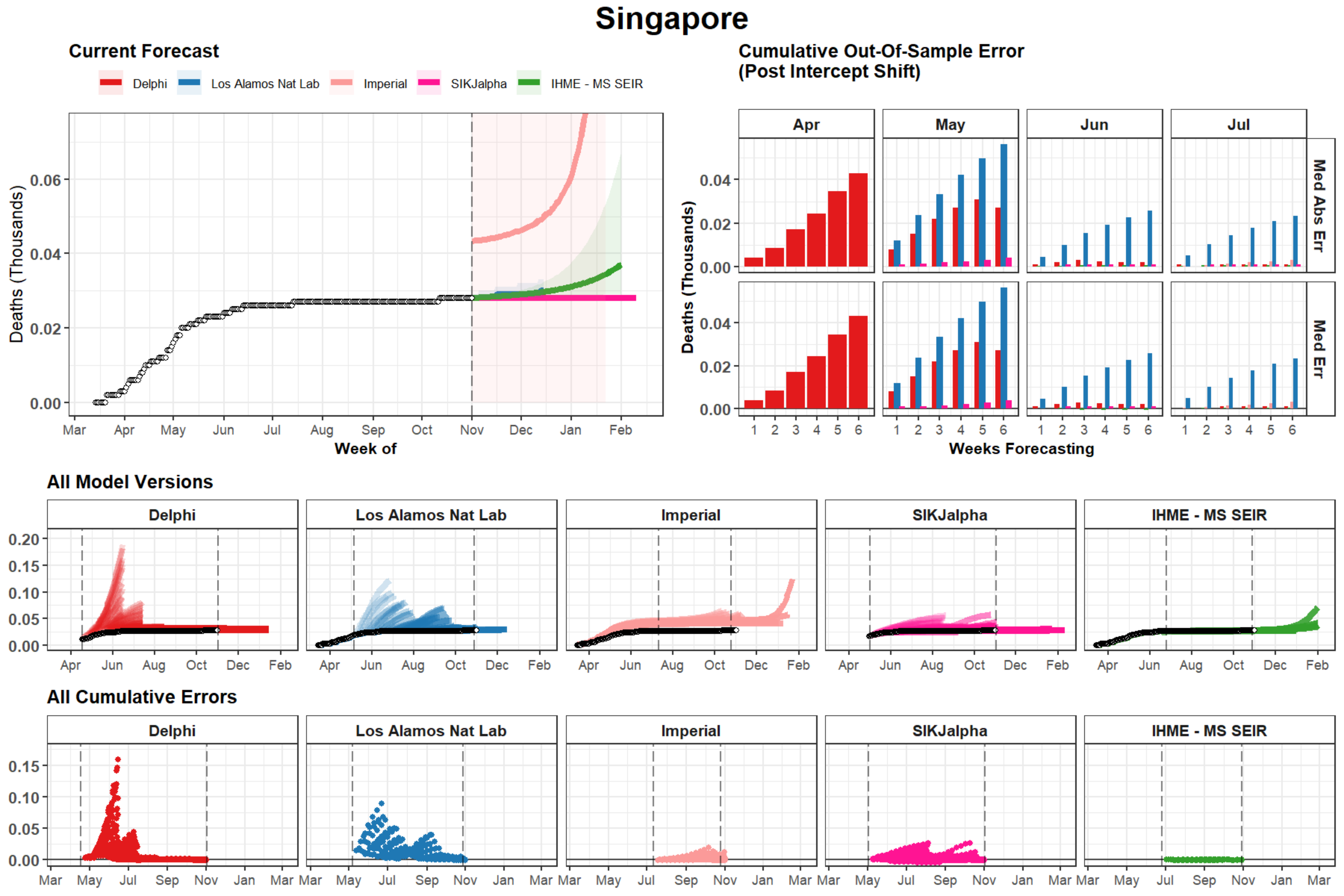

Singapore
Current Forecast
Delphi
Los Alamos Nat Lab
Imperial
SIKJalpha
IHME - MS SEIR
Deaths (Thousands)
0.06
0.04
0.02
0.00
Mar
Apr
May
Jun
Jul
Aug
Sep
Oct
Nov
Dec
Jan
Feb
Week of
Cumulative Out-Of-Sample Error
(Post Intercept Shift)
Apr
May
Jun
Jul
Med Abs Err
Med Err
Deaths (Thousands)
0.04
0.02
0.00
1 2 3 4 5 6
Weeks Forecasting
All Model Versions
Delphi
Los Alamos Nat Lab
Imperial
SIKJalpha
IHME - MS SEIR
0.20
0.15
0.10
0.05
0.00
Apr
Jun
Aug
Oct
Dec
Feb
All Cumulative Errors
Delphi
Los Alamos Nat Lab
Imperial
SIKJalpha
IHME - MS SEIR
0.15
0.10
0.05
0.00
Mar
May
Jul
Sep
Nov
Jan
Mar

Cyprus

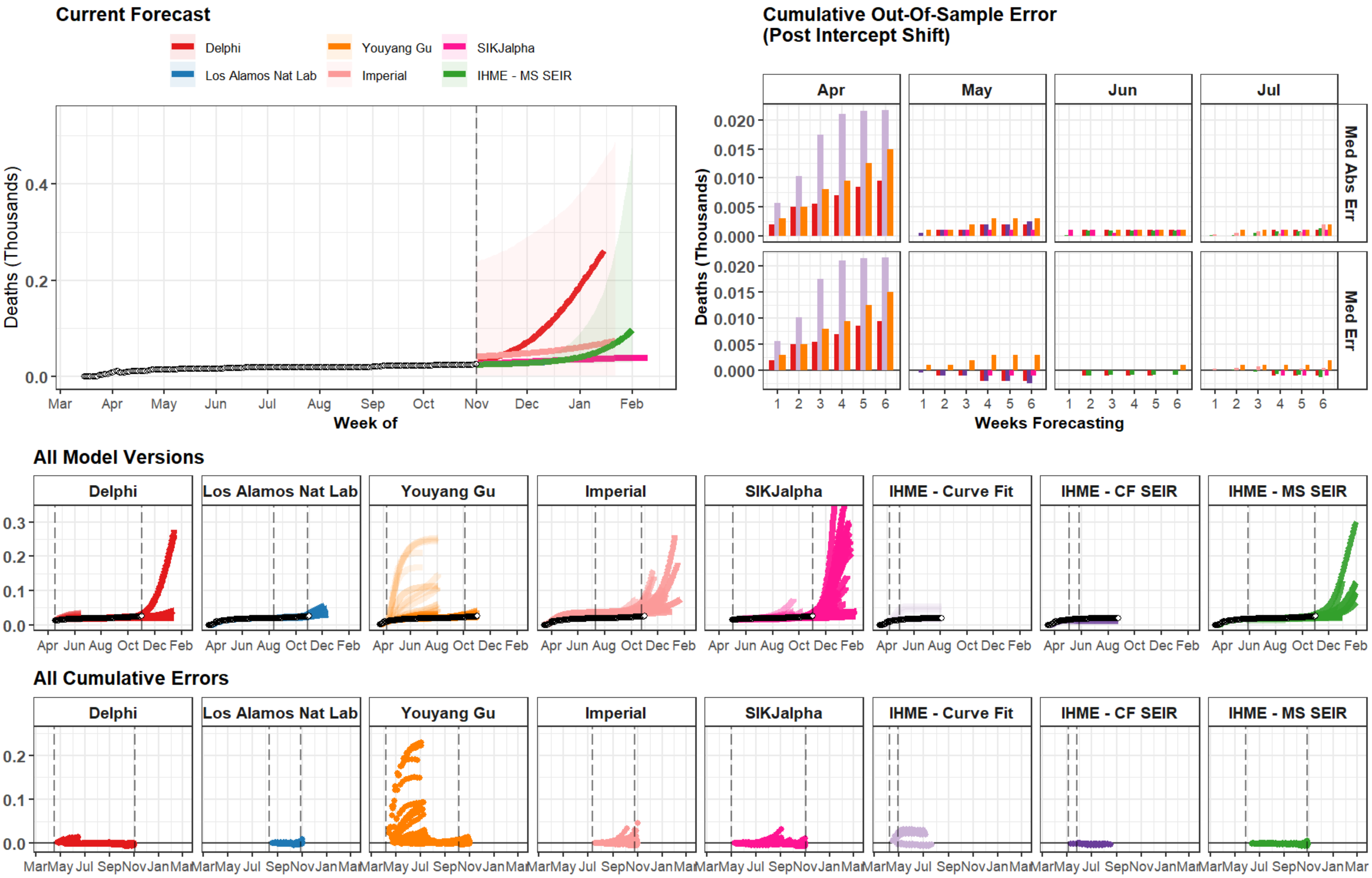

Current Forecast
Delphi
Los Alamos Nat Lab
Youyang Gu
Imperial
SIKJalpha
IHME - MS SEIR
Deaths (Thousands)
0.0
0.2
0.4
Mar
Apr
May
Jun
Jul
Aug
Sep
Oct
Nov
Dec
Jan
Feb
Week of
Cumulative Out-Of-Sample Error
(Post Intercept Shift)
Apr
May
Jun
Jul
Med Abs Err
Med Err
0.000
0.005
0.010
0.015
0.020
1 2 3 4 5 6
Weeks Forecasting
All Model Versions
Delphi
Los Alamos Nat Lab
Youyang Gu
Imperial
SIKJalpha
IHME - Curve Fit
IHME - CF SEIR
IHME - MS SEIR
0.0
0.1
0.2
0.3
Apr Jun Aug Oct Dec Feb
All Cumulative Errors
0.0
0.1
0.2
MarMay Jul SepNovJanMar

New Zealand

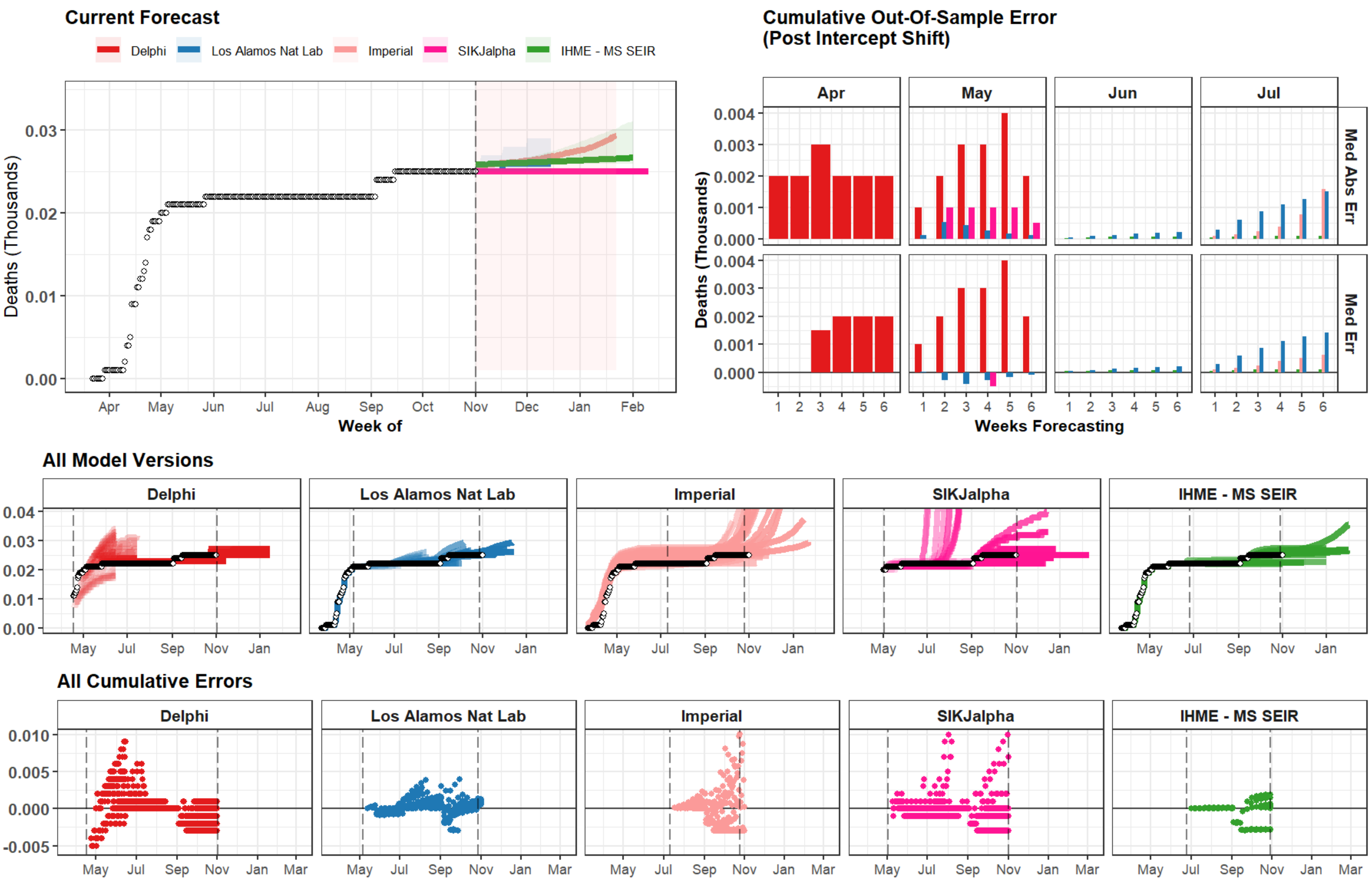

New Zealand
Current Forecast
Delphi
Los Alamos Nat Lab
Imperial
SIKJalpha
IHME - MS SEIR
Deaths (Thousands)
0.00
0.01
0.02
0.03
Apr
May
Jun
Jul
Aug
Sep
Oct
Nov
Dec
Jan
Feb
Week of
Cumulative Out-Of-Sample Error
(Post Intercept Shift)
Apr
May
Jun
Jul
Med Abs Err
Med Err
0.000
0.001
0.002
0.003
0.004
1
2
3
4
5
6
Weeks Forecasting
All Model Versions
Delphi
Los Alamos Nat Lab
Imperial
SIKJalpha
IHME - MS SEIR
0.00
0.01
0.02
0.03
0.04
May
Jul
Sep
Nov
Jan
All Cumulative Errors
Delphi
Los Alamos Nat Lab
Imperial
SIKJalpha
IHME - MS SEIR
-0.005
0.000
0.005
0.010
May
Jul
Sep
Nov
Jan
Mar

Botswana

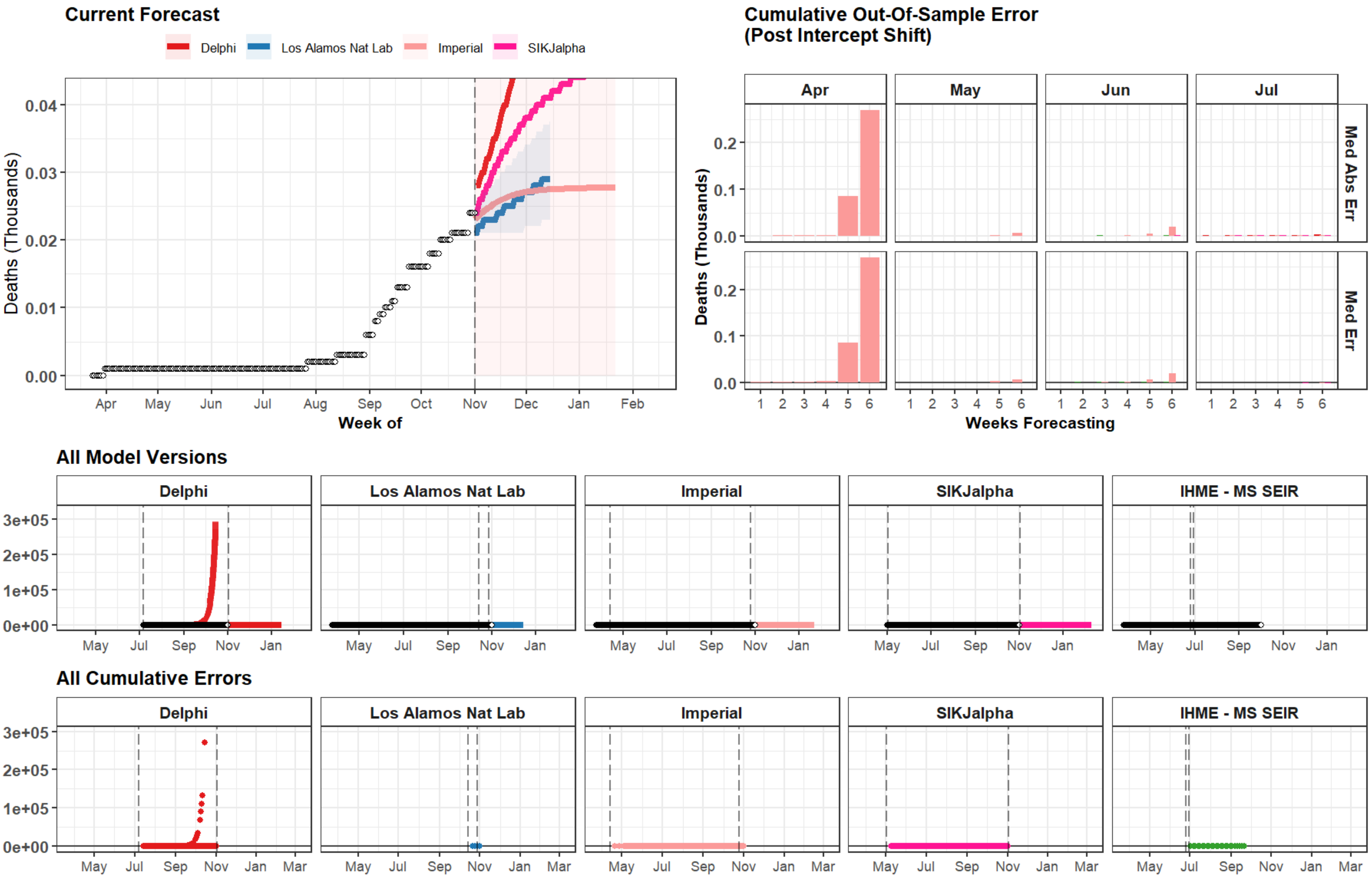
Current Forecast
Delphi
Los Alamos Nat Lab
Imperial
SIKJalpha
Deaths (Thousands)
Week of
Cumulative Out-Of-Sample Error
(Post Intercept Shift)
Apr
May
Jun
Jul
Med Abs Err
Med Err
Weeks Forecasting
All Model Versions
Delphi
Los Alamos Nat Lab
Imperial
SIKJalpha
IHME - MS SEIR
All Cumulative Errors
Delphi
Los Alamos Nat Lab
Imperial
SIKJalpha
IHME - MS SEIR

Sri Lanka

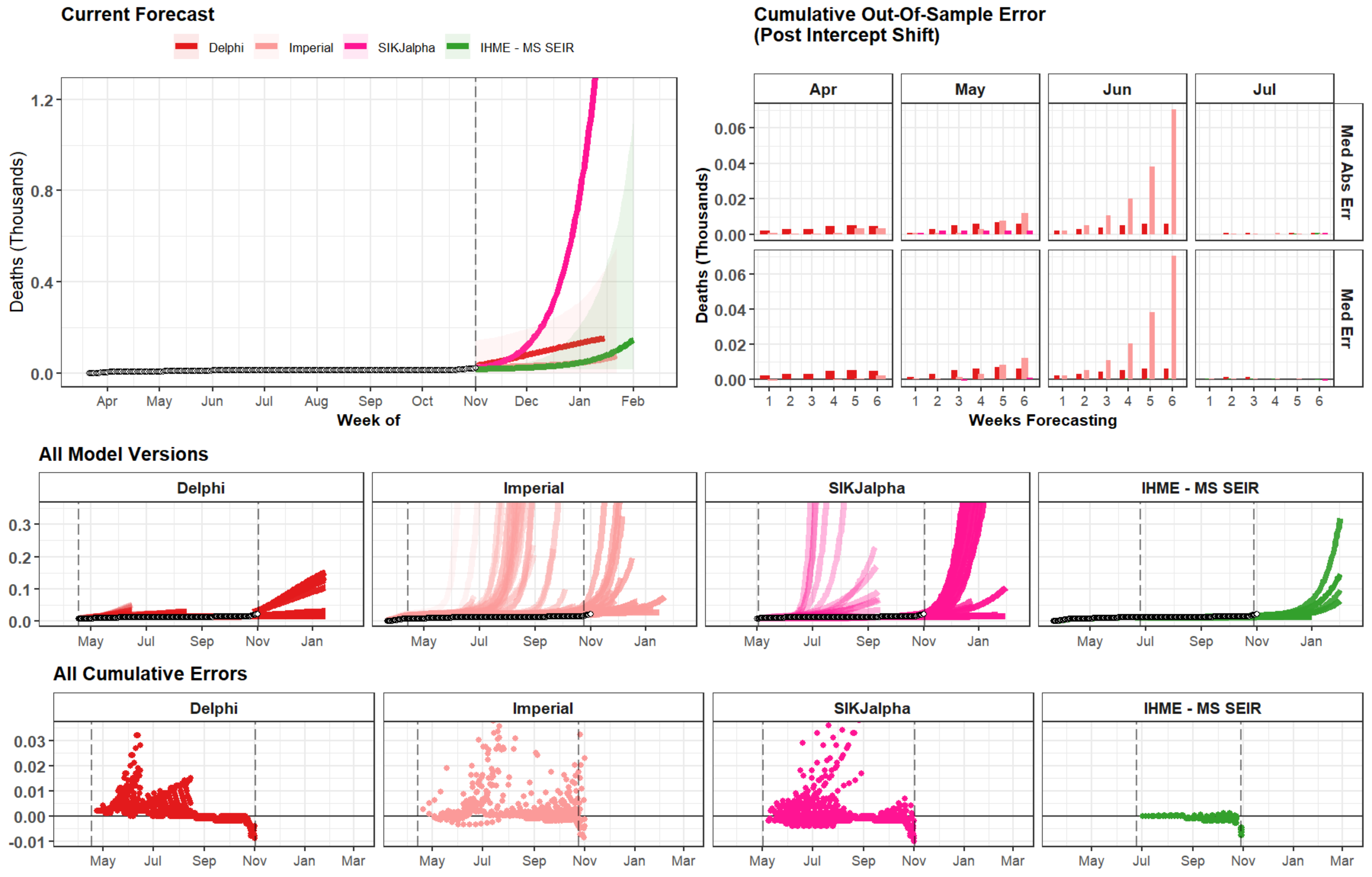

Current Forecast
Delphi
Imperial
SIKJalpha
IHME - MS SEIR
Deaths (Thousands)
1.2
0.8
0.4
0.0
Apr
May
Jun
Jul
Aug
Sep
Oct
Nov
Dec
Jan
Feb
Week of
Cumulative Out-Of-Sample Error
(Post Intercept Shift)
Apr
May
Jun
Jul
Med Abs Err
Med Err
0.06
0.04
0.02
0.00
1 2 3 4 5 6
Weeks Forecasting
All Model Versions
Delphi
Imperial
SIKJalpha
IHME - MS SEIR
0.3
0.2
0.1
0.0
May Jul Sep Nov Jan
All Cumulative Errors
Delphi
Imperial
SIKJalpha
IHME - MS SEIR
0.03
0.02
0.01
0.00
-0.01
May Jul Sep Nov Jan Mar

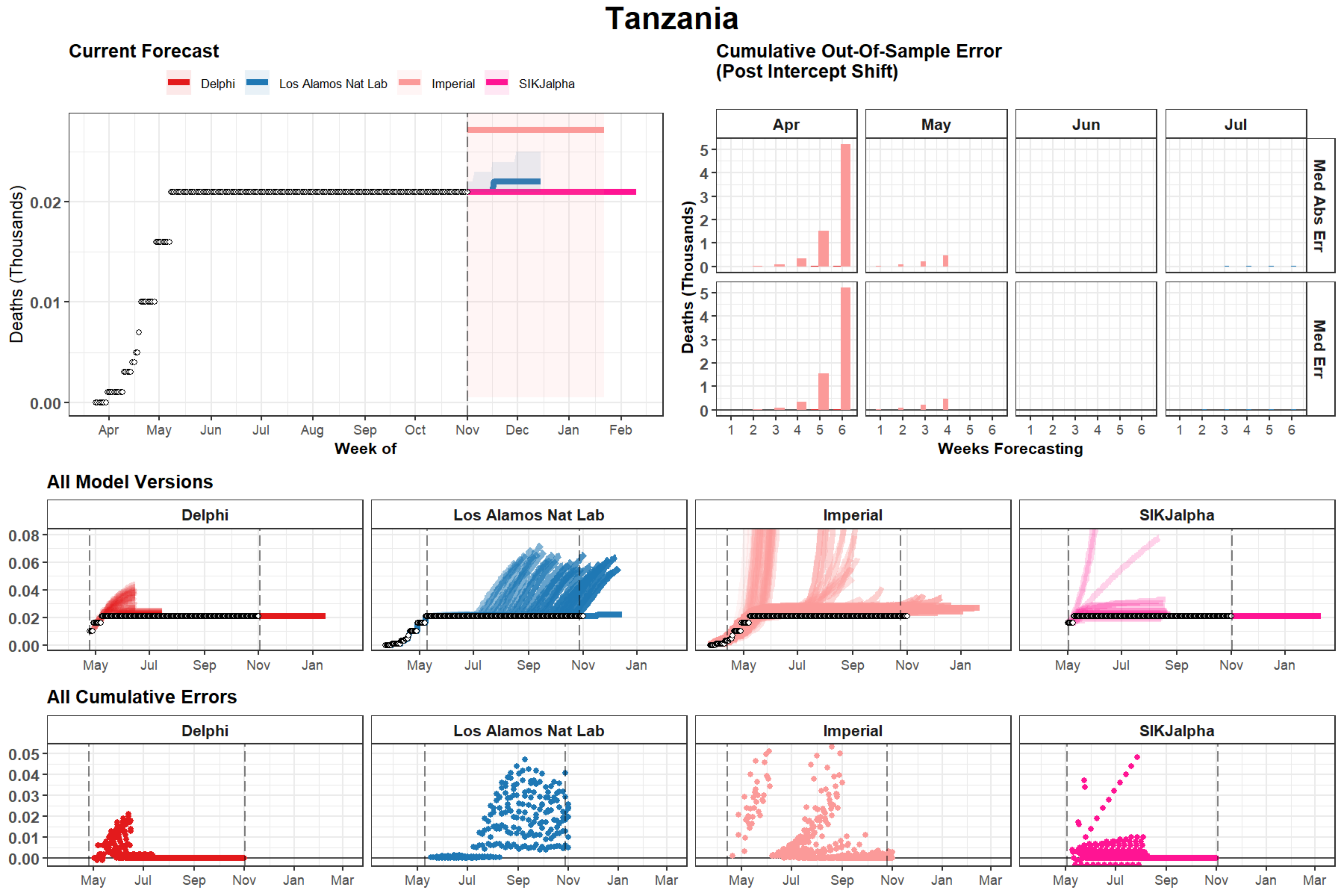

Tanzania
Current Forecast
Delphi
Los Alamos Nat Lab
Imperial
SIKJalpha
Deaths (Thousands)
0.00
0.01
0.02
Apr
May
Jun
Jul
Aug
Sep
Oct
Nov
Dec
Jan
Feb
Week of
Cumulative Out-Of-Sample Error
(Post Intercept Shift)
Apr
May
Jun
Jul
Med Abs Err
Med Err
Deaths (Thousands)
0
1
2
3
4
5
1
2
3
4
5
6
Weeks Forecasting
All Model Versions
Delphi
Los Alamos Nat Lab
Imperial
SIKJalpha
0.00
0.02
0.04
0.06
0.08
May
Jul
Sep
Nov
Jan
All Cumulative Errors
Delphi
Los Alamos Nat Lab
Imperial
SIKJalpha
0.00
0.01
0.02
0.03
0.04
0.05
May
Jul
Sep
Nov
Jan
Mar

Virgin Islands, U.S.

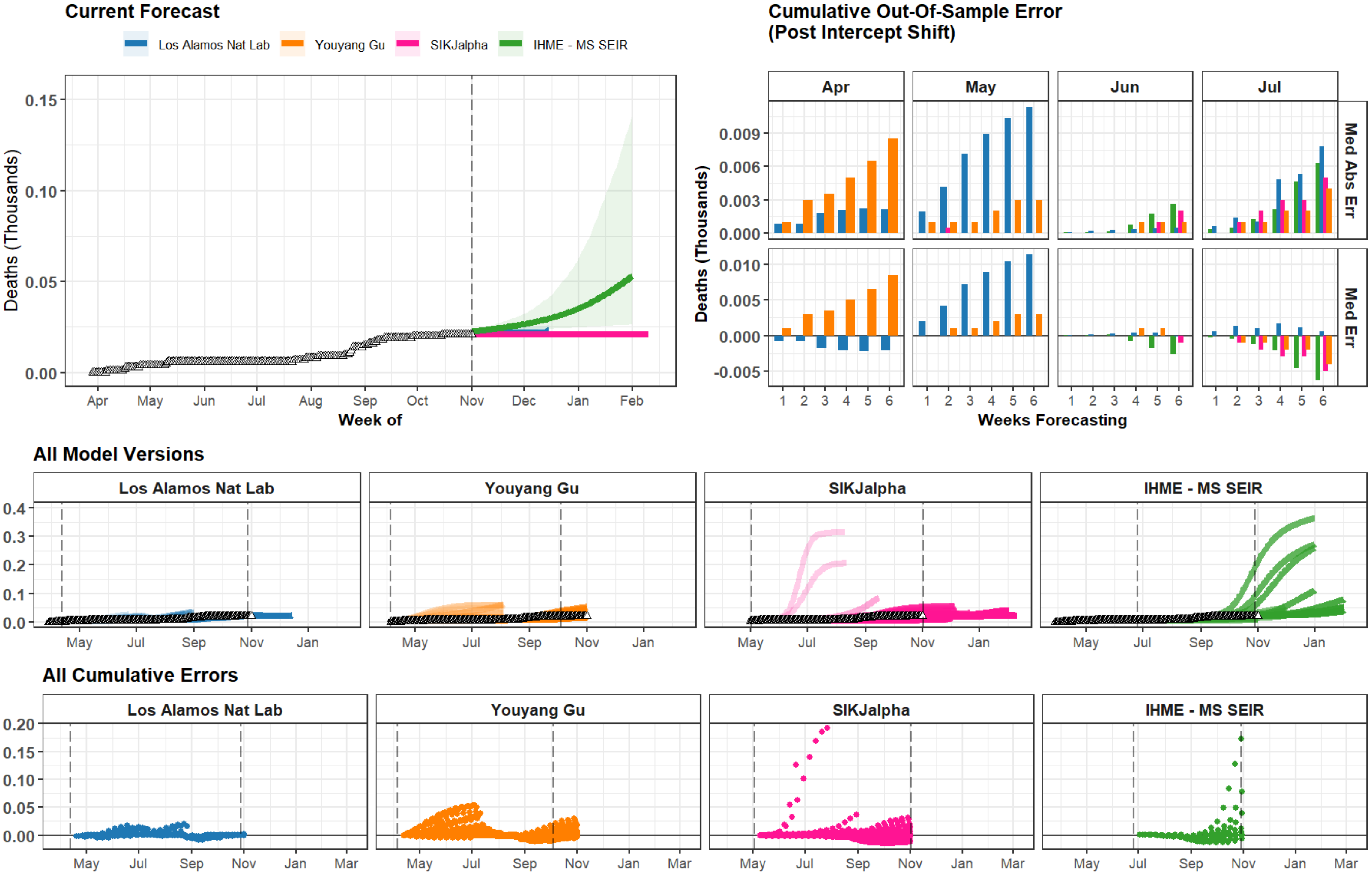

Sao Tome and Principe

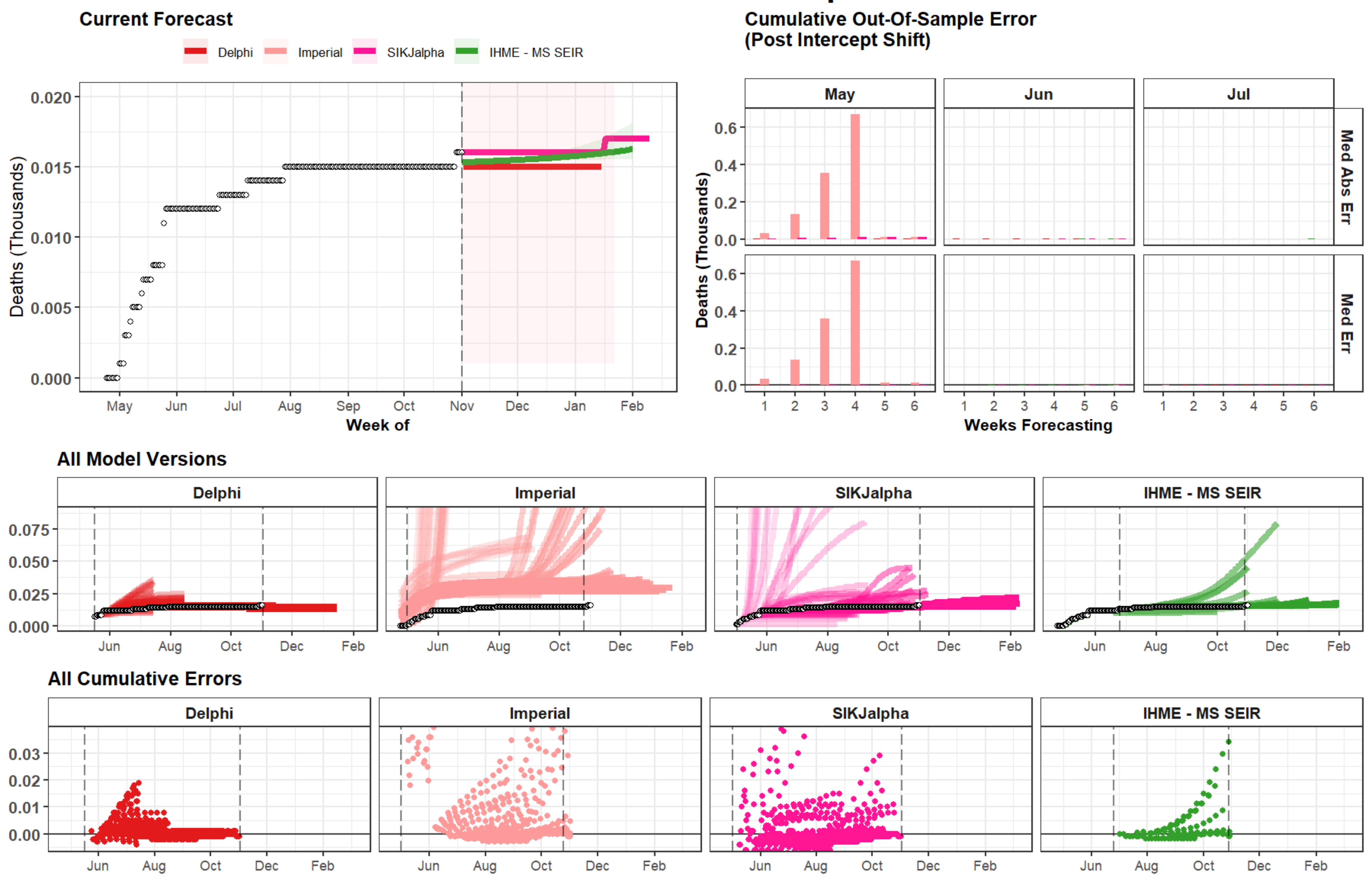

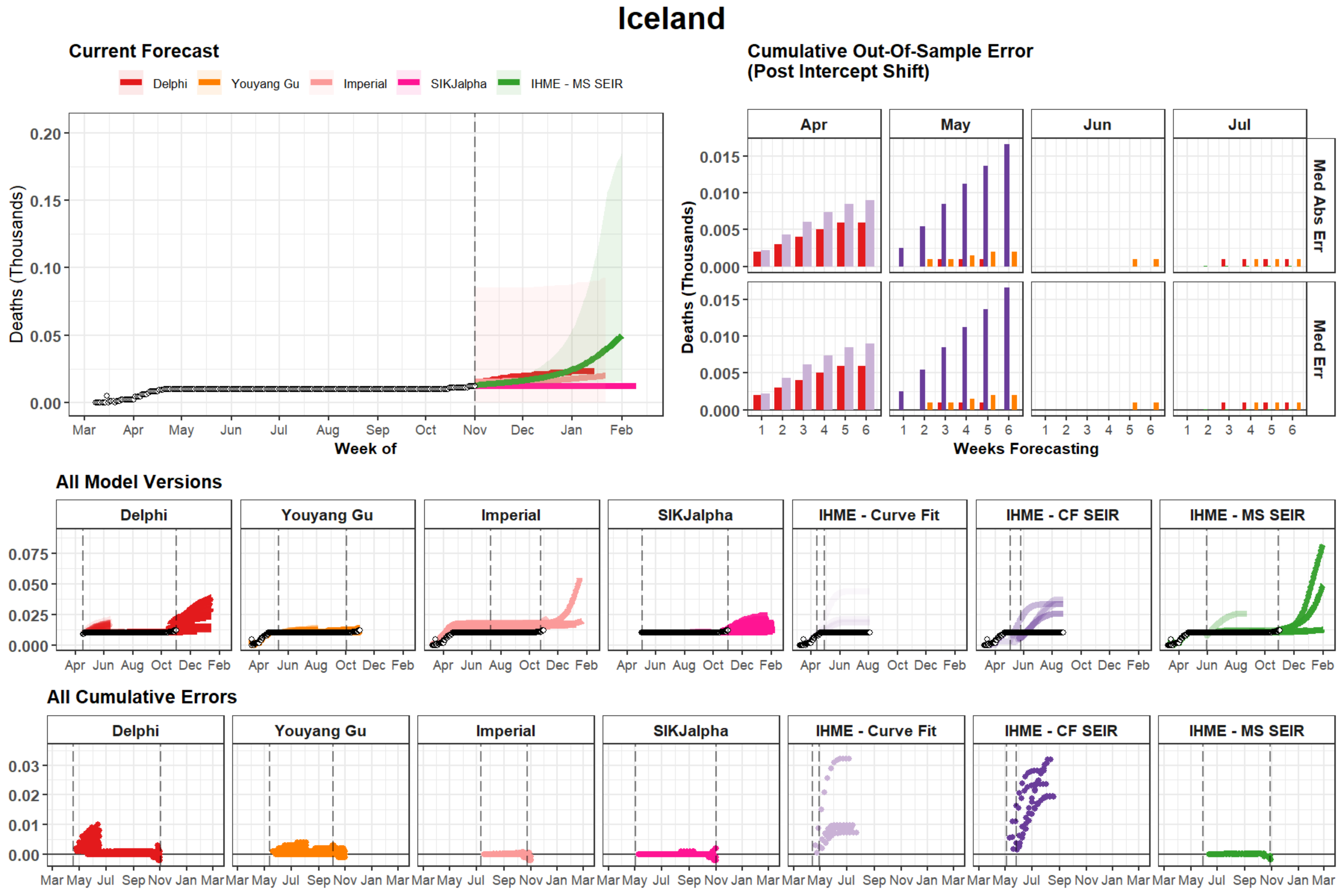

Iceland
Current Forecast
Delphi
Youyang Gu
Imperial
SIKJalpha
IHME - MS SEIR
Deaths (Thousands)
Week of
Cumulative Out-Of-Sample Error
(Post Intercept Shift)
Apr
May
Jun
Jul
Med Abs Err
Med Err
Weeks Forecasting
All Model Versions
Delphi
Youyang Gu
Imperial
SIKJalpha
IHME - Curve Fit
IHME - CF SEIR
IHME - MS SEIR
All Cumulative Errors
